# High-salt diet induces microbiome dysregulation, neuroinflammation and anxiety in the chronic period after mild repetitive closed head injury in adolescent mice

**DOI:** 10.1093/braincomms/fcae147

**Published:** 2024-05-03

**Authors:** Saef Izzy, Taha Yahya, Omar Albastaki, Tian Cao, Luke A Schwerdtfeger, Hadi Abou-El-Hassan, Kusha Chopra, Millicent N Ekwudo, Ugne Kurdeikaite, Isabelly M Verissimo, Danielle S LeServe, Toby B Lanser, Michael Aronchik, Marilia G Oliveira, Thais Moreira, Rafael Machado Rezende, Joseph El Khoury, Laura M Cox, Howard L Weiner, Ross Zafonte, Michael J Whalen

**Affiliations:** Divisions of Stroke, Cerebrovascular, and Critical Care Neurology, Department of Neurology, Brigham and Women’s Hospital, Boston, MA 02115, USA; Ann Romney Center for Neurologic Diseases, Brigham and Women’s Hospital, Harvard Medical School, Boston, MA 02115, USA; Harvard Medical School, Boston, MA 02115, USA; The Football Players Health Study at Harvard University, Boston, MA 02138, USA; Center for Immunology and Inflammatory Diseases, Massachusetts General Hospital, Boston, MA 02114, USA; Divisions of Stroke, Cerebrovascular, and Critical Care Neurology, Department of Neurology, Brigham and Women’s Hospital, Boston, MA 02115, USA; Ann Romney Center for Neurologic Diseases, Brigham and Women’s Hospital, Harvard Medical School, Boston, MA 02115, USA; Divisions of Stroke, Cerebrovascular, and Critical Care Neurology, Department of Neurology, Brigham and Women’s Hospital, Boston, MA 02115, USA; Ann Romney Center for Neurologic Diseases, Brigham and Women’s Hospital, Harvard Medical School, Boston, MA 02115, USA; Divisions of Stroke, Cerebrovascular, and Critical Care Neurology, Department of Neurology, Brigham and Women’s Hospital, Boston, MA 02115, USA; Ann Romney Center for Neurologic Diseases, Brigham and Women’s Hospital, Harvard Medical School, Boston, MA 02115, USA; Ann Romney Center for Neurologic Diseases, Brigham and Women’s Hospital, Harvard Medical School, Boston, MA 02115, USA; Ann Romney Center for Neurologic Diseases, Brigham and Women’s Hospital, Harvard Medical School, Boston, MA 02115, USA; Cancer Research Center, Massachusetts General Hospital, Boston, MA 02114, USA; Ann Romney Center for Neurologic Diseases, Brigham and Women’s Hospital, Harvard Medical School, Boston, MA 02115, USA; Ann Romney Center for Neurologic Diseases, Brigham and Women’s Hospital, Harvard Medical School, Boston, MA 02115, USA; Ann Romney Center for Neurologic Diseases, Brigham and Women’s Hospital, Harvard Medical School, Boston, MA 02115, USA; Ann Romney Center for Neurologic Diseases, Brigham and Women’s Hospital, Harvard Medical School, Boston, MA 02115, USA; Ann Romney Center for Neurologic Diseases, Brigham and Women’s Hospital, Harvard Medical School, Boston, MA 02115, USA; Ann Romney Center for Neurologic Diseases, Brigham and Women’s Hospital, Harvard Medical School, Boston, MA 02115, USA; Ann Romney Center for Neurologic Diseases, Brigham and Women’s Hospital, Harvard Medical School, Boston, MA 02115, USA; Ann Romney Center for Neurologic Diseases, Brigham and Women’s Hospital, Harvard Medical School, Boston, MA 02115, USA; Harvard Medical School, Boston, MA 02115, USA; Ann Romney Center for Neurologic Diseases, Brigham and Women’s Hospital, Harvard Medical School, Boston, MA 02115, USA; Harvard Medical School, Boston, MA 02115, USA; Harvard Medical School, Boston, MA 02115, USA; Center for Immunology and Inflammatory Diseases, Massachusetts General Hospital, Boston, MA 02114, USA; Division of Infectious Diseases, Department of Medicine, Massachusetts General Hospital, Boston, MA 02114, USA; Ann Romney Center for Neurologic Diseases, Brigham and Women’s Hospital, Harvard Medical School, Boston, MA 02115, USA; Harvard Medical School, Boston, MA 02115, USA; Ann Romney Center for Neurologic Diseases, Brigham and Women’s Hospital, Harvard Medical School, Boston, MA 02115, USA; Harvard Medical School, Boston, MA 02115, USA; Harvard Medical School, Boston, MA 02115, USA; The Football Players Health Study at Harvard University, Boston, MA 02138, USA; Department of Physical Medicine and Rehabilitation, Spaulding Rehabilitation Hospital, Massachusetts General Hospital, Brigham and Women’s Hospital, Boston, MA 02129, USA; Harvard Medical School, Boston, MA 02115, USA; The Football Players Health Study at Harvard University, Boston, MA 02138, USA; Department of Pediatrics, Massachusetts General Hospital, Boston, MA 02114, USA

**Keywords:** concussion, salt diet, microglia, neuroinflammation, microbiome

## Abstract

The associations between human concussions and subsequent sequelae of chronic neuropsychiatric and cardiovascular diseases such as hypertension have been reported; however, little is known about the underlying biological processes. We hypothesized that dietary changes, including a high-salt diet, disrupt the bidirectional gut–brain axis, resulting in worsening neuroinflammation and emergence of cardiovascular and behavioural phenotypes in the chronic period after repetitive closed head injury in adolescent mice. Adolescent mice were subjected to three daily closed head injuries, recovered for 12 weeks and then maintained on a high-salt diet or a normal diet for an additional 12 weeks. Experimental endpoints were haemodynamics, behaviour, microglial gene expression (bulk RNA sequencing), brain inflammation (brain tissue quantitative PCR) and microbiome diversity (16S RNA sequencing). High-salt diet did not affect systemic blood pressure or heart rate in sham or injured mice. High-salt diet increased anxiety-like behaviour in injured mice compared to sham mice fed with high-salt diet and injured mice fed with normal diet. Increased anxiety in injured mice that received a high-salt diet was associated with microgliosis and a proinflammatory microglial transcriptomic signature, including upregulation in interferon-gamma, interferon-beta and oxidative stress–related pathways. Accordingly, we found upregulation of tumour necrosis factor-alpha and interferon-gamma mRNA in the brain tissue of high salt diet–fed injured mice. High-salt diet had a larger effect on the gut microbiome composition than repetitive closed head injury. Increases in gut microbes in the families *Lachnospiraceae*, *Erysipelotrichaceae* and *Clostridiaceae* were positively correlated with anxiety-like behaviours. In contrast, Muribaculaceae, Acholeplasmataceae and *Lactobacillaceae* were negatively correlated with anxiety in injured mice that received a high-salt diet, a time-dependent effect. The findings suggest that high-salt diet, administered after a recovery period, may affect neurologic outcomes following mild repetitive head injury, including the development of anxiety. This effect was linked to microbiome dysregulation and an exacerbation of microglial inflammation, which may be physiological targets to prevent behavioural sequelae in the chronic period after mild repetitive head injury. The data suggest an important contribution of diet in determining long-term outcomes after mild repetitive head injury.

## Teaser

Izzy *et al*. studied the impact of a high-salt diet (HSD) on long-term cardiovascular and neuropsychiatric outcomes after repetitive closed head injury. They identified that a HSD disrupted the gut–brain axis, causing microbiome dysregulation, worsening microglial inflammation and triggering chronic anxiety-like behaviour post-repetitive head injury in adolescent mice.

## Graphical Abstract

**Graphical Abstract:**
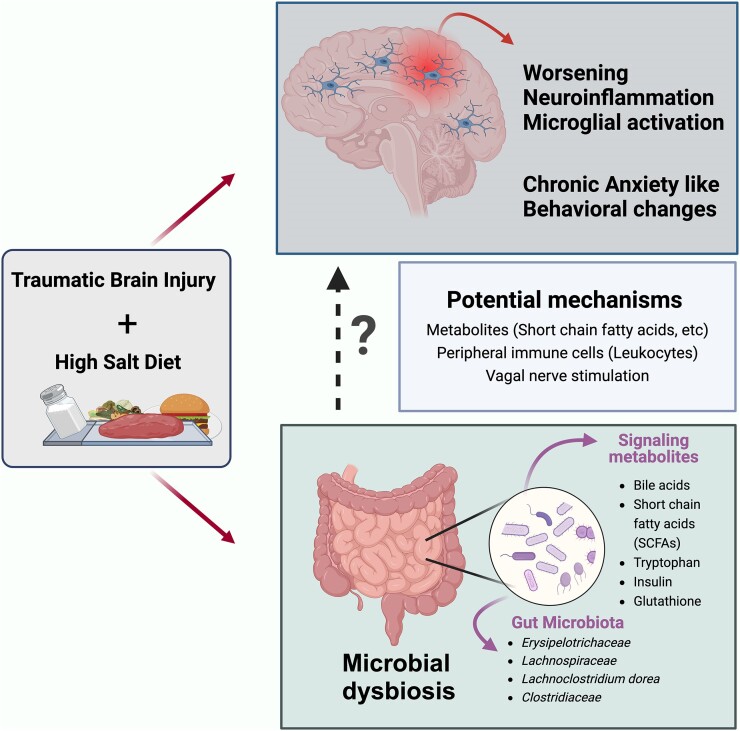

## Introduction

Concussions continue to be a prominent public health concern, with an estimated incidence of 1.6–3.8 million in the USA annually.^1^ Younger populations within the 14–19-year-old age group experience the highest rates of concussion, and nearly all athletic endeavours have some risk of concussive injury.^1-3^ Recent large-scale studies of the general population, American-style football players and military veterans demonstrated that prior traumatic brain injury (TBI) was associated with later development of neurological and psychiatric comorbidities and cardiovascular conditions, even in young patients.^4-7^ These findings indicate that brain injury may trigger progressive degenerative processes affecting the health of the brain and other organ systems and have become a central focus of public attention.^8-11^

Our work and others have demonstrated associations between TBI and higher prevalence of subsequent hypertension^4,6,12,13^ and psychiatric diseases such as anxiety.^4^ However, the pathophysiological mechanisms that underlie the progressive nature of TBI are still not clear. TBI initiates a series of neuropathological molecular and biochemical secondary injury sequelae that are long lasting and involve disruption of several biological pathways, including neuroinflammation.^14^ Other possible explanations include behavioural and lifestyle changes such as unhealthy diet and disruption to the bidirectional brain–gut axis.^15-18^ Increasing evidence suggests that sustained excess salt intake may affect brain health, beyond the well-recognized risk of hypertension.^19^

Recent preclinical studies show that high-salt diet (HSD) promotes cognitive impairment and suggest a gut-initiated adaptive immune response compromising brain function.^20,21^ High sodium intake is associated with increased inflammatory and stress responses and organ damage in patients.^22^ Increased salt consumption promotes proinflammatory interleukin-17 (IL-17)-producing helper T-cell (Th_17_) differentiation^23,24^ and inhibits the anti-inflammatory functions of regulatory T cells (FoxP3+).^25^ In addition, high sodium intake causes macrophage/microglia polarization to shift towards a classically activated proinflammatory phenotype^26,27^ and promotes the production of proinflammatory cytokines by myeloid cells in experimental models.^28,29^ There is growing evidence to support the HSD gut-initiated proinflammatory properties in the brain. In a mouse model of cerebral ischaemia, HSD promoted proinflammatory microglia polarization^30^ and exacerbated cortical blood–brain barrier disruption.^31^ A HSD has been reported to alter the gut microbiome and their short-chain fatty acids (SCFAs) in rats and to preferentially populate the gut with microbial species associated with hypertension.^32^ A growing body of literature has documented the close association between alterations in the gut microbiome with progression of neurodegenerative diseases such as Parkinson’s,^33^ amyotrophic lateral sclerosis,^34^ Alzheimer’s disease^35^ and TBI.^36^ However, the impact of HSD on the neuroinflammatory response and microbiome behavioural and cardiovascular outcomes after mild TBI has not been explored in preclinical concussion models.

Here, we tested whether HSD elevates blood pressure, alters microbiome diversity, worsens neuroinflammation and alters behavioural outcomes after repetitive closed head injury (rCHI) in mice. To investigate this, we injured adolescent mice using an established model of rCHI and HSD versus a normal diet (ND) maintained for 12 weeks.

## Materials and methods

### Experimental animals

Studies were performed using 38-day-old male C57/BL6J mice (stock #000664, Jackson Laboratories). All the procedures were performed in accordance with the NIH Guide for Care and Use of Laboratory Animals and followed protocols approved by the MGH Institutional Animal Care and Use Committee. Mice had access to food and water ad libitum and were housed on a 12-h day–night cycle in laminar flow racks in a temperature-controlled room (25°C). Investigators were blinded to study groups in all experiments. Mice were randomized to sham or rCHI at 38 (±3) days of age. Sham–injured and rCHI mice were housed in the same cage. Each cage had two sham and two rCHI mice.

### High-salt diet

HSD was introduced 60 days after rCHI. There were four experimental groups: sham mice receiving ND, sham mice receiving HSD, rCHI mice receiving ND and rCHI mice receiving HSD. ND consisted of normal chow (0.5% NaCl) and tap water ad libitum. HSD consisted of sodium-rich chow (8% NaCl) and tap water containing 1% NaCl ad libitum as previously described.^21^ The HSD was sustained for 12 weeks.

### Repetitive closed head injury model

A modified closed head injury (CHI) model was used as previously described.^37^ Mice were anaesthetized with 2.5% isoflurane in 70% N_2_O and 30% O_2_ for 90 s. Anaesthetized mice were placed on a taught KimWipe napkin and grasped by the tail. The head was placed under a 42-inch-long, 9/16-inch-diameter brass guide tube. A 1/2-inch-diameter 53-g lead cylindrical weight with a flat unbuffered surface was dropped onto the dorsal aspect of the skull directly above the right (Days 1, 3) or left (Day 2) ear between the coronal and lambdoid sutures. After impact, mice were placed supine and loss of consciousness time was recorded as time to righting reflex. Sham–injured mice received anaesthesia but no injury.

### Haemodynamics

Weekly measurements of systolic and diastolic blood pressure and heart rate were taken using the CODA non-invasive biological process (BP) system (a tail–cuff method, Kent Scientific Corporation) as previously described.^38^

### Behavioural studies

Behavioural studies were performed at 60 days post-rCHI as a baseline to ensure that there was no deficit pre-HSD administration. Mice were further tested at 4 weeks post-HSD (90 days post-rCHI), 8 weeks post-HSD (120 days post-rCHI) and 12 weeks post-HSD (150 days post-rCHI). All mice underwent a minimum of 30 min of acclimatization to the behaviour room before testing, and tests were consistently conducted between 7 a.m. and 11:30 a.m. The experimenter was blinded to the animal groups. The behavioural testing sequence commenced with the elevated plus maze and concluded with the Morris water maze (MWM). This was done to minimize the MWM-related anxiety as a confounding factor.

#### Elevated plus maze

Elevated plus maze was performed as previously described.^37^ The setup consisted of two 130 × 8 cm platforms with an 8 × 8 cm square area at their intersection. The apparatus was elevated at 60 cm above the ground. The closed arms had 10 cm walls, whereas the open arms had none. The mouse was placed in the centre of the apparatus, and the per cent time spent in the open arms was analysed by AnyMaze software. Each recorded trial was 5 min. The apparatus was cleaned with 10% ethanol between trials.

#### Open field

Mice were individually placed in housing cages with clean bedding and covered by a thin wire grid. The housing cage for the open field was 28 × 18 cm. During the open field test, mice were recorded by ceiling-mounted cameras and their movements were tracked by AnyMaze as described.^37^ Recordings lasted 30 min, and the distance covered during that time was used as a marker of overall activity.

#### Rotarod

Mice were placed on a Rotarod apparatus (Harvard Apparatus, Holliston, MA, USA), accelerating from 4 to 40 RPM in 120 s. Each trial ended when the mouse fell off the rod, and the latency to fall was manually recorded. Mice were tested for three trials per day (5-min inter-trial interval) for 3 consecutive days.

#### Morris water maze

MWM testing was performed as previously described.^39^ Each mouse was subjected to 5–7 hidden and 2 or no visible platform trials. Each mouse underwent 1–2 trials per day, and each trial consisted of four attempts with the exception of the probe trial in which each mouse was only tested once. For the hidden trials, the platform was submerged between 0.5 and 1 cm below the surface of the water and kept in the same location for all the hidden trials. For the probe trial, the platform was completely removed. The visible trials were done last, and the platform was placed 1 cm above the water surface; the platform location was changed for every visible trial. Mice were assigned four start positions around the perimeter of the pool and allowed 90 s to locate the platform, after which the mouse remained on the platform for 15 s. If the mouse did not find the platform in 90 s during the first and second hidden trials, it was guided to platform and allowed to remain there for 15 s and was given a score of 90 s. The time until the mouse mounted the platform (escape latency) was measured and recorded (AnyMaze 8.42, Stoelting, Wood Dale, IL, USA). For probe trials, the time spent in the target quadrant where the platform was removed (total 30 s swim time) was recorded.

### Preparation of brain tissue for immunohistochemistry

Mice were deeply anaesthetized with isoflurane and decapitated. The brains were removed and frozen in liquid nitrogen prior to making coronal sections (16 *μ*m) on poly-L-lysine-coated slides (Thermo Fisher Scientific) using a cryostat. The brains were cut at 0.5-mm intervals from the anterior to the posterior of the brain. For analyses using paraformaldehyde-fixed tissue, mice were transcardially perfused with PBS followed by 4% paraformaldehyde and brains were post-fixed overnight in 4% paraformaldehyde, cryoprotected in 30% sucrose overnight, frozen at –80°C and cut on a cryostat as above.

### Immunofluorescence staining

Fluorescent immunolabeling followed a standard protocol. Sections were transferred into blocking medium (0.075% Triton-X, 5% normal horse serum in 1× PBS solution) for 1 h at room temperature before applying primary antibody. The primary antibody, rabbit anti-mouse IBA1 (1:1000; Wako; RRID: AB_839506), was applied overnight followed by Cy3-conjugated anti-rabbit secondary antibodies (Jackson ImmunoResearch Laboratories, 1:300). Sections were counterstained with 10 *μ*l DAPI Mounting Medium (Vector Laboratories) and sealed by placing a glass coverslip over sections (Menzel Glaser) and coating the edges of the coverslip with clear nail polish.

### Imaging and cell quantification

Five 16-*μ*m consecutive sections of the dorsal dentate gyrus (DG) were quantified per animal, starting at anteroposterior −1.5 mm from bregma (one-in-six series, 300 *μ*m apart). All representative images were acquired using a Zeiss LSM 710 confocal microscope and processed with Zen black 2.1 (Carl Zeiss). Images were acquired using 20× objective lens, scale bar 50 *μ*m. For quantification of Iba1 positive, a full mosaic of hemisphere brain sections was acquired using a Leica DMi8 Widefield Fluorescence Microscope under a 20× lens and quantified with ImageJ software. For cell counting, ImageJ software was used and the ‘Analyze Particles’ option was used to count the cells. The region of interest within the areas of the M1 motor cortex, the hippocampal radiatum layer adjacent to pyramidal CA1, the stratum lucidum adjacent to CA3 and the DG (hereafter referred to as cortex, CA1, CA3 and DG, respectively) and the amygdala was quantified. Numbers of microglia were obtained by scanning regions at 500 × 500 *μ*m boxes at comparable sections in each animal. Data were number of IBA1^+^ cells/0.25 mm^2^. All quantifications were performed with ImageJ analysis software as previously described.^40,41^

### Quantitative polymerase chain reaction

RNA was extracted with RNeasy^®^ columns (Qiagen), cDNA was prepared and used for quantitative PCR (Applied Biosystems^™^, 437466), and the results were normalized to *Gapdh* (Mm99999915_g1). All primers and probes were from Applied Biosystems, and *IL10* (Mm01288386_m1), *Il6* (Mm00446190_m1), *TNF* (Mm00443258_m1), *Il1b* (Mm00434228_m1), *Infg* (Mm01168134_m1) and *Ccl5* (Mm01302427_m1) were used. The 2^−ΔΔCt^ method was used to calculate relative expression of each gene.

### Flow cytometry microglial sorting

For microglial cell sorting, mice were anaesthetized with CO_2_ until respiration rate slowed and transcardially perfused with 50 ml of Hanks’ balanced salt solution (HBSS). Following perfusion, the brains were homogenized using a Dounce glass tissue homogenizer. Cells were separated through Percoll (GE Healthcare Life Sciences) 30% gradient by centrifugation. Cells were isolated from the Percell layer and stained on ice for 30 min with combinations of phycoerythrin/Cy7 rat anti-mouse CD11b (Biolegend, #101216, 1:100), allophycocyanin/Cy7 rat anti-mouse CD45 (Biolegend, #103116, 1:100) in blocking buffer containing 0.2% bovine serum albumin (Sigma-Aldrich) in HBSS. Cell sorting was performed using a FACSAria III cell sorter (Becton Dickinson). Microglial cells were identified as CD45 low to intermediate/CD11b high cells.^42^ Cells were sorted directly in 1.5-mL Eppendorf tubes and stored at −80°C.

### Microglia bulk RNA sequencing

Bulk RNA sequencing (RNA-seq) was performed as previously described^43^ for samples at 12 weeks after HSD administration. Briefly, 2000 isolated CD45 low to intermediate/CD11b high cells (microglia) were lysed in 5 *µ*l TCL buffer (Qiagen) + 1% β-mercaptoethanol. Smart-Seq2 libraries were prepared and sequenced by the Broad Genomic Platform. cDNA libraries were generated from sorted cells using the Smart-Seq2 protocol. RNA-seq was performed using Illumina NextSeq500 using a High Output v2 kit to generate 2 × 38 bp reads. The processing of the bulk RNA-seq data was based on an established computational pipeline.^44^ Sequencing data were demultiplexed and provided by the Broad Institute in FASTQ format. FastQC was used to assess sequencing quality control. Trimmomatic was used for adaptor trimming of reads. Reads were then aligned to the ‘mm10’ reference genome using HISAT. The generated sequence alignment/map (SAM) files were then converted into binary alignment map files using SAMtools. StringTie was used for transcript assembly and quantification. Transcript abundances were then imported into R Studio (version 4.1.2) and converted to gene-level estimated counts using the ‘tximport’ package (version 1.22.0) from Bioconductor. Genes that achieved <10 counts summed across all samples were considered very low expressed genes and thus filtered out. Sample read counts were normalized using the variance-stabilizing transformation (VST) method from the DESeq2 (version 1.34.0) built-in VST function.^45^ These normalized sample read counts were used to plot heatmaps using ComplexHeatmap (version 2.13.1). Dot plots were generated using ggplot2 (version 3.4.0). Bar plots were generated using GraphPad Prism software for Mac.

### Differential gene expression and pathway analysis

Differential gene expression analysis was carried out with DESeq2. For comparisons of gene expression between two different sample groups, the Wald test was used with a significance cut-off of *P* < 0.05 and standard parameters. The log2 fold changes (Log2FCs) of the corresponding differentially expressed genes (DEGs) were subsequently shrunken using DESeq2’s built-in lfcshrink function. Pairwise comparisons of DEGs were visualized using DiVenn.^46^ Gene set enrichment analysis (GSEA) pathway analyses were performed through the GAGE package (version 2.44.0).^47^ For gene ontology (GO) enrichment analysis, the clusterProfiler package was used (version 4.2.2).^48^ Ingenuity pathway analysis (IPA) (https://digitalinsights.qiagen.com/products-overview/discovery-insights-portfolio/analysis-and-visualization/qiagen-ipa/) was used to identify upstream regulators (*P* < 0.05 and *Z*-score| ≥ 2) based on DEGs in a particular pairwise comparison, where input data comprised of *P*-values and Log2FCs of the DEGs. Statistical significance for all differential gene expression and pathway analyses was defined as a nominal *P*-value < 0.05.

### 16S rRNA microbiota sequencing and microbial community analysis

Faecal samples were collected from eight mice per group starting at 60 days post-rCHI prior to high salt–diet administration and were done weekly until experimental endpoint at 90 days after HSD administration [150 days post-injury (DPI)]. Caecum samples from six mice per group were collected at 90 days after salt diet administration (150 DPI). DNA was extracted using the Qiagen DNeasy PowerLyzer PowerSoil Kit (Qiagen, Hilden, Germany). The V4 16S rRNA gene was amplified with barcoded fusion primers developed by the Earth Microbiome Project.^49,50^ Paired-end sequencing was performed at the Harvard Biopolymers facility on the Illumina MiSeq. The QIIME2 pipeline^51^ was used for quality filtering of DNA sequences, demultiplexing, taxonomic assignment and calculating alpha and beta diversity; DNA demultiplexing and quality filtering were performed by DADA2, samples were aligned, and alpha and beta diversity was calculated at a depth of 1000 reads. Samples were removed if they had fewer than 1000 reads, and amplicon sequence variants (ASVs) were removed if they had fewer than 10 reads or were in fewer than two samples. A pre-trained Silva classifier was used for taxonomic assignment. To evaluate overall differences in microbial community structure between salt diet and ND in both sham and injured groups, permutational multivariate analysis of variance (PERMANOVA) tests were performed on beta diversity weighted and unweighted UniFrac distance measures. Statistical analysis of the changes in differences in relative microbial abundance was determined by linear discriminated analysis effect size (LEfSe) with the alpha set at 0.05 and the effect size set at 2. Linear discriminant analysis (LDA) scores and *P*-values were plotted in R using ggplot2, ComplexHeatmap and ColorBrewer packages.^52^ An ADONIS test in QIIME2 was used to determine the percentage of contribution to microbiome variance. To identify bacteria linked with anxiety, Spearman’s correlations were constructed in R using the stats package. Since the anxiety-like phenotype was measured using an elevated plus maze test where a lower value (per cent time spent in the open arms) suggests higher anxiety-like behaviours, the final correlation directions (positive or negative) were inversed for practical interpretation. Metagenomic content of the microbiota samples was predicted from the 16S rRNA profiles, and Kyoto Encyclopedia of Genes and Genomes (KEGG) pathway functions were categorized at level 3 using the phylogenetic investigation of communities by reconstruction of unobserved states (PICRUSt2) tool.^53^ Significant pairwise differences based on relative predicted KEGG metagenomic pathways were determined by LEfSe with the alpha set at 0.05 and the effect size set at 1. Relative abundance plots over time for specific bacteria were performed using GraphPad Prism software for Mac.

### Statistical analysis

Data are mean ± SEM. One-way ANOVA test with Tukey’s multiple comparisons was used to assess statistical significance between groups at a specific time point such as per cent time in open arm (elevated plus maze), distance travelled (open field test), per cent time in the target quadrant (probe trial), IBA-1 cell quantification and relative mRNA expression for the RT-qPCR. A two-factor repeated measures two-way ANOVA (group × time) was used to assess two independent variables including time- and study-dependent outcomes such as latency to fall (Rotarod), latency to platform (MWM) and blood pressure/heart rate (haemodynamics study). Statistical tests used are in line with other published studies.^37^ Statistical analysis for 16S rRNA sequencing data and microglia RNA-seq were expanded upon in their respective method sections. Numbers per group, significance level and statistical tests are indicated in the figure legends. No statistical methods were used to predetermine sample size. Sample sizes were chosen in accordance with previous work in the field.^20,39,54^ Statistical analyses were performed using GraphPad Prism 9 software (GraphPad Software Inc., La Jolla, CA, USA), and differences for all tests were considered significant if the *P*-value was <0.05.

## Results

### High-salt diet did not alter blood pressure or heart rate but increased anxiety-like behaviour after repetitive mild closed head injury

**Figure 1:**
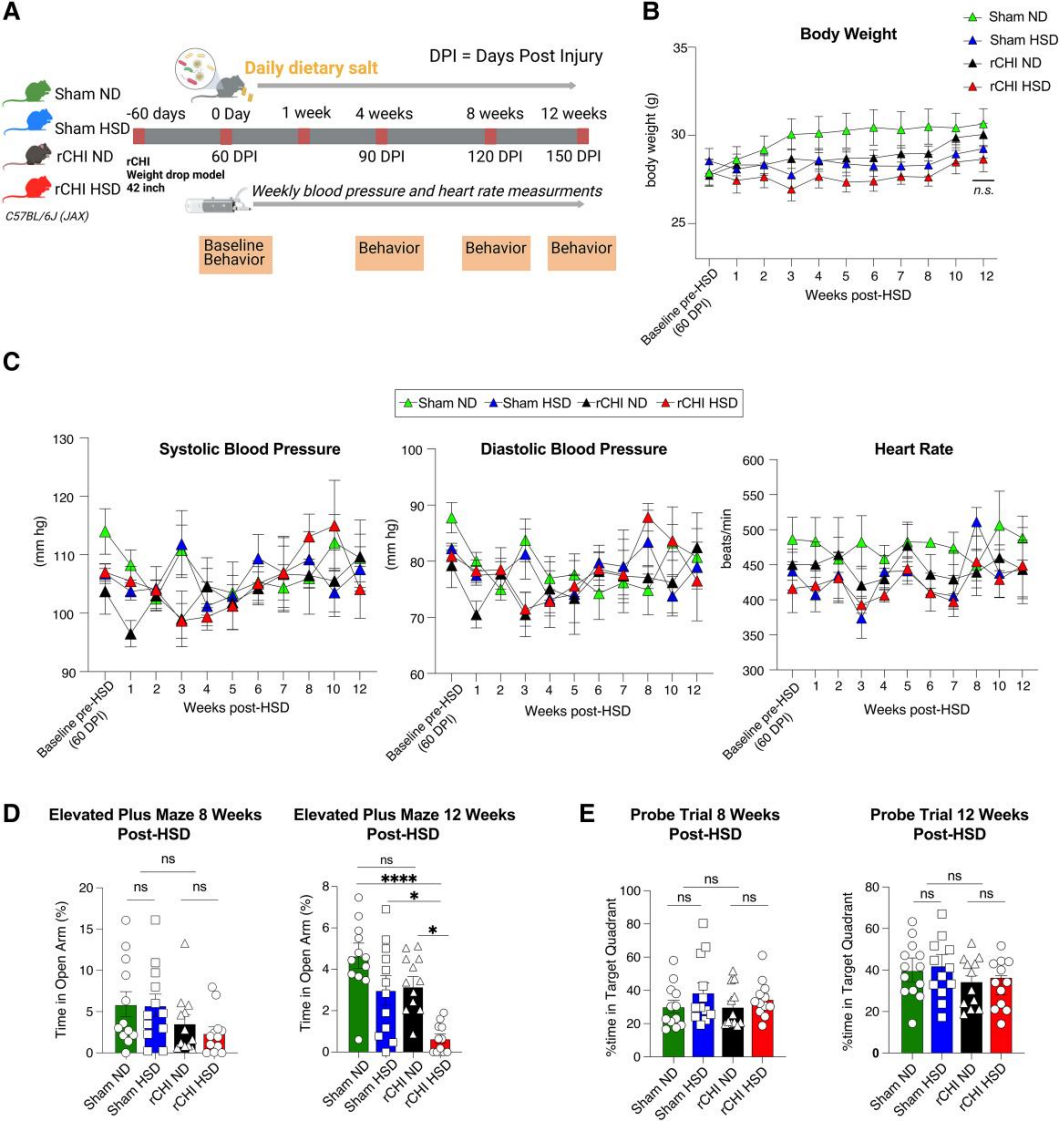
**HSD induces an anxiety-like phenotype in an rCHI model of concussion.** (**A**) Visual representation of experimental timeline of behavioural and physiological testing regimens after HSD administration. (**B**) Physiological data of weight. (**C**) Systolic/diastolic blood pressure and heart rate of the groups through 12 weeks of diet administration. Behavioural testing of anxiety-like phenotype using an (**D**) elevated plus maze and (**E**) probe trial of the MWM, at 8 and 12 weeks post-diet administration. Physiological data were analysed by a two-factor repeated measures two-way ANOVA (group × time), and the other behavioural tests were analysed by one-way ANOVA, followed by a Tukey’s multiple comparison. *n* = 12 mice/group was used for all experiments, and data are presented as mean and SEM. *P < 0.05, ***P < 0.001. ns, not significant.

Previous studies have shown an association of TBI in American-style football with development of hypertension amongst young athletes.^4,55,56^ Figure 1A shows the experimental design for rCHI and HSD to test this association experimentally. HSD mice gained less weight over time when compared with mice on a ND, independent of rCHI (Fig. 1B); however, no significant changes in systolic or diastolic blood pressure or heart rate were observed with HSD compared with ND groups in both sham and rCHI (Fig. 1C). Compared to other groups, mice in the rCHI/HSD group spent significantly less time in the open arm of the elevated plus maze (Fig. 1D), an anxiety-like phenotype, at 12 weeks after initiation of the HSD. This effect was not observed at earlier time points after rCHI (Supplementary Fig. 1A). The consumption of HSD following rCHI did not affect spatial learning and memory as shown by the MWM and probe trial testing (Fig. 1E, Supplementary Fig. 1B, C). In addition, HSD did not cause rotarod deficits or induce general locomotor and exploration deficits (assessed by an open field test) at all time points tested after rCHI (Supplementary Fig. 1D, E). Altogether, our data show that the consumption of HSD following rCHI is associated with worsening anxiety-like behaviour in the chronic period after rCHI.

### High-salt diet exacerbates chronic microglial neuroinflammatory responses following repetitive closed head injury

**Figure 2:**
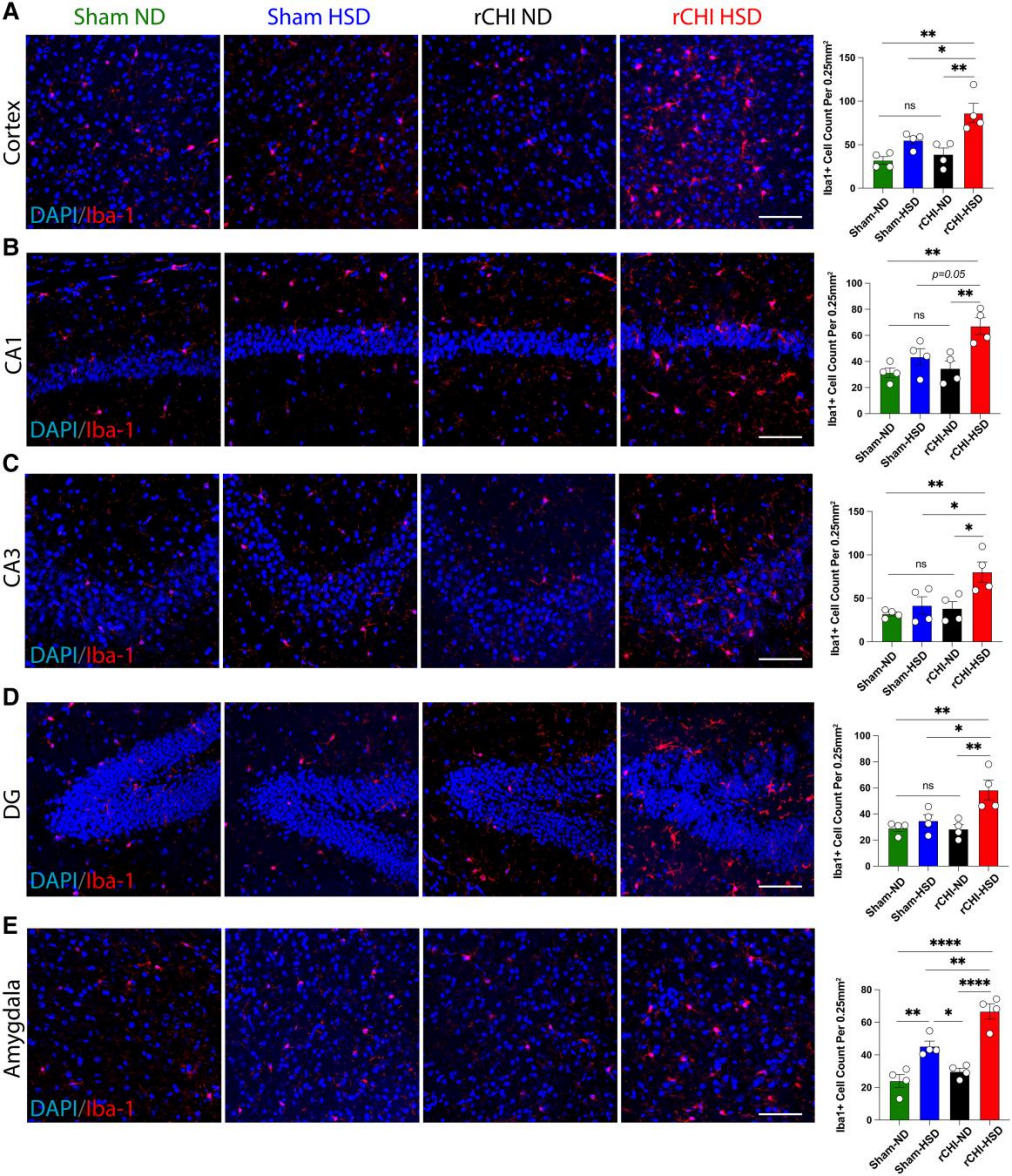
**HSD induces chronic microglial activation in an rCHI model of concussion.** Representative confocal images of immunofluorescence staining for Iba1^+^ microglia and DAPI (blue, nuclear stain) on different brain regions. Example overviews of **A** cortex, different hippocampal areas **B** CA1, **C** CA3 and **D** DG and **E** the amygdala in 12 weeks post-ND or HSD in sham and rCHI mice. Quantitative analysis of Iba1+ cells counted in CA1, CA3 and DG and the amygdala in 12 weeks post-ND or HSD in sham and rCHI mice. *n* = 4 mice/group. **P < 0.05, ***P < 0.001, ****P < 0.0001. ns, not significant by one-way ANOVA with Tukey’s multiple comparison test. Representative images’ scale bars represent 50 *µ*m.

**Figure 3:**
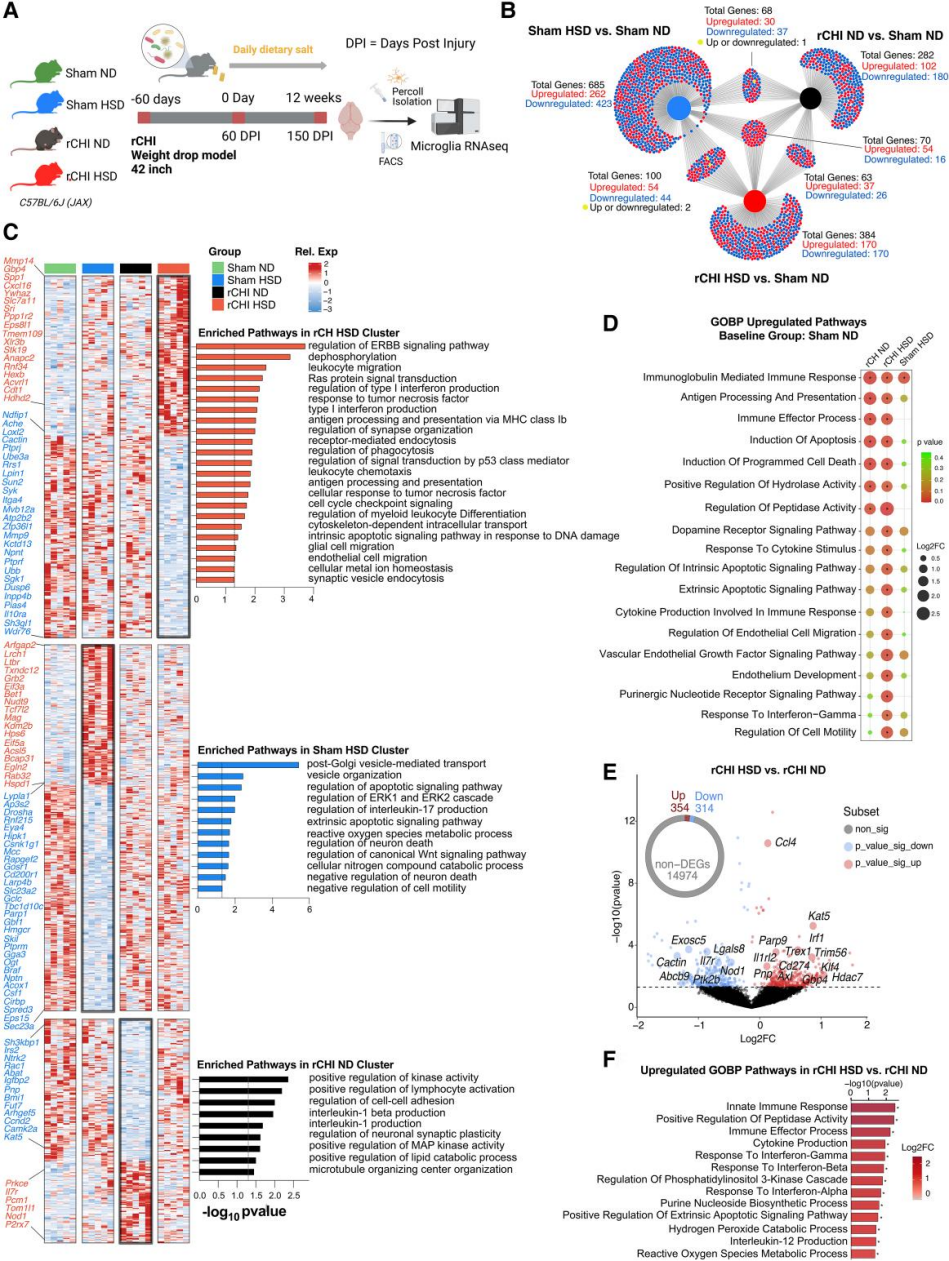
**HSD induces chronic proinflammatory microglial profile and stress response in an rCHI model of concussion.** (**A**) Visual representation of experimental timeline of microglia analysis and bulk RNA sequencing after HSD administration. (**B**) DiVenn plot showing the unique and shared DEGs (*P* < 0.05) of the following groups compared to sham ND baseline: sham HSD, rCHI ND and rCHI HSD. Directionality of gene expression is determined by Log2FCs of pairwise gene expression comparisons. Red-coloured genes represent upregulated genes. Blue-coloured genes represent downregulated genes. Yellow-coloured genes represent shared genes that are upregulated in one comparison but downregulated in the other comparison. (**C**) Heatmap of relative expression levels clustered according to the unique DEGs obtained in the following pairwise comparisons compared to sham ND baseline (from *top* to *bottom*): rCHI HSD, sham ND and rCHI ND (*n* = 5 mice/group). Enriched GO terms of each cluster are displayed on the right with selected corresponding genes labelled on the left. (**D**) Dot plot of GAGE analysis highlighting significantly upregulated GOBP pathways in the following pairwise comparisons compared to sham ND baseline: rCHI ND, rCHI HSD and rCHI ND. Dot size indicates the Log2FC of the pathway in the specific pairwise comparison (rCHI ND versus sham ND, rCHI HSD versus sham ND or sham HSD versus sham ND). Dot colour indicates significance strength of the pathway, where *P*-value < 0.05 was considered statistically significant (indicated by asterisk *). (**E**) Volcano plots showing the microglia gene expression in rCHI HSD versus rCHI ND. On the *x*-axis are the Log2FCs and the *y*-axis is the −log10 (*P*-value). Significant DEGs (*P* < 0.05) are coloured (red for upregulation and blue for downregulation). (**F**) Selected top upregulated GOBP pathways from GAGE analysis by *P*-value in rCHI HSD versus rCHI ND. Data are represented by –log_10_ (*P*-value), and increasing bar colour intensity signifies increasing Log2FCs. * *P* < 0.05.

Microglia are key players in the neuroinflammatory response to TBI, and their chronic activation after injury can lead to neurological dysfunction and neurodegeneration.^14,57^ HSD can shift macrophages/microglia towards classically activated proinflammatory phenotypes,^27^ yet the impact of HSD on the brain’s inflammatory response to rCHI is unknown, an important question given the effect of interleukin-1 signalling on anxiety and cognitive dysfunction in a 3HD adolescent rCHI model.^58^ The HSD/rCHI group had increased microgliosis in cortex (Fig. 2A), hippocampus (Fig. 2B–D) and amygdala (Fig. 2E) compared to ND/rCHI and sham groups. Therefore, we investigated the impact of HSD administration on the microglial inflammatory transcriptomic profile following rCHI and sham injury. Microglia single-cell suspensions were obtained from the mouse brains at 12 weeks after HSD administration and analysed using bulk RNA-seq (Fig. 3A, Supplementary Fig. 3A). Normalized expression counts for all genes passing quality metrics are shown in Supplementary Table 1. Several established microglia markers were highly expressed in all groups, such as *Cx3cr1*, *Hexb*, *P2ry12* and *Tmem119*, whereas we found minimal expression of non-microglia markers^43^ (Supplementary Fig. 3B). We compared the HSD-fed mice (sham and rCHI) and rCHI mice fed with ND to sham ND controls to define unique DEGs (Fig. 3B, Supplementary Table 2). To detect unique transcriptomic patterns in sham HSD, rCHI HSD and rCHI ND, we plotted the unique DEGs in each group as clusters in a heatmap (Fig. 3C, Supplementary Table 3). We found that each cluster was enriched for distinct GO terms. The cluster of unique DEGs in rCHI HSD was related to type I interferon production (*Cactin*, *Crebbp*, *Syk* and *Gbp4)*, antigen processing and presentation (*Cd1d2*, *H2-K1* and *H2-Q1*), response to *TNF* (*Cxcl16*, *Pias4* and *Syk*), regulation of phagocytosis (*Dnm2*, *Ptprj*, *Siglece* and *Syt11*), endothelial migration (*Acvrl1*, *Bsg*, *Lgmn* and *Loxl2*), synaptic organization (*Il10ra*, *Ptprf*, *Ptpro*, *Slc7a11*, *Ube3a* and *Ywhaz*) and leukocyte migration (*Cxcl16*, *Emilin1*, *Itga4*, *Mmp14*, *Mmp9*, *Ptpro* and *Spp1*). The cluster of unique DEGs in the sham HSD group was associated with regulation of neuronal death (*Cd200ra*, *Csf1*, *Egln2*, *Gclc*, *Hdac4*, *Hspd1*, *Jak2*, *Mag*, *Parp1*, *Rest* and *Tnfrsf1a)*, reactive oxygen species (ROS) metabolic processes (*Acox1*, *Eif5a*, *Grb2*, *Hdac4*, *Hspd1*, *Ier3* and *Ogt*), regulation of *IL-17* production (*Jak2* and *Parp1*), regulation of apoptotic signalling pathways (*Bcap31*, *Gclc*, *Ier3*, *Ltbr*, *Map2k5*, *Ptpn2* and *Src*) and regulation of ERK1 and ERK2 cascades (*Acta2*, *Prkd2*, *Prtm5*, *Ptpn2*, *Ptpn6* and *Rapgef2*). The cluster of unique DEGs in rCHI ND mice was mainly involved in IL-1B production (*Lilra5*, *Nod1*, *P2rx7* and *Tnfaip8*) and positive regulation of kinase activity (*Axl*, *Ccnd2*, *Fgfr1*, *Ntrk2*, *Pdcd10*, *Rac1* and *Tom1l1*).

To further evaluate unique and shared microglial pathways altered by HSD, we performed GSEA of the four groups using gene ontology biological process (GOBP) pathways. Compared to sham ND, HSD in both sham and rCHI groups was associated with a significant upregulation of immune-related pathways including immune effector processes, antigen processing and presentation, pathways involved in the regulation of hydrolase activity and apoptosis **(**Fig. 3D). However, we also found that the rCHI HSD was uniquely associated with a significant upregulation of other immune-mediated pathways (such as IFN*-*γ and cytokine responses), microglia regulation pathways (purinergic receptor signalling and cell motility) and vascular endothelial-related pathways (vascular endothelial growth factor, endothelial cell growth and development).

**Figure 4:**
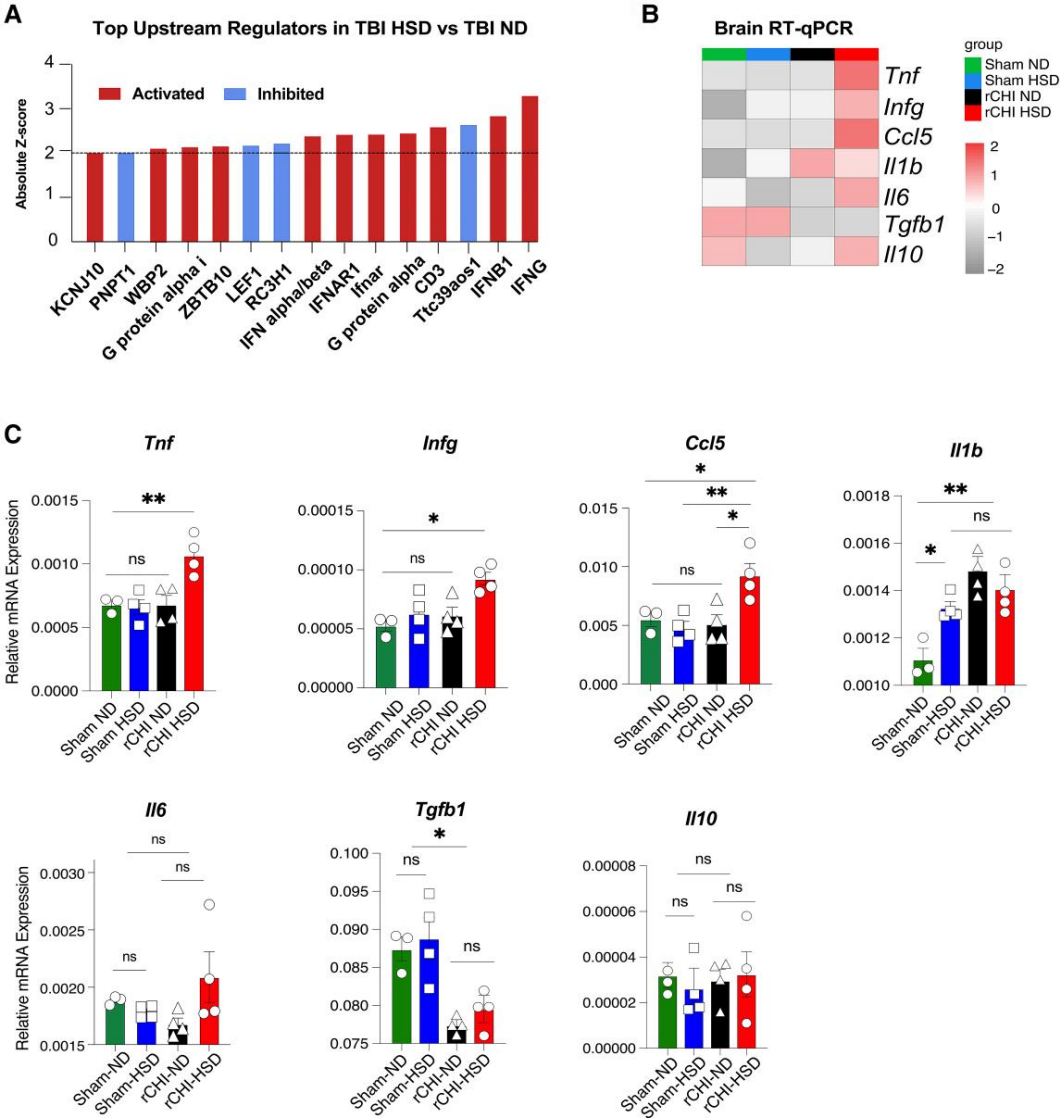
**HSD induces interferon-gamma and other proinflammatory cytokines after rCHI.** (**A**) Top IPA predicted upstream regulators for DEGs in rCHI HSD versus rCHI ND. All shown regulators were deemed significant at *P* < 0.05 and an absolute activation score ≥2. Activation scores were used to determine the activation state of the predicted upstream regulator, where an activation score ≤−2 implies the upstream regulator is inhibited and an activation score ≥2 implies that the upstream regulator is activated. (**B**) Heatmap and (**C**) bar plots of brain tissue qPCR analysed by one-way ANOVA, followed by Tukey’s multiple comparison analysis. *n* = 3–4 mice/group. Data are presented as (mean and SEM). *P < 0.05, **P < 0.01, ***P < 0.001. n.s., not significant.

We also assessed the specific effect of HSD on the microglial transcriptomic profile in the setting of rCHI by comparing rCHI HSD with rCHI ND mice. We found a total of 668 DEGs between these two groups (Fig. 3E, Supplementary Table 4). Compared to rCHI ND controls, rCHI HSD was associated with a significant upregulation of several pathways related to inflammatory processes involved in innate and adaptive immune responses (responses to IFN-γ, IFN-α and IFN-β), oxidative stress–related pathways (hydrogen peroxide catabolic process and ROS metabolic process) and cytokine response/stimulus-related pathways (*IL-12* production and cytokine production) (Fig. 3F). In line with these findings, IPA of the DEGs in rCHI HSD compared to rCHI ND revealed several top upstream regulators (*P* < 0.05 and *Z*-score| ≥ 2) predominately involved in inflammation and immune response (*Ifng*, *Ifnb1*, *Ifnar* and *Pnpt1*) (Fig. 4A). However, none of these regulators were observed in IPA of the top upstream regulators for any of the groups compared to sham ND (Supplementary Table 5).

In addition to the microglial RNA-seq results, we also found significant increases in proinflammatory cytokine mRNA including *Ifng* in the brain tissue of rCHI mice fed with HSD compared to other groups (Fig. 4B). *Tnfa* was also similarly increased in rCHI HSD mice compared to the rest of the groups. We also found increased *Il1b* and decreased *Tgfb1* in both rCHI groups compared to the sham groups, independent of diet. Altogether, our findings demonstrate that the consumption of HSD following rCHI is associated with chronic microgliosis and alteration of the microglial transcriptome towards a more proinflammatory profile.

### High-salt diet induces microbiome alterations that correlate with anxiety following repetitive TBI

**Figure 5:**
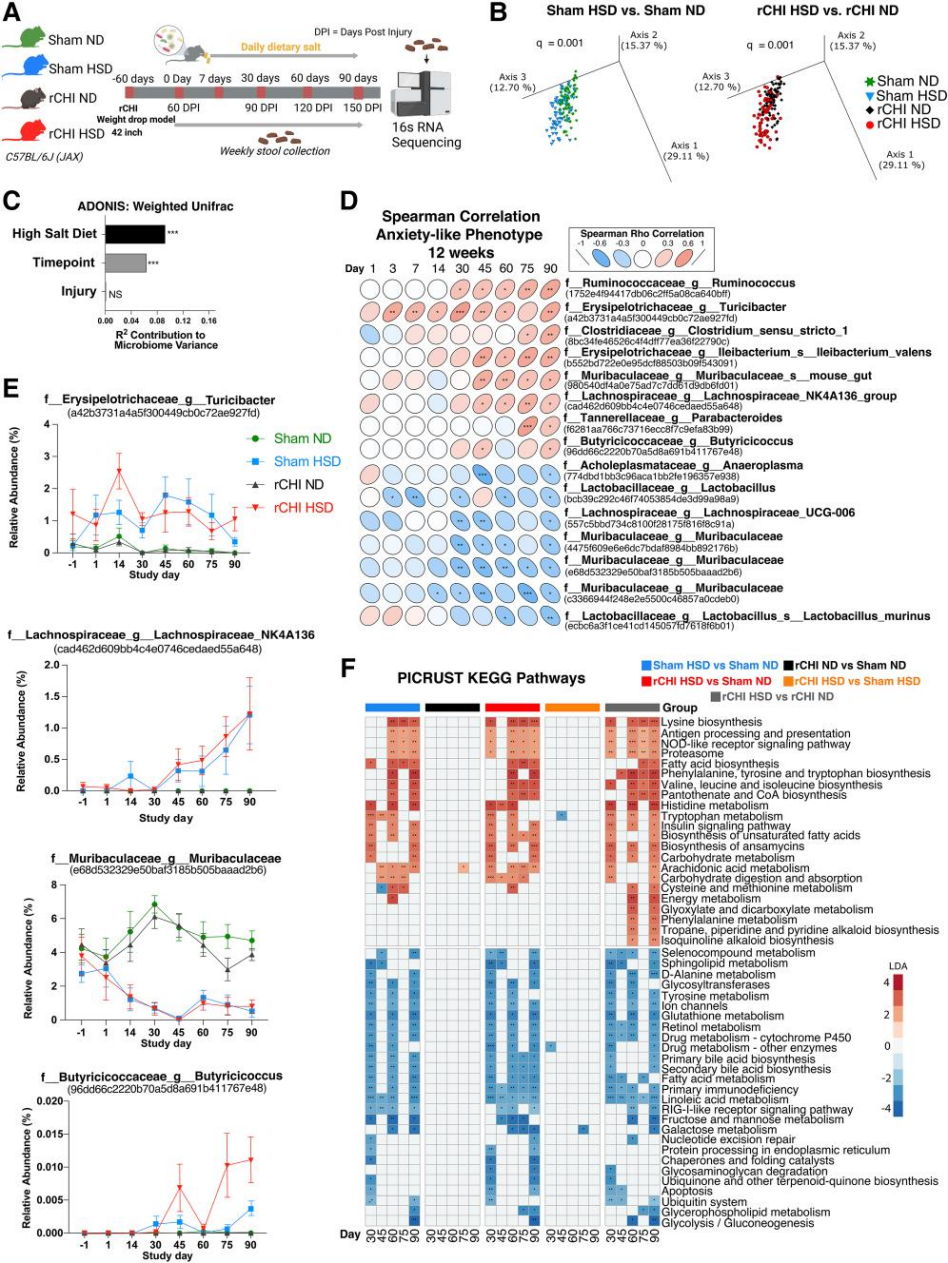
**HSD induces microbiome dysregulation in an rCHI model of concussion.** (**A**) Visual representation of experimental timeline of faecal sample collection for 16S microbiome sequencing. (**B**) Principal coordinate analysis of weighted UniFrac distances stratified by diet and injury (rCHI) microbiota structure: salt diet in sham mice (left panel, PERMANOVA: pseudo-*F* = 16.07, *P* = 0.001, *n* = 157) and by salt diet in injured (rCHI) mice (right panel, PERMANOVA: pseudo-*F* = 16.83, *P* = 0.001, *n* = 156). *P*-values and test statistics are obtained from PERMANOVA tests on beta diversity using weighted UniFrac distances. Each point represents the microbiota from one mouse. (**C**) ADONIS test using weighted UniFrac distances investigating the contribution of injury (rCHI), salt diet and time point to overall microbiome variation across faecal microbiota samples (*n* = 313) (***P < 0.001, **P > 0.001 and *P* < 0.01, *P > 0.01 and *P* < 0.05). (**D**) Spearman’s correlations between faecal microbial abundance at all time points and anxiety-like phenotype (from Fig. 1D) measured 12 weeks for each sample group (sham ND, sham HSD, rCHI ND and rCHI HSD) (***P < 0.001, **P > 0.001 and *P* < 0.01, *P > 0.01 and *P* < 0.05). (**E**) Relative abundance of selected taxa from **D** over time that were significantly correlated (Spearman’s correlation, *P* < 0.05) in at least two time points collected from faecal microbiota samples. Data are presented as mean and SEM. (**F**) Investigating microbial functional changes from faecal samples collected across 30–90 days. Significant differences based on the predicted KEGG metagenomic pathways (categorized by PICRUSt2) for each comparison were determined by LEfSe. Legend represents LDA effect size score. *n* = 7–8 mice/group for the microbiome experiments (***P < 0.001, **P > 0.001 and *P* < 0.01, *P > 0.01 and *P* < 0.05).

HSD has been shown to change the composition and diversity of the gut microbiome and increase inflammatory and stress responses in the brain.^26,27^ Altered gut–brain signalling could also contribute to development of anxiety and other mood disorders,^22,59^ but its role in rCHI-induced anxiety is unknown. To address this gap, we performed 16S rRNA sequencing on stool samples collected longitudinally at Days 0, 1, 3, 7, 14, 30, 45, 60, 75 and 90 after HSD administration (beginning 12 weeks post-rCHI) (Fig. 5A). Analysis of *β*-diversity using weighted UniFrac distances demonstrated overall microbial community structure differences between HSD and ND in both sham and rCHI groups (Fig. 5B; *P* = 0.001); however, no significant differences were found in the overall microbial community between rCHI and sham groups which received the same ND and HSD (Supplementary Fig. 3C). A weighted ADONIS test showed that HSD was the largest contributor to microbiome variance, with time point of stool collection also contributing significantly (Fig. 5C).

At the ASV level, we found a total of 23 microbes consistently (≥3 time points) altered between HSD versus ND, sham versus rCHI or both, across the 90 days sampled (Supplementary Fig. 4A). In both sham and rCHI mice, HSD decreased *Lachnoclostridium dorea* and a member of the *Ruminococcaceae* family and increased *Lachnospiraceae* family members, *Erysipelotrichaceae ileibacterium* and *Akkermansia.* The *Muribaculaceae* family is one of the most prevalent taxa in mice, and numerous *Muribaculaceae* ASVs were both decreased and increased in HSD groups compared to ND controls. Minimal bacterial species were altered in rCHI mice compared to the sham groups, independent of diet. No microbes were increased in rCHI compared to sham at more than two time points. HSD rCHI had additional microbial alterations compared to both sham ND and rCHI ND, including increased *Prevotellaceae*, two members of *Lachnospiraceae* and *Muribaculaceae*. Of note, *Prevotellaceae* was increased in rCHI HSD compared to both sham ND and rCHI ND at 7/10 time points, an effect that was increased from 5/10 time points in the sham HSD group compared to sham ND (Supplementary Fig. 4A).

While previous studies suggested high dietary salt intake as a possible behaviour modifier,^60,61^ the relationship between changes in gut microbiota and the development of anxiety-like behaviours following both rCHI and a HSD remains largely unexplored. To address this, we performed Spearman’s correlations of microbiota relative abundance with elevated plus maze measured at 12 weeks across all groups. At the ASV level, we found several microbes significantly correlated with anxiety-like behaviours at two or more independent microbiota sampling time points. Eight selected microbes were positively correlated with anxiety-like behaviours at multiple time points, including *Erysipelotrichaceae* family members (*Turibacter* and *Illebacterium* genera), *Ruminococcaceae*, *Muribaculaceae*, *Lachnospiraceae*, *Clostridiaceae*, *Tannerellaceae* and *Butyricicoccaceae*. Seven selected microbes were also negatively correlated with anxiety-like behaviours, all from the families *Muribaculaceae*, *Acholeplasmataceae*, *Lachnospiraceae* and *Lactobacillaceae* (Fig. 5D). Relative abundance plots over the course of the 3-month experiment show increased levels of microbes positively correlated with anxiety-like behaviours, including *Ruminococcaceae*, *Erysipelotrichaceae*, *Clostridiaceae* and *Lachnospiraceae* family members in HSD groups (Fig. 5E, Supplementary Fig. 3D). Plots of microbes negatively correlated with anxiety-like behaviours show elevated levels of multiple *Lactobacillaceae*, *Lachnospiraceae*, *Acholeplasmataceae* and *Muribaculaceae* members in ND groups (Fig. 5E, Supplementary Fig. 3D). The increase in two *Erysipelotrichaceae* after rCHI HSD at 15–30 days prior to development of anxiety-like behaviours (Fig. 5E, Supplementary Fig. 3D) could have contributed to the findings shown in Fig. 1D in which mice that received HSD following rCHI have increased anxiety-like behaviours.

To investigate microbial functional changes based on injury and diet, we performed predicted metagenomic analysis using PICRUSt2^53^ based on third-level KEGG pathways (Fig. 5F). LefSe testing of the predicted KEGG pathways based on the metagenomic content of the microbiota samples revealed alterations in pathways related to amino acid biosynthesis, carbohydrate metabolism and fatty acid synthesis and processing in the rCHI HSD mice at 90 days, which were not seen in sham HSD or rCHI ND groups when compared with the baseline sham ND group. However, most of the predicted metabolic changes were associated with HSD, independent of injury. Mice that received HSD showed numerous decreased metagenomic pathways related to drug metabolism, bile acid biosynthesis pathways, glutathione metabolism and sugar metabolism, among others, relative to ND mice, regardless of injury. Moreover, several metagenomic pathways involved in insulin signalling, fatty acid production, carbohydrate metabolism and biosynthesis and metabolism of amino acids (such as tryptophan) were upregulated consistently over time in mice that received HSD, independent of rCHI (Fig. 5F).

We also performed 16S rRNA sequencing on caecum samples collected at 90 days post-HSD administration (150 days post-rCHI). Consistent with stool samples, analysis of *β*-diversity using weighted UniFrac distances demonstrated overall microbial community structure differences between HSD and ND in both sham and rCHI groups (*P* < 0.05), but no differences in the overall microbial community between rCHI and sham groups, independent of diet (Supplementary Fig. 4B). A weighted ADONIS test showed HSD to be the largest contributor to microbiome variance, whereas the contribution of rCHI was insignificant (Supplementary Fig. 4C). We also found similar changes in microbiota relative abundance in the caecum samples compared to stool samples (Supplementary Fig. 4D). LEfSe testing showed that HSD administration decreased several members of *Muribaculaceae* and *Lactobacillaceae* and increased multiple *Lachnospiraceae* family members, *Prevotellaceae* and *Akkermansia* (Supplementary Fig. 4D). HSD-fed mice that received rCHI had further microbial changes compared to both ND groups (sham and rCHI), such as increases in *Marinifilaceae odoribacter*, Oscillospiraceae and two *Lachnospiraceae* family members.

Together, these data show a strong effect of HSD on microbiota composition at two anatomical sites, independent of rCHI, and unique alteration in the SCFA-producing microbes that were associated with increased anxiety phenotype in rCHI/HSD mice in the chronic period post-injury.

## Discussion

There is increasing evidence for risk of chronic neurological, psychiatric and cardiovascular comorbidities after TBI from recent human studies; however, the mechanisms driving these outcomes are still largely unknown.^12^ In this study, we aimed to understand the impact of HSD on sequelae of repetitive mild TBI in adolescent mice, including changes in haemodynamics, behaviour, brain inflammation and the gut microbiome. We found that HSD interacts with rCHI to produce an anxiety phenotype and that HSD (but not rCHI) strikingly altered the gut microbiome. These data suggest that environmental factors, such as diet, can interact with and modify the outcome of rCHI in adolescence even when such factors are presented well into adulthood (e.g. 12 weeks after injury). The data suggest that the injured adolescent brain is primed for an interaction with HSD that leads to an anxiety phenotype and increased microglial inflammation that is not induced by rCHI alone.

Excessive salt consumption has been recognized in humans as a risk factor for hypertension.^62^ However, in our study we did not find differences in blood pressure and heart rate measurements between HSD and ND groups. These negative findings could be attributed to the relatively young age of the mice and to healthy renal compensatory mechanisms. The impact of a high-salt diet in older mice and in mice with underlying comorbidities requires further investigation.

The manifestation of anxiety disorders after TBI is a strong predictor of personal, social and work dysfunction^63^; nonetheless, mechanisms responsible for development of post-traumatic anxiety are largely unexplored and remain poorly understood. There is some evidence to suggest that consuming too much salt is a potential behaviour modifier and may increase the risk of stress and anxiety.^59,64,65^ Some possible explanations include that high salt intake can disrupt the balance of electrolytes and fluids in the body, which can lead to changes in mood and behaviour. Electrolyte imbalances have been linked to increased anxiety and depression in some individuals.^66^ Additionally, salt consumption may worsen neuroinflammation and oxidative stress.^20,22^ Neuroinflammation, most specifically microglia activation, has been shown to contribute to a variety of neurological and psychiatric disorders, including anxiety, depression and cognitive impairment.^67,68^ In addition to head injury, prior studies reported that a HSD can drive macrophages/microglia towards a proinflammatory phenotype, amplifying an inflammatory response.^27-29^ Our rCHI HSD mice had chronic microglial activation in the cortex, hippocampus and amygdala, the latter recognized as a brain region involved in the interpretation of environmental threats, and play a role in generating fear and anxiety-like behaviours.^69,70^

Previous studies have shown that a HSD activates the NFAT5 transcription factor in proinflammatory macrophages/microglia, which can trigger the release of inducible nitric oxide (NO) synthase-dependent NO and proinflammatory cytokines such as tumour necrosis factor-alpha (*TNF-*α).^71,72^ A high-salt diet has been shown to impair T-cell function, which can increase the production of IFN-γ and impair the immune response.^25,73^ Similarly, we found that HSD activated microglial proinflammatory pathways such as TNF-α and IFN-γ in rCHI mice and significantly increased *Tnfa* and *Ifng* mRNAs in the brain tissue of rCHI HSD compared to other groups. The observation that salt induced a proinflammatory microglia polarization in rCHI HSD group is of translational interest, since post-TBI inflammation is one of the most frequently addressed therapeutic targets following experimental injury. In addition to microglia activation, clinical studies have demonstrated that increases in the serum levels of TNF-α and IFN-γ are associated with increased anxiety symptoms in general anxiety disorder patients.^74^ TNF-α activates the hypothalamic–pituitary–adrenal axis, leading to the production of cortisol, a stress hormone that is also involved in anxiety.^75,76^ Both TNF-α and IFN-γ have also been shown to alter neurotransmitter levels in the brain, including serotonin and dopamine, and to reduce the activity of the serotonin transporter, leading to decreased serotonin levels in the brain which is associated with increased anxiety.^77,78^ IFN-γ can also activate microglia, leading to hippocampal neuronal network dysfunction, depression-like behaviour and cognitive decline.^79,80^

The gut microbiome is altered in response to central nervous system injury,^81,82^ and manipulation of gut resident microbes is emerging as potential therapy for TBI.^83^ The gut microbiota can affect microglia and inflammatory responses in homeostasis and in disease.^84,85^ The majority of the changes that we observed in microbial community structure were driven by HSD treatment, which showed consistent alterations at multiple time points. HSD decreased *L. dorea*, a microbe shown to be decreased in multiple sclerosis.^86,87^ HSD also increased numerous microbes at multiple time points, including *Lachnospiraceae*, *Ileibacterium* (from the *Erysipelotrichaceae* family), *Akkermansia* and *Prevotellaceae*. In our study, rCHI exacerbated microbiome changes; however, without dietary alterations, there was not a consistent or robust effect.

In rCHI HSD mice, multiple microbial species were positively correlated with anxiety-like behaviour, including a *Lachnospiraceae* member, two *Erysipelotrichaceae (Turibacter* and *Illebacterium* genera), a *Clostridium* (genus of the *Clostridiaceae* family) and a *Butyricicoccaceae* member, which are all SCFA producers.^88,89^ Members of family *Lachnospiraceae* were elevated in rCHI ND mice,^90^ mirroring the increase in a *Lachnospiraceae* member in rCHI HSD mice. Numerous strains of *Lachnospiraceae* produce butyrate, a SCFA with putative immunomodulatory and anti-inflammatory functions,^91,92^ and can induce T regulatory cells.^93^ However, a subset of *Lachnospiraceae* strains adhere to the mucosa and can induce a Th_17_ response,^94^ one potential pathway that *Lachnospiraceae* could harness to contribute to the anxiety phenotype in our study. Further experimental studies of these *Lachnospiraceae* ASVs are warranted to explore their potential detrimental effects observed in our study. The *Erysipelotrichaceae* members that we showed to be positively correlated with anxiety-like behaviour spiked in abundance in the HSD groups, but more so in rCHI HSD-fed mice, prior to the onset of anxiety-like behaviours. Species within the *Erysipelotrichaceae* family that can contribute to anxiety^95^ and CNS inflammation^96^ are known to influence systemic inflammatory conditions like colitis^97^ and are highly responsive to dietary changes.^98^ The spike in abundance of *Erysipelotrichaceae* family 15+ days before the development of anxiety in our study suggests these microbes may play a potential role in driving behavioural changes.

This intricate microbiota–gut–brain communication system exerts regulatory effects through bacterial metabolites, the modulation of immune activity and interactions with enteric and vagus nerve terminals to maintain homeostasis. We found unique alterations in the gut microbiome of HSD mice, influenced by rCHI, which could affect mood and anxiety by the production of immunogenic metabolites, by neurotransmitters or by signalling via the vagus nerve.^99^ Based on PICRUSt2, we found alterations in microbiota functional genes modulated by HSD. In particular, we found alterations in several predicted microbial pathways reported in the literature to associated with anxiety such as amino acid metabolic pathways including tryptophan and insulin signaling^100^ and glutathione, an antioxidant, which has been shown to be regulated by the gut microbiota.^95,101,102^ Synthesis of secondary bile acids was also decreased, a pathway previously shown to play an anti-inflammatory role^103^ and specifically decrease activation in proinflammatory microglia profiles in animal models of multiple sclerosis.^104^ Our data also show alterations of SCFA-producing microbes in rCHI HSD mice that may be associated with anxiety. It is unclear whether SCFA is increased in mice with HSD and rCHI as this was not measured in our study, an important route of future investigation.

Taken together, we demonstrated unique alterations in the gut microbiome of HSD mice, an effect influenced only slightly by repeated TBI. Importantly, we found correlations between numerous microbes previously reported to alter microglia function with anxiety-like behaviours in the rCHI HSD mice and identified bacterial families potentially influencing anxiety-like behaviours after TBI, suggesting a way forward for targeted perturbations of gut microbiome and other microbiome-associated metabolites to improve post-TBI anxiety. Further studies are warranted to understand the mechanisms by which microbiota could alter behavioural outcomes and modulate microglia which can lead to identification of novel bacteria-derived therapeutics.

## Conclusion

The findings suggest that HSD may affect neurologic outcomes following mild repetitive head injury, including development of anxiety. This effect was linked to microbiome dysregulation and an exacerbation of microglial inflammation, which may be physiologic targets to prevent post-injury sequelae. Importantly, the HSD was administered after a recovery period, suggesting that diet may play a role in determining long-term TBI outcomes.

## Supplementary Material

fcae147_Supplementary_Data

## Data Availability

The bulk microglia RNA-seq data samples were deposited into the Sequence Read Archive (SRA) of the National Center for Biotechnology Information (NCBI), BioProject accession number PRJNA1053796. The microbiota 16S rRNA sequencing data will be available in the NCBI SRA repository under NCBI BioProject number PRJNA1053836.
