# Supplementary material for: High-salt diet induces microbiome dysregulation, neuroinflammation and anxiety in the chronic period after mild repetitive closed head injury in adolescent mice
Source: Brain Commun. 2024 May 3;6(4):fcae147. doi: 10.1093/braincomms/fcae147 (PMC11264151; doi:10.1093/braincomms/fcae147)
Supplement: fcae147_Supplementary_Data [file fcae147_supplementary_data.zip › Original_submission_manuscript.pdf]

OXFORD UNIVERSITY PRESS | Brain Communications

High salt diet induces microbiome dysregulation, neuroinflammation, and anxiety in the chronic period after mild repetitive closed head injury in adolescent mice.

Journal:	*Brain Communications*
Manuscript ID	BRAINCOM-2023-443
Manuscript Type:	Original Article
Date Submitted by the Author:	06-Oct-2023
Complete List of Authors:	Izzy, Saef; Brigham and Women's Hospital, Neurology; Harvard Medical School; Football Players Health Study at Harvard University; Massachusetts General Hospital<br>Yahya, Taha; Brigham and Women's Hospital, Department of Neurology<br>Albastaki, Omar; Brigham and Women's Hospital, Department of Neurology<br>Cao, Tian; Brigham and Women's Hospital, Department of Neurology<br>Schwerdtfeger, Luke; Brigham and Women's Hospital, Neurology<br>Abou-El-Hassan, Hadi; Brigham and Women's Hospital, Department of Neurology<br>Chopra, Kusha; Massachusetts General Hospital<br>Ekwudo, Millicent N.; Brigham and Women's Hospital, Neurology<br>Kurdeikaite, Ugne; Brigham and Women's Hospital, Neurology<br>Verissimo, Isabelly M.; Brigham and Women's Hospital, Neurology<br>LeServe, Danielle S.; Brigham and Women's Hospital, Neurology<br>Lanser, Toby B.; Brigham and Women's Hospital, Neurology<br>Aronchik, Michael; Brigham and Women's Hospital, Neurology<br>Oliveira, Marilia G.; Brigham and Women's Hospital, Neurology<br>Moreira, Thais; Harvard Medical School; Brigham and Women's Hospital, Neurology<br>Rezende, Rafael Machado; Harvard Medical School; Brigham and Women's Hospital, Neurology<br>El Khoury, Joseph; Massachusetts General Hospital; Harvard Medical School<br>Cox, Laura; Brigham and Women's Hospital; Harvard Medical School<br>Weiner, Howard L.; Harvard Medical School; Harvard Medical School<br>Zafonte, Ross; Harvard Medical School; Football Players Health Study at Harvard University; Harvard Medical School<br>Whalen, Michael ; Harvard Medical School; Massachusetts General Hospital, Department of Pediatrics
Keywords:	Neuroinflammation, Closed head injury, microbiome, Salt diet, Anxiety, Microglia

High salt diet induces microbiome dysregulation, neuroinflammation, and anxiety in the chronic period after mild repetitive closed head injury in adolescent mice.

Saef Izzy[1,2, 3, 4, 5], Taha Yahya[1,2], Omar Albastaki[1,2], Tian Cao[1,2], Luke A. Schwerdtfeger[2], Hadi Abou-El-Hassan[2], Kusha Chopra[2], Millicent N. Ekwudo[2], Ugne Kurdeikaite[2], Isabelly M. Verissimo[2], Danielle S. LeServe[2], Toby B. Lanser[2], Michael Aronchik[2], Marilia G. Oliveira[2], Thais Moreira[2,3], Rafael Machado Rezende [2,3], Joseph El Khoury[3,5,6], Laura M. Cox[2,3], Howard L. Weiner[2, 3], Ross Zafonte[3,4,7]*, Michael J. Whalen[3, 4, 8]*

[1] Department of Neurology, Divisions of Stroke, Cerebrovascular , and Critical Care Neurology, Brigham and Women's Hospital, Boston, MA, USA.

[2] Ann Romney Center for Neurologic Diseases, Brigham & Women's Hospital, Harvard Medical School, Boston, MA, USA.

[3] Harvard Medical School, Boston, MA, USA.

[4] The Football Players Health Study at Harvard University, Boston, MA, USA.

[5] Center for Immunology & Inflammatory Diseases, Massachusetts General Hospital, Boston, MA, USA.

[6] Department of Medicine, Division of Infectious Diseases, Massachusetts General Hospital, Boston, MA, USA.

[7] Spaulding Rehabilitation Hospital, Department of Physical Medicine and Rehabilitation, Massachusetts General Hospital, Brigham and Women's Hospital, Boston, MA, USA

[8] Department of Pediatrics, Massachusetts General Hospital, Boston, MA 02114, USA.

*Co-senior co-authors

Corresponding author:
Michael J. Whalen
Department of Pediatrics, Massachusetts General Hospital, Boston, MA 02114, USA.
mwhalen@mgh.harvard.edu

Word Count: 4072
Figures: 4
Supplementary Tables: 5
Supplementary Figures 3

References: 97

Key words: Concussion, salt diet, adolescent, traumatic brain injury, microglia, neuroinflammation, microbiome.

For Review Only

Abstract

Objective: Chronic neurological, psychiatric, and cardiovascular sequelae of human concussions have been reported; however, little is known about the underlying biological processes. We hypothesized that dietary changes, including a high salt diet (HSD), disrupt the bidirectional gut-brain axis, resulting in worsening neuroinflammation and behavioral phenotypes in the chronic period after repetitive closed head injury (rCHI) in adolescent mice.

Methods: Adolescent mice were subjected to three daily closed head injuries, recovered for 12 weeks, and then maintained on HSD or normal diet (ND) for an additional 12 weeks. Experimental endpoints were hemodynamics, behavior, microglial gene expression (bulk RNA sequencing), brain inflammation (brain tissue qPCR), and microbiome diversity (16S RNA sequencing).

Results: HSD did not affect systemic blood pressure or heart rate in Sham or rCHI mice. HSD increased anxiety-like behavior in rCHI mice compared to rCHI with ND and Sham injury + HSD. Increased anxiety in HSD-fed rCHI mice was associated with microgliosis and a proinflammatory microglial transcriptomic signature, including upregulation in interferon-gamma (IFN-γ), interferon beta (IFN- β) , and oxidative stress related pathways. Accordingly, we found upregulation of tumor necrosis factor-alpha (TNF-α) and IFN-γ mRNA in the brain tissue of rCHI HSD mice. HSD had a larger effect on the gut microbiome composition than rCHI. Increases in gut microbes in the families Lachnospiraceae, Erysipelotrichaceae, and Clostridiaceae were positively correlated with anxiety-like behaviors. In contrast, Muribaculaceae, Acholeplasmataceae, and Lactobacillaceae were negatively correlated with anxiety in TBI HSD-fed mice, a time-dependent effect.

Interpretations: The findings suggest that HSD, administered after a recovery period, may affect neurologic outcomes following mild repetitive head injury, including the development of anxiety. This effect was linked to microbiome dysregulation and an exacerbation of microglial inflammation, which may be physiological targets to prevent behavioral sequelae in the chronic period after repetitive mild

For Review Only

TBIs. The data suggest an important contribution of diet in determining long-term outcomes after repetitive mild TBI.

For Review Only

Introduction

Concussions continue to be a prominent public health concern, with an estimated incidence of 1.6-3.8 million in the United States annually.[1] Younger populations within the 14–19 year old age group experience the highest rates of concussion and nearly all athletic endeavors have some risk of concussive injury.[1-3] Recent large-scale studies of the general population, American-style football players, and military veterans demonstrated that prior TBI was associated with chronic neurological and psychiatric comorbidities and cardiovascular conditions such as hypertension, even in young patients.[4-7] These findings indicate that brain injury may trigger progressive degenerative processes affecting the health of the brain and other organ systems, and have become a central focus of public attention. [8-11]

The pathophysiological mechanisms that underlie the progressive nature of TBI are still not clear. TBI initiates a series of neuropathological molecular and biochemical secondary injury sequelae that are long lasting and involve disruption of several biological pathways, including neuroinflammation.[12] Other possible explanations include behavioral and lifestyle changes such as unhealthy diet and disruption to the bidirectional brain-gut axis.[13-16] Increasing evidence suggests that sustained excess salt intake may affect brain health, beyond the well-recognized risk of hypertension.[17]

Recent preclinical studies show that high salt diet (HSD) promotes cognitive impairment and suggest a gut-initiated adaptive immune response compromising brain function.[18,19] High sodium intake is associated with increased inflammatory and stress responses and organ damage in patients.[20] Increased salt consumption promotes pro-inflammatory interleukin 17 (IL17)-producing helper T (T_H17) cell differentiation[21,22] and inhibits the anti-inflammatory functions of regulatory T cells (FoxP3+).[23] In addition, high sodium intake causes macrophage/microglia polarization to shift towards a classically activated proinflammatory phenotype [24,25] and promotes the production of pro-inflammatory cytokines by myeloid cells in experimental models.[26,27] In mouse model of cerebral ischemia, HSD found to promote proinflammatory microglia polarization [28] and exacerbate cortical blood brain barrier

For Review Only

disruption.[29] Moreover, HSD also induces peripheral monocytes CD14++CD16+ expansion in humans [30] indicating increasing risks of tissue infiltration and end organ inflammation. There is growing evidence to support the HSD gut initiated pro-inflammatory properties. However, the impact of HSD on the neuroinflammatory response, microbiome, and behavioral outcomes after mild TBI has not been explored in preclinical TBI models.

Here, we tested whether HSD elevates blood pressure, alters microbiome diversity, worsens neuroinflammation, and alters behavioral outcomes after repetitive closed head injury in mice. To investigate this, we injured adolescent mice using an established model of repetitive closed head injury (rCHI) and HSD vs. a normal diet (ND) maintained for 12 weeks.

Results

HSD did not alter blood pressure or heart rate but increased anxiety-like behavior after repetitive mild CHI.

Previous studies have shown an association of TBI in American style football with development of hypertension amongst young athletes.[4,31,32] **Figure 1A** shows the experimental design for rCHI and HSD to test this association experimentally. HSD mice gained less weight over time when compared to mice on a ND, independent of rCHI (**Figure 1B**), however no significant changes in systolic or diastolic blood pressure or heart rate were observed with HSD compared with ND groups in both sham and rCHI (**Figure 1C**). Compared to other groups, mice in the rCHI/HSD group spent significantly less time in the open arm of the elevated plus maze (**Figure 1D, Supplementary Fig. 1A**), an anxiety-like phenotype, at 12 weeks after initiation of the HSD. This effect was not observed at earlier time points after rCHI. The consumption of HSD following rCHI did not affect spatial learning and memory as shown by the Morris water maze (MWM) and probe trial testing (**Figure 1E, Supplementary Fig. 1B, C**). In addition, HSD did not cause rotarod deficits or induce general locomotor and exploration deficits (assessed by an open field test) at all timepoints tested after rCHI (**Figure 1F, G, Supplementary Fig.**

1D, E). Altogether, our data show that the consumption of HSD following rCHI is associated with worsening anxiety like behavior in the chronic period after rCHI.

High salt diet exacerbates chronic microglial neuroinflammatory responses following repetitive closed head injury

Microglia are key players in the neuroinflammatory response to TBI, and their chronic activation after injury can lead to neurological dysfunction and neurodegeneration[12,33]. HSD can shift macrophages/microglia toward classically activated proinflammatory phenotypes[25], yet the impact of HSD on the brain's inflammatory response to rCHI is unknown, an important question given the effect of interleukin-1 signaling on anxiety and cognitive dysfunction in a 3HD adolescent rCHI model[34]. The HSD/rCHI group had increased microgliosis in cortex (**Figure 2A**), hippocampus (**Figure 2B-D**), and amygdala (**Figure 2E**) compared to ND/rCHI and sham groups. Therefore, we investigated the impact of HSD administration on the microglial inflammatory transcriptomic profile following rCHI and Sham injury. Microglia single-cell suspensions were obtained from the mouse brains at 12 weeks after HSD administration and analyzed using bulk RNA Sequencing **(Figure 3A, Supplementary Fig. 2A).** Normalized expression counts for all genes passing quality metrics are shown in **Supplementary Table 1**. Several established microglia markers were highly expressed in all groups, such as *Cx3cr1, Hexb, P2ry12, and Tmem119,* whereas we found minimal expression of non-microglia markers[35] **(Supplementary Fig. 2B).** We compared the HSD fed mice (Sham and rCHI) and rCHI mice fed with ND to Sham ND controls to define unique differentially expressed genes (DEGs) (Figure 3B, Supplementary Table 2). To detect unique transcriptomic patterns in Sham HSD, rCHI HSD, and rCHI ND, we plotted the unique DEGs in each group as clusters in a heatmap **(Figure 3C, Supplementary Table 3).** We found that each cluster was enriched for distinct Gene Ontology (GO) terms. The cluster of unique DEGs in rCHI HSD were related to type I interferon production (*Cactin, Crebbp, Syk, Gbp4)*, antigen processing and presentation (*Cd1d2, H2-K1, H2-Q1*), response to *TNF* (*Cxcl16, Pias4, Syk*), regulation of phagocytosis (*Dnm2, Ptprj, Siglece, Syt11*), endothelial migration (*Acvrl1, Bsg, Lgmn, Loxl2*), synaptic organization (*Il10ra, Ptprf, Ptpro, Slc7a11, Ube3a, Ywhaz*) and leukocyte migration

(*Cxcl16, Emilin1, Itga4, Mmp14, Mmp9, Ptpro, Spp1*). The cluster of unique DEGs in the Sham HSD group were associated with regulation of neuron death (*Cd200ra, Csf1, Egln2, Gclc, Hdac4, Hspd1, Jak2, Mag, Parp1, Rest, Tnfrsf1a)*, ROS metabolic processes (*Acox1, Eif5a, Grb2, Hdac4, Hspd1, Ier3, and Ogt*), regulation of *IL-17* production (*Jak2, Parp1*), regulation of apoptotic signaling pathways (*Bcap31, Gclc, Ier3, Ltbr, Map2k5, Ptpn2, Src*), and regulation of ERK1 and ERK2 cascades (*Acta2, Prkd2, Prtm5, Ptpn2, Ptpn6, Rapgef2*). The cluster of unique DEGs in rCHI ND mice were mainly involved in IL-1B production (*Lilra5, Nod1, P2rx7, Tnfaip8*), and positive regulation of kinase activity (*Axl, Ccnd2, Fgfr1, Ntrk2, Pdcd10, Rac1, Tom1l1*).

To further evaluate unique and shared microglial pathways altered by HSD, we performed gene set enrichment analysis (GSEA) of the 4 groups using GO biological process (GOBP) pathways. Compared to Sham ND, HSD in both Sham and rCHI groups was associated with a significant upregulation of immune-related pathways including immune effector processes, antigen processing and presentation, pathways involved in the regulation of hydrolase activity, and apoptosis **(Figure 3D)**. However, we also found that the rCHI HSD was uniquely associated with a significant upregulation of other immune mediated pathways (such as *IFN-γ* and cytokine responses), microglia regulation pathways (purinergic receptor signaling and cell motility), and vascular endothelial related pathways (vascular endothelial growth factor, endothelial cell growth and development).

We also assessed the specific effect of HSD on the microglial transcriptomic profile in the setting of rCHI by comparing rCHI HSD with rCHI ND mice. We found a total of 668 DEGs between these two groups **(Figure 3E. Supplementary Table 4).** Compared to rCHI ND controls, rCHI HSD was associated with a significant upregulation of several pathways related to inflammatory processes involved in innate and adaptive immune responses (responses to IFN-γ, IFN-α, and IFN-β)**,** oxidative stress related pathways (hydrogen peroxide catabolic process and ROS metabolic process) and cytokine response/stimulus related pathways (*IL-12* production, and cytokine production) (**Figure 3F)**. In line with these findings, IPA analysis of the DEGs in rCHI HSD compared to rCHI ND revealed several top upstream regulators (P <0.05 and |Z-score| ≥ 2) predominately involved in inflammation

For Review Only

and immune response (*Ifng, Ifnb1, Ifnar, Pnpt1*) **(Figure 3G)**. However, none of these regulators were observed in IPA analysis of the top upstream regulators for any of the groups compared to Sham ND (**Supplementary Table 5**).

In addition to the microglial RNA-seq results, we also found significant increases in proinflammatory cytokine mRNA including *Ifng* in the brain tissue of rCHI mice fed with HSD compared to other groups **(Figure 3H).** *Tnfa* was also similarly increased in rCHI HSD mice compared to the rest of the groups. We also found increased *Il1b* and decreased *Tgfb1* in both rCHI groups compared to the Sham groups, independent of diet. Altogether, our findings demonstrate that the consumption of HSD following rCHI is associated with chronic microgliosis and alteration of the microglial transcriptome towards a more proinflammatory profile.

High salt diet induces microbiome alterations that correlate with anxiety following repetitive TBI

HSD has been shown to change the composition and diversity of the gut microbiome and increase inflammatory and stress responses in the brain.[24,25] Altered gut-brain signaling could also contribute to development of anxiety and other mood disorders[20,36] but its role in rCHI-induced anxiety is unknown. To address this gap, we performed 16S rRNA sequencing on stool samples collected longitudinally at days 0, 1, 3, 7, 14, 30, 45, 60, 75, and 90 after HSD administration (beginning 12 weeks post-rCHI) (**Figure 4A**). Analysis of β-diversity using weighted UniFrac distances demonstrated overall microbial community structure differences between HSD and ND in both sham and rCHI groups (**Figure 4B**; p = 0.001), however, no significant differences were found in the overall microbial community between rCHI and sham groups which received the same ND and HSD (**Supplementary Fig. 2C**). A weighted ADONIS test showed that HSD was the largest contributor to microbiome variance, with timepoint of stool collection also contributing significantly (**Figure 4C**).

At the amplicon variant (ASV) level, we found a total of 23 microbes consistently (≥3 time points) altered between HSD vs. ND, sham vs. rCHI, or both, across the 90 days sampled (**Figure 4D**). In both sham and rCHI mice, HSD decreased *Lachnoclostridium dorea,* and a member of the

For Review Only

Ruminococcaceae family, and increased *Lachnospiraceae* family members, *Erysipelotrichaceae ileibacterium*, and a *Akkermansia.* The *Muribaculaceae* family is one of the most prevalent taxa in mice and numerous *Muribaculaceae* ASVs were both decreased and increased in HSD groups compared to ND controls. Minimal bacterial species were altered in rCHI mice compared to the sham groups, independent of diet. No microbes were increased in rCHI compared to sham at more than two time-points. HSD rCHI had additional microbial alterations compared to both sham ND and rCHI ND, including increased *Prevotellaceae*, two members of *Lachnospiraceae*, and *Muribaculaceae*. Of note, *Prevotellaceae* was increased in rCHI HSD compared to both Sham ND and rCHI ND at 7/10 timepoints, an effect that was increased from 5/10 timepoints in the Sham HSD group compared to Sham ND (**Figure 4D**).

While previous studies suggested high dietary salt intake as a possible behavior modifier [37,38], the relationship between changes in gut microbiota and the development of anxiety-like behaviors following both repetitive concussive head injury (rCHI) and a high-salt diet (HSD) remains largely unexplored. To address this, we performed Spearman correlations of microbiota relative abundance with elevated plus maze measured at 12 weeks across all groups. At the ASV level, we found several microbes significantly correlated with anxiety-like behaviors at 2 or more independent microbiota sampling time points. Eight selected microbes were positively correlated with anxiety-like behaviors at multiple time points, including *Erysipelotrichaceae* family members (*Turibacter* and *Illebacterium* genera), *Ruminococcaceae, Muribaculaceae, Lachnospiraceae, Clostridiaceae, Tannerellaceae* and *Butyricicoccaceae*. Seven selected microbes were also negatively correlated with anxiety-like behaviors, all from the families *Muribaculaceae*, *Acholeplasmataceae*, *Lachnospireaceae,* and *Lactobacillaceae* (**Figure 4E**). Relative abundance plots over the course of the 3-month experiment show increased levels of microbes positively correlated with anxiety-like behaviors, including *Ruminococcaceae, Erysipelotrichaceae, Clostridiaceae, and Lachnospiraceae* family members in HSD groups (**Figure 4F, Supplementary. Figure 2D**). Plots of microbes negatively correlated with anxiety-like behaviors show elevated levels of multiple *Lactobacillaceae*, *Lachnospiraceae,*

For Review Only

Acholeplasmataceae, *and Muribaculaceae* members in ND groups (**Figure 4F, Supplementary Figure 2D**). The increase in two *Erysipelotrichaceae* after rCHI HSD at 15-30 days prior to development of anxiety-like behaviors (**Figure 4F, Supplementary Figure 2D**) could have contributed to the findings shown in **Figure 1D** in which mice that received HSD following rCHI have increased anxiety-like behaviors.

To investigate microbial functional changes based on injury and diet, we performed predicted metagenomic analysis using PICRUSt2[39] based on third level KEGG pathways **(Figure 4G).** LefSe testing of the predicted KEGG pathways based on the metagenomic content of the microbiota samples revealed alterations in pathways related to amino acid biosynthesis, carbohydrate metabolism, and fatty acid synthesis and processing in the rCHI HSD mice at 90 days, which were not seen in sham HSD or rCHI ND groups when compared to the baseline sham ND group. However, most of the predicted metabolic changes were associated with HSD, independent of injury. Mice that received HSD showed numerous decreased metagenomic pathways related to drug metabolism, bile acid biosynthesis pathways, glutathione metabolism, and sugar metabolism, among others, relative to ND mice, regardless of injury. Moreover, several metagenomic pathways involved in insulin signaling, fatty acid production, carbohydrate metabolism, and biosynthesis and metabolism of amino acids (such as tryptophan) were upregulated consistently over time in mice that received HSD, independent of rCHI (**Figure 4G**).

We also performed 16S rRNA sequencing on cecum samples collected at 90 days post-HSD administration (12 weeks post-rCHI). Consistent with stool samples, analysis of β-diversity using weighted UniFrac distances demonstrated overall microbial community structure differences between HSD and ND in both sham and rCHI groups ($p < 0.05$), but no differences in the overall microbial community between rCHI and sham groups, independent of diet (**Supplementary Figure 3A**). A weighted ADONIS test showed HSD to be the largest contributor to microbiome variance, whereas the contribution of rCHI was insignificant (**Supplementary Figure 3B**). We also found similar changes in

microbiota relative abundance in the cecum samples compared to stool samples **(Supplementary Figure 3C).** LEfSe testing showed that HSD administration decreased several members of *Muribaculaceae and Lactobacillaceae,* and increased multiple *Lachnospiraceae* family members, a *Prevotellaceae,* and *Akkermansia* (**Supplementary Figure 3C)***.* HSD fed mice that received rCHI had further microbial changes compared to both ND groups (Sham and rCHI), such as increases in *Marinifilaceae Odoribacter, Oscillospiraceae,* and two *Lachnospiraceae* family members.

Together these data show a strong effect of HSD on microbiota composition at two anatomical sites, independent of rCHI, and unique alteration in the short chain fatty acid (SCFA)-producing microbes that were associated with increased anxiety phenotype in rCHI/HSD mice in the chronic period post-injury.

Discussion

There is increasing evidence for risk of chronic neurological, psychiatric, and cardiovascular comorbidities after TBI from recent human studies, however the mechanisms driving these outcomes are still largely unknown.[40] In this study, we aimed to understand the impact of HSD on sequelae of repetitive mild TBI in adolescent mice, including changes in hemodynamics, behavior, brain inflammation, and the gut microbiome. We found that HSD interacts with rCHI to produce an anxiety phenotype, and that HSD (but not rCHI) strikingly altered the gut microbiome. These data suggest that environmental factors, such as diet, can interact with and modify the outcome of rCHI in adolescence even when such factors are presented well into adulthood (e.g., 12 weeks after injury). The data suggest that the injured adolescent brain is primed for an interaction with HSD that leads to an anxiety phenotype and increased microglial inflammation that is not induced by rCHI alone.

Excessive salt consumption has been recognized in humans as a risk factor for hypertension.[41] However, in our study we did not find differences in blood pressure and heart rate measurements between HSD and ND groups. These negative findings could be attributed to the relatively young age

For Review Only

of the mice and to healthy renal compensatory mechanisms. The impact of a high salt diet in older mice and in mice with underlying comorbidities requires further investigation.

The manifestation of anxiety disorders after TBI is a strong predictor of personal, social, and work dysfunction[42]; nonetheless, mechanisms responsible for development of post-traumatic anxiety are largely unexplored and remain poorly understood. There is some evidence to suggest that consuming too much salt is a potential behavior modifier and may increase the risk of stress and anxiety.[36,43,44] Some possible explanations include that high salt intake can disrupt the balance of electrolytes and fluids in the body, which can lead to changes in mood and behavior. Electrolyte imbalances have been linked to increased anxiety and depression in some individuals.[45] Additionally, salt consumption may worsen neuroinflammation and oxidative stress.[18,20] Neuroinflammation, most specifically microglia activation, has been shown to contribute to a variety of neurological and psychiatric disorders, including anxiety, depression, and cognitive impairment.[46,47] In addition to head injury, prior studies reported that a high-salt diet can drive macrophages/microglia towards a proinflammatory phenotype, amplifying an inflammatory response.[25-27] Our rCHI HSD mice had chronic microglial activation in the cortex, hippocampus, and amygdala, the latter recognized as a brain region involved in the interpretation of environmental threats, and play a role in generating fear and anxiety-like behaviors.[48,49]

Previous studies have shown that a HSD activates the NFAT5 transcription factor in proinflammatory macrophages/microglia, which can trigger the release of inducible nitric oxide synthase (iNOS)-dependent nitric oxide (NO) and pro-inflammatory cytokines such as tumor necrosis factor alpha (*TNF-α*).[50,51] A high salt diet has been shown to impair T cell function, which can increase the production of IFN-γ and impair the immune response.[23,52] Similarly, we found that HSD activated microglial pro-inflammatory pathways such as TNF-α and IFN-γ in rCHI mice and significantly increased *Tnfa* and *Ifng* mRNAs in the brain tissue of rCHI HSD compared to other groups. The observation that salt induced a pro-inflammatory microglia polarization in rCHI HSD group is of translational interest, since post-TBI inflammation is one of the most frequently addressed therapeutic targets following

experimental injury. In addition to microglia activation, clinical studies have demonstrated that increases in the serum levels of TNF-α and IFN-γ are associated with increased anxiety symptoms in general anxiety disorder patients.[53] TNF-α activates the hypothalamic-pituitary-adrenal (HPA) axis, leading to the production of cortisol, a stress hormone that is also involved in anxiety.[54,55] Both TNF-α and IFN-γ have also been shown to alter neurotransmitter levels in the brain, including serotonin and dopamine, and to reduce the activity of the serotonin transporter, leading to decreased serotonin levels in the brain which is associated with increased anxiety.[56,57] IFN-γ can also activate microglia, leading to hippocampal neuronal network dysfunction, depression like behavior and cognitive decline. [58,59]

The gut microbiome is altered in response to central nervous system injury[60,61], and manipulation of gut resident microbes is emerging as potential therapy for TBI.[62] The gut microbiota can affect microglia and inflammatory responses in homeostasis and in disease. [63,64] The majority of the changes that we observed in microbial community structure were driven by HSD treatment, which showed consistent alterations at multiple timepoints. HSD decreased *Lachnoclostridium dorea*, a microbe shown to be decreased in multiple sclerosis[65,66]. HSD also increased numerous microbes at multiple timepoints, including *Lachnospiraceae*, *Ileibacterium* (from the *Erysipelotrichaceae family)*, *Akkermansia*, and *Prevotellaceae.* In our study, rCHI exacerbated microbiome changes, however, without dietary alterations there was not a consistent or robust effect.

In rCHI HSD mice, multiple microbial species were positively correlated with anxiety-like behavior, including a *Lachnospiraceae* member, two *Erysipelotrichaceae (Turibacter and Illebacterium genera), a Clostridium (genus of the Clostridiaceae family),* and a *Butyricicoccaceae* member, which are all SCFA producers.[67,68] Members of family *Lachnospiraceae* were elevated in rCHI ND mice[69], mirroring the increase in a *Lachnospiraceae* member in rCHI HSD mice. Numerous strains of *Lachnospiraceae* produce butyrate, a SCFA with putative immunomodulatory and anti-inflammatory functions[70 71] and can induce T regulatory cells.[72] However, a subset of *Lachnospiraceae* strains adhere to the mucosa and can induce a Th17 response[73], one potential pathway that *Lachnospiraceae* could harness to contribute to the anxiety phenotype in our study. Further experimental studies of these

For Review Only

Lachnospiraceae ASVs are warranted to explore their potential detrimental effects observed in our study. The *Erysipelotrichaceae* members that we showed to be positively correlated with anxiety-like behavior spiked in abundance in the HSD groups, but more so in rCHI HSD fed mice, prior to the onset of anxiety-like behaviors. Species within the *Erysipelotrichaceae* family that can contribute to anxiety[74], CNS inflammation[75] are known to influence systemic inflammatory conditions like colitis[76] and are highly responsive to dietary changes.[77] The spike in abundance of *Erysipelotrichaceae* family 15+ days before the development of anxiety in our study suggests these microbes may play a potential role in driving behavioral changes.

This intricate microbiota-gut-brain communication system exerts regulatory effects through bacterial metabolites, the modulation of immune activity, and interactions with enteric and vagus nerve terminals to maintain homeostasis. We found unique alterations in the gut microbiome of HSD mice, influenced by rCHI, which could affect mood and anxiety by the production of immunogenic metabolites, neurotransmitters, or by signaling via the vagus nerve.[78] Based on PICRUSt2, we found alterations in microbiota functional genes modulated by HSD. In particular, we found alterations in several predicted microbial pathways reported in the literature to associated with anxiety such as amino acid metabolic pathways including tryptophan and insulin signaling[79], and glutathione, an antioxidant, which has been shown to be regulated by the gut microbiota. [74,80,81] Synthesis of secondary bile acids were also decreased, a pathway previously shown to play an anti-inflammatory role[82] and specifically decrease activation in proinflammatory microglia profiles in animal models of multiple sclerosis.[83] Our data also show alterations of SCFA-producing microbes in rCHI HSD mice that may be associated with anxiety. It is unclear whether SCFA is increased in mice with HSD and rCHI as this was not measured in our study, an important route of future investigation.

Taken together, we demonstrated unique alterations in the gut microbiome of HSD mice, an effect influenced only slightly by repeated TBI. Importantly, we found correlations between numerous microbes previously reported to alter microglia function with anxiety like behaviors in the rCHI HSD mice and identified bacterial families potentially influencing anxiety-like behaviors after TBI, suggesting

For Review Only

a way forward for targeted perturbations of gut microbiome and other microbiome associated metabolites to improve post-TBI anxiety.

Conclusions

The findings suggest that HSD may affect neurologic outcomes following mild repetitive head injury, including development of anxiety. This effect was linked to microbiome dysregulation and an exacerbation of microglial inflammation, which may be physiologic targets to prevent post-injury sequelae. Importantly, the HSD was administered after a recovery period, suggesting that diet may play a role in determining long-term TBI outcomes**.**

For Review Only

Materials and Methods

Experimental animals

Studies were performed using 38-day old male C57/BL6J mice (Stock #000664, Jackson Laboratories). All procedures were performed in accordance with the NIH Guide for Care and Use of Laboratory Animals and followed protocols approved by the MGH Institutional Animal Care and Use Committee. Mice had access to food and water ad libitum and were housed on a 12-hour day-night cycle in laminar flow racks in a temperature-controlled room (25°C). Investigators were blinded to study groups in all experiments. Mice were randomized to sham or rCHI at 38 (+/- 3) days of age. Sham-injured and rCHI mice were housed in the same cage.

High salt diet

Sixty days after rCHI mice were separated into four groups: Sham mice receiving normal diet (ND), Sham mice receiving high salt diet (HSD), rCHI mice receiving normal diet (ND), and rCHI mice receiving a high salt diet (HSD). ND consisted of normal chow (0.5% NaCl) and tap water ad libitum.

HSD consisted of sodium-rich chow (8% NaCl) and tap water containing 1% NaCl ad libitum for 12 weeks.

Repetitive Closed head injury model (rCHI)

A modified closed head injury (CHI) model was used as previously described.[84] Mice were anaesthetized with 2.5% isoflourane in 70% N_2O and 30% O_2 for 90 seconds. Anesthetized mice were placed on a taught KimWipe napkin and grasped by the tail. The head was placed under a 42-inch long, 9/16-inch diameter brass guide tube. A 1/2-inch diameter 53g lead cylindrical weight with a flat, unbuffered surface was dropped onto dorsal aspect of the skull directly above the right (days 1, 3) or left (day 2) ear between the coronal and lambdoid sutures. After impact, mice were placed supine and loss of consciousness (LOC) time was recorded as time to righting reflex. Sham injured mice received anesthesia but no injury.

Hemodynamics

Weekly measurements of systolic and diastolic blood pressure and heart rate was done using the CODA noninvasive BP system (a tail-cuff Method, Kent Scientific Corporation) as previously described.[85]

Behavioral studies

Open field: Mice were individually placed in housing cages with clean bedding and covered by a thin wire grid. During the open field test, mice were recorded by ceiling-mounted cameras and their movements were tracked by AnyMaze as described.[84] Recordings lasted 30 minutes, and the distance covered during that time was used as a marker of overall activity.

Elevated plus maze: Elevated plus Maze was performed as previously described[84] and the percent time spent in the open arms was analyzed by Any Maze software.

For Review Only

Rotarod: Mice were placed on a Rotarod apparatus (Harvard Apparatus, Holliston, MA, USA), accelerating from 4–40 RPM in 120 seconds. Each trial ended when the mouse fell off the rod, and the latency was manually recorded. Mice were tested for 3 trials per day (1-minute inter-trial interval) for 3 consecutive days.

Morris water maze: Morris Water Maze testing was performed as previously described [86] with 7 hidden and 2 visible platform trials and 90s maximum latency to the platform. The time until the mouse mounted the platform (escape latency) was measured and recorded (AnyMaze 8.42, Stoelting, Wood Dale, IL, USA). For probe trials, the platform was removed, and the time spent in the target quadrant (total 30 seconds swim time) was recorded.

For Review Only

Preparation of brain tissue for Immunohistochemistry

Mice were deeply anesthetized with isoflurane and decapitated. The brains were removed and frozen in liquid nitrogen prior to making coronal sections (14 μm) on poly-ı-lysine-coated slide (Thermo Fisher Scientific) using a cryostat. The brains were cut at 0.5 mm intervals from the anterior to the posterior of the brain. For analyses using paraformaldehyde-fixed tissue, mice were transcardially perfused with PBS followed by 4% paraformaldehyde and brains were post- fixed overnight in 4% paraformaldehyde, cryoprotected in 30% sucrose overnight, frozen at - 80°C, and cut on a cryostat as above.

Immunofluorescence staining

Fluorescent immunolabeling followed a standard protocol. Sections were transferred into blocking medium (0.075% Triton-X, 5% normal Horse serum in 1× PBS solution) for 1 h at room temperature before applying primary antibody. The primary antibody, rabbit anti-mouse IBA1 (1:1000; Wako; RRID: AB_839506), was applied overnight followed by Cy3-conjugated anti-rabbit secondary antibodies (Jackson ImmunoResearch Laboratories, 1:300). Sections were counterstained with 10 μl

DAPI Mounting Medium (Vector Laboratories) and sealed by placing a glass coverslip over sections (Menzel Glaser) and coating the edges of the coverslip with clear nail polish.

Imaging and cell quantification

Immunostained sections were scanned using Leica DMi8 Widefield Fluorescence Microscope with 20x image, scale bar 0.5mm for microglia quantification. The representative immunostained sections were examined using a confocal microscope (LSM 710, Carl Zeiss) with 20× image, scale bar 50μm. For quantification of IBA1+ microglia, five consecutive sections of the dorsal dentate gyrus starting at approximately AP −1.5 mm were imaged. Microglia within the areas of the cortex, CA1, CA3 and dentate gyrus (DG), the amygdala were quantified. Numbers of microglia were obtained by scanning regions at 500x500 μm boxes at comparable sections in each animal. Data were number of IBA1+ cells/0.25mm^2. All quantifications were performed with Image J analysis software as previously described.[87]

Quantitative polymerase chain reaction

RNA was extracted with RNeasy® columns (Qiagen), cDNA was prepared and used for quantitative PCR (Applied Biosystems™, 437466) and the results were normalized to *Gapdh* (Mm99999915_g1). All primers and probes were from AppliedBiosystems, *IL10* (Mm01288386_m1), *Il6* (Mm00446190_m1), *TNF* (Mm00443258_m1), *Il1b* (Mm00434228_m1), *Infg* (Mm01168134_m1), and *Ccl5* (Mm01302427_m1) were used. 2-ΔΔCt method was used to calculate relative expression of each gene.

Flow cytometry microglial sorting

For microglial cell sorting, mice were anesthetized with CO_2 until respiration rate slowed and transcardially perfused with 50 mL Hanks balanced salt solution (HBSS). Following perfusion, the brains were homogenized using a dounce glass tissue homogenizer. Cells were separated through Percoll (GE Healthcare Life Sciences) 30% gradient by centrifugation. Cells were isolated from the

For Review Only

Percell layer and stained on ice for 30 min with combinations of PE/Cy7 rat anti-mouse CD11b (Biolegend, #101216, 1:100), APC/Cy7 rat anti mouse CD45 (Biolegend, #103116, 1:100) in blocking buffer containing 0.2% bovine serum albumin (BSA, Sigma-Aldrich) in HBSS. Cell sorting was performed using FACSAriaIII cell sorter (Becton Dickson). Microglial cells were identified as CD45 low to intermediate/CD11b high cells [88]. Cells were sorted directly in 1.5 mL Eppendorf tubes and stored at -80°C.

Microglia Bulk RNA-Sequencing

Bulk RNA sequencing was performed as previously described [35] for samples at 12 weeks after HSD administration. Briefly, 2,000 isolated CD45 low to intermediate/CD11b high cells (microglia) were lysed in 5ul TCL buffer + 1% β-mercaptoethanol. Smart-Seq2 libraries were prepared and sequenced by the Broad Genomic Platform. cDNA libraries were generated from sorted cells using the Smart-seq2 protocol5. RNA sequencing was performed using Illumina NextSeq500 using a High Output v2 kit to generate 2 × 38 bp reads. The processing of the bulk RNA-seq data was based on an established computational pipeline.[89] Sequencing data were demultiplexed and provided by the Broad Institute in FASTQ format. FastQC was used to assess sequencing quality control. Trimmomatic was used for adaptor trimming of reads. Reads were then aligned to the 'mm10' reference genome using HISAT. The generated SAM files were then converted into BAM files using SAMtools. StringTie was used for transcript assembly and quantification. Transcript abundances were then imported into R Studio (version 4.1.2) and converted to gene-level estimated counts using the 'tximport' package (version 1.22.0) from Bioconductor. Genes that achieved less than 10 counts summed across all samples were considered very low expressed genes and thus filtered out. Sample read counts were normalized using the variance stabilizing transformation (VST) method from the DESeq2 (version 1.34.0) built-in VST function.[90] These normalized sample read counts were used to plot heatmaps using ComplexHeatmap (version 2.13.1). Dot plots were generated using ggplot2 (version 3.4.0). Bar plots were generated using GraphPad Prism software for Mac.

Differential Gene Expression and Pathway Analysis

Differential gene expression analysis was carried out with DESeq2. Genes identified using DESeq2 that featured a nominal P value < 0.05 were considered significant differentially expressed genes (DEGs). For comparisons of gene expression between two different sample groups and for pathway analysis, the Wald Test was used with standard parameters and log2 fold-changes were subsequently shrunken using DESeq2's built-in lfcshrink function. Pairwise comparisons of DEGs were visualized using DiVenn[91]. Pathway analyses were performed through the GAGE package (version 2.44.0).[92] For GO enrichment analysis, the clusterProfiler package was used (version 4.2.2).[93] Ingenuity Pathway Analysis (IPA) was used to identify upstream regulators (P < 0.05 and |Z-score| ≥ 2) based on DEGs in a particular pairwise comparison, where input data comprised of p-values and log_2fold changes of DEGs. Statistical significance for all pathway analyses and tests was defined a nominal P value < 0.05.

16S rRNAmicrobiota sequencing and Microbial Community Analysis

Fecal samples were collected prior to salt diet treatment and over the course of the experiment. Cecum samples were collected at 90 days after salt diet administration. DNA was extracted using the Qiagen DNeasy PowerLyzer PowerSoil Kit (Qiagen, Hilden, Germany). The V4 16S rRNA gene was amplified with barcoded fusion primers developed by the Earth Microbiome Project. [94,95] Paired-end sequencing was performed at the Harvard Biopolymers facility on the Illumina MiSeq. The QIIME2 pipeline [96] was used for quality filtering of DNA sequences, demultiplexing, taxonomic assignment, and calculating alpha and beta diversity; DNA demultiplexing and quality filtering were performed by DADA2, samples were aligned, and alpha and beta-diversity were calculated at a depth of 1000 reads. Samples were removed if they had fewer than 1000 reads and ASVs were removed if they had fewer than 10 reads or were in fewer than 2 samples. A pre-trained Silva classifier was used for taxonomic assignment. To evaluate overall differences in microbial community structure between salt diet and

For Review Only

normal diet in both sham and injured groups, permutational multivariate analysis of variance (PERMANOVA) tests were performed on beta-diversity weighted and unweighted UniFrac distance measures. Statistical analysis of the changes in differences in relative microbial abundance was determined by linear discriminated analysis effect size (LEfSe), with the alpha set at 0.05 and the effect size set at 2. LDA scores and p-values were plotted in R using ggplot2, ComplexHeatmap, and ColorBrewer packages[97]. An ADONIS test in QIIME2 was used to determine the percentage of contribution to microbiome variance. To identify bacteria linked with anxiety, Spearman correlations were constructed in R using the stats package. Since the anxiety-like phenotype was measured using an elevated plus maze test where a lower value (percent of time spent in the open arms) suggests higher anxiety-like behaviors, the final correlations directions (positive or negative) were inversed for practical interpretation. Metagenomic content of the microbiota samples was predicted from the 16S rRNA profiles and KEGG pathway functions were categorized at level 3 using the phylogenetic investigation of communities by reconstruction of unobserved states (PICRUSt2) tool [39]. Significant pairwise differences based on relative predicted KEGG metagenomic pathways were determined by linear discriminant analysis effect size (LEfSe), with the alpha set at 0.05 and the effect size set at 1. Relative abundance plots over time for specific bacteria were performed using GraphPad Prism software for Mac.

Statistical analysis

Data are mean ± standard error of the mean (SEM). Student *t* test (unpaired) or One-way and Two-way ANOVA multiple comparisons tests with Tukey's multiple comparisons were used to assess statistical significance between groups. Numbers per group, significance level and statistical tests are indicated in the figure legends. Statistical analyses were performed using GraphPad Prism 9 software (GraphPad Software Inc., La Jolla, CA) and differences for all tests were considered significant if the p-value was <0.05.

Author contributions

For Review Only

SI, TY and MJW conceived the project and designed all the experiments. SI, TY, OA, TC, LMC and RZ were involved in planning and analysis of the experiments, SI, TA, TC, LAS, HA KC, MNK, UK, IMV, DSL, TBL, MA, and MGO performed the experiments. OA performed the data analysis for the RNA sequencing experiments. Data visualization and preparation of figures was performed by SI, TY, TC, LS, HA, and LMC. SI, HW, RZ provided funding. SI, TY, OA, LAS, LMC, RMR, HW, RZ, and MJW wrote the manuscript with input from all authors. JEK, HW, RZ, and MJW supervised the study.

Competing interests

Authors declare that they have no competing interests.

For Review Only

References

1. Bakhos LL, Lockhart GR, Myers R, Linakis JG. Emergency department visits for concussion in young child athletes. *Pediatrics*. Sep 2010;126(3):e550-6. doi:10.1542/peds.2009-3101
2. Mitka M. Reports of concussions from youth sports rise along with awareness of the problem. *JAMA*. Oct 27 2010;304(16):1775-6. doi:10.1001/jama.2010.1487
3. CDC. Sports and recreation-related injuries. https://www.cdc.gov/healthcommunication/toolstemplates/entertainmented/tips/SportsInjuries.html
4. Izzy S, Chen PM, Tahir Z, et al. Association of Traumatic Brain Injury With the Risk of Developing Chronic Cardiovascular, Endocrine, Neurological, and Psychiatric Disorders. *JAMA Netw Open*. Apr 1 2022;5(4):e229478. doi:10.1001/jamanetworkopen.2022.9478
5. Hilz MJ, Wang R, Markus J, et al. Severity of traumatic brain injury correlates with long-term cardiovascular autonomic dysfunction. *J Neurol*. Sep 2017;264(9):1956-1967. doi:10.1007/s00415-017-8581-1
6. Stewart IJ, Amuan ME, Wang CP, et al. Association Between Traumatic Brain Injury and Subsequent Cardiovascular Disease Among Post-9/11-Era Veterans. *JAMA Neurol*. Nov 1 2022;79(11):1122-1129. doi:10.1001/jamaneurol.2022.2682
7. Alosco ML, Mez J, Tripodis Y, et al. Age of first exposure to tackle football and chronic traumatic encephalopathy. *Ann Neurol*. May 2018;83(5):886-901. doi:10.1002/ana.25245
8. Stein CJ, MacDougall R, Quatman-Yates CC, et al. Young Athletes' Concerns About Sport-Related Concussion: The Patient's Perspective. *Clin J Sport Med*. Sep 2016;26(5):386-90. doi:10.1097/JSM.0000000000000268
9. Fishman M, Taranto E, Perlman M, Quinlan K, Benjamin HJ, Ross LF. Attitudes and Counseling Practices of Pediatricians Regarding Youth Sports Participation and Concussion Risks. *J Pediatr*. May 2017;184:19-25. doi:10.1016/j.jpeds.2017.01.048
10. Ropper AH, Gorson KC. Clinical practice. Concussion. *N Engl J Med*. Jan 11 2007;356(2):166-72. doi:10.1056/NEJMcp064645
11. Smith DH, Johnson VE, Stewart W. Chronic neuropathologies of single and repetitive TBI: substrates of dementia? *Nat Rev Neurol*. Apr 2013;9(4):211-21. doi:10.1038/nrneurol.2013.29
12. Jassam YN, Izzy S, Whalen M, McGavern DB, El Khoury J. Neuroimmunology of Traumatic Brain Injury: Time for a Paradigm Shift. *Neuron*. Sep 13 2017;95(6):1246-1265. doi:10.1016/j.neuron.2017.07.010
13. Stein MB, Jain S, Giacino JT, et al. Risk of Posttraumatic Stress Disorder and Major Depression in Civilian Patients After Mild Traumatic Brain Injury: A TRACK-TBI Study. *JAMA Psychiatry*. Mar 1 2019;76(3):249-258. doi:10.1001/jamapsychiatry.2018.4288
14. Willeumier K, Taylor DV, Amen DG. Elevated body mass in National Football League players linked to cognitive impairment and decreased prefrontal cortex and temporal pole activity. *Transl Psychiatry*. Jan 17 2012;2:e68. doi:10.1038/tp.2011.67
15. Wickwire EM, Williams SG, Roth T, et al. Sleep, Sleep Disorders, and Mild Traumatic Brain Injury. What We Know and What We Need to Know: Findings from a National Working Group. *Neurotherapeutics*. Apr 2016;13(2):403-17. doi:10.1007/s13311-016-0429-3
16. Norrie J, Heitger M, Leathem J, Anderson T, Jones R, Flett R. Mild traumatic brain injury and fatigue: a prospective longitudinal study. *Brain Inj*. 2010;24(13-14):1528-38. doi:10.3109/02699052.2010.531687
17. Farquhar WB, Edwards DG, Jurkovitz CT, Weintraub WS. Dietary sodium and health: more than just blood pressure. *J Am Coll Cardiol*. Mar 17 2015;65(10):1042-50. doi:10.1016/j.jacc.2014.12.039
18. Faraco G, Brea D, Garcia-Bonilla L, et al. Dietary salt promotes neurovascular and cognitive dysfunction through a gut-initiated TH17 response. *Nat Neurosci*. Feb 2018;21(2):240-249. doi:10.1038/s41593-017-0059-z
19. Hu L, Zhu S, Peng X, et al. High Salt Elicits Brain Inflammation and Cognitive Dysfunction, Accompanied by Alternations in the Gut Microbiota and Decreased SCFA Production. *J Alzheimers Dis*. 2020;77(2):629-640. doi:10.3233/JAD-200035
20. Gilman TL, Mitchell NC, Daws LC, Toney GM. Neuroinflammation Contributes to High Salt Intake-Augmented Neuronal Activation and Active Coping Responses to Acute Stress. *Int J Neuropsychopharmacol*. Feb 1 2019;22(2):137-142. doi:10.1093/ijnp/pyy099

For Review Only

21. Wu C, Yosef N, Thalhamer T, et al. Induction of pathogenic TH17 cells by inducible salt-sensing kinase SGK1. *Nature*. Apr 25 2013;496(7446):513-7. doi:10.1038/nature11984
22. Kleinewietfeld M, Manzel A, Titze J, et al. Sodium chloride drives autoimmune disease by the induction of pathogenic TH17 cells. *Nature*. Apr 25 2013;496(7446):518-22. doi:10.1038/nature11868
23. Hernandez AL, Kitz A, Wu C, et al. Sodium chloride inhibits the suppressive function of FOXP3+ regulatory T cells. *J Clin Invest*. Nov 2 2015;125(11):4212-22. doi:10.1172/JCI81151
24. Janakiraman M, Krishnamoorthy G. Emerging Role of Diet and Microbiota Interactions in Neuroinflammation. *Front Immunol*. 2018;9:2067. doi:10.3389/fimmu.2018.02067
25. Zhang WC, Zheng XJ, Du LJ, et al. High salt primes a specific activation state of macrophages, M(Na). *Cell Res*. Aug 2015;25(8):893-910. doi:10.1038/cr.2015.87
26. Binger KJ, Gebhardt M, Heinig M, et al. High salt reduces the activation of IL-4- and IL-13-stimulated macrophages. *J Clin Invest*. Nov 2 2015;125(11):4223-38. doi:10.1172/JCI80919
27. Hucke S, Eschborn M, Liebmann M, et al. Sodium chloride promotes pro-inflammatory macrophage polarization thereby aggravating CNS autoimmunity. *J Autoimmun*. Feb 2016;67:90-101. doi:10.1016/j.jaut.2015.11.001
28. Zhang T, Wang D, Li X, et al. Excess salt intake promotes M1 microglia polarization via a p38/MAPK/AR-dependent pathway after cerebral ischemia in mice. *Int Immunopharmacol*. Apr 2020;81:106176. doi:10.1016/j.intimp.2019.106176
29. Zhang T, Fang S, Wan C, et al. Excess salt exacerbates blood-brain barrier disruption via a p38/MAPK/SGK1-dependent pathway in permanent cerebral ischemia. *Sci Rep*. Nov 9 2015;5:16548. doi:10.1038/srep16548
30. Zhou X, Zhang L, Ji WJ, et al. Variation in dietary salt intake induces coordinated dynamics of monocyte subsets and monocyte-platelet aggregates in humans: implications in end organ inflammation. *PLoS One*. 2013;8(4):e60332. doi:10.1371/journal.pone.0060332
31. Weiner RB, Wang F, Isaacs SK, et al. Blood pressure and left ventricular hypertrophy during American-style football participation. *Circulation*. Jul 30 2013;128(5):524-31. doi:10.1161/CIRCULATIONAHA.113.003522
32. Grashow R, Tan CO, Izzy S, et al. Association Between Concussion Burden During Professional American-Style Football and Postcareer Hypertension. *Circulation*. Apr 4 2023;147(14):1112-1114. doi:10.1161/CIRCULATIONAHA.122.063767
33. Hickman S, Izzy S, Sen P, Morsett L, El Khoury J. Microglia in neurodegeneration. *Nat Neurosci*. Oct 2018;21(10):1359-1369. doi:10.1038/s41593-018-0242-x
34. Wu L, Kalish BT, Finander B, et al. Repetitive Mild Closed Head Injury in Adolescent Mice Is Associated with Impaired Proteostasis, Neuroinflammation, and Tauopathy. *J Neurosci*. Mar 23 2022;42(12):2418-2432. doi:10.1523/JNEUROSCI.0682-21.2021
35. Butovsky O, Jedrychowski MP, Moore CS, et al. Identification of a unique TGF-beta-dependent molecular and functional signature in microglia. *Nat Neurosci*. Jan 2014;17(1):131-43. doi:10.1038/nn.3599
36. Gilman TL, George CM, Andrade MA, Mitchell NC, Toney GM, Daws LC. High Salt Intake Lowers Behavioral Inhibition. *Front Behav Neurosci*. 2019;13:271. doi:10.3389/fnbeh.2019.00271
37. Beaver JN, Gilman TL. Salt as a non-caloric behavioral modifier: A review of evidence from pre-clinical studies. *Neurosci Biobehav Rev*. Apr 2022;135:104385. doi:10.1016/j.neubiorev.2021.10.007
38. Beaver JN, Weber BL, Ford MT, et al. Generalization of contextual fear is sex-specifically affected by high salt intake. *PLoS One*. 2023;18(7):e0286221. doi:10.1371/journal.pone.0286221
39. Douglas GM, Maffei VJ, Zaneveld JR, et al. PICRUSt2 for prediction of metagenome functions. *Nat Biotechnol*. Jun 2020;38(6):685-688. doi:10.1038/s41587-020-0548-6
40. Izzy S, Tahir Z, Grashow R, et al. Concussion and Risk of Chronic Medical and Behavioral Health Comorbidities. *J Neurotrauma*. Jun 1 2021;38(13):1834-1841. doi:10.1089/neu.2020.7484
41. Frisoli TM, Schmieder RE, Grodzicki T, Messerli FH. Salt and hypertension: is salt dietary reduction worth the effort? *Am J Med*. May 2012;125(5):433-9. doi:10.1016/j.amjmed.2011.10.023
42. Mallya S, Sutherland J, Pongracic S, Mainland B, Ornstein TJ. The manifestation of anxiety disorders after traumatic brain injury: a review. *J Neurotrauma*. Apr 1 2015;32(7):411-21. doi:10.1089/neu.2014.3504

For Review Only

43. Dingess PM, Thakar A, Zhang Z, Flynn FW, Brown TE. High-Salt Exposure During Perinatal Development Enhances Stress Sensitivity. *Dev Neurobiol*. Nov 2018;78(11):1131-1145. doi:10.1002/dneu.22635

44. McBride SM, Culver B, Flynn FW. Dietary sodium manipulation during critical periods in development sensitize adult offspring to amphetamines. *Am J Physiol Regul Integr Comp Physiol*. Sep 2008;295(3):R899-905. doi:10.1152/ajpregu.00186.2008

45. Torres SJ, Nowson CA, Worsley A. Dietary electrolytes are related to mood. *Br J Nutr*. Nov 2008;100(5):1038-45. doi:10.1017/S0007114508959201

46. Won E, Kim YK. Neuroinflammation-Associated Alterations of the Brain as Potential Neural Biomarkers in Anxiety Disorders. *Int J Mol Sci*. Sep 7 2020;21(18)doi:10.3390/ijms21186546

47. Wang YL, Han QQ, Gong WQ, et al. Microglial activation mediates chronic mild stress-induced depressive- and anxiety-like behavior in adult rats. *J Neuroinflammation*. Jan 17 2018;15(1):21. doi:10.1186/s12974-018-1054-3

48. Ressler KJ. Amygdala activity, fear, and anxiety: modulation by stress. *Biol Psychiatry*. Jun 15 2010;67(12):1117-9. doi:10.1016/j.biopsych.2010.04.027

49. Calhoon GG, Tye KM. Resolving the neural circuits of anxiety. *Nat Neurosci*. Oct 2015;18(10):1394-404. doi:10.1038/nn.4101

50. Bardgett ME, Holbein WW, Herrera-Rosales M, Toney GM. Ang II-salt hypertension depends on neuronal activity in the hypothalamic paraventricular nucleus but not on local actions of tumor necrosis factor-alpha. *Hypertension*. Mar 2014;63(3):527-34. doi:10.1161/HYPERTENSIONAHA.113.02429

51. Jantsch J, Schatz V, Friedrich D, et al. Cutaneous Na+ storage strengthens the antimicrobial barrier function of the skin and boosts macrophage-driven host defense. *Cell Metab*. Mar 3 2015;21(3):493-501. doi:10.1016/j.cmet.2015.02.003

52. Barbaro NR, Foss JD, Kryshtal DO, et al. Dendritic Cell Amiloride-Sensitive Channels Mediate Sodium-Induced Inflammation and Hypertension. *Cell Rep*. Oct 24 2017;21(4):1009-1020. doi:10.1016/j.celrep.2017.10.002

53. Hou R, Garner M, Holmes C, et al. Peripheral inflammatory cytokines and immune balance in Generalised Anxiety Disorder: Case-controlled study. *Brain Behav Immun*. May 2017;62:212-218. doi:10.1016/j.bbi.2017.01.021

54. Mikhaylova IV, Kuulasmaa T, Jaaskelainen J, Voutilainen R. Tumor necrosis factor-alpha regulates steroidogenesis, apoptosis, and cell viability in the human adrenocortical cell line NCI-H295R. *Endocrinology*. Jan 2007;148(1):386-92. doi:10.1210/en.2006-0726

55. Lenze EJ, Mantella RC, Shi P, et al. Elevated cortisol in older adults with generalized anxiety disorder is reduced by treatment: a placebo-controlled evaluation of escitalopram. *Am J Geriatr Psychiatry*. May 2011;19(5):482-90. doi:10.1097/JGP.0b013e3181ec806c

56. Foley KF, Pantano C, Ciolino A, Mawe GM. IFN-gamma and TNF-alpha decrease serotonin transporter function and expression in Caco2 cells. *Am J Physiol Gastrointest Liver Physiol*. Mar 2007;292(3):G779-84. doi:10.1152/ajpgi.00470.2006

57. Litteljohn D, Cummings A, Brennan A, et al. Interferon-gamma deficiency modifies the effects of a chronic stressor in mice: Implications for psychological pathology. *Brain Behav Immun*. Mar 2010;24(3):462-73. doi:10.1016/j.bbi.2009.12.001

58. Kann O, Almouhanna F, Chausse B. Interferon gamma: a master cytokine in microglia-mediated neural network dysfunction and neurodegeneration. *Trends Neurosci*. Dec 2022;45(12):913-927. doi:10.1016/j.tins.2022.10.007

59. Zhang J, He H, Qiao Y, et al. Priming of microglia with IFN-gamma impairs adult hippocampal neurogenesis and leads to depression-like behaviors and cognitive defects. *Glia*. Dec 2020;68(12):2674-2692. doi:10.1002/glia.23878

60. Bao W, Sun Y, Lin Y, Yang X, Chen Z. An integrated analysis of gut microbiota and the brain transcriptome reveals host-gut microbiota interactions following traumatic brain injury. *Brain Res*. Jan 15 2023;1799:148149. doi:10.1016/j.brainres.2022.148149

61. Treangen TJ, Wagner J, Burns MP, Villapol S. Traumatic Brain Injury in Mice Induces Acute Bacterial Dysbiosis Within the Fecal Microbiome. *Front Immunol*. 2018;9:2757. doi:10.3389/fimmu.2018.02757

For Review Only

62. Yuan B, Lu XJ, Wu Q. Gut Microbiota and Acute Central Nervous System Injury: A New Target for Therapeutic Intervention. *Front Immunol*. 2021;12:800796. doi:10.3389/fimmu.2021.800796
63. Erny D, Hrabe de Angelis AL, Jaitin D, et al. Host microbiota constantly control maturation and function of microglia in the CNS. *Nat Neurosci*. Jul 2015;18(7):965-77. doi:10.1038/nn.4030
64. Butovsky O, Weiner HL. Microglial signatures and their role in health and disease. *Nat Rev Neurosci*. Oct 2018;19(10):622-635. doi:10.1038/s41583-018-0057-5
65. Cox LM, Maghzi AH, Liu S, et al. Gut Microbiome in Progressive Multiple Sclerosis. *Ann Neurol*. Jun 2021;89(6):1195-1211. doi:10.1002/ana.26084
66. i MCEasbue, i MC. Gut microbiome of multiple sclerosis patients and paired household healthy controls reveal associations with disease risk and course. *Cell*. Sep 15 2022;185(19):3467-3486 e16. doi:10.1016/j.cell.2022.08.021
67. Lubomski M, Xu X, Holmes AJ, et al. The Gut Microbiome in Parkinson's Disease: A Longitudinal Study of the Impacts on Disease Progression and the Use of Device-Assisted Therapies. *Front Aging Neurosci*. 2022;14:875261. doi:10.3389/fnagi.2022.875261
68. Guo P, Zhang K, Ma X, He P. Clostridium species as probiotics: potentials and challenges. *J Anim Sci Biotechnol*. 2020;11:24. doi:10.1186/s40104-019-0402-1
69. You W, Zhu Y, Wei A, et al. Traumatic Brain Injury Induces Gastrointestinal Dysfunction and Dysbiosis of Gut Microbiota Accompanied by Alterations of Bile Acid Profile. *J Neurotrauma*. Jan 2022;39(1-2):227-237. doi:10.1089/neu.2020.7526
70. Liu H, Wang J, He T, et al. Butyrate: A Double-Edged Sword for Health? *Adv Nutr*. Jan 1 2018;9(1):21-29. doi:10.1093/advances/nmx009
71. Siddiqui MT, Cresci GAM. The Immunomodulatory Functions of Butyrate. *J Inflamm Res*. 2021;14:6025-6041. doi:10.2147/JIR.S300989
72. Atarashi K, Tanoue T, Shima T, et al. Induction of colonic regulatory T cells by indigenous Clostridium species. *Science*. Jan 21 2011;331(6015):337-41. doi:10.1126/science.1198469
73. Atarashi K, Tanoue T, Ando M, et al. Th17 Cell Induction by Adhesion of Microbes to Intestinal Epithelial Cells. *Cell*. Oct 8 2015;163(2):367-80. doi:10.1016/j.cell.2015.08.058
74. Wang X, Li L, Bian C, et al. Alterations and correlations of gut microbiota, fecal, and serum metabolome characteristics in a rat model of alcohol use disorder. *Front Microbiol*. 2022;13:1068825. doi:10.3389/fmicb.2022.1068825
75. Miyauchi E, Kim SW, Suda W, et al. Gut microorganisms act together to exacerbate inflammation in spinal cords. *Nature*. Sep 2020;585(7823):102-106. doi:10.1038/s41586-020-2634-9
76. Turnbaugh PJ, Ridaura VK, Faith JJ, Rey FE, Knight R, Gordon JI. The effect of diet on the human gut microbiome: a metagenomic analysis in humanized gnotobiotic mice. *Sci Transl Med*. Nov 11 2009;1(6):6ra14. doi:10.1126/scitranslmed.3000322
77. Thompson DS, Fu C, Gandhi T, et al. Differential co-expression networks of the gut microbiota are associated with depression and anxiety treatment resistance among psychiatric inpatients. *Prog Neuropsychopharmacol Biol Psychiatry*. Jan 10 2023;120:110638. doi:10.1016/j.pnpbp.2022.110638
78. Cox LM, Weiner HL. Microbiota Signaling Pathways that Influence Neurologic Disease. *Neurotherapeutics*. Jan 2018;15(1):135-145. doi:10.1007/s13311-017-0598-8
79. Soto M, Herzog C, Pacheco JA, et al. Gut microbiota modulate neurobehavior through changes in brain insulin sensitivity and metabolism. *Mol Psychiatry*. Dec 2018;23(12):2287-2301. doi:10.1038/s41380-018-0086-5
80. Mardinoglu A, Shoaie S, Bergentall M, et al. The gut microbiota modulates host amino acid and glutathione metabolism in mice. *Mol Syst Biol*. Oct 16 2015;11(10):834. doi:10.15252/msb.20156487
81. Halverson T, Alagiakrishnan K. Gut microbes in neurocognitive and mental health disorders. *Ann Med*. Dec 2020;52(8):423-443. doi:10.1080/07853890.2020.1808239
82. Bhargava P. Targeting metabolism to treat multiple sclerosis. *Neural Regen Res*. Mar 2021;16(3):502-503. doi:10.4103/1673-5374.293143
83. Romero-Ramirez L, Garcia-Rama C, Wu S, Mey J. Bile acids attenuate PKM2 pathway activation in proinflammatory microglia. *Sci Rep*. Jan 27 2022;12(1):1459. doi:10.1038/s41598-022-05408-3

For Review Only

84. Wu L, Chung JY, Saith S, et al. Repetitive head injury in adolescent mice: A role for vascular inflammation. *J Cereb Blood Flow Metab*. Nov 2019;39(11):2196-2209. doi:10.1177/0271678X18786633

85. Daugherty A, Rateri D, Hong L, Balakrishnan A. Measuring blood pressure in mice using volume pressure recording, a tail-cuff method. *J Vis Exp*. May 15 2009;(27)doi:10.3791/1291

86. Khuman J, Meehan WP, 3rd, Zhu X, et al. Tumor necrosis factor alpha and Fas receptor contribute to cognitive deficits independent of cell death after concussive traumatic brain injury in mice. *J Cereb Blood Flow Metab*. Feb 2011;31(2):778-89. doi:10.1038/jcbfm.2010.172

87. Izzy S, Brown-Whalen A, Yahya T, et al. Repetitive Traumatic Brain Injury Causes Neuroinflammation before Tau Pathology in Adolescent P301S Mice. *Int J Mol Sci*. Jan 18 2021;22(2)doi:10.3390/ijms22020907

88. Izzy S, Liu Q, Fang Z, et al. Time-Dependent Changes in Microglia Transcriptional Networks Following Traumatic Brain Injury. *Front Cell Neurosci*. 2019;13:307. doi:10.3389/fncel.2019.00307

89. Pertea M, Kim D, Pertea GM, Leek JT, Salzberg SL. Transcript-level expression analysis of RNA-seq experiments with HISAT, StringTie and Ballgown. *Nat Protoc*. Sep 2016;11(9):1650-67. doi:10.1038/nprot.2016.095

90. Love MI, Huber W, Anders S. Moderated estimation of fold change and dispersion for RNA-seq data with DESeq2. *Genome Biol*. 2014;15(12):550. doi:10.1186/s13059-014-0550-8

91. Sun L, Dong S, Ge Y, et al. DiVenn: An Interactive and Integrated Web-Based Visualization Tool for Comparing Gene Lists. *Front Genet*. 2019;10:421. doi:10.3389/fgene.2019.00421

92. Luo W, Friedman MS, Shedden K, Hankenson KD, Woolf PJ. GAGE: generally applicable gene set enrichment for pathway analysis. *BMC Bioinformatics*. May 27 2009;10:161. doi:10.1186/1471-2105-10-161

93. Yu G, Wang LG, Han Y, He QY. clusterProfiler: an R package for comparing biological themes among gene clusters. *OMICS*. May 2012;16(5):284-7. doi:10.1089/omi.2011.0118

94. Parada AE, Needham DM, Fuhrman JA. Every base matters: assessing small subunit rRNA primers for marine microbiomes with mock communities, time series and global field samples. *Environ Microbiol*. May 2016;18(5):1403-14. doi:10.1111/1462-2920.13023

95. Caporaso JG, Lauber CL, Walters WA, et al. Ultra-high-throughput microbial community analysis on the Illumina HiSeq and MiSeq platforms. *ISME J*. Aug 2012;6(8):1621-4. doi:10.1038/ismej.2012.8

96. Bolyen E, Rideout JR, Dillon MR, et al. Reproducible, interactive, scalable and extensible microbiome data science using QIIME 2. *Nat Biotechnol*. Aug 2019;37(8):852-857. doi:10.1038/s41587-019-0209-9

97. Gu Z, Eils R, Schlesner M. Complex heatmaps reveal patterns and correlations in multidimensional genomic data. *Bioinformatics*. Sep 15 2016;32(18):2847-9. doi:10.1093/bioinformatics/btw313

For Review Only

Figure 1

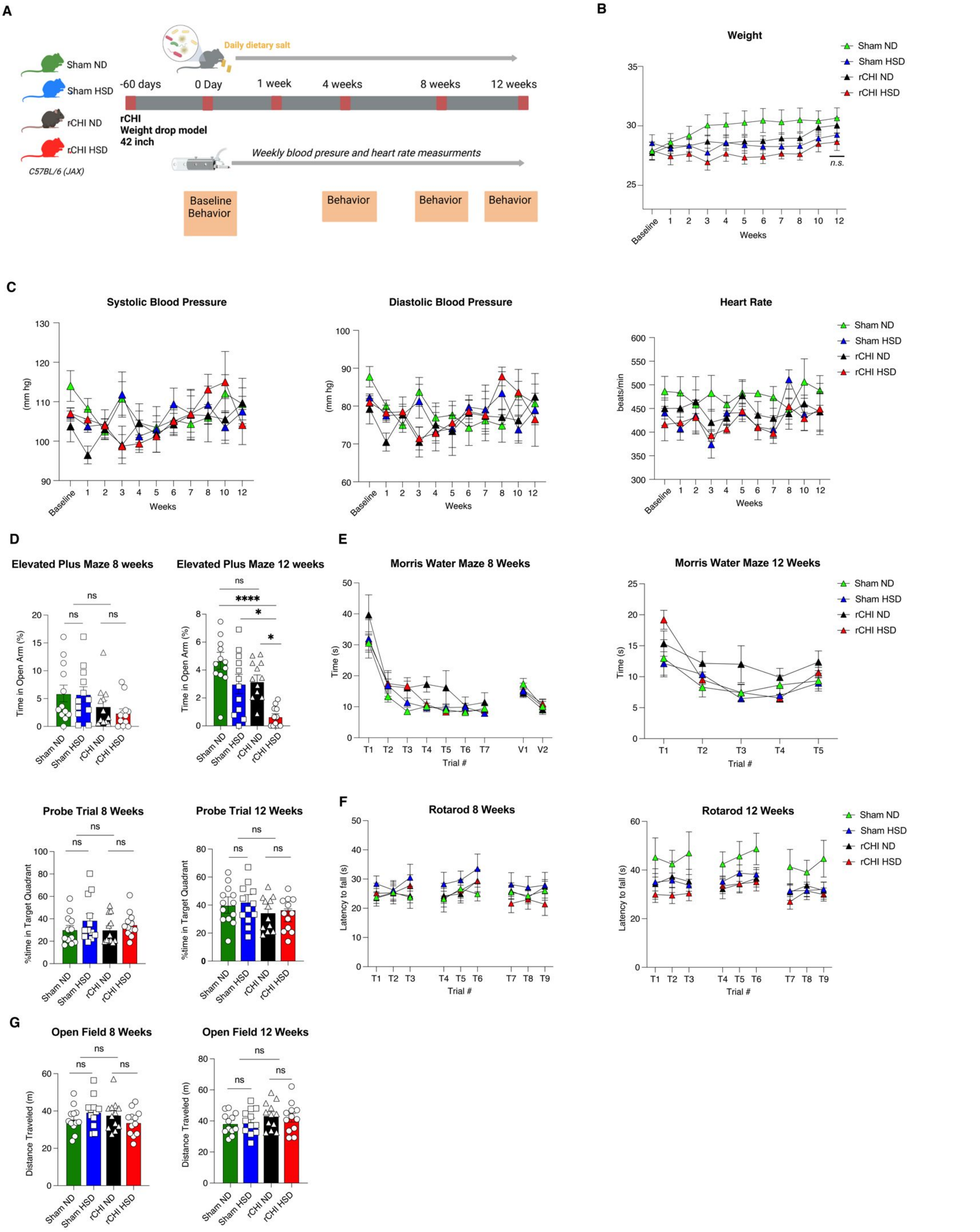
A
Sham ND
Sham HSD
rCHI ND
rCHI HSD
C57BL/6 (JAX)
Daily dietary salt
-60 days
0 Day
1 week
4 weeks
8 weeks
12 weeks
rCHI
Weight drop model
42 inch
Weekly blood presure and heart rate measurments
Baseline Behavior
Behavior
Behavior
Behavior
B
Weight
n.s.
Weeks
Baseline
C
Systolic Blood Pressure
(mm hg)
Diastolic Blood Pressure
(mm hg)
Heart Rate
beats/min
Weeks
D
Elevated Plus Maze 8 weeks
Elevated Plus Maze 12 weeks
Time in Open Arm (%)
ns
****
*
E
Morris Water Maze 8 Weeks
Morris Water Maze 12 Weeks
Time (s)
Trial #
Probe Trial 8 Weeks
Probe Trial 12 Weeks
%time in Target Quadrant
F
Rotarod 8 Weeks
Rotarod 12 Weeks
Latency to fall (s)
Trial #
G
Open Field 8 Weeks
Open Field 12 Weeks
Distance Traveled (m)

Figure 2

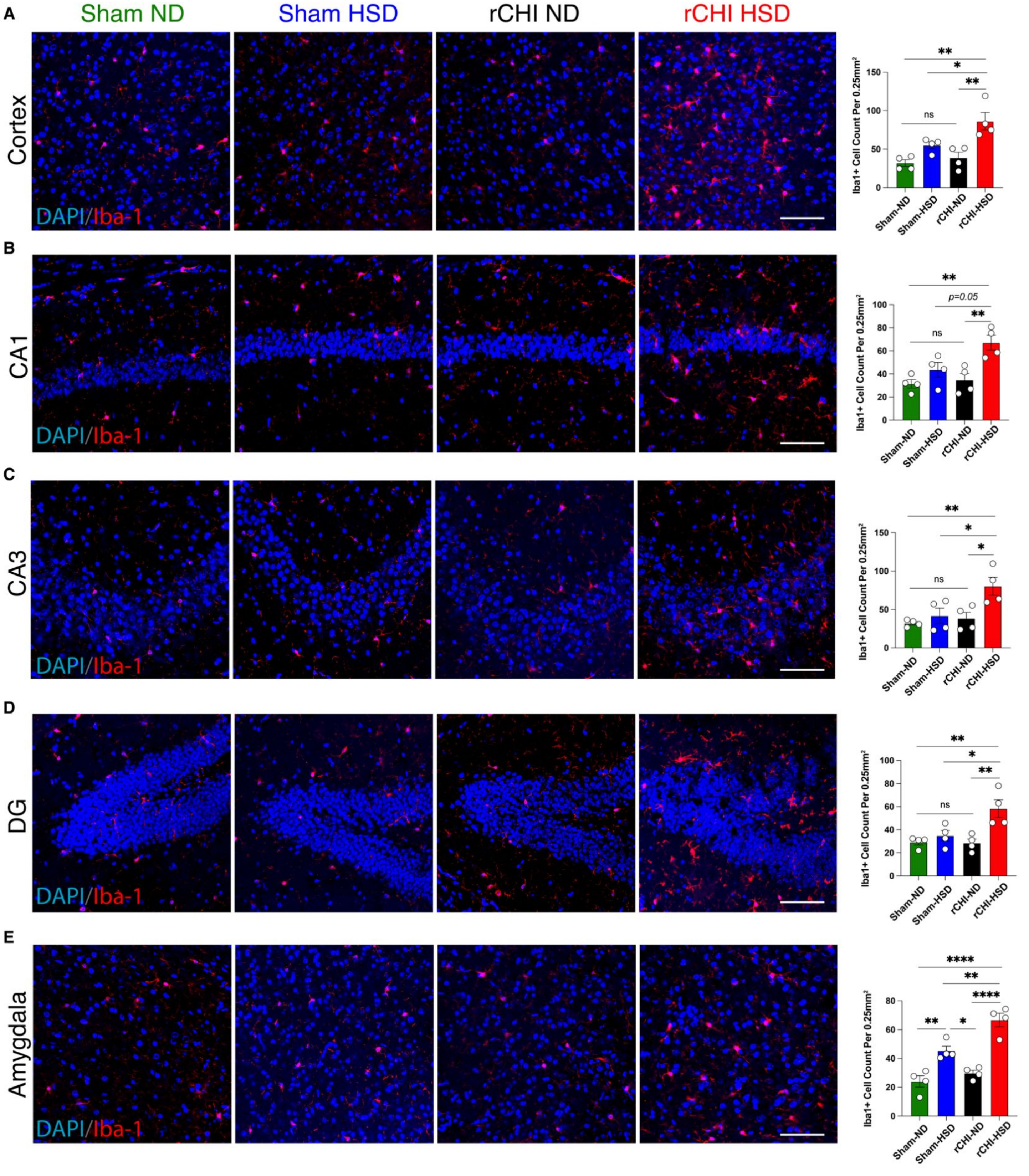
A
Sham ND
Sham HSD
rCHI ND
rCHI HSD
Cortex
DAPI/Iba-1
Iba1+ Cell Count Per 0.25mm²
Sham-ND
Sham-HSD
rCHI-ND
rCHI-HSD
ns
B
CA1
p=0.05
C
CA3
D
DG
E
Amygdala

Figure 3

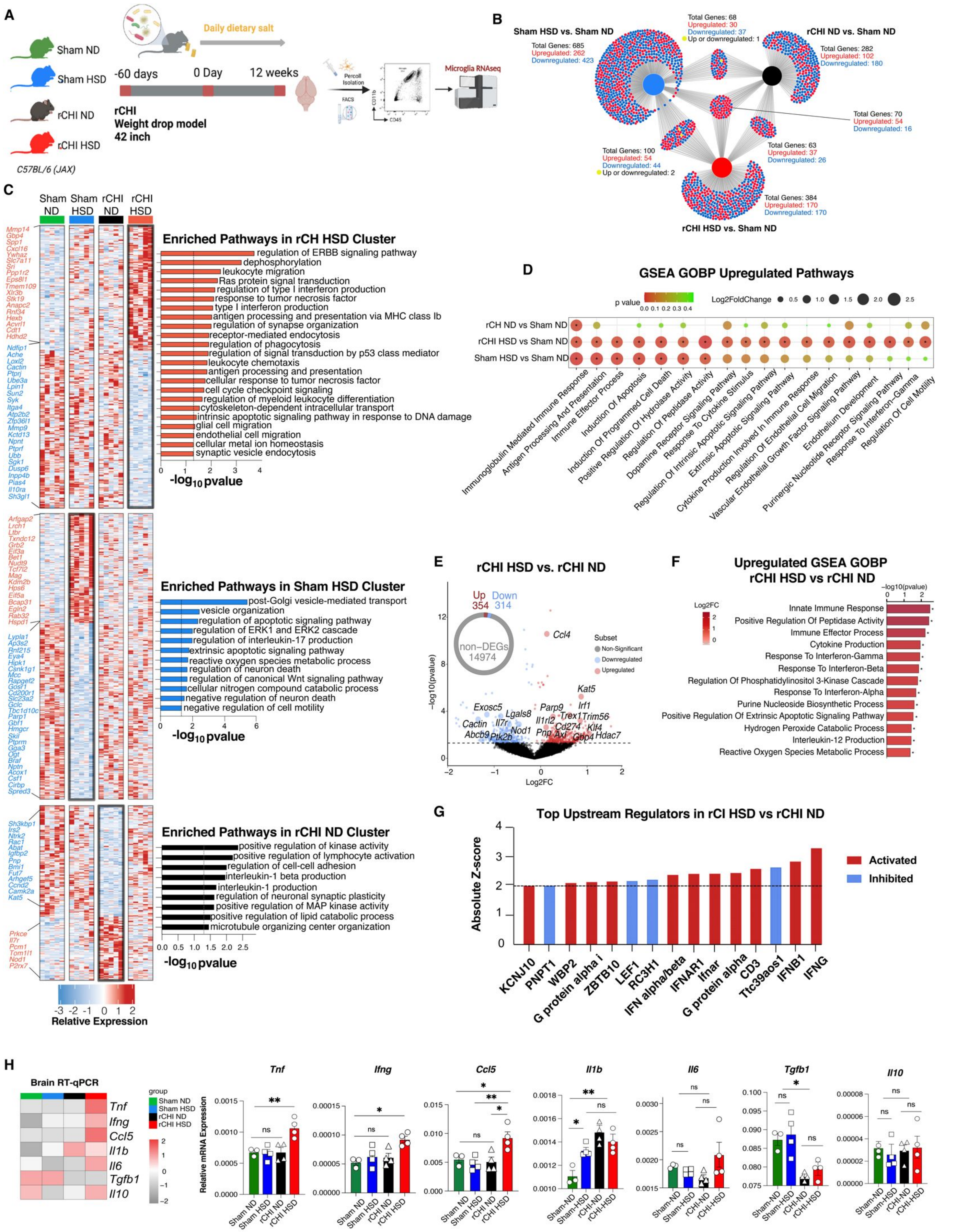
A
Sham ND
Sham HSD
rCHI ND
rCHI HSD
C57BL/6 (JAX)
Daily dietary salt
-60 days
0 Day
12 weeks
rCHI
Weight drop model
42 inch
Microglia RNAseq
B
Sham HSD vs. Sham ND
rCHI ND vs. Sham ND
rCHI HSD vs. Sham ND
Total Genes: 685
Upregulated: 262
Downregulated: 423
Total Genes: 68
Upregulated: 30
Downregulated: 37
Up or downregulated: 1
Total Genes: 282
Upregulated: 102
Downregulated: 180
Total Genes: 70
Upregulated: 54
Downregulated: 16
Total Genes: 100
Upregulated: 54
Downregulated: 44
Up or downregulated: 2
Total Genes: 63
Upregulated: 37
Downregulated: 26
Total Genes: 384
Upregulated: 170
Downregulated: 170
C
Enriched Pathways in rCH HSD Cluster
regulation of ERBB signaling pathway
dephosphorylation
leukocyte migration
Ras protein signal transduction
regulation of type I interferon production
response to tumor necrosis factor
type I interferon production
antigen processing and presentation via MHC class Ib
regulation of synapse organization
receptor-mediated endocytosis
regulation of phagocytosis
regulation of signal transduction by p53 class mediator
leukocyte chemotaxis
antigen processing and presentation
cellular response to tumor necrosis factor
cell cycle checkpoint signaling
regulation of myeloid leukocyte differentiation
cytoskeleton-dependent intracellular transport
intrinsic apoptotic signaling pathway in response to DNA damage
glial cell migration
endothelial cell migration
cellular metal ion homeostasis
synaptic vesicle endocytosis
-log10 pvalue
Enriched Pathways in Sham HSD Cluster
post-Golgi vesicle-mediated transport
vesicle organization
regulation of apoptotic signaling pathway
regulation of ERK1 and ERK2 cascade
regulation of interleukin-17 production
extrinsic apoptotic signaling pathway
reactive oxygen species metabolic process
regulation of neuron death
regulation of canonical Wnt signaling pathway
cellular nitrogen compound catabolic process
negative regulation of neuron death
negative regulation of cell motility
Enriched Pathways in rCHI ND Cluster
positive regulation of kinase activity
positive regulation of lymphocyte activation
regulation of cell-cell adhesion
interleukin-1 beta production
interleukin-1 production
regulation of neuronal synaptic plasticity
positive regulation of MAP kinase activity
positive regulation of lipid catabolic process
microtubule organizing center organization
Relative Expression
D
GSEA GOBP Upregulated Pathways
p value
Log2FoldChange
rCH ND vs Sham ND
rCHI HSD vs Sham ND
Sham HSD vs Sham ND
Immunoglobulin Mediated Immune Response
Antigen Processing And Presentation
Immune Effector Process
Induction Of Apoptosis
Induction Of Programmed Cell Death
Positive Regulation Of Hydrolase Activity
Regulation Of Peptidase Activity
Dopamine Receptor Signaling Pathway
Response To Cytokine Stimulus
Regulation Of Intrinsic Apoptotic Signaling Pathway
Extrinsic Apoptotic Signaling Pathway
Cytokine Production Involved In Immune Response
Regulation Of Endothelial Cell Migration
Vascular Endothelial Growth Factor Signaling Pathway
Endothelium Development
Purinergic Nucleotide Receptor Signaling Pathway
Response To Interferon-Gamma
Regulation Of Cell Motility
E
rCHI HSD vs. rCHI ND
Up 354
Down 314
non-DEGs 14974
Subset
Non-Significant
Downregulated
Upregulated
-log10(pvalue)
Log2FC
F
Upregulated GSEA GOBP
rCHI HSD vs rCHI ND
-log10(pvalue)
Log2FC
Innate Immune Response
Positive Regulation Of Peptidase Activity
Immune Effector Process
Cytokine Production
Response To Interferon-Gamma
Response To Interferon-Beta
Regulation Of Phosphatidylinositol 3-Kinase Cascade
Response To Interferon-Alpha
Purine Nucleoside Biosynthetic Process
Positive Regulation Of Extrinsic Apoptotic Signaling Pathway
Hydrogen Peroxide Catabolic Process
Interleukin-12 Production
Reactive Oxygen Species Metabolic Process
G
Top Upstream Regulators in rCI HSD vs rCHI ND
Absolute Z-score
Activated
Inhibited
KCNJ10
PNPT1
WBP2
G protein alpha i
ZBTB10
LEF1
RC3H1
IFN alpha/beta
IFNAR1
Ifnar
G protein alpha
CD3
Ttc39aos1
IFNB1
IFNG
H
Brain RT-qPCR
group
Sham ND
Sham HSD
rCHI ND
rCHI HSD
Tnf
Ifng
Ccl5
Il1b
Il6
Tgfb1
Il10
Relative mRNA Expression

Figure 4

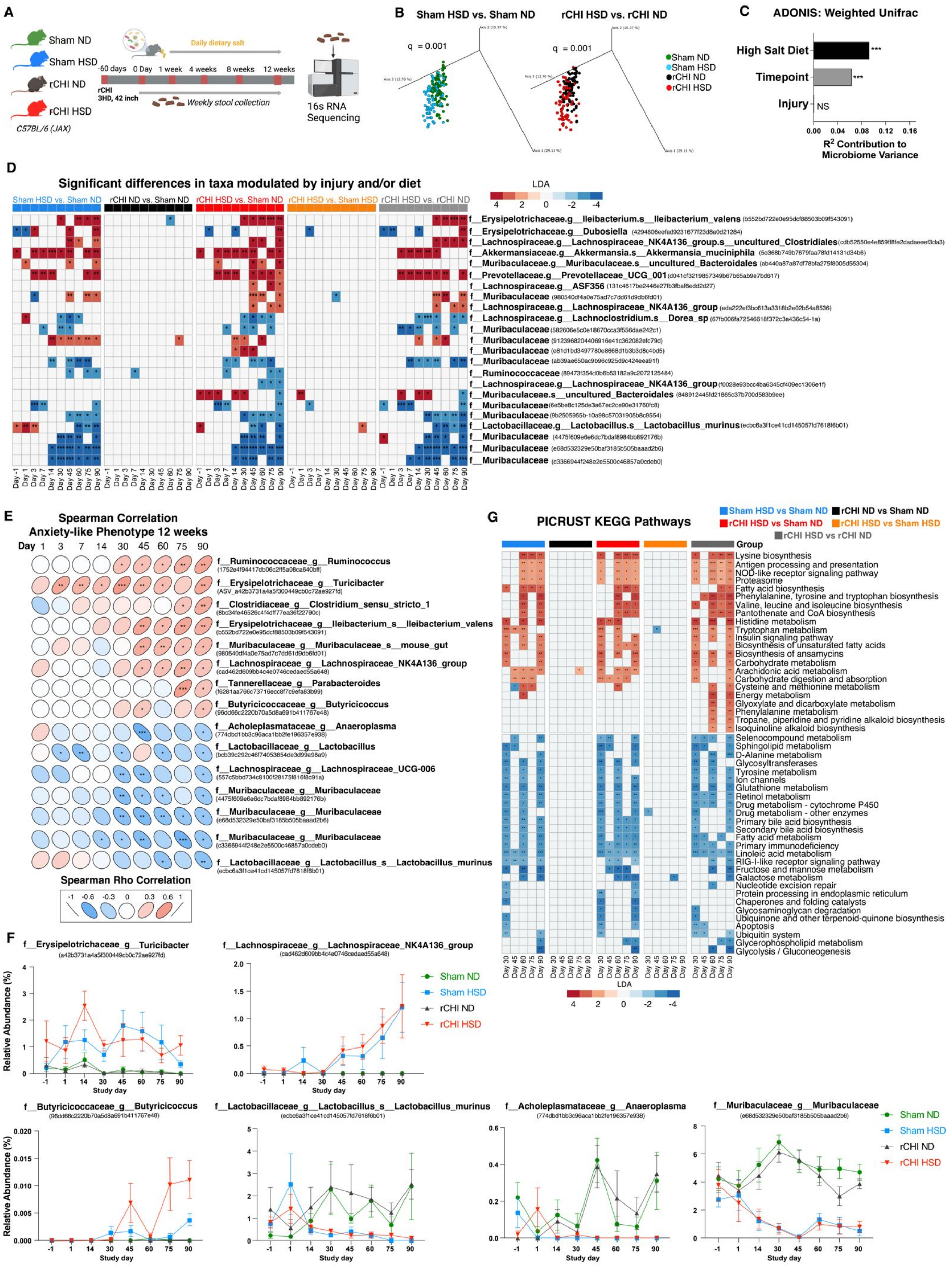
A
Sham ND
Sham HSD
rCHI ND
rCHI HSD
C57BL/6 (JAX)
Daily dietary salt
-60 days
0 Day
1 week
4 weeks
8 weeks
12 weeks
rCHI
3HD, 42 inch
Weekly stool collection
16s RNA Sequencing
B
Sham HSD vs. Sham ND
rCHI HSD vs. rCHI ND
q = 0.001
q = 0.001
Sham ND
Sham HSD
rCHI ND
rCHI HSD
C
ADONIS: Weighted Unifrac
High Salt Diet
Timepoint
Injury
NS
0.00 0.04 0.08 0.12 0.16
R2 Contribution to Microbiome Variance
D
Significant differences in taxa modulated by injury and/or diet
LDA
Sham HSD vs. Sham ND
rCHI ND vs. Sham ND
rCHI HSD vs. Sham ND
rCHI HSD vs. Sham HSD
rCHI HSD vs. rCHI ND
f__Erysipelotrichaceae.g__Ileibacterium.s__Ileibacterium_valens
f__Erysipelotrichaceae.g__Dubosiella
f__Lachnospiraceae.g__Lachnospiraceae_NK4A136_group.s__uncultured_Clostridiales
f__Akkermansiaceae.g__Akkermansia.s__Akkermansia_muciniphila
f__Muribaculaceae.g__Muribaculaceae.s__uncultured_Bacteroidales
f__Prevotellaceae.g__Prevotellaceae_UCG_001
f__Lachnospiraceae.g__ASF356
f__Muribaculaceae
f__Lachnospiraceae.g__Lachnospiraceae_NK4A136_group
f__Lachnospiraceae.g__Lachnoclostridium.s__Dorea_sp
f__Muribaculaceae
f__Muribaculaceae
f__Muribaculaceae
f__Muribaculaceae
f__Ruminococcaceae
f__Lachnospiraceae.g__Lachnospiraceae_NK4A136_group
f__Muribaculaceae.s__uncultured_Bacteroidales
f__Muribaculaceae
f__Muribaculaceae
f__Lactobacillaceae.g__Lactobacillus.s__Lactobacillus_murinus
f__Muribaculaceae
f__Muribaculaceae
f__Muribaculaceae
E
Spearman Correlation
Anxiety-like Phenotype 12 weeks
Day 1 3 7 14 30 45 60 75 90
f__Ruminococcaceae_g__Ruminococcus
f__Erysipelotrichaceae_g__Turicibacter
f__Clostridiaceae_g__Clostridium_sensu_stricto_1
f__Erysipelotrichaceae_g__Ileibacterium_s__Ileibacterium_valens
f__Muribaculaceae_g__Muribaculaceae_s__mouse_gut
f__Lachnospiraceae_g__Lachnospiraceae_NK4A136_group
f__Tannerellaceae_g__Parabacteroides
f__Butyricicoccaceae_g__Butyricicoccus
f__Acholeplasmataceae_g__Anaeroplasma
f__Lactobacillaceae_g__Lactobacillus
f__Lachnospiraceae_g__Lachnospiraceae_UCG-006
f__Muribaculaceae_g__Muribaculaceae
f__Muribaculaceae_g__Muribaculaceae
f__Muribaculaceae_g__Muribaculaceae
f__Lactobacillaceae_g__Lactobacillus_s__Lactobacillus_murinus
Spearman Rho Correlation
-1 -0.6 -0.3 0 0.3 0.6 1
G
PICRUST KEGG Pathways
Sham HSD vs Sham ND
rCHI ND vs Sham ND
rCHI HSD vs Sham ND
rCHI HSD vs Sham HSD
rCHI HSD vs rCHI ND
Group
Lysine biosynthesis
Antigen processing and presentation
NOD-like receptor signaling pathway
Proteasome
Fatty acid biosynthesis
Phenylalanine, tyrosine and tryptophan biosynthesis
Valine, leucine and isoleucine biosynthesis
Pantothenate and CoA biosynthesis
Histidine metabolism
Tryptophan metabolism
Insulin signaling pathway
Biosynthesis of unsaturated fatty acids
Biosynthesis of ansamycins
Carbohydrate metabolism
Arachidonic acid metabolism
Carbohydrate digestion and absorption
Cysteine and methionine metabolism
Energy metabolism
Glyoxylate and dicarboxylate metabolism
Phenylalanine metabolism
Tropane, piperidine and pyridine alkaloid biosynthesis
Isoquinoline alkaloid biosynthesis
Selenocompound metabolism
Sphingolipid metabolism
D-Alanine metabolism
Glycosyltransferases
Tyrosine metabolism
Ion channels
Glutathione metabolism
Retinol metabolism
Drug metabolism - cytochrome P450
Drug metabolism - other enzymes
Primary bile acid biosynthesis
Secondary bile acid biosynthesis
Fatty acid metabolism
Primary immunodeficiency
Linoleic acid metabolism
RIG-I-like receptor signaling pathway
Fructose and mannose metabolism
Galactose metabolism
Nucleotide excision repair
Protein processing in endoplasmic reticulum
Chaperones and folding catalysts
Glycosaminoglycan degradation
Ubiquinone and other terpenoid-quinone biosynthesis
Apoptosis
Ubiquitin system
Glycerophospholipid metabolism
Glycolysis / Gluconeogenesis
LDA
F
f__Erysipelotrichaceae_g__Turicibacter
f__Lachnospiraceae_g__Lachnospiraceae_NK4A136_group
Relative Abundance (%)
Study day
Sham ND
Sham HSD
rCHI ND
rCHI HSD
f__Butyricicoccaceae_g__Butyricicoccus
f__Lactobacillaceae_g__Lactobacillus_s__Lactobacillus_murinus
f__Acholeplasmataceae_g__Anaeroplasma
f__Muribaculaceae_g__Muribaculaceae

Supplementary Figure 1

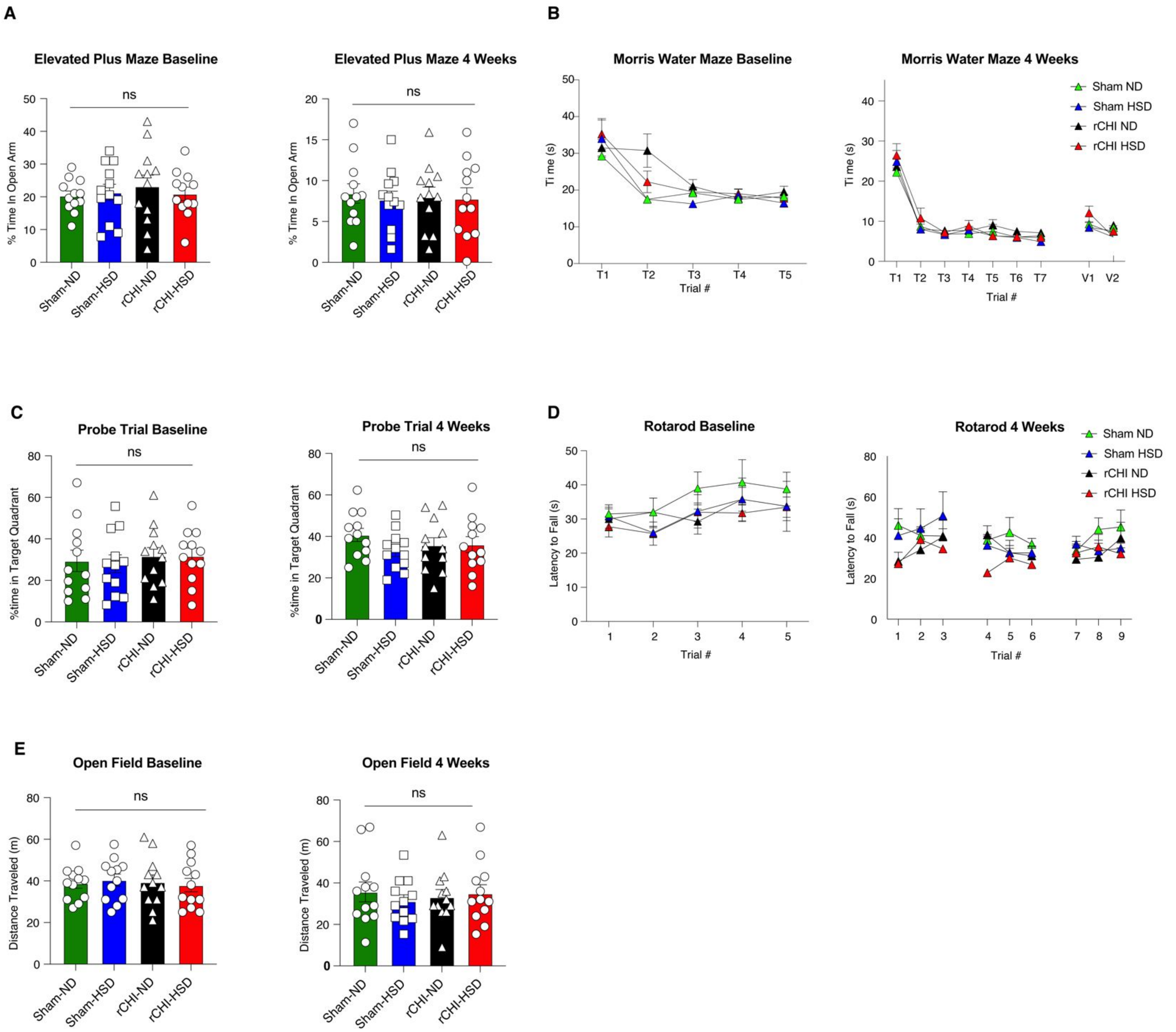

Supplementary Figure 2

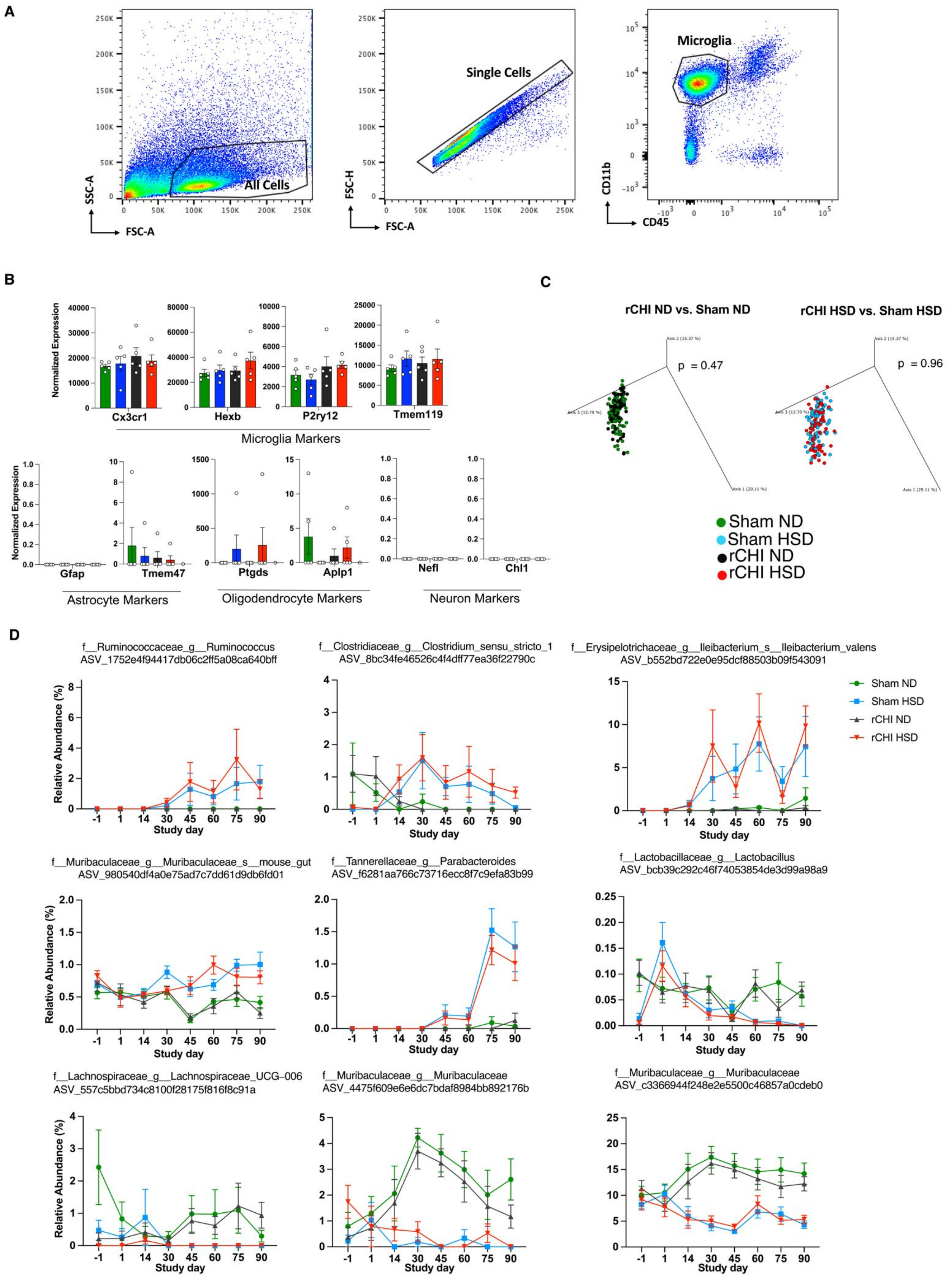
A
All Cells
SSC-A
FSC-A
Single Cells
FSC-H
FSC-A
Microglia
CD11b
CD45
B
Normalized Expression
Cx3cr1
Hexb
P2ry12
Tmem119
Microglia Markers
Gfap
Tmem47
Ptgds
Aplp1
Nefl
Chl1
Astrocyte Markers
Oligodendrocyte Markers
Neuron Markers
C
rCHI ND vs. Sham ND
rCHI HSD vs. Sham HSD
p = 0.47
p = 0.96
Axis 1 (29.11 %)
Axis 2 (15.37 %)
Axis 3 (12.70 %)
Sham ND
Sham HSD
rCHI ND
rCHI HSD
D
f__Ruminococcaceae_g__Ruminococcus
ASV_1752e4f94417db06c2ff5a08ca640bff
f__Clostridiaceae_g__Clostridium_sensu_stricto_1
ASV_8bc34fe46526c4f4dff77ea36f22790c
f__Erysipelotrichaceae_g__Ileibacterium_s__Ileibacterium_valens
ASV_b552bd722e0e95dcf88503b09f543091
f__Muribaculaceae_g__Muribaculaceae_s__mouse_gut
ASV_980540df4a0e75ad7c7dd61d9db6fd01
f__Tannerellaceae_g__Parabacteroides
ASV_f6281aa766c73716ecc8f7c9efa83b99
f__Lactobacillaceae_g__Lactobacillus
ASV_bcb39c292c46f74053854de3d99a98a9
f__Lachnospiraceae_g__Lachnospiraceae_UCG-006
ASV_557c5bbd734c8100f28175f816f8c91a
f__Muribaculaceae_g__Muribaculaceae
ASV_4475f609e6e6dc7bdaf8984bb892176b
f__Muribaculaceae_g__Muribaculaceae
ASV_c3366944f248e2e5500c46857a0cdeb0
Relative Abundance (%)
Study day
Sham ND
Sham HSD
rCHI ND
rCHI HSD

Supplementary Figure 3

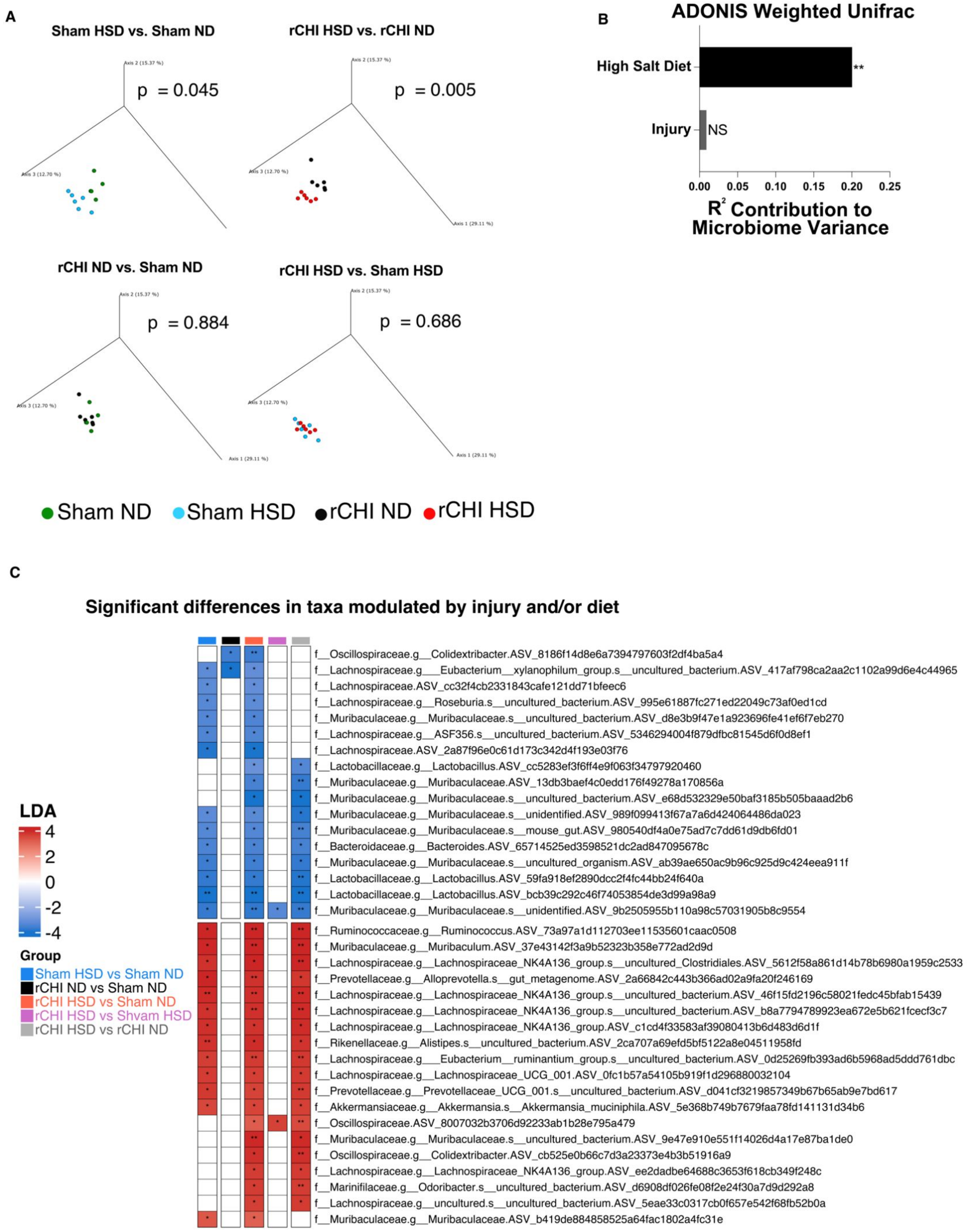

Figure Legends

Figure 1. High salt diet induces an anxiety like phenotype in a rCHI model of concussion. (A) Visual representation of experimental timeline of behavioral and physiological testing regimens after high salt diet (HSD) administration. **(B)** Physiological data of weight, **(C)** Systolic/diastolic blood pressure, and heart rate of the groups through 12 weeks of diet administration. Behavioral testing of anxiety like phenotype using an **(D)** elevated plus maze**, (E)** Morris water maze (MWM) and probe trial, **(F)** rotarod, and **(G)** open field at 8- and 12-weeks post diet administration. Physiological data, Morris water maze, and the rotarod were analyzed by two-way ANOVA and the other behavioral tests were analyzed by one-way ANOVA, followed by a Tukey's *post hoc* test. n = 12 mice/group was used for all experiments and data is presented as (mean and SEM). * p < 0.05, *** p < 0.001, ns = not significant**.**

Figure 2. High salt diet induces chronic microglial activation in a rCHI model of concussion. Representative confocal images of immunofluorescence staining for Iba1+ microglia (red), and DAPI (blue) on different brain regions. Example overviews of **(A)** cortex, different hippocampal areas **(B)** CA1, **(C)** CA3, and **(D)** dentate gyrus (DG), and **(E)** the amygdala in 12 weeks post-ND or HSD in sham and rCHI animals. Quantitative analysis of Iba1+ cells counted in CA1, CA3, and dentate gyrus (DG), and the amygdala in 12 weeks post-ND or HSD in sham and rCHI animals. n=4 mice /group. ** p < 0.05, *** p < 0.001, **** p < 0.0001 ns = not significant by one-way ANOVA with Tukey's post-hoc multiple comparison test**.** Representative images Scale bars represent 50 µm.

Figure 3. High salt diet induces chronic proinflammatory microglial profile and stress response in a rCHI model of concussion. (A) Visual representation of experimental timeline of microglia analysis and bulk RNAseq after HSD administration. **(B)** DiVenn plot showing the unique and shared differentially expressed genes (P<0.05) of the following groups compared to Sham ND baseline: Sham HSD, rCHI ND, and rCHI HSD. Directionality of gene expression is determined by log2-foldchanges of pairwise gene expression comparisons. Red-colored genes represent upregulated genes. Blue-colored genes represent downregulated genes. Yellow-colored genes represent shared genes that are up-regulated in one comparison but down-regulated in the other comparison. **(C)** Heatmap of relative expression levels clustered according to the unique DEGs obtained in the following pairwise comparisons compared to Sham ND baseline (from top to bottom): rCHI HSD, Sham ND, and rCHI ND. Enriched GO terms of each cluster are displayed on the right with selected corresponding genes labeled on the left. **(D)** Dot plot of GAGE analysis highlighting significantly upregulated GO Biological Process (BP) pathways in the following pairwise comparisons compared to Sham ND baseline: rCHI ND, rCHI HSD, and rCHI ND. Dot size indicates the $\log_2$fold change of the pathway in the specific pairwise comparison (rCHI ND vs Sham ND, rCHI HSD vs Sham ND, or Sham HSD vs Sham ND). Dot color indicates significance strength of the pathway, where p-value < 0.05 was considered statistically significant. **(E)** Volcano plots showing the microglia gene expression in rCHI HSD vs rCHI ND. On the x-axis are the log2-fold changes and the y-axis is the –log10(p-value). Significant differentially expressed genes (P <0.05) are colored (red for upregulation and blue for downregulation). **(F)** Selected top upregulated GO Biological Process (BP) pathways from GAGE analysis by p-value in rCHI HSD vs rCHI ND. Data is represented by $\log_{10}$(P) and increasing bar color intensity signifies increasing log2-foldchanges. **(G)** Top IPA predicted upstream regulators for DEGs in rCHI HSD vs rCHI ND. All shown regulators were deemed significant at P<0.05 and an absolute activation score greater than or equal to 2. Activation scores were used to determine the activation state of the predicted upstream regulator, where an activation score less than or equal to -2 implies the upstream regulator is inhibited and an activation score greater than or equal to 2 implies that the upstream regulator is activated.**(H)** Brain tissue qPCR was analyzed by one-way ANOVA, followed by Tukey *post hoc* analysis. n = 4 mice/group was used for all experiments and data is presented as (mean and SEM). * P < 0.05, ** P < 0.01, *** P < 0.001, n.s. = not significant**.**

For Review Only

Figure 4. High salt diet induces microbiome dysregulation in a rCHI model of concussion. (A) Visual representation of experimental timeline of fecal sample collection for 16s microbiome sequencing. **(B)** Principal coordinate analysis (PCoA) of weighted UniFrac distances stratified by diet and injury (rCHI) microbiota structure: salt diet in Sham mice (left panel) and by salt diet in injured (rCHI) mice (right panel). Each point represents the microbiota from one mouse. p-values are obtained from PERMANOVA tests on beta-diversity using weighted UniFrac distances. **(C)** Weighted ADONIS test on microbiota samples investigating the contribution of injury (rCHI), salt diet, and timepoint to overall microbiome variation (***= P<0.001, **= P>0.001 & P<0.01, *= P>0.01 & P<0.05). **(D)** Significant differences in taxa modulated by salt diet and/or injury phenotype over time collected from fecal microbiota samples was determined by linear discriminant analysis effect size (LEfSe). Legend represents linear discriminant analysis (LDA) effect size score. (***= P<0.001, **= P>0.001 & P<0.01, *= P>0.01 & P<0.05). **(E)** Spearman correlations between fecal microbial abundance at all timepoints and anxiety-like phenotype (from Figure 1C) measured 12 weeks for each sample group (Sham ND, Sham HSD, rCHI ND, and rCHI HSD). **(F)** Relative abundance of selected taxa from Figure 4E over time that were significantly correlated in at least two time points collected from fecal microbiota samples. Data is presented as (mean and SEM). **(G)** Investigating microbial functional changes from fecal samples collected across 30-90 days. Significant differences based on the predicted KEGG metagenomic pathways (categorized by PICRUSt2) for each comparison were determined by linear discriminant analysis effect size (LEfSe). Legend represents linear discriminant analysis (LDA) effect size score. (***= P<0.001, **= P>0.001 & P<0.01, *= P>0.01 & P<0.05).

Supplementary Figure 1. Behavioral testing of anxiety like phenotype using an **(A)** elevated plus maze**, (B)** Morris water maze (MWM), **(C)** probe trial, **(D)** rotarod, and **(E)** open field at baseline and 4 weeks post diet administration. Morris water maze, and the rotarod were analyzed by two-way ANOVA and the other behavioral tests were analyzed by one-way ANOVA, followed by a Tukey's *post hoc* test. n = 12 mice/group was used for all experiments and data is presented as (mean and SEM). ns = not significant**.**

Supplementary Figure 2. (A) Gating strategy of microglia that was sorted for RNA-sequencing **(B)** Validation of microglia RNA-sequencing data by looking into select microglia markers compared to other non-microglia markers. **C)** Principal coordinate analysis (PCoA) of weighted UniFrac distances stratified by the effect of rCHI in ND and HSD groups respectively: rCHI ND vs. Sham ND, and rCHI HSD vs. Sham HSD. Each point represents the microbiota from one mouse. p-values are obtained from PERMANOVA tests on beta-diversity using weighted UniFrac distances. **(C)** Relative abundance of taxa from Figure 4E over time that were significantly correlated in at least two time points collected from fecal microbiota samples. Data is presented as (mean and SEM).

Supplementary Figure 3. (A) Principal coordinate analysis (PCoA) of weighted UniFrac distances stratified by the following pairwise comparisons: Sham HSD vs. Sham ND, rCHI HSD vs. rCHI ND, rCHI ND vs. Sham ND, and rCHI HSD vs. Sham HSD. Each point represents the microbiota from one mouse. p-values are obtained from PERMANOVA tests on beta-diversity using weighted UniFrac distances. **(B)** Weighted ADONIS test on microbiota samples investigating the contribution of injury (rCHI) and salt die to overall microbiome variation (***= P<0.001, **= P>0.001 & P<0.01, *= P>0.01 & P<0.05). **(C)** Significant differences in taxa modulated by salt diet and/or injury phenotype collected from cecum microbiota samples determined by linear discriminant analysis effect size (LEfSe). Legend represents linear discriminant analysis (LDA) effect size score. (***= P<0.001, **= P>0.001 & P<0.01, *= P>0.01 & P<0.05**).**

For Review Only

Description of Additional Supplementary Files

Supplementary Table 1. DESeq2 normalized expression counts for all genes. List of normalized expression counts data (DESeq2) for all genes passing quality metrics.

Supplementary Table 2. List of microglia differentially expressed genes in all groups compared to Sham ND baseline. Differential gene expression was performed using DESeq2. Pairwise differential gene expression comparisons (Sham HSD vs. Sham ND, rCHI ND vs. Sham ND, and rCHI HSD vs Sham ND) were done using the Wald Test with standard parameters and log2 fold-changes were subsequently shrunken using DESeq2 built-in lfcshrink function.

Supplementary Table 3. Expression data of the unique DEGs in all pairwise comparisons compared to Sham ND baseline. Expression data (DESeq2) for the unique differentially expressed genes in the following pairwise comparisons: rCHI HSD vs. Sham ND, Sham HSD vs. Sham ND, and rCHI ND vs. Sham ND. The expression data used for heatmap visualization was produced using the variance stabilizing transformation (VST) method from the DESeq2 built-in VST function.

Supplementary Table 4. List of microglia differentially expressed genes in rCHI HSD vs. rCHI ND. Differential gene expression was performed using DESeq2. Pairwise differential gene expression comparison (rCHI HSD vs. rCHI ND) was done using the Wald Test with standard parameters and log2 fold-changes were subsequently shrunken using DESeq2 built-in lfcshrink function.

Supplementary Table 5. List of significant Ingenuity Pathway Analysis (IPA) upstream regulators in various pairwise differential gene expression comparisons. Ingenuity Pathway Analysis (IPA) was used to identify upstream regulators ($P < 0.05$ and $|Z\text{-score}| \geq 2$) based on the DEGs in the following pairwise comparisons: rCHI HSD vs. rCHI ND, Sham HSD vs. Sham ND, rCHI ND vs. Sham ND, rCHI HSD vs. Sham ND, and rCHI HSD vs. Sham HSD. The input data for IPA comprised of p-values and $\log_2$fold changes of DEGs.

For Review Only

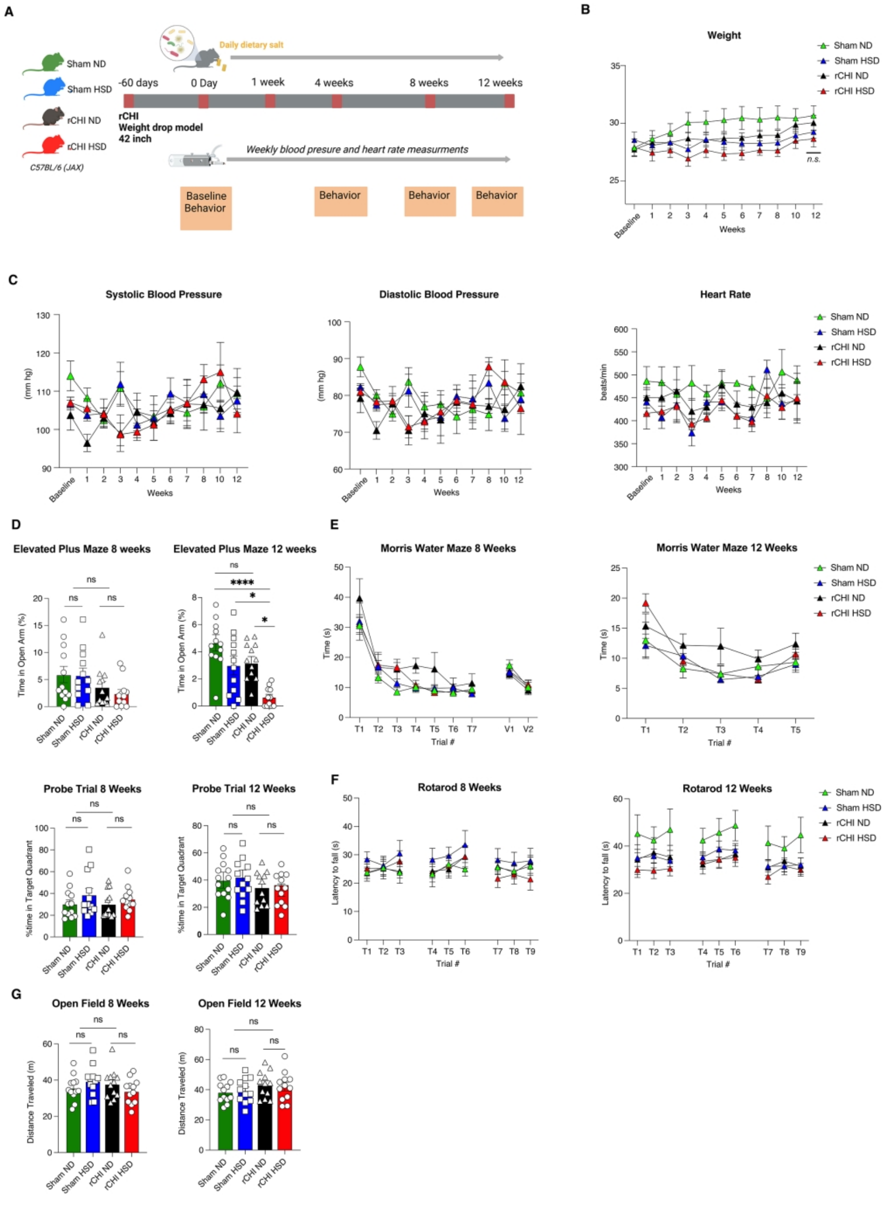

Figure 1

859x1164mm (72 x 72 DPI)

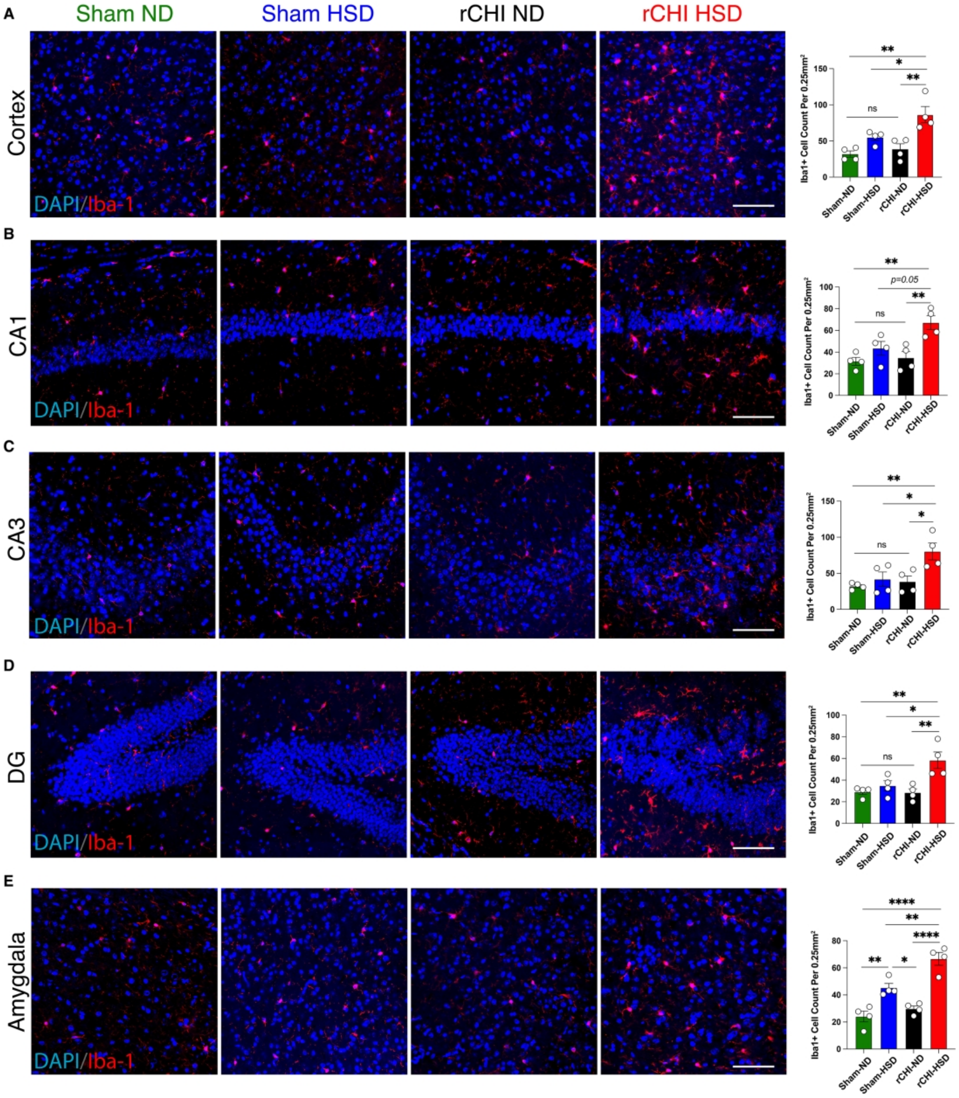

Figure 2

989x1164mm (72 x 72 DPI)

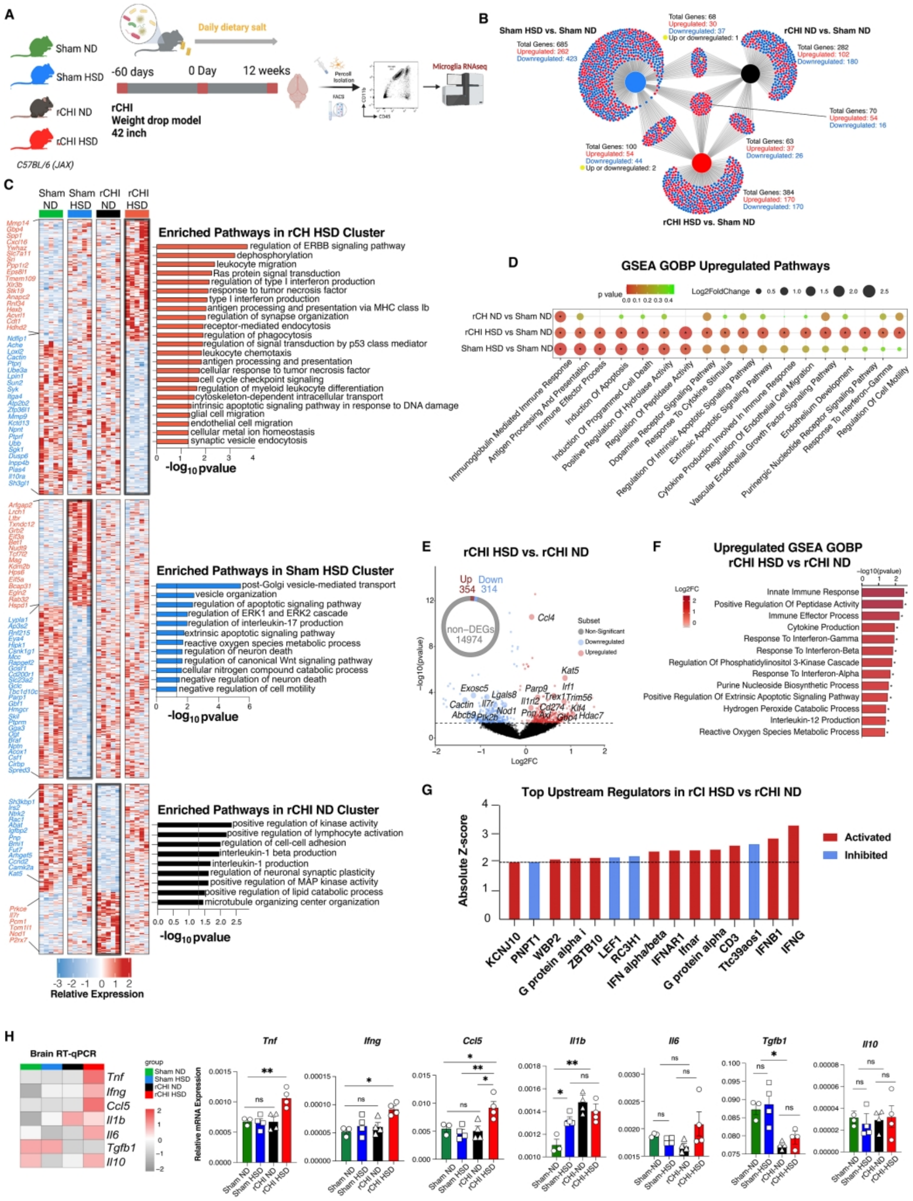

899x1190mm (72 x 72 DPI)

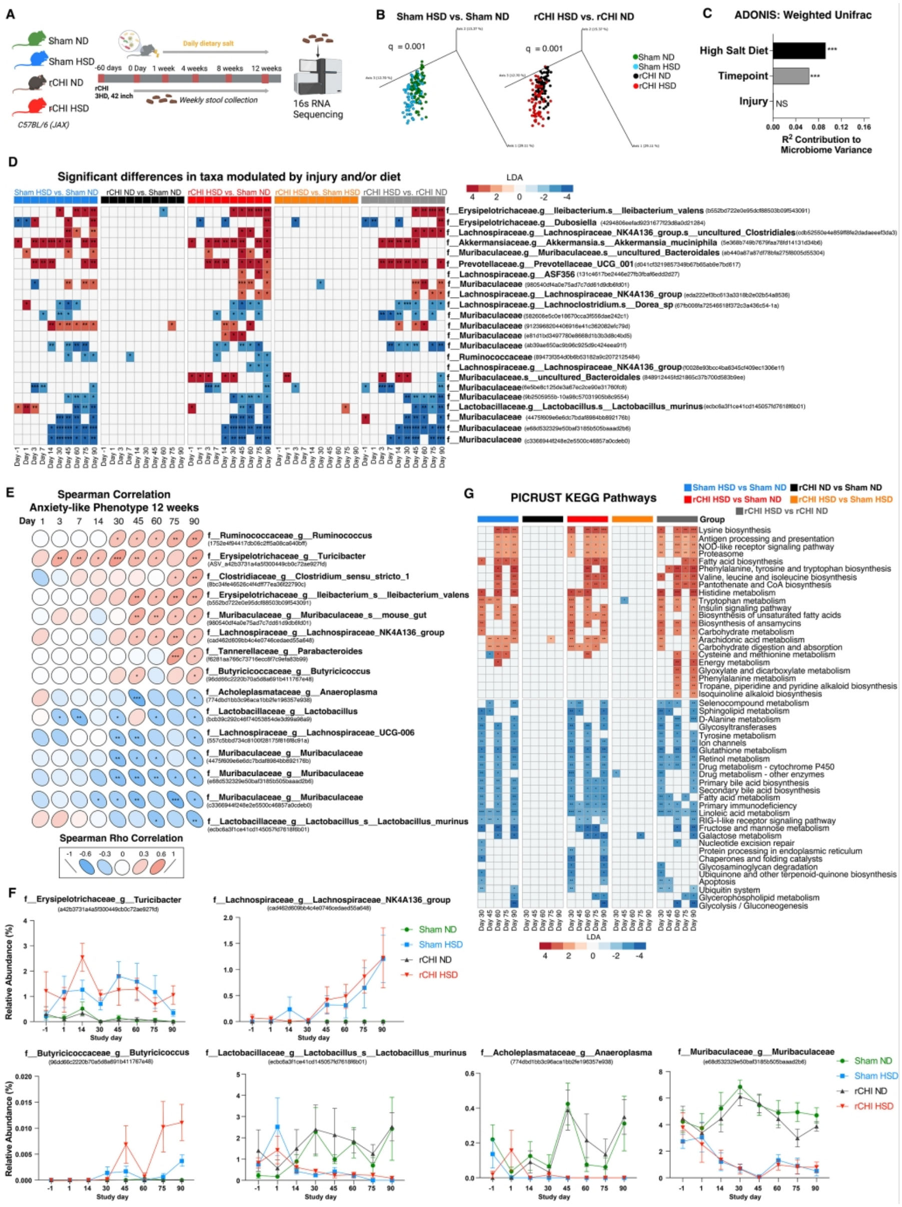

Figure 4

870x1164mm (72 x 72 DPI)

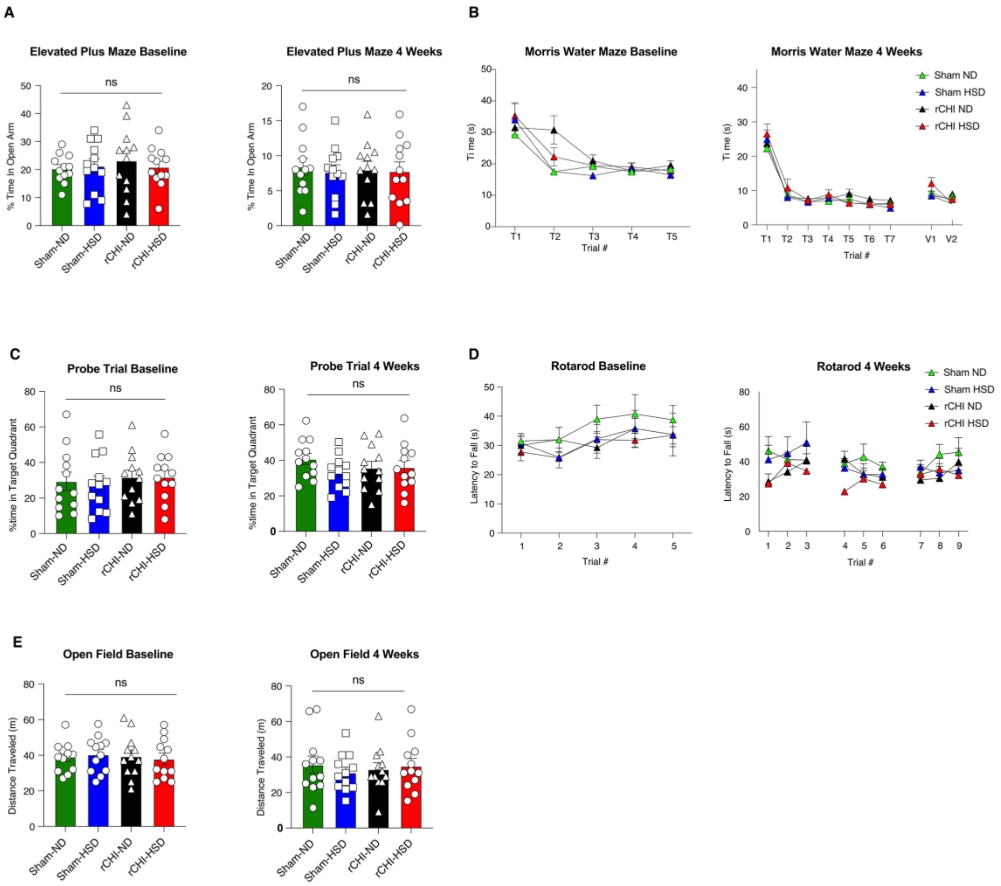

Supp_Fig1

899x875mm (72 x 72 DPI)

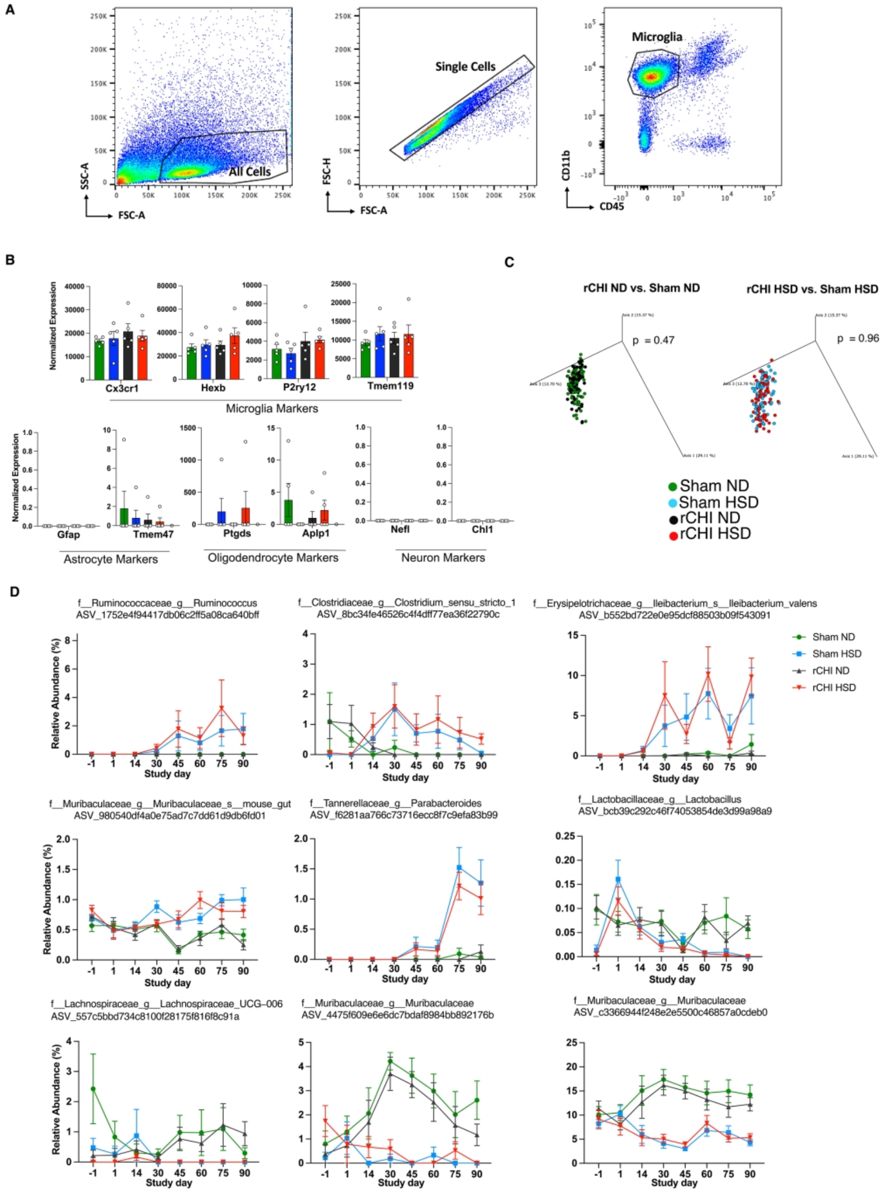

Supp_Fig2

899x1200mm (72 x 72 DPI)

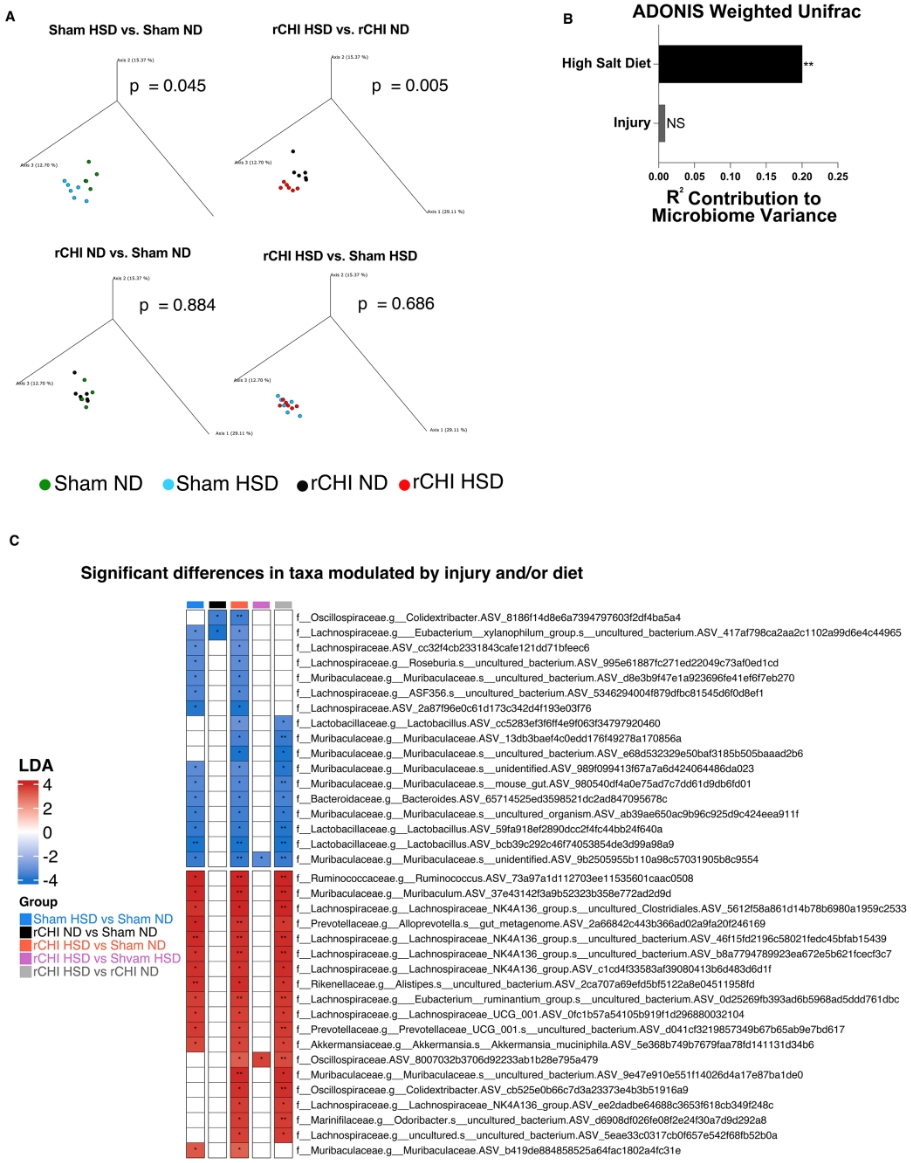

Supp_Fig3

893x1164mm (72 x 72 DPI)

Microglia
DESeq2-Normalized Expression Counts

Gene	Sham_ND_1	Sham_ND_2	Sham_ND_3	Sham_ND_4	Sham_ND_5
.	105477	91081	133124	104425	131299
0610007P14Rik	5	2	47	42	0
0610009B22Rik	61	59	77	35	32
0610009L18Rik	9	0	0	2	0
0610009O20Rik	147	0	53	80	64
0610010B08Rik	4	6	1	11	13
0610010F05Rik	24	7	25	26	26
0610010K14Rik	0	0	23	0	11
0610011F06Rik	17	0	0	0	0
0610012G03Rik	73	100	93	85	88
0610025J13Rik	0	0	0	0	0
0610030E20Rik	3	1	0	0	0
0610037L13Rik	117	91	7	127	194
0610039H22Rik	1	1	1	1	0
0610039K10Rik	1	7	1	9	9
0610040B10Rik	0	3	0	0	1
0610040J01Rik	688	372	765	529	555
1110001J03Rik	4	0	4	4	7
1110002J07Rik	0	0	0	0	0
1110002L01Rik	10	0	0	14	29
1110004E09Rik	0	17	9	0	0
1110004F10Rik	120	203	211	132	155
1110006O24Rik	12	15	21	9	10
1110008F13Rik	16	38	20	27	40
1110008L16Rik	23	6	25	25	0
1110008P14Rik	8	7	0	9	12
1110012L19Rik	21	21	39	20	28
1110018N20Rik	0	0	0	0	0
1110019D14Rik	2	8	17	13	11
1110020A21Rik	0	0	0	0	0
1110032A03Rik	61	25	46	12	47
1110034G24Rik	46	73	63	58	57
1110037F02Rik	82	60	73	121	134
1110038F14Rik	3	0	0	0	0
1110051M20Rik	230	193	279	202	271
1110059E24Rik	76	89	36	69	61
1110059G10Rik	39	37	68	34	59
1110065P20Rik	0	1	1	1	1
1190002N15Rik	10	41	18	25	16
1190007I07Rik	8	5	0	15	0
1300002E11Rik	16	15	9	40	21
1500004A13Rik	23	13	12	10	12
1500011B03Rik	44	40	33	17	14
1500011K16Rik	1	77	0	0	66
1500015A07Rik	13	20	13	14	5
1500015O10Rik	46	0	0	27	0

For Review Only

1500026H17Rik	6	0	0	0	0
1500035N22Rik	4	6	4	7	12
1600002H07Rik	44	9	28	21	19
1600010M07Rik	12	1	33	16	0
1600012H06Rik	115	126	155	125	109
1600014C10Rik	156	132	0	0	0
1600020E01Rik	4	0	0	0	15
1600023N17Rik	0	1	0	2	0
1700001C19Rik	1	4	0	5	0
1700001J11Rik	0	0	0	0	0
1700001K19Rik	1	0	0	0	0
1700001L05Rik	5	1	15	10	23
1700003F12Rik	5	0	5	5	1
1700006H20Rik	1	3	4	0	0
1700007E05Rik	0	1	0	0	0
1700007L15Rik	1	4	11	0	0
1700008J07Rik	5	4	6	6	5
1700009J07Rik	2	0	1	3	8
1700010I14Rik	17	4	15	6	13
1700010L04Rik	3	0	2	7	5
1700011L22Rik	0	0	1	4	0
1700012D01Rik	3	0	3	0	2
1700012D14Rik	0	7	0	3	7
1700015E13Rik	5	6	1	7	12
1700016A09Rik	3	0	0	3	3
1700017B05Rik	1541	682	692	1	1
1700018A23Rik	0	4	0	0	2
1700019D03Rik	2	5	1	5	0
1700020D05Rik	0	0	0	0	3
1700020I14Rik	13	24	14	36	24
1700020N01Rik	1	3	4	1	0
1700021F05Rik	17	31	34	23	26
1700022I11Rik	5	0	0	0	0
1700022N22Rik	1	0	0	2	0
1700025G04Rik	12	6	0	4	10
1700025N23Rik	3	1	11	6	14
1700028E10Rik	18	1	0	23	0
1700028J19Rik	0	0	0	0	3
1700028K03Rik	4	6	1	5	5
1700029J07Rik	34	42	17	16	33
1700030C10Rik	6	3	11	13	3
1700030J22Rik	2	2	0	3	0
1700030K09Rik	56	36	50	34	65
1700034I23Rik	2	0	0	0	0
1700034J05Rik	0	0	0	0	0
1700037C18Rik	0	0	17	9	20
1700037H04Rik	26	20	16	10	24
1700039M10Rik	1	2	0	0	6
1700040D17Rik	3	0	1	0	0
1700047I17Rik2	31	20	31	22	26

For Review Only

1700048M11Rik	0	0	0	0	0
1700049G17Rik	61	49	38	74	70
1700052K11Rik	7	4	1	7	4
1700054O19Rik	0	1	6	1	3
1700055D18Rik	24	8	12	11	7
1700061G19Rik	17	8	9	19	18
1700066B19Rik	5	11	8	0	5
1700066M21Rik	0	41	0	0	0
1700067K01Rik	0	0	0	1	0
1700073E17Rik	0	0	0	1	0
1700080G18Rik	0	4	0	3	1
1700084E18Rik	0	1	1	0	0
1700084J12Rik	3	0	0	0	0
1700086O06Rik	3	6	0	8	5
1700086P04Rik	0	0	4	3	5
1700087I21Rik	3	7	4	6	4
1700094D03Rik	0	0	52	34	46
1700095B10Rik	0	0	0	1	4
1700095J12Rik	7	3	0	4	4
1700096K18Rik	8	7	11	10	6
1700102P08Rik	3	3	4	4	4
1700110C19Rik	4	19	22	13	12
1700110I01Rik	5	2	4	4	5
1700112E06Rik	13	22	23	46	27
1700113A16Rik	0	4	2	0	6
1700120K04Rik	1	6	0	0	1
1700122E12Rik	3	0	0	3	3
1700123M08Rik	0	2	0	0	0
1700123O20Rik	31	37	33	90	77
1700125H20Rik	0	0	0	0	0
1700126G02Rik	0	0	0	0	6
1810009A15Rik	38	0	84	0	57
1810010H24Rik	2	8	10	10	4
1810011H11Rik	38	26	198	98	31
1810011O10Rik	8	14	11	10	16
1810013A23Rik	2	4	0	5	0
1810013L24Rik	57	89	116	54	71
1810014B01Rik	17	23	22	14	24
1810019D21Rik	0	0	0	0	0
1810019N24Rik	0	0	0	7	0
1810021B22Rik	0	0	0	0	0
1810022K09Rik	49	37	43	38	40
1810026B05Rik	13	54	26	39	46
1810026J23Rik	32	34	0	0	0
1810030O07Rik	158	150	224	151	167
1810032O08Rik	31	13	14	22	27
1810034E14Rik	5	2	12	5	3
1810037I17Rik	44	36	76	40	33
1810041H14Rik	0	1	2	1	3
1810043G02Rik	1	13	75	0	0

For Review Only

For Review Only

1810043H04Rik	0	0	16	4	0
1810044D09Rik	0	0	11	0	7
1810055G02Rik	4	7	0	6	0
1810058I24Rik	47	40	57	30	46
2010010A06Rik	0	0	0	0	0
2010016I18Rik	0	0	3	2	0
2010106C02Rik	0	5	1	4	7
2010107E04Rik	59	45	95	0	51
2010107G23Rik	3	6	2	2	0
2010109I03Rik	7	7	1	0	5
2010111I01Rik	80	64	70	90	119
2010204K13Rik	1	0	2	1	0
2010300C02Rik	0	5	6	4	7
2010315B03Rik	112	101	54	119	137
2010320M18Rik	15	5	11	13	7
2210013O21Rik	18	30	34	29	35
2210016F16Rik	120	110	142	126	146
2210016L21Rik	0	1	3	3	2
2210404O09Rik	21	17	7	18	9
2210408F21Rik	0	0	1	0	2
2210408I21Rik	8	5	0	12	24
2210417A02Rik	2	4	0	0	0
2210418O10Rik	20	13	21	21	11
2300009A05Rik	10	11	6	8	8
2310009A05Rik	26	42	43	37	34
2310009B15Rik	0	0	0	0	0
2310010J17Rik	0	12	1	1	2
2310011J03Rik	1	22	0	0	0
2310015A10Rik	11	20	28	21	28
2310015A16Rik	0	0	0	0	0
2310022A10Rik	57	16	23	39	38
2310022B05Rik	12	6	11	11	27
2310033P09Rik	49	45	62	32	33
2310034G01Rik	3	3	6	0	1
2310034P14Rik	2	1	0	0	0
2310035C23Rik	58	91	48	101	73
2310036O22Rik	1	25	49	45	0
2310039H08Rik	14	18	17	17	23
2310040G24Rik	6	21	28	11	14
2310047D07Rik	4	3	6	0	5
2310057M21Rik	13	21	54	40	36
2310061I04Rik	18	10	28	0	17
2310068J16Rik	0	1	0	5	0
2310075K07Rik	0	5	3	1	7
2410002F23Rik	89	72	123	74	103
2410004B18Rik	33	40	0	0	0
2410015M20Rik	28	27	69	15	22
2410016O06Rik	8	51	17	25	22
2410021H03Rik	0	0	0	0	8
2410022M11Rik	14	4	6	16	14

2410089E03Rik	5	13	39	48	26
2410131K14Rik	30	21	36	36	37
2500004C02Rik	16	21	0	15	33
2510003B16Rik	0	0	0	0	0
2510009E07Rik	141	80	90	173	158
2510039O18Rik	44	72	81	61	65
2610001J05Rik	0	0	1	1	1
2610002M06Rik	17	24	11	16	27
2610005L07Rik	94	66	76	101	105
2610008E11Rik	76	47	68	82	59
2610020C07Rik	3	10	9	17	6
2610020H08Rik	7	6	14	17	12
2610021A01Rik	21	31	23	41	55
2610027K06Rik	2	11	7	3	6
2610035D17Rik	32	21	16	25	40
2610042L04Rik	14	13	15	13	17
2610044O15Rik8	6	25	19	21	8
2610203C22Rik	18	44	24	70	0
2610301B20Rik	41	16	35	28	34
2610306M01Rik	6	6	11	13	16
2610507B11Rik	136	142	105	203	142
2610507I01Rik	4	0	0	0	0
2610524H06Rik	4	0	9	6	0
2610528A11Rik	0	0	57	19	50
2700012I20Rik	5	9	6	6	4
2700038G22Rik	5	1	10	4	7
2700046G09Rik	15	9	10	8	5
2700049A03Rik	26	22	29	60	49
2700060E02Rik	2	53	0	0	220
2700062C07Rik	5	1	0	0	0
2700070H01Rik	0	3	0	0	4
2700081O15Rik	26	12	14	12	10
2700094K13Rik	17	0	31	0	0
2700097O09Rik	34	46	48	30	43
2810001G20Rik	2	4	0	0	6
2810002D19Rik	11	21	25	31	19
2810004N23Rik	42	33	35	30	35
2810006K23Rik	66	30	85	69	89
2810013P06Rik	74	71	130	62	64
2810021J22Rik	36	52	45	49	40
2810025M15Rik	53	83	104	63	55
2810029C07Rik	2	2	0	7	4
2810039B14Rik	16	9	12	9	10
2810047C21Rik1	2	8	8	4	5
2810403A07Rik	140	104	142	161	174
2810403D21Rik	2	0	0	0	0
2810407A14Rik	0	0	0	6	0
2810408A11Rik	0	1	0	0	0
2810408B13Rik	3	7	1	3	1
2810417H13Rik	2	1	0	0	6

For Review Only

2810428I15Rik	53	53	61	37	40
2810429I04Rik	0	1	0	0	0
2810442N19Rik	3	3	0	0	0
2810454H06Rik	10	1	10	9	0
2810468N07Rik	7	1	7	0	12
2810474O19Rik	296	280	286	247	340
2900005J15Rik	26	7	20	16	29
2900026A02Rik	44	12	41	61	59
2900040C04Rik	25	0	0	2	11
2900060B14Rik	6	13	16	17	4
2900060L22Rik	0	4	5	1	0
2900076A07Rik	5	4	0	0	0
2900089D17Rik	0	0	0	0	0
2900097C17Rik	320	283	196	328	307
3000002C10Rik	4	11	1	13	15
3010026O09Rik	18	18	23	10	20
3110001I22Rik	2	18	5	10	22
3110002H16Rik	0	0	5	10	113
3110009E18Rik	4	4	0	11	10
3110035E14Rik	19	36	21	43	26
3110040N11Rik	20	39	44	27	24
3110043O21Rik	134	118	109	153	154
3110052M02Rik	55	43	60	49	64
3110053B16Rik	0	0	0	2	2
3110056K07Rik	38	1	12	24	0
3110062M04Rik	1	0	0	91	0
3110070M22Rik	1	6	0	0	6
3110082I17Rik	51	40	36	40	55
3110083C13Rik	3	1	0	0	8
3200001D21Rik	1	1	0	0	0
3300002A11Rik	3	2	0	8	1
3300002I08Rik	10	6	0	9	16
3425401B19Rik	0	2	0	0	0
3632454L22Rik	0	2	0	3	0
3830403N18Rik	2	13	0	6	0
3830406C13Rik	52	36	27	41	32
3830408C21Rik	4	0	2	6	3
4430402I18Rik	8	0	0	7	10
4632404H12Rik	15	13	23	27	37
4632415L05Rik	25	34	29	32	21
4632428C04Rik	1	0	0	0	0
4732440D04Rik	24	31	31	22	34
4732471J01Rik	13	36	20	39	56
4732491K20Rik	8	1	10	9	9
4831407H17Rik	3	0	1	1	0
4831440D22Rik	2	5	11	10	0
4831440E17Rik	3	13	4	9	7
4833411C07Rik	0	8	9	18	15
4833417C18Rik	5	5	15	11	11
4833418N02Rik	4	1	0	10	15

For Review Only

4833419F23Rik	0	0	0	0	4
4833420G17Rik	220	195	139	273	212
4833422C13Rik	0	2	1	3	0
4833438C02Rik	20	16	30	24	23
4833439L19Rik	147	107	364	191	0
4833447I15Rik	1	1	7	0	5
4921507G05Rik	3	1	0	7	4
4921507P07Rik	1	0	5	0	0
4921511C10Rik	25	26	21	36	28
4921517D16Rik	9	0	0	6	10
4921524J17Rik	1	11	14	0	18
4921531C22Rik	13	23	24	24	31
4921536K21Rik	0	0	2	2	0
4930402H24Rik	38	32	19	51	52
4930403D09Rik	4	5	3	3	3
4930404H24Rik	4	14	0	13	14
4930404I05Rik	1	2	1	0	0
4930405A10Rik	1	2	0	5	4
4930405O22Rik	4	8	14	11	9
4930412C18Rik	2	0	3	0	0
4930412L05Rik	0	0	0	0	5
4930413G21Rik	0	1	2	4	2
4930414N06Rik	0	0	32	19	8
4930426L09Rik	2	2	0	4	1
4930427A07Rik	0	2	7	3	0
4930429B21Rik	2	0	5	0	0
4930430F08Rik	3	7	12	14	11
4930432K21Rik	5	8	5	5	20
4930438A08Rik	6	0	0	0	1
4930439A04Rik	1	6	0	8	12
4930444A19Rik	29	29	20	38	30
4930445E18Rik	0	0	4	0	0
4930447K03Rik	4	8	0	9	6
4930447M23Rik	5	6	11	17	15
4930451E10Rik	0	1	1	0	0
4930451G09Rik	9	0	10	0	0
4930453L07Rik	0	0	0	0	3
4930453N24Rik	64	32	59	46	42
4930467E23Rik	4	5	13	14	8
4930469K13Rik	20	4	19	34	16
4930473A02Rik	0	4	1	3	2
4930473H19Rik	3	4	4	5	8
4930478L05Rik	3	1	0	0	0
4930480K23Rik	12	20	5	5	5
4930481A15Rik	41	34	44	25	55
4930483K19Rik	1	0	0	2	3
4930486L24Rik	0	0	3	0	0
4930487H11Rik	1	7	0	3	10
4930500A05Rik	0	0	0	6	0
4930502C15Rik	5	20	6	24	17

For Review Only

4930503E24Rik	7	0	7	4	3
4930503L19Rik	21	27	38	29	38
4930505N22Rik	1	0	1	3	0
4930506C21Rik	9	22	13	18	12
4930511A08Rik	0	0	4	2	1
4930512B01Rik	0	3	0	0	0
4930512H18Rik	0	0	0	4	0
4930513N10Rik	0	0	0	1	1
4930515G01Rik	3	0	3	1	3
4930519F09Rik	3	1	0	0	0
4930521O11Rik	7	6	8	6	10
4930522L14Rik	74	61	89	80	68
4930522P08Rik	0	0	0	0	8
4930523C07Rik	4	11	0	14	5
4930525G20Rik	1	0	0	13	0
4930526I15Rik	7	15	8	9	20
4930528A17Rik	0	0	0	1	0
4930529C04Rik	0	0	1	1	0
4930538K18Rik	0	4	4	1	0
4930539E08Rik	1	1	0	8	0
4930549G23Rik	2	0	0	6	7
4930550C14Rik	2	4	0	6	5
4930552P12Rik	0	0	0	0	2
4930555F03Rik	1	0	0	0	0
4930555G01Rik	2	1	3	1	3
4930562C15Rik	0	0	0	0	0
4930563D23Rik	0	0	0	0	0
4930563E18Rik	0	0	0	0	0
4930563E22Rik	8	13	0	5	0
4930563I02Rik	7	11	25	38	40
4930564C03Rik	1	4	1	6	2
4930565N06Rik	1	1	6	10	10
4930568A12Rik	5	0	7	0	7
4930572G02Rik	1	1	3	0	0
4930577N17Rik	8	7	6	0	8
4930579G18Rik	7	2	8	11	11
4930579G24Rik	1	1	0	10	6
4930579K19Rik	1	9	5	3	0
4930581F22Rik	10	9	17	6	9
4930590J08Rik	6	8	0	7	7
4930592I03Rik	0	0	0	6	0
4930594C11Rik	53	88	82	129	106
4930599N23Rik	0	0	2	0	0
4931402G19Rik	38	29	55	35	50
4931403G20Rik	1	1	0	0	8
4931406C07Rik	47	37	59	63	53
4931406H21Rik	99	70	82	121	110
4931406P16Rik	93	139	63	121	142
4931413K12Rik	0	3	0	2	4
4931414P19Rik	34	25	21	47	24

For Review Only

4931415C17Rik	0	0	0	4	0
4931428F04Rik	13	0	0	12	0
4931440F15Rik	9	6	17	12	11
4931440P22Rik	0	1	0	0	3
4932416H05Rik	2	1	4	9	8
4932438A13Rik	204	229	154	270	203
4932438H23Rik	0	1	0	0	0
4932443L11Rik	2	3	0	5	1
4933400F21Rik	3	6	5	2	7
4933404O12Rik	8	1	14	1	4
4933406C10Rik	0	0	1	0	0
4933406F09Rik	1	1	0	0	1
4933406I18Rik	27	12	9	24	20
4933406J10Rik	1	0	0	3	1
4933406M09Rik	4	3	0	0	0
4933407K13Rik	18	5	25	24	17
4933408J17Rik	0	0	0	0	0
4933411K16Rik	4	0	0	4	0
4933412E12Rik	7	7	19	13	12
4933413J09Rik	3	3	0	6	9
4933417D19Rik	0	1	0	8	0
4933417G07Rik	4	4	4	3	3
4933421O10Rik	37	11	7	18	39
4933423P22Rik	24	21	0	33	60
4933424M12Rik	3	1	0	0	0
4933427D14Rik	31	24	32	39	37
4933427G23Rik	1	0	1	2	1
4933431E20Rik	1	0	7	0	0
4933431G14Rik	43	12	34	20	33
4933432I03Rik	0	0	3	2	1
4933433G08Rik	1	0	0	0	4
4933433G15Rik	0	4	0	6	5
4933434E20Rik	111	1	313	124	17
4933438K21Rik	1	2	1	3	0
4933439C10Rik	16	9	9	8	15
4933439K11Rik	2	0	0	0	0
4933440N22Rik	0	0	0	1	7
5031414D18Rik	77	166	88	80	106
5031425E22Rik	18	12	20	21	12
5031425F14Rik	0	2	3	10	6
5031426D15Rik	2	1	0	0	0
5031434O11Rik	0	0	5	0	0
5031439G07Rik	56	87	94	102	92
5033403F01Rik	3	0	0	3	0
5033417F24Rik	4	0	1	0	0
5033421B08Rik	0	7	0	1	10
5430402O13Rik	1	0	2	2	5
5430405H02Rik	8	26	20	23	21
5430427O19Rik	85	124	161	106	81
5430431A17Rik	0	0	0	0	0

For Review Only

5530601H04Rik	11	0	3	13	11
5730405O15Rik	1	2	0	8	0
5730408K05Rik	3	0	0	1	0
5730409E04Rik	27	28	0	17	8
5730409K12Rik	13	12	2	11	10
5730422E09Rik	4	10	5	7	3
5730455P16Rik	145	20	59	102	59
5730480H06Rik	1	4	0	7	0
5830403F22Rik	0	0	1	2	0
5830416I19Rik	0	0	0	0	0
5830417I10Rik	0	0	110	103	94
5830432E09Rik	5	28	9	15	14
5830444B04Rik	25	29	29	20	15
5830454E08Rik	2	6	2	1	3
5930403L14Rik	3	2	0	4	1
5930403N24Rik	4	0	0	0	3
5930430L01Rik	1	1	0	1	0
6030443J06Rik	0	0	7	0	0
6030458C11Rik	77	73	90	90	106
6030468B19Rik	3	0	13	0	0
6230400D17Rik	5	2	0	0	1
6330403K07Rik	3	0	4	0	0
6330407A03Rik	32	84	59	106	82
6330408A02Rik	15	5	8	13	20
6330409D20Rik	0	0	0	0	6
6330415G19Rik	8	6	6	5	15
6330416G13Rik	186	158	194	175	220
6330418K02Rik	21	15	0	0	0
6330549D23Rik	10	14	13	19	22
6430548M08Rik	51	26	44	34	17
6430550D23Rik	1	2	0	6	14
6430562O15Rik	1	0	0	4	0
6430571L13Rik	0	0	0	0	0
6430590A07Rik	8	3	0	5	0
6530402F18Rik	16	25	9	9	12
6720489N17Rik	13	7	9	10	5
6820431F20Rik	83	82	87	112	111
8030423J24Rik	1	17	29	17	14
8030442B05Rik	8	11	13	14	22
8030453O22Rik	13	3	9	32	22
8030462N17Rik	54	65	44	50	71
8430408G22Rik	4	0	0	7	7
8430429K09Rik	4	7	7	13	10
8430431K14Rik	0	1	0	1	0
9030025P20Rik	22	33	26	24	39
9030617O03Rik	106	61	124	134	123
9030624J02Rik	176	151	235	179	145
9130011E15Rik	74	70	76	102	88
9130019O22Rik	40	16	64	36	0
9130019P16Rik	9	0	7	12	7

For Review Only

9130023H24Rik	17	38	113	53	104
9130024F11Rik	1	0	0	1	1
9130208D14Rik	8	20	10	31	20
9130221H12Rik	1	14	12	6	0
9130230L23Rik	0	0	2	0	0
9130401M01Rik	32	33	32	22	46
9230114K14Rik	4	4	4	4	0
9230116N13Rik	0	3	0	3	1
9330020H09Rik	6	5	3	2	11
9330102E08Rik	8	1	0	6	0
9330104G04Rik	0	2	1	5	0
9330133O14Rik	123	76	115	112	146
9330136K24Rik	0	0	0	0	6
9330151L19Rik	2	25	10	20	0
9330159M07Rik	8	6	7	12	12
9330198I05Rik	0	3	0	2	9
9430015G10Rik	0	0	15	9	25
9430016H08Rik	14	31	23	31	21
9430018G01Rik	2	0	0	0	0
9430020K01Rik	0	0	0	0	0
9430037G07Rik	4	1	0	4	5
9430038I01Rik	11	8	22	0	15
9430060I03Rik	13	16	32	12	18
9430083A17Rik	5	7	6	1	0
9430085M18Rik	0	1	0	0	0
9430091E24Rik	9	8	13	16	27
9530027J09Rik	4	6	0	7	11
9530034E10Rik	17	23	7	30	24
9530036M11Rik	74	67	119	117	94
9530052C20Rik	0	1	0	0	0
9530053A07Rik	1	0	0	0	3
9530057J20Rik	3	0	1	1	0
9530062K07Rik	0	0	0	0	0
9530068E07Rik	388	370	527	459	457
9530077C05Rik	2	0	4	3	1
9530082P21Rik	6	15	11	16	3
9530091C08Rik	1	2	0	0	0
9630013K17Rik	3	1	6	0	0
9630015K15Rik	1	4	0	3	0
9630028I04Rik	89	14	0	40	39
9830147E19Rik	19	13	28	39	21
9930012K11Rik	16	6	17	10	9
9930014A18Rik	3	0	11	9	7
9930021J03Rik	45	38	33	65	47
9930104L06Rik	42	45	65	39	55
9930111J21Rik1	121	130	9	75	162
9930111J21Rik2	0	0	0	0	0
A030001D20Rik	0	0	0	0	3
A130010J15Rik	49	73	59	68	60
A130051J06Rik	11	3	11	11	21

For Review Only

A130077B15Rik	6	4	0	9	4
A230046K03Rik	97	10	0	214	259
A230050P20Rik	1	0	0	0	146
A230056J06Rik	1	7	5	0	1
A230056P14Rik	13	2	20	0	10
A230072C01Rik	5	6	11	3	6
A230107N01Rik	0	0	0	0	0
A330023F24Rik	12	7	6	15	0
A330032B11Rik	0	0	0	3	6
A330035P11Rik	2	0	1	7	7
A330040F15Rik	2	4	0	0	0
A330069E16Rik	0	0	3	0	2
A430005L14Rik	1	1	0	0	43
A430010J10Rik	2	0	0	0	0
A430033K04Rik	33	59	27	42	50
A430035B10Rik	5	1	5	2	0
A430046D13Rik	28	3	16	7	25
A430057M04Rik	46	33	55	44	49
A430078I02Rik	0	0	0	1	4
A430090L17Rik	3	7	13	15	8
A430105I19Rik	0	4	0	3	7
A430105J06Rik	1	0	0	1	5
A430110L20Rik	20	10	1	12	13
A530010L16Rik	6	5	9	8	0
A530032D15Rik	5	3	7	8	4
A530040E14Rik	4	3	4	6	5
A530064D06Rik	1	1	0	0	0
A530072M11Rik	6	0	10	7	0
A530088E08Rik	43	65	82	95	79
A630001G21Rik	0	8	0	0	99
A630001O12Rik	0	4	0	8	0
A630033H20Rik	54	65	59	64	77
A630034I12Rik	1	1	0	1	1
A630066F11Rik	7	6	10	9	18
A630072M18Rik	10	6	10	13	7
A630089N07Rik	3	1	0	0	13
A730017L22Rik	4	5	6	4	7
A730020M07Rik	2	0	9	0	0
A730049H05Rik	0	1	0	1	5
A730063M14Rik	0	0	5	0	0
A730090N16Rik	0	0	0	1	0
A830009L08Rik	4	0	2	0	4
A830010M20Rik	5	13	8	18	11
A830021F12Rik	2	0	0	0	0
A830035O19Rik	0	3	1	2	3
A830052D11Rik	12	4	7	17	29
A830080D01Rik	10	48	29	44	19
A830082N09Rik	3	3	5	0	0
A930005H10Rik	3	5	6	10	10
A930006K02Rik	0	3	7	0	0

For Review Only

A930007A09Rik	4	1	12	16	13
A930013F10Rik	24	28	33	21	32
A930015D03Rik	6	0	4	0	3
A930024E05Rik	7	10	12	22	17
A930033H14Rik	3	5	7	12	3
A930041C12Rik	0	0	0	0	0
AA388235	1	0	0	4	2
AA413626	2	0	4	4	5
AA414768	9	2	6	1	6
AA415398	4	18	1	12	12
AA543186	3	11	3	5	7
AA986860	0	11	1	0	8
Aaas	0	25	54	16	19
Aacs	64	60	57	31	11
Aaed1	1	1	0	0	0
Aagab	30	2	0	0	0
Aak1	140	93	116	142	132
Aamdc	43	15	40	18	17
Aamp	350	269	476	285	353
Aanat	0	0	0	2	0
Aar2	82	88	126	84	95
Aars	296	193	287	202	271
Aars2	3	11	42	47	51
Aarsd1	33	6	80	48	0
Aasdh	36	40	25	46	35
Aasdhppt	41	68	47	64	34
Aass	5	0	0	0	0
Aatf	43	75	121	111	113
Aatk	70	20	39	58	71
AB124611	0	5	0	2	1
Abat	9	1	0	0	13
Abca1	593	642	622	1086	1104
Abca13	4	11	0	13	25
Abca17	4	0	0	0	0
Abca2	132	117	104	130	119
Abca3	220	103	171	215	203
Abca4	0	0	0	0	0
Abca7	15	27	7	0	10
Abca8a	0	1	0	0	0
Abca9	1181	1345	1179	1665	1675
Abcb10	38	67	35	60	66
Abcb1a	10	0	0	0	4
Abcb1b	64	64	33	64	57
Abcb4	52	44	9	35	35
Abcb6	58	25	67	34	68
Abcb7	42	24	20	53	36
Abcb8	29	57	55	37	40
Abcb9	19	13	25	13	24
Abcc1	92	106	125	142	127
Abcc10	70	29	36	70	87

For Review Only

Abcc3	866	449	730	795	1075
Abcc4	53	40	45	79	79
Abcc5	427	741	777	899	917
Abcd1	159	161	0	147	110
Abcd2	313	277	280	458	433
Abcd3	198	133	196	183	249
Abcd4	44	21	61	22	52
Abce1	99	96	147	144	203
Abcf1	97	101	149	91	119
Abcf2	107	117	128	100	97
Abcf3	0	91	53	30	0
Abcg1	632	551	728	607	664
Abcg2	58	80	97	96	68
Abcg3	31	26	25	46	34
Abcg4	0	2	0	4	0
Abhd10	34	41	47	32	47
Abhd11	0	0	0	0	0
Abhd12	11	24	7	25	16
Abhd13	33	100	65	31	36
Abhd14a	24	15	34	21	28
Abhd14b	97	56	94	50	82
Abhd15	0	0	243	0	305
Abhd16a	19	38	162	0	185
Abhd17a	4	6	0	10	0
Abhd17b	16	23	16	12	7
Abhd17c	7	74	35	23	35
Abhd18	7	15	14	18	19
Abhd2	121	58	84	136	100
Abhd3	33	21	33	29	25
Abhd4	287	205	278	193	257
Abhd5	46	65	40	45	66
Abhd6	65	143	196	24	170
Abhd8	43	23	31	22	34
Abi1	285	273	336	340	423
Abi2	23	20	36	87	58
Abi3	220	1644	737	33	0
Abi3bp	0	0	5	0	0
Abl1	535	511	197	282	459
Abl2	71	100	72	100	92
Ablim1	67	37	36	66	93
Ablim2	4	0	0	5	0
Abr	304	143	240	290	280
Abra	4	0	0	1	2
Abracl	24	44	41	18	31
Abt1	87	26	48	37	36
Abtb1	0	0	0	0	0
Acaa1a	162	50	12	0	226
Acaa1b	60	0	54	34	6
Acaa2	59	31	47	41	35
Acaca	22	8	9	22	22

For Review Only

Acacb	0	0	0	0	4
Acad10	26	11	19	21	35
Acad11	40	22	16	37	42
Acad12	24	17	21	42	32
Acad8	102	59	132	82	102
Acad9	64	48	53	63	55
Acadl	81	37	78	62	67
Acadm	165	145	170	143	184
Acads	58	23	25	28	56
Acadsb	67	58	72	63	86
Acadvl	21	0	0	0	0
Acap1	0	0	0	3	0
Acap2	166	244	203	291	291
Acap3	43	8	15	24	12
Acat1	81	78	127	110	87
Acat2	17	27	21	42	33
Acat3	2	11	5	17	0
Acbd3	77	42	115	70	81
Acbd4	7	6	19	12	9
Acbd5	31	59	56	92	53
Acbd6	20	25	19	18	23
Accs	39	52	70	73	67
Acd	21	0	0	0	0
Ace	17	0	0	16	3
Acer2	3	4	0	0	0
Acer3	260	256	297	286	234
Ache	31	36	17	70	51
Acin1	166	131	111	162	184
Ackr3	0	4	7	1	5
Ackr4	7	1	10	5	1
Acly	466	219	406	446	499
Acnat1	2	0	0	0	0
Aco1	69	42	59	69	130
Aco2	264	130	284	233	293
Acot1	7	4	3	0	4
Acot10	14	11	17	11	13
Acot11	15	1	14	13	0
Acot13	76	42	96	48	37
Acot2	30	21	32	35	18
Acot6	0	4	0	0	5
Acot7	11	7	0	5	8
Acot8	4	0	0	0	0
Acot9	27	0	0	0	53
Acox1	146	106	114	128	166
Acox3	381	42	86	421	462
Acoxl	0	0	0	1	4
Acp1	29	50	53	38	21
Acp2	718	677	971	790	874
Acp5	3	0	0	0	6
Acsf2	31	0	0	20	28

For Review Only

Acsf3	17	15	16	34	49
Acsl1	144	80	111	171	167
Acsl3	0	0	0	0	0
Acsl4	94	93	119	120	81
Acsl5	171	187	201	166	271
Acsl6	7	3	0	0	0
Acss1	398	316	355	395	613
Acss2	23	2	0	0	16
Acta2	10	7	10	15	8
Actb	0	6646	0	2179	2007
Actl10	0	0	0	0	1
Actl6a	104	98	100	86	88
Actn1	16	0	9	0	8
Actn3	0	0	0	0	3
Actn4	201	138	190	92	246
Actr10	143	143	108	98	107
Actr1a	310	279	282	327	347
Actr1b	0	0	0	39	0
Actr2	122	139	248	381	242
Actr3	403	294	473	358	396
Actr5	7	21	6	0	3
Actr6	25	6	35	22	17
Actr8	6	134	171	156	0
Acvr1	28	58	91	65	85
Acvr1b	5	15	6	18	23
Acvr1c	0	0	0	0	4
Acvr2a	53	44	71	64	70
Acvr2b	28	71	21	101	42
Acvrl1	62	28	71	62	26
Acy1	78	44	77	53	35
Acyp1	11	7	13	5	9
Ada	12	9	0	0	19
Adal	30	22	22	25	16
Adam10	822	859	678	998	912
Adam15	175	133	0	0	0
Adam17	527	281	343	620	624
Adam19	0	0	0	1	3
Adam1a	10	4	16	10	0
Adam1b	3	0	3	1	0
Adam22	16	34	1	19	33
Adam3	12	0	8	8	20
Adam30	7	1	0	0	3
Adam33	5	0	2	0	5
Adam4	20	12	7	31	40
Adam8	15	4	9	3	4
Adam9	116	68	80	101	87
Adamts1	49	52	0	0	0
Adamts10	62	41	44	71	112
Adamts15	0	0	0	0	0
Adamts16	97	115	89	148	128

For Review Only

Adamts2	0	1	0	0	0
Adamts6	6	5	15	9	9
Adamts7	2	0	0	0	0
Adamtsl2	19	13	17	17	15
Adamtsl4	1	0	0	0	20
Adap1	15	13	26	9	6
Adap2	6	29	0	62	0
Adar	107	88	138	152	173
Adarb1	17	18	27	45	47
Adat1	9	9	27	19	8
Adat2	0	9	0	2	5
Adat3	14	18	10	9	18
Adck1	79	60	56	79	79
Adck2	86	77	88	114	96
Adck3	14	46	26	30	45
Adck4	38	38	86	49	44
Adck5	0	65	0	0	0
Adcy1	0	0	0	0	1
Adcy10	0	0	0	0	0
Adcy3	22	14	27	0	0
Adcy5	0	0	0	0	0
Adcy6	5	9	25	15	20
Adcy7	0	187	65	349	318
Adcy8	31	18	28	31	34
Adcy9	25	60	62	68	45
Add1	204	263	227	205	271
Add2	0	4	0	1	0
Add3	147	57	86	122	49
Adgb	0	0	0	0	0
Adgra2	0	0	0	4	0
Adgra3	26	19	9	18	10
Adgre1	393	719	387	424	355
Adgre4	0	0	0	3	2
Adgre5	0	0	0	0	0
Adgrf4	0	1	0	6	0
Adgrf5	0	0	0	0	0
Adgrg1	2208	1570	2023	2536	2619
Adgrg3	0	1	0	0	9
Adgrl1	12	22	26	22	19
Adgrl2	39	35	32	43	58
Adgrl4	0	0	0	0	0
Adh5	184	142	210	112	169
Adhfe1	18	31	19	39	20
Adi1	132	67	149	97	106
Adipor1	362	423	336	468	519
Adipor2	81	70	107	86	86
Adk	0	6	6	6	18
Adnp2	24	53	58	67	52
Ado	198	184	145	101	192
Adora1	9	9	0	19	13

For Review Only

Adora2b	3	0	0	0	5
Adora3	0	354	19	458	33
Adpgk	111	78	119	56	55
Adprh	341	273	371	270	330
Adprhl2	12	28	0	0	78
Adprm	77	67	1	6	1
Adra1a	5	0	1	4	2
Adra2a	0	3	2	0	0
Adra2b	0	0	0	0	0
Adra2c	0	0	1	3	0
Adrb1	41	12	11	18	25
Adrb2	825	852	1069	939	870
Adrbk1	0	1	0	0	0
Adrbk2	44	118	124	88	95
Adrm1	0	53	10	92	7
Adsl	122	88	116	74	108
Adss	92	71	58	83	81
Adssl1	19	22	17	29	28
Adtrp	8	4	11	0	0
Aebp1	3	0	0	0	0
Aebp2	33	39	38	26	53
Aen	43	30	45	35	27
Aes	9	20	15	14	14
AF357399	18	18	12	17	0
AF529169	0	0	4	12	9
Afap1	37	33	25	45	46
Afap1l1	85	86	148	109	106
Afap1l2	0	0	0	0	0
Aff1	227	157	142	274	179
Aff3	0	0	0	0	2
Aff4	108	135	105	146	152
Afg3l1	83	64	59	107	43
Afg3l2	118	58	94	101	132
Afm	0	0	0	0	0
Afmid	31	18	36	15	24
Aftph	246	318	272	279	147
Aga	142	103	174	123	151
Agap1	21	16	23	19	22
Agap2	11	3	14	23	25
Agap3	2	6	0	43	59
Agbl2	0	0	0	0	0
Agbl3	14	6	5	9	12
Agbl5	27	20	27	28	24
Ager	2	1	0	0	0
Agfg1	35	26	43	53	52
Agfg2	28	23	26	19	24
Aggf1	43	37	44	52	63
Agk	46	31	39	46	50
Agl	42	40	31	60	61
Agmo	254	243	304	317	257

Ago1	142	183	158	179	173
Ago2	78	87	114	134	122
Ago3	79	113	97	184	242
Ago4	31	49	20	35	50
Agpat1	171	0	1	4	1
Agpat2	17	28	26	26	38
Agpat3	180	114	152	154	161
Agpat4	18	0	5	20	8
Agpat5	40	38	49	26	51
Agpat9	0	0	0	1	0
Agps	99	138	125	155	125
Agrn	32	26	0	42	55
Agtpbp1	47	27	31	50	50
Agtrap	62	59	81	60	58
Ahctf1	57	73	47	73	95
Ahcy	16	5	8	9	7
Ahcyl1	34	48	45	66	63
Ahcyl2	107	49	38	45	57
Ahdc1	31	57	56	60	56
Ahi1	9	5	24	13	29
Ahnak	0	1	28	0	0
Ahr	0	0	0	0	0
Ahrr	11	6	0	7	7
Ahsa1	145	102	184	125	144
Ahsa2	38	9	87	21	3
AI225912	7	8	21	25	36
AI314180	71	60	111	123	117
AI427809	1	0	0	0	3
AI429214	0	1	3	0	3
AI450353	26	13	11	13	20
AI463229	12	2	4	3	0
AI464131	0	0	1	3	0
AI467606	180	148	192	131	220
AI480526	17	31	16	34	29
AI481877	0	5	9	7	0
AI504432	8	1	7	5	10
AI506816	0	3	1	3	1
AI597479	42	40	52	71	78
AI607873	0	8	4	5	11
AI661453	1	8	2	3	0
AI662270	1	9	5	9	7
AI837181	0	14	125	1	14
AI839979	0	0	0	3	4
AI846148	8	5	13	11	6
AI854703	10	1	3	1	10
AI987944	67	113	126	98	121
Aida	20	12	21	20	32
Aif1	0	0	6	198	138
Aifm1	36	30	62	0	47
Aifm2	0	6	0	0	0

For Review Only

Aifm3	15	1	0	0	0
Aig1	27	25	26	35	37
Aim1	2	0	5	1	7
Aim2	24	0	34	54	170
Aimp1	48	66	78	38	43
Aimp2	0	20	35	18	38
Aip	64	96	113	49	93
Ajuba	13	9	20	9	16
AK010878	47	9	26	18	21
Ak1	162	137	0	11	216
Ak2	138	155	180	124	134
Ak3	87	54	57	63	72
Ak5	0	0	0	0	0
Ak6	34	44	35	24	40
Ak7	0	0	0	0	0
Akap1	7	52	31	14	17
Akap10	83	96	77	112	99
Akap11	31	71	36	81	42
Akap12	13	2	15	5	8
Akap13	520	432	504	649	623
Akap17b	5	2	0	8	3
Akap3	2	7	4	4	3
Akap5	12	21	39	25	12
Akap7	31	27	13	33	37
Akap8	9	0	1	0	0
Akap9	170	206	206	209	233
Akip1	4	16	77	51	71
Akirin1	205	167	142	148	114
Akirin2	64	19	37	27	14
Akna	422	110	65	228	793
Aknaos	63	34	20	51	54
Akr1a1	810	621	848	580	779
Akr1b10	135	142	166	110	126
Akr1b3	57	74	79	56	53
Akr1e1	37	22	25	43	31
Akr7a5	57	41	63	49	43
Akt1	6	0	1	3	8
Akt1s1	65	59	13	72	62
Akt2	84	44	74	51	62
Akt3	25	50	37	49	56
Aktip	52	54	42	64	81
Alad	34	13	33	5	5
Alas1	157	101	228	118	164
Alas2	0	0	0	8	0
Alcam	0	0	0	0	0
Aldh16a1	119	83	77	97	115
Aldh18a1	86	14	90	59	54
Aldh1a1	0	0	0	0	0
Aldh1a2	0	0	0	0	0
Aldh1a3	0	1	0	5	0

For Review Only

Aldh1b1	2	0	5	0	11
Aldh1l1	15	0	0	0	0
Aldh1l2	2	2	20	0	0
Aldh2	91	47	59	53	89
Aldh3a2	192	133	194	160	193
Aldh3b1	13	8	0	0	0
Aldh4a1	82	53	69	47	84
Aldh5a1	1	5	0	7	0
Aldh6a1	72	32	64	87	109
Aldh7a1	48	25	37	32	50
Aldh9a1	144	112	161	151	118
Aldoa	950	483	830	529	675
Aldoart1	19	22	28	17	36
Aldoart2	4	4	1	4	4
Aldoc	17	1	0	0	0
Alg1	175	87	191	62	50
Alg10b	5	16	20	34	24
Alg11	121	109	80	150	119
Alg12	62	58	76	55	51
Alg13	11	9	25	22	15
Alg14	40	64	67	38	36
Alg2	77	88	103	147	106
Alg3	45	20	106	40	54
Alg5	189	171	255	157	192
Alg6	19	16	9	22	41
Alg8	0	0	7	0	31
Alg9	42	41	44	38	51
Alkbh1	10	64	80	84	78
Alkbh2	5	14	0	0	0
Alkbh3	115	61	84	67	91
Alkbh3os1	4	5	0	5	0
Alkbh4	46	30	31	24	46
Alkbh5	184	111	105	194	157
Alkbh6	11	36	0	0	0
Alkbh8	40	30	29	16	36
Alms1	1	5	0	19	12
Alox15	22	8	0	14	15
Alox5	96	57	144	68	95
Alox5ap	561	667	781	472	551
Aloxe3	4	1	0	0	0
Alpk1	23	8	9	23	16
Alpk3	4	0	0	2	0
Als2	68	29	19	56	65
Als2cl	3	11	9	20	16
Als2cr11b	0	0	4	0	0
Als2cr12	2	0	0	3	3
Alyref	9	12	0	9	4
Alyref2	31	20	30	24	42
Amacr	22	26	29	30	41
Ambra1	108	126	97	143	138

For Review Only

Amd1	93	66	108	75	89
Amd2	54	54	76	56	65
Amer1	40	105	59	29	29
Amer2	16	3	7	22	15
Amfr	233	230	247	165	180
Amh	0	0	0	0	5
Amigo1	10	10	12	7	15
Amigo3	27	17	12	37	20
Ammecr1	0	4	2	4	5
Ammecr1l	119	93	123	123	122
Amn1	29	19	33	27	16
Amotl1	66	36	91	61	84
Amotl2	4	0	0	0	0
Ampd2	49	3	87	22	40
Ampd3	71	46	86	70	81
Amt	15	6	12	14	15
Amz1	3	1	0	0	0
Amz2	58	7	0	57	68
Anapc1	116	127	98	136	158
Anapc10	31	28	44	58	52
Anapc11	52	47	71	55	89
Anapc13	35	44	65	29	40
Anapc15	36	3	24	16	0
Anapc16	47	42	56	53	61
Anapc2	0	0	0	0	0
Anapc4	55	63	85	74	112
Anapc5	183	178	241	162	164
Anapc7	157	160	211	110	76
Ang	119	342	227	84	157
Angel1	49	43	49	57	93
Angel2	220	96	224	174	186
Angpt2	6	0	6	14	10
Angptl1	0	7	0	2	0
Angptl2	13	19	5	14	19
Angptl3	6	0	1	3	3
Angptl6	7	10	15	6	6
Angptl7	0	0	0	0	0
Ank	104	60	94	72	81
Ank1	0	0	2	3	0
Ank2	135	191	118	228	190
Ankdd1a	4	0	0	5	6
Ankdd1b	0	0	0	0	8
Ankfn1	0	0	3	0	0
Ankfy1	266	322	474	533	616
Ankhd1	127	122	110	146	104
Ankib1	35	16	0	0	0
Ankle1	0	0	0	4	0
Ankle2	153	172	159	199	192
Ankmy2	24	40	68	48	59
Ankra2	43	35	55	23	45

For Review Only

Ankrd10	200	236	299	183	242
Ankrd11	196	284	250	213	207
Ankrd12	92	79	107	94	90
Ankrd13a	91	131	91	68	0
Ankrd13b	2	4	20	0	17
Ankrd13c	11	21	14	25	35
Ankrd13d	6	1	40	49	23
Ankrd16	65	64	113	78	71
Ankrd17	174	157	193	177	221
Ankrd22	0	0	0	3	0
Ankrd23	13	31	47	29	34
Ankrd24	39	17	17	42	36
Ankrd26	32	33	31	41	29
Ankrd27	71	77	121	82	97
Ankrd28	5	18	15	13	30
Ankrd33b	4	6	0	0	0
Ankrd34a	2	2	0	6	5
Ankrd34b	4	0	0	3	5
Ankrd37	15	14	5	1	0
Ankrd39	66	40	52	68	71
Ankrd40	91	65	112	83	71
Ankrd42	10	0	0	0	9
Ankrd44	27	162	51	121	72
Ankrd45	4	0	6	0	0
Ankrd46	135	106	113	126	145
Ankrd49	46	78	86	54	57
Ankrd50	18	35	28	47	45
Ankrd52	133	54	83	189	209
Ankrd54	29	36	16	22	32
Ankrd55	0	3	0	3	0
Ankrd6	0	2	0	4	0
Ankrd61	0	0	0	11	4
Ankrd63	0	0	0	2	0
Ankrd7	2	3	1	0	10
Ankrd9	7	1	0	0	8
Anks1	77	79	107	104	162
Anks3	0	0	0	0	0
Anks6	25	39	34	26	29
Ankzf1	0	13	31	5	3
Anln	0	0	0	0	3
Ano10	133	79	146	71	118
Ano6	245	165	158	251	273
Ano7	0	27	0	24	0
Ano8	15	0	1	1	4
Anp32a	19	19	19	27	27
Anp32b	11	25	19	25	26
Anp32e	109	76	89	78	111
Antxr1	9	0	0	0	7
Antxr2	4	0	0	8	8
Anxa1	100	0	0	0	0

For Review Only

Anxa11	9	6	5	12	8
Anxa2	14	7	25	6	18
Anxa3	422	326	662	229	68
Anxa4	70	46	87	59	58
Anxa5	0	0	2	1	1
Anxa6	131	62	157	112	90
Anxa7	153	105	146	140	126
Anxa9	0	3	5	0	7
Aoah	4	0	0	0	0
Aoc2	3	1	3	1	4
Ap1ar	21	13	20	15	25
Ap1b1	474	214	493	306	394
Ap1g1	167	169	152	209	231
Ap1g2	142	21	75	0	102
Ap1m1	0	0	8	1	13
Ap1s1	58	50	77	55	51
Ap1s2	95	72	96	113	87
Ap2a1	108	41	32	79	71
Ap2a2	278	170	137	213	206
Ap2b1	117	98	131	177	191
Ap2m1	210	199	17	104	0
Ap2s1	69	83	131	71	95
Ap3b1	225	334	283	379	290
Ap3d1	0	0	2	3	5
Ap3m1	87	92	95	134	125
Ap3m2	61	55	84	81	48
Ap3s1	19	13	17	19	15
Ap3s2	64	72	69	93	70
Ap4b1	118	108	69	85	121
Ap4e1	24	17	23	36	43
Ap4m1	1	1	136	0	0
Ap4s1	30	28	49	27	42
Ap5b1	3	1	0	515	0
Ap5m1	76	98	98	106	149
Ap5s1	53	40	62	27	55
Ap5z1	79	0	143	128	11
Apaf1	31	26	17	59	41
Apba1	39	49	45	59	82
Apba3	1	92	0	0	0
Apbb1	54	6	45	18	32
Apbb1ip	146	285	228	393	357
Apbb2	102	80	97	149	159
Apbb3	26	40	56	72	46
Apc	146	161	148	178	175
Apc2	8	1	0	6	11
Apcdd1	0	0	0	0	0
Apeh	9	9	75	97	0
Apex1	331	67	71	1	10
Apex2	43	17	45	28	41
Aph1a	42	0	55	0	0

For Review Only

Aph1b	13	7	6	4	24
Aph1c	10	13	5	16	20
Api5	298	184	246	275	313
Apip	0	36	41	28	28
Apitd1	5	0	4	5	0
Aplf	8	1	25	12	10
Aplnr	2	0	0	0	0
Aplp1	13	0	6	0	0
Aplp2	154	86	161	155	135
Apmap	51	69	83	72	54
Apoa1bp	156	0	168	0	52
Apob	3	2	0	3	0
Apobec1	187	144	142	87	1
Apobec2	12	11	9	13	6
Apobec3	319	331	394	308	400
Apobr	90	38	61	47	42
Apod	0	0	0	0	0
Apoe	1608	1562	2696	270	300
Apold1	4	0	0	0	5
Apoo	6	0	16	0	0
Apoo-ps	10	4	14	15	10
Apool	43	29	49	24	0
Apopt1	29	26	53	23	51
App	227	178	157	222	217
Appbp2	28	44	35	72	112
Appl1	52	41	37	78	69
Appl2	36	21	40	46	29
Aprt	11	0	0	0	0
Aptx	34	44	79	53	48
Aqp1	11	2	0	5	0
Aqp11	0	3	1	0	0
Aqp4	1	0	0	4	12
Aqr	84	58	55	73	87
Araf	103	71	2	0	81
Arap1	470	351	397	528	496
Arap2	11	17	19	13	16
Arap3	52	1	0	258	0
Arcn1	260	267	265	252	286
Arel1	155	128	156	122	170
Arf1	61	763	9	360	0
Arf2	119	130	164	124	107
Arf4	361	275	439	396	442
Arf5	21	4	0	18	0
Arf6	53	54	0	0	0
Arfgap1	110	121	73	135	106
Arfgap2	47	49	26	68	29
Arfgap3	29	43	50	42	59
Arfgef1	44	60	51	79	77
Arfgef2	42	41	69	75	46
Arfip1	54	64	55	63	58

For Review Only

Arfip2	51	55	51	28	49
Arg1	1	0	0	1	0
Arg2	0	1	5	1	0
Arglu1	357	358	469	289	402
Arhgap1	68	85	193	109	150
Arhgap11a	49	37	44	56	47
Arhgap12	247	158	258	372	302
Arhgap15	9	20	14	21	18
Arhgap17	215	265	186	267	284
Arhgap18	35	31	46	53	33
Arhgap19	52	19	21	39	33
Arhgap21	26	41	20	46	47
Arhgap22	213	175	165	80	88
Arhgap23	0	2	0	9	0
Arhgap24	45	32	50	59	73
Arhgap25	445	354	413	349	402
Arhgap26	24	4	13	23	30
Arhgap27	52	1	0	54	21
Arhgap27os3	0	0	0	0	0
Arhgap29	16	18	9	19	8
Arhgap30	111	0	2	0	3
Arhgap31	127	175	121	203	183
Arhgap32	33	6	21	34	33
Arhgap33	3	5	0	0	0
Arhgap35	42	57	48	74	63
Arhgap39	32	3	0	93	217
Arhgap4	81	1	0	117	20
Arhgap44	5	2	0	0	7
Arhgap5	362	708	420	843	656
Arhgap6	5	0	0	0	0
Arhgap9	59	0	0	42	107
Arhgdia	36	260	186	595	225
Arhgdib	503	583	697	425	476
Arhgef1	84	145	136	42	12
Arhgef10	4	0	0	8	0
Arhgef10l	100	80	101	139	134
Arhgef11	14	36	23	68	59
Arhgef12	48	52	60	53	54
Arhgef15	2	1	0	0	0
Arhgef17	6	1	0	7	5
Arhgef18	33	37	51	47	70
Arhgef2	328	236	322	399	132
Arhgef25	22	6	21	19	18
Arhgef26	2	1	5	0	4
Arhgef28	0	0	0	0	2
Arhgef3	47	27	57	55	56
Arhgef39	2	0	0	0	0
Arhgef4	26	18	27	33	50
Arhgef40	174	0	183	0	175
Arhgef5	0	0	1	4	9

For Review Only

Arhgef6	209	237	322	386	316
Arhgef7	161	103	90	146	126
Arhgef9	0	0	0	7	7
Arid1a	460	624	681	720	699
Arid1b	99	108	91	137	118
Arid2	124	214	152	171	160
Arid3a	110	46	68	79	81
Arid3b	14	0	4	29	0
Arid4a	174	126	119	147	152
Arid4b	91	116	84	103	90
Arid5a	91	43	119	93	64
Arid5b	137	116	97	85	121
Arih1	70	91	145	160	113
Arih2	141	106	176	157	224
Arl1	171	204	221	150	166
Arl10	38	54	0	279	194
Arl11	231	0	7	1	1
Arl13b	19	20	29	18	34
Arl14ep	80	58	70	77	109
Arl15	28	25	23	47	54
Arl16	2	18	26	0	1
Arl2	34	29	50	43	42
Arl2bp	19	10	12	8	11
Arl3	18	15	27	8	30
Arl4a	21	13	6	2	13
Arl4c	273	188	261	267	309
Arl4d	15	3	11	14	17
Arl5a	40	74	42	50	95
Arl5b	46	58	50	58	59
Arl5c	28	16	15	26	29
Arl6	15	0	12	4	0
Arl6ip1	953	832	970	559	962
Arl6ip4	0	0	0	0	0
Arl6ip5	101	170	121	122	163
Arl6ip6	28	29	19	28	32
Arl8a	37	39	29	26	33
Arl8b	607	531	605	649	605
Armc1	72	70	101	47	48
Armc10	44	58	62	52	49
Armc3	17	20	15	9	23
Armc5	27	0	0	0	0
Armc6	4	4	43	20	25
Armc7	123	116	76	61	17
Armc8	90	52	82	106	81
Armc9	22	11	18	17	18
Armcx2	31	16	38	34	23
Armcx3	63	40	69	64	50
Armcx5	0	0	4	0	11
Armcx6	5	2	3	1	9
Armt1	21	9	34	53	57

For Review Only

Arnt	105	135	157	185	225
Arnt2	0	1	0	5	0
Arntl	33	10	62	38	0
Arpc1a	73	124	218	106	0
Arpc1b	0	524	254	21	42
Arpc2	317	403	501	403	531
Arpc3	76	119	137	114	63
Arpc4	0	0	119	1	20
Arpc5	402	393	461	323	380
Arpc5l	111	135	179	129	105
Arpin	0	1	0	4	3
Arpp19	71	76	120	80	96
Arpp21	2	1	0	0	0
Arr3	0	0	0	0	1
Arrb1	215	233	352	301	353
Arrb2	40	0	4	0	48
Arrdc1	0	0	0	0	0
Arrdc2	0	0	0	0	0
Arrdc3	441	58	57	177	272
Arrdc4	25	17	41	31	40
Arsa	304	0	1	1	3
Arsb	1001	929	1036	1101	1195
Arsg	175	156	201	160	197
Arsk	139	114	93	135	144
Arv1	57	65	81	90	64
Arvcf	217	129	219	244	288
As3mt	29	35	196	0	114
Asah1	0	0	584	1	1
Asah2	32	15	14	29	30
Asap1	242	279	214	297	274
Asap2	2	2	0	0	4
Asap3	186	127	254	171	223
Asb1	10	11	26	26	29
Asb10	57	30	51	40	45
Asb2	127	185	424	325	141
Asb3	74	64	70	69	70
Asb6	46	56	68	23	51
Asb7	55	80	56	69	98
Asb8	106	109	129	100	100
Ascc1	72	66	101	64	75
Ascc2	134	136	152	155	161
Ascc3	78	80	81	125	139
Ascl2	8	11	13	5	12
Ascl4	0	0	0	0	3
Asf1a	17	20	31	13	18
Asf1b	4	10	6	13	8
Asgr1	1	1	0	0	4
Ash1l	263	285	195	419	403
Ash2l	53	41	21	34	77
Asic1	4	0	0	0	2

For Review Only

Asic3	1	10	0	1	0
Asl	0	0	0	0	0
Asna1	23	0	9	43	29
Asns	0	0	0	0	0
Asnsd1	119	73	74	78	73
Asph	577	751	601	734	720
Asphd1	0	1	0	1	0
Asphd2	2	0	0	0	9
Aspm	1	0	9	0	0
Aspscr1	101	71	32	58	45
Asrgl1	24	8	6	10	5
Aste1	32	64	53	56	44
Asun	51	29	27	40	60
Asxl1	166	119	210	156	124
Asxl2	132	212	238	150	136
Asxl3	5	0	0	6	0
Atad1	93	171	135	228	148
Atad2	8	34	17	36	22
Atad2b	41	91	50	51	76
Atad3a	76	61	50	58	73
Atad3aos	7	2	5	9	7
Atad5	13	4	0	27	0
Atat1	83	60	129	58	72
Ate1	128	159	89	156	154
Atf1	50	42	57	40	49
Atf2	94	105	110	148	119
Atf3	0	0	0	1	0
Atf4	0	2	0	0	0
Atf5	1	0	3	2	0
Atf6	132	156	139	186	213
Atf6b	60	0	57	0	74
Atf7	271	246	344	319	346
Atf7ip	466	468	533	512	571
Atg10	43	25	42	44	47
Atg101	109	53	98	51	82
Atg12	66	116	116	114	176
Atg13	80	54	76	80	73
Atg14	101	66	116	99	109
Atg16l1	7	55	41	252	30
Atg16l2	9	15	50	0	58
Atg2a	196	35	81	148	128
Atg2b	59	75	69	87	89
Atg3	71	52	52	65	64
Atg4a	10	13	15	15	0
Atg4b	53	94	163	122	0
Atg4c	37	75	49	80	113
Atg4d	46	50	37	59	0
Atg5	0	173	182	125	173
Atg7	96	47	65	76	89
Atg9a	98	52	74	82	115

For Review Only

Athl1	38	1	23	22	16
Atic	28	21	4	25	20
Atl1	0	4	9	0	18
Atl2	60	20	29	60	67
Atl3	98	81	85	137	156
Atm	36	12	50	79	43
Atmin	67	134	124	128	127
Atn1	68	49	50	75	84
Atox1	108	101	150	0	88
Atp10a	0	0	3	0	3
Atp10b	4	0	0	0	3
Atp10d	79	40	30	71	59
Atp11a	52	70	83	82	90
Atp11b	60	68	48	76	66
Atp11c	17	39	38	54	51
Atp13a1	72	0	130	50	210
Atp13a2	141	88	0	202	223
Atp13a3	65	110	51	101	79
Atp1a1	178	69	61	104	106
Atp1a2	3	0	72	0	0
Atp1a3	24	26	26	32	25
Atp1b1	44	0	0	0	7
Atp1b2	9	0	0	0	0
Atp1b3	286	246	393	247	322
Atp2a1	0	0	14	0	4
Atp2a2	131	112	164	103	124
Atp2a3	9	24	13	34	30
Atp2b1	159	216	253	207	325
Atp2b2	9	3	4	6	14
Atp2b3	19	0	0	0	0
Atp2c1	340	459	345	432	413
Atp5a1	948	684	525	18	1
Atp5b	757	622	752	627	814
Atp5c1	0	23	15	283	11
Atp5c1-ps	6	5	4	5	4
Atp5d	0	0	72	57	0
Atp5e	72	57	112	42	0
Atp5f1	265	318	432	237	255
Atp5g1	5	1	0	0	0
Atp5g2	55	40	62	43	50
Atp5g3	152	6	48	120	107
Atp5h	32	0	72	4	0
Atp5j	188	111	202	146	151
Atp5j2	0	0	0	48	0
Atp5k	0	0	0	0	0
Atp5l	0	18	32	0	0
Atp5o	52	0	121	0	0
Atp5s	34	32	24	23	41
Atp5sl	68	54	0	0	0
ATP6	689	415	416	313	488

For Review Only

Atp6ap1	909	771	1223	738	672
Atp6ap2	658	569	0	541	312
Atp6v0a1	149	68	50	217	243
Atp6v0a2	429	385	504	489	520
Atp6v0a4	9	3	0	0	10
Atp6v0b	0	0	155	1	1
Atp6v0c	246	184	862	0	0
Atp6v0c-ps2	486	360	541	362	436
Atp6v0d1	95	118	15	42	14
Atp6v0e	200	223	306	205	277
Atp6v0e2	3	0	0	0	0
Atp6v1a	275	119	166	237	179
Atp6v1b2	382	293	424	291	319
Atp6v1c1	386	327	382	362	419
Atp6v1d	96	125	148	91	119
Atp6v1e1	179	115	223	136	127
Atp6v1f	63	180	290	153	173
Atp6v1g1	253	220	319	167	247
Atp6v1g2	7	15	16	7	10
Atp6v1h	129	83	0	152	1
Atp7a	119	172	141	259	185
Atp7b	0	1	8	0	24
ATP8	1020	768	1045	843	900
Atp8a1	202	356	301	522	502
Atp8a2	394	212	275	435	472
Atp8b1	6	6	4	4	10
Atp8b2	0	5	9	5	0
Atp8b4	0	0	0	3	0
Atp9a	9	1	0	4	1
Atp9b	221	161	176	194	212
Atpaf1	40	20	25	24	14
Atpaf2	54	37	41	32	46
Atpif1	68	1	0	0	0
Atr	25	23	40	91	33
Atraid	164	318	323	24	52
Atrip	0	1	0	6	1
Atrn	56	65	68	62	51
Atrnl1	1	4	0	0	4
Atrx	162	205	248	284	236
Atxn1	98	60	79	121	116
Atxn10	239	245	239	272	296
Atxn1l	81	68	58	53	97
Atxn2	57	84	72	79	107
Atxn2l	122	107	68	418	394
Atxn3	37	37	35	48	40
Atxn7	24	32	53	54	79
Atxn7l1	93	86	73	77	87
Atxn7l1os2	1	3	0	0	0
Atxn7l2	15	11	10	11	30
Atxn7l3	50	22	38	25	49

For Review Only

Atxn7l3b	179	164	207	167	140
AU015228	0	3	0	2	3
AU019823	19	27	14	23	33
AU021092	0	0	0	0	0
AU022252	36	40	67	28	34
AU022793	17	16	31	23	25
AU040320	183	87	122	173	198
AU040972	3	2	0	13	0
AU041133	9	5	18	44	33
Auh	31	26	24	30	24
Aup1	2	7	1	0	11
Aurka	0	10	1	2	1
Aurkaip1	0	43	0	0	0
Aurkb	0	1	0	0	0
Aven	0	4	5	4	0
Avl9	70	42	57	88	80
Avpi1	9	9	2	2	3
Avpr2	5	6	10	18	12
AW011738	1	3	0	5	11
AW046200	11	1	0	0	8
AW112010	30	25	60	34	26
AW146154	64	44	69	42	69
AW209491	40	35	67	42	63
AW549877	117	180	176	277	164
AW554918	74	32	46	77	50
Axin1	15	28	50	19	30
Axin2	0	2	0	0	0
Axl	31	30	45	79	29
AY358078	1	0	2	4	0
AY512915	0	1	0	3	0
AY702103	0	0	0	0	0
Azi2	253	169	130	168	117
Azin1	181	116	149	147	138
B130006D01Rik	45	60	42	80	68
B130034C11Rik	0	0	0	0	4
B130055M24Rik	58	48	74	56	26
B230118H07Rik	22	18	42	15	0
B230208H11Rik	6	4	3	0	0
B230217C12Rik	1	1	0	1	0
B230217O12Rik	0	0	0	3	1
B230219D22Rik	137	236	122	186	179
B230307C23Rik	13	6	15	19	13
B230311B06Rik	2	0	1	0	0
B230317F23Rik	9	3	8	1	0
B230319C09Rik	1	7	0	6	3
B230354K17Rik	16	29	34	31	19
B230369F24Rik	0	2	0	4	0
B2m	2389	3358	4415	3308	3673
B330016D10Rik	1	0	4	4	5
B3galnt1	158	124	170	136	186

For Review Only

B3galnt2	23	24	28	42	59
B3galt2	9	2	0	15	6
B3galt4	58	47	74	62	74
B3galt5	140	72	99	131	134
B3galt6	74	44	56	45	64
B3gat2	9	2	9	7	7
B3gat3	29	26	44	17	30
B3glct	23	17	28	39	15
B3gnt2	0	0	1	14	1
B3gnt3	0	0	0	4	1
B3gnt4	10	3	9	20	5
B3gnt6	1	1	13	0	0
B3gnt7	10	6	11	9	20
B3gnt8	2	5	0	5	0
B3gnt9	0	0	0	0	0
B3gntl1	78	58	96	86	83
B430010I23Rik	193	68	60	185	253
B430212C06Rik	0	0	0	3	0
B430306N03Rik	39	25	40	39	51
B430319F04Rik	0	0	2	2	4
B4galnt1	34	67	40	42	59
B4galnt2	8	6	4	7	9
B4galt1	109	62	99	224	215
B4galt3	237	0	0	0	0
B4galt4	166	456	370	357	423
B4galt5	8	20	13	13	16
B4galt6	79	68	60	67	65
B4galt7	63	30	68	41	51
B4gat1	1	1	0	0	511
B930041F14Rik	4	1	4	4	6
B930059L03Rik	4	6	10	4	9
B930095G15Rik	23	9	7	15	40
B9d1	10	5	5	7	0
B9d2	40	46	43	23	33
Babam1	43	51	62	57	49
Bace1	0	0	0	17	4
Bach1	90	177	77	185	177
Bach2	135	133	164	164	141
Bach2os	18	17	16	7	17
Bad	3	1	26	19	45
Bag1	25	27	10	21	18
Bag2	15	0	5	7	18
Bag3	46	50	52	51	62
Bag4	19	37	18	25	22
Bag5	126	72	141	80	105
Bag6	73	65	50	68	75
Bahcc1	14	19	26	21	26
Bahd1	24	25	30	38	29
Baiap2	44	32	52	33	40
Baiap2l1	29	18	16	29	38

For Review Only

Baiap3	3	4	0	4	0
Bak1	10	5	0	19	13
Bambi	7	1	9	0	5
Bambi-ps1	0	4	0	0	6
Banf1	75	38	66	39	52
Bank1	179	108	168	146	214
Banp	24	57	62	119	0
Bap1	97	74	200	116	55
Bard1	2	0	0	0	4
Basp1	31	113	65	37	33
Batf	15	13	27	15	7
Batf2	3	0	0	0	3
Batf3	0	0	0	0	0
Bax	58	0	0	0	30
Baz1a	67	58	87	51	45
Baz1b	154	102	120	149	130
Baz2a	58	85	46	100	108
Baz2b	112	158	123	189	181
Bbc3	11	8	11	5	18
Bbip1	38	21	58	0	0
Bbs1	8	0	9	0	0
Bbs10	27	18	18	33	6
Bbs12	23	30	39	38	26
Bbs2	32	24	32	32	23
Bbs4	1	5	75	39	0
Bbs5	0	0	0	0	3
Bbs7	25	31	57	30	67
Bbs9	123	74	183	105	169
Bbx	74	72	83	94	84
BC002059	26	13	48	31	47
BC002163	18	18	34	17	20
BC003331	101	100	128	82	73
BC003965	0	6	0	0	0
BC004004	185	213	240	152	196
BC005537	115	181	223	118	130
BC005561	28	100	19	33	29
BC005624	0	42	36	1	1
BC017158	18	8	65	7	63
BC017643	1	1	0	0	0
BC020402	5	7	5	7	7
BC022687	18	0	0	7	17
BC023829	55	52	89	81	93
BC024063	21	14	31	16	21
BC024978	71	17	36	18	77
BC025920	2	1	10	7	0
BC026585	61	28	29	23	55
BC028528	96	33	95	15	45
BC029214	21	10	23	12	0
BC029722	63	35	0	0	0
BC030336	26	22	19	25	12

For Review Only

BC030499	0	3	0	0	0
BC031181	97	108	120	94	138
BC031361	7	7	4	5	0
BC035044	110	344	386	267	259
BC037032	11	14	11	12	17
BC037034	15	30	0	0	0
BC037704	19	6	7	5	14
BC048403	8	22	16	18	18
BC048507	1	4	8	8	1
BC048609	1	0	6	0	0
BC049352	0	18	0	62	0
BC049715	3	3	0	1	0
BC051142	0	0	0	0	0
BC051226	0	0	0	2	0
BC051537	3	0	5	0	0
BC052040	13	25	37	19	15
BC055324	0	10	21	13	17
BC064078	0	0	0	0	0
BC065397	11	7	13	5	4
BC067074	0	0	0	0	3
BC068281	45	28	33	52	55
BC100451	18	16	7	26	9
BC106175	6	0	5	0	7
BC106179	1	3	4	2	1
BC147527	8	11	5	23	22
Bcam	0	0	0	0	0
Bcan	0	0	0	0	10
Bcap29	139	102	146	117	120
Bcap31	287	231	381	266	262
Bcar3	3	0	0	0	0
Bcas2	83	253	204	0	87
Bcas3	188	132	137	140	214
Bcas3os1	5	9	6	8	12
Bcas3os2	0	0	1	0	0
Bcat1	2	1	0	0	12
Bcat2	30	32	54	32	32
Bccip	76	53	51	45	69
Bcdin3d	41	51	51	45	35
Bckdha	15	127	0	34	28
Bckdhb	27	0	57	4	1
Bckdk	44	39	0	40	45
Bcl10	3	100	345	0	218
Bcl11a	0	0	0	6	4
Bcl2	16	20	14	6	10
Bcl2a1a	8	73	126	34	0
Bcl2a1b	170	155	25	10	245
Bcl2a1c	0	3	5	4	5
Bcl2a1d	66	63	181	20	56
Bcl2l1	607	365	453	658	600
Bcl2l11	86	74	80	111	146

For Review Only

Bcl2l12	0	0	1	1	1
Bcl2l13	96	67	111	67	80
Bcl2l2	7	13	8	16	5
Bcl3	0	4	0	0	0
Bcl6	31	53	53	47	50
Bcl6b	3	7	7	6	5
Bcl7a	11	22	10	23	32
Bcl7b	0	0	345	11	1
Bcl7c	8	11	0	13	16
Bcl9	125	103	144	150	174
Bcl9l	78	144	101	191	155
Bclaf1	230	198	140	113	324
Bco2	217	178	212	152	286
Bcor	94	122	118	140	144
Bcorl1	37	28	19	41	26
Bcr	9	11	23	9	25
Bcs1l	46	19	39	28	11
Bdh1	6	0	0	0	0
Bdp1	78	71	69	103	64
BE692007	2	2	0	3	3
Becn1	42	19	0	117	99
Bend3	12	11	16	37	29
Bend4	0	0	0	0	0
Bend5	21	29	19	5	15
Bend6	57	40	80	44	75
Best1	2	1	50	23	0
Bet1	38	40	56	39	41
Bfar	139	142	206	121	165
Bgn	0	0	0	0	0
Bhlha15	2	0	0	0	0
Bhlhb9	56	16	47	70	59
Bhlhe40	3	0	7	2	0
Bhlhe41	664	689	0	0	0
Bicd1	0	0	3	0	0
Bicd2	307	237	213	295	270
Bid	120	164	209	205	268
Bik	0	7	1	1	1
Bin1	660	729	1469	127	412
Bin2	965	802	1375	857	1097
Bin3	87	76	150	72	100
Birc2	37	60	89	74	128
Birc3	137	81	89	95	122
Birc5	2	0	7	0	5
Birc6	289	295	246	410	403
Bivm	28	19	27	22	29
Blcap	86	77	113	94	97
Blm	3	2	13	20	22
Blmh	25	71	0	157	183
Blnk	541	619	773	552	592
Bloc1s1	0	5	0	0	132

For Review Only

Bloc1s2	15	21	0	0	0
Bloc1s3	5	1	3	12	6
Bloc1s4	11	30	12	17	16
Bloc1s5	61	56	81	48	45
Bloc1s6	82	55	75	64	92
Bloc1s6os	1	0	0	1	0
Blvra	85	80	118	74	105
Blvrb	15	27	96	44	66
Blzf1	57	19	57	53	34
Bmf	148	141	201	30	120
Bmi1	20	36	0	73	73
Bmp1	20	0	8	6	10
Bmp2	0	0	0	0	5
Bmp2k	607	617	613	720	783
Bmp6	3	0	0	0	0
Bmp7	3	0	0	0	0
Bmp8b	0	0	1	1	0
Bmpr1a	21	39	36	55	77
Bmpr2	142	170	189	218	245
Bms1	32	52	44	68	123
Bmt2	23	40	21	21	49
Bmyc	188	190	270	143	203
Bnc1	0	0	0	0	0
Bnc2	0	0	4	0	9
Bnip1	19	17	23	21	23
Bnip2	144	233	287	257	218
Bnip3	41	52	74	41	33
Bnip3l	170	181	213	189	232
Bnip3l-ps	88	88	78	82	88
Bnipl	0	0	4	0	0
Bod1	18	19	17	14	12
Bod1l	93	162	88	122	109
Bola1	108	1	0	0	0
Bola3	11	4	0	9	0
Bop1	0	0	9	9	2
Bora	0	0	11	0	0
Borcs5	30	17	42	44	26
Borcs6	18	38	16	29	40
Borcs7	5	6	48	0	33
Borcs8	3	13	0	0	0
Bpgm	15	47	28	24	41
Bphl	39	32	57	34	43
Bpifb9a	0	0	0	0	0
Bpifb9b	0	0	0	0	0
Bpnt1	33	38	23	24	23
Bptf	168	209	166	205	264
Braf	199	103	107	188	130
Brap	47	26	49	75	66
Brat1	165	100	103	127	146
Brca1	0	0	0	4	0

For Review Only

Brca2	26	15	26	22	23
Brcc3	43	36	46	49	85
Brd1	82	121	105	92	94
Brd2	0	0	0	0	4
Brd3	24	95	22	61	75
Brd4	207	236	248	228	320
Brd7	205	220	157	130	144
Brd8	0	18	55	3	1
Brd9	108	86	147	100	96
Brdt	0	0	0	3	0
Bre	102	81	127	95	169
Brf1	43	183	26	94	90
Brf2	19	10	11	17	18
Bri3	13	13	17	37	29
Bri3bp	40	25	39	35	37
Brip1os	69	69	82	81	77
Brix1	48	45	49	31	37
Brk1	257	274	179	82	43
Brms1	0	0	0	26	0
Brms1l	49	64	37	34	44
Brox	85	113	86	177	133
Brpf1	109	91	124	110	150
Brpf3	23	29	38	56	20
Brs3	0	0	0	0	0
Brsk2	0	3	0	1	4
Brwd1	119	144	100	182	138
Brwd3	14	21	16	22	19
Bscl2	194	155	303	203	239
Bsdc1	102	79	81	106	104
Bsg	926	665	1050	528	676
Bsn	82	32	28	65	105
Bspry	0	0	0	0	3
Bst2	107	73	146	92	103
Btaf1	79	85	81	102	121
Btbd1	40	28	35	30	28
Btbd10	53	81	81	37	109
Btbd17	14	6	4	4	15
Btbd18	3	2	0	0	0
Btbd19	1	1	0	0	146
Btbd2	2	13	0	0	0
Btbd3	48	63	22	68	54
Btbd6	0	0	0	0	52
Btbd7	53	59	91	69	53
Btbd8	3	0	0	0	0
Btbd9	289	314	277	346	382
Btd	50	49	73	57	69
Btf3	91	97	110	79	88
Btf3l4	96	71	65	54	55
Btg1	0	0	0	0	0
Btg2	797	0	747	0	1022

For Review Only

Btg3	4	0	3	2	2
Btk	291	258	281	275	315
Btla	1	0	0	0	1
Btnl2	1	0	0	0	0
Btrc	70	51	79	97	81
Bub1	0	0	3	0	0
Bub1b	5	0	0	0	0
Bub3	189	208	240	206	214
Bud13	23	25	38	17	27
Bud31	59	45	61	44	41
Bysl	49	27	35	37	55
Bzrap1	14	34	14	31	49
Bzw1	250	209	203	208	168
Bzw2	28	29	71	24	19
C030013C21Rik	0	0	0	0	6
C030016D13Rik	0	3	4	0	4
C030034I22Rik	7	10	15	12	25
C030034L19Rik	1	1	0	0	20
C030039L03Rik	3	1	0	7	5
C130021I20Rik	0	0	0	0	2
C130026I21Rik	6	5	6	8	5
C130036L24Rik	3	0	0	5	4
C130046K22Rik	21	10	24	21	28
C130050O18Rik	117	42	39	157	156
C1d	47	57	70	53	68
C1galt1	1	4	1	7	8
C1galt1c1	80	94	101	79	90
C1qa	7922	7214	9792	6234	7928
C1qb	9155	9081	12896	7808	9876
C1qbp	55	49	83	55	57
C1qc	3530	3019	557	1124	2007
C1qtnf1	1	0	0	0	0
C1ra	7	0	0	0	9
C1rb	5	0	0	0	5
C1rl	32	37	42	19	33
C230012O17Rik	0	0	1	0	0
C230037L18Rik	1	0	5	0	0
C230057M02Rik	0	2	0	0	0
C230062I16Rik	4	0	0	0	5
C230072F16Rik	2	0	0	0	0
C230091D08Rik	13	26	10	17	15
C2cd2	31	45	41	27	77
C2cd2l	17	18	39	25	22
C2cd3	158	87	124	156	177
C2cd5	27	34	30	49	56
C3	22	7	0	8	0
C330006A16Rik	373	272	314	329	388
C330007P06Rik	51	34	63	39	76
C330013E15Rik	9	1	8	0	0
C330016L05Rik	0	2	0	0	0

For Review Only

C330018A13Rik	2	0	1	0	6
C330018D20Rik	37	12	38	35	31
C330021F23Rik	65	59	77	64	65
C3ar1	1048	576	892	939	1050
C430042M11Rik	0	0	0	1	3
C430049B03Rik	5	8	6	10	3
C4a	0	4	0	5	0
C4b	0	0	0	26	0
C530005A16Rik	3	6	0	9	9
C530008M17Rik	1	1	4	0	0
C5ar1	296	211	323	274	300
C5ar2	24	52	20	387	60
C630043F03Rik	2	0	0	0	0
C77080	7	0	5	8	0
C87436	113	92	128	143	170
C8g	0	0	11	0	0
C920006O11Rik	1	1	55	0	0
C920009B18Rik	6	2	0	6	11
C920021L13Rik	2	4	0	32	0
Caap1	29	8	5	19	1
Cab39	80	117	132	111	135
Cab39l	254	29	98	77	107
Cabin1	255	205	213	257	229
Cables1	111	62	144	116	121
Cables2	45	21	39	34	29
Cabyr	8	0	14	19	6
Cacfd1	32	27	32	23	19
Cachd1	6	0	0	0	13
Cacna1a	0	6	0	5	19
Cacna1c	1	3	0	23	10
Cacna1d	90	107	88	90	119
Cacna1e	8	4	3	6	9
Cacna1f	0	0	0	0	5
Cacna1s	9	1	16	11	0
Cacnb1	11	0	3	0	2
Cacnb2	11	29	13	23	30
Cacnb4	12	4	1	13	7
Cactin	23	28	28	30	37
Cacul1	77	100	88	104	77
Cacybp	65	69	90	74	67
Cad	0	0	50	82	97
Cadm1	295	281	264	264	295
Cadm2	2	0	0	5	0
Cage1	5	6	2	2	6
Cahm	0	3	0	4	2
Calcoco1	73	83	74	95	106
Calcrl	91	96	71	117	79
Cald1	2	0	0	0	0
Calhm2	241	221	196	218	325
Calm1	613	494	638	424	551

For Review Only

Calm2	747	809	1177	719	859
Calm3	44	40	17	29	34
Calml4	18	2	0	7	0
Calr	1	1	0	0	0
Calr3	5	0	0	2	4
Calu	208	196	408	261	311
Camk1	0	0	0	0	0
Camk1d	228	174	304	348	392
Camk2a	24	5	17	15	23
Camk2b	15	18	16	15	18
Camk2d	96	234	166	235	278
Camk2g	102	131	122	119	140
Camk2n1	50	64	40	34	40
Camk4	0	1	0	3	0
Camkk1	82	54	110	66	89
Camkk2	90	64	84	97	117
Camkmt	48	18	53	37	27
Camkv	0	3	0	0	4
Caml	109	104	96	1	2
Camp	243	118	50	85	97
Camsap1	73	120	107	147	143
Camsap2	74	46	46	58	54
Camsap3	0	0	0	2	3
Camta1	32	43	33	33	32
Camta2	14	0	1	0	0
Cand1	132	76	128	125	81
Cand2	46	19	37	60	57
Cant1	46	47	8	31	25
Canx	1752	1546	1802	1721	1742
Cap1	131	116	91	372	272
Capg	74	55	0	34	36
Capn1	0	0	0	0	0
Capn10	22	21	20	11	28
Capn11	0	0	0	0	0
Capn15	49	78	71	96	63
Capn2	13	1	0	0	25
Capn3	0	35	0	0	5
Capn5	0	2	0	0	3
Capn7	57	37	122	110	143
Capns1	15	33	85	42	32
Capns2	6	35	19	39	37
Caprin1	475	218	417	484	501
Caprin2	7	3	10	8	4
Capza1	175	166	217	158	238
Capza2	1722	1776	2355	1640	1696
Capzb	277	211	610	1	28
Car11	1	0	4	0	3
Car12	23	0	0	12	0
Car13	0	0	0	0	0
Car14	16	0	0	0	0

For Review Only

Car15	1	1	0	14	0
Car2	18	1	0	7	1
Car4	0	0	0	0	0
Car7	1	4	10	1	1
Car9	4	0	0	0	5
Card10	3	2	11	0	5
Card11	0	10	15	0	7
Card19	0	0	7	1	0
Card6	232	175	194	196	236
Card9	0	0	23	9	0
Carf	17	26	30	47	33
Carhsp1	24	37	37	26	14
Carkd	83	90	47	0	0
Carlr	0	2	0	0	3
Carm1	0	55	2	4	22
Carnmt1	5	10	2	6	10
Carns1	16	4	25	16	1
Cars	13	9	46	115	32
Cars2	0	58	39	53	33
Casc1	0	0	0	5	3
Casc3	62	116	63	108	112
Casc4	6	11	13	14	0
Casc5	0	0	0	2	0
Casd1	59	109	103	61	58
Cask	175	152	197	183	225
Caskin1	6	0	4	0	8
Caskin2	1	0	0	12	0
Casp1	15	14	111	86	89
Casp12	0	4	8	6	0
Casp2	0	0	122	0	0
Casp3	78	73	69	78	84
Casp4	18	33	58	47	48
Casp6	33	37	60	34	56
Casp7	29	24	28	23	32
Casp8	32	48	141	21	101
Casp8ap2	33	33	20	42	35
Casp9	94	69	69	124	144
Casq1	6	0	0	2	2
Casr	0	0	0	0	0
Cass4	130	132	174	207	204
Cast	83	41	62	58	67
Casz1	1	4	12	0	0
Cat	203	174	239	166	183
Catip	21	6	12	21	46
Catsper2	23	62	43	47	68
Catsperg1	33	7	0	10	0
Cav1	0	0	0	0	0
Cav2	3	3	0	18	0
Cbarp	7	0	4	1	1
Cbfa2t2	192	65	157	189	195

For Review Only

Cbfa2t3	90	113	122	119	150
Cbfb	25	30	47	27	45
Cbl	366	392	379	554	572
Cblb	66	71	25	79	70
Cbll1	44	34	59	66	101
Cbr1	11	9	0	0	0
Cbr2	0	2	0	0	0
Cbr3	15	14	26	14	24
Cbr4	26	43	47	40	36
Cbs	2	0	0	5	0
Cbwd1	6	3	10	4	10
Cbx1	33	30	43	33	44
Cbx2	9	9	0	4	4
Cbx3	64	73	81	54	58
Cbx4	54	29	42	30	26
Cbx5	50	52	46	86	71
Cbx6	21	24	27	38	31
Cbx7	13	8	2	10	22
Cbx8	22	43	43	17	39
Cby1	34	16	25	20	14
Cc2d1a	74	43	0	52	44
Cc2d1b	140	138	0	144	233
Cc2d2a	6	2	0	0	0
Ccar1	116	109	147	120	151
Ccar2	63	13	19	48	21
Ccdc102a	17	12	5	3	1
Ccdc106	1	1	0	0	0
Ccdc107	0	47	83	4	2
Ccdc108	0	0	0	0	1
Ccdc112	0	1	0	0	0
Ccdc114	6	5	0	0	0
Ccdc115	7	0	0	0	21
Ccdc116	3	1	0	0	4
Ccdc117	39	40	33	78	45
Ccdc12	95	0	0	0	62
Ccdc120	0	0	2	0	0
Ccdc122	0	0	2	0	4
Ccdc124	29	0	4	1	1
Ccdc125	4	0	6	0	16
Ccdc126	19	30	31	18	28
Ccdc127	89	61	104	76	63
Ccdc130	31	57	95	44	0
Ccdc134	35	48	40	46	47
Ccdc136	0	2	1	0	4
Ccdc137	26	22	164	91	106
Ccdc138	0	0	0	0	0
Ccdc14	12	17	21	25	13
Ccdc142	0	19	50	0	0
Ccdc146	4	24	26	23	12
Ccdc149	0	5	7	0	0

For Review Only

Ccdc15	31	30	20	36	47
Ccdc157	7	27	91	39	33
Ccdc162	23	9	57	58	45
Ccdc163	54	14	56	35	47
Ccdc166	23	30	23	23	40
Ccdc167	16	33	30	31	34
Ccdc168	1	26	21	4	0
Ccdc171	14	14	11	7	34
Ccdc173	6	0	3	1	2
Ccdc174	16	35	28	23	35
Ccdc181	28	12	20	17	34
Ccdc186	47	35	40	52	65
Ccdc188	0	0	0	5	1
Ccdc189	6	5	10	0	0
Ccdc191	36	25	20	13	26
Ccdc22	60	41	0	0	156
Ccdc25	41	18	38	39	37
Ccdc28a	6	7	11	13	8
Ccdc28b	0	0	0	0	0
Ccdc30	1	1	3	1	0
Ccdc32	48	56	54	40	31
Ccdc34	25	19	16	15	23
Ccdc36	7	2	8	7	19
Ccdc38	2	7	4	12	1
Ccdc40	0	0	0	0	5
Ccdc43	33	18	45	28	50
Ccdc47	134	114	139	157	225
Ccdc50	195	204	233	230	174
Ccdc51	31	21	18	31	39
Ccdc53	40	45	82	23	34
Ccdc57	21	10	9	6	28
Ccdc58	19	18	20	14	19
Ccdc59	27	27	44	29	30
Ccdc6	82	105	121	81	62
Ccdc61	13	8	30	0	24
Ccdc62	15	20	26	33	17
Ccdc63	0	0	0	0	0
Ccdc64	10	5	24	13	19
Ccdc66	10	52	53	56	39
Ccdc69	0	3	0	2	5
Ccdc71	105	96	0	0	0
Ccdc71l	9	26	7	16	10
Ccdc73	2	8	0	4	5
Ccdc77	12	16	31	13	15
Ccdc78	2	0	0	20	0
Ccdc8	1	2	0	0	4
Ccdc80	5	0	8	4	7
Ccdc82	41	89	51	43	60
Ccdc84	0	0	0	2	2
Ccdc85b	9	9	10	17	10

For Review Only

Ccdc85c	30	41	29	27	36
Ccdc86	72	46	68	69	92
Ccdc88a	7	22	12	48	45
Ccdc88b	1	59	0	151	0
Ccdc88c	0	2	0	0	0
Ccdc89	5	2	0	0	0
Ccdc9	15	9	12	6	13
Ccdc90b	60	7	95	55	73
Ccdc91	54	7	48	26	37
Ccdc92	0	0	3	1	0
Ccdc93	293	339	372	323	318
Ccdc94	2	1	0	0	202
Ccdc96	1	6	4	4	7
Ccdc97	195	16	74	105	1
Cchcr1	0	0	0	3	0
Ccl12	0	0	19	0	25
Ccl22	0	0	3	2	0
Ccl24	5	7	6	0	7
Ccl25	0	0	0	1	6
Ccl27a	0	0	4	1	0
Ccl27b	1	0	1	0	1
Ccl28	0	2	0	0	0
Ccl4	75	0	2	10	64
Ccl5	0	0	0	7	0
Ccl6	105	192	259	218	173
Ccl7	0	6	8	0	5
Ccl9	111	114	176	96	142
Ccm2	19	56	107	91	0
Ccm2l	0	0	0	3	8
Ccna2	15	0	15	16	0
Ccnb1	1	0	3	0	0
Ccnb2	0	0	0	0	0
Ccnc	36	56	56	46	51
Ccnd1	55	65	65	100	137
Ccnd2	10	4	13	0	1
Ccnd3	179	1	0	125	150
Ccndbp1	0	31	58	1	53
Ccne1	1	0	0	0	0
Ccnf	7	3	0	8	14
Ccng1	116	47	97	103	130
Ccng2	1	1683	0	0	0
Ccnh	67	62	56	69	67
Ccni	157	268	205	182	179
Ccnj	20	25	12	30	21
Ccnk	29	33	41	42	50
Ccnl1	147	104	205	222	214
Ccnl2	332	0	236	205	129
Ccnt1	79	73	139	121	79
Ccnt2	69	105	2	224	55
Ccny	48	90	61	127	119

For Review Only

Ccnyl1	10	9	12	9	10
Ccp110	4	10	5	17	10
Ccpg1	318	196	251	342	327
Ccpg1os	9	10	10	4	6
Ccr1	18	25	28	16	45
Ccr1l1	14	4	5	0	0
Ccr2	60	51	57	51	47
Ccr3	4	1	8	0	11
Ccr5	1047	686	0	1240	0
Ccr6	0	3	3	2	7
Ccr9	6	7	8	9	0
Ccrl2	0	20	0	102	0
Ccs	0	0	0	0	0
Ccsap	4	1	0	0	0
Ccser2	42	58	46	49	71
Cct2	231	148	238	174	170
Cct3	250	213	339	232	280
Cct4	317	279	474	321	346
Cct5	188	251	331	222	220
Cct6a	196	223	281	179	189
Cct7	336	216	408	241	284
Cct8	300	256	387	257	318
Ccz1	41	50	35	42	37
Cd101	61	31	33	37	36
Cd14	0	0	244	71	0
Cd151	75	0	7	36	17
Cd160	4	0	0	4	0
Cd163	0	3	1	0	1
Cd164	1288	1399	1839	1571	1847
Cd177	87	28	10	12	11
Cd180	47	154	177	261	221
Cd1d1	0	2	7	0	0
Cd1d2	2	1	0	0	0
Cd200	0	0	0	0	0
Cd200r1	47	31	81	63	82
Cd200r3	1	0	0	0	0
Cd200r4	3	0	0	8	0
Cd209a	0	0	0	0	0
Cd209f	0	0	0	0	0
Cd22	53	14	45	40	23
Cd226	0	1	4	0	0
Cd244	7	8	9	5	5
Cd247	0	0	3	0	0
Cd24a	8	0	0	0	0
Cd27	60	53	50	65	98
Cd274	34	21	12	25	36
Cd276	64	46	95	96	98
Cd2ap	104	167	121	112	83
Cd2bp2	52	3	0	0	0
Cd300a	375	540	564	352	547

For Review Only

For Review Only

Cd300c	0	0	0	0	0
Cd300c2	374	255	258	0	726
Cd300lb	4	2	5	0	0
Cd300ld	2	3	0	0	1
Cd300ld2	0	0	0	2	1
Cd300ld3	3	0	1	2	4
Cd300ld5	0	0	0	1	0
Cd300lf	6	4	0	0	0
Cd302	180	261	314	203	209
Cd320	39	22	14	27	0
Cd33	0	49	1	1	248
Cd34	330	0	168	162	160
Cd36	0	0	0	0	0
Cd37	17	854	0	0	0
Cd38	0	0	10	0	0
Cd3eap	25	17	20	19	24
Cd4	1	1	0	0	1
Cd40	0	2	0	0	8
Cd44	14	2	5	10	10
Cd46	10	8	0	9	8
Cd47	477	432	561	527	631
Cd48	306	269	297	235	228
Cd52	79	107	164	117	119
Cd53	1955	1838	1461	1461	1265
Cd59a	5	0	0	0	7
Cd5l	2	0	0	3	4
Cd63	0	0	55	0	0
Cd68	1264	835	931	676	0
Cd69	1	9	0	0	0
Cd72	6	14	9	0	16
Cd74	86	44	71	122	0
Cd79b	40	0	95	1	4
Cd80	50	32	48	64	51
Cd81	5371	8312	11189	5167	26
Cd82	287	440	47	326	28
Cd83	65	0	0	0	0
Cd84	504	580	872	655	684
Cd86	765	557	892	567	666
Cd9	1637	1373	3123	144	492
Cd93	8	0	0	0	0
Cd99l2	74	45	42	71	68
Cdadc1	69	46	58	70	60
Cdan1	1	16	0	147	0
Cdc123	110	131	149	87	142
Cdc14a	19	33	16	57	34
Cdc14b	17	9	0	17	7
Cdc16	65	52	95	74	59
Cdc23	111	92	91	153	101
Cdc25a	13	10	17	6	17
Cdc25b	0	1	18	0	0

Cdc26	32	59	62	40	53
Cdc27	46	30	48	79	54
Cdc34	0	0	0	12	0
Cdc37	188	193	175	1	1
Cdc37l1	105	68	81	122	122
Cdc40	102	57	83	92	78
Cdc42	1339	1327	1630	1332	1672
Cdc42bpa	27	23	20	36	20
Cdc42bpb	54	10	127	63	155
Cdc42ep1	2	16	0	0	12
Cdc42ep2	6	0	9	7	0
Cdc42ep3	40	11	63	50	44
Cdc42ep4	44	33	93	68	67
Cdc42se1	338	259	267	285	349
Cdc42se2	249	277	245	196	306
Cdc45	0	0	0	0	0
Cdc5l	65	54	96	85	79
Cdc7	10	5	14	5	14
Cdc73	9	29	30	40	38
Cdca2	3	10	0	7	3
Cdca3	0	0	4	0	0
Cdca4	190	128	180	174	172
Cdca7	66	49	74	62	68
Cdca8	0	0	2	5	0
Cdh11	2	3	3	5	5
Cdh12	3	0	0	0	1
Cdh23	197	174	189	289	268
Cdh24	0	0	1	3	0
Cdh5	1	0	4	0	0
Cdh6	6	0	0	0	0
Cdh7	0	0	0	0	4
Cdhr1	0	1	0	0	0
Cdhr4	5	0	7	0	0
Cdip1	35	0	48	5	30
Cdipt	0	0	46	1	16
Cdk1	0	0	0	0	0
Cdk10	0	1	7	6	0
Cdk11b	153	127	293	46	109
Cdk12	86	139	78	157	133
Cdk13	59	64	48	84	85
Cdk14	21	15	41	28	38
Cdk16	96	68	19	0	48
Cdk17	50	48	60	50	56
Cdk18	1	2	0	0	0
Cdk19	136	158	158	159	204
Cdk2	30	25	56	53	45
Cdk20	11	0	0	0	0
Cdk2ap1	24	26	34	28	23
Cdk2ap2	0	0	0	0	76
Cdk3-ps	0	4	0	1	2

For Review Only

Cdk4	42	15	0	0	233
Cdk5	0	35	39	10	21
Cdk5r1	51	37	32	41	41
Cdk5rap1	25	16	36	28	25
Cdk5rap2	40	41	22	35	33
Cdk5rap3	0	98	199	7	0
Cdk6	233	339	253	334	383
Cdk7	87	90	76	108	58
Cdk8	38	36	45	34	27
Cdk9	12	103	0	58	171
Cdkal1	96	92	98	110	133
Cdkl3	9	15	17	8	6
Cdkl4	10	2	10	0	5
Cdkn1b	68	80	56	87	68
Cdkn1c	11	4	3	7	12
Cdkn2aip	54	59	69	80	71
Cdkn2aipnl	136	99	125	74	115
Cdkn2b	0	4	3	1	0
Cdkn2c	3	2	5	1	10
Cdkn2d	23	17	34	13	17
Cdnf	2	0	0	0	0
Cdpf1	22	27	37	38	31
Cdr2	14	1	0	0	0
Cdr2l	15	1	8	8	0
Cds1	88	135	134	110	97
Cds2	348	172	263	372	374
Cdt1	0	0	1	1	0
Cdv3	90	68	68	98	81
Cdyl	21	18	30	20	9
Cdyl2	30	37	23	55	25
Ceacam1	87	177	128	128	86
Ceacam18	5	0	4	4	2
Ceacam2	18	53	37	40	31
Cebpa	112	166	163	76	118
Cebpd	35	37	26	53	78
Cebpg	81	217	178	133	110
Cebpz	58	48	71	51	189
Cebpzos	64	68	100	8	54
Cecr2	0	7	0	3	0
Cecr5	11	7	0	16	1
Cecr6	0	0	0	0	1
Cela1	0	0	0	0	0
Celf1	0	280	112	782	542
Celf2	392	494	538	483	589
Celf3	0	2	6	12	6
Celf4	6	7	5	14	17
Celsr1	0	2	4	6	3
Celsr2	3	0	0	0	7
Celsr3	0	0	0	0	6
Cenpa	1	0	0	8	0

For Review Only

Cenpb	30	36	49	29	41
Cenpc1	21	10	15	18	31
Cenpe	0	0	0	0	13
Cenpf	9	8	0	6	9
Cenpi	0	4	0	7	0
Cenpj	9	16	10	19	20
Cenpl	8	2	6	17	13
Cenpm	0	0	1	0	0
Cenpn	1	3	0	0	0
Cenpo	32	37	77	63	87
Cenpp	0	0	2	0	6
Cenpq	3	12	13	6	0
Cenpt	15	13	22	36	15
Cenpu	3	3	7	9	12
Cenpv	4	4	4	0	6
Cep104	83	58	44	79	88
Cep112	0	0	0	1	1
Cep112it	3	1	0	1	8
Cep120	101	75	85	114	116
Cep128	4	7	3	7	0
Cep131	28	17	2	4	16
Cep135	38	41	29	54	35
Cep152	107	88	125	110	98
Cep162	17	25	26	14	32
Cep164	39	37	51	54	40
Cep170	205	339	209	382	335
Cep170b	1	0	0	0	12
Cep19	9	5	11	8	1
Cep192	53	22	75	99	95
Cep250	92	82	122	106	88
Cep290	23	37	34	24	34
Cep295	11	33	55	74	108
Cep350	114	126	167	164	128
Cep41	12	13	19	12	10
Cep44	13	19	13	17	32
Cep57	46	76	51	59	56
Cep57l1	37	10	30	13	18
Cep63	88	72	86	83	66
Cep68	167	167	134	156	243
Cep70	14	26	19	34	34
Cep72	5	7	17	0	0
Cep76	12	1	0	0	0
Cep78	3	7	7	17	0
Cep83	5	7	0	12	0
Cep83os	17	3	15	28	35
Cep85	61	1	0	0	0
Cep85l	14	60	26	71	62
Cep89	23	21	24	18	34
Cep95	45	35	43	37	49
Cep97	24	6	14	38	29

For Review Only

Cept1	209	182	188	192	149
Cercam	6	8	7	16	11
Cerk	204	357	335	356	419
Cers1	4	1	6	3	0
Cers2	0	0	0	0	0
Cers4	7	19	0	0	0
Cers5	133	127	143	131	141
Cers6	59	100	45	105	106
Cetn2	51	33	41	31	47
Cetn3	73	77	101	62	90
Cfap126	14	0	0	4	10
Cfap20	5	22	0	28	18
Cfap36	26	17	28	26	17
Cfap43	1	4	0	5	7
Cfap46	0	3	0	0	0
Cfap52	3	0	0	0	0
Cfap69	0	0	0	1	4
Cfap74	59	85	183	190	171
Cfap97	46	31	40	55	59
Cfap99	0	1	0	0	1
Cfb	4	1	0	0	0
Cfdp1	60	47	100	60	49
Cfh	1261	1177	1208	1560	1259
Cfhr2	14	19	67	99	61
Cfl1	724	647	0	0	0
Cfl2	66	48	56	77	58
Cflar	113	169	132	215	241
Cggbp1	116	82	105	103	89
Cgn	7	0	0	2	0
Cgnl1	0	0	0	0	0
Cgref1	1	0	11	6	0
Cgrrf1	135	71	184	13	14
Ch25h	0	8	5	0	0
Chac2	6	13	14	4	7
Chad	5	7	10	3	5
Chadl	0	0	0	0	0
Chaf1a	0	3	0	4	5
Chaf1b	4	0	10	0	0
Champ1	131	129	127	157	167
Chchd1	0	0	0	0	0
Chchd10	33	0	0	0	0
Chchd2	244	157	309	183	242
Chchd3	96	78	139	82	105
Chchd4	66	37	57	39	44
Chchd5	1	1	0	0	0
Chchd6	25	18	21	0	7
Chchd7	39	23	16	25	40
Chd1	65	48	26	51	78
Chd1l	30	21	36	44	36
Chd2	109	119	108	125	161

For Review Only

Chd3	37	45	37	43	41
Chd3os	19	31	22	20	22
Chd4	84	135	81	108	95
Chd5	0	0	0	2	0
Chd6	83	141	137	177	145
Chd7	219	269	249	241	290
Chd8	73	143	83	144	169
Chd9	349	404	336	631	531
Chdh	0	4	7	5	4
Chek1	1	3	0	0	0
Chek2	45	37	26	39	37
Cherp	52	28	62	112	54
Chfr	83	70	92	106	100
Chic1	0	13	11	7	0
Chic2	6	54	13	21	25
Chid1	65	16	63	0	47
Chil1	51	10	9	14	21
Chil3	181	61	13	60	86
Chil5	6	2	0	0	0
Chka	143	118	130	158	152
Chkb	0	0	0	0	0
Chm	70	41	33	80	65
Chml	11	22	15	25	17
Chmp1a	2	1	267	0	0
Chmp1b	108	90	163	109	114
Chmp2a	0	0	183	0	0
Chmp2b	107	61	105	60	105
Chmp3	226	282	329	233	293
Chmp4b	13	4	10	11	10
Chmp5	110	104	161	97	125
Chmp6	0	79	92	18	6
Chmp7	62	74	0	46	97
Chn1	0	0	0	0	0
Chn2	226	138	142	218	210
Chordc1	94	117	164	98	122
Chp1	89	6	140	163	156
Chpf	1	0	0	0	0
Chpf2	180	152	214	134	213
Chpt1	9	22	15	28	32
Chrac1	1	1	0	318	0
Chrm1	1	0	3	0	0
Chrm5	10	11	9	7	20
Chrna2	2	10	0	11	13
Chrnb1	0	0	0	5	0
Chrnb2	3	2	4	7	0
Chst1	51	47	38	29	21
Chst10	6	1	8	2	10
Chst11	57	72	78	74	63
Chst12	87	19	175	105	109
Chst13	0	0	0	6	8

For Review Only

Chst14	65	27	61	47	76
Chst15	70	52	60	65	53
Chst2	17	7	6	2	14
Chst3	2	2	0	0	3
Chst7	118	131	214	166	156
Chst8	84	90	127	93	85
Chst9	1	3	1	0	1
Chsy1	178	174	297	188	202
Chtf18	0	0	0	0	0
Chtf8	227	0	1	5	1
Chtop	246	260	282	309	333
Chuk	72	86	68	100	109
Churc1	68	40	74	41	45
Ciao1	113	67	106	52	52
Ciapin1	70	19	0	53	81
Ciart	12	5	3	10	6
Cib1	60	35	30	73	16
Cib2	0	0	0	2	2
Cic	190	160	227	240	229
Ciita	3	2	0	1	6
Cilp2	4	11	0	0	0
Cinp	41	21	47	37	47
Cipc	81	36	44	73	64
Cir1	17	10	19	11	9
Cirbp	17	0	5	0	60
Cirh1a	55	43	36	31	27
Cisd1	56	36	57	28	42
Cisd2	94	98	161	102	142
Cisd3	10	10	16	6	5
Cisd3b	3	5	3	1	0
Cish	11	1	6	5	11
Cit	0	0	0	1	4
Cited2	1	1	0	150	0
Ciz1	213	3	0	97	84
CJ186046Rik	1	0	0	0	3
Ckap2	0	3	0	0	9
Ckap2l	13	8	6	10	10
Ckap4	142	21	24	24	24
Ckap5	69	67	102	64	77
Ckb	1426	1175	1397	0	956
Cklf	26	31	0	0	0
Cks1b	3	9	6	7	11
Cks2	10	6	4	7	7
Clasp1	93	84	78	115	111
Clasp2	526	475	673	656	588
Clasrp	55	8	113	97	0
Clcc1	132	129	164	122	145
Clcf1	0	64	77	0	1
Clcf1-pold4	1	0	0	0	0
Clcn2	0	0	0	4	0

For Review Only

Clcn3	93	87	93	135	152
Clcn4	460	333	433	431	506
Clcn5	58	71	71	100	54
Clcn6	133	66	81	105	114
Clcn7	362	214	308	312	344
Cldn1	3	1	0	0	0
Cldn12	6	3	23	34	24
Cldn15	0	0	0	0	0
Cldn2	17	0	8	0	0
Cldn22	0	0	5	0	0
Cldn34c1	1	1	0	6	4
Cldn5	0	0	0	1	0
Cldnd1	109	102	124	118	160
Clec11a	0	2	0	4	12
Clec12a	18	2	12	0	0
Clec14a	0	0	1	0	0
Clec16a	140	168	113	202	185
Clec1a	0	6	6	6	0
Clec1b	0	0	0	5	0
Clec2d	0	6	0	10	0
Clec2i	0	4	0	13	0
Clec4a1	22	5	15	10	0
Clec4a2	126	156	188	151	144
Clec4a3	227	381	423	292	342
Clec4a4	5	0	7	0	0
Clec5a	292	315	560	200	89
Clec7a	25	38	51	35	30
Clec9a	2	2	0	0	0
Clgn	28	24	28	36	51
Clic1	834	763	1120	794	846
Clic4	5	2	18	19	21
Clic5	0	1	0	0	0
Clic6	16	2	0	18	1
Clint1	77	97	95	75	87
Clip1	233	149	217	251	210
Clip2	77	42	28	72	66
Clip3	9	0	0	6	2
Clk1	0	0	3	35	1
Clk2	1	56	0	185	0
Clk2-scamp3	201	0	73	1	15
Clk3	2	2	38	329	0
Clk4	12	15	121	118	93
Clmn	3	0	0	0	0
Clmp	5	2	5	10	10
Cln3	4	1	8	24	20
Cln5	234	186	211	190	250
Cln6	36	3	25	31	36
Cln8	217	193	323	346	276
Clns1a	136	118	249	93	136
Clock	114	100	51	143	120

For Review Only

Clp1	73	53	86	42	82
Clpb	45	69	73	65	52
Clpp	86	52	0	0	0
Clptm1	270	105	252	183	188
Clptm1l	91	157	110	92	127
Clpx	105	113	140	80	111
Clspn	0	1	1	3	2
Clstn1	261	277	405	198	268
Clstn2	0	0	1	0	5
Clta	614	658	911	613	704
Cltb	67	55	86	46	68
Cltc	616	1069	581	1122	952
Clu	74	6	0	0	29
Cluap1	55	36	55	48	60
Cluh	85	102	77	84	65
Clybl	16	0	7	5	0
Cmas	17	23	24	22	10
Cmbl	2	0	0	0	0
Cmc1	16	15	25	12	8
Cmc2	16	9	8	8	10
Cmc4	7	5	14	7	0
Cmip	76	72	69	53	87
Cmklr1	117	84	127	252	233
Cmpk1	97	73	108	93	92
Cmpk2	2	2	8	0	16
Cmss1	4	3	3	7	7
Cmtm3	211	169	53	145	184
Cmtm4	98	50	79	104	69
Cmtm6	2572	1859	3041	2980	3178
Cmtm7	416	408	414	428	541
Cmtm8	0	0	9	8	2
Cmtr1	38	74	25	0	29
Cmtr2	35	19	32	20	16
Cmya5	20	40	25	59	62
Cnbd2	26	17	19	20	13
Cnbp	1	281	1198	29	441
Cndp2	368	247	518	378	365
Cnep1r1	55	57	89	64	75
Cnga2	1	1	0	0	0
Cnga4	2	2	7	4	5
Cnih1	195	150	193	123	169
Cnih4	35	101	170	133	150
Cnksr3	13	15	8	16	12
Cnn1	1	0	0	0	0
Cnn2	1	1	0	0	0
Cnn3	13	0	0	0	0
Cnnm2	26	25	21	43	19
Cnnm3	56	37	60	69	85
Cnnm4	127	128	169	119	121
Cnot1	124	185	137	202	192

For Review Only

Cnot10	117	0	122	139	57
Cnot11	29	0	1	1	1
Cnot2	293	285	308	302	354
Cnot3	110	76	54	84	119
Cnot4	0	0	1	3	4
Cnot6	39	56	41	64	68
Cnot6l	75	116	102	119	150
Cnot7	59	1	0	0	0
Cnot8	345	237	392	311	379
Cnot9	70	80	132	92	110
Cnp	233	198	225	196	226
Cnppd1	23	17	14	127	20
Cnpy2	70	145	123	72	82
Cnpy3	501	372	535	406	420
Cnpy4	26	18	49	20	45
Cnr2	3	3	155	48	0
Cnrip1	53	0	8	58	53
Cnst	68	6	27	50	27
Cntd1	4	0	0	0	3
Cntfr	0	0	0	0	0
Cntln	0	6	0	4	4
Cntn1	0	0	0	0	0
Cntnap1	0	0	4	0	1
Cntnap5b	1	1	0	1	3
Cntrl	92	122	125	72	60
Cntrob	46	32	19	23	24
Coa3	91	78	222	12	8
Coa4	46	61	56	44	36
Coa5	163	185	150	163	270
Coa6	11	16	29	18	20
Coa7	25	6	18	9	28
Coasy	1	1	281	0	112
Cobll1	0	0	0	3	0
Coch	0	0	0	0	0
Cog1	231	138	151	158	206
Cog2	49	57	96	107	100
Cog3	61	51	55	69	63
Cog4	104	43	126	32	39
Cog5	97	101	62	183	213
Cog6	92	38	52	79	69
Cog7	213	47	81	91	68
Coil	20	15	35	22	38
Col10a1	3	12	6	11	6
Col11a2	0	3	0	0	0
Col12a1	0	0	0	0	0
Col14a1	0	0	0	0	22
Col15a1	47	35	39	58	76
Col17a1	1	0	0	0	0
Col18a1	0	0	0	0	0
Col1a2	0	0	0	0	0

For Review Only

Col20a1	14	5	0	0	0
Col23a1	1	2	6	6	1
Col25a1	2	4	0	0	6
Col27a1	210	328	352	351	381
Col4a1	0	0	19	0	0
Col4a3	3	0	0	0	0
Col4a3bp	47	46	34	85	68
Col4a4	9	3	5	10	0
Col6a2	0	2	0	0	0
Col6a3	120	48	96	78	91
Col7a1	6	1	2	2	4
Col8a1	18	0	0	0	0
Col8a2	4	0	0	0	4
Col9a3	30	0	13	1	0
Colec12	0	3	0	0	0
Colgalt1	0	0	23	0	0
Colgalt2	0	1	0	0	4
Commd1	63	17	71	37	54
Commd10	74	56	89	61	69
Commd2	52	74	91	49	83
Commd3	75	125	163	123	107
Commd4	164	1	15	90	4
Commd5	45	46	0	0	0
Commd6	50	46	76	47	33
Commd7	0	50	201	21	15
Commd8	0	92	23	3	353
Commd9	88	74	116	54	79
Comp	6	2	0	0	0
Comt	721	594	1021	595	741
Comtd1	39	22	0	0	0
Copa	467	274	366	392	550
Copb1	249	207	306	252	307
Copb2	167	122	170	183	127
Cope	0	0	46	150	53
Copg1	397	147	279	210	254
Copg2	82	84	41	112	99
Coprs	33	8	21	28	24
Cops2	88	77	100	76	99
Cops3	110	144	160	121	154
Cops4	211	320	344	283	223
Cops5	81	108	100	69	68
Cops6	144	126	186	126	146
Cops7a	59	17	2	0	0
Cops7b	55	31	49	48	75
Cops8	0	0	0	0	0
Copz1	181	160	161	114	123
Copz2	4	5	2	4	4
Coq10a	35	5	24	15	5
Coq10b	30	30	49	43	43
Coq2	29	38	53	30	70

For Review Only

Coq3	19	12	24	12	22
Coq4	41	22	44	23	43
Coq5	73	71	90	61	83
Coq6	0	0	22	0	25
Coq7	27	10	23	9	9
Coq9	108	52	117	72	51
Corin	1	0	0	0	0
Coro1a	14	51	18	117	1
Coro1b	750	23	710	124	0
Coro1c	0	94	9	37	167
Coro2a	30	49	27	51	25
Coro6	1	4	0	5	0
Coro7	171	96	174	132	169
Cotl1	384	294	207	380	441
COX1	2953	1928	2749	2284	2476
Cox10	49	26	49	34	59
Cox11	22	14	0	28	21
Cox14	92	114	113	94	88
Cox15	55	28	18	33	59
Cox16	60	33	47	34	31
Cox17	0	46	73	15	11
Cox18	24	19	23	17	32
Cox19	2	10	77	0	0
COX2	682	382	712	471	504
Cox20	5	0	1	4	1
COX3	97	101	226	176	161
Cox4i1	588	280	438	373	445
Cox4i2	3	0	0	0	0
Cox5a	10	1	0	227	0
Cox5b	52	33	71	0	73
Cox6a1	170	1	0	0	0
Cox6a2	0	0	1	1	0
Cox6b1	182	114	191	117	149
Cox6c	0	0	124	0	0
Cox7a2	106	80	174	90	95
Cox7a2l	161	179	244	173	198
Cox7b	24	120	129	0	85
Cox7c	0	23	0	0	0
Cox8a	252	154	211	133	221
Cp	13	13	13	28	30
Cpd	40	55	31	68	59
Cpe	8	0	0	0	0
Cpeb2	26	64	47	61	103
Cpeb3	3	5	4	11	12
Cpeb4	50	51	26	64	44
Cplx2	5	0	0	1	1
Cpm	3	0	7	6	4
Cpn1	3	0	0	0	4
Cpn2	0	0	0	0	0
Cpne1	1	29	0	163	0

For Review Only

Cpne2	9	0	0	0	0
Cpne3	104	130	88	105	174
Cpne9	5	0	10	0	4
Cpox	11	21	15	11	8
Cpped1	38	43	46	71	90
Cpq	75	32	75	29	35
Cpsf1	1	2	0	0	0
Cpsf2	67	87	85	93	108
Cpsf3	184	178	322	258	359
Cpsf3l	0	0	8	1	1
Cpsf4	67	53	73	55	67
Cpsf4l	6	0	6	0	0
Cpsf6	116	143	129	226	197
Cpsf7	96	96	103	128	161
Cpt1a	80	42	43	84	102
Cpt1c	0	0	0	0	0
Cpt2	92	63	72	77	66
Cptp	20	29	0	0	100
Cr1l	76	257	101	68	0
Cr2	0	2	0	6	6
Cracr2a	15	11	24	25	24
Cracr2b	121	107	140	90	43
Cradd	44	48	50	39	51
Cramp1l	49	63	51	99	105
Crat	168	175	242	222	275
Crb1	1	2	0	0	6
Crb2	8	5	0	1	0
Crb3	34	42	13	18	51
Crbn	118	124	205	181	119
Crcp	64	82	83	43	50
Creb1	145	125	147	169	118
Creb3	0	0	84	1	1
Creb3l1	3	6	7	3	2
Creb3l2	166	204	205	207	231
Creb5	12	7	5	4	16
Crebbp	294	447	347	351	464
Crebl2	18	29	28	21	32
Crebrf	33	82	125	114	140
Crebzf	34	48	21	37	44
Creg1	735	573	994	700	780
Creld1	38	56	51	48	74
Creld2	193	104	185	129	172
Crem	26	13	24	13	32
Crim1	24	15	14	36	20
Crip1	0	0	0	0	0
Crip2	10	0	0	0	0
Cript	95	92	137	89	85
Crk	131	193	159	171	153
Crkl	199	177	254	288	290
Crlf2	53	26	35	31	39

For Review Only

Crlf3	381	360	386	393	424
Crls1	5	11	0	8	11
Crnde	0	0	3	1	0
Crnkl1	83	39	80	48	63
Crocc	5	0	0	15	18
Crot	86	101	91	122	75
Crtap	86	36	93	69	106
Crtc1	77	67	76	85	116
Crtc2	26	150	30	0	0
Crtc3	15	15	22	49	41
Cry1	23	26	37	19	4
Cry2	25	24	35	31	29
Cryab	6	0	0	0	10
Cryba4	0	39	55	1	25
Crybb1	37	162	197	262	107
Crybg3	33	91	81	106	74
Cryl1	317	222	353	259	310
Cryz	35	9	48	26	29
Cryzl1	43	63	65	37	63
Cs	234	197	247	248	246
Csad	96	89	180	53	90
Csde1	271	157	274	209	303
Cse1l	129	183	190	159	171
Csf1	76	18	11	27	48
Csf1r	24240	12368	35643	22117	22862
Csf2ra	3	1	0	0	0
Csf2rb	19	1	402	0	0
Csf2rb2	58	56	0	86	70
Csf3r	85	10	7	141	326
Csgalnact1	0	0	0	0	0
Csgalnact2	10	26	14	38	36
Csk	13	0	0	0	68
Csl	21	13	7	17	15
Csmd1	4	0	0	1	5
Csmd3	285	475	513	799	574
Csnk1a1	364	322	420	418	391
Csnk1d	268	174	266	221	268
Csnk1e	100	26	159	334	79
Csnk1g1	152	152	189	163	224
Csnk1g2	36	7	0	70	0
Csnk1g3	37	8	31	18	24
Csnk2a1	140	142	116	179	158
Csnk2a2	14	15	16	32	22
Csnk2b	107	86	127	98	96
Cspg5	3	0	0	0	5
Cspp1	22	22	40	45	61
Csrnp1	33	35	36	35	29
Csrnp2	32	6	16	18	17
Csrnp3	0	0	0	0	0
Csrp1	3	0	0	3	0

For Review Only

Csrp2	0	0	0	0	0
Csrp2bp	24	57	61	56	49
Cst3	55807	62069	86039	55350	67745
Cst6	0	1	0	0	0
Cst7	22	11	31	23	36
Cstb	0	72	66	1	10
Cstf1	102	59	157	123	152
Cstf2	22	24	54	57	35
Cstf2t	163	123	183	178	109
Cstf3	35	30	32	45	89
Ctage5	91	54	54	74	72
Ctbp1	6	17	0	0	0
Ctbp2	55	54	81	60	82
Ctbs	117	89	131	110	140
Ctc1	0	0	49	29	35
Ctcf	36	57	59	49	77
Ctdnep1	1	1	35	0	35
Ctdp1	78	30	35	71	88
Ctdsp1	0	0	58	0	60
Ctdsp2	0	239	41	1	1
Ctdspl	2	0	9	14	0
Ctdspl2	87	71	97	134	76
Ctif	17	15	6	22	16
Ctla2a	0	0	1	1	0
Ctnna1	188	109	136	103	141
Ctnnal1	25	61	64	47	48
Ctnnb1	19	265	205	197	0
Ctnnbip1	32	16	27	29	28
Ctnnbl1	101	60	119	59	85
Ctnnd1	231	242	215	366	227
Ctnnd2	7	18	29	27	30
Ctns	225	111	223	156	197
Ctps	17	2	38	21	15
Ctps2	145	126	134	152	156
Ctr9	51	24	104	90	109
Ctrl	0	0	0	0	0
Ctsa	3920	0	487	406	0
Ctsb	10174	5709	8640	7205	8266
Ctsc	1209	1152	1722	1204	1343
Ctsd	43326	25846	38105	28165	34468
Ctsf	0	0	0	0	0
Ctsg	0	0	0	0	0
Ctsh	210	966	2308	332	981
Ctsk	0	0	0	0	7
Ctsl	1715	1429	2294	1535	1781
Ctso	318	281	358	320	315
Ctss	11757	13219	18727	11252	12761
Ctsw	1	5	0	6	6
Ctsz	4630	2311	4593	2232	3031
Cttn	4	0	0	10	0

For Review Only

Cttnbp2nl	0	80	63	107	100
Ctu1	47	61	84	38	38
Ctu2	12	3	22	20	0
Ctxn1	3	2	4	0	1
Cuedc2	2	70	0	0	0
Cul1	225	200	256	367	304
Cul2	95	68	112	88	72
Cul3	59	58	45	52	77
Cul4a	77	119	111	140	158
Cul4b	76	16	33	60	0
Cul5	89	41	72	73	88
Cul7	0	20	32	29	39
Cul9	145	83	99	161	191
Cuta	0	0	0	0	0
Cutc	15	8	13	11	12
Cux1	306	332	279	379	382
Cux2	41	17	28	23	40
Cwc15	69	98	91	78	111
Cwc22	24	14	26	37	25
Cwc25	37	15	54	31	61
Cwc27	25	43	37	42	67
Cwf19l1	40	40	46	40	63
Cwf19l2	49	58	45	44	54
Cx3cl1	3	0	0	0	0
Cx3cr1	18497	14377	15991	18326	16752
Cxadr	1	0	0	0	0
Cxcl10	0	5	1	0	0
Cxcl12	1	1	0	27	0
Cxcl13	0	0	8	0	0
Cxcl16	0	0	1	5	1
Cxcl17	1	1	0	1	0
Cxcr2	5	1	0	0	0
Cxcr3	1	1	6	0	13
Cxcr5	0	1	0	4	2
Cxcr6	9	18	0	10	20
Cxx1a	5	5	2	10	7
Cxx1b	4	7	2	9	9
Cxx1c	40	16	38	20	22
Cxxc1	1	1	0	0	286
Cxxc4	1	0	0	1	0
Cxxc5	218	209	17	133	276
Cyb561	25	0	0	0	3
Cyb561a3	494	6	157	377	28
Cyb561d1	105	100	135	103	132
Cyb561d2	62	61	80	48	58
Cyb5a	237	206	249	214	246
Cyb5b	110	56	71	138	129
Cyb5d1	26	48	50	58	44
Cyb5d2	45	80	76	83	81
Cyb5r1	0	0	71	37	14

For Review Only

Cyb5r2	5	0	0	0	0
Cyb5r3	115	90	115	85	84
Cyb5r4	30	19	14	30	38
Cyb5rl	32	17	27	26	38
Cyba	813	569	957	573	718
Cybb	83	35	45	47	24
Cyc1	42	0	9	1	1
Cycs	40	39	74	1	22
Cyfip1	1576	1183	1816	1738	2259
Cyfip2	12	0	0	0	21
Cyhr1	118	137	147	134	178
Cyld	86	69	82	121	109
Cyp1b1	0	0	4	0	4
Cyp20a1	65	30	54	53	82
Cyp27a1	0	0	0	0	0
Cyp2d22	0	0	16	3	2
Cyp2e1	0	0	0	0	0
Cyp2j6	0	0	0	0	0
Cyp2r1	18	15	19	23	32
Cyp2t4	1	0	4	0	5
Cyp2u1	0	7	5	8	3
Cyp46a1	5	0	1	4	8
Cyp4f13	51	28	13	4	44
Cyp4f16	9	7	39	6	7
Cyp4f17	0	0	0	0	0
Cyp4f18	0	0	15	0	0
Cyp4f37	0	0	0	0	0
Cyp4v3	195	171	254	216	260
Cyp51	27	27	18	36	23
Cyr61	0	0	0	0	0
Cysltr1	188	20	241	284	158
CYTB	1928	1093	1541	1312	1557
Cyth1	106	94	119	86	146
Cyth2	31	0	0	54	0
Cyth3	164	0	0	134	138
Cyth4	349	167	592	249	833
Cytip	11	4	0	5	26
D030028A08Rik	132	76	89	104	142
D030047H15Rik	0	1	0	0	5
D030055H07Rik	2	0	1	1	0
D030056L22Rik	8	7	8	7	11
D10Jhu81e	12	30	23	16	18
D10Wsu102e	67	30	28	60	50
D11Wsu47e	75	64	79	78	124
D130007C19Rik	10	14	15	6	4
D130017N08Rik	4	3	7	17	11
D130020L05Rik	4	5	5	1	11
D130037M23Rik	1	1	0	5	1
D130040H23Rik	22	24	0	26	34
D16Ertd472e	6	9	8	9	18

For Review Only

D17H6S53E	1	23	0	0	152
D17H6S56E-5	232	0	0	0	0
D17Wsu92e	68	65	95	69	105
D1Ertd622e	137	110	126	133	122
D230017M19Rik	58	43	33	39	62
D230025D16Rik	76	82	92	100	100
D2hgdh	62	53	86	76	85
D2Wsu81e	53	48	48	38	71
D330023K18Rik	0	10	14	5	9
D330041H03Rik	3	0	0	4	0
D330045A20Rik	24	6	0	15	11
D330050I16Rik	5	0	0	0	4
D3Ertd254e	51	31	35	45	31
D3Ertd751e	29	21	23	40	33
D430020J02Rik	13	1	0	5	7
D430042O09Rik	28	56	37	65	34
D5Ertd579e	59	81	65	79	95
D5Ertd605e	23	35	18	8	19
D630033O11Rik	0	2	0	0	3
D630041G03Rik	6	1	6	1	0
D630045J12Rik	13	15	10	20	18
D6Wsu163e	149	126	141	98	114
D730005E14Rik	63	33	47	66	63
D7Ertd128e	7	0	6	5	0
D7Ertd443e	7	14	15	21	24
D830014E11Rik	3	0	0	0	0
D830030K20Rik	4	3	3	6	8
D830031N03Rik	38	48	40	80	63
D830044I16Rik	0	3	2	2	5
D830046C22Rik	3	14	9	5	12
D830050J10Rik	7	9	5	5	7
D8Ertd738e	113	5	144	0	0
D8Ertd82e	193	177	209	218	247
D930015E06Rik	119	117	111	184	219
D930016D06Rik	4	13	28	35	14
D930028M14Rik	5	3	7	4	0
D930048N14Rik	8	0	0	0	0
Daam1	16	17	16	54	33
Daam2	5	4	8	0	4
Dab2	10	3	16	32	26
Dab2ip	1	6	0	10	7
Dact1	4	0	1	0	0
Dact3	3	0	4	0	0
Dad1	211	237	279	183	275
Dag1	47	75	92	143	119
Dagla	164	112	117	144	172
Daglb	5	1	0	0	5555
Dalrd3	0	0	4	0	0
Dancr	8	18	24	0	12
Dand5	22	22	24	18	27

For Review Only

Dap	123	76	106	65	90
Dap3	25	35	49	29	39
Dapk1	10	21	21	29	14
Dapk2	5	0	0	0	0
Dapp1	262	212	329	257	330
Dars	109	141	141	104	98
Dars2	29	15	48	31	56
Daxx	8	74	62	71	0
Dazap1	8	0	0	0	0
Dazap2	0	323	828	175	24
Dbf4	14	5	11	4	11
Dbi	91	33	50	34	41
Dbndd1	0	2	0	4	10
Dbndd2	14	22	13	18	24
Dbnl	0	0	0	0	6
Dbp	4	6	0	11	2
Dbr1	25	39	32	19	45
Dbt	46	14	55	54	67
Dcaf10	32	65	24	26	23
Dcaf11	134	1	0	142	0
Dcaf12	304	264	363	362	455
Dcaf12l1	8	1	0	0	0
Dcaf13	66	70	120	53	22
Dcaf15	12	0	15	13	0
Dcaf17	52	1	44	78	30
Dcaf4	69	45	49	51	45
Dcaf5	57	60	83	80	78
Dcaf6	13	23	8	21	15
Dcaf7	269	219	247	258	292
Dcaf8	223	139	235	289	300
Dcakd	92	52	76	97	99
Dcbld2	125	147	129	129	154
Dcdc2b	4	6	6	13	5
Dchs1	0	7	8	0	0
Dck	16	22	35	37	65
Dclk1	8	0	0	3	0
Dclk2	13	1	13	9	1
Dclre1a	36	17	12	34	39
Dclre1b	92	50	43	58	72
Dclre1c	61	76	65	49	90
Dcn	0	0	0	0	0
Dcp1a	58	76	88	116	122
Dcp1b	49	52	40	27	30
Dcp2	17	30	33	31	35
Dcps	33	28	61	38	56
Dcst1	0	3	0	0	2
Dctd	16	2	29	16	14
Dctn1	159	93	103	124	155
Dctn2	0	0	2	59	247
Dctn3	6	3	1	40	10

For Review Only

Dctn4	313	269	346	352	395
Dctn5	426	456	532	452	553
Dctn6	59	59	83	17	19
Dctpp1	45	37	62	23	34
Dcun1d1	44	64	74	77	84
Dcun1d2	109	97	59	66	103
Dcun1d3	4	18	14	23	28
Dcun1d4	26	50	45	60	62
Dcun1d5	8	41	27	36	34
Dda1	193	0	165	4	153
Ddah1	1	1	0	0	0
Ddah2	100	52	72	0	72
Ddb1	356	145	233	332	320
Ddb2	10	16	0	0	0
Ddhd1	7	11	0	12	29
Ddhd2	202	183	187	203	129
Ddi2	4	34	0	62	73
Ddias	0	1	0	0	0
Ddit4l	0	1	0	0	0
Ddost	0	992	329	1	1
Ddr1	8	0	0	0	0
Ddr2	0	0	0	0	8
Ddrgk1	198	170	262	153	190
Ddt	20	19	19	21	21
Ddx1	95	110	175	125	115
Ddx10	21	90	66	44	85
Ddx11	11	3	19	35	33
Ddx17	74	370	0	0	598
Ddx18	45	37	55	30	49
Ddx19a	15	14	21	22	10
Ddx19b	20	6	25	41	16
Ddx20	46	47	63	60	57
Ddx21	88	70	86	109	111
Ddx23	29	34	19	51	49
Ddx24	249	201	207	177	187
Ddx26b	222	336	435	405	395
Ddx27	79	70	104	30	52
Ddx28	36	42	53	54	16
Ddx31	107	104	56	135	203
Ddx39	63	71	87	60	0
Ddx39b	416	274	366	307	350
Ddx3x	215	0	19	173	47
Ddx3y	93	39	89	110	107
Ddx42	147	133	142	270	127
Ddx43	0	0	0	0	0
Ddx46	91	67	82	89	116
Ddx47	12	73	183	162	78
Ddx49	0	27	0	0	0
Ddx5	0	0	2582	0	0
Ddx50	54	97	112	62	92

For Review Only

Ddx51	61	8	31	39	49
Ddx52	72	88	73	81	116
Ddx54	107	71	120	84	130
Ddx55	29	4	48	32	40
Ddx56	71	70	104	72	52
Ddx58	73	54	98	95	117
Ddx59	12	32	31	34	36
Ddx6	220	172	139	237	253
Ddx60	28	23	26	41	40
Deaf1	34	51	54	52	35
Deb1	0	0	14	0	0
Decr1	56	62	71	43	66
Decr2	20	9	41	22	57
Dedd	166	209	238	160	208
Dedd2	21	23	26	13	21
Def6	43	19	0	45	79
Def8	10	10	29	43	47
Degs1	230	165	261	170	206
Degs2	25	34	0	41	1
Dek	90	83	115	78	74
Dennd1a	131	26	43	57	75
Dennd1b	10	54	47	39	45
Dennd2a	5	22	9	12	12
Dennd2c	115	97	91	122	97
Dennd2d	24	8	4	9	0
Dennd3	6	0	22	0	11
Dennd4a	416	347	442	736	862
Dennd4b	256	150	0	241	0
Dennd4c	39	35	22	58	54
Dennd5a	175	196	236	175	183
Dennd5b	0	1	1	0	1
Dennd6a	65	98	64	107	122
Dennd6b	54	48	37	30	52
Denr	0	124	124	1	1
Depdc5	32	4	68	107	95
Depdc7	57	16	59	26	45
Deptor	42	20	35	44	40
Dera	15	35	18	30	20
Derl1	173	520	380	212	226
Derl2	182	173	187	184	169
Derl3	0	0	3	0	0
Desi1	61	28	34	39	38
Desi2	41	38	31	37	51
Det1	7	3	0	0	46
Dexi	7	40	25	23	12
Dffa	26	25	36	28	36
Dffb	36	15	29	19	8
Dfna5	10	34	14	20	8
Dgat1	70	53	52	38	51
Dgat2	7	8	8	0	8

For Review Only

Dgcr14	66	65	114	71	102
Dgcr2	316	301	219	313	372
Dgcr6	32	13	39	13	10
Dgcr8	68	96	90	75	111
Dgka	70	56	94	76	77
Dgkd	92	42	64	137	85
Dgke	22	10	15	24	20
Dgkg	0	3	6	0	1
Dgkh	38	15	25	46	27
Dgki	0	6	5	5	0
Dgkq	68	24	14	69	63
Dgkz	47	33	50	94	120
Dguok	61	7	2	17	11
Dhcr7	59	19	53	58	73
Dhdds	11	1	1	0	0
Dhdh	20	30	21	45	40
Dhfr	12	0	11	9	8
Dhh	0	0	0	0	0
Dhodh	23	23	39	27	41
Dhps	61	76	27	33	63
Dhrs1	54	52	53	41	68
Dhrs11	12	10	10	32	21
Dhrs13	0	0	0	0	0
Dhrs13os	0	0	0	0	0
Dhrs3	1117	777	1271	876	1029
Dhrs4	0	0	0	0	20
Dhrs7	154	112	180	115	126
Dhrs7b	72	108	143	91	95
Dhrsx	22	11	10	13	21
Dhtkd1	6	6	21	25	15
Dhx15	177	157	219	196	249
Dhx16	81	59	62	52	65
Dhx29	37	34	32	41	52
Dhx30	144	132	119	136	124
Dhx32	79	61	79	81	79
Dhx33	127	78	71	114	129
Dhx34	158	116	124	118	123
Dhx35	26	23	11	55	4
Dhx36	38	59	64	59	42
Dhx37	25	20	24	39	45
Dhx38	56	73	60	64	88
Dhx40	50	54	61	62	58
Dhx57	122	97	77	142	141
Dhx58	40	35	59	60	60
Dhx8	69	54	72	90	111
Dhx9	109	25	55	57	84
Diablo	0	0	0	0	0
Diaph1	6	18	25	24	15
Diaph2	50	96	84	122	124
Dicer1	63	59	59	85	100

For Review Only

Dido1	171	165	139	365	342
Diexf	44	43	23	40	55
Dimt1	0	2	40	51	0
Dip2a	3	9	35	74	64
Dip2b	377	575	469	706	600
Dip2c	3	7	14	19	30
Dirc2	46	48	63	46	80
Dis3	8	25	32	23	30
Dis3l	95	26	31	67	46
Dis3l2	60	53	83	115	111
Disc1	110	120	101	146	160
Disp1	73	25	46	46	63
Disp2	0	0	0	2	0
Dixdc1	1	0	0	0	0
Dkc1	81	29	70	62	70
Dlat	68	21	61	40	101
Dlc1	16	54	27	68	61
Dld	96	109	113	87	79
Dlec1	7	5	13	8	10
Dleu2	36	36	29	36	41
Dlg1	31	32	20	46	50
Dlg2	3	0	3	0	0
Dlg3	0	2	13	14	12
Dlg4	12	6	7	0	0
Dlg5	8	0	0	8	11
Dlgap2	0	3	0	0	0
Dlgap4	110	165	167	176	231
Dll1	3	2	0	9	5
Dlst	331	260	326	300	306
Dmap1	53	48	90	65	66
Dmpk	3	0	0	0	5
Dmrtb1	0	0	4	4	2
Dmtf1	208	127	115	214	216
Dmtn	26	14	31	19	18
Dmwd	1	0	0	0	21
Dmxl1	46	79	40	68	77
Dmxl2	31	21	16	50	50
Dna2	0	11	0	0	29
Dnaaf1	1	1	0	0	0
Dnaaf2	19	28	35	27	23
Dnaaf3	3	0	8	22	0
Dnaaf5	52	9	9	18	26
Dnah1	0	4	0	0	0
Dnah17	10	25	14	22	23
Dnah2	8	0	5	0	7
Dnah9	0	0	0	0	0
Dnaic1	0	28	0	0	0
Dnaic2	0	4	0	0	0
Dnaja1	0	0	0	4	4
Dnaja2	248	198	308	215	234

For Review Only

Dnaja3	120	138	145	116	140
Dnaja4	20	19	21	13	38
Dnajb1	0	0	0	0	0
Dnajb11	172	128	128	69	135
Dnajb12	55	54	48	58	101
Dnajb14	71	92	55	111	102
Dnajb2	42	25	36	47	47
Dnajb3	7	0	4	0	3
Dnajb4	43	44	40	19	42
Dnajb5	22	36	36	44	38
Dnajb6	97	100	79	118	111
Dnajb7	1	11	4	12	6
Dnajb9	178	161	249	218	176
Dnajc1	120	120	93	108	108
Dnajc10	117	50	118	98	142
Dnajc11	116	104	166	113	60
Dnajc12	2	13	10	6	0
Dnajc13	111	107	159	143	155
Dnajc14	208	136	190	201	218
Dnajc15	29	34	13	23	2
Dnajc16	85	103	65	88	100
Dnajc17	0	19	0	0	0
Dnajc18	140	116	169	124	144
Dnajc19	50	81	95	58	45
Dnajc19-ps	28	16	38	30	26
Dnajc2	52	40	48	22	0
Dnajc21	7	6	16	0	0
Dnajc24	38	40	60	42	40
Dnajc25	7	19	3	10	6
Dnajc27	23	13	17	24	29
Dnajc28	63	76	83	82	133
Dnajc3	303	187	271	215	213
Dnajc30	65	50	87	50	49
Dnajc4	1	2	0	0	0
Dnajc5	240	553	0	178	147
Dnajc7	11	22	171	144	0
Dnajc8	117	112	142	91	81
Dnajc9	1	1	178	0	0
Dnal1	2	2	10	0	12
Dnal4	73	64	65	45	60
Dnali1	4	0	4	0	0
Dnase1	0	0	3	0	0
Dnase1l1	12	5	0	0	0
Dnase1l3	1	5	4	8	1
Dnase2a	0	9	0	0	0
Dnd1	4	6	3	0	3
Dnhd1	0	0	0	39	0
Dnlz	21	14	0	0	0
Dnm1	2	2	0	0	0
Dnm1l	67	83	87	90	112

For Review Only

Dnm2	668	356	657	770	777
Dnm3	0	1	0	0	0
Dnmbp	60	88	48	84	67
Dnmt1	30	34	42	87	101
Dnmt3a	356	312	310	360	392
Dnmt3aos	9	0	2	4	3
Dnmt3b	8	2	45	22	0
Dnpep	72	1	0	51	0
Dnph1	5	1	2	2	2
Dnttip1	0	0	1	1	1
Dnttip2	159	106	143	100	138
Doc2g	1	1	25	0	0
Dock1	115	113	72	117	173
Dock10	296	579	590	917	564
Dock11	58	181	101	174	138
Dock2	585	310	543	642	650
Dock3	7	4	0	0	0
Dock4	291	275	279	525	484
Dock5	3	4	0	8	0
Dock6	45	12	19	31	46
Dock7	86	92	67	71	104
Dock8	914	556	569	1093	924
Dock9	87	90	72	145	112
Dok1	0	0	0	4	0
Dok2	0	4	5	6	3
Dok3	0	0	0	0	0
Dok4	0	0	4	2	2
Dolk	126	93	125	99	85
Dolpp1	14	32	0	0	0
Donson	17	7	26	31	26
Dopey1	53	114	58	140	128
Dopey2	139	78	83	141	148
Dot1l	86	69	60	96	84
Dpagt1	73	61	113	52	50
Dpcd	72	18	42	35	32
Dpf2	98	84	123	189	117
Dph1	106	0	0	0	75
Dph2	8	9	10	50	0
Dph3	33	19	48	46	37
Dph6	51	33	57	51	25
Dph7	36	5	31	27	20
Dpm1	10	2	6	0	17
Dpm1-adnp	194	267	317	265	264
Dpm2	0	0	0	0	0
Dpm3	39	41	57	26	37
Dpp3	155	106	146	105	129
Dpp7	0	0	35	0	0
Dpp8	77	108	96	143	103
Dpp9	70	49	101	83	121
Dpy19l1	39	96	67	50	50

For Review Only

Dpy19l3	131	10	42	67	52
Dpy19l4	1	207	0	0	205
Dpy30	34	26	33	27	40
Dpyd	0	0	5	0	0
Dpysl2	827	974	901	1094	1132
Dpysl5	0	6	0	0	0
Dqx1	7	11	11	10	11
Dr1	96	60	74	66	88
Dram1	0	0	0	0	0
Dram2	243	261	271	252	196
Drap1	0	0	1	2	0
Drd4	0	0	0	0	0
Drg1	167	134	205	116	151
Drg2	63	85	141	71	71
Drosha	45	38	39	54	60
Dscc1	0	0	0	0	0
Dscr3	130	137	171	142	174
Dse	40	39	59	55	70
Dsel	19	7	21	17	5
Dsn1	7	19	32	7	18
Dst	305	441	507	604	628
Dstn	245	217	285	181	204
Dstyk	114	123	86	147	113
Dtd1	48	15	41	17	25
Dtd2	6	25	53	15	38
Dtl	0	0	0	0	0
Dtna	0	0	0	0	6
Dtnb	12	4	9	23	14
Dtnbp1	16	47	22	22	21
Dtwd1	29	21	35	13	26
Dtwd2	11	7	13	4	16
Dtx2	50	53	79	62	49
Dtx3	92	35	49	128	110
Dtx3l	1	3	0	0	363
Dtx4	36	146	144	174	196
Dtymk	47	32	30	34	43
Dubr	37	16	25	24	21
Duoxa1	19	1	0	25	0
Dus1l	60	71	26	59	86
Dus2	43	14	22	19	29
Dus3l	120	83	150	121	146
Dus4l	9	4	0	8	16
Dusp1	0	0	0	0	0
Dusp10	7	0	5	8	9
Dusp11	166	232	211	243	246
Dusp12	77	78	93	75	80
Dusp13	0	0	4	1	0
Dusp16	53	29	54	49	71
Dusp18	25	18	29	9	16
Dusp19	3	5	14	0	11

For Review Only

Dusp2	9	3	0	0	0
Dusp22	103	82	98	66	111
Dusp23	7	0	4	5	10
Dusp27	46	57	67	78	34
Dusp28	11	6	0	7	0
Dusp3	322	307	312	284	355
Dusp4	3	3	1	0	0
Dusp6	0	0	0	513	0
Dusp7	0	53	251	92	50
Dusp8	12	1	0	6	6
Dut	6	0	0	2	1
Duxf3	6	7	1	4	2
Dvl1	62	50	84	40	52
Dvl2	51	14	8	20	33
Dvl3	33	10	0	0	139
Dxo	5	0	0	0	0
Dym	128	82	133	149	168
Dync1h1	307	367	391	504	436
Dync1i2	207	156	270	180	238
Dync1li1	137	102	107	125	177
Dync1li2	53	97	77	105	93
Dync2h1	12	14	35	17	30
Dync2li1	0	0	5	0	0
Dynll1	77	70	121	64	57
Dynll2	13	9	8	14	10
Dynlrb1	195	124	171	98	134
Dynlt1a	2	24	42	0	1
Dynlt1b	0	0	0	0	0
Dynlt1c	54	33	51	27	39
Dynlt1f	58	36	60	16	41
Dynlt3	66	45	90	66	62
Dyrk1a	164	149	164	175	148
Dyrk1b	142	104	100	156	149
Dyrk2	106	128	145	79	101
Dyrk4	28	29	42	27	33
Dyx1c1	1	1	0	0	18
Dzank1	0	0	0	5	20
Dzip1	2	0	0	5	1
Dzip1l	5	0	0	0	1
Dzip3	39	27	29	27	47
E030018B13Rik	930	49	164	14	428
E030024N20Rik	96	144	156	133	178
E030030I06Rik	0	0	0	0	2
E030042O20Rik	5	3	0	5	0
E130012A19Rik	3	1	4	0	1
E130102H24Rik	0	16	39	0	19
E130112N10Rik	9	11	10	14	18
E130201H02Rik	0	0	1	1	0
E130215H24Rik	3	7	5	14	11
E130307A14Rik	0	0	0	19	20

For Review Only

E130308A19Rik	42	29	30	26	43
E130309D02Rik	29	22	30	36	36
E130311K13Rik	27	16	25	29	28
E130317F20Rik	0	6	1	1	7
E230013L22Rik	13	17	9	20	26
E230016M11Rik	0	0	0	0	0
E2f1	0	0	0	0	0
E2f2	11	6	14	9	19
E2f3	82	100	100	92	111
E2f4	21	23	78	0	77
E2f5	12	20	12	15	0
E2f6	2	5	7	9	11
E2f8	0	0	0	0	0
E330009J07Rik	9	2	10	17	27
E330012B07Rik	0	0	0	0	8
E330020D12Rik	32	19	28	23	36
E330033B04Rik	95	69	75	66	125
E430024I08Rik	8	36	46	59	63
E430025E21Rik	141	82	87	125	103
E4f1	0	0	0	0	0
E530011L22Rik	2	2	1	6	0
Eaf1	71	84	79	122	101
Eapp	25	1	44	16	10
Ear2	0	5	0	1	6
Ears2	67	42	29	52	41
Ebag9	18	10	34	15	18
Ebf1	0	0	0	0	0
Ebf3	43	58	42	70	61
Ebi3	119	77	99	82	100
Ebna1bp2	26	29	47	26	55
Ebp	106	126	178	123	105
Ebpl	32	52	65	34	46
Ecd	85	92	125	77	74
Ece1	39	12	40	48	58
Ece2	15	18	38	21	31
Ech1	0	0	0	0	0
Echdc1	30	0	11	16	58
Echdc3	14	0	0	0	0
Echs1	0	0	0	0	0
Eci1	0	79	13	1	1
Eci2	132	96	149	95	110
Ecm1	0	3	0	5	0
Ecm2	0	0	0	0	0
Ecscr	315	443	836	180	205
Ecsit	48	37	56	44	38
Ect2	0	0	7	0	0
Ect2l	0	1	1	1	0
Edaradd	2	0	0	0	0
Edc3	125	80	120	97	125
Edc4	137	89	70	187	119

For Review Only

Edem1	430	622	485	759	646
Edem2	1510	19	237	0	1034
Edem3	44	73	39	62	31
Edf1	116	76	137	86	110
Edn1	6	0	11	0	5
Edn3	0	0	0	1	0
Ednrb	4	0	0	5	5
Edrf1	29	68	46	54	41
Eea1	30	45	37	69	49
Eed	122	17	72	39	42
Eef1akmt1	40	30	29	19	27
Eef1b2	12	0	4	0	5
Eef1d	25	0	2	1	0
Eef1e1	25	31	31	45	33
Eef1g	125	94	107	94	116
Eef2	0	0	0	398	65
Eef2k	417	291	510	409	465
Eef2kmt	63	112	86	0	140
Eefsec	120	65	119	98	112
Eepd1	57	44	32	56	58
Efcab1	7	0	0	0	0
Efcab11	1	1	1	1	1
Efcab12	0	0	0	0	0
Efcab14	101	135	141	122	166
Efcab2	32	2	0	1	10
Efcab6	0	0	0	0	9
Efemp1	0	0	0	0	0
Efemp2	35	0	0	0	47
Efhb	0	0	0	0	0
Efhc1	0	0	5	0	0
Efhd2	307	545	394	371	467
Efl1	62	50	59	64	75
Efna1	0	0	6	0	0
Efna4	8	0	2	0	4
Efna5	4	2	0	0	1
Efnb1	29	16	19	43	42
Efnb2	8	8	0	1	9
Efnb3	0	0	0	2	3
Efr3a	309	80	141	235	165
Efr3b	0	0	1	0	0
Efs	7	0	0	0	0
Eftud2	130	75	87	96	98
Egf	0	0	0	0	9
Egfl7	2	2	0	0	6
Egfl8	0	6	1	0	0
Egln1	12	14	38	5	31
Egln2	125	92	51	105	96
Egln3	5	0	0	3	0
Egr1	1189	0	654	0	1617
Egr2	15	10	30	38	74

For Review Only

Egr3	10	3	1	0	19
Ehbp1	18	23	12	33	20
Ehbp1l1	167	170	177	118	87
Ehd1	11	6	0	6	15
Ehd2	0	0	0	0	0
Ehd4	503	230	370	414	469
Ehhadh	5	17	0	17	14
Ehmt1	119	51	98	168	159
Ehmt2	81	74	150	42	136
Ei24	24	28	30	31	36
Eid1	12	18	18	8	14
Eid2	0	3	4	1	0
Eid2b	29	18	26	13	18
Eid3	5	3	2	4	2
Eif1a	87	93	87	96	97
Eif1ad	75	70	0	0	0
Eif1ax	30	20	33	21	15
Eif1b	135	92	100	112	112
Eif2a	77	72	82	70	108
Eif2ak1	109	129	120	125	159
Eif2ak2	43	61	45	54	84
Eif2ak3	44	90	87	68	43
Eif2ak4	53	63	40	91	78
Eif2b1	46	9	66	53	0
Eif2b2	18	89	113	36	74
Eif2b3	27	34	42	38	32
Eif2b4	0	0	15	0	0
Eif2b5	39	59	35	28	33
Eif2d	55	62	62	68	88
Eif2s1	97	115	119	141	112
Eif2s2	41	107	96	64	51
Eif2s3x	68	42	67	53	58
Eif2s3y	109	61	124	91	33
Eif3a	179	223	196	220	207
Eif3b	5	11	77	54	56
Eif3c	257	277	311	237	294
Eif3d	134	156	231	124	146
Eif3e	83	103	143	104	85
Eif3f	156	120	141	97	133
Eif3h	151	119	176	126	156
Eif3i	125	19	129	112	119
Eif3j1	24	28	19	31	27
Eif3j2	32	23	19	26	24
Eif3k	87	77	195	93	121
Eif3l	204	249	269	193	223
Eif3m	136	112	147	116	87
Eif4a2	0	0	0	0	0
Eif4a3	104	52	54	22	69
Eif4b	210	181	333	239	283
Eif4e	128	110	155	107	148

For Review Only

Eif4e2	95	69	74	59	229
Eif4e3	48	53	75	70	54
Eif4ebp1	36	42	94	35	70
Eif4ebp2	92	125	103	126	130
Eif4ebp3	17	22	1	1	1
Eif4enif1	72	98	85	73	61
Eif4g1	269	246	190	184	250
Eif4g2	464	2	487	648	74
Eif4g3	195	195	218	176	197
Eif4h	0	0	2	162	1
Eif5	0	0	76	0	0
Eif5a	337	278	431	301	346
Eif5a2	7	10	15	34	54
Eif5b	100	108	105	78	99
Eif6	92	108	148	87	118
Elac1	168	64	117	105	74
Elac2	37	34	25	49	43
Elane	0	0	0	0	0
Elavl1	216	177	195	250	258
Elavl4	7	15	15	13	8
Eldr	0	0	0	0	7
Elf1	171	153	159	210	216
Elf2	52	121	73	165	133
Elf4	129	136	181	191	194
Elfn1	0	0	5	0	0
Elfn2	0	0	0	0	2
Elk1	15	9	13	5	14
Elk3	122	150	104	179	143
Elk4	79	110	54	103	117
Ell	86	59	76	100	94
Ell2	12	3	5	3	8
Ell3	2	0	0	5	0
Elmo1	1434	1095	1456	1490	1578
Elmo2	172	188	178	222	159
Elmo3	24	15	13	19	14
Elmod2	90	65	64	101	141
Elmod3	53	25	49	39	32
Elmsan1	88	155	68	203	139
Elof1	54	0	74	7	6
Elovl1	2	1	452	353	0
Elovl2	0	0	0	2	0
Elovl5	82	53	107	97	129
Elovl6	1	4	3	2	7
Elovl7	24	0	0	0	0
Elp2	226	117	217	125	155
Elp3	74	60	78	66	68
Elp4	18	30	13	20	20
Elp5	56	50	78	52	43
Elp6	5	1	19	10	9
Emb	9	3	8	0	0

For Review Only

Emc1	136	113	146	219	210
Emc10	1	104	75	119	0
Emc2	116	50	118	94	65
Emc3	190	285	403	217	267
Emc4	95	85	111	61	84
Emc6	0	0	85	11	11
Emc7	200	172	210	151	188
Emc8	38	59	51	97	78
Emc8-1190005i06rik	0	0	15	0	0
Emc9	11	9	30	19	10
Emcn	0	0	0	0	0
Emd	5	26	0	32	17
Eme1	0	0	0	0	4
Eme2	16	0	2	3	0
Emg1	24	0	0	0	0
Emilin1	5	0	0	7	0
Emilin2	0	1	2	3	5
Eml1	0	0	0	0	0
Eml2	2	2	0	6	0
Eml3	0	1	0	0	0
Eml4	114	108	105	99	100
Eml5	15	18	16	32	44
Eml6	63	36	35	70	47
Emp2	6	7	19	14	0
Emp3	0	1	0	0	0
Emsy	112	106	100	143	156
Emx2os	0	4	0	1	1
Enah	4	3	0	7	0
Enam	0	0	0	3	3
Enc1	69	123	77	119	112
Endod1	152	149	182	270	320
Endog	0	0	0	3	2
Endov	36	7	0	52	0
Eng	588	417	668	567	666
Engase	0	102	114	57	63
Enkd1	0	0	3	0	0
Eno1	104	0	0	0	0
Eno1b	220	131	211	142	181
Eno2	3	2	0	0	0
Eno3	0	27	0	0	53
Eno4	15	38	52	69	66
Enoph1	118	72	157	90	78
Enox2	30	22	26	28	56
Enpp1	24	12	35	40	27
Enpp2	1099	28	0	284	75
Enpp4	37	27	28	64	63
Enpp5	81	15	21	37	29
Ensa	36	78	84	54	71
Enthd2	95	46	89	23	84
Entpd1	3466	2371	3243	3238	3660

For Review Only

Entpd3	2	2	0	0	0
Entpd4	43	79	0	35	51
Entpd5	22	31	17	28	33
Entpd6	71	59	59	67	100
Entpd7	10	26	9	38	33
Eny2	36	19	0	25	24
Eogt	98	27	71	88	89
Ep300	457	478	352	548	494
Ep400	330	230	255	337	317
Epas1	1	0	0	0	52
Epb41	121	36	104	124	129
Epb41l1	4	3	0	11	6
Epb41l2	2138	554	1755	2191	2279
Epb41l3	98	68	57	58	80
Epb41l4a	0	2	0	6	6
Epb41l4aos	21	17	31	17	17
Epb41l5	5	3	12	16	14
Epc1	111	21	0	175	120
Epc2	54	61	61	45	42
Epdr1	5	0	0	0	0
Epg5	46	54	47	95	96
Epha1	0	0	0	0	0
Epha2	58	44	90	52	72
Epha6	0	4	0	1	2
Ephb3	8	5	22	21	20
Ephb4	0	0	0	0	0
Ephb6	0	0	0	1	0
Ephx1	36	26	31	17	15
Epm2a	0	0	0	0	0
Epm2aip1	30	60	58	64	110
Epn1	57	7	37	86	67
Epn2	117	94	80	107	89
Epn3	5	0	0	0	0
Epor	16	3	5	0	0
Eppk1	0	4	5	8	0
Eprs	239	140	191	175	181
Eps15	143	122	126	132	147
Eps15l1	187	131	174	242	203
Eps8	8	2	9	0	0
Eps8l1	27	3	37	32	46
Eps8l2	4	0	0	0	0
Epsti1	62	47	62	60	61
Ept1	51	37	33	41	59
Eral1	0	0	0	0	0
Erap1	184	201	192	243	235
Erbb2	8	1	0	0	0
Erbb2ip	51	57	59	111	88
Erc1	1	1	15	17	0
Ercc1	23	9	34	0	35
Ercc2	41	82	76	73	98

For Review Only

Ercc3	32	17	26	49	37
Ercc4	69	47	77	92	52
Ercc5	170	105	126	73	118
Ercc6	6	14	24	21	35
Ercc6l	0	0	0	7	0
Ercc6l2	19	18	14	36	32
Ercc8	15	33	35	27	24
Erdr1	84	297	239	95	0
Ergic1	104	107	121	115	108
Ergic2	115	110	93	1	23
Ergic3	479	305	431	178	362
Erh	41	31	68	41	52
Eri1	46	40	63	57	51
Eri2	16	23	7	26	32
Eri3	116	107	109	115	121
Erich1	21	25	19	22	17
Erlec1	29	72	33	50	42
Erlin1	119	37	107	91	77
Erlin2	149	1	259	47	262
Ermap	2	21	33	40	35
Ermard	51	78	61	82	90
Ermp1	55	37	55	53	83
Ern1	15	15	19	46	43
Ero1l	41	24	16	28	37
Ero1lb	19	51	33	50	29
Erp27	0	2	1	0	0
Erp29	379	761	287	203	0
Erp44	142	125	190	146	171
Errfi1	118	65	129	124	88
Esam	0	0	0	0	54
Esco1	109	84	80	109	76
Esco2	0	0	6	0	0
Esd	72	46	73	0	62
Esf1	24	22	38	19	30
Espl1	88	64	83	82	121
Espn	0	0	3	0	0
Esr1	33	28	23	29	41
Esrp2	1	0	0	0	0
Esrra	1	1	0	4	4
Esrrg	4	0	0	5	0
Esyt1	73	65	66	101	74
Esyt2	65	71	63	92	97
Esyt3	0	0	0	6	0
Etaa1	37	19	0	23	16
Etf1	56	67	86	64	47
Etfa	69	0	65	19	7
Etfb	98	59	92	73	108
Etfdh	135	91	122	80	77
Ethe1	51	0	2	2	1
Etl4	4	4	3	6	1

For Review Only

Etnk1	99	111	132	144	132
Etohd2	24	20	21	34	48
Ets1	413	246	426	403	400
Ets2	48	33	39	82	62
Etv1	70	53	75	63	67
Etv3	103	159	109	155	171
Etv5	367	309	446	437	555
Etv6	100	71	89	147	164
Eva1a	48	24	69	39	59
Eva1b	3	23	0	0	42
Evi2a	477	671	960	276	206
Evi2b	0	0	0	0	0
Evi5	94	195	95	114	120
Evi5l	43	31	55	27	38
Evl	57	133	79	0	119
Ewsr1	60	108	102	251	190
Exd1	0	1	5	0	0
Exd2	87	60	97	84	98
Exo5	46	33	79	50	63
Exoc1	79	37	71	80	104
Exoc2	103	111	96	160	156
Exoc3	195	140	249	239	264
Exoc3l	0	3	3	0	4
Exoc3l2	0	0	0	0	0
Exoc3l4	3	1	0	5	0
Exoc4	137	144	169	225	242
Exoc5	118	52	87	118	67
Exoc6	139	217	182	237	206
Exoc6b	59	99	83	157	157
Exoc7	135	0	175	146	115
Exoc8	127	147	167	100	175
Exog	90	70	79	65	134
Exosc1	1	112	0	0	0
Exosc10	220	133	264	3	186
Exosc2	34	24	37	20	38
Exosc3	20	28	36	26	20
Exosc4	119	86	154	81	116
Exosc5	8	4	0	0	72
Exosc6	6	4	4	3	4
Exosc7	35	26	35	51	32
Exosc8	0	15	12	4	61
Exosc9	0	45	64	0	27
Exph5	2	0	0	0	0
Ext1	26	9	26	17	14
Ext2	250	141	224	199	191
Extl2	27	42	60	47	96
Extl3	607	394	463	657	627
Eya2	0	0	0	0	0
Eya3	77	86	78	91	84
Eya4	79	58	53	86	85

For Review Only

Ezh1	162	139	116	207	183
Ezh2	4	0	0	0	0
Ezr	60	1	17	11	0
F11r	1711	1492	2198	1311	1397
F13a1	11	15	27	10	0
F2	0	0	0	0	1
F2rl3	0	0	0	8	0
F3	0	10	11	0	7
F420014N23Rik	3	4	0	1	0
F420015M19Rik	151	177	104	234	204
F5	33	1	0	45	0
F630028O10Rik	0	0	8	5	7
F630048H11Rik	11	16	23	27	22
F630111L10Rik	0	2	0	0	0
F630206G17Rik	0	0	0	0	0
F730016J06Rik	0	0	0	0	0
F730043M19Rik	0	2	0	3	0
F730311O21Rik	0	1	0	0	0
F8	0	4	0	0	0
F830016B08Rik	1	0	0	4	0
F830045P16Rik	0	0	0	1	0
F830208F22Rik	4	0	0	1	2
F8a	35	12	43	22	37
F9	5	12	14	15	38
Faap100	62	59	77	75	72
Faap20	16	14	0	128	0
Faap24	25	31	11	18	35
Fabp12	0	0	0	0	0
Fadd	42	51	44	60	62
Fads1	355	0	29	50	173
Fads3	85	11	48	30	51
Fads6	2	3	0	9	15
Faf1	29	41	15	37	43
Faf2	179	121	165	230	209
Fahd1	8	7	24	19	13
Fahd2a	21	0	9	0	0
Faim	33	33	53	41	35
Fam101a	28	3	0	15	11
Fam101b	7	0	0	0	3
Fam102a	37	54	28	20	39
Fam102b	424	541	498	533	502
Fam103a1	45	79	87	34	54
Fam104a	75	58	81	66	71
Fam105a	863	1073	570	1094	1297
Fam107a	7	0	0	0	3
Fam107b	82	51	129	140	109
Fam109a	34	22	40	18	27
Fam109b	1	0	0	10	0
Fam110a	276	196	340	195	269
Fam111a	1	25	0	203	208

For Review Only

Fam114a1	35	27	47	46	23
Fam114a2	244	186	227	169	158
Fam117a	32	15	14	18	11
Fam117b	63	81	90	98	132
Fam118a	40	32	50	24	30
Fam118b	0	0	45	0	8
Fam120a	116	126	139	138	138
Fam120aos	0	4	2	5	2
Fam120b	304	204	336	247	299
Fam120c	32	47	46	64	49
Fam122a	6	9	7	8	19
Fam122b	9	27	25	14	7
Fam124a	0	0	4	0	0
Fam126a	39	74	57	82	67
Fam126b	55	40	53	99	77
Fam129a	71	75	83	106	74
Fam129b	36	19	31	28	45
Fam129c	5	4	0	1	0
Fam131a	52	36	35	45	78
Fam131b	0	0	0	0	0
Fam132b	0	0	3	0	4
Fam133b	22	40	38	32	33
Fam134a	64	69	0	0	0
Fam134b	102	192	119	96	145
Fam134c	132	69	47	107	124
Fam135a	42	24	34	45	28
Fam135b	3	0	10	5	0
Fam136a	40	46	61	54	23
Fam13a	12	5	14	12	18
Fam13b	97	113	73	89	99
Fam149a	12	11	12	12	0
Fam149b	32	33	43	23	29
Fam160a1	3	0	3	0	0
Fam160a2	122	64	101	97	114
Fam160b1	43	74	26	52	62
Fam160b2	70	78	48	72	116
Fam161a	6	9	7	9	15
Fam161b	0	0	0	5	0
Fam162a	29	19	47	31	21
Fam167a	1	0	0	0	6
Fam167b	1	1	315	0	0
Fam168a	120	286	203	237	222
Fam168b	168	190	269	215	243
Fam169a	2	0	0	8	1
Fam171a1	1	2	6	8	7
Fam172a	130	125	119	135	129
Fam173a	76	34	248	83	0
Fam173b	13	19	33	27	30
Fam174a	56	37	41	38	80
Fam175a	0	0	6	2	3

For Review Only

Fam175b	133	218	197	213	197
Fam177a	31	18	28	20	23
Fam178a	1	14	0	516	0
Fam178b	6	0	0	0	0
Fam179b	54	58	32	66	105
Fam180a	0	0	0	0	0
Fam181b	1	0	2	2	0
Fam185a	18	23	23	16	60
Fam188a	78	55	64	72	69
Fam188b	1	12	23	14	23
Fam189b	13	6	0	8	11
Fam192a	83	66	95	52	79
Fam193a	87	142	157	149	152
Fam193b	56	64	81	78	66
Fam195a	23	0	12	7	9
Fam195b	225	120	180	124	164
Fam196a	24	11	14	21	23
Fam196b	122	102	83	114	154
Fam198a	1	2	0	6	0
Fam198b	7	4	0	5	0
Fam199x	25	23	29	29	28
Fam19a1	5	6	8	5	14
Fam19a2	0	0	0	0	0
Fam19a3	0	0	2	2	2
Fam204a	26	18	29	20	33
Fam206a	54	9	46	40	31
Fam207a	74	51	48	65	68
Fam208a	113	137	105	163	158
Fam208b	138	133	81	127	170
Fam209	0	4	0	0	0
Fam20a	0	0	0	1	0
Fam20b	64	44	85	68	90
Fam20c	25	10	25	11	25
Fam21	185	110	174	125	198
Fam210a	92	91	87	155	108
Fam210b	55	40	80	22	55
Fam212a	155	242	295	182	227
Fam212b	1	5	3	16	9
Fam213a	28	7	8	9	26
Fam213b	48	0	53	26	51
Fam214a	49	73	67	62	66
Fam214b	2	2	0	41	32
Fam216a	11	15	7	9	0
Fam217b	139	36	65	124	130
Fam219a	51	22	23	57	54
Fam219b	70	216	288	0	385
Fam220a	13	45	11	15	12
Fam222a	0	1	2	2	4
Fam222b	90	57	62	50	62
Fam227a	2	2	4	0	6

For Review Only

Fam228a	0	0	1	3	0
Fam228b	1	7	0	5	8
Fam234a	90	83	124	79	66
Fam234b	117	122	98	131	80
Fam26f	19	12	50	27	39
Fam35a	75	34	21	66	53
Fam3a	0	0	27	2	2
Fam3c	70	76	59	89	60
Fam43a	9	3	0	1	15
Fam45a	129	125	158	152	114
Fam46a	81	37	39	37	211
Fam46c	265	189	331	353	427
Fam49a	44	75	34	51	57
Fam49b	326	380	243	429	490
Fam50a	1	15	0	0	84
Fam53a	103	74	136	72	114
Fam53b	211	187	317	365	177
Fam53c	21	37	37	44	44
Fam57a	5	3	4	4	0
Fam58b	104	76	142	95	96
Fam60a	15	15	21	13	18
Fam63a	43	50	38	44	49
Fam63b	68	208	110	71	79
Fam64a	0	0	0	0	0
Fam65a	93	21	88	103	31
Fam65b	16	0	13	17	26
Fam65c	2	2	0	4	4
Fam69a	74	88	98	69	71
Fam69b	8	8	0	10	8
Fam69c	0	0	0	2	7
Fam71a	0	0	0	0	4
Fam71e1	0	2	0	0	6
Fam71f2	2	0	0	0	0
Fam72a	1	1	0	0	67
Fam73a	17	20	28	19	17
Fam73b	45	1	0	33	34
Fam76a	69	34	48	56	59
Fam76b	42	78	88	87	58
Fam78a	67	33	48	75	60
Fam81a	24	0	0	4	0
Fam83d	0	0	1	0	0
Fam83g	22	23	15	43	27
Fam83h	7	13	8	16	4
Fam84b	58	33	10	40	40
Fam89a	7	4	7	7	7
Fam89b	0	144	45	0	17
Fam8a1	14	29	27	25	22
Fam91a1	304	398	354	415	389
Fam92a	1	1	15	10	11
Fam96a	69	61	61	46	68

For Review Only

Fam98a	36	25	41	43	23
Fam98b	25	3	8	0	0
Fam98c	0	2	16	0	0
Fan1	24	7	15	33	27
Fanca	0	0	0	11	0
Fancb	7	2	0	9	5
Fancc	25	32	31	41	58
Fancd2	1	2	0	21	0
Fance	77	41	38	56	52
Fancf	1	2	7	0	0
Fancg	46	19	56	66	0
Fanci	0	0	0	0	0
Fancl	3	4	33	35	0
Fancm	12	18	0	15	15
Far1	162	112	126	163	128
Far1os	19	0	0	0	5
Far2	0	0	0	0	3
Farp1	12	14	8	9	16
Farp2	0	0	0	6	13
Fars2	60	57	54	62	83
Farsa	0	0	0	0	0
Farsb	72	86	108	74	84
Fas	6	0	0	3	4
Fasn	76	14	27	0	28
Fastkd1	11	14	20	0	13
Fastkd2	22	14	23	43	66
Fastkd3	18	22	14	24	31
Fastkd5	19	28	58	25	16
Fat1	11	5	12	15	21
Fat2	4	2	0	0	0
Fat3	84	131	122	185	132
Fat4	3	2	0	4	0
Fau	480	221	450	262	256
Fbf1	60	59	60	67	81
Fbl	0	0	0	0	0
Fblim1	61	24	36	43	88
Fbln1	7	1	0	0	4
Fbrs	85	0	107	66	60
Fbrsl1	115	67	117	244	51
Fbxl12	82	74	131	95	78
Fbxl12os	6	1	5	0	12
Fbxl14	34	46	20	50	53
Fbxl15	13	19	28	11	24
Fbxl16	3	1	0	0	0
Fbxl17	42	77	42	76	89
Fbxl18	20	10	29	38	24
Fbxl19	13	10	12	11	16
Fbxl2	2	3	7	0	0
Fbxl20	105	96	98	141	155
Fbxl21	7	3	11	6	4

For Review Only

Fbxl22	8	5	9	18	7
Fbxl3	39	73	84	92	63
Fbxl4	149	86	93	130	118
Fbxl5	89	178	109	189	194
Fbxl6	0	0	1	123	114
Fbxl8	17	45	11	12	10
Fbxo10	33	20	10	44	89
Fbxo11	57	0	0	75	0
Fbxo17	11	1	0	15	0
Fbxo18	195	139	220	229	243
Fbxo21	78	16	28	47	52
Fbxo22	74	102	136	86	97
Fbxo24	2	1	3	7	1
Fbxo25	62	38	63	30	36
Fbxo28	34	43	21	41	32
Fbxo3	85	38	99	105	111
Fbxo30	33	30	37	40	54
Fbxo31	9	15	30	16	21
Fbxo32	22	44	20	40	42
Fbxo33	31	21	13	24	15
Fbxo34	78	68	93	58	67
Fbxo36	2	2	5	3	3
Fbxo38	175	160	178	235	274
Fbxo4	17	5	0	13	13
Fbxo40	21	36	26	24	28
Fbxo42	137	106	61	152	100
Fbxo44	2	3	0	6	19
Fbxo45	32	11	35	18	12
Fbxo46	59	41	74	41	39
Fbxo47	1	0	0	1	0
Fbxo48	0	7	3	9	4
Fbxo5	20	5	21	16	17
Fbxo6	42	44	49	18	100
Fbxo7	62	62	122	81	88
Fbxo8	69	36	61	50	59
Fbxo9	52	31	47	42	50
Fbxw10	14	6	15	18	10
Fbxw11	304	125	171	240	256
Fbxw17	14	16	11	10	19
Fbxw2	121	80	96	120	76
Fbxw4	99	155	204	107	101
Fbxw7	34	67	42	35	18
Fbxw8	35	24	43	29	50
Fbxw9	10	1	0	0	0
Fcer1g	2024	2110	3087	1955	2400
Fcer2a	4	0	0	0	5
Fcf1	35	0	102	0	0
Fcgr1	0	0	18	1	1
Fcgr2b	0	0	1	1	1729
Fcgr3	2778	2230	3420	1665	2066

For Review Only

Fcgr4	27	24	0	0	0
Fcgrt	141	151	268	155	163
Fcho2	124	186	79	180	140
Fchsd1	34	17	26	39	37
Fchsd2	272	413	326	444	458
Fcna	0	0	0	0	0
Fcnb	2	1	0	0	0
Fcrl1	166	84	188	122	127
Fcrlb	4	2	0	6	11
Fcrls	6939	4593	7167	5824	7484
Fdft1	47	23	45	18	34
Fdps	41	26	32	18	25
Fdx1	29	27	15	25	32
Fdxacb1	31	27	17	32	17
Fdxr	19	19	0	35	29
Fech	139	107	130	83	117
Fem1a	130	173	219	177	216
Fem1b	78	89	45	64	87
Fem1c	45	91	40	115	104
Fen1	26	20	50	31	38
Fendrr	1	0	0	0	0
Fer	139	105	103	154	169
Fer1l5	0	15	42	18	8
Fermt2	0	0	0	0	0
Fermt3	0	0	0	0	0
Fes	0	0	0	0	0
Fez2	455	358	438	322	364
Fgd2	0	0	0	0	0
Fgd3	93	75	110	115	104
Fgd4	90	49	82	126	136
Fgd5	0	3	0	0	0
Fgd6	9	0	2	8	13
Fgf1	9	9	1	4	0
Fgf11	51	27	43	45	37
Fgf12	1	0	0	1	0
Fgf13	15	12	32	16	21
Fgf14	0	0	0	0	0
Fgf2os	1	0	0	0	7
Fgfbp3	6	0	0	0	4
Fgfr1	0	0	0	0	0
Fgfr1op	55	99	114	102	93
Fgfr1op2	207	139	201	206	200
Fgfr2	6	0	0	4	0
Fgfr3	2	0	0	0	0
Fgfrl1	4	8	15	13	6
Fggy	76	96	62	59	93
Fgl2	24	20	25	36	34
Fgr	4	20	0	0	0
Fh1	81	67	114	92	82
Fhad1	26	11	23	0	29

For Review Only

Fhit	11	18	16	20	25
Fhl3	15	0	14	41	5
Fhl4	1	7	0	4	6
Fhod1	43	60	22	69	93
Fibp	0	0	0	0	0
Ficd	71	47	79	41	89
Fig4	86	73	112	77	79
Fignl1	2	0	1	9	0
Fignl2	3	0	2	4	6
Filip1l	133	105	142	124	121
Fip1l1	120	124	120	153	141
Firre	185	104	102	178	113
Fis1	117	106	158	100	107
Fitm2	4	15	6	20	12
Fiz1	119	80	70	51	67
Fkbp10	0	0	0	7	0
Fkbp14	26	13	39	22	24
Fkbp15	324	182	355	339	397
Fkbp1a	40	211	31	5	1
Fkbp1b	6	0	7	2	6
Fkbp2	11	58	62	3	1
Fkbp3	34	26	78	25	27
Fkbp4	130	149	312	103	82
Fkbp5	192	56	39	32	115
Fkbp7	4	100	75	53	0
Fkbp8	195	91	198	97	187
Fkbp9	119	104	95	74	148
Fkbpl	0	3	0	0	8
Fkrp	72	65	49	48	47
Fktn	51	30	62	54	59
Flad1	26	0	0	0	0
Flcn	476	406	578	489	410
Fli1	417	423	338	564	623
Flii	269	155	318	284	320
Flna	149	145	0	0	0
Flnb	54	63	87	81	100
Flnc	0	1	0	0	0
Flot1	0	0	0	0	0
Flot2	90	15	0	34	55
Flrt1	7	9	5	4	5
Flrt2	0	0	0	7	1
Flrt3	2	0	0	0	0
Flt1	0	0	0	0	0
Flt3	0	4	0	4	3
Flt3l	7	7	14	14	10
Flywch1	73	16	59	38	35
Fmn1	15	26	12	35	20
Fmnl1	168	94	141	251	278
Fmnl2	16	14	22	24	10
Fmnl3	320	170	248	268	180

For Review Only

Fmo1	0	0	0	0	0
Fmo2	0	0	0	0	0
Fmo5	55	68	53	80	41
Fmod	0	0	0	0	0
Fmr1	36	45	45	44	39
Fmr1nb	0	4	1	1	1
Fn1	0	9	0	0	0
Fn3k	5	8	7	5	0
Fn3krp	16	4	19	31	30
Fnbp1	67	61	106	418	722
Fnbp1l	14	24	1	56	46
Fnbp4	28	0	6	54	26
Fndc3a	85	142	150	194	146
Fndc3b	64	109	69	89	112
Fndc4	0	0	0	2	4
Fndc5	0	1	0	6	3
Fndc7	25	3	8	9	14
Fnip1	66	118	70	113	103
Fnip2	82	57	58	116	93
Fnta	44	37	14	55	69
Fntb	15	4	14	16	16
Focad	45	55	41	69	55
Folr1	19	0	1	0	0
Folr2	2	4	0	0	0
Fopnl	9	68	0	0	121
Fos	0	0	0	112	0
Fosb	13	11	7	34	3
Fosl2	34	24	20	26	24
Foxd2os	0	0	0	0	0
Foxf2	0	0	0	0	0
Foxj2	75	85	135	122	92
Foxj3	134	60	72	98	142
Foxk1	92	61	0	85	107
Foxk2	75	118	117	76	77
Foxm1	0	1	10	7	0
Foxn2	69	38	17	46	55
Foxn3	327	395	346	439	464
Foxo1	43	46	51	34	31
Foxo3	187	196	171	299	262
Foxo4	21	20	0	15	18
Foxp1	138	156	137	168	161
Foxp3	27	17	5	20	11
Foxp4	0	0	0	5	0
Foxr1	0	1	0	0	0
Foxred1	9	49	123	0	0
Foxred2	0	0	0	0	0
Fpgs	29	52	24	62	52
Fpgt	23	23	28	57	61
Fpr1	4	0	0	0	9
Fpr2	8	0	0	3	0

For Review Only

Fra10ac1	27	21	19	21	13
Fras1	6	0	3	0	0
Frat1	25	36	41	28	28
Frat2	1	1	3	2	0
Frem1	0	0	5	0	0
Frem2	0	0	0	0	0
Frg1	32	0	18	18	25
Frmd4a	867	856	812	1034	1096
Frmd4b	625	437	720	626	659
Frmd5	4	0	0	3	0
Frmd6	6	5	6	9	11
Frmd7	1	0	0	3	0
Frmd8	269	202	282	354	311
Frmd8os	2	4	0	0	3
Frmpd4	4	0	0	0	0
Frrs1	303	363	222	345	435
Frs2	74	61	42	96	73
Frs3	6	0	1	9	5
Frs3os	0	1	0	0	0
Fry	123	155	108	192	153
Fryl	113	102	139	175	262
Fscn1	907	702	576	914	966
Fsd1l	2	0	0	0	0
Fsip1	0	7	0	0	4
Fsip2	1	1	0	0	18
Fstl3	0	0	0	0	0
Fth1	1761	1136	1786	53	1469
Fto	150	65	95	156	137
Ftsj1	61	42	44	41	55
Ftsj3	59	60	86	22	34
Ftx	13	23	46	49	26
Fubp1	57	146	171	229	256
Fubp3	46	70	64	115	86
Fuca1	375	251	558	81	87
Fuca2	304	242	317	194	250
Fuk	43	30	63	40	52
Fundc1	111	121	120	108	105
Fundc2	49	6	0	28	32
Fuom	0	2	0	0	0
Furin	0	1	0	0	0
Fus	49	44	50	0	50
Fut10	29	57	41	52	41
Fut11	0	0	121	174	164
Fut4	15	8	7	11	14
Fut7	9	3	0	5	0
Fut8	122	83	76	97	95
Fuz	37	13	14	42	47
Fv1	3	4	7	10	0
Fxn	6	0	0	5	3
Fxr1	82	69	89	58	65

For Review Only

Fxr2	69	36	49	36	50
Fxyd1	7	0	0	1	1
Fxyd2	0	0	0	0	0
Fxyd4	6	0	0	0	8
Fxyd5	3	4	10	0	0
Fxyd6	0	0	0	0	0
Fyb	102	112	122	204	292
Fyco1	186	151	125	177	138
Fyn	7	7	17	9	9
Fyttd1	123	100	53	114	92
Fzd3	0	5	3	0	0
Fzd4	25	1	9	37	0
Fzd5	1	4	0	7	9
Fzd7	8	50	20	39	27
Fzd8	5	3	5	0	3
Fzr1	0	0	1	3	3
G0s2	1	0	9	0	4
G2e3	20	5	32	48	0
G370120E05Rik	9	8	0	12	11
G3bp1	223	191	295	191	303
G3bp2	344	265	402	131	301
G430095P16Rik	8	8	2	13	8
G530011O06Rik	10	65	150	67	0
G630025P09Rik	2	2	0	0	6
G6b	7	0	5	3	1
G6pc3	22	12	0	0	250
G6pd2	12	9	7	11	10
G6pdx	32	0	63	6	23
Gaa	445	249	334	336	278
Gab1	146	136	100	162	144
Gab2	228	219	262	273	392
Gab3	31	19	12	30	41
Gabarap	385	367	120	52	61
Gabarapl1	125	95	127	98	75
Gabarapl2	100	126	169	93	113
Gabbr1	61	54	50	69	46
Gabpa	48	16	46	87	78
Gabpb1	59	65	102	81	112
Gabpb2	106	49	45	94	99
Gabrb1	3	0	6	4	8
Gabrd	2	0	0	0	0
Gabrg1	0	3	0	0	0
Gadd45a	0	0	0	0	0
Gadd45b	4	0	9	7	5
Gadd45g	0	0	0	0	0
Gadd45gip1	54	66	77	60	53
Gadl1	11	0	0	7	9
Gak	0	250	150	16	60
Gal3st1	0	0	0	0	0
Gal3st4	0	155	567	0	0

For Review Only

Galc	47	25	21	38	42
Gale	13	3	0	5	14
Galk1	24	0	17	7	7
Galk2	79	34	72	55	85
Galm	37	23	38	25	28
Galns	62	12	21	8	21
Galnt1	98	100	108	110	110
Galnt10	101	77	121	98	109
Galnt11	133	79	136	86	84
Galnt12	58	66	0	0	0
Galnt13	0	1	6	3	0
Galnt14	0	0	3	2	2
Galnt16	7	0	0	9	0
Galnt2	78	115	84	117	98
Galnt3	10	15	19	6	19
Galnt4	119	95	139	213	126
Galnt6	13	6	13	6	20
Galnt7	45	54	71	70	94
Galntl6	4	0	0	0	0
Galr2	0	0	0	1	0
Galt	0	0	6	3	3
Gan	0	4	0	8	4
Ganab	0	236	151	315	293
Ganc	74	75	86	65	102
Gapdh	39	36	45	41	40
Gapdh-ps15	7	5	6	4	6
Gapdhs	2	5	1	11	0
Gapvd1	267	237	269	315	362
Gar1	0	10	0	1	2
Garem	4	0	0	5	0
Garnl3	80	70	73	87	78
Gars	168	91	170	107	152
Gart	123	87	102	109	118
Gas1	2	0	1	3	4
Gas2	4	1	0	0	8
Gas2l1	18	14	7	20	14
Gas2l3	8	6	0	13	12
Gas5	359	267	334	296	374
Gas6	3	1	442	235	0
Gas7	16	1	8	0	0
Gas8	24	41	36	20	40
Gata2	0	0	0	0	0
Gatad1	44	97	94	58	80
Gatad2a	78	67	112	129	131
Gatad2b	66	86	91	103	117
Gatb	58	41	98	63	85
Gatc	0	0	2	1	65
Gatm	248	256	360	317	368
Gatsl2	27	23	17	35	23
Gatsl3	31	18	29	14	38

Gba	0	214	69	86	5
Gba2	7	19	27	15	32
Gbas	62	46	110	58	70
Gbe1	46	33	44	46	21
Gbf1	199	157	189	183	201
Gbgt1	350	342	509	362	306
Gbp10	0	0	0	0	3
Gbp2	1	42	31	11	0
Gbp3	38	62	49	50	40
Gbp4	0	0	0	0	0
Gbp5	0	17	0	11	18
Gbp6	0	0	3	0	5
Gbp7	135	142	166	172	155
Gbp8	1	3	0	0	12
Gbp9	32	54	64	79	54
Gca	1	5	0	37	9
Gcat	0	0	0	3	6
Gcc1	44	18	57	36	56
Gcc2	32	34	40	56	71
Gcdh	46	72	60	43	38
Gch1	4	7	4	4	4
Gck	0	0	2	0	0
Gclc	0	49	82	0	26
Gclm	6	0	0	10	9
Gcn1l1	382	312	393	512	651
Gcnt1	529	360	446	652	614
Gcnt2	78	65	76	86	87
Gcnt7	10	7	9	16	6
Gcsh	25	33	30	23	40
Gda	6	0	0	0	0
Gdap10	33	44	15	61	29
Gdap1l1	0	1	0	0	0
Gdap2	96	94	78	118	111
Gde1	205	145	248	124	201
Gdf11	0	0	0	0	5
Gdf15	3	0	0	0	0
Gdf9	7	43	36	24	38
Gdi1	0	0	0	0	0
Gdi2	15	0	417	0	322
Gdpd1	17	12	26	17	11
Gdpd3	13	16	0	0	0
Gdpd5	13	1	16	8	22
Gdpgp1	49	30	33	35	20
Gem	8	1	17	10	0
Gemin2	11	8	15	9	0
Gemin4	59	28	53	77	61
Gemin5	74	35	47	59	53
Gemin6	25	27	42	28	17
Gemin7	0	73	46	0	0
Gemin8	13	11	8	14	10

For Review Only

Gen1	0	0	0	0	1
Get4	0	0	48	8	15
Gfer	12	1	0	0	37
Gfi1	0	0	0	0	0
Gfm1	64	13	86	66	75
Gfm2	498	788	396	897	735
Gfod2	22	9	14	13	12
Gfpt1	28	34	21	56	87
Gfra4	0	0	0	0	0
Gga1	103	58	52	70	111
Gga2	23	22	30	31	30
Gga3	2	379	0	245	186
Ggact	8	13	13	19	22
Ggct	2	5	6	0	9
Ggcx	35	36	96	161	107
Ggh	0	0	0	0	0
Ggn	0	0	0	1	0
Ggnbp1	0	3	20	5	16
Ggnbp2	107	85	65	94	127
Ggnbp2os	38	37	55	29	37
Ggps1	62	75	146	51	123
Ggt5	192	139	187	59	277
Ggta1	287	209	256	288	319
Ghdc	48	46	90	74	56
Ghitm	373	428	481	451	449
Ghrl	0	0	0	7	0
Gid4	58	67	51	57	72
Gid8	402	253	430	460	529
Gigyf1	121	59	0	127	151
Gigyf2	38	35	46	88	82
Gimap1	1	18	0	0	0
Gimap1os	3	11	11	7	3
Gimap5	8	11	7	6	15
Gimap6	71	81	126	47	66
Gimap9	29	38	42	28	27
Gin1	8	3	19	12	0
Ginm1	99	71	129	83	83
Gins1	8	10	5	3	0
Gins2	3	2	10	1	0
Gins3	10	11	16	11	12
Gins4	0	0	12	1	11
Gipc1	62	50	0	0	0
Git1	76	45	70	43	54
Git2	794	628	255	849	731
Gja1	2	0	0	0	0
Gja8	1	0	0	0	0
Gjb2	0	0	0	0	0
Gjb6	2	0	0	0	0
Gk	16	1	0	42	0
Gk5	1	0	13	0	16

For Review Only

Gkap1	3	11	0	12	16
Gla	6	0	0	10	7
Glb1	193	151	181	163	181
Glb1l	44	44	30	88	41
Glb1l2	5	0	3	6	0
Glcci1	21	27	17	25	21
Glce	56	22	23	63	37
Gle1	34	37	87	68	52
Glg1	63	94	113	106	116
Gli1	0	0	1	2	0
Glipr1	17	23	49	18	24
Glipr2	6	3	0	1	0
Glis2	5	3	0	0	0
Glis3	27	48	31	40	49
Glmn	16	12	0	0	0
Glmp	265	0	1	1	13
Glo1	83	49	87	72	52
Glod4	170	143	193	133	140
Glra4	2	5	7	0	0
Glrp1	19	34	43	24	14
Glrx	49	39	47	36	33
Glrx2	46	37	23	43	54
Glrx3	92	76	120	92	92
Glrx5	15	6	10	10	11
Gls	52	55	85	70	45
Gls2	0	0	0	0	0
Glt8d1	7	13	0	34	0
Glt8d2	0	0	0	0	0
Gltp	190	142	197	188	199
Gltscr1	130	123	139	181	146
Gltscr1l	161	102	136	224	164
Gltscr2	0	0	0	0	0
Glud1	212	183	165	235	216
Glyctk	0	3	14	0	0
Glyr1	351	202	333	280	299
Gm10012	17	9	13	10	11
Gm10033	57	49	62	63	54
Gm10037	0	0	0	3	6
Gm10051	67	56	71	53	59
Gm10052	173	56	93	139	149
Gm10069	7	10	9	4	4
Gm10080	36	49	74	44	48
Gm10130	11	6	12	15	15
Gm10145	8	13	19	14	18
Gm10190	1	1	0	0	3
Gm10248	4	7	1	7	0
Gm10258	0	0	0	0	0
Gm10266	0	1	0	6	5
Gm10277	2	11	7	8	26
Gm10325	7	4	4	4	7

For Review Only

Gm10336	3	11	13	5	5
Gm10362	22	27	33	22	19
Gm10369	5	20	8	24	25
Gm10384	37	41	48	34	31
Gm10390	2	1	0	1	0
Gm10406	19	15	19	21	20
Gm10408	1	1	1	4	3
Gm10409	7	6	12	8	8
Gm10410	20	11	14	14	24
Gm10416	0	2	1	4	6
Gm10419	1	2	0	0	3
Gm10432	2	1	0	5	4
Gm10433	0	0	0	0	3
Gm10451	0	1	0	3	1
Gm10499	1	0	0	0	2
Gm10509	10	13	18	27	23
Gm10532	6	0	0	4	1
Gm10538	0	0	0	0	0
Gm10548	4	0	0	4	0
Gm10575	0	0	0	1	4
Gm10578	22	27	22	33	27
Gm10584	0	0	2	2	0
Gm10603	18	3	12	11	16
Gm10614	2	0	0	0	7
Gm10638	0	2	0	3	3
Gm10651	4	1	2	2	1
Gm10654	1	1	0	0	0
Gm10677	17	14	15	19	22
Gm10693	8	1	5	4	7
Gm10698	227	169	224	200	221
Gm1070	3	1	0	4	6
Gm10705	15	15	18	14	23
Gm10767	5	2	8	1	5
Gm10768	0	1	0	0	0
Gm10778	14	11	13	13	21
Gm10785	10	4	6	7	9
Gm10790	0	0	30	0	0
Gm10791	1	6	8	0	0
Gm10804	5	1	1	3	1
Gm10825	2	1	5	1	0
Gm10847	7	2	4	10	6
Gm10851	4	3	3	0	11
Gm10910	3	0	1	0	3
Gm11007	11	14	10	24	23
Gm11110	0	0	0	0	0
Gm11127	2	0	3	0	0
Gm11201	0	1	0	12	0
Gm11335	1	0	0	0	0
Gm11423	0	0	1	1	1
Gm11457	3	6	0	8	6

For Review Only

Gm11476	0	0	0	0	0
Gm11478	144	132	185	143	141
Gm11513	0	0	1	10	0
Gm11517	1	2	3	3	1
Gm11532	5	4	0	4	3
Gm11545	25	15	33	35	30
Gm11578	7	1	1	4	6
Gm11634	3	0	3	1	2
Gm11677	0	0	0	2	3
Gm11696	12	12	15	13	18
Gm11713	6	16	12	22	19
Gm11716	3	6	4	4	3
Gm11725	10	4	7	10	16
Gm11753	146	127	151	171	203
Gm11769	8	19	15	11	41
Gm11787	5	11	0	34	168
Gm11802	4	2	0	4	4
Gm11821	4	1	0	1	2
Gm11827	4	4	2	4	9
Gm11946	0	0	0	1	1
Gm11963	0	0	0	5	0
Gm11998	2	0	0	5	0
Gm12059	1	3	0	0	9
Gm12060	3	5	7	4	10
Gm12061	0	1	0	0	8
Gm12070	59	47	59	40	51
Gm12121	1	0	2	2	2
Gm12174	132	98	143	113	111
Gm12185	18	15	20	43	25
Gm12191	61	72	77	79	72
Gm12216	7	0	5	5	0
Gm12240	0	0	0	1	2
Gm12247	1	3	6	3	1
Gm12248	0	2	0	1	5
Gm12250	16	75	39	31	50
Gm12315	9	7	5	11	6
Gm12338	54	29	55	40	48
Gm12359	3	0	1	1	6
Gm12474	13	2	1	7	17
Gm12500	0	1	0	8	0
Gm12504	10	7	6	8	11
Gm12506	0	10	0	1	2
Gm12522	18	1	25	18	17
Gm12569	6	0	0	1	0
Gm12657	28	20	33	23	28
Gm12666	1	0	1	1	0
Gm12669	59	64	103	62	60
Gm12693	15	18	21	10	18
Gm12694	0	0	0	0	0
Gm12708	11	4	5	13	10

For Review Only

Gm12781	2	0	0	0	12
Gm128	7	8	11	20	5
Gm12853	2	0	1	0	0
Gm12865	2	0	0	0	0
Gm12942	33	48	49	27	53
Gm12958	2	0	0	1	0
Gm12977	11	12	13	13	15
Gm12992	75	3	6	17	78
Gm13031	3	0	0	0	0
Gm13067	0	0	5	5	7
Gm13070	18	23	8	16	40
Gm13073	1	0	5	4	0
Gm13097	10	0	0	4	0
Gm13111	3	1	0	1	0
Gm13139	33	38	41	47	44
Gm13154	1	0	3	1	1
Gm13157	45	177	0	92	113
Gm13179	5	2	5	21	14
Gm13199	1	1	0	7	0
Gm13202	36	32	39	29	25
Gm13212	26	12	28	27	29
Gm13213	18	11	6	25	14
Gm13215	10	12	16	11	10
Gm13237	17	7	11	7	10
Gm13238	3	1	3	1	3
Gm13242	0	1	1	0	0
Gm13247	1	0	0	1	4
Gm13248	46	39	53	52	53
Gm13251	39	31	42	55	49
Gm13262	1	1	0	1	0
Gm13269	9	19	6	17	20
Gm13293	5	19	12	15	0
Gm13305	12	0	12	9	7
Gm13315	12	17	21	21	21
Gm13363	34	16	20	22	36
Gm13387	0	6	5	1	0
Gm13388	3	5	4	5	0
Gm13420	11	9	13	18	30
Gm13479	13	32	57	44	38
Gm13498	6	3	9	2	5
Gm13528	11	8	5	16	12
Gm13552	35	22	50	21	32
Gm13570	0	0	5	6	5
Gm13572	0	0	0	1	1
Gm13609	0	0	0	0	7
Gm13657	3	6	0	0	0
Gm13707	7	0	9	1	4
Gm13709	3	5	0	8	13
Gm13710	9	0	0	0	1
Gm13782	0	4	4	0	0

For Review Only

Gm13807	8	7	0	9	3
Gm13830	3	0	0	0	3
Gm13840	12	20	22	34	45
Gm13846	0	0	0	0	4
Gm13884	1	1	2	1	0
Gm13889	1	5	3	7	4
Gm14005	9	10	0	12	14
Gm14010	5	4	4	10	7
Gm14023	21	37	0	0	0
Gm14025	2	2	9	4	10
Gm14040	5	13	13	9	0
Gm14051	0	2	0	0	9
Gm14092	9	0	0	4	0
Gm14137	1	1	6	3	6
Gm14139	2	1	0	0	0
Gm14144	0	0	3	0	0
Gm14154	0	5	0	2	0
Gm14230	19	9	0	11	16
Gm14288	6	4	6	11	13
Gm14295	16	14	14	19	14
Gm14296	20	17	21	18	14
Gm14305	6	5	1	8	7
Gm14308	10	10	13	11	12
Gm14321	1	10	6	20	25
Gm14322	5	15	7	13	8
Gm14325	17	23	20	28	21
Gm14326	36	37	24	48	41
Gm14391	77	24	25	57	59
Gm14403	4	14	7	9	4
Gm14405	1	1	3	4	3
Gm14412	13	14	23	13	22
Gm14434	7	6	6	6	7
Gm14440	3	3	3	7	7
Gm14486	0	0	0	0	0
Gm14532	4	2	0	4	0
Gm14548	7	5	7	9	4
Gm14586	159	172	198	171	202
Gm14634	2	3	0	1	0
Gm14680	16	15	15	15	21
Gm14719	22	6	21	14	22
Gm14762	4	0	0	1	6
Gm15151	4	3	3	1	4
Gm15210	72	51	92	78	51
Gm15232	10	10	6	16	8
Gm15283	1	0	0	0	4
Gm15319	0	2	3	0	0
Gm15326	3	4	0	3	4
Gm15337	5	0	0	1	0
Gm15345	0	1	0	12	10
Gm15417	5	5	13	3	3

For Review Only

Gm15421	21	16	17	11	19
Gm15423	20	24	19	46	38
Gm15446	0	0	0	2	8
Gm15448	8	2	4	6	8
Gm15453	59	74	106	62	64
Gm15455	16	10	19	19	13
Gm15471	0	0	0	0	0
Gm15506	1	0	0	4	0
Gm15510	0	6	1	8	8
Gm15523	18	67	27	23	68
Gm15541	0	0	0	0	0
Gm15545	9	4	5	4	9
Gm15551	0	3	1	10	8
Gm15558	5	5	1	9	0
Gm15559	3	1	0	5	10
Gm15564	392	450	341	466	495
Gm15608	3	0	0	0	0
Gm15612	1	8	0	6	10
Gm15614	2	6	0	4	0
Gm15624	1	0	0	2	0
Gm15628	7	15	17	8	9
Gm15645	0	0	0	1	0
Gm15651	6	12	7	11	0
Gm15694	1	1	1	5	0
Gm15704	2	7	0	11	13
Gm15706	0	3	1	3	1
Gm15708	8	42	34	19	34
Gm15713	1	3	6	4	0
Gm15735	2	0	5	0	1
Gm15736	198	43	118	145	123
Gm15738	6	9	4	9	17
Gm15742	1	3	0	0	5
Gm15753	9	5	26	33	39
Gm15760	18	6	11	16	10
Gm15764	16	16	14	31	19
Gm15772	140	99	175	126	145
Gm15774	0	5	0	0	0
Gm15776	70	61	98	73	69
Gm15787	3	2	6	4	4
Gm15796	5	2	14	9	11
Gm15800	169	172	198	221	191
Gm15834	15	10	16	20	27
Gm15850	0	0	0	0	0
Gm15866	3	0	0	0	0
Gm15880	9	4	8	9	7
Gm15881	0	0	0	0	0
Gm15918	3	0	0	0	0
Gm15927	6	4	7	9	12
Gm15928	0	0	0	13	2
Gm15952	11	4	14	8	8

Gm15991	12	12	16	18	18
Gm1600	0	0	0	0	4
Gm16023	2	1	0	9	0
Gm16035	6	9	21	31	17
Gm1604a	3	3	0	6	7
Gm16062	1	3	6	4	2
Gm16116	9	8	0	23	24
Gm16118	57	91	67	92	75
Gm16120	4	11	13	10	0
Gm16124	5	6	3	3	7
Gm16146	8	8	5	15	16
Gm16150	2	1	1	0	0
Gm16160	0	3	0	0	5
Gm16168	0	0	4	0	0
Gm16206	7	2	12	5	13
Gm16231	4	0	0	0	0
Gm16246	1	0	0	0	8
Gm16283	0	0	0	0	0
Gm16287	1	6	0	5	4
Gm16299	5	2	28	30	4
Gm16322	4	4	5	7	1
Gm16348	0	0	0	0	0
Gm16351	2	2	0	0	0
Gm16386	1	4	0	10	0
Gm16432	0	0	2	3	5
Gm1647	0	3	5	0	0
Gm16486	52	27	0	0	0
Gm16523	1	2	19	4	16
Gm16525	0	0	0	1	1
Gm16573	1	1	1	0	7
Gm16576	39	17	37	32	38
Gm16578	9	16	12	39	12
Gm16599	11	8	0	10	17
Gm16617	0	1	0	2	8
Gm16638	10	2	0	11	0
Gm16675	2	0	0	1	3
Gm16702	5	5	4	3	9
Gm16731	0	5	0	0	0
Gm16740	9	0	0	2	3
Gm16759	0	0	0	0	0
Gm16793	0	0	0	1	2
Gm16794	6	10	11	7	12
Gm16835	0	1	0	4	0
Gm16845	13	9	9	15	9
Gm16853	0	0	0	8	0
Gm16861	5	3	6	23	29
Gm16867	33	17	36	53	59
Gm16876	5	27	15	23	16
Gm16897	7	0	2	3	0
Gm16907	17	13	19	16	20

For Review Only

Gm16982	5	1	1	6	12
Gm16998	5	4	4	4	1
Gm17021	0	0	0	0	0
Gm17025	10	1	7	16	22
Gm17030	0	0	0	0	5
Gm17039	22	1	4	0	0
Gm17057	4	0	0	3	3
Gm17059	8	3	3	9	12
Gm17066	68	107	76	66	101
Gm17116	0	0	3	2	0
Gm17224	3	3	0	7	0
Gm17250	0	1	0	10	2
Gm17296	3	15	15	21	39
Gm17382	2	0	0	0	0
Gm17399	4	1	0	8	14
Gm17455	6	4	0	5	1
Gm17597	2	0	0	0	0
Gm17757	5	12	12	24	8
Gm17762	1	0	0	1	0
Gm17764	0	1	0	0	0
Gm17769	0	0	0	0	0
Gm17801	0	0	2	5	4
Gm1818	31	14	30	18	25
Gm1821	0	0	0	0	0
Gm18853	4	12	13	24	9
Gm19327	36	12	71	61	76
Gm19412	1	0	3	1	2
Gm1943	6	6	13	12	14
Gm19463	0	1	0	0	6
Gm19510	0	0	0	0	2
Gm19522	1	5	0	6	13
Gm19557	10	5	0	8	12
Gm1966	3	25	6	38	28
Gm19665	8	3	9	16	1
Gm19673	2	4	0	0	0
Gm19689	4	1	1	1	1
Gm19708	6	34	14	20	29
Gm1976	0	3	9	0	0
Gm19897	18	1	9	7	11
Gm1995	2	0	1	4	4
Gm2002	12	0	11	8	7
Gm20036	3	0	3	4	0
Gm20071	30	10	13	12	13
Gm20199	23	23	0	17	73
Gm20219	6	0	4	0	4
Gm20236	17	27	20	44	31
Gm20257	12	33	53	24	35
Gm2027	2	0	0	0	0
Gm20275	7	0	0	5	0
Gm20300	10	24	26	35	29

For Review Only

Gm20337	0	0	5	0	0
Gm2036	0	0	1	3	1
Gm20385	3	2	4	5	3
Gm20482	3	10	9	7	5
Gm20492	6	0	0	0	2
Gm20501	3	0	0	1	0
Gm20511	3	0	0	3	5
Gm20522	1	1	2	0	3
Gm20544	25	4	0	18	0
Gm20594	10	15	9	16	15
Gm20597	1	0	0	0	0
Gm20604	0	18	0	0	0
Gm20605	0	0	0	0	0
Gm20616	0	1	3	7	4
Gm20636	13	12	20	12	10
Gm2065	3	1	0	0	7
Gm20655	1	0	3	5	10
Gm20717	28	12	15	14	33
Gm20750	0	1	0	2	0
Gm20783	0	0	0	0	0
Gm20875	19	8	12	8	10
Gm20939	15	12	8	18	26
Gm21057	0	5	0	5	11
Gm21119	1	3	1	1	1
Gm21145	2	2	4	4	3
Gm21284	0	2	7	0	0
Gm21451	7	10	9	20	20
Gm21596	10	5	8	5	8
Gm21685	49	61	37	50	82
Gm21811	40	77	42	0	43
Gm21885	18	29	29	22	32
Gm21948	13	18	0	0	64
Gm21949	8	0	5	0	0
Gm21975	118	78	132	201	155
Gm22	2	1	0	0	0
Gm2225	67	65	106	63	68
Gm2237	0	3	0	5	4
Gm2260	3	3	3	0	1
Gm2274	3	3	3	0	1
Gm2381	4	2	0	1	4
Gm24105	1	2	0	4	0
Gm24175	1	37	0	0	0
Gm2423	59	61	76	46	56
Gm2436	5	8	5	9	5
Gm2446	6	9	7	10	7
Gm2479	0	0	0	3	0
Gm2518	0	1	0	0	3
Gm25380	32	33	32	33	35
Gm25432	25	14	30	15	23
Gm2573	14	15	21	14	28

For Review Only

Gm26008	1	0	0	4	1
Gm26509	3	0	0	0	0
Gm26510	7	8	20	12	20
Gm26520	39	26	84	95	125
Gm26534	3	1	6	3	1
Gm26535	0	1	4	0	0
Gm26536	4	11	11	11	9
Gm26555	4	2	4	4	7
Gm26562	5	0	2	5	6
Gm26583	12	14	10	14	17
Gm26588	50	39	34	31	69
Gm26615	3	10	5	4	11
Gm26627	10	3	0	0	9
Gm26629	0	3	0	0	0
Gm26634	0	0	2	1	3
Gm26637	24	17	20	15	20
Gm26641	3	3	7	5	0
Gm26679	5	9	6	5	3
Gm26708	2	3	0	1	0
Gm26740	0	0	0	3	0
Gm26760	0	0	0	0	0
Gm26777	6	3	0	6	4
Gm26782	13	7	13	14	16
Gm26787	1	4	0	3	1
Gm26800	0	0	0	0	0
Gm26802	0	0	0	5	0
Gm26843	1	0	0	2	0
Gm26847	0	0	2	3	0
Gm26871	0	0	0	0	0
Gm26881	0	7	2	0	0
Gm26891	5	0	2	0	2
Gm26901	6	7	4	4	0
Gm26910	3	16	10	6	14
Gm26944	256	30	78	80	281
Gm26947	6	2	1	8	4
Gm27003	1	1	0	6	0
Gm27008	14	10	11	8	0
Gm27151	3	0	0	0	5
Gm27177	28	20	28	46	27
Gm27239	0	0	1	3	4
Gm27252	11	3	0	19	21
Gm28042	3	19	39	22	41
Gm2808	12	6	5	14	19
Gm2814	0	2	0	1	0
Gm28499	0	0	0	3	0
Gm28535	0	1	0	2	2
Gm28651	0	0	0	0	0
Gm28802	0	1	0	3	0
Gm28874	20	19	17	16	32
Gm2897	5	6	7	6	7

For Review Only

Gm29083	2	2	0	4	1
Gm29376	2	0	0	0	0
Gm29485	0	1	2	0	3
Gm2956	9	6	10	11	8
Gm29679	3	4	0	6	0
Gm29707	2	5	5	3	7
Gm29711	6	0	0	5	7
Gm29716	0	0	9	0	0
Gm29720	1	0	2	1	3
Gm29724	0	3	0	0	0
Gm29733	2	3	0	20	5
Gm2977	1	0	1	2	2
Gm29776	0	0	0	0	0
Gm29779	112	75	114	88	69
Gm29796	4	0	0	0	0
Gm29797	4	0	0	0	8
Gm29808	3	1	0	0	0
Gm29811	5	2	6	0	0
Gm29834	11	22	11	45	36
Gm29846	2	0	4	0	8
Gm29868	8	19	20	33	17
Gm29873	1	1	4	0	1
Gm29886	2	2	1	4	9
Gm29887	0	1	1	2	0
Gm29917	5	4	0	5	18
Gm29927	0	1	0	29	0
Gm29933	5	7	0	3	5
Gm29945	3	4	5	6	0
Gm29948	4	1	4	1	1
Gm29953	0	1	0	0	8
Gm29975	2	108	194	277	244
Gm29994	1	0	0	0	0
Gm29997	6	4	1	6	1
Gm2a	689	652	813	583	714
Gm30012	1	0	0	4	0
Gm30013	3	0	0	7	1
Gm30019	5	0	5	4	0
Gm3002	13	14	19	17	16
Gm30042	8	53	26	39	54
Gm3005	1	1	3	6	7
Gm30054	19	16	12	26	35
Gm30057	0	0	2	0	0
Gm30062	10	1	0	8	5
Gm30067	4	0	9	3	8
Gm30082	6	2	0	7	7
Gm30091	1	0	2	4	0
Gm30106	0	4	0	0	0
Gm30122	0	0	5	1	5
Gm30144	3	0	0	5	0
Gm30149	3	0	1	0	2

For Review Only

Gm30151	4	8	7	17	11
Gm30181	17	13	11	22	15
Gm30189	3	6	0	4	0
Gm30196	0	0	1	1	2
Gm30215	1	3	0	1	3
Gm30223	0	0	0	0	0
Gm30230	9	6	0	13	11
Gm30238	2	2	0	5	16
Gm30254	0	0	0	0	0
Gm30273	5	27	0	19	14
Gm30281	3	3	4	5	5
Gm30283	2	0	0	0	4
Gm30284	5	2	10	12	9
Gm30286	1	3	0	7	1
Gm30294	12	12	7	7	7
Gm30310	1	0	0	0	0
Gm30314	1	1	0	0	0
Gm30321	5	1	0	0	0
Gm30327	0	4	4	0	5
Gm30340	2	0	0	0	0
Gm30368	55	30	43	48	24
Gm30375	0	0	0	3	1
Gm30389	19	29	16	14	15
Gm30396	1	2	0	4	0
Gm30401	0	1	3	2	1
Gm30409	0	0	0	0	3
Gm30411	1	10	8	8	11
Gm30421	9	12	0	5	17
Gm30427	0	10	0	13	9
Gm30429	14	0	3	11	9
Gm30447	0	0	0	0	2
Gm30455	18	10	0	0	0
Gm30466	21	38	21	23	22
Gm30489	0	6	2	0	0
Gm30492	1	1	0	4	0
Gm3050	3	1	3	4	5
Gm30505	0	0	0	0	0
Gm30515	0	0	0	1	2
Gm30525	0	0	0	0	0
Gm30541	30	8	7	18	16
Gm30544	0	0	1	0	13
Gm3055	15	12	19	13	15
Gm30554	9	16	13	20	15
Gm30563	0	0	0	0	0
Gm30569	23	54	21	53	52
Gm30571	0	0	0	0	0
Gm30575	0	2	3	1	0
Gm30599	1	6	0	5	0
Gm30604	3	2	0	4	7
Gm30622	0	0	0	0	0

For Review Only

Gm30690	1	1	0	1	0
Gm30694	3	6	0	6	9
Gm30699	0	0	4	0	0
Gm30712	1	4	0	6	18
Gm30723	47	46	32	60	51
Gm30732	8	1	7	6	5
Gm30744	2	1	6	0	12
Gm30745	5	2	3	6	1
Gm30783	2	11	1	19	27
Gm30789	49	201	86	158	161
Gm30794	0	0	0	0	1
Gm30804	0	0	0	2	0
Gm30807	0	0	0	0	0
Gm30809	0	0	0	0	0
Gm30810	0	0	0	0	3
Gm30814	0	1	0	3	8
Gm30816	36	63	50	74	90
Gm30822	3	7	5	14	13
Gm30827	0	1	0	0	7
Gm30836	1	1	0	7	0
Gm30845	2	2	4	7	6
Gm30881	5	5	0	7	4
Gm30908	0	2	0	1	0
Gm30918	0	3	0	0	14
Gm30926	1	1	6	6	0
Gm30939	0	0	0	1	3
Gm3095	0	0	0	2	0
Gm30956	1	1	3	3	0
Gm30967	0	1	0	5	6
Gm30970	5	12	11	6	10
Gm30990	3	0	7	3	4
Gm30992	7	7	6	0	6
Gm31003	1	22	0	0	4
Gm31005	1	0	1	0	0
Gm31011	8	0	4	0	6
Gm31015	0	0	0	0	4
Gm31077	4	21	12	5	16
Gm31082	0	0	0	1	3
Gm31083	4	35	12	20	18
Gm31090	1	0	0	3	4
Gm31095	3	8	7	0	8
Gm31109	1	6	7	0	0
Gm31120	6	5	6	6	10
Gm31134	12	20	8	12	13
Gm31151	0	0	0	1	2
Gm31152	0	0	0	0	0
Gm31156	1	0	0	0	7
Gm31159	0	0	0	0	0
Gm31161	5	0	1	0	3
Gm31166	22	11	32	25	20

For Review Only

Gm31172	2	2	0	5	0
Gm31201	1	2	6	0	7
Gm31214	0	0	3	0	0
Gm31216	6	0	0	0	6
Gm31222	8	3	0	5	8
Gm31223	2	7	0	7	0
Gm31251	0	1	0	0	9
Gm31253	5	3	1	4	0
Gm31258	1	0	0	0	4
Gm31261	4	4	0	0	6
Gm31269	0	2	0	0	0
Gm31282	0	0	0	0	0
Gm31288	2	0	0	0	7
Gm31290	3	2	0	7	0
Gm31291	0	0	0	5	0
Gm31292	15	74	19	106	83
Gm31305	16	12	3	0	11
Gm31319	0	0	0	0	0
Gm31323	2	0	0	0	1
Gm31326	0	0	0	2	0
Gm31333	0	1	0	0	0
Gm3134	108	60	103	83	96
Gm31349	1	11	0	13	14
Gm31356	9	1	6	10	6
Gm31364	0	0	0	0	2
Gm31365	5	6	4	11	6
Gm31388	5	2	0	1	8
Gm31391	0	0	0	3	0
Gm31402	5	0	0	1	1
Gm31437	0	0	0	3	6
Gm31439	2	1	3	3	5
Gm31458	1	5	2	2	3
Gm31474	18	17	14	28	9
Gm31485	6	18	0	16	17
Gm31513	0	0	0	8	8
Gm31526	1	3	1	3	0
Gm31532	3	2	3	4	6
Gm31546	7	0	1	1	6
Gm31550	2	2	0	0	0
Gm31560	0	2	0	0	0
Gm31566	1	5	19	4	6
Gm31569	0	0	0	3	0
Gm31593	1	6	0	14	5
Gm31595	17	16	13	26	19
Gm31597	2	0	0	4	1
Gm31606	1	1	8	0	4
Gm31619	26	22	19	44	42
Gm31623	73	106	60	116	116
Gm3164	11	10	8	10	13
Gm31645	0	2	0	0	1

For Review Only

Gm31657	23	13	11	13	19
Gm31665	3	1	7	9	8
Gm31676	0	0	1	1	0
Gm31677	2	1	5	15	5
Gm31679	1	1	10	3	0
Gm31683	0	0	0	1	0
Gm31684	30	22	13	21	52
Gm31692	0	1	2	5	0
Gm31718	13	1	6	8	13
Gm31721	5	2	8	5	22
Gm31725	0	0	0	2	4
Gm31726	0	1	4	0	4
Gm31728	4	0	9	14	7
Gm3173	12	21	19	18	20
Gm31734	0	0	0	0	0
Gm31735	1	1	0	3	0
Gm31745	6	2	12	0	10
Gm31763	0	3	0	4	0
Gm31805	2	0	0	1	0
Gm31812	4	0	2	1	0
Gm31834	0	1	3	0	7
Gm31835	0	1	0	1	3
Gm31839	0	0	0	0	0
Gm31850	0	1	0	0	0
Gm31852	1	1	25	9	20
Gm31854	1	5	0	0	5
Gm31862	11	8	7	5	12
Gm31872	3	3	0	3	4
Gm31888	5	0	0	16	8
Gm3189	1	1	0	0	0
Gm31896	5	11	10	20	13
Gm31902	30	14	13	28	37
Gm31909	6	11	10	11	13
Gm31914	11	9	5	9	16
Gm31930	0	1	0	0	0
Gm3194	0	0	0	2	2
Gm31940	5	11	10	13	8
Gm31974	0	5	4	3	10
Gm31984	3	0	0	1	5
Gm31989	1	1	0	5	6
Gm32006	37	14	17	27	26
Gm32017	0	0	0	1	0
Gm32026	4	8	5	9	0
Gm32029	4	24	13	13	9
Gm32031	4	4	0	0	0
Gm32039	18	7	11	21	13
Gm32046	1	0	0	4	3
Gm32050	0	0	0	4	0
Gm32059	0	4	1	3	2
Gm32064	5	4	6	18	10

For Review Only

Gm32080	1	5	0	0	10
Gm32089	0	0	0	3	3
Gm32098	59	39	37	59	52
Gm32100	9	12	5	31	18
Gm32133	5	3	0	6	8
Gm32184	0	1	5	3	0
Gm3219	2	0	1	4	1
Gm32211	12	5	10	15	16
Gm32234	0	0	1	1	12
Gm32249	0	10	8	24	8
Gm32250	3	3	0	16	0
Gm32262	8	5	9	5	8
Gm32267	5	0	8	13	9
Gm32268	0	2	0	5	5
Gm32275	7	0	5	3	0
Gm32280	0	0	0	0	0
Gm32287	1	0	0	5	5
Gm32289	1	2	4	3	0
Gm32293	0	0	0	0	0
Gm32294	12	19	11	11	18
Gm3230	1	1	5	1	3
Gm32309	0	0	0	0	4
Gm32311	3	1	0	0	0
Gm32313	1	2	0	9	14
Gm32317	13	6	18	5	12
Gm32336	21	22	16	20	41
Gm32374	0	3	4	7	6
Gm32379	4	0	0	8	0
Gm32380	42	44	57	82	64
Gm3239	0	0	1	5	1
Gm32394	22	33	15	41	42
Gm32413	0	0	1	0	1
Gm32435	177	113	132	167	201
Gm32436	4	9	4	15	20
Gm32438	5	2	0	3	0
Gm3244	18	17	36	20	21
Gm32457	2	1	7	1	4
Gm32462	0	0	0	1	0
Gm32471	0	0	0	2	2
Gm32474	0	0	0	0	0
Gm3248	1	1	0	0	0
Gm32483	24	9	8	19	13
Gm32486	0	0	0	0	6
Gm32497	294	292	303	320	413
Gm3252	21	19	0	24	26
Gm32528	26	6	36	52	34
Gm32547	34	43	48	68	82
Gm3255	3	4	3	3	1
Gm32551	2	4	3	8	3
Gm32553	5	0	0	1	0

For Review Only

Gm32555	1	0	0	0	0
Gm32566	37	30	22	7	8
Gm32581	9	10	6	8	6
Gm32584	27	8	0	38	22
Gm32591	30	20	26	23	42
Gm32605	55	30	51	49	32
Gm32620	20	4	4	19	21
Gm32633	5	1	0	0	0
Gm3264	20	40	26	37	31
Gm32643	30	7	16	36	16
Gm32645	13	12	19	14	23
Gm32650	0	0	2	0	1
Gm32655	0	0	0	0	0
Gm32670	6	1	4	5	4
Gm32672	3	2	12	0	0
Gm32673	1	5	0	23	11
Gm32687	38	37	31	43	32
Gm32694	12	25	25	20	20
Gm32703	11	14	18	15	19
Gm32707	0	0	7	0	0
Gm32709	0	0	0	0	0
Gm32715	8	15	14	29	32
Gm32718	4	5	8	10	17
Gm32725	1	7	9	8	0
Gm32738	6	9	8	4	5
Gm32760	0	0	0	3	0
Gm32788	2	0	0	0	10
Gm32793	0	0	0	0	0
Gm32810	0	0	1	0	0
Gm32817	0	0	1	0	1
Gm32819	0	0	0	0	5
Gm32824	37	23	19	31	27
Gm32827	119	150	193	169	117
Gm32847	0	0	0	0	0
Gm32849	2060	2463	2149	3209	3382
Gm32853	17	33	14	21	25
Gm32854	1	0	0	3	0
Gm32856	16	31	26	31	27
Gm32861	2	0	1	1	0
Gm32882	4	0	4	3	1
Gm32898	0	2	1	4	6
Gm32899	0	7	0	8	10
Gm32900	1	0	0	0	0
Gm32904	2	4	0	1	0
Gm32908	0	0	0	5	1
Gm32934	4	1	2	3	0
Gm32940	29	44	59	46	29
Gm32952	2	0	0	4	0
Gm32992	0	3	0	3	0
Gm32999	1	0	0	0	0

For Review Only

Gm33023	23	31	33	37	16
Gm33047	26	14	15	26	40
Gm33066	0	4	0	5	0
Gm33097	2	2	0	7	0
Gm33100	2	7	6	10	0
Gm33118	1	8	10	10	6
Gm33126	0	3	0	0	2
Gm3317	35	35	39	42	52
Gm33198	4	4	3	6	12
Gm33199	0	0	2	1	2
Gm33214	0	7	5	11	5
Gm33219	0	4	0	4	0
Gm33228	0	1	0	0	0
Gm3325	26	16	16	38	25
Gm33257	0	0	0	0	2
Gm33269	3	0	0	0	3
Gm33272	40	62	51	49	93
Gm33273	8	3	7	8	13
Gm33305	12	19	13	49	55
Gm33310	13	7	0	17	5
Gm33318	0	6	1	4	2
Gm33337	0	0	0	0	0
Gm33350	4	1	7	22	1
Gm33370	26	16	32	37	28
Gm33376	2	1	1	5	4
Gm33387	12	17	17	11	17
Gm33389	12	1	5	5	12
Gm33392	0	1	0	0	12
Gm33434	15	23	26	0	1
Gm33442	1	3	0	8	5
Gm33444	10	12	16	15	5
Gm33449	3	0	0	1	5
Gm33454	0	0	0	0	0
Gm33467	1	0	0	0	6
Gm33470	0	0	0	5	0
Gm33475	10	2	0	33	17
Gm33487	2	4	7	5	6
Gm33517	43	7	11	12	35
Gm33524	0	3	0	0	0
Gm33534	3	14	0	31	22
Gm33536	0	0	0	0	1
Gm33555	0	0	0	0	4
Gm33582	7	19	6	21	18
Gm33585	0	0	0	0	0
Gm33610	5	8	11	9	6
Gm33622	43	6	6	25	43
Gm33641	0	3	10	9	0
Gm33682	13	7	14	7	29
Gm33691	3	1	0	1	0
Gm33697	44	29	57	36	26

For Review Only

Gm33721	0	7	12	0	10
Gm33723	0	3	2	2	8
Gm33729	14	16	16	14	24
Gm3373	0	0	0	1	0
Gm33733	0	4	0	5	0
Gm33746	0	0	0	0	0
Gm33747	2	2	0	3	4
Gm33764	0	0	0	4	0
Gm33767	9	21	15	19	20
Gm33771	1	2	0	0	1
Gm33786	0	0	0	0	2
Gm33795	0	0	0	0	0
Gm33804	4	4	0	0	0
Gm33813	1	0	0	6	0
Gm33821	9	9	7	18	20
Gm3383	12	13	14	16	14
Gm33832	2	0	0	1	1
Gm33856	17	22	28	47	35
Gm33862	5	8	3	7	7
Gm33864	90	68	50	89	96
Gm33869	23	46	26	38	61
Gm33877	4	8	2	9	7
Gm33885	0	2	5	3	0
Gm33922	1	1	4	7	5
Gm33926	0	0	0	0	3
Gm33933	6	6	7	10	9
Gm33937	0	0	0	0	0
Gm33938	5	1	6	16	1
Gm33940	0	0	0	0	5
Gm33971	0	4	0	0	0
Gm33989	284	310	210	400	349
Gm34058	0	0	4	0	0
Gm34076	11	9	6	8	16
Gm34079	4	6	8	14	20
Gm34087	0	0	0	0	0
Gm34102	0	7	0	9	2
Gm34121	2	2	0	4	0
Gm34137	0	0	0	0	0
Gm3414	17	15	15	12	9
Gm34156	14	1	4	13	12
Gm34159	0	0	0	5	0
Gm34168	0	4	5	2	3
Gm3417	0	0	1	2	2
Gm34178	17	13	15	31	26
Gm34186	3	11	11	17	18
Gm34189	2	3	2	3	3
Gm34197	2	0	3	6	6
Gm34218	22	10	24	52	48
Gm34220	3	2	4	12	5
Gm34223	0	4	0	0	4

For Review Only

Gm34232	0	2	0	1	0
Gm34235	10	10	6	21	15
Gm34245	3	0	0	3	3
Gm34280	251	218	203	418	441
Gm34283	4	2	4	4	0
Gm34288	8	1	0	7	7
Gm34292	0	0	0	0	0
Gm34299	0	0	0	1	0
Gm34321	2	2	0	5	0
Gm34324	0	2	7	7	0
Gm34326	1	2	1	4	0
Gm34336	1	2	4	0	0
Gm34343	0	0	0	3	1
Gm3435	13	11	13	14	13
Gm34354	1	0	0	2	0
Gm34375	17	20	12	23	21
Gm34389	0	5	0	0	3
Gm34392	0	1	0	0	0
Gm34394	3	0	0	0	0
Gm34402	6	10	0	15	9
Gm34403	4	0	3	0	0
Gm34407	3	3	5	4	7
Gm34408	25	12	7	38	35
Gm34447	0	0	0	0	0
Gm34448	3	3	0	4	0
Gm34451	2	1	5	5	1
Gm34455	0	0	15	31	31
Gm34459	0	0	0	0	0
Gm34507	0	0	0	1	1
Gm34513	0	0	0	1	0
Gm34531	5	0	0	0	0
Gm34582	0	9	1	0	0
Gm34586	1	19	11	20	0
Gm34589	71	50	135	85	49
Gm34607	0	0	0	1	0
Gm34620	4	0	0	3	1
Gm34632	0	8	5	9	13
Gm34648	0	1	0	0	16
Gm34655	5	0	0	9	0
Gm34661	0	3	1	0	1
Gm34696	1	0	4	0	0
Gm34702	1	1	0	7	7
Gm34741	0	2	0	3	5
Gm34744	0	0	0	2	0
Gm34755	4	3	6	1	3
Gm34771	4	11	8	8	16
Gm34776	0	0	0	0	0
Gm34795	7	5	2	6	3
Gm34836	29	60	47	110	109
Gm34844	1	3	0	9	22

For Review Only

Gm34847	1	0	0	0	8
Gm34849	0	4	3	2	2
Gm34854	8	16	11	16	20
Gm34857	1	0	0	0	0
Gm34858	4	1	0	3	5
Gm34861	8	1	0	0	7
Gm34865	27	27	22	30	35
Gm34868	0	0	0	0	4
Gm34872	20	14	12	28	10
Gm3488	34	33	33	49	48
Gm34907	6	0	0	2	0
Gm34917	10	12	0	7	17
Gm34921	30	35	25	47	32
Gm34934	0	1	0	0	0
Gm34945	1	5	0	6	4
Gm34979	4	1	0	8	10
Gm34980	6	13	4	7	13
Gm34982	1	3	0	0	0
Gm34983	0	2	0	6	7
Gm34997	0	1	0	4	0
Gm3500	3	4	5	3	4
Gm35002	1	1	0	0	0
Gm35021	0	0	2	0	8
Gm35029	5	11	0	0	0
Gm35035	15	21	17	15	11
Gm35060	4	4	0	11	4
Gm35071	0	0	0	1	0
Gm35074	3	0	0	0	0
Gm35089	0	1	11	0	0
Gm35102	0	3	0	0	7
Gm35113	1	16	0	13	13
Gm35117	2	17	8	18	22
Gm35135	2	0	0	4	0
Gm35145	0	0	0	0	0
Gm35150	6	0	4	0	0
Gm35154	40	17	33	7	30
Gm35166	0	0	0	0	4
Gm35169	16	13	11	27	28
Gm35188	0	1	3	0	0
Gm35200	0	4	5	2	5
Gm35242	15	0	1	6	0
Gm35243	21	14	16	33	15
Gm35247	38	25	28	27	52
Gm35248	0	0	0	0	3
Gm35280	0	0	0	4	1
Gm35285	0	0	0	0	0
Gm35290	69	86	80	110	117
Gm35295	0	0	0	0	0
Gm35314	2	2	4	0	0
Gm35315	0	3	0	0	0

For Review Only

Gm35321	0	0	0	0	6
Gm35339	1	5	11	0	9
Gm35343	5	4	1	6	1
Gm35353	0	0	2	3	2
Gm35358	1	1	0	4	0
Gm35364	0	0	1	0	0
Gm35365	2	2	7	5	7
Gm35383	0	3	0	0	0
Gm35394	0	0	0	3	0
Gm35395	1	0	2	0	0
Gm35396	0	0	0	0	0
Gm35399	2	5	6	5	7
Gm35417	19	0	0	0	0
Gm35456	0	0	0	1	2
Gm35463	1	0	0	0	0
Gm35465	1	0	0	0	0
Gm35466	2	10	1	4	13
Gm35470	0	0	0	0	0
Gm35478	8	0	4	10	11
Gm35501	1	0	0	0	3
Gm35522	3	4	7	5	6
Gm35525	0	0	0	0	0
Gm35534	15	2	39	41	36
Gm35558	0	0	0	0	0
Gm35562	0	0	0	0	0
Gm35566	15	21	23	25	16
Gm35572	3	2	1	3	4
Gm3558	5	7	9	5	7
Gm35582	4	8	4	5	13
Gm35591	1	0	0	6	0
Gm35596	15	9	16	30	20
Gm35597	7	2	5	8	11
Gm35599	2	0	0	0	0
Gm35601	3	1	0	0	0
Gm35607	0	1	0	0	2
Gm35608	0	0	0	0	0
Gm35625	0	0	1	0	0
Gm35657	1	2	4	8	0
Gm35658	8	27	15	8	30
Gm35677	4	9	6	18	15
Gm35678	1	0	0	0	2
Gm35688	0	0	0	0	0
Gm35707	12	2	0	15	19
Gm35715	18	4	10	22	19
Gm35732	2	14	16	3	15
Gm35760	0	0	0	0	0
Gm35768	37	24	25	33	26
Gm35808	1	15	1	12	11
Gm35816	3	0	6	0	0
Gm35828	10	15	14	19	48

For Review Only

Gm35831	7	12	12	14	15
Gm35835	0	0	0	1	0
Gm35853	17	1	19	5	11
Gm35856	0	0	2	4	0
Gm35866	9	19	30	22	29
Gm35887	13	1	0	1	0
Gm35906	395	370	385	494	420
Gm35908	11	0	0	2	4
Gm3591	17	0	0	3	2
Gm35911	0	0	0	3	0
Gm35934	0	0	0	4	3
Gm35959	5	12	4	0	6
Gm35970	1	2	0	10	0
Gm35999	6	7	4	9	9
Gm36003	12	20	27	22	41
Gm36025	1	0	0	0	0
Gm36027	1	17	0	15	17
Gm3604	5	1	2	27	17
Gm36043	0	0	0	5	3
Gm36046	0	1	2	1	1
Gm36048	7	0	8	11	3
Gm36055	1	0	0	1	2
Gm36109	36	32	45	70	87
Gm36117	3	1	0	10	12
Gm36118	0	0	1	0	0
Gm36148	5	2	7	0	0
Gm36163	0	0	0	1	0
Gm36166	6	5	7	5	0
Gm36167	0	1	0	5	0
Gm36181	0	0	0	0	0
Gm36184	0	3	0	0	1
Gm36188	0	0	0	0	0
Gm36195	4	1	13	18	41
Gm36198	4	5	12	10	16
Gm36208	0	3	1	3	3
Gm36220	8	17	25	14	14
Gm36227	0	0	1	0	0
Gm36229	0	0	3	0	0
Gm36243	0	0	3	2	0
Gm36267	0	1	0	0	0
Gm36279	14	38	42	50	46
Gm3629	4	3	7	6	5
Gm36297	2	0	0	0	5
Gm36299	0	2	0	0	5
Gm36304	0	0	0	0	0
Gm36311	0	0	0	0	6
Gm36315	0	4	1	0	3
Gm36328	4	4	11	7	11
Gm36330	0	0	3	2	1
Gm36338	3	0	0	0	7

For Review Only

Gm36355	0	0	0	4	7
Gm36359	0	0	0	7	0
Gm3636	53	37	61	44	48
Gm36365	317	279	426	78	327
Gm36375	4	10	23	20	27
Gm36391	1	0	0	1	2
Gm36401	10	6	7	12	6
Gm36402	15	14	11	21	27
Gm36403	2	0	0	4	0
Gm36409	0	0	3	0	0
Gm36411	0	2	0	0	0
Gm36412	3	3	0	0	0
Gm36423	5	0	0	1	18
Gm36430	7	8	6	9	10
Gm36441	2	0	0	3	3
Gm36442	3	2	7	17	7
Gm36449	3	4	6	0	0
Gm36457	7	3	0	0	2
Gm36459	4	0	0	0	4
Gm36462	3	4	8	13	3
Gm36464	0	4	0	0	9
Gm36470	0	4	0	5	4
Gm36482	6	9	7	9	7
Gm36490	10	7	8	17	20
Gm36493	0	0	3	0	2
Gm36496	1	0	0	3	10
Gm3650	6	9	0	7	10
Gm36500	7	1	0	5	0
Gm36527	76	44	48	65	130
Gm36529	0	0	1	4	0
Gm36532	0	0	0	5	0
Gm36538	0	0	0	0	2
Gm36556	7	18	9	29	15
Gm36559	1	9	8	9	5
Gm36570	0	2	0	3	5
Gm36572	5	12	0	5	6
Gm36579	1	3	0	0	14
Gm36584	3	0	0	1	0
Gm36587	47	91	25	56	45
Gm36591	7	32	0	8	23
Gm36602	3	3	0	12	15
Gm36607	8	9	25	25	17
Gm36608	1	0	0	0	0
Gm36635	3	0	6	0	6
Gm3667	0	0	1	2	1
Gm36672	3	0	0	5	5
Gm36673	0	4	2	6	4
Gm36677	3	1	0	0	9
Gm36681	58	0	28	15	39
Gm36684	1	1	0	3	0

For Review Only

Gm36693	1	0	0	3	3
Gm36704	1	0	0	1	1
Gm36712	2	10	7	4	12
Gm36722	0	0	0	2	2
Gm36738	8	5	12	12	8
Gm36743	0	0	0	0	0
Gm36756	4	1	0	0	3
Gm36774	0	7	11	0	0
Gm36778	0	0	0	0	0
Gm36790	0	0	0	2	7
Gm36796	5	21	13	23	15
Gm36800	19	21	61	31	55
Gm36816	7	7	21	17	13
Gm36818	0	1	0	3	1
Gm36826	1	3	0	8	4
Gm36839	1	1	0	0	0
Gm36851	31	34	6	18	12
Gm36853	2	8	4	11	7
Gm36856	1	0	0	3	4
Gm36864	0	6	0	1	7
Gm36874	6	10	9	11	7
Gm36877	0	0	0	2	0
Gm36884	10	10	12	12	2
Gm36885	4	4	24	24	14
Gm36897	4	0	0	0	0
Gm36901	11	1	4	3	8
Gm36906	1	1	0	0	16
Gm36908	0	0	0	2	0
Gm36911	0	0	0	2	4
Gm36913	0	0	0	0	0
Gm36917	0	1	0	0	0
Gm3696	5	3	4	4	5
Gm3704	1	0	0	7	4
Gm37053	12	7	18	22	28
Gm37125	10	17	29	9	8
Gm3716	2	7	4	4	0
Gm37168	0	0	0	4	0
Gm3718	8	7	1	6	9
Gm3739	21	20	20	35	19
Gm3740	0	0	3	1	0
Gm37416	31	21	0	0	0
Gm3764	0	0	0	1	1
Gm38137	2	2	0	0	0
Gm38293	1	0	0	6	0
Gm3837	37	39	58	41	48
Gm38396	32	38	71	40	40
Gm38414	2	0	0	3	0
Gm38418	1	3	3	4	4
Gm38422	0	0	0	0	0
Gm38424	9	3	1	25	17

For Review Only

Gm38426	4	0	0	0	0
Gm38431	126	97	135	199	195
Gm38440	1	8	3	0	0
Gm38444	3	8	6	6	12
Gm38457	1	14	5	4	4
Gm38459	0	0	0	0	0
Gm38471	3	6	8	12	7
Gm38479	46	17	19	40	72
Gm38481	11	5	5	1	3
Gm38482	5	1	5	0	3
Gm38485	20	50	32	111	111
Gm38486	0	1	3	7	0
Gm38487	0	2	0	0	4
Gm38489	13	6	14	28	20
Gm38496	10	3	9	22	0
Gm38499	2	1	5	0	0
Gm38500	16	0	11	5	4
Gm38501	1	0	0	5	6
Gm38503	0	0	2	1	5
Gm38506	0	0	2	2	0
Gm38515	0	0	7	0	1
Gm38524	15	11	7	14	24
Gm38525	4	4	13	20	0
Gm38528	0	0	0	4	0
Gm38529	11	6	6	36	38
Gm38530	0	0	0	0	5
Gm38540	0	3	0	2	3
Gm38543	21	24	32	30	21
Gm38549	0	8	6	15	12
Gm38553	1	2	1	8	6
Gm38560	0	0	4	0	0
Gm38561	1	4	0	4	8
Gm38565	2	8	8	14	19
Gm38592	9	7	9	18	7
Gm38594	0	0	0	0	1
Gm38599	15	5	5	14	8
Gm38609	13	18	19	9	17
Gm38618	0	0	0	6	4
Gm38621	0	0	0	0	1
Gm38623	2	2	1	0	14
Gm38632	53	58	48	98	96
Gm38639	14	23	26	17	26
Gm38641	7	4	18	11	9
Gm38643	0	0	0	0	3
Gm38649	12	8	12	25	32
Gm38651	2	2	0	0	1
Gm38664	2	4	11	1	0
Gm38675	0	0	7	14	14
Gm38676	3	4	6	9	6
Gm38688	3	13	8	6	9

For Review Only

Gm38690	0	1	1	3	1
Gm38708	2	0	0	5	0
Gm38709	31	10	8	23	33
Gm38718	6	7	4	3	5
Gm38720	5	3	6	2	5
Gm38729	6	0	0	4	0
Gm3873	19	19	39	23	25
Gm38745	0	0	0	2	2
Gm38752	1	1	0	17	10
Gm38767	0	0	0	0	11
Gm38781	4	1	4	4	8
Gm38782	2	1	0	0	0
Gm38785	72	35	78	83	78
Gm38803	0	0	0	2	3
Gm38804	2	1	0	4	6
Gm38805	0	0	0	0	0
Gm38832	10	5	12	0	5
Gm38834	3	6	0	0	0
Gm38849	0	0	0	0	0
Gm38850	33	48	22	26	62
Gm38852	0	3	3	2	0
Gm38859	21	5	9	18	34
Gm38865	6	34	13	18	40
Gm38871	0	0	0	0	0
Gm38880	10	5	8	8	5
Gm38882	13	3	13	3	6
Gm38883	0	6	6	0	9
Gm38907	12	6	9	0	14
Gm38908	8	4	1	12	12
Gm38914	3	0	0	0	0
Gm38918	0	0	1	0	5
Gm38927	1	3	0	1	3
Gm38935	0	0	0	1	2
Gm38939	0	0	2	1	0
Gm38948	3	7	1	4	3
Gm38951	1	1	0	7	0
Gm38952	4	0	0	0	0
Gm38956	0	4	7	0	0
Gm38957	2	0	6	9	1
Gm38967	0	0	4	1	0
Gm38968	5	0	5	7	6
Gm38978	1	4	3	15	8
Gm38983	1	1	0	5	0
Gm39000	6	6	1	4	4
Gm39002	2	0	1	1	0
Gm39006	3	0	2	2	9
Gm39010	9	0	0	2	0
Gm39012	0	1	0	2	3
Gm39019	0	0	0	3	3
Gm39038	0	0	0	0	0

For Review Only

Gm39041	0	3	1	1	2
Gm39050	0	3	3	1	0
Gm39054	0	0	3	0	2
Gm39055	3	2	0	5	1
Gm39059	0	1	4	0	0
Gm39061	5	5	0	11	0
Gm39069	1	8	12	7	10
Gm39081	0	0	0	0	2
Gm39089	3	0	0	0	0
Gm39090	7	6	3	7	0
Gm39092	0	0	0	2	1
Gm39099	0	0	0	6	0
Gm39109	3	3	5	0	0
Gm39110	1	2	7	7	0
Gm39134	0	1	4	5	0
Gm39141	4	0	2	0	0
Gm39149	0	0	0	0	0
Gm39150	10	8	5	6	5
Gm39170	5	1	5	5	1
Gm39173	59	52	42	81	54
Gm39178	0	0	0	0	6
Gm39203	1	0	0	1	0
Gm39210	0	0	0	7	3
Gm39211	0	0	2	0	0
Gm39212	0	4	1	0	5
Gm39214	11	8	11	12	23
Gm39215	1	3	5	3	0
Gm39221	13	13	13	12	0
Gm39241	1	0	0	5	0
Gm39251	3	4	9	7	12
Gm39256	23	6	11	26	17
Gm39257	6	10	1	21	13
Gm39260	6	8	3	15	11
Gm39265	0	0	0	2	4
Gm39269	10	20	32	32	28
Gm39272	70	61	50	94	79
Gm39284	0	0	2	0	0
Gm39308	2	2	4	4	0
Gm39309	7	5	5	8	5
Gm39334	7	4	0	1	0
Gm3934	47	45	53	31	41
Gm39342	6	3	0	6	6
Gm39348	10	8	0	7	10
Gm39355	0	0	11	18	21
Gm39362	0	1	0	0	0
Gm39382	0	1	1	5	1
Gm39399	2	0	0	0	0
Gm39408	1	3	4	0	0
Gm39419	15	20	19	12	23
Gm39424	4	3	6	0	6

For Review Only

Gm39425	1	2	0	0	6
Gm39443	4	9	0	12	23
Gm39446	0	0	0	0	6
Gm39454	13	21	17	16	13
Gm39463	0	0	0	5	0
Gm39467	0	0	1	2	2
Gm39469	7	0	9	53	47
Gm39474	2	1	0	5	0
Gm39478	3	0	0	0	0
Gm39491	5	0	0	5	9
Gm39497	7	10	0	7	0
Gm39499	0	0	0	2	3
Gm39503	1	1	0	11	0
Gm39518	3	54	40	34	22
Gm39526	15	6	6	7	9
Gm39552	16	9	22	21	17
Gm39556	18	5	7	11	17
Gm39565	54	28	8	62	54
Gm39584	0	0	0	6	0
Gm39585	1	6	5	5	3
Gm39603	0	0	3	0	0
Gm39606	4	0	0	0	3
Gm39618	0	1	0	3	5
Gm39624	11	8	21	24	27
Gm39627	1	2	4	8	8
Gm39642	22	20	18	47	32
Gm39656	72	72	119	113	79
Gm39662	0	0	0	0	0
Gm39664	0	0	0	4	1
Gm39666	3	15	3	7	6
Gm39667	4	0	1	1	0
Gm39668	8	4	12	11	15
Gm39673	169	125	172	139	180
Gm39679	1	3	2	0	4
Gm39688	0	0	0	0	0
Gm39701	5	3	5	4	5
Gm39703	0	2	0	4	7
Gm39712	20	23	18	39	44
Gm39715	0	0	3	0	0
Gm39751	40	20	18	44	42
Gm39761	1	1	4	0	7
Gm39762	1	5	0	4	1
Gm39772	4	0	0	2	0
Gm39785	0	0	0	0	0
Gm39786	5	0	3	0	3
Gm39796	0	0	0	0	3
Gm39807	0	0	0	0	4
Gm39828	0	4	2	0	0
Gm39847	0	0	0	0	0
Gm39860	1	1	0	6	17

For Review Only

Gm39869	9	11	5	4	6
Gm39872	1	2	1	1	7
Gm39874	0	0	0	2	0
Gm39878	9	10	5	24	17
Gm39882	35	26	24	36	50
Gm39883	4	5	0	9	22
Gm39887	0	5	0	0	4
Gm39888	0	0	0	2	0
Gm39897	20	29	14	33	35
Gm39908	8	7	8	10	13
Gm39922	1	0	0	0	3
Gm39931	0	0	0	3	5
Gm39938	2	0	0	5	0
Gm39939	214	69	92	172	237
Gm39954	33	35	38	33	45
Gm39988	19	56	39	99	57
Gm39990	0	0	0	2	6
Gm40041	3	0	1	4	2
Gm40051	0	1	1	3	0
Gm40078	36	30	29	24	42
Gm40080	48	15	23	50	32
Gm40091	5	2	7	4	9
Gm40096	5	0	0	0	0
Gm40103	7	5	6	6	8
Gm40106	5	0	0	3	0
Gm40116	4	12	0	10	15
Gm40117	3	2	0	8	1
Gm40126	13	3	10	5	5
Gm40128	0	0	1	0	1
Gm4013	0	0	0	0	0
Gm40142	0	0	0	0	7
Gm40150	3	3	9	0	0
Gm40180	0	0	4	0	0
Gm40181	4	11	19	0	10
Gm40191	3	0	6	2	0
Gm40194	2	2	3	10	12
Gm40208	103	85	64	144	188
Gm40218	8	6	7	4	12
Gm40224	2	8	5	8	0
Gm40225	0	6	0	0	0
Gm40229	0	0	0	0	4
Gm40237	0	0	0	0	1
Gm4024	209	111	184	188	186
Gm40243	1	0	0	8	5
Gm40290	1	5	0	14	0
Gm40302	1	1	0	0	0
Gm40305	1	1	0	0	9
Gm40309	159	97	118	166	143
Gm40310	3	3	3	5	10
Gm40346	43	9	4	18	29

For Review Only

Gm40352	5	2	5	0	0
Gm40378	3	1	1	3	2
Gm40385	54	15	16	56	23
Gm40386	12	29	11	42	28
Gm40397	1	4	0	0	9
Gm40402	1	1	0	1	1
Gm40406	17	39	29	60	50
Gm40409	1	1	0	0	5
Gm40417	8	8	9	11	10
Gm40443	16	10	22	14	6
Gm40448	73	68	79	73	91
Gm40454	0	1	0	0	0
Gm40462	2	5	3	1	3
Gm40466	36	28	34	45	51
Gm40468	5	5	7	8	4
Gm40474	0	2	0	1	0
Gm40475	14	20	21	40	69
Gm40491	9	0	7	4	7
Gm40496	10	5	5	8	11
Gm40498	47	31	0	0	0
Gm40525	32	17	0	0	0
Gm40527	3	0	0	5	0
Gm40528	17	25	18	36	19
Gm40531	9	7	13	13	0
Gm40543	2	5	4	9	8
Gm40556	1	0	0	4	6
Gm40559	10	0	0	6	8
Gm40564	0	0	0	1	1
Gm40571	33	18	14	36	30
Gm40573	7	0	0	0	4
Gm40578	0	0	2	2	0
Gm40579	3	4	0	0	7
Gm40582	6	4	5	4	3
Gm40591	0	1	0	3	0
Gm40637	4	3	4	0	5
Gm40645	27	0	0	0	0
Gm40668	0	6	5	6	4
Gm40670	0	3	0	0	0
Gm40671	8	4	12	13	8
Gm40680	0	0	1	0	0
Gm40696	5	5	7	6	13
Gm4070	35	90	51	121	92
Gm40715	1	0	0	0	0
Gm40720	0	0	0	0	0
Gm40721	5	3	3	6	10
Gm40743	3	0	0	0	0
Gm40776	0	0	0	0	0
Gm40781	1	0	0	4	0
Gm40782	1	4	0	4	3
Gm40799	0	0	0	0	16

For Review Only

Gm40824	12	19	11	15	15
Gm40826	0	0	0	0	5
Gm40841	114	100	99	138	179
Gm40842	33	8	10	24	26
Gm40848	1	0	0	3	3
Gm40860	34	16	20	24	31
Gm40863	5	8	5	0	0
Gm40875	2	6	1	9	5
Gm40887	8	4	1	12	4
Gm40897	3	1	0	0	4
Gm40912	2	5	22	13	11
Gm40913	21	21	31	9	17
Gm40915	2	0	4	1	0
Gm40916	4	9	7	9	8
Gm40934	0	2	0	5	0
Gm40960	2	0	3	0	0
Gm40964	0	0	0	1	0
Gm40979	3	4	0	0	0
Gm40996	2	0	0	12	0
Gm41005	1	1	0	9	16
Gm41011	0	0	2	1	0
Gm41024	9	4	0	0	0
Gm41025	0	1	0	0	0
Gm41032	6	0	0	0	0
Gm41035	7	7	4	8	4
Gm41037	3	3	2	3	0
Gm41038	3	0	0	0	0
Gm41039	3	1	0	7	0
Gm41046	20	11	16	33	18
Gm41055	0	0	0	0	0
Gm41071	65	81	120	118	120
Gm41072	0	0	0	2	0
Gm41077	16	24	19	17	25
Gm41090	1	1	0	1	2
Gm41094	0	3	1	3	0
Gm41098	29	32	29	54	45
Gm41101	2	1	0	3	5
Gm41107	322	304	203	384	402
Gm41108	0	0	0	0	0
Gm41113	1	2	5	4	4
Gm41114	2	0	0	4	7
Gm41130	21	4	0	0	0
Gm41161	1	2	0	0	0
Gm41164	5	4	0	6	11
Gm4117	0	2	0	1	0
Gm41199	1	0	1	0	5
Gm41213	0	3	1	6	2
Gm41215	0	0	0	0	4
Gm41231	4	5	0	2	1
Gm41247	2	2	4	9	6

For Review Only

Gm41252	0	0	2	0	2
Gm41254	0	4	4	0	5
Gm41273	2	0	0	1	1
Gm41280	0	0	0	3	0
Gm41283	1	0	0	0	5
Gm41284	3	0	3	1	0
Gm41285	6	1	1	7	14
Gm41287	0	4	3	5	6
Gm41291	7	7	8	14	10
Gm41292	3	6	9	13	0
Gm41298	0	3	3	2	0
Gm41299	0	0	0	0	0
Gm41308	1	1	0	0	0
Gm41330	21	9	9	15	26
Gm41338	18	19	8	29	16
Gm41343	2	0	4	0	0
Gm41344	3	0	0	0	0
Gm41358	0	0	0	0	0
Gm41361	2	2	6	11	10
Gm41362	0	0	0	0	5
Gm41368	3	9	0	14	5
Gm41377	0	0	0	5	0
Gm41389	12	2	0	10	17
Gm41395	3	2	3	6	6
Gm41406	0	0	2	2	1
Gm41410	8	5	6	9	7
Gm41414	1	1	0	1	5
Gm41442	4	4	6	16	14
Gm41453	5	11	11	12	14
Gm41476	3	3	2	4	2
Gm41485	5	0	0	0	0
Gm41489	0	0	0	4	0
Gm41491	6	9	13	8	11
Gm41498	15	11	6	10	0
Gm41514	1	0	1	2	6
Gm41523	2	1	0	1	5
Gm41525	30	20	18	36	32
Gm41526	21	17	11	25	19
Gm41549	2	0	0	0	0
Gm41553	16	41	29	31	41
Gm41556	24	15	12	22	15
Gm41569	3	1	0	0	0
Gm41572	0	0	0	5	0
Gm41590	24	3	10	25	49
Gm41598	29	31	54	20	13
Gm41604	1	4	0	3	0
Gm41607	28	25	22	52	36
Gm41610	16	17	7	13	21
Gm41611	23	10	13	24	22
Gm41620	17	16	10	12	10

For Review Only

Gm41622	3	28	25	9	25
Gm41627	0	0	0	1	0
Gm41640	19	23	12	17	38
Gm41662	11	0	11	3	3
Gm41666	0	0	0	4	4
Gm41672	7	12	10	0	12
Gm41676	0	0	0	0	0
Gm41678	0	9	9	3	7
Gm41687	0	0	0	0	0
Gm41688	9	11	0	0	0
Gm41689	5	0	0	6	3
Gm41693	20	7	8	25	28
Gm41696	3	2	5	9	19
Gm41702	0	0	1	0	5
Gm41705	3	3	0	5	5
Gm41719	1	2	0	3	0
Gm41748	4	15	11	13	8
Gm41750	1	0	6	8	0
Gm41778	0	0	0	0	1
Gm41790	0	0	1	1	1
Gm41803	0	4	0	4	1
Gm41818	0	5	0	11	0
Gm41829	2	1	0	0	0
Gm41834	0	1	1	1	1
Gm4184	12	10	18	17	13
Gm41846	3	12	7	14	10
Gm41861	0	3	0	5	0
Gm41883	1	0	1	0	4
Gm41884	3	5	0	6	6
Gm41885	89	56	0	0	0
Gm41909	1	1	8	1	5
Gm41920	0	3	3	3	0
Gm41934	3	0	0	0	0
Gm41947	1	7	8	6	8
Gm41949	64	53	93	93	127
Gm41962	4	2	0	4	3
Gm41964	1	6	0	15	6
Gm41983	1	1	1	0	0
Gm41984	0	3	3	0	0
Gm42002	19	18	19	13	29
Gm42004	6	0	0	0	0
Gm42005	13	6	14	14	4
Gm42021	1	3	0	0	1
Gm42023	3	3	1	0	3
Gm42027	64	63	12	52	57
Gm42035	217	84	140	239	132
Gm42042	27	37	82	22	88
Gm42043	44	37	45	36	56
Gm42059	0	0	0	0	4
Gm42063	0	0	0	0	3

For Review Only

Gm42074	7	15	8	15	16
Gm42078	5	1	1	4	6
Gm42084	0	0	0	0	0
Gm42085	0	1	0	0	4
Gm42090	5	1	0	8	5
Gm42094	15	21	10	15	20
Gm42102	0	1	0	0	0
Gm42111	7	5	18	8	5
Gm42127	11	24	7	28	21
Gm42135	1	2	0	1	1
Gm42139	12	8	20	17	14
Gm42145	3	2	1	4	10
Gm42147	3	3	0	3	0
Gm42158	41	8	19	13	29
Gm42161	0	0	0	0	0
Gm42162	6	7	5	5	4
Gm42166	12	13	18	15	22
Gm42198	1	2	0	0	0
Gm42199	2	8	5	6	6
Gm42202	0	2	0	0	0
Gm4221	7	2	1	6	16
Gm42219	0	3	0	1	0
Gm42226	6	5	6	10	4
Gm42232	0	0	0	3	3
Gm42244	0	0	0	4	0
Gm42250	1	3	10	9	19
Gm42253	7	1	0	5	12
Gm42259	11	16	8	18	31
Gm42267	1	0	0	1	0
Gm42278	3	0	0	0	5
Gm42282	4	7	5	12	8
Gm42307	1	5	11	6	7
Gm42309	0	2	0	0	0
Gm42326	1	5	1	16	8
Gm42338	0	3	0	4	2
Gm42343	2	4	7	7	8
Gm42344	1	1	0	7	5
Gm42351	9	12	19	28	17
Gm42355	0	1	0	14	9
Gm42357	0	1	0	1	0
Gm42362	0	24	0	0	57
Gm42364	4	10	16	10	4
Gm42368	30	34	41	39	44
Gm42372	0	5	4	0	0
Gm4258	3	5	0	19	11
Gm4285	0	10	4	4	11
Gm4349	0	0	0	0	0
Gm4419	0	0	0	4	0
Gm4432	1	1	3	7	3
Gm4450	2	2	0	3	1

For Review Only

Gm44502	123	95	0	0	115
Gm44504	0	23	0	2	1
Gm44505	7	5	1	6	9
Gm4473	3	0	0	5	1
Gm4532	0	0	1	1	1
Gm4544	0	1	4	0	10
Gm4598	2	1	3	4	3
Gm4604	32	17	31	20	18
Gm4632	0	1	0	0	0
Gm4651	1	0	0	1	2
Gm4705	22	16	30	23	17
Gm4724	4	7	6	6	8
Gm4737	14	7	11	6	7
Gm4759	0	3	1	0	0
Gm4767	13	7	9	9	16
Gm4786	13	14	20	12	15
Gm4787	1	17	0	25	1
Gm4788	44	41	75	86	60
Gm4793	11	0	10	5	11
Gm4814	0	0	0	0	0
Gm4841	0	0	0	0	0
Gm4887	8	4	3	10	10
Gm4890	1	2	4	12	0
Gm4924	54	13	23	40	47
Gm4925	4	1	0	5	5
Gm4943	3	8	5	7	6
Gm4944	36	34	37	47	40
Gm4951	4	6	10	15	9
Gm4961	0	3	5	0	0
Gm4997	6	4	5	5	4
Gm5067	0	1	1	1	0
Gm5069	4	4	0	4	0
Gm5086	0	206	0	0	105
Gm5088	5	16	13	8	14
Gm5093	16	21	20	18	19
Gm5106	0	1	0	2	0
Gm5113	7	13	6	9	6
Gm5115	3	2	1	7	4
Gm5122	0	1	0	3	0
Gm5124	0	0	1	0	0
Gm5127	0	0	0	0	0
Gm5129	2	1	6	4	8
Gm5134	11	6	8	0	10
Gm5141	24	42	29	39	60
Gm5148	120	120	163	123	133
Gm5420	6	3	0	0	0
Gm5424	1	0	5	1	0
Gm5431	41	39	0	0	0
Gm5434	8	7	6	6	12
Gm5454	38	29	43	27	38

For Review Only

Gm5464	0	7	3	3	11
Gm5481	0	0	0	1	0
Gm5486	0	0	0	0	0
Gm5512	55	39	55	43	59
Gm5563	10	0	0	0	0
Gm5566	7	8	16	7	14
Gm5590	4	0	0	0	0
Gm5593	1	0	0	0	0
Gm5595	35	12	30	30	41
Gm561	11	17	25	10	17
Gm5617	20	21	0	0	0
Gm5621	53	44	59	41	51
Gm5643	60	34	42	59	67
Gm572	0	6	0	2	9
Gm5766	1	0	0	0	0
Gm5785	9	10	7	23	23
Gm5796	6	7	9	7	7
Gm5801	12	7	13	11	10
Gm5803	17	12	11	18	19
Gm5820	3	4	4	5	0
Gm5854	7	4	9	6	6
Gm5879	151	133	189	133	156
Gm5917	11	8	9	8	8
Gm5946	7	6	1	6	1
Gm6109	5	6	7	7	9
Gm6158	20	27	27	23	26
Gm6194	5	1	2	1	1
Gm6225	2	5	3	7	5
Gm6252	46	38	54	42	46
Gm6277	1	1	0	0	0
Gm6288	0	0	0	0	0
Gm6297	3	1	0	1	0
Gm6306	1	0	0	1	2
Gm6345	0	1	1	4	2
Gm6402	30	33	0	49	47
Gm6410	1	6	0	4	1
Gm6445	1	0	0	0	1
Gm6485	137	80	118	107	163
Gm6498	4	7	11	11	4
Gm6524	10	5	7	4	5
Gm6525	1	2	1	6	1
Gm6548	126	129	146	109	149
Gm6566	7	1	0	7	0
Gm6568	12	10	8	11	7
Gm6570	13	17	17	20	15
Gm6579	4	4	4	5	1
Gm6583	20	13	20	22	39
Gm6598	3	4	0	12	7
Gm6623	20	16	22	23	11
Gm6644	51	71	90	69	82

For Review Only

Gm6654	36	32	46	31	50
Gm6710	16	10	14	25	21
Gm6712	23	52	61	56	36
Gm6713	1	2	0	0	5
Gm6745	43	55	51	36	52
Gm6750	13	7	10	5	8
Gm6756	4	1	5	2	2
Gm6793	44	50	36	41	45
Gm6815	0	1	1	0	1
Gm6904	0	0	2	0	0
Gm6978	17	18	34	20	18
Gm6988	32	39	77	51	58
Gm7020	15	7	5	5	12
Gm7030	4	0	6	0	3
Gm7072	62	60	57	52	68
Gm7099	19	16	24	27	24
Gm7102	0	0	2	2	0
Gm7104	0	1	0	4	2
Gm7120	2	12	5	6	9
Gm7265	2	0	1	3	0
Gm7285	24	21	34	19	26
Gm7334	4	7	2	3	3
Gm7361	3	0	0	1	1
Gm7367	9	13	2	8	8
Gm7429	26	26	32	34	28
Gm7443	0	2	0	0	0
Gm7444	0	1	3	3	0
Gm7609	2	1	0	1	3
Gm7694	29	35	67	92	98
Gm7710	88	102	129	91	104
Gm7846	0	4	7	1	0
Gm7854	0	1	1	2	3
Gm7855	2	1	1	3	1
Gm7866	49	41	50	31	38
Gm7889	4	0	5	5	7
Gm7931	25	16	22	13	20
Gm7936	8	12	14	8	9
Gm7972	1	1	0	12	20
Gm7977	1	1	0	0	1
Gm8013	0	0	1	4	2
Gm8069	10	13	23	9	12
Gm8096	4	3	6	4	2
Gm8149	36	34	40	67	69
Gm8203	148	162	246	155	186
Gm8210	0	0	2	0	1
Gm8234	1	1	0	1	3
Gm826	4	0	0	1	5
Gm8273	4	9	3	4	7
Gm8350	2	0	0	0	4
Gm8363	0	1	3	2	2

For Review Only

Gm8430	100	106	140	96	109
Gm8451	54	51	74	70	81
Gm8465	4	4	2	2	5
Gm8493	0	2	0	0	0
Gm8580	1	3	2	1	5
Gm8615	59	39	63	62	79
Gm8623	45	40	52	27	42
Gm8624	7	8	12	6	8
Gm8675	2	0	0	1	0
Gm8702	3	2	4	5	12
Gm8801	35	13	25	13	25
Gm884	0	4	3	4	0
Gm8909	0	0	0	1	6
Gm8942	36	30	47	30	38
Gm8979	4	7	8	6	7
Gm8989	3	4	8	4	4
Gm8994	7	9	20	10	13
Gm8995	49	160	70	147	126
Gm9054	4	3	3	2	0
Gm9079	3	3	0	0	3
Gm9159	1	6	2	3	2
Gm9199	0	0	1	0	0
Gm9222	29	8	20	47	42
Gm9234	90	91	132	76	96
Gm9385	148	74	160	104	123
Gm9457	1	0	0	2	1
Gm9465	3	1	3	5	3
Gm9484	17	13	11	25	37
Gm9530	1	0	9	7	0
Gm960	3	0	2	3	6
Gm9618	5	5	0	1	3
Gm9706	16	18	10	13	24
Gm9769	94	69	48	60	71
Gm9776	1	6	7	4	9
Gm9794	263	235	423	283	321
Gm9833	3	3	3	6	7
Gm9840	33	20	33	22	38
Gm9855	12	9	17	8	10
Gm9861	5	4	0	17	8
Gm9878	3	0	0	3	7
Gm9903	5	1	0	10	4
Gm9919	0	3	0	4	0
Gm9920	0	0	0	0	0
Gm9949	3	1	17	0	0
Gm9958	3	7	4	5	12
Gm996	76	95	75	128	87
Gm9970	0	0	0	2	1
Gmcl1	10	15	8	23	17
Gmds	14	14	23	20	33
Gmeb1	235	114	211	189	229

For Review Only

Gmeb2	29	18	15	27	34
Gmfb	138	153	135	170	112
Gmfg	0	22	0	159	134
Gmip	77	167	1	10	257
Gmnn	4	14	11	0	0
Gmppa	25	23	0	0	0
Gmppb	0	48	0	0	0
Gmpr	185	145	170	151	185
Gmpr2	79	4	146	26	171
Gmps	149	123	131	125	115
Gna11	1	0	0	0	0
Gna12	0	60	6	160	11
Gna13	377	340	295	393	400
Gna14	0	1	0	4	4
Gna15	655	663	1053	755	833
Gnai2	203	551	320	286	339
Gnai3	42	101	313	137	282
Gnal	4	6	7	11	1
Gnao1	0	1	0	1	3
Gnaq	169	159	207	166	199
Gnas	92	167	96	59	113
Gnaz	3	1	0	3	0
Gnb1	228	539	558	225	203
Gnb1l	14	27	10	14	27
Gnb2	1	0	3	4	13
Gnb4	1	0	11	4	7
Gnb5	14	12	16	13	19
Gne	96	56	76	91	132
Gng10	985	0	105	33	0
Gng11	0	0	0	0	0
Gng12	188	56	66	136	138
Gng2	188	175	225	195	212
Gng3	0	1	1	2	3
Gng5	130	120	159	127	120
Gng7	19	1	21	18	24
Gngt2	159	191	270	68	110
Gnl1	52	13	64	32	56
Gnl2	152	75	214	122	189
Gnl3	65	41	41	41	36
Gnl3l	22	32	25	43	36
Gnmt	0	0	9	0	0
Gnpat	233	138	226	222	205
Gnpda1	179	49	143	0	231
Gnpda2	41	69	79	66	43
Gnpnat1	20	29	19	30	20
Gnptab	19	21	19	52	27
Gnptg	0	0	0	0	0
Gnrh1	1	0	0	1	0
Gns	1099	1306	1249	1498	1552
Golga1	33	64	51	86	66

For Review Only

Golga2	187	140	78	166	138
Golga3	32	40	41	75	79
Golga4	113	131	128	155	110
Golga5	81	79	117	68	85
Golga7	171	120	160	99	138
Golgb1	68	98	69	104	106
Golim4	60	32	56	60	64
Golm1	3385	174	870	4239	2886
Golph3	40	34	26	49	51
Golph3l	75	66	91	84	115
Golt1b	180	228	440	307	408
Gon4l	105	94	157	176	124
Gopc	20	75	39	16	19
Gorab	12	31	29	14	31
Gorasp1	155	81	97	126	78
Gorasp2	332	210	417	238	279
Gosr1	151	71	66	124	148
Gosr2	263	24	110	160	192
Got1	58	23	83	40	56
Got2	52	30	50	43	48
Gp1ba	9	5	9	4	5
Gp5	0	0	0	3	0
Gp9	36	71	91	93	62
Gpa33	0	0	0	0	0
Gpaa1	0	0	101	13	27
Gpalpp1	37	59	39	70	71
Gpam	73	34	57	69	62
Gpank1	25	24	34	28	42
Gpat2	0	0	3	2	0
Gpat4	122	133	231	166	188
Gpatch1	48	29	29	34	60
Gpatch11	19	18	31	17	40
Gpatch2	97	92	91	98	108
Gpatch2l	112	109	110	100	121
Gpatch3	8	6	0	25	26
Gpatch4	16	14	16	4	23
Gpatch8	1	1	0	0	0
Gpbar1	1	0	2	1	3
Gpbp1	112	128	133	97	108
Gpbp1l1	31	53	33	60	45
Gpc1	1	0	0	0	0
Gpc2	23	15	0	63	97
Gpc4	4	0	0	0	0
Gpc6	5	0	0	0	0
Gpcpd1	117	123	167	147	150
Gpd1	1	1	64	57	0
Gpd1l	164	115	128	142	199
Gpd2	6	0	14	0	31
Gper1	2	1	0	0	1
Gphn	43	53	35	45	70

For Review Only

Gpi1	302	186	175	130	116
Gpkow	69	61	56	49	92
Gpm6a	27	0	0	6	18
Gpm6b	6	4	0	6	10
Gpn1	104	53	105	42	119
Gpn2	34	29	0	0	0
Gpn3	14	36	32	0	45
Gpr107	243	190	243	219	234
Gpr108	140	46	147	83	102
Gpr132	0	5	4	0	0
Gpr137	59	67	88	45	13
Gpr137b	88	0	0	0	0
Gpr137b-ps	0	0	39	69	0
Gpr137c	3	0	0	2	1
Gpr146	389	312	470	431	332
Gpr150	5	3	1	9	2
Gpr153	0	0	0	3	0
Gpr155	341	274	288	465	325
Gpr157	126	81	94	156	189
Gpr160	215	177	193	155	176
Gpr161	1	2	1	1	0
Gpr162	2	3	4	5	4
Gpr165	96	182	166	288	220
Gpr179	0	0	0	0	0
Gpr18	8	1	0	6	13
Gpr180	49	43	44	52	76
Gpr182	2	0	0	0	0
Gpr183	0	399	598	335	559
Gpr19	2	1	13	0	0
Gpr20	0	0	0	8	0
Gpr21	2	0	4	1	0
Gpr22	1	1	0	9	1
Gpr3	1	0	0	0	0
Gpr31b	1	1	0	3	0
Gpr34	3449	3425	5582	3474	4053
Gpr35	39	19	66	76	55
Gpr4	3	1	3	5	7
Gpr52	0	0	0	4	0
Gpr65	19	35	14	13	15
Gpr68	1	1	0	9	15
Gpr75	0	0	0	0	0
Gpr82	9	4	0	17	27
Gpr84	27	86	0	327	0
Gpr89	59	48	76	79	53
Gprasp1	122	103	142	76	181
Gprasp2	92	56	64	78	61
Gprc5c	7	0	0	0	0
Gprin2	0	1	0	4	5
Gps1	0	1	0	0	182
Gps2	0	109	0	0	0

For Review Only

Gpsm1	125	59	95	90	111
Gpsm2	0	0	1	2	0
Gpsm3	0	0	0	0	0
Gpt	0	0	0	4	5
Gpt2	67	27	49	25	36
Gpx1	0	158	199	0	70
Gpx3	0	0	0	0	0
Gpx4	7	6	0	0	104
Gpx7	2	4	3	0	0
Gpx8	8	0	0	0	0
Gramd1a	199	237	136	271	303
Gramd1b	53	21	44	47	67
Gramd3	0	9	1	2	3
Gramd4	17	12	35	54	45
Grap	141	240	368	63	82
Grasp	0	0	0	1	0
Grb2	83	110	137	115	92
Grcc10	0	0	187	0	96
Greb1l	0	4	7	6	7
Grhl1	0	5	0	3	0
Grhpr	28	24	28	28	21
Grik5	0	0	0	2	0
Grin2c	0	0	3	0	0
Grin3a	3	1	0	1	0
Grin3b	7	3	7	0	11
Grina	40	0	0	148	497
Grip2	5	2	8	1	1
Gripap1	0	69	116	70	101
Grk4	6	6	5	6	9
Grk5	3	7	0	16	7
Grk6	41	21	45	1	30
Grm1	0	4	0	3	0
Grm3	0	0	15	3	3
Grn	2981	0	3095	1592	997
Grpel1	0	0	34	0	0
Grpel2	34	57	66	42	115
Grsf1	47	53	28	57	46
Grtp1	5	2	8	5	1
Grwd1	39	42	50	30	56
Gsap	130	187	236	232	219
Gsdmcl-ps	2	0	5	0	0
Gsdmd	0	138	42	5	53
Gse1	100	94	117	98	137
Gsg1	37	19	43	27	17
Gsg2	19	24	39	29	22
Gsk3a	0	13	2	3	4
Gsk3b	278	282	248	292	379
Gskip	60	68	77	73	42
Gsn	39	10	8	0	0
Gspt1	137	130	127	89	113

For Review Only

Gspt2	0	0	5	0	0
Gsr	8	7	11	9	17
Gss	17	20	31	27	37
Gsta4	0	0	27	0	0
Gstcd	18	15	13	23	23
Gstk1	0	0	11	1	0
Gstm1	21	6	13	7	21
Gstm2	5	0	0	0	0
Gstm4	26	16	16	12	16
Gstm5	11	0	47	0	0
Gstm7	0	0	0	0	0
Gsto1	40	35	48	34	63
Gsto2	1	0	0	0	0
Gstp1	102	83	113	78	71
Gstp2	23	22	31	17	24
Gstt1	0	0	0	0	0
Gstt2	9	0	0	9	30
Gstt3	25	6	13	11	20
Gstz1	42	27	44	38	25
Gt(ROSA)26Sor	4	20	48	23	8
Gtdc1	57	24	47	52	49
Gtf2a1	179	121	160	221	228
Gtf2a2	44	33	55	76	63
Gtf2b	41	100	106	70	71
Gtf2e1	65	69	76	71	60
Gtf2e2	43	45	50	43	53
Gtf2f1	71	66	92	49	67
Gtf2f2	52	21	43	42	49
Gtf2h1	47	40	46	46	67
Gtf2h2	376	313	439	333	294
Gtf2h3	29	18	20	0	16
Gtf2h4	0	0	1	1	1
Gtf2h5	22	33	39	25	34
Gtf2i	278	208	259	340	338
Gtf2ird1	25	25	21	27	35
Gtf2ird2	53	41	72	58	66
Gtf3a	81	73	58	31	41
Gtf3c1	92	67	62	116	134
Gtf3c2	412	131	449	101	143
Gtf3c3	60	70	68	105	80
Gtf3c4	49	56	62	56	47
Gtf3c5	27	31	55	39	25
Gtf3c6	23	85	47	31	8
Gtpbp1	134	184	189	137	194
Gtpbp10	25	2	21	10	22
Gtpbp2	117	96	88	63	99
Gtpbp3	10	0	0	0	0
Gtpbp4	40	57	95	100	79
Gtpbp6	0	0	0	0	0
Gtpbp8	44	49	40	48	26

For Review Only

Gtse1	0	0	0	0	0
Guca1a	12	9	19	10	13
Guca1b	2	5	0	5	5
Gucd1	15	0	50	6	22
Gucy1a2	0	0	0	0	0
Gucy2f	0	0	0	0	0
Guf1	5	7	0	36	36
Guk1	32	11	53	40	41
Gusb	725	80	316	62	226
Gvin1	37	98	53	127	98
Gxylt1	66	76	53	49	89
Gyg	82	54	78	71	79
Gys1	12	19	24	31	12
Gzf1	52	84	16	61	35
Gzmm	5	1	3	0	6
H13	267	216	277	247	257
H1f0	52	39	20	29	53
H2-Aa	79	36	55	44	34
H2-Ab1	150	7	0	0	0
H2-Bl	4	3	10	3	7
H2-D1	0	0	0	0	0
H2-DMa	503	322	667	453	548
H2-DMb1	282	297	437	275	329
H2-DMb2	39	82	56	24	32
H2-Eb1	56	31	0	0	0
H2-Eb2	0	0	0	2	0
H2-K1	1041	161	503	751	186
H2-M3	1	0	0	415	0
H2-Oa	24	4	196	0	116
H2-Ob	66	5	0	156	65
H2-Q1	26	21	35	25	27
H2-Q10	2	2	0	0	0
H2-Q2	15	8	20	23	17
H2-Q4	0	21	0	28	37
H2-Q5	1	2	0	15	0
H2-Q7	26	2	0	0	0
H2-T-ps	18	15	49	20	43
H2-T10	10	10	13	9	20
H2-T22	0	0	10	10	19
H2-T23	0	40	58	33	58
H2-T24	0	0	5	0	10
H2afj	44	66	68	38	43
H2afv	40	43	0	0	0
H2afx	24	18	29	12	23
H2afy	177	165	147	165	195
H2afy3	1	2	0	5	12
H2afz	0	0	35	0	0
H3f3a	92	77	107	83	97
H3f3c	20	16	28	13	22
H60b	4	8	4	6	2

For Review Only

H6pd	94	68	102	140	132
Haao	33	26	28	32	50
Habp4	12	16	9	7	6
Hacd1	13	2	0	0	4
Hacd2	36	47	48	42	47
Hacd3	272	172	244	234	203
Hacd4	26	18	0	43	71
Hace1	15	0	9	25	13
Hacl1	0	0	4	4	9
Hadh	83	89	70	57	91
Hadha	205	187	214	161	196
Hadhb	127	84	144	116	107
Hagh	113	142	153	77	135
Haghl	12	12	36	21	0
Hap1	0	5	7	5	10
Harbi1	70	27	59	28	73
Hars	78	88	85	76	66
Hars2	43	93	134	75	96
Has3	8	3	0	10	14
Hat1	22	118	71	0	57
Haus1	14	15	19	14	24
Haus2	41	14	40	42	29
Haus3	35	40	33	39	55
Haus4	0	1	3	4	0
Haus5	23	10	0	30	0
Haus6	21	26	29	28	27
Haus7	25	12	59	27	25
Haus8	8	8	21	26	11
Havcr2	405	322	530	0	522
Hax1	0	0	12	27	0
Hbegf	18	12	15	10	8
Hbp1	262	245	406	280	304
Hbs1l	87	71	83	75	72
Hcar2	41	38	29	59	34
Hccs	40	26	28	43	32
Hcfc1	24	8	0	50	888
Hcfc1r1	0	43	0	0	0
Hcfc2	40	35	61	59	95
Hck	541	483	627	536	573
Hcls1	408	358	406	390	436
Hcn3	10	1	0	12	7
Hcst	13	0	41	21	0
Hdac1	54	58	70	40	45
Hdac10	16	0	0	0	0
Hdac11	42	63	51	59	64
Hdac2	18	26	0	24	0
Hdac3	58	240	86	98	69
Hdac4	66	58	63	62	69
Hdac5	63	42	0	132	62
Hdac6	66	19	46	30	51

For Review Only

Hdac7	0	0	0	4	0
Hdac8	41	48	41	56	37
Hdac9	18	38	33	57	34
Hdc	9	4	0	0	9
Hddc2	5	0	4	0	7
Hddc3	6	8	24	20	7
Hdgf	40	38	44	0	56
Hdgfrp2	53	14	0	135	183
Hdgfrp3	52	84	109	82	71
Hdhd2	58	47	64	59	61
Hdhd3	2	0	13	0	8
Hdlbp	351	271	350	357	334
Heatr1	95	81	96	123	119
Heatr3	48	15	62	23	58
Heatr4	2	0	0	0	0
Heatr5a	200	218	194	289	306
Heatr5b	69	46	73	50	58
Heatr6	68	67	53	110	113
Hebp1	13	24	18	28	22
Hebp2	5	0	0	2	4
Heca	31	87	63	50	73
Hectd1	130	162	166	255	198
Hectd2	6	0	0	9	0
Hectd3	201	75	94	106	62
Hecw2	0	0	0	0	4
Heg1	0	0	1	9	0
Helb	40	4	18	28	46
Hells	4	0	0	0	1
Helq	13	13	15	18	24
Helz	156	154	209	280	216
Helz2	3	5	15	12	4
Hemk1	47	20	44	17	42
Henmt1	0	0	0	2	0
Herc1	238	246	291	381	350
Herc2	469	772	559	794	1019
Herc3	9	10	4	18	17
Herc4	68	134	117	161	140
Herc6	28	28	53	51	46
Herpud1	706	573	601	179	355
Herpud2	33	31	44	57	62
Hes1	7	0	2	6	10
Hes6	0	0	0	0	0
Hes7	0	0	0	0	6
Hexa	2764	1889	2841	1837	2449
Hexb	27133	23751	38520	22170	25831
Hexdc	14	24	21	25	20
Hexim1	24	31	38	20	31
Hexim2	2	1	4	4	6
Hey1	0	0	1	0	0
Hfe	268	167	332	162	102

For Review Only

Hgf	1	1	0	0	1
Hgh1	0	0	0	0	0
Hgs	182	131	73	69	168
Hgsnat	0	0	1	3	49
Hhat	13	16	34	27	16
Hhex	288	0	26	10	1
Hhip	0	4	0	2	4
Hibadh	91	71	74	58	76
Hibch	23	11	38	23	19
Hic1	0	1	2	2	0
Hic2	13	57	28	37	43
Hid1	3	0	0	0	0
Hif1a	281	201	213	264	252
Hif1an	54	62	53	61	75
Hif3a	3	0	0	0	0
Higd1a	25	27	60	31	28
Higd1b	3	0	0	0	4
Higd2a	115	84	0	66	0
Hilpda	14	18	24	11	13
Hinfp	45	40	48	49	43
Hint1	65	35	99	53	79
Hint2	0	0	0	0	0
Hint3	13	14	19	0	19
Hip1	2	2	11	15	14
Hip1r	15	8	13	6	6
Hipk1	257	201	137	212	190
Hipk2	157	166	166	204	129
Hipk3	45	93	54	76	103
Hipk4	0	0	0	0	0
Hira	11	4	40	32	34
Hirip3	0	0	0	0	0
Hist1h1a	0	0	0	0	1
Hist1h1c	22	11	12	14	12
Hist1h1d	5	4	1	6	8
Hist1h1e	28	15	19	48	38
Hist1h2aa	1	0	2	2	1
Hist1h2ab	5	5	11	4	6
Hist1h2ac	15	13	11	9	20
Hist1h2ad	12	11	24	7	12
Hist1h2ae	10	4	8	4	7
Hist1h2af	4	3	6	2	3
Hist1h2ag	6	3	7	3	3
Hist1h2ah	6	3	9	5	4
Hist1h2ai	5	6	9	3	5
Hist1h2ak	4	5	7	4	5
Hist1h2al	12	5	12	3	10
Hist1h2an	6	5	9	4	6
Hist1h2ao	27	34	49	20	29
Hist1h2ap	22	21	31	17	20
Hist1h2bb	0	0	4	1	0

For Review Only

Hist1h2bc	33	29	58	27	45
Hist1h2be	18	16	24	26	31
Hist1h2bf	4	4	3	3	2
Hist1h2bg	43	29	40	28	49
Hist1h2bh	3	3	4	3	1
Hist1h2bj	4	4	4	4	2
Hist1h2bk	3	1	4	2	5
Hist1h2bl	0	3	1	1	2
Hist1h2bm	1	1	1	0	1
Hist1h2bn	4	1	3	2	6
Hist1h2bp	1	0	1	1	1
Hist1h2bq	4	5	4	4	1
Hist1h2br	5	4	3	4	4
Hist1h3a	3	1	3	1	6
Hist1h3b	0	0	1	1	1
Hist1h3c	3	1	3	3	6
Hist1h3d	3	3	0	2	5
Hist1h3e	18	4	3	5	16
Hist1h3f	3	3	0	2	4
Hist1h3g	0	1	0	0	2
Hist1h3i	1	0	0	1	1
Hist1h4a	1	1	1	1	4
Hist1h4b	0	0	0	1	2
Hist1h4c	7	5	4	7	13
Hist1h4d	5	4	7	11	22
Hist1h4f	0	1	0	0	1
Hist1h4h	1	3	4	3	8
Hist1h4i	8	6	7	6	9
Hist1h4j	0	0	0	0	0
Hist1h4k	1	1	0	0	4
Hist1h4m	0	1	0	0	1
Hist1h4n	2	0	0	0	3
Hist2h2aa1	55	42	56	29	60
Hist2h2aa2	38	20	38	16	38
Hist2h2ab	6	4	4	4	5
Hist2h2ac	5	1	5	2	4
Hist2h2bb	3	2	3	3	3
Hist2h2be	3	4	14	21	43
Hist2h3b	4	8	6	7	8
Hist2h3c1	3	5	4	8	6
Hist2h3c2	1	4	2	6	3
Hist2h4	3	3	1	5	4
Hist3h2a	84	70	157	57	86
Hist3h2ba	1	4	3	1	1
Hist4h4	8	3	1	5	14
Hivep1	99	81	102	144	116
Hivep2	15	15	19	25	21
Hivep3	588	411	634	630	701
Hjurp	163	144	188	230	165
Hk1	52	29	25	45	82

For Review Only

Hk2	609	483	668	570	477
Hk3	148	48	0	89	25
Hlcs	21	30	31	40	32
Hlf	17	3	23	32	12
Hltf	91	104	78	152	137
Hlx	43	16	7	23	28
Hmbox1	133	65	71	185	137
Hmbs	0	0	12	3	6
Hmces	37	35	40	39	28
Hmcn2	0	0	0	0	0
Hmg20a	142	0	157	1	71
Hmg20b	1	117	23	65	58
Hmga1	50	41	30	38	52
Hmga1-rs1	47	31	24	33	45
Hmga2	1	0	5	3	2
Hmga2-ps1	146	306	501	278	243
Hmgb1	64	93	80	71	84
Hmgb2	27	16	18	19	28
Hmgb3	0	1	0	2	0
Hmgcl	44	223	392	125	123
Hmgcr	64	52	48	66	71
Hmgcs1	84	34	77	88	90
Hmgcs2	4	0	0	0	0
Hmgn1	107	113	132	97	141
Hmgn2	0	0	0	0	0
Hmgn3	13	1	0	20	0
Hmgn5	0	2	0	0	1
Hmgxb3	102	70	98	90	134
Hmgxb4	33	39	41	60	60
Hmha1	0	0	63	1	11
Hmmr	3	0	0	0	4
Hmox1	64	43	70	40	50
Hmox2	0	630	0	24	58
Hmx2	0	0	0	2	0
Hn1	271	251	323	202	261
Hn1l	0	0	30	32	13
Hnmt	148	81	129	102	98
Hnrnpa0	37	39	42	22	23
Hnrnpa1	1	0	0	0	0
Hnrnpa2b1	0	0	4	55	0
Hnrnpa3	58	76	60	93	88
Hnrnpab	5	6	0	0	0
Hnrnpc	205	0	355	43	111
Hnrnpd	54	42	78	52	42
Hnrnpdl	32	39	78	0	30
Hnrnpf	316	255	379	309	352
Hnrnph1	0	0	0	0	0
Hnrnph2	129	132	177	174	125
Hnrnph3	0	0	0	24	0
Hnrnpk	39	632	805	570	672

For Review Only

Hnrnpl	8	13	0	94	42
Hnrnpll	13	43	13	24	41
Hnrnpm	96	131	173	94	39
Hnrnpr	165	120	98	160	154
Hnrnpu	193	379	249	244	258
Hnrnpul1	135	52	89	98	96
Hnrnpul2	28	46	57	22	43
Hoga1	10	3	4	5	4
Homer1	22	15	23	10	20
Homer3	0	13	3	14	2
Homez	9	12	33	30	49
Hook2	37	33	39	49	74
Hook3	100	249	168	239	206
Hottip	0	2	4	0	0
Hp	58	12	0	0	0
Hp1bp3	293	197	324	285	310
Hpca	3	0	0	0	0
Hpcal1	98	79	107	89	100
Hpcal4	0	0	0	0	0
Hpf1	71	74	147	76	51
Hpgd	445	650	713	666	601
Hpgds	769	579	647	553	756
Hpn	1	1	0	29	37
Hprt	136	122	203	122	191
Hps1	135	83	117	96	174
Hps3	420	342	356	584	639
Hps4	131	787	147	0	0
Hps5	94	40	75	97	111
Hps6	45	30	22	31	58
Hras	13	7	6	10	0
Hrh2	52	75	102	72	103
Hs1bp3	381	208	0	0	0
Hs2st1	38	43	32	38	53
Hs3st1	0	0	0	0	0
Hs3st2	1	0	0	2	0
Hs3st3b1	23	28	22	45	33
Hs6st1	55	94	110	81	50
Hs6st2	4	1	0	0	0
Hsbp1	255	233	302	192	286
Hsbp1l1	0	0	0	0	0
Hscb	19	15	18	14	14
Hsd11b2	5	0	0	0	4
Hsd17b1	5	8	12	5	5
Hsd17b10	43	42	73	36	48
Hsd17b11	20	25	18	29	47
Hsd17b12	128	132	138	141	160
Hsd17b4	264	250	392	234	324
Hsd17b7	3	1	0	8	10
Hsd3b4	1	2	0	1	1
Hsd3b7	0	0	190	0	0

For Review Only

Hsdl1	182	132	210	164	217
Hsdl2	9	25	0	36	27
Hsf1	1	1	0	404	0
Hsf2	37	54	32	51	49
Hsf2bp	8	13	12	16	14
Hsf5	3	0	0	0	0
Hsp90aa1	89	105	96	60	77
Hsp90ab1	0	0	1524	1016	1355
Hsp90b1	1959	1581	2124	1457	1841
Hspa12a	12	18	27	16	10
Hspa13	100	101	132	138	112
Hspa14	95	67	60	103	110
Hspa1a	5	0	0	5	15
Hspa1b	0	0	0	2	5
Hspa1l	7	0	0	4	7
Hspa2	9	3	3	4	18
Hspa4	196	152	147	163	206
Hspa4l	1	19	0	10	7
Hspa5	1640	1366	1530	762	1181
Hspa8	1730	1256	1800	499	0
Hspa9	247	173	249	164	176
Hspb1	2	0	0	0	0
Hspb11	0	6	0	0	0
Hspb3	50	67	77	36	33
Hspb6	21	2	8	8	22
Hspb8	7	0	0	0	0
Hspb9	1	0	0	0	6
Hspbap1	52	41	52	67	80
Hspbp1	67	34	65	31	56
Hspd1	73	60	93	82	71
Hspe1	15	20	61	55	0
Hspg2	6	0	0	1	1
Hsph1	37	92	56	54	60
Htatip2	18	8	19	13	10
Htatsf1	58	38	47	36	63
Htr2b	23	3	10	24	15
Htr2c	6	0	0	1	4
Htr4	0	0	0	0	0
Htr5b	0	0	0	1	2
Htra3	17	26	57	49	41
Htt	127	100	184	174	130
Hus1	10	61	24	39	31
Hus1b	1	0	5	1	0
Huwe1	411	419	453	537	584
Hvcn1	719	573	875	718	700
Hyal1	1	0	0	0	0
Hyal2	88	81	82	40	93
Hyal3	4	4	9	3	3
Hyal6	1	0	0	0	0
Hyi	0	11	0	0	13

For Review Only

Hykk	11	4	20	23	45
Hyls1	0	7	0	9	0
Hyou1	92	56	47	116	139
I830077J02Rik	46	34	0	0	0
I830127L07Rik	35	16	8	17	15
Iah1	0	63	0	0	0
Iars	30	57	153	184	202
Iars2	39	41	29	55	70
Iba57	6	4	21	15	20
Ibtk	86	20	42	29	55
Ica1	0	0	0	2	3
Ica1l	2	15	15	36	21
Icam1	481	249	443	254	409
Icam2	0	0	0	1	7
Ice1	45	86	112	104	78
Ice2	48	65	54	46	46
Ick	63	33	49	46	54
Icmt	90	99	108	133	137
Icos	1	7	0	7	0
Icosl	280	26	130	256	0
Ict1	0	46	37	12	5
Id1	1	0	13	0	0
Id3	17	1	0	0	0
Ide	129	52	58	89	134
Idh1	143	110	99	100	111
Idh2	143	387	227	34	88
Idh3a	131	75	79	56	68
Idh3b	0	143	71	68	14
Idh3g	0	0	2	0	0
Idi1	15	5	3	1	3
Idnk	0	0	51	51	53
Ids	73	102	74	128	135
Idua	351	69	193	226	255
Ier2	284	173	213	132	291
Ier3	6	0	35	0	0
Ier3ip1	148	0	186	38	2
Ier5	123	227	192	103	136
Iffo1	523	1	0	0	0
Iffo2	9	21	12	24	16
Ifi203	1	0	0	1	0
Ifi204	12	12	20	16	30
Ifi205	0	3	0	1	0
Ifi27	72	0	16	0	119
Ifi27l2a	0	3	0	0	0
Ifi30	1	1	0	0	0
Ifi44	0	2	0	0	0
Ifi47	7	0	1	8	0
Ifih1	54	58	52	57	94
Ifit1	0	12	13	11	0
Ifit2	15	11	37	27	9

For Review Only

Ifit3	1	1	159	64	0
Ifit3b	1	1	0	78	0
Ifitm1	0	0	0	0	0
Ifitm10	0	0	2	4	5
Ifitm2	5	7	6	6	0
Ifitm3	25	21	31	12	6
Ifitm6	25	5	0	0	4
Ifnar1	336	296	457	373	349
Ifnar2	253	163	206	322	202
Ifngr1	2677	2506	3565	2555	2891
Ifngr2	562	499	706	570	668
Ifrd1	60	37	55	63	28
Ifrd2	14	14	0	13	18
Ift122	0	18	0	6	27
Ift140	60	62	54	86	53
Ift172	119	60	133	79	123
Ift20	0	0	0	0	0
Ift22	19	17	27	10	17
Ift27	11	0	13	5	8
Ift43	1	2	0	11	11
Ift46	71	30	56	35	46
Ift52	89	70	109	88	92
Ift57	13	2	0	13	0
Ift74	13	15	14	16	15
Ift80	12	1	0	16	5
Ift81	10	8	6	8	10
Ift88	24	7	25	0	33
Igbp1	93	96	115	99	102
Igdcc4	5	4	2	0	0
Igf1	1	1	0	5	0
Igf1r	55	62	68	104	67
Igf2	22	0	0	1	19
Igf2bp2	18	24	16	14	18
Igf2bp3	31	24	47	49	35
Igf2r	4	0	0	0	0
Igfals	1	0	0	2	0
Igfbp2	31	0	3	7	0
Igfbp4	54	35	60	83	55
Igfn1	0	2	0	0	6
Ighmbp2	38	10	56	20	57
Igip	8	3	13	11	5
Igsf10	0	1	7	13	0
Igsf6	0	0	1	64	1
Igsf8	1	1	189	0	0
Igsf9	7	2	0	17	17
Igsf9b	3	0	2	0	4
Igtp	130	151	187	159	202
Iigp1	0	4	0	0	0
Ik	181	181	347	61	47
Ikbip	43	31	55	38	27

For Review Only

Ikbkap	86	78	81	96	116
Ikbkb	72	484	254	55	90
Ikbke	2	4	0	0	6
Ikbkg	90	108	166	181	170
Ikzf1	538	529	514	622	711
Ikzf2	19	34	21	27	30
Ikzf5	16	24	35	56	41
Il10ra	9986	59	3378	2896	0
Il10rb	240	378	379	242	263
Il11ra1	105	15	77	34	0
Il11ra2	14	0	12	9	9
Il12rb2	20	17	16	15	14
Il13ra1	379	504	446	583	553
Il15	39	54	46	75	78
Il15ra	0	7	0	24	30
Il16	641	605	630	557	676
Il17c	0	0	0	0	0
Il17ra	67	39	695	0	864
Il17rb	4	0	0	2	9
Il17rc	4	5	2	2	3
Il18	0	99	6	70	6
Il18bp	1	1	75	0	0
Il18rap	2	2	13	13	0
Il1a	138	358	358	302	287
Il1b	3	8	0	9	6
Il1bos	0	2	1	4	0
Il1r1	4	3	4	0	0
Il1r2	4	1	0	1	5
Il1rap	35	2	46	33	0
Il1rl1	20	1	0	0	15
Il1rl2	152	164	141	174	236
Il1rn	8	2	6	3	0
Il20rb	2	5	0	0	7
Il21r	302	269	367	478	525
Il27	11	11	14	8	3
Il2rg	27	9	17	17	22
Il34	0	0	0	0	8
Il3ra	0	4	0	0	0
Il4ra	1617	3	1019	0	1160
Il6	20	0	31	24	23
Il6ra	503	608	533	763	709
Il6st	710	549	686	1083	852
Il7r	65	81	52	69	71
Ildr1	23	10	27	19	22
Ildr2	39	57	45	36	8
Ilf2	61	0	62	41	0
Ilf3	237	174	250	299	239
Ilk	0	1	0	0	0
Ilkap	43	73	98	74	62
Ilvbl	227	28	3	56	29

For Review Only

Immp1l	132	24	0	0	66
Immp2l	12	5	18	11	13
Immt	224	191	290	231	300
Imp3	64	62	75	50	57
Imp4	0	0	1	0	0
Impa1	96	75	102	88	126
Impa2	11	0	0	4	14
Impact	209	138	170	205	170
Impad1	18	18	0	17	12
Impdh1	0	0	2	0	7
Impdh2	0	31	0	0	0
Impg2	0	0	1	0	0
Inadl	2	14	16	8	13
Inafm1	8	17	14	17	19
Inafm2	141	116	118	76	119
Inca1	5	3	8	4	3
Incenp	30	11	28	20	39
Inf2	0	2	0	7	10
Ing1	33	62	48	46	49
Ing2	4	22	4	9	8
Ing3	53	60	77	42	52
Ing5	64	39	78	52	78
Inhba	9	0	0	1	10
Inip	157	61	230	124	167
Ino80	19	32	54	37	37
Ino80b	9	6	0	0	0
Ino80c	63	75	108	76	87
Ino80d	103	188	126	211	198
Ino80dos	2	0	12	0	0
Ino80e	155	100	122	54	94
Inpp1	24	15	31	23	30
Inpp4a	227	202	159	256	234
Inpp4b	232	158	217	258	329
Inpp5a	1	9	14	0	0
Inpp5b	3	3	0	0	17
Inpp5d	0	0	0	3	2
Inpp5e	68	92	109	107	107
Inpp5f	75	43	50	52	81
Inpp5k	109	138	331	145	285
Inppl1	72	58	48	57	55
Insig1	51	12	15	38	39
Insig2	92	88	109	96	131
Insr	124	186	179	287	244
Ints1	17	10	0	0	0
Ints10	125	86	120	81	102
Ints12	49	33	45	66	53
Ints2	44	51	29	47	59
Ints3	136	94	121	163	184
Ints4	132	97	66	104	97
Ints5	50	50	61	65	50

For Review Only

Ints6	15	19	12	12	6
Ints7	60	40	36	61	62
Ints8	30	33	18	29	22
Ints9	23	7	79	63	70
Intu	14	7	15	19	16
Invs	18	52	38	38	40
Ip6k1	1	1	701	0	0
Ip6k2	11	6	0	0	25
Ipcef1	16	73	17	53	57
Ipmk	40	38	39	71	57
Ipo11	98	69	77	113	109
Ipo13	149	122	108	216	57
Ipo4	60	13	41	28	132
Ipo5	115	104	127	129	144
Ipo7	101	86	98	135	110
Ipo8	59	51	49	61	61
Ipo9	203	108	187	225	201
Ipp	14	12	20	23	46
Ippk	19	13	15	27	32
Iqcb1	15	6	38	25	20
Iqcc	41	25	23	29	40
Iqcd	9	2	5	6	4
Iqce	35	73	31	75	66
Iqcg	6	3	0	0	0
Iqch	4	8	5	6	5
Iqck	0	0	6	3	0
Iqgap1	21	16	16	37	15
Iqgap3	61	17	55	32	39
Iqsec1	47	38	43	50	60
Iqsec2	91	11	26	47	33
Iqsec3	0	0	5	0	0
Irak1	96	184	159	132	156
Irak2	12	14	0	160	437
Irak3	56	8	18	21	0
Irak4	1	1	0	0	0
Ireb2	101	115	102	148	123
Irf1	159	91	125	209	153
Irf2	107	113	91	166	174
Irf2bp1	35	24	50	24	19
Irf2bp2	29	17	0	216	401
Irf2bpl	192	100	110	216	203
Irf3	113	164	292	0	0
Irf4	21	31	42	36	16
Irf5	0	1	440	1	25
Irf6	22	3	0	13	19
Irf7	17	18	0	0	0
Irf8	1332	1071	1393	898	1062
Irf9	0	43	0	0	0
Irgc1	0	0	2	0	0
Irgm1	84	156	166	140	204

For Review Only

Irgm2	0	196	196	0	211
Irgq	33	42	35	48	71
Irs1	0	2	0	1	0
Irs2	74	41	50	33	70
Irs3	6	0	20	13	0
Isca1	158	93	156	107	105
Iscu	1	0	0	0	0
Isg15	0	0	3	5	11
Isg20	6	4	11	5	6
Isg20l2	51	7	22	41	71
Islr	2	0	0	9	0
Isoc1	33	22	36	28	42
Isoc2b	6	10	0	16	45
Ispd	5	0	1	2	8
Israa	14	11	8	14	15
Ist1	239	245	139	318	157
Isy1	0	48	11	9	18
Isyna1	219	202	253	178	197
Itch	150	124	138	216	165
Itfg1	303	190	288	221	285
Itfg2	45	2	32	33	19
Itga10	0	0	0	0	0
Itga3	4	0	0	0	0
Itga4	2	5	0	9	3
Itga5	52	39	68	76	72
Itga6	269	378	344	254	337
Itga9	82	87	119	142	133
Itgae	15	10	0	35	21
Itgal	3	2	0	0	0
Itgam	689	835	1019	873	1517
Itgav	156	220	150	265	256
Itgax	11	9	31	18	24
Itgb1	330	346	320	361	384
Itgb1bp1	45	48	59	44	40
Itgb1bp2	0	0	0	4	0
Itgb2	1425	1131	1652	1032	1208
Itgb2l	20	0	0	0	0
Itgb3	135	127	137	176	173
Itgb3bp	2	6	12	16	0
Itgb4	0	0	2	0	11
Itgb5	1087	1719	1353	850	1077
Itgb7	5	0	0	0	0
Itih2	0	0	0	0	0
Itih5	5	0	0	3	14
Itm2a	0	0	0	0	0
Itm2b	8043	7838	11273	7714	8920
Itm2c	1527	1201	1534	1453	1650
Itpa	25	11	50	22	27
Itpk1	96	68	107	96	97
Itpkb	44	57	58	77	79

For Review Only

Itpkc	58	64	62	44	23
Itpr1	38	5	23	43	25
Itpr2	414	288	407	517	456
Itpr3	38	36	63	49	49
Itprip	17	11	23	23	39
Itpripl1	937	673	944	718	746
Itpripl2	31	57	31	67	68
Itsn1	10	7	9	12	13
Itsn2	109	217	139	218	208
Ivd	82	53	105	35	51
Ivns1abp	72	1322	1208	2213	1865
Iws1	80	49	77	59	57
Izumo4	9	0	19	7	10
Jade1	48	31	38	41	30
Jade2	234	83	131	178	149
Jade3	2	16	4	12	6
Jag1	5	2	6	8	0
Jag2	21	14	36	23	16
Jagn1	130	118	183	114	101
Jak1	555	243	398	553	673
Jak2	63	75	73	68	55
Jak3	26	31	82	148	179
Jam2	239	278	284	285	287
Jam3	60	55	41	24	41
Jarid2	99	104	89	113	143
Jazf1	2	8	5	9	6
Jdp2	0	0	0	2	2
Jkamp	134	109	189	146	128
Jmjd1c	352	550	430	662	460
Jmjd4	20	50	44	38	74
Jmjd6	82	60	67	75	84
Jmjd7	0	0	0	7	0
Jmy	43	40	42	56	34
Josd1	46	21	51	36	42
Jph3	43	29	29	44	31
Jpx	13	19	11	7	19
Jrk	39	12	38	53	74
Jrkl	12	5	11	36	43
Jtb	0	0	0	0	0
Jun	2679	1434	2114	1708	3507
Junb	2844	2045	2585	1744	2701
Jund	31	22	29	35	37
Junos	21	10	3	11	20
Jup	111	127	197	195	185
Kalrn	11	1	4	4	0
Kank1	4	0	0	0	0
Kank2	1	0	0	3	0
Kank3	86	6	1	55	0
Kansl1	163	159	209	217	257
Kansl1l	67	103	83	128	87

For Review Only

Kansl2	48	17	38	64	105
Kansl3	166	100	106	181	152
Kantr	37	44	41	50	50
Kars	100	90	123	80	104
Kat2a	1	55	0	99	128
Kat2b	33	46	46	36	63
Kat5	28	0	0	14	42
Kat6a	208	202	163	282	263
Kat6b	91	95	91	133	124
Kat7	249	167	182	226	247
Kat8	0	70	47	9	13
Katna1	71	57	84	81	67
Katnal1	10	8	19	26	34
Katnb1	30	29	0	11	50
Katnbl1	18	24	36	35	14
Kazald1	1	5	0	7	0
Kbtbd11	1	1	5	1	0
Kbtbd12	6	0	0	0	11
Kbtbd2	192	244	232	208	330
Kbtbd3	22	26	22	32	29
Kbtbd4	102	111	85	87	124
Kbtbd6	7	4	3	8	9
Kbtbd7	78	55	42	85	85
Kbtbd8	30	5	0	31	22
Kcmf1	37	32	38	34	40
Kcna2	3	0	0	0	0
Kcna3	4	16	1	4	0
Kcna4	2	0	3	3	0
Kcnab2	3	2	4	6	0
Kcnab3	0	0	7	1	2
Kcnb1	1	9	0	6	12
Kcnb2	1	2	0	0	0
Kcnc1	0	0	0	0	0
Kcnc2	0	1	4	4	4
Kcnd1	0	0	208	91	90
Kcnd3	0	6	6	3	0
Kcne1	0	2	1	0	1
Kcne2	12	0	0	5	6
Kcng4	0	2	0	0	0
Kcnh1	2	0	0	0	6
Kcnh6	11	16	18	18	18
Kcnh7	0	0	2	0	0
Kcnip2	0	0	0	0	0
Kcnip3	71	62	97	60	105
Kcnip4	0	6	0	0	0
Kcnj10	15	9	12	21	20
Kcnj12	24	10	7	6	17
Kcnj13	62	4	7	16	0
Kcnj14	0	2	0	0	0
Kcnj16	13	3	9	9	5

For Review Only

Kcnj2	18	61	44	63	57
Kcnj8	0	0	0	2	0
Kcnj9	29	29	24	21	23
Kcnk1	7	0	0	0	4
Kcnk12	23	8	6	11	19
Kcnk6	167	179	131	202	261
Kcnk7	8	7	7	6	7
Kcnma1	27	32	7	31	38
Kcnmb3	11	1	0	13	1
Kcnn1	0	0	6	0	0
Kcnn4	9	0	12	5	11
Kcnq1ot1	195	281	235	394	328
Kcnq2	0	0	6	0	2
Kcnrg	16	16	8	28	11
Kcnt1	0	2	0	0	9
Kcnv1	3	2	0	0	0
Kcp	12	15	11	5	25
Kctd1	0	0	0	0	0
Kctd10	42	42	41	53	49
Kctd11	42	35	43	36	65
Kctd12	384	145	1593	0	0
Kctd12b	11	18	15	12	26
Kctd13	33	27	31	29	34
Kctd14	1	0	0	0	0
Kctd18	46	39	47	57	62
Kctd2	38	3	11	23	22
Kctd20	140	152	195	211	199
Kctd21	82	103	98	69	99
Kctd3	35	21	9	31	44
Kctd4	0	8	1	7	5
Kctd5	19	29	35	56	38
Kctd6	10	18	41	21	13
Kctd7	12	8	19	27	11
Kctd9	5	5	22	17	17
Kdelc1	36	71	106	54	53
Kdelc2	41	33	23	42	46
Kdelr1	112	111	54	116	111
Kdelr2	355	330	436	314	378
Kdelr3	0	5	0	4	4
Kdf1	1	0	0	0	0
Kdm1a	28	27	31	31	19
Kdm1b	56	32	36	86	72
Kdm2a	186	139	155	173	229
Kdm2b	106	76	107	145	187
Kdm3a	107	118	128	176	149
Kdm3b	159	144	210	129	188
Kdm4a	23	29	21	42	33
Kdm4b	56	90	65	86	89
Kdm4c	102	50	59	134	106
Kdm4d	0	5	0	0	2

For Review Only

Kdm5a	178	119	155	289	199
Kdm5b	55	91	56	75	116
Kdm5c	129	178	96	208	136
Kdm5d	62	63	44	70	47
Kdm6a	56	180	52	95	83
Kdm6b	78	30	29	54	84
Kdm7a	116	131	135	144	135
Kdm8	25	10	8	11	16
Kdr	3	17	10	23	16
Kdsr	66	91	64	95	80
Keap1	181	68	96	104	132
Khdc3	1	10	2	4	6
Khdrbs1	214	299	183	330	305
Khdrbs3	15	17	20	10	13
Khk	140	107	144	30	105
Khnyn	312	0	231	87	46
Khsrp	12	14	24	34	29
Kidins220	155	175	186	310	234
Kif11	0	0	0	0	0
Kif13a	59	55	60	39	72
Kif13b	126	76	91	145	142
Kif15	0	0	0	0	0
Kif16b	20	29	11	33	36
Kif1b	55	59	67	117	111
Kif1bp	19	19	22	24	32
Kif1c	12	5	14	18	7
Kif20b	0	6	10	7	0
Kif21b	508	447	340	946	764
Kif22	0	0	0	2	0
Kif23	0	0	0	5	17
Kif24	1	4	5	6	12
Kif26a	0	0	0	0	0
Kif26b	1	4	1	0	5
Kif27	0	0	1	0	8
Kif2a	29	18	25	27	20
Kif2c	0	2	0	0	0
Kif3a	40	29	44	28	30
Kif3b	62	109	85	128	106
Kif3c	19	66	11	51	41
Kif4	0	0	0	0	5
Kif5a	63	3	0	5	21
Kif5b	184	129	143	193	180
Kif5c	0	0	0	0	0
Kif6	1	0	0	0	3
Kif9	49	37	12	25	18
Kifap3	54	18	0	66	84
Kifc1	3	1	0	4	0
Kifc2	9	1	20	23	23
Kifc3	16	9	10	24	18
Kifc5b	0	3	4	4	1

For Review Only

Kin	37	32	30	25	34
Kiss1r	5	1	3	4	15
Kitl	15	31	22	51	34
Kiz	49	31	48	70	51
Kl	92	0	0	63	27
Klc1	172	201	236	223	214
Klc2	15	11	3	15	14
Klc4	111	60	123	91	90
Klf1	3	0	0	1	0
Klf10	26	17	26	25	19
Klf11	4	2	3	3	5
Klf12	86	126	107	169	169
Klf13	303	264	261	207	258
Klf15	8	0	4	3	2
Klf16	4	2	7	11	11
Klf2	2	18	0	0	0
Klf3	207	256	221	289	256
Klf4	1	12	0	0	77
Klf7	123	104	144	143	138
Klf9	4	0	0	3	4
Klhdc1	14	12	28	11	6
Klhdc10	43	60	71	47	54
Klhdc2	30	24	30	26	53
Klhdc3	156	119	0	0	0
Klhdc4	62	56	64	35	46
Klhdc8b	82	17	54	11	0
Klhdc9	2	3	0	0	6
Klhl10	148	137	149	175	131
Klhl11	34	7	24	51	62
Klhl12	6	3	25	16	45
Klhl13	0	8	0	6	10
Klhl15	14	3	5	10	0
Klhl17	67	1	0	12	0
Klhl18	89	38	8	6	0
Klhl2	38	19	0	17	0
Klhl20	38	29	35	74	51
Klhl21	89	41	35	65	52
Klhl22	129	76	95	147	103
Klhl23	25	6	30	84	69
Klhl24	239	256	276	300	209
Klhl25	144	160	158	159	191
Klhl26	51	29	31	34	37
Klhl28	46	62	40	54	54
Klhl29	2	2	0	0	6
Klhl3	29	11	16	34	38
Klhl35	7	0	13	3	2
Klhl36	113	73	99	116	124
Klhl38	44	8	19	22	37
Klhl42	32	38	36	54	46
Klhl5	48	56	46	59	64

For Review Only

Klhl6	164	300	380	201	375
Klhl7	285	222	273	277	187
Klhl8	16	13	21	10	10
Klhl9	228	213	190	294	244
Klk10	0	0	4	4	0
Klk12	4	0	0	0	0
Klk7	3	2	0	0	4
Klk8	148	204	213	119	194
Klk9	5	11	3	13	11
Klkb1	2	0	0	5	4
Klrb1b	0	1	4	0	0
Klrd1	8	6	13	9	0
Klrg2	15	5	3	7	1
Kmt2a	200	249	274	326	323
Kmt2b	41	75	15	68	72
Kmt2c	270	457	338	463	448
Kmt2d	598	659	428	1150	1131
Kmt2e	508	455	444	309	516
Kmt5a	5	26	0	21	12
Kncn	0	0	0	3	0
Kng2	0	0	0	0	14
Knop1	94	166	152	101	124
Kntc1	0	0	0	3	0
Kpna1	153	107	126	163	134
Kpna2	19	10	28	21	18
Kpna3	118	217	96	164	208
Kpna4	15	37	48	53	43
Kpna6	28	24	41	59	49
Kpnb1	155	244	275	232	170
Kptn	12	7	10	10	12
Kras	30	47	43	34	48
Krba1	61	84	94	136	88
Krcc1	156	156	157	149	153
Kremen1	28	39	29	33	47
Kri1	26	24	0	15	21
Krit1	70	92	99	104	99
Krr1	84	73	55	1	160
Krt10	5	0	10	0	0
Krt18	18	0	0	0	0
Krtcap2	46	66	140	81	117
Ksr1	22	26	22	17	38
Ksr2	1	2	0	4	3
Kti12	56	69	67	48	69
Ktn1	117	99	117	147	150
Kxd1	0	0	92	36	34
Kyat1	11	13	22	6	11
Kyat3	9	7	26	22	19
Kynu	2	0	11	0	0
L2hgdh	1	7	20	27	0
L3hypdh	3	2	13	0	7

For Review Only

L3mbtl1	0	2	0	7	0
L3mbtl2	50	43	50	75	55
L3mbtl3	17	18	17	23	27
l7Rn6	41	20	34	26	38
Lacc1	45	60	89	84	83
Lace1	12	13	19	17	0
Lactb	14	30	32	15	23
Lactb2	89	59	95	105	99
Lag3	0	0	1052	200	367
Lage3	1	1	0	0	0
Lair1	837	999	4988	386	208
Lama5	3	4	0	6	7
Lamb1	0	0	0	0	0
Lamb2	54	9	74	50	69
Lamc1	8	2	15	21	0
Lamc3	0	0	0	0	0
Lamp1	1122	1026	749	1095	1226
Lamp2	1803	1528	1755	2033	2128
Lamp3	4	2	3	3	5
Lamtor1	181	31	0	43	296
Lamtor2	4	9	0	0	0
Lamtor3	0	0	0	0	0
Lamtor4	79	70	0	53	30
Lamtor5	0	0	0	53	46
Lancl1	113	90	110	75	70
Lancl2	1	0	0	0	0
Lap3	827	587	429	0	463
Laptm4a	9	372	147	146	505
Laptm4b	155	121	132	110	157
Laptm5	6525	7601	10992	8026	9440
Large	68	126	120	101	85
Larp1	69	78	80	82	76
Larp1b	0	2	0	0	4
Larp4	39	34	28	54	41
Larp4b	175	172	182	121	167
Larp7	0	27	44	0	0
Lars	77	42	95	108	75
Lars2	1390	1524	1550	2018	2207
Las1l	56	44	56	60	39
Lasp1	570	321	559	641	741
Lat2	46	0	1	5	1
Lats1	25	78	36	67	58
Lats2	102	84	84	144	140
Layn	91	115	107	92	72
Lbh	45	19	19	38	66
Lbhd1	0	49	0	0	0
Lbp	24	0	0	0	0
Lbr	156	68	149	150	190
Lbx2	11	18	23	14	11
Lca5	12	7	0	6	10

For Review Only

Lca5l	10	5	5	9	6
Lcat	0	0	2	0	3
Lck	0	0	3	0	1
Lclat1	70	44	47	59	49
Lcmt1	37	0	10	9	1
Lcmt2	90	64	122	64	99
Lcn2	198	94	7	80	224
Lcor	471	239	211	432	483
Lcorl	27	109	28	64	50
Lcp1	1338	960	1441	1324	1614
Lcp2	493	468	625	494	421
Ldah	123	99	88	115	107
Ldb1	0	0	3	2	1
Ldha	430	385	534	323	419
Ldhal6b	0	0	0	8	0
Ldhb	942	690	1034	592	776
Ldhd	0	0	11	5	5
Ldlr	7	9	12	0	8
Ldlrad1	0	0	10	3	0
Ldlrad3	0	2	0	0	0
Ldlrad4	446	375	362	584	634
Ldlrap1	44	39	109	122	0
Ldoc1l	38	35	46	45	84
Lef1	0	0	0	0	0
Lefty1	16	13	0	36	41
Lefty2	6	6	5	5	4
Lekr1	18	1	0	0	21
Lemd2	85	0	141	23	43
Lemd3	14	23	13	14	28
Leng8	0	0	0	0	0
Leo1	25	21	40	31	33
Leprot	356	326	496	363	409
Leprotl1	232	132	306	207	217
Letm1	160	91	121	111	117
Letm2	31	15	28	43	30
Letmd1	54	22	633	0	182
Lfng	25	21	20	45	30
Lgals1	6	4	0	10	0
Lgals1-ps2	0	0	0	0	1
Lgals2	0	0	0	0	0
Lgals3	24	7	7	6	4
Lgals3bp	470	283	410	345	318
Lgals4	1	1	0	0	0
Lgals8	210	214	303	250	239
Lgals9	668	708	933	714	835
Lgalsl	10	7	1	18	8
Lgi1	6	0	0	0	1
Lgi4	23	3	7	19	17
Lgmn	9165	8173	12083	9341	8020
Lgr4	1	1	10	0	0

For Review Only

Lhfp	4	0	0	0	0
Lhfpl2	658	549	568	714	695
Lhfpl4	5	0	3	3	5
Lhpp	13	15	6	17	30
Lhx4	3	0	0	3	0
Lias	77	62	61	50	49
Lif	0	4	0	0	2
Lifr	381	294	171	815	544
Lig1	0	0	0	4	11
Lig3	32	68	44	73	62
Lig4	14	5	23	20	0
Lilra5	0	0	0	44	32
Lilra6	7	3	5	5	8
Lilrb4a	0	0	0	20	0
Lima1	51	39	77	86	49
Limch1	3	3	0	3	1
Limd1	58	86	57	65	40
Limd2	1	0	1	0	1
Limk1	65	72	46	77	90
Limk2	98	34	68	54	92
Lims1	428	491	484	488	429
Lims2	0	0	0	0	0
Lin28a	0	0	0	2	0
Lin28b	0	4	0	0	0
Lin37	34	0	0	0	0
Lin52	39	11	34	26	52
Lin54	22	3	10	15	18
Lin7b	0	0	0	0	0
Lin7c	94	103	37	80	104
Lin9	6	38	52	32	20
Lingo2	0	0	0	0	0
Lins1	11	30	19	19	53
Lipa	305	281	386	353	321
Lipc	0	0	0	0	0
Lipe	153	112	90	171	106
Liph	212	157	170	156	331
Lipo2	17	13	11	24	22
Lipo3	59	48	41	74	68
Lipt1	12	3	7	7	14
Lipt2	10	10	21	5	13
Litaf	352	271	404	304	416
Lix1	2	1	8	6	19
Lix1l	5	4	0	9	1
Llgl1	109	0	1	17	122
Llgl2	0	0	3	4	7
Llph	34	0	27	19	0
Llph-ps2	30	24	40	24	15
Lman1	149	117	178	145	199
Lman2	434	350	477	415	370
Lman2l	171	0	59	44	80

For Review Only

Lmbr1	38	44	29	40	23
Lmbr1l	24	37	47	27	36
Lmbrd1	272	215	218	245	238
Lmbrd2	10	12	17	31	32
Lmf1	121	134	0	0	0
Lmln	24	36	22	32	26
Lmna	0	0	7	2	0
Lmnb1	5	5	5	7	0
Lmnb2	27	28	14	22	13
Lmo2	53	125	126	145	133
Lmo4	33	30	24	33	27
Lmtk2	2	2	8	4	18
Lmtk3	2	2	0	0	0
Lncpint	15	4	10	13	13
Lncppara	7	0	0	0	0
Lnp	33	46	27	38	39
Lnpep	235	171	167	405	365
Lnx2	24	9	22	23	21
LOC100041034	7	6	12	13	11
LOC100041057	5	4	12	10	7
LOC100041708	2	2	0	3	6
LOC100044633	5	5	8	9	8
LOC100049077	6	3	6	4	0
LOC100503338	0	0	0	0	0
LOC100504180	7	4	0	5	5
LOC100861615	6	3	8	5	9
LOC100861749	77	154	76	129	195
LOC100861913	0	0	4	0	0
LOC100861969	10	4	8	5	7
LOC101055656	6	5	1	10	10
LOC101055663	0	0	2	2	0
LOC101055672	4	3	0	3	4
LOC101055727	6	8	4	13	10
LOC101055754	1	3	0	0	2
LOC101055758	0	1	6	0	0
LOC101055907	1	4	0	8	14
LOC101055915	31	25	37	29	31
LOC101055953	3	0	3	0	2
LOC101055995	24	19	24	29	34
LOC101056014	55935	90771	86413	107913	117410
LOC101056073	4	1	0	1	0
LOC101056115	1	2	0	1	0
LOC102631780	0	0	0	5	6
LOC102631912	48	25	51	45	42
LOC102631930	3	1	3	11	3
LOC102632031	5	0	0	0	2
LOC102632231	50	98	54	108	79
LOC102632465	12	1	0	10	9
LOC102632541	1	0	0	7	2
LOC102632594	3	0	0	0	4

For Review Only

LOC102632664	0	0	0	0	0
LOC102632739	13	9	11	8	8
LOC102632770	64	65	98	58	68
LOC102632778	0	3	0	2	1
LOC102632821	22	22	19	19	28
LOC102632901	1	0	0	4	1
LOC102632957	5	0	0	2	2
LOC102633000	0	0	1	1	0
LOC102633032	3	0	0	0	0
LOC102633149	5	6	8	29	12
LOC102633156	6	3	3	6	6
LOC102633239	8	4	5	14	6
LOC102633274	3	1	3	2	5
LOC102633497	0	1	4	3	0
LOC102633596	3	4	11	11	7
LOC102633627	8	11	17	17	10
LOC102633643	0	4	0	0	7
LOC102633880	14	8	8	19	28
LOC102633930	1	1	0	6	0
LOC102634078	32	73	38	42	43
LOC102634300	48	25	19	40	49
LOC102634333	2	1	0	0	0
LOC102634340	0	2	0	0	0
LOC102634389	1	1	1	0	0
LOC102634459	0	2	0	1	3
LOC102634481	3	0	5	0	0
LOC102634483	3	4	4	4	5
LOC102634533	3	0	4	3	6
LOC102634581	0	1	0	1	1
LOC102634683	1	3	0	5	0
LOC102634709	92	62	96	58	60
LOC102634716	5	0	0	1	4
LOC102634812	1	1	8	0	0
LOC102634873	2	1	0	4	4
LOC102634904	0	0	0	0	0
LOC102635048	36	40	49	39	41
LOC102635133	3	2	0	12	6
LOC102635154	4	4	3	3	0
LOC102635200	16	33	24	44	10
LOC102635527	3	8	41	42	44
LOC102635661	25	18	24	44	45
LOC102635786	27	17	19	50	52
LOC102635844	4	1	5	9	9
LOC102635912	8	24	13	18	9
LOC102635948	6	0	0	2	4
LOC102636299	20	23	30	25	26
LOC102636309	2	0	0	0	0
LOC102636313	1	4	4	3	0
LOC102636563	3	0	8	3	0
LOC102636700	0	0	0	3	4

For Review Only

LOC102636795	0	0	0	4	0
LOC102636907	0	0	0	2	3
LOC102637012	0	0	0	3	5
LOC102637269	4	6	6	5	4
LOC102637354	11	21	13	23	26
LOC102637515	7	7	5	8	11
LOC102637577	36	5	17	19	18
LOC102637646	0	0	8	2	2
LOC102637720	6	0	0	2	8
LOC102637763	0	0	0	0	0
LOC102637873	2	8	17	15	7
LOC102637966	0	0	0	0	2
LOC102638047	7	6	9	12	8
LOC102638183	17	15	22	19	19
LOC102638268	22	18	0	13	14
LOC102638435	2	2	3	0	4
LOC102638448	64	52	72	58	64
LOC102638515	5	1	0	0	0
LOC102638785	0	1	1	0	2
LOC102638940	38	8	55	75	112
LOC102639040	0	0	2	4	5
LOC102639044	22	22	39	26	31
LOC102639054	1	7	0	0	3
LOC102639076	5	1	0	0	0
LOC102639385	0	1	6	5	0
LOC102639505	13	8	12	23	19
LOC102639518	14	13	14	37	28
LOC102639543	1	4	0	4	1
LOC102639653	7	9	6	15	15
LOC102639683	2	11	21	20	14
LOC102639888	5	10	10	7	9
LOC102639979	7	5	7	7	0
LOC102639982	7	1	25	18	9
LOC102639987	5	4	4	6	0
LOC102640024	225	157	258	153	172
LOC102640133	23	19	14	24	21
LOC102640295	0	0	0	2	2
LOC102640359	1	0	1	0	0
LOC102640451	1	0	0	5	3
LOC102640468	9	11	26	21	11
LOC102640526	3	0	0	1	1
LOC102640673	24	8	36	34	26
LOC102640772	18	7	16	10	17
LOC102640779	3	5	2	6	7
LOC102641351	7	1	0	0	0
LOC102641859	2	0	0	0	0
LOC102641980	1	0	0	3	4
LOC102642386	1	0	0	0	0
LOC102642832	7	6	15	11	4
LOC102643083	0	0	2	0	0

For Review Only

LOC102643247	1	1	0	0	0
LOC105242405	4	12	11	8	6
LOC105242736	9	11	0	0	0
LOC105242798	0	0	1	5	0
LOC105242891	18	4	12	16	20
LOC105242920	3	0	0	5	2
LOC105243004	0	0	0	2	0
LOC105243127	9	0	0	3	9
LOC105243139	12	1	0	15	14
LOC105243194	1	8	4	5	0
LOC105243269	0	1	1	0	0
LOC105243282	0	0	0	3	0
LOC105243374	1	1	0	1	0
LOC105243453	32	9	23	20	21
LOC105243553	3	2	0	0	0
LOC105243785	0	0	2	0	5
LOC105243964	1	1	0	0	14
LOC105244007	6	20	15	19	18
LOC105244034	0	0	1	2	1
LOC105244059	0	0	0	4	0
LOC105244102	3	1	0	13	12
LOC105244124	1	0	0	0	0
LOC105244151	3	6	0	0	0
LOC105244195	0	0	0	0	0
LOC105244208	256	191	319	221	274
LOC105244251	0	3	1	5	7
LOC105244333	1	7	0	29	37
LOC105244402	1	0	0	1	1
LOC105244413	9	27	13	15	25
LOC105244416	0	0	0	0	4
LOC105244467	1	0	0	0	0
LOC105244657	35	35	30	38	38
LOC105244798	4	0	0	0	0
LOC105244993	0	0	0	3	0
LOC105245043	2	1	0	0	4
LOC105245105	0	1	0	3	3
LOC105245328	4	6	3	9	5
LOC105245359	2	0	0	0	1
LOC105245415	8	12	6	15	12
LOC105245439	279	127	216	386	402
LOC105245580	8	2	6	7	8
LOC105245651	2	1	0	4	7
LOC105245696	1	9	0	4	4
LOC105245783	22	42	36	24	41
LOC105245882	14	1	15	21	14
LOC105246016	0	0	4	0	0
LOC105246034	11	23	12	11	17
LOC105246046	0	1	9	4	0
LOC105246056	1	0	0	0	0
LOC105246114	0	0	0	2	5

For Review Only

LOC105246186	5	24	7	9	12
LOC105246245	0	1	0	0	5
LOC105246409	112	80	104	87	114
LOC105246496	0	0	0	5	3
LOC105246506	5	0	9	11	5
LOC105246668	6	2	7	12	13
LOC105246804	2	0	0	0	0
LOC105246895	2	3	7	4	5
LOC105246914	3	3	0	2	1
LOC105246961	41	54	62	83	67
LOC105246973	1	0	0	6	4
LOC105247075	2	2	3	4	3
LOC105247125	2	10	4	17	15
LOC105247188	9	6	16	17	11
LOC105247253	10	5	17	10	9
LOC105247294	10	7	6	14	14
LOC105247300	1	0	0	7	0
LOC106740	5	8	12	5	6
LOC108167320	0	4	0	24	0
LOC108167323	0	0	0	2	6
LOC108167326	0	1	0	4	0
LOC108167327	2	7	38	0	0
LOC108167334	0	1	5	1	0
LOC108167339	3	0	2	2	6
LOC108167344	55	74	84	46	87
LOC108167350	0	0	0	0	7
LOC108167355	14	25	23	14	13
LOC108167356	13	17	17	18	13
LOC108167358	1	1	0	0	10
LOC108167360	0	0	1	4	6
LOC108167365	12	8	12	15	15
LOC108167372	13	1	0	9	8
LOC108167373	1	4	7	3	0
LOC108167375	12	5	23	10	29
LOC108167376	0	3	2	5	1
LOC108167377	31	33	10	61	60
LOC108167381	4	1	0	10	5
LOC108167411	0	1	2	2	4
LOC108167413	2	6	1	6	8
LOC108167415	0	0	0	2	0
LOC108167416	4	0	0	0	0
LOC108167423	21	9	11	15	18
LOC108167428	2	2	0	0	3
LOC108167433	35	10	11	29	41
LOC108167435	19	16	6	23	26
LOC108167436	9	0	0	4	0
LOC108167437	0	0	0	5	0
LOC108167440	257	306	292	225	416
LOC108167450	23	18	16	36	29
LOC108167452	3	0	0	0	0

For Review Only

LOC108167458	2	0	0	0	0
LOC108167466	0	0	5	0	3
LOC108167482	0	1	3	8	0
LOC108167485	0	0	0	4	0
LOC108167511	220	258	284	363	514
LOC108167513	2	1	0	10	0
LOC108167514	38	23	42	28	37
LOC108167517	1	0	0	0	6
LOC108167518	30	23	24	31	26
LOC108167519	1	0	0	0	0
LOC108167523	0	0	0	3	0
LOC108167527	0	3	0	0	0
LOC108167532	4	15	18	8	23
LOC108167534	0	0	0	0	0
LOC108167536	14	39	42	58	59
LOC108167542	17	18	14	42	27
LOC108167547	0	1	1	10	14
LOC108167548	6	1	4	3	5
LOC108167549	4	1	2	5	9
LOC108167550	4	2	17	10	0
LOC108167552	0	0	0	2	2
LOC108167554	2	4	0	10	13
LOC108167555	13	6	14	0	6
LOC108167560	0	0	4	34	9
LOC108167561	0	2	0	0	7
LOC108167562	61	60	50	72	95
LOC108167564	2	2	0	0	0
LOC108167571	2	1	4	0	3
LOC108167576	1	4	2	6	7
LOC108167591	35	30	43	28	46
LOC108167597	0	1	0	0	1
LOC108167614	18	23	21	25	28
LOC108167618	0	5	0	0	0
LOC108167619	1	2	0	1	0
LOC108167626	18	29	41	58	48
LOC108167628	0	0	6	0	0
LOC108167630	1	1	0	1	0
LOC108167633	4	1	0	5	0
LOC108167637	2	1	0	3	0
LOC108167640	1	3	0	7	8
LOC108167641	1	2	7	4	5
LOC108167643	76	124	80	132	94
LOC108167645	2	0	0	0	0
LOC108167650	0	0	5	13	8
LOC108167659	1	0	0	0	0
LOC108167660	3	3	5	0	0
LOC108167669	11	3	8	7	10
LOC108167675	0	2	0	0	0
LOC108167679	2	9	0	7	7
LOC108167680	0	0	6	4	0

For Review Only

LOC108167681	0	1	0	16	0
LOC108167687	3	1	0	9	0
LOC108167690	0	0	4	1	4
LOC108167691	5	0	0	6	5
LOC108167692	2	2	0	7	5
LOC108167693	1	7	4	15	16
LOC108167700	7	11	11	10	15
LOC108167714	0	0	0	0	0
LOC108167719	0	1	1	0	0
LOC108167721	3	0	0	0	6
LOC108167725	2	2	0	0	0
LOC108167732	0	0	0	0	38
LOC108167733	12	1	7	5	14
LOC108167734	4	0	3	1	0
LOC108167735	1	2	0	8	7
LOC108167736	230	287	287	298	186
LOC108167737	15	4	5	11	13
LOC108167738	7	3	10	13	17
LOC108167745	19	20	16	36	43
LOC108167746	1	28	11	20	0
LOC108167748	272	143	220	190	311
LOC108167749	8	1	0	5	0
LOC108167751	7	0	0	3	2
LOC108167755	23	25	23	44	46
LOC108167760	10	1	0	11	1
LOC108167777	2	1	1	3	0
LOC108167794	0	2	0	10	5
LOC108167801	16	2	1	33	20
LOC108167802	9	11	9	4	12
LOC108167804	52	38	55	34	75
LOC108167806	1	1	2	2	3
LOC108167809	3	3	2	2	2
LOC108167810	0	0	35	8	15
LOC108167812	0	0	0	0	0
LOC108167814	3	3	1	6	1
LOC108167815	4	19	9	21	1
LOC108167825	3	7	16	21	11
LOC108167827	0	4	2	5	2
LOC108167844	0	0	1	0	0
LOC108167846	6	18	8	38	18
LOC108167848	10	40	24	41	26
LOC108167849	45	54	37	68	59
LOC108167875	16	12	6	15	13
LOC108167878	0	0	0	1	0
LOC108167886	7	3	7	7	10
LOC108167889	9	7	14	4	7
LOC108167890	0	3	0	0	6
LOC108167895	0	0	0	3	0
LOC108167902	9	4	3	8	16
LOC108167904	14	0	1	12	0

For Review Only

LOC108167905	18	14	12	14	25
LOC108167911	3	0	0	0	0
LOC108167915	9	12	16	38	26
LOC108167917	8	5	15	12	12
LOC108167922	84	78	104	81	75
LOC108167924	4	4	0	4	0
LOC108167926	7	11	0	0	0
LOC108167928	5	0	3	3	3
LOC108167930	4	5	0	5	4
LOC108167933	0	4	0	0	6
LOC108167939	1	0	0	28	0
LOC108167942	1	5	10	5	0
LOC108167961	0	0	0	3	1
LOC108167971	0	0	0	0	0
LOC108167986	0	0	0	1	0
LOC108167995	8	26	47	51	62
LOC108167997	23	21	41	34	15
LOC108168009	0	0	4	2	2
LOC108168017	5	0	0	0	6
LOC108168018	80	88	48	139	126
LOC108168019	0	0	0	1	1
LOC108168022	66	44	95	136	135
LOC108168025	0	0	0	7	0
LOC108168026	0	2	4	1	0
LOC108168030	10	3	0	4	5
LOC108168035	3	0	0	1	0
LOC108168043	1	1	1	3	0
LOC108168049	0	0	0	3	7
LOC108168050	13	7	10	5	13
LOC108168067	0	0	0	1	1
LOC108168071	0	0	0	0	0
LOC108168078	330	424	307	506	490
LOC108168079	0	0	0	8	0
LOC108168080	8	3	11	21	19
LOC108168082	6	13	10	13	3
LOC108168085	2	0	1	1	0
LOC108168086	3	0	0	0	6
LOC108168088	0	0	0	0	0
LOC108168091	0	2	1	0	1
LOC108168092	2	5	0	0	4
LOC108168101	36	64	88	94	47
LOC108168102	2	8	6	0	8
LOC108168108	4	11	0	15	16
LOC108168109	11	17	16	31	9
LOC108168114	48	37	60	36	50
LOC108168115	0	1	3	0	3
LOC108168144	2	2	0	5	4
LOC108168146	13	13	9	12	24
LOC108168165	1	1	0	0	5
LOC108168168	2	2	3	4	6

For Review Only

LOC108168169	2	2	3	5	6
LOC108168170	0	0	0	0	0
LOC108168171	4	5	3	6	7
LOC108168172	3	4	3	6	8
LOC108168178	0	1	0	2	2
LOC108168194	4	0	0	4	0
LOC108168201	17	10	17	53	50
LOC108168203	0	0	0	5	4
LOC108168204	2	4	0	3	8
LOC108168205	1	3	0	8	12
LOC108168207	3	4	1	5	3
LOC108168210	5	0	6	1	0
LOC108168233	37	28	67	43	56
LOC108168235	0	0	2	0	5
LOC108168238	2	3	11	9	8
LOC108168240	5	3	7	8	18
LOC108168248	0	0	0	6	0
LOC108168252	0	0	0	1	0
LOC108168254	1	4	6	0	0
LOC108168256	4	15	15	0	6
LOC108168260	1	5	4	7	4
LOC108168281	46	27	61	47	28
LOC108168283	0	0	3	5	2
LOC108168286	2	4	8	9	0
LOC108168287	6	0	0	0	4
LOC108168292	3	2	0	4	0
LOC108168293	4	7	10	8	1
LOC108168294	1	2	0	0	6
LOC108168295	19	14	8	18	23
LOC108168304	12	13	12	16	12
LOC108168305	0	0	0	0	0
LOC108168323	8	56	31	66	57
LOC108168331	3	10	8	12	0
LOC108168334	7	6	8	5	19
LOC108168335	4	3	13	0	3
LOC108168336	1	1	6	0	0
LOC108168338	17	17	17	24	13
LOC108168340	0	0	0	0	5
LOC108168342	1	0	0	11	0
LOC108168347	0	0	0	0	3
LOC108168353	14	14	0	20	30
LOC108168354	1	1	0	1	0
LOC108168358	14	10	25	20	11
LOC108168376	9	3	11	8	5
LOC108168380	4	4	11	6	11
LOC108168382	6	2	0	6	13
LOC108168389	2	0	0	0	5
LOC108168392	1	1	0	0	0
LOC108168393	1	5	0	1	5
LOC108168395	35	31	42	43	43

For Review Only

LOC108168408	5	1	0	10	12
LOC108168411	1	0	0	2	0
LOC108168412	7	16	0	15	14
LOC108168420	1	1	0	0	0
LOC108168421	7	9	7	14	15
LOC108168424	3	0	0	0	3
LOC108168427	0	0	0	0	0
LOC108168464	24	22	14	18	20
LOC108168478	5	5	6	0	4
LOC108168482	18	34	43	79	49
LOC108168534	7	8	7	6	8
LOC108168644	3	1	1	2	3
LOC108168681	1	0	0	3	0
LOC108168686	0	2	0	0	5
LOC108168688	0	1	0	0	1
LOC108168734	6	7	7	5	0
LOC108168739	28	21	18	39	54
LOC108168740	0	15	14	9	24
LOC108168747	1	0	7	0	0
LOC108168750	29	32	14	38	41
LOC108168753	0	2	9	4	5
LOC108168756	0	0	0	0	0
LOC108168762	3	0	0	0	0
LOC108168763	0	3	0	0	0
LOC108168767	0	0	0	0	0
LOC108168772	3	1	0	7	0
LOC108168774	4	3	0	0	4
LOC108168776	9	5	4	10	8
LOC108168777	3	6	0	14	12
LOC108168785	0	0	0	0	0
LOC108168795	59	27	28	42	1
LOC108168801	0	0	0	3	10
LOC108168806	3	0	1	1	0
LOC108168809	16	15	20	13	18
LOC108168810	115	76	94	124	137
LOC108168813	5	5	2	7	14
LOC108168815	0	5	2	0	2
LOC108168816	1	874	0	992	700
LOC108168817	1	0	4	0	14
LOC108168820	0	0	0	0	0
LOC108168839	12	6	9	0	9
LOC108168842	4	3	0	1	5
LOC108168843	3	4	10	19	24
LOC108168845	1	1	0	0	0
LOC108168846	0	1	0	0	0
LOC108168864	1	3	0	4	2
LOC108168868	15	8	17	23	9
LOC108168869	7	22	19	17	26
LOC108168871	0	1	4	1	3
LOC108168876	70	89	72	76	95

For Review Only

LOC108168879	11	19	25	21	30
LOC108168882	0	4	0	0	0
LOC108168883	0	0	6	0	0
LOC108168886	4	8	4	0	0
LOC108168889	4	4	4	9	8
LOC108168899	44	23	45	42	35
LOC108168900	3	0	0	4	0
LOC108168906	11	33	11	24	23
LOC108168907	47	40	27	51	71
LOC108168909	1	2	7	0	0
LOC108168924	0	4	0	8	8
LOC108168925	13	83	63	103	125
LOC108168926	4	0	3	0	0
LOC108168930	0	1	0	0	7
LOC108168933	1	19	9	13	0
LOC108168936	8	0	6	0	0
LOC108168940	19	15	13	12	5
LOC108168959	9	12	4	19	12
LOC108168974	4	5	7	3	6
LOC108168979	0	4	2	1	1
LOC108168983	4	0	0	0	3
LOC108168984	0	3	0	0	0
LOC108168986	20	9	17	23	28
LOC108168987	0	0	1	3	0
LOC108168990	0	6	0	0	0
LOC108169008	1	0	1	2	3
LOC108169018	5	0	7	0	0
LOC108169023	30	9	10	30	22
LOC108169029	7	1	9	4	12
LOC108169030	0	2	0	1	0
LOC108169038	0	0	10	1	3
LOC108169039	0	1	0	0	6
LOC108169043	1	0	0	0	0
LOC108169045	0	3	0	4	0
LOC108169046	16	41	19	34	54
LOC108169050	0	0	0	2	2
LOC108169056	16	0	6	8	14
LOC108169061	3	3	1	4	1
LOC108169069	1	2	1	0	0
LOC108169076	14	7	16	4	15
LOC108169077	2	2	1	3	3
LOC108169079	3	1	2	0	0
LOC108169093	0	0	0	0	0
LOC108169096	4	6	0	3	1
LOC108169121	3	0	4	0	0
LOC108169124	3	6	7	6	4
LOC108169128	3	0	0	4	0
LOC108169130	3	8	5	10	3
LOC108169131	0	0	4	0	1
LOC108169152	1	4	5	5	0

For Review Only

LOC108169153	3	4	3	10	1
LOC108169155	5	8	24	33	37
LOC108169159	0	0	0	4	0
LOC108169163	12	28	21	23	16
LOC108169172	28	26	13	52	33
LOC108169175	1	0	7	4	0
LOC108169177	7	6	10	13	17
LOC108169202	150	59	98	199	242
LOC108169204	3	2	0	4	4
LOC433198	0	0	11	0	0
LOC546061	0	4	2	3	2
LOC73899	10	0	0	6	8
Lonp1	17	32	14	42	30
Lonp2	142	145	170	146	199
Lonrf1	18	17	17	23	24
Lonrf3	89	61	64	100	62
Lox	3	0	0	0	0
Loxl2	6	10	5	12	11
Loxl3	32	3	80	0	140
Loxl4	5	0	13	0	1
Lpar1	5	0	0	0	0
Lpar2	4	3	0	4	3
Lpar5	171	141	171	204	303
Lpar6	486	528	661	531	561
Lpcat1	211	63	141	287	308
Lpcat2	3770	2563	4445	3314	2692
Lpcat3	49	0	0	0	0
Lpgat1	62	76	69	65	80
Lpin1	201	174	162	166	172
Lpin2	949	702	886	970	962
Lpl	12	19	38	25	10
Lpp	122	109	154	156	193
Lppos	0	0	2	2	1
Lpxn	169	195	142	157	234
Lrba	138	240	100	226	230
Lrch1	270	138	216	294	276
Lrch3	137	192	159	223	196
Lrch4	0	0	0	0	0
Lrfn1	0	0	1	0	6
Lrfn3	0	5	0	1	11
Lrg1	6	1	0	1	5
Lrguk	28	9	15	17	10
Lrif1	59	73	82	62	75
Lrig1	2	2	6	6	0
Lrig2	145	135	97	180	186
Lrig3	39	2	32	18	0
Lrmp	160	113	168	29	143
Lrp1	2035	1184	1473	2141	3258
Lrp10	30	138	0	0	0
Lrp11	1	8	0	4	17

For Review Only

Lrp12	98	87	56	45	48
Lrp3	0	0	0	0	0
Lrp4	31	35	23	43	47
Lrp5	93	45	71	143	172
Lrp6	49	67	57	90	104
Lrp8	0	0	0	3	0
Lrpap1	374	314	506	311	273
Lrpprc	151	74	95	156	137
Lrrc1	0	2	0	5	7
Lrrc14	112	0	0	0	0
Lrrc14b	5	2	9	3	3
Lrrc15	0	0	0	1	0
Lrrc16a	29	16	34	31	32
Lrrc18	25	10	14	10	11
Lrrc20	9	9	24	14	18
Lrrc25	0	173	0	76	86
Lrrc27	5	0	0	0	10
Lrrc28	16	14	19	35	42
Lrrc29	3	2	18	33	31
Lrrc3	0	1456	102	2085	2728
Lrrc32	0	0	0	0	0
Lrrc34	0	0	0	0	0
Lrrc39	54	29	43	58	52
Lrrc4	17	25	4	18	20
Lrrc40	32	19	50	32	34
Lrrc41	15	9	0	76	146
Lrrc42	0	0	0	17	3
Lrrc45	5	0	0	0	0
Lrrc47	8	20	17	18	18
Lrrc51	1	1	40	0	0
Lrrc56	11	24	19	16	16
Lrrc57	33	69	70	55	76
Lrrc58	487	537	479	572	697
Lrrc59	46	46	49	47	56
Lrrc61	120	48	55	96	105
Lrrc69	9	4	1	0	0
Lrrc7	9	3	0	8	0
Lrrc71	0	0	0	4	0
Lrrc75a	11	11	9	7	22
Lrrc8a	473	442	0	0	0
Lrrc8b	7	3	0	4	9
Lrrc8c	5	2	11	0	0
Lrrc8d	92	69	81	85	93
Lrrc9	13	3	10	11	9
Lrrcc1	15	64	44	69	47
Lrrfip1	287	190	302	251	264
Lrrfip2	36	27	25	29	52
Lrriq3	2	5	0	4	13
Lrrk1	68	91	95	117	121
Lrrn2	3	2	0	0	4

For Review Only

Lrrn3	0	4	0	5	5
Lrrn4	3	13	0	0	5
Lrrtm2	7	1	10	5	8
Lrsam1	28	37	88	72	110
Lrtm2	0	1	0	0	0
Lrwd1	100	58	120	70	105
Lsg1	88	77	85	67	119
Lsm1	0	0	11	5	1
Lsm10	46	62	76	60	58
Lsm11	3	15	8	23	13
Lsm12	57	51	8	158	92
Lsm14a	30	56	63	24	55
Lsm14b	2	22	24	7	14
Lsm2	1	45	41	42	0
Lsm3	21	49	54	37	16
Lsm4	17	1	0	0	0
Lsm6	88	63	121	93	71
Lsm7	12	0	0	0	0
Lsm8	41	25	37	38	44
Lsp1	174	124	241	205	185
Lsr	1	1	0	0	21
Lss	30	27	23	42	24
Lst1	51	149	182	12	118
Lta4h	128	85	133	84	117
Ltb	9	12	19	11	34
Ltb4r1	14	8	10	8	9
Ltb4r2	0	0	3	3	0
Ltbp3	9	0	5	7	0
Ltbp4	0	2	0	0	0
Ltbr	137	64	104	101	116
Ltc4s	391	266	388	157	188
Ltf	575	107	0	0	0
Ltn1	77	134	77	117	119
Ltv1	73	42	78	61	86
Luc7l	57	80	112	64	101
Luc7l2	152	191	99	188	182
Luc7l3	94	117	192	99	120
Lum	2	0	0	0	0
Lurap1	1	1	0	0	0
Luzp1	60	55	68	60	38
Lxn	45	31	57	42	27
Ly6a	6	5	21	1	15
Ly6c1	2	1	0	0	0
Ly6c2	8	6	0	12	0
Ly6e	2	0	877	49	767
Ly6g	27	6	5	8	8
Ly6g5b	7	1	9	0	0
Ly6g6d	0	6	6	3	14
Ly6i	1	0	0	0	0
Ly6k	0	0	0	2	2

For Review Only

Ly86	1474	2113	2767	1746	1958
Ly9	145	61	117	68	86
Ly96	28	40	0	0	0
Lyar	28	20	21	13	34
Lyl1	198	0	52	0	9
Lyn	2156	179	1345	1995	1683
Lynx1	0	0	1	0	8
Lypd6	34	9	18	15	19
Lypla1	158	75	83	102	96
Lypla2	0	106	1	1	1
Lyplal1	26	7	16	5	11
Lyrm1	11	16	7	20	38
Lyrm2	189	1	0	0	0
Lyrm4	77	64	66	59	68
Lyrm5	51	45	29	37	32
Lyrm7	4	2	4	4	4
Lyrm9	44	58	60	66	79
Lysmd1	34	22	23	38	40
Lysmd2	0	0	2	0	0
Lysmd3	0	0	0	81	0
Lysmd4	37	24	26	36	62
Lyst	157	160	112	214	141
Lyve1	0	4	0	0	0
Lyz1	106	70	76	60	67
Lyz2	1012	734	889	478	623
Lyzl4	0	0	7	1	1
Lzic	65	54	93	82	92
Lztfl1	50	48	46	45	74
Lztr1	124	118	153	115	129
Lzts1	7	2	5	3	7
Lzts2	16	7	11	14	15
Lzts3	4	1	3	1	4
M1ap	6	8	17	14	11
M6pr	641	508	766	653	668
Mab21l1	4	3	0	1	4
Macf1	1088	1859	1310	2173	2175
Macrod1	4	11	6	10	3
Macrod2	0	0	0	0	0
Mad1l1	45	38	17	66	40
Mad2l1	7	5	0	14	20
Mad2l1bp	32	18	51	37	32
Mad2l2	52	51	12	1	37
Madd	218	174	239	287	219
Maea	232	180	304	212	240
Maf	197	414	350	286	233
Maf1	0	0	2	197	0
Mafb	1069	698	960	684	935
Maff	3	6	3	5	7
Mafg	0	0	0	0	0
Mafk	96	62	60	65	58

For Review Only

Mag	78	28	80	58	60
Maged1	0	74	0	0	0
Maged2	74	54	121	83	71
Magee1	27	7	18	24	37
Magef1	17	19	61	31	40
Mageh1	3	3	0	1	0
Magi1	45	60	45	52	72
Magi3	2	15	0	8	21
Magoh	0	0	0	0	46
Magohb	6	0	0	0	0
Magt1	196	156	211	269	178
Majin	0	1	0	0	0
Mak16	47	81	86	67	50
Mal	0	0	0	0	0
Malat1	7121	7616	8161	10469	11206
Malsu1	38	36	37	24	35
Malt1	40	29	43	51	90
Mamdc4	23	15	33	13	23
Maml1	87	72	78	98	97
Maml2	445	470	493	499	485
Maml3	229	171	216	227	356
Mamld1	4	13	0	13	23
Mamstr	5	0	0	0	0
Man1a	139	221	119	120	170
Man1a2	327	312	341	372	342
Man1b1	119	3	68	0	110
Man1c1	238	214	251	203	264
Man2a1	25	12	24	28	36
Man2a2	191	196	203	258	221
Man2b1	693	0	1	29	790
Man2b2	733	528	703	697	946
Man2c1	52	47	29	0	153
Man2c1os	0	0	16	13	19
Manba	100	130	146	166	165
Manbal	46	76	71	48	66
Manea	90	62	73	111	77
Maneal	0	0	0	0	0
Manf	81	57	127	68	104
Mansc1	4	0	0	5	0
Maoa	0	0	20	10	0
Maob	0	2	0	0	0
Map10	127	8	34	73	90
Map1a	1	2	0	0	3
Map1b	7	1	18	5	1
Map1lc3a	53	43	53	31	48
Map1lc3b	0	0	330	199	191
Map1s	23	1	0	131	0
Map2k1	457	402	523	460	469
Map2k2	101	86	20	31	84
Map2k3	137	75	120	119	148

For Review Only

Map2k3os	10	2	7	0	12
Map2k4	74	53	62	53	42
Map2k5	54	58	61	72	60
Map2k6	34	24	42	36	49
Map2k7	46	47	30	44	53
Map3k1	235	214	209	326	299
Map3k10	10	8	0	4	4
Map3k11	219	179	169	194	186
Map3k12	56	35	74	72	54
Map3k13	6	2	1	0	8
Map3k14	46	86	78	89	83
Map3k15	0	0	0	0	0
Map3k19	11	4	12	22	12
Map3k2	43	66	50	95	100
Map3k3	27	122	54	133	128
Map3k4	90	68	115	153	99
Map3k5	59	142	119	97	123
Map3k7	42	56	67	78	89
Map3k9	60	76	84	90	118
Map4	688	436	387	557	634
Map4k1	5	5	8	1	0
Map4k2	0	125	40	14	253
Map4k3	52	72	64	59	59
Map4k4	108	123	156	133	215
Map4k5	34	92	23	55	75
Map6	1	0	0	4	1
Map6d1	1	2	0	3	6
Map7	14	23	37	22	38
Map7d1	50	20	0	71	68
Mapk1	93	129	92	176	177
Mapk11	0	0	0	0	0
Mapk12	0	4	0	5	0
Mapk14	1	57	931	0	0
Mapk1ip1	81	62	139	77	77
Mapk1ip1l	143	134	162	166	228
Mapk3	112	0	98	0	0
Mapk4	8	1	0	0	0
Mapk6	79	68	49	95	88
Mapk7	0	1	0	116	42
Mapk8	41	38	16	34	40
Mapk8ip1	0	0	0	3	2
Mapk8ip3	54	145	112	253	238
Mapk9	59	36	65	89	81
Mapkap1	44	46	27	58	81
Mapkapk2	48	72	130	54	59
Mapkapk3	117	78	92	76	95
Mapkapk5	0	31	0	11	19
Mapkbp1	17	42	49	53	65
Mapre1	359	344	442	332	415
Mapre2	209	186	200	212	186

For Review Only

Mapre3	28	22	6	28	24
Mapt	2	2	0	1	7
Marc2	202	193	238	139	246
March1	348	284	124	511	192
March10	3	4	5	3	9
March2	33	13	20	13	20
March3	0	0	2	2	2
March5	57	31	49	39	29
March6	91	118	123	173	171
March7	195	108	100	226	237
March8	67	54	61	91	86
March9	2	0	0	0	0
Marcks	753	1068	1217	767	913
Marcksl1	14	10	8	16	11
Marf1	130	296	79	216	204
Mark1	2	0	0	0	0
Mark2	81	95	89	112	104
Mark3	73	105	101	81	97
Mark4	27	32	20	16	23
Mars	105	37	147	35	48
Mars2	24	43	49	29	52
Marveld1	0	5	5	1	0
Marveld2	29	21	46	18	13
Mas1	0	0	0	0	9
Masp1	34	12	31	36	15
Mast1	9	4	0	25	21
Mast2	46	33	26	44	57
Mast3	103	336	147	322	268
Mast4	30	30	46	107	74
Mastl	7	4	0	0	0
Mat2a	398	485	414	508	443
Mat2b	201	134	216	134	149
Matk	101	0	157	0	0
Matn2	3	0	0	0	7
Matr3	341	227	224	253	253
Mau2	1	94	91	29	341
Mavs	214	62	295	275	156
Max	86	64	57	85	73
Maz	57	50	93	52	51
Mb21d1	84	89	67	70	110
Mb21d2	4	3	12	1	13
Mbd1	68	94	0	156	158
Mbd2	10	5	17	0	7
Mbd3	53	0	9	2	2
Mbd4	24	10	12	22	28
Mbd5	20	28	44	64	48
Mbd6	95	61	33	78	76
Mbip	43	26	46	59	46
Mblac1	7	15	5	16	6
Mblac2	1	1	120	0	0

For Review Only

Mbnl1	391	253	381	471	267
Mbnl2	218	252	296	301	213
Mbnl3	4	0	0	0	0
Mboat1	23	26	24	35	36
Mboat2	6	5	0	5	0
Mboat7	59	6	35	0	0
Mbp	107	59	75	70	57
Mbtd1	58	60	103	79	81
Mbtps1	187	157	186	198	233
Mbtps2	59	35	47	84	62
Mc1r	4	0	0	3	2
Mcat	32	10	24	26	25
Mcc	4	12	16	13	14
Mccc1	48	31	54	58	65
Mccc1os	1	2	0	4	0
Mccc2	43	39	42	44	31
Mcee	38	17	15	10	26
Mcemp1	8	3	0	0	0
Mcf2l	1	2	0	3	0
Mcfd2	369	303	367	314	348
Mcl1	1	0	0	0	0
Mcm2	11	13	12	28	25
Mcm3	194	118	186	125	174
Mcm3ap	94	72	79	106	112
Mcm4	62	31	26	47	57
Mcm5	12	15	33	22	30
Mcm6	42	32	56	57	51
Mcm7	5	5	22	1	0
Mcm9	16	9	16	13	29
Mcmbp	148	220	147	158	166
Mcmdc2	6	2	1	9	4
Mcoln1	0	0	0	0	0
Mcph1	16	7	18	27	19
Mcrs1	15	35	0	40	16
Mctp1	69	73	60	98	124
Mcts1	45	0	63	20	24
Mcts2	16	28	35	15	9
Mcu	12	13	18	15	23
Mcur1	141	178	109	174	153
Mdc1	160	141	130	170	142
Mdfi	5	0	0	9	4
Mdfic	3	1	0	8	0
Mdga1	7	0	0	8	0
Mdga2	2	2	6	1	0
Mdh1	316	170	237	205	205
Mdh2	43	23	243	241	0
Mdk	0	0	0	0	0
Mdm1	7	4	17	12	13
Mdm2	164	143	178	140	176
Mdm4	341	419	469	571	452

For Review Only

Mdn1	257	85	90	175	185
Mdp1	0	0	71	1	5
Me1	3	2	0	0	7
Me2	56	36	48	37	64
Mea1	17	0	0	0	0
Meaf6	60	69	72	73	83
Mecom	3	0	0	6	0
Mecp2	60	141	71	94	92
Mecr	13	20	23	15	27
Med1	212	219	198	276	298
Med10	37	53	72	58	89
Med11	0	0	0	0	67
Med12	151	118	128	181	168
Med12l	228	237	289	308	265
Med13	81	121	100	170	143
Med13l	114	144	155	256	188
Med14	19	32	34	75	82
Med15	75	117	137	98	121
Med16	24	17	29	27	40
Med17	154	119	158	99	88
Med18	14	7	7	24	19
Med19	23	25	24	25	21
Med20	94	94	103	124	116
Med21	50	56	65	69	74
Med22	172	294	232	0	48
Med23	118	68	88	132	143
Med24	52	37	71	87	55
Med25	69	41	62	65	78
Med26	40	20	44	41	45
Med27	43	61	58	48	47
Med28	229	163	172	166	174
Med29	39	47	44	35	55
Med30	24	23	25	22	20
Med31	45	33	24	25	22
Med4	66	45	40	1	13
Med6	34	37	57	31	44
Med7	117	127	151	95	100
Med8	0	39	0	111	0
Med9	72	35	90	49	68
Mef2a	514	820	526	862	933
Mef2c	725	921	750	887	1082
Mef2d	112	97	98	105	53
Meg3	0	1	0	0	0
Megf11	2	4	23	30	0
Megf8	102	88	119	156	150
Megf9	14	7	5	15	4
Meis1	27	13	26	15	51
Meis3	62	56	52	34	71
Melk	0	0	0	0	0
Memo1	31	70	70	56	64

For Review Only

Men1	71	51	95	54	78
Meox1	2	0	3	0	0
Mepce	42	48	96	58	30
Mertk	2257	1637	1781	2695	2732
Mesdc1	15	9	3	14	10
Mesdc2	152	210	283	201	234
Mest	14	2	7	4	1
Met	0	0	0	0	0
Metap1	134	115	88	105	152
Metap1d	128	108	108	149	150
Metap2	171	141	242	133	183
Metrn	2	0	6	6	0
Metrnl	4	5	0	0	0
Mettl1	18	4	18	18	22
Mettl10	18	15	32	20	13
Mettl13	25	13	35	28	15
Mettl14	28	40	60	40	39
Mettl15	30	5	18	27	22
Mettl16	66	60	75	87	54
Mettl17	0	25	12	27	0
Mettl18	21	0	23	12	25
Mettl2	49	28	40	32	58
Mettl20	24	4	29	6	9
Mettl21a	135	95	181	141	143
Mettl21c	0	3	2	2	1
Mettl22	25	16	25	5	23
Mettl23	0	0	0	0	0
Mettl25	55	34	50	37	52
Mettl3	52	1	0	0	0
Mettl4	28	49	45	70	59
Mettl5	29	48	57	21	30
Mettl6	37	31	58	22	44
Mettl7a1	21	46	19	42	44
Mettl7a2	6	6	3	5	5
Mettl7a3	1	3	0	3	4
Mettl8	33	30	54	30	41
Mettl9	44	32	33	38	38
Mex3a	0	5	11	3	10
Mex3b	23	18	34	15	13
Mex3c	47	46	56	25	31
Mex3d	5	5	4	3	8
Mfap1a	33	19	52	149	13
Mfap1b	65	46	74	106	63
Mfap2	0	1	0	0	4
Mfap3	214	384	566	318	499
Mfap3l	4	12	0	13	7
Mff	195	170	170	173	219
Mfge8	97	1	0	0	66
Mfhas1	38	119	79	79	82
Mfn1	34	46	27	22	34

For Review Only

Mfn2	139	30	94	71	79
Mfng	279	40	0	186	375
Mfsd1	621	434	528	538	681
Mfsd10	0	0	0	0	0
Mfsd11	0	0	28	1	1
Mfsd12	41	102	73	136	100
Mfsd14a	121	175	244	179	183
Mfsd14b	310	250	212	273	351
Mfsd2a	0	0	0	0	0
Mfsd2b	3	0	0	0	0
Mfsd3	53	41	39	28	37
Mfsd4a	22	21	18	4	9
Mfsd4b3	0	0	0	0	5
Mfsd4b4	0	0	0	0	10
Mfsd5	125	82	89	90	92
Mfsd6	1	1	0	0	0
Mfsd6l	1	0	0	0	0
Mfsd7a	6	14	15	5	6
Mfsd7b	14	36	18	19	9
Mfsd7c	2	0	0	0	7
Mfsd8	79	76	58	75	64
Mfsd9	10	10	26	15	42
Mga	130	110	91	177	155
Mgat1	396	294	349	356	438
Mgat2	68	130	75	117	154
Mgat3	0	0	8	0	0
Mgat4a	945	1081	1125	1249	1524
Mgat4b	44	38	25	11	28
Mgat5	199	253	210	322	370
Mgea5	154	194	218	192	164
Mgl2	1	2	12	0	0
Mgll	197	143	200	192	205
Mgme1	123	91	148	79	98
Mgmt	15	8	13	9	5
Mgrn1	141	14	76	116	264
Mgst1	3	1	0	0	0
Mgst3	18	17	0	0	19
Mia3	82	116	112	241	159
Miat	0	0	2	2	0
Mib1	48	57	49	49	43
Mib2	54	1	0	0	82
Mical1	95	4	77	0	95
Mical2	3	0	0	0	0
Mical3	77	70	58	83	115
Micall1	40	51	63	43	46
Micu1	108	92	121	78	81
Micu2	60	50	57	49	48
Micu3	24	55	34	22	35
Mid1	67	0	0	86	106
Mid1ip1	0	0	101	0	0

For Review Only

Midn	8	52	0	69	67
Mief1	102	94	86	103	108
Mief2	25	25	27	25	42
Mien1	0	0	39	1	1
Mier1	91	120	118	121	152
Mier2	18	29	92	24	28
Mier3	63	33	70	0	61
Mif	98	42	77	50	63
Mif4gd	6	3	26	1	17
Miip	0	18	92	27	0
Mill2	0	0	0	0	0
Milr1	41	54	39	42	30
Mina	56	29	43	54	74
Mink1	29	31	30	47	41
Minos1	34	58	91	115	0
Minpp1	174	122	158	122	89
Mios	49	14	39	57	44
Mipep	18	21	2	8	3
Mipol1	0	0	4	2	0
Mir1191	1	11	18	11	13
Mir126a	0	0	0	0	0
Mir1291	2	2	0	0	0
Mir133a-1hg	5	10	5	9	11
Mir140	6	4	3	4	3
Mir142b	9	7	7	5	3
Mir17hg	13	20	0	0	0
Mir181a-2	19	24	0	0	0
Mir181b-2	1	2	1	0	0
Mir1893	0	0	4	0	0
Mir1894	1	0	0	0	0
Mir1898	2	1	0	0	0
Mir1a-2	0	0	0	1	3
Mir20a	0	2	0	0	0
Mir223	13	1	0	0	0
Mir22hg	11	11	8	1	6
Mir24-2	3	1	2	2	1
Mir27a	0	0	1	0	2
Mir28b	0	2	1	0	1
Mir3069	1	1	1	3	0
Mir3101	4	8	6	5	3
Mir3112	1	2	0	0	1
Mir331	1	1	0	0	0
Mir339	3	1	0	0	1
Mir3474	0	2	16	1	6
Mir351	0	0	3	3	1
Mir455	2	0	0	1	0
Mir467f	1	1	0	6	0
Mir5103	1	1	0	2	3
Mir5107	0	0	3	1	16
Mir5114	44	56	86	42	26

For Review Only

Mir5116	14	2	0	0	0
Mir5122	0	0	0	0	0
Mir5123	1	2	0	6	8
Mir5129	9	4	0	0	0
Mir5130	0	0	0	2	5
Mir546	10	1	0	1	0
Mir5625	0	0	0	0	0
Mir6236	42	66	12	47	44
Mir6345	0	0	1	0	0
Mir6516	1	2	0	0	3
Mir6541	1	0	0	4	1
Mir670hg	0	2	0	0	4
Mir671	5	6	0	6	1
Mir682	41	36	52	35	38
Mir692-2	18	12	25	11	17
Mir692-3	22	12	28	13	22
Mir6920	1	1	0	1	0
Mir6934	1	1	0	0	0
Mir6948	0	0	0	0	0
Mir6983	1	0	0	2	1
Mir7021	0	0	1	1	3
Mir703	6	6	6	4	5
Mir7031	5	4	23	0	0
Mir704	4	1	4	0	0
Mir7049	1	3	1	2	3
Mir705	3	0	0	0	0
Mir7059	3	1	0	0	0
Mir7079	0	0	0	0	2
Mir7082	0	0	0	3	1
Mir7086	7	4	0	0	0
Mir7087	1	0	0	1	0
Mir7213	0	0	1	2	1
Mir7219	71	78	95	35	87
Mir7237	1	1	0	0	0
Mir7646	3	3	0	0	0
Mir7656	1	1	0	3	1
Mir7669	5	4	0	11	6
Mir7670	8	1	5	10	0
Mir7673	0	0	1	9	5
Mir7676-1	8	5	24	1	6
Mir7676-2	51	110	206	54	34
Mir8091	18	157	0	0	0
Mir8099-2	0	0	0	0	0
Mir8112	11	0	0	2	1
Mir8116	1	2	5	5	5
Mir92-1	1	2	15	21	8
Mir99ahg	31	41	18	45	22
Mirlet7c-2	0	0	0	0	3
Mirt1	4	0	0	0	0
Mis12	95	94	143	108	91

For Review Only

Mis18a	9	33	40	17	24
Mis18bp1	2	1	0	1	0
Mitd1	26	37	40	30	30
Mitf	71	125	63	114	126
Mki67	2	8	0	10	12
Mkks	37	37	41	55	39
Mkl1	93	89	113	153	122
Mkl2	50	71	66	62	98
Mkln1	1	1	0	969	0
Mknk1	155	0	2	161	339
Mknk2	1	208	27	0	0
Mkrn1	99	131	103	105	106
Mkrn2	48	49	53	57	59
Mkrn3	20	1	0	7	0
Mks1	41	18	23	30	46
Mlec	367	362	522	493	469
Mlf2	169	152	3	45	23
Mlh1	53	48	68	65	63
Mlh3	42	12	30	45	36
Mlkl	0	0	0	0	2
Mllt1	37	53	0	46	42
Mllt10	49	85	86	95	130
Mllt11	17	11	40	15	39
Mllt3	5	7	14	7	3
Mllt4	8	4	21	28	26
Mllt6	43	56	38	54	62
Mlph	169	201	0	480	345
Mlst8	68	32	52	30	37
Mlx	0	68	60	4	1
Mlxip	1	1	0	0	0
Mlxipl	0	106	1	105	913
Mlycd	64	25	30	31	28
Mmaa	74	82	102	84	125
Mmab	23	22	15	36	33
Mmachc	38	23	36	37	62
Mmadhc	40	60	135	0	103
Mmd	46	31	32	34	41
Mmgt1	56	49	55	78	111
Mmgt2	62	120	96	58	102
Mmp11	8	12	15	9	3
Mmp14	31	39	46	52	29
Mmp15	12	3	0	5	6
Mmp16	3	0	0	0	0
Mmp17	1	0	0	0	3
Mmp19	0	0	0	0	0
Mmp2	127	105	0	0	0
Mmp24	4	4	11	19	0
Mmp25	7	3	0	2	4
Mmp28	8	6	14	19	21
Mmp8	29	0	0	19	0

For Review Only

Mmp9	43	0	16	7	0
Mmrn2	7	5	6	3	1
Mms19	0	0	0	30	21
Mms22l	0	16	14	18	18
Mn1	23	0	10	3	18
Mnat1	30	30	27	23	19
Mnd1-ps	1	0	0	0	0
Mnda	0	5	0	2	3
Mndal	1	0	1	1	2
Mnt	32	22	34	55	45
Moap1	48	0	8	5	15
Mob1a	807	724	649	743	633
Mob1b	29	26	16	28	36
Mob2	0	0	53	5	0
Mob3a	305	233	345	256	314
Mob3b	6	3	7	3	3
Mob3c	1	1	0	0	0
Mob4	207	124	138	110	102
Mobp	3	0	0	6	0
Mocos	0	4	4	0	0
Mocs1	10	4	43	35	51
Mocs2	51	24	63	40	66
Mocs3	6	4	4	4	0
Mogs	23	183	0	52	0
Mok	0	0	0	8	0
Mon1a	53	43	66	40	59
Mon1b	91	43	81	90	42
Mon2	127	150	168	172	165
Morc2a	70	105	121	117	86
Morc3	370	202	0	0	0
Morf4l1	92	93	107	70	87
Morf4l1-ps1	96	95	113	83	98
Morf4l1b	79	80	105	74	79
Morf4l2	125	107	184	156	175
Morn1	42	48	0	22	15
Morn2	0	0	9	0	0
Morn3	0	8	2	4	0
Mospd1	39	19	26	30	10
Mospd2	30	67	52	64	43
Mospd3	15	5	4	14	15
Mov10	56	22	45	35	55
Mpc1	134	125	155	75	130
Mpc2	55	40	57	45	55
Mpdu1	9	2	0	45	0
Mpdz	0	0	0	0	5
Mpeg1	0	0	1150	2618	2420
Mpg	44	0	5	41	17
Mphosph10	31	44	35	24	32
Mphosph6	21	48	40	38	38
Mphosph8	13	35	37	27	0

For Review Only

Mphosph9	26	45	36	41	30
Mpi	7	1	22	0	18
Mplkip	14	22	10	29	14
Mpnd	19	8	19	0	0
Mpo	0	0	0	0	0
Mpp1	30	46	87	114	34
Mpp4	0	0	7	0	0
Mpp5	40	35	56	57	46
Mpp6	17	9	15	20	19
Mpp7	3	13	7	26	19
Mppe1	59	36	47	45	35
Mprip	94	115	152	133	64
Mpst	1	7	0	3	0
Mpv17	1	0	0	95	0
Mpv17l	16	18	20	13	17
Mpv17l2	38	23	79	19	92
Mpzl1	0	0	0	11	0
Mr1	43	38	56	60	67
Mras	41	25	55	31	47
Mrc1	32	26	29	14	26
Mrc2	38	37	33	69	81
Mre11a	32	22	28	52	50
Mrfap1	0	0	0	0	0
Mrgbp	67	41	40	46	53
Mrgpra2b	3	0	3	1	0
Mrgpre	39	19	30	39	26
Mri1	2	19	18	0	6
Mrm1	68	20	49	36	32
Mrm2	3	26	31	14	18
Mroh1	260	99	130	201	263
Mroh2a	0	0	0	4	0
Mroh6	11	2	15	24	0
Mrpl1	22	21	28	19	29
Mrpl10	68	63	102	64	86
Mrpl11	0	0	0	0	0
Mrpl12	22	49	54	41	32
Mrpl13	25	3	46	0	34
Mrpl14	27	32	7	27	44
Mrpl15	90	90	108	74	76
Mrpl16	30	48	37	38	54
Mrpl17	113	228	244	49	143
Mrpl18	51	70	102	45	59
Mrpl19	69	34	50	34	41
Mrpl2	18	3	0	37	1
Mrpl20	48	90	91	66	60
Mrpl21	34	46	37	16	26
Mrpl22	32	33	32	16	33
Mrpl23	31	46	49	10	26
Mrpl24	0	0	1	1	2
Mrpl27	18	36	42	23	23

For Review Only

Mrpl28	28	33	61	29	38
Mrpl3	81	62	94	51	44
Mrpl30	63	51	67	38	64
Mrpl32	50	44	0	0	0
Mrpl33	16	21	39	23	22
Mrpl34	1	10	76	0	0
Mrpl35	30	33	46	44	33
Mrpl36	0	0	0	0	0
Mrpl37	104	26	79	0	100
Mrpl38	58	44	0	0	0
Mrpl39	63	58	60	57	61
Mrpl4	0	0	2	1	1
Mrpl40	48	60	74	43	51
Mrpl41	28	1	0	0	0
Mrpl42	0	44	0	41	41
Mrpl43	0	0	0	0	0
Mrpl44	55	53	85	48	68
Mrpl45	51	59	65	41	28
Mrpl46	32	36	59	9	7
Mrpl47	0	0	9	7	0
Mrpl48	24	20	32	30	32
Mrpl49	122	0	1	1	71
Mrpl50	72	81	94	66	86
Mrpl51	2	4	168	0	0
Mrpl52	18	13	72	33	54
Mrpl53	11	0	0	0	0
Mrpl54	32	14	24	22	26
Mrpl55	47	0	34	1	1
Mrpl57	0	0	0	0	0
Mrpl9	83	47	98	76	99
Mrps10	27	39	49	37	23
Mrps11	5	9	0	30	40
Mrps14	12	362	0	103	0
Mrps15	0	48	35	0	1
Mrps16	35	8	0	22	34
Mrps17	0	8	0	0	0
Mrps18a	16	1	96	0	0
Mrps18b	0	0	15	0	1
Mrps18c	0	0	30	0	13
Mrps2	0	0	1	1	0
Mrps21	16	40	47	36	37
Mrps22	0	19	0	0	16
Mrps23	25	74	66	9	2
Mrps24	4	7	0	128	143
Mrps25	42	80	48	74	39
Mrps27	43	42	56	60	37
Mrps28	20	14	32	15	11
Mrps30	74	82	83	53	58
Mrps31	36	14	57	20	24
Mrps33	34	45	63	41	36

For Review Only

Mrps34	0	0	0	0	0
Mrps35	48	59	78	41	60
Mrps36	17	14	25	12	12
Mrps5	42	29	36	34	38
Mrps6	66	38	53	33	32
Mrps7	57	32	67	36	54
Mrps9	48	42	63	49	39
Mrrf	12	21	18	25	13
Mrs2	102	62	50	101	88
Mrto4	9	11	0	17	10
Mrvi1	7	1	0	3	7
Ms4a14	0	0	0	0	0
Ms4a4c	0	2	0	0	3
Ms4a6b	142	135	0	0	0
Ms4a6c	37	16	21	38	45
Ms4a6d	176	206	255	168	207
Ms4a7	0	0	0	0	0
Msantd1	8	9	4	12	16
Msantd2	13	25	69	46	31
Msantd4	37	33	59	49	51
Msh2	36	48	67	49	71
Msh3	19	12	25	14	11
Msh5	0	0	2	7	17
Msh6	64	56	90	78	45
Msi2	23	31	10	22	20
Msl1	0	0	138	1	3
Msl2	128	131	55	149	161
Msl3	70	66	95	89	66
Msl3l2	1	6	0	7	5
Mslnl	1	0	0	0	0
Msmo1	71	96	93	50	93
Msn	1551	693	956	1283	1321
Msr1	0	2	0	0	7
Msra	48	46	48	62	54
Msrb1	5	2	14	0	0
Msrb2	156	167	226	169	186
Msrb3	1	0	0	0	7
Mss51	2	1	0	0	0
Mst1	2	3	0	1	6
Msto1	0	1	114	0	0
Msx1	29	0	0	0	0
Msx1os	0	3	0	0	0
Mt1	75	41	62	18	81
Mt3	25	0	0	0	0
Mta1	22	22	14	16	35
Mta2	0	0	4	5	20
Mta3	23	35	41	55	72
Mtap	55	68	53	56	55
Mtap7d3	0	0	0	1	0
Mtbp	8	0	0	18	11

For Review Only

Mtch1	37	44	12	14	37
Mtch2	111	144	167	107	90
Mtcp1	3	9	7	5	6
Mtdh	219	483	366	338	322
Mterf1a	8	14	13	5	8
Mterf1b	6	11	10	7	1
Mterf2	0	2	0	6	0
Mterf3	35	43	99	52	55
Mterf4	31	28	48	24	41
Mtf1	24	38	0	0	0
Mtf2	70	69	58	62	104
Mtfmt	25	14	26	19	29
Mtfp1	6	4	0	8	12
Mtfr1	28	20	43	18	20
Mtfr1l	316	192	305	225	262
Mtg1	83	23	48	27	41
Mtg2	26	39	1	0	31
Mthfd1	57	43	69	57	64
Mthfd1l	13	0	16	25	0
Mthfd2	49	30	39	36	28
Mthfd2l	0	3	3	10	0
Mthfr	26	46	102	80	79
Mthfs	0	0	0	0	0
Mthfsd	58	27	35	50	47
Mthfsl	66	53	89	57	63
Mtif2	58	43	44	51	47
Mtif3	72	93	127	100	105
Mtl5	0	0	0	0	0
Mtm1	51	34	28	43	46
Mtmr1	38	26	10	25	47
Mtmr10	177	27	60	89	84
Mtmr11	8	7	18	14	11
Mtmr12	69	57	112	103	72
Mtmr14	59	44	66	66	42
Mtmr2	214	136	215	177	227
Mtmr3	176	143	255	181	194
Mtmr4	146	172	99	187	157
Mtmr6	127	315	280	456	427
Mtmr7	0	0	0	0	5
Mtmr9	70	38	36	55	70
Mto1	73	57	41	72	68
Mtor	74	121	114	130	119
Mtpap	11	60	0	41	0
Mtpn	392	215	252	356	357
Mtr	150	124	108	210	187
Mtrf1	25	15	28	9	16
Mtrf1l	17	5	8	16	20
Mtrr	26	24	29	44	37
Mtss1	98	113	72	126	169
Mtss1l	7	0	0	11	1

For Review Only

Mttp	30	11	10	28	32
Mturn	0	3	0	12	0
Mtus1	340	177	155	159	203
Mtus2	8	9	3	7	16
Mtx2	73	78	71	48	81
Mtx3	64	45	63	59	68
Muc16	7	8	16	6	5
Muc19	4	0	0	3	0
Muc5b	4	1	0	0	10
Muc6	1	4	11	18	20
Mul1	62	49	51	54	76
Mum1	70	45	78	76	86
Mus81	25	14	44	52	32
Musk	0	0	0	0	0
Mustn1	0	0	7	0	0
Mut	70	96	60	63	55
Mutyh	5	0	0	0	0
Mvb12a	5	168	0	0	0
Mvb12b	297	95	202	376	344
Mvd	20	9	22	14	27
Mvk	19	0	13	10	0
Mvp	1	1	0	182	0
Mx1	4	27	21	20	22
Mx2	11	14	35	25	27
Mxd1	81	75	42	43	79
Mxd4	8	0	0	0	0
Mxi1	69	66	79	63	67
Mxra7	1	1	0	4	6
Mxra8	5	2	5	0	0
Myadm	344	388	402	398	415
Myadml2	33	38	82	38	52
Mybbp1a	191	121	182	236	186
Mybl2	0	5	5	2	3
Mybpc3	0	11	0	0	3
Mybph	0	0	0	6	4
Myc	135	76	169	56	189
Mycbp	50	0	3	0	1
Mycbp2	397	547	543	601	602
Mycl	36	22	28	13	41
Myd88	0	0	0	0	30
Mydgf	111	97	221	0	182
Myef2	40	38	41	43	50
Myeov2	100	53	112	64	79
Myh10	28	15	10	21	0
Myh7b	0	0	0	4	4
Myh9	282	91	149	371	334
Myl12a	218	161	281	187	217
Myl12b	256	264	309	239	280
Myl2	0	0	0	0	2
Myl6	0	0	0	0	0

For Review Only

Myl9	0	0	0	0	0
Mylip	662	726	709	849	718
Mylk	0	0	0	0	0
Mynn	84	47	92	84	69
Myo10	1	1	0	0	0
Myo15	3	7	9	8	0
Myo18a	230	142	265	258	231
Myo18b	68	44	62	65	88
Myo19	24	22	13	28	24
Myo1b	143	109	73	144	211
Myo1c	126	102	154	182	172
Myo1d	26	4	22	23	0
Myo1e	57	13	58	64	58
Myo1f	596	440	582	464	609
Myo1g	48	59	101	30	63
Myo1h	0	4	0	0	0
Myo5a	22	24	13	29	20
Myo5b	0	0	0	0	0
Myo6	25	19	28	28	26
Myo7a	226	133	151	226	185
Myo9a	108	134	111	165	147
Myo9b	226	212	221	288	328
Myocd	1	0	0	6	0
Myof	0	0	0	0	0
Myom1	14	18	21	19	28
Myom3	1	0	5	0	5
Mypop	3	1	0	0	5
Mypopos	3	3	1	10	8
Myrip	4	0	1	0	0
Mysm1	247	321	310	295	205
Myt1l	0	3	0	4	0
Mzf1	6	11	7	20	13
Mzt1	50	42	70	47	47
Mzt2	0	0	10	1	1
N4bp1	106	102	168	132	150
N4bp2	10	16	16	22	16
N4bp2l1	134	138	211	114	157
N4bp2l2	134	119	159	187	189
N4bp3	4	5	11	9	0
N6amt1	0	11	15	13	11
Naa10	53	34	60	42	34
Naa15	70	64	55	90	108
Naa16	59	74	89	40	79
Naa20	1	39	0	0	0
Naa25	143	102	89	86	152
Naa30	21	18	34	46	38
Naa35	0	0	4	14	0
Naa38	0	0	0	0	0
Naa40	27	1	40	0	175
Naa50	53	41	46	81	45

For Review Only

Naa60	204	205	16	330	91
Naaa	67	72	80	67	59
Naalad2	43	106	100	68	128
Naaladl2	2	0	0	1	0
Nab1	18	11	17	18	19
Nab2	11	19	11	13	15
Nabp1	10	14	6	10	36
Nabp2	0	1	6	3	1
Naca	127	161	201	109	135
Nacad	0	1	0	4	4
Nacc1	26	29	45	44	55
Nacc2	161	151	124	148	196
Nadk	209	158	0	0	335
Nadk2	43	25	29	18	30
Nadsyn1	25	24	54	35	0
Nae1	65	46	88	31	41
Naf1	3	12	8	6	5
Naga	338	254	317	245	292
Nagk	6	17	0	0	0
Naglu	2415	100	1065	0	0
Nagpa	460	293	470	392	451
Naif1	19	31	45	60	30
Naip1	31	21	32	36	37
Naip2	203	165	243	305	253
Naip5	208	207	271	277	256
Naip6	122	48	29	57	246
Nampt	45	106	90	92	129
Nanos1	1	8	0	6	5
Nanp	21	19	24	22	17
Nans	45	46	50	44	37
Nap1l1	53	27	89	34	46
Nap1l4	185	78	296	163	160
Napa	395	255	254	22	31
Napb	11	5	7	21	8
Napepld	0	8	6	9	16
Napg	114	72	71	76	94
Naprt	13	11	0	0	0
Narf	186	104	203	219	218
Narfl	0	0	0	0	164
Nars	0	0	238	1	1
Nars2	24	20	32	24	32
Nasp	26	5	20	17	15
Nat1	13	19	4	3	11
Nat10	82	22	63	62	92
Nat14	1	3	6	0	1
Nat2	33	32	23	19	23
Nat6	92	63	87	57	76
Nat8f1	6	3	11	6	11
Nat8f4	0	0	11	5	12
Nat8l	11	34	46	18	34

For Review Only

Nat9	2	6	0	23	0
Natd1	128	77	196	135	132
Nav1	14	12	0	53	0
Nav2	367	298	200	523	413
Nav3	544	740	657	1100	723
Nbas	49	61	54	96	94
Nbea	33	60	53	62	54
Nbeal1	55	128	78	148	143
Nbeal2	175	125	124	236	216
Nbl1	6	1	7	8	13
Nbn	10	23	44	39	0
Nbr1	261	247	286	219	251
Ncam1	8	0	3	4	0
Ncan	4	1	0	0	0
Ncapd2	7	11	7	9	10
Ncapd3	23	24	0	76	89
Ncapg2	0	2	13	0	19
Ncaph	54	58	69	59	78
Ncaph2	85	0	0	163	0
Ncbp1	71	67	81	88	83
Ncbp2	49	64	102	80	68
Ncbp3	176	127	150	116	175
Ncdn	66	51	49	42	95
Nceh1	46	31	32	35	30
Ncf1	749	101	1717	0	623
Ncf2	218	360	476	263	320
Ncf4	110	21	56	25	37
Nck1	96	100	129	89	120
Nckap1	0	1	0	0	0
Nckap1l	1537	0	83	1826	558
Nckap5	3	0	0	0	0
Nckap5l	36	51	0	249	256
Nckipsd	18	0	1	2	0
Ncl	0	0	0	2	3
Ncln	120	111	117	184	63
Ncmap	0	0	0	0	0
Ncoa1	58	53	33	49	44
Ncoa2	190	142	145	210	191
Ncoa3	679	656	837	758	860
Ncoa4	262	114	115	150	137
Ncoa5	52	28	32	36	74
Ncoa6	69	80	105	95	88
Ncoa7	0	4	7	18	11
Ncor1	161	250	266	259	230
Ncor2	133	56	112	179	150
Ncs1	133	87	162	87	87
Ncstn	198	122	175	197	216
ND1	4126	2898	4034	3053	3524
ND2	308	189	269	242	269
ND3	162	69	135	70	51

For Review Only

ND4	5	9	8	5	6
ND4L	994	788	799	868	1004
ND5	3250	1234	2003	2156	2426
Ndc1	26	6	30	30	47
Nde1	121	108	108	120	109
Ndel1	161	151	142	122	147
Ndfip1	405	326	462	362	412
Ndfip2	131	94	117	101	156
Ndnf	0	0	0	0	0
Ndnl2	12	44	19	25	26
Ndor1	2	0	0	38	0
Ndrg1	2	0	0	0	0
Ndrg2	37	2	0	9	8
Ndrg3	199	129	198	189	214
Ndrg4	3	0	0	0	0
Ndst1	56	70	123	100	53
Ndst2	25	62	66	0	76
Ndufa1	73	59	65	16	9
Ndufa10	157	45	0	125	0
Ndufa11	109	2	83	0	114
Ndufa12	1	1	219	0	0
Ndufa13	160	112	171	89	122
Ndufa2	0	0	65	25	18
Ndufa3	0	71	44	10	0
Ndufa4	163	137	207	168	171
Ndufa5	68	38	68	0	0
Ndufa6	43	0	0	0	62
Ndufa7	99	8	110	60	46
Ndufa8	111	88	135	78	85
Ndufa9	88	130	182	98	161
Ndufab1	0	0	65	11	16
Ndufaf1	51	20	48	19	34
Ndufaf2	18	12	15	6	0
Ndufaf4	40	33	56	38	40
Ndufaf5	0	15	21	0	0
Ndufaf6	10	16	15	0	6
Ndufaf7	0	48	79	0	0
Ndufb10	147	61	196	0	96
Ndufb11	54	1	62	80	58
Ndufb2	30	15	36	10	7
Ndufb3	83	58	83	61	62
Ndufb4	0	21	0	19	0
Ndufb5	95	57	93	61	76
Ndufb6	13	23	82	0	34
Ndufb7	68	52	61	0	43
Ndufb8	133	65	138	90	55
Ndufb9	0	0	2	1	3
Ndufc1	28	0	72	0	0
Ndufc2	156	81	160	88	102
Ndufs1	129	127	155	119	94

For Review Only

Ndufs2	66	77	219	63	71
Ndufs3	0	0	1	0	1
Ndufs4	82	76	87	54	67
Ndufs5	34	21	32	20	25
Ndufs6	0	0	0	0	0
Ndufs7	72	117	143	51	76
Ndufs8	213	150	0	0	0
Ndufv1	0	0	6	18	0
Ndufv2	3	195	232	73	0
Ndufv3	102	70	93	82	86
Neat1	232	327	207	436	442
Neb	0	16	11	3	0
Nebl	0	0	0	0	0
Necab2	0	0	1	0	0
Necab3	0	0	0	8	0
Necap1	93	70	126	100	129
Necap2	346	235	432	274	264
Nectin2	97	73	92	55	66
Nectin3	0	1	0	0	1
Nectin4	131	99	147	153	110
Nedd1	44	21	31	27	30
Nedd4	10	1	0	16	0
Nedd4l	33	37	25	40	31
Nedd8	83	83	144	75	100
Nedd9	6	1	0	0	0
Negr1	1	5	5	6	0
Neil1	42	26	23	19	34
Neil2	0	0	0	4	0
Neil3	0	0	0	0	0
Nek1	17	37	19	37	28
Nek2	0	0	0	0	0
Nek3	12	13	12	13	15
Nek4	41	28	22	51	31
Nek5	0	0	2	6	0
Nek6	564	358	458	530	574
Nek7	85	111	134	178	127
Nek8	0	18	5	6	9
Nek9	99	72	153	121	88
Nelfa	0	0	0	0	0
Nelfb	30	12	0	0	0
Nelfcd	51	70	92	91	0
Nelfe	24	16	43	27	23
Nemf	75	86	169	119	120
Nenf	28	35	39	15	18
Neo1	4	2	0	0	16
Nepro	13	21	57	32	46
Nes	6	5	6	12	0
Net1	0	0	0	0	0
Neto1	3	3	8	0	1
Neu1	0	0	82	0	0

For Review Only

Neu3	13	14	17	20	13
Neurl1a	140	137	180	137	142
Neurl1b	7	8	5	8	5
Neurl2	4	3	7	0	4
Neurl3	0	0	0	0	0
Neurl4	81	49	106	73	102
Nexn	3	0	0	4	0
Nf1	169	220	130	206	236
Nf2	107	84	92	138	109
Nfam1	791	733	894	979	1167
Nfasc	10	0	0	0	0
Nfat5	101	118	111	223	219
Nfatc1	444	382	418	586	541
Nfatc2	142	133	158	217	231
Nfatc2ip	3	12	0	10	10
Nfatc3	93	111	99	111	152
Nfe2	3	0	0	3	0
Nfe2l1	103	78	127	146	105
Nfe2l2	548	368	643	540	475
Nfe2l3	0	1	0	5	5
Nfia	153	196	162	217	246
Nfib	0	3	0	0	0
Nfic	89	104	136	220	175
Nfil3	13	1	15	5	15
Nfix	27	22	46	33	36
Nfkb1	266	235	348	229	270
Nfkb2	69	67	41	80	18
Nfkbia	546	249	309	237	542
Nfkbib	77	54	39	0	9
Nfkbid	74	0	1	5	0
Nfkbie	1	1	171	0	84
Nfkbil1	41	18	25	15	23
Nfkbiz	119	136	123	145	85
Nfrkb	66	42	76	91	96
Nfs1	70	62	70	65	79
Nfu1	69	77	93	57	60
Nfx1	190	182	180	248	219
Nfxl1	12	58	28	25	31
Nfya	16	37	26	60	61
Nfyb	41	27	32	41	36
Nfyc	78	43	69	71	88
Ngdn	66	0	15	68	47
Ngfrap1	22	6	10	0	17
Ngly1	78	66	114	81	74
Ngp	811	162	149	237	0
Ngrn	120	84	130	73	144
Nhej1	0	0	13	1	0
Nhlrc1	1	3	2	1	5
Nhlrc2	81	126	95	120	125
Nhlrc3	34	41	40	55	62

For Review Only

Nhp2	83	56	91	81	64
Nhp2l1	66	64	65	60	96
Nhs	5	17	4	10	16
Nhsl1	1	0	1	0	0
Nhsl2	115	88	102	156	138
Nicn1	0	31	3	1	1
Nid1	0	0	0	0	0
Nid2	40	12	82	134	78
Nif3l1	42	39	45	38	33
Nifk	2	46	111	73	83
Nim1k	9	2	0	0	9
Nin	29	34	20	41	43
Ninj1	2	2	0	236	0
Ninl	39	24	24	30	61
Nip7	1	1	328	0	0
Nipa1	16	6	8	5	0
Nipa2	94	86	147	104	136
Nipal3	48	29	44	62	50
Nipbl	228	253	287	256	228
Nipsnap1	15	10	16	6	13
Nipsnap3b	51	73	78	102	61
Nisch	1	1	0	0	0
Nit1	0	0	3	0	4
Nit2	37	31	33	33	44
Nkap	32	40	41	43	43
Nkapl	1	4	5	1	3
Nkd2	0	0	0	1	4
Nkiras1	98	23	0	22	29
Nkiras2	110	1	261	0	0
Nkrf	1	8	0	7	7
Nktr	79	33	82	126	115
Nle1	8	12	0	6	13
Nlgn2	2	11	4	10	12
Nlk	21	39	23	58	54
Nln	67	30	50	67	37
Nlrc3	2	17	12	12	7
Nlrc4	0	1	0	1	0
Nlrc5	11	24	18	29	23
Nlrp1a	138	52	22	74	38
Nlrp1b	52	63	236	24	270
Nlrp1c-ps	0	22	0	220	205
Nlrp3	63	97	84	134	128
Nlrx1	34	41	55	35	49
Nmb	7	13	8	15	9
Nmd3	70	62	75	79	79
Nme1	82	43	96	56	98
Nme2	76	66	105	58	82
Nme4	6	3	7	6	8
Nme6	33	18	38	22	39
Nme7	13	4	26	0	0

For Review Only

Nmi	1	3	0	72	0
Nmnat1	15	5	9	9	8
Nmnat3	4	13	11	12	26
Nmral1	2	19	64	36	43
Nmrk1	2	2	11	8	13
Nmt1	183	148	211	152	165
Nmt2	0	6	33	0	103
Nnat	14	12	7	18	24
Nnt	92	72	68	104	124
Noa1	34	24	63	33	49
Nob1	64	17	37	16	9
Noc2l	65	18	25	24	11
Noc3l	4	11	15	14	26
Noc4l	36	2	94	0	19
Noct	2	10	10	0	0
Nod1	49	44	64	61	68
Nod2	7	5	0	14	18
Nol10	9	6	20	24	16
Nol11	51	107	74	86	88
Nol12	39	33	58	25	25
Nol4l	84	36	93	84	95
Nol6	67	97	103	102	88
Nol7	0	0	0	0	12
Nol8	24	23	29	27	29
Nol9	50	34	45	54	63
Nolc1	82	93	114	69	95
Nom1	48	58	51	64	76
Nomo1	44	53	57	73	90
Nono	149	146	90	99	0
Nop10	6	10	0	0	0
Nop14	66	51	67	61	52
Nop16	53	62	99	54	47
Nop2	0	0	0	0	226
Nop56	0	0	0	0	0
Nop58	44	61	62	62	81
Nop9	52	36	36	36	33
Nos1	3	0	0	2	2
Nos1ap	167	123	123	154	195
Nos2	0	2	0	0	0
Nos3	0	3	0	4	2
Nosip	110	105	0	80	11
Nostrin	0	0	0	0	0
Notch1	259	201	254	299	343
Notch2	687	653	544	915	860
Notch4	16	24	16	38	33
Notum	0	1	2	1	1
Nova1	15	45	34	40	47
Nova2	0	0	0	0	0
Noxo1	24	34	45	24	23
Npat	33	85	31	57	91

For Review Only

Npc1	323	326	343	426	442
Npc2	1336	819	1290	867	1084
Npcd	0	3	0	3	3
Npdc1	0	0	0	0	0
Npepl1	0	0	0	0	0
Npepps	48	43	83	111	47
Npff	0	0	0	0	0
Nphp1	14	0	11	8	10
Nphp3	7	15	16	14	20
Npl	90	67	128	60	81
Nploc4	85	66	99	63	57
Npm1	149	138	210	153	140
Npm2	6	0	1	0	8
Npm3	1	2	0	0	0
Npm3-ps1	0	4	1	2	2
Npnt	188	136	100	161	172
Npr1	5	0	1	1	2
Nprl2	0	0	25	12	0
Nprl3	45	43	28	73	80
Nptn	313	319	215	413	304
Nptxr	3	4	0	0	1
Nqo2	40	33	21	34	20
Nr0b2	1	0	0	0	0
Nr1d1	6	4	6	17	21
Nr1d2	76	29	42	120	80
Nr1h2	0	0	0	0	0
Nr2c1	23	35	52	33	27
Nr2c2	84	54	102	107	131
Nr2c2ap	10	17	18	8	10
Nr2f2	3	0	0	0	0
Nr2f6	30	17	25	29	32
Nr3c1	255	267	232	270	293
Nr3c2	50	22	38	71	83
Nr4a1	8	4	0	15	0
Nr4a2	0	0	0	2	0
Nr4a3	3	0	0	0	0
Nr6a1	1	0	11	7	4
Nradd	4	126	17	0	0
Nrarp	5	8	10	2	6
Nras	117	1	0	0	0
Nrbf2	28	33	34	28	32
Nrcam	0	0	0	0	5
Nrd1	139	39	57	7	2
Nrde2	35	38	47	47	35
Nrep	56	56	49	47	52
Nrf1	133	83	115	147	128
Nrg1	0	3	0	0	0
Nrg2	0	5	0	4	9
Nrg4	7	0	14	0	0
Nrip1	851	878	776	821	1135

For Review Only

Nrip2	0	0	0	4	0
Nrip3	1	1	2	0	3
Nrm	0	0	50	0	0
Nrn1	1	0	0	1	0
Nron	33	24	27	39	55
Nrp	5	10	12	6	10
Nrp1	472	369	493	437	438
Nrp2	48	61	66	105	100
Nrros	707	626	955	672	712
Nrxn3	0	0	4	0	0
Nsa2	52	36	46	27	38
Nsd1	219	348	185	408	397
Nsdhl	53	20	58	25	22
Nsf	100	70	99	66	59
Nsfl1c	75	87	127	61	111
Nsg1	4	0	0	4	2
Nsg2	8	0	0	0	3
Nsl1	3	3	0	8	13
Nsmaf	11	69	125	71	108
Nsmce1	68	52	56	43	35
Nsmce2	53	33	51	37	76
Nsmce4a	25	28	7	0	25
Nsmf	74	35	67	44	46
Nsrp1	42	45	52	42	42
Nsun2	72	55	83	74	89
Nsun3	34	14	18	65	34
Nsun4	47	62	54	55	80
Nsun5	49	26	6	0	25
Nsun6	40	41	38	39	42
Nsun7	0	3	1	3	2
Nt5c2	87	143	127	115	167
Nt5c3	34	46	30	27	43
Nt5c3b	44	29	41	26	35
Nt5dc1	81	41	53	46	60
Nt5dc2	9	0	0	3	10
Nt5dc3	8	3	6	8	6
Nt5m	59	29	63	31	43
Ntan1	3	0	0	0	0
Nthl1	4	13	0	24	0
Ntm	0	0	0	0	6
Ntmt1	36	30	70	59	59
Ntn1	2	5	5	0	8
Ntn3	3	4	0	3	9
Ntng2	0	0	0	7	2
Ntpcr	159	151	164	199	342
Ntrk2	20	0	0	12	6
Ntrk3	0	0	1	0	0
Nuak1	151	131	128	171	208
Nuak2	50	28	58	35	39
Nub1	125	56	92	75	122

For Review Only

Nubp1	0	0	55	6	0
Nubp2	0	0	222	42	88
Nubpl	20	18	27	0	15
Nucb1	184	13	0	837	210
Nucb2	23	14	18	27	0
Nucks1	72	59	67	98	81
Nudc	99	60	94	58	83
Nudcd1	28	25	35	14	22
Nudcd2	11	9	39	18	18
Nudcd3	225	133	193	237	226
Nudt1	8	0	11	1	5
Nudt12	2	14	0	0	0
Nudt13	68	87	57	49	57
Nudt14	11	33	1	16	25
Nudt15	2	2	1	1	0
Nudt16	0	0	0	0	30
Nudt16l1	0	0	0	0	0
Nudt18	55	58	70	31	36
Nudt19	61	40	70	34	45
Nudt2	5	4	18	9	27
Nudt21	24	21	20	26	17
Nudt22	55	34	54	29	54
Nudt3	140	106	156	119	176
Nudt4	25	66	53	32	80
Nudt5	75	67	114	69	78
Nudt6	16	12	10	13	23
Nudt7	0	11	0	8	0
Nudt9	49	39	44	56	62
Nuf2	0	0	0	0	0
Nufip1	42	36	57	30	34
Nufip2	253	217	232	296	307
Numa1	318	350	0	582	659
Numb	293	477	242	499	425
Numbl	19	0	0	0	0
Nup107	43	35	37	57	22
Nup133	61	43	56	81	72
Nup153	57	74	88	80	98
Nup155	27	32	37	60	35
Nup160	46	25	38	65	43
Nup188	85	39	58	91	89
Nup205	21	0	43	58	35
Nup210	21	3	0	44	14
Nup210l	28	15	14	22	27
Nup214	154	149	162	242	203
Nup35	25	12	17	24	28
Nup37	16	0	0	0	0
Nup43	1	9	12	4	7
Nup50	74	56	62	104	127
Nup54	39	22	42	32	29
Nup62	67	29	100	70	79

For Review Only

Nup85	0	44	0	0	68
Nup88	261	214	295	203	268
Nup93	37	30	19	0	32
Nup98	210	67	84	200	203
Nupl1	73	33	49	72	91
Nupl2	47	47	53	43	59
Nupr1	0	0	0	0	0
Nupr1l	2	0	0	0	0
Nus1	12	56	46	28	25
Nusap1	0	1	0	0	0
Nutf2	36	33	44	32	29
Nutf2-ps1	25	27	31	28	29
Nutf2-ps2	16	17	24	19	21
Nvl	86	125	85	141	142
Nwd1	5	2	0	7	0
Nwd2	0	1	0	0	0
Nxf1	6	0	0	0	0
Nxn	3	0	0	0	0
Nxpe3	1	7	9	7	1
Nxpe4	0	0	0	0	0
Nxph1	0	0	4	0	0
Nxt1	44	44	52	48	44
Nxt2	73	100	144	73	81
Nynrin	21	2	3	8	6
Oaf	5	27	28	37	44
Oard1	2	22	0	0	0
Oas1a	14	9	26	12	8
Oas1b	5	0	17	3	0
Oas1c	7	4	10	5	0
Oas1g	1	1	0	10	0
Oas2	29	1	0	12	15
Oas3	0	3	0	0	0
Oasl1	2	1	0	0	4
Oasl2	7	11	12	20	16
Oat	55	51	82	44	89
Oaz1	0	0	4	0	4
Oaz1-ps	393	336	446	280	359
Oaz2	0	0	0	0	0
Obfc1	90	33	59	62	56
Obp2b	3	0	0	2	0
Obscn	0	3	3	8	0
Ocel1	13	1	42	25	49
Ociad1	712	549	710	679	769
Ociad2	7	0	0	0	14
Ocln	18	13	26	23	11
Ocrl	27	32	34	40	36
Odc1	22	29	22	20	22
Odf2	114	88	89	68	102
Odf2l	34	24	22	30	30
Ofd1	29	16	11	20	34

For Review Only

Ogdh	424	154	237	241	303
Ogdhl	6	0	0	0	0
Ogfod1	42	72	28	39	48
Ogfod2	0	0	0	0	0
Ogfod3	50	36	45	27	54
Ogfr	53	0	0	8	4
Ogfrl1	4	80	89	30	91
Ogg1	2	1	0	0	0
Ogn	0	1	0	0	5
Ogt	415	442	473	510	487
Oip5	3	4	0	0	5
Ola1	36	43	34	25	54
Olfm2	0	2	0	9	0
Olfm4	24	2	0	13	48
Olfml2a	2	0	0	5	0
Olfml2b	216	85	185	201	158
Olfml3	5463	4866	6772	5169	6056
Olfr1029	0	0	0	2	1
Olfr1030	1	4	11	5	0
Olfr1033	8	6	12	7	0
Olfr1090	0	1	3	0	4
Olfr110	21	12	21	19	17
Olfr111	75	33	58	62	65
Olfr112	1	4	0	0	0
Olfr114	1	0	0	0	0
Olfr1264	0	0	0	0	0
Olfr1394	0	0	2	0	12
Olfr1426	3	0	0	1	0
Olfr1443	1	0	0	0	0
Olfr1444	0	0	0	1	0
Olfr1484	1	1	0	0	0
Olfr172	3	3	4	0	0
Olfr173	1	0	2	0	0
Olfr394	0	0	0	0	0
Olfr456	0	0	0	2	4
Olfr56	1	4	0	4	3
Olfr920	1	0	0	6	0
Olfr99	3	0	0	7	0
Olr1	0	1	0	0	4
Oma1	202	181	351	371	241
Omd	0	1	0	1	0
Omg	3	1	2	1	0
Opa1	82	82	89	113	79
Opa3	95	48	77	59	65
Ophn1	130	52	59	141	230
Oplah	43	20	6	21	49
Opn1sw	1	0	0	4	0
Opn3	0	1	5	5	5
Oprd1	0	0	0	1	0
Oprl1	0	0	0	0	0

For Review Only

Oprm1	15	6	14	16	18
Optn	15	17	0	15	12
Orai1	9	166	46	52	49
Orai2	308	222	287	313	313
Orai3	276	141	286	179	300
Oraov1	124	96	59	91	58
Orc1	8	13	9	18	14
Orc2	35	67	97	59	95
Orc3	121	87	199	126	192
Orc4	84	80	84	40	72
Orc5	42	74	70	61	84
Orc6	18	12	33	20	0
Orm2	0	0	0	12	27
Orm3	14	8	6	15	13
Ormdl1	118	107	185	93	93
Ormdl2	30	13	0	1	0
Ormdl3	97	88	96	92	71
Os9	124	167	70	125	130
Osbp	29	59	47	58	43
Osbpl11	199	193	159	229	217
Osbpl1a	63	36	45	63	80
Osbpl2	92	52	102	94	91
Osbpl3	31	14	19	15	36
Osbpl6	1	3	0	1	12
Osbpl7	44	37	73	48	28
Osbpl8	14	20	15	57	45
Osbpl9	98	145	173	197	230
Oser1	64	70	79	91	80
Osgep	20	0	0	0	0
Osgepl1	14	17	18	18	36
Osgin1	33	9	17	9	19
Osgin2	43	34	53	51	34
Osm	42	49	5	29	83
Ost4	44	42	73	32	40
Ostc	145	191	261	183	190
Ostf1	403	283	431	310	304
Ostm1	183	176	189	176	245
Otc	0	5	0	0	0
Otog	0	0	0	3	0
Otub1	1	1	0	0	0
Otub2	6	5	28	8	15
Otud1	8	45	25	13	49
Otud3	4	5	0	0	6
Otud4	47	68	41	71	91
Otud5	46	68	0	119	134
Otud6b	98	43	55	73	86
Otud7a	0	0	0	0	0
Otud7b	85	33	52	65	62
Otulin	21	18	0	0	0
Otx2	9	0	0	0	0

For Review Only

Ovca2	0	0	143	0	0
Ovgp1	3	1	0	0	0
Ovol1	4	1	0	0	7
Oxa1l	72	86	147	108	134
Oxct1	556	355	460	531	544
Oxld1	18	9	0	51	0
Oxnad1	12	6	14	8	20
Oxr1	104	115	102	159	167
Oxsm	39	44	71	67	88
Oxsr1	29	29	43	31	23
P2rx1	25	14	26	20	9
P2rx3	1	2	0	1	0
P2rx4	0	0	0	0	0
P2rx5	1	2	0	3	0
P2rx7	481	386	470	750	801
P2ry1	0	0	0	0	0
P2ry10	2	2	0	0	0
P2ry12	3011	1778	3761	2681	4675
P2ry13	3346	3015	4292	3456	3734
P2ry2	0	1	0	10	0
P2ry6	221	701	1142	686	912
P3h1	23	14	19	27	45
P3h2	87	138	67	202	149
P3h3	36	25	29	10	13
P3h4	11	4	8	8	5
P4ha1	0	165	447	28	199
P4hb	1275	956	1401	1034	1325
Pa2g4	120	13	176	184	0
Pabpc1	0	0	5	2	0
Pabpc4	14	11	22	11	25
Pabpc4l	2	1	0	0	0
Pabpn1	0	0	0	0	0
Pacrgl	7	3	3	6	0
Pacs1	72	100	78	97	113
Pacs2	86	147	134	138	141
Pacsin1	17	15	0	20	1
Pacsin2	325	355	517	419	453
Pacsin3	8	0	2	0	0
Padi2	181	97	144	213	236
Padi4	0	6	0	0	0
Paf1	89	69	86	58	81
Pafah1b1	126	182	87	168	132
Pafah1b2	193	93	166	179	165
Pafah1b3	0	0	0	0	9
Pafah2	46	17	18	21	13
Pag1	6	80	0	0	127
Paics	50	136	108	69	145
Paip1	47	56	51	44	72
Paip2	356	379	408	363	335
Paip2b	4	7	25	10	26

For Review Only

Pak1	141	142	195	175	147
Pak1ip1	0	0	93	78	57
Pak2	237	243	235	315	230
Pak4	46	27	37	36	44
Pak6	0	1	5	3	0
Pakap	3	0	0	0	0
Palb2	27	19	19	23	38
Pald1	737	123	1	102	139
Palld	0	7	7	3	0
Palm	2	0	0	8	0
Palmd	2	1	0	1	0
Pam	0	1	6	1	1
Pam16	1	0	0	0	0
Pan2	66	89	92	91	109
Pan3	405	472	304	419	536
Pank1	9	19	10	8	6
Pank2	48	48	70	51	61
Pank3	40	30	65	82	83
Pank4	94	86	58	79	100
Panx1	15	18	16	17	29
Paox	23	20	26	36	35
Papd4	163	117	108	182	189
Papd5	33	41	48	36	32
Papd7	32	40	11	16	28
Papln	7	6	12	36	34
Papola	219	210	244	285	299
Papolb	1	3	4	4	0
Papolg	23	70	34	75	82
Pappa	0	4	3	1	0
Pappa2	2	0	0	0	0
Papss1	194	130	197	188	189
Papss2	2	1	0	3	3
Paqr3	9	7	0	1	16
Paqr4	54	22	33	36	18
Paqr5	1	0	0	0	0
Paqr7	246	161	297	200	261
Paqr8	7	6	9	0	20
Pard3b	12	23	26	25	19
Pard3bos1	24	24	31	58	70
Pard3bos2	0	0	0	0	0
Pard6a	13	22	14	7	24
Pard6g	4	3	11	4	8
Parg	66	31	27	56	71
Park2	44	58	76	156	137
Park7	1	65	191	124	0
Parl	42	45	41	49	53
Parn	154	58	90	123	104
Parp1	141	0	3	5	1
Parp10	50	35	17	55	18
Parp11	50	46	48	65	61

For Review Only

Parp12	82	48	47	56	65
Parp14	67	88	158	150	128
Parp16	54	24	64	41	55
Parp2	0	0	0	0	40
Parp3	9	54	0	92	43
Parp4	96	15	40	84	69
Parp6	17	45	31	49	30
Parp8	159	169	153	163	173
Parp9	1	328	467	0	376
Pars2	48	14	42	30	16
Particl	32	13	11	33	40
Parva	5	1	0	8	1
Parvb	15	15	11	11	16
Parvg	682	435	592	565	685
Pask	0	0	12	0	21
Patl1	25	28	25	65	58
Patl2	0	0	0	1	2
Patz1	51	28	56	98	109
Pax6	0	0	0	4	0
Paxbp1	67	58	76	63	84
Paxip1	41	44	31	58	32
Pbdc1	33	25	28	22	20
Pbk	0	0	0	7	0
Pbld2	3	0	5	0	0
Pbrm1	88	180	116	128	104
Pbx1	26	18	27	18	25
Pbx2	16	30	28	21	12
Pbx3	28	18	14	23	13
Pbxip1	113	84	0	0	214
Pcbd2	0	5	1	1	1
Pcbp1	89	157	203	165	157
Pcbp2	145	175	124	114	125
Pcbp3	23	2	8	0	7
Pcbp4	7	0	0	0	0
Pcca	30	45	44	63	67
Pccb	45	40	41	40	24
Pcdh1	1	0	0	0	0
Pcdh12	2	0	0	0	0
Pcdh15	0	2	1	0	6
Pcdhb19	0	1	0	1	0
Pcdhb22	12	4	8	12	8
Pcdhga1	14	19	53	54	50
Pcdhga10	4	2	1	15	5
Pcdhga11	3	23	11	22	11
Pcdhga12	21	20	41	27	16
Pcdhga2	18	64	9	44	57
Pcdhga3	2	7	15	29	35
Pcdhga4	18	4	17	27	32
Pcdhga5	35	59	31	37	71
Pcdhga6	4	3	7	6	15

For Review Only

Pcdhga7	10	46	26	36	36
Pcdhga8	21	17	11	26	28
Pcdhga9	27	44	29	41	23
Pcdhgb1	19	16	28	24	31
Pcdhgb2	58	31	36	61	60
Pcdhgb4	33	29	32	63	66
Pcdhgb5	3	4	7	17	11
Pcdhgb6	26	41	8	22	17
Pcdhgb7	17	23	26	20	36
Pcdhgb8	26	6	5	24	24
Pcdhgc3	0	0	0	4	0
Pcdhgc4	9	10	8	4	0
Pcdhgc5	6	11	4	5	4
Pced1a	67	61	60	82	67
Pced1b	42	20	8	32	25
Pcf11	77	113	70	117	116
Pcgf1	15	0	41	0	0
Pcgf3	23	24	15	33	22
Pcgf5	19	18	8	18	19
Pcgf6	16	15	14	9	12
Pcid2	142	89	85	141	148
Pcif1	164	42	135	0	0
Pclo	5	0	0	0	10
Pcm1	68	71	53	88	86
Pcmt1	154	167	173	122	157
Pcmtd1	88	92	83	103	64
Pcmtd2	44	45	90	94	99
Pcna	0	0	4	2	5
Pcnp	124	131	132	106	132
Pcnt	81	41	96	105	133
Pcnx	71	73	123	77	102
Pcnx2	19	17	20	11	39
Pcnx3	66	130	82	153	236
Pcnx4	6	17	43	38	19
Pcolce	17	3	0	15	12
Pcolce2	0	0	0	14	0
Pcp4	0	0	0	0	0
Pcp4l1	19	1	0	23	0
Pcsk4	5	5	3	9	8
Pcsk7	95	58	97	119	83
Pcsk9	6	4	0	10	18
Pctp	55	18	69	35	51
Pcx	33	1	29	33	38
Pcyox1	325	153	260	265	292
Pcyt1a	46	45	77	60	72
Pcyt2	65	34	72	66	62
Pdap1	26	18	33	31	34
Pdcd1	15	13	27	13	19
Pdcd10	51	51	70	54	50
Pdcd11	53	47	62	67	81

Pdcd2	0	21	21	1	10
Pdcd2l	5	7	133	63	0
Pdcd4	70	90	61	54	68
Pdcd5	40	45	58	34	42
Pdcd6	79	84	115	66	103
Pdcd6ip	310	184	274	320	319
Pdcd7	6	12	19	12	15
Pdcl	61	40	56	84	62
Pdcl3	53	75	59	62	45
Pddc1	104	71	73	77	114
Pde10a	4	2	1	7	0
Pde12	181	105	71	63	93
Pde1a	0	0	0	0	0
Pde1b	84	80	152	20	52
Pde1c	0	4	0	3	2
Pde2a	269	152	223	243	210
Pde3a	0	1	0	0	4
Pde3b	560	969	580	973	979
Pde4a	42	17	27	46	32
Pde4b	125	55	66	106	147
Pde4d	66	48	89	78	95
Pde4dip	185	180	156	210	263
Pde6d	35	24	38	19	22
Pde6g	0	1	1	1	3
Pde7a	6	14	37	20	28
Pde7b	0	0	0	0	0
Pde8a	0	1	2	1	1
Pde8b	79	100	44	1	41
Pdgfa	43	44	45	42	50
Pdgfb	37	29	48	23	33
Pdgfc	0	3	0	1	0
Pdgfra	0	0	0	0	0
Pdgfrb	0	2	0	6	1
Pdgfrl	1	1	0	0	5
Pdha1	142	101	103	126	84
Pdhb	134	96	157	112	134
Pdhx	56	50	49	50	43
Pdia3	1877	1163	2110	1451	1675
Pdia4	693	0	11	62	3
Pdia5	0	0	0	6	1
Pdia6	527	532	752	509	538
Pdik1l	57	49	68	77	51
Pdk1	313	244	314	261	278
Pdk2	69	34	46	66	81
Pdk3	40	18	73	37	55
Pdlim2	35	36	54	45	29
Pdlim4	0	55	45	0	17
Pdlim5	68	85	83	79	124
Pdp1	18	22	14	20	15
Pdp2	112	87	97	110	114

For Review Only

Pdpk1	0	36	0	22	11
Pdpn	10	0	0	0	0
Pdpr	58	85	50	99	80
Pdrg1	0	0	0	0	0
Pds5a	104	101	67	115	113
Pds5b	65	54	44	69	73
Pdss1	3	7	20	25	23
Pdss2	11	2	40	32	53
Pdxdc1	142	103	145	194	196
Pdxk	85	16	59	81	55
Pdxp	1	0	6	0	5
Pdyn	0	0	2	2	0
Pdzd11	0	0	42	1	1
Pdzd2	6	4	14	8	12
Pdzd3	0	0	0	0	0
Pdzd4	5	2	0	8	11
Pdzd7	0	0	0	0	0
Pdzd8	86	106	122	96	99
Pdzd9	0	2	0	7	1
Pdzrn4	2	1	7	5	1
Pea15a	340	280	87	129	485
Peak1	125	197	189	234	235
Peak1os	9	23	10	29	21
Pear1	63	44	30	108	77
Pebp1	124	81	96	74	96
Pecam1	58	54	87	78	88
Pecr	69	68	91	73	69
Pef1	88	71	89	76	91
Peg10	2	10	5	10	14
Peg12	4	2	3	6	8
Peg13	106	61	40	100	105
Peg3	8	1	0	0	1
Peli1	150	150	206	185	197
Peli2	49	48	49	78	91
Peli3	39	18	12	12	7
Pelo	0	2	0	0	0
Pelp1	27	6	14	28	29
Pemt	0	0	0	0	0
Peo1	68	11	63	43	23
Pepd	261	162	243	194	221
Per1	9	1	0	100	0
Per2	2	9	17	15	29
Per3	114	140	152	120	151
Perm1	3	38	19	20	0
Perp	13	10	0	10	7
Pes1	150	112	86	105	143
Pet100	0	0	21	2	1
Pex1	83	60	48	53	45
Pex10	21	0	50	0	0
Pex11a	19	15	22	30	22

For Review Only

Pex11b	1	209	96	0	291
Pex11g	50	17	11	58	43
Pex12	53	43	80	38	35
Pex13	118	80	108	89	87
Pex14	93	73	103	92	81
Pex16	28	7	0	0	0
Pex19	90	94	0	16	95
Pex2	164	225	287	138	242
Pex26	5	11	8	17	8
Pex3	78	79	98	69	93
Pex5	97	56	105	77	78
Pex6	67	0	54	30	13
Pex7	59	43	37	45	81
Pf4	0	0	0	0	0
Pfas	0	0	1	1	34
Pfdn1	13	24	67	29	30
Pfdn2	39	30	54	29	31
Pfdn4	6	8	3	0	9
Pfdn5	0	0	0	0	0
Pfdn6	5	15	29	22	23
Pfkfb2	23	21	39	60	60
Pfkfb3	332	199	313	395	463
Pfkfb4	186	151	301	49	194
Pfkl	311	176	216	223	239
Pfkm	33	8	12	22	22
Pfkp	14	4	0	0	0
Pfn1	640	508	849	624	680
Pfn2	20	29	26	12	8
Pfpl	2	3	0	6	4
Pgam1	138	113	186	116	147
Pgam2	2	2	0	1	0
Pgam5	109	89	119	85	101
Pgap1	6	4	30	39	0
Pgap2	66	59	0	59	128
Pgap3	11	2	5	7	19
Pgbd1	17	7	6	11	10
Pgd	211	141	0	0	0
Pggt1b	107	109	128	169	108
Pgk1	83	49	66	48	45
Pgls	64	36	63	0	63
Pglyrp1	22	0	0	6	4
Pglyrp2	0	0	6	2	1
Pgm1	1	1	0	0	79
Pgm2	91	37	70	59	98
Pgm2l1	11	8	6	14	11
Pgm3	5	21	37	35	54
Pgp	7	1	0	31	43
Pgpep1	85	56	108	77	103
Pgr	1	1	0	0	0
Pgrmc1	168	118	182	160	170

For Review Only

Pgrmc2	2	56	20	18	10
Pgs1	120	76	87	128	139
Phactr1	13	11	12	18	23
Phactr2	89	83	35	117	109
Phactr4	49	66	82	79	61
Phax	65	85	93	62	61
Phb	77	68	92	47	77
Phb2	97	65	148	76	80
Phc1	19	13	29	38	32
Phc2	143	90	143	167	167
Phc3	176	347	220	349	427
Phf1	4	0	0	141	0
Phf10	43	54	62	24	39
Phf11a	6	10	8	7	9
Phf11b	19	35	26	26	29
Phf11c	0	0	0	0	0
Phf11d	0	32	4	2	0
Phf12	50	65	93	57	49
Phf13	23	9	12	35	26
Phf14	116	116	112	141	205
Phf2	13	36	11	31	52
Phf20	117	118	84	107	101
Phf20l1	76	104	104	135	153
Phf21a	77	120	112	131	128
Phf21b	3	10	0	9	3
Phf23	1	9	0	0	0
Phf24	1	0	6	15	11
Phf3	44	50	47	92	101
Phf5a	26	0	0	0	0
Phf6	2	29	44	64	35
Phf7	39	37	55	39	21
Phf8	59	58	100	105	126
Phgdh	189	96	226	155	204
Phip	181	235	209	207	286
Phka1	80	65	96	69	47
Phka2	81	145	116	159	171
Phkb	40	50	64	109	41
Phkg1	0	9	12	9	0
Phkg2	36	0	29	8	2
Phlda1	0	0	0	0	0
Phldb1	22	18	17	17	23
Phldb3	0	0	3	0	0
Phlpp1	34	12	16	15	26
Phlpp2	44	29	38	57	57
Phospho2	78	72	119	93	94
Phpt1	109	62	0	44	21
Phrf1	109	82	0	278	243
Phtf1	6	15	17	13	5
Phtf1os	31	6	26	32	14
Phtf2	41	33	32	77	58

For Review Only

Phxr4	39	82	62	48	66
Phyh	130	66	124	80	77
Phyhd1	249	203	0	445	88
Phykpl	66	19	37	20	53
Pi16	0	0	0	0	0
Pi4k2a	60	52	80	43	72
Pi4k2b	4	5	0	4	6
Pi4ka	68	45	82	65	99
Pi4kb	34	35	29	54	46
Pias1	335	281	336	293	259
Pias2	75	71	58	85	74
Pias3	117	71	98	60	103
Pias4	57	0	1	1	1
Pibf1	35	44	73	65	60
Picalm	637	671	462	521	607
Pick1	29	21	58	31	38
Pid1	221	169	180	220	184
Pidd1	22	15	31	30	53
Piezo1	51	35	37	78	72
Pif1	0	4	0	0	3
Piga	16	14	19	7	13
Pigb	48	39	62	41	49
Pigc	108	111	145	46	74
Pigf	15	6	10	19	13
Pigg	27	40	47	55	49
Pigh	59	86	122	94	89
Pigk	218	174	244	274	291
Pigl	20	17	14	22	15
Pigm	84	115	83	103	114
Pign	47	63	78	122	105
Pigo	52	4	43	23	24
Pigp	60	37	45	23	21
Pigq	300	1	66	83	0
Pigs	185	0	303	59	104
Pigt	109	28	65	126	158
Pigu	77	76	121	86	79
Pigv	132	139	213	130	142
Pigw	4	3	11	9	6
Pigx	96	97	107	54	72
Pigyl	44	22	39	26	31
Pigz	12	20	25	26	28
Pih1d1	59	44	65	22	39
Pik3ap1	590	498	657	651	713
Pik3c2a	58	79	45	74	93
Pik3c2b	39	24	13	35	32
Pik3c3	53	100	102	88	116
Pik3ca	46	92	95	63	75
Pik3cb	19	16	8	38	17
Pik3cd	239	136	135	565	214
Pik3cg	395	570	715	1212	919

For Review Only

For Review Only

Pik3ip1	40	21	29	11	25
Pik3r1	135	180	193	319	217
Pik3r2	84	1	0	63	0
Pik3r3	10	5	12	14	18
Pik3r4	123	105	112	98	99
Pik3r5	395	380	348	444	460
Pik3r6	4	22	31	19	38
Pikfyve	75	36	55	101	80
Pilra	17	27	58	41	0
Pilrb1	0	1	0	2	2
Pilrb2	6	0	0	4	0
Pim2	17	25	0	0	0
Pim3	0	34	34	8	1
Pin1	73	59	97	57	76
Pin1rt1	0	0	0	0	3
Pin4	0	0	6	0	0
Pink1	42	40	32	46	56
Pinx1	13	13	34	10	25
Pip4k2a	0	0	356	358	454
Pip4k2b	23	13	24	16	16
Pip4k2c	116	115	122	152	110
Pip5k1a	73	36	60	61	57
Pip5k1b	12	0	0	11	0
Pip5k1c	249	200	132	339	372
Pir	0	0	0	0	0
Pira1	9	5	10	6	7
Pira2	12	4	7	7	10
Pira6	4	4	0	6	8
Pirb	9	10	52	80	0
Pisd	0	195	70	0	5
Pisd-ps1	108	0	0	69	0
Pisd-ps2	0	2	0	84	72
Pisd-ps3	0	0	26	210	0
Pithd1	40	41	40	31	38
Pitpna	0	0	45	6	1
Pitpnb	134	119	123	121	153
Pitpnc1	271	264	316	369	310
Pitpnm1	146	77	172	0	252
Pitpnm2	0	0	1	0	0
Pitrm1	48	57	22	52	48
Piwil2	0	0	0	3	0
Pja1	77	52	126	58	83
Pja2	246	174	174	225	223
Pkd1	178	223	338	449	563
Pkd1l3	2	4	0	6	0
Pkd2	41	43	47	50	34
Pkd2l2	1	1	4	2	0
Pkdcc	8	6	16	11	9
Pkib	37	41	61	46	56
Pkig	126	126	174	101	103

Pkm	511	346	502	319	483
Pkmyt1	7	7	5	2	3
Pkn1	145	58	93	106	335
Pkn2	43	50	29	39	30
Pkn3	5	10	0	0	5
Pknox1	231	174	199	261	277
Pkp4	78	33	57	38	51
Pla2g12a	17	11	13	19	18
Pla2g15	1439	1168	1680	1285	1274
Pla2g16	36	2	18	7	20
Pla2g4a	130	67	162	175	138
Pla2g4c	5	0	2	1	1
Pla2g5	0	0	0	0	0
Pla2g6	33	17	30	26	13
Pla2g7	0	0	0	0	0
Plaa	87	131	98	104	90
Plac8	0	0	0	6	1
Plag1	5	1	5	13	6
Plagl2	89	72	125	97	145
Plat	0	2	0	0	0
Platr11	0	1	0	4	1
Platr25	3	3	6	5	7
Plau	79	100	77	154	172
Plaur	46	5	38	37	14
Plbd1	30	11	22	13	11
Plbd2	1	1	0	693	0
Plcb1	4	1	0	0	0
Plcb2	0	0	26	327	240
Plcb3	93	80	102	127	146
Plcb4	2	5	0	0	0
Plcd1	1	0	0	0	13
Plcd3	4	4	0	6	9
Plcd4	0	3	0	0	0
Plcg1	193	152	51	171	167
Plcg2	456	292	454	491	508
Plch1	0	0	0	0	0
Plcl1	26	41	31	33	48
Plcl2	206	209	304	206	239
Plcxd1	0	7	4	0	9
Plcxd2	12	4	12	15	12
Pld1	349	394	508	450	406
Pld2	35	46	51	55	56
Pld3	469	316	448	349	465
Pld4	2945	2126	3661	1341	2462
Plec	14	9	4	25	19
Plek	550	786	823	1043	1005
Plekha1	78	133	146	111	127
Plekha2	253	135	280	226	219
Plekha3	5	18	9	20	21
Plekha4	0	0	0	0	0

For Review Only

Plekha5	14	19	8	10	26
Plekha7	0	3	1	14	3
Plekha8	15	36	19	17	30
Plekhb1	8	0	10	4	0
Plekhb2	210	149	158	162	215
Plekhf1	2	1	8	6	0
Plekhf2	72	92	49	102	117
Plekhg1	0	0	0	0	0
Plekhg2	58	30	0	0	0
Plekhg3	0	0	0	10	0
Plekhg4	0	0	0	5	0
Plekhg5	20	13	23	43	23
Plekhg6	4	1	1	2	0
Plekhh1	7	6	3	11	4
Plekhh2	5	4	19	23	0
Plekhh3	0	5	0	5	7
Plekhj1	7	1	0	51	68
Plekhm1	101	118	69	112	94
Plekhm2	39	45	85	0	73
Plekhm3	223	98	166	281	190
Plekhn1	6	4	36	0	36
Plekho1	176	164	0	0	0
Plekho2	97	56	77	173	92
Plgrkt	257	126	23	20	16
Plin2	3	60	5	2	2
Plin3	39	22	21	20	33
Plin4	22	52	19	27	31
Plk1	0	6	0	1	1
Plk2	5	0	10	0	6
Plk3	0	34	1	41	8
Plk4	2	4	14	16	0
Pllp	0	5	0	0	0
Pln	3	9	0	12	8
Plod1	333	320	678	349	793
Plod3	347	69	88	71	0
Plp1	1	1	34	0	0
Plp2	3	0	0	4	5
Plpp1	160	180	224	215	144
Plpp2	2	11	0	36	0
Plpp3	15	21	17	6	9
Plpp5	23	6	0	0	0
Plpp6	16	17	13	20	7
Plpp7	26	36	30	23	42
Plppr3	14	35	40	32	45
Plppr4	6	17	7	9	10
Plrg1	61	89	115	73	0
Pls3	70	16	40	33	52
Plscr1	13	0	0	3	0
Plscr3	90	77	88	86	102
Plscr4	0	0	0	0	0

For Review Only

Pltp	0	0	0	0	0
Plvap	1	4	1	3	4
Plxdc1	444	255	340	353	387
Plxdc2	1	129	7148	0	0
Plxna1	38	30	9	35	32
Plxna2	1	0	0	5	1
Plxna3	22	12	25	12	43
Plxna4	163	186	180	262	314
Plxna4os1	0	0	0	0	0
Plxnb1	0	3	0	10	0
Plxnb2	2531	1	0	0	2463
Plxnb3	54	40	48	46	45
Plxnc1	1	3	6	0	5
Plxnd1	0	2	0	0	0
Pm20d1	6	0	0	0	8
Pmaip1	4	4	0	12	0
Pmel	3	2	4	0	0
Pmepa1	345	394	400	339	364
Pmf1	4	2	21	15	0
Pml	117	64	85	105	99
Pmm1	23	22	24	22	21
Pmm2	66	59	63	55	127
Pmp22	1452	1088	1480	1479	1632
Pmpca	133	121	173	101	183
Pmpcb	103	0	112	0	90
Pms1	12	8	30	18	27
Pms2	29	18	39	54	37
Pmvk	21	22	56	28	27
Pnck	0	2	0	0	0
Pnisr	68	369	67	442	430
Pnkd	31	38	45	30	29
Pnkp	114	25	123	23	61
Pnldc1	0	2	0	6	0
Pnma1	0	3	0	0	0
Pnma2	8	2	7	5	1
Pnn	0	0	94	158	0
Pno1	33	31	50	29	19
Pnp	140	0	0	0	183
Pnp2	43	37	48	46	33
Pnpla2	116	47	11	2	69
Pnpla3	3	3	4	1	14
Pnpla6	1	1	0	0	0
Pnpla7	82	105	0	0	328
Pnpla8	86	88	117	98	108
Pnpo	40	0	13	15	20
Pnpt1	49	36	41	18	3
Pnrc1	1	1	0	0	276
Pnrc2	0	0	151	0	0
Poc1a	0	9	14	1	0
Poc1b	37	36	41	52	48

For Review Only

Poc5	21	48	62	60	47
Podnl1	0	0	0	0	1
Podxl	1	5	0	5	1
Podxl2	0	0	1	4	1
Pofut1	74	70	105	86	109
Pofut2	100	86	0	0	0
Pogk	37	62	40	71	51
Poglut1	140	119	162	98	179
Pogz	96	111	108	143	115
Pola1	0	26	16	0	0
Pola2	36	38	52	29	44
Polb	63	39	79	30	49
Pold1	40	30	44	55	50
Pold2	23	29	62	33	41
Pold3	53	64	120	68	44
Pold4	38	19	0	17	21
Poldip2	238	174	241	143	164
Poldip3	263	178	256	288	341
Pole	0	0	0	0	0
Pole3	7	3	7	6	11
Pole4	28	32	45	26	30
Polg	2	2	112	104	157
Polh	17	28	22	23	16
Poli	20	15	17	22	40
Polk	37	35	43	36	67
Poll	28	3	87	37	57
Polm	8	18	0	26	0
Poln	0	0	0	0	5
Polq	1	2	0	0	1
Polr1a	132	100	138	92	127
Polr1b	62	30	77	44	60
Polr1c	49	0	93	1	2
Polr1d	106	93	125	97	128
Polr1e	12	27	23	27	19
Polr2a	127	98	153	180	183
Polr2b	17	174	133	149	0
Polr2c	45	69	97	48	66
Polr2d	43	53	0	31	39
Polr2e	189	145	257	158	173
Polr2f	0	16	14	3	1
Polr2g	0	15	46	6	29
Polr2h	42	20	56	27	37
Polr2i	40	0	1	1	0
Polr2j	79	68	0	10	7
Polr2l	26	22	29	20	36
Polr2m	89	64	97	84	99
Polr3a	74	60	77	81	95
Polr3b	101	94	74	73	150
Polr3c	61	26	51	52	74
Polr3d	25	8	31	20	49

For Review Only

Polr3e	0	0	19	0	1
Polr3f	103	44	68	69	89
Polr3g	6	3	6	15	17
Polr3gl	5	0	17	10	0
Polr3h	47	27	50	22	18
Polr3k	62	31	61	46	42
Polrmt	76	43	121	87	95
Pom121	116	94	133	79	142
Pom121l2	1	0	8	0	0
Pomc	0	0	5	4	1
Pomgnt1	23	34	0	4	9
Pomgnt2	66	40	39	41	63
Pomk	52	86	86	72	46
Pomp	135	162	262	158	185
Pomt1	0	0	15	13	18
Pomt2	112	57	65	85	122
Pon1	1	0	0	0	0
Pon2	234	201	271	210	273
Pon3	186	180	237	215	230
Pop1	13	8	19	15	19
Pop4	2	21	39	29	26
Pop5	71	0	2	1	1
Pop7	0	0	0	35	0
Por	0	0	7	1	4
Porcn	12	20	20	14	26
Postn	18	13	25	23	17
Pot1a	77	37	76	76	56
Pot1b	44	49	34	81	50
Pou2f1	46	60	56	70	71
Pou2f2	98	99	102	117	117
Pou5f2	13	24	40	14	14
Pou6f1	52	66	65	57	75
Pp2d1	0	1	0	9	9
Ppa1	12	16	33	16	14
Ppa2	0	44	126	1	4
Ppan	0	0	0	0	15
Ppara	3	0	0	3	0
Ppard	29	19	0	31	36
Ppargc1b	6	5	0	11	12
Ppat	29	23	28	41	0
Ppcdc	413	308	517	530	342
Ppcs	0	0	0	0	0
Ppfia1	45	46	44	42	61
Ppfia4	0	6	124	0	1
Ppfibp1	2	8	0	15	9
Ppfibp2	30	37	76	73	65
Pphln1	108	97	135	97	123
Ppib	0	0	850	3	53
Ppid	146	68	0	73	79
Ppie	10	22	51	0	71

For Review Only

Ppif	27	36	27	30	44
Ppifos	1	15	0	6	0
Ppig	41	65	68	41	85
Ppih	4	7	8	0	7
Ppil1	21	29	33	22	31
Ppil2	185	112	184	169	202
Ppil3	43	37	53	53	26
Ppil4	0	0	0	0	0
Ppip5k1	34	29	37	32	50
Ppip5k2	74	49	65	95	104
Ppl	14	1	0	10	9
Ppm1a	40	59	51	28	55
Ppm1b	14	30	43	29	27
Ppm1d	19	18	13	24	37
Ppm1e	3	15	8	18	21
Ppm1f	184	104	133	160	163
Ppm1g	203	153	0	0	0
Ppm1h	456	424	340	410	531
Ppm1j	0	3	0	2	6
Ppm1k	44	44	37	55	67
Ppm1l	54	76	91	147	121
Ppm1m	0	0	0	0	0
Ppm1n	0	0	0	0	3
Ppme1	124	59	58	91	62
Ppox	1	44	0	0	85
Ppp1cb	71	79	84	87	128
Ppp1cc	27	82	57	57	70
Ppp1r10	380	138	230	258	406
Ppp1r11	76	94	96	76	78
Ppp1r12a	157	152	147	134	128
Ppp1r12b	44	67	43	69	51
Ppp1r12c	9	56	0	0	167
Ppp1r13b	44	32	50	41	41
Ppp1r13l	0	0	1	1	2
Ppp1r14b	6	19	0	3	4
Ppp1r15a	0	0	0	0	0
Ppp1r15b	235	203	214	251	301
Ppp1r16a	46	40	69	45	57
Ppp1r16b	4	2	0	0	0
Ppp1r18	175	590	179	0	263
Ppp1r1a	4	1	0	4	7
Ppp1r1b	29	0	0	0	10
Ppp1r2	97	86	102	95	105
Ppp1r2-ps3	8	4	7	6	7
Ppp1r21	158	114	129	127	44
Ppp1r26	30	15	32	24	10
Ppp1r27	0	0	8	0	0
Ppp1r35	16	0	0	0	0
Ppp1r3b	243	115	191	203	249
Ppp1r3d	76	63	79	78	112

For Review Only

Ppp1r3f	2	5	9	0	9
Ppp1r3fos	1	11	16	11	7
Ppp1r7	80	57	96	59	59
Ppp1r8	23	27	18	39	27
Ppp1r9a	242	311	201	321	316
Ppp1r9b	53	91	75	66	94
Ppp2ca	102	103	103	70	104
Ppp2cb	16	35	26	20	8
Ppp2r1a	440	343	469	395	435
Ppp2r1b	124	135	122	109	123
Ppp2r2a	124	97	99	123	142
Ppp2r2c	5	0	0	0	0
Ppp2r2d	10	10	0	10	50
Ppp2r3a	13	18	35	55	32
Ppp2r3c	18	13	23	19	22
Ppp2r3d	19	15	23	17	15
Ppp2r5a	16	10	8	5	14
Ppp2r5b	19	17	28	20	33
Ppp2r5c	119	153	175	174	245
Ppp2r5d	12	6	46	0	0
Ppp2r5e	24	42	34	55	53
Ppp3ca	185	133	118	79	119
Ppp3cb	29	34	43	33	32
Ppp3cc	8	2	0	4	0
Ppp3r1	142	99	125	177	143
Ppp3r2	0	0	6	0	0
Ppp4c	0	14	0	0	0
Ppp4r1	223	227	297	285	254
Ppp4r1l-ps	29	36	29	19	30
Ppp4r2	22	30	30	23	14
Ppp4r4	0	0	3	0	0
Ppp5c	0	0	0	0	0
Ppp6c	172	149	157	168	189
Ppp6r1	43	38	28	44	45
Ppp6r2	136	117	77	117	110
Ppp6r3	228	204	239	268	307
Pprc1	119	91	103	134	72
Ppt1	1253	597	1167	1246	813
Ppt2	1	10	0	0	0
Pptc7	62	94	83	48	74
Ppwd1	19	17	13	27	14
Pqbp1	19	0	38	14	36
Pqlc1	10	12	34	17	44
Pqlc2	125	84	92	139	115
Pqlc3	17	19	13	24	26
Pradc1	0	11	12	9	11
Praf2	25	37	33	27	34
Pram1	1	1	0	0	0
Pramef8	87	105	101	73	106
Prc1	1	3	0	1	0

For Review Only

Prcc	0	17	2	11	11
Prcp	305	375	378	378	453
Prdm1	86	95	78	133	114
Prdm10	26	30	25	30	38
Prdm11	9	2	4	11	14
Prdm15	41	39	26	51	61
Prdm16	8	7	6	10	13
Prdm2	141	139	89	205	228
Prdm4	32	21	20	22	36
Prdm5	0	6	0	0	0
Prdm9	21	56	31	43	78
Prdx1	273	255	313	215	249
Prdx2	0	57	0	0	0
Prdx3	133	86	191	115	129
Prdx4	87	60	87	61	71
Prdx5	0	0	10	0	1
Prdx6	66	34	36	64	101
Prdx6b	2	0	4	0	1
Preb	6	0	66	3	0
Prelid1	113	26	214	85	0
Prelid2	0	25	4	0	0
Prelp	11	0	0	0	0
Prep	104	66	102	98	137
Prepl	33	47	57	40	58
Prex1	713	645	0	0	0
Prex2	2	1	0	0	0
Prf1	3	0	3	4	0
Prickle1	30	4	19	20	30
Prickle2	0	1	0	11	0
Prickle3	23	19	36	23	23
Prim1	0	0	0	0	0
Prim2	12	6	31	18	23
Primpol	60	64	68	63	77
Prkaa1	79	94	84	132	92
Prkaa2	1	1	0	0	19
Prkab1	471	374	511	375	457
Prkab2	16	11	33	21	43
Prkaca	52	55	62	44	42
Prkacb	200	101	90	198	183
Prkag1	118	70	131	98	83
Prkag2	17	13	10	23	23
Prkag3	0	0	0	0	0
Prkar1a	305	137	735	705	511
Prkar1b	46	39	52	42	46
Prkar2a	53	22	44	30	23
Prkar2b	2	0	0	0	0
Prkca	77	83	75	114	129
Prkcb	241	295	238	233	237
Prkcd	1183	414	219	122	1055
Prkce	40	25	29	50	48

For Review Only

Prkcg	0	0	0	0	0
Prkch	59	47	30	71	55
Prkci	9	14	15	16	13
Prkcq	11	10	6	7	12
Prkcsh	222	105	209	147	171
Prkcz	21	11	14	25	24
Prkd2	21	34	31	39	36
Prkd3	122	163	108	185	173
Prkdc	74	85	106	134	101
Prkg1	2	1	1	6	9
Prkra	67	39	56	62	72
Prkrip1	23	14	23	18	16
Prkrir	14	17	44	15	26
Prkx	90	73	107	129	150
Prlh	3	0	1	3	0
Prlr	12	0	14	0	0
Prmt1	18	9	0	0	36
Prmt2	66	50	84	72	112
Prmt3	0	0	35	50	57
Prmt5	69	37	66	48	75
Prmt6	44	42	14	45	65
Prmt7	56	41	115	62	46
Prmt9	39	25	21	28	47
Prnp	18	6	4	4	19
Prob1	0	1	0	1	1
Proca1	2	6	8	5	0
Procr	6	4	15	8	0
Prodh	20	10	15	10	14
Prok1	1	0	0	4	0
Prokr1	0	1	0	0	0
Prom1	2	0	0	0	0
Prom2	9	5	0	7	0
Prorsd1	62	89	99	44	49
Pros1	750	750	996	873	870
Prosc	47	84	54	89	83
Proser1	61	63	42	63	97
Proser2	0	3	0	0	0
Proser3	9	16	11	21	30
Prox1	32	45	20	46	54
Prox2	48	12	32	38	42
Proz	6	7	5	0	0
Prpf18	91	67	127	98	117
Prpf19	25	30	24	54	34
Prpf3	59	52	56	56	57
Prpf31	71	18	105	50	76
Prpf38a	48	39	34	22	47
Prpf38b	0	0	0	0	0
Prpf39	63	166	126	159	138
Prpf4	79	24	48	49	38
Prpf40a	99	95	130	140	145

For Review Only

Prpf40b	22	10	43	56	46
Prpf4b	109	171	127	188	194
Prpf6	116	104	99	166	161
Prpf8	301	219	227	292	357
Prps1	20	23	17	22	28
Prps1l1	1	25	20	33	10
Prps1l3	135	91	112	144	135
Prps2	129	68	86	123	107
Prpsap1	262	190	308	183	153
Prpsap2	192	112	191	150	104
Prr11	3	0	0	2	0
Prr12	30	32	31	54	65
Prr13	11	29	0	180	0
Prr14	49	113	111	146	39
Prr14l	219	349	317	408	330
Prr15	16	12	9	13	19
Prr15l	4	0	0	0	4
Prr18	1	2	0	0	7
Prr22	3	0	0	0	8
Prr3	22	19	28	20	47
Prr32	18	0	0	0	0
Prr33	3	0	0	16	6
Prr36	0	0	1	0	1
Prr5	12	5	3	9	7
Prr5l	35	14	30	16	31
Prr7	11	4	4	4	14
Prrc1	121	99	89	97	92
Prrc2a	0	0	189	468	455
Prrc2b	147	139	161	162	158
Prrc2c	214	334	242	403	418
Prrg1	3	0	0	0	0
Prrg2	21	22	27	19	29
Prrg4	1	3	3	8	0
Prrt3	4	4	6	6	5
Prrx1	0	0	0	0	0
Prrxl1	0	0	0	0	0
Prss12	13	0	0	6	0
Prss23	0	0	0	0	0
Prss27	0	0	0	0	0
Prss36	11	18	15	21	14
Prss53	8	1	16	18	0
Prss8	2	1	0	4	0
Prtn3	0	0	0	0	0
Prune	26	15	27	36	41
Prune2	100	74	64	77	94
Psap	74	533	9	1882	1406
Psat1	73	38	46	51	112
Psd	0	8	2	6	4
Psd2	0	0	0	0	0
Psd3	5	8	12	20	11

For Review Only

Psd4	0	249	1	1	1
Psen1	327	334	400	341	428
Psen2	32	32	39	20	25
Psg16	1	0	0	3	1
Psg26	3	0	0	0	8
Psip1	18	1	0	19	25
Pskh1	107	65	91	84	118
Psma1	10	62	13	0	0
Psma2	178	80	237	43	102
Psma3	110	163	171	181	124
Psma4	57	71	96	73	63
Psma5	62	50	94	74	63
Psma6	140	145	215	115	157
Psma7	215	206	251	137	120
Psma8	26	9	20	11	20
Psmb1	205	270	312	170	239
Psmb10	43	92	87	10	35
Psmb2	128	204	266	166	221
Psmb3	96	79	129	38	35
Psmb4	1	1	0	0	0
Psmb5	117	133	202	104	137
Psmb6	0	0	0	33	34
Psmb7	66	71	73	73	75
Psmb8	238	308	528	318	311
Psmb9	0	0	6	3	2
Psmc1	104	100	142	86	114
Psmc2	116	14	312	213	254
Psmc3	21	32	0	149	0
Psmc3ip	2	1	0	0	0
Psmc4	1	1	0	0	0
Psmc5	161	181	259	154	142
Psmc6	117	108	132	134	114
Psmd1	187	241	227	232	209
Psmd10	51	61	61	45	47
Psmd11	120	112	144	112	136
Psmd12	124	70	159	107	120
Psmd13	132	116	183	87	143
Psmd14	91	107	155	100	88
Psmd2	262	25	74	300	0
Psmd3	100	69	74	42	44
Psmd4	0	0	0	0	1
Psmd5	49	52	101	85	86
Psmd6	185	171	262	178	244
Psmd7	188	84	176	91	115
Psmd8	192	160	215	184	216
Psmd9	50	44	67	48	51
Psme2	0	38	77	63	83
Psme2b	177	206	189	239	134
Psme3	12	0	0	0	0
Psme4	29	45	69	69	87

For Review Only

Psmf1	102	48	123	112	61
Psmg1	40	43	15	9	0
Psmg2	12	29	62	71	0
Psmg3	14	11	30	29	26
Psmg4	18	16	20	16	23
Pspc1	92	65	44	92	106
Psph	11	31	34	26	22
Psrc1	0	8	0	0	0
Pstk	21	16	16	23	44
Pstpip1	0	0	4	2	0
Pstpip2	14	6	7	4	8
Ptafr	1156	944	1219	1121	1253
Ptar1	72	69	93	71	97
Ptbp2	35	40	47	70	56
Ptbp3	251	265	309	350	355
Ptcd1	104	109	106	99	171
Ptcd2	41	69	82	43	32
Ptcd3	103	48	75	65	70
Ptch1	34	25	54	43	38
Ptch2	0	0	0	4	0
Ptchd1	36	34	30	49	53
Ptdss1	137	107	138	139	138
Ptdss2	96	82	83	75	103
Pten	158	179	157	221	225
Pter	22	3	0	18	21
Ptgds	1	0	0	0	0
Ptger1	34	27	38	49	27
Ptger3	28	20	30	12	28
Ptger4	2	13	0	6	14
Ptges2	7	8	11	3	3
Ptges3	134	96	81	90	107
Ptgfrn	13	18	20	19	27
Ptgr1	22	0	33	0	0
Ptgr2	180	14	116	72	133
Ptgs1	1657	1770	1631	2963	216
Pth1r	0	5	6	2	3
Ptk2	3	13	1	29	11
Ptk2b	243	135	152	251	247
Ptn	1	0	0	0	0
Ptov1	4	8	0	0	0
Ptp4a1	75	40	35	46	73
Ptp4a2	437	405	466	477	541
Ptp4a3	382	265	305	377	336
Ptpa	190	173	237	213	225
Ptpdc1	15	15	6	4	15
Ptpmt1	51	68	41	40	24
Ptpn1	618	344	592	129	209
Ptpn11	26	64	23	36	33
Ptpn12	20	6	16	18	14
Ptpn14	7	6	1	1	8

For Review Only

Ptpn18	73	141	112	0	142
Ptpn2	62	111	107	46	54
Ptpn21	24	38	22	22	49
Ptpn22	0	8	6	7	6
Ptpn23	11	24	0	24	47
Ptpn4	8	15	8	26	40
Ptpn6	1	180	0	0	0
Ptpn7	0	1	0	0	0
Ptpn9	18	44	34	28	27
Ptpra	82	181	174	167	164
Ptprb	0	0	0	0	0
Ptprc	592	619	614	737	745
Ptprcap	0	2	0	3	0
Ptpre	299	84	149	403	60
Ptprf	21	1	0	1	1
Ptprg	0	0	6	0	6
Ptprh	0	6	0	2	5
Ptprj	0	24	2	69	72
Ptprm	151	137	110	202	168
Ptprn	0	0	0	0	0
Ptpro	261	176	292	258	279
Ptprr	0	0	0	0	0
Ptprs	200	169	110	192	251
Ptrf	6	0	0	0	0
Ptrh1	3	0	0	0	0
Ptrh2	88	75	85	75	96
Ptrhd1	42	24	54	51	42
Pts	0	0	0	3	4
Pttg1	72	74	167	89	69
Pttg1ip	715	680	955	645	655
Puf60	89	0	102	1	1
Pum1	100	161	114	210	140
Pum2	190	196	219	219	249
Pum3	56	27	104	44	58
Pura	56	90	62	97	99
Purb	101	145	108	114	179
Purg	1	14	5	6	12
Pus1	65	31	27	36	41
Pus10	37	51	51	40	46
Pus3	1	1	0	0	104
Pus7	23	22	15	25	35
Pus7l	15	0	0	5	16
Pvr	13	21	22	16	17
Pvrig	50	20	79	15	74
Pvt1	23	43	52	37	31
Pwp1	99	78	55	40	106
Pwp2	51	35	46	61	79
Pwwp2a	226	303	220	325	243
Pwwp2b	19	15	11	17	25
Pxdc1	30	33	22	20	36

For Review Only

Pxdn	3	0	0	0	0
Pxk	158	100	143	173	169
Pxmp4	41	38	46	26	44
Pxn	401	39	203	266	69
Pxylp1	2	0	0	0	0
Pycard	436	517	763	170	330
Pycr1	11	0	10	2	4
Pycr2	3	36	0	169	107
Pycrl	19	19	61	26	29
Pydc3	0	2	0	0	1
Pydc4	0	3	3	5	7
Pygb	213	128	157	166	138
Pygl	43	17	24	0	26
Pygm	5	0	0	0	11
Pygo2	1	14	0	244	276
Pyhin1	1	8	23	21	15
Pym1	23	15	21	12	11
Pyroxd1	64	64	56	38	74
Pyroxd2	44	63	88	73	64
Pyurf	15	12	21	14	13
Qars	16	28	242	0	0
Qdpr	79	91	164	72	124
Qk	1153	73	12	846	336
Qpct	0	0	2	0	0
Qpctl	52	29	44	32	43
Qprt	20	21	25	6	11
Qrfp	8	7	9	9	10
Qrich1	240	194	274	252	270
Qrich2	0	0	0	2	3
Qrsl1	30	31	33	24	45
Qser1	34	34	36	49	52
Qsox1	50	37	51	54	89
Qsox2	19	10	26	31	14
Qtrt1	0	0	0	0	0
Qtrtd1	3	21	0	34	0
R3hcc1	26	5	19	9	18
R3hcc1l	77	81	104	57	48
R3hdm1	115	66	108	133	101
R3hdm2	42	45	64	76	56
R3hdm4	0	51	11	39	37
R74862	20	26	0	56	58
Rab10	33	155	58	105	118
Rab10os	20	14	3	6	0
Rab11a	303	387	465	418	276
Rab11b	227	151	247	186	223
Rab11fip1	2	1	0	0	0
Rab11fip2	14	28	27	34	20
Rab11fip3	16	17	16	19	26
Rab11fip4	4	1	0	12	10
Rab11fip5	251	146	201	250	272

For Review Only

Rab12	43	112	69	79	102
Rab13	0	0	1	1	0
Rab14	8	1	715	0	202
Rab15	0	0	0	0	0
Rab18	226	115	270	158	177
Rab19	6	8	0	6	8
Rab1a	488	405	565	457	528
Rab1b	0	0	0	0	0
Rab20	156	140	151	73	65
Rab21	6	13	9	14	10
Rab22a	6	31	19	16	17
Rab23	9	7	8	13	13
Rab26	2	0	0	0	0
Rab27a	136	77	90	113	104
Rab28	20	36	44	33	19
Rab29	49	0	0	0	0
Rab2a	37	87	83	61	64
Rab2b	45	58	76	68	40
Rab30	0	0	5	2	0
Rab31	666	566	635	637	705
Rab32	156	138	198	131	208
Rab33a	0	0	0	2	0
Rab33b	72	26	64	69	65
Rab34	14	37	44	33	52
Rab35	20	0	0	0	0
Rab36	6	2	6	1	0
Rab37	0	0	0	0	0
Rab39	107	112	133	165	123
Rab3a	54	10	23	24	31
Rab3d	21	7	23	9	3
Rab3gap1	179	153	127	280	252
Rab3gap2	110	136	85	162	114
Rab3il1	80	835	802	445	511
Rab3ip	204	94	163	151	141
Rab40b	1	0	3	2	0
Rab40c	46	48	53	55	45
Rab42	23	1	0	13	6
Rab43	86	149	64	67	100
Rab44	6	0	8	0	0
Rab4a	9	18	6	6	16
Rab4b	127	107	131	98	130
Rab5a	33	93	28	50	43
Rab5b	168	110	120	231	206
Rab5c	123	93	59	129	147
Rab6a	80	47	76	73	79
Rab6b	119	232	153	230	197
Rab7	433	341	484	332	451
Rab7b	5	1	1	0	0
Rab8a	103	181	228	69	97
Rab8b	339	216	273	338	352

For Review Only

Rab9	26	29	48	41	35
Rabac1	272	283	300	183	202
Rabep1	83	83	67	106	89
Rabep2	95	88	93	79	0
Rabepk	44	44	41	76	86
Rabgap1	75	105	92	115	102
Rabgap1l	40	16	14	57	78
Rabgef1	47	30	63	37	43
Rabggta	87	56	116	80	92
Rabggtb	0	7	0	0	0
Rabif	1	1	0	0	0
Rabl2	24	23	31	17	11
Rabl3	4	17	0	10	10
Rabl6	60	68	28	54	86
Rac1	610	21	0	119	20
Rac2	0	0	125	0	81
Rac3	0	0	0	0	0
Racgap1	0	3	0	11	6
Rad1	21	24	42	30	29
Rad17	49	12	31	27	31
Rad18	0	0	0	0	7
Rad21	64	56	90	60	51
Rad23a	0	0	0	0	0
Rad23b	19	37	16	36	29
Rad50	58	53	43	56	44
Rad51	1	1	0	0	0
Rad51ap1	1	2	4	0	0
Rad51b	2	12	7	8	1
Rad51c	1	0	0	0	0
Rad51d	33	39	40	75	57
Rad52	60	42	44	45	37
Rad54l	0	0	10	3	0
Rad54l2	55	62	46	79	97
Rad9a	7	0	0	0	0
Rad9b	19	25	7	39	33
Rae1	110	91	87	6	23
Raf1	155	104	127	158	92
Rai1	181	186	149	157	199
Rai14	2	2	9	0	0
Rala	106	90	110	79	109
Ralb	332	229	382	303	328
Ralbp1	55	55	53	72	71
Ralgapa1	81	62	71	90	69
Ralgapa2	70	49	23	56	72
Ralgapb	35	91	47	78	76
Ralgds	27	11	30	26	43
Ralgps1	95	142	28	182	87
Ralgps2	38	78	114	125	130
Raly	22	48	21	48	47
Ramp1	56	56	111	79	57

For Review Only

Ramp2	0	0	5	1	6
Ran	58	6	352	248	100
Ranbp1	65	47	78	42	60
Ranbp2	60	90	47	158	107
Ranbp3	2	155	0	111	96
Ranbp3l	0	0	0	0	0
Ranbp6	44	41	57	65	56
Ranbp9	27	35	32	33	43
Rangap1	46	36	68	30	0
Rangrf	0	0	0	0	2
Rap1a	76	102	88	108	93
Rap1b	208	313	193	245	246
Rap1gap	3	1	0	0	12
Rap1gap2	60	20	65	70	79
Rap1gds1	292	358	431	515	536
Rap2a	68	66	47	57	71
Rap2b	31	28	16	31	20
Rap2c	62	75	103	85	89
Rapgef1	213	225	266	224	225
Rapgef2	63	52	67	69	86
Rapgef5	173	242	209	310	334
Rapgef6	190	257	226	297	248
Rapgefl1	8	5	5	7	13
Raph1	18	14	31	20	18
Rapsn	20	50	64	15	28
Rarb	1	0	0	0	0
Rarg	0	0	7	6	2
Rars	81	69	87	63	94
Rars2	25	17	54	29	52
Rasa1	59	80	73	85	63
Rasa2	32	27	25	44	32
Rasa3	355	239	314	354	350
Rasa4	10	0	69	606	0
Rasal2	33	20	16	22	12
Rasal3	448	3	202	0	828
Rasgef1a	0	0	0	0	0
Rasgef1b	130	66	84	88	98
Rasgrf1	0	0	0	3	3
Rasgrf2	12	22	31	36	20
Rasgrp3	541	619	667	728	715
Rasgrp4	5	24	0	20	16
Rasl10a	4	4	2	0	4
Rasl2-9	17	16	8	19	21
Rassf1	0	15	239	0	160
Rassf2	448	248	304	508	457
Rassf3	4	6	5	19	18
Rassf4	82	121	15	127	18
Rassf5	431	123	529	412	292
Rassf7	12	11	8	7	3
Rassf8	14	20	19	10	18

For Review Only

Raver2	1	3	0	1	10
Rb1	61	75	60	104	116
Rb1cc1	86	65	48	82	119
Rbak	54	44	53	40	36
Rbakdn	0	0	0	0	0
Rbbp4	128	100	119	135	114
Rbbp5	84	86	80	98	70
Rbbp6	112	140	137	108	119
Rbbp7	167	134	117	128	117
Rbbp8	18	9	8	13	7
Rbbp9	140	98	133	100	91
Rbck1	62	8	0	71	98
Rbfa	98	87	97	84	122
Rbfox1	40	51	43	58	82
Rbfox2	2	6	9	11	25
Rbfox3	7	6	1	0	11
Rbks	19	20	12	15	25
Rbl1	37	28	24	48	27
Rbl2	69	45	104	135	114
Rbm10	96	65	79	73	91
Rbm12	80	110	61	92	47
Rbm12b1	30	38	44	33	41
Rbm12b2	29	37	49	44	53
Rbm14	87	90	92	153	119
Rbm15	147	163	138	198	184
Rbm15b	8	26	28	17	29
Rbm17	46	41	58	23	62
Rbm18	149	81	137	88	102
Rbm19	71	35	47	59	75
Rbm20	2	1	0	0	0
Rbm22	77	85	55	67	59
Rbm25	151	0	109	37	0
Rbm27	78	37	56	76	110
Rbm28	68	62	79	87	98
Rbm3	1	53	73	31	10
Rbm33	219	180	153	163	204
Rbm34	35	27	28	26	50
Rbm38	72	44	96	57	55
Rbm39	225	728	291	1001	694
Rbm3os	10	0	0	5	11
Rbm4	0	59	0	0	0
Rbm41	12	31	10	32	57
Rbm42	5	6	323	0	0
Rbm43	62	52	81	40	73
Rbm45	0	0	0	10	5
Rbm47	179	124	133	227	219
Rbm48	21	36	73	40	28
Rbm4b	146	176	176	188	194
Rbm5	209	298	81	391	235
Rbm6	110	98	183	149	155

For Review Only

Rbm7	1	74	0	0	197
Rbm8a	0	8	0	0	0
Rbms1	27	11	12	25	30
Rbms2	43	33	75	47	39
Rbms3	0	0	0	5	3
Rbmx	120	112	148	152	127
Rbmx2	11	6	0	8	17
Rbmxl1	70	57	86	51	85
Rbp1	7	0	0	0	0
Rbp4	0	0	0	0	0
Rbpj	103	95	25	193	58
Rbpms	24	32	29	53	40
Rbsn	87	47	126	115	112
Rbx1	137	146	147	70	89
Rc3h1	82	156	107	147	194
Rc3h2	50	110	59	122	106
Rcan1	111	4	159	0	18
Rcan3	92	63	69	85	86
Rcbtb1	43	77	60	45	27
Rcbtb2	293	222	562	153	178
Rcc1	22	13	39	39	44
Rcc2	1	1	0	0	0
Rccd1	5	3	12	12	14
Rce1	43	0	0	0	0
Rchy1	91	59	77	57	64
Rcl1	62	74	71	65	42
Rcn1	19	18	24	18	33
Rcn2	115	113	157	127	118
Rcn3	11	7	4	9	18
Rcor1	53	57	60	58	93
Rcor3	41	52	80	39	58
Rcsd1	378	384	529	334	485
Rd3	4	0	3	0	3
Rdh1	0	2	0	0	0
Rdh10	29	20	14	29	27
Rdh11	28	20	33	39	28
Rdh12	6	0	0	0	0
Rdh13	74	54	114	65	53
Rdh14	61	57	64	58	52
Rdh5	64	1	23	0	35
Rdm1	1	1	0	119	0
Rdx	134	177	255	248	216
Rec114	8	0	11	9	3
Reck	0	5	0	0	0
Recql	45	17	40	49	40
Recql4	0	4	0	0	0
Recql5	47	46	24	53	119
Reep1	2	3	4	0	0
Reep2	0	6	0	4	4
Reep3	141	151	149	167	201

For Review Only

Reep4	0	2	0	154	0
Reep5	610	483	662	569	583
Rel	72	65	75	140	129
Rela	187	70	0	0	0
Relb	95	84	98	88	100
Rell1	30	14	15	37	34
Relt	9	10	0	38	51
Renbp	0	0	19	1	1
Repin1	68	59	69	72	91
Reps1	38	12	25	11	24
Reps2	0	0	0	0	0
Rer1	267	214	334	234	244
Rere	417	454	339	504	510
Rest	34	40	32	38	56
Retnlg	8	0	0	0	0
Retsat	55	83	74	56	55
Rev1	17	20	0	0	0
Rev3l	22	76	15	33	24
Rexo1	74	82	60	102	84
Rexo2	48	46	39	32	46
Rexo4	163	87	165	124	126
Rfc1	55	59	73	92	78
Rfc2	124	82	100	60	80
Rfc3	28	22	47	10	38
Rfc4	9	0	11	0	4
Rfc5	13	38	28	31	0
Rfesd	34	27	50	36	45
Rffl	229	156	245	237	237
Rfk	83	63	56	35	100
Rfng	26	19	36	34	46
Rft1	60	63	82	73	104
Rftn1	30	14	20	9	38
Rftn2	0	0	0	0	0
Rfwd2	31	43	26	39	18
Rfwd3	66	74	65	63	41
Rfx1	35	33	39	24	36
Rfx3	22	55	43	63	83
Rfx5	36	49	50	70	33
Rfx7	39	88	37	59	77
Rfxank	38	46	100	5	75
Rfxap	33	8	7	0	0
Rgag1	0	0	0	0	0
Rgag4	6	10	8	7	7
Rgl1	67	97	79	122	155
Rgl2	0	0	5	50	0
Rgl3	39	43	25	72	36
Rgma	0	0	0	0	0
Rgmb	126	246	175	194	230
Rgp1	0	0	2	2	15
Rgs1	0	0	0	10	14

For Review Only

Rgs10	1453	1381	2352	438	1049
Rgs11	27	18	22	11	7
Rgs12	12	5	17	30	21
Rgs14	2	20	71	0	62
Rgs18	2	2	1	0	0
Rgs19	40	116	276	190	100
Rgs2	0	0	0	0	0
Rgs3	22	10	53	20	18
Rgs5	0	0	0	0	0
Rgs7bp	24	16	30	22	20
Rgsl1	0	0	0	0	0
Rhbdd1	86	81	87	91	98
Rhbdd2	76	46	90	63	95
Rhbdd3	3	1	0	0	0
Rhbdf1	6	3	0	0	0
Rhbdf2	43	31	69	61	80
Rhbdl1	2	0	0	0	0
Rhbdl3	0	0	0	0	7
Rheb	18	54	55	59	57
Rhebl1	7	4	6	0	8
Rhno1	72	68	113	57	59
Rho	2	0	0	3	0
Rhoa	218	460	0	721	696
Rhob	544	498	265	627	860
Rhobtb1	107	114	103	130	162
Rhobtb2	20	32	29	42	38
Rhobtb3	6	0	0	0	0
Rhoc	29	27	27	20	20
Rhod	3	0	0	0	0
Rhof	0	0	5	4	0
Rhog	103	108	0	826	0
Rhoh	411	364	526	369	363
Rhoj	0	0	0	0	0
Rhoq	89	123	120	103	117
Rhot1	235	162	147	173	170
Rhot2	45	42	43	45	48
Rhox5	0	0	0	0	0
Rhpn2	5	0	0	0	0
Ribc1	0	0	0	0	0
Ric1	10	1	0	50	35
Ric3	3	5	63	0	0
Ric8a	0	61	3	1	75
Ric8b	57	55	50	57	42
Rictor	64	84	62	105	71
Rida	60	27	35	24	32
Rif1	16	72	52	57	63
Rilp	11	0	0	0	13
Rilpl1	146	146	104	91	110
Rilpl2	28	5	12	27	11
Rimbp2	0	0	0	3	0

For Review Only

Rimbp3	1	1	7	0	0
Rims3	0	0	0	0	9
Rin1	3	4	9	1	5
Rin2	550	921	1162	1046	571
Rin3	38	1	0	0	426
Ring1	37	15	6	25	19
Rinl	0	0	0	0	0
Rint1	44	34	53	38	46
Riok1	54	75	75	52	82
Riok2	62	48	46	46	72
Riok3	550	2	0	92	0
Ripk1	26	43	62	62	89
Ripk2	51	33	42	48	61
Ripk3	22	14	27	20	9
Rit1	2	0	196	0	0
Rita1	23	13	28	7	14
Rlf	3	1	0	0	0
Rlim	569	410	418	514	501
Rmdn1	66	59	60	69	66
Rmdn2	8	19	22	14	8
Rmdn3	41	27	82	48	45
Rmi1	61	63	59	57	61
Rmnd1	10	7	0	0	0
Rmnd5a	36	77	75	102	62
Rmnd5b	87	58	36	16	7
Rn7s1	9	14	11	25	14
Rn7s2	11	15	11	25	15
Rn7sk	16	22	23	28	32
Rnase10	10	3	11	12	0
Rnase12	1	15	1	0	12
Rnase4	2085	2009	3224	823	1523
Rnase6	23	49	67	48	63
Rnaseh1	0	0	28	0	32
Rnaseh2a	14	0	0	9	47
Rnaseh2b	0	14	21	11	25
Rnaseh2c	0	0	19	0	0
Rnasek	190	1	429	0	0
Rnasel	63	56	63	65	102
Rnaset2a	0	0	96	297	612
Rnaset2b	44	38	31	141	29
Rnd1	0	0	0	3	4
Rnf10	59	71	25	75	79
Rnf103	67	79	44	36	52
Rnf11	31	22	20	12	10
Rnf111	85	124	111	143	155
Rnf113a1	17	12	6	7	13
Rnf113a2	46	32	64	36	39
Rnf114	356	232	603	93	45
Rnf115	38	36	49	51	81
Rnf121	146	12	79	42	85

For Review Only

Rnf122	50	38	61	42	68
Rnf123	199	83	96	138	163
Rnf125	1	0	0	0	1
Rnf126	3	23	18	20	0
Rnf128	43	29	57	23	22
Rnf13	977	1011	1301	799	1070
Rnf130	134	199	184	122	139
Rnf135	239	117	0	139	122
Rnf138	59	58	52	72	75
Rnf139	88	135	115	141	114
Rnf14	130	107	76	108	126
Rnf141	112	85	107	107	112
Rnf144a	0	0	0	0	1
Rnf144b	26	84	41	106	60
Rnf145	207	204	125	209	263
Rnf146	150	125	189	150	126
Rnf149	47	61	48	40	41
Rnf150	18	13	34	40	24
Rnf152	4	0	0	0	0
Rnf157	5	6	25	17	26
Rnf166	66	8	203	80	72
Rnf167	0	276	113	4	9
Rnf168	20	31	24	45	31
Rnf169	200	120	241	268	228
Rnf17	0	0	29	0	0
Rnf170	51	19	41	46	35
Rnf180	267	248	261	273	329
Rnf181	0	0	117	63	1
Rnf185	81	57	105	88	103
Rnf187	135	3	54	62	88
Rnf19a	97	19	42	60	79
Rnf19b	48	63	77	48	46
Rnf2	120	120	142	162	152
Rnf20	89	92	122	107	107
Rnf213	248	210	238	333	372
Rnf214	268	181	275	213	224
Rnf215	35	30	88	0	0
Rnf216	2	105	226	107	0
Rnf219	44	12	42	32	57
Rnf220	49	91	98	54	113
Rnf222	0	0	0	5	0
Rnf223	4	1	0	0	9
Rnf225	2	0	0	0	6
Rnf24	28	33	49	70	50
Rnf25	8	107	72	45	60
Rnf26	18	35	23	14	19
Rnf31	0	0	0	0	0
Rnf32	4	0	6	1	0
Rnf34	84	64	61	79	93
Rnf38	61	127	131	111	101

For Review Only

Rnf4	0	296	61	14	2
Rnf40	53	41	0	122	0
Rnf41	105	153	141	186	165
Rnf44	112	109	122	56	194
Rnf5	0	0	0	0	0
Rnf6	171	68	146	138	87
Rnf7	133	129	152	137	101
Rnf8	50	21	18	16	13
Rnf8-cmtr1	180	154	170	242	294
Rnft1	47	30	101	68	88
Rnft2	2	1	0	6	0
Rngtt	8	0	5	7	0
Rnh1	261	104	137	159	173
Rnls	42	34	40	41	35
Rnmt	68	74	76	60	80
Rnmtl1	18	41	40	16	5
Rnpc3	51	24	55	48	43
Rnpep	214	176	231	222	271
Rnpepl1	74	125	71	99	98
Rnps1	75	67	94	81	85
Rnu12	7	1	6	7	9
Rnu1a1	1	1	1	2	3
Rnu2-10	3	2	4	7	10
Rnu3b1	4	0	4	3	4
Rnu3b2	3	2	3	7	11
Rnu3b3	3	1	2	5	7
Rnu3b4	5	3	5	10	14
Rnu5g	1	1	0	1	1
Rock1	65	69	48	64	82
Rock2	56	107	128	112	121
Rogdi	172	589	242	118	332
Rom1	12	0	0	0	9
Romo1	23	29	38	23	28
Ropn1l	0	0	0	0	0
Rora	32	23	15	21	44
Rp2	265	159	205	371	282
Rp9	25	23	28	18	16
Rpa1	57	57	99	67	49
Rpa2	57	23	36	41	28
Rpa3	3	6	0	0	0
Rpain	1	0	0	0	0
Rpap1	26	81	57	81	54
Rpap2	100	28	63	55	43
Rpap3	21	22	19	0	35
Rpe	48	34	59	47	53
Rpf1	46	93	91	76	66
Rpf2	16	24	21	25	32
Rpgr	0	5	16	16	3
Rpgrip1	0	1	0	73	0
Rpgrip1l	24	7	15	20	28

For Review Only

Rpia	0	0	1	4	0
Rpl10a	0	0	0	64	0
Rpl11	85	0	58	0	65
Rpl13	80	94	123	68	98
Rpl13-ps6	128	103	156	103	131
Rpl13a	34	32	0	0	0
Rpl14	0	0	198	139	176
Rpl14-ps1	181	148	236	171	218
Rpl17	18	13	19	18	20
Rpl17-ps10	86	62	93	74	64
Rpl17-ps8	37	29	44	33	35
Rpl18	0	0	1	1	8
Rpl21	9	0	11	5	9
Rpl22	130	87	163	124	102
Rpl23	0	0	0	0	0
Rpl23a	44	28	61	39	53
Rpl24	73	66	6	11	34
Rpl26	160	102	252	153	221
Rpl27	0	0	0	0	0
Rpl27a	0	56	0	0	0
Rpl28	56	0	0	0	0
Rpl29	31	44	63	19	20
Rpl30	8	1	0	0	64
Rpl31	21	17	24	20	19
Rpl31-ps12	15	16	16	20	19
Rpl32	189	177	186	179	166
Rpl32l	30	22	31	18	27
Rpl34	79	0	1	44	0
Rpl34-ps1	57	55	108	68	59
Rpl35	0	0	0	0	0
Rpl35a	0	0	64	0	0
Rpl36a	38	24	42	29	31
Rpl36al	0	0	40	0	29
Rpl37a	0	0	55	35	27
Rpl37rt	0	0	250	182	240
Rpl38	0	0	22	0	4
Rpl39	101	113	165	146	115
Rpl39l	0	0	0	0	0
Rpl4	243	3	0	1568	0
Rpl5	1	1	0	0	1282
Rpl6	1	2	602	0	0
Rpl7	17	14	358	350	445
Rpl7l1	1	1	1028	0	0
Rpl8	545	86	459	9	19
Rplp1	643	692	735	630	665
Rplp2	0	0	85	23	153
Rplp2-ps1	148	119	112	92	97
Rpn1	628	409	612	442	658
Rpn2	985	631	1114	757	766
Rpp14	60	56	79	40	81

For Review Only

Rpp21	2	21	0	0	14
Rpp25l	34	51	83	34	29
Rpp30	6	10	65	0	0
Rpp38	121	106	139	100	38
Rpp40	12	9	7	0	16
Rpph1	7	9	7	12	10
Rprd1a	27	51	59	83	106
Rprd1b	135	56	119	101	84
Rprd2	130	98	147	221	252
Rps10	63	64	0	14	281
Rps11	451	374	578	437	520
Rps13	63	47	0	0	0
Rps13-ps4	62	59	98	50	82
Rps14	320	357	604	345	379
Rps15	0	64	0	0	101
Rps15a	138	164	174	150	159
Rps15a-ps4	27	21	40	19	31
Rps15a-ps5	44	43	63	38	46
Rps15a-ps6	38	33	48	37	38
Rps16	132	108	226	96	90
Rps17	89	88	0	0	0
Rps19	0	0	0	120	0
Rps19-ps3	7	7	15	8	5
Rps19bp1	28	35	49	24	23
Rps2	1	3	0	0	0
Rps20	0	0	5	1	2
Rps21	0	0	92	0	0
Rps24	330	25	99	13	17
Rps26	174	0	103	35	0
Rps27l	46	39	47	32	21
Rps27rt	216	152	295	217	203
Rps28	49	58	131	48	84
Rps3	915	608	933	735	863
Rps3a1	19	21	0	0	0
Rps4l	42	49	59	49	72
Rps4x	403	525	400	168	630
Rps5	0	108	486	0	0
Rps6ka1	114	39	65	0	237
Rps6ka2	13	3	6	18	4
Rps6ka3	37	48	40	69	75
Rps6ka4	208	108	150	146	163
Rps6ka5	11	28	13	50	26
Rps6kb1	83	93	51	77	94
Rps6kb2	7	0	0	2	0
Rps6kc1	188	101	137	125	105
Rps6kl1	1	6	1	5	0
Rps7	0	55	6	8	65
Rps8	9	13	0	117	0
Rps9	398	100	446	44	155
Rpsa	148	0	108	133	128

For Review Only

Rptor	556	393	484	704	594
Rptoros	13	19	18	19	22
Rpusd1	1	1	0	0	0
Rpusd2	13	16	15	13	20
Rpusd3	8	13	20	13	18
Rpusd4	59	55	63	62	64
Rrad	10	2	24	0	0
Rraga	83	97	68	75	106
Rragc	84	104	130	81	77
Rragd	4	0	10	7	0
Rras	2	0	0	11	0
Rras2	11	21	23	13	20
Rrbp1	235	155	124	343	254
Rreb1	307	211	240	515	498
Rrm1	56	58	66	35	46
Rrm2	0	2	0	4	0
Rrm2b	57	53	39	46	38
Rrn3	87	58	89	102	116
Rrnad1	23	0	7	5	3
Rrp1	233	154	294	203	207
Rrp12	28	13	21	28	23
Rrp15	15	22	21	15	28
Rrp1b	80	78	98	77	91
Rrp36	9	0	0	0	0
Rrp7a	196	127	224	134	139
Rrp8	50	39	54	19	68
Rrp9	30	0	11	22	26
Rrs1	45	18	39	21	35
Rsad1	35	21	29	38	36
Rsad2	0	6	0	0	15
Rsbn1	65	59	61	39	67
Rsbn1l	62	38	83	62	86
Rsc1a1	3	24	38	10	14
Rsf1	65	68	42	80	70
Rsl1	4	20	14	26	33
Rsl1d1	46	319	419	0	0
Rsl24d1	73	32	64	65	56
Rslcan18	0	5	0	22	1
Rsph3a	40	31	53	31	47
Rsph3b	36	30	50	33	34
Rsph4a	0	2	0	4	0
Rsph9	8	7	0	0	4
Rspo1	10	0	3	5	8
Rspry1	71	69	96	97	71
Rsrc1	39	62	52	64	98
Rsrc2	99	109	104	105	128
Rsrp1	0	0	0	0	0
Rsu1	134	93	148	131	130
Rtca	38	37	60	59	67
Rtcb	275	205	290	241	256

For Review Only

Rtel1	0	0	8	13	0
Rtf1	52	73	21	31	25
Rtfdc1	270	245	396	254	294
Rtkn	0	0	0	0	0
Rtkn2	0	0	0	4	0
Rtn1	144	143	100	161	143
Rtn3	827	740	799	799	844
Rtn4	90	238	124	162	122
Rtn4ip1	37	37	34	48	48
Rtn4rl1	363	412	268	414	381
Rtp4	22	34	56	43	29
Rttn	6	14	17	1	0
Rubcn	142	77	171	141	183
Rufy1	63	50	50	72	104
Rufy2	17	52	35	103	90
Rufy3	34	41	37	36	49
Rundc1	100	53	71	85	62
Rundc3a	0	0	2	0	0
Runx1	184	196	139	213	268
Runx1t1	1	8	0	10	9
Runx2	3	1	1	0	11
Runx3	0	2	0	0	0
Rusc1	27	36	43	36	33
Rusc2	33	33	34	72	72
Ruvbl1	56	51	95	47	55
Ruvbl2	80	63	76	32	19
Rwdd1	22	0	49	36	35
Rwdd2a	21	16	15	8	5
Rwdd2b	4	8	18	0	13
Rwdd3	6	4	0	0	4
Rwdd4a	27	48	32	28	44
Rxfp1	11	7	10	20	13
Rxfp4	0	0	0	0	0
Rxra	106	58	148	110	90
Rxrb	31	33	89	0	0
Rxrg	0	0	2	0	0
Rybp	5	22	14	16	19
Rybp-ps	0	4	1	1	3
Ryk	14	23	25	8	9
Ryr1	0	0	0	3	0
Ryr3	3	3	8	0	0
S100a1	1	1	0	0	0
S100a10	13	19	6	8	22
S100a11	5	4	0	0	7
S100a13	18	0	27	15	11
S100a16	0	0	0	0	0
S100a6	0	0	0	0	0
S100a8	0	0	6	35	11
S100a9	614	231	95	272	204
S100b	1	0	0	4	0

For Review Only

S100pbp	24	55	28	64	71
S1pr1	1680	1	0	0	0
S1pr2	31	31	45	54	30
S1pr4	40	22	22	27	27
Saal1	36	54	58	35	37
Sac3d1	32	19	25	27	22
Sacm1l	91	68	110	114	121
Sacs	4	7	6	5	13
Sae1	100	59	106	50	76
Safb	137	114	180	137	113
Safb2	271	0	14	105	73
Sag	1	4	6	4	5
Sall1	1104	1555	1536	1677	1609
Sall2	45	47	76	52	65
Sall3	270	282	333	320	290
Sall4	0	0	0	2	1
Samd1	11	9	4	12	14
Samd10	18	13	20	19	9
Samd14	0	0	0	1	6
Samd15	1	0	4	1	0
Samd4	0	0	6	0	0
Samd4b	37	33	35	44	17
Samd8	182	140	164	194	239
Samd9l	2	5	0	5	19
Samhd1	102	102	110	87	146
Samm50	139	92	150	115	100
Samsn1	201	297	389	364	284
Sap130	157	127	111	131	197
Sap18	1	1	0	0	0
Sap18b	75	41	86	45	48
Sap30	0	11	1	2	7
Sap30bp	61	95	88	83	58
Sap30l	0	1	0	0	0
Sar1a	252	306	339	248	354
Sar1b	129	166	172	127	159
Saraf	1220	877	1326	802	942
Sardh	74	4	41	22	0
Sarm1	9	21	12	16	22
Sarnp	99	123	135	118	100
Sars	132	2	0	133	93
Sars2	19	14	13	10	17
Sart1	62	65	72	49	0
Sart3	34	27	29	38	53
Sash1	3	0	0	0	4
Sash3	527	364	599	521	487
Sass6	24	23	23	26	16
Sat1	148	145	0	303	324
Sat2	25	6	0	0	0
Satb1	4	30	34	22	10
Satb2	1	1	2	2	6

For Review Only

Sav1	0	8	1	9	9
Saysd1	67	63	100	70	97
Sbds	128	137	172	111	124
Sbf1	151	89	21	177	200
Sbf2	251	173	228	251	266
Sbk1	9	1	27	1	0
Sbno1	204	233	253	275	320
Sbno2	263	291	134	284	175
Sbsn	2	4	8	8	0
Sbspon	4	0	1	0	7
Sc5d	72	36	51	53	62
Scaf1	48	46	43	62	57
Scaf11	333	243	209	302	350
Scaf4	48	46	40	74	91
Scaf8	3	2	0	0	0
Scai	51	58	28	70	59
Scamp1	238	210	215	332	323
Scamp2	1972	1657	2268	1785	2004
Scamp3	0	33	0	204	0
Scamp4	138	128	177	112	155
Scamp5	569	195	345	524	515
Scand1	1	7	0	0	0
Scap	144	142	113	129	139
Scaper	50	24	28	50	88
Scara5	0	5	0	7	0
Scarb1	88	62	90	110	110
Scarb2	490	473	557	472	401
Scarf1	11	19	12	12	23
Scarf2	5	5	0	20	16
Scarna3b	0	0	0	0	1
Sccpdh	65	73	111	59	61
Scd2	53	20	4	66	47
Scd3	1	0	0	3	3
Scfd1	0	28	98	58	84
Scfd2	21	22	42	32	30
Scg3	0	0	0	0	0
Scimp	4	15	6	4	3
Sclt1	6	15	8	14	25
Scly	19	7	0	0	11
Scmh1	77	0	29	93	49
Scml4	0	0	0	0	0
Scn11a	1	3	0	2	0
Scn1b	9	0	1	1	1
Scn2b	0	0	0	0	0
Scn3b	4	0	0	0	0
Scn4a	0	0	0	0	0
Scn8a	2	1	0	0	0
Scnm1	0	20	33	15	31
Scnn1a	1	0	0	0	1
Scnn1b	0	3	0	0	0

For Review Only

Sco1	14	9	45	22	15
Sco2	1	1	64	0	0
Scoc	195	408	0	270	107
Scp2	138	78	114	83	101
Scpep1	373	228	355	271	306
Scrib	16	3	67	43	71
Scrn2	13	0	0	16	8
Scrn3	40	27	21	45	33
Scube1	0	4	29	19	0
Scube3	2	0	5	0	0
Scyl1	13	1	0	0	0
Scyl2	33	58	23	60	29
Scyl3	39	61	44	59	77
Sdad1	69	5	29	54	47
Sdc2	7	0	0	0	0
Sdc3	67	111	158	114	89
Sdc4	124	55	75	78	129
Sdcbp	955	971	1240	960	1170
Sdccag3	0	0	0	0	0
Sdccag8	65	46	92	102	82
Sde2	50	53	55	60	68
Sdf2	61	76	181	157	157
Sdf2l1	184	148	199	130	148
Sdf4	260	216	486	274	360
Sdha	466	462	600	495	451
Sdhaf1	36	27	37	22	25
Sdhaf2	0	0	0	12	6
Sdhaf3	1	17	13	8	20
Sdhaf4	31	27	37	34	31
Sdhb	0	201	237	76	0
Sdhc	65	52	80	40	48
Sdhd	97	64	152	85	113
Sdk1	26	32	22	48	61
Sdpr	3	0	0	1	0
Sdr42e1	15	7	25	20	21
Sebox	5	0	0	0	0
Sec11a	139	165	227	141	136
Sec11c	124	349	412	83	224
Sec13	308	196	370	246	268
Sec14l1	872	407	552	619	708
Sec14l2	3	4	0	4	0
Sec16a	260	296	304	383	378
Sec16b	37	39	33	29	20
Sec22a	30	65	36	40	53
Sec22b	140	144	178	151	154
Sec22c	2	10	5	10	16
Sec23a	67	83	47	109	75
Sec23b	148	102	106	108	182
Sec23ip	55	48	70	92	59
Sec24a	92	112	119	134	136

For Review Only

Sec24b	166	155	193	245	275
Sec24c	286	272	179	214	143
Sec24d	49	33	36	60	32
Sec31a	266	193	115	186	282
Sec31b	0	7	3	0	0
Sec61a1	563	339	438	233	749
Sec61a2	1	1	0	0	0
Sec61b	48	55	82	44	53
Sec61g	0	0	0	0	0
Sec62	160	140	132	108	128
Sec63	19	61	67	33	30
Secisbp2	72	83	105	99	63
Secisbp2l	77	153	68	161	122
Seh1l	85	78	83	66	52
Sel1l	252	151	285	337	388
Selenbp1	45	28	32	37	32
Selenbp2	16	0	8	8	7
Selk	152	152	263	146	163
Sell	0	0	5	3	0
Selm	0	0	0	0	0
Selo	9	66	14	60	71
Selplg	9927	9576	14576	9215	10768
Selt	47	51	60	66	79
Sema3b	17	0	0	0	0
Sema3c	0	0	0	0	0
Sema4a	22	2	15	11	26
Sema4b	185	84	135	197	154
Sema4c	86	55	24	34	51
Sema4d	66	438	426	375	867
Sema4g	43	58	48	85	37
Sema6a	0	0	0	6	0
Sema6b	0	0	0	0	5
Sema6c	0	1	0	1	0
Sema6d	10	19	39	57	0
Senp1	70	84	69	56	78
Senp2	152	188	195	247	233
Senp3	39	45	0	54	8
Senp5	66	55	90	96	80
Senp6	27	45	47	36	58
Senp7	55	63	80	110	103
Senp8	45	34	46	26	47
Sep15	202	293	315	224	200
Sephs1	74	53	50	48	64
Sephs2	81	87	92	59	49
Sepn1	25	20	58	19	29
Sepp1	8868	8901	11848	9194	10375
Sepsecs	3	15	11	26	11
Sept10	55	49	63	63	45
Sept11	73	30	51	73	65
Sept2	577	641	674	652	680

For Review Only

Sept4	6	3	0	17	0
Sept6	17	4	16	37	51
Sept7	216	195	199	210	199
Sept8	44	48	33	60	61
Sept9	32	13	22	29	39
Sepw1	0	10	0	0	0
Serac1	6	47	34	65	55
Serbp1	195	179	229	144	145
Serf1	0	0	0	0	0
Serf2	0	0	0	0	0
Sergef	45	39	53	38	39
Serhl	0	3	23	0	0
Serinc1	650	455	630	670	712
Serinc3	1025	1352	1220	1724	1510
Serinc4	7	0	0	6	4
Serinc5	28	9	8	30	22
Serp1	2	1	0	0	0
Serpinb1a	4	2	0	0	0
Serpinb1b	0	0	0	0	4
Serpinb6a	18	11	32	12	9
Serpinb6b	0	0	0	0	0
Serpinb8	3	1	0	0	0
Serpinb9	48	27	58	21	34
Serpind1	0	0	1	2	10
Serpine1	9	4	0	7	10
Serpine2	1578	1214	1846	1376	1610
Serpinf1	35	37	64	48	51
Serpinf2	0	1	8	3	3
Serping1	3	0	0	0	0
Serpinh1	0	0	0	0	0
Serpini1	0	1	0	2	0
Sertad1	12	17	38	16	25
Sertad2	99	104	95	111	139
Sertad3	0	0	7	1	1
Sesn1	488	477	537	474	473
Sesn2	9	2	6	14	38
Sesn3	0	0	0	3	0
Sestd1	0	0	0	0	4
Set	22	22	0	0	16
Setbp1	5	23	14	31	6
Setd1a	0	0	1	1	1
Setd1b	65	93	45	68	91
Setd2	140	175	172	235	260
Setd3	167	110	139	161	151
Setd4	17	17	22	23	8
Setd5	197	224	162	282	252
Setd6	0	0	2	4	3
Setd7	18	20	32	37	35
Setdb1	153	174	213	188	186
Setdb2	16	0	0	0	0

For Review Only

Setdb2-phf11c	5	10	39	46	47
Setmar	7	0	0	5	0
Setx	74	74	49	85	101
Sez6l	1	0	0	0	0
Sez6l2	0	0	0	0	3
Sf1	241	115	219	351	141
Sf3a1	156	104	152	170	153
Sf3a2	16	0	4	17	15
Sf3a3	113	96	117	84	106
Sf3b1	1046	736	935	1015	947
Sf3b2	388	252	381	310	346
Sf3b3	0	0	0	0	0
Sf3b4	91	29	8	7	34
Sf3b5	61	60	75	54	71
Sf3b6	41	49	66	35	56
Sfi1	25	22	26	34	55
Sfmbt1	122	122	118	152	188
Sfmbt2	4	18	8	0	12
Sfn	0	1	0	0	1
Sfpq	0	278	26	75	31
Sfr1	110	126	129	72	85
Sfswap	112	100	88	116	122
Sft2d1	27	697	0	658	781
Sft2d2	699	0	3	557	477
Sft2d3	6	7	17	26	17
Sfxn1	44	43	54	54	38
Sfxn2	38	17	27	51	51
Sfxn3	82	77	100	90	70
Sfxn4	16	23	27	14	21
Sfxn5	31	30	21	22	44
Sgcb	68	56	54	42	84
Sgcd	0	0	0	1	0
Sgce	184	173	263	165	152
Sgf29	45	45	49	16	10
Sgip1	4	2	0	0	10
Sgk1	0	0	1	14	2
Sgk2	0	0	4	0	0
Sgk3	88	127	94	152	145
Sgms1	88	86	121	106	87
Sgms2	7	0	0	0	0
Sgol1	0	3	0	6	0
Sgol2a	0	0	0	0	0
Sgpl1	1264	610	900	1014	1120
Sgpp1	48	39	34	53	34
Sgpp2	4	2	9	11	4
Sgsh	149	49	90	122	117
Sgsm2	23	13	27	19	18
Sgsm3	0	0	81	0	114
Sgta	210	195	279	170	198
Sgtb	1	5	5	10	9

For Review Only

Sh2b1	80	85	100	98	110
Sh2b2	116	120	73	80	145
Sh2b3	28	16	8	45	68
Sh2d1b1	2	5	0	5	6
Sh2d3c	170	116	224	151	156
Sh2d4b	103	27	81	74	91
Sh2d5	1	2	4	0	6
Sh2d6	8	7	16	12	11
Sh2d7	0	3	0	2	0
Sh3bgrl	30	9	29	22	21
Sh3bgrl2	9	18	9	11	13
Sh3bgrl3	410	354	481	306	413
Sh3bp1	223	173	87	259	101
Sh3bp2	88	53	16	58	241
Sh3bp4	30	1	0	13	0
Sh3bp5	79	24	50	60	56
Sh3bp5l	46	48	45	62	55
Sh3d19	6	0	0	1	0
Sh3d21	0	1	9	0	4
Sh3gl1	95	73	15	11	0
Sh3glb1	227	396	78	531	90
Sh3glb2	1	1	0	0	0
Sh3kbp1	293	242	221	296	298
Sh3pxd2a	55	71	41	76	64
Sh3pxd2b	0	2	0	1	7
Sh3rf1	9	1	0	0	0
Sh3rf3	0	0	0	0	0
Sh3tc1	105	21	107	306	404
Sh3yl1	0	0	3	0	2
Shank1	9	1	0	0	7
Shank2	3	0	0	0	1
Sharpin	0	0	0	0	40
Shb	15	23	11	17	19
Shc1	149	3	224	226	167
Shcbp1l	0	0	0	1	3
She	6	3	1	0	23
Shf	7	7	5	6	8
Shfm1	0	95	170	42	69
Shisa2	1	0	0	0	0
Shisa5	445	408	80	54	543
Shisa6	0	0	0	0	0
Shisa7	11	13	23	11	9
Shkbp1	4	5	134	93	0
Shmt1	10	8	16	13	19
Shmt2	44	26	37	29	54
Shoc2	42	29	28	55	57
Shpk	17	19	29	10	17
Shprh	73	88	64	94	132
Shq1	25	9	21	18	12
Shroom1	3	18	14	8	18

For Review Only

Shroom4	3	4	0	6	0
Shtn1	0	1	4	0	0
Siae	118	104	122	144	129
Siah1a	19	24	38	35	23
Siah1b	5	6	6	4	13
Siah2	10	10	10	9	7
Sidt2	1	2	0	116	0
Sigirr	0	0	0	0	0
Siglec1	0	9	11	4	17
Siglece	8	69	105	40	0
Siglecf	92	38	143	75	89
Siglecg	14	37	24	14	19
Siglech	2558	1885	3323	1723	5036
Sigmar1	77	69	75	85	71
Sik1	5	0	0	3	0
Sik2	116	114	115	189	177
Sik3	30	20	32	40	51
Sike1	66	69	0	83	64
Sil1	113	79	129	68	102
Simc1	51	41	40	58	78
Sin3a	74	98	87	132	155
Sin3b	94	74	97	88	142
Sipa1	360	111	203	231	336
Sipa1l1	105	106	135	229	144
Sipa1l2	299	57	363	400	49
Sipa1l3	59	42	37	114	91
Sirpa	1607	126	4	1372	1517
Sirt1	14	5	12	10	21
Sirt2	0	36	18	0	4
Sirt3	23	25	23	18	42
Sirt4	20	6	26	11	4
Sirt5	5	24	21	21	7
Sirt6	44	12	0	49	60
Sirt7	17	0	0	12	3
Siva1	27	30	50	15	4
Six3os1	1	0	0	0	3
Six5	7	3	10	5	7
Ska2	10	7	1	8	9
Ska3	0	0	0	2	0
Skap1	0	0	0	0	0
Skap2	233	202	251	265	291
Ski	326	16	97	1057	202
Skida1	3	1	7	1	2
Skil	643	811	523	745	785
Skint3	0	2	0	1	0
Skiv2l	2	1	0	0	167
Skiv2l2	76	96	72	102	114
Skp1a	155	135	173	121	158
Skp2	11	4	31	17	30
Sla	259	1033	962	933	361

For Review Only

Sla2	1	0	0	0	0
Slain2	87	41	39	58	40
Slamf1	22	16	29	26	30
Slamf6	24	144	75	0	0
Slamf7	1	1	0	0	0
Slamf8	50	104	86	79	54
Slamf9	213	238	224	154	275
Slbp	1	8	92	0	0
Slc10a1	10	4	4	3	9
Slc10a3	31	0	0	0	439
Slc10a6	4	0	0	0	4
Slc10a7	62	46	57	56	62
Slc11a1	615	477	310	249	214
Slc11a2	74	57	37	74	52
Slc12a2	93	143	127	158	115
Slc12a4	88	33	45	69	54
Slc12a5	0	1	0	4	0
Slc12a6	365	421	391	598	519
Slc12a7	70	71	85	84	76
Slc12a9	4	0	0	9	78
Slc13a2	0	0	0	0	0
Slc13a3	22	31	36	43	83
Slc13a4	4	0	0	39	0
Slc14a1	7	7	0	0	11
Slc15a2	1	30	54	0	38
Slc15a3	149	117	164	116	115
Slc15a4	374	349	401	292	263
Slc16a1	19	7	19	23	48
Slc16a10	19	50	19	36	38
Slc16a11	2	0	0	6	0
Slc16a13	47	57	73	76	56
Slc16a2	23	3	0	8	1
Slc16a3	88	45	87	101	122
Slc16a4	2	0	0	3	6
Slc16a6	475	666	799	537	477
Slc16a7	45	60	49	107	102
Slc16a8	7	1	0	0	0
Slc16a9	0	1	0	5	3
Slc17a5	85	61	57	70	88
Slc17a9	35	33	22	43	41
Slc18a1	15	10	0	11	15
Slc18a2	31	51	69	48	39
Slc18b1	22	52	30	69	81
Slc19a1	70	0	90	5	76
Slc19a2	2	7	0	10	10
Slc1a1	0	0	1	0	0
Slc1a2	14	3	6	5	8
Slc1a3	568	255	420	410	526
Slc1a4	25	55	15	16	30
Slc1a5	7	15	6	15	18

For Review Only

Slc20a1	96	8	40	49	47
Slc20a2	40	20	10	36	16
Slc22a17	1	0	0	0	0
Slc22a21	13	32	12	37	35
Slc22a23	0	0	0	0	1
Slc22a4	6	10	11	11	0
Slc22a5	58	36	45	75	30
Slc22a8	1	0	0	0	0
Slc23a1	6	2	0	0	7
Slc23a2	424	323	341	393	442
Slc23a3	0	1	0	0	7
Slc24a1	24	16	7	25	22
Slc24a3	21	30	55	32	29
Slc25a1	96	90	0	0	0
Slc25a10	29	25	52	30	50
Slc25a11	0	0	0	0	0
Slc25a12	86	69	75	59	105
Slc25a13	3	3	11	0	0
Slc25a14	20	5	23	0	11
Slc25a15	5	13	21	22	0
Slc25a16	14	10	10	11	10
Slc25a17	90	124	162	136	106
Slc25a18	0	0	0	3	0
Slc25a19	0	38	53	35	19
Slc25a2	1	1	0	0	9
Slc25a20	20	14	39	21	13
Slc25a22	0	0	0	31	0
Slc25a23	23	21	28	20	13
Slc25a25	12	17	138	87	88
Slc25a26	9	12	19	10	10
Slc25a27	37	26	20	15	38
Slc25a28	1	20	0	33	51
Slc25a29	10	20	18	14	26
Slc25a3	0	1	0	0	0
Slc25a30	11	8	0	37	65
Slc25a32	24	36	43	42	55
Slc25a33	8	12	8	15	18
Slc25a35	15	16	18	19	19
Slc25a36	206	225	140	264	233
Slc25a37	257	430	380	471	465
Slc25a38	54	48	62	30	51
Slc25a39	1	58	4	8	53
Slc25a4	285	227	269	222	188
Slc25a40	13	13	9	13	25
Slc25a42	9	6	7	11	8
Slc25a43	10	7	21	12	10
Slc25a44	41	41	106	75	102
Slc25a45	0	345	16	685	0
Slc25a46	122	89	55	109	126
Slc25a47	1	0	0	0	0

For Review Only

Slc25a5	760	705	956	693	814
Slc25a51	95	60	70	85	107
Slc25a53	1	1	0	0	0
Slc26a1	3	23	3	18	17
Slc26a10	3	0	0	0	0
Slc26a11	30	26	40	23	22
Slc26a2	89	31	67	119	93
Slc26a6	24	0	17	11	1
Slc27a1	47	35	74	58	49
Slc27a3	5	0	0	5	0
Slc27a4	42	42	41	37	47
Slc29a1	285	122	287	366	417
Slc29a2	15	10	17	12	24
Slc29a3	1650	1009	1527	1680	1953
Slc29a4	14	0	11	0	3
Slc2a1	79	59	83	52	68
Slc2a10	1	3	7	4	0
Slc2a12	21	0	0	5	0
Slc2a3	7	9	15	16	4
Slc2a4rg-ps	15	0	0	77	61
Slc2a5	0	869	172	75	265
Slc2a6	5	8	0	20	0
Slc2a8	0	0	0	0	0
Slc2a9	45	1	0	81	0
Slc30a1	15	12	17	14	9
Slc30a10	0	0	0	0	0
Slc30a4	3	26	81	53	20
Slc30a5	59	85	60	125	67
Slc30a6	34	28	45	21	59
Slc30a7	190	170	178	223	219
Slc30a9	45	119	103	99	118
Slc31a1	203	85	175	203	156
Slc31a2	1	1	0	0	0
Slc33a1	101	76	84	121	85
Slc35a1	122	73	124	120	100
Slc35a2	48	52	36	48	53
Slc35a3	32	66	34	61	93
Slc35a5	160	192	165	142	181
Slc35b1	1	1	0	0	0
Slc35b2	197	158	253	327	60
Slc35b3	54	75	74	44	85
Slc35b4	136	102	187	158	144
Slc35c1	151	53	82	125	152
Slc35c2	371	98	220	218	523
Slc35d1	18	24	29	18	13
Slc35d2	38	17	48	35	24
Slc35e1	83	55	143	115	110
Slc35e2	98	96	0	0	0
Slc35e3	31	51	28	25	32
Slc35e4	13	7	19	17	18

For Review Only

Slc35f2	0	0	0	0	10
Slc35f3	4	4	4	3	3
Slc35f5	62	114	116	106	114
Slc35f6	0	0	0	27	37
Slc35g1	0	0	0	6	0
Slc35g2	3	9	7	8	0
Slc36a1	146	218	257	179	164
Slc36a2	22	25	22	12	18
Slc36a4	39	49	18	48	36
Slc37a1	11	11	0	0	10
Slc37a2	0	133	270	0	116
Slc37a3	42	22	59	67	43
Slc37a4	0	27	0	35	89
Slc38a1	155	168	156	182	184
Slc38a10	469	307	464	466	524
Slc38a2	133	168	187	208	161
Slc38a3	18	0	0	0	0
Slc38a5	0	0	0	0	0
Slc38a6	36	29	37	61	57
Slc38a7	245	91	122	176	198
Slc38a9	172	204	233	186	195
Slc39a1	328	357	363	337	440
Slc39a10	94	91	76	144	90
Slc39a11	96	57	96	87	65
Slc39a12	58	56	55	0	52
Slc39a13	67	41	0	0	0
Slc39a14	3	21	0	21	25
Slc39a2	4	3	0	0	0
Slc39a3	124	79	122	90	86
Slc39a4	37	22	31	36	19
Slc39a6	147	58	120	95	108
Slc39a7	3	37	0	0	0
Slc39a8	54	38	56	34	58
Slc39a9	135	134	161	185	187
Slc3a2	578	160	1183	248	280
Slc40a1	24	0	0	59	97
Slc41a1	61	52	54	42	67
Slc41a3	73	48	73	56	49
Slc43a1	0	6	0	5	11
Slc43a2	467	2	0	0	375
Slc43a3	0	0	0	8	6
Slc44a1	30	19	30	43	35
Slc44a2	445	0	294	483	57
Slc45a3	0	0	0	0	0
Slc45a4	77	117	108	82	50
Slc46a1	370	316	446	222	296
Slc46a2	0	0	0	0	4
Slc46a3	41	347	231	0	175
Slc48a1	188	15	71	158	0
Slc4a10	11	0	0	0	0

For Review Only

Slc4a1ap	52	43	37	68	69
Slc4a2	117	52	55	80	85
Slc4a3	4	0	0	0	6
Slc4a4	0	0	0	0	0
Slc4a5	9	0	0	0	0
Slc4a7	80	168	90	181	180
Slc50a1	0	0	81	0	0
Slc52a2	1	1	0	0	0
Slc52a3	0	0	0	0	0
Slc5a10	21	0	23	14	23
Slc5a11	0	2	0	0	0
Slc5a3	108	121	71	177	172
Slc5a5	7	0	0	11	9
Slc5a6	39	14	50	27	41
Slc6a13	0	0	0	0	0
Slc6a20a	10	0	0	0	0
Slc6a20b	3	0	0	4	0
Slc6a6	401	292	336	424	428
Slc6a8	7	1	13	0	23
Slc6a9	1	0	0	0	0
Slc7a1	20	14	25	31	30
Slc7a10	21	0	0	0	0
Slc7a11	5	4	18	2	9
Slc7a15	8	4	11	7	4
Slc7a4	22	10	9	4	3
Slc7a5	12	21	20	25	21
Slc7a6	40	62	54	40	36
Slc7a6os	22	23	15	37	35
Slc7a7	38	43	152	207	1
Slc7a8	1261	768	1076	1447	1561
Slc8a1	291	604	390	680	713
Slc8a2	0	0	1	0	0
Slc8b1	113	77	86	73	93
Slc9a1	147	114	118	120	180
Slc9a3r1	43	54	55	36	40
Slc9a3r2	0	0	15	2	0
Slc9a5	3	0	6	6	0
Slc9a6	33	64	40	81	54
Slc9a7	50	88	67	71	95
Slc9a8	142	94	165	182	194
Slc9a9	543	432	469	593	647
Slco1a4	6	0	0	0	0
Slco1c1	6	0	0	0	0
Slco2b1	5	28	12	19	54
Slco4a1	217	132	283	200	297
Slf1	8	7	0	11	22
Slfn1	12	0	0	0	0
Slfn10-ps	12	17	19	27	26
Slfn2	40	40	54	41	34
Slfn3	2	3	0	0	0

For Review Only

Slfn4	23	0	2	8	0
Slfn5	2	6	5	6	0
Slfn8	141	130	148	184	122
Slfn9	17	19	20	14	11
Slirp	16	36	64	0	47
Slit1	5	0	0	1	0
Slk	19	33	34	28	40
Slmap	147	144	161	173	153
Slmo1	2	8	9	33	20
Slmo2	44	72	65	47	50
Sltm	92	75	122	68	86
Slu7	108	252	204	0	43
Slx4	57	35	47	73	60
Slx4ip	37	18	21	47	33
Smad1	52	48	70	71	78
Smad2	131	118	118	132	170
Smad3	383	287	282	341	363
Smad4	45	80	57	95	62
Smad5	89	137	114	123	141
Smad6	3	0	0	6	7
Smad7	75	81	75	79	127
Smagp	21	9	36	33	26
Smap1	35	62	46	35	40
Smap2	0	12	24	8	7
Smarca2	189	191	164	232	249
Smarca4	196	126	158	185	147
Smarca5	25	27	17	21	24
Smarca5-ps	3	6	4	0	3
Smarcad1	66	78	61	68	56
Smarcal1	0	0	100	94	101
Smarcb1	130	120	133	79	99
Smarcc1	24	48	39	47	46
Smarcc2	123	33	195	0	211
Smarcd1	31	16	18	30	21
Smarcd2	24	90	10	7	0
Smarcd3	0	0	0	3	2
Smarce1	52	34	43	41	68
Smc1a	137	32	122	149	51
Smc2	16	14	25	15	11
Smc3	53	39	83	87	0
Smc4	9	11	12	17	1
Smc5	5	15	24	32	42
Smc6	48	63	64	64	63
Smchd1	96	93	69	116	74
Smcr8	146	167	162	242	234
Smdt1	111	66	104	75	74
Smek1	62	62	55	107	78
Smek2	108	138	155	158	170
Smg1	206	492	253	539	473
Smg5	109	83	96	90	99

For Review Only

Smg6	90	77	81	88	122
Smg7	101	81	75	156	109
Smg8	110	54	56	51	83
Smg9	36	83	57	0	66
Smim1	0	0	1	3	1
Smim10l1	62	99	84	101	132
Smim10l2a	23	14	32	34	20
Smim11	34	36	0	0	23
Smim12	69	54	68	70	64
Smim13	15	13	22	26	31
Smim14	230	176	238	163	171
Smim15	7	58	0	0	0
Smim19	1	11	0	12	18
Smim20	61	57	55	44	66
Smim22	0	0	6	10	0
Smim24	0	6	4	9	2
Smim3	95	39	89	63	58
Smim4	9	19	22	13	12
Smim5	9	10	12	6	13
Smim6	2	0	7	8	4
Smim7	196	146	209	149	209
Smim8	27	33	28	21	26
Smkr-ps	0	0	0	0	0
Smn1	17	19	4	21	25
Smndc1	29	31	25	74	90
Smo	79	52	63	74	88
Smoc1	0	2	0	0	0
Smoc2	0	0	0	0	0
Smox	303	222	367	283	128
Smpd1	162	96	182	154	161
Smpd2	75	54	0	0	0
Smpd3	6	8	4	4	5
Smpd4	66	30	42	69	53
Smpd5	0	0	0	0	10
Smpdl3a	84	52	91	49	50
Smpdl3b	22	53	33	33	46
Sms	20	14	16	12	21
Smtn	1	0	0	0	0
Smtnl2	0	0	0	0	0
Smu1	216	182	343	213	276
Smug1	16	42	65	40	46
Smurf1	73	123	152	104	128
Smurf2	98	68	84	108	131
Smyd2	0	0	1	1	7
Smyd3	100	78	83	99	111
Smyd4	54	52	80	46	75
Smyd5	36	8	75	0	102
Snap23	138	124	129	122	159
Snap25	0	4	0	4	0
Snap29	79	64	49	76	93

For Review Only

Snap47	110	70	124	77	73
Snapc1	46	39	55	33	47
Snapc2	81	3	148	50	0
Snapc3	108	146	150	153	119
Snapc4	67	27	81	63	48
Snapc5	49	48	90	54	59
Sncaip	88	71	84	96	106
Snd1	164	163	180	217	255
Sned1	20	11	6	19	25
Snf8	0	0	2	2	4
Snhg10	5	8	5	3	0
Snhg11	3	0	0	0	0
Snhg15	12	12	8	0	0
Snhg17	35	22	45	35	26
Snhg18	0	0	0	0	0
Snhg20	56	84	105	55	53
Snhg4	4	9	6	0	6
Snhg5	0	0	0	0	0
Snhg6	21	16	0	0	0
Snhg7	20	9	27	7	0
Snhg8	47	34	40	57	64
Snhg9	0	0	0	0	0
Snip1	54	67	56	33	14
Snn	97	359	106	211	154
Snora17	0	1	0	0	0
Snora28	0	0	8	25	40
Snora30	0	0	2	0	2
Snora41	4	2	4	0	4
Snora44	0	0	1	3	3
Snora52	9	8	12	15	29
Snora73a	2	7	1	0	1
Snora78	0	6	0	0	0
Snora81	8	12	12	18	14
Snord104	18	19	32	17	18
Snord118	20	4	11	16	26
Snord12	3	0	1	1	2
Snord13	1	0	2	1	1
Snord14a	1	1	0	0	0
Snord14e	1	1	0	0	0
Snord15b	4	3	5	0	7
Snord16a	3	1	0	0	0
Snord17	15	12	14	16	12
Snord2	6	4	0	0	0
Snord33	1	1	2	3	2
Snord34	3	1	3	3	7
Snord35b	0	0	0	2	0
Snord37	2	3	3	3	3
Snord45b	3	2	0	0	0
Snord47	8	4	7	5	8
Snord52	6	9	11	0	9

For Review Only

Snord53	0	1	0	0	1
Snord55	0	1	2	3	2
Snord71	5	1	1	3	3
Snord8	0	2	1	0	3
Snord83b	0	0	4	3	7
Snord95	1	4	5	2	3
Snph	20	45	50	36	40
Snrk	191	86	174	180	197
Snrnp200	160	230	134	255	194
Snrnp25	0	16	22	15	7
Snrnp27	31	44	67	37	51
Snrnp35	23	28	30	16	43
Snrnp40	97	89	75	80	102
Snrnp48	1	1	0	0	0
Snrnp70	85	234	45	83	156
Snrpa	158	130	156	117	147
Snrpa1	0	0	0	8	0
Snrpb	0	299	49	10	1
Snrpb2	74	63	57	4	6
Snrpc	19	13	11	14	7
Snrpd1	20	52	54	34	30
Snrpd2	5	13	21	25	22
Snrpd3	0	0	12	40	25
Snrpe	0	0	50	0	18
Snrpf	14	10	26	0	0
Snrpg	22	33	21	6	21
Snta1	0	8	0	0	0
Sntb1	8	0	0	0	0
Sntb2	16	1	10	7	0
Sntg1	3	1	0	7	0
Snupn	20	35	20	19	41
Snw1	103	191	134	114	160
Snx1	172	137	198	158	193
Snx10	113	76	103	112	98
Snx11	55	45	48	57	33
Snx12	46	38	48	45	37
Snx13	157	116	137	241	233
Snx14	181	120	178	142	169
Snx15	42	38	58	33	49
Snx16	45	27	50	31	17
Snx17	86	0	36	0	0
Snx18	299	370	181	474	487
Snx19	263	51	156	287	243
Snx2	146	88	245	180	157
Snx20	150	152	157	53	61
Snx21	19	14	22	13	21
Snx22	14	12	19	12	16
Snx24	95	50	85	49	50
Snx25	7	23	35	5	18
Snx27	142	62	0	132	138

For Review Only

Snx29	390	419	401	245	454
Snx3	313	193	222	187	225
Snx30	87	59	78	93	104
Snx32	25	0	15	16	10
Snx33	13	12	15	27	0
Snx4	151	131	183	141	184
Snx5	115	1269	1086	149	429
Snx6	350	285	334	206	254
Snx7	12	7	12	0	0
Snx8	168	74	171	82	151
Snx9	7	36	43	33	42
Soat1	276	281	223	326	367
Soat2	0	0	9	0	0
Socs1	8	4	8	5	7
Socs2	3	0	0	1	3
Socs3	0	0	7	0	8
Socs4	11	49	13	36	68
Socs5	42	40	54	46	41
Socs6	124	111	160	156	13
Socs7	25	51	41	40	53
Sod1	242	253	389	115	143
Sod2	88	66	70	82	90
Sod3	6	0	0	1	0
Soga1	1892	1703	2067	2066	2310
Son	171	192	568	228	396
Sorbs1	10	10	5	16	0
Sorbs2	0	0	0	0	0
Sorbs3	0	0	0	0	9
Sord	1	2	114	113	0
Sorl1	1	216	117	176	91
Sort1	444	0	145	106	80
Sos1	67	41	69	59	59
Sos2	31	33	37	28	47
Sostdc1	21	0	0	1	0
Sowahc	467	393	460	625	671
Sox12	4	1	0	0	1
Sox13	0	1	0	12	0
Sox15	8	12	7	6	5
Sox18	0	0	0	0	0
Sox4	30	11	26	27	44
Sox6	3	0	0	5	4
Sox7	0	0	0	5	0
Sox8	1	0	0	1	0
Sox9	7	0	0	0	0
Sp1	135	93	102	180	137
Sp100	23	26	37	40	44
Sp110	49	42	73	64	78
Sp140	38	43	55	44	34
Sp2	54	54	42	46	62
Sp3	163	80	95	141	108

For Review Only

Sp3os	10	6	13	9	5
Sp4	43	32	56	59	24
Spaca1	0	0	0	0	0
Spaca6	71	47	35	83	77
Spag1	4	10	13	0	19
Spag17	0	3	1	0	0
Spag6	0	0	0	3	0
Spag8	0	0	0	0	0
Spag9	68	182	144	177	240
Sparc	3862	4254	5511	4034	4800
Sparcl1	7	1	0	0	41
Spast	44	68	71	48	34
Spata1	25	7	0	11	0
Spata13	264	573	462	819	743
Spata17	0	0	0	6	0
Spata2	74	51	64	81	93
Spata21	0	0	2	0	4
Spata24	0	0	0	0	2
Spata2l	20	50	39	57	34
Spata33	0	0	0	3	0
Spata5	54	66	59	76	58
Spata5l1	1	0	2	6	7
Spata6	32	4	14	9	30
Spata7	6	10	12	17	4
Spata9	0	0	0	0	0
Spats2	11	18	26	16	18
Spats2l	3	0	0	1	1
Spc25	0	0	0	2	0
Spcs1	129	0	108	0	0
Spcs2	426	540	570	388	520
Spcs3	258	299	351	351	444
Spdl1	0	0	0	4	0
Spdya	0	0	0	0	0
Specc1	40	283	186	416	187
Specc1l	89	117	102	117	125
Spef1	0	0	0	0	7
Spef2	2	5	0	0	0
Speg	53	41	54	32	59
Spen	47	37	47	91	100
Spg11	38	30	0	102	105
Spg20	82	183	175	217	190
Spg21	157	70	152	151	100
Spg7	33	54	32	32	41
Sphk2	46	27	55	22	71
Spi1	459	107	215	177	309
Spice1	41	20	40	26	22
Spidr	54	21	45	65	63
Spin1	70	111	79	121	100
Spint1	0	0	0	0	0
Spint2	17	0	0	7	6

For Review Only

Spire1	52	100	113	102	120
Spns1	1	1	0	0	519
Spns2	39	26	68	37	36
Spock2	0	0	1	1	0
Spon1	13	5	0	13	4
Spop	153	126	190	153	169
Spopl	43	46	49	65	48
Spp1	0	0	2	2	1
Sppl2a	357	279	379	474	505
Sppl2b	53	0	52	26	23
Sppl3	70	84	146	52	86
Spr	23	30	0	0	0
Spred1	55	88	93	68	52
Spred2	102	64	78	198	221
Spred3	4	7	4	7	6
Sprtn	50	49	54	49	83
Spry1	22	23	14	17	26
Spryd3	53	17	22	34	41
Spryd4	60	25	62	27	50
Spryd7	36	49	13	37	25
Spsb1	395	302	281	283	603
Spsb2	0	0	0	18	0
Spsb3	15	0	0	37	0
Sptan1	57	57	88	65	79
Sptb	2	0	0	13	0
Sptbn1	14	11	10	35	70
Sptbn2	2	2	3	0	10
Sptbn4	3	0	0	5	0
Sptbn5	31	19	61	81	82
Sptlc1	143	46	96	115	170
Sptlc2	291	236	337	232	246
Sptssa	108	26	256	57	48
Spty2d1	125	86	143	166	113
Spx	0	0	0	4	0
Sqle	11	1	5	9	7
Sqrdl	268	236	213	95	134
Sqstm1	919	16	1189	694	954
Sra1	102	0	111	0	0
Srbd1	86	54	78	86	100
Src	47	60	41	66	54
Srcap	331	272	255	416	368
Srcin1	0	1	1	0	2
Srd5a1	0	0	0	0	0
Srd5a3	119	105	159	132	139
Srebf1	163	41	112	58	131
Srebf2	43	46	27	55	83
Srek1	97	102	180	227	180
Srek1ip1	21	53	36	24	34
Srf	11	17	11	25	17
Srfbp1	55	44	45	35	54

For Review Only

Srgap2	57	0	0	0	0
Srgap3	13	11	6	10	0
Srgn	162	160	229	146	180
Sri	106	64	102	89	83
Srl	12	4	28	37	23
Srm	0	0	70	40	92
Srp14	138	145	186	106	151
Srp19	66	83	110	85	86
Srp54a	61	56	70	70	53
Srp54b	85	71	98	94	77
Srp54c	84	71	91	93	83
Srp68	157	105	175	127	102
Srp72	90	150	125	116	147
Srp9	97	69	83	54	47
Srpk1	44	0	40	66	26
Srpk2	80	111	70	62	138
Srpr	0	147	222	217	11
Srprb	214	222	311	182	206
Srr	33	53	33	36	89
Srrd	5	26	27	0	0
Srrm1	274	292	315	327	415
Srrm2	36	22	0	267	397
Srrt	63	74	84	95	43
Srsf1	53	97	72	71	42
Srsf10	114	120	119	155	192
Srsf11	153	10	52	15	13
Srsf2	0	0	16	0	0
Srsf3	0	1	0	0	0
Srsf4	83	149	206	112	76
Srsf5	0	0	2	0	0
Srsf6	0	138	406	0	0
Srsf7	0	0	5	8	4
Srsf9	14	66	128	89	86
Srxn1	6	10	10	3	7
Ss18	263	251	268	252	292
Ss18l1	17	51	15	35	36
Ssb	101	48	146	124	79
Ssbp1	2	103	166	0	90
Ssbp2	17	2	184	168	0
Ssbp3	37	30	35	25	42
Ssbp4	138	37	30	63	69
Ssc4d	0	0	0	0	0
Ssfa2	174	148	154	154	178
Ssh1	144	111	66	121	96
Ssh2	1422	393	1197	1317	1310
Ssh3	35	26	0	0	0
Ssna1	0	0	0	0	0
Sspn	4	9	15	13	11
Sspo	12	0	7	7	3
Ssr1	592	493	803	525	570

For Review Only

Ssr2	196	201	236	212	212
Ssr3	323	316	330	371	386
Ssr4	0	0	74	0	1
Ssrp1	77	88	84	90	109
Sssca1	29	15	0	0	0
Sstr3	0	0	0	2	0
Ssu72	127	97	178	148	205
Ssx2ip	0	0	1	4	13
St13	119	97	106	144	110
St14	71	21	45	78	48
St18	0	0	0	1	0
St3gal1	7	5	22	26	11
St3gal2	146	126	123	155	163
St3gal3	38	22	27	28	49
St3gal4	56	39	27	54	29
St3gal5	137	117	137	140	169
St3gal6	236	166	151	559	455
St5	94	53	69	107	93
St6gal1	292	167	217	244	253
St6galnac2	2	0	0	0	0
St6galnac3	0	2	0	0	13
St6galnac4	551	333	663	444	516
St6galnac6	13	24	0	0	0
St7	11	0	0	6	0
St7l	112	139	97	107	111
St8sia1	3	10	26	19	15
St8sia2	14	0	0	0	0
St8sia4	17	12	24	39	20
St8sia6	25	10	11	34	27
Stab1	72	1	0	0	1673
Stac3	0	0	0	2	0
Stag1	84	115	76	126	148
Stag2	66	60	152	325	268
Stag3	18	10	0	0	0
Stam	26	65	51	75	59
Stam2	90	79	86	98	89
Stambp	67	79	73	85	98
Stambpl1	133	120	114	110	167
Stamos	2	2	0	0	0
Star	24	20	25	22	33
Stard13	2	1	0	0	0
Stard3	583	101	501	283	363
Stard3nl	1	2	45	196	0
Stard4	1	0	0	8	9
Stard5	68	5	0	69	0
Stard6	1	6	0	7	3
Stard7	31	76	76	78	80
Stard8	168	108	175	214	157
Stard9	269	501	217	883	1224
Stat1	139	98	140	153	176

For Review Only

Stat2	32	95	44	0	76
Stat3	181	67	461	197	71
Stat5a	53	72	40	57	78
Stat5b	144	159	139	220	223
Stat6	554	302	517	501	560
Stau1	38	31	514	0	0
Stau2	11	19	9	29	29
Stbd1	5	18	10	13	5
Steap1	10	0	0	5	0
Steap2	7	0	6	0	0
Steap3	3	2	0	5	18
Stil	0	0	0	0	1
Stim1	168	130	100	154	140
Stim2	18	22	27	23	11
Stip1	0	0	51	39	0
Stk10	372	286	271	457	441
Stk11	61	56	75	52	57
Stk11ip	139	117	148	144	135
Stk16	129	83	0	0	0
Stk17b	108	81	100	104	135
Stk19	21	0	22	17	10
Stk24	81	94	87	71	72
Stk25	0	93	1	48	3
Stk26	0	0	0	7	9
Stk3	60	45	60	91	56
Stk35	28	17	7	38	30
Stk36	9	9	0	0	9
Stk38	65	43	55	102	108
Stk38l	44	35	36	38	50
Stk39	13	0	0	0	0
Stk4	70	147	90	133	124
Stk40	48	56	77	53	81
Stmn1	16	14	16	10	12
Stom	30	26	14	18	26
Stoml1	137	71	146	91	111
Stoml2	66	51	0	0	0
Ston1	0	4	3	6	4
Ston2	15	13	15	24	31
Stox2	0	3	0	0	0
Stpg1	0	0	0	0	0
Stra13	5	14	42	0	73
Strada	19	62	68	31	27
Stradb	116	0	130	83	93
Strap	101	96	186	124	167
Strbp	4	38	42	64	37
Strip1	137	99	111	107	122
Strip2	7	0	0	0	0
Strn	59	83	75	107	115
Strn3	31	56	68	44	51
Strn4	34	18	55	44	33

For Review Only

Stt3a	528	413	550	593	552
Stt3b	65	64	66	81	74
Stub1	1	1	0	0	242
Stx11	6	0	0	0	1
Stx12	134	133	130	122	149
Stx16	133	173	203	133	144
Stx17	43	65	70	69	91
Stx18	93	97	127	74	77
Stx19	0	0	1	1	4
Stx1a	14	18	18	9	12
Stx2	103	90	158	103	120
Stx3	15	26	24	12	16
Stx4a	14	10	0	0	0
Stx5a	59	0	0	0	0
Stx6	82	43	29	80	90
Stx7	348	374	454	416	343
Stx8	2	39	68	37	41
Stxbp1	47	32	44	41	51
Stxbp2	0	49	47	0	5
Stxbp3	129	130	133	111	140
Stxbp3-ps	21	35	21	37	38
Stxbp4	21	43	18	33	15
Stxbp5	96	68	82	108	123
Styx	15	22	21	18	14
Styxl1	11	4	12	11	13
Sub1	72	78	127	74	69
Sucla2	99	78	109	82	95
Suclg1	154	89	142	69	105
Suclg2	118	90	101	108	93
Suco	30	72	61	36	34
Suds3	11	27	16	15	12
Sufu	71	64	94	130	112
Sugp1	55	64	56	50	55
Sugp2	128	115	96	124	153
Sugt1	74	122	138	121	143
Sulf1	28	0	0	0	0
Sulf2	6	5	25	14	32
Sult1a1	81	0	150	50	3
Sult2b1	2	2	0	1	1
Sult4a1	5	0	5	6	0
Sult6b1	2	4	3	0	7
Sumf1	55	101	68	87	136
Sumf2	72	61	31	16	3
Sumo1	78	70	95	73	103
Sumo2	52	51	57	54	63
Sumo3	169	48	223	200	243
Sun1	128	101	121	101	162
Sun2	404	201	224	433	466
Suox	165	22	0	20	0
Supt16	104	110	65	30	82

For Review Only

Supt20	325	161	249	337	297
Supt3	4	1	0	32	28
Supt4a	0	100	137	1	1
Supt4b	35	33	69	34	25
Supt5	157	96	0	139	196
Supt6	76	69	59	140	141
Supt7l	151	24	43	55	67
Supv3l1	58	31	38	38	41
Surf1	63	33	0	0	0
Surf2	0	0	0	0	0
Surf4	796	503	681	625	784
Surf6	87	38	64	63	84
Susd1	0	9	0	10	12
Susd2	1	0	3	0	0
Susd3	817	744	810	8	14
Susd6	106	133	101	178	129
Suv39h1	32	21	48	25	53
Suv39h2	0	2	1	2	0
Suv420h1	143	149	87	177	151
Suv420h2	0	62	0	0	115
Suz12	19	24	19	33	30
Sv2a	5	51	61	57	57
Svbp	27	37	66	38	33
Svil	55	48	35	51	83
Svip	36	4	27	21	13
Swap70	150	125	204	222	192
Swi5	54	71	84	56	76
Swsap1	14	5	12	20	5
Swt1	68	53	54	67	81
Syap1	16	15	11	12	32
Sybu	6	0	0	0	0
Syce2	7	3	7	7	8
Sycp2	2	2	0	0	0
Syde1	3	1	0	0	0
Syf2	153	78	161	85	109
Syk	360	426	329	475	481
Sympk	44	17	48	76	64
Syn3	9	14	21	20	13
Syncrip	154	138	134	164	172
Syne1	20	0	0	5	3
Syne2	2	3	1	14	5
Syne4	0	0	0	7	0
Syngap1	7	15	12	21	36
Syngr1	0	740	200	1	147
Syngr2	0	0	7	0	0
Syngr3	3	0	0	0	0
Synj1	161	166	130	259	202
Synj2	38	26	35	47	50
Synj2bp	67	80	70	88	95
Synj2bp-cox16	26	51	47	51	47

For Review Only

Synm	0	0	3	1	0
Synpo	0	0	0	1	0
Synpo2	0	0	0	0	0
Synrg	4	55	107	66	91
Syp	4	0	3	0	0
Sypl	411	349	443	440	360
Sys1	80	58	70	62	40
Syt11	55	34	38	59	47
Syt12	0	0	0	0	0
Syt14	4	2	5	4	7
Syt15	1	0	0	2	0
Syt2	0	7	0	4	0
Syt3	21	16	10	13	15
Syt6	5	11	7	8	0
Syt7	0	2	0	0	5
Syt8	0	5	0	5	6
Syvn1	106	59	0	0	0
Szrd1	63	63	60	62	99
Szt2	119	83	85	151	127
T2	0	0	0	0	4
Tab1	78	57	65	73	118
Tab2	287	273	367	280	382
Tab3	8	21	10	26	24
Tac4	1	0	8	0	0
Tacc1	1543	757	940	1254	1245
Tacc2	17	8	11	7	18
Tacc3	18	21	18	26	0
Taco1	6	15	9	10	18
Tada1	92	111	147	68	24
Tada2a	28	25	30	26	38
Tada2b	12	34	28	23	23
Tada3	95	48	57	47	91
Taf1	29	53	14	53	31
Taf10	4	5	0	3	0
Taf11	10	21	26	0	17
Taf12	80	54	87	57	54
Taf13	19	23	28	21	18
Taf15	29	42	19	39	28
Taf1a	45	31	36	24	38
Taf1b	48	43	70	57	52
Taf1c	31	39	82	50	55
Taf1d	35	44	81	58	18
Taf2	70	35	39	48	43
Taf3	38	70	42	66	69
Taf4	29	44	50	40	39
Taf4b	0	2	0	5	15
Taf5	8	5	14	0	5
Taf5l	25	14	28	31	33
Taf6	49	0	37	0	17
Taf7	66	64	60	42	86

For Review Only

Taf8	42	44	67	53	67
Taf9	70	93	115	75	96
Taf9b	16	17	24	12	26
Tagap	0	0	0	53	29
Tagap1	266	146	186	128	231
Tagln2	70	38	77	61	57
Tagln3	1	0	0	0	0
Tal1	16	44	27	48	44
Taldo1	230	157	272	198	202
Tamm41	26	0	17	15	30
Tanc1	19	2	6	0	0
Tanc2	1364	1376	1460	1733	1711
Tango2	145	134	238	140	128
Tango6	15	34	34	53	46
Tank	60	37	65	59	32
Taok1	155	169	107	334	252
Taok2	22	20	59	78	0
Taok3	127	142	164	200	279
Tap1	111	141	178	122	165
Tap2	460	0	1	1	1
Tapbp	5	0	0	0	0
Tapbpl	6	19	0	23	90
Tapt1	38	71	41	27	25
Tarbp2	11	22	0	1	59
Tardbp	138	144	204	150	126
Tars	93	94	146	82	132
Tars2	24	11	1	0	25
Tarsl2	0	0	0	0	3
Tas1r3	17	9	5	20	16
Tas2r126	3	0	4	1	0
Tasp1	17	29	40	28	17
Tatdn1	10	9	17	28	37
Tatdn2	6	9	5	13	14
Tatdn3	20	27	34	20	33
Tax1bp1	211	175	272	239	291
Tax1bp3	0	0	0	0	84
Taz	21	0	43	87	82
Tbc1d1	57	10	43	43	62
Tbc1d10a	258	185	133	162	203
Tbc1d10b	23	42	42	28	22
Tbc1d10c	5	15	11	7	16
Tbc1d12	3	4	0	25	0
Tbc1d13	1	1	0	0	0
Tbc1d14	467	293	376	445	529
Tbc1d15	151	63	84	121	136
Tbc1d16	178	167	170	243	249
Tbc1d17	0	0	12	0	0
Tbc1d19	44	38	58	46	53
Tbc1d2	8	1	0	0	0
Tbc1d20	0	0	123	0	155

For Review Only

Tbc1d22a	335	241	350	352	314
Tbc1d22b	8	8	8	5	14
Tbc1d22bos	0	0	0	1	0
Tbc1d23	94	69	85	98	134
Tbc1d24	71	61	68	84	112
Tbc1d25	37	15	52	35	35
Tbc1d2b	93	101	114	105	111
Tbc1d30	0	0	0	0	3
Tbc1d31	45	78	82	108	100
Tbc1d32	11	19	27	50	31
Tbc1d4	37	28	30	37	46
Tbc1d5	340	231	304	395	336
Tbc1d7	0	30	11	16	2
Tbc1d8	176	76	126	128	136
Tbc1d8b	9	2	11	8	7
Tbc1d9	159	133	111	218	240
Tbc1d9b	379	244	295	317	406
Tbca	60	53	66	49	51
Tbcb	53	0	5	1	1
Tbcc	71	47	73	60	43
Tbccd1	99	34	103	83	123
Tbcd	17	6	9	54	69
Tbce	65	48	59	49	77
Tbcel	22	6	23	15	19
Tbck	224	143	184	280	257
Tbk1	183	171	113	233	186
Tbkbp1	45	41	35	62	63
Tbl1x	170	96	144	179	215
Tbl1xr1	119	213	144	221	251
Tbl2	94	29	121	141	0
Tbl3	0	53	47	13	18
Tbp	70	99	110	78	99
Tbpl1	143	84	255	100	209
Tbrg1	44	40	103	71	73
Tbrg3	134	121	42	117	137
Tbrg4	49	41	60	39	56
Tbx18	0	0	0	0	0
Tbx19	2	0	0	7	6
Tbx6	1	9	14	0	12
Tbxas1	747	146	653	431	354
Tc2n	4	0	0	5	0
Tcaf1	43	20	10	22	42
Tcaim	34	21	18	33	24
Tcap	2	0	0	0	0
Tcea1	60	60	88	62	79
Tcea2	55	38	25	35	62
Tceal1	41	40	43	25	38
Tceal8	27	28	33	11	27
Tceanc	48	38	21	63	77
Tceanc2	56	86	90	47	68

For Review Only

Tceb1	125	134	152	97	98
Tceb2	0	0	38	1	1
Tceb3	425	220	202	293	277
Tcerg1	79	115	53	87	90
Tcf12	30	49	32	56	39
Tcf19	3	5	0	0	7
Tcf20	153	189	146	183	206
Tcf24	0	0	0	0	1
Tcf25	578	343	439	479	675
Tcf3	110	27	27	41	42
Tcf4	333	387	352	381	495
Tcf7l2	209	129	189	191	210
Tchp	1	1	0	0	188
Tcirg1	483	239	0	0	0
Tcn2	1312	771	1401	930	1223
Tcof1	124	55	94	112	114
Tcp1	175	0	190	59	448
Tcp11	0	5	5	3	5
Tcp11l1	29	39	40	56	66
Tcp11l2	77	76	78	74	81
Tcstv1	0	4	0	0	2
Tcta	0	0	0	0	0
Tcte3	0	1	1	1	2
Tctex1d2	18	16	6	11	32
Tctex1d4	7	3	0	0	12
Tctn1	38	12	13	32	28
Tctn2	0	0	4	3	1
Tctn3	7	25	40	19	40
Tdg	18	21	26	19	14
Tdg-ps	7	7	7	5	4
Tdo2	0	1	0	0	4
Tdp1	37	41	71	60	49
Tdp2	10	34	20	16	33
Tdrd3	21	26	32	65	32
Tdrd5	0	4	0	3	0
Tdrd7	38	87	53	67	81
Tdrkh	27	29	50	56	33
Tdrp	1	0	0	1	0
Tead1	2	0	0	0	0
Tead2	16	17	14	23	13
Tead3	0	0	0	0	7
Tec	126	176	135	106	128
Tecpr1	96	85	92	112	166
Tecpr2	60	42	33	58	71
Tecr	274	206	320	248	264
Tef	143	98	170	127	128
Tefm	1	1	0	0	0
Tek	0	0	0	0	0
Tekt2	0	0	0	0	0
Telo2	43	56	73	44	74

For Review Only

Ten1	59	48	56	42	57
Tenm2	3	3	6	4	0
Tenm4	6	1	0	3	6
Tep1	66	91	27	65	100
Tepp	0	0	4	0	3
Terf1	40	37	55	34	35
Terf2	27	23	45	18	29
Terf2ip	71	52	76	87	109
Tes	52	65	91	56	69
Tesk1	29	47	0	43	36
Tesk2	32	31	22	42	33
Tet1	18	44	53	56	80
Tet2	169	314	276	399	294
Tet3	223	280	221	359	324
Tex10	104	90	123	81	130
Tex12	0	0	0	0	0
Tex14	3	1	1	5	4
Tex15	4	1	0	0	0
Tex2	55	20	62	27	57
Tex261	461	304	368	137	255
Tex264	6	86	27	106	62
Tex30	2	7	0	0	24
Tex9	1	4	10	16	5
Tfam	22	35	46	52	34
Tfap4	6	10	9	11	10
Tfb1m	13	13	12	10	34
Tfb2m	30	25	47	25	24
Tfcp2	36	49	43	47	47
Tfdp1	15	13	27	21	29
Tfdp2	19	23	22	27	16
Tfe3	452	317	332	77	322
Tfeb	36	15	56	49	48
Tfec	0	1	0	0	0
Tfg	172	182	205	167	185
Tfip11	113	93	124	86	131
Tfpi	77	57	84	68	82
Tfpt	11	5	10	11	10
Tfrc	26	8	19	29	0
Tg	0	0	0	0	0
Tgds	39	69	50	30	14
Tgfa	352	125	0	869	463
Tgfb1	1	1	0	0	0
Tgfb1i1	2	6	11	0	0
Tgfb2	14	0	0	0	0
Tgfbi	197	173	215	133	254
Tgfbr1	2003	1082	1409	2234	2239
Tgfbr2	1306	1200	1114	1430	1602
Tgfbr3	6	0	0	5	0
Tgfbr3l	0	0	4	0	3
Tgfbrap1	103	125	100	161	149

For Review Only

Tgif1	160	141	140	128	147
Tgif2	50	38	37	46	55
Tgm1	2	0	0	2	0
Tgm2	390	240	373	332	364
Tgm4	0	0	0	0	0
Tgoln1	468	299	353	433	433
Tgs1	118	299	207	238	227
Tgtp1	8	1	0	6	11
Tgtp2	3	2	0	6	11
Tha1	1	3	79	0	182
Thada	127	116	128	170	167
Thap1	20	10	26	21	24
Thap11	38	54	45	32	53
Thap2	15	16	33	34	21
Thap3	67	46	61	30	35
Thap4	22	31	27	37	44
Thap6	6	21	17	11	7
Thap7	31	55	65	33	37
Thbd	0	0	0	1	0
Thbs1	17	5	0	24	38
Thbs2	5	4	17	0	9
Them4	13	14	8	18	18
Them6	0	3	0	3	4
Themis2	334	316	402	338	360
Thg1l	13	16	20	6	20
Thnsl1	25	7	18	34	46
Thnsl2	12	8	18	7	22
Thoc1	106	57	89	50	69
Thoc2	141	122	189	237	182
Thoc3	0	0	12	2	0
Thoc5	58	73	67	57	51
Thoc6	1	1	0	0	0
Thoc7	44	40	50	41	58
Thop1	28	15	16	26	26
Thra	12	7	9	13	10
Thrap3	118	129	66	173	197
Thrb	1	4	12	6	4
Thrsp	185	246	264	190	216
Thsd1	14	5	8	12	25
Thsd4	3	2	0	0	0
Thsd7b	2	1	6	1	0
Thtpa	52	52	50	85	46
Thumpd1	89	65	144	81	114
Thumpd2	43	51	78	39	43
Thumpd3	92	146	170	139	149
Thyn1	5	0	0	0	0
Tia1	170	190	154	189	208
Tial1	98	43	74	59	54
Tiam1	11	5	0	42	0
Tiam2	1	2	4	3	0

For Review Only

Ticam1	95	110	128	120	111
Ticam2	132	159	194	76	217
Tie1	0	0	0	0	0
Tifa	82	322	323	222	42
Tifab	140	0	0	829	695
Tigar	8	3	6	7	12
Tigd2	0	0	6	8	3
Tigd3	4	4	5	7	0
Tigd5	13	1	0	0	0
Timeless	0	7	0	0	0
Timm10	28	54	52	31	50
Timm10b	0	54	58	4	0
Timm13	0	0	0	0	34
Timm17a	39	52	57	31	44
Timm17b	46	43	23	25	5
Timm21	40	22	33	6	61
Timm22	13	35	55	29	29
Timm23	22	29	28	24	21
Timm44	41	0	55	0	0
Timm50	80	52	143	59	89
Timm8a1	13	5	4	5	4
Timm8b	39	46	38	36	37
Timm9	29	12	20	13	24
Timmdc1	40	31	35	29	27
Timp2	397	313	391	308	369
Timp3	2	0	0	0	38
Tinagl1	10	0	0	1	0
Tinf2	6	14	112	36	92
Tiparp	26	36	40	58	28
Tipin	33	18	18	23	52
Tiprl	135	129	201	116	120
Tirap	54	103	108	84	81
Tjap1	81	61	62	59	40
Tjp1	158	232	204	340	337
Tjp2	43	35	26	51	51
Tk1	3	7	7	0	11
Tk2	428	21	45	88	74
Tkfc	1	3	0	0	35
Tkt	0	0	44	1	7
Tlcd1	3	0	2	0	0
Tlcd2	12	5	14	14	13
Tldc1	25	12	22	25	20
Tldc2	8	11	11	20	16
Tle1	22	21	5	8	6
Tle2	39	15	20	12	22
Tle3	141	99	119	153	155
Tle4	58	69	41	74	67
Tlk1	51	61	73	51	65
Tlk2	32	12	44	35	43
Tll2	4	0	0	0	6

For Review Only

Tln1	699	554	454	807	683
Tln2	341	193	329	419	416
Tlr1	200	0	0	0	0
Tlr11	2	4	0	0	0
Tlr12	166	123	135	183	226
Tlr13	700	482	782	651	731
Tlr2	473	0	2	1	1
Tlr3	245	141	129	183	215
Tlr4	211	282	276	353	304
Tlr5	90	19	42	164	155
Tlr6	216	146	189	179	139
Tlr7	419	476	449	448	547
Tlr8	4	3	10	0	0
Tlr9	343	0	0	0	189
Tm2d1	48	59	79	47	50
Tm2d2	201	205	313	218	240
Tm2d3	99	88	165	72	111
Tm4sf1	0	0	0	0	0
Tm6sf1	87	63	54	59	82
Tm7sf2	12	6	0	0	0
Tm7sf3	77	35	73	62	89
Tm9sf1	245	156	0	0	0
Tm9sf2	1038	745	1040	944	1084
Tm9sf3	128	187	0	0	0
Tm9sf4	150	172	245	171	188
Tma16	9	9	16	23	25
Tma7	31	23	49	24	30
Tmbim1	137	92	124	63	75
Tmbim4	35	12	0	221	226
Tmbim6	1903	1380	2661	710	1158
Tmc3	0	1	0	0	13
Tmc6	173	77	234	173	192
Tmc7	93	82	64	105	86
Tmc8	12	0	12	17	20
Tmcc1	49	47	60	54	54
Tmcc2	3	15	0	0	11
Tmcc3	204	139	199	293	396
Tmco1	197	232	299	195	200
Tmco3	123	79	72	78	70
Tmco4	61	42	32	36	40
Tmco6	31	4	0	0	43
Tmed1	1	3	0	1	0
Tmed10	433	395	351	416	454
Tmed2	284	206	274	234	281
Tmed3	408	382	494	300	415
Tmed4	194	112	257	147	144
Tmed5	271	273	372	327	327
Tmed7	171	175	133	115	131
Tmed8	29	28	0	0	0
Tmed9	230	248	107	0	83

Tmem100	170	145	202	129	166
Tmem101	22	7	25	0	25
Tmem104	500	243	382	501	327
Tmem106a	63	36	59	77	70
Tmem106b	168	147	185	200	177
Tmem106c	115	99	133	78	123
Tmem107	5	0	0	0	4
Tmem108	9	0	0	0	0
Tmem109	340	253	356	326	359
Tmem11	91	100	94	77	87
Tmem110	33	30	38	41	24
Tmem115	201	167	192	150	166
Tmem116	0	0	0	1	4
Tmem119	9125	7365	12248	8072	9512
Tmem120a	47	26	19	25	22
Tmem120b	21	6	14	15	15
Tmem123	58	83	109	89	160
Tmem126a	12	7	14	0	4
Tmem126b	29	25	47	16	26
Tmem127	0	0	329	3	96
Tmem128	92	69	143	103	107
Tmem129	96	14	17	9	46
Tmem131	107	86	90	103	91
Tmem132a	5	5	0	0	74
Tmem134	5	4	0	0	0
Tmem135	280	325	395	452	412
Tmem136	4	5	0	4	3
Tmem138	6	19	29	14	4
Tmem140	85	213	100	16	176
Tmem143	9	12	16	0	18
Tmem144	156	122	160	154	180
Tmem14a	0	0	0	0	6
Tmem14c	124	141	188	122	139
Tmem150a	14	7	0	25	0
Tmem150b	0	4	0	9	0
Tmem150c	0	0	0	1	0
Tmem151b	6	0	0	0	0
Tmem154	15	18	22	7	10
Tmem156	11	7	18	11	25
Tmem159	73	55	92	66	74
Tmem160	12	27	14	25	26
Tmem161a	106	92	8	30	142
Tmem161b	41	55	37	48	41
Tmem163	0	7	5	0	0
Tmem164	107	115	128	121	109
Tmem165	16	33	33	20	20
Tmem167	84	94	96	110	148
Tmem167b	105	42	80	76	73
Tmem168	223	145	172	266	235
Tmem17	11	7	8	12	9

For Review Only

Tmem170	28	5	28	12	5
Tmem170b	854	879	617	1041	997
Tmem173	1058	1064	1611	1269	1324
Tmem175	332	180	267	93	217
Tmem176a	0	0	27	1	1
Tmem176b	248	0	23	3	5
Tmem177	60	70	46	46	79
Tmem178b	2	1	0	0	0
Tmem179b	152	61	41	0	62
Tmem18	41	22	67	92	53
Tmem180	32	29	31	24	30
Tmem181a	105	78	124	114	119
Tmem181c-ps	44	54	27	46	52
Tmem183a	71	93	72	66	60
Tmem184b	369	127	91	253	444
Tmem184c	59	55	65	53	59
Tmem185b	257	162	191	167	215
Tmem186	1	1	0	0	0
Tmem189	27	31	40	24	19
Tmem19	93	87	131	95	149
Tmem191c	8	0	8	7	3
Tmem192	24	29	22	23	31
Tmem194	2	16	18	30	15
Tmem194b	41	29	66	26	23
Tmem198b	89	46	82	79	58
Tmem199	14	18	0	0	0
Tmem2	19	13	22	24	28
Tmem201	86	54	58	92	93
Tmem202	4	0	0	2	3
Tmem203	35	27	57	56	46
Tmem204	158	140	155	122	231
Tmem205	6	12	10	0	9
Tmem206	410	460	507	474	658
Tmem208	18	10	17	25	31
Tmem209	147	70	134	119	160
Tmem214	178	0	0	110	0
Tmem216	15	6	22	13	5
Tmem218	16	11	9	21	17
Tmem220	23	9	12	16	7
Tmem221	22	14	24	11	15
Tmem222	121	134	274	35	41
Tmem223	0	0	0	0	9
Tmem229a	0	1	0	3	0
Tmem229b	157	144	218	172	152
Tmem230	0	0	0	0	0
Tmem231	8	3	15	14	7
Tmem234	0	0	0	2	0
Tmem237	45	27	38	21	0
Tmem238	0	0	4	0	1
Tmem240	0	3	0	0	0

For Review Only

Tmem241	122	57	78	110	91
Tmem242	16	25	18	16	24
Tmem243	8	8	9	9	9
Tmem245	25	56	39	74	105
Tmem246	2	0	0	0	0
Tmem248	50	85	48	123	98
Tmem25	0	3	0	0	0
Tmem251	1	2	0	0	0
Tmem252	0	0	0	0	0
Tmem253	6	0	0	7	2
Tmem254a	12	7	13	10	13
Tmem254b	20	14	24	19	25
Tmem254c	21	14	24	19	25
Tmem255b	4	0	0	0	0
Tmem256	0	0	0	0	0
Tmem258	64	41	107	0	33
Tmem259	9	5	0	0	0
Tmem26	1	0	1	0	0
Tmem260	86	51	34	60	50
Tmem261	0	0	46	0	0
Tmem263	50	51	60	39	58
Tmem265	5	7	0	0	0
Tmem29	15	15	28	14	13
Tmem30a	227	253	172	357	322
Tmem33	178	234	302	262	241
Tmem37	176	177	222	180	175
Tmem38a	12	0	6	14	5
Tmem38b	26	14	15	34	17
Tmem39a	92	62	86	100	109
Tmem39b	13	28	22	37	25
Tmem40	8	0	4	1	0
Tmem41a	28	28	33	25	23
Tmem41b	55	63	70	64	86
Tmem42	21	24	38	30	24
Tmem43	215	152	243	198	192
Tmem44	89	44	111	86	130
Tmem45a	0	1	0	0	0
Tmem47	9	0	0	0	0
Tmem5	51	48	70	48	45
Tmem50a	767	494	826	520	662
Tmem50b	543	404	521	461	496
Tmem51	1	0	0	0	0
Tmem53	4	20	0	4	0
Tmem55a	64	51	80	53	68
Tmem55b	0	16	2	2	1
Tmem57	11	47	33	32	26
Tmem59	882	827	1244	829	977
Tmem60	105	104	92	70	70
Tmem62	14	11	14	15	12
Tmem63a	812	492	886	557	769

For Review Only

Tmem63b	30	28	15	44	41
Tmem64	70	91	70	100	72
Tmem65	37	32	32	24	32
Tmem67	0	26	21	15	38
Tmem68	123	184	150	191	181
Tmem69	28	27	30	34	31
Tmem70	97	103	89	74	93
Tmem71	20	11	48	28	24
Tmem72	5	0	0	0	0
Tmem74b	17	10	5	22	14
Tmem79	5	14	1	9	9
Tmem8	9	9	0	0	12
Tmem80	0	0	0	0	0
Tmem82	0	1	0	0	0
Tmem86a	1269	0	980	2	19
Tmem86b	13	26	32	18	21
Tmem87a	12	19	22	29	28
Tmem87b	105	99	93	96	113
Tmem88	0	0	0	0	10
Tmem8b	26	17	12	16	16
Tmem8c	47	27	51	24	33
Tmem9	62	45	85	46	69
Tmem91	3	1	5	12	19
Tmem94	83	53	70	76	100
Tmem97	33	42	42	24	19
Tmem98	9	0	0	0	0
Tmem9b	275	206	325	208	295
Tmf1	97	97	78	119	92
Tmigd3	8	9	30	7	0
Tmlhe	9	24	29	16	17
Tmod1	15	0	0	13	15
Tmod3	124	141	143	138	189
Tmpo	52	87	43	55	61
Tmprss2	0	0	0	4	5
Tmsb10	13	10	0	0	0
Tmsb15b1	6	0	0	0	0
Tmsb15l	3	6	0	0	0
Tmsb4x	1327	1704	2660	1519	1720
Tmtc1	0	1	0	8	0
Tmtc2	0	0	0	0	0
Tmtc3	56	61	67	58	64
Tmtc4	14	18	19	41	71
Tmub1	39	16	34	23	33
Tmub2	215	180	164	170	224
Tmx1	191	144	207	165	184
Tmx2	222	147	67	60	179
Tmx3	126	115	126	167	116
Tmx4	187	187	154	206	156
Tnfaip1	86	59	158	120	43
Tnfaip2	9	21	31	20	14

For Review Only

Tnfaip3	155	119	151	178	135
Tnfaip8	193	165	293	168	215
Tnfaip8l1	0	0	0	0	0
Tnfaip8l2	253	293	467	292	321
Tnfrsf10b	0	0	0	0	3
Tnfrsf11a	121	112	130	141	178
Tnfrsf12a	18	5	5	10	19
Tnfrsf13b	313	277	465	433	370
Tnfrsf14	0	0	0	0	0
Tnfrsf17	36	49	48	35	34
Tnfrsf18	0	0	3	3	2
Tnfrsf19	1	0	0	0	1
Tnfrsf1a	393	368	437	521	426
Tnfrsf1b	1098	715	1078	946	1070
Tnfrsf21	601	398	608	702	832
Tnfrsf22	8	12	5	14	17
Tnfrsf23	21	21	13	17	46
Tnfrsf26	4	6	0	0	11
Tnfrsf4	0	0	0	0	0
Tnfsf10	11	5	12	27	8
Tnfsf12	15	0	8	8	8
Tnfsf13	0	0	0	0	0
Tnfsf13b	23	4	0	42	69
Tnfsf13os	1	0	0	0	0
Tnfsf14	2	0	4	0	0
Tnfsf18	0	0	0	0	1
Tnfsf8	12	8	18	18	6
Tnfsf9	1	2	16	5	0
Tnfsfm13	11	13	0	0	0
Tnik	1	2	0	0	9
Tnip1	41	35	51	37	53
Tnk2	4	0	0	3	0
Tnks	34	58	76	45	83
Tnks1bp1	13	14	15	14	20
Tnks2	54	147	107	107	140
Tnni2	0	3	0	0	0
Tnni3	7	1	4	0	0
Tnpo1	177	300	191	342	363
Tnpo2	86	45	38	109	68
Tnpo3	135	134	209	172	234
Tnr	0	0	0	2	4
Tnrc18	101	96	92	239	212
Tnrc6a	153	137	143	156	144
Tnrc6b	190	238	189	204	217
Tnrc6c	89	121	105	109	117
Tns1	145	184	223	317	436
Tns2	0	2	0	0	0
Tns3	814	510	428	768	736
Tns4	7	22	13	7	10
Tnxb	0	0	0	0	0

For Review Only

Tob1	7	4	9	2	5
Tob2	156	0	1	149	111
Toe1	21	0	5	1	1
Tollip	208	100	222	185	139
Tom1	61	10	75	0	71
Tom1l1	62	59	81	48	80
Tom1l2	143	72	103	116	103
Tomm20	136	85	114	132	106
Tomm22	112	136	174	111	104
Tomm34	270	233	294	237	348
Tomm40	107	49	105	59	0
Tomm40l	38	37	46	19	1
Tomm5	21	29	43	22	20
Tomm6	0	0	2	0	19
Tomm6os	0	1	1	6	0
Tomm7	60	50	97	38	23
Tomm70a	55	35	28	53	64
Tomt	5	0	0	0	0
Tonsl	6	40	12	11	34
Top1	76	105	98	74	76
Top1mt	0	0	0	3	1
Top2a	0	0	0	8	8
Top2b	63	70	44	73	87
Top3a	60	51	46	106	106
Top3b	109	104	155	89	92
Topbp1	71	19	26	0	35
Topors	181	152	141	127	146
Toporsos	0	7	0	0	0
Tor1a	123	107	161	117	166
Tor1aip1	174	299	304	288	332
Tor1aip2	100	131	104	144	125
Tor1b	146	105	248	194	253
Tor2a	33	70	196	2	24
Tor3a	204	218	302	195	234
Tor4a	538	0	10	2	690
Tox4	374	168	169	233	349
Tpbgl	102	114	104	117	149
Tpcn1	123	146	154	175	169
Tpcn2	85	45	41	93	58
Tpd52	142	114	144	139	161
Tpd52l1	5	0	0	0	15
Tpd52l2	84	204	134	70	110
Tpgs1	59	62	101	69	79
Tpgs2	13	29	28	26	12
Tpi1	219	70	175	1	3
Tpk1	48	15	19	23	15
Tpm1	38	53	45	45	31
Tpm2	1	0	0	0	0
Tpm3	361	595	278	687	515
Tpm4	41	48	63	62	47

For Review Only

Tpmt	9	0	15	10	11
Tpp1	815	760	904	656	766
Tpp2	238	150	167	298	282
Tppp	241	177	218	266	258
Tppp3	2	6	0	3	0
Tpr	77	97	115	117	97
Tpra1	144	154	162	165	176
Tprgl	1	49	202	0	0
Tprkb	29	32	39	18	27
Tprn	60	48	51	48	69
Tpst1	16	0	5	9	3
Tpst2	955	740	1461	539	792
Tpx2	0	0	0	4	0
Tra2a	142	86	137	95	147
Tra2b	42	114	268	238	251
Trabd	47	57	25	0	1
Trabd2b	3	0	0	3	2
Traf1	6	0	0	5	4
Traf2	160	98	110	124	125
Traf3	130	51	67	73	124
Traf3ip1	12	9	6	8	6
Traf3ip2	0	0	0	5	7
Traf3ip3	98	116	90	90	75
Traf4	9	5	9	5	17
Traf5	108	103	149	84	125
Traf6	102	77	64	122	92
Traf7	20	32	66	170	160
Trafd1	0	0	0	0	0
Traip	0	1	6	4	0
Trak1	154	118	139	157	112
Trak2	27	33	25	42	34
Tram1	288	277	295	362	383
Tram2	19	16	0	26	24
Trank1	4	1	3	0	0
Trap1	49	88	80	60	123
Trappc10	44	49	38	78	53
Trappc11	141	138	87	135	96
Trappc12	223	131	221	211	290
Trappc13	102	82	94	90	84
Trappc2	0	10	0	0	0
Trappc2l	0	36	1	12	32
Trappc3	74	86	114	67	102
Trappc4	89	126	112	72	93
Trappc5	46	46	44	47	79
Trappc6a	30	0	0	0	0
Trappc6b	59	42	43	54	59
Trappc8	45	60	50	45	101
Trappc9	169	119	98	188	201
Trdmt1	10	13	21	20	13
Trem1	5	0	0	0	0

For Review Only

Trem2	3007	0	2620	0	724
Trem3	6	0	0	0	4
Treml1	8	7	12	11	10
Treml2	11	37	26	26	39
Trerf1	0	0	0	5	0
Trex1	0	6	41	0	1
Trf	4579	3264	5610	3738	4411
Trhde	0	0	6	0	0
Triap1	44	59	67	48	83
Trib1	152	137	120	176	183
Trib3	0	0	0	0	0
Tril	0	0	1	0	0
Trim11	36	90	150	156	55
Trim12a	11	0	15	0	0
Trim12c	58	39	37	74	58
Trim13	33	16	28	22	44
Trim14	2	12	0	0	0
Trim15	7	15	6	4	8
Trim16	5	17	5	8	15
Trim17	11	31	0	8	0
Trim2	99	82	73	116	114
Trim21	7	4	8	11	8
Trim23	49	54	56	78	67
Trim24	31	38	19	26	45
Trim25	51	95	37	73	31
Trim26	181	151	265	248	217
Trim27	72	79	48	74	116
Trim28	88	62	0	40	37
Trim3	37	47	34	54	45
Trim30a	99	64	4	36	110
Trim30b	1	4	0	0	6
Trim30c	14	2	14	25	12
Trim30d	58	195	123	127	145
Trim32	58	58	79	75	93
Trim33	25	44	47	43	55
Trim34a	60	80	109	87	85
Trim35	153	103	144	157	199
Trim36	96	100	89	156	142
Trim37	17	15	19	31	33
Trim39	10	13	36	17	0
Trim41	61	75	67	43	77
Trim43c	0	0	0	7	0
Trim44	152	187	120	208	201
Trim45	17	4	6	8	14
Trim47	1	1	0	0	0
Trim5	65	40	64	74	69
Trim56	11	24	26	37	28
Trim59	2	0	0	6	1
Trim62	5	6	0	10	14
Trim65	0	0	1	36	48

For Review Only

Trim66	1	0	5	2	0
Trim67	4	0	2	3	6
Trim68	38	18	28	36	23
Trim7	12	0	3	4	0
Trim75	0	0	0	0	0
Trim8	38	41	81	75	101
Trio	190	207	189	339	286
Triobp	27	15	37	25	28
Trip10	13	18	28	10	1
Trip11	60	108	77	138	114
Trip12	131	133	124	164	159
Trip13	0	0	0	0	0
Trip4	47	35	69	87	69
Trip6	1	6	12	0	6
Triqk	0	3	0	0	3
Trit1	63	47	88	61	61
Trmo	29	22	25	41	38
Trmt1	41	88	120	0	9
Trmt10a	36	28	52	19	31
Trmt10b	15	23	22	20	18
Trmt10c	55	57	86	41	50
Trmt11	20	25	26	20	20
Trmt12	99	50	80	76	112
Trmt13	32	18	43	32	38
Trmt1l	149	124	185	136	176
Trmt2a	1	1	0	0	0
Trmt2b	27	88	49	65	68
Trmt44	24	23	23	41	40
Trmt5	22	26	32	20	32
Trmt6	57	61	77	47	43
Trmt61a	36	33	26	28	50
Trmt61b	16	3	19	0	12
Trmu	105	0	1	6	1
Trnau1ap	100	38	48	47	0
Trnt1	72	30	60	50	74
Tro	0	1	0	0	0
Troap	1	1	0	0	1
Trove2	39	60	71	68	37
Trp53	108	97	210	132	201
Trp53bp1	96	192	112	192	183
Trp53bp2	79	54	80	79	99
Trp53cor1	29	17	14	33	44
Trp53i11	80	73	90	89	127
Trp53i13	5	0	54	48	41
Trp53inp1	14	64	25	53	66
Trp53inp2	82	46	56	53	87
Trp53rka	32	36	54	28	41
Trp53rkb	11	15	16	11	19
Trp53tg5	4	0	0	4	0
Trpc4ap	38	15	0	365	0

For Review Only

Trpm2	180	179	188	237	240
Trpm3	16	1	4	1	10
Trpm4	104	53	77	101	74
Trpm5	3	0	0	4	0
Trpm7	173	272	144	277	330
Trps1	1	3	6	13	0
Trpt1	10	0	1	2	4
Trpv2	204	70	291	129	401
Trpv4	41	9	13	17	25
Trrap	245	191	209	278	246
Trub1	69	19	40	54	60
Trub2	51	47	56	58	45
Tsacc	7	0	10	6	0
Tsc1	104	155	0	97	102
Tsc2	313	168	276	281	366
Tsc22d1	25	23	22	53	36
Tsc22d2	83	86	99	81	111
Tsc22d3	4	69	1	10	72
Tsc22d4	23	30	34	33	51
Tsen15	11	27	49	16	25
Tsen2	10	12	47	31	50
Tsen34	73	54	32	26	37
Tsen54	25	19	38	10	23
Tsfm	15	31	27	20	22
Tsg101	177	153	222	172	160
Tsga10	22	15	12	22	38
Tshb	0	0	0	3	0
Tshz1	13	26	30	13	26
Tshz2	0	0	0	0	0
Tslp	1	7	11	12	0
Tsn	159	158	224	131	192
Tsnax	125	95	143	110	155
Tspan11	0	0	0	0	0
Tspan13	365	308	393	303	365
Tspan14	1090	832	1183	954	1151
Tspan17	0	0	2	3	0
Tspan18	165	139	148	173	66
Tspan3	424	335	440	359	322
Tspan31	0	0	0	0	0
Tspan32	21	34	61	19	40
Tspan33	28	22	18	20	0
Tspan4	208	195	0	456	0
Tspan5	4	17	10	14	7
Tspan7	317	302	392	329	371
Tspan9	131	60	143	131	79
Tspear	5	0	0	0	0
Tspo	106	109	106	78	110
Tspyl1	282	300	238	223	258
Tspyl2	51	41	50	35	64
Tspyl3	52	31	44	52	36

For Review Only

Tspyl4	10	0	3	14	0
Tsr1	90	16	26	67	61
Tsr2	12	17	23	18	32
Tsr3	18	1	0	0	0
Tssc1	0	0	48	27	30
Tssc4	0	0	0	0	0
Tssk4	0	1	0	0	6
Tssk6	9	4	7	4	1
Tst	3	0	1	2	6
Tsta3	12	10	0	0	23
Tstd2	124	66	84	135	116
Tstd3	1	3	0	0	40
Ttbk1	0	3	0	6	0
Ttbk2	37	46	41	56	58
Ttc1	81	83	93	62	88
Ttc12	25	0	24	10	0
Ttc13	23	60	116	109	56
Ttc14	147	118	118	163	181
Ttc17	137	97	88	99	145
Ttc19	10	7	9	8	14
Ttc21b	9	11	33	39	40
Ttc23	4	0	1	4	6
Ttc25	0	5	0	4	5
Ttc26	6	0	0	0	0
Ttc27	37	23	42	20	8
Ttc28	197	96	154	238	171
Ttc3	108	101	126	144	101
Ttc30a1	29	19	41	32	27
Ttc30a2	11	8	14	14	18
Ttc30b	14	15	25	24	24
Ttc32	21	15	30	14	21
Ttc33	67	35	55	43	29
Ttc37	26	34	31	35	36
Ttc38	28	25	26	24	28
Ttc39a	0	0	2	5	7
Ttc39aos1	0	1	0	0	0
Ttc39b	91	130	121	157	132
Ttc4	134	114	223	169	164
Ttc41	18	7	12	17	6
Ttc5	132	111	134	84	124
Ttc7	140	161	126	201	208
Ttc7b	0	10	23	19	13
Ttc8	32	20	45	27	22
Ttc9	6	16	24	12	16
Ttc9c	0	10	0	0	73
Ttf1	76	89	114	81	69
Ttf2	13	1	31	47	47
Tti1	112	94	114	197	178
Tti2	58	16	21	22	62
Ttl	21	13	26	20	6

For Review Only

Ttll1	33	23	47	12	22
Ttll12	31	16	27	26	37
Ttll3	27	33	26	48	60
Ttll4	41	67	83	100	81
Ttll5	16	12	0	30	0
Ttll8	3	0	0	0	0
Ttll9	0	1	0	0	5
Ttn	13	0	8	12	27
Ttpal	1629	542	260	293	438
Ttr	5118	189	31	966	843
Ttyh1	7	6	6	0	15
Ttyh2	23	20	22	40	31
Ttyh3	77	61	65	135	81
Tub	1	1	0	4	3
Tuba1a	696	629	803	521	659
Tuba1b	673	506	816	528	648
Tuba1c	202	218	262	196	212
Tuba4a	19	5	9	6	7
Tubb2a	289	311	393	342	292
Tubb2a-ps2	4	7	4	5	4
Tubb2b	90	116	127	125	109
Tubb4a	6	3	0	0	6
Tubb4b	183	94	116	87	100
Tubb5	2	300	0	1847	0
Tubb6	27	22	46	27	34
Tubd1	12	5	9	0	21
Tube1	5	0	8	2	0
Tubg1	47	22	56	29	0
Tubg2	0	0	1	0	0
Tubgcp2	115	84	67	134	91
Tubgcp3	109	78	100	121	134
Tubgcp4	55	20	15	67	62
Tubgcp5	391	393	315	501	401
Tubgcp6	0	0	1	2	1
Tufm	7	0	47	0	0
Tuft1	10	6	1	13	13
Tug1	516	0	108	272	8
Tulp2	9	0	1	3	6
Tulp3	9	8	9	7	10
Tulp4	68	105	70	99	118
Tusc2	10	16	0	0	9
Tusc3	50	67	60	68	65
Tut1	131	66	136	87	94
Tvp23a	27	12	10	28	16
Tvp23b	117	135	158	87	112
Twf1	285	293	306	297	284
Twf2	0	0	0	0	157
Twistnb	80	36	114	93	0
Twsg1	20	6	17	13	33
Txlna	292	178	269	281	396

For Review Only

Txlnb	0	0	0	0	5
Txlng	35	50	61	63	50
Txn1	37	40	57	32	36
Txn2	137	92	151	80	106
Txndc11	56	52	2	42	73
Txndc12	79	68	91	62	72
Txndc15	249	212	345	215	229
Txndc16	47	106	87	74	64
Txndc17	98	87	120	44	58
Txndc2	4	1	0	1	0
Txndc5	80	84	95	84	100
Txndc9	126	137	101	100	123
Txnip	113	67	0	60	356
Txnl1	145	89	163	106	103
Txnl4a	1	1	0	0	0
Txnl4b	46	42	37	48	58
Txnrd1	84	49	39	38	55
Txnrd2	166	175	178	135	116
Txnrd3	0	6	5	4	0
Tyk2	150	88	125	271	310
Tymp	0	0	3	0	0
Tyms	38	20	43	46	54
Tyms-ps	2	5	5	1	0
Tyrobp	2275	2768	3273	2037	2400
Tysnd1	29	31	49	40	18
Tyw1	128	37	99	80	110
Tyw3	18	27	43	29	15
Tyw5	19	18	26	20	31
U2af1	16	28	46	29	30
U2af2	25	38	25	24	0
U2surp	206	195	204	207	247
Uaca	0	0	0	0	0
Uap1	52	52	46	65	66
Uap1l1	99	9	75	0	97
Uba1	387	255	365	391	543
Uba2	202	159	345	144	138
Uba3	47	13	108	92	96
Uba5	74	73	37	59	67
Uba52	104	74	0	0	0
Uba6	4	13	13	17	39
Ubac1	44	23	30	24	29
Ubac2	29	28	0	0	346
Ubald1	45	36	58	68	72
Ubald2	5	18	4	9	9
Ubap1	216	210	257	178	254
Ubap1l	0	3	0	1	0
Ubap2	69	70	50	43	40
Ubap2l	164	219	294	413	359
Ubash3b	513	429	465	481	648
Ubb	539	57	0	0	0

For Review Only

Ubc	0	0	0	0	20
Ube2a	88	97	105	72	123
Ube2b	160	129	160	152	149
Ube2c	0	0	0	0	0
Ube2cbp	0	0	0	0	0
Ube2d-ps	27	0	0	0	0
Ube2d1	26	29	25	33	50
Ube2d2a	109	0	7	50	43
Ube2d2b	11	6	4	6	9
Ube2d3	192	212	195	247	225
Ube2e1	80	95	84	86	87
Ube2e2	2	0	6	0	0
Ube2e3	32	0	47	15	240
Ube2f	130	124	149	142	146
Ube2g1	114	70	89	57	70
Ube2g2	0	0	1	1	1
Ube2h	494	399	509	389	514
Ube2i	194	167	234	185	201
Ube2j1	431	248	269	317	431
Ube2j2	28	34	28	23	29
Ube2k	58	64	102	72	66
Ube2l3	128	120	159	100	140
Ube2l6	32	24	47	30	31
Ube2m	20	0	1	1	21
Ube2n	0	0	34	0	21
Ube2o	24	27	25	14	29
Ube2q1	34	230	84	0	0
Ube2q2	39	61	33	57	26
Ube2r2	66	40	62	40	57
Ube2s	6	5	0	5	4
Ube2v1	113	75	134	83	87
Ube2v2	22	28	47	37	37
Ube2w	88	82	88	92	83
Ube2z	54	56	87	56	47
Ube3a	85	152	82	137	172
Ube3b	106	117	115	137	174
Ube3c	65	41	77	55	68
Ube4a	162	148	162	204	276
Ube4b	124	71	63	104	101
Ube4bos3	3	3	0	4	3
Ubfd1	69	101	105	85	116
Ubiad1	37	53	64	40	97
Ubl3	195	157	154	212	289
Ubl4a	0	0	0	0	10
Ubl5	0	0	2	0	0
Ubl7	60	81	55	77	64
Ublcp1	111	73	98	69	83
Ubn1	125	134	123	133	180
Ubn2	216	218	234	233	247
Ubox5	71	49	68	60	68

For Review Only

Ubp1	105	172	170	98	136
Ubqln1	113	63	82	125	125
Ubqln2	9	20	11	20	24
Ubqln4	36	28	18	33	33
Ubr1	147	118	107	170	198
Ubr2	127	135	108	187	196
Ubr3	73	67	71	107	92
Ubr4	276	297	312	454	489
Ubr5	66	102	117	129	104
Ubr7	77	79	65	82	80
Ubtd1	82	68	77	52	78
Ubtd2	17	4	8	18	18
Ubtf	138	139	120	126	137
Ubxn1	0	0	0	0	56
Ubxn11	11	2	0	6	6
Ubxn2a	26	20	13	22	27
Ubxn2b	46	45	36	49	55
Ubxn4	136	112	138	100	160
Ubxn6	101	101	210	1	1
Ubxn7	92	106	45	130	125
Ubxn8	71	53	81	73	59
Uchl3	0	44	19	0	0
Uchl4	19	23	28	26	17
Uchl5	62	77	51	60	50
Uck1	23	2	0	0	53
Uck2	0	1	6	1	0
Uckl1	3	9	13	40	49
Uckl1os	1	3	0	17	17
Ucp2	0	0	161	0	0
Uevld	19	15	9	19	21
Ufc1	0	0	1	1	1
Ufd1l	0	0	1	16	43
Ufl1	0	137	23	150	105
Ufm1	30	52	24	38	19
Ufsp1	23	18	21	14	17
Ufsp2	108	114	85	79	72
Ugcg	24	21	9	17	16
Ugdh	27	19	16	21	14
Uggt1	210	151	185	210	215
Uggt2	41	20	41	37	34
Ugp2	74	50	47	65	64
Ugt1a10	73	0	0	0	0
Ugt1a2	0	0	0	0	10
Ugt1a5	3	0	5	14	5
Ugt1a6a	2	0	0	9	18
Ugt1a6b	13	0	0	7	10
Ugt1a7c	198	268	305	212	230
Uhmk1	29	20	10	14	23
Uhrf1	3	6	0	3	0
Uhrf1bp1	245	94	126	191	241

For Review Only

Uhrf1bp1l	31	8	62	38	88
Uhrf2	30	35	53	46	51
Uimc1	66	115	80	82	98
Ulbp1	4	8	7	15	5
Ulk1	69	14	67	38	44
Ulk2	190	218	240	224	223
Ulk3	36	95	167	139	90
Ulk4	0	0	1	6	32
Umad1	20	17	57	33	40
Umodl1	0	0	0	0	0
Umps	78	60	93	73	105
Unc119b	96	39	72	94	54
Unc13a	25	8	11	12	20
Unc13b	15	27	29	12	1
Unc13d	90	54	87	89	69
Unc45a	42	25	49	29	24
Unc50	98	246	137	158	144
Unc5a	0	7	1	5	1
Unc5cl	1	4	0	4	4
Unc5d	0	0	0	0	0
Unc79	12	0	0	1	1
Unc93b1	4998	3679	5819	4163	4929
Ung	6	3	0	1	4
Unk	202	136	189	190	261
Unkl	114	32	19	83	110
Upf1	97	55	77	92	59
Upf2	23	46	46	45	40
Upf3a	18	25	14	13	25
Upf3b	23	42	45	36	36
Upk1b	365	373	549	319	412
Uprt	6	5	5	9	17
Uqcc1	97	68	126	96	90
Uqcc2	0	0	0	14	0
Uqcc3	0	0	62	36	0
Uqcr10	1	1	0	0	0
Uqcr11	0	0	0	0	63
Uqcrb	31	4	134	0	0
Uqcrc1	254	216	368	118	211
Uqcrc2	173	138	246	51	57
Uqcrfs1	128	130	211	130	143
Uqcrh	120	81	152	91	114
Uqcrh-ps1	2	3	5	6	3
Uqcrq	99	62	114	68	85
Urb1	13	25	32	48	75
Urb2	23	18	25	32	48
Urgcp	272	160	271	197	242
Uri1	21	24	16	28	42
Urm1	10	3	6	9	4
Urod	0	0	111	0	3
Uros	57	74	97	42	63

Usb1	19	31	55	49	48
Use1	77	29	73	9	43
Usf1	0	0	64	73	0
Usf2	0	0	14	1	1
Usf3	85	88	119	157	140
Ush2a	0	0	0	0	0
Ushbp1	0	0	0	0	0
Usmg5	0	0	0	0	0
Uso1	72	67	53	44	76
Usp1	10	11	8	20	6
Usp10	165	138	238	199	163
Usp11	63	33	77	65	74
Usp12	97	90	105	138	110
Usp14	118	92	164	96	129
Usp15	172	162	170	205	222
Usp16	84	78	131	72	94
Usp18	2	8	0	28	20
Usp19	16	5	0	0	296
Usp2	78	236	28	281	169
Usp20	34	18	26	54	31
Usp22	472	339	392	483	516
Usp24	142	242	243	271	307
Usp25	46	72	42	52	65
Usp27x	5	3	4	5	8
Usp28	34	42	59	59	55
Usp3	182	139	206	180	237
Usp30	23	44	36	19	27
Usp31	11	40	28	37	41
Usp32	49	60	76	36	49
Usp33	229	171	173	225	179
Usp34	94	155	155	148	155
Usp35	300	18	19	81	93
Usp36	7	54	46	0	69
Usp37	196	195	167	186	227
Usp38	124	58	0	81	0
Usp39	52	44	53	29	29
Usp4	353	92	404	288	127
Usp40	103	93	84	151	190
Usp42	53	64	39	61	72
Usp45	7	14	16	50	38
Usp46	45	13	18	26	22
Usp47	82	103	59	123	84
Usp48	262	194	228	285	321
Usp49	21	3	16	19	14
Usp5	151	89	138	141	154
Usp50	1	0	0	0	0
Usp53	2	0	0	0	0
Usp54	23	15	25	54	22
Usp6nl	37	51	49	54	44
Usp7	91	88	131	86	130

For Review Only

Usp8	320	260	312	441	394
Usp9x	56	299	109	206	179
Uspl1	82	73	138	102	126
Ust	14	8	15	12	16
Utp11l	40	45	38	20	44
Utp14a	88	57	85	86	127
Utp14b	0	0	0	6	5
Utp15	70	89	68	86	101
Utp18	9	0	10	24	18
Utp20	41	44	58	60	50
Utp23	25	39	25	22	34
Utp3	86	141	165	104	93
Utp6	29	28	55	52	34
Utrn	29	25	18	38	54
Uty	41	46	33	50	54
Uvrag	96	90	80	128	110
Uvssa	96	6	186	241	41
Uxs1	27	27	38	36	0
Uxt	0	0	0	0	0
Vac14	37	75	49	66	65
Vamp1	50	62	86	76	88
Vamp3	203	222	323	194	238
Vamp4	134	123	100	112	121
Vamp5	13	12	12	12	0
Vamp7	19	32	40	27	44
Vamp8	444	0	76	1	1
Vangl1	5	13	13	16	0
Vangl2	22	23	27	38	42
Vapa	148	147	101	166	190
Vapb	25	29	21	29	40
Vars	131	93	91	96	82
Vars2	0	0	17	38	17
Vash1	34	60	75	66	64
Vash2	4	0	0	4	4
Vasn	9	3	15	35	29
Vasp	52	33	0	0	0
Vat1	12	1	0	10	4
Vat1l	14	0	0	0	16
Vaultrc5	3	1	2	1	6
Vav1	1180	843	1259	1051	1217
Vav2	194	218	283	296	307
Vbp1	104	96	170	106	126
Vcam1	40	12	34	31	15
Vcan	3	3	13	0	0
Vcl	42	24	36	54	61
Vcp	285	204	263	241	247
Vcpip1	133	144	127	222	191
Vcpkmt	15	5	29	20	0
Vdac1	186	99	112	156	163
Vdac2	1	16	0	0	1040

For Review Only

Vdac3	89	93	118	69	90
Vegfb	16	5	0	1	0
Vezf1	75	56	46	79	103
Vezt	83	61	60	80	58
Vgll4	57	38	23	75	23
Vhl	61	156	52	126	220
Vil1	0	0	0	0	0
Vill	10	6	0	0	2
Vim	28	11	23	25	0
Vimp	194	239	389	246	236
Vipas39	125	87	145	106	167
Vipr1	24	42	47	53	32
Vipr2	0	0	0	0	4
Vis1	82	85	77	120	127
Vkorc1	33	15	240	196	0
Vkorc1l1	20	35	51	25	42
Vldlr	6	0	0	0	0
Vma21	33	39	34	50	60
Vmac	28	42	21	29	29
Vmn1r4	3	0	6	0	0
Vmn2r2	0	1	5	0	0
Vmn2r29	15	3	10	5	14
Vmn2r57	0	0	0	3	3
Vmp1	190	181	193	170	173
Vopp1	10	4	11	13	0
Vprbp	10	18	45	75	78
Vps11	186	0	40	6	18
Vps13a	40	44	34	59	48
Vps13b	173	199	201	285	265
Vps13c	37	77	55	111	92
Vps13d	123	168	130	256	237
Vps16	95	23	0	0	139
Vps18	1	1	0	1249	0
Vps25	132	132	129	171	100
Vps26a	262	176	258	191	146
Vps26b	137	97	133	166	129
Vps28	0	0	0	0	0
Vps29	99	163	149	116	127
Vps33a	214	164	138	199	171
Vps33b	55	45	49	44	68
Vps35	413	210	372	391	470
Vps36	57	76	78	47	39
Vps37a	72	78	54	89	71
Vps37b	187	183	171	224	90
Vps37c	82	59	80	84	113
Vps39	271	251	201	392	426
Vps41	115	100	133	144	210
Vps45	15	52	48	40	37
Vps4a	488	60	258	164	216
Vps4b	265	213	381	255	303

For Review Only

Vps50	104	53	78	109	93
Vps51	82	107	0	0	0
Vps52	212	120	17	127	138
Vps53	60	30	46	59	74
Vps54	59	56	51	47	87
Vps72	0	0	0	0	99
Vps8	55	33	61	102	110
Vps9d1	0	0	27	6	8
Vrk1	118	103	131	173	183
Vrk3	114	58	97	85	82
Vsig10	5	5	12	8	6
Vsig10l	19	46	19	29	41
Vsir	5653	4642	5946	5297	5783
Vstm4	3	3	0	6	0
Vta1	173	136	186	126	151
Vti1a	98	141	148	147	186
Vti1b	77	74	120	60	67
Vtn	0	0	0	0	0
Vwa1	3	6	0	0	30
Vwa3a	0	4	7	1	0
Vwa5a	137	260	391	392	257
Vwa5b2	0	0	0	0	1
Vwa7	0	0	14	13	21
Vwa8	79	54	38	98	105
Vwa9	68	64	98	73	108
Vwf	0	0	0	0	0
Wac	162	168	153	134	144
Wapl	57	124	72	104	124
Wars	119	134	159	140	155
Wars2	13	16	10	32	40
Was	70	52	18	90	63
Wasf2	557	620	610	765	871
Wasf3	8	0	0	0	0
Wash1	7	4	0	59	42
Wasl	70	88	50	62	64
Wbp1	0	0	5	0	0
Wbp11	34	39	33	62	66
Wbp1l	289	0	121	0	0
Wbp2	344	217	279	279	319
Wbp4	40	51	59	42	49
Wbp5	26	13	46	32	29
Wbscr16	1	4	0	0	8
Wbscr22	109	13	15	0	0
Wbscr27	7	3	4	9	16
Wdfy1	104	100	112	172	117
Wdfy2	215	179	183	281	222
Wdfy3	289	296	248	396	395
Wdfy4	257	240	261	345	308
Wdhd1	1	0	0	21	0
Wdpcp	58	6	0	30	29

For Review Only

Wdr1	880	590	844	733	919
Wdr11	101	80	105	118	112
Wdr12	27	37	41	36	36
Wdr13	133	132	110	116	125
Wdr18	79	72	133	41	11
Wdr19	20	11	7	13	11
Wdr20	56	69	69	86	103
Wdr20rt	2	5	6	4	9
Wdr24	50	33	75	0	43
Wdr25	45	19	16	23	31
Wdr26	163	193	163	231	225
Wdr3	63	70	55	83	73
Wdr31	0	0	0	0	0
Wdr33	105	84	119	123	184
Wdr34	13	10	8	15	17
Wdr35	36	16	7	0	16
Wdr36	107	72	98	78	105
Wdr37	106	51	72	130	118
Wdr4	94	64	76	108	84
Wdr41	122	103	139	119	117
Wdr43	97	54	105	87	91
Wdr44	48	138	57	111	99
Wdr45	53	0	75	18	31
Wdr45b	62	151	181	204	51
Wdr46	43	1	68	0	67
Wdr47	44	22	40	32	14
Wdr48	80	81	130	158	137
Wdr5	138	94	107	92	118
Wdr53	29	28	50	21	16
Wdr55	3	28	46	0	0
Wdr59	30	20	38	25	30
Wdr5b	51	43	37	34	73
Wdr6	91	71	0	0	0
Wdr60	3	25	25	25	23
Wdr61	32	40	87	96	0
Wdr62	23	23	4	22	41
Wdr7	77	191	128	256	154
Wdr70	22	16	50	21	27
Wdr73	42	21	38	36	25
Wdr74	0	0	37	8	0
Wdr75	42	76	89	61	73
Wdr76	35	15	0	19	13
Wdr77	94	73	90	111	0
Wdr78	5	0	0	5	0
Wdr81	468	269	403	575	522
Wdr82	165	158	113	167	152
Wdr83	0	0	50	35	47
Wdr83os	7	5	0	0	0
Wdr86	37	5	0	8	32
Wdr89	0	0	0	0	0

For Review Only

Wdr90	0	7	0	4	0
Wdr91	46	28	0	53	67
Wdr92	35	20	25	30	31
Wdsub1	39	32	53	34	59
Wdtc1	17	8	22	21	5
Wdyhv1	65	62	39	64	62
Wee1	0	0	2	0	0
Wfdc1	0	0	0	0	0
Wfdc21	34	0	0	0	0
Wfikkn1	21	14	11	14	5
Wfs1	67	25	50	10	60
Whrn	1	33	14	7	23
Whsc1	37	58	49	88	66
Whsc1l1	101	204	141	209	182
Wipf1	83	89	74	104	110
Wipf2	87	83	83	75	94
Wipi1	22	17	9	13	17
Wipi2	122	73	79	105	80
Wiz	52	55	35	42	50
Wls	425	372	442	396	402
Wnk1	635	729	611	833	864
Wnk3	3	1	5	6	8
Wnk4	19	9	8	5	14
Wnt2b	4	2	0	14	8
Wnt4	5	1	0	23	18
Wnt5a	1	0	0	0	0
Wrap53	1	1	0	0	0
Wrap73	1	91	87	0	0
Wrb	70	141	97	105	140
Wrn	32	9	18	25	24
Wrnip1	20	29	8	20	20
Wsb1	438	606	836	181	212
Wsb2	20	0	0	55	0
Wscd1	80	39	74	52	57
Wscd2	0	0	0	2	1
Wtap	165	200	158	198	209
Wtip	5	10	12	5	3
Wwc2	18	15	33	21	26
Wwox	27	33	27	52	51
Wwp1	30	34	28	44	59
Wwp2	61	2	0	1	16
Xab2	31	23	0	0	0
Xaf1	27	0	0	9	24
Xbp1	0	0	302	0	0
Xcr1	0	0	5	1	0
Xdh	7	0	0	0	0
Xiap	208	202	172	229	249
Xirp1	6	1	1	0	0
Xirp2	0	1	0	3	0
Xist	0	0	0	3	0

For Review Only

Xk	7	4	1	11	13
Xkr4	0	2	1	0	0
Xkr6	2	0	0	0	1
Xkr7	0	0	0	0	0
Xkr8	58	23	26	37	41
Xkrx	1	0	0	0	0
Xlr	50	62	69	45	53
Xlr3a	9	1	0	5	10
Xlr3b	6	1	1	4	10
Xlr3c	7	1	0	6	9
Xlr4a	8	0	0	1	17
Xlr4b	11	5	3	0	13
Xlr4c	5	0	0	0	9
Xndc1	19	19	14	20	13
Xntrpc	0	0	19	0	0
Xpa	20	18	20	22	37
Xpc	46	26	69	48	55
Xpnpep1	111	98	155	108	112
Xpnpep3	0	0	9	9	0
Xpo1	73	45	56	80	49
Xpo4	38	19	34	38	33
Xpo5	99	82	92	85	68
Xpo6	96	68	83	98	114
Xpo7	93	39	56	120	118
Xpot	114	70	115	132	131
Xpr1	72	99	73	84	113
Xrcc1	12	89	1	35	54
Xrcc2	22	9	6	13	20
Xrcc4	19	14	24	23	12
Xrcc5	65	31	0	45	56
Xrcc6	45	42	63	31	35
Xrcc6bp1	17	5	9	7	14
Xrn1	98	84	77	130	100
Xrn2	195	42	69	229	158
Xrra1	0	0	0	0	5
Xxylt1	19	23	21	17	9
Xylb	51	36	48	38	50
Xylt2	9	1	0	0	116
Yae1d1	68	96	77	77	82
Yaf2	48	47	48	44	52
Yap1	0	0	0	0	0
Yars	113	107	106	101	148
Yars2	15	18	10	22	39
Ybey	2	10	18	14	9
Ybx1	38	55	32	32	30
Ybx3	0	4	0	0	0
Ydjc	3	0	0	5	4
Yeats2	122	130	153	177	154
Yeats4	120	92	74	21	55
Yes1	27	23	12	54	30

For Review Only

Yif1a	98	0	108	4	10
Yif1b	259	47	215	108	0
Yipf1	141	96	164	84	112
Yipf2	0	1	0	0	8
Yipf3	189	244	271	218	171
Yipf4	74	67	73	71	49
Yipf5	124	143	155	117	138
Yipf6	71	77	85	104	76
Ykt6	107	161	165	187	175
Ylpm1	89	75	82	129	171
Yme1l1	135	152	161	146	173
Yod1	29	15	8	31	45
Ypel1	6	5	9	10	8
Ypel2	55	77	52	113	101
Ypel5	209	242	211	193	242
Yrdc	10	1	0	0	0
Ythdc1	86	73	58	101	90
Ythdc2	42	18	23	26	32
Ythdf1	39	47	40	33	18
Ythdf2	371	596	378	538	566
Ythdf3	123	153	117	163	134
Ywhab	0	0	0	1	1
Ywhae	455	477	579	450	507
Ywhag	1	1	0	0	0
Ywhah	647	598	822	600	783
Ywhaz	472	367	652	446	552
Yy2	24	18	20	21	23
Zadh2	37	15	56	57	39
Zak	23	1	1	7	33
Zbed3	63	9	0	63	51
Zbed4	15	13	22	14	13
Zbed5	72	28	66	38	41
Zbed6	76	190	64	120	107
Zbp1	0	0	2	4	0
Zbtb1	24	97	97	142	101
Zbtb10	29	3	0	5	12
Zbtb11	121	91	40	83	80
Zbtb11os1	0	0	1	1	1
Zbtb14	39	99	33	51	57
Zbtb17	99	0	40	7	37
Zbtb18	38	65	59	51	62
Zbtb2	53	27	52	64	50
Zbtb20	339	345	504	359	361
Zbtb21	96	86	95	89	53
Zbtb22	0	0	34	27	8
Zbtb24	113	32	41	108	78
Zbtb25	37	20	43	35	52
Zbtb26	30	41	29	41	38
Zbtb3	9	4	15	9	13
Zbtb32	1	2	0	8	5

For Review Only

Zbtb33	19	8	18	31	30
Zbtb34	33	44	69	80	106
Zbtb37	16	35	45	68	39
Zbtb38	91	100	59	105	154
Zbtb39	35	22	15	22	29
Zbtb4	27	1	0	233	0
Zbtb40	53	47	64	65	56
Zbtb41	11	14	21	38	19
Zbtb42	6	0	0	3	3
Zbtb43	79	67	60	76	90
Zbtb44	39	34	1	37	37
Zbtb45	10	20	23	22	22
Zbtb46	5	26	29	30	7
Zbtb48	37	39	23	26	0
Zbtb49	56	47	67	51	52
Zbtb5	50	28	49	74	64
Zbtb6	27	30	29	16	34
Zbtb7a	150	136	83	139	129
Zbtb7b	16	43	31	29	41
Zbtb8a	4	7	11	10	16
Zbtb8b	0	0	0	0	0
Zbtb8os	10	16	24	13	11
Zbtb9	58	68	94	73	93
Zc2hc1a	2	2	0	0	0
Zc2hc1c	3	9	9	1	7
Zc3h10	62	75	86	92	58
Zc3h11a	177	256	289	236	210
Zc3h12a	9	14	0	27	26
Zc3h12b	17	12	25	21	16
Zc3h12c	55	25	17	56	35
Zc3h13	46	56	81	79	75
Zc3h14	33	73	70	47	62
Zc3h15	67	63	102	76	70
Zc3h18	63	26	59	86	56
Zc3h3	47	61	41	80	55
Zc3h4	188	159	136	165	203
Zc3h6	23	5	11	7	17
Zc3h7a	69	158	93	103	114
Zc3h7b	49	52	46	85	76
Zc3h8	38	32	36	25	36
Zc3hav1	222	247	273	304	289
Zc3hav1l	1	6	0	0	0
Zc3hc1	71	36	59	37	39
Zc4h2	8	4	11	24	10
Zcchc10	5	14	9	5	12
Zcchc11	42	63	42	54	58
Zcchc14	68	49	54	71	58
Zcchc17	49	38	54	32	51
Zcchc18	1	0	0	0	0
Zcchc2	68	46	87	106	104

For Review Only

Zcchc24	48	88	48	76	73
Zcchc3	16	5	2	11	9
Zcchc4	14	19	34	25	13
Zcchc6	191	125	170	228	190
Zcchc7	0	0	0	34	0
Zcchc8	77	84	119	119	122
Zcchc9	0	0	0	4	0
Zcrb1	0	6	0	0	42
Zcwpw1	60	35	0	46	0
Zdbf2	2	0	0	1	0
Zdhhc1	27	8	25	17	12
Zdhhc12	9	0	0	0	0
Zdhhc13	71	91	120	93	86
Zdhhc14	63	43	48	67	66
Zdhhc16	6	0	8	1	2
Zdhhc17	46	30	48	81	74
Zdhhc18	26	10	26	20	25
Zdhhc20	249	256	341	292	390
Zdhhc21	23	26	27	24	29
Zdhhc22	2	0	5	6	0
Zdhhc23	5	0	0	0	1
Zdhhc24	94	94	105	88	55
Zdhhc3	91	72	86	96	108
Zdhhc4	53	112	37	46	89
Zdhhc5	27	33	23	45	35
Zdhhc6	92	1	0	0	235
Zdhhc7	94	53	69	105	118
Zdhhc8	27	31	35	40	42
Zdhhc9	99	121	125	175	158
Zeb1	68	28	62	61	75
Zeb2	432	552	470	701	603
Zeb2os	0	2	0	0	0
Zer1	20	18	29	20	28
Zf12	4	8	5	1	5
Zfa-ps	23	25	35	31	25
Zfand1	9	16	25	1	2
Zfand2a	52	43	70	41	56
Zfand2b	2	0	0	0	0
Zfand3	51	47	44	46	60
Zfand4	0	0	0	5	0
Zfand5	1	1	0	185	328
Zfand6	152	146	153	156	138
Zfas1	26	20	52	27	26
Zfat	27	26	17	18	26
Zfc3h1	98	184	119	128	136
Zfhx2	37	21	21	39	35
Zfhx3	728	807	751	948	1042
Zfp1	57	41	47	53	64
Zfp101	3	8	24	28	12
Zfp105	25	38	36	50	27

For Review Only

Zfp106	126	209	191	202	216
Zfp108	6	1	13	22	33
Zfp109	23	3	20	37	13
Zfp11	8	17	27	28	15
Zfp110	366	16	206	229	248
Zfp111	62	46	39	61	73
Zfp112	30	3	18	11	12
Zfp113	67	73	41	79	102
Zfp114	3	2	1	0	4
Zfp119a	63	32	10	33	57
Zfp119b	52	68	76	58	70
Zfp12	31	45	35	54	69
Zfp120	4	155	332	228	231
Zfp128	15	35	27	22	27
Zfp13	0	1	0	0	0
Zfp131	37	31	35	26	42
Zfp133-ps	1	0	0	1	0
Zfp14	40	14	65	28	30
Zfp141	79	28	35	37	60
Zfp142	63	72	63	89	94
Zfp143	94	148	134	161	189
Zfp146	26	49	18	23	44
Zfp148	140	153	128	183	158
Zfp157	132	119	176	153	125
Zfp160	39	65	77	96	84
Zfp169	75	88	98	113	135
Zfp174	22	18	20	14	18
Zfp180	84	8	110	66	170
Zfp182	25	52	22	53	51
Zfp184	9	3	35	16	13
Zfp185	5	0	0	0	0
Zfp189	27	18	23	35	32
Zfp2	30	30	32	64	72
Zfp202	32	16	53	29	36
Zfp207	405	509	474	500	395
Zfp212	64	47	0	0	0
Zfp213	57	43	68	47	78
Zfp217	116	224	113	217	218
Zfp219	87	92	88	85	65
Zfp229	15	11	36	12	21
Zfp235	33	52	62	63	32
Zfp236	76	123	101	106	185
Zfp239	3	1	0	0	1
Zfp24	122	98	172	168	138
Zfp248	1	8	0	15	0
Zfp251	39	7	32	29	25
Zfp26	118	71	76	184	186
Zfp260	146	152	180	209	217
Zfp266	190	173	149	234	282
Zfp27	22	24	26	19	28

For Review Only

Zfp273	26	17	24	25	1
Zfp275	58	78	67	66	118
Zfp276	33	15	37	0	316
Zfp277	103	36	94	73	95
Zfp28	40	49	44	63	35
Zfp280b	12	30	0	35	24
Zfp280c	29	34	23	63	45
Zfp280d	43	106	36	69	84
Zfp281	37	67	39	67	106
Zfp282	1	176	0	0	0
Zfp286	10	7	32	25	18
Zfp286os	1	0	0	2	0
Zfp287	13	3	9	30	31
Zfp292	153	121	143	222	185
Zfp296	1	0	13	0	0
Zfp3	36	41	13	36	24
Zfp30	24	41	25	23	44
Zfp316	31	38	43	61	47
Zfp317	89	50	77	119	116
Zfp318	39	60	60	46	27
Zfp319	131	81	49	97	105
Zfp322a	6	7	59	75	50
Zfp324	48	68	33	59	75
Zfp326	51	29	32	55	49
Zfp329	62	74	53	89	79
Zfp330	2	20	0	59	0
Zfp334	7	3	3	8	0
Zfp335	81	132	96	127	182
Zfp335os	17	35	25	29	40
Zfp33b	54	47	33	75	88
Zfp341	2	6	15	0	14
Zfp345	0	0	0	2	0
Zfp346	53	33	38	29	54
Zfp35	86	86	152	107	147
Zfp354a	20	23	21	33	33
Zfp354b	3	7	0	13	15
Zfp354c	18	11	5	14	14
Zfp358	28	14	28	24	44
Zfp36	364	167	274	67	267
Zfp362	20	18	21	20	31
Zfp367	23	24	14	22	24
Zfp369	30	15	16	44	28
Zfp36l1	0	512	1	1	3
Zfp36l2	20	186	0	263	319
Zfp36l3	3	6	1	0	9
Zfp37	37	15	14	25	32
Zfp382	17	4	16	26	33
Zfp383	6	0	27	0	26
Zfp384	12	16	0	39	33
Zfp385a	8	0	6	31	5

For Review Only

Zfp385b	0	0	0	0	0
Zfp385c	45	71	76	67	95
Zfp386	65	66	72	95	83
Zfp389	2	2	0	0	0
Zfp39	60	9	40	43	16
Zfp395	5	17	15	11	7
Zfp397	54	65	34	69	69
Zfp398	109	102	113	115	162
Zfp40	82	60	74	84	78
Zfp407	63	27	44	57	97
Zfp408	31	57	59	64	103
Zfp41	49	19	15	33	37
Zfp410	95	98	90	77	102
Zfp418	11	21	28	27	26
Zfp420	30	38	22	21	40
Zfp422	120	99	115	92	128
Zfp423	0	0	0	0	0
Zfp426	97	85	119	107	77
Zfp428	3	3	4	10	0
Zfp429	43	31	48	51	29
Zfp433	42	40	33	41	40
Zfp438	7	3	22	21	15
Zfp442	59	39	32	51	36
Zfp444	65	62	61	64	53
Zfp445	188	161	199	271	219
Zfp446	34	9	10	17	23
Zfp449	18	23	58	38	34
Zfp451	83	62	46	45	83
Zfp454	5	2	10	10	0
Zfp455	8	49	42	39	19
Zfp456	15	24	19	45	47
Zfp458	20	2	6	15	14
Zfp459	3	1	0	1	2
Zfp46	46	31	62	61	74
Zfp462	0	0	0	1	0
Zfp467	79	71	109	145	126
Zfp472	38	37	56	47	87
Zfp473	0	0	0	3	0
Zfp493	13	12	12	18	16
Zfp507	1	0	0	0	0
Zfp51	25	53	55	61	46
Zfp511	0	1	0	103	0
Zfp512	169	119	122	130	54
Zfp512b	64	78	97	56	110
Zfp513	0	0	12	12	14
Zfp516	74	46	43	107	106
Zfp518a	56	68	62	87	90
Zfp518b	3	7	8	14	18
Zfp52	81	40	51	62	84
Zfp521	1	0	0	0	0

For Review Only

For Review Only

Zfp523	15	56	26	31	14
Zfp524	47	53	52	33	54
Zfp526	17	9	10	21	11
Zfp53	30	53	36	22	31
Zfp532	251	189	208	300	311
Zfp534	3	1	1	1	3
Zfp536	0	5	0	8	5
Zfp54	11	18	9	21	19
Zfp541	0	0	0	0	4
Zfp551	15	6	1	0	19
Zfp553	65	41	80	56	42
Zfp558	6	28	27	17	41
Zfp560	29	7	25	28	36
Zfp563	38	24	46	35	27
Zfp566	21	20	55	24	14
Zfp568	32	17	9	17	42
Zfp57	11	0	15	6	4
Zfp574	127	36	78	69	130
Zfp579	56	43	0	0	0
Zfp58	48	39	54	45	46
Zfp580	13	0	8	8	11
Zfp583	13	23	20	24	18
Zfp59	25	24	58	24	20
Zfp592	296	167	190	282	365
Zfp593	12	7	24	0	16
Zfp595	11	22	17	42	44
Zfp597	40	19	25	51	43
Zfp598	23	32	51	28	39
Zfp599	10	1	0	14	0
Zfp60	17	54	37	51	21
Zfp600	1	0	1	0	1
Zfp605	19	8	7	11	18
Zfp606	109	39	49	89	73
Zfp607	19	10	24	28	32
Zfp608	65	34	49	78	78
Zfp609	27	36	24	56	60
Zfp61	38	24	49	29	40
Zfp612	11	0	12	5	0
Zfp616	0	0	0	1	4
Zfp617	22	37	58	1	103
Zfp618	0	0	2	0	0
Zfp619	20	33	43	65	67
Zfp62	195	128	144	198	238
Zfp622	8	14	15	27	21
Zfp623	49	59	73	59	64
Zfp626	15	10	9	26	39
Zfp628	19	11	3	11	7
Zfp629	52	68	52	64	97
Zfp637	90	1	0	0	0
Zfp638	78	108	143	168	192

Zfp639	14	44	0	39	24
Zfp64	58	61	69	57	68
Zfp641	17	14	6	15	14
Zfp644	38	73	38	83	78
Zfp646	99	95	154	120	109
Zfp647	32	1	14	14	23
Zfp648	0	17	0	0	0
Zfp65	27	27	57	50	34
Zfp651	7	26	12	9	10
Zfp652	95	286	0	0	0
Zfp652os	10	7	11	10	5
Zfp653	0	2	0	0	0
Zfp654	18	27	22	29	34
Zfp655	76	66	59	114	81
Zfp658	300	187	330	302	294
Zfp661	17	30	20	14	35
Zfp664	53	57	39	47	41
Zfp667	7	2	24	16	8
Zfp668	75	59	40	89	51
Zfp672	97	111	166	132	99
Zfp677	45	25	33	30	32
Zfp68	0	0	0	0	4
Zfp687	92	26	0	111	162
Zfp688	0	0	7	2	8
Zfp689	28	16	27	20	37
Zfp69	6	50	0	64	58
Zfp691	1	11	0	732	0
Zfp697	33	27	29	38	72
Zfp7	5	8	7	3	11
Zfp703	45	48	0	0	0
Zfp704	38	36	38	35	48
Zfp706	551	439	578	408	448
Zfp707	8	15	29	25	0
Zfp708	17	3	8	42	22
Zfp709	58	43	77	57	87
Zfp710	358	391	395	504	498
Zfp712	10	14	14	20	22
Zfp715	154	222	215	308	192
Zfp719	117	84	92	98	143
Zfp72	33	8	0	12	24
Zfp729a	42	50	49	77	71
Zfp729b	52	77	73	100	66
Zfp735	0	0	0	0	0
Zfp738	76	51	41	101	116
Zfp74	72	56	37	47	59
Zfp740	281	200	321	168	242
Zfp746	67	56	57	29	49
Zfp747	10	34	55	29	31
Zfp748	62	64	51	120	105
Zfp750	1	0	5	6	3

For Review Only

Zfp758	25	29	19	29	28
Zfp759	29	20	13	26	19
Zfp760	31	35	43	47	50
Zfp763	22	40	32	38	19
Zfp764	6	50	0	45	69
Zfp768	62	27	36	20	15
Zfp770	10	23	26	16	16
Zfp771	20	37	52	34	41
Zfp772	24	31	53	23	36
Zfp773	26	23	34	17	27
Zfp775	36	36	39	41	35
Zfp777	24	5	0	10	46
Zfp78	12	9	17	17	14
Zfp780b	81	79	68	78	71
Zfp781	35	23	31	27	26
Zfp783	24	10	29	5	38
Zfp784	7	17	46	13	14
Zfp786	0	0	0	1	0
Zfp787	29	21	6	23	36
Zfp788	57	29	51	44	61
Zfp790	54	46	64	80	94
Zfp791	44	6	12	29	11
Zfp799	38	19	44	55	37
Zfp800	40	70	29	49	47
Zfp808	13	27	21	32	8
Zfp809	47	101	57	79	89
Zfp81	29	23	21	51	54
Zfp810	157	154	172	171	146
Zfp811	2	7	0	0	0
Zfp819	0	0	0	0	0
Zfp82	26	22	36	18	25
Zfp820	0	1	0	1	0
Zfp821	9	17	13	4	24
Zfp825	47	44	32	66	65
Zfp827	40	29	52	46	30
Zfp830	59	61	93	69	67
Zfp831	1	0	0	3	0
Zfp839	90	113	141	77	112
Zfp84	131	40	55	97	100
Zfp846	42	62	61	43	33
Zfp85	14	11	49	28	11
Zfp850	14	7	9	10	10
Zfp85os	2	3	3	7	0
Zfp862-ps	130	86	152	152	118
Zfp865	6	1	0	85	82
Zfp866	13	69	30	104	62
Zfp867	7	33	37	57	46
Zfp868	65	89	89	86	57
Zfp869	0	38	2	31	3
Zfp87	4	10	0	207	0

For Review Only

Zfp870	47	49	46	60	23
Zfp871	190	272	161	401	328
Zfp873	45	32	50	70	54
Zfp874a	33	7	59	44	27
Zfp874b	20	14	29	29	18
Zfp879	0	2	4	1	4
Zfp882	12	16	22	33	41
Zfp9	3	0	0	2	4
Zfp90	331	0	203	263	258
Zfp91	59	48	61	75	44
Zfp93	16	9	25	24	38
Zfp930	36	49	16	25	68
Zfp931	5	18	7	18	15
Zfp932	17	20	30	26	14
Zfp933	5	20	17	24	40
Zfp934	19	16	37	30	35
Zfp935	59	40	76	70	53
Zfp936	1	0	1	1	0
Zfp937	49	19	16	28	25
Zfp939	9	12	22	30	39
Zfp94	16	0	0	12	13
Zfp940	15	1	0	0	12
Zfp942	61	44	47	54	41
Zfp943	38	47	80	96	56
Zfp944	51	46	38	49	41
Zfp945	26	32	42	38	37
Zfp946	15	22	10	16	13
Zfp947	6	1	3	3	3
Zfp948	28	14	44	17	23
Zfp949	16	19	25	19	45
Zfp950	101	184	166	221	200
Zfp951	68	73	67	77	45
Zfp952	75	60	146	107	17
Zfp953	7	8	10	9	15
Zfp954	28	29	54	48	47
Zfp955a	15	35	43	89	83
Zfp955b	36	14	44	71	48
Zfp958	23	43	56	57	17
Zfp959	56	39	49	64	48
Zfp960	29	29	25	46	55
Zfp961	47	24	62	60	30
Zfp963	74	33	54	40	34
Zfp964	44	18	44	47	34
Zfp965	4	1	3	8	10
Zfp966	13	5	12	20	16
Zfp967	3	2	1	11	8
Zfp968	3	4	1	15	12
Zfp97	28	33	27	54	54
Zfp970	14	28	16	28	20
Zfp971	3	7	9	1	12

For Review Only

Zfp973	4	1	3	7	7
Zfpl1	82	58	78	0	67
Zfpm1	11	11	13	17	13
Zfr	178	135	141	206	191
Zfr2	0	0	0	0	2
Zfx	157	164	214	192	143
Zfyve1	78	81	86	107	117
Zfyve16	22	95	32	59	40
Zfyve19	4	1	0	0	168
Zfyve21	162	0	40	1	32
Zfyve26	134	68	166	199	190
Zfyve27	145	137	162	152	125
Zfyve28	148	88	43	112	81
Zfyve9	18	7	7	16	24
Zgrf1	1	1	0	10	0
Zhx1	96	159	161	106	147
Zhx2	12	5	10	18	28
Zhx3	11	15	6	28	18
Zik1	12	39	19	13	15
Zkscan1	91	73	79	136	129
Zkscan14	49	27	87	57	61
Zkscan16	0	0	3	0	0
Zkscan17	117	79	108	111	127
Zkscan2	5	2	11	13	14
Zkscan3	76	203	274	189	173
Zkscan4	52	27	43	36	38
Zkscan5	36	35	69	51	56
Zkscan6	76	87	101	82	70
Zkscan7	28	26	24	42	37
Zkscan8	71	71	41	97	88
Zmat1	24	52	30	33	27
Zmat2	66	128	122	0	0
Zmat3	14	29	9	16	19
Zmat5	32	25	40	22	31
Zmiz1	562	439	468	668	720
Zmiz1os1	0	7	9	12	11
Zmiz2	113	117	128	114	151
Zmpste24	140	85	163	148	180
Zmym1	36	16	47	31	9
Zmym2	132	131	126	174	182
Zmym3	102	121	137	124	91
Zmym4	49	62	71	84	92
Zmym5	100	75	94	123	105
Zmym6	154	104	126	174	171
Zmynd10	0	0	0	0	0
Zmynd11	96	151	137	133	226
Zmynd15	2	15	0	24	23
Zmynd19	14	16	13	8	9
Zmynd8	183	125	162	195	212
Znf41-ps	62	48	0	0	0

For Review Only

Znfx1	228	249	285	366	333
Znhit1	0	13	1	1	17
Znhit2	43	33	76	41	39
Znhit3	34	36	42	19	17
Znhit6	15	17	29	16	26
Znrd1	14	28	32	0	21
Znrd1as	15	6	20	10	15
Znrf1	45	86	59	57	57
Znrf2	23	32	28	12	18
Znrf3	58	53	62	72	48
Zpbp	0	4	0	0	0
Zpr1	68	74	0	0	0
Zranb1	25	44	72	47	43
Zranb2	62	108	93	120	104
Zranb3	9	4	5	1	5
Zrsr1	26	23	15	21	26
Zrsr2	38	49	47	54	40
Zscan10	6	0	0	0	0
Zscan12	21	33	57	33	38
Zscan18	50	17	28	31	40
Zscan2	9	1	0	7	3
Zscan20	27	18	15	25	30
Zscan21	86	85	106	63	73
Zscan22	88	46	82	113	52
Zscan25	0	1	1	2	4
Zscan26	698	596	100	487	483
Zscan29	129	80	148	110	95
Zscan30	2	4	17	13	14
Zswim1	66	40	59	54	54
Zswim3	26	21	45	34	52
Zswim4	53	44	60	58	66
Zswim5	7	13	0	15	26
Zswim6	62	68	52	67	69
Zswim7	2	9	11	6	7
Zswim8	44	25	49	158	89
Zufsp	74	102	117	148	114
Zw10	48	60	63	74	78
Zwilch	0	9	0	0	0
Zwint	40	48	24	32	30
Zxda	16	14	11	22	21
Zxdb	13	23	5	16	23
Zxdc	45	60	37	24	57
Zyg11b	154	176	123	211	193
Zyx	0	101	110	3	6
Zzef1	267	182	163	196	238
Zzz3	139	92	88	143	112

For Review Only

Sham_HSD_1	Sham_HSD_2	Sham_HSD_3	Sham_HSD_4	Sham_HSD_5	rCHI_ND_1	rCHI_ND_2
111538	109622	94062	164477	117677	119432	94319
1	1	3	0	0	72	0
27	58	48	89	34	42	45
0	1	0	5	5	0	1
175	150	124	106	349	190	136
9	7	5	11	5	8	7
17	1	18	11	0	14	37
16	34	28	120	2	0	14
8	0	9	40	0	7	4
103	75	61	139	73	81	72
0	0	0	0	0	0	0
4	1	1	0	0	1	0
140	0	110	125	23	135	96
0	0	0	2	2	0	0
3	3	7	6	9	3	5
0	0	0	0	0	3	0
676	664	620	1011	694	594	498
6	0	9	13	0	6	3
0	0	0	2	0	0	0
24	0	1	0	60	35	1
18	16	18	25	0	9	0
176	230	184	228	251	159	88
10	3	10	11	8	7	7
41	43	34	65	47	0	0
34	4	14	14	0	1	7
9	8	4	8	0	9	0
50	24	39	59	57	26	21
2	1	0	0	0	3	2
8	24	6	7	0	6	7
1	1	0	3	0	0	0
27	34	23	31	30	39	29
53	50	59	81	67	66	66
65	52	60	123	74	95	90
0	0	0	9	0	0	5
229	208	157	327	217	286	198
16	24	63	94	52	13	31
49	41	54	91	34	49	47
0	0	1	2	2	1	0
1	6	9	19	0	6	15
7	1	18	18	13	18	8
27	7	17	62	24	45	20
11	12	16	13	6	5	18
19	22	21	47	27	26	21
1	8	1	93	0	1	1
9	18	10	11	12	6	2
39	5	3	10	0	0	0

For Review Only

0	0	0	0	0	0	1
2	3	4	8	9	3	12
4	31	19	24	55	50	1
3	1	31	42	0	32	1
164	155	171	273	111	173	80
197	151	137	0	0	206	150
12	5	0	0	0	0	0
0	0	0	0	0	0	0
0	13	2	7	28	3	19
0	0	0	0	1	2	0
0	0	0	0	7	5	0
14	4	3	5	0	2	21
0	3	0	0	0	3	0
0	1	0	0	1	0	0
3	2	0	0	5	1	0
1	0	1	0	14	5	10
9	8	6	11	30	1	6
9	3	2	9	0	7	2
24	5	7	12	31	34	20
1	1	0	0	0	0	6
0	3	0	0	0	3	0
0	0	0	0	0	0	4
6	6	0	0	4	6	0
5	1	7	5	0	4	0
1	0	0	0	0	0	3
0	788	0	1548	15	0	759
0	0	1	2	0	0	0
2	3	4	7	0	4	8
1	0	0	4	0	0	0
20	41	14	36	20	18	21
5	0	5	0	5	1	4
33	15	40	37	55	16	25
0	0	0	0	0	0	2
0	0	0	2	0	0	0
8	0	0	6	15	3	6
1	3	0	0	0	5	2
1	0	1	0	0	2	1
8	0	0	2	0	0	0
2	11	11	9	23	5	0
6	4	6	11	0	10	6
4	2	10	5	5	8	7
2	2	3	6	0	0	0
47	54	66	90	56	66	39
1	0	1	0	0	0	1
0	0	6	0	0	4	3
0	0	0	10	9	0	0
20	8	1	13	59	24	17
2	0	1	0	0	4	1
0	0	0	0	0	0	0
41	34	25	43	14	25	25

For Review Only

2	1	0	0	0	3	2
24	44	13	40	20	44	56
11	22	5	8	21	5	2
0	0	0	5	0	1	1
5	9	6	9	0	5	8
15	23	12	16	17	5	21
2	12	7	17	15	5	2
0	0	0	0	0	0	0
3	0	0	4	0	3	3
0	0	0	4	0	0	3
0	0	0	2	0	1	0
0	2	1	0	1	1	0
0	0	0	1	1	1	0
0	5	2	6	0	3	4
4	1	3	7	0	4	1
10	11	7	3	17	3	0
0	0	0	32	13	0	0
0	0	0	0	0	0	0
0	1	1	0	0	1	1
13	9	9	15	22	4	8
5	2	0	1	4	6	5
19	19	17	26	8	15	8
6	3	2	4	4	11	4
25	24	19	45	22	35	19
0	0	0	0	0	0	12
0	3	1	0	3	3	1
0	0	0	2	0	1	0
0	0	0	0	0	0	0
71	65	94	177	71	143	58
2	2	0	1	0	0	0
1	0	0	4	0	8	1
1	58	105	153	73	1	39
1	9	0	1	0	6	5
98	183	47	193	2	0	65
8	14	7	17	8	6	10
5	2	0	0	0	3	0
86	67	82	109	57	68	66
20	42	20	25	28	22	13
0	0	0	2	0	0	0
1	0	0	0	0	3	0
0	0	0	0	7	0	0
42	31	40	70	92	51	59
69	40	40	0	33	17	19
37	33	37	0	0	41	40
220	144	118	217	115	188	137
19	27	19	32	24	16	15
3	5	0	4	0	0	8
39	35	63	89	28	40	44
1	1	0	2	2	0	0
8	1	1	64	0	36	1

For Review Only

0	0	0	0	3	0	0
0	9	3	6	0	12	0
7	0	1	13	0	15	14
46	38	44	65	48	52	40
0	0	0	0	0	3	3
1	0	0	1	0	1	0
4	6	8	3	9	4	5
65	0	53	130	0	54	53
6	0	0	0	0	0	0
4	0	4	6	17	7	9
81	75	89	108	115	106	116
4	1	0	2	0	1	1
5	6	5	8	0	2	0
76	37	63	141	28	65	38
12	5	11	14	5	15	8
30	17	23	27	22	23	20
138	161	131	229	118	128	107
0	0	1	5	8	0	0
14	7	11	23	22	17	7
0	0	0	0	12	0	4
12	13	0	17	0	12	3
0	0	0	0	5	1	0
28	14	13	23	12	16	10
17	11	16	32	15	5	7
20	28	25	55	29	32	36
0	0	0	0	2	0	0
0	0	0	1	1	0	0
1	77	1	543	0	1	1
6	3	21	19	0	5	27
0	0	0	0	0	0	2
45	54	15	55	84	31	30
0	4	13	5	13	19	12
39	39	66	106	101	79	37
2	3	1	0	0	0	1
0	0	4	0	0	0	0
25	61	48	104	0	15	99
12	4	34	74	89	2	63
19	20	18	33	20	26	27
23	17	37	57	17	14	6
6	4	1	18	0	11	8
20	79	74	73	0	13	15
3	21	21	43	23	4	24
1	0	0	0	0	4	0
0	0	4	4	0	0	5
80	39	75	64	66	118	48
41	54	0	147	0	0	0
51	47	51	107	18	0	19
11	10	26	25	67	4	17
5	0	2	0	0	1	0
6	9	7	20	9	14	7

For Review Only

17	18	12	53	18	24	33
35	27	17	39	29	45	37
0	1	8	35	0	17	17
1	0	0	2	3	0	0
83	180	105	145	127	127	96
61	68	110	87	130	53	51
0	0	109	85	1	145	0
17	22	21	32	17	21	14
72	63	81	129	62	116	80
52	53	61	68	66	110	52
23	4	1	0	0	6	4
0	6	18	23	0	18	7
62	33	12	32	51	36	49
7	0	1	0	0	1	0
37	20	26	32	35	40	41
20	11	7	18	15	10	17
24	21	15	32	16	14	14
129	28	29	109	68	35	22
50	22	50	86	36	20	22
14	3	5	17	0	5	8
151	122	144	210	129	124	145
0	0	0	0	24	0	0
3	0	3	8	0	4	5
0	0	0	72	39	0	0
1	2	4	21	1	14	4
3	4	9	18	0	9	2
4	4	10	5	4	4	3
40	53	49	74	21	19	36
2	47	430	321	251	22	84
1	27	2	166	0	23	2
0	0	1	0	0	0	2
19	19	5	32	19	16	22
0	6	0	0	0	0	7
23	16	34	45	16	34	32
3	2	2	7	7	2	0
30	27	23	29	41	18	20
37	29	29	56	50	46	27
105	88	67	120	26	87	49
87	84	63	156	62	90	46
63	27	2	68	0	62	16
132	70	89	136	109	61	89
1	0	0	0	7	4	2
9	5	3	17	0	8	7
7	4	2	3	4	6	4
74	123	154	304	174	108	202
6	0	2	0	0	4	0
0	0	0	0	0	0	3
0	0	0	0	0	0	0
2	1	1	4	0	1	2
1	3	0	5	0	3	3

For Review Only

42	47	34	98	39	51	39
1	0	0	0	8	0	0
0	2	0	0	0	0	0
3	4	11	0	10	2	5
3	5	20	6	0	12	2
290	331	246	375	360	325	236
16	15	4	17	28	17	19
51	24	57	31	53	76	41
17	0	0	3	0	0	0
15	14	8	22	12	16	15
0	0	0	0	0	6	0
5	12	7	0	0	2	4
3	0	2	0	0	2	3
220	222	201	345	164	161	249
4	1	3	0	0	18	1
17	23	14	23	9	15	9
21	7	13	33	13	18	3
25	0	33	9	2	38	26
14	19	21	22	0	8	3
14	26	19	32	14	12	25
36	28	34	52	34	28	24
160	111	79	178	100	129	89
60	65	44	65	49	68	62
7	10	1	2	3	0	0
15	41	19	19	43	3	15
1	0	0	0	0	19	1
8	1	1	0	0	0	1
28	42	29	64	75	56	40
0	0	0	11	0	0	11
3	4	0	6	0	0	0
1	0	1	0	0	6	1
5	0	3	0	0	1	6
0	2	0	7	0	4	2
0	0	2	3	0	3	0
6	18	19	36	20	0	9
31	30	29	28	57	5	32
4	0	0	0	0	0	0
15	0	0	0	3	7	0
33	13	15	26	11	37	27
91	20	27	62	62	45	19
0	0	0	0	0	0	0
24	15	9	37	9	18	10
23	49	29	51	23	26	23
9	1	9	5	0	5	6
4	0	2	0	0	2	0
20	7	11	7	15	0	9
1	8	2	15	3	2	6
5	3	18	0	0	17	6
6	8	8	19	0	22	12
6	2	3	13	0	21	5

For Review Only

0	0	0	0	0	6	0
133	98	214	284	193	108	187
0	3	3	0	0	0	0
22	13	12	16	23	26	28
346	3	3	344	0	99	48
8	0	1	14	0	8	1
1	0	0	0	0	2	1
0	0	0	0	0	0	0
37	30	25	49	31	31	24
2	0	0	1	0	0	7
9	18	6	22	0	10	4
11	9	15	20	34	14	24
0	0	3	0	0	0	0
39	29	50	57	25	52	23
0	8	5	12	17	4	8
3	6	1	19	0	5	7
2	5	0	7	0	6	0
1	2	1	3	3	4	0
12	7	6	10	1	7	3
2	0	0	0	1	0	0
1	1	0	3	0	5	0
1	0	1	3	9	1	0
0	0	0	18	48	0	0
2	0	0	6	8	8	0
7	0	2	17	0	3	3
0	4	0	0	0	0	0
23	36	9	15	0	6	2
6	5	0	0	0	15	7
1	0	0	0	11	0	0
3	0	1	0	0	13	2
14	14	21	43	19	13	26
0	5	3	0	4	1	0
5	4	0	10	22	15	2
6	16	24	7	18	20	9
0	1	2	0	0	0	0
0	0	2	7	0	5	8
0	0	5	3	3	1	1
63	40	55	45	80	32	45
10	2	2	11	0	7	7
13	13	12	17	0	4	11
6	0	5	4	5	7	0
3	1	2	6	0	7	6
0	1	0	0	0	0	0
2	7	3	17	0	4	3
24	44	33	60	32	65	35
1	0	0	0	0	0	0
0	0	3	6	0	0	0
2	2	7	4	0	0	3
0	2	0	0	0	0	0
8	15	11	26	7	28	21

For Review Only

8	4	5	9	8	2	6
31	17	12	26	52	8	12
0	4	1	1	0	0	0
18	10	5	36	0	10	4
0	0	0	2	0	0	4
0	0	0	6	0	2	0
0	2	2	0	0	0	0
0	0	0	0	1	0	0
4	0	4	11	1	3	8
0	1	0	2	2	1	1
5	3	4	7	7	10	10
85	73	44	81	24	61	43
3	0	0	0	0	3	0
4	2	1	18	0	0	6
1	1	1	0	0	5	1
10	15	9	10	11	8	0
0	1	0	2	0	1	0
1	0	1	3	0	1	1
2	2	3	0	0	4	0
16	8	0	0	0	3	1
7	0	1	0	0	2	2
0	1	1	0	0	0	0
1	0	0	3	0	0	4
0	0	2	0	0	0	0
2	2	2	3	1	2	3
3	0	1	4	0	0	0
0	0	4	0	0	0	2
2	3	0	0	0	0	0
1	1	9	0	22	0	1
1	2	41	41	0	11	11
0	4	0	3	0	1	0
0	9	1	0	0	1	4
0	4	2	14	0	5	1
3	0	1	5	0	0	1
9	1	5	9	10	6	5
7	4	7	15	0	3	2
2	12	5	13	0	11	2
7	3	4	7	13	11	4
6	7	17	7	15	10	11
3	1	0	7	0	3	0
0	2	1	4	0	1	4
46	52	108	120	157	83	116
0	0	0	5	2	0	0
48	50	34	60	33	45	44
2	2	1	4	0	7	1
32	32	47	76	34	49	64
100	41	44	75	92	119	102
90	69	82	114	135	38	71
0	3	3	1	0	0	0
26	33	14	48	31	42	14

For Review Only

4	0	4	6	0	5	0
15	3	2	7	0	3	4
8	16	8	24	10	21	11
6	0	0	0	0	0	0
3	3	5	5	0	3	3
103	119	220	286	100	109	232
1	3	0	0	0	3	3
3	5	5	7	7	8	6
5	4	4	11	0	4	6
3	11	1	0	0	6	1
0	0	0	0	4	0	0
0	0	1	3	0	0	0
15	9	16	25	33	12	39
0	1	0	4	0	0	0
2	19	1	9	0	3	3
15	11	6	39	0	46	6
0	0	0	0	0	0	1
1	0	1	1	0	1	1
4	30	11	23	0	8	9
2	4	0	11	0	2	2
0	0	0	0	0	2	6
1	9	3	5	6	4	3
29	36	29	19	62	12	13
8	2	19	43	7	4	6
3	1	0	0	0	41	1
23	19	38	31	34	34	27
0	0	0	1	1	0	0
3	1	14	0	19	0	0
17	28	21	17	0	12	6
0	6	0	2	0	0	0
0	0	0	0	0	0	0
7	0	0	4	0	0	0
74	98	0	63	10	101	0
0	0	1	3	0	2	0
11	14	17	25	30	20	13
0	3	4	8	0	0	0
9	0	0	0	1	3	0
95	76	86	366	107	101	74
32	24	19	17	30	9	4
5	0	4	7	0	3	0
0	0	1	0	1	1	1
0	0	0	0	0	0	0
97	64	77	154	37	80	72
3	0	1	6	0	0	0
0	0	0	0	0	0	3
6	0	4	1	0	4	4
3	1	1	4	0	1	1
20	19	14	27	24	30	23
124	75	142	193	107	107	107
0	0	0	0	0	0	0

For Review Only

12	28	7	8	48	21	0
1	0	1	26	0	2	1
0	0	0	0	0	0	0
3	3	19	23	0	20	14
2	10	6	9	12	5	4
0	1	2	7	3	2	6
12	42	24	61	0	38	54
1	5	3	13	0	6	1
3	0	0	2	0	0	3
0	0	0	1	0	0	0
0	0	0	125	55	0	0
21	15	12	28	6	14	8
19	46	27	46	32	34	10
2	4	1	3	1	1	1
7	1	1	0	0	2	2
0	0	1	4	0	3	0
1	7	1	0	14	6	2
0	1	0	0	0	0	3
96	81	75	160	95	89	66
0	3	5	22	0	9	0
3	3	2	0	0	3	1
0	0	0	0	0	0	0
27	53	65	67	40	68	47
14	33	4	25	30	42	11
3	0	0	0	0	0	0
6	7	13	20	8	19	6
190	168	155	297	201	247	174
15	18	10	0	0	16	15
21	19	6	14	10	12	8
10	39	31	25	45	35	21
8	10	1	9	13	1	1
1	0	0	0	0	0	0
0	0	2	0	0	2	0
2	2	0	8	9	0	3
6	8	8	24	8	20	10
12	4	9	21	1	9	12
90	68	78	133	101	100	103
2	12	4	30	0	12	11
7	15	11	16	11	18	14
6	21	5	45	0	28	6
40	35	49	80	90	69	65
3	0	0	3	0	5	0
4	1	1	5	0	4	1
2	0	3	5	0	1	0
45	29	26	34	37	12	14
149	103	76	141	153	172	98
224	141	102	242	140	223	106
89	67	50	97	89	93	82
56	27	30	78	18	40	11
2	5	3	14	0	6	6

For Review Only

26	13	63	97	0	144	100
0	0	0	1	4	0	0
17	15	18	36	12	7	9
8	16	4	9	23	11	1
1	0	0	0	4	0	8
19	27	24	52	16	36	31
3	0	3	5	0	3	10
1	0	0	1	0	0	0
13	12	17	15	19	8	3
1	0	2	24	0	1	5
0	0	2	0	0	4	0
108	76	70	155	91	103	86
2	0	3	0	0	2	0
6	22	8	27	37	14	21
5	11	7	10	0	6	6
0	0	0	3	0	0	5
16	0	0	0	0	60	0
13	25	30	62	46	32	17
0	1	0	0	0	0	1
0	2	0	1	0	0	0
2	0	1	3	0	1	1
18	6	19	14	13	15	5
21	29	27	39	12	19	16
2	4	8	10	0	1	0
1	1	6	15	0	1	1
8	10	10	11	8	9	13
0	0	3	0	0	3	2
16	22	4	33	11	29	10
48	40	74	131	74	79	87
0	1	0	0	0	1	1
0	0	0	0	0	0	0
1	0	1	4	1	3	0
0	0	1	0	0	2	0
595	482	396	631	330	446	343
2	0	0	0	0	3	3
0	0	9	28	30	18	4
1	0	1	0	0	0	0
3	0	2	0	0	0	0
1	0	0	0	0	0	0
8	2	28	58	0	37	46
13	12	13	19	45	33	25
7	9	23	22	21	3	3
9	0	4	2	0	8	6
17	36	50	80	34	33	37
102	32	34	68	104	80	45
15	0	180	104	28	14	30
0	14	0	0	0	0	0
0	0	0	5	0	0	0
93	54	70	59	75	65	25
10	16	18	20	0	6	5

For Review Only

6	2	2	3	7	1	9
40	312	168	338	326	95	182
1	0	4	0	0	1	3
0	2	0	0	0	0	2
16	14	21	17	0	4	0
0	4	5	8	0	2	5
0	0	0	0	0	1	4
0	8	12	24	10	2	8
0	0	0	2	0	0	0
2	0	3	1	0	7	2
1	0	2	0	0	2	6
0	0	0	0	0	0	1
57	108	2	138	0	84	1
2	0	0	4	0	0	0
36	34	22	30	59	46	16
1	0	0	3	0	3	0
2	10	12	32	0	14	23
44	37	31	55	27	46	44
6	0	0	0	0	0	4
7	4	2	20	0	8	15
2	1	1	0	0	4	2
2	4	4	10	0	3	0
10	5	2	14	15	23	5
1	13	2	7	0	10	2
3	3	1	5	5	6	2
3	2	4	6	0	4	1
1	0	1	0	0	1	16
5	8	15	8	0	6	0
57	109	43	56	81	89	60
2	13	0	130	0	133	9
5	1	0	11	0	0	11
56	43	62	43	68	47	43
0	0	0	0	0	4	0
6	4	7	11	1	8	5
8	8	12	12	26	5	7
5	0	9	11	0	0	4
3	5	2	8	0	5	3
1	1	1	0	0	0	1
0	0	0	0	0	0	0
0	0	0	4	0	10	0
0	0	0	0	0	0	3
0	0	0	0	0	0	1
7	1	7	0	0	1	8
0	0	0	0	0	0	0
7	0	1	3	8	1	3
7	40	10	12	26	14	11
20	51	36	31	58	28	34
2	2	0	5	1	2	2
5	21	9	19	0	4	10
0	1	1	0	0	2	3

For Review Only

19	11	6	13	16	13	11
24	23	36	33	35	23	19
3	0	4	10	4	0	0
10	10	8	20	0	6	6
16	9	3	9	27	5	9
4	0	2	0	0	1	0
0	2	2	4	0	5	1
0	2	0	6	0	6	0
9	4	2	4	4	2	3
6	2	3	8	3	5	4
0	3	0	17	0	5	8
3	0	0	9	0	0	2
49	50	31	81	0	45	21
33	26	37	35	67	46	17
1	1	43	0	0	1	1
31	29	1	1268	1	18	7
121	58	104	221	99	145	103
32	31	11	45	0	13	11
401	365	347	692	358	377	265
0	0	0	3	0	0	0
154	82	72	187	75	111	89
233	259	232	362	324	335	182
82	30	29	0	43	58	13
42	111	19	118	53	50	10
56	30	32	37	32	32	18
45	45	49	87	40	50	25
3	0	0	0	0	0	0
62	68	55	214	42	126	127
42	29	26	39	22	64	34
8	0	0	2	0	0	0
7	0	2	0	0	0	0
496	570	987	1218	485	700	1062
10	0	2	0	0	6	3
1	3	0	2	3	0	0
134	84	73	136	163	129	104
247	125	119	248	158	207	193
15	0	0	0	0	0	0
3	3	0	0	7	8	0
0	0	0	0	0	0	1
1048	1331	1411	1896	1257	923	1375
73	63	42	69	14	35	8
0	0	0	0	20	0	0
57	39	18	65	73	47	29
53	52	52	36	72	50	41
72	26	38	115	17	98	9
8	27	19	25	14	16	32
53	61	20	63	57	55	37
13	8	23	7	30	53	9
120	70	118	135	137	177	60
45	29	48	57	36	99	24

For Review Only

807	748	502	992	702	1069	808
31	40	45	76	56	60	57
684	610	737	819	916	602	600
196	23	167	217	52	0	94
340	179	218	361	330	298	330
196	207	94	190	106	255	143
49	31	42	70	46	59	37
111	108	144	106	71	153	183
170	103	107	147	134	160	91
115	114	80	195	100	138	74
140	163	0	216	53	141	0
636	496	528	937	585	755	679
66	83	65	74	67	93	60
41	73	39	46	71	56	32
3	0	0	0	0	0	0
55	26	32	46	43	37	49
0	0	0	0	0	0	0
21	19	11	8	20	11	0
56	50	60	64	96	29	34
22	12	37	55	0	17	3
39	20	16	62	0	20	44
0	0	0	0	0	0	0
220	77	134	329	0	157	21
12	30	3	47	50	28	27
12	3	11	18	42	9	32
33	52	71	57	133	22	47
15	29	13	17	0	17	4
119	101	77	204	112	93	84
64	25	13	24	0	19	17
289	205	172	406	246	265	167
60	48	56	80	101	66	58
202	193	187	271	145	226	87
39	42	47	65	66	14	17
226	297	360	394	205	297	234
36	57	12	45	0	15	177
34	76	73	192	479	988	2
0	0	0	0	0	0	2
381	474	213	472	736	424	340
53	59	107	143	38	66	94
50	18	41	52	66	69	67
4	0	0	4	0	1	0
253	237	270	315	329	252	210
1	3	0	3	0	3	0
21	37	36	42	48	34	22
38	61	15	101	55	9	14
15	0	0	0	0	0	0
0	138	87	311	82	217	1
67	58	48	47	17	58	0
77	44	36	65	69	45	34
11	10	13	17	0	10	7

For Review Only

0	0	0	0	0	0	0
42	17	15	13	6	35	13
58	60	12	26	86	9	32
37	33	26	40	14	47	24
90	101	91	83	72	67	73
48	53	64	84	96	82	40
104	70	76	112	107	62	65
179	169	157	226	197	214	125
56	40	64	40	103	37	80
89	93	31	62	111	60	38
0	0	0	0	0	0	0
2	0	1	3	0	0	0
141	212	271	304	200	265	236
18	16	39	17	64	11	13
112	81	106	163	79	108	103
40	27	30	50	11	27	27
12	5	13	16	0	1	5
107	86	105	146	90	91	74
23	12	6	19	0	7	8
78	30	101	50	48	42	38
31	27	25	26	39	13	8
121	70	18	91	24	64	106
0	0	0	0	0	0	0
25	0	0	0	0	0	0
8	2	0	0	0	3	0
253	205	255	333	300	272	246
24	25	46	112	59	49	28
88	181	80	200	219	150	163
4	0	0	0	0	4	3
6	0	0	7	0	0	1
620	426	209	475	322	447	270
4	0	0	0	0	0	2
114	94	70	127	75	97	72
303	217	218	394	162	300	174
3	1	4	5	1	0	1
8	8	13	25	8	17	14
7	2	12	13	24	24	0
43	23	74	104	51	67	29
42	14	36	45	56	36	19
1	0	3	3	0	0	0
11	0	0	18	7	0	9
17	0	10	0	0	0	0
36	0	37	138	0	30	32
113	109	76	175	85	125	101
462	295	344	199	402	470	101
1	3	0	0	1	5	0
34	47	39	79	32	40	39
782	797	766	1278	671	966	691
3	0	0	0	0	0	0
18	19	13	11	34	21	0

For Review Only

21	18	3	29	57	60	19
96	94	92	150	111	139	56
5	1	0	0	0	0	0
84	79	110	156	96	87	116
266	239	231	369	257	229	153
9	0	0	0	0	0	0
334	162	235	357	312	625	241
14	10	4	0	36	7	3
3	0	10	8	0	8	7
6377	5622	0	2832	1742	0	5086
0	3	0	0	0	0	3
97	79	84	132	96	69	91
4	13	13	5	36	6	0
12	0	0	4	0	0	0
215	173	141	299	158	114	79
114	84	105	196	201	190	102
349	245	249	460	315	361	262
0	0	3	0	0	30	0
203	193	344	375	224	257	377
371	348	397	499	356	476	292
0	19	0	21	37	0	0
25	14	25	29	13	42	7
122	154	183	264	128	50	15
62	47	132	139	22	108	65
2	7	7	11	0	7	3
0	0	2	0	0	2	0
47	35	64	73	43	33	63
70	0	29	75	126	61	69
52	73	43	110	79	74	50
56	49	52	99	35	60	27
6	7	5	16	9	7	11
10	15	14	25	0	24	5
18	27	30	65	23	33	25
922	652	630	922	1115	586	891
0	106	145	0	0	769	0
663	386	344	557	440	473	377
3	1	10	1	10	0	1
3	5	4	21	0	31	7
1	0	0	0	1	1	1
16	10	23	23	24	15	29
13	13	11	22	18	8	16
0	0	0	0	0	2	0
0	0	5	1	0	0	0
38	10	9	26	12	13	27
7	1	3	13	1	8	5
89	51	74	93	98	60	56
140	30	44	0	0	115	64
81	15	53	94	0	95	4
0	1	0	0	0	1	4
74	71	64	141	92	119	84

0	0	0	11	0	0	0
9	13	5	10	0	13	9
1	0	0	0	7	0	0
22	12	12	22	34	36	13
3	1	10	0	20	1	19
31	23	20	21	22	18	7
0	0	1	78	39	28	26
144	150	163	182	180	113	100
10	30	22	55	11	41	42
21	24	5	18	0	13	7
0	0	0	5	0	0	0
9	17	2	30	13	9	6
80	50	81	122	94	59	53
94	100	90	129	115	130	96
6	35	17	75	0	40	10
77	48	64	106	83	64	51
0	0	0	0	0	72	2
0	0	0	0	0	0	2
0	0	3	0	0	4	0
20	15	0	41	0	8	6
0	0	0	6	0	0	0
13	5	11	23	0	24	16
192	215	205	49	90	253	209
25	27	34	18	26	23	46
63	45	102	85	134	84	83
146	217	248	297	360	204	218
0	0	0	0	0	0	0
146	85	37	110	82	66	60
0	0	0	0	0	0	0
0	2	1	0	7	3	0
15	10	32	37	26	9	27
460	361	0	98	190	511	366
0	0	5	0	10	9	0
1	1	1	22	0	0	0
0	0	0	0	0	0	0
0	0	0	0	0	0	0
2901	2140	1739	2908	2212	3036	1368
5	0	6	0	0	1	1
36	16	25	44	50	28	37
28	21	21	38	47	43	38
0	0	0	0	0	0	0
169	174	107	290	151	185	135
35	28	12	26	25	24	20
138	95	114	169	137	122	76
595	468	409	605	480	242	395
119	53	71	129	52	83	86
14	11	12	27	0	6	0
62	45	47	53	62	51	59
196	167	0	148	13	0	144
7	1	1	0	14	11	2

For Review Only

3	3	0	0	7	0	0
0	360	388	106	453	383	0
93	103	69	117	78	53	80
341	336	264	498	424	423	193
1	1	44	0	0	53	5
0	0	47	2	1	0	57
4	0	1	6	0	5	3
0	0	1	0	0	0	0
0	0	5	0	0	0	0
1	0	0	0	0	0	1
23	10	12	30	40	16	23
966	944	864	1512	812	972	696
1	1	0	0	41	0	0
86	68	33	144	0	99	147
0	42	79	196	85	0	47
120	87	93	152	113	125	72
71	47	59	73	101	45	44
39	18	12	56	16	44	12
0	0	0	11	0	2	2
2	0	0	0	0	0	0
38	42	55	65	52	29	38
39	34	47	75	64	67	32
35	15	13	27	61	12	11
6	4	2	9	19	8	26
1	6	9	0	0	7	0
38	19	21	78	27	41	23
88	67	79	126	83	167	65
0	0	0	0	0	0	0
206	214	199	434	163	163	267
4	0	0	0	0	4	0
102	98	126	214	165	149	138
79	69	83	85	128	86	62
140	102	95	115	49	57	59
0	0	0	0	0	7	0
15	18	23	7	2	10	29
156	440	274	390	520	163	401
181	143	135	270	74	219	103
32	6	9	36	0	12	16
11	20	6	24	0	39	3
38	37	151	100	61	11	28
0	0	0	0	0	0	0
15	8	7	19	28	35	10
33	16	22	39	24	28	43
0	0	0	0	0	0	0
36	36	34	79	22	25	45
25	45	18	28	50	17	17
27	30	29	50	28	49	21
31	27	32	50	19	31	24
58	51	22	67	85	66	83
250	233	195	345	201	291	225

For Review Only

153	159	157	234	191	145	136
96	104	114	126	91	71	75
127	147	135	199	132	76	212
8	30	42	64	44	31	30
0	0	0	1	122	0	0
39	30	17	40	64	40	26
102	152	109	142	151	150	147
19	16	0	19	6	18	11
35	31	32	71	25	60	33
0	0	0	0	34	0	0
71	150	155	172	228	102	149
67	2	2	63	0	60	8
24	26	24	52	35	30	45
65	63	30	86	130	113	73
48	63	102	108	74	64	65
7	18	7	14	21	8	20
86	26	43	65	0	23	29
80	37	56	50	107	29	56
44	70	55	88	95	70	61
25	0	7	11	0	23	9
0	2	0	0	0	0	0
0	0	0	0	0	0	0
14	0	0	0	0	4	4
196	270	156	359	201	127	99
34	8	5	15	17	4	48
19	18	22	32	9	18	18
72	117	130	156	149	102	92
0	0	1	0	0	4	0
0	1	0	0	0	1	2
7	16	7	15	29	8	24
2	1	8	11	0	2	0
0	0	0	1	0	0	0
213	215	132	307	182	205	121
15	26	25	46	6	19	22
0	1	8	5	0	2	6
3	3	1	7	0	4	5
4	0	3	1	5	3	0
76	49	35	59	22	40	52
0	7	20	25	0	10	4
0	0	0	2	10	0	0
8	15	8	31	8	5	14
0	0	56	3	4	0	58
1	5	1	0	0	0	0
9	19	17	11	26	18	8
1	0	0	1	0	5	0
124	113	97	172	133	98	104
19	16	22	23	28	22	25
0	0	0	378	870	0	0
68	41	18	81	0	45	12
0	0	0	0	0	1	2

For Review Only

6	0	0	0	28	0	1
31	26	47	39	44	31	32
0	0	0	0	0	1	5
32	103	31	77	247	215	0
53	46	68	92	35	64	42
19	42	0	58	0	34	26
62	121	64	252	133	194	136
10	24	24	17	25	13	9
17	26	15	42	22	18	24
1	63	137	365	123	171	127
179	154	164	259	132	169	94
80	76	80	69	56	34	41
1	2	1	6	0	1	1
30	35	55	79	31	14	19
0	1	1	0	0	0	2
8	29	27	17	63	31	21
45	48	84	74	88	43	99
55	31	62	74	90	40	49
5	6	3	23	0	10	3
436	460	470	789	533	547	564
4	2	6	7	0	13	2
4	2	5	6	15	2	7
14	20	10	18	8	12	21
22	15	37	39	53	40	44
0	0	0	4	0	0	0
190	235	172	365	155	209	181
29	1	19	0	44	13	46
292	265	228	134	318	119	24
64	33	18	55	112	44	0
157	157	143	546	0	147	34
37	29	14	44	16	14	16
836	653	569	1183	652	698	465
198	174	119	291	116	90	83
50	54	53	87	48	63	39
25	18	50	38	29	55	31
78	39	34	107	28	39	55
0	0	0	1	1	0	16
78	39	62	63	79	33	34
75	55	54	121	44	51	46
21	53	49	74	37	49	61
61	76	43	96	48	45	41
45	0	28	30	74	34	0
114	164	145	221	137	215	114
0	0	0	0	0	0	4
0	0	2	0	0	0	0
147	81	82	164	149	118	93
69	56	37	72	22	60	30
0	0	0	0	0	0	0
0	0	0	0	0	0	0
2	0	0	0	0	2	0

For Review Only

7	1	12	0	0	1	1
3	0	0	0	14	0	0
0	2	1	0	0	6	3
109	61	49	91	64	71	45
136	154	178	178	159	183	167
1	5	6	1	0	3	9
51	55	46	58	53	83	52
6	1	2	17	0	1	15
117	9	9	50	0	91	58
46	31	15	52	0	48	21
169	132	120	205	144	143	105
949	659	595	1220	507	795	389
16	27	23	36	23	18	17
2	3	4	3	3	2	3
0	0	2	0	0	6	0
169	135	0	225	232	225	165
10	9	23	21	6	11	7
110	108	71	139	112	140	53
56	53	46	72	66	58	38
10	26	19	16	28	4	13
54	31	30	112	23	31	34
100	124	60	102	197	165	53
37	110	42	52	85	61	45
228	167	239	278	296	295	173
30	22	26	27	13	12	11
0	0	0	0	0	0	56
35	35	39	50	66	53	51
62	46	35	29	18	102	51
12	12	10	0	0	12	11
109	69	61	69	121	129	85
0	0	5	5	0	0	3
33	32	27	37	52	63	14
197	133	131	221	124	118	104
0	44	39	0	0	0	0
16	30	49	45	48	39	19
5	6	7	8	0	0	8
12	7	2	15	0	1	14
73	103	118	136	145	153	78
621	572	571	1051	639	680	461
3	0	2	0	0	5	0
4	7	15	5	15	3	8
0	0	0	0	3	0	0
22	23	46	48	74	39	17
6	9	5	0	0	2	14
0	0	0	0	0	0	0
0	1	3	6	0	4	3
5	0	3	0	4	0	7
41	20	32	64	19	48	30
25	25	23	40	28	33	13
99	94	129	143	172	129	75

For Review Only

86	76	87	113	71	96	72
65	57	62	72	54	68	48
22	7	80	99	0	29	26
21	2	9	9	4	7	10
272	204	264	223	418	223	229
0	0	0	0	12	0	0
15	13	38	26	41	18	7
27	9	16	45	26	37	31
0	3	3	5	0	4	1
115	114	71	130	69	170	91
38	12	23	48	23	41	17
52	51	56	86	41	36	65
2	0	0	0	0	0	0
79	18	67	0	88	127	12
47	79	75	93	78	69	54
13	22	12	27	17	17	13
2	1	1	0	0	2340	1
117	94	18	109	52	15	52
124	94	117	182	133	114	120
44	28	48	65	51	48	57
49	56	66	87	116	35	81
39	31	46	70	54	52	31
27	37	35	109	0	2	2
57	69	51	91	42	64	44
0	0	0	0	0	0	0
60	32	60	102	29	133	53
195	148	149	356	237	288	166
113	143	55	233	113	202	98
155	201	167	359	106	150	113
56	36	29	49	70	79	73
197	87	172	221	112	231	146
7	8	3	8	1	7	8
0	4	0	9	0	5	0
37	8	6	20	24	16	10
0	0	4	7	0	4	5
9	6	2	0	0	17	4
0	0	0	0	14	0	0
50	64	70	95	38	44	72
0	3	0	2	0	3	0
156	111	84	189	194	132	160
1	1	0	8	0	1	6
0	0	0	0	0	0	0
0	0	0	0	2	4	0
349	433	578	350	421	716	315
112	83	123	210	128	120	109
1	1	1	0	0	1	1
5	0	3	8	0	2	2
109	142	217	196	124	158	133
45	23	40	72	18	48	51
50	34	35	74	29	11	49

For Review Only

224	213	170	380	234	251	244
217	170	204	295	326	268	235
83	111	96	165	74	95	82
107	101	78	87	174	4	103
13	14	6	11	0	6	5
24	24	26	30	39	22	37
70	49	13	30	85	5	20
82	96	90	99	116	98	83
191	194	155	351	203	199	208
0	0	0	0	0	0	0
48	20	57	51	112	71	26
40	27	20	57	19	49	39
39	41	32	52	54	31	21
85	64	40	73	128	102	36
25	1	2	9	0	10	14
1	0	4	11	1	0	0
1	0	0	1	0	2	2
4	0	0	0	0	1	3
0	0	0	11	0	0	0
50	54	38	116	39	80	35
80	50	63	100	53	80	82
3	0	0	13	0	0	0
47	45	37	126	81	144	48
1	0	0	0	0	0	2
139	119	124	202	121	130	133
48	72	79	140	116	75	51
32	26	26	43	39	32	34
138	205	84	182	71	158	50
64	80	23	69	40	18	22
2	0	4	4	7	5	1
4	1	4	7	0	1	0
5	3	2	10	0	7	2
0	1	0	3	0	0	1
2	1	5	0	0	14	1
5	1	0	14	0	0	1
119	143	95	175	90	135	103
0	0	0	85	0	0	0
26	27	14	47	38	41	22
0	0	19	0	1	5	7
0	0	0	0	0	1	2
114	76	103	158	106	134	71
134	263	255	273	151	238	106
0	0	1	49	0	41	24
0	0	9	14	12	0	0
44	42	44	42	75	31	26
26	25	15	48	9	14	19
87	61	72	104	43	115	50
0	0	0	0	0	0	0
3	0	0	5	0	3	0
16	0	0	0	77	0	47

For Review Only

13	0	12	3	17	6	0
8	4	3	8	9	4	6
502	486	378	406	89	1117	188
82	72	44	97	36	61	43
0	0	0	1	45	46	0
139	127	90	165	185	128	92
155	102	105	155	73	177	65
4	7	0	0	14	11	6
2	3	4	0	9	0	0
0	3	4	4	0	0	1
12	17	36	28	43	24	33
182	28	375	53	310	436	357
97	153	160	199	230	170	148
72	95	45	106	69	60	5
0	56	0	36	1	0	0
70	64	54	108	79	72	43
116	99	77	132	79	101	71
70	27	65	63	118	56	12
208	159	112	158	252	232	123
152	147	170	222	173	154	138
248	0	0	153	8	258	0
120	88	84	203	97	109	74
377	344	212	518	350	287	264
0	0	0	4	2	0	0
95	59	83	144	76	99	96
114	100	74	91	59	98	45
17	24	17	32	21	14	13
56	60	30	87	37	50	51
107	84	64	99	104	66	80
35	63	39	48	91	40	20
1	54	1	0	173	43	94
31	40	45	64	25	26	29
1	1	1	0	0	1	1
76	68	75	104	76	72	69
95	38	56	81	44	31	55
87	87	0	4	92	87	70
32	29	43	43	34	23	24
37	42	30	96	26	56	89
89	8	1	212	96	46	1
17	17	9	37	0	18	9
316	302	411	563	399	334	301
145	116	111	161	127	131	126
58	41	26	40	95	110	55
138	117	155	233	117	160	144
12	1	1	6	3	8	1
2	0	0	0	0	0	1
26	154	10	123	162	169	19
311	0	163	252	319	254	0
27	74	30	59	74	38	44
0	68	0	35	0	16	25

For Review Only

19	3	4	8	1	7	7
28	21	9	14	8	9	11
298	170	134	187	237	365	208
31	44	26	42	34	39	23
3	5	0	8	3	1	8
20	25	7	13	0	9	14
3	2	0	0	0	2	0
0	0	0	0	0	0	0
137	143	100	166	135	137	86
38	50	69	102	48	80	93
0	4	19	0	0	0	0
0	0	0	0	0	0	0
0	148	125	27	323	16	163
10	17	17	16	31	11	16
276	347	304	387	306	412	311
47	63	28	81	98	73	47
0	0	0	0	0	0	0
1830	1471	2159	868	3024	0	0
0	0	0	2	8	3	3
4	1	6	0	0	1	8
19	10	12	10	7	7	6
3	10	56	75	20	8	21
42	44	22	38	13	18	10
193	163	211	265	211	168	139
55	36	53	122	51	44	78
31	60	81	73	18	42	39
31	35	29	125	163	69	71
0	10	0	0	0	0	0
43	78	27	60	20	39	35
4	0	0	0	0	0	0
0	0	3	0	7	2	0
1	0	2	0	0	5	2
66	56	65	104	59	73	51
0	58	53	51	80	103	0
502	263	281	469	320	437	353
22	7	6	9	25	9	8
187	7	1	22	0	1	16
238	171	175	236	190	206	158
134	133	102	211	161	159	124
0	760	0	683	629	50	694
135	171	139	195	173	132	76
554	431	305	653	314	380	285
10	71	12	62	125	1	1
57	60	83	0	0	39	87
210	79	26	329	229	179	149
113	68	204	47	170	27	62
30	11	32	95	12	82	41
44	93	68	102	116	90	60
35	38	51	76	45	70	73
116	53	48	79	104	67	45

For Review Only

52	34	27	105	37	44	49
2	0	2	0	0	2	0
0	6	0	7	6	7	0
316	310	405	593	406	464	365
71	125	162	275	0	106	236
45	52	33	44	51	44	56
269	221	80	240	213	224	272
10	9	15	23	0	6	7
275	254	223	385	390	356	176
21	17	33	56	0	8	54
31	32	19	43	18	22	16
67	25	33	69	32	26	34
202	166	149	324	263	280	82
5	0	2	9	0	8	0
47	44	33	54	35	54	50
452	397	354	691	430	528	305
24	25	10	19	32	14	24
50	5	4	33	0	49	2
0	9	8	0	0	0	0
6	12	12	34	8	7	11
387	0	0	173	128	530	0
145	158	114	243	173	125	166
6	10	37	37	0	14	50
1	0	0	12	0	0	3
53	66	62	88	72	55	50
215	234	116	106	339	2	252
10	9	34	207	0	75	54
3	0	0	0	4	0	0
358	466	654	678	737	314	611
0	0	0	10	0	0	0
9	0	79	0	33	0	36
99	819	468	312	1185	434	525
375	705	487	1081	516	478	440
70	152	12	73	373	113	1
4	0	0	0	0	3	0
120	136	115	163	106	126	70
48	34	36	63	53	33	56
60	40	42	89	24	43	59
0	1	0	0	0	0	0
2	0	0	0	0	1	4
53	59	63	62	72	30	57
364	401	254	453	182	51	140
45	7	12	43	0	6	11
3	1	4	4	7	3	8
0	0	0	2	3	0	0
64	22	37	55	20	51	17
0	2	0	0	24	0	10
31	29	0	80	0	28	36
221	220	93	90	122	204	203
0	0	0	2	0	0	0

For Review Only

279	245	232	365	300	334	235
140	121	69	150	134	134	76
4	0	3	6	0	8	0
548	615	668	1028	445	573	585
118	73	91	183	88	117	138
109	94	155	201	105	121	112
57	54	54	92	16	53	39
0	6	3	10	0	52	0
154	180	133	219	154	144	92
65	68	101	228	117	154	73
138	82	55	171	41	112	52
108	102	92	162	143	69	84
64	67	93	155	45	69	68
123	198	135	185	168	196	138
200	165	118	299	211	199	163
135	62	228	326	204	169	3
0	0	0	1	1	0	0
24	30	30	26	21	12	14
71	39	64	75	65	67	76
18	47	24	47	23	31	38
11	22	30	22	32	19	11
48	38	19	81	20	43	30
4	4	8	0	26	4	5
25	26	24	53	18	22	0
14	9	10	22	41	11	8
344	238	344	367	411	233	287
20	5	4	9	0	2	2
44	58	72	84	53	43	53
54	46	47	99	53	52	54
28	37	20	52	21	18	22
13	8	0	0	26	9	0
1143	928	876	1035	1486	1054	775
0	0	0	0	0	0	0
107	107	176	254	165	118	129
28	26	23	47	24	25	17
40	35	34	51	56	18	27
624	518	531	729	719	624	507
80	68	59	75	68	68	48
39	45	35	88	29	83	52
13	11	20	25	13	22	18
0	0	2	0	0	0	12
92	30	4	51	0	41	19
37	140	81	91	125	27	76
91	58	93	153	52	77	64
6	22	32	58	39	22	32
47	20	23	40	13	19	35
74	81	55	77	53	61	39
0	0	0	0	0	35	0
5	7	5	13	0	2	1
48	22	24	86	0	88	18

For Review Only

417	67	138	229	0	145	9
3	1	2	0	0	0	0
21	21	24	62	36	29	12
35	161	19	222	190	156	107
1307	1213	0	1	362	661	0
565	533	557	660	631	523	400
92	134	104	155	168	75	171
1156	0	849	365	9	421	0
370	383	357	626	346	371	297
192	155	166	187	179	133	126
0	4	0	0	10	0	1
159	119	79	141	124	98	70
0	0	3	1	0	2	1
0	2	0	0	0	3	2
235	251	242	353	180	245	273
0	0	0	0	0	0	1
0	1	1	0	0	11	21
0	0	0	0	0	0	0
73	101	67	98	114	85	123
47	61	37	58	0	21	2
1	0	0	340	87	0	0
1144	996	922	1637	1067	1152	861
240	195	86	234	194	247	185
137	110	97	159	112	116	98
38	36	29	102	24	135	39
194	196	145	305	173	313	144
84	68	97	93	114	420	111
0	0	0	1	1	0	0
11	12	30	24	13	28	25
325	196	180	362	355	314	345
0	1	4	18	0	3	5
128	231	155	221	134	287	193
13	9	28	39	0	23	19
63	67	62	79	106	77	16
165	173	101	547	129	582	151
76	51	100	113	118	74	61
96	128	50	0	129	70	9
58	61	68	94	72	57	70
109	96	109	186	70	130	63
106	83	89	97	89	69	57
166	143	124	238	127	143	108
107	80	89	122	85	58	100
16	12	17	15	18	9	13
1	0	0	0	0	0	0
33	23	14	35	22	20	15
18	9	11	24	0	3	5
0	0	0	0	1	0	1
319	266	296	409	223	313	0
33	42	0	61	76	39	30
0	0	0	5	0	3	3

For Review Only

3	5	1	4	13	1	1
18	0	0	0	0	0	0
0	32	41	32	9	26	39
0	0	0	6	0	0	0
65	57	52	114	131	94	79
711	382	675	723	913	700	415
0	2	0	0	1	0	4
1	0	4	6	0	1	9
2	0	4	0	0	0	0
99	0	73	143	7	87	74
13	9	12	0	18	3	8
26	47	42	54	53	47	64
40	48	23	49	44	53	42
121	127	140	209	198	215	167
173	205	193	332	249	131	237
5	1	1	5	0	0	1
270	131	113	202	100	172	188
19	21	7	27	0	0	13
62	59	34	61	121	68	43
87	46	77	95	95	52	64
5	8	6	13	0	2	4
1	1	4	26	0	2	23
112	36	79	79	64	54	92
189	109	134	254	243	152	158
53	60	54	86	27	36	37
110	90	116	150	99	94	78
11	0	0	0	0	0	0
6	55	1	408	0	5	18
0	0	1	0	5	0	1
138	114	142	206	163	190	189
38	0	32	0	132	42	20
309	282	243	419	251	348	213
524	555	317	675	331	430	394
49	19	43	45	19	36	17
106	90	66	168	123	84	33
130	139	49	131	71	139	75
76	54	59	111	59	75	70
129	89	100	135	134	123	92
12	42	4	0	29	1	47
63	89	13	94	72	30	25
63	82	2	183	0	2	40
76	58	72	102	53	58	60
77	83	96	133	94	58	41
2	14	11	23	23	7	8
43	170	3	221	127	39	66
71	97	59	38	78	87	65
0	34	47	74	122	96	58
163	165	161	78	286	189	159
71	46	60	101	129	93	95
79	82	111	112	121	82	80

For Review Only

13	8	22	14	20	23	5
22	16	0	13	39	27	14
7	5	13	13	0	7	16
72	31	27	50	25	51	31
31	140	108	153	26	94	116
34	27	52	49	22	19	60
145	98	68	129	200	90	129
87	76	64	115	68	49	46
18	91	0	208	81	0	79
0	0	1	0	0	0	8
3	0	1	0	0	0	0
96	31	33	62	107	23	78
53	88	82	86	31	65	85
39	30	79	51	88	29	72
46	17	39	52	9	12	15
0	94	98	33	90	81	117
110	0	148	0	98	144	0
14	51	43	103	13	42	69
155	58	120	128	275	83	74
1	0	1	0	0	0	1
14	35	6	39	22	18	13
29	0	0	11	24	0	0
6	0	0	0	0	0	0
331	418	324	630	404	373	312
9	0	8	2	11	5	14
106	136	122	182	119	117	102
7	2	8	19	0	33	34
212	199	263	384	189	205	194
12	2	3	6	5	9	4
14	1	0	0	0	0	0
385	478	214	333	246	216	319
1014	789	811	550	1021	0	0
961	739	558	1237	550	899	441
0	182	8	12	6	39	153
3	5	3	10	3	10	3
244	0	56	0	93	13	171
75	56	61	147	0	76	42
296	335	340	517	347	307	225
1	1	1	105	0	1	1
54	45	46	95	36	54	46
76	177	44	130	0	57	77
0	23	116	8	10	17	40
160	121	159	248	105	130	162
79	0	0	0	0	80	0
0	0	0	45	0	0	0
0	23	21	51	0	17	19
62	43	38	218	0	23	0
18	25	17	27	28	28	19
67	53	32	0	0	45	52
557	392	459	881	516	434	608

For Review Only

1042	814	793	1478	549	1069	716
0	608	593	1018	0	725	510
137	53	108	105	223	415	109
676	444	478	558	573	494	352
0	0	4	18	0	0	7
0	0	0	247	34	0	0
352	200	0	1115	0	213	0
458	454	411	685	376	499	389
165	0	0	10	246	110	127
249	300	305	511	229	262	209
1	0	5	0	0	3	2
249	123	142	191	261	234	164
358	353	334	525	296	284	247
401	384	375	608	350	405	315
178	114	107	204	148	118	106
197	190	184	289	199	124	134
205	190	214	449	136	216	212
195	276	270	404	260	266	176
20	3	16	16	11	11	13
74	46	98	0	174	96	74
181	164	87	231	155	120	195
2	12	8	0	0	16	1
1156	710	795	1535	780	926	725
348	451	519	444	312	341	488
263	317	310	385	400	355	439
7	13	7	16	0	1	8
0	0	4	5	0	6	8
3	0	0	2	0	3	2
5	1	4	4	0	1	1
183	181	159	255	250	156	191
33	22	40	51	47	13	10
51	36	28	85	39	45	22
1	0	0	0	0	0	0
43	54	62	70	28	33	61
0	0	0	234	1	400	286
0	0	8	3	25	1	0
74	52	83	94	130	124	39
1	6	2	0	0	0	0
194	219	207	301	232	128	219
82	111	62	158	45	100	88
290	239	281	413	377	404	190
49	31	38	87	37	52	68
59	85	86	108	121	69	43
243	394	281	307	218	155	9
37	23	35	52	60	25	30
23	53	68	79	8	12	81
125	107	63	141	129	88	62
0	0	0	9	0	3	4
16	10	11	22	9	17	12
22	24	33	63	14	14	35

For Review Only

136	222	175	342	259	142	136
3	1	0	3	3	3	3
23	16	18	41	19	13	18
0	0	0	0	0	0	0
28	60	19	64	69	39	47
18	12	26	30	8	25	27
118	154	149	181	176	215	140
1	2	2	0	0	1	6
30	20	16	38	0	13	2
25	40	22	44	0	8	4
2	1	3	0	0	1	1
0	0	0	10	8	0	4
0	0	0	0	0	0	0
0	0	12	0	0	1	3
13	7	4	4	24	0	14
58	58	77	91	64	45	77
4	4	5	10	3	1	3
7	7	4	14	0	20	18
34	7	15	32	0	6	1
7	1	1	14	7	0	1
22	29	24	45	26	33	24
31	34	50	64	41	49	36
74	89	56	50	91	61	35
133	89	110	158	165	163	210
22	62	40	65	81	62	87
40	37	21	62	34	35	32
0	1	0	1	0	0	0
25	36	65	55	34	25	8
3	1	0	0	4	3	3
5	2	5	4	0	0	0
4	0	0	9	0	0	0
143	125	136	180	219	112	72
203	123	107	171	208	133	89
21	31	78	79	18	42	55
0	0	2	5	0	3	0
66	73	57	71	99	68	42
0	0	21	22	26	19	17
0	2	4	8	0	0	1
0	0	0	0	0	0	1
0	5	0	5	0	0	3
99	97	191	176	221	94	124
14	7	12	28	0	7	15
1	0	4	4	0	4	3
2	3	1	0	0	4	1
2	1	1	4	0	2	1
38	25	27	35	68	42	22
1	2	1	0	0	1	1
3340	4220	5026	6616	1811	3081	2858
3	5	0	1	0	5	0
159	139	127	202	145	160	141

For Review Only

43	29	24	33	23	29	19
7	3	4	1	7	4	10
55	67	106	93	100	60	35
165	57	76	129	66	126	101
83	80	53	62	27	46	22
2	22	26	0	36	11	4
62	27	44	58	62	31	32
24	29	29	20	8	4	9
0	0	0	1	2	0	0
0	0	0	5	0	5	3
8	7	2	7	1	1	3
1	0	1	0	0	5	1
1	19	1	1	0	1	3
2	7	13	4	17	0	1
2	1	1	0	0	2	1
82	39	50	94	59	91	68
63	78	80	143	170	69	219
1	0	0	3	0	3	0
36	33	40	57	46	57	27
0	0	0	3	0	0	1
115	63	51	122	39	59	27
3	2	2	8	0	3	2
133	458	558	338	515	403	67
0	0	206	0	0	0	0
282	493	12	464	0	272	769
18	10	12	38	7	25	12
46	80	92	66	134	44	40
58	57	44	95	65	54	45
26	10	1	577	0	2	1
0	11	4	5	10	4	0
12	4	1	10	0	1	3
16	22	6	21	18	2	30
0	3	0	0	0	2	2
62	24	56	63	63	40	25
56	56	66	125	50	48	68
0	0	5	0	20	18	6
97	87	153	155	45	59	152
112	106	99	198	106	197	113
6	10	12	19	19	13	11
45	39	17	0	23	30	27
12	11	11	25	75	4	19
14	13	9	8	23	0	0
91	142	69	146	143	78	57
42	21	16	39	43	7	12
117	72	87	161	99	90	75
83	73	65	113	106	68	73
21	17	20	33	12	10	24
28	26	14	52	18	40	27
33	32	29	25	35	23	17
28	18	21	39	13	21	14

For Review Only

0	0	0	0	0	1	0
9	9	0	10	20	0	6
5	7	4	7	2	1	4
1	0	1	2	0	0	0
75	67	51	88	141	19	77
152	142	149	217	196	207	134
49	239	95	255	45	28	27
154	165	113	40	236	222	122
1	0	1	1	0	6	4
28	54	93	123	131	49	49
27	9	21	40	22	8	20
4	6	0	2	9	6	0
0	0	0	0	10	0	0
0	0	0	4	37	0	0
51	55	55	109	48	34	58
76	50	106	160	101	120	82
88	56	72	146	66	69	105
144	135	172	216	154	121	119
17	7	6	20	8	12	8
8	23	2	120	94	30	14
0	8	5	13	0	9	15
44	10	13	36	47	41	8
30	22	49	25	38	64	22
29	19	28	43	21	22	12
2	74	39	49	0	10	34
0	9	10	10	13	14	0
34	0	0	0	0	18	15
120	144	137	154	110	125	79
69	109	81	204	34	22	75
35	11	74	52	0	28	14
31	30	15	47	24	16	29
80	110	78	157	130	77	70
0	0	17	49	0	0	0
185	142	217	320	297	243	248
116	151	181	246	192	159	146
17	62	36	39	130	29	60
0	0	0	1	1	0	0
5	156	64	108	1	3	24
1	5555	1	0	0	1	1
1	0	1	3	0	5	1
11	8	3	5	17	0	4
104	77	86	63	74	79	63
23	7	5	23	0	14	6
16	56	61	103	91	63	10
17	1	1	0	0	5	1
63	46	34	82	87	52	36
67	24	35	161	21	57	83
33	9	5	47	38	0	0
28	41	35	0	0	37	22
17	24	17	35	9	16	25

For Review Only

1	1	0	0	0	0	1
124	123	108	174	128	137	112
3	9	0	5	0	2	2
232	371	427	656	123	270	301
22	11	13	26	16	11	16
0	0	0	0	42	0	0
5	9	9	28	0	10	2
15	8	12	13	0	7	2
5	11	4	6	0	3	3
8	0	4	9	0	1	1
0	0	0	0	0	33	3
3	0	0	5	0	0	0
0	0	5	4	0	0	0
0	0	1	3	0	0	0
0	5	0	0	0	0	0
34	13	21	30	34	18	14
0	17	10	19	24	14	10
2	1	4	0	0	0	0
3	5	12	16	0	3	7
4	0	1	0	3	1	3
49	47	29	84	34	33	30
29	12	11	24	16	3	11
0	0	1	0	0	2	2
0	0	0	2	11	8	0
8	22	56	37	0	9	13
0	0	0	0	17	0	0
1	0	0	0	0	8	0
111	119	136	216	170	113	112
394	365	329	562	342	295	358
0	0	0	0	0	2	0
7	44	36	0	37	109	1
111	164	141	218	223	134	191
5	0	7	19	0	13	12
3	0	0	1	2	0	1
0	0	0	4	0	0	3
59	47	30	82	42	38	25
38	46	42	60	49	54	34
49	42	36	75	45	42	22
7	62	26	180	45	52	0
0	0	0	1	1	0	33
0	0	0	14	1	60	69
151	327	116	0	158	322	74
6	3	2	4	11	8	1
9	2	7	18	43	24	5
33	11	114	184	42	13	111
211	0	154	390	73	187	161
6	1	4	5	2	1	4
79	0	75	22	23	44	69
696	510	545	846	1087	1249	465
134	98	125	126	116	89	107

For Review Only

0	0	0	1	94	0	32
75	75	62	118	83	104	63
11	14	18	23	8	13	3
0	0	0	5	11	7	9
49	70	51	108	32	48	35
12	5	4	10	7	8	3
19	9	15	26	25	33	25
230	125	187	94	2	233	0
7	1	1	9	13	17	11
160	122	106	261	184	163	110
133	93	101	196	97	191	61
49	175	243	459	344	202	252
177	141	151	238	147	236	113
121	112	105	186	137	138	82
31	28	22	40	51	20	31
10	11	18	17	16	15	18
29	23	16	36	18	24	16
7	0	0	3	4	0	0
54	93	81	83	97	46	64
0	0	2	0	0	2	0
35	4	1	0	130	114	4
4	5	9	18	0	38	10
1	5	0	13	0	1	0
8	12	8	22	17	12	6
30	36	44	75	35	53	59
2	1	1	0	0	1	1
50	71	51	73	73	42	47
162	183	165	259	113	207	91
0	0	0	0	0	0	0
2	1	0	0	0	0	1
69	72	113	66	11	15	58
5	4	0	4	0	3	0
586	615	625	0	0	713	709
2	0	0	0	0	1	0
453	274	358	342	502	191	115
296	164	177	359	125	283	121
5	0	13	16	11	11	4
1149	905	0	1273	503	433	769
1196	884	959	1846	806	1099	633
135	95	131	139	191	133	110
136	25	61	110	21	71	37
79	56	102	102	98	101	74
1	3	1	6	0	3	4
233	286	329	480	368	248	386
30	18	15	27	17	24	33
117	75	71	137	81	139	95
8	4	10	26	0	21	14
4	42	86	0	119	235	68
616	575	445	932	351	552	501
8	7	5	0	0	5	0

For Review Only

35	30	34	0	0	24	27
0	10	8	14	8	0	4
15	22	18	45	22	13	9
55	79	52	68	84	64	45
101	91	51	145	41	51	49
0	0	4	1	4	0	0
106	69	85	124	118	115	89
35	121	16	141	0	54	71
54	39	35	51	43	39	25
16	4	53	28	0	52	148
1	3	73	0	142	1	9
20	23	19	18	13	21	10
0	4	0	0	0	0	0
572	630	666	1235	631	683	658
2	0	0	0	0	0	0
0	0	1	0	0	0	0
0	0	2	0	0	0	0
32	30	39	61	15	72	24
132	159	208	327	134	179	199
40	52	17	72	46	81	35
15	29	35	59	23	23	38
204	195	167	317	224	164	142
1	0	0	0	0	1	0
6	8	0	4	1	0	5
21	28	22	42	49	31	35
273	252	236	368	198	173	174
83	44	54	75	45	58	37
244	184	195	278	256	222	222
79	69	89	133	83	86	80
3	0	0	0	0	0	0
39	8	33	11	53	3	19
61	103	124	204	214	71	81
1	1	1	0	0	6	1
7	6	12	25	0	8	3
0	0	0	18	0	0	87
9	0	6	7	4	5	11
22	31	4	61	9	34	28
17	26	13	32	45	26	25
11	36	35	0	70	14	1
31	1	1	0	0	1	0
38	37	28	50	71	47	59
46	38	31	65	30	54	39
2	0	2	0	0	4	2
0	0	0	0	0	0	1
52	53	31	52	15	26	15
191	185	198	370	261	211	250
83	56	60	124	54	108	138
77	54	66	68	92	63	26
160	77	118	237	99	195	105
4	0	4	0	0	0	0

For Review Only

14	18	14	37	16	30	12
33	51	47	51	49	31	60
93	94	118	207	109	129	104
0	20	12	0	18	0	20
13	91	37	93	171	44	84
223	391	331	376	527	192	390
74	216	149	225	323	132	117
35	36	0	1	2	0	0
142	131	75	121	95	154	55
6	1	0	5	0	7	0
146	121	122	150	188	117	111
68	171	50	106	96	96	31
15	6	5	6	7	11	8
35	0	29	40	85	10	35
27	39	34	30	26	24	28
88	76	68	126	112	81	74
44	32	65	62	57	44	28
340	0	338	1023	245	317	253
54	0	0	18	0	0	0
37	65	47	35	95	18	41
98	126	69	102	58	121	41
98	143	158	181	190	136	101
32	37	25	42	28	27	41
1	3	0	1	0	0	0
2	6	2	7	1	4	1
100	144	108	151	131	109	118
8	7	6	19	0	36	8
259	222	155	355	140	268	204
95	99	81	172	94	124	64
923	671	696	1608	957	892	640
105	53	17	82	29	72	28
0	0	1	0	0	0	1
101	117	187	230	115	112	82
77	85	91	108	75	53	115
31	59	49	47	94	27	57
96	65	45	81	33	57	38
2	2	7	4	0	7	7
0	1	0	0	0	3	2
173	1	1	0	0	1	6
1	1	3	0	305	1	1
43	48	41	58	31	20	11
0	0	0	0	0	3	0
67	84	58	90	42	36	58
3	2	1	0	0	3	3
303	249	302	458	410	335	247
89	72	59	116	81	88	31
95	83	92	177	82	130	84
52	50	57	85	85	62	51
0	0	0	0	0	0	353
1174	131	282	711	391	1016	751

For Review Only

0	5	5	4	12	4	5
325	291	193	378	170	270	197
0	5	5	12	0	1	0
4	0	1	8	0	0	0
96	63	75	96	70	88	80
8	0	0	3	0	0	0
4	9	0	13	0	0	0
259	207	171	367	167	235	196
49	38	40	48	61	40	7
45	55	81	93	68	50	44
41	37	28	54	42	35	30
21	15	45	59	12	43	58
271	177	218	52	253	266	116
27	25	40	37	28	34	17
5	0	0	0	1	0	0
2	0	7	3	0	1	1
2	18	13	16	0	6	10
0	1	1	0	0	28	1
2	1	2	0	0	3	2
1	0	0	0	0	1	1
2	4	2	9	0	5	4
1	2	0	0	4	0	0
19	11	15	16	9	33	19
26	121	54	145	56	243	86
50	53	58	87	64	70	58
2	9	2	10	0	8	1
82	87	106	140	133	66	77
7425	7999	7893	14174	9312	9385	6863
9870	10811	11576	18387	12070	12065	9758
96	72	58	132	56	85	39
0	2383	3234	8984	2173	0	3199
0	0	0	0	0	2	0
0	9	2	16	0	7	3
0	3	0	6	0	2	0
31	27	33	41	27	66	17
0	3	0	0	0	2	0
0	0	0	0	0	6	0
1	0	4	0	0	0	1
0	0	0	6	0	0	0
1	0	0	0	0	0	0
7	11	12	22	0	16	9
41	62	49	44	38	26	28
26	23	29	32	31	32	17
126	123	117	170	124	120	104
57	39	34	51	31	28	28
1	2	5	24	0	1	7
379	281	339	608	306	419	249
47	35	67	42	32	61	35
1	2	10	0	5	2	1
0	0	2	0	0	1	0

For Review Only

3	0	2	0	0	0	1
33	27	41	41	30	33	35
68	56	79	137	65	79	66
990	726	684	521	588	843	843
0	0	2	0	0	2	0
2	2	9	8	0	3	3
0	7	0	0	3	0	0
6	14	9	0	23	0	0
14	0	3	5	0	1	2
1	0	2	0	0	0	3
401	293	250	497	188	460	172
404	340	305	474	65	40	27
2	0	0	0	0	1	0
1	1	1	1	0	1	1
176	100	119	143	108	167	100
0	1	0	0	0	1	1
1	1	1	0	0	1	1
2	3	3	9	0	2	11
4	3	3	24	0	53	4
5	5	5	0	29	1	22
121	113	131	165	93	109	115
253	7	51	156	0	14	33
242	166	157	299	275	341	142
109	215	175	194	402	250	290
36	50	45	75	32	21	31
5	4	1	5	0	13	4
21	10	13	34	12	16	7
0	0	0	0	0	0	0
9	0	10	2	1	13	10
3	2	10	36	0	14	3
71	60	80	85	70	108	83
4	0	1	2	0	0	11
16	0	0	0	0	0	0
1	17	5	0	28	1	2
0	0	11	4	19	6	11
17	13	12	23	27	16	22
18	2	11	6	7	7	10
33	29	26	54	13	34	25
92	84	88	104	94	73	73
87	161	91	189	122	74	56
0	0	0	78	48	0	0
261	247	248	453	246	269	214
1	0	0	3	0	0	0
0	0	0	4	3	0	5
0	0	11	0	10	0	0
126	109	68	111	128	113	44
54	61	37	70	24	68	42
3	2	0	0	0	0	0
266	324	212	436	238	306	233
524	529	673	898	798	460	410

For Review Only

879	894	1153	1530	807	858	896
16	15	28	45	60	28	33
18	2	0	0	0	0	3
150	595	1	2704	0	16	1062
0	0	0	0	0	0	0
273	281	158	241	130	351	68
0	0	118	64	13	0	0
263	273	280	415	205	301	281
16	13	15	15	12	10	10
21	15	12	28	11	17	17
134	211	230	258	195	149	242
89	68	95	147	41	79	130
23	24	39	91	89	51	49
5	0	0	0	0	0	0
80	68	47	55	58	65	45
69	55	104	125	67	76	61
31	49	26	43	0	33	19
3	0	0	0	0	0	0
0	0	0	64	1	0	162
91	0	61	99	128	81	126
112	133	104	126	186	128	131
52	35	47	53	46	47	35
0	0	0	2	0	0	0
36	39	40	54	31	23	29
7	0	17	0	0	0	8
110	112	169	107	127	74	92
55	36	22	44	27	35	54
3	3	3	10	0	7	3
1692	2086	813	2151	154	1422	600
194	88	215	187	353	171	99
39	0	67	164	27	0	33
0	0	0	4	0	0	0
10	22	16	38	4	0	14
0	0	0	3	6	0	0
104	70	88	108	134	112	64
7	21	9	28	11	5	2
0	0	23	20	2	0	0
0	0	1	0	0	0	0
71	186	114	0	106	12	113
62	49	98	87	150	70	75
36	17	10	29	10	15	21
388	408	332	405	292	367	312
2	3	0	15	0	4	16
224	160	202	332	165	153	150
1754	1681	1990	2545	1932	2066	1651
803	0	87	443	478	676	0
4	6	4	3	6	1	0
24	0	1	17	0	1	1
3	3	0	3	0	3	0
3	0	0	0	27	1	0

For Review Only

3	0	0	0	0	1	1
22	0	0	19	0	0	0
0	0	0	0	0	0	0
3	1	4	11	0	3	1
0	0	2	7	0	0	4
0	0	5	10	0	0	3
19	19	20	15	35	14	0
0	0	0	20	0	0	0
190	156	120	212	126	207	153
78	0	0	0	0	5	0
33	27	35	37	48	46	17
75	38	15	64	62	65	25
0	19	19	31	59	0	93
0	0	0	0	0	0	0
102	0	0	29	0	0	0
4	5	11	11	10	0	6
9	3	1	11	0	1	13
44	60	5	189	0	125	167
70	49	65	96	21	34	43
4	1	7	6	0	0	1
78	117	72	96	144	45	92
5	0	16	10	13	10	9
0	0	10	3	10	5	0
79	78	85	107	115	65	101
167	149	180	230	200	230	179
0	2	0	1	0	2	1
1	1	0	0	0	0	0
27	118	206	170	0	165	83
0	6	11	15	0	0	14
71	0	0	135	0	21	80
78	59	75	96	106	86	49
6	41	25	55	0	56	92
53	49	42	64	42	46	30
21	25	33	29	18	26	18
8	33	0	0	53	131	66
20	59	46	59	37	48	28
106	103	34	138	34	110	55
0	0	0	0	0	0	1
0	4	0	0	0	2	0
195	147	115	191	157	278	151
58	48	35	65	117	47	80
1	1	3	20	0	4	2
248	207	165	310	129	183	102
58	8	18	53	0	59	26
47	83	37	78	12	51	21
11	3	0	17	32	1	2
1	1	1	44	0	1	1
8	4	7	1	0	7	1
5	0	10	8	12	3	5
225	147	130	189	102	218	125

For Review Only

74	80	106	177	135	77	139
24	52	44	43	59	65	48
373	585	539	571	250	231	323
27	41	24	66	66	84	35
41	64	77	116	42	71	31
0	9	0	0	0	0	9
0	10	5	0	0	0	0
10	22	27	13	7	9	10
40	26	31	59	61	57	39
0	0	0	0	0	0	0
7	8	6	8	9	12	9
35	26	56	68	65	38	56
0	7	0	9	15	7	2
52	55	68	117	61	51	69
52	24	18	34	69	51	18
59	55	28	75	65	72	56
26	42	32	40	48	43	21
0	0	0	2	17	20	13
27	22	24	61	23	29	16
33	20	16	28	42	26	24
14	8	1	62	0	2	1
4	106	71	241	215	137	81
0	4	0	19	30	18	0
130	100	138	173	127	135	95
39	34	41	112	0	20	40
16	13	12	7	39	0	0
1	1	1	94	0	1	1
48	48	55	80	14	0	75
0	0	0	0	0	5	0
2	0	4	15	17	5	1
0	0	0	3	0	0	2
1	0	2	0	0	0	0
0	1	0	3	3	3	1
33	85	47	103	0	26	64
108	97	0	138	0	87	0
1	0	0	0	0	0	5
0	7	5	5	11	7	0
0	0	0	10	1	114	0
8	7	6	7	2	11	7
37	34	32	31	45	19	10
60	95	53	93	46	84	40
62	43	26	115	51	13	33
49	49	55	106	59	58	38
0	3	0	0	0	1	2
15	63	5	0	0	102	23
0	0	0	5	6	0	0
9	3	5	15	0	19	19
0	0	0	0	0	0	0
14	3	7	14	0	11	11
6	2	0	1	2	1	5

For Review Only

37	13	14	23	52	39	18
47	10	28	49	0	26	9
31	71	10	79	0	33	42
36	33	13	50	12	36	35
30	23	29	48	20	23	10
45	17	11	26	14	39	20
0	1	4	0	0	1	4
8	13	12	19	10	16	17
6	3	0	2	4	0	3
19	29	38	55	76	59	43
24	15	22	21	27	15	18
48	41	45	53	43	41	50
1	2	0	0	0	1	0
4	1	1	0	0	4	1
26	28	10	33	42	26	21
0	0	25	174	123	71	77
35	46	15	36	50	50	17
12	12	14	10	19	8	6
0	0	0	0	0	28	7
1	0	0	2	0	0	0
66	31	42	82	68	46	32
11	16	22	29	40	17	12
7	15	9	5	0	27	11
4	3	1	12	0	2	2
0	0	0	2	1	4	0
25	29	23	37	15	24	19
214	193	139	311	216	248	160
140	173	188	259	163	128	214
36	28	35	34	19	36	19
54	57	44	58	31	50	46
14	0	15	10	18	6	6
16	13	16	30	16	16	19
36	22	32	45	20	39	27
82	118	82	160	62	50	111
50	30	52	36	15	11	11
25	19	20	29	0	63	20
0	0	0	2	0	0	7
15	21	13	48	13	25	22
16	50	42	67	0	15	13
0	0	4	0	0	0	5
126	88	90	0	0	107	65
5	21	12	22	27	10	9
0	6	5	0	0	0	2
19	31	5	37	0	17	3
2	4	0	8	0	1	0
1	0	1	0	0	0	2
2	1	8	5	0	3	1
35	58	37	59	102	47	84
0	0	0	2	0	0	0
19	27	10	19	27	12	5

For Review Only

36	23	31	56	43	34	38
92	41	69	121	50	104	54
44	31	73	45	94	21	80
2	1	12	0	0	30	110
0	0	0	0	0	2	0
1	3	1	0	0	1	2
14	6	6	18	37	3	5
110	15	27	94	0	78	41
13	33	12	45	0	72	10
0	0	0	0	0	0	0
276	286	264	452	319	267	316
1	1	1	0	0	1	1
4	0	3	1	0	3	5
59	164	0	221	29	310	30
3	0	1	7	0	0	4
0	49	0	40	5	0	74
0	0	0	0	0	5	0
10	1	4	16	15	10	3
7	7	4	7	0	7	0
0	1	0	0	0	0	4
0	0	0	0	0	0	1
0	0	0	6	0	0	3
0	0	0	3	2	0	0
0	14	0	30	0	0	0
160	166	232	282	468	241	270
0	8	0	3	0	3	0
87	179	154	236	136	138	123
180	72	12	48	121	42	64
0	0	1	3	3	1	1
10	4	6	0	9	0	24
2	1	5	8	0	4	0
7	0	0	12	0	7	3
34	47	30	41	50	39	45
91	82	74	153	89	122	99
6	1	0	0	0	0	0
246	234	28	0	146	18	1
0	43	33	10	31	48	11
3	0	1	5	0	0	0
16	7	16	26	0	6	4
93	81	32	78	107	145	57
1	1	12	0	620	1	121
55	63	58	91	48	49	41
132	236	217	291	497	119	312
13	15	21	28	24	8	24
37	22	35	79	23	66	40
279	165	116	234	167	198	154
0	0	331	1	239	233	278
171	75	69	178	68	130	92
0	51	263	100	44	0	63
77	112	88	122	86	90	69

For Review Only

11	5	6	32	1	27	6
18	2	23	12	66	13	50
337	291	281	394	351	251	211
6	5	7	11	4	6	7
5	14	41	40	35	24	33
13	1	4	11	5	7	4
42	47	26	41	36	62	27
3	7	9	8	0	17	6
1112	0	0	0	0	0	0
10	5	1	10	0	6	5
7	3	2	6	0	3	5
0	0	0	0	0	0	0
0	0	0	0	1	0	0
2	0	0	0	0	0	0
42	36	50	54	51	34	47
157	234	166	369	135	206	153
332	380	302	509	230	305	214
368	403	354	617	298	463	254
256	340	330	541	330	227	158
215	239	312	419	303	186	176
289	528	272	641	262	351	235
362	384	391	522	411	255	248
31	41	46	64	76	47	59
31	20	25	38	24	32	33
0	0	0	261	0	0	0
64	74	0	102	309	170	0
0	0	1	0	0	2	0
0	2	3	0	0	0	0
2009	1603	1450	2518	1696	2179	1427
42	0	0	8	56	0	20
219	0	0	208	0	350	0
1	23	6	62	0	0	20
2	1	1	0	0	1	3
0	0	0	0	0	0	0
36	9	14	0	43	96	23
2	0	0	6	0	0	0
0	0	6	5	0	6	0
0	4	0	0	0	0	0
0	10	0	0	0	1	0
46	31	27	69	33	49	34
0	0	1	0	0	0	0
3	5	3	6	10	6	0
3	3	0	0	0	1	1
1	0	0	17	0	0	0
66	49	91	112	68	83	62
29	7	44	55	63	38	7
64	54	54	119	23	92	38
43	60	114	121	208	67	150
59	0	5	31	19	0	0
788	659	523	1125	210	619	72

For Review Only

0	0	0	0	0	4	3
316	0	290	1480	0	305	296
0	1	0	5	0	1	0
8	0	0	0	0	0	3
0	3	3	1	4	0	1
3	5	6	1	6	1	3
0	1	3	1	4	0	1
7	4	3	0	0	4	0
271	214	235	416	342	273	178
26	40	0	61	55	27	16
0	0	0	1	432	0	0
688	0	0	117	5	0	0
0	0	0	0	0	0	0
0	0	11	1522	424	0	0
0	0	0	0	0	0	0
39	13	25	19	48	21	19
0	0	0	0	0	0	0
1	1	0	0	0	3	0
3	2	4	6	1	4	9
1	5	8	8	0	2	6
531	503	532	748	489	547	471
240	319	248	475	164	223	270
109	65	91	119	77	72	142
2319	2245	594	2864	215	1567	533
9	0	0	5	0	0	0
0	1	0	6	0	4	2
0	121	0	40	0	0	0
296	743	891	1836	485	1347	285
0	0	0	0	0	0	0
4	18	20	11	21	18	4
132	121	194	111	287	0	35
86	0	0	4	1	105	0
99	21	21	79	13	45	52
10353	0	0	2879	3912	5992	7963
513	682	538	33	766	140	351
275	0	4	0	1166	0	0
531	729	496	675	747	766	718
206	575	423	1200	269	868	657
1952	0	0	828	260	2546	1301
0	1	2	0	0	0	0
67	74	49	99	51	77	44
64	52	54	91	51	31	51
1	1	1	0	0	1	7
108	79	117	206	128	116	91
8	17	23	31	14	54	47
6	6	6	7	14	5	5
75	75	81	124	58	33	52
144	66	113	98	96	108	89
10	12	15	12	24	36	10
12	2	7	23	0	20	6

For Review Only

26	54	56	95	44	55	61
14	25	41	67	16	29	70
0	0	0	0	2	0	25
0	0	334	8	10	0	0
72	91	94	125	108	87	74
95	67	67	124	80	107	74
1640	1326	1373	2337	1421	1634	1385
14	25	14	33	23	13	28
105	145	87	0	103	118	34
5	0	2	8	0	9	2
3	1	1	0	10	2	0
65	30	55	71	10	36	26
118	25	27	88	54	52	62
29	375	286	324	0	42	30
277	233	227	242	330	249	251
0	0	0	0	0	0	0
78	75	77	117	55	95	44
24	6	13	21	0	15	4
34	25	12	61	11	29	29
0	0	5	0	0	0	0
0	1	0	0	0	0	3
175	158	171	250	242	226	154
66	63	45	105	68	79	45
7	3	4	0	11	3	5
1	3	2	5	1	4	6
0	2	3	0	0	1	1
193	160	130	315	230	284	269
0	0	0	3	0	0	4
0	0	0	0	0	2	1
2	0	0	0	0	2	0
0	0	0	0	0	3	0
3	0	0	0	0	0	1
0	0	0	0	0	0	14
154	102	0	180	75	120	6
86	0	0	7	1	0	254
0	4	8	0	0	0	0
1	28	0	19	2	0	13
0	124	115	112	118	141	113
112	113	50	163	185	34	139
114	54	58	107	159	81	118
34	24	28	23	42	29	29
91	0	81	80	35	0	0
45	56	52	81	76	50	65
5	1	0	0	0	5	2
126	126	164	223	109	125	127
34	29	50	80	18	73	33
11	0	42	0	0	7	4
20	23	23	41	21	36	40
0	0	0	222	0	0	0
7	0	0	0	0	0	0

For Review Only

10	80	124	137	324	31	5
39	41	34	19	37	58	26
22	80	50	47	117	53	91
21	18	14	37	0	23	0
90	15	16	50	85	19	45
7	6	4	0	40	7	70
323	193	287	417	432	354	282
55	94	104	142	74	73	60
42	34	21	31	14	22	23
17	80	98	0	179	1	1
108	101	49	164	78	100	91
13	6	12	16	8	0	25
2	3	4	0	0	0	6
57	56	67	89	53	60	54
8	12	8	20	2	0	9
63	64	64	70	64	84	47
140	90	126	132	154	142	73
6	2	0	0	0	0	1
6	7	2	10	27	22	0
9	16	22	36	20	23	19
0	1	0	0	0	0	2
34	30	35	58	35	33	36
1	1	0	0	0	0	0
2	0	5	8	0	8	6
112	114	138	175	144	94	83
296	293	313	473	301	256	231
0	0	0	2	23	0	0
72	74	83	128	41	57	86
23	8	24	25	43	27	10
23	40	32	74	43	21	41
104	184	64	185	18	97	67
0	3	1	5	0	3	0
27	27	48	47	0	12	26
159	151	156	216	272	127	154
61	51	110	111	74	45	55
99	195	173	238	267	141	132
67	200	43	164	166	54	57
87	0	109	120	5	72	65
0	0	0	1	0	0	0
13	11	10	24	1	4	4
1	0	0	3	4	0	0
0	0	5	4	0	0	0
364	333	338	210	54	330	301
450	445	551	851	355	481	460
5	9	3	9	19	12	6
16	15	33	14	26	4	10
2	4	2	4	0	5	2
8	0	1	3	0	0	0
2	0	0	0	0	0	0
0	5	5	30	0	1	2

For Review Only

46	15	17	49	13	17	12
13	16	11	9	15	23	23
0	0	0	15	0	0	0
5	9	11	24	7	11	9
0	0	3	0	0	0	0
11	13	6	36	0	2	13
25	8	9	28	0	4	8
0	0	0	0	7	0	0
0	0	1	0	0	0	0
46	36	64	80	30	73	66
0	4	0	3	0	4	0
11	0	0	14	10	14	14
12	32	25	0	0	28	27
22	2	2	0	0	6	12
2	5	4	9	0	0	5
79	61	46	100	64	36	30
0	0	16	2	7	0	0
0	0	0	1	0	5	1
83	97	89	178	86	102	83
4	0	8	16	10	10	9
16	23	0	1	0	0	0
17	25	45	26	39	29	49
100	125	64	138	84	91	97
19	23	19	37	10	38	22
51	42	39	62	48	50	36
226	285	329	376	289	207	276
5	0	0	0	0	1	3
2	5	3	26	1	4	4
47	226	61	133	0	70	25
97	70	65	109	93	110	90
27	0	18	32	43	0	17
50	22	52	81	12	55	97
113	119	115	155	86	94	132
24	9	5	21	0	34	15
28	0	19	35	13	17	23
62	46	44	95	74	62	55
15	1	8	17	0	10	20
58	65	84	93	33	48	68
216	181	185	228	192	175	182
31	27	23	36	0	22	17
6	2	16	16	0	6	0
1	1	1	11	0	2	0
35	15	6	10	44	8	5
16	9	11	17	36	0	0
35	6	18	27	6	19	38
1	1	1	0	55	5	6
31	108	68	76	76	24	18
22	21	21	23	22	21	15
39	24	28	44	40	37	29
19	20	17	44	1	14	14

For Review Only

223	260	251	452	181	75	116
10	8	13	12	13	6	2
308	458	259	365	562	518	289
9	3	0	4	0	4	4
187	0	2	0	0	3	0
16	10	21	0	0	10	7
126	130	129	201	127	154	102
43	79	62	118	45	25	50
45	28	35	54	19	51	26
96	70	84	118	84	88	81
10	0	8	16	3	0	7
137	65	46	118	27	59	9
24	19	20	47	11	21	10
8	1	1	0	0	4	6
0	1	0	0	0	0	1
3	0	0	0	0	0	0
0	0	0	0	0	0	0
124	49	120	103	160	296	55
107	27	49	87	26	57	79
0	0	0	2	0	1	0
4	3	3	0	0	5	3
68	49	70	12	66	61	50
1327	112	100	1275	662	1291	44
21	82	28	96	41	58	54
797	743	685	0	0	939	669
66	63	31	53	42	82	15
182	189	184	188	179	122	230
96	78	92	144	108	103	97
4	0	0	5	0	0	3
1	0	0	0	0	0	0
2	12	2	15	0	9	2
97	125	126	164	22	36	0
2	0	3	0	5	0	0
11	10	12	20	9	11	8
4	21	5	12	0	6	0
0	0	1	1	0	1	0
1	3	0	0	0	0	0
4	0	0	0	0	0	0
177	112	137	155	117	142	79
0	0	0	43	47	15	30
0	0	0	0	12	0	0
318	382	236	500	163	217	170
110	62	81	139	125	124	75
73	54	63	68	100	47	36
1	1	1	0	134	1	128
30	15	0	0	40	24	0
36	31	16	45	9	22	33
32	52	46	57	51	49	45
31	72	19	52	0	27	4
112	110	109	172	142	131	104

For Review Only

68	25	21	44	71	50	37
8	20	25	17	0	16	3
91	82	57	161	148	137	109
0	0	0	0	0	0	0
118	108	124	180	95	127	153
283	345	252	392	464	260	288
65	99	124	135	131	119	107
384	381	465	615	372	422	429
6	0	0	5	0	0	0
0	0	0	0	0	2	4
39	47	46	62	15	22	30
177	73	20	41	155	41	27
88	52	68	88	73	105	78
2	0	3	0	15	9	2
10	66	13	19	94	11	40
166	57	32	109	83	15	21
13	5	19	17	41	11	10
67	0	0	243	2	65	83
1	1	2	0	0	1	0
193	137	133	228	156	104	134
85	0	0	337	0	0	57
26	31	57	52	48	43	52
9	21	25	22	38	16	15
9	0	2	0	0	4	5
139	122	124	217	116	124	137
83	0	111	2	1	22	90
70	118	100	125	104	95	67
237	244	332	387	356	316	246
10	4	13	11	25	13	22
126	111	137	188	107	149	103
0	0	127	1	2	67	108
41	95	12	75	79	4	24
0	1	0	0	0	0	0
210	173	144	209	139	106	194
114	173	121	166	103	118	96
246	30	157	173	0	137	26
1	0	1	11	0	0	5
204	205	127	367	182	147	127
18	4	18	13	17	19	17
1	1	2	0	0	1	250
6	0	1	0	0	0	0
7	14	12	17	5	0	3
15	1	3	0	0	1	8
4	0	0	0	0	0	2
4	6	3	0	10	1	7
27	20	67	64	96	38	19
1	9	0	4	0	0	3
73	50	45	71	91	46	42
23	335	95	0	164	192	10
0	0	0	0	0	0	0

For Review Only

65	71	65	99	72	57	44
96	43	44	124	49	60	59
19	12	6	20	19	0	7
0	1	0	0	0	0	0
192	228	106	353	122	151	115
110	120	60	172	156	137	95
0	0	0	0	0	0	0
203	117	232	372	157	262	234
0	0	0	0	3	0	15
0	0	0	1	219	0	1
308	247	232	384	202	274	221
58	110	146	153	145	62	82
16	34	29	103	0	58	34
52	37	72	129	89	61	58
58	4	36	86	49	32	62
13	5	11	19	0	0	5
46	0	0	171	56	133	48
27	0	0	4	0	0	0
194	127	132	365	166	319	159
2	11	8	8	0	1	6
0	7	4	4	17	0	1
24	22	43	64	21	78	35
46	49	24	48	36	82	24
7	12	18	27	13	17	11
0	0	0	0	0	31	0
55	42	37	53	98	54	33
61	45	53	97	43	37	43
132	140	118	197	109	136	128
7	8	12	11	11	10	9
0	1	4	7	3	4	3
9	0	1	9	0	4	2
6	5	3	0	0	3	4
1	1	1	0	0	1	1
106	186	46	153	297	161	10
0	5	0	0	0	1	2
0	0	0	0	0	0	6
26	2	12	13	0	12	3
23	67	21	33	134	13	36
56	46	65	86	59	69	81
1539	1696	1147	1965	1084	1657	0
239	1	1	208	162	1	91
10	1	8	7	3	6	3
0	11	3	11	7	7	5
73	84	74	102	82	70	75
637	563	680	976	617	575	606
197	104	69	97	117	48	34
134	159	115	301	136	183	102
0	108	66	65	5	61	0
0	0	0	187	119	43	38
0	6	0	0	0	0	0

For Review Only

58	57	135	152	40	124	82
531	396	403	664	681	513	417
53	48	52	89	85	70	89
66	90	81	105	100	100	61
454	279	265	519	170	250	83
0	0	0	0	0	0	0
23	2	15	12	0	6	21
0	4	0	10	0	5	4
13	0	0	4	0	0	0
2	0	0	0	0	2	3
5	2	1	3	3	3	1
0	0	0	0	0	0	0
127	100	199	319	118	245	90
4	0	7	0	0	0	5
2	2	12	0	0	0	2
0	0	0	0	0	0	0
120	158	132	279	167	106	161
0	4	9	12	0	3	0
0	0	0	0	26	0	0
0	2	6	0	11	0	0
0	1	0	0	0	1	0
8	16	6	14	41	4	15
195	145	132	272	125	166	164
298	379	311	559	198	369	324
0	0	5	0	10	5	0
365	0	104	150	333	360	282
31	23	63	61	12	31	8
2	2	1	5	0	0	0
47	52	34	85	78	34	0
891	804	960	1623	962	1127	928
9	7	5	0	32	13	1
0	0	0	7	0	0	2
14	0	1	0	0	0	0
77	80	88	136	62	75	66
210	239	262	373	291	275	184
42	31	35	47	143	97	14
0	5	0	5	0	0	0
0	0	0	1	1	112	0
1	1	1	0	0	1	1
194	186	173	72	151	0	142
114	2	25	0	61	178	6
262	213	11	13	151	88	3
4	0	2	0	0	0	0
9	6	7	6	0	2	7
28	12	10	10	0	11	21
268	201	226	267	163	155	184
11	25	32	30	0	8	3
329	284	256	465	216	378	265
168	139	129	256	31	157	143
52	77	83	141	82	66	101

For Review Only

138	72	45	115	48	76	34
70	70	48	116	98	56	69
0	0	68	52	52	71	0
569	501	237	402	396	37	215
59	110	201	148	265	59	118
100	82	86	153	114	111	79
1	1	3	0	0	1	0
453	152	166	448	530	443	397
0	0	0	0	0	0	3
723	748	706	1245	583	601	472
83	68	46	85	51	52	31
596	560	801	1073	836	543	963
70	0	7	38	0	0	0
57	24	70	104	24	36	51
94	97	64	114	115	74	84
0	8	0	7	0	6	7
26	8	29	46	43	19	36
0	0	0	0	0	0	0
0	11	16	23	10	19	19
13	5	4	19	21	9	3
7	5	6	8	0	12	7
61	66	49	119	40	62	59
112	261	159	314	184	176	161
100	80	90	153	65	118	76
17	17	3	14	0	8	0
5	5	14	11	4	2	0
238	179	0	429	251	265	149
97	71	92	139	39	53	83
3395	2429	1362	2380	1973	3998	2258
519	538	433	858	453	427	435
53	0	48	150	5	0	32
47	4	9	59	51	11	7
38	31	21	34	43	25	39
40	31	120	77	1	17	23
15	9	22	29	29	22	21
215	654	822	134	1162	898	734
532	347	337	649	262	609	277
63	69	75	78	30	50	48
1	1	0	0	0	1	1
7	2	0	0	0	0	0
163	158	170	297	206	183	96
105	103	390	258	0	107	233
6	7	10	15	0	17	2
0	2	3	5	0	0	0
1	1	76	0	0	5	1
15	0	0	0	0	0	0
27	47	46	52	40	25	21
48	42	86	98	25	48	52
153	168	92	269	95	212	153
159	136	155	198	178	120	166

For Review Only

127	95	109	276	141	146	99
0	0	50	1	114	0	0
299	279	287	380	344	267	216
131	116	54	89	166	67	72
0	0	0	1	12	0	0
33	44	52	76	36	43	54
89	117	99	149	92	96	88
0	0	0	0	0	1	0
476	402	361	573	381	303	363
117	77	37	149	29	187	125
290	258	196	402	317	317	141
37	23	28	12	19	69	65
123	133	112	228	152	125	92
417	415	349	657	721	646	265
39	39	29	52	36	31	26
159	111	17	157	0	59	3
50	60	57	67	50	47	33
41	11	6	82	0	62	18
0	0	4	2	0	0	0
0	0	0	0	0	1	0
5	0	0	5	1	1	0
7	0	0	0	0	2	0
0	7	0	1	6	11	0
0	0	3	3	0	0	3
42	50	105	123	0	28	7
23	3	2	53	0	31	17
108	101	74	62	54	101	54
42	51	38	73	55	62	31
234	160	130	173	206	265	121
11	5	9	31	5	29	18
10	19	8	36	4	6	11
3	19	1	0	0	42	1
0	0	0	6	0	0	0
6	0	3	0	0	0	0
189	157	156	181	201	119	152
113	139	71	98	35	72	33
37	65	71	87	129	67	75
92	96	43	128	103	115	90
140	172	151	232	200	157	195
49	109	68	106	140	106	24
77	14	36	93	0	22	27
49	34	40	32	49	34	9
0	2	3	11	0	5	6
4	0	0	0	0	0	0
0	0	0	0	0	0	1
12	0	8	0	0	0	0
46	25	44	45	61	31	71
0	0	0	2	3	0	0
7	1	1	0	0	0	0
0	0	0	0	0	0	0

For Review Only

14	10	4	0	0	4	17
3	1	3	4	0	2	5
3	2	3	3	0	7	7
464	538	401	556	644	237	313
0	0	0	0	0	0	0
2	2	2	1	0	0	0
75	47	64	63	34	21	57
8	1	16	0	15	3	3
0	0	0	0	0	0	0
100	68	19	140	19	102	99
6	1	0	2	15	3	1
17	0	6	0	0	3	0
11	0	0	10	0	0	0
34	2	3	10	0	3	0
0	0	0	0	0	0	0
0	0	0	0	0	0	0
1	0	0	8	0	1	1
12	25	53	82	12	62	35
58	50	53	108	64	80	51
86	48	78	105	42	64	53
113	144	164	262	90	135	117
36	32	62	233	77	21	20
32	39	40	0	0	62	36
67	32	65	102	46	90	33
38	43	173	141	82	216	0
0	0	27	0	10	0	60
118	83	40	128	93	98	69
0	0	3	32	0	5	0
1004	1071	756	1460	663	533	524
0	0	2	0	0	0	0
503	550	381	636	458	437	266
460	137	165	266	119	262	272
206	113	142	232	217	210	90
0	0	0	293	0	0	0
122	139	80	238	79	307	125
55	52	68	68	136	69	92
9	36	25	27	0	4	6
77	86	89	125	154	134	120
156	108	152	278	124	130	128
289	305	288	469	326	325	229
72	79	83	137	69	72	89
155	159	114	247	158	157	135
0	0	0	20	4	128	0
56	77	40	101	72	71	54
0	0	3	0	0	0	0
211	98	187	243	225	114	157
4	5	8	6	16	7	12
14	8	41	22	36	8	10
53	41	34	38	26	22	37
35	33	55	78	65	70	53

For Review Only

26	22	4	39	0	9	31
20	21	39	50	26	49	39
74	93	56	91	101	78	52
64	54	42	42	14	0	28
13	15	11	20	0	12	7
91	82	83	159	97	67	55
0	0	3	0	0	0	0
39	23	286	324	197	1226	0
772	0	120	94	700	791	0
150	161	100	3	2	0	75
22	31	30	43	58	41	29
0	0	10	0	10	0	0
146	142	128	184	171	161	173
309	275	206	603	493	648	368
3497	1968	2390	4690	1906	2452	2462
63	47	35	100	22	57	25
30	11	3	33	26	3	32
120	110	102	163	95	83	73
32	20	26	46	42	25	32
62	34	58	84	70	49	30
0	60	39	72	0	60	0
24	15	22	36	24	21	15
1	62	3	38	0	1	1
948	311	508	1183	232	472	792
0	7	0	18	4	11	0
324	78	195	305	451	148	247
559	352	354	672	713	560	295
0	0	0	0	0	0	0
153	6	1	0	0	1	1
12	0	35	62	0	0	34
1	1	1	0	222	1	1
0	0	0	1	1	0	0
191	122	160	280	102	136	118
125	0	91	0	0	0	0
115	91	103	189	92	93	110
184	170	227	350	204	176	195
130	50	18	157	48	29	45
0	3	0	0	0	26	0
227	178	211	295	226	209	141
11	19	18	21	8	17	19
37	41	47	96	46	54	42
0	0	0	0	0	0	0
38	40	48	83	13	12	68
8	12	12	20	10	10	12
29	31	45	43	45	25	45
0	0	5	1	6	0	0
2	0	1	0	0	6	3
3	0	0	0	0	0	0
0	6	0	0	0	0	0
33	0	30	0	0	18	14

For Review Only

7	0	0	0	6	0	0
137	93	110	178	144	108	113
8	0	0	7	0	3	5
16	20	10	15	37	4	21
116	37	60	63	38	93	28
40	33	39	58	42	38	25
2	1	1	0	0	0	1
91	92	74	100	184	100	129
359	209	240	428	169	362	190
0	169	133	13	151	236	0
71	87	67	122	93	44	66
3	0	0	2	0	0	0
82	168	142	178	77	181	98
146	70	85	131	95	82	133
121	77	45	104	69	78	56
0	0	1	0	4	0	0
85	77	58	107	44	64	48
97	42	54	91	18	5	4
248	0	0	146	36	0	196
3	2	6	6	0	10	0
30	11	16	21	30	44	17
131	51	30	95	162	89	73
29	16	50	86	24	45	43
37	67	96	140	69	67	81
218	262	120	367	143	329	240
0	0	1	0	7	0	0
4	1	0	5	0	5	3
12	44	11	34	18	50	41
146	145	176	218	184	160	133
50	75	41	115	79	97	59
118	141	135	208	155	127	121
0	77	0	239	8	274	191
5	0	0	6	0	3	5
269	208	188	330	238	164	152
8	4	9	9	10	4	6
325	330	288	609	234	230	321
23	42	23	62	41	27	28
15	80	159	147	0	21	110
20	29	56	28	56	17	27
909	934	774	1494	632	1006	587
57	56	46	79	56	60	38
159	207	179	365	126	106	100
30	14	18	21	29	31	10
8	23	32	49	13	23	14
0	0	0	0	0	0	0
7	0	0	0	0	0	0
98	124	103	208	104	98	104
136	173	140	182	250	135	120
303	251	148	349	159	190	186
30	9	21	34	94	40	30

For Review Only

442	378	326	558	246	222	297
7	9	12	19	4	16	7
0	0	4	0	7	0	0
37	62	78	119	107	63	70
13	5	4	16	4	13	12
83	109	103	125	94	67	119
66	79	75	168	68	87	62
62	100	69	158	75	74	73
49	2	41	139	52	47	28
28	54	36	64	50	22	27
14	44	20	30	26	11	6
22	30	24	42	17	29	17
4	0	0	0	0	4	0
0	60	47	21	1	0	0
236	289	246	490	374	390	212
52	80	75	150	54	31	52
286	264	293	482	336	293	268
37	26	22	56	0	6	8
63	53	39	104	77	61	48
290	209	188	289	245	364	164
167	138	80	174	115	116	84
237	152	312	335	146	106	220
172	182	112	154	49	151	138
2	15	4	23	0	16	7
25843	22036	16333	23476	15063	33410	14377
1	1	1	0	0	1	1
0	48	1	0	0	107	212
0	110	31	0	0	66	136
110	93	88	1	0	0	231
0	0	0	0	0	0	0
32	21	17	35	21	17	36
0	11	42	0	0	4	7
15	10	7	24	14	20	11
2	0	2	0	0	0	1
316	437	408	629	477	537	568
361	352	322	551	308	419	296
349	277	197	345	409	351	224
172	101	145	121	305	2	162
117	130	57	188	50	97	213
0	29	5	122	0	15	9
7	34	28	34	71	20	28
98	136	121	231	120	157	136
20	31	20	28	4	28	17
112	126	99	225	108	127	59
0	0	0	1	0	0	0
54	29	26	73	22	35	50
50	30	7	41	34	11	21
13	7	8	32	0	23	22
1	0	0	0	0	0	6
5	0	0	0	0	0	0

For Review Only

6	0	0	0	0	0	0
107	61	49	62	23	33	40
65484	61200	76702	123339	56715	74311	71533
0	1	4	0	0	0	1
38	31	40	44	20	21	20
72	70	71	167	7	0	61
139	112	91	103	126	184	99
47	34	52	77	85	105	72
129	190	80	144	206	108	136
43	35	33	46	38	91	38
72	76	68	88	115	63	89
5	7	11	0	29	3	18
83	39	41	70	92	62	93
110	75	73	174	118	130	112
0	0	0	95	16	0	0
63	52	74	87	40	61	73
1	1	1	0	48	1	14
56	87	40	57	77	35	56
0	0	0	5	9	0	0
0	228	0	1	277	0	0
2	1	0	6	0	1	5
111	154	51	141	154	116	84
35	19	15	34	27	5	12
0	0	0	1	0	0	0
128	130	78	134	230	106	87
24	37	49	62	86	36	61
12	63	421	384	240	15	68
39	30	24	32	39	24	27
113	86	72	121	136	152	68
238	109	139	291	249	257	272
14	20	35	45	76	36	34
235	213	143	266	125	221	109
15	5	9	40	29	24	37
147	147	122	234	145	152	108
74	56	33	83	0	34	5
0	0	0	0	20	0	0
4398	2849	0	1311	288	0	2369
10642	8549	6786	10387	10756	9192	5365
1504	1530	1458	2232	1102	1328	1003
43779	38938	30366	44915	48949	42533	23262
0	0	0	0	0	0	0
0	0	0	0	0	0	0
1494	2216	2326	635	294	869	126
0	0	0	0	0	0	0
2029	1753	1852	3176	1575	1778	1640
378	309	294	519	287	442	235
13552	13290	17602	22541	12344	13438	12597
11	6	0	14	0	6	0
3440	3914	4218	5689	3425	2971	2521
6	0	0	0	0	0	0

For Review Only

0	72	0	3	31	77	70
63	26	57	79	36	38	17
10	0	7	0	0	0	0
12	2	2	6	10	2	2
0	157	0	0	0	17	19
319	353	292	300	200	172	213
95	40	58	117	48	78	85
11	28	72	86	0	78	81
119	79	55	118	74	118	90
15	9	9	25	34	13	35
36	69	79	108	25	55	38
47	23	24	25	25	34	19
141	97	73	217	104	151	160
0	0	0	20	0	0	0
8	16	4	34	0	13	19
313	330	172	443	342	372	246
41	8	24	19	28	18	28
158	102	106	190	152	128	82
24	28	14	32	22	41	21
71	34	42	74	40	50	26
56	47	46	84	51	41	50
68	46	48	66	88	54	57
28	57	66	76	89	42	41
4	0	0	9	0	0	0
20764	7129	16469	24330	20116	22060	14199
4	0	1	0	0	0	0
2	3	0	0	0	3	0
0	0	0	0	0	0	0
0	4	3	7	0	0	0
0	16	0	2	1	0	0
3	1	3	0	0	0	0
0	0	0	11	0	0	0
15	1	1	10	0	15	1
0	0	0	8	0	0	1
1	2	3	12	0	2	16
10	7	1	11	13	8	12
9	9	4	11	9	7	5
18	19	16	44	20	38	35
0	1	1	0	0	1	0
1	0	0	5	0	4	1
6	83	227	445	252	258	256
30	2	0	0	9	1	4
914	136	59	769	19	133	18
124	85	109	108	128	110	48
87	53	55	98	83	73	48
255	197	205	370	229	202	263
117	58	44	119	89	160	63
57	45	32	47	51	37	31
88	75	65	132	40	128	59
256	45	0	11	0	0	177

For Review Only

0	0	0	0	0	0	10
122	107	95	157	115	134	85
35	25	26	49	41	26	32
65	48	33	34	63	16	17
710	612	724	1406	728	1046	439
25	13	16	60	0	6	17
0	0	0	5	1	0	0
7	0	46	36	12	39	0
2701	999	1132	1583	1496	1934	1717
1	0	0	0	0	0	0
148	148	120	213	163	220	136
95	66	75	107	91	83	88
0	0	0	0	0	0	0
46	44	46	63	39	56	32
0	0	0	18	5	0	0
0	0	6	0	0	0	0
0	0	0	0	0	0	0
0	0	0	0	0	0	0
19	25	15	24	34	23	9
2	2	4	0	0	0	0
4	0	0	1	10	5	11
5	0	9	16	13	6	0
58	46	58	53	13	61	43
13	22	8	23	15	22	27
0	3	0	0	0	0	0
0	0	9	0	0	0	0
0	0	0	2	0	0	0
264	174	141	272	140	288	257
45	39	49	27	56	30	11
0	0	0	0	0	0	0
237	170	175	222	70	193	154
2276	1226	1483	2673	2400	1860	1714
138	163	148	89	153	61	72
0	52	0	46	24	25	34
135	0	0	0	119	184	0
956	156	1673	409	2582	756	736
3	2	13	8	0	3	15
107	117	87	162	100	114	72
0	1	0	0	0	2	0
1	0	4	6	0	4	2
11	8	11	13	8	0	0
24	14	35	78	21	35	45
71	20	34	62	41	51	34
84	85	93	111	63	91	69
8	9	5	12	14	16	12
3	6	2	0	0	13	20
6	1	11	13	0	3	3
3	2	6	0	0	1	0
20	1	1	23	1	10	9
14	9	5	22	19	15	12

For Review Only

2	1	1	0	157	1	1
0	252	0	83	94	0	0
82	62	59	103	37	55	64
183	152	128	174	85	111	110
21	20	32	50	16	34	32
87	92	67	159	51	138	80
88	69	73	82	81	102	53
47	46	31	98	74	96	29
8	12	11	0	12	15	0
2	1	0	0	0	0	0
28	0	18	10	41	5	8
1	2	4	1	0	0	1
46	58	26	81	124	33	29
33	31	29	23	24	26	25
6	3	3	17	0	9	0
35	29	13	41	26	47	20
41	35	47	97	35	50	83
9	10	5	31	0	5	7
0	0	0	4	0	0	0
3	3	0	6	0	3	2
20	19	11	26	10	26	15
104	123	105	199	157	103	125
65	60	37	61	50	39	46
0	4	4	8	5	11	5
9	9	8	34	1	35	10
0	0	3	0	7	4	2
6	3	1	5	4	9	3
41	46	60	73	50	55	71
0	5	4	4	14	7	0
8	3	3	9	0	1	4
7	4	5	9	17	0	9
1	0	0	0	0	1	1
193	183	169	350	185	262	180
188	145	108	218	158	199	103
12	26	40	29	5	14	32
4	0	0	0	0	9	6
0	0	0	3	0	0	0
23	23	19	47	31	33	51
4	0	1	0	0	1	4
30	1	6	38	0	39	16
3	3	5	4	13	4	3
1	0	0	6	0	0	0
1	3	1	3	1	3	4
268	244	233	406	163	217	158
119	109	115	150	135	131	73
79	167	151	200	192	90	89
10	1	1	0	0	14	1
0	0	0	0	0	3	0
6	13	12	21	15	14	8
44	23	18	73	15	48	21

For Review Only

123	81	57	144	94	90	88
28	33	27	49	39	31	30
34	32	24	29	46	60	9
0	0	0	7	0	0	0
262	383	355	587	229	404	174
97	88	99	134	128	119	89
9	25	44	41	0	13	64
52	117	87	170	142	124	1
0	32	0	8	39	11	0
274	376	273	372	150	546	0
7	0	8	11	0	3	3
71	40	57	92	70	48	22
5	0	7	6	0	6	1
31	28	22	57	18	11	8
90	0	0	65	0	8	0
7	3	0	12	10	11	0
46	34	21	60	36	34	31
71	22	46	60	16	35	52
16	20	46	35	78	16	11
10	56	47	304	140	173	18
385	346	287	412	319	354	243
10	0	0	0	0	0	0
77	75	79	155	102	94	69
13	14	0	35	19	9	14
44	33	1	0	0	3	39
36	22	43	52	77	52	56
52	24	67	112	37	66	67
21	7	13	22	44	29	23
305	261	235	390	334	294	242
280	165	211	218	246	282	155
109	60	80	123	111	151	89
48	98	107	139	36	116	145
3	2	4	5	0	4	9
2	0	2	0	0	2	2
39	42	30	43	11	36	24
0	0	2	0	0	1	0
11	29	1	10	29	3	9
25	31	18	26	38	49	24
44	37	51	73	97	36	58
53	61	96	203	25	21	65
0	0	0	0	0	0	0
73	81	72	107	45	104	81
21	19	36	35	31	38	42
30	29	26	32	19	31	36
25	48	50	81	22	50	35
5	0	0	3	5	0	0
3	15	9	13	11	5	14
134	123	119	142	144	131	94
255	0	401	9	78	285	0
17	22	11	114	0	45	6

For Review Only

309	336	303	511	291	311	306
441	614	4	0	550	369	427
34	72	63	221	15	0	72
31	37	27	70	46	52	27
33	75	63	94	27	65	74
104	78	62	123	151	85	57
9	15	13	24	0	9	15
38	66	34	47	39	48	37
11	49	58	33	98	24	34
0	0	168	0	45	0	0
0	0	0	0	0	0	2
105	78	51	107	39	20	0
349	167	246	287	294	294	233
29	0	0	0	0	10	0
4	9	20	21	0	2	11
258	108	97	212	305	169	119
56	39	38	73	61	83	44
0	1	0	0	0	4	3
1	0	0	0	0	0	0
0	776	0	1	2	0	0
0	0	0	6	0	0	0
3	0	0	0	0	0	0
281	245	256	390	244	269	155
24	17	12	50	9	22	12
157	128	154	164	100	161	85
24	41	71	58	0	8	10
22	12	8	26	0	16	11
47	39	3	208	560	488	1
39	38	49	94	68	42	24
14	22	17	21	26	21	12
15	11	37	44	8	23	16
73	76	74	114	48	44	35
86	63	100	104	103	91	68
39	33	34	49	72	33	35
178	162	226	294	296	234	167
329	229	444	429	235	422	337
71	66	71	93	157	94	0
42	60	26	125	41	80	11
107	0	100	42	3	94	80
93	39	38	97	183	57	0
370	269	324	557	424	304	204
71	0	109	78	6	47	102
92	59	41	83	126	100	99
212	179	110	92	32	159	0
0	31	0	0	8	0	0
136	102	115	154	194	105	62
137	41	28	273	147	265	31
24	19	37	49	41	0	25
3194	0	0	0	56	0	0
86	80	84	82	55	72	45

For Review Only

7	3	2	0	40	24	14
72	65	85	84	72	67	90
118	72	54	138	70	100	60
22	14	27	41	0	13	48
145	117	52	136	140	109	50
102	70	59	155	56	83	47
39	6	20	72	0	44	18
113	113	198	265	90	91	196
7	24	51	46	59	25	26
53	64	50	75	75	36	64
0	0	0	0	25	0	0
47	25	53	61	71	68	47
57	38	21	48	10	48	7
258	231	225	363	106	205	121
30	44	17	38	41	21	6
103	49	18	118	55	76	23
32	18	35	52	42	55	96
201	172	134	272	164	194	190
5	13	15	56	0	10	19
94	88	102	132	69	53	69
68	34	81	72	134	48	51
14	39	85	94	0	36	28
18	22	6	14	33	20	21
142	92	65	114	131	94	86
2	1	6	16	0	8	10
1	9	2	0	0	2	1
282	221	566	790	488	432	567
238	23	0	331	121	61	13
34	21	31	54	32	17	30
172	217	117	240	138	162	185
0	3	2	0	1	0	0
68	97	115	120	122	121	67
26	29	41	59	77	43	41
117	148	0	49	17	0	0
54	41	24	119	0	128	100
48	29	42	52	19	54	21
51	35	20	39	45	25	10
39	15	11	17	41	29	13
166	141	563	147	147	170	122
199	215	165	352	201	205	147
0	0	5	0	8	2	1
29	47	47	48	62	35	28
35	22	33	69	73	52	26
10	18	2	0	54	108	3
18	11	16	12	53	10	14
25	37	31	55	37	21	24
5	29	27	34	17	21	13
22	8	22	16	22	16	19
46	58	57	102	87	80	45
11	0	0	5	0	3	1

For Review Only

97	86	82	96	81	115	71
293	337	226	470	305	351	198
15	35	11	36	19	14	6
73	71	84	83	96	50	56
88	74	73	71	54	31	67
231	40	163	51	163	48	19
16	3	13	14	4	4	12
3	0	1	2	0	3	0
24	23	37	56	23	22	37
3	2	0	0	0	3	4
77	36	25	40	47	14	30
64	23	89	7	86	117	75
73	7	7	115	2	5	16
21	26	3	70	0	42	14
3	62	1	0	25	90	20
35	23	23	28	20	21	6
3	11	6	13	0	4	2
0	0	3	7	0	0	0
26	31	14	41	30	42	20
24	23	23	73	74	50	45
89	73	85	113	83	56	56
5	18	21	25	0	14	30
3	1	12	2	12	5	0
2	1	0	10	0	1	0
1297	1102	993	1772	1002	1422	657
0	18	0	0	0	0	0
144	144	115	295	125	211	125
103	75	72	235	116	174	106
33	12	12	21	42	5	7
2	2	5	15	0	25	11
180	259	166	216	185	171	126
82	81	93	90	125	38	47
31	37	24	32	55	31	35
123	108	150	180	277	170	143
100	64	72	74	80	74	33
151	140	97	130	90	74	77
173	106	82	106	166	150	65
15	10	7	10	13	15	6
37	30	40	66	57	33	53
39	67	39	39	18	45	20
104	76	50	98	69	66	33
41	56	46	74	75	32	32
91	122	70	155	104	160	82
53	39	42	87	30	73	34
102	86	82	128	133	82	73
46	51	59	50	66	61	39
0	0	0	0	4	0	0
29	13	42	49	30	11	25
56	66	53	103	85	81	105
75	111	75	113	98	77	66

For Review Only

257	297	171	342	300	227	189
38	54	33	61	43	26	36
2	1	0	0	0	4	0
32	2	3	96	0	1	23
517	512	610	731	439	397	411
17	13	51	33	0	26	9
43	46	78	86	26	31	47
42	9	21	17	0	1	11
91	37	57	59	102	51	12
80	77	86	102	82	87	77
113	127	68	176	143	95	116
96	43	52	82	92	74	52
3	1	0	4	0	0	0
0	6	0	0	0	1	2
109	16	72	107	0	93	48
41	73	33	73	38	60	32
74	25	20	48	12	18	31
88	90	62	209	97	145	92
0	5	8	5	7	10	0
51	49	39	63	59	32	28
13	19	41	25	10	24	37
6	0	0	0	0	0	0
9	10	10	15	0	5	16
0	0	11	23	13	0	11
7	2	4	11	0	2	10
0	0	0	0	0	0	0
215	335	230	375	351	141	263
1	5	0	0	0	0	0
378	229	203	426	175	378	177
85	59	23	62	55	66	34
0	0	0	0	0	0	0
0	0	5	0	0	4	4
189	50	195	157	152	139	109
4	7	15	41	20	31	18
1	1	1	0	0	0	1
25	46	39	77	41	39	40
30	40	24	74	29	20	70
3	0	5	11	0	0	9
0	5	0	18	0	0	0
36	34	35	30	32	22	17
5	4	4	11	0	4	1
12	35	25	19	39	15	7
0	0	0	0	0	0	0
7	4	5	18	0	23	10
3	7	0	6	0	1	0
5	0	0	2	0	0	0
0	0	0	7	0	0	0
0	3	0	0	0	0	0
203	0	161	8	7	0	0
336	284	231	341	364	328	195

For Review Only

145	101	108	170	126	92	75
9	23	33	37	27	13	16
0	0	0	89	0	207	0
149	221	122	274	175	89	72
58	16	71	60	36	73	45
80	67	68	121	102	84	58
69	60	35	114	44	44	10
1	2	1	0	0	1	0
27	61	32	69	60	32	34
39	37	32	40	18	25	28
90	114	68	135	121	71	80
3	3	4	20	1	2	1
224	242	182	248	182	151	132
155	106	94	202	135	80	87
116	25	73	63	38	128	53
139	128	37	161	125	123	95
8	7	9	12	7	3	2
143	78	132	168	104	75	133
215	179	152	230	157	162	134
39	0	36	101	22	39	0
69	70	52	88	113	92	68
0	0	0	10	0	0	0
118	132	127	175	147	169	134
46	77	84	121	40	66	73
31	20	22	37	13	36	40
0	43	48	45	18	32	0
4	0	8	5	0	5	0
45	59	59	88	36	43	42
5	0	9	13	26	21	3
22	17	10	25	43	27	34
92	97	92	94	70	48	89
222	270	238	367	256	189	174
40	72	36	100	61	54	44
0	1	1	0	0	1	0
179	361	0	127	285	0	82
220	65	69	40	271	122	271
113	73	98	178	72	99	107
1	1	27	0	0	1	1
7	1	1	18	0	15	1
45	54	54	78	81	64	39
0	4	0	1	0	0	0
4	0	0	2	0	5	0
6	3	9	30	0	1	1
6	9	3	11	0	2	11
0	0	183	0	0	0	0
9	4	14	16	0	0	2
0	0	0	1	0	0	0
18	11	21	0	0	8	12
3	3	0	6	0	0	0
83	72	90	135	79	116	73

For Review Only

1056	612	464	1007	1331	953	561
0	0	0	6	0	0	0
94	55	55	143	114	120	45
55	62	50	88	69	81	49
267	339	290	523	486	278	365
5	1	7	6	10	0	1
1	2	8	0	0	1	1
37	26	6	108	0	3	8
0	5	4	3	0	0	0
0	0	0	1	39	0	0
126	132	135	206	158	132	83
0	1	1	0	0	0	0
119	66	54	130	91	109	121
481	474	408	865	486	684	626
161	130	204	161	264	77	151
609	417	502	691	576	598	556
5	0	0	0	0	3	0
250	391	573	585	331	374	491
3	0	0	0	0	0	0
18	30	24	40	56	31	30
61	79	89	144	110	73	91
1203	1099	725	927	1054	384	522
83	102	147	167	125	109	117
0	0	0	0	0	0	0
5	0	0	1	0	11	4
0	0	7	9	0	0	0
0	1	3	4	6	0	0
133	138	100	131	126	136	77
30	12	16	0	0	1	1
25	10	20	43	0	4	12
73	72	99	143	82	49	119
101	79	128	158	123	101	145
27	51	64	82	64	54	71
86	27	72	137	59	98	32
61	27	26	0	71	25	7
130	154	110	161	126	133	87
0	0	0	0	120	103	0
29	85	2	48	20	18	3
27	38	36	28	46	30	52
36	50	25	45	35	41	46
29	4	5	0	0	1	50
1	4	18	16	0	1	5
203	220	234	397	165	268	228
0	0	0	76	0	0	0
35	26	25	74	22	43	24
155	117	91	168	141	141	86
0	0	0	0	0	0	0
73	117	74	125	137	120	96
104	96	62	136	57	109	70
53	53	67	107	52	52	84

For Review Only

68	21	49	99	0	80	27
1	12	4	180	0	1	37
27	36	47	69	31	39	24
1	0	1	0	0	0	0
849	911	974	1541	817	1098	895
0	0	0	7	0	0	3
9	7	5	22	0	29	8
74	33	39	63	0	44	67
0	0	0	0	3	0	0
199	238	218	364	317	154	248
0	0	0	2	4	0	0
0	0	0	0	13	0	0
124	180	179	269	117	117	113
99	61	69	135	108	119	66
38	35	13	52	23	37	26
0	0	0	4	0	5	0
167	154	153	235	106	143	129
72	61	52	68	62	51	25
6	10	4	0	13	3	8
18	3	1	0	0	22	6
424	403	525	586	414	527	579
254	249	236	395	230	222	184
89	74	90	178	125	104	118
21	12	17	36	14	20	29
29	35	34	28	28	26	27
0	0	0	0	0	0	2
0	0	0	0	0	15	1
25	11	0	21	0	15	11
30	9	34	36	90	20	53
26	31	30	41	35	31	19
14	5	9	12	11	11	7
71	83	45	99	73	94	45
112	87	70	89	105	80	114
1	348	37	225	0	1	16
281	247	259	269	563	265	31
51	38	71	67	35	61	48
35	34	4	17	24	6	9
1	1	5	39	0	29	9
132	76	85	96	177	110	55
49	42	19	35	55	40	20
132	118	81	149	179	121	0
7	5	12	0	13	9	2
697	0	0	0	0	0	0
16	1	2	0	0	5	2
232	304	232	285	278	221	238
73	77	55	109	126	116	83
3	0	0	0	0	3	0
50	22	43	47	82	79	139
21	15	6	40	0	6	1
7	15	12	15	0	8	2

For Review Only

3	1	4	0	0	2	3
129	91	101	151	137	70	69
6	13	5	15	3	5	1
112	53	45	61	41	48	33
7	2	7	22	0	8	1
359	233	292	412	481	437	216
0	0	0	4	0	0	1
2728	0	0	0	264	0	0
153	135	58	231	180	197	174
1	0	2	0	24	5	2
0	0	0	1	0	0	0
5	0	3	10	2	4	3
55	51	56	99	41	82	49
31	32	11	20	56	7	12
3	29	6	0	186	268	30
0	0	0	14	0	0	0
182	139	104	249	96	124	104
290	376	231	609	218	371	276
235	224	189	251	232	210	123
164	136	108	148	219	154	76
90	111	61	130	215	86	122
0	24	34	0	0	0	0
0	0	0	0	0	0	0
102	146	135	207	55	55	40
14	7	13	18	12	14	12
108	152	135	202	201	136	148
1	5	3	0	0	15	5
0	0	0	38	15	0	0
35	24	35	81	21	58	35
17	20	30	73	26	40	25
96	48	81	108	50	53	66
144	126	149	259	161	169	157
98	83	105	154	73	101	146
93	100	119	192	116	94	95
23	21	32	53	50	55	33
0	1	0	0	0	18	0
6	9	8	0	0	0	3
0	0	0	0	0	0	2
1	0	0	5	0	0	0
23	23	30	48	35	42	21
177	15	92	326	270	66	229
131	158	143	241	122	181	154
0	0	3	2	0	0	0
2	1	0	13	22	2	1
1	0	0	2	5	0	1
13	0	18	29	23	0	15
4	7	11	8	6	18	13
4	1	0	2	5	0	0
3	3	5	13	8	7	8
0	0	22	0	0	3	24

For Review Only

38	28	24	39	55	13	35
24	24	28	54	14	22	45
26	24	13	40	6	29	14
0	0	0	1	1	1	0
29	18	9	20	19	20	7
0	0	0	0	0	3	0
0	0	0	3	0	15	0
12	1	2	18	0	21	5
87	63	82	141	123	155	76
11	93	23	87	0	61	5
7	5	9	25	21	22	6
3	9	21	7	27	1	11
3	0	0	6	0	0	0
7	7	7	7	5	7	18
0	0	0	0	2	0	0
27	19	35	49	14	26	23
33	113	103	158	199	91	175
71	28	23	29	12	21	21
124	48	83	112	53	116	59
0	111	80	0	0	0	0
1	6	8	6	0	2	1
85	109	62	103	94	40	70
10	90	0	95	1	24	69
1	0	3	0	0	0	0
33	38	49	95	48	35	31
6	20	33	45	45	33	22
0	0	0	0	0	0	0
40	54	32	75	35	39	56
153	85	144	179	190	163	67
39	50	47	70	54	53	48
175	125	132	233	117	91	113
34	31	44	78	48	41	35
92	54	79	123	77	118	71
57	38	31	43	58	39	38
24	37	15	59	55	44	20
0	0	15	0	66	7	10
44	45	16	7	5	22	0
0	0	0	0	0	0	0
0	0	0	0	327	0	0
0	63	0	1	64	0	0
139	56	135	211	88	108	85
0	3	0	0	0	1	4
0	1	0	0	0	0	0
653	463	476	676	533	871	518
20	36	20	64	14	39	35
0	0	3	0	0	2	0
0	0	5	3	0	0	0
0	0	1	0	0	0	3
142	108	48	104	181	208	91
128	48	0	112	22	123	100

For Review Only

445	513	579	857	843	539	604
561	17	90	72	0	1502	1
26	31	40	46	51	42	38
98	97	137	217	123	141	76
3	2	0	4	0	3	6
0	0	0	0	0	0	0
0	5	1	6	0	0	6
28	69	37	50	61	51	38
30	23	59	31	44	17	48
72	68	86	147	115	79	77
31	28	23	81	21	18	17
0	0	0	1	56	0	67
0	130	0	1	57	1	0
38	40	26	85	26	46	30
132	131	101	204	115	108	82
0	0	0	0	0	0	0
465	356	307	520	357	429	279
81	59	121	202	54	76	56
154	115	109	150	167	113	53
37	33	52	87	102	69	32
5	0	0	0	0	1	0
0	0	0	0	1	0	6
2	0	0	0	0	6	0
146	119	141	203	175	127	108
15	4	14	7	24	9	2
0	0	0	0	0	0	4
0	0	0	0	0	0	0
0	8	17	51	0	15	0
4	0	0	4	0	2	0
16	0	0	0	0	0	0
330	303	488	673	919	401	435
42	54	34	97	32	77	48
0	0	1	0	0	0	0
9	0	1	1	0	4	0
0	2	1	0	0	5	1
47	14	27	68	45	25	37
0	5	2	4	0	0	9
0	1	1	0	0	0	0
167	190	228	157	314	250	168
3	2	2	0	0	4	0
1	0	0	0	0	0	0
114	65	83	129	103	119	45
4	0	3	5	0	13	0
2	0	4	1	30	2	2
0	12	6	24	1	0	0
3	10	30	33	8	24	34
169	163	108	181	140	61	46
0	2	0	8	4	2	0
1835	367	293	540	308	736	1021
21	15	105	108	0	124	11

For Review Only

12	1	0	13	0	1	0
12	15	24	27	14	12	14
181	132	0	91	267	156	136
11	0	6	15	0	2	1
0	0	0	0	0	0	2
569	463	428	523	603	318	322
25	10	1	6	22	14	39
197	107	88	159	210	116	116
88	36	114	190	0	68	8
23	28	46	35	57	25	40
19	12	8	20	22	11	19
1	0	3	3	0	0	3
22	29	20	7	18	8	12
1	0	0	1	0	1	0
127	72	78	138	85	101	79
69	56	5	0	0	0	31
40	21	28	53	23	23	14
146	124	99	171	136	99	84
72	89	86	155	84	84	85
189	166	100	183	192	150	104
64	64	74	114	61	64	61
45	49	58	66	90	51	39
87	33	52	75	62	99	63
10	40	2	0	26	43	4
121	77	1	88	107	91	15
71	40	36	78	22	64	27
0	0	0	0	0	0	18
34	89	26	42	155	48	35
71	38	62	91	44	87	72
87	104	127	160	95	102	82
49	87	74	120	135	84	78
54	48	49	65	68	63	50
150	59	90	91	114	46	86
232	297	208	355	301	209	176
51	95	178	102	139	85	28
278	263	225	506	308	352	254
173	166	114	296	137	228	99
155	78	97	166	148	85	118
162	171	159	244	117	95	111
187	189	139	236	215	153	107
84	31	60	248	0	188	217
36	52	31	45	60	20	28
40	42	22	52	59	10	36
120	0	88	224	0	145	96
335	282	154	410	270	307	180
136	92	130	236	114	138	102
0	0	29	0	0	0	0
113	101	81	102	108	102	0
376	176	198	302	346	348	149
165	134	93	211	83	118	108

For Review Only

274	119	161	254	183	276	100
49	73	58	94	79	50	52
51	79	66	146	46	45	46
153	145	130	210	181	145	76
0	0	14	2	1	0	0
51	67	89	116	125	89	101
162	275	215	294	364	226	170
402	713	360	115	518	178	92
255	108	187	236	315	125	173
314	122	0	1	193	493	26
0	0	0	0	74	109	0
423	404	474	614	496	441	238
4	6	21	16	0	10	67
90	85	129	190	149	96	102
148	107	97	232	99	156	79
121	112	65	110	138	93	84
46	58	22	57	53	32	12
0	0	22	0	0	0	0
288	243	212	334	300	190	184
6	12	7	19	24	14	11
0	6	0	0	0	0	0
134	146	137	242	204	165	156
70	105	100	134	190	105	144
195	149	176	270	140	202	139
1	0	0	0	0	0	0
0	0	0	0	0	0	1
17	9	5	28	5	12	6
125	121	110	260	136	133	127
54	57	92	130	55	36	107
57	99	53	107	48	112	73
6	2	4	17	26	5	13
1	0	0	0	0	2	0
1514	1210	1395	1826	1561	1739	1505
183	238	149	235	225	280	115
7	6	6	9	16	6	5
110	45	69	84	77	64	67
50	26	38	64	62	34	43
151	215	135	175	157	141	54
46	60	61	121	11	0	22
1	358	71	0	0	143	4
5	0	0	3	0	0	0
105	139	107	159	0	62	26
6	4	5	5	2	4	5
8	0	0	0	0	0	0
239	135	119	172	191	212	142
71	76	67	93	96	87	60
17	16	27	36	24	28	23
76	56	50	108	51	55	66
12	8	10	19	12	4	9
10	4	3	0	0	1	0

For Review Only

239	164	177	159	213	176	43
27	26	94	0	103	9	17
111	107	93	151	156	76	108
269	250	272	486	278	360	225
105	108	88	144	85	128	67
0	118	0	168	1	61	0
192	171	215	356	196	200	179
52	29	70	64	65	62	53
23	18	0	19	0	0	0
17	5	25	41	7	40	17
0	0	0	0	0	0	0
18	6	49	50	40	22	63
0	0	5	0	0	0	2
0	0	0	2	0	0	0
0	0	53	0	70	0	0
1	3	2	15	0	2	5
0	0	3	5	0	3	7
4	0	0	0	0	0	0
1	0	2	0	0	1	1
11	1	14	0	0	3	7
78	85	118	139	181	95	107
12	11	23	39	0	18	58
59	44	41	69	52	66	46
2	11	3	20	0	16	1
0	0	0	0	110	2	1
153	114	124	149	138	83	74
0	0	1	5	0	0	0
0	1	2	0	0	0	1
0	1	0	4	0	7	0
94	83	50	148	53	73	107
145	224	163	266	109	204	237
0	0	0	1	0	0	0
68	0	4	11	47	1	3
727	662	482	1076	660	714	541
139	131	74	39	16	158	109
0	5	0	9	0	0	5
85	0	0	367	243	0	94
195	176	154	298	190	222	141
2	0	0	0	0	0	2
12	29	46	83	0	0	8
24	79	32	35	16	49	57
137	149	119	148	128	88	93
16	23	20	22	38	27	27
7	19	8	54	0	19	36
1013	33	91	360	170	70	9
63	30	43	23	38	26	33
29	7	8	44	0	33	11
53	53	40	70	52	60	68
104	77	49	104	66	78	54
3682	3338	2623	5403	2571	3835	2539

For Review Only

0	0	3	0	0	0	0
16	23	10	36	39	10	40
9	15	10	64	48	30	7
75	40	61	107	29	44	74
19	13	31	46	37	21	28
47	13	35	31	48	6	10
82	62	33	75	31	98	32
388	647	365	706	661	458	349
271	206	210	446	291	261	253
2	0	0	0	0	0	0
104	136	112	107	188	95	96
0	0	1	0	0	2	4
499	1966	2360	3539	613	1911	537
55	47	19	48	49	56	48
2	2	1	0	0	0	1
17	11	11	28	0	0	29
33	9	7	7	0	5	6
154	11	94	115	0	129	126
42	39	63	123	37	44	50
2	1	0	0	0	0	0
35	37	76	91	66	106	77
4	0	6	18	12	0	9
71	59	39	56	106	85	41
2	3	0	0	0	1	0
17	5	6	14	0	22	7
0	0	0	12	0	0	3
3	3	3	0	0	0	8
24	16	10	39	12	9	13
0	0	0	0	5	0	0
34	118	46	68	12	15	52
147	52	60	43	156	82	43
105	85	47	117	52	68	69
14	0	0	0	0	0	0
10	7	3	13	0	4	2
4	1	1	0	0	5	5
143	159	170	163	223	188	110
110	69	107	124	110	112	87
189	102	143	201	124	160	142
1	1	15	0	31	10	5
33	37	24	59	6	77	28
6	0	0	0	0	0	0
65	57	71	122	79	92	44
48	32	50	76	60	52	27
0	28	0	0	0	0	0
178	117	166	361	141	220	170
0	0	0	11	0	0	1
72	76	85	101	92	69	82
1	1	3	0	0	1	0
23	3	1	0	23	25	18
67	114	43	91	0	43	3

For Review Only

54	28	50	116	12	25	28
56	51	54	77	45	87	98
126	59	73	114	151	110	99
17	12	20	13	13	10	11
1	1	0	8	0	0	0
8	11	8	28	6	28	31
35	18	20	30	29	25	20
5	316	136	420	147	44	73
113	97	94	191	212	78	98
0	128	40	102	92	26	0
194	427	472	804	498	462	393
54	51	58	67	56	40	44
48	45	78	75	25	30	26
23	7	19	21	17	35	35
70	101	114	165	248	176	154
15	17	24	34	14	11	19
17	49	51	60	113	53	48
129	166	50	96	215	83	85
240	179	166	143	235	246	245
18	30	55	18	0	2	16
48	63	55	97	104	80	54
29	53	51	52	30	43	12
36	9	12	37	14	29	24
27	28	18	16	31	4	20
41	36	48	36	56	26	15
0	7	0	7	5	0	0
640	1239	8	799	1257	394	550
182	162	128	235	128	182	112
174	99	114	110	141	67	64
0	0	0	0	0	0	0
118	54	109	137	173	113	74
2	0	0	0	0	3	0
52	46	65	91	103	51	42
31	26	43	49	29	33	24
94	49	60	98	77	128	47
0	0	0	11	0	0	0
27	36	21	31	34	26	27
0	0	0	0	0	0	0
6	1	1	4	15	9	0
0	0	0	0	0	0	0
104	60	58	100	108	71	59
70	66	62	90	37	76	73
0	0	0	0	0	1	0
14	5	9	20	23	20	6
86	60	60	121	10	35	54
71	45	0	145	17	75	0
108	94	60	207	72	113	69
87	71	127	168	0	19	30
40	0	0	14	11	48	38
1	0	0	2	0	1	0

For Review Only

107	122	126	165	111	115	105
23	15	20	67	16	33	53
423	270	221	392	286	665	313
74	58	58	82	92	97	30
90	54	80	101	75	103	65
92	132	104	128	115	108	74
460	297	288	412	346	585	388
95	126	118	163	141	116	153
54	32	33	48	42	51	41
26	132	4	67	0	1	3
637	437	503	441	1320	615	444
0	0	0	3	4	6	0
35	130	144	119	317	133	171
48	19	27	41	42	36	21
9	76	242	112	379	58	173
42	58	496	323	99	213	41
9	7	0	12	0	2	1
97	108	68	124	83	110	66
27	34	33	71	14	113	21
68	85	88	116	60	103	77
121	114	79	115	142	144	157
222	241	230	236	182	146	105
1	0	0	0	0	0	0
3	1	5	5	8	0	0
3	6	0	0	0	0	4
156	168	120	269	116	193	218
51	140	89	86	72	47	73
143	141	168	214	169	161	180
160	145	180	187	167	126	122
135	103	122	115	162	126	91
160	135	116	184	156	120	112
83	118	54	154	78	66	70
1	1	5	280	0	1	1
23	15	163	1	8	0	127
27	29	33	59	35	27	13
81	50	39	79	38	38	18
132	112	93	173	119	111	80
22	1	16	138	98	2	10
2	1	35	9	35	8	2
33	44	45	100	34	28	44
0	0	54	1	1	21	0
0	31	0	0	16	71	0
1	3	0	0	0	1	0
15	5	7	22	0	4	10
290	248	204	326	90	120	117
80	4	18	71	0	74	14
524	370	340	668	625	538	407
0	0	0	0	0	0	0
83	65	69	121	93	92	66
55	27	48	70	50	91	49

For Review Only

175	122	130	196	164	187	163
2	0	5	0	0	0	0
28	5	2	16	25	3	2
1493	1917	1775	3298	1331	1874	796
7	8	11	0	0	0	0
0	3	0	6	0	0	0
0	0	0	2	0	0	0
7	10	7	14	20	0	11
8	1	0	0	0	2	1
35	111	125	204	107	119	161
19	0	0	0	0	0	0
0	0	0	0	26	0	0
4	24	23	31	0	22	27
3	1	0	7	0	1	0
0	2	2	0	0	1	1
0	0	0	0	0	2	2
0	0	2	0	0	0	0
1	0	0	1	0	3	3
2	0	0	0	0	1	1
0	0	12	0	0	1	1
0	0	0	0	0	2	0
0	4	0	0	6	0	0
34	27	30	65	37	29	24
25	12	23	21	21	32	24
66	67	44	81	26	50	29
46	1	1	0	0	1	9
66	20	29	47	62	29	7
0	0	0	0	4	7	0
61	38	39	66	59	68	39
172	0	59	13	603	54	0
17	17	22	26	0	31	33
11	8	6	18	0	3	7
21	18	48	53	45	14	39
188	211	171	263	168	236	148
6	5	21	16	1	17	14
0	0	0	0	14	14	11
53	44	43	86	49	35	25
1	8	2	14	0	4	9
0	0	0	0	10	0	0
26	23	41	40	71	27	34
416	417	475	728	396	486	460
35	30	37	82	32	53	70
90	87	70	117	75	67	46
1216	1399	1066	2077	1156	503	1256
5	0	0	2	6	5	0
117	66	142	87	74	131	72
22	43	26	53	81	41	10
0	3	0	0	0	1	1
300	315	198	483	268	379	198
5	8	218	0	0	1	99

For Review Only

53	14	27	37	33	46	33
249	211	229	368	302	191	82
10	3	7	8	1	7	7
103	85	74	90	75	79	90
36	15	23	57	20	36	37
0	95	0	0	0	0	0
88	105	126	226	81	110	139
0	0	3	0	8	3	0
339	254	292	408	385	293	301
43	38	44	55	49	58	43
9	4	13	10	24	9	7
16	16	5	35	5	12	22
0	0	0	0	0	0	0
54	70	34	97	25	40	68
39	36	46	56	38	49	80
79	74	73	121	59	48	70
65	13	6	36	70	37	31
2	15	5	0	0	2	1
47	48	57	51	42	44	36
0	0	0	6	0	0	0
1	0	1	0	10	5	0
38	31	31	62	42	32	33
83	79	59	0	0	59	54
86	91	165	261	290	115	152
96	122	96	148	76	45	61
13	19	24	41	43	24	24
0	2	0	0	0	2	5
62	60	68	76	10	58	6
3	2	4	0	17	10	7
48	56	99	134	150	69	74
0	6	5	24	17	6	11
11	3	10	52	0	5	48
1	1	2	0	0	0	0
53	45	41	132	18	113	42
51	40	61	76	70	32	51
100	46	39	57	53	55	58
19	6	1	19	0	8	6
23	0	14	0	24	0	5
32	34	29	40	60	29	0
3	4	0	2	0	0	0
1	28	1	0	0	3	1
170	131	202	394	199	133	184
187	165	191	313	156	240	202
0	5	0	0	0	4	0
6	3	3	0	0	0	1
127	72	105	171	153	70	112
0	61	71	91	0	93	29
38	17	23	48	28	19	38
61	60	65	72	43	49	26
0	0	24	1	1	0	0

For Review Only

238	168	87	235	70	195	78
37	33	23	40	11	21	22
1	193	1	807	0	1	3
0	6	0	0	0	0	0
26	36	76	45	35	27	84
0	0	0	0	0	0	0
0	0	3	2	0	1	0
67	21	51	47	0	6	15
57	61	53	78	63	54	59
33	1	4	19	0	4	15
7	0	5	9	12	12	7
69	71	81	129	108	86	89
67	154	118	221	70	139	91
88	58	134	29	147	92	139
19	14	13	26	7	15	16
238	174	156	278	244	152	122
18	12	19	24	0	6	15
94	95	117	152	157	59	118
0	3	0	0	0	0	3
0	0	3	2	0	0	3
13	32	25	25	20	34	22
7	9	4	9	9	11	6
0	0	0	0	0	0	0
0	0	1	0	0	0	0
22	34	31	50	20	29	26
124	73	24	76	14	90	30
32	113	65	179	119	80	45
88	81	129	185	109	115	153
109	146	124	181	164	67	80
0	0	0	5	0	0	0
0	2	0	3	0	0	2
79	35	69	68	70	64	51
15	18	20	14	9	16	1
172	91	113	160	133	211	158
115	77	90	106	64	230	75
86	35	37	63	25	57	43
240	287	191	491	262	262	187
2	6	2	5	3	7	11
22	15	10	19	20	13	2
55	43	0	74	50	47	25
18	33	38	44	18	34	34
125	1	1	0	100	80	36
9	11	4	17	0	6	0
34	131	101	107	107	14	102
58	26	21	58	41	22	54
81	620	40	253	271	363	163
11	5	9	29	63	6	6
1	0	0	1	0	0	0
63	52	59	93	112	118	55
0	0	0	0	1	4	3

For Review Only

2	2	0	4	0	0	3
5	0	1	10	0	4	3
112	92	66	145	91	104	46
83	112	81	102	127	94	56
45	39	50	49	9	28	15
64	26	44	77	63	27	17
1	14	42	4	142	22	20
55	78	94	113	89	75	94
1	3	1	15	0	4	2
130	80	185	202	75	133	101
15	63	102	1	0	46	22
360	315	315	553	222	412	194
38	68	29	74	97	35	63
307	304	449	582	455	305	423
23	55	29	142	0	33	17
46	73	84	104	77	83	73
380	124	31	615	391	211	309
71	58	28	54	19	39	30
0	0	5	10	0	0	1
99	95	109	209	114	142	77
18	19	20	27	10	16	16
54	39	48	64	45	48	20
43	64	130	140	279	63	173
5	0	0	0	0	0	3
22	43	74	133	51	59	30
3	13	15	17	0	10	9
0	4	4	0	0	1	1
80	80	87	124	64	49	71
2	8	1	0	5	3	3
0	0	0	0	0	0	0
0	0	0	0	0	0	7
0	5	6	10	0	4	4
0	0	6	0	0	0	4
3	1	15	65	0	1	1
15	22	23	33	19	23	10
7	82	1	0	24	89	31
55	85	56	61	87	32	32
53	49	78	65	75	95	64
51	70	31	62	26	23	44
23	1	4	8	21	1	0
4	1	0	3	0	0	0
23	11	13	24	12	12	38
5	0	9	8	0	4	13
12	13	6	18	0	2	5
3	9	18	11	33	6	2
0	0	144	119	45	0	102
10	29	22	35	0	22	12
338	285	315	419	490	239	414
7	3	5	20	0	0	18
80	36	50	113	84	72	40

For Review Only

16	43	33	34	35	52	28
2	3	0	22	0	5	1
6	6	0	0	0	0	0
27	25	4	18	41	38	10
0	0	0	0	0	0	0
3	9	20	13	0	4	4
31	46	67	65	65	61	43
1	0	1	0	0	21	0
35	62	47	80	77	67	24
1	0	0	1	4	4	3
32	89	2	48	0	87	10
0	0	0	0	0	0	6
9	19	91	45	0	4	6
3	16	13	19	0	5	4
104	155	132	226	202	114	140
0	0	0	0	0	0	0
0	0	0	1	8	0	0
8	10	10	18	5	12	16
0	0	0	0	5	0	0
88	49	58	103	50	67	51
0	0	0	0	0	0	0
76	92	79	106	70	63	53
0	0	0	9	0	0	4
27	17	3	47	77	17	67
10	7	19	24	13	5	11
50	34	61	70	25	23	35
46	17	39	49	22	40	14
53	65	25	37	47	46	11
28	26	14	15	0	11	4
0	0	0	0	0	0	1
110	162	131	188	106	122	162
0	0	0	0	0	0	2
404	386	272	758	236	391	375
76	64	52	117	38	59	66
0	14	0	0	46	8	5
56	21	28	47	53	35	21
27	1	1	0	23	5	2
97	133	72	55	299	97	108
18	102	108	38	43	20	246
123	96	87	130	67	121	42
1	10	4	0	0	1	5
26	16	33	24	48	14	28
32	14	13	36	16	25	26
0	1	0	1	0	0	0
33	44	64	97	53	63	59
32	22	24	34	15	43	31
15	15	18	30	10	8	18
0	1	2	0	0	1	4
116	114	111	192	132	108	98
14	1	3	1	0	5	4

For Review Only

3	5	6	0	0	5	12
50	70	51	50	69	38	73
118	74	98	167	169	157	117
177	97	161	158	157	107	124
0	24	0	51	3	142	139
32	10	16	44	38	29	3
47	45	14	57	79	84	12
62	58	0	28	22	38	46
17	4	1	24	15	23	3
188	141	160	156	200	142	160
25	39	38	36	65	47	25
106	75	61	195	62	82	88
8	2	1	6	0	5	4
55	43	30	63	47	39	28
33	19	65	103	32	32	36
102	94	92	132	33	104	56
45	60	33	48	86	40	19
84	21	15	21	80	41	28
18	20	30	49	26	33	18
22	13	41	29	49	16	37
78	67	52	103	43	73	58
2	0	0	6	0	0	5
199	199	168	263	298	193	243
20	0	13	16	0	16	12
26	16	18	20	28	19	14
114	93	67	141	99	51	104
29	15	0	16	0	1	6
23	8	15	40	15	22	20
44	30	39	65	27	45	51
6	1	0	12	0	2	3
8	0	6	6	0	2	4
5	4	19	21	0	14	13
29	35	3	45	16	22	28
111	85	75	142	68	90	60
62	48	39	107	34	60	53
71	58	45	69	54	32	35
10	9	14	20	10	13	15
210	407	142	241	362	145	159
10	17	18	13	21	17	16
140	132	76	147	121	73	74
255	148	136	100	140	50	115
38	39	23	41	30	24	31
55	38	39	87	99	68	50
1	2	1	0	0	5	1
2002	2149	2556	4188	1983	2655	2326
5	2	0	0	9	0	4
33	20	46	142	0	0	0
299	342	0	993	103	0	521
0	471	0	15	1	0	0
2975	2657	2468	4507	3728	695	2303

For Review Only

17	31	49	0	0	32	29
205	159	163	312	207	203	168
84	132	104	152	190	103	161
56	42	25	38	46	42	21
365	309	233	515	280	358	278
0	0	0	0	0	1	3
1	0	16	0	8	0	2
101	152	153	268	71	180	222
9	6	3	10	0	6	4
7890	6403	4698	11091	4545	9486	6412
30	28	26	49	29	38	28
27	21	27	46	14	0	15
33	29	14	27	34	17	11
25	16	20	43	33	26	36
1	3	3	0	19	7	13
144	103	59	113	135	126	88
153	188	206	220	130	205	130
29	64	59	69	114	41	46
77	82	50	103	46	46	103
39	22	48	86	20	50	27
0	0	0	0	0	0	0
114	89	117	193	105	118	120
23	18	0	20	27	20	19
0	0	0	0	0	0	2
0	0	0	0	0	0	0
0	0	0	36	0	0	0
452	435	400	622	432	303	318
0	0	8	33	0	8	0
77	76	107	143	155	90	84
138	90	78	96	79	57	70
1	0	0	0	0	0	1
7	10	4	3	15	0	3
0	3	12	0	15	0	3
45	31	42	105	35	28	18
0	0	2	0	0	0	2
13	16	9	37	9	24	24
0	0	0	0	0	3	1
0	1	1	0	0	1	1
0	3	0	7	0	0	4
1	1	0	12	0	0	2
67	88	86	138	50	128	105
170	163	150	253	184	222	155
0	0	0	2	0	0	0
0	0	0	5	0	3	0
15	1	2	18	0	2	2
7	9	20	64	0	50	85
33	37	22	25	56	17	20
1	3	1	0	0	1	1
129	65	50	162	48	62	74
13	0	9	20	0	15	6

For Review Only

17	10	22	41	11	14	19
0	2	3	7	0	11	1
3	0	4	13	0	0	1
72	80	50	114	91	54	55
0	11	0	0	0	0	60
63	73	44	80	52	61	46
93	74	119	82	78	82	58
5	2	0	1	0	7	4
1	0	4	4	7	4	1
145	216	185	177	218	85	110
88	111	123	167	85	127	77
101	69	59	143	54	182	137
79	94	118	232	98	123	104
15	1	14	10	0	8	5
104	97	63	105	157	35	35
1	0	0	0	0	0	0
47	36	25	35	28	25	35
318	242	209	400	234	299	286
0	0	0	24	288	260	0
10	3	5	9	0	5	0
76	42	10	73	44	14	1
34	63	23	77	66	58	88
196	485	172	574	155	177	50
54	28	57	60	68	81	45
1	2	17	100	0	11	23
191	82	190	163	169	115	120
125	151	98	206	116	127	67
0	0	0	14	0	0	4
82	37	43	105	35	54	23
70	57	41	42	80	27	62
0	35	0	8	0	0	0
511	374	389	643	449	604	417
456	464	445	738	413	516	414
362	224	249	273	274	287	185
177	124	198	0	0	153	158
88	69	93	86	92	84	79
0	0	0	0	0	0	0
77	40	109	88	273	164	145
129	17	6	63	37	23	28
9	4	11	24	19	7	10
9	0	0	0	0	0	0
2	0	0	0	0	3	1
0	1	0	0	0	1	0
0	7	0	1	0	0	0
13	20	3	22	0	8	4
65	68	30	42	110	37	70
11	30	15	17	32	24	45
152	177	210	226	298	123	207
13	23	28	35	10	17	18
315	284	147	258	234	205	167

For Review Only

0	0	0	0	0	0	0
0	0	0	0	0	0	0
59	31	48	95	30	21	63
1	0	0	0	0	0	0
35	19	38	50	15	22	41
0	0	13	13	14	10	0
0	8	0	0	0	0	0
9	1	3	3	0	3	0
32	8	80	0	51	21	9
143	143	40	117	27	84	152
21	9	20	39	7	40	54
72	0	0	2	177	127	52
119	128	185	176	173	101	116
120	77	72	119	115	80	73
0	0	0	3	1	6	0
1	0	0	6	0	2	4
6	1	6	17	0	7	8
66	56	107	97	145	101	87
49	60	72	80	62	76	40
41	33	45	94	37	31	68
15	23	21	11	9	10	20
42	62	37	50	45	30	39
15	0	0	0	0	3	0
2	3	3	9	0	1	0
0	0	85	42	94	104	0
0	0	0	262	0	0	0
16	15	17	21	29	0	21
26	31	33	30	30	12	27
0	0	0	6	0	0	0
0	0	0	0	0	0	0
103	57	153	147	45	58	85
78	131	82	106	74	94	32
1	63	16	120	186	21	94
90	76	86	148	75	83	88
1	1	1	18	0	8	9
18	21	43	45	86	47	10
294	345	389	539	312	342	390
20	32	89	73	156	20	75
205	226	290	402	243	290	270
14	13	7	19	10	9	7
122	133	121	230	170	106	132
24	3	18	21	10	23	10
0	0	0	3	11	0	0
2	3	0	6	0	0	2
0	8	64	58	0	15	36
1	1	0	0	0	1	1
74	52	52	91	28	88	12
60	23	37	54	38	45	23
3	0	0	0	0	0	0
0	0	0	0	1	0	0

For Review Only

23	0	35	64	16	26	22
0	0	0	0	2	0	0
24	51	40	44	72	58	44
5	1	3	1	4	0	0
5	0	0	0	0	0	0
2	1	1	6	0	2	2
53	39	0	87	23	55	42
708	612	694	1150	1123	833	1004
625	463	573	811	612	530	529
0	0	0	4	0	0	3
6	5	3	13	0	0	9
4	0	0	0	0	0	0
390	310	296	443	266	308	199
5	0	7	13	0	1	4
5	2	1	0	0	0	0
333	413	280	438	449	291	422
25	70	54	89	72	23	55
8	5	7	7	10	12	5
0	0	2	0	0	3	0
108	67	99	191	102	218	144
131	177	106	206	136	154	177
946	1049	572	1295	1204	1236	573
3	0	0	0	4	0	0
0	0	0	0	0	0	0
1	1	0	0	0	1	1
0	0	0	0	13	0	0
1916	1000	1492	2884	1795	1553	1102
69	131	82	142	157	74	122
51	38	37	81	37	29	25
38	45	15	16	16	55	72
15	30	13	45	50	23	35
122	289	304	293	43	130	134
80	91	51	80	90	84	43
444	11	244	859	143	231	191
336	221	225	401	287	243	236
47	72	30	63	29	50	52
98	118	110	190	91	121	88
33	6	13	33	12	20	8
0	0	0	0	0	2	2
16	23	0	0	0	0	11
55	20	69	289	0	13	12
63	40	49	67	107	114	31
0	0	0	179	68	0	0
29	19	14	7	11	10	5
6	0	3	8	0	0	0
124	88	34	81	129	51	67
27	25	19	24	18	35	32
5	3	5	1	0	8	4
6	0	2	6	0	0	2
44	45	65	146	60	63	78

For Review Only

33	47	41	63	72	55	44
7	0	1	8	0	0	0
0	0	0	2	0	5	0
0	0	0	0	2	0	5
5	3	4	11	13	1	6
0	0	0	0	0	0	0
721	687	664	1084	1450	42	0
137	73	140	168	79	130	124
9	3	12	16	0	10	4
123	0	98	19	255	106	91
0	0	0	0	6	0	4
17	25	17	14	16	6	6
7	10	3	5	7	1	7
12	12	9	18	1	9	11
4	1	3	0	1	1	1
0	0	0	2	65	65	0
1	0	2	0	0	1	2
52	5	20	43	0	2	70
8	1	1	0	13	12	8
221	203	242	359	290	312	166
322	274	349	609	467	447	379
5	7	11	12	4	5	12
10	58	47	142	69	76	106
4	1	1	0	0	0	0
0	0	0	0	0	7	0
9	7	1	0	0	12	275
11	6	8	14	7	10	10
95	81	25	195	0	0	37
466	336	221	465	330	465	235
89	168	108	237	180	157	179
282	217	188	390	222	289	291
26	46	19	40	41	27	25
485	333	442	220	1209	172	0
145	122	81	176	145	120	49
118	153	133	178	139	106	108
41	74	90	87	105	45	68
55	97	41	97	18	53	103
112	130	74	88	149	128	49
51	32	44	89	22	54	67
5	3	4	1	0	2	7
0	2	2	0	0	0	0
0	0	0	6	0	0	0
0	0	0	0	0	5	0
7	1	7	0	0	2	4
0	0	0	56	43	0	0
53	61	53	115	48	45	55
11	8	8	14	15	0	0
348	71	356	323	774	280	300
0	0	0	0	0	2	0
1318	0	0	362	1	1410	0

For Review Only

30	22	38	55	53	20	25
0	11	0	0	0	2	2
39	34	0	9	0	0	0
81	78	75	131	54	64	67
58	36	13	73	35	57	39
32	13	18	40	55	24	46
112	81	115	101	93	75	105
152	62	75	121	140	118	93
113	107	74	118	139	108	72
48	46	45	0	0	52	59
7	2	0	0	0	1	0
0	1	0	0	0	5	0
16	0	0	11	24	11	5
86	135	103	115	151	101	122
16	10	18	25	18	15	19
164	120	97	203	100	165	83
7	5	20	1	24	26	7
58	15	111	72	0	13	75
0	0	2	0	0	0	0
1	3	0	3	0	3	0
6	0	0	2	0	0	0
0	5	6	5	0	3	4
292	250	226	111	107	177	264
75	87	101	151	65	90	73
48	43	32	62	59	58	45
6	3	5	8	5	9	5
2	2	11	9	0	7	0
327	302	186	348	240	286	280
12	0	0	1	6	0	0
0	2	2	0	0	0	0
90	54	81	138	74	91	84
225	141	145	196	190	161	153
103	86	82	147	134	134	86
1	0	1	0	0	2	1
1	3	3	0	0	0	0
22	4	6	23	33	28	14
8	6	5	6	12	2	3
299	166	211	348	182	341	283
61	34	77	660	0	543	1
2	1	1	30	0	1	0
38	29	45	58	27	46	26
0	0	1	0	0	0	0
35	78	91	120	30	64	55
184	118	105	133	56	72	60
79	90	103	108	140	81	118
78	81	55	123	51	94	60
61	76	66	2	107	0	75
308	236	282	445	240	285	222
29	19	14	31	28	10	16
17	20	12	32	20	42	24

For Review Only

47	118	25	216	1	159	45
32	35	20	40	55	23	11
100	66	52	110	106	80	92
56	46	45	25	77	42	39
154	146	129	222	156	170	160
350	417	298	599	426	447	299
0	1	1	5	0	1	0
2	1	1	30	0	21	1
45	50	47	51	78	54	27
0	2	4	9	0	0	1
7	7	3	0	0	9	0
0	4	4	8	0	5	0
151	232	182	183	216	154	133
1	1	9	0	0	0	3
39	38	61	59	38	87	64
1	16	32	0	0	1	1
9	0	0	0	0	0	0
66	25	47	23	89	101	9
52	41	32	57	43	30	37
36	76	57	69	78	57	28
0	0	5	9	0	9	0
0	0	0	0	0	0	0
0	0	0	0	0	0	0
13	6	10	10	20	20	10
558	363	406	719	627	664	401
464	321	478	780	439	489	522
57	66	65	79	114	103	62
14	7	9	15	9	8	12
33	25	31	49	21	22	36
0	0	0	0	0	0	2
55	6	17	43	0	7	14
0	0	0	5	0	0	0
75	100	66	156	114	90	84
267	142	177	276	178	134	122
0	1	0	0	0	1	1
0	0	0	0	0	0	0
8	15	29	23	0	20	10
0	350	26	0	0	0	387
447	457	44	80	1253	315	266
33	42	24	22	38	30	20
11	0	0	0	0	0	0
9	12	2	8	0	9	6
31	23	24	32	51	26	30
12	6	2	13	0	12	3
6	9	10	22	4	6	5
87	51	33	56	50	69	20
72	47	44	49	81	40	52
13	13	26	41	9	23	12
0	0	0	0	0	63	0
8	7	16	33	16	19	12

For Review Only

3	0	0	6	0	0	4
35	61	32	64	89	53	0
11	15	9	0	0	16	10
0	0	0	0	19	0	0
16	35	45	72	0	32	69
357	787	602	846	687	615	600
14	13	10	42	24	21	11
50	35	31	79	25	17	39
3	0	0	0	0	0	0
88	0	73	49	167	91	78
33	24	28	50	10	20	35
2	1	8	0	0	3	1
10	17	8	32	0	3	11
5	0	8	9	0	8	3
301	28	31	0	49	120	133
12	0	0	13	0	0	0
3	0	0	4	0	5	0
5	0	5	0	0	4	8
73	89	87	191	84	36	80
49	33	52	68	63	40	37
121	154	65	162	137	127	51
173	129	153	326	48	153	165
349	355	204	275	325	326	239
79	35	35	116	29	91	31
449	553	296	734	302	493	325
0	0	0	0	0	5	0
48	47	41	88	26	56	50
529	378	290	351	572	433	206
151	105	134	163	151	73	83
69	52	80	104	43	68	66
1	1	1	293	0	1	3
5	5	4	11	8	0	10
12	7	9	22	11	4	17
41	46	78	120	60	93	61
3	20	50	69	0	20	12
11	31	26	39	10	7	7
133	117	101	150	90	118	63
0	0	14	16	3	0	8
6	0	2	12	0	6	6
10	25	16	10	27	19	8
51	44	0	19	151	43	0
0	0	0	0	0	0	0
45	91	50	92	126	46	54
790	672	710	760	440	837	510
0	0	0	0	0	1	0
1	0	0	0	2	0	4
0	0	0	0	0	0	0
0	0	0	0	0	0	0
1	1	2	0	0	1	1
1	3	1	0	0	1	4

For Review Only

1	12	0	0	32	29	14
9	0	14	0	0	3	5
155	152	164	350	231	256	144
72	39	105	80	63	55	53
2	0	0	0	0	0	0
26	9	17	44	35	21	22
28	28	42	32	49	10	32
61	65	38	83	42	91	52
114	191	117	170	226	175	110
0	0	0	0	0	1	3
40	25	24	44	17	34	32
0	1	0	0	0	0	1
1	3	0	7	9	3	8
25	7	23	56	17	46	29
0	10	0	22	19	23	0
0	0	0	32	1	0	489
94	62	65	107	49	58	48
105	134	172	204	201	232	126
1	1	1	9	0	4	2
15	7	17	45	0	6	20
46	41	39	72	49	47	41
44	55	47	54	48	16	22
102	64	143	148	110	89	96
25	4	10	14	31	5	6
62	59	71	73	88	28	72
0	0	0	0	0	3	0
12	1	220	0	124	15	4
0	2	1	0	0	0	3
218	207	188	336	255	223	135
105	180	79	272	152	142	103
174	110	105	219	139	250	137
0	0	0	0	0	0	0
227	242	227	203	183	164	156
3	4	6	9	0	0	4
404	375	187	288	332	287	198
19	13	10	19	17	11	12
43	50	46	82	35	74	37
0	1	8	12	0	2	5
64	60	61	118	56	68	54
94	61	77	205	53	156	118
5	6	4	17	0	4	1
78	33	65	103	30	56	47
23	10	27	8	0	10	13
18	18	13	25	13	15	15
1	0	6	0	1	0	1
1	0	1	7	0	0	0
1	0	0	0	0	0	0
2	1	3	4	0	0	2
9	10	9	20	0	4	25
4	0	5	4	6	3	4

For Review Only

2	4	5	10	0	7	4
24	23	19	39	25	22	27
15	32	14	42	6	10	18
65	15	46	81	76	64	38
2	5	0	0	0	4	1
17	15	24	26	30	23	25
1	0	1	3	1	2	2
4	4	8	8	9	11	9
16	8	8	15	17	26	20
1	0	0	0	0	4	3
0	0	0	1	0	2	0
1	11	0	0	0	1	2
0	1	0	0	0	6	0
3	1	1	0	0	0	0
0	3	0	0	0	1	0
27	22	28	19	17	37	14
3	0	3	7	4	5	0
0	0	0	0	0	1	4
1	2	4	13	0	0	6
0	0	0	1	0	0	8
22	23	19	43	10	18	19
0	3	6	3	2	3	3
2	13	9	0	0	16	6
1	0	1	6	0	2	2
1	1	0	0	0	0	0
0	3	3	6	6	1	1
1	2	0	0	0	0	2
9	5	19	19	11	13	11
6	4	2	7	8	9	3
238	238	203	256	135	138	142
1	1	1	5	0	3	9
17	18	18	29	17	14	12
3	5	6	5	1	9	1
0	0	0	0	4	0	0
7	10	16	7	18	15	15
7	6	3	10	12	12	5
0	0	0	22	0	0	0
0	0	2	0	0	0	1
0	5	1	1	0	2	2
1	1	3	9	0	4	8
7	4	6	10	0	5	2
7	5	4	5	0	7	0
1	0	0	0	0	0	0
30	11	15	21	18	14	12
3	0	0	3	0	0	1
3	0	0	4	0	3	1
1	5	2	8	0	0	11
0	1	4	0	4	0	0
0	0	0	1	1	17	0
3	2	5	13	0	9	11

For Review Only

0	0	0	2	0	0	0
147	139	174	248	181	149	134
0	4	2	8	0	0	3
3	0	2	1	1	2	0
0	0	0	3	0	0	3
43	29	8	26	0	39	2
2	4	1	4	6	5	6
3	0	0	0	5	0	0
1	1	0	0	0	1	0
9	21	7	16	29	6	6
17	18	7	17	20	23	14
2	3	1	8	0	2	5
2	2	5	8	0	4	4
201	203	152	294	145	194	182
45	9	9	25	0	16	35
11	6	3	0	55	340	1
0	1	0	0	0	0	0
3	0	0	0	3	0	3
0	0	1	0	0	0	5
0	1	1	3	0	3	0
2	1	1	0	0	2	0
0	0	0	6	0	5	4
1	1	0	0	0	0	0
1	5	5	7	4	8	3
1	1	6	7	0	3	0
72	54	45	82	68	56	44
1	0	0	0	0	0	0
125	106	139	194	112	105	111
28	43	34	23	30	25	39
75	61	73	141	49	87	86
6	5	6	10	0	0	3
1	0	0	1	0	1	0
2	4	1	12	1	4	4
0	4	1	0	0	0	0
9	11	55	32	45	13	11
6	4	4	17	15	11	15
58	36	47	77	55	41	49
0	10	4	7	0	0	0
1	1	4	0	0	2	1
1	1	4	6	12	3	1
7	6	13	13	8	5	6
0	0	0	0	9	0	0
1	7	1	16	0	10	4
0	0	0	4	0	0	0
30	32	27	44	22	29	24
0	1	1	1	0	0	0
56	109	67	118	80	56	72
10	18	18	25	14	8	17
0	0	0	0	0	5	0
15	6	2	12	22	8	10

For Review Only

0	0	0	0	0	0	0
5	3	13	21	0	4	2
2	0	0	0	0	2	1
3	2	5	0	0	6	0
39	35	62	85	55	39	36
0	1	0	0	0	0	0
10	8	11	23	5	18	11
1	67	82	79	119	40	43
3	3	0	2	4	0	0
11	1	4	6	14	9	4
3	17	12	21	19	19	6
0	2	0	0	0	4	0
2	3	2	6	0	0	2
0	2	0	0	0	0	0
43	28	53	51	55	33	25
4	0	1	0	1	1	0
73	15	2	107	0	63	35
7	15	8	16	17	10	11
18	1	0	0	0	2	6
30	36	39	63	25	24	28
16	14	19	23	16	17	13
9	8	22	23	7	2	10
12	10	11	20	12	15	15
10	7	6	18	4	12	11
4	4	3	7	0	0	5
0	1	0	0	0	0	1
1	0	0	0	0	1	0
55	41	48	65	79	43	47
36	23	39	49	53	49	44
0	1	5	0	0	1	1
4	5	5	23	0	9	13
0	0	3	12	0	5	6
12	13	8	20	13	14	6
24	24	20	30	28	26	20
29	19	21	17	14	15	19
0	0	0	0	2	5	7
2	9	5	8	0	5	8
9	12	22	24	20	23	25
11	37	19	40	23	29	55
9	13	1	11	8	4	1
23	10	17	24	0	4	7
27	29	26	36	39	34	21
4	1	1	7	0	7	0
5	0	0	3	0	0	0
0	0	0	0	0	0	3
2	1	0	0	0	2	0
0	7	10	1	1	0	0
2	6	13	8	0	14	12
5	0	1	0	0	0	1
1	0	1	0	0	4	3

For Review Only

4	2	0	7	13	6	1
0	0	0	0	0	3	0
35	27	16	21	11	8	8
1	0	0	0	0	3	0
0	0	0	0	2	0	0
5	1	6	11	4	3	5
3	0	10	6	0	11	3
0	2	9	6	0	5	0
45	28	35	0	0	30	29
1	0	4	0	0	23	1
6	3	0	16	0	9	2
2	5	7	1	0	0	0
3	3	2	0	6	1	1
4	7	1	1	3	6	6
0	2	0	0	5	0	0
2	2	0	0	1	1	1
0	0	0	2	1	0	0
6	5	4	10	12	6	8
6	6	6	8	4	6	10
9	13	12	16	5	13	10
22	10	12	19	10	17	6
6	4	7	10	1	4	4
13	12	10	18	11	12	7
10	6	24	20	0	3	19
8	8	9	8	14	6	7
19	29	14	26	21	17	20
30	24	21	38	63	40	35
35	29	33	52	64	69	57
4	5	7	12	22	7	11
3	1	4	1	1	3	3
8	11	11	13	14	8	8
7	6	6	11	6	6	4
6	2	5	4	1	6	4
0	1	1	0	0	0	0
1	0	0	0	0	1	2
7	6	3	5	10	11	4
163	159	212	319	111	181	186
3	1	0	0	0	3	1
19	17	23	45	32	26	28
17	5	18	26	0	10	8
1	3	1	8	0	4	1
4	1	1	4	1	2	3
85	97	67	132	75	76	75
4	2	5	7	0	10	10
0	0	0	4	0	1	1
2	0	0	0	3	0	1
0	1	0	3	0	2	3
3	3	2	0	8	3	5
1	1	3	18	0	6	2
0	6	4	7	0	9	0

For Review Only

21	12	15	29	27	18	12
27	14	33	46	18	34	43
5	5	0	2	2	7	0
7	6	3	7	13	9	3
60	39	59	96	55	87	63
15	18	13	22	16	20	16
0	0	0	0	6	0	0
0	2	2	6	0	3	0
3	0	3	10	0	7	3
6	34	22	32	0	18	6
1	0	3	0	0	0	0
4	4	1	9	6	11	2
1	6	4	6	0	5	8
6	1	3	8	0	0	5
1	11	2	11	0	3	4
404	551	540	741	859	180	457
0	0	0	2	2	3	3
1	2	1	0	0	5	3
0	0	1	0	11	0	1
6	1	3	1	0	0	0
15	4	9	12	0	7	11
1	3	0	1	4	0	1
6	0	6	14	0	11	4
1	3	1	5	0	4	1
29	7	16	11	16	1	1
3	0	0	8	0	0	1
31	31	12	44	96	47	80
2	0	0	12	0	2	0
0	1	3	0	0	1	2
98	189	59	193	0	73	205
10	15	1	17	0	7	14
2	2	2	16	0	0	0
21	26	20	36	1	11	39
10	2	7	16	19	13	9
14	28	24	28	15	11	18
122	119	129	249	109	166	160
6	8	0	0	0	6	5
68	80	80	121	57	66	58
9	7	3	7	5	2	2
15	2	3	0	0	0	4
194	232	215	345	232	180	182
6	4	11	28	0	4	25
0	0	0	0	11	0	0
0	0	0	0	0	3	0
10	7	9	17	0	9	2
12	0	0	5	0	0	0
0	0	1	0	0	0	0
14	4	6	12	3	7	4
0	0	0	5	0	0	0
8	13	7	18	0	2	7

For Review Only

15	19	20	22	6	20	19
0	0	1	0	0	0	5
12	9	3	27	0	9	19
16	5	12	18	5	13	10
0	0	2	0	1	3	3
4	9	6	10	6	6	4
8	11	5	30	10	40	10
55	49	46	104	47	114	70
1	1	1	0	0	7	9
3	3	0	8	7	6	5
6	14	9	15	6	6	4
2	0	0	0	0	1	0
0	0	0	0	1	3	0
1	0	0	0	0	1	0
1	2	7	0	0	12	8
0	0	3	0	0	1	4
0	0	1	0	0	0	1
0	0	0	1	3	0	0
2	0	2	5	0	0	1
18	13	20	8	0	33	17
7	0	3	14	0	6	16
1	1	1	0	0	1	0
0	0	0	0	0	2	0
1	0	0	8	0	1	4
5	0	0	1	1	0	4
0	4	0	5	3	2	3
0	32	0	0	0	18	33
8	8	8	9	0	2	10
1	0	0	0	1	1	0
0	2	7	6	0	3	1
28	24	14	40	25	28	27
14	10	11	25	0	51	2
5	3	6	12	9	19	7
1	14	0	3	0	1	0
8	3	8	18	0	7	26
0	0	1	0	0	4	0
8	3	3	8	0	0	2
0	0	0	0	0	0	0
1	0	1	5	9	0	0
0	3	0	0	4	0	3
0	0	0	0	2	0	1
15	5	7	10	17	11	11
0	1	0	0	0	1	0
12	7	10	13	8	9	4
0	7	1	0	0	1	0
6	16	9	17	0	22	27
65	37	48	49	60	42	35
6	9	2	10	0	14	27
0	3	1	0	7	0	4
13	10	6	18	12	12	15

3	5	2	6	0	3	2
1	8	0	5	10	5	0
0	1	5	0	0	0	0
11	4	15	1	8	11	5
0	0	6	0	7	4	0
1	2	6	6	17	0	0
1	2	2	1	0	2	1
6	16	3	16	15	9	8
48	56	121	142	92	110	96
0	0	0	17	0	0	0
2	2	0	7	0	1	6
0	3	0	1	0	0	0
6	8	23	30	0	8	6
0	0	1	8	0	0	1
1	3	3	6	0	2	1
1	2	0	10	1	4	3
0	5	0	0	0	1	0
7	9	14	11	7	3	3
0	0	0	4	0	1	0
0	0	3	1	1	0	1
1	0	0	2	5	0	1
4	0	1	0	2	6	0
8	19	27	16	13	19	8
0	0	0	0	0	0	0
7	9	15	13	5	4	3
113	46	18	90	0	36	49
5	0	1	0	3	0	3
6	8	10	12	6	14	10
4	0	0	0	0	1	0
0	0	0	0	0	0	0
12	4	0	0	9	0	0
14	8	0	10	0	2	1
10	10	25	24	8	8	12
8	13	5	11	7	9	5
2	0	2	7	0	0	0
3	0	0	4	3	0	0
24	24	18	31	0	22	20
0	0	6	0	8	6	5
17	5	7	8	28	5	4
1	2	2	0	0	0	4
12	12	7	17	14	13	5
4	4	0	1	4	4	0
19	19	8	24	0	7	2
45	14	21	55	0	36	28
3	1	2	15	0	0	2
36	25	22	33	24	20	13
21	18	21	47	11	43	29
0	0	0	0	5	1	5
0	2	2	4	0	0	1
18	37	18	24	23	38	18

For Review Only

0	0	0	13	1	0	0
5	2	1	4	4	2	3
4	2	1	1	1	3	3
7	8	1	5	6	8	3
5	0	0	7	0	0	0
3	0	0	6	0	0	1
6	5	3	7	0	0	0
0	0	0	2	7	0	3
9	1	2	51	80	3	10
14	8	8	20	1	4	8
0	0	0	0	0	0	0
27	15	0	93	52	12	13
0	0	0	18	0	0	0
0	0	0	0	0	1	0
11	8	8	20	6	12	8
0	2	0	1	0	3	1
0	3	0	0	0	1	1
20	24	21	36	27	10	11
0	0	0	0	0	0	4
2	0	0	10	0	0	1
10	8	9	21	5	14	12
26	10	18	33	12	19	18
9	0	4	4	1	10	0
1	1	1	1	0	2	1
2	3	3	6	1	4	2
1	5	2	6	3	0	0
13	7	14	15	13	12	12
5	8	8	9	8	8	7
70	25	39	54	63	28	57
54	0	0	46	45	100	32
7	23	20	40	22	28	29
1	96	7	112	27	54	15
0	0	0	0	1	0	0
141	107	115	156	146	191	188
0	1	1	12	6	1	0
51	45	86	156	41	76	79
25	0	6	1	18	6	13
0	1	1	0	5	3	0
0	1	1	0	6	3	0
4	2	3	1	1	3	3
5	0	2	0	0	0	3
3	1	1	123	0	1	1
57	57	54	84	76	54	45
9	8	4	12	0	8	7
12	10	5	9	6	9	8
1	0	0	4	0	4	4
0	0	2	0	3	2	0
34	31	34	62	41	37	42
21	22	18	42	12	20	17
25	17	18	27	13	18	21

For Review Only

1	0	0	10	0	1	0
0	0	0	7	0	0	1
12	0	9	11	10	21	20
75	93	109	43	66	79	87
1	3	7	5	0	4	5
0	7	1	7	0	0	1
12	7	13	9	17	8	7
3	1	2	7	19	1	21
3	0	3	2	5	3	0
4	14	8	9	40	16	16
40	41	32	61	39	54	45
7	8	15	4	14	2	3
2	0	0	0	0	2	1
0	0	3	0	0	2	0
0	0	3	0	0	4	0
14	16	9	27	28	27	9
2	1	0	0	0	4	0
4	2	0	8	0	5	5
1	1	3	0	0	2	1
0	0	3	0	0	0	2
1	0	1	0	0	1	0
0	1	3	6	0	1	0
24	20	30	34	39	16	14
4	2	6	4	0	3	5
0	0	0	0	0	1	0
0	5	0	6	0	0	0
0	0	0	2	0	0	0
0	0	4	0	0	0	0
6	0	0	0	0	2	4
0	0	0	0	0	0	0
3	0	0	4	0	0	0
2	1	1	0	0	1	0
6	32	28	29	47	13	4
39	34	219	45	278	282	42
5	9	11	8	0	2	9
1	3	3	11	0	4	3
12	3	4	18	0	25	5
0	0	0	0	0	0	0
14	47	47	44	61	43	25
0	1	4	0	0	1	0
4	1	3	16	0	2	24
2	22	43	0	37	15	55
10	11	6	15	10	7	10
2	0	0	4	0	5	3
1	0	2	0	1	0	0
0	1	0	4	0	0	0
0	12	0	6	0	0	0
6	2	0	7	0	4	0
10	14	16	24	17	11	21
7	3	6	6	9	7	6

For Review Only

2	0	0	0	0	1	4
0	0	0	0	0	1	0
3	3	3	3	0	3	5
9	7	6	10	13	14	6
5	1	0	0	0	0	1
10	0	0	5	1	6	6
0	0	0	0	0	0	2
0	0	0	0	0	0	2
3	1	0	2	0	1	4
0	0	2	0	0	0	0
6	7	4	12	1	7	3
4	0	0	2	2	4	3
0	3	4	3	6	0	0
120	81	48	99	121	120	40
0	0	0	1	0	4	0
2	0	1	0	0	0	0
1	3	2	3	4	1	1
1	1	4	8	0	1	1
24	8	50	40	1	17	47
6	1	0	0	0	0	0
19	25	23	35	41	39	17
0	3	3	4	0	4	2
8	0	0	6	0	7	1
0	1	0	3	0	0	0
7	4	5	14	0	3	10
1	0	1	0	0	5	5
3	5	0	1	8	0	1
1	2	5	7	0	15	3
9	4	3	10	0	4	5
3	0	0	0	0	1	0
77	162	208	282	78	255	290
1	2	1	0	0	1	0
5	3	0	6	0	0	3
965	753	752	1219	914	799	552
1	1	0	0	0	0	1
1	0	0	0	0	2	0
3	4	0	3	0	3	3
13	9	15	19	16	22	21
22	6	21	28	0	17	63
6	6	1	2	6	8	6
12	12	19	35	0	9	7
0	0	0	0	0	0	0
1	14	9	24	0	4	4
1	5	6	6	6	7	7
2	2	3	9	0	10	4
3	0	0	1	0	0	5
0	0	1	4	0	1	0
0	0	0	3	0	0	0
2	0	0	0	0	0	0
0	1	0	1	7	1	0

For Review Only

3	6	14	8	1	7	5
19	12	8	27	21	23	16
4	5	2	4	4	1	3
1	1	1	2	0	1	1
1	1	1	3	2	0	3
0	14	0	5	0	0	0
4	1	4	14	0	9	15
2	2	7	0	0	1	11
4	0	1	3	0	0	0
5	3	5	6	0	4	1
1	3	3	4	6	0	3
0	0	0	5	0	0	5
8	15	14	20	0	6	14
1	1	0	0	0	1	7
3	16	3	29	0	4	8
0	0	0	13	0	1	1
1	0	0	19	0	1	1
4	5	2	8	0	0	1
2	0	2	0	0	1	0
0	0	0	0	3	0	0
40	14	20	48	21	56	36
1	3	1	1	0	5	0
20	12	18	28	25	20	12
5	2	3	1	4	7	2
5	0	0	1	0	0	0
0	0	3	2	0	0	0
13	2	5	20	0	10	0
11	0	8	6	9	2	9
2	2	2	10	0	9	1
7	12	3	18	4	8	7
5	1	1	1	5	0	0
13	13	21	0	0	30	22
26	17	18	36	50	28	30
1	0	0	0	0	0	0
0	0	0	3	0	0	0
6	1	1	4	3	7	6
0	0	0	0	0	0	0
6	0	0	0	0	5	3
2	2	0	4	0	0	2
5	5	5	15	0	20	14
3	6	4	6	0	6	0
15	9	8	22	0	11	4
10	4	10	23	0	18	16
15	0	0	0	0	0	0
34	30	53	44	36	27	55
1	0	0	0	0	0	1
1	0	0	0	0	0	1
0	0	0	0	0	4	0
5	0	1	0	0	5	1
0	0	0	11	0	0	0

For Review Only

1	1	2	0	0	1	6
0	0	0	0	0	0	4
0	0	0	0	0	0	0
1	5	3	0	0	13	2
51	10	32	55	9	32	61
3	7	5	17	0	8	0
2	2	5	5	0	8	9
0	4	3	6	0	4	4
8	11	21	20	0	16	11
122	61	59	147	34	128	97
0	0	1	0	8	4	0
0	0	0	0	0	0	0
0	0	1	0	0	4	1
0	0	0	1	0	3	3
0	1	0	0	0	1	0
1	5	4	3	0	4	0
50	35	88	108	31	64	63
12	13	2	8	0	9	0
0	0	0	0	0	0	0
0	0	1	0	0	0	5
1	10	2	27	1	8	9
0	0	5	5	3	3	3
0	0	4	0	0	3	2
0	1	1	0	0	0	1
1	3	12	0	0	2	1
0	0	3	0	0	0	0
0	4	0	0	2	0	0
0	0	1	3	0	0	3
2	5	2	0	0	5	6
4	9	8	8	6	3	12
0	0	5	6	0	0	1
3	4	4	4	8	7	1
8	0	15	48	0	0	0
2	0	0	1	0	1	1
0	1	0	0	0	1	0
0	0	0	0	0	3	0
12	9	6	0	17	3	8
0	0	0	0	0	0	0
4	2	19	29	0	18	17
0	5	1	0	0	0	0
0	3	6	4	2	5	6
4	2	1	0	0	1	4
8	6	7	17	16	13	9
9	3	19	18	44	24	14
0	0	0	0	0	1	1
0	0	0	0	0	0	0
0	0	2	0	0	0	0
0	0	6	0	0	0	1
4	3	4	4	0	3	0
24	15	17	38	12	13	17

For Review Only

0	0	2	0	0	1	4
1	1	5	1	1	3	4
0	3	0	0	0	0	0
0	0	0	5	4	10	5
8	7	2	0	5	3	0
2	7	17	12	0	18	7
0	0	0	0	0	0	0
0	1	0	3	0	0	0
2	2	0	0	0	0	0
5	0	6	2	0	0	0
0	0	2	0	0	0	0
0	0	0	3	0	0	4
0	0	2	0	0	2	9
0	3	3	5	0	2	3
1	0	0	1	0	5	0
5	19	15	86	0	27	10
0	4	4	16	17	4	12
0	0	0	2	0	4	0
1	1	1	0	0	5	0
0	0	1	0	0	0	6
0	0	0	0	0	0	2
74	107	90	250	235	185	107
1	6	2	5	0	16	6
6	0	1	8	0	9	4
3	0	0	0	0	0	0
0	0	9	9	5	1	5
0	0	0	0	0	2	2
0	0	0	0	0	0	1
0	0	0	0	0	0	0
3	5	5	2	0	5	0
0	5	3	6	0	1	1
0	5	4	6	2	3	3
9	7	6	12	1	5	10
16	3	10	9	0	14	2
9	0	0	0	0	3	0
3	1	3	6	1	3	1
0	2	0	4	0	3	1
9	0	5	6	7	4	4
0	0	0	0	0	0	0
4	0	0	4	0	0	0
3	7	3	11	0	10	0
0	1	0	0	0	6	0
3	13	13	9	13	1	2
10	24	10	31	34	26	15
0	0	0	0	0	1	4
0	1	0	0	0	2	1
10	26	13	34	35	36	36
106	73	73	160	94	65	104
7	6	6	7	10	12	8
1	3	2	0	0	1	0

For Review Only

7	11	9	12	20	19	10
6	2	9	6	0	1	6
0	0	1	0	0	0	0
1	0	5	0	0	1	2
2	4	1	10	0	2	1
3	3	3	2	6	0	3
23	32	9	28	22	20	33
1	0	0	3	0	0	0
3	0	5	20	0	6	7
2	13	8	6	0	7	4
6	4	0	0	0	3	1
0	0	1	0	0	1	0
4	1	5	0	0	6	5
13	7	17	15	23	22	20
0	0	1	3	0	1	0
0	0	0	2	0	0	0
7	14	2	6	0	9	1
0	2	0	5	0	0	3
0	3	2	1	0	0	0
0	4	0	0	0	5	3
1	7	0	0	0	0	0
0	0	0	0	0	0	1
0	0	0	3	0	3	1
2	3	0	0	0	0	0
3	18	1	0	0	6	14
1	0	0	0	0	0	0
1	2	2	7	0	2	20
17	10	4	9	14	7	7
6	8	8	5	5	5	5
1	1	1	0	0	3	1
13	3	10	14	18	14	14
8	16	25	24	39	24	30
7	14	10	18	0	18	8
4	13	4	15	0	7	14
2	0	0	0	0	0	0
0	0	1	0	0	1	3
6	9	7	20	0	5	8
9	0	6	1	0	1	0
2	0	3	7	0	2	6
4	1	0	0	0	1	0
13	18	6	34	0	14	30
0	3	1	0	8	8	0
0	6	0	4	0	7	0
2	4	3	13	0	21	6
4	6	5	0	0	3	4
11	10	14	17	6	12	17
3	0	0	0	4	5	3
1	0	0	0	0	0	1
0	0	1	2	4	0	1
16	8	10	11	0	1	13

For Review Only

0	0	0	1	0	1	9
5	0	0	1	0	0	0
83	18	38	76	57	51	43
8	3	15	20	11	8	23
0	0	0	0	0	3	2
0	1	0	0	0	0	0
1	3	2	5	4	4	2
4	4	3	10	0	8	11
1	1	0	0	0	11	2
3	9	6	17	6	10	13
5	1	2	19	0	41	6
4	8	3	15	0	3	19
14	10	8	14	0	15	0
1	2	0	0	0	4	1
3	2	4	0	9	1	4
3	3	0	0	0	1	0
1	0	3	0	0	0	0
0	1	0	0	1	0	0
0	0	0	0	0	0	0
9	12	13	16	23	9	8
5	0	4	1	10	1	0
3	0	0	0	0	0	1
1	0	0	0	0	0	0
2	1	5	0	0	1	11
23	4	22	13	7	6	10
12	18	6	16	13	11	29
8	0	3	0	0	3	1
0	0	0	0	0	0	0
52	46	40	85	50	71	63
4	0	2	3	3	0	0
34	17	24	42	27	14	47
1	0	1	3	0	1	0
106	170	134	217	121	156	180
8	7	3	9	7	10	12
0	3	0	8	0	0	4
26	15	31	47	16	22	22
1	7	2	1	0	5	2
0	0	0	1	0	0	0
1	0	0	1	0	3	0
0	0	0	0	0	0	0
1	1	2	0	10	4	0
12	1	7	23	13	5	11
0	0	0	3	0	3	3
421	430	272	494	513	406	344
17	6	15	31	19	45	21
39	35	12	31	8	10	32
19	51	44	49	56	82	14
6	3	3	8	3	4	2
15	2	2	18	0	4	1
0	0	2	8	0	1	4

For Review Only

1	0	0	9	0	0	3
27	25	26	23	54	25	0
4	0	2	5	0	5	9
4	1	7	14	0	21	2
45	22	22	39	53	40	31
43	23	19	47	21	60	40
11	0	9	3	8	21	16
0	0	0	10	0	1	1
25	12	30	31	44	40	23
16	31	35	42	18	20	21
11	5	20	21	31	22	29
0	0	0	0	2	0	0
4	0	0	0	0	0	1
5	0	0	5	0	3	4
17	3	5	0	0	0	0
1	1	6	8	0	32	1
41	26	31	41	42	23	27
13	13	17	14	27	15	19
13	9	13	20	12	12	13
3	3	0	3	0	0	2
1	0	2	0	1	2	0
10	4	6	12	0	44	18
2	18	6	16	0	18	4
1	3	1	6	0	3	1
0	9	18	14	25	6	8
0	0	0	0	0	0	0
0	0	1	0	0	0	0
0	3	2	0	0	2	5
0	0	0	2	0	1	4
0	1	0	3	0	0	0
0	0	0	4	0	5	0
15	9	10	32	6	24	18
128	165	115	197	139	122	111
3	0	0	4	0	0	3
1895	1758	2178	3743	2443	2765	3172
31	5	5	22	0	12	14
3	0	0	0	0	0	0
11	20	13	48	15	20	22
1	1	0	0	0	2	3
3	3	5	7	0	0	0
3	5	5	0	4	3	5
7	0	8	0	0	0	7
0	0	0	0	0	3	0
1	3	5	1	4	2	0
0	0	0	1	2	16	0
1	0	4	0	1	0	0
28	37	39	43	35	15	30
0	3	0	0	0	0	0
0	0	0	3	0	0	4
0	0	2	0	0	0	0

For Review Only

15	39	21	50	23	10	23
6	14	13	23	35	40	16
0	0	0	0	0	0	0
1	11	5	15	0	0	1
13	0	0	7	0	8	6
3	8	11	15	0	6	4
0	3	0	0	0	0	0
35	37	31	48	57	50	39
4	2	5	7	5	7	9
0	0	0	0	0	0	1
4	3	4	6	0	7	3
0	0	1	0	0	0	0
0	0	0	0	0	2	3
16	16	11	38	20	17	20
1	0	0	4	0	0	5
0	5	3	4	0	0	0
12	89	54	79	39	25	56
9	3	10	8	0	5	12
33	29	34	42	32	56	25
12	2	1	6	13	7	13
3	4	0	0	0	4	0
1	0	0	0	0	0	3
13	1	1	5	0	7	14
15	22	19	29	13	21	34
1	2	0	3	0	4	2
22	17	16	17	24	9	8
3	1	5	8	0	0	11
1	0	1	0	0	4	1
5	14	51	0	53	5	10
0	3	3	7	0	2	0
14	13	9	33	18	12	27
4	0	0	0	0	2	2
5	0	0	0	0	0	0
0	4	0	0	0	4	1
5	0	0	5	0	6	0
8	6	2	23	8	13	10
0	0	2	0	0	0	4
7	2	2	13	0	3	17
0	0	1	0	1	2	0
2	9	22	34	1	5	21
0	0	0	0	0	4	0
2	2	0	0	0	0	3
19	5	7	24	33	27	23
0	1	1	0	0	0	0
11	8	14	13	15	14	3
7	22	14	26	9	9	17
9	1	13	22	0	11	6
17	3	5	20	12	17	13
3	0	2	6	7	5	2
24	46	20	66	15	30	15

For Review Only

8	1	0	0	0	8	2
7	3	4	5	0	5	0
5	10	12	6	20	9	9
0	0	0	1	0	1	1
0	0	0	0	0	0	0
0	0	3	1	2	0	0
0	0	0	0	0	0	2
0	3	0	0	0	0	0
7	14	18	18	29	31	8
3	0	1	0	0	1	2
1	0	0	0	4	0	0
0	2	0	0	0	0	0
0	5	0	0	0	0	6
2	2	1	0	0	1	6
10	14	9	24	8	16	12
13	6	18	21	30	24	14
0	4	2	0	0	6	5
42	37	39	49	54	39	46
5	3	3	11	4	6	5
21	25	18	107	0	45	130
33	28	54	52	61	52	46
9	1	3	6	8	3	0
0	13	4	0	11	2	0
3	1	1	5	0	5	1
0	0	3	0	0	1	0
7	5	7	6	9	7	7
0	0	0	0	4	0	0
9	17	4	0	0	1	1
0	0	0	0	0	0	5
0	2	0	6	0	5	1
252	82	282	337	426	304	285
0	0	14	0	0	0	0
1	3	5	5	1	2	6
1	7	1	6	4	8	2
0	0	0	0	0	0	0
0	0	0	3	0	5	0
5	1	2	9	0	0	7
0	1	0	4	0	0	0
3	17	6	25	0	15	10
7	1	1	0	17	3	7
0	0	0	0	0	0	0
4	4	1	0	0	4	0
0	1	0	0	2	0	0
42	24	15	43	0	4	5
2	4	8	1	0	3	9
2	7	4	7	0	4	1
4	0	2	0	0	11	0
54	23	12	39	50	55	36
3	4	12	17	0	24	2
0	0	0	6	7	0	4

For Review Only

0	3	0	5	0	0	0
8	10	11	24	13	18	23
0	0	0	3	0	6	0
188	292	275	426	230	275	480
3	2	1	0	0	0	1
3	2	0	0	0	0	1
0	0	0	2	2	0	0
0	0	0	0	0	5	0
0	1	0	0	0	0	1
0	0	3	0	0	5	2
0	0	0	4	0	7	2
0	1	2	12	13	0	1
4	0	0	0	0	1	0
22	18	15	30	11	9	7
6	0	0	1	0	0	0
12	13	18	19	18	20	15
0	0	1	5	0	0	1
0	0	1	0	0	0	4
1	0	4	0	0	0	0
11	1	25	14	13	14	8
0	1	0	2	2	0	0
5	7	4	4	0	1	4
1	15	11	22	0	22	19
0	0	1	4	5	1	4
3	0	2	5	1	3	0
3	0	0	4	0	1	4
25	18	25	30	0	32	0
5	0	0	0	0	0	0
0	0	0	0	5	0	0
0	0	0	0	3	0	0
0	0	0	0	0	0	0
0	0	0	4	0	0	0
3	2	23	15	0	13	5
50	69	93	140	86	134	63
0	1	3	0	1	1	3
0	0	0	0	5	3	3
2	3	18	0	0	3	2
1	1	0	0	0	1	2
1	4	2	7	0	4	2
0	0	1	9	0	1	3
0	1	0	0	0	2	0
0	2	1	0	0	2	1
0	0	5	1	0	1	1
0	0	0	0	0	0	0
4	1	3	7	1	3	4
16	14	8	3	14	4	5
0	0	0	0	0	0	0
7	0	0	4	0	6	4
32	60	91	43	54	57	18
23	10	21	1	4	4	4

For Review Only

0	0	0	0	0	0	1
5	0	8	6	2	0	6
7	7	7	13	15	17	8
0	0	0	0	0	0	0
2	11	4	6	8	1	2
4	1	5	6	11	2	0
22	30	12	29	13	13	12
2	2	1	0	0	0	1
14	11	13	35	11	18	14
36	36	28	50	56	54	41
0	1	0	0	5	0	0
3	14	6	19	21	22	4
32	34	45	49	68	42	47
0	0	0	0	0	1	3
1	1	0	0	0	7	4
9	1	5	10	0	6	9
1	0	3	0	0	1	1
0	1	0	0	0	0	0
0	7	5	0	0	10	0
0	1	0	0	0	0	1
5	3	6	6	6	7	5
3	2	1	0	0	1	4
0	0	0	0	0	0	0
3	0	0	10	0	3	5
7	22	14	23	0	8	7
0	5	3	5	0	5	6
3	0	3	0	0	0	0
0	3	2	0	0	0	2
0	1	0	4	0	2	0
0	0	3	5	2	5	4
1	10	7	0	0	16	2
9	10	18	19	0	23	5
1	0	0	0	0	4	0
0	0	0	0	0	0	3
2	0	0	4	0	0	0
9	10	11	45	0	27	1
1	0	2	0	0	0	0
38	11	17	33	20	19	14
0	0	0	2	0	1	0
0	0	4	1	0	5	0
2	3	10	7	9	2	4
28	15	28	35	41	47	26
16	16	15	38	19	40	33
0	0	0	1	0	0	1
3	0	3	3	0	0	3
0	0	6	0	0	0	0
69	126	100	137	122	71	75
3	0	0	0	0	1	3
1	6	0	0	0	0	2
7	1	0	0	0	1	1

For Review Only

4	0	0	0	0	0	0
5	9	6	17	0	6	20
7	6	6	7	10	3	6
0	0	3	0	0	0	1
8	2	6	7	0	2	1
0	0	0	3	2	1	0
1	4	3	0	0	0	0
1	3	0	0	0	1	2
0	0	0	0	0	0	0
0	0	0	2	0	0	0
1	0	3	0	0	0	2
1	2	3	5	0	3	2
0	0	0	10	0	0	0
0	0	0	2	0	0	0
2	0	2	5	0	0	0
0	0	0	4	0	0	2
1	2	6	14	6	1	15
0	2	2	6	0	0	0
14	3	4	1	14	5	6
2	0	0	1	0	0	0
5	4	0	9	0	8	5
4	0	0	8	0	0	3
28	25	37	40	0	16	56
3	0	0	5	1	0	0
0	0	0	0	0	0	0
10	30	10	19	0	8	27
2	3	3	4	0	6	8
10	5	6	7	7	10	10
13	4	10	6	10	4	5
0	1	0	0	0	0	0
12	23	19	17	0	6	27
7	6	5	1	1	3	0
1	0	0	0	0	0	0
2	0	2	0	0	1	4
0	0	0	3	0	0	0
15	0	0	0	0	0	0
2	0	0	0	0	0	0
3	1	0	0	0	1	3
18	10	18	27	13	14	20
16	5	6	10	10	5	7
0	0	0	2	4	1	0
0	0	0	0	0	0	0
7	2	21	0	10	3	11
8	11	4	31	0	4	16
15	4	8	7	3	8	5
0	0	0	0	0	2	0
22	25	16	39	12	13	9
8	12	1	23	0	10	2
4	2	2	7	0	6	0
21	11	17	19	34	31	36

For Review Only

9	4	17	14	0	9	3
2	1	0	0	0	0	0
8	10	5	6	21	32	7
0	0	0	3	0	0	0
16	10	20	22	0	68	7
0	0	7	2	14	0	3
341	320	368	661	389	348	369
4	7	0	4	16	0	6
0	0	0	0	6	0	0
1	1	0	0	0	7	1
0	3	0	0	0	6	0
0	5	6	11	0	9	3
1	1	1	0	0	1	2
7	3	6	8	4	8	6
1	10	21	20	0	36	35
1	1	20	96	0	1	1
11	7	2	14	0	1	9
4	27	5	14	1	9	32
4	0	0	0	0	3	0
3	0	0	3	0	1	0
2	3	2	7	5	5	4
1	0	1	2	0	1	0
33	31	38	74	34	70	51
2	2	2	1	0	2	6
0	1	0	4	4	0	0
0	0	0	0	0	3	2
0	2	0	3	0	0	3
0	1	1	7	0	1	5
0	0	0	0	0	1	0
0	1	0	0	0	3	0
0	0	0	0	0	1	0
0	0	0	0	0	0	0
15	19	22	15	0	24	22
35	11	4	25	0	5	9
0	0	0	0	0	5	0
7	13	14	24	6	22	15
1	0	0	3	0	0	0
0	0	0	3	0	0	0
0	6	3	0	0	1	0
0	0	4	0	0	0	0
28	49	30	38	61	52	54
5	2	2	5	6	8	8
1	0	0	0	0	0	1
0	0	4	0	0	0	2
0	0	0	0	0	0	0
0	4	0	0	0	0	0
4	0	0	0	7	0	0
2	4	0	9	0	4	9
0	0	0	0	0	0	0
6	0	0	2	5	0	4

For Review Only

3	0	0	0	0	10	3
0	3	1	0	0	4	0
47	32	54	60	81	72	57
334	243	299	265	5	374	298
20	4	2	32	0	26	35
1	0	1	1	1	1	0
5	12	5	21	7	3	8
18	15	13	33	19	26	15
0	1	0	0	0	0	2
0	1	0	0	1	0	3
2	0	2	0	5	5	0
3	0	0	5	0	2	7
3	6	1	11	3	21	2
3	5	5	7	5	8	4
0	1	0	5	0	3	3
11	9	15	16	0	3	6
0	2	0	0	0	0	0
0	0	0	0	0	0	0
1	1	0	1	0	0	0
11	7	6	22	7	11	3
0	0	0	0	0	0	0
0	0	4	2	0	10	5
11	17	2	6	17	8	5
6	2	9	17	0	3	13
1	0	0	0	0	0	0
8	5	0	13	0	0	2
11	8	6	9	8	7	11
0	0	0	0	0	4	0
77	38	69	72	21	30	31
0	1	3	0	10	0	1
14	0	0	0	0	1	0
0	0	0	0	0	0	0
11	15	11	18	7	6	13
8	2	2	10	0	14	4
0	4	2	0	0	5	2
5	1	0	0	12	7	6
0	1	1	0	0	1	0
5	0	0	5	0	0	0
43	44	38	80	76	47	37
4	5	4	19	4	1	6
4	3	5	17	0	2	15
6	19	5	17	0	14	28
0	0	0	0	0	0	3
3	0	5	1	0	0	5
0	0	0	0	1	0	0
0	2	0	0	0	5	2
4	0	3	5	6	4	3
1	0	0	0	0	0	0
64	0	49	17	97	50	50
0	1	1	0	0	1	1

For Review Only

1	0	0	3	2	0	0
2	0	0	0	0	0	0
4	3	6	4	0	3	4
4	3	0	0	0	0	0
0	9	9	11	5	12	15
0	0	0	4	0	0	3
0	0	1	1	4	0	0
3	0	0	0	0	10	13
0	0	0	0	0	3	5
0	0	0	0	0	0	4
26	37	14	42	32	21	7
23	19	51	45	40	53	32
13	15	4	26	0	2	9
3	2	0	8	0	1	0
1	2	3	0	7	12	1
1	0	0	13	0	0	0
28	11	14	21	45	20	22
8	4	4	12	3	6	6
0	0	0	3	0	5	3
1	0	0	0	0	0	2
5	7	6	34	7	5	6
0	3	0	3	0	0	3
11	10	14	22	0	0	10
46	17	8	38	0	15	14
4	0	0	4	0	0	0
5	2	0	0	0	0	2
7	1	2	20	0	20	0
3	0	0	3	0	0	0
0	0	0	0	0	0	0
4	1	0	0	0	2	0
0	1	0	0	0	0	2
4	1	6	4	6	5	5
1	1	0	0	0	4	2
1	6	21	0	0	19	4
13	22	16	34	14	13	7
1	8	8	11	14	3	10
0	3	1	0	0	5	0
5	6	0	8	9	5	5
18	9	17	23	24	33	18
0	0	0	0	0	8	0
33	24	26	0	0	33	35
0	0	0	0	1	1	2
0	1	0	0	0	3	3
3	0	0	3	0	0	3
54	44	49	75	25	51	42
55	32	48	88	21	38	67
0	2	0	0	0	4	1
1	4	4	2	6	1	1
1	1	1	0	0	0	1
1	20	9	6	26	12	25

For Review Only

0	2	0	0	0	1	0
165	155	133	227	146	155	110
0	7	5	6	1	3	6
3	3	2	6	6	14	2
14	6	11	10	32	4	8
0	0	2	1	0	3	0
2	2	2	1	0	12	6
43	18	13	43	0	26	17
9	6	8	8	14	3	2
0	3	7	6	4	3	4
137	62	18	75	64	76	38
1	2	2	6	0	4	10
0	0	0	3	0	0	0
7	27	22	26	0	11	18
2	6	15	18	1	9	3
1	0	0	0	0	7	1
6	4	4	8	17	6	12
1	1	1	1	0	1	3
5	0	0	6	0	3	1
0	0	1	0	4	0	0
0	0	0	3	0	9	4
16	5	20	39	16	20	29
7	3	35	18	0	5	22
1	0	0	1	0	0	2
64	28	2	19	39	11	23
0	0	0	0	0	0	1
0	0	0	0	0	0	0
20	34	31	28	34	22	18
12	5	5	7	4	6	6
0	2	4	0	0	0	7
3	0	0	1	0	0	0
0	0	0	0	1	1	7
12	7	2	17	0	2	3
22	14	20	20	10	14	7
0	2	0	5	0	0	0
19	11	5	13	18	9	9
29	12	6	13	29	41	11
1	4	3	1	0	4	0
0	0	0	1	4	1	3
7	2	1	0	0	2	8
66	46	48	83	54	79	68
5	26	20	18	4	11	18
12	8	13	12	7	17	10
0	0	3	0	0	2	1
16	7	5	19	16	26	21
0	0	0	1	0	0	3
1	1	2	1	0	11	2
8	13	15	4	41	20	0
4	4	3	6	0	3	4
2	0	7	0	0	1	1

For Review Only

2	1	0	1	4	4	1
0	0	0	0	0	2	0
6	10	20	17	22	22	24
4	2	10	6	13	1	5
0	3	0	4	0	1	3
3	0	7	7	7	6	0
35	18	35	53	21	35	28
0	1	0	3	0	0	1
2	1	1	1	0	3	9
10	0	0	0	0	1	1
0	2	6	4	0	3	1
2	0	1	0	0	0	0
64	68	83	102	47	70	45
0	0	0	0	0	0	3
2	3	2	0	0	1	10
0	0	1	0	4	0	1
7	0	4	4	3	5	0
2	1	7	11	0	3	2
0	0	0	0	7	0	0
26	8	22	17	8	36	29
0	6	0	0	0	1	0
3	15	23	31	0	24	8
7	14	11	35	0	43	59
1	0	0	0	0	0	2
6	6	10	21	0	2	7
1	7	7	21	0	22	5
0	0	6	6	0	4	3
12	3	2	8	36	27	18
15	4	5	28	0	19	11
0	0	2	0	0	3	0
0	0	1	0	0	0	2
0	0	4	0	0	0	0
0	5	0	2	4	0	0
0	0	0	1	5	1	5
1	0	3	5	14	5	5
2	1	1	6	0	0	1
0	0	0	4	9	0	0
1	0	2	0	0	1	0
4	2	3	14	0	12	12
2	1	0	0	0	0	4
3	0	3	0	0	1	5
16	7	2	13	5	7	7
3	1	1	5	0	1	0
6	3	0	5	10	5	5
0	2	0	0	0	0	0
7	4	0	1	0	3	0
0	0	0	0	3	0	0
0	1	3	2	3	0	0
0	0	0	0	0	0	3
1	0	0	0	0	0	0

For Review Only

0	4	0	6	0	4	0
0	3	3	6	0	0	0
3	0	4	0	0	0	0
0	3	0	0	0	1	0
1	0	1	0	0	2	7
11	2	7	15	7	5	1
6	4	10	12	0	9	0
0	0	0	2	0	0	3
1	0	0	0	0	2	0
2	1	4	6	0	0	0
0	0	0	2	3	0	3
0	0	0	0	0	0	1
3	0	0	0	0	0	0
6	1	1	0	0	0	0
4	7	1	5	0	1	0
1	1	0	5	0	0	0
0	0	2	0	6	0	0
0	4	7	11	6	4	5
3	5	3	2	5	3	3
48	37	39	81	47	42	55
0	0	0	0	0	2	0
0	0	1	0	0	2	1
0	6	1	0	0	1	3
0	0	0	0	0	0	0
0	0	0	0	0	0	5
6	12	6	12	0	12	8
1	3	4	2	0	5	0
2	3	5	16	10	7	7
2	0	0	0	0	1	1
2	8	9	8	0	5	1
5	6	9	38	15	13	26
7	6	2	23	4	2	20
1	11	7	13	15	13	3
1	0	0	0	0	3	0
35	43	16	26	7	22	18
48	62	73	88	73	88	72
3	0	0	0	0	0	0
1	1	4	0	0	4	0
3	4	4	3	6	5	0
2	3	1	0	0	1	2
37	35	36	60	52	34	33
0	6	4	6	5	8	0
1	0	11	11	11	10	8
0	0	0	27	5	0	0
0	0	0	0	0	2	4
4	2	4	4	0	4	1
3	0	4	6	0	3	4
0	0	0	0	0	0	0
15	12	15	16	0	22	5
1	3	0	0	0	2	5

For Review Only

0	2	4	0	0	5	0
3	2	2	5	0	9	5
1	0	0	0	0	0	0
12	12	22	30	27	29	19
0	0	3	0	0	0	0
0	10	0	2	15	0	4
29	0	27	28	1	0	26
4	0	1	0	0	4	1
5	2	1	0	0	0	1
2	1	13	15	0	5	3
2	8	1	7	0	2	2
0	0	0	0	0	1	0
1	1	1	9	0	12	1
21	9	27	41	0	20	11
9	10	8	10	12	9	14
16	7	5	17	18	13	21
9	5	9	26	11	25	9
11	37	35	0	123	75	2
0	0	0	0	0	0	0
4	0	5	2	3	7	9
0	0	0	3	0	2	1
0	0	0	0	5	6	1
0	2	2	0	0	4	0
8	24	7	36	0	6	35
5	1	1	0	0	2	9
23	38	35	55	21	36	26
121	88	98	122	92	66	80
0	1	0	0	0	0	0
8	0	0	0	7	1	0
8	2	8	19	23	8	3
2	4	1	7	0	5	1
9	16	7	10	0	5	23
101	85	122	135	106	159	172
0	0	0	0	0	1	0
0	0	0	0	0	0	0
5	5	4	6	9	9	3
0	3	1	3	0	2	0
22	23	47	55	44	49	20
0	1	5	0	0	0	0
35	15	34	42	29	11	34
2	0	2	3	0	0	2
5	2	1	4	6	3	4
0	0	0	2	2	0	1
0	1	0	3	0	0	1
0	0	1	6	0	0	4
0	1	0	5	0	4	0
0	0	0	0	0	0	0
8	0	0	12	0	6	8
2	0	1	0	0	2	0
9	3	2	17	10	13	10

For Review Only

5	3	15	8	19	2	7
1	6	2	0	12	4	5
0	1	1	3	5	0	0
5	11	10	13	16	17	14
26	27	20	50	24	36	34
1	4	1	12	0	15	3
1	0	0	3	0	0	1
0	4	0	2	0	0	0
19	14	31	28	15	4	12
0	5	7	0	0	3	6
1	0	0	0	0	0	0
3	0	0	0	1	0	2
0	2	0	0	0	4	0
151	86	75	158	101	85	193
9	31	19	28	34	31	29
54	30	26	102	31	104	65
0	0	0	0	0	0	0
4	1	7	8	0	4	5
0	0	0	0	5	0	4
28	16	37	50	40	21	27
53	37	17	54	13	21	31
3	5	9	5	0	5	5
0	0	0	0	0	0	0
8	3	3	7	0	8	2
0	0	0	3	0	1	0
1	2	1	6	0	2	14
4	1	1	0	0	7	5
11	17	7	19	0	3	10
0	0	4	7	0	0	1
0	0	0	2	0	0	0
2	7	3	0	0	1	1
0	0	3	4	0	5	7
2	2	0	1	0	3	2
1	5	18	7	0	3	16
0	0	8	0	0	0	1
2	2	8	12	0	16	4
85	103	31	182	127	177	123
5	4	3	11	0	9	4
4	4	0	1	0	4	0
0	0	0	0	0	0	0
0	0	0	0	0	0	0
0	1	0	0	0	1	1
137	262	172	276	305	216	204
3	0	1	10	0	4	1
2	2	19	7	0	7	2
0	20	2	5	18	2	1
1	0	0	0	0	1	7
153	188	130	277	129	147	127
2	9	0	9	11	0	12
7	3	7	25	31	29	13

For Review Only

0	5	2	8	0	0	1
1	3	1	2	2	0	1
36	0	32	10	47	59	49
13	14	43	29	17	10	16
0	1	1	1	4	0	2
0	0	4	0	0	2	0
41	59	37	39	18	35	24
3	0	0	0	0	4	2
11	5	6	21	1	19	5
6	6	6	23	0	13	10
77	60	51	134	57	61	68
0	1	0	0	0	1	1
1	7	1	15	0	0	0
32	26	42	52	58	47	36
3	3	2	5	0	5	5
0	0	0	6	0	0	0
34	14	36	39	25	25	29
5	2	3	0	6	7	4
6	5	6	7	15	6	6
57	55	38	0	0	57	30
39	34	27	0	0	44	22
0	0	3	3	5	0	3
23	18	26	31	32	19	32
8	8	3	6	5	28	1
5	9	6	7	0	4	6
1	0	1	0	0	0	0
11	10	3	10	13	2	3
0	2	2	3	0	1	0
14	30	24	34	11	14	14
1	2	0	0	8	0	1
3	0	0	0	0	0	0
1	12	1	5	11	7	0
6	7	4	3	0	1	0
2	0	2	0	0	0	0
0	3	0	0	0	0	0
0	0	0	0	0	23	0
5	3	0	0	0	5	1
2	0	0	0	0	3	2
10	8	9	13	9	6	2
0	0	0	0	0	0	0
5	15	4	9	0	8	3
37	48	85	123	55	53	65
0	1	0	2	0	0	0
0	0	0	6	0	0	3
0	0	11	0	0	3	4
0	0	0	3	1	0	0
0	1	5	0	0	0	1
2	0	1	1	0	3	5
0	0	3	0	0	4	0
0	1	1	0	0	1	1

For Review Only

8	8	8	23	0	16	4
4	0	2	4	0	1	0
56	69	78	153	74	144	125
16	16	11	18	1	11	7
1	0	1	6	1	2	4
22	17	29	28	34	37	28
3	0	3	11	7	4	7
1	0	7	0	0	0	3
1	0	6	0	0	1	0
0	0	0	0	0	0	1
17	12	20	15	0	6	7
13	8	16	40	11	33	21
0	0	3	0	0	0	2
3	8	8	11	15	8	9
1	0	3	0	0	0	1
2	0	2	1	1	0	0
5	3	6	0	0	0	4
0	0	0	0	0	0	0
1	1	0	10	0	1	1
2	22	1	0	0	1	3
0	3	1	0	0	0	0
7	17	7	0	0	11	6
0	0	0	0	0	0	2
0	0	1	0	0	1	0
5	7	5	11	9	8	5
0	0	0	0	0	0	0
3	3	3	11	0	6	0
3	1	1	0	0	0	0
4	6	4	11	0	18	6
0	0	0	0	0	4	0
35	0	0	0	0	98	0
0	0	0	0	0	6	0
22	29	37	42	41	24	18
1	0	0	1	2	1	3
0	4	4	3	5	0	3
38	35	21	57	20	33	31
1	0	2	3	3	2	4
141	258	206	328	165	112	406
1	0	0	0	4	2	0
1	1	1	0	0	6	0
0	0	1	0	0	0	2
21	16	17	0	0	20	24
4	0	5	0	0	2	1
6	0	7	0	0	9	2
3	2	1	0	0	3	3
8	0	1	0	0	0	0
3	0	0	3	2	3	0
0	0	0	6	0	0	0
0	0	0	10	0	0	4
1	10	2	5	0	5	3

For Review Only

0	0	5	0	6	0	0
0	6	3	1	0	3	0
0	2	0	0	0	0	3
1	0	0	6	0	5	0
3	1	4	7	0	3	0
0	0	3	0	0	0	1
6	3	15	7	1	4	8
6	6	1	0	18	0	1
0	6	9	7	10	10	11
2	1	3	15	0	1	11
0	0	1	3	0	0	3
0	0	0	1	0	0	0
1	2	4	1	0	0	0
23	14	14	33	8	17	19
38	11	6	15	21	10	11
6	0	11	5	10	0	5
0	0	1	4	0	0	0
2	1	0	4	0	1	0
8	0	1	6	1	1	9
0	0	0	0	0	0	0
1	4	9	13	0	2	3
0	0	2	1	0	3	1
5	4	1	6	0	2	4
0	2	6	0	0	5	4
0	1	0	2	0	0	4
8	8	5	10	13	10	6
1	0	0	0	0	0	1
1	4	2	14	0	2	15
11	4	4	8	6	7	11
4	3	4	5	5	4	1
0	0	0	2	0	0	0
2	4	2	1	0	4	2
6	16	21	18	0	9	6
13	6	14	31	24	13	2
0	6	0	2	0	1	4
1	2	2	1	0	5	4
24	15	24	57	37	38	44
10	15	22	45	15	32	15
3	4	0	8	0	0	0
8	15	2	31	0	27	24
7	10	20	16	27	10	13
2	0	1	0	0	0	0
1	0	0	7	0	3	2
19	12	22	22	19	8	26
27	25	26	33	51	32	16
0	0	0	0	0	0	0
39	26	24	46	27	31	36
4	8	2	21	0	15	3
9	6	5	32	0	24	14
4	12	7	19	0	3	9

For Review Only

8	5	13	33	0	13	22
0	3	0	6	0	0	2
16	22	29	40	31	39	20
2	4	3	0	0	8	2
0	0	3	6	0	5	5
5	9	9	15	20	0	10
0	0	0	0	0	1	0
0	2	2	0	0	9	3
0	0	0	0	0	0	7
6	6	8	0	0	9	14
0	1	7	2	0	0	0
28	14	13	25	13	9	26
0	4	4	12	0	17	18
0	0	0	0	0	0	0
6	3	2	11	1	0	5
4	1	1	3	0	0	0
7	5	12	19	0	6	5
5	0	0	0	0	2	7
0	2	0	0	0	0	5
11	0	0	1	15	0	0
1	1	0	4	0	3	0
0	0	0	0	0	0	0
2	5	0	1	0	4	0
3	0	0	0	0	1	0
15	21	16	29	16	13	13
14	6	6	14	11	5	12
0	0	0	0	0	0	0
0	0	1	0	0	0	0
2	0	5	6	0	2	3
119	90	82	0	0	131	64
1	3	0	0	0	0	11
0	0	0	0	0	0	0
0	0	0	6	0	0	0
7	3	1	8	0	5	2
102	71	87	134	59	77	105
0	0	0	0	0	0	0
3	0	5	14	0	13	9
1	2	2	3	1	3	2
0	0	1	1	0	0	0
10	10	3	18	9	13	11
0	0	0	7	2	0	0
11	7	13	12	10	5	3
5	1	0	0	0	0	0
0	0	0	0	0	0	0
13	37	19	71	12	13	56
124	261	214	146	129	104	226
30	82	61	76	89	89	59
55	25	33	49	56	63	61
0	0	0	0	0	7	0
3	0	3	0	6	3	0

For Review Only

6	13	10	19	12	11	9
3	4	4	8	4	4	4
0	0	1	6	0	0	1
1	5	0	0	0	3	0
2	4	2	1	1	6	5
10	13	5	13	41	8	7
0	1	1	1	3	1	0
5	18	2	0	0	2	9
15	17	22	21	21	20	23
0	1	2	4	0	0	1
3	16	4	20	0	24	8
1	2	0	0	0	2	0
1	0	13	0	0	0	1
21	5	7	9	0	6	19
1	4	0	0	0	0	1
7	5	5	12	12	6	6
17	18	13	23	27	12	10
0	1	1	1	8	0	2
22	8	7	8	23	8	7
0	0	1	0	0	0	2
6	3	7	7	5	10	7
2	3	0	1	0	1	2
11	4	9	7	8	10	3
3	1	0	2	3	1	0
0	2	0	0	0	4	0
9	10	11	5	18	12	20
1	0	2	0	0	1	1
11	11	9	26	29	23	8
0	1	0	0	0	2	0
2	0	2	1	0	0	2
5	6	3	5	3	5	11
6	3	8	12	0	6	1
3	0	0	0	0	0	0
13	4	3	14	0	13	19
4	3	1	2	0	0	0
0	5	8	7	0	1	5
6	5	5	11	0	1	5
24	17	21	18	0	22	3
7	2	10	1	0	6	8
2	1	1	6	0	1	3
23	24	0	0	0	0	0
19	2	5	18	14	11	7
43	35	25	54	26	41	31
0	1	0	1	0	0	0
0	2	5	28	0	4	0
0	0	0	11	0	0	8
0	16	0	0	0	0	0
0	0	0	0	0	0	3
4	4	5	5	0	7	1
0	1	0	0	0	0	1

For Review Only

0	119	100	17	197	114	0
0	0	0	0	11	0	0
15	5	7	3	17	6	16
3	3	2	0	0	3	3
0	1	1	3	0	0	0
1	2	1	0	0	2	4
0	1	6	0	0	1	0
17	19	25	39	16	26	13
3	0	1	1	0	1	0
0	0	0	4	0	0	0
22	19	26	50	22	28	27
8	9	6	13	9	9	5
6	14	2	17	22	13	13
0	0	1	0	0	1	1
4	10	9	5	13	10	11
13	11	13	23	9	12	13
2	1	20	27	0	3	2
44	48	79	68	42	66	48
6	4	0	2	2	1	1
0	0	0	0	10	0	0
0	0	0	1	0	5	3
9	7	2	14	3	4	7
3	18	1	5	0	3	4
17	15	44	38	18	31	51
0	4	0	4	1	2	4
13	7	7	17	3	5	6
33	40	45	60	34	46	29
8	9	12	19	1	21	6
1	0	3	0	0	2	0
4	4	3	11	1	5	7
1	0	0	0	3	1	1
1	1	4	0	0	0	2
0	214	0	201	0	0	0
13	9	12	10	18	20	9
19	19	19	37	15	23	15
5	0	1	2	0	0	0
7	9	12	20	0	4	1
6	4	5	1	0	2	6
5	0	0	0	0	0	0
0	0	1	1	1	0	0
0	0	0	0	0	0	0
8	7	7	0	0	5	3
1	7	12	0	0	2	22
44	20	28	49	35	35	40
141	99	149	269	124	152	155
0	0	0	0	3	6	0
1	0	0	0	0	0	1
33	45	43	0	0	57	32
7	9	10	14	6	8	3
33	32	38	50	46	50	34

For Review Only

1	4	9	1	0	2	7
1	0	1	3	1	1	0
0	3	0	0	0	6	3
43	41	44	78	47	61	47
0	0	0	0	2	0	0
13	9	13	18	5	6	8
0	0	0	0	9	5	3
0	0	0	8	0	1	1
22	7	57	31	10	36	29
17	15	16	30	24	22	16
15	15	18	0	0	25	27
49	45	45	81	53	59	44
42	29	50	84	33	51	42
0	0	10	4	4	0	3
0	0	0	1	0	1	0
23	12	19	28	11	23	14
7	4	8	10	11	10	7
12	14	11	21	9	10	12
15	14	10	23	6	16	13
3	7	5	6	1	12	1
6	5	7	11	2	6	7
155	128	168	266	113	191	126
12	9	9	11	10	9	6
3	5	4	8	10	4	4
7	7	8	15	4	10	7
20	23	25	40	7	29	21
3	0	0	2	6	1	1
0	7	3	5	0	1	3
52	35	44	81	34	48	45
1	1	27	0	147	1	1
1	0	1	0	0	0	0
1	5	1	10	0	1	1
4	0	4	1	1	0	3
1	0	1	3	4	0	6
26	0	0	0	18	33	50
2	5	4	6	0	1	1
0	3	0	1	3	1	1
128	77	101	138	107	149	98
5	4	7	16	0	5	15
3	1	3	7	9	6	3
7	3	2	12	0	2	8
138	121	123	224	115	165	134
2	2	7	10	0	7	3
10	14	8	12	8	7	9
16	15	18	35	10	18	16
3	2	3	4	4	2	3
4	10	16	17	10	9	21
4	4	7	1	0	0	0
29	28	29	25	30	12	11
77	61	71	98	51	83	46

For Review Only

42	37	26	71	29	45	46
20	20	15	17	15	17	13
56	36	30	58	72	51	56
0	3	0	0	0	0	2
42	55	37	86	46	47	33
9	8	8	16	11	10	7
3	3	1	6	2	6	3
44	37	44	77	60	43	41
0	1	0	1	0	1	1
0	0	4	0	4	4	0
21	22	15	29	21	23	17
75	57	60	113	24	69	69
8	8	7	16	1	6	3
4	3	3	7	1	5	1
56	58	80	103	51	73	50
15	15	26	30	11	17	18
0	0	3	5	4	0	0
0	3	1	0	0	0	1
4	14	6	6	13	1	2
0	0	0	0	0	1	0
12	32	28	37	43	20	22
0	4	7	5	17	4	6
0	0	0	0	0	0	0
7	9	3	10	14	12	11
30	26	33	58	19	33	34
0	0	1	0	0	1	3
1	0	0	0	1	0	0
0	2	0	0	0	1	0
56	57	60	62	29	57	98
132	78	117	153	58	101	88
3	3	7	9	1	8	0
5	4	0	5	0	0	0
1	1	0	0	1	0	2
48	30	42	79	44	52	43
6	3	7	4	3	5	0
22	15	25	40	17	22	24
15	11	14	19	8	9	6
1	1	3	0	4	14	2
0	1	1	3	1	0	0
0	0	0	1	1	0	0
11	15	18	28	9	12	5
3	7	3	3	2	1	1
55	70	55	83	54	60	34
249	213	241	361	249	197	195
1	0	1	3	1	1	0
1	1	1	1	0	1	1
4	4	4	3	0	5	0
7	4	4	3	4	4	1
0	3	1	0	1	4	3
3	1	0	0	2	4	3

For Review Only

104	90	124	196	93	112	119
78	51	65	113	52	74	66
6	5	0	6	0	4	4
0	0	0	11	0	1	2
1	4	3	5	2	1	0
61	51	51	82	46	77	48
28	28	33	76	31	49	35
7	8	8	10	6	9	8
3	2	0	3	0	0	2
5	7	6	4	1	3	1
7	6	7	26	1	21	30
4	1	0	1	0	0	0
0	0	0	8	0	9	6
40	40	36	56	26	44	30
3	5	10	8	13	4	6
3	3	6	7	4	4	4
12	12	10	20	18	16	8
90	84	143	203	177	99	75
0	1	9	5	7	3	0
0	0	0	4	1	0	0
3	0	0	1	0	3	0
0	3	0	0	1	3	0
45	44	39	49	15	15	18
92	87	114	162	115	102	91
100	120	138	258	83	110	146
0	0	0	0	2	0	0
8	3	0	0	0	0	0
55	20	19	18	0	13	8
0	2	6	0	0	5	0
1	0	0	7	0	4	3
1	1	2	3	0	0	2
4	10	7	5	18	11	6
95	116	96	143	117	42	47
3	1	9	7	3	8	4
294	203	249	566	160	301	391
8	8	4	3	0	1	3
37	20	22	75	32	29	31
15	8	8	16	10	8	8
2	2	9	9	5	6	16
6	3	0	0	0	2	3
4	7	2	5	0	7	8
0	0	0	0	0	0	1
0	0	0	0	6	0	5
4	6	1	17	7	1	1
3	8	2	6	0	5	1
103	78	78	85	117	116	73
0	0	0	0	6	3	1
28	2	26	33	60	26	58
12	51	22	34	0	17	16
140	211	175	294	151	163	178

For Review Only

16	38	12	30	31	24	27
78	109	80	136	166	140	73
3	1	26	0	0	1	2
0	15	146	462	5	0	370
9	3	5	17	0	6	0
57	58	63	0	0	25	56
60	0	44	0	0	0	0
210	179	162	288	244	255	161
25	29	196	434	57	154	83
114	94	99	177	198	190	78
9	1	1	0	95	0	1
55	54	0	17	41	51	29
233	308	285	420	408	274	359
0	0	0	0	0	0	0
936	1033	638	1546	880	909	596
177	342	555	429	1040	176	474
68	67	11	113	0	432	72
6	2	14	21	7	4	14
9	0	0	0	0	2	1
152	230	145	267	135	200	187
7	0	148	113	281	0	228
5	1	0	5	0	0	0
546	557	570	424	1070	586	231
33	20	8	29	28	24	13
14	22	12	7	30	4	7
4	1	1	3	0	1	6
33	13	8	18	16	9	12
111	81	59	112	84	69	55
0	0	1051	83	49	0	0
0	0	0	0	0	0	0
85	142	84	96	68	73	93
220	219	173	259	238	241	144
0	3	0	0	0	0	3
147	152	130	253	149	128	109
10	2	23	17	0	10	20
118	188	107	278	77	195	94
1	8	27	71	0	4	54
123	155	169	229	129	192	144
38	32	33	59	69	52	43
50	45	24	60	50	41	32
0	0	0	6	0	0	0
215	162	204	283	228	210	175
100	121	11	230	97	283	80
33	33	72	154	0	80	98
23	18	15	31	18	33	29
22	28	25	38	28	34	24
0	11	0	0	0	0	0
0	3	4	7	0	0	4
1567	1184	1560	1441	1781	1352	1205
133	80	39	54	97	32	32

For Review Only

138	116	82	160	155	105	80
61	35	22	81	49	37	58
116	135	135	215	146	110	135
100	78	56	125	104	67	66
137	147	112	198	179	93	111
89	122	100	164	105	91	110
41	24	31	62	35	51	43
3776	3182	3033	34	123	528	0
43	47	64	70	69	27	37
129	76	70	108	101	116	90
84	308	222	66	279	323	264
166	158	118	185	129	108	141
29	40	34	55	123	4	25
17	13	11	16	16	25	3
77	130	50	161	61	141	66
287	344	240	500	248	312	169
39	73	57	98	59	121	76
286	174	1	0	288	265	8
106	64	55	83	107	85	66
57	43	28	96	40	89	41
14	7	3	19	0	10	5
0	0	0	0	0	7	0
95	61	57	130	50	116	81
0	0	0	0	0	0	0
0	0	0	129	0	0	0
38	48	60	73	68	39	44
44	82	47	78	26	27	47
39	41	21	63	10	29	19
0	0	0	1	0	0	0
202	176	131	186	146	189	120
50	61	48	82	50	33	34
29	30	21	78	25	39	25
84	101	76	94	92	60	89
100	65	72	137	180	146	101
6	15	56	27	13	2	1
16	15	14	18	10	13	4
1	1	1	0	0	2	1
0	0	0	2	0	1	1
113	76	110	111	140	107	112
43	39	59	85	50	45	55
0	0	0	0	0	2	1
40	18	33	0	0	30	33
4	0	0	0	0	0	0
3	4	0	0	0	1	0
141	70	144	153	286	124	128
32	1	2	80	27	121	34
171	140	119	178	133	112	111
8	5	4	16	0	12	10
2	2	0	8	0	4	0
41	51	62	59	83	40	61

For Review Only

263	123	185	297	370	231	158
45	38	33	35	87	97	40
14	0	0	0	0	0	0
3	2	3	15	0	2	7
54	84	79	190	74	75	110
41	40	39	0	0	36	30
2	59	14	24	41	27	21
251	186	161	252	229	199	167
171	167	115	160	188	123	79
0	0	0	0	0	0	0
43	0	24	28	79	65	35
216	7	19	0	0	83	91
0	0	0	0	0	0	0
1	0	0	0	5	0	0
478	359	357	582	483	480	315
1	3	11	7	13	1	5
3	3	0	0	0	1	0
388	199	252	431	259	241	301
119	85	98	227	123	101	90
128	149	137	268	166	209	131
0	2	2	0	0	0	0
0	4	8	0	25	8	16
125	209	195	417	136	227	235
0	2	0	0	0	3	3
4	4	0	14	0	8	0
82	54	27	62	43	28	39
0	0	0	0	0	4	0
507	434	475	717	66	500	384
10	4	5	25	0	16	13
0	1	0	0	0	0	0
2	0	2	0	0	1	1
7	5	1	9	0	5	3
0	0	0	6	0	0	4
0	0	0	0	0	1	0
4712	3829	4402	6910	4860	4384	4044
39	15	3	95	0	29	41
6	7	7	16	0	0	4
0	0	3	0	0	1	1
11	23	14	55	51	24	5
4	1	1	6	0	6	17
0	0	3	0	0	1	0
5	3	19	19	7	8	8
416	2	113	792	580	970	107
63	56	35	97	36	77	70
153	123	117	154	187	103	106
84	44	41	58	99	80	33
4	0	0	0	0	3	0
0	4	0	0	0	4	0
0	0	1	0	0	5	0
0	0	0	0	0	76	4

For Review Only

119	75	42	186	84	61	107
4	0	0	3	0	0	0
8	27	0	0	132	33	34
5	8	0	0	11	0	5
22	51	19	43	0	10	10
0	0	0	0	104	127	0
0	0	0	0	19	0	0
22	0	28	27	41	3	20
0	0	0	7	0	3	0
4	0	0	0	0	0	0
153	256	194	137	205	142	27
29	6	13	38	0	30	37
0	0	7	16	0	7	7
17	17	29	68	0	16	32
0	314	259	495	480	84	243
0	0	0	3	0	0	0
147	162	155	195	226	138	104
260	0	0	227	0	0	156
1	3	2	6	0	1	0
0	0	0	0	0	3	0
22	16	22	36	0	32	7
0	0	0	0	23	0	0
0	0	0	4	0	1	4
0	0	0	3	0	0	0
10	10	0	6	0	9	5
389	453	7	0	0	0	313
11	3	4	7	0	5	6
119	96	40	0	115	101	0
4	3	6	7	0	6	0
4	3	8	8	15	20	5
13	15	10	29	36	21	37
0	0	0	2	0	0	0
3	5	5	11	0	16	0
1470	2706	0	1	1117	1613	1785
0	34	25	0	193	0	21
56	62	22	89	58	69	62
52	34	59	85	70	20	58
4	2	8	1	0	0	2
103	41	29	73	0	19	5
172	186	154	186	145	184	291
5	2	2	0	0	1	2
0	315	0	12	1	0	0
84	76	72	141	146	100	103
24	15	34	46	23	40	23
38	12	24	90	23	31	38
0	0	0	6	30	0	16
312	281	268	404	377	309	240
75	66	64	119	78	50	78
32	9	0	17	64	9	0
97	56	144	183	262	97	173

For Review Only

4	0	0	0	0	1	0
33	3	12	16	31	6	12
28	33	17	58	0	25	31
9	0	0	0	0	0	0
16	10	12	29	4	11	23
22	0	30	7	3	0	0
9	3	8	28	0	8	5
0	0	0	0	0	0	0
22	4	1	33	0	15	5
17	6	14	0	0	6	0
1	0	0	0	0	1	1
60	34	46	64	31	55	33
1	0	0	0	0	0	0
107	47	87	179	110	83	42
26	22	29	56	33	25	17
5	0	0	0	0	0	0
5	7	10	33	0	0	0
6	29	14	12	0	5	10
25	30	32	45	25	31	40
38	40	21	51	18	21	19
39	60	33	70	41	46	43
147	73	196	208	71	171	184
61	40	58	43	30	11	40
77	65	77	127	40	84	102
97	60	57	73	45	66	60
51	50	49	78	64	46	39
81	56	75	149	139	112	29
76	44	45	77	20	26	23
46	50	54	63	70	34	44
296	322	405	546	199	308	302
17	6	2	16	25	15	11
0	0	0	12	1	0	0
27	25	38	60	16	32	20
302	278	256	474	263	249	290
33	29	12	32	63	21	56
77	94	74	91	27	59	40
64	52	42	102	80	30	21
84	105	44	139	69	71	81
41	311	129	405	36	283	35
83	70	58	108	91	96	79
52	34	43	58	46	46	28
66	52	31	74	55	33	21
96	0	77	93	64	106	82
135	114	73	96	223	197	139
34	11	13	19	0	12	17
157	107	59	245	156	139	130
0	0	0	0	0	37	0
105	124	146	128	218	83	99
0	0	0	9	4	0	1
41	26	30	46	54	86	36

0	3	0	0	0	7	0
9	14	23	36	15	10	9
7	5	5	9	0	5	4
0	0	16	0	13	0	0
1	1	0	0	0	0	0
3	0	1	6	0	0	3
12	1	3	0	69	23	13
39	37	51	0	52	8	30
100	80	99	315	11	116	65
42	56	88	128	63	56	68
55	36	41	107	47	49	48
76	50	73	91	51	69	69
35	2	20	34	0	53	8
103	39	31	87	101	61	47
6	5	10	9	0	1	1
243	281	218	425	353	331	218
37	24	49	73	58	22	39
46	79	89	38	87	13	9
24	181	254	281	393	1	1
4	2	7	11	1	4	9
987	0	0	0	0	0	0
592	382	572	884	355	642	560
310	314	255	551	278	353	289
60	33	70	140	52	38	25
42	48	61	0	0	10	13
0	0	0	0	0	0	0
961	745	308	141	289	339	251
1	0	1	0	0	1	2
291	2	1	0	0	183	53
108	70	16	281	93	93	229
38	31	28	64	28	33	19
3	6	6	1	0	3	1
30	16	20	32	0	24	0
17	18	16	59	0	17	16
1	14	3	0	0	3	1
30	37	13	0	0	29	18
41	17	22	62	11	17	43
33	22	9	30	17	23	15
0	0	0	25	67	0	0
123	0	0	0	0	4	0
24	0	23	22	27	63	25
34	62	63	90	76	52	48
65	37	43	0	0	53	40
3	7	16	40	0	8	28
153	117	280	280	353	140	224
1	2	1	10	0	0	1
0	17	0	0	0	80	0
92	85	75	158	77	96	87
21	27	25	37	18	22	17
6	3	13	10	3	5	6

124	149	107	113	102	115	83
26	4	2	11	20	30	31
1	5	9	1	27	4	14
7	8	7	10	12	4	0
59	46	59	84	58	30	39
285	196	144	291	160	281	149
68	58	0	50	76	40	69
30	0	13	2	4	19	13
0	0	0	12	0	0	24
67	96	53	99	91	93	44
196	156	137	292	139	249	133
129	102	97	183	78	129	58
139	119	149	212	118	99	119
17	30	0	31	0	1	0
3	5	5	0	7	6	0
61	31	30	73	46	74	32
126	76	77	171	119	68	40
75	112	92	157	71	108	48
0	0	4	7	0	2	1
53	79	82	104	57	10	17
15	14	17	33	12	21	20
38	30	37	63	1	22	7
39	39	47	76	26	42	33
0	0	0	0	0	8	0
19	0	16	25	35	15	9
30	10	23	29	23	20	14
28	56	16	0	40	4	57
3	13	35	39	29	17	6
838	113	1939	1394	664	642	428
0	0	0	66	0	0	0
9	8	12	9	26	11	5
309	178	152	416	233	172	280
72	61	62	107	66	87	73
21	31	26	63	39	51	20
51	33	25	33	18	18	22
72	31	31	207	0	0	107
0	0	0	0	0	0	0
57	59	64	102	45	33	28
608	545	548	855	495	651	464
496	500	408	674	534	533	277
9	10	7	12	11	3	9
6	0	0	0	0	8	5
51	79	71	131	72	72	67
0	83	13	0	0	13	19
39	26	64	53	96	67	48
0	14	27	61	87	17	4
51	89	25	127	136	33	4
57	29	51	55	60	45	56
105	98	31	295	248	100	23
33	45	27	77	9	18	41

For Review Only

0	4	4	2	0	0	0
13	6	12	94	0	61	40
40	26	28	63	31	34	36
3	0	2	4	0	0	0
17	7	5	18	10	4	13
26	6	15	9	22	28	28
49	53	0	118	112	57	40
92	17	63	53	0	111	0
44	101	83	150	38	75	95
67	66	58	91	79	71	50
8	10	2	6	7	3	3
305	388	322	451	468	412	203
55	107	103	130	57	144	97
46	22	36	67	22	25	40
7	0	0	4	11	0	1
129	198	201	279	149	169	259
27	32	28	70	87	50	80
75	65	80	140	59	79	56
27	11	38	36	62	20	33
1	0	0	0	0	0	0
44	56	90	121	27	56	61
188	171	206	277	227	163	171
0	5	1	0	31	8	4
24	51	12	83	75	46	12
2	0	0	0	0	1	0
1	3	3	0	0	3	0
6	80	4	35	12	16	14
0	1	0	5	0	4	3
25	3	11	23	0	25	18
196	223	201	343	293	215	298
8	16	20	21	27	16	3
39	42	25	36	24	30	14
6	0	3	0	2	0	0
243	381	195	340	284	240	280
533	626	477	955	557	402	751
8	8	6	0	23	10	16
103	131	64	117	100	104	80
40	30	48	54	77	47	41
649	495	0	1992	141	644	384
83	37	72	81	117	37	53
3	7	14	12	5	0	0
1	0	2	0	149	1	1
3	0	3	0	0	0	0
2719	2415	1923	4004	2044	2886	1804
28957	19843	25156	44588	29984	30803	21811
41	23	10	45	10	19	10
24	12	43	58	10	26	49
5	4	5	9	0	15	2
0	0	0	0	0	0	0
160	158	534	455	148	336	41

For Review Only

2	0	4	0	0	2	2
14	0	12	51	0	0	0
212	76	0	22	521	229	99
0	98	0	1	1	0	82
4	25	19	20	5	31	14
240	0	0	1	1	328	0
0	1	0	1	1	0	3
90	84	87	123	16	38	32
38	30	14	21	24	18	16
0	0	0	0	0	0	0
15	24	28	37	15	14	37
10	0	0	0	0	0	0
242	242	193	356	164	136	174
62	60	55	83	98	60	53
0	1	2	0	0	3	1
25	97	39	91	11	36	21
0	0	0	0	0	0	0
0	121	0	137	0	79	0
25	27	10	30	54	20	14
72	56	53	103	25	36	51
91	80	94	150	65	78	85
0	8	0	25	0	0	0
13	0	6	21	14	18	13
16	1	10	9	0	1	4
3	2	3	4	0	19	2
89	23	132	270	21	47	122
153	148	191	327	159	137	179
17	40	36	138	1	69	13
1	0	0	0	0	0	0
42	11	5	71	0	67	20
0	0	0	0	0	26	0
0	0	2	0	0	3	7
29	15	11	31	23	42	20
8	0	0	8	2	8	4
50	17	37	33	13	74	68
1	1	1	3	2	3	1
11	8	8	12	7	11	12
19	11	15	24	14	24	22
22	18	16	26	12	20	17
7	6	8	11	1	10	9
7	5	4	7	3	5	4
8	6	5	10	5	7	5
10	6	6	11	9	8	9
7	7	7	10	5	10	6
9	7	7	12	5	7	8
14	8	7	15	9	18	12
10	6	7	13	6	8	7
51	36	41	77	33	52	38
37	26	27	50	26	29	27
2	3	1	3	3	4	4

For Review Only

63	51	41	87	36	45	47
31	19	23	35	8	58	48
5	4	1	5	4	5	9
59	22	29	38	28	53	52
6	4	3	6	4	5	7
3	3	4	10	5	6	7
6	4	5	9	4	7	7
1	1	0	2	2	3	3
1	3	0	6	2	1	3
5	6	5	6	9	9	6
1	0	1	3	1	3	3
9	5	4	8	5	7	10
9	5	5	11	3	9	12
3	1	2	0	1	5	6
3	0	0	1	2	1	1
1	1	4	1	3	4	3
6	1	0	5	2	9	4
4	5	4	7	8	17	16
1	3	1	2	1	4	3
1	0	0	1	2	3	0
0	0	3	1	4	1	1
3	0	2	3	0	7	2
1	0	3	2	0	3	1
11	3	6	5	7	21	21
11	16	10	21	11	26	22
1	0	0	1	0	1	1
6	1	5	5	1	7	7
9	4	12	29	1	23	19
0	0	1	0	0	1	0
2	0	2	3	0	6	1
4	7	2	5	1	11	3
4	2	1	6	3	9	2
73	35	46	73	56	101	76
47	22	32	52	36	62	49
7	4	4	6	4	11	6
6	1	3	8	5	6	8
0	6	1	1	0	3	3
45	14	14	29	0	11	46
9	8	8	8	4	4	16
11	8	5	10	5	7	13
6	4	3	6	4	4	7
0	7	0	8	0	8	7
91	154	126	200	99	149	83
1	0	0	5	0	0	4
7	1	9	4	1	7	14
119	118	124	180	117	88	88
13	13	15	22	13	28	8
560	564	634	793	635	670	616
223	161	134	317	187	295	198
55	38	29	72	91	41	36

For Review Only

557	509	464	884	462	613	464
0	91	96	0	174	70	64
34	15	26	34	20	8	18
5	11	14	20	0	23	18
114	97	89	138	109	87	138
14	14	4	29	48	18	11
86	103	114	159	101	105	125
65	0	0	0	1	59	0
30	24	37	47	40	50	17
0	0	6	0	0	0	1
225	0	0	1	2	0	114
151	3	9	134	64	80	7
53	25	38	57	51	33	33
36	32	29	48	48	25	27
3	4	1	3	3	3	5
347	392	334	659	298	352	310
75	62	58	91	54	89	69
19	10	12	38	9	26	24
1	0	1	3	0	3	0
281	209	171	314	204	261	117
50	39	36	73	41	74	65
75	67	25	46	18	92	53
4	0	0	0	0	0	0
133	106	127	160	129	122	90
0	0	0	0	20	0	0
1	1	22	53	0	1	1
0	3	3	4	0	3	0
127	135	102	112	60	64	90
79	57	65	113	104	75	51
105	0	0	1	1	40	0
0	0	1	0	0	0	3
50	83	51	112	54	53	52
1	0	0	1	205	0	0
1	0	0	0	0	0	0
312	280	272	508	308	311	165
165	0	0	0	121	0	0
103	9	125	183	0	54	112
16	10	32	45	14	21	24
0	0	9	0	155	0	14
310	0	647	1	204	0	0
53	66	72	102	83	64	60
0	0	31	0	0	0	11
212	0	177	260	12	100	153
63	53	66	83	61	34	59
52	52	19	29	64	40	32
341	309	273	401	339	389	269
0	141	0	0	192	2	0
171	158	146	207	127	161	102
0	0	0	0	0	0	0
350	790	820	249	182	168	645

For Review Only

23	2	82	0	185	44	39
26	18	10	29	28	11	24
229	103	113	232	120	147	196
150	118	120	179	148	114	95
138	252	387	350	575	181	283
152	79	131	125	229	134	58
43	34	52	58	53	31	40
15	2	3	16	0	3	10
27	16	21	27	36	12	33
23	0	0	6	74	0	37
34	44	15	30	0	19	1
54	46	47	48	42	49	24
145	226	208	240	330	204	136
0	0	0	0	0	3	0
6	0	2	0	0	5	14
295	397	314	425	442	349	200
0	0	0	4	0	0	0
113	130	79	186	151	87	86
0	0	0	0	0	14	0
76	63	71	110	27	67	53
722	765	767	949	884	647	452
863	0	379	997	0	86	437
1	6	28	0	0	31	10
160	138	205	229	123	167	143
139	102	85	163	99	135	87
579	422	475	433	401	373	446
0	0	0	330	145	0	0
147	80	78	82	109	97	66
72	60	48	81	52	68	37
12	11	18	21	28	5	5
76	85	60	107	109	84	91
379	285	283	0	0	365	304
25	27	49	48	76	23	49
0	0	0	0	0	0	0
0	0	0	2	1	3	0
41	57	30	15	23	27	13
79	75	106	146	34	82	151
6	0	0	0	0	0	0
231	310	215	476	292	311	189
0	0	0	0	0	0	1
25	15	0	16	8	19	11
0	0	3	0	0	2	1
5	5	10	0	0	2	8
46	56	39	98	54	77	41
38	9	13	49	20	16	38
140	109	130	230	52	274	128
285	400	250	455	326	281	182
4	0	5	0	9	1	3
1	1	1	0	0	1	2
0	0	0	0	0	0	0

For Review Only

368	136	147	248	100	141	109
61	33	25	36	51	18	16
2	7	1	0	0	1	1
38	39	38	63	56	49	60
18	11	6	20	26	15	16
0	0	0	1	0	1	4
182	134	108	232	130	70	81
0	0	0	3780	1649	0	0
1701	2242	1832	2826	2472	2298	1518
22	11	4	13	34	42	9
112	89	98	156	83	107	83
93	13	78	144	78	109	68
87	45	2	63	0	3	5
43	20	3	33	0	1	1
2	2	1	4	0	3	2
29	1	18	6	33	15	13
235	193	176	170	209	91	146
4	11	4	10	0	5	2
2521	2312	1478	4909	1915	238	1263
1427	3276	256	3321	756	374	301
141	130	154	211	111	152	118
4	0	0	9	0	0	0
0	0	0	0	13	0	0
39	37	46	80	79	63	27
15	5	8	13	0	6	10
1	1	1	0	29	0	0
1	1	2	0	0	1	1
33	31	81	81	25	57	44
44	32	51	94	26	85	37
158	142	72	228	66	85	46
20	152	55	65	50	18	30
4	0	0	13	0	0	0
76	204	58	131	133	32	15
19	8	15	16	14	15	6
63	24	56	34	43	38	33
23	13	11	14	0	7	21
4	0	0	0	0	0	2
1	5	0	0	0	0	0
0	0	0	3	0	0	0
39	40	24	34	36	47	29
111	112	121	180	160	123	166
40	21	54	74	0	50	47
1	2	0	1	0	1	2
401	342	417	666	283	271	423
943	710	523	1033	563	1022	463
0	3	0	7	0	3	3
84	100	49	74	73	66	36
4	4	4	5	9	11	7
0	1	1	0	14	1	0
13	5	2	3	27	21	19

For Review Only

11	35	23	22	0	9	3
6	3	1	13	1	13	7
139	152	62	128	195	191	28
26	11	7	0	0	7	14
3	0	0	10	0	3	3
74	34	17	102	0	25	6
85	26	63	189	0	335	53
67	22	61	49	44	22	43
13	14	10	16	0	13	5
9	14	7	55	0	11	6
0	0	0	0	0	0	0
7	15	16	19	0	5	7
425	365	261	648	256	503	387
0	1	0	2	0	0	4
92	62	80	139	49	104	102
80	19	22	30	92	30	58
55	47	41	100	46	59	32
156	90	108	115	101	208	51
1	4	0	0	8	2	14
39	1	2	223	219	106	123
45	43	0	30	31	0	0
0	0	0	0	0	0	1
11	0	0	0	0	0	2
66	58	52	114	53	85	122
106	59	109	179	43	144	53
67	64	127	369	262	79	126
73	65	63	108	113	68	60
178	143	9	452	0	174	132
0	444	0	129	2	70	0
4	5	10	9	13	3	4
0	0	0	100	0	0	0
112	108	119	174	147	74	103
0	0	0	0	161	332	190
565	176	118	272	288	206	253
0	0	0	0	0	1	0
162	154	0	114	118	171	146
249	141	192	230	335	156	263
22	0	1	0	0	1	0
18	15	15	31	19	13	15
0	0	2	0	0	0	0
14	26	28	32	8	12	9
0	1	2	0	0	0	0
32	68	92	119	88	61	2
0	12	9	0	1	0	0
1	400	1	761	0	1	1
0	0	0	6	0	0	0
9	11	5	12	0	6	1
40	27	82	70	73	64	19
3	26	11	16	0	23	1
36	41	23	51	14	22	16

For Review Only

3	40	59	0	0	6	1
3	2	1	0	0	1	1
0	0	0	0	0	0	0
0	0	0	5	0	1	0
0	7	0	19	0	4	3
24	28	36	54	74	20	10
0	0	0	0	12	1	3
392	397	314	500	337	422	310
279	325	243	308	252	213	199
3104	3546	2453	4851	2350	3268	1803
830	647	605	1196	515	776	391
65	62	57	99	41	30	37
20	15	15	11	17	8	0
0	18	32	1	16	0	0
83	55	53	94	51	71	45
108	71	75	138	90	108	94
0	0	0	29	0	0	0
16	16	13	31	15	21	16
21	19	15	6	45	18	12
2	10	10	8	0	8	4
30	26	53	79	65	89	44
114	94	73	119	130	107	82
0	0	4	0	0	0	0
15	20	11	18	50	16	4
4	8	11	14	0	10	4
19	4	13	18	0	0	3
17	9	22	13	0	4	0
97	100	129	162	63	90	69
2	0	0	1	9	1	1
2	0	0	0	0	1	1
79	49	57	111	68	75	75
24	1	0	14	1	4	0
22	12	13	32	20	29	15
19	47	32	37	24	42	35
2	0	0	0	0	3	5
0	0	0	0	8	0	0
22	0	0	1	0	0	0
75	69	59	92	67	74	49
3	0	2	0	0	0	0
30	19	13	32	18	35	33
15	11	16	28	40	21	5
7	6	1	12	0	1	17
17	0	0	8	4	1	0
14	35	1	0	323	1	1
8	0	0	12	0	11	5
1	0	0	3	0	0	0
198	173	207	327	139	210	126
0	3	1	0	0	1	0
161	228	0	276	41	246	137
50	23	41	61	38	26	32

For Review Only

85	43	90	133	115	103	75
103	69	53	473	0	58	27
3	0	0	0	0	6	0
126	115	160	163	78	167	112
359	456	544	740	593	576	628
26	34	24	42	56	25	24
46	46	34	42	66	43	39
718	3157	8	369	3538	5935	530
578	167	507	651	143	451	363
29	33	10	75	14	72	11
14	12	10	22	14	17	6
10	15	12	14	9	20	4
467	438	458	628	395	525	380
68	71	49	112	53	65	61
26	22	17	26	0	0	0
637	614	618	1003	624	743	663
0	2	0	0	3	0	1
1	517	366	1033	268	440	86
0	4	0	3	0	6	6
9	1	10	7	13	3	5
0	53	0	104	3	109	48
1	25	1	0	0	1	1
5	1	3	21	0	0	3
313	237	247	399	156	318	192
1	3	15	0	0	9	0
3	0	0	0	0	1	1
0	2	0	3	20	0	4
0	0	4	3	0	1	2
1	26	56	49	0	16	4
0	0	1	5	0	0	1
155	167	146	238	178	149	136
1	0	0	6	0	1	0
0	0	3	5	0	0	0
216	373	394	134	423	250	124
8	4	7	10	14	8	5
17	11	17	0	0	13	8
0	0	0	0	0	0	0
0	0	0	3	0	0	0
157	22	82	1279	994	1416	23
22	15	15	33	0	11	22
458	684	456	1031	768	622	516
807	349	613	855	943	902	459
79	50	50	110	114	90	125
24	25	20	38	37	19	12
25	23	21	64	16	8	32
48	43	0	72	0	35	0
284	204	211	476	202	364	169
0	0	0	308	0	0	80
97	92	72	174	45	60	58
38	41	20	32	24	19	10

For Review Only

1	1	5	101	0	1	3
19	6	19	3	7	3	16
295	231	249	342	216	322	191
73	60	73	109	59	70	63
0	0	65	8	8	0	0
131	85	123	197	73	129	93
7	11	11	2	7	4	0
147	131	182	238	237	249	178
10	2	24	20	34	6	22
0	0	1	3	21	11	7
0	0	0	0	0	31	0
0	0	1	0	0	3	0
1	5	20	17	0	13	4
21	11	16	30	10	8	12
98	61	121	158	48	64	71
7	0	0	5	0	6	3
29	19	22	29	0	18	27
0	0	0	0	0	8	0
32	49	25	91	67	53	58
6	11	9	15	34	3	8
89	39	57	74	75	51	40
104	25	24	87	0	177	11
0	0	0	0	0	0	0
173	289	64	48	109	55	60
22	23	27	52	15	26	19
0	0	0	0	0	7	0
89	52	82	85	110	96	85
130	131	182	198	205	124	187
6	6	8	5	6	4	8
41	87	57	192	139	72	13
59	29	35	38	55	41	11
223	228	190	318	224	148	186
196	161	210	250	232	255	238
11	2	1	17	10	1	4
0	1	0	10	0	0	6
0	129	207	21	192	0	28
90	116	89	111	119	99	75
69	31	79	82	79	91	35
54	68	84	273	0	177	0
65	69	61	56	92	23	26
23	25	26	20	36	24	15
94	112	104	125	113	100	70
175	213	200	374	155	193	203
1	1	1	0	0	1	12
83	80	80	111	125	85	109
52	51	62	62	33	48	34
17	31	48	66	62	13	51
227	100	117	157	195	152	102
147	61	121	178	109	83	80
77	81	69	115	42	47	38

For Review Only

12	23	24	29	38	33	17
62	28	36	54	65	39	43
33	38	42	57	68	27	31
54	31	33	123	0	108	83
12	5	10	19	0	19	16
57	52	45	79	30	65	52
1	332	1	0	0	1	669
10	5	1	12	6	6	1
6	16	34	42	0	4	16
42	49	66	71	55	50	37
72	67	118	123	127	92	107
177	172	99	194	49	176	117
168	20	21	144	28	35	60
119	144	82	166	102	102	132
116	112	127	152	154	111	83
47	71	40	67	76	27	49
233	151	90	193	230	210	107
6	5	17	24	0	21	40
31	34	10	42	37	40	21
3	25	18	36	0	6	23
21	23	23	25	17	20	25
4	2	6	7	20	9	15
55	46	38	80	61	57	26
0	2	0	0	0	0	3
0	0	2	0	0	3	2
8	3	0	4	0	3	0
33	22	24	49	31	15	25
43	23	24	50	24	42	32
27	35	17	59	102	61	81
18	78	53	53	132	40	20
0	0	0	0	0	0	3
33	67	199	49	138	145	101
696	103	300	0	0	1	28
20	15	12	36	0	18	2
1	1	2	334	0	1	1
71	69	150	124	67	72	94
194	184	142	297	123	49	118
14	90	132	63	214	167	61
53	67	43	58	54	44	9
1	10	416	529	0	8	2
261	204	139	154	154	69	112
157	1	1	219	172	101	11
31	48	27	34	32	27	21
26	512	49	1	1	0	199
2	6	1	9	0	15	1
10	38	33	0	0	32	7
1423	1553	1290	2177	1440	980	914
186	197	185	0	0	151	78
5	3	0	0	0	0	0
153	183	143	230	75	174	101

For Review Only

216	0	0	389	206	259	0
89	23	28	61	22	33	30
4	0	0	0	0	0	4
39	61	26	67	53	31	25
14	17	5	16	0	6	4
144	158	111	209	171	158	129
2	3	6	0	0	1	1
0	11	9	8	0	0	0
9	10	6	8	0	10	0
41	50	34	50	28	35	28
3	0	0	0	0	0	0
39	25	48	44	72	24	27
1	3	11	37	0	15	3
0	0	0	0	0	7	5
3	13	11	5	8	4	9
172	240	106	196	489	385	247
0	45	35	10	4	0	33
254	193	184	427	265	260	136
127	92	158	161	165	122	202
319	268	233	297	295	221	187
5	1	3	0	31	21	3
0	0	0	0	0	6	0
15	0	0	0	0	0	0
5	11	0	7	0	0	0
67	60	49	70	46	73	43
228	242	302	436	463	234	318
96	124	129	192	157	150	104
16	10	9	24	13	20	61
0	2	0	0	0	0	4
792	726	918	1261	504	639	575
155	137	156	279	175	182	240
35	5	24	23	0	24	6
342	314	365	277	314	156	195
34	62	25	85	93	54	69
0	0	2	0	0	3	0
1239	1174	1069	1924	1101	1532	1024
0	0	4	0	0	0	5
147	89	124	251	231	124	119
1	3	22	13	0	17	10
0	0	0	0	0	0	0
1275	2332	1906	1714	3257	865	1583
3	0	0	0	0	4	4
0	0	0	0	0	0	0
0	0	0	16	0	0	0
0	0	6	0	0	0	0
10094	9452	9175	15700	9311	10814	7087
1634	1458	1338	2302	1354	1816	1095
14	16	22	50	11	29	21
120	139	106	168	95	124	73
68	68	61	114	143	50	147

For Review Only

39	54	31	54	69	30	18
8	7	18	75	0	22	19
372	380	440	566	493	439	444
54	33	48	44	15	14	70
26	10	24	14	27	28	10
820	787	740	1277	996	904	569
45	55	59	126	26	30	49
13	1	0	2	23	25	14
105	171	199	241	225	157	153
78	64	43	39	6	72	0
3170	1154	1285	1402	2847	1336	1410
63	51	47	71	53	38	48
0	16	12	0	0	0	25
45	31	44	41	32	35	41
144	112	179	219	159	184	167
9	4	11	14	17	13	12
1	0	3	8	0	0	2
34	18	18	49	37	47	29
128	132	149	220	128	119	87
651	335	479	540	572	529	501
54	54	46	74	48	71	50
286	144	73	213	212	90	37
258	232	152	302	217	313	221
0	46	47	45	108	71	52
108	51	85	99	82	106	68
3	6	5	14	0	1	3
0	0	0	1	4	4	4
148	130	134	207	92	91	144
396	391	482	703	528	497	490
29	24	48	73	14	45	21
77	91	81	144	105	97	53
0	14	0	10	0	0	0
56	44	39	60	51	46	41
36	48	55	68	68	32	57
21	59	37	104	63	29	43
7	21	24	16	19	15	9
70	38	43	42	63	43	58
22	30	29	28	25	10	29
0	0	0	0	0	0	0
4254	1367	1359	2317	1573	3929	2582
3793	1966	2046	3327	3196	3340	2570
24	20	0	41	0	33	24
24	8	7	19	6	13	17
165	192	171	238	249	205	152
11	2	0	11	0	0	8
4	0	0	0	0	0	0
0	7	0	10	4	5	4
2	40	36	96	49	30	1
171	233	144	281	183	248	151
64	68	81	104	58	87	98

For Review Only

83	62	70	59	27	63	7
172	126	119	240	181	141	151
42	43	37	67	40	55	39
150	87	96	199	64	125	103
102	118	78	119	91	70	6
42	49	33	47	42	28	24
43	11	39	16	50	0	0
209	213	155	311	213	217	169
102	94	91	162	104	83	112
246	200	181	343	295	336	176
99	127	0	332	1	95	60
70	52	58	116	80	80	55
40	28	6	29	9	22	16
40	35	0	0	16	0	36
15	35	22	20	46	19	17
8	5	4	7	0	1	0
0	8	0	16	0	8	1
11	4	7	17	0	3	5
183	220	213	299	150	205	101
23	26	16	28	13	18	20
111	100	69	157	101	110	74
10	3	4	7	4	2	3
85	82	64	69	53	54	21
26	9	11	41	1	51	35
37	54	57	74	80	51	60
0	0	0	0	0	1	0
1	2	1	6	21	8	3
0	0	0	0	0	0	0
0	2	0	1	0	4	6
7	0	0	0	0	11	1
6	0	2	7	0	0	5
0	0	0	0	0	0	2
4	0	0	0	2	0	1
0	0	4	5	0	6	4
0	0	0	279	233	0	0
0	0	0	1	0	1	0
0	3	3	0	0	4	0
12	2	0	8	0	1	0
0	0	0	0	0	0	4
2	1	1	0	0	0	0
19	24	19	35	33	19	33
1	0	0	0	6	2	0
0	0	0	0	4	0	0
83	57	88	97	74	84	58
0	3	3	6	3	0	2
13	9	6	15	0	12	20
13	0	11	13	0	6	14
48	6	12	13	40	3	4
2	2	0	0	0	0	0
2	0	0	3	0	2	4

For Review Only

63	34	55	55	13	32	24
0	0	0	3	0	0	0
51	28	31	69	40	34	15
11	0	3	6	0	0	0
12	11	15	21	26	12	0
162	177	198	319	143	133	207
4	3	5	4	4	4	4
22	5	30	30	1	26	3
2	27	3	15	0	4	8
0	1	6	2	0	3	0
17	16	6	6	24	0	7
261	224	308	460	304	312	306
0	0	1	2	1	0	0
29	23	27	35	32	5	4
0	0	2	0	0	2	3
4	0	0	0	0	0	4
12	13	18	24	10	35	12
2	0	0	4	0	0	0
49	23	20	43	12	62	17
50	82	48	83	47	53	56
216	449	297	1870	0	0	0
9	4	36	12	42	5	33
44	27	28	54	19	24	11
3	1	0	0	0	0	0
10	12	9	64	0	55	81
14	32	35	44	67	29	27
245	236	244	208	125	126	138
104	94	85	86	125	102	72
46	35	31	41	26	10	35
9	0	1	8	5	8	1
42	20	45	67	112	19	42
21	13	32	41	15	13	25
25	11	12	29	0	5	11
6	3	9	15	0	16	5
88	66	51	47	42	27	40
29	34	23	41	50	33	11
94	63	80	182	114	89	108
317	425	400	591	338	440	319
0	0	1	4	0	3	3
0	0	0	5	0	1	0
34	42	45	58	10	34	27
106	32	39	74	25	61	60
204	163	146	261	193	222	201
263	119	146	169	247	164	116
114	66	130	182	127	150	126
176	187	190	259	224	159	193
11	16	23	35	37	58	18
64	102	72	113	151	100	77
71	38	75	106	58	87	87
0	0	3	0	0	0	0

For Review Only

270	121	102	211	154	243	251
86	63	87	103	109	83	82
123	148	120	215	181	120	204
45	16	49	109	28	34	61
12	29	60	79	0	30	57
101	61	56	69	74	69	74
78	137	135	219	75	116	127
6	3	1	10	0	44	2
8	25	8	20	45	11	7
75	76	99	122	72	96	72
187	75	94	160	230	140	91
1	1	0	2	1	0	6
125	260	204	376	287	259	206
10	15	14	14	19	5	28
155	174	140	197	193	0	122
294	1	257	1	25	0	0
19	42	24	36	35	21	9
180	125	122	248	180	275	232
3	1	1	0	0	1	4
75	46	44	68	89	32	46
123	84	88	184	22	76	108
0	6	3	10	0	0	0
35	16	29	42	44	22	22
90	63	87	112	44	63	108
33	25	55	47	80	14	34
4	8	5	19	0	7	7
4	5	0	0	0	0	1
665	377	184	983	534	714	509
0	0	0	0	0	0	0
0	0	0	0	0	0	0
8	6	1	9	0	5	11
0	3	0	2	3	0	0
1	0	1	0	0	0	0
1	0	2	0	0	7	2
33	24	24	54	35	33	18
0	0	0	0	0	6	2
39	27	43	45	53	40	34
89	55	28	120	22	98	138
14	41	84	58	0	7	22
0	0	2	0	0	3	0
1	0	0	0	25	3	0
199	189	136	306	223	131	130
0	0	0	0	3	5	0
0	0	3	0	3	0	0
0	0	0	4	50	43	40
36	40	69	81	64	40	14
1	0	1	3	0	0	0
13	5	16	27	0	19	7
6	8	13	8	0	18	4
1	2	5	5	0	1	6

For Review Only

29	23	32	64	28	30	27
12	2	9	12	0	15	0
20	19	40	34	35	26	25
45	49	38	79	16	81	36
110	0	3	44	0	3	0
245	223	164	344	204	197	192
9	22	22	16	32	8	0
125	82	69	152	73	85	71
7	1	0	2	0	0	0
21	18	17	40	10	11	17
16	1	7	11	4	8	5
100	53	179	193	60	117	122
241	246	206	374	368	378	243
1	0	0	0	3	6	0
21	17	27	16	51	14	4
25	18	24	0	0	7	12
181	256	349	367	396	198	219
4	1	3	0	0	1	1
168	120	185	242	139	182	152
5	3	4	8	0	1	1
2	11	13	16	0	32	22
26	40	59	89	22	39	58
44	35	44	48	30	26	31
190	151	132	0	0	165	94
71	0	51	23	11	69	45
35	44	3	23	68	14	8
4	2	0	0	0	2	3
236	169	100	200	157	105	100
48	5	16	54	0	10	9
9	30	8	18	0	39	25
9	3	4	13	11	6	6
5	3	4	14	0	6	15
3	3	129	0	85	8	1
17	0	25	20	35	17	0
4	4	2	21	0	4	3
78	41	51	43	27	55	26
102	56	44	49	83	32	41
118	113	117	167	136	139	115
105	38	62	92	1	30	46
176	242	273	314	274	229	219
410	249	116	195	355	148	170
39	45	36	23	82	50	47
34	38	35	52	64	35	30
1	0	2	0	0	1	0
13	10	20	35	8	24	19
8	0	0	1	4	0	5
120	125	87	187	59	148	73
35	13	5	1	30	23	6
25	57	56	84	18	35	24
48	45	64	89	32	50	57

For Review Only

235	138	288	282	197	307	158
212	240	131	173	156	169	140
11	0	13	1	0	8	6
267	197	319	344	163	158	188
1	0	0	0	0	0	3
2	1	3	0	0	4	3
1	2	0	0	0	0	0
181	200	244	360	191	180	207
14	4	2	10	0	2	4
2	0	0	0	0	0	6
0	0	0	0	0	0	5
0	0	4	0	0	7	0
1	9	5	10	21	2	10
269	235	250	450	206	218	212
49	64	39	143	61	0	77
247	388	362	535	490	345	394
1117	617	1309	1212	1361	981	976
564	423	439	500	642	380	312
15	6	9	28	11	2	15
3	0	0	0	3	4	0
0	0	0	0	0	0	0
186	143	136	197	214	89	81
3	3	3	0	2	0	3
123	106	65	123	87	114	109
23	18	27	31	19	21	16
155	124	85	169	213	240	97
20	51	58	42	102	29	65
46	32	41	69	19	35	20
174	217	197	281	247	257	176
10	0	9	26	0	18	12
29	50	23	62	129	15	66
121	71	51	110	73	129	68
145	154	138	192	234	117	104
65	43	26	43	51	45	45
27	0	0	0	28	0	0
80	91	90	91	128	121	109
110	0	68	16	1	92	71
2	4	2	5	13	0	1
15	0	0	0	0	0	0
54	16	106	212	119	184	51
24	24	11	46	34	19	36
0	0	2	0	5	0	2
65	56	41	90	42	49	25
131	115	117	214	134	137	108
127	0	0	88	0	88	73
11	11	14	14	0	20	0
6	1	9	0	18	44	11
0	0	1	0	0	1	0
20	9	14	8	0	21	14
11	0	4	0	0	15	1

For Review Only

1	3	0	0	0	0	6
71	94	66	86	92	66	46
20	35	19	44	0	5	5
41	17	29	56	17	35	30
91	97	113	116	184	57	83
12	43	11	30	20	16	12
40	18	45	43	89	7	19
81	53	51	85	91	91	81
0	984	685	0	309	442	772
3	2	3	0	93	1	1
1089	1000	1113	225	253	1117	1
7	1	1	3	0	0	4
11	0	0	0	0	0	0
23	30	18	20	14	33	51
5	8	9	15	0	12	14
0	0	0	0	0	0	0
1187	1251	1261	1751	1442	440	1042
1930	1629	1601	2524	1456	2003	879
1	5	2	1	7	3	4
77	142	62	471	95	129	2
10	31	38	273	0	16	10
0	0	0	0	0	0	0
0	0	49	73	48	0	0
0	101	67	174	83	117	0
87	66	96	123	110	86	84
15	0	1	26	0	0	0
147	675	129	531	353	302	23
318	182	102	170	243	339	154
187	182	124	249	164	124	106
10975	9329	8295	15276	8659	11404	7746
90	73	110	151	44	64	108
60	66	88	157	51	75	79
0	0	0	0	0	2	0
39	31	49	58	34	27	30
107	83	83	121	122	169	187
0	15	14	56	29	16	22
141	51	100	119	129	84	108
1527	1823	2040	3426	1778	1099	1697
52	55	48	95	51	42	45
698	643	442	794	474	701	432
0	0	53	13	19	38	0
56	64	67	61	95	35	42
110	123	92	148	156	82	167
87	104	88	155	139	113	91
35	4	38	46	15	48	39
51	0	0	0	0	49	0
13	0	0	20	0	0	0
135	70	155	131	181	184	97
16	20	8	33	13	11	13
8	4	2	13	0	2	2

For Review Only

7	7	8	5	14	2	5
1	0	0	0	0	0	0
0	0	0	0	0	0	0
47	24	30	37	61	22	17
41	0	0	119	50	53	47
107	64	96	104	84	105	43
81	0	0	80	27	28	95
253	192	316	424	447	217	505
34	8	55	37	0	7	21
1594	1447	1012	1408	1609	1779	839
522	544	501	795	595	545	450
112	71	111	214	101	145	59
30	0	0	6	1	0	0
506	358	374	698	359	336	426
0	0	0	0	0	1	0
1025	870	716	1473	878	1064	716
0	7	8	0	0	7	0
17	6	1	1	0	6	3
1	7	0	6	0	0	5
0	0	3	0	0	1	4
326	281	343	690	533	567	534
399	194	3	0	213	5	42
48	68	25	56	43	47	63
0	0	0	0	0	0	0
25	8	41	58	0	19	4
5	3	6	14	3	5	2
4	1	1	0	0	1	1
82	98	90	91	135	80	86
16	21	16	36	25	27	26
0	0	0	171	11	0	0
32	38	30	58	35	34	28
396	407	370	604	364	441	383
273	234	234	292	215	304	222
187	193	156	184	240	89	105
28	29	26	55	20	50	35
20	48	0	0	0	72	6
29	20	42	41	70	86	16
0	0	0	0	0	0	0
0	0	0	5	0	0	1
0	0	0	0	19	0	0
14	0	4	29	0	4	0
394	492	426	713	434	405	236
1	1	1	0	0	1	1
213	215	204	246	209	361	231
703	670	858	1143	720	898	773
12	3	7	8	0	10	2
4	0	0	0	0	0	0
10	31	9	19	13	16	3
10654	9162	8829	13759	9002	12097	7522
3	1	21	0	12	1	1

For Review Only

2	0	0	0	0	0	0
820	466	496	743	296	464	276
0	0	0	2	1	1	0
8	4	6	6	5	10	2
0	0	5	0	0	0	0
71	78	32	115	142	134	54
0	0	0	0	0	4	0
353	394	377	356	0	574	368
0	0	0	0	0	0	32
48	40	73	56	97	113	96
5	11	14	19	6	21	12
0	0	0	0	6	0	0
3	7	4	6	10	7	2
0	0	0	0	0	0	0
32	35	45	95	25	77	25
0	0	0	3	0	3	0
91	48	50	65	77	58	36
0	0	0	1	203	0	0
49	69	65	145	66	70	47
34	49	23	60	10	86	16
417	435	400	782	353	481	315
0	0	0	0	0	0	0
0	0	0	0	3	0	0
3	2	0	0	0	0	1
0	36	6	0	0	7	0
22	32	20	54	7	23	9
32	13	31	18	54	26	23
0	0	0	2	0	0	0
85	85	116	106	76	48	47
5	11	3	24	0	3	2
0	0	0	7	0	0	0
20	14	25	32	19	9	20
371	335	267	409	334	414	199
0	0	3	0	0	0	11
162	155	81	201	136	123	93
280	151	160	315	74	254	186
13	16	7	13	18	25	8
42	33	31	42	46	79	24
6	7	2	9	8	2	7
21	20	19	10	10	13	11
419	266	357	547	275	507	305
7	8	4	7	11	22	10
7	0	10	0	0	1	0
0	105	88	1	1	107	0
9	0	0	0	9	0	0
55	0	0	9	2	0	38
23	26	18	47	19	15	28
152	166	154	215	127	195	168
479	492	344	575	442	350	320
68	177	0	10	330	68	38

For Review Only

32	12	11	47	14	29	16
57	28	35	54	75	39	37
268	165	222	329	198	224	184
8	31	15	29	0	13	5
167	122	126	0	0	134	125
14	15	17	37	16	14	18
11	0	5	11	0	14	0
0	5	8	0	10	6	0
35	16	24	53	35	25	22
136	186	287	245	476	77	279
27	19	33	63	35	27	20
3	5	1	7	0	3	12
2	0	0	1	0	2	0
6	3	16	0	5	6	2
0	0	0	0	0	0	0
34	44	43	56	71	16	24
164	175	232	346	384	381	284
31	37	48	14	37	10	22
9	11	8	16	0	0	9
5	8	4	13	3	12	6
1	2	1	5	0	2	3
9	10	4	10	6	5	5
0	0	0	0	1	1	1
0	4	1	9	0	1	0
7	1	1	9	3	3	1
6	3	7	8	6	7	6
115	78	65	161	113	169	57
2	0	0	0	9	0	0
7	5	6	13	8	8	4
11	2	3	20	0	6	1
0	1	1	0	0	0	0
0	4	1	0	4	0	0
11	10	8	9	0	1	3
0	0	0	0	0	0	0
0	0	6	0	0	0	2
17	8	7	17	0	1	7
20	37	30	61	18	31	24
3	1	0	2	3	1	0
18	28	9	33	13	29	23
56596	65149	98164	185228	42314	54040	90167
0	2	0	0	1	2	0
0	0	3	0	0	2	1
3	3	0	2	0	0	4
42	30	48	49	0	27	48
3	3	8	8	0	0	4
0	0	0	5	0	0	1
92	83	104	164	101	82	89
9	0	3	1	0	12	8
0	0	5	4	0	3	0
0	0	0	0	2	0	0

For Review Only

0	0	4	0	0	0	0
7	11	15	25	7	19	19
90	75	61	116	93	75	55
0	0	6	4	2	0	0
5	16	3	19	0	19	3
3	0	0	0	0	0	1
0	0	0	0	0	0	0
0	0	0	0	2	0	4
0	0	2	14	0	2	0
20	12	16	38	16	29	23
6	4	5	8	3	5	4
3	11	5	5	10	12	5
4	1	3	6	2	0	1
1	0	0	0	3	0	0
11	7	13	11	17	9	7
9	8	8	21	7	20	13
0	0	0	0	0	1	3
10	5	9	17	0	7	6
9	2	1	13	0	0	2
24	31	35	65	101	68	56
25	28	35	41	45	24	33
4	0	0	0	0	2	0
2	0	0	0	0	0	1
0	2	2	0	0	0	0
0	0	1	0	0	1	0
0	3	1	0	1	1	0
4	3	1	6	2	3	3
6	0	4	7	7	1	1
0	1	1	0	0	1	0
0	2	0	16	0	9	0
70	54	69	116	64	71	77
1	1	0	3	0	0	0
1	1	1	0	1	3	6
3	1	0	0	0	2	1
2	0	0	7	0	0	0
37	28	39	84	16	31	49
4	13	5	7	0	1	1
4	3	7	9	5	8	0
11	11	4	14	6	6	10
56	10	21	59	0	25	40
31	33	38	44	19	29	42
45	56	16	55	14	19	30
0	13	0	0	0	3	0
13	14	6	15	24	14	4
0	0	3	2	1	0	3
23	27	17	34	29	25	27
1	2	0	1	0	2	0
1	3	0	2	0	0	0
0	0	1	3	0	5	1
0	3	0	2	0	0	0

For Review Only

0	0	0	0	0	0	0
0	4	4	0	0	4	3
1	0	0	0	0	6	0
6	8	6	11	5	5	4
17	23	17	38	8	6	13
5	11	6	1	3	2	3
8	9	3	9	15	15	24
4	0	0	4	6	1	1
3	1	3	2	0	1	0
0	0	1	4	0	3	1
4	5	2	14	0	8	3
0	0	0	0	0	0	5
9	6	10	15	2	8	6
19	15	21	41	12	28	17
1	5	3	9	20	12	14
2	2	2	6	0	0	2
73	54	52	99	54	71	66
0	0	0	0	0	0	0
1	0	0	3	0	0	1
24	64	14	142	0	50	91
4	0	1	0	0	5	5
20	23	19	46	21	32	21
0	0	0	0	0	0	0
0	2	4	0	0	0	1
9	0	0	0	0	5	0
11	9	14	17	4	7	20
40	33	21	36	25	25	22
2	4	6	16	0	1	0
19	10	8	14	13	11	6
8	24	13	6	0	30	14
3	5	3	14	0	8	4
7	8	5	5	8	2	5
8	11	31	47	0	16	1
7	1	0	0	0	0	2
222	249	217	362	212	204	146
21	16	8	13	13	19	12
0	0	0	2	0	3	3
0	2	2	0	0	3	0
0	1	2	3	0	0	1
12	13	12	22	11	10	22
0	0	9	0	8	6	0
49	27	21	70	26	48	38
7	11	14	14	8	14	6
5	1	0	5	2	0	5
1	1	1	0	0	121	24
2	0	0	0	0	0	0
1	0	3	0	0	0	5
1	1	0	0	1	0	0
9	12	11	18	13	8	14
0	0	6	1	0	0	0

For Review Only

1	15	1	0	0	1	2
7	9	2	4	6	9	4
15	13	6	0	0	16	10
5	0	0	0	0	1	3
6	6	20	30	0	12	10
0	1	0	2	0	0	0
0	0	0	0	0	3	1
0	0	1	3	0	0	0
10	7	10	0	0	1	1
2	8	14	7	13	5	5
1	0	0	8	0	3	3
4	0	0	0	0	0	0
2	4	1	4	0	0	1
4	22	22	20	11	3	8
0	0	0	5	0	2	3
0	0	0	0	0	6	0
1	0	1	0	0	0	1
11	16	17	25	0	7	20
4	0	3	4	0	1	3
3	0	2	0	0	0	1
2	1	5	1	0	13	1
2	0	2	4	0	0	0
0	0	1	0	0	5	0
0	0	2	5	0	3	1
266	226	313	380	322	290	241
5	0	4	6	0	1	3
13	21	50	22	0	1	25
1	1	0	1	0	3	1
28	45	26	23	46	23	7
0	0	0	0	2	1	0
0	1	0	0	0	1	1
35	31	39	63	55	33	31
1	0	0	5	0	1	1
0	0	0	0	0	0	2
1	1	0	0	0	1	2
0	3	0	0	3	1	0
7	3	0	9	11	8	4
2	1	0	0	5	0	8
3	3	3	6	0	2	1
298	182	122	318	219	346	298
7	2	3	10	0	10	6
0	1	8	6	0	1	4
9	0	6	21	11	12	5
6	16	47	41	0	17	26
1	31	11	8	1	12	8
0	4	0	0	0	0	2
10	13	10	17	34	5	10
1	2	7	9	0	2	2
0	0	0	6	0	0	0
3	1	11	6	10	5	0

For Review Only

13	39	5	33	0	5	7
4	3	0	4	0	0	0
93	111	112	170	132	97	98
0	0	0	0	0	2	7
5	18	6	3	1	5	3
6	7	22	16	0	11	5
0	4	0	0	0	0	2
0	4	1	3	0	8	0
1	0	0	3	1	0	0
51	26	36	80	25	87	61
3	4	0	5	0	0	0
1	2	1	1	3	3	1
10	2	4	16	0	12	16
2	12	11	18	0	22	10
15	12	12	23	14	17	5
9	6	14	18	16	11	12
2	2	0	1	0	2	5
5	8	9	11	0	3	6
0	18	0	0	27	0	10
4	0	4	0	0	4	0
3	0	3	7	0	1	7
2	6	19	69	39	48	30
2	2	5	1	0	2	0
3	5	3	2	5	0	3
47	63	133	82	77	84	45
2	1	4	0	0	0	0
6	27	26	52	44	15	24
25	8	18	19	25	14	7
1	1	10	0	1	0	5
1	1	2	3	0	5	8
13	4	11	14	19	2	10
3	4	4	5	0	9	1
1	1	4	7	1	3	4
51	2	42	76	26	5	30
4	3	0	1	0	0	0
16	48	46	35	46	10	31
1	0	1	0	0	7	0
1	0	0	0	6	6	0
3	4	1	5	0	3	2
0	0	0	2	2	1	1
4	0	0	0	0	0	0
5	12	8	17	0	3	1
0	0	0	0	5	1	0
8	12	7	49	0	7	41
4	12	6	24	0	2	37
1	2	0	0	0	0	0
0	1	0	0	0	0	0
152	333	374	469	664	162	533
16	16	42	51	7	15	33
0	0	0	8	0	1	0

For Review Only

3	2	2	0	0	0	2
0	1	0	0	0	6	0
2	4	8	1	1	4	2
1	8	2	1	11	2	1
271	131	202	330	179	408	363
1	0	1	6	0	1	8
31	20	26	32	38	33	28
1	1	1	0	0	1	0
15	26	35	34	10	25	19
2	0	0	0	6	0	4
0	0	0	1	0	0	3
1	2	0	0	0	0	2
16	7	9	18	0	15	24
0	0	1	0	0	0	1
27	46	27	85	24	30	34
9	14	25	28	0	9	30
5	1	1	1	0	2	8
8	4	1	7	5	1	1
9	7	3	6	11	6	10
12	2	1	9	0	1	1
0	0	7	3	0	0	0
1	1	12	16	0	4	3
2	2	2	8	15	8	8
0	0	0	17	0	0	0
5	0	1	0	0	5	2
24	74	94	107	10	11	47
2	1	0	0	0	2	2
1	2	2	3	3	3	3
6	4	6	11	9	1	6
39	35	37	66	27	36	42
4	7	0	0	5	0	1
23	25	17	27	26	17	30
0	0	0	0	1	0	0
1	0	3	1	0	2	0
23	34	59	53	19	43	41
3	3	0	0	0	0	0
2	8	1	5	0	14	1
0	1	0	0	0	3	0
0	2	1	0	0	0	0
0	0	4	11	0	6	2
1	1	7	0	0	2	0
104	155	73	164	143	94	83
2	0	1	5	0	3	3
9	6	5	7	1	0	9
1	4	4	0	0	0	0
6	7	14	7	0	2	0
2	13	8	5	3	4	9
0	0	0	6	0	0	0
2	0	6	4	0	2	1
7	2	0	0	0	0	0

For Review Only

1	6	1	8	0	1	8
1	2	7	4	0	1	2
0	0	0	0	0	6	0
0	1	4	0	0	1	7
0	3	1	0	0	1	3
4	1	1	9	0	8	1
7	2	21	18	18	10	11
0	2	4	7	0	3	0
1	4	0	3	0	0	0
2	0	3	0	0	0	4
0	0	1	1	0	4	0
0	8	0	0	0	0	0
14	0	13	11	20	19	6
0	0	0	1	4	4	0
0	1	2	0	0	1	8
174	190	213	327	365	266	350
21	4	1	12	0	4	3
14	5	6	22	11	11	19
23	39	8	52	26	17	34
1	3	8	6	0	3	9
240	149	169	271	189	225	209
0	3	0	3	11	8	0
3	0	1	7	0	6	3
34	40	48	62	56	23	19
4	2	1	8	0	4	5
2	3	12	4	0	2	0
8	0	4	7	0	3	6
14	2	1	0	0	4	28
4	12	6	17	0	6	5
43	33	15	73	28	86	44
4	1	1	4	2	3	3
1	1	3	3	3	4	1
0	0	0	43	37	0	0
2	0	0	1	0	3	2
2	0	2	5	0	3	4
6	8	9	17	0	0	8
10	3	14	15	0	20	12
0	3	4	4	0	0	0
4	1	0	0	0	0	0
13	19	11	11	10	46	20
41	38	75	48	93	21	31
52	63	28	65	30	33	28
8	1	6	12	6	14	10
1	0	0	0	0	2	0
12	5	6	15	4	10	7
3	1	4	4	7	5	10
0	0	0	2	0	0	4
0	0	0	3	0	0	0
9	2	27	8	0	7	4
7	0	6	0	0	0	3

For Review Only

19	11	11	12	8	22	28
1	0	2	0	0	4	1
1	10	15	19	0	15	34
5	6	6	12	6	13	5
90	79	81	152	80	74	74
3	1	4	5	5	0	0
4	14	8	0	0	8	17
4	0	1	0	3	2	0
1	3	10	18	5	3	6
1	0	0	0	0	0	0
1	6	1	0	0	1	1
6	1	2	5	0	6	10
0	0	0	0	0	0	0
0	0	0	1	2	0	0
2	1	1	0	0	2	1
1	20	1	69	0	6	83
9	24	18	25	24	13	36
5	4	0	0	0	0	0
5	0	1	0	0	0	2
38	48	114	102	77	58	81
0	1	2	0	0	0	1
72	45	88	130	30	148	163
0	2	0	0	0	0	0
2	0	0	7	0	7	0
2	4	3	10	0	3	2
0	0	0	3	0	0	2
0	1	2	0	1	2	0
0	0	4	0	0	1	0
12	9	12	14	14	10	8
1	0	0	0	1	0	0
0	0	0	0	4	0	3
353	447	315	709	336	387	422
0	0	0	0	0	3	0
22	7	3	8	0	9	14
13	9	7	11	8	6	15
4	0	1	11	0	4	1
0	0	0	0	0	3	0
0	6	1	0	0	0	3
0	1	1	0	0	0	0
8	1	0	10	0	2	2
80	105	60	64	81	51	39
1	5	1	8	0	3	7
1	13	3	8	0	1	2
4	14	20	17	11	22	12
42	40	42	72	27	42	43
0	0	0	2	0	0	0
1	11	6	0	13	2	0
3	11	14	9	0	11	9
4	1	1	0	0	1	1
1	3	1	1	0	5	2

For Review Only

2	3	2	1	0	5	3
0	0	3	11	0	3	6
5	3	0	5	4	7	9
4	3	4	6	4	6	10
0	0	0	0	0	1	3
1	0	0	4	0	2	0
10	17	38	45	0	24	12
2	6	1	0	0	0	0
1	0	2	4	0	0	2
0	1	3	9	0	0	1
1	2	0	1	1	5	6
1	0	0	1	0	0	5
50	30	67	100	20	64	48
0	0	0	0	0	0	5
9	6	2	4	0	9	14
6	0	12	8	0	2	5
3	0	0	0	0	0	0
1	0	0	0	3	0	1
1	2	1	0	0	4	1
25	5	7	19	0	4	2
3	4	5	7	0	1	0
12	36	30	57	0	20	33
1	1	0	1	0	0	1
8	1	4	6	0	2	0
0	0	0	0	0	0	0
13	0	0	9	4	5	8
0	7	14	0	16	0	10
0	6	1	10	0	3	1
13	18	6	12	0	14	5
13	12	11	23	8	13	11
0	0	0	0	4	1	1
46	64	61	70	0	15	38
6	8	4	14	7	12	7
12	1	19	38	19	5	15
0	9	12	6	0	8	3
1	18	1	14	0	1	1
11	14	8	36	11	19	19
0	0	0	0	0	27	0
1	2	0	0	0	0	2
0	0	0	3	0	1	5
15	1	14	16	6	17	9
1	1	0	0	0	1	1
17	21	13	24	21	18	12
10	9	13	11	7	5	8
2	6	4	9	0	2	3
1	16	1	0	0	10	1
0	0	5	0	4	3	2
2	3	3	0	0	2	2
3	3	0	1	0	0	0
28	22	8	90	0	32	17

For Review Only

1	10	0	1	0	2	0
0	0	1	0	1	0	7
10	10	3	10	6	15	15
1	1	2	0	0	0	0
1	5	12	20	0	13	14
1	3	0	0	0	1	0
0	1	3	0	0	0	2
19	17	17	13	33	14	5
0	6	3	6	0	2	3
55	61	60	61	63	23	33
7	9	5	13	13	0	16
0	3	0	3	4	3	1
1	1	0	3	0	0	3
2	1	0	0	0	4	3
0	0	1	2	7	0	0
3	0	0	8	18	14	6
15	35	12	51	75	33	40
11	8	7	15	0	4	9
2	2	0	0	0	4	3
8	2	19	26	0	29	12
3	6	6	3	0	2	4
0	1	3	4	0	2	2
1	1	0	0	0	2	2
1	0	2	0	0	0	1
0	3	2	0	0	4	0
0	1	0	0	8	1	4
0	0	0	0	0	2	1
4	9	7	10	0	10	0
4	1	5	11	0	1	3
0	0	0	5	0	2	2
35	30	12	33	0	26	13
1	9	1	0	0	1	3
0	0	0	2	1	0	5
14	16	18	27	12	13	14
135	70	83	171	64	204	217
7	0	3	7	6	11	11
0	0	0	0	0	3	0
4	109	21	0	705	43	177
1	5	0	6	0	10	10
0	0	0	0	0	1	4
5	2	11	4	0	0	9
3	2	4	4	1	3	2
5	1	1	21	0	26	12
0	1	0	1	0	1	2
0	1	0	1	0	1	2
4	0	0	3	0	3	0
8	10	13	24	8	7	24
24	5	12	27	0	18	41
0	1	0	0	0	2	1
54	42	87	89	155	88	79

For Review Only

29	20	16	40	11	20	20
5	0	0	0	0	0	0
1	0	2	0	0	4	1
0	2	0	0	0	4	2
0	13	5	13	0	9	9
23	14	27	37	18	12	36
1	0	0	4	0	0	4
12	17	19	29	0	14	5
36	28	13	44	17	30	21
3	2	2	0	7	0	2
8	4	6	0	0	3	0
38	41	37	98	9	61	77
0	0	1	8	0	0	0
2	2	2	0	0	0	1
6	2	18	16	26	7	4
0	1	2	0	0	3	0
10	20	0	17	6	13	7
12	3	15	16	15	10	10
3	5	5	8	15	11	5
0	0	3	1	0	3	3
0	0	0	4	0	0	1
0	1	0	3	1	1	0
39	12	18	52	1	5	29
0	0	5	5	0	1	0
0	6	0	0	0	0	0
0	0	4	2	0	0	0
0	0	0	4	3	1	6
19	11	9	14	6	22	6
4	1	1	20	0	4	19
0	0	0	1	0	3	0
0	0	0	5	10	0	0
0	2	4	1	0	1	0
0	3	0	0	1	0	0
0	0	0	1	0	0	0
27	24	39	37	35	22	20
3	0	0	3	0	0	3
12	7	17	0	24	5	9
1	1	3	1	0	1	3
1	0	0	0	9	5	0
15	13	7	20	19	15	23
1	3	0	1	0	1	4
0	3	0	4	0	0	0
0	0	0	0	0	0	0
0	1	0	2	9	1	0
0	0	0	0	0	0	0
6	3	2	6	12	11	5
0	0	0	4	0	3	0
5	3	5	5	6	7	4
0	3	0	0	0	1	0
2	3	5	16	3	11	1

For Review Only

4	2	11	1	21	0	12
19	46	11	28	1	15	23
0	0	0	0	0	0	1
9	11	5	24	0	14	9
14	9	32	36	23	47	18
1	1	0	0	0	1	0
4	22	6	17	6	17	6
97	243	118	263	37	175	247
0	0	1	5	0	0	5
0	0	0	0	0	0	0
0	3	4	2	0	4	1
1	0	0	0	7	3	6
20	41	28	44	68	39	36
170	132	155	237	129	191	141
2	1	6	38	0	9	37
62	55	56	125	69	85	93
0	0	3	0	0	0	0
6	0	10	8	5	7	9
158	17	10	126	69	58	38
3	1	2	10	3	3	6
0	0	0	0	0	1	0
6	0	2	3	0	0	0
317	299	147	307	106	295	110
432	462	506	961	357	588	687
277	154	197	202	114	218	83
3841	3254	3016	5246	4018	4976	2466
0	0	144	0	0	0	0
46	60	79	109	108	58	68
204	196	226	194	218	149	121
995	873	731	1286	751	851	892
28	5	19	37	0	21	15
124	137	130	214	116	163	170
1	0	0	3	9	9	0
341	391	289	614	127	305	185
185	126	182	191	207	146	259
328	353	307	335	379	251	218
116	98	191	193	162	112	175
0	0	0	0	0	0	0
3	1	4	4	0	4	0
3	0	0	7	0	3	0
2	0	2	12	0	0	4
7	9	21	20	28	12	12
63	66	75	92	86	70	57
2	1	2	5	0	13	7
146	125	107	114	126	113	106
22	11	16	26	18	18	8
0	129	124	18	92	137	106
1948	1047	1157	2708	2398	2971	2191
39	24	177	0	0	10	12
5	0	2	3	0	4	4

For Review Only

57	24	25	55	17	34	19
2	1	0	0	0	1	2
52	30	26	51	51	37	48
152	141	214	198	224	84	91
78	65	60	100	62	55	89
3	0	0	0	0	0	0
434	358	373	569	436	475	275
144	114	50	120	195	100	157
5	2	5	0	0	4	4
129	0	90	0	0	0	0
1	0	4	7	0	2	0
3	3	0	3	0	0	0
22	28	45	31	54	45	24
7	5	11	29	0	12	6
19	17	21	20	35	43	17
227	186	0	109	276	288	0
0	0	0	0	0	2	0
47	15	7	19	12	42	10
9	1	1	0	0	26	24
0	1753	1496	82	1283	0	1409
0	0	0	0	0	0	0
0	0	0	4	0	0	0
33	27	36	48	18	36	36
2	12	10	7	29	3	5
54	26	37	45	57	57	44
226	181	49	83	75	3	32
2	11	16	14	0	7	7
24	0	0	0	0	48	0
40	34	36	23	82	64	7
25	1	30	0	0	1	1
33	23	13	10	35	22	6
65	56	65	78	30	64	37
492	358	545	645	664	613	397
83	121	74	98	147	42	63
78	59	43	113	64	93	57
1	1	0	6	0	1	0
1	3	0	6	0	1	3
2	0	0	0	8	0	2
15	12	4	18	32	3	32
1	1	180	0	0	1	5
5	2	12	6	18	1	2
3	0	1	0	0	6	4
85	88	108	132	167	59	101
1	11	6	0	0	21	9
39	25	52	61	25	48	47
232	216	200	346	286	176	254
37	33	28	60	41	28	24
4	0	0	0	0	2	0
121	76	69	98	127	152	81
2	0	3	0	0	0	0

For Review Only

4	0	0	9	0	0	5
5	1	0	7	19	3	2
7	1	12	5	0	2	6
125	19	3	75	0	100	89
1	0	0	0	0	1	2
125	103	88	181	118	150	84
94	47	71	129	67	83	58
9	0	9	21	2	7	0
67	69	77	134	100	97	28
13	11	2	21	1	6	12
77	52	75	42	13	78	66
31	56	69	74	132	26	108
13	11	28	27	0	5	6
31	52	17	109	0	7	1
35	43	27	61	28	19	33
54	1	1	88	52	1	2
117	92	86	135	90	112	84
0	0	6	0	0	0	13
47	44	32	60	30	44	42
158	144	308	335	111	229	157
1	1	2	0	0	1	1
30	19	20	37	21	29	19
0	67	176	284	20	151	146
108	96	116	191	124	104	92
16	29	8	0	9	9	18
1	1	3	7	4	4	6
0	1	1	8	0	4	0
6	0	0	2	6	5	7
0	0	0	0	0	0	0
121	162	160	195	194	55	104
326	333	0	547	113	400	0
154	0	0	0	0	41	98
105	68	106	147	170	140	132
53	53	54	80	63	102	42
115	100	51	77	121	89	113
123	67	131	197	236	186	209
92	135	108	245	177	92	123
0	0	0	0	0	0	0
1	0	1	0	0	0	1
50	44	37	110	45	55	61
34	10	47	72	25	18	22
9	10	14	59	0	8	3
0	0	2	1	0	0	0
1	0	6	15	0	0	3
2227	6	7	0	892	0	11
6	0	0	10	0	0	5
7	0	1	1	0	1	7
10	0	11	8	4	12	5
1	7	0	0	6	0	1
0	5	0	3	0	0	0

For Review Only

803	1904	2565	3805	1150	2115	1944
59	56	33	108	40	43	96
48	46	45	0	0	0	0
19	17	31	47	23	23	35
0	127	0	212	0	259	0
2322	127	1740	281	179	2455	1769
3	0	0	0	0	2	3
26	21	7	21	12	31	17
46	31	3	106	0	80	142
146	139	68	9	210	0	0
7	2	8	24	0	2	6
25	14	30	23	16	8	17
7	1	1	0	249	1	1
67	67	66	96	56	56	41
22	51	31	56	63	30	18
1	0	2	5	0	6	2
45	52	58	91	44	64	75
38	48	11	58	14	27	37
0	0	0	0	4	0	0
56	0	49	73	56	40	36
23	45	42	50	29	28	22
123	134	159	196	162	58	156
0	0	0	2	0	0	0
66	78	85	103	73	58	59
657	625	641	1175	674	471	477
9	10	0	11	1	0	0
120	81	54	125	94	86	51
33	56	50	59	38	33	39
103	124	83	157	84	156	96
0	4	10	6	9	7	6
10	16	12	21	7	9	7
2	2	0	4	0	1	4
4	0	0	0	0	17	0
755	735	567	1040	546	863	466
0	1	0	0	1	0	1
1019	1374	1814	2430	1031	1282	1689
11	10	6	6	12	6	3
0	0	0	8	0	0	0
36	39	41	86	26	52	34
24	4	25	22	17	6	12
49	36	24	66	13	35	24
33	32	0	63	9	0	15
213	228	195	314	141	204	129
313	292	216	338	310	298	175
238	328	308	579	212	258	303
394	0	0	0	0	6	0
1140	946	958	1289	757	771	719
6	3	0	2	0	3	0
156	73	0	0	0	0	0
74	64	34	64	91	81	68

For Review Only

115	97	60	92	127	85	62
0	0	98	0	0	0	0
133	123	90	197	64	140	47
32	11	4	29	0	17	6
17	8	17	40	9	27	19
1	1	3	5	0	4	3
34	35	35	55	62	49	39
3	6	3	13	8	3	7
14	15	17	84	24	18	27
0	0	0	0	0	0	0
221	243	157	268	216	221	115
1	0	5	5	0	2	0
85	52	82	111	87	68	50
0	0	0	0	0	0	0
7039	8498	9431	13212	6813	7875	7741
42	27	19	76	23	13	43
45	43	49	50	46	32	56
42	37	10	31	38	24	21
46	126	75	164	138	110	77
424	326	411	591	368	472	338
267	239	228	333	166	215	273
6	1	8	11	0	2	6
0	0	0	1	0	2	2
136	71	176	157	224	153	119
350	298	412	434	405	253	347
118	41	211	151	141	63	35
278	250	236	382	195	273	229
11	45	26	32	57	16	47
257	135	123	312	233	210	206
0	1	0	1	1	2617	0
825	811	479	913	939	1045	515
11	62	22	23	90	25	14
0	0	0	14	10	0	0
149	78	106	146	92	170	92
67	72	57	135	89	90	64
62	60	59	74	68	47	54
0	0	1	0	0	0	1
136	191	133	299	107	142	75
0	3	2	0	0	0	3
4	0	4	0	0	0	0
0	0	0	0	0	0	0
40	36	41	26	1	23	33
0	0	0	6	0	0	5
28	6	1	9	0	18	4
82	44	53	93	64	55	36
0	0	0	566	253	0	0
136	271	1	581	394	301	1
552	548	409	652	554	547	371
144	182	98	443	205	46	67
165	138	94	164	174	198	98

For Review Only

7	4	2	15	0	5	11
58	50	65	95	68	57	44
64	100	75	98	110	49	61
32	60	36	36	70	45	29
26	49	40	43	49	33	30
225	141	227	367	321	335	252
5	0	15	17	0	0	0
295	159	183	251	285	182	101
46	24	44	53	85	75	83
3	5	1	8	0	9	5
160	63	91	115	170	144	95
0	0	0	0	0	0	0
21	3	13	16	4	12	20
54	97	71	112	94	63	92
115	117	118	106	213	107	102
91	150	75	127	60	170	67
130	100	114	267	135	104	93
80	61	52	109	25	49	87
65	50	83	159	63	81	50
471	288	345	730	520	658	525
0	0	0	7	0	8	0
129	127	137	91	94	133	106
41	41	38	52	64	49	48
113	109	106	212	94	171	157
31	18	59	39	75	27	33
0	0	0	3	0	2	0
0	0	1	0	0	0	0
13	9	19	18	7	20	7
110	78	65	50	90	0	12
72	105	160	178	191	123	73
0	0	0	4	0	0	2
0	0	0	0	4	4	0
1	4	1	0	0	1	1211
82	90	70	182	98	112	92
191	124	192	184	120	124	133
118	109	0	29	115	0	0
6	0	0	13	0	0	0
67	54	59	104	76	43	76
59	62	32	0	123	54	26
25	50	56	52	87	81	54
3	0	0	0	4	0	0
164	136	187	263	61	204	197
64	56	70	54	64	58	36
39	27	26	70	57	56	52
77	26	50	83	29	65	82
52	40	68	107	30	67	63
0	0	0	23	19	59	27
4	19	47	49	0	75	48
440	367	256	589	347	409	282
180	144	150	244	137	184	169

For Review Only

29	22	18	43	13	15	0
6	1	1	17	0	4	2
247	235	197	351	117	104	134
319	87	93	73	173	320	32
4	0	4	0	0	4	2
32	16	12	20	63	13	11
3	0	4	0	1	3	0
43	34	44	57	108	34	39
75	87	131	187	63	97	144
89	57	65	124	188	215	189
39	62	74	53	100	43	72
3	0	0	0	0	0	0
723	848	1085	1613	708	927	905
31	10	30	20	36	9	13
101	81	183	223	354	184	282
0	0	0	0	0	0	0
114	94	85	189	132	96	70
86	109	109	197	70	92	85
24	43	30	21	55	14	15
130	31	4	112	0	14	12
11	18	32	54	1	33	11
11	2	5	11	21	3	0
19	25	28	28	31	38	7
0	0	0	0	0	1	0
30	11	20	27	29	26	19
57	15	1	46	0	3	36
31	47	33	69	48	58	19
94	411	125	197	275	184	148
59	49	57	94	25	55	29
1	7	5	0	0	0	4
602	817	533	874	725	422	287
252	156	205	302	268	173	157
0	44	0	46	0	0	0
0	0	2	0	0	2	0
162	160	310	248	259	235	152
42	185	104	131	0	675	66
303	272	64	134	270	149	94
52	49	56	89	76	64	67
68	58	63	99	64	28	54
70	73	81	145	95	88	57
3	1	10	0	0	8	13
151	128	2	102	84	182	48
5	2	7	19	10	15	26
0	0	18	6	136	36	26
31	10	6	25	0	40	15
51	49	44	45	34	45	48
84	68	84	73	127	17	23
30	45	53	35	38	59	54
13	7	31	9	29	15	9
3	1	1	0	0	1	1

For Review Only

276	254	150	399	127	71	159
276	292	166	401	441	238	346
1	0	2	6	0	0	0
31	25	27	64	22	31	20
0	0	0	1	0	0	0
164	563	93	420	40	2	3
42	64	37	104	24	53	61
56	65	56	106	58	69	58
178	150	135	230	148	265	160
45	40	49	72	31	36	72
0	1	0	1	0	0	0
37	34	47	36	67	40	15
4	2	6	6	0	6	4
74	40	36	105	49	68	46
0	0	0	0	0	4	0
49	46	24	83	77	65	15
8	9	25	60	15	20	10
6	0	0	0	0	0	0
0	0	0	3	0	2	1
389	402	371	613	395	409	212
0	195	0	0	0	0	0
31	13	32	20	66	38	3
156	155	142	253	166	239	114
80	91	84	124	76	94	109
48	41	29	28	70	32	9
16	17	47	41	0	60	1
32	33	48	62	55	41	38
3	1	20	67	0	7	9
15	4	11	19	25	14	11
99	237	172	236	403	156	99
2	1	2	6	10	4	4
0	0	2	0	0	27	0
26	29	16	38	18	17	15
71	32	15	145	14	66	8
68	60	59	82	67	78	100
51	65	72	101	115	68	52
14	19	8	27	33	52	12
25	22	12	37	0	13	8
103	88	135	198	140	103	150
123	153	113	153	155	135	112
1	1	16	6	22	1	9
4	1	1	11	0	2	0
0	5	6	20	8	6	5
2	1	0	0	0	0	0
356	210	235	462	187	209	156
6	187	16	0	112	250	164
0	4	0	3	5	0	0
10	9	8	16	0	7	7
153	170	150	196	155	184	115
351	460	479	522	472	393	537

For Review Only

64	26	287	176	0	76	38
0	0	0	49	1	153	0
4	1	0	0	0	3	3
42	37	44	55	37	32	29
0	39	0	0	21	0	0
58	95	77	128	51	75	58
0	0	0	0	0	0	0
75	83	52	103	155	76	117
25	26	20	25	37	48	25
280	203	234	360	267	273	220
84	70	58	149	76	56	58
0	0	0	0	0	0	0
120	96	82	170	84	150	64
210	222	326	392	358	196	263
63	101	112	185	63	113	173
177	185	172	300	126	239	179
60	20	78	62	69	41	75
113	134	128	187	195	184	135
34	48	25	45	76	68	17
171	164	127	254	134	63	84
27	13	4	22	0	22	1
30	19	28	26	31	23	11
110	103	100	141	90	132	43
61	54	60	97	40	94	53
0	267	0	0	0	0	0
124	70	71	142	80	97	136
81	93	38	125	37	75	45
86	126	74	89	131	90	60
69	50	58	56	75	39	46
54	61	53	116	77	58	52
169	165	154	294	253	194	142
40	26	39	74	48	54	39
45	41	41	39	13	0	34
24	8	21	59	8	16	10
0	43	80	9	2	0	0
52	44	48	53	42	39	46
95	77	140	177	88	117	103
0	0	48	16	0	42	0
93	59	33	89	112	48	54
559	412	813	912	785	707	787
1017	654	892	1322	956	786	562
113	96	87	157	184	113	0
1	0	0	4	5	3	6
1	3	18	22	0	15	32
115	88	81	156	103	142	66
11	8	6	9	16	10	11
8	9	7	24	0	18	39
64	51	52	62	76	41	33
0	0	0	4	0	6	0
84	59	74	121	98	74	51

For Review Only

97	99	75	113	76	94	47
0	1	0	0	17	0	7
51	102	60	99	36	60	41
2048	1983	1485	2827	2381	2505	2244
10	12	9	9	30	1	13
234	287	271	394	151	182	211
11	2	1	14	0	1	0
0	3	1	0	0	0	0
72	118	90	107	44	101	27
152	120	108	137	134	49	77
253	171	250	257	257	185	140
2	1	2	0	0	2	4
1	0	2	1	20	1	14
27	13	23	21	0	7	4
13	23	15	34	27	19	17
56	28	12	34	26	42	18
42	39	63	72	77	65	36
26	3	14	26	0	12	20
73	76	64	115	86	87	55
45	0	0	0	38	35	24
23	25	1	54	10	33	16
52	23	55	98	43	55	25
10	13	16	16	13	16	6
136	147	106	158	141	126	81
0	4	0	0	0	0	0
0	27	0	0	14	0	38
14	0	0	15	0	0	0
39	37	45	59	58	55	37
1	0	1	113	0	6	0
29	32	27	72	15	42	55
24	15	34	44	24	40	38
53	50	44	70	55	43	44
50	56	47	81	55	55	53
10	7	7	10	10	6	6
4	5	3	6	4	0	3
23	30	55	81	0	34	13
28	51	54	53	34	34	23
4	4	6	6	0	16	11
16	5	24	22	6	12	20
14	10	62	44	0	25	19
7	3	4	7	0	0	1
28	127	126	146	3	130	83
83	56	70	131	62	96	40
0	0	0	0	0	3	1
111	166	322	438	487	465	666
5	2	2	1	0	5	5
192	137	190	306	171	187	152
22	10	15	0	66	10	1
86	70	66	137	194	71	102
28	8	29	34	67	22	32

For Review Only

126	90	29	117	29	109	30
176	18	8	79	187	447	46
751	493	311	568	784	812	416
15	0	0	0	0	0	0
355	45	0	1	1	0	0
139	102	120	156	184	109	100
251	139	143	262	71	131	104
226	252	206	342	118	84	233
1	0	0	0	0	0	0
0	1	6	0	0	0	0
45	25	29	83	49	60	20
6	24	8	42	11	15	1
2	0	0	0	0	2	4
0	0	0	8	0	0	0
92	163	144	168	242	47	92
1	2	0	5	0	0	2
1	2	0	0	1	2	0
10	21	10	31	5	16	5
10	23	22	31	40	17	24
2	2	0	3	0	3	0
26	21	28	37	25	40	46
3	3	28	20	0	5	20
87	118	112	167	104	129	110
549	369	374	623	608	630	286
105	123	140	191	171	63	127
1	1	0	0	0	8	3
1166	1009	1146	1916	568	1227	1036
24	34	37	48	70	8	4
290	313	237	398	301	269	390
162	140	173	237	176	181	143
0	3	2	0	0	0	0
187	178	219	292	287	259	207
147	157	111	237	105	119	85
14	0	8	27	0	0	13
235	15	75	395	0	152	159
1	0	0	21	0	1	0
20	0	15	20	0	0	11
201	174	161	225	215	183	96
0	0	0	0	0	0	0
40	41	65	63	57	54	44
32	7	7	0	0	1	4
30	7	24	28	46	179	6
0	0	0	0	0	3	0
50	82	63	101	125	51	111
33	44	35	48	91	66	44
96	121	62	175	84	84	57
74	48	76	65	91	33	42
29	11	37	35	69	41	29
0	0	84	191	90	140	66
0	0	0	120	0	0	366

For Review Only

36	16	54	77	53	51	34
94	72	62	146	45	60	55
36	16	29	26	25	40	33
0	0	0	1	1	87	0
96	117	117	181	89	114	100
6	76	34	42	24	3	13
58	55	53	81	118	39	40
71	16	6	129	0	65	81
0	59	3	0	0	3	0
0	0	0	38	64	118	0
0	0	0	0	0	0	0
35	32	60	65	27	28	28
54	27	30	68	35	58	50
48	40	41	59	72	43	19
60	1	62	176	0	117	289
158	168	155	184	210	80	110
103	45	67	46	103	41	22
16	0	19	13	7	0	17
19	16	0	2	0	0	0
27	1	1	8	0	1	25
0	0	0	0	0	0	0
3	0	1	0	0	0	2
4	7	3	8	0	5	8
1	6	0	3	0	1	4
3	8	4	12	12	5	8
0	0	0	0	0	10	27
18	18	0	0	0	25	19
1	1	1	0	0	3	2
0	0	0	0	20	0	0
0	0	0	0	0	0	0
1	0	0	0	0	0	0
0	0	4	0	0	0	0
1	1	0	0	0	1	2
0	4	3	0	0	0	3
6	3	9	14	9	10	6
5	1	1	1	2	1	0
4	0	3	0	0	0	0
0	0	1	0	0	0	1
0	0	1	0	0	0	0
8	5	7	5	6	6	1
0	0	0	0	0	0	0
0	5	3	0	0	2	4
0	1	2	1	0	0	1
7	3	4	9	0	7	7
0	0	1	0	0	4	1
0	0	0	0	0	1	0
0	0	1	0	0	3	0
0	0	0	2	0	0	0
0	5	0	0	0	21	3
28	39	48	54	72	21	63

For Review Only

8	0	2	0	0	0	1
27	21	0	0	0	0	14
2	6	5	5	6	1	11
2	2	1	0	0	0	3
0	0	0	0	8	0	0
17	2	12	30	0	14	11
0	0	0	0	0	6	0
181	161	288	43	354	6	5
0	0	0	1	0	0	3
0	2	1	0	0	0	0
1	1	0	3	0	1	1
2	0	0	0	0	2	1
2	2	2	13	1	2	2
47	35	42	80	34	38	44
20	16	16	33	16	17	17
23	21	16	36	15	22	18
0	0	3	1	0	0	2
2	0	0	0	0	2	2
0	0	0	5	0	0	0
1	1	0	1	0	1	0
0	0	0	2	1	0	0
4	2	6	5	4	2	5
1	8	2	0	0	5	1
0	1	0	1	0	0	0
3	6	0	7	6	0	3
0	0	0	0	3	3	0
0	1	2	0	0	3	1
1	1	0	0	3	0	0
2	1	0	1	1	1	1
6	4	6	0	0	10	6
1	0	0	0	3	1	1
0	1	0	2	0	0	0
36	53	63	196	94	8	103
2	2	3	0	0	4	3
3	2	1	0	1	0	3
0	3	1	3	0	1	3
11	10	5	11	9	17	2
1	9	2	9	0	7	10
0	6	2	0	0	12	8
20	0	9	1	0	12	0
2	19	1	0	1	24	1
122	143	140	0	0	208	11
2	1	1	0	0	0	1
0	8	0	2	6	6	0
2	3	3	0	0	4	3
23	10	28	4	0	7	2
25	10	20	16	14	14	28
4	0	0	4	0	0	3
0	0	0	0	6	0	0
201	175	114	154	189	154	68

For Review Only

12	28	14	39	15	13	33
2	0	0	0	0	0	2
48	26	28	30	52	38	27
64	94	104	102	54	46	103
16	3	6	16	0	9	36
50	47	57	77	38	48	28
137	98	106	160	172	145	124
85	47	58	81	99	91	102
1	5	1	0	0	1	1
0	0	121	3	3	316	52
94	66	3	512	85	1	1
112	113	126	212	175	75	99
38	45	44	65	34	69	26
1	1	3	0	0	1	2
59	19	27	48	47	41	39
425	453	348	689	410	463	426
0	144	154	73	411	0	120
54	45	50	84	33	58	66
21	7	14	37	11	31	34
6	0	0	2	0	0	0
43	26	0	57	65	41	49
72	93	85	126	83	74	80
26	41	35	41	17	38	21
4	8	6	6	10	6	7
17	40	9	26	0	13	20
43	34	39	73	22	68	41
284	534	7	225	567	305	145
63	35	50	18	103	41	25
0	0	72	67	1	60	0
2	2	0	0	0	9	0
30	0	336	1	4	420	0
46	72	21	59	139	109	19
81	93	60	148	89	96	104
51	26	21	37	58	38	8
30	47	50	49	24	48	23
38	23	1	0	97	73	25
21	43	28	54	64	39	37
60	64	56	102	54	98	63
77	91	74	203	91	91	88
7	11	14	11	17	18	11
46	27	43	86	64	35	49
7	11	4	9	0	0	0
0	0	0	0	0	0	1
0	1	2	0	0	1	0
0	1	0	20	0	6	0
146	107	96	0	0	90	93
13	0	0	0	0	7	7
0	5	0	4	11	0	0
9	15	4	23	0	32	14
10	0	0	0	0	0	0

For Review Only

12	0	0	19	0	0	3
0	4	4	4	1	5	7
71	0	0	0	0	38	0
18	17	17	35	0	0	17
4	2	0	10	0	8	4
43	20	13	54	10	22	22
1	1	1	11	0	1	1
0	9	7	6	0	0	0
0	7	4	1	0	1	0
57	34	51	71	79	76	33
0	0	45	0	0	0	0
598	602	592	1127	551	406	515
26	4	30	21	0	10	2
0	6	18	10	0	0	0
387	317	252	504	246	304	195
11	1	2	5	0	9	4
1	1	1	0	868	1	1
95	127	186	208	236	198	77
2	0	0	4	0	6	0
0	5	2	4	0	0	0
28	35	11	0	17	61	12
57	52	44	94	46	59	40
2	3	2	1	31	1	1
40	144	58	150	88	29	4
4	0	0	0	0	0	4
76	30	37	73	35	58	52
67	99	47	114	99	69	32
110	114	201	230	84	115	120
65	88	91	136	196	118	121
0	0	222	0	0	0	5
96	58	92	146	149	95	113
105	80	108	149	137	114	94
98	78	92	129	123	82	83
180	171	193	307	85	156	112
40	34	18	66	113	131	46
0	7	6	0	14	4	0
1	5	3	5	8	4	5
60	20	26	45	44	22	41
19	10	25	65	0	31	94
17	19	9	32	5	9	14
38	39	57	83	29	53	44
68	67	100	153	74	101	94
57	54	48	78	69	71	34
18	0	0	0	0	17	0
0	0	0	0	0	0	0
0	0	0	2667	2001	0	0
30	26	25	6	6	0	25
54	35	24	59	32	43	19
19	8	12	36	0	16	51
21	13	48	63	41	50	7

For Review Only

47	23	37	71	39	36	30
19	256	0	184	0	33	39
16	13	7	38	44	28	18
0	0	0	0	72	9	0
0	0	28	0	0	0	0
99	41	45	68	47	38	54
12	0	0	0	0	0	0
42	31	47	56	34	49	33
5	14	18	30	40	6	31
23	14	17	12	9	12	17
40	28	26	54	19	59	13
152	113	153	192	160	107	107
0	0	1	0	13	3	4
1	0	1	0	0	0	0
16	13	17	18	16	14	3
28	27	68	118	65	33	78
0	0	0	0	0	0	1
44	42	34	44	36	93	58
26	49	52	69	36	51	35
21	22	18	11	5	1	10
58	56	61	94	99	43	29
15	44	43	59	0	54	52
0	0	0	0	51	0	0
47	20	48	93	46	41	40
0	0	2	0	0	0	0
63	16	18	48	8	19	20
3	4	3	4	0	2	3
75	21	38	47	74	25	27
19	9	15	27	0	8	8
227	137	83	213	199	158	180
0	0	0	5	0	0	0
12	0	1	0	0	15	2
32	13	22	43	12	33	21
102	77	69	112	78	100	60
32	44	24	0	0	37	33
36	43	46	116	66	65	51
5	9	12	43	21	42	14
26	22	48	69	27	36	14
95	75	77	156	87	77	83
51	35	51	80	73	63	26
189	118	84	74	178	130	16
63	69	63	75	44	40	52
41	37	34	50	87	30	54
26	0	7	5	10	0	0
87	49	70	122	41	85	48
21	23	49	47	61	12	39
25	19	39	62	27	27	21
38	30	36	54	32	40	37
0	0	1	17	1	74	13
25	17	16	55	9	42	11

For Review Only

53	41	38	68	41	50	35
94	64	56	147	58	21	38
52	65	49	73	62	70	54
45	33	39	0	0	45	34
17	25	22	43	14	21	30
1	1	26	0	0	1	1
55	35	38	57	31	58	29
0	0	0	73	0	0	0
19	88	56	103	45	64	46
77	61	39	0	0	69	37
45	49	45	96	20	21	50
0	0	0	28	1	17	0
66	59	76	95	72	58	55
18	9	17	48	18	20	14
44	46	0	109	0	58	39
41	0	0	0	70	0	0
99	60	91	112	71	52	41
33	42	33	92	66	85	48
51	26	41	50	52	53	0
23	0	23	28	0	0	0
28	31	25	55	36	34	32
0	0	0	7	1	0	0
87	85	69	124	55	84	63
1	14	6	0	0	1	1
19	40	36	102	0	36	22
0	0	0	0	1	0	0
32	17	15	40	32	26	17
67	0	0	5	14	55	48
0	0	0	0	0	0	0
91	91	87	158	51	98	40
27	18	30	77	60	41	43
2	50	10	91	0	37	28
41	3	108	0	229	2	1
0	0	35	16	1	0	0
4	3	1	50	27	36	7
14	11	12	361	0	19	13
1	53	4	147	0	106	9
41	0	0	1	2	0	0
0	0	44	40	0	56	0
0	0	0	0	1	0	0
29	44	13	0	42	11	24
23	29	27	43	0	23	0
0	0	89	75	12	0	0
2	15	4	0	0	1	0
52	117	80	171	67	60	60
39	57	41	83	38	46	47
10	19	29	50	12	21	23
80	65	71	149	62	67	39
47	16	37	95	23	37	17
73	64	55	72	78	43	43

For Review Only

0	0	0	0	0	0	0
78	33	61	91	38	53	33
7	11	13	24	13	9	10
45	33	32	80	48	68	17
49	76	32	58	85	32	17
83	59	64	85	92	108	56
79	29	45	122	47	55	41
18	20	10	34	20	31	16
82	90	85	121	89	63	92
37	13	0	32	14	16	12
1	0	0	0	0	7	0
1	1	5	0	10	0	0
0	0	5	0	0	0	5
134	109	145	0	0	153	122
20	33	71	39	59	25	69
149	191	266	370	165	213	224
0	0	0	0	29	0	0
3	7	17	24	0	18	24
66	39	16	26	16	15	28
69	70	39	51	78	64	26
57	35	27	90	24	74	48
17	22	0	8	20	13	11
0	5	12	11	6	8	16
99	47	51	79	101	53	58
9	19	27	38	33	19	14
0	0	0	15	1	0	169
66	65	121	154	74	100	140
78	106	81	108	71	72	55
0	1	8	8	20	7	6
1	0	0	10	0	0	1
57	76	75	97	90	101	51
916	856	749	1141	1288	1426	665
1	0	1	0	0	5	0
46	50	37	72	53	60	51
0	0	2	0	6	3	7
199	207	183	306	143	179	128
1	0	0	0	0	0	0
1	0	0	0	0	7	2
1	0	0	0	0	2	0
1	1	1	0	0	1	1
10	0	0	0	0	0	0
16	0	3	12	0	0	0
82	33	0	0	57	71	57
0	0	0	0	0	0	0
0	24	30	23	48	23	34
0	0	18	0	0	0	0
48	37	66	48	60	75	54
54	60	58	81	102	82	69
0	3	0	10	0	0	4
18	0	0	0	0	0	3

95	108	84	20	302	63	98
232	147	138	270	101	108	98
2	4	0	0	5	9	7
279	165	616	453	626	265	504
9	11	2	11	8	14	5
10	6	2	6	7	7	8
4	0	0	0	0	1	0
33	24	137	69	23	79	55
45	42	22	63	32	45	31
46	39	45	0	0	42	50
62	65	52	106	86	108	87
38	35	20	44	43	40	40
21	11	7	15	0	3	1
29	18	26	39	20	28	18
234	233	206	364	174	299	193
39	22	16	0	31	10	46
36	27	0	0	56	0	0
83	59	23	67	87	65	55
0	15	13	18	3	21	10
39	29	68	59	68	40	39
0	0	6	0	0	0	1
148	76	88	76	168	91	54
0	0	0	0	0	0	0
48	39	32	64	22	55	14
46	28	56	66	51	73	75
87	67	36	76	90	56	49
111	91	110	182	104	155	113
0	0	0	2	0	0	0
17	14	7	28	0	40	17
67	14	37	53	26	26	18
80	18	79	100	0	145	8
11	0	9	6	3	7	5
166	71	61	96	48	89	64
76	82	55	71	69	72	36
229	184	145	249	190	170	168
173	182	151	248	191	163	150
93	130	86	194	197	207	66
433	364	153	283	209	400	347
0	0	2	1	0	2	3
53	31	34	56	42	32	37
82	66	63	52	63	72	69
91	80	71	151	87	120	93
6	10	1	110	39	20	183
363	309	268	466	360	374	206
105	89	157	154	190	178	144
11	14	14	15	18	21	18
8	17	6	27	0	8	11
25	32	19	48	15	40	38
78	84	80	203	156	196	135
1	2	0	0	0	1	0

For Review Only

17	10	22	30	30	18	20
2	4	0	6	0	3	4
65	78	166	189	176	134	90
11	24	9	6	16	7	14
77	85	78	88	102	38	39
27	17	47	56	24	57	28
4	11	1	5	0	0	3
1	6	1	2	0	1	0
17	1	1	0	17	1	1
9	2	5	17	0	9	24
60	56	82	75	126	62	86
92	77	50	103	133	111	79
44	19	28	64	18	56	32
0	0	0	0	0	0	0
1	0	0	0	0	0	4
85	32	46	73	123	63	43
0	0	5	22	0	8	4
1	1	4	207	89	1	1
307	317	407	493	473	345	291
20	29	6	32	0	27	3
12	14	9	23	34	18	0
1	1	1	0	0	1	1
11	31	25	32	0	23	12
35	42	31	72	11	33	27
48	38	54	35	53	24	15
1	1	0	0	37	1	3
85	50	74	139	86	67	60
6	1	3	9	0	2	2
7	0	0	0	0	0	0
296	421	299	538	270	323	292
58	21	37	63	50	66	42
148	143	137	173	219	260	136
3	0	0	0	0	1	0
0	0	0	3	0	0	0
0	10	0	0	4	0	14
153	12	98	88	9	195	139
0	29	0	5	0	0	20
500	487	597	768	430	510	510
30	17	36	17	24	6	14
0	0	0	0	0	0	0
204	580	94	346	190	205	11
64	32	37	42	43	37	32
94	86	89	143	81	52	60
11	6	20	19	10	15	3
5	5	0	8	0	6	2
287	231	108	379	295	281	239
265	188	223	435	277	297	174
290	279	252	457	353	303	225
0	0	15	0	0	0	12
5	0	0	0	0	0	7

For Review Only

0	0	0	0	0	0	0
995	1092	610	1288	400	454	501
0	0	0	0	0	2	0
68	31	61	49	64	78	32
1	1	4	0	0	0	0
4	4	4	8	1	3	10
244	191	202	346	239	287	210
66	52	33	113	90	65	70
4	23	7	23	0	28	25
50	99	94	165	197	209	173
154	93	93	187	156	219	127
7	0	11	39	0	5	7
54	46	39	57	25	45	16
715	531	318	689	584	720	404
16	39	100	83	274	138	34
0	0	0	0	0	0	2
14	6	35	17	45	15	6
6	0	0	10	0	0	0
36	12	15	40	30	17	18
179	220	142	218	355	240	153
128	95	183	159	168	80	86
166	245	212	327	311	233	275
0	1	0	0	0	0	1
0	0	0	0	0	0	0
11	17	8	13	17	25	29
1	0	0	0	0	1	0
0	0	0	0	3	0	1
6	6	11	7	3	5	3
4	0	0	0	0	0	0
265	220	199	306	290	257	211
2	0	3	0	0	0	0
14	10	7	16	10	20	8
64	48	57	65	60	58	52
0	0	0	204	1	56	70
128	149	179	246	139	182	114
26	21	14	27	33	8	32
119	79	130	214	109	138	131
184	207	137	286	127	167	168
2	2	1	9	0	6	3
17	11	14	21	18	20	11
60	47	64	72	54	68	38
58	84	94	132	48	49	61
43	66	87	137	76	68	52
1	1	1	0	238	56	1
140	85	103	161	136	123	88
18	34	42	55	14	30	26
83	0	78	0	43	58	0
0	60	0	64	0	0	0
15	3	143	0	39	99	4
75	94	56	79	82	35	36

For Review Only

252	33	167	33	336	308	36
140	87	44	161	117	86	53
87	46	98	133	103	80	121
0	0	0	0	0	0	1
19	26	12	17	36	24	18
4	28	21	23	46	19	4
40	3	8	30	42	25	26
13	0	9	17	13	9	16
171	186	102	229	147	160	120
0	5	0	4	0	0	0
52	54	29	91	6	34	52
187	163	146	196	143	140	92
236	131	0	0	205	193	0
29	25	26	22	47	21	28
47	25	26	24	12	0	26
77	77	71	62	154	82	60
7	1	4	0	12	3	0
340	254	252	556	272	353	242
57	3	14	149	119	1	74
1336	95	198	0	953	1	7
151	603	371	776	346	249	133
52	38	30	47	48	43	31
28	24	32	41	30	28	23
200	237	198	276	232	318	206
282	206	183	353	175	233	239
132	113	59	307	33	127	132
136	55	48	83	46	68	40
6	12	2	13	17	8	5
19	15	24	31	23	27	13
46	55	40	70	74	55	22
41	35	53	65	27	29	45
288	199	200	340	258	238	205
123	0	0	225	26	493	307
13	13	10	27	7	10	21
1	7	2	0	0	1	4
73	62	111	69	134	117	21
0	0	3	0	0	0	11
184	195	205	212	66	144	144
0	0	15	0	0	0	15
0	0	0	1	1	0	0
25	36	35	45	30	26	28
4	12	9	24	0	3	16
11	10	21	24	12	9	3
32	41	18	55	0	87	14
6	0	1	4	0	3	3
26	32	32	52	36	21	13
70	60	53	97	91	75	52
6	12	7	25	0	17	5
0	3	2	0	0	1	12
37	24	19	61	12	35	26

For Review Only

9	0	4	10	0	0	31
168	131	91	191	87	170	86
4	6	14	0	0	15	42
252	212	188	497	358	423	517
566	503	669	953	848	634	886
82	83	44	112	53	88	69
46	47	29	77	58	67	42
106	151	141	156	112	90	88
161	181	99	202	231	321	188
6	9	6	10	18	11	7
32	28	69	76	36	45	9
231	249	251	426	232	251	187
3	0	0	0	0	1	0
0	0	0	0	0	1	0
0	11	29	0	24	12	0
38	14	48	76	56	42	52
3	8	0	10	0	0	15
63	37	66	111	100	99	46
180	0	0	0	0	19	0
100	90	91	142	69	90	63
134	95	81	177	34	69	23
165	132	70	160	185	124	130
40	48	37	73	37	45	55
76	13	16	44	82	34	51
524	1325	619	2709	1099	1263	77
419	384	324	535	402	491	292
0	82	0	97	0	0	27
96	114	99	169	124	115	78
0	1	2	6	0	0	0
1780	1478	1407	1117	1896	1345	1026
3	0	0	1	0	0	0
469	23	39	145	333	141	50
0	0	0	1	11	0	7
0	0	0	0	0	0	0
188	248	65	204	243	375	78
0	0	3	0	0	0	0
43	31	27	74	14	33	35
123	105	182	209	226	163	218
607	780	774	1059	773	645	609
230	132	72	71	177	100	69
34	42	28	46	37	62	36
60	72	82	162	45	60	76
15	5	7	10	0	14	12
196	306	245	480	369	210	301
155	255	185	243	299	231	131
121	108	47	148	87	127	87
247	155	142	296	312	508	154
4135	2843	3131	5407	2686	3591	3661
272	224	224	445	212	253	240
92	55	56	189	19	35	94

For Review Only

5	5	5	5	79	23	116
1094	761	833	1533	819	935	934
2959	1429	1400	3315	969	2352	1838
42	62	31	48	0	13	18
146	115	86	100	118	104	56
181	192	147	273	168	203	110
459	435	362	604	592	506	356
107	160	143	172	167	97	95
0	0	2	0	0	1	0
22	16	20	66	32	32	19
0	18	0	0	0	0	0
0	0	2	0	0	1	0
48	9	1	25	0	3	1
226	197	117	219	276	274	165
0	0	0	0	0	5	0
65	50	139	134	50	78	93
56	23	3	284	0	48	0
91	65	0	129	8	77	64
1	1	242	379	0	1	9
240	1	63	132	0	72	5
1	1	1	0	0	1	1
172	120	157	255	147	128	104
0	123	69	27	46	0	0
0	0	0	0	0	0	0
203	162	169	341	133	159	152
38	0	0	64	91	79	41
43	83	48	115	96	31	80
5	76	105	161	1	107	115
94	76	57	194	64	89	84
128	113	139	228	236	158	194
35	30	40	64	5	35	29
44	24	51	55	51	26	17
0	14	20	26	12	0	13
27	33	47	75	45	29	31
0	9	9	15	0	17	0
12	17	0	25	0	0	0
0	53	63	0	83	0	0
4	1	1	0	0	2	1
63	4	165	0	114	27	123
35	0	0	33	0	32	0
88	56	91	136	57	72	66
30	0	31	46	0	0	25
64	62	97	150	65	72	51
77	10	27	102	0	11	3
78	0	0	108	72	73	54
158	100	92	216	86	79	83
0	0	0	12	1	0	0
0	0	21	0	0	0	0
124	106	108	190	80	88	139
156	71	100	241	139	184	110

For Review Only

136	201	125	389	172	219	103
0	0	0	5	5	0	0
63	65	58	113	86	65	66
26	23	16	51	26	25	27
0	0	0	0	0	0	0
76	43	60	227	111	133	72
196	211	144	0	0	165	148
0	0	0	0	10	0	36
24	114	170	289	156	1	136
113	74	86	170	52	120	42
566	161	332	266	422	274	442
0	0	5	0	0	0	6
0	0	0	0	0	0	0
4	0	0	0	0	0	5
6	0	0	0	0	4	1
89	80	117	156	33	128	79
374	301	297	588	339	437	215
116	92	63	118	75	60	64
0	0	0	0	0	3	0
139	148	103	173	107	168	81
28	51	28	29	24	34	31
11	0	0	0	0	1	1
19	53	30	74	33	20	27
109	66	125	192	98	74	121
4	0	5	7	12	3	0
2	0	3	6	0	2	0
25	12	29	26	34	42	12
0	0	0	0	0	2	0
0	0	0	0	0	0	0
25	15	27	27	39	37	34
0	0	4	0	0	0	0
10	8	10	13	32	18	20
13	26	31	46	57	59	11
9	0	6	4	0	0	1
548	394	401	675	572	719	455
154	111	147	209	148	102	167
5	11	0	0	17	14	0
116	144	132	196	92	102	126
0	0	141	0	77	39	0
67	21	23	0	0	6	12
87	82	59	138	153	83	38
23	29	42	61	7	25	22
137	76	103	152	86	98	85
44	19	35	54	10	15	26
4	9	11	17	0	0	10
26	26	28	43	33	21	14
2	4	3	0	9	11	4
2	2	0	0	0	0	0
4	3	0	0	0	0	0
0	0	0	0	0	0	0

For Review Only

6	20	6	11	0	32	5
177	174	171	265	178	205	124
7	1	4	0	5	2	3
12	1	5	7	11	1	4
0	0	0	0	0	0	2
115	73	71	93	15	94	46
0	3	0	4	6	0	4
131	109	194	245	243	210	186
126	87	68	134	125	119	70
803	1144	782	1170	1143	1101	774
5	0	0	0	0	0	0
143	153	251	295	103	102	105
523	508	373	762	593	532	561
187	253	145	297	220	202	145
1	7	1	0	13	3	5
54	132	123	131	75	97	73
3	0	1	6	5	1	0
144	157	88	111	131	104	92
678	652	515	952	359	503	453
3	0	0	0	0	3	2
159	157	200	259	306	237	159
4	0	0	0	0	0	2
168	62	119	162	178	215	135
19	14	4	19	2	7	9
37	48	47	59	52	41	36
323	356	280	493	316	336	246
79	78	72	102	108	72	0
531	243	279	491	439	392	361
75	38	0	95	80	0	73
90	0	0	29	2	0	0
4	78	30	0	0	274	1
47	30	32	38	34	11	18
158	76	42	68	99	136	69
87	94	106	119	51	97	50
82	72	100	128	114	116	55
124	67	94	132	77	83	56
194	131	170	272	193	184	181
16	24	39	32	84	24	31
20	72	75	75	126	81	47
50	23	29	45	31	36	34
63	66	48	101	36	85	49
0	73	61	167	8	0	0
18	10	10	19	12	2	10
64	84	68	104	77	73	43
61	0	28	380	408	8	222
75	73	87	144	67	66	82
8	9	12	12	19	12	3
0	6	8	11	14	3	3
98	97	104	158	115	118	105
46	18	32	42	70	63	60

66	65	86	125	97	81	63
89	91	73	134	97	79	64
4	0	5	11	0	8	2
0	1	0	0	0	0	2
136	86	68	104	128	134	141
0	40	0	74	79	0	34
0	0	0	2	0	0	0
104	16	29	62	0	41	163
53	32	36	64	30	36	35
29	60	39	132	110	92	42
5	0	4	0	0	5	0
25	24	36	30	15	17	17
130	15	13	449	0	1236	94
44	12	46	57	35	27	27
4	303	1	0	0	1	1
3	15	3	22	18	3	8
126	150	141	104	131	63	76
66	46	40	81	34	54	38
185	258	252	415	277	170	175
17	10	11	23	16	11	8
124	75	64	125	63	59	73
1	1	1	0	0	1	153
0	0	0	0	0	0	1
41	48	27	51	58	40	39
62	61	34	73	75	47	53
1	6	0	9	9	8	2
2	0	0	0	0	4	5
1	27	2	66	0	2	1
6	13	7	0	0	1	1
14	0	7	14	0	2	5
165	87	105	204	226	76	178
16	0	7	16	36	6	0
2	3	7	19	0	9	5
18	27	43	64	25	43	39
66	35	36	61	42	42	37
8	10	4	11	1	9	10
2	0	1	0	0	0	2
31	16	20	44	17	35	11
96	48	36	76	129	17	73
105	20	401	383	229	108	56
36	0	17	0	310	29	33
78	99	118	104	231	151	104
88	51	28	28	48	42	30
5	12	4	19	0	37	8
113	40	55	88	116	93	81
107	79	66	125	75	89	47
92	63	96	160	88	76	67
3	5	3	0	16	7	16
14	37	19	41	10	36	16
2	12	10	45	10	8	15

19	1	62	0	0	26	63
14	12	10	10	17	15	6
16	1	6	0	22	11	13
4	11	11	77	0	99	16
8	3	8	12	0	5	13
130	160	179	280	140	207	106
6	177	55	118	108	4	14
10	6	5	31	16	13	15
141	66	117	133	67	83	51
56	68	53	63	75	43	62
13	0	11	54	1	0	10
42	85	20	73	80	13	37
19	64	7	18	31	6	11
36	5	1	64	0	7	1
2	0	1	0	0	1	1
77	70	56	84	75	74	73
5	8	11	18	0	8	5
21	15	14	30	21	14	10
67	110	75	120	40	76	55
32	33	29	70	28	58	16
66	89	59	95	89	45	96
73	51	40	120	45	107	53
0	0	0	0	0	0	0
27	19	34	37	29	40	19
43	45	38	62	51	64	49
99	64	95	113	113	114	49
60	43	31	81	49	62	48
94	172	96	99	0	37	11
0	25	163	0	125	0	126
3	2	84	211	0	1	1
56	65	59	42	37	60	51
82	68	90	132	54	67	34
46	0	26	0	0	54	25
0	0	0	0	0	0	0
101	66	66	120	44	83	51
44	60	30	62	70	20	32
0	0	0	2	0	1	0
122	123	164	222	141	157	143
0	0	1	3	0	0	0
0	3	0	0	10	0	0
0	110	0	9	0	84	0
0	2	0	6	0	0	0
246	273	248	371	272	227	242
854	645	651	967	735	625	563
38	30	21	49	34	19	24
0	0	1	0	0	0	0
22	30	28	21	43	32	27
3	3	0	0	4	6	0
14	12	17	37	22	10	17
69	21	47	83	69	61	59

For Review Only

352	327	313	518	310	333	387
1384	1199	1053	1789	1339	1446	861
0	0	0	8	11	6	7
0	1	0	0	0	0	0
0	1	0	0	56	0	0
77	102	142	153	149	55	110
0	0	0	0	0	5	0
12	0	0	0	0	9	0
17	3	12	14	23	12	15
104	76	116	169	65	77	71
90	66	44	104	82	76	60
158	175	156	283	144	184	135
1	3	5	7	0	3	3
0	0	1	0	17	0	1
3	4	6	2	9	1	1
209	112	97	160	170	154	101
10	7	0	3	15	0	0
0	0	36	51	0	0	34
91	51	41	48	88	71	27
130	64	240	231	175	299	296
4	4	4	0	1	1	0
24	24	31	50	36	27	26
3	0	0	0	0	1	0
22	0	5	20	0	18	8
94	41	48	55	53	118	64
0	0	0	0	0	0	0
37	29	33	53	38	47	16
73	60	98	102	34	44	167
46	14	4	41	13	7	9
3	0	0	9	0	0	0
34	61	42	43	78	23	12
161	196	325	359	536	221	336
28	38	49	62	23	70	48
0	6	0	6	0	0	8
0	0	1	2	0	3	0
0	0	3	0	0	0	0
6	7	12	27	19	3	3
5	6	1	0	61	9	1
7	1	5	12	17	5	5
1	2	1	634	686	0	0
45	28	29	45	24	36	23
0	0	0	0	0	0	0
0	0	0	2	136	0	0
41	41	35	56	59	59	34
40	56	49	89	60	54	50
148	149	53	205	56	201	82
0	0	1	0	0	2	0
0	5	5	7	8	0	7
2	13	1	0	0	1	1
1014	971	427	1054	1382	1107	1280

For Review Only

6	2	0	0	0	0	0
1	0	0	0	0	7	0
0	0	98	44	0	0	0
1	0	1	0	8	0	0
53	29	43	58	19	37	33
8	12	10	24	20	8	18
426	372	364	591	525	583	394
72	58	59	72	80	87	123
764	686	702	1188	736	897	622
0	0	0	0	0	2	0
49	33	33	78	35	45	30
326	161	265	458	211	157	244
16	25	60	51	11	19	34
60	58	46	68	67	78	31
76	104	78	159	86	156	78
1	1	3	5	2	3	0
4	0	0	0	0	0	0
6	8	17	19	0	10	1
72	130	138	53	180	87	140
80	57	58	110	120	61	57
61	39	59	105	36	46	71
16	28	24	31	84	0	27
49	67	63	55	59	44	30
32	48	57	103	78	61	40
86	87	82	124	65	54	63
24	15	24	28	9	67	20
76	41	41	63	83	64	29
22	51	40	0	49	0	0
39	41	34	50	53	58	46
4	3	0	4	0	1	0
65	86	96	180	54	129	131
42	37	38	71	65	37	16
23	13	16	48	6	45	18
37	12	15	52	0	83	27
10	0	6	7	16	11	14
4	14	6	12	0	5	3
53	43	51	73	64	57	39
6	0	1	0	0	1	0
4	0	16	40	24	15	0
0	0	0	0	0	0	0
111	38	58	130	35	67	59
3	1	0	0	0	0	5
5	5	0	9	0	0	0
0	0	0	2	0	0	6
166	162	130	266	207	238	224
15	3	2	0	6	2	2
0	0	2	0	0	0	0
168	142	147	214	148	122	158
61	66	37	56	30	34	27
0	87	90	47	205	121	100

For Review Only

0	0	14	6	0	0	46
0	0	0	104	101	0	0
9	12	13	28	0	19	0
284	1215	1	344	1026	586	2
16	1	43	37	62	25	4
58	38	69	113	41	63	65
102	114	69	194	115	65	67
16	24	24	29	38	16	6
18	27	12	39	27	37	26
245	198	130	278	155	293	178
10	0	10	4	4	9	5
1	1	12	0	7	8	1
35	30	40	92	59	52	50
44	0	21	22	27	20	32
5	6	0	5	0	1	1
0	0	0	35	0	0	0
0	0	0	0	0	96	0
69	59	53	66	65	28	23
47	44	67	86	37	45	35
11	17	22	30	0	4	12
18	16	13	33	13	27	18
0	67	33	0	43	41	23
145	155	137	235	191	190	139
46	39	64	54	75	22	53
90	87	70	89	86	72	74
13	19	16	19	26	29	13
19	29	16	16	11	0	19
71	63	61	99	94	50	44
0	0	1	0	0	7	0
34	40	28	48	48	73	25
251	228	271	342	265	298	181
169	457	181	837	712	778	1
215	140	318	499	658	507	651
5	7	1	7	0	6	1
0	33	34	24	0	60	27
82	72	48	70	84	89	33
90	48	68	76	51	60	80
40	75	116	61	0	25	44
22	58	30	51	25	27	28
71	42	45	74	100	57	79
49	33	43	38	59	11	22
6	4	31	49	17	17	14
3	6	9	12	0	35	7
231	170	127	311	267	282	205
18	27	18	31	16	37	16
0	0	0	0	0	0	0
19	23	9	26	21	30	8
80	122	60	124	64	86	58
41	17	31	67	65	47	52
44	82	76	126	30	85	55

For Review Only

41	44	35	61	0	0	28
248	216	204	296	153	252	101
0	39	0	53	24	31	30
85	99	205	184	114	170	88
65	50	31	71	22	131	38
30	44	34	52	74	57	41
2	0	8	0	0	0	0
4	1	0	7	0	4	0
41	37	54	52	92	14	34
2	2	0	0	0	1	0
46	30	35	85	40	72	32
29	24	31	54	28	35	25
23	17	24	38	19	27	19
66	99	103	128	85	78	107
4	0	1	0	0	0	2
0	0	0	3	0	0	0
1	5	0	0	47	11	0
3	0	0	0	0	0	0
1	9	6	4	0	5	1
0	0	0	0	0	0	0
2	0	2	0	1	0	0
65	55	34	60	51	34	28
63	66	21	60	0	89	31
6	6	2	13	30	4	5
26	13	37	45	18	15	45
1	1	1	66	190	127	215
12	21	22	24	25	7	2
7	11	9	14	3	4	5
0	4	5	0	18	0	0
5	9	4	0	17	0	1
4	42	16	25	39	7	1
0	10	0	0	0	0	0
2	4	1	13	0	18	0
20	29	33	16	50	9	15
32	35	28	137	21	151	73
0	327	321	359	357	545	304
361	292	281	578	403	433	266
0	94	0	52	29	0	77
37	21	39	54	15	58	39
0	0	0	0	0	4	0
0	3	0	0	3	4	5
31	49	70	63	0	1	4
965	801	903	1176	956	967	635
8	0	1	0	0	0	0
20	8	21	12	18	14	14
34	35	29	43	48	32	34
26	16	17	34	45	8	18
89	101	66	102	126	66	74
13	15	30	39	14	21	29
10	11	23	34	0	20	26

For Review Only

349	194	287	306	479	400	183
0	0	0	3	0	0	0
30	20	32	63	82	45	30
10	0	2	132	0	28	0
58	43	29	70	37	30	33
176	17	20	137	0	0	27
91	107	129	5	181	86	118
4	1	1	0	25	4	9
1	0	0	0	0	0	0
267	283	395	656	240	262	403
0	0	0	0	0	0	0
41	36	58	70	59	38	17
4	0	3	0	0	2	1
50	1	4	19	0	20	7
0	0	0	0	0	0	0
165	96	126	166	176	252	142
5564	6712	4213	10608	4039	7488	4418
0	4	0	2	0	0	1
5	2	2	6	11	3	13
2	9	0	11	0	11	2
0	0	0	0	1	0	3
30	12	16	24	27	8	12
26	34	40	52	52	22	39
0	2	2	0	0	0	3
1	0	1	10	0	1	0
0	5	0	9	0	0	0
0	0	0	1	0	0	0
0	2	0	0	0	0	1
0	0	0	0	0	0	0
0	2	0	0	0	0	3
0	0	0	3	0	0	3
0	0	0	1	0	1	0
0	0	0	0	4	3	0
0	0	0	0	0	0	0
0	3	1	0	0	0	0
1	2	2	4	3	2	2
0	1	0	0	0	1	0
0	1	4	5	0	3	2
4	2	11	7	0	1	0
228	464	202	209	153	100	217
0	0	2	0	0	4	3
1	3	6	7	9	4	0
54	92	52	71	56	48	62
84	89	62	121	105	76	52
109	74	77	207	0	126	107
34	0	35	4	19	0	0
3	5	7	9	0	0	0
0	9	0	1	3	0	3
0	0	0	1	0	0	1
0	0	0	0	0	0	0

For Review Only

13	20	14	22	21	12	20
7	16	2	10	22	1	9
33	115	112	104	282	85	122
305	277	114	334	142	310	159
308	298	212	452	187	270	158
122	106	100	82	138	111	68
4	6	13	11	0	2	18
77	63	69	94	35	73	29
114	152	177	218	122	123	151
108	96	37	8	136	43	82
120	72	43	92	27	43	38
20	19	15	34	21	7	17
0	0	0	9	0	0	0
4	1	3	8	0	11	1
102	72	147	203	119	130	129
5	6	1	17	15	0	0
80	116	62	159	110	87	63
187	152	150	399	17	23	0
16	26	36	82	25	43	43
215	107	244	182	271	93	194
79	106	29	62	99	53	51
133	148	122	111	190	64	85
21	12	41	26	38	25	14
4	0	4	1	0	4	6
71	56	38	61	65	80	28
13	19	61	42	34	22	27
263	131	102	238	81	140	94
85	98	61	149	70	92	88
0	5	0	0	0	0	0
27	9	32	24	17	16	29
16	15	10	14	13	24	13
28	28	59	93	32	36	52
44	36	39	64	12	65	34
19	47	56	91	34	57	69
153	202	248	380	147	197	174
379	265	340	670	353	399	319
168	209	182	203	298	171	90
1	0	0	7	0	0	0
0	0	0	0	0	0	0
1	1	64	0	0	1	1
7	25	4	28	0	16	4
38	16	48	32	73	39	59
4	13	0	7	18	3	5
27	67	78	87	119	41	82
75	22	42	23	247	71	77
41	79	115	89	0	52	33
3	0	0	0	0	1	0
74	62	47	64	121	23	61
25	36	34	0	0	22	27
9	0	0	17	0	1	0

For Review Only

0	65	0	0	0	5	0
0	0	1	0	0	0	0
0	0	0	0	0	6	1
115	114	106	159	74	193	85
592	532	420	723	428	520	445
1	1	1	0	0	1	1
14	12	19	21	20	23	10
112	116	149	142	161	98	53
70	54	105	75	91	56	45
45	27	36	50	35	31	22
29	9	28	38	34	25	22
4	0	0	4	0	0	1
0	0	0	0	0	0	44
5	3	1	14	0	0	0
798	511	710	1009	767	810	708
0	0	0	0	0	0	0
0	0	2	1	3	1	2
3081	3665	1057	3885	1879	3646	2094
3651	2163	2758	4713	1908	4675	2624
1	0	7	0	0	0	3
1130	585	886	1624	401	1187	817
58	45	23	36	0	20	22
105	159	150	144	150	45	50
22	13	3	18	62	4	31
11	5	0	0	0	0	0
1226	1369	154	1	1202	0	0
1522	1409	1115	2056	1334	1593	845
129	158	133	221	257	203	76
0	0	0	0	0	0	0
25	17	16	18	21	23	16
1	1	0	0	0	0	4
0	38	0	4	11	0	0
0	6	9	7	12	0	0
141	45	81	131	152	86	77
121	66	117	185	199	151	126
12	0	0	15	9	0	10
462	420	357	598	353	448	263
7	0	0	0	12	0	0
163	130	79	215	237	148	116
0	0	0	4	0	0	0
135	82	93	177	113	74	72
111	106	110	220	178	128	161
128	97	94	161	107	99	170
42	0	27	1	17	0	0
14	5	2	8	0	7	11
97	2	355	2179	156	72	2059
29	134	171	315	43	187	55
46	51	58	72	79	32	49
386	337	378	583	470	324	356
23	14	6	18	0	5	6

For Review Only

244	156	200	222	122	159	111
0	0	0	172	123	0	0
264	255	160	326	252	283	333
79	46	37	86	64	64	22
0	1	0	0	0	0	7
4	0	0	6	35	0	0
23	24	6	20	17	10	11
0	28	40	285	36	25	56
0	3	0	11	0	0	7
0	0	0	0	1	3	0
2	0	0	0	0	0	0
0	0	0	1	0	0	0
1	0	1	0	0	2	1
87	76	38	83	110	117	50
436	395	477	718	561	454	502
6	6	13	18	0	6	5
82	85	36	54	115	50	84
38	9	58	68	0	14	139
76	63	0	36	32	107	72
10	16	0	20	29	17	20
41	28	34	32	34	29	22
134	157	106	141	102	69	91
35	46	49	55	63	37	49
13	23	37	55	30	28	11
22	2	26	26	0	20	41
274	220	266	327	241	192	236
3	3	1	6	1	1	2
28	38	102	49	11	14	27
0	0	0	0	0	0	0
2	0	1	0	0	2	1
195	174	202	316	120	153	124
0	2	2	5	0	2	0
2	6	5	0	23	6	19
38	20	29	52	30	25	15
1	0	0	0	0	0	0
274	178	204	428	183	274	170
1	2	25	0	0	1	1
19	38	24	23	73	31	35
10	30	61	31	27	25	39
0	2	0	0	0	1	3
13	13	12	36	12	36	13
2	8	8	5	0	0	9
40	63	51	49	91	13	21
110	69	95	95	81	143	129
1	7	69	717	0	224	2
64	34	46	60	70	62	45
135	38	72	135	25	117	34
0	0	0	1	1	0	117
19	19	8	68	9	14	35
41	36	33	61	40	57	46

For Review Only

80	57	39	64	101	40	72
53	149	214	190	41	117	78
31	23	26	44	22	51	30
0	148	0	0	0	0	0
15	86	4	119	0	47	39
80	70	36	51	57	68	39
30	48	26	53	82	55	74
106	161	152	214	189	127	146
34	13	1	0	0	1	1
27	30	29	31	36	42	14
26	29	33	32	28	27	31
9	0	0	0	0	0	0
14	19	31	25	29	14	21
600	540	505	865	520	688	426
3	18	8	17	0	11	5
33	29	24	44	48	29	50
0	0	0	0	0	0	0
57	98	74	98	62	44	65
0	0	1	0	0	0	6
61	55	71	95	59	38	62
30	34	27	60	64	50	39
40	19	25	45	40	41	21
4	2	4	7	0	5	8
0	2	2	5	0	2	2
49	115	141	156	297	177	202
23	15	24	20	43	7	37
25	21	23	33	36	19	16
10	22	23	28	51	23	9
110	0	82	0	0	0	63
13	0	0	25	1	0	0
218	321	234	335	477	138	326
68	183	88	308	164	129	41
0	6	2	13	0	13	3
1	0	2	14	0	0	2
63	36	37	64	53	77	38
58	33	26	62	72	52	32
2	0	0	0	0	0	4
0	2	0	0	0	3	0
1	0	2	0	0	4	0
0	1	0	4	0	0	0
5	2	3	12	1	16	2
34	27	72	36	0	29	70
9	1	0	4	0	0	5
11	11	11	36	0	5	34
19	23	14	31	32	35	30
3	2	14	43	0	9	11
40	1	20	21	0	9	18
41	47	28	53	0	39	10
23	50	31	59	69	32	49
16	3	6	7	5	14	4

For Review Only

38	7	18	50	20	39	6
10	21	9	13	16	14	8
18	24	16	48	0	1	6
10	12	9	32	5	30	17
32	35	8	56	7	58	13
26	63	34	34	22	15	45
2	7	11	8	18	10	19
15	15	15	21	46	24	27
3	28	8	31	0	38	22
7	9	5	20	14	14	18
1	0	0	0	3	0	3
18	5	6	18	16	7	5
3	1	2	10	13	1	4
53	73	50	76	50	48	63
10	11	16	23	27	23	33
107	50	122	119	127	99	74
4	1	0	44	0	14	1
17	12	11	29	30	8	15
12	24	21	29	25	15	25
20	0	11	17	28	6	13
116	109	86	215	147	142	144
128	5	9	224	62	78	60
0	1	1	0	0	0	1
58	58	66	111	109	99	89
111	182	178	296	95	147	120
117	52	33	86	135	92	62
92	48	94	82	69	90	79
0	0	0	0	0	0	0
126	134	143	205	125	143	113
101	82	68	160	102	114	69
86	49	94	121	90	79	72
24	45	17	34	28	23	13
210	184	181	186	243	236	151
31	9	26	20	12	16	18
10	4	0	7	0	6	0
9	1	0	0	0	0	0
20	0	10	0	0	0	0
15	0	0	16	0	0	2
0	0	3	3	0	9	5
95	122	70	117	62	92	51
6	2	3	17	0	2	27
32	58	33	43	42	51	37
73	32	1	0	0	14	4
313	287	186	279	201	233	200
83	65	66	65	68	46	59
211	49	69	151	250	151	70
32	44	30	57	77	16	22
14	11	16	28	19	35	16
67	62	70	85	124	42	81
56	66	46	84	69	60	71

For Review Only

27	20	16	50	14	21	18
244	63	40	157	73	186	6
87	48	90	121	87	68	74
45	31	38	64	26	20	36
87	63	99	140	93	97	82
355	278	193	355	381	278	217
16	27	17	22	48	23	26
92	54	49	80	98	88	68
79	45	46	126	27	59	50
97	74	107	203	77	128	73
3	4	4	4	16	1	1
27	54	79	111	0	40	11
0	0	0	0	0	0	0
0	0	85	144	11	96	87
1	0	1	0	0	0	0
255	225	118	214	230	247	200
0	2	0	0	0	0	0
470	486	808	1054	776	655	876
18	36	40	47	68	36	23
95	84	87	201	67	108	113
85	57	80	114	68	68	92
153	159	149	298	166	208	219
28	40	52	39	19	19	17
0	0	2	1	0	5	0
18	25	17	22	0	5	37
0	0	0	0	0	0	3
0	0	0	1	1	0	0
116	103	0	2	150	103	92
52	38	46	43	54	33	19
35	56	18	38	66	27	100
0	0	0	0	5	4	0
0	2	0	0	0	0	0
0	1	0	0	0	3	0
0	0	2	0	0	3	0
106	96	90	107	100	121	70
134	157	123	204	136	137	121
55	57	42	100	72	66	29
1797	2285	1538	2797	1149	2115	910
0	32	171	91	2	128	0
0	0	0	3	10	0	24
813	899	553	1286	692	785	523
45	52	43	72	62	70	46
268	134	264	243	167	169	201
126	76	37	99	13	42	20
32	42	75	47	22	44	22
42	23	37	47	41	52	31
0	0	0	84	43	154	54
56	52	100	118	55	65	75
26	15	18	29	30	19	24
158	92	83	144	175	106	70

For Review Only

18	2	13	54	31	9	25
6	0	0	0	0	0	0
42	58	67	90	67	80	85
59	0	0	0	0	0	0
66	107	112	178	82	96	107
37	80	22	108	18	21	74
1	8	3	13	0	3	2
13	7	18	48	0	62	38
179	190	154	163	164	118	81
20	65	54	83	0	37	8
3	2	9	0	4	2	7
0	0	1	0	0	0	5
61	0	0	93	1	49	0
6	7	0	8	0	6	13
14	0	0	0	0	0	0
11	9	2	21	0	5	1
0	0	0	10	0	3	0
84	121	137	176	169	108	88
0	2	4	0	0	7	3
2	0	0	0	0	2	1
592	526	302	281	787	481	403
210	236	186	349	167	237	199
20	11	9	25	6	25	14
61	0	41	0	48	66	84
141	83	96	152	134	98	50
78	84	53	94	50	95	68
88	64	91	114	107	120	59
130	80	57	144	92	98	70
3	10	4	6	0	9	10
8	4	10	8	23	8	1
102	37	35	69	69	24	88
3	0	2	7	0	1	4
181	184	179	243	98	111	162
65	73	70	107	124	44	74
17	6	20	24	46	8	16
3	2	2	0	0	2	2
6	32	20	23	45	37	15
0	0	0	8	0	0	0
65	90	51	81	109	49	25
267	315	240	419	369	313	247
166	1	1	0	0	46	2
21	6	12	28	0	24	15
95	86	126	163	210	137	170
18	11	12	39	0	9	7
9	12	9	5	14	7	0
135	87	128	200	153	121	88
19	0	0	2	0	31	17
23	5	7	83	0	25	27
24	13	0	50	0	11	0
22	5	12	34	9	26	13

For Review Only

1	6	116	0	33	347	1
83	27	25	75	32	24	38
47	42	48	87	38	34	43
76	87	75	128	63	82	52
128	112	56	131	147	104	65
25	0	18	53	0	19	8
0	38	0	0	150	138	0
188	173	245	273	163	275	143
9	14	5	15	25	8	11
85	66	70	116	67	104	58
81	77	56	112	54	62	66
64	63	60	26	191	68	56
52	43	60	101	64	25	35
0	6	11	0	0	0	0
0	0	0	1	14	0	0
51	33	34	76	19	27	42
62	39	51	89	33	49	31
0	0	10	15	0	5	0
0	0	0	0	0	0	0
1	20	56	0	0	3	34
31	38	18	33	22	64	17
416	241	244	466	254	364	333
241	153	114	305	0	206	124
258	219	225	270	172	215	124
15	4	7	33	5	3	4
22	0	0	8	18	0	3
615	630	742	831	705	748	505
45	10	11	23	42	10	24
1	1	2	1	1	6	1
207	115	131	221	100	145	122
0	0	0	0	0	0	3
144	108	78	152	97	82	72
7	1	9	44	0	54	6
105	55	84	49	105	73	2
7	6	14	13	0	7	4
14	6	19	8	10	7	5
450	150	210	0	0	126	110
75	86	111	128	150	100	131
66	50	37	108	48	68	46
9	117	189	0	97	4	54
0	0	11	2	16	0	0
0	0	0	0	0	3	4
128	1	4	156	0	4	1
57	53	63	82	53	62	56
7	16	4	10	0	8	5
28	29	3	104	0	27	2
80	6	24	62	0	1	1
116	82	75	125	54	90	36
1	1	1	3	0	0	1
234	152	174	199	190	194	118

For Review Only

21	25	17	23	84	16	19
92	97	93	156	95	72	99
24	22	17	18	36	12	21
90	48	129	91	104	136	32
49	62	54	90	52	61	65
104	41	96	138	75	72	79
87	58	62	92	67	58	64
139	77	88	111	56	51	41
12	32	13	63	0	18	27
172	146	92	204	115	187	152
171	287	213	401	231	293	305
9	8	0	168	0	1	3
42	44	38	37	68	26	52
9	8	7	12	8	10	5
26	20	36	50	35	35	29
0	1	1	0	0	0	0
56	37	0	15	43	52	33
136	46	49	99	122	86	86
24	29	19	44	21	33	28
85	85	113	200	133	175	159
8	18	42	26	12	21	20
82	88	82	169	158	104	105
72	148	175	169	214	79	112
88	72	125	122	90	110	105
14	7	6	9	6	8	3
2	7	42	0	75	17	66
6	7	8	4	7	9	7
60	81	69	131	94	73	67
44	62	0	123	0	0	27
33	13	21	51	0	27	30
46	21	21	33	30	55	18
49	108	88	103	51	118	49
231	204	174	365	192	231	151
253	236	193	272	293	175	174
47	85	48	81	74	46	34
116	89	145	183	74	135	180
60	50	69	68	81	47	88
0	5	2	8	0	0	0
53	57	56	1	0	9	0
0	4	0	0	10	0	0
11	27	39	18	4	9	6
0	0	0	0	0	0	0
13	10	14	33	5	3	26
48	71	33	61	30	46	32
102	101	74	117	64	85	46
25	24	11	100	41	21	81
604	180	109	306	482	218	11
9	16	25	13	19	5	12
16	30	27	17	29	18	14
42	48	13	27	33	16	41

For Review Only

50	47	57	85	94	45	57
73	85	115	147	114	112	50
261	190	169	693	120	18	182
53	23	13	21	47	43	57
0	0	2	0	9	0	2
52	83	64	111	49	45	56
4	1	2	12	0	0	3
94	68	96	106	98	83	77
50	30	19	80	27	66	32
264	289	245	359	259	372	210
47	84	66	117	109	65	76
66	36	92	161	0	10	73
0	90	0	20	51	71	0
39	50	47	48	33	35	67
551	654	89	799	1363	696	668
27	6	34	74	0	33	5
168	219	144	361	174	217	160
39	35	41	30	55	28	13
2	44	17	26	11	96	82
4	0	0	3	0	0	0
22	13	4	20	0	3	12
51	35	42	59	8	58	29
126	0	0	88	13	139	111
12	9	28	42	30	7	20
87	29	18	39	79	60	46
86	111	50	125	81	95	96
229	195	173	190	112	220	186
12	19	21	26	26	13	15
84	81	83	175	66	68	61
59	58	87	80	57	68	72
85	14	5	13	0	82	34
26	26	43	66	52	25	41
76	24	1	83	453	6	211
221	193	153	282	107	180	122
80	114	117	154	131	171	66
111	114	110	141	154	96	67
71	119	158	219	80	135	135
1	7	12	14	0	8	4
92	85	95	169	172	111	89
21	29	31	45	47	31	39
13	23	19	38	23	27	27
54	0	61	123	75	60	37
517	818	381	745	488	751	522
43	50	67	107	74	59	101
19	9	15	33	16	25	19
90	95	76	115	150	67	81
60	78	101	118	45	62	75
0	19	24	13	33	0	12
228	380	108	157	139	526	38
354	886	658	971	359	1046	739

For Review Only

16	13	11	25	21	16	9
236	158	199	283	157	229	249
49	19	6	88	0	5	5
2	1	12	24	0	2	8
147	110	119	176	143	71	86
365	435	335	582	388	392	351
17	31	10	17	0	7	1
112	65	58	79	137	47	111
10	10	36	62	76	56	102
3	1	0	3	0	0	0
4	0	0	2	0	0	0
31	16	18	0	0	25	21
29	53	30	16	11	0	0
84	44	42	86	37	36	50
1	0	0	3	0	0	0
0	20	0	0	0	0	0
45	55	48	65	128	34	48
10	15	15	27	15	21	28
0	0	0	431	460	0	0
19	19	19	30	28	10	16
147	97	80	119	80	160	66
81	33	37	89	60	70	55
2	1	1	0	0	0	0
235	160	140	104	106	247	179
0	0	0	0	0	0	0
15	14	2	0	16	4	2
9	8	4	8	12	12	4
1	2	3	9	4	10	1
26	33	6	0	114	4	3
239	0	168	12	48	0	109
0	0	0	0	0	0	0
2	14	1	0	0	8	2
0	0	1471	640	0	0	263
37	82	49	86	65	19	28
0	184	205	3	439	198	0
143	155	127	177	158	153	80
294	291	323	472	289	365	268
176	103	122	69	102	117	140
0	2	0	0	0	0	2
78	0	56	110	66	68	54
0	3	0	0	0	0	0
137	80	143	227	67	71	42
189	207	172	270	296	195	105
456	219	252	450	484	786	414
1	0	3	0	0	0	1
28	38	34	56	28	33	25
3	0	4	6	0	0	0
7	7	37	30	0	6	6
36	20	41	41	68	50	49
144	151	123	208	178	148	124

For Review Only

550	405	335	681	415	497	295
8	7	5	15	0	9	3
195	131	172	181	299	271	52
16	20	32	44	109	76	54
0	8	0	14	0	18	3
265	172	161	294	257	257	186
60	37	41	57	62	53	50
9	14	12	6	12	1	10
1741	1900	1305	2841	1426	1663	1008
13	20	23	30	30	8	4
121	140	147	146	126	204	111
0	0	0	4	0	0	0
0	0	0	0	0	0	0
25	18	21	32	32	27	20
0	0	0	0	0	0	0
96	125	98	101	126	89	62
0	0	0	0	0	0	0
1	1	5	16	0	6	6
100	151	110	182	83	73	90
0	0	0	0	0	0	0
0	0	0	0	0	0	2
3	3	4	9	1	4	2
123	178	64	165	69	109	66
23	9	25	44	27	29	17
9	0	0	0	0	3	11
255	653	1	0	0	1	1
0	1	1	0	0	0	2
311	162	88	25	53	173	210
155	121	80	122	128	98	53
2	0	0	10	0	0	2
0	36	14	74	0	0	29
0	0	4	7	0	4	7
1	0	3	1	10	0	0
0	179	145	137	121	143	135
510	452	378	532	430	334	300
0	0	2	5	1	0	0
31	18	31	36	125	36	84
266	171	196	403	242	272	261
7	5	4	6	4	6	11
5	18	5	16	0	2	13
479	451	488	719	450	434	427
45	47	46	74	27	33	40
599	452	352	722	478	502	371
3520	1955	1027	4669	961	1152	2509
18	14	7	14	15	11	0
1080	398	782	801	862	1112	646
84	77	75	94	44	78	38
269	198	183	224	247	236	215
12	21	13	28	0	5	7
5	4	1	0	0	3	2

For Review Only

13	22	17	24	10	4	15
5	6	1	11	0	0	4
14	24	35	30	40	34	12
5	2	8	10	0	2	4
218	220	143	162	161	195	102
2	2	3	5	0	4	10
72	132	50	97	140	33	49
0	0	0	0	0	0	0
63	41	25	0	0	67	31
3	0	0	0	0	5	0
0	0	0	0	0	4	0
25	17	28	21	60	47	54
0	0	0	0	0	0	0
0	0	8	0	0	7	0
3	9	19	0	37	7	32
8	1	0	9	0	0	2
1	110	138	0	0	12	3
49	171	70	166	177	77	70
60	14	33	129	47	170	21
186	82	200	300	251	267	192
15	0	1	0	39	15	11
214	206	198	0	0	192	169
127	110	84	221	152	192	105
0	99	223	8	634	36	0
11	82	4	68	60	3	3
27	21	27	45	32	18	19
37	24	4	36	0	23	21
0	0	0	0	0	9	4
5	0	1	15	0	4	5
30	0	0	1	40	0	39
18	8	2	1	0	2	3
0	0	0	0	0	0	0
6	10	7	8	1	1	2
847	279	617	591	394	576	331
0	35	60	0	0	0	58
1	1	2	0	0	1	1
0	0	1	0	0	0	0
195	172	201	313	242	280	147
37	1	12	36	0	1	72
12	14	9	15	17	8	8
0	0	0	0	33	2	0
6	7	30	41	46	4	30
26	24	52	71	46	44	33
39	53	12	54	0	18	65
27	19	12	13	29	1	1
76	67	71	196	81	71	64
19	34	18	29	40	8	15
0	6	0	10	0	0	0
64	82	87	135	133	140	44
0	0	0	0	0	0	0

For Review Only

39	0	0	0	0	0	0
0	0	0	2	4	0	1
477	330	301	464	501	408	224
1	1	7	0	0	43	2
16	25	7	41	12	34	12
0	4	0	0	0	0	0
28	20	17	29	0	20	0
133	214	234	341	217	246	229
0	0	0	0	0	0	0
5	0	4	0	0	10	0
354	159	1	4633	3181	346	438
71	3	31	65	0	55	5
3	1	2	0	0	0	0
0	0	0	0	0	0	0
0	0	0	0	0	0	0
1	23	9	7	0	2	1
0	0	0	0	0	0	2
353	265	383	636	365	436	398
30	9	19	39	8	11	18
94	63	86	136	57	82	45
16	16	32	37	9	18	23
42	20	49	86	0	70	34
1450	1151	1159	1911	1208	1681	1290
184	108	140	160	145	120	97
132	117	0	106	7	98	0
38	15	66	0	45	18	27
38	30	43	35	64	61	42
25	11	45	53	41	51	27
2	1	0	0	0	0	0
308	287	333	339	450	350	320
45	48	31	74	68	60	31
95	87	31	108	55	95	55
0	5	2	0	0	2	3
1	0	1	17	0	1	1
0	0	3	0	0	8	2
30	154	160	110	205	0	146
29	23	33	56	28	25	16
61	0	0	438	0	0	1
48	32	48	94	28	36	37
1	63	102	48	118	1	76
0	10	7	5	0	1	0
1	1	1	0	0	1	1
121	0	70	19	0	62	78
114	120	102	147	116	65	79
27	22	16	23	43	21	19
0	0	36	17	0	51	0
221	1	1	0	670	1	1639
0	0	0	0	0	234	0
15	0	0	10	0	8	0
51	77	50	60	70	54	46

For Review Only

49	39	29	67	19	64	38
1	0	0	0	0	4	0
0	3	3	15	0	0	0
0	5	0	1	0	6	0
68	89	68	98	78	87	86
113	120	76	0	0	120	57
56	19	91	59	89	50	60
142	162	127	171	168	147	126
72	102	105	149	83	103	107
0	20	17	30	58	0	0
75	13	52	84	13	47	30
50	62	80	101	54	43	39
48	34	50	65	31	46	25
54	30	34	56	54	45	25
54	93	91	94	70	77	56
22	37	18	0	0	0	0
245	207	170	329	190	228	104
316	266	211	288	192	442	179
0	0	0	0	0	0	0
11	9	8	14	2	6	0
57	41	38	71	26	26	26
134	6	1	0	64	66	43
15	8	19	21	38	16	16
25	23	22	30	25	29	33
22	30	23	49	20	37	27
112	50	11	62	87	61	16
1	81	3	0	0	1	1
0	0	0	0	0	0	0
3	0	2	0	0	0	1
119	61	84	132	105	117	103
26	59	39	50	44	45	47
0	0	73	17	1	62	0
108	128	110	172	87	102	98
22	13	36	41	13	28	14
123	133	108	193	273	165	201
158	203	58	100	191	197	21
40	0	0	0	0	0	0
36	33	21	80	55	70	66
208	176	237	378	171	199	205
0	25	26	10	0	29	0
52	82	83	210	37	68	63
27	15	31	44	37	35	20
0	0	0	2	1	0	0
0	0	0	46	20	91	0
29	22	28	57	16	17	29
150	130	109	178	108	70	67
76	62	69	109	84	69	70
92	29	70	96	46	84	69
76	29	66	88	127	119	84
78	17	53	47	40	34	8

For Review Only

75	0	0	16	1	0	56
47	63	42	62	44	50	50
8	4	10	8	0	14	4
0	23	7	0	0	0	0
9	29	34	27	12	6	19
53	56	53	70	49	48	30
103	77	73	75	78	122	69
122	161	108	198	57	85	97
1	0	0	0	0	0	1
3	3	3	6	0	4	0
0	28	0	32	0	15	0
52	17	25	74	11	30	24
77	47	98	105	97	46	69
190	215	188	367	222	184	237
0	24	0	0	0	0	0
42	66	65	78	99	61	87
4	0	0	0	0	0	0
296	287	183	382	231	221	200
74	227	262	416	110	213	177
7	17	26	29	0	7	6
18	25	14	39	0	30	27
0	0	0	2	61	32	0
0	0	0	14	0	34	0
237	190	0	266	67	0	156
27	21	8	42	10	17	20
21	18	15	53	0	31	9
74	64	39	70	69	57	56
40	67	31	70	50	65	33
39	68	55	132	30	62	44
58	169	124	163	216	110	115
19	35	14	33	18	26	26
65	66	57	94	86	78	54
1	9	1	0	0	1	0
16	21	14	31	27	26	14
63	0	0	1	2	60	50
0	0	0	39	9	0	0
0	1	0	0	0	0	3
6	12	19	49	52	32	16
2	14	3	1	13	22	8
37	34	23	31	0	17	11
531	252	348	781	91	516	10
0	36	0	0	0	0	0
30	40	53	74	23	28	52
272	1	0	16	14	11	206
7	10	9	10	0	4	9
18	37	42	83	0	166	105
60	93	116	164	107	109	103
537	0	472	478	36	0	498
16	75	39	119	110	59	34
35	11	33	72	23	10	58

For Review Only

34	26	39	50	31	51	28
1	4	12	5	14	5	4
76	53	82	116	79	78	46
2	0	10	12	0	1	6
36	33	24	30	37	28	27
175	107	100	230	126	171	78
49	55	41	81	23	31	24
0	0	0	0	0	0	0
16	39	17	43	12	35	24
66	69	78	136	70	51	88
3	2	1	8	0	5	9
44	36	51	86	50	44	39
15	30	10	53	100	47	52
18	13	22	42	30	25	33
14	6	7	15	0	10	27
203	56	69	182	61	151	186
248	233	193	0	0	261	177
428	407	382	774	367	421	456
0	0	0	2	0	0	0
53	56	49	66	60	54	37
99	84	88	127	62	121	131
5	3	7	0	0	0	0
0	0	0	0	0	0	0
104	100	60	139	111	109	68
76	1	13	0	0	84	1
75	93	93	100	80	88	101
28	89	143	53	196	97	84
264	153	158	336	133	454	305
92	67	69	180	123	80	84
126	145	143	227	138	135	139
30	60	27	80	16	28	58
85	27	99	234	0	3	12
29	48	31	62	64	54	50
1	0	0	1	9	0	0
4	4	13	6	36	4	9
130	0	0	0	0	0	0
198	88	178	268	111	151	254
68	37	43	61	64	36	42
1	0	0	0	0	7	0
291	141	141	283	438	46	212
1	2	7	12	0	1	1
30	0	0	24	0	0	0
109	120	65	135	142	94	77
8	8	7	8	9	8	3
176	146	165	144	382	153	0
26	27	17	28	52	22	21
0	0	0	0	0	0	0
0	0	0	0	0	0	0
204	163	107	208	100	177	104
98	79	47	96	55	49	51

For Review Only

6	5	0	9	18	6	11
8	17	3	1	0	28	6
84	51	65	104	85	117	44
39	38	37	46	66	72	11
226	221	291	324	351	264	242
87	105	72	137	120	80	81
86	103	124	181	205	57	176
12	21	20	34	28	29	27
463	442	402	653	402	486	280
172	102	108	146	101	166	86
105	121	129	181	145	143	141
4	0	0	0	0	0	0
106	18	12	60	21	9	28
17	22	21	32	24	18	34
26	24	18	26	26	36	25
21	17	18	33	14	16	20
20	0	17	8	43	18	10
44	33	20	24	19	22	29
100	166	154	151	115	187	82
0	7	14	0	62	3	2
38	39	32	57	44	21	54
85	201	126	163	385	99	188
39	35	61	78	20	36	51
4	0	11	11	12	0	3
104	132	106	141	128	94	133
0	0	0	0	0	5	0
0	31	13	0	0	8	18
291	232	248	374	231	286	214
25	7	20	32	5	22	36
18	18	28	31	27	20	32
0	0	3	0	0	0	0
0	0	94	65	0	0	0
143	98	112	191	223	184	128
38	50	64	75	73	28	55
111	88	67	149	129	107	110
244	232	224	323	232	201	260
164	115	104	169	128	133	60
653	804	1127	1263	30	1107	1029
1	5	1	0	177	1	1
81	52	80	115	101	89	56
12	29	13	22	21	17	14
14	80	0	79	0	0	70
25	30	34	54	0	99	4
105	102	126	166	135	124	96
12	28	12	22	26	16	17
11	11	8	16	6	9	7
30	30	33	55	19	19	32
1	1	1	103	0	1	1
50	56	17	95	0	138	5
0	1	6	7	0	5	7

For Review Only

7	13	9	7	16	6	9
416	351	289	695	265	463	323
91	73	96	176	108	64	53
20	19	25	58	20	28	34
12	3	8	14	28	24	5
38	56	31	67	43	31	60
8	4	5	14	4	18	7
138	188	113	206	208	80	237
40	35	18	46	46	14	22
0	0	0	0	0	0	4
14	43	44	42	65	35	33
240	175	264	399	287	260	207
0	0	0	0	0	0	0
112	127	248	215	69	171	74
96	68	87	121	96	67	70
0	85	87	15	1	0	0
92	54	41	124	61	50	42
3	0	0	1	1	3	0
14	6	0	23	0	1	0
311	1	41	571	0	50	2
0	0	0	3	1	0	0
19	0	0	0	0	0	0
94	78	109	149	243	121	210
72	26	38	43	127	61	65
847	712	653	0	0	802	757
0	0	0	0	0	0	0
3	0	0	0	0	3	5
15	19	14	7	29	8	24
0	0	0	0	0	2	1
20	18	19	23	23	29	18
0	2	0	20	0	0	5
37	8	28	19	0	17	7
61	54	35	54	68	56	64
115	107	101	173	81	63	97
0	1	1	0	0	1	1
459	399	386	573	423	388	365
55	20	11	58	0	7	4
49	42	59	87	66	41	34
111	174	80	151	173	139	148
138	98	94	167	105	134	73
18	10	10	22	8	9	10
0	0	4	9	0	0	0
749	620	529	476	639	750	521
38	27	36	37	45	45	22
55	18	42	42	58	20	32
1	0	0	0	0	0	3
73	70	127	112	125	94	95
286	220	343	376	411	193	232
1286	1318	655	360	868	73	265
51	40	36	80	88	77	60

For Review Only

0	0	0	0	0	0	0
79	29	63	84	60	31	65
18	32	14	19	45	17	18
24	0	18	35	0	9	4
178	131	79	272	88	186	97
22	19	11	30	13	16	16
61	29	50	44	73	57	43
122	104	147	192	152	102	110
75	81	119	119	65	93	139
4	2	1	6	0	5	4
62	65	53	84	48	53	64
15	19	15	47	38	37	20
53	19	33	37	28	20	24
87	120	64	116	49	84	108
0	1	0	0	0	0	1
12	0	2	0	0	0	0
21	0	16	0	67	15	0
171	26	53	142	12	21	54
0	0	0	76	48	0	0
119	70	49	123	129	121	46
47	17	15	70	12	18	33
60	60	64	99	43	70	52
33	31	28	55	29	48	24
8	4	3	0	8	4	0
2	0	0	0	0	2	2
7	2	0	8	0	5	0
3	11	1	24	0	18	2
0	21	7	9	41	16	16
0	1	3	0	0	1	3
3	0	0	0	0	0	0
1	0	0	0	42	0	0
5	10	0	3	0	6	6
72	50	68	95	65	63	60
831	764	629	792	912	997	577
70	80	55	56	51	96	65
65	66	41	87	61	48	44
3	0	0	4	0	3	0
15	15	17	24	0	15	19
33	49	43	81	20	23	35
28	21	28	54	28	43	24
0	0	0	8	0	0	0
81	144	56	70	67	45	37
15	46	29	47	71	41	77
57	66	55	102	104	76	67
58	58	81	101	16	101	60
39	37	15	90	24	30	43
33	0	0	5	5	0	0
145	57	127	139	173	97	141
52	67	65	53	138	56	74
114	136	116	172	156	88	114

For Review Only

49	58	25	97	0	0	27
165	199	168	200	226	108	141
118	111	124	172	112	167	125
293	277	286	437	311	300	341
19	18	31	29	25	42	23
20	3	4	33	0	7	2
115	132	103	166	89	108	100
116	166	83	168	174	113	102
300	267	198	333	264	107	174
157	168	132	241	165	179	121
7	3	0	3	0	3	3
37	30	29	61	70	47	71
1	15	18	0	90	8	0
198	112	5	87	86	78	116
330	335	219	393	338	391	269
10	1	8	11	9	5	11
2	0	1	0	0	0	1
5	2	3	12	7	3	6
0	0	1	1	0	5	0
43	25	16	43	23	35	23
11	1	0	0	0	0	0
11	4	1	0	8	4	9
0	0	0	3	5	1	0
0	0	0	7	25	10	10
21	14	10	43	15	16	12
4	6	1	3	0	0	0
112	57	70	144	180	117	141
0	0	0	447	204	0	0
164	167	139	213	218	123	139
229	214	220	443	232	272	250
0	2	2	1	0	0	0
39	27	20	53	43	27	22
6	0	0	3	0	0	6
10	2	10	15	3	7	7
1	0	0	0	0	0	0
3	0	3	0	0	0	0
1	2	3	10	0	2	3
2	0	0	0	0	0	0
0	0	0	4	0	0	0
23	16	16	43	16	24	10
78	11	1	58	0	1	5
0	2	1	0	0	3	0
0	0	5	0	0	0	0
23	31	16	48	35	48	38
74	105	56	93	123	85	50
1148	72	2402	3509	4053	3594	69
107	62	27	81	47	55	44
11	12	4	9	23	7	0
2	0	0	9	0	0	3
4	18	16	12	7	2	3

For Review Only

0	0	0	42	369	0	225
422	386	337	534	398	419	279
63	19	42	49	71	32	18
3	0	1	0	0	0	2
0	0	0	0	0	0	4
7	7	11	18	13	8	8
130	98	57	146	53	105	69
0	4	1	0	270	527	1
217	104	184	14	174	220	0
155	151	168	276	142	228	104
61	63	86	140	49	62	74
56	70	72	120	58	83	55
135	150	172	252	162	145	137
231	77	258	299	283	85	198
19	14	3	0	0	11	5
208	178	309	440	287	247	245
13	58	93	107	90	0	36
193	145	192	275	108	133	139
90	77	79	192	56	89	85
66	56	142	357	166	1	1
150	134	162	266	173	128	82
213	0	14	140	0	0	137
86	75	73	145	39	60	84
299	339	447	660	347	350	368
0	0	11	2	2	0	0
152	108	87	198	153	157	85
24	93	241	535	0	207	16
9	402	2	176	0	16	1
0	0	0	27	0	5	1
1	1	1	0	0	1	1
244	254	177	319	196	211	86
131	124	120	172	163	124	113
250	206	247	347	311	254	187
63	43	83	94	100	74	49
138	156	133	238	178	173	90
193	161	166	249	296	284	149
171	138	178	244	140	178	61
122	83	104	232	48	124	61
25	427	77	51	76	135	1
148	119	113	165	200	74	68
0	0	50	52	6	0	0
100	110	53	144	33	139	62
217	185	197	365	209	209	150
132	197	171	232	217	129	132
277	195	185	355	307	252	208
46	53	51	111	51	55	28
43	63	0	192	0	75	44
190	60	241	189	256	174	155
31	0	13	0	106	0	0
50	51	102	125	16	33	106

For Review Only

120	58	48	99	111	116	56
39	0	0	33	37	53	36
26	2	5	133	0	3	97
39	39	18	83	22	28	22
18	14	19	17	23	15	13
71	54	65	108	37	47	112
12	12	6	56	0	41	20
0	0	0	0	5	0	0
18	18	17	45	42	48	29
0	0	15	0	11	0	0
6	5	7	0	0	4	7
1276	1040	1088	1685	1062	1238	765
116	96	91	190	84	127	87
42	28	47	56	32	42	74
239	254	300	481	249	372	302
125	103	71	110	91	142	119
77	62	32	120	109	49	83
82	53	52	100	0	27	120
29	14	17	48	11	17	24
0	0	3	0	0	2	0
26	30	49	47	79	21	10
195	151	172	214	223	110	82
123	96	81	139	103	38	71
136	148	202	284	102	127	184
17	10	10	19	0	19	0
1	0	1	0	1009	0	0
12	39	59	70	5	41	43
16	17	17	27	29	38	23
1	2	5	0	0	0	0
0	6	0	9	3	5	4
129	144	128	188	159	70	68
13	16	32	32	18	23	13
18	26	24	41	0	0	10
98	48	34	94	0	27	187
2001	1678	1455	1761	2431	3094	25
6	1	5	0	10	10	5
11	4	2	21	0	15	9
185	107	141	219	200	316	196
0	0	0	0	0	0	0
6	13	6	0	0	7	24
68	40	38	50	32	46	45
411	375	474	615	449	563	414
349	299	294	435	471	174	290
194	208	223	308	288	257	169
0	8	9	11	26	16	5
20	17	4	67	0	14	19
354	243	0	720	1007	490	229
34	39	31	79	68	20	14
2	10	16	21	0	10	27
9	0	1	2	5	9	7

For Review Only

36	124	409	0	433	321	58
187	148	134	221	59	42	95
28	11	21	27	42	24	22
11	0	11	7	6	9	12
22	56	7	0	124	24	55
2	3	25	34	0	9	10
419	158	161	0	2926	48	1
0	0	0	8	0	0	3
21	32	34	52	36	23	29
165	161	202	250	435	80	309
3	0	0	0	0	0	0
628	453	615	912	514	608	614
3	0	0	3	0	2	2
119	85	196	78	148	111	186
6	1	3	3	1	0	3
0	0	1	0	0	0	0
0	0	0	0	0	0	6
43	158	20	30	1	0	24
125	87	55	143	104	120	202
0	0	0	0	0	0	0
302	217	267	412	346	303	242
0	1	0	0	0	0	0
84	146	113	229	295	252	226
9	0	2	0	0	1	0
2	3	0	0	0	0	0
90	63	86	134	76	77	78
54	37	43	58	57	53	32
0	0	0	0	0	0	0
112	56	103	174	103	115	70
940	461	632	1300	483	918	580
125	0	0	35	257	166	200
88	125	193	180	325	128	189
153	168	212	338	161	176	213
47	39	61	66	89	100	74
76	102	148	172	173	57	112
102	121	135	197	151	137	103
4	8	10	14	13	2	11
46	25	16	46	70	30	28
54	25	42	52	23	57	46
3	4	55	0	29	1	1
28	16	28	28	12	32	25
10	19	12	16	21	11	11
19	27	23	34	15	13	22
35	51	12	46	28	22	22
40	21	32	67	24	39	26
61	71	70	45	66	0	66
39	35	32	34	68	30	44
246	271	264	420	243	267	181
27	16	13	24	23	16	15
6	35	46	39	57	25	24

For Review Only

0	0	0	0	0	0	3
197	130	131	194	125	142	135
49	31	20	58	35	53	17
263	362	117	276	455	380	99
0	0	2	0	0	2	0
449	206	550	1120	381	592	432
0	3	1	2	0	0	4
4	261	1	305	0	1	0
52	34	45	51	21	45	23
5	5	0	0	0	0	0
3	6	9	0	11	0	0
205	174	92	214	206	180	134
33	0	23	22	0	27	12
0	2	0	0	0	0	0
24	18	1	0	0	1	1
11	33	17	42	0	5	12
9	14	13	25	4	8	19
37	48	55	70	75	66	47
60	79	102	86	39	69	82
14	12	2	15	7	21	6
86	0	18	305	0	15	27
142	131	104	146	74	98	62
82	262	91	314	286	144	91
6	0	0	0	0	0	8
54	41	35	101	36	59	38
25	13	6	31	23	9	0
11	10	1	8	7	0	4
274	302	251	322	192	174	209
0	0	0	1	0	7	0
33	32	41	58	34	33	23
23	56	39	54	56	40	43
64	66	32	84	63	68	50
24	18	15	23	9	23	7
10	0	0	0	0	6	8
14	2	2	27	0	1	1
12	19	7	27	0	8	18
70	75	47	99	79	69	54
104	99	93	164	95	87	87
63	77	57	88	62	62	53
196	122	63	10	268	116	43
8	24	11	0	0	3	1
57	112	137	151	208	79	122
24	0	0	1	40	31	0
465	319	394	583	268	467	345
199	208	166	298	164	327	150
0	1	0	0	6	0	2
28	29	8	42	0	17	37
7	13	2	19	0	13	8
9	1	0	11	0	11	2
315	222	176	243	174	211	184

For Review Only

56	70	67	99	101	66	96
0	0	3	0	6	0	0
104	39	200	484	0	429	19
4	0	0	0	0	0	0
241	193	169	201	146	232	176
2	12	2	6	9	7	8
516	521	426	748	402	475	446
307	0	0	0	210	0	0
245	133	62	193	199	75	66
9	8	26	26	0	18	7
31	9	15	27	63	3	23
19	7	14	26	12	20	14
3	0	7	3	0	0	0
154	103	102	123	124	99	58
54	25	50	60	19	32	26
0	9	24	0	0	0	0
48	89	71	97	243	46	126
49	52	39	101	37	58	75
0	0	1	0	0	0	0
734	554	661	1114	704	736	602
235	238	147	308	244	231	162
0	4	1	5	0	0	0
78	66	49	41	92	27	29
36	26	2	50	18	33	33
0	0	0	0	69	23	0
1	0	2	6	0	2	6
1	0	0	0	0	2	4
178	163	140	168	180	155	95
44	42	3	63	0	23	14
26	9	3	19	11	18	5
154	166	175	66	233	230	156
165	35	38	148	50	103	76
239	808	72	1032	15	344	553
166	173	119	175	199	132	110
4	4	3	1	0	0	0
57	39	46	94	57	62	48
3	5	7	12	25	2	11
123	89	72	41	192	4	6
0	1	0	0	0	0	0
11	16	21	20	14	13	14
131	94	123	189	142	135	116
19	30	69	73	143	30	31
215	159	168	217	149	170	99
48	99	134	166	149	115	113
88	95	82	69	48	44	50
126	142	167	295	269	205	172
411	455	392	643	310	539	305
0	0	4	0	10	1	0
118	94	113	234	180	181	125
261	274	306	394	175	307	253

43	31	51	102	38	33	57
285	200	190	506	271	305	348
100	75	112	77	112	40	60
72	37	63	90	124	215	25
107	67	45	104	36	78	86
95	46	37	83	65	126	70
57	25	19	71	43	47	49
44	59	48	58	47	55	47
99	85	60	131	70	85	66
0	7	47	0	0	4	8
1	1	1	0	375	53	1
3	19	12	38	0	48	28
6	4	4	16	32	6	10
57	46	70	56	77	34	28
189	0	0	1	92	0	0
0	204	261	212	0	0	188
35	0	0	0	0	30	0
9	0	0	0	0	3	0
29	34	34	49	52	26	40
41	50	37	68	43	29	28
3	0	1	0	0	0	1
40	33	47	57	58	46	72
0	0	0	0	0	0	39
17	35	25	37	62	45	32
43	19	27	55	103	40	44
1	0	2	5	0	0	7
3	3	6	10	0	3	11
8	6	7	12	13	6	4
0	0	2	0	0	0	0
59	41	40	71	25	33	66
33	42	41	76	48	39	21
0	0	0	0	0	0	0
56	28	52	108	72	81	122
9	0	0	33	0	0	0
17	23	20	39	36	27	27
122	94	114	21	264	0	0
161	144	119	210	112	92	101
153	123	111	205	252	105	179
7	1	0	0	0	0	1
103	93	109	163	115	56	61
410	328	294	505	245	365	213
35	86	84	135	126	89	72
62	70	64	91	37	63	55
86	13	32	48	65	39	37
70	90	88	111	152	62	57
27	19	17	32	9	24	32
153	154	142	83	88	0	50
17	112	119	183	0	130	141
16	40	65	81	69	28	39
59	59	97	115	63	63	81

For Review Only

9	0	7	3	10	10	0
134	275	313	177	0	317	19
48	73	74	96	83	72	55
32	129	56	90	58	33	132
0	4	66	0	0	0	18
0	0	0	0	19	4	6
68	34	18	52	34	48	51
29	29	36	78	24	40	48
0	47	26	82	0	56	26
7	0	0	2	8	5	0
78	94	106	159	127	94	84
228	242	399	351	472	131	310
3	0	3	0	13	2	0
72	10	54	77	1	157	37
522	557	527	571	707	564	480
56	34	89	74	117	60	56
25	21	36	46	39	13	13
74	71	63	99	79	81	98
338	201	209	264	450	263	391
49	40	45	74	31	28	53
198	165	278	329	322	264	295
210	168	202	259	209	176	204
1	3	2	7	0	4	0
23	16	15	19	20	9	28
50	31	34	48	25	51	31
0	6	2	5	0	3	0
10	9	7	5	1	7	0
173	112	65	169	22	121	54
21	2	59	75	0	34	35
41	62	150	126	144	72	55
41	23	27	48	36	37	30
457	251	210	357	398	516	308
23	4	88	704	0	1	1
15	45	29	47	41	23	31
555	1	335	775	0	1	352
0	0	0	1	1	3	0
96	60	50	84	74	109	71
0	0	0	0	0	7	0
31	26	28	31	29	23	29
643	637	675	776	489	578	349
6	6	8	43	29	14	41
0	0	0	0	0	0	0
15	7	20	12	6	16	13
35	42	0	0	0	51	26
404	172	243	404	419	542	398
4	4	18	12	0	6	14
144	175	24	178	21	79	11
433	238	101	161	202	408	179
0	7	10	16	0	3	3
9	17	18	13	28	14	10

For Review Only

0	2	1	5	0	0	6
28	51	84	91	63	73	63
43	78	101	71	101	40	32
49	33	43	42	59	28	31
0	0	0	0	10	0	0
96	196	88	111	179	107	103
100	67	77	144	79	96	65
66	116	136	188	214	178	121
153	104	156	231	188	93	123
12	12	13	22	18	15	9
87	87	96	204	114	126	86
30	128	4	0	193	205	1
74	84	81	166	85	91	106
25	23	36	42	78	74	55
15	5	13	15	0	9	15
3	1	10	9	0	0	1
8	7	10	33	35	17	22
16	14	26	32	20	15	24
109	82	51	131	85	116	78
58	89	72	80	78	83	66
119	0	98	0	73	0	69
48	38	41	44	48	26	25
38	38	54	40	24	29	29
110	105	56	134	74	48	75
188	151	148	206	127	160	149
20	20	28	53	15	32	9
62	65	56	93	44	35	37
104	119	107	113	195	116	47
57	56	50	87	43	85	53
0	0	4	0	0	7	0
32	28	52	67	195	47	72
242	112	0	0	228	141	0
41	47	72	94	51	71	71
70	99	96	133	54	76	73
12	29	3	33	0	41	15
106	124	225	208	305	110	190
12	46	42	60	77	23	50
108	96	53	138	102	112	44
760	195	241	730	335	730	197
5	0	9	9	1	0	0
0	79	0	0	0	93	0
11	18	50	49	36	23	31
38	0	14	0	177	1	1
74	56	81	100	98	89	56
0	15	10	0	25	0	0
225	80	103	217	177	252	142
70	24	45	61	43	63	41
181	161	121	305	162	180	151
214	258	185	188	67	45	106
181	93	106	161	109	184	102

For Review Only

1	22	154	604	0	109	37
86	0	61	0	0	0	0
26	18	29	38	24	9	20
45	46	47	64	40	55	41
1	0	0	0	0	0	0
104	94	116	198	184	204	94
3	0	11	8	11	8	11
80	49	67	112	60	55	46
8	0	3	0	0	0	0
0	0	0	0	0	0	0
105	0	0	8	170	109	88
18	38	34	32	62	52	14
108	112	70	126	126	107	62
216	0	156	0	92	0	0
107	131	106	217	197	187	93
50	65	87	126	43	85	31
12	52	1	0	276	466	1
110	72	60	123	109	77	54
68	36	43	54	84	49	36
127	490	213	250	113	222	152
27	63	1	63	0	50	32
2	1	2	0	156	50	5
4	5	4	12	1	2	3
0	11	0	33	0	4	0
97	84	77	130	62	51	47
50	46	57	50	49	103	27
19	14	26	29	47	23	20
115	110	145	187	61	64	130
7	4	6	12	12	8	5
40	58	60	100	48	71	66
38	42	42	52	29	20	40
406	514	461	669	324	406	256
0	0	0	0	0	0	0
0	0	0	0	0	2	2
37	11	13	31	10	28	9
31	32	43	54	51	54	32
0	11	3	15	0	0	3
48	68	87	145	51	112	67
85	99	86	112	124	70	71
48	3	5	0	0	1	1
2	1	153	0	0	19	2
164	133	190	234	161	197	167
0	8	10	16	6	7	9
2	3	0	0	0	2	9
56	32	29	36	41	57	42
2	0	0	0	0	0	0
69	65	63	41	65	72	53
6	0	0	0	0	0	1
4	2	0	0	0	4	1
178	177	217	211	221	133	141

14	0	1	0	0	1	0
614	598	510	838	678	660	441
155	64	63	88	57	125	91
124	108	0	0	0	86	69
99	98	64	90	84	86	77
20	32	13	31	21	23	16
14	45	19	78	35	70	31
0	0	75	1	1	0	17
73	67	57	92	52	88	55
18	22	20	39	10	17	19
0	0	5	0	1	0	0
307	239	231	438	324	259	275
459	354	295	605	487	379	362
51	82	41	73	69	33	30
7	0	0	0	0	0	0
88	84	31	131	107	60	74
21	18	23	0	0	17	25
6	20	16	30	119	39	36
109	102	94	99	95	73	73
65	88	58	74	84	28	34
185	166	163	240	177	205	96
62	65	74	80	65	102	95
75	72	134	165	141	133	65
36	31	0	32	4	29	21
0	10	15	23	0	0	0
8	11	5	48	36	30	30
12	35	41	30	18	29	39
360	233	158	304	217	223	223
113	99	79	84	95	36	51
64	79	40	61	72	28	19
126	105	120	98	126	90	51
21	4	10	11	9	22	8
2	0	0	5	0	0	0
15	26	31	60	25	31	30
54	73	46	95	36	40	35
31	34	43	41	54	19	18
73	64	65	117	28	39	46
30	45	17	42	16	49	60
44	39	82	95	120	24	67
55	0	83	83	5	70	50
3	0	19	17	50	5	16
0	0	0	5	0	0	0
5	6	5	7	0	0	3
77	131	119	132	190	98	85
76	0	0	0	0	0	0
90	43	30	71	66	23	33
0	0	0	0	0	0	0
109	206	263	288	367	148	209
0	0	0	1	22	0	47
0	0	0	0	0	0	0

For Review Only

1551	1504	1526	865	1476	1689	1395
7	7	5	15	0	5	13
33	8	18	1	52	23	36
19	54	37	70	29	31	75
0	1	0	0	0	0	0
139	169	80	86	156	74	122
0	0	0	108	104	0	0
27	24	39	48	32	17	30
0	0	0	0	0	0	0
31	13	11	24	40	36	28
0	0	0	4	0	0	0
98	39	65	77	119	121	66
81	71	78	62	63	29	54
1	1	0	0	0	2	1
6	1	1	16	0	3	3
69	50	39	74	34	71	37
8	0	0	0	0	15	8
0	0	0	0	0	0	6
2	8	18	0	104	116	92
2	5	7	0	13	19	6
78	90	99	138	63	57	32
1	0	0	0	0	0	1
267	78	848	1093	955	1364	182
537	304	645	598	1114	593	1369
58	78	206	171	290	224	165
20	25	43	36	55	33	42
0	0	0	7	0	2	0
24	34	22	47	20	24	10
0	1	0	0	0	3	0
1	0	0	1	0	5	5
942	81	391	1537	937	1865	68
380	394	355	708	442	381	430
0	0	1	0	0	0	0
102	100	133	146	143	160	112
241	201	158	131	262	90	130
68	44	53	81	32	53	26
3	0	0	2	0	0	0
2	0	0	0	0	0	0
0	0	4	8	0	0	0
4	4	61	39	0	36	6
5	1	5	0	44	49	1
0	124	58	39	20	0	0
67	84	51	104	48	39	46
81	58	74	103	112	66	92
13	15	34	42	0	7	18
51	20	55	56	12	36	39
0	0	5	5	0	9	0
78	66	141	135	244	111	40
40	22	8	23	41	26	15
3	0	0	1	0	3	0

For Review Only

0	0	0	0	0	0	0
1	1	1	0	0	15	2
0	0	5	0	0	5	7
1056	726	758	1157	878	930	632
132	317	3	0	68	3	13
3	12	22	16	38	18	15
0	6	0	0	0	69	37
48	32	34	61	76	69	21
85	41	57	68	104	66	34
46	44	47	70	55	46	34
53	3	34	95	244	57	49
51	91	73	85	65	21	24
47	43	55	77	21	31	33
23	10	19	28	20	13	28
0	41	0	0	1	38	25
20	5	8	24	37	23	9
1	1	1	594	0	1	35
391	409	365	574	495	438	342
58	51	62	129	48	53	64
18	38	14	33	12	20	15
69	41	42	57	38	76	16
82	58	61	100	82	52	73
9	6	7	0	0	3	10
31	47	102	76	27	63	64
0	60	0	25	1	41	0
16	9	17	22	9	11	16
15	10	18	23	7	15	15
20	20	36	47	9	22	37
0	4	2	0	43	14	5
1	1	1	0	0	1	1
2119	2015	2179	4936	264	2448	2173
64	68	54	77	20	39	60
63	0	0	35	3	53	47
16	5	34	0	0	18	15
0	17	16	31	0	18	25
0	0	0	31	0	0	0
232	24	43	599	0	267	18
44	51	60	73	51	60	88
0	0	0	76	0	0	0
0	0	48	61	0	26	208
3	1	0	0	0	0	0
113	92	92	103	198	82	56
21	40	56	78	149	21	89
8	9	36	32	100	7	17
56	135	178	146	254	74	152
8	4	2	4	0	5	1
61	40	36	53	22	39	21
460	388	381	659	76	440	316
56	68	68	76	65	42	46
80	115	33	154	0	77	53

For Review Only

70	29	49	85	32	53	29
132	116	45	110	137	138	78
3	3	0	0	0	0	0
48	15	12	20	47	1	2
55	63	59	62	30	16	39
662	939	1059	1355	902	1108	992
159	261	282	215	353	95	157
142	95	42	226	251	415	170
56	48	73	86	73	85	49
147	154	125	205	174	130	121
100	130	72	135	119	63	101
87	107	154	194	156	133	104
0	0	0	0	0	0	2
33	31	84	67	138	97	49
139	198	200	243	217	105	150
129	150	148	207	119	125	170
39	36	53	110	87	40	61
22	28	33	52	9	5	17
1	0	0	0	0	0	0
5	8	12	20	0	34	21
138	1	1	0	0	3	8
477	572	0	763	1	274	0
45	28	30	43	22	19	19
183	220	202	253	123	160	228
2	0	0	0	0	0	14
37	40	33	46	89	31	28
308	179	247	393	248	310	269
78	0	0	1	0	120	0
158	195	80	177	50	105	51
328	2	6	127	87	3	8
44	37	69	48	102	57	33
63	56	90	82	109	33	78
172	148	154	246	120	155	89
112	104	92	155	127	117	78
199	248	352	365	168	321	215
228	162	182	254	151	157	159
0	0	0	0	0	45	0
125	102	20	0	155	83	62
40	36	25	35	68	47	12
69	68	36	93	18	53	53
1	0	0	0	0	0	0
0	6	0	0	0	1	0
0	0	2	0	0	0	2
59	32	62	63	92	31	26
23	67	5	89	75	33	38
17	19	10	26	49	42	26
0	1	0	0	0	0	0
0	1	0	0	0	0	0
101	90	84	172	86	87	45
88	76	100	168	55	104	111

For Review Only

156	265	0	19	573	0	0
99	69	0	0	150	40	33
191	124	160	295	134	170	171
74	52	31	164	62	96	91
0	38	0	0	0	10	0
61	112	139	137	230	179	169
141	104	138	212	157	138	110
29	17	22	86	30	27	17
281	254	141	227	234	247	186
80	49	74	109	22	60	27
3	0	0	0	0	0	0
1	0	4	0	0	5	0
205	230	119	474	228	290	211
26	63	33	59	70	52	30
68	52	68	105	114	58	64
18	24	3	10	24	9	8
57	55	39	60	65	49	46
237	229	212	416	198	303	172
73	106	37	45	133	42	81
97	86	68	134	146	71	97
9	4	4	10	2	10	6
1	0	1	1	0	4	1
4	2	6	4	5	4	6
5	1	3	2	1	5	3
12	1	5	5	3	9	5
5	1	4	3	2	6	3
16	2	7	7	5	14	8
0	0	0	1	0	2	1
45	42	76	108	114	56	53
46	109	85	144	28	50	107
666	624	555	1542	513	720	507
3	2	2	3	0	0	0
25	28	24	43	7	0	24
10	0	0	0	0	0	0
48	21	44	42	47	15	24
169	128	235	292	244	172	246
30	32	38	59	21	13	18
67	60	59	109	108	91	57
41	32	12	44	21	30	18
2	9	15	35	0	0	1
3	1	0	0	0	1	4
83	53	28	156	27	51	28
30	52	37	85	0	29	35
29	30	23	30	42	46	0
37	39	46	66	45	36	54
62	66	74	94	35	87	46
39	26	33	45	30	37	22
9	15	9	12	13	12	0
2	0	0	0	0	1	47
24	2	3	14	0	19	11

For Review Only

0	3	0	1	0	0	4
0	0	0	0	0	0	0
101	0	0	118	0	108	123
100	76	103	151	111	85	77
141	105	136	236	143	139	123
0	3	1	0	0	1	0
0	0	0	249	274	0	0
171	193	186	313	269	209	163
20	13	13	21	26	18	20
72	58	68	133	77	72	75
34	33	34	63	36	35	36
0	0	161	37	1	0	0
10	19	30	10	48	19	0
127	112	133	222	158	98	135
0	0	0	156	0	309	0
41	26	56	88	18	49	45
0	68	0	2	1	72	0
177	189	215	485	154	293	241
0	0	0	0	0	0	10
0	0	0	0	0	0	0
0	0	0	0	0	0	0
37	56	57	69	41	39	29
0	13	21	257	0	7	26
21	28	21	33	9	22	16
16	13	17	34	11	23	15
202	167	195	322	168	193	142
12	26	20	28	24	27	20
0	0	181	169	1	176	159
71	65	96	157	64	74	87
0	82	0	82	0	0	0
0	0	34	0	0	27	0
33	19	27	58	18	32	35
0	0	0	0	31	0	0
93	77	84	66	0	112	0
0	0	0	339	84	0	0
59	0	32	19	3	0	36
131	97	160	259	77	112	175
0	0	0	4	4	0	0
102	4	42	1461	0	3386	63
1	1	52	0	0	1	22
0	5	9	0	0	3	1
7	252	369	0	355	321	422
1	171	1	0	0	6	1
689	0	397	888	147	1	565
560	455	544	1133	364	754	803
208	0	241	201	0	99	182
119	103	100	207	90	147	101
565	666	445	709	719	838	408
1196	908	640	1215	1100	1216	630
65	45	76	116	54	68	45

For Review Only

2	7	6	31	0	3	5
51	37	42	99	71	42	36
1	2	4	0	0	6	8
104	3	61	117	0	100	62
0	4	0	0	15	15	0
7	4	9	5	4	9	7
19	69	50	75	17	60	26
127	108	81	140	68	130	53
270	143	113	230	172	158	113
74	57	71	27	0	62	69
435	381	520	871	391	511	466
75	44	59	0	0	68	58
81	53	67	113	49	72	59
409	257	490	714	323	397	456
77	0	65	0	0	0	0
171	124	159	242	133	180	143
31	26	34	49	33	36	33
39	43	58	86	48	45	54
43	31	51	72	45	44	46
192	0	192	440	48	133	106
0	90	54	0	0	96	94
36	0	0	241	0	0	88
8	7	9	8	11	9	8
20	38	41	53	18	24	27
166	18	85	0	139	1	130
0	108	0	22	1	256	0
0	0	0	0	0	0	0
0	0	310	66	1	356	47
0	167	204	114	53	202	157
41	28	22	70	31	41	30
175	171	238	435	148	228	289
98	34	79	135	12	56	57
890	655	713	1502	722	818	777
18	18	18	0	0	25	22
57	55	37	123	53	66	60
707	510	481	1012	112	0	506
150	0	242	679	380	135	476
4	68	72	152	55	116	526
2	12	8	10	0	26	5
59	37	58	88	30	37	64
152	159	119	208	226	143	130
15	29	7	38	11	14	41
97	77	76	121	178	52	70
7	0	0	9	20	0	0
97	118	101	102	152	174	107
2	8	3	13	0	6	6
0	0	73	164	8	65	64
1	17	36	241	0	22	32
469	359	453	710	893	615	39
165	125	146	282	16	164	123

For Review Only

569	411	410	776	550	684	575
7	18	7	14	23	8	21
156	6	1	107	54	1	4
22	25	12	24	37	9	10
10	12	17	17	34	21	13
76	50	48	110	47	66	40
2	72	14	0	0	1	1
59	67	82	184	96	81	99
75	120	163	103	215	59	79
7	0	0	10	0	0	6
4	0	0	19	0	3	5
11	27	19	16	29	15	14
178	351	203	246	260	172	293
416	465	379	626	373	223	422
87	45	59	84	100	62	29
8	0	5	0	0	2	0
18	34	28	35	96	11	16
102	88	64	131	57	120	48
0	0	0	1	2	0	0
297	215	213	376	225	235	172
0	11	20	30	11	32	0
6	18	13	40	0	34	7
87	58	60	90	82	77	73
0	0	9	0	0	23	0
192	142	146	248	181	140	117
43	38	0	82	0	24	30
25	25	37	66	2	30	22
51	42	28	34	57	15	31
22	20	10	31	45	50	43
0	6	1	7	0	10	0
57	50	89	102	70	48	41
34	58	91	149	26	59	80
21	15	29	11	5	25	4
41	59	78	89	102	53	40
37	50	16	49	8	34	19
14	12	6	99	0	10	12
77	61	83	107	32	57	29
4	4	0	0	0	7	11
46	30	26	59	34	56	26
43	28	24	70	23	46	30
1	0	1	0	0	0	0
0	6	0	16	0	3	4
0	1	0	0	12	6	0
77	83	69	159	72	94	59
33	66	64	86	81	62	68
114	115	183	237	172	198	136
0	0	0	1277	150	0	0
154	136	96	239	122	125	121
37	62	51	69	51	45	37
247	276	239	501	232	280	214

For Review Only

0	11	0	0	0	0	23
4	58	34	61	112	58	32
345	276	281	423	275	298	209
2	0	1	0	0	0	0
0	0	0	4	0	3	0
125	171	111	158	185	132	140
948	885	740	1201	763	827	587
116	129	232	251	372	61	225
43	42	42	41	55	42	18
330	193	336	424	428	224	488
31	48	50	57	26	41	23
6	9	12	13	1	18	11
141	145	136	217	114	154	100
65	31	88	86	17	72	25
147	45	38	43	25	39	103
40	42	47	79	92	31	16
152	80	53	70	107	72	69
0	5	0	5	0	0	0
133	132	107	335	96	119	186
1	2	3	9	0	0	6
1	12	1	0	1	5	1
2	3	0	7	0	0	0
37	37	41	47	48	41	39
68	98	47	81	87	38	62
49	75	80	115	74	112	48
96	85	78	86	128	80	0
28	25	40	64	0	25	21
5	11	7	19	20	10	11
5	10	3	0	24	1	24
3	0	8	9	16	6	4
34	25	18	54	24	44	24
4	5	2	8	0	16	9
3	1	0	0	0	1	0
112	119	80	124	85	97	85
0	32	7	0	56	0	30
0	0	0	10	0	15	7
12	20	26	32	1	13	25
3	2	2	6	0	4	3
8	10	21	21	31	19	18
0	1	0	0	0	1	1
0	6	2	6	0	0	5
1	1	1	0	0	1	1
5	9	13	14	15	12	8
3	0	0	16	0	2	4
26	18	23	49	38	25	25
0	0	0	0	0	0	0
0	0	0	3	15	0	0
0	0	0	72	0	0	0
188	0	190	580	88	155	258
0	0	0	3	0	0	0

For Review Only

42	17	67	63	19	56	19
29	1	1	0	608	75	21
63	63	28	90	71	44	23
42	12	26	52	18	33	36
33	45	28	55	59	40	42
30	32	30	47	32	28	12
117	82	72	103	123	97	74
2	7	5	10	0	13	0
44	109	110	184	61	100	70
139	135	115	205	181	162	106
7	59	79	145	149	165	70
4	0	5	10	0	6	5
1086	1484	1502	2584	993	1277	1423
30	57	51	57	69	60	39
332	312	323	561	364	339	299
3	3	0	0	1	0	0
12	10	12	15	5	0	10
33	19	14	28	18	35	7
5	0	3	0	0	6	0
0	0	2	0	0	0	1
0	1	1	0	0	2	2
12	78	40	63	121	58	22
271	184	160	326	128	151	108
2	7	9	20	5	3	7
104	122	108	175	96	106	78
101	104	103	196	173	149	111
301	270	369	502	223	299	314
139	147	129	260	84	53	139
1	1	1	0	263	647	1
53	53	44	109	63	79	50
7	8	6	2	19	0	0
97	80	86	200	79	124	53
0	0	0	0	20	0	0
268	211	224	476	130	288	131
150	125	179	249	201	184	147
1151	884	829	1591	967	1452	686
6	6	8	73	0	15	64
26	10	22	25	40	19	28
122	144	107	194	144	120	107
161	156	153	167	208	297	42
26	28	17	24	43	23	21
3	38	18	73	103	31	61
35	48	49	73	76	13	44
4	0	4	9	8	3	2
668	599	412	947	234	600	490
18	26	14	38	17	25	12
105	0	105	778	0	162	188
11	0	0	0	0	13	8
9	15	21	27	1	25	36
0	0	0	1	0	0	7

For Review Only

4	7	0	10	3	3	5
89	66	98	147	32	110	54
132	149	115	264	119	173	104
165	168	139	204	201	120	92
234	185	192	299	223	225	227
1	1	2	17	0	29	3
252	265	222	417	211	297	230
303	279	171	231	391	238	270
6	1	7	4	0	1	4
0	0	1	0	0	1	0
65	20	53	53	53	49	29
59	41	42	91	89	96	34
279	206	340	341	193	166	163
53	100	57	103	74	78	58
1	1	1	0	0	1	1
37	15	56	45	39	21	49
391	282	129	281	246	171	206
2299	1841	1687	3285	1936	2454	1593
0	3	0	205	67	67	0
155	138	186	266	201	187	119
572	406	259	383	541	536	292
0	1	1	0	47	1	1
129	88	60	130	190	140	80
61	44	10	37	48	82	60
5	0	0	0	0	0	0
95	86	89	181	82	115	60
470	494	637	848	559	514	430
9	12	7	24	16	7	16
4	6	3	15	32	18	2
1	3	1	0	1	2	2
63	65	68	124	38	70	69
57	33	0	43	45	45	27
0	0	0	0	0	0	1
99	66	31	155	0	53	12
47	37	35	59	15	31	14
0	0	0	8	0	2	0
13	8	0	21	14	8	8
16	18	13	0	28	11	15
9	0	30	21	25	16	12
73	66	51	77	127	47	100
0	0	0	7	0	0	1
3	0	0	0	5	0	3
0	0	0	4	17	0	0
1	0	0	2	0	0	0
1	0	0	0	5	0	4
0	0	1	0	0	0	0
0	0	0	0	0	0	0
48	34	54	158	12	50	29
0	0	0	1	7	7	1
0	4	0	0	0	0	2

For Review Only

35	21	38	22	11	26	16
1	26	1	0	0	1	38
30	500	320	1474	189	115	558
91	77	86	201	79	87	109
417	293	233	449	379	367	242
35	42	7	0	65	51	23
0	0	20	13	27	20	11
32	35	37	62	47	74	41
20	6	19	30	0	20	18
9	0	2	7	0	0	0
1	6	1	557	0	1	1
18	20	32	38	0	2	25
48	36	62	73	83	47	62
44	7	47	73	0	43	34
1	0	0	0	0	0	0
100	130	109	204	196	163	69
81	61	75	74	56	87	34
961	1041	1089	1688	770	1053	849
0	16	0	3	5	0	0
114	86	79	137	65	90	71
74	70	48	92	80	55	35
156	345	200	356	158	84	40
241	315	206	421	199	108	116
368	315	413	639	327	415	326
574	479	447	753	519	563	404
63	35	35	58	55	24	16
57	0	0	0	7	0	0
2	12	16	17	0	9	8
28	25	32	39	48	44	27
40	225	0	0	231	0	66
17	45	37	69	20	64	14
102	51	70	207	47	161	68
35	37	14	34	42	70	51
3	0	0	0	0	0	0
34	15	52	37	59	35	23
0	0	0	0	5	0	0
296	179	153	305	161	186	134
191	301	282	496	353	337	321
214	280	254	526	353	332	230
745	453	535	734	745	660	406
0	0	4	5	0	0	0
261	255	233	404	254	155	289
30	28	24	45	48	32	21
41	43	70	57	93	58	19
175	140	122	253	141	163	120
1	2	20	0	0	3	8
35	27	21	47	27	85	43
237	137	125	237	151	105	109
76	62	61	106	45	81	74
85	110	61	112	100	99	96

For Review Only

283	266	210	329	189	179	165
312	70	87	720	287	317	73
53	23	34	63	58	41	26
112	324	123	143	346	159	171
0	0	0	0	0	8	0
614	683	421	1098	286	1022	140
1	2	1	318	0	1	71
51	52	64	107	45	35	40
0	24	0	0	0	0	0
99	163	192	228	237	117	120
47	27	43	87	64	37	41
86	121	76	154	131	86	113
126	83	88	140	137	75	107
35	60	55	51	88	61	33
247	205	198	312	255	346	220
8	21	15	81	0	43	11
10	11	10	22	0	13	0
155	130	240	334	275	274	208
0	0	0	0	0	0	0
2	1	0	21	0	0	1
46	78	31	59	83	10	36
11509	12932	9547	20451	10068	16254	8778
53	59	48	86	36	51	76
18	0	0	0	0	0	0
0	0	0	0	0	0	0
22	17	6	34	0	11	4
69	136	193	175	83	152	110
17	15	36	44	117	126	30
574	596	97	192	178	468	457
58	43	88	123	79	48	68
4	0	0	0	3	0	0
0	3	0	6	12	0	3
0	0	0	0	6	0	0
25	139	1	91	0	3	57
77	32	79	94	15	55	74
199	155	219	229	161	169	178
54	62	0	0	114	0	0
80	60	83	81	84	80	52
45	55	37	64	62	44	35
60	72	87	99	105	77	68
25	49	29	85	27	46	39
179	163	370	487	247	226	324
44	46	49	64	76	87	62
85	52	68	77	80	36	51
19	40	19	42	23	27	43
11338	10721	9679	16193	7793	10869	7714
8	4	11	27	0	7	6
45	32	54	60	32	38	38
47	54	48	106	78	83	61
485	504	595	898	427	757	528

For Review Only

0	0	0	20	0	5	0
25	12	40	28	0	7	12
186	211	159	252	241	212	135
42	61	93	95	58	33	40
22	20	18	32	22	22	24
0	0	17	0	0	0	0
11	32	15	47	0	24	17
230	197	217	283	264	114	156
0	6	0	10	0	0	0
0	0	0	0	0	0	0
30	40	40	43	0	36	28
7	3	4	42	22	63	23
725	458	567	790	497	846	562
950	753	1308	2285	795	1352	1467
0	3	5	7	0	0	4
22	15	32	26	11	8	17
1	1	1	0	1689	3	1
0	0	1	27	0	0	1
10	0	0	2	0	0	0
28	14	13	4	26	9	5
0	0	0	15	0	0	0
0	3	0	0	0	0	0
35	29	15	45	39	51	31
0	6	0	5	7	1	1
0	0	9	1	0	0	10
2013	1516	1371	2583	1296	1870	1151
32	0	0	98	19	31	59
4	0	7	4	0	2	4
0	0	0	0	0	0	0
4	0	0	0	0	0	0
3	0	0	0	0	0	0
61	3	18	42	0	27	11
118	88	103	175	138	139	95
0	47	0	3	1	0	0
533	515	479	888	521	522	439
18	18	8	39	0	19	28
0	0	0	0	5	0	0
3	1	0	0	0	0	0
11	0	23	30	25	0	29
11	15	20	20	14	15	23
0	0	0	1	110	0	0
88	36	68	111	90	66	55
123	171	261	261	234	167	176
163	186	143	171	141	135	103
6	9	16	24	5	22	11
157	129	237	271	297	198	188
0	0	6	6	2	0	6
53	27	50	49	0	8	12
221	167	160	309	151	138	176
0	0	0	15	0	0	0

For Review Only

17	16	28	0	26	15	10
13	2	7	1	0	2	6
71	89	80	104	64	56	55
0	0	0	1	7	0	0
0	5	0	0	0	0	0
142	223	283	399	208	199	244
154	115	154	192	218	197	108
21	7	0	3	0	8	5
96	119	113	179	98	102	76
988	687	1054	1088	1146	1080	884
323	282	206	372	392	386	230
0	0	0	0	0	0	0
0	0	51	20	153	0	44
44	96	61	143	98	86	60
77	51	45	108	65	34	62
6	11	35	84	17	77	23
129	133	131	190	106	117	113
4	8	11	11	28	20	20
1	1	3	5	2	3	0
82	229	128	166	571	141	285
97	113	115	189	95	102	80
131	84	51	150	40	119	67
647	655	718	1313	358	868	21
796	423	193	12	78	746	0
22	31	13	30	20	8	14
51	36	41	81	32	44	35
64	41	55	97	28	36	16
112	76	53	110	95	98	65
61	16	11	39	55	29	20
19	28	27	58	22	31	15
23	22	32	17	1	110	0
0	0	0	0	0	0	3
190	135	190	361	183	224	207
58	39	0	81	0	0	0
0	1	1	0	0	12	8
241	0	0	28	0	191	0
4	0	2	0	0	0	0
73	151	125	158	76	109	79
99	93	84	125	112	99	73
9	0	3	9	0	2	0
2	0	2	0	0	0	3
0	3	3	3	0	0	0
1394	744	719	840	575	718	622
23	54	96	74	139	36	81
0	12	6	6	0	2	3
135	25	71	91	15	133	73
13	2	16	16	0	14	19
83	72	38	163	0	63	65
216	247	161	337	281	306	132
5	21	4	0	0	4	1

For Review Only

115	90	102	162	146	113	78
115	80	102	139	189	158	93
28	15	118	33	4	4	36
20	2	6	18	1	0	3
173	162	137	254	155	215	159
147	10	87	84	0	46	109
5	0	0	0	0	1	0
10	8	9	16	0	13	18
1	0	0	4	3	1	0
21	1	4	32	0	10	14
17	3	5	17	6	2	4
493	314	472	794	400	478	349
251	156	171	248	222	66	0
217	167	140	187	86	76	163
4	0	1	0	23	8	1
62	53	52	57	95	45	36
79	59	55	91	35	49	42
5	0	0	0	0	0	0
0	7	0	3	0	0	3
0	7	0	0	66	9	3
419	381	533	869	418	17	70
1	7	1	0	169	1	1
213	263	232	336	170	168	199
55	23	45	114	86	54	81
1	4	7	1	0	2	2
0	0	0	0	16	0	0
0	1	1	1	0	3	1
271	264	16	243	96	190	111
0	7	0	2	3	0	0
1	0	1	8	0	3	1
3	1	1	0	1	0	2
0	0	0	7	0	0	0
12	10	23	30	23	19	11
19	68	89	0	71	602	63
0	1	0	0	0	3	0
3	1	2	14	0	1	5
0	3	0	5	2	6	3
95	79	105	262	39	98	103
1	0	0	0	0	0	0
521	532	397	1169	106	274	0
0	6	0	0	4	0	0
15	6	9	0	0	42	2
1	118	22	167	179	423	111
19	5	0	18	0	9	8
33	43	38	98	45	80	34
31	55	45	61	27	19	34
9	12	8	14	30	26	15
39	53	92	88	50	42	39
11	11	16	20	21	22	23
9	9	2	40	0	20	4

For Review Only

0	0	0	0	0	0	0
2	2	2	7	0	0	4
107	91	99	153	86	139	65
19	25	37	29	44	33	23
2	1	7	6	0	11	1
6	6	26	24	40	11	5
1	1	187	285	684	2	300
0	0	0	8	0	0	0
0	11	0	5	0	13	1
99	40	8	121	250	225	47
117	90	75	127	74	123	60
14	4	6	29	0	23	15
2363	2758	1400	4005	2440	2764	1522
81	104	62	102	95	71	49
0	0	0	1	0	0	3
164	139	139	238	137	174	92
26	40	47	50	30	41	24
58	37	14	90	79	43	24
93	79	53	136	66	71	100
36	74	58	35	38	18	35
87	100	84	129	116	101	156
109	95	97	154	78	127	84
272	313	253	95	196	320	201
158	61	127	186	149	169	155
240	299	200	447	497	465	182
20	66	111	92	69	56	36
5194	290	2116	848	1073	2005	15
12	10	24	16	0	5	29
3	0	0	0	114	142	93
19	14	27	28	34	42	23
4	4	17	23	18	16	12
26	11	11	29	13	16	12
1	37	1	25	0	16	4
0	0	34	0	0	0	159
40	20	30	53	24	34	0
0	0	0	3	0	4	1
10	2	0	7	0	6	4
4	6	9	21	0	7	10
0	0	0	0	0	3	1
0	0	0	0	0	4	0
240	246	280	460	268	286	240
218	354	40	239	0	551	339
0	0	3	1	0	5	1
330	413	475	725	334	463	604
4	5	1	0	0	0	8
5	1	1	0	0	1	1
84	88	72	111	83	91	45
163	200	192	264	267	168	133
21	36	25	19	0	22	13
883	425	272	397	781	1213	385

For Review Only

0	0	1	0	0	6	7
73	36	31	62	161	34	49
57	18	18	19	25	36	22
83	5	113	180	86	84	56
2	2	8	6	7	0	1
80	80	85	108	38	76	41
152	114	210	324	150	245	197
1	1	1	0	0	209	1
4	0	6	0	6	3	7
51	9	25	686	0	51	8
4	0	0	4	0	0	0
42	59	46	74	70	72	41
914	18	352	1570	209	446	533
58	46	32	129	70	64	47
101	48	151	143	46	81	188
121	67	8	99	0	45	16
0	0	0	0	0	0	0
302	445	422	590	379	419	482
66	87	45	54	52	67	28
0	4	0	0	0	63	0
0	0	1	0	0	3	0
65	44	50	59	50	58	39
1	1	0	0	0	0	0
6	8	8	14	6	10	13
30	35	13	34	0	10	2
154	140	99	300	126	242	143
417	499	450	689	133	165	160
30	23	9	10	6	16	11
22	27	33	29	34	15	23
4	0	1	5	0	2	1
29	62	46	113	27	97	39
21	0	0	5	32	0	3
120	96	62	114	133	120	70
3	5	0	4	0	0	0
495	707	630	607	633	542	497
54	56	80	123	83	82	109
6	0	0	0	0	0	0
6	0	0	2	0	0	5
48	60	61	106	38	58	77
27	33	31	67	50	31	33
5	1	2	15	0	1	3
28	29	39	47	34	77	41
37	50	39	91	19	53	29
69	65	0	92	65	100	49
11	6	18	7	16	1	4
0	2	0	0	0	0	0
0	3	0	1	1	0	2
342	373	325	523	261	368	371
14	17	9	14	75	9	77
4	26	3	21	7	8	3

For Review Only

99	68	33	91	149	58	74
22	16	7	26	11	20	12
31	1	1	64	0	1	1
16	7	32	41	1	33	26
2	2	8	12	0	1	4
13	1	3	12	0	4	4
67	11	33	42	24	42	34
0	0	0	0	0	0	0
0	0	5	6	0	0	5
290	227	259	399	371	439	253
0	0	0	0	0	1	0
9	10	3	17	0	14	27
45	44	55	40	41	39	26
92	89	100	0	0	71	55
43	28	55	59	16	30	23
0	0	0	0	0	0	0
105	92	68	120	87	93	48
32	4	2	0	24	6	1
0	9	13	16	0	8	8
8	3	9	12	0	3	2
14	7	8	13	18	11	11
107	173	77	223	70	122	102
1	0	0	6	0	4	6
42	73	0	78	0	36	52
1	1	1	4	0	1	1
41	36	33	52	34	27	21
1	1	1	0	0	0	24
12	27	21	18	30	26	6
187	56	26	111	0	91	9
3	19	5	30	0	13	3
22	28	15	49	35	15	14
4	1	33	0	0	3	58
7	9	4	17	0	13	5
0	0	12	0	0	0	0
71	13	2	23	15	17	15
21	37	24	37	39	48	28
9	11	13	11	5	12	12
18	9	14	26	9	10	16
150	186	141	305	200	147	232
378	433	335	568	281	375	312
63	42	47	86	34	33	46
64	0	0	53	12	63	58
304	137	205	365	319	249	163
11	11	25	30	0	3	27
9	9	6	17	6	12	7
8	22	3	14	0	0	8
102	72	79	86	91	136	56
16	219	0	415	477	113	0
119	106	95	100	55	45	47
0	0	1	0	0	1	1

For Review Only

797	835	811	1224	797	919	774
121	70	56	88	53	91	36
2	1	0	0	0	1	1
7	26	7	9	31	5	16
0	0	0	1	0	5	0
23	27	26	37	18	28	30
110	74	56	86	105	87	50
20	16	0	25	29	30	22
79	70	5	42	0	28	11
6	0	12	17	0	6	0
72	62	48	37	126	66	44
417	282	300	711	393	469	141
19	1	8	12	0	12	3
1873	1286	1115	2136	1174	1770	1446
15	0	0	2	0	0	0
83	64	62	109	98	69	41
0	1	1	0	0	1	0
19	0	3	0	16	0	0
10	12	8	20	31	4	5
56	58	24	0	94	0	19
501	102	26	0	7	270	69
5	2	7	32	0	1	1
0	0	16	0	0	0	2
1	1	1	0	0	15	1
16	6	10	20	5	10	11
0	0	0	0	0	0	0
47	17	41	42	0	24	56
60	136	79	118	32	78	51
36	39	28	75	25	38	31
198	203	108	346	236	209	174
65	103	101	95	49	63	46
302	89	72	216	39	153	42
1	6	676	0	525	3	1
113	44	75	92	66	109	69
66	85	108	149	104	81	61
42	20	37	89	24	38	47
44	83	71	73	114	30	69
153	91	110	131	172	172	108
12	1	48	0	0	1	1
558	269	433	469	289	12	80
94	37	47	64	83	61	45
277	138	128	138	276	202	94
96	114	66	126	148	119	55
309	266	180	165	397	566	164
13	38	18	26	0	7	4
49	22	19	30	28	48	21
181	81	45	181	27	171	66
119	136	109	0	0	115	118
37	52	36	71	56	48	17
19	16	14	23	23	12	12

For Review Only

0	0	0	0	0	0	0
4	4	5	10	5	7	1
100	77	97	109	40	76	132
0	0	0	0	27	0	0
4	1	0	4	0	1	2
1	9	1	0	0	0	1
102	173	184	396	86	216	224
13	11	5	30	12	19	9
24	22	41	56	61	30	28
20	2	15	14	30	31	0
27	191	197	0	0	110	130
7	46	21	53	1	75	29
42	55	54	0	40	16	0
168	155	197	235	241	172	171
479	337	299	382	461	454	213
195	137	161	247	185	179	129
21	0	0	0	0	0	0
0	0	0	0	0	0	0
27	30	96	95	16	63	61
191	155	127	193	246	247	192
186	157	175	273	161	214	184
413	363	360	672	400	402	361
107	139	49	164	136	80	86
83	56	54	103	51	56	45
47	15	13	80	19	59	8
53	49	51	0	0	44	75
37	3	19	29	0	17	12
6	0	1	11	0	1	1
176	117	72	147	142	132	72
49	33	21	31	24	60	36
75	40	61	68	85	137	42
1	1	1	0	0	714	1
61	49	35	34	43	20	22
208	121	115	232	134	191	127
398	480	196	410	426	479	117
26	18	23	0	0	14	0
47	47	32	93	80	48	47
56	73	59	99	80	69	44
13	0	5	11	30	0	22
3	1	7	274	0	336	16
6	5	3	7	0	8	4
37	24	26	36	17	29	33
178	0	66	136	3	807	229
0	0	0	0	0	0	0
60	25	77	136	28	83	78
404	290	324	505	445	386	281
0	1	2	0	0	0	2
11	191	27	193	116	42	32
178	35	34	63	161	12	108
11	2	2	0	0	0	0

For Review Only

66	29	59	78	52	38	53
159	90	48	115	84	66	41
0	4	2	0	0	0	0
0	0	0	0	0	0	8
12	0	0	0	0	0	0
125	176	97	183	155	107	159
0	0	0	0	0	0	0
1	1	1	359	0	7	0
0	0	0	0	0	2	0
30	22	24	36	55	29	24
0	0	0	0	0	0	0
149	195	95	240	134	98	100
4	2	0	0	0	3	0
67	21	17	61	27	32	32
0	0	0	0	0	0	0
0	0	0	0	0	0	0
7	0	2	0	4	1	0
361	316	321	472	251	408	278
2	3	12	22	0	1	7
1	0	0	0	0	1	0
22	11	12	11	27	32	17
5	0	0	0	22	0	0
12	8	11	22	11	16	8
2	0	0	0	0	8	7
9	12	0	4	41	9	7
18	7	35	17	71	8	23
24	19	47	175	25	55	65
27	24	31	38	25	19	35
0	0	256	280	1	453	281
1442	1004	766	1715	1370	1825	853
409	443	386	691	395	475	646
0	0	1	0	6	0	1
102	69	62	107	85	77	62
98	65	66	158	60	223	67
53	44	75	75	50	47	32
0	4	1	5	8	0	4
1	0	0	0	0	4	0
80	48	42	55	53	20	26
64	82	66	97	31	63	94
169	119	147	159	115	219	138
502	387	474	692	729	685	407
0	0	0	0	0	0	0
15	0	0	0	52	0	0
40	2163	228	33	3054	768	7
149	218	4	353	138	127	196
0	8	11	21	0	0	24
0	0	0	2	4	0	0
23	15	23	11	18	37	22
56	44	71	97	82	61	20
0	0	4	1	0	0	0

For Review Only

6	3	0	13	0	0	3
9	6	19	9	29	9	0
124	111	167	196	107	232	94
5	21	17	14	15	21	9
13	11	99	126	0	3	37
2	1	3	9	0	11	0
14	12	18	18	39	25	47
131	134	178	179	162	157	130
6	8	16	28	0	3	21
65	67	92	102	96	53	45
75	52	83	133	67	70	81
5	86	86	269	0	1	41
40	61	28	78	25	41	25
31	17	29	36	18	21	29
83	49	67	98	50	64	62
116	87	130	154	197	116	102
267	413	241	331	376	358	215
47	72	69	122	56	77	53
111	129	123	231	110	106	87
8	0	1	6	0	2	3
91	74	101	129	86	62	74
45	45	5	65	12	4	3
52	99	40	79	112	33	46
21	17	31	24	38	21	16
191	179	86	231	238	126	148
118	224	81	285	320	307	160
10	28	32	37	51	18	16
2	2	6	5	11	1	2
63	68	60	79	100	46	26
0	0	0	151	42	0	0
70	95	70	199	34	104	55
33	67	24	54	68	33	27
71	9	43	0	245	1	31
11	25	22	28	40	18	25
45	0	90	35	181	5	0
0	1	0	0	8	0	0
77	49	37	83	71	42	60
101	121	76	207	58	136	134
0	16	17	0	8	22	0
51	27	116	163	146	75	61
44	24	15	25	44	19	29
6	30	21	47	0	39	33
70	57	84	121	30	49	63
66	86	99	84	95	40	102
250	202	135	344	238	202	253
101	91	101	147	87	69	55
48	101	77	113	119	52	88
139	89	146	157	198	149	189
470	394	340	500	262	260	253
138	0	93	32	3	124	89

For Review Only

53	66	71	86	163	169	149
88	149	148	121	77	59	66
95	85	66	91	105	58	43
0	44	0	59	118	134	72
0	0	0	21	1	22	0
113	94	111	97	113	62	77
33	25	16	39	7	18	11
0	30	0	62	21	0	25
84	65	58	116	61	85	58
16	5	44	0	34	12	9
130	166	44	271	67	241	116
56	35	30	345	255	1	20
59	33	16	23	71	11	3
48	54	78	103	62	52	57
0	0	0	20	11	0	0
0	5	9	13	2	0	4
58	48	48	104	31	37	60
10	10	7	0	13	19	0
9	1	12	18	0	13	3
3	2	3	6	0	10	4
202	176	180	301	215	215	150
33	39	23	53	36	26	30
0	0	0	0	3	1	4
18	23	20	19	10	15	0
98	79	61	95	218	94	83
77	69	79	59	124	26	101
3	0	0	0	0	0	1
0	0	0	0	0	0	0
347	257	113	385	264	330	239
212	194	139	238	224	175	122
56	58	54	0	0	52	31
19	8	3	2	22	0	3
55	94	33	106	26	66	67
22	1	0	2	0	0	0
81	48	47	106	66	74	65
47	18	28	38	19	37	35
15	11	12	24	20	22	12
7	3	0	11	0	11	0
0	0	0	5	0	0	0
259	250	231	403	236	236	223
89	57	51	81	1	36	25
164	89	113	155	62	131	85
57	64	96	89	78	50	99
0	0	11	7	1	0	6
74	74	67	144	163	145	112
84	46	45	88	68	76	49
87	1	132	146	0	50	21
137	115	124	216	99	145	108
3	1	0	0	0	1	0
37	60	47	93	71	54	75

For Review Only

118	91	53	96	71	85	66
51	48	39	88	59	73	37
1	11	2	0	0	9	65
101	88	149	240	96	184	106
45	33	31	115	107	99	84
65	83	83	138	62	75	42
101	50	115	92	148	66	100
225	135	116	206	139	176	121
8	17	4	14	4	14	8
0	0	0	1	40	0	67
2	4	1	0	6	3	0
0	3	0	1	0	0	1
0	0	0	0	19	0	9
28	19	25	43	22	54	26
0	0	0	0	0	0	0
45	64	64	164	80	83	82
3	2	3	14	0	1	1
0	0	0	0	0	15	0
5	6	0	0	0	10	2
3	14	18	31	0	2	7
66	10	55	97	19	66	58
1	0	1	0	0	1	0
55	44	78	111	63	10	26
48	176	167	164	425	187	294
0	1	2	3	0	2	0
0	0	0	36	26	0	0
0	0	0	2	2	0	0
3	3	1	3	3	2	4
0	0	0	5	0	0	0
23	13	7	25	0	13	4
3	2	2	1	5	5	2
1	1	5	0	0	4	2
19	11	13	1	6	11	14
12	18	12	34	6	30	28
8	3	14	17	0	17	13
0	0	1	1	3	0	0
0	0	0	0	1	3	3
1	1	1	0	0	1	1
7	2	2	0	0	5	1
5	1	4	6	2	4	4
1	1	0	0	0	4	1
10	10	7	19	1	16	14
4	4	5	0	0	4	9
5	0	5	5	0	1	3
4	0	1	3	2	3	4
0	0	1	1	3	0	0
3	4	4	7	0	2	2
3	0	1	0	0	3	2
4	5	3	6	0	9	5
6	6	4	11	7	4	4

For Review Only

0	1	1	0	0	1	2
0	0	0	5	1	1	4
0	0	0	2	3	3	1
2	1	0	3	3	1	0
0	0	0	13	14	0	0
3	1	3	5	1	1	3
39	33	35	84	9	10	8
213	156	135	222	86	95	203
125	246	195	269	160	199	203
14	0	15	25	0	16	12
41	57	61	87	88	75	34
20	45	41	53	42	28	21
112	81	85	136	103	105	70
1	1	3	0	0	1	23
263	28	111	239	427	60	192
168	157	119	216	173	165	110
7	0	0	0	35	0	0
62	221	0	226	382	0	17
0	0	0	21	50	0	51
12	14	9	24	34	21	15
25	41	40	69	18	26	44
25	31	16	31	0	15	11
46	38	0	24	1	43	0
57	55	0	33	16	56	0
0	7	22	0	12	13	10
32	21	0	70	30	31	0
0	143	71	127	153	0	5
0	0	0	2	4	0	0
10	9	15	5	13	19	6
0	1	0	0	0	0	0
32	33	30	34	42	15	16
44	80	96	238	270	41	36
190	184	186	280	173	204	148
99	81	98	114	154	163	42
69	73	50	99	67	62	24
21	70	27	78	71	69	30
216	183	110	200	317	159	184
193	117	121	145	169	232	110
55	39	40	102	41	78	29
53	13	45	20	11	20	22
0	199	104	233	467	218	87
324	334	427	308	564	279	369
224	178	234	225	181	233	179
200	215	193	272	150	160	191
217	0	155	379	231	96	126
30	16	13	21	28	9	7
13	14	6	26	6	8	15
57	52	89	123	48	68	67
6	7	18	14	21	12	26
52	56	48	146	148	41	30

For Review Only

282	347	382	524	285	373	237
215	303	241	335	396	136	147
76	90	96	106	128	87	109
27	15	16	42	14	7	11
13	29	12	10	10	3	9
187	139	136	211	137	188	137
626	541	856	330	410	134	187
231	320	263	471	397	463	188
10	0	0	18	0	0	0
158	169	95	155	86	88	81
26	53	35	50	106	36	74
252	275	284	420	288	299	259
5	0	4	0	0	0	5
0	4	4	7	14	5	4
3	3	3	12	0	0	0
0	0	0	0	0	0	0
36	48	37	65	18	9	31
57	28	41	72	27	47	39
9	152	90	37	131	53	146
52	60	38	110	64	50	55
294	0	211	498	57	263	199
71	74	69	114	91	104	68
3	0	0	0	8	1	0
2097	2195	1800	3401	2121	2253	1953
753	121	261	232	105	751	159
4	1	2	0	26	2	4
3	2	0	0	0	0	0
8	0	0	0	0	1	0
202	49	12	181	0	7	36
10	166	83	0	166	80	42
181	299	458	210	1158	140	298
10	20	77	75	1	34	36
36	29	22	58	24	28	27
17	0	3	0	0	0	0
516	533	432	690	450	545	556
0	3	3	3	0	1	0
0	0	3	0	0	5	1
2	2	4	11	0	5	5
0	0	0	0	0	0	0
23	11	109	47	212	16	138
0	0	3	0	0	1	1
1	0	0	0	0	3	0
0	2	3	0	0	0	0
5	0	0	0	0	0	0
106	101	154	193	93	108	118
22	39	61	55	12	28	26
63	46	55	104	31	74	93
43	46	27	73	53	61	47
34	26	17	34	65	79	37
148	89	55	104	109	51	94

For Review Only

6	3	4	13	0	3	11
40	21	53	62	46	60	54
0	8	0	0	0	0	0
56	37	44	78	49	55	72
19	10	26	21	11	9	8
0	0	0	3	0	1	1
0	0	0	0	0	0	0
0	4	0	2	0	0	0
57	85	81	86	62	85	273
5289	5185	4253	8162	4878	5481	3545
0	0	0	0	0	1	0
54	51	55	75	45	22	53
2	2	7	0	20	16	7
658	512	428	890	346	580	361
0	0	0	0	0	0	0
129	103	77	88	128	115	57
0	0	3	1	0	5	0
5	0	0	0	5	0	0
55	37	54	68	75	115	12
0	0	1	0	0	8	2
45	46	54	53	64	50	43
4	4	4	6	8	4	1
18	13	20	8	26	15	9
1	4	7	13	24	29	3
0	3	0	0	4	0	0
5	7	18	18	3	17	13
2	2	2	0	0	0	0
1	8	0	3	12	0	10
134	126	0	135	0	133	121
442	557	435	877	569	557	461
411	410	274	368	260	326	231
4	0	5	7	0	0	7
0	0	0	11	0	0	5
154	113	166	409	57	170	219
104	81	73	121	82	102	65
0	0	0	0	0	16	0
1	0	0	0	5	0	0
57	27	49	83	69	54	50
100	75	81	108	86	85	44
82	94	104	138	126	106	77
109	117	113	125	183	242	155
254	151	111	277	86	177	76
76	50	26	44	93	68	16
78	38	15	68	17	58	29
381	374	259	606	377	240	162
31	17	13	41	20	25	19
42	49	59	45	55	48	22
102	67	108	121	82	90	88
0	0	0	0	0	0	0
20	0	3	0	0	0	0

For Review Only

88	51	73	142	91	105	50
98	8	20	88	0	55	1
44	45	43	78	32	45	52
0	0	0	1	1	1	0
4	2	2	19	0	10	1
162	120	129	222	100	161	72
41	43	40	79	73	45	66
0	0	5	1	0	1	0
367	357	442	600	279	465	369
185	0	0	116	46	148	82
63	96	145	169	80	98	93
45	30	32	0	0	27	33
66	41	80	118	132	85	118
224	59	73	158	191	120	170
0	1	1	3	0	0	5
106	48	78	54	54	27	56
38	15	10	44	19	23	10
31	50	41	36	87	55	29
24	71	25	59	0	4	20
14	18	48	58	68	38	21
492	378	330	183	505	497	356
39	23	5	34	35	46	8
76	28	0	0	0	0	0
60	30	44	70	14	34	73
6	5	0	0	0	0	2
6	10	10	15	0	19	15
1	7	0	11	0	3	0
1	6	0	5	0	0	2
56	14	36	73	0	159	83
70	89	122	146	39	194	53
276	286	214	458	230	238	190
43	153	30	90	38	123	149
125	102	120	126	113	96	77
0	0	0	0	0	0	1
10	0	6	6	0	6	4
203	0	210	600	83	220	205
1294	443	377	1431	667	1631	14
123	41	0	58	65	13	50
65	45	46	57	80	63	95
60	80	108	70	122	39	39
438	316	201	509	397	231	349
1	0	0	1	0	0	0
0	0	3	0	0	0	0
194	131	81	253	67	197	95
108	52	65	77	36	170	38
88	24	7	43	55	44	42
152	139	147	194	128	190	165
24	39	39	60	63	24	27
8	16	11	35	7	28	23
69	32	41	50	60	46	24

For Review Only

4	0	494	211	838	3	0
22	12	1	13	0	7	17
155	140	196	298	157	163	161
125	72	79	184	102	80	73
10	5	6	11	0	87	26
0	0	0	169	80	0	0
207	172	166	283	152	144	122
75	90	74	140	67	80	68
71	61	59	94	82	70	51
103	100	80	128	115	96	60
97	78	66	110	104	95	71
170	130	89	231	106	212	86
121	97	94	116	186	176	98
95	93	9	158	129	85	79
38	51	42	95	19	45	41
101	100	50	137	70	79	100
216	179	0	77	157	340	110
257	345	210	379	237	246	171
71	35	45	83	36	29	51
20	14	13	30	29	4	14
315	241	299	456	340	320	281
15	32	71	0	0	1	1105
80	75	39	51	72	65	38
46	55	87	111	76	78	73
120	104	114	141	99	145	153
39	176	41	80	17	33	155
92	226	0	61	176	242	0
0	0	0	103	36	0	0
164	85	42	89	82	84	76
0	0	0	1	0	0	0
1	64	163	638	28	0	168
0	0	249	1	157	67	12
30	21	0	80	88	56	77
1	0	10	18	0	9	0
254	247	215	309	291	330	177
14	12	11	21	32	22	27
94	134	112	229	121	134	122
103	112	6	72	110	125	66
5	119	17	367	0	2	26
27	32	25	49	42	30	31
68	43	77	80	117	44	45
3	0	0	0	0	2	1
152	112	205	214	141	144	126
73	118	74	100	117	58	62
991	843	1037	1481	1410	791	1107
26	11	9	0	0	27	22
0	0	24	0	0	0	0
34	2	4	8	0	8	2
11	0	0	0	0	13	0
377	725	537	827	346	582	431

For Review Only

263	263	169	372	213	256	177
335	333	297	429	218	453	270
0	204	7	0	17	0	0
78	81	66	123	80	89	69
22	29	17	0	0	21	8
0	6	0	0	0	0	0
211	163	146	231	205	135	153
0	6	6	7	0	5	6
187	203	136	253	174	145	96
37	33	26	44	43	43	33
0	0	0	0	0	0	0
8	25	7	8	6	3	6
136	154	188	231	268	141	137
23	33	35	64	54	53	33
63	53	37	63	57	17	25
194	211	136	248	291	112	244
248	303	273	669	205	216	243
41	61	82	66	140	103	115
199	126	222	186	244	194	125
2	0	1	22	0	0	0
0	1	0	0	0	0	0
690	443	454	702	804	754	529
18	23	12	0	0	32	19
0	0	0	8	24	0	0
153	132	111	146	194	94	58
2	2	9	15	0	3	32
0	0	0	0	0	0	0
7	11	26	16	15	10	24
24	18	22	35	14	25	27
1	158	1	2067	0	4454	52
0	0	0	0	3	0	0
80	140	172	146	143	83	123
208	400	180	243	26	26	121
0	0	0	4	37	0	18
121	116	46	76	177	60	86
54	71	105	76	71	69	58
78	67	73	134	109	100	72
118	80	130	260	128	190	128
2	1	0	5	0	2	0
35	15	15	20	30	23	27
2	0	0	0	0	0	0
380	0	0	260	0	682	279
6	167	11	314	0	1	6
1	5	0	8	0	2	6
19	93	3	98	0	61	19
0	0	0	0	5	4	4
63	38	65	70	33	37	49
192	96	50	279	59	134	145
736	0	586	290	477	655	657
142	144	166	272	161	192	157

For Review Only

53	40	91	69	54	83	5
369	275	138	547	92	903	78
74	61	42	68	68	55	60
138	133	170	230	178	154	147
386	443	360	650	294	644	409
24	0	33	73	178	35	0
20	19	32	22	42	36	6
11	12	5	32	7	7	5
10	0	0	0	0	0	0
4	4	1	5	0	2	0
1	16	14	6	14	6	0
3	2	2	0	0	2	0
171	86	93	148	158	128	121
27	33	19	31	29	15	17
0	0	0	152	11	0	0
307	358	203	527	382	390	254
36	27	53	101	35	66	50
92	116	115	138	102	98	96
135	119	31	0	0	0	0
105	97	89	128	91	90	107
18	21	22	38	13	21	16
84	62	73	141	112	81	89
0	189	0	15	233	0	127
8	0	0	6	9	12	0
48	71	46	79	51	64	44
18	46	22	38	58	9	19
13	5	0	7	0	4	4
69	91	70	67	25	126	78
32	27	42	51	43	44	39
13	1	0	0	0	0	0
88	89	113	223	69	131	119
60	92	90	105	149	57	103
11	13	11	30	25	16	16
63	16	8	32	41	14	17
160	62	70	140	184	131	96
57	67	0	0	0	14	62
0	0	3	7	0	1	3
18	27	23	46	21	10	23
2	3	0	1	0	3	2
0	2	5	0	6	0	0
11	7	28	13	0	96	11
96	80	55	91	157	83	103
0	118	87	164	27	84	62
159	145	123	186	168	128	92
19	31	61	61	0	12	24
185	131	93	196	161	124	110
9	0	0	17	0	0	0
81	63	108	95	58	34	85
46	22	48	90	66	45	50
58	88	17	132	96	73	94

For Review Only

541	557	459	667	553	638	454
69	74	69	89	126	46	88
1	1	1	0	0	1	7
1	0	1	0	0	0	0
116	89	114	174	186	177	99
153	155	188	264	142	149	146
74	75	61	98	51	51	64
88	119	52	175	78	73	77
0	0	0	0	0	0	0
16	10	17	20	11	13	7
138	150	106	197	141	125	111
22	7	8	29	46	19	12
5	2	15	28	0	1	10
0	0	0	0	0	0	0
74	111	46	124	80	31	58
416	417	427	681	450	464	324
22	33	21	70	48	67	50
42	39	40	53	48	40	45
0	152	75	34	0	0	0
56	100	126	189	58	106	111
26	21	19	46	10	18	10
19	17	13	46	30	24	23
102	106	105	135	75	86	93
17	26	15	22	35	11	23
10	10	4	13	0	6	19
41	56	85	129	55	88	63
70	87	105	162	139	137	56
115	115	117	205	137	150	128
88	81	61	108	100	119	73
26	15	61	54	108	30	54
19	21	5	22	63	9	35
85	88	65	104	76	119	119
49	46	49	105	98	112	57
133	119	85	125	151	139	111
102	96	131	185	74	108	104
31	0	2	0	0	0	0
22	15	7	0	13	33	16
0	107	76	2	30	175	99
1	4	1	6	0	0	0
8	0	4	0	0	2	0
2	3	4	4	6	4	4
68	109	116	131	78	50	59
71	83	71	182	1	0	52
103	75	84	128	96	94	76
53	58	52	83	64	57	59
244	620	61	397	0	101	19
129	50	59	84	75	177	55
534	531	445	404	366	84	204
2	9	1	106	0	1	1
127	105	119	182	108	116	65

For Review Only

233	203	235	253	319	274	254
40	20	46	0	37	38	8
0	38	0	105	36	90	88
39	27	72	81	45	35	47
0	134	107	209	136	88	111
142	52	102	119	112	114	145
22	92	29	127	0	10	79
67	35	74	47	52	26	21
1	5	56	0	0	17	20
0	0	0	0	0	0	11
722	701	567	1092	656	781	535
102	73	71	51	59	72	44
8	5	8	12	0	6	7
0	0	0	0	0	1	0
0	826	515	1235	464	0	677
99	111	88	178	99	177	79
57	33	46	76	47	38	22
0	1	5	6	0	7	0
112	77	169	158	160	116	113
0	0	0	0	0	0	75
23	14	30	28	22	19	17
0	29	37	63	0	4	36
35	49	51	93	29	44	49
57	31	69	65	76	49	62
30	16	13	13	28	13	4
214	89	196	206	97	127	160
93	41	84	111	108	69	69
8	6	17	12	8	9	10
48	51	50	75	55	53	41
20	12	28	44	26	13	24
0	0	0	0	0	0	0
0	13	9	0	0	11	5
0	0	0	6	0	5	0
0	1	0	0	0	1	0
86	141	143	203	87	108	93
365	451	272	577	356	330	313
82	47	93	55	94	31	37
3	9	3	15	0	16	20
117	148	97	195	127	140	116
11	0	0	1	21	0	0
20	13	6	26	0	1	1
0	9	0	2	0	0	6
25	22	22	33	19	12	26
448	831	782	328	399	0	0
0	0	0	28	8	0	0
1	0	0	0	0	0	1
85	145	211	245	138	121	89
24	35	72	53	12	45	44
83	57	71	156	73	75	59
35	41	49	74	76	27	47

For Review Only

0	0	3	8	3	3	2
0	0	0	0	0	0	0
0	0	0	0	0	0	0
98	118	81	89	0	29	13
0	0	0	0	4	0	1
383	330	317	563	392	422	303
73	75	85	94	55	54	43
42	33	60	91	52	47	52
0	0	0	2	0	0	3
5	6	4	6	3	3	5
1	0	1	0	0	0	0
0	2	0	1	0	0	2
27	21	21	12	19	19	8
2	5	3	16	9	2	2
0	0	2	0	0	0	0
3	1	0	0	0	4	0
102	106	63	0	0	75	69
75	67	59	106	90	76	86
36	96	57	168	28	90	77
0	0	0	0	0	0	0
88	54	38	94	114	118	56
311	288	338	473	252	292	318
10	19	44	49	0	6	15
8	0	1	0	0	1	0
1214	1444	653	1218	2174	1823	961
10	8	6	12	4	4	18
0	14	21	25	40	15	18
15	3	6	13	0	9	7
123	114	0	206	79	138	0
23	22	40	34	50	26	35
23	22	29	16	4	18	20
42	71	48	109	31	64	35
16	21	24	48	46	29	46
0	2	0	8	11	0	0
20	4	28	15	16	22	11
69	87	76	96	93	84	62
17	19	8	46	0	42	47
15	25	25	38	46	34	33
45	25	19	33	33	31	26
34	42	56	66	46	70	34
79	126	19	88	9	118	37
44	84	34	85	64	77	37
31	45	48	51	71	47	29
75	81	71	117	117	58	40
29	12	46	46	7	53	12
5	2	4	0	0	0	4
11	5	7	5	16	0	0
34	21	15	30	49	26	29
39	0	32	59	9	43	21
76	48	49	79	65	58	30

For Review Only

78	60	50	109	32	58	66
87	86	112	122	123	120	117
21	10	23	20	12	12	8
0	0	0	0	144	0	0
207	121	142	215	211	187	160
66	46	59	124	72	74	47
0	0	0	0	0	0	2
77	38	42	69	96	45	52
221	207	207	404	176	316	176
26	18	18	0	0	0	0
1	1	2	27	0	5	1
1435	1516	1500	2258	1383	1413	1696
116	69	163	240	119	164	171
21	22	18	38	20	70	32
53	40	25	66	25	34	68
68	125	260	256	57	83	226
63	21	3	0	0	1	1
155	142	182	312	155	248	113
155	106	113	173	157	177	109
0	0	0	1	153	342	177
0	0	210	0	0	0	0
47	88	47	106	0	89	4
25	50	29	52	17	30	49
32	0	26	75	63	42	32
158	120	171	296	128	150	106
158	128	104	194	154	155	109
24	31	2	0	0	4	1
0	0	0	4	0	6	0
3	5	16	30	0	12	37
1	0	0	0	0	2	3
8	42	8	19	0	5	13
9	12	47	45	0	22	35
9	13	5	16	16	12	18
18	11	24	33	26	36	20
312	172	251	310	183	251	195
0	0	0	0	0	0	0
31	250	12	1	51	114	28
75	14	71	33	98	63	42
118	165	172	265	273	130	135
29	25	60	52	87	45	53
4	2	1	8	1	16	0
3	10	16	0	0	1	1
1	78	1	0	155	1	1
477	428	256	499	528	368	424
119	63	87	85	122	132	114
180	217	182	329	233	267	206
0	0	7	0	0	0	0
38	45	52	55	58	54	49
7	0	0	0	0	1	0
46	0	0	0	49	47	0

For Review Only

424	230	222	354	423	502	274
7	5	5	19	9	5	8
0	1	0	3	0	0	0
88	84	113	119	177	164	134
90	67	53	92	50	49	65
52	63	27	31	53	27	27
94	65	101	178	131	95	92
5	5	0	1	0	0	0
113	77	63	82	146	58	44
20	13	21	18	0	0	25
37	43	55	39	63	29	16
348	304	275	453	374	299	372
0	0	27	8	25	0	27
132	85	158	151	253	174	121
5	24	11	29	0	7	9
125	128	121	246	116	93	206
383	252	224	461	317	442	241
45	64	57	110	47	53	60
91	0	46	14	41	225	0
93	76	70	146	82	24	35
83	75	41	131	30	67	93
136	29	98	27	133	16	37
58	64	33	79	23	57	45
19	7	14	24	0	4	25
212	293	234	394	306	269	148
189	166	63	141	139	274	103
59	58	55	76	118	18	53
193	125	182	247	205	185	135
124	136	171	305	146	170	158
200	43	47	207	97	90	8
116	61	73	134	25	109	48
98	67	72	128	74	105	77
148	144	91	149	183	236	147
62	77	58	151	35	115	66
34	65	83	137	72	76	80
38	24	41	66	57	60	31
0	0	0	0	0	0	0
11	4	0	3	0	0	0
3	27	6	0	8	14	2
770	166	90	116	78	271	300
4	0	0	0	0	0	0
6	28	27	38	52	15	8
29	13	27	15	25	12	10
0	1	0	0	0	0	3
69	87	68	98	102	95	68
48	34	34	71	42	33	37
33	47	28	63	56	37	40
21	23	10	33	35	17	24
32	14	10	46	37	67	38
55	44	59	76	65	58	46

For Review Only

95	70	89	165	76	113	82
0	0	31	2	1	0	0
227	320	139	180	216	233	198
131	90	67	75	141	62	65
62	26	27	102	10	38	50
14	0	2	14	13	11	3
108	106	86	247	75	132	159
1	4	0	6	0	3	1
524	419	361	643	312	454	307
75	19	55	24	135	50	22
409	414	386	727	402	310	370
221	366	176	345	400	257	158
1	3	1	0	0	1	1
0	214	5	0	0	434	29
1244	1129	855	2039	931	1395	958
142	102	91	97	136	113	56
189	209	164	125	18	183	170
0	4	5	9	0	0	6
37	49	34	66	41	44	54
71	48	66	115	35	83	65
0	0	0	2	0	0	4
9	13	6	2	0	0	0
0	0	0	1	2	0	1
0	14	24	44	20	13	20
4	0	0	19	0	8	0
34	24	20	49	22	23	17
4	0	0	0	0	0	0
34	15	16	57	0	6	7
24	15	15	30	14	20	17
7	6	5	13	7	6	8
0	1	0	0	0	0	0
79	34	44	119	24	70	55
16	18	40	25	50	4	22
33	27	28	36	45	33	48
1	0	1	3	0	0	0
49	43	80	80	26	60	60
30	25	50	91	20	58	51
1	0	0	2	0	0	0
3	1	0	0	0	0	1
9	5	9	21	5	18	16
3	0	5	5	3	6	6
100	109	102	200	70	115	68
132	151	107	143	181	151	81
30	19	17	48	19	44	54
358	320	311	501	416	348	254
109	83	102	143	151	121	132
5	1	43	124	0	1	2
0	0	0	0	0	0	0
0	0	0	3	16	0	0
72	32	14	102	9	88	46

For Review Only

55	56	48	78	55	56	48
5	3	7	0	1	3	2
12	1	2	4	0	2	2
64	84	42	130	62	52	21
0	3	0	3	0	0	0
40	41	52	63	41	39	37
23	36	51	43	51	39	28
105	80	68	93	61	108	57
66	95	71	125	88	61	63
13	97	57	118	44	3	44
17	25	38	36	23	35	18
31	23	56	75	46	51	53
228	251	255	450	132	248	314
246	237	326	424	366	152	328
84	68	93	129	94	151	70
0	0	0	0	0	2	0
17	1	0	3	16	6	9
5	0	0	0	0	0	0
55	18	43	54	66	81	33
276	379	199	498	263	166	190
141	130	220	209	150	93	39
18	19	44	40	27	8	33
10	9	5	7	0	1	1
71	54	44	59	67	30	35
2	21	12	13	45	9	2
13	30	30	18	19	8	16
29	35	29	38	25	24	18
43	76	36	56	25	53	25
26	12	27	29	16	32	24
32	30	39	43	13	8	17
123	426	53	432	483	453	332
64	119	83	100	171	66	42
0	5	0	0	0	0	0
179	139	122	253	265	236	128
124	115	67	152	150	136	76
53	79	81	106	150	104	100
9	11	8	12	0	0	0
7	8	13	23	37	17	12
7	0	0	0	0	3	0
40	31	8	43	65	53	28
14	2	2	346	0	130	1
1	1	1	0	361	1	23
12	1	5	0	11	2	1
10	0	0	0	0	0	0
157	169	220	296	153	206	135
1291	1554	1691	2073	2219	1102	1813
1536	1326	1711	1605	1344	986	1106
7	0	0	8	0	3	3
0	0	5	10	0	0	0
84	111	89	152	98	111	154

For Review Only

139	118	150	173	67	122	123
65	39	23	41	15	30	14
5	6	0	9	0	0	0
346	294	277	442	416	443	270
0	10	0	13	0	0	0
529	345	327	452	208	428	274
261	269	224	416	377	180	193
2	17	14	12	0	3	1
4	25	15	11	0	4	2
1	1	20	96	0	78	54
170	82	98	173	129	134	118
45	40	22	28	52	14	26
72	35	63	129	54	49	37
21	14	16	31	20	31	17
48	28	45	104	74	91	70
41	26	42	66	49	25	31
17	7	6	3	17	6	9
40	39	37	92	34	47	61
1	3	0	0	0	0	1
77	14	4	30	36	9	20
8	3	0	10	0	8	0
8	5	8	11	0	27	16
4	3	0	5	7	11	0
400	414	352	554	390	370	263
22	11	4	9	0	2	1
46	19	11	54	0	9	22
26	12	17	30	0	15	0
50	65	51	95	104	93	70
173	123	186	247	98	190	127
36	0	39	21	14	0	22
54	62	51	83	90	72	34
1	1	1	0	0	0	1
51	42	33	92	46	45	44
27	25	28	29	13	19	15
6	10	7	13	18	9	14
180	117	172	240	129	78	135
7	0	9	7	0	3	6
245	209	215	431	208	224	204
27	3	5	0	0	7	11
0	0	0	0	0	0	0
1	0	3	0	0	0	0
88	13	43	124	12	27	71
78	47	64	139	52	117	31
45	36	26	57	97	67	40
192	159	162	258	221	128	116
2	0	0	0	0	3	0
121	166	86	237	150	191	166
93	12	131	80	275	84	8
27	4	11	29	0	34	2
1	1	1	3	0	0	1

For Review Only

115	131	75	174	73	116	97
32	153	203	84	0	112	9
0	0	0	0	0	0	0
348	354	326	270	622	351	254
184	0	98	988	0	166	57
6	2	8	12	0	2	0
33	0	0	0	0	0	0
0	2	3	6	0	4	4
3	0	38	6	30	1	4
0	0	0	6	0	8	0
43	39	63	96	32	40	42
58	46	0	13	40	40	1
35	48	0	71	0	0	0
58	47	31	92	58	41	34
40	48	42	101	31	41	35
0	0	0	3	1	40	14
53	30	51	75	24	34	26
27	24	23	36	32	31	21
0	14	0	0	0	45	0
172	95	75	218	121	168	95
9	2	12	10	22	6	0
43	38	44	83	44	44	36
15	22	19	26	27	28	31
54	30	25	61	70	37	31
347	431	319	535	598	232	438
2	0	0	0	0	0	0
5	0	0	18	0	0	3
104	57	122	121	132	48	34
41	41	23	42	30	28	9
28	14	41	33	31	26	16
147	125	153	263	170	163	172
109	102	98	171	81	82	68
49	55	71	95	102	81	50
200	185	208	312	152	198	258
33	39	35	50	41	14	33
13	3	6	20	0	14	5
358	30	304	0	34	1	163
2	0	11	0	39	6	0
677	0	0	1	15	0	0
0	0	0	2	3	0	0
20	4	4	28	0	25	2
21	24	17	22	58	27	8
9	6	14	20	5	10	15
2	6	8	6	36	5	9
31	7	12	25	0	17	22
118	164	69	140	203	153	111
34	39	64	57	75	51	46
56	29	60	73	37	43	68
42	9	17	72	0	12	41
2	2	0	0	0	0	0

For Review Only

754	648	653	659	689	278	620
327	244	255	457	209	421	355
161	0	0	0	0	243	158
2	0	0	0	0	4	0
245	197	128	374	157	221	146
601	382	528	801	663	817	563
0	0	71	1	20	0	0
146	136	85	137	265	187	287
371	275	281	349	283	204	212
126	73	84	90	97	153	100
240	192	0	195	1	256	1
360	377	423	601	455	432	451
2	2	5	0	0	0	0
261	0	0	343	0	0	0
50	40	74	97	86	43	55
287	253	255	413	208	238	167
69	111	93	201	113	110	87
0	0	0	0	0	0	0
61	73	146	92	258	141	148
4	4	0	40	39	11	0
90	28	70	58	70	59	66
274	205	187	0	0	273	159
1143	1062	816	1121	1081	1131	630
154	154	183	0	0	135	194
185	201	186	288	212	241	162
12	23	12	28	15	19	28
42	37	43	60	36	35	27
64	94	85	152	114	140	92
24	433	401	518	321	15	422
2066	1838	0	2810	169	2106	1447
1	0	0	0	0	1	2
37	68	56	361	0	635	73
81	103	89	156	111	54	109
11	11	0	23	0	12	13
60	39	56	93	20	30	46
1	2	2	19	0	5	2
267	166	328	310	244	273	180
222	162	212	375	213	251	216
83	79	87	156	107	92	65
10	25	34	55	96	12	42
33	30	13	47	32	6	10
0	6	4	1	17	10	0
367	382	430	565	539	389	369
280	277	254	346	168	178	208
372	347	340	682	335	397	337
179	263	143	336	238	245	162
223	305	344	527	248	281	258
139	175	204	146	296	46	125
31	33	32	0	0	30	33
0	78	244	244	322	0	241

For Review Only

146	136	154	227	173	193	144
27	21	10	79	28	17	12
529	218	395	491	378	369	253
91	85	74	120	133	70	45
102	118	144	207	120	160	157
109	110	133	153	148	123	67
7	0	1	0	0	0	0
5	0	0	0	0	0	0
402	338	278	580	349	404	244
93	100	110	165	129	80	62
28	14	52	35	78	23	63
231	174	121	254	214	191	121
4	0	0	0	0	0	0
11658	12215	7749	18451	8241	13446	6294
11	18	9	51	9	19	30
16	30	4	13	36	24	14
92	102	114	98	108	76	39
7	7	2	22	0	19	9
34	19	26	32	37	10	10
0	263	0	305	8	327	266
65	111	90	218	36	117	144
108	16	0	78	186	15	88
84	67	76	125	110	76	90
9	11	1	97	0	1	6
12	4	6	61	0	9	8
420	397	277	414	403	432	229
0	0	0	0	0	4	4
14	26	0	2	3	0	29
58	63	23	216	103	220	92
13	0	15	8	10	15	10
173	123	145	170	169	176	117
8	3	0	0	9	3	0
206	175	190	294	147	171	114
3	64	1	0	0	1	1
0	0	0	0	0	1	1
2	0	1	0	0	2	0
1	0	0	0	0	1	0
9	0	7	3	12	6	3
11	8	3	8	31	24	44
81	86	61	125	64	80	68
15	18	14	46	61	43	21
112	0	109	20	31	0	77
36	38	40	83	47	53	60
1	0	0	0	0	2	2
70	130	91	128	131	125	95
14	22	43	57	42	24	29
93	74	143	122	144	122	123
104	72	57	80	120	79	58
126	152	158	113	184	311	122
12	3	14	14	3	8	7

For Review Only

4	16	9	25	0	22	4
653	832	600	1054	909	982	806
1467	1220	1238	2114	1019	1413	1307
161	106	112	351	242	207	215
0	0	728	66	72	0	0
981	0	1005	2409	4	1084	204
61	47	63	62	59	26	38
2	0	0	0	4	0	0
0	0	0	75	0	0	73
82	29	48	73	0	130	23
28	26	39	30	38	40	14
117	113	110	142	138	90	76
25	36	42	58	67	66	38
78	71	52	78	130	51	50
308	87	141	290	238	275	141
87	84	63	82	74	98	66
176	183	189	256	128	202	127
1	2	1	0	253	4	1
8	52	51	53	74	59	16
162	117	91	201	89	149	77
4	0	3	4	0	3	9
29	24	16	35	18	21	15
13	32	13	12	0	6	17
23	38	12	53	18	60	17
51	61	45	83	38	66	44
17	13	30	0	0	15	15
21	21	16	46	0	14	15
116	53	71	68	98	91	29
3	0	1	7	0	4	0
40	44	42	50	53	22	29
147	234	109	371	116	231	99
6	6	6	10	6	3	0
563	463	441	681	520	599	435
4	19	2	35	33	7	18
123	104	91	130	152	189	106
0	24	0	34	153	0	103
19	6	5	22	8	21	14
32	13	15	19	0	17	2
7	2	11	11	0	18	18
17	22	21	29	17	17	14
203	159	36	208	13	200	76
6	0	0	0	1	0	0
1	4	2	4	0	0	2
187	150	96	195	159	167	129
21	0	0	0	0	0	0
14	18	21	17	0	13	8
0	0	0	0	397	0	0
48	18	33	26	56	22	24
1	0	5	5	0	0	0
0	4	0	5	0	1	4

For Review Only

65	28	77	111	29	61	45
37	19	26	49	30	13	16
4	7	13	21	38	13	22
76	60	62	103	62	52	82
1	0	0	0	8	0	0
78	38	79	128	101	67	96
4	0	0	7	0	0	0
1	1	1	0	0	7	2
0	0	0	0	0	0	0
0	0	7	3	1	4	0
12	10	11	18	5	10	11
20	16	19	33	10	19	20
21	16	19	34	10	19	20
2	0	0	0	6	2	0
0	0	1	18	0	0	1
42	50	50	116	65	45	23
0	0	9	0	110	2	2
0	1	0	0	0	0	0
35	47	38	94	46	49	34
0	0	0	0	0	0	0
53	57	66	59	62	35	29
4	5	5	0	0	8	9
12	12	0	6	21	28	5
410	380	406	317	391	65	177
229	329	256	487	194	260	258
182	159	173	330	190	253	200
15	13	12	16	0	15	5
26	21	23	29	19	19	17
115	112	28	127	29	127	84
23	32	14	51	14	21	19
0	0	0	0	0	0	0
39	18	28	58	27	23	28
67	56	55	102	85	75	42
13	22	22	43	24	22	18
253	241	212	367	248	227	162
79	41	64	119	37	135	83
0	0	0	0	0	0	0
4	0	0	0	0	0	0
51	62	71	106	21	20	47
710	691	803	1146	871	732	575
542	544	392	802	469	525	390
0	0	0	13	0	0	0
1	0	1	0	27	19	2
83	54	53	109	53	71	56
0	0	0	20	13	0	1
33	25	13	19	53	20	27
1210	1099	987	1837	901	1194	768
72	35	65	141	150	93	104
8	21	4	13	41	9	18
910	649	549	1023	521	1004	472

For Review Only

16	23	17	40	31	37	23
57	96	76	116	79	72	94
19	44	54	71	43	27	16
28	16	17	7	59	53	23
83	234	135	195	29	131	29
15	25	4	47	0	43	14
106	107	98	169	149	81	90
29	11	27	30	11	41	37
16	1	0	0	3	2	0
14	5	7	21	5	20	12
10	6	8	6	21	17	3
2	3	8	17	1	2	4
0	0	0	0	0	0	5
3	0	0	0	0	1	0
0	398	0	858	5	1204	725
35	11	16	55	15	16	21
19	27	26	41	0	20	0
82	69	80	132	94	90	92
0	0	0	2	15	0	0
6	19	8	13	28	18	22
66	27	32	79	56	51	47
63	58	73	86	33	64	24
17	5	13	1	0	3	2
123	65	59	101	153	115	38
15	9	24	63	11	42	15
8	0	0	7	0	0	0
323	225	282	387	333	194	274
59	71	77	153	71	86	61
0	3	29	11	0	4	0
1	9	12	20	0	5	21
6	11	30	56	20	18	0
121	112	138	168	146	123	123
54	32	63	85	118	30	64
0	0	0	0	0	0	0
17	10	15	0	0	13	9
0	0	0	0	0	0	7
0	0	6	13	0	0	0
1215	1845	2762	3635	1414	1577	2282
3	0	0	0	0	0	0
0	0	0	0	0	6	0
57	48	59	92	39	50	45
19	26	23	24	32	13	17
30	24	30	45	22	40	25
187	158	193	352	143	177	150
191	168	143	282	182	226	140
142	202	122	333	161	211	58
93	53	83	149	168	123	125
101	101	136	190	286	90	186
133	95	89	134	113	98	145
15	20	15	46	28	31	17

For Review Only

235	168	151	144	183	103	90
225	227	222	370	181	158	143
4	3	1	0	0	2	0
360	430	401	642	320	353	306
0	0	0	5	0	0	0
146	85	156	195	122	83	107
11	10	3	14	7	5	6
314	220	0	364	0	0	268
0	0	0	0	0	0	49
37	63	48	87	25	59	45
0	0	3	0	0	0	0
3	0	1	0	0	0	0
523	521	397	703	497	536	352
1198	1068	790	1276	905	1134	736
698	650	341	735	470	504	503
9	11	4	18	10	16	17
23	15	16	37	20	45	17
9	1	4	13	0	2	29
0	0	0	0	0	0	0
26	2	2	17	0	16	11
21	12	18	2	50	0	0
0	0	0	0	0	0	0
11	23	155	76	0	21	43
0	0	0	0	0	0	3
2	4	0	6	11	2	5
0	0	0	3	0	0	0
11	7	4	11	14	5	8
8	3	13	11	0	1	1
0	20	5	0	0	11	13
4	0	0	0	0	0	2
46	31	39	47	36	33	34
0	0	0	0	0	4	0
41	69	50	91	69	51	46
16	8	18	21	39	15	7
63	200	243	156	332	118	183
0	0	6	0	0	0	1
8	0	7	0	11	5	0
151	284	261	337	176	143	226
56	33	49	112	29	85	35
209	176	151	197	171	173	189
0	0	0	0	2	7	0
73	127	102	211	126	88	117
140	146	168	201	176	153	153
231	144	139	382	308	239	253
112	53	73	174	190	167	96
271	329	292	382	311	225	229
4	1	0	4	0	4	0
544	449	601	612	594	275	499
25	31	7	25	37	9	5
0	0	0	0	0	4	0

For Review Only

7	0	5	5	0	3	4
0	0	0	15	1	0	125
0	0	0	1	1	0	34
203	154	114	162	199	251	103
116	48	26	115	67	108	22
67	55	69	71	77	103	105
99	127	65	160	85	108	107
97	99	100	152	144	138	125
117	133	124	207	137	118	106
341	272	185	380	234	343	189
70	150	88	133	43	9	16
0	39	0	27	49	44	41
35	25	33	71	27	33	17
34	0	0	0	41	0	0
1	2	0	4	0	5	0
65	54	50	132	32	67	0
22	15	66	64	97	22	55
0	0	0	0	0	0	0
19	10	9	0	0	19	0
73	119	86	135	152	66	84
0	0	0	0	16	0	0
0	0	0	12	6	0	0
41	34	106	72	100	37	71
84	66	47	118	62	94	142
127	117	60	182	112	87	158
2	2	7	54	56	4	2
125	178	103	162	204	141	118
14	0	6	45	0	0	8
170	105	152	223	157	165	109
261	274	323	337	501	289	239
51	137	158	317	174	117	117
137	177	69	160	133	67	79
42	38	157	50	25	41	0
246	214	201	374	137	209	128
558	0	0	1	3	0	448
340	217	196	372	389	317	161
89	90	167	204	214	110	90
107	135	100	266	133	150	147
28	47	51	100	47	56	43
165	178	128	184	188	147	109
3	0	0	0	0	0	0
88	104	86	117	168	103	100
120	64	70	160	66	76	51
39	46	11	69	54	31	11
0	0	0	54	1	0	277
9	11	9	37	0	10	24
42	42	26	67	76	49	38
0	1	2	0	12	0	0
907	772	236	931	854	445	520
89	31	35	103	26	60	36

For Review Only

14	22	6	22	0	5	7
946	827	713	1466	840	884	775
319	334	214	250	345	223	250
280	208	191	311	228	153	147
0	0	0	8	0	0	0
105	142	106	169	122	101	98
158	180	140	254	168	183	100
1	1	106	0	0	1	1
55	33	17	45	46	17	25
67	56	10	65	13	43	34
5	16	11	17	24	6	6
854	915	710	1337	607	1143	739
0	5	1	7	0	3	5
132	164	145	243	155	120	140
254	239	172	344	0	137	370
63	51	61	40	142	43	0
0	0	0	0	0	0	3
1	1	0	0	2	0	5
113	143	117	223	159	128	102
47	102	88	98	41	67	85
15	14	4	24	16	12	19
9	2	0	0	0	5	0
125	141	80	169	109	133	32
15	11	13	11	0	1	3
129	158	90	207	127	123	123
108	68	63	120	110	84	95
30	16	26	0	124	119	125
0	0	0	0	0	0	0
0	1	0	0	13	1	3
113	93	133	185	164	84	138
21	27	19	32	29	32	33
366	382	293	350	287	224	289
15	17	0	30	0	20	15
0	0	0	0	0	1	0
108	78	92	159	89	76	81
38	54	51	92	59	45	85
52	94	129	130	203	86	54
188	176	93	292	130	263	188
93	92	67	84	104	81	80
0	0	0	0	0	0	0
66	0	58	0	9	81	0
99	85	77	168	105	105	107
115	56	116	185	67	78	85
56	122	48	145	85	100	26
34	13	18	0	0	31	9
70	45	70	100	92	43	74
58	91	58	84	69	44	54
202	74	88	166	229	199	87
4	9	7	15	0	8	8
0	0	0	0	0	4	1

For Review Only

3055	2818	3020	1598	532	3080	0
0	1	0	0	0	0	3
12	14	3	10	0	5	17
33	14	41	69	26	48	28
3	1	5	11	0	2	0
0	5	51	20	0	0	0
5437	4737	4143	6901	4453	5366	3051
0	0	0	0	0	0	0
69	78	72	106	35	48	44
168	108	62	194	146	136	98
0	0	1	1	0	1	0
0	0	0	5	1	1	1
141	122	118	207	101	187	119
0	0	0	0	0	0	0
58	55	50	64	52	30	32
14	30	19	37	5	13	23
2	7	14	0	0	6	3
2	2	5	11	0	3	6
11	4	3	19	0	0	7
87	0	1	14	71	3	2
61	57	110	115	74	70	96
8	11	7	22	9	5	7
73	34	63	56	72	35	51
17	15	16	45	0	7	14
46	42	65	60	126	18	33
286	151	105	342	82	245	176
96	77	116	160	66	100	43
70	63	0	130	158	115	0
41	40	46	61	32	55	34
0	75	0	131	1	52	0
3	1	2	0	0	2	1
17	22	20	36	13	20	8
60	75	210	225	22	67	105
61	52	48	78	47	77	31
39	48	36	76	84	42	56
57	84	96	68	57	74	57
160	135	220	154	259	181	118
109	83	51	115	81	97	83
33	16	18	22	11	28	27
14	26	41	33	0	28	4
108	76	57	130	104	67	43
2	3	2	0	0	2	0
101	115	93	171	157	166	146
12	3	6	4	13	6	16
1	1	1	47	13	1	1
58	34	73	26	54	30	51
7	15	11	54	0	11	9
2	1	6	0	0	2	1
9	3	4	7	4	21	2
0	225	0	1	3	0	0

For Review Only

0	0	0	0	0	8	0
0	0	0	0	0	4	7
37	16	19	54	13	16	22
1	1	0	6	0	4	0
0	0	0	3	0	0	0
191	71	39	130	206	79	67
110	220	240	334	162	241	116
46	38	33	58	44	15	21
27	24	23	24	7	0	14
94	83	150	154	152	109	87
108	68	137	194	66	90	128
0	2	3	0	0	0	3
82	42	47	71	34	37	59
0	0	13	0	6	0	7
0	0	3	0	0	0	1
63	60	74	84	65	48	49
46	41	33	60	17	58	16
71	36	10	157	0	6	63
34	28	25	37	21	42	38
20	20	17	28	20	26	16
36	53	66	108	64	58	42
17	17	18	26	16	21	23
83	80	69	112	41	64	70
38	30	25	30	18	37	29
167	103	129	156	99	157	100
1	4	1	0	0	2	1
25	10	16	75	0	48	31
63	27	19	60	25	36	32
36	21	23	56	33	33	12
71	43	68	98	51	68	48
20	19	36	37	64	45	12
9	4	6	11	9	20	11
0	0	0	51	1	0	93
59	95	91	90	84	5	18
37	34	29	74	15	86	46
0	0	0	5	0	4	0
0	0	1	9	0	2	5
66	42	34	58	54	46	62
47	106	166	116	97	195	137
135	151	121	194	160	128	156
155	64	64	91	135	59	71
20	38	21	34	28	28	16
75	71	47	117	133	136	88
17	14	10	0	10	33	34
20	26	15	66	0	16	78
92	34	37	68	14	42	34
37	61	51	80	11	36	25
19	21	16	15	4	6	7
5	3	6	12	0	1	2
20	9	73	150	0	105	0

For Review Only

160	98	161	269	294	231	227
22	0	0	1	0	1	1
87	77	78	90	89	115	64
1	0	3	0	0	1	0
158	155	202	235	176	218	310
3	2	4	5	0	0	5
0	0	0	1	1	0	0
253	194	66	161	233	385	184
31	23	14	27	39	31	13
293	202	213	375	202	206	116
37	49	22	78	0	5	19
30	43	48	62	48	20	40
5	5	0	0	0	0	8
103	57	21	174	64	19	167
279	251	142	449	173	202	238
20	11	24	68	0	11	29
90	113	91	137	127	84	92
6	2	93	12	14	3	3
38	175	113	103	530	120	38
33	19	18	30	34	20	27
39	38	30	54	0	67	25
77	0	67	41	68	23	52
20	19	13	50	21	37	12
41	27	22	62	27	39	21
158	212	194	261	238	140	95
14	20	8	20	17	29	21
0	0	3	0	0	0	0
24	28	27	24	35	24	16
1	0	0	0	0	0	0
10	17	6	14	0	1	2
238	141	189	274	215	207	197
109	109	81	167	158	122	106
0	0	0	0	0	13	0
367	394	310	580	482	477	205
1330	1188	890	1575	1029	1131	754
6	4	0	3	0	0	0
144	85	114	173	185	118	171
610	453	319	571	554	275	309
0	0	0	0	0	0	0
27	41	33	82	37	12	61
27	14	49	23	96	1	10
1	205	53	642	174	322	55
18	10	12	17	28	6	26
447	301	315	572	217	375	271
89	126	58	148	77	148	92
1	0	0	0	0	1	0
105	74	131	262	84	92	70
326	328	314	343	489	96	142
35	32	40	45	58	29	63
37	51	56	50	83	77	21

For Review Only

2	7	0	7	0	6	3
12	7	11	125	13	43	70
43	27	22	40	42	17	24
1	0	0	0	0	0	0
0	0	0	76	38	0	0
0	0	0	0	0	0	0
2	0	0	0	0	0	0
0	1	1	3	0	5	3
4	0	0	2	0	0	0
0	23	9	49	0	22	8
112	118	68	79	78	124	89
47	1	1	0	0	8	1
3	4	0	3	0	0	9
23	35	63	63	18	17	28
126	96	79	135	58	69	56
0	0	15	21	0	17	17
41	183	80	97	65	39	55
84	130	175	132	120	77	112
69	74	65	108	217	85	173
18	16	9	21	1	3	18
5	8	15	0	0	0	8
4	2	0	0	0	1	0
3	4	3	5	0	0	0
4	2	0	0	0	4	2
43	36	28	0	53	34	34
178	123	166	310	149	253	173
129	190	94	223	189	108	147
31	34	18	51	27	28	25
9	13	8	18	15	9	10
3	23	17	34	43	14	17
14	18	28	42	15	14	27
48	46	42	105	56	38	43
14	17	37	25	0	5	10
43	19	30	61	9	47	16
3	0	4	0	0	5	0
1	2	0	0	0	2	0
72	124	77	122	111	115	124
138	71	223	122	289	80	56
6	1	6	7	0	2	3
155	102	126	224	137	107	95
155	150	120	202	259	158	170
6	4	21	40	0	11	9
36	21	25	34	32	34	45
18	18	22	20	38	23	14
0	0	0	34	0	44	18
100	74	69	125	88	106	56
17	21	20	27	0	36	49
235	104	102	151	85	65	171
49	54	8	38	63	53	15
18	25	7	43	20	14	18

For Review Only

34	25	18	15	30	22	0
28	30	18	27	29	39	16
39	44	24	70	69	73	29
53	46	55	92	25	40	81
36	0	0	44	0	0	22
2	0	0	0	0	0	0
7	0	1	0	0	0	1
8	14	0	0	0	11	10
753	172	789	310	369	945	1535
4356	391	581	4919	1237	525	151
0	1	5	13	0	4	7
33	19	33	55	14	57	13
92	84	92	143	118	61	106
0	3	3	11	0	8	2
757	752	740	1249	666	794	592
851	725	747	1388	697	843	431
274	252	215	465	256	214	203
20	3	12	25	17	10	7
431	503	449	719	464	435	231
4	3	6	7	9	3	4
159	183	144	235	188	160	94
0	0	2	6	0	2	0
186	117	98	203	175	106	94
537	1952	11	1728	0	143	1
43	59	28	86	21	47	27
14	6	5	36	0	11	0
10	0	1	3	0	6	3
0	0	21	0	114	26	9
0	0	0	0	3	0	0
84	81	68	140	92	121	81
115	86	124	134	86	115	102
51	62	69	13	120	0	0
302	316	203	349	480	428	453
0	0	1	4	4	0	0
0	0	26	0	0	11	0
15	4	15	7	23	22	7
167	0	0	0	216	0	123
0	0	0	2	3	0	4
7	5	8	10	22	7	3
110	61	71	127	138	95	60
16	25	42	39	22	2	2
72	67	83	122	30	33	41
123	99	92	184	162	167	87
11	41	9	20	13	6	13
114	126	152	173	124	122	115
312	260	274	551	215	307	245
0	0	0	0	0	0	0
11	79	190	117	94	26	43
10	13	15	18	19	12	13
368	368	275	389	327	328	193

For Review Only

3	0	0	0	0	1	0
54	70	39	55	66	47	58
42	0	39	70	23	33	0
104	101	118	169	134	140	69
63	0	43	38	63	68	0
102	83	112	135	126	87	95
213	265	352	453	295	333	224
151	103	55	66	72	63	56
117	112	91	158	112	58	60
1	1	0	0	0	2	0
120	72	116	177	127	89	77
79	19	191	137	0	8	50
386	147	144	1173	0	154	66
156	165	100	212	113	129	101
1	1	1	1541	0	392	1
80	58	43	79	87	52	41
33	41	62	38	93	27	19
158	73	107	212	42	91	156
0	0	0	1	0	0	0
371	9	18	341	64	37	330
0	5	0	0	0	0	0
32	33	75	18	52	18	26
8	4	5	6	8	0	2
2263	2200	3298	4608	2014	2563	2762
42	56	48	47	46	26	19
137	66	62	136	56	125	87
33	38	13	65	68	35	23
36	13	13	39	20	15	21
36	22	24	32	32	22	17
51	2	43	37	88	44	0
287	189	228	275	324	192	223
0	0	1	0	0	0	0
77	48	30	65	37	45	47
236	6	75	0	192	128	53
478	376	355	503	415	464	240
249	188	0	84	172	225	139
188	119	56	136	0	45	29
45	76	86	129	128	49	60
96	79	89	0	0	98	100
20	18	28	35	0	3	9
66	41	39	56	88	46	15
34	30	29	0	0	34	33
102	54	78	93	86	68	54
12	10	6	13	17	12	7
253	217	217	334	137	116	200
0	1	3	0	0	1	0
21	14	18	50	64	11	9
372	313	264	542	233	354	298
480	425	552	797	529	566	498
300	0	0	0	0	0	548

For Review Only

0	0	0	0	0	0	1
118	111	95	129	74	82	54
137	160	133	270	161	162	126
0	0	0	0	0	0	5
0	0	0	6	0	0	0
17	0	22	0	0	0	17
38	38	35	38	20	23	18
0	119	0	27	100	102	0
2	7	3	5	0	4	6
249	227	198	331	248	179	212
63	74	72	107	51	94	75
1	0	0	0	6	0	5
82	81	84	124	19	91	0
107	142	131	239	122	170	88
71	61	71	92	96	78	46
0	0	382	130	9	0	0
501	444	459	637	582	400	402
194	234	213	310	239	200	169
322	230	235	319	294	378	290
30	43	37	72	27	21	33
90	79	54	131	55	85	67
97	141	149	179	149	110	107
43	22	47	80	38	59	30
16	0	15	2	15	0	0
0	0	0	0	16	0	0
28	31	26	41	35	24	9
1	1	8	0	109	1	23
23	46	23	31	36	28	36
88	50	59	87	74	59	64
11	9	3	9	20	1	3
88	89	69	166	134	100	68
33	29	26	44	23	37	28
90	60	103	102	121	89	104
75	77	59	87	64	56	37
77	88	157	156	105	73	102
128	171	111	236	151	152	100
56	55	79	99	64	71	44
253	213	146	259	221	209	183
107	109	84	120	106	49	67
0	0	0	0	0	0	0
78	103	103	129	114	56	77
84	38	29	66	70	65	44
209	174	176	330	256	217	110
0	0	0	40	0	0	0
1	0	1	3	21	10	3
50	102	71	142	80	83	75
90	54	77	95	96	106	84
63	126	141	175	154	145	141
207	230	255	407	201	222	236
31	51	56	92	33	50	48

For Review Only

99	38	119	192	30	77	120
167	149	80	134	229	128	162
10	11	14	16	23	7	11
29	56	26	51	41	25	17
132	91	95	190	186	181	168
135	179	138	262	130	101	168
45	79	68	119	49	59	68
393	341	294	604	287	394	327
35	52	85	155	44	74	98
80	64	62	107	102	72	34
107	105	60	139	105	63	64
17	10	11	22	23	3	23
160	176	140	124	213	106	69
13	32	0	0	0	0	0
18	2	2	0	0	0	6
24	11	33	26	14	11	9
40	26	65	73	38	47	51
169	184	156	229	91	101	94
115	115	98	41	68	63	0
69	85	141	160	103	136	97
80	55	78	111	114	65	47
0	0	0	23	34	0	44
22	24	13	37	36	29	20
101	71	32	86	117	54	33
45	37	60	70	0	101	1
4	0	0	0	0	0	2
16	31	30	74	0	71	15
6	12	2	12	0	10	6
688	0	0	327	0	0	0
36	4	10	18	34	17	5
0	0	0	12	5	0	0
137	0	0	21	1	0	0
41	119	57	40	217	167	63
42	29	40	52	48	24	28
9	8	8	9	15	15	14
50	100	120	193	148	147	92
14	15	24	36	27	26	25
34	19	41	24	68	20	31
154	186	172	260	222	178	179
35	32	21	41	20	17	36
52	54	81	67	126	19	52
23	0	121	0	0	0	0
2	0	0	0	0	3	4
0	0	7	3	5	10	8
14	7	1	9	0	2	6
1	10	1	21	0	6	4
317	285	152	436	255	369	239
24	10	16	13	36	3	7
7	3	0	8	0	0	0
164	160	138	178	188	123	134

For Review Only

29	79	11	92	0	39	108
25	65	58	100	44	53	63
154	104	63	124	209	99	133
9	7	9	11	4	4	11
47	52	25	115	29	39	70
209	184	205	279	254	177	231
80	128	91	110	57	58	97
23	0	0	6	0	28	45
74	37	39	57	0	14	20
0	0	3	0	0	3	4
76	90	66	161	84	81	110
102	58	74	61	79	38	57
17	7	7	19	0	14	1
8	15	9	26	37	18	7
98	37	86	76	55	71	58
45	51	26	55	31	39	40
33	197	50	283	182	67	71
9	0	0	3	12	4	0
4	2	4	17	0	10	0
0	0	0	0	0	3	0
12	0	0	11	1	8	4
5593	4623	4144	7333	4378	6290	3385
0	0	0	0	0	0	0
227	149	140	239	144	220	198
13	101	19	39	26	52	32
108	96	74	170	54	52	65
24	40	57	62	94	58	63
21	12	38	54	24	13	18
34	23	34	79	34	36	35
438	409	334	682	413	515	415
15	9	0	13	0	5	7
72	77	48	113	88	155	85
0	0	40	0	0	0	0
0	0	0	0	0	0	0
16	2	1	415	0	1	1
65	0	46	0	0	0	0
18	22	37	0	74	2	6
208	395	202	542	131	145	131
186	130	167	233	164	157	138
155	107	151	260	118	163	160
99	85	97	158	87	112	107
7	5	5	9	1	5	4
114	71	92	176	103	102	76
53	46	28	54	73	62	37
10	10	7	27	0	9	50
261	197	158	246	206	225	155
20	38	19	35	37	23	20
9	10	19	11	30	7	14
44	0	45	1	2	44	0
46	44	78	114	72	63	38

For Review Only

51	66	51	56	14	43	17
52	0	0	77	96	95	76
0	0	0	148	0	191	0
14	0	0	1	4	0	0
85	133	129	177	68	78	111
0	0	0	0	0	0	0
0	0	0	0	0	0	0
0	0	18	0	0	0	0
39	59	46	78	50	54	44
6	49	10	11	36	5	8
220	147	140	268	215	230	111
51	37	34	59	0	92	33
119	78	100	166	125	129	107
95	77	121	172	69	155	109
121	145	181	211	172	125	155
99	77	128	119	123	106	66
5	28	29	38	0	35	11
3	1	3	0	0	2	1
255	184	446	98	164	27	279
43	51	17	41	43	56	41
518	282	371	626	379	513	363
131	188	170	389	114	168	171
31	36	78	104	148	32	114
3	2	3	3	0	2	0
57	45	52	61	52	50	39
134	160	176	193	162	165	103
33	14	22	35	0	17	24
15	66	27	30	0	5	9
26	55	46	58	57	34	52
136	93	157	205	113	177	122
105	151	140	235	124	102	112
25	2	0	145	0	22	17
72	61	38	114	117	73	68
155	185	125	191	155	160	111
60	62	4	0	0	1	4
52	64	38	76	62	27	46
329	0	0	6	322	307	221
95	105	77	162	85	89	113
69	42	62	87	56	42	48
9	23	8	31	0	31	41
28	19	8	30	40	31	27
60	62	89	106	88	72	73
261	248	238	380	354	181	237
13	3	8	26	0	10	23
163	92	128	240	84	178	67
8	0	3	0	0	3	0
0	0	4	14	0	0	2
22	18	28	28	55	47	39
39	28	38	39	43	45	41
153	88	85	130	67	87	100

For Review Only

340	268	261	89	225	335	276
206	109	191	187	355	156	77
199	80	125	157	212	98	112
12	12	23	27	21	15	4
40	50	36	72	52	56	22
67	71	63	87	49	111	102
0	1	3	0	0	0	0
90	57	70	99	103	102	64
13	0	20	23	14	12	6
31	32	28	56	38	28	58
38	28	33	42	43	23	35
99	98	89	217	113	114	116
22	65	7	50	72	77	36
37	8	43	24	64	28	33
24	34	58	75	25	36	51
89	135	119	139	132	98	127
122	82	109	201	0	136	103
9	5	46	55	16	44	51
0	5	0	5	0	6	0
68	55	49	83	129	34	95
75	60	70	71	85	77	53
272	225	245	358	228	290	127
88	98	85	136	124	131	106
16	10	12	21	15	13	7
35	46	21	28	21	34	16
467	0	0	879	8	468	0
10	7	23	18	11	8	3
38	22	21	45	10	22	13
137	227	186	302	161	111	139
27	21	46	51	84	31	59
157	128	0	34	217	50	96
0	0	0	15	33	0	0
69	74	62	72	92	71	67
4	0	9	6	0	7	0
22	10	25	29	6	25	33
60	40	67	0	0	47	44
9	4	10	10	9	2	6
21	2	0	8	0	0	0
6	1	0	1	3	0	0
986	811	1037	1839	681	1511	787
243	270	199	415	187	228	251
115	114	106	165	126	187	103
26	11	16	42	0	20	5
1	0	1	0	0	1	0
31	21	45	50	34	64	36
325	298	170	375	358	250	215
181	119	224	208	121	158	107
27	34	16	37	9	4	18
123	143	166	207	173	181	123
1	198	56	1405	776	37	1

For Review Only

92	80	77	162	67	106	65
1	6	3	0	28	1	1
65	133	87	107	88	65	43
64	53	96	126	36	44	46
18	114	14	102	95	109	28
58	50	28	44	124	42	43
0	4	0	5	5	0	1
0	0	0	8	0	0	0
8	4	13	6	0	5	8
298	300	223	326	329	307	138
151	93	90	200	77	172	68
24	21	36	32	43	26	39
0	0	0	0	0	7	0
44	118	95	116	50	109	110
20	49	242	328	251	212	6
8	31	23	59	14	28	47
0	0	0	0	0	0	0
62	58	33	61	55	47	54
12	8	50	51	0	8	40
0	0	0	0	0	0	0
0	0	0	0	0	0	0
12	2	3	7	0	12	5
0	1	0	0	0	3	3
160	239	224	222	249	185	136
25	18	18	25	25	18	25
62	27	44	81	0	26	64
0	229	109	1	463	230	105
29	27	41	65	34	29	39
278	142	163	264	131	171	336
39	50	50	102	47	59	87
127	106	172	235	166	175	198
0	108	58	214	0	39	0
1	10	1	0	0	1859	1
219	183	143	207	178	146	167
175	204	163	220	155	222	79
170	155	130	144	116	100	85
0	0	0	0	0	0	0
151	132	215	267	257	103	124
186	148	87	178	283	146	69
65	52	46	39	55	32	34
406	442	318	240	339	557	225
54	69	58	63	42	62	34
76	101	104	152	127	55	88
218	201	243	289	296	158	86
98	90	59	147	53	139	56
322	258	47	240	153	304	265
149	149	95	168	131	105	92
49	50	28	109	0	8	2
83	386	301	346	257	218	110
329	193	269	347	145	370	145

For Review Only

97	66	84	100	86	127	60
43	123	145	0	0	2	6
51	143	19	175	155	103	68
78	42	50	90	113	81	48
40	71	64	84	63	61	45
0	0	44	0	0	53	0
64	101	54	58	76	51	108
0	30	16	89	0	0	0
123	116	177	290	117	201	106
148	124	96	175	138	101	85
6	3	11	5	0	10	0
24	20	26	35	16	22	38
6183	5281	4934	9335	3350	2662	5012
3	0	0	0	0	0	0
155	151	146	261	154	158	117
93	144	217	159	205	149	104
88	71	143	141	114	49	77
0	0	0	0	0	0	0
0	2	3	18	15	5	2
7	7	0	15	11	4	6
237	222	226	369	198	247	279
0	0	0	0	0	0	3
0	0	0	15	8	0	0
63	108	66	71	38	28	40
64	62	47	129	83	90	63
0	0	0	0	0	0	0
163	219	134	209	275	108	132
64	65	100	144	46	89	117
213	171	102	219	249	210	102
49	49	26	40	24	25	31
77	0	0	44	71	66	33
596	518	603	1106	891	734	708
0	0	2	0	0	0	0
63	20	26	92	118	60	33
100	87	87	147	86	47	75
0	8	8	0	8	0	0
50	16	69	42	94	63	17
315	0	0	0	19	0	0
347	331	186	363	390	445	201
49	44	51	74	92	37	61
37	16	35	53	13	17	26
6	6	1	0	0	1	4
12	55	14	183	46	2	100
8	0	0	15	0	6	6
113	136	71	179	73	140	127
185	190	196	322	197	190	194
276	234	202	479	276	266	386
206	186	190	321	225	263	339
1	0	0	11	0	1	3
16	6	10	35	35	36	2

For Review Only

957	728	649	985	1071	894	556
118	61	73	119	98	130	85
42	49	47	49	53	24	29
208	75	130	166	214	129	125
99	70	76	153	1	0	66
18	14	8	9	42	14	23
95	94	55	118	75	102	56
6	4	6	8	5	7	4
37	22	4	48	17	74	17
29	16	10	24	52	42	23
140	159	203	314	191	198	189
76	53	34	58	89	64	40
0	0	0	0	0	0	6
131	86	141	239	94	150	88
22	5	0	27	0	10	0
34	0	0	9	0	15	0
82	130	103	99	145	109	52
99	43	73	124	121	126	115
57	88	65	108	59	106	63
168	159	103	104	101	88	84
93	72	58	81	126	107	63
66	103	110	109	163	60	106
49	51	35	28	34	40	5
293	59	55	327	51	286	68
161	6	24	82	0	2	1
25	35	19	31	1	22	18
106	102	95	133	85	95	100
113	72	88	156	204	132	47
22	32	27	58	44	43	26
1	25	3	0	0	5	14
8	13	12	24	0	6	31
59	29	37	75	47	72	51
126	51	100	0	0	108	64
24	27	29	53	0	17	28
1	13	302	180	0	26	37
30	17	0	22	3	0	24
102	93	77	240	73	122	152
19	25	23	29	14	25	24
26	33	37	56	17	25	25
0	0	0	28	18	0	0
32	26	13	66	0	163	34
8	6	2	20	18	17	5
102	42	52	121	67	126	0
0	0	6	0	15	10	2
479	374	291	644	373	401	394
166	95	165	233	124	141	186
0	0	0	67	0	0	0
8	4	0	1	0	7	6
50	9	10	21	30	8	11
0	5	0	0	0	0	6

For Review Only

0	0	0	0	2	0	0
11	21	11	40	1	38	7
21	27	19	47	22	30	42
36	52	36	43	60	30	51
12	18	37	32	38	34	26
38	31	32	92	17	69	40
0	0	0	0	5	0	0
0	0	0	0	0	0	0
12	0	0	0	0	0	0
29	6	4	25	0	20	7
27	26	16	52	0	37	25
14	45	105	6	134	7	45
66	31	62	102	44	76	65
241	106	202	173	279	117	221
76	116	76	90	136	71	110
77	86	90	122	25	49	34
23	9	12	0	33	17	0
114	121	88	115	104	60	45
35	19	35	47	58	45	45
405	327	287	517	464	501	312
599	560	659	1168	766	706	826
1	1	3	0	0	1	2
11	6	5	21	3	10	0
4	1	9	12	0	14	2
2	1	1	7	0	1	1
1	0	0	0	0	0	0
1	1	1	0	0	25	1
1	87	1	54	95	1	1
145	77	46	163	227	130	94
15	17	19	30	58	29	35
19	18	6	24	50	22	12
550	74	25	108	154	228	219
15	0	0	117	0	0	0
81	72	62	64	75	36	55
0	0	0	0	0	4	4
173	173	209	358	183	160	200
6	10	7	17	1	7	7
18	18	17	55	10	35	9
28	29	49	58	82	86	48
30	54	53	47	47	33	33
3	4	0	0	0	3	0
0	32	0	0	137	39	17
22	24	0	32	2	25	23
152	913	3	260	0	19	0
7	0	0	2	0	0	0
0	0	4	0	0	0	0
156	245	267	181	272	142	183
0	4	3	9	0	2	0
0	4	0	0	1	0	1
0	0	0	2	0	0	0

For Review Only

22	5	4	13	3	15	7
0	0	2	0	0	0	0
0	0	0	0	0	2	0
1	0	0	3	2	0	3
52	29	43	65	38	27	42
0	0	1	0	0	0	2
49	62	82	104	85	52	57
1	2	3	0	0	0	9
2	3	5	4	0	0	17
1	2	3	0	0	0	10
6	0	8	8	9	0	16
3	0	7	7	3	0	14
0	3	0	3	3	0	9
9	6	5	19	0	14	0
0	2	0	0	0	0	6
30	28	19	32	9	0	19
70	49	42	58	54	68	44
159	113	88	189	118	124	94
0	0	58	3	3	0	0
63	53	68	69	48	16	70
38	17	29	35	24	33	31
80	60	76	104	72	73	72
81	72	96	125	132	86	79
70	72	147	140	102	64	74
164	175	128	74	138	51	50
35	106	69	123	43	60	89
85	72	65	76	12	0	47
22	17	20	7	44	3	24
19	28	31	43	13	27	18
34	0	31	49	44	54	27
58	29	39	87	31	46	34
12	14	14	15	25	4	7
67	120	49	128	128	99	96
107	96	164	105	233	155	83
0	5	0	4	0	0	1
36	30	13	46	52	14	13
54	41	32	50	28	35	34
1	1	1	0	0	1	1
64	60	60	106	102	42	55
29	29	47	98	49	42	38
0	0	0	8	0	0	0
113	79	96	216	88	157	100
8	4	4	26	38	8	9
21	12	4	25	0	14	26
18	24	58	61	117	18	25
0	6	0	8	9	6	6
0	4	5	3	4	6	5
146	154	110	231	125	137	162
97	110	99	109	162	64	69
15	34	37	27	41	9	15

For Review Only

0	95	64	102	16	0	0
25	132	141	322	122	49	114
125	97	82	171	201	156	62
31	4	0	7	44	0	0
268	264	146	463	242	326	128
84	82	85	84	87	41	62
127	99	127	261	114	144	148
93	62	51	148	137	89	72
156	199	173	237	308	173	211
166	174	86	156	117	53	95
165	174	158	201	216	164	126
59	48	11	25	54	53	15
6	5	11	16	0	9	7
54	99	60	110	64	84	95
175	144	128	217	122	173	170
1	1	2	0	112	1	1
89	68	63	105	91	85	82
36	30	31	50	43	13	21
63	28	21	58	55	27	19
568	453	426	736	621	515	366
127	78	122	128	70	69	98
0	713	0	514	500	314	0
497	484	491	680	566	552	386
1	1	1	0	0	2	7
751	906	773	1212	851	607	542
593	588	570	1018	527	695	331
22	24	22	42	32	21	21
24	46	42	61	11	15	12
18	4	3	14	0	6	2
143	40	58	0	64	1	49
13	7	20	40	8	12	16
59	76	56	120	37	40	33
58	96	127	150	235	48	104
0	0	0	0	0	4	0
143	103	116	115	149	150	93
9	6	5	14	23	8	19
63	46	51	95	125	37	37
0	0	0	9	7	0	0
65	41	38	59	76	46	45
103	93	80	34	59	105	45
56	67	73	138	84	39	68
45	76	72	87	17	33	35
341	360	333	649	373	481	390
58	54	48	83	124	84	61
0	0	0	40	58	0	0
31	63	74	32	47	52	38
45	36	38	38	59	50	59
19	38	30	51	72	26	15
14	8	13	8	21	10	13
7	0	6	0	0	4	3

For Review Only

33	13	39	43	0	10	18
24	12	36	133	0	69	56
24	42	34	63	33	53	34
85	61	74	130	131	52	88
13	29	18	20	24	16	20
1	35	16	0	216	322	1
60	69	57	72	73	47	41
15	15	32	28	16	30	19
3	5	6	0	0	2	3
79	49	33	64	47	55	61
9	24	10	41	49	33	43
14	29	21	23	55	62	10
28	14	16	21	11	18	8
35	30	22	41	0	9	1
66	70	44	67	71	61	27
61	62	55	64	77	53	77
25	18	20	38	15	13	20
160	187	95	214	203	113	68
52	6	29	54	106	58	34
7	17	14	9	5	7	10
0	0	0	5	0	7	0
7	16	19	20	0	0	16
74	71	54	97	75	78	47
4	1	1	23	14	1	27
3	6	1	7	0	3	1
80	79	58	104	92	97	49
192	254	246	460	219	232	224
67	57	6	15	59	2	8
11	6	5	23	13	20	29
26	26	28	61	11	25	42
64	68	64	97	111	28	91
61	40	55	95	95	42	60
98	71	78	164	94	104	68
48	61	32	61	55	69	41
52	36	39	76	85	67	36
164	105	110	165	191	200	93
8	2	6	4	27	17	15
100	46	215	427	94	153	207
43	31	27	72	43	80	59
30	27	16	31	29	38	20
316	205	211	373	235	343	206
3	0	1	10	0	1	1
42	40	69	84	102	53	38
28	20	15	16	25	12	11
11	0	7	9	21	10	7
39	36	63	59	52	44	41
70	53	81	94	93	88	72
38	61	41	99	54	40	32
0	0	1	0	0	0	4
38	51	56	158	16	22	78

For Review Only

21	61	38	92	15	22	43
7	4	5	15	15	4	12
51	18	26	48	1	13	16
206	156	97	223	273	210	235
48	0	0	0	18	0	0
111	120	88	116	137	128	77
8	9	0	19	0	4	0
16	0	1	0	48	6	14
46	49	13	60	90	41	0
3	2	0	0	0	2	0
9	10	16	11	20	16	16
0	0	0	0	0	0	0
65	56	93	94	68	92	56
77	73	52	112	62	65	50
0	0	0	39	0	0	7
53	37	55	53	28	35	86
23	38	9	40	3	6	8
308	226	285	326	302	360	287
13	23	29	55	12	19	18
1	2	2	0	0	3	0
0	0	0	3	1	3	0
122	44	58	81	68	106	52
120	84	51	112	55	105	60
9	8	11	62	0	102	27
35	26	35	43	32	30	32
6	217	1	0	185	13	34
104	90	52	159	81	94	61
54	33	38	49	33	48	33
142	137	69	136	109	92	63
46	34	36	85	34	48	51
513	566	442	915	431	386	239
0	0	2	0	1	0	0
32	20	42	51	19	29	20
2	5	3	5	9	2	5
28	27	33	40	23	33	17
20	21	28	1	21	6	0
56	55	51	94	38	49	46
1	1	1	0	0	0	1
30	64	69	73	98	38	70
1	0	0	6	0	0	0
16	1	27	0	0	0	81
170	147	169	244	174	123	158
31	20	38	51	27	23	32
18	11	10	23	37	17	5
104	115	176	197	245	87	85
56	31	25	45	58	49	39
584	729	803	1237	1171	818	974
55	28	62	63	55	48	35
18	22	25	16	0	15	9
43	58	33	71	42	28	36

For Review Only

182	223	155	274	260	211	181
10	42	24	52	0	17	14
16	17	12	15	1	15	3
16	18	18	21	0	34	4
138	409	44	218	264	222	43
43	36	36	62	34	57	26
2	1	3	6	0	14	1
83	48	71	98	51	89	25
0	1	1	1	3	3	1
17	14	14	22	52	20	56
73	43	60	73	39	72	47
63	98	27	81	96	46	33
38	561	260	32	361	361	80
11	35	21	39	0	8	5
2	0	1	0	0	0	0
28	36	47	62	38	39	38
0	2	0	0	7	1	1
34	40	24	39	31	50	21
92	41	43	72	91	92	39
45	84	80	98	29	58	50
105	138	22	143	50	132	102
12	24	35	20	56	5	24
117	159	163	185	150	61	121
136	112	157	160	88	81	106
42	29	35	75	28	45	46
88	157	136	104	82	133	72
31	17	32	20	40	23	15
105	103	74	136	38	90	64
31	37	54	67	59	39	43
6	6	5	23	0	16	24
4	0	0	0	0	0	0
26	10	24	57	0	30	8
9	13	42	69	0	28	46
66	14	41	43	8	37	28
459	346	457	723	297	435	290
68	66	52	0	0	78	62
70	61	34	77	68	45	47
123	173	232	337	168	112	118
65	68	88	131	60	63	62
19	1	29	23	0	21	15
57	61	19	75	65	81	17
95	108	104	200	75	82	138
1	1	1	9	0	0	7
153	126	108	112	179	116	135
6	1	6	22	33	7	1
20	49	54	25	100	65	48
58	76	109	136	96	66	108
203	225	227	236	179	185	167
119	159	164	214	132	146	150
29	22	16	45	55	61	6

For Review Only

24	10	66	34	41	23	13
85	81	74	133	135	63	92
27	137	69	100	63	165	157
75	71	105	81	109	96	88
42	37	40	55	44	49	34
19	24	38	22	18	43	7
30	25	20	34	39	42	19
63	85	33	109	112	73	77
84	78	71	57	68	29	79
10	2	5	78	0	1	1
25	27	18	40	11	50	11
0	0	0	2	0	0	3
11	30	27	55	20	24	20
77	157	171	221	129	123	136
1	6	0	5	0	0	1
51	1	9	49	0	7	9
30	29	38	66	36	29	26
59	42	39	64	57	39	32
67	74	108	92	138	78	77
18	33	54	48	94	19	35
174	65	75	89	116	68	41
54	108	20	111	0	41	15
43	40	12	36	32	70	43
61	25	50	72	52	54	38
75	49	97	116	52	64	112
81	241	18	93	45	18	81
5	0	4	10	6	7	7
160	156	119	196	83	143	128
14	11	16	28	9	50	20
58	11	51	16	2	12	58
7	9	3	17	0	3	0
0	5	0	0	7	0	0
52	25	22	52	21	83	16
147	93	106	148	104	136	99
23	35	29	43	17	24	34
3	3	27	16	0	3	9
2	11	7	8	0	5	2
40	54	16	19	47	10	11
625	190	0	104	451	453	366
22	24	27	30	56	26	24
15	20	10	37	0	18	12
20	32	17	30	24	11	19
0	620	567	189	72	0	526
6	17	655	0	563	2	178
4	6	0	1	16	14	1
23	18	15	25	8	15	22
32	16	30	34	0	41	9
0	0	24	0	0	4	16
34	0	16	30	13	23	24
9	20	0	14	3	7	21

2	2	0	0	0	1	0
47	20	45	49	9	157	54
71	28	60	82	35	69	100
0	0	1	5	0	0	0
46	44	39	52	80	56	31
8	14	25	6	35	15	10
62	75	76	102	100	29	61
85	94	99	182	98	130	97
38	26	60	100	40	39	59
4	20	77	76	0	18	47
29	97	11	109	0	8	121
14	26	1	17	0	46	10
143	94	75	113	137	68	93
71	62	39	28	83	38	20
30	34	30	34	38	21	22
108	119	93	149	68	103	85
0	0	0	0	0	0	0
82	97	50	137	128	113	93
9	0	0	5	8	9	0
56	33	23	49	34	38	23
39	32	44	44	47	24	30
5	23	5	21	0	65	3
56	11	19	21	52	21	30
75	73	52	113	37	55	35
146	167	158	245	170	186	257
20	13	15	14	27	23	9
17	15	13	36	1	40	22
33	21	76	60	59	18	44
17	2	8	10	0	4	7
72	12	28	45	0	33	32
28	22	30	38	37	36	33
2	18	9	8	25	18	14
0	1	3	4	0	0	0
104	53	22	64	82	82	73
2	0	0	0	0	0	0
112	121	115	80	66	48	57
60	60	33	92	29	58	54
3	1	0	0	0	0	5
23	8	15	13	1	13	8
1	1	1	0	0	1	1
19	40	13	24	24	82	42
1	1	1	0	0	1	1
71	55	117	112	131	217	68
72	34	45	141	39	54	105
0	0	0	9	68	0	0
38	48	122	117	107	96	52
54	42	30	62	38	55	68
9	5	10	17	16	10	16
52	54	32	56	44	79	55
1	0	0	0	0	0	1

For Review Only

25	76	59	40	110	26	14
52	47	39	76	39	57	33
17	8	12	15	34	23	9
34	45	43	67	42	42	24
241	232	200	332	255	286	261
1	2	2	1	1	1	2
1	0	4	4	7	7	4
34	13	7	6	16	15	19
3	7	0	2	0	7	0
1	4	1	24	43	26	10
58	84	24	90	16	26	41
17	12	19	11	29	32	8
33	25	12	42	0	13	16
19	46	20	43	47	51	31
29	48	28	42	32	29	15
9	13	25	25	40	12	48
2	5	8	0	0	2	0
124	165	109	139	189	46	59
64	45	52	0	0	52	48
59	54	34	63	71	45	54
13	16	21	7	28	11	12
20	35	24	34	32	18	16
61	18	30	19	45	27	24
267	371	208	315	176	166	169
31	12	9	28	12	3	5
20	45	6	50	0	17	22
30	20	40	40	11	55	44
49	27	41	57	26	39	23
1	1	1	10	0	1	3
23	25	33	14	33	46	70
1	1	0	0	0	3	0
12	11	9	20	0	18	7
79	28	27	53	114	45	78
7	46	16	21	8	13	17
85	44	41	64	41	77	30
49	36	32	81	59	40	33
80	37	42	68	60	44	32
2	0	4	0	0	0	1
0	0	1	0	0	4	0
114	76	30	116	49	78	78
0	0	0	0	0	3	0
16	55	50	52	75	24	64
120	205	109	59	279	239	202
36	13	23	45	41	37	12
80	85	59	88	92	65	62
30	25	28	22	31	19	17
2	20	13	18	29	6	13
88	72	64	146	63	81	81
422	1	1	513	0	1	1
103	77	88	149	61	187	173

For Review Only

2	2	2	118	0	4	46
89	59	55	90	35	53	43
19	11	9	13	33	8	3
78	73	27	105	55	70	36
107	119	103	149	153	136	109
9	30	8	6	29	36	2
0	0	0	6	0	0	0
24	31	65	23	43	41	53
9	13	11	24	33	5	17
549	341	295	0	0	193	369
5	10	8	12	11	10	5
0	21	23	8	68	0	0
25	18	39	44	29	20	40
95	39	99	133	71	61	73
284	190	177	282	164	248	204
23	14	25	46	10	17	26
18	22	33	65	11	18	20
28	4	13	16	0	6	22
91	28	60	104	27	54	49
142	94	148	181	99	127	58
17	20	25	23	1	32	7
0	27	0	97	0	0	0
18	50	46	107	118	77	7
0	0	0	13	3	0	0
25	15	34	23	4	10	7
18	1	39	65	0	1	40
1	1	1	0	0	36	57
61	42	70	90	78	71	64
3	3	7	1	14	9	6
58	49	40	0	0	30	46
24	16	28	43	16	41	58
539	450	396	825	493	507	441
29	12	19	35	15	35	13
21	28	28	61	22	60	10
63	36	55	59	93	42	39
387	441	265	703	337	452	393
19	8	1	6	0	20	13
126	147	195	199	242	195	302
78	51	77	113	88	118	101
3	1	1	24	16	9	10
31	36	60	66	33	63	66
40	23	60	107	21	27	55
5	0	1	0	0	1	1
20	53	73	83	44	28	111
83	51	30	49	84	64	35
217	205	184	317	223	261	182
50	50	73	103	77	34	41
45	15	34	69	1	28	11
101	94	96	119	104	75	64
4	5	0	5	0	5	0

For Review Only

27	27	19	27	45	39	22
26	22	38	52	29	25	15
14	29	53	65	8	27	26
43	26	19	37	38	39	27
46	51	46	50	15	28	10
42	24	32	56	70	24	34
35	22	16	31	42	15	28
32	29	42	102	33	42	42
15	22	35	44	12	57	33
22	13	28	20	0	32	3
58	31	34	48	25	46	41
2	38	19	14	0	11	33
30	11	19	27	17	20	3
68	105	46	123	97	92	58
17	19	9	38	0	20	3
3	20	36	23	1	31	20
32	3	31	49	0	51	10
0	1	2	3	0	0	0
2	15	37	24	53	7	11
31	35	61	34	24	61	37
94	48	55	78	47	95	47
19	26	5	31	0	9	19
38	48	21	96	27	35	34
33	52	62	70	88	63	20
28	11	25	11	29	18	5
59	115	12	116	0	63	31
50	36	18	42	30	45	58
125	215	258	183	314	142	112
5	8	0	0	0	2	5
0	0	0	7	0	0	1
16	26	38	58	14	27	21
0	0	0	4	0	1	0
12	2	18	15	26	9	7
76	71	37	78	44	52	38
49	36	41	67	37	40	48
81	75	99	93	152	54	34
0	0	0	3	0	3	0
122	82	103	157	104	114	79
103	61	92	104	62	109	41
50	35	42	56	61	37	50
39	24	26	29	41	58	45
17	1	3	1	32	4	33
0	1	3	4	0	0	4
75	92	79	182	41	177	130
87	47	1	1	0	1	1
70	77	45	151	21	39	37
48	3	46	37	0	19	29
68	68	78	96	89	72	57
0	11	21	17	12	0	11
77	98	4	393	0	1	1

For Review Only

62	29	29	64	58	35	44
131	198	264	492	170	189	413
74	78	60	99	47	72	30
17	52	15	41	14	31	24
20	39	18	53	23	30	20
7	4	0	6	0	2	5
30	28	30	18	32	17	8
0	0	0	3	0	0	3
11	44	34	247	12	308	7
55	57	63	54	84	51	0
22	22	23	34	15	14	39
58	37	48	51	101	62	35
11	7	9	8	21	24	13
7	14	17	25	5	60	17
4	8	29	33	0	43	29
17	29	40	23	12	22	27
55	59	76	94	47	55	61
1	1	2	1	0	4	0
55	22	22	35	47	22	23
26	15	5	28	0	24	53
3	0	6	16	0	4	3
4	2	1	13	0	0	1
45	28	43	56	57	47	32
17	23	75	85	0	47	147
37	35	47	44	69	26	45
35	23	37	61	20	41	35
28	6	6	25	74	2	17
1	2	2	4	3	3	3
19	45	29	46	5	40	10
19	14	11	36	15	17	25
150	149	170	283	111	119	167
64	71	42	79	65	81	48
40	60	0	67	25	76	51
3	4	10	14	0	16	14
108	53	57	85	0	59	22
109	34	58	46	0	9	11
75	29	34	66	21	62	23
49	37	34	63	45	56	58
71	54	49	72	24	39	57
50	32	37	50	38	54	33
49	50	50	56	50	47	39
60	35	29	68	78	47	73
53	71	14	58	0	46	13
3	4	7	6	1	5	3
13	6	10	15	4	8	12
4	5	8	8	1	6	6
6	5	11	9	1	6	7
57	30	39	55	42	56	36
30	24	25	31	33	23	19
3	2	8	9	0	4	4

For Review Only

3	4	6	5	0	3	2
101	52	0	103	41	109	57
5	10	13	31	42	12	28
262	159	80	178	209	167	125
9	9	0	3	0	0	10
191	197	216	314	139	222	135
112	95	88	131	61	65	84
18	26	61	56	0	12	26
31	0	56	21	0	0	2
0	149	0	71	1	145	0
161	146	112	272	75	237	116
166	150	142	205	186	158	116
40	3	31	150	0	84	93
13	24	13	9	29	12	8
0	3	2	14	0	0	2
127	121	97	187	91	132	130
4	25	5	21	1	10	5
6	27	35	12	26	6	4
27	17	11	16	49	10	1
50	117	122	162	46	91	60
64	90	67	118	58	42	49
2	0	1	0	0	0	0
103	96	140	89	144	133	73
16	4	12	11	31	23	19
183	227	183	237	138	213	186
34	51	31	66	9	73	15
81	24	8	76	0	118	47
72	70	112	189	51	135	38
13	7	7	35	0	22	5
42	69	47	87	53	34	78
31	26	38	78	64	36	25
15	177	71	67	105	34	32
5	6	11	28	0	7	7
26	21	29	71	26	37	18
467	552	594	717	600	573	531
12	1	8	6	0	5	4
116	99	88	143	109	135	104
164	139	107	154	125	141	104
35	31	20	40	30	36	12
167	119	126	241	201	98	147
125	114	146	172	151	119	110
50	70	46	99	38	62	65
61	130	99	130	157	108	122
100	131	122	202	176	139	129
7	0	0	0	0	2	0
153	111	138	128	113	83	64
8	4	6	20	0	3	4
11	11	12	20	20	7	15
175	157	165	252	212	223	172
61	46	49	0	0	54	57

For Review Only

355	239	235	302	289	238	255
26	0	10	32	6	0	12
55	67	56	101	58	27	48
25	24	27	52	35	27	35
13	21	25	18	23	16	18
47	15	25	0	15	0	0
13	11	4	16	4	21	10
34	70	120	123	146	69	38
21	16	36	36	22	17	11
52	36	76	113	93	71	62
1	0	0	0	14	0	0
100	66	87	0	0	78	49
19	47	124	67	35	14	8
85	83	152	110	218	123	44
9	6	12	17	9	10	11
13	22	27	28	41	25	20
27	38	35	77	58	24	60
0	0	0	3	0	0	0
69	12	51	55	116	65	69
23	32	15	31	69	35	20
1	2	0	3	13	2	4
52	12	11	12	34	31	24
88	62	118	100	140	115	57
91	91	54	136	114	111	28
4	0	1	1	2	0	0
420	0	372	638	946	718	549
90	73	112	132	133	101	116
1	4	9	0	4	12	15
107	43	62	68	106	59	46
29	35	44	79	10	73	23
46	43	37	89	36	36	62
5	9	12	33	17	9	11
60	60	76	91	96	52	79
6	4	9	21	0	3	16
39	8	199	108	0	56	49
96	87	78	184	77	46	106
85	49	46	104	48	63	60
0	0	0	0	11	0	0
47	28	52	101	52	96	34
8	21	12	21	26	14	23
12	23	14	19	20	7	14
32	46	51	100	111	75	6
172	160	154	276	91	87	196
56	0	21	8	111	63	0
151	131	183	249	72	141	135
130	82	101	159	113	64	88

For Review Only

rCHI_ND_3	rCHI_ND_4	rCHI_ND_5	rCHI_HSD_1	rCHI_HSD_2	rCHI_HSD_3	rCHI_HSD_4
99235	84341	157746	123031	113535	111684	84015
7	5	9	1	27	7	42
33	48	84	21	31	63	46
0	4	6	0	6	0	0
115	119	330	154	138	0	119
8	8	14	7	5	7	7
17	6	26	17	13	12	11
56	50	0	10	58	0	0
0	8	31	0	0	0	0
82	97	111	69	81	76	82
1	0	3	5	0	0	0
7	1	0	1	7	1	1
99	138	236	106	111	94	0
0	0	1	0	1	0	1
5	9	4	3	1	3	0
0	0	3	0	0	0	0
588	672	894	556	584	740	458
0	6	0	0	0	6	3
0	0	6	0	0	0	0
16	1	0	1	13	1	26
0	0	8	14	0	0	0
152	200	229	143	154	176	134
10	7	15	10	6	10	14
45	32	38	24	0	43	18
12	15	67	4	18	18	1
5	1	7	2	5	6	7
41	27	36	11	43	37	23
0	0	0	3	0	0	0
5	11	17	5	5	17	10
0	0	2	6	3	0	0
28	26	39	39	6	16	32
54	59	114	52	55	61	49
71	71	111	85	92	128	102
3	5	8	0	0	0	0
225	220	340	229	234	217	197
63	31	92	8	48	18	45
46	73	72	38	39	63	52
0	0	1	0	0	0	0
3	12	0	2	14	4	12
10	17	15	4	1	2	7
32	26	14	38	16	3	16
12	9	38	7	12	10	3
10	7	16	20	15	18	20
1	1	0	1	30	1	10
8	8	22	9	6	5	5
0	5	4	1	3	0	0

For Review Only

0	0	3	0	0	0	0
5	1	0	9	8	7	2
57	15	61	10	9	17	21
14	13	26	1	5	5	5
112	110	271	187	124	126	77
135	154	0	138	154	166	128
0	0	0	0	0	0	2
0	4	0	0	3	1	0
13	5	9	11	4	2	0
0	1	0	0	0	2	2
0	0	0	1	0	0	0
12	7	22	11	2	1	18
0	1	6	3	3	3	0
0	0	0	0	1	0	0
1	0	0	0	0	0	0
0	3	0	1	3	0	4
16	1	0	8	15	10	3
4	1	4	2	1	0	1
11	7	34	18	13	4	7
3	0	6	6	1	9	0
1	0	0	0	1	0	0
3	0	1	0	0	0	0
0	0	0	0	0	0	0
1	4	9	7	0	7	1
0	0	3	1	1	0	0
627	1081	1	0	0	0	0
0	0	4	0	0	1	0
1	8	10	2	3	2	0
0	3	0	1	0	1	0
19	17	38	27	19	20	17
2	3	4	1	5	1	2
36	30	31	31	21	37	19
0	0	0	0	0	0	0
0	3	0	0	0	0	0
3	0	3	8	6	0	6
7	4	11	13	3	10	7
1	1	0	6	1	1	9
0	0	0	0	0	4	0
6	23	15	9	5	6	8
13	13	15	24	4	7	14
6	2	10	6	12	2	8
0	4	5	3	0	3	0
52	47	87	40	39	57	37
1	2	1	1	1	1	1
3	1	8	0	0	0	0
0	0	32	0	0	0	0
8	23	20	21	9	45	7
1	0	0	3	1	1	2
0	3	3	0	0	0	3
29	18	36	17	14	17	16

For Review Only

2	0	0	0	0	1	0
70	13	108	52	51	52	20
3	7	21	8	1	2	17
0	5	1	3	1	3	5
5	4	24	6	13	3	1
13	13	40	23	13	17	7
3	10	14	3	12	5	9
0	0	0	0	0	0	0
0	0	0	0	0	0	0
0	2	0	0	2	0	0
4	0	3	0	0	0	0
2	0	0	0	1	0	0
0	0	1	0	0	3	0
4	3	4	0	6	0	0
1	0	0	0	6	1	4
5	8	3	4	5	0	0
0	0	59	0	0	0	0
0	3	0	3	3	0	0
1	1	5	1	1	0	1
15	10	12	14	25	8	12
0	3	0	2	1	4	0
10	14	31	13	13	18	9
3	3	12	5	6	3	6
22	33	39	19	32	45	23
0	0	5	3	0	5	3
1	1	9	1	3	1	0
0	3	0	0	1	1	0
3	3	0	0	0	0	0
62	96	88	77	46	75	57
0	2	0	2	3	3	1
0	1	0	0	0	2	0
53	54	0	43	35	59	60
2	0	0	2	2	1	5
0	203	16	239	0	195	183
14	5	7	11	8	7	11
0	0	5	0	0	0	1
44	60	132	60	76	104	53
14	13	7	27	5	12	5
3	0	6	5	0	0	1
3	0	0	0	3	0	0
10	0	8	11	0	0	0
47	45	49	38	30	14	64
8	11	68	11	39	16	33
27	30	0	45	38	27	16
87	147	295	193	162	191	87
16	17	28	21	11	11	15
4	4	0	1	2	8	0
28	68	63	23	41	40	69
0	0	2	0	0	0	0
1	1	0	1	30	1	23

For Review Only

0	0	0	41	0	0	0
0	0	8	7	4	7	4
2	26	11	1	1	1	2
42	42	77	39	30	42	55
0	0	0	3	0	1	0
1	0	2	0	0	1	0
0	0	4	4	0	0	6
0	51	0	0	27	49	0
0	0	9	0	1	1	0
2	0	23	10	6	0	12
55	61	102	105	91	80	53
0	1	1	1	3	0	0
5	6	6	2	2	6	4
57	58	109	88	159	32	95
7	7	18	6	6	15	11
19	22	25	17	16	38	29
137	182	242	139	121	125	124
0	1	2	0	6	6	0
19	7	16	14	8	8	10
0	0	0	13	0	0	7
6	2	27	21	14	23	11
0	0	0	1	0	2	0
28	12	37	24	19	15	11
12	15	9	8	0	6	10
29	33	27	22	34	30	33
0	0	4	0	0	0	0
0	0	3	0	0	0	0
28	1	0	1	1	1	16
24	5	11	27	12	8	7
0	0	8	0	3	0	0
36	23	65	27	57	59	48
7	9	27	7	10	22	21
80	89	72	26	28	36	54
0	0	1	1	3	0	2
5	0	0	1	0	0	0
42	18	94	35	23	6	103
14	115	0	6	73	8	37
15	20	25	12	14	15	24
10	21	28	26	12	16	16
1	4	5	7	4	0	6
16	17	24	31	43	6	14
10	31	31	4	3	7	32
1	3	9	1	3	0	3
0	0	4	6	4	0	0
100	67	136	65	101	50	107
37	25	103	0	19	51	0
35	34	47	28	57	44	53
35	36	17	12	21	23	11
1	0	5	4	5	0	0
4	12	22	16	21	9	4

For Review Only

8	50	0	25	51	4	40
29	46	82	26	51	43	41
5	3	9	5	17	15	11
0	3	0	3	0	0	0
85	137	199	128	123	135	52
66	100	83	81	52	41	42
0	0	1	0	0	0	0
8	13	33	15	14	14	22
83	65	162	87	79	80	68
61	40	87	68	84	60	41
3	1	8	1	1	1	8
7	18	4	16	6	11	4
17	46	87	27	34	55	47
1	4	0	0	2	2	5
23	16	39	27	18	14	29
14	13	22	13	19	10	16
13	27	31	20	14	12	19
45	18	116	54	64	8	58
32	22	28	31	31	32	34
24	5	17	15	5	9	9
124	188	281	70	60	167	129
16	0	0	0	0	15	9
3	4	1	8	5	1	3
0	0	56	0	0	0	0
2	3	0	3	11	6	2
7	2	1	14	4	1	3
6	6	22	4	9	5	7
31	61	69	66	34	47	41
1	96	202	11	16	1	121
1	100	107	4	1	1	6
0	2	0	2	0	0	0
13	17	36	10	11	20	16
0	0	19	0	9	14	0
21	31	39	29	20	24	29
0	12	0	9	2	1	5
21	29	36	40	22	25	23
40	30	64	41	18	29	32
69	39	70	42	66	32	37
71	127	100	47	42	106	76
21	25	101	101	29	24	20
62	59	135	94	98	88	76
1	1	9	4	10	8	4
10	3	14	13	6	4	16
4	1	3	3	6	4	2
103	247	206	118	93	201	87
2	0	6	0	2	0	0
0	2	0	0	2	0	0
0	0	7	4	0	4	0
2	1	3	2	2	3	1
0	0	5	1	0	8	2

For Review Only

41	50	49	20	31	35	44
0	0	0	0	4	0	0
3	0	0	0	0	0	0
3	23	14	6	2	3	2
6	2	12	4	2	2	3
207	266	484	263	211	304	234
23	2	5	20	7	24	11
32	35	52	43	38	33	35
0	0	0	0	0	0	0
7	12	21	8	21	11	10
0	0	2	0	0	3	0
3	4	0	9	2	4	0
2	0	10	0	7	7	0
201	164	311	178	149	235	176
1	16	13	10	1	5	3
5	20	18	21	15	13	10
12	22	5	9	17	17	3
0	48	24	30	24	0	26
0	8	24	11	6	5	5
18	15	41	23	22	37	21
28	34	62	41	37	25	31
132	118	204	64	98	129	96
57	37	111	42	49	33	43
0	0	2	0	0	0	0
28	1	0	6	7	13	2
50	0	0	1	1	1	0
0	1	12	0	0	1	1
39	47	73	73	54	33	29
4	1	0	0	1	0	1
1	4	5	1	0	2	4
0	1	11	1	2	3	7
5	8	0	1	0	2	3
0	0	0	0	0	1	5
1	0	0	1	1	0	0
11	9	20	0	12	16	19
24	32	22	45	24	20	27
0	1	8	5	0	5	8
0	8	8	0	10	0	1
36	16	54	22	29	10	6
30	22	82	49	34	41	30
0	0	7	2	0	0	2
27	12	25	29	22	13	4
47	23	52	29	28	29	28
3	2	19	22	2	1	8
1	2	5	3	0	2	1
6	2	0	2	2	3	4
7	7	8	3	4	4	7
4	4	38	17	1	11	5
6	11	18	11	17	6	7
9	8	14	6	9	1	0

For Review Only

0	0	0	0	0	0	0
182	228	364	182	195	196	186
0	1	0	0	3	0	1
19	33	26	23	13	26	10
66	142	417	359	64	369	45
4	1	0	2	1	1	7
1	0	0	0	5	1	0
0	0	0	0	0	0	15
21	25	51	28	24	22	18
10	8	17	4	5	3	1
9	2	24	5	4	17	33
17	26	22	30	25	15	26
0	0	0	0	0	3	0
44	48	109	19	49	10	32
0	6	9	8	3	11	8
7	8	12	22	3	8	4
0	0	0	2	0	3	1
1	0	4	4	4	4	2
18	8	18	6	5	8	13
0	0	0	0	0	0	2
0	0	0	3	0	0	0
8	1	4	0	1	0	0
0	0	18	0	0	0	0
1	5	0	2	1	1	1
1	2	6	0	9	4	11
0	4	0	0	0	0	0
16	15	39	11	8	15	17
5	0	4	0	8	0	0
9	1	0	0	1	4	0
7	3	1	1	1	6	1
26	21	24	16	17	36	13
5	1	7	1	3	0	1
6	11	9	7	6	9	19
4	21	31	14	11	12	5
0	2	0	1	0	3	2
2	0	0	0	0	5	2
1	0	0	0	0	0	0
36	35	62	53	12	43	43
8	11	5	19	8	6	5
8	23	45	32	36	1	16
1	4	4	3	0	3	0
0	2	0	0	0	3	0
0	0	7	0	0	0	1
5	7	5	8	3	3	5
26	25	92	55	36	23	43
1	3	3	0	0	0	0
0	8	0	0	0	0	0
3	12	12	1	2	4	3
1	0	0	2	3	0	2
14	5	41	24	12	4	12

For Review Only

7	7	5	5	5	8	6
13	40	24	43	45	21	35
0	3	6	0	5	1	0
9	14	20	10	7	27	0
0	0	0	0	0	0	4
0	0	0	0	0	4	3
0	0	0	1	1	1	0
0	0	4	0	5	0	0
1	8	9	4	0	0	1
1	1	1	0	1	0	1
2	6	12	8	8	4	7
16	61	95	41	69	74	25
3	0	0	0	0	0	0
2	2	1	2	0	2	20
1	1	0	0	1	1	1
5	9	14	9	7	3	9
0	0	2	0	3	0	1
1	1	1	0	1	3	0
1	0	0	2	4	0	0
0	1	17	10	1	3	0
4	0	0	1	3	0	0
0	5	0	1	6	2	0
0	0	0	0	0	0	0
0	0	1	0	0	4	0
2	2	5	2	3	1	1
0	0	0	0	2	1	0
0	0	0	0	0	0	0
3	3	0	0	0	0	0
1	6	0	0	1	2	1
40	13	0	14	40	38	5
0	1	4	0	1	3	4
4	4	0	5	17	0	1
1	2	0	4	2	8	1
0	0	0	0	0	0	0
0	0	3	0	3	4	8
11	13	14	5	13	9	6
3	4	0	0	1	6	7
3	0	2	7	0	0	1
11	3	15	7	10	11	3
5	1	0	1	1	6	4
0	0	0	4	0	0	0
97	127	97	110	94	89	69
0	4	2	1	0	0	5
45	32	66	31	37	33	41
0	1	0	0	2	4	1
33	56	40	33	47	26	40
79	85	91	59	48	71	76
87	124	211	123	107	114	120
0	1	3	3	5	0	0
17	19	29	39	33	15	23

For Review Only

0	0	2	3	3	4	0
5	3	9	3	1	0	3
4	22	34	19	16	14	6
0	0	0	0	0	0	0
3	2	9	11	9	8	3
180	161	374	178	166	128	179
2	0	0	2	0	0	2
1	1	4	3	3	1	6
6	0	4	12	0	3	1
0	1	6	3	1	1	5
0	0	1	8	0	0	0
1	0	3	1	3	0	0
9	18	38	36	10	9	11
0	0	1	5	0	0	4
1	1	21	1	4	1	1
11	26	21	24	4	18	9
0	0	0	0	0	0	9
6	0	7	0	2	2	1
12	5	32	24	18	23	13
3	1	0	0	13	1	12
3	4	9	2	0	6	0
4	2	3	3	1	1	2
11	44	25	1	12	17	21
69	28	52	35	20	42	16
1	13	46	1	1	1	17
31	33	82	30	19	60	44
0	0	1	0	5	1	1
1	0	0	1	2	1	5
20	7	27	6	24	19	2
0	0	1	1	0	0	6
0	0	0	0	0	5	0
7	1	2	0	3	1	0
0	98	7	300	96	36	16
1	1	0	1	1	0	0
19	14	22	10	6	5	7
0	0	0	2	0	0	0
1	3	1	0	0	0	1
77	103	151	193	182	79	140
11	15	19	12	15	15	8
6	2	0	5	8	7	4
1	0	0	1	0	2	3
0	0	0	0	3	5	0
153	148	209	53	44	105	65
3	0	3	0	5	0	0
2	3	7	0	0	0	2
0	3	0	2	0	0	1
0	1	3	5	1	4	0
16	19	38	17	19	20	15
122	97	134	76	83	101	112
0	0	0	0	0	10	0

For Review Only

18	10	21	17	21	22	11
1	12	0	1	5	0	2
0	0	0	2	1	2	1
6	1	53	21	5	1	5
1	10	1	2	2	12	2
4	4	0	2	4	4	2
90	42	120	41	77	14	4
1	4	0	1	0	15	1
0	0	0	0	0	0	0
4	1	4	0	0	0	0
0	0	148	0	0	0	0
6	12	37	14	16	12	16
35	24	37	36	14	36	12
0	1	12	4	7	8	2
1	0	1	0	3	0	1
0	0	2	0	3	0	0
8	1	0	1	2	0	1
3	3	0	0	0	2	3
100	71	87	91	99	64	39
0	5	0	4	12	7	0
1	3	9	11	3	1	2
0	0	0	3	0	0	0
71	71	87	52	34	74	44
7	15	1	6	12	6	6
0	0	0	3	3	0	0
4	2	5	2	5	1	8
164	143	289	167	189	172	157
22	13	0	13	20	12	17
9	17	12	9	3	11	5
35	16	35	26	20	22	32
1	5	11	10	19	6	7
0	5	2	0	1	0	0
0	0	0	0	0	0	1
6	2	3	2	5	6	0
11	15	39	8	3	9	13
15	4	12	6	7	10	5
80	89	170	93	107	102	82
11	19	0	38	10	8	1
14	9	18	10	14	15	4
29	4	19	21	30	10	24
49	48	97	41	81	60	52
7	2	1	3	0	4	2
1	1	0	18	5	1	7
0	1	0	0	0	1	5
18	31	60	39	26	27	25
96	84	152	134	80	107	104
138	150	280	187	150	119	140
79	60	127	86	65	89	58
15	35	45	14	16	51	11
4	9	0	7	5	0	2

For Review Only

28	26	134	167	99	28	56
0	0	2	0	1	0	0
18	33	22	17	15	16	17
2	3	10	13	13	12	11
0	0	0	0	0	0	0
13	35	75	23	32	64	49
3	0	5	3	0	3	2
1	3	5	0	0	3	0
3	7	10	7	6	0	9
3	1	0	1	2	0	5
2	3	0	3	3	0	4
81	102	189	150	108	105	110
1	0	0	0	1	0	6
24	33	39	7	10	20	1
7	6	16	21	2	4	4
0	0	0	0	9	3	1
21	11	0	0	11	23	0
38	38	24	19	25	37	37
1	0	11	0	1	1	1
0	0	0	0	0	1	23
0	0	0	1	4	2	4
10	0	14	13	13	10	9
17	8	15	5	13	17	20
2	13	18	5	5	7	2
0	1	0	0	1	0	1
17	22	14	22	5	9	14
0	1	1	7	3	3	1
8	9	38	36	19	15	11
106	79	86	75	84	112	79
1	0	0	2	0	1	0
4	0	4	1	0	0	0
0	1	2	0	1	0	1
0	0	15	1	1	0	1
431	373	692	472	416	532	232
2	0	0	2	2	0	0
13	16	9	22	14	19	6
1	0	0	2	2	0	7
2	0	0	4	0	0	1
2	0	0	0	0	0	0
16	5	65	8	10	2	29
41	21	30	37	38	24	21
14	9	0	26	17	12	3
5	14	14	14	6	0	4
53	40	45	49	35	38	33
53	52	132	61	98	74	23
15	17	103	11	125	22	11
0	0	0	0	0	0	0
1	0	8	3	1	7	4
59	45	125	50	66	58	41
1	4	9	10	19	11	6

For Review Only

0	2	4	3	2	1	8
326	155	277	0	201	434	183
0	2	0	0	1	1	1
2	2	11	5	2	1	0
6	6	11	11	8	4	7
5	4	5	7	11	3	10
0	4	3	0	0	1	0
3	15	16	9	3	7	8
0	0	2	0	0	0	0
0	3	1	7	2	0	2
0	1	12	1	0	0	1
0	0	3	1	0	8	0
50	7	84	1	3	6	106
0	0	0	3	0	0	0
39	17	53	28	36	44	40
0	0	5	3	0	0	5
17	9	20	6	20	7	6
60	25	67	42	39	36	34
3	3	10	0	0	0	1
13	14	7	17	6	6	7
2	6	11	8	4	0	2
0	0	0	0	2	2	4
5	5	17	9	3	3	8
8	13	18	1	4	3	2
5	6	3	7	5	5	4
7	5	10	4	2	6	4
1	0	0	0	0	0	0
0	0	11	6	9	6	9
99	73	142	83	62	92	38
3	28	0	89	4	4	1
0	5	10	0	10	0	2
69	20	83	55	63	34	43
1	0	5	4	0	2	0
11	14	21	14	9	3	8
3	13	12	22	19	4	4
4	3	0	4	5	0	0
4	5	10	6	8	0	4
1	0	0	0	1	0	1
0	0	0	0	3	1	3
3	0	0	0	0	0	0
0	0	0	0	0	3	0
0	0	0	0	0	0	0
12	5	21	9	17	8	4
3	1	6	3	0	0	0
0	1	2	0	1	3	1
11	18	13	6	6	7	11
25	31	56	26	10	27	29
5	6	0	0	1	3	1
2	13	5	8	7	6	2
3	3	0	2	4	2	3

For Review Only

5	11	0	8	9	4	21
19	20	25	11	25	26	26
3	1	2	5	0	0	5
12	11	52	52	36	2	14
17	11	23	34	9	3	7
1	5	0	0	0	0	0
1	0	0	5	0	1	1
3	6	0	1	2	1	1
3	3	0	2	2	1	1
8	2	10	7	11	11	6
0	0	0	6	4	3	1
4	6	8	2	5	0	0
30	33	37	0	0	24	30
30	29	49	5	40	32	15
2	1	134	1	1	1	1
76	1	0	1	1	1	0
109	114	160	109	109	120	89
22	23	46	10	9	6	32
404	483	518	326	253	472	309
0	0	0	0	0	0	6
78	72	131	91	75	93	66
226	260	383	259	212	204	242
32	95	80	39	10	46	22
55	71	84	53	10	51	113
50	18	77	40	34	21	31
34	40	71	44	37	51	44
0	0	0	0	0	0	0
108	115	128	58	110	121	62
12	31	59	47	51	14	24
0	0	3	0	0	0	0
0	0	0	1	2	3	2
590	807	1258	525	700	820	888
4	0	0	3	0	5	3
0	0	4	0	3	0	0
121	84	175	115	98	147	61
251	147	428	216	158	163	147
0	0	0	0	0	1	0
8	0	24	0	0	23	0
0	0	0	0	0	0	11
1161	1380	1993	950	1103	1291	1109
28	11	142	59	62	46	58
6	10	9	0	0	0	58
52	40	97	67	60	50	44
39	45	142	53	45	0	0
38	48	68	42	42	49	43
29	36	34	12	14	28	7
31	45	105	49	49	39	45
10	14	16	7	6	8	10
159	143	302	145	170	118	82
46	28	81	45	58	46	40

For Review Only

618	667	1464	876	837	727	490
55	52	101	80	46	54	77
900	613	1322	883	947	740	666
185	97	541	0	171	0	55
258	290	578	383	526	360	266
134	242	328	158	165	90	107
24	69	24	34	34	9	36
92	92	212	80	117	29	105
112	108	161	107	63	106	121
108	123	193	104	90	111	97
0	109	118	111	79	133	0
529	676	1109	608	588	723	529
52	89	119	55	93	52	133
45	28	60	23	22	32	14
0	0	0	0	0	0	1
26	33	74	36	40	39	35
0	0	0	0	0	0	0
16	15	35	11	0	8	18
46	28	85	52	42	46	45
9	13	47	12	35	56	45
53	45	42	40	31	41	5
0	0	0	508	460	0	362
0	4	315	234	53	190	131
2	58	8	1	2	31	7
4	13	15	10	25	16	7
37	73	35	22	20	26	58
21	5	25	9	25	19	3
68	86	139	136	97	77	126
18	34	43	24	12	3	3
222	245	370	222	250	280	184
48	36	60	65	22	60	39
160	48	159	42	38	203	144
37	35	42	29	53	36	29
221	223	371	182	286	381	240
31	42	115	15	16	47	22
256	12	1021	26	228	73	4
0	0	0	0	5	0	0
311	360	663	507	496	98	214
91	62	160	61	106	56	45
33	47	71	44	28	26	83
0	0	0	0	0	0	3
230	247	308	206	144	258	202
0	0	6	3	3	1	0
27	31	43	25	16	34	34
42	5	50	8	43	58	30
0	0	0	0	0	0	0
31	0	122	93	177	0	144
47	0	0	0	54	0	0
42	32	64	35	37	32	34
18	22	35	0	7	27	10

For Review Only

0	0	0	0	6	0	0
11	15	55	22	18	23	0
26	76	63	20	28	43	10
29	9	57	38	16	32	17
85	93	132	47	62	125	54
72	49	96	41	81	84	64
67	68	120	47	79	90	39
84	150	124	84	133	120	114
21	16	88	75	11	52	30
61	38	144	67	117	65	73
44	0	0	0	0	0	0
0	3	0	0	0	3	0
279	227	468	204	207	232	153
17	23	19	9	12	43	2
86	77	130	78	98	87	113
22	42	42	11	13	31	21
4	6	0	0	5	1	0
66	91	99	67	93	93	72
12	13	9	0	12	8	8
46	43	103	46	38	57	52
8	25	38	27	12	14	10
70	132	48	59	27	58	14
0	3	0	0	0	0	0
0	1	0	0	0	0	0
4	4	0	0	4	4	21
236	286	445	214	302	220	241
65	33	62	58	85	78	62
152	126	172	99	133	94	138
2	0	0	0	0	0	8
2	7	4	4	8	2	1
307	325	805	440	369	424	262
0	0	6	0	0	0	0
73	81	115	82	39	80	67
233	241	330	258	233	252	134
1	3	2	0	1	4	2
4	15	13	11	8	8	7
8	2	0	18	6	2	6
43	84	53	40	49	47	56
27	37	58	37	50	22	49
0	0	0	0	0	0	4
0	14	3	8	10	0	9
0	0	0	0	0	0	0
22	0	0	36	0	20	0
119	84	165	96	109	104	96
134	145	334	133	396	315	262
0	0	2	0	3	0	0
46	51	63	16	29	29	34
781	829	1322	777	661	898	643
0	0	0	0	0	0	0
14	19	17	18	12	0	16

For Review Only

47	29	20	32	28	31	22
82	137	169	146	118	143	120
7	0	16	0	0	0	7
107	98	170	102	110	106	100
199	186	308	252	177	213	164
3	0	0	0	0	0	6
285	301	430	370	293	245	364
7	4	16	0	19	0	9
2	6	12	7	13	3	7
5816	8114	2277	6824	0	0	0
0	0	0	1	0	0	0
86	91	152	79	76	108	89
9	22	0	0	0	0	9
0	0	0	0	0	0	0
171	140	211	194	121	168	236
68	154	165	114	78	105	84
290	283	460	317	241	409	283
10	0	0	0	0	0	0
291	363	357	335	349	174	252
279	367	583	412	405	336	320
0	18	0	0	18	24	0
15	22	15	12	23	19	17
144	103	161	263	71	96	33
57	125	106	49	50	42	70
39	6	43	23	19	11	18
0	0	0	2	0	0	0
57	50	62	49	42	50	36
59	91	61	53	68	73	54
48	35	90	74	80	44	91
74	59	81	51	40	25	36
9	7	14	7	4	7	15
12	8	26	1	3	0	0
22	22	57	35	6	37	11
560	786	1336	749	690	791	745
0	171	0	646	0	0	0
439	343	677	517	604	346	243
3	3	0	0	0	0	0
3	10	16	10	1	6	3
0	0	0	0	0	1	0
22	16	32	9	4	8	17
18	9	0	17	0	9	11
0	2	7	0	1	1	2
0	5	8	0	5	0	7
19	10	28	12	25	14	23
7	5	8	4	1	1	4
68	69	155	100	100	75	56
37	36	0	81	84	79	40
97	16	105	6	45	55	71
0	0	8	0	0	0	0
111	112	188	105	141	86	93

For Review Only

0	0	0	2	0	3	0
2	5	29	9	19	14	19
1	1	0	0	0	0	0
21	17	20	14	18	7	8
1	1	0	0	3	1	1
25	11	32	4	5	20	15
40	6	3	0	103	0	0
90	163	198	103	105	196	128
28	22	31	8	24	48	20
4	17	23	6	10	14	0
8	0	0	0	0	0	0
11	12	6	8	3	9	6
75	80	105	89	74	98	58
130	84	190	99	81	93	52
29	6	41	60	45	40	15
58	71	79	58	52	60	65
62	78	0	0	0	0	0
0	0	4	1	4	1	1
2	0	7	1	0	0	0
0	14	55	17	27	3	2
0	0	2	0	0	1	7
18	14	30	24	3	40	21
126	211	74	167	222	194	0
22	18	66	28	39	17	8
49	96	62	57	29	56	38
196	223	320	180	267	194	231
4	0	12	3	2	1	0
93	80	214	100	30	77	77
0	0	5	0	0	0	0
0	2	5	6	1	2	4
10	16	41	10	25	11	16
327	420	1153	368	391	361	127
6	6	0	0	0	0	0
1	0	0	1	1	0	13
0	0	0	0	0	4	2
0	0	0	0	0	0	54
1649	1948	4122	2726	2194	2194	1427
1	0	0	0	0	0	1
18	14	28	28	24	38	15
35	31	47	51	30	26	46
0	0	0	0	0	0	54
70	154	164	153	127	117	133
19	24	48	20	21	35	13
93	135	194	87	127	122	96
371	283	740	381	387	402	340
64	53	125	63	49	75	78
0	13	4	0	11	8	21
28	57	76	52	43	23	45
171	0	419	201	0	211	164
8	14	0	24	2	12	1

For Review Only

0	0	0	0	0	1	0
0	391	74	382	331	0	314
81	58	129	50	56	78	51
302	334	498	294	284	383	211
80	107	123	1	1	1	21
0	64	107	69	0	0	0
0	0	3	0	0	0	1
0	0	3	1	0	1	4
1	0	9	0	0	1	0
0	1	0	2	0	3	0
14	30	29	17	19	6	14
921	709	1487	835	760	1045	747
1	10	0	0	0	21	0
92	9	131	72	7	78	23
67	78	9	75	59	85	55
72	85	166	93	115	120	73
82	56	99	53	74	54	81
15	27	47	18	29	18	25
1	4	0	2	4	0	11
0	0	43	0	0	0	30
28	37	53	33	33	47	31
43	23	56	41	43	30	34
16	6	20	20	13	14	50
16	7	26	6	9	3	5
7	2	4	1	0	7	3
28	44	48	29	17	38	52
106	109	209	96	116	103	117
0	0	0	0	0	0	12
183	200	319	144	90	247	98
0	0	3	0	0	0	0
112	166	126	72	117	117	105
0	55	78	80	38	66	56
76	66	117	66	60	51	56
0	0	0	0	0	4	0
6	0	52	28	9	0	27
235	220	196	128	114	224	109
111	129	218	173	100	60	130
7	24	23	33	6	10	22
13	6	10	23	8	16	4
72	14	46	18	2	3	11
0	0	5	0	0	6	0
8	8	27	23	4	7	12
20	20	69	31	19	26	25
0	0	0	1	0	6	0
35	46	47	34	64	47	49
12	38	38	15	28	30	26
36	40	68	28	67	31	45
31	30	39	35	36	36	26
60	58	90	28	48	28	40
206	190	311	285	266	165	177

For Review Only

133	202	297	153	150	183	137
107	119	132	97	71	107	75
126	163	225	151	114	183	108
22	29	27	27	23	26	27
165	0	14	0	0	0	0
32	43	33	15	47	19	35
118	124	205	122	115	129	84
19	0	1	0	11	13	16
53	41	76	19	56	36	30
0	0	0	0	0	0	0
147	158	158	110	109	95	120
29	10	83	11	2	21	92
24	22	48	20	43	52	31
96	73	100	54	83	47	59
81	52	123	36	43	62	58
9	10	21	10	18	8	9
7	31	50	33	10	41	73
19	26	65	34	22	36	33
63	46	66	70	47	50	27
17	2	6	12	6	5	13
0	0	0	0	0	0	31
4	4	0	2	0	0	8
4	0	19	6	0	3	0
163	172	166	143	116	157	160
51	0	17	18	16	32	41
38	12	23	18	13	19	20
99	129	156	90	81	125	117
0	1	5	3	0	0	0
0	4	0	1	0	0	3
13	7	20	12	7	10	17
6	0	10	4	1	2	2
5	0	0	0	0	5	0
204	186	263	167	173	203	115
15	49	58	17	48	10	6
9	6	8	5	3	0	8
1	2	0	4	3	8	2
0	4	3	0	1	1	0
56	38	96	20	88	71	25
13	0	16	10	3	6	5
0	1	0	3	0	1	6
14	10	7	6	5	12	5
0	0	1	51	48	0	64
1	1	0	0	0	0	0
9	13	12	11	7	12	0
3	0	2	0	1	1	4
81	98	102	63	122	94	61
12	18	21	13	7	25	24
0	0	143	0	0	0	0
0	0	87	27	38	43	45
0	0	3	6	0	0	0

For Review Only

0	0	0	0	0	0	1
41	40	32	21	29	19	22
0	1	0	9	0	2	2
134	37	13	35	11	205	167
67	32	71	52	22	49	28
26	24	40	31	31	38	21
79	140	118	70	61	120	85
11	12	14	7	15	7	10
10	23	8	8	26	17	13
146	104	270	1	166	197	49
127	121	196	107	126	165	97
55	67	167	35	42	69	77
3	3	0	0	3	0	0
42	36	26	16	25	46	35
0	0	7	0	0	0	1
12	16	10	6	20	2	13
70	96	141	30	50	81	83
70	65	59	20	37	51	55
4	5	19	9	7	3	32
506	559	830	491	481	606	391
3	0	17	2	12	6	1
5	5	20	20	5	7	2
25	4	16	14	26	5	7
34	16	48	12	32	18	33
0	0	4	0	0	0	0
222	233	286	147	161	220	175
12	37	1	40	59	2	97
160	179	378	143	155	165	124
43	58	38	39	36	18	40
1	258	370	229	467	332	252
33	43	50	24	27	24	10
606	756	912	662	601	678	652
114	190	203	127	108	151	140
53	68	71	55	44	51	42
28	23	38	31	37	25	16
35	68	75	32	38	76	33
0	0	1	0	0	0	0
44	70	77	20	33	96	9
50	68	100	57	84	54	43
52	50	135	48	52	77	54
35	68	89	60	70	63	62
0	36	15	26	25	28	31
182	172	253	147	146	130	131
1	0	0	4	0	0	0
1	0	0	0	0	2	4
54	162	148	138	76	126	81
62	43	84	34	65	51	51
0	0	0	0	0	0	14
0	0	0	1	0	0	72
2	0	0	2	3	2	3

For Review Only

3	2	0	3	3	2	0
0	0	0	0	0	0	0
3	41	10	7	1	1	1
76	59	131	51	49	82	82
90	134	291	174	138	170	119
3	5	0	2	9	6	7
40	43	77	65	38	48	34
2	1	11	2	0	1	1
5	10	124	94	77	55	35
0	27	70	16	23	43	30
126	92	166	127	140	125	96
607	687	1021	524	549	746	455
22	33	22	18	28	13	26
1	2	3	5	1	2	2
0	0	11	6	0	2	0
166	178	214	150	168	210	127
8	27	34	28	19	13	32
89	87	149	113	119	104	58
55	45	74	45	86	65	43
18	32	6	18	19	7	7
45	37	66	34	32	40	42
152	92	159	165	70	162	81
41	56	59	93	84	40	80
191	163	318	164	282	205	119
34	37	40	25	7	11	21
41	0	32	46	36	0	0
29	53	54	36	35	30	27
87	21	79	143	47	41	22
0	12	0	0	5	10	0
17	79	53	44	75	67	40
3	0	5	0	0	0	5
26	48	69	23	37	33	40
109	110	186	87	91	145	135
29	0	0	0	0	11	0
32	34	32	44	14	30	14
5	3	0	1	3	13	20
3	5	16	18	5	7	2
54	64	91	104	64	53	110
513	601	961	625	522	627	506
0	0	0	3	2	2	0
15	10	30	9	18	15	21
0	0	2	0	0	0	4
34	96	44	19	35	45	34
21	8	35	5	7	6	16
0	1	0	4	0	0	3
0	0	4	4	2	4	1
4	4	10	6	3	6	3
21	24	44	35	24	26	30
30	33	25	45	21	24	29
91	128	269	105	127	148	149

For Review Only

68	81	121	53	77	93	67
50	65	86	49	60	54	45
58	26	110	46	33	9	4
8	5	9	11	13	4	3
114	274	294	122	59	193	154
0	0	0	0	0	0	0
15	29	27	12	9	19	0
12	17	30	15	26	19	27
1	6	0	0	0	0	0
109	72	212	83	74	135	59
20	18	26	28	24	22	16
55	52	69	46	42	56	86
4	2	0	0	0	4	9
7	61	141	37	81	4	71
39	54	91	65	73	29	76
12	15	31	20	16	17	16
1	1	1139	1	1	1	1
5	77	40	48	25	46	17
140	119	234	122	127	115	99
40	37	39	35	36	54	44
88	69	102	57	84	75	71
35	58	50	40	31	41	45
39	32	34	43	43	1	18
54	49	87	65	49	42	60
0	30	0	0	71	82	41
55	83	117	78	118	60	42
185	250	360	210	183	199	193
132	118	217	106	67	170	129
276	319	245	138	114	75	80
79	62	97	49	50	52	28
229	170	397	155	193	184	96
17	11	22	2	4	9	12
0	0	8	1	3	0	0
13	8	38	11	11	3	25
4	6	5	0	4	0	10
0	4	16	8	7	1	5
0	0	10	0	0	0	0
54	59	123	54	74	53	69
1	0	5	3	0	0	0
187	130	205	124	179	196	94
4	2	0	0	0	3	10
2	4	0	1	0	0	0
0	0	0	4	0	0	0
412	463	716	510	285	311	373
134	108	182	94	106	122	105
1	39	0	1	1	1	1
0	2	0	0	0	0	5
105	152	290	108	99	203	94
91	41	79	43	52	52	42
28	33	28	32	20	34	27

For Review Only

233	252	400	199	240	285	205
259	207	370	180	183	205	141
80	67	109	67	75	88	96
31	52	101	93	20	8	74
11	12	9	0	3	26	0
23	27	44	27	4	31	8
16	1	0	68	35	41	19
55	74	156	73	137	64	70
161	158	192	124	144	241	150
0	0	0	0	0	0	9
63	31	57	57	63	16	39
13	17	47	25	27	29	19
46	42	45	16	15	35	20
97	95	164	125	62	142	86
4	22	0	8	9	5	1
1	0	5	0	2	0	2
5	0	0	0	0	0	4
0	1	0	4	4	2	0
0	0	0	0	0	12	0
54	43	74	71	72	54	48
38	70	97	75	70	46	63
0	0	6	6	6	6	3
124	149	329	95	68	138	54
0	0	0	0	0	0	0
108	111	229	110	127	121	110
33	60	70	82	61	63	106
25	51	61	47	34	24	37
168	90	167	49	138	85	83
37	32	85	27	17	34	29
7	0	8	3	0	0	3
2	0	0	2	1	4	0
2	12	5	1	2	4	6
3	0	1	0	0	0	0
4	6	14	2	1	13	2
4	1	0	3	8	5	1
77	111	165	114	107	151	100
0	0	67	0	0	0	0
16	33	15	11	41	34	18
8	19	0	0	0	0	0
0	0	4	0	0	4	3
77	104	137	124	77	120	67
157	238	429	230	119	150	166
7	0	0	0	0	0	0
0	14	1	9	0	0	0
19	54	44	36	7	9	18
9	31	40	13	15	8	30
52	46	111	50	74	70	64
0	0	0	0	0	0	0
3	0	0	5	3	6	8
0	0	0	0	0	0	13

For Review Only

12	0	20	16	8	6	6
6	3	0	1	6	1	49
0	0	121	193	253	707	248
48	30	76	42	57	53	71
0	0	1	0	0	0	0
168	105	151	74	100	154	106
128	92	177	146	89	81	84
9	7	17	0	2	10	7
0	1	0	0	0	0	2
0	1	0	0	3	3	3
14	18	24	26	14	24	15
376	212	489	430	134	95	319
228	206	220	163	154	145	111
93	41	79	23	49	23	13
215	217	5	0	186	74	0
54	75	86	68	66	53	69
73	88	153	77	96	107	70
15	98	147	76	96	87	37
145	187	267	79	105	189	110
103	182	212	122	153	155	118
189	179	192	0	0	0	162
125	103	112	58	73	148	106
270	278	512	253	300	314	317
0	0	2	0	1	0	0
93	66	121	109	120	79	94
81	47	151	74	49	38	72
14	21	18	11	11	12	12
67	64	115	65	71	53	60
70	45	191	117	102	97	65
9	29	45	27	65	58	15
2	1	203	2	31	12	254
34	34	54	34	40	30	34
692	1	0	14	1	1	1
69	110	99	68	139	60	84
74	43	129	36	80	53	26
69	94	20	0	71	68	66
45	26	62	24	34	31	53
37	29	54	55	56	38	28
7	14	54	1	8	29	1
33	67	40	14	36	29	6
244	397	374	273	275	276	218
122	157	162	105	91	107	79
88	80	133	68	51	92	31
156	155	257	124	119	123	143
7	9	16	5	4	8	7
0	0	0	0	0	0	6
29	130	21	30	110	8	127
0	297	76	84	212	301	0
13	35	28	31	46	24	27
2	40	0	0	0	0	0

For Review Only

1	6	9	4	10	2	9
13	12	31	10	11	3	7
249	141	416	296	143	159	126
34	35	0	37	0	27	32
1	0	0	0	0	1	0
6	3	10	4	16	6	9
2	1	0	7	0	0	2
5	0	0	0	0	3	0
78	125	188	112	115	109	153
58	120	176	45	107	91	58
0	0	0	26	27	0	0
0	0	5	0	3	4	0
0	178	21	151	0	188	0
3	8	8	4	8	14	6
202	240	442	355	302	309	290
54	58	103	60	79	51	29
0	0	242	0	0	0	126
1826	179	58	955	863	1121	1547
0	0	2	1	0	0	15
2	2	10	1	0	0	0
8	2	1	7	5	7	4
22	49	73	13	67	25	35
57	46	54	24	40	25	14
170	184	357	136	172	155	233
95	57	60	30	30	87	63
40	66	97	42	21	24	37
129	179	61	19	31	44	12
0	0	0	0	0	0	0
50	56	87	44	15	53	55
0	2	0	2	0	2	0
0	0	0	0	0	0	9
3	0	5	2	3	5	4
60	77	99	42	54	79	62
0	50	89	85	30	0	88
342	421	625	438	329	449	184
15	14	38	12	31	8	22
2	0	27	43	93	1	1
179	204	380	228	148	268	200
105	149	239	101	116	142	119
89	0	623	0	774	916	705
88	168	169	130	134	132	93
374	375	525	367	401	393	374
6	62	26	8	93	16	32
49	80	0	45	66	50	72
168	105	148	159	141	142	136
18	20	80	31	65	26	69
73	83	118	79	32	40	53
52	57	73	61	91	48	85
39	55	52	47	34	32	44
58	52	91	80	42	47	63

For Review Only

60	45	93	22	64	52	41
0	0	0	2	1	0	2
4	5	0	3	0	0	3
365	444	594	317	405	324	344
52	53	228	5	211	214	75
30	20	76	40	35	26	35
218	236	352	256	277	291	182
22	12	0	9	0	0	7
206	275	430	138	277	327	160
22	34	46	44	8	7	53
24	28	37	21	33	28	27
36	45	68	39	29	35	14
200	25	397	187	162	225	147
3	0	3	4	4	4	4
48	57	82	36	65	20	45
372	330	719	376	401	425	255
19	15	12	6	15	26	13
3	39	9	4	1	4	7
0	0	0	0	0	0	0
11	12	45	5	13	9	43
71	267	442	170	0	0	0
131	123	275	177	151	187	168
36	26	52	19	12	14	11
1	3	20	6	1	0	0
54	44	94	47	68	61	57
21	25	118	2	244	51	26
178	34	60	0	103	65	139
0	0	0	0	0	0	5
451	616	753	417	492	525	626
3	0	0	0	0	0	0
42	0	0	68	0	0	19
699	485	194	143	136	273	502
383	548	607	435	411	576	607
138	118	140	96	179	70	93
4	0	0	0	2	0	0
101	102	138	118	103	102	71
60	40	70	24	35	67	40
55	31	90	41	24	67	49
2	0	0	2	0	1	9
1	1	0	0	3	0	5
49	43	50	38	46	55	24
344	319	760	383	267	359	286
21	47	40	30	13	7	16
1	4	0	4	4	3	6
0	0	0	0	0	0	6
44	37	100	42	73	71	45
0	1	0	1	0	0	0
0	36	26	42	35	32	17
204	150	1001	140	190	238	117
0	0	0	4	4	4	14

For Review Only

259	347	377	289	268	400	239
98	66	210	106	96	147	88
3	1	0	2	8	2	3
605	718	973	563	474	565	483
92	121	191	86	84	114	81
147	115	287	125	125	126	122
63	62	125	50	102	15	23
28	0	44	7	7	12	0
109	144	299	101	123	182	155
95	105	117	98	80	106	69
80	68	131	71	46	152	67
70	102	172	98	92	97	100
71	84	133	128	180	67	144
136	124	197	179	124	170	99
166	215	263	162	150	185	172
298	103	346	1	2	206	272
0	19	61	0	0	0	0
23	17	14	12	16	34	9
63	46	105	87	61	51	77
40	32	44	43	25	18	20
12	18	13	0	33	29	7
28	59	72	31	40	63	39
1	3	13	24	2	9	19
25	28	33	22	12	19	22
3	10	16	0	5	7	43
181	337	431	197	305	234	200
8	1	17	7	7	5	17
48	79	116	26	48	34	58
44	66	58	28	42	35	40
12	30	27	23	17	16	32
7	0	15	12	11	12	16
199	954	1681	879	883	995	786
0	0	0	0	0	0	0
109	135	127	111	113	130	147
28	19	28	24	15	40	17
31	39	45	22	26	24	29
520	572	940	534	642	612	404
41	54	88	65	67	73	16
52	45	89	31	54	47	49
10	14	35	10	17	11	24
0	0	0	14	0	29	0
11	16	0	3	2	4	4
92	45	112	121	59	29	19
94	103	93	71	75	63	35
14	19	50	27	34	18	13
13	17	59	22	33	31	34
42	32	118	58	53	66	39
0	0	0	0	36	0	0
4	5	11	9	9	0	7
25	56	56	61	82	8	14

For Review Only

32	165	224	38	238	40	85
0	0	0	0	0	1	5
38	44	56	76	33	18	22
11	74	280	318	53	240	155
0	1169	215	0	0	1076	0
387	513	818	404	502	437	390
48	134	93	78	76	79	126
0	1128	1	0	0	0	866
346	333	565	343	351	348	368
135	118	201	81	113	139	97
0	4	0	4	0	0	10
94	107	215	101	95	107	111
0	1	0	0	2	1	0
0	2	1	1	1	3	0
227	306	387	165	238	231	341
13	0	0	0	0	0	20
10	1	0	0	0	0	3
0	0	0	0	0	8	0
59	71	217	98	132	120	90
20	18	44	5	3	29	28
235	0	1	266	0	0	0
1094	1038	1775	1012	950	1053	859
157	117	414	162	179	198	171
109	122	188	83	108	90	107
51	41	64	80	43	81	57
197	219	222	189	174	236	129
10	96	114	20	104	38	42
2141	0	129	0	0	0	1884
7	18	43	13	19	19	14
260	275	509	244	259	161	152
0	2	0	0	4	0	11
140	200	383	270	140	168	146
12	41	34	16	5	21	10
46	63	48	25	37	57	20
391	482	136	126	369	259	81
77	95	122	66	55	66	66
10	47	53	7	8	51	21
72	52	95	53	47	55	57
75	134	162	92	107	84	74
61	61	123	79	53	70	64
112	126	189	117	96	139	126
134	73	121	60	77	87	74
13	10	24	9	6	14	6
0	4	0	0	0	0	0
10	10	17	14	16	14	9
2	5	13	4	12	13	15
0	0	4	1	0	5	4
296	288	327	273	278	0	237
34	52	37	40	45	54	30
0	0	1	0	4	4	0

For Review Only

4	2	1	0	1	5	0
0	0	0	0	0	0	0
42	34	95	17	0	41	23
0	0	5	0	0	0	0
72	84	128	59	107	77	69
737	533	991	730	683	537	461
3	0	6	3	1	0	3
2	1	7	2	3	1	4
0	0	0	0	0	0	0
100	101	191	0	52	103	73
0	4	19	2	4	9	3
31	51	129	64	57	32	48
28	22	74	30	29	58	28
139	214	247	142	119	144	143
235	179	411	222	230	131	167
0	2	0	1	1	0	0
185	133	367	136	221	90	92
18	8	4	0	7	12	13
70	114	92	42	56	37	31
78	61	104	52	41	63	50
3	8	8	7	8	5	7
1	1	0	1	34	2	1
77	35	158	73	87	80	69
111	116	212	131	157	113	165
29	48	75	29	34	54	31
99	76	151	82	110	95	119
0	0	0	0	0	4	0
1	38	0	4	9	0	38
0	3	3	3	0	6	4
153	124	278	121	172	146	160
27	64	0	25	32	35	19
317	276	465	255	277	335	246
476	447	785	384	393	415	373
48	24	32	32	21	22	43
100	87	74	43	74	125	55
110	82	128	87	105	97	86
57	76	126	60	71	72	63
69	103	135	111	92	112	62
99	98	0	49	18	94	2
114	37	83	35	57	37	13
155	67	200	41	144	1	16
58	58	89	60	43	89	49
56	65	87	44	56	55	61
6	9	13	7	6	23	31
444	4	0	5	3	102	69
66	47	89	44	36	56	10
50	30	30	0	55	0	0
129	176	99	134	151	177	124
79	66	102	109	65	56	47
77	90	164	66	89	130	35

For Review Only

15	13	0	21	3	9	14
34	15	29	0	0	0	23
5	8	13	10	2	3	3
38	31	81	22	32	43	64
44	151	134	35	82	55	89
21	44	47	43	43	34	39
114	194	124	88	82	108	84
70	59	137	80	43	103	54
87	87	82	98	113	108	116
0	0	0	0	0	0	0
3	0	4	0	1	0	3
56	32	78	42	26	79	36
45	48	99	41	36	65	58
58	63	98	35	25	46	64
37	60	29	12	33	26	28
59	127	90	27	108	20	157
142	0	227	61	116	137	93
74	36	95	26	40	79	59
39	150	140	30	59	94	201
0	0	0	0	0	1	78
19	7	25	21	11	26	27
0	7	0	0	5	0	0
2	0	24	1	0	0	8
270	397	453	222	313	309	316
7	0	9	10	7	7	0
103	133	220	127	122	100	143
11	37	39	11	5	20	6
247	197	276	206	206	250	215
7	0	5	4	1	0	3
0	3	0	3	0	0	0
258	259	661	236	303	338	227
777	0	539	864	835	455	0
599	778	1128	650	546	699	542
150	22	7	137	0	35	68
5	3	8	6	5	1	7
11	0	135	181	145	9	0
38	0	88	50	0	0	87
240	385	366	182	207	350	251
1	0	0	1	1	1	1
38	38	76	55	42	53	46
3	26	0	2	5	11	41
0	0	2	16	0	0	0
109	143	204	146	107	134	200
0	0	0	59	0	0	0
0	0	0	0	0	0	0
0	0	0	15	0	0	21
0	31	0	0	14	44	32
30	13	41	28	17	17	20
44	55	0	56	54	44	47
243	432	1089	491	442	311	553

For Review Only

226	823	1104	901	814	785	742
382	0	896	0	492	621	0
461	93	504	132	163	17	152
329	438	766	496	517	323	298
0	5	15	8	0	11	0
0	1162	1	0	835	0	0
212	289	658	217	207	297	180
290	432	553	343	306	406	299
105	0	324	0	61	215	46
207	298	317	202	207	217	277
0	0	0	0	5	1	12
168	115	256	111	164	200	148
258	326	456	365	217	329	301
348	386	597	348	309	378	309
113	148	154	73	157	120	93
130	262	227	146	164	163	119
179	218	293	188	204	221	215
263	210	311	187	169	215	224
3	14	1	7	11	6	2
101	77	269	12	2	0	61
110	207	257	138	135	213	109
0	1	5	4	0	4	1
617	712	1138	841	881	871	961
366	289	707	272	297	256	266
349	295	517	279	427	330	175
13	3	16	8	5	8	7
5	4	9	11	0	0	0
0	0	0	0	0	0	0
7	0	0	1	1	6	1
172	154	258	216	190	160	124
36	21	27	18	19	23	20
32	42	71	40	52	45	34
1	0	0	0	0	0	71
33	75	68	36	39	20	41
151	244	29	234	0	129	0
0	1	4	0	0	0	40
74	65	98	60	32	98	72
0	2	20	0	0	0	6
223	227	185	134	113	229	199
41	121	146	65	81	61	90
274	327	484	335	258	233	214
31	54	87	38	45	63	50
82	61	66	51	69	103	48
226	255	370	272	239	206	207
57	30	49	50	29	38	33
39	42	59	24	54	65	59
112	63	155	80	76	65	62
0	3	0	0	1	0	2
0	11	11	10	11	25	15
42	28	51	23	19	36	26

For Review Only

178	209	296	174	192	236	198
1	0	4	3	1	5	0
24	25	29	28	17	12	19
0	0	0	0	0	0	12
59	16	34	42	47	30	40
10	10	39	13	33	18	19
191	88	305	155	155	143	100
5	2	12	1	1	9	12
8	4	0	25	33	11	18
28	10	42	23	16	18	4
392	2	1	1	3	2	7
0	4	1	0	0	7	0
0	0	0	0	0	0	0
1	1	10	0	1	0	0
5	5	4	2	4	7	0
58	68	64	48	35	31	45
4	0	9	5	5	7	1
10	5	25	9	26	12	4
4	7	8	1	3	3	3
6	2	0	1	1	3	4
33	51	39	37	16	29	43
46	47	71	29	66	44	30
31	36	57	66	23	47	51
146	107	258	179	217	193	173
33	56	102	56	43	54	43
37	31	61	20	38	35	29
1	0	0	1	0	0	4
29	35	22	40	22	32	68
0	1	3	0	3	1	1
0	0	12	4	0	0	4
0	0	0	1	0	1	1
128	138	241	129	107	110	107
107	87	179	138	137	151	142
45	63	89	44	62	41	14
3	1	0	0	2	0	0
24	24	68	76	77	25	24
22	22	23	33	19	20	18
3	0	0	2	2	0	0
0	3	0	0	0	0	1
0	0	2	3	3	0	1
114	146	158	127	113	64	115
7	27	22	9	23	0	16
3	0	6	0	0	2	0
1	1	5	3	4	1	5
5	0	4	1	1	2	1
52	39	61	23	13	30	43
0	2	0	2	2	1	3
2253	3871	5012	2269	2377	3178	4335
1	1	8	5	1	3	3
155	105	212	159	154	124	125

For Review Only

24	8	35	26	48	46	25
7	1	15	16	10	1	3
45	92	54	58	59	72	45
113	56	253	145	122	74	96
38	52	113	33	40	47	28
17	28	16	4	3	6	13
27	32	31	8	24	31	52
20	15	27	12	14	7	22
115	26	65	0	0	0	0
1	0	0	0	1	0	4
19	24	1	5	2	2	4
1	1	0	0	0	1	0
11	1	4	8	6	1	1
10	6	9	1	1	2	3
0	0	0	1	5	2	2
50	72	107	72	80	76	54
130	81	122	186	178	178	79
3	0	0	3	3	0	0
32	30	61	36	45	15	45
4	0	6	1	4	1	0
50	45	107	64	34	63	38
1	10	0	6	6	0	2
162	165	174	154	154	74	76
0	0	0	0	0	84	0
131	137	484	139	278	163	45
23	14	26	7	11	13	18
58	55	65	14	40	59	73
56	48	73	58	70	61	41
620	1	0	1	193	1	1
3	6	0	1	3	0	3
4	10	9	3	9	2	5
21	11	9	12	10	12	14
0	0	6	4	2	2	0
33	53	50	26	23	27	30
61	56	105	47	82	62	54
0	10	14	13	24	14	0
28	92	94	65	94	54	73
120	104	163	94	118	101	107
2	11	13	10	8	12	11
1	24	0	13	38	9	12
29	47	23	2	20	37	25
9	11	3	5	8	8	16
95	64	114	35	63	79	22
9	20	19	9	22	13	24
100	132	111	83	70	111	81
64	60	96	81	24	67	51
17	18	14	6	18	15	22
16	24	28	36	25	25	18
30	15	41	28	45	26	40
31	15	34	30	16	26	17

For Review Only

0	0	3	0	1	2	1
15	17	14	6	31	7	0
0	6	4	0	0	4	0
0	0	0	0	1	0	4
48	93	72	50	25	69	77
151	125	229	155	195	122	110
168	91	55	4	29	72	11
61	81	104	0	152	187	110
0	0	6	0	0	1	0
52	87	55	24	42	31	55
25	33	20	14	12	23	7
0	0	0	0	0	0	4
0	0	0	0	0	0	50
0	0	1	0	0	0	83
50	56	69	34	39	74	48
86	73	205	117	85	123	154
107	84	133	63	47	41	63
143	113	246	141	157	132	124
7	8	17	17	8	11	3
2	151	107	107	68	42	78
0	0	23	3	5	8	0
10	12	51	56	29	12	26
53	26	99	30	40	9	30
17	25	26	35	19	27	23
51	15	14	12	15	1	30
7	0	0	4	6	0	0
38	23	0	18	23	24	0
147	92	187	127	130	103	120
39	51	93	66	83	90	84
13	72	38	33	14	18	42
13	28	24	22	19	26	23
95	70	146	85	94	105	120
7	0	0	0	0	0	0
142	210	348	252	224	171	217
153	171	215	110	126	154	122
41	24	18	7	4	26	19
0	0	1	0	0	0	0
2	21	107	11	71	11	14
1	1	2017	1	1	23	1
10	4	0	3	2	2	1
4	11	4	7	12	0	5
89	56	103	67	61	85	32
15	26	36	14	31	13	40
89	9	89	83	54	32	38
1	2	19	6	10	1	1
0	46	41	46	49	39	35
30	6	106	32	113	52	50
32	8	40	12	30	6	33
47	40	0	56	50	35	40
21	6	56	28	8	8	29

For Review Only

0	3	0	1	5	0	0
86	87	157	101	82	109	107
3	2	13	0	1	2	1
149	219	424	308	192	307	297
14	14	30	5	17	5	12
0	0	0	0	0	0	0
1	17	12	3	4	7	3
14	3	26	8	26	9	8
1	2	6	2	2	8	4
0	0	0	0	2	1	1
0	0	0	0	0	0	1
2	0	0	2	0	2	2
0	0	1	0	0	5	0
4	0	0	0	0	0	0
0	3	6	0	0	1	3
7	20	16	16	11	43	7
17	13	0	12	12	5	7
2	1	0	1	0	0	0
12	4	20	9	6	11	5
0	1	0	0	0	0	0
30	30	48	37	48	67	22
9	14	26	8	13	23	8
0	0	0	0	4	0	0
3	0	0	0	7	3	3
2	2	43	15	27	25	20
0	0	0	0	0	0	0
0	0	0	1	0	0	0
135	159	141	116	135	86	94
291	309	458	252	260	271	257
0	1	0	0	0	2	4
1	1	0	1	21	27	32
107	157	228	119	158	161	152
5	6	6	9	7	3	2
0	1	1	0	0	1	0
1	0	0	6	3	0	10
45	59	60	44	49	32	42
55	57	63	29	43	61	38
29	43	42	54	26	52	34
58	0	81	0	0	178	61
0	33	26	23	31	0	0
0	37	120	0	93	58	0
145	60	308	2	13	264	3
0	5	0	6	1	2	7
26	3	35	35	19	12	14
14	48	39	22	12	1	16
147	0	16	0	157	182	128
3	4	1	5	5	3	3
60	0	11	0	73	46	57
387	485	944	951	525	525	335
92	105	214	140	75	142	55

For Review Only

0	0	1	34	36	0	0
41	84	71	46	54	85	53
3	14	22	12	12	9	14
0	4	3	0	0	0	4
41	47	85	63	43	39	55
3	5	0	4	0	1	11
21	21	40	19	29	13	18
0	188	91	0	0	111	0
9	6	5	5	9	10	1
152	164	189	122	158	149	107
106	98	194	166	149	109	65
250	280	341	195	248	245	234
102	202	239	192	218	206	106
95	147	171	124	114	121	85
17	17	34	33	18	14	27
11	8	17	9	4	11	25
20	32	56	23	37	26	4
0	0	0	0	0	1	0
57	60	119	51	54	74	94
4	1	0	2	0	0	0
34	1	0	147	60	21	144
9	26	43	18	48	5	27
0	3	0	0	0	0	0
10	10	9	15	8	8	10
51	48	66	68	40	69	33
1	1	0	1	1	109	11
34	50	62	59	39	40	31
153	137	282	99	170	161	100
0	0	0	0	0	0	17
0	4	0	0	0	3	0
25	56	133	40	32	43	26
0	0	0	3	0	0	4
539	584	0	783	727	581	522
0	0	0	0	3	0	1
162	232	421	370	130	287	210
143	241	213	161	187	148	185
0	0	2	4	0	4	0
721	706	505	518	765	649	705
1323	1046	1408	1177	885	1103	703
81	109	132	50	80	91	111
79	54	78	39	83	111	25
94	79	174	87	101	134	86
1	3	1	1	0	10	3
309	351	471	261	310	319	298
15	19	41	23	19	18	23
82	82	187	98	59	109	64
2	29	0	25	10	2	2
118	14	223	17	50	6	90
431	630	811	355	644	640	433
0	24	18	7	2	0	20

For Review Only

26	29	0	26	22	44	32
4	7	7	5	8	8	7
20	13	32	20	20	8	8
52	99	82	34	32	60	42
57	52	90	50	69	66	93
0	4	4	0	1	0	3
68	85	128	99	65	76	99
31	99	128	9	13	126	91
41	42	67	53	57	51	38
14	81	72	17	10	9	23
1	1	0	10	5	42	20
0	33	89	0	19	20	26
0	0	0	0	0	0	3
611	692	1044	605	662	673	664
0	0	0	0	0	0	25
0	0	0	0	0	0	15
1	1	0	0	0	2	0
22	40	56	28	31	56	77
192	158	263	156	181	153	186
63	37	44	30	69	110	39
21	41	58	24	31	36	33
241	254	305	192	157	244	186
0	4	8	0	0	0	0
0	0	9	0	6	6	5
19	28	38	29	27	15	30
172	247	238	246	220	184	247
39	41	75	27	51	41	32
161	170	331	190	215	169	156
80	59	127	61	80	77	64
0	0	0	0	0	0	3
19	26	14	10	8	4	9
136	129	164	101	147	128	102
1	4	0	1	1	1	1
0	0	28	6	6	3	22
123	98	6	0	0	0	0
5	0	3	6	0	4	6
42	41	41	42	38	30	18
32	25	61	18	7	54	31
36	3	0	14	10	1	6
8	17	62	12	23	17	1
34	29	39	29	20	33	18
31	40	60	26	39	34	24
0	0	0	2	4	0	0
0	0	13	2	1	3	0
30	14	63	13	15	30	17
238	222	259	158	177	187	162
119	48	192	97	127	99	68
88	67	123	71	63	71	52
93	117	234	164	127	118	92
1	4	8	0	0	0	0

For Review Only

11	18	27	25	20	19	20
38	41	58	42	56	49	67
98	114	161	81	92	103	88
0	0	6	0	0	0	0
94	80	54	40	18	78	70
326	251	431	168	260	219	234
176	157	210	151	107	124	172
0	0	12	66	0	28	0
115	105	201	94	112	69	94
3	0	0	2	2	0	6
92	195	180	102	174	100	91
135	46	268	55	38	50	52
13	6	20	8	6	12	10
24	51	0	32	31	23	23
28	19	23	23	15	14	21
72	107	154	100	84	119	88
29	39	50	41	43	33	23
251	338	103	0	171	319	277
0	0	0	0	0	0	22
15	21	36	41	13	39	29
194	79	258	124	211	97	49
86	165	214	163	109	140	78
32	34	50	20	8	41	34
0	3	0	0	3	0	0
3	3	4	0	4	5	0
93	173	192	84	67	98	110
23	13	30	9	7	14	7
208	192	341	239	178	230	178
106	102	137	104	77	122	64
651	720	1258	699	712	779	1411
44	66	82	36	43	24	37
0	0	7	5	0	2	1
102	142	140	110	109	112	102
61	93	167	68	54	90	89
20	71	60	17	47	19	40
37	15	62	43	48	43	57
0	6	13	8	2	1	1
0	1	0	4	2	2	0
1	2	0	1	1	1	1
1	1	0	1	1	1	1
38	56	133	36	24	64	78
7	5	0	5	5	0	0
62	58	121	51	50	98	87
0	2	0	1	3	4	0
322	400	430	292	272	268	198
46	72	66	73	60	67	70
85	114	161	60	75	86	102
50	53	116	64	64	50	56
0	0	0	0	0	383	0
281	277	1067	508	482	518	278

For Review Only

1	1	3	1	0	4	8
308	193	418	255	163	218	250
0	6	3	1	0	0	2
0	0	0	0	0	0	0
49	95	117	59	82	82	71
0	0	0	0	0	0	0
0	0	10	0	0	0	6
176	218	336	163	179	247	156
44	61	51	34	24	23	39
38	52	80	57	50	43	66
44	37	52	53	24	40	35
35	22	65	16	22	22	14
188	212	523	169	208	0	165
33	30	33	28	40	30	0
0	0	0	3	1	0	0
2	2	0	3	0	0	0
5	16	12	8	20	9	11
1	1	0	1	1	1	1
9	6	7	2	4	1	4
0	0	3	3	0	0	0
5	5	7	5	5	3	5
7	0	4	4	3	2	0
9	7	17	17	12	24	8
71	88	208	105	115	107	89
46	69	85	61	46	63	51
5	6	0	3	3	4	9
77	60	94	80	99	68	98
7287	8549	11061	7086	7653	7386	6302
9213	12122	14185	8698	9207	9309	8005
82	80	115	76	42	74	51
1641	3556	3164	1839	2967	555	641
1	0	0	0	1	0	6
2	9	14	2	7	0	8
0	3	0	0	5	0	2
18	30	72	25	33	28	20
1	0	0	3	0	0	2
0	0	0	0	0	0	0
2	0	5	0	2	0	0
0	0	0	4	6	0	0
0	2	11	2	0	0	0
11	5	22	14	16	4	7
33	47	51	20	18	33	23
26	39	28	18	20	23	18
144	156	176	143	105	144	87
39	30	56	25	37	53	26
3	6	1	0	2	4	0
321	293	581	323	406	303	312
47	33	76	56	39	47	11
5	6	15	4	3	2	5
0	0	0	0	4	4	2

For Review Only

0	0	0	4	0	1	0
35	23	36	21	50	31	36
55	75	111	67	66	83	73
159	675	1357	716	809	869	682
0	0	0	0	0	2	0
9	4	11	4	8	6	3
0	0	0	0	0	0	0
35	0	22	7	0	7	10
0	1	0	3	4	6	0
3	1	5	0	8	2	5
231	228	475	319	240	239	250
312	109	551	146	360	327	18
0	1	0	0	1	1	1
1	0	0	4	2	2	3
111	119	172	85	113	143	88
2	0	0	1	0	0	0
1	1	0	1	52	1	1
16	8	15	16	2	4	2
1	42	18	2	1	15	5
6	14	13	2	4	10	18
90	109	189	119	99	139	116
2	35	0	4	38	18	36
90	111	162	166	206	136	181
86	223	222	83	213	87	124
38	19	13	8	23	17	29
9	5	7	17	3	13	1
16	20	40	34	26	18	13
0	0	0	0	4	0	0
0	0	13	16	0	0	0
14	9	31	6	28	37	15
68	70	122	77	68	54	57
0	4	16	8	3	8	3
0	0	0	0	0	0	0
29	1	0	7	14	9	3
8	0	6	0	0	0	0
18	17	24	20	23	19	19
8	1	23	8	3	3	4
28	30	55	26	17	17	0
59	94	141	60	40	82	62
66	62	87	70	83	97	82
0	0	84	0	0	0	0
248	249	437	270	226	324	205
0	4	0	1	0	2	0
0	3	3	0	0	1	3
0	5	5	0	8	6	5
73	58	165	115	77	88	89
55	42	70	27	74	18	69
0	0	0	2	0	0	21
224	258	339	160	163	294	172
481	548	793	486	439	545	682

For Review Only

716	1062	1280	749	757	843	638
42	29	43	22	32	14	25
2	0	15	2	2	2	0
1	1	0	1	1	1	33
0	4	0	0	6	0	1
169	276	435	299	408	211	276
0	76	45	0	0	0	0
249	266	380	279	247	307	188
8	6	15	12	15	12	7
12	10	43	17	17	5	28
187	278	425	138	178	144	164
59	87	138	81	52	154	59
44	53	83	31	71	25	57
1	0	0	0	0	0	0
35	66	77	77	92	22	47
87	75	90	83	69	39	66
40	15	58	11	2	36	14
0	0	0	0	0	0	0
84	172	3	0	0	0	0
64	47	1	29	0	0	57
107	194	163	96	65	100	103
61	40	80	48	32	49	40
0	0	0	0	0	0	7
33	19	72	37	23	28	19
10	0	0	2	12	11	0
66	126	200	67	54	85	70
26	26	74	55	44	25	41
2	0	0	0	3	2	0
992	287	3123	2041	1713	921	669
250	230	427	379	334	76	288
37	62	53	74	58	0	48
0	0	3	0	0	9	0
12	18	21	12	15	15	17
0	5	0	0	7	0	1
98	58	142	86	47	90	49
4	12	31	9	11	15	38
0	0	65	0	52	0	13
1	4	8	0	0	0	4
106	9	199	81	34	130	82
17	60	74	75	82	50	91
18	46	62	10	37	23	14
129	402	652	304	434	409	321
7	15	19	7	5	6	6
228	163	230	158	167	213	159
1347	1454	2775	1715	1850	1782	1560
454	808	457	0	351	221	130
0	0	2	0	4	0	5
2	1	0	0	0	0	1
0	0	8	0	0	6	0
0	0	0	0	0	0	4

For Review Only

0	0	0	1	0	0	0
0	0	0	0	0	0	7
0	0	0	0	0	0	62
2	1	0	13	1	1	6
1	0	0	0	0	0	0
1	9	0	5	10	5	14
0	10	24	8	16	14	0
0	35	18	0	46	0	0
165	145	337	129	103	258	113
0	0	7	0	0	0	0
26	39	38	36	46	26	28
15	29	58	26	27	25	28
0	88	171	58	34	100	116
0	1	0	3	1	1	0
0	0	1	0	0	90	0
7	5	9	5	4	6	5
7	1	0	10	15	7	0
78	273	232	49	91	55	32
63	59	96	57	40	54	50
0	0	0	1	0	0	4
101	98	150	45	61	80	103
12	10	10	6	8	11	13
0	0	0	0	0	1	0
60	83	129	48	63	83	84
124	163	257	161	148	201	127
0	2	0	0	6	0	2
1	0	0	1	0	1	7
41	86	125	77	15	59	61
4	5	0	6	4	0	0
30	0	0	3	13	0	0
74	79	122	57	63	103	59
11	28	75	73	21	26	30
38	32	49	27	37	44	51
21	32	57	13	19	28	31
99	41	53	118	0	112	34
34	28	81	40	23	27	31
38	39	195	100	169	121	103
0	0	3	0	0	0	0
0	0	0	0	1	0	3
153	166	344	205	175	202	95
52	56	99	33	77	92	72
1	3	0	1	1	3	0
134	184	235	137	149	197	112
6	9	40	37	26	8	3
24	22	61	28	29	27	39
2	1	16	14	3	13	4
1	0	0	1	1	1	86
2	1	44	2	11	1	61
1	0	5	0	6	3	0
146	179	308	142	143	203	81

For Review Only

115	167	190	110	109	119	76
14	50	48	29	37	62	46
323	381	565	270	238	385	283
43	21	93	39	40	98	39
43	50	69	46	49	109	55
0	0	0	0	0	0	17
0	0	0	0	0	0	8
11	11	16	6	6	11	17
42	40	42	41	43	29	23
3	0	0	0	0	0	0
4	1	5	5	1	6	5
19	50	31	21	34	39	33
4	5	7	9	0	2	0
55	70	94	59	59	71	77
47	20	32	28	38	30	10
47	39	100	72	54	89	60
34	31	44	27	25	27	35
0	12	10	8	6	0	15
34	16	24	18	32	60	23
22	8	25	9	13	20	21
69	1	91	7	29	23	1
142	24	236	263	317	102	88
0	6	0	3	0	0	0
135	108	155	101	116	101	94
29	45	68	17	12	74	0
0	0	4	0	8	0	9
1	16	0	4	1	1	1
0	49	73	76	54	0	36
0	0	6	0	0	3	0
23	2	0	2	4	1	3
0	0	3	0	0	3	1
6	0	0	0	0	0	0
5	0	0	0	0	0	1
39	10	72	1	17	64	38
86	0	193	0	0	92	84
0	0	5	0	0	0	0
3	1	4	7	1	0	0
0	0	12	0	0	0	0
0	6	10	4	4	12	0
16	18	38	19	20	36	20
77	75	84	45	64	48	45
5	129	95	61	37	15	85
44	71	85	61	53	41	51
0	4	0	0	0	0	0
84	97	147	29	43	315	1
8	0	0	0	0	0	6
1	15	19	2	4	4	20
0	0	0	0	6	20	0
34	8	18	19	21	6	9
4	0	11	2	0	2	5

For Review Only

23	7	35	36	56	31	10
128	19	32	25	30	13	36
9	49	78	55	97	18	35
49	34	35	16	28	40	14
31	14	22	26	19	33	13
32	20	56	21	30	35	21
2	0	9	3	1	1	0
0	30	35	26	17	17	8
3	0	7	1	3	0	4
23	38	46	20	54	30	18
11	8	16	11	20	13	27
36	64	80	37	27	40	53
0	0	0	4	0	0	3
5	5	0	0	3	3	0
25	10	45	38	22	19	13
0	127	0	133	81	90	0
29	20	22	20	21	41	36
7	10	4	18	18	0	6
0	0	0	0	30	0	0
0	0	1	0	0	0	3
36	29	60	43	58	66	28
13	16	20	24	18	19	16
5	10	15	2	5	4	1
9	1	6	1	2	1	16
0	0	0	5	0	0	0
28	15	35	30	17	45	33
128	171	297	186	215	179	147
166	203	380	185	153	201	191
14	30	31	25	16	14	11
38	0	48	42	40	40	41
9	11	25	11	10	16	19
12	24	9	0	23	15	13
40	26	52	22	29	37	19
53	97	193	56	95	103	128
3	17	34	12	14	25	2
15	6	52	11	39	15	3
0	0	3	0	0	0	0
18	23	15	16	18	17	4
16	97	32	35	17	11	21
0	1	2	0	0	0	0
90	112	0	88	112	161	84
14	10	9	13	4	8	8
2	7	16	0	2	6	2
15	2	20	18	14	18	36
1	8	0	2	5	0	2
2	0	0	3	3	0	2
0	3	0	5	0	0	7
42	45	107	55	47	58	60
0	0	4	0	0	0	1
10	13	16	9	9	8	18

For Review Only

42	33	68	35	24	30	37
57	57	75	58	60	58	61
13	18	40	56	18	42	19
1	59	0	1	1	1	1
0	3	0	0	2	2	0
2	2	0	1	0	2	0
17	22	9	16	13	18	1
21	56	101	27	40	61	100
26	35	38	27	9	12	29
7	0	0	0	0	0	0
271	310	541	301	316	321	226
1	4	0	1	72	1	29
2	2	0	7	5	0	3
0	31	255	53	21	247	41
0	0	0	4	0	7	0
0	65	0	0	51	63	0
0	1	0	3	0	0	0
1	8	0	4	2	12	2
0	0	2	0	0	0	0
0	0	3	0	1	0	1
1	0	3	0	0	0	1
0	0	0	0	0	0	0
0	0	0	0	130	0	0
0	0	0	0	0	0	29
319	286	413	407	249	276	221
4	5	10	0	9	3	0
116	179	165	135	134	114	108
234	351	240	49	8	96	27
3	3	2	6	0	0	7
7	4	20	7	2	10	0
0	0	0	0	0	3	0
0	0	12	0	2	7	0
33	41	49	30	32	62	43
97	76	108	110	128	79	71
0	0	0	0	5	0	0
17	8	148	1	1	8	35
32	64	69	35	0	75	29
0	0	0	0	0	2	0
11	3	18	0	1	17	9
57	80	136	122	64	85	83
1	12	0	1	1	1	1
69	77	92	55	59	59	40
174	346	328	132	158	200	292
21	15	15	19	9	16	9
33	46	56	35	37	37	30
179	222	139	89	168	152	136
212	135	287	220	273	0	206
83	57	95	84	53	116	83
42	33	212	182	40	41	149
78	87	103	95	61	40	81

For Review Only

5	5	9	3	6	8	13
6	32	28	36	5	15	2
216	288	452	258	312	302	296
4	3	5	8	4	11	0
19	44	11	35	14	34	23
4	2	31	7	1	12	1
42	45	85	50	37	42	19
4	5	22	21	10	1	1
0	681	2235	1284	1241	0	0
5	0	13	11	1	0	3
2	15	14	7	0	1	2
0	15	0	18	0	18	0
6	0	0	0	11	0	0
0	0	0	2	2	0	0
34	48	60	25	33	25	49
170	264	316	184	130	142	169
203	286	404	229	195	319	254
263	391	547	307	271	366	321
267	354	324	229	240	315	264
183	214	263	166	174	333	201
273	299	420	231	244	344	278
275	294	363	289	212	323	299
32	46	65	35	30	36	23
44	33	100	34	19	39	15
705	500	0	737	0	0	0
125	82	86	120	98	154	53
0	0	0	1	0	2	0
2	2	0	0	0	1	5
1504	1829	2987	1875	1614	1805	1284
40	0	0	0	0	34	0
0	334	334	0	123	335	306
0	0	0	2	5	0	0
0	2	18	3	1	0	4
0	0	0	0	0	0	14
15	148	83	28	70	52	24
4	0	0	2	0	0	0
4	0	0	0	0	0	0
0	0	0	0	0	0	14
3	0	0	0	0	0	5
31	41	19	37	20	35	6
0	0	2	0	1	1	0
4	1	0	8	9	6	5
0	0	6	0	0	0	0
0	0	0	0	0	2	0
38	40	84	66	63	49	53
22	20	24	54	22	39	40
79	49	155	162	134	49	33
89	87	85	68	86	68	101
14	0	0	1	2	0	6
482	629	1025	630	391	664	491

For Review Only

0	0	0	0	3	0	3
280	431	863	229	424	284	234
0	4	0	3	3	11	5
0	0	0	0	0	0	5
0	1	1	0	0	0	3
0	4	4	4	0	0	3
0	1	1	1	0	0	3
8	4	0	2	5	3	4
180	307	367	307	275	242	216
26	0	31	25	23	40	30
76	0	455	0	0	1363	1062
734	538	0	578	125	0	0
0	0	0	3	0	0	9
5	45	0	5	20	26	0
0	0	0	0	1	0	10
8	36	41	35	20	24	28
0	1	3	3	0	0	0
6	0	0	8	0	0	0
3	2	0	1	0	7	3
1	6	28	4	26	3	1
525	510	895	517	462	518	452
174	239	366	173	176	209	170
98	83	114	89	86	104	129
427	2373	1480	1457	488	236	1873
0	0	0	0	0	0	7
2	0	3	1	4	0	5
0	0	0	0	0	0	0
1000	1190	573	0	0	1155	889
0	0	0	3	0	1	0
9	0	10	5	4	10	12
116	102	106	43	19	85	86
41	0	2	0	0	75	0
65	35	109	81	63	42	16
8228	9459	12600	8902	5160	9283	7777
90	13	645	3	203	21	95
266	4	990	42	0	16	231
445	711	785	820	589	498	455
406	852	854	840	608	451	599
1442	1317	2502	1544	1051	2342	1390
0	0	0	0	0	3	35
44	56	106	33	66	59	38
46	54	44	40	47	38	79
1	1	0	1	1	15	24
125	123	144	104	119	98	106
12	15	52	33	20	29	20
3	18	21	4	8	3	11
50	85	93	52	51	64	59
26	71	233	134	90	87	120
11	12	23	10	10	8	10
1	4	21	3	5	0	4

For Review Only

48	42	60	49	68	51	48
45	46	132	57	45	113	51
0	0	7	0	0	0	35
0	278	1	0	0	0	0
116	135	198	87	112	82	113
72	85	192	57	46	96	78
1152	1451	2199	1470	1479	1396	1258
13	14	16	15	21	9	26
117	4	88	16	64	120	53
1	5	8	1	13	1	5
3	1	0	16	0	8	1
86	53	70	25	34	33	26
77	87	22	76	27	36	34
3	214	364	162	309	21	285
115	239	326	181	133	223	204
0	34	0	0	0	0	0
72	57	84	88	54	72	63
1	5	40	19	3	24	5
39	30	51	32	40	32	27
0	0	17	0	0	12	7
0	0	0	0	3	0	2
120	216	277	192	145	149	169
42	56	66	34	68	68	52
8	0	6	0	0	5	4
2	3	4	3	0	3	2
0	0	3	0	0	3	0
284	116	327	226	177	206	131
0	1	0	1	0	0	0
0	1	11	2	0	1	14
2	0	0	0	0	0	0
0	0	3	1	3	0	0
0	0	0	2	0	0	0
0	0	0	0	0	0	0
0	0	0	31	260	12	43
0	0	4	0	0	0	0
0	0	0	0	0	8	0
26	6	7	0	0	0	1
119	137	134	101	156	150	85
122	51	136	122	111	123	116
99	68	89	52	68	77	22
25	57	50	26	39	22	28
0	0	95	0	86	77	0
48	36	61	35	44	59	46
5	3	0	0	0	2	3
157	130	216	128	128	166	126
48	28	103	82	62	60	35
13	0	0	0	9	15	6
26	29	30	27	18	15	22
0	0	0	0	0	0	0
0	0	7	3	1	0	0

For Review Only

83	31	236	117	18	303	419
0	38	98	43	27	27	28
17	40	69	25	17	19	39
21	33	52	41	20	32	51
39	27	36	23	42	43	30
1	10	18	17	123	123	0
442	276	534	348	235	194	256
95	60	117	93	65	59	73
18	43	61	15	19	14	35
20	105	221	25	58	1	89
106	102	138	69	103	80	59
4	6	43	8	22	6	5
9	1	21	4	0	0	2
48	78	83	51	40	56	58
7	0	8	6	8	3	9
36	44	84	62	69	67	46
67	74	162	100	94	101	55
1	1	0	2	0	0	0
8	3	0	0	5	3	16
19	23	10	7	8	10	35
0	3	0	0	0	3	0
30	36	35	24	33	15	25
0	0	0	0	0	1	0
1	1	0	1	8	3	2
122	108	227	129	130	109	90
272	364	412	255	228	337	246
0	0	1	0	31	39	0
36	120	101	42	67	76	57
12	40	34	40	19	10	15
34	30	62	52	23	35	53
68	120	194	13	113	91	42
6	0	0	4	4	0	3
3	19	40	17	19	11	35
109	143	170	45	57	172	166
17	53	78	42	44	22	22
146	134	155	109	104	180	196
67	259	53	41	58	105	59
112	0	71	0	64	78	102
0	0	0	0	0	0	8
8	7	1	18	16	13	3
0	0	0	5	0	3	4
0	0	0	0	0	0	0
336	294	217	254	297	333	0
405	560	749	467	417	400	369
8	11	0	13	0	5	13
4	8	17	3	5	3	17
0	2	0	6	0	0	4
0	0	0	0	0	0	0
0	2	0	0	3	2	0
0	1	0	3	0	1	6

For Review Only

20	23	22	35	32	17	46
15	23	27	17	11	12	0
0	0	0	0	0	0	0
9	3	17	0	4	11	11
0	1	0	0	0	0	0
2	12	22	10	1	37	4
40	10	14	8	13	3	13
0	0	2	0	0	0	0
0	4	0	0	0	0	0
73	40	100	65	61	67	43
1	0	4	6	3	0	5
8	5	7	7	6	12	8
0	15	60	22	22	0	12
2	8	9	2	6	3	0
1	5	3	0	4	0	2
73	53	66	57	46	50	58
0	0	35	18	15	0	14
0	0	0	1	3	6	0
85	115	144	78	97	103	83
4	15	15	3	5	4	9
0	0	8	0	0	0	0
22	29	41	24	26	23	23
105	56	114	104	103	128	66
18	20	41	19	11	17	4
46	59	59	24	34	45	34
160	247	384	248	206	191	263
0	1	0	0	0	0	1
27	2	0	2	4	13	13
33	40	145	22	19	146	89
75	125	151	107	76	71	64
0	20	0	25	16	36	19
67	59	114	41	77	112	40
180	160	279	56	92	163	104
11	14	27	3	11	4	6
15	22	12	27	18	17	19
67	59	94	51	50	66	41
8	14	8	10	3	17	4
62	64	130	59	91	29	42
193	204	421	116	189	56	152
10	1	29	27	9	28	6
1	2	27	1	4	28	2
1	2	47	1	26	1	2
2	14	12	11	15	17	1
16	12	13	2	5	27	8
12	8	55	44	24	14	10
22	1	0	1	1	137	1
37	54	65	31	53	37	17
17	14	34	22	16	23	18
38	33	51	33	25	38	27
56	14	27	32	11	40	10

For Review Only

206	271	363	245	252	246	216
4	8	0	19	21	2	5
236	450	419	256	262	416	255
1	1	7	4	0	0	0
0	20	0	0	0	16	0
14	5	0	12	17	4	27
134	127	232	114	115	137	105
21	51	50	74	58	49	78
30	32	70	25	19	41	36
41	105	92	51	79	69	84
3	11	5	0	7	9	4
86	11	37	13	57	2	8
21	40	21	14	13	12	32
2	2	6	5	5	1	5
0	1	5	2	1	1	0
0	0	0	0	0	0	0
0	0	0	0	1	5	0
90	79	198	270	62	246	65
91	41	179	17	65	58	28
0	0	3	0	0	0	3
2	2	0	2	2	1	2
49	62	103	53	0	65	63
9	1205	0	68	1020	1221	1091
49	26	0	60	41	81	25
681	780	0	710	692	774	621
45	53	77	56	64	61	47
95	187	281	121	120	180	131
117	104	258	90	105	131	69
0	3	4	0	0	1	0
1	1	1	0	0	0	8
5	1	0	2	2	14	1
102	0	28	0	137	100	99
0	1	0	1	0	0	14
5	9	8	2	9	6	14
4	3	11	7	7	4	16
0	0	0	0	2	0	2
0	4	6	0	4	3	0
0	0	0	0	0	1	0
87	114	263	203	98	138	91
0	0	0	0	32	0	31
0	0	0	0	0	0	0
170	231	342	155	180	264	279
119	105	125	83	84	110	81
24	48	52	20	29	78	43
1	1	0	1	1	1	17
0	25	8	0	18	0	0
19	22	35	19	16	23	0
33	30	57	37	14	31	44
43	4	71	30	34	49	13
130	105	181	140	118	82	104

For Review Only

53	23	72	29	39	44	35
7	10	24	22	6	19	5
90	90	211	100	104	146	90
5	0	0	0	0	0	0
96	111	153	141	121	117	77
286	328	434	208	329	196	107
134	110	182	75	109	135	74
445	162	624	463	368	375	379
4	7	11	8	4	6	1
2	0	0	0	0	8	0
25	18	70	14	37	43	19
54	11	35	67	80	18	121
72	83	137	73	96	102	55
4	11	6	2	3	7	9
10	31	36	21	15	45	14
11	11	78	74	32	89	52
17	11	25	14	10	14	14
27	40	5	0	0	66	42
2	1	6	1	2	2	1
117	130	130	65	105	119	103
0	60	42	81	59	0	0
32	46	120	96	65	41	59
24	14	14	3	6	27	16
217	50	0	0	0	3	0
92	159	170	112	111	103	104
3	0	46	58	0	1	0
78	114	172	67	98	84	87
225	230	301	258	185	212	222
8	15	16	5	11	12	9
86	117	154	95	106	88	132
136	0	105	131	0	0	107
55	16	83	19	28	132	13
0	4	8	0	0	1	0
91	149	303	143	121	131	149
109	84	128	73	99	134	87
210	83	192	30	98	77	158
1	1	0	1	0	0	1
138	201	231	103	176	250	129
20	32	19	19	11	15	17
5	670	0	1	1	1	8
1	0	4	0	0	1	1
9	16	3	12	5	15	5
0	5	1	3	1	3	0
0	1	11	1	4	0	4
11	0	0	0	6	0	0
11	23	37	56	37	21	60
4	4	9	4	7	7	1
40	110	51	28	52	58	49
108	104	219	175	24	103	44
0	0	0	0	0	0	0

For Review Only

47	77	97	29	56	61	37
43	62	105	56	55	74	39
0	5	14	7	4	20	9
0	0	0	0	2	0	0
185	148	200	142	188	173	126
67	135	144	125	109	87	84
0	0	5	3	3	0	0
171	221	416	228	221	186	250
0	0	0	0	0	0	0
178	23	106	0	0	0	25
248	264	429	240	278	236	234
78	103	78	53	55	105	68
103	9	81	26	37	9	77
68	67	92	66	60	85	73
46	106	74	33	70	6	38
0	1	10	11	8	7	3
66	71	13	50	46	47	76
0	0	1	0	0	0	0
145	234	213	174	136	209	161
13	1	11	7	1	6	3
0	1	0	1	0	1	0
34	57	64	60	39	41	33
25	46	119	58	62	73	48
8	12	22	9	10	18	14
143	0	31	0	0	0	0
44	39	48	33	45	49	0
50	43	71	48	15	49	48
109	97	187	105	130	116	111
3	3	7	9	8	11	0
0	3	3	3	3	1	3
3	3	0	9	2	4	4
2	0	8	0	2	3	2
1	1	0	315	1	1	1
12	80	236	70	157	163	165
0	0	0	1	0	2	2
0	0	0	0	0	0	0
1	2	41	9	4	16	2
23	39	41	13	31	10	23
49	67	114	62	68	89	42
0	1605	0	1625	0	0	0
4	3	0	10	4	101	1
7	7	10	9	3	4	4
5	5	0	0	7	9	5
80	103	137	102	86	93	42
487	575	907	595	571	555	574
210	28	189	105	13	67	18
116	126	189	168	183	141	126
0	18	89	0	0	0	59
43	0	0	0	43	0	46
5	0	5	5	0	0	0

For Review Only

85	156	159	73	130	43	90
322	470	642	523	375	545	402
56	48	118	56	70	108	62
84	62	146	93	63	81	67
119	191	431	249	46	340	231
0	0	8	0	0	0	5
9	10	0	2	1	8	51
2	0	0	0	0	0	0
0	3	1	0	0	0	0
0	0	0	1	1	1	2
0	5	0	1	0	5	1
0	0	0	0	0	0	54
180	103	213	115	104	199	168
0	4	0	3	1	1	0
7	0	13	0	2	4	2
3	0	0	0	1	0	10
152	134	168	111	149	214	88
9	8	7	8	9	3	0
0	0	0	0	0	0	0
0	0	0	5	0	0	4
0	0	0	0	1	1	0
0	12	8	5	14	4	27
125	182	253	113	129	156	157
180	280	399	224	255	288	270
0	9	8	0	0	5	9
291	283	306	327	280	329	315
42	28	35	28	22	26	39
0	4	0	2	0	1	2
17	9	0	16	39	16	22
755	1223	1382	877	839	928	707
12	5	0	12	10	15	74
1	0	0	1	0	0	10
0	2	0	4	0	0	0
77	119	138	61	76	81	69
207	172	372	242	195	192	211
67	25	69	79	75	62	14
0	0	0	0	1	0	3
0	33	12	0	0	48	0
1	141	0	1	1	1	1
0	0	108	0	149	0	0
1	363	0	59	54	2	5
6	11	138	191	142	18	22
1	0	8	4	2	0	2
5	0	16	4	4	2	18
16	8	45	25	3	2	23
163	201	376	146	174	165	153
6	13	14	16	8	15	43
246	319	453	237	315	253	200
0	149	245	138	138	136	140
59	99	121	39	122	97	63

For Review Only

67	74	102	63	55	69	41
53	60	75	69	86	48	67
0	0	0	0	0	0	0
132	93	229	17	33	14	40
61	158	121	94	91	107	126
83	79	156	84	122	105	98
5	1	6	0	0	1	0
302	384	415	206	314	323	253
0	0	0	0	0	6	0
667	742	1006	587	583	862	680
39	48	94	48	58	61	45
775	1116	1746	569	735	900	783
0	15	12	0	0	13	0
44	52	83	48	65	61	28
51	61	134	52	43	85	43
8	0	14	9	0	0	9
26	37	54	33	23	29	20
0	0	0	0	0	1	16
14	21	35	16	20	12	19
6	1	0	2	13	12	21
0	9	8	0	13	9	0
46	76	97	31	52	59	54
205	122	168	143	221	189	114
58	95	110	66	58	108	75
2	5	8	9	6	1	3
7	7	0	0	4	10	7
159	0	236	4	191	166	149
72	89	96	55	51	48	36
2058	1828	5063	3445	3349	2464	1528
352	503	746	348	422	337	401
40	0	1	0	44	48	48
22	7	67	15	35	54	4
20	25	38	14	14	17	23
33	12	54	18	29	39	9
17	20	36	11	21	11	24
363	891	1192	469	738	736	743
410	387	584	347	342	340	239
49	63	50	38	66	52	11
1	1	23	3	1	3	0
7	0	0	0	4	0	0
135	170	211	109	152	195	144
107	98	156	13	37	69	31
6	11	10	11	1	7	8
0	0	0	0	0	0	0
1	1	378	1	1	1	151
0	0	0	2	0	0	25
14	23	36	6	18	23	20
46	101	78	40	60	67	27
198	141	299	114	73	133	114
144	164	256	151	119	190	140

122	132	45	111	115	104	88
0	0	1	0	0	0	33
273	290	472	261	279	315	261
87	119	137	18	53	90	72
92	0	21	0	0	0	0
39	61	49	27	40	43	49
99	113	130	87	89	79	86
0	1	58	0	0	0	1
357	443	641	321	413	374	316
49	80	175	73	65	55	109
241	257	378	285	190	272	154
40	104	58	17	14	14	0
73	94	158	59	123	92	121
500	475	656	636	457	454	296
39	40	35	29	26	29	29
6	2	1	1	27	4	3
50	49	61	42	28	53	40
38	13	43	30	25	60	14
0	0	0	1	0	0	0
0	1	5	0	0	0	3
0	0	9	7	0	8	9
0	0	11	0	0	0	0
0	0	4	0	0	0	8
1	1	0	1	0	1	0
88	24	148	87	114	77	4
9	9	46	21	13	21	9
67	102	43	0	71	0	0
48	45	90	50	35	61	37
95	95	286	215	178	134	88
26	13	9	12	10	11	19
22	15	25	11	20	6	27
1	2	0	121	1	1	21
0	0	0	0	0	0	11
0	0	6	0	0	0	0
161	120	286	179	161	184	98
77	45	123	89	74	83	64
39	71	94	37	86	48	97
85	44	151	53	50	62	86
65	70	305	130	156	218	246
54	82	79	79	56	68	80
36	65	99	19	26	79	32
31	15	52	26	27	30	35
6	6	6	6	9	6	3
0	0	0	3	2	0	3
0	0	0	1	0	0	28
0	1	20	14	0	0	7
52	24	94	69	39	47	50
0	0	0	5	0	0	0
0	0	0	0	0	0	4
0	0	0	0	0	0	20

For Review Only

14	5	0	10	6	8	6
5	0	18	8	5	1	15
0	0	12	0	0	3	0
396	315	438	239	350	351	338
0	0	0	0	0	0	13
0	2	0	0	0	0	2
36	50	110	38	20	47	78
3	1	9	2	13	2	2
0	0	0	0	0	0	10
37	100	159	61	72	27	68
0	4	2	1	11	9	0
0	0	0	0	0	0	0
1	1	0	0	0	0	0
4	1	33	5	2	5	2
1	0	1	0	0	0	7
0	0	0	0	0	0	0
4	1	0	2	4	0	4
27	38	54	32	50	76	53
52	70	96	64	73	79	56
45	56	107	47	53	97	54
157	163	155	90	76	120	100
135	36	22	37	31	0	111
24	42	0	39	28	47	28
38	57	72	40	43	58	34
61	70	12	85	27	53	28
68	63	32	295	53	0	43
99	80	108	73	80	58	57
8	10	0	2	0	0	6
631	761	1006	763	701	689	715
0	50	0	45	0	41	0
328	386	713	401	385	419	318
209	264	396	192	133	199	134
157	186	279	227	147	183	167
0	0	6	0	0	0	0
219	213	359	214	212	173	153
46	72	24	0	82	68	62
10	23	41	8	11	33	33
70	81	114	75	80	89	81
129	136	224	144	136	114	147
256	326	483	268	320	218	288
78	112	113	74	53	55	54
159	161	207	110	127	163	133
0	20	8	71	0	0	0
35	57	50	35	43	77	45
0	5	0	24	8	2	7
122	104	265	117	160	176	159
1	0	12	0	1	7	8
7	17	12	12	18	15	8
49	22	47	53	24	41	27
41	56	64	56	30	34	42

For Review Only

17	14	31	20	2	7	20
27	36	45	39	41	24	26
75	50	120	71	94	78	61
36	0	33	0	48	31	0
18	13	19	0	0	9	12
89	121	104	64	46	78	83
3	0	0	0	0	0	5
0	1	382	1	0	27	0
736	158	3	4	0	665	193
0	78	246	116	0	0	83
44	37	54	14	22	52	20
0	0	0	0	0	6	3
125	136	179	113	125	144	108
343	302	600	464	398	311	216
1493	2028	3005	2198	1965	2099	2967
39	21	60	30	54	47	26
16	10	34	19	10	6	33
86	118	142	88	96	75	79
53	25	59	45	23	45	30
42	36	65	27	48	103	38
0	59	31	0	39	43	45
14	28	29	21	24	20	16
3	3	0	1	2	21	158
206	513	767	440	460	369	904
0	5	1	0	0	7	0
90	198	59	105	81	248	530
307	452	635	446	314	515	514
1	0	0	0	2	2	0
210	1	0	1	1	1	75
26	0	0	26	75	0	47
2	1	627	1	1	10	1021
0	0	1	6	0	0	0
118	158	210	121	119	81	161
0	68	94	0	0	0	0
67	124	119	105	85	75	163
201	211	238	211	138	219	186
32	146	134	24	19	50	193
0	0	0	0	0	0	21
149	173	286	161	151	159	203
16	19	45	10	8	15	50
60	57	67	40	38	31	64
0	0	0	0	0	0	2
44	53	87	23	49	39	92
7	5	7	4	3	4	9
32	24	79	43	44	16	46
1	0	0	0	0	0	0
2	0	4	2	9	0	5
0	0	8	2	5	0	1
0	0	4	0	0	0	0
1	22	0	1	19	1	0

For Review Only

0	0	2	0	0	0	0
74	156	145	79	85	107	128
5	0	0	3	3	0	0
13	15	8	1	9	12	5
49	50	75	63	54	54	31
26	47	66	46	41	43	74
1	1	0	0	1	7	1
110	117	156	77	88	147	26
327	234	351	312	259	236	191
0	0	5	0	131	163	117
43	82	130	56	64	88	69
0	0	0	0	0	5	0
123	184	192	132	102	69	211
99	115	164	90	65	127	72
41	54	125	55	98	54	55
0	6	7	7	0	0	0
77	64	113	45	90	49	44
49	60	67	17	1	72	1
0	0	104	0	283	105	224
2	1	0	0	5	1	0
30	18	59	28	34	23	16
41	123	89	98	22	110	71
32	58	84	44	43	37	33
87	91	81	50	46	62	63
197	202	433	234	208	272	198
2	0	0	1	0	1	7
0	0	5	3	0	0	0
53	20	79	12	24	12	41
109	157	224	212	108	163	155
55	57	88	56	48	54	48
114	138	179	108	94	173	105
0	0	1	145	0	240	0
4	6	6	3	10	0	4
188	203	310	177	185	212	138
0	4	11	10	9	7	3
300	369	457	263	277	266	229
32	27	37	22	24	40	25
85	169	135	11	47	102	101
24	38	58	37	21	17	31
699	905	1175	678	693	1061	471
21	67	43	41	54	64	33
133	140	189	148	127	155	160
15	17	28	20	9	29	17
14	13	26	14	35	23	26
0	0	0	2	0	0	19
0	0	0	0	0	0	69
75	135	117	58	86	82	94
167	209	216	72	131	141	119
207	266	413	283	265	252	129
66	63	80	56	18	60	34

For Review Only

348	349	502	301	319	336	279
5	8	10	3	3	2	8
8	0	1	6	0	0	0
66	44	63	72	64	95	51
13	7	9	10	12	7	0
23	101	294	145	110	132	98
78	85	112	65	76	104	83
99	80	144	88	107	84	61
10	13	92	4	4	26	1
35	52	31	21	35	32	34
8	22	29	11	18	6	19
44	17	62	16	24	39	29
0	0	0	0	0	0	6
39	13	1	0	0	0	0
290	313	332	240	299	298	223
70	72	52	69	76	89	60
301	333	415	317	279	300	250
17	18	78	78	67	39	13
59	52	79	63	63	59	62
273	199	483	268	209	278	206
126	78	194	192	62	84	53
127	165	429	254	264	113	179
129	67	294	73	138	217	32
29	19	63	12	32	14	23
20025	17579	32648	26833	23893	18371	9029
1	1	0	1	1	1	4
1	0	0	1	1	51	0
74	77	883	154	148	0	101
0	0	0	0	155	0	22
0	0	0	0	0	0	14
24	24	32	16	17	31	21
0	5	0	0	16	10	6
13	14	21	9	14	15	8
1	0	1	0	2	0	1
438	551	715	459	657	532	434
321	330	501	347	351	364	310
166	263	443	233	234	425	192
53	250	155	169	208	215	140
160	94	259	163	121	54	181
0	39	99	10	5	5	12
47	36	22	12	28	30	14
107	135	192	101	134	153	128
16	16	35	7	5	18	17
124	94	139	83	107	112	97
0	0	0	1	0	0	2
31	52	44	44	34	57	39
15	27	46	35	26	17	23
3	8	29	11	14	32	12
4	1	0	0	0	1	0
0	0	11	0	0	0	9

For Review Only

0	0	0	0	0	0	11
51	22	132	42	38	62	41
56953	81217	95372	52992	57105	59791	50980
0	4	0	0	0	0	0
23	20	50	25	18	32	22
0	83	9	0	69	0	56
173	96	190	106	110	144	101
16	62	106	33	30	66	53
122	111	206	241	105	144	105
28	31	94	68	76	31	49
50	42	106	50	37	60	36
9	31	0	6	8	11	8
57	63	83	58	57	32	64
101	115	126	117	115	82	72
0	0	0	0	0	0	0
64	60	115	44	42	77	45
1	103	0	1	6	4	57
62	61	95	34	32	84	35
0	0	112	0	0	0	0
0	0	1	0	0	0	0
5	4	0	4	6	22	2
109	112	158	52	62	132	88
9	10	46	16	27	39	18
0	0	8	0	0	0	23
100	141	220	154	110	121	145
42	57	44	58	27	32	48
55	122	380	8	15	14	620
29	30	58	20	18	21	11
61	82	103	45	88	122	54
179	196	491	254	240	352	271
31	40	27	59	26	41	21
120	127	192	170	149	134	134
25	30	0	6	17	17	38
125	138	210	123	156	164	126
180	53	162	141	101	134	33
0	0	0	0	0	0	0
0	0	0	0	2070	0	0
6820	7020	12663	6957	7716	9122	5780
1234	1299	1859	1032	1042	1156	1398
23948	32335	47687	34463	22318	27876	24576
0	0	0	0	0	0	0
0	0	0	0	0	7	0
597	1083	2393	743	136	1622	1215
0	0	0	3	0	5	0
1504	1766	2647	1758	1675	1801	1491
242	313	503	293	304	360	244
11384	14786	19321	10531	11358	13682	11552
0	4	4	2	6	0	0
2502	2909	4034	2828	3534	2158	2437
0	0	0	0	0	0	8

For Review Only

0	73	1	0	81	0	79
32	54	43	40	25	44	43
0	0	55	17	18	19	17
1	0	0	2	1	3	0
0	0	0	14	1	57	0
156	222	355	206	258	203	204
71	46	122	69	85	82	51
19	55	93	6	46	14	52
99	165	218	89	168	63	36
6	23	37	17	25	52	47
35	57	102	48	99	57	59
24	20	9	36	17	32	27
188	184	290	138	122	100	133
0	0	22	0	0	0	0
0	18	17	19	7	11	11
273	313	487	315	335	249	206
14	15	55	29	34	24	20
58	94	133	83	124	90	82
39	27	95	30	25	64	34
39	34	36	36	38	44	41
33	35	58	22	46	59	34
26	39	46	34	79	45	16
26	37	87	58	68	44	50
2	0	1	0	0	0	3
16959	17742	32941	18239	17411	18025	13427
3	3	1	0	0	0	2
1	2	0	6	0	3	2
1	0	0	0	1	0	41
4	0	0	5	0	0	12
52	0	41	57	0	0	161
3	5	0	0	1	1	0
1	0	0	0	0	0	0
1	1	0	0	6	1	2
5	1	0	3	0	0	5
6	3	21	9	12	1	9
5	8	13	3	8	5	10
8	7	14	4	8	8	5
22	24	25	19	28	25	12
0	1	0	3	1	5	1
0	0	0	2	3	0	3
250	9	89	237	213	246	204
0	0	37	2	4	0	6
221	110	696	1021	613	179	197
85	84	123	146	68	84	81
85	75	109	44	56	64	53
210	238	256	196	143	219	217
45	125	188	110	90	122	74
41	32	48	36	47	48	30
75	57	142	79	62	49	53
179	0	8	0	0	0	219

For Review Only

0	0	0	0	0	0	3
65	98	152	78	62	114	112
31	16	41	22	0	37	26
29	13	34	10	15	38	12
716	811	991	730	565	900	645
45	12	9	5	2	23	49
0	0	1	0	0	0	0
35	0	23	40	33	51	37
1358	1004	2760	1744	1606	1218	1134
0	0	0	0	1	0	2
171	193	321	181	116	132	133
74	95	128	74	70	91	96
0	0	0	0	0	0	10
49	48	69	55	28	67	50
0	0	65	0	0	0	0
0	0	0	0	4	7	18
0	0	0	0	0	0	12
0	0	12	1	0	1	7
20	25	30	32	29	29	19
0	0	0	0	0	0	2
4	7	12	6	7	6	0
6	20	14	6	11	5	6
42	0	54	0	49	0	29
19	6	26	8	5	24	28
0	0	5	0	0	0	3
0	0	0	0	0	3	1
0	0	6	5	0	4	0
206	142	343	247	188	156	181
16	14	61	21	31	65	24
0	0	0	0	0	0	63
191	182	362	206	190	233	160
896	1403	1990	1558	1453	1306	1646
99	71	167	88	94	112	62
5	14	0	12	26	26	0
159	0	308	139	151	0	119
1927	292	552	506	394	2090	226
1	8	20	3	10	18	1
125	117	174	90	129	105	81
0	0	0	0	2	0	0
1	0	1	5	4	7	0
9	8	16	6	4	10	6
19	20	26	18	20	12	16
37	17	90	34	50	50	32
64	59	92	58	87	49	41
4	7	11	9	9	10	4
10	9	1	1	1	1	2
3	4	9	9	1	1	5
0	1	0	0	2	2	4
17	8	55	16	27	26	30
12	15	10	11	26	11	11

For Review Only

1	1	0	5	4	2	7
0	330	250	0	265	271	0
102	53	105	46	59	64	90
61	124	196	118	73	137	88
31	15	60	31	37	40	10
66	105	160	46	60	109	62
76	69	113	79	80	72	38
30	49	48	52	59	76	55
8	7	20	5	0	0	17
0	0	0	0	0	0	0
8	3	16	25	6	9	10
4	0	9	1	0	2	1
27	96	71	45	113	36	62
35	9	44	20	8	18	19
1	10	14	3	3	1	1
38	33	51	38	21	39	27
58	113	134	73	37	55	43
5	35	35	10	25	9	6
0	1	0	0	0	2	3
0	4	0	0	0	6	2
21	5	21	10	12	15	19
127	113	201	149	119	114	98
47	41	70	28	39	54	29
6	0	5	10	5	7	3
23	15	26	17	23	14	3
0	1	0	1	0	0	3
5	4	6	8	7	4	6
41	49	93	30	42	38	72
8	4	0	0	7	8	0
0	1	0	2	8	5	6
3	2	11	7	15	4	4
0	1	27	1	1	107	0
210	158	297	228	209	204	146
170	172	364	207	161	200	94
18	33	41	21	24	28	8
0	2	11	2	2	4	2
0	0	5	0	0	0	1
19	22	34	25	20	28	49
0	0	0	0	3	0	1
13	3	44	16	6	10	54
8	5	14	5	8	1	21
0	0	0	0	0	3	0
0	0	5	1	0	1	0
236	265	347	165	189	212	181
79	167	219	82	81	154	111
115	119	174	146	115	171	68
5	1	0	1	1	8	1
8	0	0	0	0	0	0
6	9	11	0	19	8	24
26	7	38	27	18	24	20

For Review Only

81	91	155	71	84	93	75
36	44	49	37	24	48	36
25	21	75	14	26	30	38
0	0	0	0	0	0	1
406	228	389	352	393	200	301
139	80	159	111	151	92	84
22	5	75	60	60	32	21
234	16	0	12	22	3	7
6	14	31	0	0	27	0
394	452	127	261	147	440	304
4	3	0	12	6	2	0
25	53	41	45	34	32	53
0	1	0	3	5	3	0
13	27	29	14	14	14	22
0	13	0	1	0	0	0
0	7	6	6	4	13	0
38	43	59	17	32	44	39
22	27	52	46	59	48	37
10	25	24	15	18	18	39
127	15	272	246	154	99	96
316	372	486	312	224	377	228
0	0	0	0	0	0	0
0	79	70	76	64	78	69
0	22	9	19	15	16	18
46	50	128	61	50	37	42
60	39	62	41	45	46	41
61	70	55	31	31	77	58
8	28	28	3	6	12	26
293	272	428	230	206	307	179
231	235	404	301	200	356	185
68	84	191	87	94	87	83
129	101	180	63	117	47	65
5	5	6	7	4	1	9
7	1	18	2	6	0	9
74	46	82	44	12	62	19
0	0	7	0	0	0	1
7	10	14	10	1	4	7
17	21	47	20	45	13	11
46	81	103	67	53	30	45
131	29	165	48	89	80	93
0	0	0	0	0	0	202
70	53	137	113	73	100	54
13	23	104	32	46	28	20
27	46	72	12	19	32	32
20	52	52	55	38	47	21
0	0	4	0	6	0	1
11	2	0	14	8	9	21
63	123	181	88	112	115	90
0	0	0	0	314	425	262
11	14	0	0	13	70	0

For Review Only

289	281	517	317	324	285	331
430	11	729	204	345	473	315
0	65	76	56	67	76	67
28	47	44	26	22	41	59
62	45	80	37	42	51	67
16	104	84	69	76	65	66
28	23	54	10	22	6	26
39	24	92	28	68	40	45
13	44	38	13	55	41	31
0	171	4	169	150	169	116
0	0	7	1	1	1	5
80	0	160	59	60	35	0
195	252	514	288	289	345	149
0	0	0	0	0	7	22
17	3	32	1	9	17	5
192	156	267	215	277	143	134
99	40	48	18	14	17	3
0	3	0	0	5	1	0
0	0	0	0	0	0	11
0	0	1	0	0	0	0
0	0	5	0	3	0	3
0	0	0	0	0	0	8
175	179	305	163	175	220	148
13	19	14	0	14	20	28
112	99	277	103	163	77	147
23	68	73	51	45	30	34
26	7	28	54	16	25	24
26	7	0	1	91	19	1
59	36	67	50	69	57	36
14	13	29	18	20	22	14
13	32	54	32	39	18	36
43	57	52	54	46	61	33
72	95	178	88	112	97	84
29	43	29	49	0	35	36
200	211	301	160	187	156	171
347	424	643	300	247	318	263
71	76	68	69	75	93	50
62	15	50	63	46	45	52
108	111	48	121	111	0	0
34	49	95	60	68	37	10
299	393	400	317	293	266	255
100	218	328	50	239	215	33
90	110	102	90	91	105	69
181	54	342	131	69	182	136
0	0	0	0	0	0	0
130	107	154	65	74	176	125
59	229	0	193	25	205	14
0	26	39	21	0	12	20
0	0	973	2813	0	0	0
57	62	136	58	66	68	64

For Review Only

10	23	0	1	85	30	8
61	86	119	80	66	45	83
103	108	164	120	79	85	79
12	52	44	24	30	25	12
91	135	148	62	88	73	51
88	55	139	102	92	72	110
6	41	71	16	22	12	82
131	90	172	86	187	175	103
25	23	39	23	57	12	45
37	43	66	30	35	45	42
21	11	0	4	1	0	8
63	51	56	50	36	62	33
39	28	51	35	39	36	19
157	211	349	139	143	293	112
28	17	17	9	18	37	19
38	48	131	196	53	40	3
8	43	0	16	13	13	24
131	165	219	143	156	197	193
6	22	41	7	13	14	71
59	77	129	65	74	87	84
31	62	77	85	59	47	32
78	22	79	13	10	23	23
22	8	29	11	9	8	12
64	134	190	80	113	84	119
3	1	14	7	2	5	6
6	0	0	7	4	10	6
551	396	366	446	915	410	496
170	3	78	182	182	0	5
31	57	36	15	47	42	25
166	153	317	136	160	160	131
0	0	1	2	1	0	5
79	104	145	107	63	81	94
43	51	58	57	53	23	27
0	135	10	0	135	0	119
18	152	188	69	77	140	19
25	30	41	20	24	35	31
22	29	17	38	22	60	22
14	23	47	25	26	17	25
111	623	390	570	145	169	130
163	179	267	174	181	166	154
0	0	0	0	0	0	5
35	47	81	22	23	53	34
55	36	62	27	56	33	57
1	8	32	1	2	70	2
17	41	21	5	25	30	25
36	22	26	22	30	40	18
23	27	55	19	10	19	12
8	16	50	18	14	4	14
45	45	69	36	69	66	20
0	1	7	0	3	0	7

For Review Only

59	91	120	69	84	119	46
228	253	449	235	280	296	203
28	23	25	13	17	20	24
84	66	96	87	55	76	70
114	71	163	76	61	79	53
208	51	383	172	197	61	44
9	5	19	10	16	8	20
1	0	2	4	0	3	0
18	26	25	19	29	17	34
0	2	9	2	3	2	0
75	26	68	15	30	28	35
36	96	91	54	0	70	40
0	0	8	11	0	8	5
71	66	56	17	33	86	34
1	5	38	162	12	16	2
25	11	32	24	20	45	21
5	9	0	14	2	0	11
3	0	0	0	0	0	0
23	23	24	37	37	24	30
43	47	54	46	37	53	43
61	96	91	38	52	40	53
20	0	46	18	21	12	16
0	0	0	0	0	0	0
0	1	0	0	0	4	0
1009	939	1728	1135	997	1110	779
21	0	50	30	5	0	0
155	195	246	140	135	80	135
91	110	104	120	74	97	123
21	16	23	19	20	7	11
33	10	0	4	20	1	1
159	202	261	144	135	198	149
79	76	105	65	32	78	45
33	40	41	30	33	42	0
149	126	132	19	106	87	115
67	67	119	66	80	62	45
93	100	204	60	80	104	63
92	74	175	103	91	116	60
0	4	11	11	11	3	39
38	55	44	35	43	48	49
32	10	95	15	36	33	19
64	73	69	52	57	80	44
61	53	54	47	46	64	31
62	94	164	100	107	109	96
41	34	79	68	62	34	49
115	110	107	106	59	63	74
24	18	83	56	64	22	31
0	0	0	0	25	0	0
12	27	9	17	14	40	17
69	86	86	94	93	72	88
73	87	94	49	68	72	46

For Review Only

357	302	383	339	193	165	324
31	31	63	26	28	45	39
4	2	0	7	18	4	2
15	1	0	13	24	24	113
483	517	646	340	406	461	317
4	18	48	9	12	12	28
58	76	72	35	39	47	41
12	30	53	11	20	32	18
42	50	70	35	47	33	46
71	107	122	124	66	61	62
148	98	167	99	138	135	67
49	38	109	64	25	54	50
0	0	0	0	0	1	0
4	3	0	2	1	0	16
8	33	107	45	53	9	100
26	29	50	46	59	74	32
17	31	54	26	69	29	41
90	109	155	83	81	94	103
9	10	3	8	5	0	8
31	30	55	15	44	25	33
8	20	44	13	49	16	25
0	0	0	0	0	0	0
3	8	7	4	8	5	10
0	8	14	0	10	8	9
6	0	11	6	5	11	7
3	0	0	0	2	1	2
155	129	97	41	142	229	121
2	0	0	8	0	3	0
236	288	378	196	267	239	171
43	65	56	39	65	59	26
0	0	0	0	0	0	4
0	0	0	0	0	4	0
116	159	218	178	90	120	68
8	16	21	19	27	23	10
2	1	0	1	0	1	1
23	53	60	35	57	24	49
21	29	51	45	34	38	22
0	7	24	13	11	0	8
0	0	0	1	0	1	0
20	28	52	35	21	30	19
26	6	19	9	4	8	5
7	27	51	7	10	14	12
0	0	8	0	0	4	0
11	18	15	2	17	8	13
0	0	4	6	7	0	0
0	0	0	0	0	0	1
0	0	0	0	0	0	0
0	4	0	0	0	0	0
146	0	1	0	0	0	214
201	226	417	220	232	249	202

For Review Only

122	133	121	97	112	130	80
13	30	17	18	22	29	13
0	0	0	0	0	0	0
97	85	195	117	80	112	99
57	63	114	72	51	45	60
54	74	110	48	77	90	105
50	33	29	29	30	40	48
1	1	0	0	1	2	1
43	30	34	18	18	56	26
21	27	40	15	14	34	25
105	89	173	85	82	122	70
6	2	0	8	19	6	6
169	180	259	151	197	195	162
85	124	172	102	110	150	102
49	89	145	105	161	52	56
126	104	226	112	120	141	39
8	16	20	16	12	13	2
98	131	147	85	118	125	91
189	142	277	184	171	161	171
31	31	33	0	25	37	36
44	80	101	59	100	73	47
0	0	0	16	0	0	0
112	104	237	134	128	131	104
47	80	129	41	54	95	31
28	39	40	16	18	20	19
43	50	75	42	0	42	0
8	14	12	0	3	8	0
34	45	62	29	61	53	32
7	9	8	7	0	6	0
24	16	31	26	38	14	16
84	74	111	60	69	68	74
215	184	460	233	270	231	180
65	60	71	51	61	52	49
0	1	0	0	0	13	0
0	0	199	32	199	113	82
472	9	221	18	208	11	5
71	136	174	102	47	88	106
1	1	0	71	1	1	1
7	5	0	2	4	1	11
34	55	58	53	40	37	44
0	0	0	0	0	0	0
0	0	0	0	0	0	0
17	1	1	9	1	6	2
13	8	21	4	4	2	0
171	136	0	0	0	0	0
2	0	15	1	0	0	5
0	0	0	0	0	0	0
13	8	0	8	14	18	9
0	0	0	3	0	2	1
84	91	159	90	89	79	56

For Review Only

858	716	983	926	719	933	674
0	0	0	0	0	5	6
52	42	92	65	68	104	29
92	77	72	82	71	71	57
300	291	546	388	448	313	275
3	0	1	6	3	1	1
3	48	0	10	1	21	1
4	79	0	18	7	1	18
0	3	8	4	0	3	0
0	0	1	0	0	0	29
119	157	226	97	108	134	115
2	0	0	0	3	1	1
94	55	195	110	148	72	81
649	358	927	588	571	729	520
81	145	122	66	74	115	143
569	476	1055	600	680	515	446
0	2	0	5	2	1	1
259	444	668	341	199	284	296
3	2	0	0	0	3	0
20	35	34	22	26	33	53
84	71	86	70	43	75	71
814	685	988	445	562	802	466
75	100	186	78	118	102	128
0	0	152	0	0	0	0
0	8	6	5	3	6	5
0	0	0	0	0	0	0
0	3	0	0	5	3	5
96	103	190	77	93	91	53
0	60	353	38	0	0	1
30	18	33	19	6	11	37
69	105	127	91	107	76	52
78	134	227	147	94	78	112
59	62	63	49	54	47	43
68	98	133	73	83	84	87
69	47	62	13	19	19	5
168	176	246	133	72	149	72
77	0	0	0	0	0	0
126	30	0	3	7	6	2
26	21	48	31	43	41	39
59	27	88	26	45	18	40
12	13	68	14	18	4	36
3	13	22	2	6	19	4
220	203	296	151	191	249	249
0	0	0	0	0	0	0
44	40	54	47	24	21	52
126	114	181	143	115	143	73
0	0	0	0	0	0	40
110	107	174	59	85	109	126
98	49	129	61	72	90	76
45	85	120	51	52	74	62

For Review Only

30	30	64	9	16	21	37
82	130	0	16	8	1	35
23	27	34	30	20	37	47
0	0	0	0	0	0	3
709	944	1699	984	1010	1065	780
1	3	0	2	0	1	0
10	7	26	11	16	0	9
46	4	92	51	53	43	4
1	0	10	0	4	0	7
234	267	310	223	223	296	216
0	0	1	32	13	0	8
0	0	0	0	0	0	0
117	151	249	172	169	158	101
111	119	126	62	104	88	90
38	45	50	42	30	35	34
0	0	4	0	0	5	0
109	134	226	140	117	174	96
54	49	60	53	31	29	46
35	7	19	14	10	4	7
1	2	32	19	6	34	1
434	512	806	392	390	535	391
211	178	356	180	200	185	254
93	96	146	85	136	114	66
19	25	25	18	17	32	16
23	18	52	29	38	40	19
4	0	1	0	2	1	0
0	0	0	0	0	0	0
11	3	20	10	15	14	16
35	32	40	5	26	20	40
22	21	44	24	35	18	33
6	9	10	2	6	5	10
45	57	127	79	61	76	45
130	47	143	67	31	83	56
5	27	0	5	1	45	425
251	183	168	214	312	118	129
26	23	107	43	46	26	22
5	8	0	15	7	43	4
3	1	0	6	1	1	1
87	87	115	77	61	121	0
21	20	48	32	26	13	35
110	71	0	137	75	133	75
9	2	15	5	14	0	13
0	0	0	0	0	0	0
1	1	0	3	2	7	5
215	205	449	181	175	276	170
108	73	128	85	73	67	83
0	0	0	3	0	0	0
55	100	211	34	87	52	56
5	3	10	4	1	1	29
5	3	17	2	15	2	2

For Review Only

0	2	0	0	2	0	4
91	133	170	115	107	73	70
6	4	18	4	9	7	12
27	51	87	50	38	58	29
1	7	1	3	1	11	1
256	249	467	321	412	212	274
1	1	0	3	0	0	0
0	0	786	0	0	0	0
161	204	51	192	108	180	142
8	10	18	23	10	1	6
7	0	1	0	0	0	0
4	0	5	3	5	4	1
69	48	103	38	56	122	26
22	2	25	31	11	17	13
33	2	61	3	1	257	1
4	0	0	0	0	0	0
108	111	177	107	165	157	98
476	428	528	389	240	476	260
188	228	294	205	213	203	176
151	121	194	164	130	180	42
106	116	148	130	74	172	41
10	7	41	7	15	0	23
0	0	0	0	0	16	0
80	98	82	31	36	119	89
12	9	11	7	5	12	8
136	123	193	80	126	124	132
10	6	1	25	6	2	19
0	0	0	0	0	0	0
44	45	72	20	18	34	42
33	39	72	23	10	30	50
48	71	86	86	55	68	61
138	167	213	152	171	126	120
84	83	165	96	49	94	109
76	111	182	82	65	127	104
33	38	46	33	30	26	25
0	1	0	0	1	1	1
1	4	0	0	10	13	0
0	0	0	3	3	1	2
0	0	0	0	0	0	0
24	24	45	36	20	23	37
88	74	31	45	81	135	94
169	116	305	121	141	140	136
1	1	2	3	0	0	0
0	13	0	1	1	1	9
5	1	3	1	0	0	0
21	11	15	23	21	12	19
10	10	21	11	13	4	9
0	1	1	0	0	0	0
10	3	0	6	6	9	7
0	0	0	0	4	0	0

For Review Only

42	28	31	14	24	22	28
25	43	55	26	19	20	29
17	23	53	15	22	31	15
0	1	3	1	0	0	1
20	15	22	18	8	13	15
0	0	0	0	0	4	0
0	0	7	0	0	0	0
15	10	1	5	3	13	9
71	108	191	142	76	61	81
37	45	135	2	2	82	21
17	10	27	18	5	8	10
14	4	0	3	4	1	6
0	0	0	0	0	0	3
16	26	21	3	17	9	6
0	0	0	0	0	0	0
29	37	41	26	14	22	18
102	71	103	111	81	124	80
57	20	46	43	50	27	18
102	49	182	87	54	68	45
76	31	0	0	35	87	0
3	10	18	3	1	14	1
60	84	106	63	97	93	52
0	0	2	72	1	0	1
0	0	0	0	0	0	0
32	28	43	47	56	28	27
25	38	35	24	26	25	19
0	0	0	0	0	0	11
33	60	84	54	42	36	49
105	82	151	91	74	86	99
29	41	60	41	38	42	28
98	181	169	119	92	133	132
39	34	41	36	51	49	24
70	100	91	50	105	65	48
22	32	69	38	41	40	70
38	19	22	22	43	45	33
14	7	0	0	0	0	27
0	0	50	22	16	21	20
0	0	0	0	0	0	1
0	0	0	0	0	0	0
0	0	1	0	0	0	0
93	83	126	125	93	99	131
0	0	0	1	1	2	8
0	0	0	0	0	0	5
520	133	670	509	511	463	50
21	35	40	34	20	78	27
0	2	0	0	0	0	6
0	0	0	0	0	0	0
1	2	0	0	2	0	0
69	100	208	125	134	129	81
102	76	185	83	88	131	79

For Review Only

550	828	858	491	714	515	527
124	11	1788	295	90	598	11
24	62	102	22	26	42	52
91	121	188	91	89	131	117
0	4	0	1	0	0	6
0	0	0	0	0	0	9
0	2	0	7	0	0	0
37	37	119	64	38	66	20
11	30	22	23	19	29	58
33	44	86	71	60	72	58
28	0	15	29	27	38	31
0	0	9	0	98	0	0
26	0	67	0	10	0	0
53	36	57	22	49	30	34
119	123	192	99	100	99	115
0	0	0	0	0	0	0
355	348	511	342	418	455	230
66	77	136	103	73	1	48
82	112	132	135	100	112	108
73	66	113	61	115	69	50
0	0	0	0	0	0	0
0	0	0	0	0	0	0
0	1	14	0	1	0	0
118	148	219	154	110	146	138
4	4	22	5	6	1	2
0	3	0	4	0	0	0
0	0	0	0	0	0	87
16	0	38	14	15	27	32
0	0	0	0	0	0	0
0	0	5	0	0	0	0
326	831	487	210	529	319	408
43	64	80	57	55	31	51
0	0	0	0	0	0	5
0	0	6	0	0	0	0
0	0	0	2	2	1	2
49	56	61	31	8	35	27
5	3	17	1	3	6	3
0	0	0	0	0	4	0
52	139	337	144	217	201	105
4	0	0	0	0	0	3
0	3	0	0	0	1	2
84	112	216	113	79	110	65
0	4	13	4	0	4	0
1	0	6	3	3	2	48
0	0	0	5	0	0	4
18	31	24	24	7	19	11
90	92	160	85	88	99	64
5	0	0	0	4	1	0
0	252	1304	963	989	184	0
10	9	25	11	112	6	19

For Review Only

2	4	12	4	8	1	9
8	30	23	18	17	25	16
146	168	378	114	165	151	148
3	0	15	3	4	1	22
0	0	0	0	0	2	5
345	426	616	318	394	459	266
11	23	59	12	2	2	6
193	170	162	68	118	132	98
56	67	175	73	8	0	58
29	39	39	16	23	38	21
15	10	15	9	10	10	12
0	4	4	5	4	3	0
9	13	27	11	6	9	13
0	1	2	1	1	3	0
68	79	125	62	96	101	83
32	66	0	62	22	48	57
19	22	32	15	17	48	20
115	108	186	83	100	131	104
84	79	136	94	95	90	71
115	129	243	149	85	182	107
35	47	81	41	50	56	57
35	73	66	54	56	83	22
61	71	126	86	41	59	26
62	27	49	3	7	11	73
40	2	0	39	2	60	5
29	38	72	40	24	27	27
0	0	0	0	0	0	0
7	109	32	4	23	39	34
51	62	100	57	54	59	40
102	105	132	87	94	97	110
74	106	110	52	83	65	73
62	44	108	50	59	49	38
93	172	122	33	37	97	76
228	222	438	255	215	200	195
45	8	0	25	64	72	65
273	245	360	253	303	265	250
118	192	233	131	122	213	164
83	107	139	125	106	105	149
77	110	261	89	85	108	103
142	169	227	108	154	146	161
21	193	170	13	105	40	163
27	19	31	15	24	11	24
32	27	28	11	22	16	17
69	125	190	104	84	151	127
244	263	360	226	185	236	216
132	148	221	109	56	138	103
28	0	0	0	0	0	372
124	75	220	101	92	80	69
285	266	528	302	280	382	155
89	127	155	93	119	118	83

For Review Only

23	26	296	119	111	194	55
51	51	62	69	55	94	53
87	94	90	58	51	107	52
91	137	168	74	85	132	116
0	0	30	0	0	0	11
64	71	115	70	79	79	51
102	258	324	105	170	147	214
715	18	0	288	56	54	25
199	218	205	84	159	208	140
467	0	847	337	0	323	0
0	0	0	0	143	0	0
297	425	585	339	334	436	296
8	5	20	11	48	12	3
121	98	156	63	79	103	90
117	110	145	69	105	173	97
69	112	198	82	106	100	99
43	31	30	40	20	45	34
0	0	0	0	0	27	0
186	241	334	170	206	314	179
18	26	27	9	16	10	12
0	0	0	0	0	5	3
128	154	271	233	207	157	151
103	68	131	150	165	55	101
232	208	344	153	126	134	101
5	0	0	0	0	0	0
0	0	1	3	4	0	0
5	8	12	5	6	16	16
145	175	216	126	99	119	161
35	86	135	56	54	67	60
54	72	179	93	87	106	28
6	4	6	18	6	18	8
0	0	0	0	0	3	5
1401	1253	2631	1560	1482	1312	1012
134	134	357	168	168	182	191
10	16	0	7	1	13	11
59	57	133	140	103	80	30
43	45	59	40	38	39	48
112	161	225	120	72	119	55
43	64	39	52	55	55	0
116	1	294	1	2	135	107
0	0	0	1	0	0	0
98	11	123	90	24	18	149
3	0	6	3	6	5	0
0	2	0	0	0	0	19
101	151	260	126	126	129	116
72	83	133	74	61	69	39
15	13	46	15	25	27	17
37	69	70	36	47	56	43
4	21	18	4	13	9	21
2	0	0	0	0	0	2

For Review Only

212	202	413	210	194	209	160
2	269	199	1	13	10	1
93	116	117	71	73	49	105
268	252	381	297	267	270	239
66	90	109	67	50	74	49
107	108	1	66	96	0	101
178	197	314	145	177	206	151
61	87	56	95	51	68	26
0	0	0	0	5	0	0
7	29	17	21	11	31	8
0	0	0	0	0	0	34
4	6	0	26	3	0	5
0	0	4	0	0	5	0
0	0	2	0	0	0	0
0	0	0	0	0	0	0
3	1	6	6	15	5	8
0	3	4	0	4	0	0
0	0	0	0	0	0	11
4	1	5	1	0	2	4
25	0	0	0	0	10	38
120	152	168	62	93	110	107
5	30	23	10	5	41	9
60	42	94	17	40	51	34
10	2	42	18	14	4	47
3	1	0	0	2	0	16
135	123	140	109	74	113	100
4	0	0	0	0	0	0
1	1	0	0	0	0	4
3	1	9	1	4	4	0
68	90	95	63	60	65	86
153	94	385	217	303	324	144
0	1	0	0	0	7	0
10	48	85	3	93	2	40
622	656	939	662	616	673	499
0	0	21	122	130	151	74
4	0	0	0	0	0	4
0	117	211	93	115	0	107
177	192	251	174	156	176	131
0	0	5	0	0	0	2
63	47	70	27	0	39	14
50	43	76	75	30	26	23
123	115	199	80	64	124	64
21	31	60	23	18	47	39
15	19	28	25	13	11	82
26	70	408	34	0	80	33
39	10	76	48	88	57	35
30	44	41	13	14	16	20
55	54	102	53	59	52	40
65	58	120	67	62	76	10
2478	2851	5129	3294	3422	2746	2349

For Review Only

0	0	0	0	1	2	0
10	24	49	42	7	38	5
41	14	67	35	34	72	30
77	108	119	62	41	33	40
25	54	24	23	12	28	8
15	43	40	10	3	22	27
48	27	93	54	60	34	62
465	528	680	280	265	527	382
310	268	364	277	210	277	180
0	0	0	0	1	0	53
172	112	172	100	80	72	108
0	8	11	0	8	4	0
1437	2220	3074	1302	1595	1783	1782
47	30	125	34	16	52	62
2	0	0	4	0	1	5
0	22	27	25	19	13	15
6	10	9	5	11	6	7
45	1	116	57	19	34	5
37	62	45	30	28	24	46
0	0	0	1	0	0	3
69	70	131	57	45	71	81
0	0	0	9	5	2	11
70	43	131	126	105	64	39
0	0	6	0	0	1	1
4	19	23	24	27	21	4
0	0	0	0	0	0	5
0	0	16	0	0	0	0
30	17	48	30	13	41	25
0	0	1	0	0	20	0
36	62	99	97	62	47	25
153	31	119	24	63	29	40
62	74	152	67	100	62	62
0	0	0	0	0	0	0
3	4	10	0	2	0	1
0	3	5	1	8	0	0
122	201	254	128	135	147	110
129	93	217	99	131	126	92
139	187	296	193	215	178	49
3	1	24	1	1	1	46
47	41	106	16	42	130	63
0	2	0	0	0	0	8
48	48	83	60	68	36	50
40	22	28	14	24	54	23
0	0	0	0	0	0	0
171	222	242	258	182	170	121
0	0	0	1	1	0	1
69	97	134	38	71	114	69
1	2	0	1	9	1	19
43	5	0	9	7	1	3
40	50	118	86	8	107	33

For Review Only

26	20	48	57	101	32	42
105	69	169	39	67	28	53
86	104	155	63	75	79	63
17	10	26	35	20	30	7
4	1	0	6	3	0	1
17	6	34	23	30	31	20
23	9	30	27	24	15	13
115	37	122	60	141	13	111
150	164	162	100	115	189	103
0	99	114	106	71	67	111
422	506	567	335	337	163	450
45	73	62	48	43	46	44
23	45	43	41	32	38	33
18	7	60	35	29	13	7
120	105	185	166	180	121	53
13	21	43	28	16	20	17
54	68	59	17	51	33	45
134	87	156	128	70	46	108
125	183	488	49	287	18	6
22	27	96	44	31	15	39
48	67	99	84	98	88	59
63	53	98	44	38	62	51
28	35	22	25	26	13	10
25	33	53	17	19	18	11
33	36	51	37	25	31	31
1	0	3	1	2	5	4
1	635	1384	428	452	440	418
145	130	216	115	171	161	151
98	85	143	82	94	117	77
0	1	0	0	0	0	29
114	79	125	68	112	114	38
0	0	0	0	0	3	2
0	68	82	40	68	50	70
14	37	25	16	22	31	19
83	78	82	78	78	64	53
0	0	0	0	0	0	0
27	39	60	30	30	23	16
1	0	0	5	0	0	0
5	7	0	2	5	7	1
2	4	7	0	0	4	0
132	81	161	60	43	93	78
74	74	131	57	56	43	8
1	0	0	0	2	4	1
6	8	11	2	9	4	8
59	111	124	22	45	6	57
48	67	17	60	62	67	49
102	90	121	73	55	106	111
56	31	126	4	107	137	39
31	0	35	0	0	0	25
6	4	0	1	0	0	1

For Review Only

105	142	191	108	105	103	126
22	39	48	44	22	22	31
286	277	786	410	414	344	310
46	52	96	73	62	60	82
54	55	152	82	39	63	61
135	78	194	105	114	152	85
354	368	749	513	466	236	356
145	105	189	120	105	99	113
50	27	56	56	50	30	32
1	4	85	2	1	1	3
0	761	563	708	634	564	529
6	0	0	0	0	0	0
79	51	138	63	71	133	167
33	42	60	42	31	53	24
79	191	40	16	77	81	1
85	149	11	78	236	179	17
1	1	0	7	1	6	1
83	99	173	82	90	79	61
49	51	91	73	35	58	24
90	59	167	77	93	81	96
106	81	143	138	116	85	46
181	221	221	169	185	232	182
0	0	0	0	0	0	3
5	5	5	5	3	0	1
0	1	20	1	2	1	0
175	160	232	141	198	202	110
40	59	178	82	52	75	68
156	152	242	172	141	143	165
128	135	253	117	164	69	81
118	107	84	45	139	93	59
109	109	174	160	110	118	61
85	108	92	52	87	74	58
1	2	0	1	1	1	1
6	12	12	164	0	169	0
39	26	43	28	24	35	33
40	27	65	27	32	37	26
82	145	143	111	74	83	91
59	221	0	1	1	1	1
1	9	1	6	10	1	4
61	55	86	47	41	68	24
19	16	21	0	28	0	14
0	55	60	71	0	62	51
0	0	6	1	3	0	0
8	29	22	6	10	9	21
146	142	314	133	150	158	116
28	25	55	138	26	69	32
717	439	982	692	464	600	383
0	0	5	0	0	0	12
72	85	127	85	74	85	58
55	46	79	62	42	64	43

For Review Only

114	163	255	139	142	196	128
0	0	33	0	0	5	0
11	1	0	3	1	1	2
949	1894	1905	1031	1715	2039	1481
2	5	0	0	1	0	7
0	0	0	2	4	4	0
0	0	0	0	0	0	0
18	4	5	3	3	5	9
2	0	9	3	1	0	0
117	138	236	174	137	153	81
0	1	0	0	0	1	0
0	0	0	0	0	0	0
10	21	26	10	18	22	13
0	4	0	2	0	0	1
1	1	0	1	0	1	0
0	2	0	0	3	0	1
0	2	0	0	0	1	0
1	2	0	0	1	2	0
0	1	0	0	6	0	0
1	1	16	3	1	3	0
3	0	0	4	0	0	0
0	0	0	0	1	0	0
33	34	31	48	18	31	27
27	15	50	20	32	16	15
62	91	164	130	48	68	47
1	1	22	1	4	1	1
20	23	24	24	50	9	10
0	0	0	0	0	0	0
58	54	93	66	60	67	34
47	144	274	317	330	185	273
16	16	57	12	16	4	62
18	16	18	3	4	7	1
20	37	41	24	34	18	16
123	147	264	196	155	167	135
10	31	17	7	11	12	16
13	0	14	0	13	21	0
40	45	49	50	56	42	60
1	5	26	13	14	2	3
0	0	0	1	0	0	7
25	38	43	9	13	28	32
357	552	876	399	429	465	388
49	66	88	44	66	48	32
71	78	90	35	67	54	43
651	1005	1595	765	854	1014	657
0	0	0	1	0	0	0
86	72	176	105	111	94	75
28	41	46	24	41	46	39
1	5	0	1	3	3	0
238	261	372	276	232	224	186
3	111	406	1	25	7	14

For Review Only

37	29	65	20	32	33	42
206	244	352	180	245	235	157
5	19	14	9	7	7	9
89	123	138	46	81	56	55
18	26	60	30	43	21	33
0	0	51	0	0	85	0
117	142	175	99	103	143	112
0	7	1	3	4	3	0
295	225	438	311	295	304	233
30	38	51	37	24	45	51
22	10	13	1	5	8	14
13	6	19	7	13	12	5
0	0	13	0	0	0	0
37	73	69	33	37	64	28
29	34	110	42	75	68	97
68	68	119	84	65	104	55
41	27	25	35	28	40	24
2	1	22	9	10	6	12
37	29	79	28	36	48	41
17	0	0	0	0	0	0
5	0	0	8	0	1	0
36	40	41	24	32	26	34
51	90	0	67	61	75	50
121	145	181	82	188	123	208
78	88	113	67	52	75	76
33	56	70	31	22	49	38
0	0	0	5	0	2	0
41	13	121	77	52	12	24
18	15	0	1	4	12	13
107	83	134	75	90	81	89
9	4	28	0	6	17	22
50	22	73	16	36	15	27
2	1	0	0	1	0	2
141	49	92	38	96	128	28
45	76	89	32	37	55	52
58	65	83	21	53	59	27
10	2	12	3	7	12	6
6	1	20	1	5	1	3
30	41	14	30	31	49	40
0	6	12	0	9	0	0
1	66	0	1	1	1	1
233	252	113	132	99	154	161
189	236	369	175	189	259	209
3	0	0	2	0	3	1
1	5	1	6	2	9	8
90	164	176	130	88	122	62
39	104	77	67	58	89	1
33	15	16	25	21	32	39
41	45	72	32	40	36	45
0	0	1	0	0	0	0

173	200	269	129	118	190	76
26	17	34	15	13	15	12
324	214	0	1	1	1	1
0	0	0	0	0	0	2
75	29	91	49	34	58	31
0	0	0	0	0	0	15
0	0	0	0	0	0	0
28	14	13	3	2	11	7
47	51	90	67	54	56	45
4	4	0	10	7	6	0
0	10	12	10	0	2	3
56	55	60	60	42	49	75
95	109	200	156	101	164	90
65	8	191	55	61	112	72
16	8	35	10	7	18	8
138	138	206	95	149	179	128
15	15	15	2	6	6	10
125	133	202	142	116	56	78
1	0	0	0	3	0	0
0	1	7	1	0	0	0
18	24	47	25	20	23	13
8	0	3	15	2	0	8
0	0	12	0	0	0	0
0	0	0	3	0	0	0
13	20	23	25	18	23	24
33	33	150	32	28	37	23
88	83	85	97	80	94	37
120	115	197	91	105	105	113
135	76	137	82	81	103	121
0	2	0	0	1	0	0
0	0	0	0	2	0	1
51	88	90	67	62	72	68
11	14	20	7	13	10	15
142	127	269	130	129	113	88
64	77	254	59	180	173	62
46	40	84	11	48	22	54
305	307	269	253	148	267	173
11	6	10	6	9	3	2
3	5	8	4	16	10	12
28	51	0	37	41	53	32
42	64	53	28	21	43	56
63	2	0	3	5	122	1
8	7	8	0	0	6	2
30	19	127	40	91	3	123
47	21	77	21	24	67	36
273	24	374	160	12	107	320
26	32	17	37	9	7	15
0	6	0	0	1	3	0
72	70	115	48	46	44	39
4	5	0	5	5	5	0

For Review Only

1	0	1	0	1	1	4
1	3	0	0	4	7	7
70	89	137	81	57	92	91
73	55	135	81	90	106	74
45	50	23	14	15	19	12
35	54	60	50	54	34	46
157	0	51	18	152	181	0
54	63	149	48	77	63	71
7	2	1	2	0	3	17
132	120	203	139	103	85	76
15	29	54	4	1	44	89
356	294	514	285	371	284	248
34	64	66	16	45	34	47
200	524	533	312	270	163	298
52	0	136	21	25	16	40
91	127	170	112	79	85	89
529	125	415	283	265	483	110
32	48	94	25	20	54	31
1	0	16	0	5	1	23
116	109	151	119	93	116	126
17	13	39	19	15	15	16
32	45	99	38	40	44	45
88	150	138	108	73	120	78
0	0	0	1	0	2	5
65	62	32	19	95	74	70
10	1	0	9	10	8	13
2	0	8	0	0	2	2
61	72	150	87	66	82	77
3	0	3	0	4	8	3
0	0	0	0	0	0	0
0	0	0	0	0	0	0
5	0	8	0	0	4	0
0	0	8	0	0	1	0
46	7	0	19	30	1	40
15	14	28	12	17	22	23
13	1	0	2	4	160	1
49	53	40	26	25	40	34
64	75	116	44	75	45	51
42	27	60	52	47	43	43
2	0	0	2	1	0	0
0	0	3	0	0	1	0
17	45	48	23	14	9	14
4	2	21	9	12	4	4
18	41	25	18	10	19	9
7	7	10	28	12	3	8
0	0	0	0	0	178	0
9	28	32	18	8	5	26
286	379	428	261	352	375	311
12	5	0	1	1	5	10
62	50	77	68	61	66	71

For Review Only

29	37	60	25	21	38	33
3	10	13	5	3	2	5
0	0	0	0	0	0	0
35	10	26	20	15	20	21
0	0	0	0	6	0	0
10	3	18	3	8	3	8
46	59	101	51	54	47	31
1	1	31	1	12	1	3
34	63	112	35	66	51	40
3	0	0	0	4	1	3
8	26	35	5	12	11	1
0	7	0	0	0	0	0
54	18	34	9	17	8	8
0	12	18	5	11	5	4
114	120	200	127	109	187	94
0	0	0	0	0	0	0
0	0	1	5	0	5	22
9	10	22	8	9	4	23
18	0	3	27	0	0	22
32	50	100	60	65	62	34
73	0	0	0	0	0	0
77	50	87	65	96	82	64
5	3	0	0	0	0	5
51	16	53	13	50	19	10
4	23	17	6	7	13	15
46	39	54	60	63	31	35
21	37	56	15	28	25	29
39	25	29	20	27	32	10
8	6	32	0	10	7	13
0	0	0	7	4	1	0
135	116	240	126	111	117	125
0	0	0	0	2	0	2
115	431	655	305	245	386	387
64	64	135	81	45	85	43
0	12	38	0	1	4	15
54	81	80	57	27	41	45
0	3	4	0	5	0	11
129	149	111	77	89	99	88
150	92	360	29	35	112	227
113	90	174	91	77	75	53
7	1	18	1	4	4	8
35	24	53	25	42	38	11
36	21	44	20	19	19	13
1	5	0	0	0	0	0
53	79	106	53	54	47	61
19	22	43	24	19	34	21
14	12	23	5	7	11	10
0	0	0	0	2	1	6
95	158	180	129	118	100	90
4	1	12	9	2	2	2

For Review Only

10	1	12	14	8	3	1
60	98	130	70	61	60	93
123	104	207	125	152	93	72
111	131	180	152	105	145	117
0	73	1	156	0	53	0
18	14	15	8	15	17	8
92	27	103	18	74	82	41
0	75	149	39	55	0	62
5	16	0	2	1	3	1
278	139	314	210	193	216	112
45	33	66	43	37	33	42
119	84	108	98	48	110	97
2	5	3	2	0	6	0
35	39	67	34	41	39	25
33	35	53	16	21	28	41
46	58	135	96	55	116	96
41	35	29	51	25	28	25
27	32	17	7	28	6	12
34	34	33	41	24	48	22
21	20	42	13	7	18	27
64	85	112	46	65	38	42
0	6	0	5	2	0	0
151	134	249	203	209	222	141
0	9	21	9	5	6	0
23	17	25	13	12	16	16
127	88	165	65	76	113	120
2	9	8	6	4	5	4
18	21	32	22	11	23	17
37	26	53	25	41	51	24
5	0	0	0	0	3	0
4	3	10	2	5	0	6
11	6	16	7	14	15	6
43	16	44	48	43	51	50
76	98	116	60	69	106	66
62	50	71	37	43	62	53
40	35	53	46	35	38	28
13	16	21	23	10	8	11
163	210	297	160	237	146	153
22	0	24	19	21	23	26
83	99	169	121	109	83	88
68	112	161	99	109	122	82
24	32	67	31	36	24	18
62	58	52	46	15	89	35
7	1	7	4	0	1	1
1966	2893	3238	1926	2043	2081	1942
2	3	9	2	15	4	3
27	31	0	23	27	25	0
0	355	1	0	232	0	0
0	0	4	0	1195	0	0
1511	2599	2153	2336	2313	2626	2178

For Review Only

24	17	0	34	24	29	39
201	265	296	150	242	188	246
72	118	206	58	89	169	125
51	51	76	23	30	56	31
383	384	594	375	351	376	200
1	1	5	0	1	0	1
0	0	0	0	0	4	0
105	126	203	90	124	104	81
5	2	12	5	2	0	14
5229	6044	10739	6365	6035	5727	4016
30	33	48	28	30	21	17
24	36	17	27	18	24	20
27	15	43	24	20	32	22
22	17	49	40	23	17	17
14	30	27	38	6	26	18
85	122	197	124	76	89	59
180	189	320	178	151	199	101
63	70	118	91	66	45	74
51	40	105	86	23	78	41
42	31	65	48	62	58	31
0	0	0	0	0	0	10
105	102	215	194	130	117	102
25	24	11	18	14	19	18
0	0	0	0	2	0	13
0	1	0	0	0	50	0
0	0	0	0	0	0	37
358	346	506	357	346	322	268
19	0	0	0	0	0	0
83	126	127	107	139	92	104
98	67	125	75	126	95	64
0	0	0	0	0	0	13
1	0	3	10	0	6	14
0	7	9	3	2	0	3
35	41	45	29	63	51	36
1	1	0	2	2	1	0
21	21	36	18	16	28	18
4	0	0	1	1	0	0
0	1	5	5	0	0	7
0	1	0	0	0	1	0
0	8	9	0	2	0	6
74	58	125	68	93	86	96
178	240	364	190	182	203	173
0	0	0	0	1	0	0
0	0	0	0	0	0	3
10	9	0	3	6	10	24
27	62	95	26	70	30	27
40	9	32	45	18	37	29
1	0	16	3	1	1	10
79	58	101	78	51	101	94
6	17	32	13	8	0	0

For Review Only

20	30	15	13	19	19	7
43	0	0	0	5	5	13
1	1	0	2	0	0	4
73	32	45	66	37	31	0
0	0	0	3	66	0	13
79	50	123	37	63	81	19
79	82	188	99	87	31	66
6	1	11	6	2	1	3
4	0	6	8	3	4	1
124	102	233	94	89	153	83
89	85	170	91	83	90	139
136	94	320	121	100	100	14
93	157	183	105	159	132	147
3	8	10	7	12	4	2
67	98	82	56	65	33	58
0	0	0	0	0	0	5
21	31	47	17	28	35	26
366	298	544	359	282	365	228
59	0	9	241	0	252	1
1	8	8	3	3	1	0
0	0	15	14	13	0	90
29	77	58	35	35	42	48
145	134	192	74	82	271	112
37	71	194	56	24	80	16
7	22	0	1	96	2	40
167	85	83	169	92	163	36
107	111	170	76	95	102	77
1	1	0	0	10	10	1
29	51	88	54	54	59	45
19	66	56	55	54	38	32
0	62	3	0	0	0	0
426	443	767	532	529	332	361
332	410	714	373	577	401	313
235	255	484	336	208	369	187
145	175	0	180	147	191	193
64	66	86	73	39	74	65
0	0	0	0	0	0	11
0	0	155	0	132	125	109
32	28	70	42	23	14	17
15	6	13	13	2	0	13
0	1	0	1	0	0	21
0	0	6	0	0	0	0
0	0	45	1	0	0	40
0	0	0	0	5	0	0
10	7	26	17	11	13	10
21	49	39	37	36	10	38
31	18	53	17	14	26	7
272	264	395	125	144	399	148
11	9	37	23	13	20	29
179	259	425	215	277	167	158

For Review Only

0	0	0	0	0	0	24
0	0	0	0	0	0	13
52	52	79	39	45	62	56
0	0	0	0	0	0	152
30	48	66	32	44	31	17
0	10	1	6	0	0	0
0	0	0	0	0	0	62
3	5	7	0	5	5	2
23	9	48	29	28	23	6
683	133	645	757	626	323	44
11	8	44	17	7	8	69
0	96	61	90	53	63	0
149	196	288	92	141	177	147
63	102	96	51	44	65	48
4	0	3	0	0	0	7
4	6	0	2	0	4	0
6	10	33	1	1	20	12
76	132	135	49	50	83	89
102	90	66	37	46	67	27
0	75	73	28	25	56	33
13	14	28	8	8	17	12
30	68	83	45	50	37	31
0	0	15	0	0	0	0
0	1	0	0	3	0	2
74	22	72	82	67	53	82
0	0	0	0	0	0	0
20	17	13	0	0	21	23
36	21	75	12	11	20	26
0	0	0	0	1	3	1
0	0	0	0	0	0	14
118	80	139	85	85	65	85
79	119	202	50	108	64	46
84	114	129	1	64	86	4
65	97	100	57	52	100	74
7	0	0	0	2	14	0
54	27	34	12	25	34	59
335	410	615	275	314	300	293
79	53	52	27	23	61	97
213	202	319	196	179	167	174
3	8	17	10	2	5	12
113	166	234	116	138	123	173
24	5	58	10	7	9	28
0	0	10	0	0	0	0
0	0	0	2	0	0	0
54	0	164	54	61	80	0
0	3	0	1	0	1	3
21	17	36	52	69	27	55
36	19	34	55	46	35	36
0	0	0	0	0	0	0
0	0	0	0	0	0	0

For Review Only

21	22	44	28	23	25	26
0	0	0	0	0	1	0
25	12	41	36	29	57	27
0	0	2	1	0	0	0
0	1	0	0	0	0	0
0	3	0	1	0	2	5
59	38	55	41	40	46	42
841	900	1456	1019	824	1171	566
444	524	860	654	728	479	362
0	0	1	4	0	0	0
3	0	0	10	5	2	14
0	2	0	1	3	0	0
239	265	395	246	287	344	225
3	5	9	0	0	4	3
1	1	5	0	1	0	4
371	291	490	388	269	398	145
62	59	96	66	26	85	59
6	18	19	7	4	5	6
0	0	0	0	3	0	1
117	123	239	118	101	116	104
111	147	217	124	154	215	168
867	811	1542	716	757	1171	346
0	0	0	1	3	0	3
0	0	0	0	0	0	0
1	0	0	1	1	15	1
0	0	0	0	0	0	0
1248	1326	2300	1194	6	1478	1812
127	93	211	90	141	178	89
28	44	29	30	24	42	42
16	34	65	12	7	16	18
13	60	15	34	51	37	32
185	158	266	120	75	258	117
117	76	125	71	84	73	52
178	12	113	325	281	216	219
255	221	420	248	257	236	195
53	43	91	28	40	42	43
69	90	138	118	119	86	83
8	10	43	12	25	19	43
0	0	0	1	1	2	0
15	0	0	0	4	0	5
8	45	26	0	47	15	0
27	80	41	35	45	21	42
0	0	206	0	0	0	0
5	7	32	8	8	6	6
0	1	0	1	0	2	1
63	93	156	77	48	83	93
22	31	46	28	19	0	27
4	1	6	2	5	10	1
2	10	14	8	3	3	5
30	57	43	43	67	41	88

For Review Only

36	42	50	35	36	32	29
0	0	0	0	0	1	3
0	0	4	0	0	0	0
0	0	0	4	0	4	0
6	2	7	1	1	14	15
0	0	0	0	0	0	11
553	704	169	0	572	693	599
73	87	248	141	112	104	69
3	19	8	4	3	13	15
85	111	62	120	108	0	88
0	0	0	0	1	4	0
14	10	13	1	21	11	11
7	3	6	0	3	4	2
17	6	17	19	32	14	8
3	1	5	1	0	0	0
0	65	1	0	73	0	0
4	1	0	2	3	3	6
17	9	27	4	30	8	4
1	2	8	13	1	6	1
191	241	375	242	245	278	257
258	205	573	290	206	304	366
0	4	12	9	6	10	9
63	77	163	107	209	21	44
0	0	0	0	0	0	0
0	0	0	0	0	0	6
30	61	78	9	1	1	9
9	5	7	6	6	8	6
16	103	0	0	55	0	0
319	285	685	320	338	373	193
123	127	234	93	116	141	109
217	234	387	283	277	310	190
22	28	38	13	18	30	53
200	304	127	221	0	304	0
98	95	142	103	73	86	107
91	117	168	103	111	118	111
60	53	79	47	40	43	72
41	21	155	45	82	86	66
116	72	151	90	116	96	63
37	29	131	30	110	107	66
1	7	3	0	4	1	2
0	2	5	3	0	3	0
3	0	0	0	0	0	0
0	0	0	0	0	0	0
0	0	5	4	0	0	7
0	0	0	0	0	0	0
74	78	113	50	72	72	55
3	3	0	9	12	7	0
19	160	79	438	230	0	351
2	0	0	0	3	1	2
0	0	263	586	0	0	985

For Review Only

36	42	50	26	42	42	24
7	4	8	0	8	0	2
39	0	0	0	0	0	44
52	75	71	68	53	43	47
32	21	48	39	32	31	33
7	24	71	13	54	35	19
65	142	199	66	96	95	69
66	124	104	96	81	67	72
90	99	148	90	122	52	55
54	50	0	35	39	55	34
0	0	0	1	2	0	2
0	0	0	0	0	0	1
6	0	0	10	8	0	11
69	92	147	91	94	73	57
11	15	31	8	20	15	12
104	141	207	184	200	99	104
10	9	37	9	14	18	5
22	110	90	41	17	35	21
0	0	0	2	0	0	0
1	0	0	0	0	0	0
0	0	2	0	0	6	0
0	0	0	0	0	3	3
242	236	374	270	213	277	207
71	69	124	77	73	58	72
39	41	76	34	44	48	46
5	6	10	6	5	5	4
5	6	9	4	6	7	0
270	282	495	214	243	257	223
0	0	1	0	0	0	7
1	1	0	0	0	1	0
76	117	165	93	65	60	87
145	125	240	101	122	139	116
87	95	131	112	80	88	96
0	0	0	1	0	2	1
3	0	0	1	1	1	3
12	7	30	21	2	18	19
4	16	8	2	6	16	11
197	229	395	288	271	195	194
141	594	0	252	279	456	6
1	1	0	1	1	1	25
31	34	38	20	31	35	10
0	0	0	0	1	0	8
68	74	112	40	67	59	62
75	119	209	87	124	139	69
96	121	168	75	65	96	111
76	91	116	63	89	72	64
35	0	1	0	0	0	0
296	347	411	282	267	317	287
19	25	20	15	20	34	11
25	24	45	29	21	26	16

For Review Only

12	219	140	0	49	0	0
14	47	48	45	37	10	25
63	81	91	109	77	57	56
31	45	80	45	36	0	36
157	168	361	174	153	212	169
292	425	550	276	406	396	256
1	4	0	4	0	2	2
15	3	0	5	2	20	21
42	43	50	28	33	34	38
0	0	0	0	6	0	14
3	5	11	13	3	2	4
1	5	0	4	0	4	5
108	181	293	179	169	119	152
7	4	16	2	0	4	0
33	45	113	58	66	64	63
1	1	0	19	4	3	11
0	7	0	0	0	0	0
45	48	85	72	40	34	42
32	52	67	62	30	68	39
63	48	91	31	46	64	70
6	0	17	6	0	10	4
0	0	5	5	0	1	0
42	59	0	39	0	42	0
0	11	14	3	5	6	8
518	584	766	546	529	510	343
333	460	1044	756	702	513	401
58	39	95	84	112	36	55
14	5	14	3	5	7	3
21	37	50	31	27	33	21
0	0	0	2	0	0	0
23	17	90	80	35	13	10
0	5	0	0	0	1	0
96	104	110	90	95	71	106
167	191	206	127	142	178	128
3	1	0	0	0	2	2
3	0	0	0	0	0	0
52	26	44	32	5	21	17
0	0	0	0	35	0	294
211	0	0	0	111	559	1089
4	37	74	8	20	30	37
21	17	0	12	15	0	6
3	10	0	5	7	0	11
25	44	55	12	27	68	31
5	1	12	3	10	3	23
14	11	28	0	9	22	9
59	41	86	50	44	35	45
37	58	71	19	48	47	39
16	20	51	19	40	13	45
0	0	6	0	0	67	64
20	13	17	6	13	10	9

For Review Only

0	0	0	2	0	2	0
57	42	0	52	0	58	0
14	13	0	14	4	12	7
0	0	0	0	0	0	0
26	65	100	44	35	82	67
494	864	1130	530	635	390	480
15	26	13	15	18	9	2
81	59	59	34	65	20	28
0	0	7	0	0	0	3
94	97	193	85	63	102	73
21	34	52	14	33	13	36
92	113	0	1	1	147	1
5	11	10	13	12	13	37
8	0	9	7	3	8	5
33	4	57	73	34	39	31
0	0	0	0	0	0	0
0	1	9	3	0	3	1
14	6	9	9	0	17	8
77	104	86	52	56	32	94
45	43	52	50	35	16	29
79	113	148	85	111	138	95
189	171	172	182	126	167	0
213	180	515	265	332	179	247
101	61	78	64	58	56	50
282	339	491	419	346	469	347
0	0	0	2	0	1	0
51	30	62	40	25	32	34
418	199	422	355	649	355	205
0	61	163	124	67	133	77
65	47	120	100	43	59	61
1	1	0	59	2	215	1
4	4	7	4	6	7	5
15	11	24	15	13	11	12
61	81	108	77	71	178	49
18	7	41	12	45	29	15
3	19	25	22	18	8	21
57	113	153	90	88	78	132
7	6	18	6	8	0	8
1	3	5	0	0	11	2
12	10	24	6	9	32	12
49	63	12	64	64	46	0
0	63	0	0	63	69	0
106	64	62	22	35	50	41
578	0	1370	535	691	850	579
0	0	0	0	0	0	16
0	0	4	0	0	0	0
0	0	0	0	0	0	72
0	1	0	0	0	0	40
4	1	0	21	1	6	2
1	1	0	1	1	1	27

For Review Only

3	5	12	12	8	10	10
3	6	0	2	0	0	0
183	216	272	162	181	178	161
22	54	95	65	59	34	37
4	0	0	0	2	3	0
12	17	19	15	23	37	21
25	18	78	26	44	32	70
41	38	62	48	54	63	43
135	128	117	78	81	76	64
1	0	4	4	0	0	0
13	16	41	23	41	19	32
0	2	0	1	0	3	3
3	0	7	3	1	3	2
28	55	75	30	43	14	19
0	0	0	17	0	0	0
0	0	1	0	0	0	0
64	35	100	68	67	78	53
135	124	173	143	126	142	95
5	0	0	3	2	7	0
14	27	42	31	12	29	13
27	40	50	21	30	47	33
27	36	64	38	27	32	31
63	105	88	96	82	73	81
14	9	8	0	12	7	10
45	74	90	38	60	68	57
0	0	7	0	0	0	4
4	20	64	13	1	9	31
1	3	0	2	1	0	0
209	175	244	147	122	222	160
155	141	207	117	152	176	112
108	106	188	145	143	141	92
0	0	95	0	0	0	83
171	153	284	118	168	138	138
6	0	6	2	4	1	2
229	210	506	268	221	298	214
11	10	18	11	14	12	18
39	42	75	56	40	50	36
3	0	4	4	3	4	2
55	69	108	50	53	63	61
98	120	174	120	107	60	92
3	6	8	6	4	6	8
41	58	76	35	37	36	68
1	12	31	7	2	11	6
18	10	20	15	13	11	17
2	6	11	2	1	0	2
2	3	5	7	3	2	3
2	0	0	2	0	1	1
2	0	5	3	6	5	3
18	6	22	15	2	19	8
4	1	5	3	3	4	5

For Review Only

4	4	5	2	2	7	11
21	27	31	19	19	24	27
13	10	26	26	7	6	19
35	31	56	52	55	33	45
1	0	10	0	0	1	0
16	17	29	18	20	11	13
2	0	4	5	3	1	2
8	6	15	12	9	3	9
11	11	33	15	21	9	18
2	4	1	5	4	1	0
0	0	0	2	0	0	0
7	2	15	2	0	0	0
0	0	2	0	0	0	0
0	0	0	0	0	0	0
0	3	3	0	0	4	0
14	38	56	12	18	17	17
4	0	5	0	3	0	6
0	0	3	0	0	0	0
1	0	7	4	2	1	4
4	0	0	0	0	0	0
32	8	38	40	21	26	22
3	0	10	3	1	4	0
10	10	32	1	1	10	7
0	2	0	1	5	7	0
0	2	0	3	1	1	1
3	4	0	3	1	1	1
1	3	1	1	1	1	0
12	10	12	17	5	15	11
3	4	6	2	4	3	4
148	151	351	152	194	178	137
9	2	14	1	1	5	1
16	18	20	16	15	12	18
2	7	6	6	6	5	4
0	0	0	0	0	0	5
5	19	17	6	19	8	10
8	10	12	4	9	3	3
0	0	0	0	0	0	0
6	2	10	1	1	0	2
2	1	5	0	1	2	0
6	1	0	5	2	2	4
4	3	7	2	4	1	6
3	1	1	2	3	0	0
0	3	0	0	0	1	0
14	19	26	13	12	16	14
0	0	2	1	0	0	0
3	2	4	2	3	0	3
8	3	12	1	1	3	2
2	0	0	4	0	0	0
0	0	1	0	0	0	26
3	10	7	13	8	5	0

For Review Only

0	0	11	0	0	0	0
134	159	216	112	121	151	149
2	7	7	10	2	7	0
2	2	4	1	1	1	2
0	5	15	5	0	9	4
3	22	48	38	10	37	24
1	9	0	4	3	3	2
0	3	4	1	0	4	4
1	0	0	1	0	0	1
21	12	25	24	16	8	18
14	13	16	17	5	10	10
0	0	10	6	0	3	5
18	2	17	11	4	6	12
198	149	319	127	127	143	111
4	3	23	10	6	7	8
1	5	0	1	30	31	24
0	0	0	0	2	4	0
1	0	2	0	3	0	0
9	0	7	0	5	0	0
0	0	0	2	0	0	4
1	6	4	1	0	0	1
3	0	0	7	2	0	0
0	0	0	0	1	1	1
4	10	8	7	7	7	6
4	1	0	1	0	2	2
53	55	90	53	46	50	38
0	0	0	0	3	0	0
86	128	188	115	102	106	108
10	23	54	17	28	22	13
45	81	105	68	54	74	62
0	0	4	6	4	9	0
0	3	2	0	1	1	1
2	5	1	12	4	4	4
0	0	0	2	0	0	0
47	11	0	29	46	13	16
14	8	23	4	14	15	7
35	45	59	47	38	51	49
1	0	0	0	1	0	0
2	0	0	4	11	0	5
2	6	0	0	11	3	1
9	7	8	9	6	8	9
9	0	0	0	0	0	0
1	10	0	20	8	6	5
0	0	0	0	0	1	2
20	29	40	29	24	30	19
0	0	3	0	1	0	3
62	95	70	59	74	46	59
18	15	14	14	13	8	13
0	0	0	0	0	0	0
5	12	9	11	8	6	15

For Review Only

1	1	0	1	1	2	1
6	9	14	32	5	11	2
0	0	0	2	2	0	0
1	2	0	1	0	2	3
27	49	67	49	46	31	41
0	2	1	3	3	3	1
14	18	26	12	8	13	10
9	3	6	57	58	55	66
0	0	3	0	0	0	0
7	5	12	4	5	8	13
14	25	24	18	24	13	8
0	0	0	4	2	4	4
2	2	6	2	0	2	2
0	1	0	1	0	2	0
30	30	35	57	35	30	41
1	0	0	1	0	1	1
4	49	116	56	16	31	23
1	3	0	17	15	7	12
6	1	15	2	2	3	1
24	28	42	24	24	20	31
23	31	23	27	25	22	16
9	11	14	10	12	3	11
11	14	20	11	9	15	12
9	7	13	8	7	7	9
1	0	3	4	3	3	0
0	1	0	1	0	3	2
3	0	0	3	0	0	0
53	54	66	55	56	42	42
47	26	41	53	28	47	38
1	1	0	1	5	0	1
19	6	17	2	24	6	10
3	0	0	9	0	0	3
7	8	8	8	8	7	9
13	14	25	16	8	17	22
23	18	40	14	19	17	12
0	0	8	0	0	0	1
0	0	21	8	5	7	8
17	12	11	10	21	12	19
14	22	16	30	34	24	40
6	1	2	4	1	5	6
5	1	21	6	10	7	1
25	39	33	27	19	38	18
4	9	0	2	5	4	1
0	1	4	0	0	1	0
0	0	0	1	0	1	1
3	0	14	4	0	0	0
0	0	0	0	0	0	5
7	3	8	1	1	10	1
3	2	0	1	3	3	1
0	0	3	2	0	0	0

For Review Only

6	2	11	2	15	2	0
0	0	5	0	0	4	0
32	3	19	26	6	28	12
0	0	0	0	0	2	1
3	0	1	0	0	1	0
1	5	7	3	6	3	8
7	1	4	8	5	1	0
5	1	13	4	7	4	8
13	28	0	20	24	22	8
2	1	0	1	1	1	0
7	2	9	12	8	3	3
2	0	5	0	2	2	0
0	1	5	1	4	1	2
0	5	11	4	3	0	7
2	0	4	0	2	0	0
0	0	1	0	0	1	1
0	1	7	6	0	0	0
5	13	19	17	21	8	4
3	15	9	2	7	5	7
13	8	34	8	15	9	21
18	15	30	22	17	18	14
5	5	9	4	8	3	4
9	9	23	12	11	9	11
3	2	25	11	32	1	2
10	9	12	13	7	6	6
10	16	33	18	23	12	11
29	23	47	46	50	36	24
36	28	85	30	58	50	30
5	21	6	3	5	2	8
1	4	4	2	3	3	0
16	12	17	15	16	16	9
5	6	12	5	6	6	6
3	3	6	4	4	5	3
3	0	0	0	0	3	0
0	0	0	2	1	0	2
5	7	9	6	4	4	3
120	175	271	130	130	155	201
1	1	0	0	1	1	0
22	15	30	13	12	28	24
26	3	16	12	13	7	24
0	2	0	6	4	0	1
2	3	5	2	1	1	3
61	92	110	64	57	96	44
8	4	15	18	11	2	8
5	0	0	0	0	0	0
2	0	0	3	2	3	0
0	5	11	3	8	0	0
2	0	6	0	4	7	0
13	12	0	4	3	2	13
5	5	5	6	0	0	5

For Review Only

11	24	20	9	16	20	17
22	23	48	25	54	40	25
5	4	4	1	1	0	0
3	10	9	6	4	3	8
52	77	98	76	76	64	44
16	8	32	13	5	19	20
0	0	0	0	0	0	22
0	0	0	2	4	0	5
0	5	0	6	6	0	0
55	28	46	8	36	34	13
0	0	3	1	1	0	1
2	2	8	4	0	5	7
0	6	7	6	4	1	3
0	0	0	1	2	2	2
2	4	16	4	1	2	0
215	638	389	230	331	401	529
3	0	0	0	0	0	0
6	1	1	2	0	2	0
1	8	0	2	0	0	1
0	0	8	0	0	1	0
9	2	14	8	5	8	8
0	1	0	0	3	0	0
10	2	19	2	4	1	0
3	2	3	2	1	2	3
1	2	1	2	5	4	2
0	0	0	7	0	0	0
38	35	17	24	28	8	10
3	8	4	8	4	1	3
12	3	21	7	1	2	1
139	87	260	37	50	127	26
2	4	24	6	21	10	5
5	11	7	0	1	2	5
26	28	28	16	15	46	10
12	21	5	7	5	12	5
19	16	40	13	8	30	21
110	147	197	111	129	149	134
4	2	0	4	0	7	2
56	72	89	51	63	68	85
3	2	1	0	1	1	5
4	3	22	13	2	3	4
200	199	304	207	167	221	154
22	7	12	24	21	14	6
0	0	0	0	0	0	0
1	0	0	2	2	1	0
16	5	32	15	20	3	2
0	0	0	0	0	0	0
0	0	5	1	0	1	2
3	7	11	7	8	6	9
0	0	5	0	0	0	0
11	4	10	14	7	8	9

For Review Only

10	8	28	18	9	12	9
0	0	0	0	0	0	0
5	4	19	24	6	3	1
9	43	10	7	16	7	14
0	2	0	7	0	1	0
3	3	1	0	5	12	3
1	6	6	24	10	1	9
70	60	149	77	69	45	51
4	3	19	8	19	20	1
1	1	3	5	6	0	3
11	17	19	9	9	14	15
0	1	0	0	2	0	0
0	3	0	0	0	0	0
3	0	0	1	0	2	0
5	1	8	5	1	1	6
0	1	0	2	2	1	2
0	1	0	0	5	0	0
0	1	1	0	0	0	6
0	4	0	0	0	0	1
8	1	20	1	2	13	16
5	4	8	3	2	5	5
0	0	0	0	1	0	0
0	0	1	0	2	0	2
2	4	0	5	2	1	2
1	1	6	1	4	0	0
1	0	0	0	4	2	1
0	20	0	0	45	53	34
7	2	5	6	4	11	20
0	0	2	0	1	0	0
4	1	0	0	2	1	1
33	14	31	32	23	18	28
22	6	35	16	6	3	9
10	7	5	8	3	1	0
0	3	1	0	0	0	0
4	2	19	0	1	7	1
0	0	3	4	0	0	1
6	4	8	1	8	5	7
0	0	5	0	0	0	1
5	0	1	0	1	4	0
0	0	1	0	0	0	1
0	0	0	0	4	0	0
7	11	9	14	5	5	7
0	0	0	0	3	3	0
4	8	22	19	6	8	9
3	1	0	1	1	0	0
6	11	17	13	32	4	5
53	31	68	41	46	31	47
1	1	28	9	14	9	1
3	5	3	3	5	0	0
16	10	8	20	13	4	7

For Review Only

5	2	6	12	3	0	5
0	5	1	0	6	0	0
0	2	0	2	0	0	0
22	5	17	6	11	10	7
3	4	0	0	0	0	0
1	8	0	1	1	1	1
0	0	0	2	0	0	0
13	0	15	8	3	14	11
78	109	220	80	87	107	71
0	0	0	0	0	0	0
0	8	5	3	4	1	0
4	4	4	0	7	6	0
21	17	22	11	16	54	8
1	1	9	2	0	0	9
3	4	0	4	1	1	17
7	1	3	3	5	0	5
1	0	0	3	0	0	0
11	16	15	10	19	6	3
0	0	2	1	0	1	0
1	0	1	1	0	0	0
0	0	0	0	0	0	1
0	4	8	3	1	7	6
22	31	48	22	12	15	11
0	0	0	0	0	0	69
13	18	15	11	17	6	5
117	21	75	44	14	91	22
0	1	0	0	0	0	0
10	11	9	13	8	13	3
0	1	3	0	1	6	1
0	0	7	5	1	0	3
4	2	0	3	0	3	5
11	1	20	3	1	5	9
30	18	28	12	7	13	28
13	11	27	6	18	1	14
0	7	6	0	0	1	1
0	0	4	3	0	0	1
9	9	46	17	4	20	13
0	4	0	0	8	0	0
2	13	0	5	1	15	15
0	2	5	6	2	2	2
7	8	8	7	8	7	9
0	1	0	5	0	0	0
4	17	31	6	6	12	9
28	0	60	39	1	15	29
6	5	1	4	7	8	2
23	19	28	17	10	32	14
22	25	59	43	38	24	24
0	1	0	0	1	0	0
1	1	3	0	1	0	0
18	27	40	14	12	26	16

For Review Only

0	0	10	0	0	0	0
3	3	9	6	3	2	6
0	3	3	0	5	2	3
5	4	6	4	8	6	3
0	0	0	0	0	5	0
2	4	0	0	0	0	0
0	6	4	1	1	0	1
3	0	0	0	5	1	0
40	48	0	12	1	2	4
4	10	14	8	9	7	17
1	2	0	0	0	5	3
4	0	91	20	14	21	22
0	0	47	0	62	0	0
6	2	0	1	0	1	6
18	6	18	14	10	16	6
0	0	0	3	0	0	1
7	1	0	3	1	1	4
23	23	35	20	28	16	17
0	0	3	0	0	0	0
0	1	0	2	0	1	0
10	8	16	10	7	7	10
7	21	24	9	13	28	13
0	3	5	6	6	0	6
1	2	0	1	3	1	2
4	2	5	1	6	3	7
0	6	0	0	2	0	4
18	16	20	10	6	17	5
6	8	8	7	6	8	6
48	40	71	59	39	44	28
58	0	107	24	23	22	46
9	26	19	14	13	19	16
11	60	68	39	57	7	1
0	0	0	0	0	4	0
181	108	277	137	160	138	118
1	1	0	5	5	0	1
50	101	117	103	42	73	99
0	12	18	5	0	12	3
3	1	7	0	1	0	0
3	3	7	0	1	0	0
3	2	1	3	2	2	2
0	0	7	0	0	0	4
2	15	41	42	2	1	1
49	47	76	47	61	50	74
0	9	7	0	8	7	4
8	12	13	0	9	9	4
0	0	0	0	0	0	0
0	1	0	1	2	2	0
33	37	55	31	29	33	29
24	19	32	23	25	16	15
21	21	33	17	16	20	14

For Review Only

1	0	0	9	0	1	4
2	0	0	0	0	0	0
5	13	25	15	13	5	13
56	66	86	94	34	60	27
2	5	1	2	3	4	2
0	0	0	0	0	0	3
6	7	7	9	4	3	3
3	5	0	3	15	2	4
5	3	2	1	5	5	1
3	15	19	38	20	4	8
38	34	84	71	38	25	22
4	3	16	9	11	1	2
3	1	9	1	1	0	3
1	0	0	4	3	0	0
1	0	5	3	0	0	0
16	13	14	23	12	14	17
1	0	0	3	5	3	0
2	2	0	5	6	3	0
0	0	0	1	1	1	0
3	0	0	2	0	0	0
0	0	0	1	1	1	5
3	3	0	3	5	0	0
31	15	28	18	15	10	21
0	0	6	2	2	0	3
0	0	9	3	0	0	1
0	0	0	0	0	4	3
0	3	3	0	0	3	0
0	0	0	1	0	0	0
2	1	0	0	2	2	1
0	0	0	0	0	0	0
1	3	2	0	3	0	0
2	0	13	4	2	1	1
29	13	17	5	11	6	16
35	36	85	34	32	148	210
7	1	17	7	3	4	1
1	7	9	0	1	5	5
14	10	21	1	5	6	7
0	3	0	0	0	0	7
32	33	51	30	30	25	43
0	0	0	1	5	0	1
6	1	8	7	6	11	15
4	46	0	6	27	3	6
7	16	14	11	17	9	13
0	1	0	0	0	2	2
2	3	5	3	3	0	0
0	0	0	0	0	4	0
1	0	0	0	0	0	0
0	0	0	0	0	2	0
10	33	31	21	15	12	9
8	8	9	7	7	5	5

For Review Only

0	0	4	1	2	0	0
0	1	3	0	1	0	2
1	4	3	3	1	0	3
4	9	10	10	9	6	7
1	0	4	0	0	1	0
2	6	12	3	3	1	3
0	4	0	0	2	0	0
0	1	0	1	0	2	0
1	1	4	1	0	1	3
0	1	7	0	0	0	0
4	16	19	15	21	5	1
3	0	4	3	4	1	3
4	0	0	0	0	4	0
69	76	134	76	92	83	58
0	0	7	0	3	0	0
0	1	8	2	0	0	5
1	2	4	1	3	4	3
3	1	10	13	3	1	1
4	25	42	9	23	11	22
1	1	0	0	0	2	1
14	21	44	40	32	17	27
2	0	6	0	2	7	0
5	0	0	3	3	0	2
1	0	0	0	1	0	1
0	0	10	11	2	1	12
1	1	0	1	0	1	1
0	3	5	0	0	0	1
3	1	15	9	7	0	9
1	5	1	3	4	5	1
1	0	0	1	0	0	0
153	83	0	130	158	21	39
1	0	3	0	0	0	2
2	4	0	4	0	9	0
727	639	1043	737	631	727	445
0	0	0	2	4	2	0
0	5	0	3	3	1	1
0	0	0	4	0	0	0
12	17	32	20	22	12	13
3	18	18	14	13	18	5
3	3	2	3	5	5	0
46	24	22	3	23	6	8
0	0	0	0	0	0	0
1	1	14	6	10	4	8
8	7	13	5	6	4	10
0	4	6	4	8	4	2
0	0	3	0	0	0	4
0	0	3	0	0	1	0
3	5	0	3	9	2	4
2	0	0	0	0	0	4
0	1	1	0	0	6	0

For Review Only

14	12	4	5	3	3	2
12	7	21	14	9	17	8
0	0	9	5	1	2	2
1	1	2	3	0	1	0
1	6	7	0	1	1	0
0	0	0	0	0	0	0
1	1	18	19	13	1	2
1	0	8	24	4	2	0
0	0	0	0	0	0	0
13	4	24	16	6	3	9
4	4	5	8	1	4	1
1	1	0	1	0	0	0
11	8	9	2	5	7	1
0	0	0	2	1	0	0
8	1	0	1	9	13	1
1	0	0	0	1	0	5
1	0	0	1	0	1	10
0	0	14	1	1	5	1
1	0	6	5	4	4	4
0	0	0	2	2	4	0
17	37	100	61	48	21	41
0	0	8	0	5	0	0
18	15	35	13	13	8	19
3	3	7	4	10	2	1
0	0	0	0	0	0	0
0	1	0	0	1	1	0
6	3	7	11	10	6	5
0	1	3	2	1	7	1
7	5	7	11	5	1	0
9	8	26	7	3	13	14
0	0	0	0	0	0	0
15	24	0	19	30	19	9
21	7	53	43	31	27	13
0	0	1	0	0	0	0
4	4	0	0	2	1	0
3	2	9	4	6	1	4
0	0	5	0	1	1	3
1	1	5	3	0	0	0
0	2	1	0	0	0	1
6	5	22	16	11	3	11
0	0	0	8	5	0	4
5	15	21	12	17	26	13
9	4	16	21	6	1	5
0	0	8	0	0	1	0
20	21	80	43	13	15	38
0	0	0	0	1	0	22
0	3	0	0	0	0	0
0	0	4	0	0	0	1
1	0	8	2	4	6	0
0	0	0	0	0	0	0

For Review Only

0	0	8	1	1	1	4
0	5	0	6	0	1	2
0	0	14	0	0	0	0
8	0	10	1	3	1	0
35	51	57	30	25	15	12
7	4	13	8	5	6	10
5	3	6	2	9	0	2
1	1	3	6	4	7	5
22	10	28	16	12	2	3
104	45	175	78	104	141	46
0	4	0	0	4	5	2
0	0	3	0	6	0	0
0	0	0	1	2	2	0
0	3	1	3	0	0	0
0	0	4	0	0	0	4
0	0	0	0	4	5	1
66	53	93	36	37	36	66
2	1	1	6	8	3	1
0	0	0	0	4	1	3
0	1	1	4	1	0	0
3	2	0	5	7	5	17
0	3	7	4	5	5	0
0	0	0	2	1	4	2
0	0	0	0	2	1	1
4	2	5	1	1	0	0
2	0	0	0	1	2	2
4	0	3	0	0	0	0
0	1	0	0	3	5	0
0	0	6	0	2	2	0
5	10	25	10	11	11	3
1	2	0	3	6	0	4
0	5	1	2	0	0	2
0	33	8	18	0	0	0
1	1	1	0	1	0	1
4	0	0	0	0	0	0
0	0	0	0	0	3	0
8	1	13	9	5	2	15
0	0	0	0	0	0	0
15	26	57	5	11	23	14
0	0	0	0	0	0	0
1	5	5	0	0	6	5
4	2	10	2	3	0	6
6	13	10	12	9	9	6
14	18	25	4	8	6	28
0	0	0	0	3	0	0
0	1	0	4	2	0	4
1	0	0	0	0	1	0
0	0	1	0	0	0	0
0	3	4	1	0	5	0
15	15	23	32	25	20	20

For Review Only

0	0	5	0	0	1	0
4	1	0	1	2	1	7
0	1	2	0	1	0	0
5	0	6	0	4	0	0
2	1	16	14	4	2	1
5	5	22	1	4	1	1
0	1	0	2	0	2	0
5	0	0	0	0	1	0
1	0	0	0	0	3	0
0	0	1	0	0	0	0
1	0	0	3	0	4	0
0	0	6	0	5	0	0
0	0	6	1	0	0	3
3	1	6	4	1	3	3
0	4	1	1	2	2	0
97	48	58	63	134	10	9
5	3	9	9	4	11	3
1	0	4	0	0	0	0
0	3	0	0	1	2	2
0	0	3	0	0	0	0
2	0	1	2	1	0	2
116	184	193	190	185	94	75
5	2	12	2	4	12	1
5	4	1	4	4	1	4
0	0	0	3	0	1	0
6	8	5	6	0	3	6
0	4	0	0	1	0	0
0	3	0	3	0	0	0
0	0	7	0	0	0	4
6	0	8	0	0	0	0
5	2	1	2	0	2	2
3	4	6	0	3	3	4
28	11	11	2	10	7	20
3	6	27	22	5	2	1
4	0	0	5	0	0	0
2	2	4	2	1	3	3
4	4	0	1	7	1	0
0	7	5	4	4	9	4
0	1	0	6	2	0	0
0	0	0	0	0	0	0
3	5	4	9	3	18	0
0	0	5	0	0	3	0
0	3	0	9	1	12	2
10	18	22	24	26	17	18
2	0	0	0	0	0	1
0	0	0	1	4	4	3
21	19	30	25	47	27	13
92	77	153	88	89	134	65
4	5	15	11	7	4	8
0	0	4	0	0	1	0

For Review Only

17	17	32	8	20	6	7
7	3	4	3	4	7	1
1	0	3	0	1	3	0
1	1	0	9	16	3	3
1	1	1	6	3	2	7
0	0	0	0	3	0	0
16	16	21	24	6	9	6
0	1	0	3	0	5	0
2	8	4	3	8	13	12
8	6	10	15	5	11	0
0	1	4	3	3	0	0
6	0	0	0	0	2	0
10	8	1	12	11	1	1
9	17	29	17	16	13	15
1	3	1	0	0	0	0
3	0	1	0	0	0	0
0	1	22	0	10	6	5
4	0	6	0	5	3	0
0	2	0	0	0	0	2
0	0	5	4	0	0	0
0	0	0	0	0	4	0
0	0	0	1	0	2	1
0	0	3	0	0	0	0
0	0	8	1	0	0	0
6	0	5	13	20	4	12
0	0	0	0	0	0	0
1	7	25	9	8	1	2
5	1	17	2	8	4	3
1	3	3	5	11	0	4
1	1	109	1	5	1	1
19	12	14	12	9	13	6
17	16	31	23	41	16	21
11	8	13	5	7	5	8
12	9	24	2	15	7	1
2	0	0	4	1	0	1
0	0	4	0	5	0	0
7	12	13	12	6	6	7
3	1	4	5	0	5	0
3	4	5	0	0	5	3
4	1	9	1	1	4	12
9	9	22	29	10	6	29
0	1	0	3	2	0	0
0	0	0	7	0	0	4
2	2	16	4	6	5	2
0	3	0	4	12	0	4
7	7	10	14	8	6	4
0	3	0	0	1	0	0
3	2	0	0	0	2	0
0	0	2	0	0	0	0
7	2	34	26	12	6	16

For Review Only

0	0	4	0	1	0	2
0	0	0	0	0	6	0
27	17	99	54	42	59	41
4	16	32	17	15	25	21
3	1	5	9	4	2	3
0	0	0	0	0	6	3
3	5	3	1	1	3	2
20	7	12	7	6	7	10
2	4	0	4	4	3	2
11	11	13	7	14	11	12
11	2	10	3	1	2	6
4	4	21	4	12	7	7
11	8	14	7	7	10	9
0	6	0	1	2	1	0
0	0	4	7	1	1	0
0	0	0	0	0	0	0
0	0	1	4	7	0	0
0	0	0	0	4	1	4
0	1	6	5	0	0	0
15	12	19	16	9	12	8
7	4	4	7	1	3	1
0	0	0	0	2	0	0
0	0	0	1	0	6	0
1	1	18	8	1	2	7
8	14	26	10	15	11	8
21	12	23	20	11	24	8
0	0	0	3	0	0	4
0	0	0	0	1	0	0
48	34	51	77	64	32	27
3	6	1	2	3	2	1
24	24	48	22	21	38	20
1	1	0	1	0	3	0
147	150	277	106	131	146	103
14	11	14	5	13	1	6
1	0	5	1	4	0	0
19	38	29	15	32	20	21
2	4	0	3	0	3	0
0	0	8	0	0	0	0
0	0	0	0	0	0	4
0	0	0	0	16	0	0
0	1	0	2	8	6	0
3	20	32	9	16	4	24
2	0	0	0	0	0	2
288	250	487	369	407	316	194
9	19	40	30	25	4	8
27	13	36	33	39	32	27
58	49	72	75	57	25	18
5	4	13	3	5	4	1
5	2	8	7	5	3	7
0	1	0	2	0	2	0

For Review Only

0	0	0	0	0	1	1
0	29	51	24	26	15	0
2	5	1	1	1	3	3
16	4	17	39	7	13	7
40	11	31	26	35	22	15
29	43	82	35	29	21	14
0	3	35	12	20	4	15
0	2	4	2	4	0	0
24	34	46	28	30	23	21
13	19	50	7	8	38	18
15	13	27	19	8	13	25
0	0	4	1	0	0	5
0	0	4	0	1	0	0
3	2	8	3	7	1	4
1	1	19	7	3	6	1
8	3	15	2	2	13	11
50	31	62	28	39	38	34
10	22	17	24	14	12	13
12	15	24	10	20	15	13
6	3	8	0	1	4	2
0	0	0	1	1	0	2
14	29	40	11	4	2	23
11	6	14	7	13	0	5
2	0	20	11	7	16	2
0	6	4	6	5	6	13
0	0	0	0	0	0	7
1	1	0	1	0	1	1
1	0	0	1	0	3	1
0	0	0	0	0	0	0
0	0	0	0	0	3	1
0	0	0	1	1	3	1
13	21	24	13	6	15	21
145	131	162	82	134	158	93
0	0	0	0	0	0	0
2447	2248	4047	2500	2808	2081	2165
4	11	20	3	6	6	3
0	0	2	3	0	3	0
34	10	12	28	25	22	19
0	1	0	0	1	1	0
0	0	2	0	4	3	0
2	0	6	6	2	1	5
0	0	15	9	7	0	0
0	0	0	0	3	3	0
3	1	8	1	3	5	3
0	0	1	0	0	0	0
0	0	4	0	0	0	0
44	30	31	35	20	43	27
0	1	0	0	0	0	0
1	4	5	0	0	0	0
1	0	15	0	0	3	0

For Review Only

15	6	19	26	25	20	18
8	17	18	37	27	4	20
0	0	0	0	0	0	4
2	0	5	1	1	6	3
4	3	21	7	0	3	3
3	2	10	14	4	6	7
0	0	5	1	0	0	0
37	30	61	50	53	23	36
3	3	10	10	10	2	3
0	0	2	0	0	4	0
1	9	3	0	9	6	3
0	1	0	0	0	0	0
0	3	0	3	0	1	0
19	14	30	28	23	46	9
0	1	0	0	0	0	0
0	1	6	3	0	1	0
42	17	88	47	64	27	39
9	3	12	6	5	0	3
22	20	49	49	23	57	11
13	8	16	5	2	13	8
1	0	0	0	0	0	0
0	0	6	0	0	1	0
7	10	1	6	3	1	7
29	30	26	18	21	10	11
7	2	13	3	6	8	2
12	7	25	12	14	18	15
1	2	0	4	5	1	2
1	1	0	2	0	0	0
2	4	0	1	28	8	34
2	8	0	5	9	1	3
14	16	21	10	22	11	7
5	0	3	0	3	2	0
0	0	5	1	0	0	0
8	2	15	1	0	1	0
0	8	13	4	5	0	6
12	43	16	8	20	3	8
1	4	5	2	2	4	2
5	13	6	12	8	2	3
0	1	0	0	1	0	0
28	8	27	20	12	19	6
0	0	4	0	3	0	0
0	0	0	2	0	0	0
10	21	34	8	2	5	21
0	0	0	4	1	0	2
6	13	15	9	9	7	6
32	14	34	29	13	12	12
4	8	0	5	9	3	1
10	8	12	13	26	11	9
2	2	8	0	0	8	1
23	56	57	18	49	34	23

For Review Only

4	2	12	1	1	8	12
1	0	1	0	3	0	1
1	13	13	16	9	22	20
0	0	1	1	2	1	1
0	0	0	0	3	0	0
3	0	0	3	3	0	0
0	0	0	0	0	4	0
0	0	0	3	0	0	0
9	9	16	22	19	6	6
1	0	18	3	6	0	0
1	0	2	0	0	0	0
3	7	0	2	4	0	0
5	1	0	4	1	0	3
0	1	0	1	1	1	1
16	7	18	9	19	17	10
11	17	24	19	22	10	15
1	2	7	2	3	0	0
19	30	60	21	32	47	43
4	0	8	4	11	7	4
63	72	164	45	78	3	65
35	65	68	37	31	40	35
7	0	0	7	0	9	0
1	4	0	3	0	0	0
0	8	0	2	1	2	3
0	1	0	5	2	1	0
5	6	14	12	9	4	7
8	0	0	0	0	0	0
1	2	8	1	4	5	6
0	1	0	0	0	0	0
0	0	4	0	2	2	0
66	285	292	301	285	280	270
0	0	0	0	0	0	0
7	5	4	4	5	2	4
2	12	0	12	14	14	11
0	0	8	0	4	2	0
0	3	7	8	3	7	0
1	0	20	4	4	6	1
0	0	1	0	0	0	5
22	17	27	9	4	4	7
3	10	0	20	9	14	1
0	4	0	0	0	0	2
0	0	3	2	0	0	1
0	0	2	1	3	0	1
27	25	30	26	9	15	7
3	21	11	3	5	21	6
4	1	8	8	1	2	1
0	0	7	1	2	7	1
6	20	29	40	45	30	32
4	8	13	4	3	1	2
0	7	0	0	0	3	4

For Review Only

1	0	0	3	0	3	0
13	2	29	12	7	26	11
3	0	0	0	3	0	0
232	261	412	271	321	347	176
2	1	8	0	4	4	6
0	9	12	6	3	0	2
0	0	0	0	0	4	0
0	0	4	1	0	0	1
0	1	0	2	0	0	2
0	0	0	4	0	0	3
0	0	0	3	0	0	1
4	2	0	8	6	2	6
0	0	0	2	0	0	0
11	20	34	22	19	10	16
1	0	6	0	0	0	0
15	10	36	16	13	17	15
0	0	0	7	3	0	0
0	0	6	0	0	0	0
0	0	0	0	0	2	0
9	3	23	5	11	4	4
0	0	3	1	0	3	0
4	0	10	5	0	9	4
24	25	26	11	1	14	13
0	1	1	0	0	5	0
4	0	6	2	4	0	1
2	1	0	4	5	4	3
27	35	36	4	11	42	32
0	0	0	0	0	0	0
6	0	1	0	0	0	4
0	5	0	0	0	0	1
0	0	4	0	0	0	0
0	0	0	0	0	0	0
11	16	13	9	11	3	1
87	59	97	105	73	52	80
1	0	0	0	0	0	0
3	0	4	4	0	0	1
4	4	9	3	4	2	7
0	0	0	1	1	0	0
2	1	10	12	0	3	2
3	3	0	3	1	2	0
0	0	0	0	2	0	0
1	2	0	1	2	1	0
2	0	1	0	1	1	2
0	0	3	4	1	0	0
1	1	4	3	4	0	3
7	8	13	10	10	0	7
6	0	4	0	0	0	0
9	0	17	5	3	3	0
66	80	130	84	62	67	107
1	1	20	4	2	14	4

For Review Only

0	0	0	1	0	0	1
0	7	17	5	5	5	6
12	17	14	14	13	16	11
4	0	0	0	0	0	0
0	2	4	5	2	0	1
6	0	0	1	1	1	2
16	27	24	20	27	18	24
0	0	0	0	3	0	3
25	10	27	18	6	22	29
36	25	58	51	47	21	37
0	0	0	0	0	0	0
13	12	31	9	4	1	24
33	50	38	14	15	54	29
0	0	0	1	0	0	0
2	7	10	0	1	0	1
1	1	14	4	3	10	9
1	1	0	2	0	11	0
0	0	8	0	0	0	0
1	4	10	7	0	0	1
2	1	5	1	0	6	2
3	5	10	5	4	2	3
2	0	0	0	13	3	0
0	0	0	0	0	0	0
0	1	3	1	0	0	0
2	11	40	13	4	15	11
9	7	11	6	0	3	0
0	0	4	0	0	3	1
0	0	0	0	0	0	0
3	7	0	1	0	1	0
4	0	4	5	0	6	0
4	2	12	3	2	1	6
4	7	16	14	6	17	4
0	1	7	0	4	0	0
1	0	10	0	3	0	0
0	1	0	0	0	1	1
14	18	35	29	28	3	32
0	0	0	0	3	2	0
14	21	19	23	18	13	21
1	0	0	3	0	0	0
1	4	6	6	0	1	3
1	3	4	3	5	3	3
20	20	53	27	43	9	12
42	47	66	22	13	15	22
0	1	0	0	1	0	1
4	0	0	0	0	0	0
0	0	0	0	0	0	0
91	88	124	80	86	108	76
1	0	7	1	0	0	4
0	0	6	0	2	0	0
1	1	14	1	5	1	1

For Review Only

0	0	0	2	0	0	0
3	10	12	10	2	16	2
4	7	14	5	7	3	4
0	0	4	4	0	3	0
0	0	15	6	3	4	8
0	1	1	0	0	0	0
12	4	0	4	2	1	3
0	0	0	0	3	0	3
0	0	11	0	0	0	0
0	1	4	0	1	0	0
0	3	0	0	0	1	1
5	0	4	3	2	5	1
0	0	0	0	0	0	0
0	3	2	0	0	0	0
0	0	0	1	0	0	0
1	0	0	0	3	0	0
4	4	13	7	4	5	3
0	1	0	0	0	0	2
4	3	5	17	5	4	4
0	4	0	0	0	0	0
7	0	7	6	5	3	4
0	0	0	0	0	0	0
26	26	62	52	36	10	11
0	1	0	0	0	0	4
0	0	0	5	0	1	8
13	8	23	16	13	9	2
0	7	13	3	3	2	3
5	5	15	7	4	6	7
0	1	3	3	0	4	3
2	0	0	4	0	0	0
4	16	35	39	15	6	5
6	9	8	3	5	6	5
0	2	5	2	0	0	0
0	5	0	4	2	1	2
0	0	2	0	4	0	0
0	0	6	0	0	0	0
1	0	0	0	0	3	3
0	0	4	0	6	1	4
17	8	37	14	14	6	26
18	7	5	8	6	2	11
1	0	0	0	0	0	0
0	0	7	1	1	0	1
8	5	12	5	7	1	2
10	12	38	35	41	9	4
7	2	8	4	1	3	5
0	0	0	0	2	1	7
14	21	32	18	27	27	13
15	2	8	5	12	5	6
0	5	0	4	5	0	3
20	12	24	18	15	26	14

For Review Only

14	10	25	7	22	8	11
1	2	0	2	1	0	3
2	15	0	3	8	3	14
0	0	1	0	0	0	0
11	13	25	27	12	3	20
0	0	7	8	0	7	5
375	334	637	394	413	419	414
7	0	0	0	0	4	3
0	16	0	0	0	0	0
0	0	7	0	2	0	1
0	0	0	0	1	0	1
9	2	11	2	2	9	4
4	1	19	0	3	11	1
5	7	5	5	4	0	0
7	8	34	19	35	6	13
1	1	0	1	1	1	1
6	4	0	1	1	7	6
28	19	20	1	9	0	33
0	0	2	4	0	0	4
1	0	1	1	0	1	0
4	3	5	8	9	7	7
0	1	1	1	0	0	0
38	47	55	67	77	35	53
4	13	10	4	6	1	4
0	4	0	0	0	0	4
0	0	0	3	0	1	2
0	0	0	0	0	0	1
3	1	0	4	4	3	1
0	0	0	0	0	4	0
1	0	9	0	0	0	0
1	0	4	0	0	1	1
0	0	12	0	0	0	0
7	2	10	15	6	2	13
13	16	27	17	7	9	3
0	6	3	0	0	0	0
15	19	21	12	15	10	11
0	1	1	1	1	0	0
0	0	0	10	0	0	0
0	0	4	0	0	0	0
1	0	7	0	0	0	0
26	30	25	33	19	37	53
3	4	10	6	9	3	7
0	0	0	2	0	0	0
0	0	0	0	1	3	0
0	0	3	0	0	0	0
0	0	0	0	4	0	0
0	0	3	1	4	0	4
5	4	9	12	5	6	8
0	0	0	9	0	0	0
0	4	0	0	0	4	0

For Review Only

0	0	0	0	0	0	0
0	4	0	0	3	1	2
42	46	84	77	40	35	46
58	0	557	347	319	0	270
11	25	25	2	17	9	14
0	1	2	0	0	3	0
7	7	13	14	4	14	8
24	18	32	15	23	30	17
0	0	0	3	0	3	0
0	1	0	2	0	0	0
0	2	0	3	1	2	1
0	4	7	3	0	0	3
5	3	5	2	5	1	10
2	2	7	5	5	5	3
0	5	4	0	2	2	1
2	4	11	2	9	14	8
0	2	9	3	0	0	0
0	0	0	0	0	0	0
0	0	0	0	1	0	0
4	4	6	8	17	2	10
0	0	0	4	0	0	0
0	0	0	3	0	0	0
5	11	19	17	4	7	11
7	1	0	1	28	4	2
0	1	0	4	0	0	0
2	0	0	0	3	0	9
5	7	12	4	7	6	5
0	1	9	1	1	0	2
31	90	73	42	44	29	14
3	0	0	10	2	2	2
0	0	0	0	0	0	0
0	0	0	7	0	0	0
15	8	18	21	4	13	10
6	10	20	7	13	6	4
0	0	0	0	0	2	2
1	3	0	3	1	1	3
0	1	0	1	0	1	1
0	0	0	3	3	0	0
38	41	68	66	31	37	27
5	3	0	3	1	9	1
1	26	8	10	1	1	3
20	14	21	6	5	15	27
0	0	11	1	3	0	1
0	0	0	0	0	0	1
4	0	3	0	0	0	0
0	3	0	2	0	0	0
3	7	10	4	6	1	4
3	1	12	7	1	1	0
52	0	119	32	34	57	56
0	8	5	0	1	0	1

For Review Only

0	1	0	5	3	1	1
0	0	1	1	2	1	0
7	12	12	5	2	2	8
1	0	4	1	0	0	0
6	10	7	6	9	8	9
3	0	4	3	0	0	1
0	1	0	0	0	0	0
2	1	22	3	5	1	0
0	1	11	0	0	1	0
1	0	0	0	4	2	0
16	8	14	10	26	9	11
29	56	50	31	45	24	50
21	18	14	5	7	23	10
4	2	0	1	8	0	0
3	3	5	3	3	2	3
1	0	0	1	1	0	2
21	3	43	17	18	6	3
12	8	8	2	7	5	4
3	0	2	0	3	0	0
0	2	0	0	1	0	0
7	11	5	32	12	7	12
1	0	2	0	0	0	0
0	11	24	0	0	12	14
9	12	11	16	16	43	8
0	0	5	0	0	0	0
1	1	0	0	0	2	0
5	1	0	8	4	1	4
0	0	0	0	1	0	0
0	0	4	0	0	0	0
0	0	5	0	0	0	0
0	0	8	0	0	0	2
1	4	8	3	3	4	4
1	1	0	1	1	0	1
3	15	65	7	46	4	57
13	23	17	6	8	29	26
3	6	6	4	6	5	0
0	0	3	0	0	0	0
4	3	7	6	10	4	0
27	10	32	24	23	17	17
0	0	0	0	0	0	0
26	31	0	34	31	41	35
4	0	0	0	2	2	0
2	0	5	0	1	0	0
0	9	8	4	9	4	0
28	47	59	22	33	31	39
55	29	79	49	59	29	35
0	0	0	0	0	0	0
4	3	4	5	3	0	0
2	2	14	11	0	1	0
16	10	8	2	15	3	6

For Review Only

0	0	0	3	0	0	0
156	166	217	166	151	196	87
3	3	6	0	0	0	3
0	7	11	7	8	7	4
6	21	9	3	6	1	2
3	0	0	0	1	0	1
7	1	1	10	7	4	2
5	3	91	39	75	62	39
3	5	8	3	5	11	3
2	6	7	7	4	5	0
68	62	73	54	61	65	63
3	2	17	4	7	4	3
2	2	0	1	0	0	0
9	2	20	11	22	9	16
7	2	0	6	1	4	8
1	3	6	1	1	0	0
8	15	5	9	10	4	3
1	10	0	1	1	2	1
1	5	2	1	0	1	0
0	0	0	0	5	0	0
0	0	1	0	0	7	0
15	11	33	17	7	21	17
4	6	36	28	4	3	10
0	0	0	5	0	0	1
12	16	20	10	26	26	16
1	0	0	0	1	4	0
3	1	2	0	0	0	0
37	29	13	7	7	42	39
3	5	11	16	11	4	3
0	1	0	3	0	2	2
0	7	3	0	3	0	0
2	1	0	2	0	2	2
6	2	14	18	26	3	12
10	21	34	7	6	6	13
0	5	0	4	0	2	1
5	5	15	4	6	11	8
11	13	32	20	19	26	7
0	0	0	0	6	0	0
0	0	0	3	3	0	0
0	0	0	1	4	0	0
55	37	92	67	104	41	40
16	14	38	15	11	14	13
16	11	19	10	10	9	8
0	0	0	2	0	0	0
19	11	13	14	31	11	7
0	0	0	0	1	0	0
1	5	0	9	1	1	1
16	19	7	19	15	16	10
6	4	4	5	6	11	1
5	5	16	8	7	1	1

For Review Only

2	0	4	1	2	2	2
0	0	0	0	0	0	1
21	11	34	17	12	29	11
2	4	3	1	3	3	3
5	8	5	0	0	3	0
5	4	5	8	5	0	0
21	40	39	19	31	26	28
3	3	0	0	1	0	3
1	5	0	1	23	1	10
1	0	0	1	0	0	1
7	2	7	3	7	0	2
2	3	5	0	0	3	0
65	90	109	79	100	82	54
0	0	0	1	1	0	0
1	1	0	0	0	2	1
0	0	3	0	0	0	5
0	9	15	8	11	0	4
2	3	4	0	0	0	0
0	0	3	0	0	0	0
4	19	69	32	14	42	3
0	3	3	0	0	0	0
10	1	21	19	13	11	13
37	9	70	6	15	18	8
0	3	0	2	0	1	2
4	3	8	5	10	3	12
1	7	16	5	11	8	13
5	0	4	6	0	3	4
10	6	20	12	32	1	8
1	2	12	13	8	13	3
0	0	0	0	2	0	0
0	0	0	4	0	2	0
0	3	4	0	0	0	0
0	0	1	0	0	0	0
1	0	0	1	1	0	0
7	11	6	4	3	0	0
1	1	0	9	0	2	1
0	1	0	0	2	7	0
0	0	0	0	3	0	1
3	3	0	6	0	3	6
1	0	0	2	2	0	0
4	1	12	5	5	2	8
4	2	13	9	17	9	2
1	1	16	14	11	2	1
0	3	11	5	5	8	0
0	2	0	1	0	0	0
0	1	6	7	0	0	3
0	0	0	0	0	0	0
0	4	3	3	1	1	0
0	0	0	0	1	0	5
2	0	10	2	0	0	1

For Review Only

0	1	0	0	3	0	0
5	3	2	0	0	3	0
1	0	0	0	0	0	0
4	2	0	1	1	5	2
2	1	9	0	2	0	0
4	0	9	5	6	12	3
8	9	4	5	5	3	3
0	0	5	1	5	1	0
2	0	0	0	2	0	3
3	0	4	4	3	0	1
0	5	0	3	0	0	0
0	0	0	0	4	3	0
2	1	0	2	0	1	0
5	11	11	3	1	2	0
0	0	0	4	2	0	0
3	0	0	3	0	3	0
0	0	0	4	0	0	0
3	7	3	3	4	5	8
4	5	6	0	4	1	3
47	23	70	69	50	39	47
1	0	0	0	0	3	0
1	2	0	0	1	0	1
0	0	0	4	3	0	0
0	0	10	0	0	0	0
0	0	0	0	0	0	0
5	12	14	4	17	18	5
0	0	0	0	0	0	4
14	1	15	4	12	3	10
0	0	0	6	0	1	1
7	3	1	6	4	9	9
15	27	33	32	26	6	6
8	8	16	13	13	17	2
11	14	20	12	12	12	10
0	0	1	1	1	0	1
17	7	38	23	25	19	9
73	44	118	39	58	87	53
0	3	1	0	1	0	0
0	4	5	1	1	0	0
3	6	6	5	3	5	4
1	0	4	3	1	0	0
27	53	44	39	25	36	40
4	1	7	0	1	6	1
4	1	11	11	7	11	10
0	0	13	0	0	0	0
3	0	0	0	0	0	0
0	1	0	3	2	2	0
0	0	1	0	0	6	0
1	0	0	0	3	2	0
20	1	8	10	7	3	3
2	0	4	1	4	1	3

For Review Only

0	0	0	0	0	0	0
3	1	0	2	4	1	2
0	5	0	0	0	0	0
14	14	22	12	12	19	10
0	0	0	2	0	1	0
4	4	4	4	0	0	7
88	12	10	16	33	7	12
0	2	9	0	0	1	0
2	1	0	2	3	1	5
7	5	0	2	3	6	0
0	1	0	1	1	3	2
3	0	0	0	0	1	0
3	8	0	2	2	0	0
35	55	59	22	16	17	18
2	13	22	5	9	8	10
15	7	12	11	6	17	21
11	11	30	13	19	13	6
1	51	61	110	57	11	57
0	0	0	4	3	0	0
0	4	19	3	4	4	10
1	1	0	1	2	0	0
0	0	0	1	0	1	1
0	0	0	2	0	0	1
8	5	17	36	5	27	11
1	4	9	2	12	1	1
26	48	51	9	22	27	21
59	96	148	84	93	99	89
0	0	8	0	4	0	0
6	0	0	1	0	0	0
10	5	18	6	13	16	19
1	1	0	2	3	2	1
1	21	35	5	5	10	10
118	132	248	146	114	103	62
0	0	0	1	0	1	0
0	0	3	0	1	0	4
5	7	8	3	3	4	3
0	6	0	7	3	0	0
51	23	60	43	28	34	18
0	0	5	1	0	0	0
27	11	48	16	26	40	27
4	6	0	3	7	0	2
2	0	0	0	1	5	2
0	1	0	0	0	0	0
0	1	3	0	0	0	0
6	0	1	0	0	0	0
0	0	0	5	0	0	0
3	4	3	0	2	2	0
1	4	3	0	0	0	0
0	6	0	0	1	2	1
0	2	0	5	8	1	7

For Review Only

3	7	5	2	4	3	6
1	2	9	10	3	6	9
0	5	0	1	0	3	0
13	9	25	23	7	29	12
13	35	50	29	23	25	34
7	20	1	1	1	2	10
0	0	0	0	0	0	0
6	3	7	0	3	0	0
12	16	36	27	29	17	17
11	4	9	3	1	4	10
4	0	4	0	0	0	0
3	0	3	0	5	1	2
0	1	3	0	0	0	0
123	129	164	79	112	117	101
17	26	27	42	25	23	11
31	67	65	48	36	35	38
0	0	0	0	0	0	0
3	3	9	1	0	5	0
0	3	0	0	0	1	0
26	19	50	21	20	53	25
12	42	91	33	15	69	38
5	1	9	5	5	6	1
0	6	0	0	0	0	0
6	1	6	4	3	5	6
0	0	0	0	0	0	0
2	10	13	8	7	7	3
1	0	6	7	4	1	3
10	4	20	3	8	4	1
0	0	0	0	5	0	0
0	0	0	0	0	0	5
0	1	12	1	1	0	1
0	3	0	0	7	1	0
2	0	0	2	2	2	2
7	3	14	1	1	4	2
0	0	0	0	0	4	1
5	8	8	10	8	4	8
127	93	197	146	146	109	92
0	8	3	4	0	3	3
0	4	0	4	2	0	3
0	0	4	0	0	0	0
0	0	0	0	0	15	0
0	1	1	0	3	2	0
160	199	256	201	143	174	175
1	4	0	7	9	3	3
1	0	0	1	3	2	1
1	2	0	1	1	0	1
0	0	0	0	0	1	0
108	122	222	115	103	171	78
5	8	0	9	6	3	2
11	1	7	14	14	9	11

For Review Only

0	0	0	0	0	0	0
3	1	3	0	1	1	1
34	37	121	42	0	33	38
18	12	21	27	13	22	16
3	0	6	2	7	8	0
2	0	0	3	0	0	1
14	24	58	50	21	46	23
0	0	0	0	0	1	0
3	7	15	14	6	9	7
3	2	19	21	24	13	7
73	53	84	68	62	97	57
1	1	38	1	1	1	1
5	1	9	3	4	0	7
58	32	42	43	42	29	24
3	4	1	5	4	2	5
0	0	0	0	3	0	2
26	31	42	27	22	49	45
0	6	6	5	9	4	4
2	3	12	19	8	5	3
40	45	0	50	29	39	29
29	27	0	29	20	25	18
1	0	0	1	1	7	1
28	19	40	8	21	31	19
8	6	24	6	1	3	6
7	5	6	2	2	3	8
1	2	0	1	2	1	0
3	2	0	3	10	3	0
1	1	0	1	2	0	1
35	26	42	9	10	20	11
0	0	0	0	0	4	1
0	1	0	0	0	3	0
1	7	6	1	2	9	2
4	12	14	3	3	3	2
2	0	0	0	0	0	1
3	0	0	3	0	0	0
0	0	0	18	25	0	0
1	0	5	5	1	3	2
4	8	0	6	8	0	0
8	14	19	2	10	17	12
0	0	3	5	0	0	0
10	3	12	8	4	0	4
64	104	78	47	68	70	67
0	0	2	0	1	0	0
0	0	0	0	0	3	0
4	0	4	3	1	0	4
0	0	3	1	0	1	1
0	1	14	2	0	0	0
2	0	1	1	2	0	0
3	0	0	0	2	0	2
1	0	0	1	1	3	1

For Review Only

13	11	20	11	18	8	1
6	6	9	0	2	3	1
85	64	141	132	107	51	110
8	24	21	16	2	14	6
2	5	0	0	2	3	0
21	31	63	35	36	11	17
11	0	10	4	0	4	0
0	1	0	2	1	0	3
0	0	0	2	1	1	3
0	1	0	0	1	0	0
9	11	14	10	8	9	16
13	18	26	22	22	8	12
1	0	0	0	2	2	0
6	9	15	5	7	9	18
1	0	0	0	0	1	0
0	0	8	0	2	4	5
1	2	9	0	5	0	2
3	0	0	1	0	0	1
14	2	0	0	0	1	7
6	1	0	1	1	1	1
4	0	0	0	0	0	0
14	5	0	4	9	4	6
1	1	13	0	1	0	5
0	0	0	1	0	0	0
6	9	11	9	4	5	8
0	0	0	0	0	0	0
1	1	0	1	1	6	2
1	3	0	2	6	1	1
37	6	47	13	48	14	4
0	0	0	2	4	0	0
90	69	181	0	85	55	58
0	0	7	0	4	0	0
18	22	40	25	23	55	19
0	1	1	1	3	0	1
3	1	1	4	4	0	0
28	45	87	15	63	55	46
0	2	3	3	5	1	1
223	157	459	240	191	266	192
0	0	0	1	2	2	0
0	5	7	6	5	2	2
5	0	0	0	0	1	0
14	12	0	12	14	21	12
1	0	0	4	9	0	3
0	2	12	2	0	7	1
0	2	6	3	0	0	0
0	0	5	6	0	0	0
6	0	1	1	1	5	1
0	0	0	0	0	0	0
0	1	0	0	0	5	0
0	3	3	3	0	5	2

For Review Only

3	0	0	2	3	0	3
0	4	0	0	0	0	0
0	0	1	0	3	0	0
1	5	0	1	1	1	0
5	0	1	0	3	0	2
0	0	0	1	2	0	0
3	7	9	5	4	3	2
4	16	0	4	0	2	0
7	11	11	7	9	7	1
8	7	0	1	2	7	6
0	0	0	0	3	0	3
0	0	5	3	6	0	0
2	1	0	0	0	2	1
17	9	20	9	11	8	9
19	10	24	9	11	10	5
2	3	8	0	5	0	2
3	0	0	0	2	0	0
3	0	0	3	0	0	0
5	8	0	9	2	4	2
0	0	0	3	0	3	0
1	13	9	6	3	8	5
2	0	1	1	2	5	0
17	3	35	6	2	32	15
1	5	4	0	0	5	0
0	0	0	3	0	1	0
7	9	6	9	0	0	9
0	1	5	0	0	1	6
7	1	11	18	13	2	12
8	10	12	7	14	7	10
5	4	3	5	1	3	5
0	0	3	0	0	0	0
0	2	0	3	3	3	0
8	8	20	10	12	8	10
19	17	14	6	1	3	9
0	0	0	1	5	0	0
1	0	0	1	4	3	1
21	34	39	14	18	30	32
27	18	28	11	18	14	20
0	0	0	1	0	2	0
4	23	18	38	26	11	9
13	13	29	29	19	9	10
2	0	1	3	0	0	2
5	0	0	0	5	0	0
22	21	21	9	22	21	8
23	25	54	38	50	0	32
0	0	3	0	0	0	0
16	41	70	48	43	28	17
7	16	11	8	6	6	0
6	5	26	36	19	17	22
9	6	19	1	10	12	4

For Review Only

10	13	26	23	20	15	25
0	0	0	4	0	0	0
33	7	58	13	18	42	24
4	0	0	0	1	2	2
1	0	2	0	4	3	0
17	9	11	9	13	0	9
1	3	0	0	1	0	0
3	2	0	3	0	2	9
0	0	0	0	0	0	0
5	6	0	0	0	8	8
4	0	11	1	9	1	1
5	11	32	26	21	16	5
3	4	8	1	12	1	3
0	0	6	0	0	0	0
6	10	0	1	2	5	3
0	0	0	2	1	1	0
7	4	12	5	16	8	9
0	7	8	6	5	0	0
0	1	6	0	2	0	0
0	0	1	5	0	0	10
3	0	0	1	0	0	4
0	3	0	0	0	0	0
3	4	0	1	4	1	0
1	0	0	0	0	1	0
14	14	19	17	15	18	18
6	10	5	10	7	7	5
2	2	0	3	0	0	2
0	3	0	0	1	1	0
0	1	12	0	9	5	6
81	94	0	104	56	77	60
2	3	5	2	2	0	1
0	3	0	2	0	1	3
0	0	0	0	3	0	0
6	6	7	3	4	6	4
61	135	142	75	59	85	59
0	4	5	3	6	0	0
2	0	3	2	8	6	8
1	2	4	0	2	1	0
1	3	0	0	0	0	0
11	12	16	21	19	19	17
0	0	0	0	1	3	0
4	7	11	13	7	11	5
0	1	5	0	0	0	0
0	0	0	0	0	0	0
27	38	67	18	16	40	33
240	98	385	122	108	120	208
66	51	96	61	54	30	84
38	28	91	52	42	31	30
0	0	0	0	0	0	0
0	0	0	0	0	0	0

For Review Only

7	9	6	6	13	10	6
1	1	6	4	5	4	4
0	0	0	4	0	0	0
0	1	0	1	0	3	2
0	2	10	4	7	2	2
18	29	21	8	23	4	6
0	1	0	1	0	0	0
2	10	18	4	10	7	0
17	22	30	4	16	13	16
3	5	0	2	2	1	0
2	15	33	6	20	6	27
2	7	1	7	2	8	2
0	12	16	0	1	1	0
5	21	40	23	25	4	25
0	0	7	0	0	0	0
5	5	5	9	8	4	5
3	12	15	17	17	30	16
1	1	0	1	1	0	0
7	8	11	6	0	4	7
0	1	10	0	0	0	0
7	5	12	4	9	1	11
2	0	0	6	0	4	0
4	6	12	3	4	5	3
0	0	0	4	3	1	1
1	0	9	6	0	0	1
9	9	11	6	13	8	10
1	1	0	12	0	1	0
21	12	14	23	16	35	18
0	1	1	0	1	1	1
1	3	0	0	1	0	0
4	5	12	13	4	4	7
5	7	5	3	0	0	11
1	0	5	0	0	0	0
2	12	29	1	6	8	15
4	0	5	0	3	0	0
4	3	0	3	11	0	5
1	0	0	0	0	1	3
10	8	27	12	18	5	4
16	9	21	11	1	4	1
0	7	8	1	1	1	1
18	0	60	30	0	18	19
5	16	0	5	4	2	14
34	37	65	38	31	30	26
0	2	6	0	0	1	3
13	2	0	0	7	8	8
0	0	12	9	14	9	10
0	0	17	0	0	0	0
2	0	0	1	0	0	0
8	3	12	2	2	9	7
0	0	0	2	2	0	1

For Review Only

102	0	207	0	0	140	80
0	0	0	0	0	0	0
8	0	23	9	0	0	17
0	0	0	0	0	2	2
0	0	1	0	1	0	0
1	0	7	2	4	7	2
0	2	7	4	0	4	0
19	31	34	21	21	21	19
3	1	0	0	1	2	1
0	4	0	0	3	0	0
24	32	36	15	18	37	28
7	5	14	7	8	5	7
13	14	24	9	14	19	10
0	1	7	8	1	2	0
2	10	10	5	15	6	7
14	13	16	12	14	13	11
8	2	20	1	15	2	19
53	56	113	62	63	53	59
8	3	1	10	3	0	1
0	0	0	0	0	0	0
0	3	7	0	4	0	0
8	5	12	5	5	8	7
4	1	0	3	3	1	6
37	43	92	39	24	28	22
2	3	0	6	5	0	1
5	4	8	6	3	7	4
37	27	84	33	47	19	48
13	5	32	10	9	21	21
1	0	0	2	3	2	1
4	5	8	5	1	5	7
0	0	2	0	1	0	0
1	0	5	4	1	0	1
175	0	241	221	0	191	192
10	13	12	12	8	6	6
15	20	30	16	15	21	16
0	0	5	0	0	4	0
3	4	1	9	4	5	1
2	4	6	6	2	6	1
0	0	0	0	0	1	0
0	0	1	0	0	1	3
2	0	0	0	0	0	10
3	1	10	1	0	0	6
2	1	44	8	0	5	10
28	20	46	34	49	32	21
116	155	216	118	128	159	144
0	0	4	0	0	1	0
0	0	0	0	0	0	7
32	43	0	43	38	40	31
3	11	9	5	5	11	4
21	36	60	32	28	44	33

For Review Only

1	1	9	4	1	1	1
1	1	1	0	4	1	0
0	0	0	0	0	1	0
43	42	76	41	40	49	47
0	0	4	0	0	0	0
5	17	15	11	9	8	5
0	0	0	0	0	0	4
0	0	0	0	0	2	1
31	29	76	36	19	44	24
19	18	21	13	13	16	18
18	34	0	25	26	8	15
37	47	73	44	50	47	46
54	55	84	51	50	36	53
0	4	15	5	5	5	6
1	1	4	1	0	0	3
19	12	30	19	7	11	20
6	8	13	9	8	8	7
4	13	19	12	9	5	11
10	14	21	13	11	10	10
1	1	19	2	7	9	1
7	7	8	4	6	7	6
122	195	225	122	116	146	120
5	8	18	11	4	11	6
2	4	5	6	4	4	5
6	9	11	8	7	8	6
23	34	36	13	7	13	21
3	5	1	1	1	1	1
4	4	0	2	1	1	4
34	51	66	40	41	38	51
1	1	0	8	1	1	3
0	5	0	1	0	0	0
5	1	0	1	0	1	0
0	0	7	0	1	5	1
0	0	5	0	1	0	1
0	0	89	0	32	35	24
0	4	6	0	2	2	0
0	0	0	1	0	3	1
80	93	150	103	109	79	78
10	4	7	11	3	3	0
1	7	3	6	6	5	5
6	2	9	1	5	5	3
149	141	214	112	119	124	89
5	0	12	3	0	0	0
7	9	16	9	5	8	8
16	22	24	15	14	15	13
4	1	3	3	3	2	2
19	16	32	40	25	12	12
1	4	15	12	0	0	9
17	20	27	22	13	16	14
68	55	85	67	64	60	58

For Review Only

36	45	69	44	33	45	36
20	12	40	15	21	18	10
47	47	69	39	48	44	57
3	0	1	0	0	0	0
42	51	61	34	43	52	41
5	5	12	7	9	7	5
4	3	4	5	5	3	1
39	39	63	38	34	34	40
1	2	0	0	2	0	0
0	1	2	0	0	0	1
13	29	22	13	18	23	25
42	62	89	23	64	44	70
2	5	11	7	2	7	4
4	4	0	5	3	5	0
51	74	120	52	65	47	38
19	22	26	16	18	28	18
4	3	1	3	1	1	0
1	1	4	3	1	0	1
3	7	0	5	4	11	5
1	0	0	2	0	0	4
27	22	30	28	35	28	22
1	0	5	1	1	6	11
0	0	2	0	3	0	0
3	7	19	0	6	5	4
25	30	48	29	21	33	32
0	0	0	1	1	0	1
1	3	0	1	1	0	0
2	0	0	3	1	3	0
88	25	107	57	63	78	41
76	103	152	66	69	65	69
2	2	4	4	4	4	3
0	5	5	0	0	0	0
1	1	1	0	1	4	1
32	37	63	35	30	43	47
6	5	10	6	6	7	0
12	13	33	18	17	25	16
9	5	11	16	8	9	8
1	19	6	7	13	2	2
1	0	0	0	1	1	0
0	0	3	0	0	0	0
8	18	16	10	10	14	14
3	3	5	4	3	1	1
74	63	98	31	42	46	42
148	231	282	177	156	259	181
0	1	1	1	0	0	0
0	0	4	0	0	0	1
0	3	7	0	4	0	0
1	5	10	1	3	1	0
0	0	0	2	0	0	1
1	1	3	3	0	1	0

For Review Only

80	122	155	92	84	126	112
64	74	104	61	58	60	72
1	3	6	1	1	3	3
9	2	0	2	0	1	3
1	3	2	3	1	0	3
56	47	75	71	62	53	43
35	39	68	33	37	44	41
6	7	14	6	8	9	8
0	1	3	0	0	1	0
5	10	7	6	1	6	4
7	14	41	22	15	9	15
0	0	0	0	2	0	1
0	0	7	8	9	0	0
36	34	54	24	24	36	32
9	8	8	5	5	9	5
4	6	1	3	3	5	5
9	10	15	18	11	17	15
125	122	168	108	146	111	98
9	1	12	3	0	3	4
0	0	0	4	1	0	0
1	5	6	1	0	0	1
0	0	3	0	0	1	1
14	28	42	25	30	23	19
76	105	129	87	107	88	100
84	129	181	89	116	133	141
0	0	2	1	0	3	3
0	3	5	3	0	0	3
3	8	28	6	23	5	4
0	1	0	2	0	1	2
3	6	6	4	4	1	3
0	1	11	5	5	2	0
30	16	19	10	12	12	12
61	57	108	65	64	59	58
7	7	14	7	7	7	3
232	322	431	235	230	320	349
1	2	6	3	3	3	0
40	48	48	30	40	41	35
12	9	21	9	14	10	10
4	13	6	2	7	6	4
0	2	8	3	9	0	3
2	6	5	2	8	0	0
1	0	0	0	0	3	0
0	0	0	1	0	0	0
4	17	0	3	6	2	3
5	4	8	2	7	0	6
91	13	173	95	102	77	69
4	0	0	0	1	0	0
11	12	15	13	13	16	19
23	4	29	26	16	6	13
176	158	323	207	141	179	145

For Review Only

31	17	48	14	12	16	27
114	121	254	156	163	164	121
2	1	0	1	1	1	5
0	0	2	0	0	43	89
0	3	10	12	0	0	5
28	54	0	18	25	42	50
52	0	0	57	0	0	0
171	170	245	161	140	123	126
130	4	33	64	161	172	85
94	94	181	112	125	157	91
7	0	0	1	0	0	1
46	49	3	0	53	31	0
358	333	602	330	261	371	321
3	5	0	1	1	1	5
824	711	1064	731	680	1031	494
426	648	456	189	373	173	413
187	13	226	170	85	119	283
2	6	13	5	13	13	4
0	0	8	0	1	2	0
124	170	247	170	155	145	158
5	59	0	62	155	137	0
4	0	4	0	1	0	0
29	592	421	493	203	535	391
25	23	41	26	12	18	10
6	4	5	9	6	9	1
6	4	3	3	2	3	12
2	9	0	9	8	8	20
55	106	95	106	56	87	64
0	1089	47	0	0	1054	0
0	0	0	0	0	0	26
51	56	209	54	120	86	66
121	196	248	169	123	148	159
1	0	5	3	0	3	0
96	134	179	113	113	135	151
15	4	5	8	10	2	8
60	51	213	49	135	185	109
62	41	0	13	66	1	15
102	129	120	91	133	94	168
24	32	29	58	36	30	10
54	20	77	41	30	41	32
0	0	3	0	0	0	0
170	218	350	176	154	221	201
50	59	86	215	125	49	22
27	24	55	63	41	1	79
20	25	47	21	21	12	17
37	25	30	41	7	18	22
0	0	0	0	0	0	0
0	0	5	0	3	0	0
1123	1348	2034	543	1219	1372	904
52	46	156	112	61	59	67

For Review Only

127	68	216	138	136	140	98
60	81	99	58	47	88	35
135	110	185	112	90	125	86
69	64	124	87	111	81	73
86	130	179	137	128	155	151
85	91	111	72	76	68	92
41	44	88	54	52	41	66
0	3199	4652	468	3301	478	2594
47	35	56	29	35	44	54
74	99	163	92	99	123	58
102	39	596	215	406	305	98
140	143	214	104	109	131	107
24	35	25	54	7	24	78
13	21	16	7	22	11	12
101	124	223	106	82	87	48
268	357	404	311	228	262	214
66	48	100	76	57	101	70
91	160	0	6	65	23	60
50	67	112	81	45	70	35
67	54	98	53	42	66	45
3	3	11	4	6	3	2
0	1	0	0	0	0	0
82	100	115	119	58	56	62
0	0	11	0	0	0	0
0	0	0	0	0	0	0
54	65	73	45	46	55	61
14	87	158	83	47	42	61
44	58	54	13	22	34	22
0	0	0	0	0	9	0
201	183	276	209	133	199	103
29	42	66	46	28	60	38
25	22	28	34	18	56	7
51	65	151	87	75	103	59
99	132	135	75	71	155	66
10	3	23	6	25	4	4
11	18	18	16	12	4	6
1	1	914	1	1	1	236
0	0	3	0	1	3	0
81	74	135	95	111	78	63
22	56	87	35	41	47	43
1	0	3	0	0	2	0
0	20	87	14	30	37	33
0	0	0	1	2	0	0
0	0	0	0	3	2	0
127	111	228	247	217	141	192
10	8	0	5	70	1	15
92	133	217	180	110	134	99
8	9	17	8	8	7	48
3	1	0	0	2	2	6
39	32	86	66	72	27	38

For Review Only

184	117	192	39	120	225	192
52	82	114	16	31	75	73
0	2	0	1	0	0	4
5	2	13	16	10	11	9
76	150	187	92	78	129	78
31	41	0	30	25	36	32
3	5	0	2	18	62	5
211	168	325	215	185	232	142
117	139	101	177	55	83	70
0	0	0	0	0	0	1
31	48	0	0	56	15	49
99	24	0	89	0	0	0
0	0	115	0	0	0	0
1	3	0	0	3	0	0
411	397	647	292	405	441	281
11	6	11	11	5	4	0
0	0	3	0	0	0	4
172	328	615	407	380	372	249
141	125	204	142	202	129	134
152	151	184	117	164	193	140
0	0	0	2	3	0	0
13	0	10	4	0	6	4
177	231	347	229	171	212	222
0	0	6	2	4	0	1
0	4	13	0	6	12	6
35	48	58	24	44	38	21
0	0	0	0	0	0	18
351	431	385	494	500	500	416
7	1	36	19	24	1	4
0	0	0	1	2	2	5
0	4	9	4	0	0	1
4	1	6	0	4	7	3
1	0	1	2	0	0	0
1	0	0	0	0	0	0
2697	4501	6329	3690	4842	4361	3466
106	19	34	21	57	96	60
4	0	0	0	0	5	12
3	1	4	3	0	1	0
49	26	44	33	17	22	32
1	3	25	4	32	3	2
1	0	1	0	3	1	0
25	6	8	19	3	3	16
703	58	589	841	303	2	63
49	34	67	67	29	60	60
112	72	167	82	177	115	174
80	41	94	60	63	68	29
0	0	9	2	0	0	3
0	0	0	2	0	1	0
0	33	0	15	2	7	0
0	0	0	26	0	0	0

For Review Only

121	67	112	83	45	63	52
0	0	2	0	0	0	0
27	11	96	10	15	13	12
4	8	0	0	4	14	0
22	26	40	8	10	25	3
139	215	0	69	152	50	0
0	2	0	0	0	1	16
18	1	1	0	37	35	39
0	2	0	6	0	0	6
0	2	0	0	0	0	17
278	192	289	78	87	143	108
32	5	64	87	53	28	7
5	0	10	0	0	0	10
123	57	82	53	26	19	18
278	297	177	282	267	302	174
0	0	0	0	0	1	5
95	194	189	85	155	80	91
289	0	0	270	276	0	0
10	1	0	5	1	3	7
0	3	8	0	0	1	0
13	38	34	0	22	18	14
0	0	0	0	0	0	0
0	0	0	0	1	0	0
3	0	0	3	0	0	0
2	0	11	7	3	0	1
0	179	282	40	0	41	20
10	3	20	3	1	4	2
102	49	139	133	71	110	73
0	1	7	8	6	5	6
6	15	17	2	5	5	18
36	36	0	2	7	22	1
0	0	0	1	0	0	0
3	5	8	3	10	3	2
0	2605	2094	2401	2233	0	2099
0	79	0	16	17	78	77
58	39	50	79	66	67	20
21	49	60	46	34	34	40
0	7	0	2	1	0	0
21	5	47	60	26	21	17
215	145	366	228	144	204	134
0	0	0	0	2	6	0
0	0	61	0	444	0	0
107	119	145	127	107	121	85
15	21	28	24	22	13	20
49	36	31	18	48	25	28
0	23	1	13	9	0	0
232	324	511	273	286	329	286
50	114	77	63	37	53	53
0	0	0	0	0	16	47
67	151	124	52	104	71	163

For Review Only

4	0	0	0	2	2	1
12	15	11	8	13	16	12
26	35	44	1	14	30	7
0	0	0	0	0	0	11
15	18	29	22	13	20	0
0	0	9	0	16	0	22
12	7	19	6	9	17	39
0	0	0	0	0	0	8
2	4	7	12	25	12	10
0	4	0	1	0	4	0
0	0	0	0	0	0	5
41	56	75	28	37	41	23
0	3	0	5	0	0	0
97	107	94	101	86	85	86
32	27	31	23	23	23	26
0	0	0	0	0	3	7
17	14	0	12	0	0	7
2	9	17	8	11	3	23
23	37	74	59	29	22	25
24	11	36	13	15	28	33
43	51	104	44	59	45	45
101	79	304	213	257	176	111
51	65	94	47	68	41	13
52	71	117	43	63	99	59
38	39	120	57	37	56	61
33	59	53	49	47	56	34
127	96	112	70	69	99	45
26	35	43	33	51	48	21
61	65	59	34	47	42	43
235	324	463	164	249	271	288
13	11	19	6	5	36	10
23	0	1	0	0	0	0
36	39	50	18	21	42	25
258	223	466	195	251	335	251
17	18	34	20	31	41	32
43	30	107	47	46	47	59
32	26	39	36	35	53	33
129	104	178	69	59	139	65
161	97	292	219	215	75	130
58	98	102	70	82	56	75
50	49	102	41	58	62	57
30	52	53	59	40	53	36
88	94	205	89	0	99	72
189	137	308	136	244	98	114
12	5	35	12	20	7	13
24	142	93	66	125	149	61
0	0	0	0	0	0	0
98	72	166	149	105	72	138
0	1	0	0	0	9	0
45	22	96	28	46	29	18

For Review Only

1	0	9	0	0	3	0
9	19	13	13	12	20	18
1	2	8	8	5	5	2
23	0	3	26	49	0	28
0	0	12	0	0	0	7
3	0	1	3	0	0	0
5	31	31	22	30	82	26
23	38	74	7	15	27	108
652	131	325	85	103	125	39
70	109	83	48	67	76	69
63	78	56	38	45	48	42
78	63	113	56	52	53	82
11	9	41	18	22	15	5
29	61	53	13	24	20	19
2	2	8	5	0	0	1
210	302	413	208	298	290	226
17	43	67	57	23	18	41
58	51	16	17	20	29	45
164	25	0	1	1	5	14
3	10	12	9	6	10	5
0	0	0	0	0	0	0
504	621	817	503	495	378	333
301	388	428	279	285	298	278
20	24	32	24	25	78	25
48	41	0	19	16	30	35
0	3	2	0	0	4	0
224	247	821	157	231	180	138
0	3	0	1	1	3	3
33	1	228	13	2	122	28
101	111	309	178	54	269	80
27	33	45	33	32	30	36
2	3	0	7	4	2	7
11	23	19	18	15	0	24
27	0	36	31	14	14	24
1	2	18	2	5	1	18
28	25	0	42	30	30	36
27	59	70	49	42	68	23
14	14	17	16	8	14	11
0	0	26	0	0	0	0
0	3	0	102	0	5	11
0	0	34	13	11	34	0
56	46	77	43	39	55	54
40	62	0	58	45	53	75
3	22	27	28	10	22	18
188	179	171	89	86	124	216
4	18	0	2	2	2	6
0	0	0	0	0	0	0
77	89	135	91	68	106	92
20	21	33	22	14	18	16
7	0	15	0	1	8	3

For Review Only

111	104	193	85	94	114	76
22	24	39	38	20	9	18
2	1	0	13	3	4	15
4	5	0	4	3	0	0
35	52	67	24	46	31	33
209	151	380	207	212	216	146
43	68	54	72	39	0	25
15	11	53	0	21	0	16
0	0	0	24	0	0	22
71	59	115	66	76	62	100
148	183	275	136	212	163	143
86	89	167	105	115	145	115
95	129	164	89	95	94	94
20	60	34	0	5	42	0
3	0	3	5	5	6	5
24	50	82	44	44	60	38
61	112	109	82	79	93	82
64	88	74	98	95	99	47
3	3	0	9	0	4	1
14	29	121	52	99	65	27
15	21	25	22	17	0	14
63	16	41	32	34	12	14
25	29	53	29	39	48	37
0	0	0	0	0	1	0
19	16	25	18	26	8	15
22	19	37	25	30	23	14
28	27	44	59	16	8	6
21	36	0	29	3	10	12
1137	290	1409	7	441	587	153
0	0	0	0	0	0	0
14	2	10	8	8	6	9
309	362	312	165	258	214	186
70	71	114	72	60	72	58
22	24	52	57	42	19	38
20	17	53	16	29	12	13
62	53	0	205	148	0	0
0	0	19	0	26	0	0
69	63	71	56	70	56	52
509	608	829	488	478	523	382
471	413	707	485	358	459	307
10	2	12	6	1	0	4
0	14	0	0	16	0	0
44	0	26	73	66	82	35
0	11	0	0	41	26	6
54	55	62	42	69	44	23
5	17	0	0	18	15	28
106	86	238	167	20	65	42
70	60	107	55	47	29	45
279	70	21	93	1	270	90
27	41	59	100	17	37	21

For Review Only

0	0	3	3	4	3	11
6	62	46	28	32	34	44
39	10	48	32	39	43	31
0	0	0	0	0	0	0
0	4	7	8	0	4	7
20	11	37	22	12	6	24
49	33	0	28	27	0	52
100	91	165	86	1	75	49
72	113	141	54	70	66	68
50	64	114	58	73	78	56
4	13	9	9	3	6	3
296	388	650	327	356	370	283
62	74	182	115	90	62	56
31	21	62	19	15	60	60
0	1	6	2	5	0	5
187	183	396	175	146	305	242
45	90	62	47	24	54	31
55	77	114	93	120	75	61
16	51	28	13	29	24	33
0	1	0	0	0	0	0
55	53	86	50	37	50	57
191	184	270	178	101	284	119
1	1	0	0	2	2	1
136	5	173	9	57	99	83
2	0	0	3	0	3	0
1	0	24	2	5	5	34
7	18	0	4	2	4	1
0	1	0	0	0	2	0
19	1	41	13	6	23	10
192	219	334	221	247	249	141
16	9	4	16	7	1	16
24	36	59	28	16	18	25
0	0	5	0	0	0	3
364	227	448	248	240	296	212
544	786	946	621	579	703	507
0	7	13	11	3	13	27
88	125	166	87	103	158	83
52	32	43	63	36	49	41
579	82	180	507	555	626	104
73	35	50	41	78	49	44
1	3	11	0	7	7	5
0	4	0	3	2	2	1
0	2	0	0	0	0	2
1851	2143	3163	2192	2141	2161	1511
24673	27024	42421	38091	26909	39511	22048
41	15	72	41	27	37	13
37	27	56	17	24	12	32
6	1	22	9	13	4	6
0	0	3	9	0	0	0
262	203	194	193	156	161	113

For Review Only

1	0	0	0	1	0	1
13	0	0	0	10	9	0
0	203	74	196	171	216	0
0	0	50	0	0	0	0
44	24	36	22	6	21	11
0	0	1	0	0	0	56
0	3	0	0	0	0	0
29	59	109	39	39	23	34
20	17	23	14	28	14	19
0	0	4	0	0	0	4
15	16	10	15	22	30	13
0	0	0	0	2	0	0
189	221	370	154	205	232	133
61	87	87	33	64	83	42
0	0	7	1	1	0	4
20	31	40	15	24	25	31
0	0	0	0	0	0	6
0	0	0	0	90	0	0
6	17	16	26	22	9	32
29	44	85	34	21	79	40
87	67	89	49	57	78	66
0	0	0	5	0	0	35
0	13	0	13	2	11	8
4	3	19	5	12	18	17
5	1	9	1	6	6	5
107	197	161	59	121	112	173
160	160	242	155	132	116	171
37	70	95	23	45	53	158
0	0	0	4	0	2	0
25	4	0	1	40	16	22
0	0	128	33	0	25	0
0	1	9	0	1	2	1
13	16	34	11	20	32	6
4	4	6	3	7	5	1
12	27	96	18	26	36	14
0	0	2	0	0	3	1
5	7	13	4	1	10	6
6	8	41	10	11	27	5
11	10	25	10	5	21	13
2	5	18	5	2	8	2
1	3	7	3	1	5	4
4	3	8	3	4	5	5
4	9	7	3	1	8	5
3	5	8	4	3	6	5
4	4	7	5	3	8	5
3	7	22	6	11	13	5
4	7	7	3	4	7	5
21	29	58	21	17	44	33
15	28	32	12	17	31	20
1	1	3	2	0	1	1

For Review Only

23	41	62	37	29	62	63
11	18	76	16	16	47	15
3	1	12	1	4	5	3
22	25	70	40	33	41	21
3	1	8	3	3	4	1
4	4	4	5	1	4	3
3	4	6	3	1	5	3
0	3	2	0	1	1	0
1	1	3	1	1	1	0
3	3	9	1	3	5	1
0	1	1	1	0	1	0
2	5	15	2	4	3	4
3	4	16	2	4	4	4
1	1	3	0	1	2	1
0	0	2	0	0	1	3
1	2	3	0	3	5	1
0	3	8	3	3	3	3
4	10	23	5	2	5	4
1	0	4	0	0	3	0
0	0	1	0	0	0	0
1	0	0	0	0	0	1
0	2	7	2	1	2	1
0	1	3	1	1	1	3
4	17	29	9	3	6	9
7	11	10	10	10	17	15
0	0	1	1	0	0	0
1	1	6	4	0	5	6
10	5	20	2	4	6	10
0	1	3	1	2	1	0
0	1	4	1	1	1	1
2	4	11	1	1	1	1
2	4	9	1	1	2	1
30	39	120	51	54	67	27
17	27	73	25	40	43	21
3	5	12	3	5	5	1
3	6	8	1	4	7	4
8	2	4	0	5	1	1
12	7	34	11	13	30	13
3	5	23	3	4	9	5
4	4	17	3	3	5	3
1	1	7	1	1	3	0
2	1	6	7	3	2	8
110	58	155	73	66	123	86
1	1	5	0	0	1	3
4	4	13	1	5	13	2
95	91	161	107	102	91	114
17	4	13	11	13	16	16
621	524	1038	614	558	536	365
141	194	367	188	199	224	134
28	65	89	31	16	109	88

For Review Only

497	575	866	462	387	565	456
0	4	0	47	104	2	0
25	21	17	34	25	21	34
14	12	22	15	9	4	37
120	159	175	74	106	96	77
38	23	30	7	18	31	5
127	143	202	103	146	64	38
0	0	13	45	0	0	59
34	36	39	38	28	38	22
0	0	7	0	0	0	0
100	0	105	136	0	0	0
16	24	0	12	16	11	8
23	34	56	31	38	28	32
24	30	49	30	29	26	24
1	0	8	6	3	4	4
325	295	425	359	307	370	186
57	48	124	72	64	63	70
10	18	23	14	23	13	19
0	0	0	0	0	0	0
148	199	230	148	210	210	133
57	59	117	77	49	47	45
59	13	94	49	84	25	21
0	0	0	0	0	0	21
108	91	150	109	97	100	123
0	0	0	0	0	0	0
1	1	0	1	3	1	7
0	1	5	3	0	0	2
80	77	185	77	64	54	87
98	82	110	48	41	79	40
0	80	2	0	0	71	0
0	2	0	0	0	0	0
49	38	76	44	37	45	35
0	0	25	0	599	572	0
0	4	3	0	0	0	0
268	262	355	207	266	276	197
0	124	0	0	123	163	103
65	249	146	49	62	23	20
12	35	29	27	32	19	29
7	4	0	0	1	0	8
540	686	300	0	0	0	0
66	54	94	95	60	60	71
0	0	0	0	0	0	0
152	205	180	171	103	185	193
38	51	51	30	49	37	46
0	82	0	21	0	0	0
277	325	487	275	314	337	262
6	0	0	201	1	5	0
101	173	239	126	169	156	120
0	0	60	0	0	0	0
540	774	1137	803	425	737	742

For Review Only

1	7	123	8	26	1	217
8	30	10	16	13	5	9
114	0	132	185	165	155	95
106	122	246	122	170	113	95
262	370	395	316	278	262	161
133	64	150	141	132	69	34
21	54	61	35	38	53	43
6	7	16	1	4	3	2
6	17	27	19	9	19	9
23	0	10	12	26	22	27
36	33	45	26	23	32	10
39	58	45	26	45	31	22
235	204	229	177	137	170	203
0	1	0	0	0	0	0
6	0	0	3	0	23	9
296	268	516	303	339	353	276
0	0	0	0	0	3	0
104	128	124	74	107	95	81
0	0	0	0	0	0	0
52	82	88	74	0	31	69
560	880	825	541	567	807	584
672	445	631	0	818	399	0
22	3	0	9	1	3	0
129	152	200	83	187	153	129
138	124	178	137	93	112	64
472	453	948	467	480	472	85
0	0	271	926	0	0	0
80	70	112	77	53	69	61
34	33	81	39	16	35	27
5	25	19	4	7	8	1
73	102	132	96	65	60	90
247	316	0	182	308	297	280
32	42	60	22	67	35	39
0	0	0	0	0	0	24
0	0	1	5	1	0	0
24	17	64	6	32	20	12
55	84	176	31	34	93	57
0	0	0	0	0	3	0
215	231	363	230	275	277	273
0	0	0	1	1	0	0
15	0	27	21	24	28	19
3	0	4	3	0	0	0
7	2	11	1	4	5	0
43	53	57	37	33	40	60
22	27	29	12	19	13	31
92	70	277	173	176	67	115
271	328	327	246	263	348	217
2	7	0	1	12	1	10
0	1	0	0	2	0	0
0	0	0	0	0	0	0

For Review Only

179	205	357	141	166	221	119
12	29	44	3	21	10	55
63	32	0	1	1	14	1
36	40	55	29	43	42	41
5	20	14	14	16	9	18
0	0	1	0	0	0	0
67	61	107	60	73	69	74
0	0	1774	0	0	0	0
1333	1598	2811	1606	1765	1640	1859
6	1	43	11	8	12	46
93	86	151	120	121	93	90
98	92	54	70	89	93	62
0	2	14	8	1	19	2
0	0	7	1	1	11	3
1	1	12	5	2	10	4
1	6	13	7	3	2	17
155	171	308	126	151	124	161
2	5	14	9	2	15	11
1439	1822	1791	1789	1442	1724	1307
16	21	77	81	41	627	24
209	127	222	144	104	142	154
0	0	0	0	0	0	8
4	0	0	3	0	0	0
40	66	43	65	52	15	28
1	9	4	2	4	1	6
0	0	0	0	0	0	10
2	0	0	0	2	1	2
37	18	47	44	62	68	47
53	50	69	30	28	35	33
54	97	116	79	77	122	72
37	50	75	41	10	24	49
1	0	0	0	1	1	10
52	21	11	27	38	95	28
19	11	25	15	14	13	11
45	38	53	23	22	35	30
10	5	22	3	7	6	12
0	0	0	0	0	0	1
0	0	5	0	0	0	0
0	0	0	0	0	4	0
42	25	50	41	43	35	46
150	122	181	147	162	130	110
24	20	66	12	8	26	6
1	0	0	4	2	0	0
492	459	593	259	291	434	409
640	740	1236	853	729	771	430
0	0	0	0	1	0	3
78	50	85	48	49	46	49
6	6	8	5	5	0	0
0	1	0	0	1	1	4
0	12	22	0	15	0	14

For Review Only

7	15	27	43	33	14	17
3	5	9	10	0	8	9
51	44	107	61	141	86	96
9	0	0	46	23	35	23
3	7	0	2	0	8	8
0	0	0	0	0	0	0
69	267	205	46	110	46	284
35	35	69	20	36	33	45
24	23	18	4	7	6	21
24	29	30	39	13	45	24
0	4	0	0	0	4	0
12	29	16	23	6	11	11
312	349	551	472	179	286	299
6	0	9	5	0	7	20
90	86	169	43	102	89	78
68	50	52	38	43	38	19
58	38	91	59	43	30	33
119	86	251	89	92	123	80
3	2	8	2	12	2	3
44	2	252	82	56	252	230
0	46	0	35	47	48	0
0	0	0	0	0	1	10
0	0	0	0	0	0	92
67	62	143	62	93	80	64
69	82	134	74	76	74	48
151	348	325	220	291	97	80
54	98	142	72	53	70	76
165	174	146	150	16	158	0
22	0	0	0	0	0	0
3	2	9	4	4	10	3
0	0	56	0	0	0	0
64	97	148	91	67	140	139
253	240	363	212	278	297	196
97	124	481	174	178	116	129
0	0	0	2	12	1	0
0	153	10	147	152	0	157
100	143	230	104	195	106	81
1	1	334	69	0	0	1
14	24	30	19	15	13	18
2	0	0	0	0	0	4
11	8	14	18	7	16	22
1	0	1	3	0	0	0
24	37	0	96	105	37	1
0	10	16	0	0	0	0
4	1	0	1	1	1	244
0	0	0	0	0	0	3
2	1	0	5	1	1	6
29	38	61	69	53	37	64
4	1	27	13	6	6	12
20	6	28	20	9	29	19

For Review Only

3	1	0	7	1	3	4
1	1	0	2	1	1	1
0	0	0	0	0	0	26
0	0	5	1	0	0	0
0	0	0	0	4	0	26
14	19	16	14	0	21	87
3	1	0	1	0	1	2
294	322	706	324	351	409	271
199	230	340	194	217	199	163
2734	2898	4496	2815	2598	3259	2158
602	589	1027	642	651	668	438
63	49	65	27	34	40	32
20	0	16	11	14	0	8
22	0	5	0	23	0	0
41	29	99	64	58	65	38
99	81	128	95	80	101	92
0	0	0	0	0	12	9
13	21	28	6	18	22	6
17	0	17	19	17	0	16
0	0	0	0	1	5	8
41	35	35	27	51	41	49
38	65	87	79	82	102	57
3	1	0	1	1	1	0
17	51	16	5	18	19	16
19	1	42	3	25	4	10
7	7	13	9	6	8	5
7	21	37	7	19	19	13
87	76	171	90	64	116	91
0	0	3	2	0	2	6
7	0	10	0	3	0	0
45	64	80	69	64	92	67
1	3	27	1	0	3	37
21	15	28	22	26	7	14
24	29	69	43	54	27	24
0	0	0	0	2	0	0
4	0	0	4	0	0	0
0	0	0	0	0	0	16
41	50	89	63	48	74	70
0	0	0	0	0	0	0
37	24	85	22	74	57	30
17	11	17	24	10	15	14
4	3	0	3	8	1	9
1	0	1	0	49	38	16
118	36	0	1	4	19	2
8	10	0	14	9	0	6
0	0	0	6	0	0	1
150	168	287	190	138	212	168
1	0	1	3	0	1	3
157	202	92	180	0	171	157
28	46	53	41	20	24	30

For Review Only

86	82	131	81	106	77	92
345	41	531	7	460	234	152
0	0	7	0	0	5	0
109	115	198	185	81	196	96
400	549	742	481	416	514	401
52	24	34	13	26	27	13
55	23	64	53	45	28	42
373	186	5493	11	4	930	49
178	438	735	130	411	230	189
4	61	64	19	4	25	70
10	9	11	10	11	7	13
5	9	28	12	13	9	6
410	286	918	540	453	463	398
44	64	94	45	70	72	59
17	8	28	14	0	17	21
574	742	969	474	495	527	534
0	0	9	6	1	2	0
358	32	780	572	660	220	169
1	2	6	1	0	0	1
3	3	7	0	5	0	7
0	124	1	120	125	0	0
1	1	0	7	1	1	1
2	1	0	10	1	2	17
222	213	304	220	319	202	208
2	10	25	4	1	3	3
3	3	4	0	0	1	4
0	3	0	0	5	6	22
0	0	1	0	0	4	0
19	37	37	25	17	13	13
0	4	1	2	2	6	2
119	139	233	167	150	184	178
2	5	0	0	0	0	0
3	1	7	8	4	0	0
580	272	794	247	906	310	454
11	4	16	10	11	4	7
0	6	35	0	5	16	28
0	0	0	0	0	0	9
0	0	5	1	1	0	0
751	6	1582	1725	1024	327	41
12	10	33	34	25	23	12
654	693	701	557	507	821	374
811	905	1439	917	1186	811	517
79	69	157	86	51	62	45
30	10	18	31	18	28	11
18	17	56	48	15	26	35
49	58	67	34	54	69	0
197	192	307	251	205	187	250
0	0	0	0	125	0	0
113	98	121	46	79	61	61
45	35	115	29	0	14	43

For Review Only

1	8	0	74	3	1	47
1	11	26	15	8	3	5
208	207	377	244	208	250	179
38	59	96	64	60	61	39
0	0	43	180	0	43	0
86	85	155	89	87	121	64
5	0	8	0	0	10	0
132	173	280	182	154	117	148
23	15	17	3	3	10	8
0	5	1	10	3	5	14
33	0	0	0	0	0	0
0	3	5	3	0	0	1
14	9	22	2	5	3	3
15	16	23	18	11	18	17
107	89	178	70	69	85	57
0	10	10	4	6	0	0
8	1	37	9	20	25	16
1	0	0	1	4	1	11
52	67	37	35	44	40	52
14	4	8	5	3	17	20
44	53	65	30	40	50	41
68	13	128	44	18	42	53
1	1	0	0	5	0	0
168	123	194	81	72	208	81
33	40	45	36	16	38	48
0	0	0	0	0	0	0
68	75	131	47	81	69	104
151	160	219	123	146	133	154
3	9	12	3	9	0	8
56	133	19	178	22	74	55
34	28	54	25	26	21	23
207	225	299	185	137	187	168
216	309	298	174	200	124	125
9	10	0	2	14	4	1
2	0	0	4	4	0	3
164	1	171	97	54	85	0
109	99	130	79	91	84	74
47	54	105	43	66	43	32
114	31	220	137	103	156	149
62	54	90	29	37	45	36
19	30	28	10	37	26	10
87	130	164	92	75	112	74
247	200	284	130	216	192	183
1	1	441	12	1	1	1
60	78	155	88	95	117	84
47	43	79	41	37	41	42
39	24	69	44	53	56	19
140	111	182	138	95	174	77
87	107	191	78	81	83	65
60	51	101	58	53	69	36

For Review Only

12	11	16	0	10	21	19
52	34	74	47	71	24	35
30	35	24	30	34	19	0
127	10	82	45	53	51	33
1	2	16	11	18	14	4
26	63	130	41	20	56	24
1	1014	613	1	1	1	1
25	4	10	14	3	1	3
66	39	64	17	27	23	20
54	41	67	21	40	55	49
66	103	123	108	100	90	107
116	133	313	159	0	143	99
125	92	228	38	135	61	25
117	119	230	122	109	141	113
94	117	144	77	60	85	107
54	61	89	29	45	66	38
187	152	205	194	192	189	120
35	7	13	13	10	19	6
17	9	16	32	16	35	17
17	26	37	27	36	38	28
34	14	34	25	18	18	15
4	6	5	2	3	2	8
66	52	73	40	45	31	58
3	0	0	0	0	5	2
0	5	6	5	2	2	3
0	0	0	6	5	0	0
17	10	30	26	9	20	41
26	18	60	36	33	30	25
53	54	45	42	55	30	49
62	84	70	24	29	21	35
0	0	1	1	4	0	0
95	15	39	137	93	66	47
162	4	340	42	95	42	1
1	2	55	28	2	38	7
7	1	0	1	1	1	1
63	109	169	55	88	93	91
90	152	116	195	142	219	150
68	122	24	77	129	168	4
21	35	34	19	33	25	30
23	116	0	1	185	1	7
130	108	267	176	124	87	73
11	1	0	13	154	1	139
41	39	102	22	22	72	6
0	0	94	10	21	0	0
1	4	9	4	10	1	1
35	15	0	35	13	19	24
1191	1179	1625	800	1074	1189	807
126	73	0	4	22	38	38
0	0	0	0	0	0	0
133	81	223	120	106	200	122

For Review Only

191	202	0	240	190	239	190
40	58	78	26	22	48	30
0	0	0	0	3	0	3
33	43	32	27	49	36	37
15	8	8	0	4	11	6
94	136	173	82	106	121	116
1	1	0	1	0	1	1
4	10	5	0	0	1	11
5	7	14	0	13	11	0
20	30	59	16	49	40	24
0	0	0	0	0	0	8
29	59	36	19	55	19	34
1	0	0	9	5	3	4
0	0	5	0	0	6	0
12	4	15	13	12	10	4
404	246	553	361	161	174	108
37	0	173	36	48	44	0
194	227	351	289	193	340	201
249	111	378	155	168	216	98
184	212	361	197	227	202	213
4	12	0	21	26	22	37
0	2	0	0	0	0	4
0	0	0	0	0	0	6
1	3	0	0	0	0	0
75	64	87	39	44	63	45
267	327	362	247	253	325	289
81	162	175	122	123	134	85
14	24	38	25	6	6	14
0	1	0	0	0	2	0
718	1434	1211	1332	919	655	557
137	213	313	115	164	207	214
15	6	33	27	11	13	27
285	238	536	187	281	297	247
75	48	54	35	65	46	56
0	0	0	2	0	0	0
1022	1311	1880	1230	1164	977	975
0	4	0	0	0	0	0
181	216	174	161	155	202	104
2	5	0	5	1	1	12
0	0	6	0	0	0	0
663	1665	1368	460	567	881	1124
0	0	0	0	0	0	5
0	0	0	0	1	0	11
0	1	0	0	4	0	62
0	0	0	0	0	0	604
7831	9065	12687	8011	8169	9574	7713
1325	1455	2336	1385	1305	1487	1270
12	40	21	24	19	64	25
87	85	212	107	76	120	86
72	66	85	69	54	40	78

33	33	35	15	25	51	37
13	20	39	55	26	38	21
370	408	626	456	365	409	322
74	33	130	69	13	34	41
9	23	24	31	28	35	17
773	870	1105	708	644	803	601
44	70	58	27	19	64	78
11	5	15	14	10	10	12
162	161	233	154	175	143	131
47	64	103	74	59	0	52
443	2416	94	2986	53	2151	920
30	54	41	53	34	38	25
0	1	18	14	8	11	0
28	41	87	19	20	20	37
112	122	346	126	135	193	106
4	9	22	5	3	17	6
0	1	0	2	1	1	18
27	27	36	25	29	23	17
128	115	171	85	102	174	92
501	384	983	524	520	454	301
35	46	78	63	55	43	64
125	28	0	292	208	41	13
193	213	514	310	296	187	242
38	42	61	48	47	43	47
81	59	104	58	97	83	58
6	1	14	2	0	11	15
0	0	2	0	0	0	0
90	162	189	169	114	126	86
401	578	743	417	462	383	421
53	31	39	14	22	42	55
80	78	87	48	57	110	64
0	0	8	13	19	0	0
24	55	96	27	45	48	44
29	47	35	33	33	33	50
23	78	53	46	34	33	20
8	10	32	11	9	5	5
34	46	51	34	44	66	35
25	19	44	10	55	12	13
0	29	0	0	0	0	0
1403	1403	5491	2447	2133	2161	1165
1879	1725	4663	2302	2248	2328	1402
0	37	51	21	33	22	28
5	10	26	8	9	13	0
173	160	242	171	168	209	184
1	4	17	2	0	8	0
0	0	0	0	0	0	0
0	0	3	1	1	0	6
1	11	103	3	31	21	92
192	243	372	144	216	238	138
88	78	115	47	69	57	67

For Review Only

39	50	89	20	46	81	16
146	142	195	133	88	179	139
27	54	62	39	37	51	28
63	100	160	108	59	81	67
61	7	156	19	2	157	23
31	39	49	26	43	45	37
0	0	0	0	31	18	31
171	213	310	179	186	180	193
102	108	138	107	76	125	81
209	236	325	243	186	201	140
0	81	26	0	0	77	80
54	64	88	75	78	96	77
19	9	9	12	14	20	6
23	0	52	0	0	40	18
35	31	38	29	17	28	16
0	0	10	3	2	1	0
1	1	0	1	1	1	11
0	3	11	14	5	2	5
146	231	271	163	183	255	176
8	26	37	16	24	26	24
79	83	129	103	64	129	83
4	4	9	5	3	3	4
73	34	110	37	50	39	37
6	3	70	9	5	50	19
19	51	43	23	33	28	40
3	0	0	2	2	2	0
1	3	1	6	12	6	5
2	0	0	2	0	2	0
4	0	0	0	4	2	3
3	0	13	9	0	0	0
1	3	3	4	3	0	3
1	0	0	4	0	0	0
0	0	0	0	0	0	1
4	3	1	4	2	3	0
0	0	266	0	0	0	0
3	0	0	3	0	0	0
1	0	4	0	0	0	0
2	4	0	3	0	1	0
0	1	6	0	0	0	0
0	0	0	3	0	0	2
24	32	47	24	31	27	16
5	0	0	0	0	0	3
7	0	0	0	0	0	0
84	82	130	70	76	46	79
0	0	5	4	0	0	3
1	9	23	16	4	16	28
12	2	23	3	4	10	4
11	8	29	8	7	9	10
0	0	0	0	1	1	0
4	2	11	8	8	6	5

For Review Only

45	27	67	47	48	21	20
0	0	0	0	0	0	7
13	41	36	18	23	22	23
0	0	0	0	0	0	0
24	15	18	12	0	15	0
164	210	268	99	116	133	158
3	3	5	3	4	9	1
23	9	33	20	19	5	4
2	9	24	5	1	2	4
0	0	0	0	0	0	0
12	3	5	11	7	6	8
317	277	462	309	317	205	236
3	3	0	4	0	0	0
12	30	30	20	20	10	7
0	3	0	0	1	1	6
1	0	0	2	0	0	0
14	10	13	13	3	8	7
0	1	0	0	0	3	0
49	37	125	52	77	11	95
36	32	78	54	49	57	42
400	607	0	0	121	0	293
7	7	0	6	21	26	2
24	21	35	19	22	19	12
0	1	5	1	1	0	0
17	73	72	27	49	36	30
18	40	24	15	21	10	36
161	116	410	157	131	257	143
92	49	143	78	40	70	43
16	15	50	21	15	42	22
4	4	8	4	0	3	3
29	60	53	33	62	81	82
19	27	32	16	27	10	24
4	18	21	22	13	41	3
21	14	9	16	18	6	20
69	36	94	54	33	31	53
38	30	57	30	35	49	28
128	97	164	47	94	93	93
308	386	608	282	391	317	377
1	1	0	0	0	0	5
0	0	0	4	0	0	0
16	58	54	9	6	28	32
30	38	72	85	35	31	27
128	188	299	131	118	167	118
103	112	215	149	140	155	122
153	124	212	108	121	146	110
153	161	225	138	115	197	131
12	21	20	33	19	17	13
108	117	159	65	67	49	108
75	62	94	31	99	102	44
0	0	7	1	0	0	0

For Review Only

194	76	349	195	134	167	126
65	88	123	43	38	67	89
65	94	203	103	137	121	86
58	46	105	68	47	42	40
17	29	110	16	62	24	55
28	28	104	11	32	48	26
92	97	138	90	73	99	86
3	1	0	2	5	7	21
11	7	17	19	8	20	52
96	72	137	61	84	64	50
161	166	213	103	101	128	47
0	1	11	0	6	0	0
269	222	351	269	229	201	193
8	24	43	15	18	16	21
95	120	148	135	131	156	132
254	0	102	232	18	0	234
23	30	32	25	18	34	20
165	255	291	154	151	194	213
0	1	0	0	5	0	0
69	46	80	39	48	74	56
234	42	170	60	39	46	90
0	0	5	0	0	5	2
26	21	24	18	12	34	19
69	45	73	38	54	50	102
31	59	28	9	43	22	28
14	15	15	6	2	1	22
2	7	17	5	1	1	6
761	636	894	765	718	574	454
0	0	10	5	0	7	10
0	0	0	0	0	0	0
2	2	0	4	3	13	2
0	0	0	0	0	0	5
0	0	2	0	1	0	0
1	0	0	3	1	1	1
36	28	37	32	12	35	39
0	0	0	4	1	0	0
42	20	48	20	30	30	36
106	56	133	54	29	97	53
28	25	79	46	3	26	4
0	0	0	0	3	0	0
1	3	0	1	4	0	3
145	188	222	132	188	148	193
3	0	6	0	0	0	4
0	0	0	3	0	0	0
35	0	76	0	45	30	29
124	28	60	96	9	26	28
0	0	0	1	0	3	1
12	12	58	26	13	52	29
12	3	36	15	7	0	28
0	3	0	1	2	3	3

For Review Only

31	38	37	13	37	33	21
4	7	17	11	8	2	4
20	24	50	36	22	23	54
25	18	71	32	68	23	51
0	4	0	3	0	4	3
208	194	288	188	191	185	170
7	15	21	7	25	11	8
112	88	164	96	81	89	64
0	0	6	0	0	0	0
24	15	39	19	28	19	29
0	7	13	10	5	11	8
79	120	184	107	130	71	128
190	230	421	208	249	179	230
0	0	5	0	1	0	8
35	13	23	22	12	11	20
11	11	0	5	2	12	33
207	259	344	177	262	249	264
3	1	0	1	1	30	28
111	93	337	156	145	106	169
0	0	0	1	0	0	5
8	14	19	3	13	4	2
39	51	81	36	49	34	53
26	27	56	21	34	36	34
128	206	0	144	155	173	104
63	54	141	59	60	0	48
91	34	53	3	12	35	4
3	2	0	1	1	2	2
83	125	234	134	134	113	85
50	41	25	52	21	12	1
34	28	38	34	13	17	3
21	7	8	13	6	7	15
5	3	11	7	5	20	0
1	11	0	1	1	3	2
22	71	156	9	10	0	9
3	1	34	9	21	1	1
32	19	83	52	60	41	16
67	53	94	38	40	49	26
96	110	205	70	154	165	89
48	58	119	14	108	68	39
184	215	306	243	208	186	198
158	231	316	128	192	112	186
0	71	112	56	50	39	33
36	32	72	43	68	50	34
1	2	1	2	0	0	0
30	23	55	52	27	38	10
6	0	2	0	5	7	0
59	86	163	100	113	109	52
16	24	37	33	24	24	12
13	34	48	31	40	42	42
42	66	102	33	36	38	60

For Review Only

344	229	597	179	422	205	258
146	117	393	298	226	258	88
12	7	16	5	0	3	6
164	225	380	168	178	210	141
0	0	0	4	0	0	1
0	0	0	6	3	1	1
0	0	0	0	0	3	0
169	178	268	124	145	190	128
0	0	13	3	8	7	4
4	0	11	5	3	2	5
0	0	0	0	0	0	0
3	10	15	2	8	11	4
10	1	8	7	3	1	5
310	269	321	183	204	310	219
58	70	111	47	53	60	65
421	278	487	274	313	422	246
1013	496	1230	1134	363	1138	588
356	378	725	370	212	572	290
12	9	12	7	16	12	9
0	0	0	1	0	1	0
0	0	0	0	0	0	0
121	148	187	113	129	142	124
0	0	6	0	0	0	0
104	82	257	141	57	204	100
20	30	28	23	18	17	22
140	98	301	116	82	235	147
53	57	22	12	17	68	35
49	57	93	43	73	59	22
218	257	355	170	186	213	214
12	17	22	5	14	27	11
54	100	37	9	68	40	20
61	113	137	67	72	100	76
130	152	240	129	207	135	154
50	28	69	23	29	41	26
0	11	15	24	21	0	0
89	84	124	117	79	71	45
0	0	13	0	0	0	45
0	0	0	12	7	0	4
0	0	0	0	0	0	0
115	56	0	46	20	75	177
35	30	77	36	45	41	21
0	7	0	0	2	2	1
64	49	63	42	56	54	48
122	117	177	124	119	126	111
63	84	43	74	0	90	95
0	18	22	9	14	15	0
6	3	0	24	16	32	4
2	0	0	0	0	1	0
34	1	57	1	9	16	3
4	1	5	0	10	4	2

For Review Only

2	1	1	7	2	1	0
44	52	87	41	59	79	47
42	31	40	18	21	9	11
13	18	27	42	26	23	19
92	159	113	48	94	101	48
20	5	34	23	11	6	3
41	62	37	13	45	23	33
97	94	134	56	94	73	88
0	365	53	392	0	0	276
1	1	0	1	1	1	19
1009	1179	204	2	105	1056	783
0	1	6	8	1	1	1
0	0	0	0	1	0	0
34	40	64	41	44	25	105
20	1	38	12	2	5	39
0	0	0	0	0	0	21
758	1110	1694	630	805	911	871
1449	1652	2679	986	1763	1863	1292
1	0	4	6	7	4	1
482	114	502	232	207	549	11
0	28	136	40	1	11	47
0	0	0	0	63	0	63
63	83	43	41	37	19	36
87	115	103	107	0	10	85
61	82	148	95	72	80	73
1	1	0	1	1	1	1
232	97	347	29	271	171	94
279	147	735	312	352	197	157
134	140	273	101	127	135	155
8944	9131	15153	9535	9009	9809	6953
75	87	115	76	56	66	56
70	85	94	67	40	65	52
5	0	4	1	2	1	3
44	46	61	29	29	33	36
131	128	314	141	181	135	70
7	16	51	28	15	19	0
76	77	187	85	133	88	76
1387	2226	1707	1168	1452	1441	1725
49	59	60	63	34	31	47
597	522	1077	682	450	618	455
44	0	1	0	0	0	0
68	64	105	45	28	50	57
94	146	148	106	111	94	90
73	83	163	84	87	95	107
17	20	55	29	38	16	26
0	0	0	0	0	0	0
0	0	0	0	0	0	0
171	92	195	85	104	170	132
7	18	17	15	22	13	6
10	0	9	0	1	3	3

For Review Only

4	0	8	12	2	2	5
0	3	0	0	0	0	3
0	0	4	4	0	3	3
26	48	63	62	35	31	40
40	0	21	38	33	0	48
81	71	95	70	54	63	56
46	52	5	8	0	51	35
228	435	465	240	244	436	194
27	28	78	25	19	22	25
1056	1019	2390	1833	1257	1483	1137
459	474	873	503	425	582	443
112	111	159	106	107	96	100
0	0	0	0	0	30	0
298	462	535	290	317	402	395
1	1	0	2	1	0	0
734	924	1204	768	410	838	699
0	5	0	0	0	5	5
9	2	0	0	0	2	3
0	8	0	0	3	2	0
7	3	0	0	0	0	22
422	492	653	418	655	384	322
22	32	141	8	6	116	55
45	43	78	47	28	36	43
0	0	0	0	0	0	19
4	26	40	12	5	24	43
4	4	6	4	4	4	4
1	1	0	1	1	1	19
68	89	101	71	68	94	74
17	28	45	15	33	15	17
0	0	217	0	485	0	0
28	28	30	25	29	37	41
376	318	649	399	407	431	369
178	208	409	200	206	223	130
141	101	183	76	88	134	100
28	29	53	21	30	33	31
21	16	173	36	40	12	9
41	32	61	15	35	16	28
2	0	0	0	0	0	7
0	1	2	0	0	0	4
0	0	0	0	0	0	0
10	9	10	4	0	4	7
314	338	503	376	286	365	397
0	1	111	4	1	0	1
195	254	419	194	190	170	161
704	839	1239	702	737	724	711
0	2	16	11	2	8	15
0	0	1	0	0	0	0
7	14	31	14	10	11	7
9039	9417	15835	6748	6354	9147	5276
18	1	0	19	1	1	2

For Review Only

0	0	0	0	0	0	10
498	254	1063	707	376	711	398
1	7	0	0	1	5	9
2	19	15	4	12	13	22
0	0	4	3	1	0	3
64	20	63	48	55	47	71
0	0	3	0	0	0	0
137	274	760	1	149	407	218
0	0	7	0	0	0	0
51	30	127	38	60	28	33
14	6	30	6	19	3	22
0	0	1	0	0	0	0
4	5	7	5	3	2	6
0	0	0	0	0	0	0
29	40	63	84	80	50	65
0	0	0	0	0	0	6
28	39	140	22	50	63	67
0	0	93	782	0	0	0
73	50	67	64	37	61	43
18	43	98	74	83	25	55
392	421	701	433	386	424	405
0	0	0	0	0	0	17
0	6	0	0	0	0	0
0	0	0	0	0	0	0
0	3	0	0	0	0	5
51	19	41	32	20	39	30
17	8	28	5	24	14	3
0	0	1	0	0	11	0
25	59	193	57	96	77	81
25	21	0	24	16	69	2
0	0	0	0	2	5	0
17	38	21	13	23	22	26
265	333	520	307	335	306	238
0	0	10	0	0	0	0
108	52	189	104	108	79	105
166	29	277	184	188	174	173
8	12	21	12	21	11	12
32	43	76	45	80	34	30
7	5	0	8	16	4	2
15	11	18	11	4	6	0
281	324	561	378	377	308	232
2	15	0	6	10	5	9
4	1	14	4	1	2	4
92	0	150	0	0	0	72
14	7	0	0	0	10	0
31	0	2	0	30	0	28
23	26	39	25	31	16	16
106	126	275	150	178	154	119
365	384	630	422	408	473	285
172	175	18	206	125	133	0

For Review Only

22	11	78	27	34	24	43
14	23	30	17	36	69	42
156	206	406	200	225	214	214
0	29	31	0	17	21	21
148	125	0	139	145	138	115
19	15	18	19	7	19	16
0	0	11	0	5	0	8
1	8	0	3	8	0	0
28	30	53	19	31	0	24
190	240	185	131	171	67	196
37	38	41	30	20	33	23
1	11	4	2	4	3	4
2	0	0	0	0	1	0
11	11	8	7	3	5	2
0	0	12	0	0	0	5
47	57	65	39	39	38	36
283	212	437	152	378	401	341
17	15	70	34	29	18	8
13	8	12	13	10	10	7
7	9	8	11	7	8	5
3	2	3	4	4	4	2
4	9	8	5	14	10	9
1	4	4	3	0	0	1
1	3	0	6	3	2	1
4	0	6	3	4	7	3
5	7	8	5	5	5	6
91	55	146	130	133	134	63
0	6	0	2	0	0	6
4	5	6	5	8	5	6
15	3	0	0	10	10	2
3	0	1	0	0	0	3
0	3	3	5	4	0	4
8	10	9	6	3	2	3
1	0	3	0	1	0	0
2	0	3	2	0	0	1
7	9	6	1	5	3	7
24	38	47	26	31	32	32
4	0	2	1	0	1	0
32	21	48	18	31	13	20
61203	133459	84837	56768	74491	70287	110572
1	0	0	1	0	1	1
1	1	0	2	0	1	0
0	0	0	9	0	3	0
34	0	83	37	38	36	26
0	2	3	0	8	0	6
1	0	0	0	0	0	0
70	43	132	67	82	104	58
1	1	15	0	1	1	1
5	0	0	0	1	3	3
0	0	0	5	0	0	4

For Review Only

0	0	6	0	2	1	3
5	6	17	9	12	4	12
61	71	99	74	71	88	64
0	5	8	9	0	0	0
11	11	33	23	41	17	13
0	0	0	0	1	0	0
0	1	0	1	0	0	0
0	0	3	0	0	0	0
4	0	6	0	5	0	0
19	17	36	31	19	18	28
3	4	7	5	5	4	3
4	0	2	12	8	6	1
0	1	2	3	0	1	5
0	0	0	6	1	0	0
15	3	12	11	2	10	6
10	11	30	25	12	12	15
2	0	0	3	1	0	0
3	12	22	31	13	14	13
1	1	0	1	8	1	1
52	68	73	34	47	33	37
16	41	40	37	13	18	34
0	0	0	0	2	0	0
0	5	0	0	0	0	2
0	0	0	1	3	0	0
2	0	0	2	1	1	0
0	0	0	0	1	0	0
3	3	3	3	3	1	3
5	7	2	4	3	7	1
1	1	0	2	1	0	1
1	3	0	3	0	0	0
37	53	95	80	67	70	60
0	1	2	3	0	0	0
6	8	0	1	0	5	0
2	2	0	3	3	0	0
0	0	0	0	0	3	0
22	48	60	34	46	22	53
7	1	7	3	2	1	7
0	4	13	0	4	8	4
28	6	14	22	14	23	0
52	28	26	38	14	17	32
27	38	68	39	46	14	20
29	39	77	28	32	28	21
3	0	15	12	12	1	5
13	19	32	27	3	6	15
4	5	9	0	0	0	6
20	25	34	24	18	26	21
1	2	0	2	2	1	4
0	0	3	3	4	0	0
0	10	3	0	0	0	3
4	0	0	0	0	3	3

For Review Only

0	0	0	0	0	16	0
0	0	3	0	0	0	0
0	0	0	0	3	0	0
5	7	6	3	5	7	6
26	26	41	25	25	15	15
6	7	10	4	1	7	0
21	11	50	13	11	15	19
4	7	0	5	4	0	7
6	0	7	8	0	4	0
0	1	2	0	0	3	0
4	3	16	23	1	27	4
0	0	3	0	0	0	0
7	8	10	10	8	8	7
19	14	23	20	16	18	16
4	3	6	2	4	3	4
3	2	1	2	1	2	0
52	48	96	62	66	52	64
0	0	6	0	0	0	0
0	0	1	1	0	1	0
42	78	142	136	61	171	12
0	0	4	0	0	0	5
27	36	33	27	26	34	22
0	0	2	0	0	0	0
0	5	0	0	2	0	2
1	0	0	2	0	0	1
7	21	18	6	8	12	10
7	16	19	21	11	27	8
2	3	0	11	1	10	3
10	12	19	9	8	11	10
7	9	35	1	12	7	4
1	5	21	4	9	21	6
3	3	8	3	3	5	8
14	4	17	32	5	22	4
1	1	8	5	0	3	0
153	218	278	126	151	212	180
11	11	25	11	13	19	9
0	1	3	1	3	1	0
1	0	0	1	1	1	0
0	0	0	1	6	2	1
13	12	17	15	13	26	15
0	0	4	0	0	0	3
21	24	24	31	40	43	21
9	9	28	12	19	14	10
0	1	4	5	1	1	1
1	1	74	22	15	2	1
0	0	7	0	1	0	0
0	1	6	5	0	0	0
0	1	1	0	1	4	0
7	8	15	15	25	11	9
0	0	0	0	0	1	0

For Review Only

1	1	0	1	1	5	1
5	4	9	4	9	7	4
8	8	0	12	6	12	8
0	1	0	1	1	5	1
14	16	33	17	28	5	19
4	0	9	4	7	0	4
0	4	2	0	0	0	0
1	0	6	4	0	3	8
1	1	7	12	1	1	1
1	5	6	0	4	8	4
3	0	0	0	0	3	2
0	0	1	0	0	3	0
0	0	0	2	1	0	0
15	9	0	13	12	10	14
0	0	0	0	1	1	0
0	1	3	0	0	1	0
0	1	0	1	0	0	0
7	3	30	12	25	14	3
3	1	3	1	1	3	1
3	1	1	0	0	1	0
3	11	0	2	1	1	4
2	0	0	0	0	0	0
0	3	0	0	0	4	3
1	0	0	3	0	2	0
201	255	365	252	211	285	259
8	3	0	0	3	0	0
1	9	26	6	11	4	17
0	0	1	0	0	0	0
14	21	38	10	12	10	19
1	0	1	0	4	0	0
0	1	13	1	1	0	0
38	38	50	35	28	30	33
0	0	5	7	1	0	0
3	6	5	2	0	0	0
0	0	13	0	2	17	1
1	1	0	0	3	3	0
3	4	8	12	3	4	8
2	0	0	0	0	2	1
4	5	5	7	0	7	14
241	164	398	412	325	190	105
1	8	12	2	6	4	1
0	0	0	1	6	0	1
8	10	16	1	4	1	13
20	26	55	9	49	5	20
17	8	30	15	4	4	3
0	0	0	0	0	0	0
5	13	21	5	15	29	19
4	4	4	4	4	9	0
0	0	3	2	5	0	2
0	5	0	1	0	0	6

For Review Only

10	9	34	6	4	11	17
0	0	0	3	1	2	0
93	71	124	83	69	107	79
1	0	0	0	1	0	0
4	3	4	5	6	2	3
6	1	0	3	2	12	2
1	0	0	0	0	0	2
3	8	0	0	4	3	3
0	0	1	0	0	1	0
38	56	72	46	78	33	25
3	1	0	4	0	6	0
2	3	3	4	3	1	3
3	13	9	2	4	13	6
8	7	9	8	18	6	2
10	20	16	13	11	8	14
5	2	10	3	12	20	7
4	1	0	7	0	0	2
7	5	12	2	2	16	9
8	0	0	7	11	9	10
5	0	0	0	5	0	0
1	0	5	6	0	0	0
5	36	48	37	1	38	22
4	0	3	0	0	0	3
3	3	4	0	0	3	1
64	59	65	33	66	72	53
0	0	0	1	0	0	0
21	19	24	22	11	20	38
14	20	21	12	12	14	12
1	0	0	2	0	0	1
0	13	22	10	2	0	4
18	9	7	15	3	12	9
1	1	13	5	4	0	0
4	3	3	4	1	2	4
1	39	49	11	35	8	39
0	4	0	0	0	0	1
16	14	37	54	23	46	52
0	7	0	5	2	1	0
0	3	0	0	0	5	0
1	0	0	0	1	4	3
0	0	0	0	1	0	0
0	0	4	0	0	0	0
10	4	6	5	7	23	11
2	7	3	2	0	0	0
15	5	19	7	30	46	10
2	9	30	9	10	4	23
1	2	0	1	1	0	1
0	0	0	0	0	7	1
381	273	512	185	278	366	382
38	29	49	36	19	6	19
1	0	0	0	0	1	0

For Review Only

0	2	0	0	0	3	0
0	1	0	0	2	1	0
3	2	4	3	4	4	1
1	2	4	8	1	1	2
195	148	549	389	433	302	244
8	6	0	1	2	3	5
21	36	46	25	30	50	24
3	0	0	3	0	0	1
32	10	59	11	27	28	13
0	0	0	0	0	0	2
0	0	0	5	0	0	0
0	0	6	4	1	0	0
13	9	19	1	15	13	11
0	1	0	1	0	0	0
70	33	29	46	44	51	39
29	32	28	9	2	3	12
1	5	7	3	14	3	6
1	5	6	1	4	4	1
6	3	5	4	1	7	5
2	10	1	2	10	1	8
0	0	0	0	0	0	0
6	5	7	3	2	3	2
9	10	5	6	11	1	3
0	0	33	0	0	0	0
0	0	4	5	2	1	0
76	43	66	22	45	49	27
1	0	0	0	0	2	2
2	1	3	4	4	0	0
4	6	6	4	6	1	1
26	37	58	37	37	34	35
0	0	5	0	0	0	0
24	32	51	11	26	14	22
2	0	0	2	0	0	0
3	0	0	1	0	0	0
32	21	41	36	25	41	32
2	0	0	2	0	7	0
1	0	18	1	2	1	2
0	0	0	2	1	1	0
0	0	0	0	0	0	2
3	0	7	10	6	2	6
1	1	0	3	5	3	1
91	76	186	102	118	134	74
0	0	1	4	4	7	1
6	3	3	1	5	8	1
0	0	0	2	0	0	0
1	0	0	3	0	1	1
5	4	21	10	6	2	5
2	2	0	0	0	0	2
0	1	0	0	0	0	0
0	4	0	0	1	0	0

For Review Only

1	1	0	1	3	2	1
2	7	4	1	3	2	5
0	0	1	4	3	0	0
1	2	5	0	1	7	3
12	0	13	7	3	1	1
7	15	0	10	2	1	1
11	6	16	12	7	18	9
0	0	3	1	4	2	0
1	0	5	4	3	0	0
2	0	0	0	1	0	0
0	0	0	0	0	0	0
94	0	0	55	0	107	0
6	8	12	1	10	8	6
0	0	3	1	0	0	0
5	1	4	2	1	1	1
209	268	255	167	114	136	185
4	1	11	7	4	2	2
12	14	24	14	12	13	10
16	25	26	34	34	30	15
1	3	20	1	11	1	7
153	191	300	222	218	170	191
0	0	0	0	0	4	5
3	0	1	3	0	4	0
30	51	28	27	21	23	34
9	12	15	10	1	8	4
0	5	0	0	2	0	0
12	3	5	4	2	0	3
2	3	14	30	7	8	17
7	2	9	5	9	7	1
33	26	33	32	26	46	25
1	4	4	1	3	1	1
1	4	3	4	3	4	3
0	0	16	0	0	0	0
0	0	0	0	2	0	0
0	2	11	9	7	1	0
3	11	21	19	5	2	10
10	13	18	4	8	21	5
0	0	0	0	4	1	0
0	0	6	0	2	4	1
6	10	23	6	9	16	6
32	34	65	9	52	33	43
42	24	73	55	28	40	45
5	7	24	18	9	5	5
0	0	6	2	0	5	0
5	9	13	7	6	4	8
2	4	12	14	8	5	4
0	0	4	4	4	0	3
0	0	0	1	0	0	8
1	3	26	10	4	1	10
3	1	17	4	2	6	1

For Review Only

6	8	27	10	16	9	8
1	2	0	0	5	3	0
6	20	27	22	29	15	13
2	2	0	10	5	24	11
75	83	111	74	68	86	86
0	1	11	0	12	3	0
4	14	0	10	17	10	8
1	0	0	0	2	0	1
6	5	6	4	6	13	2
0	0	0	0	0	0	0
1	1	0	1	1	1	5
14	0	25	8	8	3	8
0	0	6	0	0	0	0
0	0	2	0	0	5	0
1	1	0	0	3	2	0
32	48	0	29	14	44	6
24	31	38	14	22	30	22
0	0	0	3	0	0	0
1	0	0	0	1	1	2
74	52	105	110	117	76	36
4	0	0	0	2	1	0
38	99	242	132	177	57	60
4	3	0	0	0	0	0
0	3	0	1	0	0	3
5	6	19	3	2	6	4
0	0	0	0	0	2	0
0	1	1	2	2	1	2
5	5	4	3	8	3	0
7	10	11	4	7	8	10
0	0	2	0	0	3	0
0	3	0	0	0	1	0
436	292	672	336	451	464	218
0	1	0	0	0	0	4
4	2	11	9	14	22	1
3	12	6	3	13	4	6
0	3	0	1	4	2	0
1	0	0	2	0	3	0
1	0	8	3	1	0	1
1	2	0	0	2	2	0
0	5	0	2	8	0	3
56	90	88	65	61	43	40
1	0	5	2	7	2	1
5	4	7	4	3	13	1
4	17	17	12	7	12	17
37	48	63	34	32	35	44
0	3	0	0	3	3	1
0	0	7	9	4	4	2
8	6	26	6	6	5	14
3	0	13	1	1	2	1
0	3	5	2	5	2	1

For Review Only

0	3	6	2	5	2	1
0	0	0	0	2	3	2
2	4	11	4	8	2	4
1	2	12	6	8	2	3
0	1	3	1	3	0	0
0	2	0	0	2	2	0
13	6	33	30	19	59	34
0	0	0	0	0	2	2
0	3	0	7	0	0	3
2	1	0	17	9	1	2
1	3	10	3	6	2	3
1	0	0	4	0	0	0
44	63	70	37	31	51	48
0	0	0	0	0	0	4
4	9	11	4	2	2	5
8	2	0	5	3	15	5
0	0	0	0	3	0	0
0	2	1	4	1	0	0
3	1	0	3	2	0	0
2	4	6	1	1	6	6
4	4	2	6	0	4	0
11	65	32	2	22	47	30
1	0	1	1	4	4	0
0	0	0	0	1	6	1
0	0	0	0	0	0	0
2	4	8	2	1	5	7
2	8	6	3	3	5	3
1	6	12	4	4	3	0
6	4	19	1	3	25	5
9	15	15	12	10	13	10
0	0	3	0	5	0	0
76	15	73	11	17	14	50
10	4	15	8	7	9	6
13	34	4	7	9	9	1
7	1	20	11	2	2	1
4	6	0	1	1	2	1
14	24	22	14	21	19	11
0	0	0	0	0	0	0
1	0	0	3	0	0	0
0	0	0	0	0	0	0
7	21	22	7	18	1	13
4	0	0	2	1	1	2
23	7	14	15	18	10	10
6	10	22	9	9	7	7
18	6	3	0	1	1	3
3	4	14	1	6	1	5
0	0	0	3	0	1	0
1	1	0	8	2	1	2
0	1	0	0	1	0	0
30	41	21	1	66	44	45

For Review Only

2	17	7	4	4	0	3
0	1	8	0	0	1	0
7	3	19	6	5	8	1
0	1	14	0	0	1	0
8	15	13	10	7	4	4
0	0	5	0	0	0	0
0	0	7	0	0	0	0
12	8	31	20	17	29	6
3	3	1	3	0	3	5
37	30	73	95	41	46	34
6	8	27	15	17	6	8
1	0	3	1	4	1	4
0	0	0	0	0	0	0
0	0	1	2	3	2	1
4	0	1	0	0	4	1
11	0	8	4	10	9	4
51	25	43	25	70	25	48
6	9	12	23	13	13	10
3	6	6	6	1	0	1
12	3	33	36	35	21	5
0	3	0	0	1	2	1
0	2	1	1	0	0	0
1	2	0	0	0	0	1
0	2	0	0	0	0	11
3	5	0	3	5	0	0
1	1	0	1	1	3	0
2	0	4	1	0	0	0
5	4	12	8	7	9	3
10	14	23	7	23	3	13
0	0	0	2	1	0	0
12	4	46	16	2	12	8
1	0	0	0	0	1	1
0	0	1	0	4	0	0
14	18	19	14	13	21	18
83	84	308	86	117	79	81
0	8	15	10	8	10	3
0	0	0	0	0	0	0
957	3	0	483	83	722	1
9	0	0	5	1	0	1
1	0	1	0	2	0	1
2	4	10	5	7	1	7
1	4	4	3	4	2	0
3	6	0	4	3	1	23
1	2	3	1	0	1	0
1	1	4	1	0	2	0
1	0	3	3	0	3	0
10	16	18	14	14	6	14
4	21	60	5	16	41	9
3	1	3	3	0	4	0
36	107	88	87	119	65	35

For Review Only

28	23	30	17	10	25	11
0	0	0	0	1	0	0
3	0	5	7	4	3	0
0	0	14	0	4	1	1
3	5	9	6	4	1	6
29	38	21	32	23	18	15
0	0	0	0	3	0	0
24	2	37	2	16	16	10
21	14	43	49	14	73	21
1	0	0	0	1	0	2
0	3	7	0	1	4	1
47	64	98	23	142	45	119
0	0	2	3	3	0	4
2	10	8	11	2	0	1
16	4	20	17	3	11	15
0	0	12	0	0	0	5
9	6	34	14	12	12	4
12	4	10	4	11	12	4
5	13	11	3	11	4	3
3	0	6	0	0	0	0
0	0	0	0	0	0	4
0	2	0	2	0	0	1
19	8	26	23	20	18	23
0	2	0	2	0	0	4
2	0	0	11	0	2	0
0	0	0	1	0	0	0
0	0	0	0	3	3	0
7	5	32	10	9	8	34
4	5	6	7	8	2	1
3	0	0	1	0	0	2
0	0	2	0	0	0	0
0	0	0	0	4	0	0
1	0	3	0	0	1	0
0	1	0	0	4	0	0
26	14	25	16	28	37	36
3	0	0	4	0	0	0
7	2	14	18	4	7	5
3	0	4	3	1	0	0
0	0	5	1	2	7	6
12	10	23	15	10	15	12
0	0	0	0	2	2	0
4	0	3	0	0	0	0
14	0	0	0	0	0	0
0	0	5	4	6	0	0
0	0	0	0	3	0	0
6	5	4	7	4	0	3
0	3	0	0	0	3	3
5	5	17	12	4	17	3
0	1	0	0	0	0	0
2	5	0	1	9	1	2

0	2	3	8	0	3	11
25	24	33	17	16	13	28
0	0	0	3	3	0	1
28	7	18	7	20	11	15
23	36	7	4	23	4	13
2	0	0	5	0	2	0
17	3	0	2	8	9	5
118	49	201	115	83	45	135
2	3	0	4	1	2	2
0	0	0	0	0	0	0
0	0	3	0	0	1	3
5	10	0	0	1	0	0
39	43	68	21	0	52	0
147	147	246	114	126	153	157
4	2	0	7	32	9	8
74	64	133	71	94	64	89
0	0	0	2	2	0	4
9	7	11	4	5	0	4
74	94	75	56	110	98	71
4	2	0	1	14	5	4
0	0	0	0	0	0	9
0	0	0	4	4	2	0
242	181	401	111	170	291	127
428	573	867	642	633	389	413
310	244	334	164	202	314	123
3053	3128	5689	4019	3570	3837	2604
0	0	0	3	0	0	19
91	71	68	52	41	73	51
166	151	358	84	105	142	118
728	761	1439	884	765	954	593
73	39	50	3	7	16	30
136	134	228	136	134	137	133
3	3	3	0	1	3	3
121	158	377	167	99	320	71
126	169	319	81	179	157	143
236	171	339	189	220	214	154
171	158	359	243	165	175	107
0	0	142	0	0	0	126
0	0	0	1	0	0	0
3	0	0	1	0	2	6
4	5	0	0	0	2	7
8	14	14	6	18	15	12
51	68	95	50	76	59	55
12	2	15	1	2	1	1
136	111	203	51	130	172	42
29	12	30	31	7	6	18
90	128	161	135	132	121	0
1936	1733	2287	2281	2210	1972	997
163	180	0	31	51	25	14
0	11	5	6	1	4	0

For Review Only

66	37	171	30	35	16	62
1	3	0	0	1	0	0
20	55	85	45	48	18	36
93	157	135	47	138	160	100
51	112	159	57	95	76	77
0	0	0	0	3	0	4
362	413	558	378	339	370	145
109	65	174	155	155	106	74
0	8	0	0	0	2	0
0	0	0	0	0	19	0
0	0	0	7	2	0	5
0	0	0	0	0	0	0
36	22	49	32	25	44	32
9	18	15	4	6	7	8
20	13	45	11	20	11	30
0	217	80	199	0	223	0
1	0	0	0	1	0	8
17	37	21	21	20	18	11
2	33	31	23	31	2	7
1745	1566	1738	1770	1455	1769	1230
1	1	4	4	0	0	0
0	0	0	0	0	0	6
23	34	44	67	24	17	40
27	5	9	10	10	6	8
38	24	84	35	51	46	26
10	18	294	6	160	163	2
0	4	10	3	4	0	10
7	0	0	0	0	2	0
33	29	21	9	28	7	9
1	3	21	6	1	1	3
9	8	26	21	18	14	5
54	39	95	33	57	63	50
373	483	703	413	455	479	644
20	73	66	29	55	78	41
79	52	136	66	100	44	57
4	2	12	5	1	1	1
3	0	7	3	0	1	0
1	0	0	1	0	1	6
15	14	16	11	3	18	12
1	1	1378	1	1	1	1
6	6	9	1	3	9	14
3	0	0	3	2	2	4
76	135	95	41	111	65	64
4	1	25	15	4	2	4
53	51	118	35	65	53	51
214	258	314	215	247	200	202
45	17	50	33	32	34	29
3	2	10	7	3	3	0
91	115	147	62	65	125	67
0	0	0	0	0	1	0

For Review Only

3	3	18	5	20	2	4
0	1	19	19	12	1	1
7	0	13	4	5	4	6
16	22	121	57	77	98	48
1	0	0	0	5	2	0
84	87	113	91	60	112	80
79	90	102	93	71	66	50
14	0	15	19	11	11	0
61	72	61	61	89	44	85
12	3	21	11	22	10	4
62	0	39	50	49	0	0
65	45	76	44	36	41	87
10	22	23	7	8	9	11
1	29	52	1	16	14	21
19	51	68	28	51	34	23
8	6	0	1	1	1	23
80	62	132	65	99	84	91
0	0	0	0	0	0	0
29	46	58	33	36	51	17
158	214	317	157	178	172	195
1	1	122	1	1	1	310
29	14	44	24	27	46	18
0	159	19	58	147	45	60
121	101	123	62	113	111	97
18	0	12	9	5	14	15
4	8	2	3	0	7	4
3	4	4	3	3	3	1
4	0	0	0	0	3	0
0	0	0	0	0	0	30
93	90	132	81	96	100	79
0	396	0	301	0	385	247
113	79	0	0	0	0	74
70	106	108	86	59	60	88
83	25	33	54	52	27	55
66	101	205	144	46	81	119
104	162	272	152	182	98	145
122	144	129	222	107	109	118
0	0	0	0	0	0	19
1	1	0	1	0	1	12
54	63	110	68	45	52	97
25	38	20	16	38	40	52
9	6	6	3	5	4	97
2	0	0	1	0	2	18
3	0	0	0	0	12	10
9	0	0	2	2020	5	0
5	9	0	0	0	3	7
0	2	9	7	4	0	0
9	8	14	7	6	6	3
0	0	0	0	0	0	1
0	5	0	5	0	0	0

1785	2506	2610	1814	1841	1845	1760
47	46	32	21	56	51	55
35	42	0	0	26	40	40
18	25	38	17	14	32	27
257	0	127	0	175	240	0
1061	493	3490	248	2005	233	1733
0	0	0	0	3	0	7
14	7	59	26	10	6	6
59	38	134	65	87	39	47
0	51	103	0	0	0	0
6	14	10	15	11	6	7
10	7	26	32	12	15	16
1	1	0	1	1	1	3
41	72	64	47	63	45	39
30	36	41	35	24	32	25
2	4	0	0	6	3	3
40	47	78	61	41	50	36
24	27	46	21	18	38	32
0	0	0	0	0	0	7
37	52	0	0	38	57	42
30	7	50	29	32	42	30
112	157	237	105	115	148	110
0	0	3	0	0	0	4
61	50	58	38	41	57	51
580	611	558	393	360	625	539
0	0	2	5	12	10	0
102	52	120	67	63	108	46
43	56	88	32	31	60	42
109	68	238	98	126	161	83
2	0	14	6	1	6	6
11	9	6	4	12	9	6
6	0	12	0	0	4	4
9	0	5	0	16	0	12
505	676	1089	655	668	735	470
3	1	3	0	0	0	0
1613	2164	2568	1182	1232	1463	1104
11	8	8	4	4	5	1
0	0	0	2	1	0	1
42	38	67	50	48	44	0
23	2	7	12	2	18	8
42	49	52	11	25	45	25
28	26	40	0	31	1	41
224	235	313	165	122	291	158
249	180	326	204	204	265	205
230	242	390	231	299	344	194
0	0	0	0	4	0	0
688	740	1368	613	695	753	622
3	1	3	0	0	0	3
264	19	0	0	125	84	216
61	45	81	53	50	51	30

For Review Only

44	48	89	61	87	65	59
58	92	0	0	0	10	0
79	69	157	84	83	91	64
15	8	53	22	11	66	22
32	46	43	43	49	32	36
3	6	5	6	0	0	3
57	43	58	33	59	47	43
15	1	24	12	9	9	18
13	19	0	17	10	37	16
0	4	0	0	0	0	0
231	163	320	210	198	223	176
0	0	0	1	2	1	0
69	77	132	56	63	71	81
0	0	0	0	0	0	16
7435	7504	11004	6337	7516	7913	8094
38	19	25	34	42	20	20
48	45	69	15	65	50	46
18	19	33	17	25	26	18
89	87	147	84	72	104	95
342	441	798	503	367	448	376
203	167	299	168	90	145	140
13	9	10	12	18	0	7
0	0	9	0	0	3	1
94	103	233	92	128	132	98
279	393	582	250	266	314	238
66	58	198	84	13	132	18
230	218	404	189	171	226	177
31	17	16	26	21	29	37
196	199	205	244	231	202	136
1991	0	2742	0	0	0	118
687	673	1117	748	556	836	455
45	96	38	0	18	98	34
0	0	6	0	0	0	0
127	225	255	120	109	81	54
56	75	104	65	60	97	44
76	39	122	92	102	59	62
1	1	14	0	1	0	1
80	74	155	128	121	146	81
0	0	0	0	4	2	3
0	0	0	0	5	3	28
1	1	0	0	0	0	9
30	36	107	74	67	3	15
1	0	1	2	0	0	1
2	8	27	11	6	8	9
46	42	54	31	42	30	31
0	0	508	0	0	0	0
330	1	137	192	44	73	11
396	500	810	467	485	519	374
61	84	36	77	0	96	67
109	108	155	164	112	110	91

For Review Only

2	8	5	3	6	0	5
50	40	86	44	37	38	44
67	72	104	63	70	38	40
43	57	61	41	34	49	10
36	20	80	43	30	13	46
162	227	480	327	226	240	352
0	0	17	5	6	0	10
273	218	413	159	129	226	99
57	49	80	31	71	46	67
3	5	8	4	0	1	5
65	84	126	91	74	75	51
0	0	0	0	0	0	0
13	4	22	11	6	7	9
59	77	109	55	61	79	85
108	112	123	149	180	97	30
146	105	295	181	33	87	91
115	147	120	89	96	87	108
42	50	103	29	83	97	47
102	79	111	90	87	85	83
367	371	867	433	439	481	435
0	8	9	8	0	4	0
0	63	239	126	0	91	119
52	51	75	44	55	56	53
109	190	206	108	116	137	147
26	65	46	14	31	43	29
0	0	0	0	6	1	1
0	2	0	2	0	0	2
23	18	48	28	34	33	52
7	21	83	3	13	46	25
62	161	177	121	151	158	171
0	0	0	0	0	3	3
0	0	0	4	0	3	4
1	310	1364	1	1	35	1
81	137	88	72	105	44	68
100	119	288	186	155	154	116
17	0	9	59	53	59	27
0	0	0	0	0	0	0
49	28	100	84	63	75	65
76	27	193	3	39	32	27
21	45	59	12	20	22	40
3	0	3	1	1	1	3
132	143	272	42	155	173	210
77	102	102	77	52	70	37
32	72	54	85	39	56	33
42	60	81	19	56	46	85
44	62	135	63	74	115	35
0	0	0	33	0	0	28
23	22	77	54	71	44	9
269	366	618	349	366	386	345
161	139	253	135	150	206	127

For Review Only

18	29	32	13	15	19	13
2	2	5	18	2	1	1
197	180	253	132	174	141	131
49	31	237	293	118	142	178
6	0	0	7	0	1	0
10	8	28	30	5	37	17
5	0	4	0	6	0	0
57	43	47	14	38	104	15
147	151	199	62	73	126	141
154	143	219	179	212	77	99
45	119	96	58	57	67	41
0	0	0	0	2	0	0
699	1051	1312	757	735	861	842
3	12	39	14	12	8	28
90	276	200	85	122	111	124
0	0	7	0	1	0	2
111	95	132	99	104	98	79
81	70	103	59	67	75	73
17	19	20	11	19	11	4
98	71	78	11	41	36	79
15	76	37	5	22	24	27
11	3	7	5	1	5	16
14	29	15	7	10	12	22
1	0	0	0	1	0	2
21	16	41	19	41	21	25
31	11	75	4	21	11	27
37	42	45	55	31	34	35
199	100	120	53	154	140	70
51	39	99	106	111	36	63
3	7	0	4	10	0	5
386	413	460	308	431	567	345
148	187	242	95	134	251	207
0	348	89	0	0	0	0
0	0	0	2	3	0	6
130	161	432	224	173	160	200
294	74	990	234	195	917	3
301	132	463	367	125	261	166
59	44	78	56	48	62	51
53	83	70	53	63	66	59
109	76	161	114	76	71	67
2	1	6	3	3	2	0
118	123	229	7	89	124	50
8	3	27	6	14	8	10
51	44	2	0	0	0	0
5	11	39	18	6	32	7
49	57	100	41	53	11	15
78	31	141	62	67	100	56
32	37	73	57	37	35	18
8	7	22	18	4	2	6
11	2	0	1	1	1	1

For Review Only

84	328	675	207	756	167	337
234	395	423	269	329	232	188
0	0	0	0	2	3	2
34	32	38	18	34	33	22
0	0	9	6	4	0	3
12	10	180	1	2	3	4
54	38	92	59	38	37	39
68	107	66	78	39	56	63
163	148	302	192	179	136	150
52	44	98	72	68	46	31
0	0	3	1	1	0	0
16	45	44	14	36	20	27
11	6	12	8	4	4	27
44	33	67	37	63	59	45
0	1	7	4	0	0	1
55	34	55	40	58	46	43
22	24	30	21	17	11	23
6	2	0	0	0	0	1
2	0	0	0	0	1	2
362	443	648	284	372	389	346
0	0	0	0	0	0	0
44	24	59	48	34	40	22
120	158	270	210	188	193	119
88	73	159	69	77	75	92
42	48	49	38	64	20	20
21	8	21	31	41	35	8
50	46	54	52	42	62	30
29	2	33	40	22	16	17
4	24	6	24	19	5	21
84	303	203	194	151	299	94
8	1	17	14	12	6	5
0	0	0	1	0	23	0
13	19	101	51	32	43	49
53	0	89	64	41	78	40
91	79	97	54	83	54	28
44	62	12	49	62	54	0
20	12	8	7	8	10	8
21	18	25	1	12	21	7
88	108	228	94	108	84	158
97	108	159	77	126	152	94
0	3	11	2	3	15	9
0	8	0	0	1	4	2
8	0	0	11	0	0	16
0	0	1	2	3	0	4
173	282	315	172	177	178	219
35	125	504	26	13	107	453
0	0	4	4	0	0	10
12	15	5	0	6	13	10
184	107	305	171	141	188	103
511	459	844	395	404	421	382

For Review Only

115	19	162	16	131	39	47
209	0	9	0	0	0	0
0	0	0	0	0	0	0
32	44	90	65	53	39	45
0	1	14	0	0	0	0
73	61	84	58	53	68	50
0	0	0	0	0	0	5
106	98	164	74	68	58	79
20	19	40	25	20	25	14
208	227	345	225	156	253	186
92	73	61	63	58	76	93
0	0	0	0	0	0	0
130	120	220	146	156	147	93
248	266	423	304	275	268	242
77	99	166	62	75	111	117
162	169	294	193	206	225	77
45	54	63	53	31	49	53
117	144	167	115	85	110	101
28	28	33	43	20	17	48
120	114	233	92	123	118	87
17	12	11	9	23	10	2
25	23	51	20	23	24	22
100	97	145	96	90	115	100
44	39	91	92	74	60	57
0	77	0	0	0	19	0
103	68	195	103	123	115	85
72	75	194	156	82	49	34
62	91	130	33	53	82	30
34	41	82	17	41	52	41
60	68	79	65	55	56	53
136	230	250	196	160	174	198
41	42	44	45	35	47	26
22	30	55	20	21	21	22
9	32	16	8	29	29	18
0	70	180	0	0	59	43
17	51	84	41	41	34	37
79	121	143	82	100	88	82
0	62	0	0	47	38	36
50	64	59	63	28	97	82
594	741	1240	568	678	437	724
642	1058	1531	466	798	1032	784
120	171	288	102	85	113	98
0	0	0	0	2	1	2
19	6	18	8	31	6	24
103	120	183	143	143	122	111
7	0	4	4	4	0	7
39	1	20	14	15	29	7
73	55	96	36	49	23	40
0	0	2	0	0	0	0
76	85	86	49	78	62	59

For Review Only

80	83	129	73	47	86	67
2	0	0	4	10	4	0
67	61	61	34	47	33	16
1648	1875	3290	2298	2057	2117	1270
2	2	13	8	12	6	10
153	223	412	231	220	226	203
0	3	0	9	1	1	1
0	0	0	4	0	0	2
110	104	132	78	52	78	54
123	122	257	129	137	150	118
146	160	314	224	141	219	211
4	7	8	4	0	0	0
8	2	7	1	4	4	1
12	14	27	9	9	8	11
17	11	19	31	35	20	21
14	12	70	31	10	28	26
61	37	87	38	53	59	51
39	12	29	26	13	25	5
44	76	106	45	93	60	61
0	0	0	0	0	0	14
21	20	8	19	19	31	16
37	33	49	42	40	59	43
6	8	18	19	13	8	15
118	138	175	165	148	129	94
3	0	3	0	0	1	0
16	0	30	18	28	14	0
0	0	0	0	0	0	0
33	39	62	39	42	46	38
31	0	127	3	3	6	2
29	96	103	41	36	87	67
25	42	57	25	31	22	29
37	33	72	32	38	78	29
25	35	66	36	51	27	23
6	7	10	8	6	4	5
3	0	3	3	4	3	1
1	63	25	26	4	27	12
23	47	98	30	19	31	40
9	6	14	6	3	13	2
5	15	30	10	22	9	15
6	31	41	13	14	27	15
4	4	3	8	9	3	0
47	0	44	0	26	142	25
70	66	98	70	71	108	50
0	0	3	3	0	0	0
258	153	199	260	440	147	301
7	2	14	20	5	2	0
147	186	282	171	199	156	156
3	28	123	1	12	22	223
152	100	138	96	70	100	68
18	28	29	28	12	43	38

For Review Only

78	77	217	66	57	117	65
176	148	207	115	170	60	64
391	407	901	700	591	533	323
2	0	0	0	0	0	0
20	191	286	0	0	0	91
123	111	120	77	100	135	38
239	117	277	166	128	260	76
142	215	463	190	305	237	121
0	0	0	0	0	0	9
3	1	7	1	2	4	0
39	43	87	47	43	43	59
19	15	52	18	20	37	8
0	0	0	0	1	5	0
0	0	0	0	0	0	0
105	82	142	62	153	85	56
0	4	0	0	0	3	2
6	1	17	5	1	1	1
9	0	17	16	10	8	8
23	30	26	21	9	16	10
0	3	8	2	0	0	10
31	35	78	72	54	68	39
11	6	14	19	40	36	16
102	152	213	62	124	61	146
538	374	821	577	460	544	226
49	102	114	31	92	89	128
2	0	1	3	0	1	1
1023	1083	1831	1180	1222	1169	306
11	33	37	27	19	26	33
284	248	503	215	281	256	205
146	154	212	120	130	140	155
1	0	0	0	0	0	20
186	188	271	193	196	189	114
129	95	176	58	85	139	102
13	15	20	11	0	6	8
38	52	147	124	251	196	21
0	0	0	0	0	0	9
0	32	17	0	12	0	0
168	196	257	223	108	169	172
0	0	0	0	4	1	0
46	35	100	23	36	28	50
1	15	35	1	1	1	1
39	66	139	38	36	56	60
0	3	6	0	4	2	0
78	89	141	129	74	81	39
43	57	72	22	60	69	46
72	75	114	105	80	93	75
23	48	70	53	34	35	42
31	28	54	21	17	49	35
119	28	52	0	175	91	65
0	0	44	258	0	380	0

For Review Only

19	61	50	81	1	13	30
102	75	136	64	68	116	36
15	25	57	35	23	24	9
0	0	13	29	0	0	66
115	132	147	91	116	126	94
35	41	63	17	10	86	15
0	37	82	46	54	0	24
31	67	93	17	34	52	28
1	1	13	6	0	0	1
44	42	8	144	0	129	0
0	0	0	3	0	0	4
40	49	80	38	44	21	35
45	54	78	53	36	27	49
66	63	68	32	35	39	12
33	72	218	2	29	27	102
155	118	213	130	91	111	65
57	50	38	23	22	45	40
0	0	11	0	0	0	0
0	0	1	0	0	0	0
1	13	1	4	3	19	0
1	0	12	0	0	0	8
2	0	0	1	1	1	0
6	9	8	6	1	5	5
1	1	10	0	4	0	0
11	1	11	4	4	4	7
0	0	0	0	16	0	0
17	22	0	23	32	32	20
2	2	0	1	4	1	4
0	0	0	0	0	0	0
0	0	0	0	0	0	14
0	0	0	2	2	1	1
0	0	3	1	0	2	1
2	0	4	0	1	0	0
3	1	0	5	0	2	4
4	6	13	5	7	3	7
0	0	1	1	0	1	0
0	0	0	0	0	0	1
2	2	3	0	0	1	0
1	0	0	1	0	0	0
4	5	11	8	3	4	1
0	0	0	2	1	0	2
0	2	0	0	3	4	5
3	2	3	0	0	0	0
8	4	1	5	0	1	10
0	0	2	0	3	3	0
1	0	3	1	0	0	1
1	1	0	1	1	1	1
0	1	2	1	0	0	0
4	0	4	6	1	5	1
58	90	80	74	33	55	44

For Review Only

0	0	0	10	0	16	0
0	0	0	23	0	30	0
3	3	5	5	1	3	2
5	1	0	3	2	2	1
0	0	0	0	0	0	0
11	14	0	12	10	19	11
3	0	0	0	3	0	0
13	65	38	14	23	3	44
1	0	3	3	0	1	0
2	3	1	1	0	1	2
1	1	0	1	1	2	0
0	0	0	0	3	1	1
0	0	0	24	0	2	1
22	44	61	32	43	43	47
15	19	24	13	16	21	17
15	22	22	16	17	23	15
0	0	4	0	1	0	1
0	0	0	0	1	1	0
0	0	1	0	0	0	0
0	1	3	1	0	0	0
1	1	2	0	0	0	0
6	4	9	4	4	3	5
2	1	0	16	22	15	1
0	0	1	0	0	0	0
0	1	4	3	1	1	3
2	0	0	0	0	0	1
2	2	0	1	2	2	3
0	1	3	0	0	0	1
2	3	1	0	3	3	2
8	6	0	5	6	13	5
0	0	3	1	0	0	0
0	4	5	0	0	0	0
84	104	25	61	5	11	66
1	0	1	1	1	2	1
1	2	0	3	0	2	2
0	0	2	1	0	1	0
9	4	18	10	1	13	6
9	0	18	4	5	9	4
2	5	9	2	4	0	2
7	0	1	0	0	19	0
1	99	8	2	79	15	97
174	19	0	156	155	9	154
2	0	0	1	0	3	0
0	0	0	0	0	0	0
5	3	9	5	2	2	2
13	6	20	8	6	5	7
7	14	44	10	23	12	19
5	0	1	0	0	1	0
0	3	0	3	0	0	0
117	86	156	109	80	121	88

For Review Only

12	21	12	19	22	24	11
0	0	0	0	0	3	2
37	21	54	30	22	31	63
51	69	152	62	57	73	59
1	2	37	2	3	23	9
34	48	45	33	21	67	34
149	72	205	165	120	136	90
50	36	132	58	61	58	83
1	1	0	1	1	1	7
0	52	327	64	561	0	95
189	3	0	2	1	16	1
90	159	166	111	66	107	93
48	48	92	40	48	52	41
1	4	25	10	0	4	1
31	32	71	52	35	36	30
342	476	599	407	384	468	353
0	0	126	0	135	136	134
69	65	80	50	33	44	48
25	21	35	29	31	28	18
0	0	0	0	0	0	0
17	33	61	59	17	60	16
81	83	91	92	63	105	69
31	25	50	30	16	26	24
7	10	10	4	0	3	13
11	10	22	7	14	20	36
15	46	74	46	33	36	38
109	271	267	297	450	153	3
51	36	43	0	40	68	19
43	0	1	78	0	0	0
0	0	0	2	0	0	7
0	0	1	0	285	0	387
17	17	34	15	46	11	59
62	63	109	104	77	74	87
11	43	36	39	40	28	15
29	23	42	33	28	29	18
1	68	109	63	62	66	89
35	23	68	55	43	44	34
64	92	102	60	85	42	62
75	105	133	66	105	77	84
4	8	20	20	8	4	7
35	61	60	56	55	45	59
0	4	18	0	3	1	6
0	0	0	1	0	2	0
1	0	0	2	0	0	0
0	0	0	2	1	2	0
107	109	0	121	126	134	77
0	0	18	8	19	0	0
3	5	0	0	0	5	1
9	27	31	23	14	4	11
12	0	0	0	0	0	0

For Review Only

12	6	0	0	0	0	0
1	0	13	0	1	6	16
0	0	0	0	0	0	0
12	0	13	10	17	11	13
1	8	0	6	4	5	4
15	19	28	6	24	23	16
0	1	0	0	0	1	1
6	4	6	9	0	0	0
1	0	2	4	0	0	0
45	46	61	61	43	28	23
0	53	0	0	0	0	0
691	599	1147	487	608	537	518
22	15	26	13	14	8	5
56	5	5	12	7	1	1
287	240	459	303	285	332	242
3	3	13	5	2	0	6
1	1	0	2	1	1	1
79	88	130	109	90	105	151
0	2	0	1	4	0	0
1	0	0	0	0	0	0
29	11	68	31	16	96	45
25	38	46	53	43	51	60
24	6	3	6	7	1	2
38	45	121	3	9	23	1
0	0	0	0	4	0	0
42	65	99	49	41	48	46
59	58	89	75	77	99	75
139	172	146	105	131	98	86
83	153	151	152	141	72	81
386	0	0	321	0	0	177
91	78	151	62	98	118	110
93	93	168	91	79	118	95
78	83	135	67	82	109	81
91	108	179	97	128	155	190
53	23	82	8	15	2	3
0	0	0	2	0	7	0
0	0	3	0	0	3	3
23	16	55	24	18	15	35
10	28	52	28	30	68	28
5	4	15	11	0	9	10
35	45	65	40	48	67	20
55	152	137	111	125	112	70
46	62	75	62	51	38	43
0	14	0	0	5	0	0
0	0	0	0	0	0	27
0	0	2384	0	0	0	0
0	0	86	39	22	24	32
39	25	73	27	23	36	35
12	27	37	14	24	10	57
20	29	54	39	5	28	43

For Review Only

25	51	75	33	44	38	27
0	68	9	0	0	0	17
29	13	25	28	15	9	41
30	29	7	0	21	20	8
0	0	0	0	0	74	0
117	49	51	41	46	68	57
0	0	0	0	0	0	0
46	28	58	41	46	38	51
19	22	23	15	17	29	19
1	4	27	15	11	11	13
50	33	78	30	32	38	39
159	86	215	88	66	111	163
0	4	0	1	7	1	1
1	0	0	0	0	11	70
18	9	34	17	21	17	10
66	82	77	61	43	78	62
3	0	0	0	0	0	11
33	34	57	55	60	31	30
33	46	80	43	34	46	30
37	8	66	5	7	18	31
75	37	88	57	57	93	93
3	56	62	17	12	14	34
91	0	75	322	0	0	0
35	31	58	43	28	42	37
0	0	0	0	0	0	1
27	12	61	55	21	35	29
4	2	4	2	1	22	0
41	51	27	20	28	27	33
11	37	15	3	7	16	7
231	190	369	132	194	210	110
7	0	0	0	6	0	0
1	5	10	7	15	7	1
20	20	35	28	21	15	23
59	71	109	75	91	86	61
0	0	0	0	0	0	29
67	49	60	33	56	40	46
31	15	35	24	25	56	27
31	10	72	35	8	38	27
72	99	158	92	82	100	78
57	62	65	38	49	42	51
131	54	207	87	105	152	200
50	47	95	39	48	42	85
30	39	46	54	61	15	33
15	37	0	4	14	23	0
62	71	111	42	64	53	70
30	19	20	21	17	13	20
17	39	39	25	20	32	25
36	0	52	41	24	25	30
0	0	1	8	0	0	0
28	18	51	50	31	15	22

For Review Only

54	58	46	28	27	28	36
66	74	52	39	58	40	46
43	48	76	40	46	36	56
40	45	0	27	35	45	48
28	23	45	22	36	24	24
4	33	0	1	2	1	10
44	48	58	36	25	35	23
0	0	42	0	0	0	0
42	17	80	56	6	68	22
50	50	0	57	46	65	40
31	50	80	37	73	39	54
56	0	1	0	0	13	0
37	51	75	46	47	47	48
8	8	0	1	1	2	12
36	59	60	0	59	47	0
0	41	0	0	0	0	0
49	69	57	44	42	66	64
57	43	59	49	21	57	35
27	33	50	39	0	39	25
29	22	7	0	0	27	0
29	44	54	32	25	44	20
0	0	1	0	0	0	0
51	64	117	55	70	73	77
1	1	0	1	1	1	1
23	39	0	0	50	34	47
0	0	9	0	0	0	0
19	29	33	22	22	23	30
0	27	8	51	0	0	0
0	0	0	0	0	0	0
98	114	121	64	92	74	79
58	43	42	40	33	59	44
2	78	51	21	94	9	7
1	4	138	21	1	27	238
0	13	0	0	19	0	0
37	8	0	12	7	17	59
12	0	0	5	12	0	13
2	250	93	8	3	43	4
0	0	1	28	0	0	0
32	45	0	0	0	0	41
90	0	179	0	0	0	90
9	66	49	14	22	7	90
0	0	7	0	0	0	33
0	67	0	27	78	0	0
0	9	0	2	5	3	13
39	92	107	36	48	70	65
64	42	68	43	57	41	33
19	25	34	10	14	23	26
56	51	88	67	44	71	54
13	74	49	32	39	41	20
31	35	57	39	46	38	56

For Review Only

0	0	0	29	0	0	0
32	58	83	48	59	64	57
14	17	21	15	8	21	12
27	32	52	32	34	34	34
31	39	54	34	30	48	55
77	59	103	42	24	70	53
34	66	44	18	59	56	46
7	14	14	16	21	20	13
50	79	117	62	54	54	70
13	17	29	14	20	17	10
0	0	11	0	2	2	6
8	0	0	0	0	0	0
1	0	4	3	0	0	2
165	120	0	100	180	117	128
15	18	20	26	29	33	51
152	117	189	185	192	160	141
0	0	3	0	0	0	0
1	2	33	3	15	10	10
33	32	67	13	22	42	62
71	44	53	70	41	42	30
49	46	85	51	88	58	35
0	0	39	23	15	23	21
8	0	0	11	11	12	0
77	72	132	129	35	66	63
19	17	34	10	20	16	17
0	0	1	93	0	0	0
61	99	138	49	45	99	64
63	62	135	99	79	91	68
3	4	4	0	6	6	11
0	1	0	0	0	0	0
58	49	133	50	68	79	71
1119	805	1856	1024	1205	990	670
10	0	20	0	7	0	3
43	32	60	40	33	60	51
0	10	8	2	0	3	5
144	152	233	180	209	168	112
0	0	0	0	0	0	2
8	0	6	0	0	0	1
1	6	0	2	2	2	1
3	1	0	2	0	0	0
0	1	0	0	0	2	0
0	0	3	0	0	0	0
54	0	29	49	0	77	0
0	0	0	0	0	0	0
24	20	36	13	21	26	20
61	0	19	0	0	0	68
52	49	113	56	50	66	56
59	77	99	80	50	31	47
0	2	0	2	2	0	3
13	0	23	9	16	5	9

For Review Only

66	26	22	0	0	0	71
67	143	231	136	103	90	98
7	4	10	13	4	6	5
524	864	500	271	398	399	443
10	11	16	11	12	8	5
6	6	8	6	9	2	5
0	0	0	0	1	0	0
42	70	75	76	42	60	31
21	34	51	38	31	26	26
31	43	0	41	36	60	29
48	54	74	52	54	60	71
26	30	63	19	27	31	42
8	11	21	9	1	6	14
24	16	25	10	17	33	9
190	204	400	257	229	228	177
19	13	71	38	61	7	7
0	7	24	25	29	34	22
34	63	43	48	74	41	21
17	6	15	13	23	0	10
30	22	56	37	33	31	41
1	0	0	1	1	1	0
139	66	74	87	46	69	51
0	0	0	47	0	0	0
32	46	67	46	37	30	31
69	64	88	47	49	59	78
50	44	106	33	92	49	46
92	118	161	101	100	107	99
0	0	0	0	0	0	0
9	21	33	50	9	25	3
16	21	58	9	16	30	22
28	62	148	38	40	24	16
18	4	18	15	12	6	12
74	98	165	36	62	67	61
57	45	120	61	22	56	42
166	193	325	202	176	195	177
215	212	212	144	133	199	129
173	113	162	137	83	115	105
351	215	791	391	398	368	92
4	2	1	4	3	0	0
45	49	76	32	34	61	34
76	16	124	85	59	73	20
105	94	177	108	122	80	87
18	24	145	62	117	107	19
230	222	409	283	267	336	208
163	116	204	111	102	119	144
22	4	33	14	12	24	13
1	12	17	6	2	20	16
25	9	71	37	41	41	34
69	116	161	95	104	118	117
1	1	0	1	0	0	5

For Review Only

12	31	20	36	21	14	14
2	1	0	1	11	1	7
33	181	27	109	56	41	105
0	3	14	9	6	15	4
52	59	92	41	57	48	54
38	35	44	51	45	41	37
5	2	0	1	0	1	1
0	0	5	0	1	0	0
3	0	0	1	1	1	6
13	3	13	22	5	6	7
53	77	112	35	87	18	48
68	85	100	70	95	64	72
41	25	27	63	44	39	12
0	0	0	0	0	0	10
0	0	0	0	0	1	3
39	49	76	96	43	78	39
0	7	0	4	0	0	0
44	1	0	1	1	1	1
293	335	406	297	277	357	206
11	7	25	28	14	20	12
21	16	20	10	17	7	13
23	1	0	25	1	1	1
17	14	41	30	5	4	10
35	25	44	33	13	14	20
86	45	188	19	76	88	54
1	1	0	0	1	1	1
75	90	90	56	60	69	55
6	0	0	2	1	0	15
3	0	14	0	0	7	11
384	328	663	359	350	398	260
40	48	46	68	38	61	28
189	164	314	246	196	89	152
1	3	5	1	0	3	3
0	0	0	0	0	0	0
0	0	1	0	0	10	0
55	0	229	135	169	79	47
0	0	7	0	0	0	0
440	549	920	433	432	534	437
19	22	44	10	26	13	13
0	0	0	0	0	0	0
91	197	216	73	45	41	94
34	40	84	54	44	23	45
80	107	82	49	71	81	69
3	8	47	29	18	2	10
5	1	0	2	1	3	0
271	392	396	267	138	285	189
213	193	288	237	197	238	289
255	272	408	252	252	292	291
0	0	9	0	0	0	0
0	0	0	0	7	0	2

For Review Only

0	0	0	0	0	0	27
667	586	1242	593	665	685	424
0	0	0	0	0	0	10
54	58	118	55	70	122	45
1	0	14	0	3	0	17
1	4	8	9	5	0	7
242	191	403	282	187	214	205
77	95	91	76	67	58	38
11	22	53	25	3	7	1
68	130	195	150	112	85	152
132	140	226	128	130	140	164
19	3	33	8	11	3	16
48	60	67	36	43	41	36
421	555	1073	565	565	599	392
115	37	92	44	63	155	199
0	0	0	0	0	7	0
31	16	12	10	10	7	30
0	0	0	0	2	0	0
20	37	18	19	23	31	21
181	288	309	273	155	166	63
75	142	164	97	157	82	97
261	254	309	263	272	208	209
0	0	0	1	0	1	0
0	0	0	0	5	0	10
20	21	17	18	12	9	10
0	0	0	4	2	0	4
0	10	5	3	0	0	0
0	4	6	4	2	4	5
0	2	0	1	0	0	0
264	222	477	297	228	300	196
0	0	0	0	1	0	0
4	9	9	6	18	12	11
59	50	91	46	46	56	39
0	0	1	0	0	148	0
124	109	108	86	81	140	91
17	18	13	9	14	16	8
119	144	202	179	120	193	120
140	161	269	187	133	157	126
1	0	20	3	3	3	27
18	13	30	13	21	18	17
53	38	70	42	30	48	28
46	107	115	39	60	63	69
62	74	84	48	53	94	58
1	2	222	18	17	1	66
93	92	169	93	78	151	64
23	35	21	26	7	18	42
8	131	163	0	0	0	0
0	0	0	0	0	0	0
132	22	96	18	47	13	19
54	50	66	29	49	58	38

For Review Only

49	232	156	246	275	0	26
92	81	139	89	109	65	87
67	67	52	88	57	54	91
2	2	0	0	0	0	2
15	15	25	8	19	25	33
21	9	21	23	11	9	19
8	5	8	9	11	27	37
0	0	8	0	9	0	0
114	146	248	122	99	143	130
0	3	1	6	0	0	0
39	46	54	28	21	45	2
136	137	244	120	104	135	101
0	0	537	244	111	195	116
16	19	28	10	6	22	18
0	27	40	32	28	29	0
62	73	77	71	0	71	53
4	8	7	1	2	5	2
292	325	442	283	238	292	167
1	23	0	165	1	1	1
477	24	1338	119	108	2	1
402	340	185	475	411	499	284
43	27	57	57	34	50	22
33	31	44	33	30	25	24
221	127	381	258	209	235	216
262	164	391	253	228	245	172
28	113	72	44	0	158	103
64	95	86	84	74	53	102
3	8	9	0	10	10	1
22	29	24	18	13	23	13
54	82	38	37	34	22	32
30	24	63	45	39	70	70
233	225	295	179	226	227	185
0	0	1	428	276	63	0
25	8	35	6	9	29	8
1	2	4	7	1	1	14
97	71	175	35	38	103	64
7	15	0	0	0	14	10
240	140	257	111	74	274	77
0	0	0	6	0	93	0
139	171	1	203	0	143	0
40	31	32	20	30	22	26
18	24	28	21	12	15	17
5	10	18	6	4	9	14
110	100	98	15	34	23	5
0	3	3	3	1	0	0
28	24	47	22	18	23	23
43	77	81	60	45	69	68
6	8	12	3	3	11	21
12	1	0	6	2	5	6
12	47	58	23	52	25	22

For Review Only

4	0	0	0	0	0	0
140	150	192	120	111	179	75
6	16	0	10	17	23	24
401	370	650	350	426	191	178
878	834	1149	602	701	846	568
63	66	172	46	134	56	83
51	50	55	57	49	52	52
71	76	184	59	87	142	82
131	156	324	139	274	102	82
0	7	5	5	12	4	18
56	20	51	17	59	51	3
178	325	476	233	188	262	218
3	1	6	0	0	0	0
0	1	0	2	0	0	1
10	2	0	0	14	0	14
52	44	95	90	55	18	70
3	10	0	7	10	1	0
40	88	95	65	79	63	51
75	0	29	0	0	0	37
66	70	140	96	84	97	74
73	116	82	64	55	65	42
127	142	171	140	123	158	80
42	47	57	30	28	68	15
34	31	69	61	44	63	35
484	263	506	1733	533	912	368
260	368	509	294	341	382	244
64	25	30	0	0	65	124
128	110	114	68	84	135	96
1	0	20	3	0	0	18
1382	1545	3727	1274	0	1726	862
1	0	1	0	1	0	0
395	435	411	199	142	81	6
0	0	0	0	0	0	0
0	0	0	6	0	0	0
105	114	308	121	115	154	56
0	0	5	3	2	0	0
35	39	70	13	31	19	42
180	188	246	145	108	126	115
735	697	1104	587	663	774	554
104	98	202	97	81	90	68
23	39	54	52	54	24	30
96	108	124	69	66	63	100
8	1	0	4	7	3	11
254	292	273	225	152	246	197
193	167	227	95	81	161	105
78	90	181	106	111	68	88
222	271	205	245	102	302	162
2140	3021	5422	3378	3483	2708	2868
183	256	331	217	232	218	272
11	45	59	52	109	35	152

For Review Only

5	5	8	5	5	5	8
598	779	1317	789	859	789	772
1132	1531	3473	2235	2308	1389	1558
19	4	34	35	38	33	8
109	123	186	79	74	73	90
103	170	216	123	97	144	115
397	410	609	448	423	523	295
120	105	158	84	111	98	87
0	0	0	0	0	0	26
19	39	37	16	22	7	23
26	9	1	0	0	0	4
0	0	0	0	0	0	30
6	5	33	10	3	2	14
229	139	303	190	180	223	160
0	0	0	0	0	3	0
87	81	124	84	68	56	49
86	68	0	21	58	0	8
0	66	43	48	58	0	67
385	72	328	14	99	68	1
10	8	0	1	1	3	41
111	1	0	1	1	1	4
91	137	196	85	129	115	165
110	87	0	0	126	0	134
0	80	13	0	0	0	75
86	186	204	129	142	161	178
0	46	64	50	29	55	34
6	36	45	0	71	43	8
93	112	99	10	100	1	13
74	120	124	59	76	105	148
106	213	169	180	107	157	190
46	0	10	30	40	39	35
35	34	31	19	25	33	18
16	8	15	15	0	16	11
39	41	47	53	35	39	38
0	9	25	14	9	0	9
0	8	7	11	7	0	7
0	0	0	44	29	8	41
13	410	225	1	1	23	16
26	89	0	2	2	25	68
0	27	8	1	28	30	0
51	69	123	41	61	83	81
19	0	35	0	0	0	0
54	93	91	65	60	90	86
14	13	73	20	1	58	225
58	69	57	54	54	0	53
61	213	112	76	52	76	76
0	231	1	0	0	0	0
0	0	0	13	0	0	0
72	91	163	89	98	84	120
135	125	220	122	116	110	120

For Review Only

120	187	159	22	200	215	155
0	0	1	0	0	66	0
56	61	99	75	72	71	60
21	19	33	46	27	22	46
0	0	34	0	0	0	0
121	139	162	121	124	131	79
123	155	0	171	163	162	140
0	86	0	0	0	0	0
5	125	0	75	102	62	15
56	66	104	67	70	81	51
183	125	426	250	295	219	255
5	0	7	0	0	0	0
0	0	0	0	0	0	11
0	0	6	0	0	0	0
2	0	5	0	3	2	5
57	83	149	57	90	76	98
315	372	472	325	317	343	213
62	87	157	84	56	61	41
0	0	4	0	0	0	3
139	114	220	110	94	141	84
24	20	81	12	35	24	16
0	0	10	1	1	2	21
28	25	59	46	31	30	24
82	114	123	115	86	106	104
3	0	0	2	1	4	13
0	3	0	0	0	3	1
23	25	52	28	22	26	18
0	0	0	3	4	0	0
0	0	19	0	0	0	0
8	20	43	23	25	24	15
0	5	0	3	0	2	3
20	11	22	23	5	9	19
21	27	29	40	29	48	29
0	9	0	0	3	0	0
434	489	848	466	491	452	363
119	138	227	114	118	107	147
12	17	27	0	8	17	10
84	103	223	101	155	97	117
0	0	130	0	0	61	0
16	27	0	24	27	46	4
70	78	64	79	0	75	94
21	53	31	0	32	21	30
66	114	136	118	112	95	113
29	12	51	15	6	18	28
4	6	0	0	9	2	6
24	34	15	42	52	19	55
18	4	18	2	1	1	13
0	0	1	0	0	0	11
0	0	0	1	2	0	0
0	0	0	0	0	0	0

For Review Only

5	11	14	5	10	6	5
139	123	210	170	115	179	105
2	14	21	7	2	2	6
12	5	11	5	5	5	6
0	0	11	3	1	0	0
0	86	92	85	80	99	82
4	4	0	5	2	0	0
170	177	230	147	179	116	124
90	63	125	127	101	83	86
880	711	1351	1069	750	1110	733
0	1	0	0	0	0	1
183	114	135	57	105	164	152
481	505	770	481	536	415	260
194	175	284	122	217	190	158
4	1	0	0	1	2	0
72	114	198	71	79	99	97
1	1	0	1	0	3	1
93	141	259	65	57	151	152
426	363	903	365	479	692	603
0	1	6	0	2	3	8
176	175	305	135	255	249	242
0	0	0	0	0	0	11
231	128	303	165	162	168	125
12	5	19	1	11	13	8
27	38	44	29	29	34	22
256	279	453	236	296	311	193
67	79	102	75	55	39	54
309	302	523	225	259	316	269
26	0	14	62	0	0	52
0	52	2	0	0	0	0
15	1	176	4	79	33	1
21	29	58	23	25	26	18
51	62	152	51	83	91	70
76	72	153	56	70	87	67
88	62	123	59	100	87	62
58	69	138	74	55	69	82
220	191	325	169	209	223	134
26	46	25	10	27	22	49
61	85	85	59	29	65	54
27	24	29	15	24	36	23
68	55	80	56	50	56	36
55	91	0	0	73	47	67
4	6	16	3	8	8	7
59	96	148	94	100	78	79
36	14	0	3	0	47	32
121	75	134	124	96	88	77
13	6	19	12	8	12	9
0	1	3	0	3	12	9
98	105	224	98	117	98	131
48	37	81	68	46	39	46

For Review Only

73	111	91	78	64	75	54
69	83	90	46	67	57	100
1	6	0	11	5	1	5
0	2	0	0	0	2	5
34	110	104	93	113	84	89
0	50	1	48	0	45	0
0	0	13	0	0	0	16
51	25	151	69	197	41	16
41	41	79	27	34	30	26
14	26	0	2	89	1	200
0	2	3	0	0	1	0
16	50	42	21	13	43	21
334	4	379	8	33	1	7
21	41	41	20	28	46	16
1	4	0	1	1	1	1
0	15	0	4	1	3	10
107	89	140	88	81	110	61
30	54	68	40	47	48	39
215	228	248	221	198	270	184
12	19	26	9	24	19	12
83	61	136	73	79	74	73
84	59	1812	37	1	1	18
0	0	0	7	0	0	0
14	53	40	32	34	37	23
48	43	70	36	26	54	38
1	1	3	8	6	2	5
0	2	7	1	2	2	5
9	23	0	1	36	20	1
8	153	0	1	1	4	5
4	3	0	7	5	3	1
163	138	116	83	99	78	111
14	48	28	14	14	8	0
3	10	11	14	8	10	11
29	55	44	23	51	29	25
45	31	106	42	79	51	30
11	24	33	9	4	4	2
2	0	0	1	1	0	5
5	23	31	17	15	21	20
59	53	109	52	34	16	37
375	106	70	56	199	58	159
33	22	0	30	33	33	5
125	55	141	194	165	88	31
39	26	96	68	36	21	25
5	24	36	9	4	8	3
65	79	126	95	86	63	63
68	77	92	69	63	91	61
68	113	113	75	66	78	90
5	4	5	6	0	15	7
29	18	28	20	22	38	12
2	17	49	17	0	8	8

For Review Only

50	7	108	7	1	1	4
5	3	12	9	8	9	5
3	28	39	10	14	19	21
63	43	0	1	60	16	7
2	20	0	5	2	20	5
136	214	200	128	150	163	132
0	161	0	3	3	15	23
21	17	20	17	14	8	25
55	75	162	67	88	104	65
52	46	30	25	17	44	26
17	16	12	0	10	16	16
20	14	5	31	9	12	0
19	5	0	13	18	8	10
21	5	0	152	24	23	2
1	6	12	2	0	0	4
93	57	158	51	33	57	73
7	9	17	19	15	0	7
19	24	21	24	15	10	16
101	68	144	48	58	79	61
23	34	43	38	19	25	27
78	82	164	92	89	72	77
73	112	129	101	53	40	42
0	66	0	0	0	0	0
26	21	28	22	17	32	26
43	46	69	23	44	32	37
91	113	173	77	113	79	73
52	54	58	35	39	66	47
32	14	94	36	12	41	53
112	129	882	135	141	174	136
20	88	141	1	1	13	201
45	0	46	58	60	38	47
66	88	92	51	58	71	38
13	31	0	35	0	21	0
103	0	0	1	0	0	33
78	46	123	62	64	56	67
36	22	45	25	23	31	28
0	0	0	0	0	0	4
102	140	202	101	123	93	117
3	0	0	0	3	0	0
0	5	0	0	0	0	21
0	0	67	0	124	45	0
0	0	0	0	0	3	0
268	278	369	210	271	268	199
613	697	951	459	701	511	536
22	24	29	19	13	29	28
0	0	2	0	0	6	4
24	43	45	50	10	22	23
0	0	7	5	0	0	5
34	22	14	19	21	21	32
38	32	87	75	59	27	33

For Review Only

327	414	646	289	391	385	218
872	1056	1579	864	879	1071	888
8	9	6	6	5	0	3
0	0	0	0	0	0	9
0	3	0	0	65	0	0
73	104	100	83	57	96	100
0	12	0	2	0	0	0
0	0	16	4	7	5	7
5	7	6	32	0	5	9
63	88	110	48	117	75	47
64	71	118	44	67	59	66
147	184	202	88	145	154	144
6	1	8	4	0	0	0
0	0	0	0	0	0	4
4	0	2	0	1	0	5
125	118	219	106	104	117	48
0	0	0	0	0	0	10
12	0	14	0	14	43	0
0	50	62	49	57	64	50
284	386	669	243	245	231	256
1	2	0	1	0	3	2
30	18	58	22	22	34	41
0	0	5	0	0	0	0
13	13	17	19	3	9	17
26	95	116	24	43	71	47
0	0	0	10	0	0	0
22	38	36	27	32	31	26
72	53	160	73	97	101	70
15	5	9	21	8	26	19
1	0	0	1	0	0	9
53	36	35	18	21	42	24
114	435	375	215	237	280	305
40	26	65	23	39	48	39
3	5	15	4	0	3	33
0	0	2	0	0	0	0
1	0	7	0	0	0	0
4	23	0	3	15	6	11
1	1	0	2	2	3	17
5	5	9	13	18	10	5
167	0	0	212	167	1	0
19	39	48	22	22	33	25
0	1	0	0	3	3	0
0	119	6	0	100	106	0
31	33	49	67	52	34	28
46	42	106	62	54	66	70
165	148	251	70	126	129	62
0	0	4	0	0	0	0
0	6	3	10	0	5	0
2	1	0	2	1	1	1
612	1032	1607	725	672	981	593

For Review Only

0	0	11	6	0	2	1
0	0	7	0	2	0	4
93	242	75	0	0	0	197
0	7	3	0	0	0	4
28	37	41	25	10	31	19
17	11	9	10	9	2	6
388	469	770	461	479	469	351
90	64	139	59	68	88	63
656	743	1087	771	815	544	553
2	0	3	0	5	0	0
33	32	47	34	49	30	28
173	243	275	141	200	284	379
35	29	82	36	9	33	33
51	65	132	69	66	92	39
72	90	124	76	74	90	86
0	0	2	0	4	0	5
0	0	2	0	0	0	0
1	15	25	16	27	10	2
63	69	274	145	104	145	130
57	77	42	69	66	61	0
38	32	69	35	46	63	41
0	40	12	0	9	16	26
38	59	106	45	37	36	48
39	32	52	42	63	56	52
64	98	125	61	45	52	59
6	6	87	44	74	9	30
45	78	71	81	50	58	44
22	14	41	0	0	0	41
38	26	75	35	37	44	24
0	0	0	0	1	4	3
66	151	151	107	81	122	77
30	53	50	19	48	48	33
17	21	38	19	9	34	43
33	11	67	25	62	7	27
12	10	14	3	6	6	0
3	13	18	4	5	3	13
32	47	63	20	41	42	44
1	1	0	1	1	4	1
11	4	0	7	20	20	10
0	0	0	0	0	0	34
50	67	60	51	90	42	39
0	0	0	2	3	0	3
0	0	3	9	0	0	4
4	1	0	0	0	0	3
276	277	403	262	274	313	165
0	0	0	2	3	2	6
0	2	0	2	2	1	4
164	126	215	111	97	156	121
53	28	14	28	0	41	22
48	150	241	130	108	131	114

For Review Only

71	87	96	0	61	0	0
0	0	0	0	0	0	0
13	19	25	0	0	0	15
1106	393	680	200	63	77	21
16	21	36	26	57	10	19
53	74	83	115	42	65	78
92	110	118	55	68	121	82
31	26	41	18	17	33	22
11	15	9	17	8	34	27
156	210	369	240	185	188	138
0	5	13	0	11	6	4
1	1	35	1	1	7	1
49	32	30	40	43	46	33
0	25	1	0	32	0	1
2	1	6	5	2	7	0
0	0	0	144	0	0	0
0	0	0	0	0	0	0
34	62	94	35	39	21	35
45	42	50	27	32	27	50
23	6	23	9	24	21	5
16	20	39	17	21	19	31
28	35	69	21	37	34	20
142	180	227	138	118	130	135
27	55	48	57	39	49	27
96	79	117	53	85	81	48
15	12	30	28	12	7	13
0	10	50	22	7	0	18
29	70	72	33	47	65	76
0	0	2	0	0	0	0
40	56	79	35	32	37	26
237	182	444	252	276	261	252
579	590	799	290	320	734	310
460	306	808	327	350	535	284
1	3	17	1	4	1	1
38	0	84	0	49	44	35
85	59	143	43	73	94	69
80	83	135	39	53	67	80
15	9	101	13	20	16	24
77	31	116	49	42	62	47
118	51	154	72	68	51	80
46	17	23	17	44	55	20
23	22	50	60	4	24	27
10	7	32	4	11	25	13
198	187	249	224	206	159	119
12	25	35	19	23	29	22
0	0	15	0	11	0	0
10	10	18	7	11	12	26
53	84	113	111	48	94	61
45	39	50	51	44	41	28
78	105	115	85	35	83	62

For Review Only

0	45	109	32	37	40	30
206	222	311	201	180	173	145
25	0	52	27	32	42	33
126	129	217	116	110	95	135
50	22	133	72	79	39	32
36	60	84	68	63	49	31
0	3	73	14	0	2	0
0	1	0	2	0	0	0
46	61	48	28	34	32	42
0	0	13	0	1	8	4
57	52	70	25	30	38	40
23	38	48	25	32	31	31
17	28	36	20	22	23	24
68	90	164	82	130	97	91
0	0	0	1	0	0	0
0	0	0	0	0	4	0
1	1	0	1	0	0	0
0	0	0	2	1	0	4
1	4	19	3	4	14	1
0	0	0	0	0	0	18
0	0	0	0	5	0	0
44	51	37	34	42	21	38
9	4	123	88	104	110	29
20	13	25	10	13	8	21
21	30	48	24	13	32	26
1	1	0	3	1	1	4
15	3	0	31	3	14	12
8	4	3	0	0	8	0
11	0	0	0	0	5	11
2	1	11	13	1	6	5
11	12	12	7	0	6	7
0	0	0	0	0	3	0
1	1	0	0	0	4	1
12	8	19	11	6	15	38
48	46	92	52	80	99	54
325	0	18	0	378	0	0
278	443	590	414	322	385	329
0	0	0	0	82	0	0
27	27	73	34	56	65	22
0	0	3	0	0	0	0
0	0	0	0	5	4	0
42	17	0	7	5	6	8
591	702	1110	664	622	837	532
0	0	0	0	0	2	0
15	15	37	17	8	8	36
27	27	36	25	45	28	18
18	26	17	12	9	18	16
64	62	141	90	75	72	60
11	14	46	28	24	20	15
13	12	36	16	4	19	0

For Review Only

120	243	322	201	284	271	199
0	3	5	0	0	0	0
57	51	37	21	33	40	18
17	0	124	19	36	22	0
39	48	41	24	38	25	30
0	0	21	0	0	96	0
0	89	145	68	100	53	36
1	1	17	1	4	15	2
0	0	0	0	0	0	5
421	438	564	346	403	381	340
0	0	0	0	0	0	0
33	52	57	26	52	41	34
2	4	0	4	1	5	0
16	17	18	19	1	29	7
0	0	0	1	0	0	4
123	128	309	192	165	170	118
4921	5749	8768	5970	5813	4424	4511
0	1	0	0	0	0	0
13	2	11	2	7	4	8
7	5	1	5	4	6	2
1	0	0	0	3	0	1
22	19	26	11	18	19	7
38	57	38	46	67	17	20
0	0	0	2	2	0	0
0	0	0	1	1	0	1
0	0	0	0	0	0	0
0	0	0	0	0	0	0
2	0	0	1	0	0	0
0	1	9	0	0	3	1
1	0	3	0	0	0	0
0	0	3	0	0	0	0
0	0	0	0	0	0	0
0	0	0	0	0	3	1
0	0	11	0	0	0	0
3	0	0	1	0	1	0
1	1	0	1	0	3	2
0	1	0	1	8	1	3
1	2	0	2	3	0	0
1	0	13	1	2	4	0
258	207	439	284	214	56	102
1	0	17	1	2	1	0
0	5	2	0	3	1	10
105	52	94	64	60	57	95
72	102	149	81	52	105	46
53	19	103	219	208	32	59
29	0	63	49	41	0	18
1	1	0	1	0	0	1
1	0	0	1	0	5	0
3	1	4	0	0	0	0
0	0	0	2	0	0	4

For Review Only

12	24	37	19	11	7	22
2	6	0	2	1	10	18
19	131	58	47	56	36	32
255	221	413	307	201	229	145
261	196	342	187	253	258	182
33	38	201	26	103	28	40
7	8	16	3	14	11	5
81	43	86	49	57	80	69
113	144	126	85	145	81	121
0	0	161	99	72	130	39
62	113	131	60	33	80	49
7	35	33	25	5	27	8
0	0	0	0	0	0	0
2	1	7	12	8	2	7
95	114	161	137	85	99	146
3	19	12	0	0	0	1
80	120	97	107	76	85	50
218	175	116	226	74	60	72
25	58	23	18	20	32	42
143	203	264	76	168	177	120
63	38	92	51	64	46	53
77	91	168	59	123	148	73
27	22	39	12	14	21	24
5	6	15	0	11	4	2
27	30	74	64	44	75	29
15	33	24	23	43	24	39
116	117	188	135	166	146	212
88	73	103	57	72	119	92
0	0	0	0	0	0	20
27	26	44	37	20	18	14
16	11	27	12	22	11	6
36	39	47	37	36	54	24
0	46	77	10	55	16	9
34	64	55	45	51	19	67
169	177	247	154	169	213	216
266	403	515	423	368	344	300
130	187	294	220	228	301	154
0	0	0	0	0	0	0
0	0	8	0	0	0	0
230	3	875	358	1	18	1
16	12	12	13	19	1	13
8	18	17	28	23	10	15
5	15	9	0	6	4	3
61	101	70	43	69	71	57
17	0	20	43	10	55	43
49	13	112	87	76	14	6
1	0	6	2	2	3	0
99	58	65	67	39	68	43
33	43	0	16	26	24	25
0	0	0	0	0	0	0

For Review Only

0	0	0	0	61	0	0
1	1	23	1	1	0	1
0	0	1	1	0	0	4
124	95	144	89	89	130	84
358	507	828	431	407	475	344
11	1	116	1	64	1	1
16	15	13	11	10	9	8
134	159	153	119	136	125	87
40	78	96	55	82	63	44
29	34	49	18	40	47	31
25	23	23	17	14	32	5
1	0	5	0	3	4	3
0	0	0	0	0	0	0
0	0	0	9	6	1	2
625	600	1170	443	638	680	704
0	0	0	0	0	0	11
0	0	0	2	2	0	1
3557	3030	7727	4417	3971	4005	3201
2474	2769	5397	4308	4499	2308	2190
0	0	0	0	3	0	6
923	944	1426	943	931	978	732
6	9	35	10	9	6	4
89	177	187	46	94	165	93
15	8	20	15	23	36	28
2	4	0	0	0	9	8
128	195	26	105	0	0	209
1061	1279	2191	1248	1213	1224	1031
194	269	306	3	6	83	155
0	24	10	0	0	0	0
15	11	19	22	12	14	7
0	0	0	0	0	0	2
0	0	0	0	0	0	0
3	7	9	0	9	3	0
53	110	138	75	115	102	92
96	154	149	123	109	138	131
9	0	0	12	0	0	0
389	403	601	382	405	418	324
0	0	0	0	0	0	8
197	125	237	262	125	304	108
0	0	0	0	0	0	0
43	102	99	79	91	98	40
87	188	234	67	105	96	129
149	145	310	183	136	161	148
0	0	6	28	0	0	0
10	29	36	4	16	6	12
1000	634	2536	1	2	449	386
141	60	134	92	150	150	91
61	44	44	21	65	51	49
327	313	507	293	325	434	309
13	19	15	22	27	17	3

For Review Only

131	149	299	113	121	219	145
0	0	99	0	0	0	0
271	255	559	201	210	291	112
67	34	105	55	39	32	40
0	0	0	0	0	0	0
0	0	0	0	2	0	47
14	12	22	10	25	11	24
95	232	45	7	90	131	49
5	0	5	0	0	8	7
0	0	0	0	0	0	10
0	0	0	0	0	0	4
0	0	0	0	0	3	15
1	1	42	1	1	0	0
61	97	111	110	59	76	67
325	422	668	244	312	471	347
3	3	7	4	3	15	6
53	44	65	22	56	30	62
15	68	96	60	43	47	70
54	71	224	77	72	72	50
10	25	11	18	10	10	21
24	27	50	21	30	45	17
140	105	187	120	146	69	109
33	60	78	41	23	51	43
30	37	27	10	12	19	16
3	24	44	24	26	26	11
204	239	413	260	221	282	176
4	2	1	3	4	1	4
37	48	90	15	53	36	46
0	0	2	0	0	0	1
0	0	0	0	3	0	1
126	174	235	159	185	160	187
0	0	0	0	0	1	4
12	4	11	4	19	2	18
17	26	42	24	16	21	20
0	0	0	0	3	0	19
235	234	348	269	270	195	191
9	1	11	2	1	4	13
43	10	13	5	17	17	10
69	55	96	43	34	36	41
0	0	1	2	2	0	0
9	16	17	10	13	14	7
0	0	0	3	0	0	4
55	76	57	44	29	52	39
124	44	188	137	88	80	45
5	55	0	3	1	2	278
39	35	82	37	43	54	55
70	37	133	110	95	59	65
0	0	398	425	0	169	0
14	7	33	30	42	14	34
38	50	86	70	45	64	31

For Review Only

54	42	77	31	46	67	25
99	117	162	89	72	111	94
65	60	70	66	31	38	39
0	39	62	0	0	94	0
1	18	150	1	22	123	73
46	36	110	37	41	61	35
31	40	63	37	30	25	37
104	130	146	98	131	151	90
1	1	0	1	1	996	1
34	21	60	26	24	10	24
9	14	37	10	17	22	5
0	0	0	0	0	1	7
9	13	32	11	4	5	12
541	592	1066	653	574	608	371
0	4	0	8	3	8	3
54	64	70	27	42	55	44
0	7	0	0	5	0	0
83	96	99	39	48	40	76
0	0	0	0	1	0	1
59	60	89	66	60	70	53
65	30	62	37	50	17	17
17	18	24	21	15	15	22
0	0	0	0	0	10	0
3	2	0	2	3	0	0
97	146	180	113	87	66	140
14	29	21	17	19	9	20
16	24	35	24	11	21	24
46	8	30	15	12	13	24
83	25	129	67	39	53	0
0	0	1	9	0	0	0
231	270	258	174	142	243	77
47	188	75	103	108	89	25
0	0	0	4	0	9	0
2	0	0	0	0	0	5
43	26	44	52	58	54	43
46	66	56	27	24	26	27
0	0	8	0	0	0	3
1	0	0	1	0	0	1
2	0	0	0	0	1	0
0	0	0	0	3	0	1
10	3	0	11	8	5	5
11	12	48	20	22	26	11
11	8	5	4	0	2	1
20	22	56	5	13	3	27
25	20	40	28	36	40	15
15	49	36	26	28	31	7
11	12	20	9	16	21	19
3	34	59	18	6	21	11
36	31	81	54	42	70	45
10	1	24	9	2	5	12

For Review Only

39	12	43	25	19	29	6
10	10	19	27	2	19	11
27	39	33	17	27	23	14
29	16	29	28	18	12	28
22	36	79	58	79	52	14
14	28	50	58	30	45	25
4	15	1	1	9	9	5
12	10	16	20	4	17	6
38	15	40	13	28	14	7
21	8	13	14	13	13	1
0	1	0	1	0	0	5
2	4	5	17	12	1	2
6	4	0	8	2	0	1
50	47	112	62	57	44	71
6	25	49	11	21	27	9
61	69	195	91	72	112	136
0	0	0	6	10	0	5
29	20	38	23	35	28	10
7	19	33	9	10	13	18
6	11	23	18	16	7	10
115	123	213	145	100	179	78
25	114	71	120	22	2	5
0	0	0	1	0	0	0
84	111	127	64	44	65	60
85	140	218	164	134	134	96
48	83	166	102	98	59	87
50	68	159	82	95	62	87
0	0	11	0	0	0	0
97	105	219	110	88	120	116
118	126	205	67	123	106	124
114	86	87	77	84	104	67
28	18	41	23	15	19	26
109	104	288	83	229	199	80
33	18	28	27	16	38	23
3	3	6	4	0	5	41
0	0	0	0	0	0	0
5	0	0	0	7	5	3
3	3	24	0	3	1	49
0	7	10	4	7	0	3
81	86	143	87	52	113	76
10	6	8	12	2	1	1
52	43	62	37	41	54	26
21	6	56	5	25	7	14
220	165	474	218	261	283	168
48	39	80	53	42	65	45
107	104	131	111	173	79	26
25	43	36	50	46	38	54
29	15	20	25	16	30	9
94	92	101	38	56	52	61
58	63	71	43	44	69	46

For Review Only

0	25	27	16	0	28	0
33	121	0	7	10	2	41
59	69	154	32	60	68	49
44	31	56	30	51	63	34
60	105	114	86	68	118	94
354	293	470	291	264	324	214
19	31	22	11	3	15	16
73	52	94	46	46	59	81
0	60	42	60	66	63	58
79	92	119	59	55	90	83
0	13	0	6	7	1	1
55	19	73	25	19	61	20
0	0	0	2	0	3	9
73	69	62	71	75	59	73
0	1	0	0	0	0	0
127	203	361	196	186	153	168
0	0	0	0	3	1	1
527	723	1069	683	720	556	563
24	23	30	47	40	42	18
84	105	144	33	55	113	90
64	67	120	66	81	67	64
171	140	309	163	200	205	185
20	26	61	24	20	24	16
0	1	0	0	1	0	0
6	29	28	11	9	8	32
3	0	0	0	0	0	0
0	0	3	0	0	0	0
0	0	29	0	0	0	0
45	52	45	37	29	31	24
25	32	42	50	16	73	29
0	4	0	0	0	0	0
1	0	7	0	0	0	4
0	0	0	1	0	0	9
2	0	0	0	1	0	7
56	100	164	68	105	65	118
116	131	153	106	112	130	92
34	51	69	41	49	59	37
1543	1226	2408	1515	1465	1535	1519
580	482	929	1	25	0	525
35	0	2	0	0	0	51
633	493	1035	502	618	566	609
51	60	88	58	52	54	62
209	249	430	145	260	191	199
40	30	107	51	67	65	15
31	51	69	42	43	29	51
50	25	67	44	30	26	36
82	59	108	64	70	0	30
79	105	147	76	65	90	63
20	15	35	9	24	15	18
119	57	186	173	103	116	60

For Review Only

0	4	0	12	32	13	4
0	0	0	0	0	0	0
52	53	116	71	64	53	62
33	53	0	0	0	0	0
106	82	158	68	76	84	87
50	65	96	32	46	51	56
27	27	0	10	15	1	2
44	18	35	27	27	28	30
163	143	204	167	135	116	129
51	19	127	33	29	55	145
3	1	9	0	2	5	2
0	0	0	0	0	0	0
49	56	1	84	0	0	0
9	9	7	4	3	5	14
0	0	5	0	0	0	0
7	4	17	23	1	14	2
0	0	0	0	2	1	0
87	114	162	80	140	119	92
1	0	11	7	0	1	1
2	2	0	0	4	0	2
469	417	804	72	121	0	280
170	90	377	174	225	203	160
8	35	36	12	12	32	9
24	42	155	64	61	59	59
86	86	138	89	80	93	94
58	92	145	81	53	69	107
57	72	121	81	99	97	62
79	98	150	110	78	91	96
8	1	12	4	3	7	2
7	16	5	7	18	4	2
76	62	175	61	97	88	17
0	0	11	1	1	2	3
99	211	278	140	146	113	156
77	139	114	65	58	92	51
7	11	10	11	3	2	14
0	5	0	3	0	6	3
41	34	64	19	18	36	17
0	0	7	0	0	0	0
47	87	90	44	69	62	26
160	174	378	230	270	236	221
1	92	186	1	1	1	1
14	9	25	31	18	17	4
145	181	234	130	100	132	121
1	30	22	17	5	30	1
3	3	14	2	4	7	3
123	137	164	144	129	58	111
0	0	3	0	0	0	35
23	8	30	52	41	56	17
13	0	0	4	0	0	0
15	33	20	17	13	16	22

For Review Only

37	90	320	1	1	21	63
45	18	52	55	25	17	24
27	25	59	37	74	45	23
82	96	148	120	87	78	64
95	95	150	116	118	105	55
5	0	67	5	8	0	7
0	0	0	0	0	0	75
132	189	257	172	230	139	122
20	14	16	13	9	15	20
53	78	101	74	72	61	88
88	81	137	49	106	115	72
52	81	6	61	58	0	0
69	71	83	47	56	52	47
0	0	0	0	0	0	0
0	217	1	0	0	0	0
51	23	59	39	38	42	51
20	40	63	33	48	46	30
0	0	7	8	5	0	2
0	0	0	0	144	0	0
30	1	0	1	17	4	1
55	49	69	30	27	37	54
314	308	471	431	240	306	233
197	24	376	156	167	155	0
150	196	378	222	195	210	147
30	6	0	19	9	5	17
0	7	0	0	2	3	3
656	722	854	499	553	612	575
16	6	25	11	14	12	10
1	0	3	5	3	1	1
95	164	211	88	112	143	113
2	0	5	4	0	0	0
74	91	204	88	95	107	106
2	94	0	2	1	4	2
84	3	125	5	81	87	34
0	1	6	10	5	0	7
6	10	21	8	13	6	10
159	187	0	184	245	205	114
132	111	173	135	160	113	81
53	34	61	35	59	51	37
124	4	203	29	3	83	6
0	6	4	4	1	13	30
0	3	6	9	3	0	0
3	2	0	34	1	16	1
41	77	87	73	98	54	62
2	8	10	7	6	12	5
23	7	0	63	39	18	31
16	25	35	1	1	14	7
81	78	131	82	76	92	81
0	0	5	3	0	0	0
146	152	262	114	165	167	181

For Review Only

5	42	19	22	40	11	10
131	78	119	80	65	90	84
12	10	35	22	17	23	39
18	81	182	59	47	40	119
65	51	93	64	39	55	57
79	101	155	109	53	62	84
61	61	99	53	57	63	46
75	71	104	62	54	106	70
15	50	30	34	1	53	26
133	130	244	116	115	133	86
248	276	455	258	312	256	215
9	3	0	3	8	2	1
43	25	52	20	13	41	43
6	6	8	3	6	9	10
29	25	37	35	18	40	33
0	0	0	1	0	0	7
0	0	36	0	0	39	0
83	71	95	46	53	53	74
15	15	38	20	21	14	27
112	106	179	135	135	118	115
5	19	40	16	34	21	11
73	95	189	151	83	83	111
139	202	234	72	83	135	149
80	101	205	86	103	69	94
8	0	8	0	4	8	6
16	26	120	1	2	41	3
6	8	4	9	3	0	10
58	79	81	64	72	128	85
0	30	0	0	0	28	0
2	2	0	27	113	22	18
21	27	79	60	32	14	34
74	46	87	58	81	110	34
186	239	256	209	191	234	148
274	237	324	214	181	197	172
37	65	68	50	59	60	57
181	106	273	144	115	104	84
48	62	133	45	99	61	111
5	1	0	0	5	9	2
14	0	103	0	0	0	0
0	0	2	0	0	0	11
18	17	64	5	2	13	19
0	0	4	0	0	0	0
10	17	47	16	14	22	22
44	32	65	28	33	33	26
76	102	121	109	71	89	58
24	18	79	19	33	5	20
184	72	298	175	182	659	10
11	23	27	8	14	14	14
16	25	23	16	10	20	10
38	44	60	9	61	51	28

For Review Only

53	55	64	33	58	56	56
76	92	112	96	97	97	72
299	103	52	217	2	2	137
39	17	78	27	30	26	24
4	0	3	0	2	2	0
54	63	92	51	60	65	48
0	8	0	0	0	0	3
73	86	129	53	73	80	54
45	31	71	44	48	38	23
230	306	465	279	220	229	187
69	83	106	54	58	83	81
154	8	191	17	181	42	67
0	50	58	0	0	0	0
42	34	67	68	26	53	37
507	729	656	585	476	575	597
18	81	62	19	42	19	63
123	163	263	190	154	206	150
21	24	25	25	31	66	25
3	56	114	26	39	53	0
0	0	0	0	0	0	0
3	11	22	5	15	9	9
0	51	97	44	42	50	31
125	124	148	97	119	124	91
31	11	8	9	22	11	21
72	33	86	28	57	47	16
82	88	64	90	32	86	75
186	209	356	255	234	44	159
9	12	11	12	10	3	21
104	84	98	45	69	61	70
67	90	118	52	108	50	102
6	4	56	63	32	21	9
19	41	52	26	28	28	32
1	8	0	4	2	222	186
154	178	171	182	165	153	144
102	111	165	86	93	86	88
99	124	96	51	148	116	44
133	81	161	153	119	153	83
3	5	0	4	10	8	0
90	102	59	86	86	8	88
23	48	26	23	28	18	33
20	20	33	29	27	21	7
46	55	53	43	49	42	46
483	682	1187	647	597	523	448
38	50	119	51	64	79	56
25	34	46	13	28	18	28
57	133	137	86	113	126	70
66	77	154	59	48	68	54
0	26	6	15	19	18	15
251	66	644	450	277	313	108
884	301	1142	778	346	724	722

For Review Only

22	22	15	26	14	14	15
168	222	249	227	148	113	99
70	7	0	4	13	41	2
28	1	0	9	6	2	14
89	88	162	74	91	89	77
364	441	614	367	402	386	255
8	31	19	8	19	59	60
73	65	103	37	44	128	61
11	43	61	80	27	14	30
1	0	1	0	0	0	0
4	0	4	0	0	0	3
22	23	0	8	35	25	20
24	0	33	0	17	0	0
45	68	119	95	59	77	78
0	0	0	1	3	3	0
0	0	0	0	0	0	0
14	127	45	21	56	24	58
13	25	28	12	24	15	19
0	0	471	0	0	0	0
3	31	19	15	8	8	14
158	98	234	92	92	96	43
67	72	146	52	60	81	56
0	0	0	0	0	0	1
246	10	213	171	160	139	136
0	0	0	0	0	0	11
8	6	15	10	6	3	3
5	4	11	6	5	5	7
3	3	7	8	7	4	6
34	19	0	136	1	19	57
0	0	1	0	0	40	46
0	0	0	0	0	0	0
4	3	0	6	1	1	1
0	0	182	0	0	0	0
28	26	67	27	46	42	25
0	81	13	0	130	170	0
101	95	181	161	125	142	102
270	306	481	285	298	308	261
58	154	385	112	0	194	122
0	0	6	0	0	0	5
64	57	166	60	76	63	51
0	0	0	1	5	0	1
91	143	152	79	80	139	90
120	197	344	181	271	219	227
477	216	162	147	344	278	164
2	0	0	0	0	0	0
36	37	57	28	33	43	36
0	5	2	7	0	3	0
9	17	11	4	10	6	4
41	29	51	72	58	40	36
112	146	175	53	108	142	114

For Review Only

363	372	626	366	361	438	346
4	0	14	3	0	0	0
118	439	84	162	157	74	41
24	27	71	19	23	37	84
2	3	40	11	3	0	17
215	220	363	250	241	196	164
35	48	74	36	43	44	61
1	7	7	23	5	3	1
1463	1404	2228	1258	1294	1981	980
31	10	0	5	11	8	14
140	144	204	127	100	166	114
0	0	2	1	0	0	0
0	0	11	0	0	0	0
15	18	19	26	3	24	16
0	0	0	0	0	0	38
81	74	171	73	64	81	79
0	0	0	0	0	0	0
12	7	8	2	3	5	13
107	114	173	79	96	113	70
0	0	0	0	0	0	28
0	0	0	1	5	0	0
6	2	0	6	9	3	2
103	146	166	86	70	128	58
22	20	20	27	29	25	16
0	0	0	0	0	0	8
1	1	0	1	1	1	2
0	0	0	1	1	0	4
67	270	503	196	269	0	193
87	142	187	95	109	133	89
0	1	12	3	0	2	8
0	0	0	0	0	0	0
3	0	10	6	0	9	0
0	1	0	1	0	3	0
165	143	457	124	90	106	85
397	377	725	439	505	457	309
0	0	0	4	1	1	0
35	103	51	9	33	49	42
218	256	374	200	207	277	206
14	4	15	4	4	6	6
8	10	4	6	2	8	2
351	478	692	434	361	449	351
50	45	71	39	45	72	36
390	394	602	415	405	451	267
1018	3026	3725	1040	2909	3025	2516
7	0	3	4	11	11	27
749	605	1525	823	1163	676	525
126	98	130	37	112	94	61
184	151	309	157	206	262	181
6	26	15	8	5	10	19
2	0	0	0	2	6	0

For Review Only

18	14	18	9	11	7	12
5	0	8	13	5	7	4
22	14	13	15	12	16	10
8	2	6	2	8	5	2
116	120	305	170	124	175	92
6	0	3	1	6	1	3
90	72	122	68	69	83	120
0	0	7	0	0	0	21
62	52	0	54	43	59	35
0	0	0	0	0	3	4
0	0	0	0	4	0	0
16	39	90	90	24	22	23
0	0	3	0	0	0	0
11	0	0	0	0	0	0
7	0	30	20	16	9	35
4	1	0	5	0	0	0
2	15	48	1	7	14	1
84	127	203	64	113	159	70
91	62	86	18	9	23	61
181	197	288	222	199	216	110
4	23	0	24	5	11	37
209	221	0	126	126	221	173
163	154	262	57	154	162	115
0	132	126	148	0	277	218
4	21	10	15	38	5	3
15	16	20	8	17	28	16
18	11	68	16	59	9	6
0	0	5	0	5	5	0
7	0	10	3	1	6	16
0	0	1	22	0	17	0
9	3	6	15	13	1	1
0	0	6	0	0	0	12
2	3	9	8	6	2	15
412	366	910	472	582	440	563
36	87	740	1	0	31	249
1	1	0	1	1	0	0
0	0	0	0	1	1	7
217	247	250	160	189	161	115
1	4	17	15	12	44	12
12	7	35	11	25	5	9
1	4	36	1	19	0	5
20	28	20	19	15	27	10
31	33	70	41	41	51	42
21	22	52	15	2	10	13
5	1	0	3	1	6	1
0	62	0	48	0	93	67
26	33	0	23	26	2	46
0	0	0	0	0	0	0
113	106	225	121	136	73	76
0	0	0	0	2	0	14

For Review Only

0	0	0	0	0	0	235
0	0	2	1	0	1	4
343	259	514	376	380	354	273
9235	102	10700	1844	286	1	1
12	17	26	11	18	13	24
0	0	0	0	0	0	9
16	34	43	0	10	27	23
241	188	291	200	184	242	150
0	0	0	0	0	0	0
0	0	0	2	0	0	1
5021	984	4917	173	1408	3119	15
20	58	81	40	40	45	9
2	2	11	4	4	4	7
0	0	0	0	0	2	6
0	2	0	0	0	0	0
19	2	11	3	2	7	6
0	0	0	2	0	0	0
317	428	738	350	372	329	323
18	9	28	21	15	4	13
83	74	177	127	54	109	60
23	16	33	18	13	20	12
263	83	286	26	187	118	3
1311	1292	2301	1301	1633	1286	1028
51	112	175	130	156	116	118
90	0	117	112	115	108	87
29	8	61	6	36	31	3
20	48	78	60	55	34	46
39	34	24	37	29	30	37
0	0	10	0	0	0	0
319	359	530	386	398	261	33
35	48	76	41	17	19	59
60	20	121	80	80	53	53
0	3	4	0	0	0	0
1	0	0	1	0	0	0
7	3	15	12	2	4	5
154	163	0	144	0	170	0
46	32	62	20	28	26	46
1	1	0	1	5	170	1
35	67	60	35	36	34	33
17	0	48	39	71	0	32
3	2	15	9	11	3	7
8	1	0	63	1	1	1
0	20	43	0	0	3	0
75	64	105	60	95	94	80
19	26	46	29	28	23	30
0	35	44	0	0	37	37
1	9	0	1	1	1	1
0	0	0	0	0	0	0
4	8	0	0	12	5	0
46	48	106	58	65	35	30

For Review Only

66	34	111	39	76	15	43
3	0	0	0	0	0	0
0	3	0	0	0	1	22
0	8	7	0	0	0	0
82	110	116	83	86	127	48
94	88	0	88	32	84	80
45	76	117	68	78	57	65
131	102	198	95	86	173	147
82	127	190	87	83	138	90
24	31	47	30	23	35	13
23	37	50	40	14	34	25
43	65	94	42	31	69	39
41	52	48	38	40	58	24
39	30	44	34	45	50	43
34	71	92	73	49	87	33
0	41	60	11	0	0	0
189	157	304	119	196	215	185
204	226	433	250	217	210	148
0	0	26	0	0	0	0
7	7	7	7	0	5	0
26	37	37	17	30	23	28
27	15	0	161	80	133	61
13	29	24	28	21	29	11
41	19	36	15	26	16	28
28	43	52	17	24	44	62
73	15	21	48	23	28	59
30	1	89	48	83	2	2
0	0	6	0	0	0	0
2	0	0	0	2	0	3
83	100	190	109	123	90	74
54	40	62	44	29	54	30
56	0	1	0	68	0	0
95	108	154	106	77	109	95
28	24	19	19	11	20	11
222	157	266	190	85	199	127
76	20	166	39	34	196	20
56	0	0	0	44	0	18
7	7	75	7	49	21	23
192	272	276	137	180	127	203
0	0	7	0	0	32	0
0	80	54	72	83	50	32
35	32	56	38	39	49	24
0	0	2	0	0	0	0
0	76	61	0	70	84	0
21	26	45	29	22	42	24
59	88	70	67	76	69	69
42	50	88	66	92	93	36
56	73	110	75	56	29	66
54	76	79	65	51	79	47
16	18	0	19	5	7	19

For Review Only

0	0	27	0	0	98	0
42	49	53	37	29	68	52
13	8	16	23	17	2	4
0	0	0	0	0	0	10
31	18	58	28	25	25	21
31	40	73	32	43	33	44
52	81	123	94	55	99	27
54	116	136	72	65	78	69
1	0	0	1	0	0	1
0	3	0	3	0	0	7
12	0	32	14	0	25	37
19	35	42	17	57	34	8
56	55	78	71	61	56	67
189	272	197	141	153	149	209
76	0	43	72	0	0	31
67	52	77	88	68	91	31
0	0	3	0	0	0	0
245	248	368	282	241	234	214
166	213	220	189	169	192	191
29	15	35	8	26	5	11
26	14	22	5	33	22	40
0	29	1	56	0	0	0
16	0	0	24	0	0	0
189	0	134	186	139	214	0
18	22	37	18	30	14	8
22	18	27	0	23	32	9
72	48	77	50	48	61	39
62	32	60	31	60	38	54
84	73	105	76	46	28	44
143	108	220	91	123	184	87
10	19	54	18	16	50	18
62	54	77	59	51	54	49
7	1	0	2	1	1	5
31	15	14	9	12	22	32
0	0	2	51	49	0	0
34	0	0	0	54	10	0
0	0	0	0	2	1	0
15	56	35	17	68	15	6
7	1	16	1	3	2	7
1	16	18	2	4	4	3
332	432	210	507	329	449	324
0	0	0	0	0	0	0
35	32	78	48	51	39	28
0	0	1	1	0	9	6
6	3	57	0	10	11	43
10	66	122	11	55	34	76
76	105	139	100	73	106	75
222	426	56	398	457	485	376
37	61	107	43	123	55	10
94	104	69	19	10	45	32

28	28	58	39	34	42	18
1	1	11	2	4	2	1
55	60	86	48	58	70	71
5	0	13	8	7	5	0
20	22	57	16	34	39	38
198	158	102	119	113	105	142
35	64	45	19	16	51	27
0	0	0	33	0	0	0
21	24	37	29	30	27	17
48	91	116	67	63	101	80
4	3	8	1	0	3	1
57	45	67	39	34	56	47
34	76	41	37	31	35	48
24	32	27	6	27	17	11
14	3	20	11	4	2	3
77	108	326	166	91	179	77
134	220	0	197	199	251	125
342	393	690	347	324	307	453
0	0	0	0	3	1	3
31	46	70	32	34	71	22
55	127	162	110	91	64	80
0	0	0	5	0	0	0
1	0	2	0	7	0	0
71	60	121	57	65	131	46
0	1	0	1	50	0	0
93	95	166	52	60	83	94
63	46	61	14	74	51	68
170	140	554	301	219	172	218
105	106	99	94	72	100	64
131	123	264	101	135	138	121
36	58	52	21	30	44	24
1	46	0	1	1	1	20
56	43	103	70	50	35	39
1	0	1	0	0	0	8
0	6	0	0	2	0	7
0	0	0	0	0	0	0
122	297	369	227	182	175	158
43	73	69	31	41	41	48
2	0	0	0	5	0	7
196	166	502	43	119	21	36
1	7	0	1	4	1	9
0	4	0	1	0	3	0
106	88	204	138	128	125	98
7	5	14	8	9	8	7
120	140	29	0	79	190	153
22	37	45	16	17	30	18
0	0	7	0	0	0	0
0	0	6	0	0	0	0
110	133	310	259	220	148	95
78	75	101	70	76	77	46

For Review Only

2	5	6	15	11	4	9
8	1	30	7	2	5	2
80	90	122	70	60	60	80
29	21	34	15	25	25	35
273	273	442	244	276	267	98
54	73	137	47	72	98	81
101	147	133	71	99	82	94
13	23	31	13	16	20	13
367	342	675	378	391	442	387
98	79	284	94	120	115	77
109	126	235	116	127	115	103
0	0	0	0	1	0	0
16	22	24	5	9	36	12
50	25	45	29	37	40	29
22	25	33	14	18	28	12
13	17	14	17	9	10	7
0	25	11	0	7	16	12
32	21	60	14	15	27	11
202	147	243	158	209	134	103
4	0	0	0	12	11	0
42	42	61	37	34	28	40
68	111	156	103	175	60	85
27	62	59	14	40	32	21
2	0	0	0	0	0	2
71	109	158	141	123	161	107
0	0	0	0	0	0	0
3	0	0	7	78	0	1
246	259	460	250	256	220	191
11	26	48	13	21	12	10
24	38	44	16	19	20	25
0	0	0	1	0	1	0
0	0	0	0	0	0	0
150	147	250	154	168	211	168
29	40	53	34	25	56	46
84	101	198	131	59	86	58
196	240	430	234	248	265	165
84	79	171	64	77	95	99
539	1150	404	967	58	0	548
16	1	0	1	1	1	1
51	52	102	46	59	74	50
19	14	15	6	13	13	13
63	0	25	0	69	61	68
14	22	48	7	13	28	30
85	119	126	77	109	94	114
28	12	37	9	14	21	26
3	6	7	9	13	8	11
26	39	52	20	25	36	30
1	1	0	1	70	1	1
96	24	125	116	41	42	16
0	1	12	1	0	6	5

For Review Only

11	0	23	7	6	12	8
335	267	611	328	428	400	304
91	118	65	57	46	179	67
18	28	41	19	21	36	25
7	18	9	16	10	5	11
31	29	66	44	35	21	35
7	7	12	7	7	13	10
200	118	197	85	170	118	79
9	25	24	33	32	20	18
0	0	0	0	0	0	0
44	43	35	53	59	29	20
217	289	306	194	210	258	262
0	36	0	0	0	12	101
81	142	198	105	99	115	149
66	84	138	63	83	57	81
0	0	6	0	98	0	0
33	93	83	72	57	78	72
1	1	1	1	0	1	1
17	8	15	15	4	0	56
133	55	0	1	1	714	2
0	0	7	0	25	0	25
0	0	0	0	1	2	78
185	60	119	105	64	90	94
13	78	48	98	23	18	42
692	697	0	797	756	840	591
0	2	0	1	0	2	5
0	1	1	3	0	0	0
8	11	19	17	8	8	33
0	1	0	1	0	1	1
22	24	31	20	23	25	18
3	0	0	5	0	3	8
5	15	24	38	9	35	6
61	38	129	60	101	60	23
100	99	227	69	85	131	109
22	1	0	1	1	0	1
382	394	688	408	436	357	364
48	42	21	19	25	14	7
35	51	59	53	49	62	45
143	136	321	188	215	125	38
119	72	161	102	106	95	101
17	16	32	29	7	21	14
0	0	0	0	4	0	0
584	564	1295	673	655	533	527
31	24	53	57	38	37	29
36	35	77	27	21	41	38
1	0	4	2	0	0	3
43	127	121	48	80	53	100
248	284	334	204	161	203	176
230	258	812	271	232	369	304
72	52	71	31	39	70	26

For Review Only

0	23	0	0	0	0	0
25	45	68	30	24	37	36
10	41	35	4	15	20	23
10	13	18	10	6	5	6
173	253	288	156	153	162	149
24	12	30	16	7	15	18
36	25	72	33	47	19	36
152	154	193	104	100	169	105
76	84	189	78	107	104	72
6	6	3	0	4	2	2
49	68	71	36	45	73	39
23	27	37	30	27	37	16
9	17	15	16	26	17	18
75	87	182	85	79	158	80
0	0	2	3	3	0	0
0	1	0	2	3	0	0
0	21	64	13	0	6	18
72	42	139	61	72	193	81
0	0	60	0	0	0	0
45	72	82	38	83	73	42
26	76	52	26	21	8	39
86	54	97	90	75	24	49
23	25	35	12	27	28	28
6	5	1	0	3	0	37
1	4	1	3	0	0	0
0	4	0	4	0	2	0
32	3	24	11	7	8	6
18	14	11	9	8	19	16
0	0	4	1	0	0	0
1	1	5	0	0	2	0
0	0	0	0	0	0	46
0	0	20	0	0	0	2
31	64	91	47	52	45	53
744	606	1165	593	852	919	783
20	42	141	83	64	49	99
82	44	64	25	50	47	29
1	0	4	0	0	0	0
12	21	20	12	12	0	3
42	25	43	16	12	48	34
27	30	46	26	25	20	30
0	3	0	1	0	0	6
76	83	108	55	80	101	65
17	28	20	19	20	14	46
87	48	92	31	45	68	21
35	26	124	131	27	78	71
42	23	54	28	38	41	38
0	0	11	0	0	0	0
124	75	277	149	146	138	140
99	45	89	11	37	52	29
100	161	139	81	121	138	70

For Review Only

54	67	53	29	49	17	39
153	128	255	117	177	139	150
96	135	238	112	89	125	134
278	267	460	292	204	329	195
23	22	34	25	26	18	9
22	5	1	16	10	27	24
82	130	192	118	98	145	80
91	103	219	125	129	100	108
134	115	360	155	168	270	188
106	133	210	128	116	114	123
0	1	10	0	4	0	0
28	37	68	39	52	81	33
0	1	0	7	0	16	2
37	10	298	112	39	124	89
264	359	536	277	241	326	283
1	5	4	12	12	7	5
1	4	4	0	0	1	0
9	2	9	4	6	7	8
0	0	6	5	3	0	1
32	24	44	21	32	24	22
0	0	0	0	0	0	0
16	1	7	4	2	7	3
0	4	0	4	4	3	0
12	12	14	7	15	10	6
12	21	33	21	16	30	31
0	0	10	7	9	3	1
101	144	192	55	88	95	101
0	0	674	0	0	0	0
209	172	307	116	137	188	164
387	376	464	338	255	240	234
0	0	1	2	2	0	7
23	37	35	28	33	18	28
0	0	0	3	3	0	0
3	4	11	3	6	0	3
0	0	0	3	0	2	4
0	0	4	0	0	0	0
0	2	11	1	4	4	2
0	0	0	0	0	0	30
0	0	8	0	0	0	0
24	10	42	33	16	22	21
1	1	0	10	1	1	4
0	0	0	0	1	2	2
0	0	0	0	0	9	0
41	26	69	28	34	36	30
57	109	134	109	57	73	51
0	3450	510	2772	2546	3590	1739
38	36	73	48	64	57	50
16	7	8	7	7	8	0
0	1	7	7	0	0	0
11	2	0	9	8	8	17

For Review Only

0	23	1	0	0	0	227
294	393	655	400	365	414	297
33	10	64	56	42	39	33
0	0	0	0	0	0	0
0	0	0	0	0	0	0
18	5	9	2	16	15	22
99	64	136	79	69	64	72
0	1	0	12	1	1	114
148	91	191	175	0	209	190
192	174	197	98	144	128	193
97	93	97	57	45	73	67
56	69	86	73	59	61	82
169	148	197	123	125	130	157
137	228	180	190	174	122	216
2	4	26	4	1	13	12
202	235	335	302	249	198	213
38	68	28	88	24	82	27
131	290	258	210	125	232	149
84	86	120	72	0	78	81
3	589	0	1	1	73	1
128	181	171	129	105	169	178
0	25	106	0	24	184	0
52	71	69	43	38	46	48
380	404	528	376	327	425	343
0	0	2	0	0	0	0
130	129	167	103	80	112	116
129	27	348	91	9	291	67
1	4	0	1	1	1	22
0	0	0	1	1	0	0
1	201	546	1	1	8	2
190	187	276	148	151	180	198
115	121	180	114	105	133	129
231	234	346	217	150	286	224
36	73	65	67	44	27	37
142	143	215	108	140	146	135
131	184	190	160	57	170	101
96	153	165	119	113	115	103
24	128	125	83	90	98	122
55	92	497	114	110	268	33
74	124	131	73	68	46	0
0	0	1	0	0	75	0
54	123	108	62	53	120	39
234	244	192	111	155	241	180
88	223	252	126	103	115	149
171	294	330	194	248	176	187
65	46	57	54	42	34	57
52	42	0	47	47	62	72
155	165	242	125	171	143	139
0	0	0	0	32	25	16
48	89	105	80	31	57	54

For Review Only

109	82	153	83	127	63	73
0	44	32	44	0	0	0
87	40	90	87	35	20	23
32	28	27	33	25	44	70
14	22	25	19	0	18	16
88	37	134	72	39	42	73
3	26	22	33	7	17	41
0	0	0	0	0	0	0
29	32	36	42	30	26	25
0	21	1	0	0	22	0
0	0	15	0	0	0	0
1069	1109	1715	1043	897	1320	838
75	68	169	72	75	110	103
44	39	91	60	57	40	32
311	290	514	253	247	350	231
140	84	210	58	86	155	66
38	28	74	89	56	120	43
25	11	105	13	16	87	36
29	65	37	26	49	23	27
0	0	0	3	0	0	0
50	58	28	43	28	70	41
119	153	176	157	100	141	155
85	92	64	62	71	61	57
87	187	276	117	115	195	184
8	11	21	2	18	8	6
0	0	0	0	0	0	1286
34	17	55	11	7	20	29
12	37	46	24	18	29	14
3	2	7	0	0	5	0
8	0	0	4	3	6	5
81	90	132	87	84	82	93
12	23	41	14	26	19	17
16	16	27	8	10	0	25
14	66	165	21	77	75	56
1782	1833	4712	2569	3096	2334	861
0	0	3	4	1	1	0
11	6	22	6	5	1	40
161	153	279	190	154	126	98
0	0	0	0	0	0	228
8	7	0	17	0	29	8
45	29	88	22	41	31	30
416	513	616	413	394	425	402
316	396	507	375	326	320	250
203	228	297	229	185	242	160
7	10	20	5	13	12	15
27	34	55	15	50	25	18
662	669	276	559	584	529	519
15	39	45	45	40	44	32
17	13	41	22	9	27	10
0	0	13	0	0	5	14

For Review Only

1	488	595	191	63	8	40
92	124	187	44	43	52	56
24	38	40	41	11	14	25
10	10	9	12	17	5	0
14	28	0	21	24	1	4
6	26	16	4	9	56	14
66	151	1557	128	1451	4	2
0	0	0	0	2	0	0
15	15	34	19	24	30	15
268	195	227	137	173	128	204
0	0	0	0	0	0	80
494	609	864	623	531	450	470
3	2	3	2	0	2	0
241	365	460	118	292	173	0
3	0	22	0	0	0	1
1	0	0	3	0	0	13
0	0	0	0	0	0	0
24	179	125	0	0	0	0
68	144	239	96	167	109	69
0	0	12	0	0	0	0
226	318	361	280	277	280	217
0	0	0	0	0	0	14
124	290	269	109	164	153	152
0	0	0	0	3	0	88
2	0	0	0	0	2	0
89	75	129	77	72	75	60
38	42	54	35	49	47	32
0	0	2	0	0	0	1
67	104	86	54	104	104	176
461	697	1148	773	625	679	702
0	23	32	0	0	251	0
243	150	185	54	189	201	80
179	190	234	148	151	173	196
77	43	64	57	60	39	47
85	69	127	85	95	65	117
100	141	157	89	125	117	112
4	5	4	6	8	8	4
40	40	59	24	48	31	39
22	51	94	47	21	27	30
1	2	0	1	1	31	69
27	26	59	29	17	24	0
13	18	21	3	6	9	6
10	19	16	13	18	21	9
42	14	0	32	53	0	26
40	38	44	32	35	30	23
67	80	232	76	86	76	66
66	37	111	99	77	73	46
243	260	450	262	250	253	138
10	19	34	18	7	24	21
24	21	16	24	16	10	23

For Review Only

0	0	4	0	0	0	0
138	116	248	134	84	150	101
33	27	38	26	49	42	34
181	292	422	224	126	216	74
0	2	0	1	0	2	1
44	626	425	447	427	524	417
3	8	0	5	0	0	3
1	1	0	66	215	1	1
37	35	31	12	23	28	21
2	0	0	1	0	0	2
1	3	1	10	1	6	10
172	152	309	144	104	177	123
0	14	50	0	14	14	23
0	0	0	0	3	0	6
1	92	0	1	1	27	1
16	8	46	28	1	4	44
5	8	24	10	13	12	9
54	75	93	48	36	40	42
45	63	164	46	50	38	84
17	12	14	17	9	15	15
65	4	487	2	2	26	35
122	57	112	66	118	71	75
252	160	351	90	1042	293	68
3	0	8	0	5	0	0
27	67	79	30	34	26	38
8	17	6	12	20	11	11
1	5	0	6	3	1	7
214	218	422	195	184	215	165
0	0	0	0	0	1	0
27	30	31	26	28	17	18
41	41	53	47	38	30	25
117	52	157	58	68	60	48
18	19	37	13	4	35	18
0	0	0	0	0	0	0
2	4	14	38	17	14	2
8	4	25	21	12	5	16
24	65	76	75	75	70	41
76	107	180	116	99	124	84
59	60	98	40	66	47	47
0	189	145	70	41	0	39
1	1	42	21	1	2	1
97	74	110	98	107	62	69
0	0	2	0	0	24	0
286	428	625	236	274	331	372
195	153	401	216	205	222	193
0	0	0	0	0	0	4
27	40	64	1	20	18	15
12	17	27	10	15	22	10
6	3	10	10	10	1	3
189	186	455	200	52	267	112

For Review Only

67	83	50	40	51	62	58
5	0	2	0	0	4	0
454	275	0	1	23	236	56
0	0	6	0	1	0	0
156	174	335	194	176	69	197
0	4	19	2	6	8	3
387	438	715	455	449	495	442
0	0	0	0	9	0	0
78	258	280	202	63	145	169
2	12	29	5	8	19	6
39	27	9	11	28	11	7
16	15	17	18	11	10	8
0	0	0	1	0	2	0
93	91	205	96	103	145	75
19	26	61	35	16	29	27
0	25	0	0	12	0	10
51	105	69	75	48	38	210
78	40	63	31	84	36	49
0	0	0	0	3	3	3
574	557	1008	669	644	704	453
173	165	283	154	156	238	144
0	1	0	0	0	0	0
60	86	98	75	57	43	54
21	28	10	29	33	57	33
0	0	0	0	0	23	0
4	5	4	1	2	0	1
0	0	0	0	3	0	0
118	104	249	135	119	176	96
18	9	43	9	24	18	13
13	12	27	8	5	22	21
149	0	236	166	168	196	158
53	98	218	143	113	77	66
149	183	874	1179	696	112	1220
103	141	169	170	111	155	90
0	0	0	0	0	0	0
50	45	78	84	62	53	46
7	1	10	12	18	12	3
97	38	147	43	13	112	28
0	0	0	0	0	1	0
15	7	36	10	0	9	11
106	127	205	106	112	123	111
38	106	43	13	52	37	63
97	137	226	144	133	220	96
79	157	142	87	78	134	78
42	46	129	39	43	60	44
159	224	195	164	141	250	93
318	447	615	373	360	329	307
2	1	0	0	5	0	0
139	62	183	189	79	201	71
209	237	483	226	275	216	167

For Review Only

34	38	44	31	32	54	42
204	248	300	123	190	253	215
80	77	106	51	43	78	47
45	90	233	95	1	61	101
51	52	95	33	41	68	48
61	95	119	38	40	70	56
70	27	67	38	28	20	72
37	42	80	60	30	38	35
126	112	129	88	72	104	77
10	0	0	0	0	9	23
175	1	313	1	1	10	55
14	2	41	8	8	31	13
3	19	11	23	21	25	4
74	68	100	25	64	29	39
0	0	18	0	0	87	0
88	201	100	0	269	0	0
0	0	0	0	0	0	0
0	0	0	3	2	6	2
28	39	40	41	33	26	32
23	32	50	31	40	56	50
2	8	0	0	0	5	3
56	55	78	43	47	64	59
0	5	0	0	0	30	0
18	13	30	13	12	31	43
92	23	38	42	49	66	24
0	0	0	2	0	0	2
0	4	15	0	0	0	5
4	5	7	5	2	10	5
0	0	13	0	2	1	1
38	64	73	40	54	22	44
37	26	43	48	49	45	30
0	0	9	0	0	0	0
52	54	80	53	38	43	44
0	0	0	7	0	0	0
24	24	40	33	30	5	45
65	0	11	80	82	83	0
87	132	179	111	84	112	86
238	112	215	125	133	140	94
7	12	20	0	0	2	11
97	75	86	66	92	88	69
306	264	437	283	294	310	204
73	97	85	50	64	84	94
27	76	97	50	46	49	73
44	34	54	36	21	36	18
33	115	91	41	64	114	42
24	9	39	21	19	14	40
135	0	456	161	139	102	115
9	155	165	15	123	84	120
15	56	69	41	51	43	38
65	88	94	54	68	85	60

For Review Only

0	0	0	0	0	0	48
113	91	325	79	3	110	166
40	51	78	54	50	68	49
44	111	154	78	64	63	69
53	0	151	96	8	16	60
0	0	0	0	0	0	19
30	51	78	24	44	45	53
19	49	50	26	44	38	39
55	0	69	53	25	0	43
0	7	0	0	0	0	0
70	92	131	82	104	88	88
158	250	253	177	183	185	203
5	0	0	16	2	8	0
33	25	143	72	31	27	96
522	331	619	346	443	344	368
84	69	78	30	54	37	49
42	24	24	17	23	18	21
67	92	160	49	102	99	62
168	270	372	182	194	270	100
40	73	77	58	51	83	54
224	299	302	211	217	210	246
230	262	374	258	181	243	148
5	2	0	11	2	4	0
25	26	45	21	15	22	21
24	40	50	19	18	10	32
0	3	0	1	1	0	3
3	6	9	10	3	3	7
24	42	136	129	63	66	24
27	15	45	72	23	38	46
70	82	66	47	65	77	86
26	34	62	25	24	55	33
247	267	583	314	279	247	154
443	264	0	13	0	3	191
12	25	44	15	15	22	10
1	6	185	2	371	1	295
1	3	5	0	1	0	0
62	66	193	107	62	80	41
0	0	0	0	0	0	0
19	15	28	22	25	17	23
441	613	1075	633	728	662	547
7	13	21	10	27	23	9
0	0	0	0	0	0	0
10	12	33	18	9	0	14
0	78	156	19	22	43	40
387	315	765	411	387	496	229
10	9	29	20	4	3	27
153	14	174	5	147	15	148
354	326	389	233	373	325	77
13	2	23	6	4	11	3
7	21	22	18	20	7	21

For Review Only

3	5	0	2	1	4	6
37	99	65	57	87	63	49
61	82	82	32	47	79	79
38	30	65	46	48	40	17
0	0	0	0	0	0	0
108	162	175	105	76	86	90
82	83	118	63	70	77	56
84	143	172	94	124	116	115
86	162	180	131	115	118	119
0	13	15	9	19	13	18
70	103	125	82	114	119	110
34	13	52	99	15	101	1
81	103	112	77	76	97	91
36	33	49	92	39	57	44
2	21	10	5	3	6	10
0	1	8	2	1	7	1
23	12	14	34	14	18	10
33	19	35	31	15	28	23
66	146	97	97	89	57	78
62	50	160	82	36	98	65
86	0	62	0	87	0	0
37	35	72	28	26	51	38
29	32	51	26	20	39	45
142	110	71	73	52	110	48
101	111	221	135	92	121	114
24	12	19	16	17	26	12
37	59	69	21	37	46	41
99	156	141	61	94	89	155
55	39	107	45	49	72	45
0	0	10	2	0	0	3
67	88	102	48	75	57	123
231	219	152	0	167	0	0
57	71	80	58	42	86	43
75	91	120	60	81	78	67
68	18	45	13	33	23	10
131	209	234	188	173	122	103
40	55	36	41	37	20	25
73	71	86	47	62	99	58
496	552	791	467	330	773	537
0	3	1	9	5	9	0
0	0	0	64	76	0	0
15	50	73	37	40	39	34
4	1	0	1	1	1	1
64	81	131	84	92	64	36
19	0	0	0	0	0	15
135	136	314	154	216	185	157
35	64	60	47	28	36	49
176	146	256	131	153	178	167
169	315	299	27	158	154	240
140	96	244	184	124	135	81

For Review Only

22	1	233	24	97	35	1
0	0	0	0	0	12	0
9	20	51	12	17	25	38
33	34	75	69	56	36	53
2	1	0	5	2	4	4
139	173	262	165	134	129	139
12	0	9	8	5	6	8
56	79	71	65	40	83	60
0	0	0	0	0	0	17
0	0	0	0	0	0	25
0	103	24	0	105	105	83
23	57	49	27	24	27	59
102	107	165	128	118	102	76
139	0	169	135	147	178	137
144	134	167	94	153	133	157
36	77	74	63	31	61	94
117	32	205	1	1	95	2
93	75	150	103	63	104	61
29	59	77	19	57	24	22
81	157	408	119	334	85	234
21	16	27	44	14	53	10
136	391	138	1	1	15	83
4	8	0	21	19	4	6
12	0	1	8	0	3	0
61	55	97	47	48	71	51
47	70	135	22	45	54	46
31	33	47	17	42	11	34
77	103	184	133	129	134	90
8	4	0	7	3	0	23
44	68	85	42	61	54	49
25	43	86	48	54	52	46
406	371	649	397	395	449	276
0	0	0	3	0	0	3
0	0	6	0	1	0	0
16	19	46	17	10	31	6
23	34	42	24	36	38	36
2	1	0	0	0	0	0
53	82	104	37	63	102	57
60	77	103	36	37	42	68
1	1	0	4	2	1	2
1	12	0	1	15	1	1
180	265	300	181	164	182	209
9	13	22	11	6	11	9
4	0	0	0	4	0	11
42	21	93	30	45	39	49
0	0	0	0	0	3	0
32	6	71	42	52	70	17
0	0	4	0	2	3	5
0	0	9	0	3	0	6
82	164	279	186	127	141	152

For Review Only

4	1	0	1	1	0	1
502	601	799	523	566	561	446
120	34	157	41	74	105	48
77	101	0	61	0	211	188
92	56	145	67	59	91	65
35	36	77	10	26	32	48
20	41	99	26	36	50	32
23	0	1	0	0	0	0
64	52	111	46	67	85	69
22	12	22	11	9	22	12
0	0	7	1	3	0	0
310	317	362	278	270	293	199
485	255	421	309	286	493	278
37	34	45	11	25	38	31
0	0	0	0	0	0	0
66	48	95	55	58	75	58
15	30	0	15	17	36	26
26	18	32	22	42	26	35
35	78	154	48	77	58	64
38	39	87	36	34	31	38
154	132	186	136	139	133	83
71	78	171	77	57	64	50
48	65	92	106	59	73	72
0	44	60	30	41	46	33
4	9	12	13	14	11	3
29	8	20	4	41	36	22
15	38	63	42	51	31	28
216	253	502	216	276	190	145
49	57	119	46	44	53	76
32	41	57	37	31	31	27
107	80	173	112	70	76	79
8	12	7	11	1	8	21
0	1	0	0	1	0	2
16	40	59	22	18	37	22
50	27	133	43	54	83	52
38	27	42	27	43	42	27
37	49	110	65	56	62	39
36	44	75	48	13	49	12
46	73	67	37	47	64	74
0	86	19	0	24	74	17
3	13	10	1	7	6	4
0	0	0	0	6	0	0
5	6	12	0	10	3	1
139	188	153	124	71	103	43
0	124	1	0	0	0	0
82	41	86	40	42	52	34
0	0	4	0	0	0	6
178	268	207	147	135	205	161
66	0	1	0	0	85	0
0	0	14	0	0	0	0

For Review Only

56	1661	2457	809	1321	1472	1262
8	4	28	21	13	1	9
6	7	20	30	3	11	47
55	33	64	14	46	40	57
0	0	0	0	0	0	0
173	230	125	267	66	120	285
0	0	0	0	0	0	0
32	24	40	40	27	10	27
0	0	0	0	0	0	62
12	24	25	15	31	30	16
3	3	0	0	0	0	0
61	81	95	63	102	43	59
73	59	95	61	75	62	58
1	1	0	2	1	0	0
4	6	0	5	5	15	3
42	55	86	84	48	67	42
7	0	0	0	5	12	3
2	0	0	1	0	0	1
41	47	119	55	52	88	37
8	2	16	0	7	3	2
65	58	93	53	90	82	61
1	0	1	0	2	0	0
642	297	1001	47	889	514	505
193	751	828	230	295	592	238
190	103	199	109	109	151	101
40	31	46	13	41	21	26
0	0	1	0	3	0	0
21	31	55	14	30	23	29
0	0	0	0	0	0	4
5	0	3	4	1	3	0
331	54	1129	108	190	393	18
366	419	513	369	341	412	374
0	0	0	0	0	0	9
137	112	208	98	89	122	95
88	131	177	90	126	106	85
35	48	85	33	30	68	48
0	0	5	0	0	6	0
0	0	0	1	1	0	2
0	0	0	0	0	3	0
13	11	22	6	16	74	9
92	8	35	37	11	1	8
0	81	95	174	53	44	0
49	38	68	29	55	57	41
111	87	130	40	55	48	69
5	9	21	15	12	16	17
64	29	97	63	30	28	15
0	2	0	0	0	6	0
44	136	191	131	91	186	90
9	13	25	31	15	16	25
0	0	0	0	0	0	0

For Review Only

1	0	0	1	2	0	0
1	8	0	1	1	11	1
4	7	1	2	0	0	1
775	920	1492	1093	1218	653	523
112	195	0	1	1	1	17
17	11	18	2	6	9	18
0	0	0	0	0	137	0
59	32	48	40	35	63	36
79	62	61	69	37	27	61
57	36	58	39	63	45	37
21	83	220	26	19	61	110
82	42	15	18	44	69	72
35	29	54	16	37	49	43
14	10	38	24	15	29	16
0	0	0	0	0	51	33
28	20	21	32	20	9	12
12	1	0	1	1	1	88
447	437	733	389	413	360	350
75	81	120	83	78	46	56
11	18	44	4	8	9	11
62	42	50	34	59	66	35
58	58	100	56	57	60	48
13	14	0	11	7	11	5
56	123	152	45	34	69	66
124	27	78	0	24	0	28
10	24	17	10	15	13	11
9	23	18	11	15	11	10
14	27	33	22	20	21	15
9	13	13	39	4	2	13
1	1	8	4	2	1	5
1878	2211	2280	1871	1990	1744	0
42	48	92	43	43	58	18
0	85	5	0	54	0	65
10	16	30	5	0	25	6
18	15	19	10	16	19	13
0	0	0	0	0	0	0
8	184	578	2	1	229	110
73	51	126	75	41	84	43
0	0	41	0	0	0	0
31	26	34	33	0	33	27
3	0	0	0	0	0	0
69	0	88	68	62	106	61
17	105	32	36	20	61	27
5	104	16	5	14	19	31
172	141	172	85	137	120	117
14	5	23	2	3	3	7
36	38	55	32	51	22	17
374	0	316	0	326	378	282
40	52	53	47	31	69	31
25	12	107	36	18	42	144

For Review Only

35	52	104	80	37	56	54
83	94	246	142	156	110	82
0	3	7	0	0	0	12
14	14	0	1	1	13	12
25	25	79	24	33	33	46
997	1020	1622	1155	854	764	854
103	173	187	79	129	130	133
32	15	0	7	1	41	9
59	63	107	60	68	66	39
51	132	117	109	107	108	93
103	111	236	70	68	126	112
101	112	165	83	93	101	92
2	0	0	1	2	0	2
58	94	89	61	45	41	114
221	176	315	118	77	171	163
135	143	220	103	148	117	116
38	67	62	30	38	62	72
16	49	25	19	20	15	25
1	0	0	0	0	0	2
19	6	18	26	7	21	27
75	3	0	30	2	1	4
0	379	739	196	0	391	59
25	27	34	12	19	25	22
234	206	320	140	154	225	151
0	0	0	0	0	2	2
45	33	66	49	63	85	15
211	206	546	317	259	276	232
127	0	2	120	0	0	0
111	102	148	66	63	119	73
53	32	0	14	12	8	7
65	68	115	61	55	104	41
45	70	68	33	57	46	30
122	154	267	139	177	149	107
79	125	139	86	71	109	91
242	165	339	170	142	284	208
199	110	407	234	181	229	217
0	4	0	0	3	0	2
117	30	280	7	264	404	1
38	59	43	28	31	60	33
65	73	103	4	44	41	72
0	0	0	0	6	0	0
0	2	0	0	0	0	0
0	0	4	0	0	1	4
42	61	80	78	114	30	10
33	86	0	65	21	14	67
16	7	17	11	18	12	13
0	5	0	0	0	0	22
0	2	0	0	4	1	2
55	85	144	118	86	109	62
85	92	140	71	78	108	83

For Review Only

159	0	1	167	187	227	0
0	91	195	63	16	64	7
171	140	184	119	147	149	105
49	93	33	50	14	160	149
0	0	0	0	0	11	0
80	107	149	156	74	153	91
89	134	180	99	105	170	116
32	10	38	4	17	32	31
123	142	325	248	205	230	240
61	116	101	32	65	79	81
2	0	7	0	0	9	0
15	3	27	0	0	0	9
131	242	306	162	228	281	100
34	24	66	57	40	29	35
75	74	126	87	62	78	84
18	35	36	10	12	11	8
40	53	87	50	38	64	34
209	212	304	201	218	238	143
117	42	144	111	67	53	80
95	87	96	87	60	131	80
3	4	5	1	6	6	9
0	0	4	1	3	1	0
4	3	5	4	6	5	3
0	3	4	4	3	5	1
5	7	14	5	7	6	2
1	4	7	4	5	6	1
5	7	18	7	11	7	4
0	1	0	1	3	1	0
69	82	82	28	63	65	85
145	119	128	65	54	84	70
70	134	849	571	520	616	136
4	7	17	3	6	0	3
33	29	62	26	31	25	30
0	0	0	0	0	0	0
12	24	39	19	21	33	34
262	214	263	223	228	340	165
16	30	22	21	11	9	23
50	60	122	92	85	129	72
40	35	33	14	32	50	20
0	12	0	0	0	0	0
2	1	0	3	1	1	1
54	60	113	75	89	89	40
13	56	100	8	36	33	44
0	0	0	22	0	39	0
34	33	43	49	38	65	38
41	54	90	43	43	78	62
26	23	35	22	26	26	20
3	11	8	10	13	13	9
2	0	10	0	2	0	61
18	7	32	9	8	7	0

For Review Only

0	3	3	0	0	7	0
0	0	114	0	0	0	0
81	0	83	92	0	93	0
82	107	132	65	97	106	85
108	131	179	109	106	137	144
0	0	814	21	1	57	1
0	0	0	0	0	0	0
199	225	247	166	165	175	238
13	17	17	14	13	16	19
72	78	91	60	84	86	61
36	41	48	36	41	40	32
0	0	2	0	0	0	0
7	9	13	17	14	15	16
112	113	171	111	118	150	140
0	0	0	0	0	0	0
29	50	59	41	35	46	42
78	73	237	0	68	0	0
157	213	254	182	150	224	259
0	12	104	0	10	0	13
0	0	0	0	0	0	0
0	0	0	0	0	0	0
25	15	46	17	38	28	28
0	0	0	33	0	0	22
21	0	18	18	18	17	19
17	23	23	18	14	18	17
167	171	265	124	144	175	207
28	28	38	18	19	25	28
0	27	4	0	0	0	0
70	83	112	76	50	83	108
0	0	67	74	0	0	0
28	0	0	23	19	0	0
20	37	46	42	22	33	31
0	0	0	180	0	0	0
74	0	41	0	98	0	96
0	0	286	0	0	0	0
29	0	8	0	33	49	0
91	122	203	113	96	146	156
4	3	0	0	3	0	0
12	5287	2363	3	2	3	798
1	2	0	3	1	1	229
2	0	0	0	2	0	18
199	483	393	298	239	269	430
1	2	0	1	1	74	1
530	0	1	0	544	0	0
443	705	1063	523	349	828	580
218	205	90	0	0	243	0
88	128	124	20	109	111	133
584	419	928	580	506	571	457
794	846	1339	968	735	885	571
66	84	95	62	45	75	47

For Review Only

3	18	40	3	3	12	0
39	60	50	41	55	85	35
4	12	77	0	19	29	23
116	0	170	98	88	89	107
9	0	14	16	13	0	0
1	5	6	10	5	2	3
28	58	65	99	75	41	77
56	94	212	107	54	93	75
164	167	248	191	208	210	125
0	0	181	70	77	0	64
368	548	648	423	409	533	517
66	64	0	47	51	55	58
54	74	97	55	45	79	57
301	539	565	337	375	408	489
0	0	57	0	0	0	74
120	141	237	138	124	135	157
27	26	36	21	28	19	31
46	44	70	46	50	55	42
38	40	60	34	41	40	34
166	186	182	246	155	161	171
73	97	0	78	86	52	104
0	66	0	0	0	71	46
7	8	12	5	7	5	12
37	42	57	12	32	30	14
1	1	165	2	3	1	1
0	113	1	0	250	0	0
0	0	0	0	0	0	110
0	0	123	354	0	0	292
0	190	127	0	0	0	166
23	52	51	28	20	45	23
167	227	274	160	168	234	272
28	81	122	75	12	78	181
651	942	1096	679	730	825	814
16	14	0	19	9	19	15
26	77	54	66	56	60	43
236	595	705	476	508	608	501
95	412	1	116	0	137	376
108	101	264	179	6	65	3
5	11	28	6	1	2	19
33	42	63	31	38	38	71
178	188	235	125	157	226	125
31	20	43	17	31	37	20
140	172	150	83	49	83	38
0	10	12	0	0	61	0
151	57	276	103	88	153	55
6	1	1	3	4	3	2
54	65	101	60	54	63	60
12	33	0	5	2	5	0
405	312	315	495	0	552	186
127	156	215	46	139	146	143

For Review Only

571	526	864	538	549	534	343
14	23	13	9	12	15	11
1	15	0	1	3	1	1
20	35	21	13	15	8	12
26	29	15	9	8	18	12
41	53	63	66	47	63	31
2	1	0	1	1	1	2
78	104	154	74	96	76	94
66	145	124	116	133	40	49
6	3	3	3	2	2	6
0	1	0	0	2	1	12
13	24	20	9	11	15	16
232	128	283	152	165	173	166
423	323	588	413	433	272	299
48	39	80	34	70	80	22
0	0	15	0	0	0	4
66	44	69	61	23	64	27
95	94	131	83	63	91	70
16	0	0	0	0	0	0
202	210	302	239	222	275	157
24	23	35	17	11	10	21
4	24	26	7	25	7	30
64	89	113	60	73	54	56
0	12	0	13	10	0	0
132	126	230	170	138	236	151
41	42	44	57	2	28	42
0	36	61	35	20	40	20
19	36	20	17	14	17	9
42	32	59	29	30	24	12
2	4	0	12	0	9	1
32	61	90	43	39	49	38
44	77	91	25	83	69	59
13	14	34	17	9	36	4
98	60	90	54	71	28	33
17	21	41	20	26	19	32
187	54	378	2	2	2	274
64	86	105	60	34	58	48
1	0	0	0	0	3	0
37	38	49	45	36	32	23
34	38	49	48	33	28	15
0	1	7	0	5	3	4
0	0	4	2	5	8	2
3	0	2	8	5	8	6
76	72	118	84	69	96	68
95	68	142	86	63	67	45
91	150	235	105	148	111	134
0	0	0	0	0	0	0
122	109	158	163	129	115	116
49	55	62	57	25	57	45
248	221	340	259	172	313	207

For Review Only

0	0	20	0	29	0	18
21	23	42	34	24	22	27
340	273	454	267	293	263	264
0	0	5	1	0	3	5
0	0	0	1	1	6	0
74	94	194	79	109	71	97
712	753	1352	776	763	775	519
94	229	141	99	123	140	180
47	36	67	50	31	22	44
305	394	762	292	401	234	259
30	27	33	27	33	25	47
5	14	23	14	10	10	4
161	147	206	133	106	119	103
73	33	66	46	39	45	8
28	130	129	71	28	57	41
56	47	54	37	65	62	60
68	47	120	59	50	78	52
0	0	0	0	4	0	0
234	117	283	198	172	274	142
4	3	0	2	2	3	18
0	2	0	2	1	1	2
0	2	0	0	0	0	0
18	32	68	45	37	28	35
53	49	58	25	59	23	54
53	58	88	71	44	74	62
81	90	25	74	63	0	58
31	29	39	38	34	34	32
8	5	12	7	10	8	13
2	41	21	9	13	4	3
4	15	28	14	9	1	13
45	33	76	34	19	32	30
7	5	11	25	7	13	3
0	1	3	1	0	1	0
127	104	152	88	74	75	71
0	29	112	0	3	43	50
0	9	19	10	0	5	0
13	22	41	6	10	17	23
4	7	10	1	2	4	4
7	0	7	20	12	13	17
3	2	0	4	0	0	0
3	0	0	0	0	0	0
1	1	89	1	1	1	104
9	17	15	4	15	7	18
4	0	0	1	0	0	9
20	21	30	25	27	20	29
0	0	0	0	0	0	20
0	0	0	0	0	0	0
284	0	1	0	0	389	0
168	116	5	90	0	248	144
3	0	0	0	0	0	6

For Review Only

30	48	49	22	18	41	41
1	1	908	1	3	1	1
57	46	55	27	51	60	40
14	25	66	26	22	13	19
49	56	89	41	49	46	18
26	43	59	26	23	44	27
128	128	160	98	105	82	80
19	7	28	5	21	7	9
40	58	109	64	102	79	82
127	149	152	164	65	179	96
66	44	71	0	122	0	14
0	0	0	2	0	0	6
1213	1524	2265	1340	1439	1602	1060
54	74	63	63	37	56	50
288	398	458	304	278	277	260
0	0	2	0	0	0	0
6	15	11	6	7	6	10
8	22	50	7	22	14	7
0	0	4	1	0	2	0
0	0	0	0	4	0	4
0	0	13	2	2	8	0
41	37	51	58	27	61	37
151	145	228	174	190	133	114
3	2	0	14	3	1	26
98	129	155	87	91	90	84
105	141	159	118	162	156	77
274	334	403	247	303	240	246
126	144	238	126	143	176	100
1	1	0	1	1	1	1
48	45	76	56	63	40	66
0	14	9	0	0	8	0
106	133	93	66	39	56	69
0	7	0	0	8	0	1
164	241	401	285	310	418	234
140	217	255	125	186	145	180
826	1009	1467	1056	876	943	627
10	2	24	15	12	16	2
18	11	18	17	13	29	5
111	112	158	107	102	124	93
221	105	325	83	24	54	46
26	32	51	18	11	0	18
62	30	59	40	6	58	95
35	49	57	36	49	61	35
1	0	0	0	3	5	14
244	506	733	459	580	468	166
14	22	23	15	20	29	17
141	563	391	503	567	290	0
10	0	0	0	25	0	5
15	7	42	15	7	12	14
6	0	0	1	0	0	0

For Review Only

9	6	0	2	3	3	4
117	44	162	40	68	62	119
108	163	198	128	121	137	92
132	132	133	128	130	0	112
200	237	363	205	190	199	174
3	6	0	2	2	13	1
269	214	366	225	223	242	203
42	48	130	240	281	96	241
1	1	0	2	2	1	1
0	0	0	0	2	0	5
30	42	28	59	29	24	44
65	47	97	68	51	77	41
299	180	494	227	215	207	166
62	64	98	50	38	63	32
1	1	460	1	1	1	91
29	43	87	26	22	61	43
130	146	458	346	266	190	240
1802	1972	3086	1921	1933	2016	1455
44	78	0	44	12	72	60
160	193	252	186	100	151	132
464	216	774	519	335	495	202
1	1	0	0	1	1	3
109	107	175	168	134	109	82
43	27	81	45	34	36	27
0	0	5	3	0	2	0
79	116	123	113	111	85	81
445	429	696	388	408	441	428
7	11	23	16	12	13	21
9	17	10	6	18	25	17
0	1	0	0	2	0	1
45	60	92	29	63	90	65
29	0	41	37	45	26	26
0	3	1	0	4	0	0
96	108	87	91	28	68	114
23	26	50	39	36	22	21
0	3	0	0	0	0	0
6	10	2	9	0	8	0
0	18	13	8	22	0	4
10	13	34	15	0	0	8
58	66	103	65	38	84	61
2	0	0	0	0	0	2
1	0	3	0	0	0	0
0	0	1	0	15	0	0
0	0	3	0	0	0	5
0	0	0	3	0	0	0
0	2	5	0	1	2	0
0	0	4	0	3	0	2
51	44	31	36	30	0	31
0	0	0	0	0	0	0
0	0	0	0	0	0	1

For Review Only

32	5	53	18	30	19	15
1	1	0	44	3	1	1
66	310	162	25	121	175	123
108	107	136	70	87	99	87
338	289	546	349	303	364	216
24	15	0	0	7	42	1
0	20	0	21	0	0	7
28	33	64	30	37	27	23
9	3	5	11	1	1	6
0	0	0	0	1	0	2
1	0	0	1	4	1	1
15	11	63	10	57	38	7
52	51	78	40	56	74	59
14	39	87	41	45	11	20
0	0	0	1	0	0	7
139	111	138	113	58	155	140
77	74	139	58	45	55	97
823	910	1343	797	917	1054	1032
0	0	4	0	0	0	0
67	89	106	73	74	85	66
70	56	70	61	56	56	58
101	435	0	7	33	99	90
157	159	176	148	141	134	129
360	393	552	335	353	363	338
412	553	690	509	412	478	372
18	30	41	27	25	29	35
0	0	1	0	0	0	0
6	6	0	5	15	3	5
25	33	28	16	29	26	45
260	0	281	193	231	230	242
45	54	72	49	56	46	54
90	200	140	136	63	123	103
35	34	59	38	21	35	22
0	0	0	2	0	0	8
22	20	17	23	8	32	18
1	0	0	0	0	0	0
133	191	259	109	134	212	136
81	264	343	96	290	363	262
212	316	342	260	326	334	290
510	472	1139	672	654	674	429
0	0	5	4	0	0	3
241	295	436	363	271	315	242
34	35	68	36	24	49	8
47	30	48	28	39	50	56
90	152	208	148	149	161	132
3	1	8	5	2	0	3
72	19	125	52	104	50	42
127	135	256	107	115	180	123
71	36	127	45	75	61	80
151	74	158	77	100	94	78

For Review Only

163	138	310	178	159	234	173
262	314	305	304	265	127	68
46	34	92	59	72	53	45
167	152	277	160	74	113	267
0	0	21	0	4	10	0
396	345	1140	597	712	481	181
1	1	0	1	1	1	18
55	47	60	48	49	74	59
0	0	13	0	0	0	23
117	117	192	84	119	169	160
49	68	25	40	26	15	56
79	100	147	112	97	109	48
85	83	129	69	94	114	74
31	66	89	61	63	83	64
281	230	410	270	322	361	213
32	3	46	30	20	37	69
0	9	9	13	12	13	0
222	214	266	142	199	208	179
0	0	0	0	0	0	0
1	3	0	0	3	1	22
53	66	45	15	35	34	31
10247	11560	16936	11260	10135	11215	8235
29	50	68	55	56	63	48
0	0	0	2	0	0	3
0	0	0	0	0	0	25
9	9	42	30	31	14	22
82	131	231	113	132	97	84
25	31	79	15	36	20	33
171	602	1018	246	77	616	425
66	105	89	47	43	49	55
0	0	0	0	0	0	13
4	10	0	0	0	6	7
0	7	0	0	0	0	0
25	38	65	15	5	31	3
55	52	158	15	64	13	36
145	195	346	134	177	222	159
60	0	31	29	36	36	0
46	100	119	82	51	74	59
41	38	62	32	51	47	34
61	95	151	96	64	154	82
32	52	67	36	35	18	35
151	275	233	174	261	303	197
55	37	123	52	27	55	59
54	80	115	54	53	61	61
24	32	30	20	40	12	31
7669	9867	14610	8719	8748	10699	7530
9	11	25	38	20	10	16
33	47	58	41	51	30	32
43	48	87	60	75	50	64
478	631	1033	539	550	529	570

For Review Only

0	0	0	0	7	4	18
9	35	56	10	29	25	74
167	191	294	172	207	199	188
55	46	105	47	32	46	50
13	19	33	24	11	13	40
11	21	0	0	0	0	36
36	11	56	93	42	62	18
138	120	225	134	139	169	183
0	0	4	0	0	7	13
0	0	0	0	0	0	143
0	39	73	0	45	30	33
5	18	54	33	49	10	26
436	454	1002	691	684	598	490
1064	1473	1568	840	918	1053	1292
5	0	2	4	0	0	0
9	17	39	18	18	12	25
1	1	0	1	269	3	5
0	1	0	0	0	1	0
0	0	0	0	0	0	0
12	0	10	17	12	7	32
0	0	1	0	0	3	16
0	3	0	0	0	4	2
41	16	23	17	19	27	45
7	8	0	6	0	0	6
0	0	7	0	7	0	0
1285	1513	2106	1489	1309	1545	990
77	27	0	0	36	126	50
7	0	0	0	2	3	2
0	0	0	0	0	0	33
0	0	0	0	0	0	7
0	0	2	0	0	0	0
25	8	40	49	11	22	12
82	115	151	92	101	86	120
0	0	10	0	0	0	46
499	543	702	369	391	497	440
7	9	27	23	5	55	8
0	0	0	0	0	0	18
3	0	0	0	1	2	0
0	17	5	0	26	0	0
11	14	30	18	5	7	16
0	0	1	0	41	46	46
37	83	93	67	52	35	39
178	235	213	186	163	165	135
109	127	235	136	112	161	98
11	10	25	4	16	30	10
207	264	314	136	237	141	246
0	0	4	4	0	5	6
15	28	66	50	9	10	50
161	143	217	162	128	168	114
0	0	17	0	0	26	22

For Review Only

30	14	0	26	12	0	0
1	1	1	1	1	3	0
50	73	140	52	29	69	89
0	0	0	8	0	0	0
0	0	4	0	0	0	1
269	293	521	121	283	347	223
121	97	183	145	124	181	143
0	0	29	12	10	18	5
113	93	135	85	107	91	74
651	1081	1472	747	696	874	590
248	275	484	299	280	414	260
0	0	0	12	0	13	0
27	41	113	44	90	106	0
64	69	89	63	79	102	69
57	74	86	50	47	90	73
14	15	8	70	20	44	16
167	130	230	69	152	113	86
5	12	11	3	12	1	3
0	1	0	0	0	1	0
180	0	269	66	254	27	126
111	124	140	101	38	134	64
70	60	150	60	69	70	79
546	636	1242	694	693	727	8
134	0	801	415	0	383	0
17	11	26	7	13	15	29
39	43	77	46	53	34	43
34	49	85	43	69	39	23
74	70	138	81	97	69	61
21	23	49	26	20	46	27
30	32	50	44	27	15	27
81	83	126	77	0	65	0
0	0	6	0	0	0	5
161	185	261	180	213	197	132
38	54	23	41	0	0	34
3	0	15	3	2	4	1
0	0	175	0	0	0	0
0	0	0	0	0	0	1
73	103	172	124	81	85	84
83	72	178	93	99	88	137
0	0	1	0	0	1	8
0	2	0	0	0	0	0
0	1	12	0	0	8	0
678	528	1379	701	647	792	429
16	74	36	13	26	43	64
5	3	0	4	3	2	14
60	21	204	114	26	60	126
28	7	24	10	5	8	12
69	49	97	60	52	62	41
232	241	283	207	165	247	143
3	5	0	0	3	1	0

For Review Only

100	120	132	109	105	87	63
119	114	183	131	130	30	90
21	21	61	18	15	5	6
3	2	0	5	0	1	2
180	164	287	142	183	132	155
43	25	161	61	18	7	24
0	0	0	0	2	3	0
18	8	15	12	11	0	10
4	0	3	0	0	3	0
10	8	11	9	10	9	44
2	9	20	17	7	6	6
375	443	655	385	348	373	358
122	111	342	204	196	119	161
66	139	215	0	74	22	44
0	1	0	2	1	3	12
52	73	98	96	61	68	39
37	58	107	29	32	72	40
0	0	0	2	0	3	3
0	6	6	4	8	4	8
0	0	24	3	5	5	0
429	505	561	274	382	298	195
1	3	0	1	8	1	1
195	162	360	218	230	208	212
39	41	70	79	55	71	45
1	0	9	0	0	5	4
0	0	0	0	0	0	4
0	0	4	1	1	0	0
423	48	0	266	115	170	8
0	0	0	1	0	0	0
0	0	9	1	6	6	1
3	1	0	1	0	1	0
0	0	0	0	0	0	0
20	22	43	23	23	14	22
22	1	144	28	134	5	31
1	2	0	0	0	0	2
1	0	0	3	1	1	26
0	0	1	6	0	7	0
111	117	126	89	103	97	104
0	0	9	3	1	0	0
422	238	186	247	354	279	0
0	0	0	0	0	0	0
1	11	29	6	2	2	1
42	116	217	41	30	65	11
2	10	0	6	17	13	4
49	65	93	64	27	83	48
36	33	50	36	24	49	29
29	22	12	7	7	19	5
55	82	122	97	89	54	72
25	23	42	11	33	20	14
16	10	16	3	11	16	19

For Review Only

0	0	0	0	0	0	13
2	4	0	0	0	0	0
132	102	183	115	109	129	75
34	30	35	18	22	30	40
1	3	11	3	7	2	6
18	30	24	3	15	8	19
300	310	0	122	244	1	8
0	0	1	0	0	0	9
10	11	2	0	0	0	7
114	79	292	87	484	51	46
82	81	112	96	85	68	79
34	16	46	6	17	7	11
1321	2888	2467	4397	3864	1275	2082
69	89	142	63	68	83	68
0	0	6	0	0	0	4
141	126	200	114	138	175	139
27	21	34	15	33	32	22
9	9	109	96	105	87	91
63	81	185	110	106	69	95
29	31	37	28	46	26	18
102	117	179	79	83	136	86
79	139	142	75	110	59	54
75	116	508	247	47	204	0
154	159	173	166	109	144	65
581	74	581	226	437	416	382
64	61	82	62	73	88	30
259	594	2518	422	550	251	62
8	19	32	13	5	10	4
0	121	0	0	2	26	11
30	30	39	11	26	28	29
16	27	23	10	7	10	9
11	27	41	9	21	10	19
66	5	137	70	44	13	63
0	0	199	0	0	202	0
23	19	26	28	29	0	27
0	0	0	0	0	0	1
6	1	0	2	1	0	6
4	1	18	12	11	10	13
3	0	0	0	0	0	0
4	0	4	0	0	0	0
240	233	341	199	224	281	219
178	5	549	228	625	1	159
1	0	1	0	4	0	5
569	445	1045	495	705	583	394
0	1	0	1	0	1	1
1	1	0	1	1	1	1
46	82	93	97	88	104	56
135	149	201	105	110	160	241
3	18	23	4	13	17	15
741	841	1381	884	967	560	275

For Review Only

0	1	49	1	0	0	3
24	40	65	49	54	83	67
23	21	56	32	24	19	18
11	44	137	75	7	50	1
0	2	0	1	1	5	0
60	39	104	77	33	104	43
152	222	257	187	207	144	175
1	10	0	1	1	10	1
1	3	12	3	11	0	0
29	8	113	87	39	7	6
0	0	0	0	4	0	1
39	53	68	48	66	45	56
71	382	380	575	293	497	501
47	40	80	39	61	82	41
96	114	238	104	58	111	95
21	55	103	23	27	56	22
0	0	8	5	0	1	3
433	454	689	396	349	512	276
48	46	133	96	93	45	49
6	1	0	3	0	0	0
0	0	0	1	0	0	0
35	33	76	41	37	43	68
1	1	0	0	0	0	33
0	6	11	0	5	0	5
80	17	18	13	0	47	37
93	134	239	112	160	118	115
340	201	463	217	218	328	169
12	15	41	14	8	27	69
21	25	49	24	17	18	28
2	0	6	0	2	4	6
68	48	91	74	56	64	32
0	3	3	0	0	1	22
124	100	175	110	95	114	58
1	0	0	0	0	0	11
537	608	979	567	481	318	397
70	95	121	63	90	76	59
0	0	3	0	0	0	0
3	0	4	0	0	4	0
56	60	93	43	56	56	36
36	49	54	36	56	47	25
10	0	0	1	9	1	3
36	48	96	40	68	39	48
23	59	71	61	61	51	24
65	77	54	78	51	0	0
1	2	0	2	1	9	15
0	0	0	2	1	1	3
1	0	0	5	5	0	5
269	367	633	451	356	268	266
14	24	52	17	22	15	25
11	5	0	5	5	16	3

50	38	104	83	56	80	27
20	14	28	6	13	18	12
0	1	0	1	1	1	1
10	26	34	8	20	9	30
8	0	8	2	0	2	4
8	7	7	1	10	5	2
23	29	57	70	24	26	41
0	0	0	0	0	0	86
0	2	3	0	2	0	0
292	268	482	360	319	235	318
0	0	0	1	1	0	0
16	3	30	18	13	2	3
23	35	56	29	26	43	38
96	83	0	93	62	79	70
41	57	52	24	26	25	35
0	0	0	0	81	0	0
83	71	131	71	59	57	71
7	1	22	20	6	0	8
15	4	13	14	9	5	1
1	17	9	8	4	13	4
0	22	0	4	6	8	6
108	129	132	109	93	135	46
0	2	0	0	0	0	2
28	34	31	0	64	0	46
0	1	0	1	7	3	1
18	23	39	47	38	11	31
22	38	83	4	1	1	2
9	16	26	14	22	16	15
92	88	125	150	71	59	25
7	4	0	12	7	1	1
24	22	37	29	30	37	17
2	17	75	13	17	1	34
22	12	20	24	7	15	6
0	1	0	75	15	1	67
14	48	52	10	21	64	15
35	39	34	16	27	38	43
0	12	18	16	10	7	0
17	16	31	14	26	15	9
214	181	349	206	168	198	200
401	307	600	288	372	364	265
38	37	66	36	35	38	34
53	16	170	64	31	0	17
169	237	277	183	225	199	235
5	6	50	7	39	8	18
9	5	14	7	4	6	11
4	3	13	11	17	5	9
67	42	54	90	54	84	50
314	361	0	548	423	442	32
49	51	148	78	72	119	60
1	1	28	2	1	1	0

For Review Only

613	777	1051	718	628	779	592
101	43	163	69	77	72	59
6	1	0	1	1	1	0
7	8	10	17	4	13	1
0	0	0	0	0	0	0
35	20	51	13	16	33	20
106	39	145	91	103	78	79
0	23	25	20	13	16	12
21	16	89	86	107	45	10
0	0	9	3	0	2	0
37	53	61	0	42	60	39
266	219	175	657	340	302	288
26	13	23	16	12	5	4
1245	1265	2521	1726	1504	1649	1058
0	4	0	0	0	1	0
76	69	76	62	52	60	129
13	4	15	4	6	3	4
2	0	0	0	0	4	3
13	31	6	12	14	5	32
55	44	86	71	63	77	78
0	54	77	0	0	38	0
11	8	10	6	2	16	1
0	24	0	0	0	0	0
1	1	0	1	1	1	1
8	7	17	13	7	5	19
0	0	0	0	0	0	12
18	5	58	85	8	26	30
44	93	133	47	63	24	51
44	19	62	37	29	51	45
185	204	252	172	175	228	188
55	81	164	80	56	97	85
86	170	263	230	157	122	94
1	1	0	127	1	41	6
47	78	144	80	103	60	91
80	62	71	63	148	92	74
50	32	56	34	24	44	36
81	93	77	35	47	29	41
99	130	220	156	145	101	79
1	1	0	0	1	1	1
65	396	0	11	34	39	27
34	63	73	62	67	50	24
196	217	254	135	113	212	63
146	110	198	121	84	144	90
256	297	582	454	423	153	99
8	28	43	2	14	15	12
27	26	62	38	30	22	44
125	100	123	97	94	133	18
109	124	0	146	117	142	79
22	42	39	21	29	21	40
30	14	14	7	14	11	15

For Review Only

0	0	0	0	0	0	7
1	0	5	1	0	1	4
85	52	211	179	58	80	54
0	0	0	0	0	0	0
0	3	0	4	3	2	4
4	7	0	3	1	1	0
214	223	284	111	97	252	122
13	10	8	28	14	9	16
29	35	35	7	19	21	53
0	8	30	12	7	3	11
223	0	346	130	0	0	229
20	82	87	12	38	64	45
0	58	53	0	69	75	0
116	161	217	106	200	144	96
394	340	774	626	469	527	271
149	126	282	159	202	159	176
0	0	0	0	0	0	57
0	0	0	0	0	0	91
39	39	57	27	25	50	34
137	113	265	149	124	143	98
166	181	321	156	185	137	114
372	387	583	324	370	394	320
58	67	147	109	88	75	150
63	71	152	86	70	104	96
17	139	94	18	31	46	8
54	87	0	91	44	74	84
3	14	18	1	6	2	3
0	2	0	1	0	0	0
147	90	241	126	84	101	61
42	26	86	41	8	14	18
42	78	70	54	49	75	62
1	1	0	1	1	1	1
23	35	35	27	32	30	45
145	149	272	141	169	184	136
402	353	486	356	380	503	380
0	0	181	148	0	150	135
57	48	58	50	41	34	74
56	86	117	55	53	74	28
14	6	0	0	11	10	8
89	10	363	186	87	114	1
0	1	0	3	10	0	7
17	41	40	23	13	15	23
0	63	1247	64	496	581	427
0	0	7	1	0	0	6
77	75	136	91	51	55	24
320	291	567	388	349	337	115
2	0	0	0	0	2	0
215	172	404	334	24	169	105
237	43	159	141	81	47	8
0	0	0	8	0	0	0

For Review Only

57	46	75	64	47	66	42
74	66	204	82	62	86	50
3	0	5	0	0	0	0
0	0	6	0	0	0	0
0	0	0	0	1	0	0
101	137	232	111	141	163	93
37	0	0	29	5	0	0
0	1	0	2	2	1	1
0	0	0	2	0	0	28
19	34	38	26	28	31	24
0	0	12	1	4	0	0
87	84	154	89	73	203	142
8	5	4	0	0	4	12
16	26	26	23	24	34	27
0	0	0	0	0	0	85
0	0	0	0	0	0	15
4	0	9	3	2	0	0
339	183	633	285	310	323	401
1	2	0	1	38	1	3
1	0	16	0	0	1	1
21	25	43	20	32	9	42
0	0	0	0	0	0	0
7	8	11	11	9	5	50
0	1	12	12	6	0	3
3	26	8	12	10	1	0
25	48	30	23	45	8	28
43	41	70	25	36	86	87
23	18	36	28	27	18	26
356	64	157	195	0	0	0
1108	1147	2201	1526	1484	1198	836
396	478	774	488	310	353	527
0	0	0	0	3	0	3
71	74	152	83	100	88	59
124	95	131	113	147	68	99
29	42	53	45	30	70	23
4	0	1	7	0	0	32
0	0	1	0	6	8	1
51	34	24	13	45	39	18
48	50	104	48	51	52	42
86	105	254	188	155	104	125
502	450	708	760	539	541	321
0	0	0	0	0	0	174
0	0	0	0	0	0	29
1	10	6	4	82	2441	7
80	315	0	229	16	375	49
8	8	26	9	13	12	11
0	0	0	0	0	0	0
33	2	62	17	20	16	6
62	50	63	36	40	44	67
1	0	0	0	0	1	0

For Review Only

0	0	0	0	0	0	0
6	6	6	5	1	4	18
166	94	305	144	85	153	119
8	12	46	23	7	25	11
29	50	62	1	16	65	93
0	1	0	6	2	0	1
28	27	46	19	32	23	30
146	134	252	109	153	147	91
20	30	9	10	22	14	4
53	44	101	49	51	69	54
74	95	140	79	74	113	87
57	13	229	43	232	51	4
73	33	115	43	53	101	22
13	37	45	39	14	22	28
32	86	68	66	57	59	54
105	134	188	176	114	150	109
236	277	507	239	311	251	303
58	62	106	34	54	54	76
79	96	121	117	103	124	123
1	0	0	5	0	1	7
87	98	142	71	67	85	86
45	20	19	4	13	41	8
18	79	55	36	20	34	30
1	0	20	0	17	28	1
222	232	385	174	289	197	150
184	171	240	164	145	209	140
16	38	26	12	17	37	15
2	4	4	3	4	4	10
81	32	103	66	63	56	37
0	0	120	0	0	0	0
95	34	141	168	46	102	76
26	56	31	32	25	20	44
593	57	257	3	155	245	48
20	14	34	25	12	11	24
9	20	15	26	4	45	24
0	0	0	10	5	0	0
44	49	76	42	52	46	49
113	125	153	86	62	44	79
25	20	38	13	13	19	0
46	149	150	25	4	246	82
7	2	26	6	17	16	15
34	13	48	22	10	29	40
32	41	119	49	37	78	72
85	102	179	71	56	58	77
207	213	355	147	211	214	185
76	97	110	74	61	78	87
107	97	119	38	58	55	80
114	124	221	138	112	122	165
323	283	496	252	216	437	161
100	91	248	0	0	95	89

For Review Only

50	86	98	76	62	58	50
68	89	175	36	81	99	56
45	77	87	35	60	66	63
80	135	71	99	0	0	20
0	56	1	0	0	0	0
69	125	135	74	83	75	66
37	17	22	37	20	8	8
0	24	19	0	17	28	31
61	59	93	72	43	61	64
23	34	17	1	19	21	11
187	166	334	206	184	121	105
1	21	0	17	29	29	80
44	11	31	18	2	8	16
68	58	90	72	64	60	27
0	0	17	0	0	0	0
8	4	9	0	4	7	5
73	50	97	86	71	43	58
15	5	21	12	20	9	24
20	8	29	12	5	6	9
4	7	12	5	3	10	8
173	193	319	217	205	142	149
18	34	71	40	38	16	38
0	1	0	0	0	0	0
14	11	40	19	12	0	12
117	91	115	20	13	120	140
53	75	68	43	54	79	27
4	0	3	1	0	2	0
0	0	0	0	0	0	10
233	143	308	299	155	227	126
141	148	198	155	195	182	115
46	58	0	63	72	62	42
6	0	0	8	1	0	0
35	74	51	43	27	38	19
0	0	0	0	0	0	0
43	42	108	45	69	74	33
30	21	74	33	36	21	30
11	12	20	18	10	18	17
0	0	0	0	0	6	0
0	0	0	0	0	12	0
260	339	409	249	277	314	225
37	32	73	38	8	58	11
66	89	153	75	84	89	101
81	78	120	71	81	84	87
0	15	10	11	0	13	21
131	90	136	79	94	105	55
52	53	77	66	67	50	35
36	3	97	106	135	8	21
106	123	176	81	122	113	118
0	1	9	0	3	0	2
39	51	30	12	35	72	58

For Review Only

73	73	137	53	91	55	70
41	40	57	40	43	45	38
1	39	0	12	35	1	1
117	160	262	107	142	134	127
98	42	108	71	70	84	43
48	75	87	79	57	61	61
119	52	122	75	95	45	73
180	117	277	243	131	230	120
1	9	10	14	11	5	25
0	0	0	0	21	0	25
0	2	4	4	4	1	1
0	0	12	0	0	0	11
0	0	0	0	12	0	9
29	39	59	22	35	27	19
0	0	12	0	0	0	6
100	67	99	74	90	51	81
11	4	13	7	8	5	3
0	0	0	0	0	0	0
0	4	0	10	10	5	0
7	1	14	5	12	15	6
38	38	97	60	56	33	49
2	3	0	0	1	1	0
40	51	51	19	45	38	26
173	186	306	119	102	101	152
1	0	0	1	0	0	0
0	0	35	0	0	0	0
0	1	0	0	3	1	0
3	3	6	3	3	3	2
0	0	6	0	0	0	0
25	17	28	3	6	5	29
2	2	1	4	2	1	2
2	1	0	2	4	3	2
2	7	25	5	13	1	5
6	24	34	16	22	13	11
3	11	15	14	16	11	1
0	1	1	0	0	0	0
0	0	3	0	1	1	0
0	1	0	1	1	1	0
3	2	0	1	2	1	1
1	4	6	1	4	3	1
2	3	1	1	1	0	1
5	13	22	7	8	11	5
4	3	0	2	4	2	2
0	3	1	3	3	1	4
1	4	1	0	3	1	1
0	0	1	1	0	0	4
3	1	3	3	1	3	1
2	0	0	2	1	1	0
3	6	10	3	3	8	3
6	5	11	6	9	8	1

For Review Only

0	4	0	1	1	1	2
0	0	3	4	0	1	0
0	0	1	0	1	0	0
2	2	0	1	0	1	1
0	0	3	0	0	0	0
1	3	0	0	0	3	3
38	32	30	38	17	39	35
141	121	305	117	179	150	97
147	139	395	74	169	161	151
9	17	19	13	18	12	9
61	67	65	60	30	49	46
30	36	40	24	24	30	27
91	78	144	80	65	89	78
1	5	418	1	1	1	49
56	436	222	104	127	39	26
107	151	200	124	119	148	107
0	64	0	0	0	0	0
138	0	1	0	265	236	0
0	65	36	0	74	0	76
12	20	16	11	0	20	12
22	36	43	35	19	33	35
4	33	23	7	16	19	11
0	52	49	32	0	44	43
47	54	0	36	0	0	0
0	14	19	15	0	4	0
28	34	15	32	27	40	35
0	0	26	11	77	6	0
0	0	0	0	0	0	0
3	2	27	4	2	5	3
1	0	0	0	1	0	1
18	39	38	35	23	29	16
183	390	193	4	53	85	113
176	168	279	150	158	198	145
99	124	207	129	98	134	85
41	41	43	16	50	46	39
46	23	47	47	19	53	19
93	205	304	113	297	253	181
98	113	256	119	168	121	104
41	36	60	45	41	68	32
15	21	41	10	13	16	13
429	355	368	0	91	327	326
251	39	429	367	324	280	314
142	139	364	142	208	175	169
159	188	368	194	195	202	202
188	195	167	195	158	173	116
25	15	11	15	9	13	17
7	0	10	16	11	17	10
45	43	123	49	67	46	57
15	19	20	6	19	13	18
64	18	129	60	139	153	164

For Review Only

311	236	640	363	313	260	249
231	249	323	163	181	211	238
71	72	153	97	79	80	90
0	20	2	18	20	28	5
13	3	24	1	12	11	17
140	143	297	147	142	191	120
168	783	198	126	277	827	158
244	187	380	304	242	253	266
3	0	0	0	0	0	0
109	114	160	96	76	110	87
29	40	35	32	48	60	33
229	204	417	277	237	285	188
8	0	15	3	9	0	7
8	5	0	5	8	5	2
0	0	5	4	0	0	9
0	0	0	0	0	36	0
7	17	49	22	52	19	17
42	28	80	40	37	30	53
43	62	155	4	49	19	100
39	52	64	34	24	62	42
240	236	316	222	200	260	242
74	74	107	65	65	55	62
0	3	0	1	0	0	10
1918	1954	2946	1771	1894	1964	1535
170	156	521	149	175	159	173
0	1	11	4	1	8	43
1	0	0	0	1	0	16
5	0	4	0	0	0	12
47	301	0	1	16	1	22
3	2	0	77	78	14	127
143	265	273	194	0	460	399
50	24	47	10	31	68	42
24	35	32	14	36	43	26
0	0	23	0	1	0	0
421	457	836	555	384	491	261
0	2	4	1	1	2	0
0	2	14	1	2	0	17
5	8	11	9	2	8	3
0	0	0	0	0	0	12
12	37	47	52	34	43	122
0	0	0	0	3	0	0
0	0	0	0	0	0	4
2	0	0	0	2	0	1
0	0	0	0	0	0	5
142	139	191	64	98	124	100
21	30	35	21	22	27	52
71	48	97	89	53	54	37
29	25	43	50	33	45	39
34	26	61	43	47	33	28
85	91	187	71	82	112	79

For Review Only

6	3	12	12	12	4	9
24	29	57	44	38	24	38
0	0	4	0	0	0	0
42	57	101	38	95	33	33
5	5	15	8	3	18	6
1	1	4	0	0	0	3
0	0	3	0	1	5	0
0	3	6	0	0	1	0
114	137	237	123	139	95	127
4422	4819	6566	4465	4294	4935	3704
0	0	1	2	0	2	60
40	48	105	25	41	49	40
7	0	20	3	1	1	2
567	572	1074	618	727	551	419
0	0	0	3	0	2	2
56	82	154	68	80	92	25
0	0	3	5	0	0	0
0	0	0	1	0	0	1
13	26	0	3	39	14	6
0	0	0	1	0	0	1
55	34	75	59	52	53	55
4	0	6	0	4	4	0
4	5	9	28	10	6	10
20	19	16	5	9	5	3
0	0	0	0	0	0	3
3	11	18	11	5	22	15
1	0	0	0	0	0	3
3	0	0	0	0	0	5
0	21	235	0	10	0	126
372	603	635	423	450	466	430
247	328	725	336	496	303	170
0	0	0	0	0	0	0
0	0	0	4	0	0	0
291	77	225	162	379	127	139
94	80	117	61	85	100	69
0	0	0	0	1	0	0
0	0	0	3	3	0	0
59	44	58	56	32	37	28
71	82	80	42	41	89	53
68	129	30	78	25	0	0
148	96	351	290	152	158	162
51	158	143	117	95	143	106
40	18	53	19	25	40	29
72	21	106	12	42	51	7
191	275	465	254	218	279	152
23	33	45	19	22	17	22
37	46	98	50	65	52	65
75	95	155	66	67	81	70
0	0	415	0	0	0	0
0	1	0	0	0	0	1

For Review Only

100	37	70	78	108	128	62
107	116	0	1	1	3	1
38	48	73	44	54	23	59
0	0	0	0	0	0	20
12	4	12	3	1	6	21
130	140	221	149	138	187	107
49	46	68	68	46	42	51
1	12	1	0	0	0	44
236	355	489	250	432	326	277
36	0	83	133	0	150	0
76	103	142	58	56	116	107
26	30	0	34	34	37	26
63	98	113	78	75	61	56
100	106	226	209	252	81	133
2	4	9	5	5	0	4
51	48	132	44	41	48	56
15	9	41	21	25	23	13
33	15	39	6	38	43	10
29	14	47	47	4	36	32
7	41	18	10	16	29	48
27	398	852	449	491	437	342
32	0	56	0	0	0	38
0	0	84	0	0	11	7
51	26	102	44	41	43	133
0	0	0	0	0	4	5
10	5	37	7	7	7	136
2	9	0	3	7	0	3
0	4	9	3	2	3	0
32	79	67	8	31	31	14
64	54	152	108	111	84	106
234	233	417	283	262	237	247
115	42	96	151	40	41	35
85	78	159	101	153	103	143
0	0	0	5	0	0	0
6	10	12	4	2	0	1
238	269	308	189	189	309	144
1197	520	1450	215	481	906	243
109	20	66	0	23	62	29
83	56	133	101	85	50	71
45	78	79	24	40	52	57
345	312	512	313	318	371	222
0	1	2	0	0	0	0
3	1	0	0	2	0	2
109	108	262	164	134	197	67
20	67	213	112	106	115	76
59	38	82	40	55	26	26
88	179	198	151	165	157	161
28	44	38	31	39	29	38
20	10	22	7	12	26	19
40	43	81	39	34	38	47

For Review Only

0	176	1105	1408	346	0	0
3	6	41	0	4	0	7
124	191	201	145	141	167	223
77	82	111	94	99	120	115
18	21	65	36	35	12	8
0	0	56	0	0	0	0
96	166	221	106	148	130	140
87	84	99	64	72	72	84
43	63	77	57	61	59	44
61	88	102	71	82	88	60
68	86	91	55	77	82	54
147	86	240	126	167	115	128
83	135	135	124	136	139	41
78	107	70	70	68	76	83
43	46	48	40	32	39	32
68	91	133	55	54	81	75
269	257	212	118	149	169	237
189	232	296	162	177	218	179
49	19	48	57	34	31	52
25	1	53	18	0	1	13
217	331	405	277	319	185	311
1405	877	3675	506	1	2	251
94	93	125	42	21	74	54
39	69	151	72	76	73	93
150	168	209	76	100	86	75
83	0	403	36	8	31	15
59	127	86	0	0	0	0
0	171	0	0	0	1	0
151	145	202	58	53	117	44
57	0	5	0	0	0	0
0	36	0	35	140	61	0
12	30	1	0	1	39	129
16	121	0	30	0	149	12
5	7	4	0	12	6	5
220	194	402	231	217	272	213
24	20	36	19	22	24	8
115	141	149	105	132	148	101
108	154	218	3	119	112	73
189	2	0	1	437	379	3
17	45	40	30	24	42	24
34	70	78	35	34	27	49
0	0	0	4	0	0	0
110	161	306	137	124	163	128
100	75	182	70	95	87	75
575	1262	500	779	340	1321	1426
10	13	0	24	26	8	8
0	0	0	0	0	0	0
3	11	35	12	3	1	19
9	0	18	0	0	0	10
499	500	1040	518	541	553	579

For Review Only

188	218	345	194	185	267	201
210	297	606	272	299	264	256
0	10	41	148	0	20	0
109	98	117	82	73	87	76
17	18	0	18	19	22	27
0	0	0	0	0	0	0
153	154	219	161	146	129	165
8	3	20	3	5	5	15
103	96	238	137	105	137	149
35	48	55	50	38	71	20
0	0	9	0	0	0	0
15	15	9	13	9	3	21
145	198	192	128	141	132	110
29	42	44	28	36	44	20
33	37	45	36	28	30	34
93	207	173	93	126	133	102
772	242	287	265	813	323	213
51	64	143	142	81	51	88
163	150	391	221	309	154	146
0	1	0	0	0	0	10
1	0	0	0	1	1	3
500	514	717	501	433	536	207
13	16	0	21	26	23	18
15	18	8	10	0	16	17
74	88	140	106	56	138	88
4	6	0	32	5	4	7
0	0	5	0	0	0	0
7	24	13	21	17	30	30
7	12	32	22	22	12	26
1	1	0	3	19	47	1
0	0	0	10	0	0	0
128	73	236	69	107	89	103
244	108	381	97	209	269	192
0	0	13	26	0	17	0
63	67	127	39	45	165	53
66	39	121	73	55	67	61
72	101	108	64	98	81	57
115	142	226	119	132	145	135
1	0	0	1	0	0	0
12	24	37	26	26	10	21
0	0	18	0	2	0	10
0	0	0	406	0	569	226
234	6	0	1	6	41	191
2	2	0	5	5	2	13
23	61	88	1	3	13	1
4	0	5	4	2	0	2
38	55	117	65	54	54	92
185	158	213	188	190	124	62
230	428	511	421	552	691	512
118	139	309	139	123	206	171

For Review Only

26	116	181	41	19	8	30
267	153	727	357	262	343	56
53	38	89	53	42	52	30
219	138	286	214	200	141	101
367	466	756	527	396	472	287
0	135	0	36	83	98	45
33	19	24	25	7	15	28
8	18	11	5	10	10	16
0	0	7	0	0	0	0
0	0	0	0	0	0	2
3	3	0	17	4	4	5
1	0	0	0	0	0	0
122	108	195	125	99	123	66
12	10	16	12	24	14	10
0	0	53	0	0	0	0
431	405	572	349	356	426	243
62	54	63	51	52	42	46
130	84	154	117	94	135	106
84	137	0	0	0	0	0
77	120	136	81	92	68	79
17	24	54	30	16	31	25
79	87	141	73	74	55	87
68	61	11	0	14	0	134
0	12	0	0	9	8	0
47	48	88	76	57	54	51
41	50	26	21	20	4	29
0	0	11	4	0	0	3
84	47	115	13	79	54	32
36	13	95	52	48	35	42
0	0	0	0	0	0	0
82	106	149	79	61	80	79
57	75	74	60	37	79	55
11	8	15	13	9	16	26
33	24	75	13	18	32	30
83	139	113	116	156	117	68
36	80	0	60	26	73	63
5	5	2	3	4	3	5
9	16	20	18	29	4	25
0	0	6	0	2	2	3
0	0	7	1	0	1	8
22	48	103	24	2	4	71
84	65	159	121	8	33	24
53	65	128	69	61	98	50
132	147	151	136	109	168	152
24	13	56	25	50	46	51
116	112	172	99	125	109	80
1	0	0	0	0	0	0
56	81	60	52	45	50	68
49	67	57	42	45	29	55
94	63	48	54	54	94	44

For Review Only

432	434	876	634	566	462	397
37	70	110	66	79	52	104
160	14	0	1	1	1	38
0	0	13	0	6	3	0
94	130	168	115	135	139	96
131	151	265	134	123	148	130
51	68	134	53	67	86	58
59	75	103	90	80	98	54
0	0	0	5	0	3	0
12	10	23	7	14	14	13
83	126	141	88	116	145	103
25	20	26	45	19	11	13
5	8	0	12	5	1	6
0	0	0	0	0	0	0
70	52	71	46	40	53	83
339	370	716	490	429	374	383
84	30	0	10	20	21	26
48	62	85	36	28	38	21
145	0	0	0	0	0	112
104	87	160	87	95	167	91
28	22	28	25	15	57	21
21	14	56	30	44	9	13
95	72	167	71	74	94	110
7	22	28	11	12	21	17
1	6	10	12	15	4	10
90	96	129	83	70	112	75
64	128	84	81	63	84	106
95	156	195	97	87	73	85
92	106	116	80	76	86	56
53	72	42	31	52	43	22
43	20	25	14	13	19	5
86	103	168	87	124	74	51
56	48	92	76	68	59	51
101	69	171	66	95	54	115
97	123	147	91	95	168	112
0	2	0	0	0	0	7
7	25	26	20	34	8	6
0	23	1	0	155	0	0
1	0	4	4	0	0	1
1	0	6	2	2	0	4
2	4	8	9	2	2	1
62	101	193	80	75	135	48
0	0	38	0	78	0	47
61	96	96	81	74	81	91
45	45	83	44	54	60	54
118	143	245	32	67	191	266
84	64	203	78	70	146	95
250	273	536	256	113	189	179
13	3	122	1	1	40	1
117	104	219	111	106	131	0

For Review Only

179	149	312	194	383	233	169
34	7	44	2	23	5	5
81	0	3	75	0	101	0
37	36	55	33	26	58	36
126	203	298	0	0	110	46
84	110	231	98	94	93	82
55	15	73	72	36	33	10
41	40	116	21	36	38	28
0	32	0	24	0	0	56
0	0	0	0	0	0	2
605	634	1015	617	605	729	558
62	43	126	73	50	53	24
0	3	13	3	5	14	4
0	0	3	0	0	0	4
771	889	22	817	0	0	686
134	145	272	65	84	191	119
51	49	36	23	35	39	21
2	2	3	1	0	2	4
84	84	192	77	128	131	134
47	0	0	71	4	0	0
17	36	26	18	19	28	19
34	39	44	33	24	21	20
54	21	50	32	40	30	52
53	47	81	44	50	62	13
1	9	0	30	17	15	12
199	196	311	98	199	198	139
42	92	80	67	70	81	71
2	9	9	8	4	8	13
57	64	82	42	54	76	42
8	16	33	17	28	28	26
0	0	0	0	0	0	0
8	6	24	2	9	14	0
0	2	0	0	2	0	3
1	1	20	0	1	1	5
72	92	149	83	106	144	101
372	322	407	260	208	390	264
56	50	53	71	28	47	39
1	19	10	9	10	9	12
112	133	162	111	150	126	96
0	0	1	8	0	0	16
4	8	8	2	1	5	15
0	0	0	0	5	0	0
15	5	19	24	17	12	15
480	0	107	0	0	432	0
0	127	2	0	0	0	0
0	0	0	4	3	0	0
169	135	189	114	161	162	171
19	26	90	27	15	73	36
66	74	66	55	61	156	71
60	29	80	44	33	0	58

For Review Only

0	0	0	0	1	5	7
0	0	11	0	4	0	10
0	0	0	1	0	0	4
38	56	83	23	32	104	54
4	0	5	3	1	1	4
335	386	649	458	380	438	339
58	48	104	41	42	45	57
43	44	58	60	28	66	66
0	4	7	0	0	0	4
2	3	6	4	4	3	2
0	3	3	0	0	0	0
0	0	0	0	3	3	0
25	0	21	7	11	16	10
13	7	1	3	11	5	4
0	0	1	0	0	0	5
0	0	0	3	0	0	0
101	77	0	55	87	119	40
58	70	117	56	61	68	70
63	92	163	107	50	161	122
15	0	0	0	0	0	0
74	57	67	93	117	43	64
257	254	469	257	283	329	235
4	18	35	6	12	36	23
0	1	8	1	4	1	0
903	482	1813	1630	1387	869	487
7	12	34	8	7	1	18
19	1	13	17	16	14	18
10	5	19	5	17	6	9
56	146	14	98	100	62	100
26	28	46	31	38	18	35
19	8	23	11	9	7	5
31	41	97	81	51	48	51
16	33	39	21	35	26	38
4	0	0	8	5	0	9
6	15	19	11	10	8	11
81	76	120	43	43	78	44
5	4	25	18	13	16	25
24	20	30	34	16	14	10
13	26	30	31	22	32	22
47	34	50	63	63	40	41
35	53	116	22	32	53	24
67	24	65	81	50	30	28
13	34	56	37	37	44	34
79	107	89	59	42	37	54
37	21	41	27	18	23	35
0	8	9	0	6	8	0
7	4	0	9	0	0	15
22	51	38	31	31	20	26
24	0	93	30	0	49	37
39	74	63	38	57	50	41

For Review Only

55	41	104	46	59	82	39
74	127	140	80	79	97	63
13	4	29	11	16	18	21
0	0	95	0	0	0	0
152	144	272	154	162	142	110
60	64	68	57	64	80	85
0	0	4	0	3	0	2
38	46	76	25	38	29	80
206	229	351	159	157	355	211
23	32	32	0	25	0	23
2	2	11	6	11	4	18
1290	1521	2571	1369	1280	1438	1251
164	166	289	161	158	170	133
29	21	31	15	31	20	26
35	46	72	21	46	41	54
97	318	256	103	88	87	232
1	1	113	39	24	1	21
181	245	270	206	185	152	148
140	132	250	148	164	115	140
0	249	26	0	0	388	0
10	0	816	0	307	0	0
47	64	23	11	54	128	1
33	39	56	9	36	22	14
2	33	0	34	14	32	33
113	174	234	98	194	155	138
159	102	192	135	96	127	86
50	10	1	9	3	51	8
3	0	0	0	0	0	0
3	3	31	9	8	2	12
0	3	0	0	0	0	0
11	3	47	19	29	23	35
28	31	36	15	35	4	18
7	17	6	7	11	10	0
19	26	35	19	28	22	22
157	178	292	215	198	226	193
0	0	0	0	0	0	0
0	0	8	0	12	0	14
34	50	89	57	53	33	48
201	176	288	155	138	220	129
24	35	48	30	9	58	41
9	6	0	8	3	7	7
1	2	17	1	1	5	5
12	1	0	1	1	1	4
443	345	612	464	391	315	320
52	100	153	57	72	93	92
200	159	285	179	168	239	168
0	0	0	0	0	0	0
44	40	76	36	40	42	38
1	0	1	3	0	0	1
0	36	0	61	75	70	48

For Review Only

260	279	522	338	342	277	193
11	7	10	9	11	12	9
2	3	0	1	0	3	0
69	136	159	132	102	77	57
101	53	116	51	64	54	59
31	32	31	20	19	22	28
93	120	108	85	94	137	66
0	1	8	0	0	0	4
120	107	135	78	91	118	76
25	7	0	18	14	16	15
48	19	34	29	35	21	13
363	269	586	365	281	274	242
0	0	0	0	29	16	0
179	85	187	116	71	171	118
8	12	20	13	21	5	6
170	198	186	69	152	151	76
305	243	599	342	322	332	199
38	57	85	50	42	60	62
0	0	1	0	0	0	0
67	66	62	57	45	64	46
89	67	111	84	125	69	30
39	43	129	112	20	4	87
59	50	82	84	32	27	23
7	26	55	11	12	29	14
205	210	330	292	268	232	178
140	72	326	154	77	162	112
76	44	84	73	34	60	88
186	150	198	132	169	195	144
149	236	190	142	182	145	163
95	20	184	27	118	205	172
0	0	2	83	0	93	44
67	80	75	63	63	72	55
275	124	125	144	179	73	72
84	92	72	39	43	56	107
90	57	151	48	57	94	83
33	52	41	54	31	53	37
0	0	0	3	0	0	29
0	1	0	0	4	3	0
1	1	0	2	2	9	12
124	693	865	108	136	619	62
0	0	0	0	0	0	0
25	12	26	25	18	26	42
21	24	34	20	29	13	17
0	0	12	1	0	0	0
59	68	115	84	67	62	66
32	47	40	34	20	18	43
22	36	49	37	53	35	25
27	20	44	20	30	21	33
49	34	88	73	63	22	24
67	74	114	68	81	63	41

For Review Only

99	115	144	90	81	81	84
0	0	1	0	0	0	0
180	151	513	226	424	198	191
73	46	98	50	47	27	48
13	38	99	30	39	21	105
4	0	1	9	0	12	4
147	169	263	68	154	117	170
3	3	0	0	0	3	1
502	479	695	510	403	414	206
23	90	72	10	6	37	29
396	404	483	323	307	412	324
226	210	375	266	211	184	158
1	86	0	1	1	1	11
38	333	0	160	133	1140	225
1011	1004	1476	1235	1142	894	841
120	96	151	87	75	73	91
171	0	210	184	154	241	176
0	6	5	4	3	6	0
45	43	66	50	29	61	19
73	58	134	39	77	66	44
0	0	5	0	1	0	1
0	0	12	0	0	0	0
0	1	2	3	1	0	1
18	27	22	19	24	16	19
5	1	1	3	5	0	6
16	11	27	24	26	29	14
3	5	0	0	0	0	4
62	7	11	8	15	20	70
14	12	28	24	15	13	16
9	6	15	6	8	7	5
0	3	0	0	0	0	1
32	51	86	56	33	91	50
26	18	29	12	28	18	12
53	50	69	48	17	51	37
0	0	0	0	0	0	0
38	78	79	40	58	35	30
43	28	82	60	40	55	41
0	0	0	0	0	0	14
0	0	0	0	0	0	4
12	8	14	22	18	6	10
0	0	7	6	0	0	0
120	114	154	95	75	120	88
132	88	239	114	169	152	29
43	47	66	22	14	31	40
210	221	399	336	238	379	230
135	168	266	128	125	140	124
7	1	0	1	29	8	1
0	0	0	0	0	0	56
0	0	0	0	0	0	0
63	8	95	27	62	72	39

For Review Only

39	44	81	57	39	41	37
2	0	0	3	0	0	1
0	1	7	0	1	1	0
112	32	207	104	95	22	87
5	0	0	0	0	5	0
37	35	34	26	18	28	33
23	51	46	19	40	30	22
98	73	125	51	54	90	77
53	72	120	92	66	63	64
37	48	65	26	10	33	49
25	18	43	28	24	33	25
56	61	57	54	46	27	42
225	250	361	212	201	166	208
256	234	467	269	330	343	205
86	87	158	79	94	89	79
7	0	0	0	0	0	3
1	0	3	3	5	0	1
3	0	0	2	0	0	0
30	39	44	34	36	45	39
164	222	579	328	254	388	174
37	147	0	25	6	37	6
16	1	23	2	5	24	12
4	5	7	3	16	2	2
45	58	37	44	21	69	28
2	21	24	25	22	8	21
12	9	20	10	17	17	11
35	42	77	38	24	19	26
29	17	110	49	38	32	35
36	20	67	28	34	38	13
31	20	62	10	17	24	14
406	301	608	405	187	333	209
97	50	92	69	40	117	46
0	0	0	0	0	0	0
184	147	202	198	196	227	105
89	106	142	119	109	117	100
50	77	111	50	110	98	89
0	1	0	4	11	11	0
10	15	56	17	15	37	82
0	0	11	0	0	0	0
19	21	78	48	41	40	19
7	503	0	1	194	24	1
1	1	0	1	1	191	1
0	4	0	1	0	5	7
0	0	0	0	0	0	4
201	130	369	243	134	136	190
2125	1897	2745	1955	1195	1787	1651
969	1244	2302	970	1156	868	915
0	0	13	0	0	0	20
7	4	0	0	0	5	0
116	88	179	115	78	78	98

For Review Only

123	81	199	61	66	118	147
40	31	66	25	29	24	10
0	0	0	0	7	5	0
281	345	614	321	421	283	321
0	0	0	0	0	0	1
249	215	626	338	222	250	215
223	274	375	212	260	259	159
5	2	35	4	1	8	27
10	2	39	5	1	6	25
1	14	0	5	145	8	8
121	117	172	123	167	92	93
11	24	25	18	21	31	12
62	40	58	44	24	29	20
31	19	21	25	15	21	27
60	46	68	47	35	32	27
50	34	35	25	30	44	29
13	13	20	11	5	2	6
43	37	75	39	32	59	36
1	0	0	1	0	1	10
22	7	49	16	29	20	27
0	9	12	8	8	7	34
10	0	13	8	2	9	11
0	2	0	2	1	2	0
337	343	480	330	345	350	248
5	13	7	2	10	7	9
31	3	36	40	14	38	50
11	9	15	10	15	3	8
61	89	91	55	62	50	61
114	162	268	149	253	91	171
0	0	1	0	0	34	40
59	52	91	104	55	90	44
1	2	0	1	1	1	1
23	44	47	39	50	45	56
22	25	30	30	29	40	0
6	7	7	15	6	5	14
44	158	208	143	115	152	139
5	6	10	5	8	8	1
190	236	331	211	208	301	141
5	2	32	1	2	6	41
4	3	0	0	1	0	0
2	0	0	0	0	0	4
60	25	127	128	72	44	31
95	92	88	58	104	92	97
52	45	62	31	88	46	65
133	133	173	121	180	160	143
0	2	0	3	4	0	1
141	167	330	196	163	160	119
96	129	77	92	111	99	114
4	17	40	7	30	1	0
1	1	0	1	2	1	2

For Review Only

124	86	174	104	90	101	72
70	33	122	172	16	47	17
0	0	0	0	0	0	21
288	330	347	263	180	0	247
80	106	0	113	132	189	0
8	11	3	4	3	5	7
0	40	15	0	0	0	79
2	10	0	5	0	2	1
1	1	0	1	1	1	1
0	0	0	3	0	3	0
48	49	87	39	53	31	51
66	5	72	24	31	57	0
0	54	0	0	0	46	75
40	66	59	30	39	52	46
45	51	86	0	48	0	42
0	19	51	41	57	0	41
19	58	33	23	45	25	38
21	23	39	22	25	24	21
0	24	33	9	0	14	8
88	106	107	82	69	135	51
2	5	6	5	10	9	8
36	37	57	26	41	33	37
17	30	51	12	29	25	11
30	43	43	31	36	38	24
369	371	425	253	261	428	357
0	0	0	0	0	0	38
0	0	0	0	0	0	8
10	51	0	40	44	75	216
49	38	29	29	17	69	44
17	28	56	27	37	31	45
194	158	161	117	72	124	118
61	86	92	83	69	92	63
29	43	75	30	50	67	65
167	266	294	143	176	269	329
26	37	66	21	35	43	52
4	3	12	10	3	9	5
98	1	0	12	42	21	4
1	2	0	3	7	2	0
0	0	39	0	0	0	0
0	0	0	0	0	1	1
8	12	15	18	4	9	17
19	21	30	55	41	25	11
9	5	29	18	12	13	10
9	5	13	3	8	7	9
6	7	39	4	33	6	10
83	207	240	122	156	152	67
44	77	94	49	68	64	29
37	35	105	58	50	62	30
32	36	40	23	7	19	77
0	0	0	0	0	0	0

For Review Only

418	402	1215	635	451	757	609
271	370	603	481	275	192	260
128	128	0	0	0	171	157
0	1	0	0	0	0	0
200	131	231	146	165	190	90
555	507	1058	720	703	608	492
0	0	2	0	0	0	0
118	93	367	219	212	153	61
216	232	463	276	230	351	180
76	84	200	206	117	54	42
176	197	319	235	228	0	184
268	395	780	445	467	471	308
4	0	0	0	2	0	0
0	0	287	180	0	239	88
48	48	60	56	73	34	65
225	247	404	272	241	265	211
99	163	118	83	70	140	88
0	0	0	0	0	0	9
13	52	79	154	36	41	42
0	8	0	2	1	1	0
49	42	70	62	33	38	35
184	192	0	195	212	235	172
840	751	1433	1085	895	884	727
171	150	0	58	164	187	138
167	206	315	217	213	204	126
11	17	10	10	13	17	17
28	30	38	25	26	27	36
67	67	169	95	101	116	57
44	126	0	196	34	13	12
908	1080	2053	1706	1756	1153	1400
0	0	0	0	9	0	1
62	104	319	135	455	56	126
56	58	116	73	84	72	71
18	5	14	16	15	5	0
51	46	52	21	27	49	24
4	6	1	1	2	1	4
199	182	603	313	427	293	305
195	229	285	205	245	168	212
75	44	117	75	79	80	72
50	53	29	15	39	35	36
0	26	33	32	16	21	5
0	3	0	3	0	1	1
329	371	592	382	374	334	369
168	199	411	213	234	215	177
292	413	521	370	347	389	319
171	151	223	170	196	193	163
212	331	298	284	267	215	307
81	149	213	95	163	109	93
28	24	0	20	25	18	34
0	0	0	0	0	265	221

For Review Only

118	158	254	142	129	151	135
24	58	16	13	16	68	20
237	268	801	520	418	425	296
43	81	97	96	57	100	92
115	156	216	159	103	186	113
88	106	167	76	95	149	76
1	0	3	0	0	0	0
0	0	0	0	0	0	1
302	319	572	346	325	406	331
67	89	127	102	69	108	74
21	63	54	38	35	43	39
148	198	311	177	138	209	132
0	4	0	0	0	0	0
8780	9473	14590	10864	8829	10662	6616
27	21	25	37	12	21	9
9	25	29	15	13	9	6
52	85	127	96	113	106	108
2	32	9	5	0	18	0
18	39	25	18	24	33	26
0	0	37	0	284	350	243
122	154	189	68	91	103	72
83	91	29	24	13	36	16
95	87	159	70	105	79	84
4	1	69	20	3	1	128
8	2	72	9	0	4	0
316	403	546	386	413	324	293
0	0	3	4	0	0	0
9	7	19	18	4	23	0
115	243	126	77	48	67	53
17	11	27	11	0	9	9
86	143	222	182	135	138	102
2	2	1	3	2	0	9
145	132	249	172	115	163	102
1	1	36	1	1	1	1
1	1	0	1	0	0	0
0	7	11	2	2	1	0
0	0	0	0	3	0	0
11	6	9	12	3	15	9
5	2	0	4	18	15	18
53	50	89	59	69	67	72
12	15	28	39	40	20	18
102	99	198	0	98	100	74
43	35	95	29	35	36	49
0	0	7	0	0	0	0
89	84	166	89	71	69	117
24	37	28	17	15	18	20
91	117	108	138	84	60	79
84	77	149	66	41	112	89
67	108	232	180	186	244	66
5	7	5	6	11	2	4

For Review Only

10	13	16	5	4	5	17
697	711	1630	961	662	912	417
1128	1412	1921	1086	1220	1356	998
174	117	194	158	194	85	130
0	0	1	0	0	0	0
937	0	19	184	0	1013	0
65	45	126	52	55	80	44
0	0	0	3	0	0	2
0	128	58	0	0	139	121
4	44	115	36	93	21	81
34	29	72	38	18	30	15
58	68	151	107	110	131	113
25	40	58	30	24	41	41
46	47	98	93	89	86	39
251	132	259	411	216	301	93
54	44	142	60	35	50	58
163	154	352	172	159	180	97
1	1	0	1	1	1	1
36	26	21	13	20	32	19
114	99	195	104	131	160	108
4	6	5	0	7	3	3
19	28	47	19	24	16	15
7	6	18	5	2	19	5
31	22	55	26	48	18	21
68	41	72	60	69	33	53
20	24	0	0	0	29	18
7	20	39	23	6	40	12
70	64	108	45	48	92	66
0	3	0	0	0	0	0
38	68	49	40	45	48	30
131	208	218	182	76	204	158
4	0	20	8	9	0	9
463	456	795	534	536	485	402
28	32	0	13	47	20	15
123	139	193	121	94	86	82
0	144	26	107	146	0	0
8	15	21	23	6	11	14
4	9	25	3	23	11	9
5	8	28	3	7	7	2
18	9	44	29	27	13	11
98	184	282	0	159	185	0
5	0	0	0	4	0	0
1	2	4	1	1	1	0
156	184	204	153	108	182	104
0	0	0	0	0	13	0
5	6	19	4	7	6	7
0	0	0	0	0	0	0
27	27	46	0	35	0	29
0	0	3	0	5	1	0
0	0	5	1	0	0	0

For Review Only

56	56	124	103	75	41	88
20	49	32	26	24	16	27
21	12	16	13	9	17	7
61	59	145	64	82	115	31
1	0	0	0	2	0	0
91	118	105	89	83	64	54
0	0	0	0	0	0	1
1	1	129	1	1	1	1
0	0	8	0	0	0	10
0	0	3	5	0	0	0
11	12	21	6	8	9	10
21	22	37	9	16	16	19
21	22	38	9	17	17	19
0	1	0	3	0	0	0
0	5	0	0	3	0	0
49	1	74	71	51	78	75
3	3	0	1	1	1	1
2	2	0	4	0	1	0
27	34	120	58	71	43	52
0	0	0	0	0	0	0
42	71	100	61	46	58	40
6	0	0	5	9	7	7
13	19	20	14	21	21	13
219	239	439	115	206	213	174
214	218	365	271	211	247	255
176	175	274	205	177	166	176
11	5	10	7	7	0	6
15	18	28	19	20	20	21
59	75	151	30	108	106	60
28	38	26	27	12	19	34
0	0	0	0	0	0	0
22	20	53	27	28	32	32
50	72	71	84	87	72	65
45	24	15	30	25	25	9
230	165	340	185	176	223	163
76	87	166	134	127	70	48
0	0	0	0	3	0	11
0	3	0	0	0	0	2
50	28	78	30	43	53	61
505	596	866	546	628	673	591
444	438	786	445	416	477	379
0	1	0	1	0	0	5
1	1	0	8	1	0	1
46	54	95	48	59	70	55
9	12	11	0	1	17	6
40	37	54	17	20	45	15
831	895	1401	969	958	946	866
86	87	89	91	104	66	46
11	48	6	12	18	5	6
627	640	1258	853	621	634	399

For Review Only

24	40	38	36	25	33	6
61	76	163	59	77	58	80
15	24	13	15	47	19	17
16	29	45	19	35	12	29
81	60	163	198	89	78	143
29	35	27	3	43	28	8
69	84	151	91	81	100	129
14	33	46	33	24	26	11
0	0	17	0	0	0	2
6	2	15	8	14	4	9
4	3	11	4	2	16	2
3	4	8	0	10	1	0
0	0	0	8	0	0	14
0	0	0	1	4	0	0
1073	1037	298	0	1246	1322	0
27	32	27	38	14	33	22
10	25	18	12	24	19	16
84	118	229	63	105	144	84
0	0	10	0	0	0	0
11	16	5	5	15	4	5
21	32	42	28	35	42	25
47	27	86	54	65	68	56
13	6	10	9	8	1	4
99	109	122	85	69	117	42
48	30	31	30	18	18	14
2	0	15	0	0	2	9
219	270	328	220	217	285	270
68	79	121	71	101	89	87
5	16	54	0	11	0	12
12	7	14	20	6	13	7
17	20	22	25	27	22	15
116	85	142	75	140	127	126
68	76	55	31	35	44	60
8	0	0	1	0	1	0
12	21	0	17	16	15	34
0	0	0	0	6	0	0
0	0	0	0	0	0	3
1241	1800	2034	1665	1609	1719	2251
0	0	0	10	0	2	6
0	0	0	0	0	1	3
56	39	83	38	31	45	56
18	25	31	47	51	61	34
23	14	29	20	26	25	24
113	128	226	155	119	195	86
161	195	261	260	150	189	156
68	82	239	90	83	37	85
92	126	267	138	176	192	131
145	180	254	102	172	236	176
36	142	192	148	99	135	46
21	23	18	10	30	30	22

For Review Only

155	107	237	122	125	126	79
125	195	236	208	163	261	207
0	0	0	1	3	2	0
384	415	470	252	284	443	245
4	0	3	5	1	0	3
131	84	124	84	103	98	128
7	10	10	11	20	10	6
203	270	517	306	371	0	230
75	0	0	75	0	0	175
40	29	87	43	25	22	28
0	0	0	5	0	3	0
0	0	0	0	0	0	8
388	450	753	438	397	413	352
856	893	1711	889	916	962	687
497	462	1124	752	807	696	419
11	6	7	10	5	12	5
23	23	21	16	6	28	8
1	1	22	1	12	2	2
0	4	6	3	0	0	0
12	14	41	34	10	7	39
20	0	16	15	12	18	17
0	0	12	0	0	0	0
32	1	89	50	43	71	10
0	0	8	0	0	0	2
1	0	0	0	0	7	0
0	0	0	0	5	0	3
15	20	13	14	5	17	7
1	2	0	9	4	1	3
7	12	0	10	14	8	10
1	0	1	1	0	2	0
48	29	70	26	36	50	41
0	1	4	3	3	0	1
55	68	53	44	50	88	37
29	24	38	13	18	20	35
84	174	152	101	97	102	104
0	0	0	4	1	0	0
0	0	0	0	5	0	0
262	293	424	195	179	227	241
60	23	91	64	87	51	18
223	134	333	230	146	166	141
0	0	0	0	1	1	0
244	97	205	211	189	154	95
137	137	266	113	171	120	110
239	183	271	183	146	163	183
124	145	165	81	122	100	98
304	333	297	226	168	311	227
0	2	12	0	2	0	19
400	523	731	618	455	408	362
16	9	19	15	23	13	13
0	0	8	0	0	0	3

For Review Only

0	1	9	0	3	6	8
0	164	5	175	0	183	0
1	0	2	1	0	0	0
151	124	241	179	165	146	89
49	31	110	60	64	11	38
78	93	147	64	49	40	44
78	74	246	99	86	141	121
103	112	178	109	89	125	72
125	130	159	90	85	147	95
278	250	451	252	238	303	200
41	38	73	6	24	40	8
45	14	24	0	41	0	15
18	34	46	20	49	33	35
0	29	0	47	0	49	43
1	2	0	2	1	0	0
43	0	11	50	57	72	60
34	67	38	25	89	32	20
0	0	0	7	0	0	0
10	6	38	0	13	9	0
81	76	108	49	60	102	94
0	0	1	0	0	24	32
0	0	0	0	0	0	0
52	53	78	68	46	51	46
122	77	196	97	66	67	38
134	131	298	111	130	98	70
22	9	0	5	32	17	68
119	132	209	125	115	106	74
2	5	0	0	0	0	11
122	143	175	119	126	160	123
284	181	405	304	275	211	383
81	111	125	121	162	159	126
100	233	103	227	122	149	64
0	0	147	154	99	124	37
208	145	383	332	161	252	157
0	485	219	0	0	603	500
245	178	363	317	239	395	144
176	122	167	146	107	160	75
131	122	192	170	112	178	113
71	83	104	47	54	67	52
128	125	159	108	153	85	120
0	0	0	0	0	0	4
74	86	116	80	72	99	91
66	113	44	88	66	77	14
21	32	49	34	18	49	43
0	295	13	0	0	321	0
15	14	32	3	13	9	18
53	39	73	49	30	45	70
0	0	0	0	1	0	4
902	732	1360	719	307	900	515
47	82	119	63	45	43	94

For Review Only

2	7	14	2	19	3	5
700	862	1240	628	830	636	571
172	169	385	217	293	202	68
165	266	337	197	208	208	149
0	2	0	2	0	0	0
92	103	161	92	79	105	87
184	169	254	187	175	190	140
1	1	0	1	1	1	5
31	34	51	29	28	38	28
28	43	48	32	26	33	16
6	17	6	0	9	6	8
932	861	1317	1038	340	418	762
1	2	0	1	0	4	2
114	123	187	97	128	134	140
271	192	398	5	54	230	255
0	0	14	28	51	0	0
0	0	0	0	1	0	0
1	0	0	0	0	0	0
114	120	142	143	76	162	79
48	53	157	46	60	85	60
6	12	15	13	5	5	9
0	7	9	5	6	0	3
50	124	175	149	109	137	81
8	6	16	2	8	10	3
109	104	175	97	97	129	106
81	103	149	79	79	45	107
41	223	37	41	16	206	97
6	0	0	0	0	0	0
1	3	0	11	1	5	6
127	80	154	108	134	120	47
22	36	44	26	16	33	18
175	241	483	197	242	225	294
0	10	37	0	0	13	9
0	1	4	0	1	0	0
105	113	160	93	76	85	70
43	45	64	34	45	51	40
95	107	229	129	81	197	120
191	182	257	189	160	224	109
47	64	145	87	75	65	93
0	0	64	13	0	0	16
0	0	17	0	0	82	9
93	119	157	95	94	119	66
74	81	120	73	92	67	75
61	42	60	55	62	97	71
15	27	0	30	21	14	14
31	68	77	58	52	33	36
59	62	113	38	67	63	73
197	151	248	248	105	115	114
3	21	34	29	30	7	10
0	0	0	0	0	0	0

For Review Only

0	2308	1775	3320	0	3303	0
2	0	0	0	0	3	1
4	11	20	8	4	2	7
26	24	56	29	28	27	18
3	0	0	0	1	3	4
4	0	0	5	0	68	57
3921	4249	6166	4428	4033	4728	2803
0	0	0	3	3	0	0
70	69	93	23	56	66	61
195	133	339	185	229	119	71
0	0	4	1	0	0	5
0	0	0	0	1	0	7
126	102	237	110	92	35	108
2	0	0	4	0	3	0
56	41	82	53	67	45	29
14	29	34	32	8	23	10
13	9	1	4	5	4	8
5	1	11	3	5	2	3
2	10	0	5	4	8	1
3	26	5	1	7	5	5
85	97	154	65	65	86	75
8	7	9	14	12	3	8
46	56	86	85	57	41	50
18	37	43	3	23	2	27
32	39	79	62	65	24	93
301	240	360	187	80	155	221
42	83	118	55	44	37	48
93	120	87	84	0	0	99
53	55	73	25	30	28	42
41	139	143	0	182	0	41
5	0	0	4	1	1	2
5	30	7	16	14	18	12
48	119	176	86	55	131	122
47	48	69	49	83	92	52
48	35	41	28	72	45	28
79	70	87	49	56	41	85
236	117	354	234	170	258	80
128	77	204	126	62	195	67
15	13	31	12	31	17	15
11	9	46	37	7	36	5
51	95	109	41	63	39	51
2	5	0	4	2	3	2
127	95	254	133	218	136	135
9	5	24	6	18	8	8
1	10	0	1	86	1	1
42	57	94	81	56	30	29
12	8	31	33	51	56	16
0	3	0	0	0	6	1
1	5	14	3	2	5	4
0	1	91	0	181	0	139

For Review Only

0	0	0	0	1	0	0
1	0	0	0	0	1	0
25	24	41	21	39	21	22
0	7	5	1	0	1	3
0	0	0	0	0	0	6
70	177	51	60	51	151	75
273	280	405	215	275	154	190
19	36	20	14	36	22	25
0	11	36	17	0	16	16
112	106	150	103	39	89	104
152	129	128	57	89	114	111
0	0	9	3	2	0	3
79	37	105	72	70	68	46
2	0	0	1	0	0	0
0	0	0	0	3	0	2
81	60	127	83	68	70	60
27	31	39	14	20	33	25
72	0	173	66	0	93	0
11	36	49	24	18	15	30
17	19	24	26	12	19	17
62	52	74	75	75	59	35
14	20	36	19	25	18	21
80	63	112	56	43	38	74
15	36	45	24	18	23	16
111	89	175	94	83	183	85
1	1	0	1	1	3	1
40	42	41	16	54	107	6
36	42	61	43	25	56	26
32	22	34	33	22	30	19
48	55	101	55	45	30	34
62	22	59	54	16	29	21
6	10	25	7	9	8	12
0	0	7	0	0	120	95
13	31	89	20	38	13	52
43	44	129	43	139	43	30
3	0	1	0	0	0	3
0	2	0	1	0	1	3
39	26	61	43	43	30	50
144	173	160	102	90	186	156
200	185	299	116	117	137	85
114	66	145	89	85	79	38
9	20	52	41	38	12	28
106	80	165	140	64	101	93
32	14	25	16	37	0	31
25	45	46	16	4	54	49
36	29	98	56	83	13	28
29	34	55	8	17	52	21
8	12	19	7	6	10	8
0	4	11	8	1	0	1
98	231	355	37	83	75	52

For Review Only

235	189	296	184	201	214	187
0	5	22	1	4	1	8
62	77	149	66	96	62	39
0	0	3	0	0	0	1
184	222	390	132	161	99	200
12	6	8	0	6	3	4
0	0	3	0	17	0	0
182	110	117	226	29	130	29
15	12	22	21	13	15	19
146	206	355	262	176	228	184
60	45	50	47	36	32	8
39	58	75	57	39	42	17
0	7	7	6	10	0	0
113	26	192	15	70	31	29
289	256	330	296	202	271	218
11	16	34	13	17	21	121
72	83	117	60	92	79	89
6	8	0	11	3	2	6
82	132	83	6	297	259	39
28	23	36	29	39	26	28
10	91	57	21	40	12	8
0	61	100	17	0	33	19
26	22	44	21	20	50	14
21	21	34	26	24	34	12
172	174	202	148	137	189	158
27	24	39	29	28	8	10
1	1	0	0	1	1	0
16	37	16	7	10	19	15
1	0	0	0	0	0	18
6	9	23	7	4	9	9
141	157	234	169	145	159	158
106	130	145	150	139	124	133
0	0	0	0	0	0	0
259	336	433	344	362	333	365
945	961	1557	1008	1011	994	755
0	4	6	0	0	0	0
156	54	312	211	85	98	186
302	468	555	415	352	340	309
0	244	0	0	0	0	0
35	13	28	47	20	35	22
4	7	33	50	15	63	7
217	404	82	1	104	367	126
11	13	24	7	7	9	9
342	491	633	292	310	287	219
83	95	229	118	163	122	66
1	1	13	0	0	1	0
113	125	126	92	52	79	129
223	206	273	202	221	249	195
58	37	86	47	32	54	37
44	25	69	45	52	51	22

For Review Only

0	3	5	5	4	4	5
42	71	1	8	75	29	40
12	20	10	49	14	8	17
0	2	86	0	0	14	1
0	0	23	0	0	0	0
0	31	0	0	14	0	0
0	1	0	0	11	0	1
0	0	0	0	4	1	0
0	1	2	0	4	3	0
12	1	48	5	7	0	0
47	54	153	27	80	88	49
1	1	28	1	2	1	2
0	0	9	9	0	8	0
38	23	36	37	49	37	23
93	77	144	65	43	78	67
10	0	17	16	0	21	13
45	60	227	56	44	172	117
142	128	227	97	130	115	64
96	88	153	35	126	125	131
10	7	28	5	12	10	7
4	2	0	26	11	4	0
0	1	0	1	0	2	9
1	1	2	1	3	0	4
3	0	0	0	0	2	2
0	0	31	0	48	34	0
154	204	293	195	128	148	139
113	162	181	109	129	123	91
34	32	31	27	17	35	27
15	15	13	19	8	15	11
16	39	6	34	15	28	31
19	17	26	18	21	21	13
22	18	24	36	30	17	74
31	43	38	20	9	6	16
17	24	58	21	4	28	17
0	6	0	0	4	4	0
0	0	4	0	0	0	0
108	149	201	88	92	75	111
141	117	89	69	187	251	166
8	3	8	16	10	8	2
84	117	161	135	122	91	76
193	145	239	270	154	114	46
22	27	0	8	7	14	8
22	29	56	35	23	15	23
17	23	8	6	1	18	21
0	31	83	59	0	0	75
73	52	91	54	56	82	56
33	22	76	22	41	15	42
81	123	227	142	140	109	78
20	18	29	25	32	31	24
30	12	33	12	12	36	25

For Review Only

36	20	42	33	13	34	13
20	44	72	30	23	22	47
33	44	57	56	59	61	28
113	43	126	63	35	73	89
16	16	35	17	0	17	0
0	0	0	0	0	1	0
0	0	0	0	0	0	0
1	7	0	0	10	3	5
510	576	414	3	684	54	609
291	569	210	272	170	559	220
6	9	0	5	11	0	0
24	24	53	31	24	32	48
109	113	176	122	57	95	54
0	0	0	3	0	3	3
483	728	1041	492	456	829	620
727	785	1000	501	514	872	593
231	238	327	200	158	268	239
8	14	8	8	3	13	12
406	427	501	341	269	448	251
4	4	8	8	4	7	3
149	154	175	114	103	160	76
0	0	0	0	2	2	0
87	130	157	82	76	116	91
525	85	2463	57	544	83	84
34	25	30	10	31	25	22
16	8	23	3	5	36	14
0	0	0	4	0	0	0
18	24	0	0	20	40	41
0	6	4	0	9	0	0
59	49	138	95	102	91	93
93	64	217	65	143	117	83
65	46	89	61	50	57	75
336	166	642	388	429	418	222
0	0	7	0	6	0	0
0	0	0	0	0	0	0
4	1	12	13	15	7	4
334	150	629	546	255	561	0
0	7	1	0	0	4	0
12	21	12	4	3	14	8
90	123	97	41	67	108	79
2	5	34	9	10	2	12
45	57	51	38	38	29	39
139	86	144	85	133	118	76
19	24	60	32	16	14	7
77	113	201	100	131	104	76
246	255	509	263	312	298	245
0	256	2	0	0	0	0
36	15	138	115	68	56	109
11	25	14	13	10	12	29
299	333	586	250	268	394	231

For Review Only

0	0	0	0	0	0	3
44	50	88	38	61	61	46
27	31	0	32	35	36	46
79	107	126	76	117	109	81
41	0	21	47	0	44	0
64	93	137	56	69	59	78
201	255	314	203	255	209	228
33	75	218	51	97	57	58
84	98	114	33	40	69	88
0	0	0	0	2	0	0
66	86	127	55	98	105	91
59	29	109	68	34	41	73
155	0	0	19	0	95	38
109	89	169	88	114	109	131
47	148	795	1	1	1	1
26	39	75	83	66	54	37
8	29	48	23	31	36	51
52	125	202	86	92	105	50
0	12	4	0	0	9	4
339	72	382	234	120	169	11
0	0	0	0	0	5	0
38	69	82	41	41	90	29
4	1	4	6	1	2	1
1871	2761	3329	1901	1882	2046	2029
48	34	66	21	30	23	31
66	103	118	71	65	117	71
22	88	81	35	43	58	37
12	10	11	16	21	14	33
18	38	27	19	35	26	30
34	42	70	3	38	42	29
120	176	257	157	135	179	245
0	0	0	0	2	0	20
52	40	71	43	45	35	39
144	3	268	2	252	121	1
322	269	530	351	365	425	357
175	181	468	192	197	225	174
13	58	79	125	26	44	91
51	81	76	34	60	19	81
73	94	0	90	63	84	97
12	12	23	10	28	9	6
25	33	38	17	27	69	10
0	0	629	28	0	0	19
74	71	143	68	55	76	51
6	9	20	0	6	7	7
188	188	288	194	163	208	138
0	0	10	8	0	0	0
30	49	0	55	43	15	4
264	278	496	316	367	362	242
448	422	772	454	425	433	445
0	0	2576	0	0	0	0

For Review Only

0	0	0	0	0	0	0
71	86	133	73	77	80	84
147	177	204	150	163	153	120
0	0	10	0	0	0	3
0	7	1	0	0	8	0
0	20	0	0	14	0	19
29	22	42	11	19	23	26
87	109	114	85	110	0	0
1	1	12	4	4	1	4
144	194	355	164	191	211	224
55	74	116	51	87	73	61
0	0	0	0	0	0	8
82	84	29	73	87	89	0
108	137	148	125	107	149	102
78	64	113	50	79	87	52
0	0	11	0	0	140	0
447	436	773	442	482	558	337
202	177	277	184	178	200	143
184	203	450	248	304	238	188
22	37	49	23	21	27	22
51	95	93	57	59	72	40
110	107	151	102	121	117	115
43	46	41	25	33	30	37
0	18	5	0	0	0	0
107	0	25	0	0	147	0
24	10	21	16	40	23	19
21	2	160	5	17	13	1
36	26	67	22	26	26	31
55	49	103	76	63	33	44
0	4	6	3	7	4	10
113	123	101	73	85	121	95
39	34	56	35	37	27	29
68	91	134	65	75	79	93
56	87	115	39	56	84	58
89	98	104	51	61	40	90
118	146	155	101	153	122	90
47	56	59	57	52	56	61
202	172	297	190	110	208	150
72	74	157	63	47	114	52
4	4	10	0	4	4	0
59	104	78	75	68	97	77
72	68	89	68	60	65	40
142	334	357	177	200	316	151
0	0	0	60	0	0	0
0	0	0	0	0	10	18
77	70	127	55	80	74	53
63	64	146	94	96	55	82
122	147	273	148	132	134	121
199	227	359	205	201	253	201
65	80	121	71	61	44	42

For Review Only

195	104	169	91	112	111	35
75	158	99	44	79	78	33
7	14	16	8	19	15	24
31	30	69	26	26	44	36
81	106	215	106	124	194	160
131	176	223	91	117	177	101
97	96	127	77	48	56	72
292	328	554	352	306	522	280
88	83	98	60	65	106	112
60	65	83	58	55	88	73
79	96	138	55	54	92	70
9	9	0	3	10	9	32
161	86	191	112	70	135	92
0	35	0	0	0	0	17
0	1	29	6	0	0	2
10	29	60	12	28	23	22
53	33	100	45	34	46	39
107	143	150	89	75	117	88
0	0	16	0	83	0	0
95	70	134	62	49	105	45
48	67	83	88	110	80	42
55	0	22	0	0	0	68
14	33	28	17	21	28	28
45	97	61	30	40	73	57
35	1	87	1	8	52	5
0	2	0	2	1	2	2
16	50	21	22	19	17	1
2	21	9	3	4	14	3
982	0	292	434	0	0	0
9	18	19	18	13	19	15
0	34	1	0	0	0	0
0	128	0	0	0	0	0
47	130	147	167	38	131	118
36	33	54	18	29	20	31
15	22	31	11	22	7	15
113	82	161	132	68	107	58
13	16	32	11	10	16	15
29	28	35	16	20	0	31
179	199	308	193	174	191	150
39	41	81	52	44	40	20
72	85	88	41	66	75	68
55	0	0	0	0	4	0
1	4	13	1	10	0	0
4	8	5	1	7	3	5
14	7	11	1	1	1	4
3	7	6	5	2	1	2
187	273	501	252	310	314	216
6	11	11	9	11	16	21
2	4	15	11	4	5	0
192	147	208	165	157	122	81

For Review Only

14	33	68	44	39	87	9
58	51	37	47	35	47	31
100	142	176	34	94	97	114
7	4	9	6	0	10	4
28	31	31	46	10	43	32
192	197	306	164	169	201	177
12	98	132	113	110	88	81
0	9	44	21	0	15	0
18	24	85	63	12	83	4
0	0	0	0	3	0	2
60	78	75	53	77	84	95
64	49	78	51	43	70	41
8	15	20	9	4	13	7
2	48	16	14	13	25	11
60	103	133	89	59	70	38
27	49	76	58	37	35	9
68	46	18	212	178	83	238
0	5	5	5	9	8	0
4	0	0	0	0	5	8
3	4	0	0	0	0	0
7	0	25	0	7	5	11
4449	4284	7768	5097	4544	4855	3426
0	0	5	1	0	0	0
136	144	295	144	211	182	99
36	35	88	22	42	21	69
77	73	82	47	56	38	93
27	56	69	26	29	28	69
28	26	43	21	13	10	14
39	42	34	37	22	49	39
417	450	671	477	571	371	233
6	0	7	11	0	6	7
72	86	95	92	86	100	76
0	39	0	0	0	0	0
0	0	0	0	0	0	0
1	11	0	1	1	1	150
0	68	0	0	35	35	0
5	22	0	7	9	0	27
90	424	377	208	159	177	241
135	0	214	156	163	138	146
141	171	177	93	114	115	145
52	94	161	84	88	116	108
5	2	1	7	4	3	4
72	110	123	78	72	100	96
47	69	55	24	53	35	47
8	21	28	14	18	65	7
242	221	389	164	226	233	137
34	48	50	20	31	19	15
8	16	16	0	6	5	4
0	45	5	0	33	0	0
40	60	70	68	51	48	76

For Review Only

29	35	55	23	33	73	28
71	84	21	49	33	61	72
169	0	4	0	157	194	0
17	0	1	0	0	0	0
86	173	200	97	99	131	105
0	0	10	0	0	0	0
0	3	0	0	0	0	17
0	0	0	0	0	0	0
51	94	116	45	62	61	39
12	20	9	4	8	14	11
191	172	249	214	162	179	179
42	0	111	61	65	52	35
113	119	118	85	107	69	90
69	66	201	143	128	123	84
103	114	214	174	169	157	157
60	74	130	102	83	119	77
23	1	25	12	3	6	19
7	6	722	0	2	11	3
0	60	1038	512	217	388	176
17	42	65	37	38	43	35
393	453	771	456	449	317	348
271	278	284	189	218	293	128
51	85	74	58	27	46	79
2	5	4	2	0	1	4
42	33	63	54	38	52	42
206	150	227	180	170	111	126
2	29	38	20	26	7	7
17	25	18	9	12	20	8
43	63	104	49	64	52	40
102	206	294	132	185	105	105
110	134	132	108	108	144	70
12	78	76	0	25	40	24
67	25	45	49	59	33	1
127	143	270	139	160	207	130
23	2	105	4	23	1	1
20	40	41	25	35	33	24
250	288	118	248	209	0	186
162	146	182	127	120	69	142
48	74	100	36	42	53	42
24	25	32	8	44	9	16
30	20	76	30	25	31	47
56	112	150	95	69	67	105
273	355	419	221	218	342	207
7	8	13	5	18	2	12
94	147	239	120	123	168	113
2	0	0	0	0	1	0
1	1	0	0	1	5	14
15	22	47	63	39	18	36
40	24	58	41	25	38	41
93	96	171	72	75	71	126

For Review Only

266	241	622	338	316	0	262
141	220	267	181	84	243	137
112	105	179	90	142	186	101
11	31	0	3	14	5	19
25	44	33	23	29	28	11
84	36	83	74	37	72	61
3	0	1	4	0	0	6
91	84	101	65	52	75	51
26	12	0	8	15	15	10
34	57	31	40	19	53	20
16	23	28	25	14	38	17
87	90	151	98	117	118	123
43	47	27	27	19	40	29
28	37	38	25	7	32	62
38	27	101	60	31	15	40
82	98	200	36	83	96	88
113	121	157	152	152	0	144
6	36	51	40	10	15	14
0	0	6	0	7	5	0
43	107	60	43	69	47	66
74	75	106	98	81	75	39
209	234	412	311	220	259	236
83	81	206	100	93	113	97
17	22	25	10	9	16	20
26	33	52	32	14	28	33
0	476	35	0	339	408	237
21	11	35	15	2	17	13
33	12	40	18	41	48	20
137	168	275	88	110	128	140
18	41	78	24	24	17	90
98	108	231	124	85	140	34
0	0	19	0	0	0	0
49	66	90	50	76	51	47
4	9	6	1	5	3	3
8	13	30	28	19	13	13
54	56	0	32	61	39	50
0	4	22	3	6	10	10
3	1	0	0	0	0	0
0	1	1	0	0	0	1
1008	1353	1681	991	1086	1009	738
271	276	362	193	245	262	221
91	165	164	98	110	135	115
18	13	27	6	3	24	51
0	1	0	1	0	0	0
28	43	92	45	50	53	54
229	334	364	240	194	251	268
209	132	307	135	69	113	150
33	20	38	13	24	4	6
95	138	153	96	100	129	128
111	8	0	4	210	72	2

For Review Only

56	81	125	86	61	88	126
1	2	0	2	1	0	1
51	65	131	41	56	73	69
59	43	111	46	57	54	76
18	46	27	22	20	34	41
227	41	61	193	40	41	50
0	0	0	0	0	2	3
0	0	0	0	0	0	0
0	0	14	0	0	7	47
278	298	346	289	229	189	252
118	118	204	100	109	175	120
21	27	37	27	29	66	26
0	0	0	0	0	0	0
74	60	159	103	129	54	85
51	100	306	100	24	31	299
27	34	17	23	11	44	23
1	0	0	0	0	0	0
35	34	76	53	46	48	55
5	15	82	53	14	11	32
0	0	0	0	0	0	3
4	0	0	1	0	0	0
1	7	19	4	8	15	8
3	0	0	0	0	0	0
168	187	319	105	189	199	158
6	6	36	11	14	2	24
75	63	90	14	8	107	48
0	161	169	261	0	0	0
33	38	63	48	42	42	42
196	182	378	127	180	190	97
82	54	102	76	51	94	56
161	178	264	149	113	231	203
23	36	58	66	0	0	37
5	1	0	2	58	1	1
69	211	240	91	180	113	108
172	140	355	143	155	173	191
105	116	198	90	67	117	97
0	33	17	0	0	0	0
109	207	163	110	170	122	139
217	136	234	129	140	242	130
62	33	80	47	25	34	41
210	196	501	282	367	250	171
36	32	81	34	45	45	33
61	79	119	47	48	72	101
182	129	323	142	233	168	146
73	55	158	92	91	99	72
261	234	470	279	237	280	63
112	133	198	164	159	128	146
23	31	38	43	32	90	29
60	69	361	43	30	275	9
122	259	357	182	289	194	254

For Review Only

78	80	112	72	69	68	80
0	53	0	64	0	0	0
170	77	197	53	64	66	118
87	35	94	87	62	75	58
47	50	73	56	51	40	45
38	49	170	0	0	41	0
52	74	204	151	118	74	48
10	99	0	76	33	23	0
113	172	190	128	131	156	144
84	94	153	68	118	119	77
7	3	13	3	3	19	6
18	19	47	27	22	24	22
5056	5275	8940	5545	5318	5484	4192
2	0	0	1	4	2	3
120	181	204	183	118	152	97
112	116	293	171	187	198	70
96	83	99	66	68	77	99
0	0	0	0	0	0	212
2	2	0	3	0	3	68
5	7	6	0	4	6	13
286	283	321	233	261	270	236
0	0	2	3	0	0	0
0	0	15	0	0	0	0
35	69	142	85	99	39	20
83	99	102	63	51	103	79
3	0	0	0	0	0	104
135	177	200	75	116	194	101
69	121	187	61	54	99	64
191	140	252	163	186	145	120
32	11	51	31	26	23	20
59	50	128	54	43	71	67
685	720	1208	664	940	689	726
0	0	0	0	0	0	0
7	77	0	18	15	4	7
58	67	141	48	44	68	58
0	0	0	0	0	0	0
83	87	107	22	40	40	60
169	0	103	0	0	244	0
266	236	552	218	266	293	262
50	57	76	27	51	48	72
18	29	23	31	22	28	38
1	3	0	3	2	3	1
1	60	45	8	11	29	103
0	2	0	18	9	2	1
52	141	151	104	93	101	57
179	272	339	144	146	245	202
261	240	419	319	262	374	216
212	236	273	187	252	181	143
1	6	0	1	1	1	12
5	4	0	1	23	33	6

For Review Only

673	756	1178	903	946	795	554
84	101	260	163	123	76	86
38	36	40	23	32	45	36
87	61	190	130	104	93	105
0	89	12	0	0	83	72
0	5	19	27	16	14	4
73	67	82	56	77	76	58
2	4	5	5	5	6	4
26	36	78	78	23	6	10
37	21	39	28	15	16	9
182	207	250	158	173	179	159
64	90	72	52	26	64	71
0	0	0	0	0	0	0
98	153	155	112	172	126	138
9	9	7	17	11	16	12
15	20	40	0	0	0	0
73	47	129	88	87	93	59
80	104	167	53	126	113	75
88	82	143	73	83	66	38
105	74	227	90	98	100	49
90	79	135	121	69	44	99
83	100	165	57	42	123	78
41	47	91	17	26	29	40
151	274	243	67	78	182	168
81	25	93	3	15	6	1
6	12	61	44	40	10	5
120	152	143	122	87	108	75
158	100	123	129	63	140	85
40	16	44	62	32	16	23
0	0	73	6	1	23	31
22	38	23	13	2	14	12
43	35	88	40	59	30	42
75	119	0	108	80	113	62
1	9	23	18	22	22	20
5	72	79	34	22	18	69
22	20	37	25	24	27	0
246	139	237	120	83	193	106
19	13	26	51	12	33	12
44	39	56	27	24	32	14
0	0	0	0	0	0	0
84	39	126	55	26	51	7
9	2	13	3	3	1	8
0	94	108	74	74	94	84
0	5	7	3	0	0	10
479	364	718	498	487	510	313
134	157	291	121	94	162	95
0	0	87	0	0	0	0
4	5	0	4	4	6	5
0	10	0	6	4	11	6
0	0	0	112	0	0	0

For Review Only

0	10	0	0	0	0	0
43	54	61	16	42	3	63
22	63	91	21	39	37	21
48	45	85	31	73	39	37
23	9	36	13	5	22	7
32	102	118	20	28	83	41
0	0	0	0	8	0	0
0	0	0	0	0	0	32
0	0	0	0	0	0	0
3	2	17	5	10	5	13
39	9	39	11	29	1	12
37	52	46	1	1	1	5
47	59	94	63	60	41	50
202	86	297	128	115	102	202
58	119	121	39	68	74	73
79	85	104	59	30	47	39
14	16	32	8	15	14	0
65	86	89	72	66	90	64
51	45	90	47	31	32	26
298	378	597	363	388	363	335
650	625	1170	653	655	645	753
1	9	1	0	2	11	2
5	10	8	6	7	10	8
1	6	1	2	5	2	2
2	10	0	3	1	16	1
0	0	0	0	2	0	20
1	1	137	1	1	12	1
14	8	0	28	1	2	29
159	145	105	155	128	65	91
36	24	35	15	30	19	22
14	20	10	0	8	18	22
519	454	1152	673	57	623	192
10	25	0	11	11	0	11
40	54	104	74	74	59	43
0	3	4	0	0	0	0
172	192	314	193	173	172	165
14	8	10	2	0	9	8
37	45	55	28	31	27	26
30	20	65	50	54	62	26
42	37	54	17	26	35	28
2	0	0	4	1	0	5
0	21	0	0	30	37	0
33	16	21	13	0	0	0
6	3	0	0	3	4	0
4	5	9	4	3	0	4
0	0	0	0	6	0	6
107	168	324	101	193	235	146
3	0	0	3	1	3	0
0	0	0	0	0	0	0
0	1	2	3	0	0	0

For Review Only

3	5	20	4	2	8	10
0	0	3	1	1	0	1
2	0	0	0	2	2	0
3	0	0	0	0	0	0
27	45	87	46	51	62	17
3	0	5	4	4	1	0
38	74	72	56	60	46	53
1	7	15	16	4	11	5
2	7	20	17	10	15	7
1	4	13	18	3	10	7
0	15	15	13	10	7	8
5	13	14	12	8	7	8
2	9	11	5	7	5	7
23	15	9	20	4	25	5
0	0	0	1	0	0	6
16	24	19	13	0	20	19
54	38	88	35	36	47	53
90	120	191	123	132	147	82
85	0	2	71	0	0	0
38	50	140	68	71	41	27
25	25	53	26	29	28	28
77	61	133	66	57	75	61
108	139	147	70	90	94	72
71	111	94	91	44	73	52
106	99	203	97	73	83	99
52	69	68	48	67	66	60
0	85	100	77	30	23	0
13	6	7	13	6	3	1
16	9	42	13	23	27	19
32	29	70	45	39	25	26
46	25	43	53	14	32	30
18	7	13	14	9	9	8
98	59	123	110	77	76	91
129	215	208	68	111	40	190
0	5	1	0	0	0	0
12	59	40	10	16	40	18
39	31	67	39	32	32	30
1	1	0	1	1	1	2
82	70	67	62	74	61	56
43	40	33	35	38	69	32
0	0	0	0	0	0	7
98	99	168	111	128	169	71
18	41	26	23	28	26	23
7	5	24	12	23	13	11
33	44	45	28	17	108	43
5	0	8	0	5	0	10
3	3	7	1	3	0	1
146	122	261	130	141	148	152
84	45	153	71	77	57	0
28	45	52	7	14	36	44

For Review Only

74	0	1	74	55	106	60
172	128	298	195	2	171	119
121	134	150	140	154	139	82
9	1	0	28	4	3	0
205	300	206	272	145	266	104
59	46	111	49	52	54	55
138	151	221	123	118	154	101
125	64	98	104	97	107	79
182	148	225	141	135	236	118
118	65	218	85	119	104	117
135	116	266	122	149	199	168
10	9	24	11	21	27	9
8	6	11	6	8	4	16
55	69	75	55	37	88	36
145	217	269	147	187	127	127
20	1	0	1	1	1	7
64	66	82	41	76	65	40
18	37	51	10	32	17	30
36	36	46	21	31	45	25
404	470	626	297	519	363	367
91	150	193	52	70	85	57
0	672	175	0	0	658	530
384	484	831	567	458	486	426
4	611	1237	91	1	1	1
631	733	798	550	583	691	567
365	569	792	594	523	607	519
20	18	26	23	16	26	19
20	36	99	42	43	44	95
12	4	27	14	17	7	25
20	8	106	17	11	2	2
10	12	21	13	24	16	23
32	43	62	43	32	50	57
111	149	127	49	77	108	99
0	0	0	0	0	6	0
88	99	249	125	109	134	94
1	3	31	5	8	10	18
96	58	66	52	61	23	47
0	0	17	0	0	0	0
36	53	114	36	43	71	40
89	0	315	0	73	0	71
59	88	52	38	39	60	57
40	42	71	20	31	62	34
395	353	650	362	396	400	392
72	42	81	45	70	62	115
0	0	54	0	0	0	0
47	43	85	127	40	34	25
23	26	52	46	20	31	27
33	47	40	47	25	26	55
22	13	24	7	12	15	21
0	1	4	2	2	3	0

For Review Only

7	16	47	14	2	57	15
55	133	166	13	162	156	38
40	39	35	28	60	17	28
133	78	167	110	100	130	42
20	26	47	25	14	18	9
1	1	387	3	172	1	342
54	31	50	70	39	67	32
19	21	30	9	19	28	12
0	0	12	2	0	1	3
36	28	99	63	66	57	38
22	26	39	3	12	18	16
28	26	39	9	23	17	16
12	3	43	23	25	22	22
1	2	104	17	10	53	12
43	34	57	50	44	41	25
74	33	123	43	59	90	29
21	22	27	20	21	27	12
71	173	168	57	107	78	87
33	31	54	54	58	65	29
6	3	5	17	4	5	10
4	0	1	0	0	0	0
10	13	28	11	16	13	18
70	73	81	71	75	83	46
2	3	0	1	1	32	1
2	2	5	1	5	1	7
78	57	128	83	94	58	70
208	288	415	191	217	254	196
34	13	45	7	8	12	9
18	14	8	7	11	14	8
29	23	42	33	3	23	20
63	78	61	67	65	82	66
58	54	57	46	70	73	61
74	114	95	65	51	98	72
63	53	115	72	48	74	52
65	81	94	55	51	84	20
175	89	164	101	118	125	83
4	4	32	25	29	7	11
225	117	142	80	158	109	136
45	59	89	63	48	56	64
28	25	46	32	26	30	23
223	242	371	230	230	231	198
3	1	0	1	4	5	2
49	53	60	46	34	90	36
11	18	19	10	18	22	16
9	8	10	12	4	17	7
28	36	90	42	38	54	44
70	83	126	64	39	44	74
45	36	45	53	39	42	46
0	0	8	0	0	0	0
67	164	99	46	79	99	103

For Review Only

27	74	74	33	50	46	47
6	1	14	7	17	6	10
36	10	24	22	8	7	3
173	132	237	154	166	162	189
0	0	0	0	0	24	1
107	99	111	137	94	121	45
11	2	6	0	0	6	0
19	9	24	0	22	1	11
6	35	77	53	25	36	11
0	0	7	5	2	0	0
5	22	22	15	18	13	17
0	0	0	0	0	0	9
66	74	97	67	85	99	71
52	63	84	50	56	49	46
0	7	6	45	27	51	0
13	33	39	39	41	31	54
29	23	40	13	20	36	39
257	250	419	277	284	328	161
7	31	32	20	30	27	13
4	0	0	0	0	0	0
0	0	7	3	4	0	4
54	49	150	60	66	78	46
56	63	135	59	92	65	55
50	49	117	24	23	35	75
20	35	45	24	23	25	28
45	20	273	66	12	574	1
94	94	140	69	109	111	51
39	43	68	42	28	30	25
83	153	153	121	130	148	72
48	53	61	49	66	37	53
540	534	591	518	455	521	435
0	2	3	0	1	1	0
20	16	30	22	26	23	30
10	3	11	6	3	2	5
29	30	55	24	21	37	22
0	0	38	0	39	0	0
35	44	66	43	52	39	40
1	6	107	0	1	0	1
48	69	55	30	33	74	50
4	0	0	4	2	0	2
1	6	0	32	1	2	6
131	136	266	107	141	146	115
20	19	30	32	41	39	20
25	30	46	21	17	16	19
127	119	173	107	116	149	160
17	20	36	15	18	32	31
875	897	1331	916	972	882	417
50	42	35	34	27	28	16
22	35	15	4	3	13	11
29	44	45	45	43	24	37

For Review Only

175	205	282	142	173	137	180
4	12	1	5	4	19	35
7	14	30	28	15	31	24
41	20	49	4	26	15	3
91	183	109	13	187	19	102
37	39	50	46	31	37	77
4	0	0	7	1	4	2
102	45	192	78	116	29	53
1	0	4	2	2	2	3
31	19	41	29	14	46	8
49	38	85	50	36	81	47
46	45	32	15	57	83	39
205	144	0	21	87	11	135
15	26	22	14	9	30	5
1	0	14	1	0	0	1
25	39	62	27	39	37	36
1	6	3	0	1	1	1
29	33	41	26	21	15	39
27	45	102	53	48	32	34
50	50	82	42	42	102	25
122	116	215	150	124	136	129
5	33	18	14	20	12	26
93	222	327	150	128	161	92
117	129	227	73	139	153	144
87	107	118	109	53	36	49
76	120	232	78	87	57	90
8	11	29	27	10	15	8
99	79	80	69	81	94	55
28	52	39	20	25	57	28
2	21	6	14	17	3	28
2	0	0	1	0	0	2
17	18	91	77	19	82	4
4	15	45	80	78	36	10
36	18	79	14	51	9	7
393	405	708	301	303	489	421
64	56	0	63	63	86	73
58	63	94	71	51	69	32
163	144	180	118	103	194	130
78	97	92	48	62	66	54
16	8	11	13	9	34	8
24	36	53	41	37	43	53
122	111	109	98	46	118	75
1	0	0	1	0	3	1
168	99	229	98	146	211	90
16	1	0	1	13	10	3
15	18	46	46	37	16	8
179	97	155	69	120	129	143
193	174	364	133	105	196	130
237	83	228	116	174	175	169
27	34	11	10	9	24	31

For Review Only

18	4	27	18	24	23	14
128	98	143	83	110	98	57
221	37	269	11	71	303	36
48	93	114	115	82	71	78
34	28	50	39	34	54	14
3	7	49	8	7	14	31
46	19	60	44	61	54	29
62	32	126	65	38	71	81
43	56	113	30	51	68	58
126	1	153	26	5	2	1
18	5	13	16	8	4	10
3	0	2	0	0	1	0
12	16	32	30	20	31	26
111	211	189	120	112	144	123
1	0	0	1	6	1	4
14	17	72	38	9	12	27
30	29	34	28	10	30	21
26	35	41	37	40	45	39
102	142	126	113	115	70	76
36	84	48	35	20	62	26
91	37	235	47	86	147	49
33	92	34	45	23	46	82
39	41	44	23	28	30	29
40	35	33	15	33	41	27
74	71	160	63	47	75	47
7	8	158	6	58	7	70
2	7	5	5	2	5	6
127	91	267	169	104	109	48
24	25	44	28	16	20	21
1	8	12	0	12	59	45
7	11	16	25	3	11	11
6	0	0	0	3	5	0
43	49	111	22	48	25	20
105	104	191	118	129	140	68
19	25	37	22	17	29	17
3	11	6	5	4	5	1
3	35	53	7	26	3	17
16	20	31	11	26	33	29
142	147	852	292	289	95	94
58	36	47	18	19	9	18
2	7	41	21	32	9	29
16	15	23	22	21	20	19
632	0	908	0	0	0	0
6	348	0	1	1	34	228
13	2	0	1	9	0	0
11	21	29	6	20	19	14
6	7	24	6	15	36	15
0	0	0	0	0	9	0
0	16	17	17	16	24	15
24	36	66	14	0	0	31

For Review Only

0	0	3	2	0	0	0
36	11	114	86	48	50	16
43	70	107	61	63	44	66
0	1	0	2	0	0	0
34	37	49	40	24	20	13
10	16	23	0	12	9	8
84	67	135	42	35	99	66
134	112	147	73	92	106	99
54	58	95	91	56	55	50
39	75	74	27	69	33	19
54	65	97	23	19	87	64
11	15	50	19	35	6	5
101	69	124	65	76	85	68
37	27	48	16	17	42	14
13	19	59	25	42	34	22
50	81	156	110	115	102	62
3	4	0	2	0	0	6
105	78	167	99	92	114	60
6	3	0	3	0	0	3
31	22	50	47	48	49	34
54	32	67	36	43	36	37
11	10	32	9	15	3	3
22	31	36	15	11	34	27
62	45	125	60	25	78	48
166	183	273	157	168	187	189
17	22	48	23	32	4	15
20	1	28	58	20	32	20
19	86	0	23	23	9	47
12	11	7	4	1	3	3
23	18	40	15	10	23	6
25	28	35	47	14	48	24
4	7	24	14	21	6	4
0	0	0	1	0	0	0
34	7	13	40	15	58	38
0	2	0	0	3	1	2
96	77	123	64	45	152	48
48	95	90	37	41	67	78
5	0	6	0	0	0	0
10	2	10	4	8	14	9
1	0	21	1	2	0	1
39	34	51	44	34	34	19
1	1	0	1	1	1	0
99	152	281	57	101	58	95
80	81	163	118	84	68	79
0	0	15	0	0	0	0
39	62	144	44	138	112	74
95	40	88	34	45	34	61
14	8	12	10	20	10	9
44	33	59	92	34	37	43
0	0	11	0	0	1	5

For Review Only

6	16	49	31	18	61	42
50	31	59	39	30	44	33
24	4	22	12	4	30	11
19	51	47	25	30	36	22
244	132	455	235	272	220	264
1	1	1	3	2	1	2
9	7	7	0	3	7	7
11	8	14	7	8	7	6
0	1	0	0	0	0	0
33	2	0	1	1	5	1
66	47	72	33	55	32	19
21	6	15	36	28	21	12
16	19	32	20	39	5	15
25	15	25	19	32	27	19
17	37	32	23	5	28	23
16	5	29	21	22	16	13
8	1	1	3	6	0	3
121	84	121	59	75	98	59
51	59	0	45	59	63	56
36	28	56	36	52	47	19
5	2	19	4	4	11	8
14	21	29	24	19	29	20
30	13	62	23	36	42	11
224	249	384	158	177	216	133
3	0	1	12	13	1	9
54	6	17	12	40	4	24
13	24	61	18	15	31	54
42	49	61	31	30	41	32
1	10	0	2	1	2	1
11	35	85	11	29	18	21
0	0	0	1	0	3	0
4	9	19	9	2	3	7
86	48	116	51	96	44	68
12	21	47	41	13	17	15
58	26	90	38	54	60	42
72	54	59	12	59	41	31
32	35	38	43	37	23	34
1	1	0	0	0	0	1
1	0	0	1	2	0	0
100	13	103	22	50	24	48
0	0	2	0	0	0	3
19	49	27	44	48	57	57
146	180	278	124	25	242	11
16	19	21	19	6	16	36
49	50	122	69	69	82	59
10	29	19	7	23	16	7
7	5	20	12	4	14	16
54	93	100	57	71	41	50
1	27	0	1	1	4	1
153	126	217	63	59	151	121

For Review Only

35	3	42	1	25	85	4
53	55	91	30	52	54	48
12	15	22	18	16	4	6
34	61	79	38	49	80	39
157	87	179	123	108	121	103
5	22	0	6	5	6	18
0	0	0	0	0	0	0
26	28	50	20	40	36	24
15	16	23	19	19	8	8
441	321	0	566	315	307	303
7	14	10	12	5	14	4
30	27	15	5	0	2	16
10	28	56	20	29	24	27
60	101	139	76	81	81	78
219	222	367	127	228	377	222
9	13	46	17	34	30	29
34	55	69	29	33	37	26
15	11	7	10	5	4	4
48	47	81	44	39	17	38
109	138	222	73	155	104	68
28	24	47	15	21	12	12
15	0	19	0	0	0	108
33	46	113	276	118	4	77
0	0	0	0	0	0	0
17	30	20	6	27	7	8
73	44	0	22	4	27	18
168	1	0	1	65	1	1
24	47	71	43	57	33	53
2	4	8	11	6	1	10
45	55	0	30	31	43	35
38	20	76	23	15	28	28
409	426	668	442	448	459	346
12	28	51	3	26	23	14
16	16	16	12	15	36	16
38	62	61	77	104	90	46
419	467	662	430	396	315	353
5	42	48	8	4	30	8
242	179	346	152	139	250	149
80	86	152	94	107	83	57
2	1	8	13	11	12	13
66	38	63	40	49	42	42
67	46	88	57	80	103	49
0	0	0	2	0	0	0
53	66	46	41	63	40	48
28	37	87	39	36	65	22
249	215	261	171	144	153	173
30	58	68	47	28	74	43
77	26	74	31	12	64	25
123	52	146	109	74	57	55
4	4	3	8	8	3	4

For Review Only

27	24	42	28	17	13	42
8	17	26	18	13	10	19
31	8	54	33	31	26	60
24	21	30	22	37	16	22
9	62	80	26	39	38	1
59	9	28	16	15	21	20
11	22	26	35	16	23	14
49	30	47	41	57	38	39
20	34	51	34	18	24	10
10	17	32	5	19	26	2
32	26	75	28	20	25	28
18	8	40	1	40	6	1
5	3	0	14	17	6	15
85	89	157	72	79	70	54
18	22	45	21	29	38	27
11	5	20	19	8	8	16
34	3	50	9	31	8	13
0	1	0	3	0	0	0
24	46	33	9	12	21	22
37	16	83	28	37	26	47
71	36	59	75	44	79	29
9	46	62	6	37	6	4
48	21	54	69	43	41	30
25	35	102	70	52	32	56
16	10	19	19	21	18	4
117	11	93	27	28	20	16
30	42	81	27	27	12	35
195	165	229	96	201	93	113
1	4	0	0	1	0	0
0	0	0	6	0	0	0
36	22	37	20	10	25	31
1	0	0	2	1	0	4
13	5	7	7	30	4	7
46	31	66	41	62	63	25
32	24	48	39	27	33	22
123	80	100	76	72	74	109
3	0	3	0	0	0	0
86	88	132	93	67	82	95
69	90	135	71	57	71	72
46	38	55	38	33	27	49
22	12	37	23	20	35	19
5	15	38	7	1	17	17
3	0	3	0	1	4	3
119	72	169	41	136	144	62
4	1	0	17	1	32	1
24	168	150	68	96	105	64
25	19	36	67	32	9	14
46	60	94	87	107	63	62
0	25	33	14	25	12	32
20	252	0	1	2	400	1

For Review Only

41	62	56	30	64	29	25
261	298	423	222	204	252	285
35	45	51	44	66	55	38
41	27	12	17	10	58	12
21	20	22	13	18	38	15
0	2	0	3	3	8	0
14	48	25	19	31	18	19
1	0	0	0	3	0	0
314	0	0	313	0	167	0
50	65	0	40	52	48	0
17	12	10	16	7	28	7
0	30	80	40	55	53	48
21	7	19	9	5	7	8
30	13	51	15	23	8	17
12	6	36	21	20	18	15
21	10	26	16	16	34	15
42	62	88	53	55	71	66
1	2	0	1	3	2	0
23	17	52	24	25	25	16
25	11	35	16	10	17	34
2	9	0	5	0	6	0
19	3	17	2	14	16	6
40	42	73	29	39	40	52
89	13	101	26	76	37	67
54	34	88	36	76	40	37
32	46	55	39	24	32	43
19	10	24	16	48	32	37
2	2	5	4	7	2	1
13	27	44	18	5	15	30
21	37	14	9	12	30	22
147	154	211	121	129	165	179
48	59	110	47	34	73	54
58	68	84	0	83	83	68
11	8	17	8	12	4	8
8	44	69	2	40	51	27
40	74	65	8	41	82	38
86	50	66	54	45	53	48
29	31	68	63	18	59	32
46	31	88	34	18	55	45
31	21	58	40	46	55	26
22	52	71	37	48	47	62
41	47	69	33	47	41	43
30	17	49	16	26	35	4
7	5	7	3	6	3	1
6	18	14	8	6	11	8
5	5	10	5	3	6	3
4	8	14	3	5	8	2
35	28	74	51	56	59	29
21	20	43	20	13	29	15
7	8	0	8	13	1	1

For Review Only

4	4	4	2	6	2	1
0	51	91	33	47	41	22
13	22	10	14	15	16	17
110	83	225	118	165	174	131
0	0	13	0	0	0	0
187	192	336	138	138	202	88
86	90	145	49	51	106	51
15	24	71	16	25	14	40
21	3	0	14	71	17	20
0	0	14	31	85	156	0
198	89	244	140	115	200	145
166	115	281	131	172	95	93
59	37	156	68	78	6	51
7	12	13	0	23	16	9
2	3	0	3	3	4	3
116	95	236	127	127	80	88
8	13	24	20	13	17	22
23	7	28	12	18	8	4
7	33	37	4	7	47	5
132	90	154	49	115	90	69
62	70	44	55	36	71	44
0	0	1	0	0	0	4
46	137	176	93	106	117	51
3	23	0	16	3	6	3
163	147	378	150	185	205	119
37	9	59	25	24	25	43
12	16	81	32	101	26	27
80	83	102	49	76	69	65
61	2	25	15	12	21	22
52	62	65	45	78	39	37
36	20	42	17	31	39	55
1	10	199	30	2	253	27
17	14	23	2	17	14	12
31	43	38	33	30	46	24
405	755	963	591	531	339	359
2	3	8	4	11	5	11
126	114	165	83	110	99	121
88	109	193	138	162	161	118
21	24	22	6	7	37	24
159	177	175	109	150	163	104
71	143	177	131	89	122	86
53	73	109	40	92	93	47
98	131	116	99	72	98	68
169	110	170	141	144	167	95
1	2	5	0	0	0	0
100	136	200	113	174	79	129
1	1	0	23	16	1	17
2	14	21	5	6	8	16
124	175	238	155	189	160	143
51	48	0	55	52	50	61

For Review Only

348	268	455	191	210	376	213
0	0	0	0	10	0	18
60	49	52	50	40	34	54
14	18	36	21	17	16	29
21	13	44	20	34	13	24
0	38	27	0	0	10	5
7	15	16	12	15	6	7
73	95	66	69	37	45	58
17	20	40	20	21	33	15
70	67	77	53	47	53	51
0	1	0	0	1	0	1
66	89	0	58	57	79	58
19	52	0	8	10	21	53
160	71	140	119	118	130	120
1	10	11	4	7	6	3
13	11	27	26	17	13	29
25	35	21	64	33	51	21
0	0	0	0	0	0	0
45	41	52	36	72	56	18
35	65	59	18	28	52	31
13	1	7	4	5	1	4
29	16	36	12	16	26	8
82	56	126	40	56	103	68
59	112	70	64	84	103	31
0	0	0	0	0	0	0
0	37	17	609	52	734	558
78	97	146	112	86	94	83
19	5	15	6	15	1	12
65	39	80	60	33	70	29
55	19	41	15	39	50	17
44	39	55	52	61	55	40
5	33	17	9	8	28	9
48	49	83	33	49	65	49
5	21	18	4	11	8	8
72	90	178	77	29	289	13
115	98	89	117	113	128	83
74	55	71	51	44	80	54
0	0	0	0	0	0	2
38	40	84	17	27	40	37
22	19	24	19	16	17	9
12	14	26	6	8	15	13
61	47	76	32	67	54	60
153	187	283	127	77	132	147
78	79	200	77	38	128	14
117	163	262	67	157	118	96
61	103	109	75	116	42	40

For Review Only

rCHI_HSD_5
161342
22
98
2
46
9
29
84
0
193
2
300
139
1
3
0
1262
6
3
0
35
356
16
0
20
16
39
5
19
6
84
108
124
0
441
0
121
3
20
27
35
21
66
0
5
20

For Review Only

3
7
0
0
244
0
0
6
10
3
0
0
1
0
0
6
11
0
11
4
0
0
2
0
4
813
1
6
3
30
13
51
11
2
3
0
17
2
4
46
8
0
86
0
4
21
19
0
4
64

For Review Only

0
54
6
1
9
22
8
0
0
0
0
0
1
0
5
5
57
0
1
31
1
22
7
36
3
0
0
5
167
0
0
139
1
371
22
0
147
53
2
0
0
65
56
0
288
19
6
109
0
0

For Review Only

0
0
46
95
0
2
2
172
2
0
160
4
4
147
26
48
290
8
24
0
26
4
42
21
61
5
1
0
21
0
58
9
102
0
0
91
83
45
17
11
56
8
0
1
180
157
90
15
0
7

For Review Only

45
78
9
1
162
93
116
26
131
158
0
21
47
11
65
28
44
96
48
12
178
0
1
100
10
9
12
101
0
29
0
36
0
52
14
43
50
122
163
75
204
0
22
9
95
7
0
0
4
0

For Review Only

79
0
5
6
19
450
15
72
0
22
1
0
14
367
14
56
35
4
19
32
55
157
86
1
40
0
0
62
0
7
1
21
8
0
36
70
0
3
20
51
0
15
42
0
0
5
3
0
18
7

For Review Only

0
227
5
44
483
0
0
0
34
0
30
20
0
45
7
20
7
0
9
0
0
4
7
0
0
1
40
6
7
0
25
6
18
11
1
10
0
56
15
12
3
0
0
24
55
0
0
4
0
13

For Review Only

17
17
5
9
1
0
0
0
0
3
7
89
0
0
0
7
0
0
6
10
0
0
0
3
3
0
4
0
1
46
2
9
0
0
3
10
14
7
14
7
0
112
0
71
8
70
127
135
5
35

For Review Only

4
5
9
2
8
284
0
8
15
0
1
0
11
0
7
18
0
0
40
8
0
3
21
40
52
49
3
0
30
0
2
3
54
3
14
0
0
235
17
9
0
2
152
0
0
9
5
26
231
0

For Review Only

17
0
1
38
13
4
52
8
2
0
132
27
89
9
0
2
8
0
109
0
0
0
76
27
0
3
283
0
11
65
0
0
5
4
16
19
128
22
15
27
85
0
0
6
30
136
237
118
87
6

For Review Only

136
1
24
29
0
88
10
2
13
0
4
135
4
21
10
0
40
64
0
0
4
23
37
13
0
20
0
17
94
14
0
3
0
969
0
3
1
0
4
50
35
29
4
69
76
2
0
0
95
17

For Review Only

5
271
0
0
30
14
7
0
0
0
1
1
180
0
60
0
25
83
0
17
6
0
1
11
7
9
15
18
115
78
0
69
0
9
8
5
4
0
0
0
4
1
15
3
2
12
32
1
24
0

For Review Only

15
39
1
21
14
0
6
0
11
19
0
0
38
59
0
0
161
53
779
0
150
471
95
139
72
78
4
111
33
5
0
1196
18
0
173
265
0
0
0
1658
71
3
68
44
75
25
66
13
169
69

For Review Only

1080
64
913
0
455
298
88
130
271
213
74
1260
218
46
0
64
65
9
82
55
87
326
260
75
19
78
47
132
69
457
102
349
67
483
61
226
0
774
135
43
0
347
0
67
0
0
13
31
66
22

For Review Only

0
29
38
54
159
138
88
298
63
78
0
0
325
30
204
57
18
184
26
69
19
277
30
0
0
497
106
172
0
0
476
0
144
489
6
15
9
122
68
2
18
0
119
182
499
0
86
1485
0
12

For Review Only

35
152
6
214
400
0
478
23
11
3327
6
200
7
0
388
206
690
0
665
809
3
56
258
189
28
0
66
68
128
113
8
33
33
1026
0
699
0
9
0
36
27
0
3
35
15
94
0
77
0
142

For Review Only

0
20
0
24
0
41
5
150
27
10
0
6
156
153
78
104
0
0
0
41
0
40
509
42
111
348
0
104
10
0
30
854
4
0
0
0
2971
11
37
52
0
263
43
164
507
104
15
49
0
28

For Review Only

0
4
159
485
160
176
0
2
0
0
17
1530
0
112
53
208
97
29
15
0
64
77
23
13
0
41
153
0
267
0
221
47
110
0
22
380
306
40
30
0
0
14
38
0
89
49
45
67
57
437

For Review Only

256
146
177
35
1
55
222
47
81
0
191
70
30
124
68
29
102
42
72
11
0
0
8
321
57
20
105
1
0
10
6
0
388
43
14
1
1
83
2
0
21
49
0
18
7
136
25
547
83
11

For Review Only

0
71
0
7
134
55
238
16
55
359
316
109
0
103
0
17
58
75
4
770
7
4
18
40
0
441
13
350
46
194
32
1452
346
135
92
111
1
98
123
106
122
43
304
0
5
262
108
0
0
0

For Review Only

5
0
37
166
254
0
119
0
50
53
248
1508
44
7
15
485
14
171
130
19
59
159
169
395
50
20
62
193
0
188
4
74
213
0
82
24
18
127
1181
0
12
0
50
22
2
0
16
67
64
149

For Review Only

170
126
84
11
291
0
8
32
0
175
52
75
0
70
55
24
0
137
193
72
80
86
105
131
510
152
367
293
657
80
291
8
0
11
22
12
0
114
0
217
8
0
0
716
199
290
0
172
112
67

For Review Only

464
335
187
217
21
16
0
134
322
4
57
52
34
105
36
0
0
5
5
100
117
4
77
0
227
107
39
124
82
0
6
1
0
11
9
247
0
64
0
0
201
277
0
1
28
20
145
0
0
52

For Review Only

4
14
2080
126
1
205
293
9
4
5
37
616
240
90
443
137
179
41
250
200
322
234
428
1
160
173
21
94
163
29
0
64
0
133
128
61
60
74
0
78
424
217
118
252
15
1
123
521
49
85

For Review Only

14
26
277
101
3
27
0
8
152
193
0
0
120
23
704
95
0
2237
1
0
16
82
71
277
63
70
23
0
78
9
0
0
130
162
621
16
0
350
152
734
283
864
72
0
281
168
126
123
69
120

For Review Only

94
0
3
820
333
43
331
24
333
50
62
50
376
6
65
677
24
25
1
11
23
232
39
0
82
0
285
0
639
0
24
699
1164
157
0
197
46
88
0
0
67
593
57
9
2
90
0
25
321
3

For Review Only

506
154
6
990
170
169
92
0
260
170
167
147
125
230
376
0
70
45
103
48
71
68
8
22
13
366
1
59
65
56
19
1329
16
289
55
56
1000
141
112
34
0
45
246
145
11
46
82
29
12
87

For Review Only

218
0
71
264
560
779
243
246
799
271
0
178
0
0
422
0
185
10
104
22
1
1746
261
222
137
320
258
2500
25
334
8
374
17
73
731
117
104
86
227
128
194
116
19
2
23
19
0
2
35
5

For Review Only

0
18
6
11
133
969
0
0
0
250
17
61
54
284
336
0
324
8
81
121
8
21
128
250
82
141
0
475
0
227
121
501
747
84
158
198
110
171
81
68
0
104
105
23
342
84
0
370
104
103

For Review Only

44
0
12
74
155
41
155
93
189
0
5
56
113
47
43
11
207
104
107
0
9
0
0
616
5
242
25
419
0
0
515
1246
1446
282
6
200
157
587
0
94
139
55
357
138
0
58
195
28
0
894

For Review Only

2072
1374
86
623
0
372
1450
842
26
538
0
274
711
755
301
378
425
476
35
337
172
0
1965
444
481
23
6
0
22
298
50
67
247
69
501
2
164
0
270
126
508
66
91
130
46
119
132
0
29
43

For Review Only

336
4
29
0
73
24
201
9
26
32
255
0
55
12
6
65
12
17
11
0
62
53
88
247
53
43
0
52
0
0
0
186
232
76
0
77
49
6
5
1
126
20
7
1
0
49
0
7899
3
294

For Review Only

38
8
121
182
105
26
76
27
1
0
19
0
0
7
0
102
186
3
32
0
136
9
121
0
720
33
68
115
0
1
1
28
0
89
124
4
166
173
11
93
18
12
86
14
197
115
26
47
57
44

For Review Only

0
4
2
1
103
216
209
300
0
80
44
3
43
0
93
91
97
183
14
127
8
29
50
52
68
12
75
198
111
87
50
156
0
427
262
54
2
0
0
6
8
156
48
18
6
53
92
0
0
34

For Review Only

7
203
3
648
12
0
12
16
1
0
0
0
3
2
4
18
0
6
24
3
56
18
0
5
53
0
0
227
654
1
237
232
5
4
7
100
130
91
37
2
17
521
5
8
0
489
7
109
785
176

For Review Only

1
122
27
5
100
17
31
1
16
157
135
372
365
159
26
11
46
0
100
6
219
18
0
31
84
0
90
279
0
0
94
4
0
0
299
390
7
2281
2177
155
91
220
0
480
42
132
20
149
1289
0

For Review Only

0
3
15
130
150
3
146
118
101
48
0
21
0
1179
0
0
3
76
257
64
62
420
0
0
41
525
100
302
147
0
30
151
230
22
2
13
67
21
72
152
63
59
0
0
92
356
151
84
277
0

For Review Only

23
94
220
1
80
361
274
41
230
12
221
72
16
34
54
181
94
714
0
62
235
168
42
8
5
157
25
429
213
1822
72
0
276
103
74
92
8
6
0
0
64
0
155
0
426
148
208
111
0
1380

For Review Only

4
561
1
0
169
0
0
408
99
130
75
51
264
45
0
0
18
0
7
3
7
0
29
185
106
11
151
15070
21206
151
54
13
13
6
65
1
0
0
0
0
26
48
44
248
57
0
659
76
15
0

For Review Only

0
101
146
1649
0
9
0
0
7
12
634
272
4
0
249
0
0
19
36
23
261
175
235
263
67
5
16
0
4
22
149
5
0
25
0
31
9
14
98
156
47
522
0
0
6
125
108
0
550
978

For Review Only

1897
47
10
2315
6
564
0
379
12
18
186
174
50
5
98
95
51
0
178
75
172
110
0
37
0
112
50
0
2980
163
119
0
27
0
110
28
1
0
140
105
16
449
4
383
3594
463
5
0
3
0

For Review Only

0
0
0
4
0
22
0
0
257
0
52
56
380
3
1
5
8
250
160
3
98
22
0
170
261
0
0
223
17
0
122
89
73
54
67
59
150
0
10
268
87
0
368
55
61
0
0
12
1
331

For Review Only

98
132
504
44
63
230
0
24
63
0
6
57
0
155
69
81
54
2
74
39
0
233
0
202
78
1
0
113
1
9
5
0
5
84
83
0
0
9
8
30
147
73
113
0
170
0
0
0
37
6

For Review Only

22
64
62
58
47
59
0
24
5
45
24
76
0
0
45
271
50
25
0
0
96
27
24
0
0
38
283
288
63
122
12
43
65
230
60
46
0
26
56
2
0
19
4
50
0
1
0
89
1
15

For Review Only

62
133
30
199
4
7
12
120
61
0
440
0
8
130
7
71
0
20
2
3
1
0
0
0
489
2
334
406
0
0
0
0
93
193
3
0
41
0
17
86
683
134
319
27
63
317
28
170
96
116

For Review Only

13
12
462
18
66
1
82
23
2342
5
8
0
0
0
45
378
628
616
613
471
698
597
62
49
409
100
3
0
3222
4
0
0
21
0
96
1
0
0
0
80
0
12
0
0
83
62
91
128
52
1464

For Review Only

0
1651
22
16
1
6
4
0
472
54
1
450
0
0
0
47
0
10
0
7
918
527
214
5064
0
0
0
3815
0
29
106
85
116
19963
848
0
1248
1609
5275
0
93
125
0
196
34
16
76
129
21
0

For Review Only

150
66
10
34
146
92
2947
22
189
21
0
89
118
272
418
9
142
11
42
1
0
301
85
4
3
3
195
0
0
0
0
13
8
189
1
0
13
282
81
100
54
0
75
0
254
95
0
70
0
5

For Review Only

427
67
38
73
78
65
357
189
53
0
117
3
0
131
11
109
172
10
1
51
0
40
18
14
185
405
2
185
35
56
230
3
60
253
31
348
82
277
5
22
0
2
216
814
9
1
5
0
0
0

For Review Only

55
19
0
12
0
0
50
6
11
82
0
28
60
13
6
82
1
0
154
9
8
56
250
26
77
319
0
25
121
143
0
106
170
39
59
104
31
139
229
22
7
0
13
16
12
179
19
31
46
58

For Review Only

627
22
580
9
432
0
228
83
81
197
20
229
61
11
0
6
0
47
91
0
0
106
1380
83
0
132
215
155
0
0
7
349
14
22
34
3
0
0
199
0
7
561
163
83
0
21
61
60
55
183

For Review Only

55
41
167
3
219
358
142
522
0
0
76
40
151
6
31
107
16
30
0
196
0
87
12
405
238
6
187
433
17
250
305
73
0
211
267
256
0
219
33
673
0
24
8
0
13
62
6
83
254
0

For Review Only

57
103
6
1
285
170
0
383
5
1
490
125
103
135
123
10
146
2
246
24
4
128
90
20
3
81
110
218
17
7
5
1
0
246
6
0
7
42
102
2050
230
8
19
145
1048
126
314
1
0
3

For Review Only

174
769
92
101
110
0
21
6
0
0
1
0
321
1
23
0
224
11
0
0
0
13
323
636
0
910
86
0
0
1990
33
4
0
191
304
47
0
21
0
1
121
0
0
7
17
359
33
484
218
119

For Review Only

172
118
80
0
165
156
1
605
0
1423
84
1074
18
100
85
20
56
0
36
21
20
100
212
184
7
22
0
136
5014
655
75
18
54
51
23
1952
801
114
0
0
285
322
26
0
0
0
21
120
329
261

For Review Only

185
1
462
108
1
90
156
0
865
157
509
48
213
772
60
0
122
82
2
0
1
0
0
4
153
24
180
97
206
43
27
0
0
2
309
154
85
204
200
126
102
26
5
0
0
0
49
0
0
0

For Review Only

0
0
3
499
0
0
67
1
0
114
6
0
0
0
0
7
0
117
112
120
314
88
0
121
102
32
176
30
1651
0
597
479
353
21
448
75
59
173
228
596
220
292
188
100
237
325
6
30
95
57

For Review Only

33
91
129
22
31
168
0
1
1185
89
46
3
278
586
5974
72
15
232
66
132
45
30
157
1665
2
404
716
4
441
107
0
0
301
0
228
439
179
0
384
18
82
0
60
7
48
0
0
0
0
275

For Review Only

0
209
0
17
144
118
706
135
540
485
95
1
294
135
93
0
162
128
188
0
27
110
56
85
434
0
7
99
252
130
218
154
12
255
8
441
44
158
43
1737
107
270
28
42
0
0
185
197
328
47

For Review Only

718
17
1
131
12
155
109
114
53
35
23
34
0
17
745
113
597
66
120
535
214
174
275
30
37411
580
84
544
21
0
34
0
28
0
655
689
470
23
194
0
35
248
37
201
0
85
12
36
0
0

For Review Only

0
94
137767
0
27
150
249
28
231
84
61
0
71
142
0
131
0
73
80
6
1
114
27
0
204
47
0
25
180
321
37
388
40
286
180
0
2220
14098
2786
52987
172
6
4013
0
3645
670
30378
0
7164
0

For Review Only

2
86
0
1
0
394
118
116
96
60
101
73
217
32
29
389
29
185
65
72
50
72
60
0
27519
5
0
0
0
48
10
0
0
3
8
10
11
66
0
0
731
3
1250
142
127
521
97
68
131
119

For Review Only

0
240
17
18
1555
72
11
76
2019
0
296
130
0
88
452
4
0
0
40
0
5
4
198
40
1
0
0
460
74
0
333
3054
176
89
0
1940
24
234
0
6
4
53
42
120
19
16
4
5
25
30

For Review Only

0
294
138
311
37
129
136
120
3
0
15
1
96
24
0
46
67
48
0
4
44
248
56
18
14
0
7
59
4
6
2
0
334
211
57
4
0
31
1
0
5
0
0
479
104
132
0
3
26
51

For Review Only

190
63
47
0
916
284
69
198
0
961
12
123
6
24
0
4
83
83
26
323
482
0
124
18
77
100
79
15
475
454
135
228
10
5
79
0
0
40
55
131
0
131
54
53
62
0
9
196
511
16

For Review Only

507
916
107
79
101
128
29
46
27
243
0
9
385
0
13
328
46
0
0
1144
0
0
422
30
233
97
34
0
63
28
37
113
119
25
382
553
116
110
169
0
474
416
136
158
0
203
130
72
0
122

For Review Only

46
164
135
51
170
81
39
217
13
67
0
105
73
490
61
155
68
390
32
162
75
46
27
199
27
11
649
270
50
279
0
164
75
187
102
83
57
39
1356
344
0
54
48
40
39
43
34
25
116
6

For Review Only

182
443
49
124
146
66
9
1
42
1
41
34
4
63
307
27
27
0
55
108
163
21
4
0
2248
0
318
206
15
12
267
125
21
127
112
177
164
10
67
38
162
59
175
63
134
26
23
57
117
114

For Review Only

437
65
0
0
678
22
104
36
59
107
136
65
0
0
146
63
62
197
2
43
45
2
4
3
7
0
246
0
568
72
0
4
137
8
0
37
47
0
0
52
0
11
0
22
2
2
0
0
267
415

For Review Only

247
32
102
168
132
100
66
5
81
41
116
11
365
252
172
148
34
146
346
46
125
26
206
139
62
69
19
108
0
29
151
352
102
56
125
285
228
0
24
90
0
0
33
10
0
3
5
0
0
123

For Review Only

1269
5
86
134
447
5
48
51
5
1
204
0
141
680
161
823
0
375
0
51
144
839
180
0
15
0
0
208
399
26
106
155
77
170
85
162
0
154
60
71
17
14
430
0
79
283
0
131
106
183

For Review Only

78
206
82
0
1738
4
20
85
0
535
12
0
319
189
56
0
262
52
23
11
701
460
150
58
45
1
0
23
34
62
10
98
88
503
184
93
51
0
158
67
227
19
0
12
373
157
0
81
0
13

For Review Only

0
183
5
102
20
490
3
0
432
9
7
5
168
17
39
0
205
629
404
201
138
0
0
268
26
270
24
0
77
28
172
307
143
142
42
0
16
0
0
34
254
230
4
0
1
0
15
0
8
0

For Review Only

38
45
33
0
34
8
2
20
95
77
22
0
0
6
0
54
203
40
102
0
10
83
38
0
74
25
0
74
192
61
296
58
198
34
48
0
3
8
0
1
263
9
7
1507
85
0
1
0
195
129

For Review Only

873
1296
49
159
0
0
0
52
44
142
67
0
0
71
250
0
596
185
219
114
0
7
0
205
8
0
0
18
0
1
583
86
0
1
7
21
10
3
231
8
0
150
0
6
0
49
222
0
498
58

For Review Only

0
40
155
4
9
600
10
146
0
46
21
5
36
5
202
0
62
233
166
219
84
76
106
54
0
85
0
49
116
201
145
73
240
397
144
593
340
257
154
272
288
38
37
259
600
213
0
276
317
254

For Review Only

59
117
149
163
10
147
222
556
245
23
0
701
8
156
283
200
73
75
357
13
2
205
129
204
0
3
17
236
66
120
17
7
2593
302
27
90
79
170
141
0
0
167
6
0
280
121
38
125
0
8

For Review Only

245
0
149
534
210
158
373
132
0
62
0
44
0
0
0
14
0
0
0
0
195
39
66
24
0
197
0
0
0
164
318
1
45
1245
621
6
0
406
0
148
86
191
26
41
360
98
73
75
148
5520

For Review Only

0
58
9
117
27
46
56
576
363
0
126
0
1311
37
4
33
19
159
84
0
72
13
93
0
24
0
0
72
0
108
36
153
0
19
0
236
177
186
8
178
0
77
46
0
320
0
96
0
59
106

For Review Only

166
104
124
15
0
37
20
150
191
173
963
109
67
44
199
36
67
146
0
33
89
60
23
26
50
0
2018
325
148
0
159
0
132
60
157
0
50
11
4
0
100
56
0
14
119
174
188
204
75
6

For Review Only

203
38
664
103
103
166
667
171
63
0
920
5
136
58
200
347
18
141
78
184
183
380
0
0
0
313
79
250
145
22
183
147
0
248
38
96
176
0
7
85
10
81
0
20
295
109
625
1
139
92

For Review Only

335
0
0
3751
12
0
0
6
1
128
0
4
46
0
0
0
0
0
0
0
0
0
47
30
145
0
58
0
112
37
49
22
27
272
35
26
104
8
0
37
753
129
105
1361
4
160
47
1
499
0

For Review Only

60
386
36
111
46
0
246
0
570
68
5
16
0
78
65
122
46
14
67
0
0
59
0
226
171
25
0
104
15
108
0
31
3
180
114
89
10
0
64
0
0
297
355
0
11
108
98
71
95
1

For Review Only

291
59
0
0
102
0
1
46
101
0
0
137
158
151
37
269
25
121
0
2
38
1
0
3
60
96
137
149
147
0
0
114
19
226
250
101
558
5
7
86
84
178
25
0
28
487
13
0
98
13

For Review Only

1
6
144
154
81
69
245
124
1
247
26
449
43
339
31
141
71
79
0
226
35
64
180
0
98
13
0
155
10
3
0
0
5
74
22
279
66
93
88
0
0
26
7
50
9
0
38
572
0
129

For Review Only

70
14
0
19
0
15
78
0
60
0
122
0
66
18
162
0
1
35
0
83
0
176
0
63
20
120
67
82
11
0
239
0
732
130
0
43
13
129
168
154
15
27
49
7
75
51
13
11
144
0

For Review Only

1
124
178
239
7
50
58
104
17
272
66
172
1
65
50
166
47
36
84
39
127
0
219
27
23
232
18
30
86
0
1
17
59
125
134
79
22
231
68
214
331
85
97
20
4666
7
165
50
1
4073

For Review Only

0
482
147
95
510
0
15
319
10
12565
57
83
45
43
31
184
333
98
80
96
0
198
18
0
62
1
828
0
147
141
0
2
7
56
0
27
0
0
0
0
213
336
0
0
20
85
40
86
132
0

For Review Only

36
0
0
15
0
68
149
0
1
145
144
122
302
11
124
0
76
465
212
6
51
121
226
62
119
312
211
0
135
66
86
945
680
425
0
122
0
248
112
10
9
0
0
2
19
61
32
180
36
484

For Review Only

0
0
117
0
62
1
0
10
41
269
28
6
154
88
0
4
0
105
97
30
41
70
0
0
200
0
15
31
0
0
170
86
0
206
0
40
513
95
346
21
163
29
2
0
86
1
157
55
0
2

For Review Only

50
1
91
2
0
0
163
1143
695
0
10
3
562
8
5
763
42
16
4
174
224
1151
0
0
0
0
3617
113
83
103
41
338
113
500
537
91
242
48
0
0
0
106
184
21
9
97
30
6
13
117

For Review Only

68
0
0
2
12
0
28
187
24
119
0
36
0
24
4
1
1
47
7
379
633
14
108
4
1
299
18
65
721
183
431
70
143
189
253
113
99
156
112
5
0
0
5
0
0
131
8
501
0
342

For Review Only

48
13
67
137
102
64
170
147
146
0
0
0
9
174
18
224
18
90
6
0
3
0
794
144
83
10
9
439
5
0
176
293
173
0
9
9
14
474
1860
0
90
0
151
190
131
151
3
522
32
46

For Review Only

75
16
141
54
276
563
7
47
74
18
16
10
311
6
114
33
0
70
51
152
0
0
45
9
740
644
76
6
68
0
50
5
169
330
0
4
59
0
421
52
0
9
91
15
18
51
77
35
149
20

For Review Only

0
8
0
0
116
886
20
62
0
46
49
79
25
19
164
0
2
7
71
65
159
375
489
123
929
0
74
465
184
97
286
3
27
250
36
48
277
16
7
35
65
0
73
964
0
0
0
0
25
0

For Review Only

19
21
313
104
0
35
30
83
175
0
60
6
5
22
16
410
153
307
1
47
80
43
208
4
81
0
78
0
273
150
194
0
395
0
432
32
82
0
118
178
20
112
26
27
3
0
4
0
27
4

For Review Only

8
53
16
86
1
24
3
9
16
3
2
0
0
0
3
30
0
2
0
1
52
1
9
0
0
1
0
15
7
311
0
30
12
0
21
5
0
0
4
10
1
5
0
18
0
7
0
6
31
23

For Review Only

0
235
0
4
4
50
14
0
1
10
13
0
7
236
17
0
0
0
0
0
0
7
0
10
0
107
0
221
33
156
11
1
13
0
73
9
92
4
8
0
17
0
0
0
59
0
166
37
5
13

For Review Only

0
9
0
0
57
0
24
96
0
2
17
1
9
0
71
0
176
12
16
57
45
12
25
22
4
0
0
83
68
9
20
0
17
19
41
0
16
34
32
11
7
54
0
0
0
1
2
27
18
6

For Review Only

0
0
12
0
2
4
14
3
0
0
7
0
0
6
0
0
0
22
9
19
40
8
9
0
14
14
47
55
5
1
9
5
5
6
0
8
348
0
35
45
0
5
166
23
0
0
0
4
7
9

For Review Only

32
74
8
6
128
15
0
1
0
76
0
0
0
0
0
512
3
0
0
2
15
1
0
3
0
1
32
0
0
158
15
0
41
12
23
277
0
140
0
0
343
22
0
0
13
0
0
8
4
16

For Review Only

24
0
14
14
1
16
8
95
31
4
15
0
0
0
1
0
0
3
0
14
11
11
0
0
0
3
0
10
2
0
25
13
5
0
17
0
8
0
0
0
3
18
0
11
4
24
51
0
3
18

For Review Only

3
2
0
7
0
0
5
3
141
0
0
3
20
0
5
3
0
6
1
1
0
7
28
0
8
107
4
18
5
1
0
5
15
10
0
0
35
6
12
7
16
0
21
51
7
19
40
4
0
19

For Review Only

8
4
1
12
3
0
5
2
47
13
0
0
0
0
16
0
0
28
0
0
23
23
1
1
5
1
11
17
62
64
15
75
3
219
0
175
13
0
0
6
0
0
114
16
19
0
0
63
43
37

For Review Only

0
0
0
97
8
2
5
1
3
12
76
0
6
0
0
27
6
10
0
3
16
7
29
7
0
0
0
0
0
3
0
0
9
44
11
7
36
1
46
0
8
48
21
5
0
0
0
0
14
11

For Review Only

0
0
6
13
0
1
0
0
4
0
9
2
3
123
0
0
5
0
24
0
23
1
0
2
6
0
0
18
9
0
275
0
11
1400
1
0
3
27
30
4
28
8
5
7
4
0
0
0
0
2

For Review Only

10
12
0
1
3
0
0
14
3
14
0
0
16
0
23
0
0
0
5
5
74
0
22
17
0
0
7
0
0
11
0
0
26
4
3
4
0
5
0
27
3
26
7
0
24
0
0
0
0
0

For Review Only

0
4
0
0
77
6
4
9
21
157
5
0
0
0
0
6
69
0
0
0
11
1
7
0
0
0
0
1
3
11
0
4
21
3
0
0
10
8
16
0
9
13
13
17
2
0
0
6
7
30

For Review Only

0
5
0
0
0
17
0
3
0
3
0
0
0
9
3
49
7
0
0
3
6
209
1
0
2
2
0
1
1
1
1
5
38
12
0
3
0
13
0
0
5
0
0
22
0
0
24
147
10
0

For Review Only

15
5
0
1
1
3
17
0
5
13
4
0
14
20
0
2
0
0
0
7
0
0
0
0
18
1
0
10
5
0
11
30
18
14
0
2
11
7
0
0
56
0
5
0
0
18
0
0
1
21

For Review Only

0
0
34
16
0
0
8
15
0
7
18
10
17
3
0
4
0
0
0
15
4
0
0
0
31
24
0
0
62
1
36
1
220
6
0
55
6
0
0
7
6
0
0
563
40
44
26
4
7
0

For Review Only

0
7
16
18
40
43
5
0
36
13
10
1
3
0
16
0
76
26
16
0
0
22
4
0
10
0
0
0
3
0
2
25
221
0
3223
7
2
41
1
0
4
0
0
4
1
1
60
0
0
0

For Review Only

21
11
0
0
0
5
0
53
4
2
9
1
0
17
0
0
18
7
39
20
6
0
10
29
3
24
0
0
31
8
23
5
1
0
3
10
0
6
1
24
0
0
0
5
11
32
18
15
0
51

For Review Only

9
0
13
0
0
6
0
0
8
0
0
0
0
9
25
21
4
36
5
83
77
7
0
0
6
8
0
7
0
0
220
3
8
15
0
2
0
1
22
17
0
0
0
31
10
10
0
18
3
2

For Review Only

0
8
0
507
0
0
2
0
0
0
0
0
0
22
0
18
5
0
0
5
0
7
11
0
0
0
30
5
0
0
1
0
35
132
0
0
4
0
0
4
0
0
0
2
4
10
0
4
94
6

For Review Only

0
7
17
6
0
0
45
0
39
50
0
7
69
9
1
7
0
0
3
0
5
0
0
15
17
7
3
0
0
2
16
18
0
0
0
45
0
40
0
6
14
29
24
2
0
5
121
0
0
1

For Review Only

0
28
1
0
7
1
4
8
0
0
5
0
8
0
0
0
0
0
8
0
6
0
46
0
0
23
5
12
3
0
29
7
0
0
0
0
0
0
16
10
0
0
10
11
5
0
23
21
7
25

For Review Only

21
0
32
0
53
9
578
5
3
0
3
18
0
6
20
0
14
57
0
3
7
1
72
5
0
0
0
6
0
0
0
0
1
7
0
33
2
0
0
0
68
6
0
0
7
0
2
4
0
2

For Review Only

0
5
83
502
7
1
6
31
3
2
0
1
10
0
0
6
0
8
0
6
0
9
22
9
0
0
16
0
45
0
0
3
16
15
1
7
0
5
81
8
0
25
0
0
1
0
4
0
7
0

For Review Only

0
0
8
3
23
3
0
8
0
0
11
32
13
1
4
0
20
8
1
0
25
0
5
44
0
0
8
1
3
0
0
6
0
27
41
9
2
7
29
0
0
0
4
8
80
97
6
2
8
6

For Review Only

0
219
13
0
3
0
10
40
14
13
79
4
0
26
14
0
22
0
1
0
1
22
11
0
16
0
0
85
5
0
1
0
0
18
5
11
46
4
2
6
79
28
26
1
14
4
14
9
7
0

For Review Only

0
0
26
6
1
7
65
6
0
0
3
0
188
1
1
0
4
8
0
27
6
13
44
0
10
22
5
17
11
0
0
0
1
0
6
0
0
0
11
0
0
0
0
4
3
0
0
0
0
0

For Review Only

0
1
0
0
0
7
7
1
0
1
1
0
0
0
0
5
0
18
5
63
0
0
3
0
0
26
5
1
4
9
1
8
10
3
24
95
2
1
5
10
78
0
18
14
5
4
9
0
38
4

For Review Only

8
7
0
25
0
3
27
0
0
8
12
0
0
37
20
11
13
73
0
7
0
3
0
18
0
37
136
0
1
15
0
15
185
0
2
5
0
31
0
19
6
0
0
1
0
0
0
6
0
0

For Review Only

8
0
0
24
36
5
0
0
15
8
0
0
0
171
25
51
4
4
0
25
66
8
0
8
0
8
9
17
0
3
0
12
0
7
5
14
129
6
5
0
0
3
319
0
13
0
0
196
6
7

For Review Only

0
4
18
28
0
0
56
0
9
18
119
0
0
60
9
0
38
11
5
0
0
5
31
6
12
0
6
0
26
0
0
4
11
0
2
0
3
0
4
2
5
86
3
0
5
2
0
6
6
0

For Review Only

18
0
102
25
0
44
6
0
0
3
22
34
0
6
0
0
7
7
0
0
3
0
0
10
11
0
0
6
18
0
0
0
71
2
2
62
4
255
0
0
0
0
0
1
0
0
0
0
1
0

For Review Only

0
3
0
3
0
0
5
0
4
6
0
2
7
24
13
0
0
0
0
0
12
0
16
5
3
5
0
0
9
5
0
7
24
12
3
4
28
37
0
31
16
0
0
19
21
0
46
12
33
9

For Review Only

41
0
30
7
0
11
4
11
5
0
3
11
0
0
7
0
14
0
0
0
4
0
7
0
27
13
0
0
0
0
0
0
0
3
101
0
8
1
2
32
0
15
2
1
71
471
84
57
0
0

For Review Only

14
3
0
4
1
6
1
12
30
0
9
7
10
49
0
12
16
0
6
0
11
1
6
3
0
10
0
30
0
0
12
0
0
11
0
12
0
16
7
0
0
12
49
0
0
0
0
0
5
0

For Review Only

14
0
11
0
0
0
0
48
0
0
64
5
29
0
16
27
1
82
4
0
3
9
15
55
1
10
46
19
0
6
1
1
309
25
34
0
15
6
5
2
0
3
18
40
292
7
0
0
14
63

For Review Only

1
2
0
76
0
12
4
0
69
29
0
91
80
0
1
27
10
12
25
1
16
331
15
8
13
49
5
4
94
0
10
5
2
0
0
4
1
162
8
8
5
299
3
21
32
8
14
0
41
138

For Review Only

82
21
70
4
95
11
8
66
0
0
43
143
9
10
117
39
1
3
13
0
46
6
0
8
66
0
0
5
85
169
8
1
1
88
8
38
24
1
1
1
23
7
57
424
1
1
5
2
0
4

For Review Only

241
120
8
6
3
124
70
17
0
6
24
0
0
79
7
9
15
136
7
0
0
1
31
209
256
3
0
22
0
2
5
28
133
11
582
7
67
14
12
6
14
0
0
0
1
77
0
23
47
224

For Review Only

22
188
0
10
15
0
0
317
23
165
0
12
509
7
1019
503
142
5
0
402
14
0
1006
40
7
5
38
124
184
0
125
246
0
237
11
220
95
262
84
49
11
283
299
166
40
39
0
0
1648
67

For Review Only

231
44
176
110
289
213
54
1137
75
193
72
192
46
13
195
530
127
287
108
132
13
1
219
0
268
75
61
76
0
346
92
34
117
143
0
25
0
1
188
58
0
86
6
0
210
0
217
25
5
76

For Review Only

426
77
0
18
194
0
64
344
297
0
101
0
0
1
627
6
4
462
156
400
0
4
308
0
0
40
0
1349
24
0
0
0
0
3
6693
111
9
1
47
0
5
16
1215
114
241
90
4
0
0
0

For Review Only

60
0
49
15
34
380
0
0
7
0
108
39
3
63
413
3
218
198
0
0
52
1
0
7
7
227
9
134
7
9
58
0
1
34
185
74
52
6
86
371
12
0
151
40
70
0
505
158
23
137

For Review Only

9
20
35
0
20
5
32
0
43
83
5
75
15
190
47
0
0
47
64
26
72
296
36
187
117
99
127
111
90
610
45
2
62
324
51
115
76
118
319
138
89
97
242
245
36
300
0
147
5
47

For Review Only

3
25
9
0
0
0
29
66
638
89
106
157
40
75
5
453
43
149
576
12
0
1088
637
74
0
0
771
0
167
399
62
0
41
34
1
0
92
47
152
0
17
101
0
37
229
1
45
178
37
6

For Review Only

184
13
19
18
42
446
92
3
0
204
411
231
251
30
3
56
160
182
0
145
19
60
76
3
46
24
68
25
2112
45
12
252
161
31
24
0
57
62
1151
905
0
0
174
0
71
43
0
67
305
122

For Review Only

14
71
30
0
15
44
96
81
145
117
4
494
127
27
0
309
34
136
47
0
145
211
0
0
0
11
0
0
15
278
13
50
0
351
810
19
147
61
980
75
0
0
0
4821
60624
53
77
15
0
800

For Review Only

0
0
41
1
57
385
1
91
41
0
52
0
275
106
5
67
1
236
38
111
171
30
20
0
17
293
289
80
8
48
0
0
62
10
67
3
16
16
30
12
6
10
13
15
9
20
12
69
55
4

For Review Only

116
59
8
91
8
10
7
3
3
5
2
10
10
6
0
5
5
11
2
2
0
4
5
25
39
2
10
22
3
4
10
6
133
85
12
9
16
26
9
5
3
16
209
2
20
138
20
819
298
38

For Review Only

997
270
51
25
202
15
128
24
76
0
1
0
49
38
4
613
152
38
0
330
118
68
0
245
0
0
5
162
86
113
0
72
75
0
535
48
248
81
0
4
97
0
363
82
65
616
117
323
0
2025

For Review Only

0
16
544
195
435
130
88
10
20
20
36
71
306
0
0
523
1
170
6
46
1571
15
62
359
229
508
0
131
83
18
100
0
45
0
5
33
187
0
509
10
56
0
9
85
19
366
572
7
0
0

For Review Only

386
42
0
96
18
0
186
2680
3346
0
170
216
48
24
0
8
314
32
3528
899
354
0
0
86
9
0
0
104
97
210
105
0
115
29
95
20
1
3
0
54
203
70
0
560
1643
0
164
7
0
25

For Review Only

38
27
103
0
11
220
204
42
23
33
3
13
898
17
139
91
53
158
24
0
51
0
0
143
203
391
148
507
301
14
0
125
357
144
0
413
284
320
21
0
18
1
242
0
802
0
26
42
33
28

For Review Only

0
0
0
4
7
32
12
533
470
6109
1206
93
20
2
92
177
0
23
20
6
128
125
11
31
20
12
23
185
1
0
126
3
56
56
0
0
16
102
0
48
20
18
7
0
16
0
371
0
596
42

For Review Only

109
485
1
264
665
35
46
0
898
115
21
10
662
103
3
1461
0
0
0
7
250
0
0
565
7
0
11
0
60
0
266
0
1
763
13
35
0
2
0
19
858
881
112
25
55
108
369
0
161
178

For Review Only

140
10
437
151
59
210
7
241
18
8
0
5
0
27
173
3
27
0
118
15
81
95
0
292
38
0
114
224
11
165
34
354
257
18
0
162
158
58
0
54
39
154
296
0
154
93
22
172
162
113

For Review Only

47
55
53
137
12
77
0
18
46
58
210
277
49
228
124
53
258
32
38
59
51
5
63
12
0
3
23
36
64
44
0
260
293
39
0
115
386
127
91
0
142
122
42
1
0
0
2448
0
5
274

For Review Only

428
48
10
72
18
270
469
0
18
34
0
48
0
1
23
499
4
501
221
382
28
0
0
0
97
481
140
44
0
1534
229
28
378
118
0
2629
0
206
21
0
1871
0
0
0
0
18380
2921
61
182
74

For Review Only

66
33
623
78
38
1401
111
14
203
159
1853
79
11
55
210
7
0
40
235
592
135
247
407
111
127
0
1
262
713
57
157
1
66
83
44
11
76
10
0
3899
3908
24
24
269
0
4
2
0
248
133

For Review Only

28
274
79
203
0
59
6
277
171
397
71
132
26
46
39
0
0
6
338
35
224
0
57
49
73
0
13
0
8
0
0
0
4
0
249
0
0
8
0
0
55
0
2
123
0
26
21
19
4
6

For Review Only

67
0
54
0
4
230
8
27
7
0
8
405
0
15
0
0
38
0
131
128
1521
19
26
3
83
38
322
105
63
0
39
24
31
26
77
43
164
842
4
1
106
96
218
201
162
302
21
125
105
0

For Review Only

195
97
140
75
72
31
166
62
5
182
168
1
302
38
392
180
17
267
0
95
160
0
38
122
49
7
0
822
6
0
10
0
0
0
72
0
70
140
81
0
0
257
0
0
1
106
3
18
10
5

For Review Only

70
7
27
67
0
349
14
138
2
31
28
166
431
1
31
0
358
49
207
1
41
79
63
0
159
70
0
141
11
47
36
31
41
19
21
72
104
252
98
406
367
71
51
0
44
1
176
40
69
87

For Review Only

348
386
17
333
0
0
0
402
0
0
0
8
4
385
44
395
1388
671
34
0
0
253
0
169
49
180
53
64
414
46
31
129
240
90
0
173
56
0
0
234
49
0
128
185
112
23
44
0
1
19

For Review Only

8
107
45
67
105
22
43
110
0
0
429
0
13
87
25
0
1023
1981
7
579
317
0
52
0
171
0
827
132
250
18134
181
126
0
62
269
0
105
2305
101
909
96
72
198
175
34
0
7
138
15
3

For Review Only

3
2
0
32
14
124
16
337
51
2260
948
228
6
870
0
1819
4
22
0
0
526
280
61
0
22
10
25
181
35
0
68
827
377
214
57
0
27
0
0
0
9
869
0
383
1658
12
3
6
12692
29

For Review Only

0
778
13
15
2
118
0
712
0
46
30
5
5
0
80
0
65
138
79
100
812
0
0
0
0
48
13
8
138
37
0
33
540
3
140
424
15
65
12
40
755
20
8
39
0
75
45
287
855
322

For Review Only

30
58
408
0
0
19
0
12
38
236
48
0
0
14
0
70
336
35
20
12
7
18
3
0
5
7
103
0
7
12
0
6
15
0
0
14
68
2
30
146841
4
1
2
73
0
0
116
0
4
0

For Review Only

4
36
141
0
43
0
5
1
0
47
5
14
1
0
5
18
0
24
0
67
35
0
0
0
0
0
5
4
1
0
153
0
10
0
0
91
0
7
13
58
52
35
0
5
4
39
5
0
0
0

For Review Only

0
4
0
9
33
10
12
5
8
3
0
0
15
38
4
4
132
0
0
105
2
45
0
0
0
13
19
17
11
11
1
10
0
0
396
25
2
0
0
28
2
84
19
0
86
0
0
5
30
4

For Review Only

77
12
0
1
23
0
5
0
0
16
3
0
0
28
0
0
0
14
2
0
0
0
0
0
502
2
12
0
18
0
0
49
4
0
0
0
4
4
3
210
1
0
1
43
0
0
10
6
0
4

For Review Only

31
0
145
0
12
8
0
8
1
68
4
3
11
15
21
5
4
18
20
8
5
16
0
2
199
0
31
20
0
0
7
0
1
0
0
54
4
0
0
2
4
23
0
28
13
9
1
312
56
0

For Review Only

1
0
5
1
353
11
54
4
36
0
1
0
26
10
75
18
0
5
11
0
0
0
10
0
5
87
5
7
4
72
2
25
4
0
51
5
0
0
0
0
1
149
13
4
0
19
5
3
0
0

For Review Only

0
11
0
6
1
0
5
3
2
0
4
219
15
1
0
253
0
9
28
0
382
2
9
35
18
18
10
17
8
58
2
6
30
1
1
9
14
0
0
24
34
56
7
0
10
4
2
0
12
6

For Review Only

34
0
24
15
160
3
0
0
13
0
0
10
0
0
0
21
29
0
5
97
1
124
0
1
7
0
0
10
17
2
0
580
0
10
26
0
0
1
0
17
103
0
1
15
86
0
4
18
0
1

For Review Only

1
8
0
0
0
0
39
0
0
0
0
0
114
0
6
11
0
0
0
16
2
69
0
0
0
15
3
0
20
24
0
83
19
4
10
10
31
0
0
0
20
1
21
14
0
8
0
1
0
77

For Review Only

0
0
12
0
15
0
0
23
6
31
5
0
2
0
2
10
35
18
8
32
0
0
0
0
0
0
0
12
16
0
52
0
0
36
177
7
1
1038
8
6
17
4
0
0
0
0
12
27
0
100

For Review Only

26
0
8
4
11
41
0
17
31
0
0
114
0
5
1
0
13
9
8
0
0
3
45
0
0
0
0
11
0
5
6
0
0
0
47
0
8
6
5
26
6
0
0
1
0
8
0
11
0
6

For Review Only

1

24

0

26

40

0

16

196

3

0

3

8

0

292

14

163

0

2

135

8

0

7

461

1215

471

7128

412

98

207

1554

69

235

0

579

288

326

191

0

2

0

4

15

105

0

161

47

337

2400

0

3

For Review Only

65
0
57
133
119
2
720
146
12
0
0
0
56
14
17
4
4
53
12
165
0
0
71
12
56
0
4
0
39
0
25
106
745
103
80
0
0
0
6
1315
5
0
134
16
85
317
42
0
129
0

For Review Only

0
0
0
77
0
192
139
14
155
6
33
97
28
0
71
73
186
0
90
398
0
53
299
219
31
2
3
0
0
123
407
0
145
116
177
197
200
0
25
146
75
46
0
9
0
19
9
7
1
3

For Review Only

4898
116
0
56
415
2604
0
10
181
1
24
22
0
143
54
9
67
67
0
143
28
160
0
173
1351
14
67
69
193
0
25
0
0
1341
0
2245
12
0
68
38
56
74
351
460
708
2
1748
2
0
142

For Review Only

134
0
158
21
45
7
73
5
92
0
363
7
120
0
12348
83
56
43
98
697
231
30
0
165
442
117
504
57
411
1
1001
43
9
134
134
95
0
240
5
0
0
51
0
15
89
635
0
980
51
286

For Review Only

12
72
85
54
34
281
8
247
56
1
148
26
9
92
241
156
165
109
103
597
3
10
99
234
37
0
0
55
0
131
4
5
0
148
219
118
0
73
0
88
0
256
119
100
100
115
14
85
583
345

For Review Only

34
0
360
149
7
13
2
69
142
146
80
7
2004
18
189
0
135
165
38
89
33
8
37
0
32
38
80
110
75
0
959
395
0
0
215
985
160
91
133
137
8
183
15
3
35
32
74
85
19
0

For Review Only

125
417
0
46
3
0
92
109
312
87
0
47
9
57
0
74
35
0
0
818
0
47
402
153
64
111
58
18
15
298
0
92
39
162
119
92
30
42
117
239
0
0
19
0
561
639
1
18
307
742

For Review Only

149
0
0
81
0
145
0
109
58
356
152
0
197
341
129
343
42
238
47
180
39
42
212
158
413
162
101
79
47
141
418
82
99
67
33
75
244
203
87
804
921
22
0
46
158
10
38
72
1
125

For Review Only

176
6
123
2483
6
453
0
0
220
159
408
0
11
15
45
22
72
62
102
0
55
83
24
227
0
21
0
76
0
112
67
93
89
15
5
50
48
21
21
57
14
138
109
0
204
19
338
154
124
54

For Review Only

162
161
795
21
164
146
315
294
0
5
98
33
5
0
119
0
0
22
44
0
126
48
144
978
79
0
1861
27
416
237
13
335
247
9
92
0
47
277
2
48
0
110
8
90
85
182
61
43
5
252

For Review Only

36
112
43
29
258
0
0
116
2
1
9
104
57
38
314
218
77
0
2
26
0
0
14
7
8
0
0
1
0
0
0
0
0
0
10
6
4
0
1
9
3
0
3
5
0
0
1
0
6
126

For Review Only

0
0
3
0
0
1
11
27
1
1
0
0
19
80
39
46
1
0
4
0
1
10
121
0
2
3
0
0
3
0
1
1
14
1
1
0
19
5
0
1
21
0
0
2
6
10
25
1
3
246

For Review Only

41
0
88
108
24
107
170
75
0
48
0
146
78
7
72
650
3
113
29
2
66
134
50
5
34
80
285
76
44
0
12
51
107
39
54
94
56
117
165
20
98
4
6
0
0
0
0
6
28
5

For Review Only

1
8
0
11
9
59
0
8
9
52
30
799
26
3
633
6
0
277
0
0
80
79
9
137
0
112
136
270
153
0
177
180
158
271
47
0
2
56
86
11
118
277
107
0
0
2330
21
78
62
78

For Review Only

47
126
26
9
152
153
1
78
11
7
81
212
10
0
16
61
0
111
60
15
69
57
183
45
0
69
15
73
26
214
0
6
52
176
0
88
51
37
174
101
309
121
77
50
143
47
57
99
86
49

For Review Only

97
104
117
0
48
166
95
0
109
0
98
25
101
30
100
0
138
113
64
22
44
6
145
0
88
0
41
123
27
200
89
100
217
15
58
366
136
2
57
1
92
62
32
0
146
100
54
126
61
99

For Review Only

0
121
16
44
88
159
93
35
125
43
0
3
10
0
116
444
0
16
32
125
103
0
13
99
23
1
121
167
0
0
101
1122
0
85
0
361
0
16
0
0
0
0
87
0
26
1
108
148
0
12

For Review Only

38
264
21
1693
25
16
0
188
46
0
134
68
37
34
471
83
58
83
22
74
0
110
0
85
162
111
231
8
40
29
81
8
116
79
342
285
157
375
0
64
188
127
278
432
172
34
14
47
137
0

For Review Only

34
6
203
29
77
75
0
1
0
5
68
132
92
0
0
111
19
0
444
59
16
0
21
37
92
0
120
13
11
609
88
237
0
4
1
162
25
826
24
293
321
74
180
24
8
254
461
527
8
0

For Review Only

0
1277
0
97
13
11
306
67
17
144
166
0
50
992
106
0
20
0
35
281
125
419
0
0
40
0
6
5
0
508
0
22
111
2
219
21
296
274
0
23
113
93
116
0
204
35
0
0
37
76

For Review Only

381
184
158
0
42
19
22
2
251
0
58
243
532
31
37
108
0
592
150
1504
911
52
43
316
394
121
195
1
49
108
98
469
948
23
0
71
0
369
0
116
74
43
30
71
5
41
116
19
7
51

For Review Only

0
261
0
389
1052
107
103
99
167
6
94
398
0
0
34
102
0
71
58
192
158
277
60
110
2840
736
64
232
0
252
0
118
0
3
171
0
64
192
944
199
99
135
17
333
174
167
707
7367
532
259

For Review Only

28
1805
4044
65
196
361
906
164
0
15
0
0
41
358
0
147
77
242
441
182
0
284
58
31
272
109
132
164
228
347
89
49
41
52
22
21
114
0
239
26
161
0
178
163
100
271
159
0
250
223

For Review Only

376
17
136
88
72
208
0
17
190
156
351
0
0
0
0
172
684
158
0
224
53
0
36
225
13
1
49
1
9
27
0
19
56
9
776
152
23
213
0
112
142
67
141
58
36
43
10
0
0
0

For Review Only

25
341
5
12
0
229
0
249
145
1478
0
195
663
227
0
126
1
154
1173
0
310
0
161
18
48
581
133
554
47
6
0
64
121
134
174
195
292
52
72
70
111
31
11
114
0
180
15
4
184
64

For Review Only

151
168
8
0
176
9
0
96
63
0
7
62
522
32
0
16
134
86
351
32
151
0
0
75
73
0
0
117
289
16
113
9
16
54
73
8
0
14
69
82
0
121
68
19
130
153
156
16
59
0

For Review Only

119
21
18
80
25
367
0
13
132
85
15
42
41
121
4
87
19
12
181
58
79
70
0
59
53
164
87
160
174
273
181
163
512
0
149
40
0
179
5
0
180
0
408
944
29
3
32
6
30
42

For Review Only

525
2151
5
0
0
167
0
0
0
169
103
321
0
0
4
154
0
0
47
456
11
57
0
21
83
0
46
145
58
0
54
323
44
30
0
0
0
38
9
0
70
1
1
92
125
276
0
2
8
1204

For Review Only

0
0
130
0
55
8
855
124
1342
0
102
341
80
109
182
0
0
35
111
146
87
11
82
88
77
17
107
78
72
4
209
72
55
73
12
10
70
245
35
0
158
5
0
0
528
1
0
218
118
77

For Review Only

5
308
12
0
24
152
168
29
35
401
14
0
105
62
10
37
0
51
102
21
53
77
254
62
168
33
45
81
7
73
420
710
370
15
61
114
117
64
69
61
0
22
23
299
57
8
55
121
80
153

For Review Only

197
397
64
181
81
76
0
4
70
4
103
60
47
145
0
3
0
7
0
0
0
102
121
16
12
0
28
7
3
0
0
0
12
14
162
332
697
92
78
0
0
21
1598
0
21
46
15
113
22
17

For Review Only

459
0
49
0
85
96
124
0
0
619
0
79
0
53
0
209
11348
0
1
10
0
13
31
0
0
0
0
3
0
0
0
0
1
0
1
1
5
4
0
157
0
2
168
134
127
1
0
0
0
4

For Review Only

12
25
86
420
499
59
13
118
186
50
170
34
0
1
267
22
195
401
47
177
82
156
31
6
113
34
256
253
42
62
46
65
64
163
418
816
315
0
0
1293
12
45
8
107
0
150
0
116
0
0

For Review Only

0
0
0
261
848
0
21
192
63
81
46
0
61
7
927
0
0
5277
6790
9
2193
27
95
38
24
4
2304
54
0
30
0
0
0
113
172
5
668
2
134
0
178
144
229
1
44
126
338
97
649
29

For Review Only

354
176
369
59
0
0
15
744
3
0
0
0
0
99
658
23
79
100
124
15
45
195
85
74
33
373
0
60
0
4
385
0
15
26
0
430
21
33
43
0
30
0
64
95
0
87
171
48
41
76

For Review Only

75
170
109
80
28
58
53
196
0
33
18
0
12
1011
6
56
6
52
4
101
39
49
12
4
166
26
34
24
893
1
299
67
24
8
101
75
0
4
0
0
19
47
3
17
51
28
24
35
59
0

For Review Only

1

12

53

29

71

53

8

37

38

9

3

25

0

104

18

114

0

16

19

5

202

134

0

104

332

93

81

19

226

88

173

41

213

38

29

0

0

0

5

171

8

92

27

434

79

159

55

61

121

88

For Review Only

13
201
96
107
202
435
20
108
181
182
6
149
0
206
0
307
0
755
47
118
93
306
65
0
26
6
1
1
83
160
0
0
0
12
235
283
78
3887
1109
2
1244
89
390
88
86
55
49
118
34
121

For Review Only

16
0
105
0
187
93
0
46
234
81
4
3
49
11
0
11
0
168
0
0
294
211
33
113
195
166
187
220
9
12
57
0
258
89
14
0
21
0
101
478
0
18
183
62
8
288
24
81
0
25

For Review Only

0
62
61
198
177
45
0
423
23
170
198
77
71
0
111
87
106
0
172
104
47
491
367
466
33
23
1330
19
4
273
1
162
28
73
5
29
0
185
123
206
26
0
0
168
16
63
0
161
0
373

For Review Only

31
131
38
63
79
211
127
148
62
183
376
0
66
15
82
0
20
142
31
168
23
141
102
111
19
1
4
136
248
65
50
85
454
390
94
254
117
0
2
0
20
9
25
53
172
100
328
36
28
37

For Review Only

79
209
184
58
0
113
3
107
63
572
120
169
1
71
844
51
292
50
0
3
25
61
474
30
34
190
309
16
104
106
169
65
0
492
100
231
256
11
188
72
17
54
1039
68
46
108
115
5
360
725

For Review Only

51
262
46
6
128
749
98
85
50
1
1
0
1
138
0
0
60
26
498
10
172
96
0
373
0
10
6
7
124
384
0
0
0
31
7
290
561
300
0
78
4
235
264
437
0
57
6
21
66
251

For Review Only

793
6
283
43
20
319
118
0
3237
35
303
0
0
35
10
134
3
0
122
0
0
6
160
29
0
0
0
40
145
0
0
4
0
95
604
0
50
398
9
11
798
95
920
5296
6
950
145
396
26
0

For Review Only

36
0
35
12
313
11
95
0
0
0
0
43
2
8
25
0
62
108
91
206
40
0
194
139
48
26
61
17
0
1
14
7
18
709
54
0
2
394
130
17
0
24
81
29
0
265
41
0
116
0

For Review Only

0
1
633
11510
33
2
16
283
91
10
183
84
5
18
0
34
1
666
25
114
44
246
2578
192
232
42
71
69
0
475
48
151
0
0
10
116
48
0
91
116
5
238
10
109
64
26
0
0
26
93

For Review Only

55
1
7
1
205
0
150
274
168
20
90
91
95
58
119
33
447
452
0
2
74
91
33
66
40
12
284
0
0
155
62
72
232
61
138
156
0
87
473
31
136
37
7
70
58
173
78
105
135
38

For Review Only

1
100
24
0
62
57
89
178
0
6
89
37
142
437
0
102
3
586
424
23
67
38
0
278
28
27
93
103
87
160
45
106
0
44
82
0
3
43
0
32
1107
43
73
90
19
138
114
1191
156
114

For Review Only

80
5
123
6
60
277
92
0
44
86
0
104
37
21
5
202
0
746
0
55
153
0
0
199
487
142
129
335
173
261
85
123
67
0
6
0
300
58
0
259
1
8
198
11
146
38
0
0
232
123

For Review Only

8
8
170
61
363
101
201
47
785
224
233
0
59
26
61
33
9
41
296
0
38
189
116
0
229
0
0
405
45
60
3
23
251
88
131
344
154
1808
0
94
31
109
64
235
18
13
73
0
117
7

For Review Only

7
646
122
31
13
46
21
162
39
0
61
538
0
349
143
28
112
0
0
605
6
0
138
54
0
0
5
13
0
45
14
52
68
154
0
797
39
106
178
235
29
0
667
62
55
1
81
420
1765
91

For Review Only

6
42
23
21
375
18
67
191
166
8
136
52
42
153
2
0
0
209
39
99
58
120
55
30
0
0
28
23
0
0
0
4
116
1495
174
66
4
19
70
24
5
105
38
62
161
96
1
116
79
140

For Review Only

50
201
200
569
65
27
221
179
213
249
3
43
0
18
468
6
0
9
0
44
0
7
1
4
18
0
158
236
249
401
0
52
1
1
0
0
0
0
0
34
0
0
21
31
146
1203
96
13
0
19

For Review Only

16
673
65
0
0
13
176
356
482
434
168
128
296
484
21
525
145
417
189
472
286
43
153
780
8
218
0
0
0
0
437
248
485
105
261
289
287
197
312
108
14
215
376
296
413
76
130
236
54
137

For Review Only

114
39
142
113
22
136
52
0
50
1
0
2059
169
71
428
220
136
125
46
0
67
269
180
224
22
0
45
46
0
12
186
18
79
117
5519
4
6
202
0
0
81
1022
465
361
13
58
1166
42
36
13

For Review Only

654
282
31
31
30
11
2146
0
18
229
0
940
4
251
0
0
0
1
126
0
509
0
219
0
1
145
66
0
224
1537
1
196
297
102
124
210
4
56
75
0
42
21
48
31
57
126
84
420
26
45

For Review Only

0
193
96
265
0
1192
12
0
82
16
8
267
64
0
0
0
25
87
109
36
0
217
402
7
65
26
0
333
1
47
49
74
23
27
28
33
139
137
77
80
0
120
7
875
439
0
39
25
1
182

For Review Only

132
0
702
0
444
5
1021
0
396
18
24
36
9
225
66
0
126
149
3
1190
346
0
83
81
0
8
0
213
41
34
144
106
1488
275
0
138
7
0
0
19
227
56
227
184
71
225
956
0
217
404

For Review Only

71
608
97
192
132
63
46
95
226
0
0
53
32
83
1
148
0
0
55
119
16
102
0
35
71
10
3
10
0
88
80
35
49
0
44
94
213
165
13
141
627
165
118
42
94
29
41
226
36
141

For Review Only

1
67
115
84
105
1
48
88
120
1
141
402
7
91
659
98
57
104
357
99
367
371
0
31
79
10
17
237
84
130
52
360
0
41
0
0
110
0
38
1194
20
0
22
0
496
19
402
567
12
34

For Review Only

0
93
80
68
0
211
154
179
278
36
212
0
186
74
13
0
22
38
95
108
0
52
57
148
215
24
83
218
106
5
83
154
94
179
59
222
54
158
491
11
0
36
0
147
0
207
116
229
467
174

For Review Only

0
0
18
96
6
174
11
129
31
0
7
35
102
299
183
81
0
123
55
339
82
455
10
36
129
104
25
283
9
88
58
812
0
0
22
62
20
144
110
1
172
280
8
0
70
7
70
6
0
203

For Review Only

0
1067
94
0
148
39
41
75
141
48
0
672
589
33
0
96
0
39
104
66
351
119
182
73
24
59
75
368
132
31
184
27
0
41
95
72
72
74
70
86
17
0
6
123
31
77
0
267
2
18

For Review Only

3764
24
22
102
4
487
0
28
0
45
0
108
134
295
0
91
0
1
100
13
161
0
1260
413
172
30
0
53
8
3
1575
800
0
256
224
113
0
0
0
50
0
113
140
87
40
50
5
127
47
0

For Review Only

0
24
12
1573
1004
11
0
70
91
92
173
113
78
34
0
28
0
614
138
13
128
118
34
118
167
30
31
41
10
0
5373
96
89
81
37
0
824
100
40
45
0
75
65
23
144
10
78
1118
72
212

For Review Only

77
148
11
13
100
2278
269
478
103
221
157
152
0
77
170
298
92
25
4
44
0
180
33
359
0
49
402
0
306
0
94
82
242
143
358
318
24
389
47
47
0
4
0
20
106
30
48
1
168
171

For Review Only

169
194
314
177
0
233
239
48
426
105
0
0
471
50
108
30
80
393
134
177
11
2
7
5
7
6
11
3
81
172
946
3
49
12
46
340
61
135
109
34
240
96
82
31
85
142
60
5
0
0

For Review Only

6
0
113
172
248
0
0
307
31
166
83
16
19
210
119
99
66
441
0
0
0
82
0
58
40
411
61
88
177
0
87
75
0
87
358
92
257
2
2698
0
0
801
0
337
1426
93
355
1107
1712
132

For Review Only

0
104
90
301
0
8
97
130
211
20
1017
0
118
977
0
312
55
100
78
439
0
0
14
74
174
177
0
546
153
73
479
175
1633
0
101
1705
715
391
24
71
357
49
136
3
188
11
147
262
328
361

For Review Only

853
16
0
18
24
102
53
135
147
0
0
25
371
515
93
0
29
147
1
490
0
39
103
0
335
68
55
32
23
15
59
93
49
62
76
557
150
0
67
60
0
14
5
114
78
304
0
269
54
487

For Review Only

9
48
596
7
0
129
1522
247
62
418
75
14
173
68
171
75
161
0
327
13
1
0
57
49
152
255
62
19
31
9
43
15
5
155
0
4
43
7
30
4
5
0
32
6
32
0
0
33
419
0

For Review Only

55
0
65
34
59
69
142
24
205
254
0
0
2493
96
604
2
24
17
0
3
0
86
261
8
190
262
581
271
0
102
1
222
0
591
401
2113
28
35
239
255
27
104
52
8
1032
43
914
0
22
0

For Review Only

5
166
286
141
322
22
422
735
11
0
95
80
327
71
0
40
232
3866
0
303
457
0
192
57
0
167
971
10
12
0
149
18
0
153
51
0
19
13
21
68
0
0
1
3
0
0
0
51
2
0

For Review Only

35
98
1153
212
649
0
7
67
6
0
0
37
87
51
0
150
114
2194
0
154
116
379
377
694
1016
57
0
35
69
521
124
288
54
0
41
0
401
605
511
896
0
344
49
103
262
0
74
261
113
119

For Review Only

240
130
75
311
0
1051
0
124
33
283
90
168
130
127
360
74
24
392
2
20
28
23848
110
0
0
30
159
27
282
116
0
4
0
70
80
259
16
85
54
88
60
704
83
134
65
20702
17
77
66
1144

For Review Only

0
46
392
58
16
0
53
298
6
0
64
64
1332
1871
0
36
0
0
0
32
0
0
76
1
1
3149
98
5
0
0
2
61
168
2
1024
42
0
0
24
19
1
64
252
289
29
260
0
60
240
4

For Review Only

0
21
87
0
0
401
218
9
247
1350
465
0
7
156
165
47
171
18
1
70
288
139
1789
2
6
73
48
134
45
50
74
0
406
57
1
0
0
151
135
14
0
0
1174
61
9
153
24
151
439
11

For Review Only

160
211
20
8
314
63
0
32
0
9
0
845
63
66
15
123
82
9
11
2
793
0
413
66
4
0
0
326
0
0
1
0
31
0
0
0
1
218
0
1005
2
25
0
22
137
58
27
84
53
17

For Review Only

10
0
192
50
0
12
239
0
2
272
172
32
2929
129
1
243
36
133
149
57
142
171
55
169
632
61
1382
28
231
59
14
18
38
26
51
0
4
20
6
3
419
69
2
1015
21
0
171
334
29
1614

For Review Only

0
72
54
119
1
203
478
628
4
0
6
101
1931
66
223
120
0
683
100
0
6
61
0
14
136
279
775
50
25
1
95
0
123
0
1063
100
5
0
109
75
11
93
69
0
14
0
0
560
32
15

For Review Only

60
32
0
44
1
20
96
0
0
426
0
17
81
0
72
0
135
26
32
13
21
221
3
79
0
79
0
32
109
0
37
78
29
680
24
44
11
21
327
540
85
31
413
22
10
8
132
679
184
0

For Review Only

1674
136
26
11
3
48
88
26
129
0
68
654
23
2585
0
194
7
1
25
161
661
28
0
0
14
0
38
114
64
289
104
281
1461
174
130
71
65
194
335
296
76
257
126
468
27
65
244
0
91
14

For Review Only

0
5
188
337
5
16
397
44
28
15
787
65
27
224
625
235
0
0
104
233
259
744
98
144
119
0
21
1
184
89
90
3418
74
256
466
67
72
90
16
271
17
36
5
0
145
553
0
268
271
0

For Review Only

112
107
0
0
0
186
0
0
0
35
0
174
6
32
0
0
0
555
0
0
28
0
48
0
13
29
103
52
132
1451
581
0
173
304
137
4
6
52
130
217
834
0
0
366
412
18
0
17
99
5

For Review Only

0
7
234
24
146
11
28
225
28
102
168
247
52
44
138
183
545
76
215
0
136
0
47
31
211
267
33
7
61
115
228
41
288
16
9
0
81
196
27
149
34
36
107
148
275
202
74
181
403
87

For Review Only

99
89
159
63
1
130
48
53
145
20
336
608
20
156
7
3
140
41
14
0
324
62
2
11
87
103
9
0
415
321
0
2
149
0
83
65
26
14
0
557
80
189
122
36
132
93
161
255
0
81

For Review Only

163
88
212
226
45
121
97
248
30
0
7
0
20
44
0
166
6
13
0
19
82
0
93
184
1
27
0
3
4
15
1
0
9
36
14
1
2
0
0
6
1
10
0
6
5
0
4
0
6
7

For Review Only

0
1
1
0
3
2
60
232
217
35
85
54
195
0
60
289
0
34
0
24
80
47
2
46
39
87
0
0
11
0
54
0
363
180
102
29
179
222
92
62
1065
331
205
380
401
31
20
115
25
167

For Review Only

546
360
134
36
20
291
2019
635
0
250
62
399
15
10
2
0
18
70
36
56
692
128
0
3389
242
0
0
3
237
159
117
76
55
4
604
4
0
18
0
38
0
0
6
0
189
82
141
87
50
108

For Review Only

15
53
2
70
17
0
0
0
219
9544
0
56
15
719
0
112
0
0
42
10
99
0
26
0
0
15
0
4
223
1055
490
3
0
254
145
0
9
75
92
77
192
363
56
28
307
28
68
136
0
8

For Review Only

81
0
100
1
22
245
71
63
547
0
217
0
127
110
0
88
20
33
64
61
742
0
0
109
0
11
6
0
75
214
488
180
178
0
0
668
0
173
121
95
454
1
7
293
233
33
253
70
42
80

For Review Only

0
13
390
215
45
104
315
149
104
144
141
317
239
208
64
176
631
494
89
0
374
346
112
170
180
62
34
0
116
55
0
272
0
9
428
17
265
192
396
51
64
0
234
157
1351
0
0
0
1
1162

For Review Only

483
570
65
192
0
3
210
0
249
55
0
15
248
55
77
205
954
111
226
0
0
712
0
14
172
11
0
41
30
0
3
125
260
4
114
89
127
260
0
30
0
581
0
4
172
0
69
237
377
214

For Review Only

91
557
101
213
866
0
39
24
0
0
9
0
214
31
213
523
109
230
0
175
45
128
96
0
91
39
19
148
45
0
135
96
22
31
238
0
3
12
0
0
123
61
0
239
47
247
0
109
45
197

For Review Only

965
90
0
0
202
272
89
148
1
29
226
19
0
0
116
771
69
55
336
267
58
18
180
19
24
177
175
256
192
65
27
137
129
128
259
0
48
188
0
4
3
88
124
202
98
397
141
136
0
294

For Review Only

341
37
66
77
0
140
162
51
0
21
1326
132
10
0
2041
165
51
11
168
238
49
0
91
69
42
312
150
14
91
34
5
22
0
0
272
491
58
21
236
1
0
5
14
1
13
0
203
80
179
0

For Review Only

0
0
6
121
4
716
145
134
0
7
0
9
60
13
0
1
0
106
171
0
140
518
26
0
1149
10
23
20
261
51
51
98
50
5
31
141
73
28
80
111
116
93
48
94
80
3
10
37
8
107

For Review Only

117
224
14
0
273
140
0
55
552
46
13
2473
275
28
103
232
0
239
231
961
0
418
143
74
242
244
98
0
8
1
39
55
16
30
401
0
0
60
270
64
18
13
0
592
148
318
0
60
13
0

For Review Only

468
13
1
173
89
44
147
0
103
59
36
431
0
212
26
177
431
116
125
94
128
21
116
28
356
190
75
312
298
155
215
166
332
176
100
78
0
0
29
353
0
25
49
0
120
71
75
43
33
135

For Review Only

204
1
740
87
82
11
194
0
765
34
698
314
0
0
2298
177
330
0
45
118
3
19
0
37
0
40
0
75
33
7
0
108
14
63
0
146
90
5
1
31
0
278
122
43
567
192
28
0
21
79

For Review Only

92
6
1
67
0
85
69
172
99
124
54
33
365
323
135
0
0
0
63
585
240
53
0
113
5
38
49
60
50
32
776
89
6
318
216
143
8
29
0
63
0
0
9
0
294
2203
1660
0
0
140

For Review Only

336
46
8
531
0
520
295
17
21
0
173
28
56
34
123
55
13
87
7
20
28
28
0
723
25
64
23
141
239
45
164
107
78
90
14
230
8
469
21
0
0
109
129
87
245
1
212
138
36
0

For Review Only

194
185
0
455
0
8
9
8
5
12
91
100
132
84
99
34
102
52
51
200
13
76
49
77
686
0
0
141
113
52
328
182
105
411
36
8
1119
0
19
1
22
35
8
13
52
162
90
62
45
0

For Review Only

896
478
0
0
278
796
583
184
355
119
324
557
0
0
85
500
182
1
108
0
58
0
1824
0
367
24
70
210
503
4199
0
360
107
0
78
15
350
501
172
37
46
0
688
420
800
416
538
198
0
134

For Review Only

255
60
467
123
213
171
6
0
626
164
90
338
0
20930
75
16
107
64
39
0
241
34
127
0
0
610
3
46
313
20
237
11
330
0
0
1
0
16
29
135
21
270
60
0
112
26
219
138
239
19

For Review Only

31
994
2676
213
128
2582
107
0
158
97
53
119
57
128
512
129
354
0
56
258
9
31
9
41
121
0
33
102
2
50
376
0
952
33
163
15
34
34
14
39
262
0
1
291
0
16
0
62
0
1

For Review Only

101
75
16
77
0
122
2
0
0
0
14
26
27
0
0
140
63
0
65
0
108
0
32
163
536
420
27
53
202
61
0
45
135
46
319
145
0
0
110
1236
852
0
11
99
14
29
2174
170
30
1401

For Review Only

34
112
59
21
284
70
210
43
0
11
11
0
0
20
3164
70
37
146
0
34
73
126
10
130
79
0
525
129
0
16
32
189
83
0
0
0
5
4848
6
0
64
40
65
214
454
136
139
180
183
35

For Review Only

197
382
0
832
0
144
29
0
0
86
0
0
697
1764
822
17
37
18
2
43
9
0
169
0
0
0
12
8
0
0
62
6
81
20
205
47
12
327
66
295
0
132
265
271
131
358
13
732
38
0

For Review Only

6
6
68
255
108
165
167
185
233
532
87
14
63
75
0
118
34
0
12
140
3
13
71
127
161
41
216
49
265
339
177
93
108
363
528
514
161
174
97
261
0
216
166
44
287
26
87
0
1327
131

For Review Only

0
1567
282
237
0
153
397
0
68
74
17
1696
6
289
483
13
0
4
272
130
25
5
347
12
230
139
163
5
1
116
40
338
0
0
159
69
145
272
145
0
117
194
218
180
0
72
111
192
15
8

For Review Only

3900
6
12
26
0
66
9012
0
156
138
0
0
110
0
78
32
0
0
11
14
119
8
78
13
77
338
80
52
102
14
0
35
232
103
48
165
295
128
43
38
78
0
183
20
0
95
30
5
5
1

For Review Only

0
0
44
6
2
125
261
45
27
106
247
0
96
0
8
127
78
12
59
50
121
43
108
43
201
990
94
93
35
88
27
20
29
101
117
4
0
65
337
181
93
26
99
0
86
73
68
28
0
260

For Review Only

225
0
132
0
265
0
1
68
44
267
80
33
15
130
425
56
138
48
127
54
83
2
38
58
265
41
0
47
0
9
375
234
6
690
2035
1
237
801
0
30
31
1280
12
719
188
0
219
381
68
69

For Review Only

6
111
40
0
74
71
7
3
0
0
83
0
9
65
191
24
32
147
117
18
0
8
4
0
44
265
210
49
16
40
34
74
40
41
3
0
134
173
17
208
163
32
45
27
0
112
30
167
55
42

For Review Only

48
53
63
96
19
5
3
12
5904
1784
11
74
110
4
1535
1523
473
30
878
9
307
0
236
895
61
26
9
43
0
160
156
27
470
5
0
11
166
1
6
122
20
97
226
26
249
555
2
131
18
507

For Review Only

0
90
85
212
3
156
527
126
178
0
121
174
252
277
0
104
32
208
10
0
6
118
8
5634
57
259
49
26
42
30
296
0
92
353
587
238
168
88
0
10
68
0
89
8
434
1
51
448
771
0

For Review Only

0
189
326
0
3
0
38
0
3
290
187
0
21
241
166
118
863
352
416
59
123
246
86
1
0
60
0
60
137
10
192
50
178
131
76
209
124
277
87
3
142
94
236
0
2
160
192
186
460
95

For Review Only

299
130
14
64
154
198
101
522
107
105
152
26
231
13
0
32
71
311
32
148
120
0
39
147
130
8
32
7
324
18
1
1
121
77
28
236
31
49
284
74
120
0
0
3
0
0
612
22
6
237

For Review Only

61
124
194
7
37
292
55
2
57
8
157
121
19
38
165
104
322
9
10
0
17
9489
0
265
54
178
37
25
71
992
5
207
57
0
0
261
282
436
399
286
144
12
187
43
31
381
34
7
2
89

For Review Only

101
184
0
4
175
0
0
0
137
30
285
74
181
195
190
173
36
0
325
85
690
298
63
0
68
227
56
43
91
198
154
0
69
277
119
80
52
216
80
37
53
167
350
12
200
0
7
43
67
201

For Review Only

220
180
155
34
78
158
0
133
16
51
70
181
29
37
41
109
219
70
9
123
129
523
178
33
57
1020
21
43
197
28
86
32
99
22
16
0
6
0
3
2316
408
276
34
0
62
553
187
35
287
0

For Review Only

201
0
80
124
53
64
0
1
27
475
233
41
0
223
0
58
8
104
51
0
0
11
0
396
31
76
8
60
239
80
238
50
0
399
342
158
18
298
223
77
531
134
112
320
166
241
208
101
0
473

For Review Only

172
0
164
97
95
0
111
111
234
237
13
62
10618
0
321
147
210
0
6
8
383
4
8
90
81
0
242
139
274
68
14
933
0
53
119
0
41
160
430
77
61
0
222
0
162
307
418
330
0
20

For Review Only

1401
133
88
157
130
20
149
6
33
34
313
123
5
265
24
11
137
95
127
162
116
102
15
377
0
50
169
170
70
69
22
84
0
55
173
39
163
52
67
28
107
0
147
3
625
308
87
0
28
0

For Review Only

4
60
62
69
19
102
0
0
0
37
43
8
84
149
113
138
13
210
56
623
961
0
18
5
0
0
0
0
206
29
18
916
0
140
0
331
22
51
47
41
30
0
44
428
2
0
250
0
0
0

For Review Only

13
0
0
0
111
11
121
24
33
26
17
33
19
53
0
18
63
213
2
53
47
135
131
87
141
169
151
9
45
78
79
19
118
123
0
35
81
0
101
77
0
255
7
36
49
12
3
226
130
25

For Review Only

206
259
246
0
552
114
269
138
248
105
269
27
16
111
368
0
122
19
70
655
113
0
904
0
1213
1036
33
30
21
62
28
120
129
0
120
8
65
1
91
6
111
87
749
116
54
127
43
41
29
1

For Review Only

20
115
38
89
35
0
96
23
6
71
14
18
23
38
65
59
41
124
45
28
3
39
117
22
0
127
355
17
13
45
95
104
152
80
67
179
16
543
80
65
404
10
113
37
11
56
139
88
0
134

For Review Only

71
8
39
245
0
197
0
0
113
0
33
0
166
103
41
70
61
471
23
0
0
125
113
126
51
360
110
82
156
70
743
0
63
1
47
5
83
0
124
0
0
221
53
15
179
44
987
51
47
89

For Review Only

231
28
26
20
0
102
0
99
1
37
131
86
364
41
0
74
0
42
76
109
244
15
145
264
56
160
29
122
66
20
0
44
31
24
802
0
73
198
181
45
37
191
0
203
0
34
132
239
251
43

For Review Only

62
154
357
215
65
36
60
75
102
144
6
0
20
171
0
22
42
50
167
47
106
127
95
65
135
199
8
215
50
55
14
3
61
179
46
9
15
21
169
27
30
22
2
433
13
35
45
0
23
2

For Review Only

0
87
117
0
48
12
96
152
56
76
116
46
183
45
55
220
0
109
0
89
89
0
28
104
302
32
13
104
22
68
48
1
0
59
0
106
138
6
24
0
86
0
188
125
5
58
61
7
145
0

For Review Only

52
101
15
95
342
4
8
28
7
0
89
32
35
35
33
20
1
93
0
57
5
43
40
310
7
49
77
78
0
45
0
12
67
15
67
38
79
0
3
110
0
48
84
16
117
25
23
131
73
152

For Review Only

0
118
11
60
202
49
0
63
18
0
23
3
21
129
422
41
60
23
92
130
38
0
33
67
40
95
0
87
11
0
34
925
63
30
91
626
23
250
129
0
96
90
0
93
79
398
107
66
101
8

For Review Only

52
24
37
63
85
64
43
68
46
37
60
71
26
128
52
62
31
0
16
69
139
47
42
64
37
71
39
232
0
2
58
0
14
113
54
159
0
165
198
95
56
0
1
181
0
186
43
173
61
530

For Review Only

92
431
129
54
34
11
29
2
0
84
20
39
12
42
24
41
129
0
45
42
0
34
59
143
90
76
21
4
39
32
231
112
29
24
125
76
145
83
101
79
90
105
51
3
11
4
1
88
34
11

For Review Only

3
130
17
175
2
312
104
46
165
172
229
193
96
13
0
177
18
8
0
148
109
0
167
11
252
102
99
175
14
96
41
267
32
75
687
5
236
230
34
186
236
93
185
207
6
164
1
6
283
0

For Review Only

410
5
94
40
28
40
25
106
64
85
0
0
78
204
21
33
74
4
68
39
3
53
126
116
1
213
137
13
93
85
86
17
80
26
185
181
130
0
88
23
20
67
272
10
239
77

For Review Only

Microglia
Sham HSD vs Sham ND
P < 0.05

Gene	log2FoldChange
Hnrnph1	0.333989636
Abhd15	-0.126948198
Mfsd8	-1.398018551
Chkb	0.22061624
Arrdc1	0.024511579
Mafg	0.094400813
Rsrp1	0.299713262
Eef2	-0.299259364
Rab1b	0.292205451
Flot1	0.78853472
Gpsm3	1.468295493
Oaz2	0.137935825
Rpusd1	2.464041215
Slc31a2	0.904209524
Timm21	-1.728733829
1700029J07Rik	-1.879984853
Txnl4a	0.77911263
Mrfap1	0.023690014
Ifi30	0.776255936
Calr	0.463559794
Psmb4	0.647684311
Copg1	-1.028708833
Bag3	1.008102501
Plxdc2	-0.221491768
Cacybp	0.698427169
Nptn	-0.942256458
Tbc1d13	1.688543738
Tecpr2	-1.031606308
Inpp5d	0.802815785
Fggy	-1.623113472
Braf	-0.996075377
Gm6277	1.772806814
Sec23a	-1.221551213
St13	0.60759926
Tefm	1.498651872
Ndufb10	-0.909353552
Gm36025	1.871926366
Fam214a	-1.063474011
Skil	-0.721427346
Mtx3	-0.864724409
Tfeb	1.184222298

For Review Only

Bmf	-1.725307105
Manf	0.818279753
Rinl	0.008167009
Kcnj16	-1.781455341
Atg3	0.549080062
Hsp90aa1	0.713658459
Mir7676-2	-1.441366439
Slc22a17	1.786723942
Spag9	-1.064433248
Ypel5	-0.577906494
Cant1	-1.984657227
Rps2	0.833015914
Gm32133	-1.409342793
Slco2b1	1.053357756
Larp4b	-0.729033055
Zdhhc4	-1.510371081
Atf4	0.628601034
Rdh14	0.672944567
Exosc3	0.936731583
Gm26782	1.0157963
Klf16	1.406928696
Man2b1	-0.18994907
Rabif	0.550440479
Tsc22d4	1.528293476
Zfp418	1.126257727
Zfp653	1.094838556
Zfp691	-0.672617432
Arcn1	-0.481406982
Por	0.621383972
Mapk14	-0.385528962
Lrrc59	0.937096115
Ociad1	0.394620859
Gm39673	-0.584119135
Gm16120	-1.452331762
Gm42002	-0.973264877
Rcc2	0.326283415
Iqch	-1.631408636
Xylt2	-1.700629726
Gga3	-0.862855731
Hmgxb4	0.700929117
Oaz1	0.430744014
Nup210l	-1.387670172
Ywhab	0.258151972
Rfng	0.848840635
B4galt1	1.221205514
Slc2a9	-1.469725543

For Review Only

Mat2a	0.544429045
Ahsa1	0.617604692
Csf1	-1.42883627
Epha1	1.002166287
Fam117a	-1.322404034
Gosr1	-0.815859478
I830127L07Rik	-1.62257388
Mir6236	1.566018603
Cdan1	-1.163949101
Sdf2l1	0.623999265
Zfp335os	-0.921086963
Gas5	-0.531127712
Gm9769	0.59967154
Ccdc94	-0.943038872
Gm34076	-1.36844237
Pafah2	-1.392701451
Tecr	0.427837931
D8Ertd738e	-0.613138437
Gm40302	1.658454885
Polr2m	0.528857767
Ccdc112	1.105094405
Ncor2	0.74385302
Atp2a3	-1.290049691
Gtpbp4	0.830586832
Rexo2	0.685175524
Pcbp1	0.869379814
Gm35247	-0.770406363
Gm33517	-1.466444603
Arpc2	0.367919932
Clk2	-0.794999695
Col27a1	0.581968197
Eif5a	0.407537812
Gna11	1.162206144
2700062C07Rik	0.939449438
Gm33487	-1.464978944
Kctd18	-1.234492036
Tubb2b	0.557128337
Zfp51	-0.936788223
Cacna1d	-0.50911232
Cwc15	0.532067938
0610007P14Rik	-0.919364956
Gm32824	-0.958458371
Tubb2a	0.528870012
Exoc3l2	1.115149628
Gm32211	-1.197661775
Gm31872	1.335964837

For Review Only

Gm41046	-1.291074034
Dnah17	-1.232216599
Rxfp1	-1.254807413
Gm8203	0.434135458
Eya4	-0.608050646
Fzd7	-1.222086383
Bckdhb	-0.650877292
Gm30694	-1.042143772
Gnb2	1.376596125
Haao	-1.164081217
Slc7a15	-1.163110729
Vrk3	0.522722479
1700028E10Rik	-1.216422565
Crybb1	0.867839701
Fam84b	-1.326698998
BC020402	-1.44308383
Obfc1	-0.866782122
Tmem106a	0.604018985
Zc3h7b	-0.602967054
Zfp518a	-0.728734707
Tcf7l2	0.591345295
Igip	1.08934694
Letm1	0.577735456
Tmem106b	-0.442002697
Lamb2	-1.055829947
Lrrc29	-1.158953079
Rab11fip3	-1.081375574
Ptges3	0.443236727
Dimt1	-1.099636845
Atxn1l	-0.660926721
BC005624	-1.404144688
Gm38471	-1.309986841
Hspa1b	1.37431927
Map7	-0.939117659
Mtm1	-1.245591719
Anp32a	0.946490175
Ddrgk1	0.42380955
Eps15	-0.449346262
Pepd	0.460013933
Arfgap2	1.130499544
Brwd3	-1.08350835
Calcrl	-0.879316608
Fbl	0.587659186
Med12	-0.528783953
Psmd12	0.728390222
Ufsp1	-0.885773206

For Review Only

Mlh3	-0.889545752
Rabgap1	-0.654099092
Smim19	1.332342616
Pcyt2	0.993749078
Npepps	0.75681042
Parp16	-0.736687261
1700049G17Rik	-0.965637274
1700025N23Rik	-1.218886304
Abhd14b	-1.199735597
Hip1r	-1.282043157
Ltbr	0.565526435
Srl	-1.154566743
Gm8801	-1.104297867
Grb2	0.598817531
Slc22a23	1.09589667
Usp33	-0.562272139
Zfp719	-0.480339725
Pdia6	0.435728412
Txndc12	0.471241057
Drosha	-0.628508113
Gm32715	-1.176566492
Klrd1	-1.17612934
Ptdss1	0.436235237
Acss1	-0.623247546
Glg1	0.623502873
Pnma2	-1.216141043
Rnf215	-0.507500921
Thumpd3	0.397659913
Acsl5	0.344488183
Lmo2	0.949216673
Mapkapk3	-0.705561175
Adamts16	-0.492686098
9330020H09Rik	1.115266234
AI854703	-1.254585989
Arrdc3	-1.003086475
Mboat7	1.478812378
2700097O09Rik	-0.662394071
Cpsf3l	0.416463339
Ltb4r1	-1.240399546
Lancl2	1.254080026
Nhej1	1.201297911
Gm15232	-1.121838567
Lamtor2	0.71339981
Ap3s2	-0.543643238
Polr2g	1.20052875
Rpl21	1.20423937

For Review Only

Chrac1	-0.214097179
Ercc6l2	-0.928558355
Ibtk	-1.184435019
LOC102632821	-1.099083475
Nup43	1.077740523
Pafah1b2	-0.515872031
Tcf20	-0.62044391
Ube2g2	0.479503001
Ttc41	-1.180654338
3110062M04Rik	-0.58236505
Gk	-1.076486086
Ogfod2	0.297768962
Sdhaf1	0.601931806
Sdr42e1	0.933549451
Bcap31	0.365163075
Cct8	0.348359268
D5Ertd579e	-0.666353399
Fry	-0.487677015
Magef1	-0.876240467
Pcsk4	-1.231872368
Rapgef2	-0.570558277
Zfp324	-0.791928828
Hist1h4m	1.165983445
Gm41553	-1.161467454
Epb41l1	-0.872188087
Fam227a	-0.966512466
Gclc	-0.28655196
Gm34980	-1.319274702
LOC105245415	-1.239137745
Mcc	-1.164814084
Nars	-0.194764586
Dhrs1	0.589807862
Lypla1	-1.143218777
Lrch1	0.440972355
D230017M19Rik	-0.792755691
Best1	-0.826033132
Lhpp	-1.107692691
L3mbtl2	0.487067654
Hspd1	0.63249266
Hspa9	-0.519787711
Map4k3	-0.458016626
Oxld1	-0.579271408
Ppp1r15b	-0.59693037
Gm40116	-1.173710623
Mrps33	0.528990884
Slc25a29	-1.049182644

For Review Only

Rpp30	-0.771061564
Zfp160	-0.805139786
Gm34945	-1.173899251
Rab32	0.387372117
Srsf7	0.643355354
Smc4	1.095869371
Gm30541	-1.097423286
Pex1	-1.134117664
Wdr59	-1.078324125
Zfp112	-1.29079798
Abcb7	-0.855281504
Agk	-0.500415476
Atp1b3	0.373807708
Gm10416	-1.003783843
Mis12	0.548670612
Osbpl2	0.618522248
Phf23	0.816968479
Gm11827	-0.911220468
Mrpl49	-1.176729092
Setd1a	0.617371149
Trim47	0.649665474
Bend3	-1.088611657
Slc23a2	-0.406589281
Snapc2	-0.735123268
Ogt	-0.459777491
Cfap20	1.149001484
Lrrc47	1.036986007
Rad21	-0.522569508
AW549877	-0.547191213
Golim4	-0.573387623
Ebi3	0.526738352
Pcdhga2	-1.159419427
Spred3	-1.200836673
Brf2	-0.864638557
Sparcl1	-0.469498985
Mapre2	-0.323770111
Zfp704	-0.677187041
Gbf1	-0.304303351
S100a13	0.848951823
Cables1	0.80735929
Slc9a1	-0.677888894
Slmo2	0.477982199
Abhd17a	1.035886504
Gm34186	-1.128058212
Ywhah	0.279339264
Dcbld2	-0.711682581

For Review Only

Trim44	-0.529396521
Zfp143	-0.701950527
Slc1a2	-1.263453066
3000002C10Rik	-1.089974961
Fcgr1	0.416426895
Pcdhgb1	-0.812281703
Wdfy4	-0.417767299
Eif3j1	0.656910678
Polr3f	-0.574382861
Slc5a10	0.830328527
Capns1	0.868096544
Maml2	-0.281323618
Med25	0.590269377
Plekha1	-0.688428749
Rsrc2	0.459234901
Slc39a6	-0.689948317
Srsf10	-0.352822165
Stk11ip	-0.360337815
Inpp1	0.679280271
Nkap	0.493277304
Nr2f6	0.79058024
Mphosph6	-1.050401775
Snta1	0.686565412
Syn3	-1.055516642
Dynlt1a	-0.971785174
Filip1l	0.503388819
Gm26910	1.060618067
Hsf1	-0.755044886
Orm3	-1.169270806
Dot1l	-0.531765332
Gm26627	-1.033281465
Rnase12	-1.121039534
Wdr75	-1.042600905
Chfr	-0.371700305
LOC102635200	-1.090563316
Rnf113a1	-1.109589328
LOC108168750	-1.087357488
Gm32581	-1.155618882
Dtx4	0.930687257
Atp13a3	-0.869956909
Mag	0.584058532
Usp37	-0.343241032
D430042O09Rik	-0.669029841
Tmem43	0.291003113
Clcf1-pold4	0.28497968
Gm2a	0.301476322

For Review Only

Gm32336	-0.822365529
Hnrnpa1	0.52911193
Parp1	-0.123616418
Thap1	0.745752578
Ubald1	0.466267273
Arl11	-0.437444282
Psmd11	0.318358834
Gm12474	-1.162657694
Urb2	-1.051353726
Ankrd54	0.794370124
Fbxo6	-0.893995109
Zfp638	-0.58408632
Zfp697	0.633462092
Arv1	-0.726374358
A830010M20Rik	-1.06905241
Hspa1a	1.177604506
LOC102637577	-0.925158939
Gins4	0.751041263
Ptprm	-0.636080208
Zfp664	-0.747289166
9530027J09Rik	-1.107522336
Gm39908	-1.096939256
Hmgcr	-0.430072146
Mcat	0.756663883
Tbc1d10c	-1.103723053
Specc1l	-0.350778418
Abcg3	0.625324253
Arf5	1.039984198
Egln2	0.559410226
Kdm2b	0.519303006
March7	-0.724686651
Rft1	0.511189563
Lrp5	0.699927405
Whrn	1.187399849
Gm42145	-1.06678019
Ccdc168	-1.136286385
Acox1	-0.349693668
Pik3c2b	-0.69252647
Sun1	-0.644761262
Tceb1	-0.419146899
Med26	0.591562602
Rbl1	-0.650370777
Aggf1	-0.597541401
Csnk1g1	-0.726356467
Urm1	0.963734218
Zfp40	-0.611027124

For Review Only

Parp11	-0.44396037
AW011738	1.17465286
Gm15800	0.247319093
Map2k2	1.0625327
Polr3b	-0.619928244
2900097C17Rik	-0.419912603
Bach2	-0.361127818
Eif3a	0.331295924
G6pc3	-0.499271552
Gm40841	-0.621631745
Kdm6a	-1.05563682
Limk2	-0.870180082
Nfix	0.459097767
Otub1	0.061496734
Vopp1	1.000071163
Eif4ebp2	0.390757051
Klf4	-0.886480617
Mir99ahg	-0.821875453
Strn4	0.798618469
Abhd8	0.605047991
Amigo1	0.944706985
Asah1	-0.037807223
B3gntl1	-0.403997704
Bet1	0.460345391
Card6	-0.442895665
Cd200r1	-0.998412828
Cirbp	-0.222199633
Hps6	0.603686268
Kcnj9	0.605002247
Meis1	-1.023925009
Paf1	0.494353592
Rnf130	0.588190602
Selm	0.568588141
Slfn2	0.58054716
Nudt9	0.507202665
Gramd1b	-1.016884247
Fdxr	-0.999836311
M1ap	-0.766994957
Psmd8	0.330608432
Slc2a10	-0.847032215
Zfp61	0.542254867
Ccdc47	0.365823519
Pura	0.584215076
Rogdi	0.922380193
Gm32584	-1.034655956
Bckdk	-0.478824113

For Review Only

Cep57l1	-1.034377813
Ddx46	0.536030636
Fam161b	0.923138254
Gabpb2	-0.743484431
Gm31474	-0.981009037
Gm38850	-0.956508042
Gm42199	0.959397693
Gm6583	-0.856245093
Hipk1	-0.889566833
Hp1bp3	0.327235501
Hspa5	0.7307244
Jarid2	-0.438396568
Kif1bp	0.813282696
LOC108167511	-0.6029642
Lrp12	-0.8587185
Mrps24	-1.154738813
Nt5dc1	-1.000390885
Slc25a20	0.685485651
Tex30	1.053811107
Tmem14c	0.37777579
Zfp386	-0.538342142
Zfp677	-0.83865246
Ccr2	-0.524267216
Nktr	0.681227881
Slfn5	1.079617923
Pdk1	-0.452652532
Gm41077	0.602963237
Pdcd7	0.799374338
Kidins220	-0.405488214
4921517D16Rik	-0.861151404
Gm41678	-0.954146939
Bend6	-0.510547578
Cacna1e	-1.003805048
Oasl2	0.897600366
Epb41l3	-0.719928017
Gm11725	-0.995797573
LOC108169163	-0.918773354
Tusc2	1.102203261
Mtcp1	-0.947213
Mvb12b	0.504842568
Trmt2b	-0.990537662
1110012L19Rik	0.648853381
1190002N15Rik	-1.075405635
5730455P16Rik	-1.010960513
Cct5	0.429308925
F420015M19Rik	-0.640280738

For Review Only

Fads3	-1.003081657
Fam160a2	-0.797810745
Fbxo31	0.929332717
Firre	-0.712691283
Gm32605	-0.576999904
Gm39443	-1.100402559
Herpud2	0.637890286
Hgh1	0.452282049
Kcmf1	0.590574982
LOC108167848	0.817147042
LOC108168899	-0.688405989
Lpxn	0.725316988
Rgs11	-0.993741407
Siglecg	-0.928844786
Srsf2	0.472517975
Stam	0.760406831
Taf3	0.545639756
Tra2a	0.352196419
Adck4	0.438584468
1700123O20Rik	0.634492182
Exoc6	-0.324961356
Klhl25	0.589246186
Lacc1	0.610083452
Mthfsl	-0.483000953
Nol6	-0.576841166
Ptch1	-0.732632143
Zfp658	-0.43518684
Vps29	0.496304947
Ss18l1	-0.761381311
Tob2	-0.334913467
Wasl	0.449802498
Gm30273	-1.01679017
Naaa	0.500608592
Smim14	-0.629590423
Src	0.598982838
Tceanc	-0.791652032
Zfp445	-0.319115115
Tmem154	-0.940079118
Memo1	0.445059713
Parp9	-0.563164551
Ptpmt1	-0.955139193
Zfp385c	-0.875370093
Fam53a	-0.447319935
Lipo3	-0.601620237
1700010L04Rik	-0.821104567
Irf2bp1	0.683753569

For Review Only

Letmd1	-0.904325811
Ly9	-0.724747331
Map2k5	0.446352609
Mfsd5	0.612463191
Nt5c2	-0.515885562
Pdap1	0.605963746
Sgip1	-0.790734205
Suv420h2	-0.146083731
Tead2	-0.765315891
Wwp2	-0.95106893
Zdhhc13	-0.375671152
Srbd1	-0.500948835
Gm16118	-0.46019335
Nfkb1	0.279489745
Ppip5k1	-0.575026676
Sall3	0.219689468
Triobp	0.582894388
Inf2	-0.549817112
Exosc6	1.081914678
4931406H21Rik	-0.506130387
Acta2	-0.942002078
Ap5m1	-0.465348469
B9d1	-0.972950873
Ccdc134	0.410215604
Cd101	-0.578340347
Cep78	1.014143055
Cnot8	0.299167512
Col10a1	-0.965469241
Dcdc2b	-0.932641222
E130112N10Rik	-0.725415103
Eif3h	0.306562401
Fam103a1	-0.578494487
Gemin7	-0.152480774
Gin1	0.94082273
Gm15612	-1.022323938
Gm31862	-0.965472563
Gm33864	-0.940374067
Gm34079	-0.963014423
Gm41071	-0.592698281
Gmfg	-1.222639066
Gnai3	-0.9262877
Got1	0.555607118
Hpcal1	0.354916285
Idh3g	0.242938923
Idua	-0.678898966
Ier3	-0.549386289

For Review Only

Irf8	0.330874246
LOC105244413	0.736558066
LOC108168907	-0.750875724
Lsm4	0.947373858
Mettl7a1	0.57863532
Mospd2	-0.922749475
Mpc1	-0.518805496
Mtfmt	0.532596167
Ndufa3	-0.192192547
Nfs1	0.40183633
Nt5c3b	-0.755784995
Ogg1	0.779391194
Peo1	0.727846069
Pithd1	0.586727652
Polk	-0.635598803
Psmd4	0.35418901
Ptar1	0.3828874
Rest	0.537097004
S1pr2	0.556984774
Sdhc	-0.651295057
Sept2	-0.263034695
Sgsm2	-0.896101091
Skp1a	0.43282173
Strada	0.852175409
Tbcc	0.503282224
Tmem241	-0.625669584
Tnks2	0.685814204
Tns4	0.793398698
Trappc5	0.615755381
Txnl4b	0.465583686
Ubac1	0.735630389
Vps37a	0.479570149
Ythdf3	-0.465439632
Zkscan7	-0.986766143
Zzef1	-0.498618302
Bbs5	0.730424608
Kif3b	-0.693778191
Tia1	-0.37511822
Zbtb43	-0.510181246
1110037F02Rik	-0.423095521
4930439A04Rik	-0.90928854
Cebpd	0.660353176
Gm32029	-1.021465788
Gm36328	-0.941178506
Gm40964	0.709019522
Gm42158	-0.930062436

For Review Only

Kansl1l	-0.407539343
Nop10	0.436274055
Pag1	0.503462942
Prmt5	0.574769328
Psmd3	0.837405516
Slc35a5	-0.42651685
Smpd1	0.32156021
St3gal5	0.502633636
Trim32	-0.419631868
Ankib1	-0.34648648
Pank4	-0.829667782
Tpgs2	0.729678872
Camk2g	-0.559141923
LOC108167381	-0.78955307
Mios	0.70051306
St3gal2	0.351049848
Gm29927	-0.820444609
Cmtm8	0.632347104
Cxcr6	-0.966189416
Iqcc	-0.566805912
Ccdc85b	0.707100161
Mrvi1	-0.64374471
Wsb1	-0.913822593
Zc4h2	0.699125365
Ccdc146	-0.911840794
Cyth4	0.923858702
Glt8d1	0.980441099
Gm41107	-0.596313619
Lactb2	-0.383335039
Stk40	0.536633232
Ttc3	0.386313333
Zfp574	0.605954139
Ide	-0.510352845
Marveld1	0.969333045
Mbtps1	-0.287336049
Nap1l4	0.429629127
Rad17	0.525684371
Ubr5	-0.58183357
4632415L05Rik	0.666769832
Arpp19	0.387299813
Gtf3c5	0.505155263
Zc2hc1a	0.66945159
Arid2	-0.415130304
Brd4	0.477637213
Tmlhe	-0.922671844
Gm39424	-0.973617402

For Review Only

Map3k14	0.51984117
Slc24a1	-0.931960956
2810025M15Rik	0.4429964
3830403N18Rik	0.945040829
Ank	-0.4539423
Rnf141	0.304741658
Pak4	0.55124988
Zfp330	0.879043009
Cog7	-0.939948551
Ddx56	0.528523219
Kcnh6	0.526952419
Lmln	-0.588954282
Rap1gds1	0.364651259
Slc18a2	-0.493280197
Sox4	0.934524169
Zfp729b	-0.638181256
8430429K09Rik	-1.004235336
AA415398	-0.884849421
Ap4e1	0.749951292
Diaph2	-0.46626658
Exoc6b	0.49582274
Marcksl1	0.815127623
Metap2	0.357846179
Sec24a	-0.409747449
Tmem50a	0.260292555
Ubtd1	0.401146466
2410002F23Rik	-0.531175854
Ttc39b	-0.390634223
Nfya	0.698130293
Zfp523	0.821482019
Arap1	-0.374065867
Tmem220	-0.884589782
Mbp	-0.54565181
Asb10	0.60702105
Btbd6	-0.425271797
Lin9	-0.940884489
3300002A11Rik	-0.778977235
Ech1	0.2832754
Smndc1	0.828844574
5930430L01Rik	0.870893281
6330407A03Rik	-0.558198872
LOC108167485	0.960217175
Aknaos	-0.681144498
Gm36117	-0.910309329
Tal1	0.652346579
AW146154	-0.463571347

For Review Only

2010315B03Rik	-0.666764637
LOC105246961	-0.572755383
Map1lc3a	0.421480628
Fam149b	-0.894200018
Hcls1	0.280266517
Szt2	-0.627508517
Dlgap4	0.649644267
Gm16206	-0.951095008
Txndc5	0.339214792
Tssk6	-1.009817624
Fkbp4	0.734983325
Ckap5	-0.37062253
Fyco1	-0.466777568
Smim15	0.795713798
D930028M14Rik	-0.879351662
Gm34408	-0.90704851
Fancd2	-0.519946031
Itgb5	0.623438625
Dnajc10	-0.674944214
Eif3j2	0.61155011
Prrg2	0.474610138
Tnfrsf1a	0.197127379
Zfp41	-0.913793509
Cdc14a	-0.727054151
1700113A16Rik	-0.49571128
Acbd3	0.364878088
Dhcr7	-0.880536834
Imp4	0.155927497
Noct	-0.824719068
Pcdhgb2	-0.773262267
Gltp	0.281163497
Gm14680	0.547009466
Dnaja1	0.253879419
LOC108167735	-0.904510389
Nagk	0.579769032
Sptbn1	-0.882172838
Cipc	-0.572101821
Gm40468	-0.881954963
LOC108167875	-0.80577392
Prrc2c	-0.381210964
Tbrg3	-0.576835054
1700001C19Rik	0.866113734
Acadl	0.416803803
Cyb5rl	0.658090605
Fam126b	-0.594913732
Glis3	-0.675877052

For Review Only

Hddc2	0.948192635
Snapc5	0.39123731
Appl2	0.780329263
Bud13	0.663962353
Cabyr	-0.906953945
Dleu2	0.430012021
Ptpn2	0.704374785
Ptpn6	0.465974429
Tuba1c	0.284677227
Aven	0.961723469
Trdmt1	-0.85516666
1700052K11Rik	0.929552812
Btbd17	-0.936866045
Dnaja2	0.278902293
Dyrk1b	-0.429282881
Enpp5	-0.874028485
Gm30789	-0.672585606
LOC108167915	-0.85392107
Lrrc39	-0.575680649
Rfx5	-0.65890872
Fbxw8	0.616481706
Cdc27	-0.669355624
Bbs7	-0.813544827
Mrps25	0.566866403
Ndufs2	0.72556769
Nsf	-0.4644666
Gusb	-0.897552512
Tmem70	0.335166526
Acvr1b	-0.846753587
Aldh1l2	-0.560540464
Bst2	0.399424712
Cpm	-0.803819322
Derl2	0.21330675
Edc4	-0.818676189
Lin54	0.822117658
Pisd-ps2	-1.015979908
Ppp4r1l-ps	-0.699521281
Prkd2	0.545852367
Rapgef6	-0.306472863
Sp2	-0.57894543
Tmem11	0.289410122
Wdr7	-0.572674053
Zfp111	-0.484756662
Gm41113	-0.842178433
Kpna1	-0.491333244
Nudc	0.433140426

For Review Only

2310011J03Rik	0.813235657
Mn1	-0.951603806
Gm3134	0.547441609
Gm26641	-0.891295004
Prim1	0.433065227
Ecsit	-0.608396936
Ipp	-0.855941632
0610010F05Rik	-0.850610553
2300009A05Rik	0.752700609
Alg8	-0.116352411
Ddx20	0.375045197
Gpatch1	0.418840395
Itpk1	0.332013513
Jam2	-0.335693969
Med15	0.354049552
Smap1	0.623054097
Syncrip	-0.258927682
Uspl1	0.462156102
Tango6	-0.628165461
Fancc	0.444596837
Baz1b	-0.49974727
1110006O24Rik	-0.668248904
Sept10	-0.387554402
Prpf38b	0.555412846
Dnajb11	0.425428696
Naa50	0.437833118
Sox15	-0.838578223
Trappc12	-0.391380034
Zfp266	-0.442826166
D130017N08Rik	-0.877322173
Apbb1ip	0.380068573
Polr1a	-0.331320094
LOC108167801	-0.822651662
Fyb	0.870078652
Gm33370	-0.542419987
Gm41610	-0.886082813
Gm9706	-0.738168782
Rbm38	0.47103425
Cct6a	0.349476179
Smurf2	-0.399575704
Hsph1	0.746570502
Ctnnd1	-0.423993408
Coasy	-0.624725747
Eif3b	0.914237945
Jak2	-0.370582365
Sars2	0.694099416

For Review Only

St14	-0.548056177
Bnip1	0.503350673
Ccdc14	-0.911980936
Hdac4	-0.405819043
Gm13212	-0.511675077
Parvb	0.623981501
Ube2s	0.884820963
9530036M11Rik	-0.460008716
Heatr5a	-0.410836784
Mfap3l	-0.840517887
Slk	-0.600293761
Poc1b	0.431146425
Gm20604	0.693399024
Commd8	-0.49478894
Mrps7	0.508086161
Opa1	-0.486929485
Tuba1b	0.338415141
1700095J12Rik	-1.325547796
4921507G05Rik	-0.824985033
9430085M18Rik	1.118151263
Adam30	-0.687111737
Adgre5	0.585173852
Aim1	-0.697422841
Arhgef17	-0.778812932
Baiap3	-0.762175419
Btla	0.884638422
C77080	-0.949196222
Dhrs13	0.923958065
E130317F20Rik	-0.979229932
Eid3	-1.311082019
Etl4	-0.949085062
Gm12781	-0.718032402
Gm20616	-0.731257366
Gm31388	-0.910392762
Gm31606	-0.853971251
Gm31683	0.999395868
Gm36148	-0.669860588
Gm36500	-0.680650385
Gm38561	-0.948320864
Gm41962	-0.571019186
Gna14	-0.577773649
Ispd	-0.669029336
LOC100049077	-1.370616631
LOC100503338	0.715249653
LOC108167714	0.757601601
LOC108168774	-0.832839317

For Review Only

Mboat2	-0.638051726
Mst1	-0.810522196
Pdzrn4	-0.905258264
Slamf7	1.412828723
Slc9a5	-0.713721746
Tmem136	-0.9099818
Trim67	-0.800671355
Ube4bos3	-0.82542793

For Review Only

Microglia
rCHI ND vs Sham ND
P < 0.05

Gene	log2FoldChange
Arrdc1	0.423978279
Abhd15	-0.126943493
Ccl4	-0.285478944
Chkb	0.118521497
Mafg	0.127609537
Nubp2	-0.149161306
Eef2	-0.29925057
Nisch	1.996284746
Hnrnph1	0.010899394
Gpsm3	1.533279208
Gpaa1	-0.104203703
Amz1	1.747246118
Lrrc14	-0.100296725
Flot1	0.664231249
Rpl36al	-0.06874455
Slc35f6	-0.065029605
Oaz2	0.077583665
Lpcat3	-0.033843166
Tnfrsf14	0.100926799
Mrfap1	0.072052767
Ywhag	1.412780909
Rinl	0.128151174
Psmc4	1.924972733
Rcc2	0.971264543
Txnl4a	0.813930359
Tfpt	-1.962008416
Kat5	-0.974097021
Psmb4	0.607916738
Snrnp48	1.441240356
Cant1	-2.14894371
Otub1	0.547485143
Calr	0.253152512
Parp9	-0.596566768
Wrap53	1.710103002
Rabif	0.654576188
Gtf3a	-0.931823782
Hmgxb4	0.75729066
Inpp5d	0.563285034
Gmfg	-1.512663186
Mfsd8	-0.671014173
Mrpl49	-1.248402501

For Review Only

LOC102641351	1.492270068
Cyhr1	0.461754222
2700062C07Rik	1.149858712
Tmem251	1.861265152
Cers6	-0.990324884
Rdh5	-1.37302479
Oaz1	0.425678784
Dusp16	0.993776559
Rps2	0.620786716
Plppr4	-1.466870251
Appl2	1.276285989
Kcnh6	0.809904937
Ndufaf7	-0.322040781
Pycr2	-0.415128271
D830046C22Rik	-1.508011508
Plbd2	-0.469208448
Ccdc112	1.119395089
BC005624	-1.441676547
Phf23	1.169498988
Xylt2	-1.6508432
Ywhab	0.190549237
Por	0.482384835
Slc39a8	-0.800085093
Pag1	1.049899929
Ddost	-0.205690149
Zfp653	0.803023709
4933424M12Rik	1.279311184
Dubr	-1.288273401
D17H6S53E	-0.819642453
Zfand2b	1.451421987
Crybb1	0.871951912
Eci1	-0.629380948
Unc50	-1.143546311
Uhmk1	-1.105140451
1500011K16Rik	-0.859194012
Mrps24	-1.282004148
Rida	-1.222972255
Tprgl	-0.788553598
Ctdsp2	-0.304931782
Svip	-1.203049717
Rbm42	-0.785371341
Mtfmt	0.695489229
Ogfod2	0.363242704
Tom1l1	0.590070467
Prkce	0.718145819
Nod1	0.567951105

For Review Only

Plekho2	0.705272094
Rfc1	0.448545732
Slc1a2	-1.378364086
Txnrd1	-0.960348657
Cenpb	-0.879256943
Fbl	0.533481926
Fancc	0.618562095
Zfp865	-0.807389716
Ccdc102a	-1.026339142
Apoa1bp	-0.425400141
Top3a	0.681467662
Baiap2	-0.684756927
Apbb3	0.802407789
Iws1	-0.593735974
Bmi1	-0.830033462
Zfp593	-1.154145845
Ccdc94	-0.902022157
Ftsj1	-0.626485994
Ifit3	-0.993714725
I830127L07Rik	-1.354353943
LOC108167423	-1.139387282
Serhl	1.148363948
1700029J07Rik	-1.086512696
Gm15704	-1.15268782
Strn4	0.90001793
Tsc22d3	-1.115631592
Gm34980	-1.307400312
Usp15	-0.431407891
Frs3	1.07908623
Best1	-0.820751606
LOC102633149	0.852968402
Rsrc2	0.478441417
Cables1	0.823740976
Sh3kbp1	-0.382671426
Gm12474	-1.21619635
Polr3f	-0.591487188
Hist1h4c	1.019490213
Simc1	-0.756042831
Btbd10	-0.758246743
Cars	1.199618749
Eri1	-0.617908691
Gca	-0.798945845
Igsf6	-0.623835374
Irs2	-0.664862732
Prr15	-1.051749973
Slc35a1	-0.57419607

For Review Only

Mtx3	-0.565119802
Elf4	0.367837178
Abhd17a	1.029594212
Klf4	-0.918621039
ND4	1.19060565
Nutf2	0.602735687
Cd244	-1.070627718
Cyc1	-0.89398227
Ercc4	0.551623034
Fam213a	-1.017764753
Fzd7	-1.015684076
Gm3134	0.686348001
Gm35768	-0.715270991
Got2	0.551958311
Hlcs	-0.71056921
Pnpla8	-0.389344021
Gm33023	-0.826721692
LOC105243453	-1.041578784
Nradd	-0.91439012
Phka1	-0.545593988
Gm11753	0.321782965
2900097C17Rik	-0.435200106
8430429K09Rik	-1.141218381
Adck2	0.378462557
Agk	-0.47492343
Dhx35	-1.119163442
Fam234b	-0.482403203
Klf16	0.970899466
Lmln	-0.699257278
Nhej1	1.062049034
Ntpcr	0.432511843
Pnp	-0.272075585
Psmd12	0.620474417
Rxrg	0.895004886
Slc25a46	-0.636862707
Wdr92	0.657623423
Wwp2	-0.997719545
Cd164	0.248650138
Rala	-0.410738555
Usp37	-0.340062931
Immp1l	-0.672257654
1500011B03Rik	-0.788610669
1700001C19Rik	0.908499403
1700028E10Rik	-1.039805479
1700123O20Rik	0.651820549
2010315B03Rik	-0.664274034

For Review Only

2310033P09Rik	0.585496405
2410131K14Rik	0.476785378
A130051J06Rik	-0.836543234
Abcg1	0.169703684
Actr2	0.509708898
Afg3l2	-0.446055966
Agl	0.537006002
Aip	0.59194795
Akt3	0.528819825
Anapc5	0.343575577
Ankrd37	-0.58387556
Anxa2	-0.887777441
Ap1m1	0.384812273
Apmap	0.545200419
Apoo-ps	-0.813445444
Arcn1	-0.323134914
Arfgap3	0.660337529
Arrdc3	-0.793498105
Atf4	0.162414304
Atxn10	0.29880487
AU041133	-1.032007285
Axl	-0.70506409
Azin1	-0.354987323
B3gnt2	1.182826452
Bag3	0.549214384
Bag4	-0.770605801
Bbs2	-0.504836269
Bend5	-0.799512023
Bop1	0.871875861
Brip1os	0.291888208
Brms1l	-0.727651225
C530005A16Rik	-0.904240847
Cab39l	-0.937485716
Calcrl	-0.68027064
Camk2a	-0.708879555
Car2	-0.362208538
Carm1	-0.212132433
Ccdc134	0.370751171
Ccdc181	-0.630844928
Ccdc47	0.314938022
Ccl6	0.600712722
Cdan1	-1.045130989
Cep104	-0.45119601
Cep131	-0.774426434
Cfap97	0.729401515
Cggbp1	0.339775312

For Review Only

Clcf1-pold4	0.17121644
Clic1	0.261306842
Cln6	-1.018856856
Cmas	0.671021161
Cmc2	-0.854744095
Cnnm4	0.493945093
Cnot8	0.279362482
Cox19	-0.369024495
Cpsf3l	0.226660318
Cstf2	0.707222756
Cul9	0.454836487
Cwc22	0.631671713
Cyp2d22	-0.46935276
Dapk1	0.705117625
Dcaf10	-0.861077883
Dcakd	0.388246419
Ddx6	-0.544898833
Deptor	-0.714116088
Dhdds	0.550369499
Dhdh	-0.685967923
Dimt1	-0.928400423
Dusp18	-0.952463745
Eef1akmt1	-0.716895251
Eml3	0.373635025
Eng	0.210154679
Epha1	0.416698151
Erc1	-0.783883742
Ezr	-0.949302864
F13a1	-0.85914964
F5	-0.592459348
F9	0.65290256
Fam3a	0.492367826
Fam78a	-0.522062729
Farsb	-0.393629822
Fbxl15	0.566612791
Fbxw8	0.604854292
Fcgr1	0.302884878
Fgd6	-0.839760314
Fgfr1op2	0.20934687
Fkbp2	-0.75011823
Frg1	0.916912501
Garnl3	0.371904298
Gcn1l1	0.338248472
Gins4	0.549296349
Gk	-0.953076084
Gm10509	0.629703567

For Review Only

Gm16023	0.912581317
Gm2036	0.993446589
Gm21975	0.410707174
Gm26782	0.606207997
Gm31677	-0.897722957
Gm31872	0.879593472
Gm33729	-0.75511126
Gm34076	-0.843120251
Gm34865	-0.605036455
Gm35582	-0.923100729
Gm36591	-1.13129604
Gm42002	-0.620238926
Gm5113	-1.015583051
Gnb5	-0.796581299
Grk4	-0.87926208
Grwd1	-0.854299326
Gstm4	-0.892103074
H2-DMb2	-0.693713744
H2-T24	0.739687574
Hic2	-0.7457067
Hist1h2br	0.803474064
Hjurp	0.337004613
Hmgn3	-0.620425233
Hspa8	-0.898730244
Hsph1	-0.899969892
Ibtk	-0.892486346
Igfbp2	-0.374162577
Il7r	0.518743952
Invs	0.64220488
Itpkc	-0.718117751
Jag2	0.513170129
Jarid2	-0.414157458
Kat2b	-0.420439478
Kbtbd2	-0.389500655
Kif5a	-0.915080332
Kl	-0.59217927
Krt10	-0.508128756
Lamtor2	0.44606228
Laptm5	0.234687655
Lclat1	-0.628694014
Lekr1	-0.869573991
Lilra5	-0.554309781
LOC105245415	-1.102064414
LOC108167377	-0.722614798
LOC108168810	0.582625133
LOC108168907	-0.795862802

For Review Only

Lrig1	1.042633032
Lrrc20	0.683647593
Ly6e	-0.183953021
Ly9	-0.690622575
Lyz2	-0.464995893
Maoa	-0.36154942
March1	-0.942748501
Mdc1	-0.325142751
Med15	0.397856789
Mest	-0.855039783
Mgat1	0.431026263
Mrpl24	0.630418779
Mrpl4	0.623921891
Mrpl48	0.410160203
Mrps2	0.328132992
Mrps7	0.610560729
Mta3	0.50286705
Mtif3	0.308495479
Mtus1	-0.89426981
Muc16	-1.045124399
N6amt1	0.694236947
Nap1l4	0.400240753
Ncor2	0.535018721
Nfya	0.695190505
Nipbl	-0.300075647
Nktr	0.60880384
Nop10	0.393733123
Ntrk2	-0.959452132
Nudt13	-0.531290787
Nudt3	0.283807762
Nup43	0.822602102
Oard1	0.663110664
Ogg1	0.739705937
P2rx7	0.378570775
Palb2	-0.785855938
Parp8	-0.341509037
Pcbp1	0.609509976
Pcm1	0.421537546
Pcyt2	0.732888503
Pdcd10	0.508634175
Pdcd7	0.76560055
Pde12	-1.007832929
Pex1	-0.97955958
Pisd-ps2	-1.019247466
Pla2g12a	-0.925371102
Plcxd1	0.911348414

For Review Only

Plec	-1.020366564
Plin3	-0.596152756
Pmepa1	0.240678559
Pmpca	-0.413791977
Polr3c	0.57788687
Ppp1r13b	0.449835306
Ppp2r2a	0.295297372
Prr13	-0.619397522
Prr18	0.896402164
Prune	0.520227957
Psd3	-0.972241502
Psmd8	0.319625858
Pus3	-0.525196934
Qprt	-0.799423913
Qrfp	-1.069387967
Qsox1	0.547369957
Rac1	-0.418635124
Rad52	-0.488885865
Rbm3os	-0.75034566
Relt	0.833659786
Rhoq	0.326607493
Rhot1	-0.550120669
Rpl14	-0.159414656
Rps20	0.305182224
Rpusd1	0.570399889
Rwdd3	0.896968588
Rybp-ps	0.975123025
Sall3	0.203711698
Sars2	0.811852945
Sdf4	0.312559368
Selk	0.390314461
Sept10	-0.397984381
Shmt2	0.630551955
Skiv2l2	-0.409181957
Slc5a10	0.680998415
Slc9a6	-0.680690963
Smad5	-0.342300488
Smim1	0.851902566
Snx17	0.592478797
Sparcl1	-0.44607324
Specc1l	-0.30647943
Spint2	-0.526994033
Spty2d1	-0.407572898
Srsf2	0.437702415
Stk11ip	-0.320116673
Strada	0.897008858

For Review Only

Syne1	-0.461853267
Tfdp1	0.621579783
Tfeb	0.652032982
Thg1l	-0.917535032
Tmem134	0.850307781
Tmem181a	-0.351292884
Tmem243	0.759399797
Tmem65	-0.628454426
Tmem68	-0.791384058
Tmod3	-0.336621497
Tnfaip8	-0.312477227
Tnfsf9	-0.796899578
Tns3	-0.451680034
Trappc3	0.332573868
Trim32	-0.442242166
Tsc22d4	0.828154121
Tspyl1	-0.501983216
Ttbk2	-0.682616068
Ttc21b	-1.035045492
Ttc41	-0.979035849
Ttr	-0.990291913
Ubald1	0.460842439
Wipi2	-0.439004266
Zbtb43	-0.469673123
Zc2hc1a	0.693498327
Zcchc14	0.465898163
Zfp362	0.690554418
Zfp369	-0.621330703
Zmynd15	-0.954236716
1700034J05Rik	0.714106132
2900040C04Rik	-0.451770045
2900089D17Rik	0.695330987
9430037G07Rik	-1.019493685
Abat	-0.756785764
Aoc2	-0.869813414
Arhgef5	-0.777146918
C77080	-1.003966954
Ccnd2	-0.801741102
Cnga2	1.01501981
Eid3	-1.059507661
Exoc3l2	0.877679258
Fgfr1	0.52198989
Fut7	-0.785564143
Gm11802	-0.771937716
Gm12059	-0.819932773
Gm13707	-0.794793428

For Review Only

Gm32289	-0.749298411
Gm36901	-1.000729559
Gm38422	0.793902592
Gm40887	-1.333881944
Gm40964	0.699902548
Gm42357	1.122506824
Gm4841	0.889706078
Hdc	-0.733068269
Hist1h1a	1.039205785
Hist1h4m	1.614461166
Hist1h4n	1.24241787
Kifc1	-0.753221174
LOC108167630	1.111963286
LOC108167827	-0.760912938
LOC108168286	-0.923555088
Lpar2	-1.043115278
Lrrc14b	-0.940900696
Mfsd6l	1.14132137
Neto1	-0.747161404
Nsun7	-0.728053389
Omd	1.037496024
Plp2	-0.701592316
Sla2	1.239764831
Slc22a23	1.056670298
Slc25a47	1.167417436
Tmem150c	0.990176239
Vaultrc5	-0.905490741
Zfp507	1.009355564

For Review Only

For Review Only

For Review Only

For Review Only

For Review Only

For Review Only

For Review Only

For Review Only

For Review Only

For Review Only

For Review Only

Microglia
rCHI HSD vs Sham ND
P < 0.05

Gene	log2FoldChange
Arrdc1	0.813799559
Hnrnph1	0.309190279
Mafg	0.187636011
Chkb	0.119665768
Ubb	-0.061568932
Dusp6	-0.040849622
Eef2	-0.299244407
Matk	-0.108770257
Sgk1	-0.014991034
Pisd-ps3	-0.02822925
Flot1	0.793674266
Pfdn5	0.298719666
Mrfap1	0.203843698
Cox6c	-0.077108525
Clptm1	-2.040244088
Oaz2	0.125605278
Tnfrsf14	0.19839674
Tagap	-0.084100413
Rinl	0.162482034
Cant1	-2.505209047
Slc31a2	0.835179016
Gpsm3	1.03411007
Dnase2a	-0.006254869
Otub1	0.701877366
Pnpla6	2.039437507
Ifi30	0.686962986
Rcc2	0.66809128
Pde8b	-0.598968331
Ptprj	-0.614595366
Ythdf3	-0.873596695
Cops8	1.495194819
Rab1b	0.007162119
Calr	0.290010744
Brip1os	0.533748904
Slc25a3	1.09422432
Psmb4	0.411975247
Pstpip2	-1.328761729
Bmf	-1.710562894
Mipep	-1.010477992
Inpp5d	0.591791419
Atf4	0.638651642

For Review Only

Helb	-1.497391534
Rabgap1	-0.823344964
Por	0.670366844
Psmg3	1.12867839
Zfp36l1	-0.161765366
Crybb1	0.989669427
Epha1	1.083862883
Gm32853	-1.485885998
Srsf10	-0.48467608
Rad17	0.816196493
Dhdds	1.130206557
Ube3a	-0.876731486
Ywhab	0.220746227
Gmfg	-1.474773965
Maml3	-0.622336738
LOC102633149	1.046776991
Trim47	1.058722913
Faim	0.600762532
Rbm42	-0.798908079
Muc16	-1.537426294
Ankzf1	-0.756639083
Gm35247	-0.76964335
Mcph1	1.125181276
Tefm	0.932661498
Zfp1	-0.746202432
Kcnh6	0.777138553
LOC108167679	-1.251011068
Spns1	-0.612530797
Cpsf3l	0.589033168
Mib2	-1.03865492
Plbd2	-0.467788968
Selm	1.039048987
Slc2a9	-1.426890023
Ip6k1	-0.237084193
Tsc22d4	1.300263554
Tecpr2	-0.772177226
Fam178a	-0.306803904
Fndc3b	-0.598801973
Rab40c	0.536716523
Lypla2	-0.255008435
Slco2b1	0.685167098
Xylt2	-1.597035844
Eci1	-0.629617539
Ntpcr	0.525064846
Phkg2	-0.508902524
Sri	0.462181081

For Review Only

Nup43	1.177398171
BC005624	-1.415826879
Drap1	1.57391336
LOC102643247	1.231632996
Ezr	-1.225099466
Ercc3	1.013200721
Clk2	-0.786699386
Serhl	1.277515087
Gm41077	0.764058205
Oaz1	0.239163381
Zzef1	-0.665597845
Ywhag	0.225364113
Nisch	0.268181662
Shmt2	0.753577084
Ncbp1	0.400401161
Lamtor2	0.784976529
Tmem106a	0.583420387
Plppr4	-1.268670936
Rpa1	0.584844521
Setd1a	0.7857363
Manf	0.615339807
Pnpla7	-0.617185787
Mrpl49	-1.203890148
Mbd3	-0.465004737
Esyt2	-0.757970685
Fbxo40	-0.733727035
LOC108167645	1.369511034
2900089D17Rik	0.974854568
Abcb7	-0.919500669
Gm29933	-1.074292113
Ccl6	0.697937683
Plpp7	0.613084552
Snrnp48	0.517090976
Strn4	0.940280098
LOC108168380	-1.233489157
Isyna1	0.450992218
Klf11	1.196960094
Slc7a11	1.185081187
Whrn	-0.980877741
Ammecr1	-0.970901003
Gid4	-0.611352936
Mmp9	-0.710732717
Ube2m	-0.676108596
Eps8l1	1.103156881
Gramd1a	-0.924681861
Mvb12a	-0.528011173

For Review Only

LOC102641351	0.982057019
Nhej1	1.171551817
Sf3b6	0.634827044
Eng	0.283126507
Faap20	-0.985472463
Fam3a	0.850384267
Fen1	0.717674736
Imp4	0.443928304
Nrep	0.458073035
Pdap1	0.72772566
Ppp1r2	0.453123981
Tcerg1	-0.711358117
Cnot8	0.363787062
Gm26760	1.264818833
Klf16	1.048744174
Lmln	-0.751282567
Msl2	-0.71549669
Nop10	0.683665775
Rdh5	-1.21055803
Rps2	0.32329039
Gbp4	0.901937725
4933424M12Rik	0.972428844
Fcgrt	0.477331808
Gtpbp4	0.687410593
Mast3	-0.943025293
Tmem109	0.27054303
Tpra1	0.351525712
Ercc5	-0.571531969
Gm36527	-0.888526712
Inpp4b	-0.440760923
Lurap1	1.336743242
Ogfod2	0.255216652
Pigm	-0.529284284
Ptprf	-0.825725143
Snx2	0.467162471
Tecr	0.348233731
Wdr76	-1.185438187
Ache	0.799229349
Gm8273	-1.179011846
LOC108167736	-0.590065812
Mmp14	0.584011508
Slc25a20	0.767440943
Avl9	-0.568753965
Kctd13	-0.631523312
LOC102642832	0.860936394
Gm38422	0.899659112

For Review Only

Dot1l	-0.549028888
Gm31684	-0.936502968
Mab21l1	-1.080141679
Ppl	-1.16461234
Zzz3	-0.643964329
Hccs	-0.759566893
Stx7	0.284161219
Ccdc134	0.472549057
Itga4	-0.789607007
Pef1	0.425188628
2810025M15Rik	0.523150884
Aknaos	-0.821296223
Ccdc112	0.695954999
Frg1	1.086400037
Gm36901	-1.172972797
Hexb	0.369693183
Il1rn	-0.896939523
Leprot	0.252976404
LOC102638268	-1.15343734
Loxl2	-1.048812113
Lpin1	-0.456691275
Ltb4r1	-1.12810107
Mtfmt	0.612463789
Ndufs5	0.655145282
Nfatc2ip	-1.047515845
Pisd-ps2	-1.137907438
Srsf7	0.58241499
Sun2	-0.781031616
Stk19	0.866754784
Zc2hc1a	0.875689473
Exosc5	-0.336795139
Ndfip1	0.313543671
Prkrip1	0.65793675
Rabif	0.154998824
1700096K18Rik	0.865951445
2410131K14Rik	0.504683801
2900097C17Rik	-0.43131045
6330549D23Rik	-0.852595052
Acvrl1	0.651274752
Afg3l2	-0.551318365
Anapc2	0.357316778
Car12	-0.656047755
Ccser2	-0.556161715
Cxcl16	0.668139637
Eml3	0.498348293
Gm2036	1.131507032

For Review Only

Gm30396	1.148756131
Gtf3c5	0.575900885
Hdhd2	0.347926622
Hnrnpll	-0.9402612
Iws1	-0.530886253
LOC102632465	-0.987572627
Npnt	-0.53352345
Phgdh	0.413574165
Pias4	-0.215026373
Polr2g	1.060045133
Proser1	-0.585926176
Prr18	1.081412516
Rnf34	0.4434983
Rrs1	-0.775717007
Rsrc2	0.44847311
Tprgl	-0.761362522
Zfp612	-0.975266531
Cept1	0.610421302
Cdt1	0.797176789
Syk	-0.397760283
Ssb	0.49732258
Zc3h4	-0.50514387
Fam160b2	-0.647032612
Pnrc1	-0.095637785
Gimap1	0.819203676
Cwc22	0.677216861
Gm3134	0.655728544
Mgat1	0.492345327
Atp2b2	-1.061214789
Cacnb1	-0.486418573
Cactin	-0.802869338
Capns1	0.814575297
Ccdc168	-1.122993538
Cd164	0.242265389
Ctdsp2	-0.301311721
Gm33517	-1.131743321
Gpr180	-0.607966489
Gpt2	-1.036819237
Ifit3	-0.943580009
Limd2	0.321097945
LOC108167542	-1.009061409
Mboat7	-0.390490248
N6amt1	0.749188446
Pecr	0.411922227
Phactr1	0.743175689
Prim1	0.6414951

For Review Only

Rhot1	-0.543572511
Rtn1	-0.499044357
S100a13	0.786303782
Sh3gl1	-0.932555781
Slfn4	-0.643372647
Smu1	0.32775022
St13	0.37169646
Wdfy4	-0.393364663
Ywhaz	0.346627047
Zfp518a	-0.579413502
Gm32605	-0.602992211
Il10ra	-0.652031531
Txnl4b	0.503181827
Apoo	-0.706790996
Ccdc47	0.368717363
Cep112	0.639686328
Gins4	0.699827551
Med27	0.417338907
Naaa	0.52393838
Ndufs3	0.871129668
Sf3b5	0.458791243
Spp1	0.982756635
Xlr3b	1.11852924
4930473H19Rik	-1.050115527
Gm32824	-0.724139291
Hint2	0.535409259
Tfeb	0.719729119
Cacul1	-0.411257801
Eepd1	0.632970797
Emc10	-0.597979223
Mtus1	-0.886624422
Myl12a	0.36272711
Rnf187	-0.847216511
Tgif2	-0.722057778
Xkrx	0.997537891
C2cd2	-0.629186383
Crebbp	-0.396745559
Gm38720	-1.094144637
Gm39673	-0.392680271
Metap2	0.392308246
Trappc5	0.65484758
Rrnad1	-0.736068219
Per1	-0.299700617
Igip	0.839656403
Laptm5	0.25685285
Slc39a6	-0.629009284

For Review Only

Trim27	-0.548949565
Exoc3l2	0.675290583
LOC100503338	0.774155423
Ube2g2	0.241365596
Fnip2	-0.591696901
Gm8203	0.329371493
Nadk2	-0.712019785
Zfp653	0.397260859
Neb	-0.594855356
Lgmn	-0.27549679
B230219D22Rik	-0.597102732
Fastkd2	0.714077862
Fbxw17	0.841009863
Stk16	-0.132642883
Tmx1	0.341066694
Tns2	0.824772966
Gm35732	-0.994168066
Oser1	0.472695823
Ahcyl2	-0.728032104
C330021F23Rik	0.329957769
Dip2b	-0.348402161
E430025E21Rik	-0.578212617
Epb41l3	-0.703240544
Etaa1	-0.983887042
Exoc1	0.447307424
F5	-0.59227081
Fam117b	-0.429454551
Fbl	0.28440865
Gatc	-0.206060968
Gcnt7	-0.810302405
Gm3248	0.921520102
Gm33370	-0.617329385
Gm34076	-0.923479632
Gm34945	-1.043579904
Gm35999	-0.927154379
H2-Q1	0.455295288
Hibadh	-0.712965234
Inadl	-0.964123323
Klhl13	0.994430557
Maff	-1.095242731
Manba	-0.490264285
Mapk14	-0.368061903
Mgea5	-0.276953732
Mta3	0.502695347
Nap1l4	0.449321925
Nat9	-0.389534239

For Review Only

Ncdn	-0.590831101
Ndufb10	-0.784289741
Ngp	-0.691287328
Nos1ap	-0.331315957
Phf3	0.492544513
Ralgapa2	-0.729757153
Rbm15	-0.335161716
Rrp1b	-0.325411082
Smyd2	0.962352566
Snx17	0.61576846
Sqle	-0.991884513
Tagln2	0.42273683
Vrk3	0.393314344
Wdr59	-0.92407087
Zfp442	-0.7816059
Acot2	0.630424484
Bach2	-0.337355651
Fis1	0.425429663
Gin1	0.9461024
Gm16287	-0.844327583
Gm40571	-0.725807059
Hp1bp3	0.313355517
Sdf4	0.327883805
Clcc1	0.353651853
Copb2	0.410024208
Kdm6b	-0.732978307
Mcm2	0.854101449
1500015A07Rik	-0.858991392
Hdac7	1.095922016
Mcm3	0.381048035
Usp33	-0.433006815
Brwd1	-0.4265272
Eif3e	0.431312611
Rps20	0.319698498
Slc5a10	0.721435375
Gm32029	-1.026439256
Gm9159	-0.947960095
Tstd2	-0.620109318
Hist1h2bc	0.549550441
3110062M04Rik	-0.559352044
Akt3	0.537453515
Hmg20b	-0.882862653
Rpl14	-0.159411899
Zfp704	-0.571812097
Cacna1d	-0.354907155
0610011F06Rik	-0.25117436

For Review Only

1700095J12Rik	-1.07560915
Abcb9	-0.821082901
Abhd17a	0.789210572
Acad9	0.433420496
Actr1a	0.24301437
Actr2	0.537406659
Adat3	-0.844050496
Adck1	0.354382576
Adgre5	0.565584978
Adra1a	-0.712072415
Alg3	0.657765201
Anks1	0.371987134
Ap1m1	0.438203196
Ap1s1	0.35799189
Ap2a2	-0.502553186
Arf5	0.888686296
Arl5b	-0.396773386
Arpp19	0.370263039
Atxn7l3b	0.349138556
B4galt7	0.374075313
Babam1	0.410990902
Bcl2a1a	-0.781270697
Borcs8	0.783269545
Bri3bp	-0.520578419
Bsg	0.417301809
Btd	0.404228444
Cacybp	0.350786535
Calcrl	-0.612549051
Cand1	-0.480361148
Cbl	-0.438058875
Ccnd3	-0.584965254
Cd1d2	0.888193843
Cdk2	0.590980363
Cep131	-0.782927938
Clcf1-pold4	0.135675053
Cluh	-0.450280737
Cmtm8	0.616608157
Cnppd1	-0.850933745
Cox16	0.581004179
Cryzl1	0.364964867
Ctage5	-0.483121122
Ctps2	0.200582934
D030055H07Rik	0.984246683
D3Ertd254e	0.711173301
D3Ertd751e	-0.638545431
Dapp1	0.482980325

For Review Only

Dixdc1	0.618948028
Dnaja1	0.276523859
Dnajc4	0.947784389
Dnm2	0.4496854
Eif2a	0.27883561
Emilin1	1.023095158
Ets2	0.489650817
Eva1b	-0.501139275
Exosc3	0.549751385
Fam129c	0.926325387
Fam58b	0.341796082
Fam76a	-0.49759142
Foxn3	-0.252387355
Frem2	0.652100141
Gm11423	0.747841475
Gm14025	-0.881359373
Gm15612	-1.001390519
Gm15651	-0.893130955
Gm16599	-0.877316291
Gm2260	-0.726821968
Gm2274	-0.71366424
Gm29808	0.95500947
Gm30992	-0.913948252
Gm31388	-0.809469946
Gm35358	0.90248474
Gm35582	-0.906805433
Gm36591	-0.997740036
Gm38785	0.437644784
Gm40964	0.635911638
Gm41338	-0.749374836
Gm41610	-0.881540081
Got2	0.473804591
Gpcpd1	0.512217247
Gpr19	0.867599301
Gpsm1	-0.562898021
Gzf1	-0.762256876
H2-K1	-0.798773521
H2-Ob	0.955654939
H2-T-ps	0.724301412
Hbp1	-0.436848159
Hdc	-0.731858852
Hexim2	0.923131801
Hist1h4m	0.817850759
Hsf1	-0.730363331
Hspb6	-0.919001847
I830127L07Rik	-1.037231468

For Review Only

Igtp	0.306652941
Jarid2	-0.397367403
Kansl1l	-0.385054315
Katna1	0.303980532
Kbtbd7	-0.593376946
Kctd11	0.462223855
Kctd21	-0.455607749
Kdm7a	-0.40118301
Kif2a	0.539492672
Kif5a	-0.877196081
Kl	-0.571497013
LOC102636309	0.999776503
LOC108167714	0.666456324
LOC108168392	0.934584757
LOC108168979	-0.725223097
Ly9	-0.668596275
Map2k1	0.21214444
Map4k5	-0.724315913
Mcrs1	0.911530695
Mdp1	-0.107398246
Med21	0.399123383
Mettl10	0.544044687
Mir99ahg	-0.723235672
Mrpl24	0.483595688
Mrps24	-1.111762809
Mtif3	0.280271994
Myl12b	0.200732235
Myo10	0.783130077
Nagk	0.567721053
Nat10	-0.861107801
Ncam1	-0.733130763
Ncoa2	-0.363116975
Ndufa9	0.471819869
Ndufb4	-0.182382657
Nfs1	0.381250428
Nol6	-0.523654339
Nol9	-0.389315473
Nova2	0.576492372
Ogg1	0.639642759
P3h1	-0.883484064
Paqr4	-0.565877538
Parp8	-0.332507245
Particl	-0.709057061
Pcyt2	0.700147908
Pepd	0.338113342
Phf11b	0.484723819

For Review Only

Plppr3	-0.875881664
Polr3b	-0.554016834
Pon2	0.294376411
Ppat	-0.902323724
Prkch	-0.578047859
Psmd11	0.27740895
Psmd4	0.329135107
Ptpro	0.249239915
Pygo2	-0.748371724
Rabep1	-0.460423703
Rnf145	-0.489109034
Rnf220	-0.742845272
Rpgrip1l	-0.970510534
Rpl21	0.844372546
Rufy3	0.541911971
Sar1b	0.34901233
Sec24d	0.486145275
Senp1	-0.719102535
Serf1	0.530198201
Siglece	0.955725437
Simc1	-0.59068657
Slc12a9	-0.489903045
Slc22a23	0.597292137
Slc25a53	0.864984181
Slc38a9	-0.261260445
Slc9a6	-0.661773549
Smim15	0.742390738
Smpd1	0.293602378
Snord71	-0.945880164
Snx7	-0.467362782
Specc1l	-0.304892428
Srsf4	-0.66370881
Ss18l1	-0.664115888
Ssbp4	-0.6279898
Syt11	0.483837482
Thbd	0.931811395
Thoc5	0.449734281
Tm2d2	0.271687514
Tmem150a	-0.553700952
Tmem19	0.380034406
Tmem41b	0.340320552
Tmem70	0.339702211
Tmtc3	-0.395744429
Traf3ip3	0.591892454
Trim15	-0.925939878
Trip6	-0.553748664

For Review Only

Trmt44	0.538265936
Trpm7	-0.478639947
Ubl5	0.579256545
Ufd1l	-0.093298837
Ugt1a7c	0.379189479
Usp27x	-0.982626891
Usp53	0.810458131
Uxt	0.518180404
Vat1l	-0.470545782
Vcan	-0.916798676
Vma21	0.427598967
Vps26b	-0.34638222
Wdr35	-0.596382741
Zbtb11	-0.659564393
Zbtb44	-0.859505777
Zbtb7a	-0.482696508
Zc4h2	0.67594458
Zdhhc16	0.964061001
Zfp112	-1.105969797
Zfp623	0.3289854
Zfp97	0.455630502
Zfyve1	-0.395362862
B3gnt9	0.856411029
Rtkn	0.737446177

For Review Only

rCHI HSD Cluster
Unique DEGs in rCHI HSD vs. Sham ND

Gene	Sham_ND_1	Sham_ND_2	Sham_ND_3	Sham_ND_4	Sham_ND_5	Sham_HSD_1
Frem2	7.94202038	7.94202038	7.94202038	7.94202038	7.94202038	8.07095728
Phf3	8.5614247	8.63067157	8.55717491	8.79459615	8.80953564	8.64159774
Smyd2	7.94202038	7.94202038	8.03241639	8.03219196	8.17361191	7.94202038
Syt11	8.63323637	8.51159704	8.49594131	8.62826626	8.53850584	8.52902268
Eif3e	8.78715389	8.92094326	8.9993536	8.8468408	8.73970512	9.05004817
Mmp14	8.46309648	8.55146949	8.55069191	8.58697859	8.41182964	8.55594324
Ndufa9	8.81151437	9.03656871	9.12804724	8.82115352	9.02806142	8.95304338
Med27	8.55445056	8.7011156	8.62419555	8.56206599	8.53850584	8.60633467
Cryzl1	8.55445056	8.71317579	8.66339707	8.48734968	8.63097594	8.65853119
Myl12b	9.38648683	9.46743069	9.46026215	9.28702501	9.35157056	9.42851044
C330021F23Ril	8.69217972	8.68884272	8.72570119	8.65619597	8.64162006	8.68583004
Fis1	8.93983402	8.93456737	9.05096383	8.8298088	8.83416218	8.74234432
Fcgrt	9.03317306	9.11738619	9.363863	9.03826605	9.03448307	9.20693477
Sar1b	8.98772156	9.17122963	9.09668128	8.93844461	9.02159457	9.03284796
Serf1	7.94202038	7.94202038	7.94202038	7.94202038	7.94202038	7.94202038
Psmg3	8.29323331	8.26741157	8.4348214	8.42542373	8.38706738	8.50794447
Hint2	7.94202038	7.94202038	7.94202038	7.94202038	7.94202038	7.94202038
Gbp4	7.94202038	7.94202038	7.94202038	7.94202038	7.94202038	7.94202038
Hdac7	7.94202038	7.94202038	7.94202038	8.12227563	7.94202038	7.94202038
Tns2	7.94202038	8.08100817	7.94202038	7.94202038	7.94202038	8.12430412
Myo10	8.03610127	8.04031858	7.94202038	7.94202038	7.94202038	8.0332077
Spp1	7.94202038	7.94202038	8.06983875	8.06952151	8.0296343	7.94202038
Gm11423	7.94202038	7.94202038	8.03241639	8.03219196	8.0296343	7.94202038
Thbd	7.94202038	7.94202038	7.94202038	8.03219196	7.94202038	8.0332077
Cxcl16	7.94202038	7.94202038	8.03241639	8.14351917	8.0296343	7.94202038
Dixdc1	8.03610127	7.94202038	7.94202038	7.94202038	7.94202038	7.94202038
Alg3	8.56831653	8.38002423	8.85742312	8.5087573	8.58071885	8.49342128
Clcc1	8.99930001	9.03254108	9.07082851	8.9193639	8.97498725	8.97552435
Dapp1	9.40201878	9.3205015	9.50444201	9.33327698	9.46249856	9.36043912
Ap1s1	8.65147592	8.63067157	8.72570119	8.60501026	8.56300025	8.69644988
Faim	8.47945675	8.50326399	8.5946422	8.51570698	8.45768165	8.60026035
H2-Q1	8.41964152	8.39075541	8.47388091	8.391136	8.39547674	8.5007324
Btd	8.60163044	8.6238769	8.70554857	8.61674566	8.66240089	8.79015683
Ywhaz	9.84528936	9.71233036	10.0562599	9.72999522	9.85707014	9.98731212
Kif2a	8.44618198	8.3577056	8.39224519	8.40860583	8.33270708	8.46310186
Fam58b	8.88476766	8.78700057	8.9958067	8.80798859	8.78839991	8.8351436
Myl12a	9.28260087	9.15360652	9.39534633	9.14053602	9.19326564	9.36793743
Slc7a11	8.15224289	8.13850293	8.32447998	8.06952151	8.2045402	8.25732722
Bsg	10.4695566	10.2299717	10.5163001	9.86808404	10.0325685	10.4033011
Ndufs5	8.48744472	8.39075541	8.45082017	8.34404359	8.37849082	8.40507356
Tagln2	8.71984215	8.54371805	8.72570119	8.63958273	8.59792904	8.67504275

For Review Only

Sri	8.89347732	8.71912925	8.84053261	8.7809652	8.73049415	8.94158251
Usp53	8.0750473	7.94202038	7.94202038	7.94202038	7.94202038	7.94202038
Hist1h2bc	8.47945675	8.46854779	8.62419555	8.40860583	8.52585076	8.65853119
Sf3b6	8.54024376	8.6238769	8.66881133	8.47257473	8.59224627	8.73239774
Phf11b	8.3508132	8.51980383	8.40108762	8.39995701	8.41182964	8.40507356
Plpp7	8.41964152	8.52788968	8.4348214	8.37293489	8.50630687	8.40507356
Nrep	8.63937333	8.67001091	8.56992826	8.55566982	8.56896558	8.51506123
Fbxw17	8.29323331	8.33408171	8.24134193	8.22675204	8.32287245	8.22994993
Zfp623	8.59511249	8.68884272	8.70554857	8.62826626	8.63631995	8.74726697
Ppp1r2	8.85353687	8.83924741	8.84053261	8.80798859	8.82603878	8.87771606
Phactr1	8.280517	8.26741157	8.25460081	8.32353564	8.36079479	8.38705301
Vma21	8.47945675	8.55146949	8.46631155	8.57465269	8.61466996	8.65293507
Exoc1	8.76708807	8.53585955	8.69525104	8.73851663	8.82194549	8.68583004
Eps8l1	8.42865536	8.11221209	8.48869139	8.44956944	8.53221409	8.46310186
Cd1d2	8.0750473	8.04031858	7.94202038	7.94202038	7.94202038	8.07095728
Fam129c	8.15224289	8.13850293	7.94202038	8.03219196	7.94202038	8.07095728
Mcph1	8.31735125	8.20179222	8.32447998	8.40860583	8.32287245	8.40507356
Rtkn	7.94202038	7.94202038	7.94202038	7.94202038	7.94202038	8.07095728
Gm35358	8.03610127	8.04031858	7.94202038	8.12227563	7.94202038	8.19963762
Ctps2	9.04783563	9.02035128	8.96691329	9.02808386	9.0118077	9.02237183
Uxt	7.94202038	7.94202038	7.94202038	7.94202038	7.94202038	7.94202038
Tmem109	9.58659328	9.43793231	9.56150835	9.49439361	9.52214948	9.66617292
Tmem19	8.83513458	8.84428604	8.95583036	8.80798859	8.98855207	9.07360781
Ugt1a7c	9.22354055	9.4779692	9.45121575	9.21359191	9.22796083	9.49043768
Traf3ip3	8.85807235	8.97849471	8.78757811	8.78553606	8.69240856	8.94158251
Map2k1	9.81849834	9.78531278	9.86426471	9.75469195	9.72421401	9.92497521
Dnajc4	8.03610127	8.08100817	7.94202038	7.94202038	7.94202038	7.94202038
LOC10264324	8.03610127	8.04031858	7.94202038	7.94202038	7.94202038	8.0332077
Oser1	8.68651257	8.75384776	8.73556759	8.79007957	8.716452	8.77139787
Kctd11	8.54739125	8.51980383	8.53078934	8.48001526	8.64162006	8.58166745
Anks1	8.75684959	8.80304714	8.86159106	8.8468408	9.03127785	8.91819366
Zfp97	8.43749919	8.50326399	8.40975743	8.5990584	8.58071885	8.62421082
Mcrs1	8.30549659	8.51980383	7.94202038	8.5087573	8.29167353	8.70169922
Actr1a	9.51907742	9.5064569	9.39773118	9.49656657	9.49784303	9.55973127
H2-Ob	8.69780065	8.16165221	7.94202038	9.04163532	8.64162006	8.87355778
Trmt44	8.40106353	8.41145947	8.3739998	8.51570698	8.49287238	8.65853119
B3gnt9	7.94202038	7.94202038	7.94202038	7.94202038	7.94202038	8.07095728
LOC10816839	8.03610127	8.04031858	7.94202038	7.94202038	7.94202038	8.07095728
Med21	8.60163044	8.67001091	8.66339707	8.68299269	8.6874983	8.64729097
Cdk2	8.4547119	8.43125887	8.61254153	8.59304839	8.52585076	8.47085215
Hexim2	8.0750473	8.04031858	8.12272383	8.12227563	8.15646529	8.14578597
Leprot	9.62107091	9.62084467	9.82011246	9.5721823	9.61828089	9.65459067
Gpr19	8.0750473	8.04031858	8.26731165	7.94202038	7.94202038	8.22994993
Xkrx	8.03610127	7.94202038	7.94202038	7.94202038	7.94202038	7.94202038
Xlr3b	8.17226696	8.04031858	8.03241639	8.12227563	8.21869831	8.07095728
Eif2a	8.75684959	8.76506306	8.75011935	8.68822696	8.83819286	8.70690929

For Review Only

Babam1	8.55445056	8.63739493	8.64688417	8.61674566	8.55088346	8.61830836
Emilin1	8.15224289	7.94202038	7.94202038	8.18035967	7.94202038	8.0332077
Gm38785	8.7306106	8.51980383	8.73065133	8.752939	8.70693361	8.66408047
Fen1	8.41964152	8.38002423	8.5762029	8.4416556	8.47908535	8.50794447
Fastkd2	8.38167383	8.30890035	8.3739998	8.52934701	8.64687728	8.58166745
Ercc3	8.47134264	8.34607084	8.40108762	8.56839292	8.47205105	8.60633467
H2-T-ps	8.33997962	8.32170464	8.56992826	8.34404359	8.51289973	8.5220862
Smu1	9.27684305	9.22563223	9.53438323	9.21640685	9.34219306	9.3528888
Ssb	8.87152875	8.61700876	9.00991144	8.92704725	8.71170889	8.81297517
Snx2	9.05146537	8.84929315	9.3058228	9.11907719	9.01508167	9.19232194
Stk19	8.37164039	7.94202038	8.36457263	8.3128462	8.21869831	8.32780937
LOC10263630	8.0750473	7.94202038	7.94202038	7.94202038	7.94202038	8.0332077
Pon2	9.3275706	9.2867021	9.37120912	9.2079386	9.33510616	9.44256919
Pef1	8.81151437	8.75947666	8.78299678	8.71882808	8.76666022	8.9606011
Gm26760	7.94202038	7.94202038	7.94202038	7.94202038	7.94202038	8.0332077
Lurap1	8.03610127	8.04031858	7.94202038	7.94202038	7.94202038	8.0332077
Cops8	7.94202038	7.94202038	7.94202038	7.94202038	7.94202038	7.94202038
Slc25a3	7.94202038	8.04031858	7.94202038	7.94202038	7.94202038	7.94202038
Pnpla6	8.03610127	8.04031858	7.94202038	7.94202038	7.94202038	8.0332077
Klhl13	7.94202038	8.21967477	7.94202038	8.16271545	8.21869831	8.21521951
Pfdn5	7.94202038	7.94202038	7.94202038	7.94202038	7.94202038	7.94202038
Anapc2	7.94202038	7.94202038	7.94202038	7.94202038	7.94202038	7.94202038
Slc25a53	8.03610127	8.04031858	7.94202038	7.94202038	7.94202038	8.07095728
Borcs8	8.10491559	8.29562241	7.94202038	7.94202038	7.94202038	8.4472291
Cept1	9.25643794	9.22563223	9.14639275	9.15558139	8.98855207	9.25787703
Rnf34	8.79208827	8.71912925	8.64128626	8.73364487	8.7754316	8.8438399
Adck1	8.76708807	8.6950064	8.61254153	8.73364487	8.71170889	8.74726697
D030055H07R	8.0750473	7.94202038	8.03241639	8.03219196	7.94202038	8.0332077
LOC10816764	8.0750473	7.94202038	7.94202038	7.94202038	7.94202038	8.07095728
Gimap1	8.03610127	8.3577056	7.94202038	7.94202038	7.94202038	8.0332077
Zdhhc16	8.17226696	7.94202038	8.19740715	8.03219196	8.06590615	7.94202038
Tm2d2	9.23261707	9.29911755	9.46923664	9.23036658	9.2538731	9.42141419
Igtp	8.99159767	9.11738619	9.14335887	9.05167035	9.15169109	9.18641714
Ncbp1	8.72524666	8.73669692	8.745301	8.77636651	8.73049415	8.83950361
Hexb	14.8175298	14.7531739	15.2019725	14.4116543	14.5461673	14.8211633
Isyna1	9.28546803	9.28981965	9.32636772	9.11285819	9.1374369	9.34018691
Mcm3	9.21131207	8.9870234	9.14031561	8.93086319	9.06902919	9.05345032
Tmx1	9.20204349	9.09120375	9.20234497	9.07142278	9.09937163	9.16547244
Klf11	8.13008232	8.08100817	8.0985397	8.09815136	8.13781088	8.30586539
Ets2	8.58852436	8.50326399	8.50309307	8.74816312	8.62558699	8.7172148
Nova2	7.94202038	7.94202038	7.94202038	7.94202038	7.94202038	8.09990895
Copb2	9.12463856	9.0038409	9.09028359	9.12833165	8.91120322	9.20983226
Mcm2	8.25350136	8.29562241	8.25460081	8.41709188	8.37849082	8.4472291
Dnm2	10.1516447	9.68846033	10.0631534	10.2052647	10.1597208	10.5401479
Rab40c	8.57512873	8.61700876	8.5946422	8.60501026	8.52585076	8.62421082
Drap1	7.94202038	7.94202038	8.03241639	8.06952151	7.94202038	7.94202038

For Review Only

Limd2	8.03610127	7.94202038	8.03241639	7.94202038	8.0296343	7.94202038
Tpra1	9.04419168	9.12839379	9.06425355	9.07142278	9.07517574	9.06021769
Thoc5	8.65147592	8.77060783	8.67418225	8.61674566	8.56300025	8.60633467
Tmem41b	8.63323637	8.71317579	8.6900443	8.65619597	8.74426703	8.68045778
1700096K18Ri	8.20779251	8.20179222	8.24134193	8.22675204	8.15646529	8.27014799
D3Ertd254e	8.60808027	8.48619581	8.47388091	8.54266087	8.42761354	8.55594324
Siglece	8.20779251	8.74817546	8.85323361	8.5087573	7.94202038	8.8351436
Gm30396	8.03610127	8.08100817	7.94202038	8.12227563	7.94202038	8.14578597
Stx7	9.60395794	9.7272795	9.74814912	9.67532251	9.4896249	9.69277192
Acvrl1	8.67503527	8.45948785	8.69525104	8.64516752	8.38706738	8.59412554
Mettl10	8.33997962	8.32170464	8.45082017	8.34404359	8.25733743	8.27014799
LOC10264283	8.19067125	8.18256866	8.29132606	8.24060121	8.11716757	8.21521951
Gpcpd1	8.93983402	9.00799671	9.08060589	9.01085827	8.99191131	9.00105196
Cep112	7.94202038	7.94202038	7.94202038	8.03219196	8.0296343	7.94202038
Sec24d	8.59511249	8.50326399	8.48133934	8.63394937	8.43530927	8.60026035
Acot2	8.4547119	8.39075541	8.45082017	8.47257473	8.31277085	8.52902268
Cdt1	7.94202038	7.94202038	8.03241639	8.03219196	7.94202038	7.94202038
Hdhd2	8.65147592	8.61006489	8.65793848	8.62826626	8.62015204	8.68045778
Gm3248	8.03610127	8.04031858	7.94202038	7.94202038	7.94202038	8.0332077
Pecr	8.71439619	8.74245884	8.792132	8.70369523	8.66240089	8.78551051
Ndufs3	7.94202038	7.94202038	8.03241639	7.94202038	8.0296343	7.94202038
Cox16	8.66335967	8.50326399	8.55717491	8.46502356	8.42761354	8.65293507
Ubl5	7.94202038	7.94202038	8.06983875	7.94202038	7.94202038	8.0332077
Rpa1	8.64545268	8.67634644	8.82762614	8.67240182	8.55088346	8.68045778
Katna1	8.72524666	8.67634644	8.7596622	8.74335586	8.65209254	8.69644988
B4galt7	8.68079812	8.47744782	8.67951078	8.51570698	8.56300025	8.63005896
Phgdh	9.19581707	8.88827551	9.25537021	9.03826605	9.1573356	9.27973892
Ptpro	9.39944526	9.20557184	9.42130131	9.33578884	9.34923379	9.456454
Ndfip1	9.72085635	9.62084467	9.76218429	9.57014702	9.62380619	9.77100356
Prkrip1	8.39147661	8.30890035	8.3739998	8.32353564	8.29167353	8.2943698
Rufy3	8.48744472	8.56666716	8.48869139	8.48001526	8.55088346	8.51506123
Acad9	8.68651257	8.61700876	8.5946422	8.65070489	8.58651003	8.5689455
Ache	8.46309648	8.52788968	8.31376437	8.68822696	8.56300025	8.38705301
Eepd1	8.64545268	8.58875419	8.45082017	8.61090552	8.6035597	8.49342128
Atxn7l3b	9.16409394	9.16421783	9.20234497	9.0779159	8.95773375	8.98289267
Sf3b5	8.66922285	8.6950064	8.71569667	8.5990584	8.67255208	8.54264242
Gm29808	8.10491559	8.04031858	7.94202038	7.94202038	7.94202038	8.0332077
Apoo	8.17226696	7.94202038	8.30272335	7.94202038	7.94202038	8.12430412
Cox6c	7.94202038	7.94202038	8.92940832	7.94202038	7.94202038	8.94158251
Matk	8.87152875	7.94202038	9.04761164	7.94202038	7.94202038	7.94202038
Ip6k1	8.03610127	8.04031858	10.1222914	7.94202038	7.94202038	8.0332077
Lgmn	13.2983196	13.2614914	13.5694246	13.2079342	12.9218439	13.4195281
Srsf4	8.78715389	9.10997781	9.19947654	8.87986155	8.69728409	9.08023231
Vcan	8.10491559	8.11221209	8.26731165	7.94202038	7.94202038	8.0332077
Ankzf1	7.94202038	8.29562241	8.44288721	8.14351917	8.09372556	7.94202038
Mgea5	9.08000805	9.26461678	9.23337608	9.15558139	9.0376772	9.07360781

For Review Only

Trim15	8.19067125	8.32170464	8.16326384	8.12227563	8.18956427	8.07095728
Inadl	8.0750473	8.30890035	8.30272335	8.19677454	8.25733743	8.0332077
Gm15651	8.17226696	8.28181527	8.18095171	8.24060121	7.94202038	8.16519739
Slc38a9	9.14127103	9.29602731	9.2742408	9.13749915	9.13167679	9.15023775
Maff	8.10491559	8.18256866	8.0985397	8.14351917	8.17361191	8.16519739
Rnf220	8.59511249	8.86413056	8.82327676	8.5990584	8.85804717	8.69116044
LOC10816838(	8.13008232	8.13850293	8.24134193	8.16271545	8.23215835	8.07095728
Pigm	8.79208827	8.97419951	8.75490623	8.84261679	8.86196003	8.7666341
Tmtc3	8.63937333	8.7011156	8.67418225	8.62253212	8.63631995	8.62421082
Gm35732	8.0750473	8.30890035	8.30272335	8.09815136	8.28062209	8.2943698
Rrp1b	8.77215497	8.79773552	8.82327676	8.72380123	8.76666022	8.78083557
Ercc5	9.13465193	8.93005002	8.93704021	8.70369523	8.87742635	8.94541962
Hbp1	9.40201878	9.41598086	9.66036066	9.38962786	9.4062035	9.47243985
Gm9159	8.03610127	8.18256866	8.06983875	8.09815136	8.06590615	8.09990895
Manba	8.86706792	9.03656871	9.00991144	9.07467491	9.04086033	9.02936917
Sqle	8.25350136	8.04031858	8.14402003	8.21218424	8.17361191	8.22994993
Loxl2	8.17226696	8.25232822	8.14402003	8.25382753	8.23215835	8.16519739
Exosc5	8.20779251	8.13850293	7.94202038	7.94202038	8.6775708	8.36824626
Spns1	8.03610127	8.04031858	7.94202038	7.94202038	9.80594279	8.83075953
Ufd1l	7.94202038	7.94202038	8.03241639	8.30183216	8.51289973	8.9865541
Gm35999	8.17226696	8.20179222	8.12272383	8.21218424	8.2045402	8.18303915
Gm16599	8.25350136	8.21967477	7.94202038	8.22675204	8.30237981	8.14578597
Zfp1	8.64545268	8.56666716	8.55717491	8.59304839	8.63631995	8.61235015
Esyt2	8.69217972	8.75947666	8.65243454	8.79459615	8.7926739	8.69644988
Cactin	8.39147661	8.45948785	8.41826405	8.43360924	8.47205105	8.46310186
Gm8273	8.13008232	8.23646076	8.0985397	8.12227563	8.17361191	8.18303915
Zfyve1	8.7619865	8.81356207	8.76908356	8.85938133	8.87358707	8.89006666
Dip2b	9.66485475	10.0943536	9.77433488	10.1242579	9.92790472	9.86922645
Cbl	9.64212084	9.7649001	9.60800865	9.90885872	9.88706948	9.60912054
Fam178a	8.03610127	8.30890035	7.94202038	9.84880958	7.94202038	8.0332077
Gm40571	8.47945675	8.3577056	8.27953638	8.48001526	8.41978914	8.28247836
Fnip2	8.78218727	8.67634644	8.62419555	8.89588686	8.7754316	8.57534045
Dnase2a	7.94202038	8.23646076	7.94202038	7.94202038	7.94202038	7.94202038
Stk16	8.98772156	8.82393696	7.94202038	7.94202038	7.94202038	8.97921614
Fam117b	8.68079812	8.81356207	8.78757811	8.82115352	8.92939885	8.85244269
Ptprj	7.94202038	8.42146512	8.06983875	8.68299269	8.6775708	8.53587378
Cnppd1	8.39147661	8.34607084	8.27953638	8.93844461	8.33270708	8.49342128
Emc10	8.03610127	8.92550876	8.71569667	8.90770707	7.94202038	8.41381734
Brwd1	8.94799927	9.09120375	8.83195168	9.12525659	8.95073625	8.83950361
Cacul1	8.75684959	8.90709772	8.77838756	8.8468408	8.70212555	8.8039287
Mab21l1	8.13008232	8.11221209	7.94202038	8.03219196	8.11716757	7.94202038
Rnf145	9.25053385	9.29602731	8.93323273	9.20510013	9.31113987	8.99383228
Map4k5	8.48744472	8.86901671	8.3739998	8.60501026	8.69240856	8.4472291
B230219D22Ri	9.01827346	9.39075577	8.9217079	9.13749915	9.08432138	8.8351436
Ube3a	8.79699096	9.12106925	8.75011935	8.97539079	9.06284229	8.73239774
Ccser2	8.54739125	8.68262321	8.55069191	8.56839292	8.67255208	8.52902268

For Review Only

LOC10816767!	8.0750473	8.23646076	7.94202038	8.18035967	8.17361191	8.07095728
Gm16287	8.03610127	8.18256866	7.94202038	8.14351917	8.11716757	8.07095728
Msl2	8.98382859	9.04057886	8.60663235	9.01778754	9.02806142	8.67504275
Mipep	8.33997962	8.39075541	8.06983875	8.19677454	8.09372556	8.30586539
Prkch	8.65744446	8.61006489	8.4348214	8.6934216	8.58651003	8.74234432
Avl9	8.71984215	8.57412075	8.61839545	8.77636651	8.716452	8.63005896
Nos1ap	9.12463856	9.00799671	8.92556678	9.03488446	9.13167679	8.92996779
Fam76a	8.71439619	8.51159704	8.5635863	8.61090552	8.60913958	8.61235015
Rrs1	8.56831653	8.3577056	8.50309307	8.35390548	8.45768165	8.58792851
Clptm1	9.42239466	8.93005002	9.32382107	9.12833165	9.11124409	9.95119288
Ube2m	8.36135974	7.94202038	8.03241639	8.03219196	8.34229442	8.30586539
Ssbp4	9.02202113	8.53585955	8.4348214	8.65070489	8.66240089	8.68583004
Cand1	8.99930001	8.78700057	8.94460515	8.93086319	8.72116354	8.88185346
Lpin1	9.23261707	9.19879626	9.06425355	9.07467491	9.06284229	9.204029
Rabep1	8.78715389	8.82393696	8.67418225	8.85522277	8.75778437	8.83950361
C2cd2	8.46309648	8.5959402	8.51711669	8.40860583	8.70212555	8.5220862
Eva1b	8.10491559	8.41145947	7.94202038	7.94202038	8.50630687	8.40507356
Kbtbd7	8.7619865	8.66361507	8.52399533	8.76239881	8.73970512	8.77139787
Gramd1a	9.22657498	9.39358716	8.97422517	9.36792321	9.4039805	9.04320634
Ccnd3	9.16409394	8.04031858	7.94202038	8.93086319	8.99191131	9.31954748
Sun2	9.71890025	9.2867021	9.24991529	9.7066059	9.71913248	9.89661194
Vps26b	9.01827346	8.89302096	8.9632345	9.07467491	8.91852821	9.09983349
Tmem150a	8.29323331	8.20179222	7.94202038	8.391136	7.94202038	8.09990895
1500015A07Ri	8.280517	8.38002423	8.26731165	8.27870194	8.13781088	8.21521951
Proser1	8.66922285	8.71317579	8.52399533	8.65070489	8.7926739	8.66958398
Nfatc2ip	8.10491559	8.28181527	7.94202038	8.22675204	8.21869831	8.0332077
Mast3	8.88037828	9.64381066	9.01340357	9.48566018	9.32318998	8.81297517
Helb	8.53300487	8.13850293	8.32447998	8.41709188	8.53221409	8.16519739
Syk	9.62953564	9.83297138	9.50444201	9.78061229	9.74433097	9.592887
Adat3	8.29323331	8.3577056	8.22745864	8.21218424	8.31277085	8.21521951
Itga4	8.0750473	8.16165221	7.94202038	8.21218424	8.09372556	8.14578597
6330549D23Ri	8.23905806	8.30890035	8.26731165	8.33392707	8.35165185	8.35851493
Gm38720	8.15224289	8.11221209	8.16326384	8.06952151	8.13781088	7.94202038
Ncdn	8.69780065	8.63739493	8.56992826	8.5225692	8.78410178	8.51506123
Nat10	8.78218727	8.40122805	8.65243454	8.64516752	8.77105876	8.45522941
Gpt2	8.70337638	8.45025944	8.56992826	8.391136	8.46491786	8.36824626
Pygo2	8.03610127	8.30890035	7.94202038	9.30007562	9.34219306	8.38705301
Fam160b2	8.71984215	8.79773552	8.5635863	8.69857742	8.86972975	8.83950361
Gpr180	8.59511249	8.58148203	8.53750164	8.58697859	8.69728409	8.75701406
Gm30992	8.19067125	8.20179222	8.16326384	7.94202038	8.15646529	8.09990895
Trim27	8.7306106	8.80304714	8.5635863	8.70877579	8.86972975	8.82191772
Gm29933	8.15224289	8.20179222	7.94202038	8.09815136	8.13781088	8.09990895
Zbtb7a	9.06584495	9.06037402	8.75490623	8.98259756	8.91852821	9.06693655
Gzf1	8.61446394	8.82907344	8.30272335	8.63958273	8.45768165	8.85244269
Lypla2	7.94202038	8.93456737	8.03241639	8.03219196	8.0296343	9.01885301
Mvb12a	8.15224289	9.17819253	7.94202038	7.94202038	7.94202038	8.0332077

Fndc3b	8.68651257	8.9479792	8.68479783	8.7809652	8.85411531	8.92213644
Hnrnpll	8.280517	8.58148203	8.26731165	8.38213245	8.49963208	8.40507356
Hmg20b	8.03610127	8.98276926	8.3739998	8.66164181	8.6035597	9.03631367
Bri3bp	8.53300487	8.43125887	8.50309307	8.47257473	8.47205105	8.41381734
Maml3	9.31372042	9.18854707	9.22780356	9.25502708	9.51612091	9.37290784
Gm31684	8.4547119	8.40122805	8.26731165	8.35390548	8.56896558	8.37775418
Atp2b2	8.22386465	8.11221209	8.12272383	8.16271545	8.26919051	8.25732722
D3Ertd751e	8.44618198	8.39075541	8.3739998	8.5087573	8.44288227	8.46310186
Tcerg1	8.76708807	8.97419951	8.5946422	8.77173954	8.76223557	8.96435567
Fbxo40	8.37164039	8.52788968	8.40108762	8.38213245	8.40372805	8.40507356
Kctd21	8.78218727	8.92094326	8.82327676	8.68299269	8.80115091	8.85670987
Trip6	8.03610127	8.18256866	8.25460081	7.94202038	8.15646529	7.94202038
LOC10816773(	9.31650483	9.52673916	9.40957886	9.43179709	9.10532616	9.1126818
Ncoa2	9.19893506	9.0835898	9.00640582	9.2079386	9.12005362	8.9338568
Pnrc1	8.03610127	8.04031858	7.94202038	7.94202038	9.34219306	9.2523393
Gatc	7.94202038	7.94202038	8.06983875	8.03219196	8.64162006	8.64729097
Pisd-ps3	7.94202038	7.94202038	8.40108762	9.2079386	7.94202038	7.94202038
Bcl2a1a	8.20779251	8.77060783	8.93704021	8.46502356	7.94202038	8.46310186
Ndufb4	7.94202038	8.39075541	7.94202038	8.33392707	7.94202038	8.43909474
Nat9	8.0750473	8.18256866	7.94202038	8.37293489	7.94202038	8.21521951
Gcnt7	8.23905806	8.20179222	8.2128549	8.30183216	8.15646529	8.28247836
LOC10816897(	7.94202038	8.13850293	8.06983875	8.03219196	8.0296343	7.94202038
Neb	7.94202038	8.33408171	8.24134193	8.09815136	7.94202038	7.94202038
Zfp36l1	7.94202038	9.99027377	8.03241639	8.03219196	8.09372556	7.94202038
Senp1	8.71984215	8.82907344	8.68479783	8.61090552	8.70693361	8.73239774
Trpm7	9.14456392	9.48841085	9.00288659	9.3824406	9.46249856	9.06021769
Foxn3	9.55781597	9.7710595	9.54069937	9.7174569	9.71573296	9.43790252
Crebbp	9.48138711	9.87322837	9.54279712	9.54752148	9.71573296	9.50815594
Ammecr1	7.94202038	8.13850293	8.06983875	8.12227563	8.13781088	7.94202038
Arl5b	8.57512873	8.68262321	8.5762029	8.62253212	8.60913958	8.60633467
LOC10816754(	8.32883489	8.3577056	8.27953638	8.5225692	8.39547674	8.21521951
Plppr3	8.29323331	8.51980383	8.51015037	8.44956944	8.52585076	8.50794447
Kdm7a	8.93572231	9.04057886	8.97057679	9.00036416	8.94013339	8.73738812
Gid4	8.65147592	8.73669692	8.58241219	8.61674566	8.6775708	8.5689455
Ncam1	8.20779251	7.94202038	8.0985397	8.12227563	7.94202038	8.09990895
Zfp612	8.25350136	7.94202038	8.25460081	8.14351917	7.94202038	8.07095728
Rrnad1	8.39147661	7.94202038	8.18095171	8.14351917	8.09372556	7.94202038
Snx7	8.26729451	8.20179222	8.25460081	7.94202038	7.94202038	8.22994993
Il1rn	8.20779251	8.08100817	8.16326384	8.09815136	7.94202038	8.0332077
Mmp9	8.55445056	7.94202038	8.30272335	8.18035967	7.94202038	8.25732722
Slfn4	8.39147661	7.94202038	8.06983875	8.19677454	7.94202038	8.16519739
Car12	8.39147661	7.94202038	7.94202038	8.25382753	7.94202038	8.38705301
Paqr4	8.62704025	8.40122805	8.45862639	8.48001526	8.31277085	8.5007324
Vat1l	8.29323331	7.94202038	7.94202038	7.94202038	8.29167353	8.35851493
Rnf187	9.01073162	8.11221209	8.60066643	8.64516752	8.75330618	9.51473029
Gpsm1	8.97204646	8.68884272	8.81008188	8.78553606	8.85016421	8.91819366

Rpgrip1l	8.40106353	8.20179222	8.29132606	8.34404359	8.40372805	8.38705301
E430025E21Ril	9.03317306	8.81876671	8.77374998	8.93086319	8.81783074	8.9377283
Wdr35	8.50306374	8.33408171	8.18095171	7.94202038	8.29167353	8.47085215
Kctd13	8.47945675	8.45025944	8.44288721	8.42542373	8.45033803	8.54264242
Tgif2	8.60163044	8.54371805	8.48869139	8.54920222	8.58651003	8.66958398
Rtn1	9.04419168	9.08740438	8.83195168	9.05830083	8.96812633	8.94158251
Pde8b	8.76708807	8.90709772	8.53750164	8.03219196	8.49963208	8.90625417
Ctage5	8.82577145	8.6571573	8.60066643	8.70877579	8.6775708	8.70690929
Cluh	8.79699096	8.91635315	8.72570119	8.75768405	8.64162006	8.81297517
Ngp	10.337072	9.15715626	9.0203479	9.28175929	7.94202038	8.64729097
Ahcyl2	8.89779823	8.6238769	8.49594131	8.54266087	8.59792904	8.74726697
Nadk2	8.55445056	8.43125887	8.42661608	8.32353564	8.41978914	8.43081962
Zc3h4	9.19268943	9.1464686	8.97422517	9.07142278	9.15451737	9.08023231
Zbtb11	8.95608855	8.86413056	8.51015037	8.752939	8.716452	8.65853119
Zfp442	8.65744446	8.55146949	8.45082017	8.58084728	8.46491786	8.61830836
Ap2a2	9.44240459	9.18510739	8.97785858	9.21640685	9.16294816	9.21560262
Ralgapa2	8.71984215	8.6238769	8.3739998	8.61090552	8.6775708	8.77613153
Gm41338	8.33997962	8.36901499	8.19740715	8.42542373	8.29167353	8.5007324
Npnt	9.19268943	9.06037402	8.83195168	9.05830083	9.06284229	9.21847562
Ptprf	8.37164039	8.04031858	7.94202038	8.03219196	8.0296343	8.16519739
Ubb	9.95844153	8.67634644	7.94202038	7.94202038	7.94202038	9.45184474
0610011F06Ril	8.32883489	7.94202038	7.94202038	7.94202038	7.94202038	8.19963762
Sgk1	7.94202038	7.94202038	8.03241639	8.27870194	8.06590615	9.306443
Per1	8.22386465	8.04031858	7.94202038	8.8298088	7.94202038	9.08681089
Dusp6	7.94202038	7.94202038	7.94202038	9.84394425	7.94202038	11.6228048
Faap20	8.31735125	8.30890035	7.94202038	8.94221048	7.94202038	8.55594324
Zzz3	9.02575351	8.86901671	8.77838756	8.99683886	8.85411531	8.9606011
Hccs	8.53300487	8.44085321	8.41826405	8.52934701	8.43530927	8.58792851
Adra1a	8.15224289	7.94202038	8.03241639	8.12227563	8.06590615	8.12430412
LOC10263246!	8.26729451	8.04031858	7.94202038	8.22675204	8.2045402	8.21521951
Ppl	8.29323331	8.04031858	7.94202038	8.22675204	8.2045402	8.09990895
Tstd2	8.96808402	8.73088872	8.7596622	8.96812497	8.86972975	8.89006666
Particl	8.47134264	8.29562241	8.24134193	8.45735688	8.49287238	8.40507356
Kdm6b	8.7619865	8.47744782	8.42661608	8.5990584	8.73511433	8.8438399
Phkg2	8.50306374	7.94202038	8.42661608	8.19677454	8.06590615	8.60026035
H2-K1	10.5902564	9.15360652	9.83171011	10.1817315	9.10532616	10.4438277
Ppat	8.44618198	8.41145947	8.41826405	8.51570698	7.94202038	8.49342128
P3h1	8.39147661	8.30890035	8.33489682	8.40860583	8.52585076	8.63005896
Hibadh	8.82577145	8.75947666	8.71064092	8.62253212	8.69728409	8.79477499
4930473H19Ri	8.10491559	8.13850293	8.12272383	8.14351917	8.18956427	8.09990895
Gm32853	8.32883489	8.50326399	8.27953638	8.35390548	8.37849082	8.4472291
Pnpla7	8.78218727	8.93005002	7.94202038	7.94202038	9.45826703	8.92606108
Pstpip2	8.29323331	8.18256866	8.18095171	8.12227563	8.18956427	8.16519739
Rbm15	9.05508103	9.16069332	8.98147719	9.17333936	9.09937163	9.15635975
Usp27x	8.15224289	8.11221209	8.12272383	8.14351917	8.18956427	8.09990895
Mib2	8.62704025	8.04031858	7.94202038	7.94202038	8.72584407	8.45522941

Gm36527	8.75167668	8.58875419	8.5635863	8.66164181	8.92216716	8.73239774
Hspb6	8.37164039	8.08100817	8.19740715	8.19677454	8.35165185	8.2943698
Gm2260	8.10491559	8.11221209	8.0985397	7.94202038	8.0296343	7.94202038
Gm2274	8.10491559	8.11221209	8.0985397	7.94202038	8.0296343	7.94202038
Cacnb1	8.25350136	7.94202038	8.0985397	7.94202038	8.06590615	7.94202038
Mbd3	8.62078334	7.94202038	8.2128549	8.06952151	8.06590615	7.94202038
Mdp1	7.94202038	7.94202038	8.69525104	8.03219196	8.13781088	7.94202038
Slc12a9	8.13008232	7.94202038	7.94202038	8.21218424	8.70693361	7.94202038
Gm14025	8.0750473	8.08100817	8.2128549	8.12227563	8.21869831	8.0332077
Abcb9	8.3508132	8.29562241	8.39224519	8.26650718	8.369737	8.27014799
Nol9	8.60163044	8.51159704	8.54413497	8.5990584	8.63097594	8.53587378
Inpp4b	9.322052	9.14288016	9.23059359	9.33578884	9.46038478	9.18047749
Pias4	8.64545268	7.94202038	8.03241639	8.03219196	8.0296343	7.94202038
Il10ra	13.4164223	8.68884272	11.8680724	11.6690098	7.94202038	10.1606595
Tagap	7.94202038	7.94202038	7.94202038	8.59304839	8.41182964	7.94202038
Sh3gl1	8.8443886	8.77060783	8.29132606	8.24060121	7.94202038	7.94202038
Snord71	8.15224289	8.04031858	8.03241639	8.09815136	8.09372556	7.94202038
LOC10263826[illegible]	8.38167383	8.3577056	7.94202038	8.26650718	8.26919051	8.0332077
Zbtb44	8.52567117	8.51159704	8.03241639	8.48734968	8.47205105	8.21521951
Wdr76	8.49531202	8.32170464	7.94202038	8.33392707	8.25733743	8.19963762
Etaa1	8.5107046	8.36901499	7.94202038	8.37293489	8.29167353	8.28247836

For Review Only

For Review Only

For Review Only

For Review Only

For Review Only

For Review Only

For Review Only

Sham_HSD_2	Sham_HSD_3	Sham_HSD_4	Sham_HSD_5	rCHI_ND_1	rCHI_ND_2	rCHI_ND_3
8.0375398	8.03734617	8.12784483	7.94202038	8.07053545	8.08174193	7.94202038
8.78960971	8.7243782	8.79828358	8.80374893	8.70961779	8.74079174	8.6821371
7.94202038	8.25760818	8.14271065	8.03221336	7.94202038	8.18383663	7.94202038
8.48758145	8.67271024	8.6588078	8.58712922	8.56047983	8.64768614	8.58106002
8.77419437	8.86520938	8.90229147	9.01457299	8.7687547	8.99231356	8.82336319
8.43602752	8.56243539	8.63922253	8.65636216	8.47674917	8.62741336	8.5194214
8.937712	9.03944861	9.06011838	9.27941307	9.05672903	9.27114998	8.9339267
8.6801113	8.62960285	8.74908477	8.72398244	8.627848	8.64768614	8.69451251
8.63097409	8.53329126	8.70720375	8.72398244	8.64502675	8.62051088	8.68835271
9.465956	9.39354371	9.48962034	9.55201496	9.45419625	9.36605283	9.44242847
8.64983251	8.77771577	8.81710547	8.66180921	8.7397891	8.73495507	8.66314092
8.85307109	8.95667054	9.06941697	8.82135603	8.93073128	8.93051125	8.87326967
9.11418648	9.12579139	9.23884319	9.19968025	9.19723493	9.18434884	9.28586023
8.98715122	9.17935176	9.10794935	9.1823788	9.14035421	9.10833077	9.07519072
8.17578116	7.94202038	8.18180848	7.94202038	7.94202038	7.94202038	7.94202038
8.53447614	8.34521361	8.62718148	8.36363	8.42083811	8.40363382	8.49443268
8.21184601	7.94202038	8.32050886	7.94202038	7.94202038	7.94202038	7.94202038
8.07708059	8.13256811	8.16952947	7.94202038	7.94202038	8.04083773	7.94202038
8.13295474	8.13256811	8.0493556	7.94202038	7.94202038	7.94202038	7.94202038
8.0375398	7.94202038	8.09378033	7.94202038	8.12370814	7.94202038	7.94202038
8.0375398	8.13256811	7.94202038	7.94202038	7.94202038	7.94202038	8.04025268
7.94202038	8.15502059	8.01792659	7.94202038	8.03290927	7.94202038	8.04025268
7.94202038	7.94202038	8.01792659	8.03221336	8.31578075	7.94202038	7.94202038
8.10740452	7.94202038	7.94202038	7.94202038	7.94202038	8.04083773	8.04025268
8.32305938	7.94202038	8.0493556	8.03221336	7.94202038	7.94202038	8.64358726
8.17578116	7.94202038	7.94202038	7.94202038	8.03290927	8.08174193	8.13837135
8.9249007	8.55528676	8.4862189	8.76258851	8.64502675	8.59933675	8.56625462
9.11418648	8.9441902	9.21718043	8.97199996	9.13726817	8.92131247	8.97782795
9.70076722	9.63890428	9.67517755	9.2607214	9.66494106	9.20504327	9.79228871
8.69764864	8.63593804	8.72143847	8.73382824	8.70446198	8.58480593	8.65668722
8.57075294	8.56243539	8.63922253	8.56853934	8.47674917	8.43381905	8.55871023
8.47097935	8.44399288	8.54494296	8.41720367	8.46141416	8.37125455	8.44992204
8.74237475	8.66672147	8.74908477	8.74354137	8.78282479	8.48903706	8.60260711
10.0625572	10.0309782	10.144095	9.86688848	10.124395	9.64040958	9.70696926
8.40802503	8.40708814	8.49646091	8.4726993	8.46141416	8.35988667	8.52750204
8.85774867	8.91867544	9.01463489	8.88812974	9.00130993	8.79675312	8.97782795
9.21068216	9.31356086	9.45505288	9.38275676	9.44037488	9.20504327	9.32265992
8.21184601	8.25760818	8.29719551	8.24067185	8.30468047	8.22113719	8.20161846
10.1842043	10.2148917	10.5958786	10.4175555	10.3628255	10.2036212	10.2085005
8.39829513	8.32229112	8.48102142	8.40006484	8.39468096	8.45291706	8.39045684
8.58465681	8.66672147	8.77572469	8.69875305	8.71473604	8.61353225	8.69451251

For Review Only

8.74237475	8.77771577	8.95108665	8.83857656	8.74469681	8.77486826	8.7918325
7.94202038	8.13256811	8.22561138	7.94202038	7.94202038	8.08174193	8.04025268
8.61808711	8.54804873	8.6431868	8.48014154	8.54737187	8.61353225	8.41114736
8.61808711	8.57647654	8.72143847	8.66180921	8.46913989	8.71112141	8.67586427
8.36772811	8.51039627	8.47577098	8.4726993	8.47674917	8.47129896	8.4681985
8.40802503	8.62320431	8.57656414	8.54934429	8.54070388	8.50619217	8.48583508
8.64983251	8.60361001	8.65104373	8.63411058	8.60419728	8.63424196	8.60260711
8.33471557	8.34521361	8.21532682	8.35400262	8.31578075	8.3361404	8.40092263
8.80969067	8.66672147	8.64712707	8.79479288	8.66725108	8.71112141	8.62342789
8.9668882	8.70188757	8.81088212	8.99353881	8.81020699	8.79675312	8.9339267
8.38834599	8.33392409	8.26343518	8.48014154	8.25629903	8.3931072	8.28158842
8.66210941	8.48648807	8.53083989	8.60516496	8.56047983	8.6608596	8.5194214
8.80969067	8.82268134	8.74908477	8.63411058	8.84955684	8.79675312	8.85861963
8.51928974	8.40708814	8.52123636	8.16276775	8.72987274	8.4621927	8.60962465
8.0375398	8.03734617	7.94202038	7.94202038	8.03290927	8.11311024	7.94202038
8.31102639	8.15502059	7.94202038	7.94202038	8.07053545	8.04083773	8.08091502
8.4538143	8.32229112	8.40796396	8.3236257	8.31578075	8.3236991	8.29538642
7.94202038	8.03734617	7.94202038	7.94202038	7.94202038	7.94202038	7.94202038
8.07708059	8.17530815	8.14271065	7.94202038	8.07053545	8.04083773	7.94202038
9.07129237	8.97303906	9.07403206	9.00411705	9.03633578	8.94861884	9.01556269
8.15545262	7.94202038	8.11167364	7.94202038	8.16446819	7.94202038	7.94202038
9.60486064	9.46062203	9.66589414	9.5437055	9.66494106	9.42036482	9.56284902
8.95450337	8.83711044	8.99478683	8.78115896	9.02596814	8.79675312	8.96922171
9.48087291	9.08718779	9.45664724	9.32854155	9.59612696	9.4063292	9.24123716
9.0490947	8.78284442	8.91062049	8.86784763	8.96858135	8.49768407	8.63021825
9.99960633	9.74963568	9.75814209	9.90926468	9.91154142	9.7292533	9.77201959
8.0375398	8.03734617	7.94202038	7.94202038	8.03290927	7.94202038	7.94202038
8.31102639	8.03734617	7.94202038	7.94202038	8.03290927	8.08174193	8.04025268
8.87162184	8.67864625	8.8534481	8.68840029	8.80118764	8.85393357	8.84870391
8.7946794	8.59693903	8.62718148	8.55581336	8.59814185	8.67377702	8.52750204
9.05655251	8.85595144	8.92703679	8.78573083	8.975959	8.92592423	8.7918325
8.46247064	8.53329126	8.50150825	8.52270521	8.61613381	8.53094292	8.5194214
8.47934746	8.31028213	8.84151738	8.27878152	8.67269307	8.22113719	8.65016993
9.37746175	9.38553657	9.49425368	9.47054587	9.57982009	9.46948242	9.53328256
8.7314534	8.32229112	9.17657318	8.79928395	8.80571047	9.37771188	8.9111111
8.43602752	8.35618776	8.52605893	8.39124179	8.4842469	8.49768407	8.52750204
8.0375398	8.03734617	7.94202038	7.94202038	8.07053545	8.04083773	7.94202038
8.10740452	8.1070695	7.94202038	7.94202038	8.07053545	8.08174193	8.04025268
8.63732144	8.67271024	8.68157207	8.50889015	8.81020699	8.65430576	8.58832753
8.4538143	8.61020983	8.61490875	8.3236257	8.70961779	8.50619217	8.61656411
8.13295474	8.15502059	8.16952947	7.94202038	8.29322195	8.08174193	8.18240769
9.74896078	9.67069602	9.69742953	9.57456492	9.73363668	9.75468532	9.73045025
8.13295474	8.15502059	8.32050886	7.94202038	8.30468047	8.29748128	8.20161846
7.94202038	8.03734617	7.94202038	7.94202038	7.94202038	8.08174193	8.11209808
8.10740452	8.15502059	8.09378033	7.94202038	7.94202038	8.34819167	8.08091502
8.82926513	8.81291275	8.87102028	8.75787272	8.76400532	8.83874463	8.82849652

For Review Only

8.64983251	8.70757797	8.77898878	8.57480053	8.56692462	8.74658187	8.70061788
8.10740452	8.0768069	8.23553133	7.94202038	8.07053545	8.16281037	8.11209808
8.72036359	8.79802576	8.69997674	8.55581336	8.69403455	8.59933675	8.72452052
8.38834599	8.59693903	8.63922253	8.34413843	8.57960685	8.45291706	8.57370344
8.4956873	8.67864625	8.57214987	8.39124179	8.37633837	8.52281605	8.60260711
8.4450027	8.61020983	8.74908477	8.25390127	8.39468096	8.4621927	8.44052192
8.33471557	8.38744802	8.53558024	8.24067185	8.31578075	8.58480593	8.44992204
9.39092654	9.33622162	9.40279718	9.27941307	9.28904775	9.36017627	9.45586117
9.02250965	8.93152757	9.06245167	8.91571882	8.97227787	9.00920898	8.97353528
9.29296468	9.22397593	9.15775056	9.02147745	9.06342891	9.26149891	9.14570458
8.37816259	8.38744802	8.40796396	8.2665839	8.35716075	8.3361404	8.34580159
8.07708059	7.94202038	8.01792659	7.94202038	8.07053545	7.94202038	8.04025268
9.485802	9.19230345	9.36711502	9.26609602	9.24832082	9.29018992	9.41507006
8.78450607	8.65458036	8.83850672	8.79479288	8.82793821	8.75802647	8.80248617
7.94202038	8.03734617	7.94202038	7.94202038	8.03290927	7.94202038	7.94202038
7.94202038	8.03734617	7.94202038	7.94202038	7.94202038	8.04083773	8.04025268
7.94202038	8.1070695	7.94202038	7.94202038	7.94202038	7.94202038	7.94202038
7.94202038	8.27158165	7.94202038	7.94202038	7.94202038	7.94202038	7.94202038
8.0375398	8.03734617	7.94202038	7.94202038	8.03290927	8.04083773	8.21948911
8.10740452	8.13256811	8.21532682	8.24067185	8.16446819	8.18383663	8.39045684
7.94202038	7.94202038	7.94202038	7.94202038	7.94202038	7.94202038	7.94202038
7.94202038	7.94202038	7.94202038	7.94202038	7.94202038	7.94202038	7.94202038
8.0375398	7.94202038	7.94202038	7.94202038	8.03290927	8.04083773	8.18240769
8.0375398	8.03734617	7.94202038	7.94202038	8.03290927	7.94202038	8.21948911
9.41737289	9.39088113	9.48185603	9.12243736	8.71981747	8.98374524	9.30134854
8.83408357	8.80302002	8.91886824	8.76727453	8.77816394	8.59933675	8.66314092
8.61154377	8.78793855	8.76915363	8.80374893	8.63362527	8.65430576	8.78102805
7.94202038	8.13256811	8.12784483	7.94202038	8.12370814	8.08174193	8.04025268
7.94202038	8.03734617	8.11167364	7.94202038	8.09939256	8.11311024	7.94202038
8.0375398	8.03734617	9.20113244	7.94202038	8.03290927	8.11311024	8.04025268
7.94202038	7.94202038	8.41400157	7.94202038	7.94202038	8.20316099	7.94202038
9.39892705	9.40149311	9.41939155	9.20253525	9.29436069	9.1808581	9.35823771
9.16194132	9.26673801	9.26767026	8.98283445	9.21740566	9.02579553	9.11294372
8.83408357	8.83711044	8.832451	8.68316485	8.79206141	8.71715512	8.7303724
14.4178348	14.7475591	14.9130041	14.8394855	14.8995532	14.6474187	14.8052636
9.22642157	9.19551553	9.44221602	9.35352081	9.35112051	9.06600163	9.26378697
9.10010089	9.05068322	9.11679515	9.07493083	9.29700719	8.97509381	8.99479469
9.1451513	9.05439815	9.17864284	9.12552277	9.26207343	9.08161271	9.15283848
8.0375398	8.19395436	8.1934834	8.12231837	8.19879645	8.16281037	7.94202038
8.66210941	8.66067864	8.62311693	8.79479288	8.82354286	8.48024445	8.60260711
8.10740452	7.94202038	7.94202038	8.12231837	8.16446819	7.94202038	7.94202038
8.937712	9.05068322	9.06941697	9.22787631	9.21740566	8.86390281	9.13851865
8.28567045	8.47827021	8.28074391	8.66721204	8.4989257	8.11311024	8.58832753
10.0989105	9.85297919	10.1340335	10.7621267	10.4287937	10.0815008	10.4798561
8.53447614	8.58337468	8.67028632	8.61690301	8.65065343	8.62051088	8.63021825
7.94202038	7.94202038	8.0493556	8.12231837	7.94202038	7.94202038	7.94202038

For Review Only

7.94202038	7.94202038	8.01792659	9.188179	7.94202038	7.94202038	7.94202038
9.18498119	9.04320861	9.11899403	9.08140307	9.13726817	8.91201331	9.23142062
8.68600705	8.6167406	8.62718148	8.78573083	8.70446198	8.51456759	8.68835271
8.64983251	8.6422116	8.69997674	8.76258851	8.71981747	8.5774076	8.63021825
8.22816175	8.22758337	8.23553133	8.36363	8.12370814	8.22113719	8.32145144
8.66210941	8.42590555	8.61902618	8.92727245	8.46141416	8.47129896	8.44992204
8.54191739	8.21130042	8.76584633	9.31583109	9.2593374	8.61353225	8.96922171
8.07708059	8.1070695	8.01792659	8.12231837	8.18225192	8.08174193	8.11209808
9.76849371	9.78441622	9.79341566	9.73748106	9.77446512	9.62416241	9.64958646
8.74777442	8.56243539	8.72845018	8.73382824	8.71473604	8.63424196	8.61656411
8.39829513	8.31028213	8.38295868	8.40871566	8.33702644	8.34819167	8.34580159
8.27224842	8.25760818	8.26343518	8.2665839	8.19879645	8.31082828	8.20161846
8.7314534	9.05809837	8.86520496	9.4041834	8.93459149	9.03397749	9.02373879
7.94202038	8.32229112	8.0493556	8.18041613	7.94202038	7.94202038	7.94202038
8.39829513	8.49457807	8.54028095	8.62269077	8.52021224	8.44346262	8.60260711
8.29857845	8.51039627	8.44866598	8.61106155	8.4842469	8.37125455	8.44992204
7.94202038	7.94202038	8.0493556	8.37303645	7.94202038	7.94202038	7.94202038
8.70909798	8.66067864	8.6588078	8.73382824	8.69926783	8.63424196	8.63021825
8.0375398	8.0768069	7.94202038	8.22681943	8.12370814	7.94202038	7.94202038
8.69764864	8.83711044	8.74227158	8.85959209	8.91904516	8.69270206	8.67586427
7.94202038	7.94202038	8.11167364	8.14356694	7.94202038	7.94202038	7.94202038
8.4956873	8.66067864	8.6312203	8.68840029	8.57329959	8.48024445	8.57370344
7.94202038	8.03734617	8.07346349	8.35400262	8.22901041	8.11311024	7.94202038
8.67416397	8.66672147	8.72495293	8.86373033	8.7966381	8.68014395	8.63021825
8.62456325	8.66067864	8.74908477	8.73870109	8.74469681	8.66734945	8.65668722
8.65599947	8.56949769	8.67406921	8.66180921	8.60419728	8.59933675	8.66953273
9.26022385	9.16292313	9.33735044	9.15585366	9.27564719	9.12327174	9.23797535
9.29880982	9.43273283	9.41774323	9.53743413	9.45419625	9.41477184	9.36115042
9.80289078	9.65384557	9.69742953	9.96660119	9.84570627	9.69669819	9.77406156
8.35702361	8.31028213	8.45968564	8.49471185	8.49163765	8.38232065	8.41114736
8.55651444	8.59019469	8.61076414	8.79479288	8.44559177	8.3361404	8.66953273
8.63097409	8.69615031	8.6312203	8.81260227	8.75441437	8.56232915	8.76452552
8.41754917	8.58337468	8.73539395	8.62842619	8.57329959	8.4621927	8.72452052
8.48758145	8.62320431	8.6431868	8.83857656	8.68876131	8.49768407	8.77006686
9.31328351	9.16623057	9.29572438	9.33860271	9.00130993	9.06600163	9.21150159
8.86239938	8.67864625	8.83548463	8.82135603	8.77347407	8.69889618	8.71862027
8.10740452	8.0768069	8.07346349	8.12231837	8.03290927	8.04083773	8.04025268
8.0375398	8.17530815	7.94202038	7.94202038	8.03290927	8.22113719	8.08091502
7.94202038	8.83711044	7.94202038	7.94202038	7.94202038	7.94202038	7.94202038
8.57075294	7.94202038	8.45420668	7.94202038	7.94202038	7.94202038	7.94202038
9.59146853	8.03734617	7.94202038	7.94202038	8.03290927	10.2457554	8.04025268
13.3396114	13.2831418	13.2665504	13.1580005	13.5861055	13.1622302	13.398162
8.80969067	8.55528676	8.65104373	8.7483497	8.76400532	8.79134066	9.11663871
7.94202038	8.03734617	7.94202038	7.94202038	8.03290927	7.94202038	7.94202038
7.94202038	8.35618776	7.94202038	8.03221336	8.14511998	8.20316099	8.21948911
9.04534328	9.15960465	9.08091276	9.10686137	9.13106678	9.09315477	9.09802066

For Review Only

8.07708059	8.15502059	8.1934834	7.94202038	8.09939256	8.18383663	8.16150518
8.15545262	8.36687102	8.25441514	7.94202038	8.26907835	8.13953942	8.30865559
7.94202038	8.17530815	8.22561138	7.94202038	8.24296865	8.13953942	8.25212089
9.10716941	9.16623057	9.15985963	9.05855339	9.22875529	9.23862571	9.17045164
8.10740452	7.94202038	8.0493556	7.94202038	8.09939256	7.94202038	8.11209808
8.72036359	8.51039627	8.66648202	8.3236257	8.59814185	8.65430576	8.72452052
8.17578116	8.13256811	8.16952947	7.94202038	8.07053545	8.11311024	8.35742871
8.78960971	8.79802576	8.92703679	8.66721204	8.68344724	8.70503552	8.92487351
8.59824737	8.66672147	8.66265596	8.50184812	8.57960685	8.59933675	8.66953273
8.13295474	8.21130042	8.14271065	8.0981884	8.19879645	8.16281037	8.20161846
8.66210941	8.67271024	8.65493718	8.7483497	8.72987274	8.77486826	8.71862027
8.66816378	8.74618151	8.74227158	9.02491023	8.87888053	8.90732517	8.83866429
9.17845232	9.08718779	9.42432193	9.27144315	9.10266085	9.51655792	9.57975592
7.94202038	7.94202038	8.01792659	7.94202038	8.09939256	7.94202038	8.04025268
8.77419437	8.9056261	8.84451674	8.79479288	9.09623332	8.87375142	9.02373879
7.94202038	8.17530815	8.12784483	7.94202038	8.16446819	8.13953942	8.18240769
7.94202038	8.24297584	8.15654265	8.14356694	8.18225192	8.238011	8.23626395
8.0375398	8.32229112	8.82019864	8.82135603	8.07053545	8.25396137	8.68835271
8.21184601	8.36687102	8.64712707	7.94202038	8.61019408	8.04083773	8.93841778
7.94202038	7.94202038	8.28906908	8.03221336	7.94202038	7.94202038	7.94202038
8.10740452	8.17530815	8.15654265	8.12231837	8.19879645	8.18383663	8.16150518
8.10740452	8.17530815	8.20463482	8.2122482	8.33702644	8.20316099	8.25212089
8.4450027	8.6845308	8.54028095	8.60516496	8.56692462	8.52281605	8.63021825
8.70909798	8.6845308	8.65493718	8.48747764	8.7248628	8.77486826	8.77556743
8.4538143	8.42590555	8.49646091	8.2665839	8.46913989	8.43381905	8.45914445
8.13295474	8.13256811	8.07346349	8.12231837	8.12370814	8.04083773	8.04025268
8.85774867	8.82268134	8.79828358	8.6397452	8.66725108	8.8336168	8.83866429
9.94018805	10.0922139	9.85196884	9.71783222	9.6514925	9.81205723	9.93821999
10.0579407	9.98144093	9.65385183	9.31583109	9.27564719	9.62182315	9.61285663
9.22642157	8.03734617	9.93604109	7.94202038	8.03290927	8.11311024	9.61518582
8.46247064	8.40708814	8.38295868	8.24067185	8.28136888	8.31082828	8.5194214
8.67416397	8.74079199	8.61490875	8.64533124	8.7248628	8.56232915	8.91572339
7.94202038	9.19230345	7.94202038	7.94202038	7.94202038	7.94202038	9.18775936
8.9627793	8.4699185	7.94202038	7.94202038	7.94202038	7.94202038	8.82849652
8.80969067	8.75153153	8.65493718	8.71399884	8.7397891	8.86390281	8.853677
9.11068431	8.36687102	8.35639792	8.03221336	7.94202038	8.423975	8.42114647
8.39829513	8.44399288	8.20463482	8.33401955	8.68876131	8.7290708	8.55871023
8.42687967	8.85128246	7.94202038	8.84282393	8.21432776	8.34819167	8.08091502
9.06025923	8.91434797	8.85934784	8.95364171	8.87475535	8.99231356	8.87326967
8.80471929	8.82268134	8.70720375	8.80374893	8.70961779	8.77486826	8.68835271
8.0375398	7.94202038	7.94202038	8.03221336	7.94202038	8.04083773	8.11209808
9.24192329	9.24557763	9.09452653	9.22787631	8.85804354	9.11955803	9.34650811
8.34602759	8.66672147	8.41400157	8.71399884	8.41228632	8.50619217	8.44052192
8.86702363	9.2177197	8.92974238	9.23894096	8.81020699	9.01753995	8.96922171
8.82441712	9.10492685	8.87391251	8.85125165	8.70961779	8.92131247	8.853677
8.51153646	8.61020983	8.49646091	8.58099653	8.46913989	8.61353225	8.5112199

7.94202038	8.17530815	8.09378033	7.94202038	8.07053545	8.04083773	7.94202038
7.94202038	8.0768069	8.11167364	7.94202038	7.94202038	8.04083773	7.94202038
8.70339679	8.96897535	8.86811779	8.70895368	8.83665622	9.08161271	8.70061788
7.94202038	8.35618776	8.21532682	8.18041613	7.94202038	8.34819167	7.94202038
8.4538143	8.69036509	8.6312203	8.63411058	8.44559177	8.7290708	8.43093383
8.66210941	8.76735234	8.6588078	8.65636216	8.54737187	8.79675312	8.6821371
8.9791014	9.12922565	9.04599508	8.9899849	9.05336103	9.09315477	8.91572339
8.80969067	8.64842518	8.53083989	8.77193129	8.45356668	8.49768407	8.62342789
8.55651444	8.44399288	8.38295868	8.61690301	8.29322195	8.48903706	8.36873067
9.92146621	9.35290966	9.401124	9.63780723	8.49163765	9.33634671	9.04386619
7.94202038	8.31028213	8.0493556	8.29054508	7.94202038	7.94202038	7.94202038
8.56367695	8.76735234	8.61490875	8.90006488	8.54070388	8.59933675	8.5112199
8.93346276	9.14621845	8.71790657	8.93867222	8.71473604	8.87375142	8.7303724
9.23575055	9.32211486	8.9770529	9.23065364	9.02596814	9.00501466	9.17045164
8.75845579	8.93152757	8.60239134	8.8800767	8.51320938	8.69889618	8.80775823
8.68600705	8.60361001	8.44306149	8.49471185	8.40357044	8.4621927	8.50289223
9.01477051	8.13256811	8.5587055	7.94202038	8.03290927	8.11311024	8.04025268
8.7946794	8.69615031	8.56770248	8.59320039	8.60419728	8.3931072	8.77006686
9.40687003	9.22708978	8.81710547	9.19394599	9.00130993	8.45291706	9.5029402
9.34724222	8.44399288	7.94202038	9.00761577	8.32655381	8.04083773	8.34580159
9.97189131	9.81824358	9.40446784	9.57861857	8.76400532	9.30269512	9.42883304
9.10010089	9.0048982	8.83850672	8.89610543	8.83665622	8.83874463	8.92941206
8.69764864	8.03734617	7.94202038	7.94202038	8.03290927	8.04083773	8.04025268
8.34602759	8.24297584	8.1934834	8.25390127	8.16446819	8.08174193	8.21948911
8.70909798	8.54804873	8.6431868	8.6397452	8.56692462	8.59211465	8.8181961
8.19446499	8.03734617	7.94202038	8.2665839	8.09939256	8.16281037	8.13837135
9.7568113	8.98511963	8.98469632	9.37793808	9.14035421	9.11208769	9.27960076
8.78450607	8.13256811	8.38934535	8.25390127	8.30468047	8.31082828	8.20161846
9.83268433	9.4454956	9.66189346	9.55820831	9.51438678	9.59817287	9.72196405
8.33471557	8.0768069	8.35639792	8.2665839	8.21432776	8.18383663	8.26719425
8.2582469	7.94202038	8.14271065	7.94202038	7.94202038	7.94202038	8.04025268
8.35702361	8.17530815	8.22561138	8.22681943	8.25629903	8.22113719	8.23626395
8.10740452	7.94202038	8.09378033	7.94202038	8.03290927	8.11311024	8.16150518
8.59824737	8.51813443	8.58529607	8.48747764	8.54737187	8.66734945	8.57370344
8.54926234	8.34521361	8.50150825	7.94202038	8.77816394	8.31082828	8.95175282
8.61808711	8.35618776	8.43739103	7.94202038	8.22901041	8.25396137	8.40092263
8.34602759	8.03734617	7.94202038	7.94202038	8.03290927	8.04083773	8.04025268
8.58465681	8.53329126	8.51146214	8.59320039	8.61019408	8.68645179	8.6821371
8.63732144	8.43503537	8.53558024	8.52948457	8.42083811	8.55464208	8.5194214
8.13295474	8.13256811	8.09378033	8.19683487	8.18225192	8.04083773	7.94202038
8.76898501	8.94837022	8.88538083	8.66721204	8.83665622	8.58480593	8.57370344
8.15545262	7.94202038	8.01792659	8.19683487	7.94202038	8.04083773	7.94202038
9.20750494	8.85595144	9.02682343	9.188179	8.89113387	8.74658187	8.75894258
8.53447614	8.4699185	8.6431868	8.83430642	8.64502675	8.61353225	8.4681985
9.04157666	8.71882229	8.16952947	9.20822126	7.94202038	7.94202038	7.94202038
8.0375398	8.13256811	9.0097135	8.78115896	8.03290927	8.04083773	8.58832753

For Review Only

8.76898501	8.74079199	8.75918726	8.89212713	8.74469681	8.77486826	8.71267053
8.34602759	8.24297584	8.34947941	8.41720367	8.24296865	8.423975	8.21948911
8.10740452	8.22758337	8.80775168	8.65636216	8.74469681	8.20316099	8.33382036
8.53447614	8.49457807	8.35639792	8.40006484	8.38560745	8.4621927	8.45914445
9.3610824	9.32777981	9.27898179	9.07493083	9.23157319	9.49848058	9.29208278
8.47934746	8.22758337	8.34243817	8.36363	8.34722194	8.50619217	8.33382036
8.07708059	8.1070695	8.12784483	8.14356694	8.21432776	8.13953942	8.20161846
8.47097935	8.45278715	8.30513655	8.38223614	8.40357044	8.43381905	8.5194214
8.83408357	8.71322254	8.59390351	8.9899849	8.65065343	8.7290708	8.77006686
8.32305938	8.34521361	8.28074391	8.41720367	8.33702644	8.31082828	8.41114736
8.85307109	8.80798217	8.63922253	8.9310892	8.8452797	8.76929648	8.86841549
7.94202038	8.28497683	7.94202038	8.16276775	7.94202038	8.20316099	8.08091502
9.21700703	9.28454073	9.26767026	9.5765935	9.3660956	9.68342721	9.31053417
8.90311126	9.18908112	9.01463489	9.25260718	9.07339002	9.34534437	9.21818443
8.0375398	8.03734617	7.94202038	10.0768218	8.03290927	11.2032774	8.04025268
8.76373905	8.70757797	8.0493556	8.85959209	7.94202038	8.78588955	8.5194214
7.94202038	10.9958483	9.74324478	7.94202038	7.94202038	9.47214939	7.94202038
8.2582469	8.93998992	8.95108665	8.52270521	8.26907835	8.96195609	8.30865559
7.94202038	8.4699185	8.45420668	7.94202038	7.94202038	8.43381905	8.36873067
7.94202038	8.13256811	8.18180848	7.94202038	7.94202038	8.48903706	8.13837135
8.19446499	8.22758337	8.23553133	8.2122482	8.19879645	8.28360225	8.30865559
7.94202038	8.1070695	8.01792659	7.94202038	8.09939256	8.11311024	8.11209808
7.94202038	8.15502059	7.94202038	7.94202038	7.94202038	8.18383663	8.16150518
10.1108063	10.0262667	8.96416559	8.69875305	7.94202038	10.0235157	10.1805923
8.47934746	8.77771577	8.67028632	8.29054508	8.61019408	8.78039901	8.66314092
9.10010089	9.2516675	9.07633118	9.10686137	9.23998094	9.5909844	9.23142062
9.62029526	9.70979543	9.61002289	9.46385972	9.54015528	9.76928051	9.64050968
9.58697043	9.48539153	9.703897	9.27410652	9.27294647	9.61713075	9.55797318
8.10740452	8.1070695	8.11167364	7.94202038	8.12370814	8.04083773	8.04025268
8.58465681	8.59019469	8.68899316	8.59320039	8.59202612	8.6608596	8.58832753
8.29857845	8.41659346	8.34243817	7.94202038	8.21432776	8.48024445	8.4681985
8.63097409	8.27158165	8.49646091	7.94202038	8.32655381	8.7290708	8.39045684
9.03399713	9.02425359	9.03885224	8.71399884	8.90320943	9.02989561	8.86841549
8.59149001	8.54804873	8.64712707	8.40006484	8.61613381	8.63424196	8.63693735
7.94202038	7.94202038	7.94202038	7.94202038	8.03290927	7.94202038	8.11209808
7.94202038	8.13256811	7.94202038	7.94202038	7.94202038	8.04083773	8.04025268
7.94202038	7.94202038	8.01792659	8.06955177	7.94202038	7.94202038	8.33382036
7.94202038	7.94202038	8.26343518	7.94202038	7.94202038	7.94202038	8.11209808
7.94202038	7.94202038	8.12784483	7.94202038	8.03290927	7.94202038	8.08091502
7.94202038	7.94202038	8.27220495	7.94202038	7.94202038	8.11311024	8.28158842
8.10740452	7.94202038	8.21532682	7.94202038	7.94202038	8.11311024	7.94202038
7.94202038	8.03734617	8.25441514	7.94202038	8.03290927	8.04083773	8.08091502
8.36772811	8.45278715	8.4862189	8.43372484	8.39468096	8.3236991	8.34580159
8.07708059	7.94202038	8.15654265	7.94202038	7.94202038	7.94202038	8.11209808
8.07708059	8.17530815	8.78547493	8.77193129	8.09939256	8.22113719	8.65016993
8.75845579	8.55528676	8.9563417	8.75787272	8.64502675	8.94412736	8.99898665

8.07708059	8.1070695	8.22561138	7.94202038	8.33702644	8.26912345	8.35742871
8.59824737	8.79802576	8.73539395	8.59320039	8.90320943	8.69270206	8.91572339
7.94202038	7.94202038	8.16952947	7.94202038	8.29322195	7.94202038	8.32145144
8.43602752	8.44399288	8.49646091	8.33401955	8.38560745	8.26912345	8.42114647
8.53447614	8.39737747	8.42584292	8.29054508	8.43748325	8.31082828	8.55871023
9.15525946	8.92726495	8.87966661	9.13472088	8.96486929	9.08161271	8.77556743
8.89423533	7.94202038	8.0493556	9.02147745	8.84955684	8.87375142	7.94202038
8.76373905	8.71882229	8.64712707	8.89212713	8.65623231	8.85893352	8.63021825
8.86702363	8.69615031	8.74227158	8.89212713	8.71473604	8.8336168	8.63693735
7.94202038	8.44399288	9.36365533	9.6606808	8.19879645	9.35722606	8.52750204
8.51928974	8.64842518	8.47577098	8.85959209	8.42923445	8.67377702	8.36873067
8.41754917	8.42590555	8.29719551	8.55581336	8.35716075	8.4621927	8.33382036
8.90311126	8.922981	8.89949669	9.15286228	9.18846595	8.87863169	9.20139438
8.58465681	8.6167406	8.67406921	8.9310892	8.49163765	8.53895317	8.88766264
8.2582469	8.35618776	8.28906908	8.58712922	8.35716075	8.48024445	8.40092263
9.11418648	8.93152757	8.87966661	9.32093338	9.27834098	9.013384	9.09425341
8.28567045	8.47827021	8.46510479	8.66180921	8.50611503	8.53895317	8.58832753
8.2582469	8.17530815	8.23553133	8.35400262	8.22901041	8.26912345	8.36873067
8.93346276	8.86520938	8.88538083	9.08783151	9.0431834	8.91667562	9.01556269
8.0375398	8.1070695	8.07346349	8.03221336	7.94202038	8.11311024	8.11209808
7.94202038	7.94202038	7.94202038	7.94202038	7.94202038	10.0602651	7.94202038
7.94202038	8.22758337	8.41996025	7.94202038	8.18225192	8.13953942	7.94202038
7.94202038	7.94202038	8.34243817	7.94202038	9.16169005	7.94202038	7.94202038
8.0375398	8.03734617	7.94202038	7.94202038	8.55396303	8.08174193	8.04025268
7.94202038	7.94202038	7.94202038	9.35104869	7.94202038	7.94202038	7.94202038
8.0375398	8.03734617	7.94202038	7.94202038	8.03290927	8.238011	8.04025268
8.7946794	8.88341633	8.88252866	8.88411302	8.6617645	8.85393357	8.70061788
8.48758145	8.41659346	8.37647518	8.3236257	8.32655381	8.40363382	8.37973269
7.94202038	8.03734617	8.12784483	7.94202038	8.14511998	8.11311024	7.94202038
7.94202038	8.1070695	8.01792659	7.94202038	8.25629903	8.22113719	8.04025268
8.07708059	8.03734617	8.15654265	7.94202038	8.14511998	8.238011	8.13837135
8.95865111	8.71882229	8.61076414	8.72892224	8.93459149	8.85893352	8.60962465
8.4538143	8.48648807	8.36989046	8.41720367	8.41228632	8.48903706	8.23626395
8.6801113	8.64842518	8.56770248	8.70895368	8.68876131	8.78039901	8.45914445
8.65599947	8.64842518	8.01792659	7.94202038	8.21432776	7.94202038	8.30865559
10.2839356	9.53337443	8.8294057	9.41123426	9.53376685	9.4397223	9.35531717
8.4956873	8.39737747	8.36319975	7.94202038	8.31578075	8.26912345	8.04025268
8.57774532	8.39737747	8.39563929	7.94202038	8.34722194	8.40363382	8.18240769
8.80471929	8.8178123	8.77244636	8.30191716	8.4989257	8.49768407	8.4681985
8.0375398	8.0768069	8.12784483	7.94202038	8.18225192	8.18383663	7.94202038
8.15545262	8.15502059	8.29719551	7.94202038	8.25629903	8.31082828	8.13837135
7.94202038	8.72989119	8.27220495	7.94202038	8.65065343	8.80212764	7.94202038
8.15545262	8.19395436	7.94202038	7.94202038	8.12370814	8.20316099	7.94202038
9.08580559	9.07275504	9.00724277	8.93867222	9.06342891	9.11583006	8.9111111
8.07708059	8.1070695	8.07346349	7.94202038	8.07053545	7.94202038	8.08091502
8.19446499	8.19395436	7.94202038	7.94202038	8.03290927	8.13953942	8.04025268

For Review Only

8.5269349	8.7243782	8.58094598	8.35400262	8.43748325	8.48903706	8.48583508
8.15545262	8.21130042	8.21532682	7.94202038	8.16446819	8.25396137	8.04025268
8.0375398	8.03734617	7.94202038	8.14356694	8.09939256	7.94202038	8.11209808
8.0375398	8.03734617	7.94202038	8.16276775	8.09939256	7.94202038	8.11209808
7.94202038	8.25760818	8.09378033	8.33401955	8.16446819	8.26912345	8.21948911
7.94202038	8.34521361	8.12784483	8.97199996	8.4842469	8.44346262	8.63693735
7.94202038	7.94202038	8.47046599	8.03221336	9.03976592	7.94202038	9.31053417
8.13295474	7.94202038	7.94202038	7.94202038	8.65623231	7.94202038	8.18240769
7.94202038	8.13256811	7.94202038	7.94202038	8.37633837	8.04083773	8.08091502
8.21184601	8.39737747	8.14271065	8.43372484	8.59814185	8.238011	8.25212089
8.57774532	8.5257647	8.53558024	8.58099653	8.6617645	8.62741336	8.58106002
9.12115304	9.27567709	9.1101684	9.268773	9.33848138	9.40350064	9.33166501
8.83408357	7.94202038	8.28074391	8.58099653	8.69926783	7.94202038	7.94202038
11.9221207	8.21130042	9.34442777	11.9210329	12.6158123	10.0302756	9.72409115
7.94202038	7.94202038	7.94202038	9.00060475	7.94202038	7.94202038	7.94202038
8.19446499	7.94202038	7.94202038	8.66721204	8.21432776	8.11311024	7.94202038
7.94202038	7.94202038	8.0493556	8.0981884	8.09939256	8.04083773	7.94202038
8.15545262	8.1070695	8.16952947	8.34413843	8.25629903	8.31082828	8.13837135
8.40802503	8.24297584	8.42584292	8.56853934	8.46141416	8.58480593	8.40092263
8.17578116	8.0768069	8.28074391	8.3236257	8.31578075	8.16281037	8.23626395
8.15545262	8.22758337	8.28074391	8.37303645	8.34722194	8.18383663	8.18240769

For Review Only

For Review Only

For Review Only

For Review Only

For Review Only

For Review Only

For Review Only

rCHI_ND_4	rCHI_ND_5	rCHI_HSD_1	rCHI_HSD_2	rCHI_HSD_3	rCHI_HSD_4	rCHI_HSD_5
8.10492678	7.94202038	8.04006396	7.94202038	8.07485015	8.1690622	7.94202038
8.76714329	8.614598	8.7171627	8.75934912	8.98234723	8.86336706	8.76997441
8.30552144	8.1802153	8.26657194	7.94202038	8.28001728	8.4057984	8.37227285
8.56146664	8.51259563	8.69309818	8.45583809	8.69670469	8.75688096	8.76402462
8.89786163	8.81758539	9.01358949	8.92775649	8.88777755	9.14734464	9.06713428
8.66927181	8.52222143	8.66816339	8.65857523	8.56740199	8.71351422	8.6474848
9.26823193	8.90440002	9.2158798	8.93222172	9.08887444	9.2931257	9.23867629
8.70895943	8.60638812	8.7230524	8.65857523	8.63835876	8.67409832	8.78464554
8.61450935	8.60638812	8.71122362	8.7078051	8.65640463	8.73242017	8.72096847
9.42752478	9.4021017	9.43165359	9.42541909	9.4745037	9.58555814	9.51519896
8.74652098	8.72679661	8.73468806	8.72540048	8.7859351	8.79783789	8.79903816
9.09056828	8.94200194	8.92758502	9.13834522	8.99779849	9.1396652	9.15661904
9.40979665	9.19925204	9.110809	9.39799655	9.19094247	9.46455259	9.45228023
9.27981179	9.11374165	9.01358949	9.23011822	9.04627135	9.25942617	9.32925087
7.94202038	8.09277065	7.94202038	7.94202038	8.1903034	8.30751401	8.11821235
8.43753292	8.33266479	8.50182767	8.42780437	8.56051992	8.78055669	8.69842918
7.94202038	7.94202038	8.16108418	7.94202038	7.94202038	8.53906697	8.3350262
7.94202038	7.94202038	7.94202038	8.180858	7.94202038	8.32123356	8.24681635
7.94202038	8.07258878	8.11177159	8.13710458	8.10467427	8.27836321	8.21093588
8.07505643	8.20289077	7.94202038	8.08001825	7.94202038	8.38334052	8.20118055
7.94202038	8.22372887	7.94202038	8.11100039	7.94202038	8.35963737	8.20118055
8.26731677	8.01742123	7.94202038	7.94202038	7.94202038	8.61024135	8.50963442
7.94202038	8.01742123	7.94202038	7.94202038	7.94202038	8.45754271	8.34148175
7.94202038	7.94202038	8.04006396	7.94202038	8.03596179	8.26277639	8.13230979
7.94202038	8.42265059	8.67448351	7.94202038	7.94202038	9.19221373	8.43822685
8.10492678	7.94202038	8.08064829	8.0396182	7.94202038	8.34725093	7.94202038
8.63942036	8.51742971	8.87153946	8.82294071	8.53214044	8.83671866	8.86202446
8.97605942	8.95762749	9.17517125	9.22032293	9.03162766	9.05512294	9.17916173
9.31101636	9.37028201	9.67563339	9.75543166	9.2278081	9.61082089	9.95345447
8.74652098	8.63466714	8.74043612	8.72540048	8.61979484	8.77470763	8.77293162
8.56835892	8.46698539	8.6289201	8.66492822	8.54650638	8.71987215	8.66832759
8.47949328	8.44532602	8.50182767	8.4909059	8.45395944	8.54741483	8.50516831
8.7306635	8.55013753	8.76851767	8.68975667	8.7022727	8.78055669	8.8047204
[truncated: 324,600 more chars]
