# Supplementary material for: High-salt diet induces microbiome dysregulation, neuroinflammation and anxiety in the chronic period after mild repetitive closed head injury in adolescent mice
Source: Brain Commun. 2024 May 3;6(4):fcae147. doi: 10.1093/braincomms/fcae147 (PMC11264151; doi:10.1093/braincomms/fcae147)
Supplement: fcae147_Supplementary_Data [file fcae147_supplementary_data.zip › Revision_3_manuscript.pdf]

OXFORD UNIVERSITY PRESS | Brain Communications

High salt diet induces microbiome dysregulation, neuroinflammation, and anxiety in the chronic period after mild repetitive closed head injury in adolescent mice.

Journal:	*Brain Communications*
Manuscript ID	BRAINCOM-2023-443.R3
Manuscript Type:	Original Article
Date Submitted by the Author:	26-Jan-2024
Complete List of Authors:	Izzy, Saef; Brigham and Women's Hospital, Neurology; Harvard Medical School; Football Players Health Study at Harvard University; Massachusetts General Hospital<br>Yahya, Taha; Brigham and Women’s Hospital, Department of Neurology<br>Albastaki, Omar; Brigham and Women’s Hospital, Department of Neurology<br>Cao, Tian; Brigham and Women’s Hospital, Department of Neurology<br>Schwerdtfeger, Luke; Brigham and Women's Hospital, Neurology<br>Abou-El-Hassan, Hadi; Brigham and Women’s Hospital, Department of Neurology<br>Chopra, Kusha; Massachusetts General Hospital<br>Ekwudo, Millicent N.; Brigham and Women's Hospital, Neurology<br>Kurdeikaite, Ugne; Brigham and Women's Hospital, Neurology<br>Verissimo, Isabelly M.; Brigham and Women's Hospital, Neurology<br>LeServe, Danielle S.; Brigham and Women's Hospital, Neurology<br>Lanser, Toby B.; Brigham and Women's Hospital, Neurology<br>Aronchik, Michael; Brigham and Women's Hospital, Neurology<br>Oliveira, Marilia G.; Brigham and Women's Hospital, Neurology<br>Moreira, Thais; Harvard Medical School; Brigham and Women's Hospital, Neurology<br>Rezende, Rafael Machado; Harvard Medical School; Brigham and Women's Hospital, Neurology<br>El Khoury, Joseph; Massachusetts General Hospital; Harvard Medical School<br>Cox, Laura; Brigham and Women's Hospital; Harvard Medical School<br>Weiner, Howard L.; Harvard Medical School; Harvard Medical School<br>Zafonte, Ross; Harvard Medical School; Football Players Health Study at Harvard University; Harvard Medical School<br>Whalen, Michael ; Harvard Medical School; Massachusetts General Hospital, Department of Pediatrics
Keywords:	Neuroinflammation, Closed head injury, microbiome, Salt diet, Anxiety, Microglia

SCHOLARONE™
Manuscripts

High salt diet induces microbiome dysregulation, neuroinflammation, and anxiety in the chronic period after mild repetitive closed head injury in adolescent mice.

Saef Izzy[1,2, 3, 4, 5], Taha Yahya[1,2], Omar Albastaki[1,2], Tian Cao[1,2], Luke A. Schwerdtfeger[2], Hadi Abou-El-Hassan[2], Kusha Chopra[2], Millicent N. Ekwudo[2], Ugne Kurdeikaite[2], Isabelly M. Verissimo[2], Danielle S. LeServe[2], Toby B. Lanser[2], Michael Aronchik[2], Marilia G. Oliveira[2], Thais Moreira[2,3], Rafael Machado Rezende [2,3], Joseph El Khoury[3,5,6], Laura M. Cox[2,3], Howard L. Weiner[2, 3], Ross Zafonte[3,4,7]*, Michael J. Whalen[3, 4, 8]*

[1] Department of Neurology, Divisions of Stroke, Cerebrovascular , and Critical Care Neurology, Brigham and Women's Hospital, Boston, MA, USA.

[2] Ann Romney Center for Neurologic Diseases, Brigham & Women's Hospital, Harvard Medical School, Boston, MA, USA.

[3] Harvard Medical School, Boston, MA, USA.

[4] The Football Players Health Study at Harvard University, Boston, MA, USA.

[5] Center for Immunology & Inflammatory Diseases, Massachusetts General Hospital, Boston, MA, USA.

[6] Department of Medicine, Division of Infectious Diseases, Massachusetts General Hospital, Boston, MA, USA.

[7] Spaulding Rehabilitation Hospital, Department of Physical Medicine and Rehabilitation, Massachusetts General Hospital, Brigham and Women's Hospital, Boston, MA, USA

[8] Department of Pediatrics, Massachusetts General Hospital, Boston, MA 02114, USA.

*Co-senior co-authors

Corresponding author:
Michael J. Whalen
Department of Pediatrics, Massachusetts General Hospital, Boston, MA 02114, USA.
mwhalen@mgh.harvard.edu

Word Count: 4072
Figures: 4
Supplementary Tables: 5
Supplementary Figures 3

References: 97

For Review Only

Key words: Concussion, salt diet, adolescent, traumatic brain injury, microglia, neuroinflammation, microbiome.

Abstract

Objective: The associations between human concussions and subsequent sequelae of chronic neuropsychiatric and cardiovascular such as hypertension have been reported; however, little is known about the underlying biological processes. We hypothesized that dietary changes, including a high salt diet (HSD), disrupt the bidirectional gut-brain axis, resulting in worsening neuroinflammation and emergence of cardiovascular and behavioral phenotypes in the chronic period after repetitive closed head injury (rCHI) in adolescent mice.

Methods: Adolescent mice were subjected to three daily closed head injuries, recovered for 12 weeks, and then maintained on HSD or normal diet (ND) for an additional 12 weeks. Experimental endpoints were hemodynamics, behavior, microglial gene expression (bulk RNA sequencing), brain inflammation (brain tissue qPCR), and microbiome diversity (16S RNA sequencing).

Results: HSD did not affect systemic blood pressure or heart rate in Sham or rCHI mice. HSD increased anxiety-like behavior in rCHI mice compared to rCHI with ND and Sham injury + HSD. Increased anxiety in HSD-fed rCHI mice was associated with microgliosis and a proinflammatory microglial transcriptomic signature, including upregulation in interferon-gamma (IFN-γ), interferon beta (IFN- β), and oxidative stress related pathways. Accordingly, we found upregulation of tumor necrosis factor-alpha (TNF-α) and IFN-γ mRNA in the brain tissue of rCHI HSD mice. HSD had a larger effect on the gut microbiome composition than rCHI. Increases in gut microbes in the families Lachnospiraceae, Erysipelotrichachaceae, and Clostridiaceae were positively correlated with anxiety-like behaviors. In contrast, Muribaculaceae, Acholeplasmataceae, and Lactobacillaceae were negatively correlated with anxiety in TBI HSD-fed mice, a time-dependent effect.

Interpretations: The findings suggest that HSD, administered after a recovery period, may affect neurologic outcomes following mild repetitive head injury, including the development of anxiety. This

For Review Only

effect was linked to microbiome dysregulation and an exacerbation of microglial inflammation, which may be physiological targets to prevent behavioral sequelae in the chronic period after repetitive mild TBIs. The data suggest an important contribution of diet in determining long-term outcomes after repetitive mild TBI.

For Review Only

Introduction

Concussions continue to be a prominent public health concern, with an estimated incidence of 1.6-3.8 million in the United States annually.[1] Younger populations within the 14–19 year old age group experience the highest rates of concussion and nearly all athletic endeavors have some risk of concussive injury.[1-3] Recent large-scale studies of the general population, American-style football players, and military veterans demonstrated that prior TBI was associated with later development of neurological and psychiatric comorbidities and cardiovascular conditions, even in young patients.[4-7] These findings indicate that brain injury may trigger progressive degenerative processes affecting the health of the brain and other organ systems, and have become a central focus of public attention. [8-11]

Our work and others have demonstrated associations between TBI and higher prevalence of subsequent hypertension[4,12-14] and psychiatric diseases such as anxiety.[4] However, the pathophysiological mechanisms that underlie the progressive nature of TBI are still not clear. TBI initiates a series of neuropathological molecular and biochemical secondary injury sequelae that are long lasting and involve disruption of several biological pathways, including neuroinflammation.[15] Other possible explanations include behavioral and lifestyle changes such as unhealthy diet and disruption to the bidirectional brain-gut axis.[16-19] Increasing evidence suggests that sustained excess salt intake may affect brain health, beyond the well-recognized risk of hypertension.[20]

Recent preclinical studies show that high salt diet (HSD) promotes cognitive impairment and suggest a gut-initiated adaptive immune response compromising brain function.[21,22] High sodium intake is associated with increased inflammatory and stress responses and organ damage in patients.[23] Increased salt consumption promotes pro-inflammatory interleukin 17 (IL17)-producing helper T (T_H17) cell differentiation[24,25] and inhibits the anti-inflammatory functions of regulatory T cells (FoxP3+).[26] In addition, high sodium intake causes macrophage/microglia polarization to shift towards a classically activated proinflammatory phenotype [27,28] and promotes the production of pro-inflammatory cytokines by myeloid cells in experimental models.[29,30] There is growing evidence to

For Review Only

support the HSD gut initiated pro-inflammatory properties in the brain. In a mouse model of cerebral ischemia, HSD promoted proinflammatory microglia polarization[31] and exacerbated cortical blood brain barrier disruption.[32] A HSD has been reported to alter the gut microbiome and their short chain fatty acids in rats, and to preferentially populate the gut with microbial species associated with hypertension.[33] A growing body of literature has documented the close association between alterations in the gut microbiome with progression of neurodegenerative diseases such as Parkinson's[34], ALS [35], Alzheimer's disease[36], and TBI.[37] However, the impact of HSD on the neuroinflammatory response, microbiome, behavioral and cardiovascular outcomes after mild TBI has not been explored in preclinical concussion models.

Here, we tested whether HSD elevates blood pressure, alters microbiome diversity, worsens neuroinflammation, and alters behavioral outcomes after repetitive closed head injury in mice. To investigate this, we injured adolescent mice using an established model of repetitive closed head injury (rCHI) and HSD vs. a normal diet (ND) maintained for 12 weeks.

Results

HSD did not alter blood pressure or heart rate but increased anxiety-like behavior after repetitive mild CHI.

Previous studies have shown an association of TBI in American style football with development of hypertension amongst young athletes.[4,38,39] **Figure 1A** shows the experimental design for rCHI and HSD to test this association experimentally. HSD mice gained less weight over time when compared to mice on a ND, independent of rCHI (**Figure 1B**), however no significant changes in systolic or diastolic blood pressure or heart rate were observed with HSD compared with ND groups in both sham and rCHI (**Figure 1C**). Compared to other groups, mice in the rCHI/HSD group spent significantly less time in the open arm of the elevated plus maze (**Figure 1D, Supplementary Fig.**

For Review Only

1A), an anxiety-like phenotype, at 12 weeks after initiation of the HSD. This effect was not observed at earlier time points after rCHI. The consumption of HSD following rCHI did not affect spatial learning and memory as shown by the Morris water maze (MWM) and probe trial testing (**Figure 1E, Supplementary Fig. 1B, C**). In addition, HSD did not cause rotarod deficits or induce general locomotor and exploration deficits (assessed by an open field test) at all timepoints tested after rCHI (**Figure 1F, G, Supplementary Fig. 1D, E**). Altogether, our data show that the consumption of HSD following rCHI is associated with worsening anxiety like behavior in the chronic period after rCHI.

High salt diet exacerbates chronic microglial neuroinflammatory responses following repetitive closed head injury

Microglia are key players in the neuroinflammatory response to TBI, and their chronic activation after injury can lead to neurological dysfunction and neurodegeneration[15,40]. HSD can shift macrophages/microglia toward classically activated proinflammatory phenotypes[28], yet the impact of HSD on the brain's inflammatory response to rCHI is unknown, an important question given the effect of interleukin-1 signaling on anxiety and cognitive dysfunction in a 3HD adolescent rCHI model[41]. The HSD/rCHI group had increased microgliosis in cortex (**Figure 2A**), hippocampus (**Figure 2B-D**), and amygdala (**Figure 2E**) compared to ND/rCHI and sham groups. Therefore, we investigated the impact of HSD administration on the microglial inflammatory transcriptomic profile following rCHI and Sham injury. Microglia single-cell suspensions were obtained from the mouse brains at 12 weeks after HSD administration and analyzed using bulk RNA Sequencing **(Figure 3A, Supplementary Fig. 3A).** Normalized expression counts for all genes passing quality metrics are shown in **Supplementary Table 1**. Several established microglia markers were highly expressed in all groups, such as *Cx3cr1, Hexb, P2ry12, and Tmem119,* whereas we found minimal expression of non-microglia markers[42] **(Supplementary Fig. 3B).** We compared the HSD fed mice (Sham and rCHI) and rCHI mice fed with ND to Sham ND controls to define unique differentially expressed genes (DEGs) (Figure 3B, Supplementary Table 2). To detect unique transcriptomic patterns in Sham HSD, rCHI HSD, and rCHI

ND, we plotted the unique DEGs in each group as clusters in a heatmap **(Figure 3C, Supplementary Table 3).** We found that each cluster was enriched for distinct Gene Ontology (GO) terms. The cluster of unique DEGs in rCHI HSD were related to type I interferon production (*Cactin, Crebbp, Syk, Gbp4)*, antigen processing and presentation (*Cd1d2, H2-K1, H2-Q1*), response to *TNF* (*Cxcl16, Pias4, Syk*), regulation of phagocytosis (*Dnm2, Ptprj, Siglece, Syt11*), endothelial migration (*Acvrl1, Bsg, Lgmn, Loxl2*), synaptic organization (*Il10ra, Ptprf, Ptpro, Slc7a11, Ube3a, Ywhaz*) and leukocyte migration (*Cxcl16, Emilin1, Itga4, Mmp14, Mmp9, Ptpro, Spp1*). The cluster of unique DEGs in the Sham HSD group were associated with regulation of neuron death (*Cd200ra, Csf1, Egln2, Gclc, Hdac4, Hspd1, Jak2, Mag, Parp1, Rest, Tnfrsf1a)*, ROS metabolic processes (*Acox1, Eif5a, Grb2, Hdac4, Hspd1, Ier3, and Ogt*), regulation of *IL-17* production (*Jak2, Parp1*), regulation of apoptotic signaling pathways (*Bcap31, Gclc, Ier3, Ltbr, Map2k5, Ptpn2, Src*), and regulation of ERK1 and ERK2 cascades (*Acta2, Prkd2, Prtm5, Ptpn2, Ptpn6, Rapgef2*). The cluster of unique DEGs in rCHI ND mice were mainly involved in IL-1B production (*Lilra5, Nod1, P2rx7, Tnfaip8*), and positive regulation of kinase activity (*Axl, Ccnd2, Fgfr1, Ntrk2, Pdcd10, Rac1, Tom1l1*).

To further evaluate unique and shared microglial pathways altered by HSD, we performed gene set enrichment analysis (GSEA) of the 4 groups using GO biological process (GOBP) pathways. Compared to Sham ND, HSD in both Sham and rCHI groups was associated with a significant upregulation of immune-related pathways including immune effector processes, antigen processing and presentation, pathways involved in the regulation of hydrolase activity, and apoptosis **(Figure 3D)**. However, we also found that the rCHI HSD was uniquely associated with a significant upregulation of other immune mediated pathways (such as *IFN-γ* and cytokine responses), microglia regulation pathways (purinergic receptor signaling and cell motility), and vascular endothelial related pathways (vascular endothelial growth factor, endothelial cell growth and development).

We also assessed the specific effect of HSD on the microglial transcriptomic profile in the setting of rCHI by comparing rCHI HSD with rCHI ND mice. We found a total of 668 DEGs between

For Review Only

these two groups **(Figure 3E. Supplementary Table 4).** Compared to rCHI ND controls, rCHI HSD was associated with a significant upregulation of several pathways related to inflammatory processes involved in innate and adaptive immune responses (responses to IFN-γ, IFN-α, and IFN-β)**,** oxidative stress related pathways (hydrogen peroxide catabolic process and ROS metabolic process) and cytokine response/stimulus related pathways (*IL-12* production, and cytokine production) (**Figure 3F**). In line with these findings, IPA analysis of the DEGs in rCHI HSD compared to rCHI ND revealed several top upstream regulators ($P < 0.05$ and $|Z\text{-score}| \geq 2$) predominately involved in inflammation and immune response (*Ifng, Ifnb1, Ifnar, Pnpt1*) **(Figure 3G)**. However, none of these regulators were observed in IPA analysis of the top upstream regulators for any of the groups compared to Sham ND (**Supplementary Table 5**).

In addition to the microglial RNA-seq results, we also found significant increases in proinflammatory cytokine mRNA including *Ifng* in the brain tissue of rCHI mice fed with HSD compared to other groups **(Figure 3H).** *Tnfa* was also similarly increased in rCHI HSD mice compared to the rest of the groups. We also found increased *Il1b* and decreased *Tgfb1* in both rCHI groups compared to the Sham groups, independent of diet. Altogether, our findings demonstrate that the consumption of HSD following rCHI is associated with chronic microgliosis and alteration of the microglial transcriptome towards a more proinflammatory profile.

High salt diet induces microbiome alterations that correlate with anxiety following repetitive TBI

HSD has been shown to change the composition and diversity of the gut microbiome and increase inflammatory and stress responses in the brain.[27,28] Altered gut-brain signaling could also contribute to development of anxiety and other mood disorders[23,43] but its role in rCHI-induced anxiety is unknown. To address this gap, we performed 16S rRNA sequencing on stool samples collected longitudinally at days 0, 1, 3, 7, 14, 30, 45, 60, 75, and 90 after HSD administration (beginning 12

For Review Only

weeks post-rCHI) (**Figure 4A**). Analysis of β-diversity using weighted UniFrac distances demonstrated overall microbial community structure differences between HSD and ND in both sham and rCHI groups (**Figure 4B**; $p = 0.001$), however, no significant differences were found in the overall microbial community between rCHI and sham groups which received the same ND and HSD (**Supplementary Fig. 3C**). A weighted ADONIS test showed that HSD was the largest contributor to microbiome variance, with timepoint of stool collection also contributing significantly (**Figure 4C**).

At the amplicon variant (ASV) level, we found a total of 23 microbes consistently (≥3 time points) altered between HSD vs. ND, sham vs. rCHI, or both, across the 90 days sampled (**Figure 4D**). In both sham and rCHI mice, HSD decreased *Lachnoclostridium dorea,* and a member of the *Ruminococcaceae* family*,* and increased *Lachnospiraceae* family members, *Erysipelotrichaceae ileibacterium*, and a *Akkermansia.* The *Muribaculaceae* family is one of the most prevalent taxa in mice and numerous *Muribaculaceae* ASVs were both decreased and increased in HSD groups compared to ND controls. Minimal bacterial species were altered in rCHI mice compared to the sham groups, independent of diet. No microbes were increased in rCHI compared to sham at more than two time-points. HSD rCHI had additional microbial alterations compared to both sham ND and rCHI ND, including increased *Prevotellaceae*, two members of *Lachnospiraceae*, and *Muribaculaceae*. Of note, *Prevotellaceae* was increased in rCHI HSD compared to both Sham ND and rCHI ND at 7/10 timepoints, an effect that was increased from 5/10 timepoints in the Sham HSD group compared to Sham ND (**Figure 4D**).

While previous studies suggested high dietary salt intake as a possible behavior modifier [44,45], the relationship between changes in gut microbiota and the development of anxiety-like behaviors following both repetitive concussive head injury (rCHI) and a high-salt diet (HSD) remains largely unexplored. To address this, we performed Spearman correlations of microbiota relative abundance with elevated plus maze measured at 12 weeks across all groups. At the ASV level, we found several microbes significantly correlated with anxiety-like behaviors at 2 or more independent microbiota

For Review Only

sampling time points. Eight selected microbes were positively correlated with anxiety-like behaviors at multiple time points, including *Erysipelotrichaceae* family members (*Turibacter* and *Illebacterium* genera), *Ruminococcaceae, Muribaculaceae, Lachnospiraceae, Clostridiaceae, Tannerellaceae* and *Butyricicoccaceae*. Seven selected microbes were also negatively correlated with anxiety-like behaviors, all from the families *Muribaculaceae*, *Acholeplasmataceae*, *Lachnospireaceae,* and *Lactobacillaceae* (**Figure 4E**). Relative abundance plots over the course of the 3-month experiment show increased levels of microbes positively correlated with anxiety-like behaviors, including *Ruminococcaceae, Erysipelotrichaceae, Clostridiaceae, and Lachnospiraceae* family members in HSD groups (**Figure 4F, Supplementary. Figure 3D**). Plots of microbes negatively correlated with anxiety-like behaviors show elevated levels of multiple *Lactobacillaceae*, *Lachnospiraceae, Acholeplasmataceae*, *and Muribaculaceae* members in ND groups (**Figure 4F, Supplementary Figure 2D**). The increase in two *Erysipelotrichaceae* after rCHI HSD at 15-30 days prior to development of anxiety-like behaviors (**Figure 4F, Supplementary Figure 3D**) could have contributed to the findings shown in **Figure 1D** in which mice that received HSD following rCHI have increased anxiety-like behaviors.

To investigate microbial functional changes based on injury and diet, we performed predicted metagenomic analysis using PICRUSt2[46] based on third level KEGG pathways **(Figure 4G).** LefSe testing of the predicted KEGG pathways based on the metagenomic content of the microbiota samples revealed alterations in pathways related to amino acid biosynthesis, carbohydrate metabolism, and fatty acid synthesis and processing in the rCHI HSD mice at 90 days, which were not seen in sham HSD or rCHI ND groups when compared to the baseline sham ND group. However, most of the predicted metabolic changes were associated with HSD, independent of injury. Mice that received HSD showed numerous decreased metagenomic pathways related to drug metabolism, bile acid biosynthesis pathways, glutathione metabolism, and sugar metabolism, among others, relative to ND mice, regardless of injury. Moreover, several metagenomic pathways involved in insulin signaling,

For Review Only

fatty acid production, carbohydrate metabolism, and biosynthesis and metabolism of amino acids (such as tryptophan) were upregulated consistently over time in mice that received HSD, independent of rCHI (**Figure 4G**).

We also performed 16S rRNA sequencing on cecum samples collected at 90 days post-HSD administration (150 days post-rCHI). Consistent with stool samples, analysis of β-diversity using weighted UniFrac distances demonstrated overall microbial community structure differences between HSD and ND in both sham and rCHI groups ($p < 0.05$), but no differences in the overall microbial community between rCHI and sham groups, independent of diet (**Supplementary Figure 4A**). A weighted ADONIS test showed HSD to be the largest contributor to microbiome variance, whereas the contribution of rCHI was insignificant (**Supplementary Figure 4B**). We also found similar changes in microbiota relative abundance in the cecum samples compared to stool samples **(Supplementary Figure 4C).** LEfSe testing showed that HSD administration decreased several members of *Muribaculaceae and Lactobacillaceae,* and increased multiple *Lachnospiraceae* family members, a *Prevotellaceae,* and *Akkermansia* (**Supplementary Figure 4C**). HSD fed mice that received rCHI had further microbial changes compared to both ND groups (Sham and rCHI), such as increases in *Marinifilaceae Odoribacter, Oscillospiraceae,* and two *Lachnospiraceae* family members.

Together these data show a strong effect of HSD on microbiota composition at two anatomical sites, independent of rCHI, and unique alteration in the short chain fatty acid (SCFA)-producing microbes that were associated with increased anxiety phenotype in rCHI/HSD mice in the chronic period post-injury.

Discussion

There is increasing evidence for risk of chronic neurological, psychiatric, and cardiovascular comorbidities after TBI from recent human studies, however the mechanisms driving these outcomes are still largely unknown.[12] In this study, we aimed to understand the impact of HSD on sequelae of

repetitive mild TBI in adolescent mice, including changes in hemodynamics, behavior, brain inflammation, and the gut microbiome. We found that HSD interacts with rCHI to produce an anxiety phenotype, and that HSD (but not rCHI) strikingly altered the gut microbiome. These data suggest that environmental factors, such as diet, can interact with and modify the outcome of rCHI in adolescence even when such factors are presented well into adulthood (e.g., 12 weeks after injury). The data suggest that the injured adolescent brain is primed for an interaction with HSD that leads to an anxiety phenotype and increased microglial inflammation that is not induced by rCHI alone.

Excessive salt consumption has been recognized in humans as a risk factor for hypertension.[47] However, in our study we did not find differences in blood pressure and heart rate measurements between HSD and ND groups. These negative findings could be attributed to the relatively young age of the mice and to healthy renal compensatory mechanisms. The impact of a high salt diet in older mice and in mice with underlying comorbidities requires further investigation.

The manifestation of anxiety disorders after TBI is a strong predictor of personal, social, and work dysfunction[48]; nonetheless, mechanisms responsible for development of post-traumatic anxiety are largely unexplored and remain poorly understood. There is some evidence to suggest that consuming too much salt is a potential behavior modifier and may increase the risk of stress and anxiety.[43,49,50] Some possible explanations include that high salt intake can disrupt the balance of electrolytes and fluids in the body, which can lead to changes in mood and behavior. Electrolyte imbalances have been linked to increased anxiety and depression in some individuals.[51] Additionally, salt consumption may worsen neuroinflammation and oxidative stress.[21,23] Neuroinflammation, most specifically microglia activation, has been shown to contribute to a variety of neurological and psychiatric disorders, including anxiety, depression, and cognitive impairment.[52,53] In addition to head injury, prior studies reported that a high-salt diet can drive macrophages/microglia towards a proinflammatory phenotype, amplifying an inflammatory response.[28-30] Our rCHI HSD mice had chronic microglial activation in the cortex, hippocampus, and amygdala, the latter recognized as a

For Review Only

brain region involved in the interpretation of environmental threats, and play a role in generating fear and anxiety-like behaviors.[54,55]

Previous studies have shown that a HSD activates the NFAT5 transcription factor in proinflammatory macrophages/microglia, which can trigger the release of inducible nitric oxide synthase (iNOS)-dependent nitric oxide (NO) and pro-inflammatory cytokines such as tumor necrosis factor alpha (*TNF-α*).[56,57] A high salt diet has been shown to impair T cell function, which can increase the production of IFN-γ and impair the immune response.[26,58] Similarly, we found that HSD activated microglial pro-inflammatory pathways such as TNF-α and IFN-γ in rCHI mice and significantly increased *Tnfa* and *Ifng* mRNAs in the brain tissue of rCHI HSD compared to other groups. The observation that salt induced a pro-inflammatory microglia polarization in rCHI HSD group is of translational interest, since post-TBI inflammation is one of the most frequently addressed therapeutic targets following experimental injury. In addition to microglia activation, clinical studies have demonstrated that increases in the serum levels of TNF-α and IFN-γ are associated with increased anxiety symptoms in general anxiety disorder patients.[59] TNF-α activates the hypothalamic-pituitary-adrenal (HPA) axis, leading to the production of cortisol, a stress hormone that is also involved in anxiety.[60,61] Both TNF-α and IFN-γ have also been shown to alter neurotransmitter levels in the brain, including serotonin and dopamine, and to reduce the activity of the serotonin transporter, leading to decreased serotonin levels in the brain which is associated with increased anxiety.[62,63] IFN-γ can also activate microglia, leading to hippocampal neuronal network dysfunction, depression like behavior and cognitive decline. [64,65]

The gut microbiome is altered in response to central nervous system injury[66,67], and manipulation of gut resident microbes is emerging as potential therapy for TBI.[68] The gut microbiota can affect microglia and inflammatory responses in homeostasis and in disease. [69,70] The majority of the changes that we observed in microbial community structure were driven by HSD treatment, which showed consistent alterations at multiple timepoints. HSD decreased *Lachnoclostridium dorea*, a

microbe shown to be decreased in multiple sclerosis.[71,72] HSD also increased numerous microbes at multiple timepoints, including *Lachnospiraceae*, *Ileibacterium* (from the *Erysipelotrichaceae family)*, *Akkermansia*, and *Prevotellaceae.* In our study, rCHI exacerbated microbiome changes, however, without dietary alterations there was not a consistent or robust effect.

In rCHI HSD mice, multiple microbial species were positively correlated with anxiety-like behavior, including a *Lachnospiraceae* member, two *Erysipelotrichaceae (Turibacter and Illebacterium genera), a Clostridium (genus of the Clostridiaceae family),* and a *Butyricicoccaceae* member, which are all SCFA producers.[73,74] Members of family *Lachnospiraceae* were elevated in rCHI ND mice[75], mirroring the increase in a *Lachnospiraceae* member in rCHI HSD mice. Numerous strains of *Lachnospiraceae* produce butyrate, a SCFA with putative immunomodulatory and anti-inflammatory functions[76,77] and can induce T regulatory cells.[78] However, a subset of *Lachnospiraceae* strains adhere to the mucosa and can induce a Th17 response[79], one potential pathway that *Lachnospiraceae* could harness to contribute to the anxiety phenotype in our study. Further experimental studies of these *Lachnospiraceae* ASVs are warranted to explore their potential detrimental effects observed in our study. The *Erysipelotrichaceae* members that we showed to be positively correlated with anxiety-like behavior spiked in abundance in the HSD groups, but more so in rCHI HSD fed mice, prior to the onset of anxiety-like behaviors. Species within the *Erysipelotrichaceae* family that can contribute to anxiety[80], CNS inflammation[81] are known to influence systemic inflammatory conditions like colitis[82] and are highly responsive to dietary changes.[83] The spike in abundance of *Erysipelotrichaceae* family 15+ days before the development of anxiety in our study suggests these microbes may play a potential role in driving behavioral changes.

This intricate microbiota-gut-brain communication system exerts regulatory effects through bacterial metabolites, the modulation of immune activity, and interactions with enteric and vagus nerve terminals to maintain homeostasis. We found unique alterations in the gut microbiome of HSD mice, influenced by rCHI, which could affect mood and anxiety by the production of immunogenic

For Review Only

metabolites, neurotransmitters, or by signaling via the vagus nerve.[84] Based on PICRUSt2, we found alterations in microbiota functional genes modulated by HSD. In particular, we found alterations in several predicted microbial pathways reported in the literature to associated with anxiety such as amino acid metabolic pathways including tryptophan and insulin signaling[85], and glutathione, an antioxidant, which has been shown to be regulated by the gut microbiota.[80,86,87] Synthesis of secondary bile acids were also decreased, a pathway previously shown to play an anti-inflammatory role[88] and specifically decrease activation in proinflammatory microglia profiles in animal models of multiple sclerosis.[89] Our data also show alterations of SCFA-producing microbes in rCHI HSD mice that may be associated with anxiety. It is unclear whether SCFA is increased in mice with HSD and rCHI as this was not measured in our study, an important route of future investigation.

Taken together, we demonstrated unique alterations in the gut microbiome of HSD mice, an effect influenced only slightly by repeated TBI. Importantly, we found correlations between numerous microbes previously reported to alter microglia function with anxiety like behaviors in the rCHI HSD mice and identified bacterial families potentially influencing anxiety-like behaviors after TBI, suggesting a way forward for targeted perturbations of gut microbiome and other microbiome associated metabolites to improve post-TBI anxiety. Further studies are warranted to understand the mechanisms by which microbiota could alter behavioral outcomes and modulate microglia which can lead to identification of novel bacteria-derived therapeutics.

Conclusions

The findings suggest that HSD may affect neurologic outcomes following mild repetitive head injury, including development of anxiety. This effect was linked to microbiome dysregulation and an exacerbation of microglial inflammation, which may be physiologic targets to prevent post-injury sequelae. Importantly, the HSD was administered after a recovery period, suggesting that diet may play a role in determining long-term TBI outcomes.

For Review Only

Materials and Methods

Experimental animals

Studies were performed using 38-day old male C57/BL6J mice (Stock #000664, Jackson Laboratories). All procedures were performed in accordance with the NIH Guide for Care and Use of Laboratory Animals and followed protocols approved by the MGH Institutional Animal Care and Use Committee. Mice had access to food and water ad libitum and were housed on a 12-hour day-night cycle in laminar flow racks in a temperature-controlled room (25°C). Investigators were blinded to study groups in all experiments. Mice were randomized to sham or rCHI at 38 (+/- 3) days of age. Sham-injured and rCHI mice were housed in the same cage. Each cage had 2 sham and 2 rCHI mice.

High salt diet

High salt diet (HSD) was introduced sixty days after rCHI. There were four experimental groups: Sham mice receiving normal diet (ND), Sham mice receiving HSD, rCHI mice receiving ND, and rCHI mice receiving HSD. ND consisted of normal chow (0.5% NaCl) and tap water ad libitum. HSD consisted of sodium-rich chow (8% NaCl) and tap water containing 1% NaCl ad libitum as previously described.[21] The HSD was sustained for 12 weeks.

Repetitive Closed head injury model (rCHI)

A modified closed head injury (CHI) model was used as previously described.[90] Mice were anaesthetized with 2.5% isoflourane in 70% N_2O and 30% O_2 for 90 seconds. Anesthetized mice were placed on a taught KimWipe napkin and grasped by the tail. The head was placed under a 42-inch long, 9/16-inch diameter brass guide tube. A 1/2-inch diameter 53g lead cylindrical weight with a flat, unbuffered surface was dropped onto dorsal aspect of the skull directly above the right (days 1,

For Review Only

3) or left (day 2) ear between the coronal and lambdoid sutures. After impact, mice were placed supine and loss of consciousness (LOC) time was recorded as time to righting reflex. Sham injured mice received anesthesia but no injury.

Hemodynamics

Weekly measurements of systolic and diastolic blood pressure and heart rate was done using the CODA noninvasive BP system (a tail-cuff Method, Kent Scientific Corporation) as previously described.[91]

Behavioral studies

Behavioral studies were performed at 60 days post rCHI as a baseline to ensure there were no deficits pre-HSD administration. Mice were further tested at 4 weeks post HSD (90 days post rCHI), 8 weeks post HSD (120 days post rCHI), and 12 weeks post HSD (150 days post rCHI). All mice underwent a minimum of 30 minutes of acclimatization to the behavior room before testing, and tests were consistently conducted between 7 a.m. and 11:30 a.m. The experimenter was blinded to the animal groups. The behavioral testing sequence commenced with the Elevated Plus Maze and concluded with the Morris Water Maze (MWM). This was done to minimize the MWM-related anxiety as a confounding factor.

Elevated plus maze: Elevated plus Maze was performed as previously described[90] . The setup consisted of two 130 X 8 cm platforms with an 8 X 8 cm square area at their intersection. The apparatus was elevated at 60 cm above the ground. The closed arms had 10 cm walls, whereas the open arms had none. The mouse was placed in the center of the apparatus and the percent time spent in the open arms was analyzed by Any Maze software. Each recorded trial was 5 minutes. The apparatus was cleaned with 10% ethanol between trials.

For Review Only

Open field: Mice were individually placed in housing cages with clean bedding and covered by a thin wire grid. The housing cage for the open field was 28 x 18 cm. During the open field test, mice were recorded by ceiling-mounted cameras and their movements were tracked by AnyMaze as described.[90] Recordings lasted 30 minutes, and the distance covered during that time was used as a marker of overall activity.

Rotarod: Mice were placed on a Rotarod apparatus (Harvard Apparatus, Holliston, MA, USA), accelerating from 4–40 RPM in 120 seconds. Each trial ended when the mouse fell off the rod, and the latency to fall was manually recorded. Mice were tested for 3 trials per day (5-minute inter-trial interval) for 3 consecutive days.

Morris water maze: Morris Water Maze testing was performed as previously described. [92] Each mouse was subjected to 5-7 hidden and 2 or no visible platform trials. Each mouse underwent 1-2 trials per day, each trial consisted of 4 attempts with the exception of the probe trial in which each mouse was only tested once. For the hidden trials, the platform was submerged between (0.5-1cm) below the surface of the water and was kept in the same location for all the hidden trials. For the probe trial the platform was completely removed. The visible trials were done last, and the platform was placed 1cm above the water surface, the platform location was changed for every visible trial. Mice were assigned 4 start positions around the perimeter of the pool, mice were allowed 90 seconds to locate the platform, after which the mouse remained on the platform for 15 seconds. If the mouse did not find the platform in 90 seconds during the first and second hidden trial, it was guided to platform and allowed to remain there for 15 seconds and was given a score of 90 seconds. The time until the mouse mounted the platform (escape latency) was measured and recorded (AnyMaze 8.42, Stoelting, Wood Dale, IL, USA). For probe trials, the time spent in the target quadrant where the platform was removed (total 30 seconds swim time) was recorded.

For Review Only

Preparation of brain tissue for Immunohistochemistry

Mice were deeply anesthetized with isoflurane and decapitated. The brains were removed and frozen in liquid nitrogen prior to making coronal sections (16 μm) on poly-ι-lysine-coated slide (Thermo Fisher Scientific) using a cryostat. The brains were cut at 0.5 mm intervals from the anterior to the posterior of the brain. For analyses using paraformaldehyde-fixed tissue, mice were transcardially perfused with PBS followed by 4% paraformaldehyde and brains were post- fixed overnight in 4% paraformaldehyde, cryoprotected in 30% sucrose overnight, frozen at - 80°C, and cut on a cryostat as above.

Immunofluorescence staining

Fluorescent immunolabeling followed a standard protocol. Sections were transferred into blocking medium (0.075% Triton-X, 5% normal Horse serum in 1× PBS solution) for 1 h at room temperature before applying primary antibody. The primary antibody, rabbit anti-mouse IBA1 (1:1000; Wako; RRID: AB_839506), was applied overnight followed by Cy3-conjugated anti-rabbit secondary antibodies (Jackson ImmunoResearch Laboratories, 1:300). Sections were counterstained with 10 μl DAPI Mounting Medium (Vector Laboratories) and sealed by placing a glass coverslip over sections (Menzel Glaser) and coating the edges of the coverslip with clear nail polish.

Imaging and cell quantification

Five 16 μm consecutive sections of the dorsal dentate gyrus were quantified per animal, starting at anteroposterior (AP) -1.5 mm from bregma (one in six series, 300 um apart). All representative images were acquired using a Zeiss LSM 710 confocal microscope and processed with Zen black 2.1 (Carl Zeiss). Images were acquired using 20× objective lens, scale bar 50 μm. For quantification of Iba1-positive, a full mosaic of hemisphere brain sections was acquired using a Leica DMi8 Widefield Fluorescence Microscope, under a 20× lens and quantified with ImageJ

For Review Only

software. For cell counting, ImageJ software was used and the “analyze particles” option was used to count cells. The region of interest (ROI) within the areas of the M1 motor cortex, the hippocampal radiatum layer adjacent to pyramidal CA1, the stratum lucidum adjacent to CA3, and the dentate gyrus (hereafter referred to as cortex, CA1, CA3, and DG, respectively), the amygdala was quantified. Numbers of microglia were obtained by scanning regions at 500x500 μm boxes at comparable sections in each animal. Data were number of IBA1+ cells/$0.25mm^2$ .All quantifications were performed with Image J analysis software as previously described.[93,94]

Quantitative polymerase chain reaction

RNA was extracted with RNeasy® columns (Qiagen), cDNA was prepared and used for quantitative PCR (Applied Biosystems™, 437466) and the results were normalized to *Gapdh* (Mm99999915_g1). All primers and probes were from AppliedBiosystems, *IL10* (Mm01288386_m1), *Il6* (Mm00446190_m1), *TNF* (Mm00443258_m1), *Il1b* (Mm00434228_m1), *Infg* (Mm01168134_m1), and *Ccl5* (Mm01302427_m1) were used. 2-ΔΔCt method was used to calculate relative expression of each gene.

Flow cytometry microglial sorting

For microglial cell sorting, mice were anesthetized with CO_2 until respiration rate slowed and transcardially perfused with 50 mL Hanks balanced salt solution (HBSS). Following perfusion, the brains were homogenized using a dounce glass tissue homogenizer. Cells were separated through Percoll (GE Healthcare Life Sciences) 30% gradient by centrifugation. Cells were isolated from the Percell layer and stained on ice for 30 min with combinations of PE/Cy7 rat anti-mouse CD11b (Biolegend, #101216, 1:100), APC/Cy7 rat anti mouse CD45 (Biolegend, #103116, 1:100) in blocking buffer containing 0.2% bovine serum albumin (BSA, Sigma-Aldrich) in HBSS. Cell sorting was performed using FACSAriaIII cell sorter (Becton Dickson). Microglial cells were identified as CD45 low

For Review Only

to intermediate/CD11b high cells.[95] Cells were sorted directly in 1.5 mL Eppendorf tubes and stored at -80°C.

Microglia Bulk RNA-Sequencing

Bulk RNA sequencing was performed as previously described[42] for samples at 12 weeks after HSD administration. Briefly, 2,000 isolated CD45 low to intermediate/CD11b high cells (microglia) were lysed in 5ul TCL buffer + 1% β-mercaptoethanol. Smart-Seq2 libraries were prepared and sequenced by the Broad Genomic Platform. cDNA libraries were generated from sorted cells using the Smart-seq2 protocol. RNA sequencing was performed using Illumina NextSeq500 using a High Output v2 kit to generate 2 × 38 bp reads. The processing of the bulk RNA-seq data was based on an established computational pipeline.[96] Sequencing data were demultiplexed and provided by the Broad Institute in FASTQ format. FastQC was used to assess sequencing quality control. Trimmomatic was used for adaptor trimming of reads. Reads were then aligned to the 'mm10' reference genome using HISAT. The generated SAM files were then converted into BAM files using SAMtools. StringTie was used for transcript assembly and quantification. Transcript abundances were then imported into R Studio (version 4.1.2) and converted to gene-level estimated counts using the 'tximport' package (version 1.22.0) from Bioconductor. Genes that achieved less than 10 counts summed across all samples were considered very low expressed genes and thus filtered out. Sample read counts were normalized using the variance stabilizing transformation (VST) method from the DESeq2 (version 1.34.0) built-in VST function.[97] These normalized sample read counts were used to plot heatmaps using ComplexHeatmap (version 2.13.1). Dot plots were generated using ggplot2 (version 3.4.0). Bar plots were generated using GraphPad Prism software for Mac.

Differential Gene Expression and Pathway Analysis

For Review Only

Differential gene expression analysis was carried out with DESeq2. For comparisons of gene expression between two different sample groups, the Wald Test was used with a significance cut-off of $P < 0.05$ and standard parameters. The log2 fold-changes of the corresponding differentially expressed genes (DEGs) were subsequently shrunken using DESeq2's built-in lfcshrink function. Pairwise comparisons of DEGs were visualized using DiVenn[98]. GSEA pathway analyses were performed through the GAGE package (version 2.44.0).[99] For GO enrichment analysis, the clusterProfiler package was used (version 4.2.2).[100] Ingenuity Pathway Analysis (IPA) (https://digitalinsights.qiagen.com/products-overview/discovery-insights-portfolio/analysis-and-visualization/qiagen-ipa/) was used to identify upstream regulators ($P < 0.05$ and $|Z\text{-score}| \geq 2$) based on DEGs in a particular pairwise comparison, where input data comprised of p-values and $\log_2$fold changes of the DEGs. Statistical significance for all differential gene expression and pathway analyses was defined as a nominal P value < 0.05.

For Review Only

16S rRNA microbiota sequencing and Microbial Community Analysis

Fecal samples were collected from 8 mice per group starting at 60 days post rCHI prior to high salt diet administration and was done weekly until experimental endpoint at 90 days after HSD administration (150 days post-injury). Cecum samples from 6 mice per group were collected at 90 days after salt diet administration (150 days post-injury). DNA was extracted using the Qiagen DNeasy PowerLyzer PowerSoil Kit (Qiagen, Hilden, Germany). The V4 16S rRNA gene was amplified with barcoded fusion primers developed by the Earth Microbiome Project.[101,102] Paired-end sequencing was performed at the Harvard Biopolymers facility on the Illumina MiSeq. The QIIME2 pipeline [103] was used for quality filtering of DNA sequences, demultiplexing, taxonomic assignment, and calculating alpha and beta diversity; DNA demultiplexing and quality filtering were performed by DADA2, samples were aligned, and alpha and beta-diversity were calculated at a depth of 1000 reads. Samples were removed if they had fewer than 1000 reads and ASVs were removed if they had fewer than 10 reads

or were in fewer than 2 samples. A pre-trained Silva classifier was used for taxonomic assignment. To evaluate overall differences in microbial community structure between salt diet and normal diet in both sham and injured groups, permutational multivariate analysis of variance (PERMANOVA) tests were performed on beta-diversity weighted and unweighted UniFrac distance measures. Statistical analysis of the changes in differences in relative microbial abundance was determined by linear discriminated analysis effect size (LEfSe), with the alpha set at 0.05 and the effect size set at 2. LDA scores and p-values were plotted in R using ggplot2, ComplexHeatmap, and ColorBrewer packages[104]. An ADONIS test in QIIME2 was used to determine the percentage of contribution to microbiome variance. To identify bacteria linked with anxiety, Spearman correlations were constructed in R using the stats package. Since the anxiety-like phenotype was measured using an elevated plus maze test where a lower value (percent of time spent in the open arms) suggests higher anxiety-like behaviors, the final correlations directions (positive or negative) were inversed for practical interpretation. Metagenomic content of the microbiota samples was predicted from the 16S rRNA profiles and KEGG pathway functions were categorized at level 3 using the phylogenetic investigation of communities by reconstruction of unobserved states (PICRUSt2) tool [46]. Significant pairwise differences based on relative predicted KEGG metagenomic pathways were determined by linear discriminant analysis effect size (LEfSe), with the alpha set at 0.05 and the effect size set at 1. Relative abundance plots over time for specific bacteria were performed using GraphPad Prism software for Mac.

Statistical analysis

Data are mean ± standard error of the mean (SEM). One-way ANOVA tests with Tukey's multiple comparisons was used to assess statistical significance between groups at a specific timepoint such as % time in open arm (Elevated plus maze), distance travelled (Open field test), %time in the target quadrant (Probe trial), IBA-1 cells quantification, and Relative mRNA expression for the RT-qPCR. A two-factor repeated measures two-way ANOVA (group x time) was used to assess two

For Review Only

independent variables including time and the study dependent outcomes such as latency to fall (Rotarod), latency to platform (MWM), and blood pressure/ heart rate (hemodynamics study). Statistical tests used are in line with other published studies.[90] Statistical analysis for 16S rRNA sequencing data and microglia RNA sequencing were expanded upon in their respective method sections. Numbers per group, significance level and statistical tests are indicated in the figure legends. No statistical methods were used to predetermine samples size. Sample sizes were chosen in accordance with previous work in the field.[21,92,105] Statistical analyses were performed using GraphPad Prism 9 software (GraphPad Software Inc., La Jolla, CA) and differences for all tests were considered significant if the p-value was <0.05.

Data Availability

The bulk microglia RNA-seq data samples were deposited into the Sequence Read Archive (SRA) of the National Center for Biotechnology Information (NCBI), BioProject accession number PRJNA1053796. The microbiota 16S rRNA sequencing data will be available in the NCBI SRA repository, under NCBI BioProject number PRJNA1053836.

Author contributions

SI, TY and MJW conceived the project and designed all the experiments. SI, TY, OA, TC, LMC and RZ were involved in planning and analysis of the experiments, SI, TA, TC, LAS, HA KC, MNK, UK, IMV, DSL, TBL, MA, and MGO performed the experiments. OA performed the data analysis for the RNA sequencing experiments. Data visualization and preparation of figures was performed by SI, TY, TC, LS, HA, and LMC. SI, HW, RZ provided funding. SI, TY, OA, LAS, LMC, RMR, HW, RZ, and MJW wrote the manuscript with input from all authors. JEK, HW, RZ, and MJW supervised the study.

Competing interests

Authors declare that they have no competing interests.

For Review Only

For Review Only

References

1. Bakhos LL, Lockhart GR, Myers R, Linakis JG. Emergency department visits for concussion in young child athletes. *Pediatrics*. Sep 2010;126(3):e550-6. doi:10.1542/peds.2009-3101

2. Mitka M. Reports of concussions from youth sports rise along with awareness of the problem. *JAMA*. Oct 27 2010;304(16):1775-6. doi:10.1001/jama.2010.1487

3. CDC. Sports and recreation-related injuries. https://www.cdc.gov/healthcommunication/toolstemplates/entertainmented/tips/SportsInjuries.html

4. Izzy S, Chen PM, Tahir Z, et al. Association of Traumatic Brain Injury With the Risk of Developing Chronic Cardiovascular, Endocrine, Neurological, and Psychiatric Disorders. *JAMA Netw Open*. Apr 1 2022;5(4):e229478. doi:10.1001/jamanetworkopen.2022.9478

5. Hilz MJ, Wang R, Markus J, et al. Severity of traumatic brain injury correlates with long-term cardiovascular autonomic dysfunction. *J Neurol*. Sep 2017;264(9):1956-1967. doi:10.1007/s00415-017-8581-1

6. Stewart IJ, Amuan ME, Wang CP, et al. Association Between Traumatic Brain Injury and Subsequent Cardiovascular Disease Among Post-9/11-Era Veterans. *JAMA Neurol*. Nov 1 2022;79(11):1122-1129. doi:10.1001/jamaneurol.2022.2682

7. Alosco ML, Mez J, Tripodis Y, et al. Age of first exposure to tackle football and chronic traumatic encephalopathy. *Ann Neurol*. May 2018;83(5):886-901. doi:10.1002/ana.25245

8. Stein CJ, MacDougall R, Quatman-Yates CC, et al. Young Athletes' Concerns About Sport-Related Concussion: The Patient's Perspective. *Clin J Sport Med*. Sep 2016;26(5):386-90. doi:10.1097/JSM.0000000000000268

9. Fishman M, Taranto E, Perlman M, Quinlan K, Benjamin HJ, Ross LF. Attitudes and Counseling Practices of Pediatricians Regarding Youth Sports Participation and Concussion Risks. *J Pediatr*. May 2017;184:19-25. doi:10.1016/j.jpeds.2017.01.048

10. Ropper AH, Gorson KC. Clinical practice. Concussion. *N Engl J Med*. Jan 11 2007;356(2):166-72. doi:10.1056/NEJMcp064645

11. Smith DH, Johnson VE, Stewart W. Chronic neuropathologies of single and repetitive TBI: substrates of dementia? *Nat Rev Neurol*. Apr 2013;9(4):211-21. doi:10.1038/nrneurol.2013.29

12. Izzy S, Tahir Z, Grashow R, et al. Concussion and Risk of Chronic Medical and Behavioral Health Comorbidities. *J Neurotrauma*. Jun 1 2021;38(13):1834-1841. doi:10.1089/neu.2020.7484

13. Stewart IJ, Amuan ME, Wang CP, et al. Association Between Traumatic Brain Injury and Subsequent Cardiovascular Disease Among Post-9/11-Era Veterans. *JAMA Neurol*. Sep 6 2022;doi:10.1001/jamaneurol.2022.2682

14. Izzy S, Grashow R, Radmanesh F, et al. Long-term risk of cardiovascular disease after traumatic brain injury: screening and prevention. *Lancet Neurol*. Oct 2023;22(10):959-970. doi:10.1016/S1474-4422(23)00241-7

15. Jassam YN, Izzy S, Whalen M, McGavern DB, El Khoury J. Neuroimmunology of Traumatic Brain Injury: Time for a Paradigm Shift. *Neuron*. Sep 13 2017;95(6):1246-1265. doi:10.1016/j.neuron.2017.07.010

16. Stein MB, Jain S, Giacino JT, et al. Risk of Posttraumatic Stress Disorder and Major Depression in Civilian Patients After Mild Traumatic Brain Injury: A TRACK-TBI Study. *JAMA Psychiatry*. Mar 1 2019;76(3):249-258. doi:10.1001/jamapsychiatry.2018.4288

17. Willeumier K, Taylor DV, Amen DG. Elevated body mass in National Football League players linked to cognitive impairment and decreased prefrontal cortex and temporal pole activity. *Transl Psychiatry*. Jan 17 2012;2:e68. doi:10.1038/tp.2011.67

18. Wickwire EM, Williams SG, Roth T, et al. Sleep, Sleep Disorders, and Mild Traumatic Brain Injury. What We Know and What We Need to Know: Findings from a National Working Group. *Neurotherapeutics*. Apr 2016;13(2):403-17. doi:10.1007/s13311-016-0429-3

19. Norrie J, Heitger M, Leathem J, Anderson T, Jones R, Flett R. Mild traumatic brain injury and fatigue: a prospective longitudinal study. *Brain Inj*. 2010;24(13-14):1528-38. doi:10.3109/02699052.2010.531687

For Review Only

20. Farquhar WB, Edwards DG, Jurkovitz CT, Weintraub WS. Dietary sodium and health: more than just blood pressure. *J Am Coll Cardiol*. Mar 17 2015;65(10):1042-50. doi:10.1016/j.jacc.2014.12.039
21. Faraco G, Brea D, Garcia-Bonilla L, et al. Dietary salt promotes neurovascular and cognitive dysfunction through a gut-initiated TH17 response. *Nat Neurosci*. Feb 2018;21(2):240-249. doi:10.1038/s41593-017-0059-z
22. Hu L, Zhu S, Peng X, et al. High Salt Elicits Brain Inflammation and Cognitive Dysfunction, Accompanied by Alternations in the Gut Microbiota and Decreased SCFA Production. *J Alzheimers Dis*. 2020;77(2):629-640. doi:10.3233/JAD-200035
23. Gilman TL, Mitchell NC, Daws LC, Toney GM. Neuroinflammation Contributes to High Salt Intake-Augmented Neuronal Activation and Active Coping Responses to Acute Stress. *Int J Neuropsychopharmacol*. Feb 1 2019;22(2):137-142. doi:10.1093/ijnp/pyy099
24. Wu C, Yosef N, Thalhamer T, et al. Induction of pathogenic TH17 cells by inducible salt-sensing kinase SGK1. *Nature*. Apr 25 2013;496(7446):513-7. doi:10.1038/nature11984
25. Kleinewietfeld M, Manzel A, Titze J, et al. Sodium chloride drives autoimmune disease by the induction of pathogenic TH17 cells. *Nature*. Apr 25 2013;496(7446):518-22. doi:10.1038/nature11868
26. Hernandez AL, Kitz A, Wu C, et al. Sodium chloride inhibits the suppressive function of FOXP3+ regulatory T cells. *J Clin Invest*. Nov 2 2015;125(11):4212-22. doi:10.1172/JCI81151
27. Janakiraman M, Krishnamoorthy G. Emerging Role of Diet and Microbiota Interactions in Neuroinflammation. *Front Immunol*. 2018;9:2067. doi:10.3389/fimmu.2018.02067
28. Zhang WC, Zheng XJ, Du LJ, et al. High salt primes a specific activation state of macrophages, M(Na). *Cell Res*. Aug 2015;25(8):893-910. doi:10.1038/cr.2015.87
29. Binger KJ, Gebhardt M, Heinig M, et al. High salt reduces the activation of IL-4- and IL-13-stimulated macrophages. *J Clin Invest*. Nov 2 2015;125(11):4223-38. doi:10.1172/JCI80919
30. Hucke S, Eschborn M, Liebmann M, et al. Sodium chloride promotes pro-inflammatory macrophage polarization thereby aggravating CNS autoimmunity. *J Autoimmun*. Feb 2016;67:90-101. doi:10.1016/j.jaut.2015.11.001
31. Zhang T, Wang D, Li X, et al. Excess salt intake promotes M1 microglia polarization via a p38/MAPK/AR-dependent pathway after cerebral ischemia in mice. *Int Immunopharmacol*. Apr 2020;81:106176. doi:10.1016/j.intimp.2019.106176
32. Zhang T, Fang S, Wan C, et al. Excess salt exacerbates blood-brain barrier disruption via a p38/MAPK/SGK1-dependent pathway in permanent cerebral ischemia. *Sci Rep*. Nov 9 2015;5:16548. doi:10.1038/srep16548
33. Bier A, Braun T, Khasbab R, et al. A High Salt Diet Modulates the Gut Microbiota and Short Chain Fatty Acids Production in a Salt-Sensitive Hypertension Rat Model. *Nutrients*. Aug 23 2018;10(9)doi:10.3390/nu10091154
34. Huang B, Chau SWH, Liu Y, et al. Gut microbiome dysbiosis across early Parkinson's disease, REM sleep behavior disorder and their first-degree relatives. *Nat Commun*. May 2 2023;14(1):2501. doi:10.1038/s41467-023-38248-4
35. Boddy SL, Giovannelli I, Sassani M, et al. The gut microbiome: a key player in the complexity of amyotrophic lateral sclerosis (ALS). *BMC Med*. Jan 20 2021;19(1):13. doi:10.1186/s12916-020-01885-3
36. Grabrucker S, Marizzoni M, Silajdzic E, et al. Microbiota from Alzheimer's patients induce deficits in cognition and hippocampal neurogenesis. *Brain*. Dec 1 2023;146(12):4916-4934. doi:10.1093/brain/awad303
37. Zhu CS, Grandhi R, Patterson TT, Nicholson SE. A Review of Traumatic Brain Injury and the Gut Microbiome: Insights into Novel Mechanisms of Secondary Brain Injury and Promising Targets for Neuroprotection. *Brain Sci*. Jun 19 2018;8(6)doi:10.3390/brainsci8060113
38. Weiner RB, Wang F, Isaacs SK, et al. Blood pressure and left ventricular hypertrophy during American-style football participation. *Circulation*. Jul 30 2013;128(5):524-31. doi:10.1161/CIRCULATIONAHA.113.003522

For Review Only

39. Grashow R, Tan CO, Izzy S, et al. Association Between Concussion Burden During Professional American-Style Football and Postcareer Hypertension. *Circulation*. Apr 4 2023;147(14):1112-1114. doi:10.1161/CIRCULATIONAHA.122.063767

40. Hickman S, Izzy S, Sen P, Morsett L, El Khoury J. Microglia in neurodegeneration. *Nat Neurosci*. Oct 2018;21(10):1359-1369. doi:10.1038/s41593-018-0242-x

41. Wu L, Kalish BT, Finander B, et al. Repetitive Mild Closed Head Injury in Adolescent Mice Is Associated with Impaired Proteostasis, Neuroinflammation, and Tauopathy. *J Neurosci*. Mar 23 2022;42(12):2418-2432. doi:10.1523/JNEUROSCI.0682-21.2021

42. Butovsky O, Jedrychowski MP, Moore CS, et al. Identification of a unique TGF-beta-dependent molecular and functional signature in microglia. *Nat Neurosci*. Jan 2014;17(1):131-43. doi:10.1038/nn.3599

43. Gilman TL, George CM, Andrade MA, Mitchell NC, Toney GM, Daws LC. High Salt Intake Lowers Behavioral Inhibition. *Front Behav Neurosci*. 2019;13:271. doi:10.3389/fnbeh.2019.00271

44. Beaver JN, Gilman TL. Salt as a non-caloric behavioral modifier: A review of evidence from pre-clinical studies. *Neurosci Biobehav Rev*. Apr 2022;135:104385. doi:10.1016/j.neubiorev.2021.10.007

45. Beaver JN, Weber BL, Ford MT, et al. Generalization of contextual fear is sex-specifically affected by high salt intake. *PLoS One*. 2023;18(7):e0286221. doi:10.1371/journal.pone.0286221

46. Douglas GM, Maffei VJ, Zaneveld JR, et al. PICRUSt2 for prediction of metagenome functions. *Nat Biotechnol*. Jun 2020;38(6):685-688. doi:10.1038/s41587-020-0548-6

47. Frisoli TM, Schmieder RE, Grodzicki T, Messerli FH. Salt and hypertension: is salt dietary reduction worth the effort? *Am J Med*. May 2012;125(5):433-9. doi:10.1016/j.amjmed.2011.10.023

48. Mallya S, Sutherland J, Pongracic S, Mainland B, Ornstein TJ. The manifestation of anxiety disorders after traumatic brain injury: a review. *J Neurotrauma*. Apr 1 2015;32(7):411-21. doi:10.1089/neu.2014.3504

49. Dingess PM, Thakar A, Zhang Z, Flynn FW, Brown TE. High-Salt Exposure During Perinatal Development Enhances Stress Sensitivity. *Dev Neurobiol*. Nov 2018;78(11):1131-1145. doi:10.1002/dneu.22635

50. McBride SM, Culver B, Flynn FW. Dietary sodium manipulation during critical periods in development sensitize adult offspring to amphetamines. *Am J Physiol Regul Integr Comp Physiol*. Sep 2008;295(3):R899-905. doi:10.1152/ajpregu.00186.2008

51. Torres SJ, Nowson CA, Worsley A. Dietary electrolytes are related to mood. *Br J Nutr*. Nov 2008;100(5):1038-45. doi:10.1017/S0007114508959201

52. Won E, Kim YK. Neuroinflammation-Associated Alterations of the Brain as Potential Neural Biomarkers in Anxiety Disorders. *Int J Mol Sci*. Sep 7 2020;21(18)doi:10.3390/ijms21186546

53. Wang YL, Han QQ, Gong WQ, et al. Microglial activation mediates chronic mild stress-induced depressive- and anxiety-like behavior in adult rats. *J Neuroinflammation*. Jan 17 2018;15(1):21. doi:10.1186/s12974-018-1054-3

54. Ressler KJ. Amygdala activity, fear, and anxiety: modulation by stress. *Biol Psychiatry*. Jun 15 2010;67(12):1117-9. doi:10.1016/j.biopsych.2010.04.027

55. Calhoon GG, Tye KM. Resolving the neural circuits of anxiety. *Nat Neurosci*. Oct 2015;18(10):1394-404. doi:10.1038/nn.4101

56. Bardgett ME, Holbein WW, Herrera-Rosales M, Toney GM. Ang II-salt hypertension depends on neuronal activity in the hypothalamic paraventricular nucleus but not on local actions of tumor necrosis factor-alpha. *Hypertension*. Mar 2014;63(3):527-34. doi:10.1161/HYPERTENSIONAHA.113.02429

57. Jantsch J, Schatz V, Friedrich D, et al. Cutaneous Na+ storage strengthens the antimicrobial barrier function of the skin and boosts macrophage-driven host defense. *Cell Metab*. Mar 3 2015;21(3):493-501. doi:10.1016/j.cmet.2015.02.003

58. Barbaro NR, Foss JD, Kryshtal DO, et al. Dendritic Cell Amiloride-Sensitive Channels Mediate Sodium-Induced Inflammation and Hypertension. *Cell Rep*. Oct 24 2017;21(4):1009-1020. doi:10.1016/j.celrep.2017.10.002

For Review Only

59. Hou R, Garner M, Holmes C, et al. Peripheral inflammatory cytokines and immune balance in Generalised Anxiety Disorder: Case-controlled study. *Brain Behav Immun*. May 2017;62:212-218. doi:10.1016/j.bbi.2017.01.021

60. Mikhaylova IV, Kuulasmaa T, Jaaskelainen J, Voutilainen R. Tumor necrosis factor-alpha regulates steroidogenesis, apoptosis, and cell viability in the human adrenocortical cell line NCI-H295R. *Endocrinology*. Jan 2007;148(1):386-92. doi:10.1210/en.2006-0726

61. Lenze EJ, Mantella RC, Shi P, et al. Elevated cortisol in older adults with generalized anxiety disorder is reduced by treatment: a placebo-controlled evaluation of escitalopram. *Am J Geriatr Psychiatry*. May 2011;19(5):482-90. doi:10.1097/JGP.0b013e3181ec806c

62. Foley KF, Pantano C, Ciolino A, Mawe GM. IFN-gamma and TNF-alpha decrease serotonin transporter function and expression in Caco2 cells. *Am J Physiol Gastrointest Liver Physiol*. Mar 2007;292(3):G779-84. doi:10.1152/ajpgi.00470.2006

63. Litteljohn D, Cummings A, Brennan A, et al. Interferon-gamma deficiency modifies the effects of a chronic stressor in mice: Implications for psychological pathology. *Brain Behav Immun*. Mar 2010;24(3):462-73. doi:10.1016/j.bbi.2009.12.001

64. Kann O, Almouhanna F, Chausse B. Interferon gamma: a master cytokine in microglia-mediated neural network dysfunction and neurodegeneration. *Trends Neurosci*. Dec 2022;45(12):913-927. doi:10.1016/j.tins.2022.10.007

65. Zhang J, He H, Qiao Y, et al. Priming of microglia with IFN-gamma impairs adult hippocampal neurogenesis and leads to depression-like behaviors and cognitive defects. *Glia*. Dec 2020;68(12):2674-2692. doi:10.1002/glia.23878

66. Bao W, Sun Y, Lin Y, Yang X, Chen Z. An integrated analysis of gut microbiota and the brain transcriptome reveals host-gut microbiota interactions following traumatic brain injury. *Brain Res*. Jan 15 2023;1799:148149. doi:10.1016/j.brainres.2022.148149

67. Treangen TJ, Wagner J, Burns MP, Villapol S. Traumatic Brain Injury in Mice Induces Acute Bacterial Dysbiosis Within the Fecal Microbiome. *Front Immunol*. 2018;9:2757. doi:10.3389/fimmu.2018.02757

68. Yuan B, Lu XJ, Wu Q. Gut Microbiota and Acute Central Nervous System Injury: A New Target for Therapeutic Intervention. *Front Immunol*. 2021;12:800796. doi:10.3389/fimmu.2021.800796

69. Erny D, Hrabe de Angelis AL, Jaitin D, et al. Host microbiota constantly control maturation and function of microglia in the CNS. *Nat Neurosci*. Jul 2015;18(7):965-77. doi:10.1038/nn.4030

70. Butovsky O, Weiner HL. Microglial signatures and their role in health and disease. *Nat Rev Neurosci*. Oct 2018;19(10):622-635. doi:10.1038/s41583-018-0057-5

71. Cox LM, Maghzi AH, Liu S, et al. Gut Microbiome in Progressive Multiple Sclerosis. *Ann Neurol*. Jun 2021;89(6):1195-1211. doi:10.1002/ana.26084

72. i MCEasbue, i MC. Gut microbiome of multiple sclerosis patients and paired household healthy controls reveal associations with disease risk and course. *Cell*. Sep 15 2022;185(19):3467-3486 e16. doi:10.1016/j.cell.2022.08.021

73. Lubomski M, Xu X, Holmes AJ, et al. The Gut Microbiome in Parkinson's Disease: A Longitudinal Study of the Impacts on Disease Progression and the Use of Device-Assisted Therapies. *Front Aging Neurosci*. 2022;14:875261. doi:10.3389/fnagi.2022.875261

74. Guo P, Zhang K, Ma X, He P. Clostridium species as probiotics: potentials and challenges. *J Anim Sci Biotechnol*. 2020;11:24. doi:10.1186/s40104-019-0402-1

75. You W, Zhu Y, Wei A, et al. Traumatic Brain Injury Induces Gastrointestinal Dysfunction and Dysbiosis of Gut Microbiota Accompanied by Alterations of Bile Acid Profile. *J Neurotrauma*. Jan 2022;39(1-2):227-237. doi:10.1089/neu.2020.7526

76. Liu H, Wang J, He T, et al. Butyrate: A Double-Edged Sword for Health? *Adv Nutr*. Jan 1 2018;9(1):21-29. doi:10.1093/advances/nmx009

77. Siddiqui MT, Cresci GAM. The Immunomodulatory Functions of Butyrate. *J Inflamm Res*. 2021;14:6025-6041. doi:10.2147/JIR.S300989

For Review Only

78. Atarashi K, Tanoue T, Shima T, et al. Induction of colonic regulatory T cells by indigenous Clostridium species. *Science*. Jan 21 2011;331(6015):337-41. doi:10.1126/science.1198469
79. Atarashi K, Tanoue T, Ando M, et al. Th17 Cell Induction by Adhesion of Microbes to Intestinal Epithelial Cells. *Cell*. Oct 8 2015;163(2):367-80. doi:10.1016/j.cell.2015.08.058
80. Wang X, Li L, Bian C, et al. Alterations and correlations of gut microbiota, fecal, and serum metabolome characteristics in a rat model of alcohol use disorder. *Front Microbiol*. 2022;13:1068825. doi:10.3389/fmicb.2022.1068825
81. Miyauchi E, Kim SW, Suda W, et al. Gut microorganisms act together to exacerbate inflammation in spinal cords. *Nature*. Sep 2020;585(7823):102-106. doi:10.1038/s41586-020-2634-9
82. Turnbaugh PJ, Ridaura VK, Faith JJ, Rey FE, Knight R, Gordon JI. The effect of diet on the human gut microbiome: a metagenomic analysis in humanized gnotobiotic mice. *Sci Transl Med*. Nov 11 2009;1(6):6ra14. doi:10.1126/scitranslmed.3000322
83. Thompson DS, Fu C, Gandhi T, et al. Differential co-expression networks of the gut microbiota are associated with depression and anxiety treatment resistance among psychiatric inpatients. *Prog Neuropsychopharmacol Biol Psychiatry*. Jan 10 2023;120:110638. doi:10.1016/j.pnpbp.2022.110638
84. Cox LM, Weiner HL. Microbiota Signaling Pathways that Influence Neurologic Disease. *Neurotherapeutics*. Jan 2018;15(1):135-145. doi:10.1007/s13311-017-0598-8
85. Soto M, Herzog C, Pacheco JA, et al. Gut microbiota modulate neurobehavior through changes in brain insulin sensitivity and metabolism. *Mol Psychiatry*. Dec 2018;23(12):2287-2301. doi:10.1038/s41380-018-0086-5
86. Mardinoglu A, Shoaie S, Bergentall M, et al. The gut microbiota modulates host amino acid and glutathione metabolism in mice. *Mol Syst Biol*. Oct 16 2015;11(10):834. doi:10.15252/msb.20156487
87. Halverson T, Alagiakrishnan K. Gut microbes in neurocognitive and mental health disorders. *Ann Med*. Dec 2020;52(8):423-443. doi:10.1080/07853890.2020.1808239
88. Bhargava P. Targeting metabolism to treat multiple sclerosis. *Neural Regen Res*. Mar 2021;16(3):502-503. doi:10.4103/1673-5374.293143
89. Romero-Ramirez L, Garcia-Rama C, Wu S, Mey J. Bile acids attenuate PKM2 pathway activation in proinflammatory microglia. *Sci Rep*. Jan 27 2022;12(1):1459. doi:10.1038/s41598-022-05408-3
90. Wu L, Chung JY, Saith S, et al. Repetitive head injury in adolescent mice: A role for vascular inflammation. *J Cereb Blood Flow Metab*. Nov 2019;39(11):2196-2209. doi:10.1177/0271678X18786633
91. Daugherty A, Rateri D, Hong L, Balakrishnan A. Measuring blood pressure in mice using volume pressure recording, a tail-cuff method. *J Vis Exp*. May 15 2009;(27)doi:10.3791/1291
92. Khuman J, Meehan WP, 3rd, Zhu X, et al. Tumor necrosis factor alpha and Fas receptor contribute to cognitive deficits independent of cell death after concussive traumatic brain injury in mice. *J Cereb Blood Flow Metab*. Feb 2011;31(2):778-89. doi:10.1038/jcbfm.2010.172
93. Zhao XF, Alam MM, Liao Y, et al. Targeting Microglia Using Cx3cr1-Cre Lines: Revisiting the Specificity. *eNeuro*. Jul/Aug 2019;6(4)doi:10.1523/ENEURO.0114-19.2019
94. Alam MM, Zhao XF, Liao Y, et al. Deficiency of Microglial Autophagy Increases the Density of Oligodendrocytes and Susceptibility to Severe Forms of Seizures. *eNeuro*. Jan-Feb 2021;8(1)doi:10.1523/ENEURO.0183-20.2021
95. Izzy S, Liu Q, Fang Z, et al. Time-Dependent Changes in Microglia Transcriptional Networks Following Traumatic Brain Injury. *Front Cell Neurosci*. 2019;13:307. doi:10.3389/fncel.2019.00307
96. Pertea M, Kim D, Pertea GM, Leek JT, Salzberg SL. Transcript-level expression analysis of RNA-seq experiments with HISAT, StringTie and Ballgown. *Nat Protoc*. Sep 2016;11(9):1650-67. doi:10.1038/nprot.2016.095
97. Love MI, Huber W, Anders S. Moderated estimation of fold change and dispersion for RNA-seq data with DESeq2. *Genome Biol*. 2014;15(12):550. doi:10.1186/s13059-014-0550-8
98. Sun L, Dong S, Ge Y, et al. DiVenn: An Interactive and Integrated Web-Based Visualization Tool for Comparing Gene Lists. *Front Genet*. 2019;10:421. doi:10.3389/fgene.2019.00421

99. Luo W, Friedman MS, Shedden K, Hankenson KD, Woolf PJ. GAGE: generally applicable gene set enrichment for pathway analysis. *BMC Bioinformatics*. May 27 2009;10:161. doi:10.1186/1471-2105-10-161

100. Yu G, Wang LG, Han Y, He QY. clusterProfiler: an R package for comparing biological themes among gene clusters. *OMICS*. May 2012;16(5):284-7. doi:10.1089/omi.2011.0118

101. Parada AE, Needham DM, Fuhrman JA. Every base matters: assessing small subunit rRNA primers for marine microbiomes with mock communities, time series and global field samples. *Environ Microbiol*. May 2016;18(5):1403-14. doi:10.1111/1462-2920.13023

102. Caporaso JG, Lauber CL, Walters WA, et al. Ultra-high-throughput microbial community analysis on the Illumina HiSeq and MiSeq platforms. *ISME J*. Aug 2012;6(8):1621-4. doi:10.1038/ismej.2012.8

103. Bolyen E, Rideout JR, Dillon MR, et al. Reproducible, interactive, scalable and extensible microbiome data science using QIIME 2. *Nat Biotechnol*. Aug 2019;37(8):852-857. doi:10.1038/s41587-019-0209-9

104. Gu Z, Eils R, Schlesner M. Complex heatmaps reveal patterns and correlations in multidimensional genomic data. *Bioinformatics*. Sep 15 2016;32(18):2847-9. doi:10.1093/bioinformatics/btw313

105. Abou-El-Hassan H, Rezende RM, Izzy S, et al. Vgamma1 and Vgamma4 gamma-delta T cells play opposing roles in the immunopathology of traumatic brain injury in males. *Nat Commun*. Jul 18 2023;14(1):4286. doi:10.1038/s41467-023-39857-9

For Review Only

Figure 1

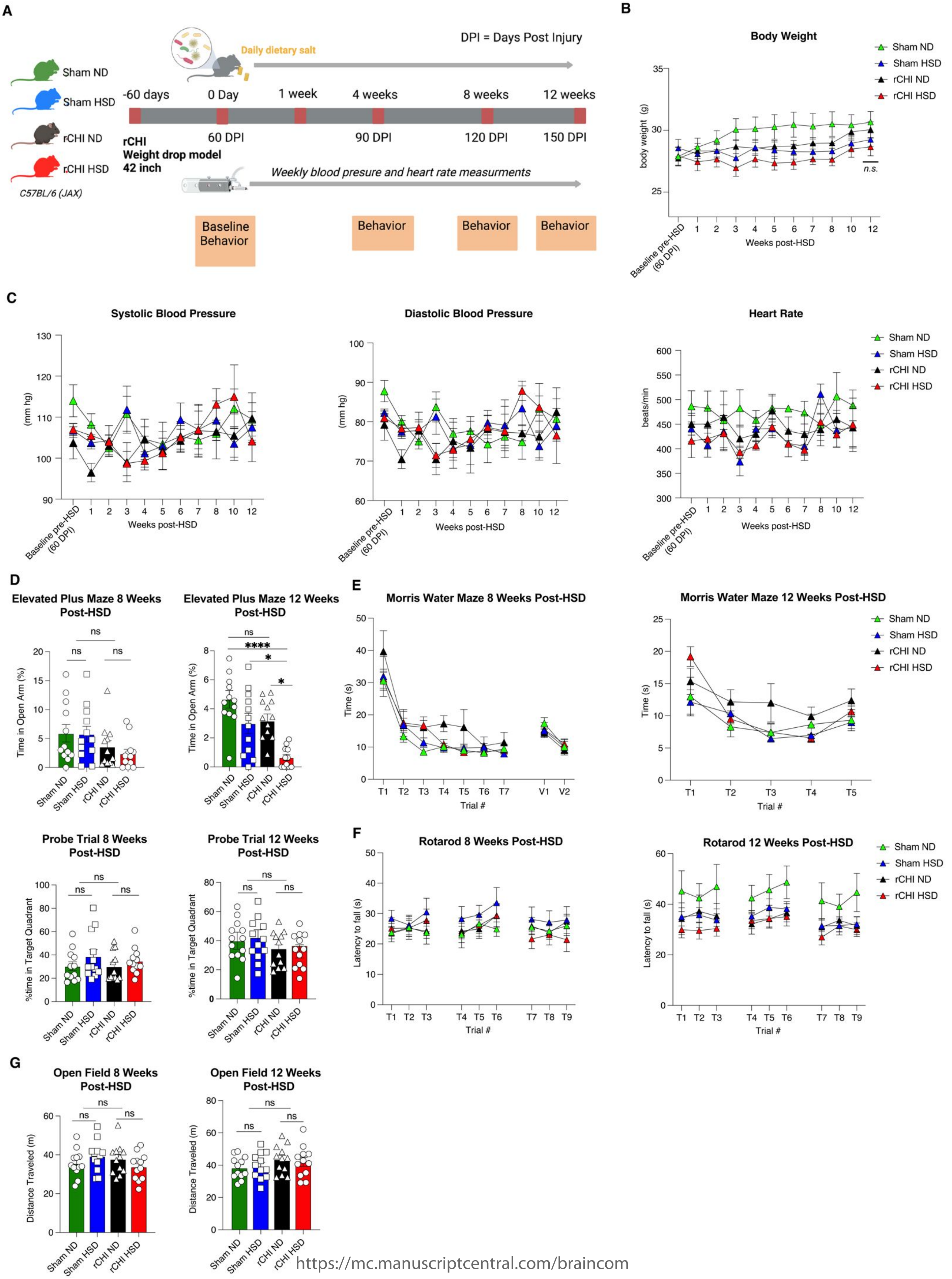
A
Sham ND
Sham HSD
rCHI ND
rCHI HSD
C57BL/6 (JAX)
Daily dietary salt
DPI = Days Post Injury
-60 days
0 Day
1 week
4 weeks
8 weeks
12 weeks
rCHI
Weight drop model
42 inch
60 DPI
90 DPI
120 DPI
150 DPI
Weekly blood presure and heart rate measurments
Baseline Behavior
Behavior
Behavior
Behavior
B
Body Weight
body weight (g)
Weeks post-HSD
Baseline pre-HSD (60 DPI)
n.s.
C
Systolic Blood Pressure
Diastolic Blood Pressure
Heart Rate
(mm hg)
beats/min
Weeks post-HSD
D
Elevated Plus Maze 8 Weeks Post-HSD
Elevated Plus Maze 12 Weeks Post-HSD
Time in Open Arm (%)
ns
****
*
E
Morris Water Maze 8 Weeks Post-HSD
Morris Water Maze 12 Weeks Post-HSD
Time (s)
Trial #
Probe Trial 8 Weeks Post-HSD
Probe Trial 12 Weeks Post-HSD
%time in Target Quadrant
F
Rotarod 8 Weeks Post-HSD
Rotarod 12 Weeks Post-HSD
Latency to fall (s)
G
Open Field 8 Weeks Post-HSD
Open Field 12 Weeks Post-HSD
Distance Traveled (m)

Figure 2

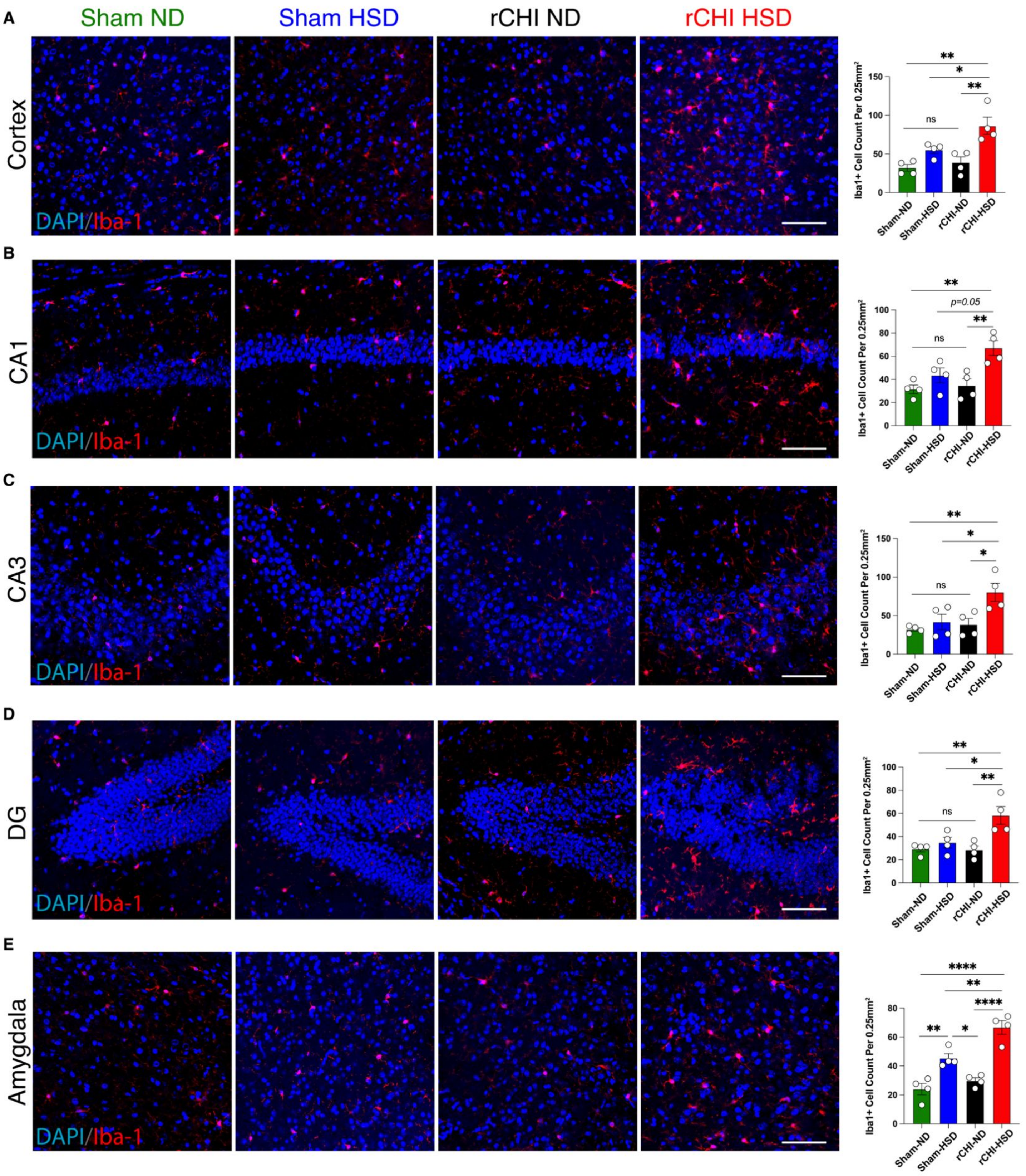
A
B
C
D
E
Sham ND
Sham HSD
rCHI ND
rCHI HSD
Cortex
CA1
CA3
DG
Amygdala
DAPI/Iba-1
Iba1+ Cell Count Per 0.25mm²
Sham-ND
Sham-HSD
rCHI-ND
rCHI-HSD
ns
p=0.05
*
**
****

Figure 3

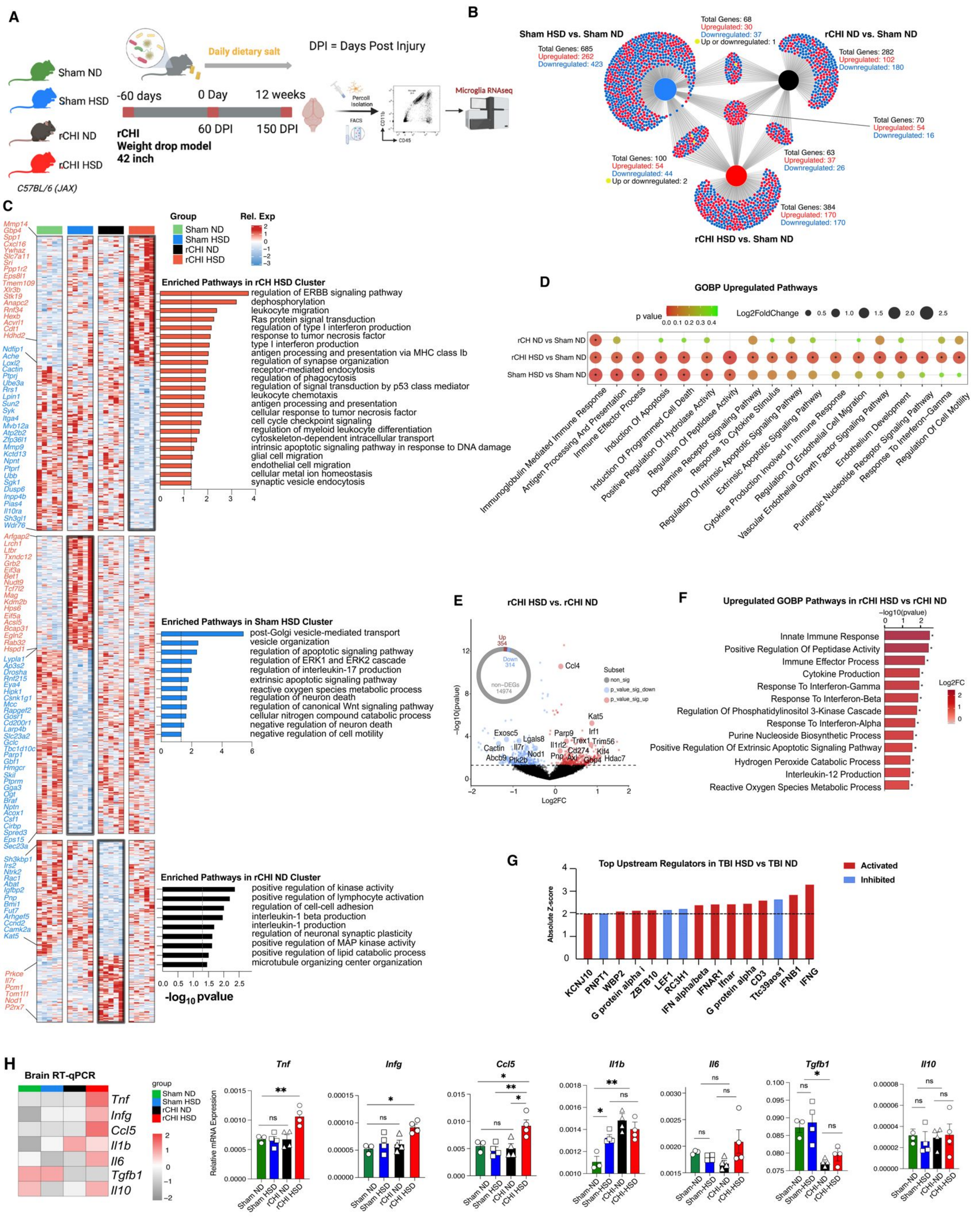
A
Sham ND
Sham HSD
rCHI ND
rCHI HSD
C57BL/6 (JAX)
Daily dietary salt
DPI = Days Post Injury
-60 days
0 Day
12 weeks
60 DPI
150 DPI
rCHI
Weight drop model
42 inch
Percoll Isolation
FACS
Microglia RNAseq
B
Sham HSD vs. Sham ND
rCHI ND vs. Sham ND
rCHI HSD vs. Sham ND
C
Group
Rel. Exp
Enriched Pathways in rCH HSD Cluster
Enriched Pathways in Sham HSD Cluster
Enriched Pathways in rCHI ND Cluster
-log10 pvalue
D
GOBP Upregulated Pathways
E
rCHI HSD vs. rCHI ND
F
Upregulated GOBP Pathways in rCHI HSD vs rCHI ND
G
Top Upstream Regulators in TBI HSD vs TBI ND
Activated
Inhibited
Absolute Z-score
H
Brain RT-qPCR
Relative mRNA Expression

878

Figure 4

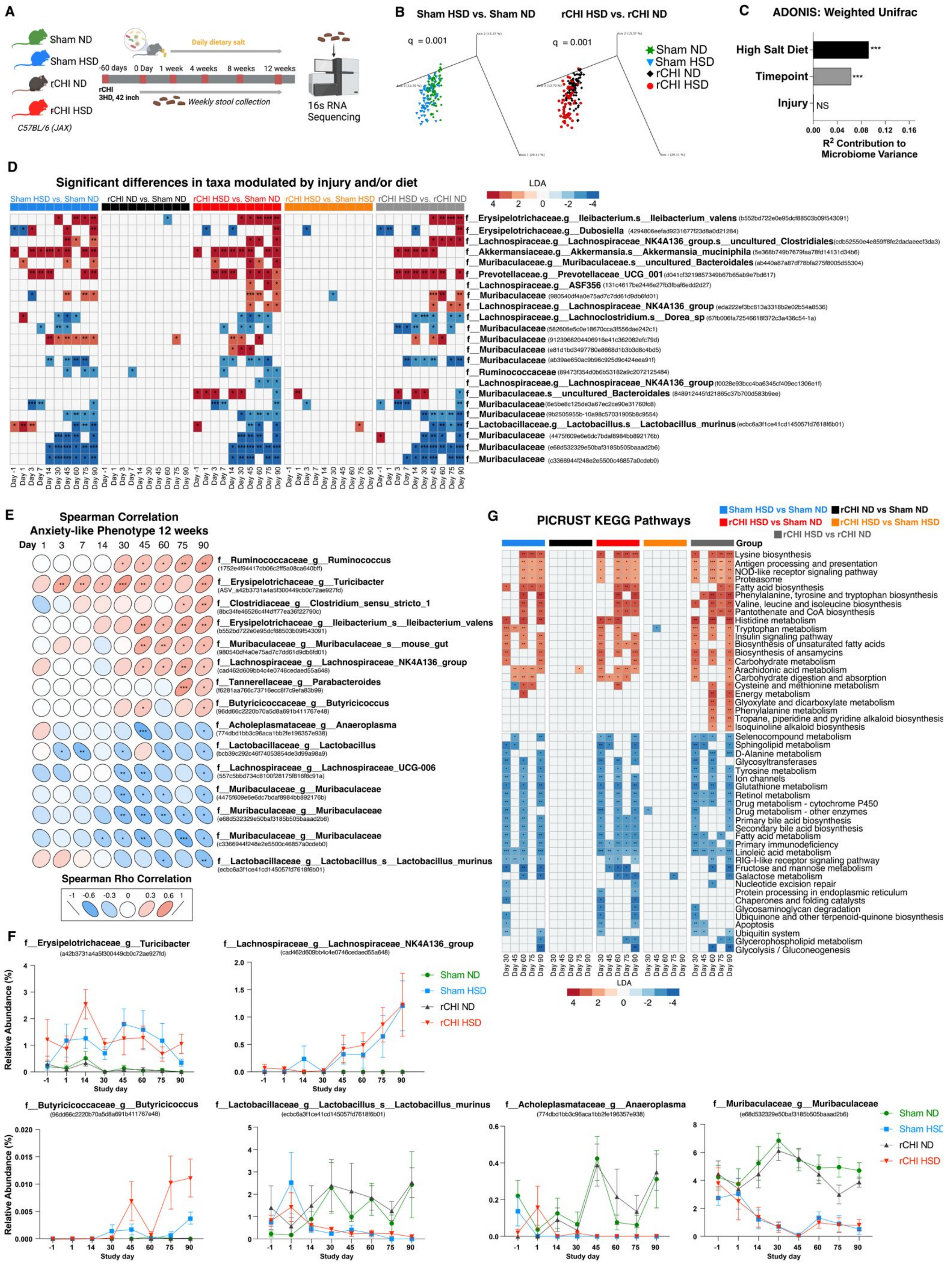
A
Sham ND
Sham HSD
rCHI ND
rCHI HSD
C57BL/6 (JAX)
Daily dietary salt
-60 days 0 Day 1 week 4 weeks 8 weeks 12 weeks
rCHI 3HD, 42 inch
Weekly stool collection
16s RNA Sequencing
B
Sham HSD vs. Sham ND
rCHI HSD vs. rCHI ND
q = 0.001
q = 0.001
Sham ND
Sham HSD
rCHI ND
rCHI HSD
C
ADONIS: Weighted Unifrac
High Salt Diet
Timepoint
Injury
NS
R2 Contribution to Microbiome Variance
D
Significant differences in taxa modulated by injury and/or diet
Sham HSD vs. Sham ND
rCHI ND vs. Sham ND
rCHI HSD vs. Sham ND
rCHI HSD vs. Sham HSD
rCHI HSD vs. rCHI ND
LDA
f__Erysipelotrichaceae.g__Ileibacterium.s__Ileibacterium_valens
f__Erysipelotrichaceae.g__Dubosiella
f__Lachnospiraceae.g__Lachnospiraceae_NK4A136_group.s__uncultured_Clostridiales
f__Akkermansiaceae.g__Akkermansia.s__Akkermansia_muciniphila
f__Muribaculaceae.g__Muribaculaceae.s__uncultured_Bacteroidales
f__Prevotellaceae.g__Prevotellaceae_UCG_001
f__Lachnospiraceae.g__ASF356
f__Muribaculaceae
f__Lachnospiraceae.g__Lachnospiraceae_NK4A136_group
f__Lachnospiraceae.g__Lachnoclostridium.s__Dorea_sp
f__Ruminococcaceae
f__Muribaculaceae.s__uncultured_Bacteroidales
f__Lactobacillaceae.g__Lactobacillus.s__Lactobacillus_murinus
E
Spearman Correlation
Anxiety-like Phenotype 12 weeks
f__Ruminococcaceae_g__Ruminococcus
f__Erysipelotrichaceae_g__Turicibacter
f__Clostridiaceae_g__Clostridium_sensu_stricto_1
f__Erysipelotrichaceae_g__Ileibacterium_s__Ileibacterium_valens
f__Muribaculaceae_g__Muribaculaceae_s__mouse_gut
f__Lachnospiraceae_g__Lachnospiraceae_NK4A136_group
f__Tannerellaceae_g__Parabacteroides
f__Butyricicoccaceae_g__Butyricicoccus
f__Acholeplasmataceae_g__Anaeroplasma
f__Lactobacillaceae_g__Lactobacillus
f__Lachnospiraceae_g__Lachnospiraceae_UCG-006
f__Muribaculaceae_g__Muribaculaceae
f__Lactobacillaceae_g__Lactobacillus_s__Lactobacillus_murinus
Spearman Rho Correlation
G
PICRUST KEGG Pathways
Sham HSD vs Sham ND
rCHI ND vs Sham ND
rCHI HSD vs Sham ND
rCHI HSD vs Sham HSD
rCHI HSD vs rCHI ND
Group
Lysine biosynthesis
Antigen processing and presentation
NOD-like receptor signaling pathway
Proteasome
Fatty acid biosynthesis
Phenylalanine, tyrosine and tryptophan biosynthesis
Valine, leucine and isoleucine biosynthesis
Pantothenate and CoA biosynthesis
Histidine metabolism
Tryptophan metabolism
Insulin signaling pathway
Biosynthesis of unsaturated fatty acids
Biosynthesis of ansamycins
Carbohydrate metabolism
Arachidonic acid metabolism
Carbohydrate digestion and absorption
Cysteine and methionine metabolism
Energy metabolism
Glyoxylate and dicarboxylate metabolism
Phenylalanine metabolism
Tropane, piperidine and pyridine alkaloid biosynthesis
Isoquinoline alkaloid biosynthesis
Selenocompound metabolism
Sphingolipid metabolism
D-Alanine metabolism
Glycosyltransferases
Tyrosine metabolism
Ion channels
Glutathione metabolism
Retinol metabolism
Drug metabolism - cytochrome P450
Drug metabolism - other enzymes
Primary bile acid biosynthesis
Secondary bile acid biosynthesis
Fatty acid metabolism
Primary immunodeficiency
Linoleic acid metabolism
RIG-I-like receptor signaling pathway
Fructose and mannose metabolism
Galactose metabolism
Nucleotide excision repair
Protein processing in endoplasmic reticulum
Chaperones and folding catalysts
Glycosaminoglycan degradation
Ubiquinone and other terpenoid-quinone biosynthesis
Apoptosis
Ubiquitin system
Glycerophospholipid metabolism
Glycolysis / Gluconeogenesis
F
f__Erysipelotrichaceae_g__Turicibacter
f__Lachnospiraceae_g__Lachnospiraceae_NK4A136_group
f__Butyricicoccaceae_g__Butyricicoccus
f__Lactobacillaceae_g__Lactobacillus_s__Lactobacillus_murinus
f__Acholeplasmataceae_g__Anaeroplasma
f__Muribaculaceae_g__Muribaculaceae
Relative Abundance (%)
Study day

879

Supplementary Figure 1

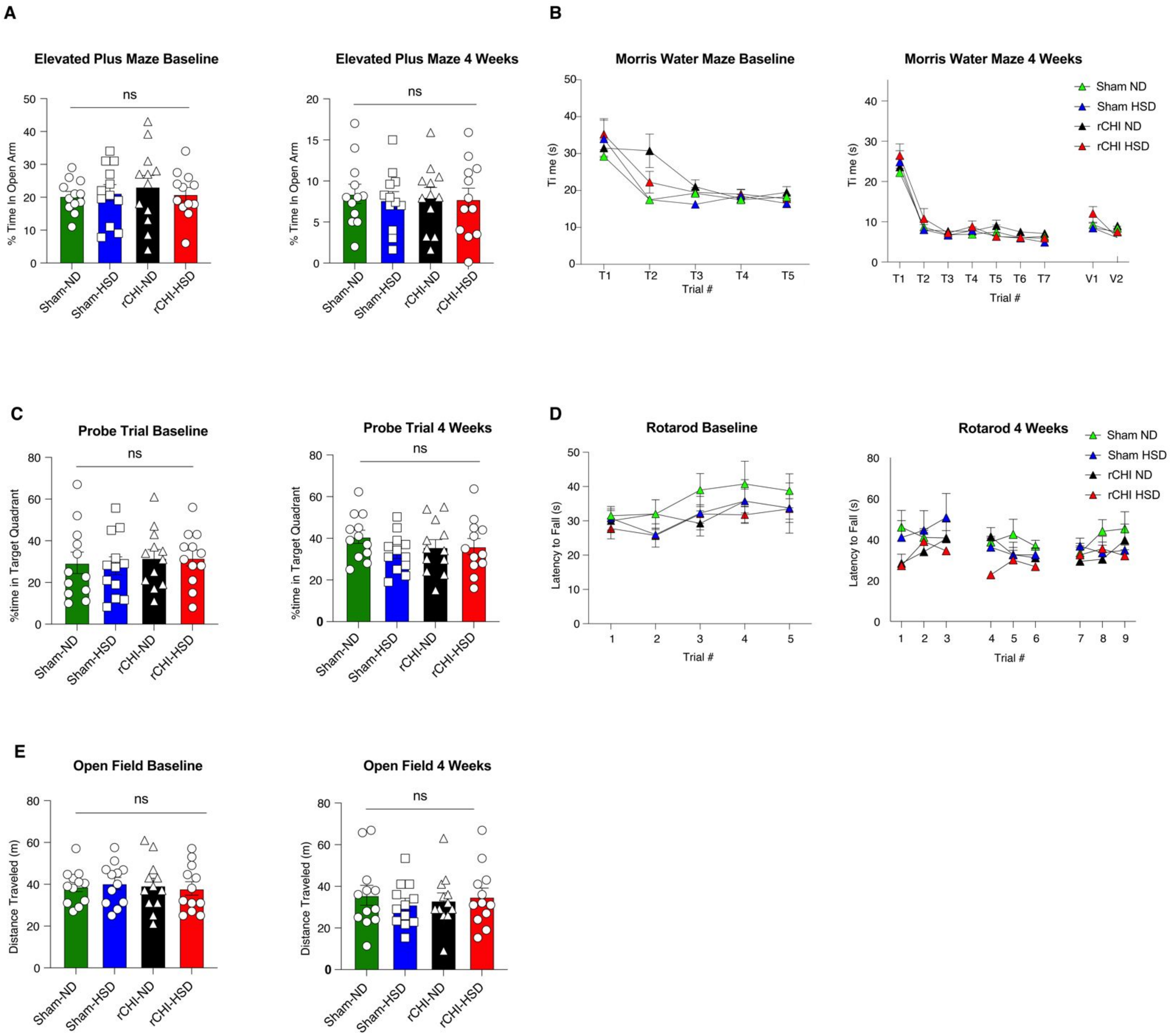

Supplementary Figure 2

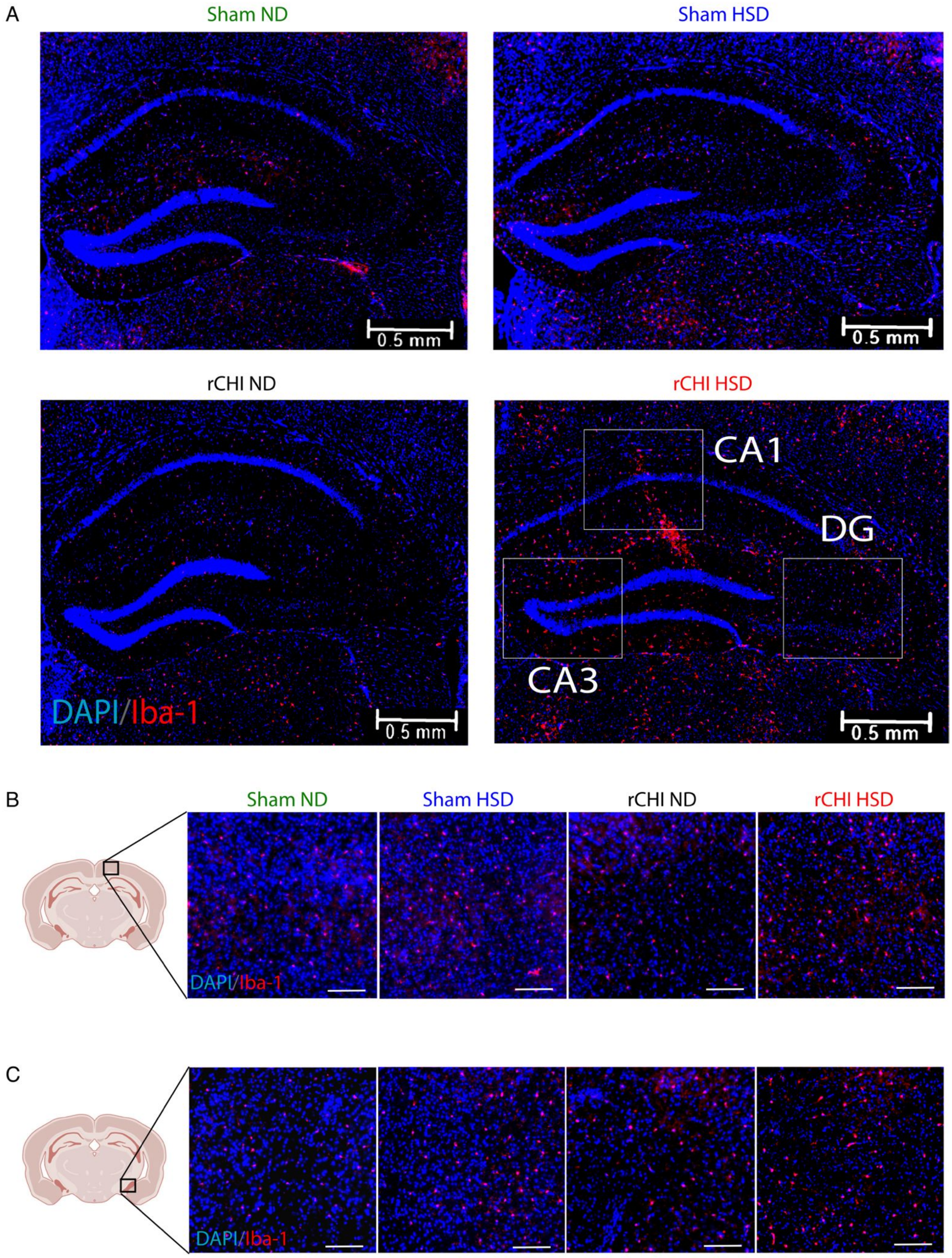

895

Supplementary Figure 3

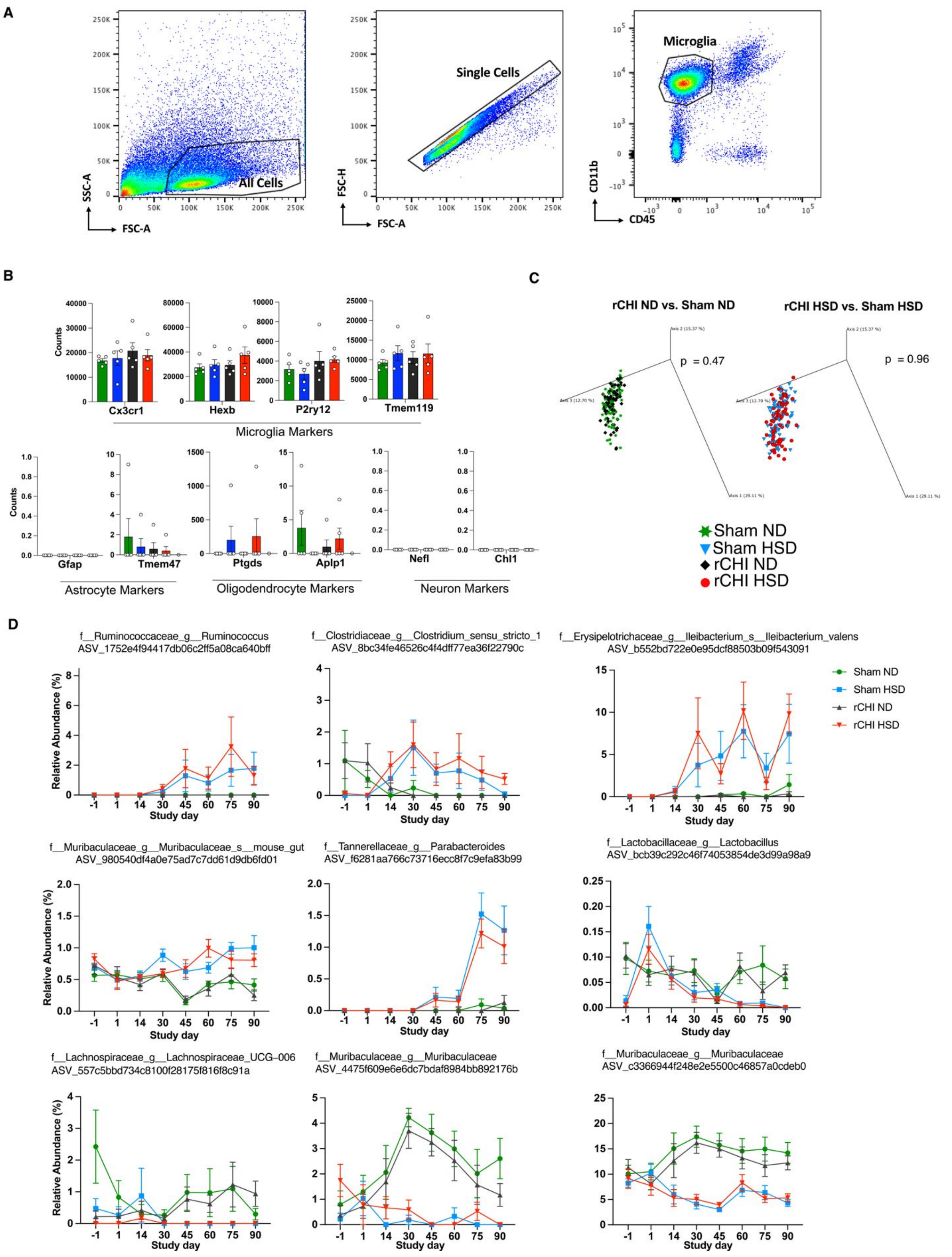

Supplementary Figure 4

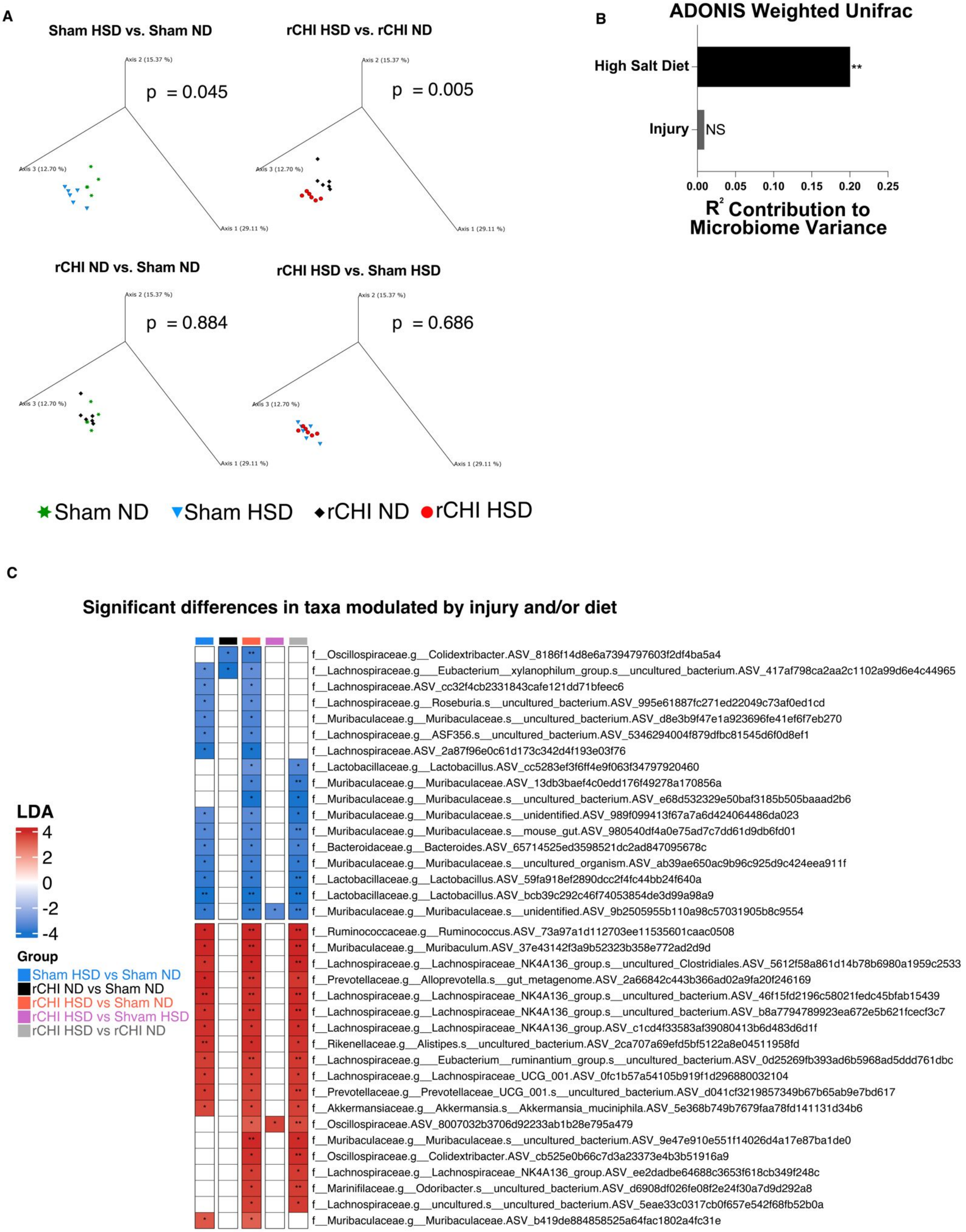

Figure Legends

Figure 1. High salt diet induces an anxiety like phenotype in a rCHI model of concussion. (A) Visual representation of experimental timeline of behavioral and physiological testing regimens after high salt diet (HSD) administration. **(B)** Physiological data of weight, **(C)** Systolic/diastolic blood pressure, and heart rate of the groups through 12 weeks of diet administration. Behavioral testing of anxiety like phenotype using an **(D)** elevated plus maze**, (E)** Morris water maze (MWM) and probe trial, **(F)** rotarod, and **(G)** open field at 8- and 12-weeks post diet administration. Physiological data, Morris water maze, and the rotarod were analyzed by a two-factor repeated measures two-way ANOVA (group x time) and the other behavioral tests were analyzed by one-way ANOVA, followed by a Tukey's multiple comparison. n = 12 mice/group was used for all experiments and data is presented as (mean and SEM). * $p < 0.05$, *** $p < 0.001$, ns = not significant**.**

Figure 2. High salt diet induces chronic microglial activation in a rCHI model of concussion. Representative confocal images of immunofluorescence staining for Iba1+ microglia (red), and DAPI (blue) on different brain regions. Example overviews of **(A)** cortex, different hippocampal areas **(B)** CA1, **(C)** CA3, and **(D)** dentate gyrus (DG), and **(E)** the amygdala in 12 weeks post-ND or HSD in sham and rCHI animals. Quantitative analysis of Iba1+ cells counted in CA1, CA3, and dentate gyrus (DG), and the amygdala in 12 weeks post-ND or HSD in sham and rCHI animals. n=4 mice /group. ** $p < 0.05$, *** $p < 0.001$, **** $p < 0.0001$ ns = not significant by one-way ANOVA with Tukey's multiple comparison test**.** Representative images Scale bars represent 50 μm.

For Review Only

Figure 3. High salt diet induces chronic proinflammatory microglial profile and stress response in a rCHI model of concussion. (A) Visual representation of experimental timeline of microglia analysis and bulk RNAseq after HSD administration. **(B)** DiVenn plot showing the unique and shared differentially expressed genes (P<0.05) of the following groups compared to Sham ND baseline: Sham HSD, rCHI ND, and rCHI HSD. Directionality of gene expression is determined by log2-foldchanges of pairwise gene expression comparisons. Red-colored genes represent upregulated genes. Blue-colored genes represent downregulated genes. Yellow-colored genes represent shared genes that are up-regulated in one comparison but down-regulated in the other comparison. **(C)** Heatmap of relative expression levels clustered according to the unique DEGs obtained in the following pairwise comparisons compared to Sham ND baseline (from top to bottom): rCHI HSD, Sham ND, and rCHI ND (n=5 mice/group). Enriched GO terms of each cluster are displayed on the right with selected corresponding genes labeled on the left.**(D)** Dot plot of GAGE analysis highlighting significantly upregulated GO Biological Process (BP) pathways in the following pairwise comparisons compared to Sham ND baseline: rCHI ND, rCHI HSD, and rCHI ND. Dot size indicates the $\log_2$fold change of the pathway in the specific pairwise comparison (rCHI ND vs Sham ND, rCHI HSD vs Sham ND, or Sham HSD vs Sham ND). Dot color indicates significance strength of the pathway, where p-value < 0.05 was considered statistically significant (indicated by asterisk *). **(E)** Volcano plots showing the microglia gene expression in rCHI HSD vs rCHI ND. On the x-axis are the log2-fold changes and the y-axis is the –log10(p-value). Significant differentially expressed genes (P <0.05) are colored (red for upregulation and blue for downregulation). **(F)** Selected top upregulated GO Biological Process (BP) pathways from GAGE analysis by p-value in rCHI HSD vs rCHI ND. Data is represented by $\log_{10}$(P) and increasing bar color intensity signifies increasing log2-foldchanges. Asterisk (*) indicates p-value < 0.05 **(G)** Top IPA predicted upstream regulators for DEGs in rCHI HSD vs rCHI ND. All shown regulators were deemed significant at P<0.05 and an absolute activation score greater than or equal

to 2. Activation scores were used to determine the activation state of the predicted upstream regulator, where an activation score less than or equal to -2 implies the upstream regulator is inhibited and an activation score greater than or equal to 2 implies that the upstream regulator is activated. **(H)** Brain tissue qPCR was analyzed by one-way ANOVA, followed by Tukey's multiple comparison analysis. n = 4 mice/group. Data is presented as (mean and SEM). * P < 0.05, ** P < 0.01, *** P < 0.001, n.s. = not significant.

Figure 4. High salt diet induces microbiome dysregulation in a rCHI model of concussion. (A) Visual representation of experimental timeline of fecal sample collection for 16S microbiome sequencing. **(B)** Principal coordinate analysis (PCoA) of weighted UniFrac distances stratified by diet and injury (rCHI) microbiota structure: salt diet in Sham mice (left panel, PERMANOVA: pseudo-F = 16.07, p =0.001, n = 157) and by salt diet in injured (rCHI) mice (right panel, PERMANOVA: pseudo-F = 16.83, p = 0.001, n = 156). p-values and test statistics are obtained from PERMANOVA tests on beta-diversity using weighted UniFrac distances. Each point represents the microbiota from one mouse **(C)** ADONIS test using weighted UniFrac distances investigating the contribution of injury (rCHI), salt diet, and timepoint to overall microbiome variation across fecal microbiota samples (n=313) (***= P<0.001, **= P>0.001 & P<0.01, *= P>0.01 & P<0.05). **(D)** Significant differences in taxa modulated by salt diet and/or injury phenotype over time collected from fecal microbiota samples was determined by linear discriminant analysis effect size (LEfSe). Legend represents linear discriminant analysis (LDA) effect size score. (***= P<0.001, **= P>0.001 & P<0.01, *= P>0.01 & P<0.05). **(E)** Spearman correlations between fecal microbial abundance at all timepoints and anxiety-like phenotype (from Figure 1C) measured 12 weeks for each sample group (Sham ND, Sham HSD, rCHI ND, and rCHI HSD) (***= P<0.001, **= P>0.001 & P<0.01, *= P>0.01 & P<0.05). **(F)** Relative abundance of selected taxa from Figure 4E over time that were significantly correlated (Spearman correlation, P < 0.05) in at least two time points collected from fecal microbiota samples. Data is presented as (mean and SEM). **(G)** Investigating microbial functional changes from fecal samples collected across 30-90 days. Significant differences based on the predicted KEGG metagenomic pathways (categorized by PICRUSt2) for each comparison were determined by linear discriminant analysis effect size (LEfSe). Legend represents linear discriminant analysis (LDA) effect size score. n = 7-8 mice/group for the microbiome experiments (***= P<0.001, **= P>0.001 & P<0.01, *= P>0.01 & P<0.05).

Supplementary Figure 1. Behavioral testing of anxiety like phenotype using an **(A)** elevated plus maze**, (B)** Morris water maze (MWM), **(C)** probe trial, **(D)** rotarod, and **(E)** open field at baseline and 4 weeks post diet administration. Morris water maze, and the rotarod were analyzed by a two-factor repeated measures two-way ANOVA (group x time) and the other behavioral tests were analyzed by one-way ANOVA, followed by Tukey's multiple comparison analysis. n = 12 mice/group was used for all experiments and data is presented as (mean and SEM). ns = not significant.

Supplementary Figure 2. Widefield images of different brain regions for microglial cell analysis. (A) Representative images of CA1, CA3, and DG and how they were chosen from the widefield microscope. **(B)** Samples of cortex and **(C)** amygdala and the region that was chosen for analysis from the widefield microscope. Sample Images scale bar is 500 um for hippocampal images and 100 um for cortex and the amygdala

Supplementary Figure 3. (A) Gating strategy of microglia that was sorted for RNA-sequencing **(B)** Validation of microglia RNA-sequencing data by looking into the expression of select microglia markers compared to other non-microglia markers. **C)** Principal coordinate analysis (PCoA) of

For Review Only

weighted UniFrac distances stratified by the effect of rCHI in ND and HSD groups respectively: rCHI ND vs. Sham ND (left panel, PERMANOVA: pseudo-F = 0.894, p = 0.47, n = 156), and rCHI HSD vs. Sham HSD (right panel, PERMANOVA: pseudo-F = 0.335, p = 0.96, n = 157). p-values and test statistics are obtained from PERMANOVA tests on beta-diversity using weighted UniFrac distances. Each point represents the microbiota from one mouse. **(C)** Relative abundance of taxa from Figure 4E over time that were significantly correlated (Spearman correlation, P <0.05) in at least two time points collected from fecal microbiota samples. Data is presented as (mean and SEM).

Supplementary Figure 4. (A) Principal coordinate analysis (PCoA) of weighted UniFrac distances stratified by the following pairwise comparisons: Sham HSD vs. Sham ND (top left panel, PERMANOVA: pseudo-F = 2.41, p = 0.045, n = 11), rCHI HSD vs. rCHI ND (top right panel, PERMANOVA: pseudo-F = 3.49, p = 0.005, n = 12), rCHI ND vs. Sham ND (bottom left panel, PERMANOVA: pseudo-F = 0.470, p = 0.884, n = 11), and rCHI HSD vs. Sham HSD (bottom right panel, PERMANOVA: pseudo-F = 0.471, p = 0.686, n = 12). p-values and test statistics are obtained from PERMANOVA tests on beta-diversity using weighted UniFrac distances. Each point represents the microbiota from one mouse. **(B)** ADONIS test using weighted UniFrac distances investigating the contribution of injury (rCHI) and salt diet to overall microbiome variation across cecum microbiota samples (n=23) (***= P<0.001, **= P>0.001 & P<0.01, *= P>0.01 & P<0.05). **(C)** Significant differences in taxa modulated by salt diet and/or injury phenotype collected from cecum microbiota samples determined by linear discriminant analysis effect size (LEfSe). Legend represents linear discriminant analysis (LDA) effect size score. n = 5-6 mice/group for the microbiome experiments (***= P<0.001, **= P>0.001 & P<0.01, *= P>0.01 & P<0.05**).**

For Review Only

Description of Additional Supplementary Files

Supplementary Table 1. DESeq2 normalized expression counts for all genes. List of normalized expression counts data (DESeq2) for all genes passing quality metrics.

Supplementary Table 2. List of microglia differentially expressed genes in all groups compared to Sham ND baseline. Differential gene expression was performed using DESeq2. Pairwise differential gene expression comparisons (Sham HSD vs. Sham ND, rCHI ND vs. Sham ND, and rCHI HSD vs Sham ND) were done using the Wald Test with standard parameters and log2 fold-changes were subsequently shrunken using DESeq2 built-in lfcshrink function.

Supplementary Table 3. Expression data of the unique DEGs in all pairwise comparisons compared to Sham ND baseline. Expression data (DESeq2) for the unique differentially expressed genes in the following pairwise comparisons: rCHI HSD vs. Sham ND, Sham HSD vs. Sham ND, and rCHI ND vs. Sham ND. The expression data used for heatmap visualization was produced using the variance stabilizing transformation (VST) method from the DESeq2 built-in VST function.

Supplementary Table 4. List of microglia differentially expressed genes in rCHI HSD vs. rCHI ND. Differential gene expression was performed using DESeq2. Pairwise differential gene

1047 expression comparison (rCHI HSD vs. rCHI ND) was done using the Wald Test with standard
1048 parameters and log2 fold-changes were subsequently shrunken using DESeq2 built-in lfcshrink
1049 function.

1051 **Supplementary Table 5. List of significant Ingenuity Pathway Analysis (IPA) upstream**
1052 **regulators in various pairwise differential gene expression comparisons.** Ingenuity Pathway
1053 Analysis (IPA) was used to identify upstream regulators (P <0.05 and |Z-score| ≥ 2) based on the
1054 DEGs in the following pairwise comparisons: rCHI HSD vs. rCHI ND, Sham HSD vs. Sham ND, rCHI
1055 ND vs. Sham ND, rCHI HSD vs. Sham ND, and rCHI HSD vs. Sham HSD. The input data for IPA
1056 comprised of p-values and log_2fold changes of DEGs.

For Review Only

Microglia
DESeq2-Normalized Expression Counts

Gene	Sham_ND_1	Sham_ND_2	Sham_ND_3
.	110344.7807	104021.7722	128568.4034
0610007P14Rik	5.230750811	2.284159643	45.39162705
0610009B22Rik	63.8151599	67.38270948	74.36500601
0610009L18Rik	9.41535146	0	0
0610009O20Rik	153.7840738	0	51.18630284
0610010B08Rik	4.184600649	6.85247893	0.965779299
0610010F05Rik	25.10760389	7.994558752	24.14448247
0610010K14Rik	0	0	22.21292387
0610011F06Rik	17.78455276	0	0
0610012G03Rik	76.36896184	114.2079822	89.81747479
0610025J13Rik	0	0	0
0610030E20Rik	3.138450487	1.142079822	0
0610037L13Rik	122.399569	103.9292638	6.760455092
0610039H22Rik	1.046150162	1.142079822	0.965779299
0610039K10Rik	1.046150162	7.994558752	0.965779299
0610040B10Rik	0	3.426239465	0
0610040J01Rik	719.7513116	424.8536937	738.8211636
1110001J03Rik	4.184600649	0	3.863117195
1110002J07Rik	0	0	0
1110002L01Rik	10.46150162	0	0
1110004E09Rik	0	19.41535697	8.69201369
1110004F10Rik	125.5380195	231.8422038	203.7794321
1110006O24Rik	12.55380195	17.13119732	20.28136528
1110008F13Rik	16.7384026	43.39903322	19.31558598
1110008L16Rik	24.06145373	6.85247893	24.14448247
1110008P14Rik	8.369201298	7.994558752	0
1110012L19Rik	21.96915341	23.98367625	37.66539266
1110018N20Rik	0	0	0
1110019D14Rik	2.092300324	9.136638573	16.41824808
1110020A21Rik	0	0	0
1110032A03Rik	63.8151599	28.55199554	44.42584775
1110034G24Rik	48.12290746	83.37182698	60.84409583
1110037F02Rik	85.7843133	68.5247893	70.50188882
1110038F14Rik	3.138450487	0	0
1110051M20Rik	240.6145373	220.4214056	269.4524244
1110059E24Rik	79.50741233	101.6451041	34.76805476
1110059G10Rik	40.79985633	42.2569534	65.67299232
1110065P20Rik	0	1.142079822	0.965779299
1190002N15Rik	10.46150162	46.82527269	17.38402738
1190007I07Rik	8.369201298	5.710399108	0
1300002E11Rik	16.7384026	17.13119732	8.69201369
1500004A13Rik	24.06145373	14.84703768	11.58935159
1500011B03Rik	46.03060714	45.68319287	31.87071686
1500011K16Rik	1.046150162	87.94014627	0
1500015A07Rik	13.59995211	22.84159643	12.55513089
1500015O10Rik	48.12290746	0	0

For Review Only

1500026H17Rik	6.276900973	0	0
1500035N22Rik	4.184600649	6.85247893	3.863117195
1600002H07Rik	46.03060714	10.27871839	27.04182037
1600010M07Rik	12.55380195	1.142079822	31.87071686
1600012H06Rik	120.3072687	143.9020575	149.6957913
1600014C10Rik	163.1994253	150.7545365	0
1600020E01Rik	4.184600649	0	0
1600023N17Rik	0	1.142079822	0
1700001C19Rik	1.046150162	4.568319287	0
1700001J11Rik	0	0	0
1700001K19Rik	1.046150162	0	0
1700001L05Rik	5.230750811	1.142079822	14.48668948
1700003F12Rik	5.230750811	0	4.828896494
1700006H20Rik	1.046150162	3.426239465	3.863117195
1700007E05Rik	0	1.142079822	0
1700007L15Rik	1.046150162	4.568319287	10.62357229
1700008J07Rik	5.230750811	4.568319287	5.794675793
1700009J07Rik	2.092300324	0	0.965779299
1700010I14Rik	17.78455276	4.568319287	14.48668948
1700010L04Rik	3.138450487	0	1.931558598
1700011L22Rik	0	0	0.965779299
1700012D01Rik	3.138450487	0	2.897337897
1700012D14Rik	0	7.994558752	0
1700015E13Rik	5.230750811	6.85247893	0.965779299
1700016A09Rik	3.138450487	0	0
1700017B05Rik	1612.1174	778.8984384	668.3192748
1700018A23Rik	0	4.568319287	0
1700019D03Rik	2.092300324	5.710399108	0.965779299
1700020D05Rik	0	0	0
1700020I14Rik	13.59995211	27.40991572	13.52091018
1700020N01Rik	1.046150162	3.426239465	3.863117195
1700021F05Rik	17.78455276	35.40447447	32.83649616
1700022I11Rik	5.230750811	0	0
1700022N22Rik	1.046150162	0	0
1700025G04Rik	12.55380195	6.85247893	0
1700025N23Rik	3.138450487	1.142079822	10.62357229
1700028E10Rik	18.83070292	1.142079822	0
1700028J19Rik	0	0	0
1700028K03Rik	4.184600649	6.85247893	0.965779299
1700029J07Rik	35.56910552	47.96735251	16.41824808
1700030C10Rik	6.276900973	3.426239465	10.62357229
1700030J22Rik	2.092300324	2.284159643	0
1700030K09Rik	58.58440909	41.11487358	48.28896494
1700034I23Rik	2.092300324	0	0
1700034J05Rik	0	0	0
1700037C18Rik	0	0	16.41824808
1700037H04Rik	27.19990422	22.84159643	15.45246878
1700039M10Rik	1.046150162	2.284159643	0
1700040D17Rik	3.138450487	0	0.965779299
1700047I17Rik2	32.43065503	22.84159643	29.93915826

For Review Only

1700048M11Rik	0	0	0
1700049G17Rik	63.8151599	55.96191126	36.69961336
1700052K11Rik	7.323051136	4.568319287	0.965779299
1700054O19Rik	0	1.142079822	5.794675793
1700055D18Rik	25.10760389	9.136638573	11.58935159
1700061G19Rik	17.78455276	9.136638573	8.69201369
1700066B19Rik	5.230750811	12.56287804	7.726234391
1700066M21Rik	0	46.82527269	0
1700067K01Rik	0	0	0
1700073E17Rik	0	0	0
1700080G18Rik	0	4.568319287	0
1700084E18Rik	0	1.142079822	0.965779299
1700084J12Rik	3.138450487	0	0
1700086O06Rik	3.138450487	6.85247893	0
1700086P04Rik	0	0	3.863117195
1700087I21Rik	3.138450487	7.994558752	3.863117195
1700094D03Rik	0	0	50.22052354
1700095B10Rik	0	0	0
1700095J12Rik	7.323051136	3.426239465	0
1700096K18Rik	8.369201298	7.994558752	10.62357229
1700102P08Rik	3.138450487	3.426239465	3.863117195
1700110C19Rik	4.184600649	21.69951661	21.24714457
1700110I01Rik	5.230750811	2.284159643	3.863117195
1700112E06Rik	13.59995211	25.12575608	22.21292387
1700113A16Rik	0	4.568319287	1.931558598
1700120K04Rik	1.046150162	6.85247893	0
1700122E12Rik	3.138450487	0	0
1700123M08Rik	0	2.284159643	0
1700123O20Rik	32.43065503	42.2569534	31.87071686
1700125H20Rik	0	0	0
1700126G02Rik	0	0	0
1810009A15Rik	39.75370617	0	81.1254611
1810010H24Rik	2.092300324	9.136638573	9.657792988
1810011H11Rik	39.75370617	29.69407536	191.2243012
1810011O10Rik	8.369201298	15.9891175	10.62357229
1810013A23Rik	2.092300324	4.568319287	0
1810013L24Rik	59.63055925	101.6451041	112.0303987
1810014B01Rik	17.78455276	26.2678359	21.24714457
1810019D21Rik	0	0	0
1810019N24Rik	0	0	0
1810021B22Rik	0	0	0
1810022K09Rik	51.26135795	42.2569534	41.52850985
1810026B05Rik	13.59995211	61.67231037	25.11026177
1810026J23Rik	33.47680519	38.83071394	0
1810030O07Rik	165.2917256	171.3119732	216.3345629
1810032O08Rik	32.43065503	14.84703768	13.52091018
1810034E14Rik	5.230750811	2.284159643	11.58935159
1810037I17Rik	46.03060714	41.11487358	73.39922671
1810041H14Rik	0	1.142079822	1.931558598
1810043G02Rik	1.046150162	14.84703768	72.43344741

For Review Only

1810043H04Rik	0	0	15.45246878
1810044D09Rik	0	0	10.62357229
1810055G02Rik	4.184600649	7.994558752	0
1810058I24Rik	49.16905763	45.68319287	55.04942003
2010010A06Rik	0	0	0
2010016I18Rik	0	0	2.897337897
2010106C02Rik	0	5.710399108	0.965779299
2010107E04Rik	61.72285957	51.39359197	91.74903339
2010107G23Rik	3.138450487	6.85247893	1.931558598
2010109I03Rik	7.323051136	7.994558752	0.965779299
2010111I01Rik	83.69201298	73.09310859	67.60455092
2010204K13Rik	1.046150162	0	1.931558598
2010300C02Rik	0	5.710399108	5.794675793
2010315B03Rik	117.1688182	115.350062	52.15208214
2010320M18Rik	15.69225243	5.710399108	10.62357229
2210013O21Rik	18.83070292	34.26239465	32.83649616
2210016F16Rik	125.5380195	125.6287804	137.1406604
2210016L21Rik	0	1.142079822	2.897337897
2210404O09Rik	21.96915341	19.41535697	6.760455092
2210408F21Rik	0	0	0.965779299
2210408I21Rik	8.369201298	5.710399108	0
2210417A02Rik	2.092300324	4.568319287	0
2210418O10Rik	20.92300324	14.84703768	20.28136528
2300009A05Rik	10.46150162	12.56287804	5.794675793
2310009A05Rik	27.19990422	47.96735251	41.52850985
2310009B15Rik	0	0	0
2310010J17Rik	0	13.70495786	0.965779299
2310011J03Rik	1.046150162	25.12575608	0
2310015A10Rik	11.50765178	22.84159643	27.04182037
2310015A16Rik	0	0	0
2310022A10Rik	59.63055925	18.27327715	22.21292387
2310022B05Rik	12.55380195	6.85247893	10.62357229
2310033P09Rik	51.26135795	51.39359197	59.87831653
2310034G01Rik	3.138450487	3.426239465	5.794675793
2310034P14Rik	2.092300324	1.142079822	0
2310035C23Rik	60.67670941	103.9292638	46.35740634
2310036O22Rik	1.046150162	28.55199554	47.32318564
2310039H08Rik	14.64610227	20.55743679	16.41824808
2310040G24Rik	6.276900973	23.98367625	27.04182037
2310047D07Rik	4.184600649	3.426239465	5.794675793
2310057M21Rik	13.59995211	23.98367625	52.15208214
2310061I04Rik	18.83070292	11.42079822	27.04182037
2310068J16Rik	0	1.142079822	0
2310075K07Rik	0	5.710399108	2.897337897
2410002F23Rik	93.10736444	82.22974716	118.7908538
2410004B18Rik	34.52295535	45.68319287	0
2410015M20Rik	29.29220454	30.83615518	66.63877162
2410016O06Rik	8.369201298	58.2460709	16.41824808
2410021H03Rik	0	0	0
2410022M11Rik	14.64610227	4.568319287	5.794675793

For Review Only

2410089E03Rik	5.230750811	14.84703768	37.66539266
2410131K14Rik	31.38450487	23.98367625	34.76805476
2500004C02Rik	16.7384026	23.98367625	0
2510003B16Rik	0	0	0
2510009E07Rik	147.5071729	91.36638573	86.9201369
2510039O18Rik	46.03060714	82.22974716	78.22812321
2610001J05Rik	0	0	0.965779299
2610002M06Rik	17.78455276	27.40991572	10.62357229
2610005L07Rik	98.33811525	75.37726823	73.39922671
2610008E11Rik	79.50741233	53.67775162	65.67299232
2610020C07Rik	3.138450487	11.42079822	8.69201369
2610020H08Rik	7.323051136	6.85247893	13.52091018
2610021A01Rik	21.96915341	35.40447447	22.21292387
2610027K06Rik	2.092300324	12.56287804	6.760455092
2610035D17Rik	33.47680519	23.98367625	15.45246878
2610042L04Rik	14.64610227	14.84703768	14.48668948
2610044O15Rik8	6.276900973	28.55199554	18.34980668
2610203C22Rik	18.83070292	50.25151215	23.17870317
2610301B20Rik	42.89215665	18.27327715	33.80227546
2610306M01Rik	6.276900973	6.85247893	10.62357229
2610507B11Rik	142.2764221	162.1753347	101.4068264
2610507I01Rik	4.184600649	0	0
2610524H06Rik	4.184600649	0	8.69201369
2610528A11Rik	0	0	55.04942003
2700012I20Rik	5.230750811	10.27871839	5.794675793
2700038G22Rik	5.230750811	1.142079822	9.657792988
2700046G09Rik	15.69225243	10.27871839	9.657792988
2700049A03Rik	27.19990422	25.12575608	28.00759967
2700060E02Rik	2.092300324	60.53023055	0
2700062C07Rik	5.230750811	1.142079822	0
2700070H01Rik	0	3.426239465	0
2700081O15Rik	27.19990422	13.70495786	13.52091018
2700094K13Rik	17.78455276	0	29.93915826
2700097O09Rik	35.56910552	52.5356718	46.35740634
2810001G20Rik	2.092300324	4.568319287	0
2810002D19Rik	11.50765178	23.98367625	24.14448247
2810004N23Rik	43.93830681	37.68863411	33.80227546
2810006K23Rik	69.04591071	34.26239465	82.0912404
2810013P06Rik	77.41511201	81.08766734	125.5513089
2810021J22Rik	37.66140584	59.38815073	43.46006845
2810025M15Rik	55.4459586	94.7926252	100.4410471
2810029C07Rik	2.092300324	2.284159643	0
2810039B14Rik	16.7384026	10.27871839	11.58935159
2810047C21Rik1	2.092300324	9.136638573	7.726234391
2810403A07Rik	146.4610227	118.7763015	137.1406604
2810403D21Rik	2.092300324	0	0
2810407A14Rik	0	0	0
2810408A11Rik	0	1.142079822	0
2810408B13Rik	3.138450487	7.994558752	0.965779299
2810417H13Rik	2.092300324	1.142079822	0

For Review Only

2810428I15Rik	55.4459586	60.53023055	58.91253723
2810429I04Rik	0	1.142079822	0
2810442N19Rik	3.138450487	3.426239465	0
2810454H06Rik	10.46150162	1.142079822	9.657792988
2810468N07Rik	7.323051136	1.142079822	6.760455092
2810474O19Rik	309.660448	319.7823501	276.2128795
2900005J15Rik	27.19990422	7.994558752	19.31558598
2900026A02Rik	46.03060714	13.70495786	39.59695125
2900040C04Rik	26.15375406	0	0
2900060B14Rik	6.276900973	14.84703768	15.45246878
2900060L22Rik	0	4.568319287	4.828896494
2900076A07Rik	5.230750811	4.568319287	0
2900089D17Rik	0	0	0
2900097C17Rik	334.7680519	323.2085895	189.2927426
3000002C10Rik	4.184600649	12.56287804	0.965779299
3010026O09Rik	18.83070292	20.55743679	22.21292387
3110001I22Rik	2.092300324	20.55743679	4.828896494
3110002H16Rik	0	0	4.828896494
3110009E18Rik	4.184600649	4.568319287	0
3110035E14Rik	19.87685308	41.11487358	20.28136528
3110040N11Rik	20.92300324	44.54111304	42.49428915
3110043O21Rik	140.1841217	134.765419	105.2699436
3110052M02Rik	57.53825892	49.10943233	57.94675793
3110053B16Rik	0	0	0
3110056K07Rik	39.75370617	1.142079822	11.58935159
3110062M04Rik	1.046150162	0	0
3110070M22Rik	1.046150162	6.85247893	0
3110082I17Rik	53.35365827	45.68319287	34.76805476
3110083C13Rik	3.138450487	1.142079822	0
3200001D21Rik	1.046150162	1.142079822	0
3300002A11Rik	3.138450487	2.284159643	0
3300002I08Rik	10.46150162	6.85247893	0
3425401B19Rik	0	2.284159643	0
3632454L22Rik	0	2.284159643	0
3830403N18Rik	2.092300324	14.84703768	0
3830406C13Rik	54.39980844	41.11487358	26.07604107
3830408C21Rik	4.184600649	0	1.931558598
4430402I18Rik	8.369201298	0	0
4632404H12Rik	15.69225243	14.84703768	22.21292387
4632415L05Rik	26.15375406	38.83071394	28.00759967
4632428C04Rik	1.046150162	0	0
4732440D04Rik	25.10760389	35.40447447	29.93915826
4732471J01Rik	13.59995211	41.11487358	19.31558598
4732491K20Rik	8.369201298	1.142079822	9.657792988
4831407H17Rik	3.138450487	0	0.965779299
4831440D22Rik	2.092300324	5.710399108	10.62357229
4831440E17Rik	3.138450487	14.84703768	3.863117195
4833411C07Rik	0	9.136638573	8.69201369
4833417C18Rik	5.230750811	5.710399108	14.48668948
4833418N02Rik	4.184600649	1.142079822	0

For Review Only

4833419F23Rik	0	0	0
4833420G17Rik	230.1530357	222.7055652	134.2433225
4833422C13Rik	0	2.284159643	0.965779299
4833438C02Rik	20.92300324	18.27327715	28.97337897
4833439L19Rik	153.7840738	122.2025409	351.5436648
4833447I15Rik	1.046150162	1.142079822	6.760455092
4921507G05Rik	3.138450487	1.142079822	0
4921507P07Rik	1.046150162	0	4.828896494
4921511C10Rik	26.15375406	29.69407536	20.28136528
4921517D16Rik	9.41535146	0	0
4921524J17Rik	1.046150162	12.56287804	13.52091018
4921531C22Rik	13.59995211	26.2678359	23.17870317
4921536K21Rik	0	0	1.931558598
4930402H24Rik	39.75370617	36.54655429	18.34980668
4930403D09Rik	4.184600649	5.710399108	2.897337897
4930404H24Rik	4.184600649	15.9891175	0
4930404I05Rik	1.046150162	2.284159643	0.965779299
4930405A10Rik	1.046150162	2.284159643	0
4930405O22Rik	4.184600649	9.136638573	13.52091018
4930412C18Rik	2.092300324	0	2.897337897
4930412L05Rik	0	0	0
4930413G21Rik	0	1.142079822	1.931558598
4930414N06Rik	0	0	30.90493756
4930426L09Rik	2.092300324	2.284159643	0
4930427A07Rik	0	2.284159643	6.760455092
4930429B21Rik	2.092300324	0	4.828896494
4930430F08Rik	3.138450487	7.994558752	11.58935159
4930432K21Rik	5.230750811	9.136638573	4.828896494
4930438A08Rik	6.276900973	0	0
4930439A04Rik	1.046150162	6.85247893	0
4930444A19Rik	30.33835471	33.12031483	19.31558598
4930445E18Rik	0	0	3.863117195
4930447K03Rik	4.184600649	9.136638573	0
4930447M23Rik	5.230750811	6.85247893	10.62357229
4930451E10Rik	0	1.142079822	0.965779299
4930451G09Rik	9.41535146	0	9.657792988
4930453L07Rik	0	0	0
4930453N24Rik	66.95361038	36.54655429	56.98097863
4930467E23Rik	4.184600649	5.710399108	12.55513089
4930469K13Rik	20.92300324	4.568319287	18.34980668
4930473A02Rik	0	4.568319287	0.965779299
4930473H19Rik	3.138450487	4.568319287	3.863117195
4930478L05Rik	3.138450487	1.142079822	0
4930480K23Rik	12.55380195	22.84159643	4.828896494
4930481A15Rik	42.89215665	38.83071394	42.49428915
4930483K19Rik	1.046150162	0	0
4930486L24Rik	0	0	2.897337897
4930487H11Rik	1.046150162	7.994558752	0
4930500A05Rik	0	0	0
4930502C15Rik	5.230750811	22.84159643	5.794675793

For Review Only

4930503E24Rik	7.323051136	0	6.760455092
4930503L19Rik	21.96915341	30.83615518	36.69961336
4930505N22Rik	1.046150162	0	0.965779299
4930506C21Rik	9.41535146	25.12575608	12.55513089
4930511A08Rik	0	0	3.863117195
4930512B01Rik	0	3.426239465	0
4930512H18Rik	0	0	0
4930513N10Rik	0	0	0
4930515G01Rik	3.138450487	0	2.897337897
4930519F09Rik	3.138450487	1.142079822	0
4930521O11Rik	7.323051136	6.85247893	7.726234391
4930522L14Rik	77.41511201	69.66686912	85.9543576
4930522P08Rik	0	0	0
4930523C07Rik	4.184600649	12.56287804	0
4930525G20Rik	1.046150162	0	0
4930526I15Rik	7.323051136	17.13119732	7.726234391
4930528A17Rik	0	0	0
4930529C04Rik	0	0	0.965779299
4930538K18Rik	0	4.568319287	3.863117195
4930539E08Rik	1.046150162	1.142079822	0
4930549G23Rik	2.092300324	0	0
4930550C14Rik	2.092300324	4.568319287	0
4930552P12Rik	0	0	0
4930555F03Rik	1.046150162	0	0
4930555G01Rik	2.092300324	1.142079822	2.897337897
4930562C15Rik	0	0	0
4930563D23Rik	0	0	0
4930563E18Rik	0	0	0
4930563E22Rik	8.369201298	14.84703768	0
4930563I02Rik	7.323051136	12.56287804	24.14448247
4930564C03Rik	1.046150162	4.568319287	0.965779299
4930565N06Rik	1.046150162	1.142079822	5.794675793
4930568A12Rik	5.230750811	0	6.760455092
4930572G02Rik	1.046150162	1.142079822	2.897337897
4930577N17Rik	8.369201298	7.994558752	5.794675793
4930579G18Rik	7.323051136	2.284159643	7.726234391
4930579G24Rik	1.046150162	1.142079822	0
4930579K19Rik	1.046150162	10.27871839	4.828896494
4930581F22Rik	10.46150162	10.27871839	16.41824808
4930590J08Rik	6.276900973	9.136638573	0
4930592I03Rik	0	0	0
4930594C11Rik	55.4459586	100.5030243	79.19390251
4930599N23Rik	0	0	1.931558598
4931402G19Rik	39.75370617	33.12031483	53.11786144
4931403G20Rik	1.046150162	1.142079822	0
4931406C07Rik	49.16905763	42.2569534	56.98097863
4931406H21Rik	103.5688661	79.94558752	79.19390251
4931406P16Rik	97.29196509	158.7490952	60.84409583
4931413K12Rik	0	3.426239465	0
4931414P19Rik	35.56910552	28.55199554	20.28136528

For Review Only

4931415C17Rik	0	0	0
4931428F04Rik	13.59995211	0	0
4931440F15Rik	9.41535146	6.85247893	16.41824808
4931440P22Rik	0	1.142079822	0
4932416H05Rik	2.092300324	1.142079822	3.863117195
4932438A13Rik	213.4146331	261.5362792	148.730012
4932438H23Rik	0	1.142079822	0
4932443L11Rik	2.092300324	3.426239465	0
4933400F21Rik	3.138450487	6.85247893	4.828896494
4933404O12Rik	8.369201298	1.142079822	13.52091018
4933406C10Rik	0	0	0.965779299
4933406F09Rik	1.046150162	1.142079822	0
4933406I18Rik	28.24605438	13.70495786	8.69201369
4933406J10Rik	1.046150162	0	0
4933406M09Rik	4.184600649	3.426239465	0
4933407K13Rik	18.83070292	5.710399108	24.14448247
4933408J17Rik	0	0	0
4933411K16Rik	4.184600649	0	0
4933412E12Rik	7.323051136	7.994558752	18.34980668
4933413J09Rik	3.138450487	3.426239465	0
4933417D19Rik	0	1.142079822	0
4933417G07Rik	4.184600649	4.568319287	3.863117195
4933421O10Rik	38.707556	12.56287804	6.760455092
4933423P22Rik	25.10760389	23.98367625	0
4933424M12Rik	3.138450487	1.142079822	0
4933427D14Rik	32.43065503	27.40991572	30.90493756
4933427G23Rik	1.046150162	0	0.965779299
4933431E20Rik	1.046150162	0	6.760455092
4933431G14Rik	44.98445698	13.70495786	32.83649616
4933432I03Rik	0	0	2.897337897
4933433G08Rik	1.046150162	0	0
4933433G15Rik	0	4.568319287	0
4933434E20Rik	116.122668	1.142079822	302.2889205
4933438K21Rik	1.046150162	2.284159643	0.965779299
4933439C10Rik	16.7384026	10.27871839	8.69201369
4933439K11Rik	2.092300324	0	0
4933440N22Rik	0	0	0
5031414D18Rik	80.55356249	189.5852504	84.9885783
5031425E22Rik	18.83070292	13.70495786	19.31558598
5031425F14Rik	0	2.284159643	2.897337897
5031426D15Rik	2.092300324	1.142079822	0
5031434O11Rik	0	0	4.828896494
5031439G07Rik	58.58440909	99.36094448	90.78325409
5033403F01Rik	3.138450487	0	0
5033417F24Rik	4.184600649	0	0.965779299
5033421B08Rik	0	7.994558752	0
5430402O13Rik	1.046150162	0	1.931558598
5430405H02Rik	8.369201298	29.69407536	19.31558598
5430427O19Rik	88.92276379	141.6178979	155.4904671
5430431A17Rik	0	0	0

For Review Only

5530601H04Rik	11.50765178	0	2.897337897
5730405O15Rik	1.046150162	2.284159643	0
5730408K05Rik	3.138450487	0	0
5730409E04Rik	28.24605438	31.97823501	0
5730409K12Rik	13.59995211	13.70495786	1.931558598
5730422E09Rik	4.184600649	11.42079822	4.828896494
5730455P16Rik	151.6917735	22.84159643	56.98097863
5730480H06Rik	1.046150162	4.568319287	0
5830403F22Rik	0	0	0.965779299
5830416I19Rik	0	0	0
5830417I10Rik	0	0	106.2357229
5830432E09Rik	5.230750811	31.97823501	8.69201369
5830444B04Rik	26.15375406	33.12031483	28.00759967
5830454E08Rik	2.092300324	6.85247893	1.931558598
5930403L14Rik	3.138450487	2.284159643	0
5930403N24Rik	4.184600649	0	0
5930430L01Rik	1.046150162	1.142079822	0
6030443J06Rik	0	0	6.760455092
6030458C11Rik	80.55356249	83.37182698	86.9201369
6030468B19Rik	3.138450487	0	12.55513089
6230400D17Rik	5.230750811	2.284159643	0
6330403K07Rik	3.138450487	0	3.863117195
6330407A03Rik	33.47680519	95.93470502	56.98097863
6330408A02Rik	15.69225243	5.710399108	7.726234391
6330409D20Rik	0	0	0
6330415G19Rik	8.369201298	6.85247893	5.794675793
6330416G13Rik	194.5839302	180.4486118	187.361184
6330418K02Rik	21.96915341	17.13119732	0
6330549D23Rik	10.46150162	15.9891175	12.55513089
6430548M08Rik	53.35365827	29.69407536	42.49428915
6430550D23Rik	1.046150162	2.284159643	0
6430562O15Rik	1.046150162	0	0
6430571L13Rik	0	0	0
6430590A07Rik	8.369201298	3.426239465	0
6530402F18Rik	16.7384026	28.55199554	8.69201369
6720489N17Rik	13.59995211	7.994558752	8.69201369
6820431F20Rik	86.83046347	93.65054538	84.022799
8030423J24Rik	1.046150162	19.41535697	28.00759967
8030442B05Rik	8.369201298	12.56287804	12.55513089
8030453O22Rik	13.59995211	3.426239465	8.69201369
8030462N17Rik	56.49210876	74.23518841	42.49428915
8430408G22Rik	4.184600649	0	0
8430429K09Rik	4.184600649	7.994558752	6.760455092
8430431K14Rik	0	1.142079822	0
9030025P20Rik	23.01530357	37.68863411	25.11026177
9030617O03Rik	110.8919172	69.66686912	119.7566331
9030624J02Rik	184.1224286	172.4540531	226.9581352
9130011E15Rik	77.41511201	79.94558752	73.39922671
9130019O22Rik	41.84600649	18.27327715	61.80987513
9130019P16Rik	9.41535146	0	6.760455092

For Review Only

9130023H24Rik	17.78455276	43.39903322	109.1330608
9130024F11Rik	1.046150162	0	0
9130208D14Rik	8.369201298	22.84159643	9.657792988
9130221H12Rik	1.046150162	15.9891175	11.58935159
9130230L23Rik	0	0	1.931558598
9130401M01Rik	33.47680519	37.68863411	30.90493756
9230114K14Rik	4.184600649	4.568319287	3.863117195
9230116N13Rik	0	3.426239465	0
9330020H09Rik	6.276900973	5.710399108	2.897337897
9330102E08Rik	8.369201298	1.142079822	0
9330104G04Rik	0	2.284159643	0.965779299
9330133O14Rik	128.67647	86.79806645	111.0646194
9330136K24Rik	0	0	0
9330151L19Rik	2.092300324	28.55199554	9.657792988
9330159M07Rik	8.369201298	6.85247893	6.760455092
9330198I05Rik	0	3.426239465	0
9430015G10Rik	0	0	14.48668948
9430016H08Rik	14.64610227	35.40447447	22.21292387
9430018G01Rik	2.092300324	0	0
9430020K01Rik	0	0	0
9430037G07Rik	4.184600649	1.142079822	0
9430038I01Rik	11.50765178	9.136638573	21.24714457
9430060I03Rik	13.59995211	18.27327715	30.90493756
9430083A17Rik	5.230750811	7.994558752	5.794675793
9430085M18Rik	0	1.142079822	0
9430091E24Rik	9.41535146	9.136638573	12.55513089
9530027J09Rik	4.184600649	6.85247893	0
9530034E10Rik	17.78455276	26.2678359	6.760455092
9530036M11Rik	77.41511201	76.51934805	114.9277366
9530052C20Rik	0	1.142079822	0
9530053A07Rik	1.046150162	0	0
9530057J20Rik	3.138450487	0	0.965779299
9530062K07Rik	0	0	0
9530068E07Rik	405.906263	422.569534	508.9656905
9530077C05Rik	2.092300324	0	3.863117195
9530082P21Rik	6.276900973	17.13119732	10.62357229
9530091C08Rik	1.046150162	2.284159643	0
9630013K17Rik	3.138450487	1.142079822	5.794675793
9630015K15Rik	1.046150162	4.568319287	0
9630028I04Rik	93.10736444	15.9891175	0
9830147E19Rik	19.87685308	14.84703768	27.04182037
9930012K11Rik	16.7384026	6.85247893	16.41824808
9930014A18Rik	3.138450487	0	10.62357229
9930021J03Rik	47.0767573	43.39903322	31.87071686
9930104L06Rik	43.93830681	51.39359197	62.77565443
9930111J21Rik1	126.5841696	148.4703768	8.69201369
9930111J21Rik2	0	0	0
A030001D20Rik	0	0	0
A130010J15Rik	51.26135795	83.37182698	56.98097863
A130051J06Rik	11.50765178	3.426239465	10.62357229

For Review Only

A130077B15Rik	6.276900973	4.568319287	0
A230046K03Rik	101.4765657	11.42079822	0
A230050P20Rik	1.046150162	0	0
A230056J06Rik	1.046150162	7.994558752	4.828896494
A230056P14Rik	13.59995211	2.284159643	19.31558598
A230072C01Rik	5.230750811	6.85247893	10.62357229
A230107N01Rik	0	0	0
A330023F24Rik	12.55380195	7.994558752	5.794675793
A330032B11Rik	0	0	0
A330035P11Rik	2.092300324	0	0.965779299
A330040F15Rik	2.092300324	4.568319287	0
A330069E16Rik	0	0	2.897337897
A430005L14Rik	1.046150162	1.142079822	0
A430010J10Rik	2.092300324	0	0
A430033K04Rik	34.52295535	67.38270948	26.07604107
A430035B10Rik	5.230750811	1.142079822	4.828896494
A430046D13Rik	29.29220454	3.426239465	15.45246878
A430057M04Rik	48.12290746	37.68863411	53.11786144
A430078I02Rik	0	0	0
A430090L17Rik	3.138450487	7.994558752	12.55513089
A430105I19Rik	0	4.568319287	0
A430105J06Rik	1.046150162	0	0
A430110L20Rik	20.92300324	11.42079822	0.965779299
A530010L16Rik	6.276900973	5.710399108	8.69201369
A530032D15Rik	5.230750811	3.426239465	6.760455092
A530040E14Rik	4.184600649	3.426239465	3.863117195
A530064D06Rik	1.046150162	1.142079822	0
A530072M11Rik	6.276900973	0	9.657792988
A530088E08Rik	44.98445698	74.23518841	79.19390251
A630001G21Rik	0	9.136638573	0
A630001O12Rik	0	4.568319287	0
A630033H20Rik	56.49210876	74.23518841	56.98097863
A630034I12Rik	1.046150162	1.142079822	0
A630066F11Rik	7.323051136	6.85247893	9.657792988
A630072M18Rik	10.46150162	6.85247893	9.657792988
A630089N07Rik	3.138450487	1.142079822	0
A730017L22Rik	4.184600649	5.710399108	5.794675793
A730020M07Rik	2.092300324	0	8.69201369
A730049H05Rik	0	1.142079822	0
A730063M14Rik	0	0	4.828896494
A730090N16Rik	0	0	0
A830009L08Rik	4.184600649	0	1.931558598
A830010M20Rik	5.230750811	14.84703768	7.726234391
A830021F12Rik	2.092300324	0	0
A830035O19Rik	0	3.426239465	0.965779299
A830052D11Rik	12.55380195	4.568319287	6.760455092
A830080D01Rik	10.46150162	54.81983144	28.00759967
A830082N09Rik	3.138450487	3.426239465	4.828896494
A930005H10Rik	3.138450487	5.710399108	5.794675793
A930006K02Rik	0	3.426239465	6.760455092

For Review Only

A930007A09Rik	4.184600649	1.142079822	11.58935159
A930013F10Rik	25.10760389	31.97823501	31.87071686
A930015D03Rik	6.276900973	0	3.863117195
A930024E05Rik	7.323051136	11.42079822	11.58935159
A930033H14Rik	3.138450487	5.710399108	6.760455092
A930041C12Rik	0	0	0
AA388235	1.046150162	0	0
AA413626	2.092300324	0	3.863117195
AA414768	9.41535146	2.284159643	5.794675793
AA415398	4.184600649	20.55743679	0.965779299
AA543186	3.138450487	12.56287804	2.897337897
AA986860	0	12.56287804	0.965779299
Aaas	0	28.55199554	52.15208214
Aacs	66.95361038	68.5247893	55.04942003
Aaed1	1.046150162	1.142079822	0
Aagab	31.38450487	2.284159643	0
Aak1	146.4610227	106.2134234	112.0303987
Aamdc	44.98445698	17.13119732	38.63117195
Aamp	366.1525568	307.219472	459.7109463
Aanat	0	0	0
Aar2	85.7843133	100.5030243	121.6881917
Aars	309.660448	220.4214056	277.1786588
Aars2	3.138450487	12.56287804	40.56273055
Aarsd1	34.52295535	6.85247893	77.26234391
Aasdh	37.66140584	45.68319287	24.14448247
Aasdhppt	42.89215665	77.66142787	45.39162705
Aass	5.230750811	0	0
Aatf	44.98445698	85.65598662	116.8592952
Aatk	73.23051136	22.84159643	37.66539266
AB124611	0	5.710399108	0
Abat	9.41535146	1.142079822	0
Abca1	620.3670462	733.2152455	600.7147239
Abca13	4.184600649	12.56287804	0
Abca17	4.184600649	0	0
Abca2	138.0918214	133.6233391	100.4410471
Abca3	230.1530357	117.6342216	165.1482601
Abca4	0	0	0
Abca7	15.69225243	30.83615518	6.760455092
Abca8a	0	1.142079822	0
Abca9	1235.503342	1536.09736	1138.653793
Abcb10	39.75370617	76.51934805	33.80227546
Abcb1a	10.46150162	0	0
Abcb1b	66.95361038	73.09310859	31.87071686
Abcb4	54.39980844	50.25151215	8.69201369
Abcb6	60.67670941	28.55199554	64.70721302
Abcb7	43.93830681	27.40991572	19.31558598
Abcb8	30.33835471	65.09854983	53.11786144
Abcb9	19.87685308	14.84703768	24.14448247
Abcc1	96.24581493	121.0604611	120.7224124
Abcc10	73.23051136	33.12031483	34.76805476

For Review Only

Abcc3	905.9660405	512.7938399	705.0188882
Abcc4	55.4459586	45.68319287	43.46006845
Abcc5	446.7061193	846.2811479	750.4105152
Abcd1	166.3378758	183.8748513	0
Abcd2	327.4450008	316.3561106	270.4182037
Abcd3	207.1377321	151.8966163	189.2927426
Abcd4	46.03060714	23.98367625	58.91253723
Abce1	103.5688661	109.6396629	141.9695569
Abcf1	101.4765657	115.350062	143.9011155
Abcf2	111.9380674	133.6233391	123.6197503
Abcf3	0	103.9292638	51.18630284
Abcg1	661.1669025	629.2859817	703.0873296
Abcg2	60.67670941	91.36638573	93.68059199
Abcg3	32.43065503	29.69407536	24.14448247
Abcg4	0	2.284159643	0
Abhd10	35.56910552	46.82527269	45.39162705
Abhd11	0	0	0
Abhd12	11.50765178	27.40991572	6.760455092
Abhd13	34.52295535	114.2079822	62.77565443
Abhd14a	25.10760389	17.13119732	32.83649616
Abhd14b	101.4765657	63.95647001	90.78325409
Abhd15	0	0	234.6843696
Abhd16a	19.87685308	43.39903322	156.4562464
Abhd17a	4.184600649	6.85247893	0
Abhd17b	16.7384026	26.2678359	15.45246878
Abhd17c	7.323051136	84.5139068	33.80227546
Abhd18	7.323051136	17.13119732	13.52091018
Abhd2	126.5841696	66.24062966	81.1254611
Abhd3	34.52295535	23.98367625	31.87071686
Abhd4	300.2450966	234.1263634	268.4866451
Abhd5	48.12290746	74.23518841	38.63117195
Abhd6	67.99976055	163.3174145	189.2927426
Abhd8	44.98445698	26.2678359	29.93915826
Abi1	298.1527962	311.7877913	324.5018444
Abi2	24.06145373	22.84159643	34.76805476
Abi3	230.1530357	1877.579227	711.7793433
Abi3bp	0	0	4.828896494
Abl1	559.6903368	583.6027889	190.2585219
Abl2	74.27666152	114.2079822	69.53610952
Ablim1	70.09206087	42.2569534	34.76805476
Ablim2	4.184600649	0	0
Abr	318.0296493	163.3174145	231.7870317
Abra	4.184600649	0	0
Abracl	25.10760389	50.25151215	39.59695125
Abt1	91.01506412	29.69407536	46.35740634
Abtb1	0	0	0
Acaa1a	169.4763263	57.10399108	11.58935159
Acaa1b	62.76900973	0	52.15208214
Acaa2	61.72285957	35.40447447	45.39162705
Acaca	23.01530357	9.136638573	8.69201369

For Review Only

Acacb	0	0	0
Acad10	27.19990422	12.56287804	18.34980668
Acad11	41.84600649	25.12575608	15.45246878
Acad12	25.10760389	19.41535697	20.28136528
Acad8	106.7073165	67.38270948	127.4828674
Acad9	66.95361038	54.81983144	51.18630284
Acadl	84.73816314	42.2569534	75.33078531
Acadm	172.6147768	165.6015741	164.1824808
Acads	60.67670941	26.2678359	24.14448247
Acadsb	70.09206087	66.24062966	69.53610952
Acadvl	21.96915341	0	0
Acap1	0	0	0
Acap2	173.6609269	278.6674765	196.0531977
Acap3	44.98445698	9.136638573	14.48668948
Acat1	84.73816314	89.08222609	122.653971
Acat2	17.78455276	30.83615518	20.28136528
Acat3	2.092300324	12.56287804	4.828896494
Acbd3	80.55356249	47.96735251	111.0646194
Acbd4	7.323051136	6.85247893	18.34980668
Acbd5	32.43065503	67.38270948	54.08364074
Acbd6	20.92300324	28.55199554	18.34980668
Accs	40.79985633	59.38815073	67.60455092
Acd	21.96915341	0	0
Ace	17.78455276	0	0
Acer2	3.138450487	4.568319287	0
Acer3	271.9990422	292.3724343	286.8364518
Ache	32.43065503	41.11487358	16.41824808
Acin1	173.6609269	149.6124566	107.2015022
Ackr3	0	4.568319287	6.760455092
Ackr4	7.323051136	1.142079822	9.657792988
Acly	487.5059756	250.1154809	392.1063953
Acnat1	2.092300324	0	0
Aco1	72.18436119	47.96735251	56.98097863
Aco2	276.1836428	148.4703768	274.2813209
Acot1	7.323051136	4.568319287	2.897337897
Acot10	14.64610227	12.56287804	16.41824808
Acot11	15.69225243	1.142079822	13.52091018
Acot13	79.50741233	47.96735251	92.71481269
Acot2	31.38450487	23.98367625	30.90493756
Acot6	0	4.568319287	0
Acot7	11.50765178	7.994558752	0
Acot8	4.184600649	0	0
Acot9	28.24605438	0	0
Acox1	152.7379237	121.0604611	110.0988401
Acox3	398.5832118	47.96735251	83.0570197
Acoxl	0	0	0
Acp1	30.33835471	57.10399108	51.18630284
Acp2	751.1358165	773.1880393	937.7716992
Acp5	3.138450487	0	0
Acsf2	32.43065503	0	0

For Review Only

Acsf3	17.78455276	17.13119732	15.45246878
Acsl1	150.6456234	91.36638573	107.2015022
Acsl3	0	0	0
Acsl4	98.33811525	106.2134234	114.9277366
Acsl5	178.8916777	213.5689267	194.1216391
Acsl6	7.323051136	3.426239465	0
Acss1	416.3677646	360.8972236	342.8516511
Acss2	24.06145373	2.284159643	0
Acta2	10.46150162	7.994558752	9.657792988
Actb	0	7590.262495	0
Actl10	0	0	0
Actl6a	108.7996169	111.9238225	96.57792988
Actn1	16.7384026	0	8.69201369
Actn3	0	0	0
Actn4	210.2761826	157.6070154	183.4980668
Actr10	149.5994732	163.3174145	104.3041643
Actr1a	324.3065503	318.6402702	272.3497623
Actr1b	0	0	0
Actr2	127.6303198	158.7490952	239.5132661
Actr3	421.5985154	335.7714676	456.8136084
Actr5	7.323051136	23.98367625	5.794675793
Actr6	26.15375406	6.85247893	33.80227546
Actr8	6.276900973	153.0386961	165.1482601
Acvr1	29.29220454	66.24062966	87.8859162
Acvr1b	5.230750811	17.13119732	5.794675793
Acvr1c	0	0	0
Acvr2a	55.4459586	50.25151215	68.57033022
Acvr2b	29.29220454	81.08766734	20.28136528
Acvrl1	64.86131006	31.97823501	68.57033022
Acy1	81.59971265	50.25151215	74.36500601
Acyp1	11.50765178	7.994558752	12.55513089
Ada	12.55380195	10.27871839	0
Adal	31.38450487	25.12575608	21.24714457
Adam10	859.9354334	981.0465668	654.7983646
Adam15	183.0762784	151.8966163	0
Adam17	551.3211355	320.9244299	331.2622995
Adam19	0	0	0
Adam1a	10.46150162	4.568319287	15.45246878
Adam1b	3.138450487	0	2.897337897
Adam22	16.7384026	38.83071394	0.965779299
Adam3	12.55380195	0	7.726234391
Adam30	7.323051136	1.142079822	0
Adam33	5.230750811	0	1.931558598
Adam4	20.92300324	13.70495786	6.760455092
Adam8	15.69225243	4.568319287	8.69201369
Adam9	121.3534188	77.66142787	77.26234391
Adamts1	51.26135795	59.38815073	0
Adamts10	64.86131006	46.82527269	42.49428915
Adamts15	0	0	0
Adamts16	101.4765657	131.3391795	85.9543576

For Review Only

Adamts2	0	1.142079822	0
Adamts6	6.276900973	5.710399108	14.48668948
Adamts7	2.092300324	0	0
Adamtsl2	19.87685308	14.84703768	16.41824808
Adamtsl4	1.046150162	0	0
Adap1	15.69225243	14.84703768	25.11026177
Adap2	6.276900973	33.12031483	0
Adar	111.9380674	100.5030243	133.2775432
Adarb1	17.78455276	20.55743679	26.07604107
Adat1	9.41535146	10.27871839	26.07604107
Adat2	0	10.27871839	0
Adat3	14.64610227	20.55743679	9.657792988
Adck1	82.64586282	68.5247893	54.08364074
Adck2	89.96891395	87.94014627	84.9885783
Adck3	14.64610227	52.5356718	25.11026177
Adck4	39.75370617	43.39903322	83.0570197
Adck5	0	74.23518841	0
Adcy1	0	0	0
Adcy10	0	0	0
Adcy3	23.01530357	15.9891175	26.07604107
Adcy5	0	0	0
Adcy6	5.230750811	10.27871839	24.14448247
Adcy7	0	213.5689267	62.77565443
Adcy8	32.43065503	20.55743679	27.04182037
Adcy9	26.15375406	68.5247893	59.87831653
Add1	213.4146331	300.3669931	219.2319008
Add2	0	4.568319287	0
Add3	153.7840738	65.09854983	83.0570197
Adgb	0	0	0
Adgra2	0	0	0
Adgra3	27.19990422	21.69951661	8.69201369
Adgre1	411.1370138	821.1553918	373.7565887
Adgre4	0	0	0
Adgre5	0	0	0
Adgrf4	0	1.142079822	0
Adgrf5	0	0	0
Adgrg1	2309.899558	1793.06532	1953.771522
Adgrg3	0	1.142079822	0
Adgrl1	12.55380195	25.12575608	25.11026177
Adgrl2	40.79985633	39.97279376	30.90493756
Adgrl4	0	0	0
Adh5	192.4916299	162.1753347	202.8136528
Adhfe1	18.83070292	35.40447447	18.34980668
Adi1	138.0918214	76.51934805	143.9011155
Adipor1	378.7063587	483.0997646	324.5018444
Adipor2	84.73816314	79.94558752	103.338385
Adk	0	6.85247893	5.794675793
Adnp2	25.10760389	60.53023055	56.01519933
Ado	207.1377321	210.1426872	140.0379983
Adora1	9.41535146	10.27871839	0

For Review Only

Adora2b	3.138450487	0	0
Adora3	0	404.2962569	18.34980668
Adpgk	116.122668	89.08222609	114.9277366
Adprh	356.7372053	311.7877913	358.3041199
Adprhl2	12.55380195	31.97823501	0
Adprm	80.55356249	76.51934805	0.965779299
Adra1a	5.230750811	0	0.965779299
Adra2a	0	3.426239465	1.931558598
Adra2b	0	0	0
Adra2c	0	0	0.965779299
Adrb1	42.89215665	13.70495786	10.62357229
Adrb2	863.0738839	973.0520081	1032.41807
Adrbk1	0	1.142079822	0
Adrbk2	46.03060714	134.765419	119.7566331
Adrm1	0	60.53023055	9.657792988
Adsl	127.6303198	100.5030243	112.0303987
Adss	96.24581493	81.08766734	56.01519933
Adssl1	19.87685308	25.12575608	16.41824808
Adtrp	8.369201298	4.568319287	10.62357229
Aebp1	3.138450487	0	0
Aebp2	34.52295535	44.54111304	36.69961336
Aen	44.98445698	34.26239465	43.46006845
Aes	9.41535146	22.84159643	14.48668948
AF357399	18.83070292	20.55743679	11.58935159
AF529169	0	0	3.863117195
Afap1	38.707556	37.68863411	24.14448247
Afap1l1	88.92276379	98.21886466	142.9353362
Afap1l2	0	0	0
Aff1	237.4760868	179.306532	137.1406604
Aff3	0	0	0
Aff4	112.9842175	154.1807759	101.4068264
Afg3l1	86.83046347	73.09310859	56.98097863
Afg3l2	123.4457191	66.24062966	90.78325409
Afm	0	0	0
Afmid	32.43065503	20.55743679	34.76805476
Aftph	257.3529399	363.1813833	262.6919693
Aga	148.553323	117.6342216	168.045598
Agap1	21.96915341	18.27327715	22.21292387
Agap2	11.50765178	3.426239465	13.52091018
Agap3	2.092300324	6.85247893	0
Agbl2	0	0	0
Agbl3	14.64610227	6.85247893	4.828896494
Agbl5	28.24605438	22.84159643	26.07604107
Ager	2.092300324	1.142079822	0
Agfg1	36.61525568	29.69407536	41.52850985
Agfg2	29.29220454	26.2678359	25.11026177
Aggf1	44.98445698	42.2569534	42.49428915
Agk	48.12290746	35.40447447	37.66539266
Agl	43.93830681	45.68319287	29.93915826
Agmo	265.7221412	277.5253967	293.5969068

For Review Only

Ago1	148.553323	209.0006074	152.5931292
Ago2	81.59971265	99.36094448	110.0988401
Ago3	82.64586282	129.0550198	93.68059199
Ago4	32.43065503	55.96191126	19.31558598
Agpat1	178.8916777	0	0.965779299
Agpat2	17.78455276	31.97823501	25.11026177
Agpat3	188.3070292	130.1970997	146.7984534
Agpat4	18.83070292	0	4.828896494
Agpat5	41.84600649	43.39903322	47.32318564
Agpat9	0	0	0
Agps	103.5688661	157.6070154	120.7224124
Agrn	33.47680519	29.69407536	0
Agtpbp1	49.16905763	30.83615518	29.93915826
Agtrap	64.86131006	67.38270948	78.22812321
Ahctf1	59.63055925	83.37182698	45.39162705
Ahcy	16.7384026	5.710399108	7.726234391
Ahcyl1	35.56910552	54.81983144	43.46006845
Ahcyl2	111.9380674	55.96191126	36.69961336
Ahdc1	32.43065503	65.09854983	54.08364074
Ahi1	9.41535146	5.710399108	23.17870317
Ahnak	0	1.142079822	27.04182037
Ahr	0	0	0
Ahrr	11.50765178	6.85247893	0
Ahsa1	151.6917735	116.4921418	177.703391
Ahsa2	39.75370617	10.27871839	84.022799
AI225912	7.323051136	9.136638573	20.28136528
AI314180	74.27666152	68.5247893	107.2015022
AI427809	1.046150162	0	0
AI429214	0	1.142079822	2.897337897
AI450353	27.19990422	14.84703768	10.62357229
AI463229	12.55380195	2.284159643	3.863117195
AI464131	0	0	0.965779299
AI467606	188.3070292	169.0278136	185.4296254
AI480526	17.78455276	35.40447447	15.45246878
AI481877	0	5.710399108	8.69201369
AI504432	8.369201298	1.142079822	6.760455092
AI506816	0	3.426239465	0.965779299
AI597479	43.93830681	45.68319287	50.22052354
AI607873	0	9.136638573	3.863117195
AI661453	1.046150162	9.136638573	1.931558598
AI662270	1.046150162	10.27871839	4.828896494
AI837181	0	15.9891175	120.7224124
AI839979	0	0	0
AI846148	8.369201298	5.710399108	12.55513089
AI854703	10.46150162	1.142079822	2.897337897
AI987944	70.09206087	129.0550198	121.6881917
Aida	20.92300324	13.70495786	20.28136528
Aif1	0	0	5.794675793
Aifm1	37.66140584	34.26239465	59.87831653
Aifm2	0	6.85247893	0

For Review Only

Aifm3	15.69225243	1.142079822	0
Aig1	28.24605438	28.55199554	25.11026177
Aim1	2.092300324	0	4.828896494
Aim2	25.10760389	0	32.83649616
Aimp1	50.21520779	75.37726823	75.33078531
Aimp2	0	22.84159643	33.80227546
Aip	66.95361038	109.6396629	109.1330608
Ajuba	13.59995211	10.27871839	19.31558598
AK010878	49.16905763	10.27871839	25.11026177
Ak1	169.4763263	156.4649356	0
Ak2	144.3687224	177.0223724	173.8402738
Ak3	91.01506412	61.67231037	55.04942003
Ak5	0	0	0
Ak6	35.56910552	50.25151215	33.80227546
Ak7	0	0	0
Akap1	7.323051136	59.38815073	29.93915826
Akap10	86.83046347	109.6396629	74.36500601
Akap11	32.43065503	81.08766734	34.76805476
Akap12	13.59995211	2.284159643	14.48668948
Akap13	543.9980844	493.378483	486.7527666
Akap17b	5.230750811	2.284159643	0
Akap3	2.092300324	7.994558752	3.863117195
Akap5	12.55380195	23.98367625	37.66539266
Akap7	32.43065503	30.83615518	12.55513089
Akap8	9.41535146	0	0.965779299
Akap9	177.8455276	235.2684433	198.9505356
Akip1	4.184600649	18.27327715	74.36500601
Akirin1	214.4607833	190.7273302	137.1406604
Akirin2	66.95361038	21.69951661	35.73383406
Akna	441.4753685	125.6287804	62.77565443
Aknaos	65.90746022	38.83071394	19.31558598
Akr1a1	847.3816314	709.2315693	818.9808454
Akr1b10	141.2302719	162.1753347	160.3193636
Akr1b3	59.63055925	84.5139068	76.29656461
Akr1e1	38.707556	25.12575608	24.14448247
Akr7a5	59.63055925	46.82527269	60.84409583
Akt1	6.276900973	0	0.965779299
Akt1s1	67.99976055	67.38270948	12.55513089
Akt2	87.87661363	50.25151215	71.46766811
Akt3	26.15375406	57.10399108	35.73383406
Aktip	54.39980844	61.67231037	40.56273055
Alad	35.56910552	14.84703768	31.87071686
Alas1	164.2455755	115.350062	220.1976801
Alas2	0	0	0
Alcam	0	0	0
Aldh16a1	124.4918693	94.7926252	74.36500601
Aldh18a1	89.96891395	15.9891175	86.9201369
Aldh1a1	0	0	0
Aldh1a2	0	0	0
Aldh1a3	0	1.142079822	0

For Review Only

Aldh1b1	2.092300324	0	4.828896494
Aldh1l1	15.69225243	0	0
Aldh1l2	2.092300324	2.284159643	19.31558598
Aldh2	95.19966476	53.67775162	56.98097863
Aldh3a2	200.8608312	151.8966163	187.361184
Aldh3b1	13.59995211	9.136638573	0
Aldh4a1	85.7843133	60.53023055	66.63877162
Aldh5a1	1.046150162	5.710399108	0
Aldh6a1	75.32281168	36.54655429	61.80987513
Aldh7a1	50.21520779	28.55199554	35.73383406
Aldh9a1	150.6456234	127.91294	155.4904671
Aldoa	993.8426541	551.6245539	801.596818
Aldoart1	19.87685308	25.12575608	27.04182037
Aldoart2	4.184600649	4.568319287	0.965779299
Aldoc	17.78455276	1.142079822	0
Alg1	183.0762784	99.36094448	184.4638461
Alg10b	5.230750811	18.27327715	19.31558598
Alg11	126.5841696	124.4867006	77.26234391
Alg12	64.86131006	66.24062966	73.39922671
Alg13	11.50765178	10.27871839	24.14448247
Alg14	41.84600649	73.09310859	64.70721302
Alg2	80.55356249	100.5030243	99.47526778
Alg3	47.0767573	22.84159643	102.3726057
Alg5	197.7223807	195.2956495	246.2737212
Alg6	19.87685308	18.27327715	8.69201369
Alg8	0	0	6.760455092
Alg9	43.93830681	46.82527269	42.49428915
Alkbh1	10.46150162	73.09310859	77.26234391
Alkbh2	5.230750811	15.9891175	0
Alkbh3	120.3072687	69.66686912	81.1254611
Alkbh3os1	4.184600649	5.710399108	0
Alkbh4	48.12290746	34.26239465	29.93915826
Alkbh5	192.4916299	126.7708602	101.4068264
Alkbh6	11.50765178	41.11487358	0
Alkbh8	41.84600649	34.26239465	28.00759967
Alms1	1.046150162	5.710399108	0
Alox15	23.01530357	9.136638573	0
Alox5	100.4304156	65.09854983	139.072219
Alox5ap	586.890241	761.767241	754.2736324
Aloxe3	4.184600649	1.142079822	0
Alpk1	24.06145373	9.136638573	8.69201369
Alpk3	4.184600649	0	0
Als2	71.13821103	33.12031483	18.34980668
Als2cl	3.138450487	12.56287804	8.69201369
Als2cr11b	0	0	3.863117195
Als2cr12	2.092300324	0	0
Alyref	9.41535146	13.70495786	0
Alyref2	32.43065503	22.84159643	28.97337897
Amacr	23.01530357	29.69407536	28.00759967
Ambra1	112.9842175	143.9020575	93.68059199

For Review Only

Amd1	97.29196509	75.37726823	104.3041643
Amd2	56.49210876	61.67231037	73.39922671
Amer1	41.84600649	119.9183813	56.98097863
Amer2	16.7384026	3.426239465	6.760455092
Amfr	243.7529878	262.678359	238.5474868
Amh	0	0	0
Amigo1	10.46150162	11.42079822	11.58935159
Amigo3	28.24605438	19.41535697	11.58935159
Ammecr1	0	4.568319287	1.931558598
Ammecr1l	124.4918693	106.2134234	118.7908538
Amn1	30.33835471	21.69951661	31.87071686
Amotl1	69.04591071	41.11487358	87.8859162
Amotl2	4.184600649	0	0
Ampd2	51.26135795	3.426239465	84.022799
Ampd3	74.27666152	52.5356718	83.0570197
Amt	15.69225243	6.85247893	11.58935159
Amz1	3.138450487	1.142079822	0
Amz2	60.67670941	7.994558752	0
Anapc1	121.3534188	145.0441374	94.64637129
Anapc10	32.43065503	31.97823501	42.49428915
Anapc11	54.39980844	53.67775162	68.57033022
Anapc13	36.61525568	50.25151215	62.77565443
Anapc15	37.66140584	3.426239465	23.17870317
Anapc16	49.16905763	47.96735251	54.08364074
Anapc2	0	0	0
Anapc4	57.53825892	71.95102876	82.0912404
Anapc5	191.4454797	203.2902083	232.752811
Anapc7	164.2455755	182.7327715	203.7794321
Ang	124.4918693	390.591299	219.2319008
Angel1	51.26135795	49.10943233	47.32318564
Angel2	230.1530357	109.6396629	216.3345629
Angpt2	6.276900973	0	5.794675793
Angptl1	0	7.994558752	0
Angptl2	13.59995211	21.69951661	4.828896494
Angptl3	6.276900973	0	0.965779299
Angptl6	7.323051136	11.42079822	14.48668948
Angptl7	0	0	0
Ank	108.7996169	68.5247893	90.78325409
Ank1	0	0	1.931558598
Ank2	141.2302719	218.1372459	113.9619573
Ankdd1a	4.184600649	0	0
Ankdd1b	0	0	0
Ankfn1	0	0	2.897337897
Ankfy1	278.2759432	367.7497026	457.7793877
Ankhd1	132.8610706	139.3337382	106.2357229
Ankib1	36.61525568	18.27327715	0
Ankle1	0	0	0
Ankle2	160.0609748	196.4377293	153.5589085
Ankmy2	25.10760389	45.68319287	65.67299232
Ankra2	44.98445698	39.97279376	53.11786144

For Review Only

Ankrd10	209.2300324	269.5308379	288.7680104
Ankrd11	205.0454318	324.3506694	241.4448247
Ankrd12	96.24581493	90.22430591	103.338385
Ankrd13a	95.19966476	149.6124566	87.8859162
Ankrd13b	2.092300324	4.568319287	19.31558598
Ankrd13c	11.50765178	23.98367625	13.52091018
Ankrd13d	6.276900973	1.142079822	38.63117195
Ankrd16	67.99976055	73.09310859	109.1330608
Ankrd17	182.0301282	179.306532	186.3954047
Ankrd22	0	0	0
Ankrd23	13.59995211	35.40447447	45.39162705
Ankrd24	40.79985633	19.41535697	16.41824808
Ankrd26	33.47680519	37.68863411	29.93915826
Ankrd27	74.27666152	87.94014627	116.8592952
Ankrd28	5.230750811	20.55743679	14.48668948
Ankrd33b	4.184600649	6.85247893	0
Ankrd34a	2.092300324	2.284159643	0
Ankrd34b	4.184600649	0	0
Ankrd37	15.69225243	15.9891175	4.828896494
Ankrd39	69.04591071	45.68319287	50.22052354
Ankrd40	95.19966476	74.23518841	108.1672815
Ankrd42	10.46150162	0	0
Ankrd44	28.24605438	185.0169311	49.25474424
Ankrd45	4.184600649	0	5.794675793
Ankrd46	141.2302719	121.0604611	109.1330608
Ankrd49	48.12290746	89.08222609	83.0570197
Ankrd50	18.83070292	39.97279376	27.04182037
Ankrd52	139.1379716	61.67231037	80.1596818
Ankrd54	30.33835471	41.11487358	15.45246878
Ankrd55	0	3.426239465	0
Ankrd6	0	2.284159643	0
Ankrd61	0	0	0
Ankrd63	0	0	0
Ankrd7	2.092300324	3.426239465	0.965779299
Ankrd9	7.323051136	1.142079822	0
Anks1	80.55356249	90.22430591	103.338385
Anks3	0	0	0
Anks6	26.15375406	44.54111304	32.83649616
Ankzf1	0	14.84703768	29.93915826
Anln	0	0	0
Ano10	139.1379716	90.22430591	141.0037776
Ano6	256.3067897	188.4431706	152.5931292
Ano7	0	30.83615518	0
Ano8	15.69225243	0	0.965779299
Anp32a	19.87685308	21.69951661	18.34980668
Anp32b	11.50765178	28.55199554	18.34980668
Anp32e	114.0303677	86.79806645	85.9543576
Antxr1	9.41535146	0	0
Antxr2	4.184600649	0	0
Anxa1	104.6150162	0	0

For Review Only

Anxa11	9.41535146	6.85247893	4.828896494
Anxa2	14.64610227	7.994558752	24.14448247
Anxa3	441.4753685	372.3180219	639.3458958
Anxa4	73.23051136	52.5356718	84.022799
Anxa5	0	0	1.931558598
Anxa6	137.0456713	70.80894894	151.6273499
Anxa7	160.0609748	119.9183813	141.0037776
Anxa9	0	3.426239465	4.828896494
Aoah	4.184600649	0	0
Aoc2	3.138450487	1.142079822	2.897337897
Ap1ar	21.96915341	14.84703768	19.31558598
Ap1b1	495.8751769	244.4050818	476.1291943
Ap1g1	174.7070771	193.0114899	146.7984534
Ap1g2	148.553323	23.98367625	72.43344741
Ap1m1	0	0	7.726234391
Ap1s1	60.67670941	57.10399108	74.36500601
Ap1s2	99.38426541	82.22974716	92.71481269
Ap2a1	112.9842175	46.82527269	30.90493756
Ap2a2	290.8297451	194.1535697	132.3117639
Ap2b1	122.399569	111.9238225	126.5170881
Ap2m1	219.6915341	227.2738845	16.41824808
Ap2s1	72.18436119	94.7926252	126.5170881
Ap3b1	235.3837865	381.4546604	273.3155416
Ap3d1	0	0	1.931558598
Ap3m1	91.01506412	105.0713436	91.74903339
Ap3m2	63.8151599	62.81439019	81.1254611
Ap3s1	19.87685308	14.84703768	16.41824808
Ap3s2	66.95361038	82.22974716	66.63877162
Ap4b1	123.4457191	123.3446207	66.63877162
Ap4e1	25.10760389	19.41535697	22.21292387
Ap4m1	1.046150162	1.142079822	131.3459846
Ap4s1	31.38450487	31.97823501	47.32318564
Ap5b1	3.138450487	1.142079822	0
Ap5m1	79.50741233	111.9238225	94.64637129
Ap5s1	55.4459586	45.68319287	59.87831653
Ap5z1	82.64586282	0	138.1064397
Apaf1	32.43065503	29.69407536	16.41824808
Apba1	40.79985633	55.96191126	43.46006845
Apba3	1.046150162	105.0713436	0
Apbb1	56.49210876	6.85247893	43.46006845
Apbb1ip	152.7379237	325.4927492	220.1976801
Apbb2	106.7073165	91.36638573	93.68059199
Apbb3	27.19990422	45.68319287	54.08364074
Apc	152.7379237	183.8748513	142.9353362
Apc2	8.369201298	1.142079822	0
Apcdd1	0	0	0
Apeh	9.41535146	10.27871839	72.43344741
Apex1	346.2757037	76.51934805	68.57033022
Apex2	44.98445698	19.41535697	43.46006845
Aph1a	43.93830681	0	53.11786144

For Review Only

Aph1b	13.59995211	7.994558752	5.794675793
Aph1c	10.46150162	14.84703768	4.828896494
Api5	311.7527483	210.1426872	237.5817075
Apip	0	41.11487358	39.59695125
Apitd1	5.230750811	0	3.863117195
Aplf	8.369201298	1.142079822	24.14448247
Aplnr	2.092300324	0	0
Aplp1	13.59995211	0	5.794675793
Aplp2	161.107125	98.21886466	155.4904671
Apmap	53.35365827	78.80350769	80.1596818
Apoa1bp	163.1994253	0	162.2509222
Apob	3.138450487	2.284159643	0
Apobec1	195.6300803	164.4594943	137.1406604
Apobec2	12.55380195	12.56287804	8.69201369
Apobec3	333.7219018	378.028421	380.5170437
Apobr	94.1535146	43.39903322	58.91253723
Apod	0	0	0
Apoe	1682.209461	1783.928681	2603.74099
Apold1	4.184600649	0	0
Apoo	6.276900973	0	15.45246878
Apoo-ps	10.46150162	4.568319287	13.52091018
Apool	44.98445698	33.12031483	47.32318564
Apopt1	30.33835471	29.69407536	51.18630284
App	237.4760868	203.2902083	151.6273499
Appbp2	29.29220454	50.25151215	33.80227546
Appl1	54.39980844	46.82527269	35.73383406
Appl2	37.66140584	23.98367625	38.63117195
Aprt	11.50765178	0	0
Aptx	35.56910552	50.25151215	76.29656461
Aqp1	11.50765178	2.284159643	0
Aqp11	0	3.426239465	0.965779299
Aqp4	1.046150162	0	0
Aqr	87.87661363	66.24062966	53.11786144
Araf	107.7534667	81.08766734	1.931558598
Arap1	491.6905763	400.8700174	383.4143816
Arap2	11.50765178	19.41535697	18.34980668
Arap3	54.39980844	1.142079822	0
Arcn1	271.9990422	304.9353124	255.9315142
Arel1	162.1532751	146.1862172	150.6615706
Arf1	63.8151599	871.4069039	8.69201369
Arf2	124.4918693	148.4703768	158.387805
Arf4	377.6602086	314.071951	423.9771122
Arf5	21.96915341	4.568319287	0
Arf6	55.4459586	61.67231037	0
Arfgap1	115.0765178	138.1916584	70.50188882
Arfgap2	49.16905763	55.96191126	25.11026177
Arfgap3	30.33835471	49.10943233	48.28896494
Arfgef1	46.03060714	68.5247893	49.25474424
Arfgef2	43.93830681	46.82527269	66.63877162
Arfip1	56.49210876	73.09310859	53.11786144

For Review Only

Arfip2	53.35365827	62.81439019	49.25474424
Arg1	1.046150162	0	0
Arg2	0	1.142079822	4.828896494
Arglu1	373.4756079	408.8645762	452.9504912
Arhgap1	71.13821103	97.07678484	186.3954047
Arhgap11a	51.26135795	42.2569534	42.49428915
Arhgap12	258.3990901	180.4486118	249.1710591
Arhgap15	9.41535146	22.84159643	13.52091018
Arhgap17	224.9222849	302.6511527	179.6349496
Arhgap18	36.61525568	35.40447447	44.42584775
Arhgap19	54.39980844	21.69951661	20.28136528
Arhgap21	27.19990422	46.82527269	19.31558598
Arhgap22	222.8299846	199.8639688	159.3535843
Arhgap23	0	2.284159643	0
Arhgap24	47.0767573	36.54655429	48.28896494
Arhgap25	465.5368222	404.2962569	398.8668504
Arhgap26	25.10760389	4.568319287	12.55513089
Arhgap27	54.39980844	1.142079822	0
Arhgap27os3	0	0	0
Arhgap29	16.7384026	20.55743679	8.69201369
Arhgap30	116.122668	0	1.931558598
Arhgap31	132.8610706	199.8639688	116.8592952
Arhgap32	34.52295535	6.85247893	20.28136528
Arhgap33	3.138450487	5.710399108	0
Arhgap35	43.93830681	65.09854983	46.35740634
Arhgap39	33.47680519	3.426239465	0
Arhgap4	84.73816314	1.142079822	0
Arhgap44	5.230750811	2.284159643	0
Arhgap5	378.7063587	808.5925137	405.6273055
Arhgap6	5.230750811	0	0
Arhgap9	61.72285957	0	0
Arhgdia	37.66140584	296.9407536	179.6349496
Arhgdib	526.2135316	665.832536	673.1481713
Arhgef1	87.87661363	165.6015741	131.3459846
Arhgef10	4.184600649	0	0
Arhgef10l	104.6150162	91.36638573	97.54370918
Arhgef11	14.64610227	41.11487358	22.21292387
Arhgef12	50.21520779	59.38815073	57.94675793
Arhgef15	2.092300324	1.142079822	0
Arhgef17	6.276900973	1.142079822	0
Arhgef18	34.52295535	42.2569534	49.25474424
Arhgef2	343.1372532	269.5308379	310.9809342
Arhgef25	23.01530357	6.85247893	20.28136528
Arhgef26	2.092300324	1.142079822	4.828896494
Arhgef28	0	0	0
Arhgef3	49.16905763	30.83615518	55.04942003
Arhgef39	2.092300324	0	0
Arhgef4	27.19990422	20.55743679	26.07604107
Arhgef40	182.0301282	0	176.7376117
Arhgef5	0	0	0.965779299

For Review Only

Arhgef6	218.6453839	270.6729177	310.9809342
Arhgef7	168.4301761	117.6342216	86.9201369
Arhgef9	0	0	0
Arid1a	481.2290746	712.6578087	657.6957025
Arid1b	103.5688661	123.3446207	87.8859162
Arid2	129.7226201	244.4050818	146.7984534
Arid3a	115.0765178	52.5356718	65.67299232
Arid3b	14.64610227	0	3.863117195
Arid4a	182.0301282	143.9020575	114.9277366
Arid4b	95.19966476	132.4812593	81.1254611
Arid5a	95.19966476	49.10943233	114.9277366
Arid5b	143.3225722	132.4812593	93.68059199
Arih1	73.23051136	103.9292638	140.0379983
Arih2	147.5071729	121.0604611	169.9771566
Arl1	178.8916777	232.9842836	213.437225
Arl10	39.75370617	61.67231037	0
Arl11	241.6606875	0	6.760455092
Arl13b	19.87685308	22.84159643	28.00759967
Arl14ep	83.69201298	66.24062966	67.60455092
Arl15	29.29220454	28.55199554	22.21292387
Arl16	2.092300324	20.55743679	25.11026177
Arl2	35.56910552	33.12031483	48.28896494
Arl2bp	19.87685308	11.42079822	11.58935159
Arl3	18.83070292	17.13119732	26.07604107
Arl4a	21.96915341	14.84703768	5.794675793
Arl4c	285.5989943	214.7110065	252.068397
Arl4d	15.69225243	3.426239465	10.62357229
Arl5a	41.84600649	84.5139068	40.56273055
Arl5b	48.12290746	66.24062966	48.28896494
Arl5c	29.29220454	18.27327715	14.48668948
Arl6	15.69225243	0	11.58935159
Arl6ip1	996.9811046	950.2104116	936.8059199
Arl6ip4	0	0	0
Arl6ip5	105.6611664	194.1535697	116.8592952
Arl6ip6	29.29220454	33.12031483	18.34980668
Arl8a	38.707556	44.54111304	28.00759967
Arl8b	635.0131485	606.4443853	584.2964758
Armc1	75.32281168	79.94558752	97.54370918
Armc10	46.03060714	66.24062966	59.87831653
Armc3	17.78455276	22.84159643	14.48668948
Armc5	28.24605438	0	0
Armc6	4.184600649	4.568319287	41.52850985
Armc7	128.67647	132.4812593	73.39922671
Armc8	94.1535146	59.38815073	79.19390251
Armc9	23.01530357	12.56287804	17.38402738
Armcx2	32.43065503	18.27327715	36.69961336
Armcx3	65.90746022	45.68319287	66.63877162
Armcx5	0	0	3.863117195
Armcx6	5.230750811	2.284159643	2.897337897
Armt1	21.96915341	10.27871839	32.83649616

For Review Only

Arnt	109.845767	154.1807759	151.6273499
Arnt2	0	1.142079822	0
Arntl	34.52295535	11.42079822	59.87831653
Arpc1a	76.36896184	141.6178979	210.5398871
Arpc1b	0	598.4498266	245.3079419
Arpc2	331.6296014	460.2581681	483.8554287
Arpc3	79.50741233	135.9074988	132.3117639
Arpc4	0	0	114.9277366
Arpc5	420.5523652	448.8373699	445.2242568
Arpc5l	116.122668	154.1807759	172.8744945
Arpin	0	1.142079822	0
Arpp19	74.27666152	86.79806645	115.8935159
Arpp21	2.092300324	1.142079822	0
Arr3	0	0	0
Arrb1	224.9222849	266.1045984	339.9543132
Arrb2	41.84600649	0	3.863117195
Arrdc1	0	0	0
Arrdc2	0	0	0
Arrdc3	461.3522215	66.24062966	55.04942003
Arrdc4	26.15375406	19.41535697	39.59695125
Arsa	318.0296493	0	0.965779299
Arsb	1047.196312	1060.992154	1000.547354
Arsg	183.0762784	178.1644522	194.1216391
Arsk	145.4148726	130.1970997	89.81747479
Arv1	59.63055925	74.23518841	78.22812321
Arvcf	227.0145852	147.328297	211.5056664
As3mt	30.33835471	39.97279376	189.2927426
Asah1	0	0	564.0151105
Asah2	33.47680519	17.13119732	13.52091018
Asap1	253.1683393	318.6402702	206.67677
Asap2	2.092300324	2.284159643	0
Asap3	194.5839302	145.0441374	245.3079419
Asb1	10.46150162	12.56287804	25.11026177
Asb10	59.63055925	34.26239465	49.25474424
Asb2	132.8610706	211.284767	409.4904227
Asb3	77.41511201	73.09310859	67.60455092
Asb6	48.12290746	63.95647001	65.67299232
Asb7	57.53825892	91.36638573	54.08364074
Asb8	110.8919172	124.4867006	124.5855296
Ascc1	75.32281168	75.37726823	97.54370918
Ascc2	140.1841217	155.3228557	146.7984534
Ascc3	81.59971265	91.36638573	78.22812321
Ascl2	8.369201298	12.56287804	12.55513089
Ascl4	0	0	0
Asf1a	17.78455276	22.84159643	29.93915826
Asf1b	4.184600649	11.42079822	5.794675793
Asgr1	1.046150162	1.142079822	0
Ash1l	275.1374927	325.4927492	188.3269633
Ash2l	55.4459586	46.82527269	20.28136528
Asic1	4.184600649	0	0

For Review Only

Asic3	1.046150162	11.42079822	0
Asl	0	0	0
Asna1	24.06145373	0	8.69201369
Asns	0	0	0
Asnsd1	124.4918693	83.37182698	71.46766811
Asph	603.6286436	857.7019461	580.4333586
Asphd1	0	1.142079822	0
Asphd2	2.092300324	0	0
Aspm	1.046150162	0	8.69201369
Aspscr1	105.6611664	81.08766734	30.90493756
Asrgl1	25.10760389	9.136638573	5.794675793
Aste1	33.47680519	73.09310859	51.18630284
Asun	53.35365827	33.12031483	26.07604107
Asxl1	173.6609269	135.9074988	202.8136528
Asxl2	138.0918214	242.1209222	229.8554731
Asxl3	5.230750811	0	0
Atad1	97.29196509	195.2956495	130.3802053
Atad2	8.369201298	38.83071394	16.41824808
Atad2b	42.89215665	103.9292638	48.28896494
Atad3a	79.50741233	69.66686912	48.28896494
Atad3aos	7.323051136	2.284159643	4.828896494
Atad5	13.59995211	4.568319287	0
Atat1	86.83046347	68.5247893	124.5855296
Ate1	133.9072208	181.5906916	85.9543576
Atf1	52.30750811	47.96735251	55.04942003
Atf2	98.33811525	119.9183813	106.2357229
Atf3	0	0	0
Atf4	0	2.284159643	0
Atf5	1.046150162	0	2.897337897
Atf6	138.0918214	178.1644522	134.2433225
Atf6b	62.76900973	0	55.04942003
Atf7	283.506694	280.9516361	332.2280788
Atf7ip	487.5059756	534.4933565	514.7603663
Atg10	44.98445698	28.55199554	40.56273055
Atg101	114.0303677	60.53023055	94.64637129
Atg12	69.04591071	132.4812593	112.0303987
Atg13	83.69201298	61.67231037	73.39922671
Atg14	105.6611664	75.37726823	112.0303987
Atg16l1	7.323051136	62.81439019	39.59695125
Atg16l2	9.41535146	17.13119732	48.28896494
Atg2a	205.0454318	39.97279376	78.22812321
Atg2b	61.72285957	85.65598662	66.63877162
Atg3	74.27666152	59.38815073	50.22052354
Atg4a	10.46150162	14.84703768	14.48668948
Atg4b	55.4459586	107.3555032	157.4220257
Atg4c	38.707556	85.65598662	47.32318564
Atg4d	48.12290746	57.10399108	35.73383406
Atg5	0	197.5798091	175.7718324
Atg7	100.4304156	53.67775162	62.77565443
Atg9a	102.5227159	59.38815073	71.46766811

For Review Only

Athl1	39.75370617	1.142079822	22.21292387
Atic	29.29220454	23.98367625	3.863117195
Atl1	0	4.568319287	8.69201369
Atl2	62.76900973	22.84159643	28.00759967
Atl3	102.5227159	92.50846555	82.0912404
Atm	37.66140584	13.70495786	48.28896494
Atmin	70.09206087	153.0386961	119.7566331
Atn1	71.13821103	55.96191126	48.28896494
Atox1	112.9842175	115.350062	144.8668948
Atp10a	0	0	2.897337897
Atp10b	4.184600649	0	0
Atp10d	82.64586282	45.68319287	28.97337897
Atp11a	54.39980844	79.94558752	80.1596818
Atp11b	62.76900973	77.66142787	46.35740634
Atp11c	17.78455276	44.54111304	36.69961336
Atp13a1	75.32281168	0	125.5513089
Atp13a2	147.5071729	100.5030243	0
Atp13a3	67.99976055	125.6287804	49.25474424
Atp1a1	186.2147289	78.80350769	58.91253723
Atp1a2	3.138450487	0	69.53610952
Atp1a3	25.10760389	29.69407536	25.11026177
Atp1b1	46.03060714	0	0
Atp1b2	9.41535146	0	0
Atp1b3	299.1989464	280.9516361	379.5512644
Atp2a1	0	0	13.52091018
Atp2a2	137.0456713	127.91294	158.387805
Atp2a3	9.41535146	27.40991572	12.55513089
Atp2b1	166.3378758	246.6892415	244.3421626
Atp2b2	9.41535146	3.426239465	3.863117195
Atp2b3	19.87685308	0	0
Atp2c1	355.6910552	524.2146381	333.1938581
Atp5a1	991.7503538	781.182598	507.0341319
Atp5b	791.9356728	710.3736491	726.2660327
Atp5c1	0	26.2678359	14.48668948
Atp5c1-ps	6.276900973	5.710399108	3.863117195
Atp5d	0	0	69.53610952
Atp5e	75.32281168	65.09854983	108.1672815
Atp5f1	277.229793	363.1813833	417.2166571
Atp5g1	5.230750811	1.142079822	0
Atp5g2	57.53825892	45.68319287	59.87831653
Atp5g3	159.0148247	6.85247893	46.35740634
Atp5h	33.47680519	0	69.53610952
Atp5j	196.6762305	126.7708602	195.0874184
Atp5j2	0	0	0
Atp5k	0	0	0
Atp5l	0	20.55743679	30.90493756
Atp5o	54.39980844	0	116.8592952
Atp5s	35.56910552	36.54655429	23.17870317
Atp5sl	71.13821103	61.67231037	0
ATP6	720.7974618	473.963126	401.7641883

For Review Only

Atp6ap1	950.9504975	880.5435425	1181.148082
Atp6ap2	688.3668068	649.8434185	0
Atp6v0a1	155.8763742	77.66142787	48.28896494
Atp6v0a2	448.7984196	439.7007313	486.7527666
Atp6v0a4	9.41535146	3.426239465	0
Atp6v0b	0	0	149.6957913
Atp6v0c	257.3529399	210.1426872	832.5017556
Atp6v0c-ps2	508.4289789	411.1487358	522.4866007
Atp6v0d1	99.38426541	134.765419	14.48668948
Atp6v0e	209.2300324	254.6838002	295.5284654
Atp6v0e2	3.138450487	0	0
Atp6v1a	287.6912946	135.9074988	160.3193636
Atp6v1b2	399.629362	334.6293877	409.4904227
Atp6v1c1	403.8139626	373.4601017	368.9276922
Atp6v1d	100.4304156	142.7599777	142.9353362
Atp6v1e1	187.260879	131.3391795	215.3687836
Atp6v1f	65.90746022	205.5743679	280.0759967
Atp6v1g1	264.675991	251.2575608	308.0835963
Atp6v1g2	7.323051136	17.13119732	15.45246878
Atp6v1h	134.9533709	94.7926252	0
Atp7a	124.4918693	196.4377293	136.1748811
Atp7b	0	1.142079822	7.726234391
ATP8	1067.073165	877.117303	1009.239367
Atp8a1	211.3223328	406.5804165	290.699569
Atp8a2	412.1831639	242.1209222	265.5893072
Atp8b1	6.276900973	6.85247893	3.863117195
Atp8b2	0	5.710399108	8.69201369
Atp8b4	0	0	0
Atp9a	9.41535146	1.142079822	0
Atp9b	231.1991859	183.8748513	169.9771566
Atpaf1	41.84600649	22.84159643	24.14448247
Atpaf2	56.49210876	42.2569534	39.59695125
Atpif1	71.13821103	1.142079822	0
Atr	26.15375406	26.2678359	38.63117195
Atraid	171.5686266	363.1813833	311.9467135
Atrip	0	1.142079822	0
Atrn	58.58440909	74.23518841	65.67299232
Atrnl1	1.046150162	4.568319287	0
Atrx	169.4763263	234.1263634	239.5132661
Atxn1	102.5227159	68.5247893	76.29656461
Atxn10	250.0298888	279.8095563	230.8212524
Atxn1l	84.73816314	77.66142787	56.01519933
Atxn2	59.63055925	95.93470502	69.53610952
Atxn2l	127.6303198	122.2025409	65.67299232
Atxn3	38.707556	42.2569534	33.80227546
Atxn7	25.10760389	36.54655429	51.18630284
Atxn7l1	97.29196509	98.21886466	70.50188882
Atxn7l1os2	1.046150162	3.426239465	0
Atxn7l2	15.69225243	12.56287804	9.657792988
Atxn7l3	52.30750811	25.12575608	36.69961336

For Review Only

Atxn7l3b	187.260879	187.3010908	199.9163149
AU015228	0	3.426239465	0
AU019823	19.87685308	30.83615518	13.52091018
AU021092	0	0	0
AU022252	37.66140584	45.68319287	64.70721302
AU022793	17.78455276	18.27327715	29.93915826
AU040320	191.4454797	99.36094448	117.8250745
AU040972	3.138450487	2.284159643	0
AU041133	9.41535146	5.710399108	17.38402738
Auh	32.43065503	29.69407536	23.17870317
Aup1	2.092300324	7.994558752	0.965779299
Aurka	0	11.42079822	0.965779299
Aurkaip1	0	49.10943233	0
Aurkb	0	1.142079822	0
Aven	0	4.568319287	4.828896494
Avl9	73.23051136	47.96735251	55.04942003
Avpi1	9.41535146	10.27871839	1.931558598
Avpr2	5.230750811	6.85247893	9.657792988
AW011738	1.046150162	3.426239465	0
AW046200	11.50765178	1.142079822	0
AW112010	31.38450487	28.55199554	57.94675793
AW146154	66.95361038	50.25151215	66.63877162
AW209491	41.84600649	39.97279376	64.70721302
AW549877	122.399569	205.5743679	169.9771566
AW554918	77.41511201	36.54655429	44.42584775
Axin1	15.69225243	31.97823501	48.28896494
Axin2	0	2.284159643	0
Axl	32.43065503	34.26239465	43.46006845
AY358078	1.046150162	0	1.931558598
AY512915	0	1.142079822	0
AY702103	0	0	0
Azi2	264.675991	193.0114899	125.5513089
Azin1	189.3531794	132.4812593	143.9011155
B130006D01Rik	47.0767573	68.5247893	40.56273055
B130034C11Rik	0	0	0
B130055M24Rik	60.67670941	54.81983144	71.46766811
B230118H07Rik	23.01530357	20.55743679	40.56273055
B230208H11Rik	6.276900973	4.568319287	2.897337897
B230217C12Rik	1.046150162	1.142079822	0
B230217O12Rik	0	0	0
B230219D22Rik	143.3225722	269.5308379	117.8250745
B230307C23Rik	13.59995211	6.85247893	14.48668948
B230311B06Rik	2.092300324	0	0.965779299
B230317F23Rik	9.41535146	3.426239465	7.726234391
B230319C09Rik	1.046150162	7.994558752	0
B230354K17Rik	16.7384026	33.12031483	32.83649616
B230369F24Rik	0	2.284159643	0
B2m	2499.252738	3835.104041	4263.915604
B330016D10Rik	1.046150162	0	3.863117195
B3galnt1	165.2917256	141.6178979	164.1824808

For Review Only

B3galnt2	24.06145373	27.40991572	27.04182037
B3galt2	9.41535146	2.284159643	0
B3galt4	60.67670941	53.67775162	71.46766811
B3galt5	146.4610227	82.22974716	95.61215059
B3galt6	77.41511201	50.25151215	54.08364074
B3gat2	9.41535146	2.284159643	8.69201369
B3gat3	30.33835471	29.69407536	42.49428915
B3glct	24.06145373	19.41535697	27.04182037
B3gnt2	0	0	0.965779299
B3gnt3	0	0	0
B3gnt4	10.46150162	3.426239465	8.69201369
B3gnt6	1.046150162	1.142079822	12.55513089
B3gnt7	10.46150162	6.85247893	10.62357229
B3gnt8	2.092300324	5.710399108	0
B3gnt9	0	0	0
B3gntl1	81.59971265	66.24062966	92.71481269
B430010I23Rik	201.9069813	77.66142787	57.94675793
B430212C06Rik	0	0	0
B430306N03Rik	40.79985633	28.55199554	38.63117195
B430319F04Rik	0	0	1.931558598
B4galnt1	35.56910552	76.51934805	38.63117195
B4galnt2	8.369201298	6.85247893	3.863117195
B4galt1	114.0303677	70.80894894	95.61215059
B4galt3	247.9375885	0	0
B4galt4	173.6609269	520.7883987	357.3383406
B4galt5	8.369201298	22.84159643	12.55513089
B4galt6	82.64586282	77.66142787	57.94675793
B4galt7	65.90746022	34.26239465	65.67299232
B4gat1	1.046150162	1.142079822	0
B930041F14Rik	4.184600649	1.142079822	3.863117195
B930059L03Rik	4.184600649	6.85247893	9.657792988
B930095G15Rik	24.06145373	10.27871839	6.760455092
B9d1	10.46150162	5.710399108	4.828896494
B9d2	41.84600649	52.5356718	41.52850985
Babam1	44.98445698	58.2460709	59.87831653
Bace1	0	0	0
Bach1	94.1535146	202.1481284	74.36500601
Bach2	141.2302719	151.8966163	158.387805
Bach2os	18.83070292	19.41535697	15.45246878
Bad	3.138450487	1.142079822	25.11026177
Bag1	26.15375406	30.83615518	9.657792988
Bag2	15.69225243	0	4.828896494
Bag3	48.12290746	57.10399108	50.22052354
Bag4	19.87685308	42.2569534	17.38402738
Bag5	131.8149204	82.22974716	136.1748811
Bag6	76.36896184	74.23518841	48.28896494
Bahcc1	14.64610227	21.69951661	25.11026177
Bahd1	25.10760389	28.55199554	28.97337897
Baiap2	46.03060714	36.54655429	50.22052354
Baiap2l1	30.33835471	20.55743679	15.45246878

For Review Only

Baiap3	3.138450487	4.568319287	0
Bak1	10.46150162	5.710399108	0
Bambi	7.323051136	1.142079822	8.69201369
Bambi-ps1	0	4.568319287	0
Banf1	78.46126217	43.39903322	63.74143372
Bank1	187.260879	123.3446207	162.2509222
Banp	25.10760389	65.09854983	59.87831653
Bap1	101.4765657	84.5139068	193.1558598
Bard1	2.092300324	0	0
Basp1	32.43065503	129.0550198	62.77565443
Batf	15.69225243	14.84703768	26.07604107
Batf2	3.138450487	0	0
Batf3	0	0	0
Bax	60.67670941	0	0
Baz1a	70.09206087	66.24062966	84.022799
Baz1b	161.107125	116.4921418	115.8935159
Baz2a	60.67670941	97.07678484	44.42584775
Baz2b	117.1688182	180.4486118	118.7908538
Bbc3	11.50765178	9.136638573	10.62357229
Bbip1	39.75370617	23.98367625	56.01519933
Bbs1	8.369201298	0	8.69201369
Bbs10	28.24605438	20.55743679	17.38402738
Bbs12	24.06145373	34.26239465	37.66539266
Bbs2	33.47680519	27.40991572	30.90493756
Bbs4	1.046150162	5.710399108	72.43344741
Bbs5	0	0	0
Bbs7	26.15375406	35.40447447	55.04942003
Bbs9	128.67647	84.5139068	176.7376117
Bbx	77.41511201	82.22974716	80.1596818
BC002059	27.19990422	14.84703768	46.35740634
BC002163	18.83070292	20.55743679	32.83649616
BC003331	105.6611664	114.2079822	123.6197503
BC003965	0	6.85247893	0
BC004004	193.53778	243.263002	231.7870317
BC005537	120.3072687	206.7164477	215.3687836
BC005561	29.29220454	114.2079822	18.34980668
BC005624	0	47.96735251	34.76805476
BC017158	18.83070292	9.136638573	62.77565443
BC017643	1.046150162	1.142079822	0
BC020402	5.230750811	7.994558752	4.828896494
BC022687	18.83070292	0	0
BC023829	57.53825892	59.38815073	85.9543576
BC024063	21.96915341	15.9891175	29.93915826
BC024978	74.27666152	19.41535697	34.76805476
BC025920	2.092300324	1.142079822	9.657792988
BC026585	63.8151599	31.97823501	28.00759967
BC028528	100.4304156	37.68863411	91.74903339
BC029214	21.96915341	11.42079822	22.21292387
BC029722	65.90746022	39.97279376	0
BC030336	27.19990422	25.12575608	18.34980668

For Review Only

BC030499	0	3.426239465	0
BC031181	101.4765657	123.3446207	115.8935159
BC031361	7.323051136	7.994558752	3.863117195
BC035044	115.0765178	392.8754587	372.7908094
BC037032	11.50765178	15.9891175	10.62357229
BC037034	15.69225243	34.26239465	0
BC037704	19.87685308	6.85247893	6.760455092
BC048403	8.369201298	25.12575608	15.45246878
BC048507	1.046150162	4.568319287	7.726234391
BC048609	1.046150162	0	5.794675793
BC049352	0	20.55743679	0
BC049715	3.138450487	3.426239465	0
BC051142	0	0	0
BC051226	0	0	0
BC051537	3.138450487	0	4.828896494
BC052040	13.59995211	28.55199554	35.73383406
BC055324	0	11.42079822	20.28136528
BC064078	0	0	0
BC065397	11.50765178	7.994558752	12.55513089
BC067074	0	0	0
BC068281	47.0767573	31.97823501	31.87071686
BC100451	18.83070292	18.27327715	6.760455092
BC106175	6.276900973	0	4.828896494
BC106179	1.046150162	3.426239465	3.863117195
BC147527	8.369201298	12.56287804	4.828896494
Bcam	0	0	0
Bcan	0	0	0
Bcap29	145.4148726	116.4921418	141.0037776
Bcap31	300.2450966	263.8204388	367.9619129
Bcar3	3.138450487	0	0
Bcas2	86.83046347	288.9461949	197.018977
Bcas3	196.6762305	150.7545365	132.3117639
Bcas3os1	5.230750811	10.27871839	5.794675793
Bcas3os2	0	0	0.965779299
Bcat1	2.092300324	1.142079822	0
Bcat2	31.38450487	36.54655429	52.15208214
Bccip	79.50741233	60.53023055	49.25474424
Bcdin3d	42.89215665	58.2460709	49.25474424
Bckdha	15.69225243	145.0441374	0
Bckdhb	28.24605438	0	55.04942003
Bckdk	46.03060714	44.54111304	0
Bcl10	3.138450487	114.2079822	333.1938581
Bcl11a	0	0	0
Bcl2	16.7384026	22.84159643	13.52091018
Bcl2a1a	8.369201298	83.37182698	121.6881917
Bcl2a1b	177.8455276	177.0223724	24.14448247
Bcl2a1c	0	3.426239465	4.828896494
Bcl2a1d	69.04591071	71.95102876	174.8060531
Bcl2l1	635.0131485	416.8591349	437.4980224
Bcl2l11	89.96891395	84.5139068	77.26234391

For Review Only

Bcl2l12	0	0	0.965779299
Bcl2l13	100.4304156	76.51934805	107.2015022
Bcl2l2	7.323051136	14.84703768	7.726234391
Bcl3	0	4.568319287	0
Bcl6	32.43065503	60.53023055	51.18630284
Bcl6b	3.138450487	7.994558752	6.760455092
Bcl7a	11.50765178	25.12575608	9.657792988
Bcl7b	0	0	333.1938581
Bcl7c	8.369201298	12.56287804	0
Bcl9	130.7687703	117.6342216	139.072219
Bcl9l	81.59971265	164.4594943	97.54370918
Bclaf1	240.6145373	226.1318047	135.2091018
Bco2	227.0145852	203.2902083	204.7452114
Bcor	98.33811525	139.3337382	113.9619573
Bcorl1	38.707556	31.97823501	18.34980668
Bcr	9.41535146	12.56287804	22.21292387
Bcs1l	48.12290746	21.69951661	37.66539266
Bdh1	6.276900973	0	0
Bdp1	81.59971265	81.08766734	66.63877162
BE692007	2.092300324	2.284159643	0
Becn1	43.93830681	21.69951661	0
Bend3	12.55380195	12.56287804	15.45246878
Bend4	0	0	0
Bend5	21.96915341	33.12031483	18.34980668
Bend6	59.63055925	45.68319287	77.26234391
Best1	2.092300324	1.142079822	48.28896494
Bet1	39.75370617	45.68319287	54.08364074
Bfar	145.4148726	162.1753347	198.9505356
Bgn	0	0	0
Bhlha15	2.092300324	0	0
Bhlhb9	58.58440909	18.27327715	45.39162705
Bhlhe40	3.138450487	0	6.760455092
Bhlhe41	694.6437077	786.8929971	0
Bicd1	0	0	2.897337897
Bicd2	321.1680998	270.6729177	205.7109907
Bid	125.5380195	187.3010908	201.8478735
Bik	0	7.994558752	0.965779299
Bin1	690.4591071	832.57619	1418.72979
Bin2	1009.534907	915.948017	1327.946536
Bin3	91.01506412	86.79806645	144.8668948
Birc2	38.707556	68.5247893	85.9543576
Birc3	143.3225722	92.50846555	85.9543576
Birc5	2.092300324	0	6.760455092
Birc6	302.3373969	336.9135474	237.5817075
Bivm	29.29220454	21.69951661	26.07604107
Blcap	89.96891395	87.94014627	109.1330608
Blm	3.138450487	2.284159643	12.55513089
Blmh	26.15375406	81.08766734	0
Blnk	565.9672378	706.9474096	746.547398
Bloc1s1	0	5.710399108	0

For Review Only

Bloc1s2	15.69225243	23.98367625	0
Bloc1s3	5.230750811	1.142079822	2.897337897
Bloc1s4	11.50765178	34.26239465	11.58935159
Bloc1s5	63.8151599	63.95647001	78.22812321
Bloc1s6	85.7843133	62.81439019	72.43344741
Bloc1s6os	1.046150162	0	0
Blvra	88.92276379	91.36638573	113.9619573
Blvrb	15.69225243	30.83615518	92.71481269
Blzf1	59.63055925	21.69951661	55.04942003
Bmf	154.830224	161.0332549	194.1216391
Bmi1	20.92300324	41.11487358	0
Bmp1	20.92300324	0	7.726234391
Bmp2	0	0	0
Bmp2k	635.0131485	704.66325	592.0227102
Bmp6	3.138450487	0	0
Bmp7	3.138450487	0	0
Bmp8b	0	0	0.965779299
Bmpr1a	21.96915341	44.54111304	34.76805476
Bmpr2	148.553323	194.1535697	182.5322875
Bms1	33.47680519	59.38815073	42.49428915
Bmt2	24.06145373	45.68319287	20.28136528
Bmyc	196.6762305	216.9951661	260.7604107
Bnc1	0	0	0
Bnc2	0	0	3.863117195
Bnip1	19.87685308	19.41535697	22.21292387
Bnip2	150.6456234	266.1045984	277.1786588
Bnip3	42.89215665	59.38815073	71.46766811
Bnip3l	177.8455276	206.7164477	205.7109907
Bnip3l-ps	92.06121428	100.5030243	75.33078531
Bnipl	0	0	3.863117195
Bod1	18.83070292	21.69951661	16.41824808
Bod1l	97.29196509	185.0169311	84.9885783
Bola1	112.9842175	1.142079822	0
Bola3	11.50765178	4.568319287	0
Bop1	0	0	8.69201369
Bora	0	0	10.62357229
Borcs5	31.38450487	19.41535697	40.56273055
Borcs6	18.83070292	43.39903322	15.45246878
Borcs7	5.230750811	6.85247893	46.35740634
Borcs8	3.138450487	14.84703768	0
Bpgm	15.69225243	53.67775162	27.04182037
Bphl	40.79985633	36.54655429	55.04942003
Bpifb9a	0	0	0
Bpifb9b	0	0	0
Bpnt1	34.52295535	43.39903322	22.21292387
Bptf	175.7532273	238.6946827	160.3193636
Braf	208.1838823	117.6342216	103.338385
Brap	49.16905763	29.69407536	47.32318564
Brat1	172.6147768	114.2079822	99.47526778
Brca1	0	0	0

For Review Only

Brca2	27.19990422	17.13119732	25.11026177
Brcc3	44.98445698	41.11487358	44.42584775
Brd1	85.7843133	138.1916584	101.4068264
Brd2	0	0	0
Brd3	25.10760389	108.4975831	21.24714457
Brd4	216.5530836	269.5308379	239.5132661
Brd7	214.4607833	251.2575608	151.6273499
Brd8	0	20.55743679	53.11786144
Brd9	112.9842175	98.21886466	141.9695569
Brdt	0	0	0
Bre	106.7073165	92.50846555	122.653971
Brf1	44.98445698	209.0006074	25.11026177
Brf2	19.87685308	11.42079822	10.62357229
Bri3	13.59995211	14.84703768	16.41824808
Bri3bp	41.84600649	28.55199554	37.66539266
Brip1os	72.18436119	78.80350769	79.19390251
Brix1	50.21520779	51.39359197	47.32318564
Brk1	268.8605917	312.9298711	172.8744945
Brms1	0	0	0
Brms1l	51.26135795	73.09310859	35.73383406
Brox	88.92276379	129.0550198	83.0570197
Brpf1	114.0303677	103.9292638	119.7566331
Brpf3	24.06145373	33.12031483	36.69961336
Brs3	0	0	0
Brsk2	0	3.426239465	0
Brwd1	124.4918693	164.4594943	96.57792988
Brwd3	14.64610227	23.98367625	15.45246878
Bscl2	202.9531315	177.0223724	292.6311276
Bsdc1	106.7073165	90.22430591	78.22812321
Bsg	968.7350502	759.4830814	1014.068264
Bsn	85.7843133	36.54655429	27.04182037
Bspry	0	0	0
Bst2	111.9380674	83.37182698	141.0037776
Btaf1	82.64586282	97.07678484	78.22812321
Btbd1	41.84600649	31.97823501	33.80227546
Btbd10	55.4459586	92.50846555	78.22812321
Btbd17	14.64610227	6.85247893	3.863117195
Btbd18	3.138450487	2.284159643	0
Btbd19	1.046150162	1.142079822	0
Btbd2	2.092300324	14.84703768	0
Btbd3	50.21520779	71.95102876	21.24714457
Btbd6	0	0	0
Btbd7	55.4459586	67.38270948	87.8859162
Btbd8	3.138450487	0	0
Btbd9	302.3373969	358.613064	267.5208658
Btd	52.30750811	55.96191126	70.50188882
Btf3	95.19966476	110.7817427	106.2357229
Btf3l4	100.4304156	81.08766734	62.77565443
Btg1	0	0	0
Btg2	833.7816793	0	721.4371362

For Review Only

Btg3	4.184600649	0	2.897337897
Btk	304.4296972	294.656594	271.383983
Btla	1.046150162	0	0
Btnl2	1.046150162	0	0
Btrc	73.23051136	58.2460709	76.29656461
Bub1	0	0	2.897337897
Bub1b	5.230750811	0	0
Bub3	197.7223807	237.5526029	231.7870317
Bud13	24.06145373	28.55199554	36.69961336
Bud31	61.72285957	51.39359197	58.91253723
Bysl	51.26135795	30.83615518	33.80227546
Bzrap1	14.64610227	38.83071394	13.52091018
Bzw1	261.5375406	238.6946827	196.0531977
Bzw2	29.29220454	33.12031483	68.57033022
C030013C21Rik	0	0	0
C030016D13Rik	0	3.426239465	3.863117195
C030034I22Rik	7.323051136	11.42079822	14.48668948
C030034L19Rik	1.046150162	1.142079822	0
C030039L03Rik	3.138450487	1.142079822	0
C130021I20Rik	0	0	0
C130026I21Rik	6.276900973	5.710399108	5.794675793
C130036L24Rik	3.138450487	0	0
C130046K22Rik	21.96915341	11.42079822	23.17870317
C130050O18Rik	122.399569	47.96735251	37.66539266
C1d	49.16905763	65.09854983	67.60455092
C1galt1	1.046150162	4.568319287	0.965779299
C1galt1c1	83.69201298	107.3555032	97.54370918
C1qa	8287.601585	8238.963833	9456.910894
C1qb	9577.504735	10371.22686	12454.68984
C1qbp	57.53825892	55.96191126	80.1596818
C1qc	3692.910073	3447.938982	537.9390695
C1qtnf1	1.046150162	0	0
C1ra	7.323051136	0	0
C1rb	5.230750811	0	0
C1rl	33.47680519	42.2569534	40.56273055
C230012O17Rik	0	0	0.965779299
C230037L18Rik	1.046150162	0	4.828896494
C230057M02Rik	0	2.284159643	0
C230062I16Rik	4.184600649	0	0
C230072F16Rik	2.092300324	0	0
C230091D08Rik	13.59995211	29.69407536	9.657792988
C2cd2	32.43065503	51.39359197	39.59695125
C2cd2l	17.78455276	20.55743679	37.66539266
C2cd3	165.2917256	99.36094448	119.7566331
C2cd5	28.24605438	38.83071394	28.97337897
C3	23.01530357	7.994558752	0
C330006A16Rik	390.2140105	310.6457115	303.2546998
C330007P06Rik	53.35365827	38.83071394	60.84409583
C330013E15Rik	9.41535146	1.142079822	7.726234391
C330016L05Rik	0	2.284159643	0

For Review Only

C330018A13Rik	2.092300324	0	0.965779299
C330018D20Rik	38.707556	13.70495786	36.69961336
C330021F23Rik	67.99976055	67.38270948	74.36500601
C3ar1	1096.36537	657.8379773	861.4751346
C430042M11Rik	0	0	0
C430049B03Rik	5.230750811	9.136638573	5.794675793
C4a	0	4.568319287	0
C4b	0	0	0
C530005A16Rik	3.138450487	6.85247893	0
C530008M17Rik	1.046150162	1.142079822	3.863117195
C5ar1	309.660448	240.9788424	311.9467135
C5ar2	25.10760389	59.38815073	19.31558598
C630043F03Rik	2.092300324	0	0
C77080	7.323051136	0	4.828896494
C87436	118.2149683	105.0713436	123.6197503
C8g	0	0	10.62357229
C920006O11Rik	1.046150162	1.142079822	53.11786144
C920009B18Rik	6.276900973	2.284159643	0
C920021L13Rik	2.092300324	4.568319287	0
Caap1	30.33835471	9.136638573	4.828896494
Cab39	83.69201298	133.6233391	127.4828674
Cab39l	265.7221412	33.12031483	94.64637129
Cabin1	266.7682914	234.1263634	205.7109907
Cables1	116.122668	70.80894894	139.072219
Cables2	47.0767573	23.98367625	37.66539266
Cabyr	8.369201298	0	13.52091018
Cacfd1	33.47680519	30.83615518	30.90493756
Cachd1	6.276900973	0	0
Cacna1a	0	6.85247893	0
Cacna1c	1.046150162	3.426239465	0
Cacna1d	94.1535146	122.2025409	84.9885783
Cacna1e	8.369201298	4.568319287	2.897337897
Cacna1f	0	0	0
Cacna1s	9.41535146	1.142079822	15.45246878
Cacnb1	11.50765178	0	2.897337897
Cacnb2	11.50765178	33.12031483	12.55513089
Cacnb4	12.55380195	4.568319287	0.965779299
Cactin	24.06145373	31.97823501	27.04182037
Cacul1	80.55356249	114.2079822	84.9885783
Cacybp	67.99976055	78.80350769	86.9201369
Cad	0	0	48.28896494
Cadm1	308.6142979	320.9244299	254.9657349
Cadm2	2.092300324	0	0
Cage1	5.230750811	6.85247893	1.931558598
Cahm	0	3.426239465	0
Calcoco1	76.36896184	94.7926252	71.46766811
Calcrl	95.19966476	109.6396629	68.57033022
Cald1	2.092300324	0	0
Calhm2	252.1221891	252.3996406	189.2927426
Calm1	641.2900495	564.1874319	616.1671927

For Review Only

Calm2	781.4741712	923.9425757	1136.722235
Calm3	46.03060714	45.68319287	16.41824808
Calml4	18.83070292	2.284159643	0
Calr	1.046150162	1.142079822	0
Calr3	5.230750811	0	0
Calu	217.5992337	223.847645	394.0379539
Camk1	0	0	0
Camk1d	238.522237	198.721889	293.5969068
Camk2a	25.10760389	5.710399108	16.41824808
Camk2b	15.69225243	20.55743679	15.45246878
Camk2d	100.4304156	267.2466783	160.3193636
Camk2g	106.7073165	149.6124566	117.8250745
Camk2n1	52.30750811	73.09310859	38.63117195
Camk4	0	1.142079822	0
Camkk1	85.7843133	61.67231037	106.2357229
Camkk2	94.1535146	73.09310859	81.1254611
Camkmt	50.21520779	20.55743679	51.18630284
Camkv	0	3.426239465	0
Caml	114.0303677	118.7763015	92.71481269
Camp	254.2144894	134.765419	48.28896494
Camsap1	76.36896184	137.0495786	103.338385
Camsap2	77.41511201	52.5356718	44.42584775
Camsap3	0	0	0
Camta1	33.47680519	49.10943233	31.87071686
Camta2	14.64610227	0	0.965779299
Cand1	138.0918214	86.79806645	123.6197503
Cand2	48.12290746	21.69951661	35.73383406
Cant1	48.12290746	53.67775162	7.726234391
Canx	1832.855084	1765.655404	1740.334297
Cap1	137.0456713	132.4812593	87.8859162
Capg	77.41511201	62.81439019	0
Capn1	0	0	0
Capn10	23.01530357	23.98367625	19.31558598
Capn11	0	0	0
Capn15	51.26135795	89.08222609	68.57033022
Capn2	13.59995211	1.142079822	0
Capn3	0	39.97279376	0
Capn5	0	2.284159643	0
Capn7	59.63055925	42.2569534	117.8250745
Capns1	15.69225243	37.68863411	82.0912404
Capns2	6.276900973	39.97279376	18.34980668
Caprin1	496.9213271	248.9734011	402.7299676
Caprin2	7.323051136	3.426239465	9.657792988
Capza1	183.0762784	189.5852504	209.5741078
Capza2	1801.470579	2028.333763	2274.410249
Capzb	289.7835949	240.9788424	589.1253723
Car11	1.046150162	0	3.863117195
Car12	24.06145373	0	0
Car13	0	0	0
Car14	16.7384026	0	0

For Review Only

Car15	1.046150162	1.142079822	0
Car2	18.83070292	1.142079822	0
Car4	0	0	0
Car7	1.046150162	4.568319287	9.657792988
Car9	4.184600649	0	0
Card10	3.138450487	2.284159643	10.62357229
Card11	0	11.42079822	14.48668948
Card19	0	0	6.760455092
Card6	242.7068376	199.8639688	187.361184
Card9	0	0	22.21292387
Carf	17.78455276	29.69407536	28.97337897
Carhsp1	25.10760389	42.2569534	35.73383406
Carkd	86.83046347	102.7871839	45.39162705
Carlr	0	2.284159643	0
Carm1	0	62.81439019	1.931558598
Carnmt1	5.230750811	11.42079822	1.931558598
Carns1	16.7384026	4.568319287	24.14448247
Cars	13.59995211	10.27871839	44.42584775
Cars2	0	66.24062966	37.66539266
Casc1	0	0	0
Casc3	64.86131006	132.4812593	60.84409583
Casc4	6.276900973	12.56287804	12.55513089
Casc5	0	0	0
Casd1	61.72285957	124.4867006	99.47526778
Cask	183.0762784	173.5961329	190.2585219
Caskin1	6.276900973	0	3.863117195
Caskin2	1.046150162	0	0
Casp1	15.69225243	15.9891175	107.2015022
Casp12	0	4.568319287	7.726234391
Casp2	0	0	117.8250745
Casp3	81.59971265	83.37182698	66.63877162
Casp4	18.83070292	37.68863411	56.01519933
Casp6	34.52295535	42.2569534	57.94675793
Casp7	30.33835471	27.40991572	27.04182037
Casp8	33.47680519	54.81983144	136.1748811
Casp8ap2	34.52295535	37.68863411	19.31558598
Casp9	98.33811525	78.80350769	66.63877162
Casq1	6.276900973	0	0
Casr	0	0	0
Cass4	135.9995211	150.7545365	168.045598
Cast	86.83046347	46.82527269	59.87831653
Casz1	1.046150162	4.568319287	11.58935159
Cat	212.3684829	198.721889	230.8212524
Catip	21.96915341	6.85247893	11.58935159
Catsper2	24.06145373	70.80894894	41.52850985
Catsperg1	34.52295535	7.994558752	0
Cav1	0	0	0
Cav2	3.138450487	3.426239465	0
Cbarp	7.323051136	0	3.863117195
Cbfa2t2	200.8608312	74.23518841	151.6273499

For Review Only

Cbfa2t3	94.1535146	129.0550198	117.8250745
Cbfb	26.15375406	34.26239465	45.39162705
Cbl	382.8909594	447.6952901	366.0303543
Cblb	69.04591071	81.08766734	24.14448247
Cbll1	46.03060714	38.83071394	56.98097863
Cbr1	11.50765178	10.27871839	0
Cbr2	0	2.284159643	0
Cbr3	15.69225243	15.9891175	25.11026177
Cbr4	27.19990422	49.10943233	45.39162705
Cbs	2.092300324	0	0
Cbwd1	6.276900973	3.426239465	9.657792988
Cbx1	34.52295535	34.26239465	41.52850985
Cbx2	9.41535146	10.27871839	0
Cbx3	66.95361038	83.37182698	78.22812321
Cbx4	56.49210876	33.12031483	40.56273055
Cbx5	52.30750811	59.38815073	44.42584775
Cbx6	21.96915341	27.40991572	26.07604107
Cbx7	13.59995211	9.136638573	1.931558598
Cbx8	23.01530357	49.10943233	41.52850985
Cby1	35.56910552	18.27327715	24.14448247
Cc2d1a	77.41511201	49.10943233	0
Cc2d1b	146.4610227	157.6070154	0
Cc2d2a	6.276900973	2.284159643	0
Ccar1	121.3534188	124.4867006	141.9695569
Ccar2	65.90746022	14.84703768	18.34980668
Ccdc102a	17.78455276	13.70495786	4.828896494
Ccdc106	1.046150162	1.142079822	0
Ccdc107	0	53.67775162	80.1596818
Ccdc108	0	0	0
Ccdc112	0	1.142079822	0
Ccdc114	6.276900973	5.710399108	0
Ccdc115	7.323051136	0	0
Ccdc116	3.138450487	1.142079822	0
Ccdc117	40.79985633	45.68319287	31.87071686
Ccdc12	99.38426541	0	0
Ccdc120	0	0	1.931558598
Ccdc122	0	0	1.931558598
Ccdc124	30.33835471	0	3.863117195
Ccdc125	4.184600649	0	5.794675793
Ccdc126	19.87685308	34.26239465	29.93915826
Ccdc127	93.10736444	69.66686912	100.4410471
Ccdc130	32.43065503	65.09854983	91.74903339
Ccdc134	36.61525568	54.81983144	38.63117195
Ccdc136	0	2.284159643	0.965779299
Ccdc137	27.19990422	25.12575608	158.387805
Ccdc138	0	0	0
Ccdc14	12.55380195	19.41535697	20.28136528
Ccdc142	0	21.69951661	48.28896494
Ccdc146	4.184600649	27.40991572	25.11026177
Ccdc149	0	5.710399108	6.760455092

For Review Only

Ccdc15	32.43065503	34.26239465	19.31558598
Ccdc157	7.323051136	30.83615518	87.8859162
Ccdc162	24.06145373	10.27871839	55.04942003
Ccdc163	56.49210876	15.9891175	54.08364074
Ccdc166	24.06145373	34.26239465	22.21292387
Ccdc167	16.7384026	37.68863411	28.97337897
Ccdc168	1.046150162	29.69407536	20.28136528
Ccdc171	14.64610227	15.9891175	10.62357229
Ccdc173	6.276900973	0	2.897337897
Ccdc174	16.7384026	39.97279376	27.04182037
Ccdc181	29.29220454	13.70495786	19.31558598
Ccdc186	49.16905763	39.97279376	38.63117195
Ccdc188	0	0	0
Ccdc189	6.276900973	5.710399108	9.657792988
Ccdc191	37.66140584	28.55199554	19.31558598
Ccdc22	62.76900973	46.82527269	0
Ccdc25	42.89215665	20.55743679	36.69961336
Ccdc28a	6.276900973	7.994558752	10.62357229
Ccdc28b	0	0	0
Ccdc30	1.046150162	1.142079822	2.897337897
Ccdc32	50.21520779	63.95647001	52.15208214
Ccdc34	26.15375406	21.69951661	15.45246878
Ccdc36	7.323051136	2.284159643	7.726234391
Ccdc38	2.092300324	7.994558752	3.863117195
Ccdc40	0	0	0
Ccdc43	34.52295535	20.55743679	43.46006845
Ccdc47	140.1841217	130.1970997	134.2433225
Ccdc50	203.9992816	232.9842836	225.0265766
Ccdc51	32.43065503	23.98367625	17.38402738
Ccdc53	41.84600649	51.39359197	79.19390251
Ccdc57	21.96915341	11.42079822	8.69201369
Ccdc58	19.87685308	20.55743679	19.31558598
Ccdc59	28.24605438	30.83615518	42.49428915
Ccdc6	85.7843133	119.9183813	116.8592952
Ccdc61	13.59995211	9.136638573	28.97337897
Ccdc62	15.69225243	22.84159643	25.11026177
Ccdc63	0	0	0
Ccdc64	10.46150162	5.710399108	23.17870317
Ccdc66	10.46150162	59.38815073	51.18630284
Ccdc69	0	3.426239465	0
Ccdc71	109.845767	109.6396629	0
Ccdc71l	9.41535146	29.69407536	6.760455092
Ccdc73	2.092300324	9.136638573	0
Ccdc77	12.55380195	18.27327715	29.93915826
Ccdc78	2.092300324	0	0
Ccdc8	1.046150162	2.284159643	0
Ccdc80	5.230750811	0	7.726234391
Ccdc82	42.89215665	101.6451041	49.25474424
Ccdc84	0	0	0
Ccdc85b	9.41535146	10.27871839	9.657792988

For Review Only

Ccdc85c	31.38450487	46.82527269	28.00759967
Ccdc86	75.32281168	52.5356718	65.67299232
Ccdc88a	7.323051136	25.12575608	11.58935159
Ccdc88b	1.046150162	67.38270948	0
Ccdc88c	0	2.284159643	0
Ccdc89	5.230750811	2.284159643	0
Ccdc9	15.69225243	10.27871839	11.58935159
Ccdc90b	62.76900973	7.994558752	91.74903339
Ccdc91	56.49210876	7.994558752	46.35740634
Ccdc92	0	0	2.897337897
Ccdc93	306.5219975	387.1650595	359.2698992
Ccdc94	2.092300324	1.142079822	0
Ccdc96	1.046150162	6.85247893	3.863117195
Ccdc97	203.9992816	18.27327715	71.46766811
Cchcr1	0	0	0
Ccl12	0	0	18.34980668
Ccl22	0	0	2.897337897
Ccl24	5.230750811	7.994558752	5.794675793
Ccl25	0	0	0
Ccl27a	0	0	3.863117195
Ccl27b	1.046150162	0	0.965779299
Ccl28	0	2.284159643	0
Ccl4	78.46126217	0	1.931558598
Ccl5	0	0	0
Ccl6	109.845767	219.2793258	250.1368384
Ccl7	0	6.85247893	7.726234391
Ccl9	116.122668	130.1970997	169.9771566
Ccm2	19.87685308	63.95647001	103.338385
Ccm2l	0	0	0
Ccna2	15.69225243	0	14.48668948
Ccnb1	1.046150162	0	2.897337897
Ccnb2	0	0	0
Ccnc	37.66140584	63.95647001	54.08364074
Ccnd1	57.53825892	74.23518841	62.77565443
Ccnd2	10.46150162	4.568319287	12.55513089
Ccnd3	187.260879	1.142079822	0
Ccndbp1	0	35.40447447	56.01519933
Ccne1	1.046150162	0	0
Ccnf	7.323051136	3.426239465	0
Ccng1	121.3534188	53.67775162	93.68059199
Ccng2	1.046150162	1922.12034	0
Ccnh	70.09206087	70.80894894	54.08364074
Ccni	164.2455755	306.0773922	197.9847563
Ccnj	20.92300324	28.55199554	11.58935159
Ccnk	30.33835471	37.68863411	39.59695125
Ccnl1	153.7840738	118.7763015	197.9847563
Ccnl2	347.3218539	0	227.9239145
Ccnt1	82.64586282	83.37182698	134.2433225
Ccnt2	72.18436119	119.9183813	1.931558598
Ccny	50.21520779	102.7871839	58.91253723

For Review Only

Ccnyl1	10.46150162	10.27871839	11.58935159
Ccp110	4.184600649	11.42079822	4.828896494
Ccpg1	332.6757516	223.847645	242.410604
Ccpg1os	9.41535146	11.42079822	9.657792988
Ccr1	18.83070292	28.55199554	27.04182037
Ccr1l1	14.64610227	4.568319287	4.828896494
Ccr2	62.76900973	58.2460709	55.04942003
Ccr3	4.184600649	1.142079822	7.726234391
Ccr5	1095.31922	783.4667577	0
Ccr6	0	3.426239465	2.897337897
Ccr9	6.276900973	7.994558752	7.726234391
Ccrl2	0	22.84159643	0
Ccs	0	0	0
Ccsap	4.184600649	1.142079822	0
Ccser2	43.93830681	66.24062966	44.42584775
Cct2	241.6606875	169.0278136	229.8554731
Cct3	261.5375406	243.263002	327.3991823
Cct4	331.6296014	318.6402702	457.7793877
Cct5	196.6762305	286.6620352	319.6729479
Cct6a	205.0454318	254.6838002	271.383983
Cct7	351.5064545	246.6892415	394.0379539
Cct8	313.8450487	292.3724343	373.7565887
Ccz1	42.89215665	57.10399108	33.80227546
Cd101	63.8151599	35.40447447	31.87071686
Cd14	0	0	235.6501489
Cd151	78.46126217	0	6.760455092
Cd160	4.184600649	0	0
Cd163	0	3.426239465	0.965779299
Cd164	1347.441409	1597.769671	1776.068131
Cd177	91.01506412	31.97823501	9.657792988
Cd180	49.16905763	175.8802925	170.9429359
Cd1d1	0	2.284159643	6.760455092
Cd1d2	2.092300324	1.142079822	0
Cd200	0	0	0
Cd200r1	49.16905763	35.40447447	78.22812321
Cd200r3	1.046150162	0	0
Cd200r4	3.138450487	0	0
Cd209a	0	0	0
Cd209f	0	0	0
Cd22	55.4459586	15.9891175	43.46006845
Cd226	0	1.142079822	3.863117195
Cd244	7.323051136	9.136638573	8.69201369
Cd247	0	0	2.897337897
Cd24a	8.369201298	0	0
Cd27	62.76900973	60.53023055	48.28896494
Cd274	35.56910552	23.98367625	11.58935159
Cd276	66.95361038	52.5356718	91.74903339
Cd2ap	108.7996169	190.7273302	116.8592952
Cd2bp2	54.39980844	3.426239465	0
Cd300a	392.3063108	616.7231037	544.6995246

For Review Only

Cd300c	0	0	0
Cd300c2	391.2601607	291.2303545	249.1710591
Cd300lb	4.184600649	2.284159643	4.828896494
Cd300ld	2.092300324	3.426239465	0
Cd300ld2	0	0	0
Cd300ld3	3.138450487	0	0.965779299
Cd300ld5	0	0	0
Cd300lf	6.276900973	4.568319287	0
Cd302	188.3070292	298.0828335	303.2546998
Cd320	40.79985633	25.12575608	13.52091018
Cd33	0	55.96191126	0.965779299
Cd34	345.2295535	0	162.2509222
Cd36	0	0	0
Cd37	17.78455276	975.3361677	0
Cd38	0	0	9.657792988
Cd3eap	26.15375406	19.41535697	19.31558598
Cd4	1.046150162	1.142079822	0
Cd40	0	2.284159643	0
Cd44	14.64610227	2.284159643	4.828896494
Cd46	10.46150162	9.136638573	0
Cd47	499.0136274	493.378483	541.8021867
Cd48	320.1219496	307.219472	286.8364518
Cd52	82.64586282	122.2025409	158.387805
Cd53	2045.223567	2099.142712	1411.003556
Cd59a	5.230750811	0	0
Cd5l	2.092300324	0	0
Cd63	0	0	53.11786144
Cd68	1322.333805	953.6366511	899.1405272
Cd69	1.046150162	10.27871839	0
Cd72	6.276900973	15.9891175	8.69201369
Cd74	89.96891395	50.25151215	68.57033022
Cd79b	41.84600649	0	91.74903339
Cd80	52.30750811	36.54655429	46.35740634
Cd81	5618.872521	9492.967478	10806.10457
Cd82	300.2450966	502.5151215	45.39162705
Cd83	67.99976055	0	0
Cd84	527.2596818	662.4062966	842.1595486
Cd86	800.3048741	636.1384607	861.4751346
Cd9	1712.547816	1568.075595	3016.12875
Cd93	8.369201298	0	0
Cd99l2	77.41511201	51.39359197	40.56273055
Cdadc1	72.18436119	52.5356718	56.01519933
Cdan1	1.046150162	18.27327715	0
Cdc123	115.0765178	149.6124566	143.9011155
Cdc14a	19.87685308	37.68863411	15.45246878
Cdc14b	17.78455276	10.27871839	0
Cdc16	67.99976055	59.38815073	91.74903339
Cdc23	116.122668	105.0713436	87.8859162
Cdc25a	13.59995211	11.42079822	16.41824808
Cdc25b	0	1.142079822	17.38402738

For Review Only

Cdc26	33.47680519	67.38270948	59.87831653
Cdc27	48.12290746	34.26239465	46.35740634
Cdc34	0	0	0
Cdc37	196.6762305	220.4214056	169.0113773
Cdc37l1	109.845767	77.66142787	78.22812321
Cdc40	106.7073165	65.09854983	80.1596818
Cdc42	1400.795067	1515.539923	1574.220257
Cdc42bpa	28.24605438	26.2678359	19.31558598
Cdc42bpb	56.49210876	11.42079822	122.653971
Cdc42ep1	2.092300324	18.27327715	0
Cdc42ep2	6.276900973	0	8.69201369
Cdc42ep3	41.84600649	12.56287804	60.84409583
Cdc42ep4	46.03060714	37.68863411	89.81747479
Cdc42se1	353.5987548	295.7986738	257.8630728
Cdc42se2	260.4913904	316.3561106	236.6159282
Cdc45	0	0	0
Cdc5l	67.99976055	61.67231037	92.71481269
Cdc7	10.46150162	5.710399108	13.52091018
Cdc73	9.41535146	33.12031483	28.97337897
Cdca2	3.138450487	11.42079822	0
Cdca3	0	0	3.863117195
Cdca4	198.7685308	146.1862172	173.8402738
Cdca7	69.04591071	55.96191126	71.46766811
Cdca8	0	0	1.931558598
Cdh11	2.092300324	3.426239465	2.897337897
Cdh12	3.138450487	0	0
Cdh23	206.091582	198.721889	182.5322875
Cdh24	0	0	0.965779299
Cdh5	1.046150162	0	3.863117195
Cdh6	6.276900973	0	0
Cdh7	0	0	0
Cdhr1	0	1.142079822	0
Cdhr4	5.230750811	0	6.760455092
Cdip1	36.61525568	0	46.35740634
Cdipt	0	0	44.42584775
Cdk1	0	0	0
Cdk10	0	1.142079822	6.760455092
Cdk11b	160.0609748	145.0441374	282.9733346
Cdk12	89.96891395	158.7490952	75.33078531
Cdk13	61.72285957	73.09310859	46.35740634
Cdk14	21.96915341	17.13119732	39.59695125
Cdk16	100.4304156	77.66142787	18.34980668
Cdk17	52.30750811	54.81983144	57.94675793
Cdk18	1.046150162	2.284159643	0
Cdk19	142.2764221	180.4486118	152.5931292
Cdk2	31.38450487	28.55199554	54.08364074
Cdk20	11.50765178	0	0
Cdk2ap1	25.10760389	29.69407536	32.83649616
Cdk2ap2	0	0	0
Cdk3-ps	0	4.568319287	0

For Review Only

Cdk4	43.93830681	17.13119732	0
Cdk5	0	39.97279376	37.66539266
Cdk5r1	53.35365827	42.2569534	30.90493756
Cdk5rap1	26.15375406	18.27327715	34.76805476
Cdk5rap2	41.84600649	46.82527269	21.24714457
Cdk5rap3	0	111.9238225	192.1900805
Cdk6	243.7529878	387.1650595	244.3421626
Cdk7	91.01506412	102.7871839	73.39922671
Cdk8	39.75370617	41.11487358	43.46006845
Cdk9	12.55380195	117.6342216	0
Cdkal1	100.4304156	105.0713436	94.64637129
Cdkl3	9.41535146	17.13119732	16.41824808
Cdkl4	10.46150162	2.284159643	9.657792988
Cdkn1b	71.13821103	91.36638573	54.08364074
Cdkn1c	11.50765178	4.568319287	2.897337897
Cdkn2aip	56.49210876	67.38270948	66.63877162
Cdkn2aipnl	142.2764221	113.0659023	120.7224124
Cdkn2b	0	4.568319287	2.897337897
Cdkn2c	3.138450487	2.284159643	4.828896494
Cdkn2d	24.06145373	19.41535697	32.83649616
Cdnf	2.092300324	0	0
Cdpf1	23.01530357	30.83615518	35.73383406
Cdr2	14.64610227	1.142079822	0
Cdr2l	15.69225243	1.142079822	7.726234391
Cds1	92.06121428	154.1807759	129.414426
Cds2	364.0602565	196.4377293	253.9999556
Cdt1	0	0	0.965779299
Cdv3	94.1535146	77.66142787	65.67299232
Cdyl	21.96915341	20.55743679	28.97337897
Cdyl2	31.38450487	42.2569534	22.21292387
Ceacam1	91.01506412	202.1481284	123.6197503
Ceacam18	5.230750811	0	3.863117195
Ceacam2	18.83070292	60.53023055	35.73383406
Cebpa	117.1688182	189.5852504	157.4220257
Cebpd	36.61525568	42.2569534	25.11026177
Cebpg	84.73816314	247.8313213	171.9087152
Cebpz	60.67670941	54.81983144	68.57033022
Cebpzos	66.95361038	77.66142787	96.57792988
Cecr2	0	7.994558752	0
Cecr5	11.50765178	7.994558752	0
Cecr6	0	0	0
Cela1	0	0	0
Celf1	0	319.7823501	108.1672815
Celf2	410.0908636	564.1874319	519.5892628
Celf3	0	2.284159643	5.794675793
Celf4	6.276900973	7.994558752	4.828896494
Celsr1	0	2.284159643	3.863117195
Celsr2	3.138450487	0	0
Celsr3	0	0	0
Cenpa	1.046150162	0	0

For Review Only

Cenpb	31.38450487	41.11487358	47.32318564
Cenpc1	21.96915341	11.42079822	14.48668948
Cenpe	0	0	0
Cenpf	9.41535146	9.136638573	0
Cenpi	0	4.568319287	0
Cenpj	9.41535146	18.27327715	9.657792988
Cenpl	8.369201298	2.284159643	5.794675793
Cenpm	0	0	0.965779299
Cenpn	1.046150162	3.426239465	0
Cenpo	33.47680519	42.2569534	74.36500601
Cenpp	0	0	1.931558598
Cenpq	3.138450487	13.70495786	12.55513089
Cenpt	15.69225243	14.84703768	21.24714457
Cenpu	3.138450487	3.426239465	6.760455092
Cenpv	4.184600649	4.568319287	3.863117195
Cep104	86.83046347	66.24062966	42.49428915
Cep112	0	0	0
Cep112it	3.138450487	1.142079822	0
Cep120	105.6611664	85.65598662	82.0912404
Cep128	4.184600649	7.994558752	2.897337897
Cep131	29.29220454	19.41535697	1.931558598
Cep135	39.75370617	46.82527269	28.00759967
Cep152	111.9380674	100.5030243	120.7224124
Cep162	17.78455276	28.55199554	25.11026177
Cep164	40.79985633	42.2569534	49.25474424
Cep170	214.4607833	387.1650595	201.8478735
Cep170b	1.046150162	0	0
Cep19	9.41535146	5.710399108	10.62357229
Cep192	55.4459586	25.12575608	72.43344741
Cep250	96.24581493	93.65054538	117.8250745
Cep290	24.06145373	42.2569534	32.83649616
Cep295	11.50765178	37.68863411	53.11786144
Cep350	119.2611185	143.9020575	161.2851429
Cep41	12.55380195	14.84703768	18.34980668
Cep44	13.59995211	21.69951661	12.55513089
Cep57	48.12290746	86.79806645	49.25474424
Cep57l1	38.707556	11.42079822	28.97337897
Cep63	92.06121428	82.22974716	83.0570197
Cep68	174.7070771	190.7273302	129.414426
Cep70	14.64610227	29.69407536	18.34980668
Cep72	5.230750811	7.994558752	16.41824808
Cep76	12.55380195	1.142079822	0
Cep78	3.138450487	7.994558752	6.760455092
Cep83	5.230750811	7.994558752	0
Cep83os	17.78455276	3.426239465	14.48668948
Cep85	63.8151599	1.142079822	0
Cep85l	14.64610227	68.5247893	25.11026177
Cep89	24.06145373	23.98367625	23.17870317
Cep95	47.0767573	39.97279376	41.52850985
Cep97	25.10760389	6.85247893	13.52091018

For Review Only

Cept1	218.6453839	207.8585275	181.5665082
Cercam	6.276900973	9.136638573	6.760455092
Cerk	213.4146331	407.7224963	323.5360651
Cers1	4.184600649	1.142079822	5.794675793
Cers2	0	0	0
Cers4	7.323051136	21.69951661	0
Cers5	139.1379716	145.0441374	138.1064397
Cers6	61.72285957	114.2079822	43.46006845
Cetn2	53.35365827	37.68863411	39.59695125
Cetn3	76.36896184	87.94014627	97.54370918
Cfap126	14.64610227	0	0
Cfap20	5.230750811	25.12575608	0
Cfap36	27.19990422	19.41535697	27.04182037
Cfap43	1.046150162	4.568319287	0
Cfap46	0	3.426239465	0
Cfap52	3.138450487	0	0
Cfap69	0	0	0
Cfap74	61.72285957	97.07678484	176.7376117
Cfap97	48.12290746	35.40447447	38.63117195
Cfap99	0	1.142079822	0
Cfb	4.184600649	1.142079822	0
Cfdp1	62.76900973	53.67775162	96.57792988
Cfh	1319.195355	1344.22795	1166.661393
Cfhr2	14.64610227	21.69951661	64.70721302
Cfl1	757.4127175	738.9256446	0
Cfl2	69.04591071	54.81983144	54.08364074
Cflar	118.2149683	193.0114899	127.4828674
Cggbp1	121.3534188	93.65054538	101.4068264
Cgn	7.323051136	0	0
Cgnl1	0	0	0
Cgref1	1.046150162	0	10.62357229
Cgrrf1	141.2302719	81.08766734	177.703391
Ch25h	0	9.136638573	4.828896494
Chac2	6.276900973	14.84703768	13.52091018
Chad	5.230750811	7.994558752	9.657792988
Chadl	0	0	0
Chaf1a	0	3.426239465	0
Chaf1b	4.184600649	0	9.657792988
Champ1	137.0456713	147.328297	122.653971
Chchd1	0	0	0
Chchd10	34.52295535	0	0
Chchd2	255.2606396	179.306532	298.4258033
Chchd3	100.4304156	89.08222609	134.2433225
Chchd4	69.04591071	42.2569534	55.04942003
Chchd5	1.046150162	1.142079822	0
Chchd6	26.15375406	20.55743679	20.28136528
Chchd7	40.79985633	26.2678359	15.45246878
Chd1	67.99976055	54.81983144	25.11026177
Chd1l	31.38450487	23.98367625	34.76805476
Chd2	114.0303677	135.9074988	104.3041643

For Review Only

Chd3	38.707556	51.39359197	35.73383406
Chd3os	19.87685308	35.40447447	21.24714457
Chd4	87.87661363	154.1807759	78.22812321
Chd5	0	0	0
Chd6	86.83046347	161.0332549	132.3117639
Chd7	229.1068855	307.219472	240.4790454
Chd8	76.36896184	163.3174145	80.1596818
Chd9	365.1064066	461.400248	324.5018444
Chdh	0	4.568319287	6.760455092
Chek1	1.046150162	3.426239465	0
Chek2	47.0767573	42.2569534	25.11026177
Cherp	54.39980844	31.97823501	59.87831653
Chfr	86.83046347	79.94558752	88.85169549
Chic1	0	14.84703768	10.62357229
Chic2	6.276900973	61.67231037	12.55513089
Chid1	67.99976055	18.27327715	60.84409583
Chil1	53.35365827	11.42079822	8.69201369
Chil3	189.3531794	69.66686912	12.55513089
Chil5	6.276900973	2.284159643	0
Chka	149.5994732	134.765419	125.5513089
Chkb	0	0	0
Chm	73.23051136	46.82527269	31.87071686
Chml	11.50765178	25.12575608	14.48668948
Chmp1a	2.092300324	1.142079822	257.8630728
Chmp1b	112.9842175	102.7871839	157.4220257
Chmp2a	0	0	176.7376117
Chmp2b	111.9380674	69.66686912	101.4068264
Chmp3	236.4299367	322.0665097	317.7413893
Chmp4b	13.59995211	4.568319287	9.657792988
Chmp5	115.0765178	118.7763015	155.4904671
Chmp6	0	90.22430591	88.85169549
Chmp7	64.86131006	84.5139068	0
Chn1	0	0	0
Chn2	236.4299367	157.6070154	137.1406604
Chordc1	98.33811525	133.6233391	158.387805
Chp1	93.10736444	6.85247893	135.2091018
Chpf	1.046150162	0	0
Chpf2	188.3070292	173.5961329	206.67677
Chpt1	9.41535146	25.12575608	14.48668948
Chrac1	1.046150162	1.142079822	0
Chrm1	1.046150162	0	2.897337897
Chrm5	10.46150162	12.56287804	8.69201369
Chrna2	2.092300324	11.42079822	0
Chrnb1	0	0	0
Chrnb2	3.138450487	2.284159643	3.863117195
Chst1	53.35365827	53.67775162	36.69961336
Chst10	6.276900973	1.142079822	7.726234391
Chst11	59.63055925	82.22974716	75.33078531
Chst12	91.01506412	21.69951661	169.0113773
Chst13	0	0	0

For Review Only

Chst14	67.99976055	30.83615518	58.91253723
Chst15	73.23051136	59.38815073	57.94675793
Chst2	17.78455276	7.994558752	5.794675793
Chst3	2.092300324	2.284159643	0
Chst7	123.4457191	149.6124566	206.67677
Chst8	87.87661363	102.7871839	122.653971
Chst9	1.046150162	3.426239465	0.965779299
Chsy1	186.2147289	198.721889	286.8364518
Chtf18	0	0	0
Chtf8	237.4760868	0	0.965779299
Chtop	257.3529399	296.9407536	272.3497623
Chuk	75.32281168	98.21886466	65.67299232
Churc1	71.13821103	45.68319287	71.46766811
Ciao1	118.2149683	76.51934805	102.3726057
Ciapin1	73.23051136	21.69951661	0
Ciart	12.55380195	5.710399108	2.897337897
Cib1	62.76900973	39.97279376	28.97337897
Cib2	0	0	0
Cic	198.7685308	182.7327715	219.2319008
Ciita	3.138450487	2.284159643	0
Cilp2	4.184600649	12.56287804	0
Cinp	42.89215665	23.98367625	45.39162705
Cipc	84.73816314	41.11487358	42.49428915
Cir1	17.78455276	11.42079822	18.34980668
Cirbp	17.78455276	0	4.828896494
Cirh1a	57.53825892	49.10943233	34.76805476
Cisd1	58.58440909	41.11487358	55.04942003
Cisd2	98.33811525	111.9238225	155.4904671
Cisd3	10.46150162	11.42079822	15.45246878
Cisd3b	3.138450487	5.710399108	2.897337897
Cish	11.50765178	1.142079822	5.794675793
Cit	0	0	0
Cited2	1.046150162	1.142079822	0
Ciz1	222.8299846	3.426239465	0
CJ186046Rik	1.046150162	0	0
Ckap2	0	3.426239465	0
Ckap2l	13.59995211	9.136638573	5.794675793
Ckap4	148.553323	23.98367625	23.17870317
Ckap5	72.18436119	76.51934805	98.50948848
Ckb	1491.810131	1341.94379	1349.19368
Cklf	27.19990422	35.40447447	0
Cks1b	3.138450487	10.27871839	5.794675793
Cks2	10.46150162	6.85247893	3.863117195
Clasp1	97.29196509	95.93470502	75.33078531
Clasp2	550.2749853	542.4879153	649.9694681
Clasrp	57.53825892	9.136638573	109.1330608
Clcc1	138.0918214	147.328297	158.387805
Clcf1	0	73.09310859	74.36500601
Clcf1-pold4	1.046150162	0	0
Clcn2	0	0	0

For Review Only

Clcn3	97.29196509	99.36094448	89.81747479
Clcn4	481.2290746	380.3125806	418.1824364
Clcn5	60.67670941	81.08766734	68.57033022
Clcn6	139.1379716	75.37726823	78.22812321
Clcn7	378.7063587	244.4050818	297.460024
Cldn1	3.138450487	1.142079822	0
Cldn12	6.276900973	3.426239465	22.21292387
Cldn15	0	0	0
Cldn2	17.78455276	0	7.726234391
Cldn22	0	0	4.828896494
Cldn34c1	1.046150162	1.142079822	0
Cldn5	0	0	0
Cldnd1	114.0303677	116.4921418	119.7566331
Clec11a	0	2.284159643	0
Clec12a	18.83070292	2.284159643	11.58935159
Clec14a	0	0	0.965779299
Clec16a	146.4610227	191.86941	109.1330608
Clec1a	0	6.85247893	5.794675793
Clec1b	0	0	0
Clec2d	0	6.85247893	0
Clec2i	0	4.568319287	0
Clec4a1	23.01530357	5.710399108	14.48668948
Clec4a2	131.8149204	178.1644522	181.5665082
Clec4a3	237.4760868	435.1324121	408.5246434
Clec4a4	5.230750811	0	6.760455092
Clec5a	305.4758474	359.7551438	540.8364074
Clec7a	26.15375406	43.39903322	49.25474424
Clec9a	2.092300324	2.284159643	0
Clgn	29.29220454	27.40991572	27.04182037
Clic1	872.4892353	871.4069039	1081.672815
Clic4	5.230750811	2.284159643	17.38402738
Clic5	0	1.142079822	0
Clic6	16.7384026	2.284159643	0
Clint1	80.55356249	110.7817427	91.74903339
Clip1	243.7529878	170.1698934	209.5741078
Clip2	80.55356249	47.96735251	27.04182037
Clip3	9.41535146	0	0
Clk1	0	0	2.897337897
Clk2	1.046150162	63.95647001	0
Clk2-scamp3	210.2761826	0	70.50188882
Clk3	2.092300324	2.284159643	36.69961336
Clk4	12.55380195	17.13119732	116.8592952
Clmn	3.138450487	0	0
Clmp	5.230750811	2.284159643	4.828896494
Cln3	4.184600649	1.142079822	7.726234391
Cln5	244.799138	212.4268468	203.7794321
Cln6	37.66140584	3.426239465	24.14448247
Cln8	227.0145852	220.4214056	311.9467135
Clns1a	142.2764221	134.765419	240.4790454
Clock	119.2611185	114.2079822	49.25474424

For Review Only

Clp1	76.36896184	60.53023055	83.0570197
Clpb	47.0767573	78.80350769	70.50188882
Clpp	89.96891395	59.38815073	0
Clptm1	282.4605438	119.9183813	243.3763833
Clptm1l	95.19966476	179.306532	106.2357229
Clpx	109.845767	129.0550198	135.2091018
Clspn	0	1.142079822	0.965779299
Clstn1	273.0451923	316.3561106	391.140616
Clstn2	0	0	0.965779299
Clta	642.3361996	751.4885227	879.8249413
Cltb	70.09206087	62.81439019	83.0570197
Cltc	644.4284999	1220.883329	561.1177726
Clu	77.41511201	6.85247893	0
Cluap1	57.53825892	41.11487358	53.11786144
Cluh	88.92276379	116.4921418	74.36500601
Clybl	16.7384026	0	6.760455092
Cmas	17.78455276	26.2678359	23.17870317
Cmbl	2.092300324	0	0
Cmc1	16.7384026	17.13119732	24.14448247
Cmc2	16.7384026	10.27871839	7.726234391
Cmc4	7.323051136	5.710399108	13.52091018
Cmip	79.50741233	82.22974716	66.63877162
Cmklr1	122.399569	95.93470502	122.653971
Cmpk1	101.4765657	83.37182698	104.3041643
Cmpk2	2.092300324	2.284159643	7.726234391
Cmss1	4.184600649	3.426239465	2.897337897
Cmtm3	220.7376842	193.0114899	51.18630284
Cmtm4	102.5227159	57.10399108	76.29656461
Cmtm6	2690.698217	2123.126388	2936.934848
Cmtm7	435.1984675	465.9685672	399.8326297
Cmtm8	0	0	8.69201369
Cmtr1	39.75370617	84.5139068	24.14448247
Cmtr2	36.61525568	21.69951661	30.90493756
Cmya5	20.92300324	45.68319287	24.14448247
Cnbd2	27.19990422	19.41535697	18.34980668
Cnbp	1.046150162	320.9244299	1157.0036
Cndp2	384.9832597	282.093716	500.2736768
Cnep1r1	57.53825892	65.09854983	85.9543576
Cnga2	1.046150162	1.142079822	0
Cnga4	2.092300324	2.284159643	6.760455092
Cnih1	203.9992816	171.3119732	186.3954047
Cnih4	36.61525568	115.350062	164.1824808
Cnksr3	13.59995211	17.13119732	7.726234391
Cnn1	1.046150162	0	0
Cnn2	1.046150162	1.142079822	0
Cnn3	13.59995211	0	0
Cnnm2	27.19990422	28.55199554	20.28136528
Cnnm3	58.58440909	42.2569534	57.94675793
Cnnm4	132.8610706	146.1862172	163.2167015
Cnot1	129.7226201	211.284767	132.3117639

For Review Only

Cnot10	122.399569	0	117.8250745
Cnot11	30.33835471	0	0.965779299
Cnot2	306.5219975	325.4927492	297.460024
Cnot3	115.0765178	86.79806645	52.15208214
Cnot4	0	0	0.965779299
Cnot6	40.79985633	63.95647001	39.59695125
Cnot6l	78.46126217	132.4812593	98.50948848
Cnot7	61.72285957	1.142079822	0
Cnot8	360.921806	270.6729177	378.5854851
Cnot9	73.23051136	91.36638573	127.4828674
Cnp	243.7529878	226.1318047	217.3003422
Cnppd1	24.06145373	19.41535697	13.52091018
Cnpy2	73.23051136	165.6015741	118.7908538
Cnpy3	524.1212313	424.8536937	516.6919249
Cnpy4	27.19990422	20.55743679	47.32318564
Cnr2	3.138450487	3.426239465	149.6957913
Cnrip1	55.4459586	0	7.726234391
Cnst	71.13821103	6.85247893	26.07604107
Cntd1	4.184600649	0	0
Cntfr	0	0	0
Cntln	0	6.85247893	0
Cntn1	0	0	0
Cntnap1	0	0	3.863117195
Cntnap5b	1.046150162	1.142079822	0
Cntrl	96.24581493	139.3337382	120.7224124
Cntrob	48.12290746	36.54655429	18.34980668
Coa3	95.19966476	89.08222609	214.4030043
Coa4	48.12290746	69.66686912	54.08364074
Coa5	170.5224764	211.284767	144.8668948
Coa6	11.50765178	18.27327715	28.00759967
Coa7	26.15375406	6.85247893	17.38402738
Coasy	1.046150162	1.142079822	271.383983
Cobll1	0	0	0
Coch	0	0	0
Cog1	241.6606875	157.6070154	145.8326741
Cog2	51.26135795	65.09854983	92.71481269
Cog3	63.8151599	58.2460709	53.11786144
Cog4	108.7996169	49.10943233	121.6881917
Cog5	101.4765657	115.350062	59.87831653
Cog6	96.24581493	43.39903322	50.22052354
Cog7	222.8299846	53.67775162	78.22812321
Coil	20.92300324	17.13119732	33.80227546
Col10a1	3.138450487	13.70495786	5.794675793
Col11a2	0	3.426239465	0
Col12a1	0	0	0
Col14a1	0	0	0
Col15a1	49.16905763	39.97279376	37.66539266
Col17a1	1.046150162	0	0
Col18a1	0	0	0
Col1a2	0	0	0

For Review Only

Col20a1	14.64610227	5.710399108	0
Col23a1	1.046150162	2.284159643	5.794675793
Col25a1	2.092300324	4.568319287	0
Col27a1	219.6915341	374.6021815	339.9543132
Col4a1	0	0	18.34980668
Col4a3	3.138450487	0	0
Col4a3bp	49.16905763	52.5356718	32.83649616
Col4a4	9.41535146	3.426239465	4.828896494
Col6a2	0	2.284159643	0
Col6a3	125.5380195	54.81983144	92.71481269
Col7a1	6.276900973	1.142079822	1.931558598
Col8a1	18.83070292	0	0
Col8a2	4.184600649	0	0
Col9a3	31.38450487	0	12.55513089
Colec12	0	3.426239465	0
Colgalt1	0	0	22.21292387
Colgalt2	0	1.142079822	0
Commd1	65.90746022	19.41535697	68.57033022
Commd10	77.41511201	63.95647001	85.9543576
Commd2	54.39980844	84.5139068	87.8859162
Commd3	78.46126217	142.7599777	157.4220257
Commd4	171.5686266	1.142079822	14.48668948
Commd5	47.0767573	52.5356718	0
Commd6	52.30750811	52.5356718	73.39922671
Commd7	0	57.10399108	194.1216391
Commd8	0	105.0713436	22.21292387
Commd9	92.06121428	84.5139068	112.0303987
Comp	6.276900973	2.284159643	0
Comt	754.274267	678.3954141	986.0606641
Comtd1	40.79985633	25.12575608	0
Copa	488.5521258	312.9298711	353.4752234
Copb1	260.4913904	236.4105231	295.5284654
Copb2	174.7070771	139.3337382	164.1824808
Cope	0	0	44.42584775
Copg1	415.3216144	167.8857338	269.4524244
Copg2	85.7843133	95.93470502	39.59695125
Coprs	34.52295535	9.136638573	20.28136528
Cops2	92.06121428	87.94014627	96.57792988
Cops3	115.0765178	164.4594943	154.5246878
Cops4	220.7376842	365.4655429	332.2280788
Cops5	84.73816314	123.3446207	96.57792988
Cops6	150.6456234	143.9020575	179.6349496
Cops7a	61.72285957	19.41535697	1.931558598
Cops7b	57.53825892	35.40447447	47.32318564
Cops8	0	0	0
Copz1	189.3531794	182.7327715	155.4904671
Copz2	4.184600649	5.710399108	1.931558598
Coq10a	36.61525568	5.710399108	23.17870317
Coq10b	31.38450487	34.26239465	47.32318564
Coq2	30.33835471	43.39903322	51.18630284

For Review Only

Coq3	19.87685308	13.70495786	23.17870317
Coq4	42.89215665	25.12575608	42.49428915
Coq5	76.36896184	81.08766734	86.9201369
Coq6	0	0	21.24714457
Coq7	28.24605438	11.42079822	22.21292387
Coq9	112.9842175	59.38815073	112.996178
Corin	1.046150162	0	0
Coro1a	14.64610227	58.2460709	17.38402738
Coro1b	784.6126217	26.2678359	685.7033022
Coro1c	0	107.3555032	8.69201369
Coro2a	31.38450487	55.96191126	26.07604107
Coro6	1.046150162	4.568319287	0
Coro7	178.8916777	109.6396629	168.045598
Cotl1	401.7216623	335.7714676	199.9163149
COX1	3089.281429	2201.929896	2654.927293
Cox10	51.26135795	29.69407536	47.32318564
Cox11	23.01530357	15.9891175	0
Cox14	96.24581493	130.1970997	109.1330608
Cox15	57.53825892	31.97823501	17.38402738
Cox16	62.76900973	37.68863411	45.39162705
Cox17	0	52.5356718	70.50188882
Cox18	25.10760389	21.69951661	22.21292387
Cox19	2.092300324	11.42079822	74.36500601
COX2	713.4744106	436.2744919	687.6348608
Cox20	5.230750811	0	0.965779299
COX3	101.4765657	115.350062	218.2661215
Cox4i1	615.1362954	319.7823501	423.0113329
Cox4i2	3.138450487	0	0
Cox5a	10.46150162	1.142079822	0
Cox5b	54.39980844	37.68863411	68.57033022
Cox6a1	177.8455276	1.142079822	0
Cox6a2	0	0	0.965779299
Cox6b1	190.3993295	130.1970997	184.4638461
Cox6c	0	0	119.7566331
Cox7a2	110.8919172	91.36638573	168.045598
Cox7a2l	168.4301761	204.4322881	235.6501489
Cox7b	25.10760389	137.0495786	124.5855296
Cox7c	0	26.2678359	0
Cox8a	263.6298409	175.8802925	203.7794321
Cp	13.59995211	14.84703768	12.55513089
Cpd	41.84600649	62.81439019	29.93915826
Cpe	8.369201298	0	0
Cpeb2	27.19990422	73.09310859	45.39162705
Cpeb3	3.138450487	5.710399108	3.863117195
Cpeb4	52.30750811	58.2460709	25.11026177
Cplx2	5.230750811	0	0
Cpm	3.138450487	0	6.760455092
Cpn1	3.138450487	0	0
Cpn2	0	0	0
Cpne1	1.046150162	33.12031483	0

For Review Only

Cpne2	9.41535146	0	0
Cpne3	108.7996169	148.4703768	84.9885783
Cpne9	5.230750811	0	9.657792988
Cpox	11.50765178	23.98367625	14.48668948
Cpped1	39.75370617	49.10943233	44.42584775
Cpq	78.46126217	36.54655429	72.43344741
Cpsf1	1.046150162	2.284159643	0
Cpsf2	70.09206087	99.36094448	82.0912404
Cpsf3	192.4916299	203.2902083	310.9809342
Cpsf3l	0	0	7.726234391
Cpsf4	70.09206087	60.53023055	70.50188882
Cpsf4l	6.276900973	0	5.794675793
Cpsf6	121.3534188	163.3174145	124.5855296
Cpsf7	100.4304156	109.6396629	99.47526778
Cpt1a	83.69201298	47.96735251	41.52850985
Cpt1c	0	0	0
Cpt2	96.24581493	71.95102876	69.53610952
Cptp	20.92300324	33.12031483	0
Cr1l	79.50741233	293.5145142	97.54370918
Cr2	0	2.284159643	0
Cracr2a	15.69225243	12.56287804	23.17870317
Cracr2b	126.5841696	122.2025409	135.2091018
Cradd	46.03060714	54.81983144	48.28896494
Cramp1l	51.26135795	71.95102876	49.25474424
Crat	175.7532273	199.8639688	233.7185903
Crb1	1.046150162	2.284159643	0
Crb2	8.369201298	5.710399108	0
Crb3	35.56910552	47.96735251	12.55513089
Crbn	123.4457191	141.6178979	197.9847563
Crcp	66.95361038	93.65054538	80.1596818
Creb1	151.6917735	142.7599777	141.9695569
Creb3	0	0	81.1254611
Creb3l1	3.138450487	6.85247893	6.760455092
Creb3l2	173.6609269	232.9842836	197.9847563
Creb5	12.55380195	7.994558752	4.828896494
Crebbp	307.5681477	510.5096803	335.1254167
Crebl2	18.83070292	33.12031483	27.04182037
Crebrf	34.52295535	93.65054538	120.7224124
Crebzf	35.56910552	54.81983144	20.28136528
Creg1	768.9203692	654.4117378	959.9846231
Creld1	39.75370617	63.95647001	49.25474424
Creld2	201.9069813	118.7763015	178.6691703
Crem	27.19990422	14.84703768	23.17870317
Crim1	25.10760389	17.13119732	13.52091018
Crip1	0	0	0
Crip2	10.46150162	0	0
Cript	99.38426541	105.0713436	132.3117639
Crk	137.0456713	220.4214056	153.5589085
Crkl	208.1838823	202.1481284	245.3079419
Crlf2	55.4459586	29.69407536	33.80227546

For Review Only

Crlf3	398.5832118	411.1487358	372.7908094
Crls1	5.230750811	12.56287804	0
Crnde	0	0	2.897337897
Crnkl1	86.83046347	44.54111304	77.26234391
Crocc	5.230750811	0	0
Crot	89.96891395	115.350062	87.8859162
Crtap	89.96891395	41.11487358	89.81747479
Crtc1	80.55356249	76.51934805	73.39922671
Crtc2	27.19990422	171.3119732	28.97337897
Crtc3	15.69225243	17.13119732	21.24714457
Cry1	24.06145373	29.69407536	35.73383406
Cry2	26.15375406	27.40991572	33.80227546
Cryab	6.276900973	0	0
Cryba4	0	44.54111304	53.11786144
Crybb1	38.707556	185.0169311	190.2585219
Crybg3	34.52295535	103.9292638	78.22812321
Cryl1	331.6296014	253.5417204	340.9200925
Cryz	36.61525568	10.27871839	46.35740634
Cryzl1	44.98445698	71.95102876	62.77565443
Cs	244.799138	224.9897249	238.5474868
Csad	100.4304156	101.6451041	173.8402738
Csde1	283.506694	179.306532	264.6235279
Cse1l	134.9533709	209.0006074	183.4980668
Csf1	79.50741233	20.55743679	10.62357229
Csf1r	25358.67993	14125.24323	34423.27155
Csf2ra	3.138450487	1.142079822	0
Csf2rb	19.87685308	1.142079822	388.2432781
Csf2rb2	60.67670941	63.95647001	0
Csf3r	88.92276379	11.42079822	6.760455092
Csgalnact1	0	0	0
Csgalnact2	10.46150162	29.69407536	13.52091018
Csk	13.59995211	0	0
Csl	21.96915341	14.84703768	6.760455092
Csmd1	4.184600649	0	0
Csmd3	298.1527962	542.4879153	495.4447803
Csnk1a1	380.7986591	367.7497026	405.6273055
Csnk1d	280.3682435	198.721889	256.8972935
Csnk1e	104.6150162	29.69407536	153.5589085
Csnk1g1	159.0148247	173.5961329	182.5322875
Csnk1g2	37.66140584	7.994558752	0
Csnk1g3	38.707556	9.136638573	29.93915826
Csnk2a1	146.4610227	162.1753347	112.0303987
Csnk2a2	14.64610227	17.13119732	15.45246878
Csnk2b	111.9380674	98.21886466	122.653971
Cspg5	3.138450487	0	0
Cspp1	23.01530357	25.12575608	38.63117195
Csrnp1	34.52295535	39.97279376	34.76805476
Csrnp2	33.47680519	6.85247893	15.45246878
Csrnp3	0	0	0
Csrp1	3.138450487	0	0

For Review Only

Csrp2	0	0	0
Csrp2bp	25.10760389	65.09854983	58.91253723
Cst3	58382.5021	70887.75245	83094.68509
Cst6	0	1.142079822	0
Cst7	23.01530357	12.56287804	29.93915826
Cstb	0	82.22974716	63.74143372
Cstf1	106.7073165	67.38270948	151.6273499
Cstf2	23.01530357	27.40991572	52.15208214
Cstf2t	170.5224764	140.4758181	176.7376117
Cstf3	36.61525568	34.26239465	30.90493756
Ctage5	95.19966476	61.67231037	52.15208214
Ctbp1	6.276900973	19.41535697	0
Ctbp2	57.53825892	61.67231037	78.22812321
Ctbs	122.399569	101.6451041	126.5170881
Ctc1	0	0	47.32318564
Ctcf	37.66140584	65.09854983	56.98097863
Ctdnep1	1.046150162	1.142079822	33.80227546
Ctdp1	81.59971265	34.26239465	33.80227546
Ctdsp1	0	0	56.01519933
Ctdsp2	0	272.9570774	39.59695125
Ctdspl	2.092300324	0	8.69201369
Ctdspl2	91.01506412	81.08766734	93.68059199
Ctif	17.78455276	17.13119732	5.794675793
Ctla2a	0	0	0.965779299
Ctnna1	196.6762305	124.4867006	131.3459846
Ctnnal1	26.15375406	69.66686912	61.80987513
Ctnnb1	19.87685308	302.6511527	197.9847563
Ctnnbip1	33.47680519	18.27327715	26.07604107
Ctnnbl1	105.6611664	68.5247893	114.9277366
Ctnnd1	241.6606875	276.3833168	207.6425493
Ctnnd2	7.323051136	20.55743679	28.00759967
Ctns	235.3837865	126.7708602	215.3687836
Ctps	17.78455276	2.284159643	36.69961336
Ctps2	151.6917735	143.9020575	129.414426
Ctr9	53.35365827	27.40991572	100.4410471
Ctrl	0	0	0
Ctsa	4100.908636	0	470.3345185
Ctsb	10643.53175	6520.133702	8344.333142
Ctsc	1264.795546	1315.675955	1663.071953
Ctsd	45325.50193	29518.19507	36801.02018
Ctsf	0	0	0
Ctsg	0	0	0
Ctsh	219.6915341	1103.249108	2229.018622
Ctsk	0	0	0
Ctsl	1794.147528	1632.032065	2215.497712
Ctso	332.6757516	320.9244299	345.748989
Ctss	12299.58746	15097.15316	18086.14893
Ctsw	1.046150162	5.710399108	0
Ctsz	4843.675251	2639.346468	4435.82432
Cttn	4.184600649	0	0

For Review Only

Cttnbp2nl	0	91.36638573	60.84409583
Ctu1	49.16905763	69.66686912	81.1254611
Ctu2	12.55380195	3.426239465	21.24714457
Ctxn1	3.138450487	2.284159643	3.863117195
Cuedc2	2.092300324	79.94558752	0
Cul1	235.3837865	228.4159643	247.2395005
Cul2	99.38426541	77.66142787	108.1672815
Cul3	61.72285957	66.24062966	43.46006845
Cul4a	80.55356249	135.9074988	107.2015022
Cul4b	79.50741233	18.27327715	31.87071686
Cul5	93.10736444	46.82527269	69.53610952
Cul7	0	22.84159643	30.90493756
Cul9	151.6917735	94.7926252	95.61215059
Cuta	0	0	0
Cutc	15.69225243	9.136638573	12.55513089
Cux1	320.1219496	379.1705008	269.4524244
Cux2	42.89215665	19.41535697	27.04182037
Cwc15	72.18436119	111.9238225	87.8859162
Cwc22	25.10760389	15.9891175	25.11026177
Cwc25	38.707556	17.13119732	52.15208214
Cwc27	26.15375406	49.10943233	35.73383406
Cwf19l1	41.84600649	45.68319287	44.42584775
Cwf19l2	51.26135795	66.24062966	43.46006845
Cx3cl1	3.138450487	0	0
Cx3cr1	19350.63955	16419.6816	15443.77677
Cxadr	1.046150162	0	0
Cxcl10	0	5.710399108	0.965779299
Cxcl12	1.046150162	1.142079822	0
Cxcl13	0	0	7.726234391
Cxcl16	0	0	0.965779299
Cxcl17	1.046150162	1.142079822	0
Cxcr2	5.230750811	1.142079822	0
Cxcr3	1.046150162	1.142079822	5.794675793
Cxcr5	0	1.142079822	0
Cxcr6	9.41535146	20.55743679	0
Cxx1a	5.230750811	5.710399108	1.931558598
Cxx1b	4.184600649	7.994558752	1.931558598
Cxx1c	41.84600649	18.27327715	36.69961336
Cxxc1	1.046150162	1.142079822	0
Cxxc4	1.046150162	0	0
Cxxc5	228.0607354	238.6946827	16.41824808
Cyb561	26.15375406	0	0
Cyb561a3	516.7981801	6.85247893	151.6273499
Cyb561d1	109.845767	114.2079822	130.3802053
Cyb561d2	64.86131006	69.66686912	77.26234391
Cyb5a	247.9375885	235.2684433	240.4790454
Cyb5b	115.0765178	63.95647001	68.57033022
Cyb5d1	27.19990422	54.81983144	48.28896494
Cyb5d2	47.0767573	91.36638573	73.39922671
Cyb5r1	0	0	68.57033022

For Review Only

Cyb5r2	5.230750811	0	0
Cyb5r3	120.3072687	102.7871839	111.0646194
Cyb5r4	31.38450487	21.69951661	13.52091018
Cyb5rl	33.47680519	19.41535697	26.07604107
Cyba	850.5200819	649.8434185	924.250789
Cybb	86.83046347	39.97279376	43.46006845
Cyc1	43.93830681	0	8.69201369
Cycs	41.84600649	44.54111304	71.46766811
Cyfip1	1648.732656	1351.080429	1753.855207
Cyfip2	12.55380195	0	0
Cyhr1	123.4457191	156.4649356	141.9695569
Cyld	89.96891395	78.80350769	79.19390251
Cyp1b1	0	0	3.863117195
Cyp20a1	67.99976055	34.26239465	52.15208214
Cyp27a1	0	0	0
Cyp2d22	0	0	15.45246878
Cyp2e1	0	0	0
Cyp2j6	0	0	0
Cyp2r1	18.83070292	17.13119732	18.34980668
Cyp2t4	1.046150162	0	3.863117195
Cyp2u1	0	7.994558752	4.828896494
Cyp46a1	5.230750811	0	0.965779299
Cyp4f13	53.35365827	31.97823501	12.55513089
Cyp4f16	9.41535146	7.994558752	37.66539266
Cyp4f17	0	0	0
Cyp4f18	0	0	14.48668948
Cyp4f37	0	0	0
Cyp4v3	203.9992816	195.2956495	245.3079419
Cyp51	28.24605438	30.83615518	17.38402738
Cyr61	0	0	0
Cysltr1	196.6762305	22.84159643	232.752811
CYTB	2016.977513	1248.293245	1488.2659
Cyth1	110.8919172	107.3555032	114.9277366
Cyth2	32.43065503	0	0
Cyth3	171.5686266	0	0
Cyth4	365.1064066	190.7273302	571.7413449
Cytip	11.50765178	4.568319287	0
D030028A08Rik	138.0918214	86.79806645	85.9543576
D030047H15Rik	0	1.142079822	0
D030055H07Rik	2.092300324	0	0.965779299
D030056L22Rik	8.369201298	7.994558752	7.726234391
D10Jhu81e	12.55380195	34.26239465	22.21292387
D10Wsu102e	70.09206087	34.26239465	27.04182037
D11Wsu47e	78.46126217	73.09310859	76.29656461
D130007C19Rik	10.46150162	15.9891175	14.48668948
D130017N08Rik	4.184600649	3.426239465	6.760455092
D130020L05Rik	4.184600649	5.710399108	4.828896494
D130037M23Rik	1.046150162	1.142079822	0
D130040H23Rik	23.01530357	27.40991572	0
D16Ertd472e	6.276900973	10.27871839	7.726234391

For Review Only

D17H6S53E	1.046150162	26.2678359	0
D17H6S56E-5	242.7068376	0	0
D17Wsu92e	71.13821103	74.23518841	91.74903339
D1Ertd622e	143.3225722	125.6287804	121.6881917
D230017M19Rik	60.67670941	49.10943233	31.87071686
D230025D16Rik	79.50741233	93.65054538	88.85169549
D2hgdh	64.86131006	60.53023055	83.0570197
D2Wsu81e	55.4459586	54.81983144	46.35740634
D330023K18Rik	0	11.42079822	13.52091018
D330041H03Rik	3.138450487	0	0
D330045A20Rik	25.10760389	6.85247893	0
D330050I16Rik	5.230750811	0	0
D3Ertd254e	53.35365827	35.40447447	33.80227546
D3Ertd751e	30.33835471	23.98367625	22.21292387
D430020J02Rik	13.59995211	1.142079822	0
D430042O09Rik	29.29220454	63.95647001	35.73383406
D5Ertd579e	61.72285957	92.50846555	62.77565443
D5Ertd605e	24.06145373	39.97279376	17.38402738
D630033O11Rik	0	2.284159643	0
D630041G03Rik	6.276900973	1.142079822	5.794675793
D630045J12Rik	13.59995211	17.13119732	9.657792988
D6Wsu163e	155.8763742	143.9020575	136.1748811
D730005E14Rik	65.90746022	37.68863411	45.39162705
D7Ertd128e	7.323051136	0	5.794675793
D7Ertd443e	7.323051136	15.9891175	14.48668948
D830014E11Rik	3.138450487	0	0
D830030K20Rik	4.184600649	3.426239465	2.897337897
D830031N03Rik	39.75370617	54.81983144	38.63117195
D830044I16Rik	0	3.426239465	1.931558598
D830046C22Rik	3.138450487	15.9891175	8.69201369
D830050J10Rik	7.323051136	10.27871839	4.828896494
D8Ertd738e	118.2149683	5.710399108	139.072219
D8Ertd82e	201.9069813	202.1481284	201.8478735
D930015E06Rik	124.4918693	133.6233391	107.2015022
D930016D06Rik	4.184600649	14.84703768	27.04182037
D930028M14Rik	5.230750811	3.426239465	6.760455092
D930048N14Rik	8.369201298	0	0
Daam1	16.7384026	19.41535697	15.45246878
Daam2	5.230750811	4.568319287	7.726234391
Dab2	10.46150162	3.426239465	15.45246878
Dab2ip	1.046150162	6.85247893	0
Dact1	4.184600649	0	0.965779299
Dact3	3.138450487	0	3.863117195
Dad1	220.7376842	270.6729177	269.4524244
Dag1	49.16905763	85.65598662	88.85169549
Dagla	171.5686266	127.91294	112.996178
Daglb	5.230750811	1.142079822	0
Dalrd3	0	0	3.863117195
Dancr	8.369201298	20.55743679	23.17870317
Dand5	23.01530357	25.12575608	23.17870317

For Review Only

Dap	128.67647	86.79806645	102.3726057
Dap3	26.15375406	39.97279376	47.32318564
Dapk1	10.46150162	23.98367625	20.28136528
Dapk2	5.230750811	0	0
Dapp1	274.0913425	242.1209222	317.7413893
Dars	114.0303677	161.0332549	136.1748811
Dars2	30.33835471	17.13119732	46.35740634
Daxx	8.369201298	84.5139068	59.87831653
Dazap1	8.369201298	0	0
Dazap2	0	368.8917824	799.6652594
Dbf4	14.64610227	5.710399108	10.62357229
Dbi	95.19966476	37.68863411	48.28896494
Dbndd1	0	2.284159643	0
Dbndd2	14.64610227	25.12575608	12.55513089
Dbnl	0	0	0
Dbp	4.184600649	6.85247893	0
Dbr1	26.15375406	44.54111304	30.90493756
Dbt	48.12290746	15.9891175	53.11786144
Dcaf10	33.47680519	74.23518841	23.17870317
Dcaf11	140.1841217	1.142079822	0
Dcaf12	318.0296493	301.5090729	350.5778855
Dcaf12l1	8.369201298	1.142079822	0
Dcaf13	69.04591071	79.94558752	115.8935159
Dcaf15	12.55380195	0	14.48668948
Dcaf17	54.39980844	1.142079822	42.49428915
Dcaf4	72.18436119	51.39359197	47.32318564
Dcaf5	59.63055925	68.5247893	80.1596818
Dcaf6	13.59995211	26.2678359	7.726234391
Dcaf7	281.4143936	250.1154809	238.5474868
Dcaf8	233.2914862	158.7490952	226.9581352
Dcakd	96.24581493	59.38815073	73.39922671
Dcbld2	130.7687703	167.8857338	124.5855296
Dcdc2b	4.184600649	6.85247893	5.794675793
Dchs1	0	7.994558752	7.726234391
Dck	16.7384026	25.12575608	33.80227546
Dclk1	8.369201298	0	0
Dclk2	13.59995211	1.142079822	12.55513089
Dclre1a	37.66140584	19.41535697	11.58935159
Dclre1b	96.24581493	57.10399108	41.52850985
Dclre1c	63.8151599	86.79806645	62.77565443
Dcn	0	0	0
Dcp1a	60.67670941	86.79806645	84.9885783
Dcp1b	51.26135795	59.38815073	38.63117195
Dcp2	17.78455276	34.26239465	31.87071686
Dcps	34.52295535	31.97823501	58.91253723
Dcst1	0	3.426239465	0
Dctd	16.7384026	2.284159643	28.00759967
Dctn1	166.3378758	106.2134234	99.47526778
Dctn2	0	0	1.931558598
Dctn3	6.276900973	3.426239465	0.965779299

For Review Only

Dctn4	327.4450008	307.219472	334.1596374
Dctn5	445.6599691	520.7883987	513.794587
Dctn6	61.72285957	67.38270948	80.1596818
Dctpp1	47.0767573	42.2569534	59.87831653
Dcun1d1	46.03060714	73.09310859	71.46766811
Dcun1d2	114.0303677	110.7817427	56.98097863
Dcun1d3	4.184600649	20.55743679	13.52091018
Dcun1d4	27.19990422	57.10399108	43.46006845
Dcun1d5	8.369201298	46.82527269	26.07604107
Dda1	201.9069813	0	159.3535843
Ddah1	1.046150162	1.142079822	0
Ddah2	104.6150162	59.38815073	69.53610952
Ddb1	372.4294578	165.6015741	225.0265766
Ddb2	10.46150162	18.27327715	0
Ddhd1	7.323051136	12.56287804	0
Ddhd2	211.3223328	209.0006074	180.6007289
Ddi2	4.184600649	38.83071394	0
Ddias	0	1.142079822	0
Ddit4l	0	1.142079822	0
Ddost	0	1132.943183	317.7413893
Ddr1	8.369201298	0	0
Ddr2	0	0	0
Ddrgk1	207.1377321	194.1535697	253.0341763
Ddt	20.92300324	21.69951661	18.34980668
Ddx1	99.38426541	125.6287804	169.0113773
Ddx10	21.96915341	102.7871839	63.74143372
Ddx11	11.50765178	3.426239465	18.34980668
Ddx17	77.41511201	422.569534	0
Ddx18	47.0767573	42.2569534	53.11786144
Ddx19a	15.69225243	15.9891175	20.28136528
Ddx19b	20.92300324	6.85247893	24.14448247
Ddx20	48.12290746	53.67775162	60.84409583
Ddx21	92.06121428	79.94558752	83.0570197
Ddx23	30.33835471	38.83071394	18.34980668
Ddx24	260.4913904	229.5580442	199.9163149
Ddx26b	232.245336	383.7388201	420.113995
Ddx27	82.64586282	79.94558752	100.4410471
Ddx28	37.66140584	47.96735251	51.18630284
Ddx31	111.9380674	118.7763015	54.08364074
Ddx39	65.90746022	81.08766734	84.022799
Ddx39b	435.1984675	312.9298711	353.4752234
Ddx3x	224.9222849	0	18.34980668
Ddx3y	97.29196509	44.54111304	85.9543576
Ddx42	153.7840738	151.8966163	137.1406604
Ddx43	0	0	0
Ddx46	95.19966476	76.51934805	79.19390251
Ddx47	12.55380195	83.37182698	176.7376117
Ddx49	0	30.83615518	0
Ddx5	0	0	2493.64215
Ddx50	56.49210876	110.7817427	108.1672815

For Review Only

Ddx51	63.8151599	9.136638573	29.93915826
Ddx52	75.32281168	100.5030243	70.50188882
Ddx54	111.9380674	81.08766734	115.8935159
Ddx55	30.33835471	4.568319287	46.35740634
Ddx56	74.27666152	79.94558752	100.4410471
Ddx58	76.36896184	61.67231037	94.64637129
Ddx59	12.55380195	36.54655429	29.93915826
Ddx6	230.1530357	196.4377293	134.2433225
Ddx60	29.29220454	26.2678359	25.11026177
Deaf1	35.56910552	58.2460709	52.15208214
Deb1	0	0	13.52091018
Decr1	58.58440909	70.80894894	68.57033022
Decr2	20.92300324	10.27871839	39.59695125
Dedd	173.6609269	238.6946827	229.8554731
Dedd2	21.96915341	26.2678359	25.11026177
Def6	44.98445698	21.69951661	0
Def8	10.46150162	11.42079822	28.00759967
Degs1	240.6145373	188.4431706	252.068397
Degs2	26.15375406	38.83071394	0
Dek	94.1535146	94.7926252	111.0646194
Dennd1a	137.0456713	29.69407536	41.52850985
Dennd1b	10.46150162	61.67231037	45.39162705
Dennd2a	5.230750811	25.12575608	8.69201369
Dennd2c	120.3072687	110.7817427	87.8859162
Dennd2d	25.10760389	9.136638573	3.863117195
Dennd3	6.276900973	0	21.24714457
Dennd4a	435.1984675	396.3016981	426.8744501
Dennd4b	267.8144415	171.3119732	0
Dennd4c	40.79985633	39.97279376	21.24714457
Dennd5a	183.0762784	223.847645	227.9239145
Dennd5b	0	1.142079822	0.965779299
Dennd6a	67.99976055	111.9238225	61.80987513
Dennd6b	56.49210876	54.81983144	35.73383406
Denr	0	141.6178979	119.7566331
Depdc5	33.47680519	4.568319287	65.67299232
Depdc7	59.63055925	18.27327715	56.98097863
Deptor	43.93830681	22.84159643	33.80227546
Dera	15.69225243	39.97279376	17.38402738
Derl1	180.9839781	593.8815073	366.9961336
Derl2	190.3993295	197.5798091	180.6007289
Derl3	0	0	2.897337897
Desi1	63.8151599	31.97823501	32.83649616
Desi2	42.89215665	43.39903322	29.93915826
Det1	7.323051136	3.426239465	0
Dexi	7.323051136	45.68319287	24.14448247
Dffa	27.19990422	28.55199554	34.76805476
Dffb	37.66140584	17.13119732	28.00759967
Dfna5	10.46150162	38.83071394	13.52091018
Dgat1	73.23051136	60.53023055	50.22052354
Dgat2	7.323051136	9.136638573	7.726234391

For Review Only

Dgcr14	69.04591071	74.23518841	110.0988401
Dgcr2	330.5834513	343.7660263	211.5056664
Dgcr6	33.47680519	14.84703768	37.66539266
Dgcr8	71.13821103	109.6396629	86.9201369
Dgka	73.23051136	63.95647001	90.78325409
Dgkd	96.24581493	47.96735251	61.80987513
Dgke	23.01530357	11.42079822	14.48668948
Dgkg	0	3.426239465	5.794675793
Dgkh	39.75370617	17.13119732	24.14448247
Dgki	0	6.85247893	4.828896494
Dgkq	71.13821103	27.40991572	13.52091018
Dgkz	49.16905763	37.68863411	48.28896494
Dguok	63.8151599	7.994558752	1.931558598
Dhcr7	61.72285957	21.69951661	51.18630284
Dhdds	11.50765178	1.142079822	0.965779299
Dhdh	20.92300324	34.26239465	20.28136528
Dhfr	12.55380195	0	10.62357229
Dhh	0	0	0
Dhodh	24.06145373	26.2678359	37.66539266
Dhps	63.8151599	86.79806645	26.07604107
Dhrs1	56.49210876	59.38815073	51.18630284
Dhrs11	12.55380195	11.42079822	9.657792988
Dhrs13	0	0	0
Dhrs13os	0	0	0
Dhrs3	1168.549731	887.3960214	1227.505489
Dhrs4	0	0	0
Dhrs7	161.107125	127.91294	173.8402738
Dhrs7b	75.32281168	123.3446207	138.1064397
Dhrsx	23.01530357	12.56287804	9.657792988
Dhtkd1	6.276900973	6.85247893	20.28136528
Dhx15	185.1685787	179.306532	211.5056664
Dhx16	84.73816314	67.38270948	59.87831653
Dhx29	38.707556	38.83071394	30.90493756
Dhx30	150.6456234	150.7545365	114.9277366
Dhx32	82.64586282	69.66686912	76.29656461
Dhx33	132.8610706	89.08222609	68.57033022
Dhx34	165.2917256	132.4812593	119.7566331
Dhx35	27.19990422	26.2678359	10.62357229
Dhx36	39.75370617	67.38270948	61.80987513
Dhx37	26.15375406	22.84159643	23.17870317
Dhx38	58.58440909	83.37182698	57.94675793
Dhx40	52.30750811	61.67231037	58.91253723
Dhx57	127.6303198	110.7817427	74.36500601
Dhx58	41.84600649	39.97279376	56.98097863
Dhx8	72.18436119	61.67231037	69.53610952
Dhx9	114.0303677	28.55199554	53.11786144
Diablo	0	0	0
Diaph1	6.276900973	20.55743679	24.14448247
Diaph2	52.30750811	109.6396629	81.1254611
Dicer1	65.90746022	67.38270948	56.98097863

For Review Only

Dido1	178.8916777	188.4431706	134.2433225
Diexf	46.03060714	49.10943233	22.21292387
Dimt1	0	2.284159643	38.63117195
Dip2a	3.138450487	10.27871839	33.80227546
Dip2b	394.3986112	656.6958975	452.9504912
Dip2c	3.138450487	7.994558752	13.52091018
Dirc2	48.12290746	54.81983144	60.84409583
Dis3	8.369201298	28.55199554	30.90493756
Dis3l	99.38426541	29.69407536	29.93915826
Dis3l2	62.76900973	60.53023055	80.1596818
Disc1	115.0765178	137.0495786	97.54370918
Disp1	76.36896184	28.55199554	44.42584775
Disp2	0	0	0
Dixdc1	1.046150162	0	0
Dkc1	84.73816314	33.12031483	67.60455092
Dlat	71.13821103	23.98367625	58.91253723
Dlc1	16.7384026	61.67231037	26.07604107
Dld	100.4304156	124.4867006	109.1330608
Dlec1	7.323051136	5.710399108	12.55513089
Dleu2	37.66140584	41.11487358	28.00759967
Dlg1	32.43065503	36.54655429	19.31558598
Dlg2	3.138450487	0	2.897337897
Dlg3	0	2.284159643	12.55513089
Dlg4	12.55380195	6.85247893	6.760455092
Dlg5	8.369201298	0	0
Dlgap2	0	3.426239465	0
Dlgap4	115.0765178	188.4431706	161.2851429
Dll1	3.138450487	2.284159643	0
Dlst	346.2757037	296.9407536	314.8440514
Dmap1	55.4459586	54.81983144	86.9201369
Dmpk	3.138450487	0	0
Dmrtb1	0	0	3.863117195
Dmtf1	217.5992337	145.0441374	111.0646194
Dmtn	27.19990422	15.9891175	29.93915826
Dmwd	1.046150162	0	0
Dmxl1	48.12290746	90.22430591	38.63117195
Dmxl2	32.43065503	23.98367625	15.45246878
Dna2	0	12.56287804	0
Dnaaf1	1.046150162	1.142079822	0
Dnaaf2	19.87685308	31.97823501	33.80227546
Dnaaf3	3.138450487	0	7.726234391
Dnaaf5	54.39980844	10.27871839	8.69201369
Dnah1	0	4.568319287	0
Dnah17	10.46150162	28.55199554	13.52091018
Dnah2	8.369201298	0	4.828896494
Dnah9	0	0	0
Dnaic1	0	31.97823501	0
Dnaic2	0	4.568319287	0
Dnaja1	0	0	0
Dnaja2	259.4452402	226.1318047	297.460024

For Review Only

Dnaja3	125.5380195	157.6070154	140.0379983
Dnaja4	20.92300324	21.69951661	20.28136528
Dnajb1	0	0	0
Dnajb11	179.9378279	146.1862172	123.6197503
Dnajb12	57.53825892	61.67231037	46.35740634
Dnajb14	74.27666152	105.0713436	53.11786144
Dnajb2	43.93830681	28.55199554	34.76805476
Dnajb3	7.323051136	0	3.863117195
Dnajb4	44.98445698	50.25151215	38.63117195
Dnajb5	23.01530357	41.11487358	34.76805476
Dnajb6	101.4765657	114.2079822	76.29656461
Dnajb7	1.046150162	12.56287804	3.863117195
Dnajb9	186.2147289	183.8748513	240.4790454
Dnajc1	125.5380195	137.0495786	89.81747479
Dnajc10	122.399569	57.10399108	113.9619573
Dnajc11	121.3534188	118.7763015	160.3193636
Dnajc12	2.092300324	14.84703768	9.657792988
Dnajc13	116.122668	122.2025409	153.5589085
Dnajc14	217.5992337	155.3228557	183.4980668
Dnajc15	30.33835471	38.83071394	12.55513089
Dnajc16	88.92276379	117.6342216	62.77565443
Dnajc17	0	21.69951661	0
Dnajc18	146.4610227	132.4812593	163.2167015
Dnajc19	52.30750811	92.50846555	91.74903339
Dnajc19-ps	29.29220454	18.27327715	36.69961336
Dnajc2	54.39980844	45.68319287	46.35740634
Dnajc21	7.323051136	6.85247893	15.45246878
Dnajc24	39.75370617	45.68319287	57.94675793
Dnajc25	7.323051136	21.69951661	2.897337897
Dnajc27	24.06145373	14.84703768	16.41824808
Dnajc28	65.90746022	86.79806645	80.1596818
Dnajc3	316.9834992	213.5689267	261.72619
Dnajc30	67.99976055	57.10399108	84.022799
Dnajc4	1.046150162	2.284159643	0
Dnajc5	251.0760389	631.5701414	0
Dnajc7	11.50765178	25.12575608	165.1482601
Dnajc8	122.399569	127.91294	137.1406604
Dnajc9	1.046150162	1.142079822	171.9087152
Dnal1	2.092300324	2.284159643	9.657792988
Dnal4	76.36896184	73.09310859	62.77565443
Dnali1	4.184600649	0	3.863117195
Dnase1	0	0	2.897337897
Dnase1l1	12.55380195	5.710399108	0
Dnase1l3	1.046150162	5.710399108	3.863117195
Dnase2a	0	10.27871839	0
Dnd1	4.184600649	6.85247893	2.897337897
Dnhd1	0	0	0
Dnlz	21.96915341	15.9891175	0
Dnm1	2.092300324	2.284159643	0
Dnm1l	70.09206087	94.7926252	84.022799

For Review Only

Dnm2	698.8283084	406.5804165	634.5169993
Dnm3	0	1.142079822	0
Dnmbp	62.76900973	100.5030243	46.35740634
Dnmt1	31.38450487	38.83071394	40.56273055
Dnmt3a	372.4294578	356.3289044	299.3915826
Dnmt3aos	9.41535146	0	1.931558598
Dnmt3b	8.369201298	2.284159643	43.46006845
Dnpep	75.32281168	1.142079822	0
Dnph1	5.230750811	1.142079822	1.931558598
Dnttip1	0	0	0.965779299
Dnttip2	166.3378758	121.0604611	138.1064397
Doc2g	1.046150162	1.142079822	24.14448247
Dock1	120.3072687	129.0550198	69.53610952
Dock10	309.660448	661.2642167	569.8097863
Dock11	60.67670941	206.7164477	97.54370918
Dock2	611.9978449	354.0447447	524.4181593
Dock3	7.323051136	4.568319287	0
Dock4	304.4296972	314.071951	269.4524244
Dock5	3.138450487	4.568319287	0
Dock6	47.0767573	13.70495786	18.34980668
Dock7	89.96891395	105.0713436	64.70721302
Dock8	956.1812483	634.9963808	549.528421
Dock9	91.01506412	102.7871839	69.53610952
Dok1	0	0	0
Dok2	0	4.568319287	4.828896494
Dok3	0	0	0
Dok4	0	0	3.863117195
Dolk	131.8149204	106.2134234	120.7224124
Dolpp1	14.64610227	36.54655429	0
Donson	17.78455276	7.994558752	25.11026177
Dopey1	55.4459586	130.1970997	56.01519933
Dopey2	145.4148726	89.08222609	80.1596818
Dot1l	89.96891395	78.80350769	57.94675793
Dpagt1	76.36896184	69.66686912	109.1330608
Dpcd	75.32281168	20.55743679	40.56273055
Dpf2	102.5227159	95.93470502	118.7908538
Dph1	110.8919172	0	0
Dph2	8.369201298	10.27871839	9.657792988
Dph3	34.52295535	21.69951661	46.35740634
Dph6	53.35365827	37.68863411	55.04942003
Dph7	37.66140584	5.710399108	29.93915826
Dpm1	10.46150162	2.284159643	5.794675793
Dpm1-adnp	202.9531315	304.9353124	306.1520377
Dpm2	0	0	0
Dpm3	40.79985633	46.82527269	55.04942003
Dpp3	162.1532751	121.0604611	141.0037776
Dpp7	0	0	33.80227546
Dpp8	80.55356249	123.3446207	92.71481269
Dpp9	73.23051136	55.96191126	97.54370918
Dpy19l1	40.79985633	109.6396629	64.70721302

For Review Only

Dpy19l3	137.0456713	11.42079822	40.56273055
Dpy19l4	1.046150162	236.4105231	0
Dpy30	35.56910552	29.69407536	31.87071686
Dpyd	0	0	4.828896494
Dpysl2	865.1661842	1112.385746	870.1671483
Dpysl5	0	6.85247893	0
Dqx1	7.323051136	12.56287804	10.62357229
Dr1	100.4304156	68.5247893	71.46766811
Dram1	0	0	0
Dram2	254.2144894	298.0828335	261.72619
Drap1	0	0	0.965779299
Drd4	0	0	0
Drg1	174.7070771	153.0386961	197.9847563
Drg2	65.90746022	97.07678484	136.1748811
Drosha	47.0767573	43.39903322	37.66539266
Dscc1	0	0	0
Dscr3	135.9995211	156.4649356	165.1482601
Dse	41.84600649	44.54111304	56.98097863
Dsel	19.87685308	7.994558752	20.28136528
Dsn1	7.323051136	21.69951661	30.90493756
Dst	319.0757995	503.6572014	489.6501045
Dstn	256.3067897	247.8313213	275.2471002
Dstyk	119.2611185	140.4758181	83.0570197
Dtd1	50.21520779	17.13119732	39.59695125
Dtd2	6.276900973	28.55199554	51.18630284
Dtl	0	0	0
Dtna	0	0	0
Dtnb	12.55380195	4.568319287	8.69201369
Dtnbp1	16.7384026	53.67775162	21.24714457
Dtwd1	30.33835471	23.98367625	33.80227546
Dtwd2	11.50765178	7.994558752	12.55513089
Dtx2	52.30750811	60.53023055	76.29656461
Dtx3	96.24581493	39.97279376	47.32318564
Dtx3l	1.046150162	3.426239465	0
Dtx4	37.66140584	166.743654	139.072219
Dtymk	49.16905763	36.54655429	28.97337897
Dubr	38.707556	18.27327715	24.14448247
Duoxa1	19.87685308	1.142079822	0
Dus1l	62.76900973	81.08766734	25.11026177
Dus2	44.98445698	15.9891175	21.24714457
Dus3l	125.5380195	94.7926252	144.8668948
Dus4l	9.41535146	4.568319287	0
Dusp1	0	0	0
Dusp10	7.323051136	0	4.828896494
Dusp11	173.6609269	264.9625186	203.7794321
Dusp12	80.55356249	89.08222609	89.81747479
Dusp13	0	0	3.863117195
Dusp16	55.4459586	33.12031483	52.15208214
Dusp18	26.15375406	20.55743679	28.00759967
Dusp19	3.138450487	5.710399108	13.52091018

For Review Only

Dusp2	9.41535146	3.426239465	0
Dusp22	107.7534667	93.65054538	94.64637129
Dusp23	7.323051136	0	3.863117195
Dusp27	48.12290746	65.09854983	64.70721302
Dusp28	11.50765178	6.85247893	0
Dusp3	336.8603522	350.6185053	301.3231412
Dusp4	3.138450487	3.426239465	0.965779299
Dusp6	0	0	0
Dusp7	0	60.53023055	242.410604
Dusp8	12.55380195	1.142079822	0
Dut	6.276900973	0	0
Duxf3	6.276900973	7.994558752	0.965779299
Dvl1	64.86131006	57.10399108	81.1254611
Dvl2	53.35365827	15.9891175	7.726234391
Dvl3	34.52295535	11.42079822	0
Dxo	5.230750811	0	0
Dym	133.9072208	93.65054538	128.4486467
Dync1h1	321.1680998	419.1432945	377.6197058
Dync1i2	216.5530836	178.1644522	260.7604107
Dync1li1	143.3225722	116.4921418	103.338385
Dync1li2	55.4459586	110.7817427	74.36500601
Dync2h1	12.55380195	15.9891175	33.80227546
Dync2li1	0	0	4.828896494
Dynll1	80.55356249	79.94558752	116.8592952
Dynll2	13.59995211	10.27871839	7.726234391
Dynlrb1	203.9992816	141.6178979	165.1482601
Dynlt1a	2.092300324	27.40991572	40.56273055
Dynlt1b	0	0	0
Dynlt1c	56.49210876	37.68863411	49.25474424
Dynlt1f	60.67670941	41.11487358	57.94675793
Dynlt3	69.04591071	51.39359197	86.9201369
Dyrk1a	171.5686266	170.1698934	158.387805
Dyrk1b	148.553323	118.7763015	96.57792988
Dyrk2	110.8919172	146.1862172	140.0379983
Dyrk4	29.29220454	33.12031483	40.56273055
Dyx1c1	1.046150162	1.142079822	0
Dzank1	0	0	0
Dzip1	2.092300324	0	0
Dzip1l	5.230750811	0	0
Dzip3	40.79985633	30.83615518	28.00759967
E030018B13Rik	972.9196509	55.96191126	158.387805
E030024N20Rik	100.4304156	164.4594943	150.6615706
E030030I06Rik	0	0	0
E030042O20Rik	5.230750811	3.426239465	0
E130012A19Rik	3.138450487	1.142079822	3.863117195
E130102H24Rik	0	18.27327715	37.66539266
E130112N10Rik	9.41535146	12.56287804	9.657792988
E130201H02Rik	0	0	0.965779299
E130215H24Rik	3.138450487	7.994558752	4.828896494
E130307A14Rik	0	0	0

For Review Only

E130308A19Rik	43.93830681	33.12031483	28.97337897
E130309D02Rik	30.33835471	25.12575608	28.97337897
E130311K13Rik	28.24605438	18.27327715	24.14448247
E130317F20Rik	0	6.85247893	0.965779299
E230013L22Rik	13.59995211	19.41535697	8.69201369
E230016M11Rik	0	0	0
E2f1	0	0	0
E2f2	11.50765178	6.85247893	13.52091018
E2f3	85.7843133	114.2079822	96.57792988
E2f4	21.96915341	26.2678359	75.33078531
E2f5	12.55380195	22.84159643	11.58935159
E2f6	2.092300324	5.710399108	6.760455092
E2f8	0	0	0
E330009J07Rik	9.41535146	2.284159643	9.657792988
E330012B07Rik	0	0	0
E330020D12Rik	33.47680519	21.69951661	27.04182037
E330033B04Rik	99.38426541	78.80350769	72.43344741
E430024I08Rik	8.369201298	41.11487358	44.42584775
E430025E21Rik	147.5071729	93.65054538	84.022799
E4f1	0	0	0
E530011L22Rik	2.092300324	2.284159643	0.965779299
Eaf1	74.27666152	95.93470502	76.29656461
Eapp	26.15375406	1.142079822	42.49428915
Ear2	0	5.710399108	0
Ears2	70.09206087	47.96735251	28.00759967
Ebag9	18.83070292	11.42079822	32.83649616
Ebf1	0	0	0
Ebf3	44.98445698	66.24062966	40.56273055
Ebi3	124.4918693	87.94014627	95.61215059
Ebna1bp2	27.19990422	33.12031483	45.39162705
Ebp	110.8919172	143.9020575	171.9087152
Ebpl	33.47680519	59.38815073	62.77565443
Ecd	88.92276379	105.0713436	120.7224124
Ece1	40.79985633	13.70495786	38.63117195
Ece2	15.69225243	20.55743679	36.69961336
Ech1	0	0	0
Echdc1	31.38450487	0	10.62357229
Echdc3	14.64610227	0	0
Echs1	0	0	0
Eci1	0	90.22430591	12.55513089
Eci2	138.0918214	109.6396629	143.9011155
Ecm1	0	3.426239465	0
Ecm2	0	0	0
Ecscr	329.5373011	505.941361	807.3914938
Ecsit	50.21520779	42.2569534	54.08364074
Ect2	0	0	6.760455092
Ect2l	0	1.142079822	0.965779299
Edaradd	2.092300324	0	0
Edc3	130.7687703	91.36638573	115.8935159
Edc4	143.3225722	101.6451041	67.60455092

For Review Only

Edem1	449.8445698	710.3736491	468.4029599
Edem2	1579.686745	21.69951661	228.8896938
Edem3	46.03060714	83.37182698	37.66539266
Edf1	121.3534188	86.79806645	132.3117639
Edn1	6.276900973	0	10.62357229
Edn3	0	0	0
Ednrb	4.184600649	0	0
Edrf1	30.33835471	77.66142787	44.42584775
Eea1	31.38450487	51.39359197	35.73383406
Eed	127.6303198	19.41535697	69.53610952
Eef1akmt1	41.84600649	34.26239465	28.00759967
Eef1b2	12.55380195	0	3.863117195
Eef1d	26.15375406	0	1.931558598
Eef1e1	26.15375406	35.40447447	29.93915826
Eef1g	130.7687703	107.3555032	103.338385
Eef2	0	0	0
Eef2k	436.2446177	332.3452281	492.5474424
Eef2kmt	65.90746022	127.91294	83.0570197
Eefsec	125.5380195	74.23518841	114.9277366
Eepd1	59.63055925	50.25151215	30.90493756
Efcab1	7.323051136	0	0
Efcab11	1.046150162	1.142079822	0.965779299
Efcab12	0	0	0
Efcab14	105.6611664	154.1807759	136.1748811
Efcab2	33.47680519	2.284159643	0
Efcab6	0	0	0
Efemp1	0	0	0
Efemp2	36.61525568	0	0
Efhb	0	0	0
Efhc1	0	0	4.828896494
Efhd2	321.1680998	622.4335028	380.5170437
Efl1	64.86131006	57.10399108	56.98097863
Efna1	0	0	5.794675793
Efna4	8.369201298	0	1.931558598
Efna5	4.184600649	2.284159643	0
Efnb1	30.33835471	18.27327715	18.34980668
Efnb2	8.369201298	9.136638573	0
Efnb3	0	0	0
Efr3a	323.2604001	91.36638573	136.1748811
Efr3b	0	0	0.965779299
Efs	7.323051136	0	0
Eftud2	135.9995211	85.65598662	84.022799
Egf	0	0	0
Egfl7	2.092300324	2.284159643	0
Egfl8	0	6.85247893	0.965779299
Egln1	12.55380195	15.9891175	36.69961336
Egln2	130.7687703	105.0713436	49.25474424
Egln3	5.230750811	0	0
Egr1	1243.872543	0	631.6196614
Egr2	15.69225243	11.42079822	28.97337897

For Review Only

Egr3	10.46150162	3.426239465	0.965779299
Ehbp1	18.83070292	26.2678359	11.58935159
Ehbp1l1	174.7070771	194.1535697	170.9429359
Ehd1	11.50765178	6.85247893	0
Ehd2	0	0	0
Ehd4	526.2135316	262.678359	357.3383406
Ehhadh	5.230750811	19.41535697	0
Ehmt1	124.4918693	58.2460709	94.64637129
Ehmt2	84.73816314	84.5139068	144.8668948
Ei24	25.10760389	31.97823501	28.97337897
Eid1	12.55380195	20.55743679	17.38402738
Eid2	0	3.426239465	3.863117195
Eid2b	30.33835471	20.55743679	25.11026177
Eid3	5.230750811	3.426239465	1.931558598
Eif1a	91.01506412	106.2134234	84.022799
Eif1ad	78.46126217	79.94558752	0
Eif1ax	31.38450487	22.84159643	31.87071686
Eif1b	141.2302719	105.0713436	96.57792988
Eif2a	80.55356249	82.22974716	79.19390251
Eif2ak1	114.0303677	147.328297	115.8935159
Eif2ak2	44.98445698	69.66686912	43.46006845
Eif2ak3	46.03060714	102.7871839	84.022799
Eif2ak4	55.4459586	71.95102876	38.63117195
Eif2b1	48.12290746	10.27871839	63.74143372
Eif2b2	18.83070292	101.6451041	109.1330608
Eif2b3	28.24605438	38.83071394	40.56273055
Eif2b4	0	0	14.48668948
Eif2b5	40.79985633	67.38270948	33.80227546
Eif2d	57.53825892	70.80894894	59.87831653
Eif2s1	101.4765657	131.3391795	114.9277366
Eif2s2	42.89215665	122.2025409	92.71481269
Eif2s3x	71.13821103	47.96735251	64.70721302
Eif2s3y	114.0303677	69.66686912	119.7566331
Eif3a	187.260879	254.6838002	189.2927426
Eif3b	5.230750811	12.56287804	74.36500601
Eif3c	268.8605917	316.3561106	300.3573619
Eif3d	140.1841217	178.1644522	223.095018
Eif3e	86.83046347	117.6342216	138.1064397
Eif3f	163.1994253	137.0495786	136.1748811
Eif3h	157.9686745	135.9074988	169.9771566
Eif3i	130.7687703	21.69951661	124.5855296
Eif3j1	25.10760389	31.97823501	18.34980668
Eif3j2	33.47680519	26.2678359	18.34980668
Eif3k	91.01506412	87.94014627	188.3269633
Eif3l	213.4146331	284.3778756	259.7946314
Eif3m	142.2764221	127.91294	141.9695569
Eif4a2	0	0	0
Eif4a3	108.7996169	59.38815073	52.15208214
Eif4b	219.6915341	206.7164477	321.6045065
Eif4e	133.9072208	125.6287804	149.6957913

For Review Only

Eif4e2	99.38426541	78.80350769	71.46766811
Eif4e3	50.21520779	60.53023055	72.43344741
Eif4ebp1	37.66140584	47.96735251	90.78325409
Eif4ebp2	96.24581493	142.7599777	99.47526778
Eif4ebp3	17.78455276	25.12575608	0.965779299
Eif4enif1	75.32281168	111.9238225	82.0912404
Eif4g1	281.4143936	280.9516361	183.4980668
Eif4g2	485.4136753	2.284159643	470.3345185
Eif4g3	203.9992816	222.7055652	210.5398871
Eif4h	0	0	1.931558598
Eif5	0	0	73.39922671
Eif5a	352.5526047	317.4981904	416.2508778
Eif5a2	7.323051136	11.42079822	14.48668948
Eif5b	104.6150162	123.3446207	101.4068264
Eif6	96.24581493	123.3446207	142.9353362
Elac1	175.7532273	73.09310859	112.996178
Elac2	38.707556	38.83071394	24.14448247
Elane	0	0	0
Elavl1	225.968435	202.1481284	188.3269633
Elavl4	7.323051136	17.13119732	14.48668948
Eldr	0	0	0
Elf1	178.8916777	174.7382127	153.5589085
Elf2	54.39980844	138.1916584	70.50188882
Elf4	134.9533709	155.3228557	174.8060531
Elfn1	0	0	4.828896494
Elfn2	0	0	0
Elk1	15.69225243	10.27871839	12.55513089
Elk3	127.6303198	171.3119732	100.4410471
Elk4	82.64586282	125.6287804	52.15208214
Ell	89.96891395	67.38270948	73.39922671
Ell2	12.55380195	3.426239465	4.828896494
Ell3	2.092300324	0	0
Elmo1	1500.179333	1250.577405	1406.174659
Elmo2	179.9378279	214.7110065	171.9087152
Elmo3	25.10760389	17.13119732	12.55513089
Elmod2	94.1535146	74.23518841	61.80987513
Elmod3	55.4459586	28.55199554	47.32318564
Elmsan1	92.06121428	177.0223724	65.67299232
Elof1	56.49210876	0	71.46766811
Elovl1	2.092300324	1.142079822	436.5322431
Elovl2	0	0	0
Elovl5	85.7843133	60.53023055	103.338385
Elovl6	1.046150162	4.568319287	2.897337897
Elovl7	25.10760389	0	0
Elp2	236.4299367	133.6233391	209.5741078
Elp3	77.41511201	68.5247893	75.33078531
Elp4	18.83070292	34.26239465	12.55513089
Elp5	58.58440909	57.10399108	75.33078531
Elp6	5.230750811	1.142079822	18.34980668
Emb	9.41535146	3.426239465	7.726234391

For Review Only

Emc1	142.2764221	129.0550198	141.0037776
Emc10	1.046150162	118.7763015	72.43344741
Emc2	121.3534188	57.10399108	113.9619573
Emc3	198.7685308	325.4927492	389.2090574
Emc4	99.38426541	97.07678484	107.2015022
Emc6	0	0	82.0912404
Emc7	209.2300324	196.4377293	202.8136528
Emc8	39.75370617	67.38270948	49.25474424
Emc8-1190005i06rik	0	0	14.48668948
Emc9	11.50765178	10.27871839	28.97337897
Emcn	0	0	0
Emd	5.230750811	29.69407536	0
Eme1	0	0	0
Eme2	16.7384026	0	1.931558598
Emg1	25.10760389	0	0
Emilin1	5.230750811	0	0
Emilin2	0	1.142079822	1.931558598
Eml1	0	0	0
Eml2	2.092300324	2.284159643	0
Eml3	0	1.142079822	0
Eml4	119.2611185	123.3446207	101.4068264
Eml5	15.69225243	20.55743679	15.45246878
Eml6	65.90746022	41.11487358	33.80227546
Emp2	6.276900973	7.994558752	18.34980668
Emp3	0	1.142079822	0
Emsy	117.1688182	121.0604611	96.57792988
Emx2os	0	4.568319287	0
Enah	4.184600649	3.426239465	0
Enam	0	0	0
Enc1	72.18436119	140.4758181	74.36500601
Endod1	159.0148247	170.1698934	175.7718324
Endog	0	0	0
Endov	37.66140584	7.994558752	0
Eng	615.1362954	476.2472856	645.1405716
Engase	0	116.4921418	110.0988401
Enkd1	0	0	2.897337897
Eno1	108.7996169	0	0
Eno1b	230.1530357	149.6124566	203.7794321
Eno2	3.138450487	2.284159643	0
Eno3	0	30.83615518	0
Eno4	15.69225243	43.39903322	50.22052354
Enoph1	123.4457191	82.22974716	151.6273499
Enox2	31.38450487	25.12575608	25.11026177
Enpp1	25.10760389	13.70495786	33.80227546
Enpp2	1149.719028	31.97823501	0
Enpp4	38.707556	30.83615518	27.04182037
Enpp5	84.73816314	17.13119732	20.28136528
Ensa	37.66140584	89.08222609	81.1254611
Enthd2	99.38426541	52.5356718	85.9543576
Entpd1	3625.956462	2707.871257	3132.022266

For Review Only

Entpd3	2.092300324	2.284159643	0
Entpd4	44.98445698	90.22430591	0
Entpd5	23.01530357	35.40447447	16.41824808
Entpd6	74.27666152	67.38270948	56.98097863
Entpd7	10.46150162	29.69407536	8.69201369
Eny2	37.66140584	21.69951661	0
Eogt	102.5227159	30.83615518	68.57033022
Ep300	478.0906241	545.9141548	339.9543132
Ep400	345.2295535	262.678359	246.2737212
Epas1	1.046150162	0	0
Epb41	126.5841696	41.11487358	100.4410471
Epb41l1	4.184600649	3.426239465	0
Epb41l2	2236.669047	632.7122212	1694.942669
Epb41l3	102.5227159	77.66142787	55.04942003
Epb41l4a	0	2.284159643	0
Epb41l4aos	21.96915341	19.41535697	29.93915826
Epb41l5	5.230750811	3.426239465	11.58935159
Epc1	116.122668	23.98367625	0
Epc2	56.49210876	69.66686912	58.91253723
Epdr1	5.230750811	0	0
Epg5	48.12290746	61.67231037	45.39162705
Epha1	0	0	0
Epha2	60.67670941	50.25151215	86.9201369
Epha6	0	4.568319287	0
Ephb3	8.369201298	5.710399108	21.24714457
Ephb4	0	0	0
Ephb6	0	0	0
Ephx1	37.66140584	29.69407536	29.93915826
Epm2a	0	0	0
Epm2aip1	31.38450487	68.5247893	56.01519933
Epn1	59.63055925	7.994558752	35.73383406
Epn2	122.399569	107.3555032	77.26234391
Epn3	5.230750811	0	0
Epor	16.7384026	3.426239465	4.828896494
Eppk1	0	4.568319287	4.828896494
Eprs	250.0298888	159.891175	184.4638461
Eps15	149.5994732	139.3337382	121.6881917
Eps15l1	195.6300803	149.6124566	168.045598
Eps8	8.369201298	2.284159643	8.69201369
Eps8l1	28.24605438	3.426239465	35.73383406
Eps8l2	4.184600649	0	0
Epsti1	64.86131006	53.67775162	59.87831653
Ept1	53.35365827	42.2569534	31.87071686
Eral1	0	0	0
Erap1	192.4916299	229.5580442	185.4296254
Erbb2	8.369201298	1.142079822	0
Erbb2ip	53.35365827	65.09854983	56.98097863
Erc1	1.046150162	1.142079822	14.48668948
Ercc1	24.06145373	10.27871839	32.83649616
Ercc2	42.89215665	93.65054538	73.39922671

For Review Only

Ercc3	33.47680519	19.41535697	25.11026177
Ercc4	72.18436119	53.67775162	74.36500601
Ercc5	177.8455276	119.9183813	121.6881917
Ercc6	6.276900973	15.9891175	23.17870317
Ercc6l	0	0	0
Ercc6l2	19.87685308	20.55743679	13.52091018
Ercc8	15.69225243	37.68863411	33.80227546
Erdr1	87.87661363	339.197707	230.8212524
Ergic1	108.7996169	122.2025409	116.8592952
Ergic2	120.3072687	125.6287804	89.81747479
Ergic3	501.1059277	348.3343456	416.2508778
Erh	42.89215665	35.40447447	65.67299232
Eri1	48.12290746	45.68319287	60.84409583
Eri2	16.7384026	26.2678359	6.760455092
Eri3	121.3534188	122.2025409	105.2699436
Erich1	21.96915341	28.55199554	18.34980668
Erlec1	30.33835471	82.22974716	31.87071686
Erlin1	124.4918693	42.2569534	103.338385
Erlin2	155.8763742	1.142079822	250.1368384
Ermap	2.092300324	23.98367625	31.87071686
Ermard	53.35365827	89.08222609	58.91253723
Ermp1	57.53825892	42.2569534	53.11786144
Ern1	15.69225243	17.13119732	18.34980668
Ero1l	42.89215665	27.40991572	15.45246878
Ero1lb	19.87685308	58.2460709	31.87071686
Erp27	0	2.284159643	0.965779299
Erp29	396.4909115	869.1227443	277.1786588
Erp44	148.553323	142.7599777	183.4980668
Errfi1	123.4457191	74.23518841	124.5855296
Esam	0	0	0
Esco1	114.0303677	95.93470502	77.26234391
Esco2	0	0	5.794675793
Esd	75.32281168	52.5356718	70.50188882
Esf1	25.10760389	25.12575608	36.69961336
Espl1	92.06121428	73.09310859	80.1596818
Espn	0	0	2.897337897
Esr1	34.52295535	31.97823501	22.21292387
Esrp2	1.046150162	0	0
Esrra	1.046150162	1.142079822	0
Esrrg	4.184600649	0	0
Esyt1	76.36896184	74.23518841	63.74143372
Esyt2	67.99976055	81.08766734	60.84409583
Esyt3	0	0	0
Etaa1	38.707556	21.69951661	0
Etf1	58.58440909	76.51934805	83.0570197
Etfa	72.18436119	0	62.77565443
Etfb	102.5227159	67.38270948	88.85169549
Etfdh	141.2302719	103.9292638	117.8250745
Ethe1	53.35365827	0	1.931558598
Etl4	4.184600649	4.568319287	2.897337897

For Review Only

Etnk1	103.5688661	126.7708602	127.4828674
Etohd2	25.10760389	22.84159643	20.28136528
Ets1	432.060017	280.9516361	411.4219813
Ets2	50.21520779	37.68863411	37.66539266
Etv1	73.23051136	60.53023055	72.43344741
Etv3	107.7534667	181.5906916	105.2699436
Etv5	383.9371095	352.9026649	430.7375673
Etv6	104.6150162	81.08766734	85.9543576
Eva1a	50.21520779	27.40991572	66.63877162
Eva1b	3.138450487	26.2678359	0
Evi2a	499.0136274	766.3355603	927.1481269
Evi2b	0	0	0
Evi5	98.33811525	222.7055652	91.74903339
Evi5l	44.98445698	35.40447447	53.11786144
Evl	59.63055925	151.8966163	76.29656461
Ewsr1	62.76900973	123.3446207	98.50948848
Exd1	0	1.142079822	4.828896494
Exd2	91.01506412	68.5247893	93.68059199
Exo5	48.12290746	37.68863411	76.29656461
Exoc1	82.64586282	42.2569534	68.57033022
Exoc2	107.7534667	126.7708602	92.71481269
Exoc3	203.9992816	159.891175	240.4790454
Exoc3l	0	3.426239465	2.897337897
Exoc3l2	0	0	0
Exoc3l4	3.138450487	1.142079822	0
Exoc4	143.3225722	164.4594943	163.2167015
Exoc5	123.4457191	59.38815073	84.022799
Exoc6	145.4148726	247.8313213	175.7718324
Exoc6b	61.72285957	113.0659023	80.1596818
Exoc7	141.2302719	0	169.0113773
Exoc8	132.8610706	167.8857338	161.2851429
Exog	94.1535146	79.94558752	76.29656461
Exosc1	1.046150162	127.91294	0
Exosc10	230.1530357	151.8966163	254.9657349
Exosc2	35.56910552	27.40991572	35.73383406
Exosc3	20.92300324	31.97823501	34.76805476
Exosc4	124.4918693	98.21886466	148.730012
Exosc5	8.369201298	4.568319287	0
Exosc6	6.276900973	4.568319287	3.863117195
Exosc7	36.61525568	29.69407536	33.80227546
Exosc8	0	17.13119732	11.58935159
Exosc9	0	51.39359197	61.80987513
Exph5	2.092300324	0	0
Ext1	27.19990422	10.27871839	25.11026177
Ext2	261.5375406	161.0332549	216.3345629
Extl2	28.24605438	47.96735251	57.94675793
Extl3	635.0131485	449.9794497	447.1558154
Eya2	0	0	0
Eya3	80.55356249	98.21886466	75.33078531
Eya4	82.64586282	66.24062966	51.18630284

For Review Only

Ezh1	169.4763263	158.7490952	112.0303987
Ezh2	4.184600649	0	0
Ezr	62.76900973	1.142079822	16.41824808
F11r	1789.962928	1703.983094	2122.782899
F13a1	11.50765178	17.13119732	26.07604107
F2	0	0	0
F2rl3	0	0	0
F3	0	11.42079822	10.62357229
F420014N23Rik	3.138450487	4.568319287	0
F420015M19Rik	157.9686745	202.1481284	100.4410471
F5	34.52295535	1.142079822	0
F630028O10Rik	0	0	7.726234391
F630048H11Rik	11.50765178	18.27327715	22.21292387
F630111L10Rik	0	2.284159643	0
F630206G17Rik	0	0	0
F730016J06Rik	0	0	0
F730043M19Rik	0	2.284159643	0
F730311O21Rik	0	1.142079822	0
F8	0	4.568319287	0
F830016B08Rik	1.046150162	0	0
F830045P16Rik	0	0	0
F830208F22Rik	4.184600649	0	0
F8a	36.61525568	13.70495786	41.52850985
F9	5.230750811	13.70495786	13.52091018
Faap100	64.86131006	67.38270948	74.36500601
Faap20	16.7384026	15.9891175	0
Faap24	26.15375406	35.40447447	10.62357229
Fabp12	0	0	0
Fadd	43.93830681	58.2460709	42.49428915
Fads1	371.3833076	0	28.00759967
Fads3	88.92276379	12.56287804	46.35740634
Fads6	2.092300324	3.426239465	0
Faf1	30.33835471	46.82527269	14.48668948
Faf2	187.260879	138.1916584	159.3535843
Fahd1	8.369201298	7.994558752	23.17870317
Fahd2a	21.96915341	0	8.69201369
Faim	34.52295535	37.68863411	51.18630284
Fam101a	29.29220454	3.426239465	0
Fam101b	7.323051136	0	0
Fam102a	38.707556	61.67231037	27.04182037
Fam102b	443.5676688	617.8651835	480.9580908
Fam103a1	47.0767573	90.22430591	84.022799
Fam104a	78.46126217	66.24062966	78.22812321
Fam105a	902.82759	1225.451649	550.4942003
Fam107a	7.323051136	0	0
Fam107b	85.7843133	58.2460709	124.5855296
Fam109a	35.56910552	25.12575608	38.63117195
Fam109b	1.046150162	0	0
Fam110a	288.7374448	223.847645	328.3649616
Fam111a	1.046150162	28.55199554	0

For Review Only

Fam114a1	36.61525568	30.83615518	45.39162705
Fam114a2	255.2606396	212.4268468	219.2319008
Fam117a	33.47680519	17.13119732	13.52091018
Fam117b	65.90746022	92.50846555	86.9201369
Fam118a	41.84600649	36.54655429	48.28896494
Fam118b	0	0	43.46006845
Fam120a	121.3534188	143.9020575	134.2433225
Fam120aos	0	4.568319287	1.931558598
Fam120b	318.0296493	232.9842836	324.5018444
Fam120c	33.47680519	53.67775162	44.42584775
Fam122a	6.276900973	10.27871839	6.760455092
Fam122b	9.41535146	30.83615518	24.14448247
Fam124a	0	0	3.863117195
Fam126a	40.79985633	84.5139068	55.04942003
Fam126b	57.53825892	45.68319287	51.18630284
Fam129a	74.27666152	85.65598662	80.1596818
Fam129b	37.66140584	21.69951661	29.93915826
Fam129c	5.230750811	4.568319287	0
Fam131a	54.39980844	41.11487358	33.80227546
Fam131b	0	0	0
Fam132b	0	0	2.897337897
Fam133b	23.01530357	45.68319287	36.69961336
Fam134a	66.95361038	78.80350769	0
Fam134b	106.7073165	219.2793258	114.9277366
Fam134c	138.0918214	78.80350769	45.39162705
Fam135a	43.93830681	27.40991572	32.83649616
Fam135b	3.138450487	0	9.657792988
Fam136a	41.84600649	52.5356718	58.91253723
Fam13a	12.55380195	5.710399108	13.52091018
Fam13b	101.4765657	129.0550198	70.50188882
Fam149a	12.55380195	12.56287804	11.58935159
Fam149b	33.47680519	37.68863411	41.52850985
Fam160a1	3.138450487	0	2.897337897
Fam160a2	127.6303198	73.09310859	97.54370918
Fam160b1	44.98445698	84.5139068	25.11026177
Fam160b2	73.23051136	89.08222609	46.35740634
Fam161a	6.276900973	10.27871839	6.760455092
Fam161b	0	0	0
Fam162a	30.33835471	21.69951661	45.39162705
Fam167a	1.046150162	0	0
Fam167b	1.046150162	1.142079822	304.2204791
Fam168a	125.5380195	326.634829	196.0531977
Fam168b	175.7532273	216.9951661	259.7946314
Fam169a	2.092300324	0	0
Fam171a1	1.046150162	2.284159643	5.794675793
Fam172a	135.9995211	142.7599777	114.9277366
Fam173a	79.50741233	38.83071394	239.5132661
Fam173b	13.59995211	21.69951661	31.87071686
Fam174a	58.58440909	42.2569534	39.59695125
Fam175a	0	0	5.794675793

For Review Only

Fam175b	139.1379716	248.9734011	190.2585219
Fam177a	32.43065503	20.55743679	27.04182037
Fam178a	1.046150162	15.9891175	0
Fam178b	6.276900973	0	0
Fam179b	56.49210876	66.24062966	30.90493756
Fam180a	0	0	0
Fam181b	1.046150162	0	1.931558598
Fam185a	18.83070292	26.2678359	22.21292387
Fam188a	81.59971265	62.81439019	61.80987513
Fam188b	1.046150162	13.70495786	22.21292387
Fam189b	13.59995211	6.85247893	0
Fam192a	86.83046347	75.37726823	91.74903339
Fam193a	91.01506412	162.1753347	151.6273499
Fam193b	58.58440909	73.09310859	78.22812321
Fam195a	24.06145373	0	11.58935159
Fam195b	235.3837865	137.0495786	173.8402738
Fam196a	25.10760389	12.56287804	13.52091018
Fam196b	127.6303198	116.4921418	80.1596818
Fam198a	1.046150162	2.284159643	0
Fam198b	7.323051136	4.568319287	0
Fam199x	26.15375406	26.2678359	28.00759967
Fam19a1	5.230750811	6.85247893	7.726234391
Fam19a2	0	0	0
Fam19a3	0	0	1.931558598
Fam204a	27.19990422	20.55743679	28.00759967
Fam206a	56.49210876	10.27871839	44.42584775
Fam207a	77.41511201	58.2460709	46.35740634
Fam208a	118.2149683	156.4649356	101.4068264
Fam208b	144.3687224	151.8966163	78.22812321
Fam209	0	4.568319287	0
Fam20a	0	0	0
Fam20b	66.95361038	50.25151215	82.0912404
Fam20c	26.15375406	11.42079822	24.14448247
Fam21	193.53778	125.6287804	168.045598
Fam210a	96.24581493	103.9292638	84.022799
Fam210b	57.53825892	45.68319287	77.26234391
Fam212a	162.1532751	276.3833168	284.9048932
Fam212b	1.046150162	5.710399108	2.897337897
Fam213a	29.29220454	7.994558752	7.726234391
Fam213b	50.21520779	0	51.18630284
Fam214a	51.26135795	83.37182698	64.70721302
Fam214b	2.092300324	2.284159643	0
Fam216a	11.50765178	17.13119732	6.760455092
Fam217b	145.4148726	41.11487358	62.77565443
Fam219a	53.35365827	25.12575608	22.21292387
Fam219b	73.23051136	246.6892415	278.1444381
Fam220a	13.59995211	51.39359197	10.62357229
Fam222a	0	1.142079822	1.931558598
Fam222b	94.1535146	65.09854983	59.87831653
Fam227a	2.092300324	2.284159643	3.863117195

For Review Only

Fam228a	0	0	0.965779299
Fam228b	1.046150162	7.994558752	0
Fam234a	94.1535146	94.7926252	119.7566331
Fam234b	122.399569	139.3337382	94.64637129
Fam26f	19.87685308	13.70495786	48.28896494
Fam35a	78.46126217	38.83071394	20.28136528
Fam3a	0	0	26.07604107
Fam3c	73.23051136	86.79806645	56.98097863
Fam43a	9.41535146	3.426239465	0
Fam45a	134.9533709	142.7599777	152.5931292
Fam46a	84.73816314	42.2569534	37.66539266
Fam46c	277.229793	215.8530863	319.6729479
Fam49a	46.03060714	85.65598662	32.83649616
Fam49b	341.0449529	433.9903322	234.6843696
Fam50a	1.046150162	17.13119732	0
Fam53a	107.7534667	84.5139068	131.3459846
Fam53b	220.7376842	213.5689267	306.1520377
Fam53c	21.96915341	42.2569534	35.73383406
Fam57a	5.230750811	3.426239465	3.863117195
Fam58b	108.7996169	86.79806645	137.1406604
Fam60a	15.69225243	17.13119732	20.28136528
Fam63a	44.98445698	57.10399108	36.69961336
Fam63b	71.13821103	237.5526029	106.2357229
Fam64a	0	0	0
Fam65a	97.29196509	23.98367625	84.9885783
Fam65b	16.7384026	0	12.55513089
Fam65c	2.092300324	2.284159643	0
Fam69a	77.41511201	100.5030243	94.64637129
Fam69b	8.369201298	9.136638573	0
Fam69c	0	0	0
Fam71a	0	0	0
Fam71e1	0	2.284159643	0
Fam71f2	2.092300324	0	0
Fam72a	1.046150162	1.142079822	0
Fam73a	17.78455276	22.84159643	27.04182037
Fam73b	47.0767573	1.142079822	0
Fam76a	72.18436119	38.83071394	46.35740634
Fam76b	43.93830681	89.08222609	84.9885783
Fam78a	70.09206087	37.68863411	46.35740634
Fam81a	25.10760389	0	0
Fam83d	0	0	0.965779299
Fam83g	23.01530357	26.2678359	14.48668948
Fam83h	7.323051136	14.84703768	7.726234391
Fam84b	60.67670941	37.68863411	9.657792988
Fam89a	7.323051136	4.568319287	6.760455092
Fam89b	0	164.4594943	43.46006845
Fam8a1	14.64610227	33.12031483	26.07604107
Fam91a1	318.0296493	454.547769	341.8858718
Fam92a	1.046150162	1.142079822	14.48668948
Fam96a	72.18436119	69.66686912	58.91253723

For Review Only

Fam98a	37.66140584	28.55199554	39.59695125
Fam98b	26.15375406	3.426239465	7.726234391
Fam98c	0	2.284159643	15.45246878
Fan1	25.10760389	7.994558752	14.48668948
Fanca	0	0	0
Fancb	7.323051136	2.284159643	0
Fancc	26.15375406	36.54655429	29.93915826
Fancd2	1.046150162	2.284159643	0
Fance	80.55356249	46.82527269	36.69961336
Fancf	1.046150162	2.284159643	6.760455092
Fancg	48.12290746	21.69951661	54.08364074
Fanci	0	0	0
Fancl	3.138450487	4.568319287	31.87071686
Fancm	12.55380195	20.55743679	0
Far1	169.4763263	127.91294	121.6881917
Far1os	19.87685308	0	0
Far2	0	0	0
Farp1	12.55380195	15.9891175	7.726234391
Farp2	0	0	0
Fars2	62.76900973	65.09854983	52.15208214
Farsa	0	0	0
Farsb	75.32281168	98.21886466	104.3041643
Fas	6.276900973	0	0
Fasn	79.50741233	15.9891175	26.07604107
Fastkd1	11.50765178	15.9891175	19.31558598
Fastkd2	23.01530357	15.9891175	22.21292387
Fastkd3	18.83070292	25.12575608	13.52091018
Fastkd5	19.87685308	31.97823501	56.01519933
Fat1	11.50765178	5.710399108	11.58935159
Fat2	4.184600649	2.284159643	0
Fat3	87.87661363	149.6124566	117.8250745
Fat4	3.138450487	2.284159643	0
Fau	502.1520779	252.3996406	434.6006845
Fbf1	62.76900973	67.38270948	57.94675793
Fbl	0	0	0
Fblim1	63.8151599	27.40991572	34.76805476
Fbln1	7.323051136	1.142079822	0
Fbrs	88.92276379	0	103.338385
Fbrsl1	120.3072687	76.51934805	112.996178
Fbxl12	85.7843133	84.5139068	126.5170881
Fbxl12os	6.276900973	1.142079822	4.828896494
Fbxl14	35.56910552	52.5356718	19.31558598
Fbxl15	13.59995211	21.69951661	27.04182037
Fbxl16	3.138450487	1.142079822	0
Fbxl17	43.93830681	87.94014627	40.56273055
Fbxl18	20.92300324	11.42079822	28.00759967
Fbxl19	13.59995211	11.42079822	11.58935159
Fbxl2	2.092300324	3.426239465	6.760455092
Fbxl20	109.845767	109.6396629	94.64637129
Fbxl21	7.323051136	3.426239465	10.62357229

For Review Only

Fbxl22	8.369201298	5.710399108	8.69201369
Fbxl3	40.79985633	83.37182698	81.1254611
Fbxl4	155.8763742	98.21886466	89.81747479
Fbxl5	93.10736444	203.2902083	105.2699436
Fbxl6	0	0	0.965779299
Fbxl8	17.78455276	51.39359197	10.62357229
Fbxo10	34.52295535	22.84159643	9.657792988
Fbxo11	59.63055925	0	0
Fbxo17	11.50765178	1.142079822	0
Fbxo18	203.9992816	158.7490952	212.4714457
Fbxo21	81.59971265	18.27327715	27.04182037
Fbxo22	77.41511201	116.4921418	131.3459846
Fbxo24	2.092300324	1.142079822	2.897337897
Fbxo25	64.86131006	43.39903322	60.84409583
Fbxo28	35.56910552	49.10943233	20.28136528
Fbxo3	88.92276379	43.39903322	95.61215059
Fbxo30	34.52295535	34.26239465	35.73383406
Fbxo31	9.41535146	17.13119732	28.97337897
Fbxo32	23.01530357	50.25151215	19.31558598
Fbxo33	32.43065503	23.98367625	12.55513089
Fbxo34	81.59971265	77.66142787	89.81747479
Fbxo36	2.092300324	2.284159643	4.828896494
Fbxo38	183.0762784	182.7327715	171.9087152
Fbxo4	17.78455276	5.710399108	0
Fbxo40	21.96915341	41.11487358	25.11026177
Fbxo42	143.3225722	121.0604611	58.91253723
Fbxo44	2.092300324	3.426239465	0
Fbxo45	33.47680519	12.56287804	33.80227546
Fbxo46	61.72285957	46.82527269	71.46766811
Fbxo47	1.046150162	0	0
Fbxo48	0	7.994558752	2.897337897
Fbxo5	20.92300324	5.710399108	20.28136528
Fbxo6	43.93830681	50.25151215	47.32318564
Fbxo7	64.86131006	70.80894894	117.8250745
Fbxo8	72.18436119	41.11487358	58.91253723
Fbxo9	54.39980844	35.40447447	45.39162705
Fbxw10	14.64610227	6.85247893	14.48668948
Fbxw11	318.0296493	142.7599777	165.1482601
Fbxw17	14.64610227	18.27327715	10.62357229
Fbxw2	126.5841696	91.36638573	92.71481269
Fbxw4	103.5688661	177.0223724	197.018977
Fbxw7	35.56910552	76.51934805	40.56273055
Fbxw8	36.61525568	27.40991572	41.52850985
Fbxw9	10.46150162	1.142079822	0
Fcer1g	2117.407928	2409.788424	2981.360696
Fcer2a	4.184600649	0	0
Fcf1	36.61525568	0	98.50948848
Fcgr1	0	0	17.38402738
Fcgr2b	0	0	0.965779299
Fcgr3	2906.205151	2546.838002	3302.965202

For Review Only

Fcgr4	28.24605438	27.40991572	0
Fcgrt	147.5071729	172.4540531	258.8288521
Fcho2	129.7226201	212.4268468	76.29656461
Fchsd1	35.56910552	19.41535697	25.11026177
Fchsd2	284.5528441	471.6789663	314.8440514
Fcna	0	0	0
Fcnb	2.092300324	1.142079822	0
Fcrl1	173.6609269	95.93470502	181.5665082
Fcrlb	4.184600649	2.284159643	0
Fcrls	7259.235976	5245.572621	6921.740235
Fdft1	49.16905763	26.2678359	43.46006845
Fdps	42.89215665	29.69407536	30.90493756
Fdx1	30.33835471	30.83615518	14.48668948
Fdxacb1	32.43065503	30.83615518	16.41824808
Fdxr	19.87685308	21.69951661	0
Fech	145.4148726	122.2025409	125.5513089
Fem1a	135.9995211	197.5798091	211.5056664
Fem1b	81.59971265	101.6451041	43.46006845
Fem1c	47.0767573	103.9292638	38.63117195
Fen1	27.19990422	22.84159643	48.28896494
Fendrr	1.046150162	0	0
Fer	145.4148726	119.9183813	99.47526778
Fer1l5	0	17.13119732	40.56273055
Fermt2	0	0	0
Fermt3	0	0	0
Fes	0	0	0
Fez2	475.9983238	408.8645762	423.0113329
Fgd2	0	0	0
Fgd3	97.29196509	85.65598662	106.2357229
Fgd4	94.1535146	55.96191126	79.19390251
Fgd5	0	3.426239465	0
Fgd6	9.41535146	0	1.931558598
Fgf1	9.41535146	10.27871839	0.965779299
Fgf11	53.35365827	30.83615518	41.52850985
Fgf12	1.046150162	0	0
Fgf13	15.69225243	13.70495786	30.90493756
Fgf14	0	0	0
Fgf2os	1.046150162	0	0
Fgfbp3	6.276900973	0	0
Fgfr1	0	0	0
Fgfr1op	57.53825892	113.0659023	110.0988401
Fgfr1op2	216.5530836	158.7490952	194.1216391
Fgfr2	6.276900973	0	0
Fgfr3	2.092300324	0	0
Fgfrl1	4.184600649	9.136638573	14.48668948
Fggy	79.50741233	109.6396629	59.87831653
Fgl2	25.10760389	22.84159643	24.14448247
Fgr	4.184600649	22.84159643	0
Fh1	84.73816314	76.51934805	110.0988401
Fhad1	27.19990422	12.56287804	22.21292387

For Review Only

Fhit	11.50765178	20.55743679	15.45246878
Fhl3	15.69225243	0	13.52091018
Fhl4	1.046150162	7.994558752	0
Fhod1	44.98445698	68.5247893	21.24714457
Fibp	0	0	0
Ficd	74.27666152	53.67775162	76.29656461
Fig4	89.96891395	83.37182698	108.1672815
Fignl1	2.092300324	0	0.965779299
Fignl2	3.138450487	0	1.931558598
Filip1l	139.1379716	119.9183813	137.1406604
Fip1l1	125.5380195	141.6178979	115.8935159
Firre	193.53778	118.7763015	98.50948848
Fis1	122.399569	121.0604611	152.5931292
Fitm2	4.184600649	17.13119732	5.794675793
Fiz1	124.4918693	91.36638573	67.60455092
Fkbp10	0	0	0
Fkbp14	27.19990422	14.84703768	37.66539266
Fkbp15	338.9526526	207.8585275	342.8516511
Fkbp1a	41.84600649	240.9788424	29.93915826
Fkbp1b	6.276900973	0	6.760455092
Fkbp2	11.50765178	66.24062966	59.87831653
Fkbp3	35.56910552	29.69407536	75.33078531
Fkbp4	135.9995211	170.1698934	301.3231412
Fkbp5	200.8608312	63.95647001	37.66539266
Fkbp7	4.184600649	114.2079822	72.43344741
Fkbp8	203.9992816	103.9292638	191.2243012
Fkbp9	124.4918693	118.7763015	91.74903339
Fkbpl	0	3.426239465	0
Fkrp	75.32281168	74.23518841	47.32318564
Fktn	53.35365827	34.26239465	59.87831653
Flad1	27.19990422	0	0
Flcn	497.9674772	463.6844076	558.2204347
Fli1	436.2446177	483.0997646	326.433403
Flii	281.4143936	177.0223724	307.117817
Flna	155.8763742	165.6015741	0
Flnb	56.49210876	71.95102876	84.022799
Flnc	0	1.142079822	0
Flot1	0	0	0
Flot2	94.1535146	17.13119732	0
Flrt1	7.323051136	10.27871839	4.828896494
Flrt2	0	0	0
Flrt3	2.092300324	0	0
Flt1	0	0	0
Flt3	0	4.568319287	0
Flt3l	7.323051136	7.994558752	13.52091018
Flywch1	76.36896184	18.27327715	56.98097863
Fmn1	15.69225243	29.69407536	11.58935159
Fmnl1	175.7532273	107.3555032	136.1748811
Fmnl2	16.7384026	15.9891175	21.24714457
Fmnl3	334.7680519	194.1535697	239.5132661

For Review Only

Fmo1	0	0	0
Fmo2	0	0	0
Fmo5	57.53825892	77.66142787	51.18630284
Fmod	0	0	0
Fmr1	37.66140584	51.39359197	43.46006845
Fmr1nb	0	4.568319287	0.965779299
Fn1	0	10.27871839	0
Fn3k	5.230750811	9.136638573	6.760455092
Fn3krp	16.7384026	4.568319287	18.34980668
Fnbp1	70.09206087	69.66686912	102.3726057
Fnbp1l	14.64610227	27.40991572	0.965779299
Fnbp4	29.29220454	0	5.794675793
Fndc3a	88.92276379	162.1753347	144.8668948
Fndc3b	66.95361038	124.4867006	66.63877162
Fndc4	0	0	0
Fndc5	0	1.142079822	0
Fndc7	26.15375406	3.426239465	7.726234391
Fnip1	69.04591071	134.765419	67.60455092
Fnip2	85.7843133	65.09854983	56.01519933
Fnta	46.03060714	42.2569534	13.52091018
Fntb	15.69225243	4.568319287	13.52091018
Focad	47.0767573	62.81439019	39.59695125
Folr1	19.87685308	0	0.965779299
Folr2	2.092300324	4.568319287	0
Fopnl	9.41535146	77.66142787	0
Fos	0	0	0
Fosb	13.59995211	12.56287804	6.760455092
Fosl2	35.56910552	27.40991572	19.31558598
Foxd2os	0	0	0
Foxf2	0	0	0
Foxj2	78.46126217	97.07678484	130.3802053
Foxj3	140.1841217	68.5247893	69.53610952
Foxk1	96.24581493	69.66686912	0
Foxk2	78.46126217	134.765419	112.996178
Foxm1	0	1.142079822	9.657792988
Foxn2	72.18436119	43.39903322	16.41824808
Foxn3	342.0911031	451.1215296	334.1596374
Foxo1	44.98445698	52.5356718	49.25474424
Foxo3	195.6300803	223.847645	165.1482601
Foxo4	21.96915341	22.84159643	0
Foxp1	144.3687224	178.1644522	132.3117639
Foxp3	28.24605438	19.41535697	4.828896494
Foxp4	0	0	0
Foxr1	0	1.142079822	0
Foxred1	9.41535146	55.96191126	118.7908538
Foxred2	0	0	0
Fpgs	30.33835471	59.38815073	23.17870317
Fpgt	24.06145373	26.2678359	27.04182037
Fpr1	4.184600649	0	0
Fpr2	8.369201298	0	0

For Review Only

Fra10ac1	28.24605438	23.98367625	18.34980668
Fras1	6.276900973	0	2.897337897
Frat1	26.15375406	41.11487358	39.59695125
Frat2	1.046150162	1.142079822	2.897337897
Frem1	0	0	4.828896494
Frem2	0	0	0
Frg1	33.47680519	0	17.38402738
Frmd4a	907.0121907	977.6203273	784.2127907
Frmd4b	653.8438514	499.0888821	695.3610952
Frmd5	4.184600649	0	0
Frmd6	6.276900973	5.710399108	5.794675793
Frmd7	1.046150162	0	0
Frmd8	281.4143936	230.700124	272.3497623
Frmd8os	2.092300324	4.568319287	0
Frmpd4	4.184600649	0	0
Frrs1	316.9834992	414.5749753	214.4030043
Frs2	77.41511201	69.66686912	40.56273055
Frs3	6.276900973	0	0.965779299
Frs3os	0	1.142079822	0
Fry	128.67647	177.0223724	104.3041643
Fryl	118.2149683	116.4921418	134.2433225
Fscn1	948.8581972	801.7400348	556.2888761
Fsd1l	2.092300324	0	0
Fsip1	0	7.994558752	0
Fsip2	1.046150162	1.142079822	0
Fstl3	0	0	0
Fth1	1842.270436	1297.402677	1724.881828
Fto	156.9225243	74.23518841	91.74903339
Ftsj1	63.8151599	47.96735251	42.49428915
Ftsj3	61.72285957	68.5247893	83.0570197
Ftx	13.59995211	26.2678359	44.42584775
Fubp1	59.63055925	166.743654	165.1482601
Fubp3	48.12290746	79.94558752	61.80987513
Fuca1	392.3063108	286.6620352	538.9048488
Fuca2	318.0296493	276.3833168	306.1520377
Fuk	44.98445698	34.26239465	60.84409583
Fundc1	116.122668	138.1916584	115.8935159
Fundc2	51.26135795	6.85247893	0
Fuom	0	2.284159643	0
Furin	0	1.142079822	0
Fus	51.26135795	50.25151215	48.28896494
Fut10	30.33835471	65.09854983	39.59695125
Fut11	0	0	116.8592952
Fut4	15.69225243	9.136638573	6.760455092
Fut7	9.41535146	3.426239465	0
Fut8	127.6303198	94.7926252	73.39922671
Fuz	38.707556	14.84703768	13.52091018
Fv1	3.138450487	4.568319287	6.760455092
Fxn	6.276900973	0	0
Fxr1	85.7843133	78.80350769	85.9543576

For Review Only

Fxr2	72.18436119	41.11487358	47.32318564
Fxyd1	7.323051136	0	0
Fxyd2	0	0	0
Fxyd4	6.276900973	0	0
Fxyd5	3.138450487	4.568319287	9.657792988
Fxyd6	0	0	0
Fyb	106.7073165	127.91294	117.8250745
Fyco1	194.5839302	172.4540531	120.7224124
Fyn	7.323051136	7.994558752	16.41824808
Fyttd1	128.67647	114.2079822	51.18630284
Fzd3	0	5.710399108	2.897337897
Fzd4	26.15375406	1.142079822	8.69201369
Fzd5	1.046150162	4.568319287	0
Fzd7	8.369201298	57.10399108	19.31558598
Fzd8	5.230750811	3.426239465	4.828896494
Fzr1	0	0	0.965779299
G0s2	1.046150162	0	8.69201369
G2e3	20.92300324	5.710399108	30.90493756
G370120E05Rik	9.41535146	9.136638573	0
G3bp1	233.2914862	218.1372459	284.9048932
G3bp2	359.8756558	302.6511527	388.2432781
G430095P16Rik	8.369201298	9.136638573	1.931558598
G530011O06Rik	10.46150162	74.23518841	144.8668948
G630025P09Rik	2.092300324	2.284159643	0
G6b	7.323051136	0	4.828896494
G6pc3	23.01530357	13.70495786	0
G6pd2	12.55380195	10.27871839	6.760455092
G6pdx	33.47680519	0	60.84409583
Gaa	465.5368222	284.3778756	322.5702858
Gab1	152.7379237	155.3228557	96.57792988
Gab2	238.522237	250.1154809	253.0341763
Gab3	32.43065503	21.69951661	11.58935159
Gabarap	402.7678125	419.1432945	115.8935159
Gabarapl1	130.7687703	108.4975831	122.653971
Gabarapl2	104.6150162	143.9020575	163.2167015
Gabbr1	63.8151599	61.67231037	48.28896494
Gabpa	50.21520779	18.27327715	44.42584775
Gabpb1	61.72285957	74.23518841	98.50948848
Gabpb2	110.8919172	55.96191126	43.46006845
Gabrb1	3.138450487	0	5.794675793
Gabrd	2.092300324	0	0
Gabrg1	0	3.426239465	0
Gadd45a	0	0	0
Gadd45b	4.184600649	0	8.69201369
Gadd45g	0	0	0
Gadd45gip1	56.49210876	75.37726823	74.36500601
Gadl1	11.50765178	0	0
Gak	0	285.5199554	144.8668948
Gal3st1	0	0	0
Gal3st4	0	177.0223724	547.5968624

For Review Only

Galc	49.16905763	28.55199554	20.28136528
Gale	13.59995211	3.426239465	0
Galk1	25.10760389	0	16.41824808
Galk2	82.64586282	38.83071394	69.53610952
Galm	38.707556	26.2678359	36.69961336
Galns	64.86131006	13.70495786	20.28136528
Galnt1	102.5227159	114.2079822	104.3041643
Galnt10	105.6611664	87.94014627	116.8592952
Galnt11	139.1379716	90.22430591	131.3459846
Galnt12	60.67670941	75.37726823	0
Galnt13	0	1.142079822	5.794675793
Galnt14	0	0	2.897337897
Galnt16	7.323051136	0	0
Galnt2	81.59971265	131.3391795	81.1254611
Galnt3	10.46150162	17.13119732	18.34980668
Galnt4	124.4918693	108.4975831	134.2433225
Galnt6	13.59995211	6.85247893	12.55513089
Galnt7	47.0767573	61.67231037	68.57033022
Galntl6	4.184600649	0	0
Galr2	0	0	0
Galt	0	0	5.794675793
Gan	0	4.568319287	0
Ganab	0	269.5308379	145.8326741
Ganc	77.41511201	85.65598662	83.0570197
Gapdh	40.79985633	41.11487358	43.46006845
Gapdh-ps15	7.323051136	5.710399108	5.794675793
Gapdhs	2.092300324	5.710399108	0.965779299
Gapvd1	279.3220933	270.6729177	259.7946314
Gar1	0	11.42079822	0
Garem	4.184600649	0	0
Garnl3	83.69201298	79.94558752	70.50188882
Gars	175.7532273	103.9292638	164.1824808
Gart	128.67647	99.36094448	98.50948848
Gas1	2.092300324	0	0.965779299
Gas2	4.184600649	1.142079822	0
Gas2l1	18.83070292	15.9891175	6.760455092
Gas2l3	8.369201298	6.85247893	0
Gas5	375.5679082	304.9353124	322.5702858
Gas6	3.138450487	1.142079822	426.8744501
Gas7	16.7384026	1.142079822	7.726234391
Gas8	25.10760389	46.82527269	34.76805476
Gata2	0	0	0
Gatad1	46.03060714	110.7817427	90.78325409
Gatad2a	81.59971265	76.51934805	108.1672815
Gatad2b	69.04591071	98.21886466	87.8859162
Gatb	60.67670941	46.82527269	94.64637129
Gatc	0	0	1.931558598
Gatm	259.4452402	292.3724343	347.6805476
Gatsl2	28.24605438	26.2678359	16.41824808
Gatsl3	32.43065503	20.55743679	28.00759967

For Review Only

Gba	0	244.4050818	66.63877162
Gba2	7.323051136	21.69951661	26.07604107
Gbas	64.86131006	52.5356718	106.2357229
Gbe1	48.12290746	37.68863411	42.49428915
Gbf1	208.1838823	179.306532	182.5322875
Gbgt1	366.1525568	390.591299	491.5816631
Gbp10	0	0	0
Gbp2	1.046150162	47.96735251	29.93915826
Gbp3	39.75370617	70.80894894	47.32318564
Gbp4	0	0	0
Gbp5	0	19.41535697	0
Gbp6	0	0	2.897337897
Gbp7	141.2302719	162.1753347	160.3193636
Gbp8	1.046150162	3.426239465	0
Gbp9	33.47680519	61.67231037	61.80987513
Gca	1.046150162	5.710399108	0
Gcat	0	0	0
Gcc1	46.03060714	20.55743679	55.04942003
Gcc2	33.47680519	38.83071394	38.63117195
Gcdh	48.12290746	82.22974716	57.94675793
Gch1	4.184600649	7.994558752	3.863117195
Gck	0	0	1.931558598
Gclc	0	55.96191126	79.19390251
Gclm	6.276900973	0	0
Gcn1l1	399.629362	356.3289044	379.5512644
Gcnt1	553.4134358	411.1487358	430.7375673
Gcnt2	81.59971265	74.23518841	73.39922671
Gcnt7	10.46150162	7.994558752	8.69201369
Gcsh	26.15375406	37.68863411	28.97337897
Gda	6.276900973	0	0
Gdap10	34.52295535	50.25151215	14.48668948
Gdap1l1	0	1.142079822	0
Gdap2	100.4304156	107.3555032	75.33078531
Gde1	214.4607833	165.6015741	239.5132661
Gdf11	0	0	0
Gdf15	3.138450487	0	0
Gdf9	7.323051136	49.10943233	34.76805476
Gdi1	0	0	0
Gdi2	15.69225243	0	402.7299676
Gdpd1	17.78455276	13.70495786	25.11026177
Gdpd3	13.59995211	18.27327715	0
Gdpd5	13.59995211	1.142079822	15.45246878
Gdpgp1	51.26135795	34.26239465	31.87071686
Gem	8.369201298	1.142079822	16.41824808
Gemin2	11.50765178	9.136638573	14.48668948
Gemin4	61.72285957	31.97823501	51.18630284
Gemin5	77.41511201	39.97279376	45.39162705
Gemin6	26.15375406	30.83615518	40.56273055
Gemin7	0	83.37182698	44.42584775
Gemin8	13.59995211	12.56287804	7.726234391

For Review Only

Gen1	0	0	0
Get4	0	0	46.35740634
Gfer	12.55380195	1.142079822	0
Gfi1	0	0	0
Gfm1	66.95361038	14.84703768	83.0570197
Gfm2	520.9827808	899.9588995	382.4486023
Gfod2	23.01530357	10.27871839	13.52091018
Gfpt1	29.29220454	38.83071394	20.28136528
Gfra4	0	0	0
Gga1	107.7534667	66.24062966	50.22052354
Gga2	24.06145373	25.12575608	28.97337897
Gga3	2.092300324	432.8482524	0
Ggact	8.369201298	14.84703768	12.55513089
Ggct	2.092300324	5.710399108	5.794675793
Ggcx	36.61525568	41.11487358	92.71481269
Ggh	0	0	0
Ggn	0	0	0
Ggnbp1	0	3.426239465	19.31558598
Ggnbp2	111.9380674	97.07678484	62.77565443
Ggnbp2os	39.75370617	42.2569534	53.11786144
Ggps1	64.86131006	85.65598662	141.0037776
Ggt5	200.8608312	158.7490952	180.6007289
Ggta1	300.2450966	238.6946827	247.2395005
Ghdc	50.21520779	52.5356718	86.9201369
Ghitm	390.2140105	488.8101637	464.5398427
Ghrl	0	0	0
Gid4	60.67670941	76.51934805	49.25474424
Gid8	420.5523652	288.9461949	415.2850985
Gigyf1	126.5841696	67.38270948	0
Gigyf2	39.75370617	39.97279376	44.42584775
Gimap1	1.046150162	20.55743679	0
Gimap1os	3.138450487	12.56287804	10.62357229
Gimap5	8.369201298	12.56287804	6.760455092
Gimap6	74.27666152	92.50846555	121.6881917
Gimap9	30.33835471	43.39903322	40.56273055
Gin1	8.369201298	3.426239465	18.34980668
Ginm1	103.5688661	81.08766734	124.5855296
Gins1	8.369201298	11.42079822	4.828896494
Gins2	3.138450487	2.284159643	9.657792988
Gins3	10.46150162	12.56287804	15.45246878
Gins4	0	0	11.58935159
Gipc1	64.86131006	57.10399108	0
Git1	79.50741233	51.39359197	67.60455092
Git2	830.6432288	717.226128	246.2737212
Gja1	2.092300324	0	0
Gja8	1.046150162	0	0
Gjb2	0	0	0
Gjb6	2.092300324	0	0
Gk	16.7384026	1.142079822	0
Gk5	1.046150162	0	12.55513089

For Review Only

Gkap1	3.138450487	12.56287804	0
Gla	6.276900973	0	0
Glb1	201.9069813	172.4540531	174.8060531
Glb1l	46.03060714	50.25151215	28.97337897
Glb1l2	5.230750811	0	2.897337897
Glcci1	21.96915341	30.83615518	16.41824808
Glce	58.58440909	25.12575608	22.21292387
Gle1	35.56910552	42.2569534	84.022799
Glg1	65.90746022	107.3555032	109.1330608
Gli1	0	0	0.965779299
Glipr1	17.78455276	26.2678359	47.32318564
Glipr2	6.276900973	3.426239465	0
Glis2	5.230750811	3.426239465	0
Glis3	28.24605438	54.81983144	29.93915826
Glmn	16.7384026	13.70495786	0
Glmp	277.229793	0	0.965779299
Glo1	86.83046347	55.96191126	84.022799
Glod4	177.8455276	163.3174145	186.3954047
Glra4	2.092300324	5.710399108	6.760455092
Glrp1	19.87685308	38.83071394	41.52850985
Glrx	51.26135795	44.54111304	45.39162705
Glrx2	48.12290746	42.2569534	22.21292387
Glrx3	96.24581493	86.79806645	115.8935159
Glrx5	15.69225243	6.85247893	9.657792988
Gls	54.39980844	62.81439019	82.0912404
Gls2	0	0	0
Glt8d1	7.323051136	14.84703768	0
Glt8d2	0	0	0
Gltp	198.7685308	162.1753347	190.2585219
Gltscr1	135.9995211	140.4758181	134.2433225
Gltscr1l	168.4301761	116.4921418	131.3459846
Gltscr2	0	0	0
Glud1	221.7838344	209.0006074	159.3535843
Glyctk	0	3.426239465	13.52091018
Glyr1	367.1987069	230.700124	321.6045065
Gm10012	17.78455276	10.27871839	12.55513089
Gm10033	59.63055925	55.96191126	59.87831653
Gm10037	0	0	0
Gm10051	70.09206087	63.95647001	68.57033022
Gm10052	180.9839781	63.95647001	89.81747479
Gm10069	7.323051136	11.42079822	8.69201369
Gm10080	37.66140584	55.96191126	71.46766811
Gm10130	11.50765178	6.85247893	11.58935159
Gm10145	8.369201298	14.84703768	18.34980668
Gm10190	1.046150162	1.142079822	0
Gm10248	4.184600649	7.994558752	0.965779299
Gm10258	0	0	0
Gm10266	0	1.142079822	0
Gm10277	2.092300324	12.56287804	6.760455092
Gm10325	7.323051136	4.568319287	3.863117195

For Review Only

Gm10336	3.138450487	12.56287804	12.55513089
Gm10362	23.01530357	30.83615518	31.87071686
Gm10369	5.230750811	22.84159643	7.726234391
Gm10384	38.707556	46.82527269	46.35740634
Gm10390	2.092300324	1.142079822	0
Gm10406	19.87685308	17.13119732	18.34980668
Gm10408	1.046150162	1.142079822	0.965779299
Gm10409	7.323051136	6.85247893	11.58935159
Gm10410	20.92300324	12.56287804	13.52091018
Gm10416	0	2.284159643	0.965779299
Gm10419	1.046150162	2.284159643	0
Gm10432	2.092300324	1.142079822	0
Gm10433	0	0	0
Gm10451	0	1.142079822	0
Gm10499	1.046150162	0	0
Gm10509	10.46150162	14.84703768	17.38402738
Gm10532	6.276900973	0	0
Gm10538	0	0	0
Gm10548	4.184600649	0	0
Gm10575	0	0	0
Gm10578	23.01530357	30.83615518	21.24714457
Gm10584	0	0	1.931558598
Gm10603	18.83070292	3.426239465	11.58935159
Gm10614	2.092300324	0	0
Gm10638	0	2.284159643	0
Gm10651	4.184600649	1.142079822	1.931558598
Gm10654	1.046150162	1.142079822	0
Gm10677	17.78455276	15.9891175	14.48668948
Gm10693	8.369201298	1.142079822	4.828896494
Gm10698	237.4760868	193.0114899	216.3345629
Gm1070	3.138450487	1.142079822	0
Gm10705	15.69225243	17.13119732	17.38402738
Gm10767	5.230750811	2.284159643	7.726234391
Gm10768	0	1.142079822	0
Gm10778	14.64610227	12.56287804	12.55513089
Gm10785	10.46150162	4.568319287	5.794675793
Gm10790	0	0	28.97337897
Gm10791	1.046150162	6.85247893	7.726234391
Gm10804	5.230750811	1.142079822	0.965779299
Gm10825	2.092300324	1.142079822	4.828896494
Gm10847	7.323051136	2.284159643	3.863117195
Gm10851	4.184600649	3.426239465	2.897337897
Gm10910	3.138450487	0	0.965779299
Gm11007	11.50765178	15.9891175	9.657792988
Gm11110	0	0	0
Gm11127	2.092300324	0	2.897337897
Gm11201	0	1.142079822	0
Gm11335	1.046150162	0	0
Gm11423	0	0	0.965779299
Gm11457	3.138450487	6.85247893	0

For Review Only

Gm11476	0	0	0
Gm11478	150.6456234	150.7545365	178.6691703
Gm11513	0	0	0.965779299
Gm11517	1.046150162	2.284159643	2.897337897
Gm11532	5.230750811	4.568319287	0
Gm11545	26.15375406	17.13119732	31.87071686
Gm11578	7.323051136	1.142079822	0.965779299
Gm11634	3.138450487	0	2.897337897
Gm11677	0	0	0
Gm11696	12.55380195	13.70495786	14.48668948
Gm11713	6.276900973	18.27327715	11.58935159
Gm11716	3.138450487	6.85247893	3.863117195
Gm11725	10.46150162	4.568319287	6.760455092
Gm11753	152.7379237	145.0441374	145.8326741
Gm11769	8.369201298	21.69951661	14.48668948
Gm11787	5.230750811	12.56287804	0
Gm11802	4.184600649	2.284159643	0
Gm11821	4.184600649	1.142079822	0
Gm11827	4.184600649	4.568319287	1.931558598
Gm11946	0	0	0
Gm11963	0	0	0
Gm11998	2.092300324	0	0
Gm12059	1.046150162	3.426239465	0
Gm12060	3.138450487	5.710399108	6.760455092
Gm12061	0	1.142079822	0
Gm12070	61.72285957	53.67775162	56.98097863
Gm12121	1.046150162	0	1.931558598
Gm12174	138.0918214	111.9238225	138.1064397
Gm12185	18.83070292	17.13119732	19.31558598
Gm12191	63.8151599	82.22974716	74.36500601
Gm12216	7.323051136	0	4.828896494
Gm12240	0	0	0
Gm12247	1.046150162	3.426239465	5.794675793
Gm12248	0	2.284159643	0
Gm12250	16.7384026	85.65598662	37.66539266
Gm12315	9.41535146	7.994558752	4.828896494
Gm12338	56.49210876	33.12031483	53.11786144
Gm12359	3.138450487	0	0.965779299
Gm12474	13.59995211	2.284159643	0.965779299
Gm12500	0	1.142079822	0
Gm12504	10.46150162	7.994558752	5.794675793
Gm12506	0	11.42079822	0
Gm12522	18.83070292	1.142079822	24.14448247
Gm12569	6.276900973	0	0
Gm12657	29.29220454	22.84159643	31.87071686
Gm12666	1.046150162	0	0.965779299
Gm12669	61.72285957	73.09310859	99.47526778
Gm12693	15.69225243	20.55743679	20.28136528
Gm12694	0	0	0
Gm12708	11.50765178	4.568319287	4.828896494

For Review Only

Gm12781	2.092300324	0	0
Gm128	7.323051136	9.136638573	10.62357229
Gm12853	2.092300324	0	0.965779299
Gm12865	2.092300324	0	0
Gm12942	34.52295535	54.81983144	47.32318564
Gm12958	2.092300324	0	0
Gm12977	11.50765178	13.70495786	12.55513089
Gm12992	78.46126217	3.426239465	5.794675793
Gm13031	3.138450487	0	0
Gm13067	0	0	4.828896494
Gm13070	18.83070292	26.2678359	7.726234391
Gm13073	1.046150162	0	4.828896494
Gm13097	10.46150162	0	0
Gm13111	3.138450487	1.142079822	0
Gm13139	34.52295535	43.39903322	39.59695125
Gm13154	1.046150162	0	2.897337897
Gm13157	47.0767573	202.1481284	0
Gm13179	5.230750811	2.284159643	4.828896494
Gm13199	1.046150162	1.142079822	0
Gm13202	37.66140584	36.54655429	37.66539266
Gm13212	27.19990422	13.70495786	27.04182037
Gm13213	18.83070292	12.56287804	5.794675793
Gm13215	10.46150162	13.70495786	15.45246878
Gm13237	17.78455276	7.994558752	10.62357229
Gm13238	3.138450487	1.142079822	2.897337897
Gm13242	0	1.142079822	0.965779299
Gm13247	1.046150162	0	0
Gm13248	48.12290746	44.54111304	51.18630284
Gm13251	40.79985633	35.40447447	40.56273055
Gm13262	1.046150162	1.142079822	0
Gm13269	9.41535146	21.69951661	5.794675793
Gm13293	5.230750811	21.69951661	11.58935159
Gm13305	12.55380195	0	11.58935159
Gm13315	12.55380195	19.41535697	20.28136528
Gm13363	35.56910552	18.27327715	19.31558598
Gm13387	0	6.85247893	4.828896494
Gm13388	3.138450487	5.710399108	3.863117195
Gm13420	11.50765178	10.27871839	12.55513089
Gm13479	13.59995211	36.54655429	55.04942003
Gm13498	6.276900973	3.426239465	8.69201369
Gm13528	11.50765178	9.136638573	4.828896494
Gm13552	36.61525568	25.12575608	48.28896494
Gm13570	0	0	4.828896494
Gm13572	0	0	0
Gm13609	0	0	0
Gm13657	3.138450487	6.85247893	0
Gm13707	7.323051136	0	8.69201369
Gm13709	3.138450487	5.710399108	0
Gm13710	9.41535146	0	0
Gm13782	0	4.568319287	3.863117195

For Review Only

Gm13807	8.369201298	7.994558752	0
Gm13830	3.138450487	0	0
Gm13840	12.55380195	22.84159643	21.24714457
Gm13846	0	0	0
Gm13884	1.046150162	1.142079822	1.931558598
Gm13889	1.046150162	5.710399108	2.897337897
Gm14005	9.41535146	11.42079822	0
Gm14010	5.230750811	4.568319287	3.863117195
Gm14023	21.96915341	42.2569534	0
Gm14025	2.092300324	2.284159643	8.69201369
Gm14040	5.230750811	14.84703768	12.55513089
Gm14051	0	2.284159643	0
Gm14092	9.41535146	0	0
Gm14137	1.046150162	1.142079822	5.794675793
Gm14139	2.092300324	1.142079822	0
Gm14144	0	0	2.897337897
Gm14154	0	5.710399108	0
Gm14230	19.87685308	10.27871839	0
Gm14288	6.276900973	4.568319287	5.794675793
Gm14295	16.7384026	15.9891175	13.52091018
Gm14296	20.92300324	19.41535697	20.28136528
Gm14305	6.276900973	5.710399108	0.965779299
Gm14308	10.46150162	11.42079822	12.55513089
Gm14321	1.046150162	11.42079822	5.794675793
Gm14322	5.230750811	17.13119732	6.760455092
Gm14325	17.78455276	26.2678359	19.31558598
Gm14326	37.66140584	42.2569534	23.17870317
Gm14391	80.55356249	27.40991572	24.14448247
Gm14403	4.184600649	15.9891175	6.760455092
Gm14405	1.046150162	1.142079822	2.897337897
Gm14412	13.59995211	15.9891175	22.21292387
Gm14434	7.323051136	6.85247893	5.794675793
Gm14440	3.138450487	3.426239465	2.897337897
Gm14486	0	0	0
Gm14532	4.184600649	2.284159643	0
Gm14548	7.323051136	5.710399108	6.760455092
Gm14586	166.3378758	196.4377293	191.2243012
Gm14634	2.092300324	3.426239465	0
Gm14680	16.7384026	17.13119732	14.48668948
Gm14719	23.01530357	6.85247893	20.28136528
Gm14762	4.184600649	0	0
Gm15151	4.184600649	3.426239465	2.897337897
Gm15210	75.32281168	58.2460709	88.85169549
Gm15232	10.46150162	11.42079822	5.794675793
Gm15283	1.046150162	0	0
Gm15319	0	2.284159643	2.897337897
Gm15326	3.138450487	4.568319287	0
Gm15337	5.230750811	0	0
Gm15345	0	1.142079822	0
Gm15417	5.230750811	5.710399108	12.55513089

For Review Only

Gm15421	21.96915341	18.27327715	16.41824808
Gm15423	20.92300324	27.40991572	18.34980668
Gm15446	0	0	0
Gm15448	8.369201298	2.284159643	3.863117195
Gm15453	61.72285957	84.5139068	102.3726057
Gm15455	16.7384026	11.42079822	18.34980668
Gm15471	0	0	0
Gm15506	1.046150162	0	0
Gm15510	0	6.85247893	0.965779299
Gm15523	18.83070292	76.51934805	26.07604107
Gm15541	0	0	0
Gm15545	9.41535146	4.568319287	4.828896494
Gm15551	0	3.426239465	0.965779299
Gm15558	5.230750811	5.710399108	0.965779299
Gm15559	3.138450487	1.142079822	0
Gm15564	410.0908636	513.9359197	329.3307409
Gm15608	3.138450487	0	0
Gm15612	1.046150162	9.136638573	0
Gm15614	2.092300324	6.85247893	0
Gm15624	1.046150162	0	0
Gm15628	7.323051136	17.13119732	16.41824808
Gm15645	0	0	0
Gm15651	6.276900973	13.70495786	6.760455092
Gm15694	1.046150162	1.142079822	0.965779299
Gm15704	2.092300324	7.994558752	0
Gm15706	0	3.426239465	0.965779299
Gm15708	8.369201298	47.96735251	32.83649616
Gm15713	1.046150162	3.426239465	5.794675793
Gm15735	2.092300324	0	4.828896494
Gm15736	207.1377321	49.10943233	113.9619573
Gm15738	6.276900973	10.27871839	3.863117195
Gm15742	1.046150162	3.426239465	0
Gm15753	9.41535146	5.710399108	25.11026177
Gm15760	18.83070292	6.85247893	10.62357229
Gm15764	16.7384026	18.27327715	13.52091018
Gm15772	146.4610227	113.0659023	169.0113773
Gm15774	0	5.710399108	0
Gm15776	73.23051136	69.66686912	94.64637129
Gm15787	3.138450487	2.284159643	5.794675793
Gm15796	5.230750811	2.284159643	13.52091018
Gm15800	176.7993774	196.4377293	191.2243012
Gm15834	15.69225243	11.42079822	15.45246878
Gm15850	0	0	0
Gm15866	3.138450487	0	0
Gm15880	9.41535146	4.568319287	7.726234391
Gm15881	0	0	0
Gm15918	3.138450487	0	0
Gm15927	6.276900973	4.568319287	6.760455092
Gm15928	0	0	0
Gm15952	11.50765178	4.568319287	13.52091018

For Review Only

Gm15991	12.55380195	13.70495786	15.45246878
Gm1600	0	0	0
Gm16023	2.092300324	1.142079822	0
Gm16035	6.276900973	10.27871839	20.28136528
Gm1604a	3.138450487	3.426239465	0
Gm16062	1.046150162	3.426239465	5.794675793
Gm16116	9.41535146	9.136638573	0
Gm16118	59.63055925	103.9292638	64.70721302
Gm16120	4.184600649	12.56287804	12.55513089
Gm16124	5.230750811	6.85247893	2.897337897
Gm16146	8.369201298	9.136638573	4.828896494
Gm16150	2.092300324	1.142079822	0.965779299
Gm16160	0	3.426239465	0
Gm16168	0	0	3.863117195
Gm16206	7.323051136	2.284159643	11.58935159
Gm16231	4.184600649	0	0
Gm16246	1.046150162	0	0
Gm16283	0	0	0
Gm16287	1.046150162	6.85247893	0
Gm16299	5.230750811	2.284159643	27.04182037
Gm16322	4.184600649	4.568319287	4.828896494
Gm16348	0	0	0
Gm16351	2.092300324	2.284159643	0
Gm16386	1.046150162	4.568319287	0
Gm16432	0	0	1.931558598
Gm1647	0	3.426239465	4.828896494
Gm16486	54.39980844	30.83615518	0
Gm16523	1.046150162	2.284159643	18.34980668
Gm16525	0	0	0
Gm16573	1.046150162	1.142079822	0.965779299
Gm16576	40.79985633	19.41535697	35.73383406
Gm16578	9.41535146	18.27327715	11.58935159
Gm16599	11.50765178	9.136638573	0
Gm16617	0	1.142079822	0
Gm16638	10.46150162	2.284159643	0
Gm16675	2.092300324	0	0
Gm16702	5.230750811	5.710399108	3.863117195
Gm16731	0	5.710399108	0
Gm16740	9.41535146	0	0
Gm16759	0	0	0
Gm16793	0	0	0
Gm16794	6.276900973	11.42079822	10.62357229
Gm16835	0	1.142079822	0
Gm16845	13.59995211	10.27871839	8.69201369
Gm16853	0	0	0
Gm16861	5.230750811	3.426239465	5.794675793
Gm16867	34.52295535	19.41535697	34.76805476
Gm16876	5.230750811	30.83615518	14.48668948
Gm16897	7.323051136	0	1.931558598
Gm16907	17.78455276	14.84703768	18.34980668

For Review Only

Gm16982	5.230750811	1.142079822	0.965779299
Gm16998	5.230750811	4.568319287	3.863117195
Gm17021	0	0	0
Gm17025	10.46150162	1.142079822	6.760455092
Gm17030	0	0	0
Gm17039	23.01530357	1.142079822	3.863117195
Gm17057	4.184600649	0	0
Gm17059	8.369201298	3.426239465	2.897337897
Gm17066	71.13821103	122.2025409	73.39922671
Gm17116	0	0	2.897337897
Gm17224	3.138450487	3.426239465	0
Gm17250	0	1.142079822	0
Gm17296	3.138450487	17.13119732	14.48668948
Gm17382	2.092300324	0	0
Gm17399	4.184600649	1.142079822	0
Gm17455	6.276900973	4.568319287	0
Gm17597	2.092300324	0	0
Gm17757	5.230750811	13.70495786	11.58935159
Gm17762	1.046150162	0	0
Gm17764	0	1.142079822	0
Gm17769	0	0	0
Gm17801	0	0	1.931558598
Gm1818	32.43065503	15.9891175	28.97337897
Gm1821	0	0	0
Gm18853	4.184600649	13.70495786	12.55513089
Gm19327	37.66140584	13.70495786	68.57033022
Gm19412	1.046150162	0	2.897337897
Gm1943	6.276900973	6.85247893	12.55513089
Gm19463	0	1.142079822	0
Gm19510	0	0	0
Gm19522	1.046150162	5.710399108	0
Gm19557	10.46150162	5.710399108	0
Gm1966	3.138450487	28.55199554	5.794675793
Gm19665	8.369201298	3.426239465	8.69201369
Gm19673	2.092300324	4.568319287	0
Gm19689	4.184600649	1.142079822	0.965779299
Gm19708	6.276900973	38.83071394	13.52091018
Gm1976	0	3.426239465	8.69201369
Gm19897	18.83070292	1.142079822	8.69201369
Gm1995	2.092300324	0	0.965779299
Gm2002	12.55380195	0	10.62357229
Gm20036	3.138450487	0	2.897337897
Gm20071	31.38450487	11.42079822	12.55513089
Gm20199	24.06145373	26.2678359	0
Gm20219	6.276900973	0	3.863117195
Gm20236	17.78455276	30.83615518	19.31558598
Gm20257	12.55380195	37.68863411	51.18630284
Gm2027	2.092300324	0	0
Gm20275	7.323051136	0	0
Gm20300	10.46150162	27.40991572	25.11026177

For Review Only

Gm20337	0	0	4.828896494
Gm2036	0	0	0.965779299
Gm20385	3.138450487	2.284159643	3.863117195
Gm20482	3.138450487	11.42079822	8.69201369
Gm20492	6.276900973	0	0
Gm20501	3.138450487	0	0
Gm20511	3.138450487	0	0
Gm20522	1.046150162	1.142079822	1.931558598
Gm20544	26.15375406	4.568319287	0
Gm20594	10.46150162	17.13119732	8.69201369
Gm20597	1.046150162	0	0
Gm20604	0	20.55743679	0
Gm20605	0	0	0
Gm20616	0	1.142079822	2.897337897
Gm20636	13.59995211	13.70495786	19.31558598
Gm2065	3.138450487	1.142079822	0
Gm20655	1.046150162	0	2.897337897
Gm20717	29.29220454	13.70495786	14.48668948
Gm20750	0	1.142079822	0
Gm20783	0	0	0
Gm20875	19.87685308	9.136638573	11.58935159
Gm20939	15.69225243	13.70495786	7.726234391
Gm21057	0	5.710399108	0
Gm21119	1.046150162	3.426239465	0.965779299
Gm21145	2.092300324	2.284159643	3.863117195
Gm21284	0	2.284159643	6.760455092
Gm21451	7.323051136	11.42079822	8.69201369
Gm21596	10.46150162	5.710399108	7.726234391
Gm21685	51.26135795	69.66686912	35.73383406
Gm21811	41.84600649	87.94014627	40.56273055
Gm21885	18.83070292	33.12031483	28.00759967
Gm21948	13.59995211	20.55743679	0
Gm21949	8.369201298	0	4.828896494
Gm21975	123.4457191	89.08222609	127.4828674
Gm22	2.092300324	1.142079822	0
Gm2225	70.09206087	74.23518841	102.3726057
Gm2237	0	3.426239465	0
Gm2260	3.138450487	3.426239465	2.897337897
Gm2274	3.138450487	3.426239465	2.897337897
Gm2381	4.184600649	2.284159643	0
Gm24105	1.046150162	2.284159643	0
Gm24175	1.046150162	42.2569534	0
Gm2423	61.72285957	69.66686912	73.39922671
Gm2436	5.230750811	9.136638573	4.828896494
Gm2446	6.276900973	10.27871839	6.760455092
Gm2479	0	0	0
Gm2518	0	1.142079822	0
Gm25380	33.47680519	37.68863411	30.90493756
Gm25432	26.15375406	15.9891175	28.97337897
Gm2573	14.64610227	17.13119732	20.28136528

For Review Only

Gm26008	1.046150162	0	0
Gm26509	3.138450487	0	0
Gm26510	7.323051136	9.136638573	19.31558598
Gm26520	40.79985633	29.69407536	81.1254611
Gm26534	3.138450487	1.142079822	5.794675793
Gm26535	0	1.142079822	3.863117195
Gm26536	4.184600649	12.56287804	10.62357229
Gm26555	4.184600649	2.284159643	3.863117195
Gm26562	5.230750811	0	1.931558598
Gm26583	12.55380195	15.9891175	9.657792988
Gm26588	52.30750811	44.54111304	32.83649616
Gm26615	3.138450487	11.42079822	4.828896494
Gm26627	10.46150162	3.426239465	0
Gm26629	0	3.426239465	0
Gm26634	0	0	1.931558598
Gm26637	25.10760389	19.41535697	19.31558598
Gm26641	3.138450487	3.426239465	6.760455092
Gm26679	5.230750811	10.27871839	5.794675793
Gm26708	2.092300324	3.426239465	0
Gm26740	0	0	0
Gm26760	0	0	0
Gm26777	6.276900973	3.426239465	0
Gm26782	13.59995211	7.994558752	12.55513089
Gm26787	1.046150162	4.568319287	0
Gm26800	0	0	0
Gm26802	0	0	0
Gm26843	1.046150162	0	0
Gm26847	0	0	1.931558598
Gm26871	0	0	0
Gm26881	0	7.994558752	1.931558598
Gm26891	5.230750811	0	1.931558598
Gm26901	6.276900973	7.994558752	3.863117195
Gm26910	3.138450487	18.27327715	9.657792988
Gm26944	267.8144415	34.26239465	75.33078531
Gm26947	6.276900973	2.284159643	0.965779299
Gm27003	1.046150162	1.142079822	0
Gm27008	14.64610227	11.42079822	10.62357229
Gm27151	3.138450487	0	0
Gm27177	29.29220454	22.84159643	27.04182037
Gm27239	0	0	0.965779299
Gm27252	11.50765178	3.426239465	0
Gm28042	3.138450487	21.69951661	37.66539266
Gm2808	12.55380195	6.85247893	4.828896494
Gm2814	0	2.284159643	0
Gm28499	0	0	0
Gm28535	0	1.142079822	0
Gm28651	0	0	0
Gm28802	0	1.142079822	0
Gm28874	20.92300324	21.69951661	16.41824808
Gm2897	5.230750811	6.85247893	6.760455092

For Review Only

Gm29083	2.092300324	2.284159643	0
Gm29376	2.092300324	0	0
Gm29485	0	1.142079822	1.931558598
Gm2956	9.41535146	6.85247893	9.657792988
Gm29679	3.138450487	4.568319287	0
Gm29707	2.092300324	5.710399108	4.828896494
Gm29711	6.276900973	0	0
Gm29716	0	0	8.69201369
Gm29720	1.046150162	0	1.931558598
Gm29724	0	3.426239465	0
Gm29733	2.092300324	3.426239465	0
Gm2977	1.046150162	0	0.965779299
Gm29776	0	0	0
Gm29779	117.1688182	85.65598662	110.0988401
Gm29796	4.184600649	0	0
Gm29797	4.184600649	0	0
Gm29808	3.138450487	1.142079822	0
Gm29811	5.230750811	2.284159643	5.794675793
Gm29834	11.50765178	25.12575608	10.62357229
Gm29846	2.092300324	0	3.863117195
Gm29868	8.369201298	21.69951661	19.31558598
Gm29873	1.046150162	1.142079822	3.863117195
Gm29886	2.092300324	2.284159643	0.965779299
Gm29887	0	1.142079822	0.965779299
Gm29917	5.230750811	4.568319287	0
Gm29927	0	1.142079822	0
Gm29933	5.230750811	7.994558752	0
Gm29945	3.138450487	4.568319287	4.828896494
Gm29948	4.184600649	1.142079822	3.863117195
Gm29953	0	1.142079822	0
Gm29975	2.092300324	123.3446207	187.361184
Gm29994	1.046150162	0	0
Gm29997	6.276900973	4.568319287	0.965779299
Gm2a	720.7974618	744.6360437	785.17857
Gm30012	1.046150162	0	0
Gm30013	3.138450487	0	0
Gm30019	5.230750811	0	4.828896494
Gm3002	13.59995211	15.9891175	18.34980668
Gm30042	8.369201298	60.53023055	25.11026177
Gm3005	1.046150162	1.142079822	2.897337897
Gm30054	19.87685308	18.27327715	11.58935159
Gm30057	0	0	1.931558598
Gm30062	10.46150162	1.142079822	0
Gm30067	4.184600649	0	8.69201369
Gm30082	6.276900973	2.284159643	0
Gm30091	1.046150162	0	1.931558598
Gm30106	0	4.568319287	0
Gm30122	0	0	4.828896494
Gm30144	3.138450487	0	0
Gm30149	3.138450487	0	0.965779299

For Review Only

Gm30151	4.184600649	9.136638573	6.760455092
Gm30181	17.78455276	14.84703768	10.62357229
Gm30189	3.138450487	6.85247893	0
Gm30196	0	0	0.965779299
Gm30215	1.046150162	3.426239465	0
Gm30223	0	0	0
Gm30230	9.41535146	6.85247893	0
Gm30238	2.092300324	2.284159643	0
Gm30254	0	0	0
Gm30273	5.230750811	30.83615518	0
Gm30281	3.138450487	3.426239465	3.863117195
Gm30283	2.092300324	0	0
Gm30284	5.230750811	2.284159643	9.657792988
Gm30286	1.046150162	3.426239465	0
Gm30294	12.55380195	13.70495786	6.760455092
Gm30310	1.046150162	0	0
Gm30314	1.046150162	1.142079822	0
Gm30321	5.230750811	1.142079822	0
Gm30327	0	4.568319287	3.863117195
Gm30340	2.092300324	0	0
Gm30368	57.53825892	34.26239465	41.52850985
Gm30375	0	0	0
Gm30389	19.87685308	33.12031483	15.45246878
Gm30396	1.046150162	2.284159643	0
Gm30401	0	1.142079822	2.897337897
Gm30409	0	0	0
Gm30411	1.046150162	11.42079822	7.726234391
Gm30421	9.41535146	13.70495786	0
Gm30427	0	11.42079822	0
Gm30429	14.64610227	0	2.897337897
Gm30447	0	0	0
Gm30455	18.83070292	11.42079822	0
Gm30466	21.96915341	43.39903322	20.28136528
Gm30489	0	6.85247893	1.931558598
Gm30492	1.046150162	1.142079822	0
Gm3050	3.138450487	1.142079822	2.897337897
Gm30505	0	0	0
Gm30515	0	0	0
Gm30525	0	0	0
Gm30541	31.38450487	9.136638573	6.760455092
Gm30544	0	0	0.965779299
Gm3055	15.69225243	13.70495786	18.34980668
Gm30554	9.41535146	18.27327715	12.55513089
Gm30563	0	0	0
Gm30569	24.06145373	61.67231037	20.28136528
Gm30571	0	0	0
Gm30575	0	2.284159643	2.897337897
Gm30599	1.046150162	6.85247893	0
Gm30604	3.138450487	2.284159643	0
Gm30622	0	0	0

Gm30690	1.046150162	1.142079822	0
Gm30694	3.138450487	6.85247893	0
Gm30699	0	0	3.863117195
Gm30712	1.046150162	4.568319287	0
Gm30723	49.16905763	52.5356718	30.90493756
Gm30732	8.369201298	1.142079822	6.760455092
Gm30744	2.092300324	1.142079822	5.794675793
Gm30745	5.230750811	2.284159643	2.897337897
Gm30783	2.092300324	12.56287804	0.965779299
Gm30789	51.26135795	229.5580442	83.0570197
Gm30794	0	0	0
Gm30804	0	0	0
Gm30807	0	0	0
Gm30809	0	0	0
Gm30810	0	0	0
Gm30814	0	1.142079822	0
Gm30816	37.66140584	71.95102876	48.28896494
Gm30822	3.138450487	7.994558752	4.828896494
Gm30827	0	1.142079822	0
Gm30836	1.046150162	1.142079822	0
Gm30845	2.092300324	2.284159643	3.863117195
Gm30881	5.230750811	5.710399108	0
Gm30908	0	2.284159643	0
Gm30918	0	3.426239465	0
Gm30926	1.046150162	1.142079822	5.794675793
Gm30939	0	0	0
Gm3095	0	0	0
Gm30956	1.046150162	1.142079822	2.897337897
Gm30967	0	1.142079822	0
Gm30970	5.230750811	13.70495786	10.62357229
Gm30990	3.138450487	0	6.760455092
Gm30992	7.323051136	7.994558752	5.794675793
Gm31003	1.046150162	25.12575608	0
Gm31005	1.046150162	0	0.965779299
Gm31011	8.369201298	0	3.863117195
Gm31015	0	0	0
Gm31077	4.184600649	23.98367625	11.58935159
Gm31082	0	0	0
Gm31083	4.184600649	39.97279376	11.58935159
Gm31090	1.046150162	0	0
Gm31095	3.138450487	9.136638573	6.760455092
Gm31109	1.046150162	6.85247893	6.760455092
Gm31120	6.276900973	5.710399108	5.794675793
Gm31134	12.55380195	22.84159643	7.726234391
Gm31151	0	0	0
Gm31152	0	0	0
Gm31156	1.046150162	0	0
Gm31159	0	0	0
Gm31161	5.230750811	0	0.965779299
Gm31166	23.01530357	12.56287804	30.90493756

For Review Only

Gm31172	2.092300324	2.284159643	0
Gm31201	1.046150162	2.284159643	5.794675793
Gm31214	0	0	2.897337897
Gm31216	6.276900973	0	0
Gm31222	8.369201298	3.426239465	0
Gm31223	2.092300324	7.994558752	0
Gm31251	0	1.142079822	0
Gm31253	5.230750811	3.426239465	0.965779299
Gm31258	1.046150162	0	0
Gm31261	4.184600649	4.568319287	0
Gm31269	0	2.284159643	0
Gm31282	0	0	0
Gm31288	2.092300324	0	0
Gm31290	3.138450487	2.284159643	0
Gm31291	0	0	0
Gm31292	15.69225243	84.5139068	18.34980668
Gm31305	16.7384026	13.70495786	2.897337897
Gm31319	0	0	0
Gm31323	2.092300324	0	0
Gm31326	0	0	0
Gm31333	0	1.142079822	0
Gm3134	112.9842175	68.5247893	99.47526778
Gm31349	1.046150162	12.56287804	0
Gm31356	9.41535146	1.142079822	5.794675793
Gm31364	0	0	0
Gm31365	5.230750811	6.85247893	3.863117195
Gm31388	5.230750811	2.284159643	0
Gm31391	0	0	0
Gm31402	5.230750811	0	0
Gm31437	0	0	0
Gm31439	2.092300324	1.142079822	2.897337897
Gm31458	1.046150162	5.710399108	1.931558598
Gm31474	18.83070292	19.41535697	13.52091018
Gm31485	6.276900973	20.55743679	0
Gm31513	0	0	0
Gm31526	1.046150162	3.426239465	0.965779299
Gm31532	3.138450487	2.284159643	2.897337897
Gm31546	7.323051136	0	0.965779299
Gm31550	2.092300324	2.284159643	0
Gm31560	0	2.284159643	0
Gm31566	1.046150162	5.710399108	18.34980668
Gm31569	0	0	0
Gm31593	1.046150162	6.85247893	0
Gm31595	17.78455276	18.27327715	12.55513089
Gm31597	2.092300324	0	0
Gm31606	1.046150162	1.142079822	7.726234391
Gm31619	27.19990422	25.12575608	18.34980668
Gm31623	76.36896184	121.0604611	57.94675793
Gm3164	11.50765178	11.42079822	7.726234391
Gm31645	0	2.284159643	0

For Review Only

Gm31657	24.06145373	14.84703768	10.62357229
Gm31665	3.138450487	1.142079822	6.760455092
Gm31676	0	0	0.965779299
Gm31677	2.092300324	1.142079822	4.828896494
Gm31679	1.046150162	1.142079822	9.657792988
Gm31683	0	0	0
Gm31684	31.38450487	25.12575608	12.55513089
Gm31692	0	1.142079822	1.931558598
Gm31718	13.59995211	1.142079822	5.794675793
Gm31721	5.230750811	2.284159643	7.726234391
Gm31725	0	0	0
Gm31726	0	1.142079822	3.863117195
Gm31728	4.184600649	0	8.69201369
Gm3173	12.55380195	23.98367625	18.34980668
Gm31734	0	0	0
Gm31735	1.046150162	1.142079822	0
Gm31745	6.276900973	2.284159643	11.58935159
Gm31763	0	3.426239465	0
Gm31805	2.092300324	0	0
Gm31812	4.184600649	0	1.931558598
Gm31834	0	1.142079822	2.897337897
Gm31835	0	1.142079822	0
Gm31839	0	0	0
Gm31850	0	1.142079822	0
Gm31852	1.046150162	1.142079822	24.14448247
Gm31854	1.046150162	5.710399108	0
Gm31862	11.50765178	9.136638573	6.760455092
Gm31872	3.138450487	3.426239465	0
Gm31888	5.230750811	0	0
Gm3189	1.046150162	1.142079822	0
Gm31896	5.230750811	12.56287804	9.657792988
Gm31902	31.38450487	15.9891175	12.55513089
Gm31909	6.276900973	12.56287804	9.657792988
Gm31914	11.50765178	10.27871839	4.828896494
Gm31930	0	1.142079822	0
Gm3194	0	0	0
Gm31940	5.230750811	12.56287804	9.657792988
Gm31974	0	5.710399108	3.863117195
Gm31984	3.138450487	0	0
Gm31989	1.046150162	1.142079822	0
Gm32006	38.707556	15.9891175	16.41824808
Gm32017	0	0	0
Gm32026	4.184600649	9.136638573	4.828896494
Gm32029	4.184600649	27.40991572	12.55513089
Gm32031	4.184600649	4.568319287	0
Gm32039	18.83070292	7.994558752	10.62357229
Gm32046	1.046150162	0	0
Gm32050	0	0	0
Gm32059	0	4.568319287	0.965779299
Gm32064	5.230750811	4.568319287	5.794675793

For Review Only

Gm32080	1.046150162	5.710399108	0
Gm32089	0	0	0
Gm32098	61.72285957	44.54111304	35.73383406
Gm32100	9.41535146	13.70495786	4.828896494
Gm32133	5.230750811	3.426239465	0
Gm32184	0	1.142079822	4.828896494
Gm3219	2.092300324	0	0.965779299
Gm32211	12.55380195	5.710399108	9.657792988
Gm32234	0	0	0.965779299
Gm32249	0	11.42079822	7.726234391
Gm32250	3.138450487	3.426239465	0
Gm32262	8.369201298	5.710399108	8.69201369
Gm32267	5.230750811	0	7.726234391
Gm32268	0	2.284159643	0
Gm32275	7.323051136	0	4.828896494
Gm32280	0	0	0
Gm32287	1.046150162	0	0
Gm32289	1.046150162	2.284159643	3.863117195
Gm32293	0	0	0
Gm32294	12.55380195	21.69951661	10.62357229
Gm3230	1.046150162	1.142079822	4.828896494
Gm32309	0	0	0
Gm32311	3.138450487	1.142079822	0
Gm32313	1.046150162	2.284159643	0
Gm32317	13.59995211	6.85247893	17.38402738
Gm32336	21.96915341	25.12575608	15.45246878
Gm32374	0	3.426239465	3.863117195
Gm32379	4.184600649	0	0
Gm32380	43.93830681	50.25151215	55.04942003
Gm3239	0	0	0.965779299
Gm32394	23.01530357	37.68863411	14.48668948
Gm32413	0	0	0.965779299
Gm32435	185.1685787	129.0550198	127.4828674
Gm32436	4.184600649	10.27871839	3.863117195
Gm32438	5.230750811	2.284159643	0
Gm3244	18.83070292	19.41535697	34.76805476
Gm32457	2.092300324	1.142079822	6.760455092
Gm32462	0	0	0
Gm32471	0	0	0
Gm32474	0	0	0
Gm3248	1.046150162	1.142079822	0
Gm32483	25.10760389	10.27871839	7.726234391
Gm32486	0	0	0
Gm32497	307.5681477	333.4873079	292.6311276
Gm3252	21.96915341	21.69951661	0
Gm32528	27.19990422	6.85247893	34.76805476
Gm32547	35.56910552	49.10943233	46.35740634
Gm3255	3.138450487	4.568319287	2.897337897
Gm32551	2.092300324	4.568319287	2.897337897
Gm32553	5.230750811	0	0

For Review Only

Gm32555	1.046150162	0	0
Gm32566	38.707556	34.26239465	21.24714457
Gm32581	9.41535146	11.42079822	5.794675793
Gm32584	28.24605438	9.136638573	0
Gm32591	31.38450487	22.84159643	25.11026177
Gm32605	57.53825892	34.26239465	49.25474424
Gm32620	20.92300324	4.568319287	3.863117195
Gm32633	5.230750811	1.142079822	0
Gm3264	20.92300324	45.68319287	25.11026177
Gm32643	31.38450487	7.994558752	15.45246878
Gm32645	13.59995211	13.70495786	18.34980668
Gm32650	0	0	1.931558598
Gm32655	0	0	0
Gm32670	6.276900973	1.142079822	3.863117195
Gm32672	3.138450487	2.284159643	11.58935159
Gm32673	1.046150162	5.710399108	0
Gm32687	39.75370617	42.2569534	29.93915826
Gm32694	12.55380195	28.55199554	24.14448247
Gm32703	11.50765178	15.9891175	17.38402738
Gm32707	0	0	6.760455092
Gm32709	0	0	0
Gm32715	8.369201298	17.13119732	13.52091018
Gm32718	4.184600649	5.710399108	7.726234391
Gm32725	1.046150162	7.994558752	8.69201369
Gm32738	6.276900973	10.27871839	7.726234391
Gm32760	0	0	0
Gm32788	2.092300324	0	0
Gm32793	0	0	0
Gm32810	0	0	0.965779299
Gm32817	0	0	0.965779299
Gm32819	0	0	0
Gm32824	38.707556	26.2678359	18.34980668
Gm32827	124.4918693	171.3119732	186.3954047
Gm32847	0	0	0
Gm32849	2155.069334	2812.942601	2075.459713
Gm32853	17.78455276	37.68863411	13.52091018
Gm32854	1.046150162	0	0
Gm32856	16.7384026	35.40447447	25.11026177
Gm32861	2.092300324	0	0.965779299
Gm32882	4.184600649	0	3.863117195
Gm32898	0	2.284159643	0.965779299
Gm32899	0	7.994558752	0
Gm32900	1.046150162	0	0
Gm32904	2.092300324	4.568319287	0
Gm32908	0	0	0
Gm32934	4.184600649	1.142079822	1.931558598
Gm32940	30.33835471	50.25151215	56.98097863
Gm32952	2.092300324	0	0
Gm32992	0	3.426239465	0
Gm32999	1.046150162	0	0

For Review Only

Gm33023	24.06145373	35.40447447	31.87071686
Gm33047	27.19990422	15.9891175	14.48668948
Gm33066	0	4.568319287	0
Gm33097	2.092300324	2.284159643	0
Gm33100	2.092300324	7.994558752	5.794675793
Gm33118	1.046150162	9.136638573	9.657792988
Gm33126	0	3.426239465	0
Gm3317	36.61525568	39.97279376	37.66539266
Gm33198	4.184600649	4.568319287	2.897337897
Gm33199	0	0	1.931558598
Gm33214	0	7.994558752	4.828896494
Gm33219	0	4.568319287	0
Gm33228	0	1.142079822	0
Gm3325	27.19990422	18.27327715	15.45246878
Gm33257	0	0	0
Gm33269	3.138450487	0	0
Gm33272	41.84600649	70.80894894	49.25474424
Gm33273	8.369201298	3.426239465	6.760455092
Gm33305	12.55380195	21.69951661	12.55513089
Gm33310	13.59995211	7.994558752	0
Gm33318	0	6.85247893	0.965779299
Gm33337	0	0	0
Gm33350	4.184600649	1.142079822	6.760455092
Gm33370	27.19990422	18.27327715	30.90493756
Gm33376	2.092300324	1.142079822	0.965779299
Gm33387	12.55380195	19.41535697	16.41824808
Gm33389	12.55380195	1.142079822	4.828896494
Gm33392	0	1.142079822	0
Gm33434	15.69225243	26.2678359	25.11026177
Gm33442	1.046150162	3.426239465	0
Gm33444	10.46150162	13.70495786	15.45246878
Gm33449	3.138450487	0	0
Gm33454	0	0	0
Gm33467	1.046150162	0	0
Gm33470	0	0	0
Gm33475	10.46150162	2.284159643	0
Gm33487	2.092300324	4.568319287	6.760455092
Gm33517	44.98445698	7.994558752	10.62357229
Gm33524	0	3.426239465	0
Gm33534	3.138450487	15.9891175	0
Gm33536	0	0	0
Gm33555	0	0	0
Gm33582	7.323051136	21.69951661	5.794675793
Gm33585	0	0	0
Gm33610	5.230750811	9.136638573	10.62357229
Gm33622	44.98445698	6.85247893	5.794675793
Gm33641	0	3.426239465	9.657792988
Gm33682	13.59995211	7.994558752	13.52091018
Gm33691	3.138450487	1.142079822	0
Gm33697	46.03060714	33.12031483	55.04942003

For Review Only

Gm33721	0	7.994558752	11.58935159
Gm33723	0	3.426239465	1.931558598
Gm33729	14.64610227	18.27327715	15.45246878
Gm3373	0	0	0
Gm33733	0	4.568319287	0
Gm33746	0	0	0
Gm33747	2.092300324	2.284159643	0
Gm33764	0	0	0
Gm33767	9.41535146	23.98367625	14.48668948
Gm33771	1.046150162	2.284159643	0
Gm33786	0	0	0
Gm33795	0	0	0
Gm33804	4.184600649	4.568319287	0
Gm33813	1.046150162	0	0
Gm33821	9.41535146	10.27871839	6.760455092
Gm3383	12.55380195	14.84703768	13.52091018
Gm33832	2.092300324	0	0
Gm33856	17.78455276	25.12575608	27.04182037
Gm33862	5.230750811	9.136638573	2.897337897
Gm33864	94.1535146	77.66142787	48.28896494
Gm33869	24.06145373	52.5356718	25.11026177
Gm33877	4.184600649	9.136638573	1.931558598
Gm33885	0	2.284159643	4.828896494
Gm33922	1.046150162	1.142079822	3.863117195
Gm33926	0	0	0
Gm33933	6.276900973	6.85247893	6.760455092
Gm33937	0	0	0
Gm33938	5.230750811	1.142079822	5.794675793
Gm33940	0	0	0
Gm33971	0	4.568319287	0
Gm33989	297.1066461	354.0447447	202.8136528
Gm34058	0	0	3.863117195
Gm34076	11.50765178	10.27871839	5.794675793
Gm34079	4.184600649	6.85247893	7.726234391
Gm34087	0	0	0
Gm34102	0	7.994558752	0
Gm34121	2.092300324	2.284159643	0
Gm34137	0	0	0
Gm3414	17.78455276	17.13119732	14.48668948
Gm34156	14.64610227	1.142079822	3.863117195
Gm34159	0	0	0
Gm34168	0	4.568319287	4.828896494
Gm3417	0	0	0.965779299
Gm34178	17.78455276	14.84703768	14.48668948
Gm34186	3.138450487	12.56287804	10.62357229
Gm34189	2.092300324	3.426239465	1.931558598
Gm34197	2.092300324	0	2.897337897
Gm34218	23.01530357	11.42079822	23.17870317
Gm34220	3.138450487	2.284159643	3.863117195
Gm34223	0	4.568319287	0

For Review Only

Gm34232	0	2.284159643	0
Gm34235	10.46150162	11.42079822	5.794675793
Gm34245	3.138450487	0	0
Gm34280	262.5836907	248.9734011	196.0531977
Gm34283	4.184600649	2.284159643	3.863117195
Gm34288	8.369201298	1.142079822	0
Gm34292	0	0	0
Gm34299	0	0	0
Gm34321	2.092300324	2.284159643	0
Gm34324	0	2.284159643	6.760455092
Gm34326	1.046150162	2.284159643	0.965779299
Gm34336	1.046150162	2.284159643	3.863117195
Gm34343	0	0	0
Gm3435	13.59995211	12.56287804	12.55513089
Gm34354	1.046150162	0	0
Gm34375	17.78455276	22.84159643	11.58935159
Gm34389	0	5.710399108	0
Gm34392	0	1.142079822	0
Gm34394	3.138450487	0	0
Gm34402	6.276900973	11.42079822	0
Gm34403	4.184600649	0	2.897337897
Gm34407	3.138450487	3.426239465	4.828896494
Gm34408	26.15375406	13.70495786	6.760455092
Gm34447	0	0	0
Gm34448	3.138450487	3.426239465	0
Gm34451	2.092300324	1.142079822	4.828896494
Gm34455	0	0	14.48668948
Gm34459	0	0	0
Gm34507	0	0	0
Gm34513	0	0	0
Gm34531	5.230750811	0	0
Gm34582	0	10.27871839	0.965779299
Gm34586	1.046150162	21.69951661	10.62357229
Gm34589	74.27666152	57.10399108	130.3802053
Gm34607	0	0	0
Gm34620	4.184600649	0	0
Gm34632	0	9.136638573	4.828896494
Gm34648	0	1.142079822	0
Gm34655	5.230750811	0	0
Gm34661	0	3.426239465	0.965779299
Gm34696	1.046150162	0	3.863117195
Gm34702	1.046150162	1.142079822	0
Gm34741	0	2.284159643	0
Gm34744	0	0	0
Gm34755	4.184600649	3.426239465	5.794675793
Gm34771	4.184600649	12.56287804	7.726234391
Gm34776	0	0	0
Gm34795	7.323051136	5.710399108	1.931558598
Gm34836	30.33835471	68.5247893	45.39162705
Gm34844	1.046150162	3.426239465	0

For Review Only

Gm34847	1.046150162	0	0
Gm34849	0	4.568319287	2.897337897
Gm34854	8.369201298	18.27327715	10.62357229
Gm34857	1.046150162	0	0
Gm34858	4.184600649	1.142079822	0
Gm34861	8.369201298	1.142079822	0
Gm34865	28.24605438	30.83615518	21.24714457
Gm34868	0	0	0
Gm34872	20.92300324	15.9891175	11.58935159
Gm3488	35.56910552	37.68863411	31.87071686
Gm34907	6.276900973	0	0
Gm34917	10.46150162	13.70495786	0
Gm34921	31.38450487	39.97279376	24.14448247
Gm34934	0	1.142079822	0
Gm34945	1.046150162	5.710399108	0
Gm34979	4.184600649	1.142079822	0
Gm34980	6.276900973	14.84703768	3.863117195
Gm34982	1.046150162	3.426239465	0
Gm34983	0	2.284159643	0
Gm34997	0	1.142079822	0
Gm3500	3.138450487	4.568319287	4.828896494
Gm35002	1.046150162	1.142079822	0
Gm35021	0	0	1.931558598
Gm35029	5.230750811	12.56287804	0
Gm35035	15.69225243	23.98367625	16.41824808
Gm35060	4.184600649	4.568319287	0
Gm35071	0	0	0
Gm35074	3.138450487	0	0
Gm35089	0	1.142079822	10.62357229
Gm35102	0	3.426239465	0
Gm35113	1.046150162	18.27327715	0
Gm35117	2.092300324	19.41535697	7.726234391
Gm35135	2.092300324	0	0
Gm35145	0	0	0
Gm35150	6.276900973	0	3.863117195
Gm35154	41.84600649	19.41535697	31.87071686
Gm35166	0	0	0
Gm35169	16.7384026	14.84703768	10.62357229
Gm35188	0	1.142079822	2.897337897
Gm35200	0	4.568319287	4.828896494
Gm35242	15.69225243	0	0.965779299
Gm35243	21.96915341	15.9891175	15.45246878
Gm35247	39.75370617	28.55199554	27.04182037
Gm35248	0	0	0
Gm35280	0	0	0
Gm35285	0	0	0
Gm35290	72.18436119	98.21886466	77.26234391
Gm35295	0	0	0
Gm35314	2.092300324	2.284159643	3.863117195
Gm35315	0	3.426239465	0

For Review Only

Gm35321	0	0	0
Gm35339	1.046150162	5.710399108	10.62357229
Gm35343	5.230750811	4.568319287	0.965779299
Gm35353	0	0	1.931558598
Gm35358	1.046150162	1.142079822	0
Gm35364	0	0	0.965779299
Gm35365	2.092300324	2.284159643	6.760455092
Gm35383	0	3.426239465	0
Gm35394	0	0	0
Gm35395	1.046150162	0	1.931558598
Gm35396	0	0	0
Gm35399	2.092300324	5.710399108	5.794675793
Gm35417	19.87685308	0	0
Gm35456	0	0	0
Gm35463	1.046150162	0	0
Gm35465	1.046150162	0	0
Gm35466	2.092300324	11.42079822	0.965779299
Gm35470	0	0	0
Gm35478	8.369201298	0	3.863117195
Gm35501	1.046150162	0	0
Gm35522	3.138450487	4.568319287	6.760455092
Gm35525	0	0	0
Gm35534	15.69225243	2.284159643	37.66539266
Gm35558	0	0	0
Gm35562	0	0	0
Gm35566	15.69225243	23.98367625	22.21292387
Gm35572	3.138450487	2.284159643	0.965779299
Gm3558	5.230750811	7.994558752	8.69201369
Gm35582	4.184600649	9.136638573	3.863117195
Gm35591	1.046150162	0	0
Gm35596	15.69225243	10.27871839	15.45246878
Gm35597	7.323051136	2.284159643	4.828896494
Gm35599	2.092300324	0	0
Gm35601	3.138450487	1.142079822	0
Gm35607	0	1.142079822	0
Gm35608	0	0	0
Gm35625	0	0	0.965779299
Gm35657	1.046150162	2.284159643	3.863117195
Gm35658	8.369201298	30.83615518	14.48668948
Gm35677	4.184600649	10.27871839	5.794675793
Gm35678	1.046150162	0	0
Gm35688	0	0	0
Gm35707	12.55380195	2.284159643	0
Gm35715	18.83070292	4.568319287	9.657792988
Gm35732	2.092300324	15.9891175	15.45246878
Gm35760	0	0	0
Gm35768	38.707556	27.40991572	24.14448247
Gm35808	1.046150162	17.13119732	0.965779299
Gm35816	3.138450487	0	5.794675793
Gm35828	10.46150162	17.13119732	13.52091018

For Review Only

Gm35831	7.323051136	13.70495786	11.58935159
Gm35835	0	0	0
Gm35853	17.78455276	1.142079822	18.34980668
Gm35856	0	0	1.931558598
Gm35866	9.41535146	21.69951661	28.97337897
Gm35887	13.59995211	1.142079822	0
Gm35906	413.2293141	422.569534	371.8250301
Gm35908	11.50765178	0	0
Gm3591	17.78455276	0	0
Gm35911	0	0	0
Gm35934	0	0	0
Gm35959	5.230750811	13.70495786	3.863117195
Gm35970	1.046150162	2.284159643	0
Gm35999	6.276900973	7.994558752	3.863117195
Gm36003	12.55380195	22.84159643	26.07604107
Gm36025	1.046150162	0	0
Gm36027	1.046150162	19.41535697	0
Gm3604	5.230750811	1.142079822	1.931558598
Gm36043	0	0	0
Gm36046	0	1.142079822	1.931558598
Gm36048	7.323051136	0	7.726234391
Gm36055	1.046150162	0	0
Gm36109	37.66140584	36.54655429	43.46006845
Gm36117	3.138450487	1.142079822	0
Gm36118	0	0	0.965779299
Gm36148	5.230750811	2.284159643	6.760455092
Gm36163	0	0	0
Gm36166	6.276900973	5.710399108	6.760455092
Gm36167	0	1.142079822	0
Gm36181	0	0	0
Gm36184	0	3.426239465	0
Gm36188	0	0	0
Gm36195	4.184600649	1.142079822	12.55513089
Gm36198	4.184600649	5.710399108	11.58935159
Gm36208	0	3.426239465	0.965779299
Gm36220	8.369201298	19.41535697	24.14448247
Gm36227	0	0	0.965779299
Gm36229	0	0	2.897337897
Gm36243	0	0	2.897337897
Gm36267	0	1.142079822	0
Gm36279	14.64610227	43.39903322	40.56273055
Gm3629	4.184600649	3.426239465	6.760455092
Gm36297	2.092300324	0	0
Gm36299	0	2.284159643	0
Gm36304	0	0	0
Gm36311	0	0	0
Gm36315	0	4.568319287	0.965779299
Gm36328	4.184600649	4.568319287	10.62357229
Gm36330	0	0	2.897337897
Gm36338	3.138450487	0	0

Gm36355	0	0	0
Gm36359	0	0	0
Gm3636	55.4459586	42.2569534	58.91253723
Gm36365	331.6296014	318.6402702	411.4219813
Gm36375	4.184600649	11.42079822	22.21292387
Gm36391	1.046150162	0	0
Gm36401	10.46150162	6.85247893	6.760455092
Gm36402	15.69225243	15.9891175	10.62357229
Gm36403	2.092300324	0	0
Gm36409	0	0	2.897337897
Gm36411	0	2.284159643	0
Gm36412	3.138450487	3.426239465	0
Gm36423	5.230750811	0	0
Gm36430	7.323051136	9.136638573	5.794675793
Gm36441	2.092300324	0	0
Gm36442	3.138450487	2.284159643	6.760455092
Gm36449	3.138450487	4.568319287	5.794675793
Gm36457	7.323051136	3.426239465	0
Gm36459	4.184600649	0	0
Gm36462	3.138450487	4.568319287	7.726234391
Gm36464	0	4.568319287	0
Gm36470	0	4.568319287	0
Gm36482	6.276900973	10.27871839	6.760455092
Gm36490	10.46150162	7.994558752	7.726234391
Gm36493	0	0	2.897337897
Gm36496	1.046150162	0	0
Gm3650	6.276900973	10.27871839	0
Gm36500	7.323051136	1.142079822	0
Gm36527	79.50741233	50.25151215	46.35740634
Gm36529	0	0	0.965779299
Gm36532	0	0	0
Gm36538	0	0	0
Gm36556	7.323051136	20.55743679	8.69201369
Gm36559	1.046150162	10.27871839	7.726234391
Gm36570	0	2.284159643	0
Gm36572	5.230750811	13.70495786	0
Gm36579	1.046150162	3.426239465	0
Gm36584	3.138450487	0	0
Gm36587	49.16905763	103.9292638	24.14448247
Gm36591	7.323051136	36.54655429	0
Gm36602	3.138450487	3.426239465	0
Gm36607	8.369201298	10.27871839	24.14448247
Gm36608	1.046150162	0	0
Gm36635	3.138450487	0	5.794675793
Gm3667	0	0	0.965779299
Gm36672	3.138450487	0	0
Gm36673	0	4.568319287	1.931558598
Gm36677	3.138450487	1.142079822	0
Gm36681	60.67670941	0	27.04182037
Gm36684	1.046150162	1.142079822	0

For Review Only

Gm36693	1.046150162	0	0
Gm36704	1.046150162	0	0
Gm36712	2.092300324	11.42079822	6.760455092
Gm36722	0	0	0
Gm36738	8.369201298	5.710399108	11.58935159
Gm36743	0	0	0
Gm36756	4.184600649	1.142079822	0
Gm36774	0	7.994558752	10.62357229
Gm36778	0	0	0
Gm36790	0	0	0
Gm36796	5.230750811	23.98367625	12.55513089
Gm36800	19.87685308	23.98367625	58.91253723
Gm36816	7.323051136	7.994558752	20.28136528
Gm36818	0	1.142079822	0
Gm36826	1.046150162	3.426239465	0
Gm36839	1.046150162	1.142079822	0
Gm36851	32.43065503	38.83071394	5.794675793
Gm36853	2.092300324	9.136638573	3.863117195
Gm36856	1.046150162	0	0
Gm36864	0	6.85247893	0
Gm36874	6.276900973	11.42079822	8.69201369
Gm36877	0	0	0
Gm36884	10.46150162	11.42079822	11.58935159
Gm36885	4.184600649	4.568319287	23.17870317
Gm36897	4.184600649	0	0
Gm36901	11.50765178	1.142079822	3.863117195
Gm36906	1.046150162	1.142079822	0
Gm36908	0	0	0
Gm36911	0	0	0
Gm36913	0	0	0
Gm36917	0	1.142079822	0
Gm3696	5.230750811	3.426239465	3.863117195
Gm3704	1.046150162	0	0
Gm37053	12.55380195	7.994558752	17.38402738
Gm37125	10.46150162	19.41535697	28.00759967
Gm3716	2.092300324	7.994558752	3.863117195
Gm37168	0	0	0
Gm3718	8.369201298	7.994558752	0.965779299
Gm3739	21.96915341	22.84159643	19.31558598
Gm3740	0	0	2.897337897
Gm37416	32.43065503	23.98367625	0
Gm3764	0	0	0
Gm38137	2.092300324	2.284159643	0
Gm38293	1.046150162	0	0
Gm3837	38.707556	44.54111304	56.01519933
Gm38396	33.47680519	43.39903322	68.57033022
Gm38414	2.092300324	0	0
Gm38418	1.046150162	3.426239465	2.897337897
Gm38422	0	0	0
Gm38424	9.41535146	3.426239465	0.965779299

For Review Only

Gm38426	4.184600649	0	0
Gm38431	131.8149204	110.7817427	130.3802053
Gm38440	1.046150162	9.136638573	2.897337897
Gm38444	3.138450487	9.136638573	5.794675793
Gm38457	1.046150162	15.9891175	4.828896494
Gm38459	0	0	0
Gm38471	3.138450487	6.85247893	7.726234391
Gm38479	48.12290746	19.41535697	18.34980668
Gm38481	11.50765178	5.710399108	4.828896494
Gm38482	5.230750811	1.142079822	4.828896494
Gm38485	20.92300324	57.10399108	30.90493756
Gm38486	0	1.142079822	2.897337897
Gm38487	0	2.284159643	0
Gm38489	13.59995211	6.85247893	13.52091018
Gm38496	10.46150162	3.426239465	8.69201369
Gm38499	2.092300324	1.142079822	4.828896494
Gm38500	16.7384026	0	10.62357229
Gm38501	1.046150162	0	0
Gm38503	0	0	1.931558598
Gm38506	0	0	1.931558598
Gm38515	0	0	6.760455092
Gm38524	15.69225243	12.56287804	6.760455092
Gm38525	4.184600649	4.568319287	12.55513089
Gm38528	0	0	0
Gm38529	11.50765178	6.85247893	5.794675793
Gm38530	0	0	0
Gm38540	0	3.426239465	0
Gm38543	21.96915341	27.40991572	30.90493756
Gm38549	0	9.136638573	5.794675793
Gm38553	1.046150162	2.284159643	0.965779299
Gm38560	0	0	3.863117195
Gm38561	1.046150162	4.568319287	0
Gm38565	2.092300324	9.136638573	7.726234391
Gm38592	9.41535146	7.994558752	8.69201369
Gm38594	0	0	0
Gm38599	15.69225243	5.710399108	4.828896494
Gm38609	13.59995211	20.55743679	18.34980668
Gm38618	0	0	0
Gm38621	0	0	0
Gm38623	2.092300324	2.284159643	0.965779299
Gm38632	55.4459586	66.24062966	46.35740634
Gm38639	14.64610227	26.2678359	25.11026177
Gm38641	7.323051136	4.568319287	17.38402738
Gm38643	0	0	0
Gm38649	12.55380195	9.136638573	11.58935159
Gm38651	2.092300324	2.284159643	0
Gm38664	2.092300324	4.568319287	10.62357229
Gm38675	0	0	6.760455092
Gm38676	3.138450487	4.568319287	5.794675793
Gm38688	3.138450487	14.84703768	7.726234391

For Review Only

Gm38690	0	1.142079822	0.965779299
Gm38708	2.092300324	0	0
Gm38709	32.43065503	11.42079822	7.726234391
Gm38718	6.276900973	7.994558752	3.863117195
Gm38720	5.230750811	3.426239465	5.794675793
Gm38729	6.276900973	0	0
Gm3873	19.87685308	21.69951661	37.66539266
Gm38745	0	0	0
Gm38752	1.046150162	1.142079822	0
Gm38767	0	0	0
Gm38781	4.184600649	1.142079822	3.863117195
Gm38782	2.092300324	1.142079822	0
Gm38785	75.32281168	39.97279376	75.33078531
Gm38803	0	0	0
Gm38804	2.092300324	1.142079822	0
Gm38805	0	0	0
Gm38832	10.46150162	5.710399108	11.58935159
Gm38834	3.138450487	6.85247893	0
Gm38849	0	0	0
Gm38850	34.52295535	54.81983144	21.24714457
Gm38852	0	3.426239465	2.897337897
Gm38859	21.96915341	5.710399108	8.69201369
Gm38865	6.276900973	38.83071394	12.55513089
Gm38871	0	0	0
Gm38880	10.46150162	5.710399108	7.726234391
Gm38882	13.59995211	3.426239465	12.55513089
Gm38883	0	6.85247893	5.794675793
Gm38907	12.55380195	6.85247893	8.69201369
Gm38908	8.369201298	4.568319287	0.965779299
Gm38914	3.138450487	0	0
Gm38918	0	0	0.965779299
Gm38927	1.046150162	3.426239465	0
Gm38935	0	0	0
Gm38939	0	0	1.931558598
Gm38948	3.138450487	7.994558752	0.965779299
Gm38951	1.046150162	1.142079822	0
Gm38952	4.184600649	0	0
Gm38956	0	4.568319287	6.760455092
Gm38957	2.092300324	0	5.794675793
Gm38967	0	0	3.863117195
Gm38968	5.230750811	0	4.828896494
Gm38978	1.046150162	4.568319287	2.897337897
Gm38983	1.046150162	1.142079822	0
Gm39000	6.276900973	6.85247893	0.965779299
Gm39002	2.092300324	0	0.965779299
Gm39006	3.138450487	0	1.931558598
Gm39010	9.41535146	0	0
Gm39012	0	1.142079822	0
Gm39019	0	0	0
Gm39038	0	0	0

Gm39041	0	3.426239465	0.965779299
Gm39050	0	3.426239465	2.897337897
Gm39054	0	0	2.897337897
Gm39055	3.138450487	2.284159643	0
Gm39059	0	1.142079822	3.863117195
Gm39061	5.230750811	5.710399108	0
Gm39069	1.046150162	9.136638573	11.58935159
Gm39081	0	0	0
Gm39089	3.138450487	0	0
Gm39090	7.323051136	6.85247893	2.897337897
Gm39092	0	0	0
Gm39099	0	0	0
Gm39109	3.138450487	3.426239465	4.828896494
Gm39110	1.046150162	2.284159643	6.760455092
Gm39134	0	1.142079822	3.863117195
Gm39141	4.184600649	0	1.931558598
Gm39149	0	0	0
Gm39150	10.46150162	9.136638573	4.828896494
Gm39170	5.230750811	1.142079822	4.828896494
Gm39173	61.72285957	59.38815073	40.56273055
Gm39178	0	0	0
Gm39203	1.046150162	0	0
Gm39210	0	0	0
Gm39211	0	0	1.931558598
Gm39212	0	4.568319287	0.965779299
Gm39214	11.50765178	9.136638573	10.62357229
Gm39215	1.046150162	3.426239465	4.828896494
Gm39221	13.59995211	14.84703768	12.55513089
Gm39241	1.046150162	0	0
Gm39251	3.138450487	4.568319287	8.69201369
Gm39256	24.06145373	6.85247893	10.62357229
Gm39257	6.276900973	11.42079822	0.965779299
Gm39260	6.276900973	9.136638573	2.897337897
Gm39265	0	0	0
Gm39269	10.46150162	22.84159643	30.90493756
Gm39272	73.23051136	69.66686912	48.28896494
Gm39284	0	0	1.931558598
Gm39308	2.092300324	2.284159643	3.863117195
Gm39309	7.323051136	5.710399108	4.828896494
Gm39334	7.323051136	4.568319287	0
Gm3934	49.16905763	51.39359197	51.18630284
Gm39342	6.276900973	3.426239465	0
Gm39348	10.46150162	9.136638573	0
Gm39355	0	0	10.62357229
Gm39362	0	1.142079822	0
Gm39382	0	1.142079822	0.965779299
Gm39399	2.092300324	0	0
Gm39408	1.046150162	3.426239465	3.863117195
Gm39419	15.69225243	22.84159643	18.34980668
Gm39424	4.184600649	3.426239465	5.794675793

For Review Only

Gm39425	1.046150162	2.284159643	0
Gm39443	4.184600649	10.27871839	0
Gm39446	0	0	0
Gm39454	13.59995211	23.98367625	16.41824808
Gm39463	0	0	0
Gm39467	0	0	0.965779299
Gm39469	7.323051136	0	8.69201369
Gm39474	2.092300324	1.142079822	0
Gm39478	3.138450487	0	0
Gm39491	5.230750811	0	0
Gm39497	7.323051136	11.42079822	0
Gm39499	0	0	0
Gm39503	1.046150162	1.142079822	0
Gm39518	3.138450487	61.67231037	38.63117195
Gm39526	15.69225243	6.85247893	5.794675793
Gm39552	16.7384026	10.27871839	21.24714457
Gm39556	18.83070292	5.710399108	6.760455092
Gm39565	56.49210876	31.97823501	7.726234391
Gm39584	0	0	0
Gm39585	1.046150162	6.85247893	4.828896494
Gm39603	0	0	2.897337897
Gm39606	4.184600649	0	0
Gm39618	0	1.142079822	0
Gm39624	11.50765178	9.136638573	20.28136528
Gm39627	1.046150162	2.284159643	3.863117195
Gm39642	23.01530357	22.84159643	17.38402738
Gm39656	75.32281168	82.22974716	114.9277366
Gm39662	0	0	0
Gm39664	0	0	0
Gm39666	3.138450487	17.13119732	2.897337897
Gm39667	4.184600649	0	0.965779299
Gm39668	8.369201298	4.568319287	11.58935159
Gm39673	176.7993774	142.7599777	166.1140394
Gm39679	1.046150162	3.426239465	1.931558598
Gm39688	0	0	0
Gm39701	5.230750811	3.426239465	4.828896494
Gm39703	0	2.284159643	0
Gm39712	20.92300324	26.2678359	17.38402738
Gm39715	0	0	2.897337897
Gm39751	41.84600649	22.84159643	17.38402738
Gm39761	1.046150162	1.142079822	3.863117195
Gm39762	1.046150162	5.710399108	0
Gm39772	4.184600649	0	0
Gm39785	0	0	0
Gm39786	5.230750811	0	2.897337897
Gm39796	0	0	0
Gm39807	0	0	0
Gm39828	0	4.568319287	1.931558598
Gm39847	0	0	0
Gm39860	1.046150162	1.142079822	0

For Review Only

Gm39869	9.41535146	12.56287804	4.828896494
Gm39872	1.046150162	2.284159643	0.965779299
Gm39874	0	0	0
Gm39878	9.41535146	11.42079822	4.828896494
Gm39882	36.61525568	29.69407536	23.17870317
Gm39883	4.184600649	5.710399108	0
Gm39887	0	5.710399108	0
Gm39888	0	0	0
Gm39897	20.92300324	33.12031483	13.52091018
Gm39908	8.369201298	7.994558752	7.726234391
Gm39922	1.046150162	0	0
Gm39931	0	0	0
Gm39938	2.092300324	0	0
Gm39939	223.8761347	78.80350769	88.85169549
Gm39954	34.52295535	39.97279376	36.69961336
Gm39988	19.87685308	63.95647001	37.66539266
Gm39990	0	0	0
Gm40041	3.138450487	0	0.965779299
Gm40051	0	1.142079822	0.965779299
Gm40078	37.66140584	34.26239465	28.00759967
Gm40080	50.21520779	17.13119732	22.21292387
Gm40091	5.230750811	2.284159643	6.760455092
Gm40096	5.230750811	0	0
Gm40103	7.323051136	5.710399108	5.794675793
Gm40106	5.230750811	0	0
Gm40116	4.184600649	13.70495786	0
Gm40117	3.138450487	2.284159643	0
Gm40126	13.59995211	3.426239465	9.657792988
Gm40128	0	0	0.965779299
Gm4013	0	0	0
Gm40142	0	0	0
Gm40150	3.138450487	3.426239465	8.69201369
Gm40180	0	0	3.863117195
Gm40181	4.184600649	12.56287804	18.34980668
Gm40191	3.138450487	0	5.794675793
Gm40194	2.092300324	2.284159643	2.897337897
Gm40208	107.7534667	97.07678484	61.80987513
Gm40218	8.369201298	6.85247893	6.760455092
Gm40224	2.092300324	9.136638573	4.828896494
Gm40225	0	6.85247893	0
Gm40229	0	0	0
Gm40237	0	0	0
Gm4024	218.6453839	126.7708602	177.703391
Gm40243	1.046150162	0	0
Gm40290	1.046150162	5.710399108	0
Gm40302	1.046150162	1.142079822	0
Gm40305	1.046150162	1.142079822	0
Gm40309	166.3378758	110.7817427	113.9619573
Gm40310	3.138450487	3.426239465	2.897337897
Gm40346	44.98445698	10.27871839	3.863117195

For Review Only

Gm40352	5.230750811	2.284159643	4.828896494
Gm40378	3.138450487	1.142079822	0.965779299
Gm40385	56.49210876	17.13119732	15.45246878
Gm40386	12.55380195	33.12031483	10.62357229
Gm40397	1.046150162	4.568319287	0
Gm40402	1.046150162	1.142079822	0
Gm40406	17.78455276	44.54111304	28.00759967
Gm40409	1.046150162	1.142079822	0
Gm40417	8.369201298	9.136638573	8.69201369
Gm40443	16.7384026	11.42079822	21.24714457
Gm40448	76.36896184	77.66142787	76.29656461
Gm40454	0	1.142079822	0
Gm40462	2.092300324	5.710399108	2.897337897
Gm40466	37.66140584	31.97823501	32.83649616
Gm40468	5.230750811	5.710399108	6.760455092
Gm40474	0	2.284159643	0
Gm40475	14.64610227	22.84159643	20.28136528
Gm40491	9.41535146	0	6.760455092
Gm40496	10.46150162	5.710399108	4.828896494
Gm40498	49.16905763	35.40447447	0
Gm40525	33.47680519	19.41535697	0
Gm40527	3.138450487	0	0
Gm40528	17.78455276	28.55199554	17.38402738
Gm40531	9.41535146	7.994558752	12.55513089
Gm40543	2.092300324	5.710399108	3.863117195
Gm40556	1.046150162	0	0
Gm40559	10.46150162	0	0
Gm40564	0	0	0
Gm40571	34.52295535	20.55743679	13.52091018
Gm40573	7.323051136	0	0
Gm40578	0	0	1.931558598
Gm40579	3.138450487	4.568319287	0
Gm40582	6.276900973	4.568319287	4.828896494
Gm40591	0	1.142079822	0
Gm40637	4.184600649	3.426239465	3.863117195
Gm40645	28.24605438	0	0
Gm40668	0	6.85247893	4.828896494
Gm40670	0	3.426239465	0
Gm40671	8.369201298	4.568319287	11.58935159
Gm40680	0	0	0.965779299
Gm40696	5.230750811	5.710399108	6.760455092
Gm4070	36.61525568	102.7871839	49.25474424
Gm40715	1.046150162	0	0
Gm40720	0	0	0
Gm40721	5.230750811	3.426239465	2.897337897
Gm40743	3.138450487	0	0
Gm40776	0	0	0
Gm40781	1.046150162	0	0
Gm40782	1.046150162	4.568319287	0
Gm40799	0	0	0

For Review Only

Gm40824	12.55380195	21.69951661	10.62357229
Gm40826	0	0	0
Gm40841	119.2611185	114.2079822	95.61215059
Gm40842	34.52295535	9.136638573	9.657792988
Gm40848	1.046150162	0	0
Gm40860	35.56910552	18.27327715	19.31558598
Gm40863	5.230750811	9.136638573	4.828896494
Gm40875	2.092300324	6.85247893	0.965779299
Gm40887	8.369201298	4.568319287	0.965779299
Gm40897	3.138450487	1.142079822	0
Gm40912	2.092300324	5.710399108	21.24714457
Gm40913	21.96915341	23.98367625	29.93915826
Gm40915	2.092300324	0	3.863117195
Gm40916	4.184600649	10.27871839	6.760455092
Gm40934	0	2.284159643	0
Gm40960	2.092300324	0	2.897337897
Gm40964	0	0	0
Gm40979	3.138450487	4.568319287	0
Gm40996	2.092300324	0	0
Gm41005	1.046150162	1.142079822	0
Gm41011	0	0	1.931558598
Gm41024	9.41535146	4.568319287	0
Gm41025	0	1.142079822	0
Gm41032	6.276900973	0	0
Gm41035	7.323051136	7.994558752	3.863117195
Gm41037	3.138450487	3.426239465	1.931558598
Gm41038	3.138450487	0	0
Gm41039	3.138450487	1.142079822	0
Gm41046	20.92300324	12.56287804	15.45246878
Gm41055	0	0	0
Gm41071	67.99976055	92.50846555	115.8935159
Gm41072	0	0	0
Gm41077	16.7384026	27.40991572	18.34980668
Gm41090	1.046150162	1.142079822	0
Gm41094	0	3.426239465	0.965779299
Gm41098	30.33835471	36.54655429	28.00759967
Gm41101	2.092300324	1.142079822	0
Gm41107	336.8603522	347.1922658	196.0531977
Gm41108	0	0	0
Gm41113	1.046150162	2.284159643	4.828896494
Gm41114	2.092300324	0	0
Gm41130	21.96915341	4.568319287	0
Gm41161	1.046150162	2.284159643	0
Gm41164	5.230750811	4.568319287	0
Gm4117	0	2.284159643	0
Gm41199	1.046150162	0	0.965779299
Gm41213	0	3.426239465	0.965779299
Gm41215	0	0	0
Gm41231	4.184600649	5.710399108	0
Gm41247	2.092300324	2.284159643	3.863117195

For Review Only

Gm41252	0	0	1.931558598
Gm41254	0	4.568319287	3.863117195
Gm41273	2.092300324	0	0
Gm41280	0	0	0
Gm41283	1.046150162	0	0
Gm41284	3.138450487	0	2.897337897
Gm41285	6.276900973	1.142079822	0.965779299
Gm41287	0	4.568319287	2.897337897
Gm41291	7.323051136	7.994558752	7.726234391
Gm41292	3.138450487	6.85247893	8.69201369
Gm41298	0	3.426239465	2.897337897
Gm41299	0	0	0
Gm41308	1.046150162	1.142079822	0
Gm41330	21.96915341	10.27871839	8.69201369
Gm41338	18.83070292	21.69951661	7.726234391
Gm41343	2.092300324	0	3.863117195
Gm41344	3.138450487	0	0
Gm41358	0	0	0
Gm41361	2.092300324	2.284159643	5.794675793
Gm41362	0	0	0
Gm41368	3.138450487	10.27871839	0
Gm41377	0	0	0
Gm41389	12.55380195	2.284159643	0
Gm41395	3.138450487	2.284159643	2.897337897
Gm41406	0	0	1.931558598
Gm41410	8.369201298	5.710399108	5.794675793
Gm41414	1.046150162	1.142079822	0
Gm41442	4.184600649	4.568319287	5.794675793
Gm41453	5.230750811	12.56287804	10.62357229
Gm41476	3.138450487	3.426239465	1.931558598
Gm41485	5.230750811	0	0
Gm41489	0	0	0
Gm41491	6.276900973	10.27871839	12.55513089
Gm41498	15.69225243	12.56287804	5.794675793
Gm41514	1.046150162	0	0.965779299
Gm41523	2.092300324	1.142079822	0
Gm41525	31.38450487	22.84159643	17.38402738
Gm41526	21.96915341	19.41535697	10.62357229
Gm41549	2.092300324	0	0
Gm41553	16.7384026	46.82527269	28.00759967
Gm41556	25.10760389	17.13119732	11.58935159
Gm41569	3.138450487	1.142079822	0
Gm41572	0	0	0
Gm41590	25.10760389	3.426239465	9.657792988
Gm41598	30.33835471	35.40447447	52.15208214
Gm41604	1.046150162	4.568319287	0
Gm41607	29.29220454	28.55199554	21.24714457
Gm41610	16.7384026	19.41535697	6.760455092
Gm41611	24.06145373	11.42079822	12.55513089
Gm41620	17.78455276	18.27327715	9.657792988

For Review Only

Gm41622	3.138450487	31.97823501	24.14448247
Gm41627	0	0	0
Gm41640	19.87685308	26.2678359	11.58935159
Gm41662	11.50765178	0	10.62357229
Gm41666	0	0	0
Gm41672	7.323051136	13.70495786	9.657792988
Gm41676	0	0	0
Gm41678	0	10.27871839	8.69201369
Gm41687	0	0	0
Gm41688	9.41535146	12.56287804	0
Gm41689	5.230750811	0	0
Gm41693	20.92300324	7.994558752	7.726234391
Gm41696	3.138450487	2.284159643	4.828896494
Gm41702	0	0	0.965779299
Gm41705	3.138450487	3.426239465	0
Gm41719	1.046150162	2.284159643	0
Gm41748	4.184600649	17.13119732	10.62357229
Gm41750	1.046150162	0	5.794675793
Gm41778	0	0	0
Gm41790	0	0	0.965779299
Gm41803	0	4.568319287	0
Gm41818	0	5.710399108	0
Gm41829	2.092300324	1.142079822	0
Gm41834	0	1.142079822	0.965779299
Gm4184	12.55380195	11.42079822	17.38402738
Gm41846	3.138450487	13.70495786	6.760455092
Gm41861	0	3.426239465	0
Gm41883	1.046150162	0	0.965779299
Gm41884	3.138450487	5.710399108	0
Gm41885	93.10736444	63.95647001	0
Gm41909	1.046150162	1.142079822	7.726234391
Gm41920	0	3.426239465	2.897337897
Gm41934	3.138450487	0	0
Gm41947	1.046150162	7.994558752	7.726234391
Gm41949	66.95361038	60.53023055	89.81747479
Gm41962	4.184600649	2.284159643	0
Gm41964	1.046150162	6.85247893	0
Gm41983	1.046150162	1.142079822	0.965779299
Gm41984	0	3.426239465	2.897337897
Gm42002	19.87685308	20.55743679	18.34980668
Gm42004	6.276900973	0	0
Gm42005	13.59995211	6.85247893	13.52091018
Gm42021	1.046150162	3.426239465	0
Gm42023	3.138450487	3.426239465	0.965779299
Gm42027	66.95361038	71.95102876	11.58935159
Gm42035	227.0145852	95.93470502	135.2091018
Gm42042	28.24605438	42.2569534	79.19390251
Gm42043	46.03060714	42.2569534	43.46006845
Gm42059	0	0	0
Gm42063	0	0	0

For Review Only

Gm42074	7.323051136	17.13119732	7.726234391
Gm42078	5.230750811	1.142079822	0.965779299
Gm42084	0	0	0
Gm42085	0	1.142079822	0
Gm42090	5.230750811	1.142079822	0
Gm42094	15.69225243	23.98367625	9.657792988
Gm42102	0	1.142079822	0
Gm42111	7.323051136	5.710399108	17.38402738
Gm42127	11.50765178	27.40991572	6.760455092
Gm42135	1.046150162	2.284159643	0
Gm42139	12.55380195	9.136638573	19.31558598
Gm42145	3.138450487	2.284159643	0.965779299
Gm42147	3.138450487	3.426239465	0
Gm42158	42.89215665	9.136638573	18.34980668
Gm42161	0	0	0
Gm42162	6.276900973	7.994558752	4.828896494
Gm42166	12.55380195	14.84703768	17.38402738
Gm42198	1.046150162	2.284159643	0
Gm42199	2.092300324	9.136638573	4.828896494
Gm42202	0	2.284159643	0
Gm4221	7.323051136	2.284159643	0.965779299
Gm42219	0	3.426239465	0
Gm42226	6.276900973	5.710399108	5.794675793
Gm42232	0	0	0
Gm42244	0	0	0
Gm42250	1.046150162	3.426239465	9.657792988
Gm42253	7.323051136	1.142079822	0
Gm42259	11.50765178	18.27327715	7.726234391
Gm42267	1.046150162	0	0
Gm42278	3.138450487	0	0
Gm42282	4.184600649	7.994558752	4.828896494
Gm42307	1.046150162	5.710399108	10.62357229
Gm42309	0	2.284159643	0
Gm42326	1.046150162	5.710399108	0.965779299
Gm42338	0	3.426239465	0
Gm42343	2.092300324	4.568319287	6.760455092
Gm42344	1.046150162	1.142079822	0
Gm42351	9.41535146	13.70495786	18.34980668
Gm42355	0	1.142079822	0
Gm42357	0	1.142079822	0
Gm42362	0	27.40991572	0
Gm42364	4.184600649	11.42079822	15.45246878
Gm42368	31.38450487	38.83071394	39.59695125
Gm42372	0	5.710399108	3.863117195
Gm4258	3.138450487	5.710399108	0
Gm4285	0	11.42079822	3.863117195
Gm4349	0	0	0
Gm4419	0	0	0
Gm4432	1.046150162	1.142079822	2.897337897
Gm4450	2.092300324	2.284159643	0

For Review Only

Gm44502	128.67647	108.4975831	0
Gm44504	0	26.2678359	0
Gm44505	7.323051136	5.710399108	0.965779299
Gm4473	3.138450487	0	0
Gm4532	0	0	0.965779299
Gm4544	0	1.142079822	3.863117195
Gm4598	2.092300324	1.142079822	2.897337897
Gm4604	33.47680519	19.41535697	29.93915826
Gm4632	0	1.142079822	0
Gm4651	1.046150162	0	0
Gm4705	23.01530357	18.27327715	28.97337897
Gm4724	4.184600649	7.994558752	5.794675793
Gm4737	14.64610227	7.994558752	10.62357229
Gm4759	0	3.426239465	0.965779299
Gm4767	13.59995211	7.994558752	8.69201369
Gm4786	13.59995211	15.9891175	19.31558598
Gm4787	1.046150162	19.41535697	0
Gm4788	46.03060714	46.82527269	72.43344741
Gm4793	11.50765178	0	9.657792988
Gm4814	0	0	0
Gm4841	0	0	0
Gm4887	8.369201298	4.568319287	2.897337897
Gm4890	1.046150162	2.284159643	3.863117195
Gm4924	56.49210876	14.84703768	22.21292387
Gm4925	4.184600649	1.142079822	0
Gm4943	3.138450487	9.136638573	4.828896494
Gm4944	37.66140584	38.83071394	35.73383406
Gm4951	4.184600649	6.85247893	9.657792988
Gm4961	0	3.426239465	4.828896494
Gm4997	6.276900973	4.568319287	4.828896494
Gm5067	0	1.142079822	0.965779299
Gm5069	4.184600649	4.568319287	0
Gm5086	0	235.2684433	0
Gm5088	5.230750811	18.27327715	12.55513089
Gm5093	16.7384026	23.98367625	19.31558598
Gm5106	0	1.142079822	0
Gm5113	7.323051136	14.84703768	5.794675793
Gm5115	3.138450487	2.284159643	0.965779299
Gm5122	0	1.142079822	0
Gm5124	0	0	0.965779299
Gm5127	0	0	0
Gm5129	2.092300324	1.142079822	5.794675793
Gm5134	11.50765178	6.85247893	7.726234391
Gm5141	25.10760389	47.96735251	28.00759967
Gm5148	125.5380195	137.0495786	157.4220257
Gm5420	6.276900973	3.426239465	0
Gm5424	1.046150162	0	4.828896494
Gm5431	42.89215665	44.54111304	0
Gm5434	8.369201298	7.994558752	5.794675793
Gm5454	39.75370617	33.12031483	41.52850985

For Review Only

Gm5464	0	7.994558752	2.897337897
Gm5481	0	0	0
Gm5486	0	0	0
Gm5512	57.53825892	44.54111304	53.11786144
Gm5563	10.46150162	0	0
Gm5566	7.323051136	9.136638573	15.45246878
Gm5590	4.184600649	0	0
Gm5593	1.046150162	0	0
Gm5595	36.61525568	13.70495786	28.97337897
Gm561	11.50765178	19.41535697	24.14448247
Gm5617	20.92300324	23.98367625	0
Gm5621	55.4459586	50.25151215	56.98097863
Gm5643	62.76900973	38.83071394	40.56273055
Gm572	0	6.85247893	0
Gm5766	1.046150162	0	0
Gm5785	9.41535146	11.42079822	6.760455092
Gm5796	6.276900973	7.994558752	8.69201369
Gm5801	12.55380195	7.994558752	12.55513089
Gm5803	17.78455276	13.70495786	10.62357229
Gm5820	3.138450487	4.568319287	3.863117195
Gm5854	7.323051136	4.568319287	8.69201369
Gm5879	157.9686745	151.8966163	182.5322875
Gm5917	11.50765178	9.136638573	8.69201369
Gm5946	7.323051136	6.85247893	0.965779299
Gm6109	5.230750811	6.85247893	6.760455092
Gm6158	20.92300324	30.83615518	26.07604107
Gm6194	5.230750811	1.142079822	1.931558598
Gm6225	2.092300324	5.710399108	2.897337897
Gm6252	48.12290746	43.39903322	52.15208214
Gm6277	1.046150162	1.142079822	0
Gm6288	0	0	0
Gm6297	3.138450487	1.142079822	0
Gm6306	1.046150162	0	0
Gm6345	0	1.142079822	0.965779299
Gm6402	31.38450487	37.68863411	0
Gm6410	1.046150162	6.85247893	0
Gm6445	1.046150162	0	0
Gm6485	143.3225722	91.36638573	113.9619573
Gm6498	4.184600649	7.994558752	10.62357229
Gm6524	10.46150162	5.710399108	6.760455092
Gm6525	1.046150162	2.284159643	0.965779299
Gm6548	131.8149204	147.328297	141.0037776
Gm6566	7.323051136	1.142079822	0
Gm6568	12.55380195	11.42079822	7.726234391
Gm6570	13.59995211	19.41535697	16.41824808
Gm6579	4.184600649	4.568319287	3.863117195
Gm6583	20.92300324	14.84703768	19.31558598
Gm6598	3.138450487	4.568319287	0
Gm6623	20.92300324	18.27327715	21.24714457
Gm6644	53.35365827	81.08766734	86.9201369

For Review Only

Gm6654	37.66140584	36.54655429	44.42584775
Gm6710	16.7384026	11.42079822	13.52091018
Gm6712	24.06145373	59.38815073	58.91253723
Gm6713	1.046150162	2.284159643	0
Gm6745	44.98445698	62.81439019	49.25474424
Gm6750	13.59995211	7.994558752	9.657792988
Gm6756	4.184600649	1.142079822	4.828896494
Gm6793	46.03060714	57.10399108	34.76805476
Gm6815	0	1.142079822	0.965779299
Gm6904	0	0	1.931558598
Gm6978	17.78455276	20.55743679	32.83649616
Gm6988	33.47680519	44.54111304	74.36500601
Gm7020	15.69225243	7.994558752	4.828896494
Gm7030	4.184600649	0	5.794675793
Gm7072	64.86131006	68.5247893	55.04942003
Gm7099	19.87685308	18.27327715	23.17870317
Gm7102	0	0	1.931558598
Gm7104	0	1.142079822	0
Gm7120	2.092300324	13.70495786	4.828896494
Gm7265	2.092300324	0	0.965779299
Gm7285	25.10760389	23.98367625	32.83649616
Gm7334	4.184600649	7.994558752	1.931558598
Gm7361	3.138450487	0	0
Gm7367	9.41535146	14.84703768	1.931558598
Gm7429	27.19990422	29.69407536	30.90493756
Gm7443	0	2.284159643	0
Gm7444	0	1.142079822	2.897337897
Gm7609	2.092300324	1.142079822	0
Gm7694	30.33835471	39.97279376	64.70721302
Gm7710	92.06121428	116.4921418	124.5855296
Gm7846	0	4.568319287	6.760455092
Gm7854	0	1.142079822	0.965779299
Gm7855	2.092300324	1.142079822	0.965779299
Gm7866	51.26135795	46.82527269	48.28896494
Gm7889	4.184600649	0	4.828896494
Gm7931	26.15375406	18.27327715	21.24714457
Gm7936	8.369201298	13.70495786	13.52091018
Gm7972	1.046150162	1.142079822	0
Gm7977	1.046150162	1.142079822	0
Gm8013	0	0	0.965779299
Gm8069	10.46150162	14.84703768	22.21292387
Gm8096	4.184600649	3.426239465	5.794675793
Gm8149	37.66140584	38.83071394	38.63117195
Gm8203	154.830224	185.0169311	237.5817075
Gm8210	0	0	1.931558598
Gm8234	1.046150162	1.142079822	0
Gm826	4.184600649	0	0
Gm8273	4.184600649	10.27871839	2.897337897
Gm8350	2.092300324	0	0
Gm8363	0	1.142079822	2.897337897

For Review Only

Gm8430	104.6150162	121.0604611	135.2091018
Gm8451	56.49210876	58.2460709	71.46766811
Gm8465	4.184600649	4.568319287	1.931558598
Gm8493	0	2.284159643	0
Gm8580	1.046150162	3.426239465	1.931558598
Gm8615	61.72285957	44.54111304	60.84409583
Gm8623	47.0767573	45.68319287	50.22052354
Gm8624	7.323051136	9.136638573	11.58935159
Gm8675	2.092300324	0	0
Gm8702	3.138450487	2.284159643	3.863117195
Gm8801	36.61525568	14.84703768	24.14448247
Gm884	0	4.568319287	2.897337897
Gm8909	0	0	0
Gm8942	37.66140584	34.26239465	45.39162705
Gm8979	4.184600649	7.994558752	7.726234391
Gm8989	3.138450487	4.568319287	7.726234391
Gm8994	7.323051136	10.27871839	19.31558598
Gm8995	51.26135795	182.7327715	67.60455092
Gm9054	4.184600649	3.426239465	2.897337897
Gm9079	3.138450487	3.426239465	0
Gm9159	1.046150162	6.85247893	1.931558598
Gm9199	0	0	0.965779299
Gm9222	30.33835471	9.136638573	19.31558598
Gm9234	94.1535146	103.9292638	127.4828674
Gm9385	154.830224	84.5139068	154.5246878
Gm9457	1.046150162	0	0
Gm9465	3.138450487	1.142079822	2.897337897
Gm9484	17.78455276	14.84703768	10.62357229
Gm9530	1.046150162	0	8.69201369
Gm960	3.138450487	0	1.931558598
Gm9618	5.230750811	5.710399108	0
Gm9706	16.7384026	20.55743679	9.657792988
Gm9769	98.33811525	78.80350769	46.35740634
Gm9776	1.046150162	6.85247893	6.760455092
Gm9794	275.1374927	268.3887581	408.5246434
Gm9833	3.138450487	3.426239465	2.897337897
Gm9840	34.52295535	22.84159643	31.87071686
Gm9855	12.55380195	10.27871839	16.41824808
Gm9861	5.230750811	4.568319287	0
Gm9878	3.138450487	0	0
Gm9903	5.230750811	1.142079822	0
Gm9919	0	3.426239465	0
Gm9920	0	0	0
Gm9949	3.138450487	1.142079822	16.41824808
Gm9958	3.138450487	7.994558752	3.863117195
Gm996	79.50741233	108.4975831	72.43344741
Gm9970	0	0	0
Gmcl1	10.46150162	17.13119732	7.726234391
Gmds	14.64610227	15.9891175	22.21292387
Gmeb1	245.8452881	130.1970997	203.7794321

For Review Only

Gmeb2	30.33835471	20.55743679	14.48668948
Gmfb	144.3687224	174.7382127	130.3802053
Gmfg	0	25.12575608	0
Gmip	80.55356249	190.7273302	0.965779299
Gmnn	4.184600649	15.9891175	10.62357229
Gmppa	26.15375406	26.2678359	0
Gmppb	0	54.81983144	0
Gmpr	193.53778	165.6015741	164.1824808
Gmpr2	82.64586282	4.568319287	141.0037776
Gmps	155.8763742	140.4758181	126.5170881
Gna11	1.046150162	0	0
Gna12	0	68.5247893	5.794675793
Gna13	394.3986112	388.3071394	284.9048932
Gna14	0	1.142079822	0
Gna15	685.2283563	757.1989218	1016.965602
Gnai2	212.3684829	629.2859817	309.0493756
Gnai3	43.93830681	115.350062	302.2889205
Gnal	4.184600649	6.85247893	6.760455092
Gnao1	0	1.142079822	0
Gnaq	176.7993774	181.5906916	199.9163149
Gnas	96.24581493	190.7273302	92.71481269
Gnaz	3.138450487	1.142079822	0
Gnb1	238.522237	615.5810239	538.9048488
Gnb1l	14.64610227	30.83615518	9.657792988
Gnb2	1.046150162	0	2.897337897
Gnb4	1.046150162	0	10.62357229
Gnb5	14.64610227	13.70495786	15.45246878
Gne	100.4304156	63.95647001	73.39922671
Gng10	1030.45791	0	101.4068264
Gng11	0	0	0
Gng12	196.6762305	63.95647001	63.74143372
Gng2	196.6762305	199.8639688	217.3003422
Gng3	0	1.142079822	0.965779299
Gng5	135.9995211	137.0495786	153.5589085
Gng7	19.87685308	1.142079822	20.28136528
Gngt2	166.3378758	218.1372459	260.7604107
Gnl1	54.39980844	14.84703768	61.80987513
Gnl2	159.0148247	85.65598662	206.67677
Gnl3	67.99976055	46.82527269	39.59695125
Gnl3l	23.01530357	36.54655429	24.14448247
Gnmt	0	0	8.69201369
Gnpat	243.7529878	157.6070154	218.2661215
Gnpda1	187.260879	55.96191126	138.1064397
Gnpda2	42.89215665	78.80350769	76.29656461
Gnpnat1	20.92300324	33.12031483	18.34980668
Gnptab	19.87685308	23.98367625	18.34980668
Gnptg	0	0	0
Gnrh1	1.046150162	0	0
Gns	1149.719028	1491.556247	1206.258344
Golga1	34.52295535	73.09310859	49.25474424

For Review Only

Golga2	195.6300803	159.891175	75.33078531
Golga3	33.47680519	45.68319287	39.59695125
Golga4	118.2149683	149.6124566	123.6197503
Golga5	84.73816314	90.22430591	112.996178
Golga7	178.8916777	137.0495786	154.5246878
Golgb1	71.13821103	111.9238225	66.63877162
Golim4	62.76900973	36.54655429	54.08364074
Golm1	3541.218299	198.721889	840.22799
Golph3	41.84600649	38.83071394	25.11026177
Golph3l	78.46126217	75.37726823	87.8859162
Golt1b	188.3070292	260.3941993	424.9428915
Gon4l	109.845767	107.3555032	151.6273499
Gopc	20.92300324	85.65598662	37.66539266
Gorab	12.55380195	35.40447447	28.00759967
Gorasp1	162.1532751	92.50846555	93.68059199
Gorasp2	347.3218539	239.8367625	402.7299676
Gosr1	157.9686745	81.08766734	63.74143372
Gosr2	275.1374927	27.40991572	106.2357229
Got1	60.67670941	26.2678359	80.1596818
Got2	54.39980844	34.26239465	48.28896494
Gp1ba	9.41535146	5.710399108	8.69201369
Gp5	0	0	0
Gp9	37.66140584	81.08766734	87.8859162
Gpa33	0	0	0
Gpaa1	0	0	97.54370918
Gpalpp1	38.707556	67.38270948	37.66539266
Gpam	76.36896184	38.83071394	55.04942003
Gpank1	26.15375406	27.40991572	32.83649616
Gpat2	0	0	2.897337897
Gpat4	127.6303198	151.8966163	223.095018
Gpatch1	50.21520779	33.12031483	28.00759967
Gpatch11	19.87685308	20.55743679	29.93915826
Gpatch2	101.4765657	105.0713436	87.8859162
Gpatch2l	117.1688182	124.4867006	106.2357229
Gpatch3	8.369201298	6.85247893	0
Gpatch4	16.7384026	15.9891175	15.45246878
Gpatch8	1.046150162	1.142079822	0
Gpbar1	1.046150162	0	1.931558598
Gpbp1	117.1688182	146.1862172	128.4486467
Gpbp1l1	32.43065503	60.53023055	31.87071686
Gpc1	1.046150162	0	0
Gpc2	24.06145373	17.13119732	0
Gpc4	4.184600649	0	0
Gpc6	5.230750811	0	0
Gpcpd1	122.399569	140.4758181	161.2851429
Gpd1	1.046150162	1.142079822	61.80987513
Gpd1l	171.5686266	131.3391795	123.6197503
Gpd2	6.276900973	0	13.52091018
Gper1	2.092300324	1.142079822	0
Gphn	44.98445698	60.53023055	33.80227546

For Review Only

Gpi1	315.937349	212.4268468	169.0113773
Gpkow	72.18436119	69.66686912	54.08364074
Gpm6a	28.24605438	0	0
Gpm6b	6.276900973	4.568319287	0
Gpn1	108.7996169	60.53023055	101.4068264
Gpn2	35.56910552	33.12031483	0
Gpn3	14.64610227	41.11487358	30.90493756
Gpr107	254.2144894	216.9951661	234.6843696
Gpr108	146.4610227	52.5356718	141.9695569
Gpr132	0	5.710399108	3.863117195
Gpr137	61.72285957	76.51934805	84.9885783
Gpr137b	92.06121428	0	0
Gpr137b-ps	0	0	37.66539266
Gpr137c	3.138450487	0	0
Gpr146	406.9524131	356.3289044	453.9162705
Gpr150	5.230750811	3.426239465	0.965779299
Gpr153	0	0	0
Gpr155	356.7372053	312.9298711	278.1444381
Gpr157	131.8149204	92.50846555	90.78325409
Gpr160	224.9222849	202.1481284	186.3954047
Gpr161	1.046150162	2.284159643	0.965779299
Gpr162	2.092300324	3.426239465	3.863117195
Gpr165	100.4304156	207.8585275	160.3193636
Gpr179	0	0	0
Gpr18	8.369201298	1.142079822	0
Gpr180	51.26135795	49.10943233	42.49428915
Gpr182	2.092300324	0	0
Gpr183	0	455.6898488	577.5360207
Gpr19	2.092300324	1.142079822	12.55513089
Gpr20	0	0	0
Gpr21	2.092300324	0	3.863117195
Gpr22	1.046150162	1.142079822	0
Gpr3	1.046150162	0	0
Gpr31b	1.046150162	1.142079822	0
Gpr34	3608.17191	3911.623389	5390.980046
Gpr35	40.79985633	21.69951661	63.74143372
Gpr4	3.138450487	1.142079822	2.897337897
Gpr52	0	0	0
Gpr65	19.87685308	39.97279376	13.52091018
Gpr68	1.046150162	1.142079822	0
Gpr75	0	0	0
Gpr82	9.41535146	4.568319287	0
Gpr84	28.24605438	98.21886466	0
Gpr89	61.72285957	54.81983144	73.39922671
Gprasp1	127.6303198	117.6342216	137.1406604
Gprasp2	96.24581493	63.95647001	61.80987513
Gprc5c	7.323051136	0	0
Gprin2	0	1.142079822	0
Gps1	0	1.142079822	0
Gps2	0	124.4867006	0

For Review Only

Gpsm1	130.7687703	67.38270948	91.74903339
Gpsm2	0	0	0.965779299
Gpsm3	0	0	0
Gpt	0	0	0
Gpt2	70.09206087	30.83615518	47.32318564
Gpx1	0	180.4486118	192.1900805
Gpx3	0	0	0
Gpx4	7.323051136	6.85247893	0
Gpx7	2.092300324	4.568319287	2.897337897
Gpx8	8.369201298	0	0
Gramd1a	208.1838823	270.6729177	131.3459846
Gramd1b	55.4459586	23.98367625	42.49428915
Gramd3	0	10.27871839	0.965779299
Gramd4	17.78455276	13.70495786	33.80227546
Grap	147.5071729	274.0991572	355.406782
Grasp	0	0	0
Grb2	86.83046347	125.6287804	132.3117639
Grcc10	0	0	180.6007289
Greb1l	0	4.568319287	6.760455092
Grhl1	0	5.710399108	0
Grhpr	29.29220454	27.40991572	27.04182037
Grik5	0	0	0
Grin2c	0	0	2.897337897
Grin3a	3.138450487	1.142079822	0
Grin3b	7.323051136	3.426239465	6.760455092
Grina	41.84600649	0	0
Grip2	5.230750811	2.284159643	7.726234391
Gripap1	0	78.80350769	112.0303987
Grk4	6.276900973	6.85247893	4.828896494
Grk5	3.138450487	7.994558752	0
Grk6	42.89215665	23.98367625	43.46006845
Grm1	0	4.568319287	0
Grm3	0	0	14.48668948
Grn	3118.573634	0	2989.08693
Grpel1	0	0	32.83649616
Grpel2	35.56910552	65.09854983	63.74143372
Grsf1	49.16905763	60.53023055	27.04182037
Grtp1	5.230750811	2.284159643	7.726234391
Grwd1	40.79985633	47.96735251	48.28896494
Gsap	135.9995211	213.5689267	227.9239145
Gsdmcl-ps	2.092300324	0	4.828896494
Gsdmd	0	157.6070154	40.56273055
Gse1	104.6150162	107.3555032	112.996178
Gsg1	38.707556	21.69951661	41.52850985
Gsg2	19.87685308	27.40991572	37.66539266
Gsk3a	0	14.84703768	1.931558598
Gsk3b	290.8297451	322.0665097	239.5132661
Gskip	62.76900973	77.66142787	74.36500601
Gsn	40.79985633	11.42079822	7.726234391
Gspt1	143.3225722	148.4703768	122.653971

For Review Only

Gspt2	0	0	4.828896494
Gsr	8.369201298	7.994558752	10.62357229
Gss	17.78455276	22.84159643	29.93915826
Gsta4	0	0	26.07604107
Gstcd	18.83070292	17.13119732	12.55513089
Gstk1	0	0	10.62357229
Gstm1	21.96915341	6.85247893	12.55513089
Gstm2	5.230750811	0	0
Gstm4	27.19990422	18.27327715	15.45246878
Gstm5	11.50765178	0	45.39162705
Gstm7	0	0	0
Gsto1	41.84600649	39.97279376	46.35740634
Gsto2	1.046150162	0	0
Gstp1	106.7073165	94.7926252	109.1330608
Gstp2	24.06145373	25.12575608	29.93915826
Gstt1	0	0	0
Gstt2	9.41535146	0	0
Gstt3	26.15375406	6.85247893	12.55513089
Gstz1	43.93830681	30.83615518	42.49428915
Gt(ROSA)26Sor	4.184600649	22.84159643	46.35740634
Gtdc1	59.63055925	27.40991572	45.39162705
Gtf2a1	187.260879	138.1916584	154.5246878
Gtf2a2	46.03060714	37.68863411	53.11786144
Gtf2b	42.89215665	114.2079822	102.3726057
Gtf2e1	67.99976055	78.80350769	73.39922671
Gtf2e2	44.98445698	51.39359197	48.28896494
Gtf2f1	74.27666152	75.37726823	88.85169549
Gtf2f2	54.39980844	23.98367625	41.52850985
Gtf2h1	49.16905763	45.68319287	44.42584775
Gtf2h2	393.352461	357.4709842	423.9771122
Gtf2h3	30.33835471	20.55743679	19.31558598
Gtf2h4	0	0	0.965779299
Gtf2h5	23.01530357	37.68863411	37.66539266
Gtf2i	290.8297451	237.5526029	250.1368384
Gtf2ird1	26.15375406	28.55199554	20.28136528
Gtf2ird2	55.4459586	46.82527269	69.53610952
Gtf3a	84.73816314	83.37182698	56.01519933
Gtf3c1	96.24581493	76.51934805	59.87831653
Gtf3c2	431.0138668	149.6124566	433.6349052
Gtf3c3	62.76900973	79.94558752	65.67299232
Gtf3c4	51.26135795	63.95647001	59.87831653
Gtf3c5	28.24605438	35.40447447	53.11786144
Gtf3c6	24.06145373	97.07678484	45.39162705
Gtpbp1	140.1841217	210.1426872	182.5322875
Gtpbp10	26.15375406	2.284159643	20.28136528
Gtpbp2	122.399569	109.6396629	84.9885783
Gtpbp3	10.46150162	0	0
Gtpbp4	41.84600649	65.09854983	91.74903339
Gtpbp6	0	0	0
Gtpbp8	46.03060714	55.96191126	38.63117195

For Review Only

Gtse1	0	0	0
Guca1a	12.55380195	10.27871839	18.34980668
Guca1b	2.092300324	5.710399108	0
Gucd1	15.69225243	0	48.28896494
Gucy1a2	0	0	0
Gucy2f	0	0	0
Guf1	5.230750811	7.994558752	0
Guk1	33.47680519	12.56287804	51.18630284
Gusb	758.4588676	91.36638573	305.1862584
Gvin1	38.707556	111.9238225	51.18630284
Gxylt1	69.04591071	86.79806645	51.18630284
Gyg	85.7843133	61.67231037	75.33078531
Gys1	12.55380195	21.69951661	23.17870317
Gzf1	54.39980844	95.93470502	15.45246878
Gzmm	5.230750811	1.142079822	2.897337897
H13	279.3220933	246.6892415	267.5208658
H1f0	54.39980844	44.54111304	19.31558598
H2-Aa	82.64586282	41.11487358	53.11786144
H2-Ab1	156.9225243	7.994558752	0
H2-Bl	4.184600649	3.426239465	9.657792988
H2-D1	0	0	0
H2-DMa	526.2135316	367.7497026	644.1747923
H2-DMb1	295.0143458	339.197707	422.0455536
H2-DMb2	40.79985633	93.65054538	54.08364074
H2-Eb1	58.58440909	35.40447447	0
H2-Eb2	0	0	0
H2-K1	1089.042319	183.8748513	485.7869873
H2-M3	1.046150162	0	0
H2-Oa	25.10760389	4.568319287	189.2927426
H2-Ob	69.04591071	5.710399108	0
H2-Q1	27.19990422	23.98367625	33.80227546
H2-Q10	2.092300324	2.284159643	0
H2-Q2	15.69225243	9.136638573	19.31558598
H2-Q4	0	23.98367625	0
H2-Q5	1.046150162	2.284159643	0
H2-Q7	27.19990422	2.284159643	0
H2-T-ps	18.83070292	17.13119732	47.32318564
H2-T10	10.46150162	11.42079822	12.55513089
H2-T22	0	0	9.657792988
H2-T23	0	45.68319287	56.01519933
H2-T24	0	0	4.828896494
H2afj	46.03060714	75.37726823	65.67299232
H2afv	41.84600649	49.10943233	0
H2afx	25.10760389	20.55743679	28.00759967
H2afy	185.1685787	188.4431706	141.9695569
H2afy3	1.046150162	2.284159643	0
H2afz	0	0	33.80227546
H3f3a	96.24581493	87.94014627	103.338385
H3f3c	20.92300324	18.27327715	27.04182037
H60b	4.184600649	9.136638573	3.863117195

For Review Only

H6pd	98.33811525	77.66142787	98.50948848
Haao	34.52295535	29.69407536	27.04182037
Habp4	12.55380195	18.27327715	8.69201369
Hacd1	13.59995211	2.284159643	0
Hacd2	37.66140584	53.67775162	46.35740634
Hacd3	284.5528441	196.4377293	235.6501489
Hacd4	27.19990422	20.55743679	0
Hace1	15.69225243	0	8.69201369
Hacl1	0	0	3.863117195
Hadh	86.83046347	101.6451041	67.60455092
Hadha	214.4607833	213.5689267	206.67677
Hadhb	132.8610706	95.93470502	139.072219
Hagh	118.2149683	162.1753347	147.7642327
Haghl	12.55380195	13.70495786	34.76805476
Hap1	0	5.710399108	6.760455092
Harbi1	73.23051136	30.83615518	56.98097863
Hars	81.59971265	100.5030243	82.0912404
Hars2	44.98445698	106.2134234	129.414426
Has3	8.369201298	3.426239465	0
Hat1	23.01530357	134.765419	68.57033022
Haus1	14.64610227	17.13119732	18.34980668
Haus2	42.89215665	15.9891175	38.63117195
Haus3	36.61525568	45.68319287	31.87071686
Haus4	0	1.142079822	2.897337897
Haus5	24.06145373	11.42079822	0
Haus6	21.96915341	29.69407536	28.00759967
Haus7	26.15375406	13.70495786	56.98097863
Haus8	8.369201298	9.136638573	20.28136528
Havcr2	423.6908157	367.7497026	511.8630284
Hax1	0	0	11.58935159
Hbegf	18.83070292	13.70495786	14.48668948
Hbp1	274.0913425	279.8095563	392.1063953
Hbs1l	91.01506412	81.08766734	80.1596818
Hcar2	42.89215665	43.39903322	28.00759967
Hccs	41.84600649	29.69407536	27.04182037
Hcfc1	25.10760389	9.136638573	0
Hcfc1r1	0	49.10943233	0
Hcfc2	41.84600649	39.97279376	58.91253723
Hck	565.9672378	551.6245539	605.5436204
Hcls1	426.8292662	408.8645762	392.1063953
Hcn3	10.46150162	1.142079822	0
Hcst	13.59995211	0	39.59695125
Hdac1	56.49210876	66.24062966	67.60455092
Hdac10	16.7384026	0	0
Hdac11	43.93830681	71.95102876	49.25474424
Hdac2	18.83070292	29.69407536	0
Hdac3	60.67670941	274.0991572	83.0570197
Hdac4	69.04591071	66.24062966	60.84409583
Hdac5	65.90746022	47.96735251	0
Hdac6	69.04591071	21.69951661	44.42584775

For Review Only

Hdac7	0	0	0
Hdac8	42.89215665	54.81983144	39.59695125
Hdac9	18.83070292	43.39903322	31.87071686
Hdc	9.41535146	4.568319287	0
Hddc2	5.230750811	0	3.863117195
Hddc3	6.276900973	9.136638573	23.17870317
Hdgf	41.84600649	43.39903322	42.49428915
Hdgfrp2	55.4459586	15.9891175	0
Hdgfrp3	54.39980844	95.93470502	105.2699436
Hdhd2	60.67670941	53.67775162	61.80987513
Hdhd3	2.092300324	0	12.55513089
Hdlbp	367.1987069	309.5036317	338.0227546
Heatr1	99.38426541	92.50846555	92.71481269
Heatr3	50.21520779	17.13119732	59.87831653
Heatr4	2.092300324	0	0
Heatr5a	209.2300324	248.9734011	187.361184
Heatr5b	72.18436119	52.5356718	70.50188882
Heatr6	71.13821103	76.51934805	51.18630284
Hebp1	13.59995211	27.40991572	17.38402738
Hebp2	5.230750811	0	0
Heca	32.43065503	99.36094448	60.84409583
Hectd1	135.9995211	185.0169311	160.3193636
Hectd2	6.276900973	0	0
Hectd3	210.2761826	85.65598662	90.78325409
Hecw2	0	0	0
Heg1	0	0	0.965779299
Helb	41.84600649	4.568319287	17.38402738
Hells	4.184600649	0	0
Helq	13.59995211	14.84703768	14.48668948
Helz	163.1994253	175.8802925	201.8478735
Helz2	3.138450487	5.710399108	14.48668948
Hemk1	49.16905763	22.84159643	42.49428915
Henmt1	0	0	0
Herc1	248.9837386	280.9516361	281.041776
Herc2	490.6444261	881.6856223	539.8706281
Herc3	9.41535146	11.42079822	3.863117195
Herc4	71.13821103	153.0386961	112.996178
Herc6	29.29220454	31.97823501	51.18630284
Herpud1	738.5820145	654.4117378	580.4333586
Herpud2	34.52295535	35.40447447	42.49428915
Hes1	7.323051136	0	1.931558598
Hes6	0	0	0
Hes7	0	0	0
Hexa	2891.559048	2157.388783	2743.778988
Hexb	28385.19235	27125.53784	37201.81859
Hexdc	14.64610227	27.40991572	20.28136528
Hexim1	25.10760389	35.40447447	36.69961336
Hexim2	2.092300324	1.142079822	3.863117195
Hey1	0	0	0.965779299
Hfe	280.3682435	190.7273302	320.6387272

For Review Only

Hgf	1.046150162	1.142079822	0
Hgh1	0	0	0
Hgs	190.3993295	149.6124566	70.50188882
Hgsnat	0	0	0.965779299
Hhat	13.59995211	18.27327715	32.83649616
Hhex	301.2912467	0	25.11026177
Hhip	0	4.568319287	0
Hibadh	95.19966476	81.08766734	71.46766811
Hibch	24.06145373	12.56287804	36.69961336
Hic1	0	1.142079822	1.931558598
Hic2	13.59995211	65.09854983	27.04182037
Hid1	3.138450487	0	0
Hif1a	293.9681956	229.5580442	205.7109907
Hif1an	56.49210876	70.80894894	51.18630284
Hif3a	3.138450487	0	0
Higd1a	26.15375406	30.83615518	57.94675793
Higd1b	3.138450487	0	0
Higd2a	120.3072687	95.93470502	0
Hilpda	14.64610227	20.55743679	23.17870317
Hinfp	47.0767573	45.68319287	46.35740634
Hint1	67.99976055	39.97279376	95.61215059
Hint2	0	0	0
Hint3	13.59995211	15.9891175	18.34980668
Hip1	2.092300324	2.284159643	10.62357229
Hip1r	15.69225243	9.136638573	12.55513089
Hipk1	268.8605917	229.5580442	132.3117639
Hipk2	164.2455755	189.5852504	160.3193636
Hipk3	47.0767573	106.2134234	52.15208214
Hipk4	0	0	0
Hira	11.50765178	4.568319287	38.63117195
Hirip3	0	0	0
Hist1h1a	0	0	0
Hist1h1c	23.01530357	12.56287804	11.58935159
Hist1h1d	5.230750811	4.568319287	0.965779299
Hist1h1e	29.29220454	17.13119732	18.34980668
Hist1h2aa	1.046150162	0	1.931558598
Hist1h2ab	5.230750811	5.710399108	10.62357229
Hist1h2ac	15.69225243	14.84703768	10.62357229
Hist1h2ad	12.55380195	12.56287804	23.17870317
Hist1h2ae	10.46150162	4.568319287	7.726234391
Hist1h2af	4.184600649	3.426239465	5.794675793
Hist1h2ag	6.276900973	3.426239465	6.760455092
Hist1h2ah	6.276900973	3.426239465	8.69201369
Hist1h2ai	5.230750811	6.85247893	8.69201369
Hist1h2ak	4.184600649	5.710399108	6.760455092
Hist1h2al	12.55380195	5.710399108	11.58935159
Hist1h2an	6.276900973	5.710399108	8.69201369
Hist1h2ao	28.24605438	38.83071394	47.32318564
Hist1h2ap	23.01530357	23.98367625	29.93915826
Hist1h2bb	0	0	3.863117195

For Review Only

Hist1h2bc	34.52295535	33.12031483	56.01519933
Hist1h2be	18.83070292	18.27327715	23.17870317
Hist1h2bf	4.184600649	4.568319287	2.897337897
Hist1h2bg	44.98445698	33.12031483	38.63117195
Hist1h2bh	3.138450487	3.426239465	3.863117195
Hist1h2bj	4.184600649	4.568319287	3.863117195
Hist1h2bk	3.138450487	1.142079822	3.863117195
Hist1h2bl	0	3.426239465	0.965779299
Hist1h2bm	1.046150162	1.142079822	0.965779299
Hist1h2bn	4.184600649	1.142079822	2.897337897
Hist1h2bp	1.046150162	0	0.965779299
Hist1h2bq	4.184600649	5.710399108	3.863117195
Hist1h2br	5.230750811	4.568319287	2.897337897
Hist1h3a	3.138450487	1.142079822	2.897337897
Hist1h3b	0	0	0.965779299
Hist1h3c	3.138450487	1.142079822	2.897337897
Hist1h3d	3.138450487	3.426239465	0
Hist1h3e	18.83070292	4.568319287	2.897337897
Hist1h3f	3.138450487	3.426239465	0
Hist1h3g	0	1.142079822	0
Hist1h3i	1.046150162	0	0
Hist1h4a	1.046150162	1.142079822	0.965779299
Hist1h4b	0	0	0
Hist1h4c	7.323051136	5.710399108	3.863117195
Hist1h4d	5.230750811	4.568319287	6.760455092
Hist1h4f	0	1.142079822	0
Hist1h4h	1.046150162	3.426239465	3.863117195
Hist1h4i	8.369201298	6.85247893	6.760455092
Hist1h4j	0	0	0
Hist1h4k	1.046150162	1.142079822	0
Hist1h4m	0	1.142079822	0
Hist1h4n	2.092300324	0	0
Hist2h2aa1	57.53825892	47.96735251	54.08364074
Hist2h2aa2	39.75370617	22.84159643	36.69961336
Hist2h2ab	6.276900973	4.568319287	3.863117195
Hist2h2ac	5.230750811	1.142079822	4.828896494
Hist2h2bb	3.138450487	2.284159643	2.897337897
Hist2h2be	3.138450487	4.568319287	13.52091018
Hist2h3b	4.184600649	9.136638573	5.794675793
Hist2h3c1	3.138450487	5.710399108	3.863117195
Hist2h3c2	1.046150162	4.568319287	1.931558598
Hist2h4	3.138450487	3.426239465	0.965779299
Hist3h2a	87.87661363	79.94558752	151.6273499
Hist3h2ba	1.046150162	4.568319287	2.897337897
Hist4h4	8.369201298	3.426239465	0.965779299
Hivep1	103.5688661	92.50846555	98.50948848
Hivep2	15.69225243	17.13119732	18.34980668
Hivep3	615.1362954	469.3948067	612.3040755
Hjurp	170.5224764	164.4594943	181.5665082
Hk1	54.39980844	33.12031483	24.14448247

For Review Only

Hk2	637.1054488	551.6245539	645.1405716
Hk3	154.830224	54.81983144	0
Hlcs	21.96915341	34.26239465	29.93915826
Hlf	17.78455276	3.426239465	22.21292387
Hltf	95.19966476	118.7763015	75.33078531
Hlx	44.98445698	18.27327715	6.760455092
Hmbox1	139.1379716	74.23518841	68.57033022
Hmbs	0	0	11.58935159
Hmces	38.707556	39.97279376	38.63117195
Hmcn2	0	0	0
Hmg20a	148.553323	0	151.6273499
Hmg20b	1.046150162	133.6233391	22.21292387
Hmga1	52.30750811	46.82527269	28.97337897
Hmga1-rs1	49.16905763	35.40447447	23.17870317
Hmga2	1.046150162	0	4.828896494
Hmga2-ps1	152.7379237	349.4764254	483.8554287
Hmgb1	66.95361038	106.2134234	77.26234391
Hmgb2	28.24605438	18.27327715	17.38402738
Hmgb3	0	1.142079822	0
Hmgcl	46.03060714	254.6838002	378.5854851
Hmgcr	66.95361038	59.38815073	46.35740634
Hmgcs1	87.87661363	38.83071394	74.36500601
Hmgcs2	4.184600649	0	0
Hmgn1	111.9380674	129.0550198	127.4828674
Hmgn2	0	0	0
Hmgn3	13.59995211	1.142079822	0
Hmgn5	0	2.284159643	0
Hmgxb3	106.7073165	79.94558752	94.64637129
Hmgxb4	34.52295535	44.54111304	39.59695125
Hmha1	0	0	60.84409583
Hmmr	3.138450487	0	0
Hmox1	66.95361038	49.10943233	67.60455092
Hmox2	0	719.5102876	0
Hmx2	0	0	0
Hn1	283.506694	286.6620352	311.9467135
Hn1l	0	0	28.97337897
Hnmt	154.830224	92.50846555	124.5855296
Hnrnpa0	38.707556	44.54111304	40.56273055
Hnrnpa1	1.046150162	0	0
Hnrnpa2b1	0	0	3.863117195
Hnrnpa3	60.67670941	86.79806645	57.94675793
Hnrnpab	5.230750811	6.85247893	0
Hnrnpc	214.4607833	0	342.8516511
Hnrnpd	56.49210876	47.96735251	75.33078531
Hnrnpdl	33.47680519	44.54111304	75.33078531
Hnrnpf	330.5834513	291.2303545	366.0303543
Hnrnph1	0	0	0
Hnrnph2	134.9533709	150.7545365	170.9429359
Hnrnph3	0	0	0
Hnrnpk	40.79985633	721.7944473	777.4523356

For Review Only

Hnrnpl	8.369201298	14.84703768	0
Hnrnpll	13.59995211	49.10943233	12.55513089
Hnrnpm	100.4304156	149.6124566	167.0798187
Hnrnpr	172.6147768	137.0495786	94.64637129
Hnrnpu	201.9069813	432.8482524	240.4790454
Hnrnpul1	141.2302719	59.38815073	85.9543576
Hnrnpul2	29.29220454	52.5356718	55.04942003
Hoga1	10.46150162	3.426239465	3.863117195
Homer1	23.01530357	17.13119732	22.21292387
Homer3	0	14.84703768	2.897337897
Homez	9.41535146	13.70495786	31.87071686
Hook2	38.707556	37.68863411	37.66539266
Hook3	104.6150162	284.3778756	162.2509222
Hottip	0	2.284159643	3.863117195
Hp	60.67670941	13.70495786	0
Hp1bp3	306.5219975	224.9897249	312.9124928
Hpca	3.138450487	0	0
Hpcal1	102.5227159	90.22430591	103.338385
Hpcal4	0	0	0
Hpf1	74.27666152	84.5139068	141.9695569
Hpgd	465.5368222	742.3518841	688.6006401
Hpgds	804.4894748	661.2642167	624.8592064
Hpn	1.046150162	1.142079822	0
Hprt	142.2764221	139.3337382	196.0531977
Hps1	141.2302719	94.7926252	112.996178
Hps3	439.3830681	390.591299	343.8174304
Hps4	137.0456713	898.8168196	141.9695569
Hps5	98.33811525	45.68319287	72.43344741
Hps6	47.0767573	34.26239465	21.24714457
Hras	13.59995211	7.994558752	5.794675793
Hrh2	54.39980844	85.65598662	98.50948848
Hs1bp3	398.5832118	237.5526029	0
Hs2st1	39.75370617	49.10943233	30.90493756
Hs3st1	0	0	0
Hs3st2	1.046150162	0	0
Hs3st3b1	24.06145373	31.97823501	21.24714457
Hs6st1	57.53825892	107.3555032	106.2357229
Hs6st2	4.184600649	1.142079822	0
Hsbp1	266.7682914	266.1045984	291.6653483
Hsbp1l1	0	0	0
Hscb	19.87685308	17.13119732	17.38402738
Hsd11b2	5.230750811	0	0
Hsd17b1	5.230750811	9.136638573	11.58935159
Hsd17b10	44.98445698	47.96735251	70.50188882
Hsd17b11	20.92300324	28.55199554	17.38402738
Hsd17b12	133.9072208	150.7545365	133.2775432
Hsd17b4	276.1836428	285.5199554	378.5854851
Hsd17b7	3.138450487	1.142079822	0
Hsd3b4	1.046150162	2.284159643	0
Hsd3b7	0	0	183.4980668

For Review Only

Hsdl1	190.3993295	150.7545365	202.8136528
Hsdl2	9.41535146	28.55199554	0
Hsf1	1.046150162	1.142079822	0
Hsf2	38.707556	61.67231037	30.90493756
Hsf2bp	8.369201298	14.84703768	11.58935159
Hsf5	3.138450487	0	0
Hsp90aa1	93.10736444	119.9183813	92.71481269
Hsp90ab1	0	0	1471.847651
Hsp90b1	2049.408168	1805.628198	2051.315231
Hspa12a	12.55380195	20.55743679	26.07604107
Hspa13	104.6150162	115.350062	127.4828674
Hspa14	99.38426541	76.51934805	57.94675793
Hspa1a	5.230750811	0	0
Hspa1b	0	0	0
Hspa1l	7.323051136	0	0
Hspa2	9.41535146	3.426239465	2.897337897
Hspa4	205.0454318	173.5961329	141.9695569
Hspa4l	1.046150162	21.69951661	0
Hspa5	1715.686266	1560.081036	1477.642327
Hspa8	1809.839781	1434.452256	1738.402738
Hspa9	258.3990901	197.5798091	240.4790454
Hspb1	2.092300324	0	0
Hspb11	0	6.85247893	0
Hspb3	52.30750811	76.51934805	74.36500601
Hspb6	21.96915341	2.284159643	7.726234391
Hspb8	7.323051136	0	0
Hspb9	1.046150162	0	0
Hspbap1	54.39980844	46.82527269	50.22052354
Hspbp1	70.09206087	38.83071394	62.77565443
Hspd1	76.36896184	68.5247893	89.81747479
Hspe1	15.69225243	22.84159643	58.91253723
Hspg2	6.276900973	0	0
Hsph1	38.707556	105.0713436	54.08364074
Htatip2	18.83070292	9.136638573	18.34980668
Htatsf1	60.67670941	43.39903322	45.39162705
Htr2b	24.06145373	3.426239465	9.657792988
Htr2c	6.276900973	0	0
Htr4	0	0	0
Htr5b	0	0	0
Htra3	17.78455276	29.69407536	55.04942003
Htt	132.8610706	114.2079822	177.703391
Hus1	10.46150162	69.66686912	23.17870317
Hus1b	1.046150162	0	4.828896494
Huwe1	429.9677167	478.5314453	437.4980224
Hvcn1	752.1819667	654.4117378	845.0568865
Hyal1	1.046150162	0	0
Hyal2	92.06121428	92.50846555	79.19390251
Hyal3	4.184600649	4.568319287	8.69201369
Hyal6	1.046150162	0	0
Hyi	0	12.56287804	0

For Review Only

Hykk	11.50765178	4.568319287	19.31558598
Hyls1	0	7.994558752	0
Hyou1	96.24581493	63.95647001	45.39162705
I830077J02Rik	48.12290746	38.83071394	0
I830127L07Rik	36.61525568	18.27327715	7.726234391
Iah1	0	71.95102876	0
Iars	31.38450487	65.09854983	147.7642327
Iars2	40.79985633	46.82527269	28.00759967
Iba57	6.276900973	4.568319287	20.28136528
Ibtk	89.96891395	22.84159643	40.56273055
Ica1	0	0	0
Ica1l	2.092300324	17.13119732	14.48668948
Icam1	503.198228	284.3778756	427.8402294
Icam2	0	0	0
Ice1	47.0767573	98.21886466	108.1672815
Ice2	50.21520779	74.23518841	52.15208214
Ick	65.90746022	37.68863411	47.32318564
Icmt	94.1535146	113.0659023	104.3041643
Icos	1.046150162	7.994558752	0
Icosl	292.9220454	29.69407536	125.5513089
Ict1	0	52.5356718	35.73383406
Id1	1.046150162	0	12.55513089
Id3	17.78455276	1.142079822	0
Ide	134.9533709	59.38815073	56.01519933
Idh1	149.5994732	125.6287804	95.61215059
Idh2	149.5994732	441.984891	219.2319008
Idh3a	137.0456713	85.65598662	76.29656461
Idh3b	0	163.3174145	68.57033022
Idh3g	0	0	1.931558598
Idi1	15.69225243	5.710399108	2.897337897
Idnk	0	0	49.25474424
Ids	76.36896184	116.4921418	71.46766811
Idua	367.1987069	78.80350769	186.3954047
Ier2	297.1066461	197.5798091	205.7109907
Ier3	6.276900973	0	33.80227546
Ier3ip1	154.830224	0	179.6349496
Ier5	128.67647	259.2521195	185.4296254
Iffo1	547.1365349	1.142079822	0
Iffo2	9.41535146	23.98367625	11.58935159
Ifi203	1.046150162	0	0
Ifi204	12.55380195	13.70495786	19.31558598
Ifi205	0	3.426239465	0
Ifi27	75.32281168	0	15.45246878
Ifi27l2a	0	3.426239465	0
Ifi30	1.046150162	1.142079822	0
Ifi44	0	2.284159643	0
Ifi47	7.323051136	0	0.965779299
Ifih1	56.49210876	66.24062966	50.22052354
Ifit1	0	13.70495786	12.55513089
Ifit2	15.69225243	12.56287804	35.73383406

For Review Only

Ifit3	1.046150162	1.142079822	153.5589085
Ifit3b	1.046150162	1.142079822	0
Ifitm1	0	0	0
Ifitm10	0	0	1.931558598
Ifitm2	5.230750811	7.994558752	5.794675793
Ifitm3	26.15375406	23.98367625	29.93915826
Ifitm6	26.15375406	5.710399108	0
Ifnar1	351.5064545	338.0556272	441.3611396
Ifnar2	264.675991	186.1590109	198.9505356
Ifngr1	2800.543984	2862.052033	3443.0032
Ifngr2	587.9363912	569.897831	681.840185
Ifrd1	62.76900973	42.2569534	53.11786144
Ifrd2	14.64610227	15.9891175	0
Ift122	0	20.55743679	0
Ift140	62.76900973	70.80894894	52.15208214
Ift172	124.4918693	68.5247893	128.4486467
Ift20	0	0	0
Ift22	19.87685308	19.41535697	26.07604107
Ift27	11.50765178	0	12.55513089
Ift43	1.046150162	2.284159643	0
Ift46	74.27666152	34.26239465	54.08364074
Ift52	93.10736444	79.94558752	105.2699436
Ift57	13.59995211	2.284159643	0
Ift74	13.59995211	17.13119732	13.52091018
Ift80	12.55380195	1.142079822	0
Ift81	10.46150162	9.136638573	5.794675793
Ift88	25.10760389	7.994558752	24.14448247
Igbp1	97.29196509	109.6396629	111.0646194
Igdcc4	5.230750811	4.568319287	1.931558598
Igf1	1.046150162	1.142079822	0
Igf1r	57.53825892	70.80894894	65.67299232
Igf2	23.01530357	0	0
Igf2bp2	18.83070292	27.40991572	15.45246878
Igf2bp3	32.43065503	27.40991572	45.39162705
Igf2r	4.184600649	0	0
Igfals	1.046150162	0	0
Igfbp2	32.43065503	0	2.897337897
Igfbp4	56.49210876	39.97279376	57.94675793
Igfn1	0	2.284159643	0
Ighmbp2	39.75370617	11.42079822	54.08364074
Igip	8.369201298	3.426239465	12.55513089
Igsf10	0	1.142079822	6.760455092
Igsf6	0	0	0.965779299
Igsf8	1.046150162	1.142079822	182.5322875
Igsf9	7.323051136	2.284159643	0
Igsf9b	3.138450487	0	1.931558598
Igtp	135.9995211	172.4540531	180.6007289
Iigp1	0	4.568319287	0
Ik	189.3531794	206.7164477	335.1254167
Ikbip	44.98445698	35.40447447	53.11786144

For Review Only

Ikbkap	89.96891395	89.08222609	78.22812321
Ikbkb	75.32281168	552.7666337	245.3079419
Ikbke	2.092300324	4.568319287	0
Ikbkg	94.1535146	123.3446207	160.3193636
Ikzf1	562.8287873	604.1602257	496.4105596
Ikzf2	19.87685308	38.83071394	20.28136528
Ikzf5	16.7384026	27.40991572	33.80227546
Il10ra	10446.85552	67.38270948	3262.402472
Il10rb	251.0760389	431.7061726	366.0303543
Il11ra1	109.845767	17.13119732	74.36500601
Il11ra2	14.64610227	0	11.58935159
Il12rb2	20.92300324	19.41535697	15.45246878
Il13ra1	396.4909115	575.6082301	430.7375673
Il15	40.79985633	61.67231037	44.42584775
Il15ra	0	7.994558752	0
Il16	670.582254	690.9582921	608.4409583
Il17c	0	0	0
Il17ra	70.09206087	44.54111304	671.2166127
Il17rb	4.184600649	0	0
Il17rc	4.184600649	5.710399108	1.931558598
Il18	0	113.0659023	5.794675793
Il18bp	1.046150162	1.142079822	72.43344741
Il18rap	2.092300324	2.284159643	12.55513089
Il1a	144.3687224	408.8645762	345.748989
Il1b	3.138450487	9.136638573	0
Il1bos	0	2.284159643	0.965779299
Il1r1	4.184600649	3.426239465	3.863117195
Il1r2	4.184600649	1.142079822	0
Il1rap	36.61525568	2.284159643	44.42584775
Il1rl1	20.92300324	1.142079822	0
Il1rl2	159.0148247	187.3010908	136.1748811
Il1rn	8.369201298	2.284159643	5.794675793
Il20rb	2.092300324	5.710399108	0
Il21r	315.937349	307.219472	354.4410027
Il27	11.50765178	12.56287804	13.52091018
Il2rg	28.24605438	10.27871839	16.41824808
Il34	0	0	0
Il3ra	0	4.568319287	0
Il4ra	1691.624812	3.426239465	984.1291055
Il6	20.92300324	0	29.93915826
Il6ra	526.2135316	694.3845316	514.7603663
Il6st	742.7666152	627.0018221	662.524599
Il7r	67.99976055	92.50846555	50.22052354
Ildr1	24.06145373	11.42079822	26.07604107
Ildr2	40.79985633	65.09854983	43.46006845
Ilf2	63.8151599	0	59.87831653
Ilf3	247.9375885	198.721889	241.4448247
Ilk	0	1.142079822	0
Ilkap	44.98445698	83.37182698	94.64637129
Ilvbl	237.4760868	31.97823501	2.897337897

For Review Only

Immp1l	138.0918214	27.40991572	0
Immp2l	12.55380195	5.710399108	17.38402738
Immt	234.3376363	218.1372459	280.0759967
Imp3	66.95361038	70.80894894	72.43344741
Imp4	0	0	0.965779299
Impa1	100.4304156	85.65598662	98.50948848
Impa2	11.50765178	0	0
Impact	218.6453839	157.6070154	164.1824808
Impad1	18.83070292	20.55743679	0
Impdh1	0	0	1.931558598
Impdh2	0	35.40447447	0
Impg2	0	0	0.965779299
Inadl	2.092300324	15.9891175	15.45246878
Inafm1	8.369201298	19.41535697	13.52091018
Inafm2	147.5071729	132.4812593	113.9619573
Inca1	5.230750811	3.426239465	7.726234391
Incenp	31.38450487	12.56287804	27.04182037
Inf2	0	2.284159643	0
Ing1	34.52295535	70.80894894	46.35740634
Ing2	4.184600649	25.12575608	3.863117195
Ing3	55.4459586	68.5247893	74.36500601
Ing5	66.95361038	44.54111304	75.33078531
Inhba	9.41535146	0	0
Inip	164.2455755	69.66686912	222.1292387
Ino80	19.87685308	36.54655429	52.15208214
Ino80b	9.41535146	6.85247893	0
Ino80c	65.90746022	85.65598662	104.3041643
Ino80d	107.7534667	214.7110065	121.6881917
Ino80dos	2.092300324	0	11.58935159
Ino80e	162.1532751	114.2079822	117.8250745
Inpp1	25.10760389	17.13119732	29.93915826
Inpp4a	237.4760868	230.700124	153.5589085
Inpp4b	242.7068376	180.4486118	209.5741078
Inpp5a	1.046150162	10.27871839	13.52091018
Inpp5b	3.138450487	3.426239465	0
Inpp5d	0	0	0
Inpp5e	71.13821103	105.0713436	105.2699436
Inpp5f	78.46126217	49.10943233	48.28896494
Inpp5k	114.0303677	157.6070154	319.6729479
Inppl1	75.32281168	66.24062966	46.35740634
Insig1	53.35365827	13.70495786	14.48668948
Insig2	96.24581493	100.5030243	105.2699436
Insr	129.7226201	212.4268468	172.8744945
Ints1	17.78455276	11.42079822	0
Ints10	130.7687703	98.21886466	115.8935159
Ints12	51.26135795	37.68863411	43.46006845
Ints2	46.03060714	58.2460709	28.00759967
Ints3	142.2764221	107.3555032	116.8592952
Ints4	138.0918214	110.7817427	63.74143372
Ints5	52.30750811	57.10399108	58.91253723

For Review Only

Ints6	15.69225243	21.69951661	11.58935159
Ints7	62.76900973	45.68319287	34.76805476
Ints8	31.38450487	37.68863411	17.38402738
Ints9	24.06145373	7.994558752	76.29656461
Intu	14.64610227	7.994558752	14.48668948
Invs	18.83070292	59.38815073	36.69961336
Ip6k1	1.046150162	1.142079822	677.0112885
Ip6k2	11.50765178	6.85247893	0
Ipcef1	16.7384026	83.37182698	16.41824808
Ipmk	41.84600649	43.39903322	37.66539266
Ipo11	102.5227159	78.80350769	74.36500601
Ipo13	155.8763742	139.3337382	104.3041643
Ipo4	62.76900973	14.84703768	39.59695125
Ipo5	120.3072687	118.7763015	122.653971
Ipo7	105.6611664	98.21886466	94.64637129
Ipo8	61.72285957	58.2460709	47.32318564
Ipo9	212.3684829	123.3446207	180.6007289
Ipp	14.64610227	13.70495786	19.31558598
Ippk	19.87685308	14.84703768	14.48668948
Iqcb1	15.69225243	6.85247893	36.69961336
Iqcc	42.89215665	28.55199554	22.21292387
Iqcd	9.41535146	2.284159643	4.828896494
Iqce	36.61525568	83.37182698	29.93915826
Iqcg	6.276900973	3.426239465	0
Iqch	4.184600649	9.136638573	4.828896494
Iqck	0	0	5.794675793
Iqgap1	21.96915341	18.27327715	15.45246878
Iqgap3	63.8151599	19.41535697	53.11786144
Iqsec1	49.16905763	43.39903322	41.52850985
Iqsec2	95.19966476	12.56287804	25.11026177
Iqsec3	0	0	4.828896494
Irak1	100.4304156	210.1426872	153.5589085
Irak2	12.55380195	15.9891175	0
Irak3	58.58440909	9.136638573	17.38402738
Irak4	1.046150162	1.142079822	0
Ireb2	105.6611664	131.3391795	98.50948848
Irf1	166.3378758	103.9292638	120.7224124
Irf2	111.9380674	129.0550198	87.8859162
Irf2bp1	36.61525568	27.40991572	48.28896494
Irf2bp2	30.33835471	19.41535697	0
Irf2bpl	200.8608312	114.2079822	106.2357229
Irf3	118.2149683	187.3010908	282.0075553
Irf4	21.96915341	35.40447447	40.56273055
Irf5	0	1.142079822	424.9428915
Irf6	23.01530357	3.426239465	0
Irf7	17.78455276	20.55743679	0
Irf8	1393.472016	1223.167489	1345.330563
Irf9	0	49.10943233	0
Irgc1	0	0	1.931558598
Irgm1	87.87661363	178.1644522	160.3193636

For Review Only

Irgm2	0	223.847645	189.2927426
Irgq	34.52295535	47.96735251	33.80227546
Irs1	0	2.284159643	0
Irs2	77.41511201	46.82527269	48.28896494
Irs3	6.276900973	0	19.31558598
Isca1	165.2917256	106.2134234	150.6615706
Iscu	1.046150162	0	0
Isg15	0	0	2.897337897
Isg20	6.276900973	4.568319287	10.62357229
Isg20l2	53.35365827	7.994558752	21.24714457
Islr	2.092300324	0	0
Isoc1	34.52295535	25.12575608	34.76805476
Isoc2b	6.276900973	11.42079822	0
Ispd	5.230750811	0	0.965779299
Israa	14.64610227	12.56287804	7.726234391
Ist1	250.0298888	279.8095563	134.2433225
Isy1	0	54.81983144	10.62357229
Isyna1	229.1068855	230.700124	244.3421626
Itch	156.9225243	141.6178979	133.2775432
Itfg1	316.9834992	216.9951661	278.1444381
Itfg2	47.0767573	2.284159643	30.90493756
Itga10	0	0	0
Itga3	4.184600649	0	0
Itga4	2.092300324	5.710399108	0
Itga5	54.39980844	44.54111304	65.67299232
Itga6	281.4143936	431.7061726	332.2280788
Itga9	85.7843133	99.36094448	114.9277366
Itgae	15.69225243	11.42079822	0
Itgal	3.138450487	2.284159643	0
Itgam	720.7974618	953.6366511	984.1291055
Itgav	163.1994253	251.2575608	144.8668948
Itgax	11.50765178	10.27871839	29.93915826
Itgb1	345.2295535	395.1596183	309.0493756
Itgb1bp1	47.0767573	54.81983144	56.98097863
Itgb1bp2	0	0	0
Itgb2	1490.763981	1291.692278	1595.467402
Itgb2l	20.92300324	0	0
Itgb3	141.2302719	145.0441374	132.3117639
Itgb3bp	2.092300324	6.85247893	11.58935159
Itgb4	0	0	1.931558598
Itgb5	1137.165226	1963.235213	1306.699391
Itgb7	5.230750811	0	0
Itih2	0	0	0
Itih5	5.230750811	0	0
Itm2a	0	0	0
Itm2b	8414.185755	8951.621642	10887.23004
Itm2c	1597.471298	1371.637866	1481.505444
Itpa	26.15375406	12.56287804	48.28896494
Itpk1	100.4304156	77.66142787	103.338385
Itpkb	46.03060714	65.09854983	56.01519933

For Review Only

Itpkc	60.67670941	73.09310859	59.87831653
Itpr1	39.75370617	5.710399108	22.21292387
Itpr2	433.1061672	328.9189886	393.0721746
Itpr3	39.75370617	41.11487358	60.84409583
Itprip	17.78455276	12.56287804	22.21292387
Itpripl1	980.242702	768.61972	911.6956581
Itpripl2	32.43065503	65.09854983	29.93915826
Itsn1	10.46150162	7.994558752	8.69201369
Itsn2	114.0303677	247.8313213	134.2433225
Ivd	85.7843133	60.53023055	101.4068264
Ivns1abp	75.32281168	1509.829524	1166.661393
Iws1	83.69201298	55.96191126	74.36500601
Izumo4	9.41535146	0	18.34980668
Jade1	50.21520779	35.40447447	36.69961336
Jade2	244.799138	94.7926252	126.5170881
Jade3	2.092300324	18.27327715	3.863117195
Jag1	5.230750811	2.284159643	5.794675793
Jag2	21.96915341	15.9891175	34.76805476
Jagn1	135.9995211	134.765419	176.7376117
Jak1	580.61334	277.5253967	384.3801609
Jak2	65.90746022	85.65598662	70.50188882
Jak3	27.19990422	35.40447447	79.19390251
Jam2	250.0298888	317.4981904	274.2813209
Jam3	62.76900973	62.81439019	39.59695125
Jarid2	103.5688661	118.7763015	85.9543576
Jazf1	2.092300324	9.136638573	4.828896494
Jdp2	0	0	0
Jkamp	140.1841217	124.4867006	182.5322875
Jmjd1c	368.2448571	628.1439019	415.2850985
Jmjd4	20.92300324	57.10399108	42.49428915
Jmjd6	85.7843133	68.5247893	64.70721302
Jmjd7	0	0	0
Jmy	44.98445698	45.68319287	40.56273055
Josd1	48.12290746	23.98367625	49.25474424
Jph3	44.98445698	33.12031483	28.00759967
Jpx	13.59995211	21.69951661	10.62357229
Jrk	40.79985633	13.70495786	36.69961336
Jrkl	12.55380195	5.710399108	10.62357229
Jtb	0	0	0
Jun	2802.636285	1637.742464	2041.657438
Junb	2975.251061	2335.553235	2496.539488
Jund	32.43065503	25.12575608	28.00759967
Junos	21.96915341	11.42079822	2.897337897
Jup	116.122668	145.0441374	190.2585219
Kalrn	11.50765178	1.142079822	3.863117195
Kank1	4.184600649	0	0
Kank2	1.046150162	0	0
Kank3	89.96891395	6.85247893	0.965779299
Kansl1	170.5224764	181.5906916	201.8478735
Kansl1l	70.09206087	117.6342216	80.1596818

For Review Only

Kansl2	50.21520779	19.41535697	36.69961336
Kansl3	173.6609269	114.2079822	102.3726057
Kantr	38.707556	50.25151215	39.59695125
Kars	104.6150162	102.7871839	118.7908538
Kat2a	1.046150162	62.81439019	0
Kat2b	34.52295535	52.5356718	44.42584775
Kat5	29.29220454	0	0
Kat6a	217.5992337	230.700124	157.4220257
Kat6b	95.19966476	108.4975831	87.8859162
Kat7	260.4913904	190.7273302	175.7718324
Kat8	0	79.94558752	45.39162705
Katna1	74.27666152	65.09854983	81.1254611
Katnal1	10.46150162	9.136638573	18.34980668
Katnb1	31.38450487	33.12031483	0
Katnbl1	18.83070292	27.40991572	34.76805476
Kazald1	1.046150162	5.710399108	0
Kbtbd11	1.046150162	1.142079822	4.828896494
Kbtbd12	6.276900973	0	0
Kbtbd2	200.8608312	278.6674765	224.0607973
Kbtbd3	23.01530357	29.69407536	21.24714457
Kbtbd4	106.7073165	126.7708602	82.0912404
Kbtbd6	7.323051136	4.568319287	2.897337897
Kbtbd7	81.59971265	62.81439019	40.56273055
Kbtbd8	31.38450487	5.710399108	0
Kcmf1	38.707556	36.54655429	36.69961336
Kcna2	3.138450487	0	0
Kcna3	4.184600649	18.27327715	0.965779299
Kcna4	2.092300324	0	2.897337897
Kcnab2	3.138450487	2.284159643	3.863117195
Kcnab3	0	0	6.760455092
Kcnb1	1.046150162	10.27871839	0
Kcnb2	1.046150162	2.284159643	0
Kcnc1	0	0	0
Kcnc2	0	1.142079822	3.863117195
Kcnd1	0	0	200.8820942
Kcnd3	0	6.85247893	5.794675793
Kcne1	0	2.284159643	0.965779299
Kcne2	12.55380195	0	0
Kcng4	0	2.284159643	0
Kcnh1	2.092300324	0	0
Kcnh6	11.50765178	18.27327715	17.38402738
Kcnh7	0	0	1.931558598
Kcnip2	0	0	0
Kcnip3	74.27666152	70.80894894	93.68059199
Kcnip4	0	6.85247893	0
Kcnj10	15.69225243	10.27871839	11.58935159
Kcnj12	25.10760389	11.42079822	6.760455092
Kcnj13	64.86131006	4.568319287	6.760455092
Kcnj14	0	2.284159643	0
Kcnj16	13.59995211	3.426239465	8.69201369

For Review Only

Kcnj2	18.83070292	69.66686912	42.49428915
Kcnj8	0	0	0
Kcnj9	30.33835471	33.12031483	23.17870317
Kcnk1	7.323051136	0	0
Kcnk12	24.06145373	9.136638573	5.794675793
Kcnk6	174.7070771	204.4322881	126.5170881
Kcnk7	8.369201298	7.994558752	6.760455092
Kcnma1	28.24605438	36.54655429	6.760455092
Kcnmb3	11.50765178	1.142079822	0
Kcnn1	0	0	5.794675793
Kcnn4	9.41535146	0	11.58935159
Kcnq1ot1	203.9992816	320.9244299	226.9581352
Kcnq2	0	0	5.794675793
Kcnrg	16.7384026	18.27327715	7.726234391
Kcnt1	0	2.284159643	0
Kcnv1	3.138450487	2.284159643	0
Kcp	12.55380195	17.13119732	10.62357229
Kctd1	0	0	0
Kctd10	43.93830681	47.96735251	39.59695125
Kctd11	43.93830681	39.97279376	41.52850985
Kctd12	401.7216623	165.6015741	1538.486423
Kctd12b	11.50765178	20.55743679	14.48668948
Kctd13	34.52295535	30.83615518	29.93915826
Kctd14	1.046150162	0	0
Kctd18	48.12290746	44.54111304	45.39162705
Kctd2	39.75370617	3.426239465	10.62357229
Kctd20	146.4610227	173.5961329	188.3269633
Kctd21	85.7843133	117.6342216	94.64637129
Kctd3	36.61525568	23.98367625	8.69201369
Kctd4	0	9.136638573	0.965779299
Kctd5	19.87685308	33.12031483	33.80227546
Kctd6	10.46150162	20.55743679	39.59695125
Kctd7	12.55380195	9.136638573	18.34980668
Kctd9	5.230750811	5.710399108	21.24714457
Kdelc1	37.66140584	81.08766734	102.3726057
Kdelc2	42.89215665	37.68863411	22.21292387
Kdelr1	117.1688182	126.7708602	52.15208214
Kdelr2	371.3833076	376.8863411	421.0797743
Kdelr3	0	5.710399108	0
Kdf1	1.046150162	0	0
Kdm1a	29.29220454	30.83615518	29.93915826
Kdm1b	58.58440909	36.54655429	34.76805476
Kdm2a	194.5839302	158.7490952	149.6957913
Kdm2b	110.8919172	86.79806645	103.338385
Kdm3a	111.9380674	134.765419	123.6197503
Kdm3b	166.3378758	164.4594943	202.8136528
Kdm4a	24.06145373	33.12031483	20.28136528
Kdm4b	58.58440909	102.7871839	62.77565443
Kdm4c	106.7073165	57.10399108	56.98097863
Kdm4d	0	5.710399108	0

For Review Only

Kdm5a	186.2147289	135.9074988	149.6957913
Kdm5b	57.53825892	103.9292638	54.08364074
Kdm5c	134.9533709	203.2902083	92.71481269
Kdm5d	64.86131006	71.95102876	42.49428915
Kdm6a	58.58440909	205.5743679	50.22052354
Kdm6b	81.59971265	34.26239465	28.00759967
Kdm7a	121.3534188	149.6124566	130.3802053
Kdm8	26.15375406	11.42079822	7.726234391
Kdr	3.138450487	19.41535697	9.657792988
Kdsr	69.04591071	103.9292638	61.80987513
Keap1	189.3531794	77.66142787	92.71481269
Khdc3	1.046150162	11.42079822	1.931558598
Khdrbs1	223.8761347	341.4818667	176.7376117
Khdrbs3	15.69225243	19.41535697	19.31558598
Khk	146.4610227	122.2025409	139.072219
Khnyn	326.3988506	0	223.095018
Khsrp	12.55380195	15.9891175	23.17870317
Kidins220	162.1532751	199.8639688	179.6349496
Kif11	0	0	0
Kif13a	61.72285957	62.81439019	57.94675793
Kif13b	131.8149204	86.79806645	87.8859162
Kif15	0	0	0
Kif16b	20.92300324	33.12031483	10.62357229
Kif1b	57.53825892	67.38270948	64.70721302
Kif1bp	19.87685308	21.69951661	21.24714457
Kif1c	12.55380195	5.710399108	13.52091018
Kif20b	0	6.85247893	9.657792988
Kif21b	531.4442824	510.5096803	328.3649616
Kif22	0	0	0
Kif23	0	0	0
Kif24	1.046150162	4.568319287	4.828896494
Kif26a	0	0	0
Kif26b	1.046150162	4.568319287	0.965779299
Kif27	0	0	0.965779299
Kif2a	30.33835471	20.55743679	24.14448247
Kif2c	0	2.284159643	0
Kif3a	41.84600649	33.12031483	42.49428915
Kif3b	64.86131006	124.4867006	82.0912404
Kif3c	19.87685308	75.37726823	10.62357229
Kif4	0	0	0
Kif5a	65.90746022	3.426239465	0
Kif5b	192.4916299	147.328297	138.1064397
Kif5c	0	0	0
Kif6	1.046150162	0	0
Kif9	51.26135795	42.2569534	11.58935159
Kifap3	56.49210876	20.55743679	0
Kifc1	3.138450487	1.142079822	0
Kifc2	9.41535146	1.142079822	19.31558598
Kifc3	16.7384026	10.27871839	9.657792988
Kifc5b	0	3.426239465	3.863117195

For Review Only

Kin	38.707556	36.54655429	28.97337897
Kiss1r	5.230750811	1.142079822	2.897337897
Kitl	15.69225243	35.40447447	21.24714457
Kiz	51.26135795	35.40447447	46.35740634
Kl	96.24581493	0	0
Klc1	179.9378279	229.5580442	227.9239145
Klc2	15.69225243	12.56287804	2.897337897
Klc4	116.122668	68.5247893	118.7908538
Klf1	3.138450487	0	0
Klf10	27.19990422	19.41535697	25.11026177
Klf11	4.184600649	2.284159643	2.897337897
Klf12	89.96891395	143.9020575	103.338385
Klf13	316.9834992	301.5090729	252.068397
Klf15	8.369201298	0	3.863117195
Klf16	4.184600649	2.284159643	6.760455092
Klf2	2.092300324	20.55743679	0
Klf3	216.5530836	292.3724343	213.437225
Klf4	1.046150162	13.70495786	0
Klf7	128.67647	118.7763015	139.072219
Klf9	4.184600649	0	0
Klhdc1	14.64610227	13.70495786	27.04182037
Klhdc10	44.98445698	68.5247893	68.57033022
Klhdc2	31.38450487	27.40991572	28.97337897
Klhdc3	163.1994253	135.9074988	0
Klhdc4	64.86131006	63.95647001	61.80987513
Klhdc8b	85.7843133	19.41535697	52.15208214
Klhdc9	2.092300324	3.426239465	0
Klhl10	154.830224	156.4649356	143.9011155
Klhl11	35.56910552	7.994558752	23.17870317
Klhl12	6.276900973	3.426239465	24.14448247
Klhl13	0	9.136638573	0
Klhl15	14.64610227	3.426239465	4.828896494
Klhl17	70.09206087	1.142079822	0
Klhl18	93.10736444	43.39903322	7.726234391
Klhl2	39.75370617	21.69951661	0
Klhl20	39.75370617	33.12031483	33.80227546
Klhl21	93.10736444	46.82527269	33.80227546
Klhl22	134.9533709	86.79806645	91.74903339
Klhl23	26.15375406	6.85247893	28.97337897
Klhl24	250.0298888	292.3724343	266.5550865
Klhl25	150.6456234	182.7327715	152.5931292
Klhl26	53.35365827	33.12031483	29.93915826
Klhl28	48.12290746	70.80894894	38.63117195
Klhl29	2.092300324	2.284159643	0
Klhl3	30.33835471	12.56287804	15.45246878
Klhl35	7.323051136	0	12.55513089
Klhl36	118.2149683	83.37182698	95.61215059
Klhl38	46.03060714	9.136638573	18.34980668
Klhl42	33.47680519	43.39903322	34.76805476
Klhl5	50.21520779	63.95647001	44.42584775

For Review Only

Klhl6	171.5686266	342.6239465	366.9961336
Klhl7	298.1527962	253.5417204	263.6577486
Klhl8	16.7384026	14.84703768	20.28136528
Klhl9	238.522237	243.263002	183.4980668
Klk10	0	0	3.863117195
Klk12	4.184600649	0	0
Klk7	3.138450487	2.284159643	0
Klk8	154.830224	232.9842836	205.7109907
Klk9	5.230750811	12.56287804	2.897337897
Klkb1	2.092300324	0	0
Klrb1b	0	1.142079822	3.863117195
Klrd1	8.369201298	6.85247893	12.55513089
Klrg2	15.69225243	5.710399108	2.897337897
Kmt2a	209.2300324	284.3778756	264.6235279
Kmt2b	42.89215665	85.65598662	14.48668948
Kmt2c	282.4605438	521.9304785	326.433403
Kmt2d	625.597797	752.6306025	413.3535399
Kmt2e	531.4442824	519.6463189	428.8060087
Kmt5a	5.230750811	29.69407536	0
Kncn	0	0	0
Kng2	0	0	0
Knop1	98.33811525	189.5852504	146.7984534
Kntc1	0	0	0
Kpna1	160.0609748	122.2025409	121.6881917
Kpna2	19.87685308	11.42079822	27.04182037
Kpna3	123.4457191	247.8313213	92.71481269
Kpna4	15.69225243	42.2569534	46.35740634
Kpna6	29.29220454	27.40991572	39.59695125
Kpnb1	162.1532751	278.6674765	265.5893072
Kptn	12.55380195	7.994558752	9.657792988
Kras	31.38450487	53.67775162	41.52850985
Krba1	63.8151599	95.93470502	90.78325409
Krcc1	163.1994253	178.1644522	151.6273499
Kremen1	29.29220454	44.54111304	28.00759967
Kri1	27.19990422	27.40991572	0
Krit1	73.23051136	105.0713436	95.61215059
Krr1	87.87661363	83.37182698	53.11786144
Krt10	5.230750811	0	9.657792988
Krt18	18.83070292	0	0
Krtcap2	48.12290746	75.37726823	135.2091018
Ksr1	23.01530357	29.69407536	21.24714457
Ksr2	1.046150162	2.284159643	0
Kti12	58.58440909	78.80350769	64.70721302
Ktn1	122.399569	113.0659023	112.996178
Kxd1	0	0	88.85169549
Kyat1	11.50765178	14.84703768	21.24714457
Kyat3	9.41535146	7.994558752	25.11026177
Kynu	2.092300324	0	10.62357229
L2hgdh	1.046150162	7.994558752	19.31558598
L3hypdh	3.138450487	2.284159643	12.55513089

For Review Only

L3mbtl1	0	2.284159643	0
L3mbtl2	52.30750811	49.10943233	48.28896494
L3mbtl3	17.78455276	20.55743679	16.41824808
l7Rn6	42.89215665	22.84159643	32.83649616
Lacc1	47.0767573	68.5247893	85.9543576
Lace1	12.55380195	14.84703768	18.34980668
Lactb	14.64610227	34.26239465	30.90493756
Lactb2	93.10736444	67.38270948	91.74903339
Lag3	0	0	1015.999822
Lage3	1.046150162	1.142079822	0
Lair1	875.6276858	1140.937742	4817.307143
Lama5	3.138450487	4.568319287	0
Lamb1	0	0	0
Lamb2	56.49210876	10.27871839	71.46766811
Lamc1	8.369201298	2.284159643	14.48668948
Lamc3	0	0	0
Lamp1	1173.780482	1171.773897	723.3686948
Lamp2	1886.208743	1745.097967	1694.942669
Lamp3	4.184600649	2.284159643	2.897337897
Lamtor1	189.3531794	35.40447447	0
Lamtor2	4.184600649	10.27871839	0
Lamtor3	0	0	0
Lamtor4	82.64586282	79.94558752	0
Lamtor5	0	0	0
Lancl1	118.2149683	102.7871839	106.2357229
Lancl2	1.046150162	0	0
Lap3	865.1661842	670.4008553	414.3193192
Laptm4a	9.41535146	424.8536937	141.9695569
Laptm4b	162.1532751	138.1916584	127.4828674
Laptm5	6826.129809	8680.948724	10615.84605
Large	71.13821103	143.9020575	115.8935159
Larp1	72.18436119	89.08222609	77.26234391
Larp1b	0	2.284159643	0
Larp4	40.79985633	38.83071394	27.04182037
Larp4b	183.0762784	196.4377293	175.7718324
Larp7	0	30.83615518	42.49428915
Lars	80.55356249	47.96735251	91.74903339
Lars2	1454.148726	1740.529648	1496.957913
Las1l	58.58440909	50.25151215	54.08364074
Lasp1	596.3055925	366.6076228	539.8706281
Lat2	48.12290746	0	0.965779299
Lats1	26.15375406	89.08222609	34.76805476
Lats2	106.7073165	95.93470502	81.1254611
Layn	95.19966476	131.3391795	103.338385
Lbh	47.0767573	21.69951661	18.34980668
Lbhd1	0	55.96191126	0
Lbp	25.10760389	0	0
Lbr	163.1994253	77.66142787	143.9011155
Lbx2	11.50765178	20.55743679	22.21292387
Lca5	12.55380195	7.994558752	0

For Review Only

Lca5l	10.46150162	5.710399108	4.828896494
Lcat	0	0	1.931558598
Lck	0	0	2.897337897
Lclat1	73.23051136	50.25151215	45.39162705
Lcmt1	38.707556	0	9.657792988
Lcmt2	94.1535146	73.09310859	117.8250745
Lcn2	207.1377321	107.3555032	6.760455092
Lcor	492.7367264	272.9570774	203.7794321
Lcorl	28.24605438	124.4867006	27.04182037
Lcp1	1399.748917	1096.396629	1391.68797
Lcp2	515.75203	534.4933565	603.6120618
Ldah	128.67647	113.0659023	84.9885783
Ldb1	0	0	2.897337897
Ldha	449.8445698	439.7007313	515.7261456
Ldhal6b	0	0	0
Ldhb	985.4734528	788.0350769	998.615795
Ldhd	0	0	10.62357229
Ldlr	7.323051136	10.27871839	11.58935159
Ldlrad1	0	0	9.657792988
Ldlrad3	0	2.284159643	0
Ldlrad4	466.5829724	428.2799331	349.6121062
Ldlrap1	46.03060714	44.54111304	105.2699436
Ldoc1l	39.75370617	39.97279376	44.42584775
Lef1	0	0	0
Lefty1	16.7384026	14.84703768	0
Lefty2	6.276900973	6.85247893	4.828896494
Lekr1	18.83070292	1.142079822	0
Lemd2	88.92276379	0	136.1748811
Lemd3	14.64610227	26.2678359	12.55513089
Leng8	0	0	0
Leo1	26.15375406	23.98367625	38.63117195
Leprot	372.4294578	372.3180219	479.0265322
Leprotl1	242.7068376	150.7545365	295.5284654
Letm1	167.384026	103.9292638	116.8592952
Letm2	32.43065503	17.13119732	27.04182037
Letmd1	56.49210876	25.12575608	611.3382962
Lfng	26.15375406	23.98367625	19.31558598
Lgals1	6.276900973	4.568319287	0
Lgals1-ps2	0	0	0
Lgals2	0	0	0
Lgals3	25.10760389	7.994558752	6.760455092
Lgals3bp	491.6905763	323.2085895	395.9695125
Lgals4	1.046150162	1.142079822	0
Lgals8	219.6915341	244.4050818	292.6311276
Lgals9	698.8283084	808.5925137	901.0720858
Lgalsl	10.46150162	7.994558752	0.965779299
Lgi1	6.276900973	0	0
Lgi4	24.06145373	3.426239465	6.760455092
Lgmn	9587.966237	9334.218382	11669.51127
Lgr4	1.046150162	1.142079822	9.657792988

For Review Only

Lhfp	4.184600649	0	0
Lhfpl2	688.3668068	627.0018221	548.5626417
Lhfpl4	5.230750811	0	2.897337897
Lhpp	13.59995211	17.13119732	5.794675793
Lhx4	3.138450487	0	0
Lias	80.55356249	70.80894894	58.91253723
Lif	0	4.568319287	0
Lifr	398.5832118	335.7714676	165.1482601
Lig1	0	0	0
Lig3	33.47680519	77.66142787	42.49428915
Lig4	14.64610227	5.710399108	22.21292387
Lilra5	0	0	0
Lilra6	7.323051136	3.426239465	4.828896494
Lilrb4a	0	0	0
Lima1	53.35365827	44.54111304	74.36500601
Limch1	3.138450487	3.426239465	0
Limd1	60.67670941	98.21886466	55.04942003
Limd2	1.046150162	0	0.965779299
Limk1	67.99976055	82.22974716	44.42584775
Limk2	102.5227159	38.83071394	65.67299232
Lims1	447.7522694	560.7611924	467.4371806
Lims2	0	0	0
Lin28a	0	0	0
Lin28b	0	4.568319287	0
Lin37	35.56910552	0	0
Lin52	40.79985633	12.56287804	32.83649616
Lin54	23.01530357	3.426239465	9.657792988
Lin7b	0	0	0
Lin7c	98.33811525	117.6342216	35.73383406
Lin9	6.276900973	43.39903322	50.22052354
Lingo2	0	0	0
Lins1	11.50765178	34.26239465	18.34980668
Lipa	319.0757995	320.9244299	372.7908094
Lipc	0	0	0
Lipe	160.0609748	127.91294	86.9201369
Liph	221.7838344	179.306532	164.1824808
Lipo2	17.78455276	14.84703768	10.62357229
Lipo3	61.72285957	54.81983144	39.59695125
Lipt1	12.55380195	3.426239465	6.760455092
Lipt2	10.46150162	11.42079822	20.28136528
Litaf	368.2448571	309.5036317	390.1748367
Lix1	2.092300324	1.142079822	7.726234391
Lix1l	5.230750811	4.568319287	0
Llgl1	114.0303677	0	0.965779299
Llgl2	0	0	2.897337897
Llph	35.56910552	0	26.07604107
Llph-ps2	31.38450487	27.40991572	38.63117195
Lman1	155.8763742	133.6233391	171.9087152
Lman2	454.0291704	399.7279376	460.6767256
Lman2l	178.8916777	0	56.98097863

For Review Only

Lmbr1	39.75370617	50.25151215	28.00759967
Lmbr1l	25.10760389	42.2569534	45.39162705
Lmbrd1	284.5528441	245.5471617	210.5398871
Lmbrd2	10.46150162	13.70495786	16.41824808
Lmf1	126.5841696	153.0386961	0
Lmln	25.10760389	41.11487358	21.24714457
Lmna	0	0	6.760455092
Lmnb1	5.230750811	5.710399108	4.828896494
Lmnb2	28.24605438	31.97823501	13.52091018
Lmo2	55.4459586	142.7599777	121.6881917
Lmo4	34.52295535	34.26239465	23.17870317
Lmtk2	2.092300324	2.284159643	7.726234391
Lmtk3	2.092300324	2.284159643	0
Lncpint	15.69225243	4.568319287	9.657792988
Lncppara	7.323051136	0	0
Lnp	34.52295535	52.5356718	26.07604107
Lnpep	245.8452881	195.2956495	161.2851429
Lnx2	25.10760389	10.27871839	21.24714457
LOC100041034	7.323051136	6.85247893	11.58935159
LOC100041057	5.230750811	4.568319287	11.58935159
LOC100041708	2.092300324	2.284159643	0
LOC100044633	5.230750811	5.710399108	7.726234391
LOC100049077	6.276900973	3.426239465	5.794675793
LOC100503338	0	0	0
LOC100504180	7.323051136	4.568319287	0
LOC100861615	6.276900973	3.426239465	7.726234391
LOC100861749	80.55356249	175.8802925	73.39922671
LOC100861913	0	0	3.863117195
LOC100861969	10.46150162	4.568319287	7.726234391
LOC101055656	6.276900973	5.710399108	0.965779299
LOC101055663	0	0	1.931558598
LOC101055672	4.184600649	3.426239465	0
LOC101055727	6.276900973	9.136638573	3.863117195
LOC101055754	1.046150162	3.426239465	0
LOC101055758	0	1.142079822	5.794675793
LOC101055907	1.046150162	4.568319287	0
LOC101055915	32.43065503	28.55199554	35.73383406
LOC101055953	3.138450487	0	2.897337897
LOC101055995	25.10760389	21.69951661	23.17870317
LOC101056014	58516.40933	103667.7275	83455.88655
LOC101056073	4.184600649	1.142079822	0
LOC101056115	1.046150162	2.284159643	0
LOC102631780	0	0	0
LOC102631912	50.21520779	28.55199554	49.25474424
LOC102631930	3.138450487	1.142079822	2.897337897
LOC102632031	5.230750811	0	0
LOC102632231	52.30750811	111.9238225	52.15208214
LOC102632465	12.55380195	1.142079822	0
LOC102632541	1.046150162	0	0
LOC102632594	3.138450487	0	0

For Review Only

LOC102632664	0	0	0
LOC102632739	13.59995211	10.27871839	10.62357229
LOC102632770	66.95361038	74.23518841	94.64637129
LOC102632778	0	3.426239465	0
LOC102632821	23.01530357	25.12575608	18.34980668
LOC102632901	1.046150162	0	0
LOC102632957	5.230750811	0	0
LOC102633000	0	0	0.965779299
LOC102633032	3.138450487	0	0
LOC102633149	5.230750811	6.85247893	7.726234391
LOC102633156	6.276900973	3.426239465	2.897337897
LOC102633239	8.369201298	4.568319287	4.828896494
LOC102633274	3.138450487	1.142079822	2.897337897
LOC102633497	0	1.142079822	3.863117195
LOC102633596	3.138450487	4.568319287	10.62357229
LOC102633627	8.369201298	12.56287804	16.41824808
LOC102633643	0	4.568319287	0
LOC102633880	14.64610227	9.136638573	7.726234391
LOC102633930	1.046150162	1.142079822	0
LOC102634078	33.47680519	83.37182698	36.69961336
LOC102634300	50.21520779	28.55199554	18.34980668
LOC102634333	2.092300324	1.142079822	0
LOC102634340	0	2.284159643	0
LOC102634389	1.046150162	1.142079822	0.965779299
LOC102634459	0	2.284159643	0
LOC102634481	3.138450487	0	4.828896494
LOC102634483	3.138450487	4.568319287	3.863117195
LOC102634533	3.138450487	0	3.863117195
LOC102634581	0	1.142079822	0
LOC102634683	1.046150162	3.426239465	0
LOC102634709	96.24581493	70.80894894	92.71481269
LOC102634716	5.230750811	0	0
LOC102634812	1.046150162	1.142079822	7.726234391
LOC102634873	2.092300324	1.142079822	0
LOC102634904	0	0	0
LOC102635048	37.66140584	45.68319287	47.32318564
LOC102635133	3.138450487	2.284159643	0
LOC102635154	4.184600649	4.568319287	2.897337897
LOC102635200	16.7384026	37.68863411	23.17870317
LOC102635527	3.138450487	9.136638573	39.59695125
LOC102635661	26.15375406	20.55743679	23.17870317
LOC102635786	28.24605438	19.41535697	18.34980668
LOC102635844	4.184600649	1.142079822	4.828896494
LOC102635912	8.369201298	27.40991572	12.55513089
LOC102635948	6.276900973	0	0
LOC102636299	20.92300324	26.2678359	28.97337897
LOC102636309	2.092300324	0	0
LOC102636313	1.046150162	4.568319287	3.863117195
LOC102636563	3.138450487	0	7.726234391
LOC102636700	0	0	0

For Review Only

LOC102636795	0	0	0
LOC102636907	0	0	0
LOC102637012	0	0	0
LOC102637269	4.184600649	6.85247893	5.794675793
LOC102637354	11.50765178	23.98367625	12.55513089
LOC102637515	7.323051136	7.994558752	4.828896494
LOC102637577	37.66140584	5.710399108	16.41824808
LOC102637646	0	0	7.726234391
LOC102637720	6.276900973	0	0
LOC102637763	0	0	0
LOC102637873	2.092300324	9.136638573	16.41824808
LOC102637966	0	0	0
LOC102638047	7.323051136	6.85247893	8.69201369
LOC102638183	17.78455276	17.13119732	21.24714457
LOC102638268	23.01530357	20.55743679	0
LOC102638435	2.092300324	2.284159643	2.897337897
LOC102638448	66.95361038	59.38815073	69.53610952
LOC102638515	5.230750811	1.142079822	0
LOC102638785	0	1.142079822	0.965779299
LOC102638940	39.75370617	9.136638573	53.11786144
LOC102639040	0	0	1.931558598
LOC102639044	23.01530357	25.12575608	37.66539266
LOC102639054	1.046150162	7.994558752	0
LOC102639076	5.230750811	1.142079822	0
LOC102639385	0	1.142079822	5.794675793
LOC102639505	13.59995211	9.136638573	11.58935159
LOC102639518	14.64610227	14.84703768	13.52091018
LOC102639543	1.046150162	4.568319287	0
LOC102639653	7.323051136	10.27871839	5.794675793
LOC102639683	2.092300324	12.56287804	20.28136528
LOC102639888	5.230750811	11.42079822	9.657792988
LOC102639979	7.323051136	5.710399108	6.760455092
LOC102639982	7.323051136	1.142079822	24.14448247
LOC102639987	5.230750811	4.568319287	3.863117195
LOC102640024	235.3837865	179.306532	249.1710591
LOC102640133	24.06145373	21.69951661	13.52091018
LOC102640295	0	0	0
LOC102640359	1.046150162	0	0.965779299
LOC102640451	1.046150162	0	0
LOC102640468	9.41535146	12.56287804	25.11026177
LOC102640526	3.138450487	0	0
LOC102640673	25.10760389	9.136638573	34.76805476
LOC102640772	18.83070292	7.994558752	15.45246878
LOC102640779	3.138450487	5.710399108	1.931558598
LOC102641351	7.323051136	1.142079822	0
LOC102641859	2.092300324	0	0
LOC102641980	1.046150162	0	0
LOC102642386	1.046150162	0	0
LOC102642832	7.323051136	6.85247893	14.48668948
LOC102643083	0	0	1.931558598

For Review Only

LOC102643247	1.046150162	1.142079822	0
LOC105242405	4.184600649	13.70495786	10.62357229
LOC105242736	9.41535146	12.56287804	0
LOC105242798	0	0	0.965779299
LOC105242891	18.83070292	4.568319287	11.58935159
LOC105242920	3.138450487	0	0
LOC105243004	0	0	0
LOC105243127	9.41535146	0	0
LOC105243139	12.55380195	1.142079822	0
LOC105243194	1.046150162	9.136638573	3.863117195
LOC105243269	0	1.142079822	0.965779299
LOC105243282	0	0	0
LOC105243374	1.046150162	1.142079822	0
LOC105243453	33.47680519	10.27871839	22.21292387
LOC105243553	3.138450487	2.284159643	0
LOC105243785	0	0	1.931558598
LOC105243964	1.046150162	1.142079822	0
LOC105244007	6.276900973	22.84159643	14.48668948
LOC105244034	0	0	0.965779299
LOC105244059	0	0	0
LOC105244102	3.138450487	1.142079822	0
LOC105244124	1.046150162	0	0
LOC105244151	3.138450487	6.85247893	0
LOC105244195	0	0	0
LOC105244208	267.8144415	218.1372459	308.0835963
LOC105244251	0	3.426239465	0.965779299
LOC105244333	1.046150162	7.994558752	0
LOC105244402	1.046150162	0	0
LOC105244413	9.41535146	30.83615518	12.55513089
LOC105244416	0	0	0
LOC105244467	1.046150162	0	0
LOC105244657	36.61525568	39.97279376	28.97337897
LOC105244798	4.184600649	0	0
LOC105244993	0	0	0
LOC105245043	2.092300324	1.142079822	0
LOC105245105	0	1.142079822	0
LOC105245328	4.184600649	6.85247893	2.897337897
LOC105245359	2.092300324	0	0
LOC105245415	8.369201298	13.70495786	5.794675793
LOC105245439	291.8758953	145.0441374	208.6083286
LOC105245580	8.369201298	2.284159643	5.794675793
LOC105245651	2.092300324	1.142079822	0
LOC105245696	1.046150162	10.27871839	0
LOC105245783	23.01530357	47.96735251	34.76805476
LOC105245882	14.64610227	1.142079822	14.48668948
LOC105246016	0	0	3.863117195
LOC105246034	11.50765178	26.2678359	11.58935159
LOC105246046	0	1.142079822	8.69201369
LOC105246056	1.046150162	0	0
LOC105246114	0	0	0

For Review Only

LOC105246186	5.230750811	27.40991572	6.760455092
LOC105246245	0	1.142079822	0
LOC105246409	117.1688182	91.36638573	100.4410471
LOC105246496	0	0	0
LOC105246506	5.230750811	0	8.69201369
LOC105246668	6.276900973	2.284159643	6.760455092
LOC105246804	2.092300324	0	0
LOC105246895	2.092300324	3.426239465	6.760455092
LOC105246914	3.138450487	3.426239465	0
LOC105246961	42.89215665	61.67231037	59.87831653
LOC105246973	1.046150162	0	0
LOC105247075	2.092300324	2.284159643	2.897337897
LOC105247125	2.092300324	11.42079822	3.863117195
LOC105247188	9.41535146	6.85247893	15.45246878
LOC105247253	10.46150162	5.710399108	16.41824808
LOC105247294	10.46150162	7.994558752	5.794675793
LOC105247300	1.046150162	0	0
LOC106740	5.230750811	9.136638573	11.58935159
LOC108167320	0	4.568319287	0
LOC108167323	0	0	0
LOC108167326	0	1.142079822	0
LOC108167327	2.092300324	7.994558752	36.69961336
LOC108167334	0	1.142079822	4.828896494
LOC108167339	3.138450487	0	1.931558598
LOC108167344	57.53825892	84.5139068	81.1254611
LOC108167350	0	0	0
LOC108167355	14.64610227	28.55199554	22.21292387
LOC108167356	13.59995211	19.41535697	16.41824808
LOC108167358	1.046150162	1.142079822	0
LOC108167360	0	0	0.965779299
LOC108167365	12.55380195	9.136638573	11.58935159
LOC108167372	13.59995211	1.142079822	0
LOC108167373	1.046150162	4.568319287	6.760455092
LOC108167375	12.55380195	5.710399108	22.21292387
LOC108167376	0	3.426239465	1.931558598
LOC108167377	32.43065503	37.68863411	9.657792988
LOC108167381	4.184600649	1.142079822	0
LOC108167411	0	1.142079822	1.931558598
LOC108167413	2.092300324	6.85247893	0.965779299
LOC108167415	0	0	0
LOC108167416	4.184600649	0	0
LOC108167423	21.96915341	10.27871839	10.62357229
LOC108167428	2.092300324	2.284159643	0
LOC108167433	36.61525568	11.42079822	10.62357229
LOC108167435	19.87685308	18.27327715	5.794675793
LOC108167436	9.41535146	0	0
LOC108167437	0	0	0
LOC108167440	268.8605917	349.4764254	282.0075553
LOC108167450	24.06145373	20.55743679	15.45246878
LOC108167452	3.138450487	0	0

For Review Only

LOC108167458	2.092300324	0	0
LOC108167466	0	0	4.828896494
LOC108167482	0	1.142079822	2.897337897
LOC108167485	0	0	0
LOC108167511	230.1530357	294.656594	274.2813209
LOC108167513	2.092300324	1.142079822	0
LOC108167514	39.75370617	26.2678359	40.56273055
LOC108167517	1.046150162	0	0
LOC108167518	31.38450487	26.2678359	23.17870317
LOC108167519	1.046150162	0	0
LOC108167523	0	0	0
LOC108167527	0	3.426239465	0
LOC108167532	4.184600649	17.13119732	17.38402738
LOC108167534	0	0	0
LOC108167536	14.64610227	44.54111304	40.56273055
LOC108167542	17.78455276	20.55743679	13.52091018
LOC108167547	0	1.142079822	0.965779299
LOC108167548	6.276900973	1.142079822	3.863117195
LOC108167549	4.184600649	1.142079822	1.931558598
LOC108167550	4.184600649	2.284159643	16.41824808
LOC108167552	0	0	0
LOC108167554	2.092300324	4.568319287	0
LOC108167555	13.59995211	6.85247893	13.52091018
LOC108167560	0	0	3.863117195
LOC108167561	0	2.284159643	0
LOC108167562	63.8151599	68.5247893	48.28896494
LOC108167564	2.092300324	2.284159643	0
LOC108167571	2.092300324	1.142079822	3.863117195
LOC108167576	1.046150162	4.568319287	1.931558598
LOC108167591	36.61525568	34.26239465	41.52850985
LOC108167597	0	1.142079822	0
LOC108167614	18.83070292	26.2678359	20.28136528
LOC108167618	0	5.710399108	0
LOC108167619	1.046150162	2.284159643	0
LOC108167626	18.83070292	33.12031483	39.59695125
LOC108167628	0	0	5.794675793
LOC108167630	1.046150162	1.142079822	0
LOC108167633	4.184600649	1.142079822	0
LOC108167637	2.092300324	1.142079822	0
LOC108167640	1.046150162	3.426239465	0
LOC108167641	1.046150162	2.284159643	6.760455092
LOC108167643	79.50741233	141.6178979	77.26234391
LOC108167645	2.092300324	0	0
LOC108167650	0	0	4.828896494
LOC108167659	1.046150162	0	0
LOC108167660	3.138450487	3.426239465	4.828896494
LOC108167669	11.50765178	3.426239465	7.726234391
LOC108167675	0	2.284159643	0
LOC108167679	2.092300324	10.27871839	0
LOC108167680	0	0	5.794675793

For Review Only

LOC108167681	0	1.142079822	0
LOC108167687	3.138450487	1.142079822	0
LOC108167690	0	0	3.863117195
LOC108167691	5.230750811	0	0
LOC108167692	2.092300324	2.284159643	0
LOC108167693	1.046150162	7.994558752	3.863117195
LOC108167700	7.323051136	12.56287804	10.62357229
LOC108167714	0	0	0
LOC108167719	0	1.142079822	0.965779299
LOC108167721	3.138450487	0	0
LOC108167725	2.092300324	2.284159643	0
LOC108167732	0	0	0
LOC108167733	12.55380195	1.142079822	6.760455092
LOC108167734	4.184600649	0	2.897337897
LOC108167735	1.046150162	2.284159643	0
LOC108167736	240.6145373	327.7769088	277.1786588
LOC108167737	15.69225243	4.568319287	4.828896494
LOC108167738	7.323051136	3.426239465	9.657792988
LOC108167745	19.87685308	22.84159643	15.45246878
LOC108167746	1.046150162	31.97823501	10.62357229
LOC108167748	284.5528441	163.3174145	212.4714457
LOC108167749	8.369201298	1.142079822	0
LOC108167751	7.323051136	0	0
LOC108167755	24.06145373	28.55199554	22.21292387
LOC108167760	10.46150162	1.142079822	0
LOC108167777	2.092300324	1.142079822	0.965779299
LOC108167794	0	2.284159643	0
LOC108167801	16.7384026	2.284159643	0.965779299
LOC108167802	9.41535146	12.56287804	8.69201369
LOC108167804	54.39980844	43.39903322	53.11786144
LOC108167806	1.046150162	1.142079822	1.931558598
LOC108167809	3.138450487	3.426239465	1.931558598
LOC108167810	0	0	33.80227546
LOC108167812	0	0	0
LOC108167814	3.138450487	3.426239465	0.965779299
LOC108167815	4.184600649	21.69951661	8.69201369
LOC108167825	3.138450487	7.994558752	15.45246878
LOC108167827	0	4.568319287	1.931558598
LOC108167844	0	0	0.965779299
LOC108167846	6.276900973	20.55743679	7.726234391
LOC108167848	10.46150162	45.68319287	23.17870317
LOC108167849	47.0767573	61.67231037	35.73383406
LOC108167875	16.7384026	13.70495786	5.794675793
LOC108167878	0	0	0
LOC108167886	7.323051136	3.426239465	6.760455092
LOC108167889	9.41535146	7.994558752	13.52091018
LOC108167890	0	3.426239465	0
LOC108167895	0	0	0
LOC108167902	9.41535146	4.568319287	2.897337897
LOC108167904	14.64610227	0	0.965779299

For Review Only

LOC108167905	18.83070292	15.9891175	11.58935159
LOC108167911	3.138450487	0	0
LOC108167915	9.41535146	13.70495786	15.45246878
LOC108167917	8.369201298	5.710399108	14.48668948
LOC108167922	87.87661363	89.08222609	100.4410471
LOC108167924	4.184600649	4.568319287	0
LOC108167926	7.323051136	12.56287804	0
LOC108167928	5.230750811	0	2.897337897
LOC108167930	4.184600649	5.710399108	0
LOC108167933	0	4.568319287	0
LOC108167939	1.046150162	0	0
LOC108167942	1.046150162	5.710399108	9.657792988
LOC108167961	0	0	0
LOC108167971	0	0	0
LOC108167986	0	0	0
LOC108167995	8.369201298	29.69407536	45.39162705
LOC108167997	24.06145373	23.98367625	39.59695125
LOC108168009	0	0	3.863117195
LOC108168017	5.230750811	0	0
LOC108168018	83.69201298	100.5030243	46.35740634
LOC108168019	0	0	0
LOC108168022	69.04591071	50.25151215	91.74903339
LOC108168025	0	0	0
LOC108168026	0	2.284159643	3.863117195
LOC108168030	10.46150162	3.426239465	0
LOC108168035	3.138450487	0	0
LOC108168043	1.046150162	1.142079822	0.965779299
LOC108168049	0	0	0
LOC108168050	13.59995211	7.994558752	9.657792988
LOC108168067	0	0	0
LOC108168071	0	0	0
LOC108168078	345.2295535	484.2418444	296.4942447
LOC108168079	0	0	0
LOC108168080	8.369201298	3.426239465	10.62357229
LOC108168082	6.276900973	14.84703768	9.657792988
LOC108168085	2.092300324	0	0.965779299
LOC108168086	3.138450487	0	0
LOC108168088	0	0	0
LOC108168091	0	2.284159643	0.965779299
LOC108168092	2.092300324	5.710399108	0
LOC108168101	37.66140584	73.09310859	84.9885783
LOC108168102	2.092300324	9.136638573	5.794675793
LOC108168108	4.184600649	12.56287804	0
LOC108168109	11.50765178	19.41535697	15.45246878
LOC108168114	50.21520779	42.2569534	57.94675793
LOC108168115	0	1.142079822	2.897337897
LOC108168144	2.092300324	2.284159643	0
LOC108168146	13.59995211	14.84703768	8.69201369
LOC108168165	1.046150162	1.142079822	0
LOC108168168	2.092300324	2.284159643	2.897337897

For Review Only

LOC108168169	2.092300324	2.284159643	2.897337897
LOC108168170	0	0	0
LOC108168171	4.184600649	5.710399108	2.897337897
LOC108168172	3.138450487	4.568319287	2.897337897
LOC108168178	0	1.142079822	0
LOC108168194	4.184600649	0	0
LOC108168201	17.78455276	11.42079822	16.41824808
LOC108168203	0	0	0
LOC108168204	2.092300324	4.568319287	0
LOC108168205	1.046150162	3.426239465	0
LOC108168207	3.138450487	4.568319287	0.965779299
LOC108168210	5.230750811	0	5.794675793
LOC108168233	38.707556	31.97823501	64.70721302
LOC108168235	0	0	1.931558598
LOC108168238	2.092300324	3.426239465	10.62357229
LOC108168240	5.230750811	3.426239465	6.760455092
LOC108168248	0	0	0
LOC108168252	0	0	0
LOC108168254	1.046150162	4.568319287	5.794675793
LOC108168256	4.184600649	17.13119732	14.48668948
LOC108168260	1.046150162	5.710399108	3.863117195
LOC108168281	48.12290746	30.83615518	58.91253723
LOC108168283	0	0	2.897337897
LOC108168286	2.092300324	4.568319287	7.726234391
LOC108168287	6.276900973	0	0
LOC108168292	3.138450487	2.284159643	0
LOC108168293	4.184600649	7.994558752	9.657792988
LOC108168294	1.046150162	2.284159643	0
LOC108168295	19.87685308	15.9891175	7.726234391
LOC108168304	12.55380195	14.84703768	11.58935159
LOC108168305	0	0	0
LOC108168323	8.369201298	63.95647001	29.93915826
LOC108168331	3.138450487	11.42079822	7.726234391
LOC108168334	7.323051136	6.85247893	7.726234391
LOC108168335	4.184600649	3.426239465	12.55513089
LOC108168336	1.046150162	1.142079822	5.794675793
LOC108168338	17.78455276	19.41535697	16.41824808
LOC108168340	0	0	0
LOC108168342	1.046150162	0	0
LOC108168347	0	0	0
LOC108168353	14.64610227	15.9891175	0
LOC108168354	1.046150162	1.142079822	0
LOC108168358	14.64610227	11.42079822	24.14448247
LOC108168376	9.41535146	3.426239465	10.62357229
LOC108168380	4.184600649	4.568319287	10.62357229
LOC108168382	6.276900973	2.284159643	0
LOC108168389	2.092300324	0	0
LOC108168392	1.046150162	1.142079822	0
LOC108168393	1.046150162	5.710399108	0
LOC108168395	36.61525568	35.40447447	40.56273055

For Review Only

LOC108168408	5.230750811	1.142079822	0
LOC108168411	1.046150162	0	0
LOC108168412	7.323051136	18.27327715	0
LOC108168420	1.046150162	1.142079822	0
LOC108168421	7.323051136	10.27871839	6.760455092
LOC108168424	3.138450487	0	0
LOC108168427	0	0	0
LOC108168464	25.10760389	25.12575608	13.52091018
LOC108168478	5.230750811	5.710399108	5.794675793
LOC108168482	18.83070292	38.83071394	41.52850985
LOC108168534	7.323051136	9.136638573	6.760455092
LOC108168644	3.138450487	1.142079822	0.965779299
LOC108168681	1.046150162	0	0
LOC108168686	0	2.284159643	0
LOC108168688	0	1.142079822	0
LOC108168734	6.276900973	7.994558752	6.760455092
LOC108168739	29.29220454	23.98367625	17.38402738
LOC108168740	0	17.13119732	13.52091018
LOC108168747	1.046150162	0	6.760455092
LOC108168750	30.33835471	36.54655429	13.52091018
LOC108168753	0	2.284159643	8.69201369
LOC108168756	0	0	0
LOC108168762	3.138450487	0	0
LOC108168763	0	3.426239465	0
LOC108168767	0	0	0
LOC108168772	3.138450487	1.142079822	0
LOC108168774	4.184600649	3.426239465	0
LOC108168776	9.41535146	5.710399108	3.863117195
LOC108168777	3.138450487	6.85247893	0
LOC108168785	0	0	0
LOC108168795	61.72285957	30.83615518	27.04182037
LOC108168801	0	0	0
LOC108168806	3.138450487	0	0.965779299
LOC108168809	16.7384026	17.13119732	19.31558598
LOC108168810	120.3072687	86.79806645	90.78325409
LOC108168813	5.230750811	5.710399108	1.931558598
LOC108168815	0	5.710399108	1.931558598
LOC108168816	1.046150162	998.1777641	0
LOC108168817	1.046150162	0	3.863117195
LOC108168820	0	0	0
LOC108168839	12.55380195	6.85247893	8.69201369
LOC108168842	4.184600649	3.426239465	0
LOC108168843	3.138450487	4.568319287	9.657792988
LOC108168845	1.046150162	1.142079822	0
LOC108168846	0	1.142079822	0
LOC108168864	1.046150162	3.426239465	0
LOC108168868	15.69225243	9.136638573	16.41824808
LOC108168869	7.323051136	25.12575608	18.34980668
LOC108168871	0	1.142079822	3.863117195
LOC108168876	73.23051136	101.6451041	69.53610952

For Review Only

LOC108168879	11.50765178	21.69951661	24.14448247
LOC108168882	0	4.568319287	0
LOC108168883	0	0	5.794675793
LOC108168886	4.184600649	9.136638573	3.863117195
LOC108168889	4.184600649	4.568319287	3.863117195
LOC108168899	46.03060714	26.2678359	43.46006845
LOC108168900	3.138450487	0	0
LOC108168906	11.50765178	37.68863411	10.62357229
LOC108168907	49.16905763	45.68319287	26.07604107
LOC108168909	1.046150162	2.284159643	6.760455092
LOC108168924	0	4.568319287	0
LOC108168925	13.59995211	94.7926252	60.84409583
LOC108168926	4.184600649	0	2.897337897
LOC108168930	0	1.142079822	0
LOC108168933	1.046150162	21.69951661	8.69201369
LOC108168936	8.369201298	0	5.794675793
LOC108168940	19.87685308	17.13119732	12.55513089
LOC108168959	9.41535146	13.70495786	3.863117195
LOC108168974	4.184600649	5.710399108	6.760455092
LOC108168979	0	4.568319287	1.931558598
LOC108168983	4.184600649	0	0
LOC108168984	0	3.426239465	0
LOC108168986	20.92300324	10.27871839	16.41824808
LOC108168987	0	0	0.965779299
LOC108168990	0	6.85247893	0
LOC108169008	1.046150162	0	0.965779299
LOC108169018	5.230750811	0	6.760455092
LOC108169023	31.38450487	10.27871839	9.657792988
LOC108169029	7.323051136	1.142079822	8.69201369
LOC108169030	0	2.284159643	0
LOC108169038	0	0	9.657792988
LOC108169039	0	1.142079822	0
LOC108169043	1.046150162	0	0
LOC108169045	0	3.426239465	0
LOC108169046	16.7384026	46.82527269	18.34980668
LOC108169050	0	0	0
LOC108169056	16.7384026	0	5.794675793
LOC108169061	3.138450487	3.426239465	0.965779299
LOC108169069	1.046150162	2.284159643	0.965779299
LOC108169076	14.64610227	7.994558752	15.45246878
LOC108169077	2.092300324	2.284159643	0.965779299
LOC108169079	3.138450487	1.142079822	1.931558598
LOC108169093	0	0	0
LOC108169096	4.184600649	6.85247893	0
LOC108169121	3.138450487	0	3.863117195
LOC108169124	3.138450487	6.85247893	6.760455092
LOC108169128	3.138450487	0	0
LOC108169130	3.138450487	9.136638573	4.828896494
LOC108169131	0	0	3.863117195
LOC108169152	1.046150162	4.568319287	4.828896494

For Review Only

LOC108169153	3.138450487	4.568319287	2.897337897
LOC108169155	5.230750811	9.136638573	23.17870317
LOC108169159	0	0	0
LOC108169163	12.55380195	31.97823501	20.28136528
LOC108169172	29.29220454	29.69407536	12.55513089
LOC108169175	1.046150162	0	6.760455092
LOC108169177	7.323051136	6.85247893	9.657792988
LOC108169202	156.9225243	67.38270948	94.64637129
LOC108169204	3.138450487	2.284159643	0
LOC433198	0	0	10.62357229
LOC546061	0	4.568319287	1.931558598
LOC73899	10.46150162	0	0
Lonp1	17.78455276	36.54655429	13.52091018
Lonp2	148.553323	165.6015741	164.1824808
Lonrf1	18.83070292	19.41535697	16.41824808
Lonrf3	93.10736444	69.66686912	61.80987513
Lox	3.138450487	0	0
Loxl2	6.276900973	11.42079822	4.828896494
Loxl3	33.47680519	3.426239465	77.26234391
Loxl4	5.230750811	0	12.55513089
Lpar1	5.230750811	0	0
Lpar2	4.184600649	3.426239465	0
Lpar5	178.8916777	161.0332549	165.1482601
Lpar6	508.4289789	603.0181458	638.3801165
Lpcat1	220.7376842	71.95102876	136.1748811
Lpcat2	3943.986112	2927.150583	4292.888983
Lpcat3	51.26135795	0	0
Lpgat1	64.86131006	86.79806645	66.63877162
Lpin1	210.2761826	198.721889	156.4562464
Lpin2	992.796504	801.7400348	855.6804588
Lpl	12.55380195	21.69951661	36.69961336
Lpp	127.6303198	124.4867006	148.730012
Lppos	0	0	1.931558598
Lpxn	176.7993774	222.7055652	137.1406604
Lrba	144.3687224	274.0991572	96.57792988
Lrch1	282.4605438	157.6070154	208.6083286
Lrch3	143.3225722	219.2793258	153.5589085
Lrch4	0	0	0
Lrfn1	0	0	0.965779299
Lrfn3	0	5.710399108	0
Lrg1	6.276900973	1.142079822	0
Lrguk	29.29220454	10.27871839	14.48668948
Lrif1	61.72285957	83.37182698	79.19390251
Lrig1	2.092300324	2.284159643	5.794675793
Lrig2	151.6917735	154.1807759	93.68059199
Lrig3	40.79985633	2.284159643	30.90493756
Lrmp	167.384026	129.0550198	162.2509222
Lrp1	2128.91558	1352.222509	1422.592907
Lrp10	31.38450487	157.6070154	0
Lrp11	1.046150162	9.136638573	0

For Review Only

Lrp12	102.5227159	99.36094448	54.08364074
Lrp3	0	0	0
Lrp4	32.43065503	39.97279376	22.21292387
Lrp5	97.29196509	51.39359197	68.57033022
Lrp6	51.26135795	76.51934805	55.04942003
Lrp8	0	0	0
Lrpap1	391.2601607	358.613064	488.6843252
Lrpprc	157.9686745	84.5139068	91.74903339
Lrrc1	0	2.284159643	0
Lrrc14	117.1688182	0	0
Lrrc14b	5.230750811	2.284159643	8.69201369
Lrrc15	0	0	0
Lrrc16a	30.33835471	18.27327715	32.83649616
Lrrc18	26.15375406	11.42079822	13.52091018
Lrrc20	9.41535146	10.27871839	23.17870317
Lrrc25	0	197.5798091	0
Lrrc27	5.230750811	0	0
Lrrc28	16.7384026	15.9891175	18.34980668
Lrrc29	3.138450487	2.284159643	17.38402738
Lrrc3	0	1662.86822	98.50948848
Lrrc32	0	0	0
Lrrc34	0	0	0
Lrrc39	56.49210876	33.12031483	41.52850985
Lrrc4	17.78455276	28.55199554	3.863117195
Lrrc40	33.47680519	21.69951661	48.28896494
Lrrc41	15.69225243	10.27871839	0
Lrrc42	0	0	0
Lrrc45	5.230750811	0	0
Lrrc47	8.369201298	22.84159643	16.41824808
Lrrc51	1.046150162	1.142079822	38.63117195
Lrrc56	11.50765178	27.40991572	18.34980668
Lrrc57	34.52295535	78.80350769	67.60455092
Lrrc58	509.475129	613.2968642	462.6082841
Lrrc59	48.12290746	52.5356718	47.32318564
Lrrc61	125.5380195	54.81983144	53.11786144
Lrrc69	9.41535146	4.568319287	0.965779299
Lrrc7	9.41535146	3.426239465	0
Lrrc71	0	0	0
Lrrc75a	11.50765178	12.56287804	8.69201369
Lrrc8a	494.8290267	504.7992812	0
Lrrc8b	7.323051136	3.426239465	0
Lrrc8c	5.230750811	2.284159643	10.62357229
Lrrc8d	96.24581493	78.80350769	78.22812321
Lrrc9	13.59995211	3.426239465	9.657792988
Lrrcc1	15.69225243	73.09310859	42.49428915
Lrrfip1	300.2450966	216.9951661	291.6653483
Lrrfip2	37.66140584	30.83615518	24.14448247
Lrriq3	2.092300324	5.710399108	0
Lrrk1	71.13821103	103.9292638	91.74903339
Lrrn2	3.138450487	2.284159643	0

For Review Only

Lrrn3	0	4.568319287	0
Lrrn4	3.138450487	14.84703768	0
Lrrtm2	7.323051136	1.142079822	9.657792988
Lrsam1	29.29220454	42.2569534	84.9885783
Lrtm2	0	1.142079822	0
Lrwd1	104.6150162	66.24062966	115.8935159
Lsg1	92.06121428	87.94014627	82.0912404
Lsm1	0	0	10.62357229
Lsm10	48.12290746	70.80894894	73.39922671
Lsm11	3.138450487	17.13119732	7.726234391
Lsm12	59.63055925	58.2460709	7.726234391
Lsm14a	31.38450487	63.95647001	60.84409583
Lsm14b	2.092300324	25.12575608	23.17870317
Lsm2	1.046150162	51.39359197	39.59695125
Lsm3	21.96915341	55.96191126	52.15208214
Lsm4	17.78455276	1.142079822	0
Lsm6	92.06121428	71.95102876	116.8592952
Lsm7	12.55380195	0	0
Lsm8	42.89215665	28.55199554	35.73383406
Lsp1	182.0301282	141.6178979	232.752811
Lsr	1.046150162	1.142079822	0
Lss	31.38450487	30.83615518	22.21292387
Lst1	53.35365827	170.1698934	175.7718324
Lta4h	133.9072208	97.07678484	128.4486467
Ltb	9.41535146	13.70495786	18.34980668
Ltb4r1	14.64610227	9.136638573	9.657792988
Ltb4r2	0	0	2.897337897
Ltbp3	9.41535146	0	4.828896494
Ltbp4	0	2.284159643	0
Ltbr	143.3225722	73.09310859	100.4410471
Ltc4s	409.0447134	303.7932326	374.722368
Ltf	601.5363433	122.2025409	0
Ltn1	80.55356249	153.0386961	74.36500601
Ltv1	76.36896184	47.96735251	75.33078531
Luc7l	59.63055925	91.36638573	108.1672815
Luc7l2	159.0148247	218.1372459	95.61215059
Luc7l3	98.33811525	133.6233391	185.4296254
Lum	2.092300324	0	0
Lurap1	1.046150162	1.142079822	0
Luzp1	62.76900973	62.81439019	65.67299232
Lxn	47.0767573	35.40447447	55.04942003
Ly6a	6.276900973	5.710399108	20.28136528
Ly6c1	2.092300324	1.142079822	0
Ly6c2	8.369201298	6.85247893	0
Ly6e	2.092300324	0	846.9884451
Ly6g	28.24605438	6.85247893	4.828896494
Ly6g5b	7.323051136	1.142079822	8.69201369
Ly6g6d	0	6.85247893	5.794675793
Ly6i	1.046150162	0	0
Ly6k	0	0	0

For Review Only

Ly86	1542.025339	2413.214663	2672.31132
Ly9	151.6917735	69.66686912	112.996178
Ly96	29.29220454	45.68319287	0
Lyar	29.29220454	22.84159643	20.28136528
Lyl1	207.1377321	0	50.22052354
Lyn	2255.49975	204.4322881	1298.973157
Lynx1	0	0	0.965779299
Lypd6	35.56910552	10.27871839	17.38402738
Lypla1	165.2917256	85.65598662	80.1596818
Lypla2	0	121.0604611	0.965779299
Lyplal1	27.19990422	7.994558752	15.45246878
Lyrm1	11.50765178	18.27327715	6.760455092
Lyrm2	197.7223807	1.142079822	0
Lyrm4	80.55356249	73.09310859	63.74143372
Lyrm5	53.35365827	51.39359197	28.00759967
Lyrm7	4.184600649	2.284159643	3.863117195
Lyrm9	46.03060714	66.24062966	57.94675793
Lysmd1	35.56910552	25.12575608	22.21292387
Lysmd2	0	0	1.931558598
Lysmd3	0	0	0
Lysmd4	38.707556	27.40991572	25.11026177
Lyst	164.2455755	182.7327715	108.1672815
Lyve1	0	4.568319287	0
Lyz1	110.8919172	79.94558752	73.39922671
Lyz2	1058.703964	838.2865891	858.5777967
Lyzl4	0	0	6.760455092
Lzic	67.99976055	61.67231037	89.81747479
Lztfl1	52.30750811	54.81983144	44.42584775
Lztr1	129.7226201	134.765419	147.7642327
Lzts1	7.323051136	2.284159643	4.828896494
Lzts2	16.7384026	7.994558752	10.62357229
Lzts3	4.184600649	1.142079822	2.897337897
M1ap	6.276900973	9.136638573	16.41824808
M6pr	670.582254	580.1765494	739.7869429
Mab21l1	4.184600649	3.426239465	0
Macf1	1138.211377	2123.126388	1265.170881
Macrod1	4.184600649	12.56287804	5.794675793
Macrod2	0	0	0
Mad1l1	47.0767573	43.39903322	16.41824808
Mad2l1	7.323051136	5.710399108	0
Mad2l1bp	33.47680519	20.55743679	49.25474424
Mad2l2	54.39980844	58.2460709	11.58935159
Madd	228.0607354	198.721889	230.8212524
Maea	242.7068376	205.5743679	293.5969068
Maf	206.091582	472.8210462	338.0227546
Maf1	0	0	1.931558598
Mafb	1118.334523	797.1717155	927.1481269
Maff	3.138450487	6.85247893	2.897337897
Mafg	0	0	0
Mafk	100.4304156	70.80894894	57.94675793

For Review Only

Mag	81.59971265	31.97823501	77.26234391
Maged1	0	84.5139068	0
Maged2	77.41511201	61.67231037	116.8592952
Magee1	28.24605438	7.994558752	17.38402738
Magef1	17.78455276	21.69951661	58.91253723
Mageh1	3.138450487	3.426239465	0
Magi1	47.0767573	68.5247893	43.46006845
Magi3	2.092300324	17.13119732	0
Magoh	0	0	0
Magohb	6.276900973	0	0
Magt1	205.0454318	178.1644522	203.7794321
Majin	0	1.142079822	0
Mak16	49.16905763	92.50846555	83.0570197
Mal	0	0	0
Malat1	7449.635305	8698.079922	7881.724858
Malsu1	39.75370617	41.11487358	35.73383406
Malt1	41.84600649	33.12031483	41.52850985
Mamdc4	24.06145373	17.13119732	31.87071686
Maml1	91.01506412	82.22974716	75.33078531
Maml2	465.5368222	536.7775162	476.1291943
Maml3	239.5683872	195.2956495	208.6083286
Mamld1	4.184600649	14.84703768	0
Mamstr	5.230750811	0	0
Man1a	145.4148726	252.3996406	114.9277366
Man1a2	342.0911031	356.3289044	329.3307409
Man1b1	124.4918693	3.426239465	65.67299232
Man1c1	248.9837386	244.4050818	242.410604
Man2a1	26.15375406	13.70495786	23.17870317
Man2a2	199.814681	223.847645	196.0531977
Man2b1	724.9820624	0	0.965779299
Man2b2	766.8280689	603.0181458	678.9428471
Man2c1	54.39980844	53.67775162	28.00759967
Man2c1os	0	0	15.45246878
Manba	104.6150162	148.4703768	141.0037776
Manbal	48.12290746	86.79806645	68.57033022
Manea	94.1535146	70.80894894	70.50188882
Maneal	0	0	0
Manf	84.73816314	65.09854983	122.653971
Mansc1	4.184600649	0	0
Maoa	0	0	19.31558598
Maob	0	2.284159643	0
Map10	132.8610706	9.136638573	32.83649616
Map1a	1.046150162	2.284159643	0
Map1b	7.323051136	1.142079822	17.38402738
Map1lc3a	55.4459586	49.10943233	51.18630284
Map1lc3b	0	0	318.7071686
Map1s	24.06145373	1.142079822	0
Map2k1	478.0906241	459.1160883	505.1025733
Map2k2	105.6611664	98.21886466	19.31558598
Map2k3	143.3225722	85.65598662	115.8935159

For Review Only

Map2k3os	10.46150162	2.284159643	6.760455092
Map2k4	77.41511201	60.53023055	59.87831653
Map2k5	56.49210876	66.24062966	58.91253723
Map2k6	35.56910552	27.40991572	40.56273055
Map2k7	48.12290746	53.67775162	28.97337897
Map3k1	245.8452881	244.4050818	201.8478735
Map3k10	10.46150162	9.136638573	0
Map3k11	229.1068855	204.4322881	163.2167015
Map3k12	58.58440909	39.97279376	71.46766811
Map3k13	6.276900973	2.284159643	0.965779299
Map3k14	48.12290746	98.21886466	75.33078531
Map3k15	0	0	0
Map3k19	11.50765178	4.568319287	11.58935159
Map3k2	44.98445698	75.37726823	48.28896494
Map3k3	28.24605438	139.3337382	52.15208214
Map3k4	94.1535146	77.66142787	111.0646194
Map3k5	61.72285957	162.1753347	114.9277366
Map3k7	43.93830681	63.95647001	64.70721302
Map3k9	62.76900973	86.79806645	81.1254611
Map4	719.7513116	497.9468022	373.7565887
Map4k1	5.230750811	5.710399108	7.726234391
Map4k2	0	142.7599777	38.63117195
Map4k3	54.39980844	82.22974716	61.80987513
Map4k4	112.9842175	140.4758181	150.6615706
Map4k5	35.56910552	105.0713436	22.21292387
Map6	1.046150162	0	0
Map6d1	1.046150162	2.284159643	0
Map7	14.64610227	26.2678359	35.73383406
Map7d1	52.30750811	22.84159643	0
Mapk1	97.29196509	147.328297	88.85169549
Mapk11	0	0	0
Mapk12	0	4.568319287	0
Mapk14	1.046150162	65.09854983	899.1405272
Mapk1ip1	84.73816314	70.80894894	134.2433225
Mapk1ip1l	149.5994732	153.0386961	156.4562464
Mapk3	117.1688182	0	94.64637129
Mapk4	8.369201298	1.142079822	0
Mapk6	82.64586282	77.66142787	47.32318564
Mapk7	0	1.142079822	0
Mapk8	42.89215665	43.39903322	15.45246878
Mapk8ip1	0	0	0
Mapk8ip3	56.49210876	165.6015741	108.1672815
Mapk9	61.72285957	41.11487358	62.77565443
Mapkap1	46.03060714	52.5356718	26.07604107
Mapkapk2	50.21520779	82.22974716	125.5513089
Mapkapk3	122.399569	89.08222609	88.85169549
Mapkapk5	0	35.40447447	0
Mapkbp1	17.78455276	47.96735251	47.32318564
Mapre1	375.5679082	392.8754587	426.8744501
Mapre2	218.6453839	212.4268468	193.1558598

For Review Only

Mapre3	29.29220454	25.12575608	5.794675793
Mapt	2.092300324	2.284159643	0
Marc2	211.3223328	220.4214056	229.8554731
March1	364.0602565	324.3506694	119.7566331
March10	3.138450487	4.568319287	4.828896494
March2	34.52295535	14.84703768	19.31558598
March3	0	0	1.931558598
March5	59.63055925	35.40447447	47.32318564
March6	95.19966476	134.765419	118.7908538
March7	203.9992816	123.3446207	96.57792988
March8	70.09206087	61.67231037	58.91253723
March9	2.092300324	0	0
Marcks	787.7510722	1219.74125	1175.353407
Marcksl1	14.64610227	11.42079822	7.726234391
Marf1	135.9995211	338.0556272	76.29656461
Mark1	2.092300324	0	0
Mark2	84.73816314	108.4975831	85.9543576
Mark3	76.36896184	119.9183813	97.54370918
Mark4	28.24605438	36.54655429	19.31558598
Mars	109.845767	42.2569534	141.9695569
Mars2	25.10760389	49.10943233	47.32318564
Marveld1	0	5.710399108	4.828896494
Marveld2	30.33835471	23.98367625	44.42584775
Mas1	0	0	0
Masp1	35.56910552	13.70495786	29.93915826
Mast1	9.41535146	4.568319287	0
Mast2	48.12290746	37.68863411	25.11026177
Mast3	107.7534667	383.7388201	141.9695569
Mast4	31.38450487	34.26239465	44.42584775
Mastl	7.323051136	4.568319287	0
Mat2a	416.3677646	553.9087135	399.8326297
Mat2b	210.2761826	153.0386961	208.6083286
Matk	105.6611664	0	151.6273499
Matn2	3.138450487	0	0
Matr3	356.7372053	259.2521195	216.3345629
Mau2	1.046150162	107.3555032	87.8859162
Mavs	223.8761347	70.80894894	284.9048932
Max	89.96891395	73.09310859	55.04942003
Maz	59.63055925	57.10399108	89.81747479
Mb21d1	87.87661363	101.6451041	64.70721302
Mb21d2	4.184600649	3.426239465	11.58935159
Mbd1	71.13821103	107.3555032	0
Mbd2	10.46150162	5.710399108	16.41824808
Mbd3	55.4459586	0	8.69201369
Mbd4	25.10760389	11.42079822	11.58935159
Mbd5	20.92300324	31.97823501	42.49428915
Mbd6	99.38426541	69.66686912	31.87071686
Mbip	44.98445698	29.69407536	44.42584775
Mblac1	7.323051136	17.13119732	4.828896494
Mblac2	1.046150162	1.142079822	115.8935159

For Review Only

Mbnl1	409.0447134	288.9461949	367.9619129
Mbnl2	228.0607354	287.8041151	285.8706725
Mbnl3	4.184600649	0	0
Mboat1	24.06145373	29.69407536	23.17870317
Mboat2	6.276900973	5.710399108	0
Mboat7	61.72285957	6.85247893	33.80227546
Mbp	111.9380674	67.38270948	72.43344741
Mbtd1	60.67670941	68.5247893	99.47526778
Mbtps1	195.6300803	179.306532	179.6349496
Mbtps2	61.72285957	39.97279376	45.39162705
Mc1r	4.184600649	0	0
Mcat	33.47680519	11.42079822	23.17870317
Mcc	4.184600649	13.70495786	15.45246878
Mccc1	50.21520779	35.40447447	52.15208214
Mccc1os	1.046150162	2.284159643	0
Mccc2	44.98445698	44.54111304	40.56273055
Mcee	39.75370617	19.41535697	14.48668948
Mcemp1	8.369201298	3.426239465	0
Mcf2l	1.046150162	2.284159643	0
Mcfd2	386.0294099	346.050186	354.4410027
Mcl1	1.046150162	0	0
Mcm2	11.50765178	14.84703768	11.58935159
Mcm3	202.9531315	134.765419	179.6349496
Mcm3ap	98.33811525	82.22974716	76.29656461
Mcm4	64.86131006	35.40447447	25.11026177
Mcm5	12.55380195	17.13119732	31.87071686
Mcm6	43.93830681	36.54655429	54.08364074
Mcm7	5.230750811	5.710399108	21.24714457
Mcm9	16.7384026	10.27871839	15.45246878
Mcmbp	154.830224	251.2575608	141.9695569
Mcmdc2	6.276900973	2.284159643	0.965779299
Mcoln1	0	0	0
Mcph1	16.7384026	7.994558752	17.38402738
Mcrs1	15.69225243	39.97279376	0
Mctp1	72.18436119	83.37182698	57.94675793
Mcts1	47.0767573	0	60.84409583
Mcts2	16.7384026	31.97823501	33.80227546
Mcu	12.55380195	14.84703768	17.38402738
Mcur1	147.5071729	203.2902083	105.2699436
Mdc1	167.384026	161.0332549	125.5513089
Mdfi	5.230750811	0	0
Mdfic	3.138450487	1.142079822	0
Mdga1	7.323051136	0	0
Mdga2	2.092300324	2.284159643	5.794675793
Mdh1	330.5834513	194.1535697	228.8896938
Mdh2	44.98445698	26.2678359	234.6843696
Mdk	0	0	0
Mdm1	7.323051136	4.568319287	16.41824808
Mdm2	171.5686266	163.3174145	171.9087152
Mdm4	356.7372053	478.5314453	452.9504912

For Review Only

Mdn1	268.8605917	97.07678484	86.9201369
Mdp1	0	0	68.57033022
Me1	3.138450487	2.284159643	0
Me2	58.58440909	41.11487358	46.35740634
Mea1	17.78455276	0	0
Meaf6	62.76900973	78.80350769	69.53610952
Mecom	3.138450487	0	0
Mecp2	62.76900973	161.0332549	68.57033022
Mecr	13.59995211	22.84159643	22.21292387
Med1	221.7838344	250.1154809	191.2243012
Med10	38.707556	60.53023055	69.53610952
Med11	0	0	0
Med12	157.9686745	134.765419	123.6197503
Med12l	238.522237	270.6729177	279.1102174
Med13	84.73816314	138.1916584	96.57792988
Med13l	119.2611185	164.4594943	149.6957913
Med14	19.87685308	36.54655429	32.83649616
Med15	78.46126217	133.6233391	132.3117639
Med16	25.10760389	19.41535697	28.00759967
Med17	161.107125	135.9074988	152.5931292
Med18	14.64610227	7.994558752	6.760455092
Med19	24.06145373	28.55199554	23.17870317
Med20	98.33811525	107.3555032	99.47526778
Med21	52.30750811	63.95647001	62.77565443
Med22	179.9378279	335.7714676	224.0607973
Med23	123.4457191	77.66142787	84.9885783
Med24	54.39980844	42.2569534	68.57033022
Med25	72.18436119	46.82527269	59.87831653
Med26	41.84600649	22.84159643	42.49428915
Med27	44.98445698	69.66686912	56.01519933
Med28	239.5683872	186.1590109	166.1140394
Med29	40.79985633	53.67775162	42.49428915
Med30	25.10760389	26.2678359	24.14448247
Med31	47.0767573	37.68863411	23.17870317
Med4	69.04591071	51.39359197	38.63117195
Med6	35.56910552	42.2569534	55.04942003
Med7	122.399569	145.0441374	145.8326741
Med8	0	44.54111304	0
Med9	75.32281168	39.97279376	86.9201369
Mef2a	537.7211834	936.5054538	507.9999112
Mef2c	758.4588676	1051.855516	724.3344741
Mef2d	117.1688182	110.7817427	94.64637129
Meg3	0	1.142079822	0
Megf11	2.092300324	4.568319287	22.21292387
Megf8	106.7073165	100.5030243	114.9277366
Megf9	14.64610227	7.994558752	4.828896494
Meis1	28.24605438	14.84703768	25.11026177
Meis3	64.86131006	63.95647001	50.22052354
Melk	0	0	0
Memo1	32.43065503	79.94558752	67.60455092

For Review Only

Men1	74.27666152	58.2460709	91.74903339
Meox1	2.092300324	0	2.897337897
Mepce	43.93830681	54.81983144	92.71481269
Mertk	2361.160916	1869.584668	1720.052931
Mesdc1	15.69225243	10.27871839	2.897337897
Mesdc2	159.0148247	239.8367625	273.3155416
Mest	14.64610227	2.284159643	6.760455092
Met	0	0	0
Metap1	140.1841217	131.3391795	84.9885783
Metap1d	133.9072208	123.3446207	104.3041643
Metap2	178.8916777	161.0332549	233.7185903
Metrn	2.092300324	0	5.794675793
Metrnl	4.184600649	5.710399108	0
Mettl1	18.83070292	4.568319287	17.38402738
Mettl10	18.83070292	17.13119732	30.90493756
Mettl13	26.15375406	14.84703768	33.80227546
Mettl14	29.29220454	45.68319287	57.94675793
Mettl15	31.38450487	5.710399108	17.38402738
Mettl16	69.04591071	68.5247893	72.43344741
Mettl17	0	28.55199554	11.58935159
Mettl18	21.96915341	0	22.21292387
Mettl2	51.26135795	31.97823501	38.63117195
Mettl20	25.10760389	4.568319287	28.00759967
Mettl21a	141.2302719	108.4975831	174.8060531
Mettl21c	0	3.426239465	1.931558598
Mettl22	26.15375406	18.27327715	24.14448247
Mettl23	0	0	0
Mettl25	57.53825892	38.83071394	48.28896494
Mettl3	54.39980844	1.142079822	0
Mettl4	29.29220454	55.96191126	43.46006845
Mettl5	30.33835471	54.81983144	55.04942003
Mettl6	38.707556	35.40447447	56.01519933
Mettl7a1	21.96915341	52.5356718	18.34980668
Mettl7a2	6.276900973	6.85247893	2.897337897
Mettl7a3	1.046150162	3.426239465	0
Mettl8	34.52295535	34.26239465	52.15208214
Mettl9	46.03060714	36.54655429	31.87071686
Mex3a	0	5.710399108	10.62357229
Mex3b	24.06145373	20.55743679	32.83649616
Mex3c	49.16905763	52.5356718	54.08364074
Mex3d	5.230750811	5.710399108	3.863117195
Mfap1a	34.52295535	21.69951661	50.22052354
Mfap1b	67.99976055	52.5356718	71.46766811
Mfap2	0	1.142079822	0
Mfap3	223.8761347	438.5586515	546.6310831
Mfap3l	4.184600649	13.70495786	0
Mff	203.9992816	194.1535697	164.1824808
Mfge8	101.4765657	1.142079822	0
Mfhas1	39.75370617	135.9074988	76.29656461
Mfn1	35.56910552	52.5356718	26.07604107

For Review Only

Mfn2	145.4148726	34.26239465	90.78325409
Mfng	291.8758953	45.68319287	0
Mfsd1	649.6592508	495.6626426	509.9314698
Mfsd10	0	0	0
Mfsd11	0	0	27.04182037
Mfsd12	42.89215665	116.4921418	70.50188882
Mfsd14a	126.5841696	199.8639688	235.6501489
Mfsd14b	324.3065503	285.5199554	204.7452114
Mfsd2a	0	0	0
Mfsd2b	3.138450487	0	0
Mfsd3	55.4459586	46.82527269	37.66539266
Mfsd4a	23.01530357	23.98367625	17.38402738
Mfsd4b3	0	0	0
Mfsd4b4	0	0	0
Mfsd5	130.7687703	93.65054538	85.9543576
Mfsd6	1.046150162	1.142079822	0
Mfsd6l	1.046150162	0	0
Mfsd7a	6.276900973	15.9891175	14.48668948
Mfsd7b	14.64610227	41.11487358	17.38402738
Mfsd7c	2.092300324	0	0
Mfsd8	82.64586282	86.79806645	56.01519933
Mfsd9	10.46150162	11.42079822	25.11026177
Mga	135.9995211	125.6287804	87.8859162
Mgat1	414.2754642	335.7714676	337.0569753
Mgat2	71.13821103	148.4703768	72.43344741
Mgat3	0	0	7.726234391
Mgat4a	988.6119033	1234.588287	1086.501711
Mgat4b	46.03060714	43.39903322	24.14448247
Mgat5	208.1838823	288.9461949	202.8136528
Mgea5	161.107125	221.5634854	210.5398871
Mgl2	1.046150162	2.284159643	11.58935159
Mgll	206.091582	163.3174145	193.1558598
Mgme1	128.67647	103.9292638	142.9353362
Mgmt	15.69225243	9.136638573	12.55513089
Mgrn1	147.5071729	15.9891175	73.39922671
Mgst1	3.138450487	1.142079822	0
Mgst3	18.83070292	19.41535697	0
Mia3	85.7843133	132.4812593	108.1672815
Miat	0	0	1.931558598
Mib1	50.21520779	65.09854983	47.32318564
Mib2	56.49210876	1.142079822	0
Mical1	99.38426541	4.568319287	74.36500601
Mical2	3.138450487	0	0
Mical3	80.55356249	79.94558752	56.01519933
Micall1	41.84600649	58.2460709	60.84409583
Micu1	112.9842175	105.0713436	116.8592952
Micu2	62.76900973	57.10399108	55.04942003
Micu3	25.10760389	62.81439019	32.83649616
Mid1	70.09206087	0	0
Mid1ip1	0	0	97.54370918

For Review Only

Midn	8.369201298	59.38815073	0
Mief1	106.7073165	107.3555032	83.0570197
Mief2	26.15375406	28.55199554	26.07604107
Mien1	0	0	37.66539266
Mier1	95.19966476	137.0495786	113.9619573
Mier2	18.83070292	33.12031483	88.85169549
Mier3	65.90746022	37.68863411	67.60455092
Mif	102.5227159	47.96735251	74.36500601
Mif4gd	6.276900973	3.426239465	25.11026177
Miip	0	20.55743679	88.85169549
Mill2	0	0	0
Milr1	42.89215665	61.67231037	37.66539266
Mina	58.58440909	33.12031483	41.52850985
Mink1	30.33835471	35.40447447	28.97337897
Minos1	35.56910552	66.24062966	87.8859162
Minpp1	182.0301282	139.3337382	152.5931292
Mios	51.26135795	15.9891175	37.66539266
Mipep	18.83070292	23.98367625	1.931558598
Mipol1	0	0	3.863117195
Mir1191	1.046150162	12.56287804	17.38402738
Mir126a	0	0	0
Mir1291	2.092300324	2.284159643	0
Mir133a-1hg	5.230750811	11.42079822	4.828896494
Mir140	6.276900973	4.568319287	2.897337897
Mir142b	9.41535146	7.994558752	6.760455092
Mir17hg	13.59995211	22.84159643	0
Mir181a-2	19.87685308	27.40991572	0
Mir181b-2	1.046150162	2.284159643	0.965779299
Mir1893	0	0	3.863117195
Mir1894	1.046150162	0	0
Mir1898	2.092300324	1.142079822	0
Mir1a-2	0	0	0
Mir20a	0	2.284159643	0
Mir223	13.59995211	1.142079822	0
Mir22hg	11.50765178	12.56287804	7.726234391
Mir24-2	3.138450487	1.142079822	1.931558598
Mir27a	0	0	0.965779299
Mir28b	0	2.284159643	0.965779299
Mir3069	1.046150162	1.142079822	0.965779299
Mir3101	4.184600649	9.136638573	5.794675793
Mir3112	1.046150162	2.284159643	0
Mir331	1.046150162	1.142079822	0
Mir339	3.138450487	1.142079822	0
Mir3474	0	2.284159643	15.45246878
Mir351	0	0	2.897337897
Mir455	2.092300324	0	0
Mir467f	1.046150162	1.142079822	0
Mir5103	1.046150162	1.142079822	0
Mir5107	0	0	2.897337897
Mir5114	46.03060714	63.95647001	83.0570197

For Review Only

Mir5116	14.64610227	2.284159643	0
Mir5122	0	0	0
Mir5123	1.046150162	2.284159643	0
Mir5129	9.41535146	4.568319287	0
Mir5130	0	0	0
Mir546	10.46150162	1.142079822	0
Mir5625	0	0	0
Mir6236	43.93830681	75.37726823	11.58935159
Mir6345	0	0	0.965779299
Mir6516	1.046150162	2.284159643	0
Mir6541	1.046150162	0	0
Mir670hg	0	2.284159643	0
Mir671	5.230750811	6.85247893	0
Mir682	42.89215665	41.11487358	50.22052354
Mir692-2	18.83070292	13.70495786	24.14448247
Mir692-3	23.01530357	13.70495786	27.04182037
Mir6920	1.046150162	1.142079822	0
Mir6934	1.046150162	1.142079822	0
Mir6948	0	0	0
Mir6983	1.046150162	0	0
Mir7021	0	0	0.965779299
Mir703	6.276900973	6.85247893	5.794675793
Mir7031	5.230750811	4.568319287	22.21292387
Mir704	4.184600649	1.142079822	3.863117195
Mir7049	1.046150162	3.426239465	0.965779299
Mir705	3.138450487	0	0
Mir7059	3.138450487	1.142079822	0
Mir7079	0	0	0
Mir7082	0	0	0
Mir7086	7.323051136	4.568319287	0
Mir7087	1.046150162	0	0
Mir7213	0	0	0.965779299
Mir7219	74.27666152	89.08222609	91.74903339
Mir7237	1.046150162	1.142079822	0
Mir7646	3.138450487	3.426239465	0
Mir7656	1.046150162	1.142079822	0
Mir7669	5.230750811	4.568319287	0
Mir7670	8.369201298	1.142079822	4.828896494
Mir7673	0	0	0.965779299
Mir7676-1	8.369201298	5.710399108	23.17870317
Mir7676-2	53.35365827	125.6287804	198.9505356
Mir8091	18.83070292	179.306532	0
Mir8099-2	0	0	0
Mir8112	11.50765178	0	0
Mir8116	1.046150162	2.284159643	4.828896494
Mir92-1	1.046150162	2.284159643	14.48668948
Mir99ahg	32.43065503	46.82527269	17.38402738
Mirlet7c-2	0	0	0
Mirt1	4.184600649	0	0
Mis12	99.38426541	107.3555032	138.1064397

For Review Only

Mis18a	9.41535146	37.68863411	38.63117195
Mis18bp1	2.092300324	1.142079822	0
Mitd1	27.19990422	42.2569534	38.63117195
Mitf	74.27666152	142.7599777	60.84409583
Mki67	2.092300324	9.136638573	0
Mkks	38.707556	42.2569534	39.59695125
Mkl1	97.29196509	101.6451041	109.1330608
Mkl2	52.30750811	81.08766734	63.74143372
Mkln1	1.046150162	1.142079822	0
Mknk1	162.1532751	0	1.931558598
Mknk2	1.046150162	237.5526029	26.07604107
Mkrn1	103.5688661	149.6124566	99.47526778
Mkrn2	50.21520779	55.96191126	51.18630284
Mkrn3	20.92300324	1.142079822	0
Mks1	42.89215665	20.55743679	22.21292387
Mlec	383.9371095	413.4328954	504.136794
Mlf2	176.7993774	173.5961329	2.897337897
Mlh1	55.4459586	54.81983144	65.67299232
Mlh3	43.93830681	13.70495786	28.97337897
Mlkl	0	0	0
Mllt1	38.707556	60.53023055	0
Mllt10	51.26135795	97.07678484	83.0570197
Mllt11	17.78455276	12.56287804	38.63117195
Mllt3	5.230750811	7.994558752	13.52091018
Mllt4	8.369201298	4.568319287	20.28136528
Mllt6	44.98445698	63.95647001	36.69961336
Mlph	176.7993774	229.5580442	0
Mlst8	71.13821103	36.54655429	50.22052354
Mlx	0	77.66142787	57.94675793
Mlxip	1.046150162	1.142079822	0
Mlxipl	0	121.0604611	0.965779299
Mlycd	66.95361038	28.55199554	28.97337897
Mmaa	77.41511201	93.65054538	98.50948848
Mmab	24.06145373	25.12575608	14.48668948
Mmachc	39.75370617	26.2678359	34.76805476
Mmadhc	41.84600649	68.5247893	130.3802053
Mmd	48.12290746	35.40447447	30.90493756
Mmgt1	58.58440909	55.96191126	53.11786144
Mmgt2	64.86131006	137.0495786	92.71481269
Mmp11	8.369201298	13.70495786	14.48668948
Mmp14	32.43065503	44.54111304	44.42584775
Mmp15	12.55380195	3.426239465	0
Mmp16	3.138450487	0	0
Mmp17	1.046150162	0	0
Mmp19	0	0	0
Mmp2	132.8610706	119.9183813	0
Mmp24	4.184600649	4.568319287	10.62357229
Mmp25	7.323051136	3.426239465	0
Mmp28	8.369201298	6.85247893	13.52091018
Mmp8	30.33835471	0	0

For Review Only

Mmp9	44.98445698	0	15.45246878
Mmrn2	7.323051136	5.710399108	5.794675793
Mms19	0	0	0
Mms22l	0	18.27327715	13.52091018
Mn1	24.06145373	0	9.657792988
Mnat1	31.38450487	34.26239465	26.07604107
Mnd1-ps	1.046150162	0	0
Mnda	0	5.710399108	0
Mndal	1.046150162	0	0.965779299
Mnt	33.47680519	25.12575608	32.83649616
Moap1	50.21520779	0	7.726234391
Mob1a	844.2431809	826.8657909	626.790765
Mob1b	30.33835471	29.69407536	15.45246878
Mob2	0	0	51.18630284
Mob3a	319.0757995	266.1045984	333.1938581
Mob3b	6.276900973	3.426239465	6.760455092
Mob3c	1.046150162	1.142079822	0
Mob4	216.5530836	141.6178979	133.2775432
Mobp	3.138450487	0	0
Mocos	0	4.568319287	3.863117195
Mocs1	10.46150162	4.568319287	41.52850985
Mocs2	53.35365827	27.40991572	60.84409583
Mocs3	6.276900973	4.568319287	3.863117195
Mogs	24.06145373	209.0006074	0
Mok	0	0	0
Mon1a	55.4459586	49.10943233	63.74143372
Mon1b	95.19966476	49.10943233	78.22812321
Mon2	132.8610706	171.3119732	162.2509222
Morc2a	73.23051136	119.9183813	116.8592952
Morc3	387.07556	230.700124	0
Morf4l1	96.24581493	106.2134234	103.338385
Morf4l1-ps1	100.4304156	108.4975831	109.1330608
Morf4l1b	82.64586282	91.36638573	101.4068264
Morf4l2	130.7687703	122.2025409	177.703391
Morn1	43.93830681	54.81983144	0
Morn2	0	0	8.69201369
Morn3	0	9.136638573	1.931558598
Mospd1	40.79985633	21.69951661	25.11026177
Mospd2	31.38450487	76.51934805	50.22052354
Mospd3	15.69225243	5.710399108	3.863117195
Mov10	58.58440909	25.12575608	43.46006845
Mpc1	140.1841217	142.7599777	149.6957913
Mpc2	57.53825892	45.68319287	55.04942003
Mpdu1	9.41535146	2.284159643	0
Mpdz	0	0	0
Mpeg1	0	0	1110.646194
Mpg	46.03060714	0	4.828896494
Mphosph10	32.43065503	50.25151215	33.80227546
Mphosph6	21.96915341	54.81983144	38.63117195
Mphosph8	13.59995211	39.97279376	35.73383406

For Review Only

Mphosph9	27.19990422	51.39359197	34.76805476
Mpi	7.323051136	1.142079822	21.24714457
Mplkip	14.64610227	25.12575608	9.657792988
Mpnd	19.87685308	9.136638573	18.34980668
Mpo	0	0	0
Mpp1	31.38450487	52.5356718	84.022799
Mpp4	0	0	6.760455092
Mpp5	41.84600649	39.97279376	54.08364074
Mpp6	17.78455276	10.27871839	14.48668948
Mpp7	3.138450487	14.84703768	6.760455092
Mppe1	61.72285957	41.11487358	45.39162705
Mprip	98.33811525	131.3391795	146.7984534
Mpst	1.046150162	7.994558752	0
Mpv17	1.046150162	0	0
Mpv17l	16.7384026	20.55743679	19.31558598
Mpv17l2	39.75370617	26.2678359	76.29656461
Mpzl1	0	0	0
Mr1	44.98445698	43.39903322	54.08364074
Mras	42.89215665	28.55199554	53.11786144
Mrc1	33.47680519	29.69407536	28.00759967
Mrc2	39.75370617	42.2569534	31.87071686
Mre11a	33.47680519	25.12575608	27.04182037
Mrfap1	0	0	0
Mrgbp	70.09206087	46.82527269	38.63117195
Mrgpra2b	3.138450487	0	2.897337897
Mrgpre	40.79985633	21.69951661	28.97337897
Mri1	2.092300324	21.69951661	17.38402738
Mrm1	71.13821103	22.84159643	47.32318564
Mrm2	3.138450487	29.69407536	29.93915826
Mroh1	271.9990422	113.0659023	125.5513089
Mroh2a	0	0	0
Mroh6	11.50765178	2.284159643	14.48668948
Mrpl1	23.01530357	23.98367625	27.04182037
Mrpl10	71.13821103	71.95102876	98.50948848
Mrpl11	0	0	0
Mrpl12	23.01530357	55.96191126	52.15208214
Mrpl13	26.15375406	3.426239465	44.42584775
Mrpl14	28.24605438	36.54655429	6.760455092
Mrpl15	94.1535146	102.7871839	104.3041643
Mrpl16	31.38450487	54.81983144	35.73383406
Mrpl17	118.2149683	260.3941993	235.6501489
Mrpl18	53.35365827	79.94558752	98.50948848
Mrpl19	72.18436119	38.83071394	48.28896494
Mrpl2	18.83070292	3.426239465	0
Mrpl20	50.21520779	102.7871839	87.8859162
Mrpl21	35.56910552	52.5356718	35.73383406
Mrpl22	33.47680519	37.68863411	30.90493756
Mrpl23	32.43065503	52.5356718	47.32318564
Mrpl24	0	0	0.965779299
Mrpl27	18.83070292	41.11487358	40.56273055

For Review Only

Mrpl28	29.29220454	37.68863411	58.91253723
Mrpl3	84.73816314	70.80894894	90.78325409
Mrpl30	65.90746022	58.2460709	64.70721302
Mrpl32	52.30750811	50.25151215	0
Mrpl33	16.7384026	23.98367625	37.66539266
Mrpl34	1.046150162	11.42079822	73.39922671
Mrpl35	31.38450487	37.68863411	44.42584775
Mrpl36	0	0	0
Mrpl37	108.7996169	29.69407536	76.29656461
Mrpl38	60.67670941	50.25151215	0
Mrpl39	65.90746022	66.24062966	57.94675793
Mrpl4	0	0	1.931558598
Mrpl40	50.21520779	68.5247893	71.46766811
Mrpl41	29.29220454	1.142079822	0
Mrpl42	0	50.25151215	0
Mrpl43	0	0	0
Mrpl44	57.53825892	60.53023055	82.0912404
Mrpl45	53.35365827	67.38270948	62.77565443
Mrpl46	33.47680519	41.11487358	56.98097863
Mrpl47	0	0	8.69201369
Mrpl48	25.10760389	22.84159643	30.90493756
Mrpl49	127.6303198	0	0.965779299
Mrpl50	75.32281168	92.50846555	90.78325409
Mrpl51	2.092300324	4.568319287	162.2509222
Mrpl52	18.83070292	14.84703768	69.53610952
Mrpl53	11.50765178	0	0
Mrpl54	33.47680519	15.9891175	23.17870317
Mrpl55	49.16905763	0	32.83649616
Mrpl57	0	0	0
Mrpl9	86.83046347	53.67775162	94.64637129
Mrps10	28.24605438	44.54111304	47.32318564
Mrps11	5.230750811	10.27871839	0
Mrps14	12.55380195	413.4328954	0
Mrps15	0	54.81983144	33.80227546
Mrps16	36.61525568	9.136638573	0
Mrps17	0	9.136638573	0
Mrps18a	16.7384026	1.142079822	92.71481269
Mrps18b	0	0	14.48668948
Mrps18c	0	0	28.97337897
Mrps2	0	0	0.965779299
Mrps21	16.7384026	45.68319287	45.39162705
Mrps22	0	21.69951661	0
Mrps23	26.15375406	84.5139068	63.74143372
Mrps24	4.184600649	7.994558752	0
Mrps25	43.93830681	91.36638573	46.35740634
Mrps27	44.98445698	47.96735251	54.08364074
Mrps28	20.92300324	15.9891175	30.90493756
Mrps30	77.41511201	93.65054538	80.1596818
Mrps31	37.66140584	15.9891175	55.04942003
Mrps33	35.56910552	51.39359197	60.84409583

For Review Only

Mrps34	0	0	0
Mrps35	50.21520779	67.38270948	75.33078531
Mrps36	17.78455276	15.9891175	24.14448247
Mrps5	43.93830681	33.12031483	34.76805476
Mrps6	69.04591071	43.39903322	51.18630284
Mrps7	59.63055925	36.54655429	64.70721302
Mrps9	50.21520779	47.96735251	60.84409583
Mrrf	12.55380195	23.98367625	17.38402738
Mrs2	106.7073165	70.80894894	48.28896494
Mrto4	9.41535146	12.56287804	0
Mrvi1	7.323051136	1.142079822	0
Ms4a14	0	0	0
Ms4a4c	0	2.284159643	0
Ms4a6b	148.553323	154.1807759	0
Ms4a6c	38.707556	18.27327715	20.28136528
Ms4a6d	184.1224286	235.2684433	246.2737212
Ms4a7	0	0	0
Msantd1	8.369201298	10.27871839	3.863117195
Msantd2	13.59995211	28.55199554	66.63877162
Msantd4	38.707556	37.68863411	56.98097863
Msh2	37.66140584	54.81983144	64.70721302
Msh3	19.87685308	13.70495786	24.14448247
Msh5	0	0	1.931558598
Msh6	66.95361038	63.95647001	86.9201369
Msi2	24.06145373	35.40447447	9.657792988
Msl1	0	0	133.2775432
Msl2	133.9072208	149.6124566	53.11786144
Msl3	73.23051136	75.37726823	91.74903339
Msl3l2	1.046150162	6.85247893	0
Mslnl	1.046150162	0	0
Msmo1	74.27666152	109.6396629	89.81747479
Msn	1622.578902	791.4613164	923.2850097
Msr1	0	2.284159643	0
Msra	50.21520779	52.5356718	46.35740634
Msrb1	5.230750811	2.284159643	13.52091018
Msrb2	163.1994253	190.7273302	218.2661215
Msrb3	1.046150162	0	0
Mss51	2.092300324	1.142079822	0
Mst1	2.092300324	3.426239465	0
Msto1	0	1.142079822	110.0988401
Msx1	30.33835471	0	0
Msx1os	0	3.426239465	0
Mt1	78.46126217	46.82527269	59.87831653
Mt3	26.15375406	0	0
Mta1	23.01530357	25.12575608	13.52091018
Mta2	0	0	3.863117195
Mta3	24.06145373	39.97279376	39.59695125
Mtap	57.53825892	77.66142787	51.18630284
Mtap7d3	0	0	0
Mtbp	8.369201298	0	0

For Review Only

Mtch1	38.707556	50.25151215	11.58935159
Mtch2	116.122668	164.4594943	161.2851429
Mtcp1	3.138450487	10.27871839	6.760455092
Mtdh	229.1068855	551.6245539	353.4752234
Mterf1a	8.369201298	15.9891175	12.55513089
Mterf1b	6.276900973	12.56287804	9.657792988
Mterf2	0	2.284159643	0
Mterf3	36.61525568	49.10943233	95.61215059
Mterf4	32.43065503	31.97823501	46.35740634
Mtf1	25.10760389	43.39903322	0
Mtf2	73.23051136	78.80350769	56.01519933
Mtfmt	26.15375406	15.9891175	25.11026177
Mtfp1	6.276900973	4.568319287	0
Mtfr1	29.29220454	22.84159643	41.52850985
Mtfr1l	330.5834513	219.2793258	294.5626861
Mtg1	86.83046347	26.2678359	46.35740634
Mtg2	27.19990422	44.54111304	0.965779299
Mthfd1	59.63055925	49.10943233	66.63877162
Mthfd1l	13.59995211	0	15.45246878
Mthfd2	51.26135795	34.26239465	37.66539266
Mthfd2l	0	3.426239465	2.897337897
Mthfr	27.19990422	52.5356718	98.50948848
Mthfs	0	0	0
Mthfsd	60.67670941	30.83615518	33.80227546
Mthfsl	69.04591071	60.53023055	85.9543576
Mtif2	60.67670941	49.10943233	42.49428915
Mtif3	75.32281168	106.2134234	122.653971
Mtl5	0	0	0
Mtm1	53.35365827	38.83071394	27.04182037
Mtmr1	39.75370617	29.69407536	9.657792988
Mtmr10	185.1685787	30.83615518	57.94675793
Mtmr11	8.369201298	7.994558752	17.38402738
Mtmr12	72.18436119	65.09854983	108.1672815
Mtmr14	61.72285957	50.25151215	63.74143372
Mtmr2	223.8761347	155.3228557	207.6425493
Mtmr3	184.1224286	163.3174145	246.2737212
Mtmr4	152.7379237	196.4377293	95.61215059
Mtmr6	132.8610706	359.7551438	270.4182037
Mtmr7	0	0	0
Mtmr9	73.23051136	43.39903322	34.76805476
Mto1	76.36896184	65.09854983	39.59695125
Mtor	77.41511201	138.1916584	110.0988401
Mtpap	11.50765178	68.5247893	0
Mtpn	410.0908636	245.5471617	243.3763833
Mtr	156.9225243	141.6178979	104.3041643
Mtrf1	26.15375406	17.13119732	27.04182037
Mtrf1l	17.78455276	5.710399108	7.726234391
Mtrr	27.19990422	27.40991572	28.00759967
Mtss1	102.5227159	129.0550198	69.53610952
Mtss1l	7.323051136	0	0

For Review Only

Mttp	31.38450487	12.56287804	9.657792988
Mturn	0	3.426239465	0
Mtus1	355.6910552	202.1481284	149.6957913
Mtus2	8.369201298	10.27871839	2.897337897
Mtx2	76.36896184	89.08222609	68.57033022
Mtx3	66.95361038	51.39359197	60.84409583
Muc16	7.323051136	9.136638573	15.45246878
Muc19	4.184600649	0	0
Muc5b	4.184600649	1.142079822	0
Muc6	1.046150162	4.568319287	10.62357229
Mul1	64.86131006	55.96191126	49.25474424
Mum1	73.23051136	51.39359197	75.33078531
Mus81	26.15375406	15.9891175	42.49428915
Musk	0	0	0
Mustn1	0	0	6.760455092
Mut	73.23051136	109.6396629	57.94675793
Mutyh	5.230750811	0	0
Mvb12a	5.230750811	191.86941	0
Mvb12b	310.7065982	108.4975831	195.0874184
Mvd	20.92300324	10.27871839	21.24714457
Mvk	19.87685308	0	12.55513089
Mvp	1.046150162	1.142079822	0
Mx1	4.184600649	30.83615518	20.28136528
Mx2	11.50765178	15.9891175	33.80227546
Mxd1	84.73816314	85.65598662	40.56273055
Mxd4	8.369201298	0	0
Mxi1	72.18436119	75.37726823	76.29656461
Mxra7	1.046150162	1.142079822	0
Mxra8	5.230750811	2.284159643	4.828896494
Myadm	359.8756558	443.1269708	388.2432781
Myadml2	34.52295535	43.39903322	79.19390251
Mybbp1a	199.814681	138.1916584	175.7718324
Mybl2	0	5.710399108	4.828896494
Mybpc3	0	12.56287804	0
Mybph	0	0	0
Myc	141.2302719	86.79806645	163.2167015
Mycbp	52.30750811	0	2.897337897
Mycbp2	415.3216144	624.7176624	524.4181593
Mycl	37.66140584	25.12575608	27.04182037
Myd88	0	0	0
Mydgf	116.122668	110.7817427	213.437225
Myef2	41.84600649	43.39903322	39.59695125
Myeov2	104.6150162	60.53023055	108.1672815
Myh10	29.29220454	17.13119732	9.657792988
Myh7b	0	0	0
Myh9	295.0143458	103.9292638	143.9011155
Myl12a	228.0607354	183.8748513	271.383983
Myl12b	267.8144415	301.5090729	298.4258033
Myl2	0	0	0
Myl6	0	0	0

For Review Only

Myl9	0	0	0
Mylip	692.5514074	829.1499505	684.7375229
Mylk	0	0	0
Mynn	87.87661363	53.67775162	88.85169549
Myo10	1.046150162	1.142079822	0
Myo15	3.138450487	7.994558752	8.69201369
Myo18a	240.6145373	162.1753347	255.9315142
Myo18b	71.13821103	50.25151215	59.87831653
Myo19	25.10760389	25.12575608	12.55513089
Myo1b	149.5994732	124.4867006	70.50188882
Myo1c	131.8149204	116.4921418	148.730012
Myo1d	27.19990422	4.568319287	21.24714457
Myo1e	59.63055925	14.84703768	56.01519933
Myo1f	623.5054967	502.5151215	562.0835519
Myo1g	50.21520779	67.38270948	97.54370918
Myo1h	0	4.568319287	0
Myo5a	23.01530357	27.40991572	12.55513089
Myo5b	0	0	0
Myo6	26.15375406	21.69951661	27.04182037
Myo7a	236.4299367	151.8966163	145.8326741
Myo9a	112.9842175	153.0386961	107.2015022
Myo9b	236.4299367	242.1209222	213.437225
Myocd	1.046150162	0	0
Myof	0	0	0
Myom1	14.64610227	20.55743679	20.28136528
Myom3	1.046150162	0	4.828896494
Mypop	3.138450487	1.142079822	0
Mypopos	3.138450487	3.426239465	0.965779299
Myrip	4.184600649	0	0.965779299
Mysm1	258.3990901	366.6076228	299.3915826
Myt1l	0	3.426239465	0
Mzf1	6.276900973	12.56287804	6.760455092
Mzt1	52.30750811	47.96735251	67.60455092
Mzt2	0	0	9.657792988
N4bp1	110.8919172	116.4921418	162.2509222
N4bp2	10.46150162	18.27327715	15.45246878
N4bp2l1	140.1841217	157.6070154	203.7794321
N4bp2l2	140.1841217	135.9074988	153.5589085
N4bp3	4.184600649	5.710399108	10.62357229
N6amt1	0	12.56287804	14.48668948
Naa10	55.4459586	38.83071394	57.94675793
Naa15	73.23051136	73.09310859	53.11786144
Naa16	61.72285957	84.5139068	85.9543576
Naa20	1.046150162	44.54111304	0
Naa25	149.5994732	116.4921418	85.9543576
Naa30	21.96915341	20.55743679	32.83649616
Naa35	0	0	3.863117195
Naa38	0	0	0
Naa40	28.24605438	1.142079822	38.63117195
Naa50	55.4459586	46.82527269	44.42584775

For Review Only

Naa60	213.4146331	234.1263634	15.45246878
Naaa	70.09206087	82.22974716	77.26234391
Naalad2	44.98445698	121.0604611	96.57792988
Naaladl2	2.092300324	0	0
Nab1	18.83070292	12.56287804	16.41824808
Nab2	11.50765178	21.69951661	10.62357229
Nabp1	10.46150162	15.9891175	5.794675793
Nabp2	0	1.142079822	5.794675793
Naca	132.8610706	183.8748513	194.1216391
Nacad	0	1.142079822	0
Nacc1	27.19990422	33.12031483	43.46006845
Nacc2	168.4301761	172.4540531	119.7566331
Nadk	218.6453839	180.4486118	0
Nadk2	44.98445698	28.55199554	28.00759967
Nadsyn1	26.15375406	27.40991572	52.15208214
Nae1	67.99976055	52.5356718	84.9885783
Naf1	3.138450487	13.70495786	7.726234391
Naga	353.5987548	290.0882747	306.1520377
Nagk	6.276900973	19.41535697	0
Naglu	2526.452642	114.2079822	1028.554953
Nagpa	481.2290746	334.6293877	453.9162705
Naif1	19.87685308	35.40447447	43.46006845
Naip1	32.43065503	23.98367625	30.90493756
Naip2	212.3684829	188.4431706	234.6843696
Naip5	217.5992337	236.4105231	261.72619
Naip6	127.6303198	54.81983144	28.00759967
Nampt	47.0767573	121.0604611	86.9201369
Nanos1	1.046150162	9.136638573	0
Nanp	21.96915341	21.69951661	23.17870317
Nans	47.0767573	52.5356718	48.28896494
Nap1l1	55.4459586	30.83615518	85.9543576
Nap1l4	193.53778	89.08222609	285.8706725
Napa	413.2293141	291.2303545	245.3079419
Napb	11.50765178	5.710399108	6.760455092
Napepld	0	9.136638573	5.794675793
Napg	119.2611185	82.22974716	68.57033022
Naprt	13.59995211	12.56287804	0
Narf	194.5839302	118.7763015	196.0531977
Narfl	0	0	0
Nars	0	0	229.8554731
Nars2	25.10760389	22.84159643	30.90493756
Nasp	27.19990422	5.710399108	19.31558598
Nat1	13.59995211	21.69951661	3.863117195
Nat10	85.7843133	25.12575608	60.84409583
Nat14	1.046150162	3.426239465	5.794675793
Nat2	34.52295535	36.54655429	22.21292387
Nat6	96.24581493	71.95102876	84.022799
Nat8f1	6.276900973	3.426239465	10.62357229
Nat8f4	0	0	10.62357229
Nat8l	11.50765178	38.83071394	44.42584775

For Review Only

Nat9	2.092300324	6.85247893	0
Natd1	133.9072208	87.94014627	189.2927426
Nav1	14.64610227	13.70495786	0
Nav2	383.9371095	340.3397869	193.1558598
Nav3	569.1056883	845.139068	634.5169993
Nbas	51.26135795	69.66686912	52.15208214
Nbea	34.52295535	68.5247893	51.18630284
Nbeal1	57.53825892	146.1862172	75.33078531
Nbeal2	183.0762784	142.7599777	119.7566331
Nbl1	6.276900973	1.142079822	6.760455092
Nbn	10.46150162	26.2678359	42.49428915
Nbr1	273.0451923	282.093716	276.2128795
Ncam1	8.369201298	0	2.897337897
Ncan	4.184600649	1.142079822	0
Ncapd2	7.323051136	12.56287804	6.760455092
Ncapd3	24.06145373	27.40991572	0
Ncapg2	0	2.284159643	12.55513089
Ncaph	56.49210876	66.24062966	66.63877162
Ncaph2	88.92276379	0	0
Ncbp1	74.27666152	76.51934805	78.22812321
Ncbp2	51.26135795	73.09310859	98.50948848
Ncbp3	184.1224286	145.0441374	144.8668948
Ncdn	69.04591071	58.2460709	47.32318564
Nceh1	48.12290746	35.40447447	30.90493756
Ncf1	783.5664715	115.350062	1658.243056
Ncf2	228.0607354	411.1487358	459.7109463
Ncf4	115.0765178	23.98367625	54.08364074
Nck1	100.4304156	114.2079822	124.5855296
Nckap1	0	1.142079822	0
Nckap1l	1607.932799	0	80.1596818
Nckap5	3.138450487	0	0
Nckap5l	37.66140584	58.2460709	0
Nckipsd	18.83070292	0	0.965779299
Ncl	0	0	0
Ncln	125.5380195	126.7708602	112.996178
Ncmap	0	0	0
Ncoa1	60.67670941	60.53023055	31.87071686
Ncoa2	198.7685308	162.1753347	140.0379983
Ncoa3	710.3359602	749.204363	808.3572731
Ncoa4	274.0913425	130.1970997	111.0646194
Ncoa5	54.39980844	31.97823501	30.90493756
Ncoa6	72.18436119	91.36638573	101.4068264
Ncoa7	0	4.568319287	6.760455092
Ncor1	168.4301761	285.5199554	256.8972935
Ncor2	139.1379716	63.95647001	108.1672815
Ncs1	139.1379716	99.36094448	156.4562464
Ncstn	207.1377321	139.3337382	169.0113773
ND1	4316.415569	3309.747323	3895.953692
ND2	322.21425	215.8530863	259.7946314
ND3	169.4763263	78.80350769	130.3802053

For Review Only

ND4	5.230750811	10.27871839	7.726234391
ND4L	1039.873261	899.9588995	771.6576598
ND5	3399.988027	1409.3265	1934.455936
Ndc1	27.19990422	6.85247893	28.97337897
Nde1	126.5841696	123.3446207	104.3041643
Ndel1	168.4301761	172.4540531	137.1406604
Ndfip1	423.6908157	372.3180219	446.1900361
Ndfip2	137.0456713	107.3555032	112.996178
Ndnf	0	0	0
Ndnl2	12.55380195	50.25151215	18.34980668
Ndor1	2.092300324	0	0
Ndrg1	2.092300324	0	0
Ndrg2	38.707556	2.284159643	0
Ndrg3	208.1838823	147.328297	191.2243012
Ndrg4	3.138450487	0	0
Ndst1	58.58440909	79.94558752	118.7908538
Ndst2	26.15375406	70.80894894	63.74143372
Ndufa1	76.36896184	67.38270948	62.77565443
Ndufa10	164.2455755	51.39359197	0
Ndufa11	114.0303677	2.284159643	80.1596818
Ndufa12	1.046150162	1.142079822	211.5056664
Ndufa13	167.384026	127.91294	165.1482601
Ndufa2	0	0	62.77565443
Ndufa3	0	81.08766734	42.49428915
Ndufa4	170.5224764	156.4649356	199.9163149
Ndufa5	71.13821103	43.39903322	65.67299232
Ndufa6	44.98445698	0	0
Ndufa7	103.5688661	9.136638573	106.2357229
Ndufa8	116.122668	100.5030243	130.3802053
Ndufa9	92.06121428	148.4703768	175.7718324
Ndufab1	0	0	62.77565443
Ndufaf1	53.35365827	22.84159643	46.35740634
Ndufaf2	18.83070292	13.70495786	14.48668948
Ndufaf4	41.84600649	37.68863411	54.08364074
Ndufaf5	0	17.13119732	20.28136528
Ndufaf6	10.46150162	18.27327715	14.48668948
Ndufaf7	0	54.81983144	76.29656461
Ndufb10	153.7840738	69.66686912	189.2927426
Ndufb11	56.49210876	1.142079822	59.87831653
Ndufb2	31.38450487	17.13119732	34.76805476
Ndufb3	86.83046347	66.24062966	80.1596818
Ndufb4	0	23.98367625	0
Ndufb5	99.38426541	65.09854983	89.81747479
Ndufb6	13.59995211	26.2678359	79.19390251
Ndufb7	71.13821103	59.38815073	58.91253723
Ndufb8	139.1379716	74.23518841	133.2775432
Ndufb9	0	0	1.931558598
Ndufc1	29.29220454	0	69.53610952
Ndufc2	163.1994253	92.50846555	154.5246878
Ndufs1	134.9533709	145.0441374	149.6957913

For Review Only

Ndufs2	69.04591071	87.94014627	211.5056664
Ndufs3	0	0	0.965779299
Ndufs4	85.7843133	86.79806645	84.022799
Ndufs5	35.56910552	23.98367625	30.90493756
Ndufs6	0	0	0
Ndufs7	75.32281168	133.6233391	138.1064397
Ndufs8	222.8299846	171.3119732	0
Ndufv1	0	0	5.794675793
Ndufv2	3.138450487	222.7055652	224.0607973
Ndufv3	106.7073165	79.94558752	89.81747479
Neat1	242.7068376	373.4601017	199.9163149
Neb	0	18.27327715	10.62357229
Nebl	0	0	0
Necab2	0	0	0.965779299
Necab3	0	0	0
Necap1	97.29196509	79.94558752	121.6881917
Necap2	361.9679561	268.3887581	417.2166571
Nectin2	101.4765657	83.37182698	88.85169549
Nectin3	0	1.142079822	0
Nectin4	137.0456713	113.0659023	141.9695569
Nedd1	46.03060714	23.98367625	29.93915826
Nedd4	10.46150162	1.142079822	0
Nedd4l	34.52295535	42.2569534	24.14448247
Nedd8	86.83046347	94.7926252	139.072219
Nedd9	6.276900973	1.142079822	0
Negr1	1.046150162	5.710399108	4.828896494
Neil1	43.93830681	29.69407536	22.21292387
Neil2	0	0	0
Neil3	0	0	0
Nek1	17.78455276	42.2569534	18.34980668
Nek2	0	0	0
Nek3	12.55380195	14.84703768	11.58935159
Nek4	42.89215665	31.97823501	21.24714457
Nek5	0	0	1.931558598
Nek6	590.0286915	408.8645762	442.3269189
Nek7	88.92276379	126.7708602	129.414426
Nek8	0	20.55743679	4.828896494
Nek9	103.5688661	82.22974716	147.7642327
Nelfa	0	0	0
Nelfb	31.38450487	13.70495786	0
Nelfcd	53.35365827	79.94558752	88.85169549
Nelfe	25.10760389	18.27327715	41.52850985
Nemf	78.46126217	98.21886466	163.2167015
Nenf	29.29220454	39.97279376	37.66539266
Neo1	4.184600649	2.284159643	0
Nepro	13.59995211	23.98367625	55.04942003
Nes	6.276900973	5.710399108	5.794675793
Net1	0	0	0
Neto1	3.138450487	3.426239465	7.726234391
Neu1	0	0	79.19390251

For Review Only

Neu3	13.59995211	15.9891175	16.41824808
Neurl1a	146.4610227	156.4649356	173.8402738
Neurl1b	7.323051136	9.136638573	4.828896494
Neurl2	4.184600649	3.426239465	6.760455092
Neurl3	0	0	0
Neurl4	84.73816314	55.96191126	102.3726057
Nexn	3.138450487	0	0
Nf1	176.7993774	251.2575608	125.5513089
Nf2	111.9380674	95.93470502	88.85169549
Nfam1	827.5047783	837.1445093	863.4066932
Nfasc	10.46150162	0	0
Nfat5	105.6611664	134.765419	107.2015022
Nfatc1	464.490672	436.2744919	403.6957469
Nfatc2	148.553323	151.8966163	152.5931292
Nfatc2ip	3.138450487	13.70495786	0
Nfatc3	97.29196509	126.7708602	95.61215059
Nfe2	3.138450487	0	0
Nfe2l1	107.7534667	89.08222609	122.653971
Nfe2l2	573.2902889	420.2853744	620.9960892
Nfe2l3	0	1.142079822	0
Nfia	160.0609748	223.847645	156.4562464
Nfib	0	3.426239465	0
Nfic	93.10736444	118.7763015	131.3459846
Nfil3	13.59995211	1.142079822	14.48668948
Nfix	28.24605438	25.12575608	44.42584775
Nfkb1	278.2759432	268.3887581	336.091196
Nfkb2	72.18436119	76.51934805	39.59695125
Nfkbia	571.1979886	284.3778756	298.4258033
Nfkbib	80.55356249	61.67231037	37.66539266
Nfkbid	77.41511201	0	0.965779299
Nfkbie	1.046150162	1.142079822	165.1482601
Nfkbil1	42.89215665	20.55743679	24.14448247
Nfkbiz	124.4918693	155.3228557	118.7908538
Nfrkb	69.04591071	47.96735251	73.39922671
Nfs1	73.23051136	70.80894894	67.60455092
Nfu1	72.18436119	87.94014627	89.81747479
Nfx1	198.7685308	207.8585275	173.8402738
Nfxl1	12.55380195	66.24062966	27.04182037
Nfya	16.7384026	42.2569534	25.11026177
Nfyb	42.89215665	30.83615518	30.90493756
Nfyc	81.59971265	49.10943233	66.63877162
Ngdn	69.04591071	0	14.48668948
Ngfrap1	23.01530357	6.85247893	9.657792988
Ngly1	81.59971265	75.37726823	110.0988401
Ngp	848.4277816	185.0169311	143.9011155
Ngrn	125.5380195	95.93470502	125.5513089
Nhej1	0	0	12.55513089
Nhlrc1	1.046150162	3.426239465	1.931558598
Nhlrc2	84.73816314	143.9020575	91.74903339
Nhlrc3	35.56910552	46.82527269	38.63117195

For Review Only

Nhp2	86.83046347	63.95647001	87.8859162
Nhp2l1	69.04591071	73.09310859	62.77565443
Nhs	5.230750811	19.41535697	3.863117195
Nhsl1	1.046150162	0	0.965779299
Nhsl2	120.3072687	100.5030243	98.50948848
Nicn1	0	35.40447447	2.897337897
Nid1	0	0	0
Nid2	41.84600649	13.70495786	79.19390251
Nif3l1	43.93830681	44.54111304	43.46006845
Nifk	2.092300324	52.5356718	107.2015022
Nim1k	9.41535146	2.284159643	0
Nin	30.33835471	38.83071394	19.31558598
Ninj1	2.092300324	2.284159643	0
Ninl	40.79985633	27.40991572	23.17870317
Nip7	1.046150162	1.142079822	316.77561
Nipa1	16.7384026	6.85247893	7.726234391
Nipa2	98.33811525	98.21886466	141.9695569
Nipal3	50.21520779	33.12031483	42.49428915
Nipbl	238.522237	288.9461949	277.1786588
Nipsnap1	15.69225243	11.42079822	15.45246878
Nipsnap3b	53.35365827	83.37182698	75.33078531
Nisch	1.046150162	1.142079822	0
Nit1	0	0	2.897337897
Nit2	38.707556	35.40447447	31.87071686
Nkap	33.47680519	45.68319287	39.59695125
Nkapl	1.046150162	4.568319287	4.828896494
Nkd2	0	0	0
Nkiras1	102.5227159	26.2678359	0
Nkiras2	115.0765178	1.142079822	252.068397
Nkrf	1.046150162	9.136638573	0
Nktr	82.64586282	37.68863411	79.19390251
Nle1	8.369201298	13.70495786	0
Nlgn2	2.092300324	12.56287804	3.863117195
Nlk	21.96915341	44.54111304	22.21292387
Nln	70.09206087	34.26239465	48.28896494
Nlrc3	2.092300324	19.41535697	11.58935159
Nlrc4	0	1.142079822	0
Nlrc5	11.50765178	27.40991572	17.38402738
Nlrp1a	144.3687224	59.38815073	21.24714457
Nlrp1b	54.39980844	71.95102876	227.9239145
Nlrp1c-ps	0	25.12575608	0
Nlrp3	65.90746022	110.7817427	81.1254611
Nlrx1	35.56910552	46.82527269	53.11786144
Nmb	7.323051136	14.84703768	7.726234391
Nmd3	73.23051136	70.80894894	72.43344741
Nme1	85.7843133	49.10943233	92.71481269
Nme2	79.50741233	75.37726823	101.4068264
Nme4	6.276900973	3.426239465	6.760455092
Nme6	34.52295535	20.55743679	36.69961336
Nme7	13.59995211	4.568319287	25.11026177

For Review Only

Nmi	1.046150162	3.426239465	0
Nmnat1	15.69225243	5.710399108	8.69201369
Nmnat3	4.184600649	14.84703768	10.62357229
Nmral1	2.092300324	21.69951661	61.80987513
Nmrk1	2.092300324	2.284159643	10.62357229
Nmt1	191.4454797	169.0278136	203.7794321
Nmt2	0	6.85247893	31.87071686
Nnat	14.64610227	13.70495786	6.760455092
Nnt	96.24581493	82.22974716	65.67299232
Noa1	35.56910552	27.40991572	60.84409583
Nob1	66.95361038	19.41535697	35.73383406
Noc2l	67.99976055	20.55743679	24.14448247
Noc3l	4.184600649	12.56287804	14.48668948
Noc4l	37.66140584	2.284159643	90.78325409
Noct	2.092300324	11.42079822	9.657792988
Nod1	51.26135795	50.25151215	61.80987513
Nod2	7.323051136	5.710399108	0
Nol10	9.41535146	6.85247893	19.31558598
Nol11	53.35365827	122.2025409	71.46766811
Nol12	40.79985633	37.68863411	56.01519933
Nol4l	87.87661363	41.11487358	89.81747479
Nol6	70.09206087	110.7817427	99.47526778
Nol7	0	0	0
Nol8	25.10760389	26.2678359	28.00759967
Nol9	52.30750811	38.83071394	43.46006845
Nolc1	85.7843133	106.2134234	110.0988401
Nom1	50.21520779	66.24062966	49.25474424
Nomo1	46.03060714	60.53023055	55.04942003
Nono	155.8763742	166.743654	86.9201369
Nop10	6.276900973	11.42079822	0
Nop14	69.04591071	58.2460709	64.70721302
Nop16	55.4459586	70.80894894	95.61215059
Nop2	0	0	0
Nop56	0	0	0
Nop58	46.03060714	69.66686912	59.87831653
Nop9	54.39980844	41.11487358	34.76805476
Nos1	3.138450487	0	0
Nos1ap	174.7070771	140.4758181	118.7908538
Nos2	0	2.284159643	0
Nos3	0	3.426239465	0
Nosip	115.0765178	119.9183813	0
Nostrin	0	0	0
Notch1	270.952892	229.5580442	245.3079419
Notch2	718.7051615	745.7781235	525.3839386
Notch4	16.7384026	27.40991572	15.45246878
Notum	0	1.142079822	1.931558598
Nova1	15.69225243	51.39359197	32.83649616
Nova2	0	0	0
Noxo1	25.10760389	38.83071394	43.46006845
Npat	34.52295535	97.07678484	29.93915826

For Review Only

Npc1	337.9065024	372.3180219	331.2622995
Npc2	1397.656617	935.3633739	1245.855296
Npcd	0	3.426239465	0
Npdc1	0	0	0
Npepl1	0	0	0
Npepps	50.21520779	49.10943233	80.1596818
Npff	0	0	0
Nphp1	14.64610227	0	10.62357229
Nphp3	7.323051136	17.13119732	15.45246878
Npl	94.1535146	76.51934805	123.6197503
Nploc4	88.92276379	75.37726823	95.61215059
Npm1	155.8763742	157.6070154	202.8136528
Npm2	6.276900973	0	0.965779299
Npm3	1.046150162	2.284159643	0
Npm3-ps1	0	4.568319287	0.965779299
Npnt	196.6762305	155.3228557	96.57792988
Npr1	5.230750811	0	0.965779299
Nprl2	0	0	24.14448247
Nprl3	47.0767573	49.10943233	27.04182037
Nptn	327.4450008	364.3234631	207.6425493
Nptxr	3.138450487	4.568319287	0
Nqo2	41.84600649	37.68863411	20.28136528
Nr0b2	1.046150162	0	0
Nr1d1	6.276900973	4.568319287	5.794675793
Nr1d2	79.50741233	33.12031483	40.56273055
Nr1h2	0	0	0
Nr2c1	24.06145373	39.97279376	50.22052354
Nr2c2	87.87661363	61.67231037	98.50948848
Nr2c2ap	10.46150162	19.41535697	17.38402738
Nr2f2	3.138450487	0	0
Nr2f6	31.38450487	19.41535697	24.14448247
Nr3c1	266.7682914	304.9353124	224.0607973
Nr3c2	52.30750811	25.12575608	36.69961336
Nr4a1	8.369201298	4.568319287	0
Nr4a2	0	0	0
Nr4a3	3.138450487	0	0
Nr6a1	1.046150162	0	10.62357229
Nradd	4.184600649	143.9020575	16.41824808
Nrarp	5.230750811	9.136638573	9.657792988
Nras	122.399569	1.142079822	0
Nrbf2	29.29220454	37.68863411	32.83649616
Nrcam	0	0	0
Nrd1	145.4148726	44.54111304	55.04942003
Nrde2	36.61525568	43.39903322	45.39162705
Nrep	58.58440909	63.95647001	47.32318564
Nrf1	139.1379716	94.7926252	111.0646194
Nrg1	0	3.426239465	0
Nrg2	0	5.710399108	0
Nrg4	7.323051136	0	13.52091018
Nrip1	890.2737881	1002.746083	749.4447359

For Review Only

Nrip2	0	0	0
Nrip3	1.046150162	1.142079822	1.931558598
Nrm	0	0	48.28896494
Nrn1	1.046150162	0	0
Nron	34.52295535	27.40991572	26.07604107
Nrp	5.230750811	11.42079822	11.58935159
Nrp1	493.7828766	421.4274542	476.1291943
Nrp2	50.21520779	69.66686912	63.74143372
Nrros	739.6281647	714.9419684	922.3192304
Nrxn3	0	0	3.863117195
Nsa2	54.39980844	41.11487358	44.42584775
Nsd1	229.1068855	397.4437779	178.6691703
Nsdhl	55.4459586	22.84159643	56.01519933
Nsf	104.6150162	79.94558752	95.61215059
Nsfl1c	78.46126217	99.36094448	122.653971
Nsg1	4.184600649	0	0
Nsg2	8.369201298	0	0
Nsl1	3.138450487	3.426239465	0
Nsmaf	11.50765178	78.80350769	120.7224124
Nsmce1	71.13821103	59.38815073	54.08364074
Nsmce2	55.4459586	37.68863411	49.25474424
Nsmce4a	26.15375406	31.97823501	6.760455092
Nsmf	77.41511201	39.97279376	64.70721302
Nsrp1	43.93830681	51.39359197	50.22052354
Nsun2	75.32281168	62.81439019	80.1596818
Nsun3	35.56910552	15.9891175	17.38402738
Nsun4	49.16905763	70.80894894	52.15208214
Nsun5	51.26135795	29.69407536	5.794675793
Nsun6	41.84600649	46.82527269	36.69961336
Nsun7	0	3.426239465	0.965779299
Nt5c2	91.01506412	163.3174145	122.653971
Nt5c3	35.56910552	52.5356718	28.97337897
Nt5c3b	46.03060714	33.12031483	39.59695125
Nt5dc1	84.73816314	46.82527269	51.18630284
Nt5dc2	9.41535146	0	0
Nt5dc3	8.369201298	3.426239465	5.794675793
Nt5m	61.72285957	33.12031483	60.84409583
Ntan1	3.138450487	0	0
Nthl1	4.184600649	14.84703768	0
Ntm	0	0	0
Ntmt1	37.66140584	34.26239465	67.60455092
Ntn1	2.092300324	5.710399108	4.828896494
Ntn3	3.138450487	4.568319287	0
Ntng2	0	0	0
Ntpcr	166.3378758	172.4540531	158.387805
Ntrk2	20.92300324	0	0
Ntrk3	0	0	0.965779299
Nuak1	157.9686745	149.6124566	123.6197503
Nuak2	52.30750811	31.97823501	56.01519933
Nub1	130.7687703	63.95647001	88.85169549

For Review Only

Nubp1	0	0	53.11786144
Nubp2	0	0	214.4030043
Nubpl	20.92300324	20.55743679	26.07604107
Nucb1	192.4916299	14.84703768	0
Nucb2	24.06145373	15.9891175	17.38402738
Nucks1	75.32281168	67.38270948	64.70721302
Nudc	103.5688661	68.5247893	90.78325409
Nudcd1	29.29220454	28.55199554	33.80227546
Nudcd2	11.50765178	10.27871839	37.66539266
Nudcd3	235.3837865	151.8966163	186.3954047
Nudt1	8.369201298	0	10.62357229
Nudt12	2.092300324	15.9891175	0
Nudt13	71.13821103	99.36094448	55.04942003
Nudt14	11.50765178	37.68863411	0.965779299
Nudt15	2.092300324	2.284159643	0.965779299
Nudt16	0	0	0
Nudt16l1	0	0	0
Nudt18	57.53825892	66.24062966	67.60455092
Nudt19	63.8151599	45.68319287	67.60455092
Nudt2	5.230750811	4.568319287	17.38402738
Nudt21	25.10760389	23.98367625	19.31558598
Nudt22	57.53825892	38.83071394	52.15208214
Nudt3	146.4610227	121.0604611	150.6615706
Nudt4	26.15375406	75.37726823	51.18630284
Nudt5	78.46126217	76.51934805	110.0988401
Nudt6	16.7384026	13.70495786	9.657792988
Nudt7	0	12.56287804	0
Nudt9	51.26135795	44.54111304	42.49428915
Nuf2	0	0	0
Nufip1	43.93830681	41.11487358	55.04942003
Nufip2	264.675991	247.8313213	224.0607973
Numa1	332.6757516	399.7279376	0
Numb	306.5219975	544.7720749	233.7185903
Numbl	19.87685308	0	0
Nup107	44.98445698	39.97279376	35.73383406
Nup133	63.8151599	49.10943233	54.08364074
Nup153	59.63055925	84.5139068	84.9885783
Nup155	28.24605438	36.54655429	35.73383406
Nup160	48.12290746	28.55199554	36.69961336
Nup188	88.92276379	44.54111304	56.01519933
Nup205	21.96915341	0	41.52850985
Nup210	21.96915341	3.426239465	0
Nup210l	29.29220454	17.13119732	13.52091018
Nup214	161.107125	170.1698934	156.4562464
Nup35	26.15375406	13.70495786	16.41824808
Nup37	16.7384026	0	0
Nup43	1.046150162	10.27871839	11.58935159
Nup50	77.41511201	63.95647001	59.87831653
Nup54	40.79985633	25.12575608	40.56273055
Nup62	70.09206087	33.12031483	96.57792988

For Review Only

Nup85	0	50.25151215	0
Nup88	273.0451923	244.4050818	284.9048932
Nup93	38.707556	34.26239465	18.34980668
Nup98	219.6915341	76.51934805	81.1254611
Nupl1	76.36896184	37.68863411	47.32318564
Nupl2	49.16905763	53.67775162	51.18630284
Nupr1	0	0	0
Nupr1l	2.092300324	0	0
Nus1	12.55380195	63.95647001	44.42584775
Nusap1	0	1.142079822	0
Nutf2	37.66140584	37.68863411	42.49428915
Nutf2-ps1	26.15375406	30.83615518	29.93915826
Nutf2-ps2	16.7384026	19.41535697	23.17870317
Nvl	89.96891395	142.7599777	82.0912404
Nwd1	5.230750811	2.284159643	0
Nwd2	0	1.142079822	0
Nxf1	6.276900973	0	0
Nxn	3.138450487	0	0
Nxpe3	1.046150162	7.994558752	8.69201369
Nxpe4	0	0	0
Nxph1	0	0	3.863117195
Nxt1	46.03060714	50.25151215	50.22052354
Nxt2	76.36896184	114.2079822	139.072219
Nynrin	21.96915341	2.284159643	2.897337897
Oaf	5.230750811	30.83615518	27.04182037
Oard1	2.092300324	25.12575608	0
Oas1a	14.64610227	10.27871839	25.11026177
Oas1b	5.230750811	0	16.41824808
Oas1c	7.323051136	4.568319287	9.657792988
Oas1g	1.046150162	1.142079822	0
Oas2	30.33835471	1.142079822	0
Oas3	0	3.426239465	0
Oasl1	2.092300324	1.142079822	0
Oasl2	7.323051136	12.56287804	11.58935159
Oat	57.53825892	58.2460709	79.19390251
Oaz1	0	0	3.863117195
Oaz1-ps	411.1370138	383.7388201	430.7375673
Oaz2	0	0	0
Obfc1	94.1535146	37.68863411	56.98097863
Obp2b	3.138450487	0	0
Obscn	0	3.426239465	2.897337897
Ocel1	13.59995211	1.142079822	40.56273055
Ociad1	744.8589155	627.0018221	685.7033022
Ociad2	7.323051136	0	0
Ocln	18.83070292	14.84703768	25.11026177
Ocrl	28.24605438	36.54655429	32.83649616
Odc1	23.01530357	33.12031483	21.24714457
Odf2	119.2611185	100.5030243	85.9543576
Odf2l	35.56910552	27.40991572	21.24714457
Ofd1	30.33835471	18.27327715	10.62357229

For Review Only

Ogdh	443.5676688	175.8802925	228.8896938
Ogdhl	6.276900973	0	0
Ogfod1	43.93830681	82.22974716	27.04182037
Ogfod2	0	0	0
Ogfod3	52.30750811	41.11487358	43.46006845
Ogfr	55.4459586	0	0
Ogfrl1	4.184600649	91.36638573	85.9543576
Ogg1	2.092300324	1.142079822	0
Ogn	0	1.142079822	0
Ogt	434.1523173	504.7992812	456.8136084
Oip5	3.138450487	4.568319287	0
Ola1	37.66140584	49.10943233	32.83649616
Olfm2	0	2.284159643	0
Olfm4	25.10760389	2.284159643	0
Olfml2a	2.092300324	0	0
Olfml2b	225.968435	97.07678484	178.6691703
Olfml3	5715.118336	5557.360412	6540.257412
Olfr1029	0	0	0
Olfr1030	1.046150162	4.568319287	10.62357229
Olfr1033	8.369201298	6.85247893	11.58935159
Olfr1090	0	1.142079822	2.897337897
Olfr110	21.96915341	13.70495786	20.28136528
Olfr111	78.46126217	37.68863411	56.01519933
Olfr112	1.046150162	4.568319287	0
Olfr114	1.046150162	0	0
Olfr1264	0	0	0
Olfr1394	0	0	1.931558598
Olfr1426	3.138450487	0	0
Olfr1443	1.046150162	0	0
Olfr1444	0	0	0
Olfr1484	1.046150162	1.142079822	0
Olfr172	3.138450487	3.426239465	3.863117195
Olfr173	1.046150162	0	1.931558598
Olfr394	0	0	0
Olfr456	0	0	0
Olfr56	1.046150162	4.568319287	0
Olfr920	1.046150162	0	0
Olfr99	3.138450487	0	0
Olr1	0	1.142079822	0
Oma1	211.3223328	206.7164477	338.9885339
Omd	0	1.142079822	0
Omg	3.138450487	1.142079822	1.931558598
Opa1	85.7843133	93.65054538	85.9543576
Opa3	99.38426541	54.81983144	74.36500601
Ophn1	135.9995211	59.38815073	56.98097863
Oplah	44.98445698	22.84159643	5.794675793
Opn1sw	1.046150162	0	0
Opn3	0	1.142079822	4.828896494
Oprd1	0	0	0
Oprl1	0	0	0

For Review Only

Oprm1	15.69225243	6.85247893	13.52091018
Optn	15.69225243	19.41535697	0
Orai1	9.41535146	189.5852504	44.42584775
Orai2	322.21425	253.5417204	277.1786588
Orai3	288.7374448	161.0332549	276.2128795
Oraov1	129.7226201	109.6396629	56.98097863
Orc1	8.369201298	14.84703768	8.69201369
Orc2	36.61525568	76.51934805	93.68059199
Orc3	126.5841696	99.36094448	192.1900805
Orc4	87.87661363	91.36638573	81.1254611
Orc5	43.93830681	84.5139068	67.60455092
Orc6	18.83070292	13.70495786	31.87071686
Orm2	0	0	0
Orm3	14.64610227	9.136638573	5.794675793
Ormdl1	123.4457191	122.2025409	178.6691703
Ormdl2	31.38450487	14.84703768	0
Ormdl3	101.4765657	100.5030243	92.71481269
Os9	129.7226201	190.7273302	67.60455092
Osbp	30.33835471	67.38270948	45.39162705
Osbpl11	208.1838823	220.4214056	153.5589085
Osbpl1a	65.90746022	41.11487358	43.46006845
Osbpl2	96.24581493	59.38815073	98.50948848
Osbpl3	32.43065503	15.9891175	18.34980668
Osbpl6	1.046150162	3.426239465	0
Osbpl7	46.03060714	42.2569534	70.50188882
Osbpl8	14.64610227	22.84159643	14.48668948
Osbpl9	102.5227159	165.6015741	167.0798187
Oser1	66.95361038	79.94558752	76.29656461
Osgep	20.92300324	0	0
Osgepl1	14.64610227	19.41535697	17.38402738
Osgin1	34.52295535	10.27871839	16.41824808
Osgin2	44.98445698	38.83071394	51.18630284
Osm	43.93830681	55.96191126	4.828896494
Ost4	46.03060714	47.96735251	70.50188882
Ostc	151.6917735	218.1372459	252.068397
Ostf1	421.5985154	323.2085895	416.2508778
Ostm1	191.4454797	201.0060486	182.5322875
Otc	0	5.710399108	0
Otog	0	0	0
Otub1	1.046150162	1.142079822	0
Otub2	6.276900973	5.710399108	27.04182037
Otud1	8.369201298	51.39359197	24.14448247
Otud3	4.184600649	5.710399108	0
Otud4	49.16905763	77.66142787	39.59695125
Otud5	48.12290746	77.66142787	0
Otud6b	102.5227159	49.10943233	53.11786144
Otud7a	0	0	0
Otud7b	88.92276379	37.68863411	50.22052354
Otulin	21.96915341	20.55743679	0
Otx2	9.41535146	0	0

For Review Only

Ovca2	0	0	138.1064397
Ovgp1	3.138450487	1.142079822	0
Ovol1	4.184600649	1.142079822	0
Oxa1l	75.32281168	98.21886466	141.9695569
Oxct1	581.6594902	405.4383367	444.2584775
Oxld1	18.83070292	10.27871839	0
Oxnad1	12.55380195	6.85247893	13.52091018
Oxr1	108.7996169	131.3391795	98.50948848
Oxsm	40.79985633	50.25151215	68.57033022
Oxsr1	30.33835471	33.12031483	41.52850985
P2rx1	26.15375406	15.9891175	25.11026177
P2rx3	1.046150162	2.284159643	0
P2rx4	0	0	0
P2rx5	1.046150162	2.284159643	0
P2rx7	503.198228	440.8428112	453.9162705
P2ry1	0	0	0
P2ry10	2.092300324	2.284159643	0
P2ry12	3149.958139	2030.617923	3632.295943
P2ry13	3500.418443	3443.370662	4145.124751
P2ry2	0	1.142079822	0
P2ry6	231.1991859	800.597955	1102.919959
P3h1	24.06145373	15.9891175	18.34980668
P3h2	91.01506412	157.6070154	64.70721302
P3h3	37.66140584	28.55199554	28.00759967
P3h4	11.50765178	4.568319287	7.726234391
P4ha1	0	188.4431706	431.7033466
P4hb	1333.841457	1091.82831	1353.056798
Pa2g4	125.5380195	14.84703768	169.9771566
Pabpc1	0	0	4.828896494
Pabpc4	14.64610227	12.56287804	21.24714457
Pabpc4l	2.092300324	1.142079822	0
Pabpn1	0	0	0
Pacrgl	7.323051136	3.426239465	2.897337897
Pacs1	75.32281168	114.2079822	75.33078531
Pacs2	89.96891395	167.8857338	129.414426
Pacsin1	17.78455276	17.13119732	0
Pacsin2	339.9988027	405.4383367	499.3078975
Pacsin3	8.369201298	0	1.931558598
Padi2	189.3531794	110.7817427	139.072219
Padi4	0	6.85247893	0
Paf1	93.10736444	78.80350769	83.0570197
Pafah1b1	131.8149204	207.8585275	84.022799
Pafah1b2	201.9069813	106.2134234	160.3193636
Pafah1b3	0	0	0
Pafah2	48.12290746	19.41535697	17.38402738
Pag1	6.276900973	91.36638573	0
Paics	52.30750811	155.3228557	104.3041643
Paip1	49.16905763	63.95647001	49.25474424
Paip2	372.4294578	432.8482524	394.0379539
Paip2b	4.184600649	7.994558752	24.14448247

For Review Only

Pak1	147.5071729	162.1753347	188.3269633
Pak1ip1	0	0	89.81747479
Pak2	247.9375885	277.5253967	226.9581352
Pak4	48.12290746	30.83615518	35.73383406
Pak6	0	1.142079822	4.828896494
Pakap	3.138450487	0	0
Palb2	28.24605438	21.69951661	18.34980668
Pald1	771.0126696	140.4758181	0.965779299
Palld	0	7.994558752	6.760455092
Palm	2.092300324	0	0
Palmd	2.092300324	1.142079822	0
Pam	0	1.142079822	5.794675793
Pam16	1.046150162	0	0
Pan2	69.04591071	101.6451041	88.85169549
Pan3	423.6908157	539.0616758	293.5969068
Pank1	9.41535146	21.69951661	9.657792988
Pank2	50.21520779	54.81983144	67.60455092
Pank3	41.84600649	34.26239465	62.77565443
Pank4	98.33811525	98.21886466	56.01519933
Panx1	15.69225243	20.55743679	15.45246878
Paox	24.06145373	22.84159643	25.11026177
Papd4	170.5224764	133.6233391	104.3041643
Papd5	34.52295535	46.82527269	46.35740634
Papd7	33.47680519	45.68319287	10.62357229
Papln	7.323051136	6.85247893	11.58935159
Papola	229.1068855	239.8367625	235.6501489
Papolb	1.046150162	3.426239465	3.863117195
Papolg	24.06145373	79.94558752	32.83649616
Pappa	0	4.568319287	2.897337897
Pappa2	2.092300324	0	0
Papss1	202.9531315	148.4703768	190.2585219
Papss2	2.092300324	1.142079822	0
Paqr3	9.41535146	7.994558752	0
Paqr4	56.49210876	25.12575608	31.87071686
Paqr5	1.046150162	0	0
Paqr7	257.3529399	183.8748513	286.8364518
Paqr8	7.323051136	6.85247893	8.69201369
Pard3b	12.55380195	26.2678359	25.11026177
Pard3bos1	25.10760389	27.40991572	29.93915826
Pard3bos2	0	0	0
Pard6a	13.59995211	25.12575608	13.52091018
Pard6g	4.184600649	3.426239465	10.62357229
Parg	69.04591071	35.40447447	26.07604107
Park2	46.03060714	66.24062966	73.39922671
Park7	1.046150162	74.23518841	184.4638461
Parl	43.93830681	51.39359197	39.59695125
Parn	161.107125	66.24062966	86.9201369
Parp1	147.5071729	0	2.897337897
Parp10	52.30750811	39.97279376	16.41824808
Parp11	52.30750811	52.5356718	46.35740634

For Review Only

Parp12	85.7843133	54.81983144	45.39162705
Parp14	70.09206087	100.5030243	152.5931292
Parp16	56.49210876	27.40991572	61.80987513
Parp2	0	0	0
Parp3	9.41535146	61.67231037	0
Parp4	100.4304156	17.13119732	38.63117195
Parp6	17.78455276	51.39359197	29.93915826
Parp8	166.3378758	193.0114899	147.7642327
Parp9	1.046150162	374.6021815	451.0189326
Pars2	50.21520779	15.9891175	40.56273055
Particl	33.47680519	14.84703768	10.62357229
Parva	5.230750811	1.142079822	0
Parvb	15.69225243	17.13119732	10.62357229
Parvg	713.4744106	496.8047224	571.7413449
Pask	0	0	11.58935159
Patl1	26.15375406	31.97823501	24.14448247
Patl2	0	0	0
Patz1	53.35365827	31.97823501	54.08364074
Pax6	0	0	0
Paxbp1	70.09206087	66.24062966	73.39922671
Paxip1	42.89215665	50.25151215	29.93915826
Pbdc1	34.52295535	28.55199554	27.04182037
Pbk	0	0	0
Pbld2	3.138450487	0	4.828896494
Pbrm1	92.06121428	205.5743679	112.0303987
Pbx1	27.19990422	20.55743679	26.07604107
Pbx2	16.7384026	34.26239465	27.04182037
Pbx3	29.29220454	20.55743679	13.52091018
Pbxip1	118.2149683	95.93470502	0
Pcbd2	0	5.710399108	0.965779299
Pcbp1	93.10736444	179.306532	196.0531977
Pcbp2	151.6917735	199.8639688	119.7566331
Pcbp3	24.06145373	2.284159643	7.726234391
Pcbp4	7.323051136	0	0
Pcca	31.38450487	51.39359197	42.49428915
Pccb	47.0767573	45.68319287	39.59695125
Pcdh1	1.046150162	0	0
Pcdh12	2.092300324	0	0
Pcdh15	0	2.284159643	0.965779299
Pcdhb19	0	1.142079822	0
Pcdhb22	12.55380195	4.568319287	7.726234391
Pcdhga1	14.64610227	21.69951661	51.18630284
Pcdhga10	4.184600649	2.284159643	0.965779299
Pcdhga11	3.138450487	26.2678359	10.62357229
Pcdhga12	21.96915341	22.84159643	39.59695125
Pcdhga2	18.83070292	73.09310859	8.69201369
Pcdhga3	2.092300324	7.994558752	14.48668948
Pcdhga4	18.83070292	4.568319287	16.41824808
Pcdhga5	36.61525568	67.38270948	29.93915826
Pcdhga6	4.184600649	3.426239465	6.760455092

For Review Only

Pcdhga7	10.46150162	52.5356718	25.11026177
Pcdhga8	21.96915341	19.41535697	10.62357229
Pcdhga9	28.24605438	50.25151215	28.00759967
Pcdhgb1	19.87685308	18.27327715	27.04182037
Pcdhgb2	60.67670941	35.40447447	34.76805476
Pcdhgb4	34.52295535	33.12031483	30.90493756
Pcdhgb5	3.138450487	4.568319287	6.760455092
Pcdhgb6	27.19990422	46.82527269	7.726234391
Pcdhgb7	17.78455276	26.2678359	25.11026177
Pcdhgb8	27.19990422	6.85247893	4.828896494
Pcdhgc3	0	0	0
Pcdhgc4	9.41535146	11.42079822	7.726234391
Pcdhgc5	6.276900973	12.56287804	3.863117195
Pced1a	70.09206087	69.66686912	57.94675793
Pced1b	43.93830681	22.84159643	7.726234391
Pcf11	80.55356249	129.0550198	67.60455092
Pcgf1	15.69225243	0	39.59695125
Pcgf3	24.06145373	27.40991572	14.48668948
Pcgf5	19.87685308	20.55743679	7.726234391
Pcgf6	16.7384026	17.13119732	13.52091018
Pcid2	148.553323	101.6451041	82.0912404
Pcif1	171.5686266	47.96735251	130.3802053
Pclo	5.230750811	0	0
Pcm1	71.13821103	81.08766734	51.18630284
Pcmt1	161.107125	190.7273302	167.0798187
Pcmtd1	92.06121428	105.0713436	80.1596818
Pcmtd2	46.03060714	51.39359197	86.9201369
Pcna	0	0	3.863117195
Pcnp	129.7226201	149.6124566	127.4828674
Pcnt	84.73816314	46.82527269	92.71481269
Pcnx	74.27666152	83.37182698	118.7908538
Pcnx2	19.87685308	19.41535697	19.31558598
Pcnx3	69.04591071	148.4703768	79.19390251
Pcnx4	6.276900973	19.41535697	41.52850985
Pcolce	17.78455276	3.426239465	0
Pcolce2	0	0	0
Pcp4	0	0	0
Pcp4l1	19.87685308	1.142079822	0
Pcsk4	5.230750811	5.710399108	2.897337897
Pcsk7	99.38426541	66.24062966	93.68059199
Pcsk9	6.276900973	4.568319287	0
Pctp	57.53825892	20.55743679	66.63877162
Pcx	34.52295535	1.142079822	28.00759967
Pcyox1	339.9988027	174.7382127	251.1026177
Pcyt1a	48.12290746	51.39359197	74.36500601
Pcyt2	67.99976055	38.83071394	69.53610952
Pdap1	27.19990422	20.55743679	31.87071686
Pdcd1	15.69225243	14.84703768	26.07604107
Pdcd10	53.35365827	58.2460709	67.60455092
Pdcd11	55.4459586	53.67775162	59.87831653

For Review Only

Pdcd2	0	23.98367625	20.28136528
Pdcd2l	5.230750811	7.994558752	128.4486467
Pdcd4	73.23051136	102.7871839	58.91253723
Pdcd5	41.84600649	51.39359197	56.01519933
Pdcd6	82.64586282	95.93470502	111.0646194
Pdcd6ip	324.3065503	210.1426872	264.6235279
Pdcd7	6.276900973	13.70495786	18.34980668
Pdcl	63.8151599	45.68319287	54.08364074
Pdcl3	55.4459586	85.65598662	56.98097863
Pddc1	108.7996169	81.08766734	70.50188882
Pde10a	4.184600649	2.284159643	0.965779299
Pde12	189.3531794	119.9183813	68.57033022
Pde1a	0	0	0
Pde1b	87.87661363	91.36638573	146.7984534
Pde1c	0	4.568319287	0
Pde2a	281.4143936	173.5961329	215.3687836
Pde3a	0	1.142079822	0
Pde3b	585.8440909	1106.675347	560.1519933
Pde4a	43.93830681	19.41535697	26.07604107
Pde4b	130.7687703	62.81439019	63.74143372
Pde4d	69.04591071	54.81983144	85.9543576
Pde4dip	193.53778	205.5743679	150.6615706
Pde6d	36.61525568	27.40991572	36.69961336
Pde6g	0	1.142079822	0.965779299
Pde7a	6.276900973	15.9891175	35.73383406
Pde7b	0	0	0
Pde8a	0	1.142079822	1.931558598
Pde8b	82.64586282	114.2079822	42.49428915
Pdgfa	44.98445698	50.25151215	43.46006845
Pdgfb	38.707556	33.12031483	46.35740634
Pdgfc	0	3.426239465	0
Pdgfra	0	0	0
Pdgfrb	0	2.284159643	0
Pdgfrl	1.046150162	1.142079822	0
Pdha1	148.553323	115.350062	99.47526778
Pdhb	140.1841217	109.6396629	151.6273499
Pdhx	58.58440909	57.10399108	47.32318564
Pdia3	1963.623855	1328.238833	2037.794321
Pdia4	724.9820624	0	10.62357229
Pdia5	0	0	0
Pdia6	551.3211355	607.5864651	726.2660327
Pdik1l	59.63055925	55.96191126	65.67299232
Pdk1	327.4450008	278.6674765	303.2546998
Pdk2	72.18436119	38.83071394	44.42584775
Pdk3	41.84600649	20.55743679	70.50188882
Pdlim2	36.61525568	41.11487358	52.15208214
Pdlim4	0	62.81439019	43.46006845
Pdlim5	71.13821103	97.07678484	80.1596818
Pdp1	18.83070292	25.12575608	13.52091018
Pdp2	117.1688182	99.36094448	93.68059199

For Review Only

Pdpk1	0	41.11487358	0
Pdpn	10.46150162	0	0
Pdpr	60.67670941	97.07678484	48.28896494
Pdrg1	0	0	0
Pds5a	108.7996169	115.350062	64.70721302
Pds5b	67.99976055	61.67231037	42.49428915
Pdss1	3.138450487	7.994558752	19.31558598
Pdss2	11.50765178	2.284159643	38.63117195
Pdxdc1	148.553323	117.6342216	140.0379983
Pdxk	88.92276379	18.27327715	56.98097863
Pdxp	1.046150162	0	5.794675793
Pdyn	0	0	1.931558598
Pdzd11	0	0	40.56273055
Pdzd2	6.276900973	4.568319287	13.52091018
Pdzd3	0	0	0
Pdzd4	5.230750811	2.284159643	0
Pdzd7	0	0	0
Pdzd8	89.96891395	121.0604611	117.8250745
Pdzd9	0	2.284159643	0
Pdzrn4	2.092300324	1.142079822	6.760455092
Pea15a	355.6910552	319.7823501	84.022799
Peak1	130.7687703	224.9897249	182.5322875
Peak1os	9.41535146	26.2678359	9.657792988
Pear1	65.90746022	50.25151215	28.97337897
Pebp1	129.7226201	92.50846555	92.71481269
Pecam1	60.67670941	61.67231037	84.022799
Pecr	72.18436119	77.66142787	87.8859162
Pef1	92.06121428	81.08766734	85.9543576
Peg10	2.092300324	11.42079822	4.828896494
Peg12	4.184600649	2.284159643	2.897337897
Peg13	110.8919172	69.66686912	38.63117195
Peg3	8.369201298	1.142079822	0
Peli1	156.9225243	171.3119732	198.9505356
Peli2	51.26135795	54.81983144	47.32318564
Peli3	40.79985633	20.55743679	11.58935159
Pelo	0	2.284159643	0
Pelp1	28.24605438	6.85247893	13.52091018
Pemt	0	0	0
Peo1	71.13821103	12.56287804	60.84409583
Pepd	273.0451923	185.0169311	234.6843696
Per1	9.41535146	1.142079822	0
Per2	2.092300324	10.27871839	16.41824808
Per3	119.2611185	159.891175	146.7984534
Perm1	3.138450487	43.39903322	18.34980668
Perp	13.59995211	11.42079822	0
Pes1	156.9225243	127.91294	83.0570197
Pet100	0	0	20.28136528
Pex1	86.83046347	68.5247893	46.35740634
Pex10	21.96915341	0	48.28896494
Pex11a	19.87685308	17.13119732	21.24714457

For Review Only

Pex11b	1.046150162	238.6946827	92.71481269
Pex11g	52.30750811	19.41535697	10.62357229
Pex12	55.4459586	49.10943233	77.26234391
Pex13	123.4457191	91.36638573	104.3041643
Pex14	97.29196509	83.37182698	99.47526778
Pex16	29.29220454	7.994558752	0
Pex19	94.1535146	107.3555032	0
Pex2	171.5686266	256.9679599	277.1786588
Pex26	5.230750811	12.56287804	7.726234391
Pex3	81.59971265	90.22430591	94.64637129
Pex5	101.4765657	63.95647001	101.4068264
Pex6	70.09206087	0	52.15208214
Pex7	61.72285957	49.10943233	35.73383406
Pf4	0	0	0
Pfas	0	0	0.965779299
Pfdn1	13.59995211	27.40991572	64.70721302
Pfdn2	40.79985633	34.26239465	52.15208214
Pfdn4	6.276900973	9.136638573	2.897337897
Pfdn5	0	0	0
Pfdn6	5.230750811	17.13119732	28.00759967
Pfkfb2	24.06145373	23.98367625	37.66539266
Pfkfb3	347.3218539	227.2738845	302.2889205
Pfkfb4	194.5839302	172.4540531	290.699569
Pfkl	325.3527005	201.0060486	208.6083286
Pfkm	34.52295535	9.136638573	11.58935159
Pfkp	14.64610227	4.568319287	0
Pfn1	669.5361038	580.1765494	819.9466247
Pfn2	20.92300324	33.12031483	25.11026177
Pfpl	2.092300324	3.426239465	0
Pgam1	144.3687224	129.0550198	179.6349496
Pgam2	2.092300324	2.284159643	0
Pgam5	114.0303677	101.6451041	114.9277366
Pgap1	6.276900973	4.568319287	28.97337897
Pgap2	69.04591071	67.38270948	0
Pgap3	11.50765178	2.284159643	4.828896494
Pgbd1	17.78455276	7.994558752	5.794675793
Pgd	220.7376842	161.0332549	0
Pggt1b	111.9380674	124.4867006	123.6197503
Pgk1	86.83046347	55.96191126	63.74143372
Pgls	66.95361038	41.11487358	60.84409583
Pglyrp1	23.01530357	0	0
Pglyrp2	0	0	5.794675793
Pgm1	1.046150162	1.142079822	0
Pgm2	95.19966476	42.2569534	67.60455092
Pgm2l1	11.50765178	9.136638573	5.794675793
Pgm3	5.230750811	23.98367625	35.73383406
Pgp	7.323051136	1.142079822	0
Pgpep1	88.92276379	63.95647001	104.3041643
Pgr	1.046150162	1.142079822	0
Pgrmc1	175.7532273	134.765419	175.7718324

For Review Only

Pgrmc2	2.092300324	63.95647001	19.31558598
Pgs1	125.5380195	86.79806645	84.022799
Phactr1	13.59995211	12.56287804	11.58935159
Phactr2	93.10736444	94.7926252	33.80227546
Phactr4	51.26135795	75.37726823	79.19390251
Phax	67.99976055	97.07678484	89.81747479
Phb	80.55356249	77.66142787	88.85169549
Phb2	101.4765657	74.23518841	142.9353362
Phc1	19.87685308	14.84703768	28.00759967
Phc2	149.5994732	102.7871839	138.1064397
Phc3	184.1224286	396.3016981	212.4714457
Phf1	4.184600649	0	0
Phf10	44.98445698	61.67231037	59.87831653
Phf11a	6.276900973	11.42079822	7.726234391
Phf11b	19.87685308	39.97279376	25.11026177
Phf11c	0	0	0
Phf11d	0	36.54655429	3.863117195
Phf12	52.30750811	74.23518841	89.81747479
Phf13	24.06145373	10.27871839	11.58935159
Phf14	121.3534188	132.4812593	108.1672815
Phf2	13.59995211	41.11487358	10.62357229
Phf20	122.399569	134.765419	81.1254611
Phf20l1	79.50741233	118.7763015	100.4410471
Phf21a	80.55356249	137.0495786	108.1672815
Phf21b	3.138450487	11.42079822	0
Phf23	1.046150162	10.27871839	0
Phf24	1.046150162	0	5.794675793
Phf3	46.03060714	57.10399108	45.39162705
Phf5a	27.19990422	0	0
Phf6	2.092300324	33.12031483	42.49428915
Phf7	40.79985633	42.2569534	53.11786144
Phf8	61.72285957	66.24062966	96.57792988
Phgdh	197.7223807	109.6396629	218.2661215
Phip	189.3531794	268.3887581	201.8478735
Phka1	83.69201298	74.23518841	92.71481269
Phka2	84.73816314	165.6015741	112.0303987
Phkb	41.84600649	57.10399108	61.80987513
Phkg1	0	10.27871839	11.58935159
Phkg2	37.66140584	0	28.00759967
Phlda1	0	0	0
Phldb1	23.01530357	20.55743679	16.41824808
Phldb3	0	0	2.897337897
Phlpp1	35.56910552	13.70495786	15.45246878
Phlpp2	46.03060714	33.12031483	36.69961336
Phospho2	81.59971265	82.22974716	114.9277366
Phpt1	114.0303677	70.80894894	0
Phrf1	114.0303677	93.65054538	0
Phtf1	6.276900973	17.13119732	16.41824808
Phtf1os	32.43065503	6.85247893	25.11026177
Phtf2	42.89215665	37.68863411	30.90493756

For Review Only

Phxr4	40.79985633	93.65054538	59.87831653
Phyh	135.9995211	75.37726823	119.7566331
Phyhd1	260.4913904	231.8422038	0
Phykpl	69.04591071	21.69951661	35.73383406
Pi16	0	0	0
Pi4k2a	62.76900973	59.38815073	77.26234391
Pi4k2b	4.184600649	5.710399108	0
Pi4ka	71.13821103	51.39359197	79.19390251
Pi4kb	35.56910552	39.97279376	28.00759967
Pias1	350.4603044	320.9244299	324.5018444
Pias2	78.46126217	81.08766734	56.01519933
Pias3	122.399569	81.08766734	94.64637129
Pias4	59.63055925	0	0.965779299
Pibf1	36.61525568	50.25151215	70.50188882
Picalm	666.3976533	766.3355603	446.1900361
Pick1	30.33835471	23.98367625	56.01519933
Pid1	231.1991859	193.0114899	173.8402738
Pidd1	23.01530357	17.13119732	29.93915826
Piezo1	53.35365827	39.97279376	35.73383406
Pif1	0	4.568319287	0
Piga	16.7384026	15.9891175	18.34980668
Pigb	50.21520779	44.54111304	59.87831653
Pigc	112.9842175	126.7708602	140.0379983
Pigf	15.69225243	6.85247893	9.657792988
Pigg	28.24605438	45.68319287	45.39162705
Pigh	61.72285957	98.21886466	117.8250745
Pigk	228.0607354	198.721889	235.6501489
Pigl	20.92300324	19.41535697	13.52091018
Pigm	87.87661363	131.3391795	80.1596818
Pign	49.16905763	71.95102876	75.33078531
Pigo	54.39980844	4.568319287	41.52850985
Pigp	62.76900973	42.2569534	43.46006845
Pigq	313.8450487	1.142079822	63.74143372
Pigs	193.53778	0	292.6311276
Pigt	114.0303677	31.97823501	62.77565443
Pigu	80.55356249	86.79806645	116.8592952
Pigv	138.0918214	158.7490952	205.7109907
Pigw	4.184600649	3.426239465	10.62357229
Pigx	100.4304156	110.7817427	103.338385
Pigyl	46.03060714	25.12575608	37.66539266
Pigz	12.55380195	22.84159643	24.14448247
Pih1d1	61.72285957	50.25151215	62.77565443
Pik3ap1	617.2285957	568.7557512	634.5169993
Pik3c2a	60.67670941	90.22430591	43.46006845
Pik3c2b	40.79985633	27.40991572	12.55513089
Pik3c3	55.4459586	114.2079822	98.50948848
Pik3ca	48.12290746	105.0713436	91.74903339
Pik3cb	19.87685308	18.27327715	7.726234391
Pik3cd	250.0298888	155.3228557	130.3802053
Pik3cg	413.2293141	650.9854983	690.5321987

For Review Only

Pik3ip1	41.84600649	23.98367625	28.00759967
Pik3r1	141.2302719	205.5743679	186.3954047
Pik3r2	87.87661363	1.142079822	0
Pik3r3	10.46150162	5.710399108	11.58935159
Pik3r4	128.67647	119.9183813	108.1672815
Pik3r5	413.2293141	433.9903322	336.091196
Pik3r6	4.184600649	25.12575608	29.93915826
Pikfyve	78.46126217	41.11487358	53.11786144
Pilra	17.78455276	30.83615518	56.01519933
Pilrb1	0	1.142079822	0
Pilrb2	6.276900973	0	0
Pim2	17.78455276	28.55199554	0
Pim3	0	38.83071394	32.83649616
Pin1	76.36896184	67.38270948	93.68059199
Pin1rt1	0	0	0
Pin4	0	0	5.794675793
Pink1	43.93830681	45.68319287	30.90493756
Pinx1	13.59995211	14.84703768	32.83649616
Pip4k2a	0	0	343.8174304
Pip4k2b	24.06145373	14.84703768	23.17870317
Pip4k2c	121.3534188	131.3391795	117.8250745
Pip5k1a	76.36896184	41.11487358	57.94675793
Pip5k1b	12.55380195	0	0
Pip5k1c	260.4913904	228.4159643	127.4828674
Pir	0	0	0
Pira1	9.41535146	5.710399108	9.657792988
Pira2	12.55380195	4.568319287	6.760455092
Pira6	4.184600649	4.568319287	0
Pirb	9.41535146	11.42079822	50.22052354
Pisd	0	222.7055652	67.60455092
Pisd-ps1	112.9842175	0	0
Pisd-ps2	0	2.284159643	0
Pisd-ps3	0	0	25.11026177
Pithd1	41.84600649	46.82527269	38.63117195
Pitpna	0	0	43.46006845
Pitpnb	140.1841217	135.9074988	118.7908538
Pitpnc1	283.506694	301.5090729	305.1862584
Pitpnm1	152.7379237	87.94014627	166.1140394
Pitpnm2	0	0	0.965779299
Pitrm1	50.21520779	65.09854983	21.24714457
Piwil2	0	0	0
Pja1	80.55356249	59.38815073	121.6881917
Pja2	257.3529399	198.721889	168.045598
Pkd1	186.2147289	254.6838002	326.433403
Pkd1l3	2.092300324	4.568319287	0
Pkd2	42.89215665	49.10943233	45.39162705
Pkd2l2	1.046150162	1.142079822	3.863117195
Pkdcc	8.369201298	6.85247893	15.45246878
Pkib	38.707556	46.82527269	58.91253723
Pkig	131.8149204	143.9020575	168.045598

For Review Only

Pkm	534.5827329	395.1596183	484.821208
Pkmyt1	7.323051136	7.994558752	4.828896494
Pkn1	151.6917735	66.24062966	89.81747479
Pkn2	44.98445698	57.10399108	28.00759967
Pkn3	5.230750811	11.42079822	0
Pknox1	241.6606875	198.721889	192.1900805
Pkp4	81.59971265	37.68863411	55.04942003
Pla2g12a	17.78455276	12.56287804	12.55513089
Pla2g15	1505.410083	1333.949232	1622.509222
Pla2g16	37.66140584	2.284159643	17.38402738
Pla2g4a	135.9995211	76.51934805	156.4562464
Pla2g4c	5.230750811	0	1.931558598
Pla2g5	0	0	0
Pla2g6	34.52295535	19.41535697	28.97337897
Pla2g7	0	0	0
Plaa	91.01506412	149.6124566	94.64637129
Plac8	0	0	0
Plag1	5.230750811	1.142079822	4.828896494
Plagl2	93.10736444	82.22974716	120.7224124
Plat	0	2.284159643	0
Platr11	0	1.142079822	0
Platr25	3.138450487	3.426239465	5.794675793
Plau	82.64586282	114.2079822	74.36500601
Plaur	48.12290746	5.710399108	36.69961336
Plbd1	31.38450487	12.56287804	21.24714457
Plbd2	1.046150162	1.142079822	0
Plcb1	4.184600649	1.142079822	0
Plcb2	0	0	25.11026177
Plcb3	97.29196509	91.36638573	98.50948848
Plcb4	2.092300324	5.710399108	0
Plcd1	1.046150162	0	0
Plcd3	4.184600649	4.568319287	0
Plcd4	0	3.426239465	0
Plcg1	201.9069813	173.5961329	49.25474424
Plcg2	477.044474	333.4873079	438.4638017
Plch1	0	0	0
Plcl1	27.19990422	46.82527269	29.93915826
Plcl2	215.5069334	238.6946827	293.5969068
Plcxd1	0	7.994558752	3.863117195
Plcxd2	12.55380195	4.568319287	11.58935159
Pld1	365.1064066	449.9794497	490.6158838
Pld2	36.61525568	52.5356718	49.25474424
Pld3	490.6444261	360.8972236	432.6691259
Pld4	3080.912228	2428.061701	3535.718013
Plec	14.64610227	10.27871839	3.863117195
Plek	575.3825892	897.6747398	794.836363
Plekha1	81.59971265	151.8966163	141.0037776
Plekha2	264.675991	154.1807759	270.4182037
Plekha3	5.230750811	20.55743679	8.69201369
Plekha4	0	0	0

For Review Only

Plekha5	14.64610227	21.69951661	7.726234391
Plekha7	0	3.426239465	0.965779299
Plekha8	15.69225243	41.11487358	18.34980668
Plekhb1	8.369201298	0	9.657792988
Plekhb2	219.6915341	170.1698934	152.5931292
Plekhf1	2.092300324	1.142079822	7.726234391
Plekhf2	75.32281168	105.0713436	47.32318564
Plekhg1	0	0	0
Plekhg2	60.67670941	34.26239465	0
Plekhg3	0	0	0
Plekhg4	0	0	0
Plekhg5	20.92300324	14.84703768	22.21292387
Plekhg6	4.184600649	1.142079822	0.965779299
Plekhh1	7.323051136	6.85247893	2.897337897
Plekhh2	5.230750811	4.568319287	18.34980668
Plekhh3	0	5.710399108	0
Plekhj1	7.323051136	1.142079822	0
Plekhm1	105.6611664	134.765419	66.63877162
Plekhm2	40.79985633	51.39359197	82.0912404
Plekhm3	233.2914862	111.9238225	160.3193636
Plekhn1	6.276900973	4.568319287	34.76805476
Plekho1	184.1224286	187.3010908	0
Plekho2	101.4765657	63.95647001	74.36500601
Plgrkt	268.8605917	143.9020575	22.21292387
Plin2	3.138450487	68.5247893	4.828896494
Plin3	40.79985633	25.12575608	20.28136528
Plin4	23.01530357	59.38815073	18.34980668
Plk1	0	6.85247893	0
Plk2	5.230750811	0	9.657792988
Plk3	0	38.83071394	0.965779299
Plk4	2.092300324	4.568319287	13.52091018
Pllp	0	5.710399108	0
Pln	3.138450487	10.27871839	0
Plod1	348.368004	365.4655429	654.7983646
Plod3	363.0141063	78.80350769	84.9885783
Plp1	1.046150162	1.142079822	32.83649616
Plp2	3.138450487	0	0
Plpp1	167.384026	205.5743679	216.3345629
Plpp2	2.092300324	12.56287804	0
Plpp3	15.69225243	23.98367625	16.41824808
Plpp5	24.06145373	6.85247893	0
Plpp6	16.7384026	19.41535697	12.55513089
Plpp7	27.19990422	41.11487358	28.97337897
Plppr3	14.64610227	39.97279376	38.63117195
Plppr4	6.276900973	19.41535697	6.760455092
Plrg1	63.8151599	101.6451041	111.0646194
Pls3	73.23051136	18.27327715	38.63117195
Plscr1	13.59995211	0	0
Plscr3	94.1535146	87.94014627	84.9885783
Plscr4	0	0	0

For Review Only

Pltp	0	0	0
Plvap	1.046150162	4.568319287	0.965779299
Plxdc1	464.490672	291.2303545	328.3649616
Plxdc2	1.046150162	147.328297	6903.390428
Plxna1	39.75370617	34.26239465	8.69201369
Plxna2	1.046150162	0	0
Plxna3	23.01530357	13.70495786	24.14448247
Plxna4	170.5224764	212.4268468	173.8402738
Plxna4os1	0	0	0
Plxnb1	0	3.426239465	0
Plxnb2	2647.806061	1.142079822	0
Plxnb3	56.49210876	45.68319287	46.35740634
Plxnc1	1.046150162	3.426239465	5.794675793
Plxnd1	0	2.284159643	0
Pm20d1	6.276900973	0	0
Pmaip1	4.184600649	4.568319287	0
Pmel	3.138450487	2.284159643	3.863117195
Pmepa1	360.921806	449.9794497	386.3117195
Pmf1	4.184600649	2.284159643	20.28136528
Pml	122.399569	73.09310859	82.0912404
Pmm1	24.06145373	25.12575608	23.17870317
Pmm2	69.04591071	67.38270948	60.84409583
Pmp22	1519.010036	1242.582846	1429.353362
Pmpca	139.1379716	138.1916584	167.0798187
Pmpcb	107.7534667	0	108.1672815
Pms1	12.55380195	9.136638573	28.97337897
Pms2	30.33835471	20.55743679	37.66539266
Pmvk	21.96915341	25.12575608	54.08364074
Pnck	0	2.284159643	0
Pnisr	71.13821103	421.4274542	64.70721302
Pnkd	32.43065503	43.39903322	43.46006845
Pnkp	119.2611185	28.55199554	118.7908538
Pnldc1	0	2.284159643	0
Pnma1	0	3.426239465	0
Pnma2	8.369201298	2.284159643	6.760455092
Pnn	0	0	90.78325409
Pno1	34.52295535	35.40447447	48.28896494
Pnp	146.4610227	0	0
Pnp2	44.98445698	42.2569534	46.35740634
Pnpla2	121.3534188	53.67775162	10.62357229
Pnpla3	3.138450487	3.426239465	3.863117195
Pnpla6	1.046150162	1.142079822	0
Pnpla7	85.7843133	119.9183813	0
Pnpla8	89.96891395	100.5030243	112.996178
Pnpo	41.84600649	0	12.55513089
Pnpt1	51.26135795	41.11487358	39.59695125
Pnrc1	1.046150162	1.142079822	0
Pnrc2	0	0	145.8326741
Poc1a	0	10.27871839	13.52091018
Poc1b	38.707556	41.11487358	39.59695125

For Review Only

Poc5	21.96915341	54.81983144	59.87831653
Podnl1	0	0	0
Podxl	1.046150162	5.710399108	0
Podxl2	0	0	0.965779299
Pofut1	77.41511201	79.94558752	101.4068264
Pofut2	104.6150162	98.21886466	0
Pogk	38.707556	70.80894894	38.63117195
Poglut1	146.4610227	135.9074988	156.4562464
Pogz	100.4304156	126.7708602	104.3041643
Pola1	0	29.69407536	15.45246878
Pola2	37.66140584	43.39903322	50.22052354
Polb	65.90746022	44.54111304	76.29656461
Pold1	41.84600649	34.26239465	42.49428915
Pold2	24.06145373	33.12031483	59.87831653
Pold3	55.4459586	73.09310859	115.8935159
Pold4	39.75370617	21.69951661	0
Poldip2	248.9837386	198.721889	232.752811
Poldip3	275.1374927	203.2902083	247.2395005
Pole	0	0	0
Pole3	7.323051136	3.426239465	6.760455092
Pole4	29.29220454	36.54655429	43.46006845
Polg	2.092300324	2.284159643	108.1672815
Polh	17.78455276	31.97823501	21.24714457
Poli	20.92300324	17.13119732	16.41824808
Polk	38.707556	39.97279376	41.52850985
Poll	29.29220454	3.426239465	84.022799
Polm	8.369201298	20.55743679	0
Poln	0	0	0
Polq	1.046150162	2.284159643	0
Polr1a	138.0918214	114.2079822	133.2775432
Polr1b	64.86131006	34.26239465	74.36500601
Polr1c	51.26135795	0	89.81747479
Polr1d	110.8919172	106.2134234	120.7224124
Polr1e	12.55380195	30.83615518	22.21292387
Polr2a	132.8610706	111.9238225	147.7642327
Polr2b	17.78455276	198.721889	128.4486467
Polr2c	47.0767573	78.80350769	93.68059199
Polr2d	44.98445698	60.53023055	0
Polr2e	197.7223807	165.6015741	248.2052798
Polr2f	0	18.27327715	13.52091018
Polr2g	0	17.13119732	44.42584775
Polr2h	43.93830681	22.84159643	54.08364074
Polr2i	41.84600649	0	0.965779299
Polr2j	82.64586282	77.66142787	0
Polr2l	27.19990422	25.12575608	28.00759967
Polr2m	93.10736444	73.09310859	93.68059199
Polr3a	77.41511201	68.5247893	74.36500601
Polr3b	105.6611664	107.3555032	71.46766811
Polr3c	63.8151599	29.69407536	49.25474424
Polr3d	26.15375406	9.136638573	29.93915826

For Review Only

Polr3e	0	0	18.34980668
Polr3f	107.7534667	50.25151215	65.67299232
Polr3g	6.276900973	3.426239465	5.794675793
Polr3gl	5.230750811	0	16.41824808
Polr3h	49.16905763	30.83615518	48.28896494
Polr3k	64.86131006	35.40447447	58.91253723
Polrmt	79.50741233	49.10943233	116.8592952
Pom121	121.3534188	107.3555032	128.4486467
Pom121l2	1.046150162	0	7.726234391
Pomc	0	0	4.828896494
Pomgnt1	24.06145373	38.83071394	0
Pomgnt2	69.04591071	45.68319287	37.66539266
Pomk	54.39980844	98.21886466	83.0570197
Pomp	141.2302719	185.0169311	253.0341763
Pomt1	0	0	14.48668948
Pomt2	117.1688182	65.09854983	62.77565443
Pon1	1.046150162	0	0
Pon2	244.799138	229.5580442	261.72619
Pon3	194.5839302	205.5743679	228.8896938
Pop1	13.59995211	9.136638573	18.34980668
Pop4	2.092300324	23.98367625	37.66539266
Pop5	74.27666152	0	1.931558598
Pop7	0	0	0
Por	0	0	6.760455092
Porcn	12.55380195	22.84159643	19.31558598
Postn	18.83070292	14.84703768	24.14448247
Pot1a	80.55356249	42.2569534	73.39922671
Pot1b	46.03060714	55.96191126	32.83649616
Pou2f1	48.12290746	68.5247893	54.08364074
Pou2f2	102.5227159	113.0659023	98.50948848
Pou5f2	13.59995211	27.40991572	38.63117195
Pou6f1	54.39980844	75.37726823	62.77565443
Pp2d1	0	1.142079822	0
Ppa1	12.55380195	18.27327715	31.87071686
Ppa2	0	50.25151215	121.6881917
Ppan	0	0	0
Ppara	3.138450487	0	0
Ppard	30.33835471	21.69951661	0
Ppargc1b	6.276900973	5.710399108	0
Ppat	30.33835471	26.2678359	27.04182037
Ppcdc	432.060017	351.7605851	499.3078975
Ppcs	0	0	0
Ppfia1	47.0767573	52.5356718	42.49428915
Ppfia4	0	6.85247893	119.7566331
Ppfibp1	2.092300324	9.136638573	0
Ppfibp2	31.38450487	42.2569534	73.39922671
Pphln1	112.9842175	110.7817427	130.3802053
Ppib	0	0	820.912404
Ppid	152.7379237	77.66142787	0
Ppie	10.46150162	25.12575608	49.25474424

For Review Only

Ppif	28.24605438	41.11487358	26.07604107
Ppifos	1.046150162	17.13119732	0
Ppig	42.89215665	74.23518841	65.67299232
Ppih	4.184600649	7.994558752	7.726234391
Ppil1	21.96915341	33.12031483	31.87071686
Ppil2	193.53778	127.91294	177.703391
Ppil3	44.98445698	42.2569534	51.18630284
Ppil4	0	0	0
Ppip5k1	35.56910552	33.12031483	35.73383406
Ppip5k2	77.41511201	55.96191126	62.77565443
Ppl	14.64610227	1.142079822	0
Ppm1a	41.84600649	67.38270948	49.25474424
Ppm1b	14.64610227	34.26239465	41.52850985
Ppm1d	19.87685308	20.55743679	12.55513089
Ppm1e	3.138450487	17.13119732	7.726234391
Ppm1f	192.4916299	118.7763015	128.4486467
Ppm1g	212.3684829	174.7382127	0
Ppm1h	477.044474	484.2418444	328.3649616
Ppm1j	0	3.426239465	0
Ppm1k	46.03060714	50.25151215	35.73383406
Ppm1l	56.49210876	86.79806645	87.8859162
Ppm1m	0	0	0
Ppm1n	0	0	0
Ppme1	129.7226201	67.38270948	56.01519933
Ppox	1.046150162	50.25151215	0
Ppp1cb	74.27666152	90.22430591	81.1254611
Ppp1cc	28.24605438	93.65054538	55.04942003
Ppp1r10	397.5370617	157.6070154	222.1292387
Ppp1r11	79.50741233	107.3555032	92.71481269
Ppp1r12a	164.2455755	173.5961329	141.9695569
Ppp1r12b	46.03060714	76.51934805	41.52850985
Ppp1r12c	9.41535146	63.95647001	0
Ppp1r13b	46.03060714	36.54655429	48.28896494
Ppp1r13l	0	0	0.965779299
Ppp1r14b	6.276900973	21.69951661	0
Ppp1r15a	0	0	0
Ppp1r15b	245.8452881	231.8422038	206.67677
Ppp1r16a	48.12290746	45.68319287	66.63877162
Ppp1r16b	4.184600649	2.284159643	0
Ppp1r18	183.0762784	673.8270948	172.8744945
Ppp1r1a	4.184600649	1.142079822	0
Ppp1r1b	30.33835471	0	0
Ppp1r2	101.4765657	98.21886466	98.50948848
Ppp1r2-ps3	8.369201298	4.568319287	6.760455092
Ppp1r21	165.2917256	130.1970997	124.5855296
Ppp1r26	31.38450487	17.13119732	30.90493756
Ppp1r27	0	0	7.726234391
Ppp1r35	16.7384026	0	0
Ppp1r3b	254.2144894	131.3391795	184.4638461
Ppp1r3d	79.50741233	71.95102876	76.29656461

For Review Only

Ppp1r3f	2.092300324	5.710399108	8.69201369
Ppp1r3fos	1.046150162	12.56287804	15.45246878
Ppp1r7	83.69201298	65.09854983	92.71481269
Ppp1r8	24.06145373	30.83615518	17.38402738
Ppp1r9a	253.1683393	355.1868245	194.1216391
Ppp1r9b	55.4459586	103.9292638	72.43344741
Ppp2ca	106.7073165	117.6342216	99.47526778
Ppp2cb	16.7384026	39.97279376	25.11026177
Ppp2r1a	460.3060714	391.7333788	452.9504912
Ppp2r1b	129.7226201	154.1807759	117.8250745
Ppp2r2a	129.7226201	110.7817427	95.61215059
Ppp2r2c	5.230750811	0	0
Ppp2r2d	10.46150162	11.42079822	0
Ppp2r3a	13.59995211	20.55743679	33.80227546
Ppp2r3c	18.83070292	14.84703768	22.21292387
Ppp2r3d	19.87685308	17.13119732	22.21292387
Ppp2r5a	16.7384026	11.42079822	7.726234391
Ppp2r5b	19.87685308	19.41535697	27.04182037
Ppp2r5c	124.4918693	174.7382127	169.0113773
Ppp2r5d	12.55380195	6.85247893	44.42584775
Ppp2r5e	25.10760389	47.96735251	32.83649616
Ppp3ca	193.53778	151.8966163	113.9619573
Ppp3cb	30.33835471	38.83071394	41.52850985
Ppp3cc	8.369201298	2.284159643	0
Ppp3r1	148.553323	113.0659023	120.7224124
Ppp3r2	0	0	5.794675793
Ppp4c	0	15.9891175	0
Ppp4r1	233.2914862	259.2521195	286.8364518
Ppp4r1l-ps	30.33835471	41.11487358	28.00759967
Ppp4r2	23.01530357	34.26239465	28.97337897
Ppp4r4	0	0	2.897337897
Ppp5c	0	0	0
Ppp6c	179.9378279	170.1698934	151.6273499
Ppp6r1	44.98445698	43.39903322	27.04182037
Ppp6r2	142.2764221	133.6233391	74.36500601
Ppp6r3	238.522237	232.9842836	230.8212524
Pprc1	124.4918693	103.9292638	99.47526778
Ppt1	1310.826153	681.8216535	1127.064442
Ppt2	1.046150162	11.42079822	0
Pptc7	64.86131006	107.3555032	80.1596818
Ppwd1	19.87685308	19.41535697	12.55513089
Pqbp1	19.87685308	0	36.69961336
Pqlc1	10.46150162	13.70495786	32.83649616
Pqlc2	130.7687703	95.93470502	88.85169549
Pqlc3	17.78455276	21.69951661	12.55513089
Pradc1	0	12.56287804	11.58935159
Praf2	26.15375406	42.2569534	31.87071686
Pram1	1.046150162	1.142079822	0
Pramef8	91.01506412	119.9183813	97.54370918
Prc1	1.046150162	3.426239465	0

For Review Only

Prcc	0	19.41535697	1.931558598
Prcp	319.0757995	428.2799331	365.064575
Prdm1	89.96891395	108.4975831	75.33078531
Prdm10	27.19990422	34.26239465	24.14448247
Prdm11	9.41535146	2.284159643	3.863117195
Prdm15	42.89215665	44.54111304	25.11026177
Prdm16	8.369201298	7.994558752	5.794675793
Prdm2	147.5071729	158.7490952	85.9543576
Prdm4	33.47680519	23.98367625	19.31558598
Prdm5	0	6.85247893	0
Prdm9	21.96915341	63.95647001	29.93915826
Prdx1	285.5989943	291.2303545	302.2889205
Prdx2	0	65.09854983	0
Prdx3	139.1379716	98.21886466	184.4638461
Prdx4	91.01506412	68.5247893	84.022799
Prdx5	0	0	9.657792988
Prdx6	69.04591071	38.83071394	34.76805476
Prdx6b	2.092300324	0	3.863117195
Preb	6.276900973	0	63.74143372
Prelid1	118.2149683	29.69407536	206.67677
Prelid2	0	28.55199554	3.863117195
Prelp	11.50765178	0	0
Prep	108.7996169	75.37726823	98.50948848
Prepl	34.52295535	53.67775162	55.04942003
Prex1	745.9050657	736.641485	0
Prex2	2.092300324	1.142079822	0
Prf1	3.138450487	0	2.897337897
Prickle1	31.38450487	4.568319287	18.34980668
Prickle2	0	1.142079822	0
Prickle3	24.06145373	21.69951661	34.76805476
Prim1	0	0	0
Prim2	12.55380195	6.85247893	29.93915826
Primpol	62.76900973	73.09310859	65.67299232
Prkaa1	82.64586282	107.3555032	81.1254611
Prkaa2	1.046150162	1.142079822	0
Prkab1	492.7367264	427.1378533	493.5132217
Prkab2	16.7384026	12.56287804	31.87071686
Prkaca	54.39980844	62.81439019	59.87831653
Prkacb	209.2300324	115.350062	86.9201369
Prkag1	123.4457191	79.94558752	126.5170881
Prkag2	17.78455276	14.84703768	9.657792988
Prkag3	0	0	0
Prkar1a	319.0757995	156.4649356	709.8477847
Prkar1b	48.12290746	44.54111304	50.22052354
Prkar2a	55.4459586	25.12575608	42.49428915
Prkar2b	2.092300324	0	0
Prkca	80.55356249	94.7926252	72.43344741
Prkcb	252.1221891	336.9135474	229.8554731
Prkcd	1237.595642	472.8210462	211.5056664
Prkce	41.84600649	28.55199554	28.00759967

For Review Only

Prkcg	0	0	0
Prkch	61.72285957	53.67775162	28.97337897
Prkci	9.41535146	15.9891175	14.48668948
Prkcq	11.50765178	11.42079822	5.794675793
Prkcsh	232.245336	119.9183813	201.8478735
Prkcz	21.96915341	12.56287804	13.52091018
Prkd2	21.96915341	38.83071394	29.93915826
Prkd3	127.6303198	186.1590109	104.3041643
Prkdc	77.41511201	97.07678484	102.3726057
Prkg1	2.092300324	1.142079822	0.965779299
Prkra	70.09206087	44.54111304	54.08364074
Prkrip1	24.06145373	15.9891175	22.21292387
Prkrir	14.64610227	19.41535697	42.49428915
Prkx	94.1535146	83.37182698	103.338385
Prlh	3.138450487	0	0.965779299
Prlr	12.55380195	0	13.52091018
Prmt1	18.83070292	10.27871839	0
Prmt2	69.04591071	57.10399108	81.1254611
Prmt3	0	0	33.80227546
Prmt5	72.18436119	42.2569534	63.74143372
Prmt6	46.03060714	47.96735251	13.52091018
Prmt7	58.58440909	46.82527269	111.0646194
Prmt9	40.79985633	28.55199554	20.28136528
Prnp	18.83070292	6.85247893	3.863117195
Prob1	0	1.142079822	0
Proca1	2.092300324	6.85247893	7.726234391
Procr	6.276900973	4.568319287	14.48668948
Prodh	20.92300324	11.42079822	14.48668948
Prok1	1.046150162	0	0
Prokr1	0	1.142079822	0
Prom1	2.092300324	0	0
Prom2	9.41535146	5.710399108	0
Prorsd1	64.86131006	101.6451041	95.61215059
Pros1	784.6126217	856.5598662	961.9161817
Prosc	49.16905763	95.93470502	52.15208214
Proser1	63.8151599	71.95102876	40.56273055
Proser2	0	3.426239465	0
Proser3	9.41535146	18.27327715	10.62357229
Prox1	33.47680519	51.39359197	19.31558598
Prox2	50.21520779	13.70495786	30.90493756
Proz	6.276900973	7.994558752	4.828896494
Prpf18	95.19966476	76.51934805	122.653971
Prpf19	26.15375406	34.26239465	23.17870317
Prpf3	61.72285957	59.38815073	54.08364074
Prpf31	74.27666152	20.55743679	101.4068264
Prpf38a	50.21520779	44.54111304	32.83649616
Prpf38b	0	0	0
Prpf39	65.90746022	189.5852504	121.6881917
Prpf4	82.64586282	27.40991572	46.35740634
Prpf40a	103.5688661	108.4975831	125.5513089

For Review Only

Prpf40b	23.01530357	11.42079822	41.52850985
Prpf4b	114.0303677	195.2956495	122.653971
Prpf6	121.3534188	118.7763015	95.61215059
Prpf8	314.8911988	250.1154809	219.2319008
Prps1	20.92300324	26.2678359	16.41824808
Prps1l1	1.046150162	28.55199554	19.31558598
Prps1l3	141.2302719	103.9292638	108.1672815
Prps2	134.9533709	77.66142787	83.0570197
Prpsap1	274.0913425	216.9951661	297.460024
Prpsap2	200.8608312	127.91294	184.4638461
Prr11	3.138450487	0	0
Prr12	31.38450487	36.54655429	29.93915826
Prr13	11.50765178	33.12031483	0
Prr14	51.26135795	129.0550198	107.2015022
Prr14l	229.1068855	398.5858578	306.1520377
Prr15	16.7384026	13.70495786	8.69201369
Prr15l	4.184600649	0	0
Prr18	1.046150162	2.284159643	0
Prr22	3.138450487	0	0
Prr3	23.01530357	21.69951661	27.04182037
Prr32	18.83070292	0	0
Prr33	3.138450487	0	0
Prr36	0	0	0.965779299
Prr5	12.55380195	5.710399108	2.897337897
Prr5l	36.61525568	15.9891175	28.97337897
Prr7	11.50765178	4.568319287	3.863117195
Prrc1	126.5841696	113.0659023	85.9543576
Prrc2a	0	0	182.5322875
Prrc2b	153.7840738	158.7490952	155.4904671
Prrc2c	223.8761347	381.4546604	233.7185903
Prrg1	3.138450487	0	0
Prrg2	21.96915341	25.12575608	26.07604107
Prrg4	1.046150162	3.426239465	2.897337897
Prrt3	4.184600649	4.568319287	5.794675793
Prrx1	0	0	0
Prrxl1	0	0	0
Prss12	13.59995211	0	0
Prss23	0	0	0
Prss27	0	0	0
Prss36	11.50765178	20.55743679	14.48668948
Prss53	8.369201298	1.142079822	15.45246878
Prss8	2.092300324	1.142079822	0
Prtn3	0	0	0
Prune	27.19990422	17.13119732	26.07604107
Prune2	104.6150162	84.5139068	61.80987513
Psap	77.41511201	608.7285449	8.69201369
Psat1	76.36896184	43.39903322	44.42584775
Psd	0	9.136638573	1.931558598
Psd2	0	0	0
Psd3	5.230750811	9.136638573	11.58935159

For Review Only

Psd4	0	284.3778756	0.965779299
Psen1	342.0911031	381.4546604	386.3117195
Psen2	33.47680519	36.54655429	37.66539266
Psg16	1.046150162	0	0
Psg26	3.138450487	0	0
Psip1	18.83070292	1.142079822	0
Pskh1	111.9380674	74.23518841	87.8859162
Psma1	10.46150162	70.80894894	12.55513089
Psma2	186.2147289	91.36638573	228.8896938
Psma3	115.0765178	186.1590109	165.1482601
Psma4	59.63055925	81.08766734	92.71481269
Psma5	64.86131006	57.10399108	90.78325409
Psma6	146.4610227	165.6015741	207.6425493
Psma7	224.9222849	235.2684433	242.410604
Psma8	27.19990422	10.27871839	19.31558598
Psmb1	214.4607833	308.3615518	301.3231412
Psmb10	44.98445698	105.0713436	84.022799
Psmb2	133.9072208	232.9842836	256.8972935
Psmb3	100.4304156	90.22430591	124.5855296
Psmb4	1.046150162	1.142079822	0
Psmb5	122.399569	151.8966163	195.0874184
Psmb6	0	0	0
Psmb7	69.04591071	81.08766734	70.50188882
Psmb8	248.9837386	351.7605851	509.9314698
Psmb9	0	0	5.794675793
Psmc1	108.7996169	114.2079822	137.1406604
Psmc2	121.3534188	15.9891175	301.3231412
Psmc3	21.96915341	36.54655429	0
Psmc3ip	2.092300324	1.142079822	0
Psmc4	1.046150162	1.142079822	0
Psmc5	168.4301761	206.7164477	250.1368384
Psmc6	122.399569	123.3446207	127.4828674
Psmd1	195.6300803	275.241237	219.2319008
Psmd10	53.35365827	69.66686912	58.91253723
Psmd11	125.5380195	127.91294	139.072219
Psmd12	129.7226201	79.94558752	153.5589085
Psmd13	138.0918214	132.4812593	176.7376117
Psmd14	95.19966476	122.2025409	149.6957913
Psmd2	274.0913425	28.55199554	71.46766811
Psmd3	104.6150162	78.80350769	71.46766811
Psmd4	0	0	0
Psmd5	51.26135795	59.38815073	97.54370918
Psmd6	193.53778	195.2956495	253.0341763
Psmd7	196.6762305	95.93470502	169.9771566
Psmd8	200.8608312	182.7327715	207.6425493
Psmd9	52.30750811	50.25151215	64.70721302
Psme2	0	43.39903322	74.36500601
Psme2b	185.1685787	235.2684433	182.5322875
Psme3	12.55380195	0	0
Psme4	30.33835471	51.39359197	66.63877162

For Review Only

Psmf1	106.7073165	54.81983144	118.7908538
Psmg1	41.84600649	49.10943233	14.48668948
Psmg2	12.55380195	33.12031483	59.87831653
Psmg3	14.64610227	12.56287804	28.97337897
Psmg4	18.83070292	18.27327715	19.31558598
Pspc1	96.24581493	74.23518841	42.49428915
Psph	11.50765178	35.40447447	32.83649616
Psrc1	0	9.136638573	0
Pstk	21.96915341	18.27327715	15.45246878
Pstpip1	0	0	3.863117195
Pstpip2	14.64610227	6.85247893	6.760455092
Ptafr	1209.349588	1078.123352	1177.284965
Ptar1	75.32281168	78.80350769	89.81747479
Ptbp2	36.61525568	45.68319287	45.39162705
Ptbp3	262.5836907	302.6511527	298.4258033
Ptcd1	108.7996169	124.4867006	102.3726057
Ptcd2	42.89215665	78.80350769	79.19390251
Ptcd3	107.7534667	54.81983144	72.43344741
Ptch1	35.56910552	28.55199554	52.15208214
Ptch2	0	0	0
Ptchd1	37.66140584	38.83071394	28.97337897
Ptdss1	143.3225722	122.2025409	133.2775432
Ptdss2	100.4304156	93.65054538	80.1596818
Pten	165.2917256	204.4322881	151.6273499
Pter	23.01530357	3.426239465	0
Ptgds	1.046150162	0	0
Ptger1	35.56910552	30.83615518	36.69961336
Ptger3	29.29220454	22.84159643	28.97337897
Ptger4	2.092300324	14.84703768	0
Ptges2	7.323051136	9.136638573	10.62357229
Ptges3	140.1841217	109.6396629	78.22812321
Ptgfrn	13.59995211	20.55743679	19.31558598
Ptgr1	23.01530357	0	31.87071686
Ptgr2	188.3070292	15.9891175	112.0303987
Ptgs1	1733.470819	2021.481284	1575.186036
Pth1r	0	5.710399108	5.794675793
Ptk2	3.138450487	14.84703768	0.965779299
Ptk2b	254.2144894	154.1807759	146.7984534
Ptn	1.046150162	0	0
Ptov1	4.184600649	9.136638573	0
Ptp4a1	78.46126217	45.68319287	33.80227546
Ptp4a2	457.1676209	462.5423278	450.0531533
Ptp4a3	399.629362	302.6511527	294.5626861
Ptpa	198.7685308	197.5798091	228.8896938
Ptpdc1	15.69225243	17.13119732	5.794675793
Ptpmt1	53.35365827	77.66142787	39.59695125
Ptpn1	646.5208003	392.8754587	571.7413449
Ptpn11	27.19990422	73.09310859	22.21292387
Ptpn12	20.92300324	6.85247893	15.45246878
Ptpn14	7.323051136	6.85247893	0.965779299

For Review Only

Ptpn18	76.36896184	161.0332549	108.1672815
Ptpn2	64.86131006	126.7708602	103.338385
Ptpn21	25.10760389	43.39903322	21.24714457
Ptpn22	0	9.136638573	5.794675793
Ptpn23	11.50765178	27.40991572	0
Ptpn4	8.369201298	17.13119732	7.726234391
Ptpn6	1.046150162	205.5743679	0
Ptpn7	0	1.142079822	0
Ptpn9	18.83070292	50.25151215	32.83649616
Ptpra	85.7843133	206.7164477	168.045598
Ptprb	0	0	0
Ptprc	619.320896	706.9474096	592.9884895
Ptprcap	0	2.284159643	0
Ptpre	312.7988985	95.93470502	143.9011155
Ptprf	21.96915341	1.142079822	0
Ptprg	0	0	5.794675793
Ptprh	0	6.85247893	0
Ptprj	0	27.40991572	1.931558598
Ptprm	157.9686745	156.4649356	106.2357229
Ptprn	0	0	0
Ptpro	273.0451923	201.0060486	282.0075553
Ptprr	0	0	0
Ptprs	209.2300324	193.0114899	106.2357229
Ptrf	6.276900973	0	0
Ptrh1	3.138450487	0	0
Ptrh2	92.06121428	85.65598662	82.0912404
Ptrhd1	43.93830681	27.40991572	52.15208214
Pts	0	0	0
Pttg1	75.32281168	84.5139068	161.2851429
Pttg1ip	747.997366	776.6142787	922.3192304
Puf60	93.10736444	0	98.50948848
Pum1	104.6150162	183.8748513	110.0988401
Pum2	198.7685308	223.847645	211.5056664
Pum3	58.58440909	30.83615518	100.4410471
Pura	58.58440909	102.7871839	59.87831653
Purb	105.6611664	165.6015741	104.3041643
Purg	1.046150162	15.9891175	4.828896494
Pus1	67.99976055	35.40447447	26.07604107
Pus10	38.707556	58.2460709	49.25474424
Pus3	1.046150162	1.142079822	0
Pus7	24.06145373	25.12575608	14.48668948
Pus7l	15.69225243	0	0
Pvr	13.59995211	23.98367625	21.24714457
Pvrig	52.30750811	22.84159643	76.29656461
Pvt1	24.06145373	49.10943233	50.22052354
Pwp1	103.5688661	89.08222609	53.11786144
Pwp2	53.35365827	39.97279376	44.42584775
Pwwp2a	236.4299367	346.050186	212.4714457
Pwwp2b	19.87685308	17.13119732	10.62357229
Pxdc1	31.38450487	37.68863411	21.24714457

For Review Only

Pxdn	3.138450487	0	0
Pxk	165.2917256	114.2079822	138.1064397
Pxmp4	42.89215665	43.39903322	44.42584775
Pxn	419.5062151	44.54111304	196.0531977
Pxylp1	2.092300324	0	0
Pycard	456.1214707	590.4552678	736.889605
Pycr1	11.50765178	0	9.657792988
Pycr2	3.138450487	41.11487358	0
Pycrl	19.87685308	21.69951661	58.91253723
Pydc3	0	2.284159643	0
Pydc4	0	3.426239465	2.897337897
Pygb	222.8299846	146.1862172	151.6273499
Pygl	44.98445698	19.41535697	23.17870317
Pygm	5.230750811	0	0
Pygo2	1.046150162	15.9891175	0
Pyhin1	1.046150162	9.136638573	22.21292387
Pym1	24.06145373	17.13119732	20.28136528
Pyroxd1	66.95361038	73.09310859	54.08364074
Pyroxd2	46.03060714	71.95102876	84.9885783
Pyurf	15.69225243	13.70495786	20.28136528
Qars	16.7384026	31.97823501	233.7185903
Qdpr	82.64586282	103.9292638	158.387805
Qk	1206.211137	83.37182698	11.58935159
Qpct	0	0	1.931558598
Qpctl	54.39980844	33.12031483	42.49428915
Qprt	20.92300324	23.98367625	24.14448247
Qrfp	8.369201298	7.994558752	8.69201369
Qrich1	251.0760389	221.5634854	264.6235279
Qrich2	0	0	0
Qrsl1	31.38450487	35.40447447	31.87071686
Qser1	35.56910552	38.83071394	34.76805476
Qsox1	52.30750811	42.2569534	49.25474424
Qsox2	19.87685308	11.42079822	25.11026177
Qtrt1	0	0	0
Qtrtd1	3.138450487	23.98367625	0
R3hcc1	27.19990422	5.710399108	18.34980668
R3hcc1l	80.55356249	92.50846555	100.4410471
R3hdm1	120.3072687	75.37726823	104.3041643
R3hdm2	43.93830681	51.39359197	61.80987513
R3hdm4	0	58.2460709	10.62357229
R74862	20.92300324	29.69407536	0
Rab10	34.52295535	177.0223724	56.01519933
Rab10os	20.92300324	15.9891175	2.897337897
Rab11a	316.9834992	441.984891	449.087374
Rab11b	237.4760868	172.4540531	238.5474868
Rab11fip1	2.092300324	1.142079822	0
Rab11fip2	14.64610227	31.97823501	26.07604107
Rab11fip3	16.7384026	19.41535697	15.45246878
Rab11fip4	4.184600649	1.142079822	0
Rab11fip5	262.5836907	166.743654	194.1216391

For Review Only

Rab12	44.98445698	127.91294	66.63877162
Rab13	0	0	0.965779299
Rab14	8.369201298	1.142079822	690.5321987
Rab15	0	0	0
Rab18	236.4299367	131.3391795	260.7604107
Rab19	6.276900973	9.136638573	0
Rab1a	510.5212792	462.5423278	545.6653038
Rab1b	0	0	0
Rab20	163.1994253	159.891175	145.8326741
Rab21	6.276900973	14.84703768	8.69201369
Rab22a	6.276900973	35.40447447	18.34980668
Rab23	9.41535146	7.994558752	7.726234391
Rab26	2.092300324	0	0
Rab27a	142.2764221	87.94014627	86.9201369
Rab28	20.92300324	41.11487358	42.49428915
Rab29	51.26135795	0	0
Rab2a	38.707556	99.36094448	80.1596818
Rab2b	47.0767573	66.24062966	73.39922671
Rab30	0	0	4.828896494
Rab31	696.7360081	646.4171791	613.2698548
Rab32	163.1994253	157.6070154	191.2243012
Rab33a	0	0	0
Rab33b	75.32281168	29.69407536	61.80987513
Rab34	14.64610227	42.2569534	42.49428915
Rab35	20.92300324	0	0
Rab36	6.276900973	2.284159643	5.794675793
Rab37	0	0	0
Rab39	111.9380674	127.91294	128.4486467
Rab3a	56.49210876	11.42079822	22.21292387
Rab3d	21.96915341	7.994558752	22.21292387
Rab3gap1	187.260879	174.7382127	122.653971
Rab3gap2	115.0765178	155.3228557	82.0912404
Rab3il1	83.69201298	953.6366511	774.5549977
Rab3ip	213.4146331	107.3555032	157.4220257
Rab40b	1.046150162	0	2.897337897
Rab40c	48.12290746	54.81983144	51.18630284
Rab42	24.06145373	1.142079822	0
Rab43	89.96891395	170.1698934	61.80987513
Rab44	6.276900973	0	7.726234391
Rab4a	9.41535146	20.55743679	5.794675793
Rab4b	132.8610706	122.2025409	126.5170881
Rab5a	34.52295535	106.2134234	27.04182037
Rab5b	175.7532273	125.6287804	115.8935159
Rab5c	128.67647	106.2134234	56.98097863
Rab6a	83.69201298	53.67775162	73.39922671
Rab6b	124.4918693	264.9625186	147.7642327
Rab7	452.9830203	389.4492192	467.4371806
Rab7b	5.230750811	1.142079822	0.965779299
Rab8a	107.7534667	206.7164477	220.1976801
Rab8b	354.644905	246.6892415	263.6577486

For Review Only

Rab9	27.19990422	33.12031483	46.35740634
Rabac1	284.5528441	323.2085895	289.7337897
Rabep1	86.83046347	94.7926252	64.70721302
Rabep2	99.38426541	100.5030243	89.81747479
Rabepk	46.03060714	50.25151215	39.59695125
Rabgap1	78.46126217	119.9183813	88.85169549
Rabgap1l	41.84600649	18.27327715	13.52091018
Rabgef1	49.16905763	34.26239465	60.84409583
Rabggta	91.01506412	63.95647001	112.0303987
Rabggtb	0	7.994558752	0
Rabif	1.046150162	1.142079822	0
Rabl2	25.10760389	26.2678359	29.93915826
Rabl3	4.184600649	19.41535697	0
Rabl6	62.76900973	77.66142787	27.04182037
Rac1	638.151599	23.98367625	0
Rac2	0	0	120.7224124
Rac3	0	0	0
Racgap1	0	3.426239465	0
Rad1	21.96915341	27.40991572	40.56273055
Rad17	51.26135795	13.70495786	29.93915826
Rad18	0	0	0
Rad21	66.95361038	63.95647001	86.9201369
Rad23a	0	0	0
Rad23b	19.87685308	42.2569534	15.45246878
Rad50	60.67670941	60.53023055	41.52850985
Rad51	1.046150162	1.142079822	0
Rad51ap1	1.046150162	2.284159643	3.863117195
Rad51b	2.092300324	13.70495786	6.760455092
Rad51c	1.046150162	0	0
Rad51d	34.52295535	44.54111304	38.63117195
Rad52	62.76900973	47.96735251	42.49428915
Rad54l	0	0	9.657792988
Rad54l2	57.53825892	70.80894894	44.42584775
Rad9a	7.323051136	0	0
Rad9b	19.87685308	28.55199554	6.760455092
Rae1	115.0765178	103.9292638	84.022799
Raf1	162.1532751	118.7763015	122.653971
Rai1	189.3531794	212.4268468	143.9011155
Rai14	2.092300324	2.284159643	8.69201369
Rala	110.8919172	102.7871839	106.2357229
Ralb	347.3218539	261.5362792	368.9276922
Ralbp1	57.53825892	62.81439019	51.18630284
Ralgapa1	84.73816314	70.80894894	68.57033022
Ralgapa2	73.23051136	55.96191126	22.21292387
Ralgapb	36.61525568	103.9292638	45.39162705
Ralgds	28.24605438	12.56287804	28.97337897
Ralgps1	99.38426541	162.1753347	27.04182037
Ralgps2	39.75370617	89.08222609	110.0988401
Raly	23.01530357	54.81983144	20.28136528
Ramp1	58.58440909	63.95647001	107.2015022

For Review Only

Ramp2	0	0	4.828896494
Ran	60.67670941	6.85247893	339.9543132
Ranbp1	67.99976055	53.67775162	75.33078531
Ranbp2	62.76900973	102.7871839	45.39162705
Ranbp3	2.092300324	177.0223724	0
Ranbp3l	0	0	0
Ranbp6	46.03060714	46.82527269	55.04942003
Ranbp9	28.24605438	39.97279376	30.90493756
Rangap1	48.12290746	41.11487358	65.67299232
Rangrf	0	0	0
Rap1a	79.50741233	116.4921418	84.9885783
Rap1b	217.5992337	357.4709842	186.3954047
Rap1gap	3.138450487	1.142079822	0
Rap1gap2	62.76900973	22.84159643	62.77565443
Rap1gds1	305.4758474	408.8645762	416.2508778
Rap2a	71.13821103	75.37726823	45.39162705
Rap2b	32.43065503	31.97823501	15.45246878
Rap2c	64.86131006	85.65598662	99.47526778
Rapgef1	222.8299846	256.9679599	256.8972935
Rapgef2	65.90746022	59.38815073	64.70721302
Rapgef5	180.9839781	276.3833168	201.8478735
Rapgef6	198.7685308	293.5145142	218.2661215
Rapgefl1	8.369201298	5.710399108	4.828896494
Raph1	18.83070292	15.9891175	29.93915826
Rapsn	20.92300324	57.10399108	61.80987513
Rarb	1.046150162	0	0
Rarg	0	0	6.760455092
Rars	84.73816314	78.80350769	84.022799
Rars2	26.15375406	19.41535697	52.15208214
Rasa1	61.72285957	91.36638573	70.50188882
Rasa2	33.47680519	30.83615518	24.14448247
Rasa3	371.3833076	272.9570774	303.2546998
Rasa4	10.46150162	0	66.63877162
Rasal2	34.52295535	22.84159643	15.45246878
Rasal3	468.6752727	3.426239465	195.0874184
Rasgef1a	0	0	0
Rasgef1b	135.9995211	75.37726823	81.1254611
Rasgrf1	0	0	0
Rasgrf2	12.55380195	25.12575608	29.93915826
Rasgrp3	565.9672378	706.9474096	644.1747923
Rasgrp4	5.230750811	27.40991572	0
Rasl10a	4.184600649	4.568319287	1.931558598
Rasl2-9	17.78455276	18.27327715	7.726234391
Rassf1	0	17.13119732	230.8212524
Rassf2	468.6752727	283.2357958	293.5969068
Rassf3	4.184600649	6.85247893	4.828896494
Rassf4	85.7843133	138.1916584	14.48668948
Rassf5	450.8907199	140.4758181	510.8972491
Rassf7	12.55380195	12.56287804	7.726234391
Rassf8	14.64610227	22.84159643	18.34980668

For Review Only

Raver2	1.046150162	3.426239465	0
Rb1	63.8151599	85.65598662	57.94675793
Rb1cc1	89.96891395	74.23518841	46.35740634
Rbak	56.49210876	50.25151215	51.18630284
Rbakdn	0	0	0
Rbbp4	133.9072208	114.2079822	114.9277366
Rbbp5	87.87661363	98.21886466	77.26234391
Rbbp6	117.1688182	159.891175	132.3117639
Rbbp7	174.7070771	153.0386961	112.996178
Rbbp8	18.83070292	10.27871839	7.726234391
Rbbp9	146.4610227	111.9238225	128.4486467
Rbck1	64.86131006	9.136638573	0
Rbfa	102.5227159	99.36094448	93.68059199
Rbfox1	41.84600649	58.2460709	41.52850985
Rbfox2	2.092300324	6.85247893	8.69201369
Rbfox3	7.323051136	6.85247893	0.965779299
Rbks	19.87685308	22.84159643	11.58935159
Rbl1	38.707556	31.97823501	23.17870317
Rbl2	72.18436119	51.39359197	100.4410471
Rbm10	100.4304156	74.23518841	76.29656461
Rbm12	83.69201298	125.6287804	58.91253723
Rbm12b1	31.38450487	43.39903322	42.49428915
Rbm12b2	30.33835471	42.2569534	47.32318564
Rbm14	91.01506412	102.7871839	88.85169549
Rbm15	153.7840738	186.1590109	133.2775432
Rbm15b	8.369201298	29.69407536	27.04182037
Rbm17	48.12290746	46.82527269	56.01519933
Rbm18	155.8763742	92.50846555	132.3117639
Rbm19	74.27666152	39.97279376	45.39162705
Rbm20	2.092300324	1.142079822	0
Rbm22	80.55356249	97.07678484	53.11786144
Rbm25	157.9686745	0	105.2699436
Rbm27	81.59971265	42.2569534	54.08364074
Rbm28	71.13821103	70.80894894	76.29656461
Rbm3	1.046150162	60.53023055	70.50188882
Rbm33	229.1068855	205.5743679	147.7642327
Rbm34	36.61525568	30.83615518	27.04182037
Rbm38	75.32281168	50.25151215	92.71481269
Rbm39	235.3837865	831.4341102	281.041776
Rbm3os	10.46150162	0	0
Rbm4	0	67.38270948	0
Rbm41	12.55380195	35.40447447	9.657792988
Rbm42	5.230750811	6.85247893	311.9467135
Rbm43	64.86131006	59.38815073	78.22812321
Rbm45	0	0	0
Rbm47	187.260879	141.6178979	128.4486467
Rbm48	21.96915341	41.11487358	70.50188882
Rbm4b	152.7379237	201.0060486	169.9771566
Rbm5	218.6453839	340.3397869	78.22812321
Rbm6	115.0765178	111.9238225	176.7376117

For Review Only

Rbm7	1.046150162	84.5139068	0
Rbm8a	0	9.136638573	0
Rbms1	28.24605438	12.56287804	11.58935159
Rbms2	44.98445698	37.68863411	72.43344741
Rbms3	0	0	0
Rbmx	125.5380195	127.91294	142.9353362
Rbmx2	11.50765178	6.85247893	0
Rbmxl1	73.23051136	65.09854983	83.0570197
Rbp1	7.323051136	0	0
Rbp4	0	0	0
Rbpj	107.7534667	108.4975831	24.14448247
Rbpms	25.10760389	36.54655429	28.00759967
Rbsn	91.01506412	53.67775162	121.6881917
Rbx1	143.3225722	166.743654	141.9695569
Rc3h1	85.7843133	178.1644522	103.338385
Rc3h2	52.30750811	125.6287804	56.98097863
Rcan1	116.122668	4.568319287	153.5589085
Rcan3	96.24581493	71.95102876	66.63877162
Rcbtb1	44.98445698	87.94014627	57.94675793
Rcbtb2	306.5219975	253.5417204	542.767966
Rcc1	23.01530357	14.84703768	37.66539266
Rcc2	1.046150162	1.142079822	0
Rccd1	5.230750811	3.426239465	11.58935159
Rce1	44.98445698	0	0
Rchy1	95.19966476	67.38270948	74.36500601
Rcl1	64.86131006	84.5139068	68.57033022
Rcn1	19.87685308	20.55743679	23.17870317
Rcn2	120.3072687	129.0550198	151.6273499
Rcn3	11.50765178	7.994558752	3.863117195
Rcor1	55.4459586	65.09854983	57.94675793
Rcor3	42.89215665	59.38815073	77.26234391
Rcsd1	395.4447613	438.5586515	510.8972491
Rd3	4.184600649	0	2.897337897
Rdh1	0	2.284159643	0
Rdh10	30.33835471	22.84159643	13.52091018
Rdh11	29.29220454	22.84159643	31.87071686
Rdh12	6.276900973	0	0
Rdh13	77.41511201	61.67231037	110.0988401
Rdh14	63.8151599	65.09854983	61.80987513
Rdh5	66.95361038	1.142079822	22.21292387
Rdm1	1.046150162	1.142079822	0
Rdx	140.1841217	202.1481284	246.2737212
Rec114	8.369201298	0	10.62357229
Reck	0	5.710399108	0
Recql	47.0767573	19.41535697	38.63117195
Recql4	0	4.568319287	0
Recql5	49.16905763	52.5356718	23.17870317
Reep1	2.092300324	3.426239465	3.863117195
Reep2	0	6.85247893	0
Reep3	147.5071729	172.4540531	143.9011155

For Review Only

Reep4	0	2.284159643	0
Reep5	638.151599	551.6245539	639.3458958
Rel	75.32281168	74.23518841	72.43344741
Rela	195.6300803	79.94558752	0
Relb	99.38426541	95.93470502	94.64637129
Rell1	31.38450487	15.9891175	14.48668948
Relt	9.41535146	11.42079822	0
Renbp	0	0	18.34980668
Repin1	71.13821103	67.38270948	66.63877162
Reps1	39.75370617	13.70495786	24.14448247
Reps2	0	0	0
Rer1	279.3220933	244.4050818	322.5702858
Rere	436.2446177	518.504239	327.3991823
Rest	35.56910552	45.68319287	30.90493756
Retnlg	8.369201298	0	0
Retsat	57.53825892	94.7926252	71.46766811
Rev1	17.78455276	22.84159643	0
Rev3l	23.01530357	86.79806645	14.48668948
Rexo1	77.41511201	93.65054538	57.94675793
Rexo2	50.21520779	52.5356718	37.66539266
Rexo4	170.5224764	99.36094448	159.3535843
Rfc1	57.53825892	67.38270948	70.50188882
Rfc2	129.7226201	93.65054538	96.57792988
Rfc3	29.29220454	25.12575608	45.39162705
Rfc4	9.41535146	0	10.62357229
Rfc5	13.59995211	43.39903322	27.04182037
Rfesd	35.56910552	30.83615518	48.28896494
Rffl	239.5683872	178.1644522	236.6159282
Rfk	86.83046347	71.95102876	54.08364074
Rfng	27.19990422	21.69951661	34.76805476
Rft1	62.76900973	71.95102876	79.19390251
Rftn1	31.38450487	15.9891175	19.31558598
Rftn2	0	0	0
Rfwd2	32.43065503	49.10943233	25.11026177
Rfwd3	69.04591071	84.5139068	62.77565443
Rfx1	36.61525568	37.68863411	37.66539266
Rfx3	23.01530357	62.81439019	41.52850985
Rfx5	37.66140584	55.96191126	48.28896494
Rfx7	40.79985633	100.5030243	35.73383406
Rfxank	39.75370617	52.5356718	96.57792988
Rfxap	34.52295535	9.136638573	6.760455092
Rgag1	0	0	0
Rgag4	6.276900973	11.42079822	7.726234391
Rgl1	70.09206087	110.7817427	76.29656461
Rgl2	0	0	4.828896494
Rgl3	40.79985633	49.10943233	24.14448247
Rgma	0	0	0
Rgmb	131.8149204	280.9516361	169.0113773
Rgp1	0	0	1.931558598
Rgs1	0	0	0

For Review Only

Rgs10	1520.056186	1577.212234	2271.512911
Rgs11	28.24605438	20.55743679	21.24714457
Rgs12	12.55380195	5.710399108	16.41824808
Rgs14	2.092300324	22.84159643	68.57033022
Rgs18	2.092300324	2.284159643	0.965779299
Rgs19	41.84600649	132.4812593	266.5550865
Rgs2	0	0	0
Rgs3	23.01530357	11.42079822	51.18630284
Rgs5	0	0	0
Rgs7bp	25.10760389	18.27327715	28.97337897
Rgsl1	0	0	0
Rhbdd1	89.96891395	92.50846555	84.022799
Rhbdd2	79.50741233	52.5356718	86.9201369
Rhbdd3	3.138450487	1.142079822	0
Rhbdf1	6.276900973	3.426239465	0
Rhbdf2	44.98445698	35.40447447	66.63877162
Rhbdl1	2.092300324	0	0
Rhbdl3	0	0	0
Rheb	18.83070292	61.67231037	53.11786144
Rhebl1	7.323051136	4.568319287	5.794675793
Rhno1	75.32281168	77.66142787	109.1330608
Rho	2.092300324	0	0
Rhoa	228.0607354	525.356718	0
Rhob	569.1056883	568.7557512	255.9315142
Rhobtb1	111.9380674	130.1970997	99.47526778
Rhobtb2	20.92300324	36.54655429	28.00759967
Rhobtb3	6.276900973	0	0
Rhoc	30.33835471	30.83615518	26.07604107
Rhod	3.138450487	0	0
Rhof	0	0	4.828896494
Rhog	107.7534667	123.3446207	0
Rhoh	429.9677167	415.7170551	507.9999112
Rhoj	0	0	0
Rhoq	93.10736444	140.4758181	115.8935159
Rhot1	245.8452881	185.0169311	141.9695569
Rhot2	47.0767573	47.96735251	41.52850985
Rhox5	0	0	0
Rhpn2	5.230750811	0	0
Ribc1	0	0	0
Ric1	10.46150162	1.142079822	0
Ric3	3.138450487	5.710399108	60.84409583
Ric8a	0	69.66686912	2.897337897
Ric8b	59.63055925	62.81439019	48.28896494
Rictor	66.95361038	95.93470502	59.87831653
Rida	62.76900973	30.83615518	33.80227546
Rif1	16.7384026	82.22974716	50.22052354
Rilp	11.50765178	0	0
Rilpl1	152.7379237	166.743654	100.4410471
Rilpl2	29.29220454	5.710399108	11.58935159
Rimbp2	0	0	0

For Review Only

Rimbp3	1.046150162	1.142079822	6.760455092
Rims3	0	0	0
Rin1	3.138450487	4.568319287	8.69201369
Rin2	575.3825892	1051.855516	1122.235545
Rin3	39.75370617	1.142079822	0
Ring1	38.707556	17.13119732	5.794675793
Rinl	0	0	0
Rint1	46.03060714	38.83071394	51.18630284
Riok1	56.49210876	85.65598662	72.43344741
Riok2	64.86131006	54.81983144	44.42584775
Riok3	575.3825892	2.284159643	0
Ripk1	27.19990422	49.10943233	59.87831653
Ripk2	53.35365827	37.68863411	40.56273055
Ripk3	23.01530357	15.9891175	26.07604107
Rit1	2.092300324	0	189.2927426
Rita1	24.06145373	14.84703768	27.04182037
Rlf	3.138450487	1.142079822	0
Rlim	595.2594423	468.2527269	403.6957469
Rmdn1	69.04591071	67.38270948	57.94675793
Rmdn2	8.369201298	21.69951661	21.24714457
Rmdn3	42.89215665	30.83615518	79.19390251
Rmi1	63.8151599	71.95102876	56.98097863
Rmnd1	10.46150162	7.994558752	0
Rmnd5a	37.66140584	87.94014627	72.43344741
Rmnd5b	91.01506412	66.24062966	34.76805476
Rn7s1	9.41535146	15.9891175	10.62357229
Rn7s2	11.50765178	17.13119732	10.62357229
Rn7sk	16.7384026	25.12575608	22.21292387
Rnase10	10.46150162	3.426239465	10.62357229
Rnase12	1.046150162	17.13119732	0.965779299
Rnase4	2181.223088	2294.438362	3113.672459
Rnase6	24.06145373	55.96191126	64.70721302
Rnaseh1	0	0	27.04182037
Rnaseh2a	14.64610227	0	0
Rnaseh2b	0	15.9891175	20.28136528
Rnaseh2c	0	0	18.34980668
Rnasek	198.7685308	1.142079822	414.3193192
Rnasel	65.90746022	63.95647001	60.84409583
Rnaset2a	0	0	92.71481269
Rnaset2b	46.03060714	43.39903322	29.93915826
Rnd1	0	0	0
Rnf10	61.72285957	81.08766734	24.14448247
Rnf103	70.09206087	90.22430591	42.49428915
Rnf11	32.43065503	25.12575608	19.31558598
Rnf111	88.92276379	141.6178979	107.2015022
Rnf113a1	17.78455276	13.70495786	5.794675793
Rnf113a2	48.12290746	36.54655429	61.80987513
Rnf114	372.4294578	264.9625186	582.3649172
Rnf115	39.75370617	41.11487358	47.32318564
Rnf121	152.7379237	13.70495786	76.29656461

For Review Only

Rnf122	52.30750811	43.39903322	58.91253723
Rnf123	208.1838823	94.7926252	92.71481269
Rnf125	1.046150162	0	0
Rnf126	3.138450487	26.2678359	17.38402738
Rnf128	44.98445698	33.12031483	55.04942003
Rnf13	1022.088709	1154.6427	1256.478868
Rnf130	140.1841217	227.2738845	177.703391
Rnf135	250.0298888	133.6233391	0
Rnf138	61.72285957	66.24062966	50.22052354
Rnf139	92.06121428	154.1807759	111.0646194
Rnf14	135.9995211	122.2025409	73.39922671
Rnf141	117.1688182	97.07678484	103.338385
Rnf144a	0	0	0
Rnf144b	27.19990422	95.93470502	39.59695125
Rnf145	216.5530836	232.9842836	120.7224124
Rnf146	156.9225243	142.7599777	182.5322875
Rnf149	49.16905763	69.66686912	46.35740634
Rnf150	18.83070292	14.84703768	32.83649616
Rnf152	4.184600649	0	0
Rnf157	5.230750811	6.85247893	24.14448247
Rnf166	69.04591071	9.136638573	196.0531977
Rnf167	0	315.2140308	109.1330608
Rnf168	20.92300324	35.40447447	23.17870317
Rnf169	209.2300324	137.0495786	232.752811
Rnf17	0	0	28.00759967
Rnf170	53.35365827	21.69951661	39.59695125
Rnf180	279.3220933	283.2357958	252.068397
Rnf181	0	0	112.996178
Rnf185	84.73816314	65.09854983	101.4068264
Rnf187	141.2302719	3.426239465	52.15208214
Rnf19a	101.4765657	21.69951661	40.56273055
Rnf19b	50.21520779	71.95102876	74.36500601
Rnf2	125.5380195	137.0495786	137.1406604
Rnf20	93.10736444	105.0713436	117.8250745
Rnf213	259.4452402	239.8367625	229.8554731
Rnf214	280.3682435	206.7164477	265.5893072
Rnf215	36.61525568	34.26239465	84.9885783
Rnf216	2.092300324	119.9183813	218.2661215
Rnf219	46.03060714	13.70495786	40.56273055
Rnf220	51.26135795	103.9292638	94.64637129
Rnf222	0	0	0
Rnf223	4.184600649	1.142079822	0
Rnf225	2.092300324	0	0
Rnf24	29.29220454	37.68863411	47.32318564
Rnf25	8.369201298	122.2025409	69.53610952
Rnf26	18.83070292	39.97279376	22.21292387
Rnf31	0	0	0
Rnf32	4.184600649	0	5.794675793
Rnf34	87.87661363	73.09310859	58.91253723
Rnf38	63.8151599	145.0441374	126.5170881

For Review Only

Rnf4	0	338.0556272	58.91253723
Rnf40	55.4459586	46.82527269	0
Rnf41	109.845767	174.7382127	136.1748811
Rnf44	117.1688182	124.4867006	117.8250745
Rnf5	0	0	0
Rnf6	178.8916777	77.66142787	141.0037776
Rnf7	139.1379716	147.328297	146.7984534
Rnf8	52.30750811	23.98367625	17.38402738
Rnf8-cmtr1	188.3070292	175.8802925	164.1824808
Rnft1	49.16905763	34.26239465	97.54370918
Rnft2	2.092300324	1.142079822	0
Rngtt	8.369201298	0	4.828896494
Rnh1	273.0451923	118.7763015	132.3117639
Rnls	43.93830681	38.83071394	38.63117195
Rnmt	71.13821103	84.5139068	73.39922671
Rnmtl1	18.83070292	46.82527269	38.63117195
Rnpc3	53.35365827	27.40991572	53.11786144
Rnpep	223.8761347	201.0060486	223.095018
Rnpepl1	77.41511201	142.7599777	68.57033022
Rnps1	78.46126217	76.51934805	90.78325409
Rnu12	7.323051136	1.142079822	5.794675793
Rnu1a1	1.046150162	1.142079822	0.965779299
Rnu2-10	3.138450487	2.284159643	3.863117195
Rnu3b1	4.184600649	0	3.863117195
Rnu3b2	3.138450487	2.284159643	2.897337897
Rnu3b3	3.138450487	1.142079822	1.931558598
Rnu3b4	5.230750811	3.426239465	4.828896494
Rnu5g	1.046150162	1.142079822	0
Rock1	67.99976055	78.80350769	46.35740634
Rock2	58.58440909	122.2025409	123.6197503
Rogdi	179.9378279	672.685015	233.7185903
Rom1	12.55380195	0	0
Romo1	24.06145373	33.12031483	36.69961336
Ropn1l	0	0	0
Rora	33.47680519	26.2678359	14.48668948
Rp2	277.229793	181.5906916	197.9847563
Rp9	26.15375406	26.2678359	27.04182037
Rpa1	59.63055925	65.09854983	95.61215059
Rpa2	59.63055925	26.2678359	34.76805476
Rpa3	3.138450487	6.85247893	0
Rpain	1.046150162	0	0
Rpap1	27.19990422	92.50846555	55.04942003
Rpap2	104.6150162	31.97823501	60.84409583
Rpap3	21.96915341	25.12575608	18.34980668
Rpe	50.21520779	38.83071394	56.98097863
Rpf1	48.12290746	106.2134234	87.8859162
Rpf2	16.7384026	27.40991572	20.28136528
Rpgr	0	5.710399108	15.45246878
Rpgrip1	0	1.142079822	0
Rpgrip1l	25.10760389	7.994558752	14.48668948

For Review Only

Rpia	0	0	0.965779299
Rpl10a	0	0	0
Rpl11	88.92276379	0	56.01519933
Rpl13	83.69201298	107.3555032	118.7908538
Rpl13-ps6	133.9072208	117.6342216	150.6615706
Rpl13a	35.56910552	36.54655429	0
Rpl14	0	0	191.2243012
Rpl14-ps1	189.3531794	169.0278136	227.9239145
Rpl17	18.83070292	14.84703768	18.34980668
Rpl17-ps10	89.96891395	70.80894894	89.81747479
Rpl17-ps8	38.707556	33.12031483	42.49428915
Rpl18	0	0	0.965779299
Rpl21	9.41535146	0	10.62357229
Rpl22	135.9995211	99.36094448	157.4220257
Rpl23	0	0	0
Rpl23a	46.03060714	31.97823501	58.91253723
Rpl24	76.36896184	75.37726823	5.794675793
Rpl26	167.384026	116.4921418	243.3763833
Rpl27	0	0	0
Rpl27a	0	63.95647001	0
Rpl28	58.58440909	0	0
Rpl29	32.43065503	50.25151215	60.84409583
Rpl30	8.369201298	1.142079822	0
Rpl31	21.96915341	19.41535697	23.17870317
Rpl31-ps12	15.69225243	18.27327715	15.45246878
Rpl32	197.7223807	202.1481284	179.6349496
Rpl32l	31.38450487	25.12575608	29.93915826
Rpl34	82.64586282	0	0.965779299
Rpl34-ps1	59.63055925	62.81439019	104.3041643
Rpl35	0	0	0
Rpl35a	0	0	61.80987513
Rpl36a	39.75370617	27.40991572	40.56273055
Rpl36al	0	0	38.63117195
Rpl37a	0	0	53.11786144
Rpl37rt	0	0	241.4448247
Rpl38	0	0	21.24714457
Rpl39	105.6611664	129.0550198	159.3535843
Rpl39l	0	0	0
Rpl4	254.2144894	3.426239465	0
Rpl5	1.046150162	1.142079822	0
Rpl6	1.046150162	2.284159643	581.3991379
Rpl7	17.78455276	15.9891175	345.748989
Rpl7l1	1.046150162	1.142079822	992.8211192
Rpl8	570.1518384	98.21886466	443.2926982
Rplp1	672.6745543	790.3192366	709.8477847
Rplp2	0	0	82.0912404
Rplp2-ps1	154.830224	135.9074988	108.1672815
Rpn1	656.9823019	467.1106471	591.0569309
Rpn2	1030.45791	720.6523675	1075.878139
Rpp14	62.76900973	63.95647001	76.29656461

For Review Only

Rpp21	2.092300324	23.98367625	0
Rpp25l	35.56910552	58.2460709	80.1596818
Rpp30	6.276900973	11.42079822	62.77565443
Rpp38	126.5841696	121.0604611	134.2433225
Rpp40	12.55380195	10.27871839	6.760455092
Rpph1	7.323051136	10.27871839	6.760455092
Rprd1a	28.24605438	58.2460709	56.98097863
Rprd1b	141.2302719	63.95647001	114.9277366
Rprd2	135.9995211	111.9238225	141.9695569
Rps10	65.90746022	73.09310859	0
Rps11	471.8137232	427.1378533	558.2204347
Rps13	65.90746022	53.67775162	0
Rps13-ps4	64.86131006	67.38270948	94.64637129
Rps14	334.7680519	407.7224963	583.3306965
Rps15	0	73.09310859	0
Rps15a	144.3687224	187.3010908	168.045598
Rps15a-ps4	28.24605438	23.98367625	38.63117195
Rps15a-ps5	46.03060714	49.10943233	60.84409583
Rps15a-ps6	39.75370617	37.68863411	46.35740634
Rps16	138.0918214	123.3446207	218.2661215
Rps17	93.10736444	100.5030243	0
Rps19	0	0	0
Rps19-ps3	7.323051136	7.994558752	14.48668948
Rps19bp1	29.29220454	39.97279376	47.32318564
Rps2	1.046150162	3.426239465	0
Rps20	0	0	4.828896494
Rps21	0	0	88.85169549
Rps24	345.2295535	28.55199554	95.61215059
Rps26	182.0301282	0	99.47526778
Rps27l	48.12290746	44.54111304	45.39162705
Rps27rt	225.968435	173.5961329	284.9048932
Rps28	51.26135795	66.24062966	126.5170881
Rps3	957.2273985	694.3845316	901.0720858
Rps3a1	19.87685308	23.98367625	0
Rps4l	43.93830681	55.96191126	56.98097863
Rps4x	421.5985154	599.5919064	386.3117195
Rps5	0	123.3446207	469.3687392
Rps6ka1	119.2611185	44.54111304	62.77565443
Rps6ka2	13.59995211	3.426239465	5.794675793
Rps6ka3	38.707556	54.81983144	38.63117195
Rps6ka4	217.5992337	123.3446207	144.8668948
Rps6ka5	11.50765178	31.97823501	12.55513089
Rps6kb1	86.83046347	106.2134234	49.25474424
Rps6kb2	7.323051136	0	0
Rps6kc1	196.6762305	115.350062	132.3117639
Rps6kl1	1.046150162	6.85247893	0.965779299
Rps7	0	62.81439019	5.794675793
Rps8	9.41535146	14.84703768	0
Rps9	416.3677646	114.2079822	430.7375673
Rpsa	154.830224	0	104.3041643

For Review Only

Rptor	581.6594902	448.8373699	467.4371806
Rptoros	13.59995211	21.69951661	17.38402738
Rpusd1	1.046150162	1.142079822	0
Rpusd2	13.59995211	18.27327715	14.48668948
Rpusd3	8.369201298	14.84703768	19.31558598
Rpusd4	61.72285957	62.81439019	60.84409583
Rrad	10.46150162	2.284159643	23.17870317
Rraga	86.83046347	110.7817427	65.67299232
Rragc	87.87661363	118.7763015	125.5513089
Rragd	4.184600649	0	9.657792988
Rras	2.092300324	0	0
Rras2	11.50765178	23.98367625	22.21292387
Rrbp1	245.8452881	177.0223724	119.7566331
Rreb1	321.1680998	240.9788424	231.7870317
Rrm1	58.58440909	66.24062966	63.74143372
Rrm2	0	2.284159643	0
Rrm2b	59.63055925	60.53023055	37.66539266
Rrn3	91.01506412	66.24062966	85.9543576
Rrnad1	24.06145373	0	6.760455092
Rrp1	243.7529878	175.8802925	283.9391139
Rrp12	29.29220454	14.84703768	20.28136528
Rrp15	15.69225243	25.12575608	20.28136528
Rrp1b	83.69201298	89.08222609	94.64637129
Rrp36	9.41535146	0	0
Rrp7a	205.0454318	145.0441374	216.3345629
Rrp8	52.30750811	44.54111304	52.15208214
Rrp9	31.38450487	0	10.62357229
Rrs1	47.0767573	20.55743679	37.66539266
Rsad1	36.61525568	23.98367625	28.00759967
Rsad2	0	6.85247893	0
Rsbn1	67.99976055	67.38270948	58.91253723
Rsbn1l	64.86131006	43.39903322	80.1596818
Rsc1a1	3.138450487	27.40991572	36.69961336
Rsf1	67.99976055	77.66142787	40.56273055
Rsl1	4.184600649	22.84159643	13.52091018
Rsl1d1	48.12290746	364.3234631	404.6615262
Rsl24d1	76.36896184	36.54655429	61.80987513
Rslcan18	0	5.710399108	0
Rsph3a	41.84600649	35.40447447	51.18630284
Rsph3b	37.66140584	34.26239465	48.28896494
Rsph4a	0	2.284159643	0
Rsph9	8.369201298	7.994558752	0
Rspo1	10.46150162	0	2.897337897
Rspry1	74.27666152	78.80350769	92.71481269
Rsrc1	40.79985633	70.80894894	50.22052354
Rsrc2	103.5688661	124.4867006	100.4410471
Rsrp1	0	0	0
Rsu1	140.1841217	106.2134234	142.9353362
Rtca	39.75370617	42.2569534	57.94675793
Rtcb	287.6912946	234.1263634	280.0759967

For Review Only

Rtel1	0	0	7.726234391
Rtf1	54.39980844	83.37182698	20.28136528
Rtfdc1	282.4605438	279.8095563	382.4486023
Rtkn	0	0	0
Rtkn2	0	0	0
Rtn1	150.6456234	163.3174145	96.57792988
Rtn3	865.1661842	845.139068	771.6576598
Rtn4	94.1535146	271.8149976	119.7566331
Rtn4ip1	38.707556	42.2569534	32.83649616
Rtn4rl1	379.7525089	470.5368865	258.8288521
Rtp4	23.01530357	38.83071394	54.08364074
Rttn	6.276900973	15.9891175	16.41824808
Rubcn	148.553323	87.94014627	165.1482601
Rufy1	65.90746022	57.10399108	48.28896494
Rufy2	17.78455276	59.38815073	33.80227546
Rufy3	35.56910552	46.82527269	35.73383406
Rundc1	104.6150162	60.53023055	68.57033022
Rundc3a	0	0	1.931558598
Runx1	192.4916299	223.847645	134.2433225
Runx1t1	1.046150162	9.136638573	0
Runx2	3.138450487	1.142079822	0.965779299
Runx3	0	2.284159643	0
Rusc1	28.24605438	41.11487358	41.52850985
Rusc2	34.52295535	37.68863411	32.83649616
Ruvbl1	58.58440909	58.2460709	91.74903339
Ruvbl2	83.69201298	71.95102876	73.39922671
Rwdd1	23.01530357	0	47.32318564
Rwdd2a	21.96915341	18.27327715	14.48668948
Rwdd2b	4.184600649	9.136638573	17.38402738
Rwdd3	6.276900973	4.568319287	0
Rwdd4a	28.24605438	54.81983144	30.90493756
Rxfp1	11.50765178	7.994558752	9.657792988
Rxfp4	0	0	0
Rxra	110.8919172	66.24062966	142.9353362
Rxrb	32.43065503	37.68863411	85.9543576
Rxrg	0	0	1.931558598
Rybp	5.230750811	25.12575608	13.52091018
Rybp-ps	0	4.568319287	0.965779299
Ryk	14.64610227	26.2678359	24.14448247
Ryr1	0	0	0
Ryr3	3.138450487	3.426239465	7.726234391
S100a1	1.046150162	1.142079822	0
S100a10	13.59995211	21.69951661	5.794675793
S100a11	5.230750811	4.568319287	0
S100a13	18.83070292	0	26.07604107
S100a16	0	0	0
S100a6	0	0	0
S100a8	0	0	5.794675793
S100a9	642.3361996	263.8204388	91.74903339
S100b	1.046150162	0	0

For Review Only

S100pbp	25.10760389	62.81439019	27.04182037
S1pr1	1757.532273	1.142079822	0
S1pr2	32.43065503	35.40447447	43.46006845
S1pr4	41.84600649	25.12575608	21.24714457
Saal1	37.66140584	61.67231037	56.01519933
Sac3d1	33.47680519	21.69951661	24.14448247
Sacm1l	95.19966476	77.66142787	106.2357229
Sacs	4.184600649	7.994558752	5.794675793
Sae1	104.6150162	67.38270948	102.3726057
Safb	143.3225722	130.1970997	173.8402738
Safb2	283.506694	0	13.52091018
Sag	1.046150162	4.568319287	5.794675793
Sall1	1154.949779	1775.934123	1483.437003
Sall2	47.0767573	53.67775162	73.39922671
Sall3	282.4605438	322.0665097	321.6045065
Sall4	0	0	0
Samd1	11.50765178	10.27871839	3.863117195
Samd10	18.83070292	14.84703768	19.31558598
Samd14	0	0	0
Samd15	1.046150162	0	3.863117195
Samd4	0	0	5.794675793
Samd4b	38.707556	37.68863411	33.80227546
Samd8	190.3993295	159.891175	158.387805
Samd9l	2.092300324	5.710399108	0
Samhd1	106.7073165	116.4921418	106.2357229
Samm50	145.4148726	105.0713436	144.8668948
Samsn1	210.2761826	339.197707	375.6881473
Sap130	164.2455755	145.0441374	107.2015022
Sap18	1.046150162	1.142079822	0
Sap18b	78.46126217	46.82527269	83.0570197
Sap30	0	12.56287804	0.965779299
Sap30bp	63.8151599	108.4975831	84.9885783
Sap30l	0	1.142079822	0
Sar1a	263.6298409	349.4764254	327.3991823
Sar1b	134.9533709	189.5852504	166.1140394
Saraf	1276.303198	1001.604004	1280.62335
Sardh	77.41511201	4.568319287	39.59695125
Sarm1	9.41535146	23.98367625	11.58935159
Sarnp	103.5688661	140.4758181	130.3802053
Sars	138.0918214	2.284159643	0
Sars2	19.87685308	15.9891175	12.55513089
Sart1	64.86131006	74.23518841	69.53610952
Sart3	35.56910552	30.83615518	28.00759967
Sash1	3.138450487	0	0
Sash3	551.3211355	415.7170551	578.5018
Sass6	25.10760389	26.2678359	22.21292387
Sat1	154.830224	165.6015741	0
Sat2	26.15375406	6.85247893	0
Satb1	4.184600649	34.26239465	32.83649616
Satb2	1.046150162	1.142079822	1.931558598

For Review Only

Sav1	0	9.136638573	0.965779299
Saysd1	70.09206087	71.95102876	96.57792988
Sbds	133.9072208	156.4649356	166.1140394
Sbf1	157.9686745	101.6451041	20.28136528
Sbf2	262.5836907	197.5798091	220.1976801
Sbk1	9.41535146	1.142079822	26.07604107
Sbno1	213.4146331	266.1045984	244.3421626
Sbno2	275.1374927	332.3452281	129.414426
Sbsn	2.092300324	4.568319287	7.726234391
Sbspon	4.184600649	0	0.965779299
Sc5d	75.32281168	41.11487358	49.25474424
Scaf1	50.21520779	52.5356718	41.52850985
Scaf11	348.368004	277.5253967	201.8478735
Scaf4	50.21520779	52.5356718	38.63117195
Scaf8	3.138450487	2.284159643	0
Scai	53.35365827	66.24062966	27.04182037
Scamp1	248.9837386	239.8367625	207.6425493
Scamp2	2063.00812	1892.426264	2190.38745
Scamp3	0	37.68863411	0
Scamp4	144.3687224	146.1862172	170.9429359
Scamp5	595.2594423	222.7055652	333.1938581
Scand1	1.046150162	7.994558752	0
Scap	150.6456234	162.1753347	109.1330608
Scaper	52.30750811	27.40991572	27.04182037
Scara5	0	5.710399108	0
Scarb1	92.06121428	70.80894894	86.9201369
Scarb2	512.6135795	540.2037556	537.9390695
Scarf1	11.50765178	21.69951661	11.58935159
Scarf2	5.230750811	5.710399108	0
Scarna3b	0	0	0
Sccpdh	67.99976055	83.37182698	107.2015022
Scd2	55.4459586	22.84159643	3.863117195
Scd3	1.046150162	0	0
Scfd1	0	31.97823501	94.64637129
Scfd2	21.96915341	25.12575608	40.56273055
Scg3	0	0	0
Scimp	4.184600649	17.13119732	5.794675793
Sclt1	6.276900973	17.13119732	7.726234391
Scly	19.87685308	7.994558752	0
Scmh1	80.55356249	0	28.00759967
Scml4	0	0	0
Scn11a	1.046150162	3.426239465	0
Scn1b	9.41535146	0	0.965779299
Scn2b	0	0	0
Scn3b	4.184600649	0	0
Scn4a	0	0	0
Scn8a	2.092300324	1.142079822	0
Scnm1	0	22.84159643	31.87071686
Scnn1a	1.046150162	0	0
Scnn1b	0	3.426239465	0

For Review Only

Sco1	14.64610227	10.27871839	43.46006845
Sco2	1.046150162	1.142079822	61.80987513
Scoc	203.9992816	465.9685672	0
Scp2	144.3687224	89.08222609	110.0988401
Scpep1	390.2140105	260.3941993	342.8516511
Scrib	16.7384026	3.426239465	64.70721302
Scrn2	13.59995211	0	0
Scrn3	41.84600649	30.83615518	20.28136528
Scube1	0	4.568319287	28.00759967
Scube3	2.092300324	0	4.828896494
Scyl1	13.59995211	1.142079822	0
Scyl2	34.52295535	66.24062966	22.21292387
Scyl3	40.79985633	69.66686912	42.49428915
Sdad1	72.18436119	5.710399108	28.00759967
Sdc2	7.323051136	0	0
Sdc3	70.09206087	126.7708602	152.5931292
Sdc4	129.7226201	62.81439019	72.43344741
Sdcbp	999.0734049	1108.959507	1197.566331
Sdccag3	0	0	0
Sdccag8	67.99976055	52.5356718	88.85169549
Sde2	52.30750811	60.53023055	53.11786144
Sdf2	63.8151599	86.79806645	174.8060531
Sdf2l1	192.4916299	169.0278136	192.1900805
Sdf4	271.9990422	246.6892415	469.3687392
Sdha	487.5059756	527.6408776	579.4675793
Sdhaf1	37.66140584	30.83615518	35.73383406
Sdhaf2	0	0	0
Sdhaf3	1.046150162	19.41535697	12.55513089
Sdhaf4	32.43065503	30.83615518	35.73383406
Sdhb	0	229.5580442	228.8896938
Sdhc	67.99976055	59.38815073	77.26234391
Sdhd	101.4765657	73.09310859	146.7984534
Sdk1	27.19990422	36.54655429	21.24714457
Sdpr	3.138450487	0	0
Sdr42e1	15.69225243	7.994558752	24.14448247
Sebox	5.230750811	0	0
Sec11a	145.4148726	188.4431706	219.2319008
Sec11c	129.7226201	398.5858578	397.9010711
Sec13	322.21425	223.847645	357.3383406
Sec14l1	912.2429415	464.8264874	533.110173
Sec14l2	3.138450487	4.568319287	0
Sec16a	271.9990422	338.0556272	293.5969068
Sec16b	38.707556	44.54111304	31.87071686
Sec22a	31.38450487	74.23518841	34.76805476
Sec22b	146.4610227	164.4594943	171.9087152
Sec22c	2.092300324	11.42079822	4.828896494
Sec23a	70.09206087	94.7926252	45.39162705
Sec23b	154.830224	116.4921418	102.3726057
Sec23ip	57.53825892	54.81983144	67.60455092
Sec24a	96.24581493	127.91294	114.9277366

For Review Only

Sec24b	173.6609269	177.0223724	186.3954047
Sec24c	299.1989464	310.6457115	172.8744945
Sec24d	51.26135795	37.68863411	34.76805476
Sec31a	278.2759432	220.4214056	111.0646194
Sec31b	0	7.994558752	2.897337897
Sec61a1	588.9825413	387.1650595	423.0113329
Sec61a2	1.046150162	1.142079822	0
Sec61b	50.21520779	62.81439019	79.19390251
Sec61g	0	0	0
Sec62	167.384026	159.891175	127.4828674
Sec63	19.87685308	69.66686912	64.70721302
Secisbp2	75.32281168	94.7926252	101.4068264
Secisbp2l	80.55356249	174.7382127	65.67299232
Seh1l	88.92276379	89.08222609	80.1596818
Sel1l	263.6298409	172.4540531	275.2471002
Selenbp1	47.0767573	31.97823501	30.90493756
Selenbp2	16.7384026	0	7.726234391
Selk	159.0148247	173.5961329	253.9999556
Sell	0	0	4.828896494
Selm	0	0	0
Selo	9.41535146	75.37726823	13.52091018
Selplg	10385.13266	10936.55637	14077.19906
Selt	49.16905763	58.2460709	57.94675793
Sema3b	17.78455276	0	0
Sema3c	0	0	0
Sema4a	23.01530357	2.284159643	14.48668948
Sema4b	193.53778	95.93470502	130.3802053
Sema4c	89.96891395	62.81439019	23.17870317
Sema4d	69.04591071	500.2309619	411.4219813
Sema4g	44.98445698	66.24062966	46.35740634
Sema6a	0	0	0
Sema6b	0	0	0
Sema6c	0	1.142079822	0
Sema6d	10.46150162	21.69951661	37.66539266
Senp1	73.23051136	95.93470502	66.63877162
Senp2	159.0148247	214.7110065	188.3269633
Senp3	40.79985633	51.39359197	0
Senp5	69.04591071	62.81439019	86.9201369
Senp6	28.24605438	51.39359197	45.39162705
Senp7	57.53825892	71.95102876	77.26234391
Senp8	47.0767573	38.83071394	44.42584775
Sep15	211.3223328	334.6293877	304.2204791
Sephs1	77.41511201	60.53023055	48.28896494
Sephs2	84.73816314	99.36094448	88.85169549
Sepn1	26.15375406	22.84159643	56.01519933
Sepp1	9277.259639	10165.65249	11442.55313
Sepsecs	3.138450487	17.13119732	10.62357229
Sept10	57.53825892	55.96191126	60.84409583
Sept11	76.36896184	34.26239465	49.25474424
Sept2	603.6286436	732.0731657	650.9352474

For Review Only

Sept4	6.276900973	3.426239465	0
Sept6	17.78455276	4.568319287	15.45246878
Sept7	225.968435	222.7055652	192.1900805
Sept8	46.03060714	54.81983144	31.87071686
Sept9	33.47680519	14.84703768	21.24714457
Sepw1	0	11.42079822	0
Serac1	6.276900973	53.67775162	32.83649616
Serbp1	203.9992816	204.4322881	221.1634594
Serf1	0	0	0
Serf2	0	0	0
Sergef	47.0767573	44.54111304	51.18630284
Serhl	0	3.426239465	22.21292387
Serinc1	679.9976055	519.6463189	608.4409583
Serinc3	1072.303916	1544.091919	1178.250745
Serinc4	7.323051136	0	0
Serinc5	29.29220454	10.27871839	7.726234391
Serp1	2.092300324	1.142079822	0
Serpinb1a	4.184600649	2.284159643	0
Serpinb1b	0	0	0
Serpinb6a	18.83070292	12.56287804	30.90493756
Serpinb6b	0	0	0
Serpinb8	3.138450487	1.142079822	0
Serpinb9	50.21520779	30.83615518	56.01519933
Serpind1	0	0	0.965779299
Serpine1	9.41535146	4.568319287	0
Serpine2	1650.824956	1386.484903	1782.828586
Serpinf1	36.61525568	42.2569534	61.80987513
Serpinf2	0	1.142079822	7.726234391
Serping1	3.138450487	0	0
Serpinh1	0	0	0
Serpini1	0	1.142079822	0
Sertad1	12.55380195	19.41535697	36.69961336
Sertad2	103.5688661	118.7763015	91.74903339
Sertad3	0	0	6.760455092
Sesn1	510.5212792	544.7720749	518.6234835
Sesn2	9.41535146	2.284159643	5.794675793
Sesn3	0	0	0
Sestd1	0	0	0
Set	23.01530357	25.12575608	0
Setbp1	5.230750811	26.2678359	13.52091018
Setd1a	0	0	0.965779299
Setd1b	67.99976055	106.2134234	43.46006845
Setd2	146.4610227	199.8639688	166.1140394
Setd3	174.7070771	125.6287804	134.2433225
Setd4	17.78455276	19.41535697	21.24714457
Setd5	206.091582	255.8258801	156.4562464
Setd6	0	0	1.931558598
Setd7	18.83070292	22.84159643	30.90493756
Setdb1	160.0609748	198.721889	205.7109907
Setdb2	16.7384026	0	0

For Review Only

Setdb2-phf11c	5.230750811	11.42079822	37.66539266
Setmar	7.323051136	0	0
Setx	77.41511201	84.5139068	47.32318564
Sez6l	1.046150162	0	0
Sez6l2	0	0	0
Sf1	252.1221891	131.3391795	211.5056664
Sf3a1	163.1994253	118.7763015	146.7984534
Sf3a2	16.7384026	0	3.863117195
Sf3a3	118.2149683	109.6396629	112.996178
Sf3b1	1094.27307	840.5707487	903.0036444
Sf3b2	405.906263	287.8041151	367.9619129
Sf3b3	0	0	0
Sf3b4	95.19966476	33.12031483	7.726234391
Sf3b5	63.8151599	68.5247893	72.43344741
Sf3b6	42.89215665	55.96191126	63.74143372
Sfi1	26.15375406	25.12575608	25.11026177
Sfmbt1	127.6303198	139.3337382	113.9619573
Sfmbt2	4.184600649	20.55743679	7.726234391
Sfn	0	1.142079822	0
Sfpq	0	317.4981904	25.11026177
Sfr1	115.0765178	143.9020575	124.5855296
Sfswap	117.1688182	114.2079822	84.9885783
Sft2d1	28.24605438	796.0296357	0
Sft2d2	731.2589634	0	2.897337897
Sft2d3	6.276900973	7.994558752	16.41824808
Sfxn1	46.03060714	49.10943233	52.15208214
Sfxn2	39.75370617	19.41535697	26.07604107
Sfxn3	85.7843133	87.94014627	96.57792988
Sfxn4	16.7384026	26.2678359	26.07604107
Sfxn5	32.43065503	34.26239465	20.28136528
Sgcb	71.13821103	63.95647001	52.15208214
Sgcd	0	0	0
Sgce	192.4916299	197.5798091	253.9999556
Sgf29	47.0767573	51.39359197	47.32318564
Sgip1	4.184600649	2.284159643	0
Sgk1	0	0	0.965779299
Sgk2	0	0	3.863117195
Sgk3	92.06121428	145.0441374	90.78325409
Sgms1	92.06121428	98.21886466	116.8592952
Sgms2	7.323051136	0	0
Sgol1	0	3.426239465	0
Sgol2a	0	0	0
Sgpl1	1322.333805	696.6686912	869.201369
Sgpp1	50.21520779	44.54111304	32.83649616
Sgpp2	4.184600649	2.284159643	8.69201369
Sgsh	155.8763742	55.96191126	86.9201369
Sgsm2	24.06145373	14.84703768	26.07604107
Sgsm3	0	0	78.22812321
Sgta	219.6915341	222.7055652	269.4524244
Sgtb	1.046150162	5.710399108	4.828896494

For Review Only

Sh2b1	83.69201298	97.07678484	96.57792988
Sh2b2	121.3534188	137.0495786	70.50188882
Sh2b3	29.29220454	18.27327715	7.726234391
Sh2d1b1	2.092300324	5.710399108	0
Sh2d3c	177.8455276	132.4812593	216.3345629
Sh2d4b	107.7534667	30.83615518	78.22812321
Sh2d5	1.046150162	2.284159643	3.863117195
Sh2d6	8.369201298	7.994558752	15.45246878
Sh2d7	0	3.426239465	0
Sh3bgrl	31.38450487	10.27871839	28.00759967
Sh3bgrl2	9.41535146	20.55743679	8.69201369
Sh3bgrl3	428.9215665	404.2962569	464.5398427
Sh3bp1	233.2914862	197.5798091	84.022799
Sh3bp2	92.06121428	60.53023055	15.45246878
Sh3bp4	31.38450487	1.142079822	0
Sh3bp5	82.64586282	27.40991572	48.28896494
Sh3bp5l	48.12290746	54.81983144	43.46006845
Sh3d19	6.276900973	0	0
Sh3d21	0	1.142079822	8.69201369
Sh3gl1	99.38426541	83.37182698	14.48668948
Sh3glb1	237.4760868	452.2636094	75.33078531
Sh3glb2	1.046150162	1.142079822	0
Sh3kbp1	306.5219975	276.3833168	213.437225
Sh3pxd2a	57.53825892	81.08766734	39.59695125
Sh3pxd2b	0	2.284159643	0
Sh3rf1	9.41535146	1.142079822	0
Sh3rf3	0	0	0
Sh3tc1	109.845767	23.98367625	103.338385
Sh3yl1	0	0	2.897337897
Shank1	9.41535146	1.142079822	0
Shank2	3.138450487	0	0
Sharpin	0	0	0
Shb	15.69225243	26.2678359	10.62357229
Shc1	155.8763742	3.426239465	216.3345629
Shcbp1l	0	0	0
She	6.276900973	3.426239465	0.965779299
Shf	7.323051136	7.994558752	4.828896494
Shfm1	0	108.4975831	164.1824808
Shisa2	1.046150162	0	0
Shisa5	465.5368222	465.9685672	77.26234391
Shisa6	0	0	0
Shisa7	11.50765178	14.84703768	22.21292387
Shkbp1	4.184600649	5.710399108	129.414426
Shmt1	10.46150162	9.136638573	15.45246878
Shmt2	46.03060714	29.69407536	35.73383406
Shoc2	43.93830681	33.12031483	27.04182037
Shpk	17.78455276	21.69951661	28.00759967
Shprh	76.36896184	100.5030243	61.80987513
Shq1	26.15375406	10.27871839	20.28136528
Shroom1	3.138450487	20.55743679	13.52091018

For Review Only

Shroom4	3.138450487	4.568319287	0
Shtn1	0	1.142079822	3.863117195
Siae	123.4457191	118.7763015	117.8250745
Siah1a	19.87685308	27.40991572	36.69961336
Siah1b	5.230750811	6.85247893	5.794675793
Siah2	10.46150162	11.42079822	9.657792988
Sidt2	1.046150162	2.284159643	0
Sigirr	0	0	0
Siglec1	0	10.27871839	10.62357229
Siglece	8.369201298	78.80350769	101.4068264
Siglecf	96.24581493	43.39903322	138.1064397
Siglecg	14.64610227	42.2569534	23.17870317
Siglech	2676.052115	2152.820464	3209.28461
Sigmar1	80.55356249	78.80350769	72.43344741
Sik1	5.230750811	0	0
Sik2	121.3534188	130.1970997	111.0646194
Sik3	31.38450487	22.84159643	30.90493756
Sike1	69.04591071	78.80350769	0
Sil1	118.2149683	90.22430591	124.5855296
Simc1	53.35365827	46.82527269	38.63117195
Sin3a	77.41511201	111.9238225	84.022799
Sin3b	98.33811525	84.5139068	93.68059199
Sipa1	376.6140584	126.7708602	196.0531977
Sipa1l1	109.845767	121.0604611	130.3802053
Sipa1l2	312.7988985	65.09854983	350.5778855
Sipa1l3	61.72285957	47.96735251	35.73383406
Sirpa	1681.163311	143.9020575	3.863117195
Sirt1	14.64610227	5.710399108	11.58935159
Sirt2	0	41.11487358	17.38402738
Sirt3	24.06145373	28.55199554	22.21292387
Sirt4	20.92300324	6.85247893	25.11026177
Sirt5	5.230750811	27.40991572	20.28136528
Sirt6	46.03060714	13.70495786	0
Sirt7	17.78455276	0	0
Siva1	28.24605438	34.26239465	48.28896494
Six3os1	1.046150162	0	0
Six5	7.323051136	3.426239465	9.657792988
Ska2	10.46150162	7.994558752	0.965779299
Ska3	0	0	0
Skap1	0	0	0
Skap2	243.7529878	230.700124	242.410604
Ski	341.0449529	18.27327715	93.68059199
Skida1	3.138450487	1.142079822	6.760455092
Skil	672.6745543	926.2267354	505.1025733
Skint3	0	2.284159643	0
Skiv2l	2.092300324	1.142079822	0
Skiv2l2	79.50741233	109.6396629	69.53610952
Skp1a	162.1532751	154.1807759	167.0798187
Skp2	11.50765178	4.568319287	29.93915826
Sla	270.952892	1179.768456	929.0796855

For Review Only

Sla2	1.046150162	0	0
Slain2	91.01506412	46.82527269	37.66539266
Slamf1	23.01530357	18.27327715	28.00759967
Slamf6	25.10760389	164.4594943	72.43344741
Slamf7	1.046150162	1.142079822	0
Slamf8	52.30750811	118.7763015	83.0570197
Slamf9	222.8299846	271.8149976	216.3345629
Slbp	1.046150162	9.136638573	88.85169549
Slc10a1	10.46150162	4.568319287	3.863117195
Slc10a3	32.43065503	0	0
Slc10a6	4.184600649	0	0
Slc10a7	64.86131006	52.5356718	55.04942003
Slc11a1	643.3823498	544.7720749	299.3915826
Slc11a2	77.41511201	65.09854983	35.73383406
Slc12a2	97.29196509	163.3174145	122.653971
Slc12a4	92.06121428	37.68863411	43.46006845
Slc12a5	0	1.142079822	0
Slc12a6	381.8448092	480.8156049	377.6197058
Slc12a7	73.23051136	81.08766734	82.0912404
Slc12a9	4.184600649	0	0
Slc13a2	0	0	0
Slc13a3	23.01530357	35.40447447	34.76805476
Slc13a4	4.184600649	0	0
Slc14a1	7.323051136	7.994558752	0
Slc15a2	1.046150162	34.26239465	52.15208214
Slc15a3	155.8763742	133.6233391	158.387805
Slc15a4	391.2601607	398.5858578	387.2774988
Slc16a1	19.87685308	7.994558752	18.34980668
Slc16a10	19.87685308	57.10399108	18.34980668
Slc16a11	2.092300324	0	0
Slc16a13	49.16905763	65.09854983	70.50188882
Slc16a2	24.06145373	3.426239465	0
Slc16a3	92.06121428	51.39359197	84.022799
Slc16a4	2.092300324	0	0
Slc16a6	496.9213271	760.6251612	771.6576598
Slc16a7	47.0767573	68.5247893	47.32318564
Slc16a8	7.323051136	1.142079822	0
Slc16a9	0	1.142079822	0
Slc17a5	88.92276379	69.66686912	55.04942003
Slc17a9	36.61525568	37.68863411	21.24714457
Slc18a1	15.69225243	11.42079822	0
Slc18a2	32.43065503	58.2460709	66.63877162
Slc18b1	23.01530357	59.38815073	28.97337897
Slc19a1	73.23051136	0	86.9201369
Slc19a2	2.092300324	7.994558752	0
Slc1a1	0	0	0.965779299
Slc1a2	14.64610227	3.426239465	5.794675793
Slc1a3	594.2132922	291.2303545	405.6273055
Slc1a4	26.15375406	62.81439019	14.48668948
Slc1a5	7.323051136	17.13119732	5.794675793

For Review Only

Slc20a1	100.4304156	9.136638573	38.63117195
Slc20a2	41.84600649	22.84159643	9.657792988
Slc22a17	1.046150162	0	0
Slc22a21	13.59995211	36.54655429	11.58935159
Slc22a23	0	0	0
Slc22a4	6.276900973	11.42079822	10.62357229
Slc22a5	60.67670941	41.11487358	43.46006845
Slc22a8	1.046150162	0	0
Slc23a1	6.276900973	2.284159643	0
Slc23a2	443.5676688	368.8917824	329.3307409
Slc23a3	0	1.142079822	0
Slc24a1	25.10760389	18.27327715	6.760455092
Slc24a3	21.96915341	34.26239465	53.11786144
Slc25a1	100.4304156	102.7871839	0
Slc25a10	30.33835471	28.55199554	50.22052354
Slc25a11	0	0	0
Slc25a12	89.96891395	78.80350769	72.43344741
Slc25a13	3.138450487	3.426239465	10.62357229
Slc25a14	20.92300324	5.710399108	22.21292387
Slc25a15	5.230750811	14.84703768	20.28136528
Slc25a16	14.64610227	11.42079822	9.657792988
Slc25a17	94.1535146	141.6178979	156.4562464
Slc25a18	0	0	0
Slc25a19	0	43.39903322	51.18630284
Slc25a2	1.046150162	1.142079822	0
Slc25a20	20.92300324	15.9891175	37.66539266
Slc25a22	0	0	0
Slc25a23	24.06145373	23.98367625	27.04182037
Slc25a25	12.55380195	19.41535697	133.2775432
Slc25a26	9.41535146	13.70495786	18.34980668
Slc25a27	38.707556	29.69407536	19.31558598
Slc25a28	1.046150162	22.84159643	0
Slc25a29	10.46150162	22.84159643	17.38402738
Slc25a3	0	1.142079822	0
Slc25a30	11.50765178	9.136638573	0
Slc25a32	25.10760389	41.11487358	41.52850985
Slc25a33	8.369201298	13.70495786	7.726234391
Slc25a35	15.69225243	18.27327715	17.38402738
Slc25a36	215.5069334	256.9679599	135.2091018
Slc25a37	268.8605917	491.0943233	366.9961336
Slc25a38	56.49210876	54.81983144	59.87831653
Slc25a39	1.046150162	66.24062966	3.863117195
Slc25a4	298.1527962	259.2521195	259.7946314
Slc25a40	13.59995211	14.84703768	8.69201369
Slc25a42	9.41535146	6.85247893	6.760455092
Slc25a43	10.46150162	7.994558752	20.28136528
Slc25a44	42.89215665	46.82527269	102.3726057
Slc25a45	0	394.0175385	15.45246878
Slc25a46	127.6303198	101.6451041	53.11786144
Slc25a47	1.046150162	0	0

For Review Only

Slc25a5	795.0741233	805.1662743	923.2850097
Slc25a51	99.38426541	68.5247893	67.60455092
Slc25a53	1.046150162	1.142079822	0
Slc26a1	3.138450487	26.2678359	2.897337897
Slc26a10	3.138450487	0	0
Slc26a11	31.38450487	29.69407536	38.63117195
Slc26a2	93.10736444	35.40447447	64.70721302
Slc26a6	25.10760389	0	16.41824808
Slc27a1	49.16905763	39.97279376	71.46766811
Slc27a3	5.230750811	0	0
Slc27a4	43.93830681	47.96735251	39.59695125
Slc29a1	298.1527962	139.3337382	277.1786588
Slc29a2	15.69225243	11.42079822	16.41824808
Slc29a3	1726.147768	1152.35854	1474.744989
Slc29a4	14.64610227	0	10.62357229
Slc2a1	82.64586282	67.38270948	80.1596818
Slc2a10	1.046150162	3.426239465	6.760455092
Slc2a12	21.96915341	0	0
Slc2a3	7.323051136	10.27871839	14.48668948
Slc2a4rg-ps	15.69225243	0	0
Slc2a5	0	992.467365	166.1140394
Slc2a6	5.230750811	9.136638573	0
Slc2a8	0	0	0
Slc2a9	47.0767573	1.142079822	0
Slc30a1	15.69225243	13.70495786	16.41824808
Slc30a10	0	0	0
Slc30a4	3.138450487	29.69407536	78.22812321
Slc30a5	61.72285957	97.07678484	57.94675793
Slc30a6	35.56910552	31.97823501	43.46006845
Slc30a7	198.7685308	194.1535697	171.9087152
Slc30a9	47.0767573	135.9074988	99.47526778
Slc31a1	212.3684829	97.07678484	169.0113773
Slc31a2	1.046150162	1.142079822	0
Slc33a1	105.6611664	86.79806645	81.1254611
Slc35a1	127.6303198	83.37182698	119.7566331
Slc35a2	50.21520779	59.38815073	34.76805476
Slc35a3	33.47680519	75.37726823	32.83649616
Slc35a5	167.384026	219.2793258	159.3535843
Slc35b1	1.046150162	1.142079822	0
Slc35b2	206.091582	180.4486118	244.3421626
Slc35b3	56.49210876	85.65598662	71.46766811
Slc35b4	142.2764221	116.4921418	180.6007289
Slc35c1	157.9686745	60.53023055	79.19390251
Slc35c2	388.1217102	111.9238225	212.4714457
Slc35d1	18.83070292	27.40991572	28.00759967
Slc35d2	39.75370617	19.41535697	46.35740634
Slc35e1	86.83046347	62.81439019	138.1064397
Slc35e2	102.5227159	109.6396629	0
Slc35e3	32.43065503	58.2460709	27.04182037
Slc35e4	13.59995211	7.994558752	18.34980668

For Review Only

Slc35f2	0	0	0
Slc35f3	4.184600649	4.568319287	3.863117195
Slc35f5	64.86131006	130.1970997	112.0303987
Slc35f6	0	0	0
Slc35g1	0	0	0
Slc35g2	3.138450487	10.27871839	6.760455092
Slc36a1	152.7379237	248.9734011	248.2052798
Slc36a2	23.01530357	28.55199554	21.24714457
Slc36a4	40.79985633	55.96191126	17.38402738
Slc37a1	11.50765178	12.56287804	0
Slc37a2	0	151.8966163	260.7604107
Slc37a3	43.93830681	25.12575608	56.98097863
Slc37a4	0	30.83615518	0
Slc38a1	162.1532751	191.86941	150.6615706
Slc38a10	490.6444261	350.6185053	448.1215947
Slc38a2	139.1379716	191.86941	180.6007289
Slc38a3	18.83070292	0	0
Slc38a5	0	0	0
Slc38a6	37.66140584	33.12031483	35.73383406
Slc38a7	256.3067897	103.9292638	117.8250745
Slc38a9	179.9378279	232.9842836	225.0265766
Slc39a1	343.1372532	407.7224963	350.5778855
Slc39a10	98.33811525	103.9292638	73.39922671
Slc39a11	100.4304156	65.09854983	92.71481269
Slc39a12	60.67670941	63.95647001	53.11786144
Slc39a13	70.09206087	46.82527269	0
Slc39a14	3.138450487	23.98367625	0
Slc39a2	4.184600649	3.426239465	0
Slc39a3	129.7226201	90.22430591	117.8250745
Slc39a4	38.707556	25.12575608	29.93915826
Slc39a6	153.7840738	66.24062966	115.8935159
Slc39a7	3.138450487	42.2569534	0
Slc39a8	56.49210876	43.39903322	54.08364074
Slc39a9	141.2302719	153.0386961	155.4904671
Slc3a2	604.6747938	182.7327715	1142.516911
Slc40a1	25.10760389	0	0
Slc41a1	63.8151599	59.38815073	52.15208214
Slc41a3	76.36896184	54.81983144	70.50188882
Slc43a1	0	6.85247893	0
Slc43a2	488.5521258	2.284159643	0
Slc43a3	0	0	0
Slc44a1	31.38450487	21.69951661	28.97337897
Slc44a2	465.5368222	0	283.9391139
Slc45a3	0	0	0
Slc45a4	80.55356249	133.6233391	104.3041643
Slc46a1	387.07556	360.8972236	430.7375673
Slc46a2	0	0	0
Slc46a3	42.89215665	396.3016981	223.095018
Slc48a1	196.6762305	17.13119732	68.57033022
Slc4a10	11.50765178	0	0

For Review Only

Slc4a1ap	54.39980844	49.10943233	35.73383406
Slc4a2	122.399569	59.38815073	53.11786144
Slc4a3	4.184600649	0	0
Slc4a4	0	0	0
Slc4a5	9.41535146	0	0
Slc4a7	83.69201298	191.86941	86.9201369
Slc50a1	0	0	78.22812321
Slc52a2	1.046150162	1.142079822	0
Slc52a3	0	0	0
Slc5a10	21.96915341	0	22.21292387
Slc5a11	0	2.284159643	0
Slc5a3	112.9842175	138.1916584	68.57033022
Slc5a5	7.323051136	0	0
Slc5a6	40.79985633	15.9891175	48.28896494
Slc6a13	0	0	0
Slc6a20a	10.46150162	0	0
Slc6a20b	3.138450487	0	0
Slc6a6	419.5062151	333.4873079	324.5018444
Slc6a8	7.323051136	1.142079822	12.55513089
Slc6a9	1.046150162	0	0
Slc7a1	20.92300324	15.9891175	24.14448247
Slc7a10	21.96915341	0	0
Slc7a11	5.230750811	4.568319287	17.38402738
Slc7a15	8.369201298	4.568319287	10.62357229
Slc7a4	23.01530357	11.42079822	8.69201369
Slc7a5	12.55380195	23.98367625	19.31558598
Slc7a6	41.84600649	70.80894894	52.15208214
Slc7a6os	23.01530357	26.2678359	14.48668948
Slc7a7	39.75370617	49.10943233	146.7984534
Slc7a8	1319.195355	877.117303	1039.178526
Slc8a1	304.4296972	689.8162123	376.6539266
Slc8a2	0	0	0.965779299
Slc8b1	118.2149683	87.94014627	83.0570197
Slc9a1	153.7840738	130.1970997	113.9619573
Slc9a3r1	44.98445698	61.67231037	53.11786144
Slc9a3r2	0	0	14.48668948
Slc9a5	3.138450487	0	5.794675793
Slc9a6	34.52295535	73.09310859	38.63117195
Slc9a7	52.30750811	100.5030243	64.70721302
Slc9a8	148.553323	107.3555032	159.3535843
Slc9a9	568.0595381	493.378483	452.9504912
Slco1a4	6.276900973	0	0
Slco1c1	6.276900973	0	0
Slco2b1	5.230750811	31.97823501	11.58935159
Slco4a1	227.0145852	150.7545365	273.3155416
Slf1	8.369201298	7.994558752	0
Slfn1	12.55380195	0	0
Slfn10-ps	12.55380195	19.41535697	18.34980668
Slfn2	41.84600649	45.68319287	52.15208214
Slfn3	2.092300324	3.426239465	0

For Review Only

Slfn4	24.06145373	0	1.931558598
Slfn5	2.092300324	6.85247893	4.828896494
Slfn8	147.5071729	148.4703768	142.9353362
Slfn9	17.78455276	21.69951661	19.31558598
Slirp	16.7384026	41.11487358	61.80987513
Slit1	5.230750811	0	0
Slk	19.87685308	37.68863411	32.83649616
Slmap	153.7840738	164.4594943	155.4904671
Slmo1	2.092300324	9.136638573	8.69201369
Slmo2	46.03060714	82.22974716	62.77565443
Sltm	96.24581493	85.65598662	117.8250745
Slu7	112.9842175	287.8041151	197.018977
Slx4	59.63055925	39.97279376	45.39162705
Slx4ip	38.707556	20.55743679	20.28136528
Smad1	54.39980844	54.81983144	67.60455092
Smad2	137.0456713	134.765419	113.9619573
Smad3	400.6755121	327.7769088	272.3497623
Smad4	47.0767573	91.36638573	55.04942003
Smad5	93.10736444	156.4649356	110.0988401
Smad6	3.138450487	0	0
Smad7	78.46126217	92.50846555	72.43344741
Smagp	21.96915341	10.27871839	34.76805476
Smap1	36.61525568	70.80894894	44.42584775
Smap2	0	13.70495786	23.17870317
Smarca2	197.7223807	218.1372459	158.387805
Smarca4	205.0454318	143.9020575	152.5931292
Smarca5	26.15375406	30.83615518	16.41824808
Smarca5-ps	3.138450487	6.85247893	3.863117195
Smarcad1	69.04591071	89.08222609	58.91253723
Smarcal1	0	0	96.57792988
Smarcb1	135.9995211	137.0495786	128.4486467
Smarcc1	25.10760389	54.81983144	37.66539266
Smarcc2	128.67647	37.68863411	188.3269633
Smarcd1	32.43065503	18.27327715	17.38402738
Smarcd2	25.10760389	102.7871839	9.657792988
Smarcd3	0	0	0
Smarce1	54.39980844	38.83071394	41.52850985
Smc1a	143.3225722	36.54655429	117.8250745
Smc2	16.7384026	15.9891175	24.14448247
Smc3	55.4459586	44.54111304	80.1596818
Smc4	9.41535146	12.56287804	11.58935159
Smc5	5.230750811	17.13119732	23.17870317
Smc6	50.21520779	71.95102876	61.80987513
Smchd1	100.4304156	106.2134234	66.63877162
Smcr8	152.7379237	190.7273302	156.4562464
Smdt1	116.122668	75.37726823	100.4410471
Smek1	64.86131006	70.80894894	53.11786144
Smek2	112.9842175	157.6070154	149.6957913
Smg1	215.5069334	561.9032723	244.3421626
Smg5	114.0303677	94.7926252	92.71481269

For Review Only

Smg6	94.1535146	87.94014627	78.22812321
Smg7	105.6611664	92.50846555	72.43344741
Smg8	115.0765178	61.67231037	54.08364074
Smg9	37.66140584	94.7926252	55.04942003
Smim1	0	0	0.965779299
Smim10l1	64.86131006	113.0659023	81.1254611
Smim10l2a	24.06145373	15.9891175	30.90493756
Smim11	35.56910552	41.11487358	0
Smim12	72.18436119	61.67231037	65.67299232
Smim13	15.69225243	14.84703768	21.24714457
Smim14	240.6145373	201.0060486	229.8554731
Smim15	7.323051136	66.24062966	0
Smim19	1.046150162	12.56287804	0
Smim20	63.8151599	65.09854983	53.11786144
Smim22	0	0	5.794675793
Smim24	0	6.85247893	3.863117195
Smim3	99.38426541	44.54111304	85.9543576
Smim4	9.41535146	21.69951661	21.24714457
Smim5	9.41535146	11.42079822	11.58935159
Smim6	2.092300324	0	6.760455092
Smim7	205.0454318	166.743654	201.8478735
Smim8	28.24605438	37.68863411	27.04182037
Smkr-ps	0	0	0
Smn1	17.78455276	21.69951661	3.863117195
Smndc1	30.33835471	35.40447447	24.14448247
Smo	82.64586282	59.38815073	60.84409583
Smoc1	0	2.284159643	0
Smoc2	0	0	0
Smox	316.9834992	253.5417204	354.4410027
Smpd1	169.4763263	109.6396629	175.7718324
Smpd2	78.46126217	61.67231037	0
Smpd3	6.276900973	9.136638573	3.863117195
Smpd4	69.04591071	34.26239465	40.56273055
Smpd5	0	0	0
Smpdl3a	87.87661363	59.38815073	87.8859162
Smpdl3b	23.01530357	60.53023055	31.87071686
Sms	20.92300324	15.9891175	15.45246878
Smtn	1.046150162	0	0
Smtnl2	0	0	0
Smu1	225.968435	207.8585275	331.2622995
Smug1	16.7384026	47.96735251	62.77565443
Smurf1	76.36896184	140.4758181	146.7984534
Smurf2	102.5227159	77.66142787	81.1254611
Smyd2	0	0	0.965779299
Smyd3	104.6150162	89.08222609	80.1596818
Smyd4	56.49210876	59.38815073	77.26234391
Smyd5	37.66140584	9.136638573	72.43344741
Snap23	144.3687224	141.6178979	124.5855296
Snap25	0	4.568319287	0
Snap29	82.64586282	73.09310859	47.32318564

For Review Only

Snap47	115.0765178	79.94558752	119.7566331
Snapc1	48.12290746	44.54111304	53.11786144
Snapc2	84.73816314	3.426239465	142.9353362
Snapc3	112.9842175	166.743654	144.8668948
Snapc4	70.09206087	30.83615518	78.22812321
Snapc5	51.26135795	54.81983144	86.9201369
Sncaip	92.06121428	81.08766734	81.1254611
Snd1	171.5686266	186.1590109	173.8402738
Sned1	20.92300324	12.56287804	5.794675793
Snf8	0	0	1.931558598
Snhg10	5.230750811	9.136638573	4.828896494
Snhg11	3.138450487	0	0
Snhg15	12.55380195	13.70495786	7.726234391
Snhg17	36.61525568	25.12575608	43.46006845
Snhg18	0	0	0
Snhg20	58.58440909	95.93470502	101.4068264
Snhg4	4.184600649	10.27871839	5.794675793
Snhg5	0	0	0
Snhg6	21.96915341	18.27327715	0
Snhg7	20.92300324	10.27871839	26.07604107
Snhg8	49.16905763	38.83071394	38.63117195
Snhg9	0	0	0
Snip1	56.49210876	76.51934805	54.08364074
Snn	101.4765657	410.006656	102.3726057
Snora17	0	1.142079822	0
Snora28	0	0	7.726234391
Snora30	0	0	1.931558598
Snora41	4.184600649	2.284159643	3.863117195
Snora44	0	0	0.965779299
Snora52	9.41535146	9.136638573	11.58935159
Snora73a	2.092300324	7.994558752	0.965779299
Snora78	0	6.85247893	0
Snora81	8.369201298	13.70495786	11.58935159
Snord104	18.83070292	21.69951661	30.90493756
Snord118	20.92300324	4.568319287	10.62357229
Snord12	3.138450487	0	0.965779299
Snord13	1.046150162	0	1.931558598
Snord14a	1.046150162	1.142079822	0
Snord14e	1.046150162	1.142079822	0
Snord15b	4.184600649	3.426239465	4.828896494
Snord16a	3.138450487	1.142079822	0
Snord17	15.69225243	13.70495786	13.52091018
Snord2	6.276900973	4.568319287	0
Snord33	1.046150162	1.142079822	1.931558598
Snord34	3.138450487	1.142079822	2.897337897
Snord35b	0	0	0
Snord37	2.092300324	3.426239465	2.897337897
Snord45b	3.138450487	2.284159643	0
Snord47	8.369201298	4.568319287	6.760455092
Snord52	6.276900973	10.27871839	10.62357229

For Review Only

Snord53	0	1.142079822	0
Snord55	0	1.142079822	1.931558598
Snord71	5.230750811	1.142079822	0.965779299
Snord8	0	2.284159643	0.965779299
Snord83b	0	0	3.863117195
Snord95	1.046150162	4.568319287	4.828896494
Snph	20.92300324	51.39359197	48.28896494
Snrk	199.814681	98.21886466	168.045598
Snrnp200	167.384026	262.678359	129.414426
Snrnp25	0	18.27327715	21.24714457
Snrnp27	32.43065503	50.25151215	64.70721302
Snrnp35	24.06145373	31.97823501	28.97337897
Snrnp40	101.4765657	101.6451041	72.43344741
Snrnp48	1.046150162	1.142079822	0
Snrnp70	88.92276379	267.2466783	43.46006845
Snrpa	165.2917256	148.4703768	150.6615706
Snrpa1	0	0	0
Snrpb	0	341.4818667	47.32318564
Snrpb2	77.41511201	71.95102876	55.04942003
Snrpc	19.87685308	14.84703768	10.62357229
Snrpd1	20.92300324	59.38815073	52.15208214
Snrpd2	5.230750811	14.84703768	20.28136528
Snrpd3	0	0	11.58935159
Snrpe	0	0	48.28896494
Snrpf	14.64610227	11.42079822	25.11026177
Snrpg	23.01530357	37.68863411	20.28136528
Snta1	0	9.136638573	0
Sntb1	8.369201298	0	0
Sntb2	16.7384026	1.142079822	9.657792988
Sntg1	3.138450487	1.142079822	0
Snupn	20.92300324	39.97279376	19.31558598
Snw1	107.7534667	218.1372459	129.414426
Snx1	179.9378279	156.4649356	191.2243012
Snx10	118.2149683	86.79806645	99.47526778
Snx11	57.53825892	51.39359197	46.35740634
Snx12	48.12290746	43.39903322	46.35740634
Snx13	164.2455755	132.4812593	132.3117639
Snx14	189.3531794	137.0495786	171.9087152
Snx15	43.93830681	43.39903322	56.01519933
Snx16	47.0767573	30.83615518	48.28896494
Snx17	89.96891395	0	34.76805476
Snx18	312.7988985	422.569534	174.8060531
Snx19	275.1374927	58.2460709	150.6615706
Snx2	152.7379237	100.5030243	236.6159282
Snx20	156.9225243	173.5961329	151.6273499
Snx21	19.87685308	15.9891175	21.24714457
Snx22	14.64610227	13.70495786	18.34980668
Snx24	99.38426541	57.10399108	82.0912404
Snx25	7.323051136	26.2678359	33.80227546
Snx27	148.553323	70.80894894	0

For Review Only

Snx29	407.9985633	478.5314453	387.2774988
Snx3	327.4450008	220.4214056	214.4030043
Snx30	91.01506412	67.38270948	75.33078531
Snx32	26.15375406	0	14.48668948
Snx33	13.59995211	13.70495786	14.48668948
Snx4	157.9686745	149.6124566	176.7376117
Snx5	120.3072687	1449.299294	1048.836319
Snx6	366.1525568	325.4927492	322.5702858
Snx7	12.55380195	7.994558752	11.58935159
Snx8	175.7532273	84.5139068	165.1482601
Snx9	7.323051136	41.11487358	41.52850985
Soat1	288.7374448	320.9244299	215.3687836
Soat2	0	0	8.69201369
Socs1	8.369201298	4.568319287	7.726234391
Socs2	3.138450487	0	0
Socs3	0	0	6.760455092
Socs4	11.50765178	55.96191126	12.55513089
Socs5	43.93830681	45.68319287	52.15208214
Socs6	129.7226201	126.7708602	154.5246878
Socs7	26.15375406	58.2460709	39.59695125
Sod1	253.1683393	288.9461949	375.6881473
Sod2	92.06121428	75.37726823	67.60455092
Sod3	6.276900973	0	0
Soga1	1979.316107	1944.961936	1996.265811
Son	178.8916777	219.2793258	548.5626417
Sorbs1	10.46150162	11.42079822	4.828896494
Sorbs2	0	0	0
Sorbs3	0	0	0
Sord	1.046150162	2.284159643	110.0988401
Sorl1	1.046150162	246.6892415	112.996178
Sort1	464.490672	0	140.0379983
Sos1	70.09206087	46.82527269	66.63877162
Sos2	32.43065503	37.68863411	35.73383406
Sostdc1	21.96915341	0	0
Sowahc	488.5521258	448.8373699	444.2584775
Sox12	4.184600649	1.142079822	0
Sox13	0	1.142079822	0
Sox15	8.369201298	13.70495786	6.760455092
Sox18	0	0	0
Sox4	31.38450487	12.56287804	25.11026177
Sox6	3.138450487	0	0
Sox7	0	0	0
Sox8	1.046150162	0	0
Sox9	7.323051136	0	0
Sp1	141.2302719	106.2134234	98.50948848
Sp100	24.06145373	29.69407536	35.73383406
Sp110	51.26135795	47.96735251	70.50188882
Sp140	39.75370617	49.10943233	53.11786144
Sp2	56.49210876	61.67231037	40.56273055
Sp3	170.5224764	91.36638573	91.74903339

For Review Only

Sp3os	10.46150162	6.85247893	12.55513089
Sp4	44.98445698	36.54655429	54.08364074
Spaca1	0	0	0
Spaca6	74.27666152	53.67775162	33.80227546
Spag1	4.184600649	11.42079822	12.55513089
Spag17	0	3.426239465	0.965779299
Spag6	0	0	0
Spag8	0	0	0
Spag9	71.13821103	207.8585275	139.072219
Sparc	4040.231927	4858.407561	5322.409716
Sparcl1	7.323051136	1.142079822	0
Spast	46.03060714	77.66142787	68.57033022
Spata1	26.15375406	7.994558752	0
Spata13	276.1836428	654.4117378	446.1900361
Spata17	0	0	0
Spata2	77.41511201	58.2460709	61.80987513
Spata21	0	0	1.931558598
Spata24	0	0	0
Spata2l	20.92300324	57.10399108	37.66539266
Spata33	0	0	0
Spata5	56.49210876	75.37726823	56.98097863
Spata5l1	1.046150162	0	1.931558598
Spata6	33.47680519	4.568319287	13.52091018
Spata7	6.276900973	11.42079822	11.58935159
Spata9	0	0	0
Spats2	11.50765178	20.55743679	25.11026177
Spats2l	3.138450487	0	0
Spc25	0	0	0
Spcs1	134.9533709	0	104.3041643
Spcs2	445.6599691	616.7231037	550.4942003
Spcs3	269.9067419	341.4818667	338.9885339
Spdl1	0	0	0
Spdya	0	0	0
Specc1	41.84600649	323.2085895	179.6349496
Specc1l	93.10736444	133.6233391	98.50948848
Spef1	0	0	0
Spef2	2.092300324	5.710399108	0
Speg	55.4459586	46.82527269	52.15208214
Spen	49.16905763	42.2569534	45.39162705
Spg11	39.75370617	34.26239465	0
Spg20	85.7843133	209.0006074	169.0113773
Spg21	164.2455755	79.94558752	146.7984534
Spg7	34.52295535	61.67231037	30.90493756
Sphk2	48.12290746	30.83615518	53.11786144
Spi1	480.1829245	122.2025409	207.6425493
Spice1	42.89215665	22.84159643	38.63117195
Spidr	56.49210876	23.98367625	43.46006845
Spin1	73.23051136	126.7708602	76.29656461
Spint1	0	0	0
Spint2	17.78455276	0	0

For Review Only

Spire1	54.39980844	114.2079822	109.1330608
Spns1	1.046150162	1.142079822	0
Spns2	40.79985633	29.69407536	65.67299232
Spock2	0	0	0.965779299
Spon1	13.59995211	5.710399108	0
Spop	160.0609748	143.9020575	183.4980668
Spopl	44.98445698	52.5356718	47.32318564
Spp1	0	0	1.931558598
Sppl2a	373.4756079	318.6402702	366.0303543
Sppl2b	55.4459586	0	50.22052354
Sppl3	73.23051136	95.93470502	141.0037776
Spr	24.06145373	34.26239465	0
Spred1	57.53825892	100.5030243	89.81747479
Spred2	106.7073165	73.09310859	75.33078531
Spred3	4.184600649	7.994558752	3.863117195
Sprtn	52.30750811	55.96191126	52.15208214
Spry1	23.01530357	26.2678359	13.52091018
Spryd3	55.4459586	19.41535697	21.24714457
Spryd4	62.76900973	28.55199554	59.87831653
Spryd7	37.66140584	55.96191126	12.55513089
Spsb1	413.2293141	344.9081061	271.383983
Spsb2	0	0	0
Spsb3	15.69225243	0	0
Sptan1	59.63055925	65.09854983	84.9885783
Sptb	2.092300324	0	0
Sptbn1	14.64610227	12.56287804	9.657792988
Sptbn2	2.092300324	2.284159643	2.897337897
Sptbn4	3.138450487	0	0
Sptbn5	32.43065503	21.69951661	58.91253723
Sptlc1	149.5994732	52.5356718	92.71481269
Sptlc2	304.4296972	269.5308379	325.4676237
Sptssa	112.9842175	29.69407536	247.2395005
Spty2d1	130.7687703	98.21886466	138.1064397
Spx	0	0	0
Sqle	11.50765178	1.142079822	4.828896494
Sqrdl	280.3682435	269.5308379	205.7109907
Sqstm1	961.4119991	18.27327715	1148.311586
Sra1	106.7073165	0	107.2015022
Srbd1	89.96891395	61.67231037	75.33078531
Src	49.16905763	68.5247893	39.59695125
Srcap	346.2757037	310.6457115	246.2737212
Srcin1	0	1.142079822	0.965779299
Srd5a1	0	0	0
Srd5a3	124.4918693	119.9183813	153.5589085
Srebf1	170.5224764	46.82527269	108.1672815
Srebf2	44.98445698	52.5356718	26.07604107
Srek1	101.4765657	116.4921418	173.8402738
Srek1ip1	21.96915341	60.53023055	34.76805476
Srf	11.50765178	19.41535697	10.62357229
Srfbp1	57.53825892	50.25151215	43.46006845

For Review Only

Srgap2	59.63055925	0	0
Srgap3	13.59995211	12.56287804	5.794675793
Srgn	169.4763263	182.7327715	221.1634594
Sri	110.8919172	73.09310859	98.50948848
Srl	12.55380195	4.568319287	27.04182037
Srm	0	0	67.60455092
Srp14	144.3687224	165.6015741	179.6349496
Srp19	69.04591071	94.7926252	106.2357229
Srp54a	63.8151599	63.95647001	67.60455092
Srp54b	88.92276379	81.08766734	94.64637129
Srp54c	87.87661363	81.08766734	87.8859162
Srp68	164.2455755	119.9183813	169.0113773
Srp72	94.1535146	171.3119732	120.7224124
Srp9	101.4765657	78.80350769	80.1596818
Srpk1	46.03060714	0	38.63117195
Srpk2	83.69201298	126.7708602	67.60455092
Srpr	0	167.8857338	214.4030043
Srprb	223.8761347	253.5417204	300.3573619
Srr	34.52295535	60.53023055	31.87071686
Srrd	5.230750811	29.69407536	26.07604107
Srrm1	286.6451445	333.4873079	304.2204791
Srrm2	37.66140584	25.12575608	0
Srrt	65.90746022	84.5139068	81.1254611
Srsf1	55.4459586	110.7817427	69.53610952
Srsf10	119.2611185	137.0495786	114.9277366
Srsf11	160.0609748	11.42079822	50.22052354
Srsf2	0	0	15.45246878
Srsf3	0	1.142079822	0
Srsf4	86.83046347	170.1698934	198.9505356
Srsf5	0	0	1.931558598
Srsf6	0	157.6070154	392.1063953
Srsf7	0	0	4.828896494
Srsf9	14.64610227	75.37726823	123.6197503
Srxn1	6.276900973	11.42079822	9.657792988
Ss18	275.1374927	286.6620352	258.8288521
Ss18l1	17.78455276	58.2460709	14.48668948
Ssb	105.6611664	54.81983144	141.0037776
Ssbp1	2.092300324	117.6342216	160.3193636
Ssbp2	17.78455276	2.284159643	177.703391
Ssbp3	38.707556	34.26239465	33.80227546
Ssbp4	144.3687224	42.2569534	28.97337897
Ssc4d	0	0	0
Ssfa2	182.0301282	169.0278136	148.730012
Ssh1	150.6456234	126.7708602	63.74143372
Ssh2	1487.625531	448.8373699	1156.037821
Ssh3	36.61525568	29.69407536	0
Ssna1	0	0	0
Sspn	4.184600649	10.27871839	14.48668948
Sspo	12.55380195	0	6.760455092
Ssr1	619.320896	563.0453521	775.520777

For Review Only

Ssr2	205.0454318	229.5580442	227.9239145
Ssr3	337.9065024	360.8972236	318.7071686
Ssr4	0	0	71.46766811
Ssrp1	80.55356249	100.5030243	81.1254611
Sssca1	30.33835471	17.13119732	0
Sstr3	0	0	0
Ssu72	132.8610706	110.7817427	171.9087152
Ssx2ip	0	0	0.965779299
St13	124.4918693	110.7817427	102.3726057
St14	74.27666152	23.98367625	43.46006845
St18	0	0	0
St3gal1	7.323051136	5.710399108	21.24714457
St3gal2	152.7379237	143.9020575	118.7908538
St3gal3	39.75370617	25.12575608	26.07604107
St3gal4	58.58440909	44.54111304	26.07604107
St3gal5	143.3225722	133.6233391	132.3117639
St3gal6	246.8914383	189.5852504	145.8326741
St5	98.33811525	60.53023055	66.63877162
St6gal1	305.4758474	190.7273302	209.5741078
St6galnac2	2.092300324	0	0
St6galnac3	0	2.284159643	0
St6galnac4	576.4287394	380.3125806	640.3116751
St6galnac6	13.59995211	27.40991572	0
St7	11.50765178	0	0
St7l	117.1688182	158.7490952	93.68059199
St8sia1	3.138450487	11.42079822	25.11026177
St8sia2	14.64610227	0	0
St8sia4	17.78455276	13.70495786	23.17870317
St8sia6	26.15375406	11.42079822	10.62357229
Stab1	75.32281168	1.142079822	0
Stac3	0	0	0
Stag1	87.87661363	131.3391795	73.39922671
Stag2	69.04591071	68.5247893	146.7984534
Stag3	18.83070292	11.42079822	0
Stam	27.19990422	74.23518841	49.25474424
Stam2	94.1535146	90.22430591	83.0570197
Stambp	70.09206087	90.22430591	70.50188882
Stambpl1	139.1379716	137.0495786	110.0988401
Stamos	2.092300324	2.284159643	0
Star	25.10760389	22.84159643	24.14448247
Stard13	2.092300324	1.142079822	0
Stard3	609.9055446	115.350062	483.8554287
Stard3nl	1.046150162	2.284159643	43.46006845
Stard4	1.046150162	0	0
Stard5	71.13821103	5.710399108	0
Stard6	1.046150162	6.85247893	0
Stard7	32.43065503	86.79806645	73.39922671
Stard8	175.7532273	123.3446207	169.0113773
Stard9	281.4143936	572.1819907	209.5741078
Stat1	145.4148726	111.9238225	135.2091018

For Review Only

Stat2	33.47680519	108.4975831	42.49428915
Stat3	189.3531794	76.51934805	445.2242568
Stat5a	55.4459586	82.22974716	38.63117195
Stat5b	150.6456234	181.5906916	134.2433225
Stat6	579.5671899	344.9081061	499.3078975
Stau1	39.75370617	35.40447447	496.4105596
Stau2	11.50765178	21.69951661	8.69201369
Stbd1	5.230750811	20.55743679	9.657792988
Steap1	10.46150162	0	0
Steap2	7.323051136	0	5.794675793
Steap3	3.138450487	2.284159643	0
Stil	0	0	0
Stim1	175.7532273	148.4703768	96.57792988
Stim2	18.83070292	25.12575608	26.07604107
Stip1	0	0	49.25474424
Stk10	389.1678604	326.634829	261.72619
Stk11	63.8151599	63.95647001	72.43344741
Stk11ip	145.4148726	133.6233391	142.9353362
Stk16	134.9533709	94.7926252	0
Stk17b	112.9842175	92.50846555	96.57792988
Stk19	21.96915341	0	21.24714457
Stk24	84.73816314	107.3555032	84.022799
Stk25	0	106.2134234	0.965779299
Stk26	0	0	0
Stk3	62.76900973	51.39359197	57.94675793
Stk35	29.29220454	19.41535697	6.760455092
Stk36	9.41535146	10.27871839	0
Stk38	67.99976055	49.10943233	53.11786144
Stk38l	46.03060714	39.97279376	34.76805476
Stk39	13.59995211	0	0
Stk4	73.23051136	167.8857338	86.9201369
Stk40	50.21520779	63.95647001	74.36500601
Stmn1	16.7384026	15.9891175	15.45246878
Stom	31.38450487	29.69407536	13.52091018
Stoml1	143.3225722	81.08766734	141.0037776
Stoml2	69.04591071	58.2460709	0
Ston1	0	4.568319287	2.897337897
Ston2	15.69225243	14.84703768	14.48668948
Stox2	0	3.426239465	0
Stpg1	0	0	0
Stra13	5.230750811	15.9891175	40.56273055
Strada	19.87685308	70.80894894	65.67299232
Stradb	121.3534188	0	125.5513089
Strap	105.6611664	109.6396629	179.6349496
Strbp	4.184600649	43.39903322	40.56273055
Strip1	143.3225722	113.0659023	107.2015022
Strip2	7.323051136	0	0
Strn	61.72285957	94.7926252	72.43344741
Strn3	32.43065503	63.95647001	65.67299232
Strn4	35.56910552	20.55743679	53.11786144

For Review Only

Stt3a	552.3672857	471.6789663	531.1786144
Stt3b	67.99976055	73.09310859	63.74143372
Stub1	1.046150162	1.142079822	0
Stx11	6.276900973	0	0
Stx12	140.1841217	151.8966163	125.5513089
Stx16	139.1379716	197.5798091	196.0531977
Stx17	44.98445698	74.23518841	67.60455092
Stx18	97.29196509	110.7817427	122.653971
Stx19	0	0	0.965779299
Stx1a	14.64610227	20.55743679	17.38402738
Stx2	107.7534667	102.7871839	152.5931292
Stx3	15.69225243	29.69407536	23.17870317
Stx4a	14.64610227	11.42079822	0
Stx5a	61.72285957	0	0
Stx6	85.7843133	49.10943233	28.00759967
Stx7	364.0602565	427.1378533	438.4638017
Stx8	2.092300324	44.54111304	65.67299232
Stxbp1	49.16905763	36.54655429	42.49428915
Stxbp2	0	55.96191126	45.39162705
Stxbp3	134.9533709	148.4703768	128.4486467
Stxbp3-ps	21.96915341	39.97279376	20.28136528
Stxbp4	21.96915341	49.10943233	17.38402738
Stxbp5	100.4304156	77.66142787	79.19390251
Styx	15.69225243	25.12575608	20.28136528
Styxl1	11.50765178	4.568319287	11.58935159
Sub1	75.32281168	89.08222609	122.653971
Sucla2	103.5688661	89.08222609	105.2699436
Suclg1	161.107125	101.6451041	137.1406604
Suclg2	123.4457191	102.7871839	97.54370918
Suco	31.38450487	82.22974716	58.91253723
Suds3	11.50765178	30.83615518	15.45246878
Sufu	74.27666152	73.09310859	90.78325409
Sugp1	57.53825892	73.09310859	54.08364074
Sugp2	133.9072208	131.3391795	92.71481269
Sugt1	77.41511201	139.3337382	133.2775432
Sulf1	29.29220454	0	0
Sulf2	6.276900973	5.710399108	24.14448247
Sult1a1	84.73816314	0	144.8668948
Sult2b1	2.092300324	2.284159643	0
Sult4a1	5.230750811	0	4.828896494
Sult6b1	2.092300324	4.568319287	2.897337897
Sumf1	57.53825892	115.350062	65.67299232
Sumf2	75.32281168	69.66686912	29.93915826
Sumo1	81.59971265	79.94558752	91.74903339
Sumo2	54.39980844	58.2460709	55.04942003
Sumo3	176.7993774	54.81983144	215.3687836
Sun1	133.9072208	115.350062	116.8592952
Sun2	422.6446655	229.5580442	216.3345629
Suox	172.6147768	25.12575608	0
Supt16	108.7996169	125.6287804	62.77565443

For Review Only

Supt20	339.9988027	183.8748513	240.4790454
Supt3	4.184600649	1.142079822	0
Supt4a	0	114.2079822	132.3117639
Supt4b	36.61525568	37.68863411	66.63877162
Supt5	164.2455755	109.6396629	0
Supt6	79.50741233	78.80350769	56.98097863
Supt7l	157.9686745	27.40991572	41.52850985
Supv3l1	60.67670941	35.40447447	36.69961336
Surf1	65.90746022	37.68863411	0
Surf2	0	0	0
Surf4	832.7355291	574.4661503	657.6957025
Surf6	91.01506412	43.39903322	61.80987513
Susd1	0	10.27871839	0
Susd2	1.046150162	0	2.897337897
Susd3	854.7046826	849.7073873	782.2812321
Susd6	110.8919172	151.8966163	97.54370918
Suv39h1	33.47680519	23.98367625	46.35740634
Suv39h2	0	2.284159643	0.965779299
Suv420h1	149.5994732	170.1698934	84.022799
Suv420h2	0	70.80894894	0
Suz12	19.87685308	27.40991572	18.34980668
Sv2a	5.230750811	58.2460709	58.91253723
Svbp	28.24605438	42.2569534	63.74143372
Svil	57.53825892	54.81983144	33.80227546
Svip	37.66140584	4.568319287	26.07604107
Swap70	156.9225243	142.7599777	197.018977
Swi5	56.49210876	81.08766734	81.1254611
Swsap1	14.64610227	5.710399108	11.58935159
Swt1	71.13821103	60.53023055	52.15208214
Syap1	16.7384026	17.13119732	10.62357229
Sybu	6.276900973	0	0
Syce2	7.323051136	3.426239465	6.760455092
Sycp2	2.092300324	2.284159643	0
Syde1	3.138450487	1.142079822	0
Syf2	160.0609748	89.08222609	155.4904671
Syk	376.6140584	486.526004	317.7413893
Sympk	46.03060714	19.41535697	46.35740634
Syn3	9.41535146	15.9891175	20.28136528
Syncrip	161.107125	157.6070154	129.414426
Syne1	20.92300324	0	0
Syne2	2.092300324	3.426239465	0.965779299
Syne4	0	0	0
Syngap1	7.323051136	17.13119732	11.58935159
Syngr1	0	845.139068	193.1558598
Syngr2	0	0	6.760455092
Syngr3	3.138450487	0	0
Synj1	168.4301761	189.5852504	125.5513089
Synj2	39.75370617	29.69407536	33.80227546
Synj2bp	70.09206087	91.36638573	67.60455092
Synj2bp-cox16	27.19990422	58.2460709	45.39162705

For Review Only

Synm	0	0	2.897337897
Synpo	0	0	0
Synpo2	0	0	0
Synrg	4.184600649	62.81439019	103.338385
Syp	4.184600649	0	2.897337897
Sypl	429.9677167	398.5858578	427.8402294
Sys1	83.69201298	66.24062966	67.60455092
Syt11	57.53825892	38.83071394	36.69961336
Syt12	0	0	0
Syt14	4.184600649	2.284159643	4.828896494
Syt15	1.046150162	0	0
Syt2	0	7.994558752	0
Syt3	21.96915341	18.27327715	9.657792988
Syt6	5.230750811	12.56287804	6.760455092
Syt7	0	2.284159643	0
Syt8	0	5.710399108	0
Syvn1	110.8919172	67.38270948	0
Szrd1	65.90746022	71.95102876	57.94675793
Szt2	124.4918693	94.7926252	82.0912404
T2	0	0	0
Tab1	81.59971265	65.09854983	62.77565443
Tab2	300.2450966	311.7877913	354.4410027
Tab3	8.369201298	23.98367625	9.657792988
Tac4	1.046150162	0	7.726234391
Tacc1	1614.2097	864.554425	907.8325409
Tacc2	17.78455276	9.136638573	10.62357229
Tacc3	18.83070292	23.98367625	17.38402738
Taco1	6.276900973	17.13119732	8.69201369
Tada1	96.24581493	126.7708602	141.9695569
Tada2a	29.29220454	28.55199554	28.97337897
Tada2b	12.55380195	38.83071394	27.04182037
Tada3	99.38426541	54.81983144	55.04942003
Taf1	30.33835471	60.53023055	13.52091018
Taf10	4.184600649	5.710399108	0
Taf11	10.46150162	23.98367625	25.11026177
Taf12	83.69201298	61.67231037	84.022799
Taf13	19.87685308	26.2678359	27.04182037
Taf15	30.33835471	47.96735251	18.34980668
Taf1a	47.0767573	35.40447447	34.76805476
Taf1b	50.21520779	49.10943233	67.60455092
Taf1c	32.43065503	44.54111304	79.19390251
Taf1d	36.61525568	50.25151215	78.22812321
Taf2	73.23051136	39.97279376	37.66539266
Taf3	39.75370617	79.94558752	40.56273055
Taf4	30.33835471	50.25151215	48.28896494
Taf4b	0	2.284159643	0
Taf5	8.369201298	5.710399108	13.52091018
Taf5l	26.15375406	15.9891175	27.04182037
Taf6	51.26135795	0	35.73383406
Taf7	69.04591071	73.09310859	57.94675793

For Review Only

Taf8	43.93830681	50.25151215	64.70721302
Taf9	73.23051136	106.2134234	111.0646194
Taf9b	16.7384026	19.41535697	23.17870317
Tagap	0	0	0
Tagap1	278.2759432	166.743654	179.6349496
Tagln2	73.23051136	43.39903322	74.36500601
Tagln3	1.046150162	0	0
Tal1	16.7384026	50.25151215	26.07604107
Taldo1	240.6145373	179.306532	262.6919693
Tamm41	27.19990422	0	16.41824808
Tanc1	19.87685308	2.284159643	5.794675793
Tanc2	1426.948821	1571.501835	1410.037776
Tango2	151.6917735	153.0386961	229.8554731
Tango6	15.69225243	38.83071394	32.83649616
Tank	62.76900973	42.2569534	62.77565443
Taok1	162.1532751	193.0114899	103.338385
Taok2	23.01530357	22.84159643	56.98097863
Taok3	132.8610706	162.1753347	158.387805
Tap1	116.122668	161.0332549	171.9087152
Tap2	481.2290746	0	0.965779299
Tapbp	5.230750811	0	0
Tapbpl	6.276900973	21.69951661	0
Tapt1	39.75370617	81.08766734	39.59695125
Tarbp2	11.50765178	25.12575608	0
Tardbp	144.3687224	164.4594943	197.018977
Tars	97.29196509	107.3555032	141.0037776
Tars2	25.10760389	12.56287804	0.965779299
Tarsl2	0	0	0
Tas1r3	17.78455276	10.27871839	4.828896494
Tas2r126	3.138450487	0	3.863117195
Tasp1	17.78455276	33.12031483	38.63117195
Tatdn1	10.46150162	10.27871839	16.41824808
Tatdn2	6.276900973	10.27871839	4.828896494
Tatdn3	20.92300324	30.83615518	32.83649616
Tax1bp1	220.7376842	199.8639688	262.6919693
Tax1bp3	0	0	0
Taz	21.96915341	0	41.52850985
Tbc1d1	59.63055925	11.42079822	41.52850985
Tbc1d10a	269.9067419	211.284767	128.4486467
Tbc1d10b	24.06145373	47.96735251	40.56273055
Tbc1d10c	5.230750811	17.13119732	10.62357229
Tbc1d12	3.138450487	4.568319287	0
Tbc1d13	1.046150162	1.142079822	0
Tbc1d14	488.5521258	334.6293877	363.1330164
Tbc1d15	157.9686745	71.95102876	81.1254611
Tbc1d16	186.2147289	190.7273302	164.1824808
Tbc1d17	0	0	11.58935159
Tbc1d19	46.03060714	43.39903322	56.01519933
Tbc1d2	8.369201298	1.142079822	0
Tbc1d20	0	0	118.7908538

For Review Only

Tbc1d22a	350.4603044	275.241237	338.0227546
Tbc1d22b	8.369201298	9.136638573	7.726234391
Tbc1d22bos	0	0	0
Tbc1d23	98.33811525	78.80350769	82.0912404
Tbc1d24	74.27666152	69.66686912	65.67299232
Tbc1d25	38.707556	17.13119732	50.22052354
Tbc1d2b	97.29196509	115.350062	110.0988401
Tbc1d30	0	0	0
Tbc1d31	47.0767573	89.08222609	79.19390251
Tbc1d32	11.50765178	21.69951661	26.07604107
Tbc1d4	38.707556	31.97823501	28.97337897
Tbc1d5	355.6910552	263.8204388	293.5969068
Tbc1d7	0	34.26239465	10.62357229
Tbc1d8	184.1224286	86.79806645	121.6881917
Tbc1d8b	9.41535146	2.284159643	10.62357229
Tbc1d9	166.3378758	151.8966163	107.2015022
Tbc1d9b	396.4909115	278.6674765	284.9048932
Tbca	62.76900973	60.53023055	63.74143372
Tbcb	55.4459586	0	4.828896494
Tbcc	74.27666152	53.67775162	70.50188882
Tbccd1	103.5688661	38.83071394	99.47526778
Tbcd	17.78455276	6.85247893	8.69201369
Tbce	67.99976055	54.81983144	56.98097863
Tbcel	23.01530357	6.85247893	22.21292387
Tbck	234.3376363	163.3174145	177.703391
Tbk1	191.4454797	195.2956495	109.1330608
Tbkbp1	47.0767573	46.82527269	33.80227546
Tbl1x	177.8455276	109.6396629	139.072219
Tbl1xr1	124.4918693	243.263002	139.072219
Tbl2	98.33811525	33.12031483	116.8592952
Tbl3	0	60.53023055	45.39162705
Tbp	73.23051136	113.0659023	106.2357229
Tbpl1	149.5994732	95.93470502	246.2737212
Tbrg1	46.03060714	45.68319287	99.47526778
Tbrg3	140.1841217	138.1916584	40.56273055
Tbrg4	51.26135795	46.82527269	57.94675793
Tbx18	0	0	0
Tbx19	2.092300324	0	0
Tbx6	1.046150162	10.27871839	13.52091018
Tbxas1	781.4741712	166.743654	630.6538821
Tc2n	4.184600649	0	0
Tcaf1	44.98445698	22.84159643	9.657792988
Tcaim	35.56910552	23.98367625	17.38402738
Tcap	2.092300324	0	0
Tcea1	62.76900973	68.5247893	84.9885783
Tcea2	57.53825892	43.39903322	24.14448247
Tceal1	42.89215665	45.68319287	41.52850985
Tceal8	28.24605438	31.97823501	31.87071686
Tceanc	50.21520779	43.39903322	20.28136528
Tceanc2	58.58440909	98.21886466	86.9201369

For Review Only

Tceb1	130.7687703	153.0386961	146.7984534
Tceb2	0	0	36.69961336
Tceb3	444.613819	251.2575608	195.0874184
Tcerg1	82.64586282	131.3391795	51.18630284
Tcf12	31.38450487	55.96191126	30.90493756
Tcf19	3.138450487	5.710399108	0
Tcf20	160.0609748	215.8530863	141.0037776
Tcf24	0	0	0
Tcf25	604.6747938	391.7333788	423.9771122
Tcf3	115.0765178	30.83615518	26.07604107
Tcf4	348.368004	441.984891	339.9543132
Tcf7l2	218.6453839	147.328297	182.5322875
Tchp	1.046150162	1.142079822	0
Tcirg1	505.2905284	272.9570774	0
Tcn2	1372.549013	880.5435425	1353.056798
Tcof1	129.7226201	62.81439019	90.78325409
Tcp1	183.0762784	0	183.4980668
Tcp11	0	5.710399108	4.828896494
Tcp11l1	30.33835471	44.54111304	38.63117195
Tcp11l2	80.55356249	86.79806645	75.33078531
Tcstv1	0	4.568319287	0
Tcta	0	0	0
Tcte3	0	1.142079822	0.965779299
Tctex1d2	18.83070292	18.27327715	5.794675793
Tctex1d4	7.323051136	3.426239465	0
Tctn1	39.75370617	13.70495786	12.55513089
Tctn2	0	0	3.863117195
Tctn3	7.323051136	28.55199554	38.63117195
Tdg	18.83070292	23.98367625	25.11026177
Tdg-ps	7.323051136	7.994558752	6.760455092
Tdo2	0	1.142079822	0
Tdp1	38.707556	46.82527269	68.57033022
Tdp2	10.46150162	38.83071394	19.31558598
Tdrd3	21.96915341	29.69407536	30.90493756
Tdrd5	0	4.568319287	0
Tdrd7	39.75370617	99.36094448	51.18630284
Tdrkh	28.24605438	33.12031483	48.28896494
Tdrp	1.046150162	0	0
Tead1	2.092300324	0	0
Tead2	16.7384026	19.41535697	13.52091018
Tead3	0	0	0
Tec	131.8149204	201.0060486	130.3802053
Tecpr1	100.4304156	97.07678484	88.85169549
Tecpr2	62.76900973	47.96735251	31.87071686
Tecr	286.6451445	235.2684433	309.0493756
Tef	149.5994732	111.9238225	164.1824808
Tefm	1.046150162	1.142079822	0
Tek	0	0	0
Tekt2	0	0	0
Telo2	44.98445698	63.95647001	70.50188882

Ten1	61.72285957	54.81983144	54.08364074
Tenm2	3.138450487	3.426239465	5.794675793
Tenm4	6.276900973	1.142079822	0
Tep1	69.04591071	103.9292638	26.07604107
Tepp	0	0	3.863117195
Terf1	41.84600649	42.2569534	53.11786144
Terf2	28.24605438	26.2678359	43.46006845
Terf2ip	74.27666152	59.38815073	73.39922671
Tes	54.39980844	74.23518841	87.8859162
Tesk1	30.33835471	53.67775162	0
Tesk2	33.47680519	35.40447447	21.24714457
Tet1	18.83070292	50.25151215	51.18630284
Tet2	176.7993774	358.613064	266.5550865
Tet3	233.2914862	319.7823501	213.437225
Tex10	108.7996169	102.7871839	118.7908538
Tex12	0	0	0
Tex14	3.138450487	1.142079822	0.965779299
Tex15	4.184600649	1.142079822	0
Tex2	57.53825892	22.84159643	59.87831653
Tex261	482.2752248	347.1922658	355.406782
Tex264	6.276900973	98.21886466	26.07604107
Tex30	2.092300324	7.994558752	0
Tex9	1.046150162	4.568319287	9.657792988
Tfam	23.01530357	39.97279376	44.42584775
Tfap4	6.276900973	11.42079822	8.69201369
Tfb1m	13.59995211	14.84703768	11.58935159
Tfb2m	31.38450487	28.55199554	45.39162705
Tfcp2	37.66140584	55.96191126	41.52850985
Tfdp1	15.69225243	14.84703768	26.07604107
Tfdp2	19.87685308	26.2678359	21.24714457
Tfe3	472.8598733	362.0393035	320.6387272
Tfeb	37.66140584	17.13119732	54.08364074
Tfec	0	1.142079822	0
Tfg	179.9378279	207.8585275	197.9847563
Tfip11	118.2149683	106.2134234	119.7566331
Tfpi	80.55356249	65.09854983	81.1254611
Tfpt	11.50765178	5.710399108	9.657792988
Tfrc	27.19990422	9.136638573	18.34980668
Tg	0	0	0
Tgds	40.79985633	78.80350769	48.28896494
Tgfa	368.2448571	142.7599777	0
Tgfb1	1.046150162	1.142079822	0
Tgfb1i1	2.092300324	6.85247893	10.62357229
Tgfb2	14.64610227	0	0
Tgfbi	206.091582	197.5798091	207.6425493
Tgfbr1	2095.438775	1235.730367	1360.783032
Tgfbr2	1366.272112	1370.495786	1075.878139
Tgfbr3	6.276900973	0	0
Tgfbr3l	0	0	3.863117195
Tgfbrap1	107.7534667	142.7599777	96.57792988

For Review Only

Tgif1	167.384026	161.0332549	135.2091018
Tgif2	52.30750811	43.39903322	35.73383406
Tgm1	2.092300324	0	0
Tgm2	407.9985633	274.0991572	360.2356785
Tgm4	0	0	0
Tgoln1	489.5982759	341.4818667	340.9200925
Tgs1	123.4457191	341.4818667	199.9163149
Tgtp1	8.369201298	1.142079822	0
Tgtp2	3.138450487	2.284159643	0
Tha1	1.046150162	3.426239465	76.29656461
Thada	132.8610706	132.4812593	123.6197503
Thap1	20.92300324	11.42079822	25.11026177
Thap11	39.75370617	61.67231037	43.46006845
Thap2	15.69225243	18.27327715	31.87071686
Thap3	70.09206087	52.5356718	58.91253723
Thap4	23.01530357	35.40447447	26.07604107
Thap6	6.276900973	23.98367625	16.41824808
Thap7	32.43065503	62.81439019	62.77565443
Thbd	0	0	0
Thbs1	17.78455276	5.710399108	0
Thbs2	5.230750811	4.568319287	16.41824808
Them4	13.59995211	15.9891175	7.726234391
Them6	0	3.426239465	0
Themis2	349.4141542	360.8972236	388.2432781
Thg1l	13.59995211	18.27327715	19.31558598
Thnsl1	26.15375406	7.994558752	17.38402738
Thnsl2	12.55380195	9.136638573	17.38402738
Thoc1	110.8919172	65.09854983	85.9543576
Thoc2	147.5071729	139.3337382	182.5322875
Thoc3	0	0	11.58935159
Thoc5	60.67670941	83.37182698	64.70721302
Thoc6	1.046150162	1.142079822	0
Thoc7	46.03060714	45.68319287	48.28896494
Thop1	29.29220454	17.13119732	15.45246878
Thra	12.55380195	7.994558752	8.69201369
Thrap3	123.4457191	147.328297	63.74143372
Thrb	1.046150162	4.568319287	11.58935159
Thrsp	193.53778	280.9516361	254.9657349
Thsd1	14.64610227	5.710399108	7.726234391
Thsd4	3.138450487	2.284159643	0
Thsd7b	2.092300324	1.142079822	5.794675793
Thtpa	54.39980844	59.38815073	48.28896494
Thumpd1	93.10736444	74.23518841	139.072219
Thumpd2	44.98445698	58.2460709	75.33078531
Thumpd3	96.24581493	166.743654	164.1824808
Thyn1	5.230750811	0	0
Tia1	177.8455276	216.9951661	148.730012
Tial1	102.5227159	49.10943233	71.46766811
Tiam1	11.50765178	5.710399108	0
Tiam2	1.046150162	2.284159643	3.863117195

For Review Only

Ticam1	99.38426541	125.6287804	123.6197503
Ticam2	138.0918214	181.5906916	187.361184
Tie1	0	0	0
Tifa	85.7843133	367.7497026	311.9467135
Tifab	146.4610227	0	0
Tigar	8.369201298	3.426239465	5.794675793
Tigd2	0	0	5.794675793
Tigd3	4.184600649	4.568319287	4.828896494
Tigd5	13.59995211	1.142079822	0
Timeless	0	7.994558752	0
Timm10	29.29220454	61.67231037	50.22052354
Timm10b	0	61.67231037	56.01519933
Timm13	0	0	0
Timm17a	40.79985633	59.38815073	55.04942003
Timm17b	48.12290746	49.10943233	22.21292387
Timm21	41.84600649	25.12575608	31.87071686
Timm22	13.59995211	39.97279376	53.11786144
Timm23	23.01530357	33.12031483	27.04182037
Timm44	42.89215665	0	53.11786144
Timm50	83.69201298	59.38815073	138.1064397
Timm8a1	13.59995211	5.710399108	3.863117195
Timm8b	40.79985633	52.5356718	36.69961336
Timm9	30.33835471	13.70495786	19.31558598
Timmdc1	41.84600649	35.40447447	33.80227546
Timp2	415.3216144	357.4709842	377.6197058
Timp3	2.092300324	0	0
Tinagl1	10.46150162	0	0
Tinf2	6.276900973	15.9891175	108.1672815
Tiparp	27.19990422	41.11487358	38.63117195
Tipin	34.52295535	20.55743679	17.38402738
Tiprl	141.2302719	147.328297	194.1216391
Tirap	56.49210876	117.6342216	104.3041643
Tjap1	84.73816314	69.66686912	59.87831653
Tjp1	165.2917256	264.9625186	197.018977
Tjp2	44.98445698	39.97279376	25.11026177
Tk1	3.138450487	7.994558752	6.760455092
Tk2	447.7522694	23.98367625	43.46006845
Tkfc	1.046150162	3.426239465	0
Tkt	0	0	42.49428915
Tlcd1	3.138450487	0	1.931558598
Tlcd2	12.55380195	5.710399108	13.52091018
Tldc1	26.15375406	13.70495786	21.24714457
Tldc2	8.369201298	12.56287804	10.62357229
Tle1	23.01530357	23.98367625	4.828896494
Tle2	40.79985633	17.13119732	19.31558598
Tle3	147.5071729	113.0659023	114.9277366
Tle4	60.67670941	78.80350769	39.59695125
Tlk1	53.35365827	69.66686912	70.50188882
Tlk2	33.47680519	13.70495786	42.49428915
Tll2	4.184600649	0	0

For Review Only

Tln1	731.2589634	632.7122212	438.4638017
Tln2	356.7372053	220.4214056	317.7413893
Tlr1	209.2300324	0	0
Tlr11	2.092300324	4.568319287	0
Tlr12	173.6609269	140.4758181	130.3802053
Tlr13	732.3051136	550.482474	755.2394117
Tlr2	494.8290267	0	1.931558598
Tlr3	256.3067897	161.0332549	124.5855296
Tlr4	220.7376842	322.0665097	266.5550865
Tlr5	94.1535146	21.69951661	40.56273055
Tlr6	225.968435	166.743654	182.5322875
Tlr7	438.336918	543.6299951	433.6349052
Tlr8	4.184600649	3.426239465	9.657792988
Tlr9	358.8295056	0	0
Tm2d1	50.21520779	67.38270948	76.29656461
Tm2d2	210.2761826	234.1263634	302.2889205
Tm2d3	103.5688661	100.5030243	159.3535843
Tm4sf1	0	0	0
Tm6sf1	91.01506412	71.95102876	52.15208214
Tm7sf2	12.55380195	6.85247893	0
Tm7sf3	80.55356249	39.97279376	70.50188882
Tm9sf1	256.3067897	178.1644522	0
Tm9sf2	1085.903868	850.8494671	1004.410471
Tm9sf3	133.9072208	213.5689267	0
Tm9sf4	156.9225243	196.4377293	236.6159282
Tma16	9.41535146	10.27871839	15.45246878
Tma7	32.43065503	26.2678359	47.32318564
Tmbim1	143.3225722	105.0713436	119.7566331
Tmbim4	36.61525568	13.70495786	0
Tmbim6	1990.823759	1576.070154	2569.938714
Tmc3	0	1.142079822	0
Tmc6	180.9839781	87.94014627	225.9923559
Tmc7	97.29196509	93.65054538	61.80987513
Tmc8	12.55380195	0	11.58935159
Tmcc1	51.26135795	53.67775162	57.94675793
Tmcc2	3.138450487	17.13119732	0
Tmcc3	213.4146331	158.7490952	192.1900805
Tmco1	206.091582	264.9625186	288.7680104
Tmco3	128.67647	90.22430591	69.53610952
Tmco4	63.8151599	47.96735251	30.90493756
Tmco6	32.43065503	4.568319287	0
Tmed1	1.046150162	3.426239465	0
Tmed10	452.9830203	451.1215296	338.9885339
Tmed2	297.1066461	235.2684433	264.6235279
Tmed3	426.8292662	436.2744919	477.0949736
Tmed4	202.9531315	127.91294	248.2052798
Tmed5	283.506694	311.7877913	359.2698992
Tmed7	178.8916777	199.8639688	128.4486467
Tmed8	30.33835471	31.97823501	0
Tmed9	240.6145373	283.2357958	103.338385

For Review Only

Tmem100	177.8455276	165.6015741	195.0874184
Tmem101	23.01530357	7.994558752	24.14448247
Tmem104	523.0750811	277.5253967	368.9276922
Tmem106a	65.90746022	41.11487358	56.98097863
Tmem106b	175.7532273	167.8857338	178.6691703
Tmem106c	120.3072687	113.0659023	128.4486467
Tmem107	5.230750811	0	0
Tmem108	9.41535146	0	0
Tmem109	355.6910552	288.9461949	343.8174304
Tmem11	95.19966476	114.2079822	90.78325409
Tmem110	34.52295535	34.26239465	36.69961336
Tmem115	210.2761826	190.7273302	185.4296254
Tmem116	0	0	0
Tmem119	9546.12023	8411.417887	11828.86485
Tmem120a	49.16905763	29.69407536	18.34980668
Tmem120b	21.96915341	6.85247893	13.52091018
Tmem123	60.67670941	94.7926252	105.2699436
Tmem126a	12.55380195	7.994558752	13.52091018
Tmem126b	30.33835471	28.55199554	45.39162705
Tmem127	0	0	317.7413893
Tmem128	96.24581493	78.80350769	138.1064397
Tmem129	100.4304156	15.9891175	16.41824808
Tmem131	111.9380674	98.21886466	86.9201369
Tmem132a	5.230750811	5.710399108	0
Tmem134	5.230750811	4.568319287	0
Tmem135	292.9220454	371.175942	381.482823
Tmem136	4.184600649	5.710399108	0
Tmem138	6.276900973	21.69951661	28.00759967
Tmem140	88.92276379	243.263002	96.57792988
Tmem143	9.41535146	13.70495786	15.45246878
Tmem144	163.1994253	139.3337382	154.5246878
Tmem14a	0	0	0
Tmem14c	129.7226201	161.0332549	181.5665082
Tmem150a	14.64610227	7.994558752	0
Tmem150b	0	4.568319287	0
Tmem150c	0	0	0
Tmem151b	6.276900973	0	0
Tmem154	15.69225243	20.55743679	21.24714457
Tmem156	11.50765178	7.994558752	17.38402738
Tmem159	76.36896184	62.81439019	88.85169549
Tmem160	12.55380195	30.83615518	13.52091018
Tmem161a	110.8919172	105.0713436	7.726234391
Tmem161b	42.89215665	62.81439019	35.73383406
Tmem163	0	7.994558752	4.828896494
Tmem164	111.9380674	131.3391795	123.6197503
Tmem165	16.7384026	37.68863411	31.87071686
Tmem167	87.87661363	107.3555032	92.71481269
Tmem167b	109.845767	47.96735251	77.26234391
Tmem168	233.2914862	165.6015741	166.1140394
Tmem17	11.50765178	7.994558752	7.726234391

For Review Only

Tmem170	29.29220454	5.710399108	27.04182037
Tmem170b	893.4122386	1003.888163	595.8858274
Tmem173	1106.826872	1215.17293	1555.87045
Tmem175	347.3218539	205.5743679	257.8630728
Tmem176a	0	0	26.07604107
Tmem176b	259.4452402	0	22.21292387
Tmem177	62.76900973	79.94558752	44.42584775
Tmem178b	2.092300324	1.142079822	0
Tmem179b	159.0148247	69.66686912	39.59695125
Tmem18	42.89215665	25.12575608	64.70721302
Tmem180	33.47680519	33.12031483	29.93915826
Tmem181a	109.845767	89.08222609	119.7566331
Tmem181c-ps	46.03060714	61.67231037	26.07604107
Tmem183a	74.27666152	106.2134234	69.53610952
Tmem184b	386.0294099	145.0441374	87.8859162
Tmem184c	61.72285957	62.81439019	62.77565443
Tmem185b	268.8605917	185.0169311	184.4638461
Tmem186	1.046150162	1.142079822	0
Tmem189	28.24605438	35.40447447	38.63117195
Tmem19	97.29196509	99.36094448	126.5170881
Tmem191c	8.369201298	0	7.726234391
Tmem192	25.10760389	33.12031483	21.24714457
Tmem194	2.092300324	18.27327715	17.38402738
Tmem194b	42.89215665	33.12031483	63.74143372
Tmem198b	93.10736444	52.5356718	79.19390251
Tmem199	14.64610227	20.55743679	0
Tmem2	19.87685308	14.84703768	21.24714457
Tmem201	89.96891395	61.67231037	56.01519933
Tmem202	4.184600649	0	0
Tmem203	36.61525568	30.83615518	55.04942003
Tmem204	165.2917256	159.891175	149.6957913
Tmem205	6.276900973	13.70495786	9.657792988
Tmem206	428.9215665	525.356718	489.6501045
Tmem208	18.83070292	11.42079822	16.41824808
Tmem209	153.7840738	79.94558752	129.414426
Tmem214	186.2147289	0	0
Tmem216	15.69225243	6.85247893	21.24714457
Tmem218	16.7384026	12.56287804	8.69201369
Tmem220	24.06145373	10.27871839	11.58935159
Tmem221	23.01530357	15.9891175	23.17870317
Tmem222	126.5841696	153.0386961	264.6235279
Tmem223	0	0	0
Tmem229a	0	1.142079822	0
Tmem229b	164.2455755	164.4594943	210.5398871
Tmem230	0	0	0
Tmem231	8.369201298	3.426239465	14.48668948
Tmem234	0	0	0
Tmem237	47.0767573	30.83615518	36.69961336
Tmem238	0	0	3.863117195
Tmem240	0	3.426239465	0

For Review Only

Tmem241	127.6303198	65.09854983	75.33078531
Tmem242	16.7384026	28.55199554	17.38402738
Tmem243	8.369201298	9.136638573	8.69201369
Tmem245	26.15375406	63.95647001	37.66539266
Tmem246	2.092300324	0	0
Tmem248	52.30750811	97.07678484	46.35740634
Tmem25	0	3.426239465	0
Tmem251	1.046150162	2.284159643	0
Tmem252	0	0	0
Tmem253	6.276900973	0	0
Tmem254a	12.55380195	7.994558752	12.55513089
Tmem254b	20.92300324	15.9891175	23.17870317
Tmem254c	21.96915341	15.9891175	23.17870317
Tmem255b	4.184600649	0	0
Tmem256	0	0	0
Tmem258	66.95361038	46.82527269	103.338385
Tmem259	9.41535146	5.710399108	0
Tmem26	1.046150162	0	0.965779299
Tmem260	89.96891395	58.2460709	32.83649616
Tmem261	0	0	44.42584775
Tmem263	52.30750811	58.2460709	57.94675793
Tmem265	5.230750811	7.994558752	0
Tmem29	15.69225243	17.13119732	27.04182037
Tmem30a	237.4760868	288.9461949	166.1140394
Tmem33	186.2147289	267.2466783	291.6653483
Tmem37	184.1224286	202.1481284	214.4030043
Tmem38a	12.55380195	0	5.794675793
Tmem38b	27.19990422	15.9891175	14.48668948
Tmem39a	96.24581493	70.80894894	83.0570197
Tmem39b	13.59995211	31.97823501	21.24714457
Tmem40	8.369201298	0	3.863117195
Tmem41a	29.29220454	31.97823501	31.87071686
Tmem41b	57.53825892	71.95102876	67.60455092
Tmem42	21.96915341	27.40991572	36.69961336
Tmem43	224.9222849	173.5961329	234.6843696
Tmem44	93.10736444	50.25151215	107.2015022
Tmem45a	0	1.142079822	0
Tmem47	9.41535146	0	0
Tmem5	53.35365827	54.81983144	67.60455092
Tmem50a	802.3971744	564.1874319	797.7337008
Tmem50b	568.0595381	461.400248	503.1710147
Tmem51	1.046150162	0	0
Tmem53	4.184600649	22.84159643	0
Tmem55a	66.95361038	58.2460709	77.26234391
Tmem55b	0	18.27327715	1.931558598
Tmem57	11.50765178	53.67775162	31.87071686
Tmem59	922.7044431	944.5000125	1201.429448
Tmem60	109.845767	118.7763015	88.85169549
Tmem62	14.64610227	12.56287804	13.52091018
Tmem63a	849.4739317	561.9032723	855.6804588

For Review Only

Tmem63b	31.38450487	31.97823501	14.48668948
Tmem64	73.23051136	103.9292638	67.60455092
Tmem65	38.707556	36.54655429	30.90493756
Tmem67	0	29.69407536	20.28136528
Tmem68	128.67647	210.1426872	144.8668948
Tmem69	29.29220454	30.83615518	28.97337897
Tmem70	101.4765657	117.6342216	85.9543576
Tmem71	20.92300324	12.56287804	46.35740634
Tmem72	5.230750811	0	0
Tmem74b	17.78455276	11.42079822	4.828896494
Tmem79	5.230750811	15.9891175	0.965779299
Tmem8	9.41535146	10.27871839	0
Tmem80	0	0	0
Tmem82	0	1.142079822	0
Tmem86a	1327.564556	0	946.4637129
Tmem86b	13.59995211	29.69407536	30.90493756
Tmem87a	12.55380195	21.69951661	21.24714457
Tmem87b	109.845767	113.0659023	89.81747479
Tmem88	0	0	0
Tmem8b	27.19990422	19.41535697	11.58935159
Tmem8c	49.16905763	30.83615518	49.25474424
Tmem9	64.86131006	51.39359197	82.0912404
Tmem91	3.138450487	1.142079822	4.828896494
Tmem94	86.83046347	60.53023055	67.60455092
Tmem97	34.52295535	47.96735251	40.56273055
Tmem98	9.41535146	0	0
Tmem9b	287.6912946	235.2684433	313.8782721
Tmf1	101.4765657	110.7817427	75.33078531
Tmigd3	8.369201298	10.27871839	28.97337897
Tmlhe	9.41535146	27.40991572	28.00759967
Tmod1	15.69225243	0	0
Tmod3	129.7226201	161.0332549	138.1064397
Tmpo	54.39980844	99.36094448	41.52850985
Tmprss2	0	0	0
Tmsb10	13.59995211	11.42079822	0
Tmsb15b1	6.276900973	0	0
Tmsb15l	3.138450487	6.85247893	0
Tmsb4x	1388.241265	1946.104016	2568.972935
Tmtc1	0	1.142079822	0
Tmtc2	0	0	0
Tmtc3	58.58440909	69.66686912	64.70721302
Tmtc4	14.64610227	20.55743679	18.34980668
Tmub1	40.79985633	18.27327715	32.83649616
Tmub2	224.9222849	205.5743679	158.387805
Tmx1	199.814681	164.4594943	199.9163149
Tmx2	232.245336	167.8857338	64.70721302
Tmx3	131.8149204	131.3391795	121.6881917
Tmx4	195.6300803	213.5689267	148.730012
Tnfaip1	89.96891395	67.38270948	152.5931292
Tnfaip2	9.41535146	23.98367625	29.93915826

For Review Only

Tnfaip3	162.1532751	135.9074988	145.8326741
Tnfaip8	201.9069813	188.4431706	282.9733346
Tnfaip8l1	0	0	0
Tnfaip8l2	264.675991	334.6293877	451.0189326
Tnfrsf10b	0	0	0
Tnfrsf11a	126.5841696	127.91294	125.5513089
Tnfrsf12a	18.83070292	5.710399108	4.828896494
Tnfrsf13b	327.4450008	316.3561106	449.087374
Tnfrsf14	0	0	0
Tnfrsf17	37.66140584	55.96191126	46.35740634
Tnfrsf18	0	0	2.897337897
Tnfrsf19	1.046150162	0	0
Tnfrsf1a	411.1370138	420.2853744	422.0455536
Tnfrsf1b	1148.672878	816.5870725	1041.110084
Tnfrsf21	628.7362475	454.547769	587.1938137
Tnfrsf22	8.369201298	13.70495786	4.828896494
Tnfrsf23	21.96915341	23.98367625	12.55513089
Tnfrsf26	4.184600649	6.85247893	0
Tnfrsf4	0	0	0
Tnfsf10	11.50765178	5.710399108	11.58935159
Tnfsf12	15.69225243	0	7.726234391
Tnfsf13	0	0	0
Tnfsf13b	24.06145373	4.568319287	0
Tnfsf13os	1.046150162	0	0
Tnfsf14	2.092300324	0	3.863117195
Tnfsf18	0	0	0
Tnfsf8	12.55380195	9.136638573	17.38402738
Tnfsf9	1.046150162	2.284159643	15.45246878
Tnfsfm13	11.50765178	14.84703768	0
Tnik	1.046150162	2.284159643	0
Tnip1	42.89215665	39.97279376	49.25474424
Tnk2	4.184600649	0	0
Tnks	35.56910552	66.24062966	73.39922671
Tnks1bp1	13.59995211	15.9891175	14.48668948
Tnks2	56.49210876	167.8857338	103.338385
Tnni2	0	3.426239465	0
Tnni3	7.323051136	1.142079822	3.863117195
Tnpo1	185.1685787	342.6239465	184.4638461
Tnpo2	89.96891395	51.39359197	36.69961336
Tnpo3	141.2302719	153.0386961	201.8478735
Tnr	0	0	0
Tnrc18	105.6611664	109.6396629	88.85169549
Tnrc6a	160.0609748	156.4649356	138.1064397
Tnrc6b	198.7685308	271.8149976	182.5322875
Tnrc6c	93.10736444	138.1916584	101.4068264
Tns1	151.6917735	210.1426872	215.3687836
Tns2	0	2.284159643	0
Tns3	851.5662321	582.460709	413.3535399
Tns4	7.323051136	25.12575608	12.55513089
Tnxb	0	0	0

For Review Only

Tob1	7.323051136	4.568319287	8.69201369
Tob2	163.1994253	0	0.965779299
Toe1	21.96915341	0	4.828896494
Tollip	217.5992337	114.2079822	214.4030043
Tom1	63.8151599	11.42079822	72.43344741
Tom1l1	64.86131006	67.38270948	78.22812321
Tom1l2	149.5994732	82.22974716	99.47526778
Tomm20	142.2764221	97.07678484	110.0988401
Tomm22	117.1688182	155.3228557	168.045598
Tomm34	282.4605438	266.1045984	283.9391139
Tomm40	111.9380674	55.96191126	101.4068264
Tomm40l	39.75370617	42.2569534	44.42584775
Tomm5	21.96915341	33.12031483	41.52850985
Tomm6	0	0	1.931558598
Tomm6os	0	1.142079822	0.965779299
Tomm7	62.76900973	57.10399108	93.68059199
Tomm70a	57.53825892	39.97279376	27.04182037
Tomt	5.230750811	0	0
Tonsl	6.276900973	45.68319287	11.58935159
Top1	79.50741233	119.9183813	94.64637129
Top1mt	0	0	0
Top2a	0	0	0
Top2b	65.90746022	79.94558752	42.49428915
Top3a	62.76900973	58.2460709	44.42584775
Top3b	114.0303677	118.7763015	149.6957913
Topbp1	74.27666152	21.69951661	25.11026177
Topors	189.3531794	173.5961329	136.1748811
Toporsos	0	7.994558752	0
Tor1a	128.67647	122.2025409	155.4904671
Tor1aip1	182.0301282	341.4818667	293.5969068
Tor1aip2	104.6150162	149.6124566	100.4410471
Tor1b	152.7379237	119.9183813	239.5132661
Tor2a	34.52295535	79.94558752	189.2927426
Tor3a	213.4146331	248.9734011	291.6653483
Tor4a	562.8287873	0	9.657792988
Tox4	391.2601607	191.86941	163.2167015
Tpbgl	106.7073165	130.1970997	100.4410471
Tpcn1	128.67647	166.743654	148.730012
Tpcn2	88.92276379	51.39359197	39.59695125
Tpd52	148.553323	130.1970997	139.072219
Tpd52l1	5.230750811	0	0
Tpd52l2	87.87661363	232.9842836	129.414426
Tpgs1	61.72285957	70.80894894	97.54370918
Tpgs2	13.59995211	33.12031483	27.04182037
Tpi1	229.1068855	79.94558752	169.0113773
Tpk1	50.21520779	17.13119732	18.34980668
Tpm1	39.75370617	60.53023055	43.46006845
Tpm2	1.046150162	0	0
Tpm3	377.6602086	679.5374939	268.4866451
Tpm4	42.89215665	54.81983144	60.84409583

For Review Only

Tpmt	9.41535146	0	14.48668948
Tpp1	852.6123822	867.9806645	873.0644862
Tpp2	248.9837386	171.3119732	161.2851429
Tppp	252.1221891	202.1481284	210.5398871
Tppp3	2.092300324	6.85247893	0
Tpr	80.55356249	110.7817427	111.0646194
Tpra1	150.6456234	175.8802925	156.4562464
Tprgl	1.046150162	55.96191126	195.0874184
Tprkb	30.33835471	36.54655429	37.66539266
Tprn	62.76900973	54.81983144	49.25474424
Tpst1	16.7384026	0	4.828896494
Tpst2	999.0734049	845.139068	1411.003556
Tpx2	0	0	0
Tra2a	148.553323	98.21886466	132.3117639
Tra2b	43.93830681	130.1970997	258.8288521
Trabd	49.16905763	65.09854983	24.14448247
Trabd2b	3.138450487	0	0
Traf1	6.276900973	0	0
Traf2	167.384026	111.9238225	106.2357229
Traf3	135.9995211	58.2460709	64.70721302
Traf3ip1	12.55380195	10.27871839	5.794675793
Traf3ip2	0	0	0
Traf3ip3	102.5227159	132.4812593	86.9201369
Traf4	9.41535146	5.710399108	8.69201369
Traf5	112.9842175	117.6342216	143.9011155
Traf6	106.7073165	87.94014627	61.80987513
Traf7	20.92300324	36.54655429	63.74143372
Trafd1	0	0	0
Traip	0	1.142079822	5.794675793
Trak1	161.107125	134.765419	134.2433225
Trak2	28.24605438	37.68863411	24.14448247
Tram1	301.2912467	316.3561106	284.9048932
Tram2	19.87685308	18.27327715	0
Trank1	4.184600649	1.142079822	2.897337897
Trap1	51.26135795	100.5030243	77.26234391
Trappc10	46.03060714	55.96191126	36.69961336
Trappc11	147.5071729	157.6070154	84.022799
Trappc12	233.2914862	149.6124566	213.437225
Trappc13	106.7073165	93.65054538	90.78325409
Trappc2	0	11.42079822	0
Trappc2l	0	41.11487358	0.965779299
Trappc3	77.41511201	98.21886466	110.0988401
Trappc4	93.10736444	143.9020575	108.1672815
Trappc5	48.12290746	52.5356718	42.49428915
Trappc6a	31.38450487	0	0
Trappc6b	61.72285957	47.96735251	41.52850985
Trappc8	47.0767573	68.5247893	48.28896494
Trappc9	176.7993774	135.9074988	94.64637129
Trdmt1	10.46150162	14.84703768	20.28136528
Trem1	5.230750811	0	0

For Review Only

Trem2	3145.773538	0	2530.341763
Trem3	6.276900973	0	0
Treml1	8.369201298	7.994558752	11.58935159
Treml2	11.50765178	42.2569534	25.11026177
Trerf1	0	0	0
Trex1	0	6.85247893	39.59695125
Trf	4790.321593	3727.748538	5418.021867
Trhde	0	0	5.794675793
Triap1	46.03060714	67.38270948	64.70721302
Trib1	159.0148247	156.4649356	115.8935159
Trib3	0	0	0
Tril	0	0	0.965779299
Trim11	37.66140584	102.7871839	144.8668948
Trim12a	11.50765178	0	14.48668948
Trim12c	60.67670941	44.54111304	35.73383406
Trim13	34.52295535	18.27327715	27.04182037
Trim14	2.092300324	13.70495786	0
Trim15	7.323051136	17.13119732	5.794675793
Trim16	5.230750811	19.41535697	4.828896494
Trim17	11.50765178	35.40447447	0
Trim2	103.5688661	93.65054538	70.50188882
Trim21	7.323051136	4.568319287	7.726234391
Trim23	51.26135795	61.67231037	54.08364074
Trim24	32.43065503	43.39903322	18.34980668
Trim25	53.35365827	108.4975831	35.73383406
Trim26	189.3531794	172.4540531	255.9315142
Trim27	75.32281168	90.22430591	46.35740634
Trim28	92.06121428	70.80894894	0
Trim3	38.707556	53.67775162	32.83649616
Trim30a	103.5688661	73.09310859	3.863117195
Trim30b	1.046150162	4.568319287	0
Trim30c	14.64610227	2.284159643	13.52091018
Trim30d	60.67670941	222.7055652	118.7908538
Trim32	60.67670941	66.24062966	76.29656461
Trim33	26.15375406	50.25151215	45.39162705
Trim34a	62.76900973	91.36638573	105.2699436
Trim35	160.0609748	117.6342216	139.072219
Trim36	100.4304156	114.2079822	85.9543576
Trim37	17.78455276	17.13119732	18.34980668
Trim39	10.46150162	14.84703768	34.76805476
Trim41	63.8151599	85.65598662	64.70721302
Trim43c	0	0	0
Trim44	159.0148247	213.5689267	115.8935159
Trim45	17.78455276	4.568319287	5.794675793
Trim47	1.046150162	1.142079822	0
Trim5	67.99976055	45.68319287	61.80987513
Trim56	11.50765178	27.40991572	25.11026177
Trim59	2.092300324	0	0
Trim62	5.230750811	6.85247893	0
Trim65	0	0	0.965779299

For Review Only

Trim66	1.046150162	0	4.828896494
Trim67	4.184600649	0	1.931558598
Trim68	39.75370617	20.55743679	27.04182037
Trim7	12.55380195	0	2.897337897
Trim75	0	0	0
Trim8	39.75370617	46.82527269	78.22812321
Trio	198.7685308	236.4105231	182.5322875
Triobp	28.24605438	17.13119732	35.73383406
Trip10	13.59995211	20.55743679	27.04182037
Trip11	62.76900973	123.3446207	74.36500601
Trip12	137.0456713	151.8966163	119.7566331
Trip13	0	0	0
Trip4	49.16905763	39.97279376	66.63877162
Trip6	1.046150162	6.85247893	11.58935159
Triqk	0	3.426239465	0
Trit1	65.90746022	53.67775162	84.9885783
Trmo	30.33835471	25.12575608	24.14448247
Trmt1	42.89215665	100.5030243	115.8935159
Trmt10a	37.66140584	31.97823501	50.22052354
Trmt10b	15.69225243	26.2678359	21.24714457
Trmt10c	57.53825892	65.09854983	83.0570197
Trmt11	20.92300324	28.55199554	25.11026177
Trmt12	103.5688661	57.10399108	77.26234391
Trmt13	33.47680519	20.55743679	41.52850985
Trmt1l	155.8763742	141.6178979	178.6691703
Trmt2a	1.046150162	1.142079822	0
Trmt2b	28.24605438	100.5030243	47.32318564
Trmt44	25.10760389	26.2678359	22.21292387
Trmt5	23.01530357	29.69407536	30.90493756
Trmt6	59.63055925	69.66686912	74.36500601
Trmt61a	37.66140584	37.68863411	25.11026177
Trmt61b	16.7384026	3.426239465	18.34980668
Trmu	109.845767	0	0.965779299
Trnau1ap	104.6150162	43.39903322	46.35740634
Trnt1	75.32281168	34.26239465	57.94675793
Tro	0	1.142079822	0
Troap	1.046150162	1.142079822	0
Trove2	40.79985633	68.5247893	68.57033022
Trp53	112.9842175	110.7817427	202.8136528
Trp53bp1	100.4304156	219.2793258	108.1672815
Trp53bp2	82.64586282	61.67231037	77.26234391
Trp53cor1	30.33835471	19.41535697	13.52091018
Trp53i11	83.69201298	83.37182698	86.9201369
Trp53i13	5.230750811	0	52.15208214
Trp53inp1	14.64610227	73.09310859	24.14448247
Trp53inp2	85.7843133	52.5356718	54.08364074
Trp53rka	33.47680519	41.11487358	52.15208214
Trp53rkb	11.50765178	17.13119732	15.45246878
Trp53tg5	4.184600649	0	0
Trpc4ap	39.75370617	17.13119732	0

For Review Only

Trpm2	188.3070292	204.4322881	181.5665082
Trpm3	16.7384026	1.142079822	3.863117195
Trpm4	108.7996169	60.53023055	74.36500601
Trpm5	3.138450487	0	0
Trpm7	180.9839781	310.6457115	139.072219
Trps1	1.046150162	3.426239465	5.794675793
Trpt1	10.46150162	0	0.965779299
Trpv2	213.4146331	79.94558752	281.041776
Trpv4	42.89215665	10.27871839	12.55513089
Trrap	256.3067897	218.1372459	201.8478735
Trub1	72.18436119	21.69951661	38.63117195
Trub2	53.35365827	53.67775162	54.08364074
Tsacc	7.323051136	0	9.657792988
Tsc1	108.7996169	177.0223724	0
Tsc2	327.4450008	191.86941	266.5550865
Tsc22d1	26.15375406	26.2678359	21.24714457
Tsc22d2	86.83046347	98.21886466	95.61215059
Tsc22d3	4.184600649	78.80350769	0.965779299
Tsc22d4	24.06145373	34.26239465	32.83649616
Tsen15	11.50765178	30.83615518	47.32318564
Tsen2	10.46150162	13.70495786	45.39162705
Tsen34	76.36896184	61.67231037	30.90493756
Tsen54	26.15375406	21.69951661	36.69961336
Tsfm	15.69225243	35.40447447	26.07604107
Tsg101	185.1685787	174.7382127	214.4030043
Tsga10	23.01530357	17.13119732	11.58935159
Tshb	0	0	0
Tshz1	13.59995211	29.69407536	28.97337897
Tshz2	0	0	0
Tslp	1.046150162	7.994558752	10.62357229
Tsn	166.3378758	180.4486118	216.3345629
Tsnax	130.7687703	108.4975831	138.1064397
Tspan11	0	0	0
Tspan13	381.8448092	351.7605851	379.5512644
Tspan14	1140.303677	950.2104116	1142.516911
Tspan17	0	0	1.931558598
Tspan18	172.6147768	158.7490952	142.9353362
Tspan3	443.5676688	382.5967403	424.9428915
Tspan31	0	0	0
Tspan32	21.96915341	38.83071394	58.91253723
Tspan33	29.29220454	25.12575608	17.38402738
Tspan4	217.5992337	222.7055652	0
Tspan5	4.184600649	19.41535697	9.657792988
Tspan7	331.6296014	344.9081061	378.5854851
Tspan9	137.0456713	68.5247893	138.1064397
Tspear	5.230750811	0	0
Tspo	110.8919172	124.4867006	102.3726057
Tspyl1	295.0143458	342.6239465	229.8554731
Tspyl2	53.35365827	46.82527269	48.28896494
Tspyl3	54.39980844	35.40447447	42.49428915

For Review Only

Tspyl4	10.46150162	0	2.897337897
Tsr1	94.1535146	18.27327715	25.11026177
Tsr2	12.55380195	19.41535697	22.21292387
Tsr3	18.83070292	1.142079822	0
Tssc1	0	0	46.35740634
Tssc4	0	0	0
Tssk4	0	1.142079822	0
Tssk6	9.41535146	4.568319287	6.760455092
Tst	3.138450487	0	0.965779299
Tsta3	12.55380195	11.42079822	0
Tstd2	129.7226201	75.37726823	81.1254611
Tstd3	1.046150162	3.426239465	0
Ttbk1	0	3.426239465	0
Ttbk2	38.707556	52.5356718	39.59695125
Ttc1	84.73816314	94.7926252	89.81747479
Ttc12	26.15375406	0	23.17870317
Ttc13	24.06145373	68.5247893	112.0303987
Ttc14	153.7840738	134.765419	113.9619573
Ttc17	143.3225722	110.7817427	84.9885783
Ttc19	10.46150162	7.994558752	8.69201369
Ttc21b	9.41535146	12.56287804	31.87071686
Ttc23	4.184600649	0	0.965779299
Ttc25	0	5.710399108	0
Ttc26	6.276900973	0	0
Ttc27	38.707556	26.2678359	40.56273055
Ttc28	206.091582	109.6396629	148.730012
Ttc3	112.9842175	115.350062	121.6881917
Ttc30a1	30.33835471	21.69951661	39.59695125
Ttc30a2	11.50765178	9.136638573	13.52091018
Ttc30b	14.64610227	17.13119732	24.14448247
Ttc32	21.96915341	17.13119732	28.97337897
Ttc33	70.09206087	39.97279376	53.11786144
Ttc37	27.19990422	38.83071394	29.93915826
Ttc38	29.29220454	28.55199554	25.11026177
Ttc39a	0	0	1.931558598
Ttc39aos1	0	1.142079822	0
Ttc39b	95.19966476	148.4703768	116.8592952
Ttc4	140.1841217	130.1970997	215.3687836
Ttc41	18.83070292	7.994558752	11.58935159
Ttc5	138.0918214	126.7708602	129.414426
Ttc7	146.4610227	183.8748513	121.6881917
Ttc7b	0	11.42079822	22.21292387
Ttc8	33.47680519	22.84159643	43.46006845
Ttc9	6.276900973	18.27327715	23.17870317
Ttc9c	0	11.42079822	0
Ttf1	79.50741233	101.6451041	110.0988401
Ttf2	13.59995211	1.142079822	29.93915826
Tti1	117.1688182	107.3555032	110.0988401
Tti2	60.67670941	18.27327715	20.28136528
Ttl	21.96915341	14.84703768	25.11026177

For Review Only

Ttll1	34.52295535	26.2678359	45.39162705
Ttll12	32.43065503	18.27327715	26.07604107
Ttll3	28.24605438	37.68863411	25.11026177
Ttll4	42.89215665	76.51934805	80.1596818
Ttll5	16.7384026	13.70495786	0
Ttll8	3.138450487	0	0
Ttll9	0	1.142079822	0
Ttn	13.59995211	0	7.726234391
Ttpal	1704.178614	619.0072633	251.1026177
Ttr	5354.19653	215.8530863	29.93915826
Ttyh1	7.323051136	6.85247893	5.794675793
Ttyh2	24.06145373	22.84159643	21.24714457
Ttyh3	80.55356249	69.66686912	62.77565443
Tub	1.046150162	1.142079822	0
Tuba1a	728.1205129	718.3682078	775.520777
Tuba1b	704.0590592	577.8923898	788.0759079
Tuba1c	211.3223328	248.9734011	253.0341763
Tuba4a	19.87685308	5.710399108	8.69201369
Tubb2a	302.3373969	355.1868245	379.5512644
Tubb2a-ps2	4.184600649	7.994558752	3.863117195
Tubb2b	94.1535146	132.4812593	122.653971
Tubb4a	6.276900973	3.426239465	0
Tubb4b	191.4454797	107.3555032	112.0303987
Tubb5	2.092300324	342.6239465	0
Tubb6	28.24605438	25.12575608	44.42584775
Tubd1	12.55380195	5.710399108	8.69201369
Tube1	5.230750811	0	7.726234391
Tubg1	49.16905763	25.12575608	54.08364074
Tubg2	0	0	0.965779299
Tubgcp2	120.3072687	95.93470502	64.70721302
Tubgcp3	114.0303677	89.08222609	96.57792988
Tubgcp4	57.53825892	22.84159643	14.48668948
Tubgcp5	409.0447134	448.8373699	304.2204791
Tubgcp6	0	0	0.965779299
Tufm	7.323051136	0	45.39162705
Tuft1	10.46150162	6.85247893	0.965779299
Tug1	539.8134837	0	104.3041643
Tulp2	9.41535146	0	0.965779299
Tulp3	9.41535146	9.136638573	8.69201369
Tulp4	71.13821103	119.9183813	67.60455092
Tusc2	10.46150162	18.27327715	0
Tusc3	52.30750811	76.51934805	57.94675793
Tut1	137.0456713	75.37726823	131.3459846
Tvp23a	28.24605438	13.70495786	9.657792988
Tvp23b	122.399569	154.1807759	152.5931292
Twf1	298.1527962	334.6293877	295.5284654
Twf2	0	0	0
Twistnb	83.69201298	41.11487358	110.0988401
Twsg1	20.92300324	6.85247893	16.41824808
Txlna	305.4758474	203.2902083	259.7946314

For Review Only

Txlnb	0	0	0
Txlng	36.61525568	57.10399108	58.91253723
Txn1	38.707556	45.68319287	55.04942003
Txn2	143.3225722	105.0713436	145.8326741
Txndc11	58.58440909	59.38815073	1.931558598
Txndc12	82.64586282	77.66142787	87.8859162
Txndc15	260.4913904	242.1209222	333.1938581
Txndc16	49.16905763	121.0604611	84.022799
Txndc17	102.5227159	99.36094448	115.8935159
Txndc2	4.184600649	1.142079822	0
Txndc5	83.69201298	95.93470502	91.74903339
Txndc9	131.8149204	156.4649356	97.54370918
Txnip	118.2149683	76.51934805	0
Txnl1	151.6917735	101.6451041	157.4220257
Txnl4a	1.046150162	1.142079822	0
Txnl4b	48.12290746	47.96735251	35.73383406
Txnrd1	87.87661363	55.96191126	37.66539266
Txnrd2	173.6609269	199.8639688	171.9087152
Txnrd3	0	6.85247893	4.828896494
Tyk2	156.9225243	100.5030243	120.7224124
Tymp	0	0	2.897337897
Tyms	39.75370617	22.84159643	41.52850985
Tyms-ps	2.092300324	5.710399108	4.828896494
Tyrobp	2379.991619	3161.276946	3160.995645
Tysnd1	30.33835471	35.40447447	47.32318564
Tyw1	133.9072208	42.2569534	95.61215059
Tyw3	18.83070292	30.83615518	41.52850985
Tyw5	19.87685308	20.55743679	25.11026177
U2af1	16.7384026	31.97823501	44.42584775
U2af2	26.15375406	43.39903322	24.14448247
U2surp	215.5069334	222.7055652	197.018977
Uaca	0	0	0
Uap1	54.39980844	59.38815073	44.42584775
Uap1l1	103.5688661	10.27871839	72.43344741
Uba1	404.8601128	291.2303545	352.5094441
Uba2	211.3223328	181.5906916	333.1938581
Uba3	49.16905763	14.84703768	104.3041643
Uba5	77.41511201	83.37182698	35.73383406
Uba52	108.7996169	84.5139068	0
Uba6	4.184600649	14.84703768	12.55513089
Ubac1	46.03060714	26.2678359	28.97337897
Ubac2	30.33835471	31.97823501	0
Ubald1	47.0767573	41.11487358	56.01519933
Ubald2	5.230750811	20.55743679	3.863117195
Ubap1	225.968435	239.8367625	248.2052798
Ubap1l	0	3.426239465	0
Ubap2	72.18436119	79.94558752	48.28896494
Ubap2l	171.5686266	250.1154809	283.9391139
Ubash3b	536.6750332	489.9522435	449.087374
Ubb	563.8749374	65.09854983	0

For Review Only

Ubc	0	0	0
Ube2a	92.06121428	110.7817427	101.4068264
Ube2b	167.384026	147.328297	154.5246878
Ube2c	0	0	0
Ube2cbp	0	0	0
Ube2d-ps	28.24605438	0	0
Ube2d1	27.19990422	33.12031483	24.14448247
Ube2d2a	114.0303677	0	6.760455092
Ube2d2b	11.50765178	6.85247893	3.863117195
Ube2d3	200.8608312	242.1209222	188.3269633
Ube2e1	83.69201298	108.4975831	81.1254611
Ube2e2	2.092300324	0	5.794675793
Ube2e3	33.47680519	0	45.39162705
Ube2f	135.9995211	141.6178979	143.9011155
Ube2g1	119.2611185	79.94558752	85.9543576
Ube2g2	0	0	0.965779299
Ube2h	516.7981801	455.6898488	491.5816631
Ube2i	202.9531315	190.7273302	225.9923559
Ube2j1	450.8907199	283.2357958	259.7946314
Ube2j2	29.29220454	38.83071394	27.04182037
Ube2k	60.67670941	73.09310859	98.50948848
Ube2l3	133.9072208	137.0495786	153.5589085
Ube2l6	33.47680519	27.40991572	45.39162705
Ube2m	20.92300324	0	0.965779299
Ube2n	0	0	32.83649616
Ube2o	25.10760389	30.83615518	24.14448247
Ube2q1	35.56910552	262.678359	81.1254611
Ube2q2	40.79985633	69.66686912	31.87071686
Ube2r2	69.04591071	45.68319287	59.87831653
Ube2s	6.276900973	5.710399108	0
Ube2v1	118.2149683	85.65598662	129.414426
Ube2v2	23.01530357	31.97823501	45.39162705
Ube2w	92.06121428	93.65054538	84.9885783
Ube2z	56.49210876	63.95647001	84.022799
Ube3a	88.92276379	173.5961329	79.19390251
Ube3b	110.8919172	133.6233391	111.0646194
Ube3c	67.99976055	46.82527269	74.36500601
Ube4a	169.4763263	169.0278136	156.4562464
Ube4b	129.7226201	81.08766734	60.84409583
Ube4bos3	3.138450487	3.426239465	0
Ubfd1	72.18436119	115.350062	101.4068264
Ubiad1	38.707556	60.53023055	61.80987513
Ubl3	203.9992816	179.306532	148.730012
Ubl4a	0	0	0
Ubl5	0	0	1.931558598
Ubl7	62.76900973	92.50846555	53.11786144
Ublcp1	116.122668	83.37182698	94.64637129
Ubn1	130.7687703	153.0386961	118.7908538
Ubn2	225.968435	248.9734011	225.9923559
Ubox5	74.27666152	55.96191126	65.67299232

Ubp1	109.845767	196.4377293	164.1824808
Ubqln1	118.2149683	71.95102876	79.19390251
Ubqln2	9.41535146	22.84159643	10.62357229
Ubqln4	37.66140584	31.97823501	17.38402738
Ubr1	153.7840738	134.765419	103.338385
Ubr2	132.8610706	154.1807759	104.3041643
Ubr3	76.36896184	76.51934805	68.57033022
Ubr4	288.7374448	339.197707	301.3231412
Ubr5	69.04591071	116.4921418	112.996178
Ubr7	80.55356249	90.22430591	62.77565443
Ubtd1	85.7843133	77.66142787	74.36500601
Ubtd2	17.78455276	4.568319287	7.726234391
Ubtf	144.3687224	158.7490952	115.8935159
Ubxn1	0	0	0
Ubxn11	11.50765178	2.284159643	0
Ubxn2a	27.19990422	22.84159643	12.55513089
Ubxn2b	48.12290746	51.39359197	34.76805476
Ubxn4	142.2764221	127.91294	133.2775432
Ubxn6	105.6611664	115.350062	202.8136528
Ubxn7	96.24581493	121.0604611	43.46006845
Ubxn8	74.27666152	60.53023055	78.22812321
Uchl3	0	50.25151215	18.34980668
Uchl4	19.87685308	26.2678359	27.04182037
Uchl5	64.86131006	87.94014627	49.25474424
Uck1	24.06145373	2.284159643	0
Uck2	0	1.142079822	5.794675793
Uckl1	3.138450487	10.27871839	12.55513089
Uckl1os	1.046150162	3.426239465	0
Ucp2	0	0	155.4904671
Uevld	19.87685308	17.13119732	8.69201369
Ufc1	0	0	0.965779299
Ufd1l	0	0	0.965779299
Ufl1	0	156.4649356	22.21292387
Ufm1	31.38450487	59.38815073	23.17870317
Ufsp1	24.06145373	20.55743679	20.28136528
Ufsp2	112.9842175	130.1970997	82.0912404
Ugcg	25.10760389	23.98367625	8.69201369
Ugdh	28.24605438	21.69951661	15.45246878
Uggt1	219.6915341	172.4540531	178.6691703
Uggt2	42.89215665	22.84159643	39.59695125
Ugp2	77.41511201	57.10399108	45.39162705
Ugt1a10	76.36896184	0	0
Ugt1a2	0	0	0
Ugt1a5	3.138450487	0	4.828896494
Ugt1a6a	2.092300324	0	0
Ugt1a6b	13.59995211	0	0
Ugt1a7c	207.1377321	306.0773922	294.5626861
Uhmk1	30.33835471	22.84159643	9.657792988
Uhrf1	3.138450487	6.85247893	0
Uhrf1bp1	256.3067897	107.3555032	121.6881917

For Review Only

Uhrf1bp1l	32.43065503	9.136638573	59.87831653
Uhrf2	31.38450487	39.97279376	51.18630284
Uimc1	69.04591071	131.3391795	77.26234391
Ulbp1	4.184600649	9.136638573	6.760455092
Ulk1	72.18436119	15.9891175	64.70721302
Ulk2	198.7685308	248.9734011	231.7870317
Ulk3	37.66140584	108.4975831	161.2851429
Ulk4	0	0	0.965779299
Umad1	20.92300324	19.41535697	55.04942003
Umodl1	0	0	0
Umps	81.59971265	68.5247893	89.81747479
Unc119b	100.4304156	44.54111304	69.53610952
Unc13a	26.15375406	9.136638573	10.62357229
Unc13b	15.69225243	30.83615518	28.00759967
Unc13d	94.1535146	61.67231037	84.022799
Unc45a	43.93830681	28.55199554	47.32318564
Unc50	102.5227159	280.9516361	132.3117639
Unc5a	0	7.994558752	0.965779299
Unc5cl	1.046150162	4.568319287	0
Unc5d	0	0	0
Unc79	12.55380195	0	0
Unc93b1	5228.658511	4201.711664	5619.86974
Ung	6.276900973	3.426239465	0
Unk	211.3223328	155.3228557	182.5322875
Unkl	119.2611185	36.54655429	18.34980668
Upf1	101.4765657	62.81439019	74.36500601
Upf2	24.06145373	52.5356718	44.42584775
Upf3a	18.83070292	28.55199554	13.52091018
Upf3b	24.06145373	47.96735251	43.46006845
Upk1b	381.8448092	425.9957735	530.2128351
Uprt	6.276900973	5.710399108	4.828896494
Uqcc1	101.4765657	77.66142787	121.6881917
Uqcc2	0	0	0
Uqcc3	0	0	59.87831653
Uqcr10	1.046150162	1.142079822	0
Uqcr11	0	0	0
Uqcrb	32.43065503	4.568319287	129.414426
Uqcrc1	265.7221412	246.6892415	355.406782
Uqcrc2	180.9839781	157.6070154	237.5817075
Uqcrfs1	133.9072208	148.4703768	203.7794321
Uqcrh	125.5380195	92.50846555	146.7984534
Uqcrh-ps1	2.092300324	3.426239465	4.828896494
Uqcrq	103.5688661	70.80894894	110.0988401
Urb1	13.59995211	28.55199554	30.90493756
Urb2	24.06145373	20.55743679	24.14448247
Urgcp	284.5528441	182.7327715	261.72619
Uri1	21.96915341	27.40991572	15.45246878
Urm1	10.46150162	3.426239465	5.794675793
Urod	0	0	107.2015022
Uros	59.63055925	84.5139068	93.68059199

For Review Only

Usb1	19.87685308	35.40447447	53.11786144
Use1	80.55356249	33.12031483	70.50188882
Usf1	0	0	61.80987513
Usf2	0	0	13.52091018
Usf3	88.92276379	100.5030243	114.9277366
Ush2a	0	0	0
Ushbp1	0	0	0
Usmg5	0	0	0
Uso1	75.32281168	76.51934805	51.18630284
Usp1	10.46150162	12.56287804	7.726234391
Usp10	172.6147768	157.6070154	229.8554731
Usp11	65.90746022	37.68863411	74.36500601
Usp12	101.4765657	102.7871839	101.4068264
Usp14	123.4457191	105.0713436	158.387805
Usp15	179.9378279	185.0169311	164.1824808
Usp16	87.87661363	89.08222609	126.5170881
Usp18	2.092300324	9.136638573	0
Usp19	16.7384026	5.710399108	0
Usp2	81.59971265	269.5308379	27.04182037
Usp20	35.56910552	20.55743679	25.11026177
Usp22	493.7828766	387.1650595	378.5854851
Usp24	148.553323	276.3833168	234.6843696
Usp25	48.12290746	82.22974716	40.56273055
Usp27x	5.230750811	3.426239465	3.863117195
Usp28	35.56910552	47.96735251	56.98097863
Usp3	190.3993295	158.7490952	198.9505356
Usp30	24.06145373	50.25151215	34.76805476
Usp31	11.50765178	45.68319287	27.04182037
Usp32	51.26135795	68.5247893	73.39922671
Usp33	239.5683872	195.2956495	167.0798187
Usp34	98.33811525	177.0223724	149.6957913
Usp35	313.8450487	20.55743679	18.34980668
Usp36	7.323051136	61.67231037	44.42584775
Usp37	205.0454318	222.7055652	161.2851429
Usp38	129.7226201	66.24062966	0
Usp39	54.39980844	50.25151215	51.18630284
Usp4	369.2910073	105.0713436	390.1748367
Usp40	107.7534667	106.2134234	81.1254611
Usp42	55.4459586	73.09310859	37.66539266
Usp45	7.323051136	15.9891175	15.45246878
Usp46	47.0767573	14.84703768	17.38402738
Usp47	85.7843133	117.6342216	56.98097863
Usp48	274.0913425	221.5634854	220.1976801
Usp49	21.96915341	3.426239465	15.45246878
Usp5	157.9686745	101.6451041	133.2775432
Usp50	1.046150162	0	0
Usp53	2.092300324	0	0
Usp54	24.06145373	17.13119732	24.14448247
Usp6nl	38.707556	58.2460709	47.32318564
Usp7	95.19966476	100.5030243	126.5170881

For Review Only

Usp8	334.7680519	296.9407536	301.3231412
Usp9x	58.58440909	341.4818667	105.2699436
Uspl1	85.7843133	83.37182698	133.2775432
Ust	14.64610227	9.136638573	14.48668948
Utp11l	41.84600649	51.39359197	36.69961336
Utp14a	92.06121428	65.09854983	82.0912404
Utp14b	0	0	0
Utp15	73.23051136	101.6451041	65.67299232
Utp18	9.41535146	0	9.657792988
Utp20	42.89215665	50.25151215	56.01519933
Utp23	26.15375406	44.54111304	24.14448247
Utp3	89.96891395	161.0332549	159.3535843
Utp6	30.33835471	31.97823501	53.11786144
Utrn	30.33835471	28.55199554	17.38402738
Uty	42.89215665	52.5356718	31.87071686
Uvrag	100.4304156	102.7871839	77.26234391
Uvssa	100.4304156	6.85247893	179.6349496
Uxs1	28.24605438	30.83615518	36.69961336
Uxt	0	0	0
Vac14	38.707556	85.65598662	47.32318564
Vamp1	52.30750811	70.80894894	83.0570197
Vamp3	212.3684829	253.5417204	311.9467135
Vamp4	140.1841217	140.4758181	96.57792988
Vamp5	13.59995211	13.70495786	11.58935159
Vamp7	19.87685308	36.54655429	38.63117195
Vamp8	464.490672	0	73.39922671
Vangl1	5.230750811	14.84703768	12.55513089
Vangl2	23.01530357	26.2678359	26.07604107
Vapa	154.830224	167.8857338	97.54370918
Vapb	26.15375406	33.12031483	20.28136528
Vars	137.0456713	106.2134234	87.8859162
Vars2	0	0	16.41824808
Vash1	35.56910552	68.5247893	72.43344741
Vash2	4.184600649	0	0
Vasn	9.41535146	3.426239465	14.48668948
Vasp	54.39980844	37.68863411	0
Vat1	12.55380195	1.142079822	0
Vat1l	14.64610227	0	0
Vaultrc5	3.138450487	1.142079822	1.931558598
Vav1	1234.457191	962.7732897	1215.916137
Vav2	202.9531315	248.9734011	273.3155416
Vbp1	108.7996169	109.6396629	164.1824808
Vcam1	41.84600649	13.70495786	32.83649616
Vcan	3.138450487	3.426239465	12.55513089
Vcl	43.93830681	27.40991572	34.76805476
Vcp	298.1527962	232.9842836	253.9999556
Vcpip1	139.1379716	164.4594943	122.653971
Vcpkmt	15.69225243	5.710399108	28.00759967
Vdac1	194.5839302	113.0659023	108.1672815
Vdac2	1.046150162	18.27327715	0

Vdac3	93.10736444	106.2134234	113.9619573
Vegfb	16.7384026	5.710399108	0
Vezf1	78.46126217	63.95647001	44.42584775
Vezt	86.83046347	69.66686912	57.94675793
Vgll4	59.63055925	43.39903322	22.21292387
Vhl	63.8151599	178.1644522	50.22052354
Vil1	0	0	0
Vill	10.46150162	6.85247893	0
Vim	29.29220454	12.56287804	22.21292387
Vimp	202.9531315	272.9570774	375.6881473
Vipas39	130.7687703	99.36094448	140.0379983
Vipr1	25.10760389	47.96735251	45.39162705
Vipr2	0	0	0
Vis1	85.7843133	97.07678484	74.36500601
Vkorc1	34.52295535	17.13119732	231.7870317
Vkorc1l1	20.92300324	39.97279376	49.25474424
Vldlr	6.276900973	0	0
Vma21	34.52295535	44.54111304	32.83649616
Vmac	29.29220454	47.96735251	20.28136528
Vmn1r4	3.138450487	0	5.794675793
Vmn2r2	0	1.142079822	4.828896494
Vmn2r29	15.69225243	3.426239465	9.657792988
Vmn2r57	0	0	0
Vmp1	198.7685308	206.7164477	186.3954047
Vopp1	10.46150162	4.568319287	10.62357229
Vprbp	10.46150162	20.55743679	43.46006845
Vps11	194.5839302	0	38.63117195
Vps13a	41.84600649	50.25151215	32.83649616
Vps13b	180.9839781	227.2738845	194.1216391
Vps13c	38.707556	87.94014627	53.11786144
Vps13d	128.67647	191.86941	125.5513089
Vps16	99.38426541	26.2678359	0
Vps18	1.046150162	1.142079822	0
Vps25	138.0918214	150.7545365	124.5855296
Vps26a	274.0913425	201.0060486	249.1710591
Vps26b	143.3225722	110.7817427	128.4486467
Vps28	0	0	0
Vps29	103.5688661	186.1590109	143.9011155
Vps33a	223.8761347	187.3010908	133.2775432
Vps33b	57.53825892	51.39359197	47.32318564
Vps35	432.060017	239.8367625	359.2698992
Vps36	59.63055925	86.79806645	75.33078531
Vps37a	75.32281168	89.08222609	52.15208214
Vps37b	195.6300803	209.0006074	165.1482601
Vps37c	85.7843133	67.38270948	77.26234391
Vps39	283.506694	286.6620352	194.1216391
Vps41	120.3072687	114.2079822	128.4486467
Vps45	15.69225243	59.38815073	46.35740634
Vps4a	510.5212792	68.5247893	249.1710591
Vps4b	277.229793	243.263002	367.9619129

For Review Only

Vps50	108.7996169	60.53023055	75.33078531
Vps51	85.7843133	122.2025409	0
Vps52	221.7838344	137.0495786	16.41824808
Vps53	62.76900973	34.26239465	44.42584775
Vps54	61.72285957	63.95647001	49.25474424
Vps72	0	0	0
Vps8	57.53825892	37.68863411	58.91253723
Vps9d1	0	0	26.07604107
Vrk1	123.4457191	117.6342216	126.5170881
Vrk3	119.2611185	66.24062966	93.68059199
Vsig10	5.230750811	5.710399108	11.58935159
Vsig10l	19.87685308	52.5356718	18.34980668
Vsir	5913.886867	5301.534532	5742.523711
Vstm4	3.138450487	3.426239465	0
Vta1	180.9839781	155.3228557	179.6349496
Vti1a	102.5227159	161.0332549	142.9353362
Vti1b	80.55356249	84.5139068	115.8935159
Vtn	0	0	0
Vwa1	3.138450487	6.85247893	0
Vwa3a	0	4.568319287	6.760455092
Vwa5a	143.3225722	296.9407536	377.6197058
Vwa5b2	0	0	0
Vwa7	0	0	13.52091018
Vwa8	82.64586282	61.67231037	36.69961336
Vwa9	71.13821103	73.09310859	94.64637129
Vwf	0	0	0
Wac	169.4763263	191.86941	147.7642327
Wapl	59.63055925	141.6178979	69.53610952
Wars	124.4918693	153.0386961	153.5589085
Wars2	13.59995211	18.27327715	9.657792988
Was	73.23051136	59.38815073	17.38402738
Wasf2	582.7056404	708.0894894	589.1253723
Wasf3	8.369201298	0	0
Wash1	7.323051136	4.568319287	0
Wasl	73.23051136	100.5030243	48.28896494
Wbp1	0	0	4.828896494
Wbp11	35.56910552	44.54111304	31.87071686
Wbp1l	302.3373969	0	116.8592952
Wbp2	359.8756558	247.8313213	269.4524244
Wbp4	41.84600649	58.2460709	56.98097863
Wbp5	27.19990422	14.84703768	44.42584775
Wbscr16	1.046150162	4.568319287	0
Wbscr22	114.0303677	14.84703768	14.48668948
Wbscr27	7.323051136	3.426239465	3.863117195
Wdfy1	108.7996169	114.2079822	108.1672815
Wdfy2	224.9222849	204.4322881	176.7376117
Wdfy3	302.3373969	338.0556272	239.5132661
Wdfy4	268.8605917	274.0991572	252.068397
Wdhd1	1.046150162	0	0
Wdpcp	60.67670941	6.85247893	0

For Review Only

Wdr1	920.6121428	673.8270948	815.1177282
Wdr11	105.6611664	91.36638573	101.4068264
Wdr12	28.24605438	42.2569534	39.59695125
Wdr13	139.1379716	150.7545365	106.2357229
Wdr18	82.64586282	82.22974716	128.4486467
Wdr19	20.92300324	12.56287804	6.760455092
Wdr20	58.58440909	78.80350769	66.63877162
Wdr20rt	2.092300324	5.710399108	5.794675793
Wdr24	52.30750811	37.68863411	72.43344741
Wdr25	47.0767573	21.69951661	15.45246878
Wdr26	170.5224764	220.4214056	157.4220257
Wdr3	65.90746022	79.94558752	53.11786144
Wdr31	0	0	0
Wdr33	109.845767	95.93470502	114.9277366
Wdr34	13.59995211	11.42079822	7.726234391
Wdr35	37.66140584	18.27327715	6.760455092
Wdr36	111.9380674	82.22974716	94.64637129
Wdr37	110.8919172	58.2460709	69.53610952
Wdr4	98.33811525	73.09310859	73.39922671
Wdr41	127.6303198	117.6342216	134.2433225
Wdr43	101.4765657	61.67231037	101.4068264
Wdr44	50.21520779	157.6070154	55.04942003
Wdr45	55.4459586	0	72.43344741
Wdr45b	64.86131006	172.4540531	174.8060531
Wdr46	44.98445698	1.142079822	65.67299232
Wdr47	46.03060714	25.12575608	38.63117195
Wdr48	83.69201298	92.50846555	125.5513089
Wdr5	144.3687224	107.3555032	103.338385
Wdr53	30.33835471	31.97823501	48.28896494
Wdr55	3.138450487	31.97823501	44.42584775
Wdr59	31.38450487	22.84159643	36.69961336
Wdr5b	53.35365827	49.10943233	35.73383406
Wdr6	95.19966476	81.08766734	0
Wdr60	3.138450487	28.55199554	24.14448247
Wdr61	33.47680519	45.68319287	84.022799
Wdr62	24.06145373	26.2678359	3.863117195
Wdr7	80.55356249	218.1372459	123.6197503
Wdr70	23.01530357	18.27327715	48.28896494
Wdr73	43.93830681	23.98367625	36.69961336
Wdr74	0	0	35.73383406
Wdr75	43.93830681	86.79806645	85.9543576
Wdr76	36.61525568	17.13119732	0
Wdr77	98.33811525	83.37182698	86.9201369
Wdr78	5.230750811	0	0
Wdr81	489.5982759	307.219472	389.2090574
Wdr82	172.6147768	180.4486118	109.1330608
Wdr83	0	0	48.28896494
Wdr83os	7.323051136	5.710399108	0
Wdr86	38.707556	5.710399108	0
Wdr89	0	0	0

For Review Only

Wdr90	0	7.994558752	0
Wdr91	48.12290746	31.97823501	0
Wdr92	36.61525568	22.84159643	24.14448247
Wdsub1	40.79985633	36.54655429	51.18630284
Wdtc1	17.78455276	9.136638573	21.24714457
Wdyhv1	67.99976055	70.80894894	37.66539266
Wee1	0	0	1.931558598
Wfdc1	0	0	0
Wfdc21	35.56910552	0	0
Wfikkn1	21.96915341	15.9891175	10.62357229
Wfs1	70.09206087	28.55199554	48.28896494
Whrn	1.046150162	37.68863411	13.52091018
Whsc1	38.707556	66.24062966	47.32318564
Whsc1l1	105.6611664	232.9842836	136.1748811
Wipf1	86.83046347	101.6451041	71.46766811
Wipf2	91.01506412	94.7926252	80.1596818
Wipi1	23.01530357	19.41535697	8.69201369
Wipi2	127.6303198	83.37182698	76.29656461
Wiz	54.39980844	62.81439019	33.80227546
Wls	444.613819	424.8536937	426.8744501
Wnk1	664.305353	832.57619	590.0911516
Wnk3	3.138450487	1.142079822	4.828896494
Wnk4	19.87685308	10.27871839	7.726234391
Wnt2b	4.184600649	2.284159643	0
Wnt4	5.230750811	1.142079822	0
Wnt5a	1.046150162	0	0
Wrap53	1.046150162	1.142079822	0
Wrap73	1.046150162	103.9292638	84.022799
Wrb	73.23051136	161.0332549	93.68059199
Wrn	33.47680519	10.27871839	17.38402738
Wrnip1	20.92300324	33.12031483	7.726234391
Wsb1	458.2137711	692.1003719	807.3914938
Wsb2	20.92300324	0	0
Wscd1	83.69201298	44.54111304	71.46766811
Wscd2	0	0	0
Wtap	172.6147768	228.4159643	152.5931292
Wtip	5.230750811	11.42079822	11.58935159
Wwc2	18.83070292	17.13119732	31.87071686
Wwox	28.24605438	37.68863411	26.07604107
Wwp1	31.38450487	38.83071394	27.04182037
Wwp2	63.8151599	2.284159643	0
Xab2	32.43065503	26.2678359	0
Xaf1	28.24605438	0	0
Xbp1	0	0	291.6653483
Xcr1	0	0	4.828896494
Xdh	7.323051136	0	0
Xiap	217.5992337	230.700124	166.1140394
Xirp1	6.276900973	1.142079822	0.965779299
Xirp2	0	1.142079822	0
Xist	0	0	0

For Review Only

Xk	7.323051136	4.568319287	0.965779299
Xkr4	0	2.284159643	0.965779299
Xkr6	2.092300324	0	0
Xkr7	0	0	0
Xkr8	60.67670941	26.2678359	25.11026177
Xkrx	1.046150162	0	0
Xlr	52.30750811	70.80894894	66.63877162
Xlr3a	9.41535146	1.142079822	0
Xlr3b	6.276900973	1.142079822	0.965779299
Xlr3c	7.323051136	1.142079822	0
Xlr4a	8.369201298	0	0
Xlr4b	11.50765178	5.710399108	2.897337897
Xlr4c	5.230750811	0	0
Xndc1	19.87685308	21.69951661	13.52091018
Xntrpc	0	0	18.34980668
Xpa	20.92300324	20.55743679	19.31558598
Xpc	48.12290746	29.69407536	66.63877162
Xpnpep1	116.122668	111.9238225	149.6957913
Xpnpep3	0	0	8.69201369
Xpo1	76.36896184	51.39359197	54.08364074
Xpo4	39.75370617	21.69951661	32.83649616
Xpo5	103.5688661	93.65054538	88.85169549
Xpo6	100.4304156	77.66142787	80.1596818
Xpo7	97.29196509	44.54111304	54.08364074
Xpot	119.2611185	79.94558752	111.0646194
Xpr1	75.32281168	113.0659023	70.50188882
Xrcc1	12.55380195	101.6451041	0.965779299
Xrcc2	23.01530357	10.27871839	5.794675793
Xrcc4	19.87685308	15.9891175	23.17870317
Xrcc5	67.99976055	35.40447447	0
Xrcc6	47.0767573	47.96735251	60.84409583
Xrcc6bp1	17.78455276	5.710399108	8.69201369
Xrn1	102.5227159	95.93470502	74.36500601
Xrn2	203.9992816	47.96735251	66.63877162
Xrra1	0	0	0
Xxylt1	19.87685308	26.2678359	20.28136528
Xylb	53.35365827	41.11487358	46.35740634
Xylt2	9.41535146	1.142079822	0
Yae1d1	71.13821103	109.6396629	74.36500601
Yaf2	50.21520779	53.67775162	46.35740634
Yap1	0	0	0
Yars	118.2149683	122.2025409	102.3726057
Yars2	15.69225243	20.55743679	9.657792988
Ybey	2.092300324	11.42079822	17.38402738
Ybx1	39.75370617	62.81439019	30.90493756
Ybx3	0	4.568319287	0
Ydjc	3.138450487	0	0
Yeats2	127.6303198	148.4703768	147.7642327
Yeats4	125.5380195	105.0713436	71.46766811
Yes1	28.24605438	26.2678359	11.58935159

For Review Only

Yif1a	102.5227159	0	104.3041643
Yif1b	270.952892	53.67775162	207.6425493
Yipf1	147.5071729	109.6396629	158.387805
Yipf2	0	1.142079822	0
Yipf3	197.7223807	278.6674765	261.72619
Yipf4	77.41511201	76.51934805	70.50188882
Yipf5	129.7226201	163.3174145	149.6957913
Yipf6	74.27666152	87.94014627	82.0912404
Ykt6	111.9380674	183.8748513	159.3535843
Ylpm1	93.10736444	85.65598662	79.19390251
Yme1l1	141.2302719	173.5961329	155.4904671
Yod1	30.33835471	17.13119732	7.726234391
Ypel1	6.276900973	5.710399108	8.69201369
Ypel2	57.53825892	87.94014627	50.22052354
Ypel5	218.6453839	276.3833168	203.7794321
Yrdc	10.46150162	1.142079822	0
Ythdc1	89.96891395	83.37182698	56.01519933
Ythdc2	43.93830681	20.55743679	22.21292387
Ythdf1	40.79985633	53.67775162	38.63117195
Ythdf2	388.1217102	680.6795737	365.064575
Ythdf3	128.67647	174.7382127	112.996178
Ywhab	0	0	0
Ywhae	475.9983238	544.7720749	559.186214
Ywhag	1.046150162	1.142079822	0
Ywhah	676.859155	682.9637334	793.8705837
Ywhaz	493.7828766	419.1432945	629.6881028
Yy2	25.10760389	20.55743679	19.31558598
Zadh2	38.707556	17.13119732	54.08364074
Zak	24.06145373	1.142079822	0.965779299
Zbed3	65.90746022	10.27871839	0
Zbed4	15.69225243	14.84703768	21.24714457
Zbed5	75.32281168	31.97823501	63.74143372
Zbed6	79.50741233	216.9951661	61.80987513
Zbp1	0	0	1.931558598
Zbtb1	25.10760389	110.7817427	93.68059199
Zbtb10	30.33835471	3.426239465	0
Zbtb11	126.5841696	103.9292638	38.63117195
Zbtb11os1	0	0	0.965779299
Zbtb14	40.79985633	113.0659023	31.87071686
Zbtb17	103.5688661	0	38.63117195
Zbtb18	39.75370617	74.23518841	56.98097863
Zbtb2	55.4459586	30.83615518	50.22052354
Zbtb20	354.644905	394.0175385	486.7527666
Zbtb21	100.4304156	98.21886466	91.74903339
Zbtb22	0	0	32.83649616
Zbtb24	118.2149683	36.54655429	39.59695125
Zbtb25	38.707556	22.84159643	41.52850985
Zbtb26	31.38450487	46.82527269	28.00759967
Zbtb3	9.41535146	4.568319287	14.48668948
Zbtb32	1.046150162	2.284159643	0

For Review Only

Zbtb33	19.87685308	9.136638573	17.38402738
Zbtb34	34.52295535	50.25151215	66.63877162
Zbtb37	16.7384026	39.97279376	43.46006845
Zbtb38	95.19966476	114.2079822	56.98097863
Zbtb39	36.61525568	25.12575608	14.48668948
Zbtb4	28.24605438	1.142079822	0
Zbtb40	55.4459586	53.67775162	61.80987513
Zbtb41	11.50765178	15.9891175	20.28136528
Zbtb42	6.276900973	0	0
Zbtb43	82.64586282	76.51934805	57.94675793
Zbtb44	40.79985633	38.83071394	0.965779299
Zbtb45	10.46150162	22.84159643	22.21292387
Zbtb46	5.230750811	29.69407536	28.00759967
Zbtb48	38.707556	44.54111304	22.21292387
Zbtb49	58.58440909	53.67775162	64.70721302
Zbtb5	52.30750811	31.97823501	47.32318564
Zbtb6	28.24605438	34.26239465	28.00759967
Zbtb7a	156.9225243	155.3228557	80.1596818
Zbtb7b	16.7384026	49.10943233	29.93915826
Zbtb8a	4.184600649	7.994558752	10.62357229
Zbtb8b	0	0	0
Zbtb8os	10.46150162	18.27327715	23.17870317
Zbtb9	60.67670941	77.66142787	90.78325409
Zc2hc1a	2.092300324	2.284159643	0
Zc2hc1c	3.138450487	10.27871839	8.69201369
Zc3h10	64.86131006	85.65598662	83.0570197
Zc3h11a	185.1685787	292.3724343	279.1102174
Zc3h12a	9.41535146	15.9891175	0
Zc3h12b	17.78455276	13.70495786	24.14448247
Zc3h12c	57.53825892	28.55199554	16.41824808
Zc3h13	48.12290746	63.95647001	78.22812321
Zc3h14	34.52295535	83.37182698	67.60455092
Zc3h15	70.09206087	71.95102876	98.50948848
Zc3h18	65.90746022	29.69407536	56.98097863
Zc3h3	49.16905763	69.66686912	39.59695125
Zc3h4	196.6762305	181.5906916	131.3459846
Zc3h6	24.06145373	5.710399108	10.62357229
Zc3h7a	72.18436119	180.4486118	89.81747479
Zc3h7b	51.26135795	59.38815073	44.42584775
Zc3h8	39.75370617	36.54655429	34.76805476
Zc3hav1	232.245336	282.093716	263.6577486
Zc3hav1l	1.046150162	6.85247893	0
Zc3hc1	74.27666152	41.11487358	56.98097863
Zc4h2	8.369201298	4.568319287	10.62357229
Zcchc10	5.230750811	15.9891175	8.69201369
Zcchc11	43.93830681	71.95102876	40.56273055
Zcchc14	71.13821103	55.96191126	52.15208214
Zcchc17	51.26135795	43.39903322	52.15208214
Zcchc18	1.046150162	0	0
Zcchc2	71.13821103	52.5356718	84.022799

Zcchc24	50.21520779	100.5030243	46.35740634
Zcchc3	16.7384026	5.710399108	1.931558598
Zcchc4	14.64610227	21.69951661	32.83649616
Zcchc6	199.814681	142.7599777	164.1824808
Zcchc7	0	0	0
Zcchc8	80.55356249	95.93470502	114.9277366
Zcchc9	0	0	0
Zcrb1	0	6.85247893	0
Zcwpw1	62.76900973	39.97279376	0
Zdbf2	2.092300324	0	0
Zdhhc1	28.24605438	9.136638573	24.14448247
Zdhhc12	9.41535146	0	0
Zdhhc13	74.27666152	103.9292638	115.8935159
Zdhhc14	65.90746022	49.10943233	46.35740634
Zdhhc16	6.276900973	0	7.726234391
Zdhhc17	48.12290746	34.26239465	46.35740634
Zdhhc18	27.19990422	11.42079822	25.11026177
Zdhhc20	260.4913904	292.3724343	329.3307409
Zdhhc21	24.06145373	29.69407536	26.07604107
Zdhhc22	2.092300324	0	4.828896494
Zdhhc23	5.230750811	0	0
Zdhhc24	98.33811525	107.3555032	101.4068264
Zdhhc3	95.19966476	82.22974716	83.0570197
Zdhhc4	55.4459586	127.91294	35.73383406
Zdhhc5	28.24605438	37.68863411	22.21292387
Zdhhc6	96.24581493	1.142079822	0
Zdhhc7	98.33811525	60.53023055	66.63877162
Zdhhc8	28.24605438	35.40447447	33.80227546
Zdhhc9	103.5688661	138.1916584	120.7224124
Zeb1	71.13821103	31.97823501	59.87831653
Zeb2	451.9368701	630.4280616	453.9162705
Zeb2os	0	2.284159643	0
Zer1	20.92300324	20.55743679	28.00759967
Zf12	4.184600649	9.136638573	4.828896494
Zfa-ps	24.06145373	28.55199554	33.80227546
Zfand1	9.41535146	18.27327715	24.14448247
Zfand2a	54.39980844	49.10943233	67.60455092
Zfand2b	2.092300324	0	0
Zfand3	53.35365827	53.67775162	42.49428915
Zfand4	0	0	0
Zfand5	1.046150162	1.142079822	0
Zfand6	159.0148247	166.743654	147.7642327
Zfas1	27.19990422	22.84159643	50.22052354
Zfat	28.24605438	29.69407536	16.41824808
Zfc3h1	102.5227159	210.1426872	114.9277366
Zfhx2	38.707556	23.98367625	20.28136528
Zfhx3	761.5973181	921.6584161	725.3002534
Zfp1	59.63055925	46.82527269	45.39162705
Zfp101	3.138450487	9.136638573	23.17870317
Zfp105	26.15375406	43.39903322	34.76805476

For Review Only

Zfp106	131.8149204	238.6946827	184.4638461
Zfp108	6.276900973	1.142079822	12.55513089
Zfp109	24.06145373	3.426239465	19.31558598
Zfp11	8.369201298	19.41535697	26.07604107
Zfp110	382.8909594	18.27327715	198.9505356
Zfp111	64.86131006	52.5356718	37.66539266
Zfp112	31.38450487	3.426239465	17.38402738
Zfp113	70.09206087	83.37182698	39.59695125
Zfp114	3.138450487	2.284159643	0.965779299
Zfp119a	65.90746022	36.54655429	9.657792988
Zfp119b	54.39980844	77.66142787	73.39922671
Zfp12	32.43065503	51.39359197	33.80227546
Zfp120	4.184600649	177.0223724	320.6387272
Zfp128	15.69225243	39.97279376	26.07604107
Zfp13	0	1.142079822	0
Zfp131	38.707556	35.40447447	33.80227546
Zfp133-ps	1.046150162	0	0
Zfp14	41.84600649	15.9891175	62.77565443
Zfp141	82.64586282	31.97823501	33.80227546
Zfp142	65.90746022	82.22974716	60.84409583
Zfp143	98.33811525	169.0278136	129.414426
Zfp146	27.19990422	55.96191126	17.38402738
Zfp148	146.4610227	174.7382127	123.6197503
Zfp157	138.0918214	135.9074988	169.9771566
Zfp160	40.79985633	74.23518841	74.36500601
Zfp169	78.46126217	100.5030243	94.64637129
Zfp174	23.01530357	20.55743679	19.31558598
Zfp180	87.87661363	9.136638573	106.2357229
Zfp182	26.15375406	59.38815073	21.24714457
Zfp184	9.41535146	3.426239465	33.80227546
Zfp185	5.230750811	0	0
Zfp189	28.24605438	20.55743679	22.21292387
Zfp2	31.38450487	34.26239465	30.90493756
Zfp202	33.47680519	18.27327715	51.18630284
Zfp207	423.6908157	581.3186292	457.7793877
Zfp212	66.95361038	53.67775162	0
Zfp213	59.63055925	49.10943233	65.67299232
Zfp217	121.3534188	255.8258801	109.1330608
Zfp219	91.01506412	105.0713436	84.9885783
Zfp229	15.69225243	12.56287804	34.76805476
Zfp235	34.52295535	59.38815073	59.87831653
Zfp236	79.50741233	140.4758181	97.54370918
Zfp239	3.138450487	1.142079822	0
Zfp24	127.6303198	111.9238225	166.1140394
Zfp248	1.046150162	9.136638573	0
Zfp251	40.79985633	7.994558752	30.90493756
Zfp26	123.4457191	81.08766734	73.39922671
Zfp260	152.7379237	173.5961329	173.8402738
Zfp266	198.7685308	197.5798091	143.9011155
Zfp27	23.01530357	27.40991572	25.11026177

For Review Only

Zfp273	27.19990422	19.41535697	23.17870317
Zfp275	60.67670941	89.08222609	64.70721302
Zfp276	34.52295535	17.13119732	35.73383406
Zfp277	107.7534667	41.11487358	90.78325409
Zfp28	41.84600649	55.96191126	42.49428915
Zfp280b	12.55380195	34.26239465	0
Zfp280c	30.33835471	38.83071394	22.21292387
Zfp280d	44.98445698	121.0604611	34.76805476
Zfp281	38.707556	76.51934805	37.66539266
Zfp282	1.046150162	201.0060486	0
Zfp286	10.46150162	7.994558752	30.90493756
Zfp286os	1.046150162	0	0
Zfp287	13.59995211	3.426239465	8.69201369
Zfp292	160.0609748	138.1916584	138.1064397
Zfp296	1.046150162	0	12.55513089
Zfp3	37.66140584	46.82527269	12.55513089
Zfp30	25.10760389	46.82527269	24.14448247
Zfp316	32.43065503	43.39903322	41.52850985
Zfp317	93.10736444	57.10399108	74.36500601
Zfp318	40.79985633	68.5247893	57.94675793
Zfp319	137.0456713	92.50846555	47.32318564
Zfp322a	6.276900973	7.994558752	56.98097863
Zfp324	50.21520779	77.66142787	31.87071686
Zfp326	53.35365827	33.12031483	30.90493756
Zfp329	64.86131006	84.5139068	51.18630284
Zfp330	2.092300324	22.84159643	0
Zfp334	7.323051136	3.426239465	2.897337897
Zfp335	84.73816314	150.7545365	92.71481269
Zfp335os	17.78455276	39.97279376	24.14448247
Zfp33b	56.49210876	53.67775162	31.87071686
Zfp341	2.092300324	6.85247893	14.48668948
Zfp345	0	0	0
Zfp346	55.4459586	37.68863411	36.69961336
Zfp35	89.96891395	98.21886466	146.7984534
Zfp354a	20.92300324	26.2678359	20.28136528
Zfp354b	3.138450487	7.994558752	0
Zfp354c	18.83070292	12.56287804	4.828896494
Zfp358	29.29220454	15.9891175	27.04182037
Zfp36	380.7986591	190.7273302	264.6235279
Zfp362	20.92300324	20.55743679	20.28136528
Zfp367	24.06145373	27.40991572	13.52091018
Zfp369	31.38450487	17.13119732	15.45246878
Zfp36l1	0	584.7448687	0.965779299
Zfp36l2	20.92300324	212.4268468	0
Zfp36l3	3.138450487	6.85247893	0.965779299
Zfp37	38.707556	17.13119732	13.52091018
Zfp382	17.78455276	4.568319287	15.45246878
Zfp383	6.276900973	0	26.07604107
Zfp384	12.55380195	18.27327715	0
Zfp385a	8.369201298	0	5.794675793

For Review Only

Zfp385b	0	0	0
Zfp385c	47.0767573	81.08766734	73.39922671
Zfp386	67.99976055	75.37726823	69.53610952
Zfp389	2.092300324	2.284159643	0
Zfp39	62.76900973	10.27871839	38.63117195
Zfp395	5.230750811	19.41535697	14.48668948
Zfp397	56.49210876	74.23518841	32.83649616
Zfp398	114.0303677	116.4921418	109.1330608
Zfp40	85.7843133	68.5247893	71.46766811
Zfp407	65.90746022	30.83615518	42.49428915
Zfp408	32.43065503	65.09854983	56.98097863
Zfp41	51.26135795	21.69951661	14.48668948
Zfp410	99.38426541	111.9238225	86.9201369
Zfp418	11.50765178	23.98367625	27.04182037
Zfp420	31.38450487	43.39903322	21.24714457
Zfp422	125.5380195	113.0659023	111.0646194
Zfp423	0	0	0
Zfp426	101.4765657	97.07678484	114.9277366
Zfp428	3.138450487	3.426239465	3.863117195
Zfp429	44.98445698	35.40447447	46.35740634
Zfp433	43.93830681	45.68319287	31.87071686
Zfp438	7.323051136	3.426239465	21.24714457
Zfp442	61.72285957	44.54111304	30.90493756
Zfp444	67.99976055	70.80894894	58.91253723
Zfp445	196.6762305	183.8748513	192.1900805
Zfp446	35.56910552	10.27871839	9.657792988
Zfp449	18.83070292	26.2678359	56.01519933
Zfp451	86.83046347	70.80894894	44.42584775
Zfp454	5.230750811	2.284159643	9.657792988
Zfp455	8.369201298	55.96191126	40.56273055
Zfp456	15.69225243	27.40991572	18.34980668
Zfp458	20.92300324	2.284159643	5.794675793
Zfp459	3.138450487	1.142079822	0
Zfp46	48.12290746	35.40447447	59.87831653
Zfp462	0	0	0
Zfp467	82.64586282	81.08766734	105.2699436
Zfp472	39.75370617	42.2569534	54.08364074
Zfp473	0	0	0
Zfp493	13.59995211	13.70495786	11.58935159
Zfp507	1.046150162	0	0
Zfp51	26.15375406	60.53023055	53.11786144
Zfp511	0	1.142079822	0
Zfp512	176.7993774	135.9074988	117.8250745
Zfp512b	66.95361038	89.08222609	93.68059199
Zfp513	0	0	11.58935159
Zfp516	77.41511201	52.5356718	41.52850985
Zfp518a	58.58440909	77.66142787	59.87831653
Zfp518b	3.138450487	7.994558752	7.726234391
Zfp52	84.73816314	45.68319287	49.25474424
Zfp521	1.046150162	0	0

For Review Only

Zfp523	15.69225243	63.95647001	25.11026177
Zfp524	49.16905763	60.53023055	50.22052354
Zfp526	17.78455276	10.27871839	9.657792988
Zfp53	31.38450487	60.53023055	34.76805476
Zfp532	262.5836907	215.8530863	200.8820942
Zfp534	3.138450487	1.142079822	0.965779299
Zfp536	0	5.710399108	0
Zfp54	11.50765178	20.55743679	8.69201369
Zfp541	0	0	0
Zfp551	15.69225243	6.85247893	0.965779299
Zfp553	67.99976055	46.82527269	77.26234391
Zfp558	6.276900973	31.97823501	26.07604107
Zfp560	30.33835471	7.994558752	24.14448247
Zfp563	39.75370617	27.40991572	44.42584775
Zfp566	21.96915341	22.84159643	53.11786144
Zfp568	33.47680519	19.41535697	8.69201369
Zfp57	11.50765178	0	14.48668948
Zfp574	132.8610706	41.11487358	75.33078531
Zfp579	58.58440909	49.10943233	0
Zfp58	50.21520779	44.54111304	52.15208214
Zfp580	13.59995211	0	7.726234391
Zfp583	13.59995211	26.2678359	19.31558598
Zfp59	26.15375406	27.40991572	56.01519933
Zfp592	309.660448	190.7273302	183.4980668
Zfp593	12.55380195	7.994558752	23.17870317
Zfp595	11.50765178	25.12575608	16.41824808
Zfp597	41.84600649	21.69951661	24.14448247
Zfp598	24.06145373	36.54655429	49.25474424
Zfp599	10.46150162	1.142079822	0
Zfp60	17.78455276	61.67231037	35.73383406
Zfp600	1.046150162	0	0.965779299
Zfp605	19.87685308	9.136638573	6.760455092
Zfp606	114.0303677	44.54111304	47.32318564
Zfp607	19.87685308	11.42079822	23.17870317
Zfp608	67.99976055	38.83071394	47.32318564
Zfp609	28.24605438	41.11487358	23.17870317
Zfp61	39.75370617	27.40991572	47.32318564
Zfp612	11.50765178	0	11.58935159
Zfp616	0	0	0
Zfp617	23.01530357	42.2569534	56.01519933
Zfp618	0	0	1.931558598
Zfp619	20.92300324	37.68863411	41.52850985
Zfp62	203.9992816	146.1862172	139.072219
Zfp622	8.369201298	15.9891175	14.48668948
Zfp623	51.26135795	67.38270948	70.50188882
Zfp626	15.69225243	11.42079822	8.69201369
Zfp628	19.87685308	12.56287804	2.897337897
Zfp629	54.39980844	77.66142787	50.22052354
Zfp637	94.1535146	1.142079822	0
Zfp638	81.59971265	123.3446207	138.1064397

For Review Only

Zfp639	14.64610227	50.25151215	0
Zfp64	60.67670941	69.66686912	66.63877162
Zfp641	17.78455276	15.9891175	5.794675793
Zfp644	39.75370617	83.37182698	36.69961336
Zfp646	103.5688661	108.4975831	148.730012
Zfp647	33.47680519	1.142079822	13.52091018
Zfp648	0	19.41535697	0
Zfp65	28.24605438	30.83615518	55.04942003
Zfp651	7.323051136	29.69407536	11.58935159
Zfp652	99.38426541	326.634829	0
Zfp652os	10.46150162	7.994558752	10.62357229
Zfp653	0	2.284159643	0
Zfp654	18.83070292	30.83615518	21.24714457
Zfp655	79.50741233	75.37726823	56.98097863
Zfp658	313.8450487	213.5689267	318.7071686
Zfp661	17.78455276	34.26239465	19.31558598
Zfp664	55.4459586	65.09854983	37.66539266
Zfp667	7.323051136	2.284159643	23.17870317
Zfp668	78.46126217	67.38270948	38.63117195
Zfp672	101.4765657	126.7708602	160.3193636
Zfp677	47.0767573	28.55199554	31.87071686
Zfp68	0	0	0
Zfp687	96.24581493	29.69407536	0
Zfp688	0	0	6.760455092
Zfp689	29.29220454	18.27327715	26.07604107
Zfp69	6.276900973	57.10399108	0
Zfp691	1.046150162	12.56287804	0
Zfp697	34.52295535	30.83615518	28.00759967
Zfp7	5.230750811	9.136638573	6.760455092
Zfp703	47.0767573	54.81983144	0
Zfp704	39.75370617	41.11487358	36.69961336
Zfp706	576.4287394	501.3730417	558.2204347
Zfp707	8.369201298	17.13119732	28.00759967
Zfp708	17.78455276	3.426239465	7.726234391
Zfp709	60.67670941	49.10943233	74.36500601
Zfp710	374.5217581	446.5532103	381.482823
Zfp712	10.46150162	15.9891175	13.52091018
Zfp715	161.107125	253.5417204	207.6425493
Zfp719	122.399569	95.93470502	88.85169549
Zfp72	34.52295535	9.136638573	0
Zfp729a	43.93830681	57.10399108	47.32318564
Zfp729b	54.39980844	87.94014627	70.50188882
Zfp735	0	0	0
Zfp738	79.50741233	58.2460709	39.59695125
Zfp74	75.32281168	63.95647001	35.73383406
Zfp740	293.9681956	228.4159643	310.0151549
Zfp746	70.09206087	63.95647001	55.04942003
Zfp747	10.46150162	38.83071394	53.11786144
Zfp748	64.86131006	73.09310859	49.25474424
Zfp750	1.046150162	0	4.828896494

For Review Only

Zfp758	26.15375406	33.12031483	18.34980668
Zfp759	30.33835471	22.84159643	12.55513089
Zfp760	32.43065503	39.97279376	41.52850985
Zfp763	23.01530357	45.68319287	30.90493756
Zfp764	6.276900973	57.10399108	0
Zfp768	64.86131006	30.83615518	34.76805476
Zfp770	10.46150162	26.2678359	25.11026177
Zfp771	20.92300324	42.2569534	50.22052354
Zfp772	25.10760389	35.40447447	51.18630284
Zfp773	27.19990422	26.2678359	32.83649616
Zfp775	37.66140584	41.11487358	37.66539266
Zfp777	25.10760389	5.710399108	0
Zfp78	12.55380195	10.27871839	16.41824808
Zfp780b	84.73816314	90.22430591	65.67299232
Zfp781	36.61525568	26.2678359	29.93915826
Zfp783	25.10760389	11.42079822	28.00759967
Zfp784	7.323051136	19.41535697	44.42584775
Zfp786	0	0	0
Zfp787	30.33835471	23.98367625	5.794675793
Zfp788	59.63055925	33.12031483	49.25474424
Zfp790	56.49210876	52.5356718	61.80987513
Zfp791	46.03060714	6.85247893	11.58935159
Zfp799	39.75370617	21.69951661	42.49428915
Zfp800	41.84600649	79.94558752	28.00759967
Zfp808	13.59995211	30.83615518	20.28136528
Zfp809	49.16905763	115.350062	55.04942003
Zfp81	30.33835471	26.2678359	20.28136528
Zfp810	164.2455755	175.8802925	166.1140394
Zfp811	2.092300324	7.994558752	0
Zfp819	0	0	0
Zfp82	27.19990422	25.12575608	34.76805476
Zfp820	0	1.142079822	0
Zfp821	9.41535146	19.41535697	12.55513089
Zfp825	49.16905763	50.25151215	30.90493756
Zfp827	41.84600649	33.12031483	50.22052354
Zfp830	61.72285957	69.66686912	89.81747479
Zfp831	1.046150162	0	0
Zfp839	94.1535146	129.0550198	136.1748811
Zfp84	137.0456713	45.68319287	53.11786144
Zfp846	43.93830681	70.80894894	58.91253723
Zfp85	14.64610227	12.56287804	47.32318564
Zfp850	14.64610227	7.994558752	8.69201369
Zfp85os	2.092300324	3.426239465	2.897337897
Zfp862-ps	135.9995211	98.21886466	146.7984534
Zfp865	6.276900973	1.142079822	0
Zfp866	13.59995211	78.80350769	28.97337897
Zfp867	7.323051136	37.68863411	35.73383406
Zfp868	67.99976055	101.6451041	85.9543576
Zfp869	0	43.39903322	1.931558598
Zfp87	4.184600649	11.42079822	0

For Review Only

Zfp870	49.16905763	55.96191126	44.42584775
Zfp871	198.7685308	310.6457115	155.4904671
Zfp873	47.0767573	36.54655429	48.28896494
Zfp874a	34.52295535	7.994558752	56.98097863
Zfp874b	20.92300324	15.9891175	28.00759967
Zfp879	0	2.284159643	3.863117195
Zfp882	12.55380195	18.27327715	21.24714457
Zfp9	3.138450487	0	0
Zfp90	346.2757037	0	196.0531977
Zfp91	61.72285957	54.81983144	58.91253723
Zfp93	16.7384026	10.27871839	24.14448247
Zfp930	37.66140584	55.96191126	15.45246878
Zfp931	5.230750811	20.55743679	6.760455092
Zfp932	17.78455276	22.84159643	28.97337897
Zfp933	5.230750811	22.84159643	16.41824808
Zfp934	19.87685308	18.27327715	35.73383406
Zfp935	61.72285957	45.68319287	73.39922671
Zfp936	1.046150162	0	0.965779299
Zfp937	51.26135795	21.69951661	15.45246878
Zfp939	9.41535146	13.70495786	21.24714457
Zfp94	16.7384026	0	0
Zfp940	15.69225243	1.142079822	0
Zfp942	63.8151599	50.25151215	45.39162705
Zfp943	39.75370617	53.67775162	77.26234391
Zfp944	53.35365827	52.5356718	36.69961336
Zfp945	27.19990422	36.54655429	40.56273055
Zfp946	15.69225243	25.12575608	9.657792988
Zfp947	6.276900973	1.142079822	2.897337897
Zfp948	29.29220454	15.9891175	42.49428915
Zfp949	16.7384026	21.69951661	24.14448247
Zfp950	105.6611664	210.1426872	160.3193636
Zfp951	71.13821103	83.37182698	64.70721302
Zfp952	78.46126217	68.5247893	141.0037776
Zfp953	7.323051136	9.136638573	9.657792988
Zfp954	29.29220454	33.12031483	52.15208214
Zfp955a	15.69225243	39.97279376	41.52850985
Zfp955b	37.66140584	15.9891175	42.49428915
Zfp958	24.06145373	49.10943233	54.08364074
Zfp959	58.58440909	44.54111304	47.32318564
Zfp960	30.33835471	33.12031483	24.14448247
Zfp961	49.16905763	27.40991572	59.87831653
Zfp963	77.41511201	37.68863411	52.15208214
Zfp964	46.03060714	20.55743679	42.49428915
Zfp965	4.184600649	1.142079822	2.897337897
Zfp966	13.59995211	5.710399108	11.58935159
Zfp967	3.138450487	2.284159643	0.965779299
Zfp968	3.138450487	4.568319287	0.965779299
Zfp97	29.29220454	37.68863411	26.07604107
Zfp970	14.64610227	31.97823501	15.45246878
Zfp971	3.138450487	7.994558752	8.69201369

For Review Only

Zfp973	4.184600649	1.142079822	2.897337897
Zfpl1	85.7843133	66.24062966	75.33078531
Zfpm1	11.50765178	12.56287804	12.55513089
Zfr	186.2147289	154.1807759	136.1748811
Zfr2	0	0	0
Zfx	164.2455755	187.3010908	206.67677
Zfyve1	81.59971265	92.50846555	83.0570197
Zfyve16	23.01530357	108.4975831	30.90493756
Zfyve19	4.184600649	1.142079822	0
Zfyve21	169.4763263	0	38.63117195
Zfyve26	140.1841217	77.66142787	160.3193636
Zfyve27	151.6917735	156.4649356	156.4562464
Zfyve28	154.830224	100.5030243	41.52850985
Zfyve9	18.83070292	7.994558752	6.760455092
Zgrf1	1.046150162	1.142079822	0
Zhx1	100.4304156	181.5906916	155.4904671
Zhx2	12.55380195	5.710399108	9.657792988
Zhx3	11.50765178	17.13119732	5.794675793
Zik1	12.55380195	44.54111304	18.34980668
Zkscan1	95.19966476	83.37182698	76.29656461
Zkscan14	51.26135795	30.83615518	84.022799
Zkscan16	0	0	2.897337897
Zkscan17	122.399569	90.22430591	104.3041643
Zkscan2	5.230750811	2.284159643	10.62357229
Zkscan3	79.50741233	231.8422038	264.6235279
Zkscan4	54.39980844	30.83615518	41.52850985
Zkscan5	37.66140584	39.97279376	66.63877162
Zkscan6	79.50741233	99.36094448	97.54370918
Zkscan7	29.29220454	29.69407536	23.17870317
Zkscan8	74.27666152	81.08766734	39.59695125
Zmat1	25.10760389	59.38815073	28.97337897
Zmat2	69.04591071	146.1862172	117.8250745
Zmat3	14.64610227	33.12031483	8.69201369
Zmat5	33.47680519	28.55199554	38.63117195
Zmiz1	587.9363912	501.3730417	451.9847119
Zmiz1os1	0	7.994558752	8.69201369
Zmiz2	118.2149683	133.6233391	123.6197503
Zmpste24	146.4610227	97.07678484	157.4220257
Zmym1	37.66140584	18.27327715	45.39162705
Zmym2	138.0918214	149.6124566	121.6881917
Zmym3	106.7073165	138.1916584	132.3117639
Zmym4	51.26135795	70.80894894	68.57033022
Zmym5	104.6150162	85.65598662	90.78325409
Zmym6	161.107125	118.7763015	121.6881917
Zmynd10	0	0	0
Zmynd11	100.4304156	172.4540531	132.3117639
Zmynd15	2.092300324	17.13119732	0
Zmynd19	14.64610227	18.27327715	12.55513089
Zmynd8	191.4454797	142.7599777	156.4562464
Znf41-ps	64.86131006	54.81983144	0

For Review Only

Znfx1	238.522237	284.3778756	275.2471002
Znhit1	0	14.84703768	0.965779299
Znhit2	44.98445698	37.68863411	73.39922671
Znhit3	35.56910552	41.11487358	40.56273055
Znhit6	15.69225243	19.41535697	28.00759967
Znrd1	14.64610227	31.97823501	30.90493756
Znrd1as	15.69225243	6.85247893	19.31558598
Znrf1	47.0767573	98.21886466	56.98097863
Znrf2	24.06145373	36.54655429	27.04182037
Znrf3	60.67670941	60.53023055	59.87831653
Zpbp	0	4.568319287	0
Zpr1	71.13821103	84.5139068	0
Zranb1	26.15375406	50.25151215	69.53610952
Zranb2	64.86131006	123.3446207	89.81747479
Zranb3	9.41535146	4.568319287	4.828896494
Zrsr1	27.19990422	26.2678359	14.48668948
Zrsr2	39.75370617	55.96191126	45.39162705
Zscan10	6.276900973	0	0
Zscan12	21.96915341	37.68863411	55.04942003
Zscan18	52.30750811	19.41535697	27.04182037
Zscan2	9.41535146	1.142079822	0
Zscan20	28.24605438	20.55743679	14.48668948
Zscan21	89.96891395	97.07678484	102.3726057
Zscan22	92.06121428	52.5356718	79.19390251
Zscan25	0	1.142079822	0.965779299
Zscan26	730.2128132	680.6795737	96.57792988
Zscan29	134.9533709	91.36638573	142.9353362
Zscan30	2.092300324	4.568319287	16.41824808
Zswim1	69.04591071	45.68319287	56.98097863
Zswim3	27.19990422	23.98367625	43.46006845
Zswim4	55.4459586	50.25151215	57.94675793
Zswim5	7.323051136	14.84703768	0
Zswim6	64.86131006	77.66142787	50.22052354
Zswim7	2.092300324	10.27871839	10.62357229
Zswim8	46.03060714	28.55199554	47.32318564
Zufsp	77.41511201	116.4921418	112.996178
Zw10	50.21520779	68.5247893	60.84409583
Zwilch	0	10.27871839	0
Zwint	41.84600649	54.81983144	23.17870317
Zxda	16.7384026	15.9891175	10.62357229
Zxdb	13.59995211	26.2678359	4.828896494
Zxdc	47.0767573	68.5247893	35.73383406
Zyg11b	161.107125	201.0060486	118.7908538
Zyx	0	115.350062	106.2357229
Zzef1	279.3220933	207.8585275	157.4220257
Zzz3	145.4148726	105.0713436	84.9885783

For Review Only

Sham_ND_4	Sham_ND_5	Sham_HSD_1	Sham_HSD_2	Sham_HSD_3
100351.193	119118.2776	109615.9334	118216.2057	101025.3385
40.36150447	0	0.982767607	1.078398548	3.222087725
33.63458706	29.03133218	26.53472539	62.54711581	51.5534036
1.921976404	0	0	1.078398548	0
76.87905614	58.06266435	171.9843312	161.7597823	133.179626
10.57087022	11.7939787	8.844908464	7.548789839	5.370146209
24.98569325	23.58795739	16.70704932	1.078398548	19.33252635
0	9.979520436	15.72428171	36.66555065	30.07281877
0	0	7.862140857	0	9.666263176
81.68399715	79.83616349	101.2250635	80.87989113	65.51578375
0	0	0	0	0
0	0	3.931070429	1.078398548	1.074029242
122.0455016	176.0024513	137.587465	0	118.1432166
0.960988202	0	0	0	0
8.648893816	8.165062175	2.948302821	3.235195645	7.518204692
0	0.907229131	0	0	0
508.3627587	503.5121674	664.3509024	716.0566362	665.8981299
3.843952807	6.350603914	5.896605643	0	9.666263176
0	0	0	0	0
13.45383482	26.30964479	23.58642257	0	1.074029242
0	0	17.68981693	17.25437678	19.33252635
126.8504426	140.6205152	172.9670989	248.0316661	197.6213805
8.648893816	9.072291305	9.827676071	3.235195645	10.74029242
25.94668145	36.28916522	40.29347189	46.37113758	36.51699422
24.02470504	0	33.41409864	4.313594194	15.03640938
8.648893816	10.88674957	8.844908464	8.627188388	4.296116967
19.21976404	25.40241565	49.13838036	25.88156516	41.88714043
0	0	1.965535214	1.078398548	0
12.49284662	9.979520436	7.862140857	25.88156516	6.444175451
0	0	0.982767607	1.078398548	0
11.53185842	42.63976914	26.53472539	36.66555065	24.70267256
55.7373157	51.71206044	52.08668318	53.91992742	63.36772526
116.2795724	121.5687035	63.87989446	56.07672452	64.44175451
0	0	0	0	0
194.1196168	245.8590944	225.053782	224.3068981	168.622591
66.30818592	55.34097696	15.72428171	25.88156516	67.66384223
32.67359886	53.5265187	48.15561275	44.21434049	57.99757905
0.960988202	0.907229131	0	0	1.074029242
24.02470504	14.51566609	0.982767607	6.470391291	9.666263176
14.41482303	0	6.87937325	1.078398548	19.33252635
38.43952807	19.05181174	26.53472539	7.548789839	18.25849711
9.609882018	10.88674957	10.81044368	12.94078258	17.18446787
16.33679943	12.70120783	18.67258454	23.72476807	22.55461408
0	59.87712262	0.982767607	8.627188388	1.074029242
13.45383482	4.536145653	8.844908464	19.41117387	10.74029242
25.94668145	0	38.32793668	5.391992742	3.222087725

For Review Only

0	0	0	0	0
6.726917412	10.88674957	1.965535214	3.235195645	4.296116967
20.18075224	17.23735348	3.931070429	33.430355	20.40655559
15.37581123	0	2.948302821	1.078398548	33.29490649
120.1235252	98.88797523	161.1738876	167.151775	183.6590003
0	0	193.6052186	162.8381808	147.1420061
0	13.60843696	11.79321129	5.391992742	0
1.921976404	0	0	0	0
4.804941009	0	0	14.01918113	2.148058484
0	0	0	0	0
0	0	0	0	0
9.609882018	20.86627	13.7587465	4.313594194	3.222087725
4.804941009	0.907229131	0	3.235195645	0
0	0	0	1.078398548	0
0	0	2.948302821	2.156797097	0
0	0	0.982767607	0	1.074029242
5.765929211	4.536145653	8.844908464	8.627188388	6.444175451
2.882964605	7.257833044	8.844908464	3.235195645	2.148058484
5.765929211	11.7939787	23.58642257	5.391992742	7.518204692
6.726917412	4.536145653	0.982767607	1.078398548	0
3.843952807	0	0	3.235195645	0
0	1.814458261	0	0	0
2.882964605	6.350603914	5.896605643	6.470391291	0
6.726917412	10.88674957	4.913838036	1.078398548	7.518204692
2.882964605	2.721687392	0.982767607	0	0
0.960988202	0.907229131	0	849.7780562	0
0	1.814458261	0	0	1.074029242
4.804941009	0	1.965535214	3.235195645	4.296116967
0	2.721687392	0.982767607	0	0
34.59557526	21.77349913	19.65535214	44.21434049	15.03640938
0.960988202	0	4.913838036	0	5.370146209
22.10272864	23.58795739	32.43133104	16.17597823	42.96116967
0	0	0	0	0
1.921976404	0	0	0	0
3.843952807	9.072291305	7.862140857	0	0
5.765929211	12.70120783	0.982767607	3.235195645	0
22.10272864	0	0.982767607	0	1.074029242
0	2.721687392	7.862140857	0	0
4.804941009	4.536145653	1.965535214	11.86238403	11.81432166
15.37581123	29.93856131	5.896605643	4.313594194	6.444175451
12.49284662	2.721687392	3.931070429	2.156797097	10.74029242
2.882964605	0	1.965535214	2.156797097	3.222087725
32.67359886	58.96989348	46.19007754	58.23352162	70.88592996
0	0	0.982767607	0	1.074029242
0	0	0	0	6.444175451
8.648893816	18.14458261	0	0	0
9.609882018	21.77349913	19.65535214	8.627188388	1.074029242
0	5.443374783	1.965535214	0	1.074029242
0	0	0	0	0
21.14174044	23.58795739	40.29347189	36.66555065	26.85073104

For Review Only

0	0	1.965535214	1.078398548	0
71.11312693	63.50603914	23.58642257	47.44953613	13.96238014
6.726917412	3.628916522	10.81044368	23.72476807	5.370146209
0.960988202	2.721687392	0	0	0
10.57087022	6.350603914	4.913838036	9.705586936	6.444175451
18.25877583	16.33012435	14.74151411	24.80316661	12.8883509
0	4.536145653	1.965535214	12.94078258	7.518204692
0	0	0	0	0
0.960988202	0	2.948302821	0	0
0.960988202	0	0	0	0
2.882964605	0.907229131	0	0	0
0	0	0	2.156797097	1.074029242
0	0	0	0	0
7.687905614	4.536145653	0	5.391992742	2.148058484
2.882964605	4.536145653	3.931070429	1.078398548	3.222087725
5.765929211	3.628916522	9.827676071	11.86238403	7.518204692
32.67359886	41.73254	0	0	0
0.960988202	3.628916522	0	0	0
3.843952807	3.628916522	0	1.078398548	1.074029242
9.609882018	5.443374783	12.77597889	9.705586936	9.666263176
3.843952807	3.628916522	4.913838036	2.156797097	0
12.49284662	10.88674957	18.67258454	20.48957242	18.25849711
3.843952807	4.536145653	5.896605643	3.235195645	2.148058484
44.20545728	24.49518652	24.56919018	25.88156516	20.40655559
0	5.443374783	0	0	0
0	0.907229131	0	3.235195645	1.074029242
2.882964605	2.721687392	0	0	0
0	0	0	0	0
86.48893816	69.85664305	69.77650011	70.09590565	100.9587487
0	0	1.965535214	2.156797097	0
0	5.443374783	0.982767607	0	0
0	51.71206044	0.982767607	62.54711581	112.7730704
9.609882018	3.628916522	0.982767607	9.705586936	0
94.17684377	28.12410305	96.3112255	197.3469344	50.47937436
9.609882018	14.51566609	7.862140857	15.09757968	7.518204692
4.804941009	0	4.913838036	2.156797097	0
51.8933629	64.41326827	84.51801421	72.25270275	88.07039782
13.45383482	21.77349913	19.65535214	45.29273904	21.48058484
0	0	0	0	0
6.726917412	0	0.982767607	0	0
0	0	0	0	0
36.51755167	36.28916522	41.2762395	33.430355	42.96116967
37.47853987	41.73254	67.81096489	43.13594194	42.96116967
0	0	36.36240146	35.5871521	39.73908194
145.1092185	151.5072648	216.2088736	155.289391	126.7354505
21.14174044	24.49518652	18.67258454	29.11676081	20.40655559
4.804941009	2.721687392	2.948302821	5.391992742	0
38.43952807	29.93856131	38.32793668	37.7439492	67.66384223
0.960988202	2.721687392	0.982767607	1.078398548	0
0	0	7.862140857	1.078398548	1.074029242

For Review Only

3.843952807	0	0	0	0
0	6.350603914	0	9.705586936	3.222087725
5.765929211	0	6.87937325	0	1.074029242
28.82964605	41.73254	45.20730993	40.97914484	47.25728664
0	0	0	0	0
1.921976404	0	0.982767607	0	0
3.843952807	6.350603914	3.931070429	6.470391291	8.592233934
0	46.26868566	63.87989446	0	56.92354981
1.921976404	0	5.896605643	0	0
0	4.536145653	3.931070429	0	4.296116967
86.48893816	107.9602665	79.60417618	80.87989113	95.58860252
0.960988202	0	3.931070429	1.078398548	0
3.843952807	6.350603914	4.913838036	6.470391291	5.370146209
114.357596	124.2903909	74.69033814	39.90074629	67.66384223
12.49284662	6.350603914	11.79321129	5.391992742	11.81432166
27.86865785	31.75301957	29.48302821	18.33277532	24.70267256
121.0845134	132.4554531	135.6219298	173.6221663	140.6978307
2.882964605	1.814458261	0	0	1.074029242
17.29778763	8.165062175	13.7587465	7.548789839	11.81432166
0	1.814458261	0	0	0
11.53185842	21.77349913	11.79321129	14.01918113	0
0	0	0	0	0
20.18075224	9.979520436	27.517493	15.09757968	13.96238014
7.687905614	7.257833044	16.70704932	11.86238403	17.18446787
35.55656347	30.84579044	19.65535214	30.19515936	26.85073104
0	0	0	0	0
0.960988202	1.814458261	0	0	0
0	0	0.982767607	83.03668823	1.074029242
20.18075224	25.40241565	5.896605643	3.235195645	22.55461408
0	0	0	0	0
37.47853987	34.47470696	44.22454232	58.23352162	16.11043863
10.57087022	24.49518652	0	4.313594194	13.96238014
30.75162246	29.93856131	38.32793668	42.05754339	70.88592996
0	0.907229131	1.965535214	3.235195645	1.074029242
0	0	0	0	4.296116967
97.05980838	66.22772653	24.56919018	65.78231146	51.5534036
43.24446908	0	11.79321129	4.313594194	36.51699422
16.33679943	20.86627	18.67258454	21.56797097	19.33252635
10.57087022	12.70120783	22.60365496	18.33277532	39.73908194
0	4.536145653	5.896605643	4.313594194	1.074029242
38.43952807	32.6602487	19.65535214	85.19348533	79.47816389
0	15.42289522	2.948302821	22.64636952	22.55461408
4.804941009	0	0.982767607	0	0
0.960988202	6.350603914	0	0	4.296116967
71.11312693	93.44460045	78.62140857	42.05754339	80.55219313
0	0	40.29347189	58.23352162	0
14.41482303	19.95904087	50.12114796	50.68473178	54.77549133
24.02470504	19.95904087	10.81044368	10.78398548	27.92476029
0	7.257833044	4.913838036	0	2.148058484
15.37581123	12.70120783	5.896605643	9.705586936	7.518204692

For Review Only

46.12743368	23.58795739	16.70704932	19.41117387	12.8883509
34.59557526	33.56747783	34.39686625	29.11676081	18.25849711
14.41482303	29.93856131	0	1.078398548	8.592233934
0	0	0.982767607	0	0
166.2509589	143.3422026	81.56971139	194.1117387	112.7730704
58.62028031	58.96989348	59.94882404	73.3311013	118.1432166
0.960988202	0.907229131	0	0	117.0691874
15.37581123	24.49518652	16.70704932	23.72476807	22.55461408
97.05980838	95.25905871	70.75926771	67.93910855	86.99636858
78.80103254	53.5265187	51.10391557	57.15512307	65.51578375
16.33679943	5.443374783	22.60365496	4.313594194	1.074029242
16.33679943	10.88674957	0	6.470391291	19.33252635
39.40051627	49.89760218	60.93159164	35.5871521	12.8883509
2.882964605	5.443374783	6.87937325	0	1.074029242
24.02470504	36.28916522	36.36240146	21.56797097	27.92476029
12.49284662	15.42289522	19.65535214	11.86238403	7.518204692
20.18075224	7.257833044	23.58642257	22.64636952	16.11043863
67.26917412	0	126.7770213	30.19515936	31.14684801
26.90766965	30.84579044	49.13838036	23.72476807	53.70146209
12.49284662	14.51566609	13.7587465	3.235195645	5.370146209
195.080605	128.8265365	148.3979087	131.5646229	154.6602108
0	0	0	0	0
5.765929211	0	2.948302821	0	3.222087725
18.25877583	45.36145653	0	0	0
5.765929211	3.628916522	0.982767607	2.156797097	4.296116967
3.843952807	6.350603914	2.948302821	4.313594194	9.666263176
7.687905614	4.536145653	3.931070429	4.313594194	10.74029242
57.65929211	44.4542274	39.31070429	57.15512307	52.62743285
0	199.5904087	1.965535214	50.68473178	461.832574
0	0	0.982767607	29.11676081	2.148058484
0	3.628916522	0	0	1.074029242
11.53185842	9.072291305	18.67258454	20.48957242	5.370146209
0	0	0	6.470391291	0
28.82964605	39.01085261	22.60365496	17.25437678	36.51699422
0	5.443374783	2.948302821	2.156797097	2.148058484
29.79063425	17.23735348	29.48302821	29.11676081	24.70267256
28.82964605	31.75301957	36.36240146	31.27355791	31.14684801
66.30818592	80.74339262	103.1905987	94.89907226	71.9599592
59.58126851	58.06266435	85.50078182	90.58547807	67.66384223
47.08842189	36.28916522	61.91435925	29.11676081	2.148058484
60.54225671	49.89760218	129.7253241	75.48789839	95.58860252
6.726917412	3.628916522	0.982767607	0	0
8.648893816	9.072291305	8.844908464	5.391992742	3.222087725
3.843952807	4.536145653	6.87937325	4.313594194	2.148058484
154.7191005	157.8578687	72.72480293	132.6430215	165.4005032
0	0	5.896605643	0	2.148058484
5.765929211	0	0	0	0
0	0	0	0	0
2.882964605	0.907229131	1.965535214	1.078398548	1.074029242
0	5.443374783	0.982767607	3.235195645	0

For Review Only

35.55656347	36.28916522	41.2762395	50.68473178	36.51699422
0	0	0.982767607	0	0
0	0	0	2.156797097	0
8.648893816	0	2.948302821	4.313594194	11.81432166
0	10.88674957	2.948302821	5.391992742	21.48058484
237.3640858	308.4579044	285.0026061	356.9499195	264.2111935
15.37581123	26.30964479	15.72428171	16.17597823	4.296116967
58.62028031	53.5265187	50.12114796	25.88156516	61.21966678
1.921976404	9.979520436	16.70704932	0	0
16.33679943	3.628916522	14.74151411	15.09757968	8.592233934
0.960988202	0	0	0	0
0	0	4.913838036	12.94078258	7.518204692
0	0	2.948302821	0	2.148058484
315.2041302	278.5193431	216.2088736	239.4044778	215.8798776
12.49284662	13.60843696	3.931070429	1.078398548	3.222087725
9.609882018	18.14458261	16.70704932	24.80316661	15.03640938
9.609882018	19.95904087	20.63811975	7.548789839	13.96238014
9.609882018	102.5168918	24.56919018	0	35.44296498
10.57087022	9.072291305	13.7587465	20.48957242	22.55461408
41.32249268	23.58795739	13.7587465	28.03836226	20.40655559
25.94668145	21.77349913	35.37963386	30.19515936	36.51699422
147.0311949	139.7132861	157.2428171	119.7022389	84.8483101
47.08842189	58.06266435	58.96605643	70.09590565	47.25728664
1.921976404	1.814458261	6.87937325	10.78398548	1.074029242
23.06371684	0	14.74151411	44.21434049	20.40655559
87.44992636	0	0.982767607	0	0
0	5.443374783	7.862140857	1.078398548	1.074029242
38.43952807	49.89760218	27.517493	45.29273904	31.14684801
0	7.257833044	0	0	0
0	0	2.948302821	4.313594194	0
7.687905614	0.907229131	0.982767607	0	1.074029242
8.648893816	14.51566609	4.913838036	0	3.222087725
0	0	0	2.156797097	0
2.882964605	0	0	0	2.148058484
5.765929211	0	5.896605643	19.41117387	20.40655559
39.40051627	29.03133218	30.46579582	32.35195645	31.14684801
5.765929211	2.721687392	3.931070429	0	0
6.726917412	9.072291305	14.74151411	0	0
25.94668145	33.56747783	32.43133104	14.01918113	16.11043863
30.75162246	19.05181174	89.43185225	21.56797097	28.99878953
0	0	0	0	0
21.14174044	30.84579044	23.58642257	16.17597823	9.666263176
37.47853987	50.80483131	22.60365496	52.84152887	31.14684801
8.648893816	8.165062175	8.844908464	1.078398548	9.666263176
0.960988202	0	3.931070429	0	2.148058484
9.609882018	0	19.65535214	7.548789839	11.81432166
8.648893816	6.350603914	0.982767607	8.627188388	2.148058484
17.29778763	13.60843696	4.913838036	3.235195645	19.33252635
10.57087022	9.979520436	5.896605643	8.627188388	8.592233934
9.609882018	13.60843696	5.896605643	2.156797097	3.222087725

For Review Only

0	3.628916522	0	0	0
262.3497791	192.3325757	130.7080917	105.6830577	229.8422577
2.882964605	0	0	3.235195645	3.222087725
23.06371684	20.86627	21.62088736	14.01918113	12.8883509
183.5487465	0	340.0375921	3.235195645	3.222087725
0	4.536145653	7.862140857	0	1.074029242
6.726917412	3.628916522	0.982767607	0	0
0	0	0	0	0
34.59557526	25.40241565	36.36240146	32.35195645	26.85073104
5.765929211	9.072291305	1.965535214	0	0
0	16.33012435	8.844908464	19.41117387	6.444175451
23.06371684	28.12410305	10.81044368	9.705586936	16.11043863
1.921976404	0	0	0	3.222087725
49.01039829	47.17591479	38.32793668	31.27355791	53.70146209
2.882964605	2.721687392	0	8.627188388	5.370146209
12.49284662	12.70120783	2.948302821	6.470391291	1.074029242
0	0	1.965535214	5.391992742	0
4.804941009	3.628916522	0.982767607	2.156797097	1.074029242
10.57087022	8.165062175	11.79321129	7.548789839	6.444175451
0	0	1.965535214	0	0
0	4.536145653	0.982767607	1.078398548	0
3.843952807	1.814458261	0.982767607	0	1.074029242
18.25877583	7.257833044	0	0	0
3.843952807	0.907229131	1.965535214	0	0
2.882964605	0	6.87937325	0	2.148058484
0	0	0	4.313594194	0
13.45383482	9.979520436	22.60365496	38.82234774	9.666263176
4.804941009	18.14458261	5.896605643	5.391992742	0
0	0.907229131	0.982767607	0	0
7.687905614	10.88674957	2.948302821	0	1.074029242
36.51755167	27.21687392	13.7587465	15.09757968	22.55461408
0	0	0	5.391992742	3.222087725
8.648893816	5.443374783	4.913838036	4.313594194	0
16.33679943	13.60843696	5.896605643	17.25437678	25.7767018
0	0	0	1.078398548	2.148058484
0	0	0	0	2.148058484
0	2.721687392	0	0	5.370146209
44.20545728	38.10362348	61.91435925	43.13594194	59.0716083
13.45383482	7.257833044	9.827676071	2.156797097	2.148058484
32.67359886	14.51566609	12.77597889	14.01918113	12.8883509
2.882964605	1.814458261	5.896605643	0	5.370146209
4.804941009	7.257833044	2.948302821	1.078398548	2.148058484
0	0	0	1.078398548	0
4.804941009	4.536145653	1.965535214	7.548789839	3.222087725
24.02470504	49.89760218	23.58642257	47.44953613	35.44296498
1.921976404	2.721687392	0.982767607	0	0
0	0	0	0	3.222087725
2.882964605	9.072291305	1.965535214	2.156797097	7.518204692
5.765929211	0	0	2.156797097	0
23.06371684	15.42289522	7.862140857	16.17597823	11.81432166

For Review Only

3.843952807	2.721687392	7.862140857	4.313594194	5.370146209
27.86865785	34.47470696	30.46579582	18.33277532	12.8883509
2.882964605	0	0	4.313594194	1.074029242
17.29778763	10.88674957	17.68981693	10.78398548	5.370146209
1.921976404	0.907229131	0	0	0
0	0	0	0	0
3.843952807	0	0	2.156797097	2.148058484
0.960988202	0.907229131	0	0	0
0.960988202	2.721687392	3.931070429	0	4.296116967
0	0	0	1.078398548	0
5.765929211	9.072291305	4.913838036	3.235195645	4.296116967
76.87905614	61.69158088	83.53524661	78.72309404	47.25728664
0	7.257833044	2.948302821	0	0
13.45383482	4.536145653	3.931070429	2.156797097	1.074029242
12.49284662	0	0.982767607	1.078398548	1.074029242
8.648893816	18.14458261	9.827676071	16.17597823	9.666263176
0.960988202	0	0	1.078398548	0
0.960988202	0	0.982767607	0	1.074029242
0.960988202	0	1.965535214	2.156797097	3.222087725
7.687905614	0	15.72428171	8.627188388	0
5.765929211	6.350603914	6.87937325	0	1.074029242
5.765929211	4.536145653	0	1.078398548	1.074029242
0	1.814458261	0.982767607	0	0
0	0	0	0	2.148058484
0.960988202	2.721687392	1.965535214	2.156797097	2.148058484
0	0	2.948302821	0	1.074029242
0	0	0	0	4.296116967
0	0	1.965535214	3.235195645	0
4.804941009	0	0.982767607	1.078398548	9.666263176
36.51755167	36.28916522	0.982767607	2.156797097	44.03519891
5.765929211	1.814458261	0	4.313594194	0
9.609882018	9.072291305	0	9.705586936	1.074029242
0	6.350603914	0	4.313594194	2.148058484
0	0	2.948302821	0	1.074029242
0	7.257833044	8.844908464	1.078398548	5.370146209
10.57087022	9.979520436	6.87937325	4.313594194	7.518204692
9.609882018	5.443374783	1.965535214	12.94078258	5.370146209
2.882964605	0	6.87937325	3.235195645	4.296116967
5.765929211	8.165062175	5.896605643	7.548789839	18.25849711
6.726917412	6.350603914	2.948302821	1.078398548	0
5.765929211	0	0	2.156797097	1.074029242
123.967478	96.16628784	45.20730993	56.07672452	115.9951581
0	0	0	0	0
33.63458706	45.36145653	47.17284514	53.91992742	36.51699422
0	7.257833044	1.965535214	2.156797097	1.074029242
60.54225671	48.08314392	31.44856343	34.50875355	50.47937436
116.2795724	99.79520436	98.27676071	44.21434049	47.25728664
116.2795724	128.8265365	88.44908464	74.40949984	88.07039782
1.921976404	3.628916522	0	3.235195645	3.222087725
45.16644548	21.77349913	25.55195779	35.5871521	15.03640938

For Review Only

3.843952807	0	3.931070429	0	4.296116967
11.53185842	0	14.74151411	3.235195645	2.148058484
11.53185842	9.979520436	7.862140857	17.25437678	8.592233934
0	2.721687392	5.896605643	0	0
8.648893816	7.257833044	2.948302821	3.235195645	5.370146209
259.4668145	184.1675135	101.2250635	128.3294273	236.2864332
0	0	0.982767607	3.235195645	0
4.804941009	0.907229131	2.948302821	5.391992742	5.370146209
1.921976404	6.350603914	4.913838036	4.313594194	4.296116967
0.960988202	3.628916522	2.948302821	11.86238403	1.074029242
0	0	0	0	0
0	0.907229131	0	0	1.074029242
23.06371684	18.14458261	14.74151411	9.705586936	17.18446787
2.882964605	0.907229131	0	1.078398548	0
0	0	1.965535214	20.48957242	1.074029242
23.06371684	15.42289522	14.74151411	11.86238403	6.444175451
0	0	0	0	0
3.843952807	0	0.982767607	0	1.074029242
12.49284662	10.88674957	3.931070429	32.35195645	11.81432166
5.765929211	8.165062175	1.965535214	4.313594194	0
7.687905614	0	0	0	0
2.882964605	2.721687392	0.982767607	9.705586936	3.222087725
17.29778763	35.38193609	28.50026061	38.82234774	31.14684801
31.71261066	54.43374783	7.862140857	2.156797097	20.40655559
0	0	2.948302821	1.078398548	0
37.47853987	33.56747783	22.60365496	20.48957242	40.81311119
1.921976404	0.907229131	0	0	0
0	0	2.948302821	1.078398548	15.03640938
19.21976404	29.93856131	16.70704932	30.19515936	22.55461408
1.921976404	0.907229131	0	6.470391291	0
0	3.628916522	0	0	0
5.765929211	4.536145653	6.87937325	0	0
119.162537	15.42289522	72.72480293	105.6830577	0
2.882964605	0	0	0	1.074029242
7.687905614	13.60843696	10.81044368	15.09757968	18.25849711
0	0	0	3.235195645	4.296116967
0.960988202	6.350603914	8.844908464	0	0
76.87905614	96.16628784	93.36292268	81.95828968	92.36651479
20.18075224	10.88674957	31.44856343	25.88156516	20.40655559
9.609882018	5.443374783	4.913838036	0	4.296116967
0	0	0	0	1.074029242
0	0	0	0	0
98.02079658	83.46508001	95.32845789	69.0175071	82.70025162
2.882964605	0	2.948302821	0	1.074029242
0	0	0	0	0
0.960988202	9.072291305	5.896605643	0	4.296116967
1.921976404	4.536145653	2.948302821	1.078398548	1.074029242
22.10272864	19.05181174	19.65535214	20.48957242	15.03640938
101.8647494	73.48555957	121.8631833	80.87989113	152.5121523
0	0	0	0	0

For Review Only

12.49284662	9.979520436	11.79321129	30.19515936	7.518204692
7.687905614	0	0.982767607	0	1.074029242
0.960988202	0	0	0	0
16.33679943	7.257833044	2.948302821	3.235195645	20.40655559
10.57087022	9.072291305	1.965535214	10.78398548	6.444175451
6.726917412	2.721687392	0	1.078398548	2.148058484
98.02079658	53.5265187	11.79321129	45.29273904	25.7767018
6.726917412	0	0.982767607	5.391992742	3.222087725
1.921976404	0	2.948302821	0	0
0	0	0	0	0
98.98178478	85.27953827	0	0	0
14.41482303	12.70120783	20.63811975	16.17597823	12.8883509
19.21976404	13.60843696	18.67258454	49.60633323	28.99878953
0.960988202	2.721687392	1.965535214	4.313594194	1.074029242
3.843952807	0.907229131	6.87937325	1.078398548	1.074029242
0	2.721687392	0	0	1.074029242
0.960988202	0	0.982767607	7.548789839	1.074029242
0	0	0	1.078398548	0
86.48893816	96.16628784	94.34569029	87.35028243	80.55219313
0	0	0	3.235195645	5.370146209
0	0.907229131	2.948302821	3.235195645	2.148058484
0	0	0	0	0
101.8647494	74.3927887	26.53472539	57.15512307	69.81190071
12.49284662	18.14458261	13.7587465	35.5871521	4.296116967
0	5.443374783	2.948302821	0	0
4.804941009	13.60843696	5.896605643	7.548789839	13.96238014
168.1729353	199.5904087	186.7258454	181.1709561	166.4745325
0	0	14.74151411	19.41117387	10.74029242
18.25877583	19.95904087	20.63811975	20.48957242	6.444175451
32.67359886	15.42289522	9.827676071	42.05754339	33.29490649
5.765929211	12.70120783	7.862140857	10.78398548	1.074029242
3.843952807	0	0.982767607	0	0
0	0	0	0	2.148058484
4.804941009	0	1.965535214	2.156797097	0
8.648893816	10.88674957	5.896605643	8.627188388	8.592233934
9.609882018	4.536145653	11.79321129	4.313594194	9.666263176
107.6306786	100.7024335	88.44908464	73.3311013	83.77428086
16.33679943	12.70120783	1.965535214	12.94078258	4.296116967
13.45383482	19.95904087	6.87937325	16.17597823	11.81432166
30.75162246	19.95904087	5.896605643	22.64636952	5.370146209
48.04941009	64.41326827	39.31070429	37.7439492	52.62743285
6.726917412	6.350603914	2.948302821	0	0
12.49284662	9.072291305	3.931070429	1.078398548	1.074029242
0.960988202	0	1.965535214	0	3.222087725
23.06371684	35.38193609	44.22454232	31.27355791	27.92476029
128.772419	111.5891831	146.4323735	111.0750505	81.62622237
172.0168881	131.5482239	220.139944	152.0541953	109.5509827
98.02079658	79.83616349	87.46631704	72.25270275	53.70146209
34.59557526	0	55.034986	29.11676081	32.22087725
11.53185842	6.350603914	1.965535214	5.391992742	3.222087725

For Review Only

50.93237469	94.35182958	25.55195779	14.01918113	67.66384223
0.960988202	0.907229131	0	0	0
29.79063425	18.14458261	16.70704932	16.17597823	19.33252635
5.765929211	0	7.862140857	17.25437678	4.296116967
0	0	0.982767607	0	0
21.14174044	41.73254	18.67258454	29.11676081	25.7767018
3.843952807	0	2.948302821	0	3.222087725
2.882964605	0.907229131	0.982767607	0	0
1.921976404	9.979520436	12.77597889	12.94078258	18.25849711
5.765929211	0	0.982767607	0	2.148058484
4.804941009	0	0	0	2.148058484
107.6306786	132.4554531	106.1389016	81.95828968	75.18204692
0	5.443374783	1.965535214	0	3.222087725
19.21976404	0	5.896605643	23.72476807	8.592233934
11.53185842	10.88674957	4.913838036	11.86238403	7.518204692
1.921976404	8.165062175	0	0	0
8.648893816	22.68072826	15.72428171	0	0
29.79063425	19.05181174	12.77597889	26.95996371	32.22087725
0	0	0	1.078398548	0
0	0	0	2.156797097	0
3.843952807	4.536145653	1.965535214	0	1.074029242
0	13.60843696	17.68981693	6.470391291	20.40655559
11.53185842	16.33012435	20.63811975	31.27355791	28.99878953
0.960988202	0	1.965535214	4.313594194	8.592233934
0	0	0.982767607	1.078398548	6.444175451
15.37581123	24.49518652	7.862140857	10.78398548	10.74029242
6.726917412	9.979520436	0	0	3.222087725
28.82964605	21.77349913	15.72428171	23.72476807	4.296116967
112.4356196	85.27953827	47.17284514	43.13594194	79.47816389
0	0	0	1.078398548	0
0	2.721687392	0	0	0
0.960988202	0	0.982767607	0	1.074029242
0	0	0	0	1.074029242
441.0935846	414.6037127	584.7467262	519.7881004	425.3155797
2.882964605	0.907229131	1.965535214	0	0
15.37581123	2.721687392	0	0	9.666263176
0	0	0.982767607	0	1.074029242
0	0	2.948302821	0	2.148058484
2.882964605	0	0.982767607	0	0
38.43952807	35.38193609	7.862140857	2.156797097	30.07281877
37.47853987	19.05181174	12.77597889	12.94078258	13.96238014
9.609882018	8.165062175	6.87937325	9.705586936	24.70267256
8.648893816	6.350603914	8.844908464	0	4.296116967
62.46423311	42.63976914	16.70704932	38.82234774	53.70146209
37.47853987	49.89760218	100.2422959	34.50875355	36.51699422
72.07411513	146.9711191	14.74151411	0	193.3252635
0	0	0	15.09757968	0
0	2.721687392	0	0	0
65.34719772	54.43374783	91.39738746	58.23352162	75.18204692
10.57087022	19.05181174	9.827676071	17.25437678	19.33252635

For Review Only

8.648893816	3.628916522	5.896605643	2.156797097	2.148058484
205.6514752	234.9723448	39.31070429	336.4603471	180.4369126
0	132.4554531	0.982767607	0	4.296116967
0	0.907229131	0	2.156797097	0
0	9.072291305	15.72428171	15.09757968	22.55461408
2.882964605	5.443374783	0	4.313594194	5.370146209
0	0	0	0	0
14.41482303	0	0	8.627188388	12.8883509
2.882964605	5.443374783	0	0	0
6.726917412	6.350603914	1.965535214	0	3.222087725
0	0	0.982767607	0	2.148058484
0	1.814458261	0	0	0
0	39.01085261	56.01775361	116.4670432	2.148058484
0	0	1.965535214	0	0
40.36150447	45.36145653	35.37963386	36.66555065	23.62864332
1.921976404	0	0.982767607	0	0
6.726917412	22.68072826	1.965535214	10.78398548	12.8883509
42.28348088	44.4542274	43.24177471	39.90074629	33.29490649
0.960988202	3.628916522	5.896605643	0	0
14.41482303	7.257833044	6.87937325	4.313594194	2.148058484
2.882964605	6.350603914	1.965535214	1.078398548	1.074029242
0.960988202	4.536145653	1.965535214	4.313594194	4.296116967
11.53185842	11.7939787	9.827676071	5.391992742	2.148058484
7.687905614	0	0.982767607	14.01918113	2.148058484
7.687905614	3.628916522	2.948302821	3.235195645	1.074029242
5.765929211	4.536145653	2.948302821	2.156797097	4.296116967
0	0	0.982767607	0	1.074029242
6.726917412	0	4.913838036	8.627188388	16.11043863
91.29387917	71.67110131	56.01775361	117.5454418	46.1832574
0	89.81568392	1.965535214	14.01918113	0
7.687905614	0	4.913838036	1.078398548	0
61.50324491	69.85664305	55.034986	46.37113758	66.58981299
0.960988202	0.907229131	0	0	0
8.648893816	16.33012435	5.896605643	4.313594194	7.518204692
12.49284662	6.350603914	7.862140857	8.627188388	12.8883509
0	11.7939787	4.913838036	0	9.666263176
3.843952807	6.350603914	2.948302821	5.391992742	2.148058484
0	0	0.982767607	1.078398548	1.074029242
0.960988202	4.536145653	0	0	0
0	0	0	0	0
0.960988202	0	0	0	0
0	3.628916522	0	0	0
17.29778763	9.979520436	6.87937325	1.078398548	7.518204692
0	0	0	0	0
1.921976404	2.721687392	6.87937325	0	1.074029242
16.33679943	26.30964479	6.87937325	43.13594194	10.74029242
42.28348088	17.23735348	19.65535214	54.99832597	38.6650527
0	0	1.965535214	2.156797097	0
9.609882018	9.072291305	4.913838036	22.64636952	9.666263176
0	0	0	1.078398548	1.074029242

For Review Only

15.37581123	11.7939787	18.67258454	11.86238403	6.444175451
20.18075224	29.03133218	23.58642257	24.80316661	38.6650527
0	2.721687392	2.948302821	0	4.296116967
21.14174044	15.42289522	9.827676071	10.78398548	8.592233934
11.53185842	2.721687392	15.72428171	9.705586936	3.222087725
0	0	3.931070429	0	2.148058484
3.843952807	1.814458261	0	2.156797097	2.148058484
3.843952807	4.536145653	0	2.156797097	0
0.960988202	5.443374783	8.844908464	4.313594194	2.148058484
11.53185842	10.88674957	5.896605643	2.156797097	3.222087725
4.804941009	6.350603914	0	3.235195645	0
0	7.257833044	2.948302821	0	0
15.37581123	17.23735348	48.15561275	53.91992742	33.29490649
29.79063425	9.979520436	32.43133104	28.03836226	39.73908194
0	0	0.982767607	1.078398548	46.1832574
0	0	30.46579582	31.27355791	1.074029242
136.4603247	119.7542452	118.9148805	62.54711581	111.6990411
17.29778763	15.42289522	31.44856343	33.430355	11.81432166
273.8816375	320.2518831	394.0898105	393.6154702	372.6881469
1.921976404	0	0	0	0
80.72300895	86.1867674	151.3462115	88.42868097	77.33010541
194.1196168	245.8590944	228.9848525	279.3052241	249.1747841
45.16644548	46.26868566	80.58694379	32.35195645	31.14684801
46.12743368	0	41.2762395	119.7022389	20.40655559
44.20545728	31.75301957	55.034986	32.35195645	34.36893574
61.50324491	30.84579044	44.22454232	48.52793468	52.62743285
0	0	2.948302821	0	0
106.6696904	102.5168918	60.93159164	73.3311013	59.0716083
55.7373157	64.41326827	41.2762395	31.27355791	27.92476029
1.921976404	0.907229131	7.862140857	0	0
0	11.7939787	6.87937325	0	2.148058484
1043.633187	1001.58096	487.4527331	614.6871726	1060.066862
12.49284662	22.68072826	9.827676071	0	2.148058484
0	0	0.982767607	3.235195645	0
124.9284662	107.9602665	131.6908594	90.58547807	78.40413465
206.6124634	184.1675135	242.743599	134.7998186	127.8094798
0	0	14.74151411	0	0
0	9.072291305	2.948302821	3.235195645	0
0	0	0	0	0
1600.045356	1519.608794	1029.940452	1435.348468	1515.45526
57.65929211	59.87712262	71.74203532	67.93910855	45.10922815
0	3.628916522	0	0	0
61.50324491	51.71206044	56.01775361	42.05754339	19.33252635
33.63458706	31.75301957	52.08668318	56.07672452	55.84952057
32.67359886	61.69158088	70.75926771	28.03836226	40.81311119
50.93237469	32.6602487	7.862140857	29.11676081	20.40655559
35.55656347	36.28916522	52.08668318	65.78231146	21.48058484
12.49284662	21.77349913	12.77597889	8.627188388	24.70267256
136.4603247	115.2180996	117.9321129	75.48789839	126.7354505
67.26917412	78.92893436	44.22454232	31.27355791	51.5534036

For Review Only

763.9856204	975.2713153	793.093459	806.6421142	539.1626794
75.91806794	71.67110131	30.46579582	43.13594194	48.33131588
863.9283934	831.9291127	672.2130433	657.8231146	791.5595512
141.2652657	99.79520436	192.622451	24.80316661	179.3628834
440.1325964	392.8302135	334.1409864	193.0333402	234.1383747
175.8608409	225.9000535	192.622451	223.2284995	100.9587487
21.14174044	47.17591479	48.15561275	33.430355	45.10922815
138.3823011	184.1675135	109.0872044	116.4670432	154.6602108
87.44992636	107.9602665	167.0704932	111.0750505	114.9211289
96.09882018	88.00122566	113.0182748	122.9374345	85.92233934
28.82964605	0	137.587465	175.7789634	0
583.3198385	602.4001427	625.0401981	534.88568	567.0874396
92.25486737	61.69158088	64.86266207	89.50707952	69.81190071
44.20545728	30.84579044	40.29347189	78.72309404	41.88714043
3.843952807	0	2.948302821	0	0
30.75162246	42.63976914	54.05221839	28.03836226	34.36893574
0	0	0	0	0
24.02470504	14.51566609	20.63811975	20.48957242	11.81432166
29.79063425	32.6602487	55.034986	53.91992742	64.44175451
20.18075224	25.40241565	21.62088736	12.94078258	39.73908194
48.04941009	74.3927887	38.32793668	21.56797097	17.18446787
0	276.7048848	0	0	0
0	167.8373891	216.2088736	83.03668823	143.9199184
9.609882018	0	11.79321129	32.35195645	3.222087725
11.53185842	6.350603914	11.79321129	3.235195645	11.81432166
22.10272864	31.75301957	32.43133104	56.07672452	76.25607616
17.29778763	17.23735348	14.74151411	31.27355791	13.96238014
130.6943954	90.72291305	116.9493452	108.9182534	82.70025162
27.86865785	22.68072826	62.89712686	26.95996371	13.96238014
185.4707229	233.1578865	284.0198385	221.0717024	184.7330296
43.24446908	59.87712262	58.96605643	51.76313033	60.14563754
23.06371684	154.2289522	198.5190566	208.1309199	200.8434682
21.14174044	30.84579044	38.32793668	45.29273904	50.47937436
326.7359886	383.7579222	222.1054792	320.2843689	386.650527
83.60597355	52.61928957	35.37963386	61.46871726	12.8883509
31.71261066	0	33.41409864	81.95828968	78.40413465
0	0	0	0	0
270.9986729	416.4181709	374.4344583	511.160912	228.7682285
96.09882018	83.46508001	52.08668318	63.62551436	114.9211289
63.42522132	84.37230914	49.13838036	19.41117387	44.03519891
4.804941009	0	3.931070429	0	0
278.6865785	254.0241565	248.6402046	255.580456	289.9878953
0.960988202	1.814458261	0.982767607	3.235195645	0
17.29778763	28.12410305	20.63811975	39.90074629	38.6650527
35.55656347	32.6602487	37.34516907	65.78231146	16.11043863
0	0	14.74151411	0	0
0	205.0337835	0	148.8189997	93.44054403
32.67359886	5.443374783	65.84542968	62.54711581	51.5534036
39.40051627	31.75301957	75.67310575	47.44953613	38.6650527
21.14174044	19.95904087	10.81044368	10.78398548	13.96238014

For Review Only

0	3.628916522	0	0	0
20.18075224	31.75301957	41.2762395	18.33277532	16.11043863
35.55656347	38.10362348	57.00052121	64.70391291	12.8883509
40.36150447	29.03133218	36.36240146	35.5871521	27.92476029
78.80103254	92.53737131	88.44908464	108.9182534	97.736661
60.54225671	49.89760218	47.17284514	57.15512307	68.73787147
59.58126851	60.78435175	102.2078311	75.48789839	81.62622237
137.4213129	166.93016	175.9154017	182.2493547	168.622591
26.90766965	50.80483131	55.034986	43.13594194	68.73787147
60.54225671	78.02170523	87.46631704	100.291065	33.29490649
0	0	0	0	0
2.882964605	0	1.965535214	0	1.074029242
279.6475667	264.003677	138.5702326	228.6204923	291.0619245
23.06371684	10.88674957	17.68981693	17.25437678	41.88714043
105.7087022	78.92893436	110.069972	87.35028243	113.8470996
40.36150447	29.93856131	39.31070429	29.11676081	32.22087725
16.33679943	0	11.79321129	5.391992742	13.96238014
67.26917412	73.48555957	105.156134	92.74227517	112.7730704
11.53185842	8.165062175	22.60365496	12.94078258	6.444175451
88.41091456	48.08314392	76.65587336	32.35195645	108.4769534
17.29778763	20.86627	30.46579582	29.11676081	26.85073104
70.15213873	60.78435175	118.9148805	75.48789839	19.33252635
0	0	0	0	0
15.37581123	2.721687392	24.56919018	0	0
0	0	7.862140857	2.156797097	0
274.8426257	212.2916165	248.6402046	221.0717024	273.8774566
67.26917412	46.26868566	23.58642257	26.95996371	49.40534512
155.6800887	166.93016	86.48354943	195.1901373	85.92233934
0.960988202	4.536145653	3.931070429	0	0
4.804941009	0.907229131	5.896605643	0	0
428.600738	452.7073361	609.3159164	459.3977816	224.4721115
0	0	3.931070429	0	0
66.30818592	117.939787	112.0355072	101.3694636	75.18204692
223.910251	265.8181352	297.778585	234.012485	234.1383747
0	3.628916522	2.948302821	1.078398548	4.296116967
10.57087022	11.7939787	7.862140857	8.627188388	13.96238014
12.49284662	0	6.87937325	2.156797097	12.8883509
46.12743368	33.56747783	42.25900711	24.80316661	79.47816389
33.63458706	16.33012435	41.2762395	15.09757968	38.6650527
0	4.536145653	0.982767607	0	3.222087725
4.804941009	7.257833044	10.81044368	0	0
0	0	16.70704932	0	10.74029242
0	48.08314392	35.37963386	0	39.73908194
123.0064898	150.6000357	111.0527396	117.5454418	81.62622237
404.5760329	419.1398583	454.0386345	318.1275718	369.4660592
0.960988202	3.628916522	0.982767607	3.235195645	0
36.51755167	19.05181174	33.41409864	50.68473178	41.88714043
759.1806794	792.9182601	768.5242688	859.4836431	822.7063992
0	5.443374783	2.948302821	0	0
19.21976404	25.40241565	17.68981693	20.48957242	13.96238014

For Review Only

32.67359886	44.4542274	20.63811975	19.41117387	3.222087725
164.3289825	151.5072648	94.34569029	101.3694636	98.81069024
0	0	4.913838036	1.078398548	0
115.3185842	73.48555957	82.552479	85.19348533	118.1432166
159.5240415	245.8590944	261.4161835	257.7372531	248.1007548
0	0	8.844908464	0	0
379.5903397	556.131457	328.2443808	174.7005649	252.3968718
0	14.51566609	13.7587465	10.78398548	4.296116967
14.41482303	7.257833044	2.948302821	0	10.74029242
2093.993292	1820.808865	6267.109031	6062.756639	0
0	0.907229131	0	3.235195645	0
82.64498535	79.83616349	95.32845789	85.19348533	90.21845631
0	7.257833044	3.931070429	14.01918113	13.96238014
0	2.721687392	11.79321129	0	0
88.41091456	223.1783661	211.2950355	186.5629489	151.4381231
94.17684377	97.07351697	112.0355072	90.58547807	112.7730704
314.243142	314.8085083	342.9858949	264.2076444	267.4332812
37.47853987	0	0	0	3.222087725
366.1365049	219.5494496	199.5018242	208.1309199	369.4660592
344.0337762	359.2627357	364.6067822	375.2826949	426.389609
0	2.721687392	0	20.48957242	0
21.14174044	15.42289522	24.56919018	15.09757968	26.85073104
149.9141595	0	119.8976481	166.0733765	196.5473512
62.46423311	77.1144761	60.93159164	50.68473178	141.7718599
17.29778763	20.86627	1.965535214	7.548789839	7.518204692
0	3.628916522	0	0	2.148058484
61.50324491	63.50603914	46.19007754	37.7439492	68.73787147
97.05980838	38.10362348	68.7937325	0	31.14684801
59.58126851	23.58795739	51.10391557	78.72309404	46.1832574
50.93237469	31.75301957	55.034986	52.84152887	55.84952057
4.804941009	8.165062175	5.896605643	7.548789839	5.370146209
0	17.23735348	9.827676071	16.17597823	15.03640938
24.02470504	14.51566609	17.68981693	29.11676081	32.22087725
959.0662254	827.392967	906.1117338	703.1158536	676.6384223
0	0	0	114.3102461	155.7342401
595.8126851	566.1109775	651.5749235	416.2618397	369.4660592
0.960988202	2.721687392	2.948302821	1.078398548	10.74029242
9.609882018	0	2.948302821	5.391992742	4.296116967
0.960988202	0	0.982767607	0	0
18.25877583	29.93856131	15.72428171	10.78398548	24.70267256
7.687905614	18.14458261	12.77597889	14.01918113	11.81432166
0	2.721687392	0	0	0
0	4.536145653	0	0	5.370146209
29.79063425	36.28916522	37.34516907	10.78398548	9.666263176
2.882964605	3.628916522	6.87937325	1.078398548	3.222087725
97.05980838	78.92893436	87.46631704	54.99832597	79.47816389
0	0	137.587465	32.35195645	47.25728664
68.23016233	101.6096626	79.60417618	16.17597823	56.92354981
0	0	0	1.078398548	0
142.2262539	116.1253287	72.72480293	76.56629694	68.73787147

0	0	0	0	0
8.648893816	8.165062175	8.844908464	14.01918113	5.370146209
0	0	0.982767607	0	0
16.33679943	13.60843696	21.62088736	12.94078258	12.8883509
0	18.14458261	2.948302821	1.078398548	10.74029242
8.648893816	5.443374783	30.46579582	24.80316661	21.48058484
59.58126851	0	0	0	1.074029242
146.0702067	156.9506396	141.5185354	161.7597823	175.0667664
43.24446908	42.63976914	9.827676071	32.35195645	23.62864332
18.25877583	7.257833044	20.63811975	25.88156516	5.370146209
1.921976404	4.536145653	0	0	0
8.648893816	16.33012435	8.844908464	18.33277532	2.148058484
75.91806794	71.67110131	78.62140857	53.91992742	86.99636858
109.552655	87.09399653	92.38015507	107.8398548	96.66263176
28.82964605	40.82531087	5.896605643	37.7439492	18.25849711
47.08842189	39.91808174	75.67310575	51.76313033	68.73787147
0	0	0	0	0
0	0.907229131	0	0	0
0	0	0	0	3.222087725
0	0	19.65535214	16.17597823	0
0	0	0	0	0
14.41482303	18.14458261	12.77597889	5.391992742	11.81432166
335.3848824	288.4988635	188.6913806	231.8556879	220.1759946
29.79063425	30.84579044	24.56919018	29.11676081	36.51699422
65.34719772	40.82531087	61.91435925	48.52793468	109.5509827
197.0025814	245.8590944	143.4840706	234.012485	266.359252
0.960988202	0	0	0	0
117.2405606	44.4542274	143.4840706	91.66387662	39.73908194
0	0	0	0	0
3.843952807	0	0	2.156797097	1.074029242
17.29778763	9.072291305	14.74151411	10.78398548	34.36893574
407.4589975	322.0663413	452.0730993	389.301876	0
2.882964605	1.814458261	0	0	5.370146209
0	0	0.982767607	1.078398548	1.074029242
5.765929211	0	0	0	0
0	0	0	0	0
2437.06608	2376.033093	2851.008828	2307.772894	1867.736851
0	8.165062175	4.913838036	0	6.444175451
21.14174044	17.23735348	35.37963386	17.25437678	26.85073104
41.32249268	52.61928957	27.517493	22.64636952	22.55461408
0	0	0	0	0
107.6306786	153.3217231	166.0877256	187.6413474	114.9211289
37.47853987	18.14458261	34.39686625	30.19515936	12.8883509
93.21585557	96.16628784	135.6219298	102.4478621	122.4393336
449.7424784	470.8519187	584.7467262	504.6905207	439.2779599
82.64498535	78.02170523	116.9493452	57.15512307	76.25607616
5.765929211	16.33012435	13.7587465	11.86238403	12.8883509
64.38620952	47.17591479	60.93159164	48.52793468	50.47937436
97.05980838	174.1879931	192.622451	180.0925576	0
18.25877583	11.7939787	6.87937325	1.078398548	1.074029242

For Review Only

0	4.536145653	2.948302821	3.235195645	0
440.1325964	29.93856131	0	388.2234774	416.7233458
53.8153393	49.89760218	91.39738746	111.0750505	74.10801768
259.4668145	299.3856131	335.123754	362.3419123	283.5437198
0	70.76387218	0.982767607	1.078398548	47.25728664
5.765929211	0.907229131	0	0	50.47937436
3.843952807	1.814458261	3.931070429	0	1.074029242
0	0	0	0	1.074029242
0	0	0	0	5.370146209
2.882964605	0	0.982767607	0	0
17.29778763	22.68072826	22.60365496	10.78398548	12.8883509
902.3679215	789.2893436	949.3535085	1018.00823	927.9612649
0	0	0.982767607	1.078398548	0
84.56696176	86.1867674	84.51801421	73.3311013	35.44296498
88.41091456	6.350603914	0	45.29273904	84.8483101
71.11312693	97.9807461	117.9321129	93.82067372	99.88471948
79.76202075	73.48555957	69.77650011	50.68473178	63.36772526
27.86865785	25.40241565	38.32793668	19.41117387	12.8883509
0	0	0	0	0
0	0	1.965535214	0	0
24.98569325	48.08314392	37.34516907	45.29273904	59.0716083
33.63458706	24.49518652	38.32793668	36.66555065	50.47937436
13.45383482	12.70120783	34.39686625	16.17597823	13.96238014
16.33679943	0	5.896605643	4.313594194	2.148058484
11.53185842	8.165062175	0.982767607	6.470391291	9.666263176
43.24446908	41.73254	37.34516907	20.48957242	22.55461408
104.747714	96.16628784	86.48354943	72.25270275	84.8483101
0	0	0	0	0
263.3107673	162.3940144	202.4501271	230.7772894	213.7318191
0	1.814458261	3.931070429	0	0
140.3042775	137.8988278	100.2422959	105.6830577	135.3276845
102.8257376	39.01085261	77.63864096	74.40949984	89.14442707
97.05980838	119.7542452	137.587465	109.9966519	102.032778
0	0	0	0	0
14.41482303	21.77349913	14.74151411	19.41117387	24.70267256
268.1157083	133.3626822	153.3117467	474.4953613	294.2840122
118.2015488	136.9915987	177.8809369	154.2109924	144.9939476
18.25877583	19.95904087	31.44856343	6.470391291	9.666263176
22.10272864	22.68072826	10.81044368	21.56797097	6.444175451
41.32249268	53.5265187	37.34516907	39.90074629	162.1784155
0	0	0	0	0
8.648893816	10.88674957	14.74151411	8.627188388	7.518204692
26.90766965	21.77349913	32.43133104	17.25437678	23.62864332
0	0	0	0	0
50.93237469	47.17591479	35.37963386	38.82234774	36.51699422
18.25877583	21.77349913	24.56919018	48.52793468	19.33252635
49.97138649	57.15543522	26.53472539	32.35195645	31.14684801
44.20545728	45.36145653	30.46579582	29.11676081	34.36893574
57.65929211	55.34097696	57.00052121	54.99832597	23.62864332
304.63326	233.1578865	245.6919018	251.2668618	209.4357021

For Review Only

172.0168881	156.9506396	150.3634439	171.4653692	168.622591
128.772419	110.6819539	94.34569029	112.153449	122.4393336
176.8218291	219.5494496	124.8114861	158.5245866	144.9939476
33.63458706	45.36145653	7.862140857	32.35195645	45.10922815
3.843952807	0.907229131	0	0	0
24.98569325	34.47470696	38.32793668	32.35195645	18.25849711
147.9921831	146.06389	100.2422959	163.9165794	117.0691874
19.21976404	7.257833044	18.67258454	17.25437678	0
24.98569325	46.26868566	34.39686625	33.430355	34.36893574
0.960988202	0	0	0	0
148.9531713	113.4036413	69.77650011	161.7597823	166.4745325
40.36150447	49.89760218	65.84542968	2.156797097	2.148058484
48.04941009	45.36145653	23.58642257	28.03836226	25.7767018
57.65929211	52.61928957	63.87989446	67.93910855	32.22087725
70.15213873	86.1867674	47.17284514	67.93910855	109.5509827
8.648893816	6.350603914	6.87937325	19.41117387	7.518204692
63.42522132	57.15543522	84.51801421	28.03836226	46.1832574
43.24446908	51.71206044	78.62140857	39.90074629	60.14563754
57.65929211	50.80483131	43.24177471	75.48789839	59.0716083
12.49284662	26.30964479	24.56919018	0	7.518204692
0	0	0	2.156797097	0
0	0	0	0	0
6.726917412	6.350603914	13.7587465	0	0
120.1235252	130.6409948	192.622451	291.1676081	167.5485617
20.18075224	2.721687392	33.41409864	8.627188388	5.370146209
24.02470504	32.6602487	18.67258454	19.41117387	23.62864332
118.2015488	106.1458083	70.75926771	126.1726302	139.6238014
0	2.721687392	0	0	1.074029242
0	2.721687392	0	1.078398548	0
12.49284662	18.14458261	6.87937325	17.25437678	7.518204692
2.882964605	0	1.965535214	1.078398548	8.592233934
2.882964605	0	0	0	0
125.8894544	199.5904087	209.3295003	231.8556879	141.7718599
32.67359886	26.30964479	14.74151411	28.03836226	26.85073104
6.726917412	0	0	1.078398548	8.592233934
4.804941009	9.072291305	2.948302821	3.235195645	1.074029242
2.882964605	0.907229131	3.931070429	0	3.222087725
68.23016233	70.76387218	74.69033814	52.84152887	37.59102346
4.804941009	9.979520436	0	7.548789839	21.48058484
2.882964605	0	0	0	0
8.648893816	6.350603914	7.862140857	16.17597823	8.592233934
0.960988202	12.70120783	0	0	60.14563754
2.882964605	3.628916522	0.982767607	5.391992742	1.074029242
10.57087022	5.443374783	8.844908464	20.48957242	18.25849711
0.960988202	9.072291305	0.982767607	0	0
94.17684377	109.7747248	121.8631833	121.859036	104.1808365
19.21976404	29.03133218	18.67258454	17.25437678	23.62864332
190.2756639	125.19762	0	0	0
0	42.63976914	66.82819729	44.21434049	19.33252635
0	0	0	0	0

For Review Only

0	0	5.896605643	0	0
33.63458706	33.56747783	30.46579582	28.03836226	50.47937436
0.960988202	6.350603914	0	0	0
51.8933629	154.2289522	31.44856343	111.0750505	33.29490649
36.51755167	39.01085261	52.08668318	49.60633323	73.03398844
17.29778763	34.47470696	18.67258454	45.29273904	0
47.08842189	84.37230914	60.93159164	130.4862244	68.73787147
8.648893816	14.51566609	9.827676071	25.88156516	25.7767018
17.29778763	19.05181174	16.70704932	28.03836226	16.11043863
10.57087022	195.9614922	0.982767607	67.93910855	147.1420061
119.162537	121.5687035	175.9154017	166.0733765	176.1407956
60.54225671	65.3204974	78.62140857	81.95828968	85.92233934
0	0	0.982767607	2.156797097	1.074029242
23.06371684	36.28916522	29.48302821	37.7439492	59.0716083
0	0	0	1.078398548	1.074029242
13.45383482	15.42289522	7.862140857	31.27355791	28.99878953
107.6306786	89.81568392	44.22454232	51.76313033	90.21845631
77.84004434	38.10362348	54.05221839	33.430355	66.58981299
4.804941009	7.257833044	4.913838036	6.470391291	3.222087725
623.6813429	565.2037483	428.4866767	496.0633323	504.7937436
7.687905614	2.721687392	3.931070429	2.156797097	6.444175451
3.843952807	2.721687392	3.931070429	2.156797097	5.370146209
24.02470504	10.88674957	13.7587465	21.56797097	10.74029242
31.71261066	33.56747783	21.62088736	16.17597823	39.73908194
0	0	0	0	0
200.8465342	211.3843874	186.7258454	253.4236589	184.7330296
49.01039829	64.41326827	28.50026061	1.078398548	20.40655559
142.2262539	103.4241209	286.9681413	285.7756153	244.8786671
25.94668145	12.70120783	62.89712686	35.5871521	19.33252635
219.10531	719.4327005	154.2945143	169.3085721	153.5861816
49.01039829	48.99037305	36.36240146	31.27355791	15.03640938
557.373157	706.7314927	821.5937196	704.1942521	611.1226386
105.7087022	114.3108704	194.5879862	187.6413474	127.8094798
53.8153393	48.08314392	49.13838036	58.23352162	56.92354981
41.32249268	28.12410305	24.56919018	19.41117387	53.70146209
47.08842189	39.01085261	76.65587336	42.05754339	36.51699422
2.882964605	7.257833044	0	0	0
69.19115053	56.24820609	76.65587336	42.05754339	66.58981299
49.01039829	56.24820609	73.70757054	59.31192017	57.99757905
47.08842189	50.80483131	20.63811975	57.15512307	52.62743285
61.50324491	73.48555957	59.94882404	81.95828968	46.1832574
4.804941009	4.536145653	44.22454232	0	30.07281877
113.3966078	148.7855774	112.0355072	176.8573619	155.7342401
7.687905614	0	0	0	0
0	0	0	0	2.148058484
93.21585557	104.33135	144.4668382	87.35028243	88.07039782
56.6983039	48.99037305	67.81096489	60.39031871	39.73908194
0	0	0	0	0
0	0	0	0	0
4.804941009	0	1.965535214	0	0

For Review Only

0	9.979520436	6.87937325	1.078398548	12.8883509
0	0	2.948302821	0	0
0	0	0	2.156797097	1.074029242
50.93237469	80.74339262	107.1216692	65.78231146	52.62743285
153.7581123	175.0952222	133.6563946	166.0733765	191.177205
0	0	0.982767607	5.391992742	6.444175451
45.16644548	76.20724696	50.12114796	59.31192017	49.40534512
6.726917412	0	5.896605643	1.078398548	2.148058484
83.60597355	98.88797523	114.98381	9.705586936	9.666263176
30.75162246	45.36145653	45.20730993	33.430355	16.11043863
145.1092185	107.0530374	166.0877256	142.3486084	128.883509
508.3627587	612.3796631	932.6464592	710.6646434	639.0473988
16.33679943	32.6602487	15.72428171	29.11676081	24.70267256
3.843952807	3.628916522	1.965535214	3.235195645	4.296116967
0	0	0	0	2.148058484
59.58126851	45.36145653	166.0877256	145.583804	0
32.67359886	21.77349913	9.827676071	9.705586936	24.70267256
144.1482303	107.9602665	108.1044368	116.4670432	76.25607616
52.8543511	46.26868566	55.034986	57.15512307	49.40534512
21.14174044	13.60843696	9.827676071	28.03836226	20.40655559
36.51755167	32.6602487	53.06945079	33.430355	32.22087725
141.2652657	96.16628784	98.27676071	133.72142	64.44175451
38.43952807	48.99037305	36.36240146	118.6238403	45.10922815
150.8751477	174.1879931	224.0710144	180.0925576	256.6929888
21.14174044	37.19639435	29.48302821	23.72476807	27.92476029
0	28.12410305	0	0	0
36.51755167	46.26868566	34.39686625	37.7439492	41.88714043
80.72300895	70.76387218	60.93159164	49.60633323	37.59102346
0	0	11.79321129	12.94078258	10.74029242
64.38620952	82.55785088	107.1216692	74.40949984	65.51578375
4.804941009	0	0	0	5.370146209
23.06371684	41.73254	32.43133104	34.50875355	28.99878953
186.4317111	142.4349735	193.6052186	143.4270069	140.6978307
0	0	0	47.44953613	41.88714043
15.37581123	32.6602487	15.72428171	32.35195645	52.62743285
18.25877583	10.88674957	4.913838036	6.470391291	7.518204692
13.45383482	13.60843696	11.79321129	7.548789839	2.148058484
65.34719772	86.1867674	71.74203532	111.0750505	126.7354505
453.5864312	499.8832509	610.298684	616.8439697	613.270697
0	0	2.948302821	0	2.148058484
22.10272864	14.51566609	3.931070429	7.548789839	16.11043863
1.921976404	0	0	0	0
53.8153393	58.96989348	21.62088736	24.80316661	49.40534512
19.21976404	14.51566609	5.896605643	9.705586936	5.370146209
0	0	0	0	0
2.882964605	2.721687392	0	1.078398548	3.222087725
8.648893816	3.628916522	4.913838036	0	3.222087725
23.06371684	38.10362348	40.29347189	21.56797097	34.36893574
28.82964605	37.19639435	24.56919018	26.95996371	24.70267256
137.4213129	125.19762	97.29399311	101.3694636	138.5497722

For Review Only

72.07411513	80.74339262	84.51801421	81.95828968	93.44054403
53.8153393	58.96989348	63.87989446	61.46871726	66.58981299
27.86865785	26.30964479	21.62088736	7.548789839	85.92233934
21.14174044	13.60843696	20.63811975	2.156797097	9.666263176
158.5630533	163.3012435	267.3127891	219.9933039	283.5437198
0	4.536145653	0	0	0
6.726917412	13.60843696	14.74151411	14.01918113	40.81311119
35.55656347	18.14458261	26.53472539	9.705586936	17.18446787
3.843952807	4.536145653	0	3.235195645	3.222087725
118.2015488	110.6819539	113.0182748	122.9374345	76.25607616
25.94668145	14.51566609	37.34516907	12.94078258	24.70267256
58.62028031	76.20724696	51.10391557	54.99832597	60.14563754
0	0	1.965535214	0	0
21.14174044	36.28916522	77.63864096	19.41117387	71.9599592
67.26917412	73.48555957	46.19007754	85.19348533	80.55219313
13.45383482	13.60843696	12.77597889	23.72476807	12.8883509
0	0	1.965535214	1.078398548	1.074029242
54.7763275	61.69158088	114.98381	101.3694636	19.33252635
130.6943954	143.3422026	121.8631833	101.3694636	125.6614213
55.7373157	47.17591479	43.24177471	30.19515936	51.5534036
52.8543511	80.74339262	48.15561275	60.39031871	70.88592996
27.86865785	36.28916522	38.32793668	33.430355	49.40534512
15.37581123	0	26.53472539	39.90074629	37.59102346
50.93237469	55.34097696	56.01775361	74.40949984	54.77549133
0	0	0	0	0
71.11312693	101.6096626	58.96605643	34.50875355	64.44175451
155.6800887	148.7855774	191.6396834	159.6029852	160.030357
105.7087022	68.94941392	111.0527396	154.2109924	59.0716083
80.72300895	142.4349735	152.3289791	216.7581082	179.3628834
54.7763275	84.37230914	55.034986	38.82234774	31.14684801
167.2119471	168.7446183	193.6052186	93.82067372	184.7330296
13.45383482	9.072291305	6.87937325	8.627188388	3.222087725
1.921976404	0	0	4.313594194	0
13.45383482	17.23735348	36.36240146	8.627188388	6.444175451
2.882964605	2.721687392	0	0	4.296116967
5.765929211	5.443374783	8.844908464	6.470391291	2.148058484
0	0	0	0	0
69.19115053	73.48555957	49.13838036	69.0175071	75.18204692
2.882964605	0	0	3.235195645	0
219.10531	172.3735348	153.3117467	119.7022389	90.21845631
4.804941009	5.443374783	0.982767607	1.078398548	0
0	7.257833044	0	0	0
0	0	0	0	0
512.2067115	558.8531444	342.9858949	466.9465715	620.7889017
140.3042775	94.35182958	110.069972	89.50707952	132.1055967
0	0	0.982767607	1.078398548	1.074029242
3.843952807	0	4.913838036	0	3.222087725
191.2366522	174.1879931	107.1216692	153.1325939	233.0643455
46.12743368	53.5265187	44.22454232	24.80316661	42.96116967
22.10272864	40.82531087	49.13838036	36.66555065	37.59102346

For Review Only

175.8608409	219.5494496	220.139944	229.6988908	182.5849711
204.690487	187.79643	213.2605707	183.3277532	219.1019653
90.33289097	81.65062175	81.56971139	119.7022389	103.1068072
65.34719772	0	105.156134	108.9182534	83.77428086
0	15.42289522	12.77597889	15.09757968	6.444175451
24.02470504	31.75301957	23.58642257	25.88156516	27.92476029
47.08842189	20.86627	68.7937325	52.84152887	13.96238014
74.95707974	64.41326827	80.58694379	103.5262607	96.66263176
170.0949117	200.4976378	187.708613	209.2093184	166.4745325
2.882964605	0	0	0	0
27.86865785	30.84579044	47.17284514	21.56797097	61.21966678
40.36150447	32.6602487	39.31070429	29.11676081	21.48058484
39.40051627	26.30964479	38.32793668	44.21434049	34.36893574
78.80103254	88.00122566	83.53524661	69.0175071	42.96116967
12.49284662	27.21687392	24.56919018	1.078398548	2.148058484
0	0	0.982767607	0	4.296116967
5.765929211	4.536145653	0.982767607	0	0
2.882964605	4.536145653	3.931070429	0	0
0.960988202	0	0	0	0
65.34719772	64.41326827	49.13838036	58.23352162	40.81311119
79.76202075	64.41326827	78.62140857	53.91992742	67.66384223
0	8.165062175	2.948302821	0	0
116.2795724	65.3204974	46.19007754	48.52793468	39.73908194
0	0	0.982767607	0	0
121.0845134	131.5482239	136.6046974	128.3294273	133.179626
51.8933629	51.71206044	47.17284514	77.64469549	84.8483101
45.16644548	40.82531087	31.44856343	28.03836226	27.92476029
181.6267701	189.6108883	135.6219298	221.0717024	90.21845631
21.14174044	29.03133218	62.89712686	86.27188388	24.70267256
2.882964605	0	1.965535214	0	4.296116967
3.843952807	0	3.931070429	1.078398548	4.296116967
10.57087022	3.628916522	4.913838036	3.235195645	2.148058484
1.921976404	0	0	1.078398548	0
0	9.072291305	1.965535214	1.078398548	5.370146209
0	7.257833044	4.913838036	1.078398548	0
99.94277298	146.9711191	116.9493452	154.2109924	102.032778
0	0	0	0	0
24.98569325	26.30964479	25.55195779	29.11676081	15.03640938
4.804941009	2.721687392	0	0	20.40655559
0	2.721687392	0	0	0
68.23016233	107.0530374	112.0355072	81.95828968	110.6250119
241.2080386	247.6735526	131.6908594	283.6188182	273.8774566
23.06371684	0	0	0	1.074029242
0.960988202	3.628916522	0	0	9.666263176
25.94668145	24.49518652	43.24177471	45.29273904	47.25728664
24.02470504	23.58795739	25.55195779	26.95996371	16.11043863
74.95707974	100.7024335	85.50078182	65.78231146	77.33010541
0	6.350603914	0	0	0
7.687905614	7.257833044	2.948302821	0	0
0	0	15.72428171	0	0

For Review Only

11.53185842	7.257833044	12.77597889	0	12.8883509
5.765929211	16.33012435	7.862140857	4.313594194	3.222087725
220.0662982	61.69158088	493.3493388	524.1016946	405.9830534
56.6983039	52.61928957	80.58694379	77.64469549	47.25728664
0.960988202	0.907229131	0	0	0
107.6306786	81.65062175	136.6046974	136.9566157	96.66263176
134.5383482	114.3108704	152.3289791	109.9966519	112.7730704
0	6.350603914	3.931070429	7.548789839	0
0	0	1.965535214	3.235195645	4.296116967
0.960988202	3.628916522	0	3.235195645	4.296116967
14.41482303	22.68072826	11.79321129	18.33277532	38.6650527
294.0623897	357.4482774	178.8637045	30.19515936	402.7609657
200.8465342	209.5699292	95.32845789	164.9949779	171.8446787
0	92.53737131	70.75926771	102.4478621	48.33131588
0.960988202	11.7939787	0	60.39031871	0
52.8543511	46.26868566	68.7937325	69.0175071	57.99757905
108.5916668	78.92893436	114.0010424	106.7614563	82.70025162
75.91806794	64.41326827	68.7937325	29.11676081	69.81190071
204.690487	186.8892009	204.4156623	171.4653692	120.2912751
170.0949117	173.2807639	149.3806763	158.5245866	182.5849711
99.94277298	0	243.7263666	0	0
68.23016233	86.1867674	117.9321129	94.89907226	90.21845631
364.2145285	263.0964479	370.5033879	370.9691007	227.6941993
2.882964605	4.536145653	0	0	0
128.772419	113.4036413	93.36292268	63.62551436	89.14442707
77.84004434	43.54699827	112.0355072	107.8398548	79.47816389
18.25877583	13.60843696	16.70704932	25.88156516	18.25849711
89.37190276	63.50603914	55.034986	64.70391291	32.22087725
81.68399715	109.7747248	105.156134	90.58547807	68.73787147
34.59557526	39.01085261	34.39686625	67.93910855	41.88714043
0	0	0.982767607	58.23352162	1.074029242
25.94668145	38.10362348	30.46579582	43.13594194	48.33131588
494.9089239	0	0.982767607	1.078398548	1.074029242
101.8647494	135.1771404	74.69033814	73.3311013	80.55219313
25.94668145	49.89760218	93.36292268	40.97914484	60.14563754
123.0064898	9.979520436	85.50078182	93.82067372	0
56.6983039	37.19639435	31.44856343	31.27355791	46.1832574
56.6983039	74.3927887	36.36240146	45.29273904	32.22087725
0	0	87.46631704	8.627188388	1.074029242
17.29778763	29.03133218	16.70704932	18.33277532	9.666263176
377.6683633	323.8807996	310.5545639	325.6763616	441.4260184
143.1872421	144.2494318	142.501303	125.0942316	119.2172458
69.19115053	41.73254	57.00052121	44.21434049	27.92476029
171.0558999	158.7650978	135.6219298	126.1726302	166.4745325
5.765929211	9.979520436	11.79321129	1.078398548	1.074029242
0	0	1.965535214	0	0
93.21585557	0	25.55195779	166.0733765	10.74029242
0.960988202	9.072291305	305.6407258	0	175.0667664
26.90766965	37.19639435	26.53472539	79.80149259	32.22087725
0	0	0	73.3311013	0

For Review Only

3.843952807	21.77349913	18.67258454	3.235195645	4.296116967
15.37581123	18.14458261	27.517493	22.64636952	9.666263176
264.2717555	283.9627179	292.8647469	183.3277532	143.9199184
26.90766965	25.40241565	30.46579582	47.44953613	27.92476029
4.804941009	0	2.948302821	5.391992742	0
11.53185842	9.072291305	19.65535214	26.95996371	7.518204692
0	0	2.948302821	2.156797097	0
0	0	0	0	0
148.9531713	122.4759326	134.6391622	154.2109924	107.4029242
69.19115053	48.99037305	37.34516907	53.91992742	74.10801768
0	47.17591479	0	4.313594194	20.40655559
2.882964605	0	0	0	0
83.60597355	0.907229131	0	159.6029852	134.2536552
12.49284662	5.443374783	9.827676071	18.33277532	18.25849711
295.9843661	362.8916522	271.2438596	374.2042963	326.5048895
45.16644548	38.10362348	46.19007754	67.93910855	30.07281877
0	0	0	0	0
259.4668145	272.1687392	1798.464721	1586.324265	2318.829133
0	4.536145653	0	0	0
0	0	3.931070429	1.078398548	6.444175451
14.41482303	9.072291305	18.67258454	10.78398548	12.8883509
23.06371684	0	2.948302821	10.78398548	60.14563754
22.10272864	46.26868566	41.2762395	47.44953613	23.62864332
213.3393808	196.8687213	189.6741482	175.7789634	226.62017
69.19115053	101.6096626	54.05221839	38.82234774	56.92354981
74.95707974	62.59881001	30.46579582	64.70391291	86.99636858
44.20545728	26.30964479	30.46579582	37.7439492	31.14684801
0	0	0	10.78398548	0
50.93237469	43.54699827	42.25900711	84.11508678	28.99878953
4.804941009	0	3.931070429	0	0
0	0	0	0	3.222087725
3.843952807	10.88674957	0.982767607	0	2.148058484
70.15213873	78.92893436	64.86266207	60.39031871	69.81190071
0	73.48555957	0	62.54711581	56.92354981
507.4017705	449.9856487	493.3493388	283.6188182	301.8022169
12.49284662	14.51566609	21.62088736	7.548789839	6.444175451
247.9349561	0	183.7775425	7.548789839	1.074029242
242.1690268	259.4675313	233.8986905	184.4061518	187.9551173
117.2405606	154.2289522	131.6908594	143.4270069	109.5509827
345.9557526	0	0	819.5828968	0
119.162537	97.07351697	132.673627	184.4061518	149.2900646
380.5513279	400.9952757	544.4532544	464.7897744	327.5789187
17.29778763	0	9.827676071	76.56629694	12.8883509
0	0	56.01775361	64.70391291	89.14442707
129.7334072	96.16628784	206.3811975	85.19348533	27.92476029
65.34719772	26.30964479	111.0527396	73.3311013	219.1019653
40.36150447	53.5265187	29.48302821	11.86238403	34.36893574
75.91806794	69.85664305	43.24177471	100.291065	73.03398844
72.07411513	41.73254	34.39686625	40.97914484	54.77549133
60.54225671	52.61928957	114.0010424	57.15512307	51.5534036

For Review Only

26.90766965	44.4542274	51.10391557	36.66555065	28.99878953
0.960988202	0	1.965535214	0	2.148058484
0.960988202	0	0	6.470391291	0
277.7255903	364.7061105	310.5545639	334.30355	434.9818429
104.747714	136.0843696	69.77650011	134.7998186	173.9927372
53.8153393	42.63976914	44.22454232	56.07672452	35.44296498
357.4876111	273.9831974	264.3644863	238.3260792	85.92233934
20.18075224	16.33012435	9.827676071	9.705586936	16.11043863
256.5838499	257.6530731	270.261092	273.9132313	239.5085209
50.93237469	29.93856131	20.63811975	18.33277532	35.44296498
37.47853987	29.93856131	30.46579582	34.50875355	20.40655559
44.20545728	42.63976914	65.84542968	26.95996371	35.44296498
76.87905614	79.83616349	198.5190566	179.014159	160.030357
8.648893816	0	4.913838036	0	2.148058484
56.6983039	66.22772653	46.19007754	47.44953613	35.44296498
335.3848824	364.7061105	444.2109584	428.1242237	380.2063516
22.10272864	27.21687392	23.58642257	26.95996371	10.74029242
51.8933629	19.05181174	49.13838036	5.391992742	4.296116967
0	0	0	9.705586936	8.592233934
18.25877583	7.257833044	5.896605643	12.94078258	12.8883509
0	2.721687392	380.331064	0	0
195.080605	166.0229309	142.501303	170.3869707	122.4393336
32.67359886	29.93856131	5.896605643	10.78398548	39.73908194
0	0	0.982767607	0	0
71.11312693	57.15543522	52.08668318	71.1743042	66.58981299
89.37190276	196.8687213	211.2950355	252.3452603	124.587392
112.4356196	18.14458261	9.827676071	9.705586936	36.51699422
0	6.350603914	2.948302821	0	0
810.1130541	595.1423096	351.8308034	502.5337236	702.4151241
0	0	0	0	0
40.36150447	97.07351697	8.844908464	0	84.8483101
571.78798	204.1265544	97.29399311	883.2084112	502.6456851
408.4199857	431.8410661	368.5378527	760.2709767	523.0522407
40.36150447	10.88674957	68.7937325	163.9165794	12.8883509
7.687905614	0	3.931070429	0	0
133.57736	121.5687035	117.9321129	146.6622026	123.5133628
65.34719772	53.5265187	47.17284514	36.66555065	38.6650527
50.93237469	48.99037305	58.96605643	43.13594194	45.10922815
0	0	0	1.078398548	0
6.726917412	4.536145653	1.965535214	0	0
45.16644548	63.50603914	52.08668318	63.62551436	67.66384223
383.4342925	119.7542452	357.727409	432.4378179	272.8034274
18.25877583	16.33012435	44.22454232	7.548789839	12.8883509
0	3.628916522	2.948302821	1.078398548	4.296116967
0	1.814458261	0	0	0
52.8543511	50.80483131	62.89712686	23.72476807	39.73908194
0	0	0	2.156797097	0
31.71261066	45.36145653	30.46579582	31.27355791	0
0	158.7650978	217.1916412	237.2476807	99.88471948
3.843952807	8.165062175	0	0	0

For Review Only

370.9414459	286.6844052	274.1921624	264.2076444	249.1747841
140.3042775	114.3108704	137.587465	130.4862244	74.10801768
6.726917412	6.350603914	3.931070429	0	3.222087725
691.9115053	634.1531622	538.5566487	663.2151073	717.4515335
131.6553836	107.0530374	115.9665776	78.72309404	97.736661
164.3289825	145.1566609	107.1216692	101.3694636	166.4745325
75.91806794	73.48555957	56.01775361	58.23352162	57.99757905
27.86865785	0	0	6.470391291	3.222087725
141.2652657	137.8988278	151.3462115	194.1117387	142.8458892
98.98178478	81.65062175	63.87989446	73.3311013	108.4769534
89.37190276	58.06266435	135.6219298	88.42868097	59.0716083
81.68399715	109.7747248	106.1389016	109.9966519	98.81069024
153.7581123	102.5168918	62.89712686	72.25270275	99.88471948
150.8751477	203.2193252	120.8804157	213.5229126	144.9939476
144.1482303	150.6000357	196.5535214	177.9357605	126.7354505
268.1157083	176.0024513	132.673627	66.86071	244.8786671
0.960988202	0.907229131	0	0	0
17.29778763	30.84579044	23.58642257	32.35195645	32.22087725
73.99609154	98.88797523	69.77650011	42.05754339	68.73787147
45.16644548	48.99037305	17.68981693	50.68473178	25.7767018
0	0.907229131	10.81044368	23.72476807	32.22087725
41.32249268	38.10362348	47.17284514	40.97914484	20.40655559
7.687905614	9.979520436	3.931070429	4.313594194	8.592233934
7.687905614	27.21687392	24.56919018	28.03836226	25.7767018
1.921976404	11.7939787	13.7587465	9.705586936	10.74029242
256.5838499	280.3338013	338.0720569	256.6588545	369.4660592
13.45383482	15.42289522	19.65535214	5.391992742	4.296116967
48.04941009	86.1867674	43.24177471	62.54711581	77.33010541
55.7373157	53.5265187	53.06945079	49.60633323	50.47937436
24.98569325	26.30964479	27.517493	39.90074629	21.48058484
3.843952807	0	12.77597889	8.627188388	0
537.1924048	872.7544236	1123.303375	1000.753853	940.8496158
0	0	0	0	0
117.2405606	147.8783483	105.156134	115.3886447	189.0291465
26.90766965	29.03133218	27.517493	28.03836226	24.70267256
24.98569325	29.93856131	39.31070429	37.7439492	36.51699422
623.6813429	548.873624	613.2469869	558.6104481	570.3095274
45.16644548	43.54699827	78.62140857	73.3311013	63.36772526
49.97138649	44.4542274	38.32793668	48.52793468	37.59102346
8.648893816	20.86627	12.77597889	11.86238403	21.48058484
0	0	0	0	2.148058484
19.21976404	22.68072826	90.41461986	32.35195645	4.296116967
58.62028031	15.42289522	36.36240146	150.9757968	86.99636858
101.8647494	73.48555957	89.43185225	62.54711581	99.88471948
16.33679943	16.33012435	5.896605643	23.72476807	34.36893574
32.67359886	20.86627	46.19007754	21.56797097	24.70267256
61.50324491	45.36145653	72.72480293	87.35028243	59.0716083
0	9.979520436	0	0	0
0.960988202	8.165062175	4.913838036	7.548789839	5.370146209
50.93237469	51.71206044	47.17284514	23.72476807	25.7767018

For Review Only

177.7828173	204.1265544	409.8140922	72.25270275	148.2160354
4.804941009	0	2.948302821	1.078398548	2.148058484
36.51755167	0	20.63811975	22.64636952	25.7767018
101.8647494	0	34.39686625	173.6221663	20.40655559
20.18075224	38.10362348	1284.477263	1308.097439	0
387.2782453	481.7386683	555.263698	574.7864263	598.2342877
109.552655	57.15543522	90.41461986	144.5054055	111.6990411
0.960988202	18.14458261	1136.079354	0	911.8508263
310.3991892	344.7470696	363.6240146	413.0266441	383.4284393
123.967478	95.25905871	188.6913806	167.151775	178.2888541
3.843952807	2.721687392	0	4.313594194	0
76.87905614	87.09399653	156.2600495	128.3294273	84.8483101
0	0	0	0	3.222087725
0	0.907229131	0	2.156797097	0
289.2574487	320.2518831	230.9503877	270.6780357	259.9150765
0	43.54699827	0	0	0
0	0	0	1.078398548	1.074029242
0	0	0	0	0
170.0949117	246.7663235	71.74203532	108.9182534	71.9599592
29.79063425	36.28916522	46.19007754	65.78231146	39.73908194
0.960988202	2.721687392	0.982767607	0	0
1058.04801	1084.138811	1124.286143	1074.084954	990.2549609
153.7581123	178.7241387	235.8642257	210.2877169	92.36651479
129.7334072	130.6409948	134.6391622	118.6238403	104.1808365
86.48893816	58.06266435	37.34516907	38.82234774	31.14684801
234.4811212	261.2819896	190.6569158	211.3661155	155.7342401
0	103.4241209	82.552479	73.3311013	104.1808365
0.960988202	0.907229131	0	0	0
27.86865785	27.21687392	10.81044368	12.94078258	32.22087725
285.4134959	248.5807818	319.3994723	211.3661155	193.3252635
0	3.628916522	0	1.078398548	4.296116967
164.3289825	202.3120961	125.7942537	249.1100647	166.4745325
24.98569325	26.30964479	12.77597889	9.705586936	30.07281877
38.43952807	40.82531087	61.91435925	72.25270275	66.58981299
312.3211656	127.9193074	162.1566552	186.5629489	108.4769534
66.30818592	63.50603914	74.69033814	54.99832597	107.4029242
22.10272864	46.26868566	94.34569029	138.0350142	53.70146209
66.30818592	88.90845479	57.00052121	65.78231146	73.03398844
96.09882018	90.72291305	107.1216692	103.5262607	117.0691874
61.50324491	68.04218479	104.1733664	89.50707952	95.58860252
148.9531713	146.06389	163.1394228	154.2109924	133.179626
120.1235252	126.1048491	105.156134	86.27188388	95.58860252
4.804941009	10.88674957	15.72428171	12.94078258	18.25849711
0	2.721687392	0.982767607	0	0
12.49284662	16.33012435	32.43133104	24.80316661	15.03640938
12.49284662	7.257833044	17.68981693	9.705586936	11.81432166
0	3.628916522	0	0	0
402.6540565	365.6133396	313.5028667	286.8540139	317.9126556
32.67359886	69.85664305	32.43133104	45.29273904	0
0	1.814458261	0	0	0

For Review Only

0.960988202	0	2.948302821	5.391992742	1.074029242
0	0	17.68981693	0	0
41.32249268	26.30964479	0	34.50875355	44.03519891
0	0	0	0	0
74.95707974	66.22772653	63.87989446	61.46871726	55.84952057
705.3653401	653.204974	698.7477687	411.9482455	724.9697382
0.960988202	0	0	2.156797097	0
0	8.165062175	0.982767607	0	4.296116967
0	0	1.965535214	0	4.296116967
55.7373157	40.82531087	97.29399311	0	78.40413465
9.609882018	4.536145653	12.77597889	9.705586936	12.8883509
53.8153393	39.91808174	25.55195779	50.68473178	45.10922815
38.43952807	54.43374783	39.31070429	51.76313033	24.70267256
149.9141595	112.4964122	118.9148805	136.9566157	150.3640938
144.1482303	123.3831618	170.018796	221.0717024	207.2876437
5.765929211	0	4.913838036	1.078398548	1.074029242
219.10531	134.2699113	265.3472539	141.2702098	121.3653043
34.59557526	19.95904087	18.67258454	22.64636952	7.518204692
49.01039829	68.94941392	60.93159164	63.62551436	36.51699422
55.7373157	66.22772653	85.50078182	49.60633323	82.70025162
8.648893816	6.350603914	4.913838036	8.627188388	6.444175451
25.94668145	0	0.982767607	1.078398548	4.296116967
55.7373157	65.3204974	110.069972	38.82234774	84.8483101
149.9141595	139.7132861	185.7430777	117.5454418	143.9199184
38.43952807	44.4542274	52.08668318	64.70391291	57.99757905
142.2262539	107.9602665	108.1044368	97.05586936	124.587392
0.960988202	0	10.81044368	0	0
0	0	5.896605643	59.31192017	1.074029242
1.921976404	0	0	0	1.074029242
178.7438055	193.2398048	135.6219298	122.9374345	152.5121523
0	67.13495566	37.34516907	0	34.36893574
306.5552364	313.9012792	303.6751906	304.1083907	260.9891057
492.0259593	518.0278335	514.9702261	598.5111944	340.4672696
42.28348088	42.63976914	48.15561275	20.48957242	46.1832574
49.01039829	74.3927887	104.1733664	97.05586936	70.88592996
109.552655	159.672327	127.7597889	149.8973982	52.62743285
76.87905614	66.22772653	74.69033814	58.23352162	63.36772526
95.13783197	98.88797523	126.7770213	95.97747081	107.4029242
242.1690268	27.21687392	11.79321129	45.29273904	4.296116967
0	52.61928957	61.91435925	95.97747081	13.96238014
142.2262539	116.1253287	61.91435925	88.42868097	2.148058484
83.60597355	80.74339262	74.69033814	62.54711581	77.33010541
62.46423311	58.06266435	75.67310575	89.50707952	103.1068072
14.41482303	0	1.965535214	15.09757968	11.81432166
117.2405606	0	42.25900711	183.3277532	3.222087725
76.87905614	102.5168918	69.77650011	104.6046592	63.36772526
56.6983039	0	0	36.66555065	50.47937436
120.1235252	156.9506396	160.19112	177.9357605	172.9187079
73.03510333	80.74339262	69.77650011	49.60633323	64.44175451
78.80103254	104.33135	77.63864096	88.42868097	119.2172458

21.14174044	14.51566609	12.77597889	8.627188388	23.62864332
24.02470504	18.14458261	21.62088736	17.25437678	0
0	16.33012435	6.87937325	5.391992742	13.96238014
57.65929211	60.78435175	70.75926771	33.430355	28.99878953
131.6553836	141.5277444	30.46579582	150.9757968	115.9951581
75.91806794	39.01085261	33.41409864	29.11676081	55.84952057
123.0064898	115.2180996	142.501303	105.6830577	73.03398844
72.07411513	76.20724696	85.50078182	81.95828968	68.73787147
0	79.83616349	17.68981693	98.13426791	0
0	2.721687392	0	0	1.074029242
0	2.721687392	2.948302821	0	1.074029242
68.23016233	53.5265187	94.34569029	33.430355	35.44296498
78.80103254	81.65062175	52.08668318	94.89907226	88.07039782
73.03510333	59.87712262	38.32793668	32.35195645	84.8483101
51.8933629	46.26868566	45.20730993	18.33277532	41.88714043
48.04941009	190.5181174	0	101.3694636	105.2548657
194.1196168	202.3120961	108.1044368	0	158.9563278
97.05980838	71.67110131	13.7587465	54.99832597	46.1832574
99.94277298	96.16628784	152.3289791	62.54711581	128.883509
0	0	0.982767607	0	1.074029242
30.75162246	22.68072826	13.7587465	37.7439492	6.444175451
0	6.350603914	28.50026061	0	0
0	0	5.896605643	0	0
237.3640858	292.12778	325.296078	450.7705933	347.9854743
0	3.628916522	8.844908464	0	8.592233934
98.98178478	112.4964122	104.1733664	146.6622026	131.0315675
32.67359886	27.21687392	6.87937325	2.156797097	8.592233934
198.9245578	294.8494674	208.3467327	214.6013111	282.4696906
5.765929211	12.70120783	11.79321129	2.156797097	3.222087725
0	0	13.7587465	1.078398548	0
415.1469032	374.6856309	378.3655287	515.4745062	229.8422577
17.29778763	0.907229131	996.5263536	850.8564547	871.0377151
602.5396025	738.4845123	944.4396705	796.9365273	599.3083169
271.9596611	9.979520436	0	196.2685358	8.592233934
4.804941009	3.628916522	2.948302821	5.391992742	3.222087725
54.7763275	0	239.7952961	0	60.14563754
40.36150447	0	73.70757054	60.39031871	65.51578375
227.7542038	231.3434283	290.8992117	361.2635137	365.1699422
0	0	0.982767607	1.078398548	1.074029242
41.32249268	45.36145653	53.06945079	48.52793468	49.40534512
115.3185842	97.07351697	74.69033814	190.8765431	47.25728664
3.843952807	0	0	24.80316661	124.587392
140.3042775	136.9915987	157.2428171	130.4862244	170.7706494
46.12743368	0	77.63864096	0	0
0	0	0	0	0
0	0	0	24.80316661	22.55461408
0	0	60.93159164	46.37113758	40.81311119
22.10272864	37.19639435	17.68981693	26.95996371	18.25849711
0	0	65.84542968	57.15512307	34.36893574
300.7893072	442.7278157	547.4015572	422.732231	492.979422

For Review Only

709.2092929	609.6579757	1024.043847	877.8164184	851.7051887
519.8946172	283.0554887	0	655.6663175	636.8993404
208.5344398	220.4566787	134.6391622	57.15512307	115.9951581
469.9232307	471.7591479	664.3509024	478.8089555	513.3859776
0	9.072291305	0	0	4.296116967
0.960988202	0.907229131	0	0	0
0	0	345.9341977	215.6797097	0
347.877729	395.5519009	450.1075641	489.592941	441.4260184
40.36150447	12.70120783	162.1566552	0	0
197.0025814	251.3024692	244.7091342	323.5195645	327.5789187
0	0	0.982767607	0	5.370146209
227.7542038	162.3940144	244.7091342	132.6430215	152.5121523
279.6475667	289.4060926	351.8308034	380.6746876	358.7257667
347.877729	380.1290057	394.0898105	414.1050426	402.7609657
87.44992636	107.9602665	174.9326341	122.9374345	114.9211289
130.6943954	115.2180996	193.6052186	204.8957242	197.6213805
147.0311949	156.9506396	201.4673595	204.8957242	229.8422577
160.4850297	224.0855952	191.6396834	297.6379994	289.9878953
6.726917412	9.072291305	19.65535214	3.235195645	17.18446787
146.0702067	0.907229131	72.72480293	49.60633323	105.2548657
248.8959443	167.8373891	177.8809369	176.8573619	93.44054403
0	21.77349913	1.965535214	12.94078258	8.592233934
810.1130541	816.5062175	1136.079354	765.6629694	853.8532472
501.6358413	455.4290235	342.0031273	486.3577454	557.4211765
418.0298678	428.2121496	258.4678807	341.8523399	332.9490649
3.843952807	9.072291305	6.87937325	14.01918113	7.518204692
4.804941009	0	0	0	4.296116967
2.882964605	0	2.948302821	0	0
3.843952807	0.907229131	4.913838036	1.078398548	4.296116967
186.4317111	192.3325757	179.8464721	195.1901373	170.7706494
23.06371684	12.70120783	32.43133104	23.72476807	42.96116967
30.75162246	41.73254	50.12114796	38.82234774	30.07281877
0	0	0.982767607	0	0
87.44992636	29.93856131	42.25900711	58.23352162	66.58981299
23.06371684	47.17591479	0	0	0
5.765929211	0.907229131	0	0	8.592233934
59.58126851	46.26868566	72.72480293	56.07672452	89.14442707
0	3.628916522	0.982767607	6.470391291	2.148058484
272.9206493	214.1060748	190.6569158	236.1692821	222.324053
116.2795724	105.2385791	80.58694379	119.7022389	66.58981299
261.3887909	268.5398226	285.0026061	257.7372531	301.8022169
50.93237469	88.00122566	48.15561275	33.430355	40.81311119
75.91806794	97.07351697	57.98328882	91.66387662	92.36651479
401.6930683	357.4482774	238.8125285	424.8890281	301.8022169
46.12743368	36.28916522	36.36240146	24.80316661	37.59102346
51.8933629	71.67110131	22.60365496	57.15512307	73.03398844
73.99609154	78.92893436	122.8459509	115.3886447	67.66384223
0	0	0	0	0
10.57087022	27.21687392	15.72428171	10.78398548	11.81432166
24.02470504	44.4542274	21.62088736	25.88156516	35.44296498

For Review Only

160.4850297	127.0120783	133.6563946	239.4044778	187.9551173
1.921976404	2.721687392	2.948302821	1.078398548	0
22.10272864	29.93856131	22.60365496	17.25437678	19.33252635
0	0	0	0	0
26.90766965	30.84579044	27.517493	64.70391291	20.40655559
22.10272864	22.68072826	17.68981693	12.94078258	27.92476029
166.2509589	179.6313678	115.9665776	166.0733765	160.030357
12.49284662	0	0.982767607	2.156797097	2.148058484
42.28348088	29.93856131	29.48302821	21.56797097	17.18446787
28.82964605	21.77349913	24.56919018	43.13594194	23.62864332
0	9.979520436	1.965535214	1.078398548	3.222087725
1.921976404	0.907229131	0	0	0
0	0	0	0	0
0	0	0	0	12.8883509
3.843952807	0	12.77597889	7.548789839	4.296116967
84.56696176	72.57833044	57.00052121	62.54711581	82.70025162
1.921976404	2.721687392	3.931070429	4.313594194	5.370146209
17.29778763	10.88674957	6.87937325	7.548789839	4.296116967
4.804941009	9.979520436	33.41409864	7.548789839	16.11043863
0	7.257833044	6.87937325	1.078398548	1.074029242
32.67359886	23.58795739	21.62088736	31.27355791	25.7767018
40.36150447	62.59881001	30.46579582	36.66555065	53.70146209
40.36150447	57.15543522	72.72480293	95.97747081	60.14563754
266.1937319	148.7855774	130.7080917	95.97747081	118.1432166
73.99609154	45.36145653	21.62088736	66.86071	42.96116967
18.25877583	27.21687392	39.31070429	39.90074629	22.55461408
0	0	0	1.078398548	0
75.91806794	26.30964479	24.56919018	38.82234774	69.81190071
3.843952807	0	2.948302821	1.078398548	0
2.882964605	0	4.913838036	2.156797097	5.370146209
0	0	3.931070429	0	0
161.4460179	106.1458083	140.5357678	134.7998186	146.0679769
141.2652657	125.19762	199.5018242	132.6430215	114.9211289
76.87905614	61.69158088	20.63811975	33.430355	83.77428086
0	3.628916522	0	0	2.148058484
53.8153393	23.58795739	64.86266207	78.72309404	61.21966678
14.41482303	0	0	0	22.55461408
0	0	0	2.156797097	4.296116967
0.960988202	0	0	0	0
2.882964605	0.907229131	0	5.391992742	0
178.7438055	162.3940144	97.29399311	104.6046592	205.1395852
18.25877583	11.7939787	13.7587465	7.548789839	12.8883509
0	0	0.982767607	0	4.296116967
0.960988202	0	1.965535214	3.235195645	1.074029242
5.765929211	2.721687392	1.965535214	1.078398548	1.074029242
29.79063425	17.23735348	37.34516907	26.95996371	28.99878953
3.843952807	0	0.982767607	2.156797097	1.074029242
3178.948971	3332.252596	3282.443808	4550.841874	5398.070969
3.843952807	4.536145653	2.948302821	5.391992742	0
130.6943954	168.7446183	156.2600495	149.8973982	136.4017137

For Review Only

40.36150447	53.5265187	42.25900711	31.27355791	25.7767018
14.41482303	5.443374783	6.87937325	3.235195645	4.296116967
59.58126851	67.13495566	54.05221839	72.25270275	113.8470996
125.8894544	121.5687035	162.1566552	61.46871726	81.62622237
43.24446908	58.06266435	81.56971139	86.27188388	56.92354981
6.726917412	6.350603914	1.965535214	23.72476807	27.92476029
16.33679943	27.21687392	60.93159164	29.11676081	47.25728664
37.47853987	13.60843696	23.58642257	31.27355791	31.14684801
13.45383482	0.907229131	0	0	0
3.843952807	0.907229131	0	0	0
19.21976404	4.536145653	7.862140857	7.548789839	2.148058484
0	0	0.982767607	0	1.074029242
8.648893816	18.14458261	0.982767607	20.48957242	1.074029242
4.804941009	0	1.965535214	7.548789839	13.96238014
0	0	1.965535214	1.078398548	1.074029242
82.64498535	75.30001783	80.58694379	42.05754339	53.70146209
177.7828173	229.52897	61.91435925	84.11508678	85.92233934
2.882964605	0	0.982767607	0	0
37.47853987	46.26868566	35.37963386	35.5871521	42.96116967
1.921976404	3.628916522	0	0	0
40.36150447	53.5265187	113.0182748	67.93910855	54.77549133
6.726917412	8.165062175	2.948302821	2.156797097	2.148058484
215.2613572	195.0542631	130.7080917	493.9065352	599.3083169
0	0	0	0	221.2500238
343.072788	383.7579222	277.1404652	531.6504844	12.8883509
12.49284662	14.51566609	17.68981693	10.78398548	12.8883509
64.38620952	58.96989348	45.20730993	86.27188388	98.81069024
39.40051627	46.26868566	57.00052121	61.46871726	47.25728664
0	463.5940857	25.55195779	10.78398548	1.074029242
3.843952807	5.443374783	0	11.86238403	4.296116967
3.843952807	8.165062175	11.79321129	4.313594194	1.074029242
14.41482303	36.28916522	15.72428171	23.72476807	6.444175451
6.726917412	0	0	3.235195645	0
22.10272864	29.93856131	60.93159164	25.88156516	60.14563754
54.7763275	44.4542274	55.034986	60.39031871	70.88592996
16.33679943	3.628916522	0	0	5.370146209
177.7828173	160.5795561	95.32845789	93.82067372	164.326474
157.6020651	127.9193074	110.069972	114.3102461	106.3288949
6.726917412	15.42289522	5.896605643	10.78398548	12.8883509
18.25877583	40.82531087	44.22454232	42.05754339	18.25849711
20.18075224	16.33012435	11.79321129	11.86238403	11.81432166
6.726917412	16.33012435	13.7587465	14.01918113	9.666263176
49.01039829	56.24820609	89.43185225	153.1325939	74.10801768
24.02470504	19.95904087	41.2762395	22.64636952	17.18446787
76.87905614	95.25905871	114.98381	77.64469549	93.44054403
65.34719772	68.04218479	81.56971139	78.72309404	69.81190071
20.18075224	23.58795739	20.63811975	18.33277532	21.48058484
36.51755167	26.30964479	27.517493	28.03836226	15.03640938
31.71261066	36.28916522	32.43133104	34.50875355	31.14684801
27.86865785	34.47470696	27.517493	19.41117387	22.55461408

3.843952807	0	0	0	0
18.25877583	11.7939787	8.844908464	9.705586936	0
0	4.536145653	4.913838036	7.548789839	4.296116967
0	5.443374783	0.982767607	0	1.074029242
37.47853987	47.17591479	73.70757054	72.25270275	54.77549133
140.3042775	194.1470339	149.3806763	153.1325939	160.030357
114.357596	0	48.15561275	257.7372531	102.032778
111.4746314	49.89760218	151.3462115	177.9357605	121.3653043
0	3.628916522	0.982767607	0	1.074029242
35.55656347	29.93856131	27.517493	58.23352162	99.88471948
14.41482303	6.350603914	26.53472539	9.705586936	22.55461408
0	2.721687392	3.931070429	6.470391291	0
0	0	0	0	0
0	27.21687392	0	0	0
49.01039829	40.82531087	50.12114796	59.31192017	59.0716083
143.1872421	117.939787	74.69033814	53.91992742	113.8470996
96.09882018	97.9807461	86.48354943	60.39031871	77.33010541
181.6267701	164.2084726	141.5185354	145.583804	184.7330296
4.804941009	16.33012435	16.70704932	7.548789839	6.444175451
0	0	7.862140857	24.80316661	2.148058484
0	0	0	8.627188388	5.370146209
31.71261066	5.443374783	43.24177471	10.78398548	13.96238014
36.51755167	23.58795739	29.48302821	23.72476807	52.62743285
30.75162246	20.86627	28.50026061	20.48957242	30.07281877
37.47853987	0	1.965535214	79.80149259	41.88714043
0	2.721687392	0	9.705586936	10.74029242
28.82964605	60.78435175	33.41409864	0	0
100.9037612	153.3217231	117.9321129	155.289391	147.1420061
90.33289097	76.20724696	67.81096489	117.5454418	86.99636858
29.79063425	42.63976914	34.39686625	11.86238403	79.47816389
16.33679943	18.14458261	30.46579582	32.35195645	16.11043863
78.80103254	66.22772653	78.62140857	118.6238403	83.77428086
0	0	0	0	18.25849711
146.0702067	177.8169096	181.8120073	153.1325939	233.0643455
113.3966078	117.939787	114.0010424	162.8381808	194.3992928
31.71261066	26.30964479	16.70704932	66.86071	38.6650527
0.960988202	0.907229131	0	0	0
6.726917412	57.15543522	4.913838036	168.2301736	68.73787147
0	0	0.982767607	5990.503937	1.074029242
6.726917412	6.350603914	0.982767607	0	1.074029242
6.726917412	15.42289522	10.81044368	8.627188388	3.222087725
77.84004434	84.37230914	102.2078311	83.03668823	92.36651479
15.37581123	19.05181174	22.60365496	7.548789839	5.370146209
17.29778763	69.85664305	15.72428171	60.39031871	65.51578375
6.726917412	0	16.70704932	1.078398548	1.074029242
22.10272864	49.89760218	61.91435925	49.60633323	36.51699422
14.41482303	40.82531087	65.84542968	25.88156516	37.59102346
11.53185842	0	32.43133104	9.705586936	5.370146209
0	0	27.517493	44.21434049	37.59102346
24.02470504	10.88674957	16.70704932	25.88156516	18.25849711

For Review Only

0	0	0.982767607	1.078398548	0
90.33289097	125.19762	121.8631833	132.6430215	115.9951581
4.804941009	0	2.948302821	9.705586936	0
256.5838499	234.9723448	228.0020849	400.0858615	458.6104862
11.53185842	15.42289522	21.62088736	11.86238403	13.96238014
0	0	0	0	0
4.804941009	12.70120783	4.913838036	9.705586936	9.666263176
17.29778763	16.33012435	14.74151411	8.627188388	12.8883509
7.687905614	0.907229131	4.913838036	11.86238403	4.296116967
0	0	7.862140857	0	4.296116967
59.58126851	0	0	0	0
0.960988202	0	2.948302821	0	0
0	0	0	0	5.370146209
1.921976404	0	0	0	1.074029242
0	0	0	5.391992742	0
18.25877583	13.60843696	33.41409864	14.01918113	22.55461408
12.49284662	15.42289522	0	18.33277532	10.74029242
0	0	1.965535214	1.078398548	4.296116967
4.804941009	3.628916522	2.948302821	5.391992742	12.8883509
0	2.721687392	3.931070429	0	1.074029242
49.97138649	49.89760218	48.15561275	50.68473178	31.14684801
24.98569325	8.165062175	28.50026061	12.94078258	11.81432166
0	6.350603914	0	0	1.074029242
1.921976404	0.907229131	0	0	0
22.10272864	19.95904087	7.862140857	23.72476807	60.14563754
0	0	0	0	0
0	9.072291305	0.982767607	0	0
112.4356196	108.8674957	109.0872044	128.3294273	146.0679769
255.6228617	237.6940322	387.2104372	393.6154702	353.3556205
0	0	0	0	0
0	78.92893436	6.87937325	47.44953613	38.6650527
134.5383482	194.1470339	109.0872044	176.8573619	151.4381231
7.687905614	10.88674957	4.913838036	0	7.518204692
0	0	2.948302821	0	0
0	10.88674957	0	0	0
30.75162246	29.03133218	57.98328882	50.68473178	32.22087725
43.24446908	62.59881001	37.34516907	49.60633323	45.10922815
43.24446908	31.75301957	48.15561275	45.29273904	38.6650527
32.67359886	25.40241565	6.87937325	66.86071	27.92476029
3.843952807	0.907229131	0	0	0
38.43952807	40.82531087	0	0	0
0	197.7759505	148.3979087	352.6363253	124.587392
5.765929211	3.628916522	5.896605643	3.235195645	2.148058484
5.765929211	9.072291305	8.844908464	2.156797097	7.518204692
32.67359886	0	32.43133104	11.86238403	122.4393336
9.609882018	222.271137	207.3639651	0	165.4005032
3.843952807	4.536145653	5.896605643	1.078398548	4.296116967
19.21976404	50.80483131	77.63864096	0	80.55219313
632.3302368	544.3374783	684.0062546	549.9832597	585.3459368
106.6696904	132.4554531	131.6908594	105.6830577	134.2536552

For Review Only

0.960988202	0.907229131	0	0	0
64.38620952	72.57833044	73.70757054	80.87989113	66.58981299
15.37581123	4.536145653	10.81044368	15.09757968	19.33252635
0	0	0	0	0
45.16644548	45.36145653	48.15561275	75.48789839	54.77549133
5.765929211	4.536145653	11.79321129	5.391992742	4.296116967
22.10272864	29.03133218	18.67258454	9.705586936	16.11043863
10.57087022	0.907229131	226.0365496	134.7998186	200.8434682
12.49284662	14.51566609	6.87937325	1.078398548	1.074029242
144.1482303	157.8578687	157.2428171	131.5646229	113.8470996
183.5487465	140.6205152	130.7080917	100.291065	108.4769534
108.5916668	293.9422383	48.15561275	188.719746	260.9891057
146.0702067	259.4675313	173.9498665	152.0541953	162.1784155
134.5383482	130.6409948	118.9148805	120.7806374	112.7730704
39.40051627	23.58795739	30.46579582	30.19515936	23.62864332
8.648893816	22.68072826	9.827676071	11.86238403	19.33252635
26.90766965	9.979520436	28.50026061	24.80316661	17.18446787
0	0	6.87937325	0	0
98.98178478	58.06266435	53.06945079	100.291065	86.99636858
2.882964605	2.721687392	0	0	2.148058484
112.4356196	89.81568392	34.39686625	4.313594194	1.074029242
35.55656347	26.30964479	3.931070429	5.391992742	9.666263176
0	0	0.982767607	5.391992742	0
4.804941009	13.60843696	7.862140857	12.94078258	8.592233934
42.28348088	68.04218479	29.48302821	38.82234774	47.25728664
22.10272864	0	1.965535214	1.078398548	1.074029242
37.47853987	37.19639435	49.13838036	76.56629694	54.77549133
116.2795724	149.6928065	159.2083524	197.3469344	177.2148249
0	0	0	0	0
0	0	1.965535214	1.078398548	0
67.26917412	53.5265187	67.81096489	77.64469549	121.3653043
1.921976404	0	4.913838036	4.313594194	0
0	0	575.9018178	663.2151073	671.2682761
0	0	1.965535214	0	0
283.4915195	244.9518652	445.193726	295.4812023	384.5024685
197.0025814	243.137407	290.8992117	176.8573619	190.1031758
0.960988202	0.907229131	4.913838036	0	13.96238014
122.0455016	373.7784018	1129.199981	975.9506864	0
823.5668889	995.2303562	1175.390058	953.3043168	1029.994043
69.19115053	90.72291305	132.673627	102.4478621	140.6978307
71.11312693	116.1253287	133.6563946	26.95996371	65.51578375
91.29387917	110.6819539	77.63864096	60.39031871	109.5509827
0	4.536145653	0.982767607	3.235195645	1.074029242
394.0051627	365.6133396	228.9848525	308.4219849	353.3556205
21.14174044	26.30964479	29.48302821	19.41117387	16.11043863
90.33289097	88.00122566	114.98381	80.87989113	76.25607616
19.21976404	19.95904087	7.862140857	4.313594194	10.74029242
150.8751477	166.0229309	3.931070429	45.29273904	92.36651479
530.4654874	537.0796453	605.384846	620.0791654	477.9430126
0	119.7542452	7.862140857	7.548789839	5.370146209

For Review Only

0	0	34.39686625	32.35195645	36.51699422
11.53185842	5.443374783	0	10.78398548	8.592233934
16.33679943	14.51566609	14.74151411	23.72476807	19.33252635
46.12743368	40.82531087	54.05221839	85.19348533	55.84952057
61.50324491	83.46508001	99.25952832	98.13426791	54.77549133
0.960988202	0	0	0	4.296116967
71.11312693	95.25905871	104.1733664	74.40949984	91.29248555
42.28348088	59.87712262	34.39686625	130.4862244	17.18446787
50.93237469	30.84579044	53.06945079	42.05754339	37.59102346
28.82964605	108.8674957	15.72428171	4.313594194	56.92354981
70.15213873	66.22772653	0.982767607	3.235195645	78.40413465
5.765929211	9.072291305	19.65535214	24.80316661	20.40655559
0	4.536145653	0	4.313594194	0
691.9115053	710.3604092	562.1430713	679.3910855	715.303475
0	0	1.965535214	0	0
0	0	0	0	1.074029242
0.960988202	0	0	0	2.148058484
52.8543511	69.85664305	31.44856343	32.35195645	41.88714043
209.495428	222.271137	129.7253241	171.4653692	223.3980823
65.34719772	111.5891831	39.31070429	56.07672452	18.25849711
20.18075224	44.4542274	14.74151411	31.27355791	37.59102346
137.4213129	184.1675135	200.4845919	210.2877169	179.3628834
0	0	0.982767607	0	0
0	8.165062175	5.896605643	8.627188388	0
20.18075224	20.86627	20.63811975	30.19515936	23.62864332
246.9739679	197.7759505	268.2955567	271.7564342	253.4709011
39.40051627	29.93856131	81.56971139	47.44953613	57.99757905
181.6267701	210.4771583	239.7952961	198.4253329	209.4357021
78.80103254	79.83616349	77.63864096	74.40949984	95.58860252
0	0	2.948302821	0	0
13.45383482	10.88674957	38.32793668	8.627188388	35.44296498
117.2405606	98.88797523	59.94882404	111.0750505	133.179626
0	0	0.982767607	1.078398548	1.074029242
8.648893816	0	6.87937325	6.470391291	12.8883509
8.648893816	1.814458261	0	0	0
0	0	8.844908464	0	6.444175451
42.28348088	23.58795739	21.62088736	33.430355	4.296116967
27.86865785	36.28916522	16.70704932	28.03836226	13.96238014
0	29.93856131	10.81044368	38.82234774	37.59102346
0	0	30.46579582	1.078398548	1.074029242
23.06371684	37.19639435	37.34516907	39.90074629	30.07281877
32.67359886	39.01085261	45.20730993	40.97914484	33.29490649
0	0	1.965535214	0	2.148058484
0	0	0	0	0
23.06371684	20.86627	51.10391557	57.15512307	33.29490649
197.0025814	239.5084905	187.708613	199.5037315	212.6577899
180.6657819	117.939787	81.56971139	60.39031871	64.44175451
72.07411513	59.87712262	75.67310575	58.23352162	70.88592996
122.0455016	132.4554531	157.2428171	83.03668823	126.7354505
3.843952807	0	3.931070429	0	4.296116967

For Review Only

21.14174044	20.86627	13.7587465	19.41117387	15.03640938
47.08842189	77.1144761	32.43133104	54.99832597	50.47937436
88.41091456	85.27953827	91.39738746	101.3694636	126.7354505
0	3.628916522	0	21.56797097	12.8883509
58.62028031	68.04218479	12.77597889	98.13426791	39.73908194
219.10531	290.3133218	219.1571764	421.6538324	355.503679
124.9284662	130.6409948	72.72480293	232.9340865	160.030357
2.882964605	0.907229131	34.39686625	38.82234774	0
96.09882018	87.09399653	139.5530002	141.2702098	80.55219313
2.882964605	0	5.896605643	1.078398548	0
91.29387917	153.3217231	143.4840706	130.4862244	131.0315675
90.33289097	81.65062175	66.82819729	184.4061518	53.70146209
16.33679943	16.33012435	14.74151411	6.470391291	5.370146209
35.55656347	26.30964479	34.39686625	0	31.14684801
33.63458706	33.56747783	26.53472539	42.05754339	36.51699422
77.84004434	69.85664305	86.48354943	81.95828968	73.03398844
29.79063425	33.56747783	43.24177471	34.50875355	69.81190071
78.80103254	39.01085261	334.1409864	0	363.0218837
24.98569325	0	53.06945079	0	0
32.67359886	39.91808174	36.36240146	70.09590565	50.47937436
170.0949117	120.6614744	96.3112255	135.8782171	74.10801768
105.7087022	136.0843696	96.3112255	154.2109924	169.6966202
53.8153393	18.14458261	31.44856343	39.90074629	26.85073104
0	0	0.982767607	3.235195645	0
0.960988202	3.628916522	1.965535214	6.470391291	2.148058484
174.8998527	125.19762	98.27676071	155.289391	115.9951581
21.14174044	17.23735348	7.862140857	7.548789839	6.444175451
195.080605	216.8277622	254.5368102	239.4044778	166.4745325
101.8647494	94.35182958	93.36292268	106.7614563	86.99636858
507.4017705	613.2868922	907.0945014	723.605426	747.5243523
62.46423311	95.25905871	103.1905987	57.15512307	18.25849711
0	2.721687392	0	0	1.074029242
88.41091456	93.44460045	99.25952832	126.1726302	200.8434682
98.02079658	109.7747248	75.67310575	91.66387662	97.736661
28.82964605	25.40241565	30.46579582	63.62551436	52.62743285
35.55656347	98.88797523	94.34569029	70.09590565	48.33131588
3.843952807	13.60843696	1.965535214	2.156797097	7.518204692
0	0	0	1.078398548	0
0	132.4554531	170.018796	1.078398548	1.074029242
0	0	0.982767607	1.078398548	3.222087725
65.34719772	48.99037305	42.25900711	51.76313033	44.03519891
0	47.17591479	0	0	0
66.30818592	48.08314392	65.84542968	90.58547807	62.29369602
0	0	2.948302821	2.156797097	1.074029242
332.5019178	346.5615279	297.778585	268.5212386	324.356831
54.7763275	62.59881001	87.46631704	77.64469549	63.36772526
75.91806794	79.83616349	93.36292268	89.50707952	98.81069024
51.8933629	49.89760218	51.10391557	53.91992742	61.21966678
0	0	0	0	0
0	927.1881714	1153.769171	141.2702098	302.8762462

For Review Only

1.921976404	1.814458261	0	5.391992742	5.370146209
264.2717555	285.7771761	319.3994723	313.8139776	207.2876437
0	0.907229131	0	5.391992742	5.370146209
0	0	3.931070429	0	1.074029242
93.21585557	73.48555957	94.34569029	67.93910855	80.55219313
0	0	7.862140857	0	0
0	0	3.931070429	9.705586936	0
197.9635696	194.1470339	254.5368102	223.2284995	183.6590003
16.33679943	24.49518652	48.15561275	40.97914484	42.96116967
42.28348088	37.19639435	44.22454232	59.31192017	86.99636858
35.55656347	49.89760218	40.29347189	39.90074629	30.07281877
29.79063425	44.4542274	20.63811975	16.17597823	48.33131588
199.885546	152.4144939	266.3300215	190.8765431	234.1383747
23.06371684	17.23735348	26.53472539	26.95996371	42.96116967
0	5.443374783	4.913838036	0	0
0	3.628916522	1.965535214	0	7.518204692
11.53185842	22.68072826	1.965535214	19.41117387	13.96238014
0	18.14458261	0	1.078398548	1.074029242
6.726917412	4.536145653	1.965535214	1.078398548	2.148058484
0	1.814458261	0.982767607	0	0
7.687905614	4.536145653	1.965535214	4.313594194	2.148058484
4.804941009	3.628916522	0.982767607	2.156797097	0
20.18075224	25.40241565	18.67258454	11.86238403	16.11043863
150.8751477	141.5277444	25.55195779	130.4862244	57.99757905
50.93237469	61.69158088	49.13838036	57.15512307	62.29369602
6.726917412	7.257833044	1.965535214	9.705586936	2.148058484
75.91806794	81.65062175	80.58694379	93.82067372	113.8470996
5990.80045	7192.512547	7297.049483	8626.109989	8477.312805
7503.395879	8959.794893	9699.916282	11658.56671	12432.9625
52.8543511	51.71206044	94.34569029	77.64469549	62.29369602
1080.150739	1820.808865	0	2569.823741	3473.410568
0	0	0	0	0
0	8.165062175	0	9.705586936	2.148058484
0	4.536145653	0	3.235195645	0
18.25877583	29.93856131	30.46579582	29.11676081	35.44296498
0	0	0	3.235195645	0
0	0	0	0	0
0	0	0.982767607	0	4.296116967
0	4.536145653	0	0	0
0	0	0.982767607	0	0
16.33679943	13.60843696	6.87937325	11.86238403	12.8883509
25.94668145	69.85664305	40.29347189	66.86071	52.62743285
24.02470504	19.95904087	25.55195779	24.80316661	31.14684801
149.9141595	160.5795561	123.8287185	132.6430215	125.6614213
47.08842189	50.80483131	56.01775361	42.05754339	36.51699422
7.687905614	0	0.982767607	2.156797097	5.370146209
316.1651184	352.0049026	372.4689231	303.0299921	364.095913
37.47853987	68.94941392	46.19007754	37.7439492	71.9599592
0	0	0.982767607	2.156797097	10.74029242
0	0	0	0	2.148058484

For Review Only

0	5.443374783	2.948302821	0	2.148058484
33.63458706	28.12410305	32.43133104	29.11676081	44.03519891
61.50324491	58.96989348	66.82819729	60.39031871	84.8483101
902.3679215	952.5905871	972.9399311	782.9173462	734.6360014
0.960988202	2.721687392	0	0	2.148058484
9.609882018	2.721687392	1.965535214	2.156797097	9.666263176
4.804941009	0	0	7.548789839	0
24.98569325	0	5.896605643	15.09757968	9.666263176
8.648893816	8.165062175	13.7587465	0	3.222087725
0	0	0.982767607	0	2.148058484
263.3107673	272.1687392	394.0898105	315.9707747	268.5073104
371.9024341	54.43374783	397.0381133	366.6555065	327.5789187
0	0	1.965535214	0	0
7.687905614	0	0.982767607	1.078398548	1.074029242
137.4213129	154.2289522	172.9670989	107.8398548	127.8094798
0	0	0	1.078398548	0
0	0	0.982767607	1.078398548	1.074029242
5.765929211	9.979520436	1.965535214	3.235195645	3.222087725
30.75162246	0	3.931070429	3.235195645	3.222087725
18.25877583	0.907229131	4.913838036	5.391992742	5.370146209
106.6696904	122.4759326	118.9148805	121.859036	140.6978307
73.99609154	97.07351697	248.6402046	7.548789839	54.77549133
246.9739679	207.7554709	237.8297609	179.014159	168.622591
111.4746314	109.7747248	107.1216692	231.8556879	187.9551173
32.67359886	26.30964479	35.37963386	53.91992742	48.33131588
18.25877583	5.443374783	4.913838036	4.313594194	1.074029242
22.10272864	17.23735348	20.63811975	10.78398548	13.96238014
0	11.7939787	0	0	0
4.804941009	17.23735348	8.844908464	0	10.74029242
22.10272864	9.072291305	2.948302821	2.156797097	10.74029242
86.48893816	107.9602665	69.77650011	64.70391291	85.92233934
5.765929211	8.165062175	3.931070429	0	1.074029242
0	4.536145653	15.72428171	0	0
10.57087022	0	0.982767607	18.33277532	5.370146209
0	1.814458261	0	0	11.81432166
22.10272864	27.21687392	16.70704932	14.01918113	12.8883509
12.49284662	6.350603914	17.68981693	2.156797097	11.81432166
28.82964605	33.56747783	32.43133104	31.27355791	27.92476029
99.94277298	69.85664305	90.41461986	90.58547807	94.51457327
71.11312693	60.78435175	85.50078182	173.6221663	97.736661
78.80103254	88.00122566	0	0	0
253.7008853	267.6325935	256.5023455	266.3644415	266.359252
4.804941009	0	0.982767607	0	0
1.921976404	5.443374783	0	0	0
3.843952807	1.814458261	0	0	11.81432166
91.29387917	96.16628784	123.8287185	117.5454418	73.03398844
112.4356196	71.67110131	53.06945079	65.78231146	39.73908194
0	0	2.948302821	2.156797097	0
209.495428	294.8494674	261.4161835	349.4011297	227.6941993
407.4589975	499.8832509	514.9702261	570.4728321	722.8216797

For Review Only

690.9505171	779.3098231	863.8527267	964.0883023	1238.355716
27.86865785	30.84579044	15.72428171	16.17597823	30.07281877
6.726917412	0	17.68981693	2.156797097	0
0	0	147.4151411	641.6471363	1.074029242
1.921976404	3.628916522	0	0	0
250.8179207	282.1482596	268.2955567	303.0299921	169.6966202
0	0	0	0	126.7354505
334.4238942	355.6338192	258.4678807	294.4028037	300.7281877
14.41482303	20.86627	15.72428171	14.01918113	16.11043863
14.41482303	16.33012435	20.63811975	16.17597823	12.8883509
225.8322274	252.2096983	131.6908594	227.5420937	247.0267256
114.357596	127.0120783	87.46631704	73.3311013	102.032778
32.67359886	36.28916522	22.60365496	25.88156516	41.88714043
2.882964605	0	4.913838036	0	0
63.42522132	80.74339262	78.62140857	73.3311013	50.47937436
93.21585557	106.1458083	67.81096489	59.31192017	111.6990411
35.55656347	24.49518652	30.46579582	52.84152887	27.92476029
0	3.628916522	2.948302821	0	0
0.960988202	1.814458261	0	0	0
81.68399715	88.00122566	89.43185225	0	65.51578375
141.2652657	129.7337657	110.069972	143.4270069	111.6990411
55.7373157	48.99037305	51.10391557	37.7439492	50.47937436
1.921976404	2.721687392	0	0	0
31.71261066	29.03133218	35.37963386	42.05754339	42.96116967
0	0	6.87937325	0	18.25849711
120.1235252	73.48555957	108.1044368	120.7806374	181.5109419
57.65929211	51.71206044	54.05221839	38.82234774	23.62864332
29.79063425	22.68072826	2.948302821	3.235195645	3.222087725
1653.860695	1580.393145	1662.842791	2249.539372	873.1857735
357.4876111	246.7663235	190.6569158	94.89907226	230.916287
32.67359886	32.6602487	38.32793668	0	71.9599592
0	0	0	0	0
10.57087022	25.40241565	9.827676071	23.72476807	17.18446787
0	0	0	0	0
92.25486737	57.15543522	102.2078311	75.48789839	94.51457327
0	22.68072826	6.87937325	22.64636952	9.666263176
0	4.536145653	0	0	24.70267256
0	2.721687392	0	0	1.074029242
105.7087022	129.7337657	69.77650011	200.58213	122.4393336
40.36150447	29.03133218	60.93159164	52.84152887	105.2548657
37.47853987	33.56747783	35.37963386	18.33277532	10.74029242
465.1182897	454.5217944	381.3138316	439.9866078	356.5777083
7.687905614	3.628916522	1.965535214	3.235195645	0
151.8361359	215.9205331	220.139944	172.5437678	216.9539068
1576.020651	1538.660605	1723.774383	1812.78796	2137.318191
0.960988202	25.40241565	789.1623885	0	93.44054403
0	2.721687392	3.931070429	6.470391291	4.296116967
11.53185842	0	23.58642257	0	1.074029242
0	0	2.948302821	3.235195645	0
0	0	2.948302821	0	0

For Review Only

13.45383482	0	2.948302821	0	0
6.726917412	0.907229131	21.62088736	0	0
0	0	0	0	0
0.960988202	0.907229131	2.948302821	1.078398548	4.296116967
0	4.536145653	0	0	2.148058484
0	4.536145653	0	0	5.370146209
0	6.350603914	18.67258454	20.48957242	21.48058484
0.960988202	0	0	0	0
188.3536875	214.1060748	186.7258454	168.2301736	128.883509
8.648893816	0	76.65587336	0	0
45.16644548	29.93856131	32.43133104	29.11676081	37.59102346
24.98569325	12.70120783	73.70757054	40.97914484	16.11043863
0	0	0	20.48957242	20.40655559
0	2.721687392	0	0	0
3.843952807	19.95904087	100.2422959	0	0
5.765929211	9.072291305	3.931070429	5.391992742	11.81432166
15.37581123	0.907229131	8.844908464	3.235195645	1.074029242
110.5136432	29.03133218	43.24177471	64.70391291	5.370146209
50.93237469	29.93856131	68.7937325	52.84152887	69.81190071
4.804941009	2.721687392	3.931070429	1.078398548	7.518204692
103.7867258	101.6096626	76.65587336	126.1726302	77.33010541
13.45383482	0	4.913838036	0	17.18446787
1.921976404	0	0	0	10.74029242
58.62028031	52.61928957	77.63864096	84.11508678	91.29248555
175.8608409	204.1265544	164.1221904	160.6813837	193.3252635
0	7.257833044	0	2.156797097	0
11.53185842	0	0.982767607	1.078398548	0
82.64498535	80.74339262	26.53472539	127.2510287	221.2500238
5.765929211	0	0	6.470391291	11.81432166
0	0	69.77650011	0	0
74.95707974	76.20724696	76.65587336	63.62551436	80.55219313
45.16644548	43.54699827	5.896605643	44.21434049	26.85073104
32.67359886	50.80483131	52.08668318	52.84152887	45.10922815
22.10272864	29.03133218	20.63811975	26.95996371	35.44296498
20.18075224	91.63014218	7.862140857	35.5871521	0
40.36150447	31.75301957	19.65535214	63.62551436	49.40534512
119.162537	130.6409948	104.1733664	111.0750505	36.51699422
1.921976404	1.814458261	0	0	0
0	0	0	4.313594194	0
198.9245578	185.0747426	191.6396834	158.5245866	123.5133628
55.7373157	60.78435175	57.00052121	51.76313033	37.59102346
0	0	0.982767607	1.078398548	3.222087725
159.5240415	166.0229309	243.7263666	223.2284995	177.2148249
20.18075224	41.73254	57.00052121	8.627188388	19.33252635
45.16644548	61.69158088	46.19007754	89.50707952	39.73908194
9.609882018	0	10.81044368	3.235195645	0
0	0	0.982767607	1.078398548	1.074029242
17.29778763	0	7.862140857	4.313594194	7.518204692
0.960988202	0.907229131	4.913838036	0	10.74029242
181.6267701	176.9096805	221.1227116	158.5245866	139.6238014

For Review Only

114.357596	136.0843696	72.72480293	86.27188388	113.8470996
25.94668145	40.82531087	23.58642257	56.07672452	47.25728664
532.3874638	518.9350627	366.5723175	630.8631508	578.9017613
75.91806794	63.50603914	26.53472539	44.21434049	25.7767018
63.42522132	91.63014218	40.29347189	69.0175071	82.70025162
0	0	0	9.705586936	0
0	0	0	10.78398548	5.370146209
13.45383482	21.77349913	9.827676071	23.72476807	28.99878953
38.43952807	32.6602487	39.31070429	28.03836226	33.29490649
4.804941009	0	0	0	0
3.843952807	9.072291305	6.87937325	8.627188388	6.444175451
31.71261066	39.91808174	34.39686625	28.03836226	60.14563754
3.843952807	3.628916522	0	7.548789839	0
51.8933629	52.61928957	51.10391557	59.31192017	73.03398844
28.82964605	23.58795739	51.10391557	25.88156516	19.33252635
82.64498535	64.41326827	57.98328882	59.31192017	30.07281877
36.51755167	28.12410305	25.55195779	45.29273904	34.36893574
9.609882018	19.95904087	0	0	0
16.33679943	35.38193609	26.53472539	23.72476807	25.7767018
19.21976404	12.70120783	32.43133104	21.56797097	17.18446787
49.97138649	39.91808174	13.7587465	8.627188388	1.074029242
138.3823011	211.3843874	3.931070429	114.3102461	76.25607616
0	0	0	4.313594194	0
115.3185842	136.9915987	127.7597889	107.8398548	148.2160354
46.12743368	19.05181174	38.32793668	36.66555065	44.03519891
2.882964605	0.907229131	15.72428171	14.01918113	12.8883509
0	0	0.982767607	1.078398548	1.074029242
3.843952807	1.814458261	47.17284514	51.76313033	59.0716083
0	0.907229131	0	0	0
0	0	1.965535214	0	4.296116967
0	0	0	0	0
0	19.05181174	0.982767607	0	2.148058484
0	3.628916522	0	1.078398548	0
74.95707974	40.82531087	32.43133104	91.66387662	50.47937436
0	56.24820609	106.1389016	104.6046592	0
0	0	0.982767607	0	0
0	3.628916522	0	7.548789839	5.370146209
0.960988202	0.907229131	0	0	0
0	14.51566609	7.862140857	7.548789839	6.444175451
17.29778763	25.40241565	36.36240146	36.66555065	34.36893574
73.03510333	57.15543522	58.96605643	102.4478621	56.92354981
42.28348088	0	60.93159164	46.37113758	27.92476029
44.20545728	42.63976914	48.15561275	52.84152887	59.0716083
0	3.628916522	0	3.235195645	0
87.44992636	96.16628784	14.74151411	67.93910855	5.370146209
0	0	0	0	0
24.02470504	11.7939787	8.844908464	3.235195645	5.370146209
0	0	0	0	0
22.10272864	10.88674957	13.7587465	3.235195645	7.518204692
0	0	5.896605643	2.156797097	0

For Review Only

34.59557526	42.63976914	36.36240146	14.01918113	15.03640938
37.47853987	29.93856131	46.19007754	10.78398548	30.07281877
55.7373157	40.82531087	30.46579582	76.56629694	10.74029242
33.63458706	42.63976914	35.37963386	35.5871521	13.96238014
22.10272864	36.28916522	29.48302821	24.80316661	31.14684801
29.79063425	30.84579044	44.22454232	18.33277532	11.81432166
3.843952807	0	0	1.078398548	4.296116967
6.726917412	30.84579044	7.862140857	14.01918113	12.8883509
0.960988202	1.814458261	5.896605643	3.235195645	0
22.10272864	31.75301957	18.67258454	31.27355791	40.81311119
16.33679943	30.84579044	23.58642257	16.17597823	23.62864332
49.97138649	58.96989348	47.17284514	44.21434049	48.33131588
4.804941009	0.907229131	0.982767607	2.156797097	0
0	0	3.931070429	1.078398548	1.074029242
12.49284662	23.58795739	25.55195779	30.19515936	10.74029242
0	141.5277444	0	0	26.85073104
37.47853987	33.56747783	34.39686625	49.60633323	16.11043863
12.49284662	7.257833044	11.79321129	12.94078258	15.03640938
0	0	0	0	0
0.960988202	0	0.982767607	0	0
38.43952807	28.12410305	64.86266207	33.430355	45.10922815
14.41482303	20.86627	10.81044368	17.25437678	23.62864332
6.726917412	17.23735348	6.87937325	16.17597823	9.666263176
11.53185842	0.907229131	3.931070429	3.235195645	1.074029242
0	4.536145653	0	0	0
26.90766965	45.36145653	24.56919018	31.27355791	24.70267256
150.8751477	204.1265544	210.3122679	208.1309199	149.2900646
221.0272864	157.8578687	137.587465	186.5629489	201.9174975
29.79063425	35.38193609	35.37963386	30.19515936	37.59102346
22.10272864	30.84579044	53.06945079	61.46871726	47.25728664
5.765929211	25.40241565	13.7587465	0	16.11043863
13.45383482	17.23735348	15.72428171	14.01918113	17.18446787
27.86865785	27.21687392	35.37963386	23.72476807	34.36893574
77.84004434	56.24820609	80.58694379	127.2510287	88.07039782
0	21.77349913	49.13838036	32.35195645	55.84952057
31.71261066	15.42289522	24.56919018	20.48957242	21.48058484
0	0	0	0	0
12.49284662	17.23735348	14.74151411	22.64636952	13.96238014
53.8153393	35.38193609	15.72428171	53.91992742	45.10922815
1.921976404	4.536145653	0	0	4.296116967
0	0	123.8287185	94.89907226	96.66263176
15.37581123	9.072291305	4.913838036	22.64636952	12.8883509
3.843952807	4.536145653	0	6.470391291	5.370146209
12.49284662	13.60843696	18.67258454	33.430355	5.370146209
19.21976404	0	1.965535214	4.313594194	0
0	3.628916522	0.982767607	0	1.074029242
3.843952807	6.350603914	1.965535214	1.078398548	8.592233934
41.32249268	54.43374783	34.39686625	62.54711581	39.73908194
1.921976404	1.814458261	0	0	0
16.33679943	9.072291305	18.67258454	29.11676081	10.74029242

For Review Only

25.94668145	32.6602487	35.37963386	24.80316661	33.29490649
66.30818592	83.46508001	90.41461986	44.21434049	74.10801768
46.12743368	40.82531087	43.24177471	33.430355	78.40413465
145.1092185	0	1.965535214	1.078398548	12.8883509
0	0	0	0	0
0	0	0.982767607	3.235195645	1.074029242
5.765929211	11.7939787	13.7587465	6.470391291	6.444175451
52.8543511	66.22772653	108.1044368	16.17597823	28.99878953
24.98569325	33.56747783	12.77597889	35.5871521	12.8883509
0.960988202	0	0	0	0
310.3991892	288.4988635	271.2438596	308.4219849	283.5437198
0	183.2602844	0.982767607	1.078398548	1.074029242
3.843952807	6.350603914	3.931070429	0	3.222087725
100.9037612	0.907229131	57.98328882	176.8573619	0
2.882964605	0	2.948302821	0	1.074029242
0	22.68072826	0	52.84152887	0
1.921976404	0	0	0	0
0	6.350603914	9.827676071	1.078398548	4.296116967
0.960988202	5.443374783	6.87937325	7.548789839	4.296116967
0.960988202	0	0	1.078398548	0
0	0.907229131	0	0	0
0	0	0	0	0
9.609882018	58.06266435	0	0	0
6.726917412	0	0	15.09757968	0
209.495428	156.9506396	157.2428171	179.014159	249.1747841
0	4.536145653	0	8.627188388	0
92.25486737	128.8265365	85.50078182	193.0333402	165.4005032
87.44992636	0	176.8981693	77.64469549	12.8883509
2.882964605	7.257833044	0	0	1.074029242
15.37581123	0	9.827676071	4.313594194	6.444175451
0	0	1.965535214	1.078398548	5.370146209
0	0	6.87937325	0	0
44.20545728	46.26868566	33.41409864	50.68473178	32.22087725
96.09882018	124.2903909	89.43185225	88.42868097	79.47816389
0	0.907229131	5.896605643	1.078398548	0
120.1235252	136.0843696	241.7608314	252.3452603	30.07281877
0.960988202	48.08314392	0	46.37113758	35.44296498
0	0	2.948302821	0	1.074029242
7.687905614	12.70120783	15.72428171	7.548789839	17.18446787
98.98178478	117.939787	91.39738746	87.35028243	34.36893574
0	0	0.982767607	1.078398548	12.8883509
66.30818592	60.78435175	54.05221839	67.93910855	62.29369602
174.8998527	162.3940144	129.7253241	254.5020574	233.0643455
28.82964605	19.05181174	12.77597889	16.17597823	22.55461408
40.36150447	45.36145653	36.36240146	23.72476807	37.59102346
213.3393808	194.1470339	274.1921624	177.9357605	124.587392
197.0025814	117.0325578	0	0	355.503679
116.2795724	71.67110131	168.0532608	80.87989113	74.10801768
215.2613572	49.89760218	0	54.99832597	282.4696906
122.0455016	107.9602665	75.67310575	120.7806374	94.51457327

For Review Only

8.648893816	9.072291305	10.81044368	5.391992742	6.444175451
16.33679943	9.072291305	17.68981693	2.156797097	24.70267256
328.657965	296.6639257	331.1926836	313.8139776	301.8022169
3.843952807	5.443374783	5.896605643	5.391992742	7.518204692
15.37581123	40.82531087	4.913838036	15.09757968	44.03519891
0	0	12.77597889	1.078398548	4.296116967
49.01039829	42.63976914	41.2762395	50.68473178	27.92476029
0	9.979520436	2.948302821	7.548789839	9.666263176
1191.62537	0	1092.837579	0	0
1.921976404	6.350603914	9.827676071	5.391992742	1.074029242
8.648893816	0	6.87937325	3.235195645	2.148058484
98.02079658	0	0	0	0
0	0	0	0	0
0	0	1.965535214	0	0
47.08842189	64.41326827	41.2762395	38.82234774	53.70146209
167.2119471	154.2289522	154.2945143	252.3452603	178.2888541
222.9492628	254.0241565	326.2788456	409.7914484	324.356831
308.4772128	313.9012792	361.6584794	434.594615	380.2063516
213.3393808	199.5904087	251.5885074	366.6555065	354.4296498
172.0168881	171.4663057	211.2950355	257.7372531	335.0971234
231.5981566	257.6530731	284.0198385	569.3944336	292.1359538
246.9739679	288.4988635	355.7618738	414.1050426	419.9454335
40.36150447	33.56747783	30.46579582	44.21434049	49.40534512
35.55656347	32.6602487	30.46579582	21.56797097	26.85073104
68.23016233	0	0	0	0
34.59557526	15.42289522	62.89712686	79.80149259	0
3.843952807	0	0	0	1.074029242
0	0.907229131	0	2.156797097	3.222087725
1509.712465	1675.652204	1974.380123	1728.672873	1557.342401
11.53185842	9.979520436	41.2762395	0	0
250.8179207	200.4976378	215.226106	0	0
0	0	0.982767607	24.80316661	6.444175451
0	0	1.965535214	1.078398548	1.074029242
0	0	0	0	0
60.54225671	74.3927887	35.37963386	9.705586936	15.03640938
0	0	1.965535214	0	0
7.687905614	0	0	0	6.444175451
0	0	0	4.313594194	0
0	0	0	10.78398548	0
38.43952807	20.86627	45.20730993	33.430355	28.99878953
0	0	0	0	1.074029242
4.804941009	4.536145653	2.948302821	5.391992742	3.222087725
0	0	2.948302821	3.235195645	0
0	0	0.982767607	0	0
62.46423311	88.90845479	64.86266207	52.84152887	97.736661
24.02470504	32.6602487	28.50026061	7.548789839	47.25728664
92.25486737	88.90845479	62.89712686	58.23352162	57.99757905
107.6306786	75.30001783	42.25900711	64.70391291	122.4393336
0	0	57.98328882	0	5.370146209
338.267847	496.2543344	774.4208744	710.6646434	561.7172934

For Review Only

0	0	0	0	0
0	658.6483488	310.5545639	0	311.4684801
0	0	0	1.078398548	0
0	0.907229131	7.862140857	0	0
1.921976404	0.907229131	0	3.235195645	3.222087725
1.921976404	3.628916522	2.948302821	5.391992742	6.444175451
0.960988202	0	0	1.078398548	3.222087725
0	0	6.87937325	4.313594194	3.222087725
195.080605	189.6108883	266.3300215	230.7772894	252.3968718
25.94668145	0	25.55195779	43.13594194	0
0.960988202	224.9928244	0	0	0
155.6800887	145.1566609	676.1441137	0	0
0	0	0	0	0
0	0	0	0	11.81432166
0	0	0	0	0
18.25877583	21.77349913	38.32793668	14.01918113	26.85073104
0	0.907229131	0	0	0
0	7.257833044	0.982767607	1.078398548	0
9.609882018	9.072291305	2.948302821	2.156797097	4.296116967
8.648893816	7.257833044	0.982767607	5.391992742	8.592233934
506.4407823	572.4615814	521.8495994	542.4344699	571.3835566
225.8322274	206.8482418	235.8642257	344.009137	266.359252
112.4356196	107.9602665	107.1216692	70.09590565	97.736661
1404.003763	1147.64485	2279.038081	2421.004741	637.9733696
0	6.350603914	8.844908464	0	0
2.882964605	3.628916522	0	1.078398548	0
0	0	0	130.4862244	0
649.6280244	0	290.8992117	801.2501215	956.9600544
0	0	0	0	0
0	14.51566609	3.931070429	19.41117387	21.48058484
117.2405606	0	129.7253241	130.4862244	208.3616729
0.960988202	3.628916522	84.51801421	0	0
61.50324491	46.26868566	97.29399311	22.64636952	22.55461408
4965.426039	23.58795739	10174.59304	0	0
313.2821538	25.40241565	504.1597825	735.46781	577.8277321
0	0	270.261092	0	4.296116967
629.4472722	620.5447253	521.8495994	786.1525418	532.7185039
544.8803104	604.2146009	202.4501271	620.0791654	454.3143693
138.3823011	446.3567322	1918.362369	0	0
0	0	0	1.078398548	2.148058484
68.23016233	61.69158088	65.84542968	79.80149259	52.62743285
67.26917412	54.43374783	62.89712686	56.07672452	57.99757905
141.2652657	0	0.982767607	1.078398548	1.074029242
83.60597355	128.8265365	106.1389016	85.19348533	125.6614213
54.7763275	30.84579044	7.862140857	18.33277532	24.70267256
16.33679943	6.350603914	5.896605643	6.470391291	6.444175451
71.11312693	53.5265187	73.70757054	80.87989113	86.99636858
147.0311949	91.63014218	141.5185354	71.1743042	121.3653043
5.765929211	15.42289522	9.827676071	12.94078258	16.11043863
0	0	11.79321129	2.156797097	7.518204692

For Review Only

38.43952807	48.08314392	25.55195779	58.23352162	60.14563754
75.91806794	48.99037305	13.7587465	26.95996371	44.03519891
11.53185842	0	0	0	0
0.960988202	0.907229131	0	0	358.7257667
117.2405606	110.6819539	70.75926771	98.13426791	100.9587487
88.41091456	70.76387218	93.36292268	72.25270275	71.9599592
1280.036285	1516.887106	1611.738876	1429.956475	1474.642149
34.59557526	18.14458261	13.7587465	26.95996371	15.03640938
60.54225671	140.6205152	103.1905987	156.3677895	93.44054403
0	10.88674957	4.913838036	0	2.148058484
6.726917412	0	2.948302821	1.078398548	1.074029242
48.04941009	39.91808174	63.87989446	32.35195645	59.0716083
65.34719772	60.78435175	115.9665776	26.95996371	28.99878953
273.8816375	316.6229666	28.50026061	404.3994557	307.1723631
188.3536875	277.6121139	272.2266272	251.2668618	243.8046379
0	0	0	0	0
81.68399715	71.67110131	76.65587336	80.87989113	82.70025162
4.804941009	12.70120783	23.58642257	6.470391291	13.96238014
38.43952807	34.47470696	33.41409864	26.95996371	12.8883509
6.726917412	2.721687392	0	0	5.370146209
0	0	0	1.078398548	0
167.2119471	156.0434105	171.9843312	170.3869707	183.6590003
59.58126851	61.69158088	64.86266207	67.93910855	48.33131588
4.804941009	0	6.87937325	3.235195645	4.296116967
4.804941009	4.536145653	0.982767607	3.235195645	2.148058484
0	0.907229131	0	2.156797097	3.222087725
277.7255903	243.137407	189.6741482	172.5437678	139.6238014
2.882964605	0	0	0	0
0	0	0	0	0
0	0	1.965535214	0	0
0	3.628916522	0	0	0
0	0	2.948302821	0	0
0	0	0	0	0
4.804941009	27.21687392	151.3462115	109.9966519	0
0.960988202	14.51566609	84.51801421	0	0
0	0	0	4.313594194	8.592233934
5.765929211	0	0.982767607	30.19515936	0
44.20545728	98.88797523	0	133.72142	123.5133628
150.8751477	120.6614744	110.069972	121.859036	53.70146209
80.72300895	77.1144761	112.0355072	58.23352162	62.29369602
26.90766965	34.47470696	33.41409864	25.88156516	30.07281877
0	43.54699827	89.43185225	0	86.99636858
48.04941009	50.80483131	44.22454232	60.39031871	55.84952057
0	0	4.913838036	1.078398548	0
152.7971241	185.0747426	123.8287185	135.8782171	176.1407956
50.93237469	40.82531087	33.41409864	31.27355791	53.70146209
0	0	10.81044368	0	45.10922815
26.90766965	20.86627	19.65535214	24.80316661	24.70267256
0	68.94941392	0	0	0
0.960988202	1.814458261	6.87937325	0	0

For Review Only

0	211.3843874	9.827676071	86.27188388	133.179626
9.609882018	19.05181174	38.32793668	44.21434049	36.51699422
39.40051627	37.19639435	21.62088736	86.27188388	53.70146209
26.90766965	22.68072826	20.63811975	19.41117387	15.03640938
33.63458706	29.93856131	88.44908464	16.17597823	17.18446787
6.726917412	0	6.87937325	6.470391291	4.296116967
320.9700594	347.468757	317.4339371	208.1309199	308.2463924
103.7867258	52.61928957	54.05221839	101.3694636	111.6990411
32.67359886	24.49518652	41.2762395	36.66555065	22.55461408
55.7373157	155.1361813	16.70704932	86.27188388	105.2548657
105.7087022	120.6614744	106.1389016	108.9182534	52.62743285
7.687905614	5.443374783	12.77597889	6.470391291	12.8883509
0	4.536145653	1.965535214	3.235195645	4.296116967
83.60597355	61.69158088	56.01775361	60.39031871	71.9599592
6.726917412	10.88674957	7.862140857	12.94078258	8.592233934
76.87905614	64.41326827	61.91435925	69.0175071	68.73787147
71.11312693	104.33135	137.587465	97.05586936	135.3276845
0.960988202	0	5.896605643	2.156797097	0
0.960988202	9.072291305	5.896605643	7.548789839	2.148058484
12.49284662	15.42289522	8.844908464	17.25437678	23.62864332
0	0	0	1.078398548	0
36.51755167	28.12410305	33.41409864	32.35195645	37.59102346
0	0	0.982767607	1.078398548	0
7.687905614	0	1.965535214	0	5.370146209
105.7087022	88.00122566	110.069972	122.9374345	148.2160354
357.4876111	339.3036948	290.8992117	315.9707747	336.1711527
0.960988202	0	0	0	0
94.17684377	73.48555957	70.75926771	79.80149259	89.14442707
19.21976404	8.165062175	22.60365496	8.627188388	25.7767018
52.8543511	22.68072826	22.60365496	43.13594194	34.36893574
123.0064898	78.02170523	102.2078311	198.4253329	68.73787147
3.843952807	1.814458261	0	3.235195645	1.074029242
38.43952807	28.12410305	26.53472539	29.11676081	51.5534036
73.03510333	107.0530374	156.2600495	162.8381808	167.5485617
50.93237469	70.76387218	59.94882404	54.99832597	118.1432166
127.8114308	99.79520436	97.29399311	210.2877169	185.8070588
49.01039829	171.4663057	65.84542968	215.6797097	46.1832574
7.687905614	48.99037305	85.50078182	0	117.0691874
2.882964605	0	0	0	0
15.37581123	0.907229131	12.77597889	11.86238403	10.74029242
0	0.907229131	0.982767607	0	0
0	0	0	0	5.370146209
751.4927738	491.7181887	357.727409	359.1067166	363.0218837
464.1573015	534.3579579	442.2454232	479.8873541	591.7901122
11.53185842	5.443374783	4.913838036	9.705586936	3.222087725
13.45383482	15.42289522	15.72428171	16.17597823	35.44296498
5.765929211	2.721687392	1.965535214	4.313594194	2.148058484
0	6.350603914	7.862140857	0	1.074029242
0	5.443374783	1.965535214	0	0
7.687905614	0	0	5.391992742	5.370146209

For Review Only

27.86865785	37.19639435	45.20730993	16.17597823	18.25849711
17.29778763	28.12410305	12.77597889	17.25437678	11.81432166
0	11.7939787	0	0	0
5.765929211	8.165062175	4.913838036	9.705586936	11.81432166
6.726917412	0	0	0	3.222087725
18.25877583	18.14458261	10.81044368	14.01918113	6.444175451
16.33679943	11.7939787	24.56919018	8.627188388	9.666263176
0	0	0	0	0
0	0	0	0	1.074029242
60.54225671	78.92893436	45.20730993	38.82234774	68.73787147
0	5.443374783	0	4.313594194	0
5.765929211	0	10.81044368	0	0
34.59557526	13.60843696	11.79321129	34.50875355	26.85073104
8.648893816	10.88674957	21.62088736	2.156797097	2.148058484
0	5.443374783	1.965535214	5.391992742	4.296116967
75.91806794	79.83616349	77.63864096	65.78231146	49.40534512
0.960988202	0.907229131	0	0	17.18446787
0.960988202	7.257833044	0	0	0
109.552655	105.2385791	81.56971139	104.6046592	95.58860252
6.726917412	0	3.931070429	0	8.592233934
3.843952807	14.51566609	15.72428171	24.80316661	0
51.8933629	31.75301957	16.70704932	26.95996371	48.33131588
105.7087022	88.90845479	98.27676071	134.7998186	68.73787147
13.45383482	29.03133218	18.67258454	24.80316661	20.40655559
51.8933629	36.28916522	50.12114796	45.29273904	41.88714043
367.0974931	303.9217587	222.1054792	307.3435863	353.3556205
0	10.88674957	4.913838036	0	0
7.687905614	0.907229131	1.965535214	5.391992742	3.222087725
95.13783197	86.1867674	46.19007754	243.718072	65.51578375
101.8647494	79.83616349	95.32845789	75.48789839	69.81190071
23.06371684	30.84579044	26.53472539	0	19.33252635
71.11312693	97.9807461	49.13838036	23.72476807	55.84952057
157.6020651	116.1253287	111.0527396	128.3294273	123.5133628
11.53185842	9.072291305	23.58642257	9.705586936	5.370146209
16.33679943	29.03133218	27.517493	0	20.40655559
56.6983039	50.80483131	60.93159164	49.60633323	47.25728664
12.49284662	16.33012435	14.74151411	1.078398548	8.592233934
79.76202075	59.87712262	57.00052121	70.09590565	90.21845631
149.9141595	220.4566787	212.2778031	195.1901373	198.6954097
32.67359886	30.84579044	30.46579582	29.11676081	24.70267256
0	0	5.896605643	2.156797097	17.18446787
0	0	0.982767607	1.078398548	1.074029242
16.33679943	0	34.39686625	16.17597823	6.444175451
11.53185842	0	15.72428171	9.705586936	11.81432166
26.90766965	31.75301957	34.39686625	6.470391291	19.33252635
0	0	0.982767607	1.078398548	1.074029242
68.23016233	56.24820609	30.46579582	116.4670432	73.03398844
17.29778763	30.84579044	21.62088736	22.64636952	22.55461408
35.55656347	44.4542274	38.32793668	25.88156516	30.07281877
36.51755167	26.30964479	18.67258454	21.56797097	18.25849711

184.5097347	135.1771404	219.1571764	280.3836226	269.5813397
15.37581123	9.979520436	9.827676071	8.627188388	13.96238014
342.1117998	380.1290057	302.692423	493.9065352	278.1735736
2.882964605	0	8.844908464	3.235195645	0
0	0	183.7775425	0	2.148058484
0	0	15.72428171	10.78398548	22.55461408
125.8894544	127.9193074	123.8287185	140.1918113	138.5497722
100.9037612	96.16628784	42.25900711	85.19348533	66.58981299
29.79063425	42.63976914	44.22454232	30.19515936	37.59102346
59.58126851	81.65062175	94.34569029	75.48789839	90.21845631
3.843952807	9.072291305	9.827676071	0	8.592233934
26.90766965	16.33012435	134.6391622	70.09590565	49.40534512
24.98569325	15.42289522	23.58642257	20.48957242	21.48058484
4.804941009	6.350603914	7.862140857	1.078398548	1.074029242
0	0	0	1.078398548	0
0	0	2.948302821	0	0
0.960988202	3.628916522	0	0	0
182.5877583	155.1361813	121.8631833	52.84152887	128.883509
52.8543511	53.5265187	105.156134	29.11676081	52.62743285
0	0.907229131	0	0	0
0	0	3.931070429	3.235195645	3.222087725
57.65929211	44.4542274	66.82819729	52.84152887	75.18204692
1499.141595	1142.201475	1304.132615	120.7806374	107.4029242
95.13783197	55.34097696	20.63811975	88.42868097	30.07281877
0	0	783.2657829	801.2501215	735.7100306
73.99609154	52.61928957	64.86266207	67.93910855	33.29490649
206.6124634	218.6422205	178.8637045	203.8173257	197.6213805
98.98178478	80.74339262	94.34569029	84.11508678	98.81069024
1.921976404	0	3.931070429	0	0
0	0	0.982767607	0	0
5.765929211	0	1.965535214	12.94078258	2.148058484
12.49284662	12.70120783	95.32845789	134.7998186	135.3276845
0	0	1.965535214	0	3.222087725
3.843952807	6.350603914	10.81044368	10.78398548	12.8883509
2.882964605	4.536145653	3.931070429	22.64636952	5.370146209
0	0	0	0	1.074029242
3.843952807	4.536145653	0.982767607	3.235195645	0
0	0	3.931070429	0	0
150.8751477	151.5072648	173.9498665	120.7806374	147.1420061
0	0	0	0	0
0	0	0	0	0
175.8608409	219.5494496	312.5200991	411.9482455	253.4709011
78.80103254	95.25905871	108.1044368	66.86071	86.99636858
37.47853987	39.91808174	71.74203532	58.23352162	67.66384223
0	0	0.982767607	1.078398548	1.074029242
0	6.350603914	29.48302821	16.17597823	0
24.02470504	36.28916522	35.37963386	33.430355	17.18446787
49.01039829	70.76387218	31.44856343	56.07672452	49.40534512
42.28348088	32.6602487	30.46579582	77.64469549	20.40655559
120.1235252	146.06389	110.069972	118.6238403	117.0691874

For Review Only

41.32249268	37.19639435	66.82819729	26.95996371	22.55461408
19.21976404	19.95904087	7.862140857	21.56797097	26.85073104
103.7867258	86.1867674	89.43185225	88.42868097	61.21966678
1.921976404	0	0	0	0
170.0949117	131.5482239	115.9665776	116.4670432	133.179626
231.5981566	263.0964479	278.1232328	372.0474992	270.6553689
138.3823011	153.3217231	63.87989446	106.7614563	133.179626
606.3835553	481.7386683	377.3827611	410.869847	499.4235974
4.804941009	3.628916522	5.896605643	0	0
0	0	0	0	0
37.47853987	33.56747783	38.32793668	50.68473178	49.40534512
107.6306786	48.99037305	173.9498665	78.72309404	21.48058484
101.8647494	90.72291305	86.48354943	56.07672452	73.03398844
6.726917412	0	1.965535214	0	3.222087725
20.18075224	22.68072826	9.827676071	71.1743042	13.96238014
0	42.63976914	163.1394228	61.46871726	34.36893574
13.45383482	19.05181174	12.77597889	5.391992742	20.40655559
57.65929211	78.02170523	65.84542968	0	0
0	0	0.982767607	1.078398548	2.148058484
151.8361359	137.8988278	189.6741482	147.7406011	142.8458892
0	0	83.53524661	0	0
76.87905614	58.96989348	25.55195779	33.430355	61.21966678
24.02470504	15.42289522	8.844908464	22.64636952	26.85073104
0	0	8.844908464	0	2.148058484
104.747714	103.4241209	136.6046974	131.5646229	133.179626
0	0	81.56971139	0	119.2172458
57.65929211	95.25905871	68.7937325	127.2510287	107.4029242
223.910251	265.8181352	232.9159229	263.1292458	356.5777083
10.57087022	9.072291305	9.827676071	4.313594194	13.96238014
93.21585557	113.4036413	123.8287185	119.7022389	147.1420061
17.29778763	5.443374783	0	0	136.4017137
44.20545728	88.00122566	40.29347189	102.4478621	12.8883509
0	0	0	1.078398548	0
209.495428	190.5181174	206.3811975	186.5629489	154.6602108
94.17684377	110.6819539	112.0355072	186.5629489	129.9575383
156.6410769	141.5277444	241.7608314	32.35195645	168.622591
0	0	0.982767607	0	1.074029242
128.772419	193.2398048	200.4845919	221.0717024	136.4017137
26.90766965	29.03133218	17.68981693	4.313594194	19.33252635
305.5942482	0	0.982767607	1.078398548	2.148058484
0	0	5.896605643	0	1.074029242
6.726917412	18.14458261	6.87937325	15.09757968	12.8883509
10.57087022	11.7939787	14.74151411	1.078398548	3.222087725
4.804941009	0	3.931070429	0	0
6.726917412	0	3.931070429	6.470391291	3.222087725
27.86865785	19.05181174	26.53472539	21.56797097	71.9599592
1.921976404	9.072291305	0.982767607	9.705586936	0
71.11312693	57.15543522	71.74203532	53.91992742	48.33131588
100.9037612	98.88797523	22.60365496	361.2635137	102.032778
5.765929211	7.257833044	0	0	0

For Review Only

45.16644548	68.94941392	63.87989446	76.56629694	69.81190071
62.46423311	48.08314392	94.34569029	46.37113758	47.25728664
1.921976404	12.70120783	18.67258454	12.94078258	6.444175451
0	2.721687392	0	1.078398548	0
159.5240415	141.5277444	188.6913806	245.874869	113.8470996
89.37190276	77.1144761	108.1044368	129.4078258	64.44175451
0	0.907229131	0	0	0
180.6657819	183.2602844	199.5018242	126.1726302	249.1747841
0	0	0	0	0
4.804941009	0.907229131	0	0	0
296.9453543	302.1073005	302.692423	266.3644415	249.1747841
96.09882018	98.88797523	57.00052121	118.6238403	156.8082693
39.40051627	40.82531087	15.72428171	36.66555065	31.14684801
49.97138649	47.17591479	51.10391557	39.90074629	77.33010541
50.93237469	73.48555957	57.00052121	4.313594194	38.6650527
9.609882018	5.443374783	12.77597889	5.391992742	11.81432166
70.15213873	14.51566609	45.20730993	0	0
1.921976404	1.814458261	26.53472539	0	0
230.6371684	207.7554709	190.6569158	136.9566157	141.7718599
0.960988202	5.443374783	1.965535214	11.86238403	8.592233934
0	0	0	7.548789839	4.296116967
35.55656347	42.63976914	23.58642257	23.72476807	46.1832574
70.15213873	58.06266435	45.20730993	52.84152887	25.7767018
10.57087022	8.165062175	6.87937325	12.94078258	19.33252635
0	54.43374783	0	0	0
29.79063425	24.49518652	54.05221839	45.29273904	39.73908194
26.90766965	38.10362348	59.94882404	48.52793468	56.92354981
98.02079658	128.8265365	129.7253241	150.9757968	126.7354505
5.765929211	4.536145653	6.87937325	8.627188388	12.8883509
0.960988202	0	0	1.078398548	4.296116967
4.804941009	9.979520436	8.844908464	0	1.074029242
0.960988202	3.628916522	5.896605643	5.391992742	3.222087725
144.1482303	0	0.982767607	1.078398548	1.074029242
93.21585557	76.20724696	104.1733664	200.58213	49.40534512
0	2.721687392	0	5.391992742	0
0	8.165062175	0	0	0
9.609882018	9.072291305	25.55195779	2.156797097	12.8883509
23.06371684	21.77349913	22.60365496	72.25270275	22.55461408
61.50324491	69.85664305	55.034986	49.60633323	69.81190071
0	867.3110488	1512.479347	1828.963938	1231.91154
0	0	234.8814581	1.078398548	1.074029242
6.726917412	9.979520436	9.827676071	1.078398548	8.592233934
6.726917412	6.350603914	0	11.86238403	3.222087725
110.5136432	100.7024335	71.74203532	90.58547807	79.47816389
630.4082604	533.4507288	626.0229657	607.1383828	730.3398844
93.21585557	0	193.6052186	112.153449	74.10801768
117.2405606	131.5482239	131.6908594	171.4653692	123.5133628
0	0.907229131	0	116.4670432	70.88592996
0	0	0	0	0
3.843952807	0	0	6.470391291	0

For Review Only

129.7334072	137.8988278	57.00052121	61.46871726	144.9939476
414.185915	459.0579401	521.8495994	427.0458252	432.8337844
96.09882018	48.99037305	52.08668318	51.76313033	55.84952057
100.9037612	103.4241209	64.86266207	97.05586936	86.99636858
299.828319	312.0868209	446.1764936	300.873195	284.6177491
0	0	0	0	0
32.67359886	21.77349913	22.60365496	2.156797097	16.11043863
0	0	0	4.313594194	0
0	0	12.77597889	0	0
0	0	1.965535214	0	0
5.765929211	3.628916522	4.913838036	2.156797097	1.074029242
0.960988202	0	0	0	0
113.3966078	145.1566609	124.8114861	107.8398548	213.7318191
3.843952807	10.88674957	3.931070429	0	7.518204692
0	0	1.965535214	2.156797097	12.8883509
0	0	0	0	0
194.1196168	167.8373891	117.9321129	170.3869707	141.7718599
5.765929211	0	0	4.313594194	9.666263176
4.804941009	0	0	0	0
9.609882018	0	0	2.156797097	6.444175451
12.49284662	0	0	1.078398548	0
9.609882018	0	7.862140857	17.25437678	6.444175451
145.1092185	130.6409948	191.6396834	156.3677895	141.7718599
280.6085549	310.2723626	292.8647469	408.7130499	334.0230942
0	0	0	0	5.370146209
192.1976404	80.74339262	358.7101766	0	111.6990411
33.63458706	27.21687392	30.46579582	24.80316661	67.66384223
0	0	1.965535214	2.156797097	1.074029242
34.59557526	46.26868566	46.19007754	56.07672452	36.51699422
763.0246322	767.5158444	875.645938	867.032433	1031.068072
18.25877583	19.05181174	8.844908464	7.548789839	5.370146209
0	0	0	0	0
17.29778763	0.907229131	13.7587465	0	1.074029242
72.07411513	78.92893436	75.67310575	86.27188388	94.51457327
241.2080386	190.5181174	206.3811975	257.7372531	281.3956613
69.19115053	59.87712262	41.2762395	33.430355	37.59102346
5.765929211	1.814458261	0	5.391992742	0
33.63458706	0.907229131	0	0	0
177.7828173	0	0.982767607	1.078398548	1.074029242
0.960988202	13.60843696	190.6569158	200.58213	185.8070588
316.1651184	0	112.0355072	2.156797097	26.85073104
113.3966078	84.37230914	257.4851131	229.6988908	11.81432166
0	0	3.931070429	0	2.148058484
9.609882018	9.072291305	8.844908464	6.470391291	7.518204692
23.06371684	18.14458261	27.517493	12.94078258	10.74029242
182.5877583	226.8072826	263.3817187	216.7581082	242.7306086
29.79063425	32.6602487	10.81044368	26.95996371	34.36893574
332.5019178	250.39524	323.3305427	306.2651878	274.9514859
89.37190276	123.3831618	165.104958	149.8973982	138.5497722
137.4213129	108.8674957	51.10391557	83.03668823	89.14442707

For Review Only

40.36150447	74.3927887	135.6219298	77.64469549	48.33131588
62.46423311	47.17591479	68.7937325	75.48789839	51.5534036
0	0	0	0	73.03398844
175.8608409	170.5590765	559.1947685	540.2776728	254.5449303
88.41091456	115.2180996	57.98328882	118.6238403	215.8798776
76.87905614	100.7024335	98.27676071	88.42868097	92.36651479
2.882964605	1.814458261	0.982767607	1.078398548	3.222087725
190.2756639	243.137407	445.193726	163.9165794	178.2888541
0	4.536145653	0	0	0
589.0857677	638.6893079	710.54098	806.6421142	758.2646447
44.20545728	61.69158088	81.56971139	73.3311013	49.40534512
1078.228762	863.6821323	585.7294939	603.9031871	860.2974226
0	26.30964479	68.7937325	0	7.518204692
46.12743368	54.43374783	56.01775361	25.88156516	75.18204692
80.72300895	58.96989348	92.38015507	104.6046592	68.73787147
4.804941009	0	0	8.627188388	0
21.14174044	9.072291305	25.55195779	8.627188388	31.14684801
0	0	0	0	0
11.53185842	7.257833044	0	11.86238403	17.18446787
7.687905614	9.072291305	12.77597889	5.391992742	4.296116967
6.726917412	0	6.87937325	5.391992742	6.444175451
50.93237469	78.92893436	59.94882404	71.1743042	52.62743285
242.1690268	211.3843874	110.069972	281.4620211	170.7706494
89.37190276	83.46508001	98.27676071	86.27188388	96.66263176
0	14.51566609	16.70704932	18.33277532	3.222087725
6.726917412	6.350603914	4.913838036	5.391992742	15.03640938
139.3432893	166.93016	233.8986905	193.0333402	0
99.94277298	62.59881001	95.32845789	76.56629694	98.81069024
2863.744841	2883.174177	3336.496026	2619.430074	1462.827827
411.3029504	490.8109596	510.0563881	580.1784191	465.0546617
7.687905614	1.814458261	52.08668318	0	51.5534036
0	26.30964479	46.19007754	4.313594194	9.666263176
19.21976404	14.51566609	37.34516907	33.430355	22.55461408
56.6983039	56.24820609	39.31070429	33.430355	128.883509
19.21976404	11.7939787	14.74151411	9.705586936	23.62864332
27.86865785	400.0880466	211.2950355	705.2726507	882.8520367
363.2535403	331.1386326	522.832367	374.2042963	361.9478545
61.50324491	68.04218479	61.91435925	74.40949984	80.55219313
0	0	0.982767607	1.078398548	0
3.843952807	4.536145653	6.87937325	2.156797097	0
118.2015488	153.3217231	160.19112	170.3869707	182.5849711
127.8114308	136.0843696	103.1905987	111.0750505	418.8714043
15.37581123	10.88674957	5.896605643	7.548789839	10.74029242
0	0	0	2.156797097	3.222087725
0	0	0.982767607	1.078398548	81.62622237
0	0	14.74151411	0	0
41.32249268	17.23735348	26.53472539	50.68473178	49.40534512
66.30818592	77.1144761	47.17284514	45.29273904	92.36651479
114.357596	109.7747248	150.3634439	181.1709561	98.81069024
194.1196168	174.1879931	156.2600495	146.6622026	166.4745325

For Review Only

133.57736	51.71206044	124.8114861	102.4478621	117.0691874
0.960988202	0.907229131	0	0	53.70146209
290.2184369	321.1591122	293.8475145	300.873195	308.2463924
80.72300895	107.9602665	128.7425565	125.0942316	57.99757905
2.882964605	3.628916522	0	0	0
61.50324491	61.69158088	32.43133104	47.44953613	55.84952057
114.357596	136.0843696	87.46631704	126.1726302	106.3288949
0	0	0	0	0
298.8673307	343.8398405	467.797381	433.5162165	387.7245563
88.41091456	99.79520436	114.98381	83.03668823	39.73908194
188.3536875	205.0337835	285.0026061	278.2268255	210.5097314
122.0455016	18.14458261	36.36240146	24.80316661	30.07281877
69.19115053	74.3927887	120.8804157	143.4270069	120.2912751
390.1612099	381.0362348	409.8140922	447.5353976	374.8362054
19.21976404	40.82531087	38.32793668	42.05754339	31.14684801
46.12743368	0	156.2600495	119.7022389	18.25849711
55.7373157	48.08314392	49.13838036	64.70391291	61.21966678
48.04941009	24.49518652	40.29347189	11.86238403	6.444175451
0	2.721687392	0	0	4.296116967
0	0	0	0	0
3.843952807	3.628916522	4.913838036	0	0
0	0	6.87937325	0	0
0	0.907229131	0	7.548789839	0
0.960988202	2.721687392	0	0	3.222087725
69.19115053	54.43374783	41.2762395	53.91992742	112.7730704
22.10272864	21.77349913	22.60365496	3.235195645	2.148058484
11.53185842	7.257833044	106.1389016	108.9182534	79.47816389
42.28348088	32.6602487	41.2762395	54.99832597	40.81311119
156.6410769	244.9518652	229.9676201	172.5437678	139.6238014
17.29778763	18.14458261	10.81044368	5.391992742	9.666263176
8.648893816	25.40241565	9.827676071	20.48957242	8.592233934
0	101.6096626	2.948302821	20.48957242	1.074029242
2.882964605	0	0	0	0
0	0	5.896605643	0	3.222087725
151.8361359	186.8892009	185.7430777	169.3085721	167.5485617
102.8257376	90.72291305	111.0527396	149.8973982	76.25607616
66.30818592	57.15543522	36.36240146	70.09590565	76.25607616
30.75162246	35.38193609	90.41461986	103.5262607	46.1832574
175.8608409	193.2398048	137.587465	185.4845503	162.1784155
75.91806794	62.59881001	48.15561275	117.5454418	73.03398844
87.44992636	61.69158088	75.67310575	15.09757968	38.6650527
21.14174044	34.47470696	48.15561275	36.66555065	42.96116967
10.57087022	5.443374783	0	2.156797097	3.222087725
0	0	3.931070429	0	0
0	0	0	0	0
0	19.95904087	11.79321129	0	8.592233934
55.7373157	68.94941392	45.20730993	26.95996371	47.25728664
0	0	0	0	0
0	0	6.87937325	1.078398548	1.074029242
0	0	0	0	0

For Review Only

0	0	13.7587465	10.78398548	4.296116967
5.765929211	0.907229131	2.948302821	1.078398548	3.222087725
0	5.443374783	2.948302821	2.156797097	3.222087725
337.3068588	345.6542987	456.0041697	580.1784191	430.6857259
0	0	0	0	0
0	0	1.965535214	2.156797097	2.148058484
81.68399715	61.69158088	73.70757054	50.68473178	68.73787147
9.609882018	0	7.862140857	1.078398548	17.18446787
0	0	0	0	0
74.95707974	82.55785088	98.27676071	73.3311013	20.40655559
1.921976404	3.628916522	5.896605643	1.078398548	0
0	0	16.70704932	0	6.444175451
0	3.628916522	10.81044368	0	0
0.960988202	0	33.41409864	2.156797097	3.222087725
0	0	0	0	0
0	0	0	0	0
0	3.628916522	0.982767607	0	0
35.55656347	48.99037305	11.79321129	26.95996371	56.92354981
58.62028031	62.59881001	57.00052121	53.91992742	56.92354981
47.08842189	75.30001783	84.51801421	51.76313033	83.77428086
118.2015488	97.07351697	111.0527396	155.289391	176.1407956
86.48893816	3.628916522	35.37963386	34.50875355	66.58981299
0	0	31.44856343	42.05754339	42.96116967
45.16644548	29.93856131	65.84542968	34.50875355	69.81190071
20.18075224	13.60843696	37.34516907	46.37113758	185.8070588
2.882964605	320.2518831	0	0	28.99878953
51.8933629	71.67110131	115.9665776	89.50707952	42.96116967
0	0	0	0	3.222087725
571.78798	672.2567857	986.6986776	1154.964845	811.9661068
0	0	0	0	2.148058484
376.7073751	498.9760218	494.3321064	593.1192017	409.2051411
242.1690268	278.5193431	452.0730993	147.7406011	177.2148249
175.8608409	115.2180996	202.4501271	121.859036	152.5121523
144.1482303	48.08314392	0	0	0
201.8075224	230.4361992	119.8976481	149.8973982	85.92233934
107.6306786	89.81568392	54.05221839	56.07672452	73.03398844
26.90766965	21.77349913	8.844908464	38.82234774	26.85073104
73.03510333	89.81568392	75.67310575	92.74227517	95.58860252
116.2795724	139.7132861	153.3117467	116.4670432	163.2524447
271.9596611	202.3120961	284.0198385	328.9115573	309.3204216
66.30818592	61.69158088	70.75926771	85.19348533	89.14442707
121.0845134	132.4554531	152.3289791	171.4653692	122.4393336
0	0	0	0	0
46.12743368	68.04218479	55.034986	83.03668823	42.96116967
0	0	0	0	3.222087725
109.552655	111.5891831	207.3639651	105.6830577	200.8434682
3.843952807	3.628916522	3.931070429	5.391992742	8.592233934
14.41482303	4.536145653	13.7587465	8.627188388	44.03519891
41.32249268	39.01085261	52.08668318	44.21434049	36.51699422
28.82964605	63.50603914	34.39686625	35.5871521	59.0716083

For Review Only

11.53185842	19.95904087	25.55195779	23.72476807	4.296116967
22.10272864	39.01085261	19.65535214	22.64636952	41.88714043
58.62028031	75.30001783	72.72480293	100.291065	60.14563754
0	22.68072826	62.89712686	58.23352162	45.10922815
8.648893816	8.165062175	12.77597889	16.17597823	11.81432166
69.19115053	46.26868566	89.43185225	88.42868097	89.14442707
0	0	0	0	3.222087725
112.4356196	0.907229131	38.32793668	24.80316661	307.1723631
119.162537	0	758.6965927	0	128.883509
35.55656347	151.5072648	147.4151411	173.6221663	107.4029242
49.01039829	22.68072826	21.62088736	33.430355	32.22087725
4.804941009	0	0	0	10.74029242
126.8504426	153.3217231	143.4840706	153.1325939	137.4757429
365.1755167	400.0880466	303.6751906	296.5596008	221.2500238
2194.897053	2246.299327	3436.738322	2122.288343	2566.929888
32.67359886	53.5265187	61.91435925	50.68473178	37.59102346
26.90766965	19.05181174	29.48302821	11.86238403	3.222087725
90.33289097	79.83616349	117.9321129	118.6238403	109.5509827
31.71261066	53.5265187	31.44856343	21.56797097	27.92476029
32.67359886	28.12410305	60.93159164	36.66555065	62.29369602
14.41482303	9.979520436	0	64.70391291	41.88714043
16.33679943	29.03133218	23.58642257	16.17597823	23.62864332
0	0	0.982767607	66.86071	3.222087725
452.625443	457.2434818	931.6636916	335.3819486	545.6068548
3.843952807	0.907229131	0	7.548789839	0
169.1339235	146.06389	318.4167047	84.11508678	209.4357021
358.4485993	403.7169631	549.3670924	379.5962891	380.2063516
0	0	0	0	0
218.1443218	0	150.3634439	6.470391291	1.074029242
0	66.22772653	11.79321129	0	37.59102346
0	0	0.982767607	1.078398548	1.074029242
0.960988202	0	0	0	0
112.4356196	135.1771404	187.708613	131.5646229	171.8446787
0	0	122.8459509	0	97.736661
86.48893816	86.1867674	113.0182748	98.13426791	110.6250119
166.2509589	179.6313678	180.8292397	183.3277532	243.8046379
0	77.1144761	127.7597889	53.91992742	19.33252635
0	0	0	3.235195645	0
127.8114308	200.4976378	223.0882468	191.9549416	226.62017
26.90766965	27.21687392	10.81044368	20.48957242	19.33252635
65.34719772	53.5265187	36.36240146	44.21434049	50.47937436
0	0	0	0	0
58.62028031	93.44460045	37.34516907	43.13594194	51.5534036
10.57087022	10.88674957	7.862140857	12.94078258	12.8883509
61.50324491	39.91808174	28.50026061	33.430355	48.33131588
0.960988202	0.907229131	0	0	5.370146209
5.765929211	3.628916522	1.965535214	0	1.074029242
0	3.628916522	2.948302821	0	0
0	0	0	6.470391291	0
156.6410769	0	32.43133104	0	32.22087725

For Review Only

0	0	6.87937325	0	0
100.9037612	157.8578687	134.6391622	100.291065	118.1432166
0	3.628916522	7.862140857	0	0
10.57087022	7.257833044	15.72428171	21.56797097	10.74029242
68.23016233	81.65062175	114.0010424	39.90074629	64.44175451
27.86865785	31.75301957	39.31070429	35.5871521	41.88714043
0	0	1.965535214	1.078398548	1.074029242
89.37190276	97.9807461	89.43185225	99.21266646	79.47816389
247.9349561	325.6952579	352.813571	225.3852966	257.767018
0.960988202	0.907229131	0	182.2493547	142.8458892
52.8543511	60.78435175	69.77650011	93.82067372	71.9599592
0	0	2.948302821	0	0
217.1833336	178.7241387	80.58694379	181.1709561	152.5121523
123.0064898	146.06389	143.4840706	75.48789839	91.29248555
80.72300895	92.53737131	118.9148805	83.03668823	48.33131588
0	0	0	0	1.074029242
73.99609154	59.87712262	83.53524661	83.03668823	62.29369602
0	90.72291305	95.32845789	45.29273904	57.99757905
65.34719772	0	243.7263666	0	0
5.765929211	5.443374783	2.948302821	2.156797097	6.444175451
24.02470504	21.77349913	29.48302821	11.86238403	17.18446787
86.48893816	39.01085261	128.7425565	54.99832597	32.22087725
37.47853987	46.26868566	28.50026061	17.25437678	53.70146209
95.13783197	95.25905871	36.36240146	72.25270275	103.1068072
213.3393808	249.4880109	214.2433384	282.5404197	128.883509
0	5.443374783	0	0	1.074029242
0.960988202	0	3.931070429	1.078398548	0
17.29778763	46.26868566	11.79321129	47.44953613	11.81432166
173.9388645	107.9602665	143.4840706	156.3677895	189.0291465
41.32249268	45.36145653	49.13838036	80.87989113	44.03519891
162.4070061	107.0530374	115.9665776	152.0541953	144.9939476
0.960988202	0.907229131	0	83.03668823	0
2.882964605	1.814458261	4.913838036	0	0
198.9245578	209.5699292	264.3644863	224.3068981	201.9174975
3.843952807	14.51566609	7.862140857	4.313594194	9.666263176
337.3068588	420.9543166	319.3994723	355.871521	309.3204216
20.18075224	29.03133218	22.60365496	45.29273904	24.70267256
109.552655	127.0120783	14.74151411	86.27188388	170.7706494
35.55656347	39.91808174	19.65535214	31.27355791	60.14563754
672.6917412	707.6387218	893.3357549	1007.224244	831.2986331
46.12743368	67.13495566	56.01775361	60.39031871	49.40534512
123.967478	156.0434105	156.2600495	223.2284995	192.2512343
12.49284662	29.03133218	29.48302821	15.09757968	19.33252635
34.59557526	18.14458261	7.862140857	24.80316661	34.36893574
0	0	0	0	0
0	0	6.87937325	0	0
85.52794996	77.1144761	96.3112255	133.72142	110.6250119
164.3289825	138.806057	133.6563946	186.5629489	150.3640938
276.7646021	263.0964479	297.778585	270.6780357	158.9563278
29.79063425	35.38193609	29.48302821	9.705586936	22.55461408

For Review Only

377.6683633	384.6651513	434.3832824	407.6346513	350.1335328
7.687905614	9.979520436	6.87937325	9.705586936	12.8883509
0.960988202	0	0	0	4.296116967
46.12743368	57.15543522	36.36240146	66.86071	83.77428086
14.41482303	16.33012435	12.77597889	5.391992742	4.296116967
117.2405606	68.04218479	81.56971139	117.5454418	110.6250119
66.30818592	96.16628784	64.86266207	85.19348533	80.55219313
81.68399715	105.2385791	60.93159164	107.8398548	74.10801768
0	0	48.15561275	2.156797097	44.03519891
47.08842189	37.19639435	27.517493	58.23352162	38.6650527
18.25877583	3.628916522	13.7587465	47.44953613	21.48058484
29.79063425	26.30964479	21.62088736	32.35195645	25.7767018
0	9.072291305	3.931070429	0	0
0.960988202	22.68072826	0	64.70391291	50.47937436
251.7789089	97.07351697	231.9331553	311.6571805	264.2111935
101.8647494	67.13495566	51.10391557	86.27188388	80.55219313
248.8959443	281.2410305	281.0715356	284.6972168	314.6905678
24.98569325	26.30964479	36.36240146	28.03836226	23.62864332
35.55656347	57.15543522	61.91435925	57.15512307	41.88714043
238.325074	223.1783661	285.0026061	225.3852966	201.9174975
50.93237469	81.65062175	164.1221904	148.8189997	85.92233934
200.8465342	274.8904266	232.9159229	163.9165794	335.0971234
152.7971241	155.1361813	169.0360284	196.2685358	120.2912751
25.94668145	43.54699827	1.965535214	16.17597823	4.296116967
21254.17606	20741.07238	25397.66327	23763.59041	17542.11961
0	0	0.982767607	1.078398548	1.074029242
0	0	0	51.76313033	1.074029242
82.64498535	63.50603914	0	118.6238403	33.29490649
135.4993364	295.7566966	108.1044368	100.291065	94.51457327
0	0	0	0	0
36.51755167	32.6602487	31.44856343	22.64636952	18.25849711
0	61.69158088	0	11.86238403	45.10922815
16.33679943	13.60843696	14.74151411	10.78398548	7.518204692
0.960988202	4.536145653	1.965535214	0	2.148058484
767.8295732	520.7495209	310.5545639	471.2601657	438.2039306
401.6930683	354.72659	354.7791062	379.5962891	345.8374158
212.3783926	243.137407	342.9858949	298.7163979	211.5837606
320.9700594	71.67110131	169.0360284	108.9182534	155.7342401
156.6410769	203.2193252	114.98381	140.1918113	61.21966678
67.26917412	0	0	31.27355791	5.370146209
17.29778763	21.77349913	6.87937325	36.66555065	30.07281877
172.0168881	143.3422026	96.3112255	146.6622026	129.9575383
30.75162246	19.95904087	19.65535214	33.430355	21.48058484
94.17684377	87.09399653	110.069972	135.8782171	106.3288949
0	4.536145653	0	0	0
43.24446908	55.34097696	53.06945079	31.27355791	27.92476029
33.63458706	26.30964479	49.13838036	32.35195645	7.518204692
17.29778763	15.42289522	12.77597889	7.548789839	8.592233934
0	0	0.982767607	0	0
2.882964605	0	4.913838036	0	0

For Review Only

0	0	5.896605643	0	0
53.8153393	44.4542274	105.156134	65.78231146	52.62743285
53190.69697	61460.23745	64355.55399	65997.99117	82380.1909
0	0	0	1.078398548	4.296116967
22.10272864	32.6602487	37.34516907	33.430355	42.96116967
0.960988202	9.072291305	70.75926771	75.48789839	76.25607616
118.2015488	137.8988278	136.6046974	120.7806374	97.736661
54.7763275	31.75301957	46.19007754	36.66555065	55.84952057
171.0558999	98.88797523	126.7770213	204.8957242	85.92233934
43.24446908	80.74339262	42.25900711	37.7439492	35.44296498
71.11312693	65.3204974	70.75926771	81.95828968	73.03398844
0	0	4.913838036	7.548789839	11.81432166
57.65929211	74.3927887	81.56971139	42.05754339	44.03519891
105.7087022	127.0120783	108.1044368	80.87989113	78.40413465
27.86865785	31.75301957	0	0	0
47.08842189	69.85664305	61.91435925	56.07672452	79.47816389
0	31.75301957	0.982767607	1.078398548	1.074029242
68.23016233	79.83616349	55.034986	93.82067372	42.96116967
0	54.43374783	0	0	0
0.960988202	0.907229131	0	245.874869	0
13.45383482	0	1.965535214	1.078398548	0
128.772419	68.94941392	109.0872044	166.0733765	54.77549133
21.14174044	14.51566609	34.39686625	20.48957242	16.11043863
0.960988202	0	0	0	0
98.98178478	127.9193074	125.7942537	140.1918113	83.77428086
45.16644548	43.54699827	23.58642257	39.90074629	52.62743285
189.3146757	0	11.79321129	67.93910855	452.1663108
27.86865785	25.40241565	38.32793668	32.35195645	25.7767018
56.6983039	77.1144761	111.0527396	92.74227517	77.33010541
351.7216818	205.9410126	233.8986905	117.5454418	149.2900646
25.94668145	27.21687392	13.7587465	21.56797097	37.59102346
149.9141595	178.7241387	230.9503877	229.6988908	153.5861816
20.18075224	13.60843696	14.74151411	5.391992742	9.666263176
146.0702067	141.5277444	144.4668382	158.5245866	131.0315675
86.48893816	98.88797523	72.72480293	60.39031871	35.44296498
0	0	0	0	0
390.1612099	0	4322.211936	3072.357465	0
6923.919994	7499.155993	10458.61288	9219.229191	7288.362435
1157.029795	1218.408722	1478.082481	1649.949779	1565.934634
27066.2327	31270.37367	43024.58307	41990.68268	32613.97196
0	0	0	0	0
0	0	0	0	0
319.048083	889.9917771	1468.254805	2389.731183	2498.192016
0	6.350603914	0	0	0
1475.11689	1615.775081	1994.035475	1890.432655	1989.102156
307.5162246	285.7771761	371.4861555	333.2251515	315.7645971
10813.03925	11577.15093	13318.46661	14331.91671	18905.06271
5.765929211	5.443374783	10.81044368	6.470391291	0
2144.925666	2749.811495	3380.720569	4220.851919	4530.255342
9.609882018	0	5.896605643	0	0

For Review Only

102.8257376	90.72291305	0	77.64469549	0
36.51755167	34.47470696	61.91435925	28.03836226	61.21966678
19.21976404	0	9.827676071	0	7.518204692
0	0.907229131	11.79321129	2.156797097	2.148058484
0	0	0	169.3085721	0
352.68267	275.7976557	313.5028667	380.6746876	313.6165386
84.56696176	65.3204974	93.36292268	43.13594194	62.29369602
49.97138649	69.85664305	10.81044368	30.19515936	77.33010541
134.5383482	143.3422026	116.9493452	85.19348533	59.0716083
57.65929211	0	14.74151411	9.705586936	9.666263176
70.15213873	79.83616349	35.37963386	74.40949984	84.8483101
27.86865785	35.38193609	46.19007754	24.80316661	25.7767018
154.7191005	173.2807639	138.5702326	104.6046592	78.40413465
0	0	0	0	0
10.57087022	10.88674957	7.862140857	17.25437678	4.296116967
364.2145285	346.5615279	307.606261	355.871521	184.7330296
22.10272864	36.28916522	40.29347189	8.627188388	25.7767018
74.95707974	100.7024335	155.2772819	109.9966519	113.8470996
35.55656347	22.68072826	23.58642257	30.19515936	15.03640938
29.79063425	55.34097696	69.77650011	36.66555065	45.10922815
40.36150447	60.78435175	55.034986	50.68473178	49.40534512
38.43952807	57.15543522	66.82819729	49.60633323	51.5534036
42.28348088	48.99037305	27.517493	61.46871726	70.88592996
0	0	3.931070429	0	0
17611.06979	15197.90239	20406.18659	7687.903252	17688.18758
0	0	3.931070429	0	1.074029242
0	0	1.965535214	3.235195645	0
25.94668145	0	0	0	0
0	0	0	4.313594194	3.222087725
4.804941009	0.907229131	0	17.25437678	0
0.960988202	0	2.948302821	1.078398548	3.222087725
0	0	0	0	0
0	11.7939787	14.74151411	1.078398548	1.074029242
3.843952807	1.814458261	0	0	0
9.609882018	18.14458261	0.982767607	2.156797097	3.222087725
9.609882018	6.350603914	9.827676071	7.548789839	1.074029242
8.648893816	8.165062175	8.844908464	9.705586936	4.296116967
19.21976404	19.95904087	17.68981693	20.48957242	17.18446787
0	259.4675313	0	1.078398548	1.074029242
0.960988202	0	0.982767607	0	0
127.8114308	250.39524	5.896605643	89.50707952	243.8046379
0	2.721687392	29.48302821	2.156797097	0
362.2925521	25.40241565	898.2495929	146.6622026	63.36772526
98.98178478	119.7542452	121.8631833	91.66387662	117.0691874
46.12743368	52.61928957	85.50078182	57.15512307	59.0716083
205.6514752	223.1783661	250.6057398	212.444514	220.1759946
132.6163718	117.0325578	114.98381	62.54711581	47.25728664
55.7373157	39.91808174	56.01775361	48.52793468	34.36893574
79.76202075	73.48555957	86.48354943	80.87989113	69.81190071
35.55656347	12.70120783	251.5885074	48.52793468	0

For Review Only

0	0	0	0	0
81.68399715	76.20724696	119.8976481	115.3886447	102.032778
28.82964605	34.47470696	34.39686625	26.95996371	27.92476029
24.98569325	34.47470696	63.87989446	51.76313033	35.44296498
550.6462396	651.3905157	697.7650011	659.9799117	777.597171
45.16644548	21.77349913	24.56919018	14.01918113	17.18446787
0.960988202	0.907229131	0	0	0
0.960988202	19.95904087	6.87937325	0	49.40534512
1670.197495	2049.430606	2654.455307	1077.32015	1215.801102
0	19.05181174	0.982767607	0	0
128.772419	161.4867852	145.4496059	159.6029852	128.883509
116.2795724	98.88797523	93.36292268	71.1743042	80.55219313
0	3.628916522	0	0	0
50.93237469	74.3927887	45.20730993	47.44953613	49.40534512
0	0	0	0	0
2.882964605	1.814458261	0	0	6.444175451
0	0	0	0	0
0	0	0	0	0
22.10272864	29.03133218	18.67258454	26.95996371	16.11043863
0	4.536145653	1.965535214	2.156797097	4.296116967
7.687905614	2.721687392	3.931070429	0	0
3.843952807	7.257833044	4.913838036	0	9.666263176
3.843952807	39.91808174	57.00052121	49.60633323	62.29369602
5.765929211	6.350603914	12.77597889	23.72476807	8.592233934
0	0	0	3.235195645	0
0	0	0	0	9.666263176
0	0	0	0	0
207.5734516	235.8795739	259.4506483	187.6413474	151.4381231
34.59557526	20.86627	44.22454232	42.05754339	52.62743285
0	0	0	0	0
272.9206493	143.3422026	232.9159229	183.3277532	187.9551173
1260.816521	1412.555756	2236.779074	1322.11662	1592.785366
82.64498535	132.4554531	135.6219298	175.7789634	158.9563278
51.8933629	0	0	56.07672452	0
128.772419	125.19762	132.673627	0	0
239.2860622	755.7218657	939.5258324	168.2301736	1796.850921
4.804941009	23.58795739	2.948302821	2.156797097	13.96238014
99.94277298	128.8265365	105.156134	126.1726302	93.44054403
0	4.536145653	0	1.078398548	0
0.960988202	0	0.982767607	0	4.296116967
6.726917412	9.979520436	10.81044368	8.627188388	11.81432166
15.37581123	16.33012435	23.58642257	15.09757968	37.59102346
57.65929211	45.36145653	69.77650011	21.56797097	36.51699422
74.95707974	112.4964122	82.552479	91.66387662	99.88471948
5.765929211	3.628916522	7.862140857	9.705586936	5.370146209
16.33679943	9.979520436	2.948302821	6.470391291	2.148058484
0.960988202	9.979520436	5.896605643	1.078398548	11.81432166
4.804941009	0.907229131	2.948302821	2.156797097	6.444175451
24.98569325	30.84579044	19.65535214	1.078398548	1.074029242
8.648893816	16.33012435	13.7587465	9.705586936	5.370146209

For Review Only

0	137.8988278	1.965535214	1.078398548	1.074029242
0	0	0	271.7564342	0
66.30818592	95.25905871	80.58694379	66.86071	63.36772526
127.8114308	110.6819539	179.8464721	163.9165794	137.4757429
37.47853987	56.24820609	20.63811975	21.56797097	34.36893574
96.09882018	90.72291305	85.50078182	99.21266646	71.9599592
73.03510333	77.1144761	86.48354943	74.40949984	78.40413465
36.51755167	64.41326827	46.19007754	49.60633323	33.29490649
4.804941009	8.165062175	7.862140857	12.94078258	11.81432166
3.843952807	0	1.965535214	1.078398548	0
14.41482303	9.979520436	27.517493	0	19.33252635
0	3.628916522	0.982767607	2.156797097	4.296116967
43.24446908	28.12410305	45.20730993	62.54711581	27.92476029
38.43952807	29.93856131	32.43133104	33.430355	31.14684801
4.804941009	6.350603914	5.896605643	3.235195645	3.222087725
62.46423311	30.84579044	34.39686625	31.27355791	13.96238014
75.91806794	86.1867674	40.29347189	37.7439492	50.47937436
7.687905614	17.23735348	8.844908464	10.78398548	5.370146209
0	2.721687392	0	0	0
0.960988202	0	2.948302821	3.235195645	0
19.21976404	16.33012435	19.65535214	20.48957242	11.81432166
94.17684377	103.4241209	102.2078311	132.6430215	112.7730704
63.42522132	57.15543522	63.87989446	64.70391291	39.73908194
4.804941009	0	0	4.313594194	4.296116967
20.18075224	21.77349913	8.844908464	9.705586936	8.592233934
0	0	0	0	3.222087725
5.765929211	7.257833044	5.896605643	3.235195645	1.074029242
76.87905614	57.15543522	40.29347189	49.60633323	64.44175451
1.921976404	4.536145653	0	5.391992742	4.296116967
4.804941009	10.88674957	7.862140857	3.235195645	3.222087725
4.804941009	6.350603914	6.87937325	4.313594194	5.370146209
0	0	0.982767607	0	0
209.495428	224.0855952	189.6741482	197.3469344	181.5109419
176.8218291	198.6831796	184.7603101	156.3677895	115.9951581
33.63458706	12.70120783	11.79321129	28.03836226	42.96116967
3.843952807	0	3.931070429	0	0
0	0	0	0	0
51.8933629	29.93856131	22.60365496	24.80316661	20.40655559
0	3.628916522	3.931070429	0	1.074029242
30.75162246	23.58795739	29.48302821	1.078398548	6.444175451
9.609882018	6.350603914	2.948302821	3.235195645	5.370146209
0	0	0.982767607	0	0
0	0	0.982767607	3.235195645	1.074029242
175.8608409	249.4880109	263.3817187	263.1292458	250.2488133
137.4213129	107.9602665	116.9493452	117.5454418	123.5133628
138.3823011	156.0434105	77.63864096	180.0925576	162.1784155
0	5039.65782	9.827676071	1.078398548	1.074029242
0	0	0	0	0
0	10.88674957	5.896605643	14.01918113	12.8883509
17.29778763	24.49518652	43.24177471	24.80316661	19.33252635

For Review Only

62.46423311	81.65062175	120.8804157	87.35028243	61.21966678
27.86865785	35.38193609	27.517493	35.5871521	28.99878953
27.86865785	12.70120783	33.41409864	34.50875355	25.7767018
0	0	0	0	0
246.9739679	299.3856131	257.4851131	413.0266441	381.2803808
99.94277298	88.90845479	95.32845789	94.89907226	106.3288949
29.79063425	50.80483131	8.844908464	26.95996371	47.25728664
68.23016233	0	51.10391557	126.1726302	93.44054403
0	0	0	34.50875355	0
168.1729353	21.77349913	269.2783244	405.4778542	293.209983
3.843952807	9.979520436	6.87937325	0	8.592233934
32.67359886	37.19639435	69.77650011	43.13594194	61.21966678
3.843952807	9.072291305	4.913838036	0	7.518204692
17.29778763	21.77349913	30.46579582	30.19515936	23.62864332
0	5.443374783	88.44908464	0	0
10.57087022	1.814458261	6.87937325	3.235195645	0
18.25877583	40.82531087	45.20730993	36.66555065	22.55461408
51.8933629	60.78435175	69.77650011	23.72476807	49.40534512
24.98569325	20.86627	15.72428171	21.56797097	49.40534512
136.4603247	0	9.827676071	60.39031871	50.47937436
347.877729	412.7892544	378.3655287	373.1258978	308.2463924
0	0	9.827676071	0	0
50.93237469	19.95904087	75.67310575	80.87989113	84.8483101
12.49284662	0	12.77597889	15.09757968	0
74.95707974	27.21687392	43.24177471	35.5871521	1.074029242
49.01039829	40.82531087	35.37963386	23.72476807	46.1832574
76.87905614	70.76387218	51.10391557	25.88156516	71.9599592
20.18075224	13.60843696	20.63811975	7.548789839	13.96238014
247.9349561	264.9109061	299.7441202	281.4620211	252.3968718
277.7255903	272.1687392	275.17493	177.9357605	226.62017
93.21585557	89.81568392	107.1216692	64.70391291	85.92233934
123.967478	139.7132861	47.17284514	105.6830577	114.9211289
12.49284662	4.536145653	2.948302821	2.156797097	4.296116967
0	0	1.965535214	0	2.148058484
35.55656347	58.96989348	38.32793668	45.29273904	32.22087725
2.882964605	0	0	0	2.148058484
8.648893816	0.907229131	10.81044368	31.27355791	1.074029242
32.67359886	35.38193609	24.56919018	33.430355	19.33252635
55.7373157	65.3204974	43.24177471	39.90074629	54.77549133
47.08842189	81.65062175	52.08668318	65.78231146	103.1068072
0	0	0	0	0
111.4746314	110.6819539	71.74203532	87.35028243	77.33010541
25.94668145	27.21687392	20.63811975	20.48957242	38.6650527
29.79063425	31.75301957	29.48302821	31.27355791	27.92476029
36.51755167	50.80483131	24.56919018	51.76313033	53.70146209
0	1.814458261	4.913838036	0	0
15.37581123	12.70120783	2.948302821	16.17597823	9.666263176
119.162537	140.6205152	131.6908594	132.6430215	127.8094798
56.6983039	224.0855952	250.6057398	0	430.6857259
38.43952807	9.072291305	16.70704932	23.72476807	11.81432166

For Review Only

338.267847	358.3555066	303.6751906	362.3419123	325.4308603
434.3666672	501.6977092	433.4005147	662.1367088	4.296116967
16.33679943	17.23735348	33.41409864	77.64469549	67.66384223
22.10272864	30.84579044	30.46579582	39.90074629	28.99878953
73.99609154	76.20724696	32.43133104	80.87989113	67.66384223
63.42522132	93.44460045	102.2078311	84.11508678	66.58981299
22.10272864	25.40241565	8.844908464	16.17597823	13.96238014
57.65929211	56.24820609	37.34516907	71.1743042	36.51699422
34.59557526	30.84579044	10.81044368	52.84152887	62.29369602
3.843952807	138.806057	0	0	180.4369126
0	0	0	0	0
0	65.3204974	103.1905987	84.11508678	54.77549133
319.048083	290.3133218	342.9858949	180.0925576	264.2111935
0	0	28.50026061	0	0
11.53185842	26.30964479	3.931070429	9.705586936	21.48058484
195.080605	117.0325578	253.5540426	116.4670432	104.1808365
59.58126851	66.22772653	55.034986	42.05754339	40.81311119
0	0	0	1.078398548	0
0	0	0.982767607	0	0
0.960988202	0.907229131	0	836.8372736	0
0	0	0	0	0
0	7.257833044	2.948302821	0	0
147.0311949	172.3735348	276.1576976	264.2076444	274.9514859
20.18075224	19.05181174	23.58642257	18.33277532	12.8883509
120.1235252	104.33135	154.2945143	138.0350142	165.4005032
42.28348088	77.1144761	23.58642257	44.21434049	76.25607616
33.63458706	29.93856131	21.62088736	12.94078258	8.592233934
0	542.5230201	46.19007754	42.05754339	3.222087725
28.82964605	44.4542274	38.32793668	40.97914484	52.62743285
21.14174044	9.072291305	13.7587465	23.72476807	18.25849711
39.40051627	14.51566609	14.74151411	11.86238403	39.73908194
57.65929211	51.71206044	71.74203532	81.95828968	79.47816389
104.747714	100.7024335	84.51801421	67.93910855	107.4029242
49.01039829	44.4542274	38.32793668	35.5871521	36.51699422
170.0949117	169.6518474	174.9326341	174.7005649	242.7306086
389.2002217	358.3555066	323.3305427	246.9532676	476.8689833
28.82964605	47.17591479	69.77650011	71.1743042	76.25607616
51.8933629	14.51566609	41.2762395	64.70391291	27.92476029
129.7334072	184.1675135	105.156134	0	107.4029242
57.65929211	0	91.39738746	42.05754339	40.81311119
295.0233779	317.5301957	363.6240146	290.0892095	347.9854743
166.2509589	42.63976914	69.77650011	0	117.0691874
105.7087022	97.07351697	90.41461986	63.62551436	44.03519891
259.4668145	115.2180996	208.3467327	193.0333402	118.1432166
0	0	0	33.430355	0
85.52794996	105.2385791	133.6563946	109.9966519	123.5133628
155.6800887	70.76387218	134.6391622	44.21434049	30.07281877
0	0	23.58642257	20.48957242	39.73908194
0	0	3138.959737	0	0
59.58126851	83.46508001	84.51801421	86.27188388	90.21845631

For Review Only

37.47853987	44.4542274	6.87937325	3.235195645	2.148058484
77.84004434	105.2385791	70.75926771	70.09590565	91.29248555
80.72300895	117.939787	115.9665776	77.64469549	57.99757905
30.75162246	36.28916522	21.62088736	15.09757968	28.99878953
69.19115053	47.17591479	142.501303	126.1726302	55.84952057
91.29387917	106.1458083	100.2422959	75.48789839	63.36772526
32.67359886	32.6602487	38.32793668	6.470391291	21.48058484
227.7542038	229.52897	111.0527396	121.859036	212.6577899
39.40051627	36.28916522	6.87937325	25.88156516	54.77549133
49.97138649	31.75301957	52.08668318	69.0175071	53.70146209
0	0	0	0	0
41.32249268	59.87712262	46.19007754	26.95996371	56.92354981
21.14174044	51.71206044	56.01775361	40.97914484	22.55461408
153.7581123	188.7036592	253.5540426	249.1100647	241.6565794
12.49284662	19.05181174	29.48302821	47.44953613	18.25849711
43.24446908	71.67110131	101.2250635	52.84152887	19.33252635
41.32249268	42.63976914	31.44856343	19.41117387	37.59102346
163.3679943	186.8892009	197.536289	185.4845503	143.9199184
39.40051627	0.907229131	4.913838036	14.01918113	16.11043863
74.95707974	67.13495566	92.38015507	94.89907226	109.5509827
54.7763275	68.04218479	66.82819729	36.66555065	86.99636858
37.47853987	40.82531087	13.7587465	42.05754339	91.29248555
11.53185842	10.88674957	17.68981693	23.72476807	6.444175451
117.2405606	88.00122566	139.5530002	99.21266646	69.81190071
8.648893816	0	1.965535214	1.078398548	6.444175451
0	9.979520436	0.982767607	9.705586936	2.148058484
707.2873165	782.0315105	277.1404652	238.3260792	607.9005508
231.5981566	0	233.8986905	24.80316661	0
55.7373157	48.99037305	33.41409864	22.64636952	33.29490649
168.1729353	166.0229309	169.0360284	234.012485	125.6614213
0	0.907229131	0	3.235195645	2.148058484
102.8257376	110.6819539	66.82819729	104.6046592	123.5133628
28.82964605	47.17591479	25.55195779	31.27355791	44.03519891
0.960988202	0.907229131	114.98381	159.6029852	0
102.8257376	86.1867674	53.06945079	44.21434049	25.7767018
24.98569325	40.82531087	47.17284514	31.27355791	45.10922815
42.28348088	36.28916522	50.12114796	37.7439492	21.48058484
28.82964605	18.14458261	38.32793668	16.17597823	11.81432166
203.7294988	205.0337835	163.1394228	152.0541953	604.6784631
176.8218291	153.3217231	195.5707538	231.8556879	177.2148249
0	0	0	0	5.370146209
37.47853987	34.47470696	28.50026061	50.68473178	50.47937436
35.55656347	46.26868566	34.39686625	23.72476807	35.44296498
0	41.73254	9.827676071	19.41117387	2.148058484
22.10272864	10.88674957	17.68981693	11.86238403	17.18446787
26.90766965	32.6602487	24.56919018	39.90074629	33.29490649
18.25877583	7.257833044	4.913838036	31.27355791	28.99878953
19.21976404	7.257833044	21.62088736	8.627188388	23.62864332
36.51755167	46.26868566	45.20730993	62.54711581	61.21966678
0	7.257833044	10.81044368	0	0

For Review Only

68.23016233	92.53737131	95.32845789	92.74227517	88.07039782
300.7893072	337.4892366	287.9509089	363.4203108	242.7306086
12.49284662	9.072291305	14.74151411	37.7439492	11.81432166
72.07411513	100.7024335	71.74203532	76.56629694	90.21845631
73.03510333	69.85664305	86.48354943	79.80149259	78.40413465
131.6553836	77.1144761	227.0193172	43.13594194	175.0667664
23.06371684	18.14458261	15.72428171	3.235195645	13.96238014
0	0.907229131	2.948302821	0	1.074029242
44.20545728	24.49518652	23.58642257	24.80316661	39.73908194
4.804941009	0	2.948302821	2.156797097	0
66.30818592	57.15543522	75.67310575	38.82234774	26.85073104
90.33289097	108.8674957	62.89712686	24.80316661	95.58860252
16.33679943	9.979520436	71.74203532	7.548789839	7.518204692
55.7373157	66.22772653	20.63811975	28.03836226	3.222087725
0	0	2.948302821	66.86071	1.074029242
43.24446908	36.28916522	34.39686625	24.80316661	24.70267256
8.648893816	7.257833044	2.948302821	11.86238403	6.444175451
0	0	0	0	3.222087725
25.94668145	37.19639435	25.55195779	33.430355	15.03640938
31.71261066	57.15543522	23.58642257	24.80316661	24.70267256
39.40051627	61.69158088	87.46631704	78.72309404	91.29248555
30.75162246	19.05181174	4.913838036	19.41117387	22.55461408
0	0	2.948302821	1.078398548	12.8883509
0	0	1.965535214	1.078398548	0
841.8256647	933.5387753	1274.649586	1188.3952	1066.511037
0	18.14458261	0	19.41117387	0
110.5136432	114.3108704	141.5185354	155.289391	123.5133628
87.44992636	86.1867674	101.2250635	80.87989113	77.33010541
12.49284662	19.05181174	32.43133104	12.94078258	12.8883509
24.02470504	13.60843696	1.965535214	2.156797097	5.370146209
188.3536875	225.9000535	176.8981693	279.3052241	178.2888541
49.97138649	58.96989348	80.58694379	87.35028243	99.88471948
39.40051627	47.17591479	30.46579582	39.90074629	25.7767018
130.6943954	112.4964122	120.8804157	116.4670432	161.1043863
77.84004434	71.67110131	98.27676071	69.0175071	77.33010541
109.552655	117.0325578	148.3979087	150.9757968	104.1808365
113.3966078	111.5891831	170.018796	114.3102461	88.07039782
52.8543511	3.628916522	14.74151411	10.78398548	7.518204692
56.6983039	38.10362348	36.36240146	32.35195645	42.96116967
37.47853987	40.82531087	38.32793668	72.25270275	41.88714043
61.50324491	79.83616349	102.2078311	81.95828968	53.70146209
59.58126851	52.61928957	40.29347189	60.39031871	49.40534512
136.4603247	127.9193074	89.43185225	131.5646229	75.18204692
57.65929211	54.43374783	52.08668318	42.05754339	45.10922815
86.48893816	100.7024335	100.2422959	92.74227517	88.07039782
54.7763275	76.20724696	45.20730993	54.99832597	63.36772526
0	0	0	0	0
23.06371684	13.60843696	28.50026061	14.01918113	45.10922815
117.2405606	112.4964122	55.034986	71.1743042	56.92354981
81.68399715	90.72291305	73.70757054	119.7022389	80.55219313

For Review Only

350.7606936	310.2723626	252.571275	320.2843689	183.6590003
38.43952807	49.89760218	37.34516907	58.23352162	35.44296498
49.01039829	0	1.965535214	1.078398548	0
71.11312693	58.06266435	31.44856343	2.156797097	3.222087725
678.4576704	544.3374783	508.0908529	552.1400568	655.1578375
18.25877583	27.21687392	16.70704932	14.01918113	54.77549133
44.20545728	72.57833044	42.25900711	49.60633323	83.77428086
22.10272864	27.21687392	41.2762395	9.705586936	22.55461408
64.38620952	41.73254	89.43185225	39.90074629	61.21966678
110.5136432	100.7024335	78.62140857	83.03668823	92.36651479
140.3042775	145.1566609	111.0527396	136.9566157	73.03398844
44.20545728	57.15543522	94.34569029	46.37113758	55.84952057
1.921976404	0	2.948302821	1.078398548	0
0	0	0	6.470391291	0
59.58126851	63.50603914	107.1216692	17.25437678	77.33010541
38.43952807	91.63014218	40.29347189	78.72309404	35.44296498
65.34719772	55.34097696	72.72480293	26.95996371	21.48058484
83.60597355	71.67110131	86.48354943	97.05586936	66.58981299
7.687905614	9.072291305	0	5.391992742	8.592233934
34.59557526	37.19639435	50.12114796	52.84152887	41.88714043
44.20545728	45.36145653	12.77597889	20.48957242	44.03519891
0	0	5.896605643	0	0
13.45383482	10.88674957	8.844908464	10.78398548	10.74029242
0	0	0	0	11.81432166
7.687905614	9.979520436	6.87937325	2.156797097	4.296116967
0	0	0	0	0
169.1339235	209.5699292	211.2950355	361.2635137	247.0267256
8.648893816	4.536145653	0.982767607	5.391992742	0
288.2964605	277.6121139	371.4861555	246.9532676	218.0279361
62.46423311	59.87712262	83.53524661	63.62551436	24.70267256
0	4.536145653	0	0	0
3.843952807	1.814458261	0	0	5.370146209
205.6514752	195.9614922	185.7430777	53.91992742	209.4357021
18.25877583	16.33012435	3.931070429	7.548789839	16.11043863
0	19.05181174	0.982767607	1.078398548	1.074029242
65.34719772	69.85664305	24.56919018	49.60633323	41.88714043
48.04941009	45.36145653	29.48302821	43.13594194	25.7767018
0	26.30964479	2.948302821	0	5.370146209
0	0	0	5.391992742	0
25.94668145	20.86627	35.37963386	36.66555065	37.59102346
21.14174044	0	4.913838036	4.313594194	4.296116967
17.29778763	23.58795739	11.79321129	37.7439492	26.85073104
0	0	0	0	0
21.14174044	20.86627	6.87937325	4.313594194	5.370146209
0	6.350603914	2.948302821	7.548789839	0
0	0	4.913838036	0	0
0	0	0	0	0
0	0	0	3.235195645	0
3.843952807	3.628916522	199.5018242	0	172.9187079
206.6124634	212.2916165	330.209916	306.2651878	248.1007548

For Review Only

111.4746314	127.0120783	142.501303	108.9182534	115.9951581
12.49284662	34.47470696	8.844908464	24.80316661	35.44296498
0	0	0	0	0
66.30818592	122.4759326	146.4323735	238.3260792	131.0315675
55.7373157	91.63014218	57.00052121	17.25437678	76.25607616
106.6696904	92.53737131	78.62140857	72.25270275	73.03398844
45.16644548	42.63976914	67.81096489	64.70391291	37.59102346
0	2.721687392	0.982767607	2.156797097	1.074029242
18.25877583	38.10362348	26.53472539	65.78231146	34.36893574
42.28348088	34.47470696	38.32793668	39.90074629	34.36893574
113.3966078	100.7024335	88.44908464	122.9374345	73.03398844
11.53185842	5.443374783	2.948302821	3.235195645	4.296116967
209.495428	159.672327	220.139944	260.9724487	195.473322
103.7867258	97.9807461	152.3289791	114.3102461	100.9587487
94.17684377	128.8265365	114.0010424	26.95996371	78.40413465
108.5916668	54.43374783	136.6046974	138.0350142	39.73908194
5.765929211	0	7.862140857	7.548789839	9.666263176
137.4213129	140.6205152	140.5357678	84.11508678	141.7718599
193.1586286	197.7759505	211.2950355	193.0333402	163.2524447
22.10272864	1.814458261	38.32793668	0	38.6650527
84.56696176	90.72291305	67.81096489	75.48789839	55.84952057
0	0	0	0	0
119.162537	130.6409948	115.9665776	142.3486084	136.4017137
55.7373157	40.82531087	45.20730993	83.03668823	90.21845631
28.82964605	23.58795739	30.46579582	21.56797097	23.62864332
21.14174044	0	0	46.37113758	51.5534036
0	0	3.931070429	0	8.592233934
40.36150447	36.28916522	44.22454232	63.62551436	63.36772526
9.609882018	5.443374783	4.913838036	0	9.666263176
23.06371684	26.30964479	21.62088736	18.33277532	10.74029242
78.80103254	120.6614744	90.41461986	104.6046592	98.81069024
206.6124634	193.2398048	218.1744088	291.1676081	255.6189595
48.04941009	44.4542274	39.31070429	77.64469549	38.6650527
0	0	0	1.078398548	1.074029242
171.0558999	133.3626822	175.9154017	389.301876	0
138.3823011	0	216.2088736	70.09590565	74.10801768
87.44992636	73.48555957	111.0527396	78.72309404	105.2548657
0	0	0.982767607	1.078398548	28.99878953
0	10.88674957	6.87937325	1.078398548	1.074029242
43.24446908	54.43374783	44.22454232	58.23352162	57.99757905
0	0	0	4.313594194	0
0	0	3.931070429	0	0
0	0	5.896605643	3.235195645	9.666263176
7.687905614	0.907229131	5.896605643	9.705586936	3.222087725
0	0	0	0	196.5473512
0	2.721687392	8.844908464	4.313594194	15.03640938
37.47853987	0	0	0	0
0	0	17.68981693	11.86238403	22.55461408
0	0	2.948302821	3.235195645	0
86.48893816	101.6096626	81.56971139	77.64469549	96.66263176

For Review Only

739.9609154	704.9170344	1037.802593	659.9799117	498.3495682
0	0	0	0	0
80.72300895	60.78435175	92.38015507	59.31192017	59.0716083
83.60597355	91.63014218	54.05221839	66.86071	53.70146209
345.9557526	355.6338192	262.3989511	365.5771079	311.4684801
3.843952807	2.721687392	4.913838036	1.078398548	7.518204692
21.14174044	0	0.982767607	2.156797097	8.592233934
49.01039829	0	36.36240146	28.03836226	6.444175451
1.921976404	1.814458261	0	5.391992742	4.296116967
0.960988202	0.907229131	0	0	0
96.09882018	125.19762	123.8287185	142.3486084	144.9939476
0	0	0	1.078398548	1.074029242
112.4356196	156.9506396	116.9493452	71.1743042	57.99757905
881.226181	511.6772296	472.711219	511.160912	438.2039306
167.2119471	125.19762	158.2255847	140.1918113	219.1019653
616.9544255	589.6989348	598.5054727	449.6921947	539.1626794
0	0	4.913838036	0	0
504.5188059	439.0988992	245.6919018	421.6538324	615.4187555
7.687905614	0	2.948302821	0	0
29.79063425	41.73254	17.68981693	32.35195645	25.7767018
68.23016233	94.35182958	59.94882404	85.19348533	95.58860252
1050.360105	838.2797166	1182.269431	1185.160005	778.6712003
139.3432893	101.6096626	81.56971139	109.9966519	157.8822985
3.843952807	0	0	0	0
5.765929211	2.721687392	4.913838036	0	0
0	0	0	0	7.518204692
1.921976404	1.814458261	0	1.078398548	3.222087725
95.13783197	77.1144761	130.7080917	148.8189997	107.4029242
0	0	29.48302821	12.94078258	17.18446787
29.79063425	23.58795739	24.56919018	10.78398548	21.48058484
134.5383482	116.1253287	71.74203532	77.64469549	106.3288949
135.4993364	134.2699113	99.25952832	85.19348533	137.4757429
92.25486737	76.20724696	26.53472539	54.99832597	68.73787147
49.97138649	45.36145653	84.51801421	29.11676081	77.33010541
33.63458706	29.03133218	59.94882404	29.11676081	27.92476029
181.6267701	106.1458083	127.7597889	166.0733765	118.1432166
0	68.04218479	0	0	0
48.04941009	0	28.50026061	91.66387662	2.148058484
44.20545728	33.56747783	26.53472539	40.97914484	38.6650527
49.01039829	22.68072826	35.37963386	53.91992742	26.85073104
25.94668145	18.14458261	28.50026061	4.313594194	5.370146209
0	15.42289522	0.982767607	4.313594194	19.33252635
254.6618735	239.5084905	199.5018242	237.2476807	251.3228426
0	0	0	0	0
24.98569325	33.56747783	34.39686625	28.03836226	26.85073104
100.9037612	117.0325578	152.3289791	126.1726302	97.736661
0	0	0	0	0
137.4213129	93.44460045	71.74203532	126.1726302	79.47816389
79.76202075	109.7747248	102.2078311	103.5262607	66.58981299
48.04941009	45.36145653	52.08668318	57.15512307	71.9599592

For Review Only

64.38620952	47.17591479	66.82819729	22.64636952	52.62743285
0	185.9819718	0.982767607	12.94078258	4.296116967
25.94668145	36.28916522	26.53472539	38.82234774	50.47937436
0	0	0.982767607	0	1.074029242
1051.321093	1026.983376	834.3696985	982.4210776	1046.104481
0	0	0	0	0
9.609882018	9.979520436	8.844908464	7.548789839	5.370146209
63.42522132	79.83616349	72.72480293	35.5871521	41.88714043
0	0	0	0	0
242.1690268	177.8169096	195.5707538	256.6588545	234.1383747
1.921976404	0	0	0	0
0	0	0	0	0
111.4746314	136.9915987	121.8631833	194.1117387	192.2512343
68.23016233	64.41326827	97.29399311	65.78231146	74.10801768
51.8933629	54.43374783	37.34516907	37.7439492	13.96238014
0	0	0	0	0
136.4603247	157.8578687	164.1221904	166.0733765	164.326474
52.8543511	63.50603914	70.75926771	65.78231146	55.84952057
16.33679943	4.536145653	5.896605643	10.78398548	4.296116967
6.726917412	16.33012435	17.68981693	3.235195645	1.074029242
580.4368739	569.739894	416.6934654	434.594615	563.8653519
173.9388645	185.0747426	249.6229722	268.5212386	253.4709011
141.2652657	102.5168918	87.46631704	79.80149259	96.66263176
16.33679943	22.68072826	20.63811975	12.94078258	18.25849711
14.41482303	34.47470696	28.50026061	37.7439492	36.51699422
0	0	0	0	0
0	5.443374783	0	0	0
22.10272864	12.70120783	24.56919018	11.86238403	0
21.14174044	19.05181174	29.48302821	9.705586936	36.51699422
12.49284662	23.58795739	25.55195779	33.430355	32.22087725
3.843952807	14.51566609	13.7587465	5.391992742	9.666263176
59.58126851	44.4542274	69.77650011	89.50707952	48.33131588
123.0064898	99.79520436	110.069972	93.82067372	75.18204692
0	329.3241744	0.982767607	375.2826949	39.73908194
167.2119471	177.8169096	276.1576976	266.3644415	278.1735736
32.67359886	39.01085261	50.12114796	40.97914484	76.25607616
23.06371684	19.05181174	34.39686625	36.66555065	4.296116967
24.02470504	0	0.982767607	1.078398548	5.370146209
56.6983039	78.02170523	129.7253241	81.95828968	91.29248555
18.25877583	26.30964479	48.15561275	45.29273904	20.40655559
116.2795724	132.4554531	129.7253241	127.2510287	86.99636858
7.687905614	14.51566609	6.87937325	5.391992742	12.8883509
0	0	684.9890222	0	0
7.687905614	8.165062175	15.72428171	1.078398548	2.148058484
233.520133	223.1783661	228.0020849	327.8331587	249.1747841
72.07411513	72.57833044	71.74203532	83.03668823	59.0716083
0.960988202	0	2.948302821	0	0
47.08842189	64.41326827	49.13838036	23.72476807	46.1832574
8.648893816	14.51566609	20.63811975	16.17597823	6.444175451
0	9.979520436	6.87937325	16.17597823	12.8883509

For Review Only

0	0	2.948302821	1.078398548	4.296116967
63.42522132	100.7024335	126.7770213	98.13426791	108.4769534
4.804941009	9.072291305	5.896605643	14.01918113	5.370146209
74.95707974	30.84579044	110.069972	57.15512307	48.33131588
6.726917412	0	6.87937325	2.156797097	7.518204692
272.9206493	322.0663413	352.813571	251.2668618	313.6165386
0	0	0	0	0
492.9869475	0	2680.990032	0	0
88.41091456	45.36145653	150.3634439	145.583804	62.29369602
5.765929211	5.443374783	0.982767607	0	2.148058484
1.921976404	0.907229131	0	0	0
3.843952807	1.814458261	4.913838036	0	3.222087725
38.43952807	47.17591479	54.05221839	54.99832597	60.14563754
19.21976404	29.93856131	30.46579582	34.50875355	11.81432166
0	126.1048491	2.948302821	31.27355791	6.444175451
0	0	0	0	0
143.1872421	152.4144939	178.8637045	149.8973982	111.6990411
484.3380537	395.5519009	285.0026061	405.4778542	248.1007548
172.9778763	215.9205331	230.9503877	241.5612749	202.9915267
120.1235252	160.5795561	161.1738876	146.6622026	115.9951581
100.9037612	84.37230914	88.44908464	119.7022389	65.51578375
16.33679943	27.21687392	0	25.88156516	36.51699422
0	0	0	0	0
61.50324491	51.71206044	100.2422959	157.4461881	144.9939476
13.45383482	9.072291305	13.7587465	7.548789839	13.96238014
94.17684377	121.5687035	106.1389016	163.9165794	144.9939476
0	0.907229131	0.982767607	5.391992742	3.222087725
0	0	0	0	0
25.94668145	35.38193609	34.39686625	25.88156516	37.59102346
15.37581123	37.19639435	16.70704932	21.56797097	32.22087725
63.42522132	56.24820609	94.34569029	51.76313033	86.99636858
168.1729353	134.2699113	141.5185354	135.8782171	160.030357
149.9141595	135.1771404	96.3112255	89.50707952	112.7730704
75.91806794	91.63014218	91.39738746	107.8398548	127.8094798
25.94668145	29.93856131	22.60365496	22.64636952	34.36893574
0	16.33012435	0	1.078398548	0
4.804941009	18.14458261	5.896605643	9.705586936	8.592233934
4.804941009	0.907229131	0	0	0
0	0.907229131	0.982767607	0	0
25.94668145	42.63976914	22.60365496	24.80316661	32.22087725
13.45383482	388.2940679	173.9498665	16.17597823	98.81069024
127.8114308	161.4867852	128.7425565	170.3869707	153.5861816
0	1.814458261	0	0	3.222087725
4.804941009	0	1.965535214	1.078398548	0
0	0.907229131	0.982767607	0	0
0	17.23735348	12.77597889	0	19.33252635
13.45383482	16.33012435	3.931070429	7.548789839	11.81432166
0.960988202	0	3.931070429	1.078398548	0
13.45383482	9.979520436	2.948302821	3.235195645	5.370146209
18.25877583	18.14458261	0	0	23.62864332

For Review Only

24.98569325	39.01085261	37.34516907	30.19515936	25.7767018
34.59557526	32.6602487	23.58642257	25.88156516	30.07281877
27.86865785	25.40241565	25.55195779	25.88156516	13.96238014
0.960988202	6.350603914	0	0	0
19.21976404	23.58795739	28.50026061	19.41117387	9.666263176
0	0	0	0	0
0	0	0	0	0
8.648893816	17.23735348	11.79321129	1.078398548	2.148058484
88.41091456	100.7024335	85.50078182	67.93910855	88.07039782
0	69.85664305	10.81044368	100.291065	24.70267256
14.41482303	0	6.87937325	5.391992742	9.666263176
8.648893816	9.979520436	2.948302821	9.705586936	22.55461408
0	0	2.948302821	0	0
16.33679943	24.49518652	6.87937325	7.548789839	7.518204692
0	7.257833044	0	0	0
22.10272864	32.6602487	26.53472539	20.48957242	37.59102346
63.42522132	113.4036413	32.43133104	121.859036	110.6250119
56.6983039	57.15543522	69.77650011	30.19515936	24.70267256
120.1235252	93.44460045	121.8631833	51.76313033	89.14442707
0	0	0	119.7022389	85.92233934
5.765929211	0	0.982767607	6.470391291	8.592233934
117.2405606	91.63014218	83.53524661	117.5454418	66.58981299
15.37581123	9.072291305	9.827676071	97.05586936	0
0.960988202	5.443374783	0.982767607	0	3.222087725
49.97138649	37.19639435	32.43133104	40.97914484	52.62743285
14.41482303	16.33012435	5.896605643	21.56797097	35.44296498
0	0	0	0	0
67.26917412	55.34097696	39.31070429	58.23352162	34.36893574
78.80103254	90.72291305	150.3634439	91.66387662	154.6602108
24.98569325	49.89760218	38.32793668	53.91992742	50.47937436
118.2015488	95.25905871	171.9843312	134.7998186	141.7718599
32.67359886	41.73254	33.41409864	33.430355	47.25728664
73.99609154	67.13495566	90.41461986	58.23352162	84.8483101
46.12743368	52.61928957	56.01775361	40.97914484	33.29490649
20.18075224	28.12410305	23.58642257	39.90074629	16.11043863
0	0	0	0	16.11043863
15.37581123	52.61928957	43.24177471	48.52793468	17.18446787
0	0	0	0	0
0	0	0	0	0
0.960988202	0.907229131	0	67.93910855	0
91.29387917	99.79520436	136.6046974	60.39031871	144.9939476
4.804941009	0	0	3.235195645	0
0	0	0	1.078398548	0
172.9778763	185.9819718	641.7472475	499.2985279	511.2379191
42.28348088	34.47470696	19.65535214	38.82234774	21.48058484
0	0	0	0	3.222087725
0.960988202	0	0	0	5.370146209
0	0	0	0	1.074029242
93.21585557	113.4036413	139.5530002	116.4670432	51.5534036
179.7047937	107.9602665	125.7942537	51.76313033	0

For Review Only

729.3900451	586.0700183	437.3315852	553.2184554	621.862931
0	938.074921	551.3326276	18.33277532	96.66263176
59.58126851	28.12410305	25.55195779	33.430355	42.96116967
82.64498535	99.79520436	96.3112255	104.6046592	147.1420061
0	4.536145653	2.948302821	2.156797097	0
0.960988202	0	0	0	0
4.804941009	4.536145653	0	5.391992742	1.074029242
51.8933629	37.19639435	27.517493	74.40949984	39.73908194
66.30818592	44.4542274	29.48302821	24.80316661	63.36772526
37.47853987	38.10362348	70.75926771	73.3311013	92.36651479
18.25877583	24.49518652	30.46579582	30.19515936	24.70267256
0	4.536145653	0	0	0
0.960988202	0	0	140.1918113	0
43.24446908	29.93856131	37.34516907	43.13594194	27.92476029
90.33289097	105.2385791	129.7253241	141.2702098	108.4769534
382.4733043	58.96989348	0	0	0
393.0441745	421.8615457	456.9869373	383.9098833	329.7269772
0	127.0120783	79.60417618	63.62551436	129.9575383
94.17684377	101.6096626	151.3462115	124.0158331	117.0691874
53.8153393	52.61928957	36.36240146	35.5871521	55.84952057
0	0	4.913838036	0	0
0.960988202	0.907229131	0	0	0
0	0	1.965535214	0	0
117.2405606	150.6000357	143.4840706	128.3294273	151.4381231
0.960988202	9.072291305	14.74151411	4.313594194	15.03640938
0	8.165062175	0	0	0
0	0	0	0	0
0	42.63976914	0	8.627188388	18.25849711
0	0	3.931070429	0	0
0	0	15.72428171	0	0
356.5266229	423.676004	324.3133104	326.7547602	524.12627
61.50324491	68.04218479	41.2762395	58.23352162	36.51699422
0	0	0	0	1.074029242
0	3.628916522	8.844908464	0	1.074029242
0	0.907229131	0	2.156797097	1.074029242
41.32249268	38.10362348	46.19007754	15.09757968	28.99878953
0.960988202	8.165062175	0	5.391992742	2.148058484
1.921976404	2.721687392	0	1.078398548	1.074029242
225.8322274	149.6928065	164.1221904	204.8957242	244.8786671
0	0	2.948302821	2.156797097	2.148058484
0	0	0.982767607	0	0
92.25486737	88.90845479	112.0355072	70.09590565	89.14442707
0	8.165062175	3.931070429	0	3.222087725
0	5.443374783	1.965535214	0	4.296116967
0	0	0	12.94078258	6.444175451
4.804941009	28.12410305	2.948302821	10.78398548	32.22087725
100.9037612	87.09399653	166.0877256	175.7789634	115.9951581
2.882964605	0	0	2.156797097	0
0	1466.989504	1803.378559	395.7722673	314.6905678
36.51755167	67.13495566	20.63811975	16.17597823	112.7730704

For Review Only

0	17.23735348	11.79321129	1.078398548	0
31.71261066	18.14458261	11.79321129	16.17597823	25.7767018
113.3966078	78.92893436	177.8809369	142.3486084	0
5.765929211	13.60843696	10.81044368	0	6.444175451
0	0	0	0	0
397.8491155	425.4904622	559.1947685	499.2985279	459.6845155
16.33679943	12.70120783	24.56919018	10.78398548	1.074029242
161.4460179	144.2494318	193.6052186	115.3886447	94.51457327
40.36150447	123.3831618	86.48354943	38.82234774	122.4393336
29.79063425	32.6602487	22.60365496	30.19515936	49.40534512
7.687905614	12.70120783	18.67258454	12.94078258	8.592233934
0.960988202	0	0.982767607	0	3.222087725
12.49284662	16.33012435	21.62088736	31.27355791	21.48058484
3.843952807	1.814458261	0.982767607	0	0
92.25486737	88.00122566	124.8114861	77.64469549	83.77428086
0	0	67.81096489	60.39031871	5.370146209
20.18075224	13.60843696	39.31070429	22.64636952	30.07281877
107.6306786	101.6096626	143.4840706	133.72142	106.3288949
67.26917412	97.9807461	70.75926771	95.97747081	92.36651479
120.1235252	144.2494318	185.7430777	179.014159	107.4029242
51.8933629	76.20724696	62.89712686	69.0175071	79.47816389
65.34719772	39.01085261	44.22454232	52.84152887	62.29369602
87.44992636	70.76387218	85.50078182	35.5871521	55.84952057
50.93237469	0	9.827676071	43.13594194	2.148058484
34.59557526	67.13495566	118.9148805	83.03668823	1.074029242
36.51755167	29.03133218	69.77650011	43.13594194	38.6650527
0	0	0	0	0
26.90766965	29.93856131	33.41409864	95.97747081	27.92476029
65.34719772	79.83616349	69.77650011	40.97914484	66.58981299
135.4993364	101.6096626	85.50078182	112.153449	136.4017137
61.50324491	46.26868566	48.15561275	93.82067372	79.47816389
50.93237469	52.61928957	53.06945079	51.76313033	52.62743285
87.44992636	29.93856131	147.4151411	63.62551436	96.66263176
211.4174044	187.79643	228.0020849	320.2843689	223.3980823
51.8933629	50.80483131	50.12114796	102.4478621	191.177205
227.7542038	266.7253644	273.2093948	283.6188182	241.6565794
119.162537	132.4554531	170.018796	179.014159	122.4393336
99.94277298	77.1144761	152.3289791	84.11508678	104.1808365
93.21585557	120.6614744	159.2083524	184.4061518	170.7706494
121.0845134	141.5277444	183.7775425	203.8173257	149.2900646
107.6306786	107.9602665	82.552479	33.430355	64.44175451
29.79063425	24.49518652	35.37963386	56.07672452	33.29490649
24.98569325	21.77349913	39.31070429	45.29273904	23.62864332
89.37190276	109.7747248	117.9321129	0	94.51457327
185.4707229	202.3120961	329.2271484	304.1083907	165.4005032
111.4746314	78.92893436	133.6563946	99.21266646	139.6238014
0	0	0	0	31.14684801
21.14174044	62.59881001	111.0527396	108.9182534	86.99636858
229.6761802	256.7458439	369.5206203	189.7981445	212.6577899
102.8257376	134.2699113	162.1566552	144.5054055	99.88471948

For Review Only

56.6983039	207.7554709	269.2783244	128.3294273	172.9187079
67.26917412	48.99037305	48.15561275	78.72309404	62.29369602
33.63458706	63.50603914	50.12114796	85.19348533	70.88592996
121.0845134	117.939787	150.3634439	156.3677895	139.6238014
0.960988202	0.907229131	0	0	15.03640938
70.15213873	55.34097696	50.12114796	72.25270275	95.58860252
176.8218291	226.8072826	159.2083524	296.5596008	230.916287
622.7203547	67.13495566	395.0725781	768.8981651	386.650527
169.1339235	178.7241387	250.6057398	116.4670432	200.8434682
155.6800887	0.907229131	308.5890286	131.5646229	0
0	0	0	0	0
289.2574487	313.9012792	415.7106978	435.6730136	509.0898606
32.67359886	48.99037305	3.931070429	6.470391291	22.55461408
74.95707974	89.81568392	88.44908464	91.66387662	138.5497722
83.60597355	107.0530374	145.4496059	115.3886447	104.1808365
100.9037612	67.13495566	118.9148805	120.7806374	69.81190071
47.08842189	39.01085261	45.20730993	62.54711581	23.62864332
0	0	0	0	23.62864332
240.2470504	234.0651157	283.0370709	262.0508473	227.6941993
12.49284662	7.257833044	5.896605643	12.94078258	7.518204692
0	6.350603914	0	6.470391291	0
201.8075224	195.9614922	131.6908594	157.4461881	147.1420061
158.5630533	120.6614744	68.7937325	113.2318476	107.4029242
183.5487465	176.0024513	191.6396834	160.6813837	189.0291465
0	0	0.982767607	0	0
0	1.814458261	0	0	0
4.804941009	12.70120783	16.70704932	9.705586936	5.370146209
172.0168881	129.7337657	122.8459509	130.4862244	118.1432166
98.98178478	106.1458083	53.06945079	61.46871726	98.81069024
96.09882018	85.27953827	56.01775361	106.7614563	56.92354981
2.882964605	7.257833044	5.896605643	2.156797097	4.296116967
4.804941009	0	0.982767607	0	0
1431.872421	1431.607568	1487.910157	1304.862244	1498.270792
213.3393808	144.2494318	179.8464721	256.6588545	160.030357
18.25877583	12.70120783	6.87937325	6.470391291	6.444175451
97.05980838	127.9193074	108.1044368	48.52793468	74.10801768
37.47853987	29.03133218	49.13838036	28.03836226	40.81311119
195.080605	126.1048491	148.3979087	231.8556879	144.9939476
6.726917412	5.443374783	45.20730993	64.70391291	65.51578375
339.2288352	0	0.982767607	386.0666803	76.25607616
1.921976404	0	4.913838036	0	0
93.21585557	117.0325578	103.1905987	149.8973982	114.9211289
1.921976404	6.350603914	5.896605643	4.313594194	5.370146209
0	0	7.862140857	0	0
120.1235252	140.6205152	234.8814581	145.583804	127.8094798
63.42522132	61.69158088	69.77650011	81.95828968	71.9599592
19.21976404	18.14458261	16.70704932	17.25437678	28.99878953
49.97138649	39.01085261	74.69033814	60.39031871	53.70146209
9.609882018	8.165062175	11.79321129	8.627188388	10.74029242
0	0	9.827676071	4.313594194	3.222087725

For Review Only

210.4564162	190.5181174	234.8814581	176.8573619	190.1031758
114.357596	0	26.53472539	28.03836226	100.9587487
90.33289097	58.96989348	109.0872044	115.3886447	99.88471948
208.5344398	242.2301779	264.3644863	269.5996371	292.1359538
58.62028031	76.20724696	103.1905987	116.4670432	94.51457327
10.57087022	9.979520436	0	127.2510287	0
145.1092185	170.5590765	188.6913806	184.4061518	230.916287
93.21585557	70.76387218	51.10391557	31.27355791	75.18204692
0	0	22.60365496	19.41117387	0
18.25877583	9.072291305	16.70704932	5.391992742	26.85073104
0	0	0	0	0
30.75162246	15.42289522	17.68981693	6.470391291	52.62743285
0	3.628916522	0	0	5.370146209
2.882964605	0	0	0	0
0	0	0	0	56.92354981
6.726917412	0	0.982767607	3.235195645	2.148058484
2.882964605	4.536145653	0	0	3.222087725
0	0	3.931070429	0	0
5.765929211	0	0.982767607	0	2.148058484
0	0	10.81044368	1.078398548	15.03640938
95.13783197	90.72291305	76.65587336	91.66387662	126.7354505
30.75162246	39.91808174	11.79321129	11.86238403	24.70267256
67.26917412	42.63976914	57.98328882	47.44953613	44.03519891
13.45383482	0	1.965535214	11.86238403	3.222087725
0	0	0	0	0
137.4213129	141.5277444	150.3634439	122.9374345	133.179626
0.960988202	0.907229131	0	0	1.074029242
6.726917412	0	0	1.078398548	2.148058484
2.882964605	2.721687392	0	1.078398548	0
114.357596	101.6096626	92.38015507	89.50707952	53.70146209
259.4668145	290.3133218	142.501303	241.5612749	175.0667664
2.882964605	1.814458261	0	0	0
49.97138649	0	66.82819729	0	4.296116967
544.8803104	604.2146009	714.4720504	713.8998391	517.6820945
54.7763275	57.15543522	136.6046974	141.2702098	79.47816389
0	0	0	5.391992742	0
0	0	83.53524661	0	0
136.4603247	164.2084726	191.6396834	189.7981445	165.4005032
0	0	1.965535214	0	0
0	48.08314392	11.79321129	31.27355791	49.40534512
66.30818592	59.87712262	23.58642257	85.19348533	34.36893574
86.48893816	70.76387218	134.6391622	160.6813837	127.8094798
26.90766965	50.80483131	15.72428171	24.80316661	21.48058484
38.43952807	24.49518652	6.87937325	20.48957242	8.592233934
272.9206493	68.04218479	995.543586	35.5871521	97.736661
61.50324491	57.15543522	61.91435925	32.35195645	46.1832574
35.55656347	26.30964479	28.50026061	7.548789839	8.592233934
51.8933629	64.41326827	52.08668318	57.15512307	42.96116967
22.10272864	76.20724696	102.2078311	83.03668823	52.62743285
3111.679797	3320.458618	3618.550329	3599.694355	2817.178701

For Review Only

0	0	0	0	3.222087725
33.63458706	46.26868566	15.72428171	24.80316661	10.74029242
26.90766965	29.93856131	8.844908464	16.17597823	10.74029242
64.38620952	90.72291305	73.70757054	43.13594194	65.51578375
36.51755167	29.93856131	18.67258454	14.01918113	33.29490649
24.02470504	21.77349913	46.19007754	14.01918113	37.59102346
84.56696176	80.74339262	80.58694379	66.86071	35.44296498
526.6215346	448.1711905	381.3138316	697.7238609	392.0206732
323.853024	287.5916344	266.3300215	222.150101	225.5461408
0	47.17591479	1.965535214	0	0
119.162537	117.0325578	102.2078311	146.6622026	120.2912751
10.57087022	5.443374783	0	0	1.074029242
2105.52515	2067.575188	490.401036	2120.131546	2534.709011
55.7373157	72.57833044	54.05221839	50.68473178	20.40655559
5.765929211	5.443374783	1.965535214	2.156797097	1.074029242
16.33679943	15.42289522	16.70704932	11.86238403	11.81432166
15.37581123	12.70120783	32.43133104	9.705586936	7.518204692
168.1729353	108.8674957	151.3462115	11.86238403	100.9587487
43.24446908	38.10362348	41.2762395	42.05754339	67.66384223
0	0	1.965535214	1.078398548	0
91.29387917	87.09399653	34.39686625	39.90074629	81.62622237
0	0	3.931070429	0	6.444175451
49.97138649	65.3204974	69.77650011	63.62551436	41.88714043
0.960988202	1.814458261	1.965535214	3.235195645	0
20.18075224	18.14458261	16.70704932	5.391992742	6.444175451
0	0	0	0	0
0.960988202	0	2.948302821	3.235195645	3.222087725
16.33679943	13.60843696	23.58642257	17.25437678	10.74029242
0	0	0	0	0
61.50324491	99.79520436	33.41409864	127.2510287	49.40534512
82.64498535	60.78435175	144.4668382	56.07672452	64.44175451
102.8257376	80.74339262	103.1905987	91.66387662	50.47937436
0	0	13.7587465	0	0
0	0	9.827676071	7.548789839	3.222087725
7.687905614	0	3.931070429	1.078398548	1.074029242
168.1729353	164.2084726	140.5357678	171.4653692	182.5849711
126.8504426	133.3626822	108.1044368	74.40949984	114.9211289
232.5591448	184.1675135	185.7430777	109.9966519	153.5861816
0	0	0.982767607	1.078398548	16.11043863
30.75162246	41.73254	32.43133104	39.90074629	25.7767018
0	0	5.896605643	0	0
57.65929211	55.34097696	63.87989446	61.46871726	76.25607616
39.40051627	53.5265187	47.17284514	34.50875355	53.70146209
0	0	0	30.19515936	0
233.520133	213.1988457	174.9326341	126.1726302	178.2888541
0	0	0	0	0
106.6696904	79.83616349	70.75926771	81.95828968	91.29248555
16.33679943	0	0.982767607	1.078398548	3.222087725
0	31.75301957	22.60365496	3.235195645	1.074029242
70.15213873	88.90845479	65.84542968	122.9374345	46.1832574

For Review Only

47.08842189	33.56747783	53.06945079	30.19515936	53.70146209
88.41091456	47.17591479	55.034986	54.99832597	57.99757905
70.15213873	107.0530374	123.8287185	63.62551436	78.40413465
20.18075224	31.75301957	16.70704932	12.94078258	21.48058484
6.726917412	0	0.982767607	1.078398548	0
34.59557526	29.03133218	7.862140857	11.86238403	8.592233934
25.94668145	21.77349913	34.39686625	19.41117387	21.48058484
91.29387917	0	4.913838036	340.7739413	146.0679769
110.5136432	97.9807461	111.0527396	104.6046592	100.9587487
0.960988202	20.86627	0	138.0350142	42.96116967
171.0558999	328.4169453	190.6569158	460.4761802	506.9418021
39.40051627	47.17591479	53.06945079	54.99832597	62.29369602
54.7763275	46.26868566	47.17284514	48.52793468	83.77428086
24.98569325	29.03133218	22.60365496	7.548789839	20.40655559
110.5136432	109.7747248	68.7937325	108.9182534	122.4393336
21.14174044	15.42289522	14.74151411	18.33277532	25.7767018
48.04941009	38.10362348	16.70704932	52.84152887	54.77549133
87.44992636	69.85664305	126.7770213	179.014159	53.70146209
45.16644548	237.6940322	235.8642257	193.0333402	178.2888541
38.43952807	31.75301957	17.68981693	32.35195645	59.0716083
78.80103254	81.65062175	47.17284514	67.93910855	59.0716083
50.93237469	75.30001783	28.50026061	57.15512307	54.77549133
44.20545728	39.01085261	35.37963386	9.705586936	12.8883509
26.90766965	33.56747783	26.53472539	30.19515936	19.33252635
48.04941009	26.30964479	40.29347189	38.82234774	51.5534036
0	0	0	7.548789839	0
195.080605	0	628.9712686	1336.135802	8.592233934
140.3042775	155.1361813	178.8637045	174.7005649	137.4757429
119.162537	79.83616349	171.0015636	106.7614563	122.4393336
0	48.99037305	0	0	0
104.747714	68.94941392	115.9665776	58.23352162	117.0691874
0	0	1.965535214	0	0
0	56.24820609	51.10391557	49.60633323	69.81190071
18.25877583	27.21687392	30.46579582	28.03836226	46.1832574
78.80103254	109.7747248	92.38015507	52.84152887	64.44175451
0	0	0	0	0
27.86865785	37.19639435	26.53472539	38.82234774	22.55461408
0	0	0	0	0
3.843952807	3.628916522	5.896605643	1.078398548	1.074029242
4.804941009	0	0	0	0
97.05980838	67.13495566	102.2078311	64.70391291	62.29369602
88.41091456	88.00122566	68.7937325	71.1743042	66.58981299
5.765929211	0	0	0	0
22.10272864	14.51566609	13.7587465	5.391992742	9.666263176
61.50324491	42.63976914	84.51801421	64.70391291	64.44175451
18.25877583	6.350603914	69.77650011	48.52793468	0
70.15213873	97.9807461	106.1389016	101.3694636	64.44175451
76.87905614	69.85664305	85.50078182	76.56629694	136.4017137
1.921976404	0.907229131	39.31070429	0	0
5.765929211	0.907229131	0.982767607	0	0

For Review Only

138.3823011	119.7542452	105.156134	131.5646229	135.3276845
32.67359886	43.54699827	22.60365496	16.17597823	21.48058484
387.2782453	362.8916522	415.7106978	291.1676081	237.3604624
78.80103254	56.24820609	72.72480293	62.54711581	62.29369602
60.54225671	60.78435175	88.44908464	58.23352162	85.92233934
148.9531713	155.1361813	90.41461986	142.3486084	111.6990411
419.9518442	503.5121674	452.0730993	320.2843689	309.3204216
141.2652657	148.7855774	93.36292268	135.8782171	126.7354505
37.47853987	53.5265187	53.06945079	34.50875355	35.44296498
0	38.10362348	25.55195779	142.3486084	4.296116967
265.2327437	186.8892009	626.0229657	471.2601657	540.2367086
0	0	0	0	0
109.552655	108.8674957	34.39686625	140.1918113	154.6602108
25.94668145	34.47470696	47.17284514	20.48957242	28.99878953
0	107.9602665	8.844908464	81.95828968	259.9150765
241.2080386	172.3735348	41.2762395	62.54711581	532.7185039
0	0	8.844908464	7.548789839	0
80.72300895	88.90845479	95.32845789	116.4670432	73.03398844
48.04941009	57.15543522	26.53472539	36.66555065	35.44296498
76.87905614	94.35182958	66.82819729	91.66387662	94.51457327
153.7581123	141.5277444	118.9148805	122.9374345	84.8483101
229.6761802	239.5084905	218.1744088	259.8940502	247.0267256
0	3.628916522	0.982767607	0	0
0	0	2.948302821	1.078398548	5.370146209
4.804941009	0	2.948302821	6.470391291	0
216.2223454	219.5494496	153.3117467	181.1709561	128.883509
113.3966078	60.78435175	50.12114796	150.9757968	95.58860252
227.7542038	186.8892009	140.5357678	152.0541953	180.4369126
150.8751477	142.4349735	157.2428171	156.3677895	193.3252635
140.3042775	104.33135	132.673627	111.0750505	131.0315675
96.09882018	158.7650978	157.2428171	145.583804	124.587392
62.46423311	121.5687035	81.56971139	127.2510287	57.99757905
0	0	0.982767607	1.078398548	5.370146209
2.882964605	168.7446183	22.60365496	16.17597823	175.0667664
19.21976404	34.47470696	26.53472539	31.27355791	35.44296498
24.98569325	18.14458261	79.60417618	53.91992742	41.88714043
77.84004434	105.2385791	129.7253241	120.7806374	99.88471948
0	65.3204974	21.62088736	1.078398548	17.18446787
2.882964605	3.628916522	1.965535214	1.078398548	37.59102346
49.01039829	29.03133218	32.43133104	47.44953613	48.33131588
3.843952807	55.34097696	0	0	57.99757905
0	24.49518652	0	33.430355	0
0	0	0.982767607	3.235195645	0
16.33679943	12.70120783	14.74151411	5.391992742	7.518204692
191.2366522	173.2807639	285.0026061	267.44284	219.1019653
45.16644548	87.09399653	78.62140857	4.313594194	19.33252635
631.3692486	568.8326648	514.9702261	399.0074629	365.1699422
0	0	0	0	0
87.44992636	76.20724696	81.56971139	70.09590565	74.10801768
82.64498535	77.1144761	54.05221839	29.11676081	51.5534036

For Review Only

198.9245578	166.0229309	171.9843312	131.5646229	139.6238014
0	0	1.965535214	0	5.370146209
10.57087022	0	27.517493	5.391992742	2.148058484
1259.855533	1267.399095	1467.272037	2067.290017	1906.401904
9.609882018	0	6.87937325	8.627188388	11.81432166
0	0.907229131	0	3.235195645	0
7.687905614	0	0	0	0
0	6.350603914	6.87937325	10.78398548	7.518204692
0.960988202	0	7.862140857	1.078398548	0
224.8712392	185.0747426	34.39686625	119.7022389	134.2536552
43.24446908	0	18.67258454	0	0
4.804941009	6.350603914	0	0	0
25.94668145	19.95904087	3.931070429	25.88156516	24.70267256
0	0	2.948302821	1.078398548	0
0	0	0	2.156797097	2.148058484
0	0	0	0	0
2.882964605	0	0	0	2.148058484
0	0	0.982767607	0	0
0	0	1.965535214	0	0
3.843952807	0	0	0	12.8883509
0.960988202	0	0	0	0
0.960988202	1.814458261	0	4.313594194	0
21.14174044	33.56747783	33.41409864	29.11676081	32.22087725
14.41482303	34.47470696	24.56919018	12.94078258	24.70267256
72.07411513	65.3204974	64.86266207	72.25270275	47.25728664
123.0064898	0	45.20730993	1.078398548	1.074029242
17.29778763	31.75301957	64.86266207	21.56797097	31.14684801
0	0	0	0	0
57.65929211	56.24820609	59.94882404	40.97914484	41.88714043
48.04941009	156.9506396	169.0360284	0	63.36772526
28.82964605	46.26868566	16.70704932	18.33277532	23.62864332
8.648893816	13.60843696	10.81044368	8.627188388	6.444175451
35.55656347	39.01085261	20.63811975	19.41117387	51.5534036
221.0272864	189.6108883	184.7603101	227.5420937	183.6590003
18.25877583	11.7939787	5.896605643	5.391992742	22.55461408
0	0	0	0	0
39.40051627	31.75301957	52.08668318	47.44953613	46.1832574
14.41482303	9.979520436	0.982767607	8.627188388	2.148058484
0	2.721687392	0	0	0
19.21976404	35.38193609	25.55195779	24.80316661	44.03519891
512.2067115	455.4290235	408.8313246	449.6921947	510.1638898
32.67359886	48.99037305	34.39686625	32.35195645	39.73908194
63.42522132	64.41326827	88.44908464	93.82067372	75.18204692
1051.321093	1176.676182	1195.04541	1508.679569	1144.915172
0	2.721687392	4.913838036	0	0
134.5383482	98.88797523	114.98381	71.1743042	152.5121523
17.29778763	24.49518652	21.62088736	46.37113758	27.92476029
9.609882018	0	0	3.235195645	0
187.3926993	244.0446361	294.8302821	339.6955428	212.6577899
195.080605	188.7036592	4.913838036	8.627188388	234.1383747

For Review Only

44.20545728	20.86627	52.08668318	15.09757968	28.99878953
162.4070061	143.3422026	244.7091342	227.5420937	245.9526964
17.29778763	9.979520436	9.827676071	3.235195645	7.518204692
94.17684377	119.7542452	101.2250635	91.66387662	79.47816389
23.06371684	27.21687392	35.37963386	16.17597823	24.70267256
0	7.257833044	0	102.4478621	0
132.6163718	125.19762	86.48354943	113.2318476	135.3276845
4.804941009	1.814458261	0	0	3.222087725
237.3640858	271.26151	333.1582188	273.9132313	313.6165386
61.50324491	44.4542274	42.25900711	40.97914484	47.25728664
7.687905614	17.23735348	8.844908464	4.313594194	13.96238014
13.45383482	6.350603914	15.72428171	17.25437678	5.370146209
0	0	0	0	0
78.80103254	60.78435175	53.06945079	75.48789839	36.51699422
95.13783197	69.85664305	38.32793668	38.82234774	49.40534512
101.8647494	67.13495566	77.63864096	79.80149259	78.40413465
26.90766965	40.82531087	63.87989446	14.01918113	6.444175451
0.960988202	0	1.965535214	16.17597823	5.370146209
43.24446908	70.76387218	46.19007754	51.76313033	61.21966678
0	0	0	0	0
0	3.628916522	0.982767607	0	1.074029242
30.75162246	29.93856131	37.34516907	33.430355	33.29490649
0	0	81.56971139	85.19348533	63.36772526
92.25486737	131.5482239	84.51801421	98.13426791	177.2148249
102.8257376	112.4964122	94.34569029	131.5646229	103.1068072
43.24446908	25.40241565	12.77597889	20.48957242	25.7767018
4.804941009	0	0	2.156797097	0
51.8933629	20.86627	60.93159164	64.70391291	73.03398844
11.53185842	16.33012435	2.948302821	2.156797097	4.296116967
85.52794996	89.81568392	47.17284514	60.39031871	106.3288949
11.53185842	0	0	6.470391291	5.370146209
22.10272864	26.30964479	10.81044368	3.235195645	10.74029242
0	0	0.982767607	1.078398548	2.148058484
93.21585557	103.4241209	52.08668318	48.52793468	44.03519891
49.97138649	56.24820609	50.12114796	43.13594194	65.51578375
69.19115053	105.2385791	98.27676071	49.60633323	41.88714043
8.648893816	13.60843696	18.67258454	6.470391291	1.074029242
4.804941009	0	22.60365496	0	15.03640938
29.79063425	19.05181174	31.44856343	36.66555065	31.14684801
0	5.443374783	2.948302821	4.313594194	0
0	0	0.982767607	30.19515936	1.074029242
227.7542038	201.404867	167.0704932	141.2702098	216.9539068
206.6124634	220.4566787	183.7775425	177.9357605	205.1395852
7.687905614	0.907229131	0	5.391992742	0
7.687905614	6.350603914	5.896605643	3.235195645	3.222087725
129.7334072	117.0325578	124.8114861	77.64469549	112.7730704
79.76202075	0	0	65.78231146	76.25607616
25.94668145	27.21687392	37.34516907	18.33277532	24.70267256
36.51755167	72.57833044	59.94882404	64.70391291	69.81190071
1.921976404	2.721687392	0	0	25.7767018

For Review Only

204.690487	178.7241387	233.8986905	181.1709561	93.44054403
19.21976404	20.86627	36.36240146	35.5871521	24.70267256
495.8699121	0	0.982767607	208.1309199	1.074029242
0	0	0	6.470391291	0
63.42522132	95.25905871	25.55195779	38.82234774	81.62622237
0	0	0	0	0
1.921976404	0	0	0	3.222087725
15.37581123	54.43374783	65.84542968	22.64636952	54.77549133
69.19115053	62.59881001	56.01775361	65.78231146	56.92354981
13.45383482	20.86627	32.43133104	1.078398548	4.296116967
7.687905614	9.979520436	6.87937325	0	5.370146209
49.97138649	71.67110131	67.81096489	76.56629694	86.99636858
143.1872421	137.8988278	65.84542968	166.0733765	126.7354505
74.95707974	59.87712262	86.48354943	62.54711581	143.9199184
6.726917412	8.165062175	18.67258454	15.09757968	13.96238014
119.162537	148.7855774	233.8986905	187.6413474	167.5485617
20.18075224	20.86627	17.68981693	12.94078258	20.40655559
109.552655	139.7132861	92.38015507	102.4478621	125.6614213
5.765929211	0	0	3.235195645	0
4.804941009	0	0	0	3.222087725
27.86865785	25.40241565	12.77597889	34.50875355	26.85073104
4.804941009	12.70120783	6.87937325	9.705586936	4.296116967
0	0	0	0	0
1.921976404	1.814458261	0	0	1.074029242
19.21976404	29.93856131	21.62088736	36.66555065	33.29490649
38.43952807	28.12410305	121.8631833	78.72309404	25.7767018
62.46423311	61.69158088	31.44856343	121.859036	69.81190071
156.6410769	143.3422026	86.48354943	87.35028243	138.5497722
122.0455016	154.2289522	107.1216692	157.4461881	133.179626
0	0	0	0	0
0.960988202	0	0	2.156797097	0
65.34719772	81.65062175	77.63864096	37.7439492	74.10801768
10.57087022	22.68072826	14.74151411	19.41117387	21.48058484
120.1235252	179.6313678	169.0360284	98.13426791	121.3653043
148.9531713	97.9807461	113.0182748	83.03668823	96.66263176
21.14174044	49.89760218	84.51801421	37.7439492	39.73908194
174.8998527	205.9410126	235.8642257	309.5003834	205.1395852
15.37581123	8.165062175	1.965535214	6.470391291	2.148058484
8.648893816	23.58795739	21.62088736	16.17597823	10.74029242
24.98569325	46.26868566	54.05221839	46.37113758	0
59.58126851	59.87712262	17.68981693	35.5871521	40.81311119
39.40051627	29.03133218	122.8459509	1.078398548	1.074029242
8.648893816	0	8.844908464	11.86238403	4.296116967
119.162537	117.939787	33.41409864	141.2702098	108.4769534
54.7763275	48.99037305	57.00052121	28.03836226	22.55461408
0	349.2832153	79.60417618	668.6071	42.96116967
14.41482303	10.88674957	10.81044368	5.391992742	9.666263176
1.921976404	3.628916522	0.982767607	0	0
48.04941009	56.24820609	61.91435925	56.07672452	63.36772526
0	5.443374783	0	0	0

For Review Only

2.882964605	0	1.965535214	2.156797097	0
4.804941009	7.257833044	4.913838036	0	1.074029242
75.91806794	59.87712262	110.069972	99.21266646	70.88592996
125.8894544	72.57833044	81.56971139	120.7806374	86.99636858
25.94668145	35.38193609	44.22454232	42.05754339	53.70146209
63.42522132	48.08314392	62.89712686	28.03836226	47.25728664
1.921976404	1.814458261	0.982767607	15.09757968	45.10922815
85.52794996	54.43374783	54.05221839	84.11508678	100.9587487
0.960988202	13.60843696	0.982767607	3.235195645	1.074029242
146.0702067	103.4241209	127.7597889	86.27188388	198.6954097
35.55656347	191.4253465	14.74151411	67.93910855	109.5509827
339.2288352	387.3868387	353.7963386	339.6955428	338.3192112
49.01039829	51.71206044	37.34516907	73.3311013	31.14684801
412.2639386	444.542274	301.7096554	327.8331587	482.2391295
0	76.20724696	22.60365496	59.31192017	31.14684801
69.19115053	103.4241209	45.20730993	78.72309404	90.21845631
350.7606936	160.5795561	373.4516907	133.72142	33.29490649
42.28348088	39.91808174	69.77650011	62.54711581	30.07281877
3.843952807	0	0	0	5.370146209
91.29387917	87.09399653	97.29399311	102.4478621	117.0691874
12.49284662	16.33012435	17.68981693	20.48957242	21.48058484
42.28348088	44.4542274	53.06945079	42.05754339	51.5534036
68.23016233	71.67110131	42.25900711	69.0175071	139.6238014
0	0	4.913838036	0	0
98.98178478	28.12410305	21.62088736	46.37113758	79.47816389
16.33679943	23.58795739	2.948302821	14.01918113	16.11043863
3.843952807	3.628916522	0	4.313594194	4.296116967
66.30818592	64.41326827	78.62140857	86.27188388	93.44054403
9.609882018	7.257833044	1.965535214	8.627188388	1.074029242
1.921976404	6.350603914	0	0	0
0	3.628916522	0	0	0
0	5.443374783	0	5.391992742	6.444175451
0	0	0	0	6.444175451
0	60.78435175	2.948302821	1.078398548	16.11043863
18.25877583	15.42289522	14.74151411	23.72476807	24.70267256
31.71261066	30.84579044	6.87937325	88.42868097	1.074029242
53.8153393	53.5265187	54.05221839	91.66387662	60.14563754
83.60597355	52.61928957	52.08668318	52.84152887	83.77428086
72.07411513	54.43374783	50.12114796	75.48789839	33.29490649
3.843952807	0	22.60365496	1.078398548	4.296116967
0	0	3.931070429	1.078398548	0
41.32249268	24.49518652	22.60365496	11.86238403	13.96238014
15.37581123	3.628916522	4.913838036	0	9.666263176
38.43952807	36.28916522	11.79321129	14.01918113	6.444175451
6.726917412	6.350603914	2.948302821	9.705586936	19.33252635
0	15.42289522	0	0	154.6602108
24.02470504	19.95904087	9.827676071	31.27355791	23.62864332
398.8101037	352.9121318	332.1754512	307.3435863	338.3192112
9.609882018	9.979520436	6.87937325	3.235195645	5.370146209
44.20545728	61.69158088	78.62140857	38.82234774	53.70146209

For Review Only

41.32249268	20.86627	15.72428171	46.37113758	35.44296498
0	0	1.965535214	3.235195645	0
0	0	5.896605643	6.470391291	0
31.71261066	24.49518652	26.53472539	26.95996371	4.296116967
10.57087022	0	0	0	0
8.648893816	4.536145653	2.948302821	9.705586936	21.48058484
39.40051627	52.61928957	30.46579582	49.60633323	71.9599592
20.18075224	0	0.982767607	0	1.074029242
53.8153393	47.17591479	34.39686625	66.86071	50.47937436
0	0	0.982767607	0	0
63.42522132	0	31.44856343	95.97747081	2.148058484
0	0	0	0	0
33.63458706	0	8.844908464	20.48957242	97.736661
14.41482303	13.60843696	2.948302821	17.25437678	13.96238014
156.6410769	116.1253287	102.2078311	167.151775	141.7718599
0	4.536145653	0	0	0
0	2.721687392	0	0	0
8.648893816	14.51566609	7.862140857	10.78398548	10.74029242
5.765929211	11.7939787	0	0	0
59.58126851	75.30001783	86.48354943	52.84152887	62.29369602
0	0	0	0	0
71.11312693	76.20724696	74.69033814	99.21266646	84.8483101
2.882964605	3.628916522	0	0	0
0	25.40241565	26.53472539	18.33277532	3.222087725
0	11.7939787	9.827676071	7.548789839	20.40655559
41.32249268	59.87712262	49.13838036	36.66555065	65.51578375
23.06371684	28.12410305	45.20730993	18.33277532	41.88714043
24.02470504	14.51566609	52.08668318	70.09590565	26.85073104
14.41482303	19.05181174	27.517493	28.03836226	15.03640938
0	0	0	0	0
177.7828173	119.7542452	108.1044368	174.7005649	140.6978307
3.843952807	0	0	0	0
251.7789089	232.2506574	397.0381133	416.2618397	292.1359538
64.38620952	73.48555957	74.69033814	69.0175071	55.84952057
0	0	0	15.09757968	0
41.32249268	79.83616349	55.034986	22.64636952	30.07281877
0	3.628916522	26.53472539	1.078398548	1.074029242
63.42522132	54.43374783	95.32845789	143.4270069	77.33010541
234.4811212	46.26868566	17.68981693	109.9966519	115.9951581
91.29387917	70.76387218	120.8804157	103.5262607	93.44054403
0	10.88674957	0.982767607	10.78398548	4.296116967
48.04941009	48.08314392	25.55195779	17.25437678	35.44296498
10.57087022	21.77349913	31.44856343	15.09757968	13.96238014
0	0	0	1.078398548	0
73.03510333	80.74339262	32.43133104	47.44953613	68.73787147
36.51755167	21.77349913	31.44856343	23.72476807	25.7767018
10.57087022	14.51566609	14.74151411	16.17597823	19.33252635
0	0	0	1.078398548	2.148058484
135.4993364	140.6205152	114.0010424	122.9374345	119.2172458
5.765929211	3.628916522	13.7587465	1.078398548	3.222087725

For Review Only

17.29778763	6.350603914	2.948302821	5.391992742	6.444175451
88.41091456	57.15543522	49.13838036	75.48789839	54.77549133
124.9284662	107.0530374	115.9665776	79.80149259	105.2548657
181.6267701	176.0024513	173.9498665	104.6046592	172.9187079
118.2015488	103.4241209	0	25.88156516	0
11.53185842	9.072291305	31.44856343	10.78398548	17.18446787
42.28348088	80.74339262	46.19007754	48.52793468	15.03640938
72.07411513	0	60.93159164	62.54711581	0
14.41482303	0	16.70704932	4.313594194	1.074029242
220.0662982	220.4566787	184.7603101	152.0541953	171.8446787
45.16644548	47.17591479	24.56919018	42.05754339	40.81311119
82.64498535	88.00122566	104.1733664	80.87989113	65.51578375
6.726917412	0.907229131	7.862140857	2.156797097	1.074029242
28.82964605	32.6602487	54.05221839	46.37113758	32.22087725
39.40051627	29.03133218	32.43133104	20.48957242	69.81190071
100.9037612	100.7024335	100.2422959	101.3694636	98.81069024
38.43952807	48.99037305	44.22454232	64.70391291	35.44296498
15.37581123	19.05181174	82.552479	22.64636952	16.11043863
38.43952807	38.10362348	17.68981693	21.56797097	32.22087725
23.06371684	13.60843696	21.62088736	14.01918113	44.03519891
55.7373157	60.78435175	76.65587336	72.25270275	55.84952057
2.882964605	2.721687392	1.965535214	0	0
225.8322274	248.5807818	195.5707538	214.6013111	180.4369126
12.49284662	11.7939787	19.65535214	0	13.96238014
23.06371684	25.40241565	25.55195779	17.25437678	19.33252635
146.0702067	90.72291305	112.0355072	100.291065	71.9599592
5.765929211	17.23735348	28.50026061	16.17597823	0
17.29778763	10.88674957	22.60365496	8.627188388	16.11043863
39.40051627	35.38193609	43.24177471	32.35195645	41.88714043
0.960988202	0	5.896605643	1.078398548	0
8.648893816	3.628916522	7.862140857	0	6.444175451
15.37581123	15.42289522	4.913838036	4.313594194	20.40655559
17.29778763	90.72291305	28.50026061	37.7439492	3.222087725
77.84004434	79.83616349	109.0872044	91.66387662	80.55219313
48.04941009	53.5265187	60.93159164	51.76313033	41.88714043
40.36150447	45.36145653	69.77650011	62.54711581	48.33131588
17.29778763	9.072291305	9.827676071	9.705586936	15.03640938
230.6371684	232.2506574	206.3811975	438.9082092	152.5121523
9.609882018	17.23735348	9.827676071	18.33277532	19.33252635
115.3185842	68.94941392	137.587465	142.3486084	81.62622237
102.8257376	91.63014218	250.6057398	159.6029852	146.0679769
33.63458706	16.33012435	37.34516907	42.05754339	24.70267256
27.86865785	45.36145653	54.05221839	40.97914484	41.88714043
0	0	0.982767607	2.156797097	1.074029242
1878.731934	2177.349913	1967.500749	2317.478481	2745.218742
0	4.536145653	4.913838036	2.156797097	0
0	0	32.43133104	21.56797097	49.40534512
0.960988202	0.907229131	293.8475145	368.8123036	0
0.960988202	1568.599167	0	507.9257163	0
1600.045356	1874.335384	2923.733631	2865.304943	2650.704169

For Review Only

0	0	16.70704932	33.430355	52.62743285
148.9531713	147.8783483	201.4673595	171.4653692	175.0667664
172.9778763	127.0120783	82.552479	142.3486084	111.6990411
37.47853987	33.56747783	55.034986	45.29273904	26.85073104
426.6787616	415.5109418	358.7101766	333.2251515	250.2488133
0	0	0	0	0
0	0	0.982767607	0	17.18446787
117.2405606	115.2180996	99.25952832	163.9165794	164.326474
5.765929211	9.979520436	8.844908464	6.470391291	3.222087725
5596.795287	6789.702813	7754.03642	6904.985906	5045.789378
17.29778763	30.84579044	29.48302821	30.19515936	27.92476029
17.29778763	22.68072826	26.53472539	22.64636952	28.99878953
24.02470504	29.03133218	32.43133104	31.27355791	15.03640938
30.75162246	15.42289522	24.56919018	17.25437678	21.48058484
33.63458706	26.30964479	0.982767607	3.235195645	3.222087725
79.76202075	106.1458083	141.5185354	111.0750505	63.36772526
170.0949117	195.9614922	150.3634439	202.7389271	221.2500238
61.50324491	78.92893436	28.50026061	69.0175071	63.36772526
110.5136432	94.35182958	75.67310575	88.42868097	53.70146209
29.79063425	34.47470696	38.32793668	23.72476807	51.5534036
0	0	0	0	0
147.9921831	153.3217231	112.0355072	95.97747081	125.6614213
17.29778763	7.257833044	22.60365496	19.41117387	0
0	0	0	0	0
0	0	0	0	0
0	0	0	0	0
309.438201	330.2314035	444.2109584	469.1033686	429.6116967
0	0	0	0	8.592233934
110.5136432	94.35182958	75.67310575	81.95828968	114.9211289
121.0845134	123.3831618	135.6219298	97.05586936	83.77428086
0	0	0.982767607	0	0
7.687905614	11.7939787	6.87937325	10.78398548	4.296116967
3.843952807	0	0	3.235195645	12.8883509
43.24446908	33.56747783	44.22454232	33.430355	45.10922815
0.960988202	0	0	0	2.148058484
15.37581123	19.05181174	12.77597889	17.25437678	9.666263176
0	0	0	0	0
0	6.350603914	0	1.078398548	1.074029242
0	3.628916522	0	3.235195645	0
0	0	0.982767607	1.078398548	0
98.02079658	84.37230914	65.84542968	94.89907226	92.36651479
197.9635696	181.4458261	167.0704932	175.7789634	161.1043863
3.843952807	0	0	0	0
0	0	0	0	0
12.49284662	5.443374783	14.74151411	1.078398548	2.148058484
56.6983039	84.37230914	6.87937325	9.705586936	21.48058484
34.59557526	30.84579044	32.43133104	39.90074629	23.62864332
0	0	0.982767607	3.235195645	1.074029242
88.41091456	74.3927887	126.7770213	70.09590565	53.70146209
0	26.30964479	12.77597889	0	9.666263176

For Review Only

19.21976404	22.68072826	16.70704932	10.78398548	23.62864332
39.40051627	4.536145653	0	2.156797097	3.222087725
3.843952807	5.443374783	2.948302821	0	4.296116967
66.30818592	84.37230914	70.75926771	86.27188388	53.70146209
0	0	0	11.86238403	0
39.40051627	80.74339262	61.91435925	78.72309404	47.25728664
73.99609154	71.67110131	91.39738746	79.80149259	127.8094798
8.648893816	0	4.913838036	2.156797097	0
3.843952807	5.443374783	0.982767607	0	4.296116967
119.162537	109.7747248	142.501303	232.9340865	198.6954097
147.0311949	127.9193074	86.48354943	119.7022389	132.1055967
171.0558999	102.5168918	99.25952832	74.40949984	63.36772526
96.09882018	97.07351697	77.63864096	101.3694636	126.7354505
19.21976404	10.88674957	14.74151411	1.078398548	15.03640938
49.01039829	60.78435175	102.2078311	104.6046592	67.66384223
6.726917412	0	0.982767607	0	0
21.14174044	21.77349913	46.19007754	38.82234774	26.85073104
325.7750004	360.1699648	312.5200991	260.9724487	224.4721115
4.804941009	0.907229131	0	0	0
1.921976404	5.443374783	9.827676071	3.235195645	5.370146209
2.882964605	0.907229131	74.69033814	45.29273904	10.74029242
24.02470504	24.49518652	33.41409864	67.93910855	24.70267256
98.98178478	74.3927887	192.622451	523.023296	184.7330296
30.75162246	104.33135	53.06945079	30.19515936	61.21966678
50.93237469	0	0.982767607	2.156797097	18.25849711
93.21585557	169.6518474	187.708613	88.42868097	204.0655559
71.11312693	134.2699113	122.8459509	162.8381808	105.2548657
0	7.257833044	0	0	0
46.12743368	42.63976914	80.58694379	39.90074629	46.1832574
51.8933629	53.5265187	68.7937325	61.46871726	44.03519891
0	0	0	37.7439492	0
469.9232307	371.9639435	502.1942472	403.3210571	417.797375
541.9973458	565.2037483	448.1420289	500.3769265	477.9430126
272.9206493	290.3133218	355.7618738	241.5612749	267.4332812
0	0	173.9498665	133.72142	212.6577899
77.84004434	90.72291305	86.48354943	74.40949984	99.88471948
0	0	0	0	0
0	0	75.67310575	43.13594194	117.0691874
32.67359886	49.89760218	126.7770213	18.33277532	6.444175451
3.843952807	4.536145653	8.844908464	4.313594194	11.81432166
6.726917412	0.907229131	8.844908464	0	0
0	0	1.965535214	0	0
0	0	0	1.078398548	0
3.843952807	2.721687392	0	7.548789839	0
13.45383482	9.072291305	12.77597889	21.56797097	3.222087725
36.51755167	31.75301957	63.87989446	73.3311013	32.22087725
33.63458706	18.14458261	10.81044368	32.35195645	16.11043863
241.2080386	252.2096983	149.3806763	190.8765431	225.5461408
23.06371684	9.072291305	12.77597889	24.80316661	30.07281877
257.5448381	163.3012435	309.5717962	306.2651878	157.8822985

0	0	0	0	0
0	0	0	0	0
76.87905614	37.19639435	57.98328882	33.430355	51.5534036
0	0	0.982767607	0	0
42.28348088	35.38193609	34.39686625	20.48957242	40.81311119
0.960988202	0.907229131	0	0	13.96238014
0	0	0	8.627188388	0
4.804941009	0	8.844908464	1.078398548	3.222087725
29.79063425	27.21687392	31.44856343	8.627188388	85.92233934
401.6930683	655.0194322	140.5357678	154.2109924	42.96116967
53.8153393	41.73254	20.63811975	9.705586936	21.48058484
51.8933629	23.58795739	70.75926771	0	0
186.4317111	132.4554531	116.9493452	138.0350142	198.6954097
85.52794996	101.6096626	117.9321129	83.03668823	77.33010541
1.921976404	3.628916522	0	0	0
5.765929211	2.721687392	0.982767607	0	0
8.648893816	12.70120783	5.896605643	1.078398548	6.444175451
108.5916668	93.44460045	64.86266207	60.39031871	114.9211289
111.4746314	84.37230914	48.15561275	64.70391291	77.33010541
52.8543511	62.59881001	40.29347189	35.5871521	48.33131588
15.37581123	14.51566609	14.74151411	24.80316661	22.55461408
66.30818592	49.89760218	41.2762395	66.86071	39.73908194
0	0	14.74151411	0	0
0	0	1.965535214	3.235195645	3.222087725
0	109.7747248	0	0	91.29248555
107.6306786	0	0	0	0
32.67359886	2.721687392	15.72428171	16.17597823	18.25849711
24.98569325	21.77349913	25.55195779	33.430355	35.44296498
0	0	0	0	0
0	0	0	0	0
117.2405606	83.46508001	101.2250635	61.46871726	164.326474
94.17684377	128.8265365	76.65587336	141.2702098	88.07039782
81.68399715	97.07351697	0.982767607	67.93910855	17.18446787
73.03510333	69.85664305	88.44908464	81.95828968	92.36651479
6.726917412	0	0.982767607	1.078398548	1.074029242
44.20545728	49.89760218	17.68981693	22.64636952	46.1832574
421.8738206	420.9543166	288.9336765	372.0474992	417.797375
32.67359886	28.12410305	19.65535214	34.50875355	95.58860252
287.3354723	237.6940322	201.4673595	243.718072	311.4684801
14.41482303	16.33012435	13.7587465	14.01918113	7.518204692
161.4460179	146.06389	119.8976481	143.4270069	129.9575383
19.21976404	9.979520436	23.58642257	3.235195645	19.33252635
4.804941009	0	0	0	0
0	0	1.965535214	3.235195645	0
0	0	0	8.627188388	68.73787147
0	0	0.982767607	1.078398548	0
59.58126851	47.17591479	72.72480293	56.07672452	55.84952057
54.7763275	55.34097696	58.96605643	24.80316661	39.73908194
0	8.165062175	2.948302821	0	0
2.882964605	0	0	0	0

For Review Only

20.18075224	11.7939787	22.60365496	0	37.59102346
0	0	0	0	0
26.90766965	25.40241565	23.58642257	54.99832597	42.96116967
1.921976404	0	4.913838036	1.078398548	3.222087725
0	0	4.913838036	0	0
0	0	1.965535214	1.078398548	1.074029242
17.29778763	22.68072826	52.08668318	42.05754339	0
993.6618006	994.3231271	695.7994659	659.9799117	745.3762938
601.5786143	597.863997	614.2297545	499.2985279	615.4187555
2.882964605	0	0	0	0
8.648893816	9.979520436	5.896605643	5.391992742	3.222087725
2.882964605	0	3.931070429	0	0
340.1898234	282.1482596	383.2793668	334.30355	317.9126556
0	2.721687392	4.913838036	0	7.518204692
0	0	4.913838036	2.156797097	1.074029242
331.5409296	394.6446718	327.2616132	445.3786005	300.7281877
92.25486737	66.22772653	24.56919018	75.48789839	57.99757905
8.648893816	4.536145653	7.862140857	5.391992742	7.518204692
0	0	0	0	2.148058484
184.5097347	138.806057	106.1389016	72.25270275	106.3288949
168.1729353	237.6940322	128.7425565	190.8765431	113.8470996
878.3432164	876.3833401	929.6981564	1131.240077	614.3447263
0	0	2.948302821	0	0
0	3.628916522	0	0	0
0	16.33012435	0.982767607	1.078398548	0
0	0	0	0	0
50.93237469	1332.719593	1882.982735	1078.398548	1602.451629
149.9141595	124.2903909	67.81096489	141.2702098	88.07039782
39.40051627	49.89760218	50.12114796	40.97914484	39.73908194
21.14174044	30.84579044	37.34516907	48.52793468	16.11043863
47.08842189	23.58795739	14.74151411	32.35195645	13.96238014
220.0662982	232.2506574	119.8976481	311.6571805	326.5048895
110.5136432	78.02170523	78.62140857	98.13426791	54.77549133
77.84004434	78.92893436	436.3488176	11.86238403	262.063135
186.4317111	226.8072826	330.209916	238.3260792	241.6565794
38.43952807	47.17591479	46.19007754	77.64469549	32.22087725
103.7867258	95.25905871	96.3112255	127.2510287	118.1432166
26.90766965	29.03133218	32.43133104	6.470391291	13.96238014
0	0	0	0	0
0	0	15.72428171	24.80316661	0
0	45.36145653	54.05221839	21.56797097	74.10801768
49.97138649	37.19639435	61.91435925	43.13594194	52.62743285
167.2119471	148.7855774	0	0	0
10.57087022	12.70120783	28.50026061	20.48957242	15.03640938
4.804941009	0	5.896605643	0	3.222087725
93.21585557	86.1867674	121.8631833	94.89907226	36.51699422
40.36150447	42.63976914	26.53472539	26.95996371	20.40655559
9.609882018	0	4.913838036	3.235195645	5.370146209
4.804941009	2.721687392	5.896605643	0	2.148058484
55.7373157	58.96989348	43.24177471	48.52793468	69.81190071

34.59557526	45.36145653	32.43133104	50.68473178	44.03519891
0.960988202	0.907229131	6.87937325	0	1.074029242
0	0	0	0	0
0	7.257833044	0	0	0
0	0	4.913838036	3.235195645	4.296116967
0	0	0	0	0
196.0415932	264.9109061	708.5754447	740.8598028	713.1554165
170.0949117	125.19762	134.6391622	78.72309404	150.3640938
8.648893816	8.165062175	8.844908464	3.235195645	12.8883509
109.552655	83.46508001	120.8804157	0	105.2548657
0	0	0	0	0
35.55656347	0	16.70704932	26.95996371	18.25849711
6.726917412	8.165062175	6.87937325	10.78398548	3.222087725
37.47853987	24.49518652	11.79321129	12.94078258	9.666263176
0	2.721687392	3.931070429	1.078398548	3.222087725
2.882964605	2.721687392	0	0	0
0	3.628916522	0.982767607	0	2.148058484
46.12743368	0	51.10391557	5.391992742	21.48058484
11.53185842	9.979520436	7.862140857	1.078398548	1.074029242
183.5487465	274.8904266	217.1916412	218.9149053	259.9150765
125.8894544	273.0759683	316.4511695	295.4812023	374.8362054
12.49284662	7.257833044	4.913838036	7.548789839	11.81432166
64.38620952	0	9.827676071	62.54711581	50.47937436
0	5.443374783	3.931070429	1.078398548	1.074029242
2.882964605	0.907229131	0	0	0
0	226.8072826	8.844908464	7.548789839	1.074029242
10.57087022	9.072291305	10.81044368	6.470391291	8.592233934
5.765929211	20.86627	93.36292268	87.35028243	26.85073104
322.8920358	252.2096983	457.9697049	362.3419123	237.3604624
155.6800887	130.6409948	87.46631704	181.1709561	115.9951581
262.3497791	355.6338192	277.1404652	234.012485	201.9174975
28.82964605	37.19639435	25.55195779	49.60633323	20.40655559
49.97138649	55.34097696	476.6422895	359.1067166	474.7209249
94.17684377	68.04218479	142.501303	131.5646229	86.99636858
89.37190276	102.5168918	115.9665776	164.9949779	142.8458892
66.30818592	41.73254	40.29347189	79.80149259	96.66263176
83.60597355	70.76387218	54.05221839	104.6046592	44.03519891
77.84004434	101.6096626	110.069972	140.1918113	79.47816389
90.33289097	89.81568392	50.12114796	34.50875355	47.25728664
3.843952807	7.257833044	4.913838036	3.235195645	4.296116967
0	0	0	2.156797097	2.148058484
0	0	0	0	0
0	0	0	0	0
6.726917412	4.536145653	6.87937325	1.078398548	7.518204692
0	0	0	0	0
57.65929211	48.08314392	52.08668318	65.78231146	56.92354981
6.726917412	8.165062175	10.81044368	8.627188388	8.592233934
15.37581123	54.43374783	342.0031273	76.56629694	382.3544101
0	0	0	0	0
0	0	1295.287706	0	0

For Review Only

36.51755167	38.10362348	29.48302821	23.72476807	40.81311119
4.804941009	12.70120783	0	11.86238403	0
6.726917412	6.350603914	38.32793668	36.66555065	0
52.8543511	77.1144761	79.60417618	84.11508678	80.55219313
24.02470504	25.40241565	57.00052121	38.82234774	13.96238014
7.687905614	19.05181174	31.44856343	14.01918113	19.33252635
105.7087022	99.79520436	110.069972	87.35028243	123.5133628
94.17684377	98.88797523	149.3806763	66.86071	80.55219313
82.64498535	76.20724696	111.0527396	115.3886447	79.47816389
0	0	47.17284514	49.60633323	48.33131588
2.882964605	0	6.87937325	2.156797097	0
1.921976404	1.814458261	0	1.078398548	0
8.648893816	0	15.72428171	0	0
112.4356196	88.90845479	84.51801421	145.583804	110.6250119
5.765929211	17.23735348	15.72428171	10.78398548	19.33252635
204.690487	114.3108704	161.1738876	129.4078258	104.1808365
5.765929211	18.14458261	6.87937325	5.391992742	21.48058484
67.26917412	85.27953827	57.00052121	16.17597823	119.2172458
0	0	0	0	2.148058484
0.960988202	0	0.982767607	3.235195645	0
2.882964605	2.721687392	5.896605643	0	0
7.687905614	3.628916522	0	5.391992742	6.444175451
302.7112836	265.8181352	286.9681413	269.5996371	242.7306086
62.46423311	92.53737131	73.70757054	93.82067372	108.4769534
39.40051627	36.28916522	47.17284514	46.37113758	34.36893574
3.843952807	5.443374783	5.896605643	3.235195645	5.370146209
10.57087022	0	1.965535214	2.156797097	11.81432166
302.7112836	328.4169453	321.3650075	325.6763616	199.769439
0.960988202	1.814458261	11.79321129	0	0
4.804941009	0	0	2.156797097	2.148058484
83.60597355	70.76387218	88.44908464	58.23352162	86.99636858
102.8257376	137.8988278	221.1227116	152.0541953	155.7342401
104.747714	107.0530374	101.2250635	92.74227517	88.07039782
2.882964605	3.628916522	0.982767607	0	1.074029242
0	7.257833044	0.982767607	3.235195645	3.222087725
19.21976404	12.70120783	21.62088736	4.313594194	6.444175451
12.49284662	10.88674957	7.862140857	6.470391291	5.370146209
284.4525077	339.3036948	293.8475145	179.014159	226.62017
225.8322274	0	59.94882404	36.66555065	82.70025162
0	0	1.965535214	1.078398548	1.074029242
19.21976404	36.28916522	37.34516907	31.27355791	48.33131588
0	0	0	0	1.074029242
55.7373157	72.57833044	34.39686625	84.11508678	97.736661
123.967478	118.8470161	180.8292397	127.2510287	112.7730704
98.98178478	106.1458083	77.63864096	97.05586936	110.6250119
60.54225671	77.1144761	76.65587336	87.35028243	59.0716083
0.960988202	58.96989348	59.94882404	81.95828968	70.88592996
304.63326	333.86032	302.692423	254.5020574	302.8762462
33.63458706	20.86627	28.50026061	20.48957242	15.03640938
13.45383482	34.47470696	16.70704932	21.56797097	12.8883509

For Review Only

82.64498535	4.536145653	46.19007754	127.2510287	26.85073104
14.41482303	29.03133218	31.44856343	37.7439492	21.48058484
55.7373157	63.50603914	98.27676071	71.1743042	55.84952057
44.20545728	19.05181174	55.034986	49.60633323	48.33131588
175.8608409	182.3530552	151.3462115	157.4461881	138.5497722
347.877729	277.6121139	343.9686625	449.6921947	320.060714
0	2.721687392	0	1.078398548	1.074029242
10.57087022	0	1.965535214	1.078398548	1.074029242
48.04941009	36.28916522	44.22454232	53.91992742	50.47937436
0	0	0	2.156797097	4.296116967
10.57087022	16.33012435	6.87937325	7.548789839	3.222087725
0	4.536145653	0	4.313594194	4.296116967
165.2899707	140.6205152	148.3979087	250.1884632	195.473322
0	10.88674957	0.982767607	1.078398548	9.666263176
75.91806794	48.99037305	38.32793668	40.97914484	65.51578375
35.55656347	8.165062175	0.982767607	17.25437678	34.36893574
2.882964605	5.443374783	8.844908464	0	0
34.59557526	50.80483131	64.86266207	26.95996371	50.47937436
53.8153393	64.41326827	51.10391557	44.21434049	34.36893574
41.32249268	34.47470696	35.37963386	81.95828968	61.21966678
3.843952807	3.628916522	0	0	5.370146209
0	0	0	0	0
0	23.58795739	0	0	0
9.609882018	8.165062175	12.77597889	6.470391291	10.74029242
492.0259593	590.606164	548.3843248	391.4586731	436.0558722
626.5643076	557.0386861	456.0041697	346.1659341	513.3859776
82.64498535	78.92893436	56.01775361	71.1743042	69.81190071
15.37581123	5.443374783	13.7587465	7.548789839	9.666263176
22.10272864	36.28916522	32.43133104	26.95996371	33.29490649
0	0	0	0	0
58.62028031	26.30964479	54.05221839	6.470391291	18.25849711
0	0	0	0	0
113.3966078	100.7024335	73.70757054	107.8398548	70.88592996
119.162537	182.3530552	262.3989511	153.1325939	190.1031758
0	4.536145653	0	1.078398548	0
0	0	0	0	0
23.06371684	34.47470696	7.862140857	16.17597823	31.14684801
0	0	0	377.439492	27.92476029
0	292.12778	439.2971204	492.8281366	47.25728664
16.33679943	9.979520436	32.43133104	45.29273904	25.7767018
0	0	10.81044368	0	0
7.687905614	19.95904087	8.844908464	12.94078258	2.148058484
33.63458706	18.14458261	30.46579582	24.80316661	25.7767018
9.609882018	0	11.79321129	6.470391291	2.148058484
8.648893816	0	5.896605643	9.705586936	10.74029242
73.99609154	55.34097696	85.50078182	54.99832597	35.44296498
56.6983039	48.08314392	70.75926771	50.68473178	47.25728664
26.90766965	15.42289522	12.77597889	14.01918113	27.92476029
0	0	0	0	0
13.45383482	9.072291305	7.862140857	7.548789839	17.18446787

For Review Only

0	0.907229131	2.948302821	0	0
7.687905614	13.60843696	34.39686625	65.78231146	34.36893574
0	33.56747783	10.81044368	16.17597823	9.666263176
0	0	0	0	0
63.42522132	68.04218479	15.72428171	37.7439492	48.33131588
862.006417	666.8134109	350.8480357	848.6996576	646.5656035
12.49284662	10.88674957	13.7587465	14.01918113	10.74029242
53.8153393	78.92893436	49.13838036	37.7439492	33.29490649
0	0	2.948302821	0	0
67.26917412	100.7024335	86.48354943	0	78.40413465
29.79063425	27.21687392	32.43133104	25.88156516	30.07281877
235.4421094	168.7446183	1.965535214	1.078398548	8.592233934
18.25877583	19.95904087	9.827676071	18.33277532	8.592233934
0	8.165062175	4.913838036	0	8.592233934
154.7191005	97.07351697	295.8130497	30.19515936	33.29490649
0	0	11.79321129	0	0
0.960988202	0	2.948302821	0	0
4.804941009	14.51566609	4.913838036	0	5.370146209
90.33289097	115.2180996	71.74203532	95.97747081	93.44054403
27.86865785	33.56747783	48.15561275	35.5871521	55.84952057
49.01039829	111.5891831	118.9148805	166.0733765	69.81190071
56.6983039	251.3024692	170.018796	139.1134128	164.326474
276.7646021	289.4060926	342.9858949	382.8314847	219.1019653
71.11312693	50.80483131	77.63864096	37.7439492	37.59102346
433.405679	407.3458796	441.2626556	596.3543973	317.9126556
6.726917412	0	0	0	0
54.7763275	65.3204974	47.17284514	50.68473178	44.03519891
442.0545728	479.9242101	519.8840642	407.6346513	311.4684801
122.0455016	136.9915987	148.3979087	113.2318476	143.9199184
84.56696176	74.3927887	67.81096489	56.07672452	85.92233934
0	0	0.982767607	1.078398548	1.074029242
6.726917412	2.721687392	4.913838036	5.391992742	4.296116967
5.765929211	13.60843696	11.79321129	7.548789839	9.666263176
45.16644548	59.87712262	40.29347189	49.60633323	83.77428086
26.90766965	24.49518652	2.948302821	21.56797097	53.70146209
11.53185842	0	10.81044368	33.430355	27.92476029
79.76202075	75.30001783	130.7080917	126.1726302	108.4769534
2.882964605	0	0	0	15.03640938
0.960988202	0	5.896605643	0	2.148058484
10.57087022	10.88674957	9.827676071	26.95996371	17.18446787
0.960988202	9.979520436	50.12114796	47.44953613	0
0	0	0	0	0
41.32249268	48.99037305	44.22454232	98.13426791	53.70146209
815.8789833	663.1844944	776.3864096	724.6838246	762.5607616
0	0	0	0	0
0	0	0.982767607	0	0
0	0	0	0	0
0	0	0	0	0
40.36150447	0	0.982767607	1.078398548	2.148058484
0	14.51566609	0.982767607	3.235195645	1.074029242

For Review Only

11.53185842	14.51566609	0.982767607	12.94078258	0
9.609882018	6.350603914	8.844908464	0	15.03640938
156.6410769	164.2084726	152.3289791	163.9165794	176.1407956
84.56696176	37.19639435	70.75926771	42.05754339	112.7730704
5.765929211	0	1.965535214	0	0
24.02470504	19.05181174	25.55195779	9.705586936	18.25849711
60.54225671	33.56747783	27.517493	30.19515936	45.10922815
65.34719772	47.17591479	59.94882404	70.09590565	40.81311119
101.8647494	105.2385791	112.0355072	205.9741228	125.6614213
1.921976404	0	0	0	0
17.29778763	21.77349913	39.31070429	26.95996371	25.7767018
0.960988202	0	0	1.078398548	0
0	0	0.982767607	3.235195645	0
38.43952807	44.4542274	24.56919018	7.548789839	24.70267256
0	0	0	10.78398548	0
0.960988202	11.7939787	0	0	0
69.19115053	47.17591479	92.38015507	66.86071	69.81190071
127.8114308	127.0120783	103.1905987	144.5054055	184.7330296
0	0	0.982767607	1.078398548	1.074029242
23.06371684	12.70120783	14.74151411	7.548789839	18.25849711
34.59557526	29.93856131	45.20730993	44.21434049	41.88714043
41.32249268	48.99037305	43.24177471	59.31192017	50.47937436
88.41091456	83.46508001	100.2422959	69.0175071	153.5861816
9.609882018	9.979520436	24.56919018	4.313594194	10.74029242
67.26917412	40.82531087	60.93159164	63.62551436	76.25607616
0	0	0	0	0
32.67359886	0	11.79321129	1.078398548	236.2864332
0	0	0	2.156797097	1.074029242
180.6657819	180.538597	214.2433384	223.2284995	201.9174975
173.9388645	132.4554531	103.1905987	194.1117387	84.8483101
215.2613572	148.7855774	171.0015636	118.6238403	112.7730704
0	0	0	0	0
225.8322274	195.9614922	223.0882468	260.9724487	243.8046379
0	0	2.948302821	4.313594194	6.444175451
269.0766965	271.26151	397.0381133	404.3994557	200.8434682
9.609882018	9.979520436	18.67258454	14.01918113	10.74029242
60.54225671	48.99037305	42.25900711	53.91992742	49.40534512
2.882964605	5.443374783	0	1.078398548	8.592233934
50.93237469	53.5265187	62.89712686	64.70391291	65.51578375
133.57736	135.1771404	92.38015507	65.78231146	82.70025162
3.843952807	3.628916522	4.913838036	6.470391291	4.296116967
42.28348088	43.54699827	76.65587336	35.5871521	69.81190071
14.41482303	13.60843696	22.60365496	10.78398548	28.99878953
13.45383482	16.33012435	17.68981693	19.41117387	13.96238014
0	2.721687392	0.982767607	0	6.444175451
6.726917412	0	0.982767607	0	1.074029242
0	0	0.982767607	0	0
5.765929211	4.536145653	1.965535214	1.078398548	3.222087725
7.687905614	23.58795739	8.844908464	10.78398548	9.666263176
3.843952807	6.350603914	3.931070429	0	5.370146209

4.804941009	4.536145653	1.965535214	4.313594194	5.370146209
21.14174044	17.23735348	23.58642257	24.80316661	20.40655559
23.06371684	22.68072826	14.74151411	34.50875355	15.03640938
32.67359886	28.12410305	63.87989446	16.17597823	49.40534512
0.960988202	0	1.965535214	5.391992742	0
20.18075224	18.14458261	16.70704932	16.17597823	25.7767018
3.843952807	2.721687392	0.982767607	0	1.074029242
7.687905614	7.257833044	3.931070429	4.313594194	8.592233934
13.45383482	21.77349913	15.72428171	8.627188388	8.592233934
3.843952807	5.443374783	0.982767607	0	0
0	2.721687392	0	0	0
4.804941009	3.628916522	0.982767607	11.86238403	0
0	2.721687392	0	1.078398548	0
2.882964605	0.907229131	2.948302821	1.078398548	1.074029242
0	1.814458261	0	3.235195645	0
25.94668145	20.86627	26.53472539	23.72476807	30.07281877
3.843952807	0.907229131	2.948302821	0	3.222087725
0	0	0	0	0
3.843952807	0	0.982767607	2.156797097	4.296116967
0.960988202	3.628916522	0	0	0
31.71261066	24.49518652	21.62088736	24.80316661	20.40655559
1.921976404	0	0	3.235195645	6.444175451
10.57087022	14.51566609	1.965535214	14.01918113	9.666263176
0	6.350603914	0.982767607	0	1.074029242
2.882964605	2.721687392	0.982767607	1.078398548	0
1.921976404	0.907229131	0	3.235195645	3.222087725
0	0	0.982767607	2.156797097	0
18.25877583	19.95904087	8.844908464	5.391992742	20.40655559
3.843952807	6.350603914	5.896605643	4.313594194	2.148058484
192.1976404	200.4976378	233.8986905	256.6588545	218.0279361
3.843952807	5.443374783	0.982767607	1.078398548	1.074029242
13.45383482	20.86627	16.70704932	19.41117387	19.33252635
0.960988202	4.536145653	2.948302821	5.391992742	6.444175451
0	0	0	0	0
12.49284662	19.05181174	6.87937325	10.78398548	17.18446787
6.726917412	8.165062175	6.87937325	6.470391291	3.222087725
0	0	0	0	0
0	0	0	0	2.148058484
2.882964605	0.907229131	0	5.391992742	1.074029242
0.960988202	0	0.982767607	1.078398548	3.222087725
9.609882018	5.443374783	6.87937325	4.313594194	6.444175451
0	9.979520436	6.87937325	5.391992742	4.296116967
0	2.721687392	0.982767607	0	0
23.06371684	20.86627	29.48302821	11.86238403	16.11043863
0	0	2.948302821	0	0
0	0	2.948302821	0	0
11.53185842	0	0.982767607	5.391992742	2.148058484
0	0	0	1.078398548	4.296116967
0.960988202	0.907229131	0	0	0
7.687905614	5.443374783	2.948302821	2.156797097	5.370146209

For Review Only

0	0	0	0	0
137.4213129	127.9193074	144.4668382	149.8973982	186.8810881
9.609882018	0	0	4.313594194	2.148058484
2.882964605	0.907229131	2.948302821	0	2.148058484
3.843952807	2.721687392	0	0	0
33.63458706	27.21687392	42.25900711	31.27355791	8.592233934
3.843952807	5.443374783	1.965535214	4.313594194	1.074029242
0.960988202	1.814458261	2.948302821	0	0
1.921976404	2.721687392	0.982767607	1.078398548	0
12.49284662	16.33012435	8.844908464	22.64636952	7.518204692
21.14174044	17.23735348	16.70704932	19.41117387	7.518204692
3.843952807	2.721687392	1.965535214	3.235195645	1.074029242
9.609882018	14.51566609	1.965535214	2.156797097	5.370146209
164.3289825	184.1675135	197.536289	218.9149053	163.2524447
10.57087022	37.19639435	44.22454232	9.705586936	9.666263176
32.67359886	152.4144939	10.81044368	6.470391291	3.222087725
3.843952807	3.628916522	0	1.078398548	0
0.960988202	1.814458261	2.948302821	0	0
3.843952807	8.165062175	0	0	1.074029242
0.960988202	0.907229131	0	1.078398548	1.074029242
4.804941009	0	1.965535214	1.078398548	1.074029242
4.804941009	0	0	0	0
0	8.165062175	0.982767607	1.078398548	0
3.843952807	9.072291305	0.982767607	5.391992742	5.370146209
0	7.257833044	0.982767607	1.078398548	6.444175451
38.43952807	46.26868566	70.75926771	58.23352162	48.33131588
1.921976404	1.814458261	0.982767607	0	0
108.5916668	100.7024335	122.8459509	114.3102461	149.2900646
41.32249268	22.68072826	27.517493	46.37113758	36.51699422
75.91806794	65.3204974	73.70757054	65.78231146	78.40413465
4.804941009	0	5.896605643	5.391992742	6.444175451
0.960988202	1.814458261	0.982767607	0	0
2.882964605	0.907229131	1.965535214	4.313594194	1.074029242
0.960988202	4.536145653	0	4.313594194	1.074029242
29.79063425	45.36145653	8.844908464	11.86238403	59.0716083
10.57087022	5.443374783	5.896605643	4.313594194	4.296116967
38.43952807	43.54699827	57.00052121	38.82234774	50.47937436
0.960988202	5.443374783	0	10.78398548	4.296116967
6.726917412	15.42289522	0.982767607	1.078398548	4.296116967
7.687905614	0	0.982767607	1.078398548	4.296116967
7.687905614	9.979520436	6.87937325	6.470391291	13.96238014
0.960988202	1.814458261	0	0	0
17.29778763	15.42289522	0.982767607	7.548789839	1.074029242
0.960988202	0	0	0	0
22.10272864	25.40241565	29.48302821	34.50875355	28.99878953
0.960988202	0	0	1.078398548	1.074029242
59.58126851	54.43374783	55.034986	117.5454418	71.9599592
9.609882018	16.33012435	9.827676071	19.41117387	19.33252635
0	0	0	0	0
12.49284662	9.072291305	14.74151411	6.470391291	2.148058484

For Review Only

0	10.88674957	0	0	0
19.21976404	4.536145653	4.913838036	3.235195645	13.96238014
0	0	1.965535214	0	0
0	0	2.948302821	2.156797097	5.370146209
25.94668145	48.08314392	38.32793668	37.7439492	66.58981299
0.960988202	0	0	1.078398548	0
12.49284662	13.60843696	9.827676071	8.627188388	11.81432166
16.33679943	70.76387218	0.982767607	72.25270275	88.07039782
0	0	2.948302821	3.235195645	0
4.804941009	6.350603914	10.81044368	1.078398548	4.296116967
15.37581123	36.28916522	2.948302821	18.33277532	12.8883509
3.843952807	0	0	2.156797097	0
3.843952807	0	1.965535214	3.235195645	2.148058484
0.960988202	0	0	2.156797097	0
45.16644548	39.91808174	42.25900711	30.19515936	56.92354981
0.960988202	0.907229131	3.931070429	0	1.074029242
88.41091456	102.5168918	71.74203532	16.17597823	2.148058484
20.18075224	12.70120783	6.87937325	16.17597823	8.592233934
6.726917412	0	17.68981693	1.078398548	0
27.86865785	22.68072826	29.48302821	38.82234774	41.88714043
25.94668145	26.30964479	15.72428171	15.09757968	20.40655559
24.02470504	12.70120783	8.844908464	8.627188388	23.62864332
10.57087022	9.072291305	11.79321129	10.78398548	11.81432166
6.726917412	9.072291305	9.827676071	7.548789839	6.444175451
0.960988202	2.721687392	3.931070429	4.313594194	3.222087725
0	0	0	1.078398548	0
0.960988202	3.628916522	0.982767607	0	0
49.97138649	48.08314392	54.05221839	44.21434049	51.5534036
52.8543511	44.4542274	35.37963386	24.80316661	41.88714043
0.960988202	0	0	1.078398548	5.370146209
16.33679943	18.14458261	3.931070429	5.391992742	5.370146209
14.41482303	0	0	0	3.222087725
8.648893816	6.350603914	11.79321129	14.01918113	8.592233934
20.18075224	19.05181174	23.58642257	25.88156516	21.48058484
21.14174044	32.6602487	28.50026061	20.48957242	22.55461408
0.960988202	0	0	0	0
4.804941009	0	1.965535214	9.705586936	5.370146209
17.29778763	27.21687392	8.844908464	12.94078258	23.62864332
42.28348088	34.47470696	10.81044368	39.90074629	20.40655559
1.921976404	4.536145653	8.844908464	14.01918113	1.074029242
15.37581123	10.88674957	22.60365496	10.78398548	18.25849711
20.18075224	29.03133218	26.53472539	31.27355791	27.92476029
5.765929211	4.536145653	3.931070429	1.078398548	1.074029242
0.960988202	0.907229131	4.913838036	0	0
0	6.350603914	0	0	0
0	0	1.965535214	1.078398548	0
0.960988202	3.628916522	0	7.548789839	10.74029242
7.687905614	11.7939787	1.965535214	6.470391291	13.96238014
0	0.907229131	4.913838036	0	1.074029242
0	0	0.982767607	0	1.074029242

For Review Only

8.648893816	2.721687392	3.931070429	2.156797097	0
0	2.721687392	0	0	0
32.67359886	40.82531087	34.39686625	29.11676081	17.18446787
0	3.628916522	0.982767607	0	0
0.960988202	0	0	0	0
6.726917412	3.628916522	4.913838036	1.078398548	6.444175451
11.53185842	12.70120783	2.948302821	0	10.74029242
9.609882018	6.350603914	0	2.156797097	9.666263176
0	0	44.22454232	30.19515936	37.59102346
3.843952807	9.072291305	0.982767607	0	4.296116967
8.648893816	0	5.896605643	3.235195645	0
0	8.165062175	1.965535214	5.391992742	7.518204692
3.843952807	0	2.948302821	3.235195645	2.148058484
2.882964605	5.443374783	3.931070429	7.548789839	1.074029242
0	0	0	2.156797097	0
0	0	1.965535214	2.156797097	0
1.921976404	0	0	0	0
10.57087022	14.51566609	5.896605643	5.391992742	4.296116967
10.57087022	11.7939787	5.896605643	6.470391291	6.444175451
18.25877583	12.70120783	8.844908464	14.01918113	12.8883509
17.29778763	12.70120783	21.62088736	10.78398548	12.8883509
7.687905614	6.350603914	5.896605643	4.313594194	7.518204692
10.57087022	10.88674957	12.77597889	12.94078258	10.74029242
19.21976404	22.68072826	9.827676071	6.470391291	25.7767018
12.49284662	7.257833044	7.862140857	8.627188388	9.666263176
26.90766965	19.05181174	18.67258454	31.27355791	15.03640938
46.12743368	37.19639435	29.48302821	25.88156516	22.55461408
54.7763275	53.5265187	34.39686625	31.27355791	35.44296498
8.648893816	3.628916522	3.931070429	5.391992742	7.518204692
3.843952807	2.721687392	2.948302821	1.078398548	4.296116967
12.49284662	19.95904087	7.862140857	11.86238403	11.81432166
5.765929211	6.350603914	6.87937325	6.470391291	6.444175451
6.726917412	6.350603914	5.896605643	2.156797097	5.370146209
0	0	0	1.078398548	1.074029242
3.843952807	0	0.982767607	0	0
8.648893816	3.628916522	6.87937325	6.470391291	3.222087725
164.3289825	183.2602844	160.19112	171.4653692	227.6941993
0.960988202	0	2.948302821	1.078398548	0
14.41482303	19.05181174	18.67258454	18.33277532	24.70267256
13.45383482	19.95904087	16.70704932	5.391992742	19.33252635
0.960988202	5.443374783	0.982767607	3.235195645	1.074029242
0.960988202	3.628916522	3.931070429	1.078398548	1.074029242
74.95707974	46.26868566	83.53524661	104.6046592	71.9599592
15.37581123	7.257833044	3.931070429	2.156797097	5.370146209
0	3.628916522	0	0	0
0	0	1.965535214	0	0
2.882964605	3.628916522	0	1.078398548	0
0.960988202	0	2.948302821	3.235195645	2.148058484
11.53185842	9.072291305	0.982767607	1.078398548	3.222087725
2.882964605	2.721687392	0	6.470391291	4.296116967

For Review Only

10.57087022	17.23735348	20.63811975	12.94078258	16.11043863
44.20545728	34.47470696	26.53472539	15.09757968	35.44296498
1.921976404	7.257833044	4.913838036	5.391992742	0
5.765929211	7.257833044	6.87937325	6.470391291	3.222087725
59.58126851	58.06266435	58.96605643	42.05754339	63.36772526
18.25877583	11.7939787	14.74151411	19.41117387	13.96238014
0	0	0	0	0
3.843952807	0	0	2.156797097	2.148058484
7.687905614	7.257833044	2.948302821	0	3.222087725
22.10272864	61.69158088	5.896605643	36.66555065	23.62864332
0	0	0.982767607	0	3.222087725
3.843952807	8.165062175	3.931070429	4.313594194	1.074029242
9.609882018	7.257833044	0.982767607	6.470391291	4.296116967
8.648893816	0	5.896605643	1.078398548	3.222087725
4.804941009	9.072291305	0.982767607	11.86238403	2.148058484
447.820502	449.0784196	397.0381133	594.1976002	579.9757905
0	0	0	0	0
5.765929211	9.072291305	0.982767607	2.156797097	1.074029242
3.843952807	0	0	0	1.074029242
1.921976404	0	5.896605643	1.078398548	3.222087725
7.687905614	8.165062175	14.74151411	4.313594194	9.666263176
0.960988202	0	0.982767607	3.235195645	0
10.57087022	0	5.896605643	0	6.444175451
4.804941009	0	0.982767607	3.235195645	1.074029242
10.57087022	11.7939787	28.50026061	7.548789839	17.18446787
2.882964605	0.907229131	2.948302821	0	0
18.25877583	30.84579044	30.46579582	33.430355	12.8883509
3.843952807	0	1.965535214	0	0
0	0.907229131	0	1.078398548	3.222087725
139.3432893	111.5891831	96.3112255	203.8173257	63.36772526
8.648893816	15.42289522	9.827676071	16.17597823	1.074029242
0	4.536145653	1.965535214	2.156797097	2.148058484
31.71261066	35.38193609	20.63811975	28.03836226	21.48058484
15.37581123	9.072291305	9.827676071	2.156797097	7.518204692
29.79063425	17.23735348	13.7587465	30.19515936	25.7767018
121.0845134	131.5482239	119.8976481	128.3294273	138.5497722
0	0	5.896605643	8.627188388	0
70.15213873	62.59881001	66.82819729	86.27188388	85.92233934
3.843952807	3.628916522	8.844908464	7.548789839	3.222087725
8.648893816	9.979520436	14.74151411	2.156797097	3.222087725
212.3783926	173.2807639	190.6569158	250.1884632	230.916287
19.21976404	24.49518652	5.896605643	4.313594194	11.81432166
0	0	0	0	0
0	0	0	0	0
8.648893816	6.350603914	9.827676071	7.548789839	9.666263176
0	0	11.79321129	0	0
0	0	0	0	1.074029242
8.648893816	10.88674957	13.7587465	4.313594194	6.444175451
12.49284662	1.814458261	0	0	0
7.687905614	7.257833044	7.862140857	14.01918113	7.518204692

For Review Only

17.29778763	16.33012435	14.74151411	20.48957242	21.48058484
0	3.628916522	0	0	1.074029242
8.648893816	0	11.79321129	9.705586936	3.222087725
29.79063425	15.42289522	15.72428171	5.391992742	12.8883509
5.765929211	6.350603914	0	0	2.148058484
3.843952807	1.814458261	3.931070429	9.705586936	6.444175451
22.10272864	21.77349913	7.862140857	11.86238403	5.370146209
88.41091456	68.04218479	54.05221839	52.84152887	49.40534512
9.609882018	0	0.982767607	1.078398548	1.074029242
2.882964605	6.350603914	2.948302821	3.235195645	0
14.41482303	14.51566609	5.896605643	15.09757968	9.666263176
0	0	1.965535214	0	0
0	4.536145653	0	0	0
0	0	0.982767607	0	0
4.804941009	11.7939787	0.982767607	2.156797097	7.518204692
0	0	0	0	3.222087725
0	7.257833044	0	0	1.074029242
0	0	0	0	0
4.804941009	3.628916522	1.965535214	0	2.148058484
28.82964605	3.628916522	17.68981693	14.01918113	21.48058484
6.726917412	0.907229131	6.87937325	0	3.222087725
0	0	0.982767607	1.078398548	1.074029242
0	0	0	0	0
9.609882018	0	0.982767607	0	0
2.882964605	4.536145653	4.913838036	0	0
0	0	0	4.313594194	0
0	0	0	34.50875355	0
3.843952807	14.51566609	7.862140857	8.627188388	8.592233934
0.960988202	0.907229131	0.982767607	0	0
0	6.350603914	0	2.156797097	7.518204692
30.75162246	34.47470696	27.517493	25.88156516	15.03640938
37.47853987	10.88674957	13.7587465	10.78398548	11.81432166
9.609882018	15.42289522	4.913838036	3.235195645	6.444175451
1.921976404	7.257833044	0.982767607	15.09757968	0
10.57087022	0	7.862140857	3.235195645	8.592233934
0.960988202	2.721687392	0	0	1.074029242
2.882964605	8.165062175	7.862140857	3.235195645	3.222087725
0	0	0	0	0
1.921976404	2.721687392	0.982767607	0	1.074029242
0	0	0	3.235195645	0
0.960988202	1.814458261	0	0	0
6.726917412	10.88674957	14.74151411	5.391992742	7.518204692
3.843952807	0	0	1.078398548	0
14.41482303	8.165062175	11.79321129	7.548789839	10.74029242
7.687905614	0	0	7.548789839	1.074029242
22.10272864	26.30964479	5.896605643	17.25437678	9.666263176
50.93237469	53.5265187	63.87989446	39.90074629	51.5534036
22.10272864	14.51566609	5.896605643	9.705586936	2.148058484
2.882964605	0	0	3.235195645	1.074029242
15.37581123	18.14458261	12.77597889	10.78398548	6.444175451

For Review Only

5.765929211	10.88674957	2.948302821	5.391992742	2.148058484
3.843952807	0.907229131	0.982767607	8.627188388	0
0	0	0	1.078398548	5.370146209
15.37581123	19.95904087	10.81044368	4.313594194	16.11043863
0	4.536145653	0	0	6.444175451
0	0	0.982767607	2.156797097	6.444175451
2.882964605	2.721687392	0.982767607	2.156797097	2.148058484
8.648893816	10.88674957	5.896605643	17.25437678	3.222087725
63.42522132	91.63014218	47.17284514	60.39031871	129.9575383
1.921976404	0	0	0	0
6.726917412	0	1.965535214	2.156797097	0
9.609882018	1.814458261	0	3.235195645	0
20.18075224	35.38193609	5.896605643	8.627188388	24.70267256
0	0	0	0	1.074029242
7.687905614	12.70120783	0.982767607	3.235195645	3.222087725
4.804941009	0.907229131	0.982767607	2.156797097	0
0	0	0	5.391992742	0
23.06371684	7.257833044	6.87937325	9.705586936	15.03640938
0.960988202	0	0	0	0
0	0	0	0	3.222087725
0	0	0.982767607	0	0
4.804941009	3.628916522	3.931070429	0	1.074029242
17.29778763	22.68072826	7.862140857	20.48957242	28.99878953
0	0	0	0	0
23.06371684	8.165062175	6.87937325	9.705586936	16.11043863
58.62028031	68.94941392	111.0527396	49.60633323	19.33252635
0.960988202	1.814458261	4.913838036	0	1.074029242
11.53185842	12.70120783	5.896605643	8.627188388	10.74029242
0	5.443374783	3.931070429	0	0
0	1.814458261	0	0	0
5.765929211	11.7939787	11.79321129	4.313594194	0
7.687905614	10.88674957	13.7587465	8.627188388	0
36.51755167	25.40241565	9.827676071	10.78398548	26.85073104
15.37581123	0.907229131	7.862140857	14.01918113	5.370146209
0	0	1.965535214	0	2.148058484
0.960988202	0.907229131	2.948302821	0	0
19.21976404	26.30964479	23.58642257	25.88156516	19.33252635
0	0	0	0	6.444175451
6.726917412	9.979520436	16.70704932	5.391992742	7.518204692
3.843952807	3.628916522	0.982767607	2.156797097	2.148058484
7.687905614	6.350603914	11.79321129	12.94078258	7.518204692
3.843952807	0	3.931070429	4.313594194	0
11.53185842	11.7939787	18.67258454	20.48957242	8.592233934
16.33679943	66.22772653	44.22454232	15.09757968	22.55461408
0	3.628916522	2.948302821	1.078398548	2.148058484
42.28348088	28.12410305	35.37963386	26.95996371	23.62864332
23.06371684	31.75301957	20.63811975	19.41117387	22.55461408
0	0	0	0	0
4.804941009	0	0	2.156797097	2.148058484
33.63458706	26.30964479	17.68981693	39.90074629	19.33252635

For Review Only

0	0	0	0	0
2.882964605	0.907229131	4.913838036	2.156797097	1.074029242
4.804941009	2.721687392	3.931070429	2.156797097	1.074029242
6.726917412	4.536145653	6.87937325	8.627188388	1.074029242
0	1.814458261	4.913838036	0	0
0.960988202	0	2.948302821	0	0
2.882964605	4.536145653	5.896605643	5.391992742	3.222087725
0	2.721687392	0	0	0
17.29778763	0	8.844908464	1.078398548	2.148058484
15.37581123	13.60843696	13.7587465	8.627188388	8.592233934
0	0	0	0	0
0	0	26.53472539	16.17597823	0
0	0	0	0	0
6.726917412	3.628916522	0	0	0
11.53185842	9.072291305	10.81044368	8.627188388	8.592233934
0	6.350603914	0	2.156797097	0
4.804941009	9.072291305	0	3.235195645	0
13.45383482	29.93856131	19.65535214	25.88156516	22.55461408
1.921976404	0	0	0	0
0	0	1.965535214	0	0
7.687905614	9.072291305	9.827676071	8.627188388	9.666263176
17.29778763	23.58795739	25.55195779	10.78398548	19.33252635
4.804941009	9.979520436	8.844908464	0	4.296116967
0.960988202	0.907229131	0.982767607	1.078398548	1.074029242
3.843952807	2.721687392	1.965535214	3.235195645	3.222087725
0	0	0.982767607	5.391992742	2.148058484
19.21976404	18.14458261	12.77597889	7.548789839	15.03640938
4.804941009	7.257833044	4.913838036	8.627188388	8.592233934
48.04941009	74.3927887	68.7937325	26.95996371	41.88714043
0	39.01085261	53.06945079	0	0
21.14174044	29.03133218	6.87937325	24.80316661	21.48058484
0	58.06266435	0.982767607	103.5262607	7.518204692
0	0	0	0	0
193.1586286	140.6205152	138.5702326	115.3886447	123.5133628
0	0	0	1.078398548	1.074029242
60.54225671	61.69158088	50.12114796	48.52793468	92.36651479
4.804941009	3.628916522	24.56919018	0	6.444175451
0	0.907229131	0	1.078398548	1.074029242
0	0.907229131	0	1.078398548	1.074029242
0.960988202	3.628916522	3.931070429	2.156797097	3.222087725
3.843952807	0	4.913838036	0	2.148058484
0	0	2.948302821	1.078398548	1.074029242
44.20545728	50.80483131	56.01775361	61.46871726	57.99757905
8.648893816	4.536145653	8.844908464	8.627188388	4.296116967
9.609882018	6.350603914	11.79321129	10.78398548	5.370146209
2.882964605	0	0.982767607	0	0
0	2.721687392	0	0	2.148058484
31.71261066	31.75301957	33.41409864	33.430355	36.51699422
14.41482303	20.86627	20.63811975	23.72476807	19.33252635
13.45383482	25.40241565	24.56919018	18.33277532	19.33252635

For Review Only

3.843952807	0.907229131	0.982767607	0	0
0	0	0	0	0
11.53185842	18.14458261	11.79321129	0	9.666263176
91.29387917	113.4036413	73.70757054	100.291065	117.0691874
2.882964605	0.907229131	0.982767607	3.235195645	7.518204692
0	0	0	7.548789839	1.074029242
10.57087022	8.165062175	11.79321129	7.548789839	13.96238014
3.843952807	6.350603914	2.948302821	1.078398548	2.148058484
4.804941009	5.443374783	2.948302821	0	3.222087725
13.45383482	15.42289522	3.931070429	15.09757968	8.592233934
29.79063425	62.59881001	39.31070429	44.21434049	34.36893574
3.843952807	9.979520436	6.87937325	8.627188388	16.11043863
0	8.165062175	1.965535214	0	0
0	0	0	0	3.222087725
0.960988202	2.721687392	0	0	3.222087725
14.41482303	18.14458261	13.7587465	17.25437678	9.666263176
4.804941009	0	1.965535214	1.078398548	0
4.804941009	2.721687392	3.931070429	2.156797097	0
0.960988202	0	0.982767607	1.078398548	3.222087725
2.882964605	0	0	0	3.222087725
0	0	0.982767607	0	1.074029242
5.765929211	3.628916522	0	1.078398548	3.222087725
13.45383482	14.51566609	23.58642257	21.56797097	32.22087725
2.882964605	0.907229131	3.931070429	2.156797097	6.444175451
0	0	0	0	0
4.804941009	0	0	5.391992742	0
1.921976404	0	0	0	0
2.882964605	0	0	0	4.296116967
0	0	5.896605643	0	0
0	0	0	0	0
0	1.814458261	2.948302821	0	0
3.843952807	0	1.965535214	1.078398548	1.074029242
5.765929211	12.70120783	5.896605643	34.50875355	30.07281877
76.87905614	254.9313857	38.32793668	36.66555065	235.2124039
7.687905614	3.628916522	4.913838036	9.705586936	11.81432166
5.765929211	0	0.982767607	3.235195645	3.222087725
7.687905614	0	11.79321129	3.235195645	4.296116967
0	4.536145653	0	0	0
44.20545728	24.49518652	13.7587465	50.68473178	50.47937436
2.882964605	3.628916522	0	1.078398548	4.296116967
18.25877583	19.05181174	3.931070429	1.078398548	3.222087725
21.14174044	37.19639435	1.965535214	23.72476807	46.1832574
13.45383482	17.23735348	9.827676071	11.86238403	6.444175451
0.960988202	0	1.965535214	0	0
2.882964605	0	0.982767607	0	2.148058484
1.921976404	1.814458261	0	1.078398548	0
0	0	0	12.94078258	0
2.882964605	0	5.896605643	2.156797097	0
15.37581123	29.03133218	9.827676071	15.09757968	17.18446787
5.765929211	6.350603914	6.87937325	3.235195645	6.444175451

For Review Only

3.843952807	0.907229131	1.965535214	0	0
0	0	0	0	0
0	2.721687392	2.948302821	3.235195645	3.222087725
10.57087022	7.257833044	8.844908464	7.548789839	6.444175451
5.765929211	0	4.913838036	1.078398548	0
2.882964605	6.350603914	9.827676071	0	0
4.804941009	6.350603914	0	0	0
0	0	0	0	0
0.960988202	2.721687392	2.948302821	1.078398548	0
0	0	0	0	2.148058484
19.21976404	4.536145653	5.896605643	7.548789839	4.296116967
1.921976404	1.814458261	3.931070429	0	0
0	0	0	3.235195645	4.296116967
84.56696176	62.59881001	117.9321129	87.35028243	51.5534036
0	0	0	0	0
0	7.257833044	1.965535214	0	1.074029242
0	0	0.982767607	3.235195645	2.148058484
0	0	0.982767607	1.078398548	4.296116967
43.24446908	32.6602487	23.58642257	8.627188388	53.70146209
0	7.257833044	5.896605643	1.078398548	0
31.71261066	15.42289522	18.67258454	26.95996371	24.70267256
0	0.907229131	0	3.235195645	3.222087725
3.843952807	8.165062175	7.862140857	0	0
1.921976404	0	0	1.078398548	0
4.804941009	16.33012435	6.87937325	4.313594194	5.370146209
27.86865785	0	0.982767607	0	1.074029242
2.882964605	4.536145653	2.948302821	5.391992742	0
5.765929211	0	0.982767607	2.156797097	5.370146209
0.960988202	0.907229131	8.844908464	4.313594194	3.222087725
0	7.257833044	2.948302821	0	0
266.1937319	221.3639079	75.67310575	174.7005649	223.3980823
0	0	0.982767607	2.156797097	1.074029242
5.765929211	0.907229131	4.913838036	3.235195645	0
560.2561216	647.7615992	948.3707409	812.034107	807.6699898
3.843952807	0	0.982767607	1.078398548	0
6.726917412	0.907229131	0.982767607	0	0
3.843952807	0	2.948302821	4.313594194	0
16.33679943	14.51566609	12.77597889	9.705586936	16.11043863
37.47853987	48.99037305	21.62088736	6.470391291	22.55461408
5.765929211	6.350603914	5.896605643	6.470391291	1.074029242
24.98569325	31.75301957	11.79321129	12.94078258	20.40655559
0	0	0	0	0
7.687905614	4.536145653	0.982767607	15.09757968	9.666263176
2.882964605	7.257833044	0.982767607	5.391992742	6.444175451
6.726917412	6.350603914	1.965535214	2.156797097	3.222087725
3.843952807	0	2.948302821	0	0
0	0	0	0	1.074029242
0.960988202	4.536145653	0	0	0
4.804941009	0	1.965535214	0	0
0	1.814458261	0	1.078398548	0

For Review Only

16.33679943	9.979520436	2.948302821	6.470391291	15.03640938
21.14174044	13.60843696	18.67258454	12.94078258	8.592233934
3.843952807	0	3.931070429	5.391992742	2.148058484
0.960988202	1.814458261	0.982767607	1.078398548	1.074029242
0.960988202	2.721687392	0.982767607	1.078398548	1.074029242
0	0	0	15.09757968	0
12.49284662	9.979520436	3.931070429	1.078398548	4.296116967
4.804941009	14.51566609	1.965535214	2.156797097	7.518204692
0	0	3.931070429	0	1.074029242
18.25877583	12.70120783	4.913838036	3.235195645	5.370146209
4.804941009	4.536145653	0.982767607	3.235195645	3.222087725
0	3.628916522	0	0	0
11.53185842	8.165062175	7.862140857	16.17597823	15.03640938
6.726917412	0.907229131	0.982767607	1.078398548	0
6.726917412	6.350603914	2.948302821	17.25437678	3.222087725
0	0	0	0	0
0	0	0.982767607	0	0
0	0	3.931070429	5.391992742	2.148058484
0	4.536145653	1.965535214	0	2.148058484
0	0	0	0	0
46.12743368	21.77349913	39.31070429	15.09757968	21.48058484
2.882964605	0.907229131	0.982767607	3.235195645	1.074029242
13.45383482	13.60843696	19.65535214	12.94078258	19.33252635
3.843952807	0	4.913838036	2.156797097	3.222087725
1.921976404	0.907229131	4.913838036	0	0
0	2.721687392	0	0	3.222087725
7.687905614	9.979520436	12.77597889	2.156797097	5.370146209
4.804941009	15.42289522	10.81044368	0	8.592233934
12.49284662	8.165062175	1.965535214	2.156797097	2.148058484
10.57087022	8.165062175	6.87937325	12.94078258	3.222087725
0	1.814458261	4.913838036	1.078398548	1.074029242
0	0	12.77597889	14.01918113	22.55461408
22.10272864	19.95904087	25.55195779	18.33277532	19.33252635
0	0	0.982767607	0	0
3.843952807	0	0	0	0
3.843952807	4.536145653	5.896605643	1.078398548	1.074029242
0	0	0	0	0
0.960988202	1.814458261	5.896605643	0	0
0	0	1.965535214	2.156797097	0
17.29778763	14.51566609	4.913838036	5.391992742	5.370146209
0	11.7939787	2.948302821	6.470391291	4.296116967
12.49284662	13.60843696	14.74151411	9.705586936	8.592233934
19.21976404	13.60843696	9.827676071	4.313594194	10.74029242
0	0	14.74151411	0	0
50.93237469	47.17591479	33.41409864	32.35195645	56.92354981
0	0	0.982767607	0	0
0.960988202	0	0.982767607	0	0
4.804941009	0	0	0	0
3.843952807	6.350603914	4.913838036	0	1.074029242
0	0	0	0	0

For Review Only

0.960988202	0	0.982767607	1.078398548	2.148058484
5.765929211	8.165062175	0	0	0
0	0	0	0	0
5.765929211	16.33012435	0.982767607	5.391992742	3.222087725
57.65929211	46.26868566	50.12114796	10.78398548	34.36893574
5.765929211	4.536145653	2.948302821	7.548789839	5.370146209
0	10.88674957	1.965535214	2.156797097	5.370146209
5.765929211	0.907229131	0	4.313594194	3.222087725
18.25877583	24.49518652	7.862140857	11.86238403	22.55461408
151.8361359	146.06389	119.8976481	65.78231146	63.36772526
0	0.907229131	0	0	1.074029242
1.921976404	0	0	0	0
0	0	0	0	1.074029242
0	0	0	0	0
0	2.721687392	0	1.078398548	0
2.882964605	7.257833044	0.982767607	5.391992742	4.296116967
71.11312693	81.65062175	49.13838036	37.7439492	94.51457327
13.45383482	11.7939787	11.79321129	14.01918113	2.148058484
0	6.350603914	0	0	0
6.726917412	0	0	0	1.074029242
6.726917412	5.443374783	0.982767607	10.78398548	2.148058484
6.726917412	3.628916522	0	0	5.370146209
0.960988202	0	0	0	4.296116967
0	12.70120783	0	1.078398548	1.074029242
5.765929211	0	0.982767607	3.235195645	12.8883509
0.960988202	2.721687392	0	0	3.222087725
1.921976404	0	0	4.313594194	0
2.882964605	0	0	0	1.074029242
4.804941009	5.443374783	1.965535214	5.391992742	2.148058484
5.765929211	9.072291305	3.931070429	9.705586936	8.592233934
2.882964605	3.628916522	0	0	5.370146209
0	5.443374783	2.948302821	4.313594194	4.296116967
0	3.628916522	7.862140857	0	16.11043863
0	0	1.965535214	0	0
0	5.443374783	0	1.078398548	0
0	3.628916522	0	0	0
4.804941009	14.51566609	11.79321129	9.705586936	6.444175451
0.960988202	2.721687392	0	0	0
19.21976404	16.33012435	3.931070429	2.156797097	20.40655559
2.882964605	3.628916522	0	5.391992742	1.074029242
0	7.257833044	0	3.235195645	6.444175451
0	0	3.931070429	2.156797097	1.074029242
5.765929211	9.072291305	7.862140857	6.470391291	7.518204692
11.53185842	11.7939787	8.844908464	3.235195645	20.40655559
0.960988202	1.814458261	0	0	0
0	0	0	0	0
0	6.350603914	0	0	2.148058484
0	0	0	0	6.444175451
0	2.721687392	3.931070429	3.235195645	4.296116967
24.02470504	18.14458261	23.58642257	16.17597823	18.25849711

For Review Only

4.804941009	0	0	0	2.148058484
0	6.350603914	0.982767607	1.078398548	5.370146209
0	0	0	3.235195645	0
0	5.443374783	0	0	0
4.804941009	7.257833044	7.862140857	7.548789839	2.148058484
6.726917412	0	1.965535214	7.548789839	18.25849711
0	8.165062175	0	0	0
3.843952807	0	0	1.078398548	0
0	3.628916522	1.965535214	2.156797097	0
0	5.443374783	4.913838036	0	6.444175451
0	0	0	0	2.148058484
0	0	0	0	0
0	6.350603914	0	0	2.148058484
6.726917412	0	0	3.235195645	3.222087725
4.804941009	0	0.982767607	0	0
101.8647494	75.30001783	4.913838036	20.48957242	16.11043863
0	9.979520436	0	4.313594194	4.296116967
0	0	0	0	0
0	0.907229131	0.982767607	1.078398548	1.074029242
1.921976404	0	0	0	1.074029242
0	0	0	0	0
79.76202075	87.09399653	72.72480293	115.3886447	96.66263176
12.49284662	12.70120783	0.982767607	6.470391291	2.148058484
9.609882018	5.443374783	5.896605643	0	1.074029242
0	1.814458261	2.948302821	0	0
10.57087022	5.443374783	0	0	9.666263176
0.960988202	7.257833044	0	0	0
2.882964605	0	0	0	0
0.960988202	0.907229131	0	0	0
2.882964605	5.443374783	2.948302821	5.391992742	5.370146209
2.882964605	4.536145653	0	5.391992742	3.222087725
1.921976404	2.721687392	0	5.391992742	4.296116967
26.90766965	8.165062175	8.844908464	7.548789839	6.444175451
15.37581123	15.42289522	15.72428171	3.235195645	10.74029242
7.687905614	7.257833044	8.844908464	0	0
2.882964605	0	2.948302821	1.078398548	3.222087725
3.843952807	5.443374783	0	2.156797097	0
0.960988202	5.443374783	8.844908464	0	5.370146209
0	0	0	0	0
0	0	3.931070429	0	0
3.843952807	5.443374783	2.948302821	7.548789839	3.222087725
2.882964605	0	0	1.078398548	0
13.45383482	4.536145653	2.948302821	14.01918113	13.96238014
24.98569325	17.23735348	9.827676071	25.88156516	10.74029242
3.843952807	0.907229131	0	0	0
0	3.628916522	0	1.078398548	0
42.28348088	38.10362348	9.827676071	28.03836226	13.96238014
111.4746314	105.2385791	104.1733664	78.72309404	78.40413465
9.609882018	11.7939787	6.87937325	6.470391291	6.444175451
0	0.907229131	0.982767607	3.235195645	2.148058484

For Review Only

12.49284662	17.23735348	6.87937325	11.86238403	9.666263176
8.648893816	7.257833044	5.896605643	2.156797097	9.666263176
0.960988202	0	0	0	1.074029242
14.41482303	4.536145653	0.982767607	0	5.370146209
2.882964605	0	1.965535214	4.313594194	1.074029242
0.960988202	0	2.948302821	3.235195645	3.222087725
20.18075224	47.17591479	22.60365496	34.50875355	9.666263176
4.804941009	0	0.982767607	0	0
7.687905614	11.7939787	2.948302821	0	5.370146209
4.804941009	19.95904087	1.965535214	14.01918113	8.592233934
1.921976404	3.628916522	5.896605643	4.313594194	0
0	3.628916522	0	0	1.074029242
13.45383482	6.350603914	3.931070429	1.078398548	5.370146209
17.29778763	18.14458261	12.77597889	7.548789839	18.25849711
0	0	0	0	1.074029242
2.882964605	0	0	0	0
0	9.072291305	6.87937325	15.09757968	2.148058484
3.843952807	0	0	2.156797097	0
0.960988202	0	0	3.235195645	2.148058484
0.960988202	0	0	4.313594194	0
0	6.350603914	0.982767607	7.548789839	0
0.960988202	2.721687392	0	0	0
0	0	0	0	0
0	0	1.965535214	3.235195645	0
8.648893816	18.14458261	2.948302821	19.41117387	1.074029242
0	4.536145653	0.982767607	0	0
4.804941009	10.88674957	0.982767607	2.156797097	2.148058484
2.882964605	3.628916522	16.70704932	10.78398548	4.296116967
15.37581123	7.257833044	5.896605643	8.627188388	8.592233934
0	0	0.982767607	1.078398548	1.074029242
19.21976404	11.7939787	12.77597889	3.235195645	10.74029242
26.90766965	33.56747783	7.862140857	17.25437678	26.85073104
10.57087022	11.7939787	6.87937325	15.09757968	10.74029242
8.648893816	14.51566609	3.931070429	14.01918113	4.296116967
0	0	1.965535214	0	0
1.921976404	1.814458261	0	0	1.074029242
12.49284662	7.257833044	5.896605643	9.705586936	7.518204692
2.882964605	9.072291305	8.844908464	0	6.444175451
0.960988202	4.536145653	1.965535214	0	3.222087725
4.804941009	5.443374783	3.931070429	1.078398548	0
25.94668145	23.58795739	12.77597889	19.41117387	6.444175451
0.960988202	0	0	3.235195645	1.074029242
8.648893816	0	0	6.470391291	0
12.49284662	8.165062175	1.965535214	4.313594194	3.222087725
0	0	3.931070429	6.470391291	5.370146209
20.18075224	11.7939787	10.81044368	10.78398548	15.03640938
3.843952807	2.721687392	2.948302821	0	0
3.843952807	0	0.982767607	0	0
2.882964605	1.814458261	0	0	1.074029242
17.29778763	9.072291305	15.72428171	8.627188388	10.74029242

0	9.072291305	0	0	0
2.882964605	2.721687392	4.913838036	0	0
56.6983039	47.17591479	81.56971139	19.41117387	40.81311119
29.79063425	16.33012435	7.862140857	3.235195645	16.11043863
5.765929211	7.257833044	0	0	0
2.882964605	0	0	1.078398548	0
3.843952807	0.907229131	0.982767607	3.235195645	2.148058484
14.41482303	14.51566609	3.931070429	4.313594194	3.222087725
0.960988202	10.88674957	0.982767607	1.078398548	0
23.06371684	7.257833044	2.948302821	9.705586936	6.444175451
15.37581123	0	4.913838036	1.078398548	2.148058484
4.804941009	7.257833044	3.931070429	8.627188388	3.222087725
12.49284662	8.165062175	13.7587465	10.78398548	8.592233934
4.804941009	4.536145653	0.982767607	2.156797097	0
2.882964605	0	2.948302821	2.156797097	4.296116967
0	0	2.948302821	3.235195645	0
4.804941009	4.536145653	0.982767607	0	3.222087725
2.882964605	0	0	1.078398548	0
0	0	0	0	0
10.57087022	16.33012435	8.844908464	12.94078258	13.96238014
0.960988202	2.721687392	4.913838036	0	4.296116967
0	3.628916522	2.948302821	0	0
0	0	0.982767607	0	0
8.648893816	12.70120783	1.965535214	1.078398548	5.370146209
4.804941009	10.88674957	22.60365496	4.313594194	23.62864332
19.21976404	37.19639435	11.79321129	19.41117387	6.444175451
6.726917412	5.443374783	7.862140857	0	3.222087725
7.687905614	0	0	0	0
78.80103254	58.06266435	51.10391557	49.60633323	42.96116967
4.804941009	0.907229131	3.931070429	0	2.148058484
39.40051627	38.10362348	33.41409864	18.33277532	25.7767018
0	0.907229131	0.982767607	0	1.074029242
160.4850297	182.3530552	104.1733664	183.3277532	143.9199184
14.41482303	18.14458261	7.862140857	7.548789839	3.222087725
2.882964605	0	0	3.235195645	0
19.21976404	19.05181174	25.55195779	16.17597823	33.29490649
0.960988202	3.628916522	0.982767607	7.548789839	2.148058484
0.960988202	0	0	0	0
1.921976404	1.814458261	0.982767607	0	0
0	0	0	0	0
0	0	0.982767607	1.078398548	2.148058484
18.25877583	11.7939787	11.79321129	1.078398548	7.518204692
0	5.443374783	0	0	0
307.5162246	374.6856309	413.7451626	463.7113758	292.1359538
23.06371684	23.58795739	16.70704932	6.470391291	16.11043863
49.97138649	30.84579044	38.32793668	37.7439492	12.8883509
65.34719772	74.3927887	18.67258454	54.99832597	47.25728664
2.882964605	0.907229131	5.896605643	3.235195645	3.222087725
7.687905614	2.721687392	14.74151411	2.156797097	2.148058484
0.960988202	0	0	0	2.148058484

0	0	0.982767607	0	0
6.726917412	7.257833044	26.53472539	26.95996371	27.92476029
7.687905614	5.443374783	3.931070429	0	2.148058484
36.51755167	19.95904087	3.931070429	1.078398548	7.518204692
22.10272864	38.10362348	44.22454232	23.72476807	23.62864332
47.08842189	29.03133218	42.25900711	24.80316661	20.40655559
18.25877583	19.05181174	10.81044368	0	9.666263176
0	0	0	0	0
35.55656347	28.12410305	24.56919018	12.94078258	32.22087725
34.59557526	14.51566609	15.72428171	33.430355	37.59102346
13.45383482	20.86627	10.81044368	5.391992742	21.48058484
0	0.907229131	0	0	0
0	0	3.931070429	0	0
4.804941009	3.628916522	4.913838036	0	0
0	0	16.70704932	3.235195645	5.370146209
22.10272864	9.979520436	0.982767607	1.078398548	6.444175451
41.32249268	29.03133218	40.29347189	28.03836226	33.29490649
19.21976404	18.14458261	12.77597889	14.01918113	18.25849711
14.41482303	17.23735348	12.77597889	9.705586936	13.96238014
0	0	2.948302821	3.235195645	0
0	0	0.982767607	0	2.148058484
27.86865785	29.03133218	9.827676071	4.313594194	6.444175451
9.609882018	15.42289522	1.965535214	19.41117387	6.444175451
7.687905614	0	0.982767607	3.235195645	1.074029242
3.843952807	4.536145653	0	9.705586936	19.33252635
2.882964605	0	0	0	0
0	9.072291305	0	0	1.074029242
0	0	0	3.235195645	2.148058484
0	0	0	0	0
0	0.907229131	0	1.078398548	0
0	4.536145653	0	0	0
29.79063425	24.49518652	14.74151411	9.705586936	10.74029242
162.4070061	106.1458083	125.7942537	177.9357605	123.5133628
0	0	2.948302821	0	0
3083.811139	3068.248919	1862.344616	1895.824648	2339.235689
20.18075224	22.68072826	30.46579582	5.391992742	5.370146209
2.882964605	0	2.948302821	0	0
29.79063425	24.49518652	10.81044368	21.56797097	13.96238014
0.960988202	0	0.982767607	1.078398548	0
2.882964605	0.907229131	2.948302821	3.235195645	5.370146209
3.843952807	5.443374783	2.948302821	5.391992742	5.370146209
7.687905614	9.072291305	6.87937325	0	8.592233934
0	0	0	0	0
0.960988202	0	0.982767607	3.235195645	5.370146209
4.804941009	0.907229131	0	0	0
2.882964605	0	0.982767607	0	4.296116967
44.20545728	26.30964479	27.517493	39.90074629	41.88714043
3.843952807	0	0	3.235195645	0
2.882964605	0	0	0	0
0	0	0	0	2.148058484

For Review Only

35.55656347	14.51566609	14.74151411	42.05754339	22.55461408
24.98569325	36.28916522	5.896605643	15.09757968	13.96238014
4.804941009	0	0	0	0
6.726917412	0	0.982767607	11.86238403	5.370146209
9.609882018	0	12.77597889	0	0
9.609882018	5.443374783	2.948302821	8.627188388	11.81432166
0	1.814458261	0	3.235195645	0
40.36150447	47.17591479	34.39686625	39.90074629	33.29490649
5.765929211	10.88674957	3.931070429	2.156797097	5.370146209
0.960988202	1.814458261	0	0	0
10.57087022	4.536145653	3.931070429	3.235195645	4.296116967
3.843952807	0	0	0	1.074029242
0	0	0	0	0
36.51755167	22.68072826	15.72428171	17.25437678	11.81432166
0	1.814458261	0.982767607	0	0
0	2.721687392	0	5.391992742	3.222087725
47.08842189	84.37230914	11.79321129	95.97747081	57.99757905
7.687905614	11.7939787	8.844908464	3.235195645	10.74029242
47.08842189	49.89760218	32.43133104	31.27355791	36.51699422
16.33679943	4.536145653	11.79321129	2.156797097	1.074029242
3.843952807	1.814458261	2.948302821	4.313594194	0
0	0	0.982767607	0	0
21.14174044	0.907229131	12.77597889	1.078398548	1.074029242
35.55656347	25.40241565	14.74151411	23.72476807	20.40655559
4.804941009	3.628916522	0.982767607	2.156797097	0
10.57087022	15.42289522	21.62088736	18.33277532	17.18446787
4.804941009	10.88674957	2.948302821	1.078398548	5.370146209
0	10.88674957	0.982767607	0	1.074029242
0	0.907229131	4.913838036	15.09757968	54.77549133
7.687905614	4.536145653	0	3.235195645	3.222087725
14.41482303	4.536145653	13.7587465	14.01918113	9.666263176
0.960988202	4.536145653	3.931070429	0	0
0	0	4.913838036	0	0
0	5.443374783	0	4.313594194	0
4.804941009	0	4.913838036	0	0
31.71261066	15.42289522	7.862140857	6.470391291	2.148058484
4.804941009	5.443374783	0	0	2.148058484
11.53185842	31.75301957	6.87937325	2.156797097	2.148058484
0	0	0	0	1.074029242
29.79063425	19.95904087	1.965535214	9.705586936	23.62864332
0	0.907229131	0	0	0
0	3.628916522	1.965535214	2.156797097	0
20.18075224	16.33012435	18.67258454	5.391992742	7.518204692
0	0	0	1.078398548	1.074029242
8.648893816	5.443374783	10.81044368	8.627188388	15.03640938
24.02470504	39.01085261	6.87937325	23.72476807	15.03640938
8.648893816	0	8.844908464	1.078398548	13.96238014
6.726917412	26.30964479	16.70704932	3.235195645	5.370146209
0.960988202	0	2.948302821	0	2.148058484
34.59557526	23.58795739	23.58642257	49.60633323	21.48058484

For Review Only

0	9.072291305	7.862140857	1.078398548	0
1.921976404	7.257833044	6.87937325	3.235195645	4.296116967
13.45383482	21.77349913	4.913838036	10.78398548	12.8883509
0.960988202	0	0	0	0
4.804941009	0	0	0	0
0	0	0	0	3.222087725
2.882964605	3.628916522	0	0	0
3.843952807	0	0	3.235195645	0
18.25877583	18.14458261	6.87937325	15.09757968	19.33252635
0	0.907229131	2.948302821	0	1.074029242
0	1.814458261	0.982767607	0	0
0	0	0	2.156797097	0
0	0	0	5.391992742	0
5.765929211	0	1.965535214	2.156797097	1.074029242
17.29778763	18.14458261	9.827676071	15.09757968	9.666263176
15.37581123	12.70120783	12.77597889	6.470391291	19.33252635
0.960988202	0.907229131	0	4.313594194	2.148058484
45.16644548	31.75301957	41.2762395	39.90074629	41.88714043
6.726917412	6.350603914	4.913838036	3.235195645	3.222087725
85.52794996	87.09399653	20.63811975	26.95996371	19.33252635
36.51755167	55.34097696	32.43133104	30.19515936	57.99757905
8.648893816	6.350603914	8.844908464	1.078398548	3.222087725
2.882964605	0	0	14.01918113	4.296116967
6.726917412	4.536145653	2.948302821	1.078398548	1.074029242
0	2.721687392	0	0	3.222087725
9.609882018	8.165062175	6.87937325	5.391992742	7.518204692
0	0	0	0	0
15.37581123	0.907229131	8.844908464	18.33277532	4.296116967
0	4.536145653	0	0	0
0	0	0	2.156797097	0
384.3952807	316.6229666	247.657437	88.42868097	302.8762462
0	0	0	0	15.03640938
7.687905614	14.51566609	0.982767607	3.235195645	5.370146209
13.45383482	18.14458261	0.982767607	7.548789839	1.074029242
0	0	0	0	0
8.648893816	1.814458261	0	0	0
3.843952807	0	4.913838036	1.078398548	2.148058484
0	0	0	1.078398548	0
11.53185842	8.165062175	2.948302821	18.33277532	6.444175451
12.49284662	10.88674957	6.87937325	1.078398548	1.074029242
4.804941009	0	0	0	0
1.921976404	2.721687392	3.931070429	4.313594194	1.074029242
1.921976404	1.814458261	0	1.078398548	0
29.79063425	23.58795739	41.2762395	25.88156516	16.11043863
16.33679943	16.33012435	1.965535214	4.313594194	8.592233934
2.882964605	2.721687392	1.965535214	7.548789839	4.296116967
5.765929211	5.443374783	3.931070429	0	2.148058484
49.97138649	43.54699827	53.06945079	24.80316661	12.8883509
11.53185842	4.536145653	2.948302821	4.313594194	12.8883509
0	3.628916522	0	0	0

For Review Only

0.960988202	0	0	3.235195645	0
20.18075224	13.60843696	7.862140857	10.78398548	11.81432166
2.882964605	2.721687392	0	0	0
401.6930683	400.0880466	184.7603101	314.8923761	295.3580415
3.843952807	0	2.948302821	2.156797097	1.074029242
6.726917412	6.350603914	2.948302821	2.156797097	0
0	0	0	0	0
0.960988202	0	0	0	0
4.804941009	0	0	1.078398548	0
6.726917412	0	0	0	3.222087725
3.843952807	0	0	0	0
0	0	0	1.078398548	2.148058484
2.882964605	0.907229131	3.931070429	0	0
13.45383482	11.7939787	21.62088736	19.41117387	16.11043863
1.921976404	0	5.896605643	0	0
22.10272864	19.05181174	11.79321129	14.01918113	19.33252635
0	2.721687392	0	0	1.074029242
0	0	0	0	1.074029242
0	0	0.982767607	0	4.296116967
14.41482303	8.165062175	10.81044368	1.078398548	26.85073104
0	0	0	1.078398548	0
3.843952807	6.350603914	4.913838036	7.548789839	4.296116967
36.51755167	31.75301957	0.982767607	16.17597823	11.81432166
0	0	0	0	1.074029242
3.843952807	0	2.948302821	0	2.148058484
4.804941009	0.907229131	2.948302821	0	0
29.79063425	28.12410305	24.56919018	19.41117387	26.85073104
0	0	4.913838036	0	0
0.960988202	0.907229131	0	0	0
0.960988202	0	0	0	0
0	0	0	0	0
0	0	0	0	0
19.21976404	0	2.948302821	2.156797097	24.70267256
81.68399715	44.4542274	49.13838036	74.40949984	99.88471948
0.960988202	0	0	1.078398548	3.222087725
2.882964605	0.907229131	0	0	0
8.648893816	11.7939787	1.965535214	3.235195645	19.33252635
0	14.51566609	0.982767607	1.078398548	0
8.648893816	0	0.982767607	4.313594194	2.148058484
0	0.907229131	0	0	1.074029242
0	0	0	1.078398548	0
6.726917412	6.350603914	0	2.156797097	1.074029242
2.882964605	4.536145653	0	0	5.370146209
1.921976404	0	0	0	0
0.960988202	2.721687392	3.931070429	1.078398548	3.222087725
7.687905614	14.51566609	15.72428171	15.09757968	8.592233934
0	0	0	0	0
5.765929211	2.721687392	6.87937325	0	0
105.7087022	98.88797523	31.44856343	64.70391291	97.736661
8.648893816	19.95904087	22.60365496	10.78398548	22.55461408

0	7.257833044	0	0	0
1.921976404	1.814458261	4.913838036	0	8.592233934
15.37581123	18.14458261	6.87937325	7.548789839	7.518204692
0	0	0	0	0
2.882964605	4.536145653	1.965535214	11.86238403	4.296116967
0	6.350603914	3.931070429	1.078398548	5.370146209
28.82964605	31.75301957	21.62088736	32.35195645	12.8883509
0	3.628916522	1.965535214	2.156797097	1.074029242
26.90766965	9.072291305	13.7587465	11.86238403	13.96238014
47.08842189	43.54699827	35.37963386	38.82234774	30.07281877
1.921976404	0	0	1.078398548	0
6.726917412	15.42289522	2.948302821	15.09757968	6.444175451
45.16644548	29.03133218	31.44856343	36.66555065	48.33131588
0	0	0	0	0
5.765929211	3.628916522	0.982767607	1.078398548	0
7.687905614	9.072291305	8.844908464	1.078398548	5.370146209
6.726917412	11.7939787	0.982767607	0	3.222087725
0	0	0	1.078398548	0
5.765929211	6.350603914	0	7.548789839	5.370146209
3.843952807	0	0	1.078398548	0
2.882964605	3.628916522	4.913838036	3.235195645	6.444175451
0	0	2.948302821	2.156797097	1.074029242
0	7.257833044	0	0	0
0	0	2.948302821	0	0
14.41482303	9.979520436	6.87937325	23.72476807	15.03640938
10.57087022	3.628916522	0	5.391992742	3.222087725
0.960988202	0	2.948302821	0	3.222087725
0	0	0	3.235195645	2.148058484
0	0	0	1.078398548	0
0	6.350603914	0	0	3.222087725
12.49284662	11.7939787	0.982767607	10.78398548	7.518204692
17.29778763	19.95904087	8.844908464	10.78398548	19.33252635
3.843952807	0	0.982767607	0	0
0	0	0	0	0
0	0	1.965535214	0	0
6.726917412	27.21687392	8.844908464	10.78398548	11.81432166
0	3.628916522	0.982767607	0	2.148058484
25.94668145	25.40241565	37.34516907	11.86238403	18.25849711
0	0	0	0	0
1.921976404	4.536145653	0	0	4.296116967
5.765929211	0	1.965535214	3.235195645	10.74029242
31.71261066	13.60843696	27.517493	16.17597823	30.07281877
25.94668145	47.17591479	15.72428171	17.25437678	16.11043863
0	2.721687392	0	0	0
3.843952807	0.907229131	2.948302821	0	3.222087725
0	0	0	0	6.444175451
105.7087022	106.1458083	67.81096489	135.8782171	107.4029242
0	0	2.948302821	0	0
0	0	0.982767607	6.470391291	0
0	0	6.87937325	1.078398548	0

For Review Only

0	5.443374783	3.931070429	0	0
0	8.165062175	4.913838036	9.705586936	6.444175451
5.765929211	0.907229131	6.87937325	6.470391291	6.444175451
2.882964605	1.814458261	0	0	3.222087725
3.843952807	0	7.862140857	2.156797097	6.444175451
0	0	0	0	0
4.804941009	6.350603914	0.982767607	4.313594194	3.222087725
0	0	0.982767607	3.235195645	0
2.882964605	0	0	0	0
0	0	0	0	0
0	0	0.982767607	0	3.222087725
4.804941009	6.350603914	0.982767607	2.156797097	3.222087725
0	0	0	0	0
0.960988202	1.814458261	0	0	0
0	0	1.965535214	0	2.148058484
0	0	0	0	0
3.843952807	11.7939787	0.982767607	2.156797097	6.444175451
0	0	0	2.156797097	2.148058484
9.609882018	9.979520436	13.7587465	3.235195645	4.296116967
0	2.721687392	1.965535214	0	0
4.804941009	5.443374783	4.913838036	4.313594194	0
0	0	3.931070429	0	0
39.40051627	32.6602487	27.517493	26.95996371	39.73908194
0	0	2.948302821	0	0
0	0	0	0	0
24.02470504	14.51566609	9.827676071	32.35195645	10.74029242
2.882964605	3.628916522	1.965535214	3.235195645	3.222087725
4.804941009	6.350603914	9.827676071	5.391992742	6.444175451
4.804941009	11.7939787	12.77597889	4.313594194	10.74029242
5.765929211	0	0	1.078398548	0
28.82964605	18.14458261	11.79321129	24.80316661	20.40655559
7.687905614	9.979520436	6.87937325	6.470391291	5.370146209
0	0	0.982767607	0	0
0	0	1.965535214	0	2.148058484
0	1.814458261	0	0	0
0	0	14.74151411	0	0
0	0	1.965535214	0	0
7.687905614	0	2.948302821	1.078398548	0
7.687905614	27.21687392	17.68981693	10.78398548	19.33252635
17.29778763	13.60843696	15.72428171	5.391992742	6.444175451
0	1.814458261	0	0	0
0	0	0	0	0
14.41482303	17.23735348	6.87937325	2.156797097	22.55461408
21.14174044	17.23735348	7.862140857	11.86238403	4.296116967
2.882964605	13.60843696	14.74151411	4.313594194	8.592233934
0	0	0	0	0
31.71261066	23.58795739	21.62088736	26.95996371	17.18446787
11.53185842	9.979520436	7.862140857	12.94078258	1.074029242
0	0	3.931070429	2.156797097	2.148058484
18.25877583	43.54699827	20.63811975	11.86238403	18.25849711

For Review Only

13.45383482	13.60843696	8.844908464	4.313594194	18.25849711
0.960988202	0	1.965535214	1.078398548	0
4.804941009	9.979520436	7.862140857	10.78398548	5.370146209
3.843952807	0	0	0	0
21.14174044	26.30964479	15.72428171	10.78398548	21.48058484
0.960988202	0	0	0	7.518204692
474.7281717	381.0362348	335.123754	345.0875355	395.242761
1.921976404	3.628916522	3.931070429	7.548789839	0
2.882964605	1.814458261	0	0	0
2.882964605	0	0.982767607	1.078398548	0
3.843952807	2.721687392	0	3.235195645	0
0	5.443374783	0	5.391992742	6.444175451
9.609882018	0	0.982767607	1.078398548	1.074029242
8.648893816	8.165062175	6.87937325	3.235195645	6.444175451
21.14174044	37.19639435	0.982767607	10.78398548	22.55461408
0	0	0.982767607	1.078398548	21.48058484
14.41482303	15.42289522	10.81044368	7.548789839	2.148058484
25.94668145	15.42289522	3.931070429	29.11676081	5.370146209
4.804941009	2.721687392	3.931070429	0	0
0.960988202	0.907229131	2.948302821	0	0
10.57087022	2.721687392	1.965535214	3.235195645	2.148058484
0.960988202	1.814458261	0.982767607	0	1.074029242
67.26917412	78.92893436	32.43133104	33.430355	40.81311119
9.609882018	10.88674957	1.965535214	2.156797097	2.148058484
0	0	0	1.078398548	0
0	0	0	0	0
0.960988202	0	0	2.156797097	0
4.804941009	0	0	1.078398548	1.074029242
4.804941009	0	0	0	0
0	0	0	1.078398548	0
0	0.907229131	0	0	0
0	0	0	0	0
17.29778763	37.19639435	14.74151411	20.48957242	23.62864332
9.609882018	14.51566609	34.39686625	11.86238403	4.296116967
2.882964605	2.721687392	0	0	0
13.45383482	12.70120783	6.87937325	14.01918113	15.03640938
0	0	0.982767607	0	0
0	0	0	0	0
1.921976404	0	0	6.470391291	3.222087725
0	0	0	0	4.296116967
48.04941009	41.73254	27.517493	52.84152887	32.22087725
5.765929211	4.536145653	4.913838036	2.156797097	2.148058484
0	4.536145653	0.982767607	0	0
0	4.536145653	0	0	4.296116967
0	0	0	0	0
0	5.443374783	0	4.313594194	0
0	2.721687392	3.931070429	0	0
6.726917412	9.979520436	1.965535214	4.313594194	0
1.921976404	0.907229131	0	0	0
0	6.350603914	5.896605643	0	0

For Review Only

3.843952807	6.350603914	2.948302821	0	0
6.726917412	0	0	3.235195645	1.074029242
42.28348088	43.54699827	46.19007754	34.50875355	57.99757905
74.95707974	296.6639257	328.2443808	262.0508473	321.1347433
19.21976404	24.49518652	19.65535214	4.313594194	2.148058484
0.960988202	1.814458261	0.982767607	0	1.074029242
11.53185842	5.443374783	4.913838036	12.94078258	5.370146209
20.18075224	24.49518652	17.68981693	16.17597823	13.96238014
3.843952807	0	0	1.078398548	0
0	0	0	1.078398548	0
0	0	1.965535214	0	2.148058484
0	0	2.948302821	0	0
0.960988202	16.33012435	2.948302821	6.470391291	1.074029242
8.648893816	9.072291305	2.948302821	5.391992742	5.370146209
2.882964605	2.721687392	0	1.078398548	0
16.33679943	6.350603914	10.81044368	9.705586936	16.11043863
0	0	0	2.156797097	0
0	1.814458261	0	0	0
0	3.628916522	0.982767607	1.078398548	0
12.49284662	2.721687392	10.81044368	7.548789839	6.444175451
0	8.165062175	0	0	0
4.804941009	3.628916522	0	0	4.296116967
8.648893816	6.350603914	10.81044368	18.33277532	2.148058484
16.33679943	18.14458261	5.896605643	2.156797097	9.666263176
0	1.814458261	0.982767607	0	0
2.882964605	9.072291305	7.862140857	5.391992742	0
6.726917412	9.072291305	10.81044368	8.627188388	6.444175451
4.804941009	0	0	0	0
62.46423311	117.939787	75.67310575	40.97914484	74.10801768
3.843952807	0	0	1.078398548	3.222087725
4.804941009	0	13.7587465	0	0
0	1.814458261	0	0	0
27.86865785	13.60843696	10.81044368	16.17597823	11.81432166
8.648893816	4.536145653	7.862140857	2.156797097	2.148058484
2.882964605	4.536145653	0	4.313594194	2.148058484
4.804941009	5.443374783	4.913838036	1.078398548	0
0	12.70120783	0	1.078398548	1.074029242
0.960988202	0	4.913838036	0	0
53.8153393	40.82531087	42.25900711	47.44953613	40.81311119
7.687905614	20.86627	3.931070429	5.391992742	4.296116967
11.53185842	13.60843696	3.931070429	3.235195645	5.370146209
24.02470504	15.42289522	5.896605643	20.48957242	5.370146209
0	0	0	0	0
0	5.443374783	2.948302821	0	5.370146209
1.921976404	0.907229131	0	0	0
4.804941009	4.536145653	0	2.156797097	0
5.765929211	3.628916522	3.931070429	0	3.222087725
0	8.165062175	0.982767607	0	0
14.41482303	35.38193609	62.89712686	0	52.62743285
2.882964605	0	0	1.078398548	1.074029242

2.882964605	2.721687392	0.982767607	0	0
0.960988202	0.907229131	1.965535214	0	0
3.843952807	10.88674957	3.931070429	3.235195645	6.444175451
1.921976404	1.814458261	3.931070429	3.235195645	0
11.53185842	7.257833044	0	9.705586936	9.666263176
0	0	0	0	0
0	2.721687392	0	0	1.074029242
0	0	2.948302821	0	0
0	0	0	0	0
1.921976404	6.350603914	0	0	0
22.10272864	13.60843696	25.55195779	39.90074629	15.03640938
29.79063425	49.89760218	22.60365496	20.48957242	54.77549133
16.33679943	11.7939787	12.77597889	16.17597823	4.296116967
2.882964605	0.907229131	2.948302821	2.156797097	0
7.687905614	3.628916522	0.982767607	2.156797097	3.222087725
0	0	0.982767607	0	0
17.29778763	10.88674957	27.517493	11.86238403	15.03640938
10.57087022	6.350603914	7.862140857	4.313594194	4.296116967
2.882964605	3.628916522	0	0	0
0.960988202	6.350603914	0.982767607	0	0
10.57087022	6.350603914	4.913838036	7.548789839	6.444175451
1.921976404	0	0	3.235195645	0
11.53185842	1.814458261	10.81044368	10.78398548	15.03640938
23.06371684	12.70120783	45.20730993	18.33277532	8.592233934
0	0	3.931070429	0	0
2.882964605	7.257833044	4.913838036	2.156797097	0
0	14.51566609	6.87937325	1.078398548	2.148058484
1.921976404	0	2.948302821	0	0
1.921976404	3.628916522	0	0	0
0	0	3.931070429	1.078398548	0
0	0	0	1.078398548	0
3.843952807	4.536145653	3.931070429	1.078398548	6.444175451
6.726917412	3.628916522	0.982767607	1.078398548	0
21.14174044	25.40241565	0.982767607	6.470391291	22.55461408
8.648893816	7.257833044	12.77597889	23.72476807	17.18446787
3.843952807	0	0.982767607	8.627188388	8.592233934
3.843952807	0	0	3.235195645	1.074029242
5.765929211	8.165062175	4.913838036	6.470391291	0
33.63458706	17.23735348	17.68981693	9.705586936	18.25849711
0.960988202	0	0	0	0
0	0	32.43133104	25.88156516	27.92476029
0.960988202	0.907229131	0	0	0
0	0	0	1.078398548	0
5.765929211	0	2.948302821	0	0
39.40051627	43.54699827	53.06945079	47.44953613	52.62743285
38.43952807	36.28916522	54.05221839	34.50875355	51.5534036
2.882964605	0	0	2.156797097	0
3.843952807	3.628916522	0.982767607	4.313594194	4.296116967
0	0	0.982767607	1.078398548	1.074029242
24.02470504	15.42289522	0.982767607	21.56797097	9.666263176

For Review Only

0	0	0	2.156797097	0
191.2366522	176.9096805	162.1566552	167.151775	142.8458892
0	0	0	7.548789839	5.370146209
5.765929211	10.88674957	2.948302821	3.235195645	2.148058484
3.843952807	3.628916522	13.7587465	6.470391291	11.81432166
0	0	0	0	2.148058484
11.53185842	6.350603914	1.965535214	2.156797097	2.148058484
38.43952807	65.3204974	42.25900711	19.41117387	13.96238014
0.960988202	2.721687392	8.844908464	6.470391291	8.592233934
0	2.721687392	0	3.235195645	7.518204692
106.6696904	100.7024335	134.6391622	66.86071	19.33252635
6.726917412	0	0.982767607	2.156797097	2.148058484
0	3.628916522	0	0	0
26.90766965	18.14458261	6.87937325	29.11676081	23.62864332
21.14174044	0	1.965535214	6.470391291	16.11043863
0	0	0.982767607	0	0
4.804941009	3.628916522	5.896605643	4.313594194	4.296116967
4.804941009	5.443374783	0.982767607	1.078398548	1.074029242
0.960988202	4.536145653	4.913838036	0	0
1.921976404	0	0	0	1.074029242
0	0.907229131	0	0	0
13.45383482	21.77349913	15.72428171	5.391992742	21.48058484
19.21976404	0	6.87937325	3.235195645	37.59102346
3.843952807	0	0.982767607	0	0
34.59557526	34.47470696	62.89712686	30.19515936	2.148058484
0	4.536145653	0	0	0
1.921976404	2.721687392	0	0	0
28.82964605	19.05181174	19.65535214	36.66555065	33.29490649
14.41482303	10.88674957	11.79321129	5.391992742	5.370146209
7.687905614	5.443374783	0	2.156797097	4.296116967
0	0	2.948302821	0	0
3.843952807	7.257833044	0	0	0
13.45383482	17.23735348	11.79321129	7.548789839	2.148058484
17.29778763	6.350603914	21.62088736	15.09757968	21.48058484
0	0.907229131	0	2.156797097	0
13.45383482	7.257833044	18.67258454	11.86238403	5.370146209
8.648893816	15.42289522	28.50026061	12.94078258	6.444175451
5.765929211	3.628916522	0.982767607	4.313594194	3.222087725
0	0.907229131	0	0	0
0	12.70120783	6.87937325	2.156797097	1.074029242
94.17684377	87.09399653	64.86266207	49.60633323	51.5534036
16.33679943	23.58795739	4.913838036	28.03836226	21.48058484
10.57087022	8.165062175	11.79321129	8.627188388	13.96238014
0	2.721687392	0	0	3.222087725
24.02470504	29.03133218	15.72428171	7.548789839	5.370146209
0	0.907229131	0	0	0
0.960988202	0	0.982767607	1.078398548	2.148058484
13.45383482	12.70120783	7.862140857	14.01918113	16.11043863
8.648893816	5.443374783	3.931070429	4.313594194	3.222087725
5.765929211	8.165062175	1.965535214	0	7.518204692

For Review Only

2.882964605	0.907229131	1.965535214	1.078398548	0
4.804941009	0	0	0	0
22.10272864	29.93856131	5.896605643	10.78398548	21.48058484
2.882964605	4.536145653	3.931070429	2.156797097	10.74029242
1.921976404	4.536145653	0	3.235195645	0
3.843952807	0	2.948302821	0	7.518204692
22.10272864	22.68072826	34.39686625	19.41117387	37.59102346
1.921976404	1.814458261	0	1.078398548	0
16.33679943	9.072291305	1.965535214	1.078398548	1.074029242
0	9.979520436	9.827676071	0	0
3.843952807	7.257833044	0	2.156797097	6.444175451
0	0	1.965535214	0	1.074029242
79.76202075	70.76387218	62.89712686	73.3311013	89.14442707
1.921976404	2.721687392	0	0	0
3.843952807	5.443374783	1.965535214	3.235195645	2.148058484
0	0	0	0	1.074029242
0	4.536145653	6.87937325	0	4.296116967
0	0	1.965535214	1.078398548	7.518204692
0	0	0	0	0
24.98569325	56.24820609	25.55195779	8.627188388	23.62864332
1.921976404	0	0	6.470391291	0
17.29778763	30.84579044	2.948302821	16.17597823	24.70267256
17.29778763	36.28916522	6.87937325	15.09757968	11.81432166
0	0	0.982767607	0	0
7.687905614	4.536145653	5.896605643	6.470391291	10.74029242
2.882964605	5.443374783	0.982767607	7.548789839	7.518204692
0	8.165062175	0	0	6.444175451
0	12.70120783	11.79321129	3.235195645	2.148058484
11.53185842	10.88674957	14.74151411	4.313594194	5.370146209
0	0	0	0	2.148058484
0	4.536145653	0	0	1.074029242
0.960988202	2.721687392	0	0	4.296116967
0.960988202	1.814458261	0	5.391992742	0
0.960988202	0	0	0	0
3.843952807	2.721687392	0.982767607	0	3.222087725
6.726917412	0	1.965535214	1.078398548	1.074029242
0	0	0	0	0
0	0	0.982767607	0	2.148058484
8.648893816	0.907229131	3.931070429	2.156797097	3.222087725
0.960988202	0	1.965535214	1.078398548	0
6.726917412	5.443374783	2.948302821	0	3.222087725
14.41482303	7.257833044	15.72428171	7.548789839	2.148058484
4.804941009	0	2.948302821	1.078398548	1.074029242
3.843952807	3.628916522	5.896605643	3.235195645	0
0.960988202	0	0	2.156797097	0
1.921976404	8.165062175	6.87937325	4.313594194	0
1.921976404	0	0	0	0
1.921976404	2.721687392	0	1.078398548	3.222087725
2.882964605	2.721687392	0	0	0
0	0	0.982767607	0	0

For Review Only

0.960988202	1.814458261	0	4.313594194	0
0.960988202	0	0	3.235195645	3.222087725
0	1.814458261	2.948302821	0	4.296116967
4.804941009	0.907229131	0	3.235195645	0
0	0	0.982767607	0	1.074029242
10.57087022	0	10.81044368	2.156797097	7.518204692
6.726917412	9.072291305	5.896605643	4.313594194	10.74029242
0	1.814458261	0	0	0
0	0	0.982767607	0	0
6.726917412	0	1.965535214	1.078398548	4.296116967
1.921976404	0.907229131	0	0	0
5.765929211	0	0	0	0
0	0	2.948302821	0	0
6.726917412	0	5.896605643	1.078398548	1.074029242
4.804941009	0	3.931070429	7.548789839	1.074029242
0	0	0.982767607	1.078398548	0
0	0	0	0	2.148058484
5.765929211	4.536145653	0	4.313594194	7.518204692
4.804941009	0.907229131	2.948302821	5.391992742	3.222087725
77.84004434	48.99037305	47.17284514	39.90074629	41.88714043
0	5.443374783	0	0	0
0.960988202	0	0	0	1.074029242
6.726917412	2.721687392	0	6.470391291	1.074029242
0	0	0	0	0
0	4.536145653	0	0	0
11.53185842	20.86627	5.896605643	12.94078258	6.444175451
2.882964605	0	0.982767607	3.235195645	4.296116967
11.53185842	0	1.965535214	3.235195645	5.370146209
4.804941009	0	1.965535214	0	0
6.726917412	10.88674957	1.965535214	8.627188388	9.666263176
24.98569325	15.42289522	4.913838036	6.470391291	9.666263176
20.18075224	11.7939787	6.87937325	6.470391291	2.148058484
14.41482303	9.979520436	0.982767607	11.86238403	7.518204692
1.921976404	3.628916522	0.982767607	0	0
30.75162246	25.40241565	34.39686625	46.37113758	17.18446787
90.33289097	71.67110131	47.17284514	66.86071	78.40413465
0	0	2.948302821	0	0
3.843952807	0	0.982767607	1.078398548	4.296116967
7.687905614	4.536145653	2.948302821	4.313594194	4.296116967
0.960988202	0	1.965535214	3.235195645	1.074029242
29.79063425	37.19639435	36.36240146	37.7439492	38.6650527
5.765929211	5.443374783	0	6.470391291	4.296116967
6.726917412	9.072291305	0.982767607	0	11.81432166
17.29778763	19.05181174	0	0	0
0	0	0	0	0
4.804941009	0.907229131	3.931070429	2.156797097	4.296116967
0	0	2.948302821	0	4.296116967
0	0	0	0	0
11.53185842	20.86627	14.74151411	12.94078258	16.11043863
0	5.443374783	0.982767607	3.235195645	0

For Review Only

0	5.443374783	0	2.156797097	4.296116967
11.53185842	20.86627	2.948302821	2.156797097	2.148058484
0	5.443374783	0.982767607	0	0
15.37581123	11.7939787	11.79321129	12.94078258	23.62864332
4.804941009	0	0	0	3.222087725
1.921976404	1.814458261	0	10.78398548	0
50.93237469	42.63976914	28.50026061	0	28.99878953
4.804941009	0	3.931070429	0	1.074029242
0	0	4.913838036	2.156797097	1.074029242
4.804941009	8.165062175	1.965535214	1.078398548	13.96238014
6.726917412	0	1.965535214	8.627188388	1.074029242
1.921976404	2.721687392	0	0	0
10.57087022	0	0.982767607	1.078398548	1.074029242
32.67359886	19.95904087	20.63811975	9.705586936	28.99878953
6.726917412	8.165062175	8.844908464	10.78398548	8.592233934
20.18075224	15.42289522	15.72428171	7.548789839	5.370146209
10.57087022	15.42289522	8.844908464	5.391992742	9.666263176
59.58126851	48.99037305	10.81044368	39.90074629	37.59102346
5.765929211	0	0	0	0
4.804941009	2.721687392	3.931070429	0	5.370146209
0	0	0	0	0
0	2.721687392	0	0	0
2.882964605	4.536145653	0	2.156797097	2.148058484
23.06371684	24.49518652	7.862140857	25.88156516	7.518204692
7.687905614	7.257833044	4.913838036	1.078398548	1.074029242
45.16644548	29.03133218	22.60365496	40.97914484	37.59102346
108.5916668	71.67110131	118.9148805	94.89907226	105.2548657
0	0	0	1.078398548	0
3.843952807	0.907229131	7.862140857	0	0
6.726917412	5.443374783	7.862140857	2.156797097	8.592233934
0.960988202	0	1.965535214	4.313594194	1.074029242
10.57087022	13.60843696	8.844908464	17.25437678	7.518204692
133.57736	163.3012435	99.25952832	91.66387662	131.0315675
0	3.628916522	0	0	0
0	0	0	0	0
3.843952807	4.536145653	4.913838036	5.391992742	4.296116967
3.843952807	6.350603914	0	3.235195645	1.074029242
37.47853987	39.91808174	21.62088736	24.80316661	50.47937436
0	0	0	1.078398548	5.370146209
42.28348088	38.10362348	34.39686625	16.17597823	36.51699422
0	6.350603914	1.965535214	0	2.148058484
3.843952807	0.907229131	4.913838036	2.156797097	1.074029242
1.921976404	0	0	0	0
0	0	0	1.078398548	0
0	2.721687392	0	0	1.074029242
0	2.721687392	0	1.078398548	0
0	3.628916522	0	0	0
0	0	7.862140857	0	0
0	0	1.965535214	0	1.074029242
5.765929211	15.42289522	8.844908464	3.235195645	2.148058484

For Review Only

3.843952807	5.443374783	4.913838036	3.235195645	16.11043863
0.960988202	6.350603914	0.982767607	6.470391291	2.148058484
1.921976404	0	0	1.078398548	1.074029242
23.06371684	15.42289522	4.913838036	11.86238403	10.74029242
34.59557526	45.36145653	25.55195779	29.11676081	21.48058484
8.648893816	19.95904087	0.982767607	4.313594194	1.074029242
0	3.628916522	0.982767607	0	0
1.921976404	0	0	4.313594194	0
31.71261066	31.75301957	18.67258454	15.09757968	33.29490649
9.609882018	11.7939787	0	5.391992742	7.518204692
0	2.721687392	0.982767607	0	0
2.882964605	4.536145653	2.948302821	0	0
4.804941009	0	0	2.156797097	0
165.2899707	215.0133039	148.3979087	92.74227517	80.55219313
31.71261066	40.82531087	8.844908464	33.430355	20.40655559
95.13783197	51.71206044	53.06945079	32.35195645	27.92476029
1.921976404	5.443374783	0	0	0
3.843952807	1.814458261	3.931070429	1.078398548	7.518204692
2.882964605	0	0	0	0
23.06371684	38.10362348	27.517493	17.25437678	39.73908194
48.04941009	29.03133218	52.08668318	39.90074629	18.25849711
3.843952807	8.165062175	2.948302821	5.391992742	9.666263176
0	0	0	0	0
5.765929211	7.257833044	7.862140857	3.235195645	3.222087725
2.882964605	0	0	0	0
9.609882018	13.60843696	0.982767607	2.156797097	1.074029242
7.687905614	0.907229131	3.931070429	1.078398548	1.074029242
4.804941009	4.536145653	10.81044368	18.33277532	7.518204692
0	0.907229131	0	0	4.296116967
0	0	0	0	0
0	6.350603914	1.965535214	7.548789839	3.222087725
0	0	0	0	3.222087725
0	0	1.965535214	2.156797097	0
0	9.072291305	0.982767607	5.391992742	19.33252635
1.921976404	0	0	0	8.592233934
9.609882018	10.88674957	1.965535214	2.156797097	8.592233934
138.3823011	170.5590765	83.53524661	111.0750505	33.29490649
3.843952807	10.88674957	4.913838036	4.313594194	3.222087725
7.687905614	0	3.931070429	4.313594194	0
0	0	0	0	0
0	3.628916522	0	0	0
0	0.907229131	0	1.078398548	0
180.6657819	168.7446183	134.6391622	282.5404197	184.7330296
7.687905614	4.536145653	2.948302821	0	1.074029242
13.45383482	0	1.965535214	2.156797097	20.40655559
0	0	0	21.56797097	2.148058484
0	8.165062175	0.982767607	0	0
159.5240415	129.7337657	150.3634439	202.7389271	139.6238014
4.804941009	9.072291305	1.965535214	9.705586936	0
17.29778763	26.30964479	6.87937325	3.235195645	7.518204692

For Review Only

0	0	0	5.391992742	2.148058484
2.882964605	1.814458261	0.982767607	3.235195645	1.074029242
53.8153393	20.86627	35.37963386	0	34.36893574
40.36150447	25.40241565	12.77597889	15.09757968	46.1832574
0	8.165062175	0	1.078398548	1.074029242
0.960988202	0.907229131	0	0	4.296116967
57.65929211	45.36145653	40.29347189	63.62551436	39.73908194
0	4.536145653	2.948302821	0	0
10.57087022	9.072291305	10.81044368	5.391992742	6.444175451
13.45383482	5.443374783	5.896605643	6.470391291	6.444175451
70.15213873	82.55785088	75.67310575	64.70391291	54.77549133
0	0	0	1.078398548	0
0.960988202	2.721687392	0.982767607	7.548789839	1.074029242
43.24446908	46.26868566	31.44856343	28.03836226	45.10922815
7.687905614	3.628916522	2.948302821	3.235195645	2.148058484
0.960988202	0	0	0	0
38.43952807	62.59881001	33.41409864	15.09757968	38.6650527
3.843952807	6.350603914	4.913838036	2.156797097	3.222087725
7.687905614	9.979520436	5.896605643	5.391992742	6.444175451
0	0	56.01775361	59.31192017	40.81311119
0	0	38.32793668	36.66555065	28.99878953
4.804941009	0	0	0	3.222087725
34.59557526	17.23735348	22.60365496	19.41117387	27.92476029
12.49284662	0	7.862140857	8.627188388	3.222087725
8.648893816	7.257833044	4.913838036	9.705586936	6.444175451
3.843952807	5.443374783	0.982767607	0	1.074029242
5.765929211	7.257833044	10.81044368	10.78398548	3.222087725
0.960988202	0.907229131	0	2.156797097	2.148058484
34.59557526	27.21687392	13.7587465	32.35195645	25.7767018
0	3.628916522	0.982767607	2.156797097	0
1.921976404	0	2.948302821	0	0
0	6.350603914	0.982767607	12.94078258	1.074029242
3.843952807	2.721687392	5.896605643	7.548789839	4.296116967
2.882964605	0	1.965535214	0	2.148058484
0	4.536145653	0	3.235195645	0
0	0	0	0	0
5.765929211	3.628916522	4.913838036	3.235195645	0
0	0	1.965535214	0	0
12.49284662	7.257833044	9.827676071	8.627188388	9.666263176
0	0	0	0	0
5.765929211	11.7939787	4.913838036	16.17597823	4.296116967
116.2795724	83.46508001	36.36240146	51.76313033	91.29248555
0	0	0	1.078398548	0
0	0	0	0	0
5.765929211	9.072291305	0	0	11.81432166
0	0	0	0	0
0	0	0	1.078398548	5.370146209
3.843952807	0	1.965535214	0	1.074029242
3.843952807	2.721687392	0	0	3.222087725
0	14.51566609	0	1.078398548	1.074029242

For Review Only

14.41482303	13.60843696	7.862140857	8.627188388	8.592233934
0	4.536145653	3.931070429	0	2.148058484
132.6163718	162.3940144	55.034986	74.40949984	83.77428086
23.06371684	23.58795739	15.72428171	17.25437678	11.81432166
2.882964605	2.721687392	0.982767607	0	1.074029242
23.06371684	28.12410305	21.62088736	18.33277532	31.14684801
0	0	2.948302821	0	3.222087725
8.648893816	4.536145653	0.982767607	0	7.518204692
11.53185842	3.628916522	0.982767607	0	6.444175451
0	3.628916522	0	0	0
12.49284662	9.979520436	16.70704932	12.94078258	21.48058484
8.648893816	15.42289522	12.77597889	8.627188388	17.18446787
0.960988202	0	0	0	3.222087725
8.648893816	7.257833044	2.948302821	8.627188388	8.592233934
4.804941009	0	0.982767607	0	3.222087725
0	0	1.965535214	0	2.148058484
0.960988202	0	4.913838036	3.235195645	6.444175451
0	0	0	0	0
11.53185842	0	0.982767607	1.078398548	0
8.648893816	14.51566609	1.965535214	23.72476807	1.074029242
0.960988202	0	0	3.235195645	1.074029242
0	0	6.87937325	18.33277532	7.518204692
0	0	0	0	0
0	0	0	0	1.074029242
7.687905614	3.628916522	4.913838036	7.548789839	5.370146209
2.882964605	0	0	0	0
0	0	2.948302821	3.235195645	3.222087725
6.726917412	0	2.948302821	1.078398548	1.074029242
31.71261066	16.33012435	3.931070429	6.470391291	4.296116967
0	0	0	0	0
113.3966078	108.8674957	34.39686625	0	0
1.921976404	0	0	0	0
16.33679943	22.68072826	21.62088736	31.27355791	39.73908194
0.960988202	1.814458261	0.982767607	0	0
2.882964605	0	0	4.313594194	4.296116967
51.8933629	40.82531087	37.34516907	37.7439492	22.55461408
2.882964605	4.536145653	0.982767607	0	2.148058484
369.0194695	364.7061105	138.5702326	278.2268255	221.2500238
0	0	0.982767607	0	0
3.843952807	3.628916522	0.982767607	1.078398548	1.074029242
3.843952807	6.350603914	0	0	1.074029242
0	0	20.63811975	17.25437678	18.25849711
0	0	3.931070429	0	5.370146209
5.765929211	9.979520436	5.896605643	0	7.518204692
0.960988202	0	2.948302821	2.156797097	1.074029242
0	4.536145653	7.862140857	0	1.074029242
5.765929211	1.814458261	2.948302821	0	0
0	3.628916522	0	0	0
1.921976404	0.907229131	0	0	0
8.648893816	5.443374783	0.982767607	10.78398548	2.148058484

For Review Only

0	1.814458261	0	0	5.370146209
0	4.536145653	0	6.470391291	3.222087725
0.960988202	0.907229131	0	2.156797097	0
2.882964605	0	0.982767607	0	0
0	4.536145653	2.948302821	1.078398548	4.296116967
0.960988202	0	0	0	3.222087725
6.726917412	12.70120783	5.896605643	3.235195645	16.11043863
4.804941009	5.443374783	5.896605643	6.470391291	1.074029242
13.45383482	9.072291305	0	6.470391291	9.666263176
12.49284662	0	1.965535214	1.078398548	3.222087725
1.921976404	0	0	0	1.074029242
0	0	0	0	0
0	0	0.982767607	2.156797097	4.296116967
14.41482303	23.58795739	22.60365496	15.09757968	15.03640938
27.86865785	14.51566609	37.34516907	11.86238403	6.444175451
0	0	5.896605643	0	11.81432166
0	0	0	0	1.074029242
0	0	1.965535214	1.078398548	0
10.57087022	9.072291305	7.862140857	0	1.074029242
0	4.536145653	0	0	0
13.45383482	4.536145653	0.982767607	4.313594194	9.666263176
4.804941009	0	0	0	2.148058484
9.609882018	15.42289522	4.913838036	4.313594194	1.074029242
5.765929211	5.443374783	0	2.156797097	6.444175451
1.921976404	0.907229131	0	1.078398548	0
8.648893816	6.350603914	7.862140857	8.627188388	5.370146209
0.960988202	4.536145653	0.982767607	0	0
15.37581123	12.70120783	0.982767607	4.313594194	2.148058484
11.53185842	12.70120783	10.81044368	4.313594194	4.296116967
3.843952807	1.814458261	3.931070429	3.235195645	4.296116967
0	0	0	0	0
3.843952807	0	1.965535214	4.313594194	2.148058484
7.687905614	9.979520436	5.896605643	17.25437678	22.55461408
9.609882018	0	12.77597889	6.470391291	15.03640938
1.921976404	5.443374783	0	6.470391291	0
0.960988202	4.536145653	0.982767607	2.156797097	2.148058484
34.59557526	29.03133218	23.58642257	16.17597823	25.7767018
24.02470504	17.23735348	9.827676071	16.17597823	23.62864332
0	0	2.948302821	4.313594194	0
29.79063425	37.19639435	7.862140857	16.17597823	2.148058484
21.14174044	13.60843696	6.87937325	10.78398548	21.48058484
0	0	1.965535214	0	1.074029242
4.804941009	0	0.982767607	0	0
24.02470504	44.4542274	18.67258454	12.94078258	23.62864332
19.21976404	11.7939787	26.53472539	26.95996371	27.92476029
2.882964605	0	0	0	0
49.97138649	32.6602487	38.32793668	28.03836226	25.7767018
12.49284662	19.05181174	3.931070429	8.627188388	2.148058484
23.06371684	19.95904087	8.844908464	6.470391291	5.370146209
11.53185842	9.072291305	3.931070429	12.94078258	7.518204692

For Review Only

8.648893816	22.68072826	7.862140857	5.391992742	13.96238014
0.960988202	0	0	3.235195645	0
16.33679943	34.47470696	15.72428171	23.72476807	31.14684801
2.882964605	2.721687392	1.965535214	4.313594194	3.222087725
3.843952807	3.628916522	0	0	3.222087725
0	10.88674957	4.913838036	9.705586936	9.666263176
0	0	0	0	0
2.882964605	6.350603914	0	2.156797097	2.148058484
0	0	0	0	0
0	0	5.896605643	6.470391291	8.592233934
5.765929211	2.721687392	0	1.078398548	7.518204692
24.02470504	25.40241565	27.517493	15.09757968	13.96238014
8.648893816	17.23735348	0	4.313594194	4.296116967
0	4.536145653	0	0	0
4.804941009	4.536145653	5.896605643	3.235195645	2.148058484
2.882964605	0	3.931070429	1.078398548	1.074029242
12.49284662	7.257833044	6.87937325	5.391992742	12.8883509
7.687905614	0	4.913838036	0	0
0	0.907229131	0	2.156797097	0
0.960988202	0.907229131	10.81044368	0	0
3.843952807	0.907229131	0.982767607	1.078398548	0
10.57087022	0	0	0	0
0	0	1.965535214	5.391992742	0
0.960988202	0.907229131	2.948302821	0	0
16.33679943	11.7939787	14.74151411	22.64636952	17.18446787
13.45383482	9.072291305	13.7587465	6.470391291	6.444175451
4.804941009	0	0	0	0
0	3.628916522	0	0	1.074029242
5.765929211	5.443374783	1.965535214	0	5.370146209
0	0	116.9493452	97.05586936	88.07039782
0.960988202	4.536145653	0.982767607	3.235195645	0
2.882964605	0	0	0	0
0	0	0	0	0
5.765929211	7.257833044	6.87937325	3.235195645	1.074029242
89.37190276	115.2180996	100.2422959	76.56629694	93.44054403
3.843952807	2.721687392	0	0	0
14.41482303	5.443374783	2.948302821	0	5.370146209
0	0	0.982767607	2.156797097	2.148058484
0	0	0	0	1.074029242
12.49284662	26.30964479	9.827676071	10.78398548	3.222087725
0	0	0	0	0
13.45383482	3.628916522	10.81044368	7.548789839	13.96238014
0	0.907229131	4.913838036	1.078398548	0
0	2.721687392	0	0	0
49.97138649	51.71206044	12.77597889	39.90074629	20.40655559
229.6761802	119.7542452	121.8631833	281.4620211	229.8422577
21.14174044	79.83616349	29.48302821	88.42868097	65.51578375
34.59557526	50.80483131	54.05221839	26.95996371	35.44296498
0	3.628916522	0	0	0
0	2.721687392	2.948302821	0	3.222087725

For Review Only

14.41482303	14.51566609	5.896605643	14.01918113	10.74029242
3.843952807	5.443374783	2.948302821	4.313594194	4.296116967
0	0	0	0	1.074029242
0	3.628916522	0.982767607	5.391992742	0
7.687905614	4.536145653	1.965535214	4.313594194	2.148058484
14.41482303	18.14458261	9.827676071	14.01918113	5.370146209
0	0	0	1.078398548	1.074029242
7.687905614	4.536145653	4.913838036	19.41117387	2.148058484
26.90766965	19.05181174	14.74151411	18.33277532	23.62864332
0.960988202	0.907229131	0	1.078398548	2.148058484
16.33679943	12.70120783	2.948302821	17.25437678	4.296116967
3.843952807	9.072291305	0.982767607	2.156797097	0
2.882964605	0	0.982767607	0	13.96238014
12.49284662	26.30964479	20.63811975	5.391992742	7.518204692
0	0	0.982767607	4.313594194	0
4.804941009	3.628916522	6.87937325	5.391992742	5.370146209
14.41482303	19.95904087	16.70704932	19.41117387	13.96238014
0	0	0	1.078398548	1.074029242
5.765929211	5.443374783	21.62088736	8.627188388	7.518204692
0	0	0	0	1.074029242
5.765929211	14.51566609	5.896605643	3.235195645	7.518204692
0.960988202	0	1.965535214	3.235195645	0
9.609882018	3.628916522	10.81044368	4.313594194	9.666263176
2.882964605	2.721687392	2.948302821	1.078398548	0
3.843952807	0	0	2.156797097	0
8.648893816	17.23735348	8.844908464	10.78398548	11.81432166
4.804941009	10.88674957	0.982767607	0	2.148058484
17.29778763	28.12410305	10.81044368	11.86238403	9.666263176
0.960988202	0	0	1.078398548	0
0	4.536145653	1.965535214	0	2.148058484
11.53185842	7.257833044	4.913838036	6.470391291	3.222087725
5.765929211	6.350603914	5.896605643	3.235195645	8.592233934
0	0	2.948302821	0	0
15.37581123	7.257833044	12.77597889	4.313594194	3.222087725
3.843952807	1.814458261	3.931070429	3.235195645	1.074029242
6.726917412	7.257833044	0	5.391992742	8.592233934
6.726917412	4.536145653	5.896605643	5.391992742	5.370146209
26.90766965	15.42289522	23.58642257	18.33277532	22.55461408
13.45383482	8.165062175	6.87937325	2.156797097	10.74029242
0.960988202	0	1.965535214	1.078398548	1.074029242
0	51.71206044	22.60365496	25.88156516	0
9.609882018	3.628916522	18.67258454	2.156797097	5.370146209
37.47853987	39.91808174	42.25900711	37.7439492	26.85073104
0	0	0	1.078398548	0
18.25877583	9.979520436	0	2.156797097	5.370146209
3.843952807	9.979520436	0	0	0
0	0	0	17.25437678	0
3.843952807	0	0	0	0
6.726917412	2.721687392	3.931070429	4.313594194	5.370146209
2.882964605	0.907229131	0	1.078398548	0

For Review Only

0	104.33135	0	128.3294273	107.4029242
1.921976404	0.907229131	0	0	0
5.765929211	8.165062175	14.74151411	5.391992742	7.518204692
4.804941009	0.907229131	2.948302821	3.235195645	2.148058484
0.960988202	0.907229131	0	1.078398548	1.074029242
0	9.072291305	0.982767607	2.156797097	1.074029242
3.843952807	2.721687392	0	1.078398548	6.444175451
19.21976404	16.33012435	16.70704932	20.48957242	26.85073104
0	0	2.948302821	0	1.074029242
0.960988202	1.814458261	0	0	0
22.10272864	15.42289522	21.62088736	20.48957242	27.92476029
5.765929211	7.257833044	7.862140857	9.705586936	6.444175451
5.765929211	6.350603914	5.896605643	15.09757968	2.148058484
0	0	0	0	1.074029242
8.648893816	14.51566609	3.931070429	10.78398548	9.666263176
11.53185842	13.60843696	12.77597889	11.86238403	13.96238014
24.02470504	0.907229131	1.965535214	1.078398548	21.48058484
82.64498535	54.43374783	43.24177471	51.76313033	84.8483101
4.804941009	9.979520436	5.896605643	4.313594194	0
0	0	0	0	0
0	0	0	0	0
9.609882018	9.072291305	8.844908464	7.548789839	2.148058484
11.53185842	0	2.948302821	19.41117387	1.074029242
38.43952807	42.63976914	16.70704932	16.17597823	47.25728664
4.804941009	4.536145653	0	4.313594194	0
6.726917412	5.443374783	12.77597889	7.548789839	7.518204692
45.16644548	36.28916522	32.43133104	43.13594194	48.33131588
14.41482303	8.165062175	7.862140857	9.705586936	12.8883509
0	0	0.982767607	0	3.222087725
4.804941009	3.628916522	3.931070429	4.313594194	3.222087725
0.960988202	0	0.982767607	0	0
3.843952807	0	0.982767607	1.078398548	4.296116967
0	95.25905871	0	230.7772894	0
7.687905614	12.70120783	12.77597889	9.705586936	12.8883509
17.29778763	17.23735348	18.67258454	20.48957242	20.40655559
1.921976404	0	4.913838036	0	1.074029242
8.648893816	5.443374783	6.87937325	9.705586936	12.8883509
6.726917412	3.628916522	5.896605643	4.313594194	5.370146209
2.882964605	0	4.913838036	0	0
0	0	0	0	1.074029242
0	0	0	0	0
3.843952807	7.257833044	7.862140857	7.548789839	7.518204692
0	9.072291305	0.982767607	7.548789839	12.8883509
37.47853987	54.43374783	43.24177471	21.56797097	30.07281877
118.2015488	120.6614744	138.5702326	106.7614563	160.030357
0	0	0	0	0
0.960988202	0	0.982767607	0	0
0	0	32.43133104	48.52793468	46.1832574
5.765929211	10.88674957	6.87937325	9.705586936	10.74029242
25.94668145	34.47470696	32.43133104	34.50875355	40.81311119

For Review Only

2.882964605	9.979520436	0.982767607	4.313594194	9.666263176
0.960988202	0	0.982767607	0	1.074029242
0	0	0	3.235195645	0
41.32249268	53.5265187	42.25900711	44.21434049	47.25728664
0	0	0	0	0
6.726917412	12.70120783	12.77597889	9.705586936	13.96238014
0	0	0	0	0
0	0	0	0	0
28.82964605	37.19639435	21.62088736	7.548789839	61.21966678
9.609882018	15.42289522	16.70704932	16.17597823	17.18446787
0	0	14.74151411	16.17597823	19.33252635
39.40051627	46.26868566	48.15561275	48.52793468	48.33131588
56.6983039	60.78435175	41.2762395	31.27355791	53.70146209
1.921976404	8.165062175	0	0	10.74029242
0	0	0	0	0
22.10272864	20.86627	22.60365496	12.94078258	20.40655559
6.726917412	6.350603914	6.87937325	4.313594194	8.592233934
10.57087022	9.072291305	11.79321129	15.09757968	11.81432166
17.29778763	17.23735348	14.74151411	15.09757968	10.74029242
4.804941009	0	2.948302821	7.548789839	5.370146209
5.765929211	5.443374783	5.896605643	5.391992742	7.518204692
127.8114308	141.5277444	152.3289791	138.0350142	180.4369126
7.687905614	7.257833044	11.79321129	9.705586936	9.666263176
5.765929211	0.907229131	2.948302821	5.391992742	4.296116967
6.726917412	8.165062175	6.87937325	7.548789839	8.592233934
22.10272864	23.58795739	19.65535214	24.80316661	26.85073104
0.960988202	0.907229131	2.948302821	0	0
6.726917412	4.536145653	0	7.548789839	3.222087725
40.36150447	41.73254	51.10391557	37.7439492	47.25728664
0	0	0.982767607	1.078398548	28.99878953
0	0	0.982767607	0	1.074029242
0.960988202	0	0.982767607	5.391992742	1.074029242
0.960988202	1.814458261	3.931070429	0	4.296116967
3.843952807	1.814458261	0.982767607	0	1.074029242
47.08842189	42.63976914	25.55195779	0	0
3.843952807	0.907229131	1.965535214	5.391992742	4.296116967
0	0.907229131	0	3.235195645	0
102.8257376	147.8783483	125.7942537	83.03668823	108.4769534
10.57087022	3.628916522	4.913838036	4.313594194	7.518204692
3.843952807	4.536145653	2.948302821	1.078398548	3.222087725
5.765929211	0.907229131	6.87937325	3.235195645	2.148058484
104.747714	135.1771404	135.6219298	130.4862244	132.1055967
6.726917412	0	1.965535214	2.156797097	7.518204692
10.57087022	6.350603914	9.827676071	15.09757968	8.592233934
19.21976404	13.60843696	15.72428171	16.17597823	19.33252635
4.804941009	0.907229131	2.948302821	2.156797097	3.222087725
21.14174044	35.38193609	3.931070429	10.78398548	17.18446787
11.53185842	6.350603914	3.931070429	4.313594194	7.518204692
22.10272864	9.979520436	28.50026061	30.19515936	31.14684801
66.30818592	74.3927887	75.67310575	65.78231146	76.25607616

For Review Only

29.79063425	45.36145653	41.2762395	39.90074629	27.92476029
24.02470504	19.05181174	19.65535214	21.56797097	16.11043863
53.8153393	32.6602487	55.034986	38.82234774	32.22087725
0	4.536145653	0	3.235195645	0
34.59557526	47.17591479	41.2762395	59.31192017	39.73908194
4.804941009	7.257833044	8.844908464	8.627188388	8.592233934
1.921976404	1.814458261	2.948302821	3.235195645	1.074029242
39.40051627	40.82531087	43.24177471	39.90074629	47.25728664
0	0.907229131	0	1.078398548	0
0	0	0	0	4.296116967
19.21976404	16.33012435	20.63811975	23.72476807	16.11043863
49.01039829	52.61928957	73.70757054	61.46871726	64.44175451
4.804941009	10.88674957	7.862140857	8.627188388	7.518204692
0	2.721687392	3.931070429	3.235195645	3.222087725
49.97138649	61.69158088	55.034986	62.54711581	85.92233934
25.94668145	21.77349913	14.74151411	16.17597823	27.92476029
1.921976404	0	0	0	3.222087725
3.843952807	1.814458261	0	3.235195645	1.074029242
5.765929211	8.165062175	3.931070429	15.09757968	6.444175451
2.882964605	0	0	0	0
18.25877583	23.58795739	11.79321129	34.50875355	30.07281877
2.882964605	2.721687392	0	4.313594194	7.518204692
0.960988202	0.907229131	0	0	0
7.687905614	7.257833044	6.87937325	9.705586936	3.222087725
32.67359886	25.40241565	29.48302821	28.03836226	35.44296498
0	0	0	0	1.074029242
2.882964605	0	0.982767607	0	0
0.960988202	2.721687392	0	2.156797097	0
88.41091456	88.90845479	55.034986	61.46871726	64.44175451
87.44992636	94.35182958	129.7253241	84.11508678	125.6614213
0.960988202	0	2.948302821	3.235195645	7.518204692
1.921976404	2.721687392	4.913838036	4.313594194	0
2.882964605	0.907229131	0.982767607	1.078398548	0
29.79063425	34.47470696	47.17284514	32.35195645	45.10922815
4.804941009	6.350603914	5.896605643	3.235195645	7.518204692
12.49284662	18.14458261	21.62088736	16.17597823	26.85073104
7.687905614	8.165062175	14.74151411	11.86238403	15.03640938
11.53185842	18.14458261	0.982767607	1.078398548	3.222087725
0	0.907229131	0	1.078398548	1.074029242
3.843952807	1.814458261	0	0	0
8.648893816	10.88674957	10.81044368	16.17597823	19.33252635
3.843952807	1.814458261	2.948302821	7.548789839	3.222087725
64.38620952	62.59881001	54.05221839	75.48789839	59.0716083
148.9531713	168.7446183	244.7091342	229.6988908	258.8410473
0	0.907229131	0.982767607	0	1.074029242
0.960988202	2.721687392	0.982767607	1.078398548	1.074029242
0.960988202	4.536145653	3.931070429	4.313594194	4.296116967
3.843952807	6.350603914	6.87937325	4.313594194	4.296116967
0	3.628916522	0	3.235195645	1.074029242
1.921976404	1.814458261	2.948302821	1.078398548	0

For Review Only

92.25486737	98.88797523	102.2078311	97.05586936	133.179626
67.26917412	73.48555957	76.65587336	54.99832597	69.81190071
1.921976404	4.536145653	5.896605643	5.391992742	0
0	0	0	0	0
0.960988202	4.536145653	0.982767607	4.313594194	3.222087725
59.58126851	71.67110131	59.94882404	54.99832597	54.77549133
25.94668145	38.10362348	27.517493	30.19515936	35.44296498
5.765929211	7.257833044	6.87937325	8.627188388	8.592233934
0.960988202	0	2.948302821	2.156797097	0
4.804941009	10.88674957	4.913838036	7.548789839	6.444175451
12.49284662	22.68072826	6.87937325	6.470391291	7.518204692
3.843952807	0	3.931070429	1.078398548	0
0.960988202	5.443374783	0	0	0
28.82964605	34.47470696	39.31070429	43.13594194	38.6650527
5.765929211	6.350603914	2.948302821	5.391992742	10.74029242
3.843952807	3.628916522	2.948302821	3.235195645	6.444175451
9.609882018	11.7939787	11.79321129	12.94078258	10.74029242
141.2652657	114.3108704	88.44908464	90.58547807	153.5861816
1.921976404	0	0	1.078398548	9.666263176
0	2.721687392	0	0	0
2.882964605	1.814458261	2.948302821	0	0
0	0	0	3.235195645	0
45.16644548	38.10362348	44.22454232	47.44953613	41.88714043
73.03510333	87.09399653	90.41461986	93.82067372	122.4393336
99.94277298	111.5891831	98.27676071	129.4078258	148.2160354
1.921976404	0.907229131	0	0	0
4.804941009	2.721687392	7.862140857	3.235195645	0
24.02470504	33.56747783	54.05221839	21.56797097	20.40655559
6.726917412	0	0	2.156797097	6.444175451
2.882964605	5.443374783	0.982767607	0	0
0.960988202	2.721687392	0.982767607	1.078398548	2.148058484
12.49284662	21.77349913	3.931070429	10.78398548	7.518204692
57.65929211	64.41326827	93.36292268	125.0942316	103.1068072
3.843952807	8.165062175	2.948302821	1.078398548	9.666263176
271.9596611	291.2205509	288.9336765	218.9149053	267.4332812
5.765929211	6.350603914	7.862140857	8.627188388	4.296116967
21.14174044	34.47470696	36.36240146	21.56797097	23.62864332
7.687905614	9.072291305	14.74151411	8.627188388	8.592233934
16.33679943	7.257833044	1.965535214	2.156797097	9.666263176
2.882964605	6.350603914	5.896605643	3.235195645	0
9.609882018	3.628916522	3.931070429	7.548789839	2.148058484
3.843952807	0	0	0	0
0	0	0	0	0
0	0	3.931070429	6.470391291	1.074029242
4.804941009	10.88674957	2.948302821	8.627188388	2.148058484
123.0064898	78.92893436	101.2250635	84.11508678	83.77428086
1.921976404	0.907229131	0	0	0
22.10272864	15.42289522	27.517493	2.156797097	27.92476029
19.21976404	29.93856131	11.79321129	54.99832597	23.62864332
181.6267701	207.7554709	137.587465	227.5420937	187.9551173

For Review Only

25.94668145	30.84579044	15.72428171	40.97914484	12.8883509
163.3679943	101.6096626	76.65587336	117.5454418	85.92233934
152.7971241	121.5687035	2.948302821	1.078398548	27.92476029
9.609882018	233.1578865	0	16.17597823	156.8082693
0	0	8.844908464	3.235195645	5.370146209
0	0	56.01775361	62.54711581	67.66384223
0	0	58.96605643	0	47.25728664
145.1092185	167.8373891	206.3811975	193.0333402	173.9927372
24.98569325	155.1361813	24.56919018	31.27355791	210.5097314
120.1235252	104.33135	112.0355072	101.3694636	106.3288949
0	0	8.844908464	1.078398548	1.074029242
153.7581123	9.979520436	54.05221839	58.23352162	0
377.6683633	362.8916522	228.9848525	332.1467529	306.0983339
3.843952807	3.628916522	0	0	0
725.5460923	755.7218657	919.8704803	1113.985701	685.2306562
274.8426257	307.5506753	173.9498665	368.8123036	596.0862292
131.6553836	255.8386148	66.82819729	72.25270275	11.81432166
10.57087022	0.907229131	5.896605643	2.156797097	15.03640938
0.960988202	2.721687392	8.844908464	0	0
159.5240415	180.538597	149.3806763	248.0316661	155.7342401
56.6983039	102.5168918	6.87937325	0	158.9563278
2.882964605	0	4.913838036	1.078398548	0
216.2223454	184.1675135	536.5911135	600.6679915	612.1966678
13.45383482	24.49518652	32.43133104	21.56797097	8.592233934
3.843952807	11.7939787	13.7587465	23.72476807	12.8883509
3.843952807	6.350603914	3.931070429	1.078398548	1.074029242
12.49284662	17.23735348	32.43133104	14.01918113	8.592233934
87.44992636	119.7542452	109.0872044	87.35028243	63.36772526
31.71261066	0	0	0	1128.804733
0	0	0	0	0
130.6943954	125.19762	83.53524661	153.1325939	90.21845631
187.3926993	192.3325757	216.2088736	236.1692821	185.8070588
1.921976404	2.721687392	0	3.235195645	0
122.0455016	108.8674957	144.4668382	163.9165794	139.6238014
17.29778763	21.77349913	9.827676071	2.156797097	24.70267256
65.34719772	99.79520436	115.9665776	202.7389271	114.9211289
30.75162246	50.80483131	0.982767607	8.627188388	28.99878953
117.2405606	171.4663057	120.8804157	167.151775	181.5109419
39.40051627	32.6602487	37.34516907	34.50875355	35.44296498
41.32249268	32.6602487	49.13838036	48.52793468	25.7767018
0	0	0	0	0
213.3393808	185.9819718	211.2950355	174.7005649	219.1019653
0	209.5699292	98.27676071	130.4862244	11.81432166
63.42522132	39.01085261	32.43133104	35.5871521	77.33010541
28.82964605	18.14458261	22.60365496	19.41117387	16.11043863
49.97138649	24.49518652	21.62088736	30.19515936	26.85073104
0	0	0	11.86238403	0
0.960988202	0	0	3.235195645	4.296116967
1439.560326	1408.019611	1539.99684	1276.823881	1675.485617
82.64498535	59.87712262	130.7080917	86.27188388	41.88714043

For Review Only

159.5240415	125.19762	135.6219298	125.0942316	88.07039782
72.07411513	71.67110131	59.94882404	37.7439492	23.62864332
148.9531713	99.79520436	114.0010424	145.583804	144.9939476
65.34719772	77.1144761	98.27676071	84.11508678	60.14563754
95.13783197	125.19762	134.6391622	158.5245866	120.2912751
99.94277298	96.16628784	87.46631704	131.5646229	107.4029242
57.65929211	58.06266435	40.29347189	25.88156516	33.29490649
4073.628987	2618.263271	3710.930485	3431.464181	3257.53069
47.08842189	46.26868566	42.25900711	50.68473178	68.73787147
80.72300895	104.33135	126.7770213	81.95828968	75.18204692
295.0233779	370.1494853	82.552479	332.1467529	238.4344917
169.1339235	112.4964122	163.1394228	170.3869707	126.7354505
15.37581123	17.23735348	28.50026061	43.13594194	36.51699422
13.45383482	28.12410305	16.70704932	14.01918113	11.81432166
121.0845134	70.76387218	75.67310575	140.1918113	53.70146209
228.715192	253.1169274	282.0543032	370.9691007	257.767018
119.162537	134.2699113	38.32793668	78.72309404	61.21966678
153.7581123	174.1879931	281.0715356	187.6413474	1.074029242
38.43952807	50.80483131	104.1733664	69.0175071	59.0716083
41.32249268	43.54699827	56.01775361	46.37113758	30.07281877
3.843952807	4.536145653	13.7587465	7.548789839	3.222087725
2.882964605	0	0	0	0
89.37190276	56.24820609	93.36292268	65.78231146	61.21966678
0	0	0	0	0
12.49284662	24.49518652	0	0	0
67.26917412	64.41326827	37.34516907	51.76313033	64.44175451
66.30818592	56.24820609	43.24177471	88.42868097	50.47937436
26.90766965	38.10362348	38.32793668	44.21434049	22.55461408
1.921976404	0	0	0	0
159.5240415	170.5590765	198.5190566	189.7981445	140.6978307
32.67359886	54.43374783	49.13838036	65.78231146	51.5534036
16.33679943	36.28916522	28.50026061	32.35195645	22.55461408
94.17684377	97.9807461	82.552479	108.9182534	81.62622237
96.09882018	109.7747248	98.27676071	70.09590565	77.33010541
24.02470504	23.58795739	5.896605643	16.17597823	60.14563754
3.843952807	20.86627	15.72428171	16.17597823	15.03640938
0	0	0.982767607	1.078398548	1.074029242
0.960988202	2.721687392	0	0	0
93.21585557	97.9807461	111.0527396	81.95828968	118.1432166
57.65929211	40.82531087	42.25900711	42.05754339	63.36772526
0	0	0	0	0
60.54225671	88.00122566	39.31070429	19.41117387	35.44296498
0	0	3.931070429	0	0
0	0	2.948302821	4.313594194	0
141.2652657	136.0843696	138.5702326	75.48789839	154.6602108
54.7763275	0	31.44856343	1.078398548	2.148058484
136.4603247	180.538597	168.0532608	150.9757968	127.8094798
0	28.12410305	7.862140857	5.391992742	4.296116967
0	0.907229131	1.965535214	2.156797097	0
43.24446908	63.50603914	40.29347189	54.99832597	66.58981299

For Review Only

124.9284662	105.2385791	258.4678807	132.6430215	198.6954097
47.08842189	83.46508001	44.22454232	40.97914484	35.44296498
5.765929211	16.33012435	13.7587465	0	0
5.765929211	9.072291305	2.948302821	2.156797097	3.222087725
40.36150447	107.9602665	53.06945079	90.58547807	84.8483101
0	0	40.29347189	43.13594194	41.88714043
0	40.82531087	1.965535214	63.62551436	15.03640938
210.4564162	212.2916165	246.6746694	200.58213	172.9187079
79.76202075	92.53737131	168.0532608	180.0925576	123.5133628
0	0	0	0	0
43.24446908	11.7939787	42.25900711	0	25.7767018
0	0	212.2778031	7.548789839	20.40655559
66.30818592	0	0	0	0
1.921976404	0.907229131	0.982767607	0	0
414.185915	301.2000713	469.7629162	387.1450789	383.4284393
8.648893816	1.814458261	0.982767607	3.235195645	11.81432166
2.882964605	0	2.948302821	3.235195645	0
446.8595138	294.8494674	381.3138316	214.6013111	270.6553689
149.9141595	171.4663057	116.9493452	91.66387662	105.2548657
148.9531713	159.672327	125.7942537	160.6813837	147.1420061
0.960988202	0	0	2.156797097	2.148058484
4.804941009	3.628916522	0	4.313594194	8.592233934
276.7646021	199.5904087	122.8459509	225.3852966	209.4357021
0	0	0	2.156797097	0
5.765929211	11.7939787	3.931070429	4.313594194	0
49.97138649	68.94941392	80.58694379	58.23352162	28.99878953
0	0	0	0	0
321.9310476	507.141084	498.2631768	468.02497	510.1638898
0	0	9.827676071	4.313594194	5.370146209
7.687905614	0	0	1.078398548	0
0.960988202	0	1.965535214	0	2.148058484
8.648893816	0.907229131	6.87937325	5.391992742	1.074029242
0	0	0	0	0
2.882964605	0	0	0	0
3338.473013	3676.999666	4630.800965	4129.188042	4727.876722
73.03510333	49.89760218	38.32793668	16.17597823	3.222087725
4.804941009	6.350603914	5.896605643	7.548789839	7.518204692
3.843952807	0	0	0	3.222087725
12.49284662	13.60843696	10.81044368	24.80316661	15.03640938
8.648893816	13.60843696	3.931070429	1.078398548	1.074029242
0		0	0	3.222087725
16.33679943	24.49518652	4.913838036	3.235195645	20.40655559
314.243142	0	408.8313246	2.156797097	121.3653043
75.91806794	48.08314392	61.91435925	60.39031871	37.59102346
73.03510333	164.2084726	150.3634439	132.6430215	125.6614213
74.95707974	55.34097696	82.552479	47.44953613	44.03519891
0	0	3.931070429	0	0
3.843952807	4.536145653	0	4.313594194	0
0	165.1157018	0	0	1.074029242
0	0	0	0	0

For Review Only

86.48893816	100.7024335	116.9493452	80.87989113	45.10922815
1.921976404	0	3.931070429	0	0
0	0	7.862140857	29.11676081	0
3.843952807	4.536145653	4.913838036	8.627188388	0
24.02470504	32.6602487	21.62088736	54.99832597	20.40655559
0	63.50603914	0	0	0
0	0	0	0	0
0	94.35182958	21.62088736	0	30.07281877
0	0	0	0	0
0	0	3.931070429	0	0
260.4278027	274.8904266	150.3634439	276.0700284	208.3616729
45.16644548	60.78435175	28.50026061	6.470391291	13.96238014
1.921976404	2.721687392	0	0	7.518204692
51.8933629	40.82531087	16.70704932	18.33277532	31.14684801
60.54225671	74.3927887	0	338.6171442	278.1735736
0.960988202	0	0	0	0
110.5136432	83.46508001	144.4668382	174.7005649	166.4745325
0	87.09399653	255.5195779	0	0
5.765929211	6.350603914	0.982767607	3.235195645	2.148058484
2.882964605	0	0	0	0
26.90766965	19.05181174	21.62088736	17.25437678	23.62864332
1.921976404	0	0	0	0
0	0	0	0	0
0.960988202	0	0	0	0
0	9.979520436	9.827676071	10.78398548	0
142.2262539	450.8928779	382.2965992	488.5145425	7.518204692
0.960988202	0.907229131	10.81044368	3.235195645	4.296116967
67.26917412	91.63014218	116.9493452	103.5262607	42.96116967
5.765929211	8.165062175	3.931070429	3.235195645	6.444175451
15.37581123	6.350603914	3.931070429	3.235195645	8.592233934
0.960988202	27.21687392	12.77597889	16.17597823	10.74029242
2.882964605	0	0	0	0
2.882964605	2.721687392	2.948302821	5.391992742	5.370146209
1529.893217	904.5074431	1444.668382	2918.146472	0
0	0	0	36.66555065	26.85073104
40.36150447	104.33135	55.034986	66.86071	23.62864332
54.7763275	41.73254	51.10391557	36.66555065	63.36772526
4.804941009	0.907229131	3.931070429	2.156797097	8.592233934
28.82964605	50.80483131	101.2250635	44.21434049	31.14684801
222.9492628	198.6831796	169.0360284	200.58213	165.4005032
0	0	4.913838036	2.156797097	2.148058484
4.804941009	48.08314392	0	339.6955428	0
94.17684377	124.2903909	82.552479	81.95828968	77.33010541
25.94668145	15.42289522	23.58642257	16.17597823	36.51699422
27.86865785	19.95904087	37.34516907	12.94078258	25.7767018
2.882964605	3.628916522	0	0	0
280.6085549	343.8398405	306.6234934	303.0299921	287.8398368
70.15213873	38.10362348	73.70757054	71.1743042	68.73787147
0	0	31.44856343	9.705586936	0
85.52794996	102.5168918	95.32845789	60.39031871	154.6602108

For Review Only

0	0	3.931070429	0	0
8.648893816	15.42289522	32.43133104	3.235195645	12.8883509
25.94668145	33.56747783	27.517493	35.5871521	18.25849711
0	0	8.844908464	0	0
22.10272864	20.86627	15.72428171	10.78398548	12.8883509
0.960988202	0	21.62088736	0	32.22087725
6.726917412	19.05181174	8.844908464	3.235195645	8.592233934
0	0	0	0	0
11.53185842	14.51566609	21.62088736	4.313594194	1.074029242
0	0	16.70704932	6.470391291	15.03640938
0	0	0.982767607	0	0
32.67359886	57.15543522	58.96605643	36.66555065	49.40534512
0	0	0.982767607	0	0
74.95707974	64.41326827	105.156134	50.68473178	93.44054403
16.33679943	21.77349913	25.55195779	23.72476807	31.14684801
0	0	4.913838036	0	0
8.648893816	27.21687392	4.913838036	7.548789839	10.74029242
10.57087022	18.14458261	5.896605643	31.27355791	15.03640938
36.51755167	22.68072826	24.56919018	32.35195645	34.36893574
22.10272864	7.257833044	37.34516907	43.13594194	22.55461408
49.97138649	44.4542274	38.32793668	64.70391291	35.44296498
212.3783926	206.8482418	144.4668382	78.72309404	210.5097314
73.03510333	57.15543522	59.94882404	43.13594194	62.29369602
67.26917412	64.41326827	75.67310575	70.09590565	82.70025162
68.23016233	54.43374783	95.32845789	64.70391291	61.21966678
41.32249268	48.08314392	50.12114796	53.91992742	52.62743285
47.08842189	60.78435175	79.60417618	60.39031871	80.55219313
40.36150447	44.4542274	74.69033814	47.44953613	48.33131588
44.20545728	60.78435175	45.20730993	53.91992742	57.99757905
320.0090712	266.7253644	290.8992117	347.2443326	434.9818429
0	14.51566609	16.70704932	6.470391291	2.148058484
0.960988202	0.907229131	0	0	0
24.02470504	30.84579044	26.53472539	26.95996371	40.81311119
326.7359886	306.6434461	296.7958174	299.7947965	274.9514859
25.94668145	31.75301957	32.43133104	31.27355791	12.8883509
55.7373157	59.87712262	75.67310575	101.3694636	79.47816389
29.79063425	37.19639435	62.89712686	56.07672452	45.10922815
111.4746314	121.5687035	82.552479	113.2318476	47.25728664
97.05980838	129.7337657	40.29347189	335.3819486	138.5497722
100.9037612	72.57833044	81.56971139	75.48789839	62.29369602
53.8153393	42.63976914	51.10391557	36.66555065	46.1832574
37.47853987	22.68072826	64.86266207	56.07672452	33.29490649
29.79063425	7.257833044	94.34569029	0	82.70025162
131.6553836	176.0024513	132.673627	122.9374345	78.40413465
9.609882018	19.95904087	33.41409864	11.86238403	13.96238014
60.54225671	89.81568392	154.2945143	115.3886447	63.36772526
0	0	0	0	0
96.09882018	71.67110131	103.1905987	133.72142	156.8082693
0	0	0	0	0
46.12743368	23.58795739	40.29347189	28.03836226	32.22087725

For Review Only

0	0	0	3.235195645	0
9.609882018	11.7939787	8.844908464	15.09757968	24.70267256
4.804941009	4.536145653	6.87937325	5.391992742	5.370146209
5.765929211	19.95904087	0	0	17.18446787
0	0	0.982767607	1.078398548	0
0	0	2.948302821	0	1.074029242
34.59557526	32.6602487	11.79321129	1.078398548	3.222087725
38.43952807	37.19639435	38.32793668	39.90074629	54.77549133
59.58126851	205.0337835	98.27676071	86.27188388	106.3288949
122.0455016	88.90845479	41.2762395	60.39031871	94.51457327
47.08842189	80.74339262	54.05221839	38.82234774	44.03519891
68.23016233	71.67110131	74.69033814	53.91992742	78.40413465
29.79063425	10.88674957	34.39686625	2.156797097	21.48058484
58.62028031	31.75301957	101.2250635	42.05754339	33.29490649
0	5.443374783	5.896605643	5.391992742	10.74029242
237.3640858	233.1578865	238.8125285	303.0299921	234.1383747
27.86865785	48.08314392	36.36240146	25.88156516	52.62743285
42.28348088	30.84579044	45.20730993	85.19348533	95.58860252
0	0	23.58642257	195.1901373	272.8034274
2.882964605	6.350603914	3.931070429	2.156797097	7.518204692
0	0	969.9916282	0	0
435.3276554	497.1615635	581.7984234	411.9482455	614.3447263
264.2717555	298.4783839	304.6579582	338.6171442	273.8774566
23.06371684	29.03133218	58.96605643	35.5871521	75.18204692
0	0	41.2762395	51.76313033	65.51578375
1.921976404	0	0	0	0
721.7021395	168.7446183	944.4396705	803.4069186	330.8010065
398.8101037	0	0.982767607	0	1.074029242
0	105.2385791	285.9853737	2.156797097	1.074029242
149.9141595	58.96989348	106.1389016	75.48789839	17.18446787
24.02470504	24.49518652	37.34516907	33.430355	30.07281877
0	0	2.948302821	6.470391291	6.444175451
22.10272864	15.42289522	29.48302821	17.25437678	21.48058484
26.90766965	33.56747783	16.70704932	19.41117387	17.18446787
14.41482303	0	0.982767607	15.09757968	3.222087725
0	0	29.48302821	39.90074629	13.96238014
19.21976404	39.01085261	40.29347189	18.33277532	23.62864332
8.648893816	18.14458261	32.43133104	23.72476807	9.666263176
9.609882018	17.23735348	0	0	0
31.71261066	52.61928957	120.8804157	0	0
0	9.072291305	23.58642257	0	24.70267256
36.51755167	39.01085261	33.41409864	66.86071	67.66384223
0	0	63.87989446	39.90074629	46.1832574
11.53185842	20.86627	2.948302821	7.548789839	17.18446787
158.5630533	176.9096805	150.3634439	126.1726302	300.7281877
4.804941009	10.88674957	0.982767607	2.156797097	1.074029242
0	0	0	18.33277532	0
79.76202075	88.00122566	90.41461986	91.66387662	80.55219313
12.49284662	19.95904087	20.63811975	29.11676081	26.85073104
5.765929211	1.814458261	5.896605643	3.235195645	13.96238014

For Review Only

134.5383482	119.7542452	121.8631833	160.6813837	114.9211289
30.75162246	45.36145653	25.55195779	4.313594194	2.148058484
6.726917412	5.443374783	0.982767607	5.391992742	9.666263176
0	3.628916522	6.87937325	8.627188388	7.518204692
40.36150447	42.63976914	57.98328882	49.60633323	63.36772526
224.8712392	184.1675135	280.088768	211.3661155	154.6602108
41.32249268	64.41326827	66.82819729	62.54711581	0
24.02470504	11.7939787	29.48302821	0	13.96238014
3.843952807	8.165062175	0	0	0
54.7763275	82.55785088	65.84542968	103.5262607	56.92354981
154.7191005	177.8169096	192.622451	168.2301736	147.1420061
111.4746314	97.07351697	126.7770213	109.9966519	104.1808365
73.99609154	122.4759326	136.6046974	128.3294273	160.030357
20.18075224	0	16.70704932	32.35195645	0
4.804941009	9.072291305	2.948302821	5.391992742	5.370146209
26.90766965	66.22772653	59.94882404	33.430355	32.22087725
73.03510333	59.87712262	123.8287185	81.95828968	82.70025162
72.07411513	87.09399653	73.70757054	120.7806374	98.81069024
9.609882018	12.70120783	0	0	4.296116967
0	51.71206044	52.08668318	85.19348533	88.07039782
13.45383482	21.77349913	14.74151411	15.09757968	18.25849711
40.36150447	26.30964479	37.34516907	32.35195645	39.73908194
37.47853987	49.89760218	38.32793668	42.05754339	50.47937436
3.843952807	0	0	0	0
28.82964605	0	18.67258454	0	17.18446787
26.90766965	24.49518652	29.48302821	10.78398548	24.70267256
25.94668145	22.68072826	27.517493	60.39031871	17.18446787
24.98569325	9.979520436	2.948302821	14.01918113	37.59102346
0	473.5736061	823.5592548	121.859036	2082.5427
25.94668145	0	0	0	0
9.609882018	7.257833044	8.844908464	8.627188388	12.8883509
269.0766965	275.7976557	303.6751906	191.9549416	163.2524447
72.07411513	65.3204974	70.75926771	65.78231146	66.58981299
56.6983039	30.84579044	20.63811975	33.430355	27.92476029
41.32249268	29.03133218	50.12114796	35.5871521	26.85073104
48.04941009	805.6194679	70.75926771	33.430355	33.29490649
0	0	0	0	0
56.6983039	86.1867674	56.01775361	63.62551436	68.73787147
515.0896761	519.8422918	597.5227051	587.7272089	588.5680245
374.7853987	395.5519009	487.4527331	539.1992742	438.2039306
11.53185842	6.350603914	8.844908464	10.78398548	7.518204692
20.18075224	0	5.896605643	0	0
38.43952807	40.82531087	50.12114796	85.19348533	76.25607616
0	0	0	89.50707952	13.96238014
56.6983039	58.06266435	38.32793668	28.03836226	68.73787147
23.06371684	0	0	15.09757968	28.99878953
94.17684377	62.59881001	50.12114796	95.97747081	26.85073104
59.58126851	62.59881001	56.01775361	31.27355791	54.77549133
126.8504426	56.24820609	103.1905987	105.6830577	33.29490649
28.82964605	46.26868566	32.43133104	48.52793468	28.99878953

For Review Only

3.843952807	0	0	4.313594194	4.296116967
53.8153393	33.56747783	12.77597889	6.470391291	12.8883509
54.7763275	30.84579044	39.31070429	28.03836226	30.07281877
0	8.165062175	2.948302821	0	2.148058484
0	6.350603914	16.70704932	7.548789839	5.370146209
19.21976404	6.350603914	25.55195779	6.470391291	16.11043863
0	50.80483131	48.15561275	57.15512307	0
129.7334072	166.0229309	90.41461986	18.33277532	67.66384223
78.80103254	64.41326827	43.24177471	108.9182534	89.14442707
56.6983039	55.34097696	65.84542968	71.1743042	62.29369602
0	7.257833044	7.862140857	10.78398548	2.148058484
343.072788	303.0145296	299.7441202	418.4186368	345.8374158
118.2015488	107.9602665	54.05221839	115.3886447	110.6250119
22.10272864	52.61928957	45.20730993	23.72476807	38.6650527
0	0	6.87937325	0	0
277.7255903	277.6121139	126.7770213	213.5229126	215.8798776
48.04941009	52.61928957	26.53472539	34.50875355	30.07281877
105.7087022	102.5168918	73.70757054	70.09590565	85.92233934
26.90766965	19.95904087	26.53472539	11.86238403	40.81311119
1.921976404	3.628916522	0.982767607	0	0
48.04941009	66.22772653	43.24177471	60.39031871	96.66263176
245.0519914	179.6313678	184.7603101	184.4061518	221.2500238
8.648893816	0	0	5.391992742	1.074029242
101.8647494	56.24820609	23.58642257	54.99832597	12.8883509
0	3.628916522	1.965535214	0	0
8.648893816	0	0.982767607	3.235195645	3.222087725
26.90766965	41.73254	5.896605643	86.27188388	4.296116967
0	0.907229131	0	1.078398548	0
17.29778763	21.77349913	24.56919018	3.235195645	11.81432166
269.0766965	195.9614922	192.622451	240.4828763	215.8798776
11.53185842	3.628916522	7.862140857	17.25437678	21.48058484
16.33679943	38.10362348	38.32793668	45.29273904	26.85073104
1.921976404	0	5.896605643	0	3.222087725
366.1365049	317.5301957	238.8125285	410.869847	209.4357021
763.0246322	924.466484	523.8151346	675.0774913	512.3119483
17.29778763	15.42289522	7.862140857	8.627188388	6.444175451
154.7191005	127.0120783	101.2250635	141.2702098	68.73787147
49.01039829	41.73254	39.31070429	32.35195645	51.5534036
172.0168881	322.0663413	637.816177	533.8072815	0
54.7763275	56.24820609	81.56971139	39.90074629	77.33010541
5.765929211	9.072291305	2.948302821	7.548789839	15.03640938
0	0	0.982767607	0	2.148058484
0	5.443374783	2.948302821	0	3.222087725
1765.335327	2221.804141	2672.145124	2604.332495	2065.358232
21305.10843	23434.63567	28458.0016	21398.6624	27018.27961
24.02470504	18.14458261	40.29347189	24.80316661	10.74029242
19.21976404	28.12410305	23.58642257	12.94078258	46.1832574
3.843952807	5.443374783	4.913838036	4.313594194	5.370146209
0	0	0	0	0
155.6800887	92.53737131	157.2428171	170.3869707	573.5316151

For Review Only

0	0.907229131	1.965535214	0	4.296116967
0	0	13.7587465	0	12.8883509
66.30818592	152.4144939	208.3467327	81.95828968	0
2.882964605	44.4542274	0	105.6830577	0
25.94668145	14.51566609	3.931070429	26.95996371	20.40655559
9.609882018	0.907229131	235.8642257	0	0
1.921976404	3.628916522	0	1.078398548	0
55.7373157	68.94941392	88.44908464	90.58547807	93.44054403
22.10272864	17.23735348	37.34516907	32.35195645	15.03640938
1.921976404	0	0	0	0
35.55656347	39.01085261	14.74151411	25.88156516	30.07281877
0	0	9.827676071	0	0
253.7008853	228.6217409	237.8297609	260.9724487	207.2876437
58.62028031	68.04218479	60.93159164	64.70391291	59.0716083
0	0	0	1.078398548	2.148058484
29.79063425	25.40241565	24.56919018	104.6046592	41.88714043
0	3.628916522	0	0	0
63.42522132	0	0	130.4862244	0
10.57087022	11.7939787	24.56919018	29.11676081	10.74029242
47.08842189	39.01085261	70.75926771	60.39031871	56.92354981
50.93237469	71.67110131	89.43185225	86.27188388	100.9587487
0	0	0	8.627188388	0
0	17.23735348	12.77597889	0	6.444175451
14.41482303	12.70120783	15.72428171	1.078398548	10.74029242
5.765929211	5.443374783	2.948302821	2.156797097	3.222087725
203.7294988	172.3735348	87.46631704	24.80316661	141.7718599
196.0415932	117.0325578	150.3634439	159.6029852	205.1395852
73.03510333	93.44460045	16.70704932	43.13594194	38.6650527
0	0	0.982767607	0	0
30.75162246	30.84579044	41.2762395	11.86238403	5.370146209
0	0	0	0	0
0	0.907229131	0	0	2.148058484
13.45383482	10.88674957	28.50026061	16.17597823	11.81432166
5.765929211	7.257833044	7.862140857	0	0
46.12743368	34.47470696	49.13838036	18.33277532	39.73908194
1.921976404	0.907229131	0.982767607	1.078398548	1.074029242
3.843952807	5.443374783	10.81044368	8.627188388	8.592233934
8.648893816	18.14458261	18.67258454	11.86238403	16.11043863
6.726917412	10.88674957	21.62088736	19.41117387	17.18446787
3.843952807	6.350603914	6.87937325	6.470391291	8.592233934
1.921976404	2.721687392	6.87937325	5.391992742	4.296116967
2.882964605	2.721687392	7.862140857	6.470391291	5.370146209
4.804941009	3.628916522	9.827676071	6.470391291	6.444175451
2.882964605	4.536145653	6.87937325	7.548789839	7.518204692
3.843952807	4.536145653	8.844908464	7.548789839	7.518204692
2.882964605	9.072291305	13.7587465	8.627188388	7.518204692
3.843952807	5.443374783	9.827676071	6.470391291	7.518204692
19.21976404	26.30964479	50.12114796	38.82234774	44.03519891
16.33679943	18.14458261	36.36240146	28.03836226	28.99878953
0.960988202	0	1.965535214	3.235195645	1.074029242

For Review Only

25.94668145	40.82531087	61.91435925	54.99832597	44.03519891
24.98569325	28.12410305	30.46579582	20.48957242	24.70267256
2.882964605	1.814458261	4.913838036	4.313594194	1.074029242
26.90766965	44.4542274	57.98328882	23.72476807	31.14684801
2.882964605	0.907229131	5.896605643	4.313594194	3.222087725
3.843952807	1.814458261	2.948302821	3.235195645	4.296116967
1.921976404	4.536145653	5.896605643	4.313594194	5.370146209
0.960988202	1.814458261	0.982767607	1.078398548	0
0	0.907229131	0.982767607	3.235195645	0
1.921976404	5.443374783	4.913838036	6.470391291	5.370146209
0.960988202	0.907229131	0.982767607	0	1.074029242
3.843952807	0.907229131	8.844908464	5.391992742	4.296116967
3.843952807	3.628916522	8.844908464	5.391992742	5.370146209
0.960988202	5.443374783	2.948302821	1.078398548	2.148058484
0.960988202	0.907229131	2.948302821	0	0
2.882964605	5.443374783	0.982767607	1.078398548	4.296116967
1.921976404	4.536145653	5.896605643	1.078398548	0
4.804941009	14.51566609	3.931070429	5.391992742	4.296116967
1.921976404	3.628916522	0.982767607	3.235195645	1.074029242
0	1.814458261	0.982767607	0	0
0.960988202	0.907229131	0	0	3.222087725
0.960988202	3.628916522	2.948302821	0	2.148058484
0.960988202	1.814458261	0.982767607	0	3.222087725
6.726917412	11.7939787	10.81044368	3.235195645	6.444175451
10.57087022	19.95904087	10.81044368	17.25437678	10.74029242
0	0.907229131	0.982767607	0	0
2.882964605	7.257833044	5.896605643	1.078398548	5.370146209
5.765929211	8.165062175	8.844908464	4.313594194	12.8883509
0	0	0	0	1.074029242
0	3.628916522	1.965535214	0	2.148058484
0	0.907229131	3.931070429	7.548789839	2.148058484
0	2.721687392	3.931070429	2.156797097	1.074029242
27.86865785	54.43374783	71.74203532	37.7439492	49.40534512
15.37581123	34.47470696	46.19007754	23.72476807	34.36893574
3.843952807	4.536145653	6.87937325	4.313594194	4.296116967
1.921976404	3.628916522	5.896605643	1.078398548	3.222087725
2.882964605	2.721687392	0	6.470391291	1.074029242
20.18075224	39.01085261	44.22454232	15.09757968	15.03640938
6.726917412	7.257833044	8.844908464	8.627188388	8.592233934
7.687905614	5.443374783	10.81044368	8.627188388	5.370146209
5.765929211	2.721687392	5.896605643	4.313594194	3.222087725
4.804941009	3.628916522	0	7.548789839	0
54.7763275	78.02170523	89.43185225	166.0733765	135.3276845
0.960988202	0.907229131	0.982767607	0	0
4.804941009	12.70120783	6.87937325	1.078398548	9.666263176
138.3823011	105.2385791	116.9493452	127.2510287	133.179626
24.02470504	19.05181174	12.77597889	14.01918113	16.11043863
605.4225671	635.9676205	550.34986	608.2167813	680.9345393
221.0272864	149.6928065	219.1571764	173.6221663	143.9199184
43.24446908	74.3927887	54.05221839	40.97914484	31.14684801

For Review Only

547.763275	432.7482953	547.4015572	548.9048612	498.3495682
85.52794996	22.68072826	0	98.13426791	103.1068072
38.43952807	29.03133218	33.41409864	16.17597823	27.92476029
30.75162246	10.88674957	4.913838036	11.86238403	15.03640938
146.0702067	124.2903909	112.0355072	104.6046592	95.58860252
22.10272864	25.40241565	13.7587465	15.09757968	4.296116967
177.7828173	124.2903909	84.51801421	111.0750505	122.4393336
2.882964605	5.443374783	63.87989446	0	0
37.47853987	25.40241565	29.48302821	25.88156516	39.73908194
0	0	0	0	6.444175451
0.960988202	64.41326827	221.1227116	0	0
62.46423311	52.61928957	148.3979087	3.235195645	9.666263176
36.51755167	47.17591479	52.08668318	26.95996371	40.81311119
31.71261066	40.82531087	35.37963386	34.50875355	31.14684801
2.882964605	1.814458261	2.948302821	4.313594194	1.074029242
267.1547201	220.4566787	341.0203597	422.732231	358.7257667
68.23016233	76.20724696	73.70757054	66.86071	62.29369602
18.25877583	25.40241565	18.67258454	10.78398548	12.8883509
1.921976404	0	0.982767607	0	1.074029242
120.1235252	111.5891831	276.1576976	225.3852966	183.6590003
63.42522132	64.41326827	49.13838036	42.05754339	38.6650527
84.56696176	81.65062175	73.70757054	72.25270275	26.85073104
0	0	3.931070429	0	0
93.21585557	127.9193074	130.7080917	114.3102461	136.4017137
0	0	0	0	0
19.21976404	0	0.982767607	1.078398548	23.62864332
0	0.907229131	0	3.235195645	3.222087725
86.48893816	121.5687035	124.8114861	145.583804	109.5509827
57.65929211	54.43374783	77.63864096	61.46871726	69.81190071
0.960988202	9.979520436	103.1905987	0	0
0	3.628916522	0	0	1.074029242
38.43952807	45.36145653	49.13838036	89.50707952	54.77549133
23.06371684	52.61928957	0.982767607	0	0
1.921976404	0	0.982767607	0	0
194.1196168	236.7868031	306.6234934	301.9515936	292.1359538
30.75162246	11.7939787	162.1566552	0	0
98.02079658	88.90845479	101.2250635	9.705586936	134.2536552
21.14174044	20.86627	15.72428171	10.78398548	34.36893574
0	0	0	0	9.666263176
52.8543511	0	304.6579582	0	694.8969194
89.37190276	79.83616349	52.08668318	71.1743042	77.33010541
0	0	0	0	33.29490649
41.32249268	100.7024335	208.3467327	0	190.1031758
49.97138649	38.10362348	61.91435925	57.15512307	70.88592996
0	27.21687392	51.10391557	56.07672452	20.40655559
296.9453543	319.3446539	335.123754	333.2251515	293.209983
0	0	0	152.0541953	0
167.2119471	113.4036413	168.0532608	170.3869707	156.8082693
23.06371684	0	0	0	0
547.763275	609.6579757	343.9686625	851.9348533	880.7039782

For Review Only

90.33289097	38.10362348	22.60365496	2.156797097	88.07039782
23.06371684	37.19639435	25.55195779	19.41117387	10.74029242
90.33289097	35.38193609	225.053782	111.0750505	121.3653043
153.7581123	139.7132861	147.4151411	127.2510287	128.883509
234.4811212	234.0651157	135.6219298	271.7564342	415.6493166
94.17684377	87.09399653	149.3806763	85.19348533	140.6978307
21.14174044	39.01085261	42.25900711	36.66555065	55.84952057
4.804941009	3.628916522	14.74151411	2.156797097	3.222087725
9.609882018	18.14458261	26.53472539	17.25437678	22.55461408
13.45383482	1.814458261	22.60365496	0	0
28.82964605	44.4542274	33.41409864	47.44953613	16.11043863
47.08842189	67.13495566	53.06945079	49.60633323	50.47937436
229.6761802	186.8892009	142.501303	243.718072	223.3980823
0	0	0	0	0
0	0	5.896605643	0	2.148058484
273.8816375	281.2410305	289.9164441	428.1242237	337.2451819
0	0	0	0	0
85.52794996	90.72291305	111.0527396	140.1918113	84.8483101
0	0	0	0	0
73.03510333	46.26868566	74.69033814	67.93910855	76.25607616
640.0181424	545.2447075	709.5582124	824.9748896	823.7804284
531.4264756	685.8652227	848.128445	0	407.0570826
27.86865785	33.56747783	0.982767607	6.470391291	30.07281877
117.2405606	173.2807639	157.2428171	148.8189997	220.1759946
92.25486737	157.8578687	136.6046974	109.9966519	91.29248555
561.2171098	579.7194144	569.0224445	455.0841874	510.1638898
0	0	0	0	0
93.21585557	100.7024335	144.4668382	86.27188388	83.77428086
29.79063425	52.61928957	70.75926771	64.70391291	51.5534036
9.609882018	0	11.79321129	11.86238403	19.33252635
69.19115053	93.44460045	74.69033814	91.66387662	64.44175451
0	0	372.4689231	307.3435863	303.9502754
36.51755167	48.08314392	24.56919018	29.11676081	52.62743285
0	0	0	0	0
1.921976404	0	0	0	0
43.24446908	29.93856131	40.29347189	61.46871726	32.22087725
77.84004434	45.36145653	77.63864096	80.87989113	113.8470996
0	0	5.896605643	0	0
184.5097347	259.4675313	227.0193172	334.30355	230.916287
0	0	0	0	0
13.45383482	12.70120783	24.56919018	16.17597823	0
0	3.628916522	0	0	3.222087725
4.804941009	4.536145653	4.913838036	5.391992742	10.74029242
34.59557526	43.54699827	45.20730993	60.39031871	41.88714043
27.86865785	42.63976914	37.34516907	9.705586936	13.96238014
135.4993364	145.1566609	137.587465	117.5454418	139.6238014
224.8712392	293.9422383	280.088768	431.3594194	268.5073104
7.687905614	9.072291305	3.931070429	0	5.370146209
0.960988202	0.907229131	0.982767607	1.078398548	1.074029242
0	0	0	0	0

For Review Only

157.6020651	196.8687213	361.6584794	146.6622026	157.8822985
34.59557526	24.49518652	59.94882404	35.5871521	26.85073104
388.2392335	0	1.965535214	7.548789839	1.074029242
49.01039829	44.4542274	37.34516907	42.05754339	40.81311119
15.37581123	12.70120783	17.68981693	11.86238403	6.444175451
0	0	0	0	0
57.65929211	69.85664305	178.8637045	144.5054055	115.9951581
976.364013	1229.295472	0	0	0
1400.15981	1670.208829	1671.6877	2417.769546	1967.621571
15.37581123	9.072291305	21.62088736	11.86238403	4.296116967
132.6163718	101.6096626	110.069972	95.97747081	105.2548657
98.98178478	99.79520436	91.39738746	14.01918113	83.77428086
4.804941009	13.60843696	85.50078182	48.52793468	2.148058484
1.921976404	4.536145653	42.25900711	21.56797097	3.222087725
3.843952807	6.350603914	1.965535214	2.156797097	1.074029242
3.843952807	16.33012435	28.50026061	1.078398548	19.33252635
156.6410769	186.8892009	230.9503877	208.1309199	189.0291465
9.609882018	6.350603914	3.931070429	11.86238403	4.296116967
732.2730097	1071.437603	2477.557138	2493.257444	1587.415219
479.5331127	0	1402.409375	3532.833645	274.9514859
157.6020651	159.672327	138.5702326	140.1918113	165.4005032
0	0	3.931070429	0	0
0	0	0	0	0
34.59557526	29.93856131	38.32793668	39.90074629	49.40534512
7.687905614	19.95904087	14.74151411	5.391992742	8.592233934
0	0	0.982767607	1.078398548	1.074029242
0	5.443374783	0.982767607	1.078398548	2.148058484
64.38620952	72.57833044	32.43133104	33.430355	86.99636858
29.79063425	50.80483131	43.24177471	34.50875355	54.77549133
78.80103254	64.41326827	155.2772819	153.1325939	77.33010541
52.8543511	0	19.65535214	163.9165794	59.0716083
0.960988202	0.907229131	3.931070429	0	0
51.8933629	54.43374783	74.69033814	219.9933039	62.29369602
12.49284662	9.072291305	18.67258454	8.627188388	16.11043863
34.59557526	57.15543522	61.91435925	25.88156516	60.14563754
23.06371684	13.60843696	22.60365496	14.01918113	11.81432166
0.960988202	3.628916522	3.931070429	0	0
0	0	0.982767607	5.391992742	0
0.960988202	1.814458261	0	0	0
47.08842189	37.19639435	38.32793668	43.13594194	25.7767018
167.2119471	117.939787	109.0872044	120.7806374	129.9575383
37.47853987	28.12410305	39.31070429	22.64636952	57.99757905
0.960988202	0	0.982767607	2.156797097	0
516.0506643	529.8218122	394.0898105	368.8123036	447.8701938
689.9895289	635.0603914	926.7498535	765.6629694	561.7172934
0	0	0	3.235195645	0
38.43952807	84.37230914	82.552479	107.8398548	52.62743285
2.882964605	2.721687392	3.931070429	4.313594194	4.296116967
0	0	0	1.078398548	1.074029242
0	11.7939787	12.77597889	5.391992742	2.148058484

For Review Only

22.10272864	40.82531087	10.81044368	37.7439492	24.70267256
8.648893816	0	5.896605643	3.235195645	1.074029242
111.4746314	126.1048491	136.6046974	163.9165794	66.58981299
0	0	25.55195779	11.86238403	7.518204692
16.33679943	13.60843696	2.948302821	0	0
0	0	72.72480293	36.66555065	18.25849711
176.8218291	183.2602844	83.53524661	28.03836226	67.66384223
52.8543511	63.50603914	65.84542968	23.72476807	65.51578375
14.41482303	18.14458261	12.77597889	15.09757968	10.74029242
27.86865785	49.89760218	8.844908464	15.09757968	7.518204692
1.921976404	2.721687392	0	0	0
34.59557526	19.05181174	6.87937325	16.17597823	17.18446787
244.0910032	371.0567144	417.676233	393.6154702	280.3216321
0.960988202	6.350603914	0	1.078398548	0
99.94277298	70.76387218	90.41461986	66.86071	85.92233934
44.20545728	41.73254	78.62140857	20.48957242	23.62864332
44.20545728	48.99037305	54.05221839	50.68473178	44.03519891
127.8114308	124.2903909	153.3117467	97.05586936	115.9951581
6.726917412	0	0.982767607	4.313594194	0
246.0129797	0	38.32793668	1.078398548	2.148058484
11.53185842	4.536145653	44.22454232	46.37113758	0
0	0	0	0	0
0	0	10.81044368	0	0
85.52794996	121.5687035	64.86266207	62.54711581	55.84952057
96.09882018	100.7024335	104.1733664	63.62551436	117.0691874
32.67359886	79.83616349	65.84542968	69.0175071	136.4017137
53.8153393	61.69158088	71.74203532	70.09590565	67.66384223
65.34719772	12.70120783	174.9326341	154.2109924	9.666263176
0	0	0	478.8089555	0
0.960988202	2.721687392	3.931070429	5.391992742	10.74029242
49.01039829	48.08314392	0	0	0
123.0064898	122.4759326	110.069972	116.4670432	127.8094798
217.1833336	231.3434283	0	0	0
126.8504426	264.003677	555.263698	189.7981445	126.7354505
0	0	0	0	0
36.51755167	1.814458261	159.2083524	166.0733765	0
98.98178478	123.3831618	244.7091342	152.0541953	206.2136144
0	0	21.62088736	0	1.074029242
23.06371684	14.51566609	17.68981693	16.17597823	16.11043863
0.960988202	0	0	0	2.148058484
15.37581123	27.21687392	13.7587465	28.03836226	30.07281877
0.960988202	0	0	1.078398548	2.148058484
0	107.9602665	31.44856343	73.3311013	98.81069024
0	0	0	12.94078258	9.666263176
0	0	0.982767607	431.3594194	1.074029242
0	0	0	0	0
7.687905614	0	8.844908464	11.86238403	5.370146209
54.7763275	85.27953827	39.31070429	29.11676081	88.07039782
10.57087022	0	2.948302821	28.03836226	11.81432166
25.94668145	8.165062175	35.37963386	44.21434049	24.70267256

For Review Only

61.50324491	0	2.948302821	43.13594194	63.36772526
74.95707974	0	2.948302821	2.156797097	1.074029242
0	0	0	0	0
3.843952807	4.536145653	0	0	0
5.765929211	0	0	7.548789839	0
11.53185842	5.443374783	23.58642257	30.19515936	38.6650527
0	3.628916522	0	0	0
358.4485993	316.6229666	385.244902	428.1242237	337.2451819
309.438201	183.2602844	274.1921624	350.4795282	260.9891057
2455.324856	2622.799416	3050.510653	3824.001253	2634.59373
547.763275	606.0290592	815.6971139	697.7238609	649.7876913
60.54225671	25.40241565	63.87989446	66.86071	61.21966678
12.49284662	16.33012435	19.65535214	16.17597823	16.11043863
5.765929211	24.49518652	0	19.41117387	34.36893574
82.64498535	48.08314392	81.56971139	59.31192017	56.92354981
75.91806794	111.5891831	106.1389016	76.56629694	80.55219313
0	0	0	0	0
9.609882018	15.42289522	15.72428171	17.25437678	13.96238014
4.804941009	7.257833044	20.63811975	20.48957242	16.11043863
10.57087022	9.979520436	1.965535214	10.78398548	10.74029242
33.63458706	41.73254	29.48302821	28.03836226	56.92354981
84.56696176	83.46508001	112.0355072	101.3694636	78.40413465
12.49284662	0	0	0	4.296116967
15.37581123	13.60843696	14.74151411	21.56797097	11.81432166
15.37581123	4.536145653	3.931070429	8.627188388	11.81432166
7.687905614	9.072291305	18.67258454	4.313594194	13.96238014
0	29.93856131	16.70704932	9.705586936	23.62864332
95.13783197	92.53737131	95.32845789	107.8398548	138.5497722
0	0	1.965535214	0	0
4.804941009	0	1.965535214	0	0
99.94277298	60.78435175	77.63864096	52.84152887	61.21966678
0.960988202	17.23735348	23.58642257	1.078398548	0
13.45383482	16.33012435	21.62088736	12.94078258	13.96238014
47.08842189	31.75301957	18.67258454	50.68473178	34.36893574
0	0	1.965535214	0	0
1.921976404	0	0	0	0
6.726917412	0	21.62088736	0	0
79.76202075	49.89760218	73.70757054	74.40949984	63.36772526
0	5.443374783	2.948302821	0	2.148058484
19.21976404	51.71206044	29.48302821	20.48957242	13.96238014
10.57087022	4.536145653	14.74151411	11.86238403	17.18446787
12.49284662	0	6.87937325	6.470391291	1.074029242
61.50324491	0.907229131	16.70704932	0	0
0	0	13.7587465	37.7439492	1.074029242
16.33679943	15.42289522	7.862140857	0	0
0	3.628916522	0.982767607	0	0
152.7971241	183.2602844	194.5879862	186.5629489	222.324053
0	0	0	3.235195645	1.074029242
58.62028031	42.63976914	158.2255847	245.874869	0
36.51755167	24.49518652	49.13838036	24.80316661	44.03519891

For Review Only

92.25486737	105.2385791	83.53524661	46.37113758	96.66263176
52.8543511	81.65062175	101.2250635	74.40949984	56.92354981
0	5.443374783	2.948302821	0	0
173.9388645	154.2289522	123.8287185	124.0158331	171.8446787
597.7346615	645.0399118	352.813571	491.7497381	584.2719075
25.94668145	27.21687392	25.55195779	36.66555065	25.7767018
53.8153393	37.19639435	45.20730993	49.60633323	36.51699422
2783.021832	0	705.6271419	3404.504217	8.592233934
232.5591448	238.6012613	568.0396769	180.0925576	544.5328256
32.67359886	0	28.50026061	35.5871521	10.74029242
8.648893816	8.165062175	13.7587465	12.94078258	10.74029242
14.41482303	12.70120783	9.827676071	16.17597823	12.8883509
560.2561216	501.6977092	458.9524725	472.3385642	491.9053927
72.07411513	70.76387218	66.82819729	76.56629694	52.62743285
23.06371684	27.21687392	25.55195779	23.72476807	18.25849711
535.2704284	613.2868922	626.0229657	662.1367088	663.7500714
0	0	0	2.156797097	0
0	783.8459688	0.982767607	557.5320496	393.0947025
1.921976404	8.165062175	0	4.313594194	0
1.921976404	2.721687392	8.844908464	1.078398548	10.74029242
67.26917412	5.443374783	0	57.15512307	0
0	0	0.982767607	26.95996371	1.074029242
12.49284662	0	4.913838036	1.078398548	3.222087725
290.2184369	260.3747605	307.606261	255.580456	265.2852227
8.648893816	5.443374783	0.982767607	3.235195645	16.11043863
3.843952807	0	2.948302821	0	0
0	0	0	2.156797097	0
0.960988202	4.536145653	0	0	4.296116967
31.71261066	0	0.982767607	28.03836226	60.14563754
0	13.60843696	0	0	1.074029242
167.2119471	214.1060748	152.3289791	180.0925576	156.8082693
2.882964605	0	0.982767607	0	0
0	6.350603914	0	0	3.222087725
459.3523604	476.2952935	212.2778031	402.2426586	423.1675213
7.687905614	2.721687392	7.862140857	4.313594194	7.518204692
16.33679943	19.95904087	16.70704932	11.86238403	18.25849711
0	7.257833044	0	0	0
0	0	0	0	0
0	1052.385791	154.2945143	23.72476807	88.07039782
23.06371684	20.86627	21.62088736	16.17597823	16.11043863
733.2339979	643.2254535	450.1075641	737.6246071	489.7573342
1040.750223	772.9592192	793.093459	376.3610934	658.3799252
66.30818592	64.41326827	77.63864096	53.91992742	53.70146209
18.25877583	19.95904087	23.58642257	26.95996371	21.48058484
34.59557526	7.257833044	24.56919018	24.80316661	22.55461408
39.40051627	0	47.17284514	46.37113758	0
287.3354723	216.8277622	279.1060004	219.9933039	226.62017
0	0	0	0	0
71.11312693	56.24820609	95.32845789	99.21266646	77.33010541
53.8153393	26.30964479	37.34516907	44.21434049	21.48058484

For Review Only

0	59.87712262	0.982767607	1.078398548	5.370146209
10.57087022	11.7939787	18.67258454	6.470391291	20.40655559
221.9882746	272.1687392	289.9164441	249.1100647	267.4332812
48.04941009	51.71206044	71.74203532	64.70391291	78.40413465
0	0	0	0	69.81190071
84.56696176	114.3108704	128.7425565	91.66387662	132.1055967
3.843952807	12.70120783	6.87937325	11.86238403	11.81432166
197.0025814	154.2289522	144.4668382	141.2702098	195.473322
16.33679943	10.88674957	9.827676071	2.156797097	25.7767018
0	6.350603914	0	0	1.074029242
0	0	0	0	0
0	0	0	0	1.074029242
7.687905614	11.7939787	0.982767607	5.391992742	21.48058484
16.33679943	17.23735348	20.63811975	11.86238403	17.18446787
73.03510333	107.9602665	96.3112255	65.78231146	129.9575383
3.843952807	2.721687392	6.87937325	0	0
19.21976404	35.38193609	28.50026061	20.48957242	23.62864332
6.726917412	9.072291305	0	0	0
44.20545728	44.4542274	31.44856343	52.84152887	26.85073104
8.648893816	7.257833044	5.896605643	11.86238403	9.666263176
40.36150447	47.17591479	87.46631704	42.05754339	61.21966678
49.97138649	70.76387218	102.2078311	26.95996371	25.7767018
0.960988202	9.072291305	0	0	0
119.162537	151.5072648	170.018796	311.6571805	68.73787147
35.55656347	33.56747783	21.62088736	24.80316661	28.99878953
0	0	0	0	0
73.03510333	78.92893436	87.46631704	56.07672452	88.07039782
202.7685106	179.6313678	127.7597889	141.2702098	195.473322
0	0	5.896605643	6.470391291	8.592233934
51.8933629	85.27953827	40.29347189	93.82067372	61.21966678
22.10272864	27.21687392	57.98328882	31.27355791	37.59102346
246.0129797	212.2916165	219.1571764	245.874869	204.0655559
247.9349561	298.4783839	192.622451	173.6221663	225.5461408
0	0	10.81044368	2.156797097	1.074029242
0	15.42289522	0	1.078398548	0
2.882964605	1.814458261	0	139.1134128	222.324053
102.8257376	97.07351697	88.44908464	125.0942316	95.58860252
49.97138649	73.48555957	67.81096489	33.430355	84.8483101
139.3432893	258.5603022	53.06945079	73.3311013	90.21845631
54.7763275	49.89760218	63.87989446	74.40949984	65.51578375
36.51755167	35.38193609	22.60365496	26.95996371	27.92476029
92.25486737	118.8470161	92.38015507	120.7806374	111.6990411
275.8036139	221.3639079	171.9843312	229.6988908	214.8058484
0	0	0.982767607	1.078398548	1.074029242
77.84004434	92.53737131	81.56971139	86.27188388	85.92233934
63.42522132	48.08314392	51.10391557	54.99832597	66.58981299
45.16644548	53.5265187	16.70704932	33.430355	51.5534036
156.6410769	166.93016	223.0882468	107.8398548	125.6614213
99.94277298	88.00122566	144.4668382	65.78231146	129.9575383
62.46423311	45.36145653	75.67310575	87.35028243	74.10801768

11.53185842	5.443374783	11.79321129	24.80316661	25.7767018
58.62028031	56.24820609	60.93159164	30.19515936	38.6650527
27.86865785	19.95904087	32.43133104	40.97914484	45.10922815
60.54225671	63.50603914	53.06945079	33.430355	35.44296498
18.25877583	14.51566609	11.79321129	5.391992742	10.74029242
36.51755167	36.28916522	56.01775361	56.07672452	48.33131588
0	0	0.982767607	358.0283181	1.074029242
0	22.68072826	9.827676071	5.391992742	1.074029242
50.93237469	51.71206044	5.896605643	17.25437678	36.51699422
68.23016233	51.71206044	41.2762395	52.84152887	70.88592996
108.5916668	98.88797523	70.75926771	72.25270275	126.7354505
207.5734516	51.71206044	173.9498665	185.4845503	106.3288949
26.90766965	119.7542452	165.104958	21.56797097	22.55461408
123.967478	130.6409948	116.9493452	155.289391	88.07039782
129.7334072	99.79520436	114.0010424	120.7806374	136.4017137
58.62028031	55.34097696	46.19007754	76.56629694	42.96116967
216.2223454	182.3530552	228.9848525	162.8381808	96.66263176
22.10272864	41.73254	5.896605643	5.391992742	18.25849711
25.94668145	29.03133218	30.46579582	36.66555065	10.74029242
24.02470504	18.14458261	2.948302821	26.95996371	19.33252635
27.86865785	36.28916522	20.63811975	24.80316661	24.70267256
5.765929211	3.628916522	3.931070429	2.156797097	6.444175451
72.07411513	59.87712262	54.05221839	49.60633323	40.81311119
0	0	0	2.156797097	0
5.765929211	4.536145653	0	0	2.148058484
2.882964605	0	7.862140857	3.235195645	0
35.55656347	13.60843696	32.43133104	23.72476807	25.7767018
30.75162246	35.38193609	42.25900711	24.80316661	25.7767018
48.04941009	54.43374783	26.53472539	37.7439492	18.25849711
45.16644548	29.93856131	17.68981693	84.11508678	56.92354981
0	0	0	0	0
126.8504426	141.5277444	32.43133104	72.25270275	213.7318191
153.7581123	396.45913	684.0062546	111.0750505	322.2087725
20.18075224	0	19.65535214	16.17597823	12.8883509
0	0	0.982767607	1.078398548	2.148058484
142.2262539	111.5891831	69.77650011	74.40949984	161.1043863
200.8465342	138.806057	190.6569158	198.4253329	152.5121523
159.5240415	157.8578687	13.7587465	97.05586936	141.7718599
23.06371684	17.23735348	52.08668318	72.25270275	46.1832574
207.5734516	363.7988813	0.982767607	10.78398548	446.7961646
207.5734516	184.1675135	256.5023455	219.9933039	149.2900646
0	0	154.2945143	1.078398548	1.074029242
34.59557526	14.51566609	30.46579582	51.76313033	28.99878953
0.960988202	22.68072826	25.55195779	552.1400568	52.62743285
12.49284662	17.23735348	1.965535214	6.470391291	1.074029242
0	0	9.827676071	40.97914484	35.44296498
862.9674052	963.4773366	1398.478305	1674.752946	1385.497722
0	0	182.7947749	212.444514	198.6954097
0	0	4.913838036	3.235195645	0
134.5383482	185.0747426	150.3634439	197.3469344	153.5861816

For Review Only

0	191.4253465	212.2778031	0	0
46.12743368	64.41326827	87.46631704	24.80316661	30.07281877
0.960988202	0	3.931070429	0	0
31.71261066	63.50603914	38.32793668	65.78231146	27.92476029
12.49284662	0	13.7587465	18.33277532	5.370146209
102.8257376	95.25905871	141.5185354	170.3869707	119.2172458
0	0	1.965535214	3.235195645	6.444175451
4.804941009	9.979520436	0	11.86238403	9.666263176
4.804941009	5.443374783	8.844908464	10.78398548	6.444175451
39.40051627	64.41326827	40.29347189	53.91992742	36.51699422
8.648893816	0	2.948302821	0	0
26.90766965	38.10362348	38.32793668	26.95996371	51.5534036
15.37581123	40.82531087	0.982767607	3.235195645	11.81432166
1.921976404	7.257833044	0	0	0
13.45383482	13.60843696	2.948302821	14.01918113	11.81432166
305.5942482	142.4349735	169.0360284	258.8156516	113.8470996
8.648893816	16.33012435	0	48.52793468	37.59102346
171.0558999	178.7241387	249.6229722	208.1309199	197.6213805
207.5734516	149.6928065	124.8114861	99.21266646	169.6966202
212.3783926	258.5603022	313.5028667	289.010811	250.2488133
31.71261066	17.23735348	4.913838036	1.078398548	3.222087725
0	0	0	0	0
0	0	14.74151411	0	0
8.648893816	2.721687392	4.913838036	11.86238403	0
73.03510333	65.3204974	65.84542968	64.70391291	52.62743285
244.0910032	305.736217	224.0710144	260.9724487	324.356831
136.4603247	120.6614744	94.34569029	133.72142	138.5497722
33.63458706	19.05181174	15.72428171	10.78398548	9.666263176
0	0	0	2.156797097	0
838.9427001	1376.266591	778.3519449	782.9173462	985.9588439
254.6618735	232.2506574	152.3289791	147.7406011	167.5485617
17.29778763	21.77349913	34.39686625	5.391992742	25.7767018
346.9167408	348.3759861	336.1065216	338.6171442	392.0206732
42.28348088	36.28916522	33.41409864	66.86071	26.85073104
3.843952807	0	0	0	2.148058484
991.7398242	1095.93279	1217.649065	1266.039896	1148.137259
0	0	0	0	4.296116967
169.1339235	156.9506396	144.4668382	95.97747081	133.179626
15.37581123	0	0.982767607	3.235195645	23.62864332
0	9.979520436	0	0	0
816.8399715	977.0857736	1253.028699	2514.825415	2047.099735
0	0	2.948302821	0	0
0	0	0	0	0
2.882964605	12.70120783	0	0	0
0	0	0	0	6.444175451
7413.062988	8092.483844	9920.056226	10193.02308	9854.218293
1396.315857	1496.928065	1605.84227	1572.305084	1437.051125
21.14174044	24.49518652	13.7587465	17.25437678	23.62864332
92.25486737	88.00122566	117.9321129	149.8973982	113.8470996
73.99609154	71.67110131	66.82819729	73.3311013	65.51578375

For Review Only

42.28348088	20.86627	38.32793668	58.23352162	33.29490649
41.32249268	22.68072826	7.862140857	7.548789839	19.33252635
496.8309003	413.6964835	365.5895499	409.7914484	472.5728664
47.08842189	44.4542274	53.06945079	35.5871521	51.5534036
22.10272864	35.38193609	25.55195779	10.78398548	25.7767018
689.9895289	676.7929314	805.8694379	848.6996576	794.7816389
64.38620952	61.69158088	44.22454232	59.31192017	63.36772526
11.53185842	11.7939787	12.77597889	1.078398548	0
209.495428	188.7036592	103.1905987	184.4061518	213.7318191
33.63458706	46.26868566	76.65587336	69.0175071	46.1832574
2126.666891	1691.982328	3115.373315	1244.471925	1380.127576
56.6983039	51.71206044	61.91435925	54.99832597	50.47937436
6.726917412	9.072291305	0	17.25437678	12.8883509
39.40051627	27.21687392	44.22454232	33.430355	47.25728664
171.0558999	135.1771404	141.5185354	120.7806374	192.2512343
11.53185842	5.443374783	8.844908464	4.313594194	11.81432166
7.687905614	0	0.982767607	0	3.222087725
22.10272864	14.51566609	33.41409864	19.41117387	19.33252635
109.552655	91.63014218	125.7942537	142.3486084	160.030357
531.4264756	610.5652048	639.7817122	361.2635137	514.4600068
65.34719772	49.89760218	53.06945079	58.23352162	49.40534512
142.2262539	162.3940144	281.0715356	155.289391	78.40413465
273.8816375	260.3747605	253.5540426	250.1884632	163.2524447
23.06371684	37.19639435	0	49.60633323	50.47937436
108.5916668	129.7337657	106.1389016	54.99832597	91.29248555
8.648893816	5.443374783	2.948302821	6.470391291	5.370146209
1.921976404	1.814458261	0	0	0
140.3042775	116.1253287	145.4496059	140.1918113	143.9199184
636.1741896	417.3254	389.1759724	421.6538324	517.6820945
36.51755167	67.13495566	28.50026061	25.88156516	51.5534036
72.07411513	76.20724696	75.67310575	98.13426791	86.99636858
6.726917412	0	0	15.09757968	0
53.8153393	30.84579044	55.034986	47.44953613	41.88714043
34.59557526	38.10362348	35.37963386	51.76313033	59.0716083
42.28348088	28.12410305	20.63811975	63.62551436	39.73908194
6.726917412	17.23735348	6.87937325	22.64636952	25.7767018
50.93237469	67.13495566	68.7937325	40.97914484	46.1832574
34.59557526	39.01085261	21.62088736	32.35195645	31.14684801
0	0	0	0	0
1641.367849	3181.652561	4180.693401	1474.170816	1459.60574
1675.963424	2450.425882	3727.637534	2120.131546	2197.463829
33.63458706	33.56747783	23.58642257	21.56797097	0
10.57087022	18.14458261	23.58642257	8.627188388	7.518204692
187.3926993	167.8373891	162.1566552	207.0525213	183.6590003
3.843952807	0	10.81044368	2.156797097	0
0	0	3.931070429	0	0
2.882964605	0	0	7.548789839	0
52.8543511	0	1.965535214	43.13594194	38.6650527
208.5344398	233.1578865	168.0532608	251.2668618	154.6602108
123.0064898	78.92893436	62.89712686	73.3311013	86.99636858

For Review Only

61.50324491	95.25905871	81.56971139	66.86071	75.18204692
173.9388645	137.8988278	169.0360284	135.8782171	127.8094798
48.04941009	45.36145653	41.2762395	46.37113758	39.73908194
76.87905614	94.35182958	147.4151411	93.82067372	103.1068072
95.13783197	116.1253287	100.2422959	127.2510287	83.77428086
34.59557526	57.15543522	41.2762395	52.84152887	35.44296498
13.45383482	38.10362348	42.25900711	11.86238403	41.88714043
270.9986729	238.6012613	205.3984299	229.6988908	166.4745325
127.8114308	112.4964122	100.2422959	101.3694636	97.736661
217.1833336	224.0855952	241.7608314	215.6797097	194.3992928
8.648893816	11.7939787	97.29399311	136.9566157	0
77.84004434	60.78435175	68.7937325	56.07672452	62.29369602
24.98569325	30.84579044	39.31070429	30.19515936	6.444175451
10.57087022	45.36145653	39.31070429	37.7439492	0
33.63458706	12.70120783	14.74151411	37.7439492	23.62864332
6.726917412	0	7.862140857	5.391992742	4.296116967
0.960988202	0	0	8.627188388	0
0	9.979520436	10.81044368	4.313594194	7.518204692
199.885546	299.3856131	179.8464721	237.2476807	228.7682285
30.75162246	26.30964479	22.60365496	28.03836226	17.18446787
83.60597355	112.4964122	109.0872044	107.8398548	74.10801768
7.687905614	8.165062175	9.827676071	3.235195645	4.296116967
81.68399715	77.1144761	83.53524661	88.42868097	68.73787147
29.79063425	19.95904087	25.55195779	9.705586936	11.81432166
32.67359886	36.28916522	36.36240146	58.23352162	61.21966678
0	0	0	0	0
3.843952807	0	0.982767607	2.156797097	1.074029242
2.882964605	0	0	0	0
5.765929211	0	0	2.156797097	0
0.960988202	1.814458261	6.87937325	0	0
5.765929211	10.88674957	5.896605643	0	2.148058484
0	0	0	0	0
0	0	3.931070429	0	0
3.843952807	3.628916522	0	0	4.296116967
87.44992636	81.65062175	0	0	0
2.882964605	0	0	0	0
0	0.907229131	0	3.235195645	3.222087725
4.804941009	5.443374783	11.79321129	2.156797097	0
0	0	0	0	0
0	5.443374783	1.965535214	1.078398548	1.074029242
17.29778763	16.33012435	18.67258454	25.88156516	20.40655559
0	0	0.982767607	0	0
0	0	0	0	0
57.65929211	95.25905871	81.56971139	61.46871726	94.51457327
0	0	0	3.235195645	3.222087725
20.18075224	18.14458261	12.77597889	9.705586936	6.444175451
5.765929211	15.42289522	12.77597889	0	11.81432166
15.37581123	0	47.17284514	6.470391291	12.8883509
0	0	1.965535214	2.156797097	0
8.648893816	4.536145653	1.965535214	0	0

For Review Only

60.54225671	51.71206044	61.91435925	36.66555065	59.0716083
1.921976404	0	0	0	0
20.18075224	20.86627	50.12114796	30.19515936	33.29490649
0	3.628916522	10.81044368	0	3.222087725
10.57087022	17.23735348	11.79321129	11.86238403	16.11043863
194.1196168	236.7868031	159.2083524	190.8765431	212.6577899
5.765929211	6.350603914	3.931070429	3.235195645	5.370146209
29.79063425	34.47470696	21.62088736	5.391992742	32.22087725
12.49284662	0.907229131	1.965535214	29.11676081	3.222087725
0	0	0	1.078398548	6.444175451
4.804941009	9.979520436	16.70704932	17.25437678	6.444175451
378.6293515	297.5711548	256.5023455	241.5612749	330.8010065
0	1.814458261	0	0	1.074029242
26.90766965	9.979520436	28.50026061	24.80316661	28.99878953
0	8.165062175	0	0	2.148058484
0	0	3.931070429	0	0
4.804941009	22.68072826	11.79321129	14.01918113	19.33252635
0	0	1.965535214	0	0
50.93237469	44.4542274	48.15561275	24.80316661	21.48058484
34.59557526	58.96989348	49.13838036	88.42868097	51.5534036
0	0	212.2778031	484.2009483	318.9866848
11.53185842	23.58795739	8.844908464	4.313594194	38.6650527
27.86865785	30.84579044	43.24177471	29.11676081	30.07281877
0	0	2.948302821	1.078398548	0
54.7763275	56.24820609	9.827676071	12.94078258	9.666263176
22.10272864	19.95904087	13.7587465	34.50875355	37.59102346
202.7685106	180.538597	240.7780637	254.5020574	262.063135
66.30818592	89.81568392	102.2078311	101.3694636	91.29248555
29.79063425	39.91808174	45.20730993	37.7439492	33.29490649
6.726917412	4.536145653	8.844908464	0	1.074029242
53.8153393	34.47470696	41.2762395	21.56797097	48.33131588
20.18075224	11.7939787	20.63811975	14.01918113	34.36893574
25.94668145	9.979520436	24.56919018	11.86238403	12.8883509
16.33679943	15.42289522	5.896605643	3.235195645	9.666263176
51.8933629	48.08314392	86.48354943	71.1743042	54.77549133
40.36150447	41.73254	28.50026061	36.66555065	24.70267256
111.4746314	100.7024335	92.38015507	67.93910855	85.92233934
301.7502954	342.9326113	311.5373315	458.3193831	429.6116967
3.843952807	3.628916522	0	0	1.074029242
0	0	0	0	0
29.79063425	17.23735348	33.41409864	45.29273904	48.33131588
82.64498535	65.3204974	104.1733664	34.50875355	41.88714043
166.2509589	207.7554709	200.4845919	175.7789634	156.8082693
139.3432893	169.6518474	258.4678807	128.3294273	156.8082693
169.1339235	135.1771404	112.0355072	71.1743042	139.6238014
123.967478	170.5590765	172.9670989	201.6605286	204.0655559
40.36150447	29.93856131	10.81044368	17.25437678	24.70267256
82.64498535	80.74339262	62.89712686	109.9966519	77.33010541
128.772419	96.16628784	69.77650011	40.97914484	80.55219313
0	1.814458261	0	0	3.222087725

For Review Only

277.7255903	180.538597	265.3472539	130.4862244	109.5509827
72.07411513	105.2385791	84.51801421	67.93910855	93.44054403
199.885546	123.3831618	120.8804157	159.6029852	128.883509
67.26917412	42.63976914	44.22454232	17.25437678	52.62743285
91.29387917	75.30001783	11.79321129	31.27355791	64.44175451
51.8933629	76.20724696	99.25952832	65.78231146	60.14563754
138.3823011	122.4759326	76.65587336	147.7406011	144.9939476
10.57087022	14.51566609	5.896605643	3.235195645	1.074029242
22.10272864	14.51566609	7.862140857	26.95996371	8.592233934
91.29387917	72.57833044	73.70757054	81.95828968	106.3288949
99.94277298	119.7542452	183.7775425	80.87989113	100.9587487
3.843952807	5.443374783	0.982767607	1.078398548	0
317.1261066	276.7048848	122.8459509	280.3836226	219.1019653
9.609882018	11.7939787	9.827676071	16.17597823	15.03640938
28.82964605	95.25905871	152.3289791	187.6413474	150.3640938
83.60597355	41.73254	288.9336765	1.078398548	276.0255151
32.67359886	26.30964479	18.67258454	45.29273904	25.7767018
297.9063425	212.2916165	176.8981693	134.7998186	131.0315675
0	0	2.948302821	1.078398548	1.074029242
37.47853987	65.3204974	73.70757054	49.60633323	47.25728664
139.3432893	128.8265365	120.8804157	90.58547807	94.51457327
0	0	0	6.470391291	3.222087725
31.71261066	32.6602487	34.39686625	17.25437678	31.14684801
112.4356196	100.7024335	88.44908464	67.93910855	93.44054403
23.06371684	29.03133218	32.43133104	26.95996371	59.0716083
17.29778763	6.350603914	3.931070429	8.627188388	5.370146209
6.726917412	0	3.931070429	5.391992742	0
909.0948389	693.1230557	653.5404587	406.5562528	197.6213805
1.921976404	0	0	0	0
4.804941009	15.42289522	0	0	0
5.765929211	10.88674957	7.862140857	6.470391291	1.074029242
0	0	0	3.235195645	0
0	4.536145653	0.982767607	0	1.074029242
0	7.257833044	0.982767607	0	2.148058484
25.94668145	18.14458261	32.43133104	25.88156516	25.7767018
0	0	0	0	0
26.90766965	27.21687392	38.32793668	29.11676081	46.1832574
123.0064898	96.16628784	87.46631704	59.31192017	30.07281877
49.01039829	37.19639435	13.7587465	44.21434049	90.21845631
0	4.536145653	0	0	2.148058484
4.804941009	19.05181174	0.982767607	0	0
185.4707229	163.3012435	195.5707538	203.8173257	146.0679769
0	0	0	0	0
0	2.721687392	0	0	3.222087725
24.02470504	16.33012435	0	0	0
63.42522132	76.20724696	35.37963386	43.13594194	74.10801768
3.843952807	0	0.982767607	0	1.074029242
22.10272864	20.86627	12.77597889	5.391992742	17.18446787
23.06371684	16.33012435	5.896605643	8.627188388	13.96238014
3.843952807	0.907229131	0.982767607	2.156797097	5.370146209

For Review Only

24.02470504	30.84579044	28.50026061	24.80316661	34.36893574
3.843952807	13.60843696	11.79321129	2.156797097	9.666263176
49.01039829	30.84579044	19.65535214	20.48957242	42.96116967
67.26917412	46.26868566	44.22454232	52.84152887	40.81311119
60.54225671	24.49518652	108.1044368	0	3.222087725
214.300369	194.1470339	240.7780637	240.4828763	176.1407956
14.41482303	12.70120783	8.844908464	23.72476807	23.62864332
87.44992636	81.65062175	122.8459509	88.42868097	74.10801768
0.960988202	0	6.87937325	1.078398548	0
24.02470504	17.23735348	20.63811975	19.41117387	18.25849711
2.882964605	4.536145653	15.72428171	1.078398548	7.518204692
162.4070061	153.3217231	98.27676071	57.15512307	192.2512343
198.9245578	234.0651157	236.8469933	265.2860429	221.2500238
2.882964605	1.814458261	0.982767607	0	0
10.57087022	9.979520436	20.63811975	18.33277532	28.99878953
0	0	24.56919018	19.41117387	25.7767018
277.7255903	232.2506574	177.8809369	276.0700284	374.8362054
0	69.85664305	3.931070429	1.078398548	3.222087725
137.4213129	125.19762	165.104958	129.4078258	198.6954097
2.882964605	3.628916522	4.913838036	3.235195645	4.296116967
10.57087022	5.443374783	1.965535214	11.86238403	13.96238014
45.16644548	48.99037305	25.55195779	43.13594194	63.36772526
24.98569325	48.08314392	43.24177471	37.7439492	47.25728664
0	0	186.7258454	162.8381808	141.7718599
33.63458706	41.73254	69.77650011	0	54.77549133
10.57087022	0	34.39686625	47.44953613	3.222087725
0	5.443374783	3.931070429	2.156797097	0
168.1729353	118.8470161	231.9331553	182.2493547	107.4029242
49.01039829	56.24820609	47.17284514	5.391992742	17.18446787
15.37581123	40.82531087	8.844908464	32.35195645	8.592233934
5.765929211	9.072291305	8.844908464	3.235195645	4.296116967
9.609882018	0	4.913838036	3.235195645	4.296116967
11.53185842	0	2.948302821	3.235195645	138.5497722
5.765929211	0	16.70704932	0	26.85073104
16.33679943	0	3.931070429	4.313594194	2.148058484
71.11312693	46.26868566	76.65587336	44.21434049	54.77549133
62.46423311	47.17591479	100.2422959	60.39031871	47.25728664
141.2652657	93.44460045	115.9665776	121.859036	125.6614213
80.72300895	62.59881001	103.1905987	40.97914484	66.58981299
288.2964605	189.6108883	172.9670989	260.9724487	293.209983
152.7971241	173.2807639	402.9347189	268.5212386	124.587392
32.67359886	33.56747783	38.32793668	48.52793468	38.6650527
51.8933629	48.99037305	33.41409864	40.97914484	37.59102346
0	5.443374783	0.982767607	0	2.148058484
32.67359886	34.47470696	12.77597889	10.78398548	21.48058484
2.882964605	1.814458261	7.862140857	0	0
111.4746314	112.4964122	117.9321129	134.7998186	93.44054403
21.14174044	33.56747783	34.39686625	14.01918113	5.370146209
51.8933629	41.73254	24.56919018	61.46871726	60.14563754
56.6983039	58.06266435	47.17284514	48.52793468	68.73787147

For Review Only

193.1586286	340.210924	230.9503877	148.8189997	309.3204216
266.1937319	169.6518474	208.3467327	258.8156516	140.6978307
9.609882018	9.072291305	10.81044368	0	13.96238014
282.5305313	221.3639079	262.3989511	212.444514	342.6153281
3.843952807	0	0.982767607	0	0
0	0	1.965535214	1.078398548	3.222087725
0	3.628916522	0.982767607	2.156797097	0
114.357596	176.0024513	177.8809369	215.6797097	262.063135
12.49284662	9.979520436	13.7587465	4.313594194	2.148058484
4.804941009	3.628916522	1.965535214	0	0
0	0	0	0	0
8.648893816	0	0	0	4.296116967
6.726917412	0.907229131	0.982767607	9.705586936	5.370146209
313.2821538	293.0350092	264.3644863	253.4236589	268.5073104
65.34719772	65.3204974	48.15561275	69.0175071	41.88714043
444.9375374	406.4386505	242.743599	418.4186368	388.7985855
1105.136432	1026.076147	1097.751417	665.3719044	1405.904277
296.9453543	468.1302314	554.2809304	456.162586	471.4988371
20.18075224	10.88674957	14.74151411	6.470391291	9.666263176
2.882964605	0	2.948302821	0	0
0	12.70120783	0	0	0
97.05980838	112.4964122	182.7947749	154.2109924	146.0679769
2.882964605	0	2.948302821	3.235195645	3.222087725
156.6410769	121.5687035	120.8804157	114.3102461	69.81190071
20.18075224	16.33012435	22.60365496	19.41117387	28.99878953
157.6020651	188.7036592	152.3289791	133.72142	91.29248555
50.93237469	39.01085261	19.65535214	54.99832597	62.29369602
56.6983039	44.4542274	45.20730993	34.50875355	44.03519891
222.9492628	154.2289522	171.0015636	234.012485	211.5837606
9.609882018	10.88674957	9.827676071	0	9.666263176
32.67359886	43.54699827	28.50026061	53.91992742	24.70267256
130.6943954	79.83616349	118.9148805	76.56629694	54.77549133
143.1872421	138.806057	142.501303	166.0733765	148.2160354
31.71261066	42.63976914	63.87989446	46.37113758	27.92476029
14.41482303	19.05181174	26.53472539	0	0
99.94277298	89.81568392	78.62140857	98.13426791	96.66263176
0.960988202	145.1566609	108.1044368	0	73.03398844
0	0	1.965535214	4.313594194	2.148058484
0	0	14.74151411	0	0
77.84004434	106.1458083	53.06945079	17.25437678	113.8470996
16.33679943	34.47470696	23.58642257	25.88156516	11.81432166
3.843952807	2.721687392	0	0	2.148058484
46.12743368	62.59881001	63.87989446	60.39031871	44.03519891
141.2652657	136.0843696	128.7425565	124.0158331	125.6614213
34.59557526	30.84579044	124.8114861	0	0
5.765929211	9.979520436	10.81044368	11.86238403	15.03640938
21.14174044	17.23735348	5.896605643	1.078398548	9.666263176
0	0	0	0	1.074029242
25.94668145	0	19.65535214	9.705586936	15.03640938
0	6.350603914	10.81044368	0	4.296116967

For Review Only

6.726917412	0	0.982767607	3.235195645	0
72.07411513	49.89760218	69.77650011	101.3694636	70.88592996
22.10272864	24.49518652	19.65535214	37.7439492	20.40655559
24.98569325	34.47470696	40.29347189	18.33277532	31.14684801
80.72300895	75.30001783	89.43185225	104.6046592	121.3653043
16.33679943	0	11.79321129	46.37113758	11.81432166
14.41482303	20.86627	39.31070429	19.41117387	48.33131588
100.9037612	89.81568392	79.60417618	57.15512307	54.77549133
192.1976404	332.9530909	0	1061.144172	735.7100306
0	0	2.948302821	2.156797097	3.222087725
370.9414459	188.7036592	1070.233924	1078.398548	1195.394546
5.765929211	6.350603914	6.87937325	1.078398548	1.074029242
0	0	10.81044368	0	0
48.04941009	62.59881001	22.60365496	32.35195645	19.33252635
20.18075224	0	4.913838036	8.627188388	9.666263176
0	0	0	0	0
1052.282081	1112.262914	1166.54515	1349.076584	1354.350874
1953.689014	1930.58359	1896.741482	1756.711235	1719.520816
2.882964605	4.536145653	0.982767607	5.391992742	2.148058484
41.32249268	268.5398226	75.67310575	153.1325939	66.58981299
0	0	9.827676071	33.430355	40.81311119
0	0	0	0	0
50.93237469	27.21687392	0	0	52.62743285
50.93237469	41.73254	0	108.9182534	71.9599592
72.07411513	63.50603914	85.50078182	71.1743042	103.1068072
0	0	14.74151411	0	1.074029242
0	420.0470874	144.4668382	727.9190202	138.5497722
140.3042775	458.1507109	312.5200991	196.2685358	109.5509827
105.7087022	142.4349735	183.7775425	196.2685358	133.179626
7712.891307	8564.242992	10785.87449	10060.38006	8909.07256
97.05980838	77.1144761	88.44908464	78.72309404	118.1432166
78.80103254	68.94941392	58.96605643	71.1743042	94.51457327
0	3.628916522	0	0	0
51.8933629	37.19639435	38.32793668	33.430355	52.62743285
116.2795724	151.5072648	105.156134	89.50707952	89.14442707
0	0	0	16.17597823	15.03640938
103.7867258	68.04218479	138.5702326	54.99832597	107.4029242
1939.274191	2002.254691	1500.686136	1965.920554	2191.019653
57.65929211	35.38193609	51.10391557	59.31192017	51.5534036
615.9934373	672.2567857	685.9717898	693.4102667	474.7209249
4.804941009	0.907229131	0	0	56.92354981
64.38620952	52.61928957	55.034986	69.0175071	71.9599592
138.3823011	127.0120783	108.1044368	132.6430215	98.81069024
88.41091456	65.3204974	85.50078182	112.153449	94.51457327
36.51755167	59.87712262	34.39686625	4.313594194	40.81311119
0	0	50.12114796	0	0
0	0	12.77597889	0	0
144.1482303	172.3735348	132.673627	75.48789839	166.4745325
13.45383482	9.979520436	15.72428171	21.56797097	8.592233934
5.765929211	9.072291305	7.862140857	4.313594194	2.148058484

For Review Only

8.648893816	5.443374783	6.87937325	7.548789839	8.592233934
0	2.721687392	0.982767607	0	0
0	0.907229131	0	0	0
56.6983039	44.4542274	46.19007754	25.88156516	32.22087725
8.648893816	0.907229131	40.29347189	0	0
61.50324491	89.81568392	105.156134	69.0175071	103.1068072
76.87905614	203.2193252	79.60417618	0	0
415.1469032	438.19167	248.6402046	207.0525213	339.3932404
61.50324491	45.36145653	33.41409864	8.627188388	59.0716083
1272.348379	1464.267817	1566.531566	1560.4427	1086.917593
474.7281717	381.943464	513.0046909	586.6488104	538.0886501
110.5136432	97.07351697	110.069972	76.56629694	119.2172458
1.921976404	0.907229131	29.48302821	0	0
310.3991892	380.1290057	497.2804092	386.0666803	401.6869364
7.687905614	0	0	0	0
568.9050154	704.0098053	1007.336797	938.2067372	769.0049371
4.804941009	4.536145653	0	7.548789839	8.592233934
0	7.257833044	16.70704932	6.470391291	1.074029242
2.882964605	0	0.982767607	7.548789839	0
0	0	0	0	3.222087725
561.2171098	575.1832688	320.3822399	303.0299921	368.3920299
117.2405606	0	392.1242752	209.2093184	3.222087725
43.24446908	76.20724696	47.17284514	73.3311013	26.85073104
0	0	0	0	0
34.59557526	37.19639435	24.56919018	8.627188388	44.03519891
4.804941009	3.628916522	4.913838036	3.235195645	6.444175451
0	19.05181174	3.931070429	1.078398548	1.074029242
22.10272864	39.01085261	80.58694379	105.6830577	96.66263176
13.45383482	25.40241565	15.72428171	22.64636952	17.18446787
0	0	0	0	0
29.79063425	29.93856131	31.44856343	40.97914484	32.22087725
348.8387172	371.0567144	389.1759724	438.9082092	397.3908194
198.9245578	196.8687213	268.2955567	252.3452603	251.3228426
106.6696904	106.1458083	183.7775425	208.1309199	167.5485617
41.32249268	27.21687392	27.517493	31.27355791	27.92476029
0	165.1157018	19.65535214	51.76313033	0
43.24446908	27.21687392	28.50026061	21.56797097	45.10922815
9.609882018	0	0	0	0
0	0.907229131	0	0	0
0	0	0	0	0
5.765929211	3.628916522	13.7587465	0	4.296116967
331.5409296	288.4988635	387.2104372	530.5720858	457.536457
0	0	0.982767607	1.078398548	1.074029242
240.2470504	216.8277622	209.3295003	231.8556879	219.1019653
686.1455761	757.536324	690.8856278	722.5270275	921.5170894
17.29778763	7.257833044	11.79321129	3.235195645	7.518204692
0	0.907229131	3.931070429	0	0
18.25877583	15.42289522	9.827676071	33.430355	9.666263176
8976.590793	7275.977627	10470.40609	9880.287501	9482.604175
0	0	2.948302821	1.078398548	22.55461408

For Review Only

0	0	1.965535214	0	0
686.1455761	630.5242457	805.8694379	502.5337236	532.7185039
2.882964605	4.536145653	0	0	0
16.33679943	27.21687392	7.862140857	4.313594194	6.444175451
2.882964605	0	0	0	5.370146209
48.04941009	44.4542274	69.77650011	84.11508678	34.36893574
0	1.814458261	0	0	0
783.2053844	493.532647	346.9169653	424.8890281	404.9090241
3.843952807	9.979520436	0	0	0
70.15213873	56.24820609	47.17284514	43.13594194	78.40413465
19.21976404	0	4.913838036	11.86238403	15.03640938
42.28348088	29.03133218	0	0	0
4.804941009	7.257833044	2.948302821	7.548789839	4.296116967
19.21976404	0	0	0	0
82.64498535	44.4542274	31.44856343	37.7439492	48.33131588
2.882964605	0.907229131	0	0	0
62.46423311	36.28916522	89.43185225	51.76313033	53.70146209
0	0.907229131	0	0	0
73.99609154	81.65062175	48.15561275	74.40949984	69.81190071
51.8933629	83.46508001	33.41409864	52.84152887	24.70267256
468.9622425	389.201297	409.8140922	469.1033686	429.6116967
0	0	0	0	0
1.921976404	0	0	0	0
0	0	2.948302821	2.156797097	0
0	0	0	38.82234774	6.444175451
24.98569325	47.17591479	21.62088736	34.50875355	21.48058484
14.41482303	16.33012435	31.44856343	14.01918113	33.29490649
0	0	0	0	0
76.87905614	94.35182958	83.53524661	91.66387662	124.587392
30.75162246	18.14458261	4.913838036	11.86238403	3.222087725
0	0	0	0	0
18.25877583	48.08314392	19.65535214	15.09757968	26.85073104
339.2288352	291.2205509	364.6067822	361.2635137	286.7658075
0	0	0	0	3.222087725
164.3289825	96.16628784	159.2083524	167.151775	86.99636858
149.9141595	300.2928422	275.17493	162.8381808	171.8446787
23.06371684	19.95904087	12.77597889	17.25437678	7.518204692
71.11312693	61.69158088	41.2762395	35.5871521	33.29490649
6.726917412	12.70120783	5.896605643	7.548789839	2.148058484
4.804941009	11.7939787	20.63811975	21.56797097	20.40655559
292.1404133	377.4073183	411.7796274	286.8540139	383.4284393
5.765929211	17.23735348	6.87937325	8.627188388	4.296116967
8.648893816	0.907229131	6.87937325	0	10.74029242
16.33679943	110.6819539	0	113.2318476	94.51457327
3.843952807	6.350603914	8.844908464	0	0
18.25877583	0	54.05221839	0	0
23.06371684	13.60843696	22.60365496	28.03836226	19.33252635
139.3432893	180.538597	149.3806763	179.014159	165.4005032
398.8101037	335.6747783	470.7456838	530.5720858	369.4660592
42.28348088	72.57833044	66.82819729	190.8765431	0

For Review Only

38.43952807	20.86627	31.44856343	12.94078258	11.81432166
25.94668145	32.6602487	56.01775361	30.19515936	37.59102346
235.4421094	215.9205331	263.3817187	177.9357605	238.4344917
29.79063425	29.03133218	7.862140857	33.430355	16.11043863
0	0	164.1221904	131.5646229	135.3276845
30.75162246	23.58795739	13.7587465	16.17597823	18.25849711
1.921976404	0	10.81044368	0	5.370146209
6.726917412	0	0	5.391992742	8.592233934
21.14174044	11.7939787	34.39686625	17.25437678	25.7767018
139.3432893	120.6614744	133.6563946	200.58213	308.2463924
31.71261066	24.49518652	26.53472539	20.48957242	35.44296498
3.843952807	16.33012435	2.948302821	5.391992742	1.074029242
0	0	1.965535214	0	0
12.49284662	11.7939787	5.896605643	3.235195645	17.18446787
0	0	0	0	0
36.51755167	35.38193609	33.41409864	47.44953613	46.1832574
389.2002217	331.1386326	161.1738876	188.719746	249.1747841
22.10272864	19.05181174	30.46579582	39.90074629	51.5534036
12.49284662	9.979520436	8.844908464	11.86238403	8.592233934
9.609882018	6.350603914	4.913838036	8.627188388	4.296116967
2.882964605	5.443374783	0.982767607	2.156797097	1.074029242
8.648893816	7.257833044	8.844908464	10.78398548	4.296116967
3.843952807	0	0	0	0
0	0	0	4.313594194	1.074029242
4.804941009	4.536145653	6.87937325	1.078398548	1.074029242
4.804941009	8.165062175	5.896605643	3.235195645	7.518204692
123.967478	176.9096805	113.0182748	84.11508678	69.81190071
0	0	1.965535214	0	0
4.804941009	6.350603914	6.87937325	5.391992742	6.444175451
9.609882018	9.072291305	10.81044368	2.156797097	3.222087725
1.921976404	0	0	1.078398548	1.074029242
2.882964605	3.628916522	0	4.313594194	1.074029242
12.49284662	9.072291305	10.81044368	10.78398548	8.592233934
0	1.814458261	0	0	0
0	0	0	0	6.444175451
7.687905614	12.70120783	16.70704932	8.627188388	7.518204692
27.86865785	28.12410305	19.65535214	39.90074629	32.22087725
0	1.814458261	2.948302821	1.078398548	0
27.86865785	30.84579044	17.68981693	30.19515936	9.666263176
103703.1198	106517.7722	55620.71549	70256.58703	105431.0065
0.960988202	0	0	2.156797097	0
0.960988202	0	0	0	3.222087725
4.804941009	5.443374783	2.948302821	3.235195645	0
43.24446908	38.10362348	41.2762395	32.35195645	51.5534036
10.57087022	2.721687392	2.948302821	3.235195645	8.592233934
0	1.814458261	0	0	0
103.7867258	71.67110131	90.41461986	89.50707952	111.6990411
9.609882018	8.165062175	8.844908464	0	3.222087725
6.726917412	1.814458261	0	0	5.370146209
0	3.628916522	0	0	0

For Review Only

0	0	0	0	4.296116967
7.687905614	7.257833044	6.87937325	11.86238403	16.11043863
55.7373157	61.69158088	88.44908464	80.87989113	65.51578375
1.921976404	0.907229131	0	0	6.444175451
18.25877583	25.40241565	4.913838036	17.25437678	3.222087725
3.843952807	0.907229131	2.948302821	0	0
1.921976404	1.814458261	0	0	0
0.960988202	0	0	0	0
0	0	0	0	2.148058484
27.86865785	10.88674957	19.65535214	12.94078258	17.18446787
5.765929211	5.443374783	5.896605643	4.313594194	5.370146209
13.45383482	5.443374783	2.948302821	11.86238403	5.370146209
1.921976404	4.536145653	3.931070429	1.078398548	3.222087725
2.882964605	0	0.982767607	0	0
10.57087022	6.350603914	10.81044368	7.548789839	13.96238014
16.33679943	9.072291305	8.844908464	8.627188388	8.592233934
0	6.350603914	0	0	0
18.25877583	25.40241565	9.827676071	5.391992742	9.666263176
5.765929211	0	8.844908464	2.156797097	1.074029242
40.36150447	39.01085261	23.58642257	33.430355	37.59102346
38.43952807	44.4542274	24.56919018	30.19515936	37.59102346
0	0	3.931070429	0	0
0	0	1.965535214	0	0
0	0	0	2.156797097	2.148058484
0.960988202	2.721687392	0	0	1.074029242
0	0	0	3.235195645	1.074029242
3.843952807	4.536145653	3.931070429	3.235195645	1.074029242
2.882964605	5.443374783	5.896605643	0	4.296116967
0.960988202	0.907229131	0	1.078398548	1.074029242
4.804941009	0	0	2.156797097	0
55.7373157	54.43374783	68.7937325	58.23352162	74.10801768
0.960988202	3.628916522	0.982767607	1.078398548	0
0	0	0.982767607	1.078398548	1.074029242
3.843952807	3.628916522	2.948302821	1.078398548	0
0	0	1.965535214	0	0
37.47853987	37.19639435	36.36240146	30.19515936	41.88714043
11.53185842	5.443374783	3.931070429	14.01918113	5.370146209
2.882964605	0	3.931070429	3.235195645	7.518204692
42.28348088	9.072291305	10.81044368	11.86238403	4.296116967
40.36150447	39.91808174	55.034986	10.78398548	22.55461408
42.28348088	40.82531087	30.46579582	35.5871521	40.81311119
48.04941009	47.17591479	44.22454232	60.39031871	17.18446787
8.648893816	8.165062175	0	14.01918113	0
17.29778763	8.165062175	12.77597889	15.09757968	6.444175451
1.921976404	3.628916522	0	0	3.222087725
24.02470504	23.58795739	22.60365496	29.11676081	18.25849711
0	0	0.982767607	2.156797097	0
2.882964605	0	0.982767607	3.235195645	0
2.882964605	0	0	0	1.074029242
2.882964605	3.628916522	0	3.235195645	0

For Review Only

3.843952807	0	0	0	0
1.921976404	2.721687392	0	4.313594194	4.296116967
2.882964605	4.536145653	0.982767607	0	0
4.804941009	3.628916522	5.896605643	8.627188388	6.444175451
22.10272864	23.58795739	16.70704932	24.80316661	18.25849711
7.687905614	9.979520436	4.913838036	11.86238403	6.444175451
18.25877583	16.33012435	7.862140857	9.705586936	3.222087725
1.921976404	1.814458261	3.931070429	0	0
1.921976404	7.257833044	2.948302821	1.078398548	3.222087725
0	0	0	0	1.074029242
14.41482303	6.350603914	3.931070429	5.391992742	2.148058484
0	1.814458261	0	0	0
11.53185842	7.257833044	8.844908464	6.470391291	10.74029242
18.25877583	17.23735348	18.67258454	16.17597823	22.55461408
12.49284662	12.70120783	0.982767607	5.391992742	3.222087725
0	3.628916522	1.965535214	2.156797097	2.148058484
55.7373157	58.06266435	71.74203532	58.23352162	55.84952057
0	0	0	0	0
0	1.814458261	0.982767607	0	0
72.07411513	101.6096626	23.58642257	69.0175071	15.03640938
3.843952807	4.536145653	3.931070429	0	1.074029242
24.98569325	28.12410305	19.65535214	24.80316661	20.40655559
0	2.721687392	0	0	0
0	0	0	2.156797097	4.296116967
4.804941009	0	8.844908464	0	0
22.10272864	17.23735348	10.81044368	9.705586936	15.03640938
35.55656347	25.40241565	39.31070429	35.5871521	22.55461408
3.843952807	0.907229131	1.965535214	4.313594194	6.444175451
14.41482303	13.60843696	18.67258454	10.78398548	8.592233934
19.21976404	12.70120783	7.862140857	25.88156516	13.96238014
6.726917412	8.165062175	2.948302821	5.391992742	3.222087725
6.726917412	0	6.87937325	8.627188388	5.370146209
17.29778763	8.165062175	7.862140857	11.86238403	33.29490649
5.765929211	0	6.87937325	1.078398548	0
147.0311949	156.0434105	218.1744088	268.5212386	233.0643455
23.06371684	19.05181174	20.63811975	17.25437678	8.592233934
1.921976404	1.814458261	0	0	0
0	0	0	2.156797097	2.148058484
4.804941009	2.721687392	0	1.078398548	2.148058484
20.18075224	9.979520436	11.79321129	14.01918113	12.8883509
0.960988202	0.907229131	0	0	9.666263176
32.67359886	23.58795739	48.15561275	29.11676081	22.55461408
9.609882018	15.42289522	6.87937325	11.86238403	15.03640938
5.765929211	6.350603914	4.913838036	1.078398548	0
0	0	0.982767607	1.078398548	1.074029242
0	0	1.965535214	0	0
2.882964605	3.628916522	0.982767607	0	3.222087725
0	0	0.982767607	1.078398548	0
10.57087022	3.628916522	8.844908464	12.94078258	11.81432166
0	0	0	0	6.444175451

For Review Only

0	0	0.982767607	16.17597823	1.074029242
7.687905614	5.443374783	6.87937325	9.705586936	2.148058484
0	0	14.74151411	14.01918113	6.444175451
4.804941009	0	4.913838036	0	0
15.37581123	18.14458261	5.896605643	6.470391291	21.48058484
4.804941009	1.814458261	0	1.078398548	0
1.921976404	0	0	0	0
2.882964605	8.165062175	0	0	1.074029242
14.41482303	12.70120783	9.827676071	7.548789839	10.74029242
4.804941009	0	1.965535214	8.627188388	15.03640938
0	0	0.982767607	0	0
2.882964605	0	3.931070429	0	0
0.960988202	0	1.965535214	4.313594194	1.074029242
19.21976404	19.05181174	3.931070429	23.72476807	23.62864332
0	0	0	0	0
0	4.536145653	0	0	0
0	12.70120783	0.982767607	0	1.074029242
18.25877583	16.33012435	10.81044368	17.25437678	18.25849711
1.921976404	0.907229131	3.931070429	0	3.222087725
3.843952807	0	2.948302821	0	2.148058484
12.49284662	10.88674957	1.965535214	1.078398548	5.370146209
0	0	1.965535214	0	2.148058484
0	0	0	0	1.074029242
0	0	0	0	2.148058484
212.3783926	248.5807818	261.4161835	243.718072	336.1711527
4.804941009	6.350603914	4.913838036	0	4.296116967
27.86865785	33.56747783	12.77597889	22.64636952	53.70146209
0.960988202	0.907229131	0.982767607	1.078398548	0
14.41482303	22.68072826	27.517493	48.52793468	27.92476029
0	3.628916522	0	0	0
0	0	0	1.078398548	0
36.51755167	34.47470696	34.39686625	33.430355	41.88714043
0	0	0.982767607	0	0
2.882964605	0	0	0	0
0	3.628916522	0.982767607	1.078398548	0
2.882964605	2.721687392	0	3.235195645	0
8.648893816	4.536145653	6.87937325	3.235195645	0
0	0.907229131	1.965535214	1.078398548	0
14.41482303	10.88674957	2.948302821	3.235195645	3.222087725
370.9414459	364.7061105	292.8647469	196.2685358	131.0315675
6.726917412	7.257833044	6.87937325	2.156797097	3.222087725
3.843952807	6.350603914	0	1.078398548	8.592233934
3.843952807	3.628916522	8.844908464	0	6.444175451
23.06371684	37.19639435	5.896605643	17.25437678	50.47937436
20.18075224	12.70120783	0.982767607	33.430355	11.81432166
0	0	0	4.313594194	0
10.57087022	15.42289522	9.827676071	14.01918113	10.74029242
3.843952807	0	0.982767607	2.156797097	7.518204692
0	0	0	0	0
1.921976404	4.536145653	2.948302821	1.078398548	11.81432166

For Review Only

8.648893816	10.88674957	12.77597889	42.05754339	5.370146209
0	4.536145653	3.931070429	3.235195645	0
83.60597355	103.4241209	91.39738746	119.7022389	120.2912751
4.804941009	2.721687392	0	0	0
10.57087022	4.536145653	4.913838036	19.41117387	6.444175451
11.53185842	11.7939787	5.896605643	7.548789839	23.62864332
0	0	0	4.313594194	0
3.843952807	4.536145653	0	4.313594194	1.074029242
1.921976404	0.907229131	0.982767607	0	0
79.76202075	60.78435175	50.12114796	28.03836226	38.6650527
5.765929211	3.628916522	2.948302821	4.313594194	0
3.843952807	2.721687392	0.982767607	2.156797097	1.074029242
16.33679943	13.60843696	9.827676071	2.156797097	4.296116967
16.33679943	9.979520436	1.965535214	12.94078258	11.81432166
9.609882018	8.165062175	14.74151411	12.94078258	12.8883509
13.45383482	12.70120783	8.844908464	6.470391291	15.03640938
6.726917412	0	1.965535214	2.156797097	0
4.804941009	5.443374783	4.913838036	8.627188388	9.666263176
23.06371684	0	0	19.41117387	0
1.921976404	5.443374783	3.931070429	0	4.296116967
3.843952807	0	2.948302821	0	3.222087725
0	0	1.965535214	6.470391291	20.40655559
0.960988202	0	1.965535214	2.156797097	5.370146209
1.921976404	5.443374783	2.948302821	5.391992742	3.222087725
44.20545728	78.92893436	46.19007754	67.93910855	142.8458892
0	6.350603914	1.965535214	1.078398548	4.296116967
13.45383482	11.7939787	5.896605643	29.11676081	27.92476029
17.29778763	11.7939787	24.56919018	8.627188388	19.33252635
0	9.072291305	0.982767607	1.078398548	10.74029242
3.843952807	5.443374783	0.982767607	1.078398548	2.148058484
14.41482303	13.60843696	12.77597889	4.313594194	11.81432166
8.648893816	7.257833044	2.948302821	4.313594194	4.296116967
2.882964605	0	0.982767607	1.078398548	4.296116967
9.609882018	26.30964479	50.12114796	2.156797097	45.10922815
4.804941009	0.907229131	3.931070429	3.235195645	0
58.62028031	54.43374783	15.72428171	51.76313033	49.40534512
9.609882018	4.536145653	0.982767607	0	1.074029242
1.921976404	3.628916522	0.982767607	0	0
5.765929211	7.257833044	2.948302821	4.313594194	1.074029242
1.921976404	0	0	0	0
0	0	3.931070429	0	0
14.41482303	16.33012435	4.913838036	12.94078258	8.592233934
0	2.721687392	0	0	0
27.86865785	37.19639435	7.862140857	12.94078258	7.518204692
22.10272864	23.58795739	3.931070429	12.94078258	6.444175451
3.843952807	0	0.982767607	2.156797097	0
4.804941009	0	0	1.078398548	0
216.2223454	377.4073183	149.3806763	359.1067166	401.6869364
34.59557526	26.30964479	15.72428171	17.25437678	45.10922815
0	0	0	0	0

For Review Only

0	0	2.948302821	2.156797097	2.148058484
0	2.721687392	0	1.078398548	0
7.687905614	0	1.965535214	4.313594194	8.592233934
3.843952807	0	0.982767607	8.627188388	2.148058484
348.8387172	466.3157731	266.3300215	141.2702098	216.9539068
9.609882018	0	0.982767607	0	1.074029242
26.90766965	33.56747783	30.46579582	21.56797097	27.92476029
0	5.443374783	0.982767607	1.078398548	1.074029242
29.79063425	23.58795739	14.74151411	28.03836226	37.59102346
0	0	1.965535214	0	0
2.882964605	0	0	0	0
0	0	0.982767607	2.156797097	0
7.687905614	20.86627	15.72428171	7.548789839	9.666263176
0	0	0	0	1.074029242
55.7373157	53.5265187	26.53472539	49.60633323	28.99878953
40.36150447	24.49518652	8.844908464	15.09757968	26.85073104
9.609882018	12.70120783	4.913838036	1.078398548	1.074029242
2.882964605	4.536145653	7.862140857	4.313594194	1.074029242
4.804941009	8.165062175	8.844908464	7.548789839	3.222087725
9.609882018	0	11.79321129	2.156797097	1.074029242
1.921976404	1.814458261	0	0	7.518204692
9.609882018	11.7939787	0.982767607	1.078398548	12.8883509
0	5.443374783	1.965535214	2.156797097	2.148058484
32.67359886	8.165062175	0	0	0
0	6.350603914	4.913838036	0	1.074029242
69.19115053	86.1867674	23.58642257	79.80149259	100.9587487
0	0	1.965535214	1.078398548	0
0	2.721687392	0.982767607	2.156797097	2.148058484
5.765929211	6.350603914	5.896605643	4.313594194	6.444175451
26.90766965	41.73254	38.32793668	37.7439492	39.73908194
0	0.907229131	3.931070429	7.548789839	0
24.02470504	25.40241565	22.60365496	26.95996371	18.25849711
0	0	0	0	0
0.960988202	0	0.982767607	0	3.222087725
55.7373157	43.54699827	22.60365496	36.66555065	63.36772526
0	0	2.948302821	3.235195645	0
0.960988202	0	1.965535214	8.627188388	1.074029242
4.804941009	0	0	1.078398548	0
2.882964605	0	0	2.156797097	1.074029242
6.726917412	7.257833044	0	0	4.296116967
3.843952807	4.536145653	0.982767607	1.078398548	7.518204692
126.8504426	85.27953827	102.2078311	167.151775	78.40413465
0	0	1.965535214	0	1.074029242
12.49284662	7.257833044	8.844908464	6.470391291	5.370146209
0	0	0.982767607	4.313594194	4.296116967
0	0	5.896605643	7.548789839	15.03640938
6.726917412	9.072291305	1.965535214	14.01918113	8.592233934
0	0	0	0	0
6.726917412	6.350603914	1.965535214	0	6.444175451
3.843952807	0	6.87937325	2.156797097	0

For Review Only

15.37581123	0	0.982767607	6.470391291	1.074029242
8.648893816	0	0.982767607	2.156797097	7.518204692
0.960988202	3.628916522	0	0	0
5.765929211	4.536145653	0	1.078398548	4.296116967
6.726917412	4.536145653	0	3.235195645	1.074029242
14.41482303	14.51566609	3.931070429	1.078398548	1.074029242
9.609882018	13.60843696	6.87937325	2.156797097	22.55461408
0	0	0	2.156797097	4.296116967
0	0	0.982767607	4.313594194	0
0	5.443374783	1.965535214	0	3.222087725
0	0	0	0	1.074029242
0	34.47470696	0	8.627188388	0
4.804941009	12.70120783	13.7587465	0	13.96238014
0.960988202	0	0	0	0
7.687905614	6.350603914	0	1.078398548	2.148058484
286.3744841	168.7446183	171.0015636	204.8957242	228.7682285
10.57087022	11.7939787	20.63811975	4.313594194	1.074029242
12.49284662	15.42289522	13.7587465	5.391992742	6.444175451
34.59557526	39.01085261	22.60365496	42.05754339	8.592233934
19.21976404	0	0.982767607	3.235195645	8.592233934
182.5877583	282.1482596	235.8642257	160.6813837	181.5109419
4.804941009	0	0	3.235195645	0
2.882964605	1.814458261	2.948302821	0	1.074029242
42.28348088	41.73254	33.41409864	43.13594194	51.5534036
10.57087022	0.907229131	3.931070429	2.156797097	1.074029242
2.882964605	0	1.965535214	3.235195645	12.8883509
9.609882018	4.536145653	7.862140857	0	4.296116967
31.71261066	18.14458261	13.7587465	2.156797097	1.074029242
3.843952807	10.88674957	3.931070429	12.94078258	6.444175451
32.67359886	68.04218479	42.25900711	35.5871521	16.11043863
1.921976404	2.721687392	3.931070429	1.078398548	1.074029242
1.921976404	1.814458261	0.982767607	1.078398548	3.222087725
7.687905614	13.60843696	0	0	0
0	0	1.965535214	0	0
5.765929211	0.907229131	1.965535214	0	2.148058484
20.18075224	0.907229131	5.896605643	8.627188388	9.666263176
20.18075224	9.979520436	9.827676071	3.235195645	15.03640938
4.804941009	1.814458261	0	3.235195645	4.296116967
0	0	3.931070429	1.078398548	0
36.51755167	16.33012435	12.77597889	20.48957242	11.81432166
39.40051627	23.58795739	40.29347189	40.97914484	80.55219313
65.34719772	53.5265187	51.10391557	67.93910855	30.07281877
14.41482303	11.7939787	7.862140857	1.078398548	6.444175451
0.960988202	0	0.982767607	0	0
6.726917412	9.072291305	11.79321129	5.391992742	6.444175451
3.843952807	6.350603914	2.948302821	1.078398548	4.296116967
0	5.443374783	0	0	0
2.882964605	0	0	0	0
7.687905614	14.51566609	8.844908464	2.156797097	28.99878953
11.53185842	0	6.87937325	0	6.444175451

For Review Only

13.45383482	22.68072826	18.67258454	11.86238403	11.81432166
0	0	0.982767607	0	2.148058484
36.51755167	23.58795739	0.982767607	10.78398548	16.11043863
11.53185842	10.88674957	4.913838036	6.470391291	6.444175451
77.84004434	68.04218479	88.44908464	85.19348533	86.99636858
3.843952807	0	2.948302821	1.078398548	4.296116967
0	0	3.931070429	15.09757968	8.592233934
2.882964605	2.721687392	3.931070429	0	1.074029242
4.804941009	3.628916522	0.982767607	3.235195645	10.74029242
0	5.443374783	0.982767607	0	0
26.90766965	0	0.982767607	6.470391291	1.074029242
4.804941009	0	5.896605643	1.078398548	2.148058484
2.882964605	0.907229131	0	0	0
0	0	0	0	0
0.960988202	0	1.965535214	1.078398548	1.074029242
49.01039829	56.24820609	0.982767607	21.56797097	1.074029242
32.67359886	13.60843696	8.844908464	25.88156516	19.33252635
1.921976404	1.814458261	4.913838036	4.313594194	0
0	5.443374783	4.913838036	0	1.074029242
133.57736	114.3108704	37.34516907	51.76313033	122.4393336
0.960988202	0.907229131	0	1.078398548	2.148058484
130.6943954	122.4759326	70.75926771	48.52793468	94.51457327
6.726917412	0	0	2.156797097	0
0.960988202	0	1.965535214	0	0
3.843952807	4.536145653	1.965535214	4.313594194	3.222087725
0.960988202	0	0	0	0
2.882964605	0	0	1.078398548	2.148058484
2.882964605	6.350603914	0	0	4.296116967
4.804941009	11.7939787	11.79321129	9.705586936	12.8883509
0.960988202	0.907229131	0.982767607	0	0
0	0	0	0	0
486.2600301	444.542274	346.9169653	482.0441512	338.3192112
7.687905614	0	0	0	0
20.18075224	17.23735348	21.62088736	7.548789839	3.222087725
12.49284662	2.721687392	12.77597889	9.705586936	7.518204692
0.960988202	0	3.931070429	0	1.074029242
0	5.443374783	0	0	0
0	0	0	6.470391291	1.074029242
0	0.907229131	0	1.078398548	1.074029242
0	3.628916522	7.862140857	1.078398548	0
90.33289097	42.63976914	78.62140857	113.2318476	64.44175451
0	7.257833044	0.982767607	5.391992742	1.074029242
14.41482303	14.51566609	0.982767607	14.01918113	3.222087725
29.79063425	8.165062175	3.931070429	15.09757968	21.48058484
34.59557526	45.36145653	41.2762395	43.13594194	45.10922815
0	2.721687392	0	0	0
4.804941009	3.628916522	0.982767607	11.86238403	6.444175451
11.53185842	21.77349913	2.948302821	11.86238403	15.03640938
0	4.536145653	3.931070429	1.078398548	1.074029242
3.843952807	5.443374783	0.982767607	3.235195645	1.074029242

For Review Only

4.804941009	5.443374783	1.965535214	3.235195645	2.148058484
0	0	0	0	3.222087725
5.765929211	6.350603914	4.913838036	3.235195645	0
5.765929211	7.257833044	3.931070429	3.235195645	4.296116967
1.921976404	1.814458261	0	0	0
3.843952807	0	0.982767607	0	0
50.93237469	45.36145653	9.827676071	18.33277532	40.81311119
4.804941009	3.628916522	1.965535214	6.470391291	1.074029242
2.882964605	7.257833044	0.982767607	0	2.148058484
7.687905614	10.88674957	0	1.078398548	3.222087725
4.804941009	2.721687392	0.982767607	2.156797097	0
0.960988202	0	0.982767607	0	0
41.32249268	50.80483131	49.13838036	32.35195645	71.9599592
0	4.536145653	0	0	0
8.648893816	7.257833044	8.844908464	6.470391291	2.148058484
7.687905614	16.33012435	5.896605643	0	12.8883509
5.765929211	0	2.948302821	0	0
0.960988202	0	0.982767607	0	0
0	0	0.982767607	2.156797097	1.074029242
0	5.443374783	24.56919018	5.391992742	7.518204692
6.726917412	3.628916522	2.948302821	4.313594194	5.370146209
45.16644548	25.40241565	11.79321129	38.82234774	32.22087725
4.804941009	1.814458261	0.982767607	1.078398548	0
8.648893816	0	7.862140857	1.078398548	4.296116967
0	3.628916522	0	0	0
3.843952807	0	12.77597889	0	0
7.687905614	0.907229131	0	7.548789839	15.03640938
0	5.443374783	0	6.470391291	1.074029242
17.29778763	20.86627	12.77597889	19.41117387	6.444175451
15.37581123	10.88674957	12.77597889	12.94078258	11.81432166
0	0	0	0	0
63.42522132	51.71206044	45.20730993	69.0175071	65.51578375
11.53185842	0	5.896605643	8.627188388	4.296116967
4.804941009	17.23735348	11.79321129	1.078398548	20.40655559
0	2.721687392	0	9.705586936	12.8883509
0	0	0.982767607	19.41117387	1.074029242
23.06371684	11.7939787	10.81044368	15.09757968	8.592233934
0	4.536145653	0	0	0
10.57087022	0	0.982767607	2.156797097	0
0	2.721687392	0	0	0
19.21976404	27.21687392	14.74151411	1.078398548	15.03640938
0.960988202	0	0.982767607	1.078398548	0
19.21976404	9.979520436	16.70704932	22.64636952	13.96238014
7.687905614	4.536145653	9.827676071	9.705586936	13.96238014
5.765929211	9.979520436	1.965535214	6.470391291	4.296116967
5.765929211	11.7939787	0.982767607	17.25437678	1.074029242
0	4.536145653	0	0	5.370146209
0	0	1.965535214	3.235195645	3.222087725
0.960988202	4.536145653	2.948302821	3.235195645	0
41.32249268	39.01085261	27.517493	23.72476807	8.592233934

9.609882018	10.88674957	0.982767607	10.78398548	0
1.921976404	0	0	0	1.074029242
14.41482303	12.70120783	9.827676071	10.78398548	3.222087725
0	0	0.982767607	1.078398548	2.148058484
13.45383482	13.60843696	0.982767607	5.391992742	12.8883509
0	2.721687392	0.982767607	3.235195645	0
0	0	0	1.078398548	3.222087725
17.29778763	18.14458261	18.67258454	18.33277532	18.25849711
0	3.628916522	0	6.470391291	3.222087725
75.91806794	44.4542274	54.05221839	65.78231146	64.44175451
5.765929211	7.257833044	6.87937325	9.705586936	5.370146209
1.921976404	2.721687392	0	3.235195645	0
2.882964605	0	0.982767607	1.078398548	0
0	4.536145653	1.965535214	1.078398548	0
0	0.907229131	0	0	1.074029242
4.804941009	0	2.948302821	0	0
37.47853987	48.99037305	14.74151411	37.7439492	12.8883509
8.648893816	21.77349913	10.81044368	8.627188388	7.518204692
0	0	1.965535214	2.156797097	0
36.51755167	37.19639435	7.862140857	2.156797097	20.40655559
3.843952807	4.536145653	2.948302821	6.470391291	6.444175451
0	0	0	1.078398548	3.222087725
0	0	0.982767607	1.078398548	0
0	0	0.982767607	0	2.148058484
0	0	0	3.235195645	2.148058484
6.726917412	0	0	1.078398548	0
0	3.628916522	0	0	0
9.609882018	7.257833044	3.931070429	9.705586936	7.518204692
13.45383482	10.88674957	3.931070429	1.078398548	5.370146209
0	0	0	0	0
40.36150447	0.907229131	34.39686625	32.35195645	12.8883509
2.882964605	9.072291305	0.982767607	9.705586936	1.074029242
0.960988202	0	0	0	0
12.49284662	16.33012435	13.7587465	17.25437678	19.33252635
119.162537	124.2903909	132.673627	75.48789839	89.14442707
6.726917412	12.70120783	6.87937325	0	3.222087725
0	1.814458261	0	0	0
953.3002961	635.0603914	3.931070429	117.5454418	22.55461408
0	12.70120783	0.982767607	5.391992742	0
0	0	0	0	0
0	8.165062175	4.913838036	2.156797097	11.81432166
0.960988202	4.536145653	2.948302821	2.156797097	4.296116967
18.25877583	21.77349913	4.913838036	1.078398548	1.074029242
0	0	0	1.078398548	0
0	0	0	1.078398548	0
3.843952807	1.814458261	3.931070429	0	0
22.10272864	8.165062175	7.862140857	10.78398548	13.96238014
16.33679943	23.58795739	23.58642257	5.391992742	12.8883509
0.960988202	2.721687392	0	1.078398548	0
73.03510333	86.1867674	53.06945079	45.29273904	93.44054403

For Review Only

20.18075224	27.21687392	28.50026061	21.56797097	17.18446787
0	0	4.913838036	0	0
0	0	0.982767607	0	2.148058484
0	0	0	2.156797097	0
8.648893816	7.257833044	0	14.01918113	5.370146209
40.36150447	31.75301957	22.60365496	15.09757968	28.99878953
3.843952807	0	0.982767607	0	0
23.06371684	20.86627	11.79321129	18.33277532	20.40655559
49.01039829	64.41326827	35.37963386	30.19515936	13.96238014
0	0	2.948302821	2.156797097	2.148058484
7.687905614	7.257833044	7.862140857	4.313594194	6.444175451
98.98178478	113.4036413	37.34516907	44.21434049	39.73908194
0	0	0	0	1.074029242
0	6.350603914	1.965535214	2.156797097	2.148058484
12.49284662	0	5.896605643	2.156797097	19.33252635
0	0	0	1.078398548	2.148058484
11.53185842	4.536145653	9.827676071	21.56797097	0
18.25877583	10.88674957	11.79321129	3.235195645	16.11043863
2.882964605	5.443374783	2.948302821	5.391992742	5.370146209
0.960988202	0.907229131	0	0	3.222087725
0	2.721687392	0	0	0
0	0	0	1.078398548	0
22.10272864	25.40241565	38.32793668	12.94078258	19.33252635
2.882964605	0	0	0	5.370146209
0	0	0	6.470391291	0
1.921976404	2.721687392	0	0	4.296116967
0	0	0	0	0
28.82964605	19.95904087	18.67258454	11.86238403	9.666263176
3.843952807	10.88674957	3.931070429	1.078398548	1.074029242
0.960988202	0	0	0	0
0.960988202	2.721687392	0	0	0
0	5.443374783	0	2.156797097	4.296116967
0	0	0	3.235195645	0
3.843952807	0	0	0	0
32.67359886	48.99037305	26.53472539	25.88156516	41.88714043
1.921976404	1.814458261	2.948302821	0	0
7.687905614	12.70120783	11.79321129	7.548789839	18.25849711
3.843952807	0.907229131	0.982767607	1.078398548	3.222087725
0	0	0.982767607	0	0
3.843952807	13.60843696	14.74151411	14.01918113	7.518204692
2.882964605	2.721687392	0.982767607	3.235195645	0
0	0	0	3.235195645	0
0	0	0	0	0
2.882964605	0.907229131	0	1.078398548	0
0	0	0	0	0
5.765929211	3.628916522	5.896605643	3.235195645	2.148058484
3.843952807	0	0	0	0
9.609882018	2.721687392	4.913838036	3.235195645	5.370146209
0	0.907229131	0	3.235195645	0
4.804941009	0	1.965535214	3.235195645	5.370146209

For Review Only

9.609882018	0.907229131	3.931070429	2.156797097	11.81432166
31.71261066	33.56747783	18.67258454	49.60633323	11.81432166
3.843952807	0	0	0	0
22.10272864	14.51566609	8.844908464	11.86238403	5.370146209
49.97138649	29.93856131	13.7587465	9.705586936	34.36893574
3.843952807	0	0.982767607	1.078398548	0
12.49284662	15.42289522	3.931070429	23.72476807	6.444175451
191.2366522	219.5494496	95.32845789	262.0508473	126.7354505
3.843952807	3.628916522	0	0	1.074029242
0	0	0	0	0
2.882964605	1.814458261	0	3.235195645	4.296116967
5.765929211	7.257833044	0.982767607	0	0
40.36150447	27.21687392	19.65535214	44.21434049	30.07281877
140.3042775	180.538597	167.0704932	142.3486084	166.4745325
22.10272864	21.77349913	1.965535214	1.078398548	6.444175451
96.09882018	56.24820609	60.93159164	59.31192017	60.14563754
0	0	0	0	3.222087725
11.53185842	9.979520436	5.896605643	0	10.74029242
0	127.0120783	155.2772819	18.33277532	10.74029242
0	0.907229131	2.948302821	1.078398548	2.148058484
0	0	0	0	0
3.843952807	2.721687392	5.896605643	0	2.148058484
196.0415932	274.8904266	311.5373315	322.441166	157.8822985
510.2847351	508.9555422	424.5556063	498.2201294	543.4587963
275.8036139	279.4265722	272.2266272	166.0733765	211.5837606
3184.714901	2442.260819	3774.810379	3509.108877	3239.272193
0	0	0	0	154.6602108
62.46423311	72.57833044	45.20730993	64.70391291	84.8483101
159.5240415	156.0434105	200.4845919	211.3661155	242.7306086
932.1585557	872.7544236	977.8537691	941.4419328	785.1153757
24.02470504	9.072291305	27.517493	5.391992742	20.40655559
149.9141595	175.0952222	121.8631833	147.7406011	139.6238014
1.921976404	0.907229131	0.982767607	0	0
150.8751477	212.2916165	335.123754	421.6538324	310.3944509
217.1833336	208.6627	181.8120073	135.8782171	195.473322
282.5305313	250.39524	322.3477751	380.6746876	329.7269772
214.300369	177.8169096	114.0010424	105.6830577	205.1395852
0	0	0	0	0
0	5.443374783	2.948302821	1.078398548	4.296116967
0.960988202	9.979520436	2.948302821	0	0
0.960988202	4.536145653	1.965535214	0	2.148058484
16.33679943	9.072291305	6.87937325	9.705586936	22.55461408
59.58126851	68.04218479	61.91435925	71.1743042	80.55219313
5.765929211	0	1.965535214	1.078398548	2.148058484
172.9778763	168.7446183	143.4840706	134.7998186	114.9211289
17.29778763	0	21.62088736	11.86238403	17.18446787
27.86865785	129.7337657	0	139.1134128	133.179626
2057.47574	2955.752507	1914.431299	1129.08328	1242.651833
0	0	38.32793668	25.88156516	190.1031758
3.843952807	15.42289522	4.913838036	0	2.148058484

For Review Only

43.24446908	43.54699827	56.01775361	25.88156516	26.85073104
0	0	1.965535214	1.078398548	0
41.32249268	42.63976914	51.10391557	32.35195645	27.92476029
137.4213129	156.0434105	149.3806763	152.0541953	229.8422577
86.48893816	94.35182958	76.65587336	70.09590565	64.44175451
2.882964605	0	2.948302821	0	0
298.8673307	247.6735526	426.5211415	386.0666803	400.6129072
149.9141595	124.2903909	141.5185354	122.9374345	53.70146209
4.804941009	6.350603914	4.913838036	2.156797097	5.370146209
0	0	126.7770213	0	96.66263176
2.882964605	2.721687392	0.982767607	0	4.296116967
0.960988202	0	2.948302821	3.235195645	0
29.79063425	29.03133218	21.62088736	30.19515936	48.33131588
9.609882018	9.979520436	6.87937325	5.391992742	11.81432166
13.45383482	16.33012435	18.67258454	18.33277532	22.55461408
73.03510333	78.02170523	223.0882468	200.58213	0
0	9.072291305	0	0	0
33.63458706	38.10362348	46.19007754	16.17597823	7.518204692
31.71261066	28.12410305	8.844908464	1.078398548	1.074029242
2003.660401	2474.921068	0	1890.432655	1606.747746
0	0	0	0	0
0	0	0	0	0
55.7373157	47.17591479	32.43133104	29.11676081	38.6650527
17.29778763	18.14458261	1.965535214	12.94078258	10.74029242
30.75162246	30.84579044	53.06945079	28.03836226	39.73908194
73.03510333	132.4554531	222.1054792	195.1901373	52.62743285
16.33679943	2.721687392	1.965535214	11.86238403	17.18446787
0	0	23.58642257	0	0
17.29778763	16.33012435	39.31070429	36.66555065	38.6650527
0	0	24.56919018	1.078398548	32.22087725
15.37581123	14.51566609	32.43133104	24.80316661	13.96238014
52.8543511	68.94941392	63.87989446	60.39031871	69.81190071
549.6852514	632.338704	483.5216627	386.0666803	585.3459368
45.16644548	50.80483131	81.56971139	130.4862244	79.47816389
92.25486737	95.25905871	76.65587336	63.62551436	46.1832574
0	0	0.982767607	1.078398548	0
7.687905614	0	0.982767607	3.235195645	0
3.843952807	0	1.965535214	0	0
6.726917412	19.95904087	14.74151411	12.94078258	4.296116967
0	0	0.982767607	1.078398548	193.3252635
3.843952807	8.165062175	4.913838036	2.156797097	12.8883509
0	0	2.948302821	0	1.074029242
81.68399715	84.37230914	83.53524661	94.89907226	115.9951581
10.57087022	8.165062175	0.982767607	11.86238403	6.444175451
66.30818592	42.63976914	38.32793668	26.95996371	55.84952057
241.2080386	239.5084905	228.0020849	232.9340865	214.8058484
27.86865785	47.17591479	36.36240146	35.5871521	30.07281877
3.843952807	11.7939787	3.931070429	0	0
112.4356196	109.7747248	118.9148805	81.95828968	74.10801768
0	3.628916522	1.965535214	0	3.222087725

For Review Only

4.804941009	4.536145653	3.931070429	0	0
0	4.536145653	4.913838036	1.078398548	0
4.804941009	7.257833044	6.87937325	1.078398548	12.8883509
69.19115053	99.79520436	122.8459509	20.48957242	3.222087725
0	0	0.982767607	0	0
67.26917412	95.25905871	122.8459509	111.0750505	94.51457327
64.38620952	107.9602665	92.38015507	50.68473178	76.25607616
4.804941009	0.907229131	8.844908464	0	9.666263176
57.65929211	52.61928957	65.84542968	74.40949984	82.70025162
22.10272864	11.7939787	12.77597889	11.86238403	2.148058484
151.8361359	83.46508001	75.67310575	56.07672452	80.55219313
23.06371684	49.89760218	30.46579582	60.39031871	74.10801768
6.726917412	12.70120783	12.77597889	11.86238403	30.07281877
40.36150447	0	30.46579582	56.07672452	18.25849711
35.55656347	14.51566609	34.39686625	46.37113758	28.99878953
0	0	53.06945079	1.078398548	1.074029242
89.37190276	64.41326827	114.98381	99.21266646	92.36651479
0	0	0	0	6.444175451
36.51755167	39.91808174	46.19007754	47.44953613	34.36893574
197.0025814	167.8373891	155.2772819	155.289391	330.8010065
0	19.05181174	0.982767607	1.078398548	2.148058484
40.36150447	21.77349913	29.48302821	20.48957242	21.48058484
11.53185842	107.0530374	0	72.25270275	189.0291465
80.72300895	106.1458083	106.1389016	103.5262607	124.587392
10.57087022	30.84579044	15.72428171	31.27355791	8.592233934
7.687905614	8.165062175	0.982767607	1.078398548	3.222087725
2.882964605	0	0	1.078398548	1.074029242
6.726917412	0	5.896605643	0	0
0	0	0	0	0
97.05980838	105.2385791	118.9148805	174.7005649	171.8446787
150.8751477	170.5590765	320.3822399	359.1067166	0
0	0	151.3462115	0	0
112.4356196	107.9602665	103.1905987	73.3311013	113.8470996
58.62028031	78.02170523	52.08668318	57.15512307	57.99757905
61.50324491	91.63014218	113.0182748	107.8398548	54.77549133
180.6657819	165.1157018	120.8804157	72.25270275	140.6978307
95.13783197	108.8674957	90.41461986	145.583804	115.9951581
0	0	0	0	0
0	0	0.982767607	0	1.074029242
57.65929211	34.47470696	49.13838036	47.44953613	39.73908194
40.36150447	24.49518652	33.41409864	10.78398548	50.47937436
0.960988202	13.60843696	8.844908464	10.78398548	15.03640938
0	0	0	0	2.148058484
11.53185842	0	0.982767607	0	6.444175451
47.08842189	695.8447431	2188.623461	6.470391291	7.518204692
7.687905614	7.257833044	5.896605643	0	0
0	0	6.87937325	0	1.074029242
2.882964605	12.70120783	9.827676071	0	11.81432166
0	0	0.982767607	7.548789839	0
1.921976404	1.814458261	0	5.391992742	0

For Review Only

1677.8854	1776.354638	789.1623885	2053.270836	2754.885005
65.34719772	78.02170523	57.98328882	60.39031871	35.44296498
0	0	47.17284514	49.60633323	48.33131588
12.49284662	30.84579044	18.67258454	18.33277532	33.29490649
0	8.165062175	0	136.9566157	0
1917.171463	1526.866627	2281.986384	136.9566157	1868.810881
0	7.257833044	2.948302821	0	0
14.41482303	17.23735348	25.55195779	22.64636952	7.518204692
98.02079658	87.09399653	45.20730993	33.430355	3.222087725
0.960988202	0.907229131	143.4840706	149.8973982	73.03398844
4.804941009	9.979520436	6.87937325	2.156797097	8.592233934
19.21976404	34.47470696	24.56919018	15.09757968	32.22087725
0	0	6.87937325	1.078398548	1.074029242
56.6983039	61.69158088	65.84542968	72.25270275	70.88592996
35.55656347	29.03133218	21.62088736	54.99832597	33.29490649
3.843952807	3.628916522	0.982767607	0	2.148058484
63.42522132	71.67110131	44.22454232	56.07672452	62.29369602
36.51755167	36.28916522	37.34516907	51.76313033	11.81432166
0	0	0	0	0
77.84004434	0	55.034986	0	52.62743285
34.59557526	56.24820609	22.60365496	48.52793468	45.10922815
205.6514752	127.9193074	120.8804157	144.5054055	170.7706494
0	0	0	0	0
57.65929211	60.78435175	64.86266207	84.11508678	91.29248555
459.3523604	565.2037483	645.6783179	673.9990928	688.452744
0.960988202	0.907229131	8.844908464	10.78398548	0
78.80103254	83.46508001	117.9321129	87.35028243	57.99757905
43.24446908	67.13495566	32.43133104	60.39031871	53.70146209
110.5136432	117.0325578	101.2250635	133.72142	89.14442707
2.882964605	6.350603914	0	4.313594194	10.74029242
13.45383482	13.60843696	9.827676071	17.25437678	12.8883509
0.960988202	3.628916522	1.965535214	2.156797097	0
13.45383482	9.979520436	3.931070429	0	0
627.5252958	606.0290592	741.9895434	792.6229331	608.9745801
0.960988202	3.628916522	0	1.078398548	0
2088.227362	1973.223359	1001.440192	1481.719606	1948.289045
9.609882018	2.721687392	10.81044368	10.78398548	6.444175451
0	0	0	0	0
63.42522132	36.28916522	35.37963386	42.05754339	44.03519891
13.45383482	18.14458261	23.58642257	4.313594194	26.85073104
35.55656347	29.03133218	48.15561275	38.82234774	25.7767018
0.960988202	33.56747783	32.43133104	34.50875355	0
275.8036139	198.6831796	209.3295003	245.874869	209.4357021
203.7294988	217.7349913	307.606261	314.8923761	231.9903162
274.8426257	211.3843874	233.8986905	353.7147239	330.8010065
189.3146757	0	387.2104372	0	0
657.31593	848.259237	1120.355072	1020.165027	1028.920014
4.804941009	6.350603914	5.896605643	3.235195645	0
0	0	153.3117467	78.72309404	0
62.46423311	52.61928957	72.72480293	69.0175071	36.51699422

For Review Only

55.7373157	54.43374783	113.0182748	104.6046592	64.44175451
0	0	0	0	105.2548657
79.76202075	64.41326827	130.7080917	132.6430215	96.66263176
23.06371684	33.56747783	31.44856343	11.86238403	4.296116967
29.79063425	36.28916522	16.70704932	8.627188388	18.25849711
0.960988202	0	0.982767607	1.078398548	3.222087725
49.97138649	65.3204974	33.41409864	37.7439492	37.59102346
7.687905614	19.05181174	2.948302821	6.470391291	3.222087725
0	41.73254	13.7587465	16.17597823	18.25849711
0	0	0	0	0
258.5058263	161.4867852	217.1916412	262.0508473	168.622591
0	0	0.982767607	0	5.370146209
64.38620952	45.36145653	83.53524661	56.07672452	88.07039782
0	0	0	0	0
10060.58548	10166.40964	6917.701187	9164.230865	10129.16978
23.06371684	31.75301957	41.2762395	29.11676081	20.40655559
49.01039829	81.65062175	44.22454232	46.37113758	52.62743285
12.49284662	20.86627	41.2762395	39.90074629	10.74029242
94.17684377	88.00122566	45.20730993	135.8782171	80.55219313
479.5331127	440.0061283	416.6934654	351.5579268	441.4260184
218.1443218	322.9735705	262.3989511	257.7372531	244.8786671
12.49284662	20.86627	5.896605643	1.078398548	8.592233934
0	0	0	0	0
115.3185842	154.2289522	133.6563946	76.56629694	189.0291465
357.4876111	310.2723626	343.9686625	321.3627674	442.5000476
0	99.79520436	115.9665776	44.21434049	226.62017
195.080605	239.5084905	273.2093948	269.5996371	253.4709011
26.90766965	32.6602487	10.81044368	48.52793468	27.92476029
247.9349561	200.4976378	252.571275	145.583804	132.1055967
27.86865785	716.7110131	0	1.078398548	0
669.8087766	858.2387575	810.7832759	874.5812228	514.4600068
0	138.806057	10.81044368	66.86071	23.62864332
12.49284662	17.23735348	0	0	0
159.5240415	149.6928065	146.4323735	84.11508678	113.8470996
46.12743368	59.87712262	65.84542968	77.64469549	61.21966678
106.6696904	69.85664305	60.93159164	64.70391291	63.36772526
0	0	0	0	1.074029242
65.34719772	94.35182958	133.6563946	205.9741228	142.8458892
4.804941009	0	0	3.235195645	2.148058484
9.609882018	0	3.931070429	0	4.296116967
0	0	0	0	0
70.15213873	81.65062175	39.31070429	38.82234774	44.03519891
0	2.721687392	0	0	0
4.804941009	0.907229131	27.517493	6.470391291	1.074029242
29.79063425	43.54699827	80.58694379	47.44953613	56.92354981
191.2366522	173.2807639	0	0	0
125.8894544	0	133.6563946	292.2460066	1.074029242
442.0545728	425.4904622	542.4877191	590.9624046	439.2779599
29.79063425	76.20724696	141.5185354	196.2685358	105.2548657
114.357596	134.2699113	162.1566552	148.8189997	100.9587487

For Review Only

0	10.88674957	6.87937325	4.313594194	2.148058484
50.93237469	38.10362348	57.00052121	53.91992742	69.81190071
69.19115053	54.43374783	62.89712686	107.8398548	80.55219313
34.59557526	44.4542274	31.44856343	64.70391291	38.6650527
42.28348088	48.08314392	25.55195779	52.84152887	42.96116967
313.2821538	271.26151	221.1227116	152.0541953	243.8046379
3.843952807	3.628916522	4.913838036	0	16.11043863
186.4317111	168.7446183	289.9164441	171.4653692	196.5473512
69.19115053	48.99037305	45.20730993	25.88156516	47.25728664
0	7.257833044	2.948302821	5.391992742	1.074029242
85.52794996	75.30001783	157.2428171	67.93910855	97.736661
0	0	0	0	0
21.14174044	10.88674957	20.63811975	3.235195645	13.96238014
91.29387917	90.72291305	53.06945079	104.6046592	76.25607616
127.8114308	116.1253287	113.0182748	126.1726302	126.7354505
147.0311949	89.81568392	89.43185225	161.7597823	80.55219313
93.21585557	111.5891831	127.7597889	107.8398548	122.4393336
74.95707974	80.74339262	78.62140857	65.78231146	55.84952057
86.48893816	107.0530374	63.87989446	53.91992742	89.14442707
535.2704284	575.1832688	462.883543	310.578782	370.5400884
0.960988202	0	0	0	0
13.45383482	229.52897	126.7770213	136.9566157	147.1420061
56.6983039	53.5265187	40.29347189	44.21434049	40.81311119
127.8114308	195.0542631	111.0527396	117.5454418	113.8470996
52.8543511	68.04218479	30.46579582	19.41117387	63.36772526
3.843952807	0.907229131	0	0	0
2.882964605	5.443374783	0	0	1.074029242
21.14174044	34.47470696	12.77597889	9.705586936	20.40655559
68.23016233	61.69158088	108.1044368	84.11508678	69.81190071
169.1339235	160.5795561	70.75926771	113.2318476	171.8446787
0	0	0	0	0
4.804941009	0	0	0	0
0	0	0.982767607	4.313594194	1.074029242
73.99609154	69.85664305	80.58694379	97.05586936	75.18204692
159.5240415	206.8482418	187.708613	133.72142	206.2136144
0	0	115.9665776	117.5454418	0
0	0	5.896605643	0	0
91.29387917	79.83616349	65.84542968	58.23352162	63.36772526
111.4746314	38.10362348	57.98328882	66.86071	34.36893574
32.67359886	36.28916522	24.56919018	53.91992742	60.14563754
2.882964605	1.814458261	2.948302821	0	0
243.130015	215.9205331	161.1738876	146.6622026	200.8434682
85.52794996	73.48555957	62.89712686	60.39031871	75.18204692
55.7373157	73.48555957	38.32793668	29.11676081	27.92476029
51.8933629	53.5265187	75.67310575	28.03836226	53.70146209
73.03510333	86.1867674	51.10391557	43.13594194	73.03398844
10.57087022	17.23735348	0	0	0
50.93237469	58.96989348	3.931070429	20.48957242	50.47937436
319.048083	376.5000892	432.4177471	395.7722673	274.9514859
203.7294988	168.7446183	176.8981693	155.289391	161.1043863

For Review Only

26.90766965	21.77349913	28.50026061	23.72476807	19.33252635
0.960988202	6.350603914	5.896605643	1.078398548	1.074029242
133.57736	223.1783661	242.743599	253.4236589	211.5837606
491.0649711	174.1879931	313.5028667	93.82067372	99.88471948
2.882964605	8.165062175	3.931070429	0	4.296116967
12.49284662	18.14458261	31.44856343	17.25437678	12.8883509
1.921976404	1.814458261	2.948302821	0	4.296116967
37.47853987	26.30964479	42.25900711	36.66555065	47.25728664
166.2509589	155.1361813	73.70757054	93.82067372	140.6978307
217.1833336	215.0133039	87.46631704	61.46871726	69.81190071
87.44992636	78.02170523	38.32793668	66.86071	79.47816389
0	0	2.948302821	0	0
737.0779508	828.3001962	710.54098	914.4819691	1165.321727
15.37581123	9.979520436	30.46579582	10.78398548	32.22087725
207.5734516	185.0747426	99.25952832	87.35028243	196.5473512
0	0	0	0	0
107.6306786	94.35182958	112.0355072	101.3694636	91.29248555
77.84004434	88.00122566	84.51801421	117.5454418	117.0691874
15.37581123	20.86627	23.58642257	46.37113758	32.22087725
33.63458706	43.54699827	127.7597889	33.430355	4.296116967
27.86865785	47.17591479	10.81044368	19.41117387	34.36893574
0.960988202	0	10.81044368	2.156797097	5.370146209
17.29778763	11.7939787	18.67258454	26.95996371	30.07281877
0	8.165062175	0	0	0
34.59557526	13.60843696	29.48302821	11.86238403	21.48058484
24.02470504	19.05181174	56.01775361	16.17597823	1.074029242
42.28348088	51.71206044	30.46579582	50.68473178	35.44296498
309.438201	243.137407	92.38015507	443.2218034	134.2536552
102.8257376	67.13495566	57.98328882	52.84152887	61.21966678
0	0	0.982767607	7.548789839	5.370146209
488.1820065	401.9025048	591.6260995	881.0516141	572.4575859
128.772419	135.1771404	247.657437	168.2301736	220.1759946
0	0	0	47.44953613	0
0	6.350603914	0	0	2.148058484
243.130015	229.52897	159.2083524	172.5437678	332.9490649
27.86865785	309.3651335	41.2762395	199.5037315	111.6990411
264.2717555	141.5277444	297.778585	293.3244052	68.73787147
81.68399715	66.22772653	51.10391557	52.84152887	60.14563754
49.97138649	46.26868566	66.82819729	62.54711581	67.66384223
67.26917412	99.79520436	68.7937325	78.72309404	86.99636858
0.960988202	11.7939787	2.948302821	1.078398548	10.74029242
149.9141595	143.3422026	148.3979087	138.0350142	2.148058484
0	6.350603914	4.913838036	2.156797097	7.518204692
1.921976404	1.814458261	0	0	19.33252635
21.14174044	25.40241565	30.46579582	10.78398548	6.444175451
61.50324491	43.54699827	50.12114796	52.84152887	47.25728664
74.95707974	68.94941392	82.552479	73.3311013	90.21845631
56.6983039	41.73254	29.48302821	48.52793468	56.92354981
15.37581123	5.443374783	12.77597889	7.548789839	33.29490649
0	0	2.948302821	1.078398548	1.074029242

For Review Only

452.625443	242.2301779	271.2438596	273.9132313	161.1043863
289.2574487	193.2398048	271.2438596	314.8923761	178.2888541
0	0	0.982767607	0	2.148058484
33.63458706	32.6602487	30.46579582	26.95996371	28.99878953
4.804941009	0	0	0	0
0	0	161.1738876	607.1383828	99.88471948
67.26917412	51.71206044	41.2762395	69.0175071	39.73908194
75.91806794	73.48555957	55.034986	70.09590565	60.14563754
190.2756639	211.3843874	174.9326341	161.7597823	144.9939476
80.72300895	56.24820609	44.22454232	43.13594194	52.62743285
2.882964605	1.814458261	0	1.078398548	0
24.98569325	22.68072826	36.36240146	36.66555065	50.47937436
12.49284662	12.70120783	3.931070429	2.156797097	6.444175451
55.7373157	58.96989348	72.72480293	43.13594194	38.6650527
3.843952807	0	0	0	0
42.28348088	28.12410305	48.15561275	49.60633323	25.7767018
9.609882018	23.58795739	7.862140857	9.705586936	26.85073104
0	0	5.896605643	0	0
2.882964605	0	0	0	0
301.7502954	315.7157374	382.2965992	433.5162165	398.4648487
0	0	0	210.2877169	0
26.90766965	22.68072826	30.46579582	14.01918113	34.36893574
120.1235252	157.8578687	153.3117467	167.151775	152.5121523
101.8647494	101.6096626	78.62140857	98.13426791	90.21845631
45.16644548	51.71206044	47.17284514	44.21434049	31.14684801
21.14174044	27.21687392	15.72428171	18.33277532	50.47937436
54.7763275	46.26868566	31.44856343	35.5871521	51.5534036
0.960988202	0	2.948302821	1.078398548	21.48058484
12.49284662	26.30964479	14.74151411	4.313594194	11.81432166
151.8361359	150.6000357	97.29399311	255.580456	184.7330296
8.648893816	3.628916522	1.965535214	1.078398548	2.148058484
0	0	0	0	2.148058484
25.94668145	17.23735348	25.55195779	31.27355791	17.18446787
38.43952807	14.51566609	69.77650011	34.50875355	16.11043863
94.17684377	112.4964122	66.82819729	64.70391291	63.36772526
19.21976404	21.77349913	50.12114796	70.09590565	77.33010541
14.41482303	8.165062175	13.7587465	20.48957242	8.592233934
14.41482303	20.86627	24.56919018	23.72476807	12.8883509
167.2119471	138.806057	101.2250635	94.89907226	144.9939476
163.3679943	128.8265365	120.8804157	164.9949779	121.3653043
8.648893816	3.628916522	0.982767607	1.078398548	17.18446787
7.687905614	0	3.931070429	1.078398548	1.074029242
7.687905614	0	0	5.391992742	6.444175451
0.960988202	0	1.965535214	1.078398548	0
197.0025814	185.9819718	349.8652681	226.4636952	252.3968718
231.5981566	0	5.896605643	201.6605286	17.18446787
0	0	0	4.313594194	0
11.53185842	11.7939787	9.827676071	9.705586936	8.592233934
134.5383482	159.672327	150.3634439	183.3277532	161.1043863
548.7242632	410.067567	344.9514301	496.0633323	514.4600068

For Review Only

168.1729353	167.8373891	62.89712686	28.03836226	308.2463924
0.960988202	4.536145653	0	0	0
0	6.350603914	3.931070429	1.078398548	0
35.55656347	58.06266435	41.2762395	39.90074629	47.25728664
0	0	0	42.05754339	0
70.15213873	75.30001783	57.00052121	102.4478621	82.70025162
5.765929211	0	0	0	0
90.33289097	83.46508001	73.70757054	89.50707952	55.84952057
14.41482303	24.49518652	24.56919018	28.03836226	21.48058484
265.2327437	270.3542809	275.17493	218.9149053	251.3228426
55.7373157	80.74339262	82.552479	75.48789839	62.29369602
0	60.78435175	0	0	0
173.9388645	152.4144939	117.9321129	103.5262607	88.07039782
295.9843661	240.4157196	206.3811975	239.4044778	350.1335328
163.3679943	129.7337657	61.91435925	108.9182534	120.2912751
246.0129797	170.5590765	173.9498665	199.5037315	184.7330296
72.07411513	74.3927887	58.96605643	21.56797097	83.77428086
94.17684377	109.7747248	111.0527396	144.5054055	137.4757429
25.94668145	36.28916522	33.41409864	51.76313033	26.85073104
95.13783197	79.83616349	168.0532608	176.8573619	136.4017137
23.06371684	17.23735348	26.53472539	14.01918113	4.296116967
24.02470504	19.05181174	29.48302821	20.48957242	30.07281877
119.162537	105.2385791	108.1044368	111.0750505	107.4029242
66.30818592	67.13495566	59.94882404	58.23352162	64.44175451
0	43.54699827	0	287.9324124	0
126.8504426	129.7337657	121.8631833	75.48789839	76.25607616
83.60597355	49.89760218	79.60417618	100.291065	40.81311119
62.46423311	70.76387218	84.51801421	135.8782171	79.47816389
39.40051627	40.82531087	67.81096489	53.91992742	62.29369602
46.12743368	42.63976914	53.06945079	65.78231146	56.92354981
159.5240415	157.8578687	166.0877256	177.9357605	165.4005032
33.63458706	49.89760218	39.31070429	28.03836226	41.88714043
21.14174044	18.14458261	44.22454232	44.21434049	44.03519891
24.02470504	19.95904087	23.58642257	8.627188388	22.55461408
0.960988202	11.7939787	0	46.37113758	85.92233934
29.79063425	39.91808174	51.10391557	47.44953613	51.5534036
91.29387917	90.72291305	93.36292268	83.03668823	150.3640938
106.6696904	0	0	0	51.5534036
47.08842189	61.69158088	91.39738746	63.62551436	35.44296498
828.3718299	846.4447788	549.3670924	444.300202	873.1857735
852.396535	981.6219192	999.4746565	705.2726507	958.0340836
100.9037612	48.08314392	111.0527396	103.5262607	93.44054403
0	0	0.982767607	0	0
28.82964605	0	0.982767607	3.235195645	19.33252635
149.9141595	136.0843696	113.0182748	94.89907226	86.99636858
14.41482303	3.628916522	10.81044368	8.627188388	6.444175451
14.41482303	46.26868566	7.862140857	9.705586936	7.518204692
32.67359886	64.41326827	62.89712686	54.99832597	55.84952057
0	0	0	0	0
53.8153393	58.06266435	82.552479	63.62551436	79.47816389

For Review Only

51.8933629	70.76387218	95.32845789	106.7614563	80.55219313
0	0	0	1.078398548	0
55.7373157	27.21687392	50.12114796	109.9966519	64.44175451
2589.863204	2478.549985	2012.708059	2138.464322	1594.933424
13.45383482	9.072291305	9.827676071	12.94078258	9.666263176
193.1586286	212.2916165	229.9676201	309.5003834	291.0619245
3.843952807	0.907229131	10.81044368	2.156797097	1.074029242
0	0	0	3.235195645	1.074029242
100.9037612	137.8988278	70.75926771	127.2510287	96.66263176
143.1872421	136.0843696	149.3806763	129.4078258	115.9951581
127.8114308	166.0229309	248.6402046	184.4061518	268.5073104
5.765929211	0	1.965535214	1.078398548	2.148058484
0	0	0.982767607	0	2.148058484
17.29778763	19.95904087	26.53472539	14.01918113	24.70267256
19.21976404	11.7939787	12.77597889	24.80316661	16.11043863
26.90766965	13.60843696	55.034986	30.19515936	12.8883509
38.43952807	35.38193609	41.2762395	42.05754339	67.66384223
25.94668145	19.95904087	25.55195779	3.235195645	15.03640938
83.60597355	48.99037305	71.74203532	81.95828968	68.73787147
25.94668145	0	44.22454232	0	0
11.53185842	22.68072826	22.60365496	26.95996371	1.074029242
30.75162246	52.61928957	51.10391557	24.80316661	59.0716083
5.765929211	8.165062175	9.827676071	14.01918113	17.18446787
135.4993364	129.7337657	133.6563946	158.5245866	113.8470996
1.921976404	0.907229131	0	4.313594194	0
4.804941009	20.86627	0	29.11676081	0
0	0	13.7587465	0	0
35.55656347	47.17591479	38.32793668	39.90074629	48.33131588
0	0	0.982767607	0	1.074029242
67.26917412	53.5265187	28.50026061	34.50875355	28.99878953
20.18075224	27.21687392	23.58642257	16.17597823	36.51699422
21.14174044	39.91808174	52.08668318	53.91992742	47.25728664
40.36150447	39.91808174	49.13838036	60.39031871	50.47937436
4.804941009	4.536145653	9.827676071	7.548789839	7.518204692
2.882964605	3.628916522	3.931070429	5.391992742	3.222087725
28.82964605	37.19639435	22.60365496	32.35195645	59.0716083
36.51755167	34.47470696	27.517493	54.99832597	57.99757905
2.882964605	9.072291305	3.931070429	4.313594194	6.444175451
14.41482303	11.7939787	15.72428171	5.391992742	25.7767018
24.02470504	28.12410305	13.7587465	10.78398548	66.58981299
2.882964605	7.257833044	6.87937325	3.235195645	4.296116967
143.1872421	11.7939787	27.517493	136.9566157	135.3276845
101.8647494	57.15543522	81.56971139	60.39031871	75.18204692
0	3.628916522	0	0	0
305.5942482	452.7073361	109.0872044	179.014159	345.8374158
12.49284662	6.350603914	4.913838036	2.156797097	2.148058484
166.2509589	198.6831796	188.6913806	147.7406011	204.0655559
0	59.87712262	21.62088736	10.78398548	16.11043863
75.91806794	74.3927887	84.51801421	75.48789839	70.88592996
21.14174044	30.84579044	27.517493	8.627188388	31.14684801

For Review Only

68.23016233	71.67110131	123.8287185	97.05586936	31.14684801
178.7438055	340.210924	172.9670989	19.41117387	8.592233934
517.0116525	617.8230379	738.058473	531.6504844	334.0230942
0	0	14.74151411	0	0
0.960988202	0.907229131	348.8825005	48.52793468	0
130.6943954	90.72291305	136.6046974	109.9966519	128.883509
172.0168881	166.0229309	246.6746694	149.8973982	153.5861816
262.3497791	318.4374248	222.1054792	271.7564342	221.2500238
0	0	0.982767607	0	0
0	0	0	1.078398548	6.444175451
26.90766965	33.56747783	44.22454232	26.95996371	31.14684801
3.843952807	8.165062175	5.896605643	25.88156516	8.592233934
0	4.536145653	1.965535214	0	0
0	9.072291305	0	0	0
86.48893816	83.46508001	90.41461986	175.7789634	154.6602108
0	0	0.982767607	2.156797097	0
0	0	0.982767607	2.156797097	0
4.804941009	5.443374783	9.827676071	22.64636952	10.74029242
18.25877583	8.165062175	9.827676071	24.80316661	23.62864332
0	6.350603914	1.965535214	2.156797097	0
72.07411513	58.06266435	25.55195779	22.64636952	30.07281877
14.41482303	38.10362348	2.948302821	3.235195645	30.07281877
170.0949117	140.6205152	85.50078182	127.2510287	120.2912751
342.1117998	397.3663592	539.5394163	397.9290644	401.6869364
112.4356196	139.7132861	103.1905987	132.6430215	150.3640938
0	0	0.982767607	1.078398548	0
1200.274264	1382.617195	1145.90703	1088.104135	1230.837511
10.57087022	25.40241565	23.58642257	36.66555065	39.73908194
309.438201	335.6747783	285.0026061	337.5387457	254.5449303
184.5097347	148.7855774	159.2083524	150.9757968	185.8070588
0	0	0	3.235195645	2.148058484
184.5097347	185.9819718	183.7775425	191.9549416	235.2124039
75.91806794	88.90845479	144.4668382	169.3085721	119.2172458
8.648893816	4.536145653	13.7587465	0	8.592233934
111.4746314	239.5084905	230.9503877	16.17597823	80.55219313
0	0	0.982767607	0	0
0	17.23735348	19.65535214	0	16.11043863
231.5981566	144.2494318	197.536289	187.6413474	172.9187079
1.921976404	0	0	0	0
47.08842189	39.01085261	39.31070429	44.21434049	69.81190071
0	74.3927887	31.44856343	7.548789839	7.518204692
0	86.1867674	29.48302821	7.548789839	25.7767018
0	0	0	0	0
79.76202075	104.33135	49.13838036	88.42868097	67.66384223
41.32249268	41.73254	32.43133104	47.44953613	37.59102346
74.95707974	73.48555957	94.34569029	130.4862244	66.58981299
47.08842189	43.54699827	72.72480293	51.76313033	81.62622237
21.14174044	31.75301957	28.50026061	11.86238403	39.73908194
82.64498535	96.16628784	0	0	90.21845631
0	0	0	0	0

66.30818592	60.78435175	35.37963386	17.25437678	57.99757905
98.98178478	97.9807461	92.38015507	77.64469549	66.58981299
24.02470504	38.10362348	35.37963386	17.25437678	31.14684801
0.960988202	0.907229131	0	0	0
116.2795724	137.8988278	94.34569029	126.1726302	125.6614213
23.06371684	25.40241565	5.896605643	81.95828968	36.51699422
0	55.34097696	57.00052121	59.31192017	56.92354981
48.04941009	57.15543522	69.77650011	17.25437678	6.444175451
0.960988202	15.42289522	0	63.62551436	3.222087725
25.94668145	0	0	0	0
0	0	0	0	0
40.36150447	27.21687392	34.39686625	34.50875355	64.44175451
51.8933629	67.13495566	53.06945079	29.11676081	32.22087725
45.16644548	37.19639435	47.17284514	43.13594194	44.03519891
110.5136432	0	58.96605643	1.078398548	66.58981299
117.2405606	80.74339262	155.2772819	181.1709561	166.4745325
54.7763275	39.91808174	101.2250635	48.52793468	71.9599592
7.687905614	2.721687392	15.72428171	0	20.40655559
1.921976404	0	18.67258454	17.25437678	0
10.57087022	11.7939787	26.53472539	1.078398548	1.074029242
0	0	0	0	0
0	0	2.948302821	0	1.074029242
8.648893816	9.979520436	3.931070429	7.548789839	3.222087725
3.843952807	2.721687392	0.982767607	6.470391291	0
4.804941009	2.721687392	2.948302821	8.627188388	4.296116967
0	0	0	0	0
0	0	17.68981693	19.41117387	0
0	0	0.982767607	1.078398548	1.074029242
0	0	0	0	0
0	0	0	0	0
0	0	0.982767607	0	0
0.960988202	2.721687392	0	0	4.296116967
0	0	0.982767607	1.078398548	0
0	0	0	4.313594194	3.222087725
0.960988202	5.443374783	5.896605643	3.235195645	9.666263176
1.921976404	0.907229131	4.913838036	1.078398548	1.074029242
0	1.814458261	3.931070429	0	3.222087725
0	0.907229131	0	0	1.074029242
2.882964605	0	0	0	1.074029242
4.804941009	2.721687392	7.862140857	5.391992742	7.518204692
0	0.907229131	0	0	0
0	0	0	5.391992742	3.222087725
0	0.907229131	0	1.078398548	2.148058484
0.960988202	5.443374783	6.87937325	3.235195645	4.296116967
2.882964605	0.907229131	0	0	1.074029242
0.960988202	0	0	0	0
5.765929211	0	0	0	1.074029242
1.921976404	2.721687392	0	0	0
0.960988202	14.51566609	0	5.391992742	0
40.36150447	23.58795739	27.517493	42.05754339	51.5534036

0	0	7.862140857	0	2.148058484
0	0	26.53472539	22.64636952	0
5.765929211	7.257833044	1.965535214	6.470391291	5.370146209
0	0	1.965535214	2.156797097	1.074029242
1.921976404	4.536145653	0	0	0
0.960988202	0	16.70704932	2.156797097	12.8883509
0	0	0	0	0
45.16644548	39.91808174	177.8809369	173.6221663	309.3204216
0	0	0	0	0
0	2.721687392	0	2.156797097	1.074029242
3.843952807	0.907229131	0.982767607	1.078398548	0
0	3.628916522	1.965535214	0	0
5.765929211	0.907229131	1.965535214	2.156797097	2.148058484
33.63458706	34.47470696	46.19007754	37.7439492	45.10922815
10.57087022	15.42289522	19.65535214	17.25437678	17.18446787
12.49284662	19.95904087	22.60365496	22.64636952	17.18446787
0.960988202	0	0	0	3.222087725
0	0	1.965535214	0	0
0	0	0	0	0
1.921976404	0.907229131	0.982767607	1.078398548	0
0.960988202	2.721687392	0	0	0
3.843952807	4.536145653	3.931070429	2.156797097	6.444175451
0	0	0.982767607	8.627188388	2.148058484
0	0	0	1.078398548	0
1.921976404	2.721687392	2.948302821	6.470391291	0
0	0	0	0	0
0	0	0	1.078398548	2.148058484
0	1.814458261	0.982767607	1.078398548	0
2.882964605	0.907229131	1.965535214	1.078398548	0
0	0	5.896605643	4.313594194	6.444175451
0.960988202	0	0.982767607	0	0
1.921976404	0.907229131	0	1.078398548	0
33.63458706	78.92893436	35.37963386	57.15512307	67.66384223
0	0	1.965535214	2.156797097	3.222087725
0	0	2.948302821	2.156797097	1.074029242
2.882964605	0.907229131	0	3.235195645	1.074029242
10.57087022	5.443374783	10.81044368	10.78398548	5.370146209
9.609882018	0	0.982767607	9.705586936	2.148058484
8.648893816	4.536145653	0	6.470391291	2.148058484
0.960988202	5.443374783	19.65535214	0	9.666263176
51.8933629	30.84579044	1.965535214	20.48957242	1.074029242
0	0	119.8976481	154.2109924	150.3640938
0	0	1.965535214	1.078398548	1.074029242
1.921976404	0.907229131	0	8.627188388	0
4.804941009	4.536145653	1.965535214	3.235195645	3.222087725
20.18075224	7.257833044	22.60365496	10.78398548	30.07281877
43.24446908	19.95904087	24.56919018	10.78398548	21.48058484
0	2.721687392	3.931070429	0	0
0	0	0	0	0
103.7867258	82.55785088	197.536289	188.719746	122.4393336

For Review Only

16.33679943	21.77349913	11.79321129	30.19515936	15.03640938
0.960988202	0	1.965535214	0	0
28.82964605	27.21687392	47.17284514	28.03836226	30.07281877
109.552655	114.3108704	62.89712686	101.3694636	111.6990411
9.609882018	10.88674957	15.72428171	3.235195645	6.444175451
52.8543511	35.38193609	49.13838036	50.68473178	61.21966678
147.0311949	110.6819539	134.6391622	105.6830577	113.8470996
59.58126851	88.90845479	83.53524661	50.68473178	62.29369602
931.1975675	0	0.982767607	5.391992742	1.074029242
154.7191005	307.5506753	0	0	129.9575383
0	0	92.38015507	71.1743042	3.222087725
100.9037612	96.16628784	110.069972	121.859036	135.3276845
54.7763275	53.5265187	37.34516907	48.52793468	47.25728664
6.726917412	0	0.982767607	1.078398548	3.222087725
28.82964605	41.73254	57.98328882	20.48957242	28.99878953
473.7671835	425.4904622	417.676233	488.5145425	373.7621761
43.24446908	20.86627	0	155.289391	165.4005032
62.46423311	57.15543522	53.06945079	48.52793468	53.70146209
43.24446908	32.6602487	20.63811975	7.548789839	15.03640938
0	1.814458261	5.896605643	0	0
44.20545728	38.10362348	42.25900711	28.03836226	0
91.29387917	117.939787	70.75926771	100.291065	91.29248555
14.41482303	35.38193609	25.55195779	44.21434049	37.59102346
6.726917412	2.721687392	3.931070429	8.627188388	6.444175451
26.90766965	23.58795739	16.70704932	43.13594194	9.666263176
51.8933629	56.24820609	42.25900711	36.66555065	41.88714043
461.2743368	312.99405	279.1060004	575.8648249	7.518204692
28.82964605	33.56747783	61.91435925	37.7439492	53.70146209
3.843952807	0.907229131	0	0	77.33010541
0	0	1.965535214	2.156797097	0
100.9037612	828.3001962	29.48302821	0	360.8738252
29.79063425	25.40241565	45.20730993	77.64469549	22.55461408
80.72300895	113.4036413	79.60417618	100.291065	64.44175451
34.59557526	29.93856131	50.12114796	28.03836226	22.55461408
35.55656347	56.24820609	29.48302821	50.68473178	53.70146209
0	93.44460045	37.34516907	24.80316661	1.074029242
32.67359886	37.19639435	20.63811975	46.37113758	30.07281877
74.95707974	100.7024335	58.96605643	69.0175071	60.14563754
55.7373157	92.53737131	75.67310575	98.13426791	79.47816389
8.648893816	2.721687392	6.87937325	11.86238403	15.03640938
49.97138649	26.30964479	45.20730993	29.11676081	46.1832574
4.804941009	5.443374783	6.87937325	11.86238403	4.296116967
0	0	0	0	0
0	2.721687392	0	1.078398548	2.148058484
0	0	0	1.078398548	0
0	0	143.4840706	115.3886447	103.1068072
18.25877583	0	12.77597889	0	0
1.921976404	3.628916522	0	5.391992742	0
18.25877583	19.05181174	8.844908464	16.17597823	4.296116967
18.25877583	0	9.827676071	0	0

For Review Only

6.726917412	0	11.79321129	0	0
2.882964605	0.907229131	0	4.313594194	4.296116967
28.82964605	19.05181174	69.77650011	0	0
17.29778763	16.33012435	17.68981693	18.33277532	18.25849711
2.882964605	16.33012435	3.931070429	2.156797097	0
22.10272864	17.23735348	42.25900711	21.56797097	13.96238014
0	0	0.982767607	1.078398548	1.074029242
1.921976404	2.721687392	0	9.705586936	7.518204692
0.960988202	1.814458261	0	7.548789839	4.296116967
52.8543511	40.82531087	56.01775361	36.66555065	54.77549133
4.804941009	13.60843696	0	0	48.33131588
714.0142339	574.2760396	587.6950291	649.1959262	635.8253111
26.90766965	32.6602487	25.55195779	4.313594194	32.22087725
4.804941009	0	0	6.470391291	19.33252635
246.0129797	284.869947	380.331064	341.8523399	270.6553689
2.882964605	2.721687392	10.81044368	1.078398548	2.148058484
0	0	0.982767607	1.078398548	1.074029242
105.7087022	92.53737131	93.36292268	136.9566157	199.769439
5.765929211	0	1.965535214	0	0
0	0	0	5.391992742	2.148058484
33.63458706	46.26868566	27.517493	37.7439492	11.81432166
38.43952807	59.87712262	56.01775361	56.07672452	47.25728664
3.843952807	0	1.965535214	3.235195645	2.148058484
49.97138649	0	39.31070429	155.289391	62.29369602
7.687905614	0	3.931070429	0	0
38.43952807	53.5265187	74.69033814	32.35195645	39.73908194
86.48893816	38.10362348	65.84542968	106.7614563	50.47937436
165.2899707	149.6928065	108.1044368	122.9374345	215.8798776
112.4356196	78.02170523	63.87989446	94.89907226	97.736661
0	0	0	0	238.4344917
67.26917412	78.92893436	94.34569029	62.54711581	98.81069024
79.76202075	88.90845479	103.1905987	86.27188388	115.9951581
71.11312693	71.67110131	96.3112255	84.11508678	98.81069024
149.9141595	158.7650978	176.8981693	184.4061518	207.2876437
21.14174044	13.60843696	39.31070429	36.66555065	19.33252635
0	0	0	7.548789839	6.444175451
3.843952807	0	0.982767607	5.391992742	3.222087725
28.82964605	9.072291305	58.96605643	21.56797097	27.92476029
61.50324491	39.01085261	18.67258454	10.78398548	26.85073104
13.45383482	13.60843696	16.70704932	20.48957242	9.666263176
33.63458706	49.89760218	37.34516907	42.05754339	61.21966678
72.07411513	117.939787	66.82819729	72.25270275	107.4029242
43.24446908	49.89760218	56.01775361	58.23352162	51.5534036
43.24446908	0	17.68981693	0	0
0	4.536145653	0	0	0
2515.867112	2195.494496	0	0	0
39.40051627	15.42289522	29.48302821	28.03836226	26.85073104
23.06371684	29.03133218	53.06945079	37.7439492	25.7767018
36.51755167	34.47470696	18.67258454	8.627188388	12.8883509
25.94668145	0	20.63811975	14.01918113	51.5534036

For Review Only

39.40051627	27.21687392	46.19007754	24.80316661	39.73908194
0	16.33012435	18.67258454	276.0700284	0
27.86865785	12.70120783	15.72428171	14.01918113	7.518204692
0	0	0	0	0
0	0	0	0	30.07281877
109.552655	30.84579044	97.29399311	44.21434049	48.33131588
0	0	11.79321129	0	0
54.7763275	41.73254	41.2762395	33.430355	50.47937436
19.21976404	17.23735348	4.913838036	15.09757968	19.33252635
24.98569325	17.23735348	22.60365496	15.09757968	18.25849711
43.24446908	31.75301957	39.31070429	30.19515936	27.92476029
127.8114308	58.06266435	149.3806763	121.859036	164.326474
2.882964605	0	0	0	1.074029242
91.29387917	0	0.982767607	0	1.074029242
12.49284662	15.42289522	15.72428171	14.01918113	18.25849711
18.25877583	83.46508001	27.517493	29.11676081	73.03398844
10.57087022	0	0	0	0
57.65929211	60.78435175	43.24177471	45.29273904	36.51699422
29.79063425	42.63976914	25.55195779	52.84152887	55.84952057
13.45383482	23.58795739	20.63811975	23.72476807	19.33252635
66.30818592	73.48555957	57.00052121	60.39031871	65.51578375
49.97138649	45.36145653	14.74151411	47.44953613	46.1832574
0	0	0	0	0
44.20545728	48.08314392	46.19007754	21.56797097	51.5534036
0.960988202	0	0	0	2.148058484
37.47853987	23.58795739	61.91435925	17.25437678	19.33252635
0	5.443374783	2.948302821	4.313594194	3.222087725
34.59557526	29.03133218	73.70757054	22.64636952	40.81311119
13.45383482	16.33012435	18.67258454	9.705586936	16.11043863
193.1586286	238.6012613	223.0882468	147.7406011	89.14442707
3.843952807	0	0	0	0
23.06371684	0	11.79321129	0	1.074029242
18.25877583	26.30964479	31.44856343	14.01918113	23.62864332
61.50324491	78.02170523	100.2422959	83.03668823	74.10801768
0	0	31.44856343	47.44953613	25.7767018
39.40051627	29.03133218	35.37963386	46.37113758	49.40534512
0	30.84579044	4.913838036	9.705586936	12.8883509
25.94668145	39.91808174	25.55195779	23.72476807	51.5534036
71.11312693	68.94941392	93.36292268	80.87989113	82.70025162
36.51755167	48.99037305	50.12114796	37.7439492	54.77549133
47.08842189	129.7337657	185.7430777	127.2510287	90.21845631
43.24446908	53.5265187	61.91435925	74.40949984	67.66384223
32.67359886	37.19639435	40.29347189	39.90074629	36.51699422
35.55656347	0.907229131	25.55195779	0	7.518204692
63.42522132	54.43374783	85.50078182	52.84152887	75.18204692
15.37581123	23.58795739	20.63811975	24.80316661	52.62743285
15.37581123	29.93856131	24.56919018	20.48957242	41.88714043
9.609882018	23.58795739	37.34516907	32.35195645	38.6650527
0.960988202	1.814458261	0	0	1.074029242
22.10272864	20.86627	24.56919018	18.33277532	17.18446787

For Review Only

27.86865785	34.47470696	52.08668318	44.21434049	40.81311119
49.01039829	39.91808174	92.38015507	69.0175071	60.14563754
36.51755167	58.06266435	51.10391557	70.09590565	52.62743285
0	0	44.22454232	35.5871521	41.88714043
22.10272864	19.95904087	16.70704932	26.95996371	23.62864332
0	0	0.982767607	1.078398548	27.92476029
42.28348088	29.93856131	54.05221839	37.7439492	40.81311119
0	0	0	0	0
0	90.72291305	18.67258454	94.89907226	60.14563754
0	0	75.67310575	65.78231146	41.88714043
54.7763275	55.34097696	44.22454232	52.84152887	48.33131588
0.960988202	0.907229131	0	0	0
41.32249268	46.26868566	64.86266207	63.62551436	81.62622237
0	0	17.68981693	9.705586936	18.25849711
39.40051627	37.19639435	43.24177471	49.60633323	0
0	0	40.29347189	0	0
46.12743368	61.69158088	97.29399311	64.70391291	97.736661
39.40051627	25.40241565	32.43133104	45.29273904	35.44296498
8.648893816	6.350603914	50.12114796	28.03836226	44.03519891
6.726917412	0	22.60365496	0	24.70267256
28.82964605	29.03133218	27.517493	33.430355	26.85073104
0.960988202	64.41326827	0	0	0
63.42522132	78.02170523	85.50078182	91.66387662	74.10801768
0	0	0.982767607	15.09757968	6.444175451
31.71261066	48.99037305	18.67258454	43.13594194	38.6650527
0	0	0	0	0
21.14174044	23.58795739	31.44856343	18.33277532	16.11043863
0.960988202	0.907229131	65.84542968	0	0
0	0	0	0	0
73.03510333	89.81568392	89.43185225	98.13426791	93.44054403
35.55656347	20.86627	26.53472539	19.41117387	32.22087725
28.82964605	36.28916522	1.965535214	53.91992742	10.74029242
98.98178478	0	40.29347189	3.235195645	115.9951581
0	0.907229131	0	0	37.59102346
21.14174044	30.84579044	3.931070429	3.235195645	1.074029242
0	0	13.7587465	11.86238403	12.8883509
0	0	0.982767607	57.15512307	4.296116967
0	0.907229131	40.29347189	0	0
0	11.7939787	0	0	47.25728664
0.960988202	0	0	0	0
34.59557526	33.56747783	28.50026061	47.44953613	13.96238014
0	14.51566609	22.60365496	31.27355791	28.99878953
8.648893816	1.814458261	0	0	95.58860252
123.0064898	129.7337657	1.965535214	16.17597823	4.296116967
71.11312693	35.38193609	51.10391557	126.1726302	85.92233934
57.65929211	33.56747783	38.32793668	61.46871726	44.03519891
14.41482303	9.979520436	9.827676071	20.48957242	31.14684801
50.93237469	52.61928957	78.62140857	70.09590565	76.25607616
19.21976404	21.77349913	46.19007754	17.25437678	39.73908194
39.40051627	32.6602487	71.74203532	69.0175071	59.0716083

For Review Only

0	0	0	0	0
39.40051627	54.43374783	76.65587336	35.5871521	65.51578375
11.53185842	10.88674957	6.87937325	11.86238403	13.96238014
32.67359886	34.47470696	44.22454232	35.5871521	34.36893574
31.71261066	29.03133218	48.15561275	81.95828968	34.36893574
34.59557526	48.99037305	81.56971139	63.62551436	68.73787147
47.08842189	35.38193609	77.63864096	31.27355791	48.33131588
24.02470504	11.7939787	17.68981693	21.56797097	10.74029242
97.05980838	79.83616349	80.58694379	97.05586936	91.29248555
16.33679943	9.072291305	36.36240146	14.01918113	0
2.882964605	6.350603914	0.982767607	0	0
0	0	0.982767607	1.078398548	5.370146209
0	2.721687392	0	0	5.370146209
0	0	131.6908594	117.5454418	155.7342401
36.51755167	40.82531087	19.65535214	35.5871521	76.25607616
161.4460179	187.79643	146.4323735	205.9741228	285.6917783
0	0	0	0	0
11.53185842	14.51566609	2.948302821	7.548789839	18.25849711
44.20545728	28.12410305	64.86266207	42.05754339	17.18446787
47.08842189	46.26868566	67.81096489	75.48789839	41.88714043
47.08842189	64.41326827	56.01775361	37.7439492	28.99878953
13.45383482	9.979520436	16.70704932	23.72476807	0
6.726917412	15.42289522	0	5.391992742	12.8883509
74.95707974	40.82531087	97.29399311	50.68473178	54.77549133
21.14174044	18.14458261	8.844908464	20.48957242	28.99878953
0.960988202	2.721687392	0	0	0
143.1872421	146.06389	64.86266207	70.09590565	129.9575383
85.52794996	59.87712262	76.65587336	114.3102461	86.99636858
6.726917412	4.536145653	0	1.078398548	8.592233934
0	0	0.982767607	0	0
48.04941009	84.37230914	56.01775361	81.95828968	80.55219313
1232.947863	1198.449681	900.2151281	923.1091575	804.4479021
0	6.350603914	0.982767607	0	1.074029242
59.58126851	48.99037305	45.20730993	53.91992742	39.73908194
0	0	0	0	2.148058484
162.4070061	168.7446183	195.5707538	223.2284995	196.5473512
0	6.350603914	0.982767607	0	0
0	0	0.982767607	0	0
0.960988202	5.443374783	0.982767607	0	0
0	0	0.982767607	1.078398548	1.074029242
0	0	9.827676071	0	0
0	0	15.72428171	0	3.222087725
17.29778763	73.48555957	80.58694379	35.5871521	0
0	0	0	0	0
15.37581123	31.75301957	0	25.88156516	32.22087725
4.804941009	18.14458261	0	0	19.33252635
52.8543511	65.3204974	47.17284514	39.90074629	70.88592996
53.8153393	49.89760218	53.06945079	64.70391291	62.29369602
0.960988202	0	0	3.235195645	0
17.29778763	9.979520436	17.68981693	0	0

For Review Only

13.45383482	33.56747783	93.36292268	116.4670432	90.21845631
102.8257376	81.65062175	228.0020849	158.5245866	148.2160354
4.804941009	5.443374783	1.965535214	4.313594194	0
324.8140122	292.12778	274.1921624	177.9357605	661.6020129
4.804941009	7.257833044	8.844908464	11.86238403	2.148058484
6.726917412	0.907229131	9.827676071	6.470391291	2.148058484
5.765929211	0	3.931070429	0	0
49.97138649	49.89760218	32.43133104	25.88156516	147.1420061
23.06371684	37.19639435	44.22454232	45.29273904	23.62864332
0	0	45.20730993	42.05754339	48.33131588
59.58126851	94.35182958	60.93159164	70.09590565	55.84952057
18.25877583	26.30964479	37.34516907	37.7439492	21.48058484
7.687905614	10.88674957	20.63811975	11.86238403	7.518204692
17.29778763	18.14458261	28.50026061	19.41117387	27.92476029
216.2223454	237.6940322	229.9676201	251.2668618	221.2500238
25.94668145	37.19639435	38.32793668	23.72476807	17.18446787
0	28.12410305	35.37963386	29.11676081	0
54.7763275	58.06266435	81.56971139	63.62551436	24.70267256
24.02470504	0	0	16.17597823	13.96238014
34.59557526	25.40241565	38.32793668	31.27355791	73.03398844
9.609882018	0	0	0	6.444175451
76.87905614	71.67110131	145.4496059	81.95828968	94.51457327
0	0	0	0	0
48.04941009	42.63976914	47.17284514	42.05754339	34.36893574
54.7763275	57.15543522	45.20730993	30.19515936	60.14563754
49.01039829	42.63976914	85.50078182	72.25270275	38.6650527
96.09882018	95.25905871	109.0872044	98.13426791	118.1432166
0	0	0	0	0
41.32249268	41.73254	16.70704932	15.09757968	7.518204692
24.02470504	42.63976914	65.84542968	15.09757968	39.73908194
85.52794996	76.20724696	78.62140857	19.41117387	84.8483101
13.45383482	9.979520436	10.81044368	0	9.666263176
98.98178478	65.3204974	163.1394228	76.56629694	65.51578375
63.42522132	38.10362348	74.69033814	88.42868097	59.0716083
170.0949117	205.9410126	225.053782	198.4253329	155.7342401
173.9388645	176.0024513	170.018796	196.2685358	162.1784155
179.7047937	142.4349735	91.39738746	140.1918113	92.36651479
438.21062	387.3868387	425.5383739	392.5370716	164.326474
0	4.536145653	0	0	2.148058484
52.8543511	63.50603914	52.08668318	33.430355	36.51699422
69.19115053	61.69158088	80.58694379	71.1743042	67.66384223
124.9284662	107.9602665	89.43185225	86.27188388	76.25607616
39.40051627	0	5.896605643	10.78398548	1.074029242
342.1117998	323.8807996	356.7446414	333.2251515	287.8398368
201.8075224	169.6518474	103.1905987	95.97747081	168.622591
8.648893816	14.51566609	10.81044368	15.09757968	15.03640938
15.37581123	18.14458261	7.862140857	18.33277532	6.444175451
42.28348088	33.56747783	24.56919018	34.50875355	20.40655559
121.0845134	153.3217231	76.65587336	90.58547807	85.92233934
10.57087022	0.907229131	0.982767607	2.156797097	0

For Review Only

26.90766965	29.03133218	16.70704932	10.78398548	23.62864332
11.53185842	0	1.965535214	4.313594194	0
152.7971241	184.1675135	63.87989446	84.11508678	178.2888541
6.726917412	14.51566609	10.81044368	25.88156516	9.666263176
46.12743368	73.48555957	75.67310575	91.66387662	83.77428086
56.6983039	61.69158088	26.53472539	18.33277532	50.47937436
5.765929211	4.536145653	3.931070429	11.86238403	1.074029242
2.882964605	0	0.982767607	6.470391291	1.074029242
0	9.072291305	16.70704932	1.078398548	1.074029242
17.29778763	18.14458261	8.844908464	2.156797097	5.370146209
51.8933629	68.94941392	58.96605643	60.39031871	88.07039782
73.03510333	78.02170523	90.41461986	83.03668823	53.70146209
49.97138649	29.03133218	43.24177471	20.48957242	30.07281877
0	0	0	0	0
0	0	0.982767607	0	0
60.54225671	49.89760218	83.53524661	34.50875355	49.40534512
0	0	0	0	5.370146209
0	0	0.982767607	1.078398548	4.296116967
361.3315639	312.0868209	301.7096554	341.8523399	437.1299014
13.45383482	24.49518652	19.65535214	31.27355791	6.444175451
9.609882018	0	11.79321129	15.09757968	9.666263176
174.8998527	0	0.982767607	1.078398548	1.074029242
19.21976404	19.95904087	10.81044368	33.430355	26.85073104
24.02470504	24.49518652	34.39686625	45.29273904	33.29490649
41.32249268	71.67110131	47.17284514	40.97914484	57.99757905
0	0	0.982767607	1.078398548	0
60.54225671	60.78435175	83.53524661	53.91992742	79.47816389
3.843952807	5.443374783	5.896605643	1.078398548	3.222087725
0	0	6.87937325	0	0
382.4733043	376.5000892	290.8992117	454.0057889	321.1347433
36.51755167	47.17591479	57.00052121	22.64636952	39.73908194
226.7932156	168.7446183	145.4496059	154.2109924	147.1420061
1.921976404	2.721687392	2.948302821	0	0
0	2.721687392	0	0	0
5.765929211	3.628916522	0	10.78398548	0
53.8153393	171.4663057	150.3634439	12.94078258	105.2548657
0	0.907229131	0	31.27355791	0
577.5539093	546.1519366	491.3838036	525.1800931	641.1954573
12.49284662	37.19639435	29.48302821	18.33277532	38.6650527
0	27.21687392	0	0	0
0	165.1157018	200.4845919	625.4711581	100.9587487
41.32249268	45.36145653	62.89712686	34.50875355	39.73908194
61.50324491	71.67110131	92.38015507	92.74227517	95.58860252
20.18075224	0	10.81044368	6.470391291	21.48058484
3.843952807	3.628916522	4.913838036	5.391992742	0
356.5266229	303.0145296	282.0543032	249.1100647	115.9951581
179.7047937	196.8687213	260.4334159	202.7389271	239.5085209
229.6761802	254.0241565	285.0026061	300.873195	270.6553689
0	1.814458261	0	0	16.11043863
0	0	4.913838036	0	0

For Review Only

0	0	0	0	0
815.8789833	651.3905157	977.8537691	1177.611215	655.1578375
0	0	0	0	0
80.72300895	62.59881001	66.82819729	33.430355	65.51578375
0	0	0.982767607	1.078398548	4.296116967
7.687905614	0	3.931070429	4.313594194	4.296116967
247.9349561	209.5699292	239.7952961	205.9741228	216.9539068
62.46423311	79.83616349	64.86266207	56.07672452	35.44296498
26.90766965	21.77349913	3.931070429	24.80316661	7.518204692
138.3823011	191.4253465	49.13838036	106.7614563	100.9587487
174.8998527	156.0434105	151.3462115	100.291065	99.88471948
22.10272864	0	6.87937325	0	11.81432166
61.50324491	52.61928957	53.06945079	49.60633323	41.88714043
445.8985256	552.5025405	702.6788391	572.6296292	341.5412989
28.82964605	57.15543522	15.72428171	42.05754339	107.4029242
0	0	0	0	0
27.86865785	18.14458261	13.7587465	6.470391291	37.59102346
0	0	5.896605643	0	0
26.90766965	23.58795739	35.37963386	12.94078258	16.11043863
217.1833336	167.8373891	175.9154017	237.2476807	152.5121523
158.5630533	133.3626822	125.7942537	102.4478621	196.5473512
276.7646021	297.5711548	163.1394228	264.2076444	227.6941993
5.765929211	0	0	1.078398548	0
0	0	0	0	0
18.25877583	25.40241565	10.81044368	18.33277532	8.592233934
0	4.536145653	0.982767607	0	0
0	4.536145653	0	0	0
9.609882018	7.257833044	5.896605643	6.470391291	11.81432166
0	0	3.931070429	0	0
283.4915195	185.9819718	260.4334159	237.2476807	213.7318191
3.843952807	0	1.965535214	0	3.222087725
19.21976404	11.7939787	13.7587465	10.78398548	7.518204692
45.16644548	42.63976914	62.89712686	51.76313033	61.21966678
0.960988202	0.907229131	0	0	0
126.8504426	136.0843696	125.7942537	160.6813837	192.2512343
21.14174044	14.51566609	25.55195779	22.64636952	15.03640938
109.552655	142.4349735	116.9493452	85.19348533	139.6238014
179.7047937	171.4663057	180.8292397	223.2284995	147.1420061
8.648893816	0	1.965535214	2.156797097	1.074029242
12.49284662	9.979520436	16.70704932	11.86238403	15.03640938
40.36150447	30.84579044	58.96605643	50.68473178	68.73787147
86.48893816	97.9807461	57.00052121	90.58547807	100.9587487
38.43952807	71.67110131	42.25900711	71.1743042	93.44054403
0	0	0.982767607	1.078398548	1.074029242
82.64498535	137.8988278	137.587465	91.66387662	110.6250119
44.20545728	34.47470696	17.68981693	36.66555065	45.10922815
13.45383482	0	81.56971139	0	83.77428086
0	0	0	64.70391291	0
0	158.7650978	14.74151411	3.235195645	153.5861816
77.84004434	40.82531087	73.70757054	101.3694636	60.14563754

For Review Only

317.1261066	82.55785088	247.657437	35.5871521	179.3628834
64.38620952	53.5265187	137.587465	93.82067372	47.25728664
65.34719772	116.1253287	85.50078182	49.60633323	105.2548657
0.960988202	0	0	0	0
17.29778763	17.23735348	18.67258454	28.03836226	12.8883509
12.49284662	13.60843696	3.931070429	30.19515936	22.55461408
9.609882018	32.6602487	39.31070429	3.235195645	8.592233934
2.882964605	0.907229131	12.77597889	0	9.666263176
104.747714	122.4759326	168.0532608	200.58213	109.5509827
3.843952807	3.628916522	0	5.391992742	0
42.28348088	49.89760218	51.10391557	58.23352162	31.14684801
142.2262539	177.8169096	183.7775425	175.7789634	156.8082693
0	303.9217587	231.9331553	141.2702098	0
17.29778763	27.21687392	28.50026061	26.95996371	27.92476029
33.63458706	0	46.19007754	26.95996371	27.92476029
29.79063425	37.19639435	75.67310575	83.03668823	76.25607616
5.765929211	4.536145653	6.87937325	1.078398548	4.296116967
235.4421094	264.9109061	334.1409864	273.9132313	270.6553689
0	0	56.01775361	3.235195645	15.03640938
0	0	1312.977523	102.4478621	212.6577899
376.7073751	409.1603379	148.3979087	650.2743247	398.4648487
57.65929211	27.21687392	51.10391557	40.97914484	32.22087725
34.59557526	33.56747783	27.517493	25.88156516	34.36893574
293.1014015	229.52897	196.5535214	255.580456	212.6577899
266.1937319	232.2506574	277.1404652	222.150101	196.5473512
54.7763275	223.1783661	129.7253241	121.859036	63.36772526
88.41091456	117.0325578	133.6563946	59.31192017	51.5534036
5.765929211	4.536145653	5.896605643	12.94078258	2.148058484
21.14174044	15.42289522	18.67258454	16.17597823	25.7767018
42.28348088	33.56747783	45.20730993	59.31192017	42.96116967
32.67359886	41.73254	40.29347189	37.7439492	56.92354981
156.6410769	145.1566609	283.0370709	214.6013111	214.8058484
21.14174044	28.12410305	120.8804157	0	0
20.18075224	7.257833044	12.77597889	14.01918113	10.74029242
8.648893816	14.51566609	0.982767607	7.548789839	2.148058484
73.03510333	85.27953827	71.74203532	66.86071	119.2172458
0	0	0	0	3.222087725
210.4564162	197.7759505	180.8292397	210.2877169	220.1759946
0	148.7855774	0	0	16.11043863
0.960988202	0.907229131	0	0	0
23.06371684	29.03133218	24.56919018	38.82234774	37.59102346
16.33679943	13.60843696	3.931070429	12.94078258	9.666263176
2.882964605	9.979520436	10.81044368	10.78398548	22.55461408
59.58126851	83.46508001	31.44856343	44.21434049	19.33252635
0	0.907229131	5.896605643	0	1.074029242
18.25877583	20.86627	25.55195779	34.50875355	34.36893574
54.7763275	68.94941392	68.7937325	64.70391291	56.92354981
5.765929211	9.979520436	5.896605643	12.94078258	7.518204692
4.804941009	10.88674957	0	3.235195645	2.148058484
17.29778763	30.84579044	36.36240146	25.88156516	20.40655559

For Review Only

22.10272864	0	8.844908464	0	4.296116967
129.7334072	119.7542452	165.104958	141.2702098	97.736661
50.93237469	0	3.931070429	6.470391291	15.03640938
502.5968295	374.6856309	247.657437	228.6204923	201.9174975
1057.087022	655.9266614	556.2464656	542.4344699	718.5255627
92.25486737	85.27953827	80.58694379	89.50707952	47.25728664
59.58126851	48.99037305	45.20730993	50.68473178	31.14684801
142.2262539	129.7337657	104.1733664	162.8381808	151.4381231
226.7932156	195.9614922	158.2255847	195.1901373	106.3288949
7.687905614	11.7939787	5.896605643	9.705586936	6.444175451
37.47853987	0	31.44856343	30.19515936	74.10801768
210.4564162	227.7145118	227.0193172	268.5212386	269.5813397
3.843952807	0	2.948302821	0	0
0	0	0	0	0
8.648893816	9.072291305	0	11.86238403	31.14684801
73.03510333	80.74339262	37.34516907	15.09757968	51.5534036
0	17.23735348	2.948302821	8.627188388	0
56.6983039	70.76387218	61.91435925	39.90074629	70.88592996
156.6410769	0	176.8981693	0	0
84.56696176	75.30001783	98.27676071	97.05586936	97.736661
76.87905614	61.69158088	131.6908594	102.4478621	86.99636858
111.4746314	158.7650978	162.1566552	142.3486084	75.18204692
40.36150447	86.1867674	39.31070429	51.76313033	39.73908194
33.63458706	27.21687392	74.69033814	14.01918113	17.18446787
0	565.2037483	514.9702261	1428.878077	664.8241006
252.7398971	290.3133218	411.7796274	414.1050426	347.9854743
24.02470504	33.56747783	0	88.42868097	0
85.52794996	108.8674957	94.34569029	122.9374345	106.3288949
0	0	0	1.078398548	2.148058484
1754.764456	506.2338548	1749.326341	1593.873055	1511.159143
0	0	2.948302821	0	0
239.2860622	232.2506574	460.9180077	24.80316661	41.88714043
1.921976404	0	0	0	0
1.921976404	2.721687392	0	0	0
176.8218291	57.15543522	184.7603101	267.44284	69.81190071
0	0	0	0	3.222087725
47.08842189	39.91808174	42.25900711	33.430355	28.99878953
201.8075224	173.2807639	120.8804157	113.2318476	195.473322
728.4290569	780.2170523	596.5399375	841.1508678	831.2986331
144.1482303	124.2903909	226.0365496	142.3486084	77.33010541
34.59557526	67.13495566	33.41409864	45.29273904	30.07281877
91.29387917	79.83616349	58.96605643	77.64469549	88.07039782
17.29778763	9.979520436	14.74151411	5.391992742	7.518204692
248.8959443	208.6627	192.622451	329.9899558	263.1371642
172.0168881	136.0843696	152.3289791	274.9916299	198.6954097
83.60597355	78.92893436	118.9148805	116.4670432	50.47937436
189.3146757	195.9614922	242.743599	167.151775	152.5121523
2933.89698	3197.075456	4063.744056	3065.887073	3362.785556
232.5591448	244.0446361	267.3127891	241.5612749	240.5825502
67.26917412	46.26868566	90.41461986	59.31192017	60.14563754

For Review Only

4.804941009	5.443374783	4.913838036	5.391992742	5.370146209
834.1377591	910.8580471	1075.147762	820.6612954	894.6663584
2071.890563	2200.937871	2908.00935	1541.031526	1503.640938
28.82964605	42.63976914	41.2762395	66.86071	33.29490649
115.3185842	98.88797523	143.4840706	124.0158331	92.36651479
117.2405606	133.3626822	177.8809369	207.0525213	157.8822985
347.877729	373.7784018	451.0903317	469.1033686	388.7985855
97.05980838	141.5277444	105.156134	172.5437678	153.5861816
0	0	0	0	2.148058484
24.02470504	23.58795739	21.62088736	17.25437678	21.48058484
36.51755167	0	0	19.41117387	0
0	0	0	0	2.148058484
8.648893816	7.257833044	47.17284514	9.705586936	1.074029242
181.6267701	194.1470339	222.1054792	212.444514	125.6614213
0	0	0	0	0
96.09882018	48.08314392	63.87989446	53.91992742	149.2900646
0	68.94941392	55.034986	24.80316661	3.222087725
15.37581123	8.165062175	89.43185225	70.09590565	0
120.1235252	0	0.982767607	1.078398548	259.9150765
0	103.4241209	235.8642257	1.078398548	67.66384223
0	0	0.982767607	1.078398548	1.074029242
85.52794996	110.6819539	169.0360284	129.4078258	168.622591
24.02470504	16.33012435	0	132.6430215	74.10801768
9.609882018	0	0	0	0
161.4460179	155.1361813	199.5018242	174.7005649	181.5109419
0	0	37.34516907	0	0
0	56.24820609	42.25900711	89.50707952	51.5534036
57.65929211	41.73254	4.913838036	81.95828968	112.7730704
74.95707974	77.1144761	92.38015507	81.95828968	61.21966678
94.17684377	146.06389	125.7942537	121.859036	149.2900646
10.57087022	14.51566609	34.39686625	32.35195645	42.96116967
18.25877583	30.84579044	43.24177471	25.88156516	54.77549133
5.765929211	0	0	15.09757968	21.48058484
36.51755167	36.28916522	26.53472539	35.5871521	50.47937436
0	0	0	9.705586936	9.666263176
0	5.443374783	11.79321129	18.33277532	0
0	0	0	57.15512307	67.66384223
0	87.09399653	3.931070429	1.078398548	1.074029242
76.87905614	52.61928957	61.91435925	4.313594194	177.2148249
9.609882018	6.350603914	34.39686625	0	0
58.62028031	56.24820609	86.48354943	60.39031871	97.736661
18.25877583	0	29.48302821	0	33.29490649
58.62028031	68.94941392	62.89712686	66.86071	104.1808365
0	30.84579044	75.67310575	10.78398548	28.99878953
0	39.01085261	76.65587336	0	0
86.48893816	49.89760218	155.2772819	107.8398548	98.81069024
0.960988202	2.721687392	0	0	0
0	0	0	0	22.55461408
84.56696176	92.53737131	121.8631833	114.3102461	115.9951581
114.357596	85.27953827	153.3117467	76.56629694	107.4029242

For Review Only

60.54225671	64.41326827	133.6563946	216.7581082	134.2536552
0	0.907229131	0	0	0
51.8933629	60.78435175	61.91435925	70.09590565	62.29369602
19.21976404	22.68072826	25.55195779	24.80316661	17.18446787
0	0	0	0	0
49.01039829	68.94941392	74.69033814	46.37113758	64.44175451
0	0	192.622451	227.5420937	154.6602108
17.29778763	0	0	0	0
70.15213873	0	23.58642257	122.9374345	182.5849711
78.80103254	78.02170523	111.0527396	79.80149259	92.36651479
418.990856	400.9952757	556.2464656	173.6221663	356.5777083
2.882964605	0	0	0	5.370146209
0	0	0	0	0
0	0	3.931070429	0	0
7.687905614	0	5.896605643	0	0
96.09882018	117.0325578	87.46631704	86.27188388	125.6614213
263.3107673	239.5084905	367.5550851	324.5979631	318.9866848
52.8543511	59.87712262	114.0010424	99.21266646	67.66384223
0	0.907229131	0	0	0
147.0311949	99.79520436	136.6046974	159.6029852	110.6250119
25.94668145	27.21687392	27.517493	54.99832597	30.07281877
15.37581123	0	10.81044368	0	0
38.43952807	28.12410305	18.67258454	57.15512307	32.22087725
72.07411513	90.72291305	107.1216692	71.1743042	134.2536552
0	0	3.931070429	0	5.370146209
5.765929211	0	1.965535214	0	3.222087725
18.25877583	30.84579044	24.56919018	12.94078258	31.14684801
3.843952807	0	0	0	0
0	0	0	0	0
35.55656347	25.40241565	24.56919018	16.17597823	28.99878953
0	0	0	0	4.296116967
12.49284662	13.60843696	9.827676071	8.627188388	10.74029242
49.01039829	28.12410305	12.77597889	28.03836226	33.29490649
5.765929211	0	8.844908464	0	6.444175451
509.3237469	520.7495209	538.5566487	424.8890281	430.6857259
171.0558999	115.2180996	151.3462115	119.7022389	157.8822985
5.765929211	8.165062175	4.913838036	11.86238403	0
116.2795724	79.83616349	114.0010424	155.289391	141.7718599
0	0	0	0	151.4381231
0	0	65.84542968	22.64636952	24.70267256
87.44992636	0	85.50078182	88.42868097	63.36772526
25.94668145	20.86627	22.60365496	31.27355791	45.10922815
114.357596	108.8674957	134.6391622	81.95828968	110.6250119
14.41482303	16.33012435	43.24177471	20.48957242	37.59102346
0	14.51566609	3.931070429	9.705586936	11.81432166
30.75162246	41.73254	25.55195779	28.03836226	30.07281877
11.53185842	0	1.965535214	4.313594194	3.222087725
0	0	1.965535214	2.156797097	0
0	0.907229131	3.931070429	3.235195645	0
0	0	0	0	0

For Review Only

19.21976404	11.7939787	5.896605643	21.56797097	6.444175451
131.6553836	128.8265365	173.9498665	187.6413474	183.6590003
7.687905614	4.536145653	6.87937325	1.078398548	4.296116967
0	3.628916522	11.79321129	1.078398548	5.370146209
0	0	0	0	0
70.15213873	92.53737131	113.0182748	78.72309404	76.25607616
3.843952807	0	0	3.235195645	0
197.9635696	214.1060748	128.7425565	117.5454418	208.3616729
132.6163718	98.88797523	123.8287185	93.82067372	73.03398844
940.8074495	1058.736395	789.1623885	1233.687939	839.8908671
0	0	4.913838036	0	0
214.300369	198.6831796	140.5357678	164.9949779	269.5813397
563.1390862	490.8109596	513.9874585	547.8264626	400.6129072
208.5344398	209.5699292	183.7775425	272.8348328	155.7342401
9.609882018	9.072291305	0.982767607	7.548789839	1.074029242
106.6696904	137.8988278	53.06945079	142.3486084	132.1055967
2.882964605	0	2.948302821	0	1.074029242
140.3042775	95.25905871	141.5185354	169.3085721	94.51457327
518.933629	430.933837	666.3164376	703.1158536	553.1250595
4.804941009	4.536145653	2.948302821	0	0
208.5344398	223.1783661	156.2600495	169.3085721	214.8058484
0	0	3.931070429	0	0
211.4174044	158.7650978	165.104958	66.86071	127.8094798
4.804941009	13.60843696	18.67258454	15.09757968	4.296116967
31.71261066	32.6602487	36.36240146	51.76313033	50.47937436
220.0662982	244.9518652	317.4339371	383.9098833	300.7281877
76.87905614	16.33012435	77.63864096	84.11508678	77.33010541
227.7542038	491.7181887	521.8495994	262.0508473	299.6541584
0	8.165062175	73.70757054	40.97914484	0
4.804941009	0	88.44908464	0	0
0	76.20724696	3.931070429	84.11508678	32.22087725
14.41482303	20.86627	46.19007754	32.35195645	34.36893574
139.3432893	77.1144761	155.2772819	81.95828968	45.10922815
87.44992636	87.09399653	85.50078182	101.3694636	113.8470996
62.46423311	71.67110131	80.58694379	77.64469549	107.4029242
54.7763275	54.43374783	121.8631833	72.25270275	100.9587487
238.325074	198.6831796	190.6569158	141.2702098	182.5849711
24.02470504	28.12410305	15.72428171	25.88156516	41.88714043
57.65929211	55.34097696	19.65535214	77.64469549	80.55219313
39.40051627	32.6602487	49.13838036	24.80316661	31.14684801
68.23016233	79.83616349	61.91435925	71.1743042	51.5534036
65.34719772	42.63976914	0	78.72309404	65.51578375
0	15.42289522	17.68981693	10.78398548	10.74029242
77.84004434	67.13495566	62.89712686	90.58547807	73.03398844
227.7542038	0	59.94882404	0	30.07281877
70.15213873	130.6409948	73.70757054	78.72309404	93.44054403
0.960988202	0	7.862140857	9.705586936	12.8883509
0.960988202	4.536145653	0	6.470391291	8.592233934
115.3185842	113.4036413	96.3112255	104.6046592	111.6990411
52.8543511	56.24820609	45.20730993	19.41117387	34.36893574

For Review Only

77.84004434	58.06266435	64.86266207	70.09590565	92.36651479
57.65929211	87.09399653	87.46631704	98.13426791	78.40413465
9.609882018	14.51566609	3.931070429	0	5.370146209
0	0	0	1.078398548	0
149.9141595	125.19762	133.6563946	92.74227517	73.03398844
0.960988202	0.907229131	0	43.13594194	0
0	0	0	0	0
128.772419	70.76387218	102.2078311	17.25437678	31.14684801
36.51755167	29.93856131	52.08668318	34.50875355	38.6650527
70.15213873	75.30001783	28.50026061	64.70391291	41.88714043
0	8.165062175	4.913838036	0	4.296116967
39.40051627	39.01085261	24.56919018	25.88156516	38.6650527
226.7932156	0	127.7597889	16.17597823	13.96238014
28.82964605	55.34097696	43.24177471	12.94078258	49.40534512
0	0	3.931070429	326.7547602	1.074029242
4.804941009	0	2.948302821	16.17597823	3.222087725
99.94277298	123.3831618	123.8287185	161.7597823	151.4381231
59.58126851	45.36145653	64.86266207	49.60633323	42.96116967
246.0129797	206.8482418	181.8120073	278.2268255	270.6553689
5.765929211	11.7939787	16.70704932	10.78398548	11.81432166
98.02079658	55.34097696	121.8631833	80.87989113	68.73787147
0	0	0.982767607	1.078398548	1.074029242
0	3.628916522	0	0	0
31.71261066	39.91808174	40.29347189	51.76313033	28.99878953
41.32249268	39.01085261	60.93159164	65.78231146	36.51699422
0.960988202	2.721687392	0.982767607	6.470391291	0
0.960988202	3.628916522	1.965535214	0	0
21.14174044	26.30964479	0.982767607	29.11676081	2.148058484
0	0	5.896605643	14.01918113	7.518204692
6.726917412	6.350603914	13.7587465	0	7.518204692
121.0845134	104.33135	162.1566552	93.82067372	112.7730704
5.765929211	11.7939787	15.72428171	0	7.518204692
9.609882018	10.88674957	1.965535214	3.235195645	7.518204692
55.7373157	48.99037305	17.68981693	29.11676081	46.1832574
64.38620952	33.56747783	64.86266207	37.7439492	38.6650527
11.53185842	6.350603914	7.862140857	10.78398548	4.296116967
0.960988202	0	1.965535214	0	1.074029242
27.86865785	20.86627	30.46579582	17.25437678	21.48058484
71.11312693	34.47470696	94.34569029	51.76313033	38.6650527
23.06371684	244.9518652	103.1905987	21.56797097	430.6857259
211.4174044	185.9819718	35.37963386	0	18.25849711
128.772419	116.1253287	76.65587336	106.7614563	126.7354505
33.63458706	44.4542274	86.48354943	54.99832597	30.07281877
14.41482303	8.165062175	4.913838036	12.94078258	4.296116967
75.91806794	71.67110131	111.0527396	43.13594194	59.0716083
53.8153393	88.90845479	105.156134	85.19348533	70.88592996
55.7373157	74.3927887	90.41461986	67.93910855	103.1068072
5.765929211	7.257833044	2.948302821	5.391992742	3.222087725
21.14174044	35.38193609	13.7587465	39.90074629	20.40655559
0	0	1.965535214	12.94078258	10.74029242

For Review Only

69.19115053	0	18.67258454	1.078398548	66.58981299
8.648893816	7.257833044	13.7587465	12.94078258	10.74029242
11.53185842	23.58795739	15.72428171	1.078398548	6.444175451
34.59557526	39.01085261	3.931070429	11.86238403	11.81432166
7.687905614	11.7939787	7.862140857	3.235195645	8.592233934
146.0702067	149.6928065	127.7597889	172.5437678	192.2512343
0	93.44460045	5.896605643	190.8765431	59.0716083
17.29778763	21.77349913	9.827676071	6.470391291	5.370146209
99.94277298	112.4964122	138.5702326	71.1743042	125.6614213
31.71261066	44.4542274	55.034986	73.3311013	56.92354981
15.37581123	8.165062175	12.77597889	0	11.81432166
23.06371684	9.979520436	41.2762395	91.66387662	21.48058484
13.45383482	23.58795739	18.67258454	69.0175071	7.518204692
0	17.23735348	35.37963386	5.391992742	1.074029242
0	0	1.965535214	0	1.074029242
58.62028031	61.69158088	75.67310575	75.48789839	60.14563754
13.45383482	16.33012435	4.913838036	8.627188388	11.81432166
23.06371684	14.51566609	20.63811975	16.17597823	15.03640938
82.64498535	79.83616349	65.84542968	118.6238403	80.55219313
24.02470504	22.68072826	31.44856343	35.5871521	31.14684801
80.72300895	86.1867674	64.86266207	95.97747081	63.36772526
98.02079658	79.83616349	71.74203532	54.99832597	42.96116967
0	10.88674957	0	0	0
25.94668145	26.30964479	26.53472539	20.48957242	36.51699422
51.8933629	57.15543522	42.25900711	48.52793468	40.81311119
66.30818592	86.1867674	97.29399311	69.0175071	102.032778
61.50324491	68.94941392	58.96605643	46.37113758	33.29490649
70.15213873	81.65062175	92.38015507	185.4845503	103.1068072
95.13783197	0	0	26.95996371	175.0667664
0	0	2.948302821	2.156797097	90.21845631
58.62028031	47.17591479	55.034986	70.09590565	63.36772526
51.8933629	42.63976914	80.58694379	73.3311013	96.66263176
0	205.0337835	45.20730993	0	27.92476029
0	0	0	0	0
59.58126851	73.48555957	99.25952832	71.1743042	70.88592996
34.59557526	29.93856131	43.24177471	64.70391291	32.22087725
1.921976404	1.814458261	0	0	0
147.9921831	176.9096805	119.8976481	132.6430215	176.1407956
0	0	0	0	1.074029242
3.843952807	1.814458261	0	3.235195645	0
76.87905614	9.979520436	0	118.6238403	0
0	0	0	2.156797097	0
287.3354723	311.1795918	241.7608314	294.4028037	266.359252
879.3042046	780.2170523	839.2835365	695.5670638	699.1930364
36.51755167	29.93856131	37.34516907	32.35195645	22.55461408
0.960988202	0.907229131	0	0	1.074029242
38.43952807	42.63976914	21.62088736	32.35195645	30.07281877
0	0	2.948302821	3.235195645	0
23.06371684	20.86627	13.7587465	12.94078258	18.25849711
54.7763275	82.55785088	67.81096489	22.64636952	50.47937436

For Review Only

409.380974	400.9952757	345.9341977	352.6363253	336.1711527
833.1767709	983.4363775	1360.150368	1292.99986	1130.952792
2.882964605	2.721687392	0	0	0
0	0	0	1.078398548	0
0	0	0	1.078398548	0
106.6696904	42.63976914	75.67310575	109.9966519	152.5121523
0	0	0	0	0
7.687905614	9.072291305	11.79321129	0	0
13.45383482	18.14458261	16.70704932	3.235195645	12.8883509
57.65929211	73.48555957	102.2078311	81.95828968	124.587392
60.54225671	51.71206044	88.44908464	71.1743042	47.25728664
147.0311949	127.0120783	155.2772819	188.719746	167.5485617
0	7.257833044	0.982767607	3.235195645	5.370146209
0	0	0	0	1.074029242
1.921976404	1.814458261	2.948302821	4.313594194	6.444175451
154.7191005	156.0434105	205.3984299	120.7806374	104.1808365
0.960988202	1.814458261	9.827676071	7.548789839	0
11.53185842	0	0	0	38.6650527
70.15213873	72.57833044	89.43185225	54.99832597	44.03519891
396.8881273	275.7976557	127.7597889	69.0175071	257.767018
0	0.907229131	3.931070429	4.313594194	4.296116967
32.67359886	18.14458261	23.58642257	25.88156516	33.29490649
0	0	2.948302821	0	0
16.33679943	19.05181174	21.62088736	0	5.370146209
115.3185842	72.57833044	92.38015507	44.21434049	51.5534036
0	0	0	0	0
31.71261066	24.49518652	36.36240146	31.27355791	35.44296498
102.8257376	118.8470161	71.74203532	64.70391291	105.2548657
7.687905614	9.072291305	45.20730993	15.09757968	4.296116967
0	0	2.948302821	0	0
27.86865785	29.03133218	33.41409864	65.78231146	45.10922815
259.4668145	265.8181352	158.2255847	211.3661155	349.0595036
68.23016233	75.30001783	27.517493	40.97914484	52.62743285
14.41482303	0	0	6.470391291	0
1.921976404	0	0	0	1.074029242
0	0	0	0	3.222087725
6.726917412	3.628916522	5.896605643	7.548789839	12.8883509
0	0	4.913838036	6.470391291	1.074029242
1.921976404	5.443374783	6.87937325	1.078398548	5.370146209
0	0	0.982767607	2.156797097	1.074029242
26.90766965	29.03133218	44.22454232	30.19515936	31.14684801
0	4.536145653	0	0	0
6.726917412	1.814458261	0	0	0
45.16644548	31.75301957	40.29347189	44.21434049	37.59102346
45.16644548	47.17591479	39.31070429	60.39031871	52.62743285
141.2652657	116.1253287	145.4496059	160.6813837	56.92354981
0	0	0	0	1.074029242
3.843952807	8.165062175	0	5.391992742	5.370146209
0	0	1.965535214	14.01918113	1.074029242
788.9713136	1029.705063	996.5263536	1047.124991	458.6104862

For Review Only

3.843952807	0	5.896605643	2.156797097	0
0	2.721687392	0.982767607	0	0
0	0	0	0	105.2548657
0.960988202	0	0.982767607	0	1.074029242
37.47853987	49.89760218	52.08668318	31.27355791	46.1832574
5.765929211	9.072291305	7.862140857	12.94078258	10.74029242
419.9518442	397.3663592	418.6590006	401.16426	390.946644
100.9037612	90.72291305	70.75926771	62.54711581	63.36772526
645.7840716	645.9471409	750.8344519	739.7814042	753.9685277
0	0	0	0	0
25.94668145	34.47470696	48.15561275	35.5871521	35.44296498
392.0831863	360.1699648	320.3822399	173.6221663	284.6177491
24.02470504	19.95904087	15.72428171	26.95996371	64.44175451
63.42522132	53.5265187	58.96605643	62.54711581	49.40534512
58.62028031	100.7024335	74.69033814	112.153449	83.77428086
3.843952807	1.814458261	0.982767607	1.078398548	3.222087725
0	2.721687392	3.931070429	0	0
7.687905614	11.7939787	5.896605643	8.627188388	18.25849711
68.23016233	97.9807461	70.75926771	140.1918113	148.2160354
41.32249268	31.75301957	78.62140857	61.46871726	62.29369602
35.55656347	68.94941392	59.94882404	42.05754339	63.36772526
0	22.68072826	15.72428171	30.19515936	25.7767018
42.28348088	41.73254	48.15561275	72.25270275	67.66384223
40.36150447	38.10362348	31.44856343	51.76313033	61.21966678
71.11312693	80.74339262	84.51801421	93.82067372	88.07039782
62.46423311	30.84579044	23.58642257	16.17597823	25.7767018
52.8543511	72.57833044	74.69033814	44.21434049	44.03519891
0	22.68072826	21.62088736	54.99832597	42.96116967
37.47853987	38.10362348	38.32793668	44.21434049	36.51699422
2.882964605	1.814458261	3.931070429	3.235195645	0
110.5136432	151.5072648	63.87989446	92.74227517	103.1068072
25.94668145	39.01085261	41.2762395	39.90074629	40.81311119
24.98569325	31.75301957	22.60365496	14.01918113	17.18446787
44.20545728	54.43374783	36.36240146	12.94078258	16.11043863
2.882964605	9.072291305	9.827676071	0	6.444175451
7.687905614	5.443374783	3.931070429	15.09757968	6.444175451
29.79063425	39.01085261	52.08668318	46.37113758	54.77549133
0	0	5.896605643	0	1.074029242
23.06371684	0	3.931070429	0	17.18446787
0	5.443374783	0	0	0
56.6983039	53.5265187	109.0872044	40.97914484	62.29369602
0	7.257833044	2.948302821	1.078398548	0
2.882964605	8.165062175	4.913838036	5.391992742	0
6.726917412	1.814458261	0	0	0
191.2366522	310.2723626	163.1394228	174.7005649	139.6238014
11.53185842	5.443374783	14.74151411	3.235195645	2.148058484
0	0	0	0	2.148058484
164.3289825	188.7036592	165.104958	153.1325939	157.8822985
33.63458706	35.38193609	59.94882404	71.1743042	39.73908194
72.07411513	110.6819539	0	93.82067372	96.66263176

5.765929211	0	0	0	15.03640938
40.36150447	79.83616349	0	0	0
0	13.60843696	8.844908464	12.94078258	13.96238014
804.3471249	190.5181174	279.1060004	1310.254236	1.074029242
25.94668145	0	15.72428171	1.078398548	46.1832574
94.17684377	73.48555957	57.00052121	40.97914484	74.10801768
55.7373157	75.30001783	100.2422959	122.9374345	74.10801768
13.45383482	19.95904087	15.72428171	25.88156516	25.7767018
17.29778763	16.33012435	17.68981693	29.11676081	12.8883509
227.7542038	205.0337835	240.7780637	213.5229126	139.6238014
0.960988202	4.536145653	9.827676071	0	10.74029242
0	0	0.982767607	1.078398548	12.8883509
47.08842189	51.71206044	34.39686625	32.35195645	42.96116967
15.37581123	22.68072826	43.24177471	0	22.55461408
0.960988202	0	4.913838036	6.470391291	0
0	27.21687392	0	0	0
0	0	0	0	0
29.79063425	32.6602487	67.81096489	63.62551436	56.92354981
32.67359886	40.82531087	46.19007754	47.44953613	71.9599592
8.648893816	24.49518652	10.81044368	18.33277532	23.62864332
24.98569325	15.42289522	17.68981693	17.25437678	13.96238014
27.86865785	48.99037305	0	72.25270275	35.44296498
114.357596	159.672327	142.501303	167.151775	147.1420061
30.75162246	72.57833044	45.20730993	42.05754339	68.73787147
66.30818592	70.76387218	88.44908464	93.82067372	75.18204692
12.49284662	20.86627	12.77597889	20.48957242	17.18446787
7.687905614	0	18.67258454	31.27355791	17.18446787
53.8153393	56.24820609	69.77650011	67.93910855	65.51578375
0	0	0	0	1.074029242
28.82964605	30.84579044	33.41409864	43.13594194	30.07281877
284.4525077	278.5193431	246.6746694	245.874869	291.0619245
559.2951334	597.863997	166.0877256	492.8281366	194.3992928
479.5331127	385.5723805	211.2950355	150.9757968	341.5412989
0	0	4.913838036	7.548789839	1.074029242
54.7763275	19.95904087	0	35.5871521	36.51699422
77.84004434	65.3204974	80.58694379	77.64469549	51.5534036
76.87905614	88.90845479	88.44908464	51.76313033	73.03398844
57.65929211	31.75301957	39.31070429	80.87989113	124.587392
62.46423311	39.01085261	21.62088736	62.54711581	32.22087725
87.44992636	80.74339262	69.77650011	45.29273904	48.33131588
55.7373157	31.75301957	48.15561275	35.5871521	46.1832574
42.28348088	12.70120783	5.896605643	4.313594194	33.29490649
21.14174044	24.49518652	2.948302821	6.470391291	9.666263176
232.5591448	184.1675135	227.0193172	183.3277532	136.4017137
23.06371684	25.40241565	17.68981693	29.11676081	19.33252635
0	0	0	0	0
3.843952807	6.350603914	18.67258454	24.80316661	9.666263176
99.94277298	115.2180996	78.62140857	131.5646229	64.44175451
30.75162246	26.30964479	40.29347189	18.33277532	33.29490649
67.26917412	71.67110131	43.24177471	88.42868097	81.62622237

For Review Only

0	61.69158088	40.29347189	47.44953613	37.59102346
195.080605	243.137407	243.7263666	232.9340865	219.1019653
0	29.03133218	0	42.05754339	0
192.1976404	184.1675135	83.53524661	106.7614563	220.1759946
69.19115053	82.55785088	63.87989446	53.91992742	33.29490649
41.32249268	53.5265187	29.48302821	47.44953613	36.51699422
0	0	1.965535214	0	8.592233934
0	0	3.931070429	1.078398548	0
26.90766965	22.68072826	40.29347189	39.90074629	57.99757905
0	0	1.965535214	2.156797097	0
30.75162246	26.30964479	45.20730993	32.35195645	37.59102346
26.90766965	26.30964479	28.50026061	25.88156516	33.29490649
18.25877583	19.05181174	22.60365496	18.33277532	25.7767018
135.4993364	128.8265365	64.86266207	106.7614563	110.6250119
6.726917412	0	3.931070429	0	1.074029242
0	0	0	0	0
0	0	0.982767607	5.391992742	0
0	0	2.948302821	0	0
6.726917412	0.907229131	0.982767607	9.705586936	6.444175451
0	0	0	0	0
0	0	1.965535214	0	2.148058484
46.12743368	39.91808174	63.87989446	59.31192017	36.51699422
70.15213873	73.48555957	61.91435925	71.1743042	22.55461408
7.687905614	5.443374783	5.896605643	6.470391291	2.148058484
35.55656347	39.91808174	25.55195779	14.01918113	39.73908194
0	0	0.982767607	1.078398548	1.074029242
11.53185842	7.257833044	11.79321129	22.64636952	23.62864332
2.882964605	0	6.87937325	11.86238403	9.666263176
4.804941009	0	0	4.313594194	5.370146209
9.609882018	0	4.913838036	9.705586936	4.296116967
11.53185842	13.60843696	3.931070429	45.29273904	17.18446787
0	0	0	10.78398548	0
0	3.628916522	1.965535214	4.313594194	1.074029242
19.21976404	14.51566609	19.65535214	31.27355791	35.44296498
42.28348088	80.74339262	31.44856343	37.7439492	30.07281877
0	3.628916522	0	352.6363253	344.7633866
269.0766965	325.6952579	354.7791062	314.8923761	301.8022169
0	0	0	101.3694636	0
59.58126851	50.80483131	36.36240146	22.64636952	41.88714043
1.921976404	0	0	0	0
7.687905614	0	0	3.235195645	0
24.02470504	44.4542274	30.46579582	52.84152887	75.18204692
652.510989	697.6592014	948.3707409	863.7972373	969.8484053
0	12.70120783	7.862140857	0	1.074029242
22.10272864	9.979520436	19.65535214	8.627188388	22.55461408
38.43952807	32.6602487	33.41409864	37.7439492	31.14684801
19.21976404	19.95904087	25.55195779	17.25437678	18.25849711
65.34719772	92.53737131	87.46631704	108.9182534	70.88592996
28.82964605	27.21687392	12.77597889	16.17597823	32.22087725
19.21976404	30.84579044	9.827676071	11.86238403	24.70267256

For Review Only

231.5981566	274.8904266	342.9858949	209.2093184	308.2463924
0	0	0	0	0
37.47853987	43.54699827	29.48302821	21.56797097	34.36893574
0	0	9.827676071	0	2.148058484
25.94668145	48.99037305	57.00052121	46.37113758	31.14684801
7.687905614	3.628916522	172.9670989	18.33277532	21.48058484
28.82964605	82.55785088	89.43185225	115.3886447	138.5497722
0	0	3.931070429	1.078398548	1.074029242
0	4.536145653	0.982767607	0	0
490.1039829	441.8205866	262.3989511	305.1867892	424.2415505
0	4.536145653	0	0	0
24.02470504	48.99037305	40.29347189	38.82234774	62.29369602
8.648893816	0	3.931070429	0	3.222087725
12.49284662	43.54699827	49.13838036	1.078398548	4.296116967
4.804941009	0	0	0	0
193.1586286	143.3422026	162.1566552	103.5262607	135.3276845
4967.348015	5494.179615	5468.118966	7238.211057	4524.885196
1.921976404	0.907229131	0	4.313594194	0
4.804941009	0	4.913838036	2.156797097	2.148058484
6.726917412	0	1.965535214	9.705586936	0
0	3.628916522	0	0	0
18.25877583	15.42289522	29.48302821	12.94078258	17.18446787
59.58126851	58.96989348	25.55195779	36.66555065	42.96116967
0	0	0	2.156797097	2.148058484
0	0	0.982767607	0	1.074029242
0	0	0	5.391992742	0
0	10.88674957	0	0	0
0.960988202	0	0	2.156797097	0
0	0	0	0	0
0.960988202	0	0	2.156797097	0
0	0	0	0	0
0	0	0	0	0
0	0	0	0	0
0	0	0	0	0
1.921976404	3.628916522	0	3.235195645	1.074029242
3.843952807	2.721687392	0.982767607	2.156797097	2.148058484
5.765929211	0	0	1.078398548	0
6.726917412	0	0	1.078398548	4.296116967
0	3.628916522	3.931070429	2.156797097	11.81432166
356.5266229	218.6422205	224.0710144	500.3769265	216.9539068
0.960988202	0	0	0	2.148058484
0.960988202	0	0.982767607	3.235195645	6.444175451
108.5916668	71.67110131	53.06945079	99.21266646	55.84952057
56.6983039	58.96989348	82.552479	95.97747081	66.58981299
135.4993364	208.6627	107.1216692	79.80149259	82.70025162
20.18075224	44.4542274	33.41409864	0	37.59102346
3.843952807	0	2.948302821	5.391992742	7.518204692
4.804941009	4.536145653	0	9.705586936	0
0.960988202	0	0	0	0
0	0	0	0	0

For Review Only

15.37581123	16.33012435	12.77597889	21.56797097	15.03640938
14.41482303	10.88674957	6.87937325	17.25437678	2.148058484
49.97138649	44.4542274	32.43133104	124.0158331	120.2912751
300.7893072	283.9627179	299.7441202	298.7163979	122.4393336
172.0168881	272.1687392	302.692423	321.3627674	227.6941993
87.44992636	52.61928957	119.8976481	114.3102461	107.4029242
17.29778763	12.70120783	3.931070429	6.470391291	13.96238014
56.6983039	86.1867674	75.67310575	67.93910855	74.10801768
121.0845134	174.1879931	112.0355072	163.9165794	190.1031758
38.43952807	65.3204974	106.1389016	103.5262607	39.73908194
58.62028031	76.20724696	117.9321129	77.64469549	46.1832574
19.21976404	0	19.65535214	20.48957242	16.11043863
11.53185842	24.49518652	0	0	0
14.41482303	11.7939787	3.931070429	1.078398548	3.222087725
89.37190276	84.37230914	100.2422959	77.64469549	157.8822985
0.960988202	0	4.913838036	6.470391291	1.074029242
88.41091456	64.41326827	78.62140857	125.0942316	66.58981299
120.1235252	117.939787	183.7775425	163.9165794	161.1043863
55.7373157	39.01085261	15.72428171	28.03836226	38.6650527
220.0662982	196.8687213	211.2950355	115.3886447	262.063135
60.54225671	72.57833044	77.63864096	114.3102461	31.14684801
90.33289097	82.55785088	130.7080917	159.6029852	131.0315675
14.41482303	32.6602487	20.63811975	12.94078258	44.03519891
0.960988202	10.88674957	3.931070429	0	4.296116967
46.12743368	25.40241565	69.77650011	60.39031871	40.81311119
54.7763275	40.82531087	12.77597889	20.48957242	65.51578375
189.3146757	208.6627	258.4678807	141.2702098	109.5509827
87.44992636	72.57833044	83.53524661	105.6830577	65.51578375
0	0	0	5.391992742	0
17.29778763	32.6602487	26.53472539	9.705586936	34.36893574
8.648893816	17.23735348	15.72428171	16.17597823	10.74029242
49.01039829	30.84579044	27.517493	30.19515936	63.36772526
27.86865785	75.30001783	43.24177471	38.82234774	41.88714043
30.75162246	36.28916522	18.67258454	50.68473178	60.14563754
175.8608409	172.3735348	150.3634439	217.8365068	266.359252
297.9063425	275.7976557	372.4689231	285.7756153	365.1699422
169.1339235	222.271137	165.104958	225.3852966	195.473322
0	0	0.982767607	0	0
2.882964605	0	0	0	0
0	0	0.982767607	1.078398548	68.73787147
7.687905614	13.60843696	6.87937325	26.95996371	4.296116967
12.49284662	44.4542274	37.34516907	17.25437678	51.5534036
0	5.443374783	3.931070429	14.01918113	0
68.23016233	82.55785088	26.53472539	72.25270275	83.77428086
114.357596	121.5687035	73.70757054	23.72476807	45.10922815
70.15213873	78.02170523	40.29347189	85.19348533	123.5133628
0	0	2.948302821	0	0
62.46423311	56.24820609	72.72480293	66.86071	50.47937436
0	0	24.56919018	38.82234774	36.51699422
0	0	8.844908464	0	0

For Review Only

0	0	0	70.09590565	0
0	0	0	0	1.074029242
0	6.350603914	0	0	0
103.7867258	121.5687035	113.0182748	122.9374345	113.8470996
510.2847351	493.532647	581.7984234	573.7080278	451.0922815
49.01039829	0	0.982767607	1.078398548	1.074029242
7.687905614	18.14458261	13.7587465	12.94078258	20.40655559
152.7971241	151.5072648	110.069972	125.0942316	160.030357
64.38620952	79.83616349	68.7937325	58.23352162	112.7730704
29.79063425	20.86627	44.22454232	29.11676081	38.6650527
19.21976404	8.165062175	28.50026061	9.705586936	30.07281877
0.960988202	0	3.931070429	0	0
0	0	0	0	0
2.882964605	0	4.913838036	3.235195645	1.074029242
720.7411513	726.6905336	784.2485505	551.0616583	762.5607616
0	0	0	0	0
0	0	0	0	2.148058484
2576.409369	4241.296185	3027.906998	3952.33068	1135.248909
3321.175225	3387.593573	3588.084534	2332.57606	2962.172649
9.609882018	0	0.982767607	0	7.518204692
659.2379064	827.392967	1110.527396	630.8631508	951.5899082
25.94668145	40.82531087	57.00052121	48.52793468	24.70267256
194.1196168	135.1771404	103.1905987	171.4653692	161.1043863
9.609882018	11.7939787	21.62088736	14.01918113	3.222087725
7.687905614	4.536145653	10.81044368	5.391992742	0
26.90766965	180.538597	1204.873086	1476.327613	165.4005032
993.6618006	1202.078598	1495.772298	1519.463555	1197.542605
176.8218291	0	126.7770213	170.3869707	142.8458892
1.921976404	0	0	0	0
10.57087022	22.68072826	24.56919018	18.33277532	17.18446787
0	0	0.982767607	1.078398548	0
0	0	0	40.97914484	0
5.765929211	0	0	6.470391291	9.666263176
93.21585557	102.5168918	138.5702326	48.52793468	86.99636858
132.6163718	127.9193074	118.9148805	71.1743042	125.6614213
19.21976404	0.907229131	11.79321129	0	0
402.6540565	410.9747961	454.0386345	452.9273904	383.4284393
0	0	6.87937325	0	0
204.690487	214.1060748	160.19112	140.1918113	84.8483101
0	0	0	0	0
55.7373157	73.48555957	132.673627	88.42868097	99.88471948
161.4460179	119.7542452	109.0872044	114.3102461	118.1432166
172.0168881	149.6928065	125.7942537	104.6046592	100.9587487
0	8.165062175	41.2762395	0	28.99878953
20.18075224	11.7939787	13.7587465	5.391992742	2.148058484
0	115.2180996	95.32845789	2.156797097	381.2803808
66.30818592	131.5482239	28.50026061	144.5054055	183.6590003
42.28348088	65.3204974	45.20730993	54.99832597	62.29369602
348.8387172	303.9217587	379.3482964	363.4203108	405.9830534
9.609882018	23.58795739	22.60365496	15.09757968	6.444175451

For Review Only

168.1729353	133.3626822	239.7952961	168.2301736	214.8058484
74.95707974	51.71206044	0	0	0
302.7112836	208.6627	259.4506483	274.9916299	171.8446787
34.59557526	39.91808174	77.63864096	49.60633323	39.73908194
2.882964605	0	0	1.078398548	0
0	0	3.931070429	0	0
22.10272864	34.47470696	22.60365496	25.88156516	6.444175451
98.02079658	126.1048491	0	30.19515936	42.96116967
2.882964605	0	0	3.235195645	0
7.687905614	0	0	0	0
0.960988202	0	1.965535214	0	0
0.960988202	0.907229131	0	0	0
0	0	0.982767607	0	1.074029242
87.44992636	98.88797523	85.50078182	81.95828968	40.81311119
402.6540565	486.274814	428.4866767	425.9674266	512.3119483
7.687905614	5.443374783	5.896605643	6.470391291	13.96238014
49.01039829	55.34097696	80.58694379	91.66387662	38.6650527
78.80103254	75.30001783	37.34516907	9.705586936	62.29369602
75.91806794	90.72291305	74.69033814	67.93910855	0
16.33679943	26.30964479	9.827676071	17.25437678	0
34.59557526	31.75301957	40.29347189	30.19515936	36.51699422
174.8998527	171.4663057	131.6908594	169.3085721	113.8470996
34.59557526	29.03133218	34.39686625	49.60633323	52.62743285
15.37581123	25.40241565	12.77597889	24.80316661	39.73908194
34.59557526	30.84579044	21.62088736	2.156797097	27.92476029
273.8816375	271.26151	269.2783244	237.2476807	285.6917783
3.843952807	0	2.948302821	3.235195645	1.074029242
72.07411513	74.3927887	27.517493	40.97914484	109.5509827
0.960988202	0	0	0	0
0	0	1.965535214	0	1.074029242
180.6657819	171.4663057	191.6396834	187.6413474	216.9539068
2.882964605	2.721687392	0	2.156797097	2.148058484
0.960988202	14.51566609	1.965535214	6.470391291	5.370146209
34.59557526	16.33012435	37.34516907	21.56797097	31.14684801
0	0	0.982767607	0	0
192.1976404	236.7868031	269.2783244	191.9549416	219.1019653
0	18.14458261	0.982767607	2.156797097	26.85073104
24.02470504	17.23735348	18.67258454	40.97914484	25.7767018
55.7373157	63.50603914	9.827676071	32.35195645	65.51578375
0	0	0	2.156797097	0
6.726917412	21.77349913	12.77597889	14.01918113	12.8883509
3.843952807	7.257833044	1.965535214	8.627188388	8.592233934
53.8153393	64.41326827	39.31070429	67.93910855	54.77549133
149.9141595	124.2903909	108.1044368	74.40949984	102.032778
119.162537	0	0.982767607	7.548789839	74.10801768
47.08842189	48.08314392	62.89712686	36.66555065	49.40534512
118.2015488	94.35182958	132.673627	40.97914484	77.33010541
4.804941009	0.907229131	0	0	0
52.8543511	16.33012435	18.67258454	20.48957242	8.592233934
62.46423311	55.34097696	40.29347189	38.82234774	35.44296498

For Review Only

53.8153393	58.96989348	78.62140857	61.46871726	41.88714043
144.1482303	116.1253287	52.08668318	160.6813837	229.8422577
39.40051627	49.89760218	30.46579582	24.80316661	27.92476029
0	36.28916522	0	159.6029852	0
88.41091456	39.01085261	14.74151411	92.74227517	4.296116967
80.72300895	62.59881001	78.62140857	75.48789839	38.6650527
47.08842189	27.21687392	29.48302821	51.76313033	27.92476029
156.6410769	156.9506396	104.1733664	173.6221663	163.2524447
0	341.1181531	33.41409864	14.01918113	1.074029242
28.82964605	14.51566609	26.53472539	32.35195645	31.14684801
31.71261066	36.28916522	25.55195779	31.27355791	35.44296498
7.687905614	0.907229131	8.844908464	0	0
10.57087022	14.51566609	13.7587465	20.48957242	33.29490649
542.958334	621.4519544	589.6605643	582.3352162	542.3847671
0	19.05181174	2.948302821	19.41117387	8.592233934
62.46423311	52.61928957	32.43133104	31.27355791	25.7767018
0.960988202	1.814458261	0	0	0
94.17684377	98.88797523	56.01775361	105.6830577	79.47816389
3.843952807	0	0	0	1.074029242
60.54225671	76.20724696	59.94882404	59.31192017	76.25607616
55.7373157	29.03133218	29.48302821	36.66555065	28.99878953
21.14174044	18.14458261	39.31070429	20.48957242	26.85073104
6.726917412	0	3.931070429	2.156797097	4.296116967
0	0	0	2.156797097	2.148058484
123.0064898	94.35182958	48.15561275	124.0158331	151.4381231
17.29778763	22.68072826	22.60365496	16.17597823	25.7767018
20.18075224	10.88674957	24.56919018	22.64636952	24.70267256
22.10272864	11.7939787	9.827676071	23.72476807	24.70267256
0	194.1470339	108.1044368	0	88.07039782
0.960988202	0.907229131	12.77597889	0	0
158.5630533	142.4349735	214.2433384	346.1659341	251.3228426
109.552655	113.4036413	66.82819729	197.3469344	94.51457327
0	6.350603914	0	6.470391291	2.148058484
0	0	0.982767607	0	2.148058484
60.54225671	60.78435175	61.91435925	38.82234774	39.73908194
38.43952807	21.77349913	57.00052121	35.5871521	27.92476029
0	0	1.965535214	0	0
0	0	0	2.156797097	0
0	5.443374783	0.982767607	0	2.148058484
0.960988202	0	0	1.078398548	0
11.53185842	7.257833044	4.913838036	2.156797097	3.222087725
51.8933629	45.36145653	33.41409864	29.11676081	77.33010541
14.41482303	4.536145653	8.844908464	1.078398548	0
21.14174044	9.979520436	10.81044368	11.86238403	11.81432166
25.94668145	14.51566609	18.67258454	24.80316661	15.03640938
42.28348088	51.71206044	2.948302821	2.156797097	15.03640938
27.86865785	31.75301957	39.31070429	1.078398548	21.48058484
25.94668145	29.03133218	40.29347189	50.68473178	30.07281877
35.55656347	64.41326827	22.60365496	53.91992742	33.29490649
5.765929211	13.60843696	15.72428171	3.235195645	6.444175451

For Review Only

34.59557526	32.6602487	37.34516907	7.548789839	19.33252635
24.98569325	25.40241565	9.827676071	22.64636952	9.666263176
39.40051627	20.86627	17.68981693	25.88156516	17.18446787
23.06371684	28.12410305	9.827676071	12.94078258	9.666263176
58.62028031	54.43374783	31.44856343	37.7439492	8.592233934
60.54225671	59.87712262	25.55195779	67.93910855	36.51699422
16.33679943	9.979520436	1.965535214	7.548789839	11.81432166
21.14174044	15.42289522	14.74151411	16.17597823	16.11043863
19.21976404	32.6602487	2.948302821	30.19515936	8.592233934
23.06371684	21.77349913	6.87937325	9.705586936	5.370146209
3.843952807	0	0.982767607	0	0
3.843952807	0	17.68981693	5.391992742	6.444175451
4.804941009	3.628916522	2.948302821	1.078398548	2.148058484
78.80103254	60.78435175	52.08668318	78.72309404	53.70146209
30.75162246	22.68072826	9.827676071	11.86238403	17.18446787
112.4356196	105.2385791	105.156134	53.91992742	131.0315675
0	0	3.931070429	1.078398548	0
31.71261066	19.95904087	16.70704932	12.94078258	11.81432166
17.29778763	17.23735348	11.79321129	25.88156516	22.55461408
8.648893816	10.88674957	19.65535214	0	11.81432166
135.4993364	134.2699113	114.0010424	117.5454418	92.36651479
0	0	125.7942537	5.391992742	9.666263176
0	9.072291305	0	1.078398548	1.074029242
84.56696176	78.02170523	57.00052121	62.54711581	70.88592996
117.2405606	142.4349735	109.0872044	196.2685358	191.177205
98.98178478	58.06266435	114.98381	56.07672452	35.44296498
90.33289097	89.81568392	90.41461986	51.76313033	100.9587487
1.921976404	4.536145653	0	0	0
101.8647494	119.7542452	123.8287185	144.5054055	153.5861816
100.9037612	120.6614744	99.25952832	88.42868097	73.03398844
73.99609154	92.53737131	84.51801421	52.84152887	100.9587487
10.57087022	35.38193609	23.58642257	48.52793468	18.25849711
147.0311949	214.1060748	206.3811975	198.4253329	194.3992928
36.51755167	17.23735348	30.46579582	9.705586936	27.92476029
14.41482303	10.88674957	9.827676071	4.313594194	0
13.45383482	0	8.844908464	1.078398548	0
0	0	19.65535214	0	10.74029242
22.10272864	0	14.74151411	0	0
8.648893816	7.257833044	0	0	3.222087725
114.357596	75.30001783	93.36292268	131.5646229	75.18204692
9.609882018	16.33012435	5.896605643	2.156797097	3.222087725
33.63458706	46.26868566	31.44856343	62.54711581	35.44296498
31.71261066	34.47470696	71.74203532	34.50875355	1.074029242
254.6618735	264.9109061	307.606261	309.5003834	199.769439
57.65929211	65.3204974	81.56971139	70.09590565	70.88592996
63.42522132	56.24820609	207.3639651	52.84152887	74.10801768
29.79063425	30.84579044	31.44856343	47.44953613	32.22087725
12.49284662	17.23735348	13.7587465	11.86238403	17.18446787
51.8933629	45.36145653	65.84542968	66.86071	75.18204692
64.38620952	73.48555957	55.034986	71.1743042	49.40534512

For Review Only

0.960988202	9.072291305	26.53472539	21.56797097	17.18446787
60.54225671	0	239.7952961	67.93910855	42.96116967
51.8933629	61.69158088	85.50078182	51.76313033	96.66263176
32.67359886	38.10362348	44.22454232	33.430355	40.81311119
63.42522132	93.44460045	85.50078182	67.93910855	106.3288949
307.5162246	289.4060926	348.8825005	299.7947965	207.2876437
11.53185842	13.60843696	15.72428171	29.11676081	18.25849711
80.72300895	56.24820609	90.41461986	58.23352162	52.62743285
59.58126851	40.82531087	77.63864096	48.52793468	49.40534512
73.99609154	103.4241209	95.32845789	79.80149259	114.9211289
6.726917412	0	2.948302821	4.313594194	4.296116967
60.54225671	84.37230914	26.53472539	58.23352162	84.8483101
0	0	0	0	0
19.21976404	47.17591479	0	0	91.29248555
2.882964605	1.814458261	0.982767607	0	1.074029242
233.520133	190.5181174	250.6057398	242.6396734	126.7354505
0	3.628916522	0	2.156797097	0
935.0415203	888.1773188	461.9007754	524.1016946	867.8156273
44.20545728	29.03133218	17.68981693	38.82234774	42.96116967
101.8647494	133.3626822	93.36292268	90.58547807	93.44054403
74.95707974	86.1867674	83.53524661	61.46871726	85.92233934
201.8075224	238.6012613	150.3634439	171.4653692	160.030357
18.25877583	19.95904087	27.517493	43.13594194	55.84952057
0.960988202	2.721687392	0	0	2.148058484
19.21976404	25.40241565	17.68981693	26.95996371	18.25849711
0	0	0	0	0
0.960988202	0.907229131	0	0	0
0.960988202	37.19639435	114.0010424	111.0750505	0
40.36150447	45.36145653	51.10391557	40.97914484	49.40534512
22.10272864	29.93856131	34.39686625	60.39031871	19.33252635
0.960988202	0	0	0	0
0	0	0	2.156797097	0
5.765929211	0.907229131	0	1.078398548	0
0	4.536145653	0	0	2.148058484
121.0845134	76.20724696	104.1733664	103.5262607	96.66263176
107.6306786	121.5687035	131.6908594	169.3085721	132.1055967
48.04941009	39.01085261	54.05221839	61.46871726	45.10922815
1394.393881	1519.608794	1766.03339	2464.140683	1651.856974
59.58126851	2.721687392	0	34.50875355	183.6590003
5.765929211	0.907229131	0	0	0
489.1429947	488.0892722	798.9900646	969.4802951	593.9381707
73.99609154	46.26868566	44.22454232	56.07672452	46.1832574
250.8179207	252.2096983	263.3817187	144.5054055	283.5437198
63.42522132	73.48555957	123.8287185	81.95828968	39.73908194
35.55656347	49.89760218	31.44856343	45.29273904	80.55219313
43.24446908	26.30964479	41.2762395	24.80316661	39.73908194
0	15.42289522	0	0	0
75.91806794	112.4964122	55.034986	56.07672452	107.4029242
19.21976404	13.60843696	25.55195779	16.17597823	19.33252635
105.7087022	103.4241209	155.2772819	99.21266646	89.14442707

For Review Only

21.14174044	9.979520436	17.68981693	2.156797097	13.96238014
0	0	5.896605643	0	0
95.13783197	72.57833044	41.2762395	62.54711581	71.9599592
0	0	57.98328882	0	0
110.5136432	102.5168918	64.86266207	115.3886447	120.2912751
66.30818592	66.22772653	36.36240146	86.27188388	23.62864332
24.02470504	20.86627	0.982767607	8.627188388	3.222087725
30.75162246	48.08314392	12.77597889	7.548789839	19.33252635
186.4317111	177.8169096	175.9154017	204.8957242	165.4005032
77.84004434	49.89760218	19.65535214	70.09590565	57.99757905
0	4.536145653	2.948302821	2.156797097	9.666263176
1.921976404	0	0	0	1.074029242
0.960988202	0.907229131	59.94882404	0	0
7.687905614	10.88674957	5.896605643	7.548789839	0
0	0	13.7587465	0	0
7.687905614	9.979520436	10.81044368	9.705586936	2.148058484
0	0	0	0	0
92.25486737	89.81568392	82.552479	130.4862244	147.1420061
6.726917412	0.907229131	0	2.156797097	4.296116967
4.804941009	0.907229131	1.965535214	0	0
123.967478	440.0061283	581.7984234	567.2376365	324.356831
224.8712392	213.1988457	206.3811975	254.5020574	199.769439
27.86865785	19.05181174	19.65535214	11.86238403	9.666263176
103.7867258	69.85664305	59.94882404	0	44.03519891
71.11312693	87.09399653	138.5702326	89.50707952	103.1068072
74.95707974	79.83616349	76.65587336	90.58547807	56.92354981
70.15213873	62.59881001	86.48354943	69.0175071	97.736661
73.03510333	82.55785088	127.7597889	86.27188388	61.21966678
9.609882018	12.70120783	2.948302821	10.78398548	4.296116967
5.765929211	7.257833044	7.862140857	4.313594194	10.74029242
96.09882018	95.25905871	100.2422959	39.90074629	37.59102346
0	0.907229131	2.948302821	0	2.148058484
177.7828173	178.7241387	177.8809369	198.4253329	192.2512343
74.95707974	82.55785088	63.87989446	78.72309404	75.18204692
11.53185842	6.350603914	16.70704932	6.470391291	21.48058484
0	0	2.948302821	2.156797097	2.148058484
26.90766965	26.30964479	5.896605643	34.50875355	21.48058484
0	0	0	0	0
41.32249268	20.86627	63.87989446	97.05586936	54.77549133
186.4317111	200.4976378	262.3989511	339.6955428	257.767018
96.09882018	0	163.1394228	1.078398548	1.074029242
14.41482303	26.30964479	20.63811975	6.470391291	12.8883509
115.3185842	136.9915987	93.36292268	92.74227517	135.3276845
19.21976404	0	17.68981693	11.86238403	12.8883509
9.609882018	6.350603914	8.844908464	12.94078258	9.666263176
100.9037612	129.7337657	132.673627	93.82067372	137.4757429
1.921976404	0.907229131	18.67258454	0	0
50.93237469	40.82531087	22.60365496	5.391992742	7.518204692
0	0	23.58642257	14.01918113	0
28.82964605	19.95904087	21.62088736	5.391992742	12.8883509

For Review Only

0	264.003677	0.982767607	6.470391291	124.587392
55.7373157	39.01085261	81.56971139	29.11676081	26.85073104
36.51755167	31.75301957	46.19007754	45.29273904	51.5534036
85.52794996	78.92893436	74.69033814	93.82067372	80.55219313
88.41091456	73.48555957	125.7942537	120.7806374	60.14563754
0	0	24.56919018	0	19.33252635
15.37581123	86.1867674	0	40.97914484	0
132.6163718	219.5494496	184.7603101	186.5629489	263.1371642
16.33679943	7.257833044	8.844908464	15.09757968	5.370146209
66.30818592	84.37230914	83.53524661	71.1743042	75.18204692
73.99609154	70.76387218	79.60417618	83.03668823	60.14563754
28.82964605	11.7939787	62.89712686	67.93910855	64.44175451
43.24446908	73.48555957	51.10391557	46.37113758	64.44175451
0	0	0	6.470391291	11.81432166
0.960988202	30.84579044	0	0	0
27.86865785	27.21687392	50.12114796	35.5871521	36.51699422
27.86865785	28.12410305	60.93159164	42.05754339	54.77549133
0	8.165062175	0	0	10.74029242
0	0	0	0	0
21.14174044	20.86627	0.982767607	21.56797097	60.14563754
57.65929211	54.43374783	30.46579582	40.97914484	19.33252635
379.5903397	420.0470874	408.8313246	259.8940502	262.063135
47.08842189	176.0024513	236.8469933	164.9949779	122.4393336
214.300369	216.8277622	253.5540426	236.1692821	241.6565794
21.14174044	19.95904087	14.74151411	4.313594194	7.518204692
0	0	21.62088736	0	0
599.6566379	616.9158088	604.4020784	679.3910855	796.9296974
11.53185842	7.257833044	44.22454232	10.78398548	11.81432166
5.765929211	3.628916522	0.982767607	1.078398548	2.148058484
111.4746314	133.3626822	203.4328947	124.0158331	140.6978307
0.960988202	0	0	0	0
81.68399715	91.63014218	141.5185354	116.4670432	83.77428086
37.47853987	0	6.87937325	1.078398548	9.666263176
56.6983039	116.1253287	103.1905987	59.31192017	90.21845631
6.726917412	17.23735348	6.87937325	6.470391291	15.03640938
10.57087022	9.072291305	13.7587465	6.470391291	20.40655559
0	0	442.2454232	161.7597823	225.5461408
162.4070061	97.9807461	73.70757054	92.74227517	119.2172458
46.12743368	40.82531087	64.86266207	53.91992742	39.73908194
0	57.15543522	8.844908464	126.1726302	202.9915267
5.765929211	3.628916522	0	0	11.81432166
1.921976404	0.907229131	0	0	0
0	71.67110131	125.7942537	1.078398548	4.296116967
56.6983039	88.90845479	56.01775361	57.15512307	67.66384223
13.45383482	9.979520436	6.87937325	17.25437678	4.296116967
33.63458706	48.99037305	27.517493	31.27355791	3.222087725
29.79063425	39.01085261	78.62140857	6.470391291	25.7767018
73.99609154	93.44460045	114.0010424	88.42868097	80.55219313
0	0	0.982767607	1.078398548	1.074029242
153.7581123	154.2289522	229.9676201	163.9165794	186.8810881

For Review Only

17.29778763	9.072291305	20.63811975	26.95996371	18.25849711
123.0064898	126.1048491	90.41461986	104.6046592	99.88471948
17.29778763	20.86627	23.58642257	23.72476807	18.25849711
112.4356196	98.88797523	88.44908464	51.76313033	138.5497722
75.91806794	55.34097696	48.15561275	66.86071	57.99757905
59.58126851	55.34097696	102.2078311	44.21434049	103.1068072
45.16644548	69.85664305	85.50078182	62.54711581	66.58981299
73.03510333	72.57833044	136.6046974	83.03668823	94.51457327
36.51755167	29.03133218	11.79321129	34.50875355	13.96238014
160.4850297	151.5072648	169.0360284	157.4461881	98.81069024
335.3848824	387.3868387	168.0532608	309.5003834	228.7682285
135.4993364	0	8.844908464	8.627188388	0
23.06371684	35.38193609	41.2762395	47.44953613	40.81311119
6.726917412	8.165062175	8.844908464	8.627188388	7.518204692
24.98569325	26.30964479	25.55195779	21.56797097	38.6650527
0	0	0	1.078398548	1.074029242
1.921976404	0	55.034986	39.90074629	0
54.7763275	44.4542274	133.6563946	49.60633323	52.62743285
33.63458706	23.58795739	23.58642257	31.27355791	20.40655559
135.4993364	185.9819718	83.53524661	91.66387662	121.3653043
29.79063425	47.17591479	7.862140857	19.41117387	45.10922815
102.8257376	91.63014218	80.58694379	94.89907226	88.07039782
129.7334072	138.806057	70.75926771	159.6029852	187.9551173
125.8894544	116.1253287	86.48354943	77.64469549	134.2536552
8.648893816	2.721687392	13.7587465	7.548789839	6.444175451
0	0	1.965535214	7.548789839	45.10922815
14.41482303	9.979520436	5.896605643	7.548789839	8.592233934
88.41091456	91.63014218	58.96605643	87.35028243	74.10801768
0	0	43.24177471	66.86071	0
61.50324491	31.75301957	32.43133104	14.01918113	22.55461408
37.47853987	19.05181174	45.20730993	22.64636952	22.55461408
100.9037612	114.3108704	48.15561275	116.4670432	94.51457327
148.9531713	185.0747426	227.0193172	219.9933039	186.8810881
198.9245578	259.4675313	248.6402046	254.5020574	207.2876437
66.30818592	42.63976914	46.19007754	91.66387662	51.5534036
152.7971241	155.1361813	114.0010424	95.97747081	155.7342401
104.747714	37.19639435	58.96605643	53.91992742	74.10801768
8.648893816	0	0	5.391992742	2.148058484
7.687905614	1.814458261	52.08668318	61.46871726	60.14563754
0	0	0	4.313594194	0
16.33679943	20.86627	10.81044368	29.11676081	41.88714043
0	0	0	0	0
14.41482303	23.58795739	12.77597889	10.78398548	15.03640938
54.7763275	51.71206044	47.17284514	76.56629694	35.44296498
89.37190276	85.27953827	100.2422959	108.9182534	79.47816389
42.28348088	19.05181174	24.56919018	25.88156516	11.81432166
267.1547201	220.4566787	593.5916347	194.1117387	117.0691874
12.49284662	4.536145653	8.844908464	17.25437678	26.85073104
30.75162246	12.70120783	15.72428171	32.35195645	28.99878953
73.99609154	52.61928957	41.2762395	51.76313033	13.96238014

For Review Only

46.12743368	59.87712262	49.13838036	50.68473178	61.21966678
76.87905614	69.85664305	71.74203532	91.66387662	123.5133628
427.6397498	79.83616349	256.5023455	204.8957242	181.5109419
19.21976404	48.08314392	52.08668318	24.80316661	13.96238014
0	0	0	0	2.148058484
41.32249268	65.3204974	51.10391557	89.50707952	68.73787147
3.843952807	5.443374783	3.931070429	1.078398548	2.148058484
62.46423311	89.81568392	92.38015507	73.3311013	103.1068072
51.8933629	41.73254	49.13838036	32.35195645	20.40655559
281.5695431	234.9723448	259.4506483	311.6571805	263.1371642
81.68399715	67.13495566	46.19007754	90.58547807	70.88592996
57.65929211	93.44460045	64.86266207	38.82234774	98.81069024
0.960988202	0.907229131	0	97.05586936	0
62.46423311	54.43374783	38.32793668	53.91992742	50.47937436
500.6748531	550.6880822	541.5049515	705.2726507	95.58860252
29.79063425	34.47470696	26.53472539	6.470391291	36.51699422
211.4174044	166.93016	165.104958	236.1692821	154.6602108
28.82964605	48.08314392	38.32793668	37.7439492	44.03519891
74.95707974	65.3204974	1.965535214	47.44953613	18.25849711
0	2.721687392	3.931070429	0	0
6.726917412	11.7939787	21.62088736	14.01918113	4.296116967
39.40051627	44.4542274	50.12114796	37.7439492	45.10922815
44.20545728	67.13495566	123.8287185	0	0
18.25877583	11.7939787	11.79321129	9.705586936	30.07281877
52.8543511	44.4542274	85.50078182	31.27355791	19.33252635
90.33289097	80.74339262	84.51801421	119.7022389	53.70146209
263.3107673	264.003677	225.053782	210.2877169	185.8070588
21.14174044	13.60843696	11.79321129	20.48957242	22.55461408
98.98178478	103.4241209	82.552479	87.35028243	89.14442707
117.2405606	95.25905871	57.98328882	62.54711581	93.44054403
22.10272864	21.77349913	83.53524661	15.09757968	5.370146209
22.10272864	19.05181174	25.55195779	28.03836226	46.1832574
79.76202075	0	74.69033814	25.88156516	1.074029242
56.6983039	94.35182958	217.1916412	208.1309199	164.326474
121.0845134	143.3422026	78.62140857	122.9374345	125.6614213
82.64498535	71.67110131	109.0872044	122.9374345	118.1432166
124.9284662	128.8265365	69.77650011	128.3294273	169.6966202
8.648893816	5.443374783	0.982767607	7.548789839	12.8883509
51.8933629	65.3204974	90.41461986	91.66387662	102.032778
24.98569325	28.12410305	20.63811975	31.27355791	33.29490649
24.98569325	25.40241565	12.77597889	24.80316661	20.40655559
21.14174044	35.38193609	53.06945079	0	65.51578375
625.6033193	646.8543701	508.0908529	882.1300126	409.2051411
71.11312693	84.37230914	42.25900711	53.91992742	71.9599592
33.63458706	29.03133218	18.67258454	9.705586936	16.11043863
84.56696176	105.2385791	88.44908464	102.4478621	81.62622237
60.54225671	68.04218479	58.96605643	84.11508678	108.4769534
36.51755167	15.42289522	0	20.48957242	25.7767018
542.958334	194.1470339	224.0710144	409.7914484	115.9951581
1164.717701	833.743571	347.8997329	955.4611139	706.7112411

For Review Only

10.57087022	22.68072826	15.72428171	14.01918113	11.81432166
306.5552364	196.8687213	231.9331553	170.3869707	213.7318191
60.54225671	0	48.15561275	20.48957242	6.444175451
13.45383482	16.33012435	1.965535214	1.078398548	12.8883509
94.17684377	89.81568392	144.4668382	118.6238403	127.8094798
426.6787616	417.3254	358.7101766	469.1033686	359.799796
18.25877583	34.47470696	16.70704932	33.430355	10.74029242
97.05980838	72.57833044	110.069972	70.09590565	62.29369602
39.40051627	0	9.827676071	10.78398548	38.6650527
1.921976404	1.814458261	2.948302821	1.078398548	0
3.843952807	0	3.931070429	0	0
0	0	30.46579582	17.25437678	19.33252635
7.687905614	0.907229131	28.50026061	57.15512307	32.22087725
54.7763275	68.94941392	82.552479	47.44953613	45.10922815
0	2.721687392	0.982767607	0	0
0	0	0	21.56797097	0
44.20545728	50.80483131	44.22454232	59.31192017	51.5534036
9.609882018	22.68072826	9.827676071	16.17597823	16.11043863
344.0337762	411.8820253	0	0	0
15.37581123	14.51566609	18.67258454	20.48957242	20.40655559
146.0702067	99.79520436	144.4668382	104.6046592	85.92233934
58.62028031	51.71206044	79.60417618	35.5871521	39.73908194
10.57087022	0	1.965535214	1.078398548	1.074029242
325.7750004	337.4892366	230.9503877	172.5437678	150.3640938
0	0	0	0	0
5.765929211	6.350603914	14.74151411	15.09757968	2.148058484
6.726917412	9.072291305	8.844908464	8.627188388	4.296116967
5.765929211	7.257833044	0.982767607	2.156797097	3.222087725
76.87905614	0	25.55195779	35.5871521	6.444175451
0	4.536145653	234.8814581	0	180.4369126
66.30818592	0	0	0	0
80.72300895	65.3204974	1.965535214	15.09757968	1.074029242
201.8075224	0	0	0	1579.897015
29.79063425	34.47470696	36.36240146	88.42868097	52.62743285
5.765929211	0.907229131	0	198.4253329	220.1759946
116.2795724	138.806057	140.5357678	167.151775	136.4017137
354.6046465	281.2410305	288.9336765	313.8139776	346.9114451
0	228.6217409	172.9670989	111.0750505	131.0315675
0	0	0	2.156797097	0
49.97138649	43.54699827	76.65587336	0	60.14563754
2.882964605	0	0	3.235195645	0
55.7373157	75.30001783	134.6391622	86.27188388	153.5861816
216.2223454	202.3120961	185.7430777	223.2284995	184.7330296
431.4837026	510.7700005	448.1420289	236.1692821	270.6553689
5.765929211	0	0.982767607	0	3.222087725
48.04941009	30.84579044	27.517493	40.97914484	36.51699422
1.921976404	0	2.948302821	0	4.296116967
10.57087022	8.165062175	6.87937325	7.548789839	39.73908194
44.20545728	50.80483131	35.37963386	21.56797097	44.03519891
97.05980838	93.44460045	141.5185354	162.8381808	132.1055967

For Review Only

306.5552364	438.19167	540.5221839	436.7514121	359.799796
1.921976404	2.721687392	7.862140857	7.548789839	5.370146209
101.8647494	303.9217587	191.6396834	141.2702098	184.7330296
37.47853987	27.21687392	15.72428171	21.56797097	34.36893574
0	4.536145653	0	8.627188388	0
250.8179207	251.3024692	260.4334159	185.4845503	172.9187079
36.51755167	46.26868566	58.96605643	39.90074629	44.03519891
18.25877583	16.33012435	8.844908464	15.09757968	12.8883509
1234.869839	1155.809912	1710.998404	2048.957242	1401.60816
6.726917412	18.14458261	12.77597889	21.56797097	24.70267256
168.1729353	125.19762	118.9148805	150.9757968	157.8822985
0.960988202	0.907229131	0	0	0
0	0	0	0	0
24.98569325	11.7939787	24.56919018	19.41117387	22.55461408
0	0	0	0	0
99.94277298	81.65062175	94.34569029	134.7998186	105.2548657
5.765929211	0.907229131	0	0	0
12.49284662	5.443374783	0.982767607	1.078398548	5.370146209
93.21585557	131.5482239	98.27676071	162.8381808	118.1432166
0	0	0	0	0
3.843952807	0.907229131	0	0	0
4.804941009	6.350603914	2.948302821	3.235195645	4.296116967
147.9921831	156.0434105	120.8804157	191.9549416	68.73787147
35.55656347	12.70120783	22.60365496	9.705586936	26.85073104
12.49284662	9.979520436	8.844908464	0	0
665.9648238	0	250.6057398	704.1942521	1.074029242
0	0	0	1.078398548	1.074029242
314.243142	217.7349913	305.6407258	174.7005649	94.51457327
122.0455016	132.4554531	152.3289791	130.4862244	85.92233934
0	0	1.965535214	0	0
0	11.7939787	0	38.82234774	15.03640938
5.765929211	8.165062175	0	0	4.296116967
0	0	0.982767607	0	3.222087725
164.3289825	151.5072648	0	193.0333402	155.7342401
471.8452071	460.8723983	501.2114796	487.4361439	405.9830534
0	0	0	0	2.148058484
31.71261066	43.54699827	30.46579582	19.41117387	33.29490649
197.9635696	216.8277622	261.4161835	184.4061518	210.5097314
0	8.165062175	6.87937325	5.391992742	4.296116967
14.41482303	10.88674957	4.913838036	19.41117387	5.370146209
432.4446908	368.335027	470.7456838	486.3577454	524.12627
52.8543511	50.80483131	44.22454232	50.68473178	49.40534512
335.3848824	421.8615457	588.6777967	487.4361439	378.0582931
1288.685179	2233.598119	3459.341977	2108.269162	1103.028031
24.02470504	17.23735348	17.68981693	15.09757968	7.518204692
1002.310694	911.7652762	1061.389016	429.2026223	839.8908671
106.6696904	115.2180996	82.552479	83.03668823	80.55219313
217.1833336	198.6831796	264.3644863	213.5229126	196.5473512
19.21976404	19.05181174	11.79321129	22.64636952	13.96238014
0	0	4.913838036	4.313594194	1.074029242

For Review Only

9.609882018	23.58795739	12.77597889	23.72476807	18.25849711
13.45383482	2.721687392	4.913838036	6.470391291	1.074029242
16.33679943	27.21687392	13.7587465	25.88156516	37.59102346
3.843952807	0	4.913838036	2.156797097	8.592233934
155.6800887	195.0542631	214.2433384	237.2476807	153.5861816
5.765929211	0	1.965535214	2.156797097	3.222087725
98.02079658	106.1458083	70.75926771	142.3486084	53.70146209
0	0	0	0	0
0	0	61.91435925	44.21434049	26.85073104
9.609882018	0	2.948302821	0	0
4.804941009	0	0	0	0
41.32249268	20.86627	24.56919018	18.33277532	30.07281877
1.921976404	0	0	0	0
10.57087022	3.628916522	0	0	8.592233934
22.10272864	0	2.948302821	9.705586936	20.40655559
4.804941009	6.350603914	7.862140857	1.078398548	0
49.01039829	61.69158088	0.982767607	118.6238403	148.2160354
107.6306786	85.27953827	48.15561275	184.4061518	75.18204692
0	66.22772653	58.96605643	15.09757968	35.44296498
270.0376847	172.3735348	182.7947749	88.42868097	214.8058484
0	32.6602487	14.74151411	0	1.074029242
0	0	210.3122679	222.150101	212.6577899
166.2509589	83.46508001	124.8114861	118.6238403	90.21845631
19.21976404	14.51566609	0	106.7614563	239.5085209
1.921976404	1.814458261	10.81044368	88.42868097	4.296116967
19.21976404	29.93856131	26.53472539	22.64636952	28.99878953
25.94668145	28.12410305	36.36240146	25.88156516	4.296116967
0.960988202	0.907229131	0	0	0
0	5.443374783	4.913838036	0	1.074029242
39.40051627	7.257833044	29.48302821	0	0
15.37581123	0	17.68981693	8.627188388	2.148058484
0	0	0	0	0
11.53185842	7.257833044	5.896605643	10.78398548	7.518204692
335.3848824	719.4327005	832.4041632	300.873195	662.6760422
68.23016233	0	0	37.7439492	64.44175451
0	0	0.982767607	1.078398548	2.148058484
3.843952807	4.536145653	0	0	1.074029242
206.6124634	130.6409948	191.6396834	185.4845503	215.8798776
34.59557526	0	36.36240146	1.078398548	12.8883509
5.765929211	8.165062175	11.79321129	15.09757968	9.666263176
0	0	0	0	0
19.21976404	6.350603914	5.896605643	7.548789839	32.22087725
22.10272864	38.10362348	25.55195779	25.88156516	55.84952057
30.75162246	40.82531087	38.32793668	57.15512307	12.8883509
8.648893816	9.072291305	26.53472539	20.48957242	12.8883509
70.15213873	0	74.69033814	72.25270275	76.25607616
31.71261066	47.17591479	18.67258454	36.66555065	19.33252635
2.882964605	0	0	6.470391291	0
82.64498535	92.53737131	62.89712686	88.42868097	93.44054403
0	0	0	0	0

For Review Only

0	0	38.32793668	0	0
2.882964605	3.628916522	0	0	0
339.2288352	351.0976735	468.7801486	355.871521	323.2828018
0	0	0.982767607	1.078398548	7.518204692
33.63458706	29.03133218	15.72428171	26.95996371	7.518204692
4.804941009	0.907229131	0	4.313594194	0
11.53185842	39.01085261	27.517493	21.56797097	18.25849711
251.7789089	284.869947	130.7080917	230.7772894	251.3228426
0	0	0	0	0
9.609882018	0	4.913838036	0	4.296116967
0	2234.505349	347.8997329	171.4653692	1.074029242
44.20545728	40.82531087	69.77650011	3.235195645	33.29490649
0	4.536145653	2.948302821	1.078398548	2.148058484
0	0	0	0	0
0	7.257833044	0	0	0
11.53185842	0	0.982767607	24.80316661	9.666263176
0	0	0	0	0
325.7750004	330.2314035	346.9169653	285.7756153	411.3531996
14.41482303	0	29.48302821	9.705586936	20.40655559
100.9037612	89.81568392	92.38015507	67.93910855	92.36651479
21.14174044	19.05181174	15.72428171	17.25437678	34.36893574
52.8543511	115.2180996	41.2762395	21.56797097	52.62743285
1421.30155	1480.597941	1425.01303	1241.236729	1244.799891
97.05980838	166.0229309	180.8292397	116.4670432	150.3640938
0	81.65062175	129.7253241	126.1726302	0
17.29778763	24.49518652	37.34516907	16.17597823	70.88592996
51.8933629	33.56747783	37.34516907	32.35195645	46.1832574
26.90766965	24.49518652	24.56919018	11.86238403	48.33131588
0	0	1.965535214	1.078398548	0
424.7567852	390.1085261	302.692423	309.5003834	357.6517375
28.82964605	26.30964479	44.22454232	51.76313033	33.29490649
22.10272864	55.34097696	93.36292268	93.82067372	33.29490649
5.765929211	0	0	5.391992742	2.148058484
0	0	0.982767607	0	1.074029242
4.804941009	0.907229131	0	0	3.222087725
151.8361359	0	29.48302821	166.0733765	171.8446787
27.86865785	17.23735348	28.50026061	24.80316661	35.44296498
0	166.0229309	59.94882404	0	0
44.20545728	29.93856131	47.17284514	34.50875355	51.5534036
1.921976404	62.59881001	0.982767607	67.93910855	109.5509827
0.960988202	12.70120783	0	10.78398548	7.518204692
0	0	0.982767607	1.078398548	1.074029242
0	297.5711548	118.9148805	0	75.18204692
94.17684377	97.9807461	112.0355072	129.4078258	109.5509827
14.41482303	18.14458261	26.53472539	23.72476807	17.18446787
17.29778763	2.721687392	0	0	38.6650527
0	250.39524	217.1916412	1.078398548	1.074029242
0	0	0	0	0
0.960988202	0	14.74151411	0	0
49.97138649	43.54699827	50.12114796	83.03668823	53.70146209

For Review Only

57.65929211	42.63976914	48.15561275	42.05754339	31.14684801
0	0.907229131	0.982767607	0	0
4.804941009	0.907229131	0	3.235195645	3.222087725
3.843952807	0.907229131	0	5.391992742	0
82.64498535	98.88797523	66.82819729	95.97747081	73.03398844
0	0	111.0527396	129.4078258	81.62622237
68.23016233	46.26868566	55.034986	20.48957242	97.736661
94.17684377	162.3940144	139.5530002	174.7005649	136.4017137
137.4213129	104.33135	70.75926771	109.9966519	112.7730704
0	0	0	21.56797097	18.25849711
27.86865785	39.91808174	73.70757054	14.01918113	55.84952057
28.82964605	44.4542274	49.13838036	66.86071	85.92233934
52.8543511	45.36145653	47.17284514	36.66555065	53.70146209
31.71261066	37.19639435	53.06945079	32.35195645	36.51699422
65.34719772	39.91808174	53.06945079	100.291065	97.736661
16.33679943	19.05181174	21.62088736	39.90074629	19.33252635
137.4213129	148.7855774	240.7780637	223.2284995	182.5849711
276.7646021	309.3651335	310.5545639	286.8540139	226.62017
0	0	0	0	0
5.765929211	9.979520436	10.81044368	9.705586936	8.592233934
24.98569325	27.21687392	56.01775361	44.21434049	40.81311119
99.94277298	142.4349735	131.6908594	6.470391291	1.074029242
22.10272864	14.51566609	14.74151411	8.627188388	20.40655559
21.14174044	36.28916522	24.56919018	24.80316661	23.62864332
34.59557526	60.78435175	21.62088736	32.35195645	24.70267256
35.55656347	51.71206044	110.069972	53.91992742	11.81432166
24.98569325	0	0.982767607	87.35028243	3.222087725
0	4.536145653	0	0	0
0	0.907229131	2.948302821	0	2.148058484
88.41091456	115.2180996	116.9493452	65.78231146	90.21845631
42.28348088	54.43374783	25.55195779	63.62551436	41.88714043
0.960988202	1.814458261	0	0	78.40413465
93.21585557	116.1253287	106.1389016	138.0350142	118.1432166
25.94668145	17.23735348	21.62088736	14.01918113	38.6650527
172.9778763	166.0229309	120.8804157	143.4270069	115.9951581
143.1872421	0	155.2772819	218.9149053	62.29369602
46.12743368	59.87712262	39.31070429	0	0
29.79063425	35.38193609	35.37963386	35.5871521	22.55461408
151.8361359	156.9506396	204.4156623	189.7981445	254.5449303
2.882964605	0.907229131	0	26.95996371	27.92476029
5.765929211	26.30964479	51.10391557	88.42868097	89.14442707
25.94668145	33.56747783	26.53472539	16.17597823	33.29490649
0.960988202	0	0	0	0
9.609882018	6.350603914	0	0	0
19.21976404	32.6602487	28.50026061	23.72476807	30.07281877
80.72300895	89.81568392	147.4151411	140.1918113	117.0691874
77.84004434	86.1867674	74.69033814	66.86071	74.10801768
70.15213873	136.0843696	90.41461986	31.27355791	75.18204692
49.97138649	67.13495566	74.69033814	31.27355791	70.88592996
19.21976404	44.4542274	76.65587336	18.33277532	56.92354981

0	0.907229131	73.70757054	0	0
66.30818592	80.74339262	46.19007754	67.93910855	45.10922815
14.41482303	15.42289522	7.862140857	4.313594194	10.74029242
9.609882018	0	0	24.80316661	7.518204692
21.14174044	16.33012435	8.844908464	31.27355791	36.51699422
44.20545728	38.10362348	52.08668318	60.39031871	56.92354981
83.60597355	86.1867674	101.2250635	83.03668823	78.40413465
75.91806794	128.8265365	119.8976481	173.6221663	115.9951581
0	0	0.982767607	0	0
3.843952807	0.907229131	2.948302821	3.235195645	3.222087725
3.843952807	8.165062175	0	30.19515936	0
39.40051627	57.15543522	51.10391557	18.33277532	26.85073104
69.19115053	41.73254	75.67310575	50.68473178	105.2548657
151.8361359	167.8373891	186.7258454	231.8556879	201.9174975
12.49284662	16.33012435	0	25.88156516	0
81.68399715	110.6819539	41.2762395	71.1743042	69.81190071
0	0	3.931070429	0	0
201.8075224	247.6735526	290.8992117	309.5003834	196.5473512
206.6124634	208.6627	72.72480293	244.7964705	281.3956613
14.41482303	17.23735348	6.87937325	18.33277532	27.92476029
27.86865785	23.58795739	17.68981693	26.95996371	15.03640938
0.960988202	0.907229131	0	0	0
33.63458706	0	0	0	0
0.960988202	3.628916522	232.9159229	204.8957242	0
13.45383482	23.58795739	26.53472539	22.64636952	8.592233934
22.10272864	15.42289522	20.63811975	19.41117387	16.11043863
73.03510333	50.80483131	72.72480293	69.0175071	41.88714043
77.84004434	45.36145653	39.31070429	72.25270275	33.29490649
67.26917412	64.41326827	38.32793668	73.3311013	59.0716083
112.4356196	106.1458083	57.00052121	182.2493547	133.179626
13.45383482	12.70120783	18.67258454	37.7439492	15.03640938
54.7763275	68.04218479	63.87989446	71.1743042	61.21966678
8.648893816	8.165062175	0.982767607	9.705586936	1.074029242
15.37581123	12.70120783	15.72428171	22.64636952	15.03640938
0.960988202	3.628916522	61.91435925	0	0
0	13.60843696	0	0	0
2.882964605	0	0	1.078398548	0
29.79063425	32.6602487	5.896605643	12.94078258	20.40655559
10.57087022	10.88674957	1.965535214	15.09757968	3.222087725
39.40051627	0	36.36240146	36.66555065	24.70267256
509.3237469	310.2723626	521.8495994	271.7564342	373.7621761
0	0	0	38.82234774	0
40.36150447	55.34097696	29.48302821	43.13594194	56.92354981
0	0.907229131	267.3127891	1.078398548	0
14.41482303	8.165062175	6.87937325	10.78398548	9.666263176
70.15213873	58.96989348	17.68981693	39.90074629	45.10922815
93.21585557	111.5891831	58.96605643	100.291065	124.587392
2.882964605	48.08314392	527.746205	0	506.9418021
70.15213873	71.67110131	15.72428171	80.87989113	41.88714043
0	64.41326827	34.39686625	11.86238403	35.44296498

28.82964605	39.91808174	33.41409864	28.03836226	41.88714043
5.765929211	0	0.982767607	4.313594194	12.8883509
39.40051627	77.1144761	74.69033814	57.15512307	88.07039782
0	6.350603914	1.965535214	0	10.74029242
21.14174044	28.12410305	35.37963386	35.5871521	25.7767018
162.4070061	183.2602844	171.9843312	115.3886447	107.4029242
50.93237469	23.58795739	48.15561275	59.31192017	44.03519891
0	0	0	0	0
30.75162246	45.36145653	15.72428171	42.05754339	18.25849711
91.29387917	94.35182958	64.86266207	74.40949984	83.77428086
9.609882018	8.165062175	2.948302821	2.156797097	1.074029242
26.90766965	49.89760218	43.24177471	38.82234774	54.77549133
27.86865785	24.49518652	14.74151411	32.35195645	10.74029242
23.06371684	33.56747783	17.68981693	14.01918113	23.62864332
17.29778763	19.05181174	13.7587465	6.470391291	7.518204692
153.7581123	147.8783483	199.5018242	60.39031871	74.10801768
0	0	243.7263666	251.2668618	207.2876437
394.0051627	481.7386683	420.6245359	438.9082092	410.2791704
1.921976404	5.443374783	0	0	0
52.8543511	60.78435175	52.08668318	60.39031871	52.62743285
141.2652657	109.7747248	97.29399311	90.58547807	94.51457327
0	0	4.913838036	3.235195645	7.518204692
0	2.721687392	0	0	0
87.44992636	56.24820609	102.2078311	107.8398548	64.44175451
0	77.1144761	74.69033814	1.078398548	13.96238014
83.60597355	116.1253287	73.70757054	100.291065	99.88471948
54.7763275	63.50603914	27.517493	95.97747081	153.5861816
247.9349561	368.335027	259.4506483	164.9949779	169.6966202
73.03510333	70.76387218	90.41461986	72.25270275	74.10801768
128.772419	116.1253287	123.8287185	156.3677895	153.5861816
66.30818592	46.26868566	29.48302821	64.70391291	28.99878953
0	151.5072648	83.53524661	29.11676081	106.3288949
39.40051627	37.19639435	28.50026061	51.76313033	33.29490649
0.960988202	1.814458261	0.982767607	0	0
2.882964605	3.628916522	3.931070429	4.313594194	13.96238014
0	0	127.7597889	0	0
241.2080386	273.0759683	194.5879862	94.89907226	191.177205
43.24446908	51.71206044	66.82819729	39.90074629	46.1832574
0	0	0.982767607	0	0
0	238.6012613	285.9853737	152.0541953	151.4381231
3.843952807	6.350603914	0.982767607	2.156797097	7.518204692
0	9.072291305	29.48302821	0	0
91.29387917	95.25905871	107.1216692	129.4078258	69.81190071
5.765929211	6.350603914	7.862140857	8.627188388	7.518204692
122.0455016	39.91808174	172.9670989	157.4461881	177.2148249
23.06371684	9.072291305	25.55195779	29.11676081	18.25849711
0	0	0	0	0
0	0	0	0	0
195.080605	225.9000535	200.4845919	175.7789634	114.9211289
74.95707974	101.6096626	96.3112255	85.19348533	50.47937436

For Review Only

0	8.165062175	5.896605643	5.391992742	0
10.57087022	6.350603914	7.862140857	18.33277532	3.222087725
56.6983039	53.5265187	82.552479	54.99832597	69.81190071
37.47853987	24.49518652	38.32793668	40.97914484	39.73908194
308.4772128	286.6844052	222.1054792	238.3260792	312.5425094
63.42522132	85.27953827	85.50078182	113.2318476	77.33010541
67.26917412	94.35182958	84.51801421	111.0750505	133.179626
19.21976404	7.257833044	11.79321129	22.64636952	21.48058484
379.5903397	394.6446718	455.0214021	476.6521584	431.7597552
104.747714	111.5891831	169.0360284	109.9966519	115.9951581
118.2015488	128.8265365	103.1905987	130.4862244	138.5497722
0	0	3.931070429	0	0
9.609882018	45.36145653	104.1733664	19.41117387	12.8883509
52.8543511	29.03133218	16.70704932	23.72476807	22.55461408
18.25877583	19.95904087	25.55195779	25.88156516	19.33252635
16.33679943	13.60843696	20.63811975	18.33277532	19.33252635
4.804941009	12.70120783	19.65535214	0	18.25849711
19.21976404	29.93856131	43.24177471	35.5871521	21.48058484
167.2119471	222.271137	98.27676071	179.014159	165.4005032
0	0	0	7.548789839	15.03640938
52.8543511	48.08314392	37.34516907	42.05754339	34.36893574
75.91806794	107.9602665	83.53524661	216.7581082	135.3276845
31.71261066	29.03133218	38.32793668	37.7439492	65.51578375
3.843952807	0	3.931070429	0	11.81432166
170.0949117	129.7337657	102.2078311	142.3486084	113.8470996
0	0	0	0	0
0	0	0	33.430355	13.96238014
273.8816375	230.4361992	285.9853737	250.1884632	266.359252
18.25877583	27.21687392	24.56919018	7.548789839	21.48058484
22.10272864	12.70120783	17.68981693	19.41117387	30.07281877
0	0	0	0	3.222087725
0	0	0	0	100.9587487
161.4460179	171.4663057	140.5357678	105.6830577	120.2912751
42.28348088	40.82531087	37.34516907	53.91992742	68.73787147
112.4356196	99.79520436	109.0872044	94.89907226	71.9599592
257.5448381	278.5193431	239.7952961	250.1884632	240.5825502
128.772419	65.3204974	161.1738876	124.0158331	111.6990411
1197.391299	737.5772831	641.7472475	867.032433	1210.430955
0	0	0.982767607	5.391992742	1.074029242
46.12743368	67.13495566	79.60417618	56.07672452	85.92233934
25.94668145	12.70120783	11.79321129	31.27355791	13.96238014
13.45383482	32.6602487	13.7587465	86.27188388	0
16.33679943	39.91808174	24.56919018	32.35195645	36.51699422
133.57736	104.33135	103.1905987	109.9966519	135.3276845
23.06371684	23.58795739	11.79321129	30.19515936	12.8883509
8.648893816	9.979520436	10.81044368	11.86238403	8.592233934
25.94668145	30.84579044	29.48302821	32.35195645	35.44296498
0	0	0.982767607	1.078398548	1.074029242
70.15213873	96.16628784	49.13838036	60.39031871	18.25849711
0.960988202	0	0	1.078398548	6.444175451

For Review Only

10.57087022	9.979520436	6.87937325	14.01918113	9.666263176
363.2535403	410.9747961	408.8313246	378.5178905	310.3944509
127.8114308	103.4241209	89.43185225	78.72309404	103.1068072
28.82964605	34.47470696	19.65535214	20.48957242	26.85073104
10.57087022	12.70120783	11.79321129	3.235195645	8.592233934
49.01039829	55.34097696	37.34516907	60.39031871	33.29490649
9.609882018	11.7939787	7.862140857	4.313594194	5.370146209
197.0025814	206.8482418	135.6219298	202.7389271	121.3653043
21.14174044	32.6602487	39.31070429	37.7439492	19.33252635
0	0	0	0	0
41.32249268	70.76387218	13.7587465	46.37113758	47.25728664
206.6124634	225.9000535	235.8642257	188.719746	283.5437198
0	0	0	0	0
110.5136432	117.0325578	110.069972	136.9566157	266.359252
58.62028031	64.41326827	94.34569029	73.3311013	93.44054403
0	0.907229131	0	91.66387662	93.44054403
61.50324491	91.63014218	90.41461986	58.23352162	44.03519891
0	0.907229131	2.948302821	0	0
2.882964605	0	13.7587465	6.470391291	0
81.68399715	0	305.6407258	1.078398548	44.03519891
0	0	0	0	0
0	0	18.67258454	0	0
94.17684377	124.2903909	92.38015507	84.11508678	117.0691874
38.43952807	52.61928957	70.75926771	28.03836226	40.81311119
0	0	832.4041632	767.8197665	701.3410949
0	0	0	0	0
3.843952807	0	2.948302821	0	0
19.21976404	27.21687392	14.74151411	20.48957242	15.03640938
10.57087022	0	0	0	0
22.10272864	20.86627	19.65535214	19.41117387	20.40655559
0	0	0	2.156797097	0
17.29778763	20.86627	36.36240146	8.627188388	30.07281877
60.54225671	69.85664305	59.94882404	58.23352162	37.59102346
126.8504426	83.46508001	113.0182748	115.3886447	108.4769534
0	17.23735348	0	1.078398548	1.074029242
360.3705757	414.6037127	451.0903317	430.2810208	414.5752873
20.18075224	39.01085261	54.05221839	21.56797097	11.81432166
42.28348088	38.10362348	48.15561275	45.29273904	63.36772526
190.2756639	166.0229309	109.0872044	187.6413474	85.92233934
94.17684377	75.30001783	135.6219298	105.6830577	100.9587487
22.10272864	20.86627	17.68981693	10.78398548	10.74029242
0	0	0	0	4.296116967
677.4966822	463.5940857	736.0929377	668.6071	568.1614689
40.36150447	41.73254	37.34516907	29.11676081	38.6650527
28.82964605	20.86627	54.05221839	19.41117387	45.10922815
0	0	0.982767607	0	0
109.552655	117.0325578	71.74203532	75.48789839	136.4017137
223.910251	215.0133039	281.0715356	237.2476807	368.3920299
117.2405606	957.1267327	1263.839143	1421.329287	703.4891533
48.04941009	43.54699827	50.12114796	43.13594194	38.6650527

For Review Only

0	0	0	0	0
68.23016233	49.89760218	77.63864096	31.27355791	67.66384223
15.37581123	11.7939787	17.68981693	34.50875355	15.03640938
6.726917412	10.88674957	23.58642257	0	19.33252635
141.2652657	155.1361813	174.9326341	141.2702098	84.8483101
24.02470504	21.77349913	21.62088736	20.48957242	11.81432166
37.47853987	32.6602487	59.94882404	31.27355791	53.70146209
177.7828173	156.9506396	119.8976481	112.153449	157.8822985
128.772419	91.63014218	73.70757054	87.35028243	127.8094798
5.765929211	8.165062175	3.931070429	2.156797097	1.074029242
59.58126851	65.3204974	60.93159164	70.09590565	56.92354981
17.29778763	14.51566609	14.74151411	20.48957242	16.11043863
14.41482303	23.58795739	52.08668318	20.48957242	35.44296498
123.967478	136.0843696	85.50078182	129.4078258	68.73787147
2.882964605	0	0	1.078398548	0
0	0	11.79321129	0	2.148058484
0	32.6602487	20.63811975	0	17.18446787
69.19115053	101.6096626	168.0532608	28.03836226	56.92354981
48.04941009	51.71206044	0	0	0
46.12743368	68.04218479	116.9493452	75.48789839	52.62743285
43.24446908	58.96989348	46.19007754	18.33277532	16.11043863
59.58126851	41.73254	58.96605643	64.70391291	68.73787147
26.90766965	42.63976914	32.43133104	33.430355	30.07281877
3.843952807	17.23735348	7.862140857	4.313594194	3.222087725
0.960988202	0.907229131	1.965535214	0	0
4.804941009	0	6.87937325	2.156797097	0
7.687905614	0	2.948302821	11.86238403	1.074029242
9.609882018	12.70120783	0	22.64636952	7.518204692
3.843952807	0	0	1.078398548	3.222087725
0	0	2.948302821	0	0
0	0	0.982767607	0	0
6.726917412	0	4.913838036	10.78398548	0
42.28348088	44.4542274	70.75926771	53.91992742	73.03398844
838.9427001	789.2893436	816.6798815	823.896491	675.5643931
85.52794996	75.30001783	68.7937325	86.27188388	59.0716083
60.54225671	88.00122566	63.87989446	71.1743042	44.03519891
0	0	2.948302821	0	0
20.18075224	27.21687392	14.74151411	16.17597823	18.25849711
44.20545728	48.99037305	32.43133104	52.84152887	46.1832574
36.51755167	38.10362348	27.517493	22.64636952	30.07281877
0	0	0	0	0
94.17684377	106.1458083	79.60417618	155.289391	60.14563754
51.8933629	30.84579044	14.74151411	49.60633323	31.14684801
53.8153393	51.71206044	56.01775361	71.1743042	59.0716083
48.04941009	68.94941392	57.00052121	62.54711581	86.99636858
21.14174044	42.63976914	38.32793668	39.90074629	16.11043863
0	0	32.43133104	0	0
152.7971241	125.19762	142.501303	61.46871726	136.4017137
47.08842189	34.47470696	51.10391557	72.25270275	69.81190071
134.5383482	131.5482239	112.0355072	146.6622026	124.587392

For Review Only

53.8153393	41.73254	48.15561275	62.54711581	26.85073104
180.6657819	176.0024513	162.1566552	214.6013111	180.4369126
159.5240415	146.06389	115.9665776	119.7022389	133.179626
280.6085549	323.8807996	287.9509089	298.7163979	307.1723631
21.14174044	25.40241565	18.67258454	19.41117387	33.29490649
31.71261066	9.072291305	19.65535214	3.235195645	4.296116967
138.3823011	122.4759326	113.0182748	142.3486084	110.6250119
118.2015488	97.07351697	114.0010424	179.014159	89.14442707
175.8608409	138.806057	294.8302821	287.9324124	212.6577899
144.1482303	94.35182958	154.2945143	181.1709561	141.7718599
1.921976404	0	6.87937325	3.235195645	0
51.8933629	58.96989348	36.36240146	32.35195645	31.14684801
172.9778763	0	0.982767607	16.17597823	19.33252635
140.3042775	35.38193609	194.5879862	120.7806374	5.370146209
392.0831863	299.3856131	324.3133104	361.2635137	235.2124039
12.49284662	17.23735348	9.827676071	1.078398548	8.592233934
0	3.628916522	1.965535214	0	1.074029242
0	6.350603914	4.913838036	2.156797097	3.222087725
0	7.257833044	0	0	1.074029242
19.21976404	42.63976914	42.25900711	26.95996371	17.18446787
0	0	10.81044368	1.078398548	0
15.37581123	5.443374783	10.81044368	4.313594194	1.074029242
0	0.907229131	0	0	0
8.648893816	6.350603914	0	0	0
15.37581123	28.12410305	20.63811975	15.09757968	10.74029242
3.843952807	12.70120783	3.931070429	6.470391291	1.074029242
93.21585557	83.46508001	110.069972	61.46871726	75.18204692
449.7424784	412.7892544	0	0	0
155.6800887	143.3422026	161.1738876	180.0925576	149.2900646
387.2782453	379.2217766	225.053782	230.7772894	236.2864332
0	0	0	2.156797097	2.148058484
18.25877583	26.30964479	38.32793668	29.11676081	21.48058484
7.687905614	0	5.896605643	0	0
5.765929211	4.536145653	9.827676071	2.156797097	10.74029242
0	0	0.982767607	0	0
0	0	2.948302821	0	3.222087725
5.765929211	0	0.982767607	2.156797097	3.222087725
0	0	1.965535214	0	0
0	0	0	0	0
20.18075224	12.70120783	22.60365496	17.25437678	17.18446787
17.29778763	0	76.65587336	11.86238403	1.074029242
3.843952807	0	0	2.156797097	1.074029242
0	0	0	0	5.370146209
34.59557526	37.19639435	22.60365496	33.430355	17.18446787
73.99609154	85.27953827	72.72480293	113.2318476	60.14563754
1808.579796	1275.564158	1128.217213	77.64469549	2579.818239
49.01039829	101.6096626	105.156134	66.86071	28.99878953
5.765929211	3.628916522	10.81044368	12.94078258	4.296116967
0	0	1.965535214	0	0
19.21976404	9.979520436	3.931070429	19.41117387	17.18446787

0.960988202	0.907229131	0	0	0
327.6969768	388.2940679	414.7279302	416.2618397	361.9478545
19.21976404	22.68072826	61.91435925	20.48957242	45.10922815
2.882964605	0.907229131	2.948302821	0	1.074029242
0	7.257833044	0	0	0
18.25877583	22.68072826	6.87937325	7.548789839	11.81432166
80.72300895	107.0530374	127.7597889	105.6830577	61.21966678
0	0	0	4.313594194	1.074029242
41.32249268	92.53737131	213.2605707	112.153449	197.6213805
173.9388645	112.4964122	152.3289791	162.8381808	180.4369126
70.15213873	57.15543522	59.94882404	67.93910855	92.36651479
71.11312693	57.15543522	55.034986	75.48789839	77.33010541
110.5136432	142.4349735	132.673627	161.7597823	184.7330296
131.6553836	108.8674957	227.0193172	83.03668823	277.0995444
10.57087022	18.14458261	18.67258454	15.09757968	3.222087725
163.3679943	216.8277622	204.4156623	191.9549416	331.8750357
9.609882018	31.75301957	12.77597889	62.54711581	99.88471948
159.5240415	200.4976378	189.6741482	156.3677895	206.2136144
36.51755167	31.75301957	88.44908464	83.03668823	84.8483101
0	0	64.86266207	60.39031871	152.5121523
99.94277298	124.2903909	147.4151411	144.5054055	173.9927372
31.71261066	30.84579044	209.3295003	0	15.03640938
70.15213873	68.04218479	84.51801421	80.87989113	78.40413465
305.5942482	282.1482596	293.8475145	365.5771079	480.0910711
2.882964605	1.814458261	0	0	11.81432166
82.64498535	103.4241209	149.3806763	116.4670432	93.44054403
204.690487	230.4361992	23.58642257	100.291065	258.8410473
143.1872421	0	8.844908464	433.5162165	2.148058484
0	0	0	0	0
0	0	0.982767607	1.078398548	1.074029242
147.9921831	128.8265365	239.7952961	273.9132313	190.1031758
128.772419	103.4241209	128.7425565	133.72142	128.883509
222.9492628	189.6108883	245.6919018	222.150101	265.2852227
43.24446908	42.63976914	61.91435925	46.37113758	89.14442707
107.6306786	123.3831618	135.6219298	168.2301736	142.8458892
102.8257376	108.8674957	189.6741482	173.6221663	178.2888541
83.60597355	129.7337657	168.0532608	148.8189997	191.177205
96.09882018	79.83616349	119.8976481	89.50707952	111.6990411
288.2964605	0	24.56919018	460.4761802	82.70025162
40.36150447	39.91808174	145.4496059	128.3294273	121.3653043
0	0.907229131	0	0	53.70146209
81.68399715	78.02170523	98.27676071	118.6238403	56.92354981
171.0558999	221.3639079	213.2605707	199.5037315	211.5837606
87.44992636	104.33135	129.7253241	212.444514	183.6590003
176.8218291	195.9614922	272.2266272	210.2877169	198.6954097
46.12743368	46.26868566	45.20730993	57.15512307	54.77549133
60.54225671	75.30001783	42.25900711	67.93910855	0
229.6761802	121.5687035	186.7258454	64.70391291	258.8410473
0	0	30.46579582	0	13.96238014
66.30818592	78.92893436	49.13838036	54.99832597	109.5509827

For Review Only

107.6306786	55.34097696	117.9321129	62.54711581	51.5534036
8.648893816	0	38.32793668	0	0
68.23016233	0	25.55195779	2.156797097	5.370146209
27.86865785	23.58795739	38.32793668	42.05754339	19.33252635
15.37581123	20.86627	17.68981693	15.09757968	20.40655559
88.41091456	96.16628784	69.77650011	58.23352162	69.81190071
24.98569325	19.95904087	11.79321129	12.94078258	6.444175451
0	0	0	0	0
22.10272864	39.91808174	17.68981693	19.41117387	18.25849711
1.921976404	0	0	0	16.11043863
3.843952807	7.257833044	5.896605643	5.391992742	7.518204692
1077.267774	1136.758101	1254.011467	1121.53449	1168.543815
68.23016233	88.00122566	114.0010424	103.5262607	97.736661
67.26917412	50.80483131	41.2762395	30.19515936	50.47937436
336.3458706	322.0663413	234.8814581	273.9132313	322.2087725
95.13783197	155.1361813	122.8459509	111.0750505	76.25607616
41.32249268	29.03133218	75.67310575	66.86071	34.36893574
62.46423311	63.50603914	80.58694379	57.15512307	55.84952057
41.32249268	34.47470696	28.50026061	15.09757968	18.25849711
3.843952807	0	0	0	3.222087725
47.08842189	48.08314392	25.55195779	32.35195645	52.62743285
133.57736	125.19762	191.6396834	162.8381808	184.7330296
72.07411513	93.44460045	120.8804157	103.5262607	86.99636858
212.3783926	204.1265544	133.6563946	159.6029852	216.9539068
17.29778763	19.05181174	16.70704932	10.78398548	10.74029242
0	0	0.982767607	0	1.074029242
47.08842189	24.49518652	11.79321129	42.05754339	63.36772526
11.53185842	25.40241565	15.72428171	18.33277532	18.25849711
5.765929211	12.70120783	0.982767607	2.156797097	5.370146209
2.882964605	2.721687392	0	6.470391291	0
86.48893816	97.07351697	126.7770213	155.289391	137.4757429
18.25877583	24.49518652	12.77597889	17.25437678	34.36893574
0	0	17.68981693	28.03836226	25.7767018
69.19115053	120.6614744	96.3112255	51.76313033	36.51699422
2847.408042	195.9614922	1966.517982	1809.552764	1562.712547
1.921976404	2.721687392	5.896605643	1.078398548	5.370146209
27.86865785	9.979520436	10.81044368	4.313594194	2.148058484
241.2080386	224.0855952	181.8120073	115.3886447	151.4381231
0	0	0	0	0
0	0	5.896605643	14.01918113	6.444175451
44.20545728	66.22772653	66.82819729	43.13594194	40.81311119
458.3913722	490.8109596	403.9174865	404.3994557	509.0898606
362.2925521	304.8289879	342.9858949	322.441166	315.7645971
204.690487	204.1265544	190.6569158	224.3068981	239.5085209
3.843952807	13.60843696	0	8.627188388	9.666263176
38.43952807	21.77349913	19.65535214	18.33277532	4.296116967
123.967478	189.6108883	347.8997329	262.0508473	0
34.59557526	29.93856131	33.41409864	42.05754339	33.29490649
17.29778763	12.70120783	1.965535214	10.78398548	17.18446787
0.960988202	7.257833044	8.844908464	0	1.074029242

0	128.8265365	35.37963386	133.72142	439.2779599
44.20545728	48.99037305	183.7775425	159.6029852	143.9199184
21.14174044	44.4542274	27.517493	11.86238403	22.55461408
6.726917412	5.443374783	10.81044368	0	11.81432166
23.06371684	42.63976914	21.62088736	60.39031871	7.518204692
24.98569325	36.28916522	1.965535214	3.235195645	26.85073104
0	0	411.7796274	170.3869707	172.9187079
0	0	0	0	0
26.90766965	24.49518652	20.63811975	34.50875355	36.51699422
160.4850297	148.7855774	162.1566552	173.6221663	216.9539068
0	0	2.948302821	0	0
708.2483047	675.8857022	617.1780573	488.5145425	660.5279837
2.882964605	0	2.948302821	0	0
387.2782453	54.43374783	116.9493452	91.66387662	210.5097314
0.960988202	0.907229131	5.896605643	1.078398548	3.222087725
0	5.443374783	0	0	1.074029242
1.921976404	4.536145653	0	0	0
66.30818592	65.3204974	42.25900711	170.3869707	21.48058484
194.1196168	152.4144939	122.8459509	93.82067372	59.0716083
0	0	0	0	0
247.9349561	253.1169274	296.7958174	234.012485	286.7658075
0	0	0	1.078398548	0
184.5097347	227.7145118	82.552479	157.4461881	121.3653043
0	0	8.844908464	0	2.148058484
0	0	1.965535214	3.235195645	0
72.07411513	87.09399653	88.44908464	67.93910855	92.36651479
49.01039829	38.10362348	53.06945079	39.90074629	46.1832574
2.882964605	3.628916522	0	0	0
85.52794996	62.59881001	110.069972	60.39031871	110.6250119
619.8373901	594.2350805	923.8015507	497.1417308	678.7864808
0.960988202	0.907229131	122.8459509	0	0
201.8075224	127.0120783	86.48354943	134.7998186	207.2876437
210.4564162	225.9000535	150.3634439	181.1709561	227.6941993
42.28348088	52.61928957	46.19007754	42.05754339	65.51578375
93.21585557	89.81568392	74.69033814	109.9966519	158.9563278
109.552655	162.3940144	100.2422959	130.4862244	144.9939476
5.765929211	10.88674957	3.931070429	8.627188388	10.74029242
34.59557526	37.19639435	45.20730993	26.95996371	17.18446787
38.43952807	41.73254	53.06945079	26.95996371	45.10922815
0	94.35182958	2.948302821	4.313594194	59.0716083
24.02470504	31.75301957	27.517493	17.25437678	30.07281877
4.804941009	14.51566609	9.827676071	20.48957242	12.8883509
15.37581123	15.42289522	18.67258454	29.11676081	24.70267256
14.41482303	67.13495566	34.39686625	54.99832597	12.8883509
35.55656347	28.12410305	39.31070429	22.64636952	34.36893574
38.43952807	96.16628784	59.94882404	76.56629694	75.18204692
58.62028031	71.67110131	38.32793668	37.7439492	34.36893574
312.3211656	220.4566787	241.7608314	292.2460066	283.5437198
16.33679943	22.68072826	26.53472539	17.25437678	13.96238014
19.21976404	32.6602487	5.896605643	37.7439492	49.40534512

For Review Only

0	0	0	0	0
166.2509589	153.3217231	193.6052186	140.1918113	140.6978307
24.98569325	39.91808174	48.15561275	33.430355	21.48058484
255.6228617	62.59881001	258.4678807	390.3802745	125.6614213
0	0	0	0	2.148058484
163.3679943	299.3856131	441.2626556	222.150101	590.716083
1.921976404	3.628916522	0	3.235195645	1.074029242
162.4070061	97.07351697	3.931070429	281.4620211	1.074029242
24.98569325	26.30964479	51.10391557	36.66555065	48.33131588
0	0.907229131	4.913838036	5.391992742	0
4.804941009	6.350603914	2.948302821	6.470391291	9.666263176
159.5240415	125.19762	201.4673595	187.6413474	98.81069024
0	23.58795739	32.43133104	0	24.70267256
0	9.979520436	0	2.156797097	0
234.4811212	250.39524	23.58642257	19.41117387	1.074029242
20.18075224	13.60843696	10.81044368	35.5871521	18.25849711
11.53185842	9.979520436	8.844908464	15.09757968	13.96238014
36.51755167	67.13495566	36.36240146	51.76313033	59.0716083
70.15213873	58.06266435	58.96605643	85.19348533	109.5509827
13.45383482	11.7939787	13.7587465	12.94078258	2.148058484
0	0	84.51801421	0	19.33252635
69.19115053	112.4964122	139.5530002	141.2702098	111.6990411
812.9960187	304.8289879	80.58694379	282.5404197	97.736661
0	0	5.896605643	0	0
30.75162246	39.01085261	53.06945079	44.21434049	37.59102346
5.765929211	9.979520436	24.56919018	14.01918113	6.444175451
8.648893816	9.072291305	10.81044368	10.78398548	1.074029242
242.1690268	244.9518652	269.2783244	325.6763616	269.5813397
1.921976404	2.721687392	0	0	0
23.06371684	40.82531087	32.43133104	34.50875355	44.03519891
47.08842189	47.17591479	22.60365496	60.39031871	41.88714043
51.8933629	80.74339262	62.89712686	71.1743042	34.36893574
29.79063425	12.70120783	23.58642257	19.41117387	16.11043863
0	0	9.827676071	0	0
32.67359886	0	13.7587465	2.156797097	2.148058484
8.648893816	16.33012435	11.79321129	20.48957242	7.518204692
54.7763275	43.54699827	68.7937325	80.87989113	50.47937436
127.8114308	91.63014218	102.2078311	106.7614563	99.88471948
73.03510333	50.80483131	61.91435925	83.03668823	61.21966678
37.47853987	33.56747783	192.622451	131.5646229	67.66384223
53.8153393	52.61928957	7.862140857	25.88156516	11.81432166
100.9037612	107.0530374	56.01775361	120.7806374	147.1420061
5.765929211	0	23.58642257	0	0
401.6930683	250.39524	456.9869373	344.009137	423.1675213
178.7438055	202.3120961	195.5707538	224.3068981	178.2888541
0	0	0	1.078398548	0
32.67359886	18.14458261	27.517493	31.27355791	8.592233934
18.25877583	23.58795739	6.87937325	14.01918113	2.148058484
11.53185842	9.072291305	8.844908464	1.078398548	0
240.2470504	246.7663235	309.5717962	239.4044778	189.0291465

For Review Only

75.91806794	92.53737131	55.034986	75.48789839	71.9599592
0.960988202	0	0	0	3.222087725
0	183.2602844	102.2078311	42.05754339	214.8058484
0	0	3.931070429	0	0
151.8361359	160.5795561	236.8469933	208.1309199	181.5109419
5.765929211	7.257833044	1.965535214	12.94078258	2.148058484
439.1716082	479.0169809	507.1080853	561.8456437	457.536457
0	0	301.7096554	0	0
70.15213873	58.96989348	240.7780637	143.4270069	66.58981299
13.45383482	9.072291305	8.844908464	8.627188388	27.92476029
15.37581123	15.42289522	30.46579582	9.705586936	16.11043863
12.49284662	11.7939787	18.67258454	7.548789839	15.03640938
0	0	2.948302821	0	7.518204692
108.5916668	94.35182958	151.3462115	111.0750505	109.5509827
31.71261066	17.23735348	53.06945079	26.95996371	53.70146209
0	0	0	9.705586936	25.7767018
58.62028031	58.06266435	47.17284514	95.97747081	76.25607616
65.34719772	36.28916522	48.15561275	56.07672452	41.88714043
1.921976404	0	0	0	1.074029242
612.1494845	639.596537	721.3514236	597.4327958	709.9333288
125.8894544	188.7036592	230.9503877	256.6588545	157.8822985
1.921976404	0	0	4.313594194	1.074029242
66.30818592	58.96989348	76.65587336	71.1743042	52.62743285
31.71261066	47.17591479	35.37963386	28.03836226	2.148058484
0	0	0	0	0
0.960988202	0	0.982767607	0	2.148058484
0	0	0.982767607	0	0
158.5630533	111.5891831	174.9326341	175.7789634	150.3640938
23.06371684	28.12410305	43.24177471	45.29273904	3.222087725
8.648893816	2.721687392	25.55195779	9.705586936	3.222087725
269.0766965	228.6217409	151.3462115	179.014159	187.9551173
155.6800887	103.4241209	162.1566552	37.7439492	40.81311119
427.6397498	463.5940857	234.8814581	871.3460272	77.33010541
145.1092185	127.9193074	163.1394228	186.5629489	127.8094798
1.921976404	0	3.931070429	4.313594194	3.222087725
52.8543511	40.82531087	56.01775361	42.05754339	49.40534512
12.49284662	5.443374783	2.948302821	5.391992742	7.518204692
64.38620952	90.72291305	120.8804157	95.97747081	77.33010541
0	0	0	1.078398548	0
5.765929211	14.51566609	10.81044368	17.25437678	22.55461408
94.17684377	117.939787	128.7425565	101.3694636	132.1055967
48.04941009	39.01085261	18.67258454	32.35195645	74.10801768
221.9882746	186.8892009	211.2950355	171.4653692	180.4369126
123.967478	133.3626822	47.17284514	106.7614563	143.9199184
70.15213873	71.67110131	86.48354943	102.4478621	88.07039782
221.0272864	178.7241387	123.8287185	153.1325939	179.3628834
319.048083	409.1603379	403.9174865	490.6713395	421.0194628
0	0	0	0	4.296116967
66.30818592	88.00122566	115.9665776	101.3694636	121.3653043
324.8140122	319.3446539	256.5023455	295.4812023	328.652948

For Review Only

39.40051627	31.75301957	42.25900711	33.430355	54.77549133
175.8608409	183.2602844	280.088768	215.6797097	204.0655559
101.8647494	80.74339262	98.27676071	80.87989113	120.2912751
75.91806794	0	70.75926771	39.90074629	67.66384223
73.03510333	78.02170523	105.156134	72.25270275	48.33131588
110.5136432	92.53737131	93.36292268	49.60633323	39.73908194
54.7763275	70.76387218	56.01775361	26.95996371	20.40655559
35.55656347	39.01085261	43.24177471	63.62551436	51.5534036
76.87905614	83.46508001	97.29399311	91.66387662	64.44175451
0	0	0	7.548789839	50.47937436
0	0	0.982767607	1.078398548	1.074029242
16.33679943	9.979520436	2.948302821	20.48957242	12.8883509
9.609882018	9.072291305	5.896605643	4.313594194	4.296116967
51.8933629	78.02170523	56.01775361	49.60633323	75.18204692
114.357596	18.14458261	185.7430777	0	0
0	73.48555957	0	219.9933039	280.3216321
0	0	34.39686625	0	0
10.57087022	5.443374783	8.844908464	0	0
28.82964605	26.30964479	28.50026061	36.66555065	36.51699422
25.94668145	28.12410305	40.29347189	53.91992742	39.73908194
0	6.350603914	2.948302821	0	1.074029242
57.65929211	46.26868566	39.31070429	35.5871521	50.47937436
0	0	0	0	0
34.59557526	26.30964479	16.70704932	37.7439492	26.85073104
53.8153393	39.91808174	42.25900711	20.48957242	28.99878953
0	0	0.982767607	0	2.148058484
0	0	2.948302821	3.235195645	6.444175451
7.687905614	0.907229131	7.862140857	6.470391291	7.518204692
0	0	0	0	2.148058484
72.07411513	51.71206044	57.98328882	44.21434049	42.96116967
43.24446908	33.56747783	32.43133104	45.29273904	44.03519891
2.882964605	0	0	0	0
75.91806794	88.00122566	55.034986	30.19515936	55.84952057
0	0	8.844908464	0	0
37.47853987	29.93856131	16.70704932	24.80316661	21.48058484
5.765929211	20.86627	119.8976481	101.3694636	122.4393336
151.8361359	83.46508001	158.2255847	155.289391	127.8094798
150.8751477	180.538597	150.3634439	132.6430215	119.2172458
0	0	6.87937325	1.078398548	0
75.91806794	98.88797523	101.2250635	100.291065	117.0691874
291.1794251	297.5711548	402.9347189	353.7147239	315.7645971
69.19115053	64.41326827	34.39686625	92.74227517	90.21845631
86.48893816	62.59881001	60.93159164	75.48789839	68.73787147
53.8153393	65.3204974	84.51801421	14.01918113	34.36893574
74.95707974	68.94941392	68.7937325	97.05586936	94.51457327
24.98569325	39.01085261	26.53472539	20.48957242	18.25849711
174.8998527	78.92893436	150.3634439	166.0733765	152.5121523
120.1235252	117.939787	16.70704932	120.7806374	127.8094798
46.12743368	42.63976914	15.72428171	43.13594194	69.81190071
75.91806794	51.71206044	57.98328882	63.62551436	104.1808365

For Review Only

0.960988202	5.443374783	8.844908464	0	7.518204692
238.325074	90.72291305	131.6908594	296.5596008	336.1711527
40.36150447	54.43374783	47.17284514	78.72309404	79.47816389
151.8361359	97.07351697	31.44856343	139.1134128	60.14563754
106.6696904	87.09399653	0	4.313594194	70.88592996
0	0	0	0	0
62.46423311	50.80483131	66.82819729	36.66555065	19.33252635
31.71261066	39.01085261	28.50026061	31.27355791	38.6650527
28.82964605	0	0	50.68473178	27.92476029
0	1.814458261	6.87937325	0	0
103.7867258	84.37230914	76.65587336	101.3694636	113.8470996
235.4421094	223.1783661	224.0710144	260.9724487	428.5376675
0	10.88674957	2.948302821	0	3.222087725
67.26917412	71.67110131	70.75926771	10.78398548	57.99757905
494.9089239	486.274814	513.0046909	600.6679915	566.0134104
54.7763275	64.41326827	55.034986	36.66555065	95.58860252
29.79063425	18.14458261	24.56919018	22.64636952	38.6650527
81.68399715	80.74339262	72.72480293	76.56629694	67.66384223
215.2613572	204.1265544	332.1754512	216.7581082	224.4721115
66.30818592	78.02170523	48.15561275	43.13594194	48.33131588
297.9063425	303.0145296	194.5879862	177.9357605	298.5801292
285.4134959	224.9928244	206.3811975	181.1709561	216.9539068
6.726917412	11.7939787	0.982767607	3.235195645	2.148058484
19.21976404	16.33012435	22.60365496	17.25437678	16.11043863
14.41482303	25.40241565	49.13838036	33.430355	36.51699422
0	0	0	6.470391291	2.148058484
5.765929211	1.814458261	9.827676071	9.705586936	7.518204692
60.54225671	85.27953827	170.018796	120.7806374	69.81190071
27.86865785	47.17591479	20.63811975	2.156797097	63.36772526
81.68399715	57.15543522	40.29347189	66.86071	161.1043863
42.28348088	29.03133218	40.29347189	24.80316661	28.99878953
340.1898234	317.5301957	449.1247965	270.6780357	225.5461408
582.3588503	0	22.60365496	4.313594194	94.51457327
21.14174044	10.88674957	14.74151411	48.52793468	31.14684801
0	751.1857201	545.436022	1.078398548	359.799796
0	0	0	0	0
84.56696176	88.90845479	94.34569029	64.70391291	53.70146209
2.882964605	2.721687392	0	0	0
34.59557526	18.14458261	30.46579582	28.03836226	30.07281877
699.5994109	648.6688283	631.9195714	686.9398754	724.9697382
19.21976404	14.51566609	5.896605643	6.470391291	8.592233934
0	3.628916522	0	0	0
18.25877583	19.05181174	14.74151411	7.548789839	21.48058484
0	145.1566609	34.39686625	45.29273904	0
488.1820065	414.6037127	397.0381133	185.4845503	260.9891057
18.25877583	16.33012435	3.931070429	4.313594194	19.33252635
122.0455016	16.33012435	141.5185354	188.719746	25.7767018
395.9271391	264.9109061	425.5383739	256.6588545	108.4769534
6.726917412	2.721687392	0	7.548789839	10.74029242
9.609882018	16.33012435	8.844908464	18.33277532	19.33252635

For Review Only

0.960988202	9.072291305	0	2.156797097	1.074029242
99.94277298	105.2385791	27.517493	54.99832597	90.21845631
78.80103254	107.9602665	42.25900711	84.11508678	108.4769534
38.43952807	32.6602487	48.15561275	35.5871521	46.1832574
0	0	0	0	0
129.7334072	103.4241209	94.34569029	211.3661155	94.51457327
94.17684377	63.50603914	98.27676071	72.25270275	82.70025162
103.7867258	107.9602665	64.86266207	125.0942316	146.0679769
123.0064898	106.1458083	150.3634439	112.153449	167.5485617
12.49284662	6.350603914	11.79321129	12.94078258	13.96238014
96.09882018	82.55785088	85.50078182	93.82067372	103.1068072
68.23016233	88.90845479	29.48302821	138.0350142	4.296116967
80.72300895	110.6819539	72.72480293	90.58547807	86.99636858
55.7373157	74.3927887	24.56919018	24.80316661	38.6650527
10.57087022	22.68072826	14.74151411	5.391992742	13.96238014
0	9.979520436	2.948302821	1.078398548	10.74029242
14.41482303	22.68072826	7.862140857	7.548789839	10.74029242
46.12743368	24.49518652	15.72428171	15.09757968	27.92476029
129.7334072	103.4241209	107.1216692	88.42868097	54.77549133
70.15213873	82.55785088	57.00052121	95.97747081	77.33010541
88.41091456	42.63976914	116.9493452	0	105.2548657
31.71261066	37.19639435	47.17284514	40.97914484	44.03519891
42.28348088	48.08314392	37.34516907	40.97914484	57.99757905
147.0311949	107.9602665	108.1044368	113.2318476	60.14563754
190.2756639	166.93016	184.7603101	162.8381808	158.9563278
16.33679943	26.30964479	19.65535214	21.56797097	30.07281877
22.10272864	56.24820609	60.93159164	70.09590565	60.14563754
84.56696176	92.53737131	102.2078311	128.3294273	114.9211289
56.6983039	68.04218479	56.01775361	60.39031871	53.70146209
0	0	0	0	4.296116967
64.38620952	53.5265187	31.44856343	30.19515936	55.84952057
35.55656347	0	237.8297609	120.7806374	0
73.03510333	99.79520436	40.29347189	50.68473178	77.33010541
83.60597355	88.90845479	68.7937325	106.7614563	103.1068072
29.79063425	9.072291305	11.79321129	31.27355791	3.222087725
156.6410769	185.0747426	104.1733664	133.72142	241.6565794
24.98569325	45.36145653	11.79321129	49.60633323	45.10922815
54.7763275	49.89760218	106.1389016	103.5262607	56.92354981
961.94919	629.6170166	746.9033814	210.2877169	258.8410473
4.804941009	9.979520436	4.913838036	0	9.666263176
0	0	0	85.19348533	0
30.75162246	51.71206044	10.81044368	19.41117387	53.70146209
0	0	37.34516907	0	15.03640938
38.43952807	66.22772653	72.72480293	60.39031871	86.99636858
9.609882018	4.536145653	0	16.17597823	10.74029242
218.1443218	198.6831796	221.1227116	86.27188388	110.6250119
38.43952807	25.40241565	68.7937325	25.88156516	48.33131588
180.6657819	176.0024513	177.8809369	173.6221663	129.9575383
375.7463869	213.1988457	210.3122679	278.2268255	198.6954097
143.1872421	140.6205152	177.8809369	100.291065	113.8470996

For Review Only

0	178.7241387	0.982767607	23.72476807	165.4005032
0	0	84.51801421	0	65.51578375
24.02470504	27.21687392	25.55195779	19.41117387	31.14684801
45.16644548	35.38193609	44.22454232	49.60633323	50.47937436
4.804941009	2.721687392	0.982767607	0	0
146.0702067	115.2180996	102.2078311	101.3694636	124.587392
7.687905614	15.42289522	2.948302821	0	11.81432166
49.01039829	77.1144761	78.62140857	52.84152887	71.9599592
0	0	7.862140857	0	3.222087725
0	0	0	0	0
185.4707229	52.61928957	103.1905987	0	0
50.93237469	36.28916522	17.68981693	40.97914484	36.51699422
110.5136432	101.6096626	106.1389016	120.7806374	75.18204692
67.26917412	80.74339262	212.2778031	0	167.5485617
141.2652657	176.0024513	105.156134	141.2702098	113.8470996
117.2405606	96.16628784	49.13838036	70.09590565	93.44054403
0	16.33012435	11.79321129	56.07672452	1.074029242
81.68399715	78.02170523	108.1044368	77.64469549	64.44175451
43.24446908	24.49518652	66.82819729	38.82234774	46.1832574
147.0311949	161.4867852	124.8114861	528.4152887	228.7682285
37.47853987	39.91808174	26.53472539	67.93910855	1.074029242
0	0	1.965535214	1.078398548	2.148058484
11.53185842	12.70120783	3.931070429	5.391992742	4.296116967
0	0	0	11.86238403	0
54.7763275	58.06266435	95.32845789	90.58547807	82.70025162
62.46423311	38.10362348	49.13838036	49.60633323	61.21966678
17.29778763	29.93856131	18.67258454	15.09757968	27.92476029
122.0455016	107.0530374	113.0182748	118.6238403	155.7342401
8.648893816	16.33012435	6.87937325	4.313594194	6.444175451
55.7373157	84.37230914	39.31070429	62.54711581	64.44175451
37.47853987	52.61928957	37.34516907	45.29273904	45.10922815
320.9700594	440.0061283	399.0036485	554.2968539	495.1274804
0	2.721687392	0	0	0
0	0	0	0	0
27.86865785	24.49518652	36.36240146	11.86238403	13.96238014
37.47853987	25.40241565	30.46579582	34.50875355	46.1832574
0	0	0	11.86238403	3.222087725
62.46423311	48.08314392	47.17284514	73.3311013	93.44054403
55.7373157	47.17591479	83.53524661	106.7614563	92.36651479
0	31.75301957	47.17284514	3.235195645	5.370146209
114.357596	0	1.965535214	1.078398548	164.326474
238.325074	195.9614922	161.1738876	143.4270069	204.0655559
8.648893816	2.721687392	0	8.627188388	10.74029242
0	0	1.965535214	3.235195645	0
47.08842189	36.28916522	55.034986	34.50875355	31.14684801
0	0	1.965535214	0	0
50.93237469	107.9602665	67.81096489	70.09590565	67.66384223
0	0	5.896605643	0	0
3.843952807	3.628916522	3.931070429	2.156797097	0
160.4850297	182.3530552	174.9326341	190.8765431	233.0643455

147.9921831	0	13.7587465	0	1.074029242
546.8022868	528.9145831	603.4193108	644.882332	547.7549133
134.5383482	117.0325578	152.3289791	69.0175071	67.66384223
0	0	121.8631833	116.4670432	0
84.56696176	90.72291305	97.29399311	105.6830577	68.73787147
35.55656347	30.84579044	19.65535214	34.50875355	13.96238014
36.51755167	46.26868566	13.7587465	48.52793468	20.40655559
0.960988202	0.907229131	0	0	80.55219313
69.19115053	82.55785088	71.74203532	72.25270275	61.21966678
10.57087022	21.77349913	17.68981693	23.72476807	21.48058484
0	0	0	0	5.370146209
224.8712392	221.3639079	301.7096554	257.7372531	248.1007548
484.3380537	462.6868566	451.0903317	381.7530862	316.8386263
36.51755167	50.80483131	50.12114796	88.42868097	44.03519891
0	0	6.87937325	0	0
53.8153393	49.89760218	86.48354943	90.58547807	33.29490649
0	0	20.63811975	19.41117387	24.70267256
31.71261066	21.77349913	5.896605643	21.56797097	17.18446787
98.02079658	76.20724696	107.1216692	109.9966519	100.9587487
30.75162246	41.73254	63.87989446	94.89907226	62.29369602
119.162537	114.3108704	181.8120073	179.014159	175.0667664
88.41091456	70.76387218	60.93159164	70.09590565	79.47816389
57.65929211	72.57833044	73.70757054	77.64469549	143.9199184
9.609882018	34.47470696	35.37963386	33.430355	0
0	3.628916522	0	10.78398548	16.11043863
29.79063425	0	7.862140857	11.86238403	5.370146209
34.59557526	40.82531087	11.79321129	37.7439492	44.03519891
227.7542038	215.0133039	353.7963386	251.2668618	169.6966202
33.63458706	90.72291305	111.0527396	106.7614563	84.8483101
32.67359886	41.73254	62.89712686	85.19348533	42.96116967
70.15213873	94.35182958	123.8287185	113.2318476	128.883509
8.648893816	34.47470696	20.63811975	4.313594194	10.74029242
0	0	1.965535214	0	0
37.47853987	16.33012435	14.74151411	28.03836226	33.29490649
60.54225671	37.19639435	53.06945079	78.72309404	49.40534512
23.06371684	32.6602487	30.46579582	36.66555065	46.1832574
60.54225671	75.30001783	71.74203532	69.0175071	69.81190071
67.26917412	29.93856131	29.48302821	48.52793468	18.25849711
56.6983039	69.85664305	43.24177471	42.05754339	88.07039782
4.804941009	68.04218479	54.05221839	0	89.14442707
0	0	2.948302821	0	20.40655559
0	0	0	0	0
6.726917412	6.350603914	4.913838036	6.470391291	5.370146209
117.2405606	140.6205152	75.67310575	141.2702098	127.8094798
48.04941009	0	74.69033814	0	0
69.19115053	32.6602487	88.44908464	46.37113758	32.22087725
0	0	0	0	0
186.4317111	208.6627	107.1216692	222.150101	282.4696906
1.921976404	13.60843696	0	0	0
9.609882018	12.70120783	0	0	0

420.9128324	951.6833579	1524.272559	1621.911417	1638.968623
10.57087022	6.350603914	6.87937325	7.548789839	5.370146209
28.82964605	19.05181174	32.43133104	8.627188388	19.33252635
0	56.24820609	18.67258454	58.23352162	39.73908194
0	0	0	1.078398548	0
182.5877583	90.72291305	136.6046974	182.2493547	85.92233934
0	0	0	0	0
19.21976404	16.33012435	26.53472539	25.88156516	41.88714043
0	0	0	0	0
21.14174044	18.14458261	30.46579582	14.01918113	11.81432166
0	0	0	0	0
87.44992636	88.90845479	96.3112255	42.05754339	69.81190071
60.54225671	86.1867674	79.60417618	76.56629694	83.77428086
0	0	0.982767607	1.078398548	0
0	0	5.896605643	1.078398548	1.074029242
58.62028031	72.57833044	67.81096489	53.91992742	41.88714043
0	0	7.862140857	0	0
0	6.350603914	0	0	0
56.6983039	51.71206044	1.965535214	8.627188388	19.33252635
0	7.257833044	1.965535214	5.391992742	7.518204692
54.7763275	53.5265187	76.65587336	97.05586936	106.3288949
2.882964605	0	0.982767607	0	0
692.8724935	631.4314749	262.3989511	84.11508678	910.776797
602.5396025	780.2170523	527.746205	327.8331587	692.7488609
124.9284662	146.9711191	57.00052121	84.11508678	221.2500238
40.36150447	34.47470696	19.65535214	26.95996371	46.1832574
0	0	0	0	0
19.21976404	18.14458261	23.58642257	36.66555065	23.62864332
0	0	0	1.078398548	0
3.843952807	0	0.982767607	0	0
793.7762547	0	925.7670859	87.35028243	419.9454335
354.6046465	329.3241744	373.4516907	424.8890281	381.2803808
0	0	0	0	1.074029242
98.98178478	106.1458083	100.2422959	107.8398548	142.8458892
166.2509589	154.2289522	236.8469933	216.7581082	169.6966202
43.24446908	43.54699827	66.82819729	47.44953613	56.92354981
0	0	2.948302821	0	0
0	0	1.965535214	0	0
0	0	0	0	4.296116967
48.04941009	31.75301957	3.931070429	4.313594194	65.51578375
0	0	4.913838036	1.078398548	5.370146209
0.960988202	68.04218479	0	133.72142	62.29369602
54.7763275	38.10362348	65.84542968	90.58547807	54.77549133
100.9037612	64.41326827	79.60417618	62.54711581	79.47816389
23.06371684	29.03133218	12.77597889	16.17597823	36.51699422
54.7763275	57.15543522	50.12114796	21.56797097	59.0716083
0	11.7939787	0	0	5.370146209
87.44992636	99.79520436	76.65587336	71.1743042	151.4381231
25.94668145	9.979520436	39.31070429	23.72476807	8.592233934
2.882964605	0	2.948302821	0	0

For Review Only

0	0	0	0	0
0	8.165062175	0.982767607	1.078398548	1.074029242
0.960988202	4.536145653	0	0	5.370146209
1005.193659	518.0278335	1037.802593	782.9173462	814.1141653
0	386.4796096	129.7253241	341.8523399	3.222087725
24.02470504	17.23735348	2.948302821	12.94078258	23.62864332
0	0	0	6.470391291	0
36.51755167	41.73254	47.17284514	34.50875355	36.51699422
49.97138649	74.3927887	83.53524661	44.21434049	61.21966678
44.20545728	65.3204974	45.20730993	47.44953613	50.47937436
88.41091456	0	52.08668318	3.235195645	36.51699422
59.58126851	80.74339262	50.12114796	98.13426791	78.40413465
46.12743368	55.34097696	46.19007754	46.37113758	59.0716083
19.21976404	8.165062175	22.60365496	10.78398548	20.40655559
0	0	0	44.21434049	0
6.726917412	12.70120783	19.65535214	5.391992742	8.592233934
0	0	0.982767607	1.078398548	1.074029242
493.9479357	454.5217944	384.2621344	441.0650063	392.0206732
66.30818592	59.87712262	57.00052121	54.99832597	66.58981299
13.45383482	7.257833044	17.68981693	40.97914484	15.03640938
46.12743368	40.82531087	67.81096489	44.21434049	45.10922815
54.7763275	55.34097696	80.58694379	62.54711581	65.51578375
0	0	8.844908464	6.470391291	7.518204692
98.02079658	56.24820609	30.46579582	50.68473178	109.5509827
15.37581123	6.350603914	0	64.70391291	0
24.02470504	12.70120783	15.72428171	9.705586936	18.25849711
24.02470504	13.60843696	14.74151411	10.78398548	19.33252635
26.90766965	29.03133218	19.65535214	21.56797097	38.6650527
11.53185842	0	0	4.313594194	2.148058484
0	10.88674957	0.982767607	1.078398548	1.074029242
790.8932901	1381.709966	2082.48456	2172.973075	2340.309718
46.12743368	57.15543522	62.89712686	73.3311013	57.99757905
0	29.03133218	61.91435925	0	0
8.648893816	42.63976914	15.72428171	5.391992742	36.51699422
10.57087022	22.68072826	0	18.33277532	17.18446787
0	0	0	0	0
0	0	228.0020849	25.88156516	46.1832574
62.46423311	92.53737131	43.24177471	54.99832597	64.44175451
285.4134959	555.2242279	0	0	0
135.4993364	26.30964479	0	0	51.5534036
2.882964605	3.628916522	2.948302821	1.078398548	0
72.07411513	71.67110131	111.0527396	99.21266646	98.81069024
34.59557526	47.17591479	20.63811975	43.13594194	60.14563754
11.53185842	9.072291305	7.862140857	9.705586936	38.6650527
137.4213129	140.6205152	55.034986	145.583804	191.177205
6.726917412	11.7939787	7.862140857	4.313594194	2.148058484
34.59557526	35.38193609	59.94882404	43.13594194	38.6650527
89.37190276	40.82531087	452.0730993	418.4186368	409.2051411
49.01039829	73.48555957	55.034986	73.3311013	73.03398844
40.36150447	77.1144761	78.62140857	124.0158331	35.44296498

For Review Only

40.36150447	61.69158088	68.7937325	31.27355791	52.62743285
132.6163718	147.8783483	129.7253241	125.0942316	48.33131588
0	0.907229131	2.948302821	3.235195645	0
19.21976404	0	47.17284514	16.17597823	12.8883509
22.10272864	19.95904087	54.05221839	67.93910855	63.36772526
767.8295732	970.7351697	650.5921559	1012.616237	1137.396967
117.2405606	126.1048491	156.2600495	281.4620211	302.8762462
133.57736	110.6819539	139.5530002	102.4478621	45.10922815
69.19115053	68.04218479	55.034986	51.76313033	78.40413465
135.4993364	103.4241209	144.4668382	166.0733765	134.2536552
103.7867258	114.3108704	98.27676071	140.1918113	77.33010541
102.8257376	101.6096626	85.50078182	115.3886447	165.4005032
0	0.907229131	0	0	0
101.8647494	54.43374783	32.43133104	33.430355	90.21845631
200.8465342	238.6012613	136.6046974	213.5229126	214.8058484
144.1482303	114.3108704	126.7770213	161.7597823	158.9563278
38.43952807	37.19639435	38.32793668	38.82234774	56.92354981
38.43952807	21.77349913	21.62088736	30.19515936	35.44296498
0	0	0.982767607	0	0
16.33679943	23.58795739	4.913838036	8.627188388	12.8883509
76.87905614	65.3204974	135.6219298	1.078398548	1.074029242
3.843952807	8.165062175	468.7801486	616.8439697	0
43.24446908	28.12410305	44.22454232	30.19515936	32.22087725
257.5448381	206.8482418	179.8464721	237.2476807	216.9539068
0	0	1.965535214	0	0
44.20545728	31.75301957	36.36240146	43.13594194	35.44296498
262.3497791	298.4783839	302.692423	193.0333402	265.2852227
60.54225671	0.907229131	76.65587336	0	0
84.56696176	93.44460045	155.2772819	210.2877169	85.92233934
59.58126851	79.83616349	322.3477751	2.156797097	6.444175451
57.65929211	71.67110131	43.24177471	39.90074629	74.10801768
46.12743368	41.73254	61.91435925	60.39031871	96.66263176
155.6800887	137.8988278	169.0360284	159.6029852	165.4005032
102.8257376	97.07351697	110.069972	112.153449	98.81069024
320.0090712	337.4892366	195.5707538	267.44284	378.0582931
204.690487	203.2193252	224.0710144	174.7005649	195.473322
0	0	0	0	0
102.8257376	0	122.8459509	109.9966519	21.48058484
30.75162246	51.71206044	39.31070429	38.82234774	26.85073104
51.8933629	102.5168918	67.81096489	73.3311013	38.6650527
4.804941009	0	0.982767607	0	0
0	8.165062175	0	6.470391291	0
0	5.443374783	0	0	2.148058484
67.26917412	45.36145653	57.98328882	34.50875355	66.58981299
43.24446908	54.43374783	22.60365496	72.25270275	5.370146209
13.45383482	17.23735348	16.70704932	20.48957242	10.74029242
0	0	0	1.078398548	0
0.960988202	0	0	1.078398548	0
75.91806794	84.37230914	99.25952832	97.05586936	90.21845631
106.6696904	91.63014218	86.48354943	81.95828968	107.4029242

For Review Only

13.45383482	1.814458261	153.3117467	285.7756153	0
117.2405606	0	97.29399311	74.40949984	0
178.7438055	149.6928065	187.708613	133.72142	171.8446787
53.8153393	176.0024513	72.72480293	56.07672452	33.29490649
0	0	0	40.97914484	0
132.6163718	78.92893436	59.94882404	120.7806374	149.2900646
131.6553836	91.63014218	138.5702326	112.153449	148.2160354
15.37581123	11.7939787	28.50026061	18.33277532	23.62864332
232.5591448	266.7253644	276.1576976	273.9132313	151.4381231
65.34719772	79.83616349	78.62140857	52.84152887	79.47816389
5.765929211	0	2.948302821	0	0
6.726917412	0	0.982767607	0	4.296116967
152.7971241	156.9506396	201.4673595	248.0316661	127.8094798
39.40051627	31.75301957	25.55195779	67.93910855	35.44296498
57.65929211	72.57833044	66.82819729	56.07672452	73.03398844
15.37581123	4.536145653	17.68981693	25.88156516	3.222087725
46.12743368	39.01085261	56.01775361	59.31192017	41.88714043
213.3393808	245.8590944	232.9159229	246.9532676	227.6941993
95.13783197	88.90845479	71.74203532	114.3102461	39.73908194
77.84004434	77.1144761	95.32845789	92.74227517	73.03398844
6.726917412	8.165062175	8.844908464	4.313594194	4.296116967
1.921976404	2.721687392	0.982767607	0	1.074029242
6.726917412	9.072291305	3.931070429	2.156797097	6.444175451
2.882964605	3.628916522	4.913838036	1.078398548	3.222087725
6.726917412	9.979520436	11.79321129	1.078398548	5.370146209
4.804941009	6.350603914	4.913838036	1.078398548	4.296116967
9.609882018	12.70120783	15.72428171	2.156797097	7.518204692
0.960988202	0.907229131	0	0	0
61.50324491	74.3927887	44.22454232	45.29273904	81.62622237
107.6306786	109.7747248	45.20730993	117.5454418	91.29248555
113.3966078	301.2000713	654.5232264	672.9206942	596.0862292
0	8.165062175	2.948302821	2.156797097	2.148058484
22.10272864	25.40241565	24.56919018	30.19515936	25.7767018
0	0	9.827676071	0	0
20.18075224	39.91808174	47.17284514	22.64636952	47.25728664
356.5266229	255.8386148	166.0877256	138.0350142	252.3968718
17.29778763	14.51566609	29.48302821	34.50875355	40.81311119
64.38620952	44.4542274	65.84542968	64.70391291	63.36772526
39.40051627	25.40241565	40.29347189	34.50875355	12.8883509
0	0	1.965535214	9.705586936	16.11043863
0	0	2.948302821	1.078398548	0
77.84004434	48.99037305	81.56971139	57.15512307	30.07281877
52.8543511	39.01085261	29.48302821	56.07672452	39.73908194
0	31.75301957	28.50026061	32.35195645	24.70267256
45.16644548	48.08314392	36.36240146	42.05754339	49.40534512
73.03510333	59.87712262	60.93159164	71.1743042	79.47816389
24.02470504	29.03133218	38.32793668	28.03836226	35.44296498
15.37581123	2.721687392	8.844908464	16.17597823	9.666263176
70.15213873	0	1.965535214	0	0
19.21976404	25.40241565	23.58642257	2.156797097	3.222087725

For Review Only

3.843952807	0	0	3.235195645	0
61.50324491	0	0	0	0
0	58.96989348	99.25952832	0	0
65.34719772	88.90845479	98.27676071	81.95828968	110.6250119
98.98178478	118.8470161	138.5702326	113.2318476	146.0679769
0	0	0	3.235195645	1.074029242
133.57736	159.672327	0	0	0
164.3289825	197.7759505	168.0532608	208.1309199	199.769439
17.29778763	18.14458261	19.65535214	14.01918113	13.96238014
71.11312693	58.06266435	70.75926771	62.54711581	73.03398844
31.71261066	31.75301957	33.41409864	35.5871521	36.51699422
0.960988202	7.257833044	0	0	172.9187079
4.804941009	8.165062175	9.827676071	20.48957242	32.22087725
119.162537	92.53737131	124.8114861	120.7806374	142.8458892
0	0	0	0	0
37.47853987	48.08314392	40.29347189	28.03836226	60.14563754
10.57087022	30.84579044	0	73.3311013	0
147.0311949	200.4976378	173.9498665	203.8173257	230.916287
0	0	0	0	0
0	0	0	0	0
0	0	0	0	0
18.25877583	18.14458261	36.36240146	60.39031871	61.21966678
0	58.06266435	0	14.01918113	22.55461408
19.21976404	17.23735348	20.63811975	30.19515936	22.55461408
19.21976404	17.23735348	15.72428171	14.01918113	18.25849711
172.0168881	150.6000357	198.5190566	180.0925576	209.4357021
17.29778763	24.49518652	11.79321129	28.03836226	21.48058484
42.28348088	0	0	0	194.3992928
65.34719772	53.5265187	69.77650011	70.09590565	103.1068072
0	0	0	88.42868097	0
0	0	0	0	36.51699422
27.86865785	28.12410305	32.43133104	20.48957242	28.99878953
0	26.30964479	0	0	0
33.63458706	24.49518652	91.39738746	83.03668823	90.21845631
174.8998527	217.7349913	0	0	0
0	3.628916522	57.98328882	0	34.36893574
140.3042775	104.33135	128.7425565	104.6046592	171.8446787
0	0	0	0	0
1506.8295	0	100.2422959	4.313594194	45.10922815
0	1163.067745	0.982767607	1.078398548	55.84952057
0	0	0	5.391992742	9.666263176
336.3458706	403.7169631	6.87937325	271.7564342	396.3167902
0	0	0.982767607	184.4061518	1.074029242
8.648893816	17.23735348	677.1268813	0	426.389609
605.4225671	603.3073718	550.34986	490.6713395	584.2719075
22.10272864	138.806057	204.4156623	0	258.8410473
88.41091456	88.00122566	116.9493452	111.0750505	107.4029242
424.7567852	596.9567679	555.263698	718.2134333	477.9430126
727.4680687	694.937514	1175.390058	979.185882	687.3787147
38.43952807	73.48555957	63.87989446	48.52793468	81.62622237

For Review Only

0	12.70120783	1.965535214	7.548789839	6.444175451
32.67359886	26.30964479	50.12114796	39.90074629	45.10922815
0	0	0.982767607	2.156797097	4.296116967
96.09882018	34.47470696	102.2078311	3.235195645	65.51578375
0	14.51566609	0	4.313594194	0
11.53185842	9.072291305	6.87937325	4.313594194	9.666263176
79.76202075	96.16628784	18.67258454	74.40949984	53.70146209
97.05980838	76.20724696	124.8114861	116.4670432	86.99636858
212.3783926	228.6217409	265.3472539	154.2109924	121.3653043
13.45383482	254.9313857	72.72480293	61.46871726	76.25607616
419.9518442	471.7591479	427.5039091	410.869847	558.4952057
0	0	73.70757054	47.44953613	63.36772526
48.04941009	74.3927887	79.60417618	57.15512307	71.9599592
331.5409296	343.8398405	401.9519513	277.148427	526.2743285
0	91.63014218	75.67310575	0	69.81190071
144.1482303	144.2494318	168.0532608	133.72142	170.7706494
18.25877583	28.12410305	30.46579582	28.03836226	36.51699422
36.51755167	41.73254	38.32793668	46.37113758	62.29369602
35.55656347	34.47470696	42.25900711	33.430355	54.77549133
92.25486737	81.65062175	188.6913806	0	206.2136144
0	0	0	97.05586936	57.99757905
115.3185842	0	35.37963386	0	0
7.687905614	4.536145653	7.862140857	7.548789839	9.666263176
23.06371684	20.86627	19.65535214	40.97914484	44.03519891
0	0	163.1394228	19.41117387	91.29248555
0.960988202	1.814458261	0	116.4670432	0
0	0	0	0	0
12.49284662	15.42289522	0	0	332.9490649
33.63458706	0	0	180.0925576	219.1019653
30.75162246	19.05181174	40.29347189	30.19515936	23.62864332
208.5344398	184.1675135	171.9843312	184.4061518	255.6189595
46.12743368	76.20724696	96.3112255	36.66555065	84.8483101
706.3263283	782.9387397	874.6631704	706.3510492	765.7828494
0	0	17.68981693	19.41117387	19.33252635
47.08842189	65.3204974	56.01775361	59.31192017	39.73908194
161.4460179	571.5543522	694.8166982	549.9832597	516.6080653
0	0	147.4151411	0	259.9150765
0	215.0133039	3.931070429	73.3311013	77.33010541
17.29778763	3.628916522	1.965535214	12.94078258	8.592233934
66.30818592	68.04218479	57.98328882	39.90074629	62.29369602
140.3042775	147.8783483	149.3806763	171.4653692	127.8094798
48.04941009	23.58795739	14.74151411	31.27355791	7.518204692
73.99609154	85.27953827	95.32845789	83.03668823	81.62622237
1.921976404	0	6.87937325	0	0
120.1235252	95.25905871	95.32845789	127.2510287	108.4769534
4.804941009	0	1.965535214	8.627188388	3.222087725
7.687905614	58.96989348	0	0	78.40413465
112.4356196	0	0.982767607	18.33277532	38.6650527
42.28348088	140.6205152	460.9180077	387.1450789	486.5352465
127.8114308	116.1253287	162.1566552	134.7998186	156.8082693

For Review Only

676.535694	538.8941035	559.1947685	443.2218034	440.3519891
18.25877583	19.95904087	6.87937325	19.41117387	7.518204692
0	0	153.3117467	6.470391291	1.074029242
12.49284662	18.14458261	21.62088736	26.95996371	12.8883509
12.49284662	16.33012435	9.827676071	12.94078258	18.25849711
59.58126851	58.06266435	74.69033814	53.91992742	51.5534036
0	0	1.965535214	77.64469549	15.03640938
72.07411513	96.16628784	57.98328882	72.25270275	88.07039782
77.84004434	69.85664305	73.70757054	129.4078258	175.0667664
6.726917412	0	6.87937325	0	0
10.57087022	0	3.931070429	0	0
12.49284662	18.14458261	10.81044368	29.11676081	20.40655559
329.6189532	230.4361992	174.9326341	378.5178905	218.0279361
494.9089239	451.800107	408.8313246	501.455325	407.0570826
33.63458706	41.73254	85.50078182	48.52793468	63.36772526
3.843952807	0	7.862140857	0	5.370146209
44.20545728	34.47470696	17.68981693	36.66555065	30.07281877
98.02079658	105.2385791	100.2422959	94.89907226	68.73787147
4.804941009	2.721687392	0	0	0
195.080605	187.79643	291.8819793	231.8556879	228.7682285
26.90766965	20.86627	0	11.86238403	21.48058484
14.41482303	25.40241565	5.896605643	19.41117387	13.96238014
73.99609154	82.55785088	85.50078182	62.54711581	64.44175451
0	0	0	0	9.666263176
128.772419	126.1048491	188.6913806	153.1325939	156.8082693
18.25877583	61.69158088	42.25900711	40.97914484	0
21.14174044	23.58795739	24.56919018	26.95996371	39.73908194
20.18075224	31.75301957	50.12114796	45.29273904	30.07281877
36.51755167	32.6602487	21.62088736	21.56797097	10.74029242
0	13.60843696	0	6.470391291	1.074029242
37.47853987	60.78435175	56.01775361	53.91992742	95.58860252
59.58126851	78.02170523	33.41409864	62.54711581	97.736661
9.609882018	12.70120783	20.63811975	16.17597823	31.14684801
76.87905614	63.50603914	40.29347189	63.62551436	83.77428086
24.98569325	29.93856131	36.36240146	53.91992742	17.18446787
0	0	13.7587465	12.94078258	6.444175451
62.46423311	50.80483131	75.67310575	65.78231146	89.14442707
21.14174044	0.907229131	3.931070429	4.313594194	0
29.79063425	42.63976914	45.20730993	32.35195645	27.92476029
31.71261066	30.84579044	42.25900711	30.19515936	25.7767018
3.843952807	0	0.982767607	0	1.074029242
0	3.628916522	0	6.470391291	0
4.804941009	7.257833044	0	1.078398548	0
93.21585557	64.41326827	75.67310575	89.50707952	74.10801768
61.50324491	88.90845479	32.43133104	71.1743042	68.73787147
100.9037612	116.1253287	112.0355072	124.0158331	196.5473512
0	0	0	0	0
125.8894544	117.939787	151.3462115	146.6622026	103.1068072
56.6983039	60.78435175	36.36240146	66.86071	54.77549133
231.5981566	232.2506574	242.743599	297.6379994	256.6929888

For Review Only

12.49284662	0	0	11.86238403	0
29.79063425	22.68072826	3.931070429	62.54711581	36.51699422
244.0910032	266.7253644	339.0548245	297.6379994	301.8022169
0	0	1.965535214	0	1.074029242
3.843952807	0	0	0	0
154.7191005	129.7337657	122.8459509	184.4061518	119.2172458
767.8295732	765.7013862	931.6636916	954.3827154	794.7816389
155.6800887	110.6819539	114.0010424	139.1134128	249.1747841
46.12743368	43.54699827	42.25900711	45.29273904	45.10922815
397.8491155	345.6542987	324.3133104	208.1309199	360.8738252
41.32249268	26.30964479	30.46579582	51.76313033	53.70146209
0.960988202	0	5.896605643	9.705586936	12.8883509
135.4993364	166.0229309	138.5702326	156.3677895	146.0679769
69.19115053	94.35182958	63.87989446	33.430355	94.51457327
98.98178478	81.65062175	144.4668382	48.52793468	40.81311119
34.59557526	44.4542274	39.31070429	45.29273904	50.47937436
81.68399715	56.24820609	149.3806763	86.27188388	56.92354981
0	0	0	5.391992742	0
204.690487	243.137407	130.7080917	142.3486084	114.9211289
9.609882018	8.165062175	0.982767607	2.156797097	3.222087725
0	9.979520436	0.982767607	12.94078258	1.074029242
0	0	1.965535214	3.235195645	0
34.59557526	29.93856131	36.36240146	39.90074629	44.03519891
69.19115053	65.3204974	66.82819729	105.6830577	50.47937436
45.16644548	49.89760218	48.15561275	80.87989113	85.92233934
30.75162246	17.23735348	94.34569029	91.66387662	83.77428086
34.59557526	31.75301957	27.517493	26.95996371	42.96116967
7.687905614	4.536145653	4.913838036	11.86238403	7.518204692
0	11.7939787	4.913838036	10.78398548	3.222087725
0	3.628916522	2.948302821	0	8.592233934
26.90766965	39.91808174	33.41409864	26.95996371	19.33252635
19.21976404	11.7939787	3.931070429	5.391992742	2.148058484
0	0	2.948302821	1.078398548	0
105.7087022	81.65062175	110.069972	128.3294273	85.92233934
0	0	0	34.50875355	7.518204692
0	0	0	0	0
15.37581123	17.23735348	11.79321129	21.56797097	27.92476029
0.960988202	2.721687392	2.948302821	2.156797097	2.148058484
7.687905614	8.165062175	7.862140857	10.78398548	22.55461408
2.882964605	0	0	1.078398548	0
0	0	0	6.470391291	2.148058484
0	0	0.982767607	1.078398548	1.074029242
7.687905614	19.95904087	4.913838036	9.705586936	13.96238014
0	6.350603914	2.948302821	0	0
14.41482303	9.979520436	25.55195779	19.41117387	24.70267256
0	0	0	0	0
0	0	0	0	0
33.63458706	9.979520436	0	0	0
261.3887909	185.0747426	184.7603101	0	204.0655559
3.843952807	0	0	0	0

For Review Only

61.50324491	64.41326827	41.2762395	18.33277532	71.9599592
0	0	28.50026061	1.078398548	1.074029242
51.8933629	27.21687392	61.91435925	67.93910855	30.07281877
25.94668145	24.49518652	41.2762395	12.94078258	27.92476029
33.63458706	33.56747783	32.43133104	48.52793468	30.07281877
25.94668145	19.95904087	29.48302821	34.50875355	32.22087725
109.552655	109.7747248	114.98381	88.42868097	77.33010541
4.804941009	11.7939787	1.965535214	7.548789839	5.370146209
48.04941009	68.94941392	43.24177471	117.5454418	118.1432166
131.6553836	102.5168918	136.6046974	145.583804	123.5133628
100.9037612	66.22772653	6.87937325	63.62551436	84.8483101
3.843952807	4.536145653	3.931070429	0	5.370146209
1611.577214	1459.731671	1067.285621	1600.343446	1613.191921
49.97138649	58.96989348	29.48302821	61.46871726	54.77549133
307.5162246	263.0964479	326.2788456	336.4603471	346.9114451
1.921976404	0.907229131	2.948302821	3.235195645	0
11.53185842	12.70120783	11.79321129	10.78398548	12.8883509
18.25877583	8.165062175	32.43133104	20.48957242	15.03640938
0.960988202	5.443374783	4.913838036	0	3.222087725
0.960988202	0	0	0	2.148058484
0	0	0	1.078398548	1.074029242
42.28348088	15.42289522	11.79321129	84.11508678	42.96116967
186.4317111	216.8277622	266.3300215	198.4253329	171.8446787
4.804941009	17.23735348	1.965535214	7.548789839	9.666263176
83.60597355	132.4554531	102.2078311	131.5646229	115.9951581
110.5136432	90.72291305	99.25952832	112.153449	110.6250119
349.7997054	257.6530731	295.8130497	291.1676081	396.3167902
125.8894544	178.7241387	136.6046974	158.5245866	138.5497722
0	0	0.982767607	1.078398548	1.074029242
43.24446908	43.54699827	52.08668318	57.15512307	47.25728664
1.921976404	6.350603914	6.87937325	8.627188388	6.444175451
79.76202075	52.61928957	95.32845789	86.27188388	92.36651479
0	0	0	0	0
238.325074	321.1591122	263.3817187	227.5420937	240.5825502
122.0455016	144.2494318	147.4151411	134.7998186	192.2512343
770.7125378	854.609841	1131.165516	953.3043168	890.3702414
21.14174044	0	5.896605643	6.470391291	8.592233934
15.37581123	19.95904087	25.55195779	10.78398548	23.62864332
113.3966078	90.72291305	119.8976481	155.289391	114.9211289
127.8114308	84.37230914	158.2255847	168.2301736	164.326474
9.609882018	15.42289522	25.55195779	30.19515936	18.25849711
47.08842189	0	2.948302821	40.97914484	19.33252635
36.51755167	48.08314392	34.39686625	51.76313033	52.62743285
0	3.628916522	3.931070429	0	4.296116967
500.6748531	441.8205866	656.4887616	645.9607305	442.5000476
24.98569325	14.51566609	17.68981693	28.03836226	15.03640938
291.1794251	293.9422383	103.1905987	0	112.7730704
0	0	10.81044368	0	0
21.14174044	9.072291305	8.844908464	16.17597823	22.55461408
1.921976404	5.443374783	0	0	0

For Review Only

8.648893816	8.165062175	3.931070429	7.548789839	0
67.26917412	88.00122566	87.46631704	71.1743042	105.2548657
106.6696904	112.4964122	129.7253241	160.6813837	123.5133628
170.0949117	181.4458261	162.1566552	181.1709561	149.2900646
241.2080386	241.3229487	229.9676201	199.5037315	206.2136144
0.960988202	0	0.982767607	1.078398548	2.148058484
264.2717555	290.3133218	247.657437	285.7756153	238.4344917
272.9206493	158.7650978	297.778585	300.873195	183.6590003
7.687905614	0	5.896605643	1.078398548	7.518204692
0	6.350603914	0	0	1.074029242
50.93237469	56.24820609	63.87989446	21.56797097	56.92354981
59.58126851	51.71206044	57.98328882	44.21434049	45.10922815
290.2184369	317.5301957	274.1921624	222.150101	365.1699422
71.11312693	82.55785088	52.08668318	107.8398548	61.21966678
0	0	0.982767607	1.078398548	1.074029242
67.26917412	53.5265187	36.36240146	16.17597823	60.14563754
319.048083	293.0350092	384.2621344	304.1083907	138.5497722
1715.36394	1818.087178	2259.382729	1985.331728	1811.887331
196.0415932	0	0	3.235195645	0
107.6306786	140.6205152	152.3289791	148.8189997	199.769439
503.5578177	467.2230022	562.1430713	437.8298107	278.1735736
0	0	0	1.078398548	1.074029242
123.967478	126.1048491	126.7770213	94.89907226	64.44175451
48.04941009	79.83616349	59.94882404	47.44953613	10.74029242
6.726917412	0	4.913838036	0	0
105.7087022	99.79520436	93.36292268	92.74227517	95.58860252
453.5864312	363.7988813	461.9007754	532.7288829	684.156627
11.53185842	20.86627	8.844908464	12.94078258	7.518204692
19.21976404	14.51566609	3.931070429	6.470391291	3.222087725
0	0.907229131	0.982767607	3.235195645	1.074029242
56.6983039	55.34097696	61.91435925	70.09590565	73.03398844
63.42522132	42.63976914	56.01775361	35.5871521	0
2.882964605	2.721687392	0	0	0
55.7373157	76.20724696	97.29399311	71.1743042	33.29490649
30.75162246	27.21687392	46.19007754	39.90074629	37.59102346
0	0	0	0	0
3.843952807	2.721687392	12.77597889	8.627188388	0
13.45383482	22.68072826	15.72428171	19.41117387	13.96238014
0	9.979520436	8.844908464	0	32.22087725
89.37190276	44.4542274	71.74203532	71.1743042	54.77549133
0	0	0	0	0
1.921976404	0	2.948302821	0	0
0.960988202	0.907229131	0	0	0
0	0	0.982767607	0	0
0	0	0.982767607	0	0
0	0	0	0	1.074029242
0	0	0	0	0
14.41482303	28.12410305	47.17284514	36.66555065	57.99757905
0	0.907229131	0	0	0
0	0	0	4.313594194	0

21.14174044	13.60843696	34.39686625	22.64636952	40.81311119
0	0	0.982767607	28.03836226	1.074029242
259.4668145	97.07351697	29.48302821	539.1992742	343.6893574
79.76202075	91.63014218	89.43185225	83.03668823	92.36651479
260.4278027	277.6121139	409.8140922	315.9707747	250.2488133
41.32249268	64.41326827	34.39686625	45.29273904	7.518204692
15.37581123	7.257833044	0	0	21.48058484
43.24446908	29.93856131	31.44856343	37.7439492	39.73908194
18.25877583	0	19.65535214	6.470391291	20.40655559
0	0	8.844908464	0	2.148058484
0	0	0.982767607	6.470391291	1.074029242
57.65929211	26.30964479	17.68981693	21.56797097	34.36893574
56.6983039	69.85664305	47.17284514	38.82234774	66.58981299
51.8933629	42.63976914	43.24177471	7.548789839	50.47937436
0	0	0.982767607	0	0
109.552655	80.74339262	98.27676071	140.1918113	117.0691874
74.95707974	117.0325578	79.60417618	65.78231146	80.55219313
922.5486737	1061.458083	944.4396705	1122.612889	1169.617844
0	0	0	17.25437678	0
98.02079658	74.3927887	112.0355072	92.74227517	84.8483101
57.65929211	61.69158088	72.72480293	75.48789839	51.5534036
150.8751477	142.4349735	153.3117467	372.0474992	214.8058484
124.9284662	134.2699113	236.8469933	339.6955428	221.2500238
263.3107673	326.602487	361.6584794	339.6955428	443.5740768
475.6891599	409.1603379	564.1086065	516.5529047	480.0910711
21.14174044	22.68072826	61.91435925	37.7439492	37.59102346
11.53185842	5.443374783	56.01775361	0	0
7.687905614	18.14458261	1.965535214	12.94078258	17.18446787
32.67359886	28.12410305	27.517493	26.95996371	34.36893574
73.03510333	0	39.31070429	242.6396734	0
38.43952807	43.54699827	16.70704932	48.52793468	39.73908194
81.68399715	102.5168918	100.2422959	54.99832597	75.18204692
46.12743368	55.34097696	34.39686625	39.90074629	15.03640938
0.960988202	0	2.948302821	0	0
19.21976404	19.05181174	33.41409864	16.17597823	55.84952057
0	0	0	0	0
135.4993364	123.3831618	290.8992117	193.0333402	164.326474
79.76202075	203.2193252	187.708613	324.5979631	302.8762462
236.4030976	243.137407	210.3122679	301.9515936	272.8034274
594.8516969	642.3182244	732.1618673	488.5145425	574.6056443
3.843952807	0	0	0	4.296116967
368.0584813	342.9326113	256.5023455	274.9916299	250.2488133
27.86865785	18.14458261	29.48302821	30.19515936	25.7767018
38.43952807	48.08314392	40.29347189	46.37113758	75.18204692
145.1092185	139.7132861	171.9843312	150.9757968	131.0315675
9.609882018	14.51566609	0.982767607	2.156797097	21.48058484
104.747714	68.04218479	34.39686625	29.11676081	22.55461408
103.7867258	165.1157018	232.9159229	147.7406011	134.2536552
88.41091456	53.5265187	74.69033814	66.86071	65.51578375
128.772419	123.3831618	83.53524661	118.6238403	65.51578375

For Review Only

235.4421094	249.4880109	278.1232328	286.8540139	225.5461408
205.6514752	129.7337657	306.6234934	75.48789839	93.44054403
57.65929211	29.03133218	52.08668318	24.80316661	36.51699422
178.7438055	255.8386148	110.069972	349.4011297	132.1055967
0	0	0	0	0
223.910251	679.5146188	603.4193108	736.5462086	452.1663108
0	0	0.982767607	2.156797097	1.074029242
42.28348088	48.08314392	50.12114796	56.07672452	68.73787147
0	0	0	25.88156516	0
103.7867258	116.1253287	97.29399311	175.7789634	206.2136144
31.71261066	27.21687392	46.19007754	29.11676081	46.1832574
95.13783197	57.15543522	84.51801421	130.4862244	81.62622237
154.7191005	110.6819539	123.8287185	89.50707952	94.51457327
63.42522132	47.17591479	34.39686625	64.70391291	59.0716083
323.853024	352.0049026	242.743599	221.0717024	212.6577899
35.55656347	29.03133218	7.862140857	22.64636952	16.11043863
7.687905614	6.350603914	9.827676071	11.86238403	10.74029242
140.3042775	147.8783483	152.3289791	140.1918113	257.767018
2.882964605	0	0	0	0
0	0	1.965535214	1.078398548	0
57.65929211	64.41326827	45.20730993	84.11508678	33.29490649
8855.506279	9769.043278	11310.67239	13945.85003	10253.75717
63.42522132	71.67110131	52.08668318	63.62551436	51.5534036
0	0	17.68981693	0	0
0	0	0	0	0
10.57087022	23.58795739	21.62088736	18.33277532	6.444175451
189.3146757	139.7132861	67.81096489	146.6622026	207.2876437
32.67359886	46.26868566	16.70704932	16.17597823	38.6650527
360.3705757	786.5676562	564.1086065	642.7255349	104.1808365
81.68399715	33.56747783	57.00052121	46.37113758	94.51457327
5.765929211	0	3.931070429	0	0
0	4.536145653	0	3.235195645	0
0.960988202	0	0	0	0
54.7763275	0	24.56919018	149.8973982	1.074029242
53.8153393	70.76387218	75.67310575	34.50875355	84.8483101
237.3640858	211.3843874	195.5707538	167.151775	235.2124039
51.8933629	7.257833044	53.06945079	66.86071	0
92.25486737	72.57833044	78.62140857	64.70391291	89.14442707
34.59557526	52.61928957	44.22454232	59.31192017	39.73908194
105.7087022	93.44460045	58.96605643	77.64469549	93.44054403
24.98569325	42.63976914	24.56919018	52.84152887	31.14684801
215.2613572	181.4458261	175.9154017	175.7789634	397.3908194
46.12743368	58.06266435	43.24177471	49.60633323	52.62743285
56.6983039	44.4542274	83.53524661	56.07672452	73.03398844
18.25877583	26.30964479	18.67258454	43.13594194	20.40655559
8835.325527	9412.502229	11142.61913	11561.51084	10395.52903
24.98569325	9.979520436	7.862140857	4.313594194	11.81432166
60.54225671	40.82531087	44.22454232	34.50875355	57.99757905
70.15213873	58.96989348	46.19007754	58.23352162	51.5534036
626.5643076	616.9158088	476.6422895	543.5128684	639.0473988

16.33679943	0	0	0	0
35.55656347	46.26868566	24.56919018	12.94078258	42.96116967
201.8075224	180.538597	182.7947749	227.5420937	170.7706494
57.65929211	55.34097696	41.2762395	65.78231146	99.88471948
27.86865785	35.38193609	21.62088736	21.56797097	19.33252635
0	0	0	0	18.25849711
62.46423311	49.89760218	10.81044368	34.50875355	16.11043863
138.3823011	131.5482239	226.0365496	212.444514	233.0643455
0	0	0	6.470391291	0
0	0	0	0	0
36.51755167	35.38193609	29.48302821	43.13594194	42.96116967
0	0	6.87937325	3.235195645	4.296116967
643.8620952	645.9471409	712.5065152	493.9065352	608.9745801
1656.74366	1369.915987	933.6292268	812.034107	1404.830248
5.765929211	3.628916522	0	3.235195645	5.370146209
28.82964605	19.95904087	21.62088736	16.17597823	34.36893574
0	0	0.982767607	1.078398548	1.074029242
0	0	0	0	1.074029242
0	3.628916522	9.827676071	0	0
11.53185842	8.165062175	27.517493	15.09757968	13.96238014
0	0	0	0	0
0	0	0	3.235195645	0
20.18075224	30.84579044	34.39686625	31.27355791	16.11043863
1.921976404	9.072291305	0	6.470391291	0
6.726917412	9.072291305	0	0	9.666263176
1322.319766	1460.6389	1978.311193	1634.852199	1472.49409
46.12743368	46.26868566	31.44856343	0	0
2.882964605	2.721687392	3.931070429	0	7.518204692
0	0	0	0	0
0	0	3.931070429	0	0
1.921976404	0	2.948302821	0	0
15.37581123	22.68072826	59.94882404	3.235195645	19.33252635
106.6696904	126.1048491	115.9665776	94.89907226	110.6250119
0.960988202	0.907229131	0	50.68473178	0
455.5084076	429.1193787	523.8151346	555.3752525	514.4600068
13.45383482	34.47470696	17.68981693	19.41117387	8.592233934
2.882964605	0	0	0	0
0	3.628916522	2.948302821	1.078398548	0
0	14.51566609	10.81044368	0	24.70267256
29.79063425	5.443374783	10.81044368	16.17597823	21.48058484
0.960988202	0.907229131	0	0	0
65.34719772	82.55785088	86.48354943	38.82234774	73.03398844
225.8322274	235.8795739	120.8804157	184.4061518	280.3216321
154.7191005	136.9915987	160.19112	200.58213	153.5861816
22.10272864	7.257833044	5.896605643	9.705586936	17.18446787
270.9986729	228.6217409	154.2945143	139.1134128	254.5449303
3.843952807	2.721687392	0	0	6.444175451
35.55656347	31.75301957	52.08668318	29.11676081	53.70146209
180.6657819	168.7446183	217.1916412	180.0925576	171.8446787
0	0	0	0	0

For Review Only

44.20545728	42.63976914	16.70704932	17.25437678	30.07281877
4.804941009	0	12.77597889	2.156797097	7.518204692
81.68399715	91.63014218	69.77650011	95.97747081	85.92233934
0	0	0	0	0
0	2.721687392	0	5.391992742	0
337.3068588	127.9193074	139.5530002	240.4828763	303.9502754
163.3679943	138.806057	151.3462115	124.0158331	165.4005032
16.33679943	13.60843696	20.63811975	7.548789839	0
80.72300895	96.16628784	94.34569029	128.3294273	121.3653043
975.4030248	859.1459866	970.9743959	740.8598028	1132.026821
297.9063425	313.9012792	317.4339371	304.1083907	221.2500238
0	0	0	0	0
6.726917412	30.84579044	0	0	54.77549133
51.8933629	64.41326827	43.24177471	103.5262607	65.51578375
33.63458706	50.80483131	75.67310575	54.99832597	48.33131588
32.67359886	49.89760218	5.896605643	11.86238403	37.59102346
146.0702067	170.5590765	126.7770213	143.4270069	140.6978307
0	10.88674957	3.931070429	8.627188388	11.81432166
0	0.907229131	0.982767607	1.078398548	3.222087725
72.07411513	28.12410305	80.58694379	246.9532676	137.4757429
69.19115053	77.1144761	95.32845789	121.859036	123.5133628
111.4746314	110.6819539	128.7425565	90.58547807	54.77549133
632.3302368	708.5459509	635.8506418	706.3510492	771.1529956
535.2704284	432.7482953	782.2830153	456.162586	207.2876437
24.98569325	15.42289522	21.62088736	33.430355	13.96238014
51.8933629	34.47470696	50.12114796	38.82234774	44.03519891
49.01039829	46.26868566	62.89712686	44.21434049	59.0716083
86.48893816	63.50603914	110.069972	81.95828968	56.92354981
13.45383482	19.05181174	59.94882404	17.25437678	11.81432166
21.14174044	39.91808174	18.67258454	30.19515936	28.99878953
40.36150447	76.20724696	22.60365496	23.72476807	34.36893574
0.960988202	0	0	0	0
158.5630533	137.8988278	186.7258454	145.583804	204.0655559
15.37581123	9.072291305	57.00052121	42.05754339	0
0	9.072291305	0	1.078398548	1.074029242
13.45383482	1.814458261	236.8469933	0	0
0	0	3.931070429	0	2.148058484
146.0702067	131.5482239	71.74203532	162.8381808	134.2536552
101.8647494	78.92893436	97.29399311	100.291065	90.21845631
0	0	8.844908464	0	3.222087725
5.765929211	0	1.965535214	0	2.148058484
0	0	0	3.235195645	3.222087725
974.4420366	1016.096626	1369.978044	802.3285201	772.2270248
50.93237469	30.84579044	22.60365496	58.23352162	103.1068072
10.57087022	3.628916522	0	12.94078258	6.444175451
117.2405606	106.1458083	132.673627	26.95996371	76.25607616
18.25877583	16.33012435	12.77597889	2.156797097	17.18446787
0	103.4241209	81.56971139	77.64469549	40.81311119
163.3679943	179.6313678	212.2778031	266.3644415	172.9187079
9.609882018	8.165062175	4.913838036	22.64636952	4.296116967

94.17684377	99.79520436	113.0182748	97.05586936	109.5509827
76.87905614	131.5482239	113.0182748	86.27188388	109.5509827
43.24446908	61.69158088	27.517493	16.17597823	126.7354505
4.804941009	5.443374783	19.65535214	2.156797097	6.444175451
145.1092185	141.5277444	170.018796	174.7005649	147.1420061
71.11312693	82.55785088	144.4668382	10.78398548	93.44054403
0	5.443374783	4.913838036	0	0
11.53185842	9.979520436	9.827676071	8.627188388	9.666263176
1.921976404	0	0.982767607	0	0
21.14174044	19.05181174	20.63811975	1.078398548	4.296116967
10.57087022	11.7939787	16.70704932	3.235195645	5.370146209
294.0623897	374.6856309	484.5044303	338.6171442	506.9418021
248.8959443	91.63014218	246.6746694	168.2301736	183.6590003
55.7373157	218.6422205	213.2605707	180.0925576	150.3640938
12.49284662	0	3.931070429	0	1.074029242
57.65929211	50.80483131	60.93159164	57.15512307	55.84952057
59.58126851	49.89760218	77.63864096	63.62551436	59.0716083
0.960988202	0	4.913838036	0	0
0	3.628916522	0	7.548789839	0
10.57087022	0	0	7.548789839	0
510.2847351	81.65062175	411.7796274	410.869847	572.4575859
0	0	0.982767607	7.548789839	1.074029242
284.4525077	270.3542809	209.3295003	283.6188182	249.1747841
73.03510333	58.06266435	54.05221839	24.80316661	48.33131588
0.960988202	6.350603914	0.982767607	4.313594194	7.518204692
0	0	0	0	0
0	0	0	1.078398548	1.074029242
294.0623897	366.5205687	266.3300215	284.6972168	17.18446787
0	1.814458261	0	7.548789839	0
0	6.350603914	0.982767607	0	1.074029242
0	0.907229131	2.948302821	1.078398548	1.074029242
0	36.28916522	0	0	0
16.33679943	17.23735348	11.79321129	10.78398548	24.70267256
217.1833336	151.5072648	18.67258454	73.3311013	95.58860252
0.960988202	2.721687392	0	1.078398548	0
0	20.86627	2.948302821	1.078398548	2.148058484
5.765929211	7.257833044	0	3.235195645	0
40.36150447	62.59881001	93.36292268	85.19348533	112.7730704
0	0	0.982767607	0	0
51.8933629	492.6254179	512.0219233	573.7080278	426.389609
0	0	0	6.470391291	0
10.57087022	8.165062175	14.74151411	6.470391291	9.666263176
89.37190276	0	0.982767607	127.2510287	23.62864332
12.49284662	17.23735348	18.67258454	5.391992742	0
27.86865785	48.99037305	32.43133104	46.37113758	40.81311119
52.8543511	51.71206044	30.46579582	59.31192017	48.33131588
9.609882018	15.42289522	8.844908464	12.94078258	8.592233934
90.33289097	119.7542452	38.32793668	57.15512307	98.81069024
17.29778763	10.88674957	10.81044368	11.86238403	17.18446787
7.687905614	16.33012435	8.844908464	9.705586936	2.148058484

5.765929211	0	0	0	0
0	0	1.965535214	2.156797097	2.148058484
138.3823011	117.0325578	105.156134	98.13426791	106.3288949
33.63458706	20.86627	18.67258454	26.95996371	39.73908194
3.843952807	11.7939787	1.965535214	1.078398548	7.518204692
8.648893816	6.350603914	5.896605643	6.470391291	27.92476029
111.4746314	0	0.982767607	1.078398548	200.8434682
0	0	0	0	0
3.843952807	15.42289522	0	11.86238403	0
38.43952807	0	97.29399311	43.13594194	8.592233934
72.07411513	80.74339262	114.98381	97.05586936	80.55219313
13.45383482	17.23735348	13.7587465	4.313594194	6.444175451
1655.782672	4568.805901	2322.279856	2974.223197	1503.640938
81.68399715	64.41326827	79.60417618	112.153449	66.58981299
2.882964605	0	0	0	0
181.6267701	160.5795561	161.1738876	149.8973982	149.2900646
38.43952807	46.26868566	25.55195779	43.13594194	50.47937436
79.76202075	58.06266435	57.00052121	39.90074629	15.03640938
65.34719772	92.53737131	91.39738746	85.19348533	56.92354981
55.7373157	70.76387218	35.37963386	79.80149259	62.29369602
126.8504426	140.6205152	85.50078182	107.8398548	90.21845631
84.56696176	128.8265365	107.1216692	102.4478621	104.1808365
221.9882746	304.8289879	267.3127891	337.5387457	271.7293982
220.0662982	130.6409948	155.2772819	65.78231146	136.4017137
384.3952807	44.4542274	235.8642257	322.441166	214.8058484
109.552655	82.55785088	19.65535214	71.1743042	119.2172458
1318.475813	1376.266591	5104.494951	312.7355791	2272.645876
9.609882018	19.05181174	11.79321129	10.78398548	25.7767018
0	3.628916522	2.948302821	0	0
17.29778763	38.10362348	18.67258454	15.09757968	28.99878953
10.57087022	3.628916522	3.931070429	4.313594194	18.25849711
20.18075224	6.350603914	25.55195779	11.86238403	11.81432166
47.08842189	54.43374783	0.982767607	39.90074629	1.074029242
11.53185842	2.721687392	0	0	36.51699422
14.41482303	3.628916522	39.31070429	21.56797097	32.22087725
0	2.721687392	0	0	0
4.804941009	6.350603914	9.827676071	2.156797097	0
7.687905614	8.165062175	3.931070429	6.470391291	9.666263176
1.921976404	0	0	0	0
0	0	0	0	0
254.6618735	264.003677	235.8642257	265.2860429	300.7281877
1015.764529	183.2602844	214.2433384	381.7530862	42.96116967
0.960988202	1.814458261	0	0	3.222087725
715.9362103	712.1748675	324.3133104	445.3786005	510.1638898
0.960988202	0	3.931070429	5.391992742	1.074029242
0	151.5072648	4.913838036	1.078398548	1.074029242
98.02079658	103.4241209	82.552479	94.89907226	77.33010541
116.2795724	143.3422026	160.19112	215.6797097	206.2136144
16.33679943	27.21687392	20.63811975	38.82234774	26.85073104
896.6019922	327.5097161	867.7837971	458.3193831	292.1359538

For Review Only

0	0	0	0	1.074029242
55.7373157	36.28916522	71.74203532	38.82234774	33.29490649
24.98569325	27.21687392	56.01775361	19.41117387	19.33252635
0	0	81.56971139	5.391992742	121.3653043
0	0	1.965535214	2.156797097	8.592233934
75.91806794	48.99037305	78.62140857	86.27188388	91.29248555
147.9921831	249.4880109	149.3806763	122.9374345	225.5461408
0	0	0.982767607	1.078398548	1.074029242
2.882964605	8.165062175	3.931070429	0	6.444175451
0	398.2735883	50.12114796	9.705586936	26.85073104
0	3.628916522	3.931070429	0	0
53.8153393	56.24820609	41.2762395	63.62551436	49.40534512
239.2860622	194.1470339	898.2495929	19.41117387	378.0582931
71.11312693	47.17591479	57.00052121	49.60633323	34.36893574
151.8361359	104.33135	99.25952832	51.76313033	162.1784155
66.30818592	48.99037305	118.9148805	72.25270275	8.592233934
3.843952807	0	0	0	0
574.6709447	470.8519187	296.7958174	479.8873541	453.24034
80.72300895	68.94941392	64.86266207	93.82067372	48.33131588
8.648893816	70.76387218	0	4.313594194	0
0	0	0	0	1.074029242
41.32249268	75.30001783	63.87989446	47.44953613	53.70146209
37.47853987	0	0.982767607	1.078398548	0
0	9.979520436	5.896605643	8.627188388	8.592233934
0	34.47470696	29.48302821	37.7439492	13.96238014
111.4746314	104.33135	151.3462115	150.9757968	106.3288949
280.6085549	238.6012613	409.8140922	538.1208757	483.3131588
22.10272864	43.54699827	29.48302821	24.80316661	9.666263176
34.59557526	34.47470696	21.62088736	29.11676081	35.44296498
5.765929211	0	3.931070429	0	1.074029242
73.03510333	50.80483131	28.50026061	66.86071	49.40534512
7.687905614	0.907229131	20.63811975	0	0
97.05980838	110.6819539	117.9321129	103.5262607	66.58981299
2.882964605	5.443374783	2.948302821	5.391992742	0
516.0506643	432.7482953	486.4699655	762.4277738	676.6384223
102.8257376	92.53737131	53.06945079	60.39031871	85.92233934
0	0	5.896605643	0	0
4.804941009	2.721687392	5.896605643	0	0
67.26917412	79.83616349	47.17284514	64.70391291	65.51578375
41.32249268	37.19639435	26.53472539	35.5871521	33.29490649
10.57087022	13.60843696	4.913838036	1.078398548	2.148058484
46.12743368	35.38193609	27.517493	31.27355791	41.88714043
66.30818592	73.48555957	36.36240146	53.91992742	41.88714043
4.804941009	68.94941392	67.81096489	70.09590565	0
9.609882018	9.072291305	10.81044368	6.470391291	19.33252635
0	0	0	2.156797097	0
4.804941009	7.257833044	0	3.235195645	0
394.0051627	477.2025227	336.1065216	402.2426586	349.0595036
15.37581123	27.21687392	13.7587465	18.33277532	9.666263176
14.41482303	16.33012435	3.931070429	28.03836226	3.222087725

For Review Only

47.08842189	42.63976914	97.29399311	73.3311013	35.44296498
34.59557526	14.51566609	21.62088736	17.25437678	7.518204692
0	0	30.46579582	1.078398548	1.074029242
35.55656347	31.75301957	15.72428171	7.548789839	34.36893574
0	0.907229131	1.965535214	2.156797097	8.592233934
10.57087022	0	12.77597889	1.078398548	3.222087725
72.07411513	27.21687392	65.84542968	11.86238403	35.44296498
0	0	0	0	0
0	6.350603914	0	0	5.370146209
377.6683633	400.9952757	285.0026061	244.7964705	278.1735736
0	6.350603914	0	0	0
24.02470504	19.95904087	8.844908464	10.78398548	3.222087725
30.75162246	26.30964479	44.22454232	47.44953613	59.0716083
0	0	90.41461986	95.97747081	107.4029242
28.82964605	45.36145653	42.25900711	30.19515936	59.0716083
0	0	0	0	0
56.6983039	95.25905871	103.1905987	99.21266646	73.03398844
0	0	31.44856343	4.313594194	2.148058484
0	9.979520436	0	9.705586936	13.96238014
21.14174044	0	7.862140857	3.235195645	9.666263176
10.57087022	9.072291305	13.7587465	7.548789839	8.592233934
130.6943954	96.16628784	105.156134	186.5629489	82.70025162
2.882964605	0	0.982767607	0	0
33.63458706	17.23735348	41.2762395	78.72309404	0
0	8.165062175	0.982767607	1.078398548	1.074029242
20.18075224	11.7939787	40.29347189	38.82234774	35.44296498
29.79063425	0	0.982767607	1.078398548	1.074029242
19.21976404	11.7939787	11.79321129	29.11676081	22.55461408
83.60597355	79.83616349	183.7775425	60.39031871	27.92476029
9.609882018	9.072291305	2.948302821	20.48957242	5.370146209
14.41482303	34.47470696	21.62088736	30.19515936	16.11043863
31.71261066	46.26868566	3.931070429	1.078398548	35.44296498
13.45383482	23.58795739	6.87937325	9.705586936	4.296116967
0	0	0	0	12.8883509
35.55656347	58.96989348	69.77650011	14.01918113	2.148058484
40.36150447	49.89760218	20.63811975	39.90074629	25.7767018
14.41482303	16.33012435	8.844908464	11.86238403	13.96238014
18.25877583	17.23735348	17.68981693	9.705586936	15.03640938
253.7008853	211.3843874	147.4151411	200.58213	151.4381231
452.625443	421.8615457	371.4861555	466.9465715	359.799796
28.82964605	46.26868566	61.91435925	45.29273904	50.47937436
7.687905614	48.08314392	62.89712686	0	0
213.3393808	170.5590765	298.7613526	147.7406011	220.1759946
12.49284662	22.68072826	10.81044368	11.86238403	26.85073104
10.57087022	7.257833044	8.844908464	9.705586936	6.444175451
11.53185842	9.072291305	7.862140857	23.72476807	3.222087725
72.07411513	92.53737131	100.2422959	77.64469549	84.8483101
658.2769182	0	15.72428171	236.1692821	0
104.747714	114.3108704	116.9493452	114.3102461	102.032778
0	0	0	0	1.074029242

For Review Only

665.9648238	738.4845123	783.2657829	900.462788	871.0377151
81.68399715	97.07351697	118.9148805	75.48789839	60.14563754
0	0	1.965535214	1.078398548	0
17.29778763	15.42289522	6.87937325	28.03836226	7.518204692
0	0	0	0	0
22.10272864	19.95904087	22.60365496	29.11676081	27.92476029
114.357596	84.37230914	108.1044368	79.80149259	60.14563754
10.57087022	0.907229131	19.65535214	17.25437678	0
55.7373157	44.4542274	77.63864096	75.48789839	5.370146209
4.804941009	0	5.896605643	0	12.8883509
35.55656347	42.63976914	70.75926771	66.86071	51.5534036
351.7216818	378.3145474	409.8140922	304.1083907	322.2087725
11.53185842	21.77349913	18.67258454	1.078398548	8.592233934
1614.460179	1771.818492	1840.723728	1386.820533	1197.542605
0	2.721687392	14.74151411	0	0
49.97138649	61.69158088	81.56971139	69.0175071	66.58981299
3.843952807	0	0	1.078398548	1.074029242
4.804941009	0	18.67258454	0	3.222087725
15.37581123	3.628916522	9.827676071	12.94078258	8.592233934
73.99609154	55.34097696	55.034986	62.54711581	25.7767018
72.07411513	240.4157196	492.3665712	109.9966519	27.92476029
19.21976404	0	4.913838036	2.156797097	7.518204692
0	0	0	0	17.18446787
77.84004434	0	0.982767607	1.078398548	1.074029242
13.45383482	8.165062175	15.72428171	6.470391291	10.74029242
0	0	0	0	0
50.93237469	18.14458261	46.19007754	18.33277532	44.03519891
120.1235252	60.78435175	58.96605643	146.6622026	84.8483101
20.18075224	53.5265187	35.37963386	42.05754339	30.07281877
214.300369	198.6831796	194.5879862	218.9149053	115.9951581
95.13783197	107.0530374	63.87989446	111.0750505	108.4769534
195.080605	141.5277444	296.7958174	95.97747081	77.33010541
0	0	0.982767607	6.470391291	726.0437674
116.2795724	77.1144761	111.0527396	47.44953613	80.55219313
115.3185842	90.72291305	64.86266207	91.66387662	115.9951581
46.12743368	48.08314392	41.2762395	21.56797097	39.73908194
58.62028031	84.37230914	43.24177471	89.50707952	76.25607616
136.4603247	164.2084726	150.3634439	98.13426791	118.1432166
0	0	11.79321129	1.078398548	51.5534036
314.243142	54.43374783	548.3843248	290.0892095	465.0546617
42.28348088	77.1144761	92.38015507	39.90074629	50.47937436
151.8361359	130.6409948	272.2266272	148.8189997	137.4757429
120.1235252	137.8988278	94.34569029	122.9374345	70.88592996
209.495428	474.4808353	303.6751906	286.8540139	193.3252635
17.29778763	11.7939787	12.77597889	40.97914484	19.33252635
33.63458706	21.77349913	48.15561275	23.72476807	20.40655559
110.5136432	99.79520436	177.8809369	87.35028243	48.33131588
0	0	116.9493452	146.6622026	117.0691874
24.02470504	29.03133218	36.36240146	56.07672452	38.6650527
16.33679943	16.33012435	18.67258454	17.25437678	15.03640938

For Review Only

0	9.072291305	0	0	0
2.882964605	2.721687392	3.931070429	4.313594194	5.370146209
101.8647494	103.4241209	98.27676071	83.03668823	104.1808365
25.94668145	33.56747783	0	0	0
5.765929211	0	3.931070429	1.078398548	0
7.687905614	0	0.982767607	9.705586936	1.074029242
172.0168881	148.7855774	100.2422959	186.5629489	197.6213805
11.53185842	16.33012435	12.77597889	11.86238403	5.370146209
46.12743368	32.6602487	23.58642257	23.72476807	44.03519891
0	9.072291305	19.65535214	2.156797097	16.11043863
0	105.2385791	26.53472539	205.9741228	211.5837606
64.38620952	39.01085261	6.87937325	49.60633323	22.55461408
33.63458706	80.74339262	41.2762395	59.31192017	57.99757905
174.8998527	166.93016	165.104958	167.151775	211.5837606
447.820502	475.3880644	470.7456838	363.4203108	321.1347433
199.885546	146.06389	191.6396834	147.7406011	172.9187079
0	0	20.63811975	0	0
0	0	0	0	0
58.62028031	51.71206044	26.53472539	32.35195645	103.1068072
169.1339235	179.6313678	187.708613	167.151775	136.4017137
178.7438055	176.9096805	182.7947749	169.3085721	187.9551173
323.853024	399.1808174	405.8830217	391.4586731	386.650527
138.3823011	81.65062175	105.156134	149.8973982	52.62743285
83.60597355	58.96989348	81.56971139	60.39031871	57.99757905
0	47.17591479	46.19007754	16.17597823	13.96238014
0	0	52.08668318	52.84152887	54.77549133
20.18075224	22.68072826	36.36240146	3.235195645	20.40655559
0	0	5.896605643	0	1.074029242
86.48893816	78.02170523	172.9670989	126.1726302	77.33010541
34.59557526	17.23735348	48.15561275	35.5871521	22.55461408
91.29387917	97.9807461	73.70757054	43.13594194	65.51578375
0	0	0.982767607	1.078398548	1.074029242
32.67359886	52.61928957	59.94882404	52.84152887	37.59102346
177.7828173	169.6518474	204.4156623	130.4862244	123.5133628
238.325074	254.0241565	391.1415076	517.6313033	210.5097314
56.6983039	88.00122566	25.55195779	19.41117387	24.70267256
40.36150447	60.78435175	46.19007754	50.68473178	34.36893574
53.8153393	44.4542274	55.034986	78.72309404	63.36772526
4.804941009	9.979520436	12.77597889	0	5.370146209
0	340.210924	2.948302821	1.078398548	7.518204692
7.687905614	5.443374783	5.896605643	5.391992742	3.222087725
41.32249268	31.75301957	36.36240146	25.88156516	27.92476029
464.1573015	51.71206044	174.9326341	0	70.88592996
0	0	0	0	0
78.80103254	45.36145653	58.96605643	26.95996371	82.70025162
213.3393808	268.5398226	397.0381133	312.7355791	347.9854743
0	3.628916522	0	1.078398548	2.148058484
0	158.7650978	10.81044368	205.9741228	28.99878953
151.8361359	0	174.9326341	37.7439492	36.51699422
0	0	10.81044368	2.156797097	2.148058484

For Review Only

65.34719772	62.59881001	64.86266207	31.27355791	63.36772526
76.87905614	77.1144761	156.2600495	97.05586936	51.5534036
0	5.443374783	0	4.313594194	2.148058484
0	0	0	0	0
0	0	11.79321129	0	0
173.9388645	163.3012435	122.8459509	189.7981445	104.1808365
0	0	0	0	0
0	0	0.982767607	1.078398548	1.074029242
0	0	0	0	0
13.45383482	20.86627	29.48302821	23.72476807	25.7767018
0	0	0	0	0
170.0949117	156.0434105	146.4323735	210.2877169	102.032778
10.57087022	8.165062175	3.931070429	2.156797097	0
25.94668145	37.19639435	65.84542968	22.64636952	18.25849711
0	0	0	0	0
0	0	0	0	0
3.843952807	0	6.87937325	0	2.148058484
407.4589975	388.2940679	354.7791062	340.7739413	344.7633866
0	20.86627	1.965535214	3.235195645	12.8883509
0	0	0.982767607	0	0
29.79063425	27.21687392	21.62088736	11.86238403	12.8883509
0	0	4.913838036	0	0
1.921976404	8.165062175	11.79321129	8.627188388	11.81432166
6.726917412	3.628916522	1.965535214	0	0
3.843952807	2.721687392	8.844908464	12.94078258	0
24.02470504	19.05181174	17.68981693	7.548789839	37.59102346
38.43952807	32.6602487	23.58642257	20.48957242	50.47937436
35.55656347	31.75301957	26.53472539	25.88156516	33.29490649
198.9245578	0.907229131	0	0	274.9514859
1390.549928	1416.184673	1417.150889	1082.712143	822.7063992
653.4719772	646.8543701	401.9519513	477.730557	414.5752873
0	0	0	0	1.074029242
70.15213873	84.37230914	100.2422959	74.40949984	66.58981299
115.3185842	163.3012435	96.3112255	70.09590565	70.88592996
34.59557526	36.28916522	52.08668318	47.44953613	80.55219313
1.921976404	0	0	4.313594194	1.074029242
5.765929211	0	0.982767607	0	0
77.84004434	48.99037305	78.62140857	51.76313033	45.10922815
68.23016233	86.1867674	62.89712686	88.42868097	70.88592996
174.8998527	176.0024513	166.0877256	128.3294273	157.8822985
569.8660036	586.9772475	493.3493388	417.3402383	509.0898606
0	0	0	0	0
0	0	14.74151411	0	0
18.25877583	48.99037305	39.31070429	2332.57606	244.8786671
192.1976404	269.4470518	146.4323735	235.0908836	4.296116967
10.57087022	19.95904087	0	8.627188388	11.81432166
0	0	0	0	0
25.94668145	23.58795739	22.60365496	16.17597823	24.70267256
39.40051627	30.84579044	55.034986	47.44953613	76.25607616
0	0	0	0	4.296116967

For Review Only

7.687905614	0	5.896605643	3.235195645	0
5.765929211	0	8.844908464	6.470391291	20.40655559
176.8218291	110.6819539	121.8631833	119.7022389	179.3628834
13.45383482	9.979520436	4.913838036	22.64636952	18.25849711
0	42.63976914	12.77597889	11.86238403	106.3288949
0.960988202	0	1.965535214	1.078398548	3.222087725
26.90766965	36.28916522	13.7587465	12.94078258	19.33252635
166.2509589	138.806057	128.7425565	144.5054055	191.177205
31.71261066	18.14458261	5.896605643	8.627188388	17.18446787
45.16644548	45.36145653	63.87989446	72.25270275	98.81069024
65.34719772	78.02170523	73.70757054	56.07672452	89.14442707
0	39.01085261	4.913838036	92.74227517	92.36651479
70.15213873	54.43374783	39.31070429	65.78231146	30.07281877
45.16644548	29.93856131	30.46579582	18.33277532	31.14684801
68.23016233	70.76387218	81.56971139	52.84152887	71.9599592
126.8504426	154.2289522	114.0010424	93.82067372	139.6238014
327.6969768	329.3241744	262.3989511	445.3786005	258.8410473
91.29387917	56.24820609	46.19007754	77.64469549	74.10801768
118.2015488	127.9193074	109.0872044	139.1134128	132.1055967
5.765929211	6.350603914	7.862140857	0	1.074029242
75.91806794	115.2180996	89.43185225	79.80149259	108.4769534
31.71261066	23.58795739	44.22454232	48.52793468	5.370146209
33.63458706	36.28916522	51.10391557	106.7614563	42.96116967
7.687905614	6.350603914	20.63811975	18.33277532	33.29490649
222.9492628	225.9000535	187.708613	193.0333402	92.36651479
177.7828173	133.3626822	115.9665776	241.5612749	86.99636858
20.18075224	21.77349913	9.827676071	30.19515936	34.36893574
0	2.721687392	1.965535214	2.156797097	6.444175451
65.34719772	50.80483131	61.91435925	73.3311013	64.44175451
90.33289097	91.63014218	0	0	0
75.91806794	89.81568392	68.7937325	102.4478621	75.18204692
45.16644548	41.73254	32.43133104	72.25270275	25.7767018
0	191.4253465	69.77650011	9.705586936	46.1832574
28.82964605	19.05181174	10.81044368	26.95996371	23.62864332
6.726917412	0	44.22454232	0	96.66263176
2.882964605	1.814458261	0	1.078398548	0
39.40051627	61.69158088	75.67310575	52.84152887	39.73908194
143.1872421	46.26868566	99.25952832	130.4862244	81.62622237
14.41482303	9.979520436	0	17.25437678	18.25849711
83.60597355	0	50.12114796	29.11676081	124.587392
16.33679943	0.907229131	43.24177471	25.88156516	16.11043863
30.75162246	38.10362348	5.896605643	32.35195645	22.55461408
61.50324491	57.15543522	68.7937325	61.46871726	90.21845631
111.4746314	67.13495566	64.86266207	92.74227517	106.3288949
232.5591448	212.2916165	245.6919018	217.8365068	144.9939476
72.07411513	67.13495566	99.25952832	98.13426791	108.4769534
102.8257376	70.76387218	47.17284514	108.9182534	82.70025162
151.8361359	154.2289522	136.6046974	95.97747081	156.8082693
517.9726408	429.1193787	461.9007754	424.8890281	365.1699422
86.48893816	89.81568392	135.6219298	0	99.88471948

For Review Only

84.56696176	110.6819539	52.08668318	71.1743042	76.25607616
149.9141595	98.88797523	86.48354943	160.6813837	158.9563278
49.01039829	75.30001783	93.36292268	91.66387662	70.88592996
0	59.87712262	0	47.44953613	0
2.882964605	0.907229131	0	0	0
97.05980838	119.7542452	111.0527396	101.3694636	119.2172458
32.67359886	18.14458261	32.43133104	26.95996371	17.18446787
0	20.86627	0	32.35195645	0
67.26917412	58.06266435	82.552479	70.09590565	62.29369602
24.98569325	28.12410305	15.72428171	5.391992742	47.25728664
156.6410769	155.1361813	127.7597889	179.014159	47.25728664
0	0	55.034986	37.7439492	32.22087725
11.53185842	16.33012435	57.98328882	35.5871521	17.18446787
42.28348088	59.87712262	47.17284514	58.23352162	83.77428086
9.609882018	0	0	0	0
8.648893816	1.814458261	0	5.391992742	9.666263176
60.54225671	52.61928957	57.00052121	51.76313033	51.5534036
12.49284662	10.88674957	9.827676071	10.78398548	7.518204692
5.765929211	11.7939787	8.844908464	1.078398548	12.8883509
7.687905614	3.628916522	2.948302821	2.156797097	3.222087725
143.1872421	189.6108883	198.5190566	189.7981445	193.3252635
20.18075224	23.58795739	32.43133104	42.05754339	24.70267256
0	0	0	0	0
20.18075224	22.68072826	17.68981693	24.80316661	21.48058484
71.11312693	81.65062175	96.3112255	85.19348533	65.51578375
71.11312693	79.83616349	75.67310575	74.40949984	84.8483101
0	0	2.948302821	0	0
0	0	0	0	0
271.9596611	116.1253287	341.0203597	277.148427	121.3653043
147.9921831	146.06389	208.3467327	209.2093184	149.2900646
0	0	55.034986	62.54711581	57.99757905
3.843952807	4.536145653	18.67258454	8.627188388	3.222087725
66.30818592	48.08314392	54.05221839	101.3694636	35.44296498
0	9.072291305	21.62088736	1.078398548	0
47.08842189	45.36145653	79.60417618	51.76313033	50.47937436
31.71261066	41.73254	46.19007754	19.41117387	30.07281877
11.53185842	19.05181174	14.74151411	11.86238403	12.8883509
0	0	6.87937325	3.235195645	0
0	0	0	0	0
204.690487	250.39524	254.5368102	269.5996371	248.1007548
38.43952807	41.73254	87.46631704	61.46871726	54.77549133
99.94277298	116.1253287	161.1738876	95.97747081	121.3653043
103.7867258	118.8470161	56.01775361	69.0175071	103.1068072
0.960988202	6.350603914	0	0	11.81432166
95.13783197	100.7024335	72.72480293	79.80149259	71.9599592
44.20545728	68.04218479	82.552479	49.60633323	48.33131588
0	92.53737131	85.50078182	1.078398548	141.7718599
117.2405606	144.2494318	134.6391622	124.0158331	133.179626
3.843952807	0	2.948302821	1.078398548	0
73.03510333	84.37230914	36.36240146	64.70391291	50.47937436

For Review Only

73.99609154	66.22772653	115.9665776	98.13426791	56.92354981
31.71261066	42.63976914	50.12114796	51.76313033	41.88714043
48.04941009	0	0.982767607	11.86238403	2.148058484
147.0311949	107.9602665	99.25952832	94.89907226	160.030357
60.54225671	43.54699827	44.22454232	35.5871521	33.29490649
51.8933629	53.5265187	63.87989446	89.50707952	89.14442707
92.25486737	96.16628784	99.25952832	53.91992742	123.5133628
208.5344398	231.3434283	221.1227116	145.583804	124.587392
18.25877583	22.68072826	7.862140857	18.33277532	4.296116967
1.921976404	3.628916522	0	0	0
2.882964605	0	1.965535214	4.313594194	1.074029242
0	0	0	3.235195645	0
0	0	0	0	0
33.63458706	23.58795739	27.517493	20.48957242	26.85073104
0	0	0	0	0
52.8543511	48.08314392	44.22454232	69.0175071	68.73787147
0	5.443374783	2.948302821	2.156797097	3.222087725
0	0	0	0	0
0	0	4.913838036	6.470391291	0
6.726917412	0	2.948302821	15.09757968	19.33252635
54.7763275	58.06266435	64.86266207	10.78398548	59.0716083
0	0	0.982767607	0	1.074029242
31.71261066	12.70120783	54.05221839	47.44953613	83.77428086
202.7685106	139.7132861	47.17284514	189.7981445	179.3628834
0	0	0	1.078398548	2.148058484
24.02470504	36.28916522	0	0	0
0	1.814458261	0	0	0
0	3.628916522	2.948302821	3.235195645	1.074029242
2.882964605	2.721687392	0	0	0
14.41482303	26.30964479	22.60365496	14.01918113	7.518204692
0	0.907229131	2.948302821	2.156797097	2.148058484
0	0	0.982767607	1.078398548	5.370146209
17.29778763	12.70120783	18.67258454	11.86238403	13.96238014
16.33679943	16.33012435	11.79321129	19.41117387	12.8883509
15.37581123	23.58795739	7.862140857	3.235195645	15.03640938
0.960988202	1.814458261	0	0	1.074029242
0.960988202	0.907229131	0	0	0
0	0	0.982767607	1.078398548	1.074029242
0	0	6.87937325	2.156797097	2.148058484
0	6.350603914	4.913838036	1.078398548	4.296116967
0	0	0.982767607	1.078398548	0
15.37581123	10.88674957	9.827676071	10.78398548	7.518204692
0	0	3.931070429	4.313594194	5.370146209
2.882964605	1.814458261	4.913838036	0	5.370146209
2.882964605	6.350603914	3.931070429	0	1.074029242
1.921976404	0	0	0	1.074029242
2.882964605	2.721687392	2.948302821	4.313594194	4.296116967
0	0	2.948302821	0	1.074029242
4.804941009	7.257833044	3.931070429	5.391992742	3.222087725
0	8.165062175	5.896605643	6.470391291	4.296116967

For Review Only

0	0.907229131	0	1.078398548	1.074029242
2.882964605	1.814458261	0	0	0
2.882964605	2.721687392	0	0	0
0	2.721687392	1.965535214	1.078398548	0
2.882964605	6.350603914	0	0	0
1.921976404	2.721687392	2.948302821	1.078398548	3.222087725
34.59557526	36.28916522	38.32793668	35.5871521	37.59102346
172.9778763	178.7241387	209.3295003	168.2301736	144.9939476
245.0519914	176.0024513	122.8459509	265.2860429	209.4357021
14.41482303	6.350603914	13.7587465	0	16.11043863
35.55656347	46.26868566	40.29347189	61.46871726	65.51578375
15.37581123	39.01085261	19.65535214	48.52793468	44.03519891
76.87905614	92.53737131	110.069972	87.35028243	91.29248555
0	0	0.982767607	1.078398548	3.222087725
79.76202075	141.5277444	258.4678807	30.19515936	119.2172458
112.4356196	133.3626822	165.104958	169.3085721	127.8094798
7.687905614	0	6.87937325	0	0
9.609882018	0.907229131	60.93159164	238.3260792	0
3.843952807	5.443374783	0	0	0
13.45383482	6.350603914	11.79321129	15.09757968	9.666263176
32.67359886	27.21687392	24.56919018	44.21434049	42.96116967
24.02470504	19.95904087	24.56919018	33.430355	17.18446787
38.43952807	22.68072826	45.20730993	40.97914484	0
0	16.33012435	56.01775361	59.31192017	0
0	0	0	7.548789839	23.62864332
5.765929211	19.05181174	31.44856343	22.64636952	0
0	0	0	154.2109924	76.25607616
0	0	0	0	0
6.726917412	0	9.827676071	9.705586936	16.11043863
6.726917412	0	0	1.078398548	0
18.25877583	37.19639435	31.44856343	35.5871521	32.22087725
109.552655	145.1566609	43.24177471	86.27188388	103.1068072
151.8361359	175.0952222	186.7258454	198.4253329	199.769439
107.6306786	88.90845479	97.29399311	87.35028243	105.2548657
54.7763275	29.93856131	67.81096489	78.72309404	53.70146209
43.24446908	33.56747783	20.63811975	75.48789839	28.99878953
231.5981566	211.3843874	212.2778031	197.3469344	118.1432166
136.4603247	153.3217231	189.6741482	126.1726302	129.9575383
31.71261066	44.4542274	54.05221839	42.05754339	42.96116967
29.79063425	15.42289522	52.08668318	14.01918113	48.33131588
0	0	0	214.6013111	111.6990411
455.5084076	441.8205866	318.4167047	360.1851152	458.6104862
275.8036139	220.4566787	220.139944	191.9549416	251.3228426
172.9778763	142.4349735	196.5535214	231.8556879	207.2876437
50.93237469	55.34097696	213.2605707	0	166.4745325
12.49284662	19.05181174	29.48302821	17.25437678	13.96238014
11.53185842	14.51566609	12.77597889	15.09757968	6.444175451
47.08842189	45.36145653	56.01775361	56.07672452	95.58860252
4.804941009	16.33012435	5.896605643	7.548789839	19.33252635
126.8504426	125.19762	51.10391557	60.39031871	51.5534036

For Review Only

235.4421094	411.8820253	277.1404652	374.2042963	410.2791704
179.7047937	204.1265544	211.2950355	326.7547602	258.8410473
89.37190276	94.35182958	74.69033814	97.05586936	103.1068072
15.37581123	9.072291305	26.53472539	16.17597823	17.18446787
25.94668145	0	12.77597889	31.27355791	12.8883509
135.4993364	166.93016	183.7775425	149.8973982	146.0679769
143.1872421	389.201297	615.2125221	583.4136147	919.3690309
197.9635696	230.4361992	227.0193172	345.0875355	282.4696906
0	0	9.827676071	0	0
78.80103254	136.9915987	155.2772819	182.2493547	102.032778
31.71261066	38.10362348	25.55195779	57.15512307	37.59102346
313.2821538	332.9530909	247.657437	296.5596008	305.0243047
0	0	4.913838036	0	4.296116967
4.804941009	6.350603914	0	4.313594194	4.296116967
0.960988202	2.721687392	2.948302821	3.235195645	3.222087725
0	7.257833044	0	0	0
34.59557526	61.69158088	35.37963386	51.76313033	39.73908194
44.20545728	37.19639435	56.01775361	30.19515936	44.03519891
149.9141595	11.7939787	8.844908464	163.9165794	96.66263176
38.43952807	48.08314392	51.10391557	64.70391291	40.81311119
110.5136432	129.7337657	288.9336765	0	226.62017
78.80103254	81.65062175	69.77650011	79.80149259	74.10801768
0.960988202	0	2.948302821	0	0
1985.401625	2095.699292	2060.863672	2367.084814	1933.252635
219.10531	359.2627357	740.0240082	130.4862244	280.3216321
15.37581123	0	3.931070429	1.078398548	2.148058484
0	0	2.948302821	2.156797097	0
0	8.165062175	7.862140857	0	0
108.5916668	0	198.5190566	52.84152887	12.8883509
169.1339235	82.55785088	9.827676071	179.014159	89.14442707
101.8647494	72.57833044	177.8809369	322.441166	491.9053927
56.6983039	53.5265187	9.827676071	21.56797097	82.70025162
26.90766965	42.63976914	35.37963386	31.27355791	23.62864332
0.960988202	0	16.70704932	0	3.222087725
600.6176261	608.7507466	507.1080853	574.7864263	463.9806324
0	0.907229131	0	3.235195645	3.222087725
11.53185842	0	0	0	3.222087725
5.765929211	4.536145653	1.965535214	2.156797097	4.296116967
0	0	0	0	0
25.94668145	39.91808174	22.60365496	11.86238403	117.0691874
4.804941009	3.628916522	0	0	3.222087725
4.804941009	0	0.982767607	0	0
0.960988202	0	0	2.156797097	3.222087725
0	0	4.913838036	0	0
172.9778763	124.2903909	104.1733664	108.9182534	165.4005032
38.43952807	39.91808174	21.62088736	42.05754339	65.51578375
61.50324491	70.76387218	61.91435925	49.60633323	59.0716083
42.28348088	30.84579044	42.25900711	49.60633323	28.99878953
44.20545728	56.24820609	33.41409864	28.03836226	18.25849711
135.4993364	97.9807461	145.4496059	95.97747081	59.0716083

For Review Only

8.648893816	4.536145653	5.896605643	3.235195645	4.296116967
56.6983039	21.77349913	39.31070429	22.64636952	56.92354981
0	0	0	8.627188388	0
79.76202075	69.85664305	55.034986	39.90074629	47.25728664
0	17.23735348	18.67258454	10.78398548	27.92476029
0	0	0	0	0
2.882964605	0	0	0	0
0	0	0	4.313594194	0
170.0949117	217.7349913	56.01775361	91.66387662	86.99636858
3876.626406	4354.699827	5197.857874	5591.496474	4567.846365
0	37.19639435	0	0	0
46.12743368	30.84579044	53.06945079	54.99832597	59.0716083
10.57087022	0	1.965535214	2.156797097	7.518204692
787.0493372	674.071244	646.6610855	552.1400568	459.6845155
5.765929211	0	0	0	0
77.84004434	84.37230914	126.7770213	111.0750505	82.70025162
0	3.628916522	0	0	3.222087725
0	1.814458261	4.913838036	0	0
54.7763275	30.84579044	54.05221839	39.90074629	57.99757905
2.882964605	0	0	0	1.074029242
73.03510333	52.61928957	44.22454232	49.60633323	57.99757905
5.765929211	6.350603914	3.931070429	4.313594194	4.296116967
8.648893816	27.21687392	17.68981693	14.01918113	21.48058484
16.33679943	3.628916522	0.982767607	4.313594194	7.518204692
0	0	0	3.235195645	0
15.37581123	16.33012435	4.913838036	7.548789839	19.33252635
0.960988202	0.907229131	1.965535214	2.156797097	2.148058484
1.921976404	0	0.982767607	8.627188388	0
0	0	131.6908594	135.8782171	0
372.8634223	471.7591479	434.3832824	600.6679915	467.2027202
337.3068588	402.809734	403.9174865	442.1434049	294.2840122
3.843952807	0	3.931070429	0	5.370146209
0	0	0	0	0
399.7710919	169.6518474	151.3462115	121.859036	178.2888541
112.4356196	113.4036413	102.2078311	87.35028243	78.40413465
0	6.350603914	0	0	0
0	0	0.982767607	0	0
30.75162246	53.5265187	56.01775361	29.11676081	52.62743285
87.44992636	90.72291305	98.27676071	80.87989113	86.99636858
98.02079658	95.25905871	80.58694379	101.3694636	111.6990411
208.5344398	172.3735348	107.1216692	126.1726302	121.3653043
145.1092185	90.72291305	249.6229722	162.8381808	119.2172458
30.75162246	37.19639435	74.69033814	53.91992742	27.92476029
21.14174044	64.41326827	76.65587336	40.97914484	16.11043863
170.0949117	280.3338013	374.4344583	403.3210571	278.1735736
24.98569325	19.95904087	30.46579582	18.33277532	13.96238014
62.46423311	57.15543522	41.2762395	52.84152887	63.36772526
116.2795724	90.72291305	100.2422959	72.25270275	115.9951581
0	0	0	0	0
6.726917412	5.443374783	19.65535214	0	3.222087725

For Review Only

98.02079658	108.8674957	86.48354943	54.99832597	78.40413465
0	470.8519187	96.3112255	8.627188388	21.48058484
35.55656347	32.6602487	43.24177471	48.52793468	46.1832574
0.960988202	0	0	0	0
12.49284662	3.628916522	3.931070429	2.156797097	2.148058484
147.0311949	153.3217231	159.2083524	129.4078258	138.5497722
62.46423311	43.54699827	40.29347189	46.37113758	42.96116967
1.921976404	0.907229131	0	0	5.370146209
455.5084076	458.1507109	360.6757118	384.9882818	474.7209249
24.98569325	20.86627	181.8120073	0	0
49.97138649	78.02170523	61.91435925	103.5262607	155.7342401
0	0	44.22454232	32.35195645	34.36893574
65.34719772	47.17591479	64.86266207	44.21434049	85.92233934
190.2756639	200.4976378	220.139944	63.62551436	78.40413465
6.726917412	5.443374783	0	1.078398548	1.074029242
47.08842189	75.30001783	104.1733664	51.76313033	83.77428086
16.33679943	23.58795739	37.34516907	16.17597823	10.74029242
32.67359886	37.19639435	30.46579582	53.91992742	44.03519891
25.94668145	45.36145653	23.58642257	76.56629694	26.85073104
35.55656347	22.68072826	13.7587465	19.41117387	51.5534036
271.9596611	547.0591657	483.5216627	407.6346513	354.4296498
17.29778763	0	38.32793668	24.80316661	5.370146209
35.55656347	0	74.69033814	30.19515936	0
62.46423311	71.67110131	58.96605643	32.35195645	47.25728664
12.49284662	0	5.896605643	5.391992742	0
33.63458706	63.50603914	5.896605643	10.78398548	10.74029242
0	9.072291305	0.982767607	7.548789839	0
4.804941009	0	0.982767607	6.470391291	0
77.84004434	74.3927887	55.034986	15.09757968	38.6650527
110.5136432	154.2289522	68.7937325	95.97747081	131.0315675
222.9492628	223.1783661	271.2438596	308.4219849	229.8422577
54.7763275	43.54699827	42.25900711	164.9949779	32.22087725
159.5240415	102.5168918	122.8459509	109.9966519	128.883509
3.843952807	0	0	0	0
8.648893816	6.350603914	9.827676071	0	6.444175451
91.29387917	121.5687035	199.5018242	0	225.5461408
666.925812	865.4965905	1271.701284	477.730557	404.9090241
0	0	120.8804157	44.21434049	0
82.64498535	90.72291305	63.87989446	48.52793468	49.40534512
63.42522132	48.99037305	58.96605643	86.27188388	115.9951581
399.7710919	333.86032	430.4522119	340.7739413	215.8798776
0	1.814458261	0.982767607	0	0
0	0	0	0	3.222087725
126.8504426	126.1048491	190.6569158	141.2702098	86.99636858
55.7373157	118.8470161	106.1389016	56.07672452	69.81190071
52.8543511	75.30001783	86.48354943	25.88156516	7.518204692
218.1443218	163.3012435	149.3806763	149.8973982	157.8822985
23.06371684	30.84579044	23.58642257	42.05754339	41.88714043
24.02470504	15.42289522	7.862140857	17.25437678	11.81432166
33.63458706	48.99037305	67.81096489	34.50875355	44.03519891

For Review Only

0	0	3.931070429	0	530.5704454
9.609882018	0	21.62088736	12.94078258	1.074029242
140.3042775	163.3012435	152.3289791	150.9757968	210.5097314
85.52794996	75.30001783	122.8459509	77.64469549	84.8483101
35.55656347	20.86627	9.827676071	5.391992742	6.444175451
38.43952807	83.46508001	0	0	0
101.8647494	136.9915987	203.4328947	185.4845503	178.2888541
81.68399715	78.02170523	73.70757054	97.05586936	79.47816389
67.26917412	48.08314392	69.77650011	65.78231146	63.36772526
90.33289097	69.85664305	101.2250635	107.8398548	85.92233934
89.37190276	75.30001783	95.32845789	84.11508678	70.88592996
122.0455016	92.53737131	167.0704932	140.1918113	95.58860252
111.4746314	133.3626822	118.9148805	104.6046592	100.9587487
51.8933629	42.63976914	93.36292268	100.291065	9.666263176
63.42522132	23.58795739	37.34516907	54.99832597	45.10922815
59.58126851	125.19762	99.25952832	107.8398548	53.70146209
208.5344398	9.979520436	212.2778031	193.0333402	0
174.8998527	186.8892009	252.571275	372.0474992	225.5461408
34.59557526	80.74339262	69.77650011	37.7439492	48.33131588
0	0	19.65535214	15.09757968	13.96238014
314.243142	376.5000892	309.5717962	259.8940502	321.1347433
256.5838499	360.1699648	14.74151411	34.50875355	76.25607616
91.29387917	39.01085261	78.62140857	80.87989113	41.88714043
68.23016233	38.10362348	45.20730993	59.31192017	93.44054403
148.9531713	174.1879931	117.9321129	112.153449	122.4393336
14.41482303	11.7939787	38.32793668	189.7981445	44.03519891
0	0	90.41461986	243.718072	0
0	0	0	0	0
107.6306786	68.94941392	161.1738876	91.66387662	45.10922815
0	0	0	0	0
0	0	0.982767607	69.0175071	175.0667664
7.687905614	3.628916522	0	0	267.4332812
85.52794996	78.02170523	29.48302821	22.64636952	0
2.882964605	6.350603914	0.982767607	0	10.74029242
242.1690268	264.9109061	249.6229722	266.3644415	230.916287
33.63458706	32.6602487	13.7587465	12.94078258	11.81432166
119.162537	71.67110131	92.38015507	144.5054055	120.2912751
0	81.65062175	101.2250635	120.7806374	6.444175451
161.4460179	0	4.913838036	128.3294273	18.25849711
24.02470504	38.10362348	26.53472539	34.50875355	26.85073104
60.54225671	62.59881001	66.82819729	46.37113758	82.70025162
0	0	2.948302821	0	0
147.9921831	161.4867852	149.3806763	120.7806374	220.1759946
116.2795724	87.09399653	71.74203532	127.2510287	79.47816389
1265.621462	1188.470161	973.9226987	909.0899764	1113.768324
0	0	25.55195779	11.86238403	9.666263176
0	0	0	0	25.7767018
12.49284662	9.979520436	33.41409864	2.156797097	4.296116967
6.726917412	2.721687392	10.81044368	0	0
504.5188059	517.1206044	370.5033879	781.8389476	576.7537028

For Review Only

203.7294988	192.3325757	258.4678807	283.6188182	181.5109419
356.5266229	350.1904444	329.2271484	359.1067166	318.9866848
0	0.907229131	0	219.9933039	7.518204692
86.48893816	98.88797523	76.65587336	87.35028243	70.88592996
0	0	21.62088736	31.27355791	18.25849711
1.921976404	0	0	6.470391291	0
142.2262539	185.9819718	207.3639651	175.7789634	156.8082693
3.843952807	11.7939787	0	6.470391291	6.444175451
138.3823011	99.79520436	183.7775425	218.9149053	146.0679769
74.95707974	43.54699827	36.36240146	35.5871521	27.92476029
0.960988202	0	0	0	0
24.98569325	9.979520436	7.862140857	26.95996371	7.518204692
148.9531713	147.8783483	133.6563946	166.0733765	201.9174975
26.90766965	44.4542274	22.60365496	35.5871521	37.59102346
51.8933629	26.30964479	61.91435925	57.15512307	39.73908194
134.5383482	153.3217231	190.6569158	227.5420937	146.0679769
537.1924048	412.7892544	243.7263666	326.7547602	293.209983
102.8257376	84.37230914	40.29347189	65.78231146	88.07039782
234.4811212	229.52897	195.5707538	135.8782171	238.4344917
0	0	1.965535214	0	1.074029242
0	11.7939787	0	1.078398548	0
426.6787616	468.1302314	678.1096489	477.730557	487.6092758
0	0	17.68981693	24.80316661	12.8883509
5.765929211	0	0	0	0
102.8257376	100.7024335	150.3634439	142.3486084	119.2172458
18.25877583	13.60843696	1.965535214	2.156797097	9.666263176
0	0	0	0	0
37.47853987	18.14458261	6.87937325	11.86238403	27.92476029
32.67359886	24.49518652	23.58642257	19.41117387	23.62864332
0	1517.794335	0.982767607	170.3869707	1.074029242
1.921976404	0	0	0	0
121.0845134	134.2699113	78.62140857	150.9757968	184.7330296
312.3211656	243.137407	204.4156623	431.3594194	193.3252635
0	0	0	0	0
72.07411513	53.5265187	118.9148805	125.0942316	49.40534512
94.17684377	80.74339262	53.06945079	76.56629694	112.7730704
81.68399715	88.90845479	76.65587336	72.25270275	78.40413465
105.7087022	151.5072648	115.9665776	86.27188388	139.6238014
0	0	1.965535214	1.078398548	0
21.14174044	29.93856131	34.39686625	16.17597823	16.11043863
0	0	1.965535214	0	0
271.9596611	329.3241744	373.4516907	0	0
188.3536875	0	5.896605643	180.0925576	11.81432166
7.687905614	8.165062175	0.982767607	5.391992742	0
66.30818592	0	18.67258454	100.291065	3.222087725
6.726917412	2.721687392	0	0	0
74.95707974	72.57833044	61.91435925	40.97914484	69.81190071
205.6514752	142.4349735	188.6913806	103.5262607	53.70146209
848.5525822	1110.448456	723.3169589	0	629.3811357
147.0311949	159.672327	139.5530002	155.289391	178.2888541

For Review Only

0	68.94941392	52.08668318	43.13594194	97.736661
189.3146757	64.41326827	362.641247	296.5596008	148.2160354
54.7763275	70.76387218	72.72480293	65.78231146	45.10922815
211.4174044	202.3120961	135.6219298	143.4270069	182.5849711
481.4550891	508.0483131	379.3482964	477.730557	386.650527
0	0	23.58642257	0	35.44296498
27.86865785	26.30964479	19.65535214	20.48957242	34.36893574
12.49284662	4.536145653	10.81044368	12.94078258	5.370146209
4.804941009	0	9.827676071	0	0
0	0	3.931070429	4.313594194	1.074029242
4.804941009	16.33012435	0.982767607	17.25437678	15.03640938
0	0.907229131	2.948302821	2.156797097	2.148058484
147.9921831	127.0120783	168.0532608	92.74227517	99.88471948
22.10272864	9.979520436	26.53472539	35.5871521	20.40655559
37.47853987	0	0	0	0
439.1716082	400.0880466	301.7096554	386.0666803	218.0279361
49.97138649	51.71206044	35.37963386	29.11676081	56.92354981
138.3823011	122.4759326	90.41461986	125.0942316	123.5133628
0	0	132.673627	128.3294273	33.29490649
99.94277298	122.4759326	103.1905987	104.6046592	95.58860252
16.33679943	9.072291305	17.68981693	22.64636952	23.62864332
68.23016233	65.3204974	82.552479	66.86071	78.40413465
46.12743368	2.721687392	0	203.8173257	0
6.726917412	8.165062175	7.862140857	0	0
87.44992636	50.80483131	47.17284514	76.56629694	49.40534512
36.51755167	27.21687392	17.68981693	49.60633323	23.62864332
0	8.165062175	12.77597889	5.391992742	0
98.02079658	97.9807461	67.81096489	98.13426791	75.18204692
36.51755167	45.36145653	31.44856343	29.11676081	45.10922815
0	0	12.77597889	1.078398548	0
127.8114308	112.4964122	86.48354943	95.97747081	121.3653043
50.93237469	73.48555957	58.96605643	99.21266646	96.66263176
9.609882018	10.88674957	10.81044368	14.01918113	11.81432166
17.29778763	23.58795739	61.91435925	17.25437678	8.592233934
87.44992636	100.7024335	157.2428171	66.86071	75.18204692
0	0	56.01775361	72.25270275	0
5.765929211	3.628916522	0	0	3.222087725
23.06371684	28.12410305	17.68981693	29.11676081	24.70267256
0	0	1.965535214	3.235195645	0
0	0	0	2.156797097	5.370146209
0	66.22772653	10.81044368	7.548789839	30.07281877
29.79063425	24.49518652	94.34569029	86.27188388	59.0716083
79.76202075	84.37230914	0	127.2510287	93.44054403
119.162537	151.5072648	156.2600495	156.3677895	132.1055967
61.50324491	33.56747783	18.67258454	33.430355	65.51578375
102.8257376	110.6819539	181.8120073	141.2702098	99.88471948
0	0	8.844908464	0	0
102.8257376	104.33135	79.60417618	67.93910855	115.9951581
42.28348088	46.26868566	45.20730993	23.72476807	51.5534036
42.28348088	29.93856131	57.00052121	94.89907226	18.25849711

For Review Only

569.8660036	500.7904801	531.6772755	600.6679915	492.979422
77.84004434	67.13495566	67.81096489	79.80149259	74.10801768
0	219.5494496	0.982767607	1.078398548	1.074029242
0	0.907229131	0.982767607	0	1.074029242
117.2405606	135.1771404	114.0010424	95.97747081	122.4393336
127.8114308	130.6409948	150.3634439	167.151775	201.9174975
66.30818592	82.55785088	72.72480293	80.87989113	65.51578375
71.11312693	69.85664305	86.48354943	128.3294273	55.84952057
0.960988202	3.628916522	0	0	0
8.648893816	10.88674957	15.72428171	10.78398548	18.25849711
98.98178478	108.8674957	135.6219298	161.7597823	113.8470996
11.53185842	14.51566609	21.62088736	7.548789839	8.592233934
0	0	4.913838036	2.156797097	16.11043863
0	0	0	0	0
76.87905614	81.65062175	72.72480293	119.7022389	49.40534512
399.7710919	311.1795918	408.8313246	449.6921947	458.6104862
35.55656347	37.19639435	21.62088736	35.5871521	22.55461408
39.40051627	46.26868566	41.2762395	42.05754339	42.96116967
0	4.536145653	0	163.9165794	80.55219313
106.6696904	127.0120783	55.034986	107.8398548	135.3276845
35.55656347	34.47470696	25.55195779	22.64636952	20.40655559
31.71261066	13.60843696	18.67258454	18.33277532	13.96238014
103.7867258	111.5891831	100.2422959	114.3102461	112.7730704
17.29778763	12.70120783	16.70704932	28.03836226	16.11043863
10.57087022	11.7939787	9.827676071	10.78398548	4.296116967
71.11312693	62.59881001	40.29347189	60.39031871	91.29248555
78.80103254	86.1867674	68.7937325	93.82067372	112.7730704
66.30818592	95.25905871	113.0182748	124.0158331	125.6614213
103.7867258	84.37230914	86.48354943	87.35028243	65.51578375
34.59557526	30.84579044	25.55195779	16.17597823	65.51578375
14.41482303	10.88674957	18.67258454	22.64636952	5.370146209
124.9284662	101.6096626	83.53524661	94.89907226	69.81190071
48.04941009	49.89760218	48.15561275	49.60633323	52.62743285
119.162537	138.806057	130.7080917	128.3294273	91.29248555
116.2795724	129.7337657	100.2422959	103.5262607	140.6978307
0	0	30.46579582	0	2.148058484
13.45383482	29.03133218	21.62088736	16.17597823	7.518204692
48.04941009	2.721687392	0	115.3886447	81.62622237
0.960988202	0.907229131	0.982767607	4.313594194	1.074029242
5.765929211	0	7.862140857	0	4.296116967
0	6.350603914	1.965535214	3.235195645	4.296116967
83.60597355	123.3831618	66.82819729	117.5454418	124.587392
15.37581123	2.721687392	69.77650011	89.50707952	76.25607616
70.15213873	93.44460045	101.2250635	80.87989113	90.21845631
51.8933629	57.15543522	52.08668318	62.54711581	55.84952057
192.1976404	220.4566787	239.7952961	668.6071	65.51578375
97.05980838	146.9711191	126.7770213	53.91992742	63.36772526
416.1078914	422.7687748	524.7979022	572.6296292	477.9430126
19.21976404	0	1.965535214	9.705586936	1.074029242
28.82964605	74.3927887	124.8114861	113.2318476	127.8094798

For Review Only

323.853024	269.4470518	228.9848525	218.9149053	252.3968718
30.75162246	25.40241565	39.31070429	21.56797097	49.40534512
0.960988202	0.907229131	0	40.97914484	0
32.67359886	22.68072826	38.32793668	29.11676081	77.33010541
133.57736	177.8169096	0	144.5054055	114.9211289
134.5383482	127.9193074	139.5530002	56.07672452	109.5509827
52.8543511	60.78435175	21.62088736	99.21266646	31.14684801
36.51755167	37.19639435	65.84542968	37.7439492	79.47816389
0	0	0.982767607	5.391992742	60.14563754
0	0	0	0	0
600.6176261	711.2676383	709.5582124	755.9573825	608.9745801
60.54225671	76.20724696	100.2422959	78.72309404	76.25607616
9.609882018	10.88674957	7.862140857	5.391992742	8.592233934
0	0	0	0	0
7.687905614	12.70120783	0	890.757201	553.1250595
171.0558999	117.0325578	97.29399311	119.7022389	94.51457327
24.02470504	48.08314392	56.01775361	35.5871521	49.40534512
1.921976404	0	0	1.078398548	5.370146209
170.0949117	136.9915987	110.069972	83.03668823	181.5109419
0	104.33135	0	0	0
31.71261066	27.21687392	22.60365496	15.09757968	32.22087725
54.7763275	51.71206044	0	31.27355791	39.73908194
36.51755167	29.93856131	34.39686625	52.84152887	54.77549133
49.01039829	75.30001783	56.01775361	33.430355	74.10801768
20.18075224	11.7939787	29.48302821	17.25437678	13.96238014
213.3393808	174.1879931	210.3122679	95.97747081	210.5097314
53.8153393	68.94941392	91.39738746	44.21434049	90.21845631
19.21976404	4.536145653	7.862140857	6.470391291	18.25849711
64.38620952	73.48555957	47.17284514	54.99832597	53.70146209
11.53185842	29.03133218	19.65535214	12.94078258	30.07281877
0	0	0	0	0
6.726917412	7.257833044	0	14.01918113	9.666263176
0	0	0	0	0
0	0	0	1.078398548	0
81.68399715	98.88797523	84.51801421	152.0541953	153.5861816
456.4693958	436.3772118	358.7101766	486.3577454	292.1359538
73.03510333	58.06266435	80.58694379	50.68473178	99.88471948
19.21976404	11.7939787	2.948302821	9.705586936	3.222087725
157.6020651	156.0434105	114.98381	159.6029852	104.1808365
4.804941009	2.721687392	10.81044368	0	0
13.45383482	4.536145653	19.65535214	14.01918113	6.444175451
6.726917412	0	0	9.705586936	0
20.18075224	32.6602487	24.56919018	23.72476807	23.62864332
0.960988202	133.3626822	440.279888	896.1491938	839.8908671
0	0	0	0	0
0	0	0.982767607	0	0
248.8959443	183.2602844	83.53524661	156.3677895	226.62017
45.16644548	45.36145653	23.58642257	37.7439492	77.33010541
84.56696176	86.1867674	81.56971139	61.46871726	76.25607616
49.01039829	42.63976914	34.39686625	44.21434049	52.62743285

For Review Only

0.960988202	0	0	0	3.222087725
0.960988202	0	0	0	0
0	0	0	0	0
63.42522132	82.55785088	96.3112255	127.2510287	86.99636858
0	0	0	0	0
422.8348088	326.602487	376.3999935	355.871521	340.4672696
59.58126851	36.28916522	71.74203532	80.87989113	91.29248555
56.6983039	42.63976914	41.2762395	35.5871521	64.44175451
0	0	0	0	0
3.843952807	6.350603914	4.913838036	6.470391291	4.296116967
1.921976404	0	0.982767607	0	1.074029242
3.843952807	0	0	2.156797097	0
12.49284662	13.60843696	26.53472539	22.64636952	22.55461408
7.687905614	0	1.965535214	5.391992742	3.222087725
0	4.536145653	0	0	2.148058484
4.804941009	5.443374783	2.948302821	1.078398548	0
0	0	100.2422959	114.3102461	67.66384223
59.58126851	89.81568392	73.70757054	72.25270275	63.36772526
145.1092185	115.2180996	35.37963386	103.5262607	61.21966678
0	3.628916522	0	0	0
70.15213873	107.0530374	86.48354943	58.23352162	40.81311119
269.0766965	346.5615279	305.6407258	310.578782	363.0218837
24.98569325	21.77349913	9.827676071	20.48957242	47.25728664
0	0	7.862140857	0	1.074029242
1205.079205	1129.500268	1193.079875	1557.207504	701.3410949
6.726917412	16.33012435	9.827676071	8.627188388	6.444175451
24.98569325	0	0	15.09757968	22.55461408
9.609882018	16.33012435	14.74151411	3.235195645	6.444175451
65.34719772	21.77349913	120.8804157	122.9374345	0
24.98569325	34.47470696	22.60365496	23.72476807	42.96116967
22.10272864	20.86627	22.60365496	23.72476807	31.14684801
45.16644548	82.55785088	41.2762395	76.56629694	51.5534036
50.93237469	28.12410305	15.72428171	22.64636952	25.7767018
2.882964605	0	0	2.156797097	0
0	15.42289522	19.65535214	4.313594194	30.07281877
54.7763275	48.99037305	67.81096489	93.82067372	81.62622237
20.18075224	16.33012435	16.70704932	20.48957242	8.592233934
37.47853987	25.40241565	14.74151411	26.95996371	26.85073104
23.06371684	34.47470696	44.22454232	26.95996371	20.40655559
54.7763275	47.17591479	33.41409864	45.29273904	60.14563754
48.04941009	49.89760218	77.63864096	135.8782171	20.40655559
55.7373157	16.33012435	43.24177471	90.58547807	36.51699422
46.12743368	39.01085261	30.46579582	48.52793468	51.5534036
63.42522132	62.59881001	73.70757054	87.35028243	76.25607616
38.43952807	35.38193609	28.50026061	12.94078258	49.40534512
4.804941009	13.60843696	4.913838036	2.156797097	4.296116967
0	4.536145653	10.81044368	5.391992742	7.518204692
29.79063425	29.93856131	33.41409864	22.64636952	16.11043863
0	15.42289522	38.32793668	0	34.36893574
40.36150447	78.02170523	74.69033814	51.76313033	52.62743285

For Review Only

50.93237469	60.78435175	76.65587336	64.70391291	53.70146209
72.07411513	87.09399653	85.50078182	92.74227517	120.2912751
11.53185842	23.58795739	20.63811975	10.78398548	24.70267256
50.93237469	26.30964479	0	0	0
123.0064898	209.5699292	203.4328947	130.4862244	152.5121523
58.62028031	51.71206044	64.86266207	49.60633323	63.36772526
0	0	0	0	0
46.12743368	39.91808174	75.67310575	40.97914484	45.10922815
190.2756639	183.2602844	217.1916412	223.2284995	222.324053
14.41482303	27.21687392	25.55195779	19.41117387	19.33252635
0	0	0.982767607	1.078398548	2.148058484
1665.392554	1552.269042	1410.271516	1634.852199	1611.043863
134.5383482	116.1253287	114.0010424	74.40949984	175.0667664
50.93237469	41.73254	20.63811975	23.72476807	19.33252635
56.6983039	29.03133218	52.08668318	43.13594194	26.85073104
320.9700594	228.6217409	66.82819729	134.7998186	279.2476029
74.95707974	0	61.91435925	22.64636952	3.222087725
192.1976404	253.1169274	152.3289791	153.1325939	195.473322
117.2405606	149.6928065	152.3289791	114.3102461	121.3653043
0.960988202	0.907229131	0	0	0
0	0	0	0	225.5461408
22.10272864	81.65062175	46.19007754	94.89907226	50.47937436
25.94668145	22.68072826	24.56919018	53.91992742	31.14684801
0.960988202	53.5265187	31.44856343	0	27.92476029
144.1482303	114.3108704	155.2772819	129.4078258	183.6590003
78.80103254	119.7542452	155.2772819	138.0350142	111.6990411
0	22.68072826	23.58642257	33.430355	2.148058484
0	2.721687392	0	0	0
19.21976404	14.51566609	2.948302821	5.391992742	17.18446787
0.960988202	0	0.982767607	0	0
26.90766965	15.42289522	7.862140857	45.29273904	8.592233934
26.90766965	33.56747783	8.844908464	12.94078258	50.47937436
12.49284662	12.70120783	8.844908464	14.01918113	5.370146209
19.21976404	29.93856131	17.68981693	11.86238403	25.7767018
229.6761802	264.003677	306.6234934	185.4845503	269.5813397
0	76.20724696	0	0	0
83.60597355	74.3927887	30.46579582	269.5996371	12.8883509
41.32249268	56.24820609	73.70757054	15.09757968	76.25607616
155.6800887	184.1675135	115.9665776	177.9357605	184.7330296
26.90766965	19.95904087	28.50026061	26.95996371	64.44175451
6.726917412	14.51566609	3.931070429	2.156797097	1.074029242
24.02470504	0	2.948302821	10.78398548	17.18446787
0	0	0.982767607	84.11508678	1.074029242
427.6397498	479.9242101	468.7801486	461.5545787	274.9514859
116.2795724	123.3831618	116.9493452	67.93910855	93.44054403
233.520133	225.9000535	176.8981693	234.012485	195.473322
0	0	0	0	7.518204692
44.20545728	48.08314392	37.34516907	48.52793468	55.84952057
0	0	6.87937325	0	0
0	140.6205152	45.20730993	0	0

For Review Only

338.267847	284.869947	416.6934654	248.0316661	238.4344917
4.804941009	12.70120783	6.87937325	5.391992742	5.370146209
0.960988202	0	0	1.078398548	0
94.17684377	121.5687035	86.48354943	90.58547807	121.3653043
80.72300895	101.6096626	88.44908464	72.25270275	56.92354981
33.63458706	31.75301957	51.10391557	67.93910855	28.99878953
100.9037612	100.7024335	92.38015507	70.09590565	108.4769534
0	2.721687392	4.913838036	5.391992742	0
103.7867258	90.72291305	111.0527396	83.03668823	67.66384223
48.04941009	28.12410305	19.65535214	14.01918113	22.55461408
35.55656347	41.73254	36.36240146	46.37113758	59.0716083
379.5903397	304.8289879	342.0031273	327.8331587	295.3580415
15.37581123	1.814458261	0	0	28.99878953
123.0064898	123.3831618	129.7253241	91.66387662	169.6966202
7.687905614	6.350603914	4.913838036	25.88156516	11.81432166
209.495428	217.7349913	122.8459509	138.0350142	129.9575383
304.63326	368.335027	376.3999935	271.7564342	240.5825502
47.08842189	46.26868566	44.22454232	69.0175071	61.21966678
0.960988202	0.907229131	89.43185225	0	49.40534512
57.65929211	39.01085261	91.39738746	81.95828968	75.18204692
79.76202075	111.5891831	81.56971139	80.87989113	44.03519891
51.8933629	62.59881001	133.6563946	31.27355791	105.2548657
47.08842189	69.85664305	57.00052121	69.0175071	35.44296498
14.41482303	17.23735348	18.67258454	7.548789839	15.03640938
269.0766965	233.1578865	208.3467327	315.9707747	251.3228426
223.910251	168.7446183	185.7430777	179.014159	67.66384223
59.58126851	57.15543522	57.98328882	62.54711581	59.0716083
172.0168881	195.0542631	189.6741482	134.7998186	195.473322
212.3783926	227.7145118	121.8631833	146.6622026	183.6590003
135.4993364	0	196.5535214	46.37113758	50.47937436
12.49284662	16.33012435	114.0010424	65.78231146	78.40413465
74.95707974	89.81568392	96.3112255	72.25270275	77.33010541
96.09882018	189.6108883	145.4496059	155.289391	97.736661
68.23016233	66.22772653	60.93159164	83.03668823	62.29369602
112.4356196	124.2903909	33.41409864	70.09590565	89.14442707
37.47853987	50.80483131	37.34516907	25.88156516	44.03519891
0	0	0	0	0
6.726917412	5.443374783	10.81044368	4.313594194	0
0	10.88674957	2.948302821	29.11676081	6.444175451
414.185915	321.1591122	756.7310575	179.014159	96.66263176
4.804941009	0	3.931070429	0	0
21.14174044	38.10362348	5.896605643	30.19515936	28.99878953
31.71261066	21.77349913	28.50026061	14.01918113	28.99878953
0	0	0	1.078398548	0
59.58126851	71.67110131	67.81096489	93.82067372	73.03398844
33.63458706	56.24820609	47.17284514	36.66555065	36.51699422
24.02470504	34.47470696	32.43133104	50.68473178	30.07281877
10.57087022	24.49518652	20.63811975	24.80316661	10.74029242
60.54225671	69.85664305	31.44856343	15.09757968	10.74029242
45.16644548	61.69158088	54.05221839	47.44953613	63.36772526

93.21585557	88.90845479	93.36292268	75.48789839	95.58860252
0.960988202	0.907229131	0	0	33.29490649
281.5695431	251.3024692	223.0882468	345.0875355	149.2900646
83.60597355	81.65062175	128.7425565	97.05586936	71.9599592
53.8153393	35.38193609	60.93159164	28.03836226	28.99878953
0	6.350603914	13.7587465	0	2.148058484
175.8608409	186.8892009	106.1389016	114.3102461	92.36651479
0	0.907229131	0.982767607	4.313594194	0
460.3133486	612.3796631	514.9702261	451.8489918	387.7245563
39.40051627	38.10362348	73.70757054	20.48957242	59.0716083
366.1365049	449.0784196	401.9519513	446.4569991	414.5752873
183.5487465	190.5181174	217.1916412	394.6938687	189.0291465
0	170.5590765	0.982767607	3.235195645	1.074029242
0	0	0	230.7772894	5.370146209
893.7190276	1109.541227	1222.562903	1217.511961	918.2950017
107.6306786	103.4241209	139.5530002	109.9966519	97.736661
56.6983039	406.4386505	185.7430777	225.3852966	176.1407956
2.882964605	4.536145653	0	4.313594194	5.370146209
53.8153393	59.87712262	36.36240146	52.84152887	36.51699422
71.11312693	73.48555957	69.77650011	51.76313033	70.88592996
0	1.814458261	0	0	0
0	0	8.844908464	14.01918113	6.444175451
0.960988202	1.814458261	0	0	0
10.57087022	29.03133218	0	15.09757968	25.7767018
0	10.88674957	3.931070429	0	0
30.75162246	25.40241565	33.41409864	25.88156516	21.48058484
2.882964605	0.907229131	3.931070429	0	0
18.25877583	36.28916522	33.41409864	16.17597823	17.18446787
18.25877583	12.70120783	23.58642257	16.17597823	16.11043863
4.804941009	3.628916522	6.87937325	6.470391291	5.370146209
0	3.628916522	0	1.078398548	0
57.65929211	44.4542274	77.63864096	36.66555065	47.25728664
15.37581123	29.93856131	15.72428171	19.41117387	42.96116967
62.46423311	29.03133218	32.43133104	29.11676081	30.07281877
2.882964605	0	0.982767607	0	1.074029242
64.38620952	73.48555957	48.15561275	46.37113758	85.92233934
53.8153393	29.93856131	29.48302821	26.95996371	53.70146209
0.960988202	0	0.982767607	0	0
0	0	2.948302821	1.078398548	0
22.10272864	11.7939787	8.844908464	5.391992742	9.666263176
0	6.350603914	2.948302821	0	5.370146209
101.8647494	116.1253287	98.27676071	117.5454418	109.5509827
107.6306786	150.6000357	129.7253241	162.8381808	114.9211289
55.7373157	64.41326827	29.48302821	20.48957242	18.25849711
238.325074	239.5084905	351.8308034	345.0875355	334.0230942
122.0455016	116.1253287	107.1216692	89.50707952	109.5509827
0	0	4.913838036	1.078398548	46.1832574
0	0	0	0	0
0	0	0	0	0
42.28348088	67.13495566	70.75926771	34.50875355	15.03640938

For Review Only

40.36150447	51.71206044	54.05221839	60.39031871	51.5534036
3.843952807	0	4.913838036	3.235195645	7.518204692
2.882964605	5.443374783	11.79321129	1.078398548	2.148058484
62.46423311	90.72291305	62.89712686	90.58547807	45.10922815
0	2.721687392	0	3.235195645	0
32.67359886	31.75301957	39.31070429	44.21434049	55.84952057
17.29778763	26.30964479	22.60365496	38.82234774	54.77549133
83.60597355	98.88797523	103.1905987	86.27188388	73.03398844
53.8153393	62.59881001	64.86266207	102.4478621	76.25607616
41.32249268	32.6602487	12.77597889	104.6046592	61.21966678
40.36150447	29.93856131	16.70704932	26.95996371	40.81311119
53.8153393	72.57833044	30.46579582	24.80316661	60.14563754
383.4342925	266.7253644	224.0710144	270.6780357	273.8774566
344.9947644	293.9422383	241.7608314	255.580456	350.1335328
77.84004434	117.939787	82.552479	73.3311013	99.88471948
0	0	0	0	0
4.804941009	3.628916522	16.70704932	1.078398548	0
0	0	4.913838036	0	0
25.94668145	51.71206044	54.05221839	19.41117387	46.1832574
131.6553836	231.3434283	271.2438596	408.7130499	213.7318191
101.8647494	56.24820609	138.5702326	140.1918113	236.2864332
0	21.77349913	17.68981693	20.48957242	47.25728664
15.37581123	4.536145653	9.827676071	9.705586936	5.370146209
49.97138649	30.84579044	69.77650011	58.23352162	47.25728664
10.57087022	9.072291305	1.965535214	22.64636952	12.8883509
9.609882018	30.84579044	12.77597889	32.35195645	32.22087725
24.02470504	21.77349913	28.50026061	37.7439492	31.14684801
45.16644548	42.63976914	42.25900711	81.95828968	38.6650527
20.18075224	26.30964479	25.55195779	12.94078258	28.99878953
25.94668145	14.51566609	31.44856343	32.35195645	41.88714043
73.99609154	292.12778	120.8804157	459.3977816	56.92354981
47.08842189	43.54699827	62.89712686	128.3294273	89.14442707
0	0	0	5.391992742	0
160.4850297	167.8373891	175.9154017	149.8973982	131.0315675
82.64498535	118.8470161	121.8631833	124.0158331	71.9599592
65.34719772	74.3927887	52.08668318	85.19348533	86.99636858
10.57087022	9.072291305	8.844908464	11.86238403	8.592233934
27.86865785	0	6.87937325	8.627188388	13.96238014
0	0	6.87937325	0	0
28.82964605	12.70120783	39.31070429	33.430355	8.592233934
835.0987473	420.0470874	13.7587465	2.156797097	2.148058484
0	0	0.982767607	1.078398548	1.074029242
0	0	11.79321129	1.078398548	5.370146209
0	0	9.827676071	0	0
127.8114308	230.4361992	154.2945143	182.2493547	236.2864332
2146.847643	2031.286023	1268.752981	1675.831344	1816.183448
1374.213129	1453.381067	1509.531045	1429.956475	1837.664033
4.804941009	0	6.87937325	0	0
0	2.721687392	0	0	5.370146209
154.7191005	135.1771404	82.552479	119.7022389	95.58860252

For Review Only

123.0064898	133.3626822	136.6046974	127.2510287	161.1043863
44.20545728	49.89760218	63.87989446	42.05754339	24.70267256
1.921976404	0	4.913838036	6.470391291	0
319.048083	330.2314035	340.0375921	317.0491732	297.5061
0	0	0	10.78398548	0
416.1078914	392.8302135	519.8840642	372.0474992	351.2075621
228.715192	205.9410126	256.5023455	290.0892095	240.5825502
5.765929211	9.979520436	1.965535214	18.33277532	15.03640938
5.765929211	9.979520436	3.931070429	26.95996371	16.11043863
0	165.1157018	0.982767607	1.078398548	21.48058484
163.3679943	151.5072648	167.0704932	88.42868097	105.2548657
20.18075224	21.77349913	44.22454232	43.13594194	23.62864332
30.75162246	48.08314392	70.75926771	37.7439492	67.66384223
32.67359886	19.05181174	20.63811975	15.09757968	17.18446787
28.82964605	31.75301957	47.17284514	30.19515936	48.33131588
35.55656347	39.91808174	40.29347189	28.03836226	45.10922815
10.57087022	6.350603914	16.70704932	7.548789839	6.444175451
31.71261066	33.56747783	39.31070429	42.05754339	39.73908194
0.960988202	0	0.982767607	3.235195645	0
23.06371684	34.47470696	75.67310575	15.09757968	4.296116967
0	8.165062175	7.862140857	3.235195645	0
17.29778763	16.33012435	7.862140857	5.391992742	8.592233934
2.882964605	3.628916522	3.931070429	3.235195645	0
324.8140122	326.602487	393.1070429	446.4569991	378.0582931
5.765929211	18.14458261	21.62088736	11.86238403	4.296116967
32.67359886	41.73254	45.20730993	20.48957242	11.81432166
6.726917412	19.95904087	25.55195779	12.94078258	18.25849711
48.04941009	62.59881001	49.13838036	70.09590565	54.77549133
227.7542038	165.1157018	170.018796	132.6430215	199.769439
1.921976404	0	35.37963386	0	41.88714043
54.7763275	46.26868566	53.06945079	66.86071	54.77549133
0	0	0.982767607	1.078398548	1.074029242
39.40051627	52.61928957	50.12114796	45.29273904	35.44296498
24.98569325	23.58795739	26.53472539	26.95996371	30.07281877
12.49284662	9.072291305	5.896605643	10.78398548	7.518204692
166.2509589	178.7241387	176.8981693	126.1726302	184.7330296
5.765929211	3.628916522	6.87937325	0	9.666263176
182.5877583	195.9614922	240.7780637	225.3852966	230.916287
11.53185842	22.68072826	26.53472539	3.235195645	5.370146209
0	0	0	0	0
0.960988202	0	0.982767607	0	3.222087725
81.68399715	41.73254	86.48354943	14.01918113	46.1832574
77.84004434	103.4241209	76.65587336	50.68473178	68.73787147
37.47853987	39.01085261	44.22454232	38.82234774	27.92476029
133.57736	135.1771404	188.6913806	171.4653692	173.9927372
0	0	1.965535214	0	0
181.6267701	188.7036592	118.9148805	179.014159	92.36651479
56.6983039	48.99037305	91.39738746	12.94078258	140.6978307
40.36150447	0	26.53472539	4.313594194	11.81432166
2.882964605	0	0.982767607	1.078398548	1.074029242

For Review Only

115.3185842	100.7024335	113.0182748	141.2702098	80.55219313
73.03510333	196.8687213	31.44856343	164.9949779	218.0279361
0	0	0	0	0
213.3393808	38.10362348	342.0031273	381.7530862	350.1335328
796.6592193	630.5242457	180.8292397	0	105.2548657
6.726917412	10.88674957	5.896605643	2.156797097	8.592233934
7.687905614	2.721687392	32.43133104	0	0
6.726917412	0	0	2.156797097	3.222087725
0	0	2.948302821	0	40.81311119
0	0	0	0	0
29.79063425	45.36145653	42.25900711	42.05754339	67.66384223
3.843952807	0	57.00052121	49.60633323	0
0	30.84579044	34.39686625	51.76313033	0
29.79063425	39.91808174	57.00052121	50.68473178	33.29490649
24.02470504	4.536145653	39.31070429	51.76313033	45.10922815
5.765929211	55.34097696	0	0	0
27.86865785	26.30964479	52.08668318	32.35195645	54.77549133
23.06371684	19.05181174	26.53472539	25.88156516	24.70267256
0	0	0	15.09757968	0
56.6983039	80.74339262	169.0360284	102.4478621	80.55219313
4.804941009	3.628916522	8.844908464	2.156797097	12.8883509
34.59557526	33.56747783	42.25900711	40.97914484	47.25728664
12.49284662	21.77349913	14.74151411	23.72476807	20.40655559
27.86865785	24.49518652	53.06945079	32.35195645	26.85073104
295.9843661	334.7675492	341.0203597	464.7897744	342.6153281
0	34.47470696	1.965535214	0	0
0.960988202	0	4.913838036	0	0
34.59557526	83.46508001	102.2078311	61.46871726	131.0315675
55.7373157	25.40241565	40.29347189	44.21434049	24.70267256
22.10272864	47.17591479	27.517493	15.09757968	44.03519891
111.4746314	108.8674957	144.4668382	134.7998186	164.326474
80.72300895	73.48555957	107.1216692	109.9966519	105.2548657
56.6983039	36.28916522	48.15561275	59.31192017	76.25607616
326.7359886	305.736217	196.5535214	199.5037315	223.3980823
49.01039829	46.26868566	32.43133104	42.05754339	37.59102346
0	9.979520436	12.77597889	3.235195645	6.444175451
84.56696176	67.13495566	351.8308034	32.35195645	326.5048895
0	31.75301957	1.965535214	0	11.81432166
0.960988202	6.350603914	665.33367	0	0
0	0	0	0	0
13.45383482	11.7939787	19.65535214	4.313594194	4.296116967
24.02470504	18.14458261	20.63811975	25.88156516	18.25849711
19.21976404	14.51566609	8.844908464	6.470391291	15.03640938
7.687905614	5.443374783	1.965535214	6.470391291	8.592233934
11.53185842	19.95904087	30.46579582	7.548789839	12.8883509
147.0311949	140.6205152	115.9665776	176.8573619	74.10801768
71.11312693	60.78435175	33.41409864	42.05754339	68.73787147
49.01039829	58.96989348	55.034986	31.27355791	64.44175451
33.63458706	39.01085261	41.2762395	9.705586936	18.25849711
0	5.443374783	1.965535214	2.156797097	0

For Review Only

775.5174788	619.6374962	741.0067758	698.8022594	701.3410949
402.6540565	377.4073183	321.3650075	263.1292458	273.8774566
0	0	158.2255847	0	0
0	0	1.965535214	0	0
175.8608409	205.0337835	240.7780637	212.444514	137.4757429
625.6033193	663.1844944	590.6433319	411.9482455	567.0874396
0.960988202	0.907229131	0	0	76.25607616
175.8608409	195.0542631	143.4840706	146.6622026	91.29248555
339.2288352	275.7976557	364.6067822	296.5596008	301.8022169
157.6020651	140.6205152	123.8287185	78.72309404	90.21845631
172.0168881	126.1048491	235.8642257	207.0525213	0
430.5227144	496.2543344	353.7963386	406.5562528	454.3143693
0	0	1.965535214	2.156797097	5.370146209
0	171.4663057	256.5023455	0	0
45.16644548	45.36145653	49.13838036	43.13594194	79.47816389
209.495428	217.7349913	282.0543032	272.8348328	273.8774566
69.19115053	100.7024335	67.81096489	119.7022389	99.88471948
0	0	0	0	0
56.6983039	74.3927887	59.94882404	78.72309404	156.8082693
0	0	3.931070429	4.313594194	0
59.58126851	80.74339262	88.44908464	30.19515936	75.18204692
0	0	269.2783244	221.0717024	200.8434682
907.1728625	983.4363775	1123.303375	1145.259258	876.4078613
0	0	151.3462115	166.0733765	196.5473512
164.3289825	170.5590765	181.8120073	216.7581082	199.769439
22.10272864	22.68072826	11.79321129	24.80316661	12.8883509
23.06371684	27.21687392	41.2762395	39.90074629	46.1832574
60.54225671	68.04218479	62.89712686	101.3694636	91.29248555
212.3783926	205.0337835	23.58642257	466.9465715	430.6857259
682.3016233	1050.571333	2030.397876	1982.096532	0
0	11.7939787	0.982767607	0	0
166.2509589	174.1879931	36.36240146	73.3311013	60.14563754
100.9037612	78.02170523	79.60417618	111.0750505	95.58860252
16.33679943	18.14458261	10.81044368	11.86238403	0
51.8933629	48.99037305	58.96605643	42.05754339	60.14563754
0	9.979520436	0.982767607	2.156797097	2.148058484
281.5695431	359.2627357	262.3989511	179.014159	352.2815913
187.3926993	181.4458261	218.1744088	174.7005649	227.6941993
74.95707974	63.50603914	81.56971139	85.19348533	93.44054403
34.59557526	36.28916522	9.827676071	26.95996371	36.51699422
0	39.01085261	32.43133104	32.35195645	13.96238014
0.960988202	0	0	6.470391291	4.296116967
399.7710919	411.8820253	360.6757118	411.9482455	461.832574
224.8712392	254.9313857	275.17493	298.7163979	272.8034274
288.2964605	376.5000892	365.5895499	374.2042963	365.1699422
141.2652657	130.6409948	175.9154017	283.6188182	153.5861816
314.243142	296.6639257	219.1571764	328.9115573	369.4660592
110.5136432	118.8470161	136.6046974	188.719746	219.1019653
0	0	30.46579582	35.5871521	34.36893574
0	75.30001783	0	84.11508678	262.063135

For Review Only

123.967478	150.6000357	143.4840706	146.6622026	165.4005032
0	22.68072826	26.53472539	22.64636952	10.74029242
481.4550891	296.6639257	519.8840642	235.0908836	424.2415505
73.99609154	63.50603914	89.43185225	91.66387662	79.47816389
192.1976404	160.5795561	100.2422959	127.2510287	154.6602108
74.95707974	111.5891831	107.1216692	118.6238403	142.8458892
0	3.628916522	6.87937325	0	1.074029242
0	0	4.913838036	0	0
313.2821538	325.6952579	395.0725781	364.4987094	298.5801292
73.99609154	78.92893436	91.39738746	107.8398548	118.1432166
39.40051627	21.77349913	27.517493	15.09757968	55.84952057
144.1482303	150.6000357	227.0193172	187.6413474	129.9575383
0.960988202	3.628916522	3.931070429	0	0
7757.096765	8629.56349	11457.10476	13172.63827	8322.652594
24.02470504	19.95904087	10.81044368	19.41117387	9.666263176
14.41482303	13.60843696	15.72428171	32.35195645	4.296116967
85.52794996	145.1566609	90.41461986	109.9966519	122.4393336
0	3.628916522	6.87937325	7.548789839	2.148058484
15.37581123	23.58795739	33.41409864	20.48957242	27.92476029
2.882964605	87.09399653	0	283.6188182	0
98.98178478	97.07351697	63.87989446	119.7022389	96.66263176
8.648893816	41.73254	106.1389016	17.25437678	0
98.98178478	82.55785088	82.552479	72.25270275	81.62622237
0	67.13495566	8.844908464	11.86238403	1.074029242
0	0	11.79321129	4.313594194	6.444175451
434.3666672	373.7784018	412.762395	428.1242237	297.5061
3.843952807	2.721687392	0	0	0
13.45383482	3.628916522	13.7587465	28.03836226	0
15.37581123	159.672327	57.00052121	67.93910855	24.70267256
0	16.33012435	12.77597889	0	16.11043863
147.9921831	163.3012435	170.018796	132.6430215	155.7342401
0	5.443374783	7.862140857	3.235195645	0
117.2405606	126.1048491	202.4501271	188.719746	204.0655559
24.02470504	0	2.948302821	69.0175071	1.074029242
8.648893816	0	0	0	0
0.960988202	0	1.965535214	0	1.074029242
0	0	0.982767607	0	0
6.726917412	9.072291305	8.844908464	0	7.518204692
10.57087022	22.68072826	10.81044368	8.627188388	3.222087725
63.42522132	67.13495566	79.60417618	92.74227517	65.51578375
24.02470504	23.58795739	14.74151411	19.41117387	15.03640938
28.82964605	128.8265365	110.069972	0	117.0691874
46.12743368	37.19639435	35.37963386	40.97914484	42.96116967
0	0	0.982767607	0	0
116.2795724	98.88797523	68.7937325	140.1918113	97.736661
19.21976404	18.14458261	13.7587465	23.72476807	46.1832574
105.7087022	134.2699113	91.39738746	79.80149259	153.5861816
73.03510333	66.22772653	102.2078311	77.64469549	61.21966678
255.6228617	213.1988457	123.8287185	163.9165794	169.6966202
11.53185842	8.165062175	11.79321129	3.235195645	15.03640938

11.53185842	4.536145653	3.931070429	17.25437678	9.666263176
1000.388718	904.5074431	641.7472475	897.2275923	644.4175451
1219.494028	1201.171369	1441.72008	1315.646229	1329.648201
89.37190276	196.8687213	158.2255847	114.3102461	120.2912751
0.960988202	0.907229131	0	0	781.893288
2.882964605	4.536145653	964.0950226	0	1079.399388
44.20545728	71.67110131	59.94882404	50.68473178	67.66384223
0	0	1.965535214	0	0
0	56.24820609	0	0	0
88.41091456	48.08314392	80.58694379	31.27355791	51.5534036
23.06371684	27.21687392	27.517493	28.03836226	41.88714043
109.552655	107.9602665	114.98381	121.859036	118.1432166
44.20545728	47.17591479	24.56919018	38.82234774	45.10922815
63.42522132	54.43374783	76.65587336	76.56629694	55.84952057
243.130015	402.809734	302.692423	93.82067372	151.4381231
50.93237469	53.5265187	85.50078182	90.58547807	67.66384223
160.4850297	195.0542631	172.9670989	197.3469344	202.9915267
0	0	0.982767607	2.156797097	1.074029242
23.06371684	17.23735348	7.862140857	56.07672452	54.77549133
91.29387917	135.1771404	159.2083524	126.1726302	97.736661
6.726917412	2.721687392	3.931070429	0	3.222087725
22.10272864	28.12410305	28.50026061	25.88156516	17.18446787
28.82964605	13.60843696	12.77597889	34.50875355	13.96238014
24.98569325	20.86627	22.60365496	40.97914484	12.8883509
75.91806794	52.61928957	50.12114796	65.78231146	48.33131588
0	0	16.70704932	14.01918113	32.22087725
23.06371684	25.40241565	20.63811975	22.64636952	17.18446787
88.41091456	84.37230914	114.0010424	57.15512307	76.25607616
1.921976404	2.721687392	2.948302821	0	1.074029242
53.8153393	41.73254	39.31070429	47.44953613	45.10922815
117.2405606	209.5699292	144.4668382	252.3452603	117.0691874
0	8.165062175	5.896605643	6.470391291	6.444175451
455.5084076	596.9567679	553.2981628	499.2985279	473.6468956
24.02470504	28.12410305	3.931070429	20.48957242	2.148058484
114.357596	145.1566609	120.8804157	112.153449	97.736661
105.7087022	0	0	25.88156516	0
12.49284662	4.536145653	18.67258454	6.470391291	5.370146209
20.18075224	15.42289522	31.44856343	14.01918113	16.11043863
15.37581123	6.350603914	6.87937325	2.156797097	11.81432166
10.57087022	13.60843696	16.70704932	23.72476807	22.55461408
33.63458706	37.19639435	199.5018242	171.4653692	38.6650527
0	8.165062175	5.896605643	0	0
2.882964605	0	0.982767607	4.313594194	2.148058484
165.2899707	137.8988278	183.7775425	161.7597823	103.1068072
0	0	20.63811975	0	0
13.45383482	6.350603914	13.7587465	19.41117387	22.55461408
1.921976404	0	0	0	0
20.18075224	0	47.17284514	19.41117387	35.44296498
0	0.907229131	0.982767607	0	5.370146209
0	0	0	4.313594194	0

For Review Only

105.7087022	82.55785088	63.87989446	30.19515936	82.70025162
15.37581123	21.77349913	36.36240146	20.48957242	27.92476029
8.648893816	8.165062175	3.931070429	7.548789839	13.96238014
71.11312693	95.25905871	74.69033814	64.70391291	66.58981299
0	0	0.982767607	0	0
118.2015488	88.90845479	76.65587336	40.97914484	84.8483101
0	0	3.931070429	0	0
0	0	0.982767607	1.078398548	1.074029242
0	0	0	0	0
6.726917412	1.814458261	0	0	7.518204692
9.609882018	11.7939787	11.79321129	10.78398548	11.81432166
18.25877583	22.68072826	19.65535214	17.25437678	20.40655559
18.25877583	22.68072826	20.63811975	17.25437678	20.40655559
0	0	1.965535214	0	0
0	0	0	0	1.074029242
0	29.93856131	41.2762395	53.91992742	53.70146209
0	0	0	0	9.666263176
0	0	0	1.078398548	0
57.65929211	45.36145653	34.39686625	50.68473178	40.81311119
0	0	0	0	0
37.47853987	52.61928957	52.08668318	61.46871726	70.88592996
0	0	3.931070429	5.391992742	5.370146209
13.45383482	11.7939787	11.79321129	12.94078258	0
343.072788	292.12778	402.9347189	409.7914484	436.0558722
251.7789089	218.6422205	225.053782	354.7931224	274.9514859
172.9778763	158.7650978	178.8637045	171.4653692	185.8070588
13.45383482	4.536145653	14.74151411	14.01918113	12.8883509
32.67359886	15.42289522	25.55195779	22.64636952	24.70267256
96.09882018	98.88797523	113.0182748	120.7806374	30.07281877
35.55656347	22.68072826	22.60365496	34.50875355	15.03640938
0.960988202	0	0	0	0
24.02470504	20.86627	38.32793668	19.41117387	30.07281877
61.50324491	78.02170523	65.84542968	60.39031871	59.0716083
28.82964605	21.77349913	12.77597889	23.72476807	23.62864332
190.2756639	174.1879931	248.6402046	259.8940502	227.6941993
82.64498535	117.939787	77.63864096	44.21434049	68.73787147
0	0	0	0	0
0	0	3.931070429	0	0
46.12743368	40.82531087	50.12114796	66.86071	76.25607616
499.7138649	600.5856844	697.7650011	745.173397	862.4454811
443.015561	449.9856487	532.6600431	586.6488104	421.0194628
0	0	0	0	0
3.843952807	0	0.982767607	0	1.074029242
50.93237469	61.69158088	81.56971139	58.23352162	56.92354981
1.921976404	0.907229131	0	0	0
30.75162246	23.58795739	32.43133104	26.95996371	13.96238014
796.6592193	886.3628605	1189.148805	1185.160005	1060.066862
67.26917412	63.50603914	70.75926771	37.7439492	69.81190071
14.41482303	10.88674957	7.862140857	22.64636952	4.296116967
535.2704284	697.6592014	894.3185225	699.8806579	589.6420537

For Review Only

42.28348088	37.19639435	15.72428171	24.80316661	18.25849711
96.09882018	65.3204974	56.01775361	103.5262607	81.62622237
23.06371684	29.03133218	18.67258454	47.44953613	57.99757905
14.41482303	34.47470696	27.517493	17.25437678	18.25849711
183.5487465	164.2084726	81.56971139	252.3452603	144.9939476
32.67359886	28.12410305	14.74151411	26.95996371	4.296116967
71.11312693	84.37230914	104.1733664	115.3886447	105.2548657
26.90766965	21.77349913	28.50026061	11.86238403	28.99878953
0	0	15.72428171	1.078398548	0
21.14174044	12.70120783	13.7587465	5.391992742	7.518204692
8.648893816	8.165062175	9.827676071	6.470391291	8.592233934
0	10.88674957	1.965535214	3.235195645	8.592233934
0	0	0	0	0
0	0	2.948302821	0	0
1.921976404	17.23735348	0	429.2026223	0
17.29778763	19.05181174	34.39686625	11.86238403	17.18446787
27.86865785	25.40241565	18.67258454	29.11676081	27.92476029
92.25486737	102.5168918	80.58694379	74.40949984	85.92233934
0	9.072291305	0	0	0
15.37581123	14.51566609	5.896605643	20.48957242	8.592233934
23.06371684	29.93856131	64.86266207	29.11676081	34.36893574
44.20545728	62.59881001	61.91435925	62.54711581	78.40413465
11.53185842	17.23735348	16.70704932	5.391992742	13.96238014
73.03510333	90.72291305	120.8804157	70.09590565	63.36772526
23.06371684	17.23735348	14.74151411	9.705586936	25.7767018
0	0	7.862140857	0	0
199.885546	267.6325935	317.4339371	242.6396734	302.8762462
114.357596	83.46508001	57.98328882	76.56629694	82.70025162
6.726917412	0	0	3.235195645	31.14684801
15.37581123	15.42289522	0.982767607	9.705586936	12.8883509
12.49284662	13.60843696	5.896605643	11.86238403	32.22087725
132.6163718	171.4663057	118.9148805	120.7806374	148.2160354
52.8543511	55.34097696	53.06945079	34.50875355	67.66384223
3.843952807	4.536145653	0	0	0
0	0	16.70704932	10.78398548	16.11043863
0	0	0	0	0
0	0	0	0	6.444175451
1459.741078	1560.434105	1194.062643	1989.645322	2966.468766
7.687905614	0	2.948302821	0	0
0	0	0	0	0
55.7373157	58.06266435	56.01775361	51.76313033	63.36772526
39.40051627	64.41326827	18.67258454	28.03836226	24.70267256
22.10272864	29.93856131	29.48302821	25.88156516	32.22087725
163.3679943	203.2193252	183.7775425	170.3869707	207.2876437
158.5630533	166.93016	187.708613	181.1709561	153.5861816
57.65929211	162.3940144	139.5530002	217.8365068	131.0315675
160.4850297	105.2385791	91.39738746	57.15512307	89.14442707
197.9635696	141.5277444	99.25952832	108.9182534	146.0679769
115.3185842	39.01085261	130.7080917	102.4478621	95.58860252
19.21976404	12.70120783	14.74151411	21.56797097	16.11043863

For Review Only

171.0558999	122.4759326	230.9503877	181.1709561	162.1784155
161.4460179	195.0542631	221.1227116	244.7964705	238.4344917
0	0	3.931070429	3.235195645	1.074029242
280.6085549	291.2205509	353.7963386	463.7113758	430.6857259
0	2.721687392	0	0	0
135.4993364	161.4867852	143.4840706	91.66387662	167.5485617
9.609882018	17.23735348	10.81044368	10.78398548	3.222087725
416.1078914	335.6747783	308.5890286	237.2476807	0
0	0	0	0	0
33.63458706	30.84579044	36.36240146	67.93910855	51.5534036
2.882964605	1.814458261	0	0	3.222087725
0	0.907229131	2.948302821	0	1.074029242
500.6748531	386.4796096	513.9874585	561.8456437	426.389609
909.0948389	970.7351697	1177.355593	1151.72965	848.483101
674.6137176	754.8146366	685.9717898	700.9590565	366.2439714
13.45383482	15.42289522	8.844908464	11.86238403	4.296116967
16.33679943	41.73254	22.60365496	16.17597823	17.18446787
0	9.979520436	8.844908464	1.078398548	4.296116967
0	0	0	0	0
25.94668145	7.257833044	25.55195779	2.156797097	2.148058484
7.687905614	7.257833044	20.63811975	12.94078258	19.33252635
0	0	0	0	0
40.36150447	62.59881001	10.81044368	24.80316661	166.4745325
0	0	0	0	0
0	0	1.965535214	4.313594194	0
0	0.907229131	0	0	0
17.29778763	5.443374783	10.81044368	7.548789839	4.296116967
4.804941009	0	7.862140857	3.235195645	13.96238014
0	0	0	21.56797097	5.370146209
0	8.165062175	3.931070429	0	0
35.55656347	48.08314392	45.20730993	33.430355	41.88714043
2.882964605	0	0	0	0
43.24446908	75.30001783	40.29347189	74.40949984	53.70146209
13.45383482	18.14458261	15.72428171	8.627188388	19.33252635
102.8257376	127.0120783	61.91435925	215.6797097	260.9891057
0	0	0	0	6.444175451
0	0	7.862140857	0	7.518204692
328.657965	329.3241744	148.3979087	306.2651878	280.3216321
104.747714	61.69158088	55.034986	35.5871521	52.62743285
165.2899707	212.2916165	205.3984299	189.7981445	162.1784155
1.921976404	3.628916522	0	0	0
229.6761802	192.3325757	71.74203532	136.9566157	109.5509827
149.9141595	130.6409948	137.587465	157.4461881	180.4369126
196.0415932	196.8687213	227.0193172	155.289391	149.2900646
104.747714	106.1458083	110.069972	57.15512307	78.40413465
304.63326	395.5519009	266.3300215	354.7931224	313.6165386
0	0	3.931070429	1.078398548	0
738.038939	667.7206401	534.6255783	484.2009483	645.4915743
6.726917412	9.072291305	24.56919018	33.430355	7.518204692
0	0	0	0	0

For Review Only

1.921976404	4.536145653	6.87937325	0	5.370146209
143.1872421	100.7024335	0	0	0
0.960988202	0.907229131	0	0	0
177.7828173	126.1048491	199.5018242	166.0733765	122.4393336
0	64.41326827	114.0010424	51.76313033	27.92476029
46.12743368	72.57833044	65.84542968	59.31192017	74.10801768
111.4746314	93.44460045	97.29399311	136.9566157	69.81190071
126.8504426	96.16628784	95.32845789	106.7614563	107.4029242
106.6696904	94.35182958	114.98381	143.4270069	133.179626
227.7542038	315.7157374	335.123754	293.3244052	198.6954097
56.6983039	0	68.7937325	161.7597823	94.51457327
18.25877583	0.907229131	0	42.05754339	0
21.14174044	18.14458261	34.39686625	26.95996371	35.44296498
0	17.23735348	33.41409864	0	0
5.765929211	0	0.982767607	2.156797097	0
36.51755167	20.86627	63.87989446	58.23352162	53.70146209
50.93237469	58.06266435	21.62088736	16.17597823	70.88592996
0	0	0	0	0
10.57087022	30.84579044	18.67258454	10.78398548	9.666263176
71.11312693	68.94941392	71.74203532	128.3294273	92.36651479
2.882964605	0.907229131	0	0	0
7.687905614	7.257833044	0	0	0
70.15213873	78.92893436	40.29347189	36.66555065	113.8470996
101.8647494	96.16628784	82.552479	71.1743042	50.47937436
85.52794996	83.46508001	124.8114861	126.1726302	64.44175451
0	31.75301957	1.965535214	2.156797097	7.518204692
122.0455016	132.4554531	122.8459509	191.9549416	110.6250119
0	0	13.7587465	0	6.444175451
112.4356196	150.6000357	167.0704932	113.2318476	163.2524447
276.7646021	301.2000713	256.5023455	295.4812023	346.9114451
138.3823011	113.4036413	50.12114796	147.7406011	169.6966202
186.4317111	229.52897	134.6391622	190.8765431	74.10801768
1.921976404	21.77349913	41.2762395	40.97914484	168.622591
187.3926993	212.2916165	241.7608314	230.7772894	215.8798776
1.921976404	625.9881001	548.3843248	0	0
223.910251	316.6229666	334.1409864	234.012485	210.5097314
112.4356196	135.1771404	87.46631704	97.05586936	179.3628834
168.1729353	153.3217231	105.156134	145.583804	107.4029242
89.37190276	52.61928957	27.517493	50.68473178	54.77549133
133.57736	146.06389	162.1566552	191.9549416	137.4757429
0	13.60843696	2.948302821	0	0
67.26917412	99.79520436	86.48354943	112.153449	92.36651479
66.30818592	71.67110131	117.9321129	69.0175071	75.18204692
24.98569325	10.88674957	38.32793668	49.60633323	11.81432166
0.960988202	2.721687392	0	0	0
22.10272864	13.60843696	8.844908464	11.86238403	9.666263176
43.24446908	28.12410305	41.2762395	45.29273904	27.92476029
0	0	0	1.078398548	2.148058484
660.1988946	467.2230022	891.3702197	832.5236794	253.4709011
59.58126851	42.63976914	87.46631704	33.430355	37.59102346

For Review Only

9.609882018	9.979520436	13.7587465	23.72476807	6.444175451
630.4082604	694.937514	929.6981564	891.8355996	765.7828494
286.3744841	255.8386148	313.5028667	360.1851152	229.8422577
255.6228617	234.0651157	275.17493	224.3068981	205.1395852
2.882964605	0	0	0	0
112.4356196	88.00122566	103.1905987	153.1325939	113.8470996
158.5630533	159.672327	155.2772819	194.1117387	150.3640938
0	0	0.982767607	1.078398548	113.8470996
17.29778763	24.49518652	54.05221839	35.5871521	18.25849711
46.12743368	62.59881001	65.84542968	60.39031871	10.74029242
8.648893816	2.721687392	4.913838036	17.25437678	11.81432166
517.9726408	718.5254714	839.2835365	986.7346718	762.5607616
3.843952807	0	0	5.391992742	1.074029242
91.29387917	133.3626822	129.7253241	176.8573619	155.7342401
228.715192	227.7145118	249.6229722	257.7372531	184.7330296
0	0.907229131	61.91435925	54.99832597	65.51578375
2.882964605	1.814458261	0	0	0
4.804941009	3.628916522	0.982767607	1.078398548	0
119.162537	113.4036413	111.0527396	154.2109924	125.6614213
70.15213873	112.4964122	46.19007754	109.9966519	94.51457327
7.687905614	5.443374783	14.74151411	15.09757968	4.296116967
4.804941009	6.350603914	8.844908464	2.156797097	0
86.48893816	68.04218479	122.8459509	152.0541953	85.92233934
4.804941009	15.42289522	14.74151411	11.86238403	13.96238014
80.72300895	113.4036413	126.7770213	170.3869707	96.66263176
117.2405606	83.46508001	106.1389016	73.3311013	67.66384223
163.3679943	145.1566609	29.48302821	17.25437678	27.92476029
0	0	0	0	0
3.843952807	0	0	1.078398548	0
150.8751477	101.6096626	111.0527396	100.291065	142.8458892
40.36150447	30.84579044	20.63811975	29.11676081	20.40655559
347.877729	347.468757	359.6929442	411.9482455	314.6905678
24.98569325	21.77349913	14.74151411	18.33277532	0
0	0	0	0	0
57.65929211	111.5891831	106.1389016	84.11508678	98.81069024
74.95707974	48.08314392	37.34516907	58.23352162	54.77549133
129.7334072	87.09399653	51.10391557	101.3694636	138.5497722
202.7685106	263.0964479	184.7603101	189.7981445	99.88471948
86.48893816	76.20724696	91.39738746	99.21266646	71.9599592
0	0	0	0	0
11.53185842	29.03133218	64.86266207	0	62.29369602
64.38620952	92.53737131	97.29399311	91.66387662	82.70025162
69.19115053	84.37230914	113.0182748	60.39031871	124.587392
45.16644548	71.67110131	55.034986	131.5646229	51.5534036
0	0	33.41409864	14.01918113	19.33252635
51.8933629	53.5265187	68.7937325	48.52793468	75.18204692
43.24446908	91.63014218	57.00052121	98.13426791	62.29369602
180.6657819	182.3530552	198.5190566	79.80149259	94.51457327
19.21976404	11.7939787	3.931070429	9.705586936	7.518204692
0	0	0	0	0

For Review Only

0	656.8338905	3002.35504	3038.92711	3243.56831
0	3.628916522	0	1.078398548	0
10.57087022	9.072291305	11.79321129	15.09757968	3.222087725
24.98569325	35.38193609	32.43133104	15.09757968	44.03519891
4.804941009	0	2.948302821	1.078398548	5.370146209
0	0.907229131	0	5.391992742	54.77549133
3592.173898	4001.787695	5343.30748	5108.373924	4449.703149
0	0	0	0	0
46.12743368	75.30001783	67.81096489	84.11508678	77.33010541
169.1339235	166.0229309	165.104958	116.4670432	66.58981299
0	0	0	0	1.074029242
0	0	0	0	0
149.9141595	49.89760218	138.5702326	131.5646229	126.7354505
0	0	0	0	0
71.11312693	52.61928957	57.00052121	59.31192017	53.70146209
21.14174044	39.91808174	13.7587465	32.35195645	20.40655559
0	0	1.965535214	7.548789839	15.03640938
3.843952807	7.257833044	1.965535214	2.156797097	5.370146209
7.687905614	13.60843696	10.81044368	4.313594194	3.222087725
7.687905614	0	85.50078182	0	1.074029242
111.4746314	103.4241209	59.94882404	61.46871726	118.1432166
10.57087022	7.257833044	7.862140857	11.86238403	7.518204692
74.95707974	60.78435175	71.74203532	36.66555065	67.66384223
24.98569325	40.82531087	16.70704932	16.17597823	17.18446787
70.15213873	28.12410305	45.20730993	45.29273904	69.81190071
238.325074	196.8687213	281.0715356	162.8381808	112.7730704
71.11312693	105.2385791	94.34569029	83.03668823	124.587392
38.43952807	33.56747783	68.7937325	67.93910855	0
51.8933629	40.82531087	40.29347189	43.13594194	49.40534512
34.59557526	99.79520436	0	80.87989113	0
0	5.443374783	2.948302821	1.078398548	2.148058484
24.02470504	10.88674957	16.70704932	23.72476807	21.48058484
122.0455016	131.5482239	58.96605643	80.87989113	225.5461408
72.07411513	84.37230914	59.94882404	56.07672452	51.5534036
41.32249268	49.89760218	38.32793668	51.76313033	38.6650527
83.60597355	77.1144761	56.01775361	90.58547807	103.1068072
150.8751477	180.538597	157.2428171	145.583804	236.2864332
149.9141595	128.8265365	107.1216692	89.50707952	54.77549133
29.79063425	29.93856131	32.43133104	17.25437678	19.33252635
16.33679943	0	13.7587465	28.03836226	44.03519891
41.32249268	69.85664305	106.1389016	81.95828968	61.21966678
6.726917412	0	1.965535214	3.235195645	2.148058484
199.885546	182.3530552	99.25952832	124.0158331	99.88471948
7.687905614	12.70120783	11.79321129	3.235195645	6.444175451
0	0	0.982767607	1.078398548	1.074029242
71.11312693	62.59881001	57.00052121	36.66555065	78.40413465
35.55656347	25.40241565	6.87937325	16.17597823	11.81432166
5.765929211	0.907229131	1.965535214	1.078398548	6.444175451
9.609882018	12.70120783	8.844908464	3.235195645	4.296116967
34.59557526	43.54699827	0	242.6396734	0

For Review Only

1.921976404	0	0	0	0
2.882964605	5.443374783	0	0	0
34.59557526	20.86627	36.36240146	17.25437678	20.40655559
3.843952807	0	0.982767607	1.078398548	0
0	0	0	0	0
72.07411513	91.63014218	187.708613	76.56629694	41.88714043
325.7750004	259.4675313	108.1044368	237.2476807	257.767018
24.02470504	25.40241565	45.20730993	40.97914484	35.44296498
9.609882018	0.907229131	26.53472539	25.88156516	24.70267256
132.6163718	103.4241209	92.38015507	89.50707952	161.1043863
157.6020651	144.2494318	106.1389016	73.3311013	147.1420061
0	0	0	2.156797097	3.222087725
83.60597355	62.59881001	80.58694379	45.29273904	50.47937436
0	5.443374783	0	0	13.96238014
0	2.721687392	0	0	3.222087725
58.62028031	55.34097696	61.91435925	64.70391291	79.47816389
39.40051627	34.47470696	45.20730993	44.21434049	35.44296498
0	8.165062175	69.77650011	38.82234774	10.74029242
18.25877583	28.12410305	33.41409864	30.19515936	26.85073104
19.21976404	16.33012435	19.65535214	21.56797097	18.25849711
39.40051627	45.36145653	35.37963386	57.15512307	70.88592996
19.21976404	18.14458261	16.70704932	18.33277532	19.33252635
73.03510333	101.6096626	81.56971139	86.27188388	74.10801768
30.75162246	34.47470696	37.34516907	32.35195645	26.85073104
130.6943954	159.672327	164.1221904	111.0750505	138.5497722
0	0	0.982767607	4.313594194	1.074029242
62.46423311	61.69158088	24.56919018	10.78398548	17.18446787
39.40051627	36.28916522	61.91435925	29.11676081	20.40655559
19.21976404	29.03133218	35.37963386	22.64636952	24.70267256
45.16644548	39.01085261	69.77650011	46.37113758	73.03398844
26.90766965	45.36145653	19.65535214	20.48957242	38.6650527
0	10.88674957	8.844908464	4.313594194	6.444175451
5.765929211	0.907229131	0	0	0
45.16644548	0	57.98328882	102.4478621	97.736661
48.04941009	67.13495566	36.36240146	36.66555065	31.14684801
0	0	0	0	0
0	0.907229131	0	0	1.074029242
65.34719772	33.56747783	64.86266207	45.29273904	36.51699422
126.8504426	182.3530552	46.19007754	114.3102461	178.2888541
184.5097347	166.0229309	132.673627	162.8381808	129.9575383
75.91806794	89.81568392	152.3289791	69.0175071	68.73787147
31.71261066	39.91808174	19.65535214	40.97914484	22.55461408
85.52794996	115.2180996	73.70757054	76.56629694	50.47937436
46.12743368	37.19639435	16.70704932	15.09757968	10.74029242
50.93237469	59.87712262	19.65535214	28.03836226	16.11043863
50.93237469	78.92893436	90.41461986	36.66555065	39.73908194
26.90766965	37.19639435	36.36240146	65.78231146	54.77549133
10.57087022	17.23735348	18.67258454	22.64636952	17.18446787
3.843952807	0	4.913838036	3.235195645	6.444175451
350.7606936	0	19.65535214	9.705586936	78.40413465

For Review Only

227.7542038	217.7349913	157.2428171	105.6830577	172.9187079
0.960988202	9.072291305	21.62088736	0	0
97.05980838	67.13495566	85.50078182	83.03668823	83.77428086
3.843952807	0	0.982767607	0	3.222087725
266.1937319	299.3856131	155.2772819	167.151775	216.9539068
12.49284662	0	2.948302821	2.156797097	4.296116967
1.921976404	3.628916522	0	0	0
123.967478	363.7988813	248.6402046	209.2093184	70.88592996
16.33679943	22.68072826	30.46579582	24.80316661	15.03640938
267.1547201	223.1783661	287.9509089	217.8365068	228.7682285
51.8933629	54.43374783	36.36240146	52.84152887	23.62864332
55.7373157	40.82531087	29.48302821	46.37113758	51.5534036
5.765929211	0	4.913838036	5.391992742	0
93.21585557	92.53737131	101.2250635	61.46871726	22.55461408
270.0376847	332.0458618	274.1921624	270.6780357	152.5121523
50.93237469	32.6602487	19.65535214	11.86238403	25.7767018
77.84004434	100.7024335	88.44908464	121.859036	97.736661
9.609882018	65.3204974	5.896605643	2.156797097	99.88471948
31.71261066	46.26868566	37.34516907	188.719746	121.3653043
15.37581123	22.68072826	32.43133104	20.48957242	19.33252635
29.79063425	45.36145653	38.32793668	40.97914484	32.22087725
24.98569325	33.56747783	75.67310575	0	71.9599592
9.609882018	20.86627	19.65535214	20.48957242	13.96238014
19.21976404	19.95904087	40.29347189	29.11676081	23.62864332
165.2899707	145.1566609	155.2772819	228.6204923	208.3616729
21.14174044	34.47470696	13.7587465	21.56797097	8.592233934
2.882964605	0	0	0	3.222087725
12.49284662	23.58795739	23.58642257	30.19515936	28.99878953
0	0	0.982767607	0	0
11.53185842	0	9.827676071	18.33277532	6.444175451
125.8894544	174.1879931	233.8986905	152.0541953	202.9915267
105.7087022	140.6205152	107.1216692	117.5454418	86.99636858
0	0	0	0	0
291.1794251	331.1386326	360.6757118	424.8890281	332.9490649
916.7827445	1044.220729	1307.080917	1281.137476	955.8860252
2.882964605	0	5.896605643	4.313594194	0
166.2509589	59.87712262	141.5185354	91.66387662	122.4393336
344.9947644	292.12778	599.4882404	488.5145425	342.6153281
0	0	0	0	0
18.25877583	36.28916522	26.53472539	44.21434049	35.44296498
19.21976404	0	26.53472539	15.09757968	52.62743285
438.21062	0	0.982767607	221.0717024	56.92354981
13.45383482	6.350603914	17.68981693	10.78398548	12.8883509
316.1651184	336.5820074	439.2971204	324.5979631	338.3192112
125.8894544	71.67110131	87.46631704	135.8782171	62.29369602
0	0	0.982767607	0	0
74.95707974	99.79520436	103.1905987	79.80149259	140.6978307
214.300369	234.0651157	320.3822399	353.7147239	337.2451819
33.63458706	58.06266435	34.39686625	34.50875355	42.96116967
49.97138649	32.6602487	36.36240146	54.99832597	60.14563754

For Review Only

13.45383482	0	1.965535214	7.548789839	0
64.38620952	55.34097696	11.79321129	7.548789839	11.81432166
17.29778763	29.03133218	42.25900711	29.11676081	23.62864332
0	0	0.982767607	0	0
25.94668145	27.21687392	0	0	0
0	0	0	0	0
0	5.443374783	1.965535214	0	0
3.843952807	0.907229131	0	1.078398548	1.074029242
1.921976404	5.443374783	3.931070429	0	0
0	20.86627	0	24.80316661	9.666263176
129.7334072	105.2385791	110.069972	127.2510287	73.03398844
0	36.28916522	46.19007754	1.078398548	1.074029242
5.765929211	0	2.948302821	4.313594194	0
53.8153393	52.61928957	22.60365496	37.7439492	67.66384223
59.58126851	79.83616349	123.8287185	103.5262607	84.8483101
9.609882018	0	0	0	16.11043863
104.747714	50.80483131	40.29347189	197.3469344	85.92233934
156.6410769	164.2084726	82.552479	140.1918113	187.9551173
95.13783197	131.5482239	67.81096489	79.80149259	69.81190071
7.687905614	12.70120783	17.68981693	17.25437678	9.666263176
37.47853987	36.28916522	4.913838036	8.627188388	16.11043863
3.843952807	5.443374783	3.931070429	2.156797097	0
3.843952807	4.536145653	2.948302821	4.313594194	3.222087725
0	0	3.931070429	2.156797097	0
19.21976404	7.257833044	42.25900711	38.82234774	30.07281877
228.715192	155.1361813	174.9326341	132.6430215	178.2888541
138.3823011	91.63014218	126.7770213	204.8957242	100.9587487
30.75162246	24.49518652	30.46579582	36.66555065	19.33252635
13.45383482	16.33012435	8.844908464	14.01918113	8.592233934
23.06371684	21.77349913	2.948302821	24.80316661	18.25849711
13.45383482	19.05181174	13.7587465	19.41117387	30.07281877
41.32249268	26.30964479	47.17284514	49.60633323	45.10922815
33.63458706	32.6602487	13.7587465	18.33277532	39.73908194
23.06371684	25.40241565	42.25900711	20.48957242	32.22087725
4.804941009	6.350603914	2.948302821	0	4.296116967
0	0	0.982767607	2.156797097	0
150.8751477	119.7542452	70.75926771	133.72142	82.70025162
162.4070061	148.7855774	135.6219298	76.56629694	239.5085209
16.33679943	5.443374783	5.896605643	1.078398548	6.444175451
80.72300895	112.4964122	152.3289791	109.9966519	135.3276845
193.1586286	188.7036592	152.3289791	161.7597823	128.883509
18.25877583	11.7939787	5.896605643	4.313594194	22.55461408
25.94668145	19.95904087	35.37963386	22.64636952	26.85073104
11.53185842	14.51566609	17.68981693	19.41117387	23.62864332
0	66.22772653	0	0	0
77.84004434	62.59881001	98.27676071	79.80149259	74.10801768
45.16644548	42.63976914	16.70704932	22.64636952	21.48058484
189.3146757	161.4867852	230.9503877	112.153449	109.5509827
21.14174044	56.24820609	48.15561275	58.23352162	8.592233934
19.21976404	5.443374783	17.68981693	26.95996371	7.518204692

For Review Only

11.53185842	19.95904087	33.41409864	26.95996371	19.33252635
24.98569325	33.56747783	27.517493	32.35195645	19.33252635
46.12743368	54.43374783	38.32793668	47.44953613	25.7767018
96.09882018	73.48555957	52.08668318	49.60633323	59.0716083
28.82964605	0	35.37963386	0	0
0	0	1.965535214	0	0
0	4.536145653	6.87937325	0	1.074029242
11.53185842	24.49518652	7.862140857	15.09757968	0
281.5695431	397.3663592	740.0240082	185.4845503	847.4090717
928.3146029	764.794157	4280.935697	421.6538324	624.0109895
0	13.60843696	0	1.078398548	5.370146209
38.43952807	28.12410305	32.43133104	20.48957242	35.44296498
129.7334072	73.48555957	90.41461986	90.58547807	98.81069024
3.843952807	2.721687392	0	3.235195645	3.222087725
500.6748531	597.863997	743.9550786	810.9557084	794.7816389
507.4017705	587.8844766	836.3352337	781.8389476	802.2998436
188.3536875	192.3325757	269.2783244	271.7564342	230.916287
5.765929211	6.350603914	19.65535214	3.235195645	12.8883509
328.657965	264.9109061	423.5728387	542.4344699	482.2391295
4.804941009	3.628916522	3.931070429	3.235195645	6.444175451
120.1235252	98.88797523	156.2600495	197.3469344	154.6602108
0	5.443374783	0	0	2.148058484
83.60597355	90.72291305	182.7947749	126.1726302	105.2548657
1774.945209	0	527.746205	2105.033967	11.81432166
25.94668145	30.84579044	42.25900711	63.62551436	30.07281877
0	19.05181174	13.7587465	6.470391291	5.370146209
1.921976404	0	9.827676071	0	1.074029242
27.86865785	0	0	0	22.55461408
0	0	0	0	0
128.772419	82.55785088	82.552479	87.35028243	73.03398844
116.2795724	121.5687035	113.0182748	92.74227517	133.179626
64.38620952	56.24820609	50.12114796	66.86071	74.10801768
481.4550891	363.7988813	296.7958174	340.7739413	218.0279361
1.921976404	0.907229131	0	0	1.074029242
0	0	0	0	27.92476029
12.49284662	11.7939787	14.74151411	4.313594194	16.11043863
261.3887909	7.257833044	164.1221904	0	0
2.882964605	5.443374783	0	0	0
6.726917412	9.072291305	6.87937325	5.391992742	8.592233934
95.13783197	107.0530374	108.1044368	65.78231146	76.25607616
0	8.165062175	15.72428171	26.95996371	45.10922815
65.34719772	58.96989348	70.75926771	72.25270275	89.14442707
83.60597355	85.27953827	120.8804157	106.7614563	98.81069024
26.90766965	14.51566609	10.81044368	44.21434049	9.666263176
83.60597355	101.6096626	112.0355072	135.8782171	163.2524447
285.4134959	257.6530731	306.6234934	280.3836226	294.2840122
0	142.4349735	0	0	0
89.37190276	0	10.81044368	85.19348533	204.0655559
12.49284662	29.93856131	9.827676071	14.01918113	16.11043863
270.0376847	359.2627357	361.6584794	396.8506658	295.3580415

For Review Only

0	4.536145653	2.948302821	0	0
60.54225671	45.36145653	53.06945079	75.48789839	41.88714043
30.75162246	32.6602487	41.2762395	0	41.88714043
76.87905614	96.16628784	102.2078311	108.9182534	126.7354505
40.36150447	66.22772653	61.91435925	0	46.1832574
59.58126851	65.3204974	100.2422959	89.50707952	120.2912751
206.6124634	207.7554709	209.3295003	285.7756153	378.0582931
71.11312693	58.06266435	148.3979087	111.0750505	59.0716083
42.28348088	52.61928957	114.98381	120.7806374	97.736661
0.960988202	0	0.982767607	1.078398548	0
80.72300895	90.72291305	117.9321129	77.64469549	124.587392
96.09882018	111.5891831	77.63864096	20.48957242	205.1395852
57.65929211	322.9735705	379.3482964	158.5245866	154.6602108
101.8647494	93.44460045	153.3117467	177.9357605	107.4029242
0	0	0.982767607	1.078398548	1.074029242
46.12743368	52.61928957	78.62140857	62.54711581	46.1832574
36.51755167	49.89760218	32.43133104	44.21434049	66.58981299
129.7334072	105.2385791	155.2772819	78.72309404	114.9211289
3.843952807	0	0	0	0
260.4278027	281.2410305	364.6067822	9.705586936	19.33252635
0	0	0	5.391992742	0
44.20545728	48.99037305	31.44856343	35.5871521	80.55219313
0.960988202	0	7.862140857	4.313594194	5.370146209
1957.532967	2177.349913	2224.003095	2372.476807	3542.148439
38.43952807	16.33012435	41.2762395	60.39031871	51.5534036
76.87905614	99.79520436	134.6391622	71.1743042	66.58981299
27.86865785	13.60843696	32.43133104	40.97914484	13.96238014
19.21976404	28.12410305	35.37963386	14.01918113	13.96238014
27.86865785	27.21687392	35.37963386	23.72476807	25.7767018
23.06371684	0	50.12114796	2.156797097	46.1832574
198.9245578	224.0855952	282.0543032	203.8173257	244.8786671
0	0	0	0	1.074029242
62.46423311	59.87712262	75.67310575	51.76313033	32.22087725
0	88.00122566	231.9331553	6.470391291	80.55219313
375.7463869	492.6254179	469.7629162	405.4778542	381.2803808
138.3823011	125.19762	244.7091342	202.7389271	0
88.41091456	87.09399653	184.7603101	128.3294273	60.14563754
56.6983039	60.78435175	44.22454232	81.95828968	92.36651479
0	0	94.34569029	85.19348533	95.58860252
16.33679943	35.38193609	19.65535214	19.41117387	30.07281877
23.06371684	26.30964479	64.86266207	44.21434049	41.88714043
0	313.9012792	33.41409864	32.35195645	31.14684801
65.34719772	65.3204974	100.2422959	58.23352162	83.77428086
8.648893816	8.165062175	11.79321129	10.78398548	6.444175451
171.0558999	230.4361992	248.6402046	234.012485	233.0643455
0.960988202	0	0	1.078398548	3.222087725
41.32249268	36.28916522	20.63811975	15.09757968	19.33252635
396.8881273	325.6952579	365.5895499	337.5387457	283.5437198
462.235325	587.8844766	471.7284514	458.3193831	592.8641414
0	0	294.8302821	0	0

For Review Only

0	18.14458261	0	0	0
69.19115053	111.5891831	115.9665776	119.7022389	102.032778
146.0702067	135.1771404	134.6391622	172.5437678	142.8458892
0	0	0	0	0
0	0	0	0	0
0	0	16.70704932	0	23.62864332
31.71261066	45.36145653	37.34516907	40.97914484	37.59102346
48.04941009	39.01085261	0	128.3294273	0
5.765929211	8.165062175	1.965535214	7.548789839	3.222087725
237.3640858	204.1265544	244.7091342	244.7964705	212.6577899
82.64498535	78.92893436	61.91435925	79.80149259	77.33010541
0	0	0.982767607	0	0
14.41482303	217.7349913	80.58694379	87.35028243	90.21845631
136.4603247	132.4554531	105.156134	153.1325939	140.6978307
54.7763275	63.50603914	69.77650011	65.78231146	76.25607616
0.960988202	0.907229131	0	0	410.2791704
373.8244105	466.3157731	492.3665712	478.8089555	492.979422
177.7828173	182.3530552	190.6569158	252.3452603	228.7682285
304.63326	391.0157553	316.4511695	248.0316661	252.3968718
22.10272864	26.30964479	29.48302821	46.37113758	39.73908194
69.19115053	59.87712262	88.44908464	85.19348533	57.99757905
96.09882018	127.0120783	95.32845789	152.0541953	160.030357
28.82964605	28.12410305	42.25900711	23.72476807	50.47937436
0.960988202	19.05181174	15.72428171	0	16.11043863
0	19.05181174	0	0	0
13.45383482	26.30964479	27.517493	33.430355	27.92476029
0	0	0.982767607	1.078398548	8.592233934
54.7763275	23.58795739	22.60365496	49.60633323	24.70267256
38.43952807	51.71206044	86.48354943	53.91992742	63.36772526
4.804941009	3.628916522	10.81044368	9.705586936	3.222087725
79.76202075	78.92893436	86.48354943	95.97747081	74.10801768
35.55656347	33.56747783	32.43133104	31.27355791	27.92476029
88.41091456	75.30001783	88.44908464	64.70391291	110.6250119
53.8153393	42.63976914	73.70757054	83.03668823	63.36772526
131.6553836	156.0434105	75.67310575	94.89907226	168.622591
131.6553836	157.8578687	125.7942537	184.4061518	119.2172458
52.8543511	61.69158088	55.034986	59.31192017	84.8483101
196.0415932	250.39524	248.6402046	229.6988908	156.8082693
99.94277298	91.63014218	105.156134	117.5454418	90.21845631
3.843952807	2.721687392	0	0	0
81.68399715	105.2385791	76.65587336	111.0750505	110.6250119
38.43952807	88.00122566	82.552479	40.97914484	31.14684801
203.7294988	262.1892187	205.3984299	187.6413474	189.0291465
0	9.072291305	0	0	0
0	0	0.982767607	0	1.074029242
73.99609154	58.06266435	49.13838036	109.9966519	76.25607616
66.30818592	75.30001783	88.44908464	58.23352162	82.70025162
127.8114308	163.3012435	61.91435925	135.8782171	151.4381231
223.910251	224.0855952	203.4328947	248.0316661	273.8774566
57.65929211	61.69158088	30.46579582	54.99832597	60.14563754

For Review Only

94.17684377	123.3831618	97.29399311	40.97914484	127.8094798
120.1235252	113.4036413	164.1221904	160.6813837	85.92233934
19.21976404	21.77349913	9.827676071	11.86238403	15.03640938
31.71261066	29.93856131	28.50026061	60.39031871	27.92476029
163.3679943	179.6313678	129.7253241	98.13426791	102.032778
179.7047937	177.8169096	132.673627	193.0333402	148.2160354
102.8257376	83.46508001	44.22454232	85.19348533	73.03398844
436.2886436	443.6350448	386.2276696	367.733905	315.7645971
123.967478	94.35182958	34.39686625	56.07672452	91.29248555
78.80103254	72.57833044	78.62140857	69.0175071	66.58981299
49.97138649	70.76387218	105.156134	113.2318476	64.44175451
17.29778763	16.33012435	16.70704932	10.78398548	11.81432166
121.0845134	124.2903909	157.2428171	189.7981445	150.3640938
0	50.80483131	12.77597889	34.50875355	0
5.765929211	5.443374783	17.68981693	2.156797097	2.148058484
21.14174044	24.49518652	23.58642257	11.86238403	35.44296498
47.08842189	49.89760218	39.31070429	28.03836226	69.81190071
96.09882018	145.1566609	166.0877256	198.4253329	167.5485617
0.960988202	0.907229131	113.0182748	124.0158331	105.2548657
124.9284662	113.4036413	67.81096489	91.66387662	151.4381231
70.15213873	53.5265187	78.62140857	59.31192017	83.77428086
0	0	0	0	0
24.98569325	15.42289522	21.62088736	25.88156516	13.96238014
57.65929211	45.36145653	99.25952832	76.56629694	34.36893574
0	48.08314392	44.22454232	39.90074629	64.44175451
0.960988202	0	3.931070429	0	0
38.43952807	44.4542274	15.72428171	33.430355	32.22087725
16.33679943	15.42289522	5.896605643	12.94078258	2.148058484
0	0	676.1441137	0	0
18.25877583	19.05181174	35.37963386	4.313594194	10.74029242
0.960988202	0.907229131	0	0	0
15.37581123	39.01085261	134.6391622	0	0
144.1482303	95.25905871	40.29347189	128.3294273	61.21966678
36.51755167	17.23735348	41.2762395	31.27355791	42.96116967
13.45383482	15.42289522	8.844908464	8.627188388	8.592233934
75.91806794	65.3204974	49.13838036	107.8398548	128.883509
16.33679943	14.51566609	13.7587465	16.17597823	25.7767018
20.18075224	12.70120783	33.41409864	20.48957242	44.03519891
201.8075224	195.0542631	151.3462115	200.58213	184.7330296
35.55656347	30.84579044	34.39686625	34.50875355	22.55461408
62.46423311	58.06266435	51.10391557	58.23352162	86.99636858
0	0	22.60365496	0	129.9575383
0	9.072291305	1.965535214	0	0
13.45383482	4.536145653	0	0	7.518204692
8.648893816	16.33012435	13.7587465	7.548789839	1.074029242
6.726917412	9.072291305	0.982767607	10.78398548	1.074029242
203.7294988	208.6627	311.5373315	307.3435863	163.2524447
13.45383482	20.86627	23.58642257	10.78398548	17.18446787
2.882964605	0	6.87937325	3.235195645	0
183.5487465	218.6422205	161.1738876	172.5437678	148.2160354

For Review Only

36.51755167	79.83616349	28.50026061	85.19348533	11.81432166
44.20545728	46.26868566	24.56919018	70.09590565	62.29369602
78.80103254	88.90845479	151.3462115	112.153449	67.66384223
14.41482303	4.536145653	8.844908464	7.548789839	9.666263176
36.51755167	39.91808174	46.19007754	56.07672452	26.85073104
215.2613572	202.3120961	205.3984299	198.4253329	220.1759946
133.57736	81.65062175	78.62140857	138.0350142	97.736661
5.765929211	29.03133218	22.60365496	0	0
31.71261066	36.28916522	72.72480293	39.90074629	41.88714043
0	0	0	0	3.222087725
70.15213873	95.25905871	74.69033814	97.05586936	70.88592996
90.33289097	48.99037305	100.2422959	62.54711581	79.47816389
11.53185842	18.14458261	16.70704932	7.548789839	7.518204692
11.53185842	0.907229131	7.862140857	16.17597823	9.666263176
85.52794996	62.59881001	96.3112255	39.90074629	92.36651479
27.86865785	21.77349913	44.22454232	54.99832597	27.92476029
151.8361359	130.6409948	32.43133104	212.444514	53.70146209
4.804941009	0.907229131	8.844908464	0	0
3.843952807	3.628916522	3.931070429	2.156797097	4.296116967
0	0	0	0	0
0.960988202	0.907229131	11.79321129	0	0
4000.593884	4471.732384	5496.619227	4985.43649	4450.777178
0.960988202	3.628916522	0	0	0
182.5877583	236.7868031	223.0882468	160.6813837	150.3640938
79.76202075	99.79520436	12.77597889	108.9182534	20.40655559
88.41091456	53.5265187	106.1389016	103.5262607	79.47816389
43.24446908	36.28916522	23.58642257	43.13594194	61.21966678
12.49284662	22.68072826	20.63811975	12.94078258	40.81311119
34.59557526	32.6602487	33.41409864	24.80316661	36.51699422
306.5552364	373.7784018	430.4522119	441.0650063	358.7257667
8.648893816	15.42289522	14.74151411	9.705586936	0
92.25486737	81.65062175	70.75926771	83.03668823	51.5534036
13.45383482	0	0	0	42.96116967
34.59557526	0	0	0	0
0	0	15.72428171	2.156797097	1.074029242
0	57.15543522	63.87989446	0	49.40534512
0	0	17.68981693	23.72476807	39.73908194
113.3966078	191.4253465	204.4156623	425.9674266	216.9539068
49.01039829	51.71206044	182.7947749	140.1918113	179.3628834
124.9284662	129.7337657	152.3289791	115.3886447	162.1784155
87.44992636	103.4241209	97.29399311	91.66387662	104.1808365
5.765929211	2.721687392	6.87937325	5.391992742	5.370146209
65.34719772	77.1144761	112.0355072	76.56629694	98.81069024
46.12743368	68.04218479	52.08668318	49.60633323	30.07281877
30.75162246	43.54699827	9.827676071	10.78398548	7.518204692
189.3146757	219.5494496	256.5023455	212.444514	169.6966202
26.90766965	38.10362348	19.65535214	40.97914484	20.40655559
8.648893816	3.628916522	8.844908464	10.78398548	20.40655559
0	2.721687392	43.24177471	0	48.33131588
40.36150447	57.15543522	45.20730993	47.44953613	83.77428086

For Review Only

47.08842189	43.54699827	50.12114796	71.1743042	54.77549133
8.648893816	39.01085261	51.10391557	0	0
70.15213873	0	0	0	0
0.960988202	0.907229131	13.7587465	0	0
150.8751477	127.0120783	83.53524661	143.4270069	138.5497722
0	0	0	0	0
0	0	0	0	0
0	0	0	0	19.33252635
42.28348088	68.94941392	38.32793668	63.62551436	49.40534512
19.21976404	5.443374783	5.896605643	52.84152887	10.74029242
191.2366522	147.8783483	216.2088736	158.5245866	150.3640938
62.46423311	67.13495566	50.12114796	39.90074629	36.51699422
132.6163718	99.79520436	116.9493452	84.11508678	107.4029242
92.25486737	117.0325578	93.36292268	83.03668823	129.9575383
197.0025814	201.404867	118.9148805	156.3677895	194.3992928
69.19115053	85.27953827	97.29399311	83.03668823	137.4757429
26.90766965	18.14458261	4.913838036	30.19515936	31.14684801
0	268.5398226	2.948302821	1.078398548	3.222087725
270.0376847	153.3217231	250.6057398	198.4253329	479.0170418
51.8933629	28.12410305	42.25900711	54.99832597	18.25849711
464.1573015	468.1302314	509.0736205	304.1083907	398.4648487
260.4278027	278.5193431	128.7425565	202.7389271	182.5849711
49.97138649	58.96989348	30.46579582	38.82234774	83.77428086
4.804941009	7.257833044	2.948302821	2.156797097	3.222087725
56.6983039	49.89760218	56.01775361	48.52793468	55.84952057
172.9778763	215.0133039	131.6908594	172.5437678	189.0291465
18.25877583	24.49518652	32.43133104	15.09757968	23.62864332
35.55656347	37.19639435	14.74151411	71.1743042	28.99878953
34.59557526	44.4542274	25.55195779	59.31192017	49.40534512
216.2223454	162.3940144	133.6563946	100.291065	168.622591
142.2262539	140.6205152	103.1905987	162.8381808	150.3640938
77.84004434	84.37230914	24.56919018	2.156797097	0
0	62.59881001	70.75926771	65.78231146	40.81311119
178.7438055	205.9410126	152.3289791	199.5037315	134.2536552
77.84004434	0	58.96605643	66.86071	4.296116967
27.86865785	26.30964479	51.10391557	69.0175071	40.81311119
276.7646021	115.2180996	323.3305427	0	0
145.1092185	172.3735348	93.36292268	113.2318476	82.70025162
58.62028031	65.3204974	67.81096489	45.29273904	66.58981299
48.04941009	34.47470696	8.844908464	24.80316661	8.592233934
24.98569325	19.95904087	27.517493	20.48957242	8.592233934
118.2015488	76.20724696	58.96605643	66.86071	95.58860252
273.8816375	291.2205509	256.5023455	267.44284	255.6189595
18.25877583	12.70120783	12.77597889	3.235195645	8.592233934
135.4993364	139.7132861	160.19112	99.21266646	137.4757429
0	0	7.862140857	0	3.222087725
0	0	0	0	4.296116967
51.8933629	19.95904087	21.62088736	19.41117387	30.07281877
51.8933629	39.91808174	38.32793668	30.19515936	40.81311119
82.64498535	117.939787	150.3634439	94.89907226	91.29248555

For Review Only

423.795797	357.4482774	334.1409864	289.010811	280.3216321
197.9635696	162.3940144	202.4501271	117.5454418	205.1395852
98.02079658	114.3108704	195.5707538	86.27188388	134.2536552
11.53185842	14.51566609	11.79321129	12.94078258	24.70267256
19.21976404	39.91808174	39.31070429	53.91992742	38.6650527
82.64498535	115.2180996	65.84542968	76.56629694	67.66384223
5.765929211	4.536145653	0	1.078398548	3.222087725
82.64498535	91.63014218	88.44908464	61.46871726	75.18204692
23.06371684	16.33012435	12.77597889	0	21.48058484
57.65929211	45.36145653	30.46579582	34.50875355	30.07281877
21.14174044	30.84579044	37.34516907	30.19515936	35.44296498
99.94277298	84.37230914	97.29399311	105.6830577	95.58860252
49.97138649	30.84579044	21.62088736	70.09590565	7.518204692
36.51755167	48.99037305	36.36240146	8.627188388	46.1832574
48.04941009	48.99037305	23.58642257	36.66555065	62.29369602
123.0064898	99.79520436	87.46631704	145.583804	127.8094798
231.5981566	37.19639435	119.8976481	88.42868097	117.0691874
34.59557526	0	8.844908464	5.391992742	49.40534512
0	0	0	5.391992742	0
63.42522132	58.96989348	66.82819729	59.31192017	52.62743285
73.03510333	79.83616349	73.70757054	64.70391291	75.18204692
186.4317111	215.9205331	267.3127891	242.6396734	263.1371642
107.6306786	109.7747248	86.48354943	105.6830577	91.29248555
11.53185842	0	15.72428171	10.78398548	12.8883509
25.94668145	39.91808174	34.39686625	49.60633323	22.55461408
0.960988202	0.907229131	458.9524725	0	0
15.37581123	0	9.827676071	7.548789839	24.70267256
36.51755167	38.10362348	37.34516907	23.72476807	22.55461408
159.5240415	172.3735348	134.6391622	244.7964705	199.769439
27.86865785	36.28916522	26.53472539	22.64636952	49.40534512
92.25486737	74.3927887	154.2945143	138.0350142	0
36.51755167	15.42289522	0	0	0
63.42522132	58.06266435	67.81096489	79.80149259	66.58981299
3.843952807	3.628916522	3.931070429	0	9.666263176
33.63458706	26.30964479	21.62088736	10.78398548	26.85073104
0	0	58.96605643	43.13594194	71.9599592
9.609882018	3.628916522	8.844908464	4.313594194	10.74029242
0	14.51566609	20.63811975	2.156797097	0
0.960988202	5.443374783	5.896605643	1.078398548	0
1009.9986	1104.097852	969.0088606	874.5812228	1113.768324
284.4525077	278.5193431	238.8125285	291.1676081	213.7318191
101.8647494	114.3108704	113.0182748	122.9374345	113.8470996
29.79063425	13.60843696	25.55195779	11.86238403	17.18446787
0	0	0.982767607	0	1.074029242
51.8933629	55.34097696	30.46579582	22.64636952	48.33131588
231.5981566	224.0855952	319.3994723	321.3627674	182.5849711
213.3393808	173.2807639	177.8809369	128.3294273	240.5825502
19.21976404	0	26.53472539	36.66555065	17.18446787
149.9141595	147.8783483	120.8804157	154.2109924	178.2888541
0	943.5182958	0.982767607	213.5229126	60.14563754

For Review Only

66.30818592	81.65062175	90.41461986	86.27188388	82.70025162
0.960988202	0	0.982767607	6.470391291	3.222087725
75.91806794	93.44460045	63.87989446	143.4270069	93.44054403
76.87905614	52.61928957	62.89712686	57.15512307	103.1068072
72.07411513	20.86627	17.68981693	122.9374345	15.03640938
121.0845134	199.5904087	57.00052121	53.91992742	30.07281877
0	0	0	4.313594194	0
0	1.814458261	0	0	0
24.02470504	0	7.862140857	4.313594194	13.96238014
236.4030976	214.1060748	292.8647469	323.5195645	239.5085209
101.8647494	151.5072648	148.3979087	100.291065	96.66263176
50.93237469	29.03133218	23.58642257	22.64636952	38.6650527
0	3.628916522	0	0	0
115.3185842	115.2180996	43.24177471	127.2510287	102.032778
188.3536875	0	19.65535214	52.84152887	259.9150765
24.02470504	38.10362348	7.862140857	33.430355	24.70267256
0	0	0	0	0
48.04941009	54.43374783	60.93159164	62.54711581	35.44296498
27.86865785	26.30964479	11.79321129	8.627188388	53.70146209
0	0	0	0	0
0	0	0	0	0
4.804941009	12.70120783	11.79321129	2.156797097	3.222087725
2.882964605	2.721687392	0	1.078398548	0
163.3679943	156.9506396	157.2428171	257.7372531	240.5825502
12.49284662	0	24.56919018	19.41117387	19.33252635
72.07411513	70.76387218	60.93159164	29.11676081	47.25728664
5.765929211	16.33012435	0	246.9532676	117.0691874
56.6983039	43.54699827	28.50026061	29.11676081	44.03519891
273.8816375	240.4157196	273.2093948	153.1325939	175.0667664
106.6696904	83.46508001	38.32793668	53.91992742	53.70146209
246.0129797	215.0133039	124.8114861	114.3102461	184.7330296
0	126.1048491	0	116.4670432	62.29369602
1200.274264	0	0.982767607	10.78398548	1.074029242
164.3289825	90.72291305	215.226106	197.3469344	153.5861816
183.5487465	132.4554531	171.9843312	219.9933039	175.0667664
159.5240415	117.0325578	167.0704932	167.151775	139.6238014
0	0	0	0	0
111.4746314	115.2180996	148.3979087	142.3486084	230.916287
191.2366522	155.1361813	182.7947749	159.6029852	93.44054403
42.28348088	61.69158088	63.87989446	56.07672452	49.40534512
375.7463869	426.3976914	399.0036485	476.6521584	341.5412989
45.16644548	35.38193609	53.06945079	74.40949984	62.29369602
85.52794996	64.41326827	74.69033814	108.9182534	111.6990411
215.2613572	81.65062175	214.2433384	216.7581082	260.9891057
80.72300895	102.5168918	96.3112255	97.05586936	63.36772526
376.7073751	386.4796096	316.4511695	278.2268255	50.47937436
138.3823011	190.5181174	146.4323735	160.6813837	102.032778
38.43952807	33.56747783	48.15561275	53.91992742	30.07281877
157.6020651	195.9614922	81.56971139	416.2618397	323.2828018
245.0519914	274.8904266	323.3305427	208.1309199	288.913866

For Review Only

104.747714	84.37230914	95.32845789	71.1743042	90.21845631
0	0	42.25900711	132.6430215	155.7342401
122.0455016	125.19762	50.12114796	154.2109924	20.40655559
56.6983039	67.13495566	76.65587336	45.29273904	53.70146209
45.16644548	78.92893436	39.31070429	76.56629694	68.73787147
0	89.81568392	0	0	47.25728664
98.02079658	99.79520436	62.89712686	108.9182534	57.99757905
5.765929211	7.257833044	0	32.35195645	17.18446787
166.2509589	166.0229309	120.8804157	125.0942316	190.1031758
81.68399715	74.3927887	145.4496059	133.72142	103.1068072
7.687905614	5.443374783	5.896605643	3.235195645	11.81432166
27.86865785	37.19639435	23.58642257	21.56797097	27.92476029
5090.354505	5246.506062	6076.452115	5695.022734	5299.260279
5.765929211	0	2.948302821	0	0
121.0845134	136.9915987	152.3289791	162.8381808	156.8082693
141.2652657	168.7446183	91.39738746	155.289391	233.0643455
57.65929211	60.78435175	86.48354943	76.56629694	153.5861816
0	0	0	0	0
0	27.21687392	0	2.156797097	3.222087725
0.960988202	0	6.87937325	7.548789839	0
376.7073751	233.1578865	232.9159229	239.4044778	242.7306086
0	0.907229131	0	0	0
12.49284662	19.05181174	0	0	0
94.17684377	95.25905871	61.91435925	116.4670432	70.88592996
70.15213873	97.9807461	62.89712686	66.86071	50.47937436
0	0	0	0	0
128.772419	130.6409948	160.19112	236.1692821	143.9199184
99.94277298	112.4964122	62.89712686	70.09590565	107.4029242
134.5383482	140.6205152	209.3295003	184.4061518	109.5509827
30.75162246	36.28916522	48.15561275	52.84152887	27.92476029
86.48893816	57.15543522	75.67310575	0	0
735.1559743	790.1965727	585.7294939	558.6104481	647.6396328
0	0	0	0	2.148058484
56.6983039	38.10362348	61.91435925	21.56797097	27.92476029
59.58126851	58.06266435	98.27676071	93.82067372	93.44054403
0	0	0	8.627188388	8.592233934
59.58126851	59.87712262	49.13838036	17.25437678	74.10801768
0	0	309.5717962	0	0
268.1157083	289.4060926	341.0203597	356.9499195	199.769439
40.36150447	44.4542274	48.15561275	47.44953613	54.77549133
30.75162246	26.30964479	36.36240146	17.25437678	37.59102346
0	7.257833044	5.896605643	6.470391291	1.074029242
0	0	11.79321129	59.31192017	15.03640938
8.648893816	14.51566609	7.862140857	0	0
165.2899707	106.1458083	111.0527396	146.6622026	76.25607616
270.0376847	201.404867	181.8120073	204.8957242	210.5097314
380.5513279	358.3555066	271.2438596	252.3452603	216.9539068
331.5409296	279.4265722	202.4501271	200.58213	204.0655559
20.18075224	0	0.982767607	0	0
28.82964605	26.30964479	15.72428171	6.470391291	10.74029242

For Review Only

704.4043519	833.743571	940.5086	785.0741433	697.0449779
113.3966078	101.6096626	115.9665776	65.78231146	78.40413465
34.59557526	32.6602487	41.2762395	52.84152887	50.47937436
111.4746314	113.4036413	204.4156623	80.87989113	139.6238014
39.40051627	9.979520436	97.29399311	75.48789839	81.62622237
12.49284662	9.979520436	17.68981693	15.09757968	8.592233934
82.64498535	93.44460045	93.36292268	101.3694636	59.0716083
3.843952807	8.165062175	5.896605643	4.313594194	6.444175451
0	39.01085261	36.36240146	23.72476807	4.296116967
22.10272864	28.12410305	28.50026061	17.25437678	10.74029242
221.9882746	204.1265544	137.587465	171.4653692	218.0279361
79.76202075	66.22772653	74.69033814	57.15512307	36.51699422
0	0	0	0	0
118.2015488	166.93016	128.7425565	92.74227517	151.4381231
14.41482303	15.42289522	21.62088736	5.391992742	0
0	14.51566609	33.41409864	0	0
74.95707974	95.25905871	80.58694379	140.1918113	110.6250119
124.9284662	107.0530374	97.29399311	46.37113758	78.40413465
103.7867258	76.20724696	56.01775361	94.89907226	69.81190071
114.357596	106.1458083	165.104958	171.4653692	110.6250119
83.60597355	82.55785088	91.39738746	77.64469549	62.29369602
106.6696904	89.81568392	64.86266207	111.0750505	118.1432166
17.29778763	28.12410305	48.15561275	54.99832597	37.59102346
196.0415932	46.26868566	287.9509089	63.62551436	59.0716083
0	60.78435175	158.2255847	6.470391291	25.7767018
30.75162246	12.70120783	24.56919018	37.7439492	20.40655559
151.8361359	124.2903909	104.1733664	109.9966519	102.032778
88.41091456	107.0530374	111.0527396	77.64469549	94.51457327
20.18075224	14.51566609	21.62088736	34.50875355	28.99878953
0	0	0.982767607	26.95996371	3.222087725
24.02470504	27.21687392	7.862140857	14.01918113	12.8883509
32.67359886	66.22772653	57.98328882	31.27355791	39.73908194
0	0	123.8287185	54.99832597	107.4029242
24.02470504	20.86627	23.58642257	29.11676081	31.14684801
92.25486737	0	0.982767607	14.01918113	324.356831
21.14174044	37.19639435	29.48302821	18.33277532	0
246.0129797	139.7132861	100.2422959	100.291065	82.70025162
20.18075224	24.49518652	18.67258454	26.95996371	24.70267256
34.59557526	22.68072826	25.55195779	35.5871521	39.73908194
7.687905614	0	0	0	0
58.62028031	66.22772653	31.44856343	28.03836226	13.96238014
18.25877583	11.7939787	7.862140857	6.470391291	2.148058484
106.6696904	0	100.2422959	45.29273904	55.84952057
4.804941009	0	0	0	6.444175451
552.568216	473.5736061	470.7456838	403.3210571	312.5425094
160.4850297	137.8988278	163.1394228	102.4478621	177.2148249
33.63458706	42.63976914	0	0	0
0	0	7.862140857	4.313594194	0
7.687905614	29.03133218	49.13838036	9.705586936	10.74029242
0	0	0	5.391992742	0

3.843952807	0	0	0	0
50.93237469	60.78435175	10.81044368	22.64636952	11.81432166
28.82964605	28.12410305	20.63811975	29.11676081	20.40655559
32.67359886	53.5265187	35.37963386	56.07672452	38.6650527
20.18075224	4.536145653	11.79321129	19.41117387	39.73908194
61.50324491	56.24820609	37.34516907	33.430355	34.36893574
0	0	0	0	0
0	0	0	0	0
0	0	11.79321129	0	0
13.45383482	4.536145653	28.50026061	6.470391291	4.296116967
9.609882018	54.43374783	26.53472539	28.03836226	17.18446787
6.726917412	20.86627	13.7587465	48.52793468	112.7730704
84.56696176	59.87712262	64.86266207	33.430355	66.58981299
200.8465342	165.1157018	236.8469933	114.3102461	216.9539068
99.94277298	99.79520436	74.69033814	125.0942316	81.62622237
72.07411513	85.27953827	75.67310575	92.74227517	96.66263176
12.49284662	15.42289522	22.60365496	9.705586936	12.8883509
100.9037612	72.57833044	112.0355072	130.4862244	94.51457327
40.36150447	45.36145653	34.39686625	20.48957242	37.59102346
380.5513279	364.7061105	398.0208809	352.6363253	308.2463924
800.5031721	783.8459688	588.6777967	603.9031871	707.7852703
5.765929211	7.257833044	0.982767607	1.078398548	3.222087725
4.804941009	12.70120783	10.81044368	6.470391291	5.370146209
13.45383482	7.257833044	3.931070429	1.078398548	9.666263176
22.10272864	16.33012435	1.965535214	1.078398548	1.074029242
0	0	0.982767607	0	0
0	0	0.982767607	1.078398548	1.074029242
0	0	0.982767607	93.82067372	1.074029242
100.9037612	127.0120783	142.501303	83.03668823	49.40534512
24.02470504	21.77349913	14.74151411	18.33277532	20.40655559
19.21976404	18.14458261	18.67258454	19.41117387	6.444175451
173.9388645	192.3325757	540.5221839	79.80149259	26.85073104
52.8543511	0	14.74151411	0	0
49.97138649	51.71206044	79.60417618	77.64469549	66.58981299
1.921976404	0.907229131	0	0	0
190.2756639	189.6108883	170.018796	186.5629489	224.4721115
4.804941009	2.721687392	5.896605643	10.78398548	7.518204692
20.18075224	23.58795739	17.68981693	19.41117387	18.25849711
49.97138649	46.26868566	27.517493	31.27355791	52.62743285
42.28348088	53.5265187	29.48302821	58.23352162	56.92354981
0.960988202	14.51566609	2.948302821	4.313594194	0
0	0	0	34.50875355	0
8.648893816	21.77349913	21.62088736	25.88156516	0
0	0	149.3806763	984.5778747	3.222087725
0.960988202	0	6.87937325	0	0
0	0	0	0	4.296116967
220.0662982	225.9000535	153.3117467	264.2076444	286.7658075
0	0	0	4.313594194	3.222087725
2.882964605	0	0	4.313594194	0
2.882964605	0	0	0	0

For Review Only

10.57087022	11.7939787	21.62088736	5.391992742	4.296116967
0	0	0	0	2.148058484
0	0.907229131	0	0	0
0	0	0.982767607	0	0
35.55656347	37.19639435	51.10391557	31.27355791	46.1832574
0	0	0	0	1.074029242
43.24446908	48.08314392	48.15561275	66.86071	88.07039782
4.804941009	9.072291305	0.982767607	2.156797097	3.222087725
3.843952807	9.072291305	1.965535214	3.235195645	5.370146209
5.765929211	8.165062175	0.982767607	2.156797097	3.222087725
0.960988202	15.42289522	5.896605643	0	8.592233934
0	11.7939787	2.948302821	0	7.518204692
0	8.165062175	0	3.235195645	0
19.21976404	11.7939787	8.844908464	6.470391291	5.370146209
0	0	0	2.156797097	0
21.14174044	33.56747783	29.48302821	30.19515936	20.40655559
46.12743368	49.89760218	68.7937325	52.84152887	45.10922815
103.7867258	101.6096626	156.2600495	121.859036	94.51457327
8.648893816	0	0	0	62.29369602
76.87905614	44.4542274	61.91435925	57.15512307	73.03398844
36.51755167	29.93856131	37.34516907	18.33277532	31.14684801
81.68399715	61.69158088	78.62140857	64.70391291	81.62622237
94.17684377	103.4241209	79.60417618	77.64469549	103.1068072
115.3185842	107.0530374	68.7937325	77.64469549	157.8822985
126.8504426	118.8470161	161.1738876	188.719746	137.4757429
80.72300895	102.5168918	34.39686625	114.3102461	74.10801768
33.63458706	48.99037305	83.53524661	77.64469549	69.81190071
12.49284662	18.14458261	21.62088736	18.33277532	21.48058484
22.10272864	10.88674957	18.67258454	30.19515936	33.29490649
43.24446908	50.80483131	33.41409864	0	33.29490649
29.79063425	31.75301957	57.00052121	31.27355791	41.88714043
6.726917412	12.70120783	11.79321129	15.09757968	15.03640938
124.9284662	90.72291305	65.84542968	129.4078258	52.62743285
220.0662982	143.3422026	105.156134	103.5262607	176.1407956
0	4.536145653	0	5.391992742	0
16.33679943	8.165062175	35.37963386	32.35195645	13.96238014
36.51755167	45.36145653	53.06945079	44.21434049	34.36893574
0	105.2385791	0.982767607	1.078398548	1.074029242
73.99609154	74.3927887	62.89712686	64.70391291	64.44175451
42.28348088	47.17591479	28.50026061	31.27355791	50.47937436
0	0	0	0	0
97.05980838	134.2699113	111.0527396	85.19348533	103.1068072
21.14174044	35.38193609	7.862140857	4.313594194	4.296116967
13.45383482	8.165062175	20.63811975	12.94078258	4.296116967
30.75162246	27.21687392	17.68981693	25.88156516	62.29369602
0	0	0	6.470391291	0
4.804941009	3.628916522	0	4.313594194	5.370146209
170.0949117	139.7132861	143.4840706	166.0733765	118.1432166
20.18075224	49.89760218	95.32845789	118.6238403	106.3288949
51.8933629	27.21687392	14.74151411	36.66555065	39.73908194

For Review Only

3.843952807	9.072291305	0	102.4478621	68.73787147
103.7867258	0	24.56919018	142.3486084	151.4381231
80.72300895	101.6096626	122.8459509	104.6046592	88.07039782
0	7.257833044	30.46579582	4.313594194	0
209.495428	155.1361813	263.3817187	284.6972168	156.8082693
68.23016233	44.4542274	82.552479	88.42868097	91.29248555
112.4356196	125.19762	124.8114861	106.7614563	136.4017137
99.94277298	68.94941392	91.39738746	66.86071	54.77549133
179.7047937	158.7650978	153.3117467	214.6013111	185.8070588
123.967478	155.1361813	163.1394228	187.6413474	92.36651479
140.3042775	156.9506396	162.1566552	187.6413474	169.6966202
29.79063425	40.82531087	57.98328882	51.76313033	11.81432166
9.609882018	7.257833044	5.896605643	5.391992742	11.81432166
108.5916668	91.63014218	53.06945079	106.7614563	64.44175451
185.4707229	219.5494496	171.9843312	155.289391	137.4757429
0	0	0.982767607	1.078398548	2.148058484
97.05980838	81.65062175	87.46631704	73.3311013	67.66384223
24.98569325	29.03133218	35.37963386	32.35195645	33.29490649
31.71261066	16.33012435	61.91435925	30.19515936	22.55461408
517.0116525	513.4916879	558.2120009	488.5145425	457.536457
156.6410769	121.5687035	124.8114861	84.11508678	131.0315675
0.960988202	0.907229131	0	768.8981651	0
432.4446908	459.9651692	488.4355007	521.9448975	527.3483577
0	0	0.982767607	1.078398548	1.074029242
576.5929211	710.3604092	738.058473	977.0290849	830.2246039
428.600738	500.7904801	582.781191	634.0983465	612.1966678
20.18075224	20.86627	21.62088736	25.88156516	23.62864332
54.7763275	35.38193609	23.58642257	49.60633323	45.10922815
6.726917412	29.93856131	17.68981693	4.313594194	3.222087725
60.54225671	46.26868566	140.5357678	43.13594194	62.29369602
13.45383482	11.7939787	12.77597889	7.548789839	21.48058484
36.51755167	37.19639435	57.98328882	81.95828968	60.14563754
115.3185842	97.07351697	57.00052121	103.5262607	136.4017137
3.843952807	0	0	0	0
136.4603247	91.63014218	140.5357678	111.0750505	124.587392
4.804941009	10.88674957	8.844908464	6.470391291	5.370146209
79.76202075	72.57833044	61.91435925	49.60633323	54.77549133
0.960988202	0.907229131	0	0	0
49.01039829	51.71206044	63.87989446	44.21434049	40.81311119
6.726917412	33.56747783	101.2250635	100.291065	85.92233934
49.01039829	56.24820609	55.034986	72.25270275	78.40413465
61.50324491	45.36145653	44.22454232	81.95828968	77.33010541
344.9947644	327.5097161	335.123754	388.2234774	357.6517375
85.52794996	48.08314392	57.00052121	58.23352162	51.5534036
25.94668145	7.257833044	0	0	0
103.7867258	70.76387218	30.46579582	67.93910855	79.47816389
33.63458706	47.17591479	44.22454232	38.82234774	40.81311119
39.40051627	34.47470696	18.67258454	40.97914484	32.22087725
8.648893816	11.7939787	13.7587465	8.627188388	13.96238014
7.687905614	4.536145653	6.87937325	0	6.444175451

29.79063425	27.21687392	32.43133104	14.01918113	41.88714043
76.87905614	96.16628784	23.58642257	12.94078258	38.6650527
65.34719772	35.38193609	23.58642257	45.29273904	36.51699422
100.9037612	139.7132861	83.53524661	65.78231146	79.47816389
21.14174044	26.30964479	12.77597889	31.27355791	19.33252635
223.910251	0	0.982767607	37.7439492	17.18446787
62.46423311	50.80483131	58.96605643	74.40949984	61.21966678
36.51755167	17.23735348	14.74151411	16.17597823	34.36893574
2.882964605	2.721687392	2.948302821	5.391992742	6.444175451
73.03510333	81.65062175	77.63864096	52.84152887	35.44296498
35.55656347	33.56747783	8.844908464	25.88156516	10.74029242
21.14174044	19.95904087	13.7587465	31.27355791	22.55461408
28.82964605	6.350603914	27.517493	15.09757968	17.18446787
24.98569325	0	34.39686625	32.35195645	23.62864332
49.01039829	47.17591479	64.86266207	75.48789839	47.25728664
71.11312693	58.06266435	59.94882404	66.86071	59.0716083
15.37581123	30.84579044	24.56919018	19.41117387	21.48058484
133.57736	117.0325578	157.2428171	201.6605286	102.032778
27.86865785	37.19639435	51.10391557	6.470391291	31.14684801
9.609882018	14.51566609	6.87937325	18.33277532	15.03640938
0	0	0	0	0
12.49284662	9.979520436	6.87937325	17.25437678	20.40655559
70.15213873	84.37230914	72.72480293	76.56629694	57.99757905
0	0	3.931070429	1.078398548	1.074029242
0.960988202	6.350603914	2.948302821	6.470391291	1.074029242
88.41091456	52.61928957	78.62140857	85.19348533	62.29369602
226.7932156	190.5181174	188.6913806	273.9132313	264.2111935
25.94668145	23.58795739	65.84542968	61.46871726	6.444175451
20.18075224	14.51566609	10.81044368	6.470391291	5.370146209
53.8153393	31.75301957	25.55195779	28.03836226	30.07281877
75.91806794	68.04218479	62.89712686	73.3311013	68.73787147
45.16644548	56.24820609	59.94882404	43.13594194	59.0716083
73.03510333	63.50603914	96.3112255	76.56629694	83.77428086
82.64498535	50.80483131	47.17284514	65.78231146	34.36893574
76.87905614	49.89760218	51.10391557	38.82234774	41.88714043
158.5630533	184.1675135	161.1738876	113.2318476	118.1432166
6.726917412	15.42289522	7.862140857	2.156797097	6.444175451
98.98178478	103.4241209	98.27676071	49.60633323	230.916287
81.68399715	68.94941392	42.25900711	33.430355	28.99878953
24.02470504	32.6602487	29.48302821	29.11676081	17.18446787
292.1404133	262.1892187	310.5545639	221.0717024	226.62017
0	0	2.948302821	0	1.074029242
35.55656347	35.38193609	41.2762395	43.13594194	74.10801768
23.06371684	9.072291305	27.517493	21.56797097	16.11043863
4.804941009	10.88674957	10.81044368	0	7.518204692
51.8933629	52.61928957	38.32793668	38.82234774	67.66384223
68.23016233	52.61928957	68.7937325	57.15512307	86.99636858
30.75162246	46.26868566	37.34516907	65.78231146	44.03519891
0	0	0	0	1.074029242
101.8647494	94.35182958	37.34516907	54.99832597	60.14563754

For Review Only

73.03510333	66.22772653	20.63811975	65.78231146	40.81311119
10.57087022	8.165062175	6.87937325	4.313594194	5.370146209
24.02470504	11.7939787	50.12114796	19.41117387	27.92476029
219.10531	172.3735348	202.4501271	168.2301736	104.1808365
32.67359886	0	47.17284514	0	0
114.357596	110.6819539	109.0872044	129.4078258	94.51457327
3.843952807	0	7.862140857	9.705586936	0
0	38.10362348	15.72428171	0	1.074029242
44.20545728	0	45.20730993	52.84152887	13.96238014
0.960988202	0	2.948302821	2.156797097	0
16.33679943	10.88674957	8.844908464	10.78398548	17.18446787
0	0	0	0	0
89.37190276	78.02170523	63.87989446	60.39031871	99.88471948
64.38620952	59.87712262	75.67310575	78.72309404	55.84952057
0.960988202	1.814458261	0	0	0
77.84004434	67.13495566	52.08668318	39.90074629	59.0716083
19.21976404	22.68072826	22.60365496	40.97914484	9.666263176
280.6085549	353.8193609	302.692423	243.718072	306.0983339
23.06371684	26.30964479	12.77597889	24.80316661	31.14684801
5.765929211	0	0.982767607	2.156797097	2.148058484
0	0.907229131	0	0	0
84.56696176	49.89760218	119.8976481	47.44953613	62.29369602
92.25486737	97.9807461	117.9321129	90.58547807	54.77549133
44.20545728	80.74339262	8.844908464	8.627188388	11.81432166
43.24446908	31.75301957	34.39686625	28.03836226	37.59102346
0	213.1988457	5.896605643	234.012485	1.074029242
100.9037612	107.0530374	102.2078311	97.05586936	55.84952057
38.43952807	38.10362348	53.06945079	35.5871521	40.81311119
168.1729353	143.3422026	139.5530002	147.7406011	74.10801768
58.62028031	68.04218479	45.20730993	36.66555065	38.6650527
673.6527294	547.0591657	504.1597825	610.3735784	474.7209249
0	0	0	0	2.148058484
19.21976404	25.40241565	31.44856343	21.56797097	45.10922815
0.960988202	4.536145653	1.965535214	5.391992742	3.222087725
29.79063425	22.68072826	27.517493	29.11676081	35.44296498
0.960988202	1.814458261	19.65535214	22.64636952	30.07281877
39.40051627	50.80483131	55.034986	59.31192017	54.77549133
0	0	0.982767607	1.078398548	1.074029242
44.20545728	54.43374783	29.48302821	69.0175071	74.10801768
4.804941009	0	0.982767607	0	0
177.7828173	297.5711548	15.72428171	1.078398548	28.99878953
149.9141595	125.19762	167.0704932	158.5245866	181.5109419
25.94668145	23.58795739	30.46579582	21.56797097	40.81311119
17.29778763	23.58795739	17.68981693	11.86238403	10.74029242
123.0064898	123.3831618	102.2078311	124.0158331	189.0291465
37.47853987	31.75301957	55.034986	33.430355	26.85073104
911.0168153	945.332754	573.9362826	786.1525418	862.4454811
50.93237469	58.06266435	54.05221839	30.19515936	66.58981299
26.90766965	10.88674957	17.68981693	23.72476807	26.85073104
48.04941009	24.49518652	42.25900711	62.54711581	35.44296498

For Review Only

194.1196168	195.9614922	178.8637045	240.4828763	166.4745325
21.14174044	29.93856131	9.827676071	45.29273904	25.7767018
35.55656347	11.7939787	15.72428171	18.33277532	12.8883509
26.90766965	13.60843696	15.72428171	19.41117387	19.33252635
220.0662982	224.9928244	135.6219298	441.0650063	47.25728664
58.62028031	66.22772653	42.25900711	38.82234774	38.6650527
10.57087022	10.88674957	1.965535214	1.078398548	3.222087725
75.91806794	92.53737131	81.56971139	51.76313033	76.25607616
0	3.628916522	0	1.078398548	1.074029242
31.71261066	51.71206044	16.70704932	15.09757968	15.03640938
55.7373157	63.50603914	71.74203532	46.37113758	64.44175451
51.8933629	62.59881001	61.91435925	105.6830577	28.99878953
219.10531	209.5699292	37.34516907	604.9815857	279.2476029
21.14174044	24.49518652	10.81044368	37.7439492	22.55461408
0	0	1.965535214	0	1.074029242
24.98569325	38.10362348	27.517493	38.82234774	50.47937436
0.960988202	0	0	2.156797097	0
26.90766965	27.21687392	33.41409864	43.13594194	25.7767018
35.55656347	54.43374783	90.41461986	44.21434049	46.1832574
85.52794996	85.27953827	44.22454232	90.58547807	85.92233934
154.7191005	171.4663057	103.1905987	148.8189997	23.62864332
22.10272864	39.91808174	11.79321129	25.88156516	37.59102346
175.8608409	143.3422026	114.98381	171.4653692	175.0667664
147.0311949	113.4036413	133.6563946	120.7806374	168.622591
92.25486737	76.20724696	41.2762395	31.27355791	37.59102346
108.5916668	122.4759326	86.48354943	169.3085721	146.0679769
13.45383482	16.33012435	30.46579582	18.33277532	34.36893574
63.42522132	154.2289522	103.1905987	111.0750505	79.47816389
50.93237469	46.26868566	30.46579582	39.90074629	57.99757905
15.37581123	11.7939787	5.896605643	6.470391291	5.370146209
0	0	3.931070429	0	0
33.63458706	29.03133218	25.55195779	10.78398548	25.7767018
61.50324491	65.3204974	8.844908464	14.01918113	45.10922815
27.86865785	32.6602487	64.86266207	15.09757968	44.03519891
480.4941009	358.3555066	451.0903317	373.1258978	490.8313635
0	0	66.82819729	71.1743042	55.84952057
45.16644548	70.76387218	68.7937325	65.78231146	36.51699422
208.5344398	197.7759505	120.8804157	186.5629489	249.1747841
81.68399715	58.96989348	63.87989446	73.3311013	94.51457327
11.53185842	19.05181174	18.67258454	1.078398548	31.14684801
60.54225671	29.03133218	56.01775361	65.78231146	20.40655559
101.8647494	167.8373891	93.36292268	116.4670432	111.6990411
0	0.907229131	0.982767607	1.078398548	1.074029242
161.4460179	125.19762	150.3634439	135.8782171	115.9951581
14.41482303	0	5.896605643	1.078398548	6.444175451
27.86865785	22.68072826	19.65535214	52.84152887	57.99757905
176.8218291	168.7446183	57.00052121	81.95828968	117.0691874
200.8465342	196.8687213	199.5018242	242.6396734	243.8046379
224.8712392	255.8386148	116.9493452	171.4653692	176.1407956
18.25877583	25.40241565	28.50026061	23.72476807	17.18446787

For Review Only

24.02470504	0.907229131	23.58642257	10.78398548	70.88592996
63.42522132	107.0530374	83.53524661	87.35028243	79.47816389
0	286.6844052	26.53472539	147.7406011	74.10801768
70.15213873	86.1867674	73.70757054	76.56629694	112.7730704
60.54225671	31.75301957	41.2762395	39.90074629	42.96116967
33.63458706	21.77349913	18.67258454	25.88156516	40.81311119
60.54225671	40.82531087	29.48302821	26.95996371	21.48058484
66.30818592	76.20724696	61.91435925	91.66387662	35.44296498
64.38620952	96.16628784	82.552479	84.11508678	76.25607616
0	0	9.827676071	2.156797097	5.370146209
24.02470504	16.33012435	24.56919018	29.11676081	19.33252635
1.921976404	0	0	0	0
28.82964605	28.12410305	10.81044368	32.35195645	28.99878953
213.3393808	167.8373891	75.67310575	169.3085721	183.6590003
0	0	0.982767607	6.470391291	0
34.59557526	21.77349913	50.12114796	1.078398548	9.666263176
22.10272864	39.91808174	29.48302821	31.27355791	40.81311119
58.62028031	42.63976914	57.98328882	45.29273904	41.88714043
114.357596	105.2385791	65.84542968	79.80149259	115.9951581
44.20545728	24.49518652	17.68981693	35.5871521	57.99757905
93.21585557	95.25905871	171.0015636	70.09590565	80.55219313
72.07411513	45.36145653	53.06945079	116.4670432	21.48058484
56.6983039	68.04218479	42.25900711	43.13594194	12.8883509
52.8543511	44.4542274	59.94882404	26.95996371	53.70146209
85.52794996	71.67110131	73.70757054	52.84152887	104.1808365
56.6983039	0	79.60417618	259.8940502	19.33252635
7.687905614	0	4.913838036	0	4.296116967
122.0455016	165.1157018	157.2428171	168.2301736	127.8094798
27.86865785	36.28916522	13.7587465	11.86238403	17.18446787
72.07411513	79.83616349	57.00052121	11.86238403	54.77549133
0	12.70120783	6.87937325	9.705586936	3.222087725
1.921976404	0	0	5.391992742	0
27.86865785	48.99037305	51.10391557	26.95996371	23.62864332
102.8257376	133.3626822	144.4668382	100.291065	113.8470996
31.71261066	29.93856131	22.60365496	37.7439492	31.14684801
12.49284662	13.60843696	2.948302821	3.235195645	28.99878953
13.45383482	12.70120783	1.965535214	11.86238403	7.518204692
23.06371684	39.91808174	39.31070429	58.23352162	17.18446787
64.38620952	242.2301779	614.2297545	204.8957242	0
19.21976404	28.12410305	21.62088736	25.88156516	28.99878953
21.14174044	21.77349913	14.74151411	21.56797097	10.74029242
42.28348088	25.40241565	19.65535214	34.50875355	18.25849711
0.960988202	2.721687392	0	668.6071	608.9745801
252.7398971	289.4060926	5.896605643	18.33277532	703.4891533
0	8.165062175	3.931070429	6.470391291	0
24.02470504	29.03133218	22.60365496	19.41117387	16.11043863
24.98569325	29.93856131	31.44856343	17.25437678	32.22087725
0	23.58795739	0	0	25.7767018
37.47853987	29.93856131	33.41409864	0	17.18446787
29.79063425	4.536145653	8.844908464	21.56797097	0

For Review Only

0	0	1.965535214	2.156797097	0
64.38620952	86.1867674	46.19007754	21.56797097	48.33131588
91.29387917	75.30001783	69.77650011	30.19515936	64.44175451
0	0	0	0	1.074029242
41.32249268	14.51566609	45.20730993	47.44953613	41.88714043
10.57087022	6.350603914	7.862140857	15.09757968	26.85073104
66.30818592	62.59881001	60.93159164	80.87989113	81.62622237
110.5136432	146.9711191	83.53524661	101.3694636	106.3288949
80.72300895	70.76387218	37.34516907	28.03836226	64.44175451
54.7763275	88.00122566	3.931070429	21.56797097	82.70025162
61.50324491	93.44460045	28.50026061	104.6046592	11.81432166
31.71261066	33.56747783	13.7587465	28.03836226	1.074029242
73.99609154	92.53737131	140.5357678	101.3694636	80.55219313
25.94668145	23.58795739	69.77650011	66.86071	41.88714043
20.18075224	36.28916522	29.48302821	36.66555065	32.22087725
88.41091456	116.1253287	106.1389016	128.3294273	99.88471948
0	0	0	0	0
102.8257376	69.85664305	80.58694379	104.6046592	53.70146209
9.609882018	0	8.844908464	0	0
49.01039829	26.30964479	55.034986	35.5871521	24.70267256
39.40051627	36.28916522	38.32793668	34.50875355	47.25728664
20.18075224	13.60843696	4.913838036	24.80316661	5.370146209
49.01039829	32.6602487	55.034986	11.86238403	20.40655559
61.50324491	48.08314392	73.70757054	78.72309404	55.84952057
260.4278027	198.6831796	143.4840706	180.0925576	169.6966202
16.33679943	20.86627	19.65535214	14.01918113	16.11043863
36.51755167	30.84579044	16.70704932	16.17597823	13.96238014
43.24446908	75.30001783	32.43133104	22.64636952	81.62622237
9.609882018	0	16.70704932	2.156797097	8.592233934
37.47853987	17.23735348	70.75926771	12.94078258	30.07281877
43.24446908	42.63976914	27.517493	23.72476807	32.22087725
14.41482303	12.70120783	1.965535214	19.41117387	9.666263176
0.960988202	1.814458261	0	1.078398548	3.222087725
58.62028031	67.13495566	102.2078311	57.15512307	23.62864332
0.960988202	0	1.965535214	0	0
139.3432893	114.3108704	110.069972	130.4862244	123.5133628
45.16644548	78.92893436	58.96605643	64.70391291	35.44296498
2.882964605	0	2.948302821	1.078398548	0
17.29778763	14.51566609	22.60365496	8.627188388	16.11043863
0	0	0.982767607	1.078398548	1.074029242
58.62028031	41.73254	18.67258454	43.13594194	13.96238014
98.98178478	0	0.982767607	1.078398548	1.074029242
124.9284662	48.99037305	69.77650011	59.31192017	125.6614213
53.8153393	99.79520436	70.75926771	36.66555065	48.33131588
11.53185842	12.70120783	0	0	0
102.8257376	96.16628784	37.34516907	51.76313033	131.0315675
83.60597355	81.65062175	53.06945079	45.29273904	32.22087725
13.45383482	16.33012435	8.844908464	5.391992742	10.74029242
59.58126851	76.20724696	51.10391557	58.23352162	34.36893574
0	0	0.982767607	0	0

For Review Only

29.79063425	12.70120783	24.56919018	81.95828968	63.36772526
31.71261066	48.99037305	51.10391557	50.68473178	41.88714043
20.18075224	9.979520436	16.70704932	8.627188388	12.8883509
21.14174044	28.12410305	33.41409864	48.52793468	46.1832574
288.2964605	282.1482596	236.8469933	250.1884632	214.8058484
0.960988202	2.721687392	0.982767607	2.156797097	2.148058484
7.687905614	4.536145653	0.982767607	0	4.296116967
20.18075224	17.23735348	33.41409864	14.01918113	7.518204692
0	3.628916522	2.948302821	7.548789839	0
0	17.23735348	0.982767607	4.313594194	1.074029242
53.8153393	38.10362348	57.00052121	90.58547807	25.7767018
16.33679943	37.19639435	16.70704932	12.94078258	20.40655559
26.90766965	32.6602487	32.43133104	26.95996371	12.8883509
33.63458706	24.49518652	18.67258454	49.60633323	21.48058484
23.06371684	12.70120783	28.50026061	51.76313033	30.07281877
16.33679943	38.10362348	8.844908464	14.01918113	26.85073104
5.765929211	3.628916522	1.965535214	5.391992742	8.592233934
66.30818592	117.939787	121.8631833	177.9357605	117.0691874
0	0	62.89712686	48.52793468	55.84952057
43.24446908	41.73254	57.98328882	58.23352162	36.51699422
7.687905614	9.979520436	12.77597889	17.25437678	22.55461408
23.06371684	16.33012435	19.65535214	37.7439492	25.7767018
23.06371684	18.14458261	59.94882404	19.41117387	32.22087725
270.9986729	331.1386326	262.3989511	400.0858615	223.3980823
0	14.51566609	30.46579582	12.94078258	9.666263176
40.36150447	39.91808174	19.65535214	48.52793468	6.444175451
49.01039829	39.01085261	29.48302821	21.56797097	42.96116967
26.90766965	35.38193609	48.15561275	29.11676081	44.03519891
13.45383482	0	0.982767607	1.078398548	1.074029242
49.01039829	19.05181174	22.60365496	26.95996371	35.44296498
0	0.907229131	0.982767607	1.078398548	0
10.57087022	16.33012435	11.79321129	11.86238403	9.666263176
85.52794996	66.22772653	77.63864096	30.19515936	28.99878953
26.90766965	29.03133218	6.87937325	49.60633323	17.18446787
74.95707974	70.76387218	83.53524661	47.44953613	44.03519891
53.8153393	54.43374783	48.15561275	38.82234774	34.36893574
27.86865785	36.28916522	78.62140857	39.90074629	45.10922815
4.804941009	0	1.965535214	0	4.296116967
0.960988202	3.628916522	0	0	1.074029242
0.960988202	93.44460045	112.0355072	81.95828968	32.22087725
0	0	0	0	0
62.46423311	60.78435175	15.72428171	59.31192017	53.70146209
190.2756639	215.9205331	117.9321129	221.0717024	117.0691874
25.94668145	19.05181174	35.37963386	14.01918113	24.70267256
56.6983039	58.06266435	78.62140857	91.66387662	63.36772526
24.98569325	35.38193609	29.48302821	26.95996371	30.07281877
10.57087022	6.350603914	1.965535214	21.56797097	13.96238014
61.50324491	88.00122566	86.48354943	77.64469549	68.73787147
0	0	414.7279302	1.078398548	1.074029242
161.4460179	174.1879931	101.2250635	83.03668823	94.51457327

For Review Only

37.47853987	21.77349913	1.965535214	2.156797097	2.148058484
54.7763275	61.69158088	87.46631704	63.62551436	59.0716083
14.41482303	12.70120783	18.67258454	11.86238403	9.666263176
79.76202075	70.76387218	76.65587336	78.72309404	28.99878953
115.3185842	98.88797523	105.156134	128.3294273	110.6250119
13.45383482	20.86627	8.844908464	32.35195645	8.592233934
0	0	0	0	0
48.04941009	30.84579044	23.58642257	33.430355	69.81190071
8.648893816	9.072291305	8.844908464	14.01918113	11.81432166
0	0	539.5394163	367.733905	316.8386263
9.609882018	4.536145653	4.913838036	10.78398548	8.592233934
0	0	0	22.64636952	24.70267256
27.86865785	30.84579044	24.56919018	19.41117387	41.88714043
109.552655	73.48555957	93.36292268	42.05754339	106.3288949
290.2184369	266.7253644	279.1060004	204.8957242	190.1031758
13.45383482	31.75301957	22.60365496	15.09757968	26.85073104
45.16644548	37.19639435	17.68981693	23.72476807	35.44296498
15.37581123	7.257833044	27.517493	4.313594194	13.96238014
85.52794996	46.26868566	89.43185225	30.19515936	64.44175451
126.8504426	89.81568392	139.5530002	101.3694636	158.9563278
28.82964605	29.03133218	16.70704932	21.56797097	26.85073104
0	3.628916522	0	29.11676081	0
106.6696904	146.9711191	17.68981693	53.91992742	49.40534512
1.921976404	7.257833044	0	0	0
19.21976404	33.56747783	24.56919018	16.17597823	36.51699422
61.50324491	52.61928957	17.68981693	1.078398548	41.88714043
703.4433637	0	0.982767607	1.078398548	1.074029242
36.51755167	65.3204974	59.94882404	45.29273904	75.18204692
2.882964605	9.979520436	2.948302821	3.235195645	7.518204692
0	0	57.00052121	52.84152887	42.96116967
33.63458706	43.54699827	23.58642257	17.25437678	30.07281877
392.0831863	406.4386505	529.7117402	485.2793468	425.3155797
24.02470504	0	28.50026061	12.94078258	20.40655559
40.36150447	19.95904087	20.63811975	30.19515936	30.07281877
54.7763275	78.92893436	61.91435925	38.82234774	59.0716083
484.3380537	451.800107	380.331064	475.5737599	284.6177491
19.21976404	19.95904087	18.67258454	8.627188388	1.074029242
295.9843661	174.1879931	123.8287185	158.5245866	209.4357021
94.17684377	129.7337657	76.65587336	54.99832597	82.70025162
11.53185842	21.77349913	2.948302821	1.078398548	1.074029242
73.99609154	64.41326827	30.46579582	38.82234774	64.44175451
96.09882018	59.87712262	39.31070429	24.80316661	64.44175451
0	0	4.913838036	0	1.074029242
97.05980838	105.2385791	19.65535214	57.15512307	78.40413465
45.16644548	53.5265187	81.56971139	54.99832597	32.22087725
161.4460179	219.5494496	213.2605707	221.0717024	197.6213805
27.86865785	44.4542274	49.13838036	53.91992742	78.40413465
27.86865785	28.12410305	44.22454232	16.17597823	36.51699422
115.3185842	95.25905871	99.25952832	101.3694636	103.1068072
5.765929211	2.721687392	3.931070429	5.391992742	0

For Review Only

27.86865785	25.40241565	26.53472539	29.11676081	20.40655559
24.98569325	17.23735348	25.55195779	23.72476807	40.81311119
45.16644548	45.36145653	13.7587465	31.27355791	56.92354981
36.51755167	17.23735348	42.25900711	28.03836226	20.40655559
43.24446908	62.59881001	45.20730993	54.99832597	49.40534512
19.21976404	13.60843696	41.2762395	25.88156516	34.36893574
15.37581123	14.51566609	34.39686625	23.72476807	17.18446787
32.67359886	37.19639435	31.44856343	31.27355791	45.10922815
22.10272864	32.6602487	14.74151411	23.72476807	37.59102346
16.33679943	24.49518652	21.62088736	14.01918113	30.07281877
39.40051627	31.75301957	57.00052121	33.430355	36.51699422
9.609882018	41.73254	1.965535214	40.97914484	20.40655559
16.33679943	12.70120783	29.48302821	11.86238403	20.40655559
74.95707974	64.41326827	66.82819729	113.2318476	49.40534512
25.94668145	23.58795739	16.70704932	20.48957242	9.666263176
4.804941009	34.47470696	2.948302821	21.56797097	38.6650527
12.49284662	12.70120783	31.44856343	3.235195645	33.29490649
0.960988202	0	0	1.078398548	2.148058484
22.10272864	32.6602487	1.965535214	16.17597823	39.73908194
42.28348088	55.34097696	30.46579582	37.7439492	65.51578375
76.87905614	85.27953827	92.38015507	51.76313033	59.0716083
27.86865785	9.979520436	18.67258454	28.03836226	5.370146209
52.8543511	33.56747783	37.34516907	51.76313033	22.55461408
47.08842189	42.63976914	32.43133104	56.07672452	66.58981299
30.75162246	7.257833044	27.517493	11.86238403	26.85073104
75.91806794	80.74339262	57.98328882	124.0158331	12.8883509
49.01039829	48.99037305	49.13838036	38.82234774	19.33252635
164.3289825	132.4554531	122.8459509	231.8556879	277.0995444
0	0	4.913838036	8.627188388	0
0	0	0	0	0
17.29778763	22.68072826	15.72428171	28.03836226	40.81311119
0.960988202	0	0	0	0
3.843952807	21.77349913	11.79321129	2.156797097	19.33252635
63.42522132	58.96989348	74.69033814	76.56629694	39.73908194
44.20545728	27.21687392	48.15561275	38.82234774	44.03519891
66.30818592	60.78435175	79.60417618	80.87989113	106.3288949
2.882964605	0	0	0	0
73.99609154	101.6096626	119.8976481	88.42868097	110.6250119
93.21585557	90.72291305	101.2250635	65.78231146	98.81069024
41.32249268	29.93856131	49.13838036	37.7439492	45.10922815
26.90766965	9.979520436	38.32793668	25.88156516	27.92476029
9.609882018	9.072291305	16.70704932	1.078398548	3.222087725
6.726917412	0	0	1.078398548	3.222087725
146.0702067	107.0530374	73.70757054	99.21266646	84.8483101
81.68399715	74.3927887	85.50078182	50.68473178	1.074029242
99.94277298	56.24820609	68.7937325	83.03668823	48.33131588
54.7763275	41.73254	47.17284514	3.235195645	49.40534512
82.64498535	51.71206044	66.82819729	73.3311013	83.77428086
29.79063425	2.721687392	0	11.86238403	22.55461408
198.9245578	0	75.67310575	105.6830577	4.296116967

For Review Only

57.65929211	20.86627	60.93159164	31.27355791	31.14684801
385.3562689	297.5711548	128.7425565	213.5229126	283.5437198
67.26917412	48.99037305	72.72480293	84.11508678	64.44175451
42.28348088	24.49518652	16.70704932	56.07672452	16.11043863
27.86865785	16.33012435	19.65535214	42.05754339	19.33252635
0.960988202	3.628916522	6.87937325	4.313594194	0
31.71261066	37.19639435	29.48302821	30.19515936	32.22087725
1.921976404	3.628916522	0	0	0
252.7398971	234.0651157	10.81044368	47.44953613	36.51699422
72.07411513	39.91808174	54.05221839	61.46871726	67.66384223
23.06371684	34.47470696	21.62088736	23.72476807	24.70267256
24.02470504	61.69158088	57.00052121	39.90074629	51.5534036
17.29778763	13.60843696	10.81044368	7.548789839	9.666263176
24.98569325	12.70120783	6.87937325	15.09757968	18.25849711
23.06371684	36.28916522	3.931070429	8.627188388	31.14684801
28.82964605	31.75301957	16.70704932	31.27355791	42.96116967
67.26917412	48.08314392	54.05221839	63.62551436	81.62622237
0.960988202	0	0.982767607	1.078398548	2.148058484
26.90766965	22.68072826	54.05221839	23.72476807	23.62864332
28.82964605	35.38193609	25.55195779	16.17597823	5.370146209
11.53185842	11.7939787	2.948302821	0	6.444175451
0	10.88674957	3.931070429	2.156797097	1.074029242
51.8933629	37.19639435	44.22454232	30.19515936	46.1832574
92.25486737	50.80483131	16.70704932	24.80316661	80.55219313
47.08842189	37.19639435	36.36240146	37.7439492	50.47937436
36.51755167	33.56747783	34.39686625	24.80316661	39.73908194
15.37581123	11.7939787	27.517493	6.470391291	6.444175451
2.882964605	2.721687392	0.982767607	2.156797097	2.148058484
16.33679943	20.86627	18.67258454	48.52793468	31.14684801
18.25877583	40.82531087	18.67258454	15.09757968	11.81432166
212.3783926	181.4458261	147.4151411	160.6813837	182.5849711
73.99609154	40.82531087	62.89712686	76.56629694	45.10922815
102.8257376	15.42289522	39.31070429	64.70391291	0
8.648893816	13.60843696	2.948302821	4.313594194	10.74029242
46.12743368	42.63976914	106.1389016	57.15512307	61.21966678
85.52794996	75.30001783	107.1216692	36.66555065	62.29369602
68.23016233	43.54699827	73.70757054	31.27355791	36.51699422
54.7763275	15.42289522	48.15561275	39.90074629	36.51699422
61.50324491	43.54699827	69.77650011	58.23352162	52.62743285
44.20545728	49.89760218	49.13838036	34.50875355	39.73908194
57.65929211	27.21687392	48.15561275	53.91992742	53.70146209
38.43952807	30.84579044	58.96605643	37.7439492	31.14684801
45.16644548	30.84579044	52.08668318	76.56629694	15.03640938
7.687905614	9.072291305	2.948302821	4.313594194	7.518204692
19.21976404	14.51566609	12.77597889	6.470391291	10.74029242
10.57087022	7.257833044	3.931070429	5.391992742	8.592233934
14.41482303	10.88674957	5.896605643	5.391992742	11.81432166
51.8933629	48.99037305	56.01775361	32.35195645	41.88714043
26.90766965	18.14458261	29.48302821	25.88156516	26.85073104
0.960988202	10.88674957	2.948302821	2.156797097	8.592233934

6.726917412	6.350603914	2.948302821	4.313594194	6.444175451
0	60.78435175	99.25952832	56.07672452	0
16.33679943	11.7939787	4.913838036	10.78398548	13.96238014
197.9635696	173.2807639	257.4851131	171.4653692	85.92233934
0	1.814458261	8.844908464	9.705586936	0
184.5097347	129.7337657	187.708613	212.444514	231.9903162
102.8257376	106.1458083	110.069972	102.4478621	94.51457327
56.6983039	36.28916522	17.68981693	28.03836226	65.51578375
0	152.4144939	30.46579582	0	60.14563754
0.960988202	29.03133218	0	160.6813837	0
191.2366522	172.3735348	158.2255847	157.4461881	120.2912751
146.0702067	113.4036413	163.1394228	161.7597823	152.5121523
107.6306786	73.48555957	39.31070429	3.235195645	33.29490649
15.37581123	21.77349913	12.77597889	25.88156516	13.96238014
9.609882018	0	0	3.235195645	2.148058484
101.8647494	133.3626822	124.8114861	130.4862244	104.1808365
17.29778763	25.40241565	3.931070429	26.95996371	5.370146209
26.90766965	16.33012435	5.896605643	29.11676081	37.59102346
12.49284662	13.60843696	26.53472539	18.33277532	11.81432166
130.6943954	117.0325578	49.13838036	126.1726302	131.0315675
54.7763275	55.34097696	62.89712686	97.05586936	71.9599592
0	0	1.965535214	0	1.074029242
106.6696904	115.2180996	101.2250635	103.5262607	150.3640938
12.49284662	12.70120783	15.72428171	4.313594194	12.8883509
181.6267701	156.9506396	179.8464721	244.7964705	196.5473512
34.59557526	34.47470696	33.41409864	54.99832597	33.29490649
49.01039829	50.80483131	79.60417618	25.88156516	8.592233934
78.80103254	63.50603914	70.75926771	75.48789839	120.2912751
40.36150447	33.56747783	12.77597889	7.548789839	7.518204692
93.21585557	79.83616349	41.2762395	74.40949984	50.47937436
31.71261066	24.49518652	30.46579582	28.03836226	40.81311119
0	0	14.74151411	190.8765431	76.25607616
15.37581123	17.23735348	4.913838036	6.470391291	11.81432166
21.14174044	28.12410305	25.55195779	22.64636952	31.14684801
641.9401188	653.204974	458.9524725	595.2759987	637.9733696
11.53185842	9.979520436	11.79321129	1.078398548	8.592233934
109.552655	136.9915987	114.0010424	106.7614563	94.51457327
142.2262539	163.3012435	161.1738876	149.8973982	114.9211289
29.79063425	8.165062175	34.39686625	33.430355	21.48058484
167.2119471	165.1157018	164.1221904	128.3294273	135.3276845
119.162537	82.55785088	122.8459509	122.9374345	156.8082693
80.72300895	83.46508001	49.13838036	75.48789839	49.40534512
118.2015488	95.25905871	59.94882404	140.1918113	106.3288949
167.2119471	155.1361813	98.27676071	141.2702098	131.0315675
0	0	6.87937325	0	0
127.8114308	205.0337835	150.3634439	119.7022389	148.2160354
23.06371684	20.86627	7.862140857	4.313594194	6.444175451
7.687905614	8.165062175	10.81044368	11.86238403	12.8883509
187.3926993	192.3325757	171.9843312	169.3085721	177.2148249
0	0	59.94882404	49.60633323	52.62743285

For Review Only

351.7216818	302.1073005	348.8825005	257.7372531	252.3968718
0.960988202	15.42289522	25.55195779	0	10.74029242
39.40051627	35.38193609	54.05221839	72.25270275	60.14563754
18.25877583	15.42289522	24.56919018	25.88156516	28.99878953
15.37581123	23.58795739	12.77597889	22.64636952	26.85073104
0	19.05181174	46.19007754	16.17597823	26.85073104
9.609882018	13.60843696	12.77597889	11.86238403	4.296116967
54.7763275	51.71206044	33.41409864	75.48789839	128.883509
11.53185842	16.33012435	20.63811975	17.25437678	38.6650527
69.19115053	43.54699827	51.10391557	38.82234774	81.62622237
0	0	0.982767607	0	0
0	0	98.27676071	71.1743042	93.44054403
45.16644548	39.01085261	18.67258454	50.68473178	133.179626
115.3185842	94.35182958	83.53524661	89.50707952	163.2524447
0.960988202	4.536145653	8.844908464	6.470391291	12.8883509
20.18075224	23.58795739	12.77597889	23.72476807	28.99878953
51.8933629	36.28916522	26.53472539	40.97914484	37.59102346
0	0	0	0	0
31.71261066	34.47470696	67.81096489	12.94078258	54.77549133
29.79063425	36.28916522	22.60365496	34.50875355	16.11043863
6.726917412	2.721687392	0.982767607	2.156797097	0
24.02470504	27.21687392	51.10391557	12.94078258	11.81432166
60.54225671	66.22772653	86.48354943	66.86071	126.7354505
108.5916668	47.17591479	89.43185225	98.13426791	57.99757905
1.921976404	3.628916522	3.931070429	0	1.074029242
468.0012543	438.19167	412.762395	0	399.5388779
105.7087022	86.1867674	88.44908464	78.72309404	120.2912751
12.49284662	12.70120783	0.982767607	4.313594194	9.666263176
51.8933629	48.99037305	105.156134	46.37113758	66.58981299
32.67359886	47.17591479	28.50026061	37.7439492	47.25728664
55.7373157	59.87712262	45.20730993	46.37113758	39.73908194
14.41482303	23.58795739	4.913838036	9.705586936	12.8883509
64.38620952	62.59881001	58.96605643	64.70391291	81.62622237
5.765929211	6.350603914	5.896605643	4.313594194	9.666263176
151.8361359	80.74339262	38.32793668	8.627188388	213.7318191
142.2262539	103.4241209	94.34569029	93.82067372	83.77428086
71.11312693	70.76387218	83.53524661	52.84152887	49.40534512
0	0	0	0	0
30.75162246	27.21687392	46.19007754	30.19515936	55.84952057
21.14174044	19.05181174	7.862140857	22.64636952	12.8883509
15.37581123	20.86627	11.79321129	24.80316661	15.03640938
23.06371684	51.71206044	31.44856343	49.60633323	54.77549133
202.7685106	175.0952222	169.0360284	172.5437678	165.4005032
2.882964605	5.443374783	55.034986	0	22.55461408
188.3536875	215.9205331	148.3979087	141.2702098	196.5473512
137.4213129	101.6096626	127.7597889	88.42868097	108.4769534

For Review Only

Sham_HSD_4	Sham_HSD_5	rCHI_ND_1	rCHI_ND_2	rCHI_ND_3
111994.6522	113139.9162	116606.6402	108861.0992	113182.3128
0	0	70.29672196	0	7.983838258
60.6013245	32.6891164	41.00642114	51.93809798	37.63809465
3.404568792	4.807222999	0	1.154179955	0
72.1768584	335.5441654	185.5052385	156.9684739	131.1630571
7.490051343	4.807222999	7.810746884	8.079259686	9.124386581
7.490051343	0	13.66880705	42.70465834	19.38932148
81.70965101	1.9228892	0	16.15851937	63.87070607
27.23655034	0	6.834403524	4.61671982	0
94.64701242	70.18545579	79.0838122	83.10095677	93.52496245
0	0	0	0	1.140548323
0	0	0.976343361	0	7.983838258
85.11421981	22.1132258	131.8063537	110.8012757	112.9142839
1.361827517	1.9228892	0	0	0
4.085482551	8.653001399	2.929030082	5.770899775	5.702741613
0	0	2.929030082	0	0
688.4038098	667.2425523	579.9479562	574.7816176	670.6424137
8.85187886	0	5.858060163	3.462539865	0
1.361827517	0	0	0	0
0	57.68667599	34.17201762	1.154179955	18.24877316
17.02284396	0	8.787090245	0	0
155.2483369	241.3225946	155.2385943	101.567836	173.363345
7.490051343	7.691556799	6.834403524	8.079259686	11.40548323
44.2593943	45.18789619	0	0	51.32467452
9.532792618	0	0.976343361	8.079259686	13.68657987
5.447310068	0	8.787090245	0	5.702741613
40.17391175	54.80234219	25.38492737	24.23777906	46.76248123
0	0	2.929030082	2.30835991	0
4.766396309	0	5.858060163	8.079259686	5.702741613
2.042741275	0	0	0	0
21.10832651	28.843338	38.07739106	33.4712187	31.93535303
55.15401443	64.41678819	64.4386618	76.17587704	61.58960942
83.75239229	71.14690039	92.75261925	103.876196	80.97893091
6.128223826	0	0	5.770899775	3.421644968
222.658799	208.6334782	279.2342011	228.5276311	256.6233726
64.00589329	49.99511919	12.69246369	35.77957861	71.85454432
61.96315202	32.6891164	47.84082467	54.24645789	52.46522284
1.361827517	1.9228892	0.976343361	0	0
12.93736141	0	5.858060163	17.31269933	3.421644968
12.25644765	12.4987798	17.57418049	9.233439641	11.40548323
42.21665302	23.0746704	43.93545122	23.0835991	36.49754632
8.85187886	5.768667599	4.881716803	20.77523919	13.68657987
32.00294665	25.9590042	25.38492737	24.23777906	11.40548323
63.32497954	0	0.976343361	1.154179955	1.140548323
7.490051343	11.5373352	5.858060163	2.30835991	9.124386581
6.809137585	0	0	0	0

For Review Only

0	0	0	1.154179955	0
5.447310068	8.653001399	2.929030082	13.85015946	5.702741613
16.3419302	52.87945299	48.81716803	1.154179955	65.01125439
28.59837785	0	31.24298754	1.154179955	15.96767652
185.8894561	106.7203506	168.9074014	92.33439641	127.7414121
0	0	201.1267323	173.1269933	153.9740236
0	0	0	0	0
0	0	0	0	0
4.766396309	26.9204488	2.929030082	21.92941915	14.82712819
0	0.9614446	1.952686721	0	0
0	6.730112199	4.881716803	0	0
3.404568792	0	1.952686721	24.23777906	13.68657987
0	0	2.929030082	0	0
0	0.9614446	0	0	0
0	4.807222999	0.976343361	0	1.140548323
0	13.4602244	4.881716803	11.54179955	0
7.490051343	28.843338	0.976343361	6.925079731	18.24877316
6.128223826	0	6.834403524	2.30835991	4.56219329
8.170965101	29.8047826	33.19567426	23.0835991	12.54603155
0	0	0	6.925079731	3.421644968
0	0	2.929030082	0	1.140548323
0	0	0	4.61671982	3.421644968
0	3.845778399	5.858060163	0	0
3.404568792	0	3.905373442	0	1.140548323
0	0	0	3.462539865	0
1054.054498	14.421669	0	876.0225859	715.1237983
1.361827517	0	0	0	0
4.766396309	0	3.905373442	9.233439641	1.140548323
2.723655034	0	0	0	0
24.5128953	19.228892	17.57418049	24.23777906	21.67041813
0	4.807222999	0.976343361	4.61671982	2.281096645
25.19380906	52.87945299	15.62149377	28.85449888	41.05973961
0	0	0	2.30835991	0
1.361827517	0	0	0	0
4.085482551	14.421669	2.929030082	6.925079731	3.421644968
0	0	4.881716803	2.30835991	7.983838258
0	0	1.952686721	1.154179955	1.140548323
1.361827517	0	0	0	0
6.128223826	22.1132258	4.881716803	0	6.843289936
7.490051343	0	9.763433606	6.925079731	14.82712819
3.404568792	4.807222999	7.810746884	8.079259686	6.843289936
4.085482551	0	0	0	0
61.28223826	53.84089759	64.4386618	45.01301825	59.30851278
0	0	0	1.154179955	1.140548323
0	0	3.905373442	3.462539865	3.421644968
6.809137585	8.653001399	0	0	0
8.85187886	56.72523139	23.43224065	19.62105924	9.124386581
0	0	3.905373442	1.154179955	1.140548323
0	0	0	0	0
29.27929161	13.4602244	24.40858401	28.85449888	33.07590136

For Review Only

0	0	2.929030082	2.30835991	2.281096645
27.23655034	19.228892	42.95910786	64.63407749	79.83838258
5.447310068	20.1903366	4.881716803	2.30835991	3.421644968
3.404568792	0	0.976343361	1.154179955	0
6.128223826	0	4.881716803	9.233439641	5.702741613
10.89462014	16.3445582	4.881716803	24.23777906	14.82712819
11.57553389	14.421669	4.881716803	2.30835991	3.421644968
0	0	0	0	0
2.723655034	0	2.929030082	3.462539865	0
2.723655034	0	0	3.462539865	0
1.361827517	0	0.976343361	0	4.56219329
0	0.9614446	0.976343361	0	2.281096645
0.680913758	0.9614446	0.976343361	0	0
4.085482551	0	2.929030082	4.61671982	4.56219329
4.766396309	0	3.905373442	1.154179955	1.140548323
2.042741275	16.3445582	2.929030082	0	5.702741613
21.78924027	12.4987798	0	0	0
0	0	0	0	0
0	0	0.976343361	1.154179955	1.140548323
10.21370638	21.1517812	3.905373442	9.233439641	17.10822484
0.680913758	3.845778399	5.858060163	5.770899775	0
17.70375772	7.691556799	14.64515041	9.233439641	11.40548323
2.723655034	3.845778399	10.73977697	4.61671982	3.421644968
30.64111913	21.1517812	34.17201762	21.92941915	25.0920631
0	0	0	13.85015946	0
0	2.8843338	2.929030082	1.154179955	1.140548323
1.361827517	0	0.976343361	0	0
0	0	0	0	3.421644968
120.5217352	68.26256659	139.6171006	66.9424374	70.713996
0.680913758	0	0	0	0
2.723655034	0	7.810746884	1.154179955	0
104.179805	70.18545579	0.976343361	45.01301825	60.4490611
0.680913758	0	5.858060163	5.770899775	2.281096645
131.4163554	1.9228892	0	75.02169708	0
11.57553389	7.691556799	5.858060163	11.54179955	15.96767652
0	0	2.929030082	0	0
74.21959967	54.80234219	66.39134852	76.17587704	50.18412619
17.02284396	26.9204488	21.47955393	15.00433942	15.96767652
1.361827517	0	0	0	3.421644968
0	0	2.929030082	0	3.421644968
0	6.730112199	0	0	11.40548323
47.66396309	88.45290319	49.79351139	68.09661735	53.60577116
0	31.7276718	16.59783713	21.92941915	9.124386581
0	0	40.03007778	46.1671982	30.79480471
147.7582856	110.566129	183.5525518	158.1226538	99.22770407
21.78924027	23.0746704	15.62149377	17.31269933	18.24877316
2.723655034	0	0	9.233439641	4.56219329
60.6013245	26.9204488	39.05373442	50.78391802	31.93535303
1.361827517	1.9228892	0	0	0
43.57848054	0	35.14836098	1.154179955	1.140548323

For Review Only

0	2.8843338	0	0	0
4.085482551	0	11.71612033	0	0
8.85187886	0	14.64515041	16.15851937	2.281096645
44.2593943	46.14934079	50.76985475	46.1671982	47.90302955
0	0	2.929030082	3.462539865	0
0.680913758	0	0.976343361	0	1.140548323
2.042741275	8.653001399	3.905373442	5.770899775	0
88.5187886	0	52.72254147	61.17153762	0
0	0	0	0	0
4.085482551	16.3445582	6.834403524	10.3876196	2.281096645
73.53868591	110.566129	103.4923962	133.8848748	62.73015774
1.361827517	0	0.976343361	1.154179955	0
5.447310068	0	1.952686721	0	5.702741613
96.00883994	26.9204488	63.46231844	43.85883829	65.01125439
9.532792618	4.807222999	14.64515041	9.233439641	7.983838258
18.38467148	21.1517812	22.45589729	23.0835991	21.67041813
155.9292507	113.4504628	124.9719502	123.4972552	156.2551202
3.404568792	7.691556799	0	0	0
15.66101644	21.1517812	16.59783713	8.079259686	21.67041813
0	11.5373352	0	4.61671982	0
11.57553389	0	11.71612033	3.462539865	6.843289936
0	4.807222999	0.976343361	0	0
15.66101644	11.5373352	15.62149377	11.54179955	31.93535303
21.78924027	14.421669	4.881716803	8.079259686	13.68657987
37.45025671	27.8818934	31.24298754	41.55047838	33.07590136
0	1.9228892	0	0	0
0.680913758	0.9614446	0	0	0
369.7361708	0	0.976343361	1.154179955	31.93535303
12.93736141	0	4.881716803	31.16285879	27.37315974
0	0	0	2.30835991	0
37.45025671	80.76134639	30.26664418	34.62539865	41.05973961
3.404568792	12.4987798	18.55052385	13.85015946	7.983838258
72.1768584	97.10590459	77.13112548	42.70465834	91.24386581
0	0	0	1.154179955	0
0	0	0	0	5.702741613
70.81503088	0	14.64515041	114.2638156	47.90302955
50.38761813	85.56856939	1.952686721	72.71333717	15.96767652
22.47015403	19.228892	25.38492737	31.16285879	17.10822484
38.81208423	16.3445582	13.66880705	6.925079731	11.40548323
12.25644765	0	10.73977697	9.233439641	1.140548323
49.70670437	0	12.69246369	17.31269933	18.24877316
29.27929161	22.1132258	3.905373442	27.70031892	11.40548323
0	0	3.905373442	0	1.140548323
2.723655034	0	0	5.770899775	0
43.57848054	63.45534359	115.2085165	55.40063784	114.0548323
100.0943225	0	0	0	42.20028794
72.85777215	17.3060028	0	21.92941915	39.91919129
17.02284396	64.41678819	3.905373442	19.62105924	39.91919129
0	0	0.976343361	0	1.140548323
13.61827517	8.653001399	13.66880705	8.079259686	4.56219329

For Review Only

36.0884292	17.3060028	23.43224065	38.08793852	9.124386581
26.55563658	27.8818934	43.93545122	42.70465834	33.07590136
23.83198155	0	16.59783713	19.62105924	5.702741613
1.361827517	2.8843338	0	0	0
98.73249498	122.1034642	123.9956068	110.8012757	96.94660742
59.23949699	124.987798	51.74619811	58.86317771	75.27618929
57.87766947	0.9614446	141.5697873	0	0
21.78924027	16.3445582	20.50321057	16.15851937	9.124386581
87.83787484	59.60956519	113.2558298	92.33439641	94.66551078
46.30213557	63.45534359	107.3977697	60.01735766	69.57344768
0	0	5.858060163	4.61671982	3.421644968
15.66101644	0	17.57418049	8.079259686	7.983838258
21.78924027	49.03367459	35.14836098	56.5548178	19.38932148
0	0	0.976343361	0	1.140548323
21.78924027	33.650561	39.05373442	47.32137816	26.23261142
12.25644765	14.421669	9.763433606	19.62105924	15.96767652
21.78924027	15.3831136	13.66880705	16.15851937	14.82712819
74.21959967	65.37823279	34.17201762	25.39195901	51.32467452
58.55858323	34.61200559	19.52686721	25.39195901	36.49754632
11.57553389	0	4.881716803	9.233439641	27.37315974
142.9918893	124.0263534	121.0665767	167.3560935	141.427992
0	23.0746704	0	0	18.24877316
5.447310068	0	3.905373442	5.770899775	3.421644968
49.02579061	37.49633939	0	0	0
14.29918893	0.9614446	13.66880705	4.61671982	2.281096645
12.25644765	0	8.787090245	2.30835991	7.983838258
3.404568792	3.845778399	3.905373442	3.462539865	6.843289936
50.38761813	20.1903366	18.55052385	41.55047838	35.356998
218.5733165	241.3225946	21.47955393	96.95111623	1.140548323
113.0316839	0	22.4558729	2.30835991	1.140548323
0	0	0	2.30835991	0
21.78924027	18.2674474	15.62149377	25.39195901	14.82712819
0	0	0	8.079259686	0
30.64111913	15.3831136	33.19567426	36.93375856	23.95151477
4.766396309	6.730112199	1.952686721	0	0
19.746499	39.41922859	17.57418049	23.0835991	23.95151477
38.13117047	48.07222999	44.91179459	31.16285879	45.6219329
81.70965101	24.9975596	84.94187237	56.5548178	78.69783426
106.2225463	59.60956519	87.87090245	53.09227793	80.97893091
46.30213557	0	60.53328835	18.46687928	23.95151477
92.60427115	104.7974614	59.55694499	102.722016	70.713996
0	6.730112199	3.905373442	2.30835991	1.140548323
11.57553389	0	7.810746884	8.079259686	11.40548323
2.042741275	3.845778399	5.858060163	4.61671982	4.56219329
206.9977826	167.2913604	105.4450829	233.1443509	117.4764772
0	0	3.905373442	0	2.281096645
0	0	0	3.462539865	0
0	0	0	0	0
2.723655034	0	0.976343361	2.30835991	2.281096645
3.404568792	0	2.929030082	3.462539865	0

For Review Only

66.72954833	37.49633939	49.79351139	45.01301825	46.76248123
0	7.691556799	0	0	0
0	0	0	0	3.421644968
0	9.614445999	1.952686721	5.770899775	3.421644968
4.085482551	0	11.71612033	2.30835991	6.843289936
255.3426594	346.1200559	317.3115922	272.3864694	236.0935028
11.57553389	26.9204488	16.59783713	21.92941915	26.23261142
21.10832651	50.95656379	74.2020954	47.32137816	36.49754632
2.042741275	0	0	0	0
14.98010269	11.5373352	15.62149377	17.31269933	7.983838258
0	0	5.858060163	0	0
0	0	1.952686721	4.61671982	3.421644968
0	0	1.952686721	3.462539865	2.281096645
234.9152467	157.6769144	157.191281	287.3908088	229.2502128
0	0	17.57418049	1.154179955	1.140548323
15.66101644	8.653001399	14.64515041	10.3876196	5.702741613
22.47015403	12.4987798	17.57418049	3.462539865	13.68657987
6.128223826	1.9228892	37.1010477	30.00867883	0
14.98010269	0	7.810746884	3.462539865	0
21.78924027	13.4602244	11.71612033	28.85449888	20.52986981
35.40751544	32.6891164	27.3376141	27.70031892	31.93535303
121.202649	96.14445999	125.9482935	102.722016	150.5523786
44.2593943	47.11078539	66.39134852	71.55915722	65.01125439
1.361827517	2.8843338	0	0	0
12.93736141	41.34211779	2.929030082	17.31269933	31.93535303
0	0	18.55052385	1.154179955	57.02741613
0	0	0	1.154179955	0
43.57848054	72.10834499	54.67522819	46.1671982	44.48138458
7.490051343	0	0	12.69597951	4.56219329
4.085482551	0	0	0	1.140548323
0	0	5.858060163	1.154179955	0
0	0	0.976343361	6.925079731	5.702741613
4.766396309	0	3.905373442	2.30835991	0
2.042741275	0	2.929030082	0	1.140548323
24.5128953	19.228892	0	10.3876196	12.54603155
19.06558524	54.80234219	4.881716803	36.93375856	27.37315974
0	0	0	0	0
0	2.8843338	6.834403524	0	0
17.70375772	10.5758906	36.12470434	31.16285879	41.05973961
42.21665302	59.60956519	43.93545122	21.92941915	34.21644968
0	0	0	0	0
25.19380906	8.653001399	17.57418049	11.54179955	30.79480471
34.72660168	22.1132258	25.38492737	26.54613897	53.60577116
3.404568792	0	4.881716803	6.925079731	3.421644968
0	0	1.952686721	0	1.140548323
4.766396309	14.421669	0	10.3876196	6.843289936
10.21370638	2.8843338	1.952686721	6.925079731	7.983838258
0	0	16.59783713	6.925079731	4.56219329
12.93736141	0	21.47955393	13.85015946	6.843289936
8.85187886	0	20.50321057	5.770899775	10.2649349

For Review Only

0	0	5.858060163	0	0
193.3795074	185.5588078	105.4450829	215.8316516	207.5797947
0	0	0	0	0
10.89462014	22.1132258	25.38492737	32.31703874	21.67041813
234.2343329	0	96.65799269	55.40063784	75.27618929
9.532792618	0	7.810746884	1.154179955	4.56219329
0	0	1.952686721	1.154179955	1.140548323
0	0	0	0	0
33.36477416	29.8047826	30.26664418	27.70031892	23.95151477
0.680913758	0	0	8.079259686	11.40548323
14.98010269	0	9.763433606	4.61671982	10.2649349
13.61827517	32.6891164	13.66880705	27.70031892	19.38932148
0	0	0	0	0
38.81208423	24.036115	50.76985475	26.54613897	50.18412619
8.170965101	16.3445582	3.905373442	9.233439641	0
12.93736141	0	4.881716803	8.079259686	7.983838258
4.766396309	0	5.858060163	0	0
2.042741275	2.8843338	3.905373442	0	1.140548323
6.809137585	0.9614446	6.834403524	3.462539865	20.52986981
0	0.9614446	0	0	0
2.042741275	0	4.881716803	0	0
2.042741275	8.653001399	0.976343361	0	9.124386581
12.25644765	46.14934079	0	0	0
4.085482551	7.691556799	7.810746884	0	1.140548323
11.57553389	0	2.929030082	3.462539865	1.140548323
0	0	0	0	0
10.21370638	0	5.858060163	2.30835991	18.24877316
0	0	14.64515041	8.079259686	5.702741613
0	10.5758906	0	0	10.2649349
0	0	12.69246369	2.30835991	7.983838258
29.27929161	18.2674474	12.69246369	30.00867883	29.65425639
0	3.845778399	0.976343361	0	5.702741613
6.809137585	21.1517812	14.64515041	2.30835991	6.843289936
4.766396309	17.3060028	19.52686721	10.3876196	4.56219329
0	0	0	0	0
4.766396309	0	4.881716803	9.233439641	2.281096645
2.042741275	2.8843338	0.976343361	1.154179955	1.140548323
30.64111913	76.91556799	31.24298754	51.93809798	41.05973961
7.490051343	0	6.834403524	8.079259686	9.124386581
11.57553389	0	3.905373442	12.69597951	9.124386581
2.723655034	4.807222999	6.834403524	0	1.140548323
4.085482551	0	6.834403524	6.925079731	0
0	0	0	0	0
11.57553389	0	3.905373442	3.462539865	5.702741613
40.85482551	30.7662272	63.46231844	40.39629843	29.65425639
0	0	0	0	1.140548323
4.085482551	0	0	0	0
2.723655034	0	0	3.462539865	3.421644968
0	0	0	0	1.140548323
17.70375772	6.730112199	27.3376141	24.23777906	15.96767652

6.128223826	7.691556799	1.952686721	6.925079731	7.983838258
17.70375772	49.99511919	7.810746884	13.85015946	14.82712819
0.680913758	0	0	0	0
24.5128953	0	9.763433606	4.61671982	10.2649349
1.361827517	0	0	4.61671982	0
4.085482551	0	1.952686721	0	0
0	0	0	0	0
0	0.9614446	0	0	0
7.490051343	0.9614446	2.929030082	9.233439641	1.140548323
1.361827517	1.9228892	0.976343361	1.154179955	1.140548323
4.766396309	6.730112199	9.763433606	11.54179955	2.281096645
55.15401443	23.0746704	59.55694499	49.62973807	18.24877316
0	0	2.929030082	0	3.421644968
12.25644765	0	0	6.925079731	2.281096645
0	0	4.881716803	1.154179955	1.140548323
6.809137585	10.5758906	7.810746884	0	5.702741613
1.361827517	0	0.976343361	0	0
2.042741275	0	0.976343361	1.154179955	1.140548323
0	0	3.905373442	0	1.140548323
0	0	2.929030082	1.154179955	0
0	0	1.952686721	2.30835991	4.56219329
0	0	0	0	0
2.042741275	0	0	4.61671982	0
0	0	0	0	0
2.042741275	0.9614446	1.952686721	3.462539865	2.281096645
2.723655034	0	0	0	0
0	0	0	2.30835991	0
0	0	0	0	3.421644968
0	21.1517812	0	1.154179955	1.140548323
27.9174641	0	10.73977697	12.69597951	45.6219329
2.042741275	0	0.976343361	0	0
0	0	0.976343361	4.61671982	4.56219329
9.532792618	0	4.881716803	1.154179955	1.140548323
3.404568792	0	0	1.154179955	0
6.128223826	9.614445999	5.858060163	5.770899775	0
10.21370638	0	2.929030082	2.30835991	12.54603155
8.85187886	0	10.73977697	2.30835991	3.421644968
4.766396309	12.4987798	10.73977697	4.61671982	3.421644968
4.766396309	14.421669	9.763433606	12.69597951	12.54603155
4.766396309	0	2.929030082	0	5.702741613
2.723655034	0	0.976343361	4.61671982	0
81.70965101	150.9468022	81.03649893	133.8848748	110.6331873
3.404568792	1.9228892	0	0	0
40.85482551	31.7276718	43.93545122	50.78391802	51.32467452
2.723655034	0	6.834403524	1.154179955	0
51.74944564	32.6891164	47.84082467	73.86751713	37.63809465
51.06853188	88.45290319	116.1848599	117.7263554	90.10331749
77.62416846	129.795021	37.1010477	81.94677681	99.22770407
0.680913758	0	0	0	0
32.68386041	29.8047826	41.00642114	16.15851937	19.38932148

For Review Only

4.085482551	0	4.881716803	0	0
4.766396309	0	2.929030082	4.61671982	5.702741613
16.3419302	9.614445999	20.50321057	12.69597951	4.56219329
0	0	0	0	0
3.404568792	0	2.929030082	3.462539865	3.421644968
194.7413349	96.14445999	106.4214263	267.7697496	205.2986981
0	0	2.929030082	3.462539865	2.281096645
4.766396309	6.730112199	7.810746884	6.925079731	1.140548323
7.490051343	0	3.905373442	6.925079731	6.843289936
0	0	5.858060163	1.154179955	0
0	3.845778399	0	0	0
2.042741275	0	0	0	1.140548323
17.02284396	31.7276718	11.71612033	45.01301825	10.2649349
2.723655034	0	0	0	0
6.128223826	0	2.929030082	3.462539865	1.140548323
26.55563658	0	44.91179459	6.925079731	12.54603155
0	0	0	1.154179955	0
0.680913758	0	0.976343361	1.154179955	6.843289936
15.66101644	0	7.810746884	10.3876196	13.68657987
7.490051343	0	1.952686721	2.30835991	3.421644968
0	0	1.952686721	6.925079731	3.421644968
3.404568792	5.768667599	3.905373442	3.462539865	4.56219329
12.93736141	59.60956519	11.71612033	15.00433942	12.54603155
29.27929161	6.730112199	3.905373442	6.925079731	78.69783426
0	0	40.03007778	1.154179955	1.140548323
21.10832651	32.6891164	33.19567426	31.16285879	35.356998
0.680913758	0.9614446	0	0	0
0	18.2674474	0	0	1.140548323
11.57553389	0	11.71612033	6.925079731	22.81096645
1.361827517	0	0	0	0
0	0	0	0	0
2.723655034	0	0	0	7.983838258
42.89756678	9.614445999	98.61067942	0	0
2.042741275	0	1.952686721	0	1.140548323
17.02284396	28.843338	19.52686721	15.00433942	21.67041813
5.447310068	0	0	0	0
0	0.9614446	2.929030082	0	1.140548323
249.2144356	102.8745722	98.61067942	85.40931668	87.82222084
11.57553389	28.843338	8.787090245	4.61671982	12.54603155
4.766396309	0	2.929030082	0	6.843289936
0	0.9614446	0.976343361	1.154179955	1.140548323
0	0	0	0	0
104.8607188	35.57345019	78.10746884	83.10095677	174.5038934
4.085482551	0	0	0	3.421644968
0	0	0	3.462539865	2.281096645
0.680913758	0	3.905373442	4.61671982	0
2.723655034	0	0.976343361	1.154179955	0
18.38467148	23.0746704	29.29030082	26.54613897	18.24877316
131.4163554	102.8745722	104.4687396	123.4972552	139.1468954
0	0	0	0	0

For Review Only

5.447310068	46.14934079	20.50321057	0	20.52986981
17.70375772	0	1.952686721	1.154179955	1.140548323
0	0	0	0	0
15.66101644	0	19.52686721	16.15851937	6.843289936
6.128223826	11.5373352	4.881716803	4.61671982	1.140548323
4.766396309	2.8843338	1.952686721	6.925079731	4.56219329
41.53573927	0	37.1010477	62.32571757	102.649349
8.85187886	0	5.858060163	1.154179955	1.140548323
1.361827517	0	0	3.462539865	0
0.680913758	0	0	0	4.56219329
85.11421981	52.87945299	0	0	0
19.06558524	5.768667599	13.66880705	9.233439641	6.843289936
31.32203289	30.7662272	33.19567426	11.54179955	39.91919129
2.042741275	0.9614446	0.976343361	1.154179955	0
0	0	1.952686721	2.30835991	1.140548323
2.723655034	0	2.929030082	0	0
0	13.4602244	5.858060163	2.30835991	9.124386581
0	0	0	3.462539865	3.421644968
108.9462014	91.33723699	86.89455909	76.17587704	114.0548323
14.98010269	0	8.787090245	0	0
0	0	2.929030082	1.154179955	1.140548323
0	0	0	0	0
45.62122182	38.45778399	66.39134852	54.24645789	80.97893091
17.02284396	28.843338	41.00642114	12.69597951	7.983838258
0	0	0	0	0
13.61827517	7.691556799	18.55052385	6.925079731	4.56219329
202.2313863	193.2503646	241.1568101	200.8273122	187.0499249
0	0	15.62149377	17.31269933	25.0920631
9.532792618	9.614445999	11.71612033	9.233439641	10.2649349
17.02284396	43.26500699	34.17201762	24.23777906	39.91919129
6.128223826	12.4987798	0.976343361	1.154179955	1.140548323
0	0	0	0	0
0	0	1.952686721	0	0
5.447310068	8.653001399	0	3.462539865	6.843289936
16.3419302	7.691556799	19.52686721	11.54179955	12.54603155
14.29918893	0.9614446	8.787090245	13.85015946	17.10822484
90.56152987	97.10590459	97.63433606	118.8805354	91.24386581
20.42741275	0	11.71612033	12.69597951	12.54603155
10.89462014	10.5758906	17.57418049	16.15851937	15.96767652
30.64111913	0	27.3376141	6.925079731	33.07590136
54.47310068	86.53001399	67.36769188	75.02169708	55.88686781
2.042741275	0	4.881716803	0	7.983838258
3.404568792	0	3.905373442	1.154179955	1.140548323
3.404568792	0	0.976343361	0	0
23.15106779	35.57345019	11.71612033	16.15851937	20.52986981
96.00883994	147.1010238	167.931058	113.1096356	109.492639
164.7811295	134.602244	217.7245694	122.3430752	157.3956685
66.04863457	85.56856939	90.79993253	94.64275632	90.10331749
53.11127316	17.3060028	39.05373442	12.69597951	17.10822484
9.532792618	0	5.858060163	6.925079731	4.56219329

For Review Only

66.04863457	0	140.5934439	115.4179955	31.93535303
0.680913758	3.845778399	0	0	0
24.5128953	11.5373352	6.834403524	10.3876196	20.52986981
6.128223826	22.1132258	10.73977697	1.154179955	2.281096645
0	3.845778399	0	9.233439641	0
35.40751544	15.3831136	35.14836098	35.77957861	14.82712819
3.404568792	0	2.929030082	11.54179955	3.421644968
0.680913758	0	0	0	1.140548323
10.21370638	18.2674474	7.810746884	3.462539865	3.421644968
16.3419302	0	0.976343361	5.770899775	3.421644968
0	0	3.905373442	0	2.281096645
105.5416326	87.49145859	100.5633661	99.25947614	92.38441413
0	0	1.952686721	0	1.140548323
18.38467148	35.57345019	13.66880705	24.23777906	27.37315974
6.809137585	0	5.858060163	6.925079731	7.983838258
2.042741275	0	0	5.770899775	0
0	0	58.58060163	0	23.95151477
42.21665302	44.22645159	31.24298754	19.62105924	43.34083626
0	0	0	1.154179955	1.140548323
0.680913758	0	0	0	0
2.042741275	0	0.976343361	1.154179955	0
9.532792618	12.4987798	14.64515041	5.770899775	11.40548323
26.55563658	11.5373352	18.55052385	18.46687928	19.38932148
6.809137585	0	0.976343361	0	2.281096645
10.21370638	0	0.976343361	1.154179955	0
7.490051343	7.691556799	8.787090245	15.00433942	19.38932148
0	0	2.929030082	2.30835991	0
22.47015403	10.5758906	28.31395746	11.54179955	9.124386581
89.19970236	71.14690039	77.13112548	100.4136561	120.8981222
0	0	0.976343361	1.154179955	1.140548323
0	0	0	0	4.56219329
2.723655034	0.9614446	2.929030082	0	0
0	0	1.952686721	0	0
429.6565816	317.276718	435.4491388	395.8837246	491.576327
0	0	2.929030082	3.462539865	2.281096645
19.06558524	28.843338	17.57418049	4.61671982	14.82712819
0	0	0	0	1.140548323
0	0	0	0	2.281096645
0	0	0	0	2.281096645
39.49299799	0	36.12470434	53.09227793	18.24877316
12.93736141	43.26500699	32.2193309	28.85449888	46.76248123
14.98010269	20.1903366	2.929030082	3.462539865	15.96767652
1.361827517	0	7.810746884	6.925079731	5.702741613
54.47310068	32.6891164	32.2193309	42.70465834	60.4490611
46.30213557	99.99023839	78.10746884	51.93809798	60.4490611
70.81503088	26.9204488	13.66880705	34.62539865	17.10822484
0	0	0	0	0
3.404568792	0	0	0	1.140548323
40.17391175	72.10834499	63.46231844	28.85449888	67.29235103
13.61827517	0	5.858060163	5.770899775	1.140548323

For Review Only

2.042741275	6.730112199	0.976343361	10.3876196	0
230.1488504	313.4309396	92.75261925	210.0607518	371.8187532
0	0	0.976343361	3.462539865	0
0	0	0	2.30835991	2.281096645
11.57553389	0	3.905373442	0	6.843289936
5.447310068	0	1.952686721	5.770899775	5.702741613
0	0	0.976343361	4.61671982	0
16.3419302	9.614445999	1.952686721	9.233439641	3.421644968
1.361827517	0	0	0	0
0.680913758	0	6.834403524	2.30835991	0
0	0	1.952686721	6.925079731	0
0	0	0	1.154179955	0
93.96609867	0	82.01284229	1.154179955	57.02741613
2.723655034	0	0	0	0
20.42741275	56.72523139	44.91179459	18.46687928	44.48138458
2.042741275	0	2.929030082	0	0
21.78924027	0	13.66880705	26.54613897	19.38932148
37.45025671	25.9590042	44.91179459	50.78391802	68.43289936
0	0	0	4.61671982	3.421644968
13.61827517	0	7.810746884	17.31269933	14.82712819
0	0	3.905373442	2.30835991	2.281096645
6.809137585	0	2.929030082	0	0
9.532792618	14.421669	22.45589729	5.770899775	5.702741613
4.766396309	0	9.763433606	2.30835991	9.124386581
3.404568792	4.807222999	5.858060163	2.30835991	5.702741613
4.085482551	0	3.905373442	1.154179955	7.983838258
0	0	0.976343361	18.46687928	1.140548323
5.447310068	0	5.858060163	0	0
38.13117047	77.87701259	86.89455909	69.25079731	112.9142839
88.5187886	0	129.853667	10.3876196	3.421644968
7.490051343	0	0	12.69597951	0
29.27929161	65.37823279	45.88813795	49.62973807	78.69783426
0	0	3.905373442	0	1.140548323
7.490051343	0.9614446	7.810746884	5.770899775	12.54603155
8.170965101	24.9975596	4.881716803	8.079259686	3.421644968
7.490051343	0	0	4.61671982	4.56219329
5.447310068	0	4.881716803	3.462539865	4.56219329
0	0	0	1.154179955	1.140548323
0	0	0	0	0
2.723655034	0	9.763433606	0	3.421644968
0	0	0	3.462539865	0
0	0	0	1.154179955	0
0	0	0.976343361	9.233439641	13.68657987
0	0	0	0	3.421644968
2.042741275	7.691556799	0.976343361	3.462539865	0
8.170965101	24.9975596	13.66880705	12.69597951	12.54603155
21.10832651	55.76378679	27.3376141	39.24211847	28.51370807
3.404568792	0.9614446	1.952686721	2.30835991	5.702741613
12.93736141	0	3.905373442	11.54179955	2.281096645
0	0	1.952686721	3.462539865	3.421644968

For Review Only

8.85187886	15.3831136	12.69246369	12.69597951	5.702741613
22.47015403	33.650561	22.45589729	21.92941915	21.67041813
6.809137585	3.845778399	0	0	3.421644968
13.61827517	0	5.858060163	6.925079731	13.68657987
6.128223826	25.9590042	4.881716803	10.3876196	19.38932148
0	0	0.976343361	0	1.140548323
2.723655034	0	4.881716803	1.154179955	1.140548323
4.085482551	0	5.858060163	0	3.421644968
2.723655034	3.845778399	1.952686721	3.462539865	3.421644968
5.447310068	2.8843338	4.881716803	4.61671982	9.124386581
11.57553389	0	4.881716803	9.233439641	0
6.128223826	0	0	2.30835991	4.56219329
55.15401443	0	43.93545122	24.23777906	34.21644968
23.83198155	64.41678819	44.91179459	19.62105924	34.21644968
0	0	0.976343361	1.154179955	2.281096645
863.3986457	0.9614446	17.57418049	8.079259686	86.68167252
150.4819406	95.18301539	141.5697873	118.8805354	124.3197672
30.64111913	0	12.69246369	12.69597951	25.0920631
471.1923208	344.1971667	368.0814469	305.8576881	460.7815223
2.042741275	0	0	0	0
127.3308728	72.10834499	108.374113	102.722016	88.96276916
246.4907806	311.5080504	327.0750258	210.0607518	257.7639209
0	41.34211779	56.62791491	15.00433942	36.49754632
80.3478235	50.95656379	48.81716803	11.54179955	62.73015774
25.19380906	30.7662272	31.24298754	20.77523919	57.02741613
59.23949699	38.45778399	48.81716803	28.85449888	38.77864297
0	0	0	0	0
145.7155443	40.38067319	123.0192634	146.5808543	123.1792188
26.55563658	21.1517812	62.48597508	39.24211847	13.68657987
1.361827517	0	0	0	0
0	0	0	0	0
829.3529578	466.3006309	683.4403524	1225.739112	672.9235103
0	0	5.858060163	3.462539865	4.56219329
1.361827517	2.8843338	0	0	0
92.60427115	156.7154698	125.9482935	120.0347153	138.006347
168.8666121	151.9082468	202.1030756	222.7567313	286.277629
0	0	0	0	0
0	6.730112199	7.810746884	0	9.124386581
0	0	0	1.154179955	0
1291.012486	1208.535862	901.1649218	1586.997438	1324.176603
46.98304933	13.4602244	34.17201762	9.233439641	31.93535303
0	19.228892	0	0	6.843289936
44.2593943	70.18545579	45.88813795	33.4712187	59.30851278
24.5128953	69.22401119	48.81716803	47.32137816	44.48138458
78.30508222	16.3445582	95.68164933	10.3876196	43.34083626
17.02284396	13.4602244	15.62149377	36.93375856	33.07590136
42.89756678	54.80234219	53.69888483	42.70465834	35.356998
4.766396309	28.843338	51.74619811	10.3876196	11.40548323
91.92335739	131.7179102	172.8127748	69.25079731	181.3471833
38.81208423	34.61200559	96.65799269	27.70031892	52.46522284

For Review Only

675.4664484	674.9341091	1043.711052	932.5774037	704.8588634
51.74944564	53.84089759	58.58060163	65.78825744	62.73015774
557.6683682	880.6832535	587.7587031	692.5079731	1026.49349
147.7582856	49.99511919	0	108.4929158	211.0014397
245.8098668	317.276718	290.9503214	380.8793852	294.2614672
129.3736141	101.9131276	248.9675569	165.0477336	152.8334752
47.66396309	44.22645159	57.60425827	42.70465834	27.37315974
72.1768584	68.26256659	149.3805342	211.2149318	104.9304457
100.0943225	128.8335764	156.2149377	105.0303759	127.7414121
132.7781829	96.14445999	134.7353838	85.40931668	123.1792188
147.0773718	50.95656379	137.6644138	0	0
638.0161917	562.4450909	737.1392372	783.6881895	603.3500627
50.38761813	64.41678819	90.79993253	69.25079731	59.30851278
31.32203289	68.26256659	54.67522819	36.93375856	51.32467452
0	0	0	0	0
31.32203289	41.34211779	36.12470434	56.5548178	29.65425639
0	0	0	0	0
5.447310068	19.228892	10.73977697	0	18.24877316
43.57848054	92.29868159	28.31395746	39.24211847	52.46522284
37.45025671	0	16.59783713	3.462539865	10.2649349
42.21665302	0	19.52686721	50.78391802	60.4490611
0	0	0	0	0
224.0206265	0	153.2859076	24.23777906	0
32.00294665	48.07222999	27.3376141	31.16285879	2.281096645
12.25644765	40.38067319	8.787090245	36.93375856	4.56219329
38.81208423	127.8721318	21.47955393	54.24645789	42.20028794
11.57553389	0	16.59783713	4.61671982	23.95151477
138.9064067	107.6817952	90.79993253	96.95111623	77.55728594
16.3419302	0	18.55052385	19.62105924	20.52986981
276.4509859	236.5153716	258.7309905	192.7480525	253.2017276
54.47310068	97.10590459	64.4386618	66.9424374	54.74631949
184.5276285	139.409467	220.6535995	100.4136561	182.4877316
44.2593943	63.45534359	13.66880705	19.62105924	42.20028794
268.2800208	197.096143	289.9739781	270.0781095	252.0611793
30.64111913	0	14.64515041	204.2898521	35.356998
130.7354416	460.5319633	964.6272402	2.30835991	291.9803706
0	0	0	2.30835991	0
321.391294	707.6232255	413.9695849	392.4211847	354.7105283
97.37066746	36.53489479	64.4386618	108.4929158	103.7898974
35.40751544	63.45534359	67.36769188	77.33005699	37.63809465
2.723655034	0	0.976343361	0	0
214.4878339	316.3152734	246.0385269	242.3777906	262.3261142
2.042741275	0	2.929030082	0	0
28.59837785	46.14934079	33.19567426	25.39195901	30.79480471
68.7722896	52.87945299	8.787090245	16.15851937	47.90302955
0	0	0	0	0
211.7641789	78.83845719	211.8665092	1.154179955	35.356998
32.00294665	16.3445582	56.62791491	0	53.60577116
44.2593943	66.33967739	43.93545122	39.24211847	47.90302955
11.57553389	0	9.763433606	8.079259686	20.52986981

For Review Only

0	0	0	0	0
8.85187886	5.768667599	34.17201762	15.00433942	12.54603155
17.70375772	82.68423559	8.787090245	36.93375856	29.65425639
27.23655034	13.4602244	45.88813795	27.70031892	33.07590136
56.51584195	69.22401119	65.41500516	84.25513672	96.94660742
57.19675571	92.29868159	80.06015557	46.1671982	82.11947923
76.26234095	102.8745722	60.53328835	75.02169708	76.41673761
153.8865094	189.4045862	208.9374792	144.2724944	95.8060591
27.23655034	99.02879379	36.12470434	92.33439641	23.95151477
42.21665302	106.7203506	58.58060163	43.85883829	69.57344768
0	0	0	0	50.18412619
2.042741275	0	0	0	0
206.9977826	192.28892	258.7309905	272.3864694	318.212982
11.57553389	61.53245439	10.73977697	15.00433942	19.38932148
110.9889426	75.95412339	105.4450829	118.8805354	98.08715574
34.04568792	10.5758906	26.36127073	31.16285879	25.0920631
10.89462014	0	0.976343361	5.770899775	4.56219329
99.41340873	86.53001399	88.84724581	85.40931668	75.27618929
12.93736141	0	6.834403524	9.233439641	13.68657987
34.04568792	46.14934079	41.00642114	43.85883829	52.46522284
17.70375772	37.49633939	12.69246369	9.233439641	9.124386581
61.96315202	23.0746704	62.48597508	122.3430752	79.83838258
0	0	0	0	0
0	0	0	0	0
0	0	2.929030082	0	4.56219329
226.7442816	288.43338	265.5653941	283.928269	269.1694041
76.26234095	56.72523139	47.84082467	32.31703874	74.13564097
136.1827517	210.5563674	146.4515041	188.1313327	173.363345
0	0	3.905373442	3.462539865	2.281096645
4.766396309	0	0	1.154179955	2.281096645
323.4340353	309.5851612	436.4254822	311.6285879	350.148335
0	0	0	2.30835991	0
86.47604732	72.10834499	94.70530597	83.10095677	83.26002755
268.2800208	155.7540252	292.9030082	200.8273122	265.7477592
3.404568792	0.9614446	0	1.154179955	1.140548323
17.02284396	7.691556799	16.59783713	16.15851937	4.56219329
8.85187886	23.0746704	23.43224065	0	9.124386581
70.81503088	49.03367459	65.41500516	33.4712187	49.04357787
30.64111913	53.84089759	35.14836098	21.92941915	30.79480471
2.042741275	0	0	0	0
12.25644765	6.730112199	0	10.3876196	0
0	0	0	0	0
93.96609867	0	29.29030082	36.93375856	25.0920631
119.1599077	81.72279099	122.0429201	116.5721755	135.7252504
135.5018379	386.5007291	458.8813795	116.5721755	152.8334752
0	0.9614446	4.881716803	0	0
53.79218692	30.7662272	39.05373442	45.01301825	52.46522284
870.2077833	645.1293265	943.1476863	797.538349	890.76824
0	0	0	0	0
7.490051343	32.6891164	20.50321057	0	15.96767652

For Review Only

19.746499	54.80234219	58.58060163	21.92941915	53.60577116
102.1370638	106.7203506	135.7117271	64.63407749	93.52496245
0	0	0	0	7.983838258
106.2225463	92.29868159	84.94187237	133.8848748	122.0386705
251.2571769	247.0912622	223.5826296	176.5895331	226.9691162
0	0	0	0	3.421644968
243.0862118	299.9707152	610.2146003	278.1573692	325.0562719
0	34.61200559	6.834403524	3.462539865	7.983838258
5.447310068	0	7.810746884	8.079259686	2.281096645
1928.347764	1674.836493	0	5870.159252	6633.429044
0	0	0	3.462539865	0
89.88061612	92.29868159	67.36769188	105.0303759	98.08715574
3.404568792	34.61200559	5.858060163	0	10.2649349
2.723655034	0	0	0	0
203.5932138	151.9082468	111.3031431	91.18021645	195.0337632
133.4590967	193.2503646	185.5052385	117.7263554	77.55728594
313.2203289	302.855049	352.4599532	302.3951482	330.7590136
0	0	29.29030082	0	11.40548323
255.3426594	215.3635904	250.9202437	435.1258431	331.8995619
339.7759655	342.2742776	464.7394396	337.0205469	318.212982
14.29918893	35.57345019	0	0	0
19.746499	12.4987798	41.00642114	8.079259686	17.10822484
179.7612322	123.0649088	48.81716803	17.31269933	164.2389585
94.64701242	21.1517812	105.4450829	75.02169708	65.01125439
7.490051343	0	6.834403524	3.462539865	44.48138458
0	0	1.952686721	0	0
49.70670437	41.34211779	32.2193309	72.71333717	65.01125439
51.06853188	121.1420196	59.55694499	79.6384169	67.29235103
74.90051343	75.95412339	72.24940868	57.70899775	54.74631949
67.41046209	33.650561	58.58060163	31.16285879	84.40057587
10.89462014	8.653001399	6.834403524	12.69597951	10.2649349
17.02284396	0	23.43224065	5.770899775	13.68657987
44.2593943	22.1132258	32.2193309	28.85449888	25.0920631
627.8024853	1072.010729	572.1372093	1028.37434	638.7070607
0	0	750.8080443	0	0
379.2689635	423.0356239	461.8104095	435.1258431	500.7007136
0.680913758	9.614445999	0	1.154179955	3.421644968
14.29918893	0	30.26664418	8.079259686	3.421644968
0	0.9614446	0.976343361	1.154179955	0
15.66101644	23.0746704	14.64515041	33.4712187	25.0920631
14.98010269	17.3060028	7.810746884	18.46687928	20.52986981
0	0	1.952686721	0	0
0.680913758	0	0	0	0
17.70375772	11.5373352	12.69246369	31.16285879	21.67041813
8.85187886	0.9614446	7.810746884	5.770899775	7.983838258
63.32497954	94.22157079	58.58060163	64.63407749	77.55728594
0	0	112.2794865	73.86751713	42.20028794
64.00589329	0	92.75261925	4.61671982	110.6331873
0	0	0.976343361	4.61671982	0
96.00883994	88.45290319	116.1848599	96.95111623	126.6008638

For Review Only

7.490051343	0	0	0	0
6.809137585	0	12.69246369	10.3876196	2.281096645
0	6.730112199	0	0	1.140548323
14.98010269	32.6891164	35.14836098	15.00433942	23.95151477
0	19.228892	0.976343361	21.92941915	1.140548323
14.29918893	21.1517812	17.57418049	8.079259686	28.51370807
53.11127316	37.49633939	27.3376141	30.00867883	45.6219329
123.926304	173.060028	110.3267997	115.4179955	102.649349
37.45025671	10.5758906	40.03007778	48.47555811	31.93535303
12.25644765	0	12.69246369	8.079259686	4.56219329
3.404568792	0	0	0	9.124386581
20.42741275	12.4987798	8.787090245	6.925079731	12.54603155
83.07147853	90.37579239	57.60425827	61.17153762	85.5411242
87.83787484	110.566129	126.9246369	110.8012757	148.2712819
51.06853188	0	39.05373442	11.54179955	33.07590136
72.1768584	79.79990179	62.48597508	58.86317771	66.15180271
0	0	70.29672196	2.30835991	70.713996
0	0	0	2.30835991	0
0	0	3.905373442	0	2.281096645
27.9174641	0	7.810746884	6.925079731	0
4.085482551	0	0	0	0
15.66101644	0	23.43224065	18.46687928	20.52986981
33.36477416	86.53001399	247.0148702	241.2236106	143.7090886
12.25644765	24.9975596	22.45589729	53.09227793	25.0920631
57.87766947	128.8335764	82.01284229	95.79693627	55.88686781
202.2313863	346.1200559	199.1740456	251.6112302	223.5474712
0	0	0	0	4.56219329
74.90051343	78.83845719	64.4386618	69.25079731	106.070994
0	0	0	0	0
0	6.730112199	2.929030082	0	0
25.19380906	24.9975596	8.787090245	31.16285879	11.40548323
66.72954833	182.674474	498.9114572	422.4298636	372.9593015
0	9.614445999	8.787090245	0	6.843289936
14.98010269	0	0	0	1.140548323
0	0	0	0	0
0	0	0	0	0
1980.09721	2126.715455	2964.178443	1578.918179	1880.764184
0	0	0.976343361	1.154179955	1.140548323
29.96020537	48.07222999	27.3376141	42.70465834	20.52986981
25.87472282	45.18789619	41.9827645	43.85883829	39.91919129
0	0	0	0	0
197.46499	145.1781346	180.6235217	155.8142939	79.83838258
17.70375772	24.036115	23.43224065	23.0835991	21.67041813
115.0744252	131.7179102	119.11389	87.71767659	106.070994
411.9528239	461.4934079	236.2750933	455.9010823	423.1434277
87.83787484	49.99511919	81.03649893	99.25947614	72.99509265
18.38467148	0	5.858060163	0	0
36.0884292	59.60956519	49.79351139	68.09661735	31.93535303
100.7752363	12.4987798	0	166.2019135	195.0337632
0	13.4602244	10.73977697	2.30835991	9.124386581

For Review Only

0	6.730112199	0	0	0
72.1768584	435.5344037	373.9395071	0	0
79.66690974	74.99267879	51.74619811	92.33439641	92.38441413
339.0950517	407.6525103	412.9932415	222.7567313	344.4455934
0	0	51.74619811	5.770899775	91.24386581
1.361827517	0.9614446	0	65.78825744	0
4.085482551	0	4.881716803	3.462539865	0
0	0	0	0	0
0	0	0	0	1.140548323
0	0	0	1.154179955	0
20.42741275	38.45778399	15.62149377	26.54613897	15.96767652
1029.541603	780.6930151	949.0057465	803.3092487	1050.445005
0	39.41922859	0	0	1.140548323
98.05158122	0	96.65799269	169.6644534	104.9304457
133.4590967	81.72279099	0	54.24645789	76.41673761
103.4988913	108.6432398	122.0429201	83.10095677	82.11947923
49.70670437	97.10590459	43.93545122	50.78391802	93.52496245
38.13117047	15.3831136	42.95910786	13.85015946	17.10822484
7.490051343	0	1.952686721	2.30835991	1.140548323
0	0	0	0	0
44.2593943	49.99511919	28.31395746	43.85883829	31.93535303
51.06853188	61.53245439	65.41500516	36.93375856	49.04357787
18.38467148	58.64812059	11.71612033	12.69597951	18.24877316
6.128223826	18.2674474	7.810746884	30.00867883	18.24877316
0	0	6.834403524	0	7.983838258
53.11127316	25.9590042	40.03007778	26.54613897	31.93535303
85.79513356	79.79990179	163.0493412	75.02169708	120.8981222
0	0	0	0	0
295.5165712	156.7154698	159.1439678	308.166048	208.720343
0	0	3.905373442	0	0
145.7155443	158.638359	145.4751607	159.2768338	127.7414121
57.87766947	123.0649088	83.96552901	71.55915722	0
78.30508222	47.11078539	55.65157155	68.09661735	86.68167252
0	0	6.834403524	0	0
4.766396309	1.9228892	9.763433606	33.4712187	6.843289936
265.5563658	499.9511919	159.1439678	462.826162	268.0288558
183.8467148	71.14690039	213.819196	118.8805354	126.6008638
24.5128953	0	11.71612033	18.46687928	7.983838258
16.3419302	0	38.07739106	3.462539865	14.82712819
68.09137585	58.64812059	10.73977697	32.31703874	82.11947923
0	0	0	0	0
12.93736141	26.9204488	34.17201762	11.54179955	9.124386581
26.55563658	23.0746704	27.3376141	49.62973807	22.81096645
0	0	0	0	0
53.79218692	21.1517812	24.40858401	51.93809798	39.91919129
19.06558524	48.07222999	16.59783713	19.62105924	13.68657987
34.04568792	26.9204488	47.84082467	24.23777906	41.05973961
34.04568792	18.2674474	30.26664418	27.70031892	35.356998
45.62122182	81.72279099	64.4386618	95.79693627	68.43289936
234.9152467	193.2503646	284.1159179	259.6904899	234.9529545

For Review Only

159.3338195	183.6359186	141.5697873	156.9684739	151.6929269
85.79513356	87.49145859	69.3203786	86.56349663	122.0386705
135.5018379	126.9106872	74.2020954	244.6861505	143.7090886
43.57848054	42.30356239	30.26664418	34.62539865	25.0920631
0.680913758	117.2962412	0	0	188.1904732
27.23655034	61.53245439	39.05373442	30.00867883	36.49754632
96.6897537	145.1781346	146.4515041	169.6644534	134.5847021
12.93736141	5.768667599	17.57418049	12.69597951	21.67041813
48.34487685	24.036115	58.58060163	38.08793852	60.4490611
0	32.6891164	0	0	0
117.1171665	219.2093688	99.58702278	171.9728133	167.6606034
42.89756678	0	58.58060163	9.233439641	33.07590136
35.40751544	33.650561	29.29030082	51.93809798	27.37315974
58.55858323	124.987798	110.3267997	84.25513672	109.492639
73.53868591	71.14690039	62.48597508	75.02169708	92.38441413
9.532792618	20.1903366	7.810746884	23.0835991	10.2649349
44.2593943	0	22.45589729	33.4712187	7.983838258
34.04568792	102.8745722	28.31395746	64.63407749	21.67041813
59.92041074	91.33723699	68.34403524	70.40497726	71.85454432
7.490051343	0	22.45589729	10.3876196	19.38932148
0	0	0	0	0
0	0	0	0	4.56219329
0	0	3.905373442	4.61671982	4.56219329
244.4480393	193.2503646	123.9956068	114.2638156	185.9093766
10.21370638	16.3445582	3.905373442	55.40063784	58.16796445
21.78924027	8.653001399	17.57418049	20.77523919	43.34083626
106.2225463	143.2552454	99.58702278	106.1845559	112.9142839
0	0	3.905373442	0	0
0	0	0.976343361	2.30835991	0
10.21370638	27.8818934	7.810746884	27.70031892	14.82712819
7.490051343	0	1.952686721	0	6.843289936
0.680913758	0	0	0	5.702741613
209.0405238	174.9829172	200.1503889	139.6557746	232.6718578
31.32203289	5.768667599	18.55052385	25.39195901	17.10822484
3.404568792	0	1.952686721	6.925079731	10.2649349
4.766396309	0	3.905373442	5.770899775	1.140548323
0.680913758	4.807222999	2.929030082	0	0
40.17391175	21.1517812	39.05373442	60.01735766	63.87070607
17.02284396	0	9.763433606	4.61671982	14.82712819
1.361827517	9.614445999	0	0	0
21.10832651	7.691556799	4.881716803	16.15851937	15.96767652
2.042741275	3.845778399	0	66.9424374	0
0	0	0	0	1.140548323
7.490051343	24.9975596	17.57418049	9.233439641	10.2649349
0.680913758	0	4.881716803	0	3.421644968
117.1171665	127.8721318	95.68164933	120.0347153	92.38441413
15.66101644	26.9204488	21.47955393	28.85449888	13.68657987
257.3854007	836.4568019	0	0	0
55.15401443	0	43.93545122	13.85015946	0
0	0	0.976343361	2.30835991	0

For Review Only

0	26.9204488	0	1.154179955	0
26.55563658	42.30356239	30.26664418	36.93375856	46.76248123
0	0	0.976343361	5.770899775	0
52.4303594	237.4768162	209.9138225	0	152.8334752
62.64406578	33.650561	62.48597508	48.47555811	76.41673761
39.49299799	0	33.19567426	30.00867883	29.65425639
171.5902671	127.8721318	189.4106119	156.9684739	90.10331749
11.57553389	24.036115	12.69246369	10.3876196	12.54603155
28.59837785	21.1517812	17.57418049	27.70031892	11.40548323
248.5335218	118.2576858	166.9547147	146.5808543	166.5200551
176.3566634	126.9106872	165.0020279	108.4929158	144.849637
46.98304933	53.84089759	33.19567426	47.32137816	62.73015774
4.085482551	0	0.976343361	1.154179955	3.421644968
53.79218692	29.8047826	13.66880705	21.92941915	47.90302955
0	0	0	2.30835991	0
11.57553389	60.57100979	30.26664418	24.23777906	13.68657987
50.38761813	84.60712479	41.9827645	114.2638156	79.83838258
50.38761813	86.53001399	39.05373442	56.5548178	79.83838258
15.66101644	0	9.763433606	3.462539865	4.56219329
537.2409554	512.4499717	534.0598182	650.9574947	577.1174512
4.766396309	0	12.69246369	2.30835991	3.421644968
4.085482551	14.421669	1.952686721	8.079259686	5.702741613
12.25644765	7.691556799	11.71612033	24.23777906	28.51370807
26.55563658	50.95656379	39.05373442	50.78391802	38.77864297
2.723655034	0	0	0	0
248.5335218	149.023913	204.0557624	208.9065719	253.2017276
0	42.30356239	12.69246369	53.09227793	13.68657987
91.24244363	305.7393828	116.1848599	27.70031892	182.4877316
37.45025671	107.6817952	42.95910786	0	49.04357787
371.7789121	0	143.522474	39.24211847	1.140548323
29.96020537	15.3831136	13.66880705	18.46687928	37.63809465
805.5209762	626.8618791	681.4876657	536.6936791	691.1722835
198.1459037	111.5275736	87.87090245	95.79693627	130.0225088
59.23949699	46.14934079	61.50963171	45.01301825	60.4490611
25.87472282	27.8818934	53.69888483	35.77957861	31.93535303
72.85777215	26.9204488	38.07739106	63.47989753	39.91919129
0.680913758	0.9614446	0	18.46687928	0
42.89756678	75.95412339	32.2193309	39.24211847	50.18412619
82.39056477	42.30356239	49.79351139	53.09227793	57.02741613
50.38761813	35.57345019	47.84082467	70.40497726	59.30851278
65.36772081	46.14934079	43.93545122	47.32137816	39.91919129
20.42741275	71.14690039	33.19567426	0	0
150.4819406	131.7179102	209.9138225	131.5765149	207.5797947
0	0	0	4.61671982	1.140548323
0	0	0	0	1.140548323
111.6698564	143.2552454	115.2085165	107.3387358	61.58960942
49.02579061	21.1517812	58.58060163	34.62539865	70.713996
0	0	0	0	0
0	0	0	0	0
0	0	1.952686721	0	2.281096645

For Review Only

0	0	0.976343361	1.154179955	3.421644968
0	13.4602244	0	0	0
0	0	5.858060163	3.462539865	3.421644968
61.96315202	61.53245439	69.3203786	51.93809798	86.68167252
121.202649	152.8696914	178.670835	192.7480525	102.649349
0.680913758	0	2.929030082	10.3876196	3.421644968
39.49299799	50.95656379	81.03649893	60.01735766	45.6219329
11.57553389	0	0.976343361	17.31269933	2.281096645
34.04568792	0	88.84724581	66.9424374	5.702741613
35.40751544	0	46.86448131	24.23777906	0
139.5873205	138.4480224	139.6171006	121.1888953	143.7090886
830.7147853	487.4524121	776.1929716	448.9760025	692.3128318
24.5128953	22.1132258	17.57418049	19.62105924	25.0920631
2.042741275	2.8843338	1.952686721	3.462539865	1.140548323
0	0	5.858060163	0	0
153.2055957	223.0551472	219.6772561	190.4396926	189.3310216
14.29918893	5.768667599	10.73977697	8.079259686	9.124386581
94.64701242	107.6817952	136.6880705	61.17153762	101.5088007
49.02579061	63.45534359	56.62791491	43.85883829	62.73015774
10.89462014	26.9204488	3.905373442	15.00433942	20.52986981
76.26234095	22.1132258	30.26664418	39.24211847	51.32467452
69.45320336	189.4045862	161.0966545	61.17153762	173.363345
35.40751544	81.72279099	59.55694499	51.93809798	46.76248123
189.2940248	284.5876016	288.0212914	199.6731322	217.8447296
18.38467148	12.4987798	11.71612033	12.69597951	38.77864297
0	0	0	64.63407749	46.76248123
34.04568792	63.45534359	51.74619811	58.86317771	33.07590136
19.746499	17.3060028	99.58702278	58.86317771	99.22770407
0	0	11.71612033	12.69597951	0
46.98304933	116.3347966	125.9482935	98.10529618	19.38932148
3.404568792	0	0	3.462539865	3.421644968
25.19380906	49.99511919	61.50963171	16.15851937	29.65425639
150.4819406	119.2191304	115.2085165	120.0347153	124.3197672
0	0	0	0	33.07590136
30.64111913	46.14934079	38.07739106	21.92941915	36.49754632
5.447310068	0	0	9.233439641	5.702741613
10.21370638	0	0.976343361	16.15851937	3.421644968
92.60427115	139.409467	149.3805342	90.0260365	61.58960942
715.6403601	614.3630993	663.9134852	532.0769593	585.1012895
0	0	4.881716803	0	0
3.404568792	14.421669	2.929030082	9.233439641	17.10822484
0	2.8843338	0	0	0
32.68386041	71.14690039	38.07739106	19.62105924	38.77864297
0	0	1.952686721	16.15851937	23.95151477
0	0	0	0	0
4.085482551	0	3.905373442	3.462539865	0
0	3.845778399	0	8.079259686	4.56219329
43.57848054	18.2674474	46.86448131	34.62539865	23.95151477
27.23655034	26.9204488	32.2193309	15.00433942	34.21644968
97.37066746	165.3684712	125.9482935	86.56349663	103.7898974

For Review Only

76.9432547	68.26256659	93.72896261	83.10095677	77.55728594
49.02579061	51.91800839	66.39134852	55.40063784	57.02741613
67.41046209	0	28.31395746	30.00867883	66.15180271
6.128223826	3.845778399	6.834403524	11.54179955	9.124386581
151.8437681	401.8838427	217.7245694	264.3072097	130.0225088
0	11.5373352	0	0	0
17.70375772	39.41922859	17.57418049	8.079259686	17.10822484
30.64111913	24.9975596	36.12470434	35.77957861	13.68657987
3.404568792	0	3.905373442	1.154179955	1.140548323
88.5187886	66.33967739	165.9783713	105.0303759	124.3197672
32.68386041	22.1132258	40.03007778	19.62105924	22.81096645
58.55858323	39.41922859	35.14836098	75.02169708	62.73015774
0	0	0	0	4.56219329
0	84.60712479	123.9956068	13.85015946	7.983838258
63.32497954	74.99267879	67.36769188	62.32571757	44.48138458
18.38467148	16.3445582	16.59783713	15.00433942	13.68657987
0	0	2284.643464	1.154179955	1.140548323
74.21959967	49.99511919	14.64515041	60.01735766	5.702741613
123.926304	127.8721318	111.3031431	138.5015946	159.6767652
44.2593943	49.03367459	46.86448131	65.78825744	45.6219329
59.23949699	111.5275736	34.17201762	93.48857636	100.3682524
47.66396309	51.91800839	50.76985475	35.77957861	39.91919129
74.21959967	0	1.952686721	2.30835991	44.48138458
61.96315202	40.38067319	62.48597508	50.78391802	61.58960942
0	0	0	0	0
69.45320336	27.8818934	129.853667	61.17153762	62.73015774
242.405298	227.8623702	281.1868878	191.5938725	211.0014397
158.6529057	108.6432398	197.2213588	113.1096356	150.5523786
244.4480393	101.9131276	146.4515041	130.4223349	314.791337
33.36477416	67.30112199	77.13112548	84.25513672	90.10331749
150.4819406	107.6817952	225.5353163	168.5102734	261.1855659
5.447310068	0.9614446	6.834403524	9.233439641	19.38932148
6.128223826	0	4.881716803	0	0
13.61827517	23.0746704	15.62149377	11.54179955	14.82712819
4.766396309	0	3.905373442	5.770899775	4.56219329
0	0	16.59783713	4.61671982	0
0	13.4602244	0	0	0
64.68680705	36.53489479	42.95910786	83.10095677	61.58960942
1.361827517	0	2.929030082	0	1.140548323
128.6927003	186.5202524	128.8773236	184.6687928	213.2825363
5.447310068	0	0.976343361	6.925079731	4.56219329
0	0	0	0	2.281096645
0	1.9228892	3.905373442	0	0
238.3198155	404.7681765	699.0618462	363.5666859	469.9059089
142.9918893	123.0649088	117.1612033	125.8056151	152.8334752
0	0	0.976343361	1.154179955	1.140548323
5.447310068	0	1.952686721	2.30835991	0
133.4590967	119.2191304	154.262251	153.505934	119.7575739
49.02579061	17.3060028	46.86448131	58.86317771	103.7898974
50.38761813	27.8818934	10.73977697	56.5548178	31.93535303

For Review Only

258.7472282	224.9780364	245.0621835	281.619909	265.7477592
200.8695587	313.4309396	261.6600206	271.2322894	295.4020156
112.3507701	71.14690039	92.75261925	94.64275632	91.24386581
59.23949699	167.2913604	3.905373442	118.8805354	35.356998
7.490051343	0	5.858060163	5.770899775	12.54603155
20.42741275	37.49633939	21.47955393	42.70465834	26.23261142
20.42741275	81.72279099	4.881716803	23.0835991	18.24877316
67.41046209	111.5275736	95.68164933	95.79693627	62.73015774
239.0007292	195.1732538	194.2923288	240.0694307	183.6282799
0	0	0	0	0
34.72660168	107.6817952	69.3203786	30.00867883	71.85454432
38.81208423	18.2674474	47.84082467	45.01301825	14.82712819
35.40751544	51.91800839	30.26664418	24.23777906	52.46522284
49.70670437	123.0649088	99.58702278	41.55047838	110.6331873
6.128223826	0	9.763433606	16.15851937	4.56219329
7.490051343	0.9614446	0	0	1.140548323
0.680913758	0	1.952686721	2.30835991	5.702741613
0	0	0.976343361	3.462539865	0
7.490051343	0	0	0	0
78.98599598	37.49633939	78.10746884	40.39629843	61.58960942
68.09137585	50.95656379	78.10746884	94.64275632	43.34083626
8.85187886	0	0	0	0
85.79513356	77.87701259	140.5934439	55.40063784	141.427992
0	0	0	2.30835991	0
137.5445792	116.3347966	126.9246369	153.505934	123.1792188
95.32792618	111.5275736	73.22575204	58.86317771	37.63809465
29.27929161	37.49633939	31.24298754	39.24211847	28.51370807
123.926304	68.26256659	154.262251	57.70899775	191.6121182
46.98304933	38.45778399	17.57418049	25.39195901	42.20028794
2.723655034	6.730112199	4.881716803	1.154179955	7.983838258
4.766396309	0	0.976343361	0	2.281096645
6.809137585	0	6.834403524	2.30835991	2.281096645
2.042741275	0	0	1.154179955	3.421644968
0	0	13.66880705	1.154179955	4.56219329
9.532792618	0	0	1.154179955	4.56219329
119.1599077	86.53001399	131.8063537	118.8805354	87.82222084
57.87766947	0	0	0	0
32.00294665	36.53489479	40.03007778	25.39195901	18.24877316
0	0.9614446	4.881716803	8.079259686	9.124386581
0	0	0.976343361	2.30835991	0
107.5843738	101.9131276	130.8300103	81.94677681	87.82222084
185.8894561	145.1781346	232.3697198	122.3430752	179.0660866
33.36477416	0	40.03007778	27.70031892	7.983838258
9.532792618	11.5373352	0	0	0
28.59837785	72.10834499	30.26664418	30.00867883	21.67041813
32.68386041	8.653001399	13.66880705	21.92941915	10.2649349
70.81503088	41.34211779	112.2794865	57.70899775	59.30851278
0	0	0	0	0
3.404568792	0	2.929030082	0	3.421644968
0	74.03123419	0	54.24645789	0

For Review Only

2.042741275	16.3445582	5.858060163	0	13.68657987
5.447310068	8.653001399	3.905373442	6.925079731	6.843289936
276.4509859	85.56856939	1090.575534	216.9858316	0
66.04863457	34.61200559	59.55694499	49.62973807	54.74631949
0.680913758	43.26500699	44.91179459	0	0
112.3507701	177.867251	124.9719502	106.1845559	191.6121182
105.5416326	70.18545579	172.8127748	75.02169708	145.9901853
0	13.4602244	10.73977697	6.925079731	10.2649349
0	8.653001399	0	0	0
2.723655034	0	0	1.154179955	0
19.06558524	41.34211779	23.43224065	38.08793852	15.96767652
36.0884292	298.047826	425.6857052	412.042244	428.8461693
135.5018379	221.132258	165.9783713	170.8186334	260.0450176
72.1768584	66.33967739	58.58060163	5.770899775	106.070994
24.5128953	0.9614446	0	0	245.2178894
73.53868591	75.95412339	70.29672196	49.62973807	61.58960942
89.88061612	75.95412339	98.61067942	81.94677681	83.26002755
42.89756678	113.4504628	54.67522819	13.85015946	17.10822484
107.5843738	242.2840392	226.5116596	141.9641345	165.3795068
151.1628544	166.3299158	150.3568775	159.2768338	117.4764772
104.179805	7.691556799	251.896587	0	215.563633
138.225493	93.26012619	106.4214263	85.40931668	142.5685403
352.7133269	336.50561	280.2105445	304.7035081	307.9480471
2.723655034	1.9228892	0	0	0
98.05158122	73.06978959	96.65799269	110.8012757	106.070994
61.96315202	56.72523139	95.68164933	51.93809798	92.38441413
21.78924027	20.1903366	13.66880705	15.00433942	15.96767652
59.23949699	35.57345019	48.81716803	58.86317771	76.41673761
67.41046209	99.99023839	64.4386618	92.33439641	79.83838258
32.68386041	87.49145859	39.05373442	23.0835991	10.2649349
0	166.3299158	41.9827645	108.4929158	2.281096645
43.57848054	24.036115	25.38492737	33.4712187	38.77864297
0	0	0.976343361	1.154179955	789.2594392
70.81503088	73.06978959	70.29672196	79.6384169	78.69783426
55.15401443	42.30356239	30.26664418	63.47989753	84.40057587
2.723655034	88.45290319	84.94187237	80.79259686	78.69783426
29.27929161	32.6891164	22.45589729	27.70031892	51.32467452
65.36772081	24.9975596	54.67522819	102.722016	42.20028794
144.3537168	92.29868159	44.91179459	1.154179955	7.983838258
25.19380906	0	17.57418049	10.3876196	37.63809465
383.354446	383.6163953	326.0986824	347.4081665	278.2937907
109.6271151	122.1034642	127.9009802	145.4266743	139.1468954
27.23655034	91.33723699	107.3977697	63.47989753	100.3682524
158.6529057	112.4890182	156.2149377	166.2019135	177.9255383
4.085482551	2.8843338	7.810746884	1.154179955	7.983838258
0	0	0	1.154179955	0
83.75239229	155.7540252	165.0020279	21.92941915	33.07590136
171.5902671	306.7008274	247.9912136	0	0
40.17391175	71.14690039	37.1010477	50.78391802	14.82712819
23.83198155	0	15.62149377	28.85449888	2.281096645

For Review Only

5.447310068	0.9614446	6.834403524	8.079259686	1.140548323
9.532792618	7.691556799	8.787090245	12.69597951	14.82712819
127.3308728	227.8623702	356.3653266	240.0694307	283.9965323
28.59837785	32.6891164	38.07739106	26.54613897	38.77864297
5.447310068	2.8843338	0.976343361	9.233439641	1.140548323
8.85187886	0	8.787090245	16.15851937	6.843289936
0	0	1.952686721	0	2.281096645
0	0	0	0	5.702741613
113.0316839	129.795021	133.7590404	99.25947614	88.96276916
69.45320336	46.14934079	78.10746884	107.3387358	66.15180271
0	0	0	0	0
0	0	0	0	0
18.38467148	310.5466058	15.62149377	188.1313327	0
10.89462014	29.8047826	10.73977697	18.46687928	3.421644968
263.5136245	294.2020476	402.2534645	358.949966	230.3907612
55.15401443	94.22157079	71.27306532	54.24645789	61.58960942
0	0	0	0	0
591.0331423	2907.40847	0	0	2082.641237
1.361827517	7.691556799	2.929030082	3.462539865	0
0	0	0.976343361	9.233439641	2.281096645
6.809137585	6.730112199	6.834403524	6.925079731	9.124386581
51.06853188	19.228892	7.810746884	24.23777906	25.0920631
25.87472282	12.4987798	17.57418049	11.54179955	65.01125439
180.442146	202.8648106	164.0256846	160.4310138	193.8932148
83.07147853	49.03367459	42.95910786	90.0260365	108.3520906
49.70670437	17.3060028	41.00642114	45.01301825	45.6219329
85.11421981	156.7154698	67.36769188	81.94677681	147.1307336
0	0	0	0	0
40.85482551	19.228892	38.07739106	40.39629843	57.02741613
0	0	0	0	0
0	6.730112199	1.952686721	0	0
0	0	4.881716803	2.30835991	3.421644968
70.81503088	56.72523139	71.27306532	58.86317771	68.43289936
34.72660168	76.91556799	100.5633661	0	0
319.3485527	307.662272	426.6620486	407.4255241	390.0675263
6.128223826	24.036115	8.787090245	9.233439641	17.10822484
14.98010269	0	0.976343361	18.46687928	2.281096645
160.695647	182.674474	201.1267323	182.3604329	204.1581497
143.672803	154.7925806	155.2385943	143.1183144	119.7575739
465.064097	604.7486533	48.81716803	801.0008888	101.5088007
132.7781829	166.3299158	128.8773236	87.71767659	100.3682524
444.6366843	301.8936044	371.010477	328.9412872	426.5650727
42.21665302	120.180575	0.976343361	1.154179955	6.843289936
0	0	38.07739106	100.4136561	55.88686781
224.0206265	220.1708134	174.7654615	171.9728133	191.6121182
32.00294665	163.445582	26.36127073	71.55915722	20.52986981
64.68680705	11.5373352	80.06015557	47.32137816	83.26002755
69.45320336	111.5275736	87.87090245	69.25079731	59.30851278
51.74944564	43.26500699	68.34403524	84.25513672	44.48138458
53.79218692	99.99023839	65.41500516	51.93809798	66.15180271

For Review Only

71.49594464	35.57345019	42.95910786	56.5548178	68.43289936
0	0	1.952686721	0	0
4.766396309	5.768667599	6.834403524	0	4.56219329
403.7818588	390.3465075	453.0233193	421.2756836	416.3001378
187.2512836	0	103.4923962	272.3864694	59.30851278
29.96020537	49.03367459	42.95910786	64.63407749	34.21644968
163.419302	204.7876998	218.7009128	313.9369478	248.6395343
15.66101644	0	5.858060163	8.079259686	25.0920631
262.151797	374.9633939	347.5782364	203.1356721	234.9529545
38.13117047	0	7.810746884	62.32571757	25.0920631
29.27929161	17.3060028	21.47955393	18.46687928	27.37315974
46.98304933	30.7662272	25.38492737	39.24211847	41.05973961
220.6160577	252.8599298	273.376141	94.64275632	228.1096645
6.128223826	0	7.810746884	0	3.421644968
36.76934296	33.650561	52.72254147	57.70899775	54.74631949
470.5114071	413.4211779	515.5092944	352.0248863	424.283976
12.93736141	30.7662272	13.66880705	27.70031892	21.67041813
22.47015403	0	47.84082467	2.30835991	3.421644968
0	0	0	0	0
23.15106779	7.691556799	6.834403524	12.69597951	12.54603155
117.7980802	123.0649088	517.4619811	0	80.97893091
165.4620433	166.3299158	122.0429201	191.5938725	149.4118303
25.19380906	0	13.66880705	57.70899775	41.05973961
8.170965101	0	0	3.462539865	1.140548323
59.92041074	69.22401119	53.69888483	57.70899775	61.58960942
72.1768584	325.9297194	1.952686721	290.8533487	23.95151477
140.949148	0	73.22575204	62.32571757	203.0176014
0	3.845778399	0	0	0
461.6595282	708.5846701	306.5718152	705.2039526	514.3872935
6.809137585	0	0	0	3.421644968
0	31.7276718	0	41.55047838	47.90302955
212.4450926	1139.311851	423.7330185	605.9444764	797.2432775
736.0677729	496.1054135	466.6921263	507.8391802	436.8300076
49.70670437	358.6188357	110.3267997	1.154179955	157.3956685
0	0	2.929030082	0	4.56219329
110.9889426	101.9131276	123.0192634	80.79259686	115.1953806
42.89756678	50.95656379	32.2193309	64.63407749	68.43289936
60.6013245	23.0746704	41.9827645	68.09661735	62.73015774
0	0	0	0	2.281096645
0	0	0.976343361	4.61671982	1.140548323
42.21665302	69.22401119	29.29030082	65.78825744	55.88686781
308.4539326	174.9829172	49.79351139	161.5851937	392.348623
29.27929161	0	5.858060163	12.69597951	23.95151477
2.723655034	6.730112199	2.929030082	9.233439641	1.140548323
1.361827517	2.8843338	0	0	0
37.45025671	19.228892	49.79351139	19.62105924	50.18412619
0	23.0746704	0	11.54179955	0
54.47310068	0	27.3376141	41.55047838	0
61.28223826	117.2962412	199.1740456	234.2985309	232.6718578
1.361827517	0	0	0	0

For Review Only

248.5335218	288.43338	326.0986824	271.2322894	295.4020156
102.1370638	128.8335764	130.8300103	87.71767659	111.7737356
4.085482551	0	7.810746884	0	3.421644968
699.9793437	427.8428469	559.4447456	675.1952737	690.0317352
124.6072178	84.60712479	114.2321732	159.2768338	104.9304457
136.8636654	100.951683	118.1375466	129.268155	167.6606034
62.64406578	15.3831136	51.74619811	45.01301825	71.85454432
6.809137585	0	50.76985475	0	31.93535303
149.1201131	148.0624684	140.5934439	106.1845559	124.3197672
155.2483369	112.4890182	150.3568775	84.25513672	108.3520906
116.4362527	39.41922859	109.3504564	60.01735766	91.24386581
110.3080289	137.4865778	67.36769188	96.95111623	79.83838258
105.5416326	43.26500699	67.36769188	78.48423695	80.97893091
125.9690453	161.5226928	191.3632987	159.2768338	155.1145719
203.5932138	202.8648106	194.2923288	188.1313327	189.3310216
221.9778853	196.1346984	165.0020279	3.462539865	339.8834001
0.680913758	0.9614446	0	0	0
17.70375772	20.1903366	11.71612033	16.15851937	26.23261142
51.06853188	62.49389899	65.41500516	87.71767659	71.85454432
32.00294665	22.1132258	30.26664418	43.85883829	45.6219329
14.98010269	30.7662272	18.55052385	12.69597951	13.68657987
55.15401443	19.228892	41.9827645	34.62539865	31.93535303
0	24.9975596	3.905373442	5.770899775	1.140548323
36.0884292	17.3060028	21.47955393	0	28.51370807
14.98010269	39.41922859	10.73977697	9.233439641	3.421644968
249.8953494	395.1537305	227.488003	331.2496471	206.4392464
6.128223826	0	1.952686721	2.30835991	9.124386581
57.19675571	50.95656379	41.9827645	61.17153762	54.74631949
67.41046209	50.95656379	50.76985475	62.32571757	50.18412619
35.40751544	20.1903366	17.57418049	25.39195901	13.68657987
0	24.9975596	8.787090245	0	7.983838258
704.74574	1428.706675	1029.065902	894.4894652	226.9691162
0	0	0	0	0
172.9520946	158.638359	115.2085165	148.8892142	124.3197672
32.00294665	23.0746704	24.40858401	19.62105924	31.93535303
34.72660168	53.84089759	17.57418049	31.16285879	35.356998
496.3861299	691.2786673	609.238257	585.1692372	593.0851278
51.06853188	65.37823279	66.39134852	55.40063784	46.76248123
59.92041074	27.8818934	81.03649893	60.01735766	59.30851278
17.02284396	12.4987798	21.47955393	20.77523919	11.40548323
0	0	0	13.85015946	0
34.72660168	0	40.03007778	21.92941915	12.54603155
61.96315202	120.180575	26.36127073	87.71767659	104.9304457
104.179805	49.99511919	75.17843876	73.86751713	107.2115423
39.49299799	37.49633939	21.47955393	36.93375856	15.96767652
27.23655034	12.4987798	18.55052385	40.39629843	14.82712819
52.4303594	50.95656379	59.55694499	45.01301825	47.90302955
0	0	34.17201762	0	0
8.85187886	0	1.952686721	1.154179955	4.56219329
58.55858323	0	85.91821573	20.77523919	28.51370807

For Review Only

155.9292507	0	141.5697873	10.3876196	36.49754632
0	0	0	0	0
42.21665302	34.61200559	28.31395746	13.85015946	43.34083626
151.1628544	182.674474	152.3095642	123.4972552	12.54603155
0.680913758	348.0429451	645.3629613	0	0
449.4030806	606.6715425	510.6275776	461.671982	441.3922008
105.5416326	161.5226928	73.22575204	197.3647723	54.74631949
248.5335218	8.653001399	411.0405548	0	0
426.2520128	332.6598316	362.2233868	342.7914467	394.6297196
127.3308728	172.0985834	129.853667	145.4266743	153.9740236
0	9.614445999	0	1.154179955	0
96.00883994	119.2191304	95.68164933	80.79259686	107.2115423
0.680913758	0	1.952686721	1.154179955	0
0	0	2.929030082	2.30835991	0
240.3625567	173.060028	239.2041233	315.0911277	258.9044692
0	0	0	1.154179955	14.82712819
0	0	10.73977697	24.23777906	11.40548323
0	0	0	0	0
66.72954833	109.6046844	82.98918565	141.9641345	67.29235103
39.49299799	0	20.50321057	2.30835991	22.81096645
231.5106779	83.64568019	0	0	268.0288558
1114.655823	1025.861388	1124.747551	993.7489413	1247.759865
159.3338195	186.5202524	241.1568101	213.5232917	179.0660866
108.2652876	107.6817952	113.2558298	113.1096356	124.3197672
69.45320336	23.0746704	131.8063537	45.01301825	58.16796445
207.6786963	166.3299158	305.5954719	166.2019135	224.6880196
63.32497954	109.6046844	410.0642114	128.113975	11.40548323
0.680913758	0.9614446	0	0	2441.913959
16.3419302	12.4987798	27.3376141	28.85449888	7.983838258
246.4907806	341.312833	306.5718152	398.1920845	296.5425639
12.25644765	0	2.929030082	5.770899775	0
150.4819406	128.8335764	280.2105445	222.7567313	159.6767652
26.55563658	0	22.45589729	21.92941915	13.68657987
53.79218692	101.9131276	75.17843876	18.46687928	52.46522284
372.4598259	124.0263534	568.2318358	174.2811732	445.9543941
76.9432547	113.4504628	72.24940868	70.40497726	87.82222084
0	124.0263534	68.34403524	10.3876196	11.40548323
64.00589329	69.22401119	55.65157155	80.79259686	82.11947923
126.6499591	67.30112199	126.9246369	72.71333717	85.5411242
66.04863457	85.56856939	67.36769188	65.78825744	69.57344768
162.0574745	122.1034642	139.6171006	124.6514351	127.7414121
83.07147853	81.72279099	56.62791491	115.4179955	152.8334752
10.21370638	17.3060028	8.787090245	15.00433942	14.82712819
0	0	0	0	0
23.83198155	21.1517812	19.52686721	17.31269933	11.40548323
16.3419302	0	2.929030082	5.770899775	2.281096645
0	0.9614446	0	1.154179955	0
278.4937272	214.4021458	305.5954719	0	337.6023035
41.53573927	73.06978959	38.07739106	34.62539865	38.77864297
3.404568792	0	2.929030082	3.462539865	0

For Review Only

2.723655034	12.4987798	0.976343361	1.154179955	4.56219329
0	0	0	0	0
21.78924027	8.653001399	25.38492737	45.01301825	47.90302955
4.085482551	0	0	0	0
77.62416846	125.9492426	91.77627589	91.18021645	82.11947923
492.3006474	877.7989197	683.4403524	478.9846814	840.5841138
0	0.9614446	0	4.61671982	3.421644968
4.085482551	0	0.976343361	10.3876196	2.281096645
0	0	0	0	0
97.37066746	6.730112199	84.94187237	85.40931668	114.0548323
0	17.3060028	2.929030082	9.233439641	0
36.76934296	50.95656379	45.88813795	73.86751713	35.356998
33.36477416	42.30356239	51.74619811	48.47555811	31.93535303
142.3109755	190.3660308	209.9138225	192.7480525	158.5362168
226.0633678	239.3997054	127.9009802	273.5406494	268.0288558
3.404568792	0	0	1.154179955	0
137.5445792	96.14445999	167.931058	216.9858316	211.0014397
18.38467148	0	0	15.00433942	20.52986981
41.53573927	116.3347966	66.39134852	49.62973807	79.83838258
64.68680705	91.33723699	50.76985475	73.86751713	88.96276916
8.85187886	0	1.952686721	4.61671982	3.421644968
17.70375772	0	1.952686721	26.54613897	1.140548323
53.79218692	61.53245439	52.72254147	106.1845559	87.82222084
172.9520946	233.6310378	148.4041908	182.3604329	126.6008638
58.55858323	25.9590042	35.14836098	42.70465834	33.07590136
102.1370638	95.18301539	91.77627589	90.0260365	112.9142839
0	0	0	0	0
277.8128134	0	4.881716803	20.77523919	1.140548323
0	4.807222999	0	1.154179955	0
140.2682342	156.7154698	185.5052385	218.1400115	174.5038934
0	126.9106872	41.00642114	23.0835991	30.79480471
285.3028648	241.3225946	339.7674895	245.8403304	361.5538183
459.616787	318.2381626	419.827645	454.7469023	542.9010016
30.64111913	18.2674474	35.14836098	19.62105924	54.74631949
114.3935114	118.2576858	82.01284229	38.08793852	114.0548323
89.19970236	68.26256659	135.7117271	86.56349663	125.4603155
75.58142719	56.72523139	73.22575204	80.79259686	65.01125439
91.92335739	128.8335764	120.0902333	106.1845559	78.69783426
0	27.8818934	0.976343361	54.24645789	112.9142839
64.00589329	69.22401119	29.29030082	28.85449888	130.0225088
124.6072178	0	1.952686721	46.1671982	176.78499
69.45320336	50.95656379	56.62791491	69.25079731	66.15180271
90.56152987	90.37579239	56.62791491	47.32137816	63.87070607
15.66101644	22.1132258	6.834403524	9.233439641	6.843289936
150.4819406	122.1034642	38.07739106	76.17587704	506.4034552
25.87472282	74.99267879	84.94187237	75.02169708	75.27618929
50.38761813	117.2962412	93.72896261	66.9424374	57.02741613
53.11127316	274.9731556	184.5288951	183.5146129	147.1307336
68.7722896	124.0263534	90.79993253	109.6470957	90.10331749
76.26234095	116.3347966	80.06015557	92.33439641	87.82222084

For Review Only

9.532792618	19.228892	22.45589729	5.770899775	17.10822484
8.85187886	37.49633939	26.36127073	16.15851937	38.77864297
8.85187886	0	6.834403524	18.46687928	5.702741613
34.04568792	24.036115	49.79351139	35.77957861	43.34083626
104.179805	24.9975596	91.77627589	133.8848748	50.18412619
33.36477416	21.1517812	18.55052385	69.25079731	23.95151477
87.83787484	192.28892	87.87090245	148.8892142	130.0225088
78.30508222	65.37823279	47.84082467	53.09227793	79.83838258
141.6300618	77.87701259	0	91.18021645	99.22770407
0	0	0	9.233439641	0
0	0	0	0	3.421644968
42.21665302	102.8745722	22.45589729	90.0260365	63.87070607
58.55858323	29.8047826	63.46231844	98.10529618	51.32467452
34.72660168	84.60712479	28.31395746	83.10095677	66.15180271
35.40751544	8.653001399	11.71612033	17.31269933	42.20028794
22.47015403	86.53001399	79.0838122	135.0390547	67.29235103
0	94.22157079	140.5934439	0	161.9578618
70.13411712	12.4987798	41.00642114	79.6384169	84.40057587
87.15696108	264.397265	81.03649893	85.40931668	44.48138458
0	0	0	1.154179955	0
26.55563658	21.1517812	17.57418049	15.00433942	21.67041813
7.490051343	23.0746704	0	0	0
0	0	0	0	2.281096645
428.9756678	388.4236183	364.1760735	360.104146	307.9480471
1.361827517	10.5758906	4.881716803	16.15851937	7.983838258
123.926304	114.4119074	114.2321732	117.7263554	117.4764772
12.93736141	0	32.2193309	39.24211847	12.54603155
261.4708832	181.7130294	200.1503889	223.9109113	281.7154357
4.085482551	4.807222999	8.787090245	4.61671982	7.983838258
0	0	0	0	0
226.7442816	236.5153716	210.8901659	368.1834057	294.2614672
374.5025671	981.6349365	0	0	886.2060467
842.2903192	528.7945299	877.7326811	508.9933602	683.1884452
8.170965101	5.768667599	38.07739106	176.5895331	171.0822484
6.809137585	2.8843338	9.763433606	3.462539865	5.702741613
0	89.41434779	12.69246369	197.3647723	12.54603155
100.0943225	0	74.2020954	48.47555811	43.34083626
352.0324131	333.6212762	299.7374117	259.6904899	273.7315974
71.49594464	0	0.976343361	1.154179955	1.140548323
64.68680705	34.61200559	52.72254147	53.09227793	43.34083626
88.5187886	0	55.65157155	88.87185654	3.421644968
5.447310068	9.614445999	16.59783713	46.1671982	0
168.8666121	100.951683	126.9246369	186.9771527	124.3197672
0	0	78.10746884	0	0
30.64111913	0	0	0	0
34.72660168	0	16.59783713	21.92941915	0
148.4391993	0	22.45589729	0	0
18.38467148	26.9204488	27.3376141	21.92941915	34.21644968
0	0	43.93545122	60.01735766	50.18412619
599.8850212	496.1054135	423.7330185	701.7414127	277.1532424

For Review Only

1006.390535	527.8330853	1043.711052	826.3928478	257.7639209
693.1702061	0	707.8489364	588.6317771	435.6894592
71.49594464	214.4021458	405.1824946	125.8056151	525.7927767
379.9498772	550.9077557	482.3136201	406.2713442	375.2403981
12.25644765	0	0	8.079259686	0
168.1856983	32.6891164	0	0	0
759.2188407	0	207.9611358	0	241.7962444
466.4259245	361.5031695	487.1953369	448.9760025	330.7590136
6.809137585	236.5153716	107.3977697	146.5808543	119.7575739
347.9469306	220.1708134	255.8019605	241.2236106	236.0935028
0	0	2.929030082	2.30835991	0
130.0545279	250.9370406	228.4643464	189.2855126	191.6121182
357.4797232	284.5876016	277.2815144	285.0824489	294.2614672
413.9955651	336.50561	395.419061	363.5666859	396.9108163
138.9064067	142.2938008	115.2085165	122.3430752	128.8819605
196.7840762	191.3274754	121.0665767	154.660114	148.2712819
305.7302775	130.7564656	210.8901659	244.6861505	204.1581497
275.0891584	249.975596	259.7073339	203.1356721	299.9642088
10.89462014	10.5758906	10.73977697	15.00433942	3.421644968
0	167.2913604	93.72896261	85.40931668	115.1953806
157.2910782	149.023913	117.1612033	225.0650912	125.4603155
0	0	15.62149377	1.154179955	0
1045.202619	749.9267879	904.0939519	836.7804674	703.718315
302.3257088	299.9707152	332.9330859	563.2398181	417.4406861
262.151797	384.5778399	346.601893	506.6850003	398.0513646
10.89462014	0	0.976343361	9.233439641	14.82712819
3.404568792	0	5.858060163	9.233439641	5.702741613
1.361827517	0	2.929030082	2.30835991	0
2.723655034	0	0.976343361	1.154179955	7.983838258
173.6330084	240.36115	152.3095642	220.4483714	196.1743115
34.72660168	45.18789619	12.69246369	11.54179955	41.05973961
57.87766947	37.49633939	43.93545122	25.39195901	36.49754632
0	0	0	0	1.140548323
47.66396309	26.9204488	32.2193309	70.40497726	37.63809465
159.3338195	0.9614446	390.5373442	330.0954672	172.2227967
2.042741275	24.036115	0.976343361	0	0
64.00589329	124.987798	121.0665767	45.01301825	84.40057587
0	0	0	0	0
204.9550413	223.0551472	124.9719502	252.7654102	254.3422759
107.5843738	43.26500699	97.63433606	101.567836	46.76248123
281.2173822	362.4646141	394.4427177	219.2941915	312.5102404
59.23949699	35.57345019	50.76985475	78.48423695	35.356998
73.53868591	116.3347966	67.36769188	49.62973807	93.52496245
209.0405238	209.5949228	151.3332209	10.3876196	257.7639209
35.40751544	57.68667599	24.40858401	34.62539865	65.01125439
53.79218692	7.691556799	11.71612033	93.48857636	44.48138458
96.00883994	124.0263534	85.91821573	71.55915722	127.7414121
6.128223826	0	2.929030082	4.61671982	0
14.98010269	8.653001399	16.59783713	13.85015946	0
42.89756678	13.4602244	13.66880705	40.39629843	47.90302955

For Review Only

232.8725054	249.0141514	138.6407572	156.9684739	203.0176014
2.042741275	2.8843338	2.929030082	3.462539865	1.140548323
27.9174641	18.2674474	12.69246369	20.77523919	27.37315974
0	0	0	0	0
43.57848054	66.33967739	38.07739106	54.24645789	67.29235103
20.42741275	7.691556799	24.40858401	31.16285879	11.40548323
123.2453903	169.2142496	209.9138225	161.5851937	217.8447296
0	0	0.976343361	6.925079731	5.702741613
25.87472282	0	12.69246369	2.30835991	9.124386581
29.96020537	0	7.810746884	4.61671982	31.93535303
0	0	0.976343361	1.154179955	447.0949425
6.809137585	7.691556799	0	4.61671982	0
0	0	0	0	0
0	0	0.976343361	3.462539865	1.140548323
2.723655034	23.0746704	0	16.15851937	5.702741613
61.96315202	61.53245439	43.93545122	88.87185654	66.15180271
6.809137585	2.8843338	0.976343361	3.462539865	4.56219329
9.532792618	0	19.52686721	20.77523919	11.40548323
21.78924027	0	5.858060163	1.154179955	4.56219329
9.532792618	6.730112199	0	1.154179955	6.843289936
30.64111913	24.9975596	32.2193309	27.70031892	37.63809465
43.57848054	39.41922859	47.84082467	41.55047838	52.46522284
34.04568792	87.49145859	59.55694499	40.39629843	35.356998
107.5843738	158.638359	159.1439678	242.3777906	166.5200551
44.2593943	77.87701259	60.53328835	100.4136561	37.63809465
42.21665302	32.6891164	34.17201762	36.93375856	42.20028794
0.680913758	0	0	0	1.140548323
37.45025671	32.6891164	24.40858401	9.233439641	33.07590136
0	3.845778399	2.929030082	3.462539865	0
2.723655034	0	0	0	0
6.128223826	0	0	0	0
122.5644765	210.5563674	109.3504564	83.10095677	145.9901853
116.4362527	199.9804768	129.853667	102.722016	122.0386705
53.79218692	17.3060028	41.00642114	63.47989753	51.32467452
3.404568792	0	2.929030082	0	3.421644968
48.34487685	95.18301539	66.39134852	48.47555811	27.37315974
14.98010269	24.9975596	18.55052385	19.62105924	25.0920631
5.447310068	0	0	1.154179955	3.421644968
0	0	0	1.154179955	0
3.404568792	0	0	3.462539865	0
119.8408215	212.4792566	91.77627589	143.1183144	130.0225088
19.06558524	0	6.834403524	17.31269933	7.983838258
2.723655034	0	3.905373442	3.462539865	3.421644968
0	0	3.905373442	1.154179955	1.140548323
2.723655034	0	1.952686721	1.154179955	5.702741613
23.83198155	65.37823279	41.00642114	25.39195901	59.30851278
0	0	0.976343361	1.154179955	0
4504.925426	1741.17617	3008.113894	3298.646312	2569.655371
0.680913758	0	4.881716803	0	1.140548323
137.5445792	139.409467	156.2149377	162.7393737	176.78499

For Review Only

22.47015403	22.1132258	28.31395746	21.92941915	27.37315974
0.680913758	6.730112199	3.905373442	11.54179955	7.983838258
63.32497954	96.14445999	58.58060163	40.39629843	51.32467452
87.83787484	63.45534359	123.0192634	116.5721755	128.8819605
42.21665302	25.9590042	44.91179459	25.39195901	43.34083626
0	34.61200559	10.73977697	4.61671982	19.38932148
39.49299799	59.60956519	30.26664418	36.93375856	30.79480471
13.61827517	7.691556799	3.905373442	10.3876196	22.81096645
0.680913758	1.9228892	0	0	131.1630571
3.404568792	0	4.881716803	3.462539865	1.140548323
4.766396309	0.9614446	0.976343361	3.462539865	21.67041813
0	0	4.881716803	1.154179955	1.140548323
0.680913758	0	0.976343361	3.462539865	12.54603155
2.723655034	16.3445582	0	1.154179955	11.40548323
0	0	1.952686721	1.154179955	0
64.00589329	56.72523139	88.84724581	78.48423695	57.02741613
97.37066746	163.445582	67.36769188	252.7654102	148.2712819
2.042741275	0	2.929030082	0	3.421644968
38.81208423	44.22645159	55.65157155	31.16285879	36.49754632
2.042741275	0	0	1.154179955	4.56219329
83.07147853	37.49633939	57.60425827	31.16285879	57.02741613
5.447310068	0	2.929030082	2.30835991	1.140548323
230.1488504	495.1439689	393.4663743	77.33005699	184.7688283
0	0	0	0	0
315.9439839	0	265.5653941	887.5643855	149.4118303
25.87472282	6.730112199	24.40858401	13.85015946	26.23261142
44.94030806	128.8335764	42.95910786	46.1671982	66.15180271
64.68680705	62.49389899	52.72254147	51.93809798	63.87070607
392.8872386	0	1.952686721	1.154179955	707.13996
3.404568792	9.614445999	3.905373442	0	3.421644968
6.809137585	0	0.976343361	3.462539865	4.56219329
14.29918893	17.3060028	1.952686721	34.62539865	23.95151477
0	0	1.952686721	2.30835991	0
42.89756678	60.57100979	39.05373442	28.85449888	37.63809465
85.11421981	48.07222999	46.86448131	78.48423695	69.57344768
0	19.228892	17.57418049	6.925079731	0
105.5416326	43.26500699	57.60425827	175.4353532	31.93535303
134.8209242	101.9131276	192.339642	130.4223349	136.8657987
12.93736141	18.2674474	12.69246369	12.69597951	2.281096645
0	22.1132258	29.29030082	31.16285879	1.140548323
17.02284396	72.10834499	3.905373442	21.92941915	33.07590136
5.447310068	22.1132258	0	0	10.2649349
99.41340873	137.4865778	76.15478212	65.78825744	108.3520906
26.55563658	41.34211779	6.834403524	13.85015946	10.2649349
109.6271151	95.18301539	87.87090245	86.56349663	114.0548323
76.9432547	101.9131276	66.39134852	84.25513672	72.99509265
22.47015403	11.5373352	9.763433606	27.70031892	19.38932148
35.40751544	17.3060028	39.05373442	31.16285879	18.24877316
17.02284396	33.650561	22.45589729	19.62105924	34.21644968
26.55563658	12.4987798	20.50321057	16.15851937	35.356998

For Review Only

0	0	0.976343361	0	0
6.809137585	19.228892	0	6.925079731	17.10822484
4.766396309	1.9228892	0.976343361	4.61671982	0
1.361827517	0	0	0	0
59.92041074	135.5636886	18.55052385	88.87185654	54.74631949
147.7582856	188.4431416	202.1030756	154.660114	172.2227967
173.6330084	43.26500699	27.3376141	31.16285879	191.6121182
27.23655034	226.9009256	216.748226	140.8099545	69.57344768
0.680913758	0	5.858060163	4.61671982	0
83.75239229	125.9492426	47.84082467	56.5548178	59.30851278
27.23655034	21.1517812	7.810746884	23.0835991	28.51370807
1.361827517	8.653001399	5.858060163	0	0
0	9.614445999	0	0	0
2.723655034	35.57345019	0	0	0
74.21959967	46.14934079	33.19567426	66.9424374	57.02741613
108.9462014	97.10590459	117.1612033	94.64275632	98.08715574
99.41340873	63.45534359	67.36769188	121.1888953	122.0386705
147.0773718	148.0624684	118.1375466	137.3474147	163.0984101
13.61827517	7.691556799	11.71612033	9.233439641	7.983838258
81.70965101	90.37579239	29.29030082	16.15851937	2.281096645
8.85187886	0	8.787090245	17.31269933	0
24.5128953	45.18789619	40.03007778	9.233439641	11.40548323
17.02284396	36.53489479	62.48597508	25.39195901	60.4490611
29.27929161	20.1903366	21.47955393	13.85015946	19.38932148
33.36477416	0	9.763433606	39.24211847	58.16796445
6.809137585	12.4987798	13.66880705	0	7.983838258
0	0	17.57418049	17.31269933	43.34083626
104.8607188	105.758906	122.0429201	91.18021645	167.6606034
138.9064067	32.6891164	21.47955393	86.56349663	44.48138458
35.40751544	0	27.3376141	16.15851937	14.82712819
32.00294665	23.0746704	15.62149377	33.4712187	14.82712819
106.9034601	124.987798	75.17843876	80.79259686	108.3520906
33.36477416	0	0	0	7.983838258
217.8924027	285.5490462	237.2514366	286.2366289	161.9578618
167.5047846	184.5973632	155.2385943	168.5102734	174.5038934
26.55563658	124.987798	28.31395746	69.25079731	46.76248123
0.680913758	0.9614446	0	0	0
73.53868591	0.9614446	2.929030082	27.70031892	2.281096645
0	0	0.976343361	1.154179955	1.140548323
2.042741275	0	4.881716803	1.154179955	11.40548323
3.404568792	16.3445582	0	4.61671982	4.56219329
42.89756678	71.14690039	77.13112548	72.71333717	101.5088007
15.66101644	0	13.66880705	6.925079731	17.10822484
70.13411712	87.49145859	61.50963171	11.54179955	101.5088007
0	0	4.881716803	1.154179955	1.140548323
55.83492819	83.64568019	50.76985475	41.55047838	0
109.6271151	20.1903366	55.65157155	95.79693627	34.21644968
32.00294665	36.53489479	0	0	36.49754632
0	0	36.12470434	25.39195901	53.60577116
23.83198155	8.653001399	15.62149377	28.85449888	23.95151477

For Review Only

0	0	0	1.154179955	0
118.478994	123.0649088	133.7590404	129.268155	98.08715574
3.404568792	0	1.952686721	2.30835991	3.421644968
446.6794255	118.2576858	263.6127073	347.4081665	169.9417001
17.70375772	15.3831136	10.73977697	18.46687928	15.96767652
0	40.38067319	0	0	0
19.06558524	0	9.763433606	2.30835991	1.140548323
8.85187886	0	6.834403524	2.30835991	15.96767652
4.085482551	0	2.929030082	3.462539865	1.140548323
6.128223826	0	0.976343361	1.154179955	0
0	0	32.2193309	3.462539865	0
3.404568792	0	0	0	2.281096645
2.723655034	0	0	0	0
2.042741275	0	0	0	4.56219329
0	0	0	0	0
20.42741275	32.6891164	17.57418049	16.15851937	7.983838258
12.93736141	23.0746704	13.66880705	11.54179955	19.38932148
0	0	0	0	2.281096645
10.89462014	0	2.929030082	8.079259686	13.68657987
0	2.8843338	0.976343361	3.462539865	0
57.19675571	32.6891164	32.2193309	34.62539865	34.21644968
16.3419302	15.3831136	2.929030082	12.69597951	10.2649349
0	0	1.952686721	2.30835991	0
1.361827517	10.5758906	7.810746884	0	3.421644968
25.19380906	0	8.787090245	15.00433942	2.281096645
0	16.3445582	0	0	0
0	0	7.810746884	0	0
147.0773718	163.445582	110.3267997	129.268155	153.9740236
382.6735322	328.8140532	288.0212914	413.1964239	331.8995619
0	0	1.952686721	0	0
0	35.57345019	106.4214263	1.154179955	1.140548323
148.4391993	214.4021458	130.8300103	220.4483714	122.0386705
12.93736141	0	12.69246369	13.85015946	5.702741613
0.680913758	1.9228892	0	1.154179955	0
2.723655034	0	0	3.462539865	1.140548323
55.83492819	40.38067319	37.1010477	28.85449888	51.32467452
40.85482551	47.11078539	52.72254147	39.24211847	62.73015774
51.06853188	43.26500699	41.00642114	25.39195901	33.07590136
122.5644765	43.26500699	50.76985475	0	66.15180271
0.680913758	0.9614446	0	38.08793852	0
9.532792618	0.9614446	58.58060163	79.6384169	0
0	151.9082468	314.3825621	85.40931668	165.3795068
2.723655034	10.5758906	7.810746884	1.154179955	0
12.25644765	41.34211779	23.43224065	5.770899775	29.65425639
125.2881316	40.38067319	12.69246369	128.113975	15.96767652
265.5563658	70.18545579	182.5762084	185.8229728	167.6606034
3.404568792	1.9228892	0.976343361	4.61671982	3.421644968
14.98010269	22.1132258	42.95910786	79.6384169	68.43289936
576.0530396	1045.09028	1219.452857	536.6936791	441.3922008
85.79513356	111.5275736	86.89455909	123.4972552	104.9304457

For Review Only

0.680913758	90.37579239	0	36.93375856	0
80.3478235	79.79990179	101.5397095	72.71333717	46.76248123
15.66101644	7.691556799	12.69246369	3.462539865	3.421644968
3.404568792	10.5758906	6.834403524	10.3876196	0
73.53868591	30.7662272	46.86448131	40.39629843	46.76248123
6.809137585	6.730112199	7.810746884	3.462539865	3.421644968
17.70375772	24.036115	32.2193309	28.85449888	23.95151477
64.00589329	1.9228892	227.488003	0	0
6.128223826	12.4987798	16.59783713	12.69597951	10.2649349
177.718491	176.9058064	159.1439678	126.9597951	173.363345
133.4590967	93.26012619	186.4815819	70.40497726	120.8981222
312.5394151	330.7369424	197.2213588	290.8533487	285.1370807
162.0574745	141.3323562	230.4170331	130.4223349	116.3359289
126.6499591	131.7179102	134.7353838	94.64275632	108.3520906
27.23655034	49.03367459	19.52686721	35.77957861	19.38932148
11.57553389	15.3831136	14.64515041	20.77523919	12.54603155
24.5128953	17.3060028	23.43224065	18.46687928	22.81096645
2.042741275	3.845778399	0	0	0
56.51584195	93.26012619	44.91179459	73.86751713	65.01125439
0	0	1.952686721	0	4.56219329
0	124.987798	111.3031431	4.61671982	38.77864297
12.25644765	0	37.1010477	11.54179955	10.2649349
8.85187886	0	0.976343361	0	0
14.98010269	16.3445582	11.71612033	6.925079731	11.40548323
51.06853188	33.650561	51.74619811	68.09661735	58.16796445
0	0	0.976343361	1.154179955	1.140548323
49.70670437	70.18545579	41.00642114	54.24645789	38.77864297
176.3566634	108.6432398	202.1030756	105.0303759	174.5038934
0	0	0	0	0
0	0	0	1.154179955	0
44.94030806	10.5758906	14.64515041	66.9424374	28.51370807
2.723655034	0	2.929030082	0	0
0	0	696.1328161	818.3135882	614.7555459
0	0	0.976343361	0	0
232.8725054	482.6451891	186.4815819	132.7306948	184.7688283
244.4480393	120.180575	276.305171	139.6557746	163.0984101
10.89462014	10.5758906	10.73977697	4.61671982	0
866.8032145	483.6066337	422.7566751	887.5643855	822.3353406
1256.966798	774.9243475	1073.001353	730.5959116	1508.945431
94.64701242	183.6359186	129.853667	126.9597951	92.38441413
74.90051343	20.1903366	69.3203786	42.70465834	90.10331749
69.45320336	94.22157079	98.61067942	85.40931668	107.2115423
4.085482551	0	2.929030082	4.61671982	1.140548323
326.8386041	353.8116127	242.1331534	445.5134627	352.4294317
18.38467148	16.3445582	23.43224065	38.08793852	17.10822484
93.28518491	77.87701259	135.7117271	109.6470957	93.52496245
17.70375772	0	20.50321057	16.15851937	2.281096645
0	114.4119074	229.4406897	78.48423695	134.5847021
634.6116229	337.4670546	538.941535	578.2441575	491.576327
0	0	4.881716803	0	0

For Review Only

0	0	23.43224065	31.16285879	29.65425639
9.532792618	7.691556799	0	4.61671982	4.56219329
30.64111913	21.1517812	12.69246369	10.3876196	22.81096645
46.30213557	80.76134639	62.48597508	51.93809798	59.30851278
98.73249498	39.41922859	49.79351139	56.5548178	65.01125439
0.680913758	3.845778399	0	0	0
84.43330605	113.4504628	112.2794865	102.722016	77.55728594
96.00883994	0	52.72254147	81.94677681	35.356998
34.72660168	41.34211779	38.07739106	28.85449888	46.76248123
19.06558524	0	50.76985475	170.8186334	15.96767652
0	136.5251332	0.976343361	10.3876196	1.140548323
12.25644765	12.4987798	20.50321057	11.54179955	0
0	0	0	0	0
840.9284917	606.6715425	666.8425153	759.4504104	696.8750251
0	0	0	0	0
0	0	0	0	0
0	0	0	0	1.140548323
41.53573927	14.421669	70.29672196	27.70031892	25.0920631
222.658799	128.8335764	174.7654615	229.6818111	218.9852779
49.02579061	44.22645159	79.0838122	40.39629843	71.85454432
40.17391175	22.1132258	22.45589729	43.85883829	23.95151477
215.8496614	215.3635904	160.1203111	163.8935536	274.8721457
0	0	0.976343361	0	0
2.723655034	0.9614446	0	5.770899775	0
28.59837785	47.11078539	30.26664418	40.39629843	21.67041813
250.5762631	190.3660308	168.9074014	200.8273122	196.1743115
51.06853188	43.26500699	56.62791491	42.70465834	44.48138458
189.2940248	246.1298176	216.748226	256.22795	183.6282799
90.56152987	79.79990179	83.96552901	92.33439641	91.24386581
0	0	0	0	0
7.490051343	50.95656379	2.929030082	21.92941915	21.67041813
138.9064067	205.7491444	69.3203786	93.48857636	155.1145719
0	0	5.858060163	1.154179955	1.140548323
17.02284396	0	7.810746884	3.462539865	0
12.25644765	0	0	100.4136561	140.2874437
4.766396309	3.845778399	4.881716803	12.69597951	5.702741613
41.53573927	8.653001399	33.19567426	32.31703874	47.90302955
21.78924027	43.26500699	25.38492737	28.85449888	36.49754632
0	67.30112199	13.66880705	1.154179955	41.05973961
0	0	0.976343361	0	9.124386581
34.04568792	68.26256659	45.88813795	68.09661735	38.77864297
44.2593943	28.843338	52.72254147	45.01301825	35.356998
0	0	3.905373442	2.30835991	0
0	0	0	1.154179955	0
35.40751544	14.421669	25.38492737	17.31269933	34.21644968
251.9380906	250.9370406	206.0084491	288.5449888	271.4505008
84.43330605	51.91800839	105.4450829	159.2768338	135.7252504
46.30213557	88.45290319	61.50963171	30.00867883	100.3682524
161.3765608	95.18301539	190.3869553	121.1888953	106.070994
0	0	0	0	1.140548323

For Review Only

25.19380906	15.3831136	29.29030082	13.85015946	12.54603155
34.72660168	47.11078539	30.26664418	69.25079731	43.34083626
140.949148	104.7974614	125.9482935	120.0347153	111.7737356
0	17.3060028	0	23.0835991	0
63.32497954	164.4070266	42.95910786	96.95111623	107.2115423
256.0235732	506.6813041	187.4579252	450.1301825	371.8187532
153.2055957	310.5466058	128.8773236	135.0390547	200.7365048
0.680913758	1.9228892	0	0	0
82.39056477	91.33723699	150.3568775	63.47989753	131.1630571
3.404568792	0	6.834403524	0	3.421644968
102.1370638	180.7515848	114.2321732	128.113975	104.9304457
72.1768584	92.29868159	93.72896261	35.77957861	153.9740236
4.085482551	6.730112199	10.73977697	9.233439641	14.82712819
27.23655034	81.72279099	9.763433606	40.39629843	27.37315974
20.42741275	24.9975596	23.43224065	32.31703874	31.93535303
85.79513356	107.6817952	79.0838122	85.40931668	82.11947923
42.21665302	54.80234219	42.95910786	32.31703874	33.07590136
696.5747749	235.553927	309.5008453	292.0075286	286.277629
12.25644765	0	0	0	0
23.83198155	91.33723699	17.57418049	47.32137816	17.10822484
69.45320336	55.76378679	118.1375466	47.32137816	221.2663746
123.2453903	182.674474	132.782697	116.5721755	98.08715574
28.59837785	26.9204488	26.36127073	47.32137816	36.49754632
0.680913758	0	0	0	0
4.766396309	0.9614446	3.905373442	1.154179955	3.421644968
102.8179775	125.9492426	106.4214263	136.1932347	106.070994
12.93736141	0	35.14836098	9.233439641	26.23261142
241.7243842	134.602244	261.6600206	235.4527108	237.2340511
117.1171665	90.37579239	121.0665767	73.86751713	120.8981222
1094.909324	920.1024821	870.8982776	738.6751713	742.496958
55.83492819	27.8818934	70.29672196	32.31703874	50.18412619
0	0	0	1.154179955	0
156.6101644	110.566129	109.3504564	94.64275632	116.3359289
73.53868591	72.10834499	51.74619811	132.7306948	69.57344768
32.00294665	90.37579239	26.36127073	65.78825744	22.81096645
55.15401443	31.7276718	55.65157155	43.85883829	42.20028794
2.723655034	0	6.834403524	8.079259686	0
0	0	2.929030082	2.30835991	0
0	0	0.976343361	6.925079731	1.140548323
0	293.240603	0.976343361	1.154179955	1.140548323
39.49299799	29.8047826	19.52686721	12.69597951	43.34083626
0	0	2.929030082	0	7.983838258
61.28223826	40.38067319	35.14836098	66.9424374	70.713996
0	0	2.929030082	3.462539865	0
311.8585014	394.1922859	327.0750258	285.0824489	367.2565599
78.98599598	77.87701259	85.91821573	35.77957861	52.46522284
120.5217352	78.83845719	126.9246369	96.95111623	96.94660742
57.87766947	81.72279099	60.53328835	58.86317771	57.02741613
0	0	0	407.4255241	0
484.1296823	375.9248385	991.9648543	866.7891463	320.4940787

For Review Only

2.723655034	11.5373352	3.905373442	5.770899775	1.140548323
257.3854007	163.445582	263.6127073	227.3734512	351.2888834
8.170965101	0	0.976343361	0	0
5.447310068	0	0	0	0
65.36772081	67.30112199	85.91821573	92.33439641	55.88686781
2.042741275	0	0	0	0
8.85187886	0	0	0	0
249.8953494	160.5612482	229.4406897	226.2192712	200.7365048
32.68386041	58.64812059	39.05373442	8.079259686	50.18412619
63.32497954	65.37823279	48.81716803	50.78391802	43.34083626
36.76934296	40.38067319	34.17201762	34.62539865	50.18412619
40.17391175	11.5373352	41.9827645	66.9424374	39.91919129
35.40751544	243.2454838	259.7073339	133.8848748	214.4230847
25.19380906	26.9204488	33.19567426	19.62105924	37.63809465
0	0.9614446	0	0	0
2.042741275	0	0.976343361	1.154179955	2.281096645
10.89462014	0	5.858060163	11.54179955	5.702741613
0	0	27.3376141	1.154179955	1.140548323
0	0	2.929030082	2.30835991	10.2649349
0	0	0.976343361	1.154179955	0
6.128223826	0	4.881716803	4.61671982	5.702741613
0	3.845778399	0	0	7.983838258
10.89462014	8.653001399	32.2193309	21.92941915	10.2649349
98.73249498	53.84089759	237.2514366	99.25947614	80.97893091
59.23949699	61.53245439	68.34403524	66.9424374	52.46522284
6.809137585	0	7.810746884	1.154179955	5.702741613
95.32792618	127.8721318	64.4386618	88.87185654	87.82222084
9651.271612	8952.972114	9162.982439	7921.137032	8311.175627
12519.96128	11604.63632	11779.58265	11262.488	10507.8717
89.88061612	53.84089759	82.98918565	45.01301825	93.52496245
6117.329206	2089.219115	0	3692.221676	1871.639797
0	0	1.952686721	0	1.140548323
10.89462014	0	6.834403524	3.462539865	2.281096645
4.085482551	0	1.952686721	0	0
27.9174641	25.9590042	64.4386618	19.62105924	20.52986981
0	0	1.952686721	0	1.140548323
0	0	5.858060163	0	0
0	0	0	1.154179955	2.281096645
4.085482551	0	0	0	0
0	0	0	0	0
14.98010269	0	15.62149377	10.3876196	12.54603155
29.96020537	36.53489479	25.38492737	32.31703874	37.63809465
21.78924027	29.8047826	31.24298754	19.62105924	29.65425639
115.7553389	119.2191304	117.1612033	120.0347153	164.2389585
34.72660168	29.8047826	27.3376141	32.31703874	44.48138458
16.3419302	0	0.976343361	8.079259686	3.421644968
413.9955651	294.2020476	409.0878681	287.3908088	366.1160116
28.59837785	30.7662272	59.55694499	40.39629843	53.60577116
0	4.807222999	1.952686721	1.154179955	5.702741613
0	0	0.976343361	0	0

For Review Only

0	0	0	1.154179955	0
27.9174641	28.843338	32.2193309	40.39629843	39.91919129
93.28518491	62.49389899	77.13112548	76.17587704	62.73015774
354.7560682	565.3294247	823.0574529	972.9737021	181.3471833
0	0	1.952686721	0	0
5.447310068	0	2.929030082	3.462539865	10.2649349
0	2.8843338	0	0	0
0	22.1132258	0	0	39.91919129
3.404568792	0	0.976343361	2.30835991	0
0	0	0	3.462539865	3.421644968
338.4141379	180.7515848	449.1179459	198.5189523	263.4666625
322.7531215	62.49389899	39.05373442	31.16285879	355.8510767
0	0	0.976343361	0	0
0.680913758	0	0.976343361	1.154179955	1.140548323
97.37066746	103.8360168	163.0493412	115.4179955	126.6008638
0	0	0.976343361	1.154179955	2.281096645
0	0	0.976343361	1.154179955	1.140548323
6.128223826	0	1.952686721	12.69597951	18.24877316
16.3419302	0	51.74619811	4.61671982	1.140548323
0	27.8818934	0.976343361	25.39195901	6.843289936
112.3507701	89.41434779	106.4214263	132.7306948	102.649349
106.2225463	0	13.66880705	38.08793852	2.281096645
203.5932138	264.397265	332.9330859	163.8935536	102.649349
132.0972691	386.5007291	244.0858401	334.712187	98.08715574
51.06853188	30.7662272	20.50321057	35.77957861	43.34083626
3.404568792	0	12.69246369	4.61671982	10.2649349
23.15106779	11.5373352	15.62149377	8.079259686	18.24877316
0	0	0	0	0
1.361827517	0.9614446	12.69246369	11.54179955	0
24.5128953	0	13.66880705	3.462539865	15.96767652
57.87766947	67.30112199	105.4450829	95.79693627	77.55728594
1.361827517	0	0	12.69597951	0
0	0	0	0	0
0	26.9204488	0.976343361	2.30835991	33.07590136
2.723655034	18.2674474	5.858060163	12.69597951	9.124386581
15.66101644	25.9590042	15.62149377	25.39195901	20.52986981
4.085482551	6.730112199	6.834403524	11.54179955	9.124386581
36.76934296	12.4987798	33.19567426	28.85449888	31.93535303
70.81503088	90.37579239	71.27306532	84.25513672	67.29235103
128.6927003	117.2962412	72.24940868	64.63407749	75.27618929
53.11127316	46.14934079	0	0	0
308.4539326	236.5153716	262.636364	246.9945104	282.855984
2.042741275	0	0	0	0
2.723655034	2.8843338	0	5.770899775	0
0	9.614445999	0	0	0
75.58142719	123.0649088	110.3267997	50.78391802	83.26002755
47.66396309	23.0746704	66.39134852	48.47555811	62.73015774
0	0	0	0	0
296.8783987	228.8238148	298.7610683	268.9239295	255.4828243
611.4605551	767.2327907	449.1179459	473.2137816	548.6037432

For Review Only

1041.79805	775.8857921	837.7026034	1034.14524	816.632599
30.64111913	57.68667599	27.3376141	38.08793852	47.90302955
0	0	0	3.462539865	2.281096645
1841.190803	0	15.62149377	1225.739112	1.140548323
0	0	0	0	0
164.1002158	124.987798	342.6965196	78.48423695	192.7526665
43.57848054	12.4987798	0	0	0
282.5792098	197.096143	293.8793515	324.3245674	283.9965323
10.21370638	11.5373352	9.763433606	11.54179955	9.124386581
19.06558524	10.5758906	16.59783713	19.62105924	13.68657987
175.6757497	187.481697	145.4751607	279.3115491	213.2825363
100.0943225	39.41922859	77.13112548	150.0433942	67.29235103
61.96315202	85.56856939	49.79351139	56.5548178	50.18412619
0	0	0	0	1.140548323
37.45025671	55.76378679	63.46231844	51.93809798	39.91919129
85.11421981	64.41678819	74.2020954	70.40497726	99.22770407
29.27929161	0	32.2193309	21.92941915	45.6219329
0	0	0	0	0
43.57848054	0.9614446	0	186.9771527	95.8060591
67.41046209	123.0649088	79.0838122	145.4266743	72.99509265
85.79513356	178.8286956	124.9719502	151.1975741	122.0386705
36.0884292	44.22645159	45.88813795	40.39629843	69.57344768
1.361827517	0	0	0	0
36.76934296	29.8047826	22.45589729	33.4712187	37.63809465
0	0	0	9.233439641	11.40548323
72.85777215	122.1034642	72.24940868	106.1845559	75.27618929
29.96020537	25.9590042	34.17201762	62.32571757	29.65425639
6.809137585	0	6.834403524	3.462539865	2.281096645
1464.645494	148.0624684	1388.360259	692.5079731	1131.423936
127.3308728	339.3899438	166.9547147	114.2638156	285.1370807
111.6698564	25.9590042	0	38.08793852	42.20028794
2.723655034	0	0	0	0
25.87472282	3.845778399	0	16.15851937	13.68657987
2.042741275	5.768667599	0	0	0
73.53868591	128.8335764	109.3504564	73.86751713	111.7737356
19.06558524	10.5758906	4.881716803	2.30835991	4.56219329
13.61827517	1.9228892	0	0	0
0	0	0	0	1.140548323
0	101.9131276	11.71612033	130.4223349	120.8981222
59.23949699	144.21669	68.34403524	86.56349663	19.38932148
19.746499	9.614445999	14.64515041	24.23777906	20.52986981
275.7700722	280.7418232	358.3180133	360.104146	147.1307336
10.21370638	0	3.905373442	18.46687928	7.983838258
226.0633678	158.638359	149.3805342	173.1269933	260.0450176
1732.925515	1857.510967	2017.125383	1905.551106	1536.318591
301.644795	459.5705187	660.0081117	0	517.8089385
2.042741275	5.768667599	0.976343361	0	0
11.57553389	0	0.976343361	1.154179955	2.281096645
2.042741275	0	2.929030082	0	0
0	25.9590042	0.976343361	0	0

For Review Only

0	0	0.976343361	1.154179955	0
12.93736141	0	0	0	0
0	0	0	0	0
7.490051343	0	2.929030082	1.154179955	2.281096645
4.766396309	0	0	4.61671982	1.140548323
6.809137585	0	0	3.462539865	1.140548323
10.21370638	33.650561	13.66880705	0	0
13.61827517	0	0	0	0
144.3537168	121.1420196	202.1030756	176.5895331	188.1904732
0	0	4.881716803	0	0
25.19380906	46.14934079	44.91179459	19.62105924	29.65425639
43.57848054	59.60956519	63.46231844	28.85449888	17.10822484
21.10832651	56.72523139	0	107.3387358	0
0	0	0	0	0
19.746499	0	0	0	0
7.490051343	9.614445999	0	6.925079731	7.983838258
7.490051343	0	0.976343361	15.00433942	7.983838258
128.6927003	0	122.0429201	192.7480525	88.96276916
65.36772081	20.1903366	33.19567426	49.62973807	71.85454432
4.085482551	0	0	1.154179955	0
65.36772081	138.4480224	43.93545122	106.1845559	115.1953806
6.809137585	12.4987798	9.763433606	10.3876196	13.68657987
2.042741275	9.614445999	4.881716803	0	0
72.85777215	110.566129	63.46231844	116.5721755	68.43289936
156.6101644	192.28892	224.5589729	206.598212	141.427992
0.680913758	0	1.952686721	1.154179955	0
0	0	0	0	1.140548323
115.7553389	0	161.0966545	95.79693627	46.76248123
10.21370638	0	0	16.15851937	4.56219329
91.92335739	0	20.50321057	92.33439641	34.21644968
65.36772081	101.9131276	83.96552901	56.5548178	84.40057587
37.45025671	0	54.67522819	106.1845559	12.54603155
43.57848054	40.38067319	44.91179459	34.62539865	43.34083626
19.746499	17.3060028	25.38492737	20.77523919	23.95151477
0	50.95656379	127.9009802	76.17587704	112.9142839
40.17391175	35.57345019	46.86448131	32.31703874	38.77864297
93.96609867	32.6891164	107.3977697	63.47989753	43.34083626
0	0	0	1.154179955	0
0	0	1.952686721	0	0
130.0545279	150.9468022	271.4234542	174.2811732	174.5038934
44.2593943	112.4890182	45.88813795	92.33439641	59.30851278
13.61827517	0	3.905373442	2.30835991	1.140548323
211.0832651	124.0263534	178.670835	117.7263554	152.8334752
36.0884292	0	57.60425827	30.00867883	6.843289936
53.11127316	11.5373352	49.79351139	24.23777906	27.37315974
11.57553389	30.7662272	0.976343361	2.30835991	2.281096645
29.96020537	0	0.976343361	1.154179955	1.140548323
0.680913758	0	6.834403524	1.154179955	2.281096645
5.447310068	11.5373352	2.929030082	5.770899775	1.140548323
128.6927003	98.06734919	212.8428526	144.2724944	166.5200551

For Review Only

120.5217352	129.795021	75.17843876	160.4310138	131.1630571
29.27929161	56.72523139	63.46231844	55.40063784	15.96767652
388.8017561	240.36115	225.5353163	372.8001255	368.3971082
44.94030806	63.45534359	82.01284229	40.39629843	49.04357787
78.98599598	40.38067319	69.3203786	35.77957861	49.04357787
0	0	0	10.3876196	0
0	0	0	0	0
8.85187886	6.730112199	8.787090245	11.54179955	12.54603155
40.17391175	58.64812059	55.65157155	45.01301825	47.90302955
0	0	0	0	3.421644968
5.447310068	8.653001399	11.71612033	10.3876196	4.56219329
46.30213557	62.49389899	37.1010477	64.63407749	21.67041813
6.128223826	14.421669	6.834403524	2.30835991	4.56219329
79.66690974	58.64812059	49.79351139	79.6384169	62.73015774
23.15106779	66.33967739	49.79351139	20.77523919	53.60577116
51.06853188	62.49389899	70.29672196	64.63407749	53.60577116
27.23655034	46.14934079	41.9827645	24.23777906	38.77864297
1.361827517	16.3445582	19.52686721	15.00433942	0
41.53573927	22.1132258	28.31395746	18.46687928	38.77864297
19.06558524	40.38067319	25.38492737	27.70031892	25.0920631
42.21665302	0	1.952686721	1.154179955	78.69783426
164.1002158	206.710589	133.7590404	93.48857636	161.9578618
12.93736141	28.843338	17.57418049	0	0
117.7980802	122.1034642	131.8063537	109.6470957	153.9740236
76.26234095	0	19.52686721	46.1671982	33.07590136
4.766396309	37.49633939	0	0	0
64.00589329	0	0.976343361	1.154179955	1.140548323
54.47310068	13.4602244	0	86.56349663	0
0	0	4.881716803	0	0
10.21370638	16.3445582	4.881716803	1.154179955	26.23261142
2.042741275	0	0	2.30835991	0
0	0	0	0	6.843289936
2.042741275	2.8843338	2.929030082	1.154179955	5.702741613
70.13411712	0	25.38492737	73.86751713	44.48138458
93.96609867	0	84.94187237	0	98.08715574
0	0	0	5.770899775	0
3.404568792	10.5758906	6.834403524	0	3.421644968
6.809137585	0.9614446	111.3031431	0	0
4.766396309	1.9228892	10.73977697	8.079259686	0
21.10832651	43.26500699	18.55052385	11.54179955	18.24877316
63.32497954	44.22645159	82.01284229	46.1671982	87.82222084
78.30508222	49.03367459	12.69246369	38.08793852	5.702741613
72.1768584	56.72523139	56.62791491	43.85883829	50.18412619
0	0	0.976343361	2.30835991	0
0	0	99.58702278	26.54613897	95.8060591
3.404568792	5.768667599	0	0	9.124386581
10.21370638	0	18.55052385	21.92941915	1.140548323
0	0	0	0	0
9.532792618	0	10.73977697	12.69597951	38.77864297
0.680913758	1.9228892	0.976343361	5.770899775	4.56219329

For Review Only

15.66101644	49.99511919	38.07739106	20.77523919	26.23261142
33.36477416	0	25.38492737	10.3876196	145.9901853
53.79218692	0	32.2193309	48.47555811	10.2649349
34.04568792	11.5373352	35.14836098	40.39629843	55.88686781
32.68386041	19.228892	22.45589729	11.54179955	35.356998
17.70375772	13.4602244	38.07739106	23.0835991	36.49754632
0	0	0.976343361	4.61671982	2.281096645
12.93736141	9.614445999	15.62149377	19.62105924	0
1.361827517	3.845778399	0	3.462539865	3.421644968
37.45025671	73.06978959	57.60425827	49.62973807	26.23261142
14.29918893	25.9590042	14.64515041	20.77523919	12.54603155
36.0884292	41.34211779	40.03007778	57.70899775	41.05973961
0	0	0.976343361	0	0
0	0	3.905373442	1.154179955	5.702741613
22.47015403	40.38067319	25.38492737	24.23777906	28.51370807
118.478994	118.2576858	69.3203786	88.87185654	0
24.5128953	48.07222999	48.81716803	19.62105924	33.07590136
6.809137585	18.2674474	7.810746884	6.925079731	7.983838258
0	0	27.3376141	8.079259686	0
1.361827517	0	0	0	0
55.83492819	65.37823279	44.91179459	36.93375856	41.05973961
19.746499	38.45778399	16.59783713	13.85015946	14.82712819
3.404568792	0	26.36127073	12.69597951	5.702741613
8.170965101	0	1.952686721	2.30835991	10.2649349
1.361827517	0.9614446	3.905373442	0	0
25.19380906	14.421669	23.43224065	21.92941915	31.93535303
211.7641789	207.6720336	242.1331534	184.6687928	145.9901853
176.3566634	156.7154698	124.9719502	246.9945104	189.3310216
23.15106779	18.2674474	35.14836098	21.92941915	15.96767652
39.49299799	29.8047826	48.81716803	53.09227793	43.34083626
6.809137585	17.3060028	5.858060163	6.925079731	10.2649349
20.42741275	15.3831136	15.62149377	21.92941915	13.68657987
30.64111913	19.228892	38.07739106	31.16285879	45.6219329
108.9462014	59.60956519	48.81716803	128.113975	60.4490611
24.5128953	14.421669	10.73977697	12.69597951	3.421644968
19.746499	0	61.50963171	23.0835991	17.10822484
1.361827517	0	0	8.079259686	0
32.68386041	12.4987798	24.40858401	25.39195901	20.52986981
45.62122182	0	14.64515041	15.00433942	18.24877316
0	0	0	5.770899775	0
0	0	104.4687396	75.02169708	102.649349
14.98010269	25.9590042	9.763433606	10.3876196	15.96767652
0	0	0	2.30835991	2.281096645
25.19380906	0	16.59783713	3.462539865	17.10822484
5.447310068	0	0.976343361	0	1.140548323
0	0	0	2.30835991	2.281096645
3.404568792	0	2.929030082	1.154179955	0
40.17391175	98.06734919	45.88813795	96.95111623	47.90302955
1.361827517	0	0	0	0
12.93736141	25.9590042	11.71612033	5.770899775	11.40548323

For Review Only

38.13117047	41.34211779	33.19567426	43.85883829	47.90302955
82.39056477	48.07222999	101.5397095	62.32571757	65.01125439
30.64111913	90.37579239	20.50321057	92.33439641	14.82712819
0	0	29.29030082	126.9597951	1.140548323
0	0	1.952686721	0	0
0	0	0.976343361	2.30835991	2.281096645
12.25644765	35.57345019	2.929030082	5.770899775	19.38932148
64.00589329	0	76.15478212	47.32137816	23.95151477
30.64111913	0	70.29672196	11.54179955	29.65425639
0	0	0	0	7.983838258
307.7730188	306.7008274	260.6836773	364.7208658	309.0885954
0	0	0.976343361	1.154179955	1.140548323
0.680913758	0	2.929030082	5.770899775	2.281096645
150.4819406	27.8818934	302.6664418	34.62539865	0
4.766396309	0	0	4.61671982	0
27.23655034	4.807222999	0	85.40931668	0
0	0	4.881716803	0	0
10.89462014	14.421669	9.763433606	3.462539865	1.140548323
4.766396309	0	6.834403524	0	0
0	0	0	4.61671982	0
0	0	0	1.154179955	1.140548323
4.085482551	0	0	3.462539865	0
2.042741275	1.9228892	0	0	0
20.42741275	0	0	0	0
192.0176799	449.9560727	235.2987499	311.6285879	363.8349149
2.042741275	0	2.929030082	0	4.56219329
160.695647	130.7564656	134.7353838	141.9641345	132.3036054
32.68386041	116.3347966	41.00642114	73.86751713	266.8883075
2.042741275	2.8843338	0.976343361	1.154179955	3.421644968
0	8.653001399	0	27.70031892	7.983838258
5.447310068	0	3.905373442	0	0
8.170965101	0	6.834403524	3.462539865	0
27.9174641	48.07222999	38.07739106	51.93809798	37.63809465
104.179805	85.56856939	119.11389	114.2638156	110.6331873
0	0	0	0	0
0	140.3709116	17.57418049	1.154179955	19.38932148
6.809137585	29.8047826	46.86448131	12.69597951	36.49754632
3.404568792	0	0	0	0
17.70375772	0	5.858060163	4.61671982	12.54603155
53.11127316	102.8745722	141.5697873	65.78825744	65.01125439
0	596.0956519	0.976343361	139.6557746	1.140548323
61.96315202	46.14934079	47.84082467	47.32137816	78.69783426
198.1459037	477.8379661	116.1848599	360.104146	198.4554081
19.06558524	23.0746704	7.810746884	27.70031892	23.95151477
53.79218692	22.1132258	64.4386618	46.1671982	37.63809465
159.3338195	160.5612482	193.3159854	177.7437131	204.1581497
0.680913758	229.7852594	227.488003	320.8620275	241.7962444
121.202649	65.37823279	126.9246369	106.1845559	94.66551078
68.09137585	42.30356239	0	72.71333717	47.90302955
83.07147853	82.68423559	87.87090245	79.6384169	88.96276916

For Review Only

21.78924027	0.9614446	26.36127073	6.925079731	5.702741613
8.170965101	63.45534359	12.69246369	57.70899775	6.843289936
268.2800208	337.4670546	245.0621835	243.5319705	246.3584377
7.490051343	3.845778399	5.858060163	8.079259686	4.56219329
27.23655034	33.650561	23.43224065	38.08793852	21.67041813
7.490051343	4.807222999	6.834403524	4.61671982	4.56219329
27.9174641	34.61200559	60.53328835	31.16285879	47.90302955
5.447310068	0	16.59783713	6.925079731	4.56219329
0	0	0	0	0
6.809137585	0	5.858060163	5.770899775	5.702741613
4.085482551	0	2.929030082	5.770899775	2.281096645
0	0	0	0	0
0	0.9614446	0	0	6.843289936
0	0	0	0	0
36.76934296	49.03367459	33.19567426	54.24645789	38.77864297
251.2571769	129.795021	201.1267323	176.5895331	193.8932148
346.5851031	221.132258	297.784725	246.9945104	231.5313095
420.123789	286.5104908	452.0469759	293.1617086	299.9642088
368.3743433	317.276718	221.6299428	182.3604329	304.5264021
285.3028648	291.3177138	181.5998651	203.1356721	208.720343
436.4657192	251.8984852	342.6965196	271.2322894	311.3696921
355.4369819	395.1537305	248.9675569	286.2366289	313.6507887
43.57848054	73.06978959	45.88813795	68.09661735	36.49754632
25.87472282	23.0746704	31.24298754	38.08793852	50.18412619
177.718491	0	0	0	804.0865674
69.45320336	297.0863814	165.9783713	0	142.5685403
0	0	1.952686721	0	0
0	0	0	0	2.281096645
1714.540844	1630.610041	2127.452183	1647.014796	1715.384677
5.447310068	53.84089759	0	23.0835991	45.6219329
141.6300618	0	341.7201762	0	0
42.21665302	0	0	23.0835991	0
0	0	0.976343361	3.462539865	0
0	0	0	0	0
0	41.34211779	93.72896261	26.54613897	17.10822484
4.085482551	0	0	0	4.56219329
3.404568792	0	5.858060163	0	4.56219329
0	0	0	0	0
0	0	0.976343361	0	3.421644968
46.98304933	31.7276718	47.84082467	39.24211847	35.356998
0	0	0	0	0
4.085482551	9.614445999	5.858060163	0	4.56219329
0	0	0.976343361	1.154179955	0
11.57553389	0	0	0	0
76.26234095	65.37823279	81.03649893	71.55915722	43.34083626
37.45025671	60.57100979	37.1010477	8.079259686	25.0920631
81.02873726	22.1132258	89.82358917	43.85883829	90.10331749
82.39056477	199.9804768	65.41500516	173.1269933	101.5088007
21.10832651	18.2674474	0	0	15.96767652
766.0279783	201.903366	604.3565402	83.10095677	549.7442915

For Review Only

0	0	3.905373442	3.462539865	0
1007.752363	0	297.784725	341.6372667	319.3535303
3.404568792	0	0.976343361	0	0
0	0	0	3.462539865	0
0.680913758	3.845778399	0	1.154179955	0
0.680913758	5.768667599	0.976343361	3.462539865	0
0.680913758	3.845778399	0	1.154179955	0
0	0	3.905373442	0	9.124386581
283.2601235	328.8140532	266.5417374	205.444032	205.2986981
41.53573927	52.87945299	26.36127073	18.46687928	29.65425639
0.680913758	415.3440671	0	0	86.68167252
79.66690974	4.807222999	0	0	837.1624688
0	0	0	0	0
1036.35074	407.6525103	0	0	5.702741613
0	0	0	0	0
12.93736141	46.14934079	20.50321057	21.92941915	9.124386581
0	0	0	0	0
0	0	2.929030082	0	6.843289936
4.085482551	0.9614446	3.905373442	10.3876196	3.421644968
5.447310068	0	1.952686721	6.925079731	1.140548323
509.3234913	470.1464093	534.0598182	543.6187588	598.7878694
323.4340353	157.6769144	217.7245694	311.6285879	198.4554081
81.02873726	74.03123419	70.29672196	163.8935536	111.7737356
1950.137004	206.710589	1529.930046	615.1779161	487.0141338
3.404568792	0	0	0	0
4.085482551	0	3.905373442	2.30835991	2.281096645
27.23655034	0	0	0	0
1250.157661	466.3006309	1315.134507	328.9412872	1140.548323
0	0	0	0	0
7.490051343	20.1903366	17.57418049	4.61671982	10.2649349
75.58142719	275.9346002	0	40.39629843	132.3036054
2.723655034	0.9614446	102.5160529	0	46.76248123
53.79218692	12.4987798	43.93545122	60.01735766	74.13564097
1960.350711	3761.171275	5850.249416	9190.734982	9384.431598
22.47015403	736.4665635	136.6880705	405.1171642	102.649349
0	1121.044403	0	0	303.3858538
459.616787	718.1991161	747.8790142	828.7012078	507.5440036
817.0965101	258.6285974	847.466037	758.2962305	463.062619
563.796592	249.975596	2485.770196	1501.588122	1644.670681
0	0	0	0	0
67.41046209	49.03367459	75.17843876	50.78391802	50.18412619
61.96315202	49.03367459	30.26664418	58.86317771	52.46522284
0	0	0.976343361	8.079259686	1.140548323
140.2682342	123.0649088	113.2558298	105.0303759	142.5685403
21.10832651	13.4602244	52.72254147	54.24645789	13.68657987
4.766396309	13.4602244	4.881716803	5.770899775	3.421644968
84.43330605	55.76378679	32.2193309	60.01735766	57.02741613
66.72954833	92.29868159	105.4450829	102.722016	29.65425639
8.170965101	23.0746704	35.14836098	11.54179955	12.54603155
15.66101644	0	19.52686721	6.925079731	1.140548323

For Review Only

64.68680705	42.30356239	53.69888483	70.40497726	54.74631949
45.62122182	15.3831136	28.31395746	80.79259686	51.32467452
0	1.9228892	0	28.85449888	0
5.447310068	9.614445999	0	0	0
85.11421981	103.8360168	84.94187237	85.40931668	132.3036054
84.43330605	76.91556799	104.4687396	85.40931668	82.11947923
1591.295453	1366.212776	1595.345051	1598.539238	1313.911668
22.47015403	22.1132258	12.69246369	32.31703874	14.82712819
0	99.02879379	115.2085165	39.24211847	133.4441537
5.447310068	0	8.787090245	2.30835991	1.140548323
0	9.614445999	1.952686721	0	3.421644968
48.34487685	9.614445999	35.14836098	30.00867883	98.08715574
59.92041074	51.91800839	50.76985475	71.55915722	87.82222084
220.6160577	0	41.00642114	34.62539865	3.421644968
164.7811295	317.276718	243.1094968	289.6991687	131.1630571
0	0	0	0	0
79.66690974	52.87945299	92.75261925	50.78391802	82.11947923
14.29918893	0	14.64515041	4.61671982	1.140548323
41.53573927	10.5758906	28.31395746	33.4712187	44.48138458
0	0	0	0	0
0	0	0	3.462539865	0
170.2284396	232.6695932	220.6535995	177.7437131	136.8657987
71.49594464	65.37823279	77.13112548	51.93809798	47.90302955
0	10.5758906	2.929030082	5.770899775	9.124386581
3.404568792	0.9614446	3.905373442	6.925079731	2.281096645
0	0	0.976343361	1.154179955	0
214.4878339	221.132258	277.2815144	310.4744079	323.9157236
2.042741275	0	0	4.61671982	0
0	0	1.952686721	1.154179955	0
0	0	1.952686721	0	2.281096645
0	0	2.929030082	0	0
0	0	0	1.154179955	0
0	0	0	16.15851937	0
122.5644765	72.10834499	117.1612033	6.925079731	0
4.766396309	0.9614446	0	293.1617086	0
0	0	0	0	0
12.93736141	1.9228892	0	15.00433942	29.65425639
76.26234095	113.4504628	137.6644138	130.4223349	135.7252504
110.9889426	177.867251	33.19567426	160.4310138	139.1468954
72.85777215	152.8696914	79.0838122	136.1932347	112.9142839
15.66101644	40.38067319	28.31395746	33.4712187	28.51370807
54.47310068	33.650561	0	0	0
55.15401443	73.06978959	48.81716803	75.02169708	54.74631949
0	0	4.881716803	2.30835991	5.702741613
151.8437681	104.7974614	122.0429201	146.5808543	179.0660866
54.47310068	17.3060028	71.27306532	38.08793852	54.74631949
0	0	6.834403524	4.61671982	14.82712819
27.9174641	20.1903366	35.14836098	46.1671982	29.65425639
151.1628544	0	0	0	0
0	0	0	0	0

For Review Only

93.28518491	311.5080504	30.26664418	5.770899775	94.66551078
12.93736141	35.57345019	56.62791491	30.00867883	0
32.00294665	112.4890182	51.74619811	105.0303759	19.38932148
25.19380906	0	22.45589729	0	23.95151477
34.04568792	81.72279099	18.55052385	51.93809798	44.48138458
0	38.45778399	6.834403524	80.79259686	1.140548323
283.9410373	415.3440671	345.6255496	325.4787473	504.1223586
96.6897537	71.14690039	71.27306532	69.25079731	108.3520906
21.10832651	13.4602244	21.47955393	26.54613897	20.52986981
0	172.0985834	0.976343361	1.154179955	22.81096645
111.6698564	74.99267879	97.63433606	105.0303759	120.8981222
10.89462014	7.691556799	0	28.85449888	4.56219329
0	0	0	6.925079731	10.2649349
60.6013245	50.95656379	58.58060163	62.32571757	54.74631949
13.61827517	1.9228892	0	10.3876196	7.983838258
47.66396309	61.53245439	82.01284229	54.24645789	41.05973961
89.88061612	148.0624684	138.6407572	84.25513672	76.41673761
0	0	0	1.154179955	1.140548323
6.809137585	25.9590042	21.47955393	0	9.124386581
24.5128953	19.228892	22.45589729	21.92941915	21.67041813
0	0	0	2.30835991	0
39.49299799	33.650561	32.2193309	41.55047838	34.21644968
0	0	0	0	0
5.447310068	0	7.810746884	6.925079731	1.140548323
119.1599077	138.4480224	91.77627589	95.79693627	139.1468954
322.0722077	289.3948246	249.9439003	266.6155696	310.2291437
1.361827517	22.1132258	0	0	0
87.15696108	39.41922859	55.65157155	99.25947614	41.05973961
17.02284396	41.34211779	26.36127073	11.54179955	13.68657987
50.38761813	41.34211779	20.50321057	47.32137816	38.77864297
125.9690453	17.3060028	94.70530597	77.33005699	77.55728594
3.404568792	0	2.929030082	0	6.843289936
32.00294665	0	11.71612033	30.00867883	3.421644968
147.0773718	261.5129312	123.9956068	177.7437131	124.3197672
75.58142719	71.14690039	43.93545122	63.47989753	19.38932148
162.0574745	256.7057082	137.6644138	152.3517541	166.5200551
111.6698564	159.5998036	52.72254147	65.78825744	76.41673761
81.70965101	4.807222999	70.29672196	75.02169708	127.7414121
0.680913758	0	0	0	0
16.3419302	0.9614446	3.905373442	4.61671982	9.124386581
2.042741275	3.845778399	0	0	0
2.723655034	0	0	0	0
142.9918893	51.91800839	322.193309	347.4081665	383.2242364
579.4576084	341.312833	469.6211564	530.9227793	461.9220707
6.128223826	18.2674474	11.71612033	6.925079731	9.124386581
9.532792618	24.9975596	3.905373442	11.54179955	4.56219329
2.723655034	0	4.881716803	2.30835991	0
2.042741275	0	0	0	0
0	0	0	0	0
20.42741275	0	0.976343361	2.30835991	0

For Review Only

33.36477416	12.4987798	16.59783713	13.85015946	22.81096645
6.128223826	14.421669	22.45589729	26.54613897	17.10822484
10.21370638	0	0	0	0
16.3419302	6.730112199	10.73977697	10.3876196	10.2649349
0	0	0	0	0
24.5128953	0	1.952686721	15.00433942	2.281096645
19.06558524	0	3.905373442	9.233439641	45.6219329
0	6.730112199	0	0	0
0	0	0	0	0
54.47310068	28.843338	71.27306532	76.17587704	83.26002755
2.042741275	0	3.905373442	0	1.140548323
9.532792618	9.614445999	13.66880705	16.15851937	9.124386581
0	0	27.3376141	31.16285879	0
0	0	5.858060163	13.85015946	2.281096645
6.128223826	0	0	5.770899775	1.140548323
68.09137585	61.53245439	35.14836098	34.62539865	83.26002755
1.361827517	6.730112199	0	0	0
0.680913758	0	4.881716803	1.154179955	0
121.202649	82.68423559	99.58702278	95.79693627	96.94660742
10.89462014	9.614445999	9.763433606	10.3876196	4.56219329
0.680913758	0	0	0	0
17.70375772	37.49633939	28.31395746	56.5548178	25.0920631
93.96609867	80.76134639	88.84724581	111.9554556	119.7575739
25.19380906	9.614445999	37.1010477	25.39195901	20.52986981
42.21665302	46.14934079	48.81716803	41.55047838	52.46522284
256.0235732	277.8574894	202.1030756	318.5536676	182.4877316
0	0	0.976343361	3.462539865	0
17.70375772	0.9614446	3.905373442	4.61671982	30.79480471
90.56152987	0	68.34403524	28.85449888	37.63809465
74.21959967	89.41434779	107.3977697	103.876196	85.5411242
21.78924027	41.34211779	0	19.62105924	0
55.15401443	11.5373352	53.69888483	111.9554556	76.41673761
105.5416326	82.68423559	91.77627589	152.3517541	205.2986981
14.29918893	0	33.19567426	17.31269933	12.54603155
23.83198155	12.4987798	16.59783713	26.54613897	17.10822484
64.68680705	71.14690039	60.53328835	63.47989753	76.41673761
11.57553389	0	9.763433606	23.0835991	9.124386581
63.32497954	31.7276718	46.86448131	78.48423695	70.713996
155.2483369	184.5973632	170.8600881	210.0607518	220.1258263
24.5128953	0	21.47955393	19.62105924	11.40548323
10.89462014	0	5.858060163	0	1.140548323
7.490051343	0	1.952686721	0	1.140548323
6.809137585	42.30356239	7.810746884	5.770899775	2.281096645
11.57553389	34.61200559	0	0	18.24877316
18.38467148	5.768667599	18.55052385	43.85883829	13.68657987
0	52.87945299	4.881716803	6.925079731	25.0920631
51.74944564	73.06978959	23.43224065	20.77523919	42.20028794
15.66101644	21.1517812	20.50321057	17.31269933	19.38932148
29.96020537	38.45778399	36.12470434	33.4712187	43.34083626
29.96020537	0.9614446	13.66880705	16.15851937	63.87070607

For Review Only

307.7730188	174.0214726	73.22575204	133.8848748	234.9529545
8.170965101	12.4987798	5.858060163	2.30835991	4.56219329
248.5335218	540.3318651	505.7458608	333.558007	269.1694041
2.723655034	0	3.905373442	4.61671982	1.140548323
0	0	2.929030082	0	0
0	0	9.763433606	8.079259686	15.96767652
136.8636654	122.1034642	150.3568775	117.7263554	152.8334752
80.3478235	43.26500699	24.40858401	57.70899775	23.95151477
36.76934296	18.2674474	49.79351139	30.00867883	34.21644968
80.3478235	80.76134639	85.91821573	93.48857636	46.76248123
10.89462014	2.8843338	0	8.079259686	3.421644968
80.3478235	25.9590042	57.60425827	10.3876196	98.08715574
32.00294665	10.5758906	20.50321057	11.54179955	23.95151477
0	0	3.905373442	6.925079731	2.281096645
0	0	0	1.154179955	0
0	0	0	0	0
0	0	0	0	0
70.13411712	153.831136	288.9976347	63.47989753	102.649349
59.23949699	24.9975596	55.65157155	91.18021645	103.7898974
1.361827517	0	0.976343361	0	0
0	0	4.881716803	3.462539865	2.281096645
8.170965101	63.45534359	59.55694499	57.70899775	55.88686781
868.165042	636.4763251	1260.459278	50.78391802	10.2649349
65.36772081	39.41922859	56.62791491	62.32571757	55.88686781
0	0	916.7864156	772.14639	776.7134077
36.0884292	40.38067319	80.06015557	17.31269933	51.32467452
128.0117866	172.0985834	119.11389	265.4613897	108.3520906
98.05158122	103.8360168	100.5633661	111.9554556	133.4441537
3.404568792	0	0	3.462539865	0
0	0	0	0	1.140548323
10.21370638	0	8.787090245	2.30835991	5.702741613
111.6698564	21.1517812	35.14836098	0	116.3359289
0	4.807222999	0	0	0
13.61827517	8.653001399	10.73977697	9.233439641	5.702741613
8.170965101	0	5.858060163	0	4.56219329
0.680913758	0	0.976343361	0	0
0	0	0	0	0
0	0	0	0	0
105.5416326	112.4890182	138.6407572	91.18021645	99.22770407
29.27929161	45.18789619	14.64515041	34.62539865	0
0	11.5373352	0	0	0
340.4568792	156.7154698	211.8665092	196.2105924	193.8932148
94.64701242	120.180575	121.0665767	86.56349663	135.7252504
46.30213557	96.14445999	45.88813795	41.55047838	27.37315974
0	128.8335764	0.976343361	147.7350343	1.140548323
0	38.45778399	23.43224065	0	0
30.64111913	8.653001399	21.47955393	38.08793852	21.67041813
38.81208423	49.03367459	47.84082467	51.93809798	37.63809465
35.40751544	0	26.36127073	4.61671982	49.04357787
117.1171665	136.5251332	127.9009802	120.0347153	148.2712819

For Review Only

29.96020537	68.26256659	48.81716803	42.70465834	60.4490611
11.57553389	0	15.62149377	3.462539865	7.983838258
109.6271151	142.2938008	133.7590404	125.8056151	102.649349
0	0	0	0	5.702741613
122.5644765	91.33723699	123.9956068	176.5895331	109.492639
266.9181933	446.1102943	253.8492737	332.4038271	326.1968203
91.92335739	125.9492426	116.1848599	123.4972552	152.8334752
418.7619614	357.6573911	412.0168982	495.1432007	507.5440036
3.404568792	0	0	0	4.56219329
0	0	1.952686721	4.61671982	2.281096645
42.21665302	14.421669	21.47955393	34.62539865	28.51370807
27.9174641	149.023913	40.03007778	31.16285879	61.58960942
59.92041074	70.18545579	102.5160529	90.0260365	82.11947923
0	14.421669	8.787090245	2.30835991	4.56219329
12.93736141	90.37579239	10.73977697	46.1671982	11.40548323
74.21959967	79.79990179	14.64515041	24.23777906	12.54603155
11.57553389	39.41922859	10.73977697	11.54179955	19.38932148
165.4620433	1.9228892	63.46231844	95.79693627	30.79480471
0	0	0.976343361	0	2.281096645
155.2483369	149.9853576	101.5397095	154.660114	133.4441537
229.4679366	0	0	65.78825744	0
35.40751544	46.14934079	41.9827645	60.01735766	36.49754632
14.98010269	36.53489479	15.62149377	17.31269933	27.37315974
0	0	3.905373442	5.770899775	247.498986
147.7582856	111.5275736	121.0665767	158.1226538	104.9304457
1.361827517	0.9614446	21.47955393	103.876196	3.421644968
85.11421981	99.99023839	92.75261925	77.33005699	88.96276916
263.5136245	342.2742776	308.5245019	283.928269	256.6233726
7.490051343	24.036115	12.69246369	25.39195901	9.124386581
128.0117866	102.8745722	145.4751607	118.8805354	98.08715574
0.680913758	1.9228892	65.41500516	124.6514351	155.1145719
51.06853188	75.95412339	3.905373442	27.70031892	62.73015774
0	0	0	0	0
142.3109755	133.6407994	103.4923962	223.9109113	103.7898974
113.0316839	99.02879379	115.2085165	110.8012757	124.3197672
117.7980802	0	133.7590404	30.00867883	239.5151477
7.490051343	0	0	5.770899775	1.140548323
249.8953494	174.9829172	143.522474	146.5808543	157.3956685
8.85187886	16.3445582	18.55052385	19.62105924	22.81096645
0	0	0.976343361	288.5449888	5.702741613
0	0	0	0	1.140548323
11.57553389	4.807222999	0	3.462539865	10.2649349
0	0	0.976343361	9.233439641	0
0	0	0	2.30835991	0
0	9.614445999	0.976343361	8.079259686	12.54603155
43.57848054	92.29868159	37.1010477	21.92941915	12.54603155
2.723655034	0	0	3.462539865	4.56219329
48.34487685	87.49145859	44.91179459	48.47555811	45.6219329
0	157.6769144	187.4579252	11.54179955	123.1792188
0	0	0	0	0

For Review Only

67.41046209	69.22401119	55.65157155	50.78391802	53.60577116
84.43330605	47.11078539	58.58060163	68.09661735	49.04357787
13.61827517	18.2674474	0	8.079259686	0
0	0	0	0	0
240.3625567	117.2962412	147.4278474	132.7306948	211.0014397
117.1171665	149.9853576	133.7590404	109.6470957	76.41673761
0	0	0	0	0
253.2999181	150.9468022	255.8019605	270.0781095	195.0337632
0	2.8843338	0	17.31269933	0
0.680913758	210.5563674	0	1.154179955	203.0176014
261.4708832	194.2118092	267.5180808	255.0737701	282.855984
104.179805	139.409467	60.53328835	94.64275632	88.96276916
70.13411712	0	56.62791491	39.24211847	117.4764772
87.83787484	85.56856939	59.55694499	66.9424374	77.55728594
58.55858323	47.11078539	31.24298754	71.55915722	52.46522284
12.93736141	0	0	5.770899775	0
116.4362527	53.84089759	129.853667	55.40063784	75.27618929
2.723655034	0	0	0	0
248.5335218	159.5998036	311.453532	183.5146129	165.3795068
5.447310068	0	0.976343361	6.925079731	14.82712819
2.723655034	16.3445582	0	1.154179955	0
43.57848054	20.1903366	76.15478212	40.39629843	38.77864297
32.68386041	34.61200559	80.06015557	27.70031892	28.51370807
18.38467148	12.4987798	16.59783713	12.69597951	9.124386581
0	0	30.26664418	0	163.0984101
36.0884292	94.22157079	52.72254147	38.08793852	50.18412619
66.04863457	41.34211779	36.12470434	49.62973807	57.02741613
134.1400104	104.7974614	132.782697	147.7350343	124.3197672
7.490051343	10.5758906	9.763433606	10.3876196	3.421644968
4.766396309	2.8843338	3.905373442	3.462539865	0
6.128223826	0	3.905373442	2.30835991	3.421644968
0	0	2.929030082	4.61671982	2.281096645
0	0	0.976343361	1.154179955	1.140548323
104.179805	285.5490462	157.191281	11.54179955	13.68657987
0	0	0.976343361	2.30835991	0
0	0	0	6.925079731	0
8.85187886	0	11.71612033	3.462539865	1.140548323
22.47015403	128.8335764	12.69246369	41.55047838	26.23261142
58.55858323	56.72523139	67.36769188	93.48857636	55.88686781
1337.995535	1042.205946	1617.800948	0	0
141.6300618	155.7540252	0.976343361	105.0303759	4.56219329
4.766396309	2.8843338	5.858060163	3.462539865	7.983838258
7.490051343	6.730112199	6.834403524	5.770899775	5.702741613
69.45320336	78.83845719	68.34403524	86.56349663	91.24386581
664.5718282	593.2113181	561.3974323	699.4330528	555.4470331
66.04863457	112.4890182	46.86448131	39.24211847	239.5151477
204.9550413	130.7564656	178.670835	117.7263554	132.3036054
44.2593943	4.807222999	59.55694499	0	0
127.3308728	114.4119074	41.9827645	43.85883829	49.04357787
0	0	0	0	5.702741613

For Review Only

103.4988913	38.45778399	121.0665767	94.64275632	96.94660742
452.1267356	654.7437725	500.864144	481.2930413	367.2565599
60.6013245	81.72279099	68.34403524	102.722016	63.87070607
71.49594464	96.14445999	97.63433606	70.40497726	95.8060591
353.3942406	163.445582	244.0858401	95.79693627	135.7252504
0	0	0	0	0
8.170965101	0	5.858060163	24.23777906	10.2649349
6.809137585	0	4.881716803	4.61671982	2.281096645
2.723655034	0	0	0	0
0	0	1.952686721	3.462539865	0
2.042741275	2.8843338	2.929030082	1.154179955	0
0	0	0	0	0
217.2114889	113.4504628	239.2041233	103.876196	205.2986981
0	0	0	5.770899775	0
0	0	0	2.30835991	7.983838258
0	0	0	0	3.421644968
189.9749386	160.5612482	103.4923962	185.8229728	173.363345
8.170965101	0	2.929030082	0	10.2649349
0	24.9975596	0	0	0
0	10.5758906	0	0	0
0	0	0.976343361	0	0
9.532792618	39.41922859	3.905373442	17.31269933	0
185.2085423	120.180575	162.0729979	189.2855126	142.5685403
380.630791	190.3660308	360.2707	373.9543054	205.2986981
0	9.614445999	4.881716803	0	0
102.1370638	320.1610518	351.4836098	325.4787473	331.8995619
41.53573927	11.5373352	30.26664418	9.233439641	47.90302955
3.404568792	0	0	0	0
57.87766947	74.99267879	33.19567426	0	19.38932148
1105.12303	924.9097051	1100.338967	1071.078998	861.1139836
0	30.7662272	12.69246369	1.154179955	13.68657987
4.766396309	0	0	2.30835991	1.140548323
0	0	0	0	0
92.60427115	59.60956519	73.22575204	76.17587704	87.82222084
253.9808319	279.7803786	268.4944242	212.3691117	236.0935028
32.00294665	137.4865778	94.70530597	16.15851937	76.41673761
3.404568792	0	0	0	0
0.680913758	0.9614446	109.3504564	0	0
0	0	0.976343361	1.154179955	1.140548323
49.02579061	145.1781346	0	163.8935536	0
0	58.64812059	173.7891182	6.925079731	1.140548323
8.85187886	145.1781346	85.91821573	3.462539865	6.843289936
0	0	0	0	1.140548323
4.085482551	0	1.952686721	8.079259686	5.702741613
6.809137585	0	10.73977697	24.23777906	18.24877316
181.8039735	156.7154698	151.3332209	212.3691117	185.9093766
20.42741275	0	7.810746884	3.462539865	6.843289936
316.6248977	207.6720336	369.0577903	305.8576881	280.5748874
174.3139222	29.8047826	153.2859076	165.0477336	0
96.00883994	78.83845719	64.4386618	116.5721755	67.29235103

78.30508222	46.14934079	74.2020954	39.24211847	76.41673761
78.98599598	94.22157079	54.67522819	79.6384169	60.4490611
35.40751544	49.99511919	69.3203786	0	0
273.7273309	380.7320615	36.12470434	248.1486903	150.5523786
100.7752363	254.782819	57.60425827	136.1932347	69.57344768
104.179805	109.6046844	108.374113	91.18021645	94.66551078
0	0	0.976343361	0	5.702741613
305.0493638	509.5656379	432.5201087	458.2094422	344.4455934
0	0	0	3.462539865	0
847.7376293	560.5222017	586.7823597	544.7729388	760.7457312
57.87766947	49.03367459	50.76985475	35.77957861	44.48138458
730.6204628	803.7676855	530.1544448	1111.475297	883.92495
25.87472282	0	0	0	0
70.81503088	23.0746704	35.14836098	58.86317771	50.18412619
77.62416846	110.566129	72.24940868	96.95111623	58.16796445
4.766396309	0	5.858060163	8.079259686	9.124386581
31.32203289	41.34211779	18.55052385	41.55047838	29.65425639
0	0	0	0	0
15.66101644	9.614445999	18.55052385	21.92941915	15.96767652
12.93736141	20.1903366	8.787090245	3.462539865	6.843289936
5.447310068	0	11.71612033	8.079259686	0
81.02873726	38.45778399	60.53328835	68.09661735	52.46522284
213.8069202	176.9058064	171.8364315	185.8229728	233.8124061
104.179805	62.49389899	115.2085165	87.71767659	66.15180271
9.532792618	0	7.810746884	0	2.281096645
7.490051343	3.845778399	1.952686721	0	7.983838258
292.1120024	241.3225946	258.7309905	171.9728133	181.3471833
94.64701242	37.49633939	51.74619811	95.79693627	82.11947923
1620.574745	1896.930196	3903.420755	2606.138339	2347.248448
584.2240048	435.5344037	416.898615	502.0682805	401.4730096
102.1370638	4.807222999	0	36.93375856	45.6219329
40.17391175	49.03367459	10.73977697	8.079259686	25.0920631
23.15106779	41.34211779	24.40858401	45.01301825	22.81096645
52.4303594	0.9614446	16.59783713	26.54613897	37.63809465
19.746499	27.8818934	21.47955393	24.23777906	19.38932148
91.24244363	1117.198625	876.7563378	847.168087	414.0190411
441.9130292	251.8984852	594.5931066	319.7078476	467.6248123
53.11127316	28.843338	48.81716803	55.40063784	55.88686781
0	0	0.976343361	1.154179955	1.140548323
0	0	0	0	7.983838258
202.2313863	198.0575876	178.670835	110.8012757	153.9740236
175.6757497	0	104.4687396	268.9239295	122.0386705
10.21370638	0	16.59783713	2.30835991	6.843289936
3.404568792	0	0	0	0
0	0	4.881716803	1.154179955	1.140548323
0	0	0	0	0
35.40751544	38.45778399	24.40858401	24.23777906	15.96767652
66.72954833	24.036115	46.86448131	60.01735766	52.46522284
183.165801	91.33723699	206.9847924	176.5895331	225.8285679
134.8209242	171.1371388	117.1612033	191.5938725	164.2389585

For Review Only

187.9321973	135.5636886	142.5461306	114.2638156	139.1468954
0.680913758	109.6046844	0	0	0
258.7472282	330.7369424	260.6836773	249.3028703	311.3696921
60.6013245	159.5998036	65.41500516	83.10095677	99.22770407
0.680913758	11.5373352	0	0	104.9304457
51.74944564	34.61200559	41.9827645	62.32571757	44.48138458
101.45615	88.45290319	93.72896261	101.567836	112.9142839
0	0	0.976343361	0	0
390.1635836	366.3103925	295.8320382	418.9673237	407.1757512
101.45615	27.8818934	182.5762084	144.2724944	55.88686781
273.7273309	304.7779382	309.5008453	162.7393737	274.8721457
8.170965101	18.2674474	67.36769188	75.02169708	45.6219329
155.2483369	146.1395792	122.0429201	106.1845559	83.26002755
447.3603393	693.2015565	630.7178109	305.8576881	570.2741613
35.40751544	34.61200559	30.26664418	30.00867883	44.48138458
106.9034601	0	57.60425827	3.462539865	6.843289936
45.62122182	48.07222999	45.88813795	38.08793852	57.02741613
55.83492819	0	60.53328835	20.77523919	43.34083626
1.361827517	0	0	0	0
0	0	0.976343361	0	0
3.404568792	0.9614446	0.976343361	0	0
0	0	1.952686721	0	0
0.680913758	5.768667599	10.73977697	0	0
2.042741275	0	0	3.462539865	1.140548323
83.75239229	0	27.3376141	8.079259686	100.3682524
36.0884292	0	30.26664418	19.62105924	10.2649349
42.21665302	51.91800839	98.61067942	62.32571757	76.41673761
49.70670437	52.87945299	60.53328835	35.77957861	54.74631949
117.7980802	198.0575876	258.7309905	139.6557746	108.3520906
21.10832651	4.807222999	28.31395746	20.77523919	29.65425639
24.5128953	3.845778399	5.858060163	12.69597951	25.0920631
0	0	41.00642114	1.154179955	1.140548323
4.085482551	0	0	0	0
0	0	0	0	0
123.2453903	193.2503646	116.1848599	175.4353532	183.6282799
66.72954833	33.650561	70.29672196	38.08793852	87.82222084
59.23949699	124.0263534	65.41500516	86.56349663	44.48138458
87.15696108	99.02879379	112.2794865	103.876196	96.94660742
157.971992	192.28892	153.2859076	225.0650912	74.13564097
72.1768584	134.602244	103.4923962	27.70031892	61.58960942
63.32497954	0	21.47955393	31.16285879	41.05973961
21.78924027	47.11078539	33.19567426	10.3876196	35.356998
7.490051343	0	4.881716803	6.925079731	6.843289936
0	0	0	0	0
0	0	0	1.154179955	0
0	0	0	0	0
30.64111913	58.64812059	30.26664418	81.94677681	59.30851278
1.361827517	2.8843338	0	0	0
0	0	0	0	0
0	0	0	0	0

For Review Only

0	0	3.905373442	19.62105924	15.96767652
2.723655034	0	1.952686721	5.770899775	5.702741613
2.042741275	0	6.834403524	8.079259686	0
378.5880497	619.1703223	231.3933765	361.2583259	451.6571358
0	0	0	0	0
0.680913758	0	0	0	0
42.89756678	32.6891164	20.50321057	65.78825744	41.05973961
0	14.421669	2.929030082	3.462539865	3.421644968
0	0	0	0	0
95.32792618	18.2674474	99.58702278	114.2638156	42.20028794
1.361827517	14.421669	2.929030082	1.154179955	0
0	0	2.929030082	0	0
6.809137585	0	0	0	1.140548323
6.809137585	0	2.929030082	0	4.56219329
0	0	0	0	1.140548323
0	0	0	0	0
5.447310068	0	0.976343361	1.154179955	4.56219329
55.83492819	11.5373352	60.53328835	40.39629843	30.79480471
73.53868591	61.53245439	78.10746884	58.86317771	59.30851278
71.49594464	40.38067319	62.48597508	61.17153762	51.32467452
178.3994047	86.53001399	131.8063537	135.0390547	179.0660866
158.6529057	74.03123419	20.50321057	23.0835991	153.9740236
0	0	60.53328835	41.55047838	27.37315974
69.45320336	44.22645159	87.87090245	38.08793852	43.34083626
96.00883994	78.83845719	210.8901659	0	69.57344768
0	9.614445999	0	69.25079731	77.55728594
87.15696108	89.41434779	95.68164933	79.6384169	112.9142839
21.78924027	0	4.881716803	0	9.124386581
994.1340873	637.4377697	520.3910112	604.7902965	719.6859916
0	0	0	0	0
433.0611504	440.3416267	426.6620486	307.0118681	374.0998498
181.1230597	114.4119074	255.8019605	313.9369478	238.3745994
157.971992	208.6334782	205.0321057	103.876196	179.0660866
199.5077312	0	0	0	0
162.0574745	75.95412339	299.7374117	144.2724944	249.7800827
46.30213557	130.7564656	67.36769188	106.1845559	52.46522284
18.38467148	0	3.905373442	6.925079731	11.40548323
85.11421981	148.0624684	130.8300103	138.5015946	79.83838258
189.2940248	119.2191304	126.9246369	147.7350343	147.1307336
319.3485527	313.4309396	317.3115922	264.3072097	291.9803706
93.28518491	66.33967739	70.29672196	102.722016	88.96276916
168.1856983	151.9082468	153.2859076	155.8142939	181.3471833
13.61827517	3.845778399	124.9719502	0	0
68.7722896	69.22401119	69.3203786	62.32571757	39.91919129
0	0	0	0	0
165.4620433	216.325035	111.3031431	181.2062529	139.1468954
4.085482551	15.3831136	6.834403524	13.85015946	1.140548323
14.98010269	34.61200559	7.810746884	11.54179955	7.983838258
25.87472282	24.9975596	21.47955393	42.70465834	55.88686781
53.11127316	62.49389899	68.34403524	61.17153762	46.76248123

For Review Only

26.55563658	0	8.787090245	35.77957861	19.38932148
34.04568792	24.9975596	47.84082467	45.01301825	30.79480471
61.96315202	97.10590459	76.15478212	60.01735766	85.5411242
28.59837785	13.4602244	0	32.31703874	41.05973961
13.61827517	0	11.71612033	8.079259686	20.52986981
108.2652876	93.26012619	65.41500516	63.47989753	101.5088007
0	0	0	0	3.421644968
220.6160577	189.4045862	1196.99696	0	0
64.00589329	673.0112199	772.2875982	0	839.4435654
2.042741275	1.9228892	0	86.56349663	0
29.27929161	55.76378679	40.03007778	33.4712187	50.18412619
0	9.614445999	0	0	0
125.2881316	164.4070266	157.191281	199.6731322	142.5685403
410.5909963	473.9921877	632.6704976	424.7382235	391.2080747
3193.485527	1832.513407	2393.99392	2841.591049	1702.838646
68.09137585	21.1517812	55.65157155	28.85449888	44.48138458
22.47015403	24.9975596	2.929030082	36.93375856	18.24877316
110.9889426	91.33723699	81.03649893	84.25513672	98.08715574
31.32203289	40.38067319	24.40858401	36.93375856	60.4490611
57.19675571	67.30112199	47.84082467	34.62539865	47.90302955
49.02579061	0	58.58060163	0	0
24.5128953	23.0746704	20.50321057	17.31269933	15.96767652
25.87472282	0	0.976343361	1.154179955	3.421644968
805.5209762	223.0551472	460.8340662	914.1105244	234.9529545
12.25644765	3.845778399	10.73977697	0	0
207.6786963	433.6115145	144.4988174	285.0824489	102.649349
457.5740457	685.5099997	546.7522819	340.4830868	350.148335
0	0	0	0	1.140548323
0	0	0.976343361	1.154179955	239.5151477
42.21665302	0	0	39.24211847	29.65425639
0	213.4407012	0.976343361	1.154179955	2.281096645
0.680913758	0.9614446	0	0	0
190.6558524	98.06734919	132.782697	136.1932347	134.5847021
0	0	0	0	0
128.6927003	88.45290319	90.79993253	126.9597951	76.41673761
238.3198155	196.1346984	171.8364315	225.0650912	229.2502128
106.9034601	46.14934079	28.31395746	51.93809798	36.49754632
0	0	25.38492737	0	0
200.8695587	217.2864796	204.0557624	162.7393737	169.9417001
14.29918893	7.691556799	16.59783713	21.92941915	18.24877316
65.36772081	44.22645159	52.72254147	48.47555811	68.43289936
0	0	0	0	0
56.51584195	12.4987798	11.71612033	78.48423695	50.18412619
13.61827517	9.614445999	9.763433606	13.85015946	7.983838258
29.27929161	43.26500699	24.40858401	51.93809798	36.49754632
0.680913758	5.768667599	0	0	1.140548323
0	0	5.858060163	3.462539865	2.281096645
0	0	0	0	0
0	0	0	0	0
0	0	17.57418049	16.15851937	1.140548323

For Review Only

0	5.768667599	0	0	0
121.202649	138.4480224	105.4450829	130.4223349	84.40057587
4.766396309	0	2.929030082	5.770899775	5.702741613
10.21370638	35.57345019	3.905373442	24.23777906	14.82712819
42.89756678	36.53489479	90.79993253	32.31703874	55.88686781
39.49299799	40.38067319	37.1010477	28.85449888	29.65425639
0	0	0	1.154179955	1.140548323
68.09137585	176.9058064	97.63433606	148.8892142	125.4603155
291.4310886	162.4841374	353.4362965	219.2941915	372.9593015
8.85187886	145.1781346	230.4170331	0	0
83.07147853	89.41434779	42.95910786	76.17587704	49.04357787
1.361827517	0	0	0	0
121.202649	74.03123419	176.7181483	113.1096356	140.2874437
89.19970236	91.33723699	80.06015557	153.505934	112.9142839
70.81503088	66.33967739	76.15478212	64.63407749	46.76248123
0	3.845778399	0	0	0
72.85777215	42.30356239	62.48597508	55.40063784	87.82222084
61.96315202	17.3060028	4.881716803	4.61671982	55.88686781
99.41340873	34.61200559	0	226.2192712	0
4.085482551	0	9.763433606	0	2.281096645
14.29918893	28.843338	42.95910786	19.62105924	34.21644968
64.68680705	155.7540252	86.89455909	84.25513672	46.76248123
58.55858323	23.0746704	43.93545122	49.62973807	36.49754632
95.32792618	66.33967739	65.41500516	93.48857636	99.22770407
249.8953494	137.4865778	321.2169656	277.0031892	224.6880196
0	6.730112199	0	0	2.281096645
3.404568792	0	4.881716803	3.462539865	0
23.15106779	17.3060028	48.81716803	47.32137816	60.4490611
148.4391993	176.9058064	156.2149377	153.505934	124.3197672
78.30508222	75.95412339	94.70530597	68.09661735	62.73015774
141.6300618	149.023913	123.9956068	139.6557746	130.0225088
162.7383883	7.691556799	267.5180808	220.4483714	0
4.085482551	0	2.929030082	5.770899775	4.56219329
224.7015403	228.8238148	160.1203111	175.4353532	214.4230847
6.128223826	9.614445999	3.905373442	6.925079731	0
414.6764789	224.9780364	224.5589729	370.4917656	342.1644968
42.21665302	39.41922859	26.36127073	32.31703874	36.49754632
100.0943225	0	20.50321057	126.9597951	96.94660742
19.06558524	53.84089759	16.59783713	31.16285879	27.37315974
1017.285155	607.6329871	982.2014207	677.5036336	797.2432775
53.79218692	53.84089759	58.58060163	43.85883829	23.95151477
248.5335218	121.1420196	103.4923962	115.4179955	151.6929269
14.29918893	27.8818934	30.26664418	11.54179955	17.10822484
33.36477416	12.4987798	22.45589729	16.15851937	15.96767652
0	0	0	0	0
0	0	0	0	0
141.6300618	99.99023839	95.68164933	120.0347153	85.5411242
123.926304	240.36115	131.8063537	138.5015946	190.4715699
237.6389017	152.8696914	185.5052385	214.6774716	236.0935028
23.15106779	90.37579239	39.05373442	34.62539865	75.27618929

For Review Only

379.9498772	236.5153716	216.748226	342.7914467	396.9108163
12.93736141	3.845778399	15.62149377	8.079259686	5.702741613
0	6.730112199	0	0	9.124386581
81.02873726	102.8745722	61.50963171	80.79259686	75.27618929
10.89462014	3.845778399	12.69246369	13.85015946	14.82712819
85.11421981	90.37579239	65.41500516	137.3474147	26.23261142
114.3935114	65.37823279	84.94187237	71.55915722	88.96276916
107.5843738	72.10834499	72.24940868	84.25513672	112.9142839
94.64701242	49.99511919	45.88813795	32.31703874	11.40548323
43.57848054	48.07222999	21.47955393	31.16285879	39.91919129
20.42741275	24.9975596	10.73977697	6.925079731	9.124386581
28.59837785	16.3445582	28.31395746	19.62105924	50.18412619
0	0	3.905373442	0	0
14.29918893	0.9614446	0	0	44.48138458
333.6477416	359.5802803	380.7739106	244.6861505	330.7590136
102.1370638	51.91800839	30.26664418	60.01735766	79.83838258
328.2004316	323.0453856	286.0686046	309.320228	343.3050451
38.13117047	0	5.858060163	9.233439641	19.38932148
70.81503088	74.03123419	59.55694499	55.40063784	67.29235103
196.7840762	235.553927	355.3889832	189.2855126	311.3696921
118.478994	110.566129	113.2558298	96.95111623	143.7090886
228.1061091	140.3709116	103.4923962	253.9195901	144.849637
104.8607188	47.11078539	147.4278474	159.2768338	147.1307336
15.66101644	0	15.62149377	8.079259686	33.07590136
15985.13139	14482.24001	32619.63168	16593.64521	22839.48016
0	0	0.976343361	1.154179955	1.140548323
0	0	104.4687396	244.6861505	1.140548323
0	0	64.4386618	156.9684739	84.40057587
0.680913758	0	0	266.6155696	0
0	0	0	0	0
23.83198155	20.1903366	16.59783713	41.55047838	27.37315974
0	0	3.905373442	8.079259686	0
16.3419302	13.4602244	19.52686721	12.69597951	14.82712819
0	0	0	1.154179955	1.140548323
428.2947541	458.6090741	524.2963846	655.5742145	499.5601653
375.1834809	296.1249368	409.0878681	341.6372667	366.1160116
234.9152467	393.2308413	342.6965196	258.5363099	189.3310216
82.39056477	293.240603	1.952686721	186.9771527	60.4490611
128.0117866	48.07222999	94.70530597	245.8403304	182.4877316
83.07147853	0	14.64515041	10.3876196	0
23.15106779	68.26256659	19.52686721	32.31703874	53.60577116
157.2910782	115.373352	153.2859076	156.9684739	122.0386705
19.06558524	3.845778399	27.3376141	19.62105924	18.24877316
153.2055957	103.8360168	123.9956068	68.09661735	141.427992
0.680913758	0	0	0	0
49.70670437	21.1517812	34.17201762	57.70899775	35.356998
27.9174641	32.6891164	10.73977697	24.23777906	17.10822484
21.78924027	0	22.45589729	25.39195901	3.421644968
0	0	0	6.925079731	4.56219329
0	0	0	0	0

For Review Only

0	0	0	0	0
42.21665302	22.1132258	32.2193309	46.1671982	58.16796445
83983.22205	54528.33048	72553.05147	82561.95473	64957.64862
0	0	0	1.154179955	0
29.96020537	19.228892	20.50321057	23.0835991	26.23261142
113.7125977	6.730112199	0	70.40497726	0
70.13411712	121.1420196	179.6471783	114.2638156	197.3148598
52.4303594	81.72279099	102.5160529	83.10095677	18.24877316
98.05158122	198.0575876	105.4450829	156.9684739	139.1468954
31.32203289	36.53489479	88.84724581	43.85883829	31.93535303
59.92041074	110.566129	61.50963171	102.722016	57.02741613
0	27.8818934	2.929030082	20.77523919	10.2649349
47.66396309	88.45290319	60.53328835	107.3387358	65.01125439
118.478994	113.4504628	126.9246369	129.268155	115.1953806
64.68680705	15.3831136	0	0	0
59.23949699	38.45778399	59.55694499	84.25513672	72.99509265
0	46.14934079	0.976343361	16.15851937	1.140548323
38.81208423	74.03123419	34.17201762	64.63407749	70.713996
3.404568792	8.653001399	0	0	0
0.680913758	266.3201542	0	0	0
4.085482551	0	0.976343361	5.770899775	5.702741613
96.00883994	148.0624684	113.2558298	96.95111623	124.3197672
23.15106779	25.9590042	4.881716803	13.85015946	10.2649349
0.680913758	0	0	0	0
91.24244363	221.132258	103.4923962	100.4136561	114.0548323
42.21665302	82.68423559	35.14836098	70.40497726	47.90302955
261.4708832	230.746704	14.64515041	78.48423695	62.73015774
21.78924027	37.49633939	23.43224065	31.16285879	33.07590136
82.39056477	130.7564656	148.4041908	78.48423695	69.57344768
198.1459037	239.3997054	250.9202437	313.9369478	204.1581497
30.64111913	73.06978959	35.14836098	39.24211847	35.356998
181.1230597	120.180575	215.7718827	125.8056151	136.8657987
27.23655034	27.8818934	23.43224065	42.70465834	28.51370807
159.3338195	139.409467	148.4041908	124.6514351	142.5685403
56.51584195	0	33.19567426	5.770899775	205.2986981
0	19.228892	0	0	0
892.6779373	276.8960448	0	2734.252314	0
7072.651209	10341.29812	8974.54817	6192.175459	7778.53956
1519.799509	1059.511949	1296.583983	1157.642495	1407.43663
30583.24146	47061.75172	41526.81215	26848.53412	27313.85123
0	0	0	0	0
0	0	0	0	0
432.3802366	282.6647124	848.4423803	145.4266743	680.9073486
0	0	0	0	0
2162.582097	1514.275245	1735.938495	1892.855126	1715.384677
353.3942406	275.9346002	431.5437654	271.2322894	276.0126941
15348.47703	11868.07214	13120.10208	14539.20489	12984.0021
9.532792618	0	5.858060163	0	0
3873.718372	3292.947755	2900.716124	2909.687667	2853.651903
0	0	0	0	0

For Review Only

2.042741275	29.8047826	75.17843876	80.79259686	0
53.79218692	34.61200559	37.1010477	19.62105924	36.49754632
0	0	0	0	0
4.085482551	9.614445999	1.952686721	2.30835991	1.140548323
0	0	16.59783713	21.92941915	0
204.2741275	192.28892	167.931058	245.8403304	177.9255383
79.66690974	46.14934079	76.15478212	98.10529618	80.97893091
58.55858323	0	76.15478212	93.48857636	21.67041813
80.3478235	71.14690039	115.2085165	103.876196	112.9142839
17.02284396	32.6891164	12.69246369	40.39629843	6.843289936
73.53868591	24.036115	53.69888483	43.85883829	39.91919129
17.02284396	24.036115	33.19567426	21.92941915	27.37315974
147.7582856	99.99023839	147.4278474	184.6687928	214.4230847
13.61827517	0	0	0	0
23.15106779	0	12.69246369	21.92941915	0
301.644795	328.8140532	363.1997301	283.928269	311.3696921
12.93736141	26.9204488	17.57418049	32.31703874	15.96767652
129.3736141	146.1395792	124.9719502	94.64275632	66.15180271
21.78924027	21.1517812	40.03007778	24.23777906	44.48138458
50.38761813	38.45778399	48.81716803	30.00867883	44.48138458
57.19675571	49.03367459	40.03007778	57.70899775	37.63809465
44.94030806	84.60712479	52.72254147	65.78825744	29.65425639
51.74944564	85.56856939	41.00642114	47.32137816	29.65425639
6.128223826	0	0	0	2.281096645
16566.63174	19340.41957	21538.13453	16388.20118	19342.559
0	0	0	0	3.421644968
0	0	2.929030082	0	1.140548323
0	0	0	0	1.140548323
4.766396309	0	0	0	4.56219329
1.361827517	0.9614446	0	0	59.30851278
0	0	0	0	3.421644968
7.490051343	0	0	0	1.140548323
6.809137585	0	14.64515041	1.154179955	1.140548323
5.447310068	0	0	1.154179955	5.702741613
8.170965101	0	1.952686721	18.46687928	6.843289936
7.490051343	12.4987798	7.810746884	13.85015946	5.702741613
7.490051343	8.653001399	6.834403524	5.770899775	9.124386581
29.96020537	19.228892	37.1010477	40.39629843	25.0920631
0	0	0.976343361	0	0
3.404568792	0	3.905373442	1.154179955	0
303.0066225	242.2840392	251.896587	295.4700685	285.1370807
0	8.653001399	0.976343361	4.61671982	0
523.6226802	18.2674474	129.853667	20.77523919	252.0611793
73.53868591	123.0649088	107.3977697	55.40063784	96.94660742
66.72954833	79.79990179	71.27306532	55.40063784	96.94660742
251.9380906	220.1708134	197.2213588	303.5493282	239.5151477
81.02873726	85.56856939	156.2149377	72.71333717	51.32467452
32.00294665	49.03367459	36.12470434	35.77957861	46.76248123
89.88061612	38.45778399	124.9719502	68.09661735	85.5411242
7.490051343	0	0	204.2898521	204.1581497

For Review Only

0	0	0	11.54179955	0
106.9034601	110.566129	130.8300103	98.10529618	74.13564097
33.36477416	39.41922859	25.38492737	36.93375856	35.356998
23.15106779	60.57100979	15.62149377	19.62105924	33.07590136
957.3647444	699.9316687	1021.255155	506.6850003	816.632599
40.85482551	0	5.858060163	19.62105924	51.32467452
3.404568792	0.9614446	0	0	0
24.5128953	11.5373352	38.07739106	0	39.91919129
1077.88648	1438.321121	1888.248059	1981.726983	1548.864622
0	0	0	0	0
145.0346305	156.7154698	214.7955393	156.9684739	195.0337632
72.85777215	87.49145859	81.03649893	101.567836	84.40057587
0	0	0	0	0
42.89756678	37.49633939	54.67522819	36.93375856	55.88686781
12.25644765	4.807222999	0	0	0
0	0	0	0	0
0	0	0	0	0
0	0	0	0	0
16.3419302	32.6891164	22.45589729	10.3876196	22.81096645
0	0	0	0	0
0.680913758	9.614445999	4.881716803	12.69597951	4.56219329
10.89462014	12.4987798	5.858060163	0	6.843289936
36.0884292	12.4987798	59.55694499	49.62973807	47.90302955
15.66101644	14.421669	21.47955393	31.16285879	21.67041813
0	0	0	0	0
0	0	0	0	0
1.361827517	0	0	0	0
185.2085423	134.602244	281.1868878	296.6242485	234.9529545
18.38467148	53.84089759	29.29030082	12.69597951	18.24877316
0	0	0	0	0
151.1628544	67.30112199	188.4342686	177.7437131	217.8447296
1820.082476	2307.46704	1815.998651	1978.264443	1021.931297
60.6013245	147.1010238	59.55694499	83.10095677	112.9142839
31.32203289	23.0746704	24.40858401	39.24211847	5.702741613
0	114.4119074	179.6471783	0	181.3471833
278.4937272	2482.449957	738.1155806	849.4764469	2197.836618
5.447310068	0	2.929030082	17.31269933	1.140548323
110.3080289	96.14445999	111.3031431	83.10095677	142.5685403
0	0	1.952686721	0	0
4.085482551	0	3.905373442	2.30835991	1.140548323
8.85187886	7.691556799	0	0	10.2649349
53.11127316	20.1903366	34.17201762	51.93809798	21.67041813
42.21665302	39.41922859	49.79351139	39.24211847	42.20028794
75.58142719	60.57100979	88.84724581	79.6384169	72.99509265
8.170965101	13.4602244	15.62149377	13.85015946	4.56219329
0	0	12.69246369	23.0835991	11.40548323
8.85187886	0	2.929030082	3.462539865	3.421644968
0	0	0.976343361	0	0
15.66101644	0.9614446	9.763433606	10.3876196	19.38932148
14.98010269	18.2674474	14.64515041	13.85015946	13.68657987

For Review Only

0	150.9468022	0.976343361	1.154179955	1.140548323
56.51584195	90.37579239	0	0	0
70.13411712	35.57345019	53.69888483	73.86751713	116.3359289
118.478994	81.72279099	108.374113	126.9597951	69.57344768
34.04568792	15.3831136	33.19567426	36.93375856	35.356998
108.2652876	49.03367459	134.7353838	92.33439641	75.27618929
55.83492819	77.87701259	99.58702278	61.17153762	86.68167252
66.72954833	71.14690039	93.72896261	33.4712187	34.21644968
0	11.5373352	14.64515041	0	9.124386581
0	0	0	0	0
6.809137585	39.41922859	4.881716803	9.233439641	9.124386581
0.680913758	0	0	1.154179955	4.56219329
55.15401443	119.2191304	32.2193309	33.4712187	30.79480471
15.66101644	23.0746704	25.38492737	28.85449888	39.91919129
11.57553389	0	8.787090245	0	1.140548323
27.9174641	24.9975596	45.88813795	23.0835991	43.34083626
66.04863457	33.650561	48.81716803	95.79693627	66.15180271
21.10832651	0	4.881716803	8.079259686	5.702741613
2.723655034	0	0	0	0
4.085482551	0	2.929030082	2.30835991	0
17.70375772	9.614445999	25.38492737	17.31269933	23.95151477
135.5018379	150.9468022	100.5633661	144.2724944	144.849637
41.53573927	48.07222999	38.07739106	53.09227793	53.60577116
5.447310068	4.807222999	10.73977697	5.770899775	6.843289936
23.15106779	0.9614446	34.17201762	11.54179955	26.23261142
0	6.730112199	3.905373442	2.30835991	0
3.404568792	3.845778399	8.787090245	3.462539865	5.702741613
49.70670437	48.07222999	53.69888483	81.94677681	46.76248123
2.723655034	13.4602244	6.834403524	0	9.124386581
6.128223826	0	0.976343361	4.61671982	0
6.128223826	16.3445582	0	10.3876196	3.421644968
0	0	0.976343361	1.154179955	0
238.3198155	177.867251	255.8019605	207.7523919	239.5151477
148.4391993	151.9082468	194.2923288	118.8805354	193.8932148
19.746499	4.807222999	13.66880705	36.93375856	20.52986981
0	0	8.787090245	6.925079731	0
2.042741275	0	0	0	0
[truncated: 2,577,961 more chars]
