# Supplementary material for: High-salt diet induces microbiome dysregulation, neuroinflammation and anxiety in the chronic period after mild repetitive closed head injury in adolescent mice
Source: Brain Commun. 2024 May 3;6(4):fcae147. doi: 10.1093/braincomms/fcae147 (PMC11264151; doi:10.1093/braincomms/fcae147)
Supplement: fcae147_Supplementary_Data [file fcae147_supplementary_data.zip › Supplementary_figures.docx]

**
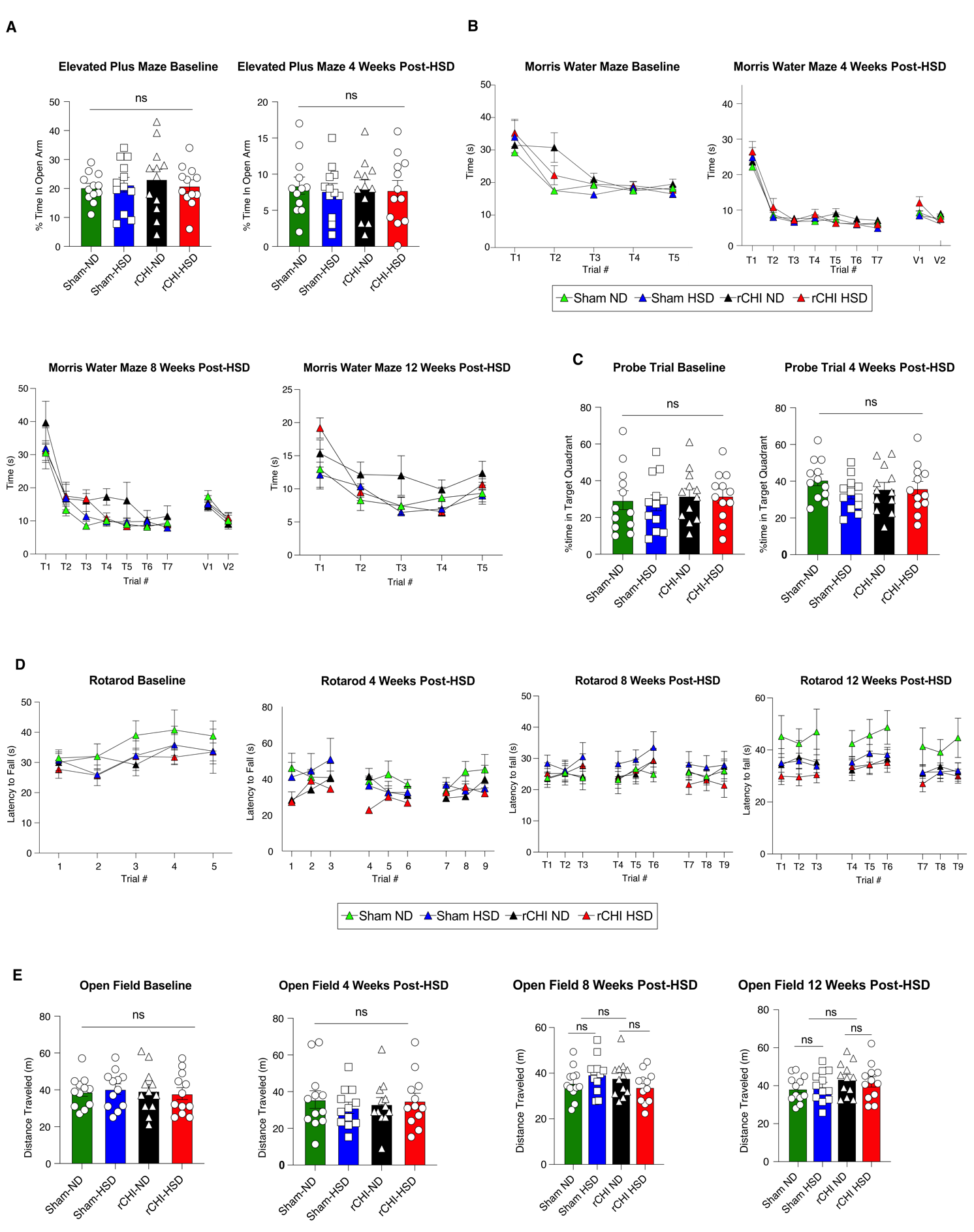
**

**Supplementary Figure 1.** Behavioral testing of anxiety like phenotype using an **(A)** elevated plus maze at baseline and 4 weeks post diet administration, **(B)** Morris water maze (MWM) at baseline, 4 weeks, 8 weeks, and 12 weeks post diet administration, **(C)**probe trial at baseline and 4 weeks post diet administration, and **(D)** rotarod at baseline, 4 weeks, 8 weeks, and 12 weeks post diet administration. Behavioral tests of **(E)** open field at baseline, 4 weeks, 8 weeks, and 12 weeks post diet administration. Morris water maze, and the rotarod were analyzed by a two-factor repeated measures two-way ANOVA (group x time) and the other behavioral tests were analyzed by one-way ANOVA, followed by Tukey’s multiple comparison analysis. n = 12 mice/group was used for all experiments and data is presented as (mean and SEM). ns = not significant**.** HSD = High Salt Diet, ND = Normal Diet, and rCHI = repetitive Closed Head Injury.


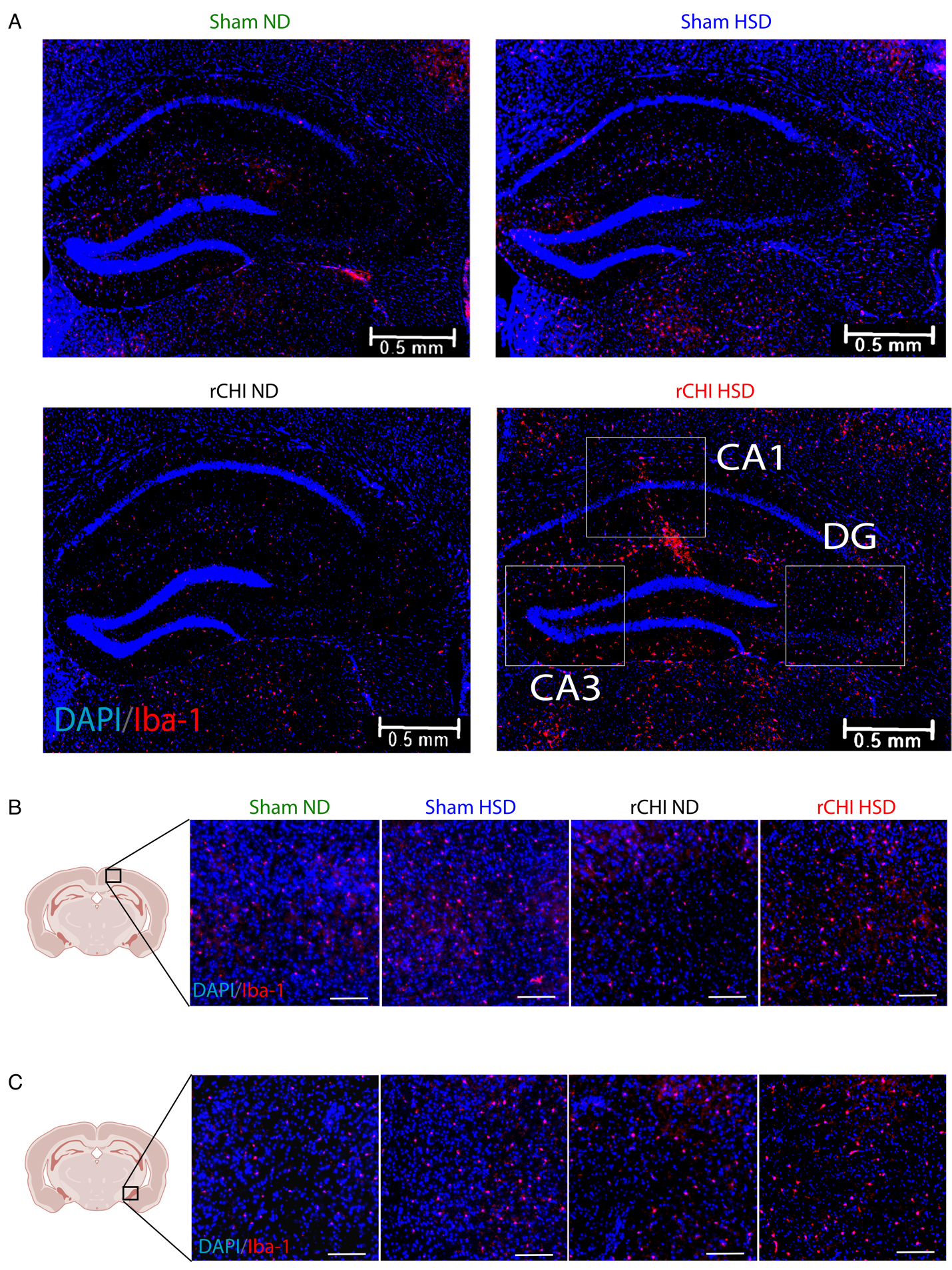


**Supplementary Figure 2. Widefield images of different brain regions for microglial cell analysis. (A)** Representative images of CA1, CA3, and dentate gyrus (DG) and how they were chosen from the widefield microscope. **(B)** Samples of cortex and **(C)** amygdala and the region that was chosen for analysis from the widefield microscope. Sample Images scale bar is 500 um for hippocampal images and 100 um for cortex and the amygdala. HSD = High Salt Diet, ND = Normal Diet, and rCHI = repetitive Closed Head Injury


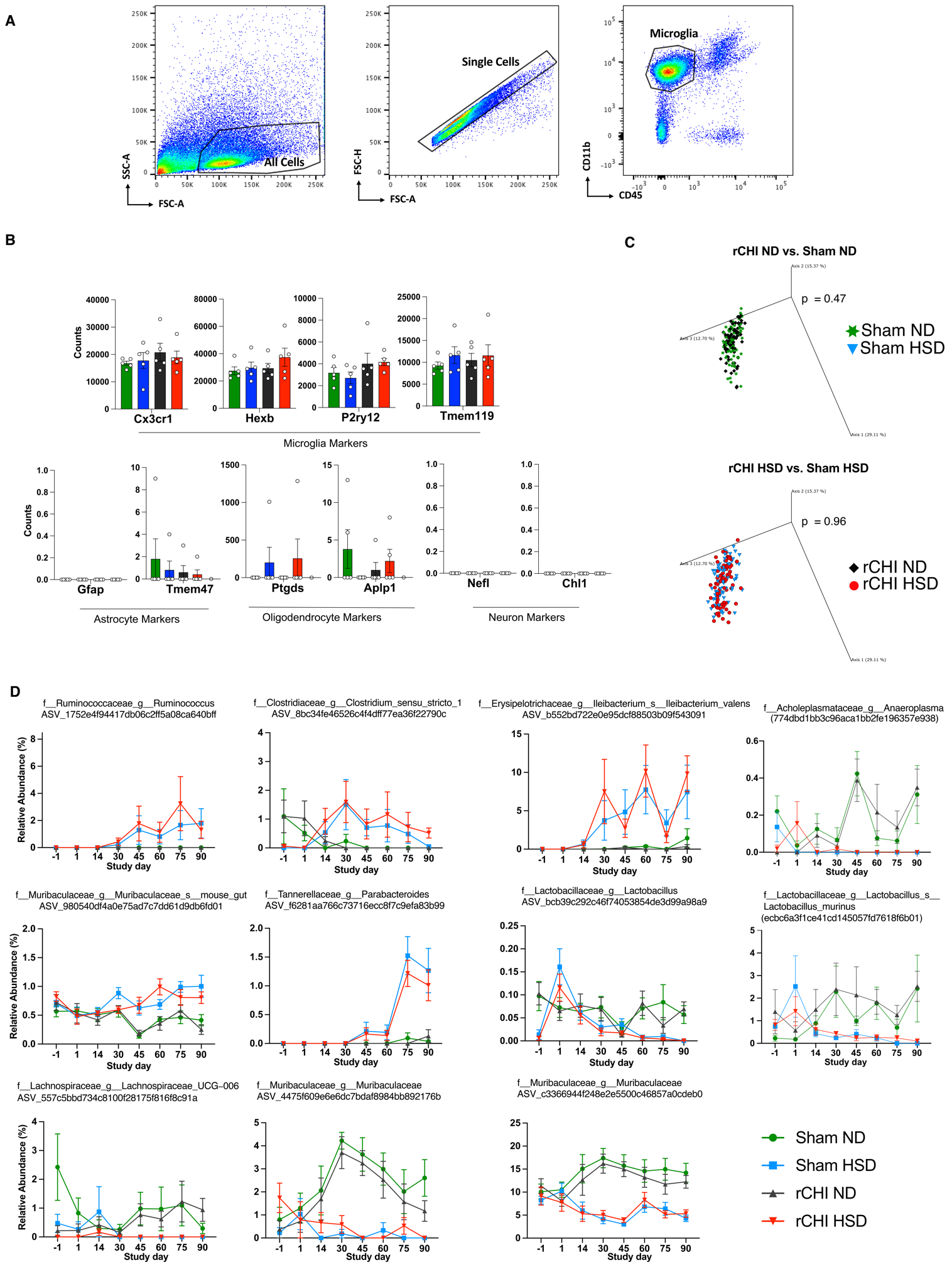


**Supplementary Figure 3. (A)** Gating strategy of microglia that was sorted for RNA-sequencing. **(B)** Validation of microglia RNA-sequencing data by looking into the expression of select microglia markers compared to other non-microglia markers. **C)** Principal coordinate analysis (PCoA) of weighted UniFrac distances stratified by the effect of rCHI in ND and HSD groups respectively: rCHI ND vs. Sham ND (left panel, PERMANOVA: pseudo-F = 0.894, p = 0.47, n = 156), and rCHI HSD vs. Sham HSD (right panel, PERMANOVA: pseudo-F = 0.335, p = 0.96, n = 157). p-values and test statistics are obtained from PERMANOVA tests on beta-diversity using weighted UniFrac distances. Each point represents the microbiota from one mouse. HSD = High Salt Diet, ND = Normal Diet, and rCHI = repetitive Closed Head Injury**. (D)** Relative abundance of taxa from Figure 5D over time that were significantly correlated (Spearman correlation, P <0.05) in at least two time points collected from fecal microbiota samples. Data is presented as (mean and SEM).


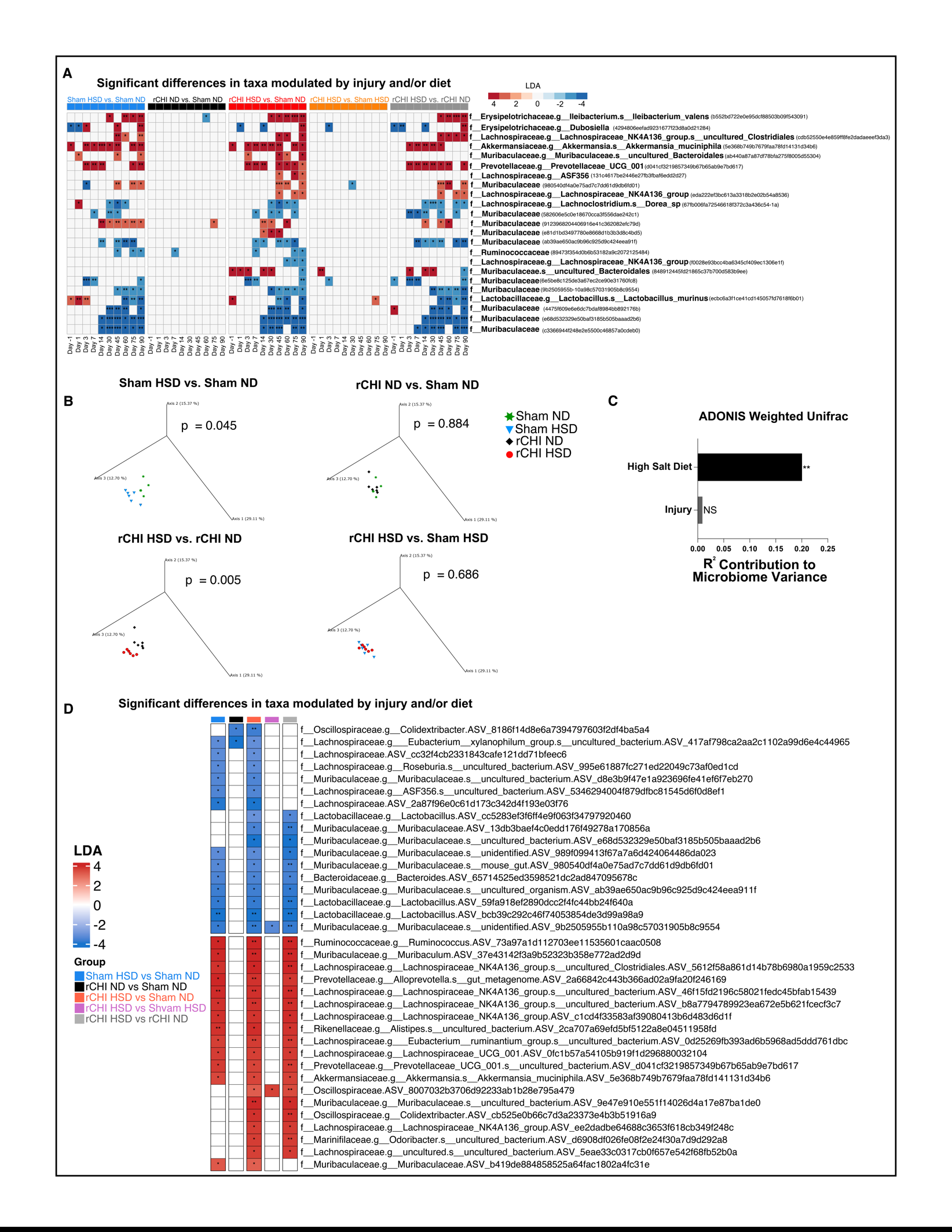


**Supplementary Figure 4. (A)** Significant differences in taxa modulated by salt diet and/or injury phenotype over time collected from fecal microbiota samples was determined by linear discriminant analysis effect size (LEfSe). Legend represents linear discriminant analysis (LDA) effect size score. (***= P<0.001, **= P>0.001 & P<0.01, *= P>0.01 & P<0.05). HSD = High Salt Diet, ND = Normal Diet, and rCHI = repetitive Closed Head Injury. **(B)** Principal coordinate analysis (PCoA) of weighted UniFrac distances (based on fecal microbiota samples) stratified by the following pairwise comparisons: Sham HSD vs. Sham ND (top left panel, PERMANOVA: pseudo-F = 2.41, p = 0.045, n = 11), rCHI HSD vs. rCHI ND (top right panel, PERMANOVA: pseudo-F = 3.49, p = 0.005, n = 12), rCHI ND vs. Sham ND (bottom left panel, PERMANOVA: pseudo-F = 0.470, p = 0.884, n = 11), and rCHI HSD vs. Sham HSD (bottom right panel, PERMANOVA: pseudo-F = 0.471, p = 0.686, n = 12). p-values and test statistics are obtained from PERMANOVA tests on beta-diversity using weighted UniFrac distances. Each point represents the microbiota from one mouse. **(C)** ADONIS test using weighted UniFrac distances investigating the contribution of injury (rCHI) and salt diet to overall microbiome variation across cecum microbiota samples (n=23) (***= P<0.001, **= P>0.001 & P<0.01, *= P>0.01 & P<0.05). **(D)** Significant differences in taxa modulated by salt diet and/or injury phenotype collected from cecum microbiota samples determined by linear discriminant analysis effect size (LEfSe). Legend represents linear discriminant analysis (LDA) effect size score. n = 5-6 mice/group for the microbiome experiments (***= P<0.001, **= P>0.001 & P<0.01, *= P>0.01 & P<0.05**).**
