# Supplementary material for: High-salt diet induces microbiome dysregulation, neuroinflammation and anxiety in the chronic period after mild repetitive closed head injury in adolescent mice
Source: Brain Commun. 2024 May 3;6(4):fcae147. doi: 10.1093/braincomms/fcae147 (PMC11264151; doi:10.1093/braincomms/fcae147)
Supplement: fcae147_Supplementary_Data [file fcae147_supplementary_data.zip › Supplementary_table_legends.docx]

**Description of Additional Supplementary Files**

**Supplementary Table 1. DESeq2 normalized expression counts for all genes**. List of normalized expression counts data (DESeq2) for all genes passing quality metrics.

**Supplementary Table 2. List of microglia differentially expressed genes in all groups compared to Sham ND baseline.** Differential gene expression was performed using DESeq2. Pairwise differential gene expression comparisons (Sham HSD vs. Sham ND, rCHI ND vs. Sham ND, and rCHI HSD vs Sham ND) were done using the Wald Test with standard parameters and log2 fold-changes were subsequently shrunken using DESeq2 built-in lfcshrink function.

**Supplementary Table 3. Expression data of the unique DEGs in all pairwise comparisons compared to Sham ND baseline.** Expression data (DESeq2) for the unique differentially expressed genes in the following pairwise comparisons: rCHI HSD vs. Sham ND, Sham HSD vs. Sham ND, and rCHI ND vs. Sham ND. The expression data used for heatmap visualization was produced using the variance stabilizing transformation (VST) method from the DESeq2 built-in VST function.

**Supplementary Table 4. List of microglia differentially expressed genes in rCHI HSD vs. rCHI ND.** Differential gene expression was performed using DESeq2. Pairwise differential gene expression comparison (rCHI HSD vs. rCHI ND) was done using the Wald Test with standard parameters and log2 fold-changes were subsequently shrunken using DESeq2 built-in lfcshrink function.

**Supplementary Table 5. List of significant Ingenuity Pathway Analysis (IPA) upstream regulators in various pairwise differential gene expression comparisons.** Ingenuity Pathway Analysis (IPA) was used to identify upstream regulators **(P <0.05 and |Z-score|** ≥ **2)** based on the DEGs in the following pairwise comparisons: rCHI HSD vs. rCHI ND, Sham HSD vs. Sham ND, rCHI ND vs. Sham ND, rCHI HSD vs. Sham ND, and rCHI HSD vs. Sham HSD. The input data for IPA comprised of p-values and log_2_fold changes of DEGs.
