# Supplementary material for: High-salt diet induces microbiome dysregulation, neuroinflammation and anxiety in the chronic period after mild repetitive closed head injury in adolescent mice
Source: Brain Commun. 2024 May 3;6(4):fcae147. doi: 10.1093/braincomms/fcae147 (PMC11264151; doi:10.1093/braincomms/fcae147)
Supplement: fcae147_Supplementary_Data [file Original_submission_decision_letter.pdf › Original_submission_decision_letter.pdf]

From: brcoms.editorialoffice@oup.com
To: MWHALEN@mgh.harvard.edu
CC:
BCC:
Subject: Brain Communications - Decision on Manuscript ID BRAINCOM-2023-443
Body: 14-Nov-2023

Dear Dr. Whalen,

Thank you for your recent submission BRAINCOM-2023-443 entitled "High salt diet induces microbiome dysregulation, neuroinflammation, and anxiety in the chronic period after mild repetitive closed head injury in adolescent mice." to Brain Communications.

Your manuscript has been reviewed, and while the reviewers and editorial team appreciate many aspects of the paper, they raise some concerns that you will need to address in order for the paper to be considered for publication.

If you can address these concerns, I invite you to submit a revised manuscript and a response to each of the reviewers' comments. The reviewer(s)' comments are included at the bottom of this message.

In addition to the reviewers' comments, please also address our Scientific Editor's comments added below to comply with our requirements for presentation of scientific data at Brain Communications.

Please format your revised manuscript according to our Instructions to Authors
https://academic.oup.com/braincomms/pages/General_Instructions
A summary of key points is attached to help with this. Please note that there can be substantial delays to publication if you don't resubmit in line with our requirements.

To revise your manuscript, click the link below or log into https://mc.manuscriptcentral.com/braincom and enter your Author Centre, where you will find your manuscript title listed under "Manuscripts with Decisions." Under "Actions," click on "Create a Revision." Your manuscript number will be appended to denote a revision.

*** PLEASE NOTE: This is a two-step process. After clicking on the link, you will be directed to a webpage to confirm. ***

https://mc.manuscriptcentral.com/braincom?URL_MASK=6786dc41c0144bc6954b4ae98e457588

Please upload a revised manuscript with changes tracked with track changes or in coloured text.

When submitting your revised manuscript, you will be asked to respond to the reviewer(s) comments. Please be as specific as possible in your response to all of the comments.

The editorial team at Brain Communications aims to increase transparency in scientific publishing and to demonstrate the value that peer review brings to the scientific process. Please therefore consider whether you are willing to have reviewer(s)' comments on your paper and your response published as supplementary data with the paper if it is accepted. This is entirely optional and will only be done if both the authors and reviewers agree. While submitting your revision there will be an opportunity to review or update the answer you gave previously, if necessary.

IMPORTANT: Your original files are available to you when you upload your revised manuscript. Please delete any redundant files before completing the submission.

Because we are trying to facilitate timely publication of manuscripts submitted to Brain Communications, we would appreciate your revised manuscript as soon as possible. If it is not possible for you to resubmit within 3 months, the option to submit a revision will expire, and we would then consider your revised paper as a new submission. However, if you need a little more time, please get in touch and we can extend this deadline for you.

Once again, thank you for submitting your manuscript to Brain Communications and I look forward to receiving your revision.

Best wishes

Dr. Rakez Kayed
Associate Editor, Brain Communications

Professor Tara Spires-Jones
Founding Editor, Brain Communications

Reviewer(s)' Comments to Author:

Reviewer: 1 **Hubbard, Brad**

Comments to the Author
The study investigated the impact of a high salt diet (HSD) on adolescent mice with repetitive closed head injuries (rCHI). The results showed that HSD worsened neuroinflammation, increased anxiety-like behavior, and disrupted the gut-brain axis, suggesting that dietary changes may play a role in chronic neurological and psychiatric sequelae following head injuries. This study is novel and imparts new findings in diet-induced detrimental outcomes after mild TBI. Intriguing evidence is presented to suggest that even if rCHI does not result in

chronic abnormalities, rCHI may predispose the brain for worsened outcomes when presented with a diet-induced insult, such as high salt diet. Further, the HSD was initiated 12 weeks after rCHI, which highlights that HSD does not have to be present during the immediate post-injury process. Further strengths include longitudinal microbiome and behavioral profiling as well as investigation of apthology in multiple brain regions.

There are just a few minor concerns:
Please expand on some areas of Methodology, especially behavioral assays.
n=4/group for several of the outcomes measures appears underpowered. Please comment on any power analysis or add as a limitation.
Was there any a priori decision to conduct behavioral or fecal microbiome analysis as a repeated one-way or two-way ANOVA? This would strengthen the analysis of these measures.

Reviewer: 2 **Bachstetter, Adam**

Comments to the Author
The introduction notes that prior TBI was associated with hypertension; however, it was unclear as written the direction of this association (that is, does TBI cause hypertension or is hypertension exacerbating TBI outcomes). Using a repetitive weight drop model of mTBI in male mice on a high salt diet, the research finds interactive effects of TBI and a high salt diet on anxiety-like behaviors and on microglial changes. The paper's findings might be impactful; however, as presented, the paper is difficult to evaluate. There are severe areas of the paper that need to be clarified.

Major concerns.

1) The association between TBI and hypertension is not clearly articulated in the current form, nor is the purpose of the study. Furthermore, there needs to be better alignment between the abstract and the introduction regarding the study's objectives. Both sections require revision. It appears that the study's objective is to evaluate if TBI is increasing the risk for hypertension, so they are stressing mice on a halt salt diet to see if there is a greater risk for hypertension, which they don't find. However, the paper needs to be clear about the goal so the readers don't need to make assumptions about the purpose of the study.

2) The rationale for studying the gut microbiome must be better articulated in the introduction.

3) Generally, all methods are too short and do not provide sufficient detail for evaluating the results.

4) No reference is provided for the model of a high salt diet used and the physiological relevance to people.

5) The timeline in Figure 1 is confusing. When was the TBI introduced compared to the diet? Was it 2 months before the diet changes? If it was, that might get to the question of whether TBI leads to hypertension or whether hypertension at the time of TBI leads to worse outcomes. What the study is trying to model needs to be clarified. It would be helpful to include time post-injury instead of or in addition to the time when the diet was introduced.

6) Were the same cohort tested repeatedly in the different behavioral assays? This will affect the outcomes of the behavioral assays, and methods need to be better described.

7) statistical methods are unclear and possibly incorrect. For instance, a one-way and two-way ANOVA are mentioned, but it doesn't state if the data were evaluated for repeated measures.

8) sampling methods for IHC are unclear. How many regions of interest were selected for each animal? How were those selected? Was the experimenter blind to treatment conditions? The figure in the paper shows confocal images, but the results were generated with widefield images. Examples of the widefield images should be included as supplemental data.

9) it is interesting that microglia numbers increased. How was this quantified? Microglia often hypertrophy after injury, but with a mild injury, it is less likely to have recruitment of cells or proliferation of microglia. Is the greater number of microglia associated with proliferation, or is it an artifact of the quantification methods? From the FACS sorting experiments, can you look at microglia numbers in those samples?

10) what is the sample size of the microbiome experiments?

11) How were animals housed for the microbiome experiments?

Comments from the Scientific Editor:

-Please provide details of the statistical analysis performed, the experimental unit (n= mice, cells, wells, plate downs, etc)) and the number of data-points for each group analysed in these plots, and if the number is <10 show the single data-points. Please note our policy on pseudoreplication, we do not accept analyses where individual cells are the experimental unit when there is likely biological variability between animals, culture replicates, etc. Please see instructions for authors for more details. For this type of data, we require either that a mean/median be taken per animal/culture replicate followed by statistical analyses or that more sophisticated analyses are performed such as mixed effects modelling which can account for random variables.

-Please supply a graphical abstract. A graphical abstract is a visual representation of the central finding or methodology of the paper. It will be displayed in the table of contents. Authors should strive to make the graphical abstract informative, interesting, visually appealing, and straightforward (no caption needed, no abbreviations except gene names). The technical requirements for the graphical abstracts are:
- Size: 120 x 120 mm (300-600dpi) or 3000 x 3000 pixels
- Font: Arial 12-16 pt
- File type: tiff or PDF
- Aspect ratio: 1:1 preferred

-Please add an Abbreviated Summary (up to 50 words) of your paper that captures the main purpose and conclusions of the work. The summary should state the main results written in the third person ('[1st author surname] et al. report that ...') and conclusion. This summary will be used in the contents list online and should not contain any abbreviations other than those on

the accepted abbreviations list and accepted gene/protein names, making it accessible to the non-specialist. Please upload it as a Word document. Allowed abbreviations: AIDS; ANOVA; AMPA; ATP; A,T,C,G; CNS; CSF; CT; DNA; ECG; EEG; EMG; GABA; HIV; MRI; NMDA; PET; PCR; RNA.

-Please include the page numbers corresponding to the items listed in the reporting guideline checklist.

-Please include a paragraph about data availability. We strongly encourage data sharing either as supplementary information or uploads to repositories which you can link in the paper.
