# Supplementary material for: High-salt diet induces microbiome dysregulation, neuroinflammation and anxiety in the chronic period after mild repetitive closed head injury in adolescent mice
Source: Brain Commun. 2024 May 3;6(4):fcae147. doi: 10.1093/braincomms/fcae147 (PMC11264151; doi:10.1093/braincomms/fcae147)
Supplement: fcae147_Supplementary_Data [file Original_submission_Response_to_reviewers_comments.pdf › Original_submission_Response_to_reviewers_comments.pdf]

Point by point response to the reviewers:
We thank the reviewers for taking the time to read and thoroughly review our manuscript. We have incorporated their feedback into our revised manuscript, which we believe has strengthened the manuscript considerably. We hope our study is now suitable for publication in Brain Communications.

Reviewer #1

The study investigated the impact of a high salt diet (HSD) on adolescent mice with repetitive closed head injuries (rCHI). The results showed that HSD worsened neuroinflammation, increased anxiety-like behavior, and disrupted the gut-brain axis, suggesting that dietary changes may play a role in chronic neurological and psychiatric sequelae following head injuries.

1. This study is novel and imparts new findings in diet-induced detrimental outcomes after mild TBI. Intriguing evidence is presented to suggest that even if rCHI does not result in chronic abnormalities, rCHI may predispose the brain for worsened outcomes when presented with a diet-induced insult, such as high salt diet. Further, the HSD was initiated 12 weeks after rCHI, which highlights that HSD does not have to be present during the immediate post-injury process. Further strengths include longitudinal microbiome and behavioral profiling as well as investigation of pathology in multiple brain regions.

Response: We thank the reviewer for these positive comments and interest in our work.

There are just a few minor concerns:

2. Please expand on some areas of Methodology, especially behavioral assays.
n=4/group for several of the outcome's measures appears underpowered. Please comment on any power analysis or add as a limitation.

Response: We thank the reviewer for these important comments. As requested, we have expanded on the methodology sections of the manuscript including the time of the day when the animals were tested and have added more experimental detail to the description of the behavioral assays. We referenced the behavior tests used in the manuscript and we added more references to support the high salt diet model used in our study. We also acknowledged the limitation of not performing a power analysis and supported our approach by citing some of our and others previous work (PMID 37463881, 31645758, and 20940727) to justify the lower number of mice used in immunohistochemistry (IBA-1, others) and microglial RNA sequencing experiments.

Please refer to Page 17 line (430-467) for expanded methods on the behavioral assays

Please refer to Page 24 line (588-602) for our statement regarding the statistical power of experiments.

3. Was there any a priori decision to conduct behavioral or fecal microbiome analysis as a repeated one-way or two-way ANOVA? This would strengthen the analysis of these measures.

Response: We thank the reviewer for this important comment. Regarding our behavioral experiments, in line with other published studies (PMID 30001646), we performed a two-factor repeated measures two-way ANOVA (group x time) to assess two independent variables including time and the study dependent outcome such as latency to fall (rotarod), latency to platform (MWM) and blood pressure/heart rate (hemodynamics study). In line with other published literature (PMID: 30001646), we also used one-way ANOVA followed by Tukey's multiple comparisons to compare the means of multiple groups at one specific timepoint such as time in the open arm (elevated plus maze), distance traveled (open field test), and time in the target quadrant (probe trial-MWM). Please refer to Page 24 Line (589-602). Regarding microbiome analysis, in line with published literature by our group and others (PMID: 31784610, 37463881, 36113426, 36253847), we used PERMANOVA for the analysis of overall microbiome community differences between groups to account for phylogenetic and compositional information in our study. Please refer to Page 23 Line (570-587).
Reviewer #2

The introduction notes that prior TBI was associated with hypertension; however, it was unclear as written the direction of this association (that is, does TBI cause hypertension or is hypertension exacerbating TBI outcomes). Using a repetitive weight drop model of mTBI in male mice on a high salt diet, the research finds interactive effects of TBI and a high salt diet on anxiety-like behaviors and on microglial changes. The paper's findings might be impactful; however, as presented, the paper is difficult to evaluate. There are severe areas of the paper that need to be clarified.

Major concerns.

1. The association between TBI and hypertension is not clearly articulated in the current form, nor is the purpose of the study. Furthermore, there needs to be better alignment between the abstract and the introduction regarding the study's objectives. Both sections require revision. It appears that the study's objective is to evaluate if TBI is increasing the risk for hypertension, so they are stressing mice on a halt salt diet to see if there is a greater risk for hypertension, which they don't find. However, the paper needs to be clear about the goal, so the readers don't need to make assumptions about the purpose of the study.

Response: We thank the reviewer for this comment. Our study was done to evaluate the potential impact of rCHI and HSD on the development of hypertension as well as worsening neuroinflammation and understand whether their synergistic effect would predispose the brain for worsened cardiovascular and behavioral outcomes. Per the reviewer suggestion, we have revised the introduction section to clarify the purpose for this study, and we also clarified that TBI appears to lead to hypertension in the clinical studies. Please refer to Page 4 Line (104).

2. The rationale for studying the gut microbiome must be better articulated in the introduction.

Response: We have now added a section to the Introduction better explaining the rationale to study the microbiome. Please refer to Page 5 Line (119).

3. Generally, all methods are too short and do not provide sufficient detail for evaluating the results.

4. No reference is provided for the model of a high salt diet used and the physiological relevance to people

Response: As requested, we have expanded on the methodology sections of the manuscript and elaborated on the methods of the behavioral assays (Please see response to Reviewer #1). The HSD model used in the current study was first published in Nature Neuroscience by the Iadecola group (PMID ##); this paper is now referenced in the test of the Methods section. Although the Iadecola paper acknowledged that the amount of salt intake in their study is greater than that which a human would be expected to ingest, the high salt dose is accepted in the literature for the purposes of modeling and predicting outcome that might occur over years with a lower level of sustained salt intake in a human.

5. The timeline in Figure 1 is confusing. When was the TBI introduced compared to the diet? Was it 2 months before the diet changes? If it was, that might get to the question of whether TBI leads to hypertension or whether hypertension at the time of TBI leads to worse outcomes. What the study is trying to model needs to be clarified. It would be helpful to include time post-injury instead of or in addition to the time when the diet was introduced.

Response: Thank you for this important comment. We acknowledge the error in the timeline representation in Figure 1, and we have rectified it in our revised manuscript. Concerning the timing of repeated closed head injury (rCHI) in comparison to HSD, we initiated HSD 60 days after rCHI. To avoid any further confusion about the timeline in our study, we have revised the text to make it more clear to the reviewers and readership. Our study was done to evaluate the potential impact of rCHI and HSD on the development hypertension and worsening neuroinflammation and understand whether their synergistic effect would predispose the brain for worsened cardiovascular and behavioral outcomes.

6. Were the same cohort tested repeatedly in the different behavioral assays? This will affect the outcomes of the behavioral assays, and methods need to be better described.

Response: We thank the reviewer for this comment. We used the same cohorts repeatedly in different behavioral assays to detect degenerative changes over time- a goal that could not be achieved with certainty using separate cohorts for each time point. While it is true that repeat testing can change the outcome of some behavioral tests (e.g. reversal trials in the MWM will lead to reduced overall latency to the platform because of procedural learning but group differences will still be detectable in our experience), all of our behavioral experiments included a control group (Sham ND) to support the validity of our results. Notably, we designed our behavioral testing sequence to commence with the Elevated Plus Maze and conclude with the Morris Water Maze (MWM), strategically minimizing MWW-related anxiety as a confounding factor. In response to the request, we have expanded the methodology section, providing additional details about the timeline of our behavioral assays and the testing environment under the Behavioral studies section on Page 17 Line 430 of the manuscript.

7. Statistical methods are unclear and possibly incorrect. For instance, a one-way and two-way ANOVA are mentioned, but it doesn't state if the data were evaluated for repeated measures.

Response: We thank the reviewer for this comment. We have revised our statistical methods section and clarified the analytic testing used for each experiment. Regarding our behavioral experiments, in line with other published studies (PMID 30001646), we performed a two factor repeated measures two-way ANOVA (group x time) to assess two independent variables including time and the study dependent outcome such as latency to fall (rotarod), latency to platform (MWM) and blood pressure/ heart rate (hemodynamics study). We used one-way ANOVA followed by Tukey's multiple comparisons to compare the means of multiple groups when the test only had a single time point even if it was done several times over the course of the studies. This included time in the open arm (Elevated plus maze), distance traveled (Open field test), and time in the target quadrant (Probe trial-MWM).
Statistical analysis for 16S rRNA sequencing data and microglia RNA sequencing are expanded upon in their respective methods sections. Differences were considered statistically significant with a p value of less than 0.05.

8. Sampling methods for IHC are unclear. How many regions of interest were selected for each animal? How were those selected? Was the experimenter blind to treatment conditions? The figure in the paper shows confocal images, but the results were generated with widefield images. Examples of the widefield images should be included as supplemental data.

Response: We thank the reviewer for this comment. We have added the widefield images in Supplementary Figure 2 and describe how the different brain regions were chosen for the analysis in the Methods.

9. It is interesting that microglia numbers increased. How was this quantified? Microglia often hypertrophy after injury, but with a mild injury, it is less likely to have recruitment of cells or proliferation of microglia. Is the greater number of microglia associated with proliferation, or is it an artifact of the quantification methods? From the FACS sorting experiments, can you look at microglia numbers in those samples?

Response: We thank the reviewer for this comment. We obtained the numbers of microglia by scanning regions at 500x500 µm boxes for 6 consecutive sections at comparable depth in each animal. Data were number of IBA1+ cells/0.25mm2. In line with the published literature (PMID 33472865) and (PMID 31201215). All quantifications were performed with Image J analysis software as previously described. We have confidence in the validity of our quantification methods and do not perceive any artifacts. Our group (Dr. Whalen, Dr. El Khoury, Dr. Weiner) have advanced expertise in studying microglia biology in neurological diseases. We updated the methodology section of our manuscript to address the reviewer's comment. As we mentioned in our methodology section, we sorted microglia from the whole brain, so our FACS data might not reflect the changes we observed in our IHC section (hippocampus, amygdala, and cortex).

10. What is the sample size of the microbiome experiments?

Response: We thank the reviewer for this comment. Regarding the microbiome experiments, we collected weekly stool samples from (n=8) mice per group condition. We have added the sample size to the legend of Figure 4 Page 41 Line (983) and the 16S rRNA microbiota sequencing and Microbial Community Analysis of the methods Page 23 Line (558-560).

11. How were animals housed for the microbiome experiments?

Response: We thank the reviewer for this comment. Mice were co-housed in cages of 4-animals per cage, with 2 being sham and 2 rCHI and 60 days post post rCHI the HSD or ND was introduced to the cages. We have clarified this in the methodology section on Page 16 Line (407-409).
