# Supplementary material for: High-salt diet induces microbiome dysregulation, neuroinflammation and anxiety in the chronic period after mild repetitive closed head injury in adolescent mice
Source: Brain Commun. 2024 May 3;6(4):fcae147. doi: 10.1093/braincomms/fcae147 (PMC11264151; doi:10.1093/braincomms/fcae147)
Supplement: fcae147_Supplementary_Data [file Revision_1_ Response_to_reviewers_comments.pdf › Revision_1_ Response_to_reviewers_comments.pdf]

Point by point response to the Scientific Editor:
We sincerely thank the reviewers for provisionally accepting our study for publication in Brain Communications. We also thank the Scientific Editor for their valuable comments which we have addressed in our revised manuscript as detailed below.

Scientific Editor Comments:

1. Fig4b-c,f, Supp Fig3-4: please report in the figure legend the test used and where missing and relevant test statistic values alongside p values (for example F or t values for ANOVAs and t-tests). Also please different symbols to make the figure colour-blind friendly.

Response: We thank the Scientific Editor for these comments. We have updated the legends for these figures to reflect the tests used alongside p-values and relevant test statistic values. We have also updated the visualizations of the PCoA plots in Figure 4b, Supplementary Figure 3c, and Supplementary Figure 4a by using different symbols denoting different groups as recommended. The legend for Figure 4c had already stated the test used (ADONIS) and the accompanying p-value ranges as exhibited by the asterisks in the figure.

2. Fig4c: please, report in the legend the number of single data-points and what each data-point represents.

Response: We thank the Scientific Editor for this feedback. Upon reviewing the figure legends, we believe this comment refers to Figure 4b, not Figure 4c. We have updated the legends for all PCoA plots (Figure 4b, Supplementary Figure 3c, and Supplementary Figure 4a) to report the number of individual data points and clarify what each data point represents.

3. Supp Fig4b: please, report in the legend the number of single data-points.

Response: We thank the Scientific Editor for this feedback. Upon reviewing the figure legends, we believe this comment refers to Supplementary Figure 4a, not Supplementary Figure 4b We have updated the legend for the PCoA plot in Supplementary Figure 4a to report the number of individual data points and define what each data point represents, which we also did for all the other PCoA plots (Figure 4b, Supplementary Figure 3c).

4. Please supply a graphical abstract. A graphical abstract is a visual representation of the central finding or methodology of the paper. It will be displayed in the table of contents. Authors should strive to make the graphical abstract informative, interesting, visually appealing, and straightforward (no caption needed, no abbreviations except gene names). The technical requirements for the graphical abstracts are:
- Size: 120 x 120 mm (300-600dpi) or 3000 x 3000 pixels
- Font: Arial 12-16 pt
- File type: tiff or PDF
- Aspect ratio: 1:1 preferred

Response: We thank the Scientific Editor for this comment. We have submitted the graphical

abstract in the last submission. We are also kindly attaching it below:

5. Please add an Abbreviated Summary (up to 50 words) of your paper that captures the main purpose and conclusions of the work. The summary should state the main results written in the third person ('[1st author surname] et al. report that ...') and conclusion. This summary will be used in the contents list online and should not contain any abbreviations other than those on the accepted abbreviations list and accepted gene/protein names, making it accessible to the non-specialist. Please upload it as a Word document. Allowed abbreviations: AIDS; ANOVA; AMPA; ATP; A,T,C,G; CNS; CSF; CT; DNA; ECG; EEG; EMG; GABA; HIV; MRI; NMDA; PET; PCR; RNA.

Response: We thank the Scientific Editor for this comment. We have submitted an abbreviated summary previously, and we are kindly pasting it below:

Izzy et al. studied the impact of a high salt diet (HSD) on long-term cardiovascular and neuropsychiatric outcomes after repetitive closed head injury (rCHI). They identified that HSD disrupted the gut-brain axis, causing microbiome dysregulation, worsening microglial inflammation, and triggering anxiety-like behavior in chronic period after rCHI in adolescent mice.
