# Supplementary material for: High-salt diet induces microbiome dysregulation, neuroinflammation and anxiety in the chronic period after mild repetitive closed head injury in adolescent mice
Source: Brain Commun. 2024 May 3;6(4):fcae147. doi: 10.1093/braincomms/fcae147 (PMC11264151; doi:10.1093/braincomms/fcae147)
Supplement: fcae147_Supplementary_Data [file Revision_2_ Response_to_reviewers_comments.pdf › Revision_2_ Response_to_reviewers_comments.pdf]

Point by point response to the Scientific Editor:
We sincerely thank the reviewers for provisionally accepting our study for publication in Brain Communications. We also thank the Scientific Editor for their valuable comments which we have addressed in our revised manuscript as detailed below.

Scientific Editor Comments:

1. Fig4c and Supp Fig4b: please report in the figure legend the test used, and, please, show the single data-points since the number is <10 .

Response: We thank the Scientific Editor for these comments. The legend for both figures had already stated the test used (ADONIS test). We implemented the ADONIS statistical test to identify which factors are associated with differences in overall microbiome variation across all samples, in line with published literature by our group and others (PMIDs: 35272713, 36113426, 33876477). In the context of our work, the ADONIS test specifically accounts for the contribution of high salt diet administration, injury, and/or timepoint (in the case for fecal samples) on the variation in microbiota composition across all samples (n = 313 for fecal samples in total in Figure 4c, n = 23 for cecum samples in total for Supplementary Figure 4b). We have updated the legends for these figures accordingly to clearly state the test used and the total number of data-points.
