# Supplementary material for: High-salt diet induces microbiome dysregulation, neuroinflammation and anxiety in the chronic period after mild repetitive closed head injury in adolescent mice
Source: Brain Commun. 2024 May 3;6(4):fcae147. doi: 10.1093/braincomms/fcae147 (PMC11264151; doi:10.1093/braincomms/fcae147)
Supplement: fcae147_Supplementary_Data [file Revision_3_ Response_to_reviewers_comments.pdf › Revision_3_ Response_to_reviewers_comments.pdf]

We thank the scientific editor for this valuable comment. We think there is some sort of confusion regarding the ADONIS statistical testing used in our manuscript. As you may be aware, ADONIS fundamentally operates by 1) computing aggregated centroids for each user-defined group based on variables of interest (such as diet, injury, or timepoint), and 2) permuting and testing for separation between these group centroids. Therefore, the key experimental units remain the grouping factors themselves provided in the formula – in our case diet, injury, and timepoint. The individual samples/measurements are not being treated as the experimental unit here, however, ADONIS asks whether differences between these user-defined variable groups better explain variation between multidimensional averages than expected by chance alone. For example, for the high salt diet variable, the ADONIS test essentially asks: "do the centroids for high-salt vs normal diet groups explain more variation between aggregates than random noise?"

The way we have discussed the results of the ADONIS test is consistent with published literature by our group and others (PMIDs: 35272713, 36113426, 33876477). If our response doesn't help clearing the confusion about the ADONIS test and the reported results, we are happy to remove Figures 4C and Supplementary Figure 4B, and instead mention the statistical results in the manuscript with further elaboration on the ADONIS interpretation.

For more details on the computational inner-workings of ADONIS please see the links below.
- https://rdrr.io/rforge/vegan/man/adonis.html
- https://chrischizinski.github.io/rstats/adonis/
- https://onlinelibrary.wiley.com/doi/full/10.1002/9781118445112.stat07841
