# Supplementary material for: High-salt diet induces microbiome dysregulation, neuroinflammation and anxiety in the chronic period after mild repetitive closed head injury in adolescent mice
Source: Brain Commun. 2024 May 3;6(4):fcae147. doi: 10.1093/braincomms/fcae147 (PMC11264151; doi:10.1093/braincomms/fcae147)
Supplement: fcae147_Supplementary_Data [file Revision_3_decision_letter.pdf › Revision_3_decision_letter.pdf]

From: brcoms.editorialoffice@oup.com
To: MWHALEN@mgh.harvard.edu
CC:
BCC:
Subject: Brain Communications - Decision on Manuscript ID BRAINCOM-2023-443.R3
Body: 07-Feb-2024

Dear Dr. Whalen,

Thank you for your recent submission BRAINCOM-2023-443.R3
entitled "High salt diet induces microbiome dysregulation, neuroinflammation, and anxiety in the chronic period after mild repetitive closed head injury in adolescent mice." to Brain Communications.

I am pleased to let you know that we would like to provisionally accept your manuscript entitled "High salt diet induces microbiome dysregulation, neuroinflammation, and anxiety in the chronic period after mild repetitive closed head injury in adolescent mice." for publication in Brain Communications if you can comply with the requests below around data analysis. Further peer-review will not be necessary.
Please would you respond to the comments shown below from reviewers and/or our Scientific Editor (regarding our requirements for presentation of scientific data at Brain Communications) and submit a revised version.

Please format your revised manuscript according to our Instructions to Authors
https://academic.oup.com/braincomms/pages/General_Instructions
A summary of key points is attached to help with this. Please note that there can be substantial delays to publication if you don't resubmit in line with our requirements.

To revise your manuscript, click the link below or log into
https://mc.manuscriptcentral.com/braincom and enter your Author Centre, where you will find your manuscript title listed under "Manuscripts with Decisions." Under "Actions," click on "Create a Revision." Your manuscript number will be appended to denote a revision.

*** PLEASE NOTE: This is a two-step process. After clicking on the link, you will be directed to a webpage to confirm. ***

https://mc.manuscriptcentral.com/braincom?URL_MASK=d8243b39fff84bc6b72f1709fd77d5d
2

Please upload a revised manuscript with the changes you have made shown either with "track changes" or in coloured text.

When submitting your revised manuscript, you will be asked to respond to the reviewer(s) and

editor's comments. Please be as specific as possible in your response to all of the comments.

The editorial team at Brain Communications also aims to increase transparency in scientific publishing and to demonstrate the value that peer review brings to the scientific process. Please therefore consider whether you are willing to have reviewer comments on your paper and your response published as supplementary data with the paper if it is accepted. This is entirely optional and will only be done if both the authors and reviewers agree. When submitting your revision you will have the opportunity to update the answer you entered previously.

IMPORTANT: Your original files are available to you when you upload your revised manuscript. Please delete any redundant files before completing the submission.

Because we are trying to facilitate timely publication of manuscripts submitted to Brain Communications, we would appreciate your revised manuscript as soon as possible. If it is not possible for you to submit your revision within 3 months, the option to submit a revision will expire, and we will consider your revised paper as a new submission. However, if you need a little more time, please get in touch and we can extend this deadline for you.

Once again, thank you for submitting your manuscript to Brain Communications and I look forward to receiving your revision.

Best wishes

Dr. Manuela Marescotti
Scientific Editor, Brain Communications

Professor Tara Spires-Jones
Founding Editor, Brain Communications

Comments from the Scientific Editor:

- thank you for reporting the number of data-points in the Fig4c and Supp Fig4b, but I still see in the two legends that the number of animals is <10. lease note: We do not accept analyses where individual measurements are the experimental unit when there is likely to be biological variability between animals, culture replicates, etc. For this type of data, we require either that a mean or median is taken per animal/culture replicate followed by statistical analyses, or that more sophisticated analyses are performed such as mixed effects modelling which can account for random effects. Please, clarify this point for me.
